# ﻿Studies in phylogeny and divergence times of *Irpicaceae* and *Meripilaceae* (*Polyporales*, *Basidiomycota*), with an emphasis on *Ceriporia* and *Meripilus* including ten new species

**DOI:** 10.3897/imafungus.16.161336

**Published:** 2025-10-15

**Authors:** Chao-Ge Wang, Ying-Da Wu, Xin Zhang, Yu-Cheng Dai, Zhen-Hao Li, Yuan Yuan

**Affiliations:** 1 State Key Laboratory of Efficient Production of Forest Resources, School of Ecology and Nature Conservation, Beijing Forestry University, Beijing 100083, China Beijing Forestry University Beijing China; 2 Key Laboratory of Forest and Grassland Fire Risk Prevention, Ministry of Emergency Management, China Fire and Rescue Institute, Beijing 102202, China China Fire and Rescue Institute Beijing China; 3 Zhejiang Key Laboratory of Biological Breeding and Exploitation of Edible and Medicinal Mushrooms, Jinhua 321200, Zhejiang, China Zhejiang Key Laboratory of Biological Breeding and Exploitation of Edible and Medicinal Mushrooms Jinhua China; 4 Zhejiang Shouxiangu Pharmaceutical Co., Ltd, Jinhua 321000, Zhejiang, China Zhejiang Shouxiangu Pharmaceutical Co., Ltd Jinhua China

**Keywords:** *
Meruliopsis
*, molecular clock, *
Physisporinus
*, taxonomy, wood-decaying fungi

## Abstract

Species in *Ceriporia* and *Meripilus* are important wood-decaying fungi causing white rot on both angiosperm and gymnosperm wood. Morphologically, the two genera share similar micromorphology: a monomitic hyphal system of cyanophilous generative hyphae bearing simple septa. Phylogenetic and morphological analyses of *Ceriporia* and other related genera in *Irpicaceae* and *Meripilus* in *Meripilaceae* were carried out. *Ceriporia* is characterized by mostly resupinate basidiomata with a white to brightly colored pore surface when fresh, usually without changing color when bruised, and cylindrical to allantoid basidiospores. *Meripilus* is similar to *Ceriporia*, but it has resupinate, effused-reflexed to pileate basidiomata, sometimes with an erubescent pore surface when bruised, and ellipsoid to globose basidiospores. Phylogenies of species in the two genera were reconstructed with multiple-loci DNA sequences, including ITS, nLSU, nSSU, TEF1, RPB1, and RPB2, as well as two combined datasets: ITS+nLSU+TEF1+RPB1+nSSU for *Ceriporia* and ITS+nLSU+TEF1+RPB2+nSSU for *Meripilus*. Three new species of *Ceriporia*, one new species of *Meruliopsis*, and six new species of *Meripilus* are described and illustrated. Moreover, the evolutionary times of the *Polyporales*, including *Irpicaceae* and *Meripilaceae*, were revealed based on the conserved regions of three-loci DNA sequences (ITS+nLSU+TEF1). *Irpicaceae* and *Meripilaceae* are estimated to have emerged at the junction of the early and late Cretaceous, with mean crown ages of 108.9 Myr and 97.23 Myr, respectively. Bayesian evolutionary analysis shows that the divergence of *Ceriporia* emerged with a mean crown age of 83.61 Myr [95% highest posterior density (HPD): 65.25–106.35 Myr], which occurred during the late Cretaceous; the initial diversification of *Meripilus* also occurred during the late Cretaceous, with a mean crown age of 81.38 Myr [95% HPD: 61.89–105.78 Myr].

## ﻿Introduction

Wood-decaying fungi are capable of causing white or brown rot in wood, playing a crucial role as decomposers in forest ecosystems. *Polyporales* is one of the main orders of wood-decaying fungi, including more than 15 families (Justo et al. 2017). Many studies focus on certain families in the *Polyporales*, such as *Polyporaceae* Fr. ex Corda, *Fomitopsidaceae* Jülich, and *Ganodermataceae* Donk, which contain many taxa and have high medicinal and ecological value (Ji et al. 2022; Sun et al. 2022; Liu et al. 2023; Spirin et al. 2024). In contrast, *Irpicaceae* Spirin & Zmitr., especially the poroid species, and *Meripilaceae* Jülich mostly have unimpressive basidiomata with light-colored hymenophores, and thus they have received less research attention.

*Irpicaceae*, typified by *Irpex* Fr., was established by Spirin in 2003 (Spirin 2003). It belongs to the phlebioid clade within the *Polyporales* Gäum. and includes thirteen genera, viz., *Byssomerulius* Parmasto, *Ceriporia* Donk, *Crystallicutis* El-Gharabawy, *Cytidiella* Pouzar, *Efibula* Sheng H. Wu, Leal-Dutra & G.W. Griff., *Gloeoporus* Mont., *Irpex*, *Leptoporus* Quél., *Meruliopsis* Bondartsev, *Phanerochaetella* C.C. Chen & Sheng H. Wu, *Raduliporus* Spirin & Zmitr., *Resiniporus* Zmitr., and *Trametopsis* Tomšovský, with variable hymenophores, including corticioid and irpicoid species, and resupinate to pileate polypores (Chen et al. 2021).

Previous phylogenetic studies (Binder et al. 2013; Justo et al. 2017; Chen et al. 2020, 2021) determined that the genus *Ceriporia* is polyphyletic. Three species—*Phanerochaete allantospora* Burds. & Gilb., *Candelabrochaete langloisii* (Pat.) Boidin, and *Candelabrochaete septocystidia* (Burt) Burds.—were proposed to be combined into *Ceriporia* based on similar morphology and correlative phylogeny (Wang et al. 2023). Although two species accepted in *Leptoporus* Quél., *L. mollis* (Pers.) Quél. and *L. submollis* B.K. Cui & Shun Liu, are nested in the *Ceriporia* clade, the genus causes brown rot in dead gymnosperm wood, while *Ceriporia* causes white rot in dead angiosperm and gymnosperm wood (Ryvarden and Gilbertson 1993; Chen et al. 2020; Liu et al. 2023; Wang et al. 2023). Therefore, *Leptoporus* and *Ceriporia* represent two separate genera because of their different ecological habits (Wang et al. 2023). Wang et al. (2023) clarified the relationship between *Ceriporia* and related genera by reconstructing the phylogenies of *Irpicaceae* using five-loci DNA sequences and amended the definition of *Ceriporia*.

*Meripilaceae* was established in 1982 as belonging to the *Polyporales* and contains three genera, viz., *Meripilus* P. Karst., *Physisporinus* P. Karst., and *Spongipellis* Pat. (Wang and Dai 2022). Wang et al. (2024) discussed the phylogenetic relationship of *Meripilus* and *Physisporinus* using multigene phylogenetic analyses and indicated that *Meripilus* is a separate genus nested in the *Physisporinus* clade. In addition, the status of three confusing species—*Physisporinus vitreus* (Pers.) P. Karst., *P. sanguinolentus* (Alb. & Schwein.) Pilát, and *P. expallescens* (P. Karst.) Pilát—was discussed (Wang et al. 2024). Recently, Westphalen et al. (2025) transferred all species of *Physisporinus* into *Meripilus* to propose a monophyletic classification of genera within *Meripilaceae*. In addition, *Henningsia* Möller was also combined into *Meripilus* based on morphological and phylogenetic evidence (Westphalen et al. 2025). *Spongipellis* traditionally contains four species: *S. delectans* (Peck) Murrill, *S. litschaueri* Lohwag, *S. spumeus* (Sowerby) Pat., and *S. unicolor* (Fr.) Murrill. The type species, *S. spumeus*, formed a separate clade addressed in *Meripilaceae* (Spirin et al. 2022; Wang and Dai 2022; Miettinen et al. 2023), while differing from the other three species nested in *Cerrenaceae* (Wang and Dai 2022; Spirin et al. 2022; Miettinen et al. 2023).

Morphologically, *Ceriporia* has resupinate basidiomata with a white to brightly colored pore surface when fresh, usually without changing color when bruised; a monomitic hyphal system of cyanophilous generative hyphae bearing simple septa; and cylindrical to allantoid, hyaline, thin-walled basidiospores that are negative in Melzer’s reagent and Cotton Blue (Wang et al. 2023). *Meripilus* is characterized by annual, resupinate, effused-reflexed to pileate basidiomata, sometimes with a white pore surface when fresh and erubescent when bruised, or with a brightly colored pore surface; a monomitic hyphal system of cyanophilous generative hyphae bearing simple septa; and ellipsoid to globose, hyaline, thin- to slightly thick-walled basidiospores that are negative in Melzer’s reagent and mostly negative in Cotton Blue (Wang et al. 2024). Thus, much of the micromorphology of *Ceriporia* and *Meripilus* overlaps, and sometimes it is difficult to distinguish them macroscopically.

Comparative analyses of nucleotide sequences have concluded that molecular evolution occurs at a relatively constant rate, thereby giving rise to the concept of the molecular clock (Zuckerkandl 1962; Zuckerkandl and Pauling 1965). By using fossil records or geological events as calibration points in conjunction with phylogenetic trees as the foundational framework, estimation of divergence times among different taxonomic groups has become a widely adopted methodology in evolutionary biology (Avise and Johns 1999; Yang et al. 2007; Havill et al. 2008; Lockwood et al. 2013; Zhao et al. 2016, 2017; Varga et al. 2019; Ji et al. 2022). Molecular divergence times of fungi should reflect their different taxonomic ranks according to the geological ages when they evolved (Hennig 1966; Zhao et al. 2016). *Ceriporia* and *Meripilus* belong to different families, *Irpicaceae* and *Meripilaceae*, respectively, but have similar macroscopic morphological characteristics. In addition, the molecular divergence times of *Ceriporia* and *Meripilus*, and of *Irpicaceae* and *Meripilaceae*, need to be analyzed in an attempt to reveal the reasons for differences between them.

Poroid fungi in the *Irpicaceae*, especially *Ceriporia* and related genera, including *Meruliopsis* and *Gloeoporus*, have long been neglected. Early studies (Jia et al. 2014; Miettinen et al. 2016; Yuan et al. 2017) insufficiently addressed species diversity and taxonomic classification until Chen et al. (2020) proved the independence of *Ceriporia* and related genera by phylogenetic studies and sorted out the taxonomic status of the relevant species. Wang et al. (2023) clarified the taxonomy and phylogeny of *Ceriporia* and other related taxa in *Irpicaceae* with more specimens from around the world, especially from China, amended their definitions, studied morphologically confusing species, and speculated on the divergence time of *Irpicaceae*. *Meripilaceae*, as a small family in *Polyporales*, included two unimpressive genera, *Meripilus* and *Spongipellis* (Wang and Dai 2022; Wang et al. 2024; Westphalen et al. 2025). Although phylogenetic and morphological analyses of *Meripilaceae* have been carried out (Wang et al. 2024), more relevant specimens are being continuously collected in China, and there is a lack of research on the divergence time of *Meripilaceae*. In addition, *Ceriporia* and *Meripilus* have similar morphological characteristics and ecological habits, with broad-leaved trees being the most common hosts, but they belong to different families and have not been analyzed together. In this study, the morphology, phylogeny, and divergence times of *Ceriporia* and *Meripilus* are investigated. In addition, three new species of *Ceriporia*, one new species of *Meruliopsis*, and six new species of *Meripilus* are described and illustrated.

## ﻿Materials and methods

### ﻿Morphological studies

The studied specimens were deposited in the Fungarium of the Institute of Microbiology, Beijing Forestry University (BJFC). Morphological descriptions are based on field notes and voucher specimens. The microscopic analysis follows Wu et al. (2022) and Wang et al. (2023). Sections were studied at a magnification of up to 1000× using a Nikon Eclipse 80i microscope with phase contrast illumination. Microscopic features and measurements were made from slide preparations stained with Cotton Blue and Melzer’s reagent. Spores were measured from sections cut from the tubes. To represent the variation in spore size, 5% of measurements were excluded from each end of the range and are given in parentheses. In the description: KOH = 5% potassium hydroxide, IKI = Melzer’s reagent, IKI– = neither amyloid nor dextrinoid, CB = Cotton Blue, CB+ = cyanophilous in Cotton Blue, CB– = acyanophilous in Cotton Blue, L = arithmetic average of spore length, W = arithmetic average of spore width, Q = L/W ratios, and n = number of basidiospores measured from a given number of specimens. Color terms follow Anonymous (1969) and Petersen (1996).

### ﻿DNA extraction, amplification, and sequencing

A CTAB rapid plant genome extraction kit DN14 (Aidlab Biotechnologies Co., Ltd, Beijing) was used to obtain DNA from dried specimens and to perform the polymerase chain reaction (PCR) according to the manufacturer’s instructions with some modifications (Sun et al. 2020; Wang et al. 2023). The internal transcribed spacer (ITS) and large subunit nuclear ribosomal RNA gene (nLSU) were amplified using the primer pairs ITS5/ITS4 and LR0R/LR7, respectively (White et al. 1990; Hopple and Vilgalys 1999) (https://sites.duke.edu/vilgalyslab/rdna_primers_for_fungi/). The small subunit nuclear ribosomal RNA gene (nSSU) region was amplified with primer pairs NS1 and NS4 (White et al. 1990). Part of TEF1 was amplified with primer pairs EF1-983F and EF1-1567R (Rehner and Buckley 2005). RPB1 was amplified with primer pairs RPB1-Af and RPB1-Cr (Matheny et al. 2002). RPB2 was amplified with primer pairs fRPB2-5F and fRPB2-7CR (Matheny 2005).

The PCR procedure for ITS and TEF1 was as follows: initial denaturation at 95 °C for 3 min, followed by 34 cycles at 94 °C for 40 s, 54 °C for ITS and 54 °C for TEF1 for 45 s, and 72 °C for 1 min, with a final extension at 72 °C for 10 min. The PCR procedure for nLSU and nSSU was as follows: initial denaturation at 94 °C for 1 min, followed by 34 cycles of denaturation at 94 °C for 30 s, annealing at 50 °C for nLSU and 52 °C for nSSU for 1 min, and extension at 72 °C for 1.5 min, with a final extension at 72 °C for 10 min. The PCR procedure for RPB1 and RPB2 was as follows: initial denaturation at 94 °C for 2 min, followed by 10 cycles at 94 °C for 45 s, 60 °C for 45 s, and 72 °C for 1.5 min, then followed by 37 cycles at 94 °C for 45 s, 52 °C for 1 min, and 72 °C for 1.5 min, with a final extension at 72 °C. The PCR products were purified and sequenced at the Beijing Genomics Institute (BGI), China, with the same primers used in PCR. Newly generated sequences were deposited in GenBank. All sequences analyzed in this study are listed in Table 1.

**Table 1.** Taxa information and GenBank accession numbers of the sequences used in this study.

Species name	Sample no.	Location	GenBank accession No.						References
			ITS	nLSU	nSSU	TEF1	RPB1	RPB2	
*Agaricus campestris*	LAPAG370	—	KM657927	KR006607	—	KR006636	—	—	Zhou et al. (2016)
*Amylocorticium cebennense*	CFMR: HHB-2808	USA	GU187505	GU187561	GU187612	GU187675	GU187439	—	Binder et al. (2010)
*Antella americana*	HHB-4100-Sp	USA	EU232186	KP135196	—	—	—	—	Unpublished
*Aphanobasidium pseudotsugae*	CFMR: HHB-822	USA	GU187509	GU187567	GU187620	GU187695	GU187455	—	Binder et al. (2010)
*Athelia epiphylla*	CFMR: FP-100564	USA	GU187501	GU187558	GU187613	GU187676	GU187440	—	Binder et al. (2010)
*Bjerkandera adusta*	Dai 14516	China	MW507097	MW520204	OQ509532	OQ559572	OQ542973	—	Wang et al. (2021)
*B. fumosa*	Dai 21100	China	MW507109	MW520211	OQ509533	OQ559573	OQ542974	—	Wang et al. (2021)
*Boletopsis leucomelaena*	AFTOL-ID 1527	USA	DQ484064	DQ154112	DQ435797	GU187763	GU187494	—	Matheny et al. (2006a)
*Boletus edulis*	HMJAU4637	—	JN563894	KF112455	—	KF112202	KF112586	—	Feng et al. (2012)
*Bondarzewia tibetica*	Yu 56	China	KT693203	KT693205	—	KX066148	KX066158	—	Unpublished
*Byssomerulius corium*	FP-102382	USA	KP135007	KP135230	—	—	KP134802	—	Floudas and Hibbett (2015)
*Callistosporium graminicolor*	AFTOL-ID 978	USA	DQ484065	AY745702	AY752974	GU187761	GU187493	—	Matheny et al. (2006a)
*Ceriporia allantoidea*	Cui 8097	China	KC182780	—	—	—	—	—	Jia et al. (2014)
*C. allantoidea*	Dai 8110 (holotype)	China	KC182767	KC182784	—	—	—	—	Jia et al. (2014)
*C. allantospora*	RLG-10478 (holotype)	USA	KP135039	—	—	—	—	—	Floudas and Hibbett (2015)
*C. arbuscula*	GC 1708–338 (holotype)	China	LC427008	LC427040	—	—	LC427058	—	Chen et al. (2020)
*C. arbuscula*	GC 1708-340	China	LC427009	LC427041	—	—	LC427068	—	Chen et al. (2020)
*C. arbuscula*	Dai 26109	China	PQ650033ª	PQ642628ª	PQ644088ª	PQ662349ª	PQ662329ª	—	Present study
*C. arbuscula*	Dai 26107	China	PQ650034ª	PQ642629ª	PQ644089ª	PQ662350ª	PQ662330ª	—	Present study
***C. armeniaca***	**Dai 24678A (holotype)**	**China**	**PQ650035ª**	**PQ642630ª**	**PQ644090ª**	**PQ662351ª**	**PQ662331ª**	—	**Present study**
*C. aurantiocarnescens*	JV 0105/10	Czechia	KX236482	KX236482	—	—	—	—	Spirin et al. (2016)
*C. aurantiocarnescens*	Dai 17951	China	MW491774	MW491764	—	—	—	—	Unpublished
*C. bresadolae*	Rivoire 3701	France	KX236467	KX236467	—	—	—	—	Spirin et al. (2016)
*C. bresadolae*	Dai 24539	China	OQ476820	OQ476765	OQ509535	OQ559575	OQ542975	—	Wang et al. (2023)
*C. bresadolae*	Dai 24541	China	OQ476821	OQ476766	OQ509536	OQ559576	OR090875	—	Wang et al. (2023)
*C. bresadolae*	VS 4018	Russia	KX236466	—	—	—	—	—	Spirin et al. (2016)
*C. bubalinomarginata*	Dai 17937	China	MW491775	MW491765	—	—	—	—	Chen et al. (2022)
*C. bubalinomarginata*	Dai 12113	China	OQ476822	OQ476768	OQ509538	—	—	—	Wang et al. (2023)
Ceriporia cf. mellita	GC 1508-71	China	LC427022	LC427044	—	—	LC427067	—	Chen et al. (2020)
Ceriporia cf. mellita	WEI 17-024	China	LC427024	LC427046	—	—	—	—	Chen et al. (2020)
Ceriporia cf. mellita	GC 1608-7	Japan	LC427023	LC427045	—	—	LC427060	—	Chen et al. (2020)
Ceriporia cf. mellita	Dai 8168	China	KC182768	KC182785	—	OQ559578	—	—	Jia et al. (2014)
Ceriporia cf. mellita	Dai 27083	China	PQ650036ª	PQ642631ª	PQ644091ª	—	—	—	Present study
Ceriporia cf. mellita	Dai 27085	China	PQ650037ª	PQ642632ª	—	—	—	—	Present study
*C. crassa*	Dai 22034 (holotype)	China	OQ476823	OQ476769	OQ509539	OQ559579	OQ542977	—	Wang et al. (2023)
*C. crassa*	JV 1008/41-J	USA	PP485892ª	—	—	—	—	—	Present study
***C. crassiparietata***	**Dai 26986**	**China**	**PQ650038ª**	**PQ642633ª**	**PQ644092ª**	**PQ662352ª**	**PQ662332ª**	—	**Present stud**y
***C. crassiparietata***	**Dai 26988**	**China**	**PQ650039ª**	—		**PQ662353ª**	**PQ662333ª**	—	**Present study**
***C. crassiparietata***	**Dai 25079 (holotype)**	**China**	**PQ650040ª**	**PQ642634ª**	**PQ644093ª**	**PQ662354ª**		—	**Present study**
***C. crassiparietata***	**Dai 7759**	**China**	KC182777	—	—	—	—	—	Jia et al. (2014)
*C. daedaleoides*	Dai 16779 (holotype)	Thailand	KY825130	OR088493	OR095704	—	—	—	Wang et al. (2023)
*C. eucalypti*	Dai 18675 (holotype)	Australia	MW491779	MW491769	OR095705	OQ559580	—	—	Chen et al. (2022); Wang et al. (2023)
*C. excelsa*	Yuan 2747	China	KC182778	—	—	—	—	—	Jia et al. (2014)
*C. excelsa*	Yuan 2744	China	KC182773	—	—	—	—	—	Jia et al. (2014)
*C. gossypina*	Dai 23392 (holotype)	China	OQ476824	OQ476770	OQ509540	OQ559581	OR090876	—	Wang et al. (2023)
*C. gossypina*	Dai 26113	China	PQ650041ª	PQ642667ª	PQ644094ª	PQ662355ª	PQ662334ª	—	Present study
*C. griseoviolascens*	JV0110/26	Czechia	KX236487	KX236487	—	—	—	—	Spirin et al. (2016)
*C. griseoviolascens*	Dai 13202	France	OQ476825	OQ476771	OQ509541	OQ559582	—	—	Wang et al. (2023)
*C. griseoviolascens*	Dai 27053	China	PQ650042ª	PQ642635ª	PQ644095ª	PQ662356ª	PQ662335ª	—	Present study
*C. griseoviolascens*	Dai 27054	China	PQ650043ª	PQ642636ª	PQ644096ª	PQ662357ª	—	—	Present study
*C. hinnulea*	Cui 11291 (holotype)	China	OQ476826	OQ476772	OQ509542	OQ559583	OQ542978	—	Wang et al. (2023)
*C. humilis*	Dai 7642	China	KC182775	—	OR095706	OR113375	—	—	Jia et al. (2014); Wang et al. (2023)
*C. humilis*	Spirin 4706 (holotype)	Russia	KX752608	—	—	—	—	—	Miettinen et al. (2016)
*C. langloisii*	FP-110343-sp	USA	KY948793	KY948886	—	—	KY948981	—	Justo et al. (2017)
*C. macrospora*	Cui 6740 (holotype)	China	OQ476827	—	OQ509543	—	—	—	Wang et al. (2023)
*C. macrospora*	Dai 24695	China	OR086075	OR088494	OR095707	—	OR090877	—	Wang et al. (2023)
“Ceriporia aff. macrospora"	MEL 2382688	Australia	KP013052	—	—	—	—	—	Unpublished
*C. manzanitae*	Ryvarden 21832 (holotype)	USA	KX236478	KX236478	—	—	—	—	Spirin et al. (2016)
*C. mellita*	BR 4865	France	KX236485	KX236485	—	—	—	—	Spirin et al. (2016)
*C. mellita*	Dai 19118	China	OQ476831	OQ476776	OQ509547	—	—	—	Wang et al. (2023)
*C. mellita*	Dai 18486A	China	OQ476832	OQ476777	OR095708	—	—	—	Wang et al. (2023)
*C. mpurii*	Dai 24426	China	OQ476833	OQ476778	OR095709	—	OR090878	—	Wang et al. (2023)
*C. mpurii*	He 6687	China	OQ476834	OQ476779	OR095710	OR113376	OR090879	—	Wang et al. (2023)
*C. mpurii*	Miettinen 14381 (holotype)	Indonesia	KX752603	KX752603	—	—	—	—	Miettinen et al. (2016)
*C. occidentalis*	Spirin 8558	USA	KX236475	KX236475	—	—	—	—	Spirin et al. (2016)
*C. occidentalis*	JV 1105/12-J	USA	KX236473	KX236473	—	—	—	—	Spirin et al. (2016)
*C. orientalis*	Li 1045	China	JX623946	—	OR095711	—	—	—	Jia et al. (2014); Wang et al. (2023)
*C. orientalis*	Dai 13400 (holotype)	China	OQ476835	OQ476780	OQ509548	OQ559585	OQ542979	—	Wang et al. (2023)
*C. orientalis*	Dai 25794	China	PQ650044ª	PQ642637ª	PQ644098ª	PQ662359ª	—	—	Present study
*C. pierii*	Dai 23500	China	OQ476836	OQ476781	OQ509549	OQ559586	OR090880	—	Wang et al. (2023)
*C. pierii*	Dai 23499	China	OQ476837	OQ476782	OQ509550	OQ559587	OQ542980	—	Wang et al. (2023)
*C. pierii*	Rivoire 1161 (holotype)	France	KX752604	KX752604	—	—	—	—	Miettinen et al. (2016)
*C. pseudospissa*	Dai 24566 (holotype)	China	OQ476838	OQ476783	OR095712	OR113377	—	—	Wang et al. (2023)
*C. pseudospissa*	Yuan 5965	China	KC182772	KC182783	—	—	—	—	Jia et al. (2014)
*C. punctata*	Dai 15899 (holotype)	China	OQ476839	OQ476784	OQ509551	OQ559588	OQ542981	—	Wang et al. (2023)
*C. punctata*	Dai 15904	China	OQ476840	OR088495	OQ509552	OQ559589	OQ542982	—	Wang et al. (2023)
*C. punctata*	Yuan 438	China	PQ650045ª	—	—	—	—	—	Present study
*C. punctata*	Yuan 439	China	PQ650046ª	—	—	—	—	—	Present study
*C. punicans*	Dai 13376	China	OQ476841	OQ476785	OQ509553	OQ559590	OQ542983	—	Wang et al. (2023)
*C. punicans*	JV 0808/30 (holotype)	USA	KX236479	KX236479	—	—	—	—	Spirin et al. (2016)
*C. purpurea*	Dai 22445	China	OQ476842	OQ476786	OQ509554	OQ559591	—	—	Wang et al. (2023)
*C. purpurea*	Dai 16368	China	KX494577	KX494581	OQ509555	OQ559592	OQ542984	—	Wang et al. (2023)
*C. purpurea*	Rivoire 4413 (neotype)	France	KX236461	KX236461	—	—	—	—	Yuan et al. (2017)
*C. reticulata*	Li 1316	China	JX623947	—	OR095713	—	—	—	Jia et al. (2014); Wang et al. (2023)
*C. reticulata*	KHL11981	Norway	JX109845	JX109845	—	—	—	—	Binder et al. (2013)
“*C. reticulata*"	RLG-11354-Sp	USA	KP135041	KP135204	—	—	KP134794	—	Floudas and Hibbett (2015)
“*C. reticulata*"	Dai 27072	China	PQ650047ª	PQ642638ª	PQ644099ª	PQ662360ª	PQ662337ª	—	Present study
*C. septocystidia*	RLG-9759-sp	USA	MZ636934	GQ470631	—	MZ913692	MZ748442	—	Chen et al. (2021)
*C. septocystidia*	RMJ-119-sp	USA	KY948783	—	—	—	KY948959	—	Justo et al. (2017)
*C. sericea*	Spirin 4944 (holotype)	Russia	KX752609	KX752609	—	—	—	—	Miettinen et al. (2016)
*C. sericea*	Dai 27086	China	PQ650048ª	PQ642639ª	PQ644100ª	PQ662361ª	—	—	Present study
*C. sericea*	Dai 26044	China	PQ650049ª	PQ642640ª	PQ644101ª	PQ662362ª	PQ662338ª	—	Present study
*C. sinospissa*	Cui 11282 (holotype)	China	OQ476843	OQ476787	OQ509556	OQ559593	—	—	Wang et al. (2023)
*C. sinospissa*	Dai 10477	China	KC182769	KC182781	—	—	—	—	Jia et al. (2014)
*C. sinospissa*	Dai 16831	China	OQ476844	OQ476788	OQ509557	OQ559594	—	—	Wang et al. (2023)
*C. sinoviridans*	Dai 13621A (holotype)	China	MW491781	MW491771	OQ509558	—	—	—	Chen et al. (2022)
*C. sinoviridans*	Li 1046	China	KC182776	—	—	—	—	—	Jia et al. (2014)
*C. sordescens*	Miettinen 15492.2 (holotype)	USA	KX752606	KX752606	—	—	—	—	Miettinen et al. (2016)
*Ceriporia* sp.	Dai 26805	China	PP479793ª	PP479793ª	PQ644097ª	PQ662358ª	PQ662336ª	—	Present study
*C. spissa*	JV 0108/6	USA	KX236483	KX236483	—	—	—	—	Spirin et al. (2016)
*C. spissa*	Dai 19164	Canada	OQ476845	OQ476789	OQ509559	—	—	—	Wang et al. (2023)
*C. subbadia*	Dai 15062	China	OQ476846	—	OQ509560	—	—	—	Wang et al. (2023)
*C. subviridans*	Cui 8012 (holotype)	China	KC182774	—	OR095714	OR113378	—	—	Jia et al. (2014); Wang et al. (2023)
*C. subviridans*	GC 1704-54	China	LC427026	LC427048	—	—	—	—	Chen et al. (2020)
*C. torpida*	Murdoch 90 (holotype)	Finland	KX236477	KX236477	—	—	—	—	Spirin et al. (2016)
*C. triumphalis*	Kout 18 (holotype)	Spain	KX236476	KX236476	—	—	—	—	Spirin et al. (2016)
*C. viridans*	Dai 17003	China	OQ476847	OQ476790	OQ509561	—	—	—	Wang et al. (2023)
*C. viridans*	Miettinen 11701	Netherlands	KX752600	KX752600	—	—	—	—	Miettinen et al. (2016)
*C. viridans*	Yuan 5702	Netherlands	KC182779	—	—	—	—	—	Jia et al. (2014)
***C. wuyiana***	**Dai 24998 (holotype)**	**China**	**PQ650050ª**	**PQ642641ª**	**PQ644102ª**	**PQ662363ª**	**PQ662339ª**	—	**Present study**
*Cerrena albocinnamomea*	Dai 12892	China	KC485522	KC485539	—	—	—	—	Yuan (2014)
*C. albocinnamomea*	Dai 12955	China	KC485521	KC485538	—	—	—	—	Yuan (2014)
*C. aurantiopora*	NIBRFG0000102423	Rep. Korea	FJ821532	FJ821521	—	—	—	—	Lee and Lim (2010)
*C. aurantiopora*	SNU-m 03110102	Rep. Korea	FJ821531	FJ821520	—	—	—	—	Lee and Lim (2010)
*C. consors*	F20080702KCM29	Rep. Korea	FJ821527	FJ821516	—	—	—	—	Lee and Lim (2010)
*C. consors*	F20080208 LYW10	Rep. Korea	FJ821528	FJ821517	—	—	—	FJ821543	Lee and Lim (2010)
*C. unicolor*	KHL-GB	Sweden	JQ031127	JQ031127	—	JX109891	—	JX109863	Sjoekvist et al. (2012)
*C. unicolor*	FD 299	USA	KP135304	KP135209	—	—	—	—	Floudas and Hibbett (2015)
*C. zonata*	Dai 7821	China	KC485529	KC485547	—	—	—	—	Yuan (2014)
*C. zonata*	Dai 7359	China	KC485528	KC485546	—	—	—	—	Yuan (2014)
*Cotylidia* sp.	MB5	—	AY854079	AY629317	AY705958	AY885148	AY864868	—	Unpublished
*Crystallicutis serpens*	HHB-15692-Sp	USA	KP135031	KP135200	—	—	KP134785	—	Floudas and Hibbett (2015)
*Crystallicutis* sp.	Miettinen-16854.3	USA	KY948742	KY948890	—	—	KY948964	—	Justo et al. (2017)
*Crystallicutis* sp.	Dai 6090	China	JX623934	JX644066	—	—	—	—	Jia et al. (2014)
*Cytidiella albida*	GB-1833	Spain	KY948748	KY948889	—	MZ913675	KY948960	—	Justo et al. (2017)
*C. nitidula*	T-407	Canada	KY948747	—	—	MZ913676	KY948961	—	Justo et al. (2017)
*Efibula americana*	FP-102165	USA	KP135016	KP135256	—	MZ913669	KP134808	—	Floudas and Hibbett (2015)
*Flaviporus minutus*	Dai 16222	China	KY131881	KY131938	—	—	—	—	Wu et al. (2017)
*F. minutus*	Dai 16240	China	KY131883	KY131940	—	—	—	—	Wu et al. (2017)
*Gloeophyllum sepiarium*	Wilcox-3BB	USA	HM536091	HM536061	HM536062	HM536110	—	—	Garcia-Sandoval et al. (2011)
*Gloeoporus africanus*	O 918063	Uganda	MG572763	MG572747	—	—	—	—	Jung et al. (2018)
*G. africanus*	O 918572 (holotype)	Uganda	MG572764	MG572748	—	—	—	—	Jung et al. (2018)
*G. citrinoalbus*	Dai 16238 (holotype)	China	KU360396	KU360404	OR095715	—	—	—	Yuan et al. (2016); Wang et al. (2023)
*G. citrinoalbus*	Dai 19547	Sri Lanka	OQ476849	OQ476792	OQ509563	OQ559595	OQ542985	—	Yuan et al. (2016); Wang et al. (2023)
*G. citrinoalbus*	Dai 15293	China	OQ476850	OQ476793	OQ509564	OQ559596	—	—	Yuan et al. (2016); Wang et al. (2023)
*G. dichrous*	Dai 23626	China	OQ476851	OQ476794	OQ509565	OQ559597	OQ542986	—	Wang et al. (2023)
*G. dichrous*	Dai 23260	China	OQ476852	OQ476795	OQ509566	OQ559598	OQ542987	—	Wang et al. (2023)
*G. dichrous*	Dai 22633	China	OQ476853	OQ476796	OQ509567	OQ559599	OQ542988	—	Wang et al. (2023)
*G. hainanensis*	Dai 15259	China	KU360403	KU360410	OQ509568	OQ559600	—	—	Yuan et al. (2016); Wang et al. (2023)
*G. hainanensis*	Yuan 4397	China	KU360400	KU360409	—	—	—	—	Yuan et al. (2016)
*G. hainanensis*	Dai 15268 (holotype)	China	KU360401	KU360411	OQ509569	OQ559601	OQ542989	—	Yuan et al. (2016); Wang et al. (2023)
*G. orientalis*	Cui 17922	China	OQ476854	OQ476797	OQ509570	OQ559602	—	—	Wang et al. (2023)
*G. orientalis*	Dai 18536A	China	OQ476856	—	—	—	—	—	Wang et al. (2023)
*G. pannocinctus*	FP 135015	USA	MG572755	MG572739	—	—	—	—	Jung et al. (2018)
*G. pannocinctus*	L-15726-Sp	USA	KP135060	KP135214	—	—	KP134867	—	Jung et al. (2018)
*G. septatus*	Dai 22221 (holotype)	China	OQ476857	OQ476798	OQ509572	OQ559604	OQ542990	—	Wang et al. (2023)
*G. thelephoroides*	BZ-2896	Belize	MG572757	MG572741	—	—	—	—	Jung et al. (2018)
*G. thelephoroides*	JV 1808/26	French Guiana	OQ476858	OQ476799	OQ509573	OQ559605	—	—	Wang et al. (2023)
*G. variiformis*	Dai 20655	China	OQ476859	OQ476801	OQ509574	OQ559606	OQ542991	—	Wang et al. (2023)
*G. variiformis*	Dai 22225 (holotype)	China	OQ476860	OQ476802	OQ509575	OQ559607	OQ542992	—	Wang et al. (2023)
*Hapalopilus ochraceolateritius*	Miettinen-16992.1	USA	KY948741	KY948891	—	—	KY948965	—	Justo et al. (2017)
*Hydnochaete duportii*	AFTOL-ID 666	—	DQ404386	AY635770	AY662669	DQ435793	—	—	Matheny et al. (2006a)
*Hyphoderma praetermissum*	AFTOL-ID 518	—	AY854081	AY700185	AY707094	AY885150	AY864871	—	Matheny et al. (2006a)
*Irpex flavus*	WHC 1381	China	LC427029	LC427052	—	—	LC427064	—	Chen et al. (2020)
*I. laceratus*	Dai 16433	China	OQ476861	OQ476803	OQ509576	OQ559608	OQ542993	—	Wang et al. (2023)
*I. laceratus*	PBU 0048	Thailand	KC570339	KU760725	—	—	—	—	Permpornsakul et al. (2016)
*I. laceratus*	Dai 21940	China	OQ476862	OQ476804	OQ509577	OQ559609	OQ542994	—	Wang et al. (2023)
*I. laceratus*	SFFPS MZ-340 (holotype)	—	AB091675	—	—	—	—	—	Suhara et al. (2003)
*I. lacteus*	Dai 11230	China	OQ476863	OQ476805	OQ509578	OQ559610	OQ542995	—	Wang et al. (2023)
*I. lacteus*	FD-9	—	KP135026	KP135224	—	—	KP134806	—	Floudas and Hibbett (2015)
*I. latemarginatus*	FP-55521-T	USA	KP135024	KP135202	—	—	KP134805	—	Floudas and Hibbett (2015)
*I. latemarginatus*	Marcin Piatek 4.IX.1997	Poland	KX752592	KX752592	—	—	—	—	Miettinen et al. (2016)
*I. rosettiformis*	Meijer 3729	Brazil	JN649346	JN649346	—	—	—	—	Sjökvist et al. (2012)
*Irpiciporus pachyodon*	SP-Lgt	Italy	AY849307	—	—	—	—	—	Pilotti et al. (2005)
*I. pachyodon*	PRM 846564	Czechia	HQ728293	HQ729003	—	—	—	—	Tomšovský (2012)
*I. sinuosus*	PW 17/171	Thailand	MK589288	—	—	—	—	—	Thamvithayakorn et al. (2019)
*I. sinuosus*	Dai 12234	China	KX161649	KX161658	—	OM982699	—	—	Unpublished
*I. xuchilensis*	Ryvarden 44669	Ecuador	KX161650	KX161659	—	—	—	—	Unpublished
*Jaapia argillacea*	CBS: 252.74	Netherlands	GU187524	GU187581	—	GU187711	—	—	Binder et al. (2010)
*Junghuhnia fimbriatella*	Miettinen 2091	Russia	JN710555	JN710555	—	—	—	—	Miettinen et al. (2012)
*Lactarius deceptivus*	AFTOL-ID 682	USA	AY854089	AY631899	AY707093	AY885158	AY864883	—	Matheny et al. (2006a)
*Leptoporus mollis*	RLG-7163	USA	KY948794	MZ637155	—	MZ913693	KY948956	—	Justo et al. (2017)
*L. mollis*	Dai 21062	Belarus	MW377302	MW377381	—	MW337129	—	—	Liu et al. (2023)
*L. submollis*	Dai 20182 (paratype)	China	ON468434	ON468246	—	ON468452	ON468448	—	Liu et al. (2023)
*L. submollis*	Cui 18379 (paratype)	China	ON468433	ON468245	—	ON468451	ON468447	—	Liu et al. (2023)
*Leptosporomyces raunkiaeri*	CFMR: HHB-7628	USA	GU187528	GU187588	GU187640	GU187719	GU187471	—	Binder et al. (2010)
*Macrohyporia dictyopora*	PBU 0051	Thailand	KC570331	KU760726	—	—	—	—	Permpornsakul et al. (2016)
***Meripilus albomarginatus***	**Dai 19796 (holotype)**	**China**	**PQ650057ª**	**PQ642661ª**	**PQ644116ª**	**PQ662374ª**	—	—	**Present study**
***M. albomarginatus***	**Dai 25241**	**China**	**PQ650058ª**	**PQ642662ª**	**PQ644117ª**	**PQ662375ª**	—	**PQ662347ª**	**Present study**
***M. albomarginatus***	**Dai 24711**	**China**	**PQ650059ª**	—	—	—	—	—	**Present stud**y
*M. albostygius*	Kout 1807/15.1 (holotype)	Puerto Rico	OM669892	OM669976	OM670027	—	—	OM810070	Wang et al. (2024)
*M. albostygius*	Kout 1508/18.1	Puerto Rico	OM669893	—	—	—	—	—	Wang et al. (2024)
*M. albostygius*	RP 185	Brazil	KP859303	—	—	—	—	—	Wang et al. (2024)
*M. brasiliensis*	RP 215	Brazil	PP259422	PP259408	—	—	—	—	Westphalen et al. 2025
*M. brasiliensis*	RP 200	Brazil	PP259421	PP259407	—	—	—	—	Westphalen et al. 2025
*M. caesiomarginatus*	Dai 19793	China	OM669891	OM669975	OM670024	OM810094	—	OM810067	Wang et al. (2024)
*M. castanopsidis*	Dai 20396 (holotype)	China	MT309485	MT309470	OM670025	OM810095	—	OM810068	Wang et al. (2024)
*M. castanopsidis*	Dai 20397	China	MT309486	MT309472	OM670026	OM810096	—	OM810069	Wang et al. (2024)
*M. castanopsidis*	Dai 11693	China	KY131865	KY131922	—	—	—	—	Wang et al. (2024)
*M. cinereus*	Cui 3266	China	KY131844	KY131903	—	—	—	—	Wu et al. (2017)
*M. cinereus*	Dai 22427	China	PQ650060ª	PQ642648ª	—	—	—	—	Present study
*M. cinereus*	Dai 24688	China	PQ650061ª	PQ642649ª	PQ644108ª	PQ662368ª	—	PQ662343ª	Present study
*M. cinereus*	Dai 17581	China	PQ650062ª	PQ642650ª	—	—	—	—	Present study
*M. cinereus*	Dai 24690	China	PQ650063ª	PQ642651ª	PQ644109ª	PQ662369ª	—	—	Present study
*M. concrescens*	MV 690	Brazil	PP259426	PP259411	—	—	—	—	Westphalen et al. (2025)
*M. crataegi*	Dai 15499	China	KY131846	KY131905	OM670028	OM810097	—	OM810071	Wu et al. (2017); Wang et al. (2024)
*M. crataegi*	Dai 15497 (holotype)	China	KY131845	KY131904	OM670029	OM810098	—	OM810072	Wu et al. (2017); Wang et al. (2024)
*M. crocatus*	Dai 12800	USA	KY131869	KY131925	—	—	—	—	Wu et al. (2017)
*M. crocatus*	Dai 15917	China	KY131870	KY131926	OM670063	OM810124	—	—	Wu et al. (2017); Wang et al. (2024)
*M. crocatus*	JV 0509/40	USA	OM669894	OM669977	OM670030	OM810099	—	—	Wang et al. (2024)
*M. crocatus*	DLL 2009-061	USA	JQ673152	—	—	—	—	—	Brazee et al. (2012)
*M. crocatus*	MJ 19/09	Slovakia	JQ409466	OM669978	—	—	—	—	Vampola and Vlasák (2012)
*M. crocatus*	JV 0808/33	USA	OM669895	OM669979	OM670031	OM810100	—	—	Wang et al. (2024)
***M. crystallinus***	**Cui 10491 (holotype)**	**China**	**PQ650064ª**	—	—	—	—	—	**Present stud**y
***M. crystallinus***	**Cui 10475**	**China**	**PQ650065ª**	**PQ642647ª**	—	—	—	—	**Present stud**y
*M. dollingerii*	Dollinger 880	USA	OM669897	—	OM670032	OM810101	—	OM810073	Wang et al. (2024)
*M. dollingerii*	Dollinger 1000	USA	OM669899	OM669982	OM670034	—	—	OM810075	Wang et al. (2024)
***M. emarginatus***	**Dai 24682A (holotype)**	**China**	**PQ650066ª**	**PQ642652ª**	**PQ644110ª**	**PQ662370ª**	—	**PQ662344ª**	**Present study**
***M. emarginatus***	**Dai 24683A**	**China**	**PQ650067ª**	**PQ642653ª**	**PQ644111ª**	**PQ662371ª**	—	**PQ662345ª**	**Present study**
***M. emarginatus***	**Dai 16971**	**China**	**PQ650068ª**	**PQ642654ª**	—	—	—	—	**Present study**
***M. emarginatus***	**Dai 24694A**	**China**	**PQ650069ª**	**PQ642655ª**	**PQ644112ª**	**PQ662372ª**	—	**PQ662346ª**	**Present study**
***M. emarginatus***	**Dai 26696**	**China**	**PQ650070ª**	**PQ642656ª**	—	—	—	—	**Present stud**y
*M. eminens*	Dai 11400	China	KY131852	KY131909	OM670035	OM810103	—	—	Wu et al. (2017), Wang et al. (2024)
*M. eminens*	Dai 20832	China	MT279689	MT279689	OM670036	OM810104	—	—	Wang et al. (2024)
*M. eminens*	Dai 22472	China	OM669900	OM669983	OM670037	OM810105	—	—	Wang et al. (2024)
*M. eminens*	Dai 20868	China	MT840117	MT840135	—	—	—	—	Wang et al. (2024)
*M. expallescens*	Dai 21060	Belarus	MT840130	MT840148	OM670077	—	—	OM810090	Chen and Dai (2021), Wang et al. (2024)
*M. expallescens*	MJ 332/94	Czechia	OM669935	—	—	—	—	—	Wang et al. (2024)
*M. expallescens*	MJ 642/93	Czechia	OM669936	—	—	—	—	—	Wang et al. (2024)
*M. galapagensis*	MV 513	Brazil	PP259430	PP259414	—	PP239252	PP239254	—	Westphalen et al. (2025)
*M. giganteus*	CBS 421.48	Germany	MH856418	—	—	—	—	—	Vu et al. (2019)
*M. giganteus*	FP-100460-Sp	Netherland	KP135306	—	—	—	—	—	Floudas and Hibbett (2015)
*M. giganteus*	Cui 9202	UK	OM669888	OM669973	OM670021	—	—	—	Wang et al. (2024)
*M. giganteus*	Cui 9203	UK	OM669889	—	OM670022	—	—	—	Wang et al. (2024)
*M. giganteus*	FP-135344-Sp	UK	KP135307	KP135228	—	—	—	—	Floudas and Hibbett (2015)
*M. furcatus*	TAA 150972 (holotype)	Russia	KY131853	KY131910	—	—	—	—	Wu et al. (2017)
*M. lavendulus*	Dai 9925	China	KY131858	KY131915	—	—	—	—	Wu et al. (2017)
*M. lavendulus*	Dai 13587A (holotype)	China	KY131859	KY131916	OM670038	OM810106	—	—	Wu et al. (2017)
*M. lineatus*	Dai 17986	China	MT840121	MT840139	OM670039	OM810107	—	OM810076	Chen and Dai (2021), Wang et al. (2024)
*M. lineatus*	Dai 18281	Vietnam	MT840123	MT840141	OM670040	OM810108	—	OM810077	Chen and Dai (2021), Wang et al. (2024)
*M. lineatus*	JV 1008/18	USA	OM669902	OM669985	OM670042	—	—	—	Wang et al. (2024)
*M. lineatus*	JV 1407/37	Costa Rica	OM669903	OM669986	OM670043	OM810109	—	OM810078	Wang et al. (2024)
*M. longicystidius*	PDD 70600 (holotype)	New Zealand	KY131863	—	—	—	—	—	Wu et al. (2017)
*M. longicystidius*	Cui 16630	Australia	MT279747	MT279912	—	—	—	—	Unpublished
***M. malayanus***	**Dai 18529 (holotype)**	**Malaysia**	**PQ650071ª**	**PQ642657ª**	**PQ644113ª**	—	—	—	**Present stud**y
*M. minutissimus*	JV 1704/83 (holotype)	Costa Rica	OM669905	OM669987	—	—	—	—	Wang et al. (2024)
*M. neovitreus*	JV 0509/47	USA	OM669906	OM669988	OM670045	OM810110	—	OM810080	Wang et al. (2024)
*M. neovitreus*	JV 0509/127	USA	OM669907	OM669989	OM670046	OM810111	—	OM810081	Wang et al. (2024)
*M. neovitreus*	JV 1009/59 (holotype)	USA	OM669908	OM669990	OM670047	OM810112	—	OM810082	Wang et al. (2024)
*M. neovitreus*	JV 0709/188	USA	OM971904	OM971890	—	—	—	—	Wang et al. (2024)
***M. niveomarginatus***	**Dai 26373**	**China**	**PQ642624ª**	—	—	—	—	—	**Present study**
***M. niveomarginatus***	**Dai 18268**	**China**	**PQ642625ª**	**PQ642658ª**	—	—	—	—	**Present study**
***M. niveomarginatus***	**Dai 18540A (holotype)**	**China**	**PQ642626ª**	**PQ642659ª**	**PQ644114ª**	**PQ662373ª**	—	—	**Present study**
***M. niveomarginatus***	**Dai 17695**	**China**	**PQ642627ª**	**PQ642660ª**	**PQ644115ª**	—	—	—	**Present study**
***M. noncontusus***	**Dai 24718**	**China**	**PQ650072ª**	—	—	—	—	—	**Present study**
*M. obscurus*	MCW 590	Brazil	PP259431	—	—	PP239253	—	—	Westphalen et al. 2025
*M. obscurus*	MCW 722	Brazil	PP259432	—	—	—	—	—	Westphalen et al. 2025
*M. pouzarii*	Dai 21043	Belarus	MT840124	MT840142	OM670048	OM810113	—	OM810083	Unpublished, Wang et al. (2024)
*M. pouzarii*	JV 0511/23	Czechia	JQ409465	KY131921	OM670049	OM810114	—	—	Vampola and Vlasák (2012), Wang et al. (2024)
*M. pouzarii*	MJ 27/04	Slovakia	JQ409462	—	—	—	—	—	Wang et al. (2024)
*M. revolubilis*	MCW 702	Brazil	PP259434	PP259418	—	—	—	—	Westphalen et al. 2025
*M. rhododendri*	Dai 22272	China	OM669916	OM669995	OM670054	OM810118	—	—	Wang et al. (2024)
*M. rhododendri*	Dai 22279 (holotype)	China	OM669917	OM669996	OM670055	—	—	—	Wang et al. (2024)
*M. rigidus*	JV 1704/79 (holotype)	Costa Rica	OM669918	OM669997	OM670056	OM810119	—	OM810084	Wang et al. (2024)
*M. rigidus*	F 2061	Mexico	KU747872	—	—	—	—	—	Wang et al. (2024)
*M. robledoi*	HCFC 1095	Argentina	PP259428	PP259412	—	—	—	—	Westphalen et al. 2025
*M. roseus*	Dai 19877 (holotype)	China	MT840126	MT840144	OM670057	—	—	OM810085	Wang et al. (2024)
*M. sanguinolentus*	Dai 20976	Belarus	MT840118	MT840136	OM670058	—	—	—	Wang et al. (2024)
*M. sanguinolentus*	Dai 21030	Belarus	MT309482	—	—	—	—	—	Wang et al. (2024)
*M. sanguinolentus*	JV 1310/11	Czechia	OM669920	OM669998	OM670059	OM810120	—	OM810086	Wang et al. (2024)
*M. sanguinolentus*	JV 1610/2-Tejklova	Czechia	OM669921	OM669999	OM670060	OM810121	—	—	Wang et al. (2024)
*M. sanguinolentus*	MJ 39/00	Slovakia	OM669922	OM670000	—	—	—	—	Wang et al. (2024)
*M. sanguinolentus*	MJ 111/04	Czechia	OM669923	—	OM670061	OM810122	—	—	Wang et al. (2024)
*M. stillicidiorum*	Cui 16620 (holotype)	Australia	OM669927	OM670004	—	—	—	—	Wang et al. (2024)
*M. stillicidiorum*	HCFC 1088	Australia	PP259435	PP259416					
*M. srilankensis*	Dai 19535 (holotype)	Sri Lanka	OM669924	OM670001	OM670062	OM810123	—	—	Wang et al. (2024)
*M. subfurcatus*	Dai 2105	China	KY131854	KY131911	—	—	—	—	Wang et al. (2024)
*M. subfurcatus*	Dai 2544 (holotype)	China	KY131855	KY131912	—	—	—	—	Wang et al. (2024)
*M. subfurcatus*	Dai 11313	China	KY131856	KY131913	—	—	—	—	Wang et al. (2024)
*M. subfurcatus*	Dai 25999	China	PQ650073ª	PQ642663ª	PQ644118ª	—	—	—	Wang et al. (2024)
*M. subfurcatus*	Dai 26167	China	PQ650074ª	PQ642664ª	PQ644119ª	PQ662376ª	—	PQ662348ª	Wang et al. (2024)
*M. sublineatus*	Dai 20523 (holotype)	China	MT309462	MT309468	—	OM810125	—	—	Wang et al. (2024)
*M. sublineatus*	Dai 17885	Singapore	MT309460	MT309466	—	OM810126	—	—	Wang et al. (2024)
*M. sublineatus*	Dai 19639	Sri Lanka	MT309461	MT309467	OM670064	OM810127	—	OM810087	Wang et al. (2024)
*M. sublineatus*	Dai 22598	China	OM669925	OM670002	OM670065	OM810128	—	—	Wang et al. (2024)
*M. sublineatus*	Dai 17553	China	OM669926	OM670003	OM670066	—	—	—	Wang et al. (2024)
*M. sulphureus*	Dai 17839 (holotype)	China	MG132179	MG132181	OM670067	OM810129	—	—	Dai and Dai (2018)
*M. sulphureus*	Dai 17841	China	MG132180	MG132182	OM670068	OM810130	—	—	Dai and Dai (2018)
*M. sumstinei*	Russell 5913	USA	MN906088	—	—	—	—	—	Unpublished
*M. tamilnaduensis*	MKDM01a	India	OQ553780	OQ553784	—	—	—	—	Crous et al. (2023)
*M. tamilnaduensis*	MKDM01 (holotype)	India	OQ553779	OQ553783	—	—	—	—	Crous et al. (2023)
*M. tamilnaduensis*	A164FB3	Singapore	OQ569361	—	—	—	—	—	Crous et al. (2023)
*M. tibeticus*	Cui 9588	China	KY131873	KY131929	OM670069	OM810131	—	—	Wu et al. (2017), Wang et al. (2024)
*M. tibeticus*	Cui 9381 (holotype)	China	KY131871	KY131927	OM670070	—	—	—	Wu et al. (2017), Wang et al. (2024)
*M. vinctus*	Cui 16903	Puerto Rico	MT840129	MT840147	OM670074	OM810135	—	OM810088	Chen and Dai (2021), Wang et al. (2024)
*M. vinctus*	JV 1407/36	Costa Rica	OM669933	OM670008	OM670075	OM810136	—	—	Wang et al. (2024)
*M. vinctus*	Kout 1807/3	Puerto Rico	OM669934	OM670009	OM670076	OM810137	—	OM810089	Wang et al. (2024)
*M. vitreosanguineus*	MJ 144/95	Czechia	OM669937	OM670010	—	—	—	—	Wang et al. (2024)
*M. vitreosanguineus*	Kout 0609/1	Czechia	OM669938	—	—	—	—	—	Wang et al. (2024)
*M. vitreosanguineus*	JV 0909/3 (holotype)	Czechia	OM669939	OM670011	OM670078	—	—	—	Wang et al. (2024)
*M. vitreus*	Miettinen-13591	Finland	KY948731	KY948870	—	—	—	—	Justo et al. (2017)
*M. vitreus*	Dai 12685	Czechia	MT840115	MT840133	OM670071	OM810132	—	—	Chen and Dai (2021), Wang et al. (2024)
*M. vitreus*	Cui 10340	China	KY131848	—	—	OM810133	—	—	Wu et al. (2017), Wang et al. (2024)
*M. vitreus*	Cui 10341	China	KY131849	KY131907	OM670072	—	—	—	Wu et al. (2017), Wang et al. (2024)
*M. vitreus*	JV 0110/48	Czechia	OM669931	OM670005	OM670073	OM810134	—	—	Wang et al. (2024)
*M. vitreus*	MJ 129/04	Czechia	OM669932	OM670006	—	—	—	—	Wang et al. (2024)
*M. yunnanensis*	CLZhao 21583	China	OP852341	OP852343	—	—	—	—	Cai et al. (2023)
*M. yunnanensis*	CLZhao 21647 (holotype)	China	OP852340	OP852342	—	—	—	—	Cai et al. (2023)
*Meripilus* sp.	TUFC 100564	Japan	LC643683	LC643708	—	—	—	—	Shino et al. (2022)
*Meripilus* sp. 1	JV 0309/45	USA	OM669911	—	OM670051	OM810116	—	—	Wang et al. (2024)
*Meripilus* sp. 1	JV 0709/83	USA	OM669912	OM669992	OM670052	OM810117	—	—	Wang et al. (2024)
*Meripilus* sp. 1	JV 0308/66	USA	OM669910	—	OM670050	OM810115	—	—	Wang et al. (2024)
*Meripilus* sp. 2	MJ 6003-Beneschova	Czechia	OM669919	—	—	—	—	—	Wang et al. (2024)
*Meripilus* sp. 2	CWU 3874	Ukraine	OM971903	OM971889	—	—	—	—	Wang et al. (2024)
*Meripilus* sp. 3	JV 8908/19	Czechia	OM669913	—	—	—	—	—	Wang et al. (2024)
*Meripilus* sp. 3	JV 1310/15-1	Czechia	OM669914	OM669993	—	—	—	—	Wang et al. (2024)
*Meripilus* sp. 3	MJ 4738-53/02	Czechia	OM669915	OM669994	OM670053	—	—	—	Wang et al. (2024)
*Meruliopsis albomellea*	Dai 15205 (holotype)	China	KX494574	KX494578		—	—	—	Yuan et al. (2017)
*M. albomellea*	Dai 15223	China	KX494575	KX494579	OR095716	—	—	—	Yuan et al. (2017); Wang et al. (2023)
*M. albostramineus*	HHB-10729	USA	KP135051	KP135229	—	—	KP134787	—	Floudas and Hibbett (2015)
*M. bambusicola*	Dai 21944 (holotype)	China	OQ476864	OQ476806	OQ509579	OQ559611	OQ542996	—	Wang et al. (2023)
*M. crassitunicata*	Dai 10833	China	JX623935	JX644064	OQ509580	—	—	—	Jia et al. (2014); Wang et al. (2023)
*M. crassitunicata*	CHWC 1506-46	China	LC427010	LC427034	—	—	LC427055	—	Chen et al. (2020)
*M. crassitunicata*	Dai 9995	China	JX623905	—	—	—	—	—	Jia et al. (2014)
*M. crystallinus*	Dai 26052	China	PQ650051ª	PQ642642ª	PQ644103ª	PQ662364ª	PQ662340ª	—	Present study
*M. crystallinus*	He 7477	China	PQ650052ª	PQ642668ª	—	—	—	—	Present study
*M. crystallinus*	Dai 26217	China	PQ650053ª	PQ642643ª	PQ644104ª	PQ662365ª	PQ662341ª	—	Present study
*M. cystidiata*	ICN 139059	Brazil	MG572754	MG572738	—	—	—	—	Jung et al. (2018)
*M. cystidiata*	776308	Brazil	MG572749	MG572733	—	—	—	—	Jung et al. (2018)
*M. faginea*	LEF-334408 (holotype)	Russia	MW673659	MW673660	—	—	—	—	Unpublished
*M. leptocystidiata*	Li 1011	China	JX623898	JX644049	—	—	—	—	Jia et al. (2014)
*M. leptocystidiata*	Wu 1708-43 (holotype)	China	LC427013	LC427033	—	—	LC427070	—	Chen et al. (2020)
*M. marginata*	Cui 6878 (holotype)	China	JX623943	JX644057	—	—	—	—	Wang et al. (2023)
*M. marginata*	Dai 14737	China	OQ476865	OQ476807	OQ509581	OR113379	—	—	Wang et al. (2023)
*M. marginata*	Cui 11626	China	OQ476866	OQ476808	OQ509582	—	—	—	Wang et al. (2023)
*M. marginata*	Wei 3388	China	OQ476867	—	—	—	—	—	Wang et al. (2023)
*M. nanlingensis*	Dai 13414	China	OQ476868	OQ476809	OQ509583	OQ559612	OQ542997	—	Wang et al. (2023)
*M. nanlingensis*	Dai 17172	China	OQ476869	OQ476810	OQ509584	OQ559613	—	—	Wang et al. (2023)
*M. nanlingensis*	Dai 8173 (holotype)	China	JX623942	JX644053	OQ509585	—	—	—	Jia et al. (2014); Wang et al. (2023)
*M. parvispora*	CHWC 1505-129	China	LC427015	LC427051	—	—	LC427066	—	Chen et al. (2020)
*M. parvispora*	Wu 1209-58 (holotype)	China	LC427017	LC427039	—	—	LC427065	—	Chen et al. (2020)
*M. pseudocystidiata*	Dai 3204	China	—	JX644056	—	—	—	—	Jia et al. (2014)
*M. pseudocystidiata*	Dai 18405	Vietnam	OQ476870	OQ476811	OQ509586	OQ559614	OQ542998	—	Wang et al. (2023)
*M. pseudocystidiata*	Li 1704 (holotype)	China	JX623944	—	—	—	—	—	Jia et al. (2014)
*Meruliopsis* sp.	FD-278	USA	KP135057	KP135205	—	—	KP134796	—	Floudas and Hibbett (2015)
***M. rhizomorpha***	**Dai 25742**	**China**	**PQ650054ª**	**PQ642644ª**	**PQ644105ª**	—	—	—	**Present stud**y
***M. rhizomorpha***	**Dai 24733 (holotype)**	**China**	**PQ650055ª**	**PQ642645ª**	**PQ644106ª**	**PQ662366ª**	—	—	**Present study**
***M. rhizomorpha***	**Dai 25816**	**China**	**PQ650056ª**	**PQ642646ª**	**PQ644107ª**	**PQ662367ª**	**PQ662342ª**	—	**Present study**
*M. rosea*	Dai 18640A (holotype)	Australia	OQ476871	OQ476812	OQ509587	OQ559615	OQ542999	—	Wang et al. (2023)
*M. tarda*	Dai 10226	China	JX623945	—	—	—	—	—	Jia et al. (2014)
*M. tarda*	LE 247365	Russia	KF856503	KF856506	—	—	—	—	Zmitrovich and Malysheva (2014)
*M. taxicola*	GC 1704-60	China	LC427028	LC427050	—	—	LC427063	—	Chen et al. (2020)
*M. taxicola*	Dai 21878	China	OQ476872	OQ476813	OQ509588	OQ559616	OQ543000	—	Wang et al. (2023)
*M. taxicola*	Dai 22625	China	OL457966	OL457436	OQ509589	OQ559617	OQ543001	—	Wang et al. (2023)
*M. taxicola*	Dai 22636	China	OQ476873	OQ476814	OQ509590	OQ559618	OR090882	—	Wang et al. (2023)
*M. taxicola*	Dai 17248	China	OQ476874	OQ476815	OQ509591	OQ559619	—	—	Wang et al. (2023)
*M. variegata*	Li 1780 (holotype)	China	JX623936	JX644065		—	—	—	Jia et al. (2014)
*M. variegata*	Dai 19791	China	OQ476875	OQ476816	OQ509592	—	—	—	Wang et al. (2023)
*M. variegata*	Dai 19886	China	OQ476876	OQ476817	OQ509593	OQ559620	—	—	Wang et al. (2023)
*Neolentinus adhaerens*	DAOM 214911	—	HM536096	HM536071	HM536072	HM536117	—	—	Garcia-Sandoval et al. (2011)
*Phanerochaete* s.l. sp.	RLG-13408-Sp	USA	KP135020	KP135257	—	—	KP134801	—	Floudas and Hibbett (2015)
*Phanerochaetella exilis*	HHB-6988	USA	KP135001	KP135236	—	—	KP134799	—	Floudas and Hibbett (2015)
*Phanerochaetella* sp.	HHB-11463	USA	KP134994	KP135235	—	—	KP134797	—	Floudas and Hibbett (2015)
*P. xerophila*	HHB-8509-Sp	USA	KP134996	KP135259	—	—	KP134800	—	Floudas and Hibbett (2015)
*Podoserpula ailaoshanensis*	ZJL2015015	China	KU324484	KU324487	KU324491	KU324494	—	—	Zhou et al. (2016)
*Pseudolagarobasidium acaciicola*	CBS 115543	South Africa	DQ517883	—	—	—	—	—	Wood and Ginns (2006)
*P. acaciicola*	CBS 115544	South Africa	DQ517882	—	—	—	—	—	Wood and Ginns (2006)
*P. baiyunshanense*	Han 405 (holotype)	China	MT428549	MT428547	—	—	—	—	Han et al. (2021)
*P. baiyunshanense*	Han 406	China	MT428550	MT428548	—	—	—	—	Han et al. (2021)
*P. belizense*	VPB 197	Brazil	KJ832058	—	—	—	—	—	Martin et al. (2015)
*P. belizense*	CFMR: DCL04-31	Belize	JQ070173	—	—	—	—	—	Martin et al. (2015)
*Pseudospongipellis delectans*	OSM-F925	Czechia	HQ728296	HQ729006	—	—	—	—	Tomšovský (2012)
*P. delectans*	BRNM 686401	Czechia	HQ728295	HQ729005	—	—	—	—	Tomšovský (2012)
*P. delectans*	MUcc 838	Czechia	HQ728294	HQ729004	—	—	—	—	Tomšovský (2012)
*P. litschaueri*	Dai 20266	China	OM971908	OM971893	OM971929	—	—	—	Wang and Dai (2022)
*P. litschaueri*	Dai 13845	China	OM971911	OM971895	OM971931	OM982700	—	—	Wang and Dai (2022)
*P. litschaueri*	BRNM 67093	Czechia	HQ728303	HQ729013	—	—	—	—	Tomšovský (2012)
*P. unicolor*	CFMRcc-FP-59199-T	USA	HQ728310	HQ729012	—	—	—	—	Tomšovský (2012)
*P. unicolor*	CFMRcc-FP-71791-T	USA	HQ728313	HQ729011	—	—	—	—	Tomšovský Tomšovský (2012)
*Raduliporus aneirinus*	HHB-15629-Sp	USA	KP135023	KP135207	—	—	KP134795	—	Floudas and Hibbett (2015)
*Radulodon americanus*	CFMR-HHB 11240	USA	JQ070174	—	—	—	—	—	Nakasone and Lindner (2012)
*R. americanus*	RLG 6350	USA	JQ070175	—	—	—	—	—	Nakasone and Lindner (2012)
*R. casearius*	HHB-9567-sp	USA	KY948752	KY948871	—	—	—	—	Justo et al. (2017)
*R. casearius*	KRT-Iso-26	USA	MN430944	—	—	—	—	—	Unpublished
*R. erikssonii*	CBS 126044	Sweden	MH864059	MH875514	—	—	—	—	Vu et al. (2019)
*R. erikssonii*	X 3536	Norway	KY415963	KY415963	—	—	—	—	Kotiranta et al. (2017)
*R. yunnanensis*	He 6183	China	OM971915	OM971897	OM971935	OM982704	—	—	Wang and Dai (2022)
*R. yunnanensis*	Cui 17979 (holotype)	China	OM971917	OM971898	OM971937	OM982706	—	—	Wang and Dai (2022)
*Resiniporus pseudogilvescens*	Wu 1209-46	China	KY688203	MZ637268	—	MZ913713	MZ748436	—	Chen et al. (2018)
*R. resinascens*	BRNM 710169	Czechia	FJ496675	FJ496698	—	—	—	—	Tomšovský et al. (2010)
"*Rigidoporus hypobrunneus*"	Cui 16874	China	PQ650075ª	PQ642665ª	—	—	—	—	Present study
“*R. hypobrunneus*"	JV 1712/13-J	Martinique	OM669886	OM669970	—	—	—	—	Wang et al. (2024)
“*R. hypobrunneus*"	Dai 10569	China	KY131879	KY131936	—	—	—	—	Wu et al. (2017)
“*R. hypobrunneus*"	Dai 19451	China	PQ650076ª	PQ642666ª	—	—	—	—	Present study
“*R. hypobrunneus*"	CM 108b	Cameroon	KJ831816	—	—	—	—	—	Unpublished
“*R. hypobrunneus*"	Dai 10503	China	KY131878	KY131935	—	—	—	—	Wu et al. (2017)
*Russula emeticicolor*	FH12253	Germany	KT934011	KT933872	—	—	KT957382	—	Looney et al. (2016)
*Schizophyllum radiatum*	AFTOL-ID-516	Panama	AY571060	AY571023	AY705952	—	DQ447939	—	Binder et al. (2006)
*Serpula himantioides*	MUCL: 30528	Belgium	GU187545	GU187600	GU187651	GU187748	GU187480	—	Binder et al. (2010)
*Spongipellis quercicola*	Dai 20899	China	OM971920	—	OM971939	OM982702	—	—	Wang and Dai (2022)
*S. quercicola*	Cui 10009 (holotype)	China	OM971919	OM971899	OM971938	OM982701	—	—	Wang and Dai (2022)
*S. sibirica*	Dai 1723	China	OM971921	—	—	—	—	—	Wang and Dai (2022)
*S. spumeus*	BRNM 734877	Czechia	HQ728283	HQ729018	—	—	—	—	Tomšovský (2012)
*S. spumeus*	BRNM 712630	Czechia	HQ728288	HQ729019	—	—	—	—	Tomšovský (2012)
*S. spumeus*	He 6736	China	OM971924	OM971900	—	—	—	—	Wang and Dai (2022)
*S. spumeus*	Dai 20901	China	OM971927	OM971901	OM971940	OM982703	—	—	Wang and Dai (2022)
*Steccherinum tenue*	KHL 12316	USA	JN710598	JN710598	—	—	—	—	Miettinen et al. (2012)
*Tomentella* sp.	AFTOL-ID 1016	USA	DQ835998	DQ835997	DQ092920	—	—	—	Matheny et al. (2006b)
*Trametes ochracea*	HHB 13445	USA	JN164954	JN164812	—	—	—	—	Justo and Hibbett (2011)
*Trametopsis cervina*	AJ-185	USA	JN165020	JN164796	—	—	JN164839	—	Justo and Hibbett (2011)
*T. cervina*	Cui 9985	China	—	OQ476818	—	—	—	—	Wang et al. (2023)

ª Newly generated sequences in this study. **Bold** = new taxa.

### ﻿Sequence alignment

Sequences generated in this study were aligned with additional sequences downloaded from GenBank using BioEdit (Hall 1999) and ClustalX (Thompson et al. 1997). The final ITS, nLSU, RPB1, RPB2, TEF1, and nSSU datasets were subsequently aligned using MAFFT v.7 under the E-INS-i strategy with no cost for opening gaps and equal cost for transformations (command line: mafft –genafpair –maxiterate 1000) (Katoh and Standley 2013) and visualized in BioEdit. Alignments were spliced and transformed into formats in Mesquite v.3.2 (Maddison and Maddison 2017). Multiple sequence alignments were trimmed by trimAI v.1.2 using the -htmlout -gt 0.8 -st option to deal with gaps when necessary (Capella-Gutierrez et al. 2009).

### ﻿Phylogenetic analyses

A combined matrix was reconstructed for phylogenetic analyses: a 5-gene dataset (ITS+nLSU+TEF1+RPB1+nSSU) was used to determine the phylogenetic position of the new species of *Ceriporia*. In addition, a 5-gene dataset (ITS+nLSU+TEF1+RPB2+nSSU) was used to determine the phylogenetic position of the new species of *Meripilus*. The sequence alignments and the retrieved topologies were deposited in TreeBase (http://www.treebase.org), under accession ID: 32348 (Reviewer access URL: http://purl.org/phylo/treebase/phylows/study/TB2:S32348?x-access-code=28c9481ac7c399614f499481eb27d278&format=html. Sequences from GenBank of *Bjerkandera adusta* (Willd.) P. Karst. and *B. fumosa* (Pers.) P. Karst. were used as outgroups for *Ceriporia*, while those of *Trametes ochracea* (Pers.) Gilb. & Ryvarden were used as outgroups for *Meripilus* (Wu et al. 2017; Chen et al. 2020). The phylogenetic analyses followed the approach of Han et al. (2016) and Zhu et al. (2019). Maximum likelihood (ML) and Bayesian inference (BI) analyses were performed based on the two datasets. The best-fit evolutionary model was selected by the Akaike Information Criterion (AIC) in MrModeltest 2.2 (Nylander 2004) after scoring 24 models of evolution in PAUP* version 4.0b10 (Swofford 2002).

Sequences were analyzed using ML with RAxML-HPC2 through the CIPRES Science Gateway (Miller et al. 2009). Branch support (BT) for ML analysis was determined by 1000 bootstrap replicates. Bayesian phylogenetic inference and Bayesian posterior probabilities (BPP) were computed with MrBayes 3.1.2 (Ronquist and Huelsenbeck 2003). Four Markov chains were run for 8,000,000 generations for the 5-gene datasets of *Ceriporia* and 5,000,000 generations for the 5-gene datasets of *Meripilus* until the split deviation frequency value was less than 0.01; trees were sampled every 100 generations. The first 25% of the sampled trees were discarded as burn-in, and the remainder was used to reconstruct a majority rule consensus tree and calculate BPP of the clades. All trees were viewed in FigTree v.1.4.3 (http://tree.bio.ed.ac.uk/software/figtree/). Branches that received bootstrap support of MP ≥ 75%, ML ≥ 75%, and BPP ≥ 0.95 were considered significantly supported. MP and ML bootstrap supports ≥ 50% and BPP ≥ 0.90 are presented on the ML topologies.

### ﻿Divergence time estimation

We used two fossil calibrations, *Archaeomarasmius leggetti* Hibbett et al. and *Quatsinoporites cranhamii* Smith et al., in estimating divergence times of *Ceriporia* in *Irpicaceae* and *Meripilus* in *Meripilaceae*. *A. leggetti* was recorded at 94–90 Myr (Hibbett et al. 1997) as the representative of the minimum age of *Tricholomataceae* R. Heim ex Pouzar belonging to the *Agaricales*. *Q. cranhamii*, found in marine calcareous concretions on Vancouver Island, was considered to be the representative of the minimum divergence time of the *Hymenochaetales* at 113 Myr (Smith et al. 2004). Divergence times were estimated using BEAST v2.6.5 (Bouckaert et al. 2014) based on a dataset of ITS+nLSU+TEF1. The GTR+G+I substitution model was selected as the best-fit model using MrModeltest 2 v.2.4 (Nylander 2004). An XML file was executed using BEAUti v2. The clock model was set to an uncorrelated lognormal relaxed clock (Drummond et al. 2006; Lepage et al. 2007). The Yule process speciation was used as the tree prior (Gernhard 2008).

For calibration, we specified a gamma distribution prior (scale = 20, shape = 1) on the *Agaricales* (offset = 90 Myr) and *Hymenochaetales* (offset = 125 Myr) clades (Sánchez-Ramírez et al. 2014; Zhao et al. 2016, 2017; Wang et al. 2023). All the ucld.mean parameters for different genes were set to uniform. We ran Monte Carlo Markov chains of 100 million generations, logging states every 1000 generations. The resulting log file was confirmed with convergence of the chains using Tracer v1.6 (Rambaut et al. 2013; http://tree.bio.ed.ac.uk/software/tracer/). An ultrametric maximum clade credibility (MCC) tree was summarized using TreeAnnotator v2.6.5, discarding 20% of states as burn-in and annotating clades with ≥ 0.8 posterior probability. FigTree v1.4.3 (http://tree.bio.ed.ac.uk/software/figtree/) was used to visualize the resulting tree and to obtain the means and 95% highest posterior density (HPD) (Drummond and Rambaut 2007). A 95% HPD marks the shortest interval that contains 95% of the values sampled.

## ﻿Results

### ﻿Molecular phylogeny

**Figure 1.**
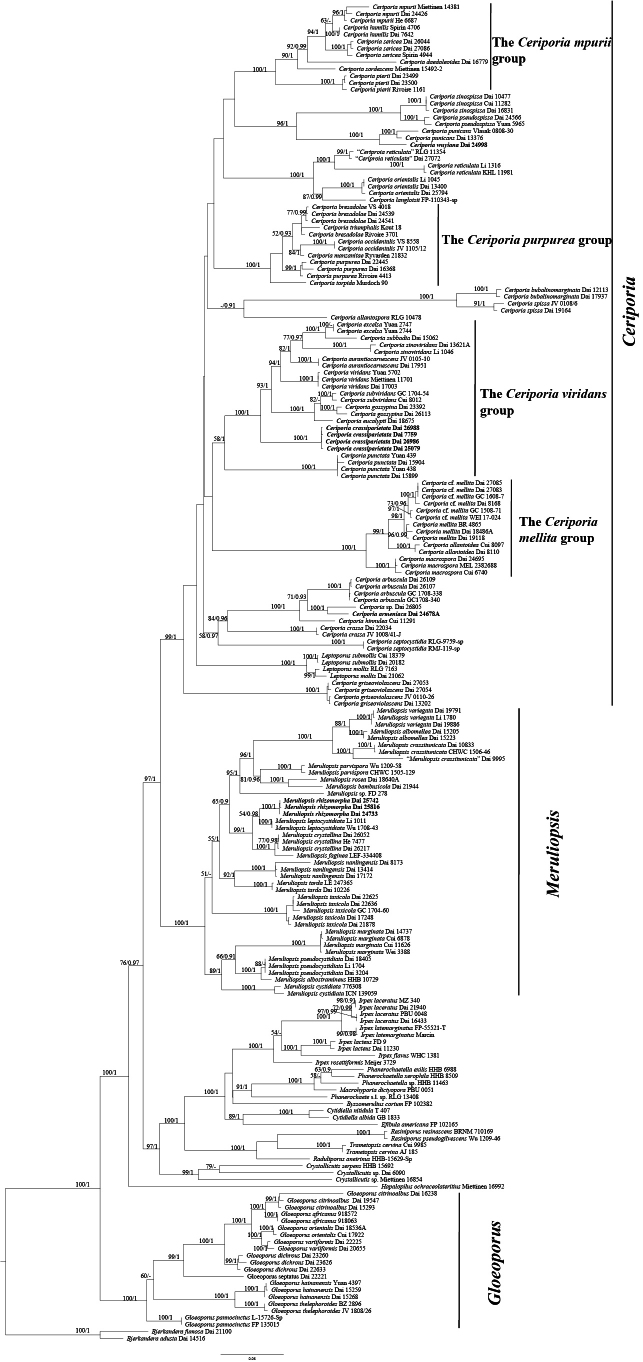
ML analysis of *Irpicaceae* based on the dataset of ITS+nLSU+TEF1+RPB1+nSSU. ML bootstrap values higher than 50% and Bayesian posterior probability values more than 0.90 are shown. New taxa are in bold.

The combined 5-gene dataset (ITS+nLSU+TEF1+RPB1+nSSU) included sequences from 194 samples representing 93 taxa in the phylogeny of *Ceriporia*. The dataset had an aligned length of 4807 characters, of which 2802 (58%) characters were constant, 293 (6%) were variable and parsimony-uninformative, and 1712 (36%) were parsimony-informative. The phylogenetic reconstruction performed with ML and BI analyses for two combined datasets showed similar topology and few differences in statistical support. The best-fit model applied in the Bayesian analysis was GTR+I+G, lset nst = 6, rates = invgamma, and prset statefreqpr = dirichlet (1, 1, 1, 1). Bayesian analysis resulted in a nearly congruent topology with an average standard deviation of split frequencies = 0.008965 compared to the ML analysis, and thus only the ML tree is provided (Fig. 1).

**Figure 2.**
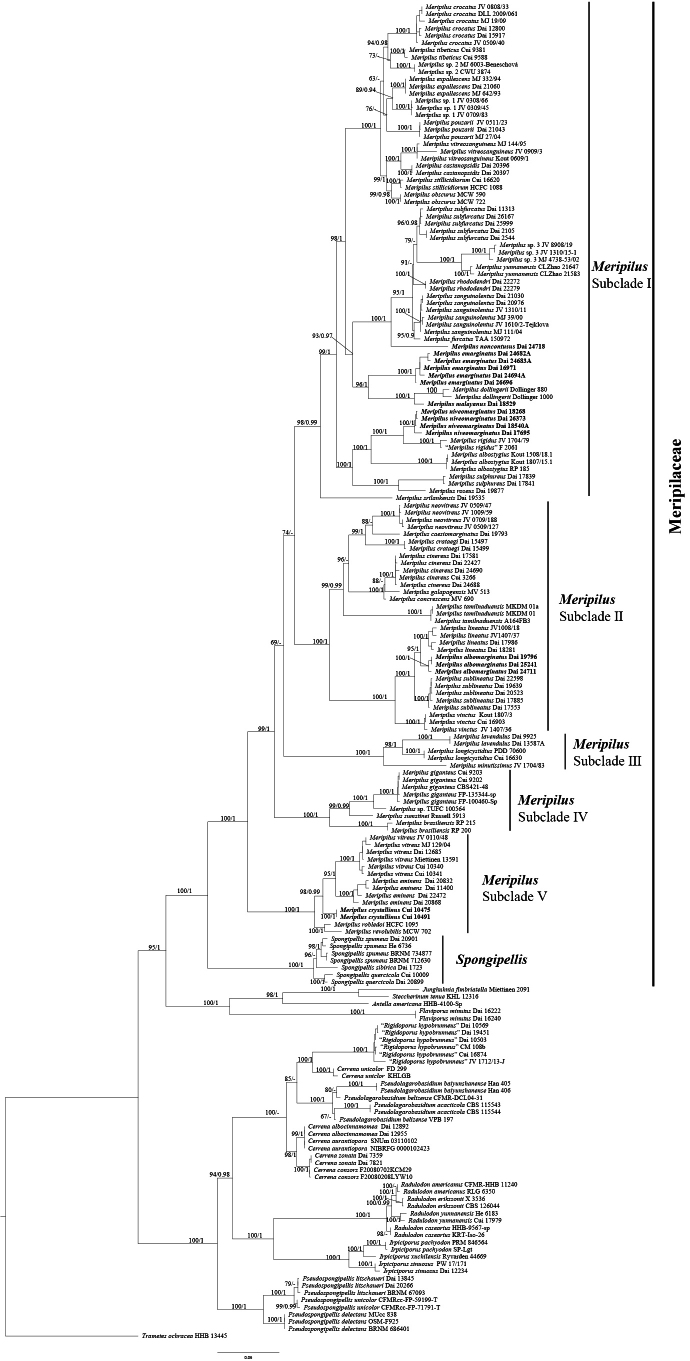
ML analysis of *Meripilaceae* based on the dataset of ITS+nLSU+TEF1+RPB2+nSSU. ML bootstrap values higher than 50% and Bayesian posterior probability values more than 0.90 are shown. New taxa are in bold.

Similarly, the combined 5-gene dataset (ITS+nLSU+TEF1+RPB2+nSSU) included sequences from 185 samples representing 77 taxa in the phylogeny of *Meripilus*. The dataset had an aligned length of 4758 characters, of which 3076 (65%) characters were constant, 267 (5%) were variable and parsimony-uninformative, and 1415 (30%) were parsimony-informative. The phylogenetic reconstruction performed with ML and BI analyses for two combined datasets showed similar topology and few differences in statistical support. The best-fit model applied in the Bayesian analysis was GTR+I+G, lset nst = 6, rates = invgamma, and prset statefreqpr = dirichlet (1, 1, 1, 1). Bayesian analysis resulted in a nearly congruent topology with an average standard deviation of split frequencies = 0.005200 compared to the ML analysis, and thus only the ML tree is provided (Fig. 2).

In the molecular phylogenetic analyses, four new well-supported lineages in *Irpicaceae* were formed: *Ceriporia armeniaca*, *C. crassiparietata*, *C. wuyiana*, and *Meruliopsis rhizomorpha*. In addition, four distinct groups were nested in the *Ceriporia* clade: the *C. mellita* group, the *C. pierii* group, the *C. purpurea* group, and the *C. viridans* group; these groups agree with a previous study by Wang et al. (2023). *Ceriporia armeniaca* and *C. wuyiana* were not included in any groups. *Ceriporia crassiparietata* nested in the *C. viridans* group along with eight other species. *Meruliopsis rhizomorpha* was closely related to *M. leptocystidiata* but differed in morphology. The molecular phylogenetic analyses of *Meripilaceae*, which include two genera (*Meripilus* and *Spongipellis*), showed that *Meripilus* was divided into five groups, with six new well-supported lineages nested within: *M. albomarginatus*, *M. crystallinus*, *M. emarginatus*, *M. malayanus*, *M. niveomarginatus*, and *M. noncontusus*. Four new species (*M. emarginatus*, *M. malayanus*, *M. niveomarginatus*, and *M. noncontusus*) nested in Subclade I; *M. albomarginatus* nested in Subclade II; and *M. crystallinus* nested in Subclade V.

### ﻿Divergence time estimation

**Figure 3.**
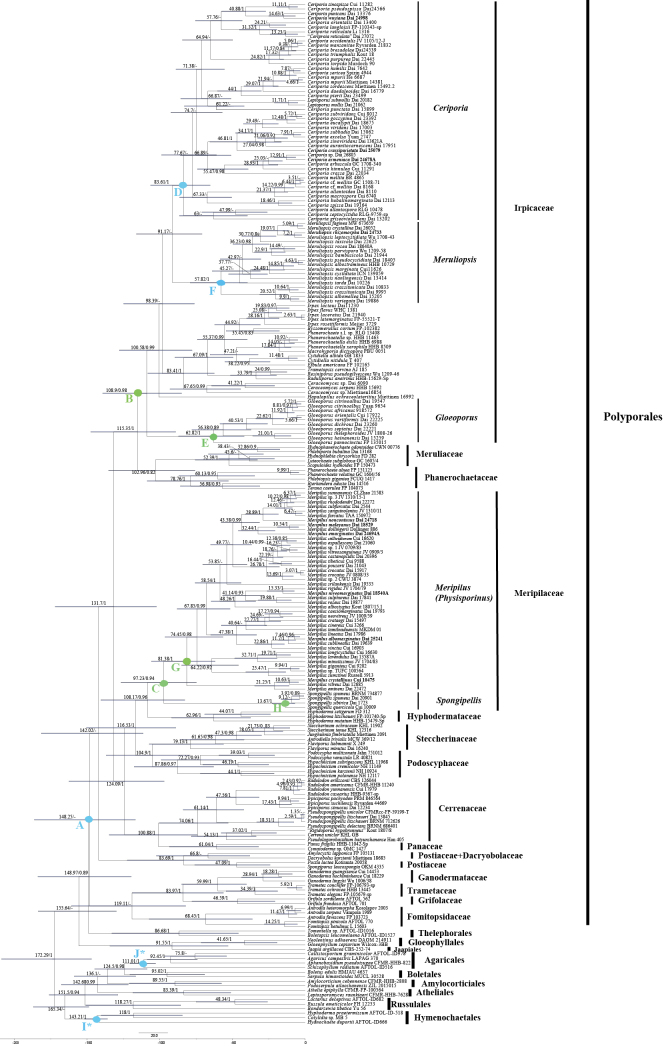
Divergence time estimation of *Irpicaceae* and *Meripilaceae* from Bayesian evolutionary analysis sampling tree based on the conserved regions of three DNA fragments (ITS+nLSU+TEF1). Posterior probabilities are not less than 0.80, and the mean ages (Myr) of each node are annotated. The 95% highest posterior densities of divergence time estimation are marked by horizontal bars.

The MCMC tree (Fig. 3) shows that the ancestor of the *Polyporales* evolved during the late Jurassic at 148.25 Myr [95% HPD of 116.72–186.39 Myr], which is largely consistent with the divergence time of the *Polyporales* by Ji et al. (2022). The two main clades, the *Irpicaceae* main clade and the *Meripilaceae* main clade, had strong support (0.98 PP; 0.94 PP, Fig. 5).

The divergence time of the *Irpicaceae* main clade emerged with a mean stem age of 115.35 Myr [95% HPD of 88.98–147.46 Myr] and a mean crown age of 108.9 Myr [95% HPD of 84.92–139.28 Myr], which belongs to the early Cretaceous period. The initial diversification of the *Meripilaceae* main clade occurred during the late Cretaceous with a mean stem age of 108.17 Myr [95% HPD of 82.75–140.53 Myr] and a mean crown age of 97.23 Myr [95% HPD of 74.77–126.72 Myr]. Their divergence times mostly overlapped. Three clades in the *Irpicaceae* are the *Ceriporia* clade, the *Meruliopsis* clade, and the *Gloeoporus* clade, and two clades in the *Meripilaceae* are the *Meripilus* clade and the *Spongipellis* clade. The divergence time of the *Ceriporia* clade emerged with a mean stem age of 91.17 Myr [95% HPD of 70.92–116.08 Myr] and a mean crown age of 83.61 Myr [95% HPD of 65.25–106.35 Myr], which belongs to the late Cretaceous period. The divergence time of the *Meripilus* clade emerged with a mean stem age of 97.23 Myr [95% HPD of 74.77–126.72 Myr] and a mean crown age of 81.38 Myr [95% HPD of 61.89–105.78 Myr], which belongs to the late Cretaceous period. The divergence times of the main nodes are shown in Fig. 3 and summarized in Table 2. The international chronostratigraphic chart follows Cohen et al. (2013; updated) (URL: http://www.stratigraphy.org/ICSchart/ChronostratChart2022-10.pdf).

**Table 2.** Estimated divergence times of the main nodes.

Node	Means of stem age (Mya)/95% HPD (Mya)/posterior probabilities	Means of crown age (Mya)/95% HPD (Mya)/posterior probabilities	Period
A: *Polyporales*	148.97/119.64–183.87/0.89	148.25/116.72–186.39/–	late Jurassic
B: the *Irpicaceae* main clade	115.35/88.98–147.46/1	108.9/84.92–139.28/0.98	early Cretaceous
C: the *Meripilaceae* main clade	108.17/82.75–140.53/0.96	97.23/74.77–126.72/0.94	late Cretaceous
D: the *Ceriporia* clade	91.17/70.92–116.08/-	83.61/65.25–106.35/1	late Cretaceous
E: the *Gloeoporus* clade	108.9/88.98–147.46/0.98	62.82/41.96–87.11/1	Paleogene
F: the *Meruliopsis* clade	91.17/70.92–116.08/-	57.82/39.69–79.07/1	Paleogene
G: the *Meripilus* clade	97.23/74.77–126.72/0.94	81.38/61.89–105.78/1	late Cretaceous
H: the *Spongipellis* clade	97.23/74.77–126.72/0.94	13.67/7.07–22.73/1	Neogene
I*: *Hymenochaetales* (Calibration point)	165.34/148.75–187.06/–	143.21/135.91–151.74/1	Early Cretaceous
J*: *Agaricales* (Calibration point)	124.5/108.65–144.81/0.98	111.01/102.83–120.44/1	Early Cretaceous

Hyphen “–” represents a posterior probability (PP) < 0.8.

### ﻿Taxonomy

#### ﻿ Ceriporia

Taxon classificationAnimaliaPolyporalesIrpicaceae

Donk, Rev. Niederl. Homob. Aphyll. 2: 170 (1933).

7952FE58-48F7-5B91-9AD6-057FDC140054

##### Type species.

*Ceriporia viridans* (Berk. & Broome) Donk [as ‘Ceraporia’], Meded. Bot. Mus. Herb. Rijks Univ. Utrecht 9: 171 (1933).

##### Description.

For a detailed description of this genus, see Wang et al. (2023).

#### ﻿ Ceriporia armeniaca

Taxon classificationAnimaliaPolyporalesIrpicaceae

Y.C. Dai, Chao G. Wang & Yuan Yuan sp. nov.

553EB31C-CA8E-51AA-A457-C8D7AFF775DC

856725

Figs 4, 5

##### Etymology.

*Armeniaca* (Lat.): refers to the species having an apricot pore surface when dry.

**Figure 4.**
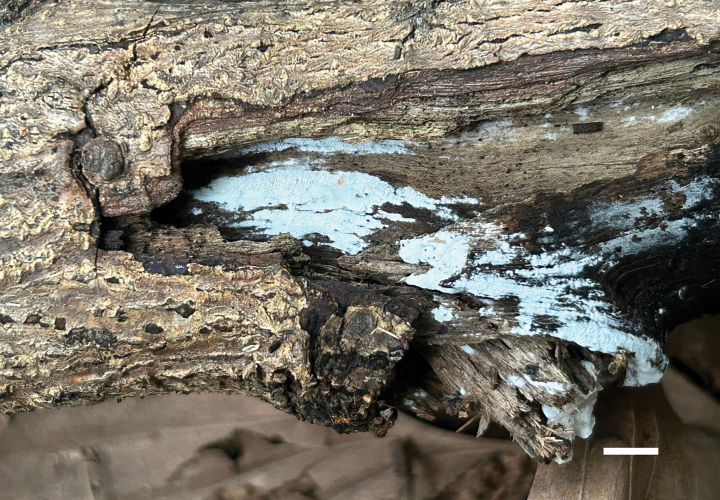
Basidiomata of *Ceriporia armeniaca* (holotype, Dai 24678A). Scale bar: 1 cm.

##### Diagnosis.

Differs from other *Ceriporia* species by resupinate basidiomata with a white pore surface when fresh, apricot when dry, round to angular pores of 5–7 per mm, subicular hyphae distinctly wider than tramal hyphae, lunate to allantoid basidiospores measuring 4–4.5 × 2–2.2 µm.

**Figure 5.**
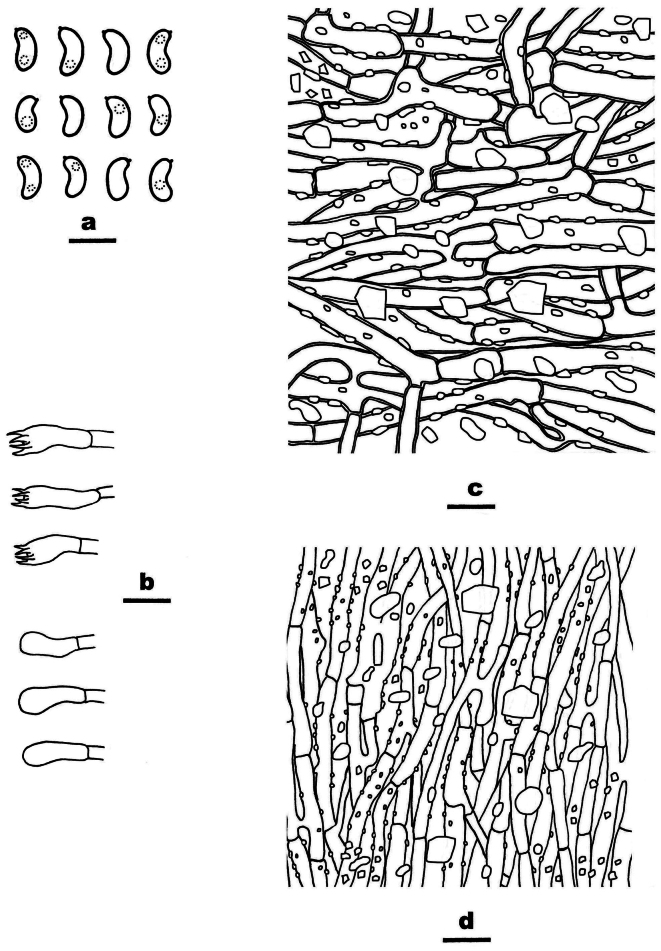
Microscopic structures of *Ceriporia armeniaca* (drawn from the holotype, Dai 24678A) a basidiospores b basidia and basidioles c hyphae from subiculum d hyphae from trama. Scale bars: 5 µm (a); 10 µm (b–d).

##### Type.

CHINA • Guangdong Province, Guangzhou, Baiyunshan Forest Park, on fallen angiosperm branch, 18 April 2023, Dai 24678A (BJFC042232, holotype).

##### Description.

Basidiomata annual, resupinate, soft, without odor or taste when fresh, consistently soft when fresh and dry, up to 5 cm long, 3 cm wide, and 0.2 mm thick at the center. Pore surface white when fresh, becoming apricot upon drying; sterile margin indistinct to almost lacking; pores round to angular, 5–7 per mm; dissepiments thin, lacerate. Subiculum very thin to almost absent. Tubes concolorous with pore surface, soft when dry, up to 0.2 mm long. Hyphal system monomitic; generative hyphae simple septate, hyaline, IKI−, CB+; tissues becoming orange-brown in KOH. Subicular hyphae slightly thick-walled with a wide lumen, abundantly covered with small to large hyaline crystals and oily substances, sometimes encrusted with fine crystals and oily substances, frequently branched at more or less a right angle, straight, slightly interwoven, 5–7 µm in diam. Tramal hyphae thin-walled with a wide lumen, abundantly covered with rhombic or irregular hyaline crystals and olive oily substances, sometimes encrusted with fine crystals and oily substances, frequently branched, straight to slightly flexuous, subparallel along the tubes, agglutinated, 3–4 µm in diam. Cystidia and cystidioles absent. Basidia clavate, with four sterigmata and a simple basal septum, 11–15.5 × 4–6 µm; basidioles clavate to pyriform, smaller than basidia. Basidiospores lunate to allantoid, hyaline, thin-walled, smooth, sometimes with one or two small guttules, IKI−, CB−, 4–4.5(–5) × 2–2.2 µm, L = 4.19 µm, W = 2.09 µm, Q = 2.01 (n = 30/1).

##### Notes.

*Ceriporia armeniaca* is closely related to *C. arbuscula* C.C. Chen & Sheng H. Wu and *C. hinnulea* Y.C. Dai, Chao G. Wang & Yuan Yuan, but *C. arbuscula* has a yellowish brown to pale brown pore surface when dry, short and tortuously branched and narrower subicular hyphae (2–5 μm diam. vs. 5–7 µm in diam., Chen et al. 2020), and smaller basidiospores (3–3.5 × 1–1.5 μm vs. 4–4.5 × 2–2.2 µm, Chen et al. 2020); *Ceriporia hinnulea* is distinguished from *C. armeniaca* by a fawn to cinnamon pore surface when dry, narrower subicular hyphae without crystals or oily substances (3–5 μm diam. vs. 5–7 µm in diam., Wang et al. 2023), and relatively smaller basidiospores (3.5–4 × 2–2.1 µm vs. 4–4.5 × 2–2.2 µm, Wang et al. 2023). In addition, these three species form three independent lineages in the *Ceriporia* clade (Fig. 1).

*Ceriporia alba*M. Pieri & B. Rivoire, *C. camaresiana* (Bourdot & Galzin) Bondartsev & Singer and *C. rhodella* (Fr.) Donk all have white pore surfaces when fresh. However, the first two have bigger pores (3–4 per mm in *C. alba*, 1–3 per mm in *C. camaresiana* vs. 5–7 per mm, Ryvarden and Gilbertson 1993; Pieri and Rivoire 1997) and bigger basidiospores (5.5–7 × 2–2.5 μm in *C. alba*, 5–6 × 2–3 μm in *C. camaresiana* vs. 4–4.5 × 2–2.2 µm, Ryvarden and Gilbertson 1993; Pieri and Rivoire 1997); *C. rhodella* has narrower basidiospores (3.5–4 × 1.5–2 μm vs. 4–4.5 × 2–2.2 µm, Lombard and Gilbertson 1965).

#### ﻿ Ceriporia crassiparietata

Taxon classificationAnimaliaPolyporalesIrpicaceae

Y.C. Dai, Chao G. Wang & Yuan Yuan, sp. nov .

5B7F27E7-B946-5990-BDF3-4C447E456D36

856727

Figs 6, 7

##### Etymology.

*Crassiparietata* (Lat.): refers to the species having a pore surface with thick dissepiments.

**Figure 6.**
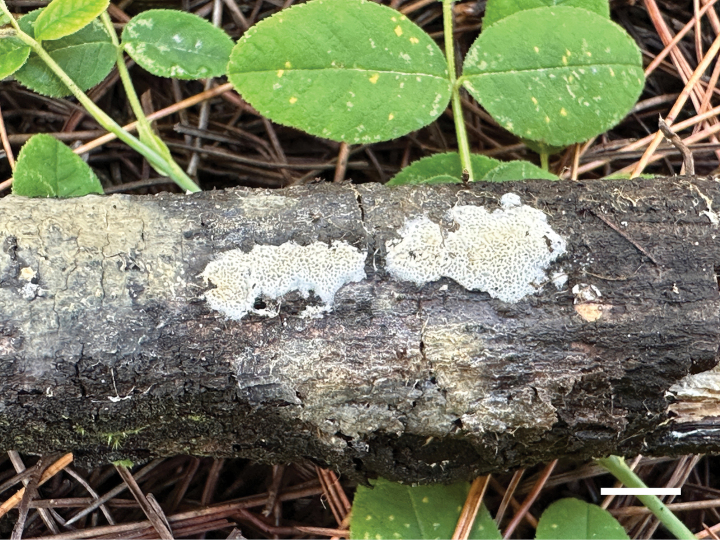
Basidiomata of *Ceriporia crassiparietata* (holotype, Dai 25079). Scale bar: 1 cm.

##### Diagnosis.

Differs from other *Ceriporia* species by resupinate basidiomata with white to light yellow pore surface when fresh, cream to orange-yellow when dry, angular to irregular or sinuous pores of 4–5 per mm, distinctly thick dissepiments, subicular hyphae distinctly wider than tramal hyphae, lunate to allantoid basidiospores measuring 4–4.4 × 2.1–2.3 µm.

**Figure 7.**
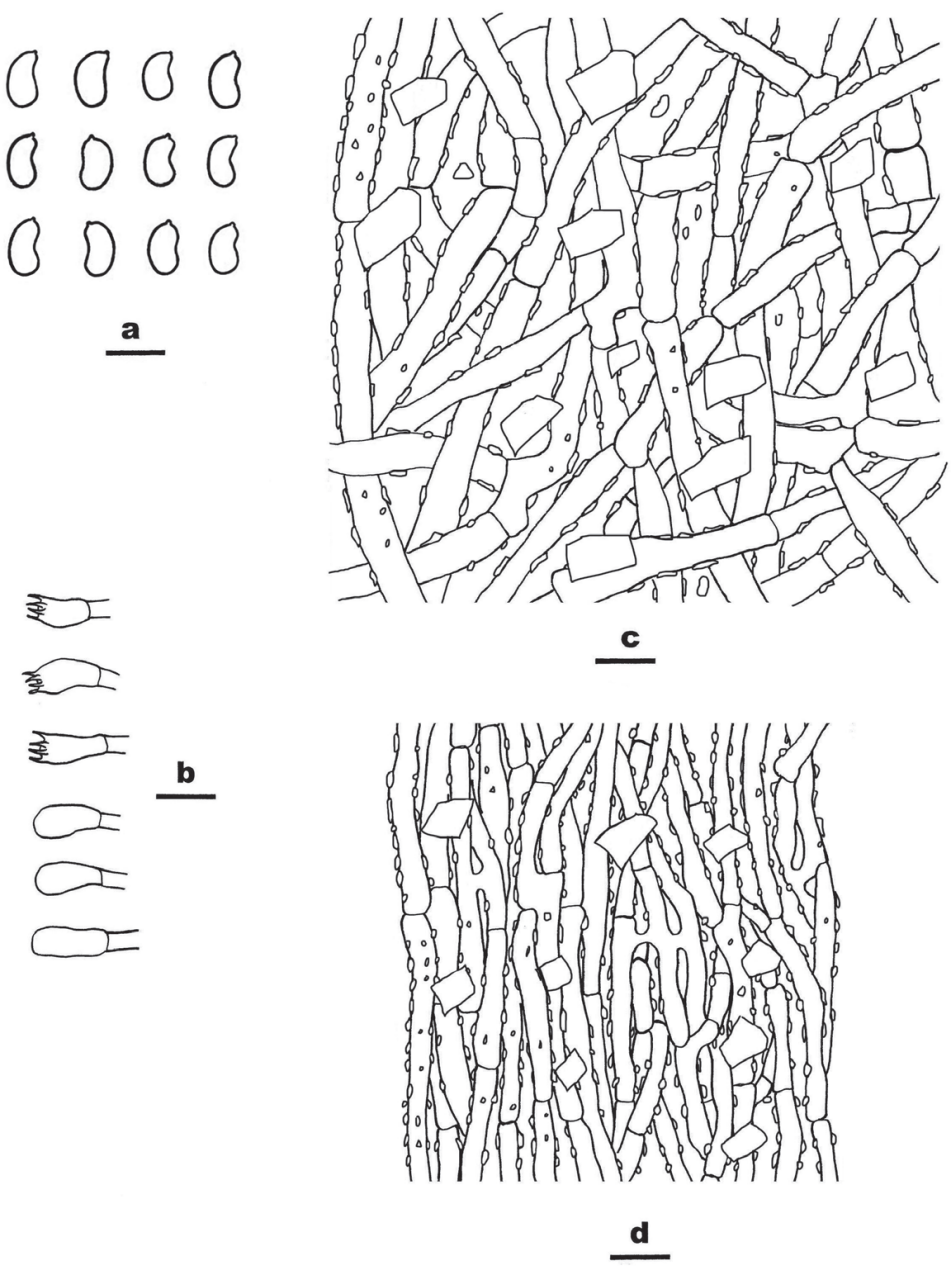
Microscopic structures of *Ceriporia crassiparietata* (drawn from the holotype, Dai 25079). a basidiospores b basidia and basidioles c hyphae from subiculum d hyphae from trama. Scale bars: 5 µm (a); 10 µm (b–d).

##### Type.

CHINA • Zhejiang Province, Wuyi County, Dahongyan Forest Park, on rotten angiosperm wood, 19 June 2023, Dai 25079 (BJFC042632, holotype).

##### Description.

Basidiomata annual, resupinate, soft, without odor or taste when fresh, consistently soft when fresh and dry, up to 3 cm long, 1 cm wide, and 0.2 mm thick at the center. Pore surface white to light yellow when fresh, becoming cream to orange-yellow upon drying; sterile margin indistinct to almost lacking; pores angular to irregular or sinuous, 4–5 per mm; dissepiments thick, entire. Subiculum very thin to almost absent. Tubes concolorous with pore surface, soft when dry, up to 0.2 mm long. Hyphal system monomitic; generative hyphae simple septate, hyaline, IKI−, CB+; tissues unchanged in KOH. Subicular hyphae thin-walled with a wide lumen, abundantly covered with large, rhombic, hyaline crystals and some oily substances, encrusted with fine crystals, frequently branched at more or less a right angle, slightly flexuous, interwoven, 5–6 µm in diam. Tramal hyphae thin-walled with a wide lumen, abundantly covered with rhombic hyaline or pale orange crystals and oily substances, encrusted with fine crystals, frequently branched, straight to slightly flexuous, subparallel along the tubes, agglutinated, 3–4.5 µm in diam. Cystidia and cystidioles absent. Basidia mostly barrel-shaped to short clavate, with four sterigmata and a simple basal septum, 9–11 × 5 µm; basidioles of similar shape to basidia, but smaller. Basidiospores lunate to allantoid, hyaline, thin-walled, smooth, IKI−, CB−, (3.8–)4–4.4(–4.6) × (2–)2.1–2.3(–2.5) µm, L = 4.08 µm, W = 2.19 µm, Q = 1.85–1.89 (n = 60/2).

##### Additional specimens examined.

CHINA • Liaoning Province, Fengcheng County, Tongyuanbao, on *Quercus*, 27 August 2006, Dai 7759 (BJFC010215); Xizang Autonomous Region, Linzhi, Chayu County, fallen branch of *Rosaceae*, 27 October 2023, Dai 26986 (BJFC044538), Dai 26988 (BJFC044540).

##### Notes.

*Ceriporia crassiparietata* is closely related to the *Ceriporia viridans* group by sharing resupinate basidiomata with a cream, cinnamon buff, pinkish, lilac to apricot orange pore surface; subicular hyphae wider than tramal hyphae; hyphae frequently branched at a right angl; and oblong-ellipsoid, short cylindrical, lunate to allantoid basidiospores mostly wider than 1.5 µm (Wang et al. 2023). *Ceriporia eucalypti* Y.C. Dai & Jia J. Chen, *C. gossypina* Y.C. Dai, Chao G. Wang & Yuan Yuan and *C. subviridans* Y.C. Dai, Chao G. Wang & Yuan Yuan are similar to *C. crassiparietata* by having almost the same size of pores (3–5 per mm or 4–5 per mm). However, *C. eucalypti* has narrower basidiospores (4–4.4 × 1.1–1.4 µm vs. 4–4.4 × 2.1–2.3 µm, Chen et al. 2022); *C. gossypina* has a white, buff to deep olive pore surface when fresh and relatively smaller basidiospores (3.5–4 × 1.8–2 µm vs. 4–4.4 × 2.1–2.3 µm, Wang et al. 2023); and *C. subviridans* has a peach to apricot orange pore surface when dry, wider subicular hyphae (4.5–9 µm in diam. vs. 5–6 µm in diam., Wang et al. 2023), and relatively smaller basidiospores (3.3–3.7 × 1.8–2 µm vs. 4–4.4 × 2.1–2.3 µm, Wang et al. 2023).

*Ceriporia cystidiata* Ryvarden & Iturr., *C. microspora* I. Lindblad & Ryvarden and *C. otakou* (G. Cunn.) P.K. Buchanan & Ryvarden share white, cream to isabelline basidiomata with *C. crassiparietata*. However, *C. cystidiata* is known from *C. crassiparietata* by tubular encrusted cystidia and narrower allantoid basidiospores (4–4.5 × 1 µm vs. 4–4.4 × 2.1–2.3 µm, Ryvarden and Iturriaga 2003). *Ceriporia microspora* is easily distinguished from *C. crassiparietata* by smaller ellipsoid basidiospores (3–3.5 × 1.5–2 µm vs. 4–4.4 × 2.1–2.3 µm, Lindblad and Ryvarden 1999). *Ceriporia otakou* differs from *C. crassiparietata* by larger pores (1–3 per mm vs. 4–5 per mm, Cunningham 1947) and bigger ovoid to ellipsoid basidiospores (4.5–6 × 2–2.5 µm vs. 4–4.4 × 2.1–2.3 µm, Cunningham 1947).

#### ﻿ Ceriporia wuyiana

Taxon classificationAnimaliaPolyporalesIrpicaceae

Y.C. Dai, Chao G. Wang & Yuan Yuan sp. nov.

8A5B7E5E-55ED-55BA-87EF-5533D2ABC047

856728

Figs 8, 9

##### Etymology.

*Wuyiana* (Lat.): refers to the species being found in Wuyi County, Zhengjiang Prov., East China.

**Figure 8.**
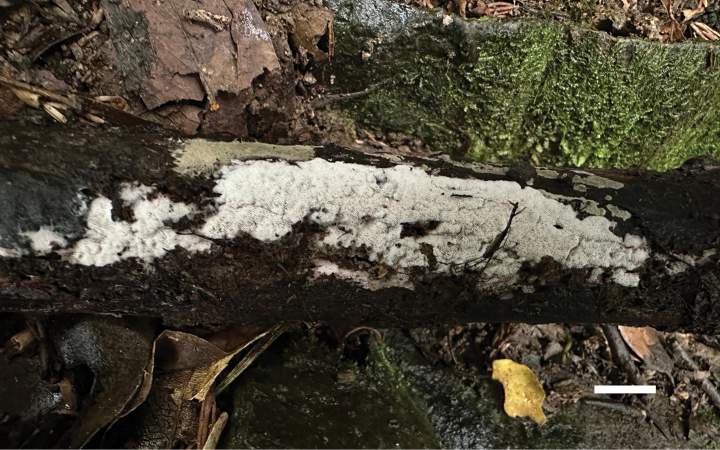
Basidiomata of *Ceriporia wuyiana* (holotype, Dai 24998). Scale bar: 1 cm.

##### Diagnosis.

Differs from other *Ceriporia* species by resupinate basidiomata with a white to cream pore surface when fresh, clay pink to pale lavender when dry, round to angular pores of 5–6 per mm, subicular hyphae relatively wider than tramal hyphae, allantoid to lunate basidiospores measuring 4.3–5 × 1.7–2 µm.

**Figure 9.**
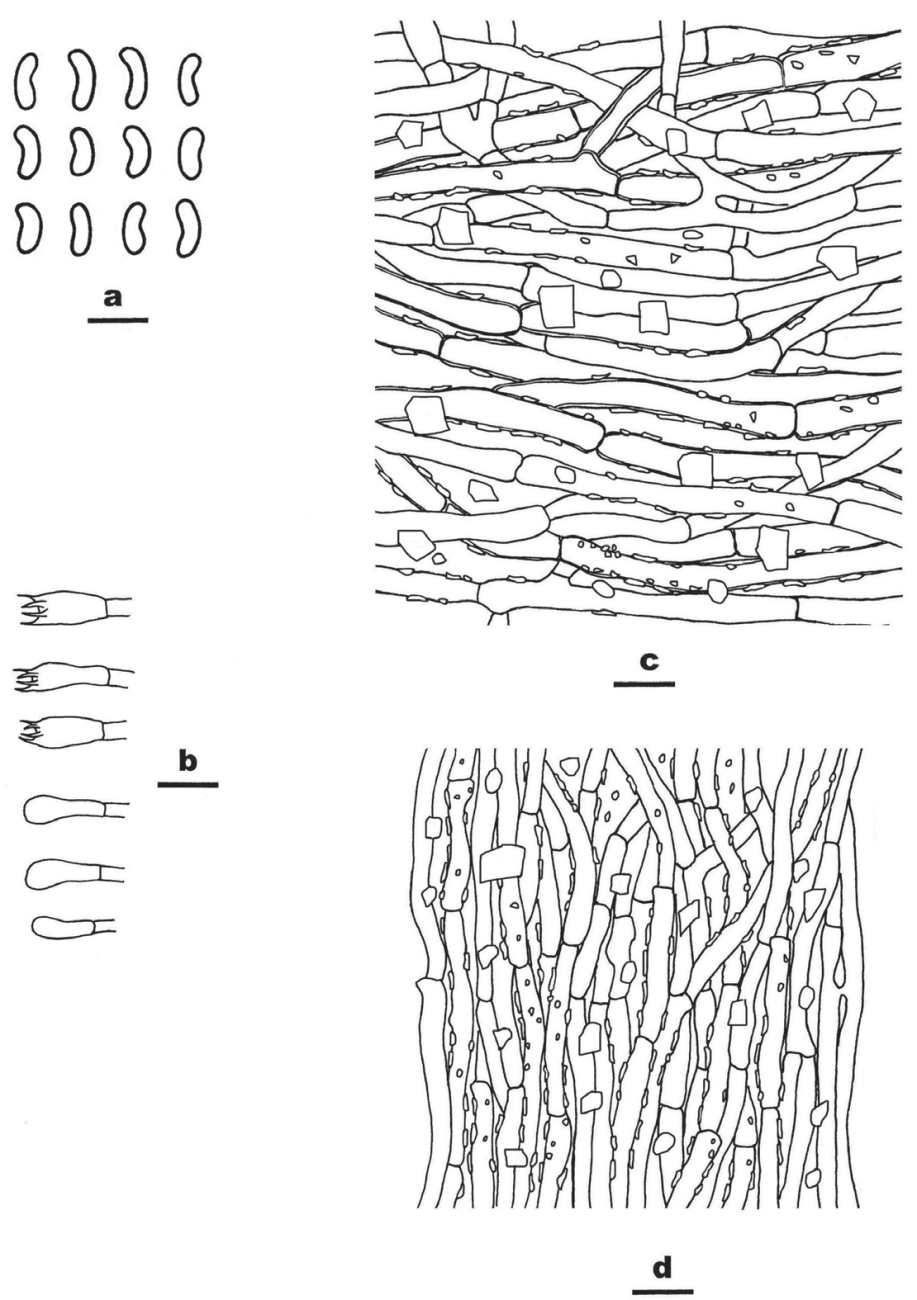
Microscopic structures of *Ceriporia wuyiana* (drawn from the holotype, Dai 24998). a basidiospores b basidia and basidioles c hyphae from subiculum d hyphae from trama. Scale bars: 5 µm (a); 10 µm (b–d).

##### Type.

CHINA • Zhejiang Province, Wuyi County, Guodong Forest Park, on rotten angiosperm wood, 19 June 2023, Dai 24998 (BJFC042551, holotype).

##### Description.

Basidiomata annual, resupinate, soft, without odor or taste when fresh, soft when dry, up to 6 cm long, 2.5 cm wide, and 0.2 mm thick at the center. Pore surface white to cream when fresh, becoming clay pink to pale lavender upon drying; sterile margin indistinct to almost lacking; pores round to angular, 5–6 per mm; dissepiments thin, lacerate. Subiculum very thin to almost absent. Tubes concolorous with pore surface, soft when dry, up to 0.2 mm long. Hyphal system monomitic; generative hyphae simple septate, hyaline, IKI−, CB+; tissues becoming orange-brown in KOH. Subicular hyphae thin- to slightly thick-walled with a wide lumen, abundantly covered with small, rhombic, hyaline crystals and oily substances, sometimes encrusted with fine crystals, frequently branched at more or less a right angle, straight, slightly interwoven, 4–5 µm in diam. Tramal hyphae thin-walled with a wide lumen, abundantly covered with rhombic or irregular pale orange crystals and oily substances, sometimes encrusted with fine crystals, frequently branched, straight to slightly flexuous, subparallel along the tubes, agglutinated, 3–4 µm in diam. Cystidia and cystidioles absent. Basidia barrel-shaped to somewhat pyriform, with four sterigmata and a simple basal septum, 9–12.5 × 4–5 µm; basidioles of similar shape to basidia, but smaller. Basidiospores allantoid to lunate, hyaline, thin-walled, smooth, IKI−, CB−, (4.1–)4.3–5 × (1.5–)1.7–2 µm, L = 4.58 µm, W = 1.83 µm, Q = 2.51 (n = 30/1).

##### Notes.

*Ceriporia wuyiana* is similar and related to *C. punicans* Vlasák & Spirin by subicular hyphae relatively wider than tramal hyphae and almost the same size as basidiospores (4.1–5.3 × 1.7–2.1 µm vs. 4.3–5 × 1.7–2 µm, Spirin et al. 2016). However, the latter has a white or pale pink pore surface when fresh, orange to pinkish orange when dry, and entire dissepiments (Spirin et al. 2016).

#### ﻿ Meruliopsis

Taxon classificationAnimaliaPolyporalesMeripilaceae

Bondartsev, in Parmasto, Izv. Akad. Nauk Estonsk. SSR, Ser. Bio l. 8: 274 (1959).

9453B8E6-2900-581A-B538-1ACC9797869D

##### Type species.

*Meruliopsis taxicola* (Pers.) Bondartsev, in Parmasto, Eesti NSV Tead. Akad. Toim., Biol. seer 8(4): 274 (1959).

##### Description.

For a detailed description of this genus, see Wang et al. (2023).

#### ﻿ Meruliopsis rhizomorpha

Taxon classificationAnimaliaPolyporalesMeripilaceae

Y.C. Dai, Chao G. Wang & Yuan Yuan sp. nov.

9D57F82E-FF1C-5BAE-AFD6-C31C7A93981A

856729

Figs 10, 11

##### Etymology.

*Rhizomorpha* (Lat.): refers to the species having rose-colored rhizomorphs when fresh.

**Figure 10.**
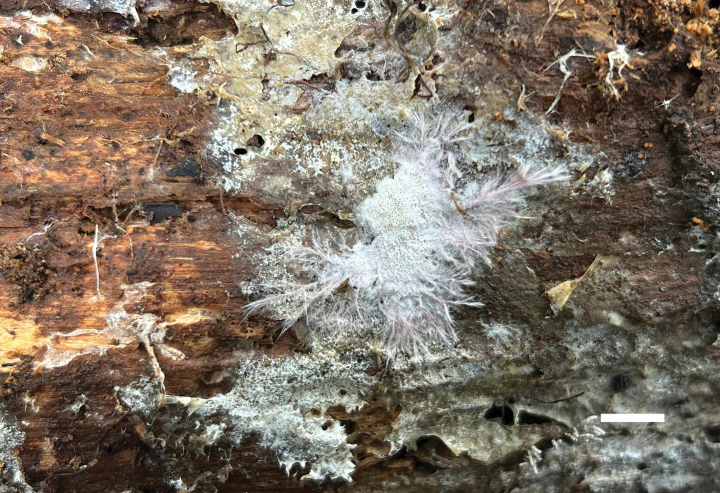
Basidiomata of *Meruliopsis rhizomorpha* (holotype, Dai 24733). Scale bar: 1 cm.

##### Diagnosis.

Differs from other *Meruliopsis* species by resupinate basidiomata with a white pore surface when fresh, rose-colored rhizomorphs, round to angular pores of 2–4 per mm, subicular hyphae distinctly wider than tramal hyphae, smooth hymenial cystidia, ellipsoid to broadly ellipsoid basidiospores measuring 3.8–4.2 × 2.3–2.7 µm.

**Figure 11.**
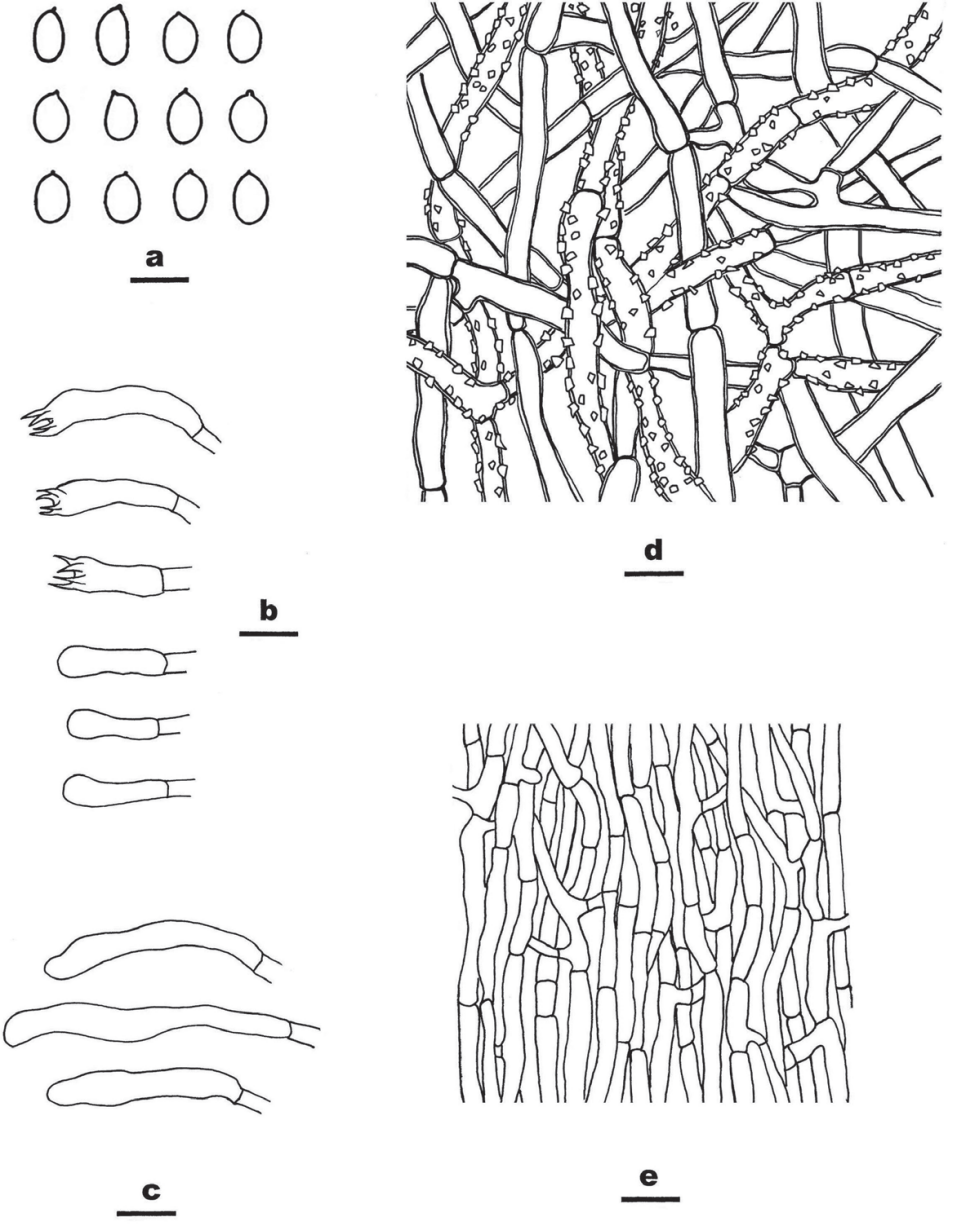
Microscopic structures of *Meruliopsis rhizomorpha* (drawn from the holotype, Dai 24733). a basidiospores b basidia and basidioles c hymenial cystidia d hyphae from subiculum e hyphae from trama. Scale bars: 5 µm (a); 10 µm (b–e).

##### Type.

CHINA • Hunan Province, Changsha, Yuelushan Forest Park, on fallen angiosperm branch, 18 April 2023, Dai 24733 (BJFC042287, holotype).

##### Description.

Basidiomata annual, resupinate, soft, without odor or taste when fresh, soft when dry, up to 15 cm long, 6 cm wide, and 0.2 mm thick at the center. Pore surface white when fresh, becoming white to cream upon drying; sterile margin white when fresh and dry, up to 2 mm wide, and with rose-colored rhizomorphs when fresh; pores round to angular, 2–4 per mm; dissepiments thin to slightly thick, entire to slightly lacerate. Subiculum white, soft when dry, up to 0.1 mm thick. Tubes concolorous with pore surface, soft when dry, up to 0.1 mm long. Hyphal system monomitic; generative hyphae simple septate, hyaline, IKI−, weakly CB+; tissues unchanged in KOH. Subicular hyphae slightly thick-walled with a wide lumen, sometimes encrusted with fine crystals, frequently branched at more or less a right angle, straight to more or less flexuous, interwoven, 4–7 µm in diam. Tramal hyphae thin-walled with a wide lumen, frequently branched, straight to slightly flexuous, subparallel along the tubes, agglutinated, 2.5–3 µm in diam. Hymenial cystidia present, clavate, thin-walled, smooth, 30–46 × 5–5.5 µm; cystidioles absent. Basidia mostly clavate, sometimes constricted at middle, with four sterigmata and a simple basal septum, 16–27 × 5–6 µm; basidioles of similar shape to basidia, but smaller. Large stellate crystal agglomerations present among the tube trama. Basidiospores ellipsoid to broadly ellipsoid, hyaline, thin-walled, smooth, IKI−, CB−, (3.6–)3.8–4.2(–4.6) × (2–)2.3–2.7(–2.9) µm, L = 3.95 µm, W = 2.53 µm, Q = 1.55–1.59 (n = 90/3).

##### Additional specimens examined.

CHINA • Guizhou Province, Guiyang, Qianlingshan Park, fallen branch of *Pinus massoniana*, 21 August 2023, Dai 25816 (BJFC043365), Dai 25742 (BJFC043291).

##### Notes.

*Meruliopsis rhizomorpha* is similar and closely related to *M. leptocystidiata* C.C. Chen & Sheng H. Wu by the white to cream pore surface, encrusted subicular hyphae, and smooth hymenial cystidia. However, the latter has smaller pores (4–5 per mm vs. 2–4 per mm, Chen et al. 2020) and narrower basidiospores (3–4 × 1.5–2 µm vs. 3.8–4.2 × 2.3–2.7 µm, Chen et al. 2020).

*Meruliopsis faginea* Volobuev & Ismailov also has almost the same size pores as *M. rhizomorpha* (3–4 per mm in *M. faginea*, 2–4 per mm in *M. rhizomorpha*, Crous et al. 2021) and long hymenial cystidia, but it differs from *M. rhizomorpha* by lacking rhizomorphs, having a pale grayish brown pore surface with light pinkish brown tints when fresh, a cottony to fimbriate sterile margin, and slimmer suballantoid to ellipsoid basidiospores (4–4.9 × 2–2.3 µm vs. 3.8–4.2 × 2.3–2.7 µm, Crous et al. 2021).

In Wang et al. (2023), the sequences of three species, *M. ambigua* (Berk.) Ginns, *M. bella* (Berk. & M.A. Curtis) Ginns, and *M. miniata* (Wakef.) Ginns are unavailable. However, *M. ambigua* has effused-reflexed basidiomata and a dark purple to violet-brown pore surface (Ginns 1976), which differs from *M. rhizomorpha*. Though *M. bella* and *M. miniata* both have resupinate basidiomata and almost the same pore sizes (2–4 per mm in *M. bella*, 2–3 per mm in *M. miniata*, Ginns 1971; Ginns 1976), the rose-colored rhizomorphs are absent.

#### ﻿ Meripilus

Taxon classificationAnimaliaPolyporalesMeripilaceae

P. Karst., Bidr. Känn. Finl. Nat. Folk 37: 33 (1882)

D9080072-8288-5FE2-B009-D87ACB9619E5

18044

##### Type species.

*Meripilus giganteus* (Pers.) P. Karst., Bidr. Känn. Finl. Nat. Folk 37: 33 (1882). For a detailed description of this genus, see Wang et al. (2024) and Westphalen et al. (2025).

#### ﻿ Meripilus albomarginatus

Taxon classificationAnimaliaPolyporalesMeripilaceae

Y.C. Dai, Xin Zhang, Chao G. Wang & Yuan Yuan sp. nov.

2ABBF704-1396-5AD3-A10A-DF89F3435A5E

856730

Figs 12, 13

##### Etymology.

*Albomarginatus* (Lat.): refers to the species having a white pileal margin when fresh.

**Figure 12.**
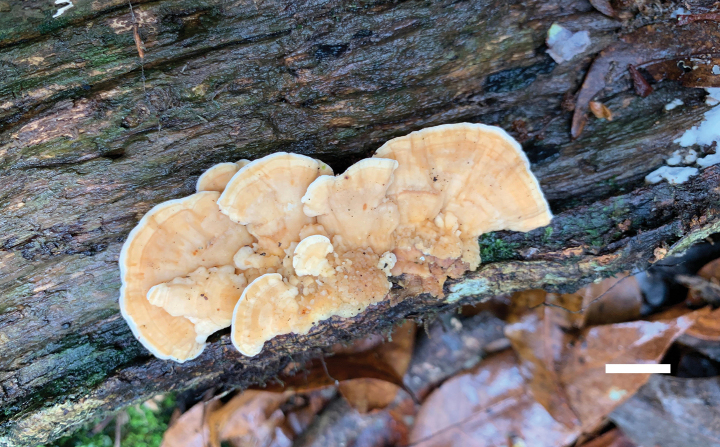
Basidiomata of *Meripilus albomarginatus* (holotype, Dai 19796). Scale bar: 1 cm.

##### Diagnosis.

Differs from other *Meripilus* species by effused-reflexed to pileate basidiomata with buff yellow to cinnamon buff and concentrically zonate-sulcate pileal surface when dry, thick-walled and apically encrusted hyphoid cystidia, subglobose basidiospores measuring 5.2–6.2 × 4.6–5.7 µm.

**Figure 13.**
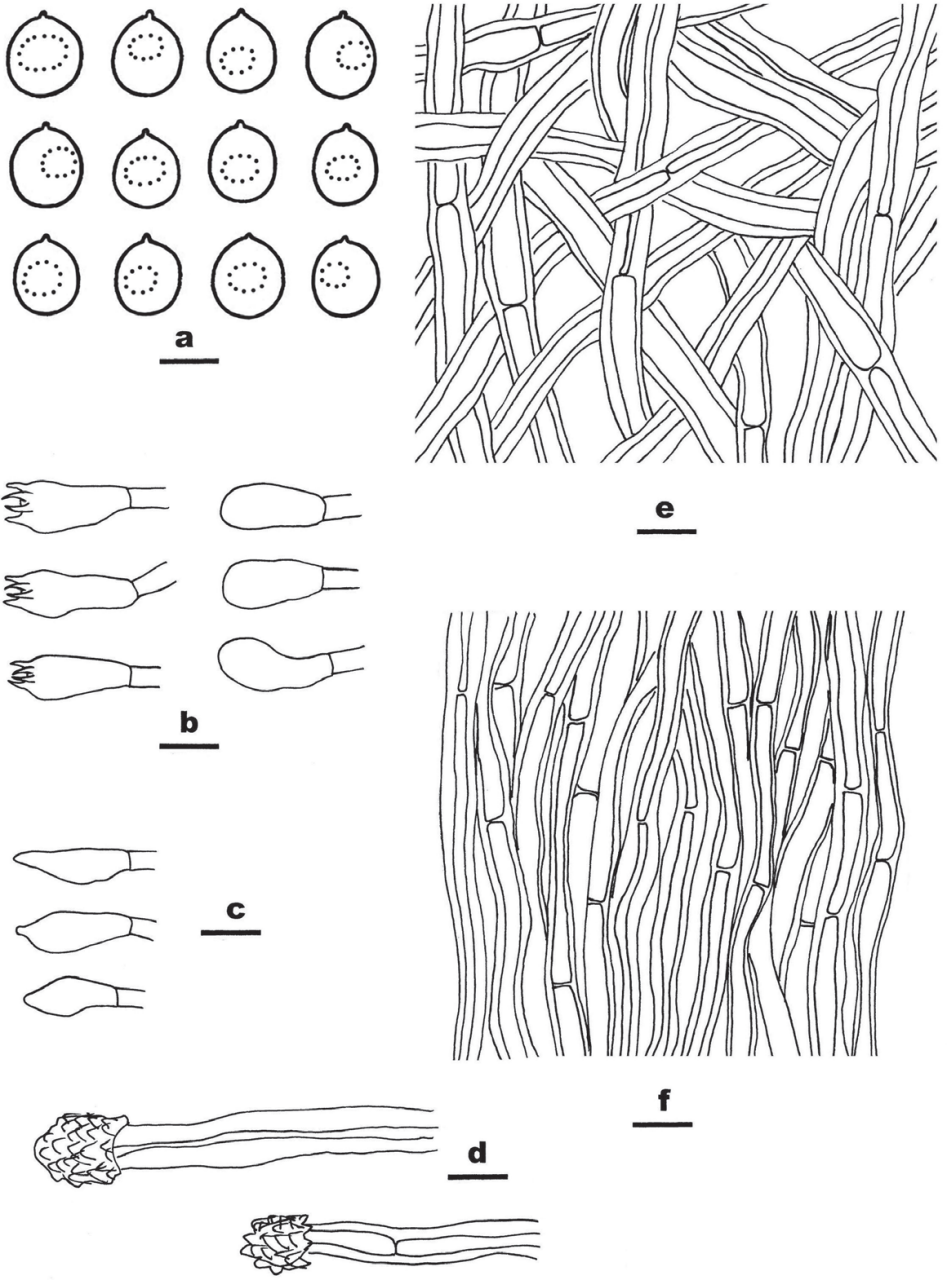
Microscopic structures of *Meripilus albomarginatus* (drawn from the holotype, Dai 19796). a basidiospores b basidia and basidioles c cystidioles d hyphoid cystidia e hyphae from context f hyphae from trama. Scale bars: 5 µm (a); 10 µm (b–f).

##### Type.

CHINA • Yunnan Province, Honghe, Pingbian County, Daweishan National Forest Park, on fallen angiosperm trunk, 26 June 2019, Dai 19796 (BJFC031471, holotype).

##### Description.

Basidiomata annual, effused-reflexed to pileate, imbricate, soft corky, and without odor or taste when fresh, becoming hard corky upon drying, up to 3 cm long and 2.5 cm wide when resupinate; pileus flabelliform, projecting up to 2 cm, 3.5 cm wide, and 1.6 mm thick at the base. Pileal surface orange-yellow to yellow-brown when fresh, becoming buff yellow to cinnamon buff, velutinate, concentrically zonate, and radially sulcate upon drying; margin sharp, thinning out, white when fresh, buff yellow and slightly curved when dry. Pore surface cream to pinkish buff when fresh, unchanged after bruising, clay buff to grayish brown when dry; sterile margin distinct, thinning out, white when fresh, cream to buff when dry; pores angular, 7–8 per mm; dissepiments thin, entire to slightly lacerate. Subiculum buff yellow, soft corky, up to 0.6 mm thick. Tubes concolorous with pore surface, corky when dry, up to 1 mm long. Hyphal system monomitic; generative hyphae simple septate, hyaline, smooth, IKI−, CB+; tissues unchanged in KOH. Contextual hyphae distinctly thick-walled to almost solid, unbranched, straight, loosely interwoven, 5–9 µm in diam. Tramal hyphae distinctly thick-walled with a median lumen, unbranched, slightly flexuous, subparallel along the tubes, agglutinated, 4–8 µm in diam. Hyphoid cystidia present, arising from tramal hyphae and completely embedded in trama, not projecting from the hymenium and sometimes projecting from the dissepiment edge, thick-walled with swollen tips, apically encrusted, 9–12 µm in diam. at the apex. Hymenial cystidia absent; cystidioles fusoid to mamillate, thin-walled, smooth, 14–17 × 6–6.5 µm; basidia barrel-shaped to somewhat pyriform, with four sterigmata and a simple basal septum, 17–20 × 8–9 µm; basidioles of similar shape to basidia, but smaller. Basidiospores subglobose, hyaline, thin-walled, smooth, with one medium or small guttule, IKI−, CB−, (5–)5.2–6.2(–6.5) × (4.5–)4.6–5.7(–6) µm, L = 5.79 µm, W = 5.19 µm, Q = 1.11–1.12 (n = 60/2).

##### Additional specimens examined.

CHINA • Guangdong Province, Guangzhou, Tianluhu Forest Park, on root of dead bamboo, 20 April 2023, Dai 24711 (BJFC042265). • Yunnan Province, Yuxi, Shimenxia Forest Park, on dead bamboo, 29 June 2023, Dai 25241 (BJFC042792).

##### Notes.

*Meripilus albomarginatus* is similar to *M. lineatus* (Pers.) Westph. & Rajchenb. and *M. sublineatus* (Y.C. Dai, Yuan Yuan & Chao G. Wang) Westph. & Rajchenb. in morphology by having effused-reflexed to pileate basidiomata with concentrically zonate-sulcate pileal surface, clay buff to gray-brown pore surface when dry, thick-walled and apically encrusted hyphoid cystidia and almost the same size as basidiospores (5–6 µm in *M. lineatus*; 4.8–5.6 × 4.5–5.2 µm in *M. sublineatus*; 5.2–6.2 × 4.6–5.7 µm in *M. albomarginatus*, Ryvarden and Gilbertson 1994; Wang et al. 2024). However, *M. lineatus* has a pinkish-buff to reddish-brown pileal surface and a bright orange-red pore surface when fresh (Ryvarden and Johansen 1980), and *M. sublineatus* has a normally azonate pileal surface and ventricose thin-walled hymenial cystidia bearing fine crystals (Wang et al. 2024).

*Meripilus albomarginatus* forms an independent lineage in the *Meripilus* clade (Fig. 2) and groups with *M. lineatus* and *M. sublineatus* in a joint subclade (Fig. 2). However, there are 20 base pair differences in ITS sequences between *Meripilus albomarginatus* and *M. lineatus*, which account for a 3% nucleotide difference in the ITS regions.

Four recorded synonyms of *Meripilus lineatus* (*Physisporinus lineatus*), viz. *Polyporus zonalis* Berk. (Sri Lanka), *P. pusiolus* Ces. (Malaysia), *P. punctatus* Jungh. (Indonesia), and *P. epilinteus* Berk. & Broome (Sri Lanka), were originally described from Asia. *Polyporus zonalis* has a pinkish-buff to reddish-brown upper surface, concolorous margin, and white to dingy livid gray pore surface (Pegler and Waterston 1968); *P. punctatus* has a cloudy-dirty and pale red pore surface and very small pores (Junghuhn 1838); and *P. pusiolus* and *P. epilinteus* have resupinate basidiomata (Berkeley and Broome 1873; Cesati 1879). The above four species are different from *Meripilus lineatus*, *M. sublineatus* and *M. albomarginatus* in morphology.

#### ﻿ Meripilus crystallinus

Taxon classificationAnimaliaPolyporalesMeripilaceae

Y.C. Dai, Chao G. Wang & Yuan Yuan, sp. nov .

3A6D2884-43E4-5DAC-A470-4106864D4D58

856731

Figs 14, 15

##### Etymology.

*Crystallinus* (Lat.): refers to the species having hyphoid cystidia with crystals.

**Figure 14.**
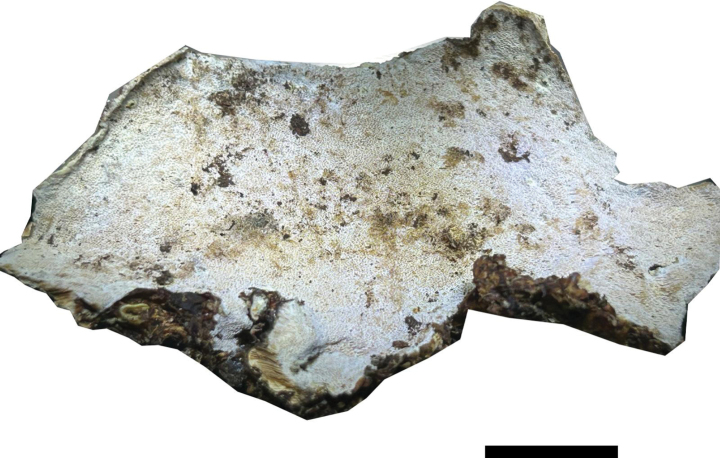
Basidiomata of *Meripilus crystallinus* (holotype, Cui 10491). Scale bar: 1 cm.

##### Diagnosis.

Differs from other *Meripilus* species by resupinate basidiomata with buff yellow, salmon to clay pink pore surface when dry, angular pores of 6–8 per mm, thin-walled and apically encrusted hyphae at the dissepiment edge, broadly ellipsoid to ovoid and slightly thick-walled basidiospores measuring 4.3–5 × 4–4.5 µm.

**Figure 15.**
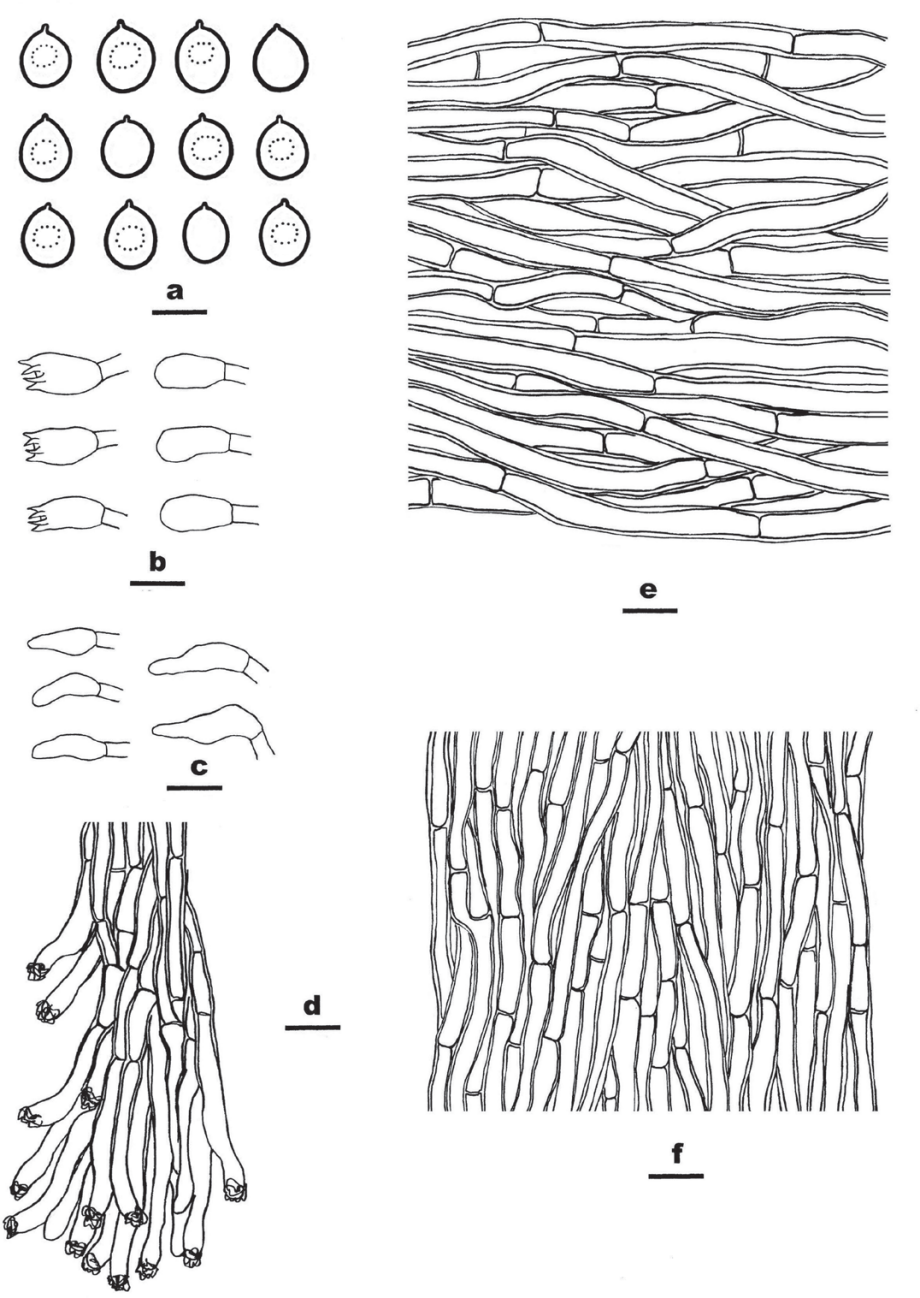
Microscopic structures of *Meripilus crystallinus* (drawn from the holotype, Cui 10491). a basidiospores b basidia and basidioles c cystidioles d hyphoid cystidia-like hyphae at the dissepiment edge e hyphae from subiculum f hyphae from trama. Scale bars: 5 µm (a); 10 µm (b–f).

##### Type.

CHINA • Yunnan Province, Weixi County, Laojunshan Nature Reserve, on fallen trunk of *Picea*, 22 September 2011, Cui 10491 (BJFC011386, holotype).

##### Description.

Basidiomata annual, resupinate, soft to ceraceous, and without odor or taste when fresh, becoming brittle upon drying, up to 10 cm long, 6 cm wide, and 3.2 mm thick at the center. Pore surface buff yellow, salmon to clay pink when dry; sterile margin almost absent; pores angular, 6–8 per mm; dissepiments thin, entire to slightly lacerate. Subiculum very thin to almost absent, up to 0.2 mm thick. Tubes concolorous with pore surface, brittle when dry, up to 3 mm long. Hyphal system monomitic; generative hyphae simple septate, hyaline to yellowish brown, smooth, IKI−, CB+; tissues unchanged in KOH. Subicular hyphae slightly thick-walled with a wide lumen, unbranched, moderately simple septate, slightly flexuous, loosely interwoven, agglutinated, 4–7 µm in diam. Tramal hyphae slightly thick- to thick-walled with a wide lumen, occasionally branched, frequently simple septate, slightly flexuous, subparallel along the tubes, agglutinated, 3–5 µm in diam.; some thin-walled hyphae at the dissepiment edge bearing crystals at the tips and resembling hyphoid cystidia. Hymenial cystidia absent; cystidioles fusoid, thin-walled, smooth, 12–18 × 4–5 µm; basidia barrel-shaped, with four sterigmata and a simple basal septum, 11–12 × 5.5–6 µm; basidioles of similar shape to basidia, but smaller. Basidiospores broadly ellipsoid to ovoid, hyaline, slightly thick-walled, smooth, sometimes with one medium guttule, IKI−, weakly CB+, (4.2–)4.3–5 × 4–4.5 µm, L = 4.75 µm, W = 4.15 µm, Q = 1.13–1.15 (n = 60/2).

##### Additional specimen examined.

CHINA • Yunnan Province, Weixi County, Laojunshan Nature Reserve, on fallen trunk of *Picea*, 22 September 2011, Cui 10475 (BJFC011370).

##### Notes.

*Meripilus crystallinus* is similar and related to *M. eminens* (Y.C. Dai) Rajchenb. & Westph. by having annual and resupinate basidiomata, the absence of a sterile margin, angular and almost the same size pores (7–8 per mm in *M. eminens*; 6–8 per mm in *M. crystallinus*, Dai 1998), and almost the same size as basidiospores (4.2–6 × 3.9–5.2 µm in *M. eminens*; 4.3–5 × 4–4.5 µm in *M. crystallinus*, Dai 1998). However, *M. eminens* has thin- to fairly thick-walled tramal hyphae, and thick-walled and apically encrusted hyphoid cystidia penetrating above the hymenial surface (Dai 1998). *Meripilus vitreus* (Pers.) Rajchenb. & Westph. is phylogenetically related to *M. crystallinus* (Fig. 4), but *M. vitreus* has thick-walled hyphoid cystidia with coarse crystals in most specimens, relatively bigger pores (5–6 per mm vs. 6–8 per mm), and thin-walled basidiospores (5–5.5 × 4–4.5 µm vs. 4.3–5 × 4–4.5 µm, Wang et al. 2024). In addition, there are 20 base pair differences in ITS sequences between these two species, which accounts for a 3% nucleotide difference in the ITS regions.

*Meripilus crystallinus* and *M. stillicidiorum* (Speg.) Rajchenb. & Westph. share the pinkish buff, buff yellow, salmon to clay pink pore surface, the absence of sterile margin, and almost the same pore size (5–7 per mm in *M. stillicidiorum*; 6–8 per mm in *M. crystallinus*, Wang et al. 2024). However, *M. stillicidiorum* lacks any kind of cystidia, relatively larger basidiospores (5–5.7 × 4–4.8 µm vs. 4.3–5 × 4–4.5 µm, Wang et al. 2024), and so far, is only known from the type locality in Australia. *Meripilus revolubilis* Westph. & R.M. Silveira and *M. robledoi* Rajchenb. & Westph. were described with the resupinate basidiomata, white, cream to isabelline pore surface, thick-walled encrusted cystidia, which are similar to *M. crystallinus* (Westphalen et al. 2025). Phylogenetically, *Meripilus crystallinus*, *M. eminens*, *M. revolubilis*, *M. robledoi*, and *M. vitreus* form a distinct joint clade and share similar morphological characters.

#### ﻿ Meripilus emarginatus

Taxon classificationAnimaliaPolyporalesMeripilaceae

Y.C. Dai, Chao G. Wang & Yuan Yuan sp. nov.

33FB8212-05B9-540E-8804-EA882774676E

856732

Figs 16, 17

##### Etymology.

*Emarginatus* (Lat.): refers to the basidiomata of species lacking a sterile margin.

**Figure 16.**
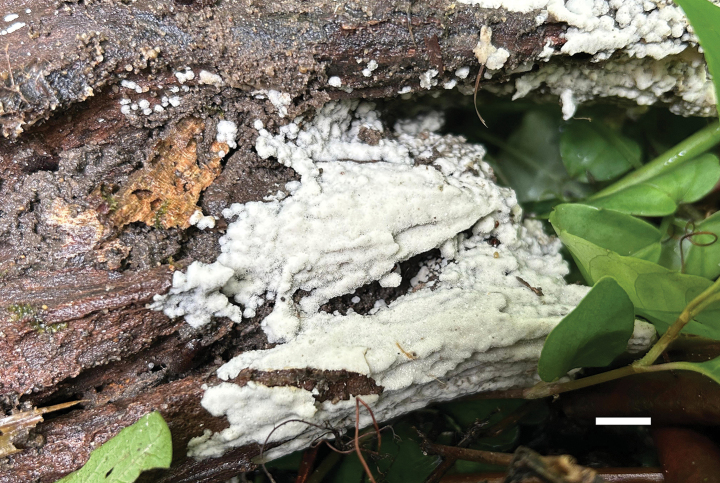
Basidiomata of *Meripilus emarginatus* (holotype, Dai 24682A). Scale bar: 1 cm.

##### Diagnosis.

Differs from other *Meripilus* species by resupinate basidiomata with a white pore surface when fresh, round to angular pores of 6–7 per mm, thick-walled and apically encrusted hyphoid cystidia, thin-walled and smooth hymenial cystidia, subglobose to globose basidiospores measuring 4.8–5.2 × 4.5–5.2 µm.

**Figure 17.**
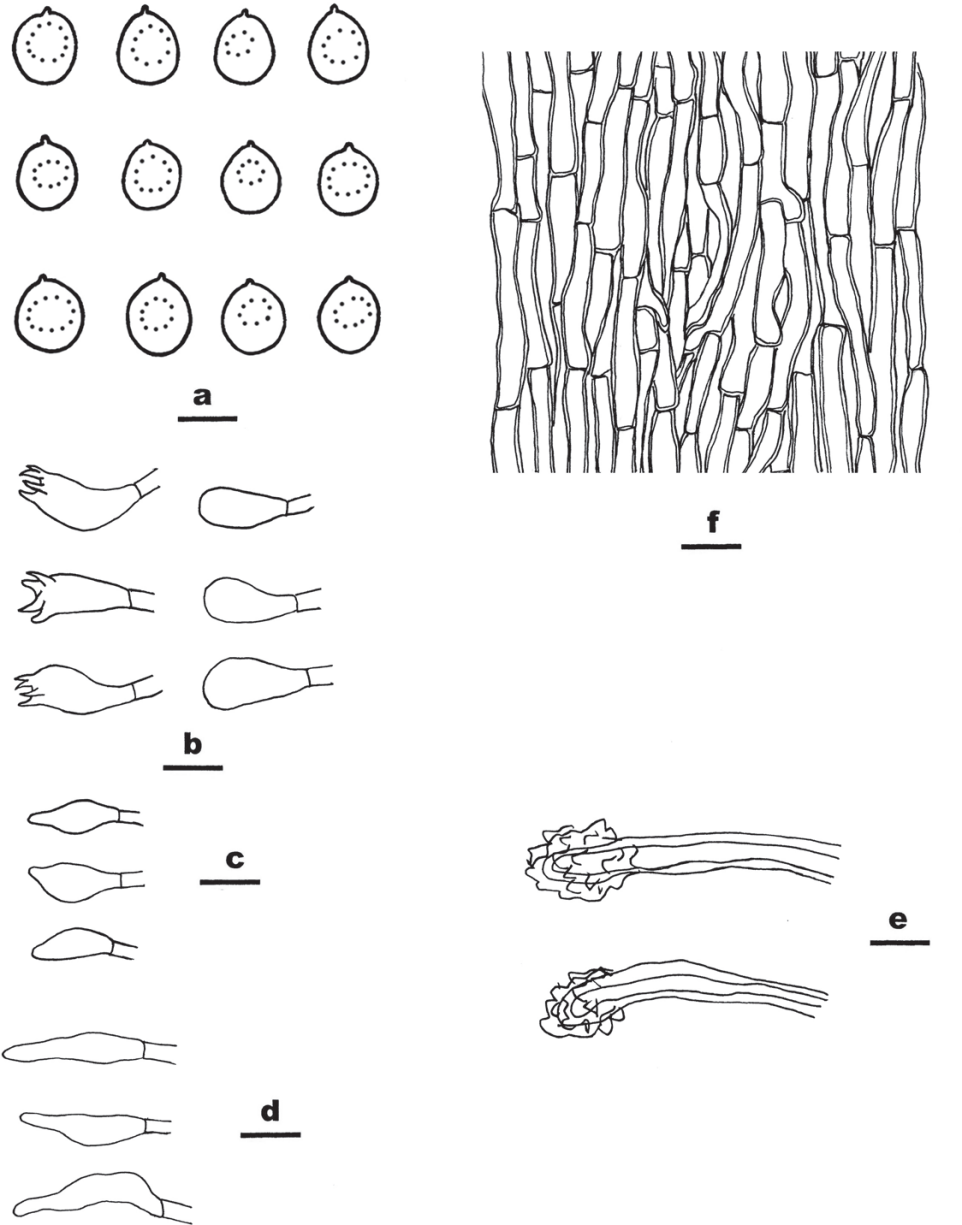
Microscopic structures of *Meripilus emarginatus* (drawn from the holotype, Dai 24682A). a basidiospores b basidia and basidioles c cystidioles d hymenial cystidia e hyphoid cystidia f hyphae from trama. Scale bars: 5 µm (a); 10 µm (b–f).

##### Type.

CHINA • Guangdong Province, Guangzhou, Baiyunshan Forest Park, on rotten angiosperm wood, 18 April 2023, Dai 24682A (BJFC042236, holotype).

##### Description.

Basidiomata annual, resupinate, soft to ceraceous, and without odor or taste when fresh, becoming fragile upon drying, up to 8 cm long, 5 cm wide, and 0.6 mm thick at the center. Pore surface white when fresh, unchanged after bruising, pinkish buff to clay buff when dry; sterile margin absent; pores round to angular, 6–7 per mm; dissepiments thin, slightly lacerate. Subiculum very thin to almost absent. Tubes concolorous with pore surface, fragile when dry, up to 0.6 mm long. Hyphal system monomitic; generative hyphae simple septate, hyaline to pale yellowish, smooth, IKI−, moderately CB+; tissues unchanged in KOH. Tramal hyphae slightly thick-walled with a wide lumen, occasionally branched, frequently simple septate, slightly flexuous, subparallel along the tubes, agglutinated, 4–5 µm in diam. Hyphoid cystidia present, arising from tramal hyphae and completely embedded in trama, not projecting from the hymenium, sometimes projecting from the dissepiment edge, thick-walled with swollen tips, apically encrusted, 6–12 µm in diam. at the apex; hymenial cystidia present, fusoid, thin-walled, smooth, 20–25 × 4.5–5.5 µm; cystidioles fusoid, thin-walled, smooth, 13–14 × 5–6 µm; basidia barrel-shaped to capitate, with four sterigmata and a simple basal septum, 15–17 × 7–8 µm; basidioles mostly pyriform, smaller. Crystals present among the hymenium and tube trama. Basidiospores subglobose to globose, hyaline, thin-walled, smooth, sometimes with one small or large guttule, IKI−, weakly CB+, (4.5–)4.8–5.2(–5.5) × 4.5–5.2(–5.5) µm, L = 5.11 µm, W = 4.90 µm, Q = 1.03–1.05 (n = 90/3).

##### Additional specimens examined.

CHINA • Fujian Province, Fuding County, Tailao Mts., on fallen trunk of *Cunninghamia*, 22 August 2016, Dai 16971 (BJFC023076); • Guangdong Province, Guangzhou, Baiyunshan Forest Park, on rotten angiosperm wood, 18 April 2023, Dai 24683A (BJFC042237), Maofengshan Forest Park, on rotten angiosperm wood, 19 April 2023, Dai 24694A (BJFC042248); • Xizang Autonomous Region, Linzhi, Motuo County, on dead *Miscanthus*, 24 October 2023, Dai 26696 (BJFC044246).

##### Notes.

*Meripilus emarginatus* is similar to *M. albostygius* (Berk. & M.A. Curtis) Westph. & Rajchenb., *M. eminens*, *M. rigidus* (Y.C. Dai, Chao G. Wang & Vlasák) Westph. & Rajchenb., *M. srilankensis*, and *M. sulphureus* (Y.C. Dai, Yuan Yuan & Chao G. Wang) Westph. & Rajchenb. in micromorphology by the thick-walled hyphoid cystidia and subglobose basidiospores. However, *M. albostygius* has a red to violet pore surface when fresh, smaller pores (8–10 per mm vs. 6–7 per mm, Wang et al. 2024), and smaller basidiospores (4–4.7 × 3.2–4 µm vs. 4.8–5.2 × 4.5–5.2 µm, Wang et al. 2024), and to date is only known from Central and South America; *M. eminens* also has ceraceous basidiomata with a white pore surface when fresh, almost the same size angular pores (7–8 per mm vs. 6–7 per mm, Dai 1998) and basidiospores (4.2–6 × 3.9–5.2 µm vs. 4.8–5.2 × 4.5–5.2 µm, Dai 1998), but, it differs from *M. emarginatus* by the absence of thin-walled and smooth hymenial cystidia; *M. rigidus* differs from *M. emarginatus* by a brown-red pore surface when fresh, smaller pores (10–12 per mm vs. 6–7 per mm, Wang et al. 2024), smaller basidiospores (4–4.6 × 3.2–4 µm vs. 4.8–5.2 × 4.5–5.2 µm, Wang et al. 2024), and, to date, is only known from Central America; *M. srilankensis* is distinguished from *M. emarginatus* by distinctly thick-walled and wider tramal hyphae (3.8–8 µm in diam. vs. 4–5 µm in diam., Wang et al. 2024) and the absence of hymenial cystidia; *M. sulphureus* differs from *M. emarginatus* by a sulphur yellow pore surface when fresh and relatively smaller basidiospores (4–5 × 3.5–4 µm vs. 4.8–5.2 × 4.5–5.2 µm, Dai and Dai 2018). In macro-morphology, *Meripilus emarginatus* has an unchanged pore surface.

Phylogenetically, *Meripilus emarginatus* forms an independent lineage nested in the *Meripilus* clade (100% ML, 1.00 BPP, Figs 3, 4). However, it grouped with *M. dollingerii* (Y.C. Dai, Chao G. Wang & Vlasák) Westph. & Rajchenb. and *M. malayanus* in a joint subclade (90% ML, 1.00 BPP, Fig. 4). Although *Meripilus dollingerii* and *M. emarginus* share thin-walled smooth hymenial cystidia and almost the same size as basidiospores (4.5–5.5 × 4–5 µm in *M. dollingerii*, 4.8–5.2 × 4.5–5.2 µm in *M. emarginatus*, Wang et al. 2024), *M. dollingerii* has a pinkish to red pore surface when fresh and thin-walled apically encrusted hyphoid cystidia. *Meripilus malayanus* also has thick-walled and apically encrusted hyphoid cystidia, but it differs from *M. emarginatus* by hard corky basidiomata when dry, a grayish brown pore surface, and the presence of a sterile margin.

#### ﻿ Meripilus malayanus

Taxon classificationAnimaliaPolyporalesMeripilaceae

Y.C. Dai, Chao G. Wang & Yuan Yuan sp. nov.

F940BC4A-FEA2-5E83-9DF4-75966749C72E

856733

Figs 18, 19

##### Etymology.

*Malayanus* (Lat.): refers to the species being found in Malaysia.

**Figure 18.**
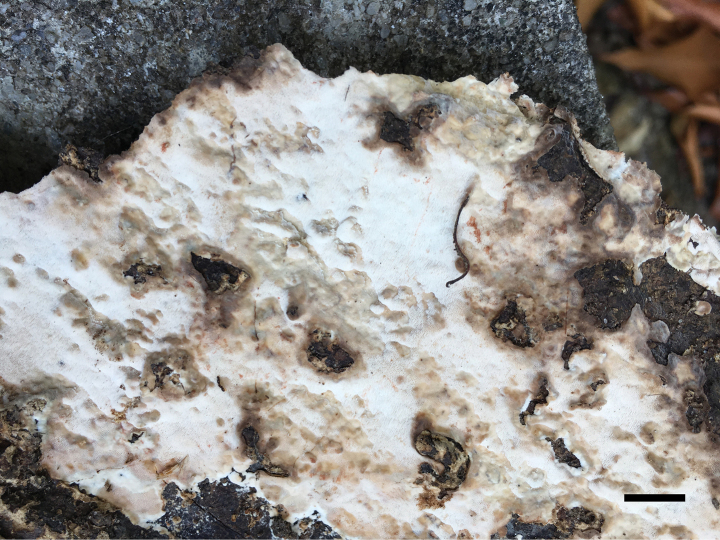
Basidiomata of *Meripilus malayanus* (holotype, Dai 18529). Scale bar: 1 cm.

##### Diagnosis.

Differs from other *Meripilus* species by resupinate nodulose basidiomata with a honey buff to grayish brown pore surface when dry, angular and sometimes elongated pores of 7–8 per mm, thick-walled and apically encrusted hyphoid cystidia, subglobose basidiospores measuring 5–5.5 × 4.5–5 µm.

**Figure 19.**
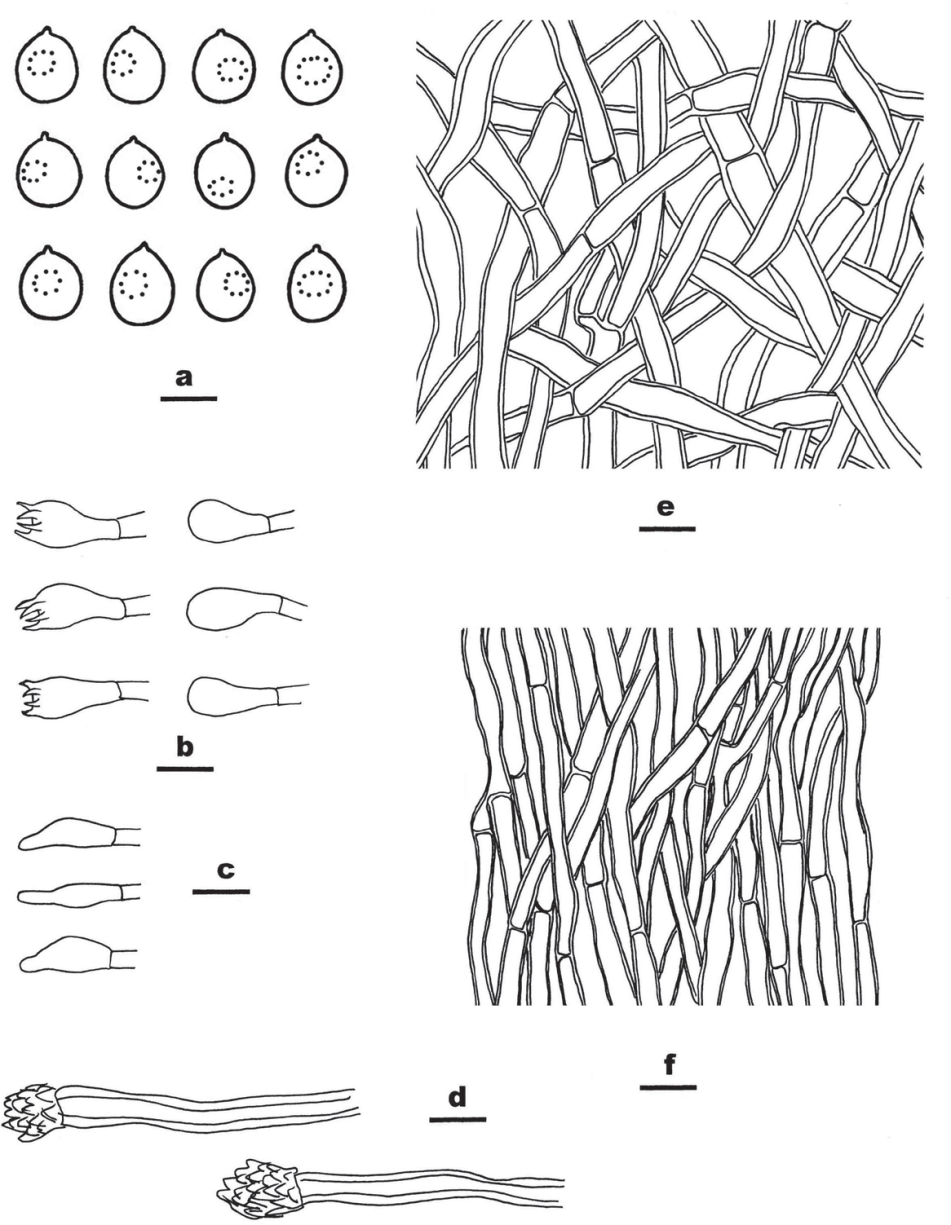
Microscopic structures of *Meripilus malayanus* (drawn from the holotype, Dai 18529). a basidiospores b basidia and basidioles c cystidioles d hyphoid cystidia e hyphae from subiculum f hyphae from trama. Scale bars: 5 µm (a); 10 µm (b–f).

##### Type.

MALAYSIA • Selangor, Taman Botani Negara Shah Alam, on fallen angiosperm trunk, 12 April 2018, Dai 18529 (BJFC026818, holotype).

##### Description.

*Basidiomata* annual, resupinate, nodulose, soft corky, and without odor or taste when fresh, becoming hard corky upon drying, up to 15 cm long, 6 cm wide, and 0.8 mm thick at the center. Pore surface white to cream when fresh, unchanged after bruising, honey buff to grayish brown when dry; sterile margin thinning out, grayish brown when dry; pores angular, sometimes elongated, 7–8 per mm; dissepiments thin, slightly lacerate. Subiculum pinkish buff, corky, up to 0.3 mm thick. Tubes concolorous with pore surface, hard corky when dry, up to 0.5 mm long. Hyphal system monomitic; generative hyphae simple septate, smooth, IKI−, CB+; tissues becoming reddish brown in KOH. Subicular hyphae distinctly thick-walled with a wide lumen, rarely branched and simple septate, more or less flexuous, interwoven, 5–8 µm in diam. Tramal hyphae slightly thick-walled with a wide lumen, occasionally branched, moderately simple septate, straight, subparallel along the tubes, agglutinated, 3.5–4.8 µm in diam. Hyphoid cystidia present, arising from tramal hyphae and completely embedded in trama, not projecting from the hymenium or dissepiment edge, thick-walled with swollen tips, apically encrusted, 7–9 µm in diam. at the apex. Hymenial cystidia absent; cystidioles fusoid, thin-walled, smooth, 14–17 × 4–5 µm; basidia barrel-shaped to capitate, with four sterigmata and a simple basal septum, 14–17 × 7–8 µm; basidioles of similar shape to basidia, but smaller. Basidiospores subglobose, hyaline, thin-walled, smooth, with one medium or small guttule, IKI−, CB−, 5–5.5(–6) × (4.2–) 4.5–5(–5.5) µm, L = 5.20 µm, W = 4.68 µm, Q = 1.11 (n = 30/1).

##### Notes.

*Meripilus malayanus* is similar to *M. rigidus* in morphology by having resupinate and hard corky to rigid basidiomata with a honey buff to grayish brown or deep olive pore surface when dry, distinctly thick-walled subicular hyphae, and thick-walled and apically encrusted hyphoid cystidia.

However, the latter has round and smaller pores (10–12 per mm vs. 7–8 per mm, Wang et al. 2024), smaller basidiospores (4–4.6 × 3.2–4 µm vs. 5–5.5 × 4.5–5 µm, Wang et al. 2024), and so far, it is only known from the type locality in Central America.

In the phylogenetic analyses, *Meripilus malayanus* is closely related to *M. dollingerii*, but *M. dollingerii* has thin-walled and apically encrusted hyphoid cystidia and thin-walled smooth hymenial cystidia (Wang et al. 2024).

*Rigidoporus adnatus* Corner and *Polyporus pellicula* Jungh. occur in Southeast Asia and have resupinate basidiomata. However, *Rigidoporus adnatus* has smaller basidiospores (2.5–3.2 × 1.7–2 µm vs. 5–5.5 × 4.5–5 µm, Corner 1987), and *Polyporus pellicula* has larger pores (1−3 per mm vs. 7–8 per mm, Teixeira 1992).

#### ﻿ Meripilus niveomarginatus

Taxon classificationAnimaliaPolyporalesMeripilaceae

Y.C. Dai, Chao G. Wang & Yuan Yuan sp. nov.

32D63492-81C1-5D4A-A7AE-1EA7E8B9DA73

856734

Figs 20, 21

##### Etymology.

*Niveomarginatus* (Lat.): refers to the species having a white sterile margin when fresh.

**Figure 20.**
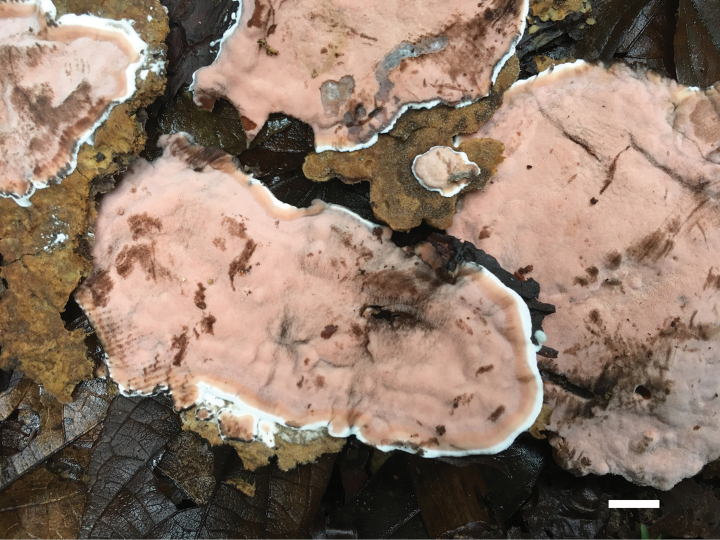
Basidiomata of *Meripilus niveomarginatus* (holotype, Dai 18540A). Scale bar: 1 cm.

##### Diagnosis.

Differs from other *Meripilus* species by resupinate basidiomata with orange-yellow to peach when fresh, white sterile margin when fresh, angular to irregular pores of 6–8 per mm, thick-walled and apically encrusted hyphoid cystidia, broadly ellipsoid basidiospores measuring 4.2–5.2 × 4–4.6 µm.

**Figure 21.**
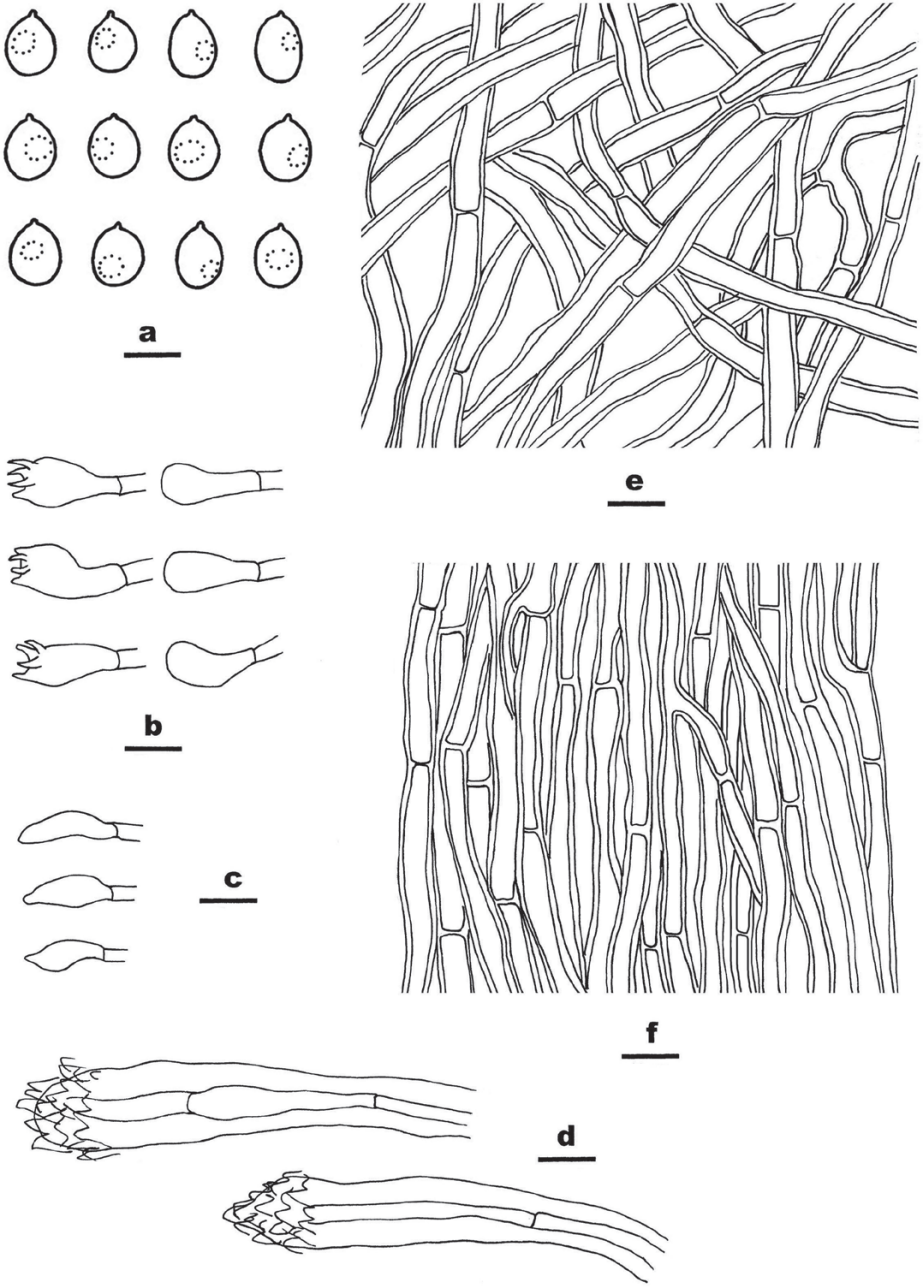
Microscopic structures of *Meripilus niveomarginatus* (drawn from the holotype, Dai 18540A). a basidiospores b basidia and basidioles c cystidioles d hyphoid cystidia e hyphae from subiculum f hyphae from trama. Scale bars: 5 µm (a); 10 µm (b–f).

##### Type.

CHINA • Guangdong Province, Zhaoqing, Dinghushan Nature Reserve, on rotten wood of *Pinus massoniana*, 28 April 2018, Dai 18540A (BJFC027008, holotype).

##### Description.

*Basidiomata* annual, resupinate, soft corky, and without odor or taste when fresh, becoming hard corky to somewhat rigid upon drying, up to 15 cm long, 6 cm wide, and 4 mm thick at center. Pore surface brownish vinaceous, orange-yellow to peach when fresh, becoming reddish brown after bruising, pinkish buff, vinaceous gray, dark brown to black when dry; sterile margin distinct, thinning out, white when fresh, cream to olivaceous buff when dry, up to 2 mm wide; pores angular to irregular, 6–8 per mm; dissepiments thin, slightly lacerate. Subiculum cream, corky, up to 1 mm thick. Tubes paler than pore surface, cinnamon buff, hard corky when dry, up to 3 mm long. Hyphal system monomitic; generative hyphae simple septate, hyaline, smooth, IKI−, CB+; tissues becoming dark brown in KOH. Subicular hyphae distinctly thick-walled with a wide lumen, rarely branched and simple septate, more or less flexuous, interwoven, 4–8 µm in diam. Tramal hyphae distinctly thick-walled with a wide lumen, occasionally branched, moderately simple septate, slightly flexuous, subparallel along the tubes, agglutinated, 4–6 µm in diam. Hyphoid cystidia present, arising from tramal hyphae and completely embedded in trama, not projecting from the hymenium, sometimes projecting from the dissepiment edge, thick-walled with swollen tips, apically encrusted, 10–15 µm in diam. at the apex. Hymenial cystidia absent; cystidioles fusoid, thin-walled, smooth, 13–14 × 4–5 µm; basidia barrel-shaped to capitate, with four sterigmata and a simple basal septum, 14–17 × 7–9 µm; basidioles of similar shape to basidia, but smaller. Basidiospores broadly ellipsoid, hyaline, thin-walled, smooth, with one medium or small guttule, IKI−, weakly CB+, (4–)4.2–5.2(–5.5) × (3.8–)4–4.6(–4.8) µm, L = 4.78 µm, W = 4.16 µm, Q = 1.13–1.17 (n = 90/3).

##### Additional specimens examined.

CHINA • Fujian Province, Quanzhou, Qingyuanshan, on fallen trunk of *Pinus massoniana*, 23 September 2017, Dai 18268 (BJFC025793); • Hainan Province, Baisha County, Yinggeling Nature Reserve, on rotten wood of *Pinus latteri*, 10 June 2017, Dai 17695 (BJFC025227); • Zhejiang Province, Wuyi County, on rotten wood of *Pinus massoniana*, 13 October 2023, Dai 26373 (BJFC043923).

##### Notes.

*Meripilus niveomarginatus* is similar and related to *M. rigidus* by the resupinate and rigid basidiomata with a pinkish buff to vinaceous gray or dark brown pore surface when dry, distinctly thick-walled generative hyphae, and thick-walled and apically encrusted hyphoid cystidia. However, the latter has round and smaller pores (10–12 per mm vs. 6–8 per mm, Wang et al. 2024), relatively smaller basidiospores (4–4.6 × 3.2–4 µm vs. 4.2–5.2 × 4–4.6 µm, Wang et al. 2024), and so far, it is only known from Central and South America. Similarly, *Meripilus albostygius* and *M. sulphureus* also have rigid basidiomata and thick-walled and apically encrusted hyphoid, but *M. albostygius* has smaller basidiospores (4–4.7 × 3.2–4 µm vs. 4.2–5.2 × 4–4.6 µm, Wang et al. 2024) and, to date, only occurs in Central America; *M. sulphureus* has a sulphurous pore surface when fresh and narrower basidiospores (4–5 × 3.5–4 µm vs. 4.2–5.2 × 4–4.6 µm, Dai and Dai 2018). *Meripilus roseus* (Jia J. Chen & Y.C. Dai) Westph. & Rajchenb. and *M. niveomarginatus* share a rose or brownish vinaceous pore surface when fresh. However, the former has an almost lacking sterile margin and smaller basidiospores (3.5–4.1 × 3.1–3.8 µm vs. 4.2–5.2 × 4–4.6 µm, Chen and Dai 2021).

*Poria endoxantha* Petch was originally described from Sri Lanka, and it is characterized by a rose-pink to salmon-pink pore surface when fresh, becoming brown or blackish brown upon bruising, thick-walled hyphoid cystidia embedded in trama, globose basidiospores of 5–7 µm in diam., and growth on angiosperm wood in the tropics (Petch 1922; Lowe 1966). *Meripilus niveomarginatus* is similar to *Poria endoxantha* in morphology, but the latter has pale yellowish-brown subicular hyphae that are obviously wider than tramal hyphae, while *M. niveomarginatus* has almost uniform hyphae in subiculum and trama. In addition, *Poria endoxantha* has bigger basidiospores (5–7 µm vs. 4.2–5.2 × 4–4.6 µm, Lowe 1966) and growth on angiosperm wood in the tropics, differing from *M. niveomarginatus*.

#### ﻿ Meripilus noncontusus

Taxon classificationAnimaliaPolyporalesMeripilaceae

Y.C. Dai, Chao G. Wang & Yuan Yuan sp. nov.

C84EB0FB-54ED-53C8-B783-DF262DA31764

856735

Figs 22, 23

##### Etymology.

*Noncontusus* (Lat.): refers to the species having pores without color changing when bruised.

**Figure 22.**
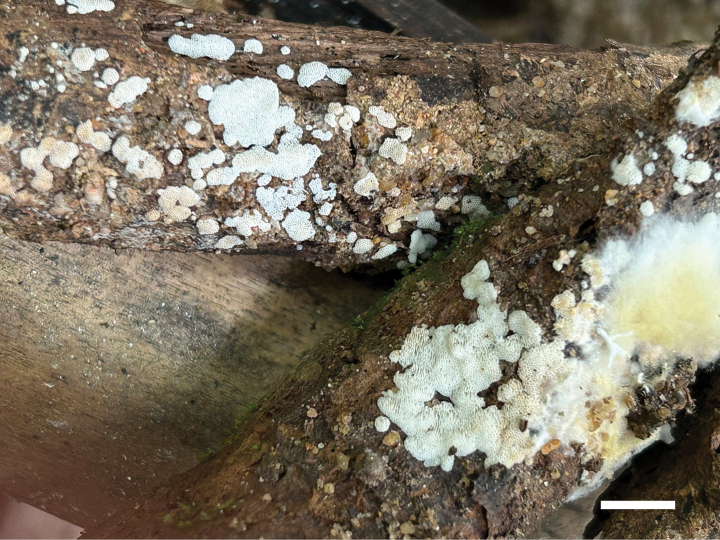
Basidiomata of *Meripilus noncontusus* (holotype, Dai 24718). Scale bar: 1 cm.

##### Diagnosis.

Differs from other *Meripilus* species by resupinate basidiomata with a white pore surface when fresh, the absence of sterile margin, angular pores of 5–6 per mm, slightly thick-walled and apically encrusted hyphoid cystidia at the dissepiment edge, broadly ellipsoid to subglobose basidiospores measuring 5–5.5 × 4.2–4.7 µm.

**Figure 23.**
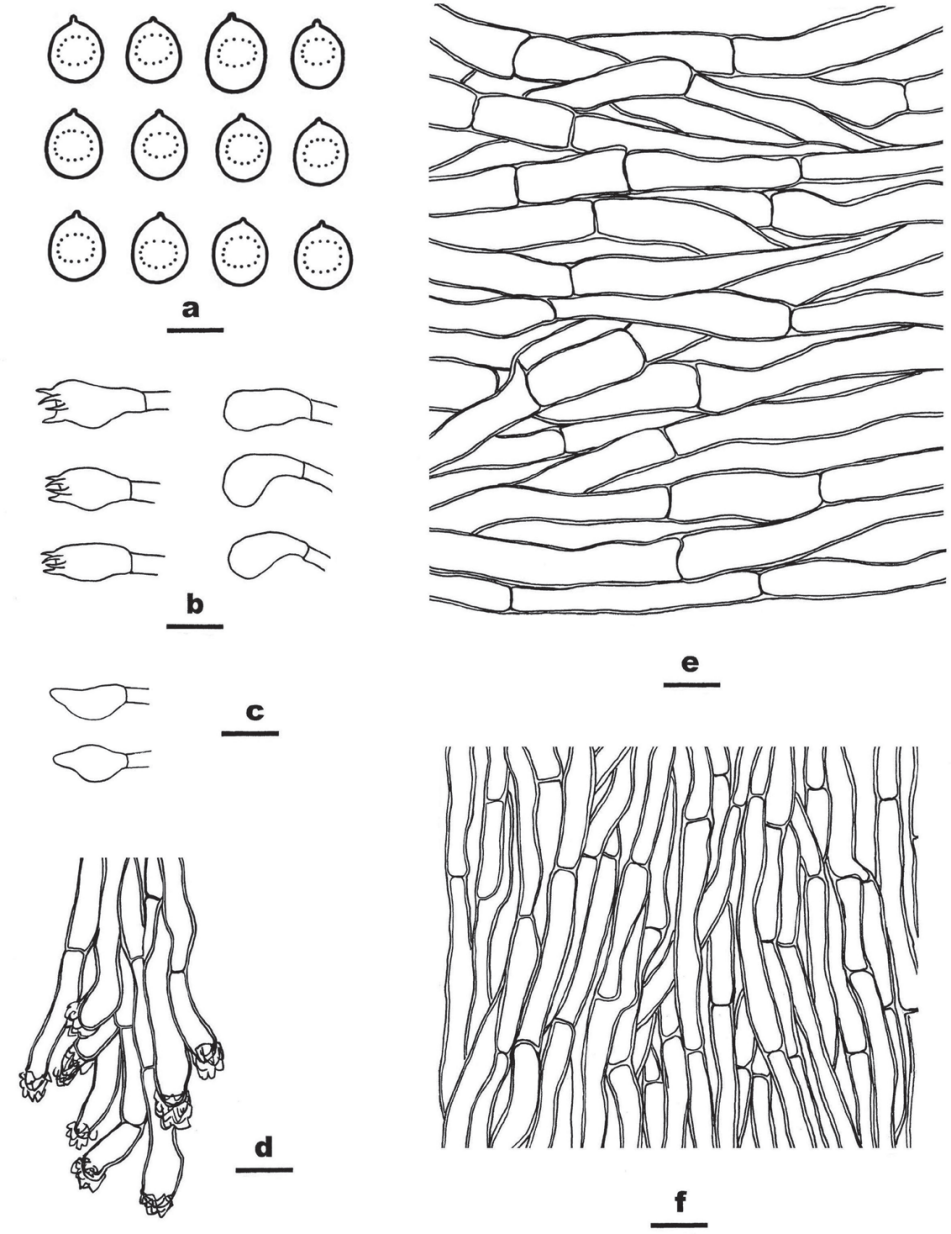
Microscopic structures of *Meripilus noncontusus* (drawn from the holotype, Dai 24718). a basidiospores b basidia and basidioles c cystidioles d hyphoid cystidia-like hyphae at the dissepiment edge e hyphae from subiculum f hyphae from trama. Scale bars: 5 µm (a); 10 µm (b–f).

##### Type.

CHINA • Guangdong Province, Guangzhou, Baiyunshan Forest Park, on fallen angiosperm trunk, 20 April 2023, Dai 24718 (BJFC042272, holotype).

##### Description.

Basidiomata annual, resupinate, soft to ceraceous, and without odor or taste when fresh, becoming fragile upon drying, usually in small patches up to 2 cm long, 1 cm wide, and 0.7 mm thick at the center. Pore surface white to pinkish buff when fresh, unchanged after bruising, pinkish buff to buff yellow when dry; sterile margin absent; pores angular, 5–6 per mm; dissepiments thin, slightly lacerate. Subiculum pinkish buff, fragile, up to 0.2 mm thick. Tubes concolorous with pore surface, fragile when dry, up to 0.5 mm long. Hyphal system monomitic; generative hyphae simple septate, hyaline, smooth, IKI−, CB+; tissues unchanged in KOH. Subicular hyphae slightly thick-walled with a wide lumen, rarely branched, frequently simple septate, slightly flexuous, loosely interwoven, agglutinated, 5–8 µm in diam. Tramal hyphae slightly thick-walled with a wide lumen, occasionally branched, slightly flexuous, subparallel along the tubes, agglutinated, 3–6 µm in diam.; some slightly thick-walled hyphae at the dissepiment edge bearing crystals at tips and resembling hyphoid cystidia. Hymenial cystidia absent; cystidioles fusoid, thin-walled, smooth, 11–12 × 5.5–6 µm; basidia barrel-shaped to capitate, with four sterigmata and a simple basal septum, 12–14 × 6.5–8 µm; basidioles of similar shape to basidia, but smaller. Basidiospores broadly ellipsoid to subglobose, hyaline, thin-walled, smooth, with one medium guttule, IKI−, CB−, (4.8–)5–5.5(–5.6) × (4–)4.2–4.7(–4.8) µm, L = 5.13 µm, W = 4.45 µm, Q = 1.15 (n = 30/1).

##### Notes.

In our phylogenetic analyses, *Meripilus noncontusus* is closely related to the erubescent species complex of *Meripilus*, viz., *M. furcatus* (Núñez & Ryvarden) Westph. & Rajchenb., *M. rhododendri* (Y.C. Dai, Yuan Yuan & Chao G. Wang) Westph. & Rajchenb., *M. sanguinolentus* (Alb. & Schwein.) Rajchenb. & Westph., *M. subfurcatus* (Y.C. Dai, Yuan Yuan & Chao G. Wang) Westph. & Rajchenb. and *M. yunnanensis* (C.L. Zhao) Westph. & Rajchenb., and these species nested in a joint subclade (Fig. 2). They all have a white pore surface when fresh, but the first three species have an erubescent pore surface when bruised. *Meripilus furcatus* differs from *M. noncontusus* by thin-walled and forked hymenial cystidia; *M. yunnanensis* also has thin-walled hyphoid cystidia at the dissepiment edge, but it has bigger pores (2–3 per mm vs. 5–6 per mm, Cai et al. 2023).

Morphologically, *Meripilus emarginatus* is similar to *M. noncontusus*, sharing ceraceous white basidiomata without color change after being bruised and almost the same size of angular pores (6–7 per mm in *M. emarginatus*, 5–6 per mm in *M. noncontusus*, this study). However, *M. emarginatus* has thin-walled smooth hymenial cystidia and distinctly thick-walled hyphoid cystidia bearing coarse crystals.

### ﻿Combinations

#### ﻿ Meripilus africanus

Taxon classificationAnimaliaPolyporalesMeripilaceae

(Decock & Ryvarden) Y.C. Dai, Chao G. Wang & Yuan Yuan comb. nov.

5CA47E44-8D79-54A0-A108-6F95D821C09D

860610

##### Basionym.

*Physisporinus africanus* Decock & Ryvarden, Syn. Fung. (Oslo) 44: 16 (2021).

#### ﻿ Meripilus cataractus

Taxon classificationAnimaliaPolyporalesMeripilaceae

(Ryvarden) Y.C. Dai, Chao G. Wang & Yuan Yuan comb. nov.

4F0573F5-03EF-5C5E-88B7-9C4EB126F5E2

860611

##### Basionym.

*Physisporinus cataractus* Ryvarden, Syn. Fung. (Oslo) 39: 68 (2019).

#### ﻿ Meripilus expallescens

Taxon classificationAnimaliaPolyporalesMeripilaceae

(P. Karst.) Y.C. Dai, Chao G. Wang & Yuan Yuan comb. nov.

0E46294C-CE88-503B-8EFF-74A1BE7EE39D

860613

≡ Physisporinus expallescens (P. Karst.) Pilát, in Kavina & Pilát, Atlas Champ. l’Europe, III, Polyporaceae (Praha) 1: 250 (1938).

##### Basionym.

*Caloporus expallescens* P. Karst., Meddn Soc. Fauna Flora fenn. 9: 110 (1883).

#### ﻿ Meripilus resinosus

Taxon classificationAnimaliaPolyporalesMeripilaceae

(Ipulet & Ryvarden) Y.C. Dai, Chao G. Wang & Yuan Yuan comb. nov.

0E073E15-9DA2-5721-8AA2-3FD2E41048C0

860612

##### Basionym.

*Physisporinus resinosus* Ipulet & Ryvarden, Syn. Fung. (Oslo) 20: 97 (2005).

#### ﻿ Meripilus vinctus

Taxon classificationAnimaliaPolyporalesMeripilaceae

(Berk.) Y.C. Dai, Chao G. Wang & Yuan Yuan comb. nov.

7792CE90-BA2A-57EE-ABD5-210273E58AD6

860648

≡ Physisporinus vinctus (Berk.) Murrill, Mycologia 34(5): 595 (1942)

##### Basionym.

*Polyporus vinctus* Berk., Ann. Mag. nat. Hist., Ser. 2 9: 196 (1852)

### ﻿Key to distinct groups of *Ceriporia*

**Table:** 

1	Almost uniform hyphae in subiculum and trama	**2**
–	Subicular hyphae wider than tramal hyphae	**3**
2	Allantoid basidiospores less than 2 µm in width	**the *Ceriporia mellita* group**
–	Short cylindrical to allantoid basidiospores mostly longer than 5 µm in length	**the *Ceriporia purpurea* group**
3	Fan-shaped, rhombic, or corolliform crystals present	**the *Ceriporia pierii* group**
–	Fan-shaped, rhombic, or corolliform crystals absent	**the *Ceriporia viridans* group**

### ﻿Key to accepted taxa of Meripilus (Physisporinus)

**Table:** 

1	Basidiomata effused-reflexed to pileate or stipitate	**2**
–	Basidiomata resupinate	**17**
2	Cystidia absent	**3**
–	Cystidia present	**7**
3	Basidiospores subglobose, < 5 μm in length	**4**
–	Basidiospores subglobose, > 5 μm in length	**6**
4	Pores 12–15 per mm	***M. minutissimus***
–	Pores 5–12 per mm	**5**
5	Pores 5–8 per mm, pore surface white to ochraceous when fresh	***M. africanus***
–	Pores 8–12 per mm, pore surface yellowish when fresh	***M. brasiliensis***
6	Pore surface grayish white when fresh	***M. giganteus***
–	Pore surface golden brown when fresh	***M. sumstinei***
7	Thin-walled hyphoid cystidia present	**8**
–	Thin-walled hyphoid cystidia absent and thick-walled hyphoid cystidia present	**11**
8	Hymenial cystidia present; American species	***M. neovitreus***
–	Hymenial cystidia absent; Asian species	**9**
9	Pores 5–6 per mm	***M. cinereus***
–	Pores 6–8 per mm	**10**
10	Sterile margin almost lacking	***M. crataegi***
–	Sterile margin distinct caesious to bluish gray when fresh	***M. caesiomarginatus***
11	Basidiomata completely pileate	**12**
–	Basidiomata effused-reflexed	**13**
12	Pore surface lavender when fresh; pores 9–10 per mm	***M. lavendulus***
–	Pore surface light brown when fresh; pores 6–8 per mm	***M. tamilnaduensis***
13	Pileal surface distinctly sulcate and zonate	**14**
–	Pileal surface indistinctly sulcate and zonate	**15**
14	Sterile margin distinct, white when fresh	***M. albomarginatus***
–	Sterile margin indistinct	***M. lineatus***
15	Thick-walled hyphoid cystidia present in both context and trama	***M. longicystidius***
–	Thick-walled hyphoid cystidia present in trama only	**16**
16	Pileal surface salmon to clay pink when dry	***M. vinctus***
–	Pileal surface olivaceous buff to bluish gray when dry	***M. sublineatus***
17	Pore surface becoming bright red upon bruising	**18**
–	Pore surface unchanged or becoming brown tones rather than bright red upon bruising	**24**
18	Thin-walled hyphoid and apically encrusted cystidia present	**19**
–	Thin-walled hyphoid and apically encrusted cystidia absent	**21**
19	Pores > 7 per mm	***M.* sp. 2**
–	Pores ≤ 7 per mm	**20**
20	Pore surface smoky gray to pinkish brown when dry	***M. pouzarii***
–	Pore surface buff yellow to clay yellow when dry	***M.* sp**. **3**
21	Tramal hyphae sometimes encrusted with crystals	***M. subfurcatus***
–	Tramal hyphae smooth	**22**
22	Pores 8–10 per mm	***M.* sp. 1**
–	Pores 3–7 per mm	**23**
23	Basidia clavate, 20–35 × 8–10 µm	***M. rhododendri***
–	Basidia broadly clavate, 12–23 × 6.5–8 µm	***M. sanguinolentus***
24	Cystidia absent	**25**
–	Cystidia present	**30**
25	Pores 3–4 per mm	***M. vitreosanguineus***
–	Pores 5–10 per mm	**26**
26	African species	**27**
–	Asian, European, American, and Oceanian species	**28**
27	Basidiospores globose 2.5–3 µm in diam	***M. resinosus***
–	Basidiospores globose 4–5 µm in diam	***M. cataractus***
28	Pore surface light pinkish to dark fawn when fresh	***M. crocatus***
–	Pore surface cream to white when fresh	**29**
29	Basidiospores subglobose, 3.9–4.8 × 3.4–4.2	***M. obscurus***
–	Basidiospores broadly ellipsoid to subglobose, 5–5.7 × 4–4.8	***M. stillicidiorum***
30	Basidiomata perennial	***M. roseus***
–	Basidiomata annual	**31**
31	Hyphoid cystidia thick-walled, completely embedded in trama	**32**
–	Hyphoid cystidia absent or thin-walled or thick-walled and projecting from hymenium or the dissepiment edge	**33**
32	Basidiospores 5–5.5 × 4.5–5 µm	***M. malayanus***
–	Basidiospores 4–4.7 × 3.2–4 µm	***M. albostygius***
33	Pores 2–3 per mm	**34**
–	Pores 4–12 per mm	**35**
34	Forked hymenial cystidia present	***M. furcatus***
–	Forked hymenial cystidia absent	***M. yunnanensis***
35	Hyphoid cystidia only thick-walled or thick- and thin-walled or absent	**36**
–	Hyphoid cystidia only thin-walled	**45**
36	Pores 10–12 per mm	***M. rigidus***
–	Pores 5–9 per mm	**37**
37	Pore surface sulphur yellow when fresh	***M. sulphureus***
–	Pore surface white to isabelline or buff yellow, pale brown to peach when fresh	**38**
38	Pore surface often nodulose	***M. vitreus***
–	Pores surface not nodulose	**39**
39	Thin-walled and smooth hymenial cystidia present	***M. emarginatus***
–	Thin-walled and smooth hymenial cystidia absent	**40**
40	Pore surface becoming reddish brown or pale brown upon bruising	**41**
–	Pore surface unchanged upon bruising	**42**
41	Pore surface vinaceous gray, dark brown to black when dry	***M. niveomarginatus***
–	Pore surface cream to buff when dry	***M. eminens***
42	Basidiospores < 4.5 µm in length	***M. revolubilis***
–	Basidiospores ≥ 4.5 µm in length	**43**
43	South American species	***M. robledoi***
–	Asian species	**44**
44	Pores round	***M. srilankensis***
–	Pores angular	***M. noncontusus***
45	Hymenial cystidia present	**46**
–	Hymenial cystidia absent	**47**
46	Basidiospores ovoid, Q=1.22–1.28	***M. castanopsidis***
–	Basidiospores broadly ellipsoid to subglobose, Q=1.15–1.20	***M. dollingerii***
47	Basidiospores thick-walled	***M. crystallinus***
–	Basidiospores thin-walled	**48**
48	Basidiomata juicy when fresh, easily separated from substrate	***M. tibeticus***
–	Basidiomata waxy when fresh, not easily separated from substrate	***M. expallescens***

## ﻿Discussion

*Ceriporia* and *Meripilus* belong to *Irpicaceae* and *Meripilaceae*, respectively, and they are distantly related in phylogeny but very similar in morphology, with mostly light-colored hymenophores, a monomitic hyphal system, and cyanophilous generative hyphae bearing simple septa. In the phylogenetic analyses of *Irpicaceae* (Fig. 1), four distinct groups are nested in the *Ceriporia* clade: the *C. mellita* group, the *C. pierii* group, the *C. purpurea* group, and the *C. viridans* group; they are coincident with the previous study (Wang et al. 2023). *Ceriporia armeniaca* and *C. wuyiana* are not included in any groups. *Ceriporia crassiparietata* nested in the *C. viridans* group, which involved nine species, and the group has resupinate basidiomata with a white to light yellow pore surface when fresh, subicular hyphae wider than tramal hyphae, hyphae frequently branched at a right angle, and lunate to allantoid basidiospores wider than 1.5 µm. *Meripilus* is divided into five groups, as mentioned in the previous study by Wang et al. (2024). Four new species, *M. emarginatus*, *M. malayanus*, *M. niveomarginatus*, and *M. noncontusus*, nested in Subclade I; among them, *M. emarginatus* and *M. noncontusus* have soft to ceraceous basidiomata with a white pore surface when fresh but without reddening when bruised, while *M. malayanus* and *M. niveomarginatus* have hard, corky to rigid basidiomata with a fuscous pore surface when dry and thick-walled, encrusted hyphoid cystidia. *Meripilus albomarginatus* nested in Subclade II, and it has resupinate to effused-reflexed basidiomata, a white sterile margin, distinctly thick-walled to almost solid contextual hyphae, and thick-walled, encrusted hyphoid cystidia, which fit the common characteristics of the other species in Subclade II. *Meripilus crystallinus* is nested in Subclade V, and it has resupinate, soft to ceraceous basidiomata with a white to cream pore surface when fresh, thin-walled and encrusted hyphoid cystidia, and slightly thick-walled basidiospores.

*Leptoporus* has effused-reflexed to pileate basidiomata, a poroid hymenophore, cylindric to allantoid basidiospores, and causes brown rot in gymnosperm wood (Ryvarden and Melo 2017; Liu et al. 2023). However, *Ceriporia* has resupinate basidiomata with a poroid or smooth hymenophore, cylindrical to allantoid basidiospores, and is associated with white rot. In addition, *Leptoporus* is only distantly related to any other taxa of *Ceriporia* (Fig. 1). Thus, it is also regarded as a separate genus in our study. In phylogeny, although four distinct groups are nested in *Ceriporia* with similar characters, separating them into smaller genera is still not justified. Some species addressed in the *Ceriporia* clade lack distinct morphological characteristics and scatter across the clade without high support in the phylogeny. In our previous study (Wang et al. 2024), *Physisporinus* was divided into five groups in the phylogenetic analyses, and *Meripilus*, including two species sequences, was considered a separate genus nested in *Physisporinus*. Morphologically, species of *Physisporinus* contain various types of basidiomata, viz., resupinate, effused-reflexed, and pileate, ellipsoid to globose basidiospores, and hyphoid cystidia that may be present or absent, while *Meripilus* only has large, multi-pileate, fleshy basidiomata. We did not further process the relationships between *Physisporinus* and *Meripilus* due to the differences in macromorphology in Wang et al. (2024). However, *Meripilus* nested in the *Physisporinus* clade in all previous studies (Wang and Dai 2022; Wang et al. 2024; Westphalen et al. 2025). In addition, Westphalen et al. (2025) also mentioned that species of the five clades in *Physisporinus* are overlapping in morphology and transferred all species of *Physisporinus* to *Meripilus*. At present, a proposal to merge the two genera (*Physisporinus* and *Meripilus*) is a reasonable approach.

Karsten (1889) established the genus *Physisporinus* and segregated it from *Poria* based on the “fruit-layer separated from basal layer” (Donk 1966). The type species of the genus, *Poria vitrea* Pers. sensu P. Karst., seems to be an undetermined species because of confusing labels by Karsten on herbarium specimens (Donk 1960, 1966). Accordingly, Donk (1967) transferred *Poria vitrea* and other typical species of *Physisporinus* to *Rigidoporus* Murrill, which is not yet acceptable. Donk (1967) also indicated that the type specimen of *Physisporinus vitreus*, *Poria vitrea* Pers. 1796 sensu Pers., may not exist, and mycologists have often confused the species with *Polyporus undatus* Pers. 1825. Donk (1967) wrote that the qualifications are “undulata, subinterrupta; poris obliquis,” and this conception appears to be the same as *Poria vitrea* described in Lowe (1966) and *Poria undata* described in Bourdot and Galzin (1928), and the critical feature, thick-walled, encrusted cystidia, was not mentioned. We studied many specimens of *Physisporinus undatus* and concluded that the cystidia feature is physiologically determined—thin- or thick-walled, or sometimes not present at all. All of these are possible, and globose, smaller basidiospores and a somewhat nodulose character of older basidiomata are important, which correspond to *Poria vitrea*. Moreover, Karsten (1883) described *Caloporus expallescens* P. Karst., which Lowe (1956) determined to be aberrant (not changing color) *Physisporinus sanguinolentus*, but Pilát (1938) regarded it as a good species similar to *P. vitreus*. He studied the type and added a new feature that *C. expallescens* does not detach from the substrate. We observed this feature in three samples: MJ 642/93 (Czechia), MJ 332/94 (Czechia), and Dai 21060 (Belarus). Thus, we still follow the concept of Wang et al. (2024) that *P. undatus* is a synonym of *P. vitreus* and *P. expallescens* is a good species in this study.

*Meripilus lineatus* (*Physisporinus lineatus*) has effused-reflexed to pileate or stipitate basidiomata, a concentrically zonate-sulcate upper surface, thick-walled hyphoid cystidia with or without crystals, and subglobose to globose basidiospores (Ryvarden 1972; Ryvarden 2015; Ryvarden and Melo 2017; Wang et al. 2024). *Meripilus albomarginatus*, *M. lineatus*, *M. sublineatus*, and *M. vinctus* form a sister clade in our phylogeny, and they share thick-walled hyphoid cystidia with crystals. In Wang et al. (2024), eight specimens from the subtropical and tropical zones formed a well-supported distinct lineage and are characterized by pileate basidiomata, thick-walled, encrusted hyphoid cystidia, and thin-walled hymenial cystidia with or without crystals; these characters are consistent with the definition of *M. lineatus*. *Meripilus vinctus* (*Physisporinus vinctus*) is similar to *M. lineatus* in micromorphology by having thick-walled, encrusted hyphoid cystidia and thin-walled, mammillate hymenial cystidia. However, *M. vinctus* has mostly resupinate basidiomata and a cinnamon-orange to buff pore surface, while *M. lineatus* has mostly pileate basidiomata with rich coloration and a distinct concentrically zonate-sulcate pileal surface in macromorphology. Thus, the samples JV 0610/B5, JV 1407/36, JV 0610/A31B, Kout 1807/3, and Cui 16903 were considered as *M. vinctus* (*Physisporinus vinctus*) in Wang et al. (2024). Up to now, the thin-walled hymenial cystidia remain an uncertain character distinguishing *M. lineatus*.

Species of *Ceriporia* and *Meripilus* grow on different angiosperm and gymnosperm trees, causing a white rot ecology. The host trees of these fungi are summarized in Table 3. Up to now, 67 species are accepted under *Ceriporia*, including three new species in the present study: 53 species grow on angiosperm wood, four species grow on gymnosperm wood, and nine species grow on both angiosperm and gymnosperm wood. Moreover, the substrate of *Ceriporia albobrunnea* Ryvarden & Iturr. was recorded as wood without indicating angiosperm or gymnosperm (Ryvarden and Iturriaga 2003). Forty-nine taxa are accepted in *Meripilus*, including six new species in the present study: 28 grow on angiosperm wood or bamboo, six taxa grow on gymnosperm wood, 14 taxa grow on both angiosperm and gymnosperm wood or *Miscanthus*, and one species, *M. rigidus*, grows on ferns. Species in both genera exhibit distinct growth advantages on angiosperm wood, which is largely related to the evolution of plants. In studies of plant origins, the Paleocene epoch of the Paleogene period is typically recognized as a critical phase for the emergence of angiosperm-endemic genera. During this interval, the climate remained warm, continuing Cretaceous patterns, but became more humid, thus creating favorable conditions for biological development (Wang 1989). In addition, most species of both genera evolved from the Paleogene in our dating analyses.

**Table 3.** The type locality, host trees and main morphological characteristics of accepted species in *Ceriporia* and *Meripilus*.

Species	DNA sequences	Type locality	Basidiomata	Shape of basdiospores	Size of basidiospores (μm)	Host trees	References
*Ceriporia allantoidea*	＋	China: Hunan	Resupinate	Allantoid	4.5–5 × 1.1–1.5	Angiosperm (undetermined)	Wang et al. (2023)
*C. allantospora*	＋	USA	Resupinate	Allantoid	10–11.5 × 2.5–3	Angiosperm (*Platanus*, *Sycamore*)	Gilbertson et al. (1976)
*C. arbuscula*	＋	China: Taiwan	Resupinate	Cylindrical to slightly curved	3–3.5 × 1–1.5	Gymnosperm (*Pinus*)	Chen et al. (2020)
***C. armeniaca***	＋	**China: Guangdong**	**Resupinate**	**Lunate to allantoid**	**4–4.5 × 2–2.2**	**Angiosperm (undetermined)**	**Present study**
*C. aurantiocarnescens*	＋	Germany	Resupinate	Short cylindrical and moderately curved	3.2–4 × 1.8–2.2	Angiosperm (*Populus*)	Wang et al. (2023)
*C. bresadolae*	＋	France	Resupinate to effused-reflexed	Allantoid	5.9–8.2 × 1.8–2.3	Angiosperm (*Quercus*, *Ulmus*, *Rosaceae*) and gymnosperm (*Pinus*, *Picea*, *Juniperus*)	Spirin et al. (2016), Wang et al. 2023
*C. bubalinomarginata*	＋	China: Henan	Resupinate	Allantoid	3.5–4.3 × 1–1.2	Angiosperm (undetermined)	Jia et al. (2014)
*C. crassa*	＋	China: Hainan	Resupinate	Allantoid	3.8–4.1 × 1.2–1.6	Gymnosperm (*Pinus*)	Wang et al. (2023)
***C. crassiparietata***	＋	**China: Zhejiang**	**Resupinate**	**Lunate to allantoid**	**4–4.4 × 2.1–2.3**	**Angiosperm (*Quercus*, Rosaceae)**	**Present study**
*C. daedaleoides*	＋	Thailand	Resupinate	Ellipsoid to slightly curved	3.7–4.1 × 2–2.3	Angiosperm (undetermined)	Wang et al. (2023)
*C. eucalypti*	＋	Australia: Victoria	Resupinate	Allantoid	4–4.4 × 1.1–1.4	Angiosperm (*Eucalyptus*)	Chen et al. (2022)
*C. excelsa*	＋	Sweden	Resupinate	Lunate to short cylindrical, moderately curved	3.6–4.2 × 2–2.2	Angiosperm (*Betula*, *Fagus*, *Fraxinus*, *Salix*, *Viburnum*) and gymnosperm (*Picea*)	Wang et al. (2023)
*C. gossypina*	＋	China: Xizang	Resupinate	Allantoid	3.5–4 × 1.8–2	Angiosperm (undetermined)	Wang et al. (2023)
*C. griseoviolascens*	＋	France	Resupinate	Bean-shaped	5–6.1 × 2.5–3.1	Angiosperm (*Prunus*, *Arbutus*, *Quercus*, *Populus*)	Spirin et al. (2016)
*C. hinnulea*	＋	China: Fujian	Resupinate	Lunate to allantoid	3.5–4 × 2–2.1	Angiosperm (undetermined)	Wang et al. (2023)
*C. humilis*	＋	Russia	Resupinate	Narrowly ellipsoid to cylindrical	3.2–4.2 × 1.9–2.2	Angiosperm (*Quercus*)	Miettinen et al. (2016)
*C. langloisii*	＋	USA: Virginia	Resupinate	Ellipsoid to broadly allantoid	7–9.5 × 3–4	Angiosperm (undetermined)	Burdsall (1984)
*C. macrospora*	＋	China: Hainan	Resupinate	Allantoid	5–7.2 × 1.6–2	Angiosperm (undetermined)	Wang et al. (2023)
*C. manzanitae*	＋	USA: California	Resupinate	Cylindrical to allantoid	5.1–6.2 × 2.2–2.7	Angiosperm (*Arctostaphylos*)	Spirin et al. (2016)
*C. mellita*	＋	France	Resupinate	Allantoid	5–6 × 1.5–2	Angiosperm (*Quercus*, *Populus*)	Wang et al. (2023)
*C. mpurii*	＋	Indonesia	Resupinate	Ellipsoid to narrowly ellipsoid	2.8–3.9 × 2–2.3	Angiosperm (*Spondias*, *Prunus*, *Populus*)	Spirin et al. (2016)
*C. occidentalis*	＋	USA: Washington	Resupinate	Allantoid	5.1–7.1 × 1.8–2.2	Angiosperm (*Platanus*, *Umbellularia*, *Corylus*)	Spirin et al. (2016)
*C. orientalis*	＋	China: Zhejiang	Resupinate	Lunate to allantoid	5.4–6.5 × 2.8–3.1	Angiosperm (undetermined)	Wang et al. (2023)
*C. pierii*	＋	France	Resupinate	Ellipsoid to rarely cylindrical	4.1–5.4 × 2.4–3.1	Angiosperm (*Populus*)	Miettinen et al. (2016)
*C. pseudospissa*	＋	China: Beijing	Resupinate	Allantoid	5–7.2 × 1.6–2.1	Angiosperm (undetermined)	Wang et al. (2023)
*C. punctata*	＋	China: Xinjiang	Resupinate	Allantoid	4–5 × 1.7–2.1	Angiosperm (*Populus*)	Wang et al. (2023)
*C. punicans*	＋	USA: Pennsylvania	Resupinate	Short cylindrical, slightly curved	4.1–5.3 × 1.7–2.1	Angiosperm (undetermined)	Spirin et al. (2016)
*C. purpurea*	＋	France	Resupinate	Allantoid	5–8.4 × 1.7–2.3	Angiosperm (*Corylus*, *Populus*, *Alnus*, *Salix*, *Quercus*, *Fraxinus*, *Acer*, *Tilia*, *Pyracantha*)	Spirin et al. (2016)
*C. reticulata*	＋	Germany	Resupinate	Allantoid	7.5–9 × 3–3.5	Angiosperm (Acacia, Alnus, Acer, Betula etc.) and gymnosperm (*Picea*)	Domanski (1963)
*C. septocystidia*	＋	Jamaica	Resupinate	Allantoid	4.5–6.5 × 1.5–2	Angiosperm (*Populus*) and gymnosperm (undetermined)	Burdsall (1984)
*C. sericea*	＋	Russia: Khabarovsk	Resupinate	Cylindrical to slightly curved	3.9–4.8 × 2.2–2.7	Angiosperm (Tilia) and gymnosperm (*Picea*, *Pinus*)	Miettinen et al. (2016)
*C. sinospissa*	＋	China: Fujian	Resupinate	Allantoid	5–5.8 × 1.5–2	Angiosperm (undetermined)	Wang et al. (2023)
*C. sino-viridans*	＋	China: Hainan	Resupinate	Lunate to allantoid	3–3.5 × 1.7–2.2	Angiosperm (undetermined)	Chen et al. (2022)
*C. sordescens*	＋	USA: New York	Resupinate	Ellipsoid to narrowly ellipsoid	3.3–4.2 × 2.1–2.5	Angiosperm (*Acer*)	Miettinen et al. (2016)
*C. spissa*	＋	USA: Carolina	Resupinate	Allantoid	4.2–4.8 × 1.3–1.6 (Dai 19164)	Angiosperm (undetermined) and gymnosperm (*Pinus*)	Rajchenberg (1983), present study
*C. subbadia*	＋	USA: Alabama	Resupinate	Cylindrical to allantoid	4.5–5.8 × 2–2.6	Angiosperm (*Fagus*)	Wang et al. (2023)
*C. subviridans*	＋	China: Yunnan	Resupinate	Lunate to allantoid	3.3–3.7 × 1.8–2	Angiosperm (undetermined)	Wang et al. (2023)
*C. torpida*	＋	Finland	Resupinate	Short cylindrical, slightly to moderately curved	4.3–5.7 × 1.9–2.3	Angiosperm (*Fagus*, *Salix*)	Spirin et al. (2016)
*C. triumphalis*	＋	Spain: Canary Island	Resupinate	Short cylindrical to allantoid	4.1–5 × 1.8–2.1	Angiosperm (undetermined)	Spirin et al. (2016)
***C. wuyiana***	＋	**China: Zhejiang**	**Resupinate**	**Lunate to allantoid**	**4.3–5 × 1.7–2**	**Angiosperm (undetermined)**	**Present study**
*C. viridans* (type species)	＋	UK	Resupinate	Cylindrical to allantoid	4–4.6 × 1.7–2.1 (Dai 17003)	Angiosperm (*Padus*, *Populus*)	Ryvarden and Gilbertson (1993), Wang et al. (2023)
*Ceriporia alania*	–	USA: Hawaiian Island	Resupinate	Narrowly allantoid	7.5–10 × 2–2.5	Angiosperm (*Metrosideros*)	Gilbertson and Hemmes (2004)
*Ceriporia alba*	–	France	Resupinate	Cylindrical to allantoid	5.5–7 × 2–2.5	Angiosperm (undetermined)	Pieri and Rivoire (1997)
*Ceriporia albobrunnea*	–	Venezuela	Resupinate	Cylindrical	4–4.5 × 1.5	Unknown	Ryvarden and Iturriaga (2003)
*Ceriporia amazonica*	–	Brazil: Amapá	Resupinate	Ellipsoid	3 × 2	Angiosperm (undetermined)	Soares et al. (2014)
*Ceriporia angulata*	–	Brazil: Amazonas	Resupinate	Oblong-ellipsoid	4–4.5 × 1.7–2.2	Angiosperm (undetermined)	Gomes-Silva et al. (2012)
*Ceriporia aurea*	–	Venezuela	Resupinate	Cylindrical to allantoid	4–5 × 2	Angiosperm (undetermined)	Ryvarden (2014)
*Ceriporia camaresiana*	–	France	Resupinate	Cylindrical to sub-allantoid	5–6 × 2–3	Angiosperm (*Betula*. *Eucalyptus*, *Prunus*) and gymnosperm (*Picea*)	Ryvarden and Gilbertson (1993)
*Ceriporia citrina*	–	Costa Rica	Resupinate	Oblong-ellipsoid to subcylindrical	7–8 × 3.2–3.5	Angiosperm (undetermined)	Mata and Ryvarden (2010)
*Ceriporia cystidiata*	–	Venezuela	Resupinate	Allantoid	4–4.5 × 1	Angiosperm (undetermined)	Ryvarden and Iturriaga (2003)
*Ceriporia dentipora*	–	Ecuador	Resupinate	Oblong-ellipsoid to cylindrical	5–6 × 2.5–3	Angiosperm (undetermined)	Læssøe and Ryvarden (2010)
*Ceriporia ellipsospora*	–	*Seychelles*	Resupinate	Ellipsoid	3–4 × 2.5–2.8	Angiosperm (undetermined)	Ryvarden (2018)
*Ceriporia ferrugineocincta*	–	USA: Florida	Resupinate	Subcylindrical	3.5–5 × 2–3	Angiosperm (*Quercus*) and gymnosperm (undetermined)	Ryvarden and Johansen (1980)
*Ceriporia incrustata*	–	Costa Rica	Resupinate	Ellipsoid	3–3.5 × 1.8–2	Angiosperm (undetermined)	Mata and Ryvarden (2010)
*Ceriporia kenyensis*	–	Kenya	Resupinate	Cylindrical	3–4 × 1–1.2	Angiosperm (undetermined)	Decock et al. (2021)
*Ceriporia leptoderma*	–	Sri Lanka	Resupinate	Broadly ellipsoid	5–6 × 3–4	Angiosperm (undetermined)	Ryvarden and Johansen (1980)
*Ceriporia microspora*	–	Costa Rica	Resupinate	Ellipsoid	3–3.5 × 1.5–2	Angiosperm (*Quercus*, *Manilkara*)	Lindblad and Ryvarden (1999)
*Ceriporia otakou*	–	New Zealand	Resupinate	Ovoid to ellipsoid or pip-shaped	4.5–6 × 2–2.5	Angiosperm (*Nothofagus*)	Cunningham (1947)
*Ceriporia retamoana*	–	Argentina: Chubut	Resupinate	Cylindrical, slightly curved	4.5–5 × 1.2–1.5	Angiosperm (*Diostea*)	Rajchenberg (2000)
*Ceriporia rhodella*	–	Sweden	Resupinate	Short cylindrical, slightly curved	3.5–4 × 1.5–2	Angiosperm (*Alnus*, *Populus*, *Fagus*) and gymnosperm (undetermined)	Lombard and Gilbertson (1965)
*Ceriporia rubescens*	–	Sri Lanka	Resupinate	Cylindrical	4–5 × 1.5–2	Angiosperm (undetermined)	Ryvarden (2015)
*Ceriporia straminea*	–	Bolivia	Resupinate	Ellipsoid	4.5–5.5 × 2.5–2.8	Angiosperm (undetermined)	Ryvarden (2014)
*Ceriporia subpudorina*	–	—	Resupinate	Ovoid to ellipsoid, slightly narrowed	5–6 × 3–3.5	Angiosperm (*Salicis*)	Bondartsev (1953)
*Ceriporia subspissa*	–	Guyana	Resupinate	Ellipsoid	5.5–6.5 × 3–3.5	Angiosperm (undetermined)	Aime et al. (2007)
*Ceriporia totara*	–	New Zealand: Auckland	Resupinate	Ovoid to subglobose	2.5–3 × 1.7–2.5	Gymnosperm (*Podocarpus*, *Dacrydium*)	Buchanan and Ryvarden (1988)
*Ceriporia vermicularis*	–	France: Réunion	Resupinate	Cylindrical to allantoid	6–7 × 0.8–1	Angiosperm (undetermined)	Pieri and Rivoire (1997)
*Ceriporia violacea*	–	Sweden	Resupinate	Oblong-ellipsoid	3.5–4.5 × 2–2.4	Gymnosperm (*Pinus*)	Ryvarden and Melo (2017)
***Meripilus albomarginatus***	＋	**China: Yunnan**	**Annual, effused-reflexed to pileate**	**Subglobose**	**5.2–6.2 × 4.6–5.7**	**Angiosperm (undetermined and bamboo)**	**Present study**
*M. albostygius*	＋	USA: Puerto Rico	Annual, resupinate	Boadly ellipsoid to subglobose	4–4.7 × 3.2–4	Angiosperm (undetermined)	Wang et al. (2024)
*M. brasiliensis*	＋	Brazil	Annual, pileate	Subglobose	3–5 × 3–4	Angiosperm (undetermined)	Westphalen et al. 2025
*M. caesiomarginatus*	＋	China: Yunnan	Annual, resupinate to effused-reflexed	Broadly ovoid	4–4.8 × 3.3–4.1	Angiosperm (undetermined)	Wang et al. (2024)
*M. castanopsidis*	＋	China: Yunann	Annual, resupinate	Ovoid	4.8–5.6 × 3.8–4.3	Angiosperm (*Castanopsis*) and gymnosperm (*Dacrydium*)	Chen and Dai (2021)
*M. cinereus*	＋	Japan	Annual, effused-reflexed to pileate	Globose	5–6	Angiosperm (*Fagus*, bamboo, *Lithocarpus*) and gymnosperm (*Pinus*)	Núñez and Ryvarden (1999)
*M. crataegi*	＋	China: Tianjin	Annual, effused-reflexed	Broadly ellipsoid to subglobose	4.2–5 × 3.2–4.2	Angiosperm (*Crataegus*, *Lonicera*)	Wu et al. (2017)
*M. crocatus*	＋	Tunisia	Annual to perennial, resupinate	Ovoid to subglobose	3.5–5.5 × 3.5–5	Angiosperm (*Betula*) and gymnosperm (*Abies*, *Picea*)	Ryvarden (1983), Ryvarden and Melo (2017)
***M. crystallinus***	＋	**China: Yunnan**	**Annual, resupinate**	**Broadly ellipsoid to ovoid**	**4.3–5 × 4–4.**5	**Gymnosperm (*Picea*)**	**Present study**
*M. dollingerii*	＋	USA: Florida	Annual, resupinate	Broadly ellipsoid to subglobose	4.5–5.5 × 4–5	Angiosperm (undetermined) and gymnosperm (*Pinus*)	Wang et al. (2024)
***M. emarginatus***	＋	**China: Guangdong**	**Annual, resupinat**e	**Subglobose to globose**	**4.8–5.2 × 4.5–5.2**	**Angiosperm (*Miscanthus* and other undetermined wood) and gymnosperm (*Cunninghamia*)**	**Present study**
*M. eminens*	＋	China: Jilin	Annual, resupinate	Globose	4.2–6 × 3.9–5.2	Angiosperm (*Quercus*, *Populus*)	Dai (1998)
*M. expallescens*	＋	Finland	Annual, resupinate	Ovoid to globose	5.5–6.5 × 4.5–5.2	Gymnosperm (*Picea*, *Pinus*)	Wang et al. (2024)
*M. furcatus*	＋	Russia	Annual, resupinate	Globose	4.5–5.5	Angiosperm (*Alnus*)	Núñez et al. (2001)
*M. giganteus* (type species)	＋	Sweden	Annual, multipileate	Subglobose, broadly ovoid to broadly ellipsoid	6–6.5 × 5.5–6	Angiosperm (Quercus, Fagus)	Larsen and Lombard (1988)
*M. lavendulus*	＋	China: Hainan	Annual, pileate	Globose	4.2–5 × 4–5	Angiosperm (undetermined)	Wu et al. (2017)
*M. lineatus*	＋	Hungary	Annual, resupinate, effused-reflexed to pileate or with a stipe	Globose	5–6	Angiosperm (*Khaya*, *Ginkgo*, *Magnolia*, *Quercus*, *Cocos*, bamboo) and gymnosperm (*Metasequoia*)	Ryvarden (1972), Ryvarden and Johansen (1980), Ryvarden and Gilbertson (1994)
*M. longicystidius*	＋	New Zealand	Annual, resupinate to effused-reflexed	Globose	4.2–5.7	Angiosperm (*Eucalyptus*, *Nothofagus*)	Buchanan and Ryvarden (2000)
***M. malayanus***	＋	**Malaysia**	**Annual, resupinate**	**Subglobose**	**5–5.5 × 4.5**–5	**Angiosperm (undetermined)**	**Present study**
*M. minutissimus*	＋	Costa Rica	Annual, resupinate to effused-reflexed	Subglobose to globose	4.1–4.6 × 4–4.6	Angiosperm (undetermined)	Wang et al. (2024)
*M. neovitreus*	＋	USA: New Jersey	Annual, resupinate to effused-reflexed	Broadly ellipsoid to subglobose	4.9–6 × 4–4.8	Angiosperm (undetermined)	Wang et al. (2024)
***M. niveomarginatus***	＋	**China: Guangdong**	**Annual, resupinate**	**Broadly ellipsoid**	**4.2–5.2 × 4–4.6**	**Gymnosperm (*Pinus massoniana*, *P. latteri*)**	**Present study**
***M. noncontusus***	＋	**China: Guangdong**	**Annual, resupinate**	**Broadly ellipsoid to subglobose**	**5–5.5 × 4.2–4.7**	**Angiosperm (undetermined)**	**Present study**
*M. obscurus*	＋	Brazil	Annual, resupinate	Subglobose	3.9–4.8 × 3.4–4.2	Angiosperm (undetermined) and gymnosperm (*Araucaria angustifolia*)	Westphalen et al. 2025
*M. pouzarii*	＋	Slovakia	Annual, resupinate	Broadly ellipsoid to ovoid	4.7–6.3 × 4–4.6	Angiosperm (*Alnus*, *Fagus*, *Ulmus*, *Salix*) and gymnosperm (*Picea*)	Vampola and Vlasák (2012)
*M. revolubilis*	＋	Brazil	Annual, resupinate	Subglobose to globose	3.4–4.4 × 3.2–4	Angiosperm (undetermined)	Westphalen et al. 2025
*M. rhododendri*	＋	China: Guizhou	Annual, resupinate	Subglobose	5.3–6.3 × 5–5.5	Angiosperm (*Rhododendron*)	Wang et al. (2024)
*M. rigidus*	＋	Costa Rica	Annual, resupinate	Broadly ellipsoid to subglobose	4–4.6 × 3.2–4	Fern (*Tectaria*)	Wang et al. (2024)
*M. robledoi*	＋	Argentina	Annual, resupinate	Subglobose to globose	4.5–5.2 × 3.2–4	Angiosperm (*Nothofagus*) and gymnosperm (*Araucaria araucana*)	Westphalen et al. 2025
*M. roseus*	＋	China: Yunnan	Perennial, resupinate	Subglobose	3.5–4.1 × 3.1–3.8	Angiosperm (undetermined)	Chen and Dai (2021)
*M. sanguinolentus*	＋	Germany	Annual or biennial, resupinate	Ovoid to subglobose	6–7 × 5–6	Angiosperm (*Quercus*, *Alnus*) and gymnosperm (*Picea*, *Abies*)	Wang et al. (2024)
*M. srilankensis*	＋	Sri Lanka	Annual, resupinate	Broadly ellipsoid to subglobose	5–5.8 × 4–4.8	Angiosperm (undetermined)	Wang et al. (2024)
*M. stillicidiorum*	＋	Australia	Annual, resupinate	Broadly ellipsoid to subglobose	5–5.7 × 4–4.8	Angiosperm (*Eucalyptus*)	Wang et al. (2024)
*M. subfurcatus*	＋	China: Jilin	Annual, resupinate	Broadly ellipsoid to subglobose	5.3–6.4 × 4.5–5.6	Angiosperm (*Larix*) and gymnosperm (*Picea*)	Wang et al. (2024)
*M. sublineatus*	＋	China: Yunnan	Annual, resupinate to effused-reflexed	Subglobose to globose	4.8–5.6 × 4.5–5.2	Angiosperm (bamboo and other undetermined wood)	Wang et al. (2024)
*M. sulphureus*	＋	Singapore	Annual, resupinate	Subglobose to globose	4–5 × 3.5–4	Angiosperm (undetermined)	Dai and Dai (2018)
*M. sumstinei*	＋	USA	Annual, multipileate	Subglobose to broadly ellipsoid	5–5.5 × 4–4.5	Angiosperm (undetermined)	Larsen and Lombard (1988)
*M. tamilnaduensis*	＋	India	Annual, Pileate	broadly ellipsoid to subglobose	4.5–5.8 × 4–5	Angiosperm (*Azadirachta*)	Crous et al. (2023)
*M. tibeticus*	＋	China: Xizang	Annual, resupinate	Broadly ellipsoid	4.8–5.5 × 3.7–4.3	Gymnosperm (*Abies*, *Pinus*, *Picea*)	Wu et al. (2017)
*M. vinctus*	＋	Dominican Republic	Perennial, resupinate to effused-reflexed	Ovoid to subglobose	4–5.5 × 3–4	Angiosperm (undetermined) and gymnosperm (*Picea*)	Ryvarden (1972), Setliff (1972), Ryvarden and Johansen (1980)
*M. vitreosanguineus*	＋	Czechia	Annual, resupinate	Broadly ellipsoid to subglobose	5–6.1 × 4–5	Gymnosperm (*Pinus*, *Picea*, *Abies*)	Wang et al. (2024)
*M. vitreus*	＋	Finland	Annual, resupinate	Ovoid to globose	5–5.5 × 4–4.5	Angiosperm (*Fagus*, *Ulmus*) and gymnosperm (*Pinus*, *Picea*, *Abies*)	Wang et al. (2024)
*M. yunnanensis*	＋	China: Yunnan	Annual, resupinate	subglobose	4–5.5 × 3.5–5	Angiosperm (undetermined)	Cai et al. (2023)
*Meripilus* sp. 1	＋	USA: California	Annual, resupinate	Ellipsoid to broadly ellipsoid	5–6 × 3.8–4.5	Gymnosperm (*Picea*, *Thuja*)	Wang et al. (2024)
*Meripilus* sp. 2	＋	Czechia	Annual, resupinate	Broadly ellipsoid	4.6–5.2 × 3.8–4.2	Angiosperm (*Alnus*)	Wang et al. (2024)
*Meripilus* sp. 3	＋	Czechia	Annual, resupinate	Broadly ellipsoid or ovoid	5.5–6.4 × 4.7–5.2	Angiosperm (*Alnus*) and gymnosperm (*Picea*)	Wang et al. (2024)
*Meripilus africanus*	–	São Tomé	Annual, effused-reflexed to pileate	Subglobose	3–3.5 × 2.5–3	Angiosperm (*Olea capensis*)	Decock and Ryvarden (2021)
*M. cataractus*	–	Zimbabwe	Annual, resupinate	Globose	4–5	Angiosperm (undetermined)	Ryvarden (2019)
*M. resinosus*	–	Uganda	Annual, resupinate	Globose	2.5–3	Angiosperm (undetermined)	Ipulet and Ryvarden (2005)

Abbreviations used: + = Present; – = Absent. **Bold** = new taxa.

In the dating analyses, we estimated the divergence times of studied taxa using crown ages, as stated in Varga et al. (2019). The divergence time of the *Polyporales*, including *Irpicaceae* and *Meripilaceae*, with a mean crown age of 148.25 Myr, occurred during the late Jurassic, which aligns with the divergence times of ordinal-level taxonomic units in *Agaricomycetes* during the Jurassic period (Varga et al. 2019). Our result is consistent with the divergence time ranges of orders proposed by Zhao et al. (2017) and Ji et al. (2022). *Irpicaceae* and *Meripilaceae* are estimated to have emerged at the junction of the early and late Cretaceous, with mean crown ages of 108.9 Myr and 97.23 Myr, respectively. *Ceriporia* and *Meripilus* are estimated at 83.61 Myr and 81.38 Myr, respectively, emerging in the late Cretaceous.

The rapid diversification of angiosperms, along with numerous animal and insect groups during the mid- to late Cretaceous, combined with the successful proliferation of flowering plants around 100 million years ago, led to terrestrial biodiversity exceeding marine biodiversity for the first time in Earth’s geological history. Following their emergence in the late Jurassic, angiosperms underwent explosive radiation during the early Cretaceous, ultimately displacing gymnosperm-dominated forests primarily composed of conifers and cycads (Ramírez-Barahona et al. 2020). The late Cretaceous mass extinction event caused catastrophic devastation to plant communities while simultaneously triggering a surge in saprotrophic organisms such as fungi—non-photosynthetic life forms that derived nutrients by decomposing dead plant matter—though this flourishing of decomposers proved short-lived in the post-apocalyptic ecosystem. *Ceriporia* and *Meripilus* likely emerged after this mass extinction. Species in *Ceriporia* occurred between around 63 Myr (*C. griseoviolascens*) and 5 Myr (*C. occidentalis* and *C. manzanitae*), and they all evolved from the Paleogene, becoming more prevalent in the Neogene. Similarly, species in *Meripilus* occurred between around 54 Myr (*M. srilankensis*) and 6 Myr (*M. yunnanensis* and *Meripilus* sp. 3), and they also evolved from the Paleogene, becoming more prevalent in the Neogene. In addition, the hosts of both genera are angiosperm wood. In studies of plant evolution, the Paleocene epoch of the Paleogene period stands as a critical phase for the origination of angiosperm-endemic genera. During this interval, the climate maintained Cretaceous-like warmth while becoming more humid than the Cretaceous baseline, creating favorable climatic conditions that drove biotic proliferation (Wang 1989). Thus, we speculate that the timing and evolutionary process of the two genera are somewhat coincident.

*Ceriporia* is estimated to have emerged in the early Cretaceous, and species in it occurred between 6.2 and 103.33 Myr, and they all evolved from the early Cretaceous, becoming more prevalent in the Neogene (Wang et al. 2023). Among them, *C. griseoviolascens* and *Leptoporus* are estimated at 103.33 Myr and 88.4 Myr, respectively. In our study, the divergence time of the *Ceriporia* clade evolved at 83.61 Myr in the late Cretaceous, posterior to the results of Wang et al. (2023). In addition, species in *Ceriporia* occurred between around 63 Myr (*C. griseoviolascens*) and 5 Myr (*C. occidentalis* and *C. manzanitae*). The phylogenies of *C. griseoviolascens* and *Leptoporus* are unstable in *Ceriporia*, which makes a difference for their divergence times. In the dating analyses, the divergence times of *Polyporales*, including taxonomic classes, were estimated using more comprehensive species sequences.

Climatic records across geological ages reveal that the Mesozoic Era—namely the Triassic, Jurassic, and Cretaceous periods—was characterized by globally warm yet predominantly arid conditions. The Cenozoic Era witnessed a climatic shift, transitioning into a more humid and progressively warmer environment, culminating in the climatically frigid conditions of the Quaternary Ice Age. From the late Cretaceous to the Eocene of the Paleogene period, the Qinghai-Tibet Plateau began experiencing crustal shortening, thickening, and progressive uplift, driven by the ongoing collision and accretion between the Indian and Eurasian plates, which facilitated the region’s sustained elevation during this orogenic phase (Li 1995). Most species of *Ceriporia* and *Meripilus* emerged during this period. Dynamically shifting climatic regimes and evolving topography exert both direct and indirect controls on floristic distribution patterns, accelerating diversification. The Hengduan Mountains and eastern Himalayan floristic regions constitute the primary convergence zone for northern temperate flora, serving as the modern distribution center for numerous critical alpine plant lineages; significantly, the Sino-Himalayan Floristic Subkingdom is recognized as the evolutionary cradle of Northern Hemisphere temperate flora (Sun 2002). The reason for this phenomenon also lies in the change of the climate in the Arctic region during the Cenozoic Tertiary. From the late Cretaceous to the Paleogene, the Arctic sustained a temperate climate that supported thermophilic temperate flora; as the climate cooled, these plants underwent progressive southward migration (Zachos et al. 2001). Studies revealed that during the Eocene of the Paleogene period, East Asia hosted a luxuriant thermophilic flora, which was subsequently supplanted by temperate thermophilic coniferous-broadleaved mixed forests by the Miocene. Intriguingly, the most representative extant refugia of Paleogene floral elements are now localized in Southeast Asia (Tiffney 1985; Budantsev 1992, 1994). However, the species of *Ceriporia* and *Meripilus* distributed in Southeast Asia account for only a minority at present. In addition to species diversity, the origin, dispersal, and evolution of *Ceriporia* and *Meripilus* will be the focus of further studies by collecting more ecological information on the species.

*Spongipellis* is a genus that usually has pileate basidiomata, and although it is addressed in the *Meripilaceae* main clade, it occurred during the Neogene period with a mean crown age of 13.67 Myr. Nevertheless, the divergence time of *Meripilus*, with a mean crown age of 81.38 Myr, occurred during the late Cretaceous. Perhaps *Spongipellis* will be treated as an independent family, but this is outside the scope of our current study.

## Supplementary Material

XML Treatment for Ceriporia

XML Treatment for Ceriporia armeniaca

XML Treatment for Ceriporia crassiparietata

XML Treatment for Ceriporia wuyiana

XML Treatment for Meruliopsis

XML Treatment for Meruliopsis rhizomorpha

XML Treatment for Meripilus

XML Treatment for Meripilus albomarginatus

XML Treatment for Meripilus crystallinus

XML Treatment for Meripilus emarginatus

XML Treatment for Meripilus malayanus

XML Treatment for Meripilus niveomarginatus

XML Treatment for Meripilus noncontusus

XML Treatment for Meripilus africanus

XML Treatment for Meripilus cataractus

XML Treatment for Meripilus expallescens

XML Treatment for Meripilus resinosus

XML Treatment for Meripilus vinctus

## ﻿Additional information

### Use of AI

No use of AI was reported.

### Adherence to national and international regulations

All the fungal strains used in this study have been legally obtained, respecting the Convention on Biological Diversity (Rio Convention).

### Author ORCIDs

Chao-Ge Wang https://orcid.org/0000-0003-4381-5720

Ying-Da Wu https://orcid.org/0000-0003-1295-4015

Xin Zhang https://orcid.org/0009-0005-8363-7852

Yu-Cheng Dai https://orcid.org/0000-0002-6523-0320

Yuan Yuan https://orcid.org/0000-0001-6674-9848

### Data availability

All of the data that support the findings of this study are available in the main text or Supplementary Information.

## Supplementary materials

Supplementary material 1 Alignment for Fig. 1 Data type pdf https://binary.pensoft.net/file/1437925This dataset is made available under the Open Database License (http://opendatacommons.org/licenses/odbl/1.0/). The Open Database License (ODbL) is a license agreement intended to allow users to freely share, modify, and use this Dataset while maintaining this same freedom for others, provided that the original source and author(s) are credited.Chao-Ge Wang, Ying-Da Wu, Xin Zhang, Yu-Cheng Dai, Zhen-Hao Li, Yuan Yuan

Supplementary material 2 Alignment for Fig. 2 Data type pdf https://binary.pensoft.net/file/1437926This dataset is made available under the Open Database License (http://opendatacommons.org/licenses/odbl/1.0/). The Open Database License (ODbL) is a license agreement intended to allow users to freely share, modify, and use this Dataset while maintaining this same freedom for others, provided that the original source and author(s) are credited.Chao-Ge Wang, Ying-Da Wu, Xin Zhang, Yu-Cheng Dai, Zhen-Hao Li, Yuan Yuan
