# Supplementary material for: ﻿Studies in phylogeny and divergence times of Irpicaceae and Meripilaceae (Polyporales, Basidiomycota), with an emphasis on Ceriporia and Meripilus including ten new species
Source: IMA Fungus. 2025 Oct 15;16:e161336. doi: 10.3897/imafungus.16.161336 (PMC12547424; doi:10.3897/imafungus.16.161336)
Supplement: Supplementary material 1 — Alignment for Fig. 1 [file imafungus-16-e161336-s001.pdf]

MATRIX

Dai15205_Ceriporia_albomellea TGAGTTTT---GAATGGGTTGTTGCTGGCCTTGTA----TTGAAGGCA-T

Dai15223_Ceriporia_albomellea TGAGTTTT---GAATGGGTTGTTGCTGGCCTTGTA----TTGAAGGCA-T

Li1780_Ceriporia_variegata TGAGTTTT---GAATGGGTTGTTGCTGGTCTTGTA-----TAAAGGCA-T

Dai19791_Ceriporia_variegata TGAGTTTT---GAATGGGTTGTTGCTGGTCTTGTA-----TAAAGGCA-T

Dai19886 TGAGTTTT---GAATGGGTTGTTGCTGGTCTTGTA-----TAAAGGCA-T

Dai10833_Ceriporia_crassitunic TGAGTTTT---GAACGGGTTGTTGCTGGCCTTGTT----ATGAAGGCA-T

CHWC1506_46Meruliopsis_crassit TGAGTTTT---GAACGGGTTGTTGCTGGCCTTGTT----ATGAAGGCA-T

Dai9995_Ceriporia_crassitunica TGAGTTTT---GAACGGGTTGTTGCTGGCCTTGTA-----TAAAGGCA-T

Wu1209_58_Meruliopsis_parvispo CGAGTT--TTGAAACAAGTTGTAGCTGGCC-TTTC-CACAGGAAGGCA-T

CHWC1505_129_Meruliopsis_parvi CGAGTT--TTGAAACAAGTTGTAGCTGGYC?TTTY-CACAGGAAGGCA-T

Dai21944 CGAGTT--TTTGAACGGGTTGTTGCTGGC--TTTC-TA-AAGAGAGCA-T

830Dai18640A --

GC1704_60_Meruliopsis_taxicola -------------------------GGCC-TTTC-----ACAAGGCA-T

Dai22625 CGAGTT--TTG-AACGGGTTGTAGCTGGCC-TTTC-----ACAAGGCA-T

Dai22636 CGAGTT--TTGAAACGGGTTGTAGCTGGCC-TTTC-----ACAAGGCA-T

Dai21878 CGAGTT--TTGAAACGGGTTGTAGCTGGCC-TTTC-----ACAAGGCA-T

1169Dai17248 CGAGTT--TTGAAACGGGTTGTAGCTGGCC-TTTC-----ACAAGGCA-T

Wu1708_43_Meruliopsis_leptocys --CATT

Li1011 CGAGTT--TTGA-ACGGGTTGTTGCTGGCC-TTTC-----ACAGGGCATT

ZX95Dai25742Meruliopsis_leptoc --

WCG1306Dai24733 CGAGTT--TTGAAACGGGTTGTCGCTGGCC-TTTC-----ACAAGGCATT

LXL99Dai25816 --

WCG1559Dai26052Meruliopsis CGAGTT--TTGAAACAGGTTGTCGCTGGCC-TTTC-----ACGAGGCATT

He7477 CGAGTT--TTG-AACAGGTTGTCGCTGGCC-TTTC-----ACGAGGCATT

HLX243Dai26217 CGAGTT--TTGAAACAGGTTGTCGCTGGCC-TTTC-----ACGAGGCATT

RussiaMW673659Meruliopsis_fagi CGAGTT--TTGAAACGGGTTGTCGCTGGCC-TTTC-----ACGAGGCATT

FD278 CGAGTT--TTGAAACGGGTTGTCGCTGGCC-TTTC-----ACGAGGCA-T

Dai10226_Ceriporia_tarda TGAGTTTTTTGAACGGGTTGTAGCTGGCC-TTTT-----ACGGGGCA-T

LE247365 ---GGGGCA-T

Dai8173_Meruliopsis_nanlingens TGAGTT--TTG-AACGGGTTGTAGCTGGCC-TTTC----CATAGGGCA-T

860Dai17172 TGAGTT--TTGAAACGGGTTGTAGCTGGCC-TTTC----CATAGGGCA-T

879Dai13414 TGAGTT--TTGAAACGGGTTGTAGCTGGCC-TTTC----CATAGGGCA-T

Li_1704_Meruliopsis_pseudocyst CGAGTTTATTGAAACAGGTTGTCGCTGGCCTTTTTTACAAAAAGGCA-T

833Dai18405 ------------------------------------TACAAAAAGGCA-T

HHB_10729_Meruliopsis_albostra CGAGTTTATTGAAACAGGTTGTCGCTGGCC-TTTTGAATAGAAAGGCA-T

Cui6878_Ceriporia_pseudocystid CGAGTTTATTGAAACAAGTTGTCGCTGGTT-TCTT-TAACAAGAAACA-T

869Dai14737 CGAGTTTATTGAAACAAGTTGTCGCTGGTT-TCTT-TAACAAGAAACA-T

876Cui11626 CGAGTTTATTGAAACAAGTTGTCGCTGGTT-TCTT-TAACAAGAAACA-T

1199WEI3388 CGAGTTTATTGAAACAAGTTGTCGCTGGTT-TCTT-TAACAAGAAACA-T

776308_Meruliopsis_cystidiata CGAGTTTT---GAAAGGGTTGTCGCTGGCC-TTTG-TATTTGAAGGCATT

ICN139059_Meruliopsis_cystidia CGAGTTTT---GAAAGGGTTGTCGCTGGCC-TTTG-TATTTGAAGGTATC

HHB15692Ceraceomyces_serpens CGAGTTTT---GAACAGGTTGTAGCTGGCC-TTT-----AGCGAGGCA-T

HHB_15629_Sp_Ceriporiopsis_ane CGAGTTTTTTGAACGGGTTGTAGCTGGCC-TTTTA---ATCGAGGCA-T

AJ185Trametopsis_cervina CGAGTTT-ATTGGACGGGTTGTCGCTGGCC-TTT------ACCGGCAA-T

FD9Irpex_lacteus CGAGTTTT---GAACGGGTTGTAGCTGGCC-TCT-----CACGAGGCA-T

908Dai11230 CGAGTTTT---GAACGGGTTGTAGCTGGCC-TCT-----CACGAGGCA-T

FP55521TEmmia_lacerata CGAGTTTT---GAACGGGTTGTAGCTGGCC-TTT-----AACGAGGCA-T

PBU0048Ceriporia_cystidiata CGAGTTTT---GAACGGGTTGTAGCTGGCC-TTT-----AACGAGGTA-T

MZ340C_lacerataT ---GTTTT---GAACGGGTTGTAGCTGGCC-TTT-----AACGAGGTA-T

Dai21940 CGAGTTTT---GAACGGGTTGTAGCTGGCC-TTT-----AACGAGGTA-T

847Dai16433 CGAGTTTT---GAACGGGTTGTAGCTGGCC-TTT-----AACGAGGTA-T

MarcinEmmia_latemarginatus --------------GGGTTGTAGCTGGCC-TTT-----AACGAGGCA-T

Meijer3729Hydnopolyporus_fimbr CGAGTTTT---GAACGGGTTGTCGCTGGCC-TTT-----CACGAGGCA-T

RLG13408Phanerochaete_sp CGAGTTTTTT-GAACGGGTTGTAGCTGGCC-TTT-----CGCGAGGCA-T

WHC1381Flavodon_flavus CGAGTTTT---GAACGGGTTGTAGCTGACC-TTT-----TTTGGGGTA-T

GB1833Phlebia_albida CGAGTTTTTT-GAACGGGTTGTAGCTGGCC-TTT-----CACGAGGCA-T

T407Phlebia_nitidula CGAGTTTTTT-GAACGGGTTGTAGCTGGCC-TTT-----CACGAGGCA-T

HHB6988Phanerochaete_exilis CGAGTTT-TT-GAACGGGTTGTCGCTGGCC-TTT-----CACGAGGCATT

HHB8509Phanerochaetella_xeroph TGAGTTT-TT-GAACGGGTTGTCGCTGGCC-TTT-----CACGAGGCATT

PBU0051Macrohyporia_dictyopora CGAGTTT-TT-GAACGGGTTGTCGCTGGCC-TTT-----CACAAGGCATT

HHB11463Phanerochaete_sp CGAGTTTATT-GAACGGGTTGTCGCTGGCC-TTT-----CACGAGGCATT

FP102382Byssomerulius_corium CGAGTTTTTT-GAACGGGTTGTCGCTGGCC-TTT-----CACGAGGCA-T

FP102165Efibula_americana CGAGTTT-TT-GAACGGGTTGTAGCTGGCC-T--TG-TAACAGAGGCA-T

Murdoch90Ceriporia_torpida CGAGTTTTT--GAACAGGTTGTAGCTGGCC-TCTCA-C----GAGGTAAT

Rivoire4413_Ceriporia_purpurea CGAGTTTTT--GAACGGGTTGTAGCTGACC-TCTCA-AACGGGAGGTA-C

Kout_18_Ceriporia_triumphalis CGAGTTCTT--GAACGGGTTGTAGCTGACC-TCTCA-AACGGGAGGTA-C

Rivoire3701_Ceriporia_bresadol CGAGTTTTT--GAACGGGTTGTAGCTGACC-TCTCA-C----GAGGTA-C

VS4018 CGAGTTTTT--GAACAGGTTGTAGCTGACC-TCTCA-C----GAGGTA-T

Ryvarden21832_Ceriporia_manzan CGAGTTTTT--GAACGGGTTGTAGCTGACC-TCTCA-C---TGAGGTA-C

Dai24539 CGAGTTTTT--GAACGGGTTGTAGCTGATC-TCTCA-C----GAGGTA-T

Dai24541 CGAGTTTTT--GAACGGGTTGTAGCTGACC-TCTCA-C----GAGGTA-T

JV1105_12_Ceriporia_occidental CGAGTTTTT--GAACGGGTTGTAGCTGACC-TCTCA-C---TGAGGTA-T

VS8558Ceriporia_occidentalis CGAGTTTTT--GAACGGGTTGTAGCTGACC-TCTCA-C---TGAGGTA-T

Dai22445 CGAGTTTTT--GAACGGGTTGTAGCTGACC-TCTCA-AACGGGAGGTA-C

846Dai16368 CGAGTTTTTG-GAACGGGTTGTAGCTGACC-TCTCA-A-ACGGAGGTA-T

Dai17951_Ceriporia_aurantiocar TGAGTTTT---GAACGGGTTGTAGCTGGCC-TATA-----AAAAGGCA-T

Miettinen_11701C_viridans -------------ACGGGTTGTAGCTGGCC-TTTT-----ACAAGGCA-T

JV0105_10Ceriporia_aurantiocar TGAGTTTT---GAACGGGTTGTAGCTGGCC-TATA-----AAAAGGCA-T

Yuan5702C_viridans TGAGTTTT---GAACGGGTTGTAGCTGGCC-TTTT-----ACAAGGCA-T

858Dai17003 TGAGTTTT---GAACGGGTTGTAGCTGGCC-TTTT-----ACAAGGCA-T

Yuan2747_Ceriporia_viridans TGAGTTTT---GAACGGGTTGTAGCTGGCC-TTAT-----AAAAGGCA-T

Yuan2744C_viridans TGAGTTTT---GAACGGGTTGTAGCTGGCC-TTAT-----AAAAGGCA-T

Li1046C_viridans CGAGTTTT---GAACGGGTTGGAGCTGGCC-TTTA-----AGAAGGCA-T

865C_sinoviridans CGAGTTTT---GACTCGGTTGGAGCTGGCC-TTTA-----AGAAGGCA-T

871Dai15062 TACGACTT---TAACTGAATGTAGCTGGCC-TTAT-----AAAAGGCA-T

Dai7642_Ceriporia_humilis CGAGTTTTT--G-ACGGGTTGTAGCTGGCC-TCTT--ACCGAGGTAAA-C

Spirin4706_Ceriporia_humilis CGAGTTTTT--GAACGGGTTGTAGCTGGCC-TCTT--ACCGAGGTAAA-C

Spirin4944_Ceriporia_sericea CGAGTTTT---GAACGGGTTGTAGCTGRCC-TCTC--ACCGAGGTAAA-C

WCG1547Dai26044ceriporia CGAGTTTT---GAACGGGTTGTAGCTGGCC-TCTC--ACCGAGGTAAA-C

ZZW1558Dai27086 ---

Miettinen14381_Ceriporia_mpuri CGAGTTTTT--GAACAGGTTGTAGCTGGCC-TCTTC-GTCGAGGTAAT-C

Miettinen15492_2_Ceriporia_sor CGAGTTTTT--GAACGGGTTGTAGCTGACC-TCTC--

-ACGAGGT-AT-C

He6687 CGAGTTTTT--GAACAGGTTGTAGCTGGCC-TCTTC-ACCGAGGTAAT-C

ZH53Dai24426 CGAGTTTTT--GAACAGGTTGTAGCTGGCC-TCTTC-ACCGAGGTAAT-C

Vlasak0808_30_Ceriporia_punica CGAGTTTTT--GAACAGGTTGTAGCTGACC-TCTT----GCGAGGTAC-T

887Dai13376 CGAGTTTTT--GAACAGGTTGTAGCTGACC-TCTT----GCGAGGTAC-T

WCG1443Dai24998 TGAGTTTTT--GAACAGGTTGTAGCTGGCC-TCTAT---TCGGAGGTAAT

0108_6Ceriporia_spissa CGAGTTCTT--TGTTAGGTTGTAGCTGACC-TCT---TACCGGAGGTA-T

Dai19164 CGAGTT-TT--TGTCAAGTTGTAGCTGACC-TCT---TACCGGAGGTA-T

Dai17937_Ceriporia_bubalinomar TGAGTT-TT--TGTTAGGTTGTAGCTGACC-TCT---GCATGGAGGTA-T

903Dai12113 CGAGTT-TT--TGTTAGGTTGTAGCTGACC-TCT---GTATGGAGGTA-T

LZB929Dai25079 TGAGTTT----TGACGGGTTGTAGCTGACC-TTT---ACAAAAAGGTA-T

LX45Dai26988 TGAGTTTT---GAACGGGTTGTAGCTGACC-TTT---ACAAAAAGGTA-T

LX43Dai26986 TGAGTTTT---GAACGGGTTGTAGCTGACC-TTT---ACAAAAGGGTA-T

Dai7759Ceriporia TGAGTTTT---GAACGGGTTGTAGCTGACC-TTT---ACAAAAAGGTA-T

Cui8012_Ceriporia_viridans TGAGTTTT---GAACGGGTTGTAGCTGGCC-TA----A-TAAAAGGCA-T

GC1704_54Ceriporia_viridans TGAGTTTT---GAACGGGTTGTAGCTGGCC-TA----A-CAAAAGGCA-T

Dai23392 TGAGTTTT---G-ACGGGTTGTAGCTGGCC-TA----A-ACAAAGGCA-T

WCG1585Dai26113Ceriproia TGAGTTTT---GAACGGGTTGTAGCTGGCC-TA----A-ACAAAGGCA-T

Dai18675C_eucalypti TGAGTTTT---GAACGGGTTGTAGCTGGCC-TA----ATAAAAAGGCA-T

Dai22034 CGAGTTT-TT-GGACAGGTTGTTGCTGACT-TC------TATGAGGTA-T

JV1008_41JTardaFLORIDAKeys CGAGTTT-TT-GGACAGGTTGTCGCTGACT-TC------TATGAGGTA-T

Rivoire1161_Ceriporia_pierii CGAGTTTT---GAACGGGTTGTAGCTGGCC-TCT-----GACGAGGTATT

Dai23499C_pierii CGAGTTTT---GAACGGGTTGTAGCTGGCC-TCT---

--GACGAGGTATT
Dai23500 CGAGTTTT---GAACGGGTTGTAGCTGGCC-TCT-----GACGAGGTATT
841Dai15899 CGAGTTTT---GAACGGGTTGTAGCTGACC-TCT--TAAGAAGAGGTA-T
842Dai15904 CGAGTTTT---GAACGGGTTGTAGCTGACC-TCT--TAAGAAGAGGTA-T
LZB1066xinjiang --
LZB1065xinjiang --
851Dai16779 -----------GAAGGGTCTAAAGGTGGCC-TT----------------
RMJ119sp_Candelabrochaete_sept TGAGTTTT---GAACGGGTTGTCGCTGGCC-TCT-----GACGAGGCA-T
RLG9759spCandelabrochaete_sept -------------------TGTCGCTGGCC-TCT-----GACGAGGCA-T
RLG10478Phanerochaete_allantos CGAGTCTT---GAACGGGTTGTAGCTGGCT-TCTCA---ATGGAGGCA-T
Dai19118_Ceriporia_spissa TGAGCTT----GAACAGGTTGTAGCTGGCTCT------GAAAGGAGCA-T
Dai18486A TGAGCTT----GAACAGGTTGTAGCTGGCTCT------GAAAGGAGCA-T
WEI17_024_Ceriporia_mellita TGAGCTT----GAACAGGTTGTAGCTGGCTCT------GAAAGGAGTA-C
GC1508_71Ceriporia_mellita TGAGCTT----GAACAGGTTGTAGCTGGCTCT------GAAAGGAGTA-C
GC1608_7_Ceriporia_mellita TGAGCTT----GAACAGGTTGTAGCTGGCTCT------GAAAGGGGCA-T
ZZW1557Dai27085 TGAGCTT----GAACAGGTTGTAGCTGGCTCT------GAAAGGGGCA-T
ZZW1554Dai27083 TGAGCTT----GAACAGGTTGTAGCTGGCTCT------GAAAGGGGCA-T
Dai8168 TGAGCTT----GAACAGGTTGTAGCTGGCTCT------GAAAGGAGTA-T
BR4865C_mellita TGAGCTT----GAACAGGTTGTAGCTGGCTCT------GAAAGGAGCA-T
MEL2382688Ceriporia_sp --
Dai8110 TGAGTTT----GAACAAGTTGTAGCTGGCTCT------GAAAGGAGCA-T
Cui8097 TGAGTTT----GAACAAGTTGTAGCTGGCTCT------GAAAGGAGCA-T
909Cui6740 TGAGTTT----GAACAGGTTGTAGCTGGCTCT------AACAAGAGCA-T
W1258Dai24695 TGAGTTT----GAACAGGTTGTAGCTGGCTCT------AACAAGAGCA-T

JV0110_26_Ceriporia_griseoviol CGAGTTCTTT-GAACAGGTTGTAGCTGGCCTTCTGACAACAGGAGGCA-T

896Dai13202 CGAGTTCTTT-GAACAGGTTGTAGCTGGCCTTCTGACAACAGGAGGCA-T

LWY393Dai27053C_griseoviolasce CGAGTTCTTT-GAACAGGTTGTAGCTGGCCTTCTGACAACAGGAGGCA-T

LWY394DAI27054 CGAGTTCTTT-GAACAGGTTGTAGCTGGCCTTCTGACAACAGGAGGCA-T

FP135015G_pannocinctus CGAGTTC-TT-GAACAGGTTGTYGCTGGCC-TTT------ACAGGGTA-T

L15726SpG_pannocinctus CGAGTTC-TT-GAACAGGTTGTCGCTGGCC-TTT------ACAGGGTA-T

Dai22221 CGAGTTC-TT-GAACAGGTTGTAGCTGGCC-TTC------ACAGGGTA-T

Dai22633 --------------------------------T------TAAGAGCA-T

Dai23260 CGAGTTC-TT-GAACGGGCTGTAGCTGGCTTTCT------TAAGAGCA-T

Dai23626 CGAGTTC-TT-GGACGGGTTGTAGCTGGCTTTTT------TAAGAGCA-T

Dai16238G_citrinoalbus CGAGTTT-TT-GAACGGGTTGTCGCTGGC--TTT------TCGAAGCA-T

1175Dai15293 CGAGTTT-TT-GAACGGGTTGTCGCTGGC--TTT------TCGAAGTA-T

Dai19547 CGAGTTT-TT-GAACGGGTTGTCGCTGGC--TTT------TCGAAGCA-T

918063G_africanus CGAGTTT-TT-GAACGGGTTGTCGCTGGC--TTT------TCGAAGCA-T

918572G_africanus CGAGTTT-TT-GAACGGGTTGTCGCTGGC--TTT------TCGAAGCA-T

Dai18536A CGAGTTC-TT-GAACGGGTTGTCGCTGGC--TTT------TAGAAGCA-T

1164Cui17922 CGAGTTC-TT-GAACGGGTTGTCGCTGGC--TTT------TAGAAGCA-T

Dai22225 CGAGTTC-TT-GAACGGGTTGTCGCTGGC--TTT------TAGAAGTA-T

1163Dai20655 ---

Yuan4397G_hainanensis CGAGTTC-TT-GAACGGGTTGCTGCTGACTTTAT------TGGGAGTA-T

1176Dai15268 ---

1177Dai15259 CGAGTTC-TT-GAACGGGTTGCTGCTGACTTTAT------TGGGAGTA-T

BZ2896G_thelephoroides CGAGTTC-TT-GAACGGGTTGCTGCTGACTCT--------AACGAGTA-T

1166JV1808_26 CGAGTTC-TT-GAACGGGTTGCTGCTGACTCT--

------AACGAGTA-T

Miettinen16992Hapalopilus_ochr TGAGTTAT---GGACAGGTTGTTGCTGRCCTT------TTTTAAGGTA-T

GC1708_338_Ceriporia_arbuscula -------------------GTTGCTGGCC-TC------AACGAGGCA-T

WCG1555Dai26107Ceriporia ---

GC1708_340_Ceriporia_arbuscula TGAGTCTT---GGACAGGTTGTTGCTGGCC-TC-----AAACGAGGCA-T

WCG1556Dai26109Ceriporia TGAGTCTT---GGACAGGTTGTTGCTGGCC-TC-----AAACGAGGCA-T

883Cui11291 TGAGTTCT---GAACAGGTTGTTGCTGACC-TCTA-AATAAGGGGGTA-T

HLX320Dai26805 AGAGTTCT---GAACAGGTTGTTGCTGACC-TC-----TAATGGGGTA-T

WCG1266Dai24678A TGAGTTCT---GAACGGGTTGTTGCTGGCC-TC-----TAACGGGGTA-T

Dai6090_Ceriporia_sulphuricolo CGAGTTTTTTTGAACAGTGTATAGCTGGTCTTCTA-AGGTGTGAGGCA-C

RLG_11354_Ceriproia_reticulata CGAGTTTT---GAACAGGTTGTAGCTGGCC-TCTT----AATGAGGTA-T

ZZW1543Dai27072 CGAGTTTT---GAACAGGTTGTAGCTGGCC-TCTT----AACGAGGTA-T

Li1316_Ceriporia_reticulata CGAGTTTT---GAACAGGTTGTAGCTGGCC-TCTT-----ACGAGGTA-T

KHL11981Ceriporia_reticulata CCAGTTTT---GAACAGGTTGTAGCTGGCC-TCTT-----ACGAGGTA-T

FP110343sp_Candelabrochaete_la CGAGTTTT---GAACAGGTTGTAGCTGGCC-TTTC-----ACAAGGCA-T

Li1045_Ceriporia_reticulata CGAGTTCT---GAACGGGTTGTAGCTGGCC-TCTT-TAATTGGAGGTA-T

ZX136Dai25794ceriporia CGAGTTCT---GAACGGGTTGTAGCTGGCC-TCTT-TAATTGGAGGTA-T

892Dai13400 CGAGTTCT---GAACGGGTTGTAGCTGGCC-TCTT-TAATTGGAGGTA-T

RLG7163Leptoporus_mollis CGAGTTATTT-GAACAGGTTGTAGCTGGCC-TCTT---ACTTGAGGCA-T

Dai21062Leptoporus_mollis CGAGTTATTT-GAACAGGTTGTAGCTGGCC-TCTT---ACTTGAGGCA-T

Dai20182Leptoporus_submollis CGAGTTTTTT-GAACAGGTTGTAGCTGGCC-TCTT---ACTTGAGGCA-T

Cui18379Leptoporus_submollis CGAGTTTTTT-GAACAGGTTGTAGCTGGCC-TCTT---ACTTGAGGCA-T

Wu1209_46Resiniporus_pseudogil TGAGTTTATTGAAACAGGTTGTAGCTGGCCTTTT------GTTAGGCACT

BRNM710169Resiniporus_resinasc
TGAGTTTATTGAAACAGGTTGTAGCTGGCCTTTT------GTTAGGCACT
Dai14516Bjerkandera_adusta CGAGTTTT---GAATGGGTTGTCTGCGGCT--------CGCAAGGGCA-T
Dai21100Bjerkandera_fumosa CGAGTTTT---GAACGGGTTGTCTGCGGCT--------CGCGAGAGCA-T
Miettinen16854Ceraceomyces_sp
TGAGTTTATTGGAGCAAGTTGTAGCTGGTCTTGTGT---TATGAGGCATC
Dai10477C_spissa TGAGTTTT---GAACAGGTTGTAGCTGGCC-TCT-----AACGAGGTAAT
855Dai16831 TGAGTTTT---GAACAGGTTGTAGCTGGCC-TCT-----AACGAGGTAAT
882Cui11282 TGAGTTTT---GAACAGGTTGTAGCTGGCC-TCT-----AACGAGGTAAT
Dai24566 TGAGTTCT---GAACAGGTTGTAGCTGGCC-CCT-----AACGAGGTAAT
Yuan5965 TGAGTTCT---GAACAGGTTGTAGCTGGCC-TCT-----AACGAGGTAAT
Dai3204 --
1194CUI9985 --

Dai15205_Ceriporia_albomellea GTGCACGCCTAGCTCA--TCCGCTC-TTTACCTCTGTGCATTTTATGTAG
Dai15223_Ceriporia_albomellea GTGCACGCCTAGCTCA--TCCGCTC-TTTACCTCTGTGCATTTTATGTAG
Li1780_Ceriporia_variegata GTGCACGCCTGGCTCA--TCCGCTC-TTTACCTCTGTGCATTTTTTGTAG
Dai19791_Ceriporia_variegata GTGCACGCCTGGCTCA--TCCGCTC-TTTACCTCTGTGCATTTTTTGTAG
Dai19886 GTGCACGCCTGGCTCA--TCCGCTC-TTTACCTCTGTGCATTTTTTGTAG
Dai10833_Ceriporia_crassitunic GTGCACGCCTGACTCA--TCCACTC-TTTACCTTTGTGCATTTTTTGTAG
CHWC1506_46Meruliopsis_crassit GTGCACGCCTGACTCA--TCCACTC-TTTACCTTTGTGCATTTTTTGTAG
Dai9995_Ceriporia_crassitunica GTGCACGCCTGGCTCA--TCCACTC-TCTACCTTTGTGCATTTTTTGTAG
Wu1209_58_Meruliopsis_parvispo GTGCTCGCTTGGCTCA--TCCACTC-TCTACCTCTGTGCATTTTTTGTAG
CHWC1505_129_Meruliopsis_parvi GTGCACGCTTGGCTCA--TCCACTC-TCTACCTCTGTGCATTTTTTGTAG
Dai21944 GTGCACGCCTGGCTCA--TCCACTCTTCAACCTCTGTGCATTTTTTGTAG

830Dai18640A --TTTTTGGTGG

GC1704_60_Meruliopsis_taxicola GTGCACGCTCGGCTCA-TTCCACTCTTCAACCTCTGTGCACTCTTTGTAA

Dai22625 GTGCACGCTCGGCTCA-TTCCACTCTTCAACCTCTGTGCACTCTTTGTAA

Dai22636 GTGCACGCTCGGCTCA-TTCCACTCTTCAACCTCTGTGCACTCTTTGTAA

Dai21878 GTGCACGCCCGGCTCA-TTCCACTCTTCAACCTCTGTGCACTCTTTGTAA

1169Dai17248 GTGCACGCTCGGCTCA-TTCCACTCTTCAACCTCTGTGCACTCTTCGTAA

Wu1708_43_Meruliopsis_leptocys GTGCACGCCTGGCTCA--TCCACTCTTCAACCTCTGTGCACTTTTTGTAG

Li1011 GTGCACGCCTGGCTCA--TCCACTCTTCAACCTCTGTGCACTTTTTGTAG

ZX95Dai25742Meruliopsis_leptoc --AAA

WCG1306Dai24733 GTGCACGCCTGGCTCA--TCCACTCTTCAACCTCTGTGCACTTTTTGAAA

LXL99Dai25816 ---AAAA

WCG1559Dai26052Meruliopsis GTGCACGCCTGGCTCA--TCCACTC-TCCACCTCTGTGCACTTTTTGTAG

He7477 GTGCACGCCTGGCTCA--TCCACTC-TCCACCTCTGTGCACTTTTTGTAG

HLX243Dai26217 GTGCACGCCTGGCTCA--TCCACTC-TCCACCTCTGTGCACTTTTTGTAG

RussiaMW673659Meruliopsis_fagi GTGCACGCCTGGCTCA--TCCACTC-TCCACCTCTGTGCACTTTTTGTAG

FD278 GTGCACGCCTGGCTCA--TCCACTCTTCTACCTCTGTGCACTTTTTGTAG

Dai10226_Ceriporia_tarda GTGCACGCCTGGCTCA--TCCACTCTTCAACCTCTGTGCACTTTTTGTAG

LE247365 GTGCACGCCTGGCTCA--TCCACTCTTCAACCTCTGTGCACTTTTTGTAG

Dai8173_Meruliopsis_nanlingens GTGCACGCCTGGCTCA-TTCCACTC-TCAACCTCTGTGAACTTTTTGTAG

860Dai17172 GTGCACACCTGGCTCA-TTCCACTC-TCAACCTCTGTGAACTTTTTGTAG

879Dai13414 GTGCACGCCTGGCTCA-TTCCACTC-TCAACCTCTGTGAACTTTTTGTAG

Li_1704_Meruliopsis_pseudocyst GTGCACGCTTGGCTCA--TCCATTC-TCAACCTCTGTGCACTTTTTGTAG

833Dai18405 GTGCACGCTTGGCTCA--TCCATTC-TCAACCTCTGTGCACTTTTTGTAG

HHB_10729_Meruliopsis_albostra GTGCACGCTTGGCTCA--TCCACTC-

TTAACCACTGTGCACTTTTTGTAG

Cui6878_Ceriporia_pseudocystid GTGCACGCTTGGCTCA--TCCACTC-TCAACCTCTGTGCACTCTTTGTAG

869Dai14737 GTGCACGCTTGGCTCA--TCCACTC-TCAACCTCTGTGCACTCTTTGTAG

876Cui11626 GTGCACGCTTGGCTCA--TCCACTC-TCAACCTCTGTGCACTCTTTGTAG

1199WEI3388 GTGCACGCTTGGCTCA--TCCACTC-TCAACCTTTGTGCACTCTTTGTAG

776308_Meruliopsis_cystidiata GTGCACGCCTGGCTCA--TCCACTC-TCAACCTCTGTGCACTTTKTGTAG

ICN139059_Meruliopsis_cystidia GTGCACGCCTGGCTCA--TCCACTC-TCAACCTCTGTGCACTTTTTGTAG

HHB15692Ceraceomyces_serpens GTGCACGCCTGGCTCA--TCCACTCTCTTACCTCTGTGCACCCTTTAAAG

HHB_15629_Sp_Ceriporiopsis_ane GTGCACGCTCGGCTCAATTCCACTC-TTAACCACTGTGCACTTTTTGTAA

AJ185Trametopsis_cervina GTGCACGCCTGGCTCAATTCCACTCTTTAACCACTGTGCACTTTTTGTAA

FD9Irpex_lacteus GTGCACGCCTGGCTCA--TCCACTC-TTAACCTCTGTGCACTTTATGTAA

908Dai11230 GTGCACGCCTGGCTCA--TCCACTC-TTAACCTCTGTGCACTTTATGTAA

FP55521TEmmia_lacerata GTGCACACCTGGCTCA--TCCACTC-TCAACCTCTGTGCACTTTATGTAA

PBU0048Ceriporia_cystidiata GTGCACGCCTGGCTCA--TCCACTC-TCAACCTCTGTGCACTTTATGTAA

MZ340C_laceratаT GTGCACGCCTGGCTCA--TCCACTC-TCAACCTCTGTGCACTTTATGTAA

Dai21940 GTGCACGCCTGGCTCA--TCCACTC-TCAACCTCTGTGCACTTTATGTAA

847Dai16433 GTGCACGCCTGGCTCA--TCCACTC-TCAACCTCTGTGCACTTTATGTAA

MarcinEmmia_latemarginatus GTGCACACCTGGCTCA--TCCACTC-TCAACCTCTGTGCACTTTATGTAA

Meijer3729Hydnopolyporus_fimbr GTGCACACCTGGCTCA--TCCACTC-TCAACCTCTGTGCACTTTATGTAA

RLG13408Phanerochaete_sp GTGCACGCCTGGCTCA--TCCACTC-TCAACCTCTGTGCACTTTATGTAA

WHC1381Flavodon_flavus GTGCACGCCTGGCTCA--TCCACTC-TCAACCTCTGTGCACTTCATGTAA

GB1833Phlebia_albida GTGCACACTCGGCTCA--TCCATTC-TCAACCTCTGTGCACCCCTTGTAA

T407Phlebia_nitidula GTGCACACTCGGCTCA--TCCACTC-

TCAACCTCTGTGCACCCTTTGTAA
HHB6988Phanerochaete_exilis GTGCACGCCTGGCTCA--
TCCACTCTTCAACCTCTGTGCACTTACTGTAG
HHB8509Phanerochaetella_xeroph GTGCACGCCTGGCTCA--
TCCACTCCTCAACCTCTGTGCACTTACTGTAA
PBU0051Macrohyporia_dictyopora GTGCACGCCTGGCTCA--
TCCACTCCTCAACCTCTGTGAACTTATAGTAA
HHB11463Phanerochaete_sp GTGCACGCTCGGCTCA--
TCCACTCTTCAACCTCTGTGCACTTACTGTAA
FP102382Byssomerulius_corium GTGCACGCCTGGCTCA--TCCACTC-
TCAACCTCTGTGCACCCTATGTAA
FP102165Efibula_americana GTGCACGCCTGGCTCA--
TCCACTCTTTAACCCCTGTGCACCATATGTAG
Murdoch90Ceriporia_torpida GTGCACACCTGGCTCA-TTCCACTC-
TCAACCTCTGTGCACTACTCGTAG
Rivoire4413_Ceriporia_purpurea GTGCACGCCTGGCTCA-TTCCACTC-
TCAACCCCTGTGCACTTCTCGTAG
Kout_18_Ceriporia_triumphalis GTGCACGCCTGGCTCA-TTCCACTC-
TCAACCCCTGTGCACTTTTCGTAG
Rivoire3701_Ceriporia_bresadol GTGCACGCCYGGCTCA-TTCCACTC-
TCAACCCCTGTGCACTTCTCGTAG
VS4018 GTGCACACCTGGCTCA-TTCCACTC-
TCAACCCCTGTGCACTTCTCGTAG
Ryvarden21832_Ceriporia_manzan GTGCACGCCTGGCTCA-TTCCACTC-
TCAACCCCTGTGCACTTCTCGTAG
Dai24539 GTGCACACCTGGCTCA-TTCCACTC-
TCAACCCCTGTGCACTTCTCGTAG
Dai24541 GTGCACACCTGGCTCA-TTCCACTC-
TCAACCCCTGTGCACTTCTCGTAG
JV1105_12_Ceriporia_occidental GTGCACGCCTGGCTCA-TTCCACTC-
TCAACCCCYGTGCACTTCTCGTAG
VS8558Ceriporia_occidentalis GTGCACGCCTGGCTCA-TTCCACTC-
TCAACCCCTGTGCACTTCTCGTAG
Dai22445 GTGCACGCCTGGCTCA-TTCCACTC-
TCAACCCCTGTGCACTTCTCGTAG
846Dai16368 GTGCACGCCTGGCTCA-TTCCACTC-
TCAACCCCTGTGCACTTCTCGTAG
Dai17951_Ceriporia_aurantiocar GTGCACGCCTGGCTCA--TTCACTC-
TAAATCTTTGTGCACTTTTTGTAG
Miettinen_11701C_viridans GTGCACGCCTGGCTCA--TCCACTC-
TAAACCTCTGTGCACTTTCTGTAG
JV0105_10Ceriporia_aurantiocar GTGCACGCCTGGCTCA--TTCACTC-
TAAATCTTTGTGCACTTTTTGTAG
Yuan5702C_viridans GTGCACGCCTGGCTCA--TCCACTC-

TAAACCTCTGTGCACTTTCTGTAG
858Dai17003 GTGCACGCCTGGCTCA--TCCACTC-
TAAACCTCTGTGCACTTTCTGTAG
Yuan2747_Ceriporia_viridans GTGCACGCCTGGCTCA--
TTTACTCTTAAACTTCTGTGCACTTTTTGTAG
Yuan2744C_viridans GTGCACGCCTGGCTCA--
TTTACTCTTAAACTTCTGTGCACTTTTTGTAG
Li1046C_viridans GTGCTCACCTGGCTCA--
TCCACTCATTAGCTTCTGTGCACTTTTTGTAG
865C_sinoviridans GTGCTCACCTGGCTCA--
TCCACTCATTAGCTTCTGTGCACTTTTTGTAG
871Dai15062 GTGCACGCCTGACTCA--TCTACTC-
TTAACTTCTGTGCACTTTTTGTAG
Dai7642_Ceriporia_humilis GTGCACGCCTGGCTCA-
TTCCACTCTTCAACCTCTGTGCACCTTGTGTAG
Spirin4706_Ceriporia_humilis GTGCACGCCTGGCTCA-
TTCCACTCTTCAACCTCTGTGCACCTTGTGTAG
Spirin4944_Ceriporia_sericea GTGCACGCCTGGCTCA-
TTCCACTCTTCAACCTCTGTGCACCTCATGTAG
WCG1547Dai26044ceriporia GTGCACGCCTGGCTCA-
TTCCACTCTTCAACCTCTGTGCACCTCACGTAG
ZZW1558Dai27086 --
Miettinen14381_Ceriporia_mpuri GTGCACGCCTGGCTCA-
TTCCACTCTTCAACCTCTGTGCACCTTATGTAG
Miettinen15492_2_Ceriporia_sor GTGCACGCCTGGCTCA-TTCCACTC-
TCAACCTCTGTGCACCTTTTGTAG
He6687 GTGCACGCCTGGCTCA-
TTCCACTCTTCAACCTCTGTGCACCTTGTGTAG
ZH53Dai24426 GTGCACGCCTGGCTCA-
TTCCACTCTTCAACCTCTGTGCACCTTGTGTAG
Vlasak0808_30_Ceriporia_punica GTGCACGCCTGGCTCA-TTCCACTC-
TTAACCTCTGTGCACTTCTTGTAA
887Dai13376 GTGCACGCCTGGCTCA-TTCCACTC-
TTAACCTCTGTGCACTTCTTGTAA
WCG1443Dai24998 GTGCACGCCTGGCTCA-TTCCACTC-
TCAACCTCTGTGCACTTCTTGTAA
0108_6Ceriporia_spissa GTGCACGCTTGACTTA-
TCCAACTCTTCAACCTTTGTGCACCTTTTGTAA
Dai19164 GTGCACGCTTGACTCA-
TCCAACTCTTCAACCTTTGTGCACTTTTTGTAA
Dai17937_Ceriporia_bubalinomar GTGCACACTTGACTTT-
TCCAACTCTTCAACCTTTGTGCACTTTTTGTAA
903Dai12113 GTGCACACTTGACTTT-
TCCAACTCTTCAACCTTTGTGCACTTTTTGTAA

LZB929Dai25079 GTGCACGCCTGGCTCA--TCCACTC-TCAACCTTTGTGCACCTTTTGTAG
LX45Dai26988 GTGCACGCCTGGCTCA--TCCACTC-TCAACCTTTGTGCACCTTTTGTAG
LX43Dai26986 GTGCACGCCTGGCTCA--TCCACTC-TCAACCTTTGTGCACCTTTTGTAG
Dai7759Ceriporia GTGCACGCCTGGCTCA--TCTACTC-TCAACCTTTGTGCACCTTTTGTAG
Cui8012_Ceriporia_viridans GTGCACGCCTGGCTCA--TCCACTC-TCAACCACTGTGCACTTTATGTAA
GC1704_54Ceriporia_viridans GTGCACGCCTGGCTCA--TCCACTC-TCAACCACTGTGCACTTTATGTAA
Dai23392 GTGCACGCCTGGCTCA--TCCACTC-TCAACCACTGTGCACTTTATGTAA
WCG1585Dai26113Ceriproia GTGCACGCCTGGCTCA--TCCACTC-TCAACCACTGTGCACTTTATGTAG
Dai18675C_eucalypti GTGCACGCCTGGCTCA--TCCACTC-TCAACCACTGTGCACTTTTTGTAG
Dai22034 GTGCACACCTGGCTCA-ATCCACTC-TCAACCTCTGTGCACTTTTTGTAG
JV1008_41JTardaFLORIDAKeys GTGCACACCTGGCTCA-ATCCACTC-TCAACCTCTGTGCACTTTTTGTAG
Rivoire1161_Ceriporia_pierii GTGCACGCCTGGCTCA-TTCCACTC-TCAACCCCTGTGCACCTTTTGTAG
Dai23499C_pierii GTGCACGCCTGGCTCA-TTCCACTC-TCAACCCCTGTGCACCTTTTGTAG
Dai23500 GTGCACGCCTGGCTCA-TTCCACTC-TCAACCCCTGTGCACCTTTTGTAG
841Dai15899 GTGCACGCCTGGCTCA--TCCACTCTTCAACCTCTGTGCACTTTTTGTAG
842Dai15904 GTGCACGCCTGGCTCA--TCCACTCTTCAACCTCTGTGCACTTTTTGTAG
LZB1066xinjiang --
LZB1065xinjiang --
851Dai16779 ---CAA
RMJ119sp_Candelabrochaete_sept GTGCACGCCTGGCTCA--TCCACACTCAAACCACCGTGCACCCTTTGTAA
RLG9759spCandelabrochaete_sept GTGCACGCCTGGCTCA--TCCACACTCAAACCACCGTGCACCCTTTGTAA
RLG10478Phanerochaete_allantos GTGCACGCCTGGCTCA--TCCACTC-TCAACCTCTGTGCACCCTTTGTAG
Dai19118_Ceriporia_spissa GTGCACGCCTGGCTCA--TCCACTCTTTAACCTTTGTGCACTCTTTGCAA
Dai18486A GTGCACGCCTGGCTCA--

TCCACTCTTTAACCTTTGTGCACTCTTTGCAA

WEI17_024_Ceriporia_mellita GTGCACGCCTGGCTCA--
TCCACTCTTTAACCTTTGTGCACTCTTTGCAA

GC1508_71Ceriporia_mellita GTGCACGCCTGGCTCA--
TCCACTCTTTAACCTTTGTGCACTCTTTGCAA

GC1608_7_Ceriporia_mellita GTGCACGCCTGGCTCA--
TCCACTCTTTAACCTTTGTGCACTCTTTGCAA

ZZW1557Dai27085 GTGCACGCCTGGCTCA--
TCCACTCTTTAACCTTTGTGCACTCTTTGCAA

ZZW1554Dai27083 GTGCACGCCTGGCTCA--
TCCACTCTTTAACCTTTGTGCACTCTTTGCAA

Dai8168 GTGCACGCCTGGCTCA--
TCCACTCTTTAACCTTTGTGCACTCTTTGCAA

BR4865C_mellita GTGCACGCCTGGCTCA--
TCCACTCTTTAACCTTTGTGCACTCTTTGCAA

MEL2382688Ceriporia_sp --

Dai8110 GTGCACGCTTGGCTCA--
TCCACTTTTTAACCTTTGTGCACTCTTTGCAA

Cui8097 GTGCACGCTTGGCTCA--
TCCACTTTTTAACCTTTGTGCACTCTTTGCAA

909Cui6740 GTGCACACCTGGCTCA--
TCCATTCTTTAACCTTTGTACACTCTTTGCAA

W1258Dai24695 GTGCACACCTGGCTCA--
TCCATTCTTTAACCTTTGTGCACTCTTTGCAA

JV0110_26_Ceriporia_griseoviol GTGCACGCCTGGCTCA-
TTCCACTCTTCAACCCCTGTGCACTTTCTGTAG

896Dai13202 GTGCACGCCTGGCTCA-
TTCCACTTTTCAACCCCTGTGCACTTTCTGTAG

LWY393Dai27053C_griseoviolasce GTGCACGCCTGGCTCA-
TTCCACTCTTCAACCCCTGTGCACTTTCTGTAG

LWY394DAI27054 GTGCACGCCTGGCTCA-
TTCCACTCTTCAACCCCTGTGCACTTTCTGTAG

FP135015G_pannocinctus
GTGCACACCTGGCTCAATTTCCACTCTTAACCTCTGTGCACTCTTTGTAG

L15726SpG_pannocinctus
GTGCACACCTGGCTCAATTTCCACTCTTAACCTCTGTGCACTCTTTGTAG

Dai22221 GTGCACGCCTGGCTCAA-
TCCACTCTTAAACCTCTGTGCACTTATTGTAG

Dai22633
GTGCACGCCTGGCTCAATTCCACTCCTTAACCTCTGTGCACTTATTGTAG

Dai23260
GTGCACGCCTGGCTCAATTCCACTCCTTAACCTCTGTGCACTTATTGTAG

Dai23626
GTGCACGCCTGGCTCAATTCCACTCCTTAACCTCTGTGCACTTATTGTAG

Dai16238G_citrinoalbus
GTGCACGCCTGGCTCAATCCACTCCTTTAACCTCTGTGCACTTATTGTAG
1175Dai15293
GTGCACGCCTGGCTCAATCCACTCCTTTAACCTCTGTGCACTTATTGTAG
Dai19547
GTGCACGCCTGGCTCAATCCACTCCTTTAACCTCTGTGCACTTATTGTAG
918063G_africanus
GTGCACGCCTGGCTCAATCCACTCCTTCAACCTCTGTGCACTTATTGTAG
918572G_africanus
GTGCACGCCTGGCTCAATCCACTCCTTCAACCTCTGTGCACTTATTGTAG
Dai18536A
GTGCACGCCCGGCTCAATCCACTCCTTTAACCTCTGTGCACTTATTGTAG
1164Cui17922
GTGCACGCCCGGCTCAATCCACTCCTTTAACCTCTGTGCACTTATTGTAG
Dai22225
GTGCACGCCCGGCTCAATCCACTCCTTTAACCTCTGTGCACTTATTGTAG
1163Dai20655 --
Yuan4397G_hainanensis
GTGCACACCTGGCTCAATTCCACTCTTTAACCCCTGTGCACTTTTTGTAG
1176Dai15268 --
1177Dai15259
GTGCACACCTGGCTCAATTCCACTCTTTAACCCCTGTGCACTTTTTGTAG
BZ2896G_thelephoroides
GTGCACACCTGGCTCAATTCCACTCTTTAACCTCTGTGCACTTTTTGTAG
1166JV1808_26
GTGCACACCTGGCTCAATTCCACTCTTTAACCTCTGTGCACTTTTTGTAG
Miettinen16992Hapalopilus_ochr GTGCACACTTGGCTAT--TTTACTC-
TCAACCTCTGTGCACTATTTGTAG
GC1708_338_Ceriporia_arbuscula GTGCACGCCTGGCTCA--
TCCACTCTTTTACCTCTGTGCACTATTCGTAG
WCG1555Dai26107Ceriporia --
GC1708_340_Ceriporia_arbuscula GTGCACGCCTGGCTCA--
TCCACTCTTTTACCTCTGTGCACTATTCGTAG
WCG1556Dai26109Ceriporia GTGCACGCCTGGCTCA--
TCCACTCTTTTATCTCTGTGCACTATTCGTAG
883Cui11291 GTGCACGCCTGGCTCA--
TCCACTCTTTTACCTCTGTGCACCATTTGTAG
HLX320Dai26805 GTGCACGCTTGGCTCA--
TCCACTCTTTTACCTCTGTGCACTATTTGTAG
WCG1266Dai24678A GTGCACGCCTGGCTCA--
TCCACTCTTTTACCTCTGTGCACTATTTGTAA
Dai6090_Ceriporia_sulphuricolo
GTGCACGCCTGGCTTCAATCCACTCTTCAACCTCTGTGCACTATTTGTAG
RLG_11354_Ceriproia_reticulata GTGCACGCTTGGCTCA--

TCCACTCTTCAACCCCTGTGCACTTTTCATAG

ZZW1543Dai27072 GTGCACGCTTGGCTCA--TCCACTCTTCAACCCCTGTGCACTTTTCATAG

Li1316_Ceriporia_reticulata GTGCACGCCTGGCTCA--TCCACTCTTCAACCCCTGTGCACTTTTCATAG

KHL11981Ceriporia_reticulata GTGCACGCCTGGCTCA--TCCACTCTTCAACCCCTGTGCACTTTTCATAG

FP110343sp_Candelabrochaete_la GTGCACGCTTGGCTCA--TCCACTCTTTAACCCCTGTGCACTTTTCATGG

Li1045_Ceriporia_reticulata GTGCACGCCTGGCTCA--TCCACTCTTTAACCCCTGTGCACTTTTCATGG

ZX136Dai25794ceriporia GTGCACGCCTGGCTCA--TCCACTCTTTAACCCCTGTGCACTTTTCATGG

892Dai13400 GTGCACGCCTGGCTCA--TCCACTCTTTAACCCCTGTGCACTTTTCATGG

RLG7163Leptoporus_mollis GTGCACGCTTGGCTCA--TCCACTCTCTAACCCCTGTGCACTTTTTGTAG

Dai21062Leptoporus_mollis GTGCACGCTTGGCTCA--TCCACTCTCCAACCCCTGTGCACTTTTCGTAG

Dai20182Leptoporus_submollis GTGCACGCTTGGCTCA--TCCACTCTCCAACCCCTGTGCACTTTTCGTAG

Cui18379Leptoporus_submollis GTGCACGCTTGGCTCA--TCCACTCTCCAACCCCTGTGCACTTTTCGTAG

Wu1209_46Resiniporus_pseudogil GTGCACGCCTGGATCAATTCCACTCTTCAACCACTGTGCACTTTTTGTAA

BRNM710169Resiniporus_resinasc GTGCACGCCTGGATCAATTCCACTCTTCAACCACTGTGCACTTTTTGTAA

Dai14516Bjerkandera_adusta GTGCACGCCTGTCTCA--TCCACTC-TCAACTTCTGTGCACTTTTCATAG

Dai21100Bjerkandera_fumosa GTGCACGCCTGTCTTC-ATCCACTC-TCCACTTCTGTGCACTTTTCATAG

Miettinen16854Ceraceomyces_sp GTGCACGCTTGATTCATTTATAATCTTCAACCTCTGTGCACTTTTTGTAG

Dai10477C_spissa GTGCACACTTGGCTCA-TTTCACTC-TCAACCTCTGTGCACTCTT-GTAA

855Dai16831 GTGCACACTTGGCTCA-TTTCACTC-TCAACCTCTGTGCACTCTT-GTAA

882Cui11282 GTGCACACTTGGCTCA-TTTCACTC-TCAACCTCTGTGCACTCTT-GTAA

Dai24566 GTGCACACTTGGCTCA-TTTCACTC-TCAACCTCTGTGCACTCTT-GTAA

Yuan5965 GTGCACACTTGGCTCA-TTTCACTC-TCAACCTCTGTGCACTCTT-GTAA

Dai3204 --

1194CUI9985 ---

Dai15205_Ceriporia_albomellea G---AA----CTGG-------------TTTTGAAGC---TAAC-----
--

Dai15223_Ceriporia_albomellea G---AA----CTGG-------------TTTTGAAGC---TAAT------
-

Li1780_Ceriporia_variegata G---AA----TTGG-------------TTTTGAAGC---TGAT------
-

Dai19791_Ceriporia_variegata G---AA----TTGG-------------TTTTGAAGC---TGAT------
-

Dai19886 G---AA----TTGG-------------TTTTGAAGC---TGAT-

Dai10833_Ceriporia_crassitunic G---AA----TTGG------T------TTTTGAAGC---TGAT-------

CHWC1506_46Meruliopsis_crassit G---AA----TTGG------T------TTTTGAAGC---TGAT---
----

Dai9995_Ceriporia_crassitunica G---AA----TTGG-------------TTTTGAAGC---TAATG-----
-

Wu1209_58_Meruliopsis_parvispo A---AA-TGGTT-----------------TTGAAGC---AGA----
CGC-

CHWC1505_129_Meruliopsis_parvi A---AA-TGGTT-----------------TTGAAGC---AGA----
CGC-

Dai21944 G---AT-TGGTT-----------------TTGAAGC---AGA----
TGT-

830Dai18640A A---AT-TGGTT-----------------TTGAAGC---GGG----
CGT-

GC1704_60_Meruliopsis_taxicola G---AAATGACC-----------------GGGACGC---TGG----
CGC-

Dai22625 G---AAATGACC-----------------GGGACGC---TGG--
--CGC-

Dai22636 G---AAATGACC-----------------GGGACGC---TGG--
--CGC-

Dai21878 G---AAATGACC-----------------GGGACGC---TGG--
--CGC-

1169Dai17248 G---AAATGACC-----------------GGGACGC---TGG--
--CGC-

Wu1708_43_Meruliopsis_leptocys G---AAATGGGT-----------------TTGAAGC---TGA----
TGC-

Li1011 G---AAATGGGT-----------------TTGAAGC---TGA---
-TGC-

ZX95Dai25742Meruliopsis_leptoc A---AAATGGGT-----------------TTGAAGC---TGA----
TGC-

WCG1306Dai24733 A---AAATGGGT-----------------TTGAAGC---
TGA----TGC-

LXL99Dai25816 A---AA-TGGGT-----------------TTGAAGC---TGA--
--TGC-
WCG1559Dai26052Meruliopsis G---AA-TGGGT-----------------TTGAAGC---TGA---
-TGT-
He7477 G---AA-TGGGT-----------------TTGAAGC---TGA---
-TGT-
HLX243Dai26217 G---AA-TGGGT-----------------TTGAAGC---TGA--
--TGC-
RussiaMW673659Meruliopsis_fagi G---AA-TGGGC-----------------TTGAAGC---TGA----
TGC-
FD278 G---AATTGGTT----------------TTGAAAGC---TGG-
---CGT-
Dai10226_Ceriporia_tarda G---AA-CGGACAATTT------TTATTTCTGAAGC---
TGA----CTTT
LE247365 G---AA-CGGACAATTT------TTATTTCTGAAGC--
-TGA----CTTT
Dai8173_Meruliopsis_nanlingens A---AA-CGGTC----------------TTGGAAGC---TGG----
CGA-
860Dai17172 A---AA-CGGTC----------------TTGGAAGC---TGG--
--CGA-
879Dai13414 A---AA-CGGTC----------------TTGGAAGC---TGG--
--CGA-
Li_1704_Meruliopsis_pseudocyst A---AA-TGGTCAG-------------------GGC---TGG----
CGC-
833Dai18405 A---AA-TGGTCAG-------------------GGC---TGG----
CGC-
HHB_10729_Meruliopsis_albostra G---AA-CGGTCAG-------------------GAT---TGG----
CGC-
Cui6878_Ceriporia_pseudocystid G---AA-TGGTTGG------------------GGCA---TGG----
CGC-
869Dai14737 G---AA-TGGTTGG------------------GGCA---TGG----
CGC-
876Cui11626 G---AA-TGGTTGG------------------GGCA---TGG----
CGC-
1199WEI3388 G---AA-TGGTTGG------------------GGCA---TGG---
-CGC-
776308_Meruliopsis_cystidiata G---AA-CGGT---------------------GGA---
TGACCTCTGG-
ICN139059_Meruliopsis_cystidia G---AA-CGGT---------------------GGA---CGA-
CTCTGG-
HHB15692Ceraceomyces_serpens A---AA-AGGTTAC--------------------------------CGC-
HHB_15629_Sp_Ceriporiopsis_ane G-----ATCGGT-----------------TGAAGGA--------------
AJ185Trametopsis_cervina G-----ATTGGC-----------------TGTAGGG---GAAT---
TAT-

FD9Irpex_lacteus GAAAAAATGG-------------------TGGAAGC---T-------
---

908Dai11230 GAAAAAATGG-------------------TGGAAGC---T-----

FP55521TEmmia_lacerata G---AAACGG-------------------TGTAAGC---CAGC---
----

PBU0048Ceriporia_cystidiata G---AAACGG-------------------TGTAAGC---CAGC------
-

MZ340C_lacerataT G---AAACGG-------------------TGTAAGC---CAGC--
-----

Dai21940 G---AAACGG-------------------TGTAAGC---CAGC--

847Dai16433 G---AAACGG-------------------TGTAAGC---CAGC--

MarcinEmmia_latemarginatus G---AAACGG-------------------TGTAAGC---CAGC----
---

Meijer3729Hydnopolyporus_fimbr G---AAACGG-------------------TGTAAGC---CAGC----
---

RLG13408Phanerochaete_sp G---AAATGGTT-----------------TGAAAGC---CAGC-
--CTT-

WHC1381Flavodon_flavus G--AGAACGG-------------------TGTAAGC---TGAT-
--T-T-

GB1833Phlebia_albida G---AAAT--TG-----------------TTGAAAC---CA-------
G-

T407Phlebia_nitidula G---AAATTGTG-----------------TCGAAAC---CAGC---
TGG-

HHB6988Phanerochaete_exilis G---AAACGGTT-----------------GATGGGC---TGAC---
----

HHB8509Phanerochaetella_xeroph G---AAATGGT------------------CGAAGGC---TGAC---
----

PBU0051Macrohyporia_dictyopora G---AAATGRTC-----------------GA--AGC---CGAC----
---

HHB11463Phanerochaete_sp G---AAATGGT------------------CGAGGGC---TGAC--
-----

FP102382Byssomerulius_corium G---AATCGGTG-----------------GACGTGC---TGGC--
-T-C-

FP102165Efibula_americana G---AA-----------------------TGAACGG---TGAA---
CGT-

Murdoch90Ceriporia_torpida G---AGGGA-----TTAGATGGA--GAGCTGATTGC---
AAAG---TC--

Rivoire4413_Ceriporia_purpurea G---AAGGACTAAATTAGGCGGA--
GAGCTGACTGA---AAAG---TC--

Kout_18_Ceriporia_triumphalis G---
AAGGATTTAATCTGACGGGGAGAGCTGATTGC---AAAG---TC--

Rivoire3701_Ceriporia_bresadol G----AGGATTTAATCAGACGGA--GAGCTGATTGC---AAAG---TC--
VS4018 G----AGGA-TTAATCAGACGGA--GAGCTGATTGA---AAAG---TC--
Ryvarden21832_Ceriporia_manzan G----AGGA--CAATTAGGCGGAC-AAGCTGRCTGC---AAAG---TC--
Dai24539 G----AGGA-TTAATCAGACGGA--GAGCTGATTGA---AAAG---TC--
Dai24541 G----AGGA-TTAATCAGACGGA--GAGCTGATTGA---AAAG---TC--
JV1105_12_Ceriporia_occidental G----AGGACAATATTAGGCGGGGAGAATTGATTGC---AAAG---TC--
VS8558Ceriporia_occidentalis G----AGGACAATATTAGGCGGGGAGAATTGATTGC---AAAG---TC--
Dai22445 G---AAGGACTAAATTGGGCGGA--GAGCTGACTGA---AAAG---TT--
846Dai16368 G---AAGGACTAAATTAGGCGGA--GAGCTGACTGA---AAAG---TC--
Dai17951_Ceriporia_aurantiocar G---AATGA--------------------CAGATGC---TGAC---TT--
Miettinen_11701C_viridans G---AATGA--------------------TGAATAT---TGAC---TT--
JV0105_10Ceriporia_aurantiocar G---AATGA--------------------CAGATGC---TGAC---TT--
Yuan5702C_viridans G---AATGA--------------------TGAATAT---TGAC---TT--
858Dai17003 G---AATGA--------------------TGAATAT---TGAC---TT--
Yuan2747_Ceriporia_viridans A---AGGGA--------------------CAGATGC---TGAC---TT--
Yuan2744C_viridans A---AGGGA--------------------CAGATGC---TGAC---TT--
Li1046C_viridans G---AATGA--------------------CAAATGC---TGAC---TT--
865C_sinoviridans G---AATGA--------------------CAAATGC---TGAC---TT--
871Dai15062 A---AGGGA--------------------CAAATGC---TGAC---TT--
Dai7642_Ceriporia_humilis G---AG---------ATGACCGAGGGGGC--CTTCG---AAAG---TT--
Spirin4706_Ceriporia_humilis G---AG---------ATGACCGAGGGGGC--CTTCG---AAAG---TT--
Spirin4944_Ceriporia_sericea A---AG---------ACGACCGAGGAGGC--CTTCG---AAAG---TC--
WCG1547Dai26044ceriporia A---AG---------ATGACCGAGGAGGC--CTTCG---AAAG---TC--
ZZW1558Dai27086 --

Miettinen14381_Ceriporia_mpuri G---AGA--------ACGACTGAGGAGGC--CTTCG---AAAG---TT--

Miettinen15492_2_Ceriporia_sor G---AG---------ACGGTGGACGAGGC--CTTCG---AAAG---TC--

He6687 G---AG---------ATGACCGAGGGGGC--CTTCG---AAAG---TT--

ZH53Dai24426 G---AG---------ATGACCGAGGAGGC--CTTCG---AAAG---TT--

Vlasak0808_30_Ceriporia_punica G---AG--------TTCCGCGGAC-------------------------

887Dai13376 G---AG--------TTCCGCGGAT-------------------------

WCG1443Dai24998 G---AG--------TTCCGCGGAT-------------------------

0108_6Ceriporia_spissa G---GAGTGG-------------------CGAACGT---CGGC---TTC-

Dai19164 G---AAGTGG-------------------CGAACGT---CGGC---TTC-

Dai17937_Ceriporia_bubalinomar G---AAGTGG-------------------TGAATGT---TGAC---TT--

903Dai12113 G---GAGTGG-------------------TGAATGT---TGAC---TT--

LZB929Dai25079 G---AGTCA--------------------CAGATAC---TGAT---GA--

LX45Dai26988 G---AGTCA--------------------CAGATAC---TGAT---GA--

LX43Dai26986 G---AGTCA--------------------CAGATAC---TGAT---GA--

Dai7759Ceriporia G---AGTCA--------------------CAGATAC---TGAT---GA--

Cui8012_Ceriporia_viridans G---AA---------ATGATGGATGTTGGCAGTT----------------

GC1704_54Ceriporia_viridans G---AA---------ATGACGGATGTTGGCAGTT----------------

Dai23392 G---AA---------ATGACAGATGATGACAGTT----------------

WCG1585Dai26113Ceriproia G---AA---------ATGACAGATGATGACAATT----------------

Dai18675C_eucalypti G---AA---------ATGACAGATGTTGGCAGTT----------------

Dai22034 G---AATCAGA------------------CAGATGC---TTGA---TGT-

JV1008_41JTardaFLORIDAKeys G---AATCAGA------------------CAGATGC---TTGA---TGT-

Rivoire1161_Ceriporia_pierii A---AG--------ATGGGTAGAGGAGGT-------------C---TT--

Dai23499C_pierii A---A---------ATGGGCAGAGGAGGT------------C---
TT--
Dai23500 A---A---------ATGGGCAGAGGAGGT------------C--
-TT--
841Dai15899 G---AA----------------------TGAACAG---AAGC---
TGA-
842Dai15904 G---AA----------------------TGAACAG---AAGC---
TGA-
LZB1066xinjiang --
LZB1065xinjiang --
851Dai16779 G---AA--
RMJ119sp_Candelabrochaete_sept G---AA----------------------CCGAAAC---CGAC---
TCT-
RLG9759spCandelabrochaete_sept G---AA----------------------CCGAAAC---CGAC---
TCT-
RLG10478Phanerochaete_allantos G-TCGG----------------------TGGATGC---TGACGAA-
---
Dai19118_Ceriporia_spissa G---AG--------------------CTTGGTAAACATTGGT---C-
--
Dai18486A G---AG--------------------CTTGGTAAACATTGGT--
-C---
WEI17_024_Ceriporia_mellita G---AG--------------------CTTGGTAAACATTGGT---
C---
GC1508_71Ceriporia_mellita G---AG--------------------CTTGGTAAACATTGGT---
C---
GC1608_7_Ceriporia_mellita G---AG--------------------CTTGGTAAACATTGGT---
C---
ZZW1557Dai27085 G---AG--------------------
CTTGGTAAACATTGGT---C---
ZZW1554Dai27083 G---AG--------------------
CTTGGTAAACATTGGT---C---
Dai8168 G---AG--------------------CTTGGTAAACATTGGT--
-C---
BR4865C_mellita G---AG--------------------CTTGGTAAACATTGGT---
C---
MEL2382688Ceriporia_sp --------------------------------AAACATTGGT---C---
Dai8110 G---AG--------------------CTTGGTCAACATTGGT--
-C---
Cui8097 G---AG--------------------CTTGGTCAACATTGGT--
-C---
909Cui6740 G---AG--------------------TCTGGTAAACATTGGT--
-C---
W1258Dai24695 G---AG--------------------TCTGGTAAACATTGGT-
--C---

JV0110_26_Ceriporia_griseoviol G---GA--------------------ATCGAACGGCGGTAAG---CAG-

896Dai13202 G---GA--------------------ATCGAACGGCGGTAAG---CAG-

LWY393Dai27053C_griseoviolasce G---GA--------------------ATCGAACGGCGGTAAG---CAG-

LWY394DAI27054 G---GA--------------------ATCGAACGGCGGTAAG---CAG-

FP135015G_pannocinctus G-TCGG----------------------TTGATGC-----------TG

L15726SpG_pannocinctus G-TCGG----------------------TTGATGC-----------TG

Dai22221 G-TCGA----------------------TGGAGGC-----------TG

Dai22633 G-TCGG----------------------TGGAGGC-----------TG

Dai23260 G-TCGG----------------------TGGAGGC-----------TG

Dai23626 G-TCGG----------------------TGGAGGC-----------TG

Dai16238G_citrinoalbus A-TTGG----------------------TGAATAAA--TGGGCTTTCAA

1175Dai15293 A-TTGG----------------------TGAATAAA--TGGGCTTTCAA

Dai19547 A-TTGG----------------------TGAATAAA--TGGGCTTTCAA

918063G_africanus A-CTGG----------------------TGAATGAA--TGGGCCTTTTG

918572G_africanus A-CTGG----------------------TGAATGAA--TGGGCCTTTTG

Dai18536A A-TGGG----------------------TGAAGGAA--TGGGTGCAAAA

1164Cui17922 A-TGGG----------------------TGAAGGAA--TGGGTGCAAAA

Dai22225 A-TGGG----------------------TGAAGGAA--TGGGTGCAAAA

1163Dai20655 ---AA

Yuan4397G_hainanensis G-TTGG----------------------TGAATGAC--TGGCATTT-TT

1176Dai15268 --

1177Dai15259 G-TTGG----------------------TGAATGAC--TGGCAATTTTT

BZ2896G_thelephoroides G-TTGG----------------------TGAAAGAC--TGGCTTT----

1166JV1808_26 G-TTGG----------------------TGAAAGGC--TGGCTTT----

Miettinen16992Hapalopilus_ochr GATTGA--------------------GTCGTTGGC---TGGC---TGTT

GC1708_338_Ceriporia_arbuscula GG--AA------------------------------TTTGAC-------

WCG1555Dai26107Ceriporia ---

GC1708_340_Ceriporia_arbuscula GG--AA------------------------------TTTGAC-------

WCG1556Dai26109Ceriporia GG--AA------------------------------TTTGAC-------

883Cui11291 GG--AA------------------------------TTTGAT-------

HLX320Dai26805 GG--AA------------------------------TGTGAT-------

WCG1266Dai24678A GG--AA------------------------------TGGGAT-------

Dai6090_Ceriporia_sulphuricolo GG--AA----------------------CGGAGGC---TGCC-------

RLG_11354_Ceriproia_reticulata GAAAAG--------------------GCTGGGAATA--C----------

ZZW1543Dai27072 GAAAAG--------------------GCTGGGAATA--C----------

Li1316_Ceriporia_reticulata AAAGAG--------------------GCTGGGAACA--T----------

KHL11981Ceriporia_reticulata AAAGAG--------------------GCTGGGAACA--T----------

FP110343sp_Candelabrochaete_la ATAAAG--------------------GCTGAGGATC--TA---------

Li1045_Ceriporia_reticulata AAGAGG--------------------GCTGAGAAGG--TAGTG-ATAC-

ZX136Dai25794ceriporia AAGAGA--------------------GCTGAGAAGG--TAGTG-ATAC-

892Dai13400 AAGAGG--------------------GCTGAGAAGG--TAGTG-ATAC-

RLG7163Leptoporus_mollis AAGAAA--------------------TCAAGATGTA--AAA--------

Dai21062Leptoporus_mollis AAGAAA--------------------TCAAGATGTA--AAA--------

Dai20182Leptoporus_submollis AAGAAA--------------------TCAAGACGTA--AAA--------

Cui18379Leptoporus_submollis AAGAAA--------------------TCAAGATGTA--AAA--------

Wu1209_46Resiniporus_pseudogil GAGTAA--------------GATTTTTCTGTTGCTGGTAGTA------

BRNM710169Resiniporus_resinasc GAGTAA--------------GATTTTTCTGTTGCTGGTAGTA------

Dai14516Bjerkandera_adusta G-CCGG------------------CTTGTGGGTG--GTTCGCGCACTT-

Dai21100Bjerkandera_fumosa G-CCGG------------------CTTGTGGGTGCAGTTCGCGTGTGT-

Miettinen16854Ceraceomyces_sp A---AA------------------TGACTGAAAGC---TGACT------

Dai10477C_spissa G---AA----------------------TAGGTGATATCAGTCAA----

855Dai16831 G---AA----------------------

TAGGTGATATCAGTCAA----
882Cui11282 G---AA----------------------
TAGGTGATATCAGTCAA----
Dai24566 G---AA----------------------
TGGGTGATATCAGCCAA----
Yuan5965 G---AA----------------------
TGGGTGATATTAGCCTT----
Dai3204 --
1194CUI9985 --

Dai15205_Ceriporia_albomellea --GCAAGTT-GGTGAAATA--AACCTTT-------
CCTATG--TTTTAAT
Dai15223_Ceriporia_albomellea --GCAAGTT-GGTGAAATA--AACCTTT-------
CCTATG--TTTTAAT
Li1780_Ceriporia_variegata --GCAAGTT-GGCGAAGT---AACCTTT-------CCTATG-
-TTTTAAT
Dai19791_Ceriporia_variegata --GCAAGTT-GGCGAAGT---AACCTTT-------
CCTATG--TTTTAAT
Dai19886 --GCAAGTT-GGCGAAGT---AACCTTT-------
CCTATG--TTTTAAT
Dai10833_Ceriporia_crassitunic --GCAAGTT-AGTGAAAGT--AACCTTT-------CCTATG-
-TTTTAAT
CHWC1506_46Meruliopsis_crassit --GCAAGTT-AGTGAAAGT--AACCTTT-------
CCTATG--TTTTAAT
Dai9995_Ceriporia_crassitunica --ATCAATT-AGCGA------AGCCTTT-------CCTATG--
TTTTAAT
Wu1209_58_Meruliopsis_parvispo ----AAGTC-AGCAGA-----TAGCCTT-------TCTATG--
-TTTTAC
CHWC1505_129_Meruliopsis_parvi ----AAGTC-RGCAGA-----TAGCCTT-------
TCTATG---TTTTAC
Dai21944 TAAAAAGTC-TGTAGATGGC---CTTT--------
CCTATG---TTTTAT
830Dai18640A ----AAGTC-TGCAGATAGC---CTTTT-------
CCTATG---TTTTAC
GC1704_60_Meruliopsis_taxicola ----AAGTC-AGTTGAAAAG---TCTTT-------CTTATG-
--TTTTAT
Dai22625 ----AAGTC-AGTTGAAAAG---TCTTT-------
CTTATG---TTTTAT
Dai22636 ----AAGTC-AGTTGAAAAG---TCTTT-------
CTTATG---TTTTAT
Dai21878 ----AAGTC-AGTTGAAAAG---TCTTT-------
CTTATG---TTTTAT
1169Dai17248 ----AAGTC-AGTTGAAAAG---TCTTT-------

CTTATG---TTTTAT

Wu1708_43_Meruliopsis_leptocys ----AARTC-AGTAGATGCC---CCTTT-------CCTATG--TTTTTAC

Li1011 ----AAATC-AGTAGATGCC---CCTTT-------CCTATG--TTTTTAC

ZX95Dai25742Meruliopsis_leptoc ----AAGTC-AGTAGATGCCTTTTCTTT-------CCTATG--TTTTTAT

WCG1306Dai24733 ----AAGTC-AGTAGATGCCTTTTCTTT-------CCTATG--TTTTTAT

LXL99Dai25816 ----AAGTC-AGTAGATGCCTTTTCTTT-------CCTATG--TTTTTAT

WCG1559Dai26052Meruliopsis ----AAGTC-AGTCGATATG--CCCTTT-------TCTATG--TTTTTAC

He7477 ----AAGTC-AGTCGATATG--CCCTTT-------TCTATG--TTTTTAC

HLX243Dai26217 ----AAGTC-AGTCGATATG--CCCTTT-------TCTATG--TTTTTAC

RussiaMW673659Meruliopsis_fagi ----AAGTC-AGTCGATATG--CCCCTT-------TCTATG--TTTTTAC

FD278 ----AAGTC-AGTCGATTGCCGGACTT--------TCTATGTTTTTTTAC

Dai10226_Ceriporia_tarda TATAAAGTT-AGTGGAAGAAAAATCCTT-------TCTACG--TTTTTAA

LE247365 TATAAAGTT-AGTGGAAGAAAAATCCTT-------TCTACG--TTTTTAA

Dai8173_Meruliopsis_nanlingens TAAAAAGTT-GGTAGAAAAG---ACCTT-------TCTATGTATTATTAT

860Dai17172 TAAAAAGTT-GGTAGAAAAG---ACCTT-------TCTATGTATTATTAT

879Dai13414 TAAAAAGTT-GGTAGAAAAG---ACCTT-------TCTATGTATTATTAT

Li_1704_Meruliopsis_pseudocyst ----AAGTC-AGTCAGAAAA----CCTT-------TCTATG--TTTTTAT

833Dai18405 ----AAGTC-AGTCAGAAAA----CCTT-------TCTATG--TTTTTAT

HHB_10729_Meruliopsis_albostra ----AAGTC-GGTCAGAAAA----CCTT-------TCTATG--TTTTTAT

Cui6878_Ceriporia_pseudocystid ----AAGTCAAGTCAGAAAA---GCCTT-------CCTATG--TTTTTAT

869Dai14737 ----AAGTCAAGTCAGAAAA---GCCTT-------CCTATG--TTTTTAT

876Cui11626 ----AAGTCAAGTCAGAAAA---GCCTT-------CCTATG--TTTTTAT

1199WEI3388 ----AAGTCAAGTCAGAAAA---GCCTT-------

CCTATG--TTTTTAT
776308_Meruliopsis_cystidiata ----GGGTC-------GGAA---GCCTT-------CCTATG--TTTTTAT
ICN139059_Meruliopsis_cystidia ----GGGTC-------AGAA---GCCTT-------CTTATG--TTTTTAT
HHB15692Ceraceomyces_serpens ----TAGCC-GGTTGAAGGC-CTCTTTA------TCCTATG---TTTTAT
HHB_15629_Sp_Ceriporiopsis_ane --------C-TTTGTGTAATAAAGCCTT------TCTTATG----TTTAC
AJ185Trametopsis_cervina -ATTTACTC-TCTTGGTAACAAGGCCTC------TCTTATG----TTTAC
FD9Irpex_lacteus ---TCCAGG-ATCTCGCGAGAGGTCTTCGGTCTTTCTTATG---TTTTAC
908Dai11230 ---TCCAGG-ATCTCGCGAGAGGTCTTCGGTCTTTCTTATG---TTTTAC
FP55521TEmmia_lacerata -TATTTAAT-AGTTGGTAACAAGCCTTT-------CTTATG----TTTAC
PBU0048Ceriporia_cystidiata -TATTTAAT-AGTCGGTAATAAGCCTTT-------CTTATG----TTTAC
MZ340C_lacerataT -TATTTATT-AGTTGGTAATAAGCCTTT-------CTTATG----TTTAC
Dai21940 -TATTTATT-AGTTGGTAATAAGCCTTT-------CTTATG----TTTAC
847Dai16433 -TATTTAAT-AGTCGGTAATAAGCCTTT-------CTTATG----TTTAC
MarcinEmmia_latemarginatus -TATTTAAT-AGTTGGTRACAAGCCTTT-------CTTATG----TTTAC
Meijer3729Hydnopolyporus_fimbr --TTTAGTT-AGTTGGTCATAAGCCTTT-------CTTATG---TTTTAC
RLG13408Phanerochaete_sp -CATTGGTT-GGTTGAT----GACCTTT-------CTTATG---TTTTAC
WHC1381Flavodon_flavus -TGTAAAGA-AGTCAGTGACAAGYCTTC------TCTTGCG---TTTTAC
GB1833Phlebia_albida -CTTTTGTT-AGCTGGTGG-AAGCCGTT------TCTTATG----TTTAT
T407Phlebia_nitidula -CTTTTGTT-AGCTGGTGGAAAGCTTTT------TCTTATG----TTTAT
HHB6988Phanerochaete_exilis --TTTCATT-GGTCGGTTTTGAGCCTTT-------CTTATG---TTTTAC
HHB8509Phanerochaetella_xeroph --TTTAATC-GGTCAGCTTGAAGCCTTT-------CTTATG---TTTTAC
PBU0051Macrohyporia_dictyopora --TTTAATC-GGTTGG--TGAAGCCTTT-------CTTATG---TTTTAT
HHB11463Phanerochaete_sp -TGTTAATTCGGTCGGTTTGAAGCCGTG------

TCTTACG---TTTTAC
FP102382Byssomerulius_corium -TTTGAGTC-GGTAAG---AAAGCCTTT------
TCTTATG---TTTTAC
FP102165Efibula_americana -ATCTGCTT-TGCGGGTGCTGAAGCCTTAGT---
TCTTATG---TTTTAC
Murdoch90Ceriporia_torpida -----AGTT-CAGA-------
AGTCTTTTGTCTTTCCTATGTTTTTTAC
Rivoire4413_Ceriporia_purpurea -----AGTT-TGCA-------AGTCTTTTATCCTTCCTATG-
TTTTTTAC
Kout_18_Ceriporia_triumphalis ----TAGTT-CACACC-----
AGTCTCTCGACCGTCCTATG--TTTTTAC
Rivoire3701_Ceriporia_bresadol -----AGTT-TACA-------AGTCTTTCGATTTTCCTATG--
TTTTTAC
VS4018 -----AGTT-TACA-------
AGTCTTTCGACTTTCCTATG--TTTTTAC
Ryvarden21832_Ceriporia_manzan -----AGTT-TACA-------
AGTCTTTTGACCTTCCTATG--TTTTTAC
Dai24539 -----AGTT-TACA-------
AGTCTTTCGACTTTCCTATG--TTTTTAC
Dai24541 -----AGTT-TACA-------
AGTCTTTCGACTTTCCTATG--TTTTTAC
JV1105_12_Ceriporia_occidental -----AGTT-CACG-------AAGTCTTTTACCTTCCTATG-
-TTTTTAC
VS8558Ceriporia_occidentalis -----AGTT-CACG-------AAGTCTTTTACCTTCCTATG-
-TTTTTAC
Dai22445 -----AGTT-TGCA-------
AGTCTTTTATCCTTCCTATG-TTTTTTAC
846Dai16368 -----AGCT-TGCA-------
AGTCTGTTATCCTTTCTATG--TTTTTAC
Dai17951_Ceriporia_aurantiocar ----TTGTC-AGTTGA-----AGTTTTT-------TCTATG--
TTTTTAT
Miettinen_11701C_viridans ----GTGTC-AGTGGA-----AATCTTT-------CCTATG--
TTTTTAT
JV0105_10Ceriporia_aurantiocar ----TTGTC-AGTTGA-----AGTTTTT-------TCTATG--
TTTTTAT
Yuan5702C_viridans ----GTGTC-AGTGGA-----AATCTTT-------CCTATG--
TTTTTAT
858Dai17003 ----GTGTC-AGTGGA-----AATCTTT-------
CCTATG--TTTTTAT
Yuan2747_Ceriporia_viridans ----TTGTC-AGTAGA-----AGTCTTT-------TCTATG--
TTTTTCT
Yuan2744C_viridans ----TTGTC-AGTAGA-----AGTCTTT-------TCTATG--
TTTTTCT
Li1046C_viridans ----TTGTC-AGTGGA-----AGTCTTT-------CCTATG--

TTTTTCT

865C_sinoviridans ----TTGTC-AGTGGA-----AGTCTTT-------CCTATG--TTTTTCT

871Dai15062 ----TTGTC-AGTAAA-----TGTCTTT-------TCTATG--TTTTTCT

Dai7642_Ceriporia_humilis -----GGTC-TTCA-G-----AGTCTCC-------CCTAC-GTTTTTTAC

Spirin4706_Ceriporia_humilis -----GGTC-TTCA-G-----AGTCTCC-------CCTAC-GTTTTTTAC

Spirin4944_Ceriporia_sericea -----GGCC-TTCA-G-----AGTCTCT-------TCTAYGTCTTTTTAC

WCG1547Dai26044ceriporia -----GGCC-TTCA-G-----AGTCTCT-------TCTAT-TTTTTTTAC

ZZW1558Dai27086 --TTTTTTTAC

Miettinen14381_Ceriporia_mpuri -----GGTC-TTCA-A-----AGTCTCTC-----GCCTACG--TTTTTTT

Miettinen15492_2_Ceriporia_sor -----GGCC-TTTG-A-----AGCCTTT-------CTTACG--TTTTTAT

He6687 -----GGTC-TTCA-A-----AGTCTCA-------CCTACGTTTTTTTAC

ZH53Dai24426 -----GGTC-TTCG-A-----AGTCTCG-------CCTACGTTTTTTTAC

Vlasak0808_30_Ceriporia_punica -----GGTC-AGCGTC-----AGTTGATCTTGACTCTTATG--TTTTCAC

887Dai13376 -----GGTC-AGCGTC-----AGTTGATCTTGACTCTTATG--TTTTCAT

WCG1443Dai24998 -----GATC-AGCGTC-----AGTTGATGTTGACTCTTATG--TTTTCAT

0108_6Ceriporia_spissa --TTCAGTC-GACTGC-----AGTCTCC-----TTCTTACGTCTTTTCAT

Dai19164 --TTCAGTC-GACTGC-----AGTCTCC-----TTCTTACGTCTTTTCAT

Dai17937_Ceriporia_bubalinomar --TGAAGTC-AACTGT-----AG-CTTC-----TTCTTACGTCTTT--AT

903Dai12113 --TGAAGTC-AACTGT-----AG-CTTC-----TTCTTACGTCTTT--AT

LZB929Dai25079 ----AAATC-AGTTGA-----TGCTGCT-------TCTATG--TTTTTAT

LX45Dai26988 ----AAATC-AGTTGA-----TGCTGCT-------TCTATG--TTTTTAT

LX43Dai26986 ----AAATC-AGTTGA-----TGCTGCT-------TCTATG--TTTTTAT

Dai7759Ceriporia ----AAATC-AGTTGA-----TGCTGCT-------TCTATG--TTTTTAT

Cui8012_Ceriporia_viridans -----TGTC-AGTTGA-----AGTCTTT-------CTTATG--TTTTTAT

GC1704_54Ceriporia_viridans -----TGTC-AGTTGA-----AGTCTTT-------CTTATG--TTTTTAT

Dai23392 -----TGTC-AGCGGA-----AGTCTTT-------CTTATG--TTTTTAT

WCG1585Dai26113Ceriproia -----TGTC-AGCGGA-----AGTCTTT-------CTTATG--TTTTTAT

Dai18675C_eucalypti -----TGTC-AGCTGA-----AGTCTTT-------CTTATG--TTTTTAT

Dai22034 AAAATATCA-AGTTGA-----AGTCTTT-------CCTATG--TTTTTAT

JV1008_41JTardaFLORIDAKeys AAAATATCA-AGTTGA-----AGTCTTT-------CCTATG--TTTTTAT

Rivoire1161_Ceriporia_pierii ----TTGAT-CTCTGA-----AGCCCCT---CTCTTCTATG--TTTTTAT

Dai23499C_pierii ----TTGAT-CTCTGA-----AGCCCCT---CTCTTCTATG--TTTTTAC

Dai23500 ----TTGAT-CTCTGA-----AGCCCCT---CTCTTCTATG--TTTTTAC

841Dai15899 -TGAAAGTC-AGTGGA-----AGTCTTT-------TCTATG-TTTTTTAC

842Dai15904 -TGAAAGTC-AGTGGA-----AGTCTTT-------TCTATG-TTTTTTAC

LZB1066xinjiang --TTTTTAC

LZB1065xinjiang --TTTTTAC

851Dai16779 ---------------------AGTCTCT--CTCTTCTTATG-TCTTTTAT

RMJ119sp_Candelabrochaete_sept -------TC-GTCGGATTTCAAGTCCTC-----TGCTTACGTTTTTTGAT

RLG9759spCandelabrochaete_sept -------TC-GTCGGATTTCAAGTCCTC-----TGCTTACGTTTTTTGAT

RLG10478Phanerochaete_allantos ----------AGTCGGCGTGAAGCCGCT-------CCTACG---TTTTAT

Dai19118_Ceriporia_spissa TTTCATGGCTGGTGAA-----TATCCTCTC----TCTTGTG--TTTTTAT

Dai18486A TTTCATGGCTGGTG-A-----TATCCTCTC----TCTTGTG--TTTTTAT

WEI17_024_Ceriporia_mellita TTTCATGGCTAGTG-A-----TCTCCTCTC----TCTTGTG--TTTTTAT

GC1508_71Ceriporia_mellita TCTCACGGCTAGTG-A-----TCTCCTCTC----TCTTGTG--TTTTTAT

GC1608_7_Ceriporia_mellita TTTCATGGCTAGTG-A-----TCTCCTCTC----TCTTGTG--TTTTTAT

ZZW1557Dai27085 TTTCATGGCTAGTG-A-----TCTCCTCTC----
TCTTGTG--TTTTTAT
ZZW1554Dai27083 TTTCATGGCTAGTG-A-----TCTCCTCTC----
TCTTGTG--TTTTTAT
Dai8168 TTTCACGGCTAGTG-A-----TCTCCTCTC----
TCTTGTG--TTTTTAT
BR4865C_mellita TTTCATGGCTGGTG-A-----TATCCTCTC----
TCTTGTG--TTTTTAT
MEL2382688Ceriporia_sp TTCAGTGGCTAGTG-A-----CATCCTTGC----
TCTTGTG--TTTTTAT
Dai8110 TTTCACGGCTAGTG-A-----TTTCCTCTC----
TCTTGTG--TTTTTAT
Cui8097 TTTCACGGCTAGTG-A-----TTTCCTCTC----
TCTTGTG--TTTTTAT
909Cui6740 TTCAGTGGCTAGTG-A-----CATCCTTGC----
TCTTGTG--TTTTTAT
W1258Dai24695 TTCAGTGGCTAGTG-A-----CATCCTTGC----
TCTTGTG--TTTTTAT
JV0110_26_Ceriporia_griseoviol GTGAAAGCCTGATG-A-----AGCCTTTGC----
TCTTATG--TTTTTAT
896Dai13202 GTGAAAGCCTGATG-A-----AGCCTTTGC----
TCTTATG--TTTTTAT
LWY393Dai27053C_griseoviolasce GTGAAAGCCTGATG-A-----AGCCTTTGC----
TCTTATG--TTTTTAT
LWY394DAI27054 GTGAAAGCCTGATG-A-----AGCCTTTGC----
TCTTATG--TTTTTAT
FP135015G_pannocinctus GCGCAAGTC-AGTTTG-----AAGCCTT-------
CCTATG---TTTTAT
L15726SpG_pannocinctus GCGCAAGTC-AGTTTG-----AAGCCTT-------
CCTATG---TTTTAT
Dai22221 GCGCAAGTC-AGTTTG-----AAACCTT-------
CCTATG---TTTTAT
Dai22633 GCGCAAGTC-AGTTTG-----AAGCCTT-------
CCTATG---TTTTAT
Dai23260 GCGCAAGTC-AGTTTG-----AAGCCTT-------
CCTATG---TTTTAT
Dai23626 GCGCAAGTC-AGTTTG-----AAGCCTT-------
CCTATG---TTTTAT
Dai16238G_citrinoalbus ACCCTAG---TTTTTG-----AAGCCTC-------TCTATG-
TTTTTAAT
1175Dai15293 GCCCTAG---TTTTTG-----AAGCCTC-------
TCTATG-TTTTTAAT
Dai19547 GCCCCAG---TTTTTG-----AAGCCTC-------
TCTATG-TTTTTAAT

918063G_africanus GCCCTAG----TTTTG-----AAGCCTC-------TCTATG-TTTTTAAT

918572G_africanus GCCCTAG----TTTTG-----AAGCCTC-------TCTATG-TTTTTAAT

Dai18536A GCCCGGG----TTCTG-----AAGCCTT-------TCTATGTTTTTTAAT

1164Cui17922 GCCCGGG----TTCTG-----AAGCCTT-------TCTATGTTTTTTAAT

Dai22225 GCCCTAG----TTCTG-----AAGCCTT-------TCTATG-TTTTTAAT

1163Dai20655 GCCCTAG----TTCTG-----AAGCCTT-------TCTATG-TTTTTAAT

Yuan4397G_hainanensis ACAAATGTC-AGTTTG-----AAGCCTT-------CCTATG---TTTTAT

1176Dai15268 ----ATGTC-AGTTTG-----AAGCCTT-------CCTATG---TTTTAT

1177Dai15259 ACAAATGTC-AGTTTG-----AAGCCTT-------CCTATG---TTTTAT

BZ2896G_thelephoroides ----AAGTC-GGTCTG-----AGGCCTT-------CCTATG---TTTTAT

1166JV1808_26 ----AAGTC-GGTCTG-----AGGCCTT-------CCTATG---TTTTAT

Miettinen16992Hapalopilus_ochr TGTTAGGTC-AGCTAA-----AGGCTCTATC--TTCCTATG--TTTTTAT

GC1708_338_Ceriporia_arbuscula -AGAAAGCCTATGGGCTGAAA-GTCTTA------TCTTATGATTTTTTAC

WCG1555Dai26107Ceriporia ---TTTTTTAC

GC1708_340_Ceriporia_arbuscula -AGAAAGCCTATGGGCTGAAA-GTCTTA------TCTTATGATTTTTTAC

WCG1556Dai26109Ceriporia -AGAAAGCCTATGGGCTGAAA-GTCTTA------TCTTATGATTTTTTAC

883Cui11291 -GAAAAGCTTTATTGCTGGAA-GTCTTT------TCTTATG--TTTTTAT

HLX320Dai26805 -GAAAAGCTTCATTGCTTGCA-TTCATT------TCTTATG--TTTTAAT

WCG1266Dai24678A -GAAAAGCTTAATTGCTGGAAGTCCGCT------TCTTATG--TTTTCAT

Dai6090_Ceriporia_sulphuricolo ----------AGTCAGCTCAGAGTCGTC----TCTTCTATG--TTTTTAC

RLG_11354_Ceriproia_reticulata ----TTCTCAGTTCAA-----TGTCTGC-------TCTGTGTRATTTTTA

ZZW1543Dai27072 ----TTCTCAGTTCAA-----TGTCTGC-------TCTGTGTAATTTTTA

Li1316_Ceriporia_reticulata ----TTCTCAGTTCGA-----TGTTTGC-------

TCTGTGTAATTTTTA
KHL11981Ceriporia_reticulata ----TTCTCAGTTCGA-----TGTTTGC-------TCTGTGTAATTTTTA
FP110343sp_Candelabrochaete_la ----TTCTCAGTTTGA-----CGTTTGT-------TCTGTGTAATTTTTC
Li1045_Ceriporia_reticulata --TTCTCTCAGTTTAA-----TGTCTAC-------TCTGTGTAATTT---
ZX136Dai25794ceriporia --TTCTCTCAGTTTAA-----TGTCTAC-------TCTGTGTAATTT---
892Dai13400 --TTCTCTCAGTTTAA-----TGTCTAC-------TCTGTGTAATTT---
RLG7163Leptoporus_mollis ----AATCTTGGTGGA-----AGTCTTT-------TCTATG-TTTTTTCT
Dai21062Leptoporus_mollis ----AATCTTGGTGGA-----AGTCTTT-------TCTATG-TTTTTTCT
Dai20182Leptoporus_submollis ----AATCTTGGTGGA-----AGTCTTT-------TCTATGTTTTTTTCT
Cui18379Leptoporus_submollis ----AATCTTGGTGGA-----AGTCTTT-------TCTATGTTTTTTTCT
Wu1209_46Resiniporus_pseudogil GCAAAAATT------------GGCCTCT-------CTTATG-----TTAC
BRNM710169Resiniporus_resinasc GCAAAAATT------------GGCCTCT-------CTTATG-----TTAC
Dai14516Bjerkandera_adusta GTAGGTGTC-------------------------CTTATG-CTTTAT--
Dai21100Bjerkandera_fumosa CTGTAGGTC-------------------------TTTATG-CTTTATAC
Miettinen16854Ceraceomyces_sp CTGTCAGTC------------CGTCTTT-------TCTATG---CTTTAT
Dai10477C_spissa ----------GGCTGATTTTGAGCTCTT-------CTTATG-TTTTCAAT
855Dai16831 ----------GGCTGATTTTGAGCTTTT-------CTTATG-TTTTCAAT
882Cui11282 ----------GGCTGATTTTGAGCTCTT-------CTTATG-TTTTCAAT
Dai24566 ----------GGCTGATTTTGAGCTCTT-------CTTATG--TTTTAAT
Yuan5965 ----------GGCTGATTTTGAGCTCTT-------CTTATG--TTTTAAT
Dai3204 ---
1194CUI9985 ---

Dai15205_Ceriporia_albomellea TATAAACACTTTA-GTTATAGAATGTTT----

ATATGC--AGATAATGCA

Dai15223_Ceriporia_albomellea TATAAACACTTTA-GTTATAGAATGTTT----ATATGC--AGATAATGCA

Li1780_Ceriporia_variegata TATAAACGCTTTA-GTTATAGAATGTTT----ATATGC--AGATAATGCA

Dai19791_Ceriporia_variegata TATAAACGCTTTA-GTTATAGAATGTTT----ATATGC--AGATAATGCA

Dai19886 TATAAACGCTTTA-GTTATAGAATGTTT----ATATGC--AGATAATGCA

Dai10833_Ceriporia_crassitunic TAAAAACACTTTG-GTTATAGAATGTTT----GTATGC--AGATAATGCA

CHWC1506_46Meruliopsis_crassit TAAAAACACTTTG-GTTATAGAATGTTT----GTATGC--AGATAATGCA

Dai9995_Ceriporia_crassitunica TAAAAACAATTCA-GTCATAGAATGTTT----GTATGT--GCATAATGCA

Wu1209_58_Meruliopsis_parvispo TACAAACGCTTCA-GTTATAGAATG-TT-T--ACTTGC--GAATAACGCA

CHWC1505_129_Meruliopsis_parvi TACAAACGCTTCA-GTTATAGAATG-TT-T--ACTTGC--GAATAACGCA

Dai21944 TACAAACGCTTCA-GTTATAGAATG-TT-T--ATTTGC--GAATAACGCA

830Dai18640A TACAAACGCTTCA-GTTATAGAATG-TT-T--ACTTGC--GAATAACGCA

GC1704_60_Meruliopsis_taxicola TACACACGCTTCA-GTTATAGAATG-TT-T--ACTTGC---GATAACGCA

Dai22625 TACACACGCTTCA-GTTATAGAATG-TT-T--ACTTGC---GATAACGCA

Dai22636 TACACACGCTTCA-GTTATAGAATG-TT-T--ACTTGC---GATAACGCA

Dai21878 TACACACGCTTCA-GTTATAGAATG-TT-T--ACTTGC---GATAACGCA

1169Dai17248 TACACACGCTTCA-GTTATAGAATG-TT-T--ACTTGC---GATAACGCA

Wu1708_43_Meruliopsis_leptocys TACCAACGCTTCA-GTTATAGAATG-TT-T--ACTTGC--GGATAACGCA

Li1011 TACCAACGCTTCA-GTTATAGAATG-TT-T--ACTTGC--GGATAACGCA

ZX95Dai25742Meruliopsis_leptoc TACAAACGCTTCA-GTTATAGAATG-TT-T--ACTTGC--AGATAATGCA

WCG1306Dai24733 TACAAACGCTTCA-GTTATAGAATG-TT-T--ACTTGC--AGATAATGCA

LXL99Dai25816 TACAAACGCTTCA-GTTATAGAATG-TT-T--ACTTGC--AGATAATGCA

WCG1559Dai26052Meruliopsis TACACACGCTTCA-GTTATAGAATG-TT-T--

ACTTGC--GGATAACGCA
He7477 TACACACGCTTCA-GTTATAGAATG-TT-T--
ACTTGC--GGATAACGCA
HLX243Dai26217 TACACACGCTTCA-GTTATAGAATG-TT-T--
ACTTGC--GGATAACGCA
RussiaMW673659Meruliopsis_fagi TACACACGCTTTA-GTTATAGAATG-TT-G--
ACTTGC--GGATAACGCA
FD278 TACAAACGCTTCA-GTTATAGAATG-TCAT--
ACTTGC--GAATAACGCA
Dai10226_Ceriporia_tarda TACAAACGCTTCA-GTTATAGAATG-TT-T--
ACTTGC--AGATAATGCA
LE247365 TACAAACGCTTCA-GTTATAGAATG-TT-T--
ACTTGC--AGATAATGCA
Dai8173_Meruliopsis_nanlingens TACAAACGTTTCA-GTTATAGAATG-TT-T--
ACTTGC--GAATAACGCA
860Dai17172 TACAAACGTTTCA-GTTATAGAATG-TT-T--
ACTTGC--GAATAACGCA
879Dai13414 TACAAACGTTTCA-GTTATAGAATG-TT-T--
ACTTGC--GAATAACGCA
Li_1704_Meruliopsis_pseudocyst TACAAACGCTTCA-GTTATAGAATG-TT-G--
ACTTGCA-AAATAATGCA
833Dai18405 TACAAACGCTTCA-GTTATAGAATG-TT-G--
ACTTGCA-AAATAATGCA
HHB_10729_Meruliopsis_albostra TACAAACGCTTCA-GTTATAGAATG-TC-A--
ACTTGCA-AAATAATGCA
Cui6878_Ceriporia_pseudocystid TATAAATGCTTCA-GTTATAGAATG-TT-G--
ACTTGCA-ACATAATGCA
869Dai14737 TATAAACGCTTCA-GTTATAGAATG-TT-G--
ACTTGCA-ACATAATGCA
876Cui11626 TATAAACGCTTCA-GTTATAGAATG-TT-G--
ACTTGCA-ACATAATGCA
1199WEI3388 TATAAACGCTTCA-GTTATAGAATG-TT-G--
ACTTGCA-ACATAATGCA
776308_Meruliopsis_cystidiata TACAAACGCTTCA-GTTATAGAATG-TT-A--
ATTTGC--GGATAACGCA
ICN139059_Meruliopsis_cystidia TACAAACGCTTCA-GTTATAGAATG-TT-A--
ATTTGC--GGATAACGCA
HHB15692Ceraceomyces_serpens TACAAACGCTTCA-GTATCAGAATG-TTTA--
TCTTGC----GTAACGCA
HHB_15629_Sp_Ceriporiopsis_ane TACAAACGCTTCA-GTTATAGAATG-TC-T--
ATTTGTGT--ATAACACA
AJ185Trametopsis_cervina TACAAACGCTTCA-GTTATAGAATG-TCAT--
ACTTGTGT--ATAACACA
FD9Irpex_lacteus TACAAACGCTTCA-GTTATAGAATG-T--C--

AACTGTGT--ATAACACA
908Dai11230 TACAAACGCTTCA-GTTATAGAATG-T--C--
AACTGTGT--ATAACACA
FP55521TEmmia_lacerata TACAAACGCTTCA-GTTATAGAATG-T--T--
TACTGTGT--ATAACACA
PBU0048Ceriporia_cystidiata TACAAACGCTTCA-GTTATAGAATG-T--T--
TACTGTGT--ATAACACA
MZ340C_lacerataT TACAAACGCTTCA-GTTATAGAATG-T--T--
TACTGTGT--ATAACACA
Dai21940 TACAAACGCTTCA-GTTATAGAATG-T--T--
TACTGTGT--ATAACACA
847Dai16433 TACAAACGCTTCA-GTTATAGAATG-T--T--
TACTGTGT--ATAACACA
MarcinEmmia_latemarginatus TACAAACGCTTCA-GTTATAGAATG-T--T--
TACTGTGT--ATAACACA
Meijer3729Hydnopolyporus_fimbr TACAAACGTTTCA-GTTCTAGAATG-TCTT--
TACTGTGT--ATAACACA
RLG13408Phanerochaete_sp TACAAACGTCTCA-GTTATAGAATG-T--T--
TACTGTGT--ATAACACA
WHC1381Flavodon_flavus CACAAACACTTCA-GTTATAGAATG-T--T--
TACTGTGT--ATAACACA
GB1833Phlebia_albida TACAAACGCTTCA-GTTTTAGAATG-T--C--
TACTGTGT--ATAACACA
T407Phlebia_nitidula TACAAACGCTTCA-GTTTTAGAATG-T--T--
TACTGTGT--ATAACACA
HHB6988Phanerochaete_exilis TACAAACGCTTCA-GTTATAGAATG-TT-T--
TCATGTGT--ATAACACA
HHB8509Phanerochaetella_xeroph TACAAACGCTTCA-GTTATAGAATG-TC-T--
TACTGTGT--ATAACACA
PBU0051Macrohyporia_dictyopora TACAAACGCTTCA-GTTATAGAATG-T--T--
TACTGTGT--ATAACACA
HHB11463Phanerochaete_sp TACAAACGCTTCA-GTTATAGAATG-TT----
TACTGTGT--ATAACACA
FP102382Byssomerulius_corium TACAAACGCTTCA-GTTATAGAATG-T--T--
TACTGTGT--ATAACACA
FP102165Efibula_americana TACAAACGCTTCA-GTTTTAGAATG-T--T--
TACTGTGT--ATAACACA
Murdoch90Ceriporia_torpida TACAAACGCTTCA-GTTACAGAATG-TTTA-
CCTTTGCGT--GTAACGCA
Rivoire4413_Ceriporia_purpurea TACAAACGCTTCA-GTTAAAGAATG-TCTT-
ACTTTGCGT--ACACCGCA
Kout_18_Ceriporia_triumphalis TACAAACGCTTCA-GTTACAGAATG-TCTT-
ACTTTGCGT-AACATCGCA
Rivoire3701_Ceriporia_bresadol TACAAACGCTTCA-GTTACAGAATG-TCTT-

ACTTTGCGT--ACAACGCA
VS4018 TACAAACGCTTCA-GTTACAGAATG-TCTT-
ACTTTGCGT--ACAACGCA
Ryvarden21832_Ceriporia_manzan TACAAACGCTTCA-GTTACAGAATG-TCTT-
ACTTTGCGT--ACAACGCA
Dai24539 TACAAACGCTTCA-GTTACAGAATG-TCTT-
ACTTTGCGT--ACAACGCA
Dai24541 TACAAACGCTTCA-GTTACAGAATG-TCTT-
ACTTTGCGT--ACAACGCA
JV1105_12_Ceriporia_occidental TACAAACGCTTCA-GTTACAGAATG-TCTT-
ACTGTGCGT--ACATCGCA
VS8558Ceriporia_occidentalis TACAAACGCTTCA-GTTACAGAATG-TCTT-
ACTGTGCGT--ACATCGCA
Dai22445 TACAAACGCTTCA-GTTAAAGAATG-TCTT-
ACTTTGCGT--ACACCGCA
846Dai16368 CACAAACGCTTCA-GTTAAAGAATG-TCTT-
ACTTTGCGT--ACACCGCA
Dai17951_Ceriporia_aurantiocar CACAAACATTTCA-GTATCAGAATG-TAA---
CTTTGCAT--ATAATGCA
Miettinen_11701C_viridans TACAAACGCTTTA-GTAACAGAATG-TAA---
CTTTGCAT--ATAATGCA
JV0105_10Ceriporia_aurantiocar YACAAAMATTTCA-GTATCAGAATG-TAA---
CTTTGCAT--ATAATGCA
Yuan5702C_viridans TACAAACGCTTTA-GTAACAGAATG-TAA---
CTTTGCAT--GTAATGCA
858Dai17003 TACAAACGCTTTA-GTAACAGAATG-TAA---
CTTTGCAT--ATAATGCA
Yuan2747_Ceriporia_viridans TACAAATGCTTCA-GTAACAGAATG-TCA---
CTTTGCAT--ATAATGCA
Yuan2744C_viridans TACAAATGCTTCA-GTAACAGAATG-TCA---
CTTTGCAT--ATAATGCA
Li1046C_viridans TACAAATGTTTTA-GTATCAGAATG-TAA---
ATTTGCAT--ATAATGCA
865C_sinoviridans TACAAATGTTTTA-GTATCAGAATG-TAA---
ATTTGCAT--ATAATGCA
871Dai15062 TACAAACGCTTCA-GTAACAGAATG-TCA---
CTTTGCAT--ATAATGCA
Dai7642_Ceriporia_humilis TACAAACGCTTCA-GTCAATGAATG-TAAT--
CTCTGCGAA--AAACGCA
Spirin4706_Ceriporia_humilis TACAAACGCTTCA-GTCAATGAATG-TAAT--
CTCTGCGAA--AAACGCA
Spirin4944_Ceriporia_sericea TACAAACGCTTCA-GTCAATGAATG-TAA---
CTCTGCGAA--AAACGCA
WCG1547Dai26044ceriporia TACAAACGCTTCA-GTCAATGAATG-TAA---

CTCTGCGAA--AAACGCA
ZZW1558Dai27086 TACAAACGCTTCA-GTCAATGAATG-TAA---
CTCTGCGAA--AAACGCA
Miettinen14381_Ceriporia_mpuri TTTA---
Miettinen15492_2_Ceriporia_sor CACAAACGCTTCA-GTTAAAGAATG-TCT---
GTCTGCGG----AACGCA
He6687 TATAAACGCTTCA-GTCAATGAATG-TAAT--
CTCTGCGAAAAAAACGCA
ZH53Dai24426 TATAAACGCTTCA-GTCAATGAATG-TAAT--
CTCTGCGAAATAAACGCA
Vlasak0808_30_Ceriporia_punica TACAAACATCT---GTATAAGAATG-TAA---
TCTTGCGT--CTAACGCA
887Dai13376 TACAAACATTT---GTATAAGAATG-TAA---
TCTTGCGT--CTAACGCA
WCG1443Dai24998 TACAAACATCT---GTATAAGAATG-TAA---
TTGTGCGT--CTAACGCA
0108_6Ceriporia_spissa CACAAACGTCTCA-GTCACAGAATG-
TCATTACTTTGCGG--ATAACGCG
Dai19164 CACAAACGTCTCA-GTCACAGAATG-
TCATTACTTTGCGG--ATAACGCG
Dai17937_Ceriporia_bubalinomar CACAAATATCTCA-GTAACAGAATG-
TTCTTACTTTGCGG--TTAACGCG
903Dai12113 CACAAATATCTCA-GTAACAGAATG-
TTCTTACTTTGCGG--TTAACGCG
LZB929Dai25079 CACACACGCTTCA-GTTATAGAATG-TAT---
CTCTGCGG--ATAACGCA
LX45Dai26988 CACACACGCTTCA-GTTATAGAATG-TAT---
CTCTGCGG--ATAACGCA
LX43Dai26986 CACACACGCTTCA-GTTATAGAATG-TAT---
CTCTGCGG--ATAACGCA
Dai7759Ceriporia CACACACGCTTCA-GTTATAGAATG-TAT---
CTCTGCGG--ATAACGCA
Cui8012_Ceriporia_viridans AACCAACATCATA-GTAACAGAATG-TAC---
AATTGCAT--ATAATGCA
GC1704_54Ceriporia_viridans AACCAACATCATA-GTAACAGAATG-TAC---
AATTGCAT--ATAATGCA
Dai23392 TACCAACATCATA-GTAACAGAATG-TTC---
AATTGCAT--ATAATGCA
WCG1585Dai26113Ceriproia TACCAACATCATA-GTAACAGAATG-TTC---
AATTGCAT--ATAATGCA
Dai18675C_eucalypti TATCAACATCATA-GTAAAAGAATG-TAA---
CATTGCAT--ATAATGCA
Dai22034 TACAAACA--TCA-GTAACAGAATG-TTT--
ACTTTGCAT--ATAATGCA

JV1008_41JTardaFLORIDAKeys TACAAACA--TCA-GTAACAGAATG-TTT--ACTTTGCAT--ATAATGCA
Rivoire1161_Ceriporia_pierii TACCAACGCTTCA-GTTAATGAATG-TCA---TTTTGCGG--ATAACGCA
Dai23499C_pierii TACCAACGCTTCA-GTTAATGAATG-TCA---TTTTGCGG--ATAACGCA
Dai23500 TACCAACGCTTCA-GTTAATGAATG-TCA---TTTTGCGG--ATAACGCA
841Dai15899 CACAAACGCATCA-GTCAAAGAATG-TTA---ATTTGCG--AATAACGCA
842Dai15904 CACAAACGCATCA-GTCAAAGAATG-TTA---ATTTGCG--AATAACGCA
LZB1066xinjiang CACAAACGCATCA-GTCAAAGAATG-TTA---ATTTGCG--AATAACGCA
LZB1065xinjiang CACAAACGCATCA-GTCAAAGAATG-TTA---ATTTGCG--AATAACGCA
851Dai16779 TATAAACGC-TCA-GTCAATGAATG-TAT---CTCTGCG--GATAACGCA
RMJ119sp_Candelabrochaete_sept CACAAACGATTCA-GTCAACGAATG-TCT---TCTTGCGAAAATAACGCA
RLG9759spCandelabrochaete_sept CACAAACGATTCA-GTCAACGAATG-TCT---TCTTGCGAAAATAACGCA
RLG10478Phanerochaete_allantos CACAAACATCTCA-GTCTCAGAATGT------ACTTGCA--GATAATGCA
Dai19118_Ceriporia_spissa CACAAACATGTCATGTTGTAGAATG-GTCT--TTATGC--AGATAATGCA
Dai18486A CACAAACATGTCATGTTGTAGAATG-GTCT--TTATGC--AGATAATGCA
WEI17_024_Ceriporia_mellita CACAAACATGTCATGTTGTAGAATG-GTCT--TTATGC--AGATAATGCA
GC1508_71Ceriporia_mellita CACAAACATGTCATGTTGTAGAATG-GTCT--TTATGC--AGATAATGCA
GC1608_7_Ceriporia_mellita CACAAACATATCATGTTGTAGAATG-GTCT--TTATGC--AGATGATGCA
ZZW1557Dai27085 CACAAACATATCATGTTGTAGAATG-GTCT--TTATGC--AGATGATGCA
ZZW1554Dai27083 CACAAACATATCATGTTGTAGAATG-GTCT--TTATGC--AGATGATGCA
Dai8168 CACAAACATATCATGTTGTAGAATG-GTCT--TTATGC--AGATGATGCA
BR4865C_mellita CACAAACATGTCATGTTGTAGAATG-GTCT--TTATGC--AGATAATGCA
MEL2382688Ceriporia_sp CACAAACGTATTATGTTGTGGAATG-TTCT--TCGTGC--AAATAATGCA

Dai8110 CACAAACATATCATGTTGTAGAACG-GTTT--TTGTGC--AAACGATGCA

Cui8097 CACAAACATATTATGTTGTAGAACG-GTTT--TTGTGC--AAACGATGCA

909Cui6740 CACAAACGTATTATGTTGTGGAATG-TTCT--TCATGC--AAATAATGCA

W1258Dai24695 CACAAACGTATTATGTTGTGGAATG-TTCT--TCGTGC--AAATAATGCA

JV0110_26_Ceriporia_griseoviol CACCAACATCTCA-GTCGCAGAATG-------TCATGT--TA--CATATA

896Dai13202 CACCAACATCTCA-GTCGCAGAATG-------TCATGT--TA--CATATA

LWY393Dai27053C_griseoviolasce CACCAACATCTCA-GTCGCAGAATG-------TCATGT--TA--CATATA

LWY394DAI27054 CACCAACATCTCA-GTCGCAGAATG-------TCATGT--TA--CATATA

FP135015G_pannocinctus TACAAACGCTTCA-GTTATAGAATG-T--TTACTTTGC-T--ATAACGCA

L15726SpG_pannocinctus TACAAACGCTTCA-GTTATAGAATG-T--TTACTTTGC-T--ATAACGCA

Dai22221 TACAAACGCTTCA-GTTATAGAATG-T--TTACTTTGC-T--ATAACGCA

Dai22633 TACAAACGTATCA-GTTATAGAATG-TCTT-ACTTTGC-T--ATAATGCA

Dai23260 TACAAACGTATCA-GTTATAGAATG-TCTT-ACTTTGC-T--ATAATGCA

Dai23626 TACAAACGTATCA-GTTATAGAATG-TCTT-ACTTTGC-T--ATAATGCA

Dai16238G_citrinoalbus CATAAACGTATTA-GTTATAGAATG-TCTTTACTTTGC-T--ATAATGCA

1175Dai15293 TATAAACGTATCA-GTTATAGAATG-TCTTTACTTTGC-T--ATAATGCA

Dai19547 CATAAACGTATTA-GTTATAGAATG-TCTTTACTTTGC-T--ATAATGCA

918063G_africanus CATAAACGTATCA-GTTATAGAATG-TCTTTACTTTGC-T--ATAATGCA

918572G_africanus CATAAACGTATCA-GTTATAGAATG-TCTTTACTTTGC-T--ATAATGCA

Dai18536A TACAAACGTATCA-GTCGAAGAATG-TCTTTACTTTGC-T--ATAACGCA

1164Cui17922 TACAAACGTATCA-GTCGAAGAATG-TCTTTACTTTGC-T--ATAACGCA

Dai22225 TACAAACGTATCA-GTCTAAGAATG-TCTTTACTTTGC-T--ATAACGCA

1163Dai20655 TACAAACGTATCA-GTCTAAGAATG-TCTTTACTTTGC-T--ATAACGCA

Yuan4397G_hainanensis TACAAACGCTTCA-GTTATAGAATG-TTTT--ACTTGC-T--ATAACGCA

1176Dai15268 TACAAACGCTTCA-GTTATAGAATG-TTTT--ACTTGC-T--ATAACGCA

1177Dai15259 TACAAACGCTTCA-GTTATAGAATG-TTTT--ACTTGC-T--ATAACGCA

BZ2896G_thelephoroides TATAAACGCTTCA-GTTATAGAATG-TTTT-AYTYTGC-T--ATAACGCA

1166JV1808_26 TATAAACGCTTCA-GTTATAGAATG-TTTA--CTTTGC-T--ATAACGCA

Miettinen16992Hapalopilus_ochr AATAAACGTTTTA-GTTTGTGAATGTTT----ACTTGCGT----AACGCA

GC1708_338_Ceriporia_arbuscula TACAAACACTAGATGTCAATGAATGTTTATT-ACTTGCG-AAATAACGCA

WCG1555Dai26107Ceriporia TACAAACACTAGATGTCAATGAATGTTTATT-ACTTGCG-AAATAACGCA

GC1708_340_Ceriporia_arbuscula TACAAACACTAGATGTCAATGAATGTTTATT-ACTTGCG-AAATAACGCA

WCG1556Dai26109Ceriporia TACAAACACTAGATGTCAATGAATGTTTATT-ACTTGCG-AAATAACGCA

883Cui11291 TACACACATTTGAAGTCGAAGAATGTTTATT-ACTTGCG--GATAACGCA

HLX320Dai26805 TATAAACACTTGAAGTTAAAGAATGGTTATT-A-TTGCA-G-ATAATGCA

WCG1266Dai24678A CACAAACACTTGAAGTCAAAGAATGGTTATT-ACTTGCA-GAATAATGCA

Dai6090_Ceriporia_sulphuricolo TACAAACGCTTCA-GTGTCAGGATGCTCAT-ATCGTGCGT----AACGCA

RLG_11354_Ceriproia_reticulata TACCAACTCTTTT-GTATATGAATG-TCTT---CTTGC--GGATAACGC-

ZZW1543Dai27072 TACCAACTCTTTT-GTATATGAATG-TCTT---CTTGC--GGATAACGC-

Li1316_Ceriporia_reticulata TACCAACTC--TT-GTATATGAATG-TCTT---CTTGC--AGATAATGCA

KHL11981Ceriporia_reticulata TACCAACTC--TT-GTATATGAATG-TCTT---CTTGC--AGATAATGCA

FP110343sp_Candelabrochaete_la TACCAACTC--TT-GTTTATGAATG-TCTT---TTTGC--GGATAACGCA

Li1045_Ceriporia_reticulata TACCAACTCTTTT-GTAAATGAATG-TCTT--ACTTGC--GGATAACGCA

ZX136Dai25794ceriporia TACCAACTCTTTT-GTAAATGAATG-TCTT--ACTTGC--GGATAACGCA

892Dai13400 TACCAACTCTTTT-GTAAATGAATG-TCTT--ACTTGC--GGATAACGCA

RLG7163Leptoporus_mollis TACAAACTT--CA-GTTTAAGAATG-TCTA---CTTGC--AGACAATGCA

Dai21062Leptoporus_mollis TACAAACTT--CA-GTTTAAGAATG-TCTA---CTTGC--AAACAATGCA

Dai20182Leptoporus_submollis TACAAACTT--CA-GTTTAAGAATG-TCTA---CTTGC--AAATAATGCA

Cui18379Leptoporus_submollis TACAAACTT--CA-GTTTAAGAATG-TCTA---CTTGC--AAATAATGCA

Wu1209_46Resiniporus_pseudogil CACAAACACATCA-GTTTCAGAATG-TCAT--TGTTGTTTTTATAACACC

BRNM710169Resiniporus_resinasc CACAAACACATCA-GTTTCAGAATG-TCAT--TGTTGTTTTTATAACACC

Dai14516Bjerkandera_adusta TACAAACGATTCA-GTTTTAGAATG-TCAT-ACTTTGC-T--ATAACGCA

Dai21100Bjerkandera_fumosa TACAAACGAATCA-GTTTTAGAATG-TCAT-ACTTTGC-T--ATAACGCA

Miettinen16854Ceraceomyces_sp TACAACCATCTT--GTTACAGAATGT-----TAATTGGGT--ATAACATG

Dai10477C_spissa CATATACAACT-A-TTGTTAGAATG-TT---GAACTGCATCCTTGATGCA

855Dai16831 CATATACAACT-A-TTGTTAGAATG-TT---GAACTGCATCCTTGATGCA

882Cui11282 CATATACAACT-A-TTGTTAGAATG-TT---GAACTGCATCCTTGATGCA

Dai24566 CACATACAACT-A-TTGTTAGAATG-TT---AACTTGCA---TTAATGCA

Yuan5965 CACATACAACT-A-TTGTTAGAATG-TT---GACTTGCA---TTAATGCA

Dai3204 --

1194CUI9985 --

Dai15205_Ceriporia_albomellea T-TTAATTATACAACTTTCAGCAACGGATCTCTTGGCTCTTGCATCGATG

Dai15223_Ceriporia_albomellea T-TTAATTATACAACTTTCAGCAACGGATCTCTTGGCTCTTGCATCGATG

Li1780_Ceriporia_variegata T-TTAATTATACAACTTTCAGCAACGGATCTCTTGGCTCTTGCATCGATG

Dai19791_Ceriporia_variegata T-TTAATTATACAACTTTCAGCAACGGATCTCTTGGCTCTTGCATCGATG

Dai19886 T-TTAATTATACAACTTTCAGCAACGGATCTCTTGGCTCTTGCATCGATG

Dai10833_Ceriporia_crassitunic T-
TTAATTATACAACTTTCAGCAACGGATCTCTTGGCTCTTGCATCGATG
CHWC1506_46Meruliopsis_crassit T-
TTAATTATACAACTTTCAGCAACGGATCTCTTGGCTCTTGCATCGATG
Dai9995_Ceriporia_crassitunica T-
TTAATTATACAACTTTCAGCAACGGATCTCTTGGCTCTTGCATCGATG
Wu1209_58_Meruliopsis_parvispo T-TT--
ATATACAACTTTCAGCAACGGATCTCTTGGCTCTCGCATCGATG
CHWC1505_129_Meruliopsis_parvi T-TT--
ATATACAACTTTCAGCAACGGATCTCTTGGCTCTCGCATCGATG
Dai21944 T-TT--
ATATACAACTTTCAGCAACGGATCTCTTGGCTCTCGCATCGATG
830Dai18640A T-TT--
ATATACAACTTTCAGCAACGGATCTCTTGGCTCTCGCATCGATG
GC1704_60_Meruliopsis_taxicola T-TT--
ATATACAACTTTCAGCAACGGATCTCTTGGCTCTCGCATCGATG
Dai22625 T-TT--
ATATACAACTTTCAGCAACGGATCTCTTGGCTCTCGCATCGATG
Dai22636 T-TT--
ATATACAACTTTCAGCAACGGATCTCTTGGCTCTCGCATCGATG
Dai21878 T-TT--
ATATACAACTTTCAGCAACGGATCTCTTGGCTCTCGCATCGATG
1169Dai17248 T-TT--
ATATACAACTTTCAGCAACGGATCTCTTGGCTCTCGCATCGATG
Wu1708_43_Meruliopsis_leptocys T-TT--
ATATACAACTTTCAGCAACGGATCTCTTGGCTCTCGCATCGATG
Li1011 T-TT--
ATATACAACTTTCAGCAACGGATCTCTTGGCTCTCGCATCGATG
ZX95Dai25742Meruliopsis_leptoc T-TT--
ATATACAACTTTCAGCAACGGATCTCTTGGCTCTCGCATCGATG
WCG1306Dai24733 T-TT--
ATATACAACTTTCAGCAACGGATCTCTTGGCTCTCGCATCGATG
LXL99Dai25816 T-TT--
ATATACAACTTTCAGCAACGGATCTCTTGGCTCTCGCATCGATG
WCG1559Dai26052Meruliopsis T-TT--
ATATACAACTTTCAGCAACGGATCTCTTGGCTCTCGCATCGATG
He7477 T-TT--
ATATACAACTTTCAGCAACGGATCTCTTGGCTCTCGCATCGATG
HLX243Dai26217 T-TT--
ATATACAACTTTCAGCAACGGATCTCTTGGCTCTCGCATCGATG
RussiaMW673659Meruliopsis_fagi T-TT--
ATATACAACTTTCAGCAACGGATCTCTTGGCTCTCGCATCGATG
FD278 T-TT--
ATATACAACTTTCAGCAACGGATCTCTTGGCTCTCGCATCGATG

Dai10226_Ceriporia_tarda T-TT--
ATATACAACTTTCAGCAACGGATCTCTTGGCTCTCGCATCGATG
LE247365 T-TT--
ATATACAACTTTCAGCAACGGATCTCTTGGCTCTCGCATCGATG
Dai8173_Meruliopsis_nanlingens T-TT--
ATATACAACTTTCAGCAACGGATCTCTTGGCTCTCGCATCGATG
860Dai17172 T-TT--
ATATACAACTTTCAGCAACGGATCTCTTGGCTCTCGCATCGATG
879Dai13414 T-TT--
ATATACAACTTTCAGCAACGGATCTCTTGGCTCTCGCATCGATG
Li_1704_Meruliopsis_pseudocyst T-TT--
ATATACAACTTTCAGCAACGGATCTCTTGGCTCTCGCATCGATG
833Dai18405 T-TT--
ATATACAACTTTCAGCAACGGATCTCTTGGCTCTCGCATCGATG
HHB_10729_Meruliopsis_albostra T-TT--
ATATACAACTTTCAGCAACGGATCTCTTGGCTCTCGCATCGATG
Cui6878_Ceriporia_pseudocystid T-TT--
ATATACAACTTTCAGCAACGGATCTCTTGGCTCTCGCATCGATG
869Dai14737 T-TT--
ATATACAACTTTCAGCAACGGATCTCTTGGCTCTCGCATCGATG
876Cui11626 T-TT--
ATATACAACTTTCAGCAACGGATCTCTTGGCTCTCGCATCGATG
1199WEI3388 T-TT--
ATATACAACTTTCAGCAACGGATCTCTTGGCTCTCGCATCGATG
776308_Meruliopsis_cystidiata T-TT--
ATATACAACTTTCAGCAACGGATCTCTTGGCTCTCGCATCGATG
ICN139059_Meruliopsis_cystidia T-TT--
ATATACAACTTTCAGCAACGGATCTCTTGGCTCTCGCATCGATG
HHB15692Ceraceomyces_serpens T-TA--
TAATACAACTTTCAGCAACGGATCTCTTGGCTCTCGCATCGATG
HHB_15629_Sp_Ceriporiopsis_ane T-TT--
ATATACAACTTTCAGCAACGGATCTCTTGGCTCTCGCATCGATG
AJ185Trametopsis_cervina T-TT--
ATATACAACTTTCAGCAACGGATCTCTTGGCTCTCGCATCGATG
FD9Irpex_lacteus T-TT--
ATATACAACTTTCAGCAACGGATCTCTTGGCTCTCGCATCGATG
908Dai11230 T-TT--
ATATACAACTTTCAGCAACGGATCTCTTGGCTCTCGCATCGATG
FP55521TEmmia_lacerata A-TT--
ATATACAACTTTCAGCAACGGATCTCTTGGCTCTCGCATCGATG
PBU0048Ceriporia_cystidiata A-TT--
ATATACAACTTTCAGCAACGGATCTCTTGGCTCTCGCATCGATG
MZ340C_lacerataT A-TT--
ATATACAACTTTCAGCAACGGATCTCTTGGCTCTCGCATCGATG

Dai21940 A-TT--
ATATACAACTTTCAGCAACGGATCTCTTGGCTCTCGCATCGATG
847Dai16433 A-TT--
ATATACAACTTTCAGCAACGGATCTCTTGGCTCTCGCATCGATG
MarcinEmmia_latemarginatus A-TT--
ATATACAACTTTCAGCAACGGATCTCTTGGCTCTCGCATCGATG
Meijer3729Hydnopolyporus_fimbr ATTT--
ATATACAACTTTCAGCAACGGATCTCTTGGCTCTCGCATCGATG
RLG13408Phanerochaete_sp ATTT--
ATATACAACTTTCAGCAACGGATCTCTTGGCTCTCGCATCGATG
WHC1381Flavodon_flavus A-TT--
ATATACAACTTTCAGCAACGGATCTCTTGGCTCTCGCATCGATG
GB1833Phlebia_albida A--T--
ATATACAACTTTCAGCAACGGATCTCTTGGCTCTCGCATCGATG
T407Phlebia_nitidula A--T--
ATATACAACTTTCAGCAACGGATCTCTTGGCTCTCGCATCGATG
HHB6988Phanerochaete_exilis A--T--
ATATACAACTTTCAGCAACGGATCTCTTGGCTCTCGCATCGATG
HHB8509Phanerochaetella_xeroph A--T--
ATATACAACTTTCAGCAACGGATCTCTTGGCTCTCGCATCGATG
PBU0051Macrohyporia_dictyopora A--T--
ATATACAACTTTCAGCAACGGATCTCTTGGCTCTCGCATCGATG
HHB11463Phanerochaete_sp A--T--
ATATACAACTTTCAGCAACGGATCTCTTGGCTCTCGCATCGATG
FP102382Byssomerulius_corium A--T--
ATATACAACTTTCAGCAACGGATCTCTTGGCTCTCGCATCGATG
FP102165Efibula_americana A-----
ATATACAACTTTCAGCAACGGATCTCTTGGCTCTCGCATCGATG
Murdoch90Ceriporia_torpida T-
TTGAATATACAACTTTCAACAACGGATCTCTTGGCTCTCGCATCGATG
Rivoire4413_Ceriporia_purpurea T-
TTTAACATACAACTTTCAACAACGGATCTCTTGGCTCTCGCATCGATG
Kout_18_Ceriporia_triumphalis T-
TTTAATATACAACTTTCAACAACGGATCTCTTGGCTCTCGCATCGATG
Rivoire3701_Ceriporia_bresadol T-
TTTAATATACAACTTTCAACAACGGATCTCTTGGCTCTCGCATCGATG
VS4018 T-
TTTAATATACAACTTTCAACAACGGATCTCTTGGCTCTCGCATCGATG
Ryvarden21832_Ceriporia_manzan T-
TTTAATATACAACTTTCAACAACGGATCTCTTGGCTCTCGCATCGATG
Dai24539 T-
TTTAATATACAACTTTCAACAACGGATCTCTTGGCTCTCGCATCGATG
Dai24541 T-
TTTAATATACAACTTTCAACAACGGATCTCTTGGCTCTCGCATCGATG

JV1105_12_Ceriporia_occidental T-
TTTAATATACAACTTTCAACAACGGATCTCTTGGCTCTCGCATCGATG

VS8558Ceriporia_occidentalis T-
TTTAATATACAACTTTCAACAACGGATCTCTTGGCTCTCGCATCGATG

Dai22445 T-
TTTAACATACAACTTTCAACAACGGATCTCTTGGCTCTCGCATCGATG

846Dai16368 T-
TTTAACATACAACTTTCAACAACGGATCTCTTGGCTCTCGCATCGATG

Dai17951_Ceriporia_aurantiocar T-T---
ATATACAACTTTCAGCAACGGATCTCTTGGCTCTCGCATCGATG

Miettinen_11701C_viridans T-T---
ATATACAACTTTCAGCAACGGATCTCTTGGCTCTCGCATCGATG

JV0105_10Ceriporia_aurantiocar T-T---
ATATACAACTTTCAGCAACGGATCTCTTGGCTCTCGCATCGATG

Yuan5702C_viridans T-T---
ATATACAACTTTCAGCAACGGATCTCTTGGCTCTCGCATCGATG

858Dai17003 T-T---
ATATACAACTTTCAGCAACGGATCTCTTGGCTCTCGCATCGATG

Yuan2747_Ceriporia_viridans T-T---
ATATATAACTTTCAGCAACGGATCTCTTGGCTCTCGCATCGATG

Yuan2744C_viridans T-T---
ATATATAACTTTCAGCAACGGATCTCTTGGCTCTCGCATCGATG

Li1046C_viridans T-T---
ATATACAACTTTCAGCAACGGATCTCTTGGCTCTCGCATCGATG

865C_sinoviridans T-T---
ATATACAACTTTCAGCAACGGATCTCTTGGCTCTCGCATCGATG

871Dai15062 T-T---
ATATACAACTTTCAGCAACGGATCTCTTGGCTCTCGCATCGATG

Dai7642_Ceriporia_humilis T-CT--
ATATACAACTTTCAGCAACGGATCTCTTGGCTCTCGCATCGATG

Spirin4706_Ceriporia_humilis T-CT--
ATATACAACTTTCAGCAACGGATCTCTTGGCTCTCGCATCGATG

Spirin4944_Ceriporia_sericea T-CT--
ATATACAACTTTCAGCAACGGATCTCTTGGCTCTCGCATCGATG

WCG1547Dai26044ceriporia T-CT--
ATATACAACTTTCAGCAACGGATCTCTTGGCTCTCGCATCGATG

ZZW1558Dai27086 T-CT--
ATATACAACTTTCAGCAACGGATCTCTTGGCTCTCGCATCGATG

Miettinen14381_Ceriporia_mpuri ---

Miettinen15492_2_Ceriporia_sor T-CT--
ATATACAACTTTCAGCAACGGATCTCTTGGCTCTCGCATCGATG

He6687 T-CT--
ATATACAACTTTCAGCAACGGATCTCTTGGCTCTCGCATCGATG

ZH53Dai24426 T-CT--

ATATACAACTTTCAGCAACGGATCTCTTGGCTCTCGCATCGATG
Vlasak0808_30_Ceriporia_punica T-TT--
GTATACAACTTTCAGCAACGGATCTCTTGGCTCTCGCATCGATG
887Dai13376 T-TT--
GTATACAACTTTCAGCAACGGATCTCTTGGCTCTCGCATCGATG
WCG1443Dai24998 T-TT--
GTATACAACTTTCAGCAACGGATCTCTTGGCTCTCGCATCGATG
0108_6Ceriporia_spissa A-
TGTTAAATACAACTTTCAGCAACGGATCTCTTGGCTCTCGCATCGATG
Dai19164 A-
TGTTAAATACAACTTTCAGCAACGGATCTCTTGGCTCTCGCATCGATG
Dai17937_Ceriporia_bubalinomar A-
TGTTAAATACAACTTTCAGCAACGGATCTCTTGGCTCTCGCATCGATG
903Dai12113 A-
TGTTAAATACAACTTTCAGCAACGGATCTCTTGGCTCTCGCATCGATG
LZB929Dai25079 A-CT--
ATATACAACTTTCAGCAACGGATCTCTTGGCTCTCGCATCGATG
LX45Dai26988 A-CT--
ATATACAACTTTCAGCAACGGATCTCTTGGCTCTCGCATCGATG
LX43Dai26986 A-CT--
ATATACAACTTTCAGCAACGGATCTCTTGGCTCTCGCATCGATG
Dai7759Ceriporia A-CT--
ATATACAACTTTCAGCAACGGATCTCTTGGCTCTCGCATCGATG
Cui8012_Ceriporia_viridans T-TT--
ATATACAACTTTCAGCAACGGATCTCTTGGCTCTCGCATCGATG
GC1704_54Ceriporia_viridans T-TT--
ATATACAACTTTCAGCAACGGATCTCTTGGCTCTCGCATCGATG
Dai23392 T-TT--
ATATACAACTTTCAGCAACGGATCTCTTGGCTCTCGCATCGATG
WCG1585Dai26113Ceriproia T-CT--
ATATACAACTTTCAGCAACGGATCTCTTGGCTCTCGCATCGATG
Dai18675C_eucalypti T-T---
ATATACAACTTTCAGCAACGGATCTCTTGGCTCTCGCATCGATG
Dai22034 T-TA--
TAATACAACTTTCAGCAACGGATCTCTTGGCTCTCGCATCGATG
JV1008_41JTardaFLORIDAKeys T-TA--
TAATACAACTTTCAGCAACGGATCTCTTGGCTCTCGCATCGATG
Rivoire1161_Ceriporia_pierii T-TA--
ATATACAACTTTCAGCAACGGATCTCTTGGCTCTCGCATCGATG
Dai23499C_pierii T-TA--
ATATACAACTTTCAGCAACGGATCTCTTGGCTCTCGCATCGATG
Dai23500 T-TA--
ATATACAACTTTCAGCAACGGATCTCTTGGCTCTCGCATCGATG
841Dai15899 T-TA--

TAATACAACTTTCAGCAACGGATCTCTTGGCTCTCGCATCGATG

842Dai15904 T-TA--

TAATACAACTTTCAGCAACGGATCTCTTGGCTCTCGCATCGATG

LZB1066xinjiang T-TA--

TAATACAACTTTCAGCAACGGATCTCTTGGCTCTCGCATCGATG

LZB1065xinjiang T-TA--

TAATACAACTTTCAGCAACGGATCTCTTGGCTCTCGCATCGATG

851Dai16779 T-CT--

ATATACAACTTTCAGCAACGGATCTCTTGGCTCTCGCATCGATG

RMJ119sp_Candelabrochaete_sept T-CA--

TAATACAACTTTCAGCAACGGATCTCTTGGCTCTCGCATCGATG

RLG9759spCandelabrochaete_sept T-CA--

TAATACAACTTTCAGCAACGGATCTCTTGGCTCTCGCATCGATG

RLG10478Phanerochaete_allantos T-T---

ATATACAACTTTCAGCAACGGATCTCTTGGCTCTCGCATCGATG

Dai19118_Ceriporia_spissa

TGTTGAATAAACAACTTTCAGCAACGGATCTCTTGGCTCTCGCATCGATG

Dai18486A

TGTTGAATAAACAACTTTCAGCAACGGATCTCTTGGCTCTCGCATCGATG

WEI17_024_Ceriporia_mellita

TGTTGAATAAACAACTTTCAGCAACGGATCTCTTGGCTCTCGCATCGATG

GC1508_71Ceriporia_mellita

TGTTGAATAAACAACTTTCAGCAACGGATCTCTTGGCTCTCGCATCGATG

GC1608_7_Ceriporia_mellita

TGTTGAATAAACAACTTTCAGCAACGGATCTCTTGGCTCTCGCATCGATG

ZZW1557Dai27085

TGTTGAATAAACAACTTTCAGCAACGGATCTCTTGGCTCTCGCATCGATG

ZZW1554Dai27083

TGTTGAATAAACAACTTTCAGCAACGGATCTCTTGGCTCTCGCATCGATG

Dai8168

TGTTGAATAAACAACTTTCAGCAACGGATCTCTTGGCTCTCGCATCGATG

BR4865C_mellita

TGTTGAATAAACAACTTTCAGCAACGGATCTCTTGGCTCTCGCATCGATG

MEL2382688Ceriporia_sp

TGTTAAATGTACAACTTTCAGCAACGGATCTCTTGGCTCTCGCATCGATG

Dai8110

CGTTGAATATACAACTTTCAGCAACGGATCTCTTGGCTCTCGCATCGATG

Cui8097

CGTTGAATATACAACTTTCAGCAACGGATCTCTTGGCTCTCGCATCGATG

909Cui6740

TGTTAAATATACAACTTTCAGCAACGGATCTCTTGGCTCTCGCATCGATG

W1258Dai24695

TGTTAAATATACAACTTTCAGCAACGGATCTCTTGGCTCTCGCATCGATG

JV0110_26_Ceriporia_griseoviol

TGTTGTACCAACAACTTTCAGCAACGGATCTCTTGGCTCTCGCATCGATG

896Dai13202
TGTTGTACCAACAACTTTCAGCAACGGATCTCTTGGCTCTCGCATCGATG

LWY393Dai27053C_griseoviolasce
TGTTGTACCAACAACTTTCAGCAACGGATCTCTTGGCTCTCGCATCGATG

LWY394DAI27054
TGTTGTACCAACAACTTTCAGCAACGGATCTCTTGGCTCTCGCATCGATG

FP135015G_pannocinctus AAT---
ATATACAACTTTCAGCAACGGATCTCTTGGCTCTCGCATCGATG

L15726SpG_pannocinctus AAT---
ATATACAACTTTCAGCAACGGATCTCTTGGCTCTCGCATCGATG

Dai22221 AAT---
ATATACAACTTTCAGCAACGGATCTCTTGGCTCTCGCATCGATG

Dai22633 ATT---
ATATACAACTTTCAGCAACGGATCTCTTGGCTCTCGCATCGATG

Dai23260 ATT---
ATATACAACTTTCAGCAACGGATCTCTTGGCTCTCGCATCGATG

Dai23626 ATT---
ATATACAACTTTCAGCAACGGATCTCTTGGCTCTCGCATCGATG

Dai16238G_citrinoalbus ATT---
ATATACAACTTTCAGCAACGGATCTCTTGGCTCTCGCATCGATG

1175Dai15293 ATT---
ATATACAACTTTCAGCAACGGATCTCTTGGCTCTCGCATCGATG

Dai19547 ATG---
ATATACAACTTTCAGCAACGGATCTCTTGGCTCTCGCATCGATG

918063G_africanus ATT---
ATATACAACTTTCAGCAACGGATCTCTTGGCTCTCGCATCGATG

918572G_africanus ATT---
ATATACAACTTTCAGCAACGGATCTCTTGGCTCTCGCATCGATG

Dai18536A ATT---
ATATACAACTTTCAGCAACGGATCTCTTGGCTCTCGCATCGATG

1164Cui17922 ATT---
ATATACAACTTTCAGCAACGGATCTCTTGGCTCTCGCATCGATG

Dai22225 ATT---
ATATACAACTTTCAGCAACGGATCTCTTGGCTCTCGCATCGATG

1163Dai20655 ATT---
ATATACAACTTTCAGCAACGGATCTCTTGGCTCTCGCATCGATG

Yuan4397G_hainanensis A-TT--
ATATACAACTTTCAGCAACGGATCTCTTGGCTCTCGCATCGATG

1176Dai15268 A-TT--
ATATACAACTTTCAGCAACGGATCTCTTGGCTCTCGCATCGATG

1177Dai15259 A-TT--
ATATACAACTTTCAGCAACGGATCTCTTGGCTCTCGCATCGATG

BZ2896G_thelephoroides ATT---

ATATACAACTTTCAGCAACGGATCTCTTGGCTCTCGCATCGATG

1166JV1808_26 ATT---

ATATACAACTTTCAGCAACGGATCTCTTGGCTCTCGCATCGATG

Miettinen16992Hapalopilus_ochr T-TA--

ATGTACAACTTTCAGCAACGGATCTCTTGGCTCTCGCATCGATG

GC1708_338_Ceriporia_arbuscula T-TA--

TAATACAACTTTCAGCAACGGATCTCTTGGCTCTCGCATCGATG

WCG1555Dai26107Ceriporia T-TA--

TAATACAACTTTCAGCAACGGATCTCTTGGCTCTCGCATCGATG

GC1708_340_Ceriporia_arbuscula T-TA--

TAATACAACTTTCAGCAACGGATCTCTTGGCTCTCGCATCGATG

WCG1556Dai26109Ceriporia T-TA--

TAATACAACTTTCAGCAACGGATCTCTTGGCTCTCGCATCGATG

883Cui11291 T-TA--

TAATACAACTTTCAGCAACGGATCTCTTGGCTCTCGCATCGATG

HLX320Dai26805 T-TA--

TAATACAACTTTCAGCAACGGATCTCTTGGCTCTCGCATCGATG

WCG1266Dai24678A T-TA--

TAATACAACTTTCAGCAACGGATCTCTTGGCTCTCGCATCGATG

Dai6090_Ceriporia_sulphuricolo T-TT--

ATATACAACTTTCAGCAACGGATCTCTTGGCTCTCGCATCGATG

RLG_11354_Ceriproia_reticulata AATT--

ATAAATAACTTTCAGCAACGGATCTCTTGGCTCTCGCATCGATG

ZZW1543Dai27072 AACT--

ATAAATAACTTTCAGCAACGGATCTCTTGGCTCTCGCATCGATG

Li1316_Ceriporia_reticulata AACA--

ATAAATAACTTTCAGCAACGGATCTCTTGGCTCTCGCATCGATG

KHL11981Ceriporia_reticulata AACA--

ATAAATAACTTTCAGCAACGGATCTCTTGGCTCTCGCATCGATG

FP110343sp_Candelabrochaete_la AACT--

ATAAATAACTTTCAGCAACGGATCTCTTGGCTCTCGCATCGATG

Li1045_Ceriporia_reticulata AACT--

ATAAATAACTTTCAGCAACGGATCTCTTGGCTCTCGCATCGATG

ZX136Dai25794ceriporia AACT--

ATAAATAACTTTCAGCAACGGATCTCTTGGCTCTCGCATCGATG

892Dai13400 AACT--

ATAAATAACTTTCAGCAACGGATCTCTTGGCTCTCGCATCGATG

RLG7163Leptoporus_mollis AATT--

ATATACAACTTTCAGCAACGGATCTCTTGGCTCTCGCATCGATG

Dai21062Leptoporus_mollis AATT--

ATATACAACTTTCAGCAACGGATCTCTTGGCTCTCGCATCGATG

Dai20182Leptoporus_submollis ATT---

ATATACAACTTTCAGCAACGGATCTCTTGGCTCTCGCATCGATG

Cui18379Leptoporus_submollis ATT---

ATATACAACTTTCAGCAACGGATCTCTTGGCTCTCGCATCGATG
Wu1209_46Resiniporus_pseudogil TGT---
ATATACAACTTTCAGCAACGGATCTCTTGGCTCTCGCATCGATG
BRNM710169Resiniporus_resinasc TGT---
ATATACAACTTTCAGCAACGGATCTCTTGGCTCTCGCATCGATG
Dai14516Bjerkandera_adusta A-TT--
ATATACAACTTTCAGCAACGGATCTCTTGGCTCTCGCATCGATG
Dai21100Bjerkandera_fumosa A-TT--
ATATACAACTTTCAGCAACGGATCTCTTGGCTCTCGCATCGATG
Miettinen16854Ceraceomyces_sp G-C-----
ATACAACTTTCAGCAACGGATCTCTTGGCTCTCGCATCGATG
Dai10477C_spissa
TGTAAAATATATAACTTTCAGCAACGGATCTCTTGGCTCTCGCATCGATG
855Dai16831
TGTAAAATATATAACTTTCAGCAACGGATCTCTTGGCTCTCGCATCGATG
882Cui11282
TGTAAAATATATAACTTTCAGCAACGGATCTCTTGGCTCTCGCATCGATG
Dai24566
TGTAATATATATAACTTTCAGCAACGGATCTCTTGGCTCTCGCATCGATG
Yuan5965
TGTAATATATATAACTTTCAGCAACGGATCTCTTGGCTCTCGCATCGATG
Dai3204 --
1194CUI9985 --

Dai15205_Ceriporia_albomellea
AAGAACGCAGCGAAATGCGATAAGTAATGTGAATTGCAGAATTCAGTGAA
Dai15223_Ceriporia_albomellea
AAGAACGCAGCGAAATGCGATAAGTAATGTGAATTGCAGAATTCAGTGAA
Li1780_Ceriporia_variegata
AAGAACGCAGCGAAATGCGATAAGTAATGTGAATTGCAGAATTCAGTGAA
Dai19791_Ceriporia_variegata
AAGAACGCAGCGAAATGCGATAAGTAATGTGAATTGCAGAATTCAGTGAA
Dai19886
AAGAACGCAGCGAAATGCGATAAGTAATGTGAATTGCAGAATTCAGTGAA
Dai10833_Ceriporia_crassitunic
AAGAACGCAGCGAAATGCGATAAGTAATGTGAATTGCAGAATTCAGTGAA
CHWC1506_46Meruliopsis_crassit
AAGAACGCAGCGAAATGCGATAAGTAATGTGAATTGCAGAATTCAGTGAA
Dai9995_Ceriporia_crassitunica
AAGAACGCAGCGAAATGCGATAAGTAATGTGAATTGCAGAATTCAGTGAA
Wu1209_58_Meruliopsis_parvispo
AAGAACGCAGCGAAATGCGATAAGTAATGTGAATTGCAGAATTCAGTGAA
CHWC1505_129_Meruliopsis_parvi

AAGAACGCAGCGAAATGCGATAAGTAATGTGAATTGCAGAATTCAGTGAA

Dai21944

AAGAACGCAGCGAAATGCGATAAGTAATGTGAATTGCAGAATTCAGTGAA

830Dai18640A

AAGAACGCAGCGAAATGCGATAAGTAATGTGAATTGCAGAATTCAGTGAA

GC1704_60_Meruliopsis_taxicola

AAGAACGCAGCGAAATGCGATAAGTAATGTGAATTGCAGAATTCAGTGAA

Dai22625

AAGAACGCAGCGAAATGCGATAAGTAATGTGAATTGCAGAATTCAGTGAA

Dai22636

AAGAACGCAGCGAAATGCGATAAGTAATGTGAATTGCAGAATTCAGTGAA

Dai21878

AAGAACGCAGCGAAATGCGATAAGTAATGTGAATTGCAGAATTCAGTGAA

1169Dai17248

AAGAACGCAGCGAAATGCGATAAGTAATGTGAATTGCAGAATTCAGTGAA

Wu1708_43_Meruliopsis_leptocys

AAGAACGCAGCGAAATGCGATAAGTAATGTGAATTGCAGAATTCAGTGAA

Li1011

AAGAACGCAGCGAAATGCGATAAGTAATGTGAATTGCAGAATTCAGTGAA

ZX95Dai25742Meruliopsis_leptoc

AAGAACGCAGCGAAATGCGATAAGTAATGTGAATTGCAGAATTCAGTGAA

WCG1306Dai24733

AAGAACGCAGCGAAATGCGATAAGTAATGTGAATTGCAGAATTCAGTGAA

LXL99Dai25816

AAGAACGCAGCGAAATGCGATAAGTAATGTGAATTGCAGAATTCAGTGAA

WCG1559Dai26052Meruliopsis

AAGAACGCAGCGAAATGCGATAAGTAATGTGAATTGCAGAATTCAGTGAA

He7477

AAGAACGCAGCGAAATGCGATAAGTAATGTGAATTGCAGAATTCAGTGAA

HLX243Dai26217

AAGAACGCAGCGAAATGCGATAAGTAATGTGAATTGCAGAATTCAGTGAA

RussiaMW673659Meruliopsis_fagi

AAGAACGCAGCGAAATGCGATAAGTAATGTGAATTGCAGAATTCAGTGAA

FD278

AAGAACGCAGCGAAATGCGATAAGTAATGTGAATTGCAGAATTCAGTGAA

Dai10226_Ceriporia_tarda

AAGAACGCAGCGAAATGCGATAAGTAATGTGAATTGCAGAATTCAGTGAA

LE247365

AAGAACGCAGCGAAATGCGATAAGTAATGTGAATTGCAGAATTCAGTGAA

Dai8173_Meruliopsis_nanlingens

AAGAACGCAGCGAAATGCGATAAGTAATGTGAATTGCAGAATTCAGTGAA

860Dai17172

AAGAACGCAGCGAAATGCGATAAGTAATGTGAATTGCAGAATTCAGTGAA

879Dai13414

AAGAACGCAGCGAAATGCGATAAGTAATGTGAATTGCAGAATTCAGTGAA
Li_1704_Meruliopsis_pseudocyst
AAGAACGCAGCGAAATGCGATAAGTAATGTGAATTGCAGAATTCAGTGAA
833Dai18405
AAGAACGCAGCGAAATGCGATAAGTAATGTGAATTGCAGAATTCAGTGAA
HHB_10729_Meruliopsis_albostra
AAGAACGCAGCGAAATGCGATAAGTAATGTGAATTGCAGAATTCAGTGAA
Cui6878_Ceriporia_pseudocystid
AAGAACGCAGCGAAATGCGATAAGTAATGTGAATTGCAGAATTCAGTGAA
869Dai14737
AAGAACGCAGCGAAATGCGATAAGTAATGTGAATTGCAGAATTCAGTGAA
876Cui11626
AAGAACGCAGCGAAATGCGATAAGTAATGTGAATTGCAGAATTCAGTGAA
1199WEI3388
AAGAACGCAGCGAAATGCGATAAGTAATGTGAATTGCAGAATTCAGTGAA
776308_Meruliopsis_cystidiata
AAGAACGCAGCGAAATGCGATAAGTAATGTGAATTGCAGAATTCAGTGAA
ICN139059_Meruliopsis_cystidia
AAGAACGCAGCGAAATGCGATAAGTAATGTGAATTGCAGAATTCAGTGAA
HHB15692Ceraceomyces_serpens
AAGAACGCAGCGAAATGCGATAAGTAATGTGAATTGCAGAATTCAGTGAA
HHB_15629_Sp_Ceriporiopsis_ane
AAGAACGCAGCGAAATGCGATAAGTAATGTGAATTGCAGAATTCAGTGAA
AJ185Trametopsis_cervina
AAGAACGCAGCGAAATGCGATAAGTAATGTGAATTGCAGAATTCAGTGAA
FD9Irpex_lacteus
AAGAACGCAGCGAAATGCGATAAGTAATGTGAATTGCAGAATTCAGTGAA
908Dai11230
AAGAACGCAGCGAAATGCGATAAGTAATGTGAATTGCAGAATTCAGTGAA
FP55521TEmmia_lacerata
AAGAACGCAGCGAAATGCGATAAGTAATGTGAATTGCAGAATTCAGTGAA
PBU0048Ceriporia_cystidiata
AAGAACGCAGCGAAATGCGATAAGTAATGTGAATTGCAGAATTCAGTGAA
MZ340C_lacerataT
AAGAACGCAGCGAAATGCGATAAGTAATGTGAATTGCAGAATTCAGTGAA
Dai21940
AAGAACGCAGCGAAATGCGATAAGTAATGTGAATTGCAGAATTCAGTGAA
847Dai16433
AAGAACGCAGCGAAATGCGATAAGTAATGTGAATTGCAGAATTCAGTGAA
MarcinEmmia_latemarginatus
AAGAACGCAGCGAAATGCGATAAGTAATGTGAATTGCAGAATTCAGTGAA
Meijer3729Hydnopolyporus_fimbr
AAGAACGCAGCGAAATGCGATAAGTAATGTGAATTGCAGAATTCAGTGAA
RLG13408Phanerochaete_sp

AAGAACGCAGCGAAATGCGATAAGTAATGTGAATTGCAGAATTCAGTGAA
WHC1381Flavodon_flavus
AAGAACGCAGCGAAATGCGATAAGTAATGTGAATTGCAGAATTCAGTGAA
GB1833Phlebia_albida
AAGAACGCAGCGAAATGCGATAAGTAATGTGAATTGCAGAATTCAGTGAA
T407Phlebia_nitidula
AAGAACGCAGCGAAATGCGATAAGTAATGTGAATTGCAGAATTCAGTGAA
HHB6988Phanerochaete_exilis
AAGAACGCAGCGAAATGCGATAAGTAATGTGAATTGCAGAATTCAGTGAA
HHB8509Phanerochaetella_xeroph
AAGAACGCAGCGAAATGCGATAAGTAATGTGAATTGCAGAATTCAGTGAA
PBU0051Macrohyporia_dictyopora
AAGAACGCAGCGAAATGCGATAAGTAATGTGAATTGCAGAATTCAGTGAA
HHB11463Phanerochaete_sp
AAGAACGCAGCGAAATGCGATAAGTAATGTGAATTGCAGAATTCAGTGAA
FP102382Byssomerulius_corium
AAGAACGCAGCGAAATGCGATAAGTAATGTGAATTGCAGAATTCAGTGAA
FP102165Efibula_americana
AAGAACGCAGCGAAATGCGATAAGTAATGTGAATTGCAGAATTCAGTGAA
Murdoch90Ceriporia_torpida
AAGAACGCAGCGAAATGCGATAAGTAATGTGAATTGCAGAATTCAGTGAA
Rivoire4413_Ceriporia_purpurea
AAGAACGCAGCGAAATGCGATAAGTAATGTGAATTGCAGAATTCAGTGAA
Kout_18_Ceriporia_triumphalis
AAGAACGCAGCGAAATGCGATAAGTAATGTGAATTGCAGAATTCAGTGAA
Rivoire3701_Ceriporia_bresadol
AAGAACGCAGCGAAATGCGATAAGTAATGTGAATTGCAGAATTCAGTGAA
VS4018
AAGAACGCAGCGAAATGCGATAAGTAATGTGAATTGCAGAATTCAGTGAA
Ryvarden21832_Ceriporia_manzan
AAGAACGCAGCGAAATGCGATAAGTAATGTGAATTGCAGAATTCAGTGAA
Dai24539
AAGAACGCAGCGAAATGCGATAAGTAATGTGAATTGCAGAATTCAGTGAA
Dai24541
AAGAACGCAGCGAAATGCGATAAGTAATGTGAATTGCAGAATTCAGTGAA
JV1105_12_Ceriporia_occidental
AAGAACGCAGCGAAATGCGATAAGTAATGTGAATTGCAGAATTCAGTGAA
VS8558Ceriporia_occidentalis
AAGAACGCAGCGAAATGCGATAAGTAATGTGAATTGCAGAATTCAGTGAA
Dai22445
AAGAACGCAGCGAAATGCGATAAGTAATGTGAATTGCAGAATTCAGTGAA
846Dai16368
AAGAACGCAGCGAAATGCGATAAGTAATGTGAATTGCAGAATTCAGTGAA
Dai17951_Ceriporia_aurantiocar

AAGAACGCAGCGAAATGCGATAAGTAATGTGAATTGCAGAATTCAGTGAA

Miettinen_11701C_viridans

AAGAACGCAGCGAAATGCGATAAGTAATGTGAATTGCAGAATTCAGTGAA

JV0105_10Ceriporia_aurantiocar

AAGAACGCAGCGAAATGCGATAAGTAATGTGAATTGCAGAATTCAGTGAA

Yuan5702C_viridans

AAGAACGCAGCGAAATGCGATAAGTAATGTGAATTGCAGAATTCAGTGAA

858Dai17003

AAGAACGCAGCGAAATGCGATAAGTAATGTGAATTGCAGAATTCAGTGAA

Yuan2747_Ceriporia_viridans

AAGAACGCAGCGAAATGCGATAAGTAATGTGAATTGCAGAATTCAGTGAA

Yuan2744C_viridans

AAGAACGCAGCGAAATGCGATAAGTAATGTGAATTGCAGAATTCAGTGAA

Li1046C_viridans

AAGAACGCAGCGAAATGCGATAAGTAATGTGAATTGCAGAATTCAGTGAA

865C_sinoviridans

AAGAACGCAGCGAAATGCGATAAGTAATGTGAATTGCAGAATTCAGTGAA

871Dai15062

AAGAACGCAGCGAAATGCGATAAGTAATGTGAATTGCAGAATTCAGTGAA

Dai7642_Ceriporia_humilis

AAGAACGCAGCGAAATGCGATAAGTAATGTGAATTGCAGAATTCAGTGAA

Spirin4706_Ceriporia_humilis

AAGAACGCAGCGAAATGCGATAAGTAATGTGAATTGCAGAATTCAGTGAA

Spirin4944_Ceriporia_sericea

AAGAACGCAGCGAAATGCGATAAGTAATGTGAATTGCAGAATTCAGTGAA

WCG1547Dai26044ceriporia

AAGAACGCAGCGAAATGCGATAAGTAATGTGAATTGCAGAATTCAGTGAA

ZZW1558Dai27086

AAGAACGCAGCGAAATGCGATAAGTAATGTGAATTGCAGAATTCAGTGAA

Miettinen14381_Ceriporia_mpuri --

Miettinen15492_2_Ceriporia_sor

AAGAACGCAGCGAAATGCGATAAGTAATGTGAATTGCAGAATTCAGTGAA

He6687

AAGAACGCAGCGAAATGCGATAAGTAATGTGAATTGCAGAATTCAGTGAA

ZH53Dai24426

AAGAACGCAGCGAAATGCTATAAGTAATGTGAATTGCAGAATTCAGTGAA

Vlasak0808_30_Ceriporia_punica

AAGAACGCAGCGAAATGCGATAAGTAATGTGAATTGCAGAATTCAGTGAA

887Dai13376

AAGAACGCAGCGAAATGCGATAAGTAATGTGAATTGCAGAATTCAGTGAA

WCG1443Dai24998

AAGAACGCAGCGAAATGCGATAAGTAATGTGAATTGCAGAATTCAGTGAA

0108_6Ceriporia_spissa

AAGAACGCAGCGAAATGCGATAAGTAATGTGAATTGCAGAATTCAGTGAA

Dai19164

AAGAACGCAGCGAAATGCGATAAGTAATGTGAATTGCAGAATTCAGTGAA

Dai17937_Ceriporia_bubalinomar

AAGAACGCAGCGAAATGCGATAAGTAATGTGAATTGCAGAATTCAGTGAA

903Dai12113

AAGAACGCAGCGAAATGCGATAAGTAATGTGAATTGCAGAATTCAGTGAA

LZB929Dai25079

AAGAACGCAGCGAAATGCGATAAGTAATGTGAATTGCAGAATTCAGTGAA

LX45Dai26988

AAGAACGCAGCGAAATGCGATAAGTAATGTGAATTGCAGAATTCAGTGAA

LX43Dai26986

AAGAACGCAGCGAAATGCGATAAGTAATGTGAATTGCAGAATTCAGTGAA

Dai7759Ceriporia

AAGAACGCAGCGAAATGCGATAAGTAATGTGAATTGCAGAATTCAGTGAA

Cui8012_Ceriporia_viridans

AAGAACGCAGCGAAATGCGATAAGTAATGTGAATTGCAGAATTCAGTGAA

GC1704_54Ceriporia_viridans

AAGAACGCAGCGAAATGTGATAAGTAATGTGAATTGCAGAATTCAGTGAA

Dai23392

AAGAACGCAGCGAAATGCGATAAGTAATGTGAATTGCAGAATTCAGTGAA

WCG1585Dai26113Ceriproia

AAGAACGCAGCGAAATGCGATAAGTAATGTGAATTGCAGAATTCAGTGAA

Dai18675C_eucalypti

AAGAACGCAGCGAAATGCGATAAGTAATGTGAATTGCAGAATTCAGTGAA

Dai22034

AAGAACGCAGCGAAATGCGATAAGTAATGTGAATTGCAGAATTCAGTGAA

JV1008_41JTardaFLORIDAKeys

AAGAACGCAGCGAAATGCGATAAGTAATGTGAATTGCAGAATTCAGTGAA

Rivoire1161_Ceriporia_pierii

AAGAACGCAGCGAAATGCGATAAGTAATGTGAATTGCAGAATTCAGTGAA

Dai23499C_pierii

AAGAACGCAGCGAAATGCGATAAGTAATGTGAATTGCAGAATTCAGTGAA

Dai23500

AAGAACGCAGCGAAATGCGATAAGTAATGTGAATTGCAGAATTCAGTGAA

841Dai15899

AAGAACGCAGCGAAATGCGATAAGTAATGTGAATTGCAGAATTCAGTGAA

842Dai15904

AAGAACGCAGCGAAATGCGATAAGTAATGTGAATTGCAGAATTCAGTGAA

LZB1066xinjiang

AAGAACGCAGCGAAATGCGATAAGTAATGTGAATTGCAGAATTCAGTGAA

LZB1065xinjiang

AAGAACGCAGCGAAATGCGATAAGTAATGTGAATTGCAGAATTCAGTGAA

851Dai16779

AAGAACGCAGCGAAATGCGATAAGTAATGTGAATTGCAGAATTCAGTGAA

RMJ119sp_Candelabrochaete_sept
AAGAACGCAGCGAAATGCGATAAGTAATGTGAATTGCAGAATTCAGTGAA
RLG9759spCandelabrochaete_sept
AAGAACGCAGCGAAATGCGATAAGTAATGTGAATTGCAGAATTCAGTGAA
RLG10478Phanerochaete_allantos
AAGAACGCAGCGAAATGCGATAAGTAATGTGAATTGCAGAATTCAGTGAA
Dai19118_Ceriporia_spissa
AAGAACGCAGCGAAATGCGATAAGTAATGTGAATTGCAGAATTCAGTGAA
Dai18486A
AAGAACGCAGCGAAATGCGATAAGTAATGTGAATTGCAGAATTCAGTGAA
WEI17_024_Ceriporia_mellita
AAGAACGCAGCGAAATGCGATAAGTAATGTGAATTGCAGAATTCAGTGAA
GC1508_71Ceriporia_mellita
AAGAACGCAGCGAAATGCGATAAGTAATGTGAATTGCAGAATTCAGTGAA
GC1608_7_Ceriporia_mellita
AAGAACGCAGCGAAATGCGATAAGTAATGTGAATTGCAGAATTCAGTGAA
ZZW1557Dai27085
AAGAACGCAGCGAAATGCGATAAGTAATGTGAATTGCAGAATTCAGTGAA
ZZW1554Dai27083
AAGAACGCAGCGAAATGCGATAAGTAATGTGAATTGCAGAATTCAGTGAA
Dai8168
AAGAACGCAGCGAAATGCGATAAGTAATGTGAATTGCAGAATTCAGTGAA
BR4865C_mellita
AAGAACGCAGCGAAATGCGATAAGTAATGTGAATTGCAGAATTCAGTGAA
MEL2382688Ceriporia_sp
AAGAACGCAGCGAAATGCGATAAGTAATGTGAATTGCAGAATTCAGTGAA
Dai8110
AAGAACGCAGCGAAATGCGATAAGTAATGTGAATTGCAGAATTCAGTGAA
Cui8097
AAGAACGCAGCGAAATGCGATAAGTAATGTGAATTGCAGAATTCAGTGAA
909Cui6740
AAGAACGCAGCGAAATGCGATAAGTAATGTGAATTGCAGAATTCAGTGAA
W1258Dai24695
AAGAACGCAGCGAAATGCGATAAGTAATGTGAATTGCAGAATTCAGTGAA
JV0110_26_Ceriporia_griseoviol
AAGAACGCAGCGAAATGCGATAAGTAATGTGAATTGCAGAATTCAGTGAA
896Dai13202
AAGAACGCAGCGAAATGCGATAAGTAATGTGAATTGCAGAATTCAGTGAA
LWY393Dai27053C_griseoviolasce
AAGAACGCAGCGAAATGCGATAAGTAATGTGAATTGCAGAATTCAGTGAA
LWY394DAI27054
AAGAACGCAGCGAAATGCGATAAGTAATGTGAATTGCAGAATTCAGTGAA
FP135015G_pannocinctus
AAGAACGCAGCGAAATGCGATAAGTAATGTGAATTGCAGAATTCAGTGAA

L15726SpG_pannocinctus
AAGAACGCAGCGAAATGCGATAAGTAATGTGAATTGCAGAATTCAGTGAA
Dai22221
AAGAACGCAGCGAAATGCGATAAGTAATGTGAATTGCAGAATTCAGTGAA
Dai22633
AAGAACGCAGCGAAATGCGATAAGTAATGTGAATTGCAGAATTCAGTGAA
Dai23260
AAGAACGCAGCGAAATGCGATAAGTAATGTGAATTGCAGAATTCAGTGAA
Dai23626
AAGAACGCAGCGAAATGCGATAAGTAATGTGAATTGCAGAATTCAGTGAA
Dai16238G_citrinoalbus
AAGAACGCAGCGAAATGCGATAAGTAATGTGAATTGCAGAATTCAGTGAA
1175Dai15293
AAGAACGCAGCGAAATGCGATAAGTAATGTGAATTGCAGAATTCAGTGAA
Dai19547
AAGAACGCAGCGAAATGCGATAAGTAATGTGAATTGCAGAATTCAGTGAA
918063G_africanus
AAGAACGCAGCGAAATGCGATAAGTAATGTGAATTGCAGAATTCAGTGAA
918572G_africanus
AAGAACGCAGCGAAATGCGATAAGTAATGTGAATTGCAGAATTCAGTGAA
Dai18536A
AAGAACGCAGCGAAATGCGATAAGTAATGTGAATTGCAGAATTCAGTGAA
1164Cui17922
AAGAACGCAGCGAAATGCGATAAGTAATGTGAATTGCAGAATTCAGTGAA
Dai22225
AAGAACGCAGCGAAATGCGATAAGTAATGTGAATTGCAGAATTCAGTGAA
1163Dai20655
AAGAACGCAGCGAAATGCGATAAGTAATGTGAATTGCAGAATTCAGTGAA
Yuan4397G_hainanensis
AAGAACGCAGCGAAATGCGATAAGTAATGTGAATTGCAGAATTCAGTGAA
1176Dai15268
AAGAACGCAGCGAAATGCGATAAGTAATGTGAATTGCAGAATTCAGTGAA
1177Dai15259
AAGAACGCAGCGAAATGCGATAAGTAATGTGAATTGCAGAATTCAGTGAA
BZ2896G_thelephoroides
AAGAACGCAGCGAAATGCGATAAGTAATGTGAATTGCAGAATTCAGTGAA
1166JV1808_26
AAGAACGCAGCGAAATGCGATAAGTAATGTGAATTGCAGAATTCAGTGAA
Miettinen16992Hapalopilus_ochr
AAGAACGCAGCGAAATGCGATAAGTAATGTGAATTGCAGAATTCAGTGAA
GC1708_338_Ceriporia_arbuscula
AAGAACGCAGCGAAATGCGATAAGTAATGTGAATTGCAGAATTCAGTGAA
WCG1555Dai26107Ceriporia
AAGAACGCAGCGAAATGCGATAAGTAATGTGAATTGCAGAATTCAGTGAA

GC1708_340_Ceriporia_arbuscula
AAGAACGCAGCGAAATGCGATAAGTAATGTGAATTGCAGAATTCAGTGAA
WCG1556Dai26109Ceriporia
AAGAACGCAGCGAAATGCGATAAGTAATGTGAATTGCAGAATTCAGTGAA
883Cui11291
AAGAACGCAGCGAAATGCGATAAGTAATGTGAATTGCAGAATTCAGTGAA
HLX320Dai26805
AAGAACGCAGCGAAATGCGATAAGTAATGTGAATTGCAGAATTCAGTGAA
WCG1266Dai24678A
AAGAACGCAGCGAAATGCGATAAGTAATGTGAATTGCAGAATTCAGTGAA
Dai6090_Ceriporia_sulphuricolo
AAGAACGCAGCGAAATGCGATAAGTAATGTGAATTGCAGAATTCAGTGAA
RLG_11354_Ceriproia_reticulata
AAGAACGCAGCGAAATGCGATAAGTAATGTGAATTGCAGAATTCAGTGAA
ZZW1543Dai27072
AAGAACGCAGCGAAATGCGATAAGTAATGTGAATTGCAGAATTCAGTGAA
Li1316_Ceriporia_reticulata
AAGAACGCAGCGAAATGCGATAAGTAATGTGAATTGCAGAATTCAGTGAA
KHL11981Ceriporia_reticulata
AAGAACGCAGCGAAATGCGATAAGTAATGTGAATTGCAGAATTCAGTGAA
FP110343sp_Candelabrochaete_la
AAGAACGCAGCGAAATGCGATAAGTAATGTGAATTGCAGAATTCAGTGAA
Li1045_Ceriporia_reticulata
AAGAACGCAGCGAAATGCGATAAGTAATGTGAATTGCAGAATTCAGTGAA
ZX136Dai25794ceriporia
AAGAACGCAGCGAAATGCGATAAGTAATGTGAATTGCAGAATTCAGTGAA
892Dai13400
AAGAACGCAGCGAAATGCGATAAGTAATGTGAATTGCAGAATTCAGTGAA
RLG7163Leptoporus_mollis
AAGAACGCAGCGAAATGCGATAAGTAATGTGAATTGCAGAATTCAGTGAA
Dai21062Leptoporus_mollis
AAGAACGCAGCGAAATGCGATAAGTAATGTGAATTGCAGAATTCAGTGAA
Dai20182Leptoporus_submollis
AAGAACGCAGCGAAATGCGATAAGTAATGTGAATTGCAGAATTCAGTGAA
Cui18379Leptoporus_submollis
AAGAACGCAGCGAAATGCGATAAGTAATGTGAATTGCAGAATTCAGTGAA
Wu1209_46Resiniporus_pseudogil
AAGAACGCAGCGAAATGCGATAAGTAATGTGAATTGCAGAATTCAGTGAA
BRNM710169Resiniporus_resinasc
AAGAACGCAGCGAAATGCGATAAGTAATGTGAATTGCAGAATTCAGTGAA
Dai14516Bjerkandera_adusta
AAGAACGCAGCGAAATGCGATAAGTAATGTGAATTGCAGAATTCAGTGAA
Dai21100Bjerkandera_fumosa
AAGAACGCAGCGAAATGCGATAAGTAATGTGAATTGCAGAATTCAGTGAA

Miettinen16854Ceraceomyces_sp
AAGAACGCAGCGAAATGCGATAAGTAATGTGAATTGCAGAATTCAGTGAA
Dai10477C_spissa
AAGAACGCAGCGAAATGCGATAAGTAATGTGAATTGCAGAATTCAGTGAA
855Dai16831
AAGAACGCAGCGAAATGCGATAAGTAATGTGAATTGCAGAATTCAGTGAA
882Cui11282
AAGAACGCAGCGAAATGCGATAAGTAATGTGAATTGCAGAATTCAGTGAA
Dai24566
AAGAACGCAGCGAAATGCGATAAGTAATGTGAATTGCAGAATTCAGTGAA
Yuan5965
AAGAACGCAGCGAAATGCGATAAGTAATGTGAATTGCAGAATTCAGTGAA
Dai3204 --
1194CUI9985 --

Dai15205_Ceriporia_albomellea
TCATCGAATCTTTGAACGCACCTTGCGCTCCTTGGTATTCCGAGGAGCAT
Dai15223_Ceriporia_albomellea
TCATCGAATCTTTGAACGCACCTTGCGCTCCTTGGTATTCCGAGGAGCAT
Li1780_Ceriporia_variegata
TCATCGAATCTTTGAACGCACCTTGCGCTCCTTGGTATTCCGAGGAGCAT
Dai19791_Ceriporia_variegata
TCATCGAATCTTTGAACGCACCTTGCGCTCCTTGGTATTCCGAGGAGCAT
Dai19886
TCATCGAATCTTTGAACGCACCTTGCGCTCCTTGGTATTCCGAGGAGCAT
Dai10833_Ceriporia_crassitunic
TCATCGAATCTTTGAACGCACCTTGCGCTCCTTGGTATTCCGAGGAGCAT
CHWC1506_46Meruliopsis_crassit
TCATCGAATCTTTGAACGCACCTTGCGCTCCTTGGTATTCCGAGGAGCAT
Dai9995_Ceriporia_crassitunica
TCATCGAATCTTTGAACGCACCTTGCGCTCCTTGGTATTCCGAGGAGCAT
Wu1209_58_Meruliopsis_parvispo
TCATCGAATCTTTGAACGCACCTTGCGCTCCTTGGTATTCCGAGGAGCAT
CHWC1505_129_Meruliopsis_parvi
TCATCGAATCTTTGAACGCACCTTGCGCTCCTTGGTATTCCGAGGAGCAT
Dai21944
TCATCGAATCTTTGAACGCACCTTGCGCTCCTTGGTATTCCGAGGAGCAT
830Dai18640A
TCATCGAATCTTTGAACGCACCTTGCGCTCCTTGGTATTCCGAGGAGCAT
GC1704_60_Meruliopsis_taxicola
TCATCGAATCTTTGAACGCACCTTGCGCTCCTTGGTATTCCGAGGAGCAT
Dai22625
TCATCGAATCTTTGAACGCACCTTGCGCTCCTTGGTATTCCGAGGAGCAT

Dai22636

TCATCGAATCTTTGAACGCACCTTGCGCTCCTTGGTATTCCGAGGAGCAT

Dai21878

TCATCGAATCTTTGAACGCACCTTGCGCTCCTTGGTATTCCGAGGAGCAT

1169Dai17248

TCATCGAATCTTTGAACGCACCTTGCGCTCCTTGGTATTCCGAGGAGCAT

Wu1708_43_Meruliopsis_leptocys

TCATCGAATCTTTGAACGCACCTTGCGCTCCTTGGTATTCCGAGGAGCAT

Li1011

TCATCGAATCTTTGAACGCACCTTGCGCTCCTTGGTATTCCGAGGAGCAT

ZX95Dai25742Meruliopsis_leptoc

TCATCGAATCTTTGAACGCACCTTGCGCTCCTTGGTATTCCGAGGAGCAT

WCG1306Dai24733

TCATCGAATCTTTGAACGCACCTTGCGCTCCTTGGTATTCCGAGGAGCAT

LXL99Dai25816

TCATCGAATCTTTGAACGCACCTTGCGCTCCTTGGTATTCCGAGGAGCAT

WCG1559Dai26052Meruliopsis

TCATCGAATCTTTGAACGCACCTTGCGCTCCTTGGTATTCCGAGGAGCAT

He7477

TCATCGAATCTTTGAACGCACCTTGCGCTCCTTGGTATTCCGAGGAGCAT

HLX243Dai26217

TCATCGAATCTTTGAACGCACCTTGCGCTCCTTGGTATTCCGAGGAGCAT

RussiaMW673659Meruliopsis_fagi

TCATCGAATCTTTGAACGCACCTTGCGCTCCTTGGTATTCCGAGGAGCAT

FD278

TCATCGAATCTTTGAACGCACCTTGCGCTCCTTGGTATTCCGAGGAGCAT

Dai10226_Ceriporia_tarda

TCATCGAATCTTTGAACGCACCTTGCGCTCCTTGGTATTCCGAGGAGCAT

LE247365

TCATCGAATCTTTGAACGCACCTTGCGCTCCTTGGTATTCCGAGGAGCAT

Dai8173_Meruliopsis_nanlingens

TCATCGAATCTTTGAACGCACCTTGCGCTCCTTGGTATTCCGAGGAGCAT

860Dai17172

TCATCGAATCTTTGAACGCACCTTGCGCTCCTTGGTATTCCGAGGAGCAT

879Dai13414

TCATCGAATCTTTGAACGCACCTTGCGCTCCTTGGTATTCCGAGGAGCAT

Li_1704_Meruliopsis_pseudocyst

TCATCGAATCTTTGAACGCACCTTGCGCTCCTTGGTATTCCGAGGAGCAT

833Dai18405

TCATCGAATCTTTGAACGCACCTTGCGCTCCTTGGTATTCCGAGGAGCAT

HHB_10729_Meruliopsis_albostra

TCATCGAATCTTTGAACGCACCTTGCGCTCCTTGGTATTCCGAGGAGCAT

Cui6878_Ceriporia_pseudocystid

TCATCGAATCTTTGAACGCACCTTGCGCTCCTTGGTATTCCGAGGAGCAT

869Dai14737
TCATCGAATCTTTGAACGCACCTTGCGCTCCTTGGTATTCCGAGGAGCAT
876Cui11626
TCATCGAATCTTTGAACGCACCTTGCGCTCCTTGGTATTCCGAGGAGCAT
1199WEI3388
TCATCGAATCTTTGAACGCACCTTGCGCTCCTTGGTATTCCGAGGAGCAT
776308_Meruliopsis_cystidiata
TCATCGAATCTTTGAACGCACCTTGCGCTCCTTGGTATTCCGAGGAGCAT
ICN139059_Meruliopsis_cystidia
TCATCGAATCTTTGAACGCACCTTGCGCTCCTTGGTATTCCGAGGAGCAT
HHB15692Ceraceomyces_serpens
TCATCGAATCTTTGAACGCACCTTGCGCTCCTTGGTATTCCGAGGAGCAT
HHB_15629_Sp_Ceriporiopsis_ane
TCATCGAATCTTTGAACGCACCTTGCGCTCCTTGGTATTCCGAGGAGCAT
AJ185Trametopsis_cervina
TCATCGAATCTTTGAACGCACCTTGCGCTCCTTGGTATTCCGAGGAGCAT
FD9Irpex_lacteus
TCATCGAATCTTTGAACGCACCTTGCACTCCTTGGTATTCCGAGGAGTAT
908Dai11230
TCATCGAATCTTTGAACGCACCTTGCACTCCTTGGTATTCCGAGGAGTAT
FP55521TEmmia_lacerata
TCATCGAATCTTTGAACGCACCTTGCACTCCTTGGTATTCCGAGGAGTAT
PBU0048Ceriporia_cystidiata
TCATCGAATCTTTGAACGCACCTTGCACTCCTTGGTATTCCGAGGAGTAT
MZ340C_lacerataT
TCATCGAATCTTTGAACGCACCTTGCACTCCTTGGTATTCCGAGGAGTAT
Dai21940
TCATCGAATCTTTGAACGCACCTTGCACTCCTTGGTATTCCGAGGAGTAT
847Dai16433
TCATCGAATCTTTGAACGCACCTTGCACTCCTTGGTATTCCGAGGAGTAT
MarcinEmmia_latemarginatus
TCATCGAATCTTTGAACGCACCTTGCACTCCTTGGTATTCCGAGGAGTAT
Meijer3729Hydnopolyporus_fimbr
TCATCGAATCTTTGAACGCACCTTGCACTCCTTGGCATTCCGAGGAGTAT
RLG13408Phanerochaete_sp
TCATCGAATCTTTGAACGCACCTTGCGCTCCTTGGTATTCCGAGGAGCAT
WHC1381Flavodon_flavus
TCATCGAATCTTTGAACGCACCTTGCACTCCTTGGTATTCCGAGGAGTAT
GB1833Phlebia_albida
TCATCGAATCTTTGAACGCACCTTGCGCTCCTTGGTATTCCGAGGAGCAT
T407Phlebia_nitidula
TCATCGAATCTTTGAACGCACCTTGCGCTCCTTGGTATTCCGAGGAGCAT
HHB6988Phanerochaete_exilis
TCATCGAATCTTTGAACGCACCTTGCGCTCCTTGGTATTCCATGGAGCAT

HHB8509Phanerochaetella_xeroph
TCATCGAATCTTTGAACGCACCTTGCGCTCCTTGGTATTCCTTGGAGCAT
PBU0051Macrohyporia_dictyopora
TCATCGAATCTTTGAACGCACCTTGCGCTCCTTGGTATTCCTCGGAGCAT
HHB11463Phanerochaete_sp
TCATCGAATCTTTGAACGCACCTTGCGCTCCTTGGTATTCCTTGGAGCAT
FP102382Byssomerulius_corium
TCATCGAATCTTTGAACGCACCTTGCGCTCCTTGGCATTCCGAGGAGCAT
FP102165Efibula_americana
TCATCGAATCTTTGAACGCACCTTGCGCTCCTTGGTATTCCGAGGAGCAT
Murdoch90Ceriporia_torpida
TCATCGAATCTTTGAACGCACCTTGCGCTCCTTGGCATTCCTTGGAGCAT
Rivoire4413_Ceriporia_purpurea
TCATCGAATCTTTGAACGCACCTTGCGCTCCTTGGTATTCCTTGGAGCAT
Kout_18_Ceriporia_triumphalis
TCATCGAATCTTTGAACGCACCTTGCGCTCCTTGGTATTCCTTGGAGCAT
Rivoire3701_Ceriporia_bresadol
TCATCGAATCTTTGAACGCACCTTGCGCTCCTTGGTATTCCTTGGAGCAT
VS4018
TCATCGAATCTTTGAACGCACCTTGCGCTCCTTGGTATTCCTTGGAGCAT
Ryvarden21832_Ceriporia_manzan
TCATCGAATCTTTGAACGCACCTTGCGCTCCTTGGTATTCCTTGGAGCAT
Dai24539
TCATCGAATCTTTGAACGCACCTTGCGCTCCTTGGTATTCCTTGGAGCAT
Dai24541
TCATCGAATCTTTGAACGCACCTTGCGCTCCTTGGTATTCCTTGGAGCAT
JV1105_12_Ceriporia_occidental
TCATCGAATCTTTGAACGCACCTTGCGCTCCTTGGTATTCCTTGGAGCAT
VS8558Ceriporia_occidentalis
TCATCGAATCTTTGAACGCACCTTGCGCTCCTTGGTATTCCTTGGAGCAT
Dai22445
TCATCGAATCTTTGAACGCACCTTGCGCTCCTTGGTATTCCTTGGAGCAT
846Dai16368
TCATCGAATCTTTGAACGCACCTTGCGCTCCTTGGTATTCCTTGGAGCAT
Dai17951_Ceriporia_aurantiocar
TCATCGAATCTTTGAACGCACCTTGCGCTCCTTGGTATTCCTTGGAGCAT
Miettinen_11701C_viridans
TCATCGAATCTTTGAACGCACCTTGCGCTCCTTGGTATTCCTCGGAGCAT
JV0105_10Ceriporia_aurantiocar
TCATCGAATCTTTGAACGCACCTTGCGCTCCTTGGTATTCCTTGGAGCAT
Yuan5702C_viridans
TCATCGAATCTTTGAACGCACCTTGCGCTCCTTGGTATTCCTCGGAGCAT
858Dai17003
TCATCGAATCTTTGAACGCACCTTGCGCTCCTTGGTATTCCTCGGAGCAT

Yuan2747_Ceriporia_viridans
TCATCGAATTTTTGAACGCACCTTGCGCTCCTTGGTATTCCTCGGAGCAT
Yuan2744C_viridans
TCATCGAATTTTTGAACGCACCTTGCGCTCCTTGGTATTCCTCGGAGCAT
Li1046C_viridans
TCATCGAATCTTTGAACGCACCTTGCGCTCCTTGGTATTCCTCGGAGCAT
865C_sinoviridans
TCATCGAATCTTTGAACGCACCTTGCGCTCCTTGGTATTCCTCGGAGCAT
871Dai15062
TCATCGAATCTTTGAACGCACCTTGCGCTCCTTGGTATTCCTCGGAGCAT
Dai7642_Ceriporia_humilis
TCATCGAATCTTTGAACGCACCTTGCGCTCCTTGGTATTCCTCGGAGCAT
Spirin4706_Ceriporia_humilis
TCATCGAATCTTTGAACGCACCTTGCGCTCCTTGGTATTCCTCGGAGCAT
Spirin4944_Ceriporia_sericea
TCATCGAATCTTTGAACGCACCTTGCGCTCCTTGGTATTCCTCGGAGCAT
WCG1547Dai26044ceriporia
TCATCGAATCTTTGAACGCACCTTGCGCTCCTTGGTATTCCTCGGAGCAT
ZZW1558Dai27086
TCATCGAATCTTTGAACGCACCTTGCGCTCCTTGGTATTCCTCGGAGCAT
Miettinen14381_Ceriporia_mpuri --
Miettinen15492_2_Ceriporia_sor
TCATCGAATCTTTGAACGCACCTTGCGCTCCTTGGTATTCCTCGGAGCAT
He6687
TCATCGAATCTTTGAACGCACCTTGCGCTCCTTGGTATTCCTCGGAGCAT
ZH53Dai24426
TCATCGAATCTTTGAACGCACCTTGCGCTCCTTGGTATTCCTCGGAGCAT
Vlasak0808_30_Ceriporia_punica
TCATCGAATCTTTGAACGCACCTTGCGCTCCTTGGTATTCCTCGGAGCAT
887Dai13376
TCATCGAATCTTTGAACGCACCTTGCGCTCCTTGGTATTCCTCGGAGCAT
WCG1443Dai24998
TCATCGAATCTTTGAACGCACCTTGCGCTCCTTGGTATTCCTTGGAGCAT
0108_6Ceriporia_spissa
TCATCGAATCTTTGAACGCACCTTGCGCTCCTTGGTATTCCTCGGAGCAT
Dai19164
TCATCGAATCTTTGAACGCACCTTGCGCTCCTTGGTATTCCTCGGAGCAT
Dai17937_Ceriporia_bubalinomar
TCATCGAATCTTTGAACGCACCTTGCGCTCCTTGGTATTCCTTGGAGCAT
903Dai12113
TCATCGAATCTTTGAACGCACCTTGCGCTCCTTGGTATTCCTTGGAGCAT
LZB929Dai25079
TCATCGAATCTTTGAACGCACCTTGCGCTCCTTGGTATTCCTCGGAGCAT
LX45Dai26988

TCATCGAATCTTTGAACGCACCTTGCGCTCCTTGGTATTCCTCGGAGCAT

LX43Dai26986

TCATCGAATCTTTGAACGCACCTTGCGCTCCTTGGTATTCCTCGGAGCAT

Dai7759Ceriporia

TCATCGAATCTTTGAACGCACCTTGCGCTCCTTGGTATTCCTCGGAGCAT

Cui8012_Ceriporia_viridans

TCATCGAATCTTTGAACGCACCTTGCGCTCCTTGGTATTCCTCGGAGCAT

GC1704_54Ceriporia_viridans

TCATCGAATCTTTGAACGCACCTTGCGCTCCTTGGTATTCCTCGGAGCAT

Dai23392

TCATCGAATCTTTGAACGCACCTTGCGCTCCTTGGTATTCCTCGGAGCAT

WCG1585Dai26113Ceriproia

TCATCGAATCTTTGAACGCACCTTGCGCTCCTTGGTATTCCTCGGAGCAT

Dai18675C_eucalypti

TCATCGAATCTTTGAACGCACCTTGCGCTCCTTGGTATTCCTCGGAGCAT

Dai22034

TCATCGAATCTTTGAACGCACCTTGCGCTCCTAGGTATTCCTTGGAGCAT

JV1008_41JTardaFLORIDAKeys

TCATCGAATCTTTGAACGCACCTTGCGCTCCTAGGTATTCCTTGGAGCAT

Rivoire1161_Ceriporia_pierii

TCATCGAATCTTTGAACGCACCTTGCGCTCCTTGGTATTCCTCGGAGCAT

Dai23499C_pierii

TCATCGAATCTTTGAACGCACCTTGCGCTCCTTGGTATTCCTCGGAGCAT

Dai23500

TCATCGAATCTTTGAACGCACCTTGCGCTCCTTGGTATTCCTCGGAGCAT

841Dai15899

TCATCGAATCTTTGAACGCACCTTGCGCTCCTGGGCATTCCTAGGAGCAT

842Dai15904

TCATCGAATCTTTGAACGCACCTTGCGCTCCTGGGCATTCCTAGGAGCAT

LZB1066xinjiang

TCATCGAATCTTTGAACGCACCTTGCGCTCCTGGGCATTCCTAGGAGCAT

LZB1065xinjiang

TCATCGAATCTTTGAACGCACCTTGCGCTCCTGGGCATTCCTAGGAGCAT

851Dai16779

TCATCGAATCTTTGAACGCACCTTGCGCTCCTTGGTATTCCTCGGAGCAT

RMJ119sp_Candelabrochaete_sept

TCATCGAATCTTTGAACGCACCTTGCGCTCCTCGGTATTCCTTGGAGCAT

RLG9759spCandelabrochaete_sept

TCATCGAATCTTTGAACGCACCTTGCGCTCCTCGGTATTCCTTGGAGCAT

RLG10478Phanerochaete_allantos

TCATCGAATCTTTGAACGCACCTTGCGCTCCTTGGTATTCCTCGGAGCAT

Dai19118_Ceriporia_spissa

TCATCGAATCTTTGAACGCACCTTGCGCTCCTTGGTATTCCTTGGAGCAT

Dai18486A

TCATCGAATCTTTGAACGCACCTTGCGCTCCTTGGTATTCCTTGGAGCAT
WEI17_024_Ceriporia_mellita
TCATCGAATCTTTGAACGCACCTTGCGCTCCTTGGTATTCCTTGGAGCAT
GC1508_71Ceriporia_mellita
TCATCGAATCTTTGAACGCACCTTGCGCTCCTTGGTATTCCTTGGAGCAT
GC1608_7_Ceriporia_mellita
TCATCGAATCTTTGAACGCACCTTGCGCTCCTTGGTATTCCTTGGAGCAT
ZZW1557Dai27085
TCATCGAATCTTTGAACGCACCTTGCGCTCCTTGGTATTCCTTGGAGCAT
ZZW1554Dai27083
TCATCGAATCTTTGAACGCACCTTGCGCTCCTTGGTATTCCTTGGAGCAT
Dai8168
TCATCGAATCTTTGAACGCACCTTGCGCTCCTTGGTATTCCTTGGAGCAT
BR4865C_mellita
TCATCGAATCTTTGAACGCACCTTGCGCTCCTTGGTATTCCTTGGAGCAT
MEL2382688Ceriporia_sp
TCATCGAATCTTTGAACGCACCTTGCGCTCCTTGGTATTCCTTGGAGCAT
Dai8110
TCATCGAATCTTTGAACGCACCTTGCGCTCCTTGGTATTCCTTGGAGCAT
Cui8097
TCATCGAATCTTTGAACGCACCTTGCGCTCCTTGGTATTCCTTGGAGCAT
909Cui6740
TCATCGAATCTTTGAACGCACCTTGCGCTCCTTGGTATTCCTTGGAGCAT
W1258Dai24695
TCATCGAATCTTTGAACGCACCTTGCGCTCCTTGGTATTCCTTGGAGCAT
JV0110_26_Ceriporia_griseoviol
TCATCGAATCTTTGAACGCACCTTGCGCCCCTTGGTATTCCTTGGAGCAT
896Dai13202
TCATCGAATCTTTGAACGCACCTTGCGCCCCTTGGTATTCCTTGGAGCAT
LWY393Dai27053C_griseoviolasce
TCATCGAATCTTTGAACGCACCTTGCGCCCCTTGGTATTCCTTGGAGCAT
LWY394DAI27054
TCATCGAATCTTTGAACGCACCTTGCGCCCCTTGGTATTCCTTGGAGCAT
FP135015G_pannocinctus
TCATCGAATCTTTGAACGCACCTTGCGCCCCTTGGTATTCCGAGGGGCAT
L15726SpG_pannocinctus
TCATCGAATCTTTGAACGCACCTTGCGCCCCTTGGTATTCCGAGGGGCAT
Dai22221
TCATCGAATCTTTGAACGCACCTTGCGCCCCTTGGTATTCCGAGGGGCAT
Dai22633
TCATCGAATCTTTGAACGCACCTTGCACCCCTTGGTATTCCGAGGGGTAT
Dai23260
TCATCGAATCTTTGAACGCACCTTGCACCCCTTGGTATTCCGAGGGGTAT
Dai23626

TCATCGAATCTTTGAACGCACCTTGCACCCCTTGGTATTCCGAGGGGTAT
Dai16238G_citrinoalbus
TCATCGAATCTTTGAACGCACCTTGCACCCCTTGGTATTCCGAGGGGTAT
1175Dai15293
TCATCGAATCTTTGAACGCACCTTGCACCCCTTGGTATTCCGAGGGGTAT
Dai19547
TCATCGAATCTTTGAACGCACCTTGCACCCCTTGGTATTCCGAGGGGTAT
918063G_africanus
TCATCGAATCTTTGAACGCACCTTGCACCCCTTGGTATTCCGAGGGGTAT
918572G_africanus
TCATCGAATCTTTGAACGCACCTTGCACCCCTTGGTATTCCGAGGGGTAT
Dai18536A
TCATCGAATCTTTGAACGCACCTTGCACCCCTTGGTATTCCGAGGGGTAT
1164Cui17922
TCATCGAATCTTTGAACGCACCTTGCACCCCTTGGTATTCCGAGGGGTAT
Dai22225
TCATCGAATCTTTGAACGCACCTTGCACCCCTTGGTATTCCGAGGGGTAT
1163Dai20655
TCATCGAATCTTTGAACGCACCTTGCACCCCTTGGTATTCCGAGGGGTAT
Yuan4397G_hainanensis
TCATCGAATCTTTGAACGCACCTTGCGCCCTTTGGTATTCCGAAGGGCAT
1176Dai15268
TCATCGAATCTTTGAACGCACCTTGCGCCCTTTGGTATTCCGAAGGGCAT
1177Dai15259
TCATCGAATCTTTGAACGCACCTTGCGCCCTTTGGTATTCCGAAGGGCAT
BZ2896G_thelephoroides
TCATCGAATCTTTGAACGCACCTTGCGCCCCTTGGTATTCCGAGGGGCAT
1166JV1808_26
TCATCGAATCTTTGAACGCACCTTGCGCCCCTTGGTATTCCGAGGGGCAT
Miettinen16992Hapalopilus_ochr
TCATCGAATCTTTGAACGCAACTTGCGCCCTTTGGCATTCCGAGGGGCAC
GC1708_338_Ceriporia_arbuscula
TCATCGAATCTTTGAACGCACCTTGCGCTCCTTGGTATTCCTCGGAGCAT
WCG1555Dai26107Ceriporia
TCATCGAATCTTTGAACGCACCTTGCGCTCCTTGGTATTCCTCGGAGCAT
GC1708_340_Ceriporia_arbuscula
TCATCGAATCTTTGAACGCACCTTGCGCTCCTTGGTATTCCTCGGAGCAT
WCG1556Dai26109Ceriporia
TCATCGAATCTTTGAACGCACCTTGCGCTCCTTGGTATTCCTCGGAGCAT
883Cui11291
TCATCGAATCTTTGAACGCACCTTGCGCTCCTTGGTATTCCTTGGAGCAT
HLX320Dai26805
TCATCGAATCTTTGAACGCACCTTGCGCTCCTTGGTATTCCTCGGAGCAT
WCG1266Dai24678A

TCATCGAATCTTTGAACGCACCTTGCGCTCCTTGGCATTCCTTGGAGCAT
Dai6090_Ceriporia_sulphuricolo
TCATCGAATCTTTGAACGCACCTTGCGCTCCTTGGTATTCTGAGGAGCAT
RLG_11354_Ceriproia_reticulata
TCATCGAATCTTTGAACGCACCTTGCGCTCCTTGGTATTCCTTGGAGCAT
ZZW1543Dai27072
TCATCGAATCTTTGAACGCACCTTGCGCTCCTTGGTATTCCTTGGAGCAT
Li1316_Ceriporia_reticulata
TCATCGAATCTTTGAACGCACCTTGCGCTCCTTGGTATTCCTTGGAGCAT
KHL11981Ceriporia_reticulata
TCATCGAATCTTTGAACGCACCTTGCGCTCCTTGGTATTCCTTGGAGCAT
FP110343sp_Candelabrochaete_la
TCATCGAATCTTTGAACGCACCTTGCGCTCCTTGGTATTCCTTGGAGCAT
Li1045_Ceriporia_reticulata
TCATCGAATCTTTGAACGCACCTTGCGCTCCTTGGTATTCCTTGGAGCAT
ZX136Dai25794ceriporia
TCATCGAATCTTTGAACGCACCTTGCGCTCCTTGGTATTCCTTGGAGCAT
892Dai13400
TCATCGAATCTTTGAACGCACCTTGCGCTCCTTGGTATTCCTTGGAGCAT
RLG7163Leptoporus_mollis
TCATCGAATCTTTGAACGCACCTTGCGCTCCTTGGTATTCCTTGGAGCAT
Dai21062Leptoporus_mollis
TCATCGAATCTTTGAACGCACCTTGCGCTCCTTGGTATTCCTTGGAGCAT
Dai20182Leptoporus_submollis
TCATCGAATCTTTGAACGCACCTTGCGCTCCTTGGTATTCCTTGGAGCAT
Cui18379Leptoporus_submollis
TCATCGAATCTTTGAACGCACCTTGCGCTCCTTGGTATTCCTTGGAGCAT
Wu1209_46Resiniporus_pseudogil
TCATCGAATCTTTGAACGCACCTTGCGCTCCTTGGTATTCCGAGGAGCAT
BRNM710169Resiniporus_resinasc
TCATCGAATCTTTGAACGCACCTTGCGCTCCTTGGTATTCCGAGGAGCAT
Dai14516Bjerkandera_adusta
TCATCGAATCTTTGAACGCACCTTGCGCTCCTTGGTATTCCGAGGAGCAT
Dai21100Bjerkandera_fumosa
TCATCGAATCTTTGAACGCACCTTGCGCTCCTTGGTATTCCGAGGAGCAT
Miettinen16854Ceraceomyces_sp
TCATCGAATCTTTGAACGCACCTTGCGCTCTTTGGTATTCCGAAGAGCAT
Dai10477C_spissa
TCATCGAATCTTTGAACGCACCTTGCGCTCCTTGGTATTCCTTGGAGCAT
855Dai16831
TCATCGAATCTTTGAACGCACCTTGCGCTCCTTGGTATTCCTTGGAGCAT
882Cui11282
TCATCGAATCTTTGAACGCACCTTGCGCTCCTTGGTATTCCTTGGAGCAT
Dai24566

TCATCGAATCTTTGAACGCACCTTGCGCTCCTTGGTATTCCTTGGAGCAT

Yuan5965

TCATCGAATCTTTGAACGCACCTTGCGCTCCTTGGTATTCCTTGGAGCAT

Dai3204 --

1194CUI9985 --

Dai15205_Ceriporia_albomellea GCCTGTTTGAGTATCATGAAAATCTCAACTC-CT---------------A

Dai15223_Ceriporia_albomellea GCCTGTTTGAGTATCATGAAAATCTCAACTC-CT---------------A

Li1780_Ceriporia_variegata GCCTGTTTGAGTATCATGAAAATCTCAACTT-CT---------------A

Dai19791_Ceriporia_variegata GCCTGTTTGAGTATCATGAAAATCTCAACTT-CT---------------A

Dai19886 GCCTGTTTGAGTATCATGAAAATCTCAACTT-CT---------------A

Dai10833_Ceriporia_crassitunic GCCTGTTTGAGTATCATGGAAACCTCAACTT-CTAGAAACTTTTTGATAA

CHWC1506_46Meruliopsis_crassit GCCTGTTTGAGTATCATGGAAACCTCAACTT-CTAGAAAATTTTTGATAA

Dai9995_Ceriporia_crassitunica GCCTGTTTGAGTATCATGGAAATCTCAACTT-CTAGAAATGTTTAATAAA

Wu1209_58_Meruliopsis_parvispo GCCTGTTTGAGTATCATGGAATTCTCAACTC-CCAAATTTTTT----TAA

CHWC1505_129_Meruliopsis_parvi GCCTGTTTGAGTATCATGGAATTCTCAACTC-CCAATTTTTTT-----AA

Dai21944 GCCTGTTTGAGTATCATGGAATTCTCAACTC-CCTTATTTTTT----TAA

830Dai18640A GCCTGTTTGAGTATCATGGAATTCTCAACTC-CCAAATTTTTT----TAA

GC1704_60_Meruliopsis_taxicola GCCTGTTTGAGTATCATGGAATTCTCAACTC-CCAAATTTTTT---TTAA

Dai22625 GCCTGTTTGAGTATCATGGAATTCTCAACTC-CCAAATTTTTT---TTAA

Dai22636 GCCTGTTTGAGTATCATGGAATTCTCAACTC-CCAAATTTTTT---TTAA

Dai21878 GCCTGTTTGAGTATCATGGAATTCTCAACTC-CCAAATTTTTT----TAA

1169Dai17248 GCCTGTTTGAGTATCATGGAATTCTCAACTC-CCAAATTTTTT----TAA

Wu1708_43_Meruliopsis_leptocys GCCTGTTTGAGTATCATGGAATTCTCAACTC-CTAAATTTT----TGTTA

Li1011 GCCTGTTTGAGTATCATGGAATTCTCAACTC-

CTAAATTTT----TGTTA
ZX95Dai25742Meruliopsis_leptoc GCCTGTTTGAGTATCATGGAATTCTCAACTC-
CTAAATTTT----TGTTA
WCG1306Dai24733 GCCTGTTTGAGTATCATGGAATTCTCAACTC-
CTAAATTTT----TGTTA
LXL99Dai25816 GCCTGTTTGAGTATCATGGAATTCTCAACTC-
CTAAATTTT----TGTTA
WCG1559Dai26052Meruliopsis GCCTGTTTGAGTATCATGGAATTCTCAACTC-
CCAAATTTTTTAATAAAA
He7477 GCCTGTTTGAGTATCATGGAATTCTCAACTC-
CCAAATTTTTTAATAAAA
HLX243Dai26217 GCCTGTTTGAGTATCATGGAATTCTCAACTC-
CCAAATTTTTTAATAAAA
RussiaMW673659Meruliopsis_fagi GCCTGTTTGAGTATCATGGAATTCTCAACTC-
CCCAAATTTTT--TAATA
FD278 GCCTGTTTGAGTATCATGGAATTCTCAACTC-
CCAAAATTTTTTGAAACA
Dai10226_Ceriporia_tarda GCCTGTTTGAGTATCATGGAATTCTCAACTC-
CTAAATTTATT-------
LE247365 GCCTGTTTGAGTATCATGGAATTCTCAACTC-
CTAAATTTATT-------
Dai8173_Meruliopsis_nanlingens GCCTGTTTGAGTATCATGGAATTCTCAACTC-
CTGATTTTTTT------A
860Dai17172 GCCTGTTTGAGTATCATGGAATTCTCAACTC-
CTGATTTTTTT------A
879Dai13414 GCCTGTTTGAGTATCATGGAATTCTCAACTC-
CTGATTTTTTT------A
Li_1704_Meruliopsis_pseudocyst GCCTGTTTGAGTATCATGGAATTCTCAACTC-
CTCAATTTTTT--TT-AA
833Dai18405 GCCTGTTTGAGTATCATGGAATTCTCAACTC-
CTCAATTTTTTT----AA
HHB_10729_Meruliopsis_albostra GCCTGTTTGAGTATCATGGAATTCTCAACTC-
CTCAATTTTTT--TT-AT
Cui6878_Ceriporia_pseudocystid GCCTGTTTGAGTATCATGGAATTCTCAACTC-
CTTAATTTTTTT----AA
869Dai14737 GCCTGTTTGAGTATCATGGAATTCTCAACTC-
CTTAATTTTTTT----AA
876Cui11626 GCCTGTTTGAGTATCATGGAATTCTCAACTC-
CTTAATTTTTTT----AA
1199WEI3388 GCCTGTTTGAGTATCATGGAATTCTCAACTC-
CTTAATTTTTTT----AA
776308_Meruliopsis_cystidiata GCCTGTTTGAGTATCATGGAAATCTCAACTC-
CTTGATTTTTT--TTAAA
ICN139059_Meruliopsis_cystidia GCCTGTTTGAGTATCATGGAAATCTCAACTC-

CTTGATTTTTT--TAAAA
HHB15692Ceraceomyces_serpens GCCTGTTTGAGTGTCATGGAATTCTCAACCC-AAAGAATTTTTT-TT---
HHB_15629_Sp_Ceriporiopsis_ane GCCTGTTTGAGTGTCATGGAATTCTCAACCC-TTAGAATTTATTTC---
AJ185Trametopsis_cervina GCCTGTTTGAGTGTCATGGAATTCTCAACTC-ATTAGATTTTTTTCT---
FD9Irpex_lacteus GCCTGTTTGAGTCTCATGGTATTCTCRACCC-CTAAATTTTTGTAATGAA
908Dai11230 GCCTGTTTGAGTCTCATGGTATTCTCAACCC-CTAAATTTTTGTAATGAA
FP55521TEmmia_lacerata GCCTGTTTGAGTCTCATGGAATTCTCAACCC-CTAAA-TTTTGTAATG-A
PBU0048Ceriporia_cystidiata GCCTGTTTGAGTCTCATGGAATTCTCAACCC-CTAAA-TTTTGTAATG-A
MZ340C_lacerataT GCCTGTTTGAGTCTCATGGAATTCTCAACCC-CTAAA-TTTTGTAATG-A
Dai21940 GCCTGTTTGAGTCTCATGGAATTCTCAACCC-CTAAA-TTTTGTAATG-A
847Dai16433 GCCTGTTTGAGTCTCATGGAATTCTCAACCC-CTAAA-TTTTGTAATG-A
MarcinEmmia_latemarginatus GCCTGTTTGAGTCTCATGGAATTCTCAACCC-CTAAA-TTTTGTAATG-A
Meijer3729Hydnopolyporus_fimbr GCCTGTTTGAGTCTCATGGAATTCTCAACCC-CTAAATTTTTGTAATGAA
RLG13408Phanerochaete_sp GCCTGTTTGAGTGTCATGGAATTCTCAACCC-TCAAA-TTTTTTAATCAA
WHC1381Flavodon_flavus GCCTGTTTGAGTCTCATGGTATTCTCAACCC-CTAAATTTTTCTAGTGAA
GB1833Phlebia_albida GCCTGTTTGAGTGTCATGGAATTCTCAACCT-TCAA--TTTTGTTA----
T407Phlebia_nitidula GCCTGTTTGAGTGTCATGGAATTCTCAACCT-TCAA--TTTTATTA----
HHB6988Phanerochaete_exilis GCCTGTTTGAGTGTCATGGTATTCTCAATCC-CT-AGATTTTGTTATGA-
HHB8509Phanerochaetella_xeroph GCCTGTTTGAGTGTCATGGAATTCTCAATCC-TC-AGATTTTGTCATGA-
PBU0051Macrohyporia_dictyopora GCCTGTTTGAGTGTCATGGAATTCTCAATCC-CT-AGATTTTGTAATGA-
HHB11463Phanerochaete_sp GCCTGTTTGAGTGTCATGGAATTCTCAATCC-CTAAGATTTTGTAATGA-
FP102382Byssomerulius_corium GCCTGTTTGAGTATCATGAAATTCTCAACTC-CCCATTTTTATTAAT---
FP102165Efibula_americana GCCTGTTTGAGTGTCGTGAAATTCTCAATCC-

CTTAGATTTCATTAATGA
Murdoch90Ceriporia_torpida GCCTGTTTGAGTATCATGAAATTCTCAACTC-
TCAAGTTTC--TTAACAG
Rivoire4413_Ceriporia_purpurea GCCTGTTTGAGTATCATGAAATTCTCAACTC-
CCAAGTTTTCTTTAACAA
Kout_18_Ceriporia_triumphalis GCCTGTTTGAGTATCATGAAATTCTCAACTC-
CCAAGTTTC--TTAACGG
Rivoire3701_Ceriporia_bresadol GCCTGTTTGAGTATCATGAAATTCTCAACTC-
CCAAGTTTTC-TTAACGG
VS4018 GCCTGTTTGAGTATCATGAAATTCTCAACTC-
CCAAGTTTTC-TTAACGG
Ryvarden21832_Ceriporia_manzan GCCTGTTTGAGTATCATGAAATTCTCAACTC-
CCAAGTTTTCTTTAACGG
Dai24539 GCCTGTTTGAGTATCATGAAATTCTCAACTC-
CCAAGTTTTC-TTAACGG
Dai24541 GCCTGTTTGAGTATCATGAAATTCTCAACTC-
CCAAGTTTTC-TTAACGG
JV1105_12_Ceriporia_occidental GCCTGTTTGAGTATCATGAAATTCTCAACTC-
CCAAGTTTC--TTCACAG
VS8558Ceriporia_occidentalis GCCTGTTTGAGTATCATGAAATTCTCAACTC-
CCAAGTTTC--TTCACAG
Dai22445 GCCTGTTTGAGTATCATGAAATTCTCAACTC-
CCAAGTTTTCT-------
846Dai16368 GCCTGTTTGAGTATCATGAAATTCTCAACTC-
CCAAGTTTTCTTTAACAA
Dai17951_Ceriporia_aurantiocar GCCTGTTTGAGTATCATGGAATTCTCAACTC-
CCAAGTTTCTTATGA---
Miettinen_11701C_viridans GCCTGTTTGAGTATCATGGAATTCTCAACTC-
TCAAGTTTCTTAGGA---
JV0105_10Ceriporia_aurantiocar GCCTGTTTGAGTATCATGGAATTCTCAACTC-
CCAAGTTTCTTATGA---
Yuan5702C_viridans GCCTGTTTGAGTATCATGGAATTCTCAACTC-
TCAAGTTTCTTAGGA---
858Dai17003 GCCTGTTTGAGTATCATGGAATTCTCAACTC-
TCAAGTTTCTTAGGA---
Yuan2747_Ceriporia_viridans GCCTGTTTGAGTATCATGGAATTATCAACTC-
TCAAGTTTCTTATGA---
Yuan2744C_viridans GCCTGTTTGAGTATCATGGAATTATCAACTC-
TCAAGTTTCTTATGA---
Li1046C_viridans GCCTGTTTGAGTATCATGGAATTCTCAACTC-
TCAGTTTTCTTACGA---
865C_sinoviridans GCCTGTTTGAGTATCATGGAATTCTCAACTC-
TCAGTTTTCTTACGA---
871Dai15062 GCCTGTTTGAGTATCATGGAATTATCAACTC-

TCAAGTTTCTTATGA---

Dai7642_Ceriporia_humilis GCCTGTTTGAGTATCATGGAATTCTCAACTC-CCAAGTTTCTTA-CTGA-

Spirin4706_Ceriporia_humilis GCCTGTTTGAGTATCATGGAATTCTCAACTC-CCAAGTTTCTTA-CTGA-

Spirin4944_Ceriporia_sericea GCCTGTTTGAGTATCATGGAATTCTCAACTC-CCAAGTTTCTTACCTGA-

WCG1547Dai26044ceriporia GCCTGTTTGAGTATCATGGAATTCTCAACTC-CCAAGTTTCTTACCTGA-

ZZW1558Dai27086 GCCTGTTTGAGTATCATGGAATTCTCAACTC-CCAAGTTTCTTACCTGA-

Miettinen14381_Ceriporia_mpuri --

Miettinen15492_2_Ceriporia_sor GCCTGTTTGAGTATCATGGAATTCTCAACTC-CCGAGTTTCTTCATTGA-

He6687 GCCTGTTTGAGTATCATGGAATTCTCAACTC-CCAAGTTTCTTA-ACGA-

ZH53Dai24426 GCCTGTTTGAGTATCATGGAATTCTCAACTC-CCAAGTTTCTTA-ACGA-

Vlasak0808_30_Ceriporia_punica GCCTGTTTGAGTATCATGGAATTCTCAACCGCTCAGGTTTTCTTTATTGA

887Dai13376 GCCTGTTTGAGTATCATGGAATTCTCAACCGCTCAGGTTTTCTTAATCGA

WCG1443Dai24998 GCCTGTTTGAGTATCATGGAATTCTCAACCCTTCAGGTTTTGTTAAATCG

0108_6Ceriporia_spissa GCCTGTTTGAGTATCATGAAAATCTCAAACT-TGATGTGTTTTTTTTAA-

Dai19164 GCCTGTTTGAGTATCATGAAAATCTCAAACT-TGATGTGTTTTTTTTAA-

Dai17937_Ceriporia_bubalinomar GCCTGTTTGAGTATCATGAAAATCTCAAGAT-TGATGTG-TTTTGCTAA-

903Dai12113 GCCTGTTTGAGTATCATGAAAATCTCAAGAT-TGATGTG-TTTTGCTAA-

LZB929Dai25079 GCCTGTTTGAGTATCATGGAATTCTCAACTC-CCAAGTTTCTTATGA---

LX45Dai26988 GCCTGTTTGAGTATCATGGAATTCTCAACTC-CCAAGTTTCTTATGA---

LX43Dai26986 GCCTGTTTGAGTATCATGGAATTCTCAACTC-CCAAGTTTCTTATGA---

Dai7759Ceriporia GCCTGTTTGAGTATCATGGAATTCTCAACTC-CCAAGTTTCTTATGA---

Cui8012_Ceriporia_viridans GCCTGTTTGAGTATCATGGAATTCTCAACTC-CCAAGTTTCTTTTGA---

GC1704_54Ceriporia_viridans GCCTGTTTGAGTATCATGGAATTCTCAACTC-CCAAGTTTCTTCTGA---

Dai23392 GCCTGTTTGAGTATCATGGAATTCTCAACTC-CCAAGTTTCTTTTGA---

WCG1585Dai26113Ceriproia GCCTGTTTGAGTATCATGGAATTCTCAACTC-CCAAGTTTCTTTTGA---

Dai18675C_eucalypti GCCTGTTTGAGTATCATGGAATTCTCAACTC-CCAAGTTTCTTGTGA---

Dai22034 GCCTGTTTGAGTATCATGAAATTCTCAACTC-CCAAGTT-TCTTACT---

JV1008_41JTardaFLORIDAKeys GCCTGTTTGAGTATCATGAAATTCTCAACTC-CCAAGTT-TCTTACT---

Rivoire1161_Ceriporia_pierii GCCTGTTTGAGTATCATGGAATTCTCAACTC-CCCAGTTTCTTATTGA--

Dai23499C_pierii GCCTGTTTGAGTATCATGGAATTCTCAACTC-CCCAGTTTCTTATTGA--

Dai23500 GCCTGTTTGAGTATCATGGAATTCTCAACTC-CCCAGTTTCTTATTGA--

841Dai15899 GCCTGTTTGAGTATCATGGAATTCTCAACTC-ATCAGCTTTTTCTTATGA

842Dai15904 GCCTGTTTGAGTATCATGGAATTCTCAACTC-ATCAGCTTTTTCTTATGA

LZB1066xinjiang GCCTGTTTGAGTATCATGGAATTCTCAACTC-ATCAGCTTTTTCTTATGA

LZB1065xinjiang GCCTGTTTGAGTATCATGGAATTCTCAACTC-ATCAGCTTTTTCTTATGA

851Dai16779 GCCTGTTTGAGTATCATGGAATTCTCAACTC-TCCAGTTTTTTTAAAG-

RMJ119sp_Candelabrochaete_sept GCCTGTTTGAGTATCATGAAATTCTCAACTC-CCGAATTTCTTGTCGA--

RLG9759spCandelabrochaete_sept GCCTGTTTGAGTATCATGAAATTCTCAACTC-CCGAATTTCTTGTCGA--

RLG10478Phanerochaete_allantos GCCTGTTTGAGTATCATGGAATTCTCAACTC-TCAAGTTTTCT-------

Dai19118_Ceriporia_spissa GCCTGTTTGAGTATCATGAGACTCTCAACTTGCTACATTTTTGTATT-GA

Dai18486A GCCTGTTTGAGTATCATGAGACTCTCAACTTGCTACATTTTTGTATT-GA

WEI17_024_Ceriporia_mellita GCCTGTTTGAGTATCATGAGACTCTCACCTTGCTACATTTTTGTATT-GA

GC1508_71Ceriporia_mellita GCCTGTTTGAGTATCATGAGACTCTCAACTTGCTACATTTTTGTATT-GA

GC1608_7_Ceriporia_mellita GCCTGTTTGAGTATCATGAGACTCTCAACTTGCTACATTTTTGTATT-GA

ZZW1557Dai27085 GCCTGTTTGAGTATCATGAGACTCTCAACTTGCTACATTTTTGTATT-GA

ZZW1554Dai27083
GCCTGTTTGAGTATCATGAGACTCTCAACTTGCTACATTTTTGTATT-GA
Dai8168
GCCTGTTTGAGTATCATGAGACTCTCAACTTGCTACATTTTTGTATT-GA
BR4865C_mellita
GCCTGTTTGAGTATCATGAGACTCTCAACTTGCTACGTTTTTGTATT-GA
MEL2382688Ceriporia_sp
GCCTGTTTGAGAATCATGAGACTCTCAACTTGCTACATTTTTATGTTAAA
Dai8110
GCCTGTTTGAGTATCATGAGACTCTCAACTTGCTACATTTTTGTATT-GA
Cui8097
GCCTGTTTGAGTATCATGAGACTCTCAACTTGCTACATTTTTGTATT-GA
909Cui6740
GCCTGTTTGAGAATCATGAGACTCTCAACTTGCTACATTTTTATGTTAAA
W1258Dai24695
GCCTGTTTGAGAATCATGAGACTCTCAACTTGCTACATTTTTATGTTAAA
JV0110_26_Ceriporia_griseoviol GCCTGTTTGAGTATCATGGAAATCTCAACTC-CTGAGCTTAATTGTT---
896Dai13202 GCCTGTTTGAGTATCATGGAAATCTCAACTC-CTGAGCTTAATTGTT---
LWY393Dai27053C_griseoviolasce GCCTGTTTGAGTATCATGGAAATCTCAACTC-CTGAGCTTAATTGTT---
LWY394DAI27054
GCCTGTTTGAGTATCATGGAAATCTCAACTC-CTGAGCTTAATTGTT---
FP135015G_pannocinctus GCCTGTTTGAGTGTCATGGAATTCTCATCCC-CTAAAKTT----------
L15726SpG_pannocinctus GCCTGTTTGAGTGTCATGGAATTCTCATCCC-CTAAAKTT----------
Dai22221 GCCTGTTTGAGTGTCATGGAATTCTCAACCC-CCAAATTCTTTATTG---
Dai22633 GCCTGTTTGAGTGTCATGGTATTCTCAACCC-CCAAATTCTTTATTG---
Dai23260 GCCTGTTTGAGTGTCATGGTATTCTCAACCC-CCAAATTCTTTATTG---
Dai23626 GCCTGTTTGAGTGTCATGGTATTCTCAACCC-CCAAATTCTTTATTG---
Dai16238G_citrinoalbus GCCTGTTTGAGTGTCATGGTATTCTCAACCC-CCAAATTCTTTATCG---
1175Dai15293 GCCTGTTTGAGTGTCATGGTATTCTCAACCC-CCAAATTCTTTATCG---
Dai19547 GCCTGTTTGAGTGTCATGGTATTCTCAACCC-CCAAATTCTTTATCG---
918063G_africanus GCCTGTTTGAGTGTCATGGTATTCTCAACCC-CCAAATTCTTTATTG---

918572G_africanus GCCTGTTTGAGTGTCATGGTATTCTCAACCC-
CCAAATTCTTTATTG---
Dai18536A GCCTGTTTGAGTGTCATGGTATTCTCAACCC-
CCAAATTCTTTATTG---
1164Cui17922 GCCTGTTTGAGTGTCATGGTATTCTCAACCC-
CCAAATTCTTTATTG---
Dai22225 GCCTGTTTGAGTGTCATGGTATTCTCAACCC-
CCAAATTCTTTATTG---
1163Dai20655 GCCTGTTTGAGTGTCATGGTATTCTCAACCC-
CCAAATTCTTTATTG---
Yuan4397G_hainanensis GCCTGTTTGAGTGTCATGGAATTCTCAAACC-
CCCAATTTATTT------
1176Dai15268 GCCTGTTTGAGTGTCATGGAATTCTCAAACC-
CCCAATTTATTT------
1177Dai15259 GCCTGTTTGAGTGTCATGGAATTCTCAAACC-
CCCAATTTATTTT-----
BZ2896G_thelephoroides GCCTGTTTGAGTGTCATGGAATTCTCAAACC-
CCCAATTTATTT------
1166JV1808_26 GCCTGTTTGAGTGTCATGGAATTCTCAAACC-
CCCAATTTATTT------
Miettinen16992Hapalopilus_ochr ACCTGTTTGAGTATCATGGAATTCTCAATCT-
CTTAAATTCTGTATG---
GC1708_338_Ceriporia_arbuscula
GCCTGTTTGAGTATCATGGAAATCTCAACTCTCTAGATTTTTC------
WCG1555Dai26107Ceriporia
GCCTGTTTGAGTATCATGGAAATCTCAACTCTCTAGATTTTTC------
GC1708_340_Ceriporia_arbuscula
GCCTGTTTGAGTATCATGGAAATCTCAACTCTCTAGATTTTTC------
WCG1556Dai26109Ceriporia
GCCTGTTTGAGTATCATGGAAATCTCAACTCTCTAGATTTTTT-------
883Cui11291
GCCTGTTTGAGTATCATGGAATTCTCAACTCTCTAGATTTTTC-------
HLX320Dai26805
GCCTGTTTGAGTATCATGGAATTCTCAACTCATTAGAATTGTTTTTTGGT
WCG1266Dai24678A
GCCTGTTTGAGTATCATGGAATTCTCAACTTGCTAGATTTTTTCTTC---
Dai6090_Ceriporia_sulphuricolo GCCTGTTTGAGTGTCATGGAATTCTCAACCC-
CTAGGTTTGTTCTTG---
RLG_11354_Ceriproia_reticulata GCCTGTTTGAGTATCATGGAATTCTCAACTC-
CCAAGTTTCTTAT-----
ZZW1543Dai27072 GCCTGTTTGAGTATCATGGAATTCTCAACTC-
CCAAGTTTCTT-------
Li1316_Ceriporia_reticulata GCCTGTTTGAGTATCATGGAAATCTCAACTC-
CCAAGTTTT---------

KHL11981Ceriporia_reticulata GCCTGTTTGAGTATCATGGAAATCTCAACTC-CCAAGTTTT---------

FP110343sp_Candelabrochaete_la GCCTGTTTGAGTATCATGGAATTCTCAACTC-CCAAGTTTCTTA------

Li1045_Ceriporia_reticulata GCCTGTTTGAGTATCATGGAATTCTCAACTC-CCAAGTTTCTTAT-----

ZX136Dai25794ceriporia GCCTGTTTGAGTATCATGGAATTCTCAACTC-CCAAGTTTCTTAT-----

892Dai13400 GCCTGTTTGAGTATCATGGAATTCTCAACTC-CCAAGTTTCTTAT-----

RLG7163Leptoporus_mollis GCCTGTTTGAGTATCATGGAATTATCAACCC-CCAAATTTTTCCCCTCCG

Dai21062Leptoporus_mollis GCCTGTTTGAGTATCATGGAATTATCAACCC-CCAAATTTTTCCCCTCCG

Dai20182Leptoporus_submollis GCCTGTTTGAGTATCATGGAATTATCAACCC-CCAAATTTTTCCCCTCCG

Cui18379Leptoporus_submollis GCCTGTTTGAGTATCATGGAATTATCAACCC-CCAAATTTTTCCCCTCCG

Wu1209_46Resiniporus_pseudogil GCCTGTTTGAGTGTCATGGAATTCTCAACTTGTTAGAATTTT--------

BRNM710169Resiniporus_resinasc GCCTGTTTGAGTGTCATGGAATTCTCAACTTGTTAGAATTTT--------

Dai14516Bjerkandera_adusta GCCTGTTTGAGTCTCATGGAATTCTCAACCT-TCAGCTTTATTGATGAAG

Dai21100Bjerkandera_fumosa GCCTGTTTGAGTCTCATGGAATTCTCAACCT-TTGACTTTGTTGTCAGAG

Miettinen16854Ceraceomyces_sp GCCTGTTTGAGTGTCATGGACTTCTCAAAAA-CAGGATTTTTTTTTTGG

Dai10477C_spissa GCCTGTTTGAGTATCATGAAATTCTCATGCCTACAAATTTTCTTACG---

855Dai16831 GCCTGTTTGAGTATCATGAAATTCTCATGCCTACAAATTTTCTTACG---

882Cui11282 GCCTGTTTGAGTATCATGAAATTCTCATGCCTACAAATTTTCTTACG---

Dai24566 GCCTGTTTGAGCATCATGAAATTCTCATGTCTACAAATTTTCTCATG---

Yuan5965 GCCTGTTTGAGCATCATGAAATTCTCATGTCTACAAATTTTCTCATG---

Dai3204 --

1194CUI9985 --

Dai15205_Ceriporia_albomellea A----AGTTTTCAGGGGC-TTGGATATGGA-GGT-TGTGTTG---GCTCT

Dai15223_Ceriporia_albomellea A----AGTTTTCAGGGGC-TTGGATATGGA-GGT-TGTGTTG---GCTCT
Li1780_Ceriporia_variegata A----AGTTTTTAGGAGC-TTGGATATGGA-GGT-TGTGTTG---GCTCT
Dai19791_Ceriporia_variegata A----AGTTTTTAGGAGC-TTGGATATGGA-GGT-TGTGTTG---GCTCT
Dai19886 A----AGTTTTTAGGAGC-TTGGATATGGA-GGT-TGTGTTG---GCTCT
Dai10833_Ceriporia_crassitunic A----AGTTTTCAGAAGC-TTGGATGTGGAGGGT-TGTGTTG---GCTTT
CHWC1506_46Meruliopsis_crassit A----AGTTTTCAGAAGC-TTGGATGTGGAGGGT-TGTGTTG---GCTTT
Dai9995_Ceriporia_crassitunica A----TATTTTCAGAAGC-TTGGATGTGGA-GGC-TGTGTTG---GCTCT
Wu1209_58_Meruliopsis_parvispo TTAAAAGTT-TGGTGAGC-TTGGATGTGGAGGATCAGTGTTG---GCTCA
CHWC1505_129_Meruliopsis_parvi TTAAAAGTT-TGGTGAGC-TTGGATGTGGAGGATCAGTGTTG---GCTCA
Dai21944 TTAAAAGTG-AGGTGAGC-TTGGATGTGGAGGATTTGTGTCG---GCTAT
830Dai18640A TTAAAAATT-TGGTGAGC-TTGGATGTGGAGGATTAGTGTCG---GCTCC
GC1704_60_Meruliopsis_taxicola TCGAAAGTT-TGGTGAGC-TTGGACTTGGAGGT--TGTGTCG---GCTCC
Dai22625 TCGAAAGTT-TGGTGAGC-TTGGACTTGGAGGT--CGTGTCG---GCTCC
Dai22636 TCGAAAGTT-TGGTGAGC-TTGGACTTGGAGGT--CGTGTCG---GCTCC
Dai21878 TCGAAAGTT-TGGTGAGC-TTGGACTTGGAGGT--CGTGTCG---GCTCC
1169Dai17248 TCGAAAGTT-TGGTGAGC-TTGGACTTGGAGGT--CGTGTCG---GCTCC
Wu1708_43_Meruliopsis_leptocys TCAAAAATT-TGGTGAGC-TTGGACTTGGAGGC--TGTGTCG---GCTC-
Li1011 TCAAAAATT-TGGTGAGC-TTGGACTTGGAGGC--TGTGTCG---GCTC-
ZX95Dai25742Meruliopsis_leptoc TCAAAAGTT-TAGTGAGC-TTGGATTTGGAGGC--CGTGTCG---GCTC-
WCG1306Dai24733 TCAAAAGTT-TAGTGAGC-TTGGATTTGGAGGC--CGTGTCG---GCTC-
LXL99Dai25816 TCAAAAGTT-TAGTGAGC-TTGGATTTGGAGGC--CGTGTCG---GCTC-
WCG1559Dai26052Meruliopsis TAAAAAATC-TGGTGAGC-TTGGACTTGGAGGC--TGTGTCG---GCTCT

He7477 TAAAAAATC-TGGTGAGC-TTGGACTTGGAGGC--TGTGTCG---GCTCT

HLX243Dai26217 TAAAAAATC-TGGTGAGC-TTGGACTTGGAGGC--TGTGTCG---GCTCT

RussiaMW673659Meruliopsis_fagi ATAAAAATC-TGGTGAGC-TTGGACTTGGAGGT--TGTGTCG---GCTCT

FD278 TAAAAATTC-TGGTGAGC-TTGGAAGTGGAGGCT-TGTGTCG---GCTTT

Dai10226_Ceriporia_tarda -------TT-TAGTGAGC-TTGGATTTGGAGGC--TGTGTCG---GCTTT

LE247365 -------TT-TAGTGAGC-TTGGATTTGGAGGC--TGTGTCG---GCTTT

Dai8173_Meruliopsis_nanlingens AATAAAAAT-TGGTGAGC-TTGGATTTGGAGGCT-TGTGTCG---GCTTT

860Dai17172 AATAAAAAT-TGGTGAGC-TTGGATTTGGAGGCT-TGTGTCG---GCTTT

879Dai13414 AATAAAAAT-TGGTGAGC-TTGGATTTGGAGGCT-TGTGTCG---GCTTT

Li_1704_Meruliopsis_pseudocyst TTAAAAAAT-GAGTGAGC-TTGGATTTGGAGGC--TGTGTTG---GCTTT

833Dai18405 TTAAAAAAT-GAGTGAGC-TTGGATTTGGAGGC--TGTGTTG---GCTTT

HHB_10729_Meruliopsis_albostra GAAAAAAAT-GAGTGAGC-TTGGATTTGGAGGC--TGTGTTG---GCTTT

Cui6878_Ceriporia_pseudocystid TTAAAAATT-GAGTGAGC-TTGGATTTGGAGGCT-TGTGTTG---GCTTT

869Dai14737 TTAAAAATT-GAGTGAGC-TTGGATTTGGAGGCT-TGTGTTG---GCTTT

876Cui11626 TTAAAAATT-GAGTGAGC-TTGGATTTGGAGGCT-TGTGTTG---GCTTT

1199WEI3388 TTAAAAATT-GAGTGAGC-TTGGATTTGGAGGCT-TGTGTTG---GCTTT

776308_Meruliopsis_cystidiata ACAAAAATT-GAGTGAGC-TTGGAATTGGAGGT--TGTGTTG---GCTTT

ICN139059_Meruliopsis_cystidia CAAAAAATT-GAGTGAGC-TTGGAATTGGAGGT--TGTGTTG---GCGTT

HHB15692Ceraceomyces_serpens ------ATT-CTGCGGGC-TTGGACTTGGAGGCT-TGTGTCG---GCTCT

HHB_15629_Sp_Ceriporiopsis_ane ----------TAATGGGC-TTGGACTTGGAGGCT-TGTGTCG---GCTGC

AJ185Trametopsis_cervina ----------AATGCAGC-TTGGACTTGGAGG-T-TGTGTCG---GTTTT

FD9Irpex_lacteus -----GGTT-TAGCGGGC-TTGGACTTGGAGG-T-TGTGTCG---GCC--

908Dai11230 -----GGTT-TAGCGGGC-TTGGACTTGGAGG-T-TGTGTCG---GCC--
FP55521TEmmia_lacerata -----AGTT-TAGTGGGC-TTGGACTTGGAGG-T-TGTGTCG---GC---
PBU0048Ceriporia_cystidiata -----AGTT-TAGTGGGC-TTGGACTTGGAGG-T-TGTGTCG---GC---
MZ340C_lacerataT -----AGTT-TAGTGGGC-TTGGACTTGGAGG-T-TGTGTCG---GC---
Dai21940 -----AGTT-TAGTGGGC-TTGGACTTGGAGG-T-TGTGTCG---GC---
847Dai16433 -----AGTT-TAGTGGGC-TTGGACTTGGAGG-T-TGTGTCG---GC---
MarcinEmmia_latemarginatus -----AGTT-TAGTGGGC-TTGGACTTGGAGG-T-TGTGTCG---GC---
Meijer3729Hydnopolyporus_fimbr -----AGTT-TAGTGGGC-TTGGACTTGGAGG-T-TGTGTCG---GTT--
RLG13408Phanerochaete_sp -----AGTT-TGGTGGGC-TTGGACTTGGAGG-T-CGTGTCG---GCT--
WHC1381Flavodon_flavus -----GATT-TGGTGGGC-TTGGACTTGGAGG-T-TGTGTTG---GCT--
GB1833Phlebia_albida ----------TTGGAGGC-TTGGATATGGAGG-T-TGTGTCG---GAT--
T407Phlebia_nitidula ----------TTGTAGGC-TTGGATGTGGAGG-T-TGTGTCG---GATC-
HHB6988Phanerochaete_exilis -----AGTC-TAGCGGAC-TTGGACTTGGAGG-T-TGTGTCGGCGGCT--
HHB8509Phanerochaetella_xeroph -----AGTC-TGGCGGAC-TTGGACTTGGAGG-T-CGTGTCG---GCT--
PBU0051Macrohyporia_dictyopora -----AGTC-TAGTGGAC-TTGGACTTGGAGG-T-CGTGTCG---GCT--
HHB11463Phanerochaete_sp -----AGTCTTAGTGGAC-TTGGACTTGGAGG-T-CGTGTCG---GCT--
FP102382Byssomerulius_corium ----------GGGCGAGC-TTGGACTTGGAGG-T-CGTGTCG---GCTAT
FP102165Efibula_americana -----AGTCTAATGGGGC-TTGGACTTGGAGG-C-TGTGTCG---GCT--
Murdoch90Ceriporia_torpida A----GACC-TGGGGAGC-TTGGACTTGGAGGCT-TGTGCTG---GTTTC
Rivoire4413_Ceriporia_purpurea G----AACC-TGGTGAGC-TTGGACTTGGAGGCT-TGTGCTG---GTTCC
Kout_18_Ceriporia_triumphalis A----GACC-TGGTGAGC-TTGGACTTGGAGGCT-TGTGCTG---GTTTC
Rivoire3701_Ceriporia_bresadol A----AACA-TGGCGAGC-TTGGACTTGGAGGCT-TGTGCTG---GTTTC

VS4018 A----AACA-TGGTGAGC-TTGGACTTGGAGGCT-TGTGCTG---GTTAC

Ryvarden21832_Ceriporia_manzan A----AACA-TGGTGAGC-TTGGACTTGGAGGTT-TGTGCTG---GTTTC

Dai24539 A----AACA-TGGTGAGC-TTGGACTTGGAGGCT-TGTGCTG---GTTAC

Dai24541 A----AACA-TGGTGAGC-TTGGACTTGGAGGCT-TGTGCTG---GTTTC

JV1105_12_Ceriporia_occidental A----GACG-TGGTGAGC-TTGGACTTGGAGGCT-TGTGCTG---GTTCC

VS8558Ceriporia_occidentalis A----GACG-TGGTGAGC-TTGGACTTGGAGGCT-TGTGCTG---GTTCC

Dai22445 ---

846Dai16368 G----GACC-TGGCGAGC-TTGGACTTGGAGGTT-TGTGCTG---GTTCT

Dai17951_Ceriporia_aurantiocar -----AGCC-TGGTGAGC-TTGGACTTGGAGG-C-TGTGCAG---GCTT-

Miettinen_11701C_viridans -----AACT-TGGTGAGC-TTGGATTTGGAGG-T-TGTGCAG---GCTT-

JV0105_10Ceriporia_aurantiocar -----AGCC-TGGTGAGC-TTGGACTTGGAGG-C-TGTGCAG---GCTT-

Yuan5702C_viridans -----AACT-TGGTGAGC-TTGGATTTGGAGG-T-TGTGCAG---GCTT-

858Dai17003 -----AACT-TGGTGAGC-TTGGATTTGGAGG-T-TGTGCAG---GCTT-

Yuan2747_Ceriporia_viridans -----AGCC-TGGTGAGC-TTGGACTTGGAGG-C-TGTGCGG---GCTT-

Yuan2744C_viridans -----AGCC-TGGTGAGC-TTGGACTTGGAGG-C-TGTGCGG---GCTT-

Li1046C_viridans -----AACC-TGTTGAGC-TTGGACTTGGAGG-C-TGTGCGG---GCTT-

865C_sinoviridans -----AACC-TGTTGAGC-TTGGACTTGGAGG-C-TGTGCGG---GCTT-

871Dai15062 -----AGCC-TGATGAGC-TTGGACTTGGAGG-C-TGTGCAG---GCTT-

Dai7642_Ceriporia_humilis -----AGCTGTGGTGAGC-TTGGACTTGGAGG-T-CGTGTCG---GTCT-

Spirin4706_Ceriporia_humilis -----AGCTGTGGTGAGC-TTGGACTTGGAGG-T-CGTGTCG---GTCT-

Spirin4944_Ceriporia_sericea -----AGCTGTGGTGAGC-TTGGACTTGGAGG-T-CGTGTCG---GTCT-

WCG1547Dai26044ceriporia -----AGCTGTGGTGAGC-TTGGACTTGGAGG-T-CGTGTCG---GTCT-

ZZW1558Dai27086 -----AGCTGTGGTGAGC-TTGGACTTGGAGG-

T-TGTGTCG---GTCT-

Miettinen14381_Ceriporia_mpuri ---

Miettinen15492_2_Ceriporia_sor -----AGCTTCGGCGAGC-TTGGACTTGGAGGCT-CGTGTCG---GTCT-

He6687 -----AGCTTTGGTGAGC-TTGGACTTGGAGG-T-CGTGTCG---GTCT-

ZH53Dai24426 -----AGCTTCGGTGAGC-TTGGACTTGGAGG-T-CGTGTCG---CTCT-

Vlasak0808_30_Ceriporia_punica -----AACTTTGATCGGC-TTGGAGTTGGAGG-T-TGTGCAG---GGTTT

887Dai13376 -----AACT-TGGTCGGC-TTGGAGTTGGAGG-T-CGTGCAG---GGTTT

WCG1443Dai24998 -----AAGCTTGATTGGC-TTGGAGTTGGAGG-T-CGTGCAG---GGTTT

0108_6Ceriporia_spissa -----AACA-TGTCAAGA-TTGGACTTGGAGGGC-AGTGTCG---GCTT-

Dai19164 -----AACA-TGTCAAGA-TTGGACTTGGAGGGC-AGTGTCG---GCTT-

Dai17937_Ceriporia_bubalinomar -----AACA-CATCAAGA-TTGGACTTGGAGGGC-AGTGTCG---GCTG-

903Dai12113 -----AACA-CATCAAGA-TTGGACTTGGAGGGC-AGTGTCG---GCTG-

LZB929Dai25079 -----AGCC-TGGTGAGC-TTGGACTTGGAGG-T-TGTGCTG---GCTG-

LX45Dai26988 -----AGCC-TGGTGAGC-TTGGACTTGGAGG-T-TGTGCTG---GCTG-

LX43Dai26986 -----AGCC-TGGTGAGC-TTGGACTTGGAGG-T-TGTGCTG---GCTG-

Dai7759Ceriporia -----AGCC-TGGTGAGC-TTGGACTTGGAGG-T-TGTGCTG---GCTG-

Cui8012_Ceriporia_viridans -----AGCA-TGGTGAGC-TTGGACTTGGAGG-C-TGTGCAG---GCTT-

GC1704_54Ceriporia_viridans -----AGCA-TGGTGAGC-TTGGACTTGGAGG-C-TGTGCAG---GCTT-

Dai23392 -----AGCATTGGTGAGC-TTGGACTTGGAGG-T-TGTGCAG---GCTT-

WCG1585Dai26113Ceriproia -----AGCA-TGGTGAGC-TTGGACTTGGAGG-T-TGTGCAG---GCTT-

Dai18675C_eucalypti -----AGCA-TGGTGAGC-TTGGACTTGGAGG-T-TGTGCAG---GCTT-

Dai22034 ----------TGGTGAGC-TTGGATTTGGAGGCT-TGTGCTG---GCTT-

JV1008_41JTardaFLORIDAKeys ----------TGGTGAGC-TTGGATTTGGAGGCT-TGTGCTG---GCTT-

Rivoire1161_Ceriporia_pierii -----AGCTGTGGTGAGC-TTGGATTTGGAGG-T-TGTGTTG---GTCT-
Dai23499C_pierii -----AGCTGTGGTGAGC-TTGGATTTGGAGG-T-TGTGTTG---GTCT-
Dai23500 -----AGCTGTGGTGAGC-TTGGATTTGGAGG-T-TGTGTTG---GTCT-
841Dai15899 -AAAAGGTC-TGGCGATG-TTGGATGTGGAGG-T-TGTGCAG---GCCCC
842Dai15904 -AAAAGGTC-TGGCGATG-TTGGATGTGGAGG-T-TGTGCAG---GCCCC
LZB1066xinjiang -AAAAGGTC-TGGCGATG-TTGGATGTGGAGG-T-TGTGCAG---GCCCC
LZB1065xinjiang -AAAAGGTC-TGGCGATG-TTGGATGTGGAGG-T-TGTGCAG---GCCCC
851Dai16779 -----AGCTGTGATGAGC-TTGGACTTGGAGG-T-CGTGTCG---GTTTC
RMJ119sp_Candelabrochaete_sept -----AGTT-CGGTGAGC-TTGGACTTGGAGG-T-CGTGCTG---GCTCG
RLG9759spCandelabrochaete_sept -----AGTT-CGGTGAGC-TTGGACTTGGAGG-T-CGTGCTG---GCTCG
RLG10478Phanerochaete_allantos -----GACT-TGACGAGC-TTGGACTTGGAGG-T-TGTGCCG---GCTCT
Dai19118_Ceriporia_spissa -----AGTG-TGGCAAGCGTTGGACTTGGAGG--TCGTGCTGGTTACTTC
Dai18486A -----AGTG-TGGCAAGCGTTGGACTTGGAGG--TCGTGCTGGTTGCTTC
WEI17_024_Ceriporia_mellita -----AGTG-TGGCAAGCGTTGGACTTGGAGG--CCGTGCTGGTTGCTTC
GC1508_71Ceriporia_mellita -----AGTG-TGGCAAGCGTTGGACTTGGAGG--CCGTGCTGGTTGCTTC
GC1608_7_Ceriporia_mellita -----AGTG-TCGCAAGCGTTGGACTTGGAGG--CTGTGCTGGTTGCTTC
ZZW1557Dai27085 -----AGTG-TCGCAAGCGTTGGACTTGGAGG--CTGTGCTGGTTGCTTC
ZZW1554Dai27083 -----AGTG-TCGCAAGCGTTGGACTTGGAGG--CTGTGCTGGTTGCTTC
Dai8168 -----AGTG-TCGCAAGCGTTGGACTTGGAGG--CTGTGCTGGTTGCTTC
BR4865C_mellita -----AGTG-TGGCAAGCGTTGGACTTGGAGG--TCGTGCTGGTTGCTTC
MEL2382688Ceriporia_sp -----AGTG-CAGCAAGCATTGGACTTGGAGG--TTGTGCTGGTTGCTCT
Dai8110 -----AGTG-CAGCAAGCGTTGGACTTGGAGG--TTGTGCTGGCTGCTTT

Cui8097 -----AGTG-TAGCAAGTGTTGGACTTGGAGG--TTGTGCTGGCTGCTTT

909Cui6740 -----AATG-CAGCAAGCATTGGACTTGGAGG--TTGTGCTGGTTGCTCT

W1258Dai24695 -----AGTG-CAGCAAGCATTGGACTTGGAGG--TTGTGCTGGTTGCTCT

JV0110_26_Ceriporia_griseoviol ----------CAGCGAGC-TTGGACTTGGAGGTGTTGTGCTG---GCTGT

896Dai13202 ----------CAGCGAGC-TTGGACTTGGAGGTGTTGTGCTG---GCTGT

LWY393Dai27053C_griseoviolasce ----------CGGCGAGC-TTGGACTTGGAGGTGTTGTGCTG---GCTGT

LWY394DAI27054 ----------CGGCGAGC-TTGGACTTGGAGGTGTTGTGCTG---GCTGT

FP135015G_pannocinctus -----ATTTTTGGTGGGC-TTGGATGTGGA-GGT-TGTGCCG---GTTTG

L15726SpG_pannocinctus -----ATTTTTGGTGGGC-TTGGATGTGGA-GGT-TGTGCCG---GTTTG

Dai22221 -----AGTCTTGGTGGGC-TTGGACTTGGA-GGT-CGTGCTG---GCTTT

Dai22633 -----AGTCTTGGTGGGC-TTGGACTTGGA-GGT-TGTGCTG---GATTT

Dai23260 -----AGTCTTGGTGGGC-TTGGACTTGGA-GGT-TGTGCTG---GATTT

Dai23626 -----AGTCTTGGTGGGC-TTGGACTTGGA-GGT-TGTGCTG---GATTT

Dai16238G_citrinoalbus -----AGTTGTGGTGGGC-TTGGATTTGGA-GGC-TGTGCTG---GCTTT

1175Dai15293 -----AGTTGTGGTGGGC-TTGGATTTGGA-GGC-TGTGCTG---GCTTT

Dai19547 -----AGTTGTGGTGGGC-TTGGATTTGGA-GGC-TGTGCTG---GCTTT

918063G_africanus -----AATTCTGGCGGGC-TTGGATTTGGA-GGC-TGTGCTG---GCTTT

918572G_africanus -----AATTCTGGCGGGC-TTGGATTTGGA-GGC-TGTGCTG---GCTTT

Dai18536A -----AGTTCTGGCGGGC-TTGGACTTGGA-GGT-TGTGCTG---GCTTT

1164Cui17922 -----AGTTCTGGCGGGC-TTGGACTTGGA-GGT-TGTGCTG---GCTTC

Dai22225 -----AGTTCTGGCGGGC-TTGGACTTGGA-GGC-TGTGCTG---GCTTT

1163Dai20655 -----AGTTCTGGCGGGC-TTGGACTTGGAGGGT-TGTGCTG---GCTTT

Yuan4397G_hainanensis ----------TGGTGGGCTTTGGATTTGGAGGCT-TGTGCTGGTGGCTTT

1176Dai15268 ----------TGGTGGGCTTTGGATTTGGAGGCT-TGTGCTGGTGGCTTT

1177Dai15259 ----------TGGTGGGCTTTGGATTTGGAGGCT-TGTGCTGGT-GCTTT

BZ2896G_thelephoroides ----------TGGTGGGCTTTGGACTTGGAGGCT-TGTGCTG---GCTTT

1166JV1808_26 ----------TGGTGGGCTTTGGACTTGGAGGCT-TGTGCTG---GCTTT

Miettinen16992Hapalopilus_ochr -----AGTTTTAGAGGAC-TTGGATCTGGAGG--CTGTGTCGAT-GCCAT

GC1708_338_Ceriporia_arbuscula ----------TAGTGAGC-TTGGATATGGAGGCT-TGTGCTG---GCTGC

WCG1555Dai26107Ceriporia ----------TAGTGAGC-TTGGATATGGAGGCT-TGTGCTG---GCTGC

GC1708_340_Ceriporia_arbuscula ----------TAGTGAGC-TTGGATATGGAGGCT-TGTGCTG---GCTGC

WCG1556Dai26109Ceriporia --

883Cui11291 ----------TAGAGAGC-TTGGATTTGGAGGTC-TGTGCTG---GCTTA

HLX320Dai26805 GAAAGAATTTATTGAGC-TTGGATTTGGAGGCT-TGTGCTG---GCGCT

WCG1266Dai24678A ----------TAGTAAGC-TTGGACTTGGAGGCC-TGTGCTG---GCTAT

Dai6090_Ceriporia_sulphuricolo ----------GGGCGGGC-TTGGACTTGGAGGCA-TGTGTCG---GCTCT

RLG_11354_Ceriproia_reticulata --AGAAGCT-TGGTGAGC-TTGGACTTGGAGGATTTGTGCTG---GCTAT

ZZW1543Dai27072 --AGAAGCA-TGGTGAGC-TTGGACTTGGAGGATTTGTGCTG---GCTAT

Li1316_Ceriporia_reticulata -------------------TTT---------------------------

KHL11981Ceriporia_reticulata -------------------TTT---------------------------

FP110343sp_Candelabrochaete_la --GGAAACA-TGGTGAGC-TTGGACTTGGAGGATCTGTGCTG---GCTAG

Li1045_Ceriporia_reticulata --GGAGACA-TGGTGAGC-TTGGACTTGGAGGATTTGTGCTG---GCTAT

ZX136Dai25794ceriporia --GGAGACA-TGGTGAGC-TTGGACTTGGAGGATTTGTGCTG---GCTAT

892Dai13400 --GGAGACA-TGGTGAGC-TTGGACTTGGAGGATTTGTGCTG---GCTAT

RLG7163Leptoporus_mollis GGAGTCATG-TGTGGGGC-TTGGACTTAGAGGTT-TATGCTG--TGCTAT

Dai21062Leptoporus_mollis GAAGTCATC-TGTGGGGC-TTGGACTTAGAGGTT-

TATGCTG--TGCTAT

Dai20182Leptoporus_submollis GGAGACACG-CGTGGGGC-TTGGACTTAGAGGTT-TATGCTG--TGCTAT

Cui18379Leptoporus_submollis GGAGACACG-CGTGGGGC-TTGGACTTAGAGGTT-TATGCTG--TGCTAT

Wu1209_46Resiniporus_pseudogil -----ATTC-TAATAAGC-TTGGACTTGGAGGCT-TGTGTCG---GCTTA

BRNM710169Resiniporus_resinasc -----GTTC-TAATAAGC-TTGGACTTGGAGGCT-TGTGTCG---GCTTA

Dai14516Bjerkandera_adusta G---------------C-TTGGACTTGGAGG--TCGTGCCG---GCTCT

Dai21100Bjerkandera_fumosa G---------------C-TTGGACTTGGAGG--TCGTGTCG---GCTCT

Miettinen16854Ceraceomyces_sp T---------TGTTGTTT-TTGGACTTGGAGG--TCGTGTCG---GCTAC

Dai10477C_spissa -----AATCTTGTCTGGC-ATGGATGTGGAGGCTTTGTGCTG---GCTTT

855Dai16831 -----AATCTTGTCTGGC-ATGGATGTGGAGGCTTTGTGCTG---GCTTT

882Cui11282 -----AATCTTGTCTGGC-ATGGATGTGGAGGCTTTGTGCTG---GCTTT

Dai24566 -----AATCTTGTTTGAC-GTGGATTTGGAGGTTTTGTGCTG---GCTTT

Yuan5965 -----AATCTTGTTTGAC-GTGGATTTGGAGGTTGTGTGCTG---GCTTT

Dai3204 ---

1194CUI9985 ---

Dai15205_Ceriporia_albomellea TCTT--------TGAGTCGACTCCTCTGAAATACATTAGCGTGAACCTGT

Dai15223_Ceriporia_albomellea TCTT--------TGAGTCGACTCCTCTGAAATACATTAGCGTGAACCTGT

Li1780_Ceriporia_variegata TGTT--------TCAGTCGACTCCTCTGAAATACATTAGCGTGAACCTGT

Dai19791_Ceriporia_variegata TGTT--------TCAGTCGACTCCTCTGAAATACATTAGCGTGAACCTGT

Dai19886 TGTT--------TCAGTCGACTCCTCTGAAATACATTAGCGTGAACCTGT

Dai10833_Ceriporia_crassitunic TGTT--------TTAGTCGACTCCTCTGAAATATATTAGCGTGAACCTTT

CHWC1506_46Meruliopsis_crassit TGTT--------TTAGTCGACTCCTCTGAAATATATTAGCGTGAACCTTT

Dai9995_Ceriporia_crassitunica TGTG--------

TTAGTCGACTCCTCTGAAATACATTAGTGTGAACCTAT
Wu1209_58_Meruliopsis_parvispo TTATTT------
AGAGTCGACTCCTCTGAAATACATTAGCGTGAATCT-T
CHWC1505_129_Meruliopsis_parvi TTATTT------
AGAGTCGACTCCTCTGAAATACATTAGCGTGAATCT-T
Dai21944 CTGTC--------
TAGTCGACTCCTCTGAAATACATTAGCGTGAATCT-T
830Dai18640A TCGTC--------
GAGTCGACTCCTCTGAAATACATTAGCGTGAATCT-T
GC1704_60_Meruliopsis_taxicola TTCGG--------
GAGTGGACTCCTCTGAAATTTATTAGTGTGAATCT-T
Dai22625 TTCGG--------
GAGTGGACTCCTCTGAAATTTATTAGTGTGAATCT-T
Dai22636 TTCGG--------
GAGTGGACTCCTCTGAAATTTATTAGTGTGAATCT-T
Dai21878 TTCGG--------
GAGTGGACTCCTCTGAAATGTATTAGTGTGAATCT-T
1169Dai17248 TTCGG--------
GAGTGGACTCCTCTGAAATGTATTAGTGTGAATCT-T
Wu1708_43_Meruliopsis_leptocys TAGTC---------
AGTCGACTCCTCTGAAATGCATTAGTGTGAATCT-T
Li1011 TAGTC---------
AGTCGACTCCTCTGAAATGCATTAGTGTGAATCT-T
ZX95Dai25742Meruliopsis_leptoc TAGTC---------
AGTCGACTCCTCTGAAATGCATTAGTGTGAATCT-T
WCG1306Dai24733 TAGTC---------
AGTCGACTCCTCTGAAATGCATTAGTGTGAATCT-T
LXL99Dai25816 TAGTC---------
AGTCGACTCCTCTGAAATGCATTAGTGTGAATCT-T
WCG1559Dai26052Meruliopsis TAGTC---------
AGTCGACTCCTCTGAAATGCATTAGTGTGAATCT-T
He7477 TAGTC---------
AGTCGACTCCTCTGAAATGCATTAGTGTGAATCT-T
HLX243Dai26217 TAGTC---------
AGTCGACTCCTCTGAAATGCATTAGTGTGAATCT-T
RussiaMW673659Meruliopsis_fagi TAGTC---------
AGTCGACTCCTCTGAAATGCATTAGTGTGAATCT-T
FD278 TAATT--------
AAATCGACTCCTCTGAAATGCATTAGCGTGAATCT-T
Dai10226_Ceriporia_tarda TTATT--------
GAGTCGACTCCTCTGAAATGCATTAGCGTGAATGT-T
LE247365 TTATT--------
GAGTCGACTCCTCTAAAATGCATTAGCGTGAATGT-T
Dai8173_Meruliopsis_nanlingens TTCTT---

GTTAAGAGTCGACTCCTCTGAAATGGATTAGCCTGAGTCT-T
860Dai17172 TTATT---
GTTAAGAGTCGACTCCTCTGAAATGGATTAGCGTGAGTCT-T
879Dai13414 TTATT---
GTTAAGAGTCGACTCCTCTGAAATGGATTAGCGTGAGTCT-T
Li_1704_Meruliopsis_pseudocyst TGTAATAA----
AAAATCAACTCCTCTGAAATAGATTAGCGTGAATCT-T
833Dai18405 TGTAATAA----
AAAATCAACTCCTCTGAAATAGATTAGCGTGAATCT-T
HHB_10729_Meruliopsis_albostra TGTAATAA----
AAAATCAACTCCTCTGAAATGGATTAGCGTGAATCT-T
Cui6878_Ceriporia_pseudocystid TGTAACAA----
AAAGTCAACTCCTCTGAAATAGATTAGCGTGAATCT-T
869Dai14737 TGTAACAA----
AAAGTCAACTCCTCTGAAATAGATTAGCGTGAATCT-T
876Cui11626 TGTAACAA----
AAAGTCAACTCCTCTGAAATAGATTAGCGTGAATCT-T
1199WEI3388 TGTAACAA----
AAAGTCAACTCCTCTGAAATAGATTAGCGTGAATCT-T
776308_Meruliopsis_cystidiata --------------
GGTCAACTCCTCTGAAATATATTAGCGTGAATCT-T
ICN139059_Meruliopsis_cystidia --------------
AGTCAACTCCTCTGAAATGTATTAGCGTGAATCT-T
HHB15692Ceraceomyces_serpens AGC---------
TCAGTCGACTCCTCTAAAATGTATTAGCGTGAATCT-T
HHB_15629_Sp_Ceriporiopsis_ane TTGC--------
TCAGTCGACTCCTCTTAAATGCATTAGCGTGAATCT-T
AJ185Trametopsis_cervina TA----------
ATAATCGACTCCTCTTAAATGCATTAGCGTGAATCT-T
FD9Irpex_lacteus --------
CTCGCYGGTCGACTCCTCTGAAATGCATTAGCGTGAATCT-T
908Dai11230 --------
CTTGTCGGTCGACTCCTCTGAAATGCATTAGCGTGAATCT-T
FP55521TEmmia_lacerata ----------
TTCTAGTCGACTCCTCTGAAATGCATTAGCGTGAATCT-T
PBU0048Ceriporia_cystidiata ----------
TTCTAGTCGACTCCTCTGAAATGTATTAGCGTGAATCT-T
MZ340C_lacerataT ----------
TTCTAGTCGACTCCTCTGAAATGCATTAGCGTGAATCT-T
Dai21940 ----------
TTCTAGTCGACTCCTCTGAAATGCATTAGCGTGAATCT-T
847Dai16433 ----------
TTCTAGTCGACTCCTCTGAAATGTATTAGCGTGAATCT-T
MarcinEmmia_latemarginatus ----------

TTCTAGTCGACTCCTCTGAAATGCATTAGCGTGAATCT-T

Meijer3729Hydnopolyporus_fimbr ---------

GTCTCAGTCGACTCCTCTGAAAAGCATTAGCGTGAATCT-T

RLG13408Phanerochaete_sp --------

CTCGTCAGTCGACTCCTCTTAAATGCATTAGCGTGAATGT-T

WHC1381Flavodon_flavus --------

CTCGCTAGTCGACTCCTCTGAAATACATTAGTGTGAACCT-T

GB1833Phlebia_albida --------

CTAGTCATTCGACTCCTCTCAAATGCATTAGCGTGAATCT-T

T407Phlebia_nitidula --------

CCAGTTATTCGACTCCTCTTAAATGCATTAGCGTGAATCT-T

HHB6988Phanerochaete_exilis --------

CTAGTCAGTCGACTCCTCTTAAATGTATTAGCGTGAATGT-T

HHB8509Phanerochaetella_xeroph --------

CTTGTCAGTCGACTCCTCTTAAATGCATTAGCGTGAATGT-T

PBU0051Macrohyporia_dictyopora --------

CTAGTCAGTCGACTCCTCTTAAATGCATTAGCGTGAATGT-T

HHB11463Phanerochaete_sp --------

CTTGTCAGTCGACTCCTCTTAAATGCATTAGCGTGAATGT-T

FP102382Byssomerulius_corium T----------

GTAGTCGACTCCTCTTAAATGTATTAGCGTGAATCT-T

FP102165Efibula_americana --------

CTAGTCAGCTGACTCCTCTTAAATGCATTAGCGTGAATCTTT

Murdoch90Ceriporia_torpida ATCT------

TTGAAATCAGCTCCTCTGAAATGTATTAGCGTGAATCT-T

Rivoire4413_Ceriporia_purpurea TGTT----

ACTCGGAATCAGCTCCTCTGAAATGTATTAGCGTGAATCT-T

Kout_18_Ceriporia_triumphalis TTAA------

TCGAAGTCAGCTCCTCTGAAATGCATTAGCGTGAATCT-T

Rivoire3701_Ceriporia_bresadol TTAA------

TCGAAATCAGCTCCTCTGAAATGCATTAGCGTGAATCT-T

VS4018 TTAA------

TCGAAATCAGCTCCTCTGAAATGCATTAGCGTGAATCT-T

Ryvarden21832_Ceriporia_manzan TTAA------

TCGAAATCAGCTCCTCTGAAATGCATTAGCGTGAATCT-T

Dai24539 TTAA------

TCGAAATCAGCTCCTCTGAAATGCATTAGCGTGAATCT-T

Dai24541 TTAA------

TCGAAATCAGCTCCTCTGAAATGCATTAGCGTGAATCT-T

JV1105_12_Ceriporia_occidental TTAC------

TCGGAATCAGCTCCTCTGAAATGCATTAGCGTGAATCT-T

VS8558Ceriporia_occidentalis TTAC------

TCGGAATCAGCTCCTCTGAAATGCATTAGCGTGAATCT-T

Dai22445 ---

846Dai16368 GTTA-----
CTCGGAATCAGCTCCTCTGAAATGCATTAGCGTGAATCT-T

Dai17951_Ceriporia_aurantiocar --------
TGTATAAGCCAGCTCCTCTGAAATGTATTAGCGTGAACCT-T

Miettinen_11701C_viridans --------
TTAACAAGTCTGCTCCTCTGAAATGTATTAGTGTGAATCT-T

JV0105_10Ceriporia_aurantiocar --------
TGTATAAGCCAGCTCCTCTGAAATGTATTAGCGTGAACCT-T

Yuan5702C_viridans --------
TTAACAAGTCTGCTCCTCTGAAATGTATTAGTGTGAATCT-T

858Dai17003 --------
TTAACAAGTCTGCTCCTCTGAAATGTATTAGTGTGAATCT-T

Yuan2747_Ceriporia_viridans ---------
TTATAAGTCAGCTCCTCTGAAATATATTAGTGTGAATCT-T

Yuan2744C_viridans ---------
TTATAAGTCAGCTCCTCTGAAATATATTAGTGTGAATCT-T

Li1046C_viridans --------
GTAATAAGTCAGCTCCTCTAAAATGCATTAGCGTGAATTT-T

865C_sinoviridans --------
GTAATAAGTCAGCTCCTCTAAAATGCATTAGCGTGAATTT-T

871Dai15062 --------
TTTATAAGTCAGCTCCTCTGAAATATATTAGTGTGAATCT-T

Dai7642_Ceriporia_humilis --------GCTTCAG-
TCGACTCCTCTGAAATACATTAGCGTGAACCT-T

Spirin4706_Ceriporia_humilis --------GCTTCAG-
TCGACTCCTCTGAAATACATTAGCGTGAACCT-T

Spirin4944_Ceriporia_sericea --------GCTTCAG-
TCGACTCCTCTGAAATGTATTAGCGTGAACCT-T

WCG1547Dai26044ceriporia --------GCTTCAG-
TCGACTCCTCTGAAATGCATTAGCGTGAACCT-T

ZZW1558Dai27086 --------GCTTCAG-
TCGACTCCTCTGAAATGCATTAGCGTGAACCT-T

Miettinen14381_Ceriporia_mpuri --

Miettinen15492_2_Ceriporia_sor --------
GCTTCAGATCGACTCCTCTGAAATGCATTAGCGTGAATCT-T

He6687 --------GCTTCAG-
TCGACTCCTCTGAAATGCATTAGCGTGAACCT-T

ZH53Dai24426 --------GCTACAC-
TCGACTCCTCTGAAATGCATTAGCGTGAATCT-T

Vlasak0808_30_Ceriporia_punica C-------
TGTGAAACCCAGCTCCTCTGAAATGCATTAGCGTGAATCC-T

887Dai13376 CTCTCT--
TGTGAAACTCAGCTCCTCTGAAATACATTAGCGTGAATCT-T

WCG1443Dai24998 CTC---------

AAACTCAGCTCCTCTGAAAAGCATTAGCGTGAATCT-T

0108_6Ceriporia_spissa ---------

GAAGAAGTCGGCTCCTCTGAAATGCATCAGCGTGAATCT-T

Dai19164 ---------

GAAGAAGTCGGCTCCTCTGAAATGCATCAGCGTGAATCT-T

Dai17937_Ceriporia_bubalinomar ---------

AATAACGTCGACTCCTCTGAAATGCATCAGCGTGAATCT-T

903Dai12113 ---------

AATAACGTCGACTCCTCTGAAATGCGTCAGCGTGAATCT-T

LZB929Dai25079 ----------

AACAAGTCAGCTCCTCTGAAATGCATTAGTGTGAAATC-T

LX45Dai26988 ----------

AACAAGTCAGCTCCTCTGAAATGCATTAGTGTGAAATC-T

LX43Dai26986 ----------

AACAAGTCAGCTCCTCTGAAATGCATTAGTGTGAAATC-T

Dai7759Ceriporia ----------

AACAAGTCAGCTCCTCTGAAATGCATTAGTGTGAAATC-T

Cui8012_Ceriporia_viridans --------

GTTTCGAGTCTGCTCCTCTGAAATGAATTAGTATGAATCT-T

GC1704_54Ceriporia_viridans --------

GTTTCAAGTCTGCTCCTCTGAAATGAATTAGTATGAATCT-T

Dai23392 --------

GTTTCAAGTCTGCTCCTCTGAAATGAATTAGTATGAATCT-T

WCG1585Dai26113Ceriproia --------

GTCTCAAGTCTGCTCCTCTGAAATGAATTAGTATGAATCT-T

Dai18675C_eucalypti --------

GTTCCAAGTCTGCTCCTCTGAAATGAATTAGTATGAATCT-T

Dai22034 --------

TTGAAAGTCAAGCTCCTCTAAAATGCATTAGCGTGAATCT-T

JV1008_41JTardaFLORIDAKeys --------

TTGAAAGTCAAGCTCCTCTAAAATGCATTAGCGTGAATCT-T

Rivoire1161_Ceriporia_pierii --------

GTCTCTGATCAACTCCTCTGAAATGCATTAGTGTGAATCT-C

Dai23499C_pierii --------

GTCTCTGATCAACTCCTCTGAAATGCATTAGTGTGAATCT-C

Dai23500 --------

GTCTCTGATCAACTCCTCTGAAATGCATTAGTGTGAATCT-C

841Dai15899 GCCCCC--

TTTGTGGGTCAGCTCCTCTAAAATGTATTAGCGTGAATCT-T

842Dai15904 GCCCCC--

TTTGTGGGTCAGCTCCTCTAAAATGTATTAGCGTGAATCT-T

LZB1066xinjiang GCCCCC--

TTTGTGGGTCAGCTCCTCTAAAATGTATTAGCGTGAATCT-T

LZB1065xinjiang GCCCCC--

TTTGTGGGTCAGCTCCTCTAAAATGTATTAGCGTGAATCT-T
851Dai16779 ACT-----
TTTTGAAATCGACTCCTCTGAAATGCATTAGCGTGAATCT-T
RMJ119sp_Candelabrochaete_sept TC----------
GCGGTCAGCTCCTCTGAAATGCATTAGCGTGAACCT-T
RLG9759spCandelabrochaete_sept TC----------
GCGGTCAGCTCCTCTGAAATGCATTAGCGTGAACCT-T
RLG10478Phanerochaete_allantos TG----------
CTAGTCGGCTCCTCTGAAATGCATTAGCGTGAATCT-T
Dai19118_Ceriporia_spissa GCTTG--A----T-
GACTAGCTCCTCTGAAATGCATCAGCATGAACTTAT
Dai18486A GCTTG--A----T-
GACTAGCTCCTCTGAAATGCATCAGCATGAACTTAT
WEI17_024_Ceriporia_mellita GCTTG--A----T-
GGCTAGCTCCTCTGAAATGCATCAGCATGAACTTAT
GC1508_71Ceriporia_mellita GCTTG--A----T-
GGCTAGCTCCTCTGAAATGCATCAGCATGAACTTAT
GC1608_7_Ceriporia_mellita GCTTG--A----T-
GGCTAGCTCCTCTGAAATGCATCAGCATGAACTTAT
ZZW1557Dai27085 GCTTG--A----T-
GGCTAGCTCCTCTGAAATGCATCAGCATGAACTTAT
ZZW1554Dai27083 GCTTG--A----T-
GGCTAGCTCCTCTGAAATGCATCAGCATGAACTTAT
Dai8168 GCTTG--A----
TGGGCTAGCTCCTCTGAAATGCATCAGCATGAACTTAT
BR4865C_mellita GCTTG--A----T-
GACTAGCTCCTCTGAAATGCATCAGCATGAACTTAT
MEL2382688Ceriporia_sp CCTTG--A----T---
CTAGCTCCTCTGAAATAAATCAGCGTGAACTTAT
Dai8110 GTTTGAAA----T-
AGCTAGCTCCTCTGAAATGTATCAGCATGAACTTAT
Cui8097 GTTTGAAA----T-
AGCTAGCTCCTCTGAAATGTATCAGCATGAACTTAT
909Cui6740 TCTTG--A----T---
CTAGCTCCTCTGAAATAAATCAGCGTGAACTTAT
W1258Dai24695 CCTTG--A----T---
CTAGCTCCTCTGAAATAAATCAGCGTGAACTTAT
JV0110_26_Ceriporia_griseoviol CTCATGA-------
AGTCAGCTCCTCTGAAATGCATTAGTGTGAACCT-T
896Dai13202 CTCATGA-------
AGTCAGCTCCTCTGAAATGCATTAGTGTGAACCT-T
LWY393Dai27053C_griseoviolasce CTCATGA-------
AGTCAGCTCCTCTGAAATGCATTAGTGTGAACCT-T
LWY394DAI27054 CTCATGA-------

AGTCAGCTCCTCTGAAATGCATTAGTGTGAACCT-T
FP135015G_pannocinctus TTAAA---------
AGCCGGCTCCTCTGAAATGCATTAGTGTGAATCT-T
L15726SpG_pannocinctus TTAAA---------
AGCCGGCTCCTCTGAAATGCATTAGTGTGAATCT-T
Dai22221 AATTA---------
GTTCGGCTCCTCTTAAATACATTAGTGTGAATCT-T
Dai22633 TTATT---------
ATTCAGCTCCTCTGAAATATATTAGTGTGAATCT-T
Dai23260 TTATT---------
ATTCAGCTCCTCTGAAATATATTAGTGTGAATCT-T
Dai23626 TTATT---------
ATTCAGCTCCTCTGAAATATATTAGTGTGAATCT-T
Dai16238G_citrinoalbus --------------
GTTCAGCTCCTCTGAAATATATTAGTGTGAATCT-T
1175Dai15293 --------------
GTTCAGCTCCTCTGAAATATATTAGTGTGAATCT-T
Dai19547 --------------
GTTCAGCTCCTCTAAAATATATTAGTGTGAATCT-T
918063G_africanus --------------
GTTCAGCTCCTCTGAAATATATTAGTGTGAATCT-T
918572G_africanus --------------
GTTCAGCTCCTCTGAAATATATTAGTGTGAATCT-T
Dai18536A --------------
GTTCAGCTCCTCTAAAATATATTAGTGTGAATCT-T
1164Cui17922 --------------
GTTCAGCTCCTCTAAAATATATTAGTGTGAATCT-T
Dai22225 --------------
GTTCAGCTCCTCTAAAATATATTAGCGTGAATCT-T
1163Dai20655 --------------
GTTCAGCTCCTCTAAAATATATTAGCGTGAATCT-T
Yuan4397G_hainanensis TTTAATTA----
GTGTTCAGCTCCTCTGAAATGCATTAGTGTGAATCT-T
1176Dai15268 TTTAATTA----
GTGTTCAGCTCCTCTGAAATGCATTAGTGTGAATCT-T
1177Dai15259 TTTAAT---
BZ2896G_thelephoroides TTAAATT-----
AGCTTCAGCTCCTCTGAAATACATTAGTGTGAATCT-T
1166JV1808_26 TTAAATT-----
AGCTTCAGCTCCTCTGAAATACATTAGTGTGAATCT-T
Miettinen16992Hapalopilus_ochr G-----------
TTAGTCGACTCCTCTGAAATGTATTAGTATGAATCT-T
GC1708_338_Ceriporia_arbuscula TT-----------
AGCTAGCTCCTCTGAAATATAGTAGCGTGAATCC-T

WCG1555Dai26107Ceriporia TT-----------
AGCTAGCTCCTCTGAAATATAGTAGCGTGAATTC-T

GC1708_340_Ceriporia_arbuscula TT-----------
AGCTAGCTCCTCTGAAATATAGTAGCGTGAATCC-T

WCG1556Dai26109Ceriporia --

883Cui11291 TTATAA------
CAAGTCAGCTCCTCTAAAATATATTAGCGTGAATCC-T

HLX320Dai26805
ATATATAATATACATGCTGGCTCCTCTAAAATGAATTAGCATAAATTC-T

WCG1266Dai24678A AATAATA-----
TAGGCTGGCTCCTCTAAAATGCATTAGCATAAATCC-T

Dai6090_Ceriporia_sulphuricolo CG----------
TCAGCCGACTCCTCTGAAATGTATTAGCGTGAACCT-T

RLG_11354_Ceriproia_reticulata TAAT--------
CTAGTCGGCTCCTCTGAAATGCATTAGCATGAATTT-T

ZZW1543Dai27072 TAAT--------
CTAGTCGGCTCCTCTGAAATGCATTAGCATGAATTT-T

Li1316_Ceriporia_reticulata --

KHL11981Ceriporia_reticulata --

FP110343sp_Candelabrochaete_la TAAT--------
CAAGTCAGCTCCTCTGAAATGTATTAGCATAAATCA-T

Li1045_Ceriporia_reticulata C-----------
TTAGTCGGCTCCTCTGAAATGTATTAGCATGAATCT-T

ZX136Dai25794ceriporia C-----------
TTAGTCGGCTCCTCTGAAATGTATTAGCATGAATCT-T

892Dai13400 C-----------
TTAGTCGGCTCCTCTGAAATGTATTAGCATGAATCT-T

RLG7163Leptoporus_mollis CTT---------
CCCCTCAGCTCCTCTCAAATGCATTAGCGTGAACTT-T

Dai21062Leptoporus_mollis CTT---------
TCCCTCAGCTCCTCTCAAATGCATTAGCGTGAACTT-T

Dai20182Leptoporus_submollis CTT---------
TTGCTCAGCTCCTCTCAAATGCATTAGCGTGAACTT-T

Cui18379Leptoporus_submollis CTT---------
TTGCTCAGCTCCTCTCAAATGCATTAGCGTGAACTT-T

Wu1209_46Resiniporus_pseudogil TG----------
CAGCCCGACTCCTCTTAAATTCAGTAGCGTGAATTTGT

BRNM710169Resiniporus_resinasc TA----------
CAGCCTGACTCCTCTTAAATTCAGTAGCGTGAATTTGT

Dai14516Bjerkandera_adusta C-----------
GTAGTCGGCTCCTCTGAAATGCATTAGTGCGAACGT-T

Dai21100Bjerkandera_fumosa C-----------
GTAGTCGACTCCTCTGAAATGCATTAGTGCGAACGT-T

Miettinen16854Ceraceomyces_sp TAGCTT------

TTAGCAGACTCCTCTGAAATGTATTAGTATGAACCT-T

Dai10477C_spissa CTTT--------

GAAGTCGGCTCCTCTGAAATAAATCAGCATGAATCT-T

855Dai16831 CTTT--------

GAAGTCGGCTCCTCTGAAATAAATCAGCATGAATCT-T

882Cui11282 CTTT--------

GAAGTCGGCTCCTCTGAAATAAATCAGCATGAATCT-T

Dai24566 GTTT--------

GAAGTCGGCTCCTCTGAAATACATCAGCATGAATCT-T

Yuan5965 GTTT--------

GAAGTCGGCTCCTCTGAAATACATCAGCATGAATCT-T

Dai3204 --

1194CUI9985 --

Dai15205_Ceriporia_albomellea

GTGGATCATCCTTCAATGTGATAATTGTCTGCATTGTGGT---TGTGAAG

Dai15223_Ceriporia_albomellea

GTGGATCATCCTTCAATGTGATAATTGTCTGCATTGTGGT---TGTGAAG

Li1780_Ceriporia_variegata

ATGGATCATCCTTCAATGTGATAATTGTCTGCATTGTGGT---TGTGAAG

Dai19791_Ceriporia_variegata

ATGGATCATCCTTCAATGTGATAATTGTCTGCATTGTGGT---TGTGAAG

Dai19886

ATGGATCATCCTTCAATGTGATAATTGTCTGCATTGTGGT---TGTGAAG

Dai10833_Ceriporia_crassitunic GTGGATC-

ATCTTCAATGTGATAATTGTCTGCATTGTAGT---TGTGAGG

CHWC1506_46Meruliopsis_crassit GTGGATC-

ATCTTCAATGTGATAATTGTCTGCATTGTAGT---TGTGAGG

Dai9995_Ceriporia_crassitunica GTGGATC-

ATCTTCAATGTGATAATTGTCTGCATTGTAGT---TGTGAGG

Wu1209_58_Meruliopsis_parvispo ACGGATC-

GCCTTCAGTGTGATAATTGTCTACTCTGTGGT---CGTGAAG

CHWC1505_129_Meruliopsis_parvi ACGGATC-

GCCTTCAGTGTGATAATTGTCTACTCTGTGGT---CGTGAAG

Dai21944 ACGGATC-

GCCTTCAGTGTGATAATTGTCTACGCTGTGGC---TGTGAAG

830Dai18640A ACGGATC-

GCCTTCAGTGTGATAATTGTCTACGCTGTGGC---TGTGAAG

GC1704_60_Meruliopsis_taxicola ACTGATC-

GCCTTCAGTGTGATAATTGTCTACGCTGTGGT---CGAGAAG

Dai22625 ACTGATC-

GCCTTCAGTGTGATAATTGTCTACGCTGTGGT---CGAGAAG

Dai22636 ACTGATC-

GCCTTCAGTGTGATAATTGTCTACGCTGTGGT---CGAGAAG
Dai21878 ACTGATC-
GCCTTCAGTGTGATAATTGTCTACGCTGTGGT---CGAGAAG
1169Dai17248 ACTGATC-
GCCTTCAGTGTGATAATTGTCTACGCTGTGGT---CGAGAAG
Wu1708_43_Meruliopsis_leptocys ACTGATC-
GCCTTCAGTGTGATAATTGTCTACGCTGTTGT---TGTGAAG
Li1011 ACTGATC-
GCCTTCAGTGTGATAATTGTCTACGCTGTTGT---TGTGAAG
ZX95Dai25742Meruliopsis_leptoc ACTGATC-
GCCTTCAGTGTGATAATTGTCTACGCTGTGGT---TGTGAAG
WCG1306Dai24733 ACTGATC-
GCCTTCAGTGTGATAATTGTCTACGCTGTGGT---TGTGAAG
LXL99Dai25816 ACTGATC-
GCCTTCAGTGTGATAATTGTCTACGCTGTGGT---TGTGAAG
WCG1559Dai26052Meruliopsis ACTGATC-
GCCTTCAGTGTGATAATTGTCTACGCTGTGGT---TGTGACG
He7477 ACTGATC-
GCCTTCAGTGTGATAATTGTCTACGCTGTGGT---TGTGACG
HLX243Dai26217 ACTGATC-
GCCTTCAGTGTGATAATTGTCTACGCTGTGGT---TGTGACG
RussiaMW673659Meruliopsis_fagi ACTGATC-
GCCTTCAGTGTGATAATTGTCTACGCTGTGGT---GGTGAAG
FD278 ACGGATC-
GCCTTCAGTGTGATAATTGTCTACGCTGTGGT---TGTGAAG
Dai10226_Ceriporia_tarda ATGGATC-
GCCTTCAGTGTGATAATTGTCTACGCTGTGGT---TGTGAAA
LE247365 ATGGATC-
GCCTTCAGTGTGATAATTGTCTACGCTGTGGT---TGTGAAA
Dai8173_Meruliopsis_nanlingens ACGAATC-
GCCTTCAGTGTGATAATTGTCTACGCTGTGGC---TGTGACG
860Dai17172 ACGAATC-
GCCTTCAGTGTGATAATTGTCTACGCTGTGGC---TGTGACG
879Dai13414 ACGAATC-
GCCTTCAGTGTGATAATTGTCTACGCTGTGGC---TGTGACG
Li_1704_Meruliopsis_pseudocyst ACGGATC-
GCCTTCAGTGTGATAATTGTCTACGCTGTGGC---TGTGAAG
833Dai18405 ACGGATC-
GCCTTCAGTGTGATAATTGTCTACGCTGTGGC---TGTGAAG
HHB_10729_Meruliopsis_albostra ACGGATC-
GCCTTCAGTGTGATAATTGTCTACGCTGTGGC---TGTGAAG
Cui6878_Ceriporia_pseudocystid ACGGATC-
GCCTTCAGTGTGATAATTGTCTACGCTGTGGC---CGTGAAG
869Dai14737 ACGGATC-

GCCTTCAGTGTGATAATTGTCTACGCTGTGGC---CGTGAAG

876Cui11626 ACGGATC-

GCCTTCAGTGTGATAATTGTCTACGCTGTGGC---CGTGAAG

1199WEI3388 ACGGATC-

GCCTTCAGTGTGATAATTGTCTACGCTGTGGC---CGTGAAG

776308_Meruliopsis_cystidiata ACGGATC-

GCCTTCAGTGTGATAATTGTCTACGCTGTGGT---TGTGAAG

ICN139059_Meruliopsis_cystidia ACGGATC-

GCCTTCAGTGTGATAATTGTCTACGCTGTGGT---TGTGAAG

HHB15692Ceraceomyces_serpens ACGGATC-

GCCTTCAGTGTGATAATTATCTGCGCTGTGGC---TGTGAAG

HHB_15629_Sp_Ceriporiopsis_ane ACGGATC-

GCCTTCAGTGTGATAATTATCTGCGCTGTGGTG---TTGAAG

AJ185Trametopsis_cervina ACGGATC-

GCCTTCAGTGTGATAATTATCTACGCTGTGGTG----TGAAG

FD9Irpex_lacteus ACGGATC-

GCCTTCAGTGTGATAATTATCTGCGCTGTGGTG---TTGAAG

908Dai11230 ACGGATC-

GCCTTCAGTGTGATAATTATCTGCGCTGTGGTG---TTGAAG

FP55521TEmmia_lacerata ACGGATC-

GCCTTCAGTGTGATAATTATCTGCGCTGTGGTG---TTGAAG

PBU0048Ceriporia_cystidiata ACGGATC-

GCCTTCAGTGTGATAATTATCTGCGCTGTGGTG---TTGAAG

MZ340C_lacerataT ACGGATC-

GCCTTCAGTGTGATAATTATCTGCGCTGTGGTG---TTGAAG

Dai21940 ACGGATC-

GCCTTCAGTGTGATAATTATCTGCGCTGTGGTG---TTGAAG

847Dai16433 ACGGATC-

GCCTTCAGTGTGATAATTATCTGCGCTGTGGTG---TTGAAG

MarcinEmmia_latemarginatus ACGGATC-

GCCTTCAGTGTGATAATTATCTGCGCTGTGGTG---TTGAAG

Meijer3729Hydnopolyporus_fimbr ACGGATC-

GCCTTCAGTGTGATAATTATCTGCGCTGTGGTG---TTGAAG

RLG13408Phanerochaete_sp ACGGATC-

GCCTTCAGTGTGATAATTATCTGCGCTGTGGTG---TTGAAG

WHC1381Flavodon_flavus ACGGATC-

GCCTTCAGTGTGATAATTATCTGCGCTGTGGTG---GTGAAG

GB1833Phlebia_albida ACGGATC-

ATCTTCAGTGTGATAATTATCTGCGCTGTGGTG---TTGAAG

T407Phlebia_nitidula ACGAATC-

ATCTTCAGTGTGATAATTATCTGCGCTGTGGTG---TTGAAG

HHB6988Phanerochaete_exilis ACGGATC-

GCCTTCAGTGTGATAATTATCTGCGCTGTGGTG--TTTGAAG

HHB8509Phanerochaetella_xeroph ACGGATC-

GCCTTCAGTGTGATAATTATCTGCGCTGTGGTG---TTGAAG

PBU0051Macrohyporia_dictyopora ACGGATC-

GCCTTCAGTGTGATAATTATCTGCGCTGTGGTG---TTGAAG

HHB11463Phanerochaete_sp ACGGATC-

GCCTTCAGTGTGATAATTATCTGCGCTGTGGTG---TTGAAG

FP102382Byssomerulius_corium ACGGATC-

GCCTTCAGTGTGATAATTATCTGCGCTGTGGTG---TTGAAG

FP102165Efibula_americana ACGGATC-

GCCTTCAGTGTGATAATTATCTGCGCTGCGGTG---TTGAAG

Murdoch90Ceriporia_torpida ACGGATC-

GCCTTCAGCGTGATAATTGTCTACGTTGTGGC---TGTGAAG

Rivoire4413_Ceriporia_purpurea ACGGATC-

GCCTTCAGCGTGATAATTGTCTACGTTGTGGC---TGTGAAG

Kout_18_Ceriporia_triumphalis ACGGATC-

GCCTTCAGCGTGATAATTGTCTACGTTGTGGC---TGTGAAG

Rivoire3701_Ceriporia_bresadol ACGGATC-

GCCTTCAGCGTGATAATTGTCTACGTTGTGGC---TGTGAAG

VS4018 ACGGATC-

GCCTTCAGCGTGATAATTGTCTACGTTGTGGC---TGTGAAG

Ryvarden21832_Ceriporia_manzan ACGGATC-

GCCTTCAGCGTGATAATTGTCTACGTTGTGGC---TGTGAAG

Dai24539 ACGGATC-

GCCTTCAGCGTGATAATTGTCTACGTTGTGGC---TGTGAAG

Dai24541 ACGGATC-

GCCTTCAGCGTGATAATTGTCTACGTTGTGGC---TGTGAAG

JV1105_12_Ceriporia_occidental ACGGATC-

GCCTTCAGCGTGATAATTGTCTACGTTGTGGC---TGTGAAG

VS8558Ceriporia_occidentalis ACGGATC-

GCCTTCAGCGTGATAATTGTCTACGTTGTGGC---TGTGAAG

Dai22445 --

846Dai16368 ACGGATC-

GCCTTCAGCGTGATAATTGTCTACGTTGTGGC---TGTGAAG

Dai17951_Ceriporia_aurantiocar ACGGATC-

GCCTTCAGTGTGATAAATGTCTACGCTGTGGC---TGTGAAG

Miettinen_11701C_viridans ACAGATC-

GCCTTCAGTGTGATAATTGTCTACGCTGTGG----TGTGAAG

JV0105_10Ceriporia_aurantiocar ACGGATC-

GCCTYCAGTGTGATAAATGTCTACGCTGTGGC---TGTGAAG

Yuan5702C_viridans ACAGATC-

GCCTTCAGTGTGATAATTGTCTACGCTGTGG----TGTGAAG

858Dai17003 ACAGATC-

GCCTTCAGTGTGATAATTGTCTACGCTGTGG----TGTGAAG

Yuan2747_Ceriporia_viridans ACAGATC-

GCCTTCAGCGTGATAAATGTCTACGTTGTGGC---TGTGAAG

Yuan2744C_viridans ACAGATC-
GCCTTCAGCGTGATAAATGTCTACGTTGTGGC---TGTGAAG
Li1046C_viridans ACAGATC-
GCCTTCAATGTGATAAATGTCTACGTTGTGGC---TGTGAAG
865C_sinoviridans ACAGATC-
GCCTTCAATGTGATAAATGTCTACGTTGTGGC---TGTGAAG
871Dai15062 ACCGATC-
GCCTTCAGCGTGATAAATGTCTACGTTGTGGC---TGTGAAG
Dai7642_Ceriporia_humilis ACGGATC-
ACCTTCAACGTGATAATTGTCTGCGTTGTGGT---TGTGAAG
Spirin4706_Ceriporia_humilis ACGGATC-
ACCTTCAACGTGATAATTGTCTGCGTTGTGGT---TGTGAAG
Spirin4944_Ceriporia_sericea GCGGATC-
ATCTTCAACGTGATAATTGTCTGCGTTGTGGT---TGTGAAG
WCG1547Dai26044ceriporia ACGGATC-
ATCTTCAACGTGATAATTGTCTGCGTTGTGGT---TGTGAAG
ZZW1558Dai27086 ACGGATC-
ATCTTCAACGTGATAATTGTCTGCGTTGTGGT---TGTGAAG
Miettinen14381_Ceriporia_mpuri ---
Miettinen15492_2_Ceriporia_sor ACGGATC-
ATCTTCAACGTGATAATTGTCTGCGTTGTGGC---TGTGAAG
He6687
ACGGATCTACCTTCAACGTGATAATTGTCTGCGTTGTGGT---CGTGAAG
ZH53Dai24426 ACGGATCTACCTTCAACGT-------------------------

Vlasak0808_30_Ceriporia_punica ACCGATC-
ACTTTCAACGTGATAATTATCTGCGTTGTGAG---TGTGAAG
887Dai13376 ACTGATC-
ACTTTCAACGTGATAATTATCTGCGTTGTGAG---TGTGAAG
WCG1443Dai24998 ACCGATC-
ACTTTCAACGTGATAATTATCTGCGTTGTGGG---TGTGAAG
0108_6Ceriporia_spissa
ACGGATCATCTTTCAGCGTGATAAATTTCTGCGCTGTGGCA--TGTGAAG
Dai19164
ACGGATCATCTTTCAGCGTGATAAATTTCTGCGCTGTGGCA--TGTGAAG
Dai17937_Ceriporia_bubalinomar
ACGGATCATCTTTCAGCGTGATAAATTTCTGCGCTGCAGCT--TGTGAAG
903Dai12113
ACGGATCATCTTTCAGCGTGATAAATTTCTGCGCTGCAGCT--TGTGAAG
LZB929Dai25079 ACGGATC-
ACTTTCAGTGTGATAATTATCTGCGCTGTCTG---TGTGAAG
LX45Dai26988 ACGGATC-
ACTTTCAGTGTGATAATTATCTGCGCTGTCTG---TGTGAAG
LX43Dai26986 ACGGATC-

ACTTTCAGTGTGATAATTATCTGCGCTGTCTG---TGTGAAG

Dai7759Ceriporia ACGGATC-

ACTTTCAGTGTGATAATTATCTGCGCTGTCTG---TGTGAAG

Cui8012_Ceriporia_viridans ATGGATC-

GCCGTCAGTGTGATAATTGTCTATGCTGTGGC---TGTGAAG

GC1704_54Ceriporia_viridans ATGGATC-

GCCGTCAGTGTGATAATTGTCTATGCTGTGGC---TGTGAAG

Dai23392 ATGGATC-

GCCGTCAGTGTGATAATTGTCTATGCTGTGGC---TGTGAAG

WCG1585Dai26113Ceriproia ATGGATC-

GCCGTCAGTGTGATAATTGTCTATGCTGTGGC---TGTGAAG

Dai18675C_eucalypti ACGGATC-

GCTCTCAGCGGGATAATTGTCTATGCTGTGGC---TGTGAAG

Dai22034 ACGGATC-

GCCTTCAGTGTGATAATTGTCTACGCTGTGGC----------

JV1008_41JTardaFLORIDAKeys ACGGATC-

GCCTTCAGTGTGATAATTGTCTACGCTGTGGC---TGTGAAG

Rivoire1161_Ceriporia_pierii ACGGATC-

GCCTTCAACGTGATAATTGTCTGCGTTGTGGT---TGTGAAG

Dai23499C_pierii ACGGATC-

GCCTTCAACGTGATAATTGTCTGCGTTGTGGT---CGTGAAG

Dai23500 ACGGATC-

GCCTTCAACGTGATAATTGTCTGCGTTGTGGT---CGTGAAG

841Dai15899 ACGGATC-

GTCATCAGCGTGATAATTGTCTACGTTGTGGC---TGTGAAG

842Dai15904 ACGGATC-

GTCATCAGCGTGATAATTGTCTACGTTGTGGC---TGTGAAG

LZB1066xinjiang ACGGATC-

GTCATCAGCGTGATAATTGTCTACGTTGTGGC---TGTGAAG

LZB1065xinjiang ACGGATC-

GTCATCAGCGTGATAATTGTCTACGTTGTGGC---TGTGAAG

851Dai16779 ACGGATC-

ACCTTCAACGTGATAATTGTCTGCGTTGTGGTTG-TGTGAAG

RMJ119sp_Candelabrochaete_sept

ACGGAATCGCCTTCAGCGTGATAATTGTCGTCGCTGTGGTG---GTGAAG

RLG9759spCandelabrochaete_sept

ACGGAATCGCCTTCAGCGTGATAATTGTCGTCGCTGTGGTG---GTGAAG

RLG10478Phanerochaete_allantos ACTGATC-

GCCTTCAGCGTGATAATTGTCTGCGCTGTGGA---CGTGACG

Dai19118_Ceriporia_spissa

GTGAATCAACTTTCAGTGTGATAATTTTCTGCGCTGTAGGTTGTGTAGAT

Dai18486A

GTGAATCAACTTTCAGTGTGATAATTTTCTGCGCTGTAGGTTGTGTAGAT

WEI17_024_Ceriporia_mellita

GTGAATCAACTTTCAGTGTGATAATTTTCTGCGCTGTAGGTTGTGCAGAT
GC1508_71Ceriporia_mellita
GTGAATCAACTTTCAGTGTGATAATTTTCTGCGCTGTAGGTTGTGCAGAT
GC1608_7_Ceriporia_mellita
GTGAATCAACTTTCAGTGTGATAATTTTCTGCGCTGTAGGTTGTGCAGAT
ZZW1557Dai27085
GTGAATCAACTTTCAGTGTGATAATTTTCTGCGCTGTAGGTTGTGCAGAT
ZZW1554Dai27083
GTGAATCAACTTTCAGTGTGATAATTTTCTGCGCTGTAGGTTGTGCAGAT
Dai8168
GTGAATCAACTTTCAGTGTGATAATTTTCTGCGCTGTAGGTTGTGCAGAT
BR4865C_mellita
GTGAATCAACTTTCAGTGTGATAATTTTCTGCGCTGTAGGTTGTGTAGAT
MEL2382688Ceriporia_sp
GTGAATCAACTTTCAGTGTGATAATTTTCTGCGCTGTGGGTTGTGTGCAT
Dai8110
GTGAATCAACTTTCAGTGTGATAATCTTCTACGCTGTATGTTGC------
Cui8097
GTGAATCAACTTTCAGTGTGATAATCTTCTACGCTGTATGTTGC------
909Cui6740
GTGAATCAACTTTCAGTGTGATAATTTTCTGCGCTGTGGGTTGTGTGCAT
W1258Dai24695
GTGAATCAACTTTCAGTGTGATAATTTTCTGCGCTGTGGGTTGTGTGCAT
JV0110_26_Ceriporia_griseoviol ACCGATC-
GCCCTCAGTGTGATAATTGTCTGCGCTGTG----ATGTGAA-
896Dai13202 ACCGATC-
GCCCTCAGTGTGATAATTGTCTGCGCTGTG----ATGTGAA-
LWY393Dai27053C_griseoviolasce ACCGATC-
GCCCTCAGTGTGATAATTGTCTGCGCTGTG----ATGTGAA-
LWY394DAI27054 ACCGATC-
GCCCTCAGTGTGATAATTGTCTGCGCTGTG----ATGTGAA-
FP135015G_pannocinctus ACTGATC-
GCCTTCAGTGTGATAATTGTCTACGCTGTGGT---TGTGAAG
L15726SpG_pannocinctus ACTGATC-
GCCTTCAGTGTGATAATTGTCTACGCTGTGGT---TGTGAAG
Dai22221 ACCGATC-
GCCTTCAGTGTGATAATTGTCTACGCTGTGG----TGTGAAG
Dai22633 ACGGATC-
GCCTTCAGTGTGATAATTGTCTACGCTGTGGT---TGTGAAG
Dai23260 ACGGATC-
GCCTTCAGTGTGATAATTGTCTACGCTGTGGT---TGTGAAG
Dai23626 ACGGATC-
GCCTTCAGTGTGATAATTGTCTACGCTGTGGT---TGTGAAG
Dai16238G_citrinoalbus ATGGATC-

GCCTTCAGTGTGATAATTGTCTACGCTGTGGT---TGTGAAG

1175Dai15293 ATGGATC-
GCCTTCAGTGTGATAATTGTCTACGCTGTGGT---TGTGAAG

Dai19547 ATGGATC-
GCCTTCAGTGTGATAATTGTCTACGCTGTGGT---TGTGAAG

918063G_africanus ACGGATC-
GCCTTCAGTGTGATAATTGTCTACGCTGTGGT---TGTGAAG

918572G_africanus ACGGATC-
GCCTTCAGTGTGATAATTGTCTACGCTGTGGT---TGTGAAG

Dai18536A ACGGATC-
GCCTTCAGTGTGATAATTGTCTACGCTGTTTT---CGTGAAG

1164Cui17922 ACGGATC-
GCCTTCAGTGTGATAATTGTCTACGCTGTTTT---CGTGAAG

Dai22225 ACGGATC-
GCCTTCAGTGTGATAATTGTCTACGCTGTTTT---CGTGAAG

1163Dai20655 ACGGATC-
GCCTTCAGTGTGATAATTGTCTACGCTGTTTT---CGTGAAG

Yuan4397G_hainanensis ACTGGTC-
ACTATCAGTGTGATAATTATCTGCGCTGTTGT---GATTCAG

1176Dai15268 ACTGGTC-
ACTATCAGTGTGATAATTATCTGCGCTGTTGT---GATTCAG

1177Dai15259 --

BZ2896G_thelephoroides ACTGGCC-
ACTATCAGTGTGATAATTATCTGCGCTGTTGT---GATTCAG

1166JV1808_26 ACTGGCC-
ACTATCAGTGTGATAATTATCTGCGCTGTTGT---GATTCAG

Miettinen16992Hapalopilus_ochr ATGGATC-
ATCTTCAGTATGATAATTGTCTATGCTGTGGTG----TGAAG

GC1708_338_Ceriporia_arbuscula ATGCATT-
GCTTTCAGCAGGATAATTATCTATGTTGTGGCAT---TGAAG

WCG1555Dai26107Ceriporia ATGCATT-
GCTTTCAGCAGGATAATTATCTATGTTGTGGCAT---TGAAG

GC1708_340_Ceriporia_arbuscula ATGCATT-
GCTTTCAGCAGGATAATTATCTATGTTGTGGCAT---TGAAG

WCG1556Dai26109Ceriporia --

883Cui11291 ACAGATT-
GCCTTCAGCAGGATAATTGTCTATGTTGTTGCTC---TGGAG

HLX320Dai26805 ATACATT-
GCTTTCAGCAGGATAATTGTCTATGTTGTGGCTT---TGAAG

WCG1266Dai24678A ATACAAT-
GCTTTCAGCAGGATAATTGTCTATGTTGTGGCTT---TGAAG

Dai6090_Ceriporia_sulphuricolo ACTGACC-
GCCTTCAGCGTGATAATTATCTGCGCTGTGGTGC-CGTGAAG

RLG_11354_Ceriproia_reticulata GC-GATC-

ATCTTCAGTGTGATAATTGTCTACGCTGTGRA---TGTGAAC
ZZW1543Dai27072 GC-AATC-
ATCTTCAGTGTGATAATTGTCTACGCTGTGGA---TGTGAAC
Li1316_Ceriporia_reticulata --
KHL11981Ceriporia_reticulata --
FP110343sp_Candelabrochaete_la GC-AATC-
ATCCTCAGTGTGATAATTGTCTACGCTGTGAA---TGTGAAC
Li1045_Ceriporia_reticulata GC-GATC-
ATCCTCAGTGTGATAATTATCTGCGCTGTGAA---TGTGAAC
ZX136Dai25794ceriporia GC-GATC-
ATCCTCAGTGTGATAATTATCTGCGCTGTGAA---TGTGAAC
892Dai13400 GC-GATC-
ATCCTCAGTGTGATAATTATCTGCGCTGTGAA---TGTGAAC
RLG7163Leptoporus_mollis ACACATT-
GCCTTCAGCGTGATAATCGTCTACGTTGTCGCAT-TATGT-A
Dai21062Leptoporus_mollis ACACATT-
GCCTACAGCGTGATAATCATCTACGTTGTCGCAT-TATGT-A
Dai20182Leptoporus_submollis ACACATT-
GCCTTCAGCGTGATAATTGTCTACGTTGTCGCAC-TATGT-A
Cui18379Leptoporus_submollis ACACATT-
GCCTTCAGCGTGATAATTGTCTACGTTGTCGCAC-TATGT-A
Wu1209_46Resiniporus_pseudogil ATGGATC-
GCTTTCAGTGTGATAATTATCTACGCTGTGGTGT---TGAAG
BRNM710169Resiniporus_resinasc ATGGATC-
GCTTTCAGTGTGATAATTATCTACGCTGTGGTGT---TGAAG
Dai14516Bjerkandera_adusta ACCAGCC--
GCTTCAGCGTGATAATTATCTGCGTTGCTGTGGGTATTCAG
Dai21100Bjerkandera_fumosa ACCAGCC--
GCTTCAGCGTGATAATTATCTGCGTTGCTGTGGGTATGCAG
Miettinen16854Ceraceomyces_sp
ACGGATCACTTATCAGTGTGATAATTATCTGTGCTGTATT---TGTGAAG
Dai10477C_spissa ACTGATC-
ATTCTCAATGTGATAAATTTCTGCGTTGTG----ATGTGATG
855Dai16831 ACTGATC-
ATTCTCAATGTGATAAATTTCTGCGTTGTG----ATGTGATG
882Cui11282 ACTGATC-
ATTCTCAATGTGATAAATTTCTGCGTTGTG----ATGTGATG
Dai24566 ACTGATC-
ATTCTCAATGTGATAAATTTCTGCGTTGTG----GTGTGATG
Yuan5965 ACTGATC-
ATTCTCAATGTGATAAATTTCTGCGTTGTG----GTGTGATG
Dai3204 --
1194CUI9985 --

Dai15205_Ceriporia_albomellea TGGTT-ATATTCGTTCATGCTTATAATTCTCTTATT--AAAGAGATATTT
Dai15223_Ceriporia_albomellea TGGTT-ATATTCGTTCATGCTTATAATTCTCTTATT--AAAGAGATATTT
Li1780_Ceriporia_variegata TGGTT-ATGTTTGTTCATGCTTATAATTCTCTTACT--GAAGAGATATAT
Dai19791_Ceriporia_variegata TGGTT-ATGTTTGTTCATGCTTATAATTCTCTTACT--GAAGAGATATAT
Dai19886 TGGTT-ATGTTTGTTCATGCTTATAATTCTCTTACT--GAAGAGATATAT
Dai10833_Ceriporia_crassitunic TGCT--ATATTTGTTCATGCTTATAATGCTCTTTTT--GAAGAGACATTT
CHWC1506_46Meruliopsis_crassit TGCT--ATATTTGTTCATGCTTATAATGCTCTTTTT--GAAGAGACATTT
Dai9995_Ceriporia_crassitunica TGTGTTATATTTGTTCATGCTTATAATTCTCTTATT--GAAGAGACATTT
Wu1209_58_Meruliopsis_parvispo T-ATC--TATGTGTTCATGCTTATAACTGTCTCTC-----TGAGACAATT
CHWC1505_129_Meruliopsis_parvi T-ATC--TATGTGTTCATGCTTATAACTGTCTCTC-----TGAGACAATT
Dai21944 TGATT--TATGTATTCATGCTTATAATCGTCTTTTT--ATGAAGACAATT
830Dai18640A TAATT--TATGCATTCATGCTTACAATCCTCTCTTTTCTAAGAGACCATT
GC1704_60_Meruliopsis_taxicola TATTT-TTAT--ATTCATGCTTACAACCGTCTCTTG--ACTGAGACAATT
Dai22625 TATTT-TTAT--ATTCATGCTTACAACCGTCTCTTG--ACTGAGACAATT
Dai22636 TATTT-TTAT--ATTCATGCTTACAACCGTCTCTTG--ACTGAGACAATT
Dai21878 TATTT-TTAT--ATTCATGCTTACAACCGTCTCTTG--ACTGAGACAATT
1169Dai17248 TATTT-TTAT--ATTCATGCTTACAACCGTCTCTTG--ACTGAGACAATT
Wu1708_43_Meruliopsis_leptocys T-ATC--TATATATTCATGCTTATAACTGTCTCTTC---TAGAGACAA--
Li1011 T-ATC--TATATATTCATGCTTATAACTGTCTCTTC---TAGAGACAA--
ZX95Dai25742Meruliopsis_leptoc TAATC--TATGTATTCATGCTTATAACTGTCTCTTC---TAGAGACAAT-
WCG1306Dai24733 TAATC--TATGTATTCATGCTTATAACTGTCTCTTC---TAGAGACAAT-
LXL99Dai25816 TAATC--

TATGTATTCATGCTTATAACTGTCTCTTC---TAGAGACAAT-

WCG1559Dai26052Meruliopsis TATCT--CATATATTCATGCTTATAACTGTCTCTT-----AGAGACAATT

He7477 TATCT--CATATATTCATGCTTATAACTGTCTCTT-----AGAGACAATT

HLX243Dai26217 TATCT--TATATATTCATGCTTATAACTGTCTCTT-----AGAGACAATT

RussiaMW673659Meruliopsis_fagi TATCT--CATATATTCATGCTTATAACTGTCTCTCA--TTAGAGACAATG

FD278 TAAAT-TTATATGTCCATGCTTATAATCGTCTCTC-----TGAGACAAAT

Dai10226_Ceriporia_tarda TAAAA-ATATATATTCATGCTTACAATCGTCTCCT-----AGAGACAATT

LE247365 TAAAA-ATATATATTCATGCTTACAATCGTCTCCT-----AGAGACAATT

Dai8173_Meruliopsis_nanlingens T-ATA--AATATATTCATGCTTATAATCGTCTCTT-----GAAGACAAT-

860Dai17172 T-ATA--AATATATTCATGCTTATAATCGTCTCTT-----GAAGACAAT-

879Dai13414 T-ATA--AATATATTCATGCTTATAATCGTCTCTT-----GAAGACAAT-

Li_1704_Meruliopsis_pseudocyst TGATT--TATATGTTCATGCTTACAATCGTCTCTTT---AAGAGACAATT

833Dai18405 TGATTTATATATGTTCATGCTTACAATCGTCTCTTT---AAGAGACAATT

HHB_10729_Meruliopsis_albostra TGATT--TATATGTTCATGCTTACAATCGTCTCTTT--TAAGAGACAATT

Cui6878_Ceriporia_pseudocystid TGA----AATATGTTCATGCTTATAATTGTCTCTT----ATGAGACAATT

869Dai14737 TGA----AATATGTTCATGCTTATAATTGTCTCTT----ATGAGACAATT

876Cui11626 TGA----AATATGTTCATGCTTATAATCGTCTCTT----ATGAGACAATT

1199WEI3388 TGA----AATATGTTCATGCTTATAATCGTCTCTT----ATGAGACAATT

776308_Meruliopsis_cystidiata TGAWT--TTTTAATTCATGCTTATAATCGTCTCTT----ATGAGACAATT

ICN139059_Meruliopsis_cystidia TGATT--TTTTAATTCATGCTTATAATCGTCTCTT----ATGAGACAATT

HHB15692Ceraceomyces_serpens TATTT-AATTG-ACTCATGCTTACAATCGTCTCTTG----GGAGACAGTT

HHB_15629_Sp_Ceriporiopsis_ane TATTT-TTAT--ATTCGTGCTTATAATCGTCTCCTT--ACCGAGACAATT

AJ185Trametopsis_cervina TATCA-TTAT--GTTCATGCTTATAATCGTCTGTTC--

-ATCAGACAATT

FD9Irpex_lacteus TATTT-ATGGT-GTTCATGCTTCGAACCGTCTCCTT--GCCGAGACAAT-

908Dai11230 TATTT-ATGGT-GTTCATGCTTCGAACCGTCTCCTT--GCCGAGACAAT-

FP55521TEmmia_lacerata TATTT-ATTA--GTTCATGCTTATAGTCGTCTCTTA--CC-GAGACAAT-

PBU0048Ceriporia_cystidiata TATTT-ATTA--GTTCATGCTTATAGTCGTCTCTTA--CC-GAGACAAT-

MZ340C_lacerataT TATTT-ATTA--GTTCATGCTTATAATCGTCTCTTA--CC-GAGACAAT-

Dai21940 TATTT-ATTA--GTTCATGCTTATAATCGTCTCTTA--CC-GAGACAAT-

847Dai16433 TATTT-ATTA--GTTCATGCTTATAGTCGTCTCTTA--CC-GAGACAAT-

MarcinEmmia_latemarginatus TATTT-ATTA--GTTCATGCTTATAGTCGTCTCTTA--CC-GAGACAAT-

Meijer3729Hydnopolyporus_fimbr TATTT-ATTA--GTTCATGCTTATAATCGTCTCTGA--CTGGAGACAAT-

RLG13408Phanerochaete_sp TATTT-ATTG--GTTCATGCTTACAACCGTCTGTTA--TC--GGACAAC-

WHC1381Flavodon_flavus TATTT-ATTGT-ATTCACGCTTCTAACCGTCTCTCC--TTCGAGACAAA-

GB1833Phlebia_albida TATTT-TATT--GATCATGCTTATAATCGTCCTTTA--CC--GGACAGC-

T407Phlebia_nitidula TA-TT-TATT--GATCATGCTTACAATCGTCCTTTA--CC--GGACAAC-

HHB6988Phanerochaete_exilis TATTT-TGTA---TTCGTGCTTACAATCGTCTCCTA-----GAGACAAT-

HHB8509Phanerochaetella_xeroph TATTT-TGTA---TTCGTGCTTACAGTCGTCTCGTT-----GAGACAA--

PBU0051Macrohyporia_dictyopora TATTT-TGTA---TTCGTGCTTACAATAGTCTCTTG-----CAGACAA--

HHB11463Phanerochaete_sp TATTT-TGTA---TTCGTGCTTACAATCGTCTCTCT-----GTGGAG---

FP102382Byssomerulius_corium TATTT-CATA--GTTCGTGCTTATAATCGTCTCGCA-----AGAGACA--

FP102165Efibula_americana TATTT-TTTTATGTTCATGCTTCTAATCGTCTCTCA--C--GAGACAAAT

Murdoch90Ceriporia_torpida TATTT-GTGT--GTTCGTGCTTACAATCATCTTGTA----AAAGATAAC-

Rivoire4413_Ceriporia_purpurea TATTT-GTGT--GTTCACGCTTACAGTCATCTCGCA----AGAGATAAA-

Kout_18_Ceriporia_triumphalis TATTT-GTGT--GTTCACGCTTACAGTCATCTCGCA--

--AGAGATAAAC

Rivoire3701_Ceriporia_bresadol TATTT-GTGT--GTTCACGCTTACAGTCATCTCGCA----AGAGATAAAC

VS4018 TATTT-GTGT--GTTCACGCTTACAGTCATCTCGCA----AGAGATAAAC

Ryvarden21832_Ceriporia_manzan TATTT-GTGT--GTTCACGCTTACAGTCATCTCGCA----AGAGATAAAC

Dai24539 TATTT-GTGT--GTTCACGCTTACAGTCATCTCGCA----AGAGATAAA-

Dai24541 TATTT-GTGT--GTTCACGCTTACAGTCATCTCGCA----AGAGATAAAC

JV1105_12_Ceriporia_occidental TATTT-GTGT--GTTCACGCTTACAGTCATCTCGCA----AGAGATAAAC

VS8558Ceriporia_occidentalis TATTT-GTGT--GTTCACGCTTACAGTCATCTCGCA----AGAGATAAAC

Dai22445 -TTTT--

846Dai16368 TATTT-GTGT--GTTCACGCTTACAGTCATCTCGCA----AGAGATAAC-

Dai17951_Ceriporia_aurantiocar TATTT-ATAT--GTTCATGCTTACAATTGTCTTTCT-----GAGACAAT-

Miettinen_11701C_viridans TATTT-ATGT--GTTCATGCTTCTAATTGTCTTTAT------TGACAAT-

JV0105_10Ceriporia_aurantiocar TATTT-ATAT--GTTCATGCTTACAATTGTCTTTCT-----GAGACAAT-

Yuan5702C_viridans TATTT-ATGT--GTTCATGCTTCTAATTGTCTTTAT------TGACAAT-

858Dai17003 TATTT-ATGT--GTTCATGCTTCTAATTGTCTTTAT------TGACAAT-

Yuan2747_Ceriporia_viridans TATTT-ATAT--GTTCATGCTTATAATTGTCTTCTT------AGACAAT-

Yuan2744C_viridans TATTT-ATAT--GTTCATGCTTATAATTGTCTTCTT------AGACAAT-

Li1046C_viridans TATTT-ATGT--GTTCATGCTTACAATTGTCTCTTT------AGACAAG-

865C_sinoviridans TATTT-ATGT--GTTCATGCTTACAATTGTCTCTTT------AGACAAG-

871Dai15062 TATTT-ATAT--GTTCATGCTTATAATTGTCTTCTT------AGACAAA-

Dai7642_Ceriporia_humilis TACTT-GTGT--GTTCATGCTTACAGTCGTCTTCTTAT--GGAGACACT-

Spirin4706_Ceriporia_humilis TACTT-GTGT--GTTCATGCTTACAGTCGTCTTCTTAT--GGAGACACT-

Spirin4944_Ceriporia_sericea TACTT-GTGC--GTTCATGCTTACAGTCGTCTCTTAAC--TGAGACACT-

WCG1547Dai26044ceriporia TGCTT-GTGC--GTTCATGCTTACAGTCGTCTCTTAAC--CGAGACACT-

ZZW1558Dai27086 TGCTT-GTGC--GTTCATGCTCACAGTCGTCTCTTAAC--CGAGACACT-

Miettinen14381_Ceriporia_mpuri --CTT-GTGC--GTTCACGCTTACAGTCGTCTTCTTGTAAAGAGACACT-

Miettinen15492_2_Ceriporia_sor TCTTT-GAGT--GTTCACGCTTACAGTCGTCTCTTC----AGAGACAAC-

He6687 TACTC-GTGT--GTTCACGCTTACAGTCGTCTTCTTGTAAAGAGACACT-

ZH53Dai24426 --

Vlasak0808_30_Ceriporia_punica TACTC-GTGTG-GTTGACGCTTATAAACGTCTTCAA--TTGAAGATAGT-

887Dai13376 TACTT-GTGTG-GTTGACGCTTATAAATGTCTTCAA--TTGAAGATAAT-

WCG1443Dai24998 TACTT-GCATG-GTTGACGCTTCTAATTGTCTTCAA--TCGAAGATAAT-

0108_6Ceriporia_spissa TATTT-GTTGT-GTTCATGCTTACAGTTGTCTYGTG---AGGCAACAACT

Dai19164 TATTT-GTTGT-GTTCATGCTTACAGTTGTCTCGTG---AGGCAACAACT

Dai17937_Ceriporia_bubalinomar TAT----TTCT-GTTCATGCTTATGGTTATCTTTAG---AGATAACAA-T

903Dai12113 TAT----TTCT-GTTCATGCTTATGGTTGTCTTTAG---AGATAACAA-T

LZB929Dai25079 TATTC-ATAT--GTTCATGCTTATAATTGTCTTGTT--ACAAAGACA---

LX45Dai26988 TATTC-ATAT--GTTCATGCTTATAATTGTCTTGTT--ACAAAGACA---

LX43Dai26986 TATTC-ATAT--GTTCATGCTTATAATTGTCTTGTT--ACAAAGACA---

Dai7759Ceriporia TATTC-ATAT--GTTCATGCTTATAATTGTCTTGTT--ACAAAGACA---

Cui8012_Ceriporia_viridans TATTT-TCTAT-GTTCATACTTATAATTGTCTATCC-----AAGACATT-

GC1704_54Ceriporia_viridans TATTT-TCTAT-GTTCATACTTATAATTGTCTATCC-----AAGACATT-

Dai23392 TATTT-CTCGT-ATTCATACTTAGAATTGTCTCAGT-----GAGACAGT-

WCG1585Dai26113Ceriproia TATTT-CTCGT-ATTCATACTTAGAATTGTCTCAGT-----GAGACAGT-

Dai18675C_eucalypti TATTT-TATAT-GTTCATACTTATAATTGTCTATCT------AGACATT-

Dai22034 --

JV1008_41JTardaFLORIDAKeys TATTT-ATGT--GTTCATGCTTATAATCGTCTCT-------AAGACAAT-

Rivoire1161_Ceriporia_pierii TATTT-ATGG--ATTCATGCTTATAACTGTCTCTTA-----GAGACAAT-

Dai23499C_pierii TATTT-ATGC--ATTCATGCTTATAACTGTCTCTTA-----GAGACAAT-

Dai23500 TATTT-ATGC--ATTCATGCTTATAACTGTCTCTTA-----GAGACAAT-

841Dai15899 TATTT-ATTGC-GTTCATGCTTACAGTCGTCTCTTA----TGAGACAGCT

842Dai15904 TATTT-ATTGC-GTTCATGCTTACAGTCGTCTCTTA----TGAGACAGCT

LZB1066xinjiang TATTT-ATTGC-GTTCATGCTTACAGTCGTCTCTTA----TGAGACAGCT

LZB1065xinjiang TATTT-ATTGC-GTTCATGCTTACAGTCGTCTCTTA----TGAGACAGCT

851Dai16779 TACTT-GTGC--ATTCATGCTTACAGTCGTCTCCTT---GAGAGGCTACT

RMJ119sp_Candelabrochaete_sept TACTC-GCGT--GTTCGTGCTTACAGTCGTCCTTCG------GGACAATT

RLG9759spCandelabrochaete_sept TACTC-GCGT--GTTCGTGCTTACAGTCGTCCTTCG------GGACAATT

RLG10478Phanerochaete_allantos TACTC-GTGT--GTTCATGCTTACAGTCGTCTCTTG-----GAGACAACT

Dai19118_Ceriporia_spissa CACCTCTGTTG--CTCATGCTTAGAGTTGTCTCTTT------CGAGACTT

Dai18486A CACCTCTGTTG--CTCATGCTTAGAGCTGTCTCTTT------CGAGACTT

WEI17_024_Ceriporia_mellita CACCTCTGTTG--CTCATGCTTAGAGTTGTCTCTT-------GGAGACTT

GC1508_71Ceriporia_mellita CACCTCTGTTG--CTCATGCTTAGAGTTGTCTCTT-------GGAGACTT

GC1608_7_Ceriporia_mellita CACCTCTGTTG--CTCATGCTTAGAGTTGTCTCTT-------GGAGACTT

ZZW1557Dai27085 CACCTCTGTTG--CTCATGCTTAGAGTTGTCTCTT-------GGAGACTT

ZZW1554Dai27083 CACCTCTGTTG--CTCATGCTTAGAGTTGTCTCTT-------GGAGACTT

Dai8168 CACCTCTGTTG--CTCATGCTTAGAGTTGTCTCTT-------GGAGACTT

BR4865C_mellita CACCTCTGTTG--CTCATGCTTAGAGTTGTCTCTTT------CGAGACTT

MEL2382688Ceriporia_sp CACCTCTGGTGGTTTCGCGCTTAGAGTTGTCTC-TT------AGAGACTT

Dai8110 --CTTCTGTCG--CTCATGCTTAGAGTTGTCTCTT-------AGAGACTT

Cui8097 --CTTCTGTCG--CTCATGCTTAGAGTTGTCTCTT-------AGAGACTT

909Cui6740 CACCTCTGGTGGTTTCGCGCTTAGAGTTGTCTCTT-------AGAGACTT

W1258Dai24695 CACCTCTGGTGGTTTCGCGCTTAGAGTTGTCTCTT-------AGAGACTT

JV0110_26_Ceriporia_griseoviol -AGTGCTCGCGTGTTCATGCTTGAAACTGTCTCTCTTAGTCGAGAYGAAC

896Dai13202 -AGTGCTCGCGTGTTCATGCTTGAAACTGTCTCTCTTAGTCGAGACGAAC

LWY393Dai27053C_griseoviolasce -AGTGCTCGCGTGTTCATGCTTGAAACTGTCTCTCTTAGTTGAGACGAAC

LWY394DAI27054 -AGTGCTCGCGTGTTCATGCTTGAAACTGTCTCTCTTAGTTGAGACGAAC

FP135015G_pannocinctus TAA---ATTAATGTTCATGCTTCTAATCGTCTCTTTT---TGAGACAATT

L15726SpG_pannocinctus TAA---ATTAATGTTCATGCTTCTAATCGTCTCTTTT---TGAGACAATT

Dai22221 TAT---AATTGTATTCATGCTTCTAACTGTCTTTTAA--TTGAGACAACT

Dai22633 TAT---ATTTGTATTCATGCTTCTAATCGTCTCTTA---ATGAGACAATT

Dai23260 TAT---ATTTGTATTCATGCTTCTAATCGTCTCTTG---ATGAGACAATT

Dai23626 TAT---ATTTGTATTCATGCTTCTAATCGTCTCTTA---CTGAGACAATT

Dai16238G_citrinoalbus TATAA-ACTTGTATTCATGCTTCTAATCGTCTTTTAA--ACAAGACACTT

1175Dai15293 TATAA-ACTTGTATTCATGCTTCTAATCGTCTTTTAA--ACAAGACACTT

Dai19547 TATAA-ACTTGTATTCATGCTTCTAATCGTCTTTTAA--ACAAGACACTT

918063G_africanus TATAA-ATCTGTATTCATGCTTCTAATCGTCTTTTTA--CTAGGACAATT

918572G_africanus TATAA-ATCTGTATTCATGCTTCTAATCGTCTTTTTA--CTAGGACAATT

Dai18536A TATAA-AATTGTATTCATGCTTCTAATCGTCTTTCTA--ATAAGACAATT

1164Cui17922 TATAA-AATTGTATTCATGCTTCTAATCGTCTTTCTA--ATAAGACAATT

Dai22225 TATAA-AATTGGATTCATGCTTCTAATCGTCTTTGTA--CTAAGACAATT

1163Dai20655 TATAA-AATTGGATTCATGCTTCTAATCGTCTTTGTA--CTAAGACAATT

Yuan4397G_hainanensis TAT---TTATATGTTCATGCTTCTAATCGTCTCGTTTACATGAGACAATT

1176Dai15268 TAT---TTATATGTTCATGCTTCTAATCGTCTCGTTTACATGAGACAATT

1177Dai15259 --

BZ2896G_thelephoroides TAT---TTAAATGTTCATGCTTCTAATCGTCTTYTT---ACGAGACAAT-

1166JV1808_26 TAT---TTAAATGTTCATGCTTCTAATCGTCTTCTT---ACGAGACAAT-

Miettinen16992Hapalopilus_ochr TATAT-TTATTTATTCATGCTTTGAATCCTCTCTTA--GAAGAGGTAATT

GC1708_338_Ceriporia_arbuscula A-----------ATTTATGCTTATAGCTGTCTTGTTTTTACAAGATATGC

WCG1555Dai26107Ceriporia A-----------ATTTATGCTTATAGCTGTCTTGTTTTTACAAGATATGC

GC1708_340_Ceriporia_arbuscula A-----------ATTTATGCTTATAGCTGTCTTGTTTTTACAAGATATGC

WCG1556Dai26109Ceriporia --

883Cui11291 A-----------ATTTATGCTTATAGTTGTCTTTGA--ATGAGGACAATT

HLX320Dai26805 A-----------ATTTATGCTTACAGTTGTCTTATTC-AATGAGACAATT

WCG1266Dai24678A A-----------ATTTATGCTTATAGCTGTCTTACT--ATCAAGACAAAT

Dai6090_Ceriporia_sulphuricolo TGCTTAAGCTCTGATCATGCTTACGATTATCTTTTTCTGAGGAGAGGACG

RLG_11354_Ceriproia_reticulata A-----ATTTATATTTATGCTTATAGTCGTCTTTTA-----AAGACACTT

ZZW1543Dai27072 A-----ATTTATATTTATGCTTATAGTCGTCTTTTA-----AAGACACTT

Li1316_Ceriporia_reticulata --

KHL11981Ceriporia_reticulata --

FP110343sp_Candelabrochaete_la A-----TTTTATGTTTATGCTTATAGTCGTCTTTTT--ATTAAGACACTT

Li1045_Ceriporia_reticulata A------TCTATGTTTATGCTTATAGTCGTCTTTTT--ATTAAGACACTT

ZX136Dai25794ceriporia A------TCTATGTTTATGCTTATAGTCGTCTTTTT--ATTAAGACACTT

892Dai13400 A------TCTATGTTTATGCTTATAGTCGTCTTTTT--ATTAAGACACTT

RLG7163Leptoporus_mollis T-----TCATACGTTCTCGCTTACAATCGTCTCTCT-----GAGACATTT

Dai21062Leptoporus_mollis T-----TCATACGTTCTCGCTTACAATCGTCTCTCT-----GAGACATTT

Dai20182Leptoporus_submollis T-----TCATACGTTCTCGCTTACAATCGTCTCTCT-----G--------

Cui18379Leptoporus_submollis T-----TCATACGTTCTCGCTTACAATCGTCTCTCT-----GAGACATTT

Wu1209_46Resiniporus_pseudogil TATTAATTAT--GTTCGTGCTTATAACAGTCTCTTG--ACTGAGACAATT

BRNM710169Resiniporus_resinasc TATTAATTAT--GTTCATGCTTATAATAGTCTCTTG--ACTGAGACAATT

Dai14516Bjerkandera_adusta T----------GTTCACGCTTCTAACCGTC--------TTCGGACAAAT

Dai21100Bjerkandera_fumosa T----------GTTCGCGCTTCTAACCGTC--------TTCGGACAACA

Miettinen16854Ceraceomyces_sp TATTT-ATGT--GCTCATGCTTACAATCGTTTCTTT--AAAGAGACGACA

Dai10477C_spissa TACTT-GAGTG-GTCCATGCTTTTAATTATCTTTGATTTTGAAGATGATC

855Dai16831 TACTT-GAGTG-GTCCATGCTTTTAATTATCTTTGATTTTGAAGATGATC

882Cui11282 TACTT-GAGTG-GTCCATGCTTTTAATTATCTTTGATTTTGAAGATGATC

Dai24566 TACTT-GAGTG-GTACATGCTTTTCATTATC-------TTGAAGATGATC

Yuan5965 TACTT-GAGTG-ATACATGCTTTTCATTATC-------TTGAAGATGATC

Dai3204 --

1194CUI9985 --

Dai15205_Ceriporia_albomellea TT------TATGACTTCGTAACGGCGA-GTGAAGCGGGAAGAGCTCAAAT

Dai15223_Ceriporia_albomellea TT------TATGACTTCGTAACGGCGA-GTGAAGCGGGAAGAGCTCAAAT

Li1780_Ceriporia_variegata TT-----GTATGACTTCGTAACGGCGA-GTGAAGCGGGAAGAGCTCAAAT

Dai19791_Ceriporia_variegata TT-----GTATGACTTCGTAACGGCGA-GTGAAGCGGGAAGAGCTCAAAT

Dai19886 T---------------GTAACGGCGA-GTGAAGCGGGAAGAGCTCAAAT

Dai10833_Ceriporia_crassitunic TT------TATGGCTTTGTAACGGCGA-GTGAAGCGGGAAGAGCTCAAAT

CHWC1506_46Meruliopsis_crassit TT------TATGGCTTTGTAACGGCGA-GTGAAGCGGGAAGAGCTCAAAT

Dai9995_Ceriporia_crassitunica TT------TATAACCTT--------------------------------
Wu1209_58_Meruliopsis_parvispo ---ATTT-TATGACTTCGTAACGGCGA-GTGAAGCGGGATGAGCTCAAAT
CHWC1505_129_Meruliopsis_parvi ---ATTT-TATGACTTC----CGGCGA-GTGAAGCGGGATGAGCTCAAAT
Dai21944 ---TTCT-TATGACTTCGTAACGGCGA-GTGAAGCGGGAAGAGCTCAAAT
830Dai18640A ---TTCT-TATGACTTCGTAACGGCGA-GTGAAGCGGGAAGAGCTCAAAT
GC1704_60_Meruliopsis_taxicola ----TTT-TATGACTTCGTAACGGCGA-GTGAAGCGGGAAGAGCTCAAAT
Dai22625 ----TTT-TATGACTTCGTAACGGCGA-GTGAAGCGGGAAGAGCTCAAAT
Dai22636 ----TTT-TATGACTTCGTAACGGCGA-GTGAAGCGGGAAGAGCTCAAAT
Dai21878 ----TTT-TATGACTTCGTAACGGCGA-GTGAAGCGGGAAGAGCTCAAAT
1169Dai17248 ----TTT-TATGACTTCGTAACGGCGA-GTGAAGCGGGAAGAGCTCAAAT
Wu1708_43_Meruliopsis_leptocys ---ATTT-TATGACTTC-------------GAAGCGGGAAGAGCTCAAAT
Li1011 ---ATTT-TATGACTTCGTAACGGCGA-GTGAAGCGGGAAGAGCTCAAAT
ZX95Dai25742Meruliopsis_leptoc ---ATTT-TATGACTTCGTAACGGCGA-GTGAAGCGGGAAGAGCTCAAAT
WCG1306Dai24733 ---ATTT-TATGACTTCGTAACGGCGA-GTGAAGCGGGAAGAGCTCAAAT
LXL99Dai25816 ---ATTT-TATGACTTCGTAACGGCGA-GTGAAGCGGGAAGAGCTCAAAT
WCG1559Dai26052Meruliopsis --AATTT-TATGACTTCGTAACGGCGA-GTGAAGCGGGAAGAGCTCAAAT
He7477 --AATTT-TATGACTTCGTAACGGCGA-GTGAAGCGGGAAGAGCTCAAAT
HLX243Dai26217 --AATTT-TATGACTTCGTAACGGCGA-GTGAAGCGGGAAGAGCTCAAAT
RussiaMW673659Meruliopsis_fagi --AATTT-AATGACTTCGTAACGGCGA-GTGAAGCGGGAAGAGCTCAAAT
FD278 TT------TATGACTTC--AACGGCGA-GTGAAGCGGGAAAAGCTCAAAT
Dai10226_Ceriporia_tarda T-------TATGACTTC--------------------------------
LE247365 T-------TATGACTTC--------------------------------
Dai8173_Meruliopsis_nanlingens -----TT-TATGACTTCGTAACGGCGA-GTGAAGCGGGAAGAGCTCAAAT
860Dai17172 -----TT-TATGACTTCGTAACGGCGA-

GTGAAGCGGGAAGAGCTCAAAT
879Dai13414 -----TT-TATGACTTCGTAACGGCGA-
GTGAAGCGGGAAGAGCTCAAAT
Li_1704_Meruliopsis_pseudocyst A-AATTT-TATGACTTC--------------------------------
833Dai18405 A-AATTT-TATGACTTC-------------------------------T
HHB_10729_Meruliopsis_albostra A-AATTTATATGACTTCGTAACGGCGA-
GTGAAGCGGGAAGAGCTCAAAT
Cui6878_Ceriporia_pseudocystid T-CATTTATATGACTTCGTAACGGCGA-
GTGAAGCGGGAAGAGCTCAAAT
869Dai14737 T-CATTTATATGACTTCGTAACGGCGA-
GTGAAGCGGGAAGAGCTCAAAT
876Cui11626 T-CATTTATATGACTTCGTAACGGCGA-
GTGAAGCGGGAAGAGCTCAAAT
1199WEI3388 T-CATTTATATGACTTC-------------------------------
-
776308_Meruliopsis_cystidiata T-ATC---TATGACTTCGTAACGGCGA-
GTGAAGCGGGAAAAGCTCAAAT
ICN139059_Meruliopsis_cystidia T-ATC---TATGACTTCGTAACGGCGA-
GTGAAGCGGGAAAAGCTCAAAT
HHB15692Ceraceomyces_serpens T------ATATGACATCGTAACTGCGA-
GTGAAGCGGGAAAAGCTCAAAT
HHB_15629_Sp_Ceriporiopsis_ane A-TCA-T-TATGACATCGTAACTGCGA-
GTGAAGCGGGAAAAGCTCAAAT
AJ185Trametopsis_cervina TA----T-TATGACATCGTAACTGCGA-
GTGAAGCGGGAAAAGCTCAAAT
FD9Irpex_lacteus --CA--T-TTGACAATCGTAACTGCGA-
GTGAAGCGGGAAAAGCTCAAAT
908Dai11230 --CA--T-TTGACAATCGTAACTGCGA-
GTGAAGCGGGAAAAGCTCAAAT
FP55521TEmmia_lacerata --T---T-ATGACAATC-TAACTGCGA-
GTGAAGCGGGAAAAGCTCAAAT
PBU0048Ceriporia_cystidiata --T---T-ATGACAATCGTAACTGCGA-
GTGAAGCGGGAAAAGCTCAAAT
MZ340C_lacerataT --T---T-ATGACAATC--------------------------------
Dai21940 --T---T-ATGACAATCGTAACTGCGA-
GTGAAGCGGGAAAAGCTCAAAT
847Dai16433 --T---T-ATGACAATCGTAACTGCGA-
GTGAAGCGGGAAAAGCTCAAAT
MarcinEmmia_latemarginatus --T---T-ATGACAATCGTAACTGCGA-
GTGAAGCGGGAAAAGCTCAAAT
Meijer3729Hydnopolyporus_fimbr --TA--T-TTGACAATCGTAACTGCGA-
GTGAAGCGGGAAAAGCTCAAAT
RLG13408Phanerochaete_sp --T---T-ATGACAATC--------------
AGCGGGAAAAGCTCAAAT

WHC1381Flavodon_flavus --TAC-T-TTGACAATCGTAACTGCGA-GTGAAGCGGGAAAAGCTCAAAT

GB1833Phlebia_albida --T---C-TTGACAATCGTAACTGCGA-GTGAAGCGGGAAAAGCTCAAAT

T407Phlebia_nitidula --T---A-TTGACAATC--------------------------------

HHB6988Phanerochaete_exilis --TTA-C-TTGACAATCGTAACTGCGA-GTGAAGCGGGAAAAGCTCAAAT

HHB8509Phanerochaetella_xeroph --TTA-C-TTGACAATCGTAACTGCGA-GTGAAGCGGGAAAAGCTCAAAT

PBU0051Macrohyporia_dictyopora --TTA-T-CTGACAATCGTAACTGCGA-GTGAAGCGGGAAAAGCTCAAAT

HHB11463Phanerochaete_sp -----------------GTAACTGCGA-GTGAAGCGGGAAAAGCTCAAAT

FP102382Byssomerulius_corium ---AC-C-TTGACAATC-TAACTGCGA-GTGAAGCGGGAAAAGCTCAAAT

FP102165Efibula_americana --CAC-T-TTGACAATCGTAACTGCGA-GTGAAGCGGGAAAAGCTCAAAT

Murdoch90Ceriporia_torpida ------T-TTTAACTTCGTAACTGCGA-GTGAAGCGGGAAAAGCTCAAAT

Rivoire4413_Ceriporia_purpurea A-C-T-T-TTCAACTTCGTAACTGCGA-GTGAAGCGGGAAAAGCTCAAAT

Kout_18_Ceriporia_triumphalis A-C-T-T-TTCAACTTCGTAACTGCGA-GTGAAGCGGGAAAAGCTCAAAT

Rivoire3701_Ceriporia_bresadol A-C-T-T-TTCAACTTCGTAACTGCGA-GTGAAGCGGGAAAAGCTCAAAT

VS4018 A-CTT-T-TTCAACTTC--------------------------------

Ryvarden21832_Ceriporia_manzan A-CTT-T-TTCAACTTCGTAACTGCGA-GTGAAGCGGGAAAAGCTCAAAT

Dai24539 -----------------GTAACTGCGA-GTGAAGCGGGAAAAGCTCAAAT

Dai24541 A-CTT-T-TTCAACTTCGTAACTGCGA-GTGAAGCGGGAAAAGCTCAAAT

JV1105_12_Ceriporia_occidental T-T-T-T-TTCAACTTCGTAACTGCGA-GTGAAGCGGGAAAAGCTCAAAT

VS8558Ceriporia_occidentalis T-T-T-T-TTCAACTTCGTAACTGCGA-GTGAAGCGGGAAAAGCTCAAAT

Dai22445 -----------------GTAACTGCGA-GTGAAGCGGGAAAAGCTCAAAT

846Dai16368 A-C-T-T-TTCAACTTCGTAACTGCGA-GTGAAGCGGGAAAAGCTCAAAT

Dai17951_Ceriporia_aurantiocar ------C-TTTAACTTCGTAACTGCGA-GTGAAGCGGGAAAAGCTCAAAT

Miettinen_11701C_viridans ------C-TTTGACTTCGTAACTGCGA-GTGAAGCGGGAAAAGCTCAAAT

JV0105_10Ceriporia_aurantiocar ------C-TTTAACTTCGTAACTGCGA-GTGAAGCGGGAAAAGCTCAAAT

Yuan5702C_viridans ------C-TTTGACTTC--------------------------------

858Dai17003 ------C-TTTGACTTC--------------------------------

Yuan2747_Ceriporia_viridans ------C-TTTAACTTC--------------------------------

Yuan2744C_viridans ------C-TTTAACTTC--------------------------------

Li1046C_viridans ------C-TTTAACTTC--------------------------------

865C_sinoviridans ------C-TTTAACTTC--------------------------------

871Dai15062 ------C-TTTAACTTC--------------------------------

Dai7642_Ceriporia_humilis ----T-T-CTTGACTTC--------------------------------

Spirin4706_Ceriporia_humilis ----T-T-CTTGACTTC--------------------------------

Spirin4944_Ceriporia_sericea ----TAT-CTTGACTTCGTAACTGCGA-GTGAAGCGGGAAGAGCTCAAAT

WCG1547Dai26044ceriporia ----C-T-CTTGACTTCGTAACTGCGA-GTGAAGCGGGAAGAGCTCAAAT

ZZW1558Dai27086 ----C-T-CTTGACTTCGTAACTGCGA-GTGAAGCGGGAAGAGCTCAAAT

Miettinen14381_Ceriporia_mpuri ----Y-T-CTTGACTTCGTAACTGCGA-GTGAAGCGGGAAGAGCTCAAAT

Miettinen15492_2_Ceriporia_sor ------T-TCTGACTTCGTAACTGCGA-GTGAAGCGGGAAAAGCTCAAAT

He6687 ----T-T-CTTGACTTCGTAACTGCGA-GTGAAGCGGGAAGAGCTCAAAT

ZH53Dai24426 -----------------GTAACTGCGA-GTGAAGCGGGAAGAGCTCAAAT

Vlasak0808_30_Ceriporia_punica ----T-A-TCTGACTTCGTAACTGCGA-GTGAAGCGGGAAAAGCTCAAAT

887Dai13376 ----C-A-TCTGACTTCGTAACTGCGA-GTGAAGCGGGAAAAGCTCAAAT

WCG1443Dai24998 ----T-A-TCTGACTTCGTAACTGCGAGGTGAAGCGGGAAAAGCTCAAAT

0108_6Ceriporia_spissa T-TTT-T-TATGACTTCGTAACTGCGA-GTGAAGCGGGAAAAGCTCAAAT

Dai19164 T-TTT-T-TATGACTTCGTAACTGCGA-GTGAAGCGGGAAAAGCTCAAAT

Dai17937_Ceriporia_bubalinomar A-TTT-T-TATGACTTCGTAACTGCGA-GTGAAGCGGGAAAAGCTCAAAT

903Dai12113 A-TTT-T-TATGACTTCGTAACTGCGA-GTGAAGCGGGAAAAGCTCAAAT

LZB929Dai25079 ------C-TTTGACTTCGTAACTGCGA-GTGAAGCGGGAAAAGCTCAAAT

LX45Dai26988 ------C-TTTGACTTC--------------------------------

LX43Dai26986 ------C-TTTGACTTCGTAACTGCGA-GTGAAGCGGGAAAAGCTCAAAT

Dai7759Ceriporia ------C-TTTGACTTC---------------------------------
Cui8012_Ceriporia_viridans ---AT-A-TTTGACTTC---------------------------------
GC1704_54Ceriporia_viridans ---AT-A-TTTGACTTC------------
TGAAGCGGGAAAAGCTCAAAT
Dai23392 ---AT-A--TTGACCTCGTAACTGCGA-
GTGAAGCGGGAAAAGCTCAAAT
WCG1585Dai26113Ceriproia ---AT-A-TTTGACTTCGTAACTGCGA-
GTGAAGCGGGAAAAGCTCAAAT
Dai18675C_eucalypti ---AT-A-TTTGACTTCGTAACTGCGA-
GTGAAGCGGGAAAAGCTCAAAT
Dai22034 -----------------GTAACTGCGA-
GTGAAGCGGGAAGAGCTCAAAT
JV1008_41JTardaFLORIDAKeys ----T-T-CTTAACTTC---------------------------------
Rivoire1161_Ceriporia_pierii ------C-TTTGACTTC---------------------------------
Dai23499C_pierii ------C-TTTGACTTCGTAACTGCGA-
GTGAAGCGGGAAAAGCTCAAAT
Dai23500 ------C-TTTGACTTCGTAACTGCGA-
GTGAAGCGGGAAAAGCTCAAAT
841Dai15899 ------C-TTCGACTTCGTAACTGCGA-
GTGAAGCGGGAAAAGCTCAAAT
842Dai15904 ------C-TTCGACTTC---------------------------------
LZB1066xinjiang ------C-TTCGACTTC---------------------------------
LZB1065xinjiang ------C-TTCGACTTC---------------------------------
851Dai16779 T-------CTTGACTTC--AACTGCGA-
GTGAAGCGGGAAGAGCTCAAAT
RMJ119sp_Candelabrochaete_sept ------T-CTTAACTTC---------------------------------
RLG9759spCandelabrochaete_sept ------T-CTTAACTTCGTAACTGCGA-
GTGAAGCGGGAAGAGCTCAAAT
RLG10478Phanerochaete_allantos ------T-TATAACTTC---------------------------------
Dai19118_Ceriporia_spissa ------TAATTGATTTCGTAACTGCGA-
GTGAAGCGGGAAGAGCTCAAAT
Dai18486A ------TAATTGATTTCGTAACTGCGA-
GTGAAGCGGGAAGAGCTCAAAT
WEI17_024_Ceriporia_mellita ------TAATTGATTTCGTAACTGCGA-
GTGAAGCGGGAAGAGCTCAAAT
GC1508_71Ceriporia_mellita ------TAATTGATTTC-------------------------GCTCAAAT
GC1608_7_Ceriporia_mellita ------TAATTGATTTCGTAACTGCGA-
GTGAAGCGGGAAGAGCTCAAAT
ZZW1557Dai27085 ------TAATTGATTTCGTAACTGCGA-
GTGAAGCGGGAAGAGCTCAAAT
ZZW1554Dai27083 ------TAATTGATTTCGTAACTGCGA-
GTGAAGCGGGAAGAGCTCAAAT
Dai8168 ------TAATTGACTTCGTAACTGCGA-
GTGAAGCGGGAAGAGCTCAAAT

BR4865C_mellita ------TAATTGATTTCGTAACTGCGA-
GTGAAGCGGGAAGAGCTCAAAT
MEL2382688Ceriporia_sp ------TAATTGATTTC--------------------------------
Dai8110 ------TAATCGATTTCGTAACTGCGA-
GTGAAGCGGGAAGAGCTCAAAT
Cui8097 ------TAATCGATTTC--------------------------------
909Cui6740 ------TAATTGATTTC--------------------------------
W1258Dai24695 ------TAATTGATTTCGTAACTGCGA-
GTGAAGCGGGAAGAGCTCAAAT
JV0110_26_Ceriporia_griseoviol T-----T-CATGACCTTGTAAGTGCGA-
GTGAAGCGGGAAAAGCTCAAAT
896Dai13202 T-----T-CATGACCTTGTAACTGCGA-
GTGAAGCGGGAAAAGCTCAAAT
LWY393Dai27053C_griseoviolasce T-----T-CATGACCTTGTAACTGCGA-
GTGAAGCGGGAAAAGCTCAAAT
LWY394DAI27054 T-----T-CATGACCTTGTAACTGCGA-
GTGAAGCGGGAAAAGCTCAAAT
FP135015G_pannocinctus A-----C-TTTGACATCGTAACTGCGA-
GTGAAGCGGGAAAAGCTCAAAT
L15726SpG_pannocinctus A-----C-TTTGACATCGTAACTGCGA-
GTGAAGCGGGAAAAGCTCAAAT
Dai22221 A--C--C-TTTGACATCGTAACTGCGA-
GTGAAGCGGGAAAAGCTCAAAT
Dai22633 A--CATC-TTTGACATCGTAACTGCGA-
GTGAAGCGGGAAAAGCTCAAAT
Dai23260 A--CATC-TTTGACATCGTAACTGCGA-
GTGAAGCGGGAAAAGCTCAAAT
Dai23626 A--CATC-TTTGACATCGTAACTGCGA-
GTGAAGCGGGAAAAGCTCAAAT
Dai16238G_citrinoalbus A--ACCA-T--------GTAACTGCGA-
GTGAAGCGGGAAAAGCTCAAAT
1175Dai15293 A--ACCA-TT-GACATCGTAACTGCGA-
GTGAAGCGGGAAAAGCTCAAAT
Dai19547 A--ACCA-TTTGACATCGTAACTGCGA-
GTGAAGCGGGAAAAGCTCAAAT
918063G_africanus A--ACCA-TTTGACATCGTAACTGCGA-
GTGAAGCGGGAAAAGCTCAAAT
918572G_africanus A--ACCA-TTTGACATCGTAACTGCGA-
GTGAAGCGGGAAAAGCTCAAAT
Dai18536A A--ACTC-TTTGACATC--------------------------------
1164Cui17922 A--ACTC--TTGACATC-------------
AAGCGGGAAAAGCTCAAAT
Dai22225 A--ACTA-TTTGACATCGTAACTGCGA-
GTGAAGCGGGAAAAGCTCAAAT

1163Dai20655 A--ACTA--TTGACATC---AGCCCGA---------
GGAAAAGCTCAAAT
Yuan4397G_hainanensis A-ATAAC-T--------GTAACTGCGA-
GTGAAGCGGGAAGAGCTCAAAT
1176Dai15268 A-ATAAC-TTTGACATCGTAACTGCGA-
GTGAAGCGGGAAGAGCTCAAAT
1177Dai15259 -----------------GTAACTGCGA-
GTGAAGCGGGAAGAGCTCAAAT
BZ2896G_thelephoroides ---TAAC-TTTGACATCGTAACTGCGA-
GTGAAGCGGGAAGAGCTCAAAT
1166JV1808_26 ---TAAC-TTTGACATCGTAACTGCGA-
GTGAAGCGGGAAGAGCTCAAAT
Miettinen16992Hapalopilus_ochr A-----C-TTTGCAATCGTAACTGCGA-
GTGAAGCGGGAAAAGCTCAAAT
GC1708_338_Ceriporia_arbuscula AT----T-TTTGTACTTGTAACTGCGA-
GTGAAGCGGGAAGAGCTCAAAT
WCG1555Dai26107Ceriporia AT----T-TTTGTACTTGTAACTGCGA-
GTGAAGCGGGAAGAGCTCAAAT
GC1708_340_Ceriporia_arbuscula AT----T-TTTGTACTTGTAACTGCGA-
GTGAAGCGGGAAGAGCTCAAAT
WCG1556Dai26109Ceriporia -----------------GTAACTGCGA-
GTGAAGCGGGAAGAGCTCAAAT
883Cui11291 T-------TATCAACTTGTAACTGCGA-
GTGAAGCGGGAAGAGCTCAAAT
HLX320Dai26805 T-AATTT-TCTA-ATCTGTAACTGCGA-
GTGAAGCGGGAAGAGCTCAAAT
WCG1266Dai24678A A---TTT-TCTA-ATCTGTAACTGCGA-
GTGAAGCGGGAAGAGCTCAAAT
Dai6090_Ceriporia_sulphuricolo CCTATTTATATGACATCGTAACTGCGA-
GTGAAGCGGGAAAAGCTCAAAT
RLG_11354_Ceriproia_reticulata T-------CTTGACTTCGTAACTGCGA-
GTGAAGCGGGAAAAGCTCAAAT
ZZW1543Dai27072 T-------CTTGACTTCGTAACTGCGA-
GTGAAGCGGGAAAAGCTCAAAT
Li1316_Ceriporia_reticulata ---
KHL11981Ceriporia_reticulata -----------------GTAACTGCGA-
GTGAAGCGGGAAAAGCTCAAAT
FP110343sp_Candelabrochaete_la T-------T-TGACTTCGTAACTGCGA-
GTGAAGCGGGAAAAGCTCAAAT
Li1045_Ceriporia_reticulata T-------T-GTACTTC---------------------------------
ZX136Dai25794ceriporia T-------T-GTACTTCGTAACTGCGA-
GTGAAGCGGGAAAAGCTCAAAT
892Dai13400 T-------T-GTACTTCGTAACTGCGA-
GTGAAGCGGGAAAAGCTCAAAT

RLG7163Leptoporus_mollis TT------TCTAACTTCGTAACTGCGA-GTGAACCGGGAAAAGCTCAAAT

Dai21062Leptoporus_mollis TT------TCTAACTTCGTAACTGCGA-GTGAACCGGGAAAAGCTCAAAT

Dai20182Leptoporus_submollis ----------------GTAACTGCGA-GTGAACCGGGAAAAGCTCAAAT

Cui18379Leptoporus_submollis TT------T--------GTAACTGCGA-GTGAACCGGGAAAAGCTCAAAT

Wu1209_46Resiniporus_pseudogil --CTCACATATGACATC--------------------------------

BRNM710169Resiniporus_resinasc --CTCACATATGACATCGTAACTGCGA-GTGAAGCGGGAAAAGCTCAAAT

Dai14516Bjerkandera_adusta T-------TCTGAACTCGTAACTGCGA-GTGAAGCGGGAAAAGCTCAAAT

Dai21100Bjerkandera_fumosa T-----T-TCTAAACTCGTAACTGCGA-GTGAAGCGGGAAAAGCTCAAAT

Miettinen16854Ceraceomyces_sp GC----T-TATGACATCGTAACTGCGA-GTGAAGCGGGAAAAGCTCAAAT

Dai10477C_spissa AC----C-TTTG-CTTCGTAACTGCGA-GTGAAGCGGGAAAAGCTCAAAT

855Dai16831 AC----C-TTTG-CTTCGTAACTGCGA-GTGAAGCGGGAAAAGCTCAAAT

882Cui11282 AC----C-TTTG-CTTCGTAACTGCGA-GTGAAGCGGGAAAAGCTCAAAT

Dai24566 AT----T-TTTG-CTTCGTAACTGCGA-GTGAAGCGGGAAAAGCTCAAAT

Yuan5965 AT----T-TCTG-CTTCGTAACTGCGA-GTGAAGCGGGAAAAGCTCAAAT

Dai3204 ----------------GTAACGGCGA-GTGAAGCGGGAAGAGCTCAAAT

1194CUI9985 ----------------GTAACTGCGA-GTGAAGCGGGAAAAGCTCAAAT

Dai15205_Ceriporia_albomellea TTAAAATCTGACAGTCTTTGGCTGTTCGAGTTGTATTCTAGAGAAGTGTT

Dai15223_Ceriporia_albomellea TTAAAATCTGACAGTCTTTGGCTGTTCGAGTTGTATTCTAGAGAAGTGTT

Li1780_Ceriporia_variegata TTAAAATCTGACAGTCTTTGGCTGTTCGAGTTGTATTCTAGAGAAGTGTT

Dai19791_Ceriporia_variegata TTAAAATCTGACAGTCTTTGGCTGTTCGAGTTGTATTCTAGAGAAGTGTT

Dai19886 TTAAAATCTGACAGTCTTTGGCTGTTCGAGTTGTATTCTAGAGAAGTGTT

Dai10833_Ceriporia_crassitunic

TTAAAATCTAACAGTCTTTGGCTGTTCGAGTTGTATTCTAGAGAAGTGTT

CHWC1506_46Meruliopsis_crassit

TTAAAATCTAACAGTCTTTGGCTGTTCGAGTTGTATTCTAGAGAAGTGTT

Dai9995_Ceriporia_crassitunica --

Wu1209_58_Meruliopsis_parvispo

TTAAAATCTGGCAGTCTTTGGCTGTCCGAGTTGTATTCTAGAGAAGTGTT

CHWC1505_129_Meruliopsis_parvi

TTAAAATCTGGCAGTCTTTGGCTGTCCGAGTTGTATTCTAGAGAAGTGTT

Dai21944

TTAAAATCTGGCAGTCTTTGGCTGTCCGAGTTGTATTCTAGAGAAGTGTT

830Dai18640A

TTAAAATCTGGCAGTCTTTGGCTGTCCGAGTTGTATTCTAGAGAAGTGTT

GC1704_60_Meruliopsis_taxicola

TTAAAATCTGGCGGTCTTTGGCTGTCCGAGTTGTATTCTAGAGAAGTGTT

Dai22625

TTAAAATCTGGCGGTCTTTGGCTGTCCGAGTTGTATTCTAGAGAAGTGTT

Dai22636

TTAAAATCTGGCGGTCTTTGGCTGTCCGAGTTGTATTCTAGAGAAGTGTT

Dai21878

TTAAAATCTGGCGGTCTTTGGCCGTCCGAGTTGTATTCTAGAGAAGTGTT

1169Dai17248

TTAAAATCTGGCGGTCTTTGGCCGTCCGAGTTGTATTCTAGAGAAGTGTT

Wu1708_43_Meruliopsis_leptocys

TTAAAATCTGATAGTCTTTGGCTGTCCGAGTTGTAGTCTAGAGAAGTGTT

Li1011

TTAAAATCTGATAGTCTTTGGCTGTCCGAGTTGTAGTCTAGAGAAGTGTT

ZX95Dai25742Meruliopsis_leptoc

TTAAAATCTGATAGTCTTTGGCTGTCCGAGTTGTAGTCTAGAGAAGTGTT

WCG1306Dai24733

TTAAAATCTGATAGTCTTTGGCTGTCCGAGTTGTAGTCTAGAGAAGTGTT

LXL99Dai25816

TTAAAATCTGATAGTCTTTGGCTGTCCGAGTTGTAGTCTAGAGAAGTGTT

WCG1559Dai26052Meruliopsis

TTAAAATCTGACAGTCTTTGGCTGTCCGAGTTGTAGTCTAGAGAAGTGTT

He7477

TTAAAATCTGACAGTCTTTGGCTGTCCGAGTTGTAGTCTAGAGAAGTGTT

HLX243Dai26217

TTAAAATCTGACAGTCTTTGGCTGTCCGAGTTGTAGTCTAGAGAAGTGTT

RussiaMW673659Meruliopsis_fagi

TTAAAATCTGACTGTCTTTGGCTGTCCGAGTTGTAGTCTAGAGAAGTGTT

FD278 TTAAAATCTGGCGG----

TGGCCGTCCGAGTTGTATTCTAGAGAAGTGCT

Dai10226_Ceriporia_tarda --

LE247365 --

Dai8173_Meruliopsis_nanlingens
TTAAAATCTGACAGTCTTTGATTGTCCGAGTTGTAGTCTAGAGAAGTGTT
860Dai17172
TTAAAATCTGACAGTCTTTGATTGTCCGAGTTGTAGTCTAGAGAAGTGTT
879Dai13414
TTAAAATCTGACAGTCTTTGATTGTCCGAGTTGTAGTCTAGAGAAGTGTT
Li_1704_Meruliopsis_pseudocyst --
833Dai18405
TTAAAATCTGGCGGTCTTTGGCTGTCCGAGTTGTAGTCTAGAGAAGTGTT
HHB_10729_Meruliopsis_albostra
TTAAAATCTGGCAGTCTTTGGCTGTCCGAGTTGTAGTCTAGAGAAGTGTT
Cui6878_Ceriporia_pseudocystid
TTAAAATCTGGCAGTCTTTGGCTGTCCGAGTTGTAGTCTAGAGAAGTGTT
869Dai14737
TTAAAATCTGGCAGTCTTTGGCTGTCCGAGTTGTAGTCTAGAGAAGTGTT
876Cui11626
TTAAAATCTGGCAGTCTTTGGCTGTCCGAGTTGTAGTCTAGAGAAGTGTT
1199WEI3388 --
776308_Meruliopsis_cystidiata
TTAAAATCTGGCAGTCTTTGATTGTCCGAGTTGTAGTCTAGAGAAGTGTT
ICN139059_Meruliopsis_cystidia
TTAAAATCTGGCAGTCTTTGATTGTCCGAGTTGTAGTCTAGAGAAGTGTT
HHB15692Ceraceomyces_serpens TTAAAATCTGGCGG-----
GGTCGTCCGAGTTGTAGTCTAGAGAAGTGTC
HHB_15629_Sp_Ceriporiopsis_ane TTAAAATCTGGCAG-------
TTGTCCGAGTTGTAGTCTAGAGAAGCGTT
AJ185Trametopsis_cervina TTAAAATCTGGCAG-
CTTTTGTTGCCCGAGTTGTAGTCTAGAGAAGCGTC
FD9Irpex_lacteus TTAAAATCTGGCGG-----
GGTCGTCCGAGTTGTATTCTAGAGAAGTGTT
908Dai11230 TTAAAATCTGGCGG-
CTTTGGTCGTCCGAGTTGTATTCTAGAGAAGTGTT
FP55521TEmmia_lacerata TTAAAATCTGGCGG-
CTTTGGTCGTCCGAGTTGTATCCTAGAGAAGTGTT
PBU0048Ceriporia_cystidiata TTAAAATCTGGCGG-
CTTTGGTCGTCCGAGTTGTATCCTAGAGAAGTGTT
MZ340C_lacerataT --
Dai21940 TTAAAATCTGGCGG-
CTTTGGTCGTCCGAGTTGTATCCTAGAGAAGTGTT
847Dai16433 TTAAAATCTGGCGG-
CTTTGGTCGTCCGAGTTGTATCCTAGAGAAGTGTT
MarcinEmmia_latemarginatus TTAAAATCTGGCGG-
CTTTGGTCGTCCGAGTTGTATCCTAGAGAAGTGTT
Meijer3729Hydnopolyporus_fimbr TTAAAATCTGGCGG-

CTTTGGTCGTCCGAGTTGTATTCTAGAGAAGTGTT

RLG13408Phanerochaete_sp TTAAAATCTGGCGG-----
GGTCGTCCGAGTTGTATTCTAGAGAAGTGTT

WHC1381Flavodon_flavus TTAAAATCTGGCGG-
CTTTGGTCGTCCGAGTTGTATTCTAGAGAAGTGTT

GB1833Phlebia_albida TTAAAATCTGGCGG-
CTTTGGTCGTCCGAGTTGTATTCTAGAGAAGTGTT

T407Phlebia_nitidula ---

HHB6988Phanerochaete_exilis TTAAAATCTGGCGG-
CTTTGGTCGTCCGAGTTGTATTCTAGAGAAGCGTT

HHB8509Phanerochaetella_xeroph TTAAAATCTGACGG-
CTTTGGCCGTCCGAGTTGTATTCTAGAGAAGCGTT

PBU0051Macrohyporia_dictyopora TTAAAATCTGGCGG-
CTTTGGTCGTCCGAGTTGTATTCTAGAGAAGCGTT

HHB11463Phanerochaete_sp TTAAAATCTGGCGG-
CTTTGGTCGTCCGAGTTGTATTCTAGAGAAGCGTT

FP102382Byssomerulius_corium TTAAAATCTGGCGG-
CTTTTGTCGTCCGAGTTGTATTCTAGAGAAGCGTT

FP102165Efibula_americana TTAAAATCTGGCGG-
CTTTGGTCGTCCGAGTTGTAATCTAGAGAAGTGTC

Murdoch90Ceriporia_torpida
TTAAAATCTGGCAGTCTTTGATTGTCCGAGTTGTATTCTAGAGAAGTGTC

Rivoire4413_Ceriporia_purpurea
TTAAAATCTGGCAGTCTTTGATTGTCCGAGTTGTATTCTAGAGAAGTGTT

Kout_18_Ceriporia_triumphalis
TTAAAATCTGGCAGTCTTTGATTGTCCGAGTTGTATTCTAGAGAAGTGTC

Rivoire3701_Ceriporia_bresadol
TTAAAATCTGGCAGTCTTTGATTGTCCGAGTTGTATTCTAGAGAAGTGTC

VS4018 ---

Ryvarden21832_Ceriporia_manzan
TTAAAATCTGGCAGTCTTTGATTGTCCGAGTTGTATTCTAGAGAAGTGTC

Dai24539
TTAAAATCTGGCAGTCTTTGATTGTCCGAGTTGTATTCTAGAGAAGTGTC

Dai24541
TTAAAATCTGGCAGTCTTTGATTGTCCGAGTTGTATTCTAGAGAAGTGTC

JV1105_12_Ceriporia_occidental
TTAAAATCTGGCAGTCTTTGATTGTCCGAGTTGTATTCTAGAGAAGTGTC

VS8558Ceriporia_occidentalis
TTAAAATCTGGCAGTCTTTGATTGTCCGAGTTGTATTCTAGAGAAGTGTC

Dai22445
TTAAAATCTGGCAGTCTTTGATTGTCCGAGTTGTATTCTAGAGAAGTGTT

846Dai16368
TTAAAATCTGGCAGTCTTTGATTGTCCGAGTTGTATTCTAGAGAAGTGTT

Dai17951_Ceriporia_aurantiocar

TTAAAATCTGGCAGTCTTTGGCTGTCCGAATTGTATTCTAAAGAAGCGTT

Miettinen_11701C_viridans

TTAAAATCTGACGGTCTTTGGCTGTCCGAATTGTATTCTAAAGAAGCGTT

JV0105_10Ceriporia_aurantiocar

TTAAAATCTGGCAGTCTTTGGCTGTCCGAATTGTATTCTAAAGAAGCGTT

Yuan5702C_viridans --

858Dai17003 --

Yuan2747_Ceriporia_viridans --

Yuan2744C_viridans --

Li1046C_viridans --

865C_sinoviridans --

871Dai15062 --

Dai7642_Ceriporia_humilis --

Spirin4706_Ceriporia_humilis --

Spirin4944_Ceriporia_sericea

TTAAAATCTGGCGGCCTCCGGTCGTCCGAGTTGTAGTCTAGAGAAGCGTC

WCG1547Dai26044ceriporia

TTAAAATCTGGCGGCCTCCGGTCGTCCGAGTTGTAGTCTAGAGAAGCGTC

ZZW1558Dai27086

TTAAAATCTGGCGGCCTCCGGTCGTCCGAGTTGTAGTCTAGAGAAGCGTC

Miettinen14381_Ceriporia_mpuri

TTAAAATCTGGCGGTCTCCGATCGTCCGAGTTGTAGTCTAGAGAAGCGTC

Miettinen15492_2_Ceriporia_sor

TTAAAATCTGGCGGTCTTTGGCCGTCCGAGTTGTAGTCTAGAGAAGCGTC

He6687

TTAAAATCTGGCGGTCTCTGATCGTCCGAGTTGTAGTCTAGAGAAGCGTC

ZH53Dai24426

TTAAAATCTGGCGGTCTCTGATCGTCCGAGTTGTAGTCTAGAGAAGCGTC

Vlasak0808_30_Ceriporia_punica

TTAAAATCTGGCAGTCTTTGGTTGCCCGAGTTGTATTCTAGAGAAGTGTT

887Dai13376

TTAAAATCTGGCAGTCTTTGGTTGCCCGAGTTGTATTCTAGAGAAGTGTT

WCG1443Dai24998

TTAAAATCTGGCAGTCTTTGGTTGCCCGAGTTGTATTCTAGAGAAGTGTT

0108_6Ceriporia_spissa

TTAAAATCTGGCAGTCTTTGATTGTCCGAGTTGTAGTCTAGAGAAGTGTC

Dai19164

TTAAAATCTGACAGTCTTTGATTGTCCGAGTTGTAGTCTAGAGAAGTGTC

Dai17937_Ceriporia_bubalinomar

TTAAAATCTGACAGTCTTTGATTGTCCGAGTTGTAGTCTAGAGAAGTGTC

903Dai12113

TTAAAATCTGACAGTCTTTGATTGTCCGAGTTGTAGTCTAGAGAAGTGTC

LZB929Dai25079

TTAAAATCTGGCGGTCTTTGGCTGTCCGAATTGTATTCTAGAGAAGTGTT

LX45Dai26988 --

LX43Dai26986

TTAAAATCTGGCGGTCTTTGGCTGTCCGAATTGTGTTCTAGAGAAGTGTT

Dai7759Ceriporia --

Cui8012_Ceriporia_viridans --

GC1704_54Ceriporia_viridans

TTAAAATCTGGCAGTCTTTGGCTGTCCGAATTGTATTCTAGAGAAGTGTT

Dai23392

TTAAAATCTGGCAGTCTTTGGCTGTCCGAATTGTATTCTAGAGAAGTGTT

WCG1585Dai26113Ceriproia

TTAAAATCTGGCAGTCTTTGGCTGTCCGAATTGTATTCTAGAGAAGTGTT

Dai18675C_eucalypti

TTAAAATCTGGCGGTCTTTGGCTGTCCGAATTGTATTCTAGAGAAGTGTT

Dai22034

TTAAAATCTGGCAGTCTTTGGCTGTCCGAGTTGTATTCTAGAGAAGTGTT

JV1008_41JTardaFLORIDAKeys --

Rivoire1161_Ceriporia_pierii --

Dai23499C_pierii

TTAAAATCTGGCGGTCTTTGGCCGTCCGAGTTGTAGTCTAGAGAAGTGTT

Dai23500

TTAAAATCTGGCGGTCTTTGGCCGTCCGAGTTGTAGTCTAGAGAAGTGTT

841Dai15899

TTAAAATCTGGCGGTTTTTTGCCGTCCGAGTTGTAGTCTAGAGAAGTGTT

842Dai15904 --

LZB1066xinjiang --

LZB1065xinjiang --

851Dai16779

TTAAAATCTGGCGGTCTCTGGCCGTCCGAGTTGTAGTCTAGAGAAGCGTC

RMJ119sp_Candelabrochaete_sept --

RLG9759spCandelabrochaete_sept TTAAAATCTGGCAG-CTTTGGTTGTCCGAGTTGTAGTCTAGAGAAGCGTT

RLG10478Phanerochaete_allantos --

Dai19118_Ceriporia_spissa

TTAAAATCTGGCAGTCTTTGATTGCCCGAGTTGTAGCCTAGAGAAGTGTC

Dai18486A

TTAAAATCTGGCAGTCTTTGATTGCCCGAGTTGTAGCCTAGAGAAGTGTC

WEI17_024_Ceriporia_mellita

TTAAAATCTGGCAGTCTTTGATTGCCCGAGTTGTAGCCTAGAGAAGTGTC

GC1508_71Ceriporia_mellita

TTAAAATCTGGCAGTCTTTGATTGCCCGAGTTGTAGCCTAGAGAAGTGTC

GC1608_7_Ceriporia_mellita

TTAAAATCTGGCAGTCTTTGATTGCCCGAGTTGTAGCCTAGAGAAGTGTC

ZZW1557Dai27085

TTAAAATCTGGCAGTCTTTGATTGCCCGAGTTGTAGCCTAGAGAAGTGTC

ZZW1554Dai27083
TTAAAATCTGGCAGTCTTTGATTGCCCGAGTTGTAGCCTAGAGAAGTGTC
Dai8168
TTAAAATCTGGCAGTCTTTGATTGCCCGAGTTGTAGCCTAGAGAAGTGTC
BR4865C_mellita
TTAAAATCTGGCAGTCTTTGATTGCCCGAGTTGTAGCCTAGAGAAGTGTC
MEL2382688Ceriporia_sp --
Dai8110
TTAAAATCTGGCAGTCTTTGATTGCCCGAGTTGTAGCCTAGAGAAGTGTC
Cui8097 --
909Cui6740 --
W1258Dai24695
TTAAAATCTGGCAGTCTTTGATTGTCCGAGTTGTAGCCTAGAGAAGTGTC
JV0110_26_Ceriporia_griseoviol
TTAAAATCTGGCAGTCTTTGGCTGTCCGAGTTGTAGTCTAGAGAAGCGTC
896Dai13202
TTAAAATCTGGCAGTCTTTGGCTGTCCGAGTTGTAGTCTAGAGAAGCGTC
LWY393Dai27053C_griseoviolasce
TTAAAATCTGGCAGTCTTTGGCTGTCCGAGTTGTAGTCTAGAGAAGCGTC
LWY394DAI27054
TTAAAATCTGGCAGTCTTTGGCTGTCCGAGTTGTAGTCTAGAGAAGCGTC
FP135015G_pannocinctus
TTAAAATCTGRCAGTCTTTGGCTGTCCGAGTTGTAGTCTAGAGAAGTGTT
L15726SpG_pannocinctus
TTAAAATCTGGCAGTCTTTGGCTGTCCGAGTTGTAGTCTAGAGAAGTGTT
Dai22221
TTAAAATCTGGCAGTCTTTGATTGTCCGAGTTGTAGTCTAGAGAAGTGTT
Dai22633
TTAAAATCTGGCAGTCTTTGATTGTCCGAGTTGTAGTCTAGAGAAGCATT
Dai23260
TTAAAATCTGGCATTCTTTGATTGTCCGAGTTGTAGTCTAGAGAAGCATT
Dai23626
TTAAAATCTGGCAGTCTTCGATTGTCCGAGTTGTAGTCTAGAGAAGCATT
Dai16238G_citrinoalbus
TTAAAATCTGGCAGTCTTTGATTGTCCGAGTTGTAGTCTAGAGAAGCATT
1175Dai15293
TTAAAATCTGGCAGTCTTTGATTGTCCGAGTTGTAGTCTAGAGAAGCATT
Dai19547
TTAAAATCTGGCAGTCTTTGATTGTCCGAGTTGTAGTCTAGAGAAGCATT
918063G_africanus
TTAAAATCTGGCAGTCTTCGATTGTCCGAGTTGTAGTCTAGAGAAGCATT
918572G_africanus
TTAAAATCTGGCAGTCTTCGATTGTCCGAGTTGTAGTCTAGAGAAGCATT
Dai18536A --

1164Cui17922
TTAAAATCTGGCAGTCTTTGATTGTCCGAGTTGTAGTCTAGAGAAGCATT
Dai22225
TTAAAATCTGGCAGTCTTTGATTGTCCGAGTTGTAGTCTAGAGAAGCATT
1163Dai20655
TTAAAATCCGGCAGTCTTTGATTGTCCGAGTTGTAGTCTAGAGAAGCATT
Yuan4397G_hainanensis
TTAAAATCTGGCAGTCTTTGATTGTCCGAGTTGTAGTCTAGAGAAGCGTT
1176Dai15268
TTAAAATCTGGCAGTCTTTGATTGTCCGAGTTGTAGTCTAGAGAAGCGTT
1177Dai15259
TTAAAATCTGGCAGTCTTTGATTGTCCGAGTTGTAGTCTAGAGAAGCGTT
BZ2896G_thelephoroides
TTAAAATCTGGCAGTCTTTGATTGTCCGAGTTGTAGTCTAGAGAAGCGTT
1166JV1808_26
TTAAAATCTGGCAGTCTTTGATTGTCCGAGTTGTAGTCTAGAGAAGCGTT
Miettinen16992Hapalopilus_ochr TTAAAATCTGGCGG--TTTTGCCGTCCGAGTTGTAGTCTAGAGAAGTGTT
GC1708_338_Ceriporia_arbuscula
TTAAAATCTGGCAGTCTTTGGCTGTCCGAGTTGTATTCTAGAGAAGTGTT
WCG1555Dai26107Ceriporia
TTAAAATCTGGCAGTCTTTGGCTGTCCGAGTTGTATTCTAGAGAAGTGTT
GC1708_340_Ceriporia_arbuscula
TTAAAATCTGGCAGTCTTTGGCTGTCCGAGTTGTATTCTAGAGAAGTGTT
WCG1556Dai26109Ceriporia
TTAAAATCTGGCAGTCTTTGGCTGTCCGAGTTGTATTCTAGAGAAGTGTT
883Cui11291
TTAAAATCTGACAGTCTTTGGCTGTCCGAGTTGTATTCTAGAGAAGTGTT
HLX320Dai26805 TTAAAATCTGGCAG-CTTCAGCTGTCCGAGTTGTATTCTAGAGAAGTGCT
WCG1266Dai24678A TTAAAATCTGGCAG-CTTTAGCTGTCCGAGTTGTATTCTAGAGAAGTGTC
Dai6090_Ceriporia_sulphuricolo TTAAAATCTGGCGG-TTTTGGCCGTCCGAGTTGTATTCTAGAGAAGCGTC
RLG_11354_Ceriproia_reticulata TTAAAATCTGGCAGTCT-TGGCTGTCCGAGTTGTATTCTAGAGAAGTGCT
ZZW1543Dai27072 TTAAAATCTGGCAGTCT-TGGCTGTCCGAGTTGTATTCTAGAGAAGTGCT
Li1316_Ceriporia_reticulata --
KHL11981Ceriporia_reticulata
TTAAAATCTGGCAGCCTTGTGTTGTCCGAGTTGTATTCTAGAGAAGTGCT
FP110343sp_Candelabrochaete_la TTAAAATCTGGCAGTCT-TGGCTGTCCGAGTTGTATTCTAGAGAAGTGTT
Li1045_Ceriporia_reticulata --

ZX136Dai25794ceriporia TTAAAATCTGGCAGCCT-TGGTTGTCCGAGTTGTAGTCTAGAGAAGTGCT

892Dai13400 TTAAAATCTGGCAGCCT-TGGTTGTCCGAGTTGTAGTCTAGAGAAGTGCT

RLG7163Leptoporus_mollis TTAAAATCTGGCAGCATTTCGCTGTCCGAGTTGTAGTCTAGAGAAGCGTT

Dai21062Leptoporus_mollis TTAAAATCTGGCAGCATTTCGCTGTCCGAGTTGTAGTCTAGAGAAGCGTT

Dai20182Leptoporus_submollis TTAAAATCTGGCAGCATTTCGTTGTCCGAGTTGTAGTCTAGAGAAGCGTT

Cui18379Leptoporus_submollis TTAAAATCTGGCAGCATTTCGTTGTCCGAGTTGTAGTCTAGAGAAGCGTT

Wu1209_46Resiniporus_pseudogil ---

BRNM710169Resiniporus_resinasc TTAAAATCTGGTAGCTTTCAGTTGCCCGAGTTGTAGTCTAGAGAAGCGTC

Dai14516Bjerkandera_adusta TTAAAATCTGGCGGTCTTCGGCCGTCCGAGTTGTAATCTGGAGAAGCGTT

Dai21100Bjerkandera_fumosa TTAAAATCTGGCGGTCTTCGGCCGTCCGAGTTGTAATCTGGAGAAGCGTC

Miettinen16854Ceraceomyces_sp TTAAAATCTGACGG-TTTTGGCCGTCCGAGTTGTATTCTAGAGAAGTGTT

Dai10477C_spissa TTAAAATCTGACTGCCTTTGGCTGTCCGAGTTGTAGTCTAGAGAAGTGTT

855Dai16831 TTAAAATCTGACTGCCTTTGGCTGTCCGAGTTGTAGTCTAGAGAAGTGTT

882Cui11282 TTAAAATCTGACTGCCTTTGGCTGTCCGAGTTGTAGTCTAGAGAAGTGTT

Dai24566 TTAAAATCTGACTGCCTTTGGCTGTCCGAGTTGTAGTCTAGAGAAGTGTT

Yuan5965 TTAAAATCTGACTGCCTTTGGCTGTCCGAGTTGTAGTCTAGAGAAGTGTT

Dai3204 TTAAAATCTGGCGGTCTTTGGCTGTCCGAGTTGTAGTCTAGAGAAGTGTT

1194CUI9985 TTAAAATCTGGCAG-CTTTTGTTGCCCGAGTTGTAGTCTAGAGAAGCGTC

Dai15205_Ceriporia_albomellea TTCTGTGCTGGACCGTGTACAAGTCTCTTGGAACAGAGCGTCATAGAGGG

Dai15223_Ceriporia_albomellea TTCTGTGCTGGACCGTGTACAAGTCTCTTGGAACAGAGCGTCATAGAGGG

Li1780_Ceriporia_variegata TTCTGTGCTGGACCGTGTACAAGTCTCTTGGAACAGAGCGTCATAGAGGG

Dai19791_Ceriporia_variegata

TTCTGTGCTGGACCGTGTACAAGTCTCTTGGAACAGAGCGTCATAGAGGG
Dai19886
TTCTGTGCTGGACCGTGTACAAGTCTCTTGGAACAGAGCGTCATAGAGGG
Dai10833_Ceriporia_crassitunic
TTCTGTGCTGGACCGTGTACAAGTCTCTTGGAACAGAGCGTCATAGAGGG
CHWC1506_46Meruliopsis_crassit
TTCTGTGCTGGACCGTGTACAAGTCTCTTGGAACAGAGCGTCATAGAGGG
Dai9995_Ceriporia_crassitunica --
Wu1209_58_Meruliopsis_parvispo
TTCCGCGCTGGACCGTGTACAAGTCTCTTGGAACAGAGCGTCATAGAGGG
CHWC1505_129_Meruliopsis_parvi
TTCCGCGCTGGACCGTGTACAAGTCTCTTGGAACAGAGCGTCATAGAGGG
Dai21944
TTCCGCGCTGGACCGTGTACAAGTCTCTTGGAACAGAGCGTCATAGAGGG
830Dai18640A
TTCCGCGTTGGACCGTGTACAAGTCTCTTGGAACAGAGCGTCATAGAGGG
GC1704_60_Meruliopsis_taxicola
TTCCGCGCTGGACCGTGTACAAGTCTCTTGGAACAGAGCGTCATAGAGGG
Dai22625
TTCCGCGCTGGACCGTGTACAAGTCTCTTGGAACAGAGCGTCATAGAGGG
Dai22636
TTCCGCGCTGGACCGTGTACAAGTCTCTTGGAACAGAGCGTCATAGAGGG
Dai21878
TTCCGCGCTGGACCGTGTACAAGTCTCTTGGAACAGAGCGTCATAGAGGG
1169Dai17248
TTCCGCGCTGGACCGTGTACAAGTCTCTTGGAACAGAGCGTCATAGAGGG
Wu1708_43_Meruliopsis_leptocys
TTCCGCGTTGGACCGTGTACAAGTCTCTTGGAACAGAGCGTCATAGAGGG
Li1011
TTCCGCGTTGGACCGTGTACAAGTCTCTTGGAACAGAGCGTCATAGAGGG
ZX95Dai25742Meruliopsis_leptoc
TTCCGCGTTGGACCGTGTACAAGTCTCTTGGAACAGAGCGTCATAGAGGG
WCG1306Dai24733
TTCCGCGTTGGACCGTGTACAAGTCTCTTGGAACAGAGCGTCATAGAGGG
LXL99Dai25816
TTCCGCGTTGGACCGTGTACAAGTCTCTTGGAACAGAGCGTCATAGAGGG
WCG1559Dai26052Meruliopsis
TTCCGCGTTGGACCGTGTACAAGTCTCTTGGAACAGAGCGTCATAGAGGG
He7477
TTCCGCGTTGGACCGTGTACAAGTCTCTTGGAACAGAGCGTCATAGAGGG
HLX243Dai26217
TTCCGCGTTGGACCGTGTACAAGTCTCTTGGAACAGAGCGTCATAGAGGG
RussiaMW673659Meruliopsis_fagi
TTCCGCGTTGGACCGTGTACAAGTCTCTTGGAACAGAGCGTCATAGAGGG

FD278
TTCCGCGCTGGACCGTGTACAAGTCTCTTGGAACAGAGCGTCATAGAGGG
Dai10226_Ceriporia_tarda --
LE247365 ----
GCGTTGGACCGTGTACAAGTCTCTTGGAACAGAGCGTCATAGAGGG
Dai8173_Meruliopsis_nanlingens
TTCCGCGCTGGACCGTGTACAAGTCTCTTGGAACAGAGCGTCATAGAGGG
860Dai17172
TTCCGCGCTGGACCGTGTACAAGTCTCTTGGAACAGAGCGTCATAGAGGG
879Dai13414
TTCCGCGCTGGACCGTGTACAAGTCTCTTGGAACAGAGCGTCATAGAGGG
Li_1704_Meruliopsis_pseudocyst --
833Dai18405
TTCCGCGTTGGACCGTGTACAAGTCTCTTGGAACAGAGCGTCATAGAGGG
HHB_10729_Meruliopsis_albostra
TTCCGCGTTGGACCGTGTACAAGTCTCTTGGAACAGAGCGTCATAGAGGG
Cui6878_Ceriporia_pseudocystid
TTCCGCGCTGGACCGTGTACAAGTCTCTTGGAACAGAGCGTCATAGAGGG
869Dai14737
TTCCGCGCTGGACCGTGTACAAGTCTCTTGGAACAGAGCGTCATAGAGGG
876Cui11626
TTCCGCGCTGGACCGTGTACAAGTCTCTTGGAACAGAGCGTCATAGAGGG
1199WEI3388 --
776308_Meruliopsis_cystidiata
TTCCGCGCTGGACCGTGTACAAGTCTCTTGGAACAGAGCGTCATAGAGGG
ICN139059_Meruliopsis_cystidia
TTCCGCGCTGGACCGTGTACAAGTCTCTTGGAACAGAGCGTCATAGAGGG
HHB15692Ceraceomyces_serpens
TTCCGCGCCGGACCGTGTACAAGTCTCTTGGAACAGAGCGTCATAGAGGG
HHB_15629_Sp_Ceriporiopsis_ane
TTCCGCGTTGGACCGTGTACAAGTCTCTTGGAACAGAGCGTCATAGAGGG
AJ185Trametopsis_cervina
TTCCGCGTTGGACCGTGTATAAGTCTCTTGGAACAGAGCGTCATAGAGGG
FD9Irpex_lacteus
TTCCGCGTTGGACCGTGTATAAGTCTCTTGGAATAGAGCGTCATAGAGGG
908Dai11230
TTCCGCGTTGGACCGTGTATAAGTCTCTTGGAATAGAGCGTCATAGAGGG
FP55521TEmmia_lacerata
TTCCGCGTTGGACCGTGTATAAGTCTCTTGGAACAGAGCGTCATAGAGGG
PBU0048Ceriporia_cystidiata
TTCCGCGTTGGACCGTGTATAAGTCTCTTGGAACAGAGCGTCATAGAGGG
MZ340C_lacerataT --
Dai21940
TTCCGCGTTGGACCGTGTATAAGTCTCTTGGAACAGAGCGTCATAGAGGG

847Dai16433
TTCCGCGTTGGACCGTGTATAAGTCTCTTGGAACAGAGCGTCATAGAGGG
MarcinEmmia_latemarginatus
TTCCGCGTTGGACCGTGTATAAGTCTCTTGGAACAGAGCGTCATAGAGGG
Meijer3729Hydnopolyporus_fimbr
TTCCGCGTTGGACCGTGTATAAGTCTCTTGGAACAGAGCGTCATAGAGGG
RLG13408Phanerochaete_sp
TTCCGCGCTGGACCGTGTACAAGTCTCTTGGAACAGAGCGTCATAGAGGG
WHC1381Flavodon_flavus
TTCCGCGTTGGACCGTGTATAAGTCTCTTGGAACAGAGCGTCATAGAGGG
GB1833Phlebia_albida
TTCCGCGTTGGACCGTGTACAAGTCTCTTGGAACAGAGCGTCATAGAGGG
T407Phlebia_nitidula --
HHB6988Phanerochaete_exilis
TTCCGCGTTGGACCGTGTACAAGTCTCTTGGAACAGAGCGTCATAGAGGG
HHB8509Phanerochaetella_xeroph
TTCCGCGCTGGACCGTGTACAAGTCTCTTGGAACAGAGCGTCATAGAGGG
PBU0051Macrohyporia_dictyopora
TTCCGCGTTGGACCGTGTACAAGTCTCTTGGAACAGAGCGTCATAGAGGG
HHB11463Phanerochaete_sp
TTCCGCGTTGGACCGTGTACAAGTCTCTTGGAATAGAGCGTCATAGAGGG
FP102382Byssomerulius_corium
TTCCGCGTTGGACCGTGTACAAGTCTCTTGGAACAGAGCGTCATAGAGGG
FP102165Efibula_americana
TTCCGCGCTGGACCGTGTATAAGTCTCTTGGAACAGAGCGTCATAGAGGG
Murdoch90Ceriporia_torpida
TTCCGCGTTGGACCGTGTACAAGTCTCTTGGAACAGAGCGTCATAGAGGG
Rivoire4413_Ceriporia_purpurea
TTCCGCGTTGGACCGTGTACAAGTCTCTTGGAACAGAGCGTCATAGAGGG
Kout_18_Ceriporia_triumphalis
TTCCGCGTTGGACCGTGTACAAGTCTCTTGGAACAGAGCGTCATAGAGGG
Rivoire3701_Ceriporia_bresadol
TTCCGCGTTGGACCGTGTACAAGTCTCTTGGAACAGAGCGTCATAGAGGG
VS4018 --
Ryvarden21832_Ceriporia_manzan
TTCCGCGTTGGACCGTGTACAAGTCTCTTGGAACAGAGCGTCATAGAGGG
Dai24539
TTCCGCGTTGGACCGTGTACAAGTCTCTTGGAACAGAGCGTCATAGAGGG
Dai24541
TTCCGCGTTGGACCGTGTACAAGTCTCTTGGAACAGAGCGTCATAGAGGG
JV1105_12_Ceriporia_occidental
TTCCGCGTTGGACCGTGTACAAGTCTCTTGGAACAGAGCGTCATAGAGGG
VS8558Ceriporia_occidentalis
TTCCGCGTTGGACCGTGTACAAGTCTCTTGGAACAGAGCGTCATAGAGGG

Dai22445
TTCCGCGTTGGACCGTGTACAAGTCTCTTGGAACAGAGCGTCATAGAGGG
846Dai16368
TTCCGCGTTGGACCGTGTACAAGTCTCTTGGAACAGAGCGTCATAGAGGG
Dai17951_Ceriporia_aurantiocar
TTCTGTGCTGGACCGTGTACAAGTCTCTTGGAACAGAGCGTCATAGAGGG
Miettinen_11701C_viridans
TTCTGTGCTGGACCGTGTACAAGTCTCTTGGAACAGAGCGTCGTAGAGGG
JV0105_10Ceriporia_aurantiocar
TTCTGTGCTGGACCGTGTACAAGTCTCTTGGAACAGAGCGTCATAGAGGG
Yuan5702C_viridans --
858Dai17003 --
Yuan2747_Ceriporia_viridans --
Yuan2744C_viridans --
Li1046C_viridans --
865C_sinoviridans --
871Dai15062 --
Dai7642_Ceriporia_humilis --
Spirin4706_Ceriporia_humilis --
Spirin4944_Ceriporia_sericea
TTCCGCGTTGGACCGTGTACAAGTCTCTTGGAACAGAGCGTCGTAGAGGG
WCG1547Dai26044ceriporia
TTCCGCGTTGGACCGTGTACAAGTCTCTTGGAACAGAGCGTCGTAGAGGG
ZZW1558Dai27086
TTCCGCGTTGGACCGTGTACAAGTCTCTTGGAACAGAGCGTCGTAGAGGG
Miettinen14381_Ceriporia_mpuri
TTCCGTGCTGGACCGTGTACAAGTCTCTTGGAACAGAGCGTCATAGAGGG
Miettinen15492_2_Ceriporia_sor
TTCCGCGCTGGACCGTGTACAAGTCTCTTGGAACAGAGCGTCATAGAGGG
He6687
TTCCGTGCTGGACCGTGTACAAGTCTCTTGGAACAGAGCGTCATAGAGGG
ZH53Dai24426
TTCCGTGCTGGACCGTGTACAAGTCTCTTGGAACAGAGCGTCATAGAGGG
Vlasak0808_30_Ceriporia_punica
TTCCGTGCCGGACCGTGTACAAGTCTCTTGGAACAGAGCGTCATGGAGGG
887Dai13376
TTCCGTGCCGGACCGTGTACAAGTCTCTTGGAACAGAGCGTCATAGAGGG
WCG1443Dai24998
TTCTGTGCCGGACCGTGTACAAGTCTCTTGGAACAGAGCGTCATAGAGGG
0108_6Ceriporia_spissa
TTCCGTGCTGGACCGTGTACAAGTCCCTTGGAACAGGGCGTCACAGAGGG
Dai19164
TTCCGTGCTGGACCGTGTACAAGTCCCTTGGAACAGGGCGTCACAGAGGG
Dai17937_Ceriporia_bubalinomar

TTCCGTGCTGGACCGTGTACAAGTCCCTTGGAACAGGGCCTCATAGAGGG

903Dai12113

TTCCGTGCTGGACCGTGTACAAGTCCCTTGGAACAGGGCGTCATAGAGGG

LZB929Dai25079

TTCTGTGCTGGACCGTGTACAAGTCTCTTGGAACAGAGCGTCATAGAGGG

LX45Dai26988 --

LX43Dai26986

TTCTGTGCTGGACCGTGTACAAGTCTCTTGGAACAGAGCGTCATAGAGGG

Dai7759Ceriporia --

Cui8012_Ceriporia_viridans --

GC1704_54Ceriporia_viridans

TTCTGTGCTGGACCGTGTACAAGTCTCTTGGAACAGAGCGTCATAGAGGG

Dai23392

TTCTGTGCTGGACCGTGTACAAGTCTCTTGGAACAGAGCGTCATAGAGGG

WCG1585Dai26113Ceriproia

TTCTGTGCTGGACCGTGTACAAGTCTCTTGGAACAGAGCGTCATAGAGGG

Dai18675C_eucalypti

TTCTGTGCTGGACCGTGTACAAGTCTCTTGGAACAGAGCGTCATAGAGGG

Dai22034

TTCCGCGCTGGACCGTGTACAAGTCTCTTGGAACAGAGCGTCATAGAGGG

JV1008_41JTardaFLORIDAKeys --

Rivoire1161_Ceriporia_pierii --

Dai23499C_pierii

TTCCGTGCTGGACCGTGTACAAGTCTCTTGGAACAGAGCGTCATAGAGGG

Dai23500

TTCCGTGCTGGACCGTGTACAAGTCTCTTGGAACAGAGCGTCATAGAGGG

841Dai15899

TTCCGCGCTGGACCGTGTACAAGTCTCTTGGAACAGAGCGTCATAGAGGG

842Dai15904 --

LZB1066xinjiang --

LZB1065xinjiang --

851Dai16779

TTCCGCGCTGGACCGTGTACAAGTCTCTTGGAACAGAGCGTCGTAGAGGG

RMJ119sp_Candelabrochaete_sept --

RLG9759spCandelabrochaete_sept

TTCCGCGCTGGACCGTGTACAAGTCTCTTGGAACAGAGCGTCACAGAGGG

RLG10478Phanerochaete_allantos --

Dai19118_Ceriporia_spissa

TTCCGCGCTGGACCGTGTACAAGTCTCTTGGAACAGAGCATCATAGAGGG

Dai18486A

TTCCGCACTGGACCGTGTACAAGTCTCTTGGAACAGAGCATCATAGAGGG

WEI17_024_Ceriporia_mellita

TTCCGCGCTGGACCGTGTACAAGTCTCTTGGAACAGAGCATCATAGAGGG

GC1508_71Ceriporia_mellita

TTCCGCGCTGGACCGTGTACAAGTCTCTTGGAACAGAGCATCATAGAGGG
GC1608_7_Ceriporia_mellita
TTCCGCGCTGGACCGTGTACAAGTCTCTTGGAACAGAGCATCATAGAGGG
ZZW1557Dai27085
TTCCGCGCTGGACCGTGTACAAGTCTCTTGGAACAGAGCATCATAGAGGG
ZZW1554Dai27083
TTCCGCGCTGGACCGTGTACAAGTCTCTTGGAACAGAGCATCATAGAGGG
Dai8168
TTCCGCGCTGGACCGTGTACAAGTCTCTTGGAACAGAGCATCATAGAGGG
BR4865C_mellita
TTCCGCGCTGGACCGTGTACAAGTCTCTTGGAACAGAGCATCATAGAGGG
MEL2382688Ceriporia_sp --
Dai8110
TTCCGCGCTGGACCGTGTACAAGTCTCTTGGAACAGAGCATCATAGAGGG
Cui8097 --
909Cui6740 --
W1258Dai24695
TTCCGCGCTGGACCATGTACAAGTCTCTTGGAACAGAGCATCATAGAGGG
JV0110_26_Ceriporia_griseoviol
TTCCGCGCTGGACCGTGTACAAGTCTCTTGGAACAGAGCGTCGTAGAGGG
896Dai13202
TTCCGCGCTGGACCGTGTACAAGTCTCTTGGAACAGAGCGTCGTAGAGGG
LWY393Dai27053C_griseoviolasce
TTCCGCGCTGGACCGTGTACAAGTCTCTTGGAACAGAGTGTCGTAGAGGG
LWY394DAI27054
TTCCGCGCTGGACCGTGTACAAGTCTCTTGGAACAGAGTGTCGTAGAGGG
FP135015G_pannocinctus
TTCCGCGCTGGACCGTGTACAAGTCTCTTGGAACAGAGCGTCATAGAGGG
L15726SpG_pannocinctus
TTCCGCGCTGGACCGTGTACAAGTCTCTTGGAACAGAGCGTCATAGAGGG
Dai22221
TTCCGCGCTGGACCGTGTACAAGTCTCTTGGAACAGAGCGTCATAGAGGG
Dai22633
TTCCGCGCTGGACCGTGTACAAGTCTCTTGGAATAGAGCGTCATAGAGGG
Dai23260
TTCCGCGCTGGACCGTGTACAAGTCTCTTGGAATAGAGCGTCATAGAGGG
Dai23626
TTCCGCGCTGGACCGTGTACAAGTCTCTTGGAATAGAGCGTCATAGAGGG
Dai16238G_citrinoalbus
TTCCGCGTTGGACCGTGTACAAGTCTCTTGGAATAGAGCGTCATAGAGGG
1175Dai15293
TTCCGCGTTGGACCGTGTACAAGTCTCTTGGAATAGAGCGTCATAGAGGG
Dai19547
TTCCGCGTTGGACCGTGTACAAGTCTCTTGGAATAGAGCGTCATAGAGGG

918063G_africanus
TTCCGCGTTGGACCGTGTACAAGTCTCTTGGAATAGAGCGTCATAGAGGG
918572G_africanus
TTCCGCGTTGGACCGTGTACAAGTCTCTTGGAATAGAGCGTCATAGAGGG
Dai18536A --
1164Cui17922
TTCCGCGTTGGACCGTGTACAAGTCTCTTGGAATAGAGCGTCATAGAGGG
Dai22225
TTCCGCGTTGGACCGTGTACAAGTCTCTTGGAATAGAGCGTCATAGAGGG
1163Dai20655
TTCCGCGTTGGACCGTGTACAAGTCTCTTGGAATAGAGCGTCATAGAGGG
Yuan4397G_hainanensis
TTCCGCGTTGGACCGTGTACAAGTCTCTTGGAACAGAGCGTCATAGAGGG
1176Dai15268
TTCCGCGTTGGACCGTGTACAAGTCTCTTGGAACAGAGCGTCATAGAGGG
1177Dai15259
TTCCGCGTTGGACCGTGTACAAGTCTCTTGGAACAGAGCGTCATAGAGGG
BZ2896G_thelephoroides
TTCCGCGTTGGACCGTGTACAAGTCTCTTGGAACAGAGCGTCATAGAGGG
1166JV1808_26
TTCCGCGTTGGACCGTGTACAAGTCTCTTGGAACAGAGCGTCATAGAGGG
Miettinen16992Hapalopilus_ochr
TTCCGTGCTAGACCGTGTATAAGTCTTTTGGAATAGGGCATCATAGAGGG
GC1708_338_Ceriporia_arbuscula
TTCCGCGCTAGACCGTGTACAAGTCTCTTGGAACAGAGCGTCATAGAGGG
WCG1555Dai26107Ceriporia
TTCCGCGCTAGACCGTGTACAAGTCTCTTGGAACAGAGCGTCATAGAGGG
GC1708_340_Ceriporia_arbuscula
TTCCGCGCTAGACCGTGTACAAGTCTCTTGGAACAGAGCGTCATAGAGGG
WCG1556Dai26109Ceriporia
TTCCGCGCTAGACCGTGTACAAGTCTCTTGGAACAGAGCGTCATAGAGGG
883Cui11291
TTCCGCGCTAGACCGTGTACAAGTCTCTTGGAACAGAGCGTCATAGAGGG
HLX320Dai26805
TTCCGTGCTAGACCGTGTACAAGTCTCTTGGAACAGAGCGTCATAGAGGG
WCG1266Dai24678A
TTCCGTGCTAGACCGTGTACAAGTCTCTTGGAACAGAGCGTCATAGAGGG
Dai6090_Ceriporia_sulphuricolo
TTCCGCGCTGGACCGTGTACAAGTCTCTTGGAACAGAGCGTCGTAGAGGG
RLG_11354_Ceriproia_reticulata
TTCTGTGCTGGACCGTGTACAAGTCTCTTGGAACAGAGCGTCATAGAGGG
ZZW1543Dai27072
TTCTGTGCTGGACCGTGTACAAGTCTCTTGGAACAGAGCGTCATAGAGGG
Li1316_Ceriporia_reticulata --

KHL11981Ceriporia_reticulata
TTCTGTGCTGGACCGTGTACAAGTCTCTTGGAACAGAGCGTCATAGAGGG
FP110343sp_Candelabrochaete_la
TTCTGTGCTGGACCGTGTACAAGTCTCTTGGAACAGAGCGTCATAGAGGG
Li1045_Ceriporia_reticulata --
ZX136Dai25794ceriporia
TTCTGTGCTGGACCGTGTACAAGTCTCTTGGAACAGAGCGTCATAGAGGG
892Dai13400
TTCTGTGCTGGACCGTGTACAAGTCTCTTGGAACAGAGCGTCATAGAGGG
RLG7163Leptoporus_mollis
TTCCGCCCTGGACCGTGTACAAGTCTCTTGGAACAGAGCGTCATAGAGGG
Dai21062Leptoporus_mollis
TTCCGCTCTGGACCGTGTACAAGTCTCTTGGAACAGAGCGTCATAGAGGG
Dai20182Leptoporus_submollis
TTCCGCGCTGGACCGTGTACAAGTCTCTTGGAACAGAGCGTCATAGAGGG
Cui18379Leptoporus_submollis
TTCCGCGCTGGACCGTGTACAAGTCTCTTGGAACAGAGCGTCATAGAGGG
Wu1209_46Resiniporus_pseudogil -
TCCGTGTTGGACCGTGTATAAGTCTCTTGGAACAGAGCGTCATAGAGGG
BRNM710169Resiniporus_resinasc
TTCCGTGTTGGACCGTGTATAAGTCTCTTGGAACAGAGCGTCATAGAGGG
Dai14516Bjerkandera_adusta
TTCCGCGCTGGACCGTGTACAAGTCTCTTGGAACAGAGCGTCATAGAGGG
Dai21100Bjerkandera_fumosa
TTCCGCGCTGGACCGTGTACAAGTCTCTTGGAACAGAGCGTCACAGAGGG
Miettinen16854Ceraceomyces_sp
ATCTGTGCTGGACCGTGTACAAGTCTCTTGGAACAGAGCGTCACAGAGGG
Dai10477C_spissa
TTCTGTGTCGGACCGTGTACAAGTCCCTTGGAACAGGGCGTCATAGAGGG
855Dai16831
TTCTGTGTCGGACCGTGTACAAGTCCCTTGGAACAGGGCGTCATAGAGGG
882Cui11282
TTCTGTGTCGGACCGTGTACAAGTCCCTTGGAACAGGGCGTCATAGAGGG
Dai24566
TTCTGTGTCGGACCGTGTACAAGTCCCTTGGAACAGGGCGTCATAGAGGG
Yuan5965
TTCTGTGTCGGACCGTGTACAAGTCCCTTGGAACAGGGCGTCATAGAGGG
Dai3204
TTCCGCGTTGGACCGTGTACAAGTCTCTTGGAACAGAGCGTCATAGAGGG
1194CUI9985
TTCCGCGTTGGACCGTGTATAAGTCTCTTGGAACAGAGCGTCATAGAGGG

Dai15205_Ceriporia_albomellea

TGAGAATCCCGTCTTTGACACGGACTGCCAGTGCTATGTGATGCGCTCTC
Dai15223_Ceriporia_albomellea
TGAGAATCCCGTCTTTGACACGGACTGCCAGTGCTATGTGATGCGCTCTC
Li1780_Ceriporia_variegata
TGAGAATCCCGTCTTTGACACGGACTACCAGTGCTTTGTGATGCGCTCTC
Dai19791_Ceriporia_variegata
TGAGAATCCCGTCTTTGACACGGACTACCAGTGCTTTGTGATGCGCTCTC
Dai19886
TGAGAATCCCGTCTTTGACACGGACTACCAGTGCTTTGTGATGCGCTCTC
Dai10833_Ceriporia_crassitunic
TGAGAATCCCGTCTTTGACACGGACTACCAGTGCTATGTGATGCGCTCTC
CHWC1506_46Meruliopsis_crassit
TGAGAATCCCGTCTTTGACACGGACTACCAGTGCTATGTGATGCGCTCTC
Dai9995_Ceriporia_crassitunica --
Wu1209_58_Meruliopsis_parvispo
TGAGAATCCCGTCTTTGACACGGACTACCAGTGCTTTGTGATGCGCTCTC
CHWC1505_129_Meruliopsis_parvi
TGAGAATCCCGTCTTTGACACGGACTACCAGTGCTTTGTGATGCGCTCTC
Dai21944
TGAGAATCCCGTCTTTGACACGGACTACCAGTGCTTTGTGATGCGCTCTC
830Dai18640A
TGAGAATCCCGTCTTTGACACGGACTACCAGTGCTTTGTGATGCGCTCTC
GC1704_60_Meruliopsis_taxicola
TGAGAATCCCGTCTTTGACACGGACTACCAGTGCTTTGTGATGCGCTCTC
Dai22625
TGAGAATCCCGTCTTTGACACGGACTACCAGTGCTTTGTGATGCGCTCTC
Dai22636
TGAGAATCCCGTCTTTGACACGGACTACCAGTGCTTTGTGATGCGCTCTC
Dai21878
TGAGAATCCCGTCTTTGACACGGACTACCAGTGCTTTGTGATGCGCTCTC
1169Dai17248
TGAGAATCCCGTCTTTGACACGGACTACCAGTGCTTTGTGATGCGCTCTC
Wu1708_43_Meruliopsis_leptocys
TGAGAATCCCGTCTTTGACACGGACTACCAATGCTTTGTGATGCGCTCTC
Li1011
TGAGAATCCCGTCTTTGACACGGACTACCAATGCTTTGTGATGCGCTCTC
ZX95Dai25742Meruliopsis_leptoc
TGAGAATCCCGTCTTTGACACGGACTACCAATGCTTTGTGATGCGCTCTC
WCG1306Dai24733
TGAGAATCCCGTCTTTGACACGGACTACCAATGCTTTGTGATGCGCTCTC
LXL99Dai25816
TGAGAATCCCGTCTTTGACACGGACTACCAATGCTTTGTGATGCGCTCTC
WCG1559Dai26052Meruliopsis
TGAGAATCCCGTCTTTGACACGGACTACCAATGCTTTGTGATGCGCTCTC

He7477
TGAGAATCCCGTCTTTGACACGGACTACCAATGCTTTGTGATGCGCTCTC
HLX243Dai26217
TGAGAATCCCGTCTTTGACACGGACTACCAATGCTTTGTGATGCGCTCTC
RussiaMW673659Meruliopsis_fagi
TGAGAATCCCGTCTTTGACACGGACTACCAATGCTTTGTGATGCGCTCTC
FD278
TGAGAATCCCGTCTTTGACACGGACAACCAGTGCTTTGTGATGCGCTCTC
Dai10226_Ceriporia_tarda --
LE247365
TGAGAATCCCGTCTTTGACACGGACTACCAATGCTTTGTGATGCGCTCTC
Dai8173_Meruliopsis_nanlingens
TGAGAATCCCGTCTTTGACACGGACTACCAGTGCTTTGTGATGCGCTCTC
860Dai17172
TGAGAATCCCGTCTTTGACACGGACTACCAGTGCTTTGTGATGCGCTCTC
879Dai13414
TGAGAATCCCGTCTTTGACACGGACTACCAGTGCTTTGTGATGCGCTCTC
Li_1704_Meruliopsis_pseudocyst --
833Dai18405
TGAGAATCCCGTCTTTGACACGGACTACCAATGCTTTGTGATGCGCTCTC
HHB_10729_Meruliopsis_albostra
TGAGAATCCCGTCTTTGACACGGACTACCAATGCTTTGTGATGCGCTCTC
Cui6878_Ceriporia_pseudocystid
TGAGAATCCCGTCTTTGACACGGACTACCAGTGCTTTGTGATGCGCTCTC
869Dai14737
TGAGAATCCCGTCTTTGACACGGACTACCAGTGCTTTGTGATGCGCTCTC
876Cui11626
TGAGAATCCCGTCTTTGACACGGACTACCAGTGCTTTGTGATGCGCTCTC
1199WEI3388 --
776308_Meruliopsis_cystidiata
TGAGAATCCCGTCTTTGACACGGACTACCAGTGCTTTGTGATGCGCTCTC
ICN139059_Meruliopsis_cystidia
TGAGAATCCCGTCTTTGACACGGACTACCAGTGCTTTGTGATGCGCTCTC
HHB15692Ceraceomyces_serpens
TGAGAATCCCGTCTTTGACACGGACTACCGGTGCTTTGTGATGCGCTCTC
HHB_15629_Sp_Ceriporiopsis_ane
TGAGAATCCCGTCTTTGACACGGACTACCAATGCTATGTGATGCGCTCTC
AJ185Trametopsis_cervina
TGAGAATCCCGTCTTTAACACGGACTACCAGTGCTTTGTGATGCGCTCTC
FD9Irpex_lacteus
TGAGAATCCCGTCTTTGACACGGACTACCAATGCTTTGTGATACACTCTC
908Dai11230
TGAGAATCCCGTCTTTGACACGGACTACCAATGCTTTGTGATACACTCTC
FP55521TEmmia_lacerata

TGAGAATCCCGTCTTTGACACGGACTACCAATGCTTTGTGATACACTCTC

PBU0048Ceriporia_cystidiata

TGAGAATCCCGTCTTTGACACGGACTACCAATGCTTTGTGATACACTCTC

MZ340C_lacerataT --

Dai21940

TGAGAATCCCGTCTTTGACACGGACTACCAATGCTTTGTGATACACTCTC

847Dai16433

TGAGAATCCCGTCTTTGACACGGACTACCAATGCTTTGTGATACACTCTC

MarcinEmmia_latemarginatus

TGAGAATCCCGTCTTTGACACGGACTACCAATGCTTTGTGATACACTCTC

Meijer3729Hydnopolyporus_fimbr

TGAGAATCCCGTCTTTGACACGGACTACCAATGCTTTGTGATGCGCTCTC

RLG13408Phanerochaete_sp

TGAGAATCCCGTCTTTGACACGGACTACCAGTGCTTTGTGATGCGCTCTC

WHC1381Flavodon_flavus

TGAGAATCCCGTCTTTGACACGGACTACCAATGCTTTGTGATACACTCTC

GB1833Phlebia_albida

TGAGAATCCCGTCTTTGACACGGACTACCAATGCTTTGTGATGCGCTCTC

T407Phlebia_nitidula --

HHB6988Phanerochaete_exilis

TGAGAATCCCGTCTTTGACACGGACTACCAGTGCTTTGTGATGCGCTCTC

HHB8509Phanerochaetella_xeroph

TGAGAATCCCGTCTTTGACACGGACTACCAGTGCTTTGTGATGCGCTCTC

PBU0051Macrohyporia_dictyopora

TGAGAATCCCGTCTTTGACACGGACTACCAGTGCTTTGTGATGCGCTCTC

HHB11463Phanerochaete_sp

TGAGAATCCCGTCTTTGACACGGACTACCAATGCTTTGTGATGCGCTCTC

FP102382Byssomerulius_corium

TGAGAATCCCGTCTTTGACACGGACTACCAGTGCTTTGTGATGCGCTCTC

FP102165Efibula_americana

TGAGAATCCCGTCTTTGACACGGACTACCAGTGCTTTGTGATGCGCTCTC

Murdoch90Ceriporia_torpida

TGAGAATCCCGTCTTTGACACGGACTACCAGTGCTTTGTGATGCGCTCTC

Rivoire4413_Ceriporia_purpurea

TGAGAATCCCGTCTTTGACACGGACTACCAATGCTTTGTGATGCGCTCTC

Kout_18_Ceriporia_triumphalis

TGAGAATCCCGTCTTTGACACGGACTACCAGTGCTTTGTGATGCGCTCTC

Rivoire3701_Ceriporia_bresadol

TGAGAATCCCGTCTTTGACACGGACTACCAGTGCTTTGTGATGCGCTCTC

VS4018 --

Ryvarden21832_Ceriporia_manzan

TGAGAATCCCGTCTTTGACACGGACTACCAGTGCTTTGTGATGCGCTCTC

Dai24539

TGAGAATCCCGTCTTTGACACGGACTACCAGTGCTTTGTGATGCGCTCTC

Dai24541
TGAGAATCCCGTCTTTGACACGGACTACCAGTGCTTTGTGATGCGCTCTC
JV1105_12_Ceriporia_occidental
TGAGAATCCCGTCTTTGACACGGACTACCAGTGCTTTGTGATGCGCTCTC
VS8558Ceriporia_occidentalis
TGAGAATCCCGTCTTTGACACGGACTACCAGTGCTTTGTGATGCGCTCTC
Dai22445
TGAGAATCCCGTCTTTGACACGGACTACCAATGCTTTGTGATGCGCTCTC
846Dai16368
TGAGAATCCCGTCTTTGACACGGACTACCAATGCTTTGTGATGCGCTCTC
Dai17951_Ceriporia_aurantiocar
TGAGAATCCCGTCTTTGACATGGACTACCAGTGCTATGTGATGCGCTCTC
Miettinen_11701C_viridans
TGAGAATCCCGTCTTTGACATGGACTACCAGTGCTATGTGATGCGCTCTC
JV0105_10Ceriporia_aurantiocar
TGAGAATCCCGTCTTTGACATGGACTACCAGTGCTATGTGATGCGCTCTC
Yuan5702C_viridans --
858Dai17003 --
Yuan2747_Ceriporia_viridans --
Yuan2744C_viridans --
Li1046C_viridans --
865C_sinoviridans --
871Dai15062 --
Dai7642_Ceriporia_humilis --
Spirin4706_Ceriporia_humilis --
Spirin4944_Ceriporia_sericea
TGAGAATCCCGTCTTTGACACGGACTACCAGTGCTTTGTGATGCGCTCTC
WCG1547Dai26044ceriporia
TGAGAATCCCGTCTTTGACACGGACTACCAGTGCTTTGTGATGCGCTCTC
ZZW1558Dai27086
TGAGAATCCCGTCTTTGACACGGACTACCAGTGCTTTGTGATGCGCTCTC
Miettinen14381_Ceriporia_mpuri
TGAGAATCCCGTCTTTGACACGGACTACCAGTGCTTTGTGATGCGCTCTC
Miettinen15492_2_Ceriporia_sor
TGAGAATCCCGTCTTTGACACGGACTACCAGTGCTTTGTGATGCGCTCTC
He6687
TGAGAATCCCGTCTTTGACACGGACTACCAGTGCTTTGTGATGCGCTCTC
ZH53Dai24426
TGAGAATCCCGTCTTTGACACGGACTACCAGTGCTTTGTGATGCGCTCTC
Vlasak0808_30_Ceriporia_punica
TGAGAATCCCGTCTTTGACACGGACTACCGGTGCTTTGTGATGCGCTCTC
887Dai13376
TGAGAATCCCGTCTTTGACACGGACTACCGGTGCTTTGTGATGCGCTCTC
WCG1443Dai24998

TGAGAATCCCGTCTTTGACACGGACTACCGGTGCTTTGTGATGCGCTCTC

0108_6Ceriporia_spissa

TGAGAATCCCGTCTTTGACACGGACTACCAGTGCTTTGTGATGCACTCTC

Dai19164

TGAGAATCCCGTCTTTGACACGGACTACCAGTGCTTTGTGATGCACTCTC

Dai17937_Ceriporia_bubalinomar

TGAGAATCCCGTCTTTGACACGGACTACCAGTGCTTTGTGATGCACTCTC

903Dai12113

TGAGAATCCCGTCTTTGACACGGACTACCAGTGCTTTGTGATGCACTCTC

LZB929Dai25079

TGAGAATCCCGTCTTTGACACGGACTACCAGTGCTATGTGATGCGCTCTC

LX45Dai26988 --

LX43Dai26986

TGAGAATCCCGTCTTTGACACGGACTACCAGTGCTATGTGATGCGCTCTC

Dai7759Ceriporia --

Cui8012_Ceriporia_viridans --

GC1704_54Ceriporia_viridans

TGAGAATCCCGTCTTTGACACGGACTACCAGTGCTTTGTGATGCGCTCTC

Dai23392

TGAGAATCCCGTCTTTGACACGGACTACCAGTGCTTTGTGATGCACTCTC

WCG1585Dai26113Ceriproia

TGAGAATCCCGTCTTTGACACGGACTACCAGTGCTTTGTGATGCACTCTC

Dai18675C_eucalypti

TGAGAATCCCGTCTTTGACACGGACTACCAGTGCTTTGTGATGCGCTCTC

Dai22034

TGAGAATCCCGTCTTTGACACGGACTACCAGTGCTTTGTGATGCGCTCTC

JV1008_41JTardaFLORIDAKeys --

Rivoire1161_Ceriporia_pierii --

Dai23499C_pierii

TGAGAATCCCGTCTTTGACACGGACTACCAGTGCTTTGTGATGCGCTCTC

Dai23500

TGAGAATCCCGTCTTTGACACGGACTACCAGTGCTTTGTGATGCGCTCTC

841Dai15899

TGAGAATCCCGTCTTTGACACGGACTACCAGTGCTTTGTGATGCGCTCTC

842Dai15904 --

LZB1066xinjiang --

LZB1065xinjiang --

851Dai16779

TGAGAATCCCGTCTTTGACACGGACTACCAGTGCTTTGTGATGCGCTCTC

RMJ119sp_Candelabrochaete_sept --

RLG9759spCandelabrochaete_sept

TGAGAATCCCGTCTTTGACACGGACTACCAGTGCTTTGTGATGCGCTCTC

RLG10478Phanerochaete_allantos --

Dai19118_Ceriporia_spissa

TGAGAATCCCGTCTTTGACACGGACTACCAGTGCTTTGTGATGCGCTCTC
Dai18486A
TGAGAATCCCGTCTTTGACACGGACTACCAGTGCTTTGTGATGCGCTCTC
WEI17_024_Ceriporia_mellita
TGAGAATCCCGTCTTTGACACGGACTACCAGTGCTTTGTGATGCGCTCTC
GC1508_71Ceriporia_mellita
TGAGAATCCCGTCTTTGACACGGACTACCAGTGCTTTGTGATGCGCTCTC
GC1608_7_Ceriporia_mellita
TGAGAATCCCGTCTTTGACACGGACTACCAGTGCTTTGTGATGCGCTCTC
ZZW1557Dai27085
TGAGAATCCCGTCTTTGACACGGACTACCAGTGCTTTGTGATGCGCTCTC
ZZW1554Dai27083
TGAGAATCCCGTCTTTGACACGGACTACCAGTGCTTTGTGATGCGCTCTC
Dai8168
TGAGAATCCCGTCTTTGACACGGACTACCAGTGCTTTGTGATGCGCTCTC
BR4865C_mellita
TGAGAATCCCGTCTTTGACACGGACTACCAGTGCTTTGTGATGCGCTCTC
MEL2382688Ceriporia_sp --
Dai8110
TGAGAATCCCGTCTTTGACATGGACTACCAGTGCTTTGTGATGCGCTCTC
Cui8097 --
909Cui6740 --
W1258Dai24695
TGAGAATCCCGTCTTTGACATGGACTACCAGTGCTTTGTGATGCGCTCTC
JV0110_26_Ceriporia_griseoviol
TGAGAATCCCGTCTCTGACACGGACTACCAGTGCTTTGTGATGCGCTCTC
896Dai13202
TGAGAATCCCGTCTCTGACACGGACTACCAGTGCTTTGTGATGCGCTCTC
LWY393Dai27053C_griseoviolasce
TGAGAATCCCGTCTCTGACACGGACTACCAGTGCTTTGTGATGCGCTCTC
LWY394DAI27054
TGAGAATCCCGTCTCTGACACGGACTACCAGTGCTTTGTGATGCGCTCTC
FP135015G_pannocinctus
TGAGAATCCCGTCCATGACACGGACTACCAGTGCTTTGTGATGCGCTCTC
L15726SpG_pannocinctus
TGAGAATCCCGTCCATGACACGGACTACCAGTGCTTTGTGATGCGCTCTC
Dai22221
TGAGAATCCCGTCCATGACACGGACTACCAGTGCTTTGTGATGCGCTCTC
Dai22633
TGAGAATCCCGTCCATGACACGGACTACCAGTGCTTTGTGATGTGCTCTC
Dai23260
TGAGAATCCCGTCCATGACACGGACTACCAGTGCTTTGTGATGTGCTCTC
Dai23626
TGAGAATCCCGTCCATGACACGGACTACCAGTGCTTTGTGATGTGCTCTC

Dai16238G_citrinoalbus
TGAGAATCCCGTCCATGACACGGACTACCAGTGCTTTGTGATGTGCTCTC
1175Dai15293
TGAGAATCCCGTCCATGACACGGACTACCAGTGCTTTGTGATGTGCTCTC
Dai19547
TGAGAATCCCGTCCATGACACGGACTACCAGTGCTTTGTGATGTGCTCTC
918063G_africanus
TGAGAATCCCGTCCATGACACGGACTACCAGTGCTTTGTGATGTGCTCTC
918572G_africanus
TGAGAATCCCGTCCATGACACGGACTACCAGTGCTTTGTGATGTGCTCTC
Dai18536A --
1164Cui17922
TGAGAATCCCGTCCATGACACGGACTACCAGTGCTTTGTGATGTGCTCTC
Dai22225
TGAGAATCCCGTCCATGACACGGACTACCAGTGCTTTGTGATGTGCTCTC
1163Dai20655
TGAGAATCCCGTCCATGACACGGACTACCAGTGCTTTGTGATGTGCTCTC
Yuan4397G_hainanensis
TGAGAATCCCGTCCATGACACGGACTACCAATGCTTTGTGATGCGCTCTC
1176Dai15268
TGAGAATCCCGTCCATGACACGGACTACCAATGCTTTGTGATGCGCTCTC
1177Dai15259
TGAGAATCCCGTCCATGACACGGACTACCAATGCTTTGTGATGCGCTCTC
BZ2896G_thelephoroides
TGAGAATCCCGTCCATGACACGGACTACCAATGCTTTGTGATGCGCTCTC
1166JV1808_26
TGAGAATCCCGTCCATGACACGGACTACCAATGCTTTGTGATGCGCTCTC
Miettinen16992Hapalopilus_ochr
TGAGAATCCCGTCTTTGACACGGACTACTAGTACTTTGTGATGCGCTCTC
GC1708_338_Ceriporia_arbuscula
TGAGAATCCCGTCTTTGACACGGACTACTAGTGCTTTGTGATGCGCTCTC
WCG1555Dai26107Ceriporia
TGAGAATCCCGTCTTTGACACGGACTACTAGTGCTTTGTGATGCGCTCTC
GC1708_340_Ceriporia_arbuscula
TGAGAATCCCGTCTTTGACACGGACTACTAGTGCTTTGTGATGCGCTCTC
WCG1556Dai26109Ceriporia
TGAGAATCCCGTCTTTGACACGGACTACTAGTGCTTTGTGATGCGCTCTC
883Cui11291
TGAGAATCCCGTCTTTGACACGGACTACTAGTGCTTTGTGATGCGCTCTC
HLX320Dai26805
TGAGAATCCCGTCTTTGACACGGACTACTAGTGCTTTGTGATGCGCTCTC
WCG1266Dai24678A
TGAGAATCCCGTCTTTGACACGGACTACTAGTGCTTTGTGATGCGCTCTC
Dai6090_Ceriporia_sulphuricolo

TGAGAATCCCGTCTTTGACACGGACTGCCAGTGCTTTGTGATGCGCTTTC

RLG_11354_Ceriproia_reticulata

TGAGAATCCCGTCTTTGACACGGACTACCAGTGCTATGTGATGCGCTCTC

ZZW1543Dai27072

TGAGAATCCCGTCTTTGACACGGACTACCAGTGCTATGTGATGCGCTCTC

Li1316_Ceriporia_reticulata --

KHL11981Ceriporia_reticulata

TGAGAATCCCGTCTTTGACACGGACTACCAGTGCTATGTGATGCGCTCTC

FP110343sp_Candelabrochaete_la

TGAGAATCCCGTCTTTGACACGGACTACCAGTGCTATGTGATGCGCTCTC

Li1045_Ceriporia_reticulata --

ZX136Dai25794ceriporia

TGAGAATCCCGTCTTTGACACGGACTACCAGTGCTATGTGATGCGCTCTC

892Dai13400

TGAGAATCCCGTCTTTGACACGGACTACCAGTGCTATGTGATGCGCTCTC

RLG7163Leptoporus_mollis

TGAGAATCCCGTCTTTGACACGGACTACCAGTGCTTTGTGATGCGCTCTC

Dai21062Leptoporus_mollis

TGAGAATCCCGTCTTTGACACGGACTACCAGTGCTTTGTGATGCGCTCTC

Dai20182Leptoporus_submollis

TGAGAATCCCGTCTTTGACACGGACTACCAGTGCTTTGTGATGCGCTCTC

Cui18379Leptoporus_submollis

TGAGAATCCCGTCTTTGACACGGACTGCCAGTGCTTTGTGATGCGCTCTC

Wu1209_46Resiniporus_pseudogil

TGAGAATCCCGTCTTTAACATGGACTACCAATGCTTTGTGATGCGCTCTC

BRNM710169Resiniporus_resinasc

TGAGAATCCCGTCTTTAACATGGACTACCAATGCTTTGTGATGCGCTCTC

Dai14516Bjerkandera_adusta

TGAGAATCCCGTCTTTGACACGGACTACCAGTGCTATGTGATGCGCTCTC

Dai21100Bjerkandera_fumosa

TGAGAATCCCGTCTTTGACACGGACTACCAGTGCTATGTGATGCGCTCTC

Miettinen16854Ceraceomyces_sp

TGAGAATCCCGTCTTTGACACGGACTGCCGGTGCTTTGTGTTGCACTTTC

Dai10477C_spissa

TGAGAATCCCGTCTTTGACACGGACTACCGGTGCTTTGTGATGCGCTTTC

855Dai16831

TGAGAATCCCGTCTTTGACACGGACTACCGGTGCTTTGTGATGCGCTTTC

882Cui11282

TGAGAATCCCGTCTTTGACACGGACTACCGGTGCTTTGTGATGCGCTTTC

Dai24566

TGAGAATCCCGTCTTTGACACGGACTACCGGTGCTTTGTGATGCGCTTTC

Yuan5965

TGAGAATCCCGTCTTTGACACGGACTACCGGTGCTTTGTGATGCGCTTTC

Dai3204

TGAGAATCCCGTCTTTGACACGGACTACCAATGCTTTGTGATGCGCTCTC
1194CUI9985
TGAGAATCCCGTCTTTAACACGGACTACCAGTGCTTTGTGATGCGCTCTC

Dai15205_Ceriporia_albomellea
AAAGAGTCGAGTTGTTTGGGAATGCAGCTCAAAATGGGTGGTAAATTCCA
Dai15223_Ceriporia_albomellea
AAAGAGTCGAGTTGTTTGGGAATGCAGCTCAAAATGGGTGGTAAATTCCA
Li1780_Ceriporia_variegata
AAAGAGTCGAGTTGTTTGGGAATGCAGCTCAAAATGGGTGGTAAATTCCA
Dai19791_Ceriporia_variegata
AAAGAGTCGAGTTGTTTGGGAATGCAGCTCAAAATGGGTGGTAAATTCCA
Dai19886
AAAGAGTCGAGTTGTTTGGGAATGCAGCTCAAAATGGGTGGTAAATTCCA
Dai10833_Ceriporia_crassitunic
AAAGAGTCGAGTTGTTTGGGAATGCAGCTCAAAATGGGTGGTAAATTCCA
CHWC1506_46Meruliopsis_crassit
AAAGAGTCGAGTTGTTTGGGAATGCAGCTCAAAATGGGTGGTAAATTCCA
Dai9995_Ceriporia_crassitunica --
Wu1209_58_Meruliopsis_parvispo
AAAGAGTCGAGTTGTTTGGGAATGCAGCTCAAAATGGGTGGTAAATTCCA
CHWC1505_129_Meruliopsis_parvi
AAAGAGTCGAGTTGTTTGGGAATGCAGCTCAAAATGGGTGGTAAATTCCA
Dai21944
AAAGAGTCGAGTTGTTTGGGAATGCAGCTCAAAATGGGTGGTAAATTCCA
830Dai18640A
AAAGAGTCGAGTTGTTTGGGAATGCAGCTCAAAATGGGTGGTAAATTCCA
GC1704_60_Meruliopsis_taxicola
AAAGAGTCGAGTTGTTTGGGAATGCAGCTCAAAATGGGTGGTAAATTCCA
Dai22625
AAAGAGTCGAGTTGTTTGGGAATGCAGCTCAAAATGGGTGGTAAATTCCA
Dai22636
AAAGAGTCGAGTTGTTTGGGAATGCAGCTCAAAATGGGTGGTAAATTCCA
Dai21878
AAAGAGTCGAGTTGTTTGGGAATGCAGCTCAAAATGGGTGGTAAATTCCA
1169Dai17248
AAAGAGTCGAGTTGTTTGGGAATGCAGCTCAAAATGGGTGGTAAATTCCA
Wu1708_43_Meruliopsis_leptocys
AAAGAGTCGAGTTGTTTGGGAATGCAGCTCAAAATGGGTGGTAAATTCCA
Li1011
AAAGAGTCGAGTTGTTTGGGAATGCAGCTCAAAATGGGTGGTAAATTCCA
ZX95Dai25742Meruliopsis_leptoc
AAAGAGTCGAGTTGTTTGGGAATGCAGCTCAAAATGGGTGGTAAATTCCA

WCG1306Dai24733
AAAGAGTCGAGTTGTTTGGGAATGCAGCTCAAAATGGGTGGTAAATTCCA
LXL99Dai25816
AAAGAGTCGAGTTGTTTGGGAATGCAGCTCAAAATGGGTGGTAAATTCCA
WCG1559Dai26052Meruliopsis
AAAGAGTCGAGTTGTTTGGGAATGCAGCTCAAAATGGGTGGTAAATTCCA
He7477
AAAGAGTCGAGTTGTTTGGGAATGCAGCTCAAAATGGGTGGTAAATTCCA
HLX243Dai26217
AAAGAGTCGAGTTGTTTGGGAATGCAGCTCAAAATGGGTGGTAAATTCCA
RussiaMW673659Meruliopsis_fagi
AAAGAGTCGAGTTGTTTGGGAATGCAGCTCAAAATGGGTGGTAAATTCCA
FD278
AAAGAGTCGAGTTGTTTGGGAATGCAGCTCAAAATGGGTGGTAAATTCCA
Dai10226_Ceriporia_tarda --
LE247365
AAAGAGTCGAGTTGTTTGGGAATGCAGCTCAAAATGGGTGGTAAATTCCA
Dai8173_Meruliopsis_nanlingens
AAAGAGTCGAGTTGTTTGGGAATGCAGCTCAAAATGGGTGGTAAATTCCA
860Dai17172
AAAGAGTCGAGTTGTTTGGGAATGCAGCTCAAAATGGGTGGTAAATTCCA
879Dai13414
AAAGAGTCGAGTTGTTTGGGAATGCAGCTCAAAATGGGTGGTAAATTCCA
Li_1704_Meruliopsis_pseudocyst --
833Dai18405
AAAGAGTCGAGTTGTTTGGGAATGCAGCTCAAAATGGGTGGTAAATTCCA
HHB_10729_Meruliopsis_albostra
AAAGAGTCGAGTTGTTTGGGAATGCAGCTCAAAATGGGTGGTAAATTCCA
Cui6878_Ceriporia_pseudocystid
AAAGAGTCGAGTTGTTTGGGAATGCAGCTCAAAATGGGTGGTAAATTCCA
869Dai14737
AAAGAGTCGAGTTGTTTGGGAATGCAGCTCAAAATGGGTGGTAAATTCCA
876Cui11626
AAAGAGTCGAGTTGTTTGGGAATGCAGCTCAAAATGGGTGGTAAATTCCA
1199WEI3388 --
776308_Meruliopsis_cystidiata
AAAGAGTCGAGTTGTTTGGGAATGCAGCTCAAAATGGGTGGTAAATTCCA
ICN139059_Meruliopsis_cystidia
AAAGAGTCGAGTTGTTTGGGAATGCAGCTCAAAATGGGTGGTAAATTCCA
HHB15692Ceraceomyces_serpens
AAAGAGTCGAGTTGTTTGGGAATGCAGCTCAAAATGGGTGGTAAATTCCA
HHB_15629_Sp_Ceriporiopsis_ane
AAAGAGTCGAGTTGTTTGGGAATGCAGCTCAAAATGGGTGGTAAATTCCA
AJ185Trametopsis_cervina

AAAGAGTCGAGTTGTTTGGGAATGCAGCTCAAAATGGGTGGTAAATTCCA

FD9Irpex_lacteus

AAAGAGTCGAGTTGTTTGGGAATGCAGCTCAAAATGGGTGGTAAATTCCA

908Dai11230

AAAGAGTCGAGTTGTTTGGGAATGCAGCTCAAAATGGGTGGTAAATTCCA

FP55521TEmmia_lacerata

AAAGAGTCGAGTTGTTTGGGAATGCAGCTCAAAATGGGTGGTAAATTCCA

PBU0048Ceriporia_cystidiata

AAAGAGTCGAGTTGTTTGGGAATGCAGCTCAAAATGGGTGGTAAATTCCA

MZ340C_laceratаT --

Dai21940

AAAGAGTCGAGTTGTTTGGGAATGCAGCTCAAAATGGGTGGTAAATTCCA

847Dai16433

AAAGAGTCGAGTTGTTTGGGAATGCAGCTCAAAATGGGTGGTAAATTCCA

MarcinEmmia_latemarginatus

AAAGAGTCGAGTTGTTTGGGAATGCAGCTCAAAATGGGTGGTAAATTCCA

Meijer3729Hydnopolyporus_fimbr

AAAGAGTCGAGTTGTTTGGGAATGCAGCTCAAAATGGGTGGTAAATTCCA

RLG13408Phanerochaete_sp

AAAGAGTCGAGTTGTTTGGGAATGCAGCTCAAAATGGGTGGTAAATTCCA

WHC1381Flavodon_flavus

AAAGAGTCGAGTTGTTTGGGAATGCAGCTCAAAATGGGTGGTAAATTCCA

GB1833Phlebia_albida

AAAGAGTCGAGTTGTTTGGGAATGCAGCTCAAAATGGGTGGTAAATTCCA

T407Phlebia_nitidula --

HHB6988Phanerochaete_exilis

AAAGAGTCGAGTTGTTTGGGAATGCAGCTCAAAATGGGTGGTAAATTCCA

HHB8509Phanerochaetella_xeroph

AAAGAGTCGAGTTGTTTGGGAATGCAGCTCAAAATGGGTGGTAAATTCCA

PBU0051Macrohyporia_dictyopora

AAAGAGTCGAGTTGTTTGGGAATGCAGCTCAAAATGGGTGGTAAATTCCA

HHB11463Phanerochaete_sp

AAAGAGTCGAGTTGTTTGGGAATGCAGCTCAAAATGGGTGGTAAATTCCA

FP102382Byssomerulius_corium

AAAGAGTCGAGTTGTTTGGGAATGCAGCTCAAAATGGGTGGTAAATTCCA

FP102165Efibula_americana

AAAGAGTCGAGTTGTTTGGGAATGCAGCTCAAAATGGGTGGTAAATTCCA

Murdoch90Ceriporia_torpida

AAAGAGTCGAGTTGTTTGGGAATGCAGCTCAAAATGGGTGGTAAATTCCA

Rivoire4413_Ceriporia_purpurea

AAAGAGTCGAGTTGTTTGGGAATGCAGCTCAAAATGGGTGGTAAATTCCA

Kout_18_Ceriporia_triumphalis

AAAGAGTCGAGTTGTTTGGGAATGCAGCTCAAAATGGGTGGTAAATTCCA

Rivoire3701_Ceriporia_bresadol

AAAGAGTCGAGTTGTTTGGGAATGCAGCTCAAAATGGGTGGTAAATTCCA

VS4018 --

Ryvarden21832_Ceriporia_manzan

AAAGAGTCGAGTTGTTTGGGAATGCAGCTCAAAATGGGTGGTAAATTCCA

Dai24539

AAAGAGTCGAGTTGTTTGGGAATGCAGCTCAAAATGGGTGGTAAATTCCA

Dai24541

AAAGAGTCGAGTTGTTTGGGAATGCAGCTCAAAATGGGTGGTAAATTCCA

JV1105_12_Ceriporia_occidental

AAAGAGTCGAGTTGTTTGGGAATGCAGCTCAAAATGGGTGGTAAATTCCA

VS8558Ceriporia_occidentalis

AAAGAGTCGAGTTGTTTGGGAATGCAGCTCAAAATGGGTGGTAAATTCCA

Dai22445

AAAGAGTCGAGTTGTTTGGGAATGCAGCTCAAAATGGGTGGTAAATTCCA

846Dai16368

AAAGAGTCGAGTTGTTTGGGAATGCAGCTCAAAATGGGTGGTAAATTCCA

Dai17951_Ceriporia_aurantiocar

AAAGAGTCGAGTTGTTTGGGAATGCAGCTCAAAATGGGTGGTAAATTCCA

Miettinen_11701C_viridans

AAAGAGTCGAGTTGTTTGGGAATGCAGCTCAAAATGGGTGGTAAATTCCA

JV0105_10Ceriporia_aurantiocar

AAAGAGTCGAGTTGTTTGGGAATGCAGCTCAAAATGGGTGGTAAATTCCA

Yuan5702C_viridans --

858Dai17003 --

Yuan2747_Ceriporia_viridans --

Yuan2744C_viridans --

Li1046C_viridans --

865C_sinoviridans --

871Dai15062 --

Dai7642_Ceriporia_humilis --

Spirin4706_Ceriporia_humilis --

Spirin4944_Ceriporia_sericea

AAAGAGTCGAGTTGTTTGGGAATGCAGCTCAAAATGGGTGGTAAATTCCA

WCG1547Dai26044ceriporia

AAAGAGTCGAGTTGTTTGGGAATGCAGCTCAAAATGGGTGGTAAATTCCA

ZZW1558Dai27086

AAAGAGTCGAGTTGTTTGGGAATGCAGCTCAAAATGGGTGGTAAATTCCA

Miettinen14381_Ceriporia_mpuri

AAAGAGTCGAGTTGTTTGGGAATGCAGCTCAAAATGGGTGGTAAATTCCA

Miettinen15492_2_Ceriporia_sor

AAAGAGTCGAGTTGTTTGGGAATGCAGCTCAAAATGGGTGGTAAATTCCA

He6687

AAAGAGTCGAGTTGTTTGGGAATGCAGCTCAAAATGGGTGGTAAATTCCA

ZH53Dai24426

AAAGAGTCGAGTTGTTTGGGAATGCAGCTCAAAATGGGTGGTAAATTCCA

Vlasak0808_30_Ceriporia_punica

AAAGAGTCGAGTTGTTTGGGAATGCAGCTCAAAATGGGTGGTAAATTCCA

887Dai13376

AAAGAGTCGAGTTGTTTGGGAATGCAGCTCAAAATGGGTGGTAAATTCCA

WCG1443Dai24998

AAAGAGTCGAGTTGTTTGGGAATGCAGCTCAAAATGGGTGGTAAATTCCA

0108_6Ceriporia_spissa

AAAGAGTCGAGTTGTTTGGGAATGCAGCTCAAAATGGGTGGTAAATTCCA

Dai19164

AAAGAGTCGAGTTGTTTGGGAATGCAGCTCAAAATGGGTGGTAAATTCCA

Dai17937_Ceriporia_bubalinomar

AAAGAGTCGAGTTGTTTGGGAATGCAGCTCAAAATGGGTGGTAAATTCCA

903Dai12113

AAAGAGTCGAGTTGTTTGGGAATGCAGCTCAAAATGGGTGGTAAATTCCA

LZB929Dai25079

AAAGAGTCGAGTTGTTTGGGAATGCAGCTCAAAATGGGTGGTAAATTCCA

LX45Dai26988 --

LX43Dai26986

AAAGAGTCGAGTTGTTTGGGAATGCAGCTCAAAATGGGTGGTAAATTCCA

Dai7759Ceriporia --

Cui8012_Ceriporia_viridans --

GC1704_54Ceriporia_viridans

AAAGAGTCGAGTTGTTTGGGAATGCAGCTCAAAATGGGTGGTAAATTCCA

Dai23392

AAAGAGTCGAGTTGTTTGGGAATGCAGCTCAAAATGGGTGGTAAATTCCA

WCG1585Dai26113Ceriproia

AAAGAGTCGAGTTGTTTGGGAATGCAGCTCAAAATGGGTGGTAAATTCCA

Dai18675C_eucalypti

AAAGAGTCGAGTTGTTTGGGAATGCAGCTCAAAATGGGTGGTAAATTCCA

Dai22034

AAAGAGTCGAGTTGTTTGGGAATGCAGCTCAAAATGGGTGGTAAATTCCA

JV1008_41JTardaFLORIDAKeys --

Rivoire1161_Ceriporia_pierii --

Dai23499C_pierii

AAAGAGTCGAGTTGTTTGGGAATGCAGCTCAAAATGGGTGGTAAATTCCA

Dai23500

AAAGAGTCGAGTTGTTTGGGAATGCAGCTCAAAATGGGTGGTAAATTCCA

841Dai15899

AAAGAGTCGAGTTGTTTGGGAATGCAGCTCAAAATGGGTGGTAAATTCCA

842Dai15904 --

LZB1066xinjiang --

LZB1065xinjiang --

851Dai16779

AAAGAGTCGAGTTGTTTGGGAATGCAGCTCAAAATGGGTGGTAAATTCCA
RMJ119sp_Candelabrochaete_sept --
RLG9759spCandelabrochaete_sept
AAAGAGTCGAGTTGTTTGGGAATGCAGCTCAAAATGGGTGGTAAATTCCA
RLG10478Phanerochaete_allantos --
Dai19118_Ceriporia_spissa
AAAGAGTCGAGTTGTTTGGGAATGCAGCTCAAAATGGGTGGTAAATTCCA
Dai18486A
AAAGAGTCGAGTTGTTTGGGAATGCAGCTCAAAATGGGTGGTAAATTCCA
WEI17_024_Ceriporia_mellita
AAAGAGTCGAGTTGTTTGGGAATGCAGCTCAAAATGGGTGGTAAATTCCA
GC1508_71Ceriporia_mellita
AAAGAGTCGAGTTGTTTGGGAATGCAGCTCAAAATGGGTGGTAAATTCCA
GC1608_7_Ceriporia_mellita
AAAGAGTCGAGTTGTTTGGGAATGCAGCTCAAAATGGGTGGTAAATTCCA
ZZW1557Dai27085
AAAGAGTCGAGTTGTTTGGGAATGCAGCTCAAAATGGGTGGTAAATTCCA
ZZW1554Dai27083
AAAGAGTCGAGTTGTTTGGGAATGCAGCTCAAAATGGGTGGTAAATTCCA
Dai8168
AAAGAGTCGAGTTGTTTGGGAATGCAGCTCAAAATGGGTGGTAAATTCCA
BR4865C_mellita
AAAGAGTCGAGTTGTTTGGGAATGCAGCTCAAAATGGGTGGTAAATTCCA
MEL2382688Ceriporia_sp --
Dai8110
AAAGAGTCGAGTTGTTTGGGAATGCAGCTCAAAATGGGTGGTAAATTCCA
Cui8097 --
909Cui6740 --
W1258Dai24695
AAAGAGTCGAGTTGTTTGGGAATGCAGCTCAAAATGGGTGGTAAATTCCA
JV0110_26_Ceriporia_griseoviol
AAAGAGTCGAGTTGTTTGGGAATGCAGCTCAAAACGGGTGGTAAATTCCA
896Dai13202
AAAGAGTCGAGTTGTTTGGGAATGCAGCTCAAAACGGGTGGTAAATTCCA
LWY393Dai27053C_griseoviolasce
AAAGAGTCGAGTTGTTTGGGAATGCAGCTCAAAACGGGTGGTAAATTCCA
LWY394DAI27054
AAAGAGTCGAGTTGTTTGGGAATGCAGCTCAAAACGGGTGGTAAATTCCA
FP135015G_pannocinctus
AAAGAGTCGAGTTGTTTGGGAATGCAGCTCAAAATGGGTGGTAAATTCCA
L15726SpG_pannocinctus
AAAGAGTCGAGTTGTTTGGGAATGCAGCTCAAAATGGGTGGTAAATTCCA
Dai22221
AAAGAGTCGAGTTGTTTGGGAATGCAGCTCAAAATGGGTGGTAAATTCCA

Dai22633
AAAGAGTCGAGTTGTTTGGGAATGCAGCTCAAAATGGGTGGTAAATTCCA
Dai23260
AAAGAGTCGAGTTGTTTGGGAATGCAGCTCAAAATGGGTGGTAAATTCCA
Dai23626
AAAGAGTCGAGTTGTTTGGGAATGCAGCTCAAAATGGGTGGTAAATTCCA
Dai16238G_citrinoalbus
AAAGAGTCGAGTTGTTTGGGAATGCAGCTCAAAATGGGTGGTAAATTCCA
1175Dai15293
AAAGAGTCGAGTTGTTTGGGAATGCAGCTCAAAATGGGTGGTAAATTCCA
Dai19547
AAAGAGTCGAGTTGTTTGGGAATGCAGCTCAAAATGGGTGGTAAATTCCA
918063G_africanus
AAAGAGTCGAGTTGTTTGGGAATGCAGCTCAAAATGGGTGGTAAATTCCA
918572G_africanus
AAAGAGTCGAGTTGTTTGGGAATGCAGCTCAAAATGGGTGGTAAATTCCA
Dai18536A --
1164Cui17922
AAAGAGTCGAGTTGTTTGGGAATGCAGCTCAAAATGGGTGGTAAATTCCA
Dai22225
AAAGAGTCGAGTTGTTTGGGAATGCAGCTCAAAATGGGTGGTAAATTCCA
1163Dai20655
AAAGAGTCGAGTTGTTTGGGAATGCAGCTCAAAATGGGTGGTAAATTCCA
Yuan4397G_hainanensis
AAAGAGTCGAGTTGTTTGGGAATGCAGCTCAAAATGGGTGGTAAATTCCA
1176Dai15268
AAAGAGTCGAGTTGTTTGGGAATGCAGCTCAAAATGGGTGGTAAATTCCA
1177Dai15259
AAAGAGTCGAGTTGTTTGGGAATGCAGCTCAAAATGGGTGGTAAATTCCA
BZ2896G_thelephoroides
AAAGAGTCGAGTTGTTTGGGAATGCAGCTCAAAATGGGTGGTAAATTCCA
1166JV1808_26
AAAGAGTCGAGTTGTTTGGGAATGCAGCTCAAAATGGGTGGTAAATTCCA
Miettinen16992Hapalopilus_ochr
AAAGAGTCGAGTTGTTTGGGAATGCAGCTCAAAATGGGTGGTAGATTCCA
GC1708_338_Ceriporia_arbuscula
AAAGAGTCGAGTTGTTTGGGAATGCAGCTCAAAATGGGTGGTAAATTCCA
WCG1555Dai26107Ceriporia
AAAGAGTCGAGTTGTTTGGGAATGCAGCTCAAAATGGGTGGTAAATTCCA
GC1708_340_Ceriporia_arbuscula
AAAGAGTCGAGTTGTTTGGGAATGCAGCTCAAAATGGGTGGTAAATTCCA
WCG1556Dai26109Ceriporia
AAAGAGTCGAGTTGTTTGGGAATGCAGCTCAAAATGGGTGGTAAATTCCA
883Cui11291

AAAGAGTCGAGTTGTTTGGGAATGCAGCTCAAAATGGGTGGTAAATTCCA

HLX320Dai26805

AAAGAGTCGAGTTGTTTGGGAATGCAGCTCAAAATGGGTGGTAAATTCCA

WCG1266Dai24678A

AAAGAGTCGAGTTGTTTGGGAATGCAGCTCAAAATGGGTGGTAAATTCCA

Dai6090_Ceriporia_sulphuricolo

AAAGAGTCGAGTTGTTTGGGAATGCAGCTCAAAATGGGTGGTAAATTCCA

RLG_11354_Ceriproia_reticulata

AAAGAGTCGAGTTGTTTGGGAATGCAGCTCAAAATGGGTGGTAAATTCCA

ZZW1543Dai27072

AAAGAGTCGAGTTGTTTGGGAATGCAGCTCAAAATGGGTGGTAAATTCCA

Li1316_Ceriporia_reticulata --

KHL11981Ceriporia_reticulata

AAAGAGTCGAGTTGTTTGGGAATGCAGCTCAAAATGGGTGGTAAATTCCA

FP110343sp_Candelabrochaete_la

AAAGAGTCGAGTTGTTTGGGAATGCAGCTCAAAATGGGTGGTAAATTCCA

Li1045_Ceriporia_reticulata --

ZX136Dai25794ceriporia

AAAGAGTCGAGTTGTTTGGGAATGCAGCTCAAAATGGGTGGTAAATTCCA

892Dai13400

AAAGAGTCGAGTTGTTTGGGAATGCAGCTCAAAATGGGTGGTAAATTCCA

RLG7163Leptoporus_mollis

AAAGAGTCGAGTTGTTTGGGAATGCAGCTCAAAATGGGTGGTAAATTCCA

Dai21062Leptoporus_mollis

AAAGAGTCGAGTTGTTTGGGAATGCAGCTCAAAATGGGTGGTAAATTCCA

Dai20182Leptoporus_submollis

AAAGAGTCGAGTTGTTTGGGAATGCAGCTCAAAATGGGTGGTAAATTCCA

Cui18379Leptoporus_submollis

AAAGAGTCGAGTTGTTTGGGAATGCAGCTCAAAATGGGTGGTAAATTCCA

Wu1209_46Resiniporus_pseudogil

AAAGAGTCGAGTTGTTTGGGAATGCAGCTCAAAATGGGTGGTAAATTCCA

BRNM710169Resiniporus_resinasc

AAAGAGTCGAGTTGTTTGGGAATGCAGCTCAAAATGGGTGGTAAATTCCA

Dai14516Bjerkandera_adusta

GATGAGTCGAGTTGTTTGGGAATGCAGCTCAAAATGGGTGGTAAATTCCA

Dai21100Bjerkandera_fumosa

GACGAGTCGAGTTGTTTGGGAATGCAGCTCAAAATGGGTGGTAAATTCCA

Miettinen16854Ceraceomyces_sp

AAAGAGTCGAGTTGTTTGGGAATGCAGCTCAAAATGGGTGGTAAATTCCA

Dai10477C_spissa

AAAGAGTCGAGTTGTTTGGGAATGCAGCTCAAAATGGGTGGTAAATTCCA

855Dai16831

AAAGAGTCGAGTTGTTTGGGAATGCAGCTCAAAATGGGTGGTAAATTCCA

882Cui11282

AAAGAGTCGAGTTGTTTGGGAATGCAGCTCAAAATGGGTGGTAAATTCCA
Dai24566
AAAGAGTCGAGTTGTTTGGGAATGCAGCTCAAAATGGGTGGTAAATTCCA
Yuan5965
AAAGAGTCGAGTTGTTTGGGAATGCAGCTCAAAATGGGTGGTAAATTCCA
Dai3204
AAAGAGTCGAGTTGTTTGGGAATGCAGCTCAAAATGGGTGGTAAATTCCA
1194CUI9985
AAAGAGTCGAGTTGTTTGGGAATGCAGCTCAAAATGGGTGGTAAATTCCA

Dai15205_Ceriporia_albomellea
TCTAAAGCTAAATATTGGCGAGAGACCGATAGCGAACAAGTACCGTGAGG
Dai15223_Ceriporia_albomellea
TCTAAAGCTAAATATTGGCGAGAGACCGATAGCGAACAAGTACCGTGAGG
Li1780_Ceriporia_variegata
TCTAAAGCTAAATATTGGCGAGAGACCGATAGCGAACAAGTACCGTGAGG
Dai19791_Ceriporia_variegata
TCTAAAGCTAAATATTGGCGAGAGACCGATAGCGAACAAGTACCGTGAGG
Dai19886
TCTAAAGCTAAATATTGGCGAGAGACCGATAGCGAACAAGTACCGTGAGG
Dai10833_Ceriporia_crassitunic
TCTAAAGCTAAATATTGGCGAGAGACCGATAGCGAACAAGTACCGTGAGG
CHWC1506_46Meruliopsis_crassit
TCTAAAGCTAAATATTGGCGAGAGACCGATAGCGAACAAGTACCGTGAGG
Dai9995_Ceriporia_crassitunica --
Wu1209_58_Meruliopsis_parvispo
TCTAAAGCTAAATATTGGCGAGAGACCGATAGCGAACAAGTACCGTGAGG
CHWC1505_129_Meruliopsis_parvi
TCTAAAGCTAAATATTGGCGAGAGACCGATAGCGAACAAGTACCGTGAGG
Dai21944
TCTAAAGCTAAATATTGGCGAGAGACCGATAGCGAACAAGTACCGTGAGG
830Dai18640A
TCTAAAGCTAAATATTGGCGAGAGACCGATAGCGAACAAGTACCGTGAGG
GC1704_60_Meruliopsis_taxicola
TCTAAAGCTAAATATTGGCGAGAGACCGATAGCGAACAAGTACCGTGAGG
Dai22625
TCTAAAGCTAAATATTGGCGAGAGACCGATAGCGAACAAGTACCGTGAGG
Dai22636
TCTAAAGCTAAATATTGGCGAGAGACCGATAGCGAACAAGTACCGTGAGG
Dai21878
TCTAAAGCTAAATATTGGCGAGAGACCGATAGCGAACAAGTACCGTGAGG
1169Dai17248
TCTAAAGCTAAATATTGGCGAGAGACCGATAGCGAACAAGTACCGTGAGG

Wu1708_43_Meruliopsis_leptocys
TCTAAAGCTAAATATTGGCGAGAGACCGATAGCGAACAAGTACCGTGAGG
Li1011
TCTAAAGCTAAATATTGGCGAGAGACCGATAGCGAACAAGTACCGTGAGG
ZX95Dai25742Meruliopsis_leptoc
TCTAAAGCTAAATATTGGCGAGAGACCGATAGCGAACAAGTACCGTGAGG
WCG1306Dai24733
TCTAAAGCTAAATATTGGCGAGAGACCGATAGCGAACAAGTACCGTGAGG
LXL99Dai25816
TCTAAAGCTAAATATTGGCGAGAGACCGATAGCGAACAAGTACCGTGAGG
WCG1559Dai26052Meruliopsis
TCTAAAGCTAAATATTGGCGAGAGACCGATAGCGAACAAGTACCGTGAGG
He7477
TCTAAAGCTAAATATTGGCGAGAGACCGATAGCGAACAAGTACCGTGAGG
HLX243Dai26217
TCTAAAGCTAAATATTGGCGAGAGACCGATAGCGAACAAGTACCGTGAGG
RussiaMW673659Meruliopsis_fagi
TCTAAAGCTAAATATTGGCGAGAGACCGATAGCGAACAAGTACCGTGAGG
FD278
TCTAAAGCTAAATATTGGCGAGAGACCGATAGCGAACAAGTACCGTGAGG
Dai10226_Ceriporia_tarda --
LE247365
TCTAAAGCTAAATATTGGCGAGAGACCGATAGCGAACAAGTACCGTGAGG
Dai8173_Meruliopsis_nanlingens
TCTAAAGCTAAATATTGGCGAGAGACCGATAGCGAACAAGTACCGTGAGG
860Dai17172
TCTAAAGCTAAATATTGGCGAGAGACCGATAGCGAACAAGTACCGTGAGG
879Dai13414
TCTAAAGCTAAATATTGGCGAGAGACCGATAGCGAACAAGTACCGTGAGG
Li_1704_Meruliopsis_pseudocyst --
833Dai18405
TCTAAAGCTAAATATTGGCGAGAGACCGATAGCGAACAAGTACCGTGAGG
HHB_10729_Meruliopsis_albostra
TCTAAAGCTAAATATTGGCGAGAGACCGATAGCGAACAAGTACCGTGAGG
Cui6878_Ceriporia_pseudocystid
TCTAAAGCTAAATATTGGCGAGAGACCGATAGCGAACAAGTACCGTGAGG
869Dai14737
TCTAAAGCTAAATATTGGCGAGAGACCGATAGCGAACAAGTACCGTGAGG
876Cui11626
TCTAAAGCTAAATATTGGCGAGAGACCGATAGCGAACAAGTACCGTGAGG
1199WEI3388 --
776308_Meruliopsis_cystidiata
TCTAAAGCTAAATATTGGCGAGAGACCGATAGCGAACAAGTACCGTGAGG
ICN139059_Meruliopsis_cystidia

TCTAAAGCTAAATATTGGCGAGAGACCGATAGCGAACAAGTACCGTGAGG

HHB15692Ceraceomyces_serpens

TCTAAAGCTAAATATTGGCGAGAGACCGATAGCGAACAAGTACCGTGAGG

HHB_15629_Sp_Ceriporiopsis_ane

TCTAAAGCTAAATATTGGCGAGAGACCGATAGCGAACAAGTACCGTGAGG

AJ185Trametopsis_cervina

TCTAAAGCTAAATATTGGCGAGAGACCGATAGCGAACAAGTACCGTGAGG

FD9Irpex_lacteus

TCTAAAGCTAAATATTGGCGAGAGACCGATAGCGAACAAGTACCGTGAGG

908Dai11230

TCTAAAGCTAAATATTGGCGAGAGACCGATAGCGAACAAGTACCGTGAGG

FP55521TEmmia_lacerata

TCTAAAGCTAAATATTGGCGAGAGACCGATAGCGAACAAGTACCGTGAGG

PBU0048Ceriporia_cystidiata

TCTAAAGCTAAATATTGGCGAGAGACCGATAGCGAACAAGTACCGTGAGG

MZ340C_laceratаT --

Dai21940

TCTAAAGCTAAATATTGGCGAGAGACCGATAGCGAACAAGTACCGTGAGG

847Dai16433

TCTAAAGCTAAATATTGGCGAGAGACCGATAGCGAACAAGTACCGTGAGG

MarcinEmmia_latemarginatus

TCTAAAGCTAAATATTGGCGAGAGACCGATAGCGAACAAGTACCGTGAGG

Meijer3729Hydnopolyporus_fimbr

TCTAAAGCTAAATATTGGCGAGAGACCGATAGCGAACAAGTACCGTGAGG

RLG13408Phanerochaete_sp

TCTAAAGCTAAATATTGGCGAGAGACCGATAGCGAACAAGTACCGTGAGG

WHC1381Flavodon_flavus

TCTAAAGCTAAATATTGGCGAGAGACCGATAGCGAACAAGTACCGTGAGG

GB1833Phlebia_albida

TCTAAAGCTAAATATTGGCGAGAGACCGATAGCGAACAAGTACCGTGAGG

T407Phlebia_nitidula --

HHB6988Phanerochaete_exilis

TCTAAAGCTAAATATTGGCGAGAGACCGATAGCGAACAAGTACCGTGAGG

HHB8509Phanerochaetella_xeroph

TCTAAAGCTAAATATTGGCGAGAGACCGATAGCGAACAAGTACCGTGAGG

PBU0051Macrohyporia_dictyopora

TCTAAAGCTAAATATTGGCGAGAGACCGATAGCGAACAAGTACCGTGAGG

HHB11463Phanerochaete_sp

TCTAAAGCTAAATATTGGCGAGAGACCGATAGCGAACAAGTACCGTGAGG

FP102382Byssomerulius_corium

TCTAAAGCTAAATATTGGCGAGAGACCGATAGCGAACAAGTACCGTGAGG

FP102165Efibula_americana

TCTAAAGCTAAATATTGGCGAGAGACCGATAGCGAACAAGTACCGTGAGG

Murdoch90Ceriporia_torpida

TCTAAAGCTAAATATTGGCGAGAGACCGATAGCGAACAAGTACCGTGAGG
Rivoire4413_Ceriporia_purpurea
TCTAAAGCTAAATATTGGCGAGAGACCGATAGCGAACAAGTACCGTGAGG
Kout_18_Ceriporia_triumphalis
TCTAAAGCTAAATATTGGCGAGAGACCGATAGCGAACAAGTACCGTGAGG
Rivoire3701_Ceriporia_bresadol
TCTAAAGCTAAATATTGGCGAGAGACCGATAGCGAACAAGTACCGTGAGG
VS4018 --
Ryvarden21832_Ceriporia_manzan
TCTAAAGCTAAATATTGGCGAGAGACCGATAGCGAACAAGTACCGTGAGG
Dai24539
TCTAAAGCTAAATATTGGCGAGAGACCGATAGCGAACAAGTACCGTGAGG
Dai24541
TCTAAAGCTAAATATTGGCGAGAGACCGATAGCGAACAAGTACCGTGAGG
JV1105_12_Ceriporia_occidental
TCTAAAGCTAAATATTGGCGAGAGACCGATAGCGAACAAGTACCGTGAGG
VS8558Ceriporia_occidentalis
TCTAAAGCTAAATATTGGCGAGAGACCGATAGCGAACAAGTACCGTGAGG
Dai22445
TCTAAAGCTAAATATTGGCGAGAGACCGATAGCGAACAAGTACCGTGAGG
846Dai16368
TCTAAAGCTAAATATTGGCGAGAGACCGATAGCGAACAAGTACCGTGAGG
Dai17951_Ceriporia_aurantiocar
TCTAAAGCTAAATATTGGCGAGAGACCGATAGCGAACAAGTACCGTGAGG
Miettinen_11701C_viridans
TCTAAAGCTAAATATTGGCGAGAGACCGATAGCGAACAAGTACCGTGAGG
JV0105_10Ceriporia_aurantiocar
TCTAAAGCTAAATATTGGCGAGAGACCGATAGCGAACAAGTACCGTGAGG
Yuan5702C_viridans --
858Dai17003 --
Yuan2747_Ceriporia_viridans --
Yuan2744C_viridans --
Li1046C_viridans --
865C_sinoviridans --
871Dai15062 --
Dai7642_Ceriporia_humilis --
Spirin4706_Ceriporia_humilis --
Spirin4944_Ceriporia_sericea
TCTAAAGCTAAATATTGGCGAGAGACCGATAGCGAACAAGTACCGTGAGG
WCG1547Dai26044ceriporia
TCTAAAGCTAAATATTGGCGAGAGACCGATAGCGAACAAGTACCGTGAGG
ZZW1558Dai27086
TCTAAAGCTAAATATTGGCGAGAGACCGATAGCGAACAAGTACCGTGAGG
Miettinen14381_Ceriporia_mpuri

TCTAAAGCTAAATATTGGCGAGAGACCGATAGCGAACAAGTACCGTGAGG
Miettinen15492_2_Ceriporia_sor
TCTAAAGCTAAATATTGGCGAGAGACCGATAGCGAACAAGTACCGTGAGG
He6687
TCTAAAGCTAAATATTGGCGAGAGACCGATAGCGAACAAGTACCGTGAGG
ZH53Dai24426
TCTAAAGCTAAATATTGGCGAGAGACCGATAGCGAACAAGTACCGTGAGG
Vlasak0808_30_Ceriporia_punica
TCTAAAGCTAAATATTGGCGAGAGACCGATAGCGAACAAGTACCGTGAGG
887Dai13376
TCTAAAGCTAAATATTGGCGAGAGACCGATAGCGAACAAGTACCGTGAGG
WCG1443Dai24998
TCTAAAGCTAAATATTGGCGAGAGACCGATAGCGAACAAGTACCGTGAGG
0108_6Ceriporia_spissa
TCTAAAGCTAAATATTGGCGAGAGACCGATAGCGAACAAGTACCGTGAGG
Dai19164
TCTAAAGCTAAATATTGGCGAGAGACCGATAGCGAACAAGTACCGTGAGG
Dai17937_Ceriporia_bubalinomar
TCTAAAGCTAAATATTGGCGAGAGACCGATAGCGAACAAGTACCGTGAGG
903Dai12113
TCTAAAGCTAAATATTGGCGAGAGACCGATAGCGAACAAGTACCGTGAGG
LZB929Dai25079
TCTAAAGCTAAATATTGGCGAGAGACCGATAGCGAACAAGTACCGTGAGG
LX45Dai26988 --
LX43Dai26986
TCTAAAGCTAAATATTGGCGAGAGACCGATAGCGAACAAGTACCGTGAGG
Dai7759Ceriporia --
Cui8012_Ceriporia_viridans --
GC1704_54Ceriporia_viridans
TCTAAAGCTAAATATTGGCGAGAGACCGATAGCGAACAAGTACCGTGAGG
Dai23392
TCTAAAGCTAAATATTGGCGAGAGACCGATAGCGAACAAGTACCGTGAGG
WCG1585Dai26113Ceriproia
TCTAAAGCTAAATATTGGCGAGAGACCGATAGCGAACAAGTACCGTGAGG
Dai18675C_eucalypti
TCTAAAGCTAAATATTGGCGAGAGACCGATAGCGAACAAGTACCGTGAGG
Dai22034
TCTAAAGCTAAATATTGGCGAGAGACCGATAGCGAACAAGTACCGTGAGG
JV1008_41JTardaFLORIDAKeys --
Rivoire1161_Ceriporia_pierii --
Dai23499C_pierii
TCTAAAGCTAAATATTGGCGAGAGACCGATAGCGAACAAGTACCGTGAGG
Dai23500
TCTAAAGCTAAATATTGGCGAGAGACCGATAGCGAACAAGTACCGTGAGG

841Dai15899
TCTAAAGCTAAATATTGGCGAGAGACCGATAGCGAACAAGTACCGTGAGG
842Dai15904 --
LZB1066xinjiang --
LZB1065xinjiang --
851Dai16779
TCTAAAGCTAAATATTGGCGAGAGACCGATAGCGAACAAGTACCGTGAGG
RMJ119sp_Candelabrochaete_sept --
RLG9759spCandelabrochaete_sept
TCTAAAGCTAAATATTGGCGAGAGACCGATAGCGAACAAGTACCGTTAGG
RLG10478Phanerochaete_allantos --
Dai19118_Ceriporia_spissa
TCTAAAGCTAAATATTGGCGAGAGACCGATAGCGAACAAGTACCGTGAGG
Dai18486A
TCTAAAGCTAAATATTGGCGAGAGACCGATAGCGAACAAGTACCGTGAGG
WEI17_024_Ceriporia_mellita
TCTAAAGCTAAATATTGGCGAGAGACCGATAGCGAACAAGTACCGTGAGG
GC1508_71Ceriporia_mellita
TCTAAAGCTAAATATTGGCGAGAGACCGATAGCGAACAAGTACCGTGAGG
GC1608_7_Ceriporia_mellita
TCTAAAGCTAAATATTGGCGAGAGACCGATAGCGAACAAGTACCGTGAGG
ZZW1557Dai27085
TCTAAAGCTAAATATTGGCGAGAGACCGATAGCGAACAAGTACCGTGAGG
ZZW1554Dai27083
TCTAAAGCTAAATATTGGCGAGAGACCGATAGCGAACAAGTACCGTGAGG
Dai8168
TCTAAAGCTAAATATTGGCGAGAGACCGATAGCGAACAAGTACCGTGAGG
BR4865C_mellita
TCTAAAGCTAAATATTGGCGAGAGACCGATAGCGAACAAGTACCGTGAGG
MEL2382688Ceriporia_sp --
Dai8110
TCTAAAGCTAAATATTGGCGAGAGACCGATAGCGAACAAGTACCGTGAGG
Cui8097 --
909Cui6740 --
W1258Dai24695
TCTAAAGCTAAATATTGGCGAGAGACCGATAGCGAACAAGTACCGTGAGG
JV0110_26_Ceriporia_griseoviol
TCTAAAGCTAAATATTGGCGAGAGACCGATAGCGAACAAGTACCGTGAGG
896Dai13202
TCTAAAGCTAAATATTGGCGAGAGACCGATAGCGAACAAGTACCGTGAGG
LWY393Dai27053C_griseoviolasce
TCTAAAGCTAAATATTGGCGAGAGACCGATAGCGAACAAGTACCGTGAGG
LWY394DAI27054
TCTAAAGCTAAATATTGGCGAGAGACCGATAGCGAACAAGTACCGTGAGG

FP135015G_pannocinctus
TCTAAAGCTAAATATTGGCGAGAGACCGATAGCGAACAAGTACCGTGAGG
L15726SpG_pannocinctus
TCTAAAGCTAAATATTGGCGAGAGACCGATAGCGAACAAGTACCGTGAGG
Dai22221
TCTAAAGCTAAATATTGGCGAGAGACCGATAGCGAACAAGTACCGTGAGG
Dai22633
TCTAAAGCTAAATATTGGCGAGAGACCGATAGCGAACAAGTACCGTGAGG
Dai23260
TCTAAAGCTAAATATTGGCGAGAGACCGATAGCGAACAAGTACCGTGAGG
Dai23626
TCTAAAGCTAAATATTGGCGAGAGACCGATAGCGAACAAGTACCGTGAGG
Dai16238G_citrinoalbus
TCTAAAGCTAAATATTGGCGAGAGACGGATAGCGA-----CAGTACGTGA
1175Dai15293
TCTAAAGCTAAATATTGGCGAGAGACCGATAGCGAACAAGTACCGTGAGG
Dai19547
TCTAAAGCTAAATATTGGCGAGAGACCGATAGCGAACAAGTACCGTGAGG
918063G_africanus
TCTAAAGCTAAATATTGGCGAGAGACCGATAGCGAACAAGTACCGTGAGG
918572G_africanus
TCTAAAGCTAAATATTGGCGAGAGACCGATAGCGAACAAGTACCGTGAGG
Dai18536A --
1164Cui17922
TCTAAAGCTAAATATTGGCGAGAGACCGATAGCGAACAAGTACCGTGAGG
Dai22225
TCTAAAGCTAAATATTGGCGAGAGACCGATAGCGAACAAGTACCGTGAGG
1163Dai20655
TCTAAAGCTAAATATTGGCGAGAGACCGATAGCGAACAAGTACCGTGAGG
Yuan4397G_hainanensis
TCTAAAGCTAAATATTGGCGAGAGACCGATAGCGAACAAGTACCGTGAGG
1176Dai15268
TCTAAAGCTAAATATTGGCGAGAGACCGATAGCGAACAAGTACCGTGAGG
1177Dai15259
TCTAAAGCTAAATATTGGCGAGAGACCGATAGCGAACAAGTACCGTGAGG
BZ2896G_thelephoroides
TCTAAAGCTAAATATTGGCGAGAGACCGATAGCGAACAAGTACCGTGAGG
1166JV1808_26
TCTAAAGCTAAATATTGGCGAGAGACCGATAGCGAACAAGTACCGTGAGG
Miettinen16992Hapalopilus_ochr
TCTAAAGCTAAATATTGGCGAGAGACCGATAGCGAACAAGTACCGTGAGG
GC1708_338_Ceriporia_arbuscula
TCTAAAGCTAAATATTGGCGAGAGACCGATAGCGAACAAGTACCGTGAGG
WCG1555Dai26107Ceriporia

TCTAAAGCTAAATATTGGCGAGAGACCGATAGCGAACAAGTACCGTGAGG

GC1708_340_Ceriporia_arbuscula

TCTAAAGCTAAATATTGGCGAGAGACCGATAGCGAACAAGTACCGTGAGG

WCG1556Dai26109Ceriporia

TCTAAAGCTAAATATTGGCGAGAGACCGATAGCGAACAAGTACCGTGAGG

883Cui11291

TCTAAAGCTAAATATTGGCGAGAGACCGATAGCGAACAAGTACCGTGAGG

HLX320Dai26805

TCTAAAGCTAAATATTGGCGAGAGACCGATAGCGAACAAGTACCGTGAGG

WCG1266Dai24678A

TCTAAAGCTAAATATTGGCGAGAGACCGATAGCGAACAAGTACCGTGAGG

Dai6090_Ceriporia_sulphuricolo

TCTAAAGCTAAATATTGGCGAGAGACCGATAGCGAACAAGTACCGTGAGG

RLG_11354_Ceriproia_reticulata

TCTAAAGCTAAATATTGGCGAGAGACCGATAGCGAACAAGTACCGTGAGG

ZZW1543Dai27072

TCTAAAGCTAAATATTGGCGAGAGACCGATAGCGAACAAGTACCGTGAGG

Li1316_Ceriporia_reticulata --

KHL11981Ceriporia_reticulata

TCTAAAGCTAAATATTGGCGAGAGACCGATAGCGAACAAGTACCGTGAGG

FP110343sp_Candelabrochaete_la

TCTAAAGCTAAATATTGGCGAGAGACCGATAGCGAACAAGTACCGTGAGG

Li1045_Ceriporia_reticulata --

ZX136Dai25794ceriporia

TCTAAAGCTAAATATTGGCGAGAGACCGATAGCGAACAAGTACCGTGAGG

892Dai13400

TCTAAAGCTAAATATTGGCGAGAGACCGATAGCGAACAAGTACCGTGAGG

RLG7163Leptoporus_mollis

TCTAAAGCTAAATATTGGCGAGAGACCGATAGCGAACAAGTACCGTGAGG

Dai21062Leptoporus_mollis

TCTAAAGCTAAATATTGGCGAGAGACCGATAGCGAACAAGTACCGTGAGG

Dai20182Leptoporus_submollis

TCTAAAGCTAAATATTGGCGAGAGACCGATAGCGAACAAGTACCGTGAGG

Cui18379Leptoporus_submollis

TCTAAAGCTAAATATTGGCGAGAGACCGATAGCGAACAAGTACCGTGAGG

Wu1209_46Resiniporus_pseudogil

TCTAAAGCTAAATATTGGCGAGAGACCGATAGCGAACAAGTACCGTGAGG

BRNM710169Resiniporus_resinasc

TCTAAAGCTAAATATTGGCGAGAGACCGATAGCGAACAAGTACCGTGAGG

Dai14516Bjerkandera_adusta

TCTAAAGCTAAATATTGGCGAGAGACCGATAGCGAACAAGTACCGTGAGG

Dai21100Bjerkandera_fumosa

TCTAAAGCTAAATATTGGCGAGAGACCGATAGCGAACAAGTACCGTGAGG

Miettinen16854Ceraceomyces_sp

TCTAAAGCTAAATATTGGCGAGAGACCGATAGCGAACAAGTACCGTGAGG

Dai10477C_spissa

TCTAAAGCTAAATATTGGCGAGAGACCGATAGCGAACAAGTACCGTGAGG

855Dai16831

TCTAAAGCTAAATATTGGCGAGAGACCGATAGCGAACAAGTACCGTGAGG

882Cui11282

TCTAAAGCTAAATATTGGCGAGAGACCGATAGCGAACAAGTACCGTGAGG

Dai24566

TCTAAAGCTAAATATTGGCGAGAGACCGATAGCGAACAAGTACCGTGAGG

Yuan5965

TCTAAAGCTAAATATTGGCGAGAGACCGATAGCGAACAAGTACCGTGAGG

Dai3204

TCTAAAGCTAAATATTGGCGAGAGACCGATAGCGAACAAGTACCGTGAGG

1194CUI9985

TCTAAAGCTAAATATTGGCGAGAGACCGATAGCGAACAAGTACCGGGAGG

Dai15205_Ceriporia_albomellea GAAAGATGAAAAGCACTTTGGAAAGAGAG-TAAACAGTACGTGAAATTGC

Dai15223_Ceriporia_albomellea

GAAAGATGAAAAGCACTTTGGAAAGAGAGTTAAACAGTACGTGAAATTGC

Li1780_Ceriporia_variegata

GAAAGATGAAAAGCACTTTGGAAAGAGAGTTAAACAGTACGTGAAATTGC

Dai19791_Ceriporia_variegata

GAAAGATGAAAAGCACTTTGGAAAGAGAGTTAAACAGTACGTGAAATTGC

Dai19886

GAAAGATGAAAAGCACTTTGGAAAGAGAGTTAAACAGTACGTGAAATTGC

Dai10833_Ceriporia_crassitunic

GAAAGATGAAAAGCACTTTGGAAAGAGAGTTAAACAGTACGTGAAATTGC

CHWC1506_46Meruliopsis_crassit

GAAAGATGAAAAGCACTTTGGAAAGAGAGTTAAACAGTACGTGAAATTGC

Dai9995_Ceriporia_crassitunica --

Wu1209_58_Meruliopsis_parvispo

GAAAGATGAAAAGCACTTTGGAAAGAGAGTTAAACAGTACGTGAAATTGC

CHWC1505_129_Meruliopsis_parvi

GAAAGATGAAAAGCACTTTGGAAAGAGAGTTAAACAGTACGTGAAATTGC

Dai21944

GAAAGATGAAAAGCACTTTGGAAAGAGAGTTAAACAGTACGTGAAATTGC

830Dai18640A

GAAAGATGAAAAGCACTTTGGAAAGAGAGTTAAACAGTACGTGAAATTGC

GC1704_60_Meruliopsis_taxicola

GAAAGATGAAAAGCACTTTGGAAAGAGAGTTAAACAGTACGTGAAATTGC

Dai22625

GAAAGATGAAAAGCACTTTGGAAAGAGAGTTAAACAGTACGTGAAATTGC

Dai22636
GAAAGATGAAAAGCACTTTGGAAAGAGAGTTAAACAGTACGTGAAATTGC
Dai21878
GAAAGATGAAAAGCACTTTGGAAAGAGAGTTAAACAGTACGTGAAATTGC
1169Dai17248
GAAAGATGAAAAGCACTTTGGAAAGAGAGTTAAACAGTACGTGAAATTGC
Wu1708_43_Meruliopsis_leptocys
GAAAGATGAAAAGCACTTTGGAAAGAGAGTTAAACAGTACGTGAAATTGC
Li1011
GAAAGATGAAAAGCACTTTGGAAAGAGAGTTAAACAGTACGTGAAATTGC
ZX95Dai25742Meruliopsis_leptoc
GAAAGATGAAAAGCACTTTGGAAAGAGAGTTAAACAGTACGTGAAATTGC
WCG1306Dai24733
GAAAGATGAAAAGCACTTTGGAAAGAGAGTTAAACAGTACGTGAAATTGC
LXL99Dai25816
GAAAGATGAAAAGCACTTTGGAAAGAGAGTTAAACAGTACGTGAAATTGC
WCG1559Dai26052Meruliopsis
GAAAGATGAAAAGCACTTTGGAAAGAGAGTTAAACAGTACGTGAAATTGC
He7477
GAAAGATGAAAAGCACTTTGGAAAGAGAGTTAAACAGTACGTGAAATTGC
HLX243Dai26217
GAAAGATGAAAAGCACTTTGGAAAGAGAGTTAAACAGTACGTGAAATTGC
RussiaMW673659Meruliopsis_fagi
GAAAGATGAAAAGCACTTTGGAAAGAGAGTTAAACAGTACGTGAAATTGC
FD278
GAAAGATGAAAAGCACTTTGGAAAGAGAGTTAAACAGTACGTGAAATTGC
Dai10226_Ceriporia_tarda --
LE247365
GAAAGATGAAAAGCACTTTGGAAAGAGAGTTAAACAGTACGTGAAATTGC
Dai8173_Meruliopsis_nanlingens
GAAAGATGAAAAGCACTTTGGAAAGAGAGTTAAACAGTACGTGAAATTGC
860Dai17172
GAAAGATGAAAAGCACTTTGGAAAGAGAGTTAAACAGTACGTGAAATTGC
879Dai13414
GAAAGATGAAAAGCACTTTGGAAAGAGAGTTAAACAGTACGTGAAATTGC
Li_1704_Meruliopsis_pseudocyst --
833Dai18405
GAAAGATGAAAAGCACTTTGGAAAGAGAGTTAAACAGTACGTGAAATTGC
HHB_10729_Meruliopsis_albostra
GAAAGATGAAAAGCACTTTGGAAAGAGAGTTAAACAGTACGTGAAATTGC
Cui6878_Ceriporia_pseudocystid
GAAAGATGAAAAGCACTTTGGAAAGAGAGTTAAACAGTACGTGAAATTGC
869Dai14737
GAAAGATGAAAAGCACTTTGGAAAGAGAGTTAAACAGTACGTGAAATTGC

876Cui11626

GAAAGATGAAAAGCACTTTGGAAAGAGAGTTAAACAGTACGTGAAATTGC

1199WEI3388 --

776308_Meruliopsis_cystidiata

GAAAGATGAAAAGCACTTTGGAAAGAGAGTTAAACAGTACGTGAAATTGC

ICN139059_Meruliopsis_cystidia

GAAAGATGAAAAGCACTTTGGAAAGAGAGTTAAACAGTACGTGAAATTGC

HHB15692Ceraceomyces_serpens

GAAAGATGAAAAGCACTTTGGAAAGAGAGTTAAACAGTACGTGAAATTGC

HHB_15629_Sp_Ceriporiopsis_ane

GAAAGATGAAAAGCACTTTGGAAAGAGAGTTAAACAGTACGTGAAATTGC

AJ185Trametopsis_cervina

GAAAGATGAAAAGCACTTTGGAAAGAGAGTTAAACAGTACGTGAAATTGC

FD9Irpex_lacteus

GAAAGATGAAAAGCACTTTGGAAAGAGAGTTAAACAGTACGTGAAATTGC

908Dai11230

GAAAGATGAAAAGCACTTTGGAAAGAGAGTTAAACAGTACGTGAAATTGC

FP55521TEmmia_lacerata

GAAAGATGAAAAGCACTTTGGAAAGAGAGTTAAACAGTACGTGAAATTGC

PBU0048Ceriporia_cystidiata

GAAAGATGAAAAGCACTTTGGAAAGAGAGTTAAACAGTACGTGAAATTGC

MZ340C_lacerataT --

Dai21940

GAAAGATGAAAAGCACTTTGGAAAGAGAGTTAAACAGTACGTGAAATTGC

847Dai16433

GAAAGATGAAAAGCACTTTGGAAAGAGAGTTAAACAGTACGTGAAATTGC

MarcinEmmia_latemarginatus

GAAAGATGAAAAGCACTTTGGAAAGAGAGTTAAACAGTACGTGAAATTGC

Meijer3729Hydnopolyporus_fimbr

GAAAGATGAAAAGCACTTTGGAAAGAGAGTTAAACAGTACGTGAAATTGC

RLG13408Phanerochaete_sp

GAAAGATGAAAAGCACTTTGGAAAGAGAGTTAAACAGTACGTGAAATTGC

WHC1381Flavodon_flavus

GAAAGATGAAAAGCACTTTGGAAAGAGAGTTAAACAGTACGTGAAATTGC

GB1833Phlebia_albida

GAAAGATGAAAAGCACTTTGGAAAGAGAGTTAAACAGTACGTGAAATTGC

T407Phlebia_nitidula --

HHB6988Phanerochaete_exilis

GAAAGATGAAAAGCACTTTGGAAAGAGAGTTAAACAGTACGTGAAATTGC

HHB8509Phanerochaetella_xeroph

GAAAGATGAAAAGCACTTTGGAAAGAGAGTTAAACAGTACGTGAAATTGC

PBU0051Macrohyporia_dictyopora

GAAAGATGAAAAGCACTTTGGAAAGAGAGTTAAACAGTACGTGAAATTGC

HHB11463Phanerochaete_sp

GAAAGATGAAAAGCACTTTGGAAAGAGAGTTAAACAGTACGTGAAATTGC
FP102382Byssomerulius_corium
GAAAGATGAAAAGCACTTTGGAAAGAGAGTTAAACAGTACGTGAAATTGC
FP102165Efibula_americana
GAAAGATGAAAAGCACTTTGGAAAGAGAGTTAAACAGTACGTGAAATTGC
Murdoch90Ceriporia_torpida
GAAAGATGAAAAGCACTTTGGAAAGAGAGTTAAACAGTACGTGAAATTGT
Rivoire4413_Ceriporia_purpurea
GAAAGATGAAAAGCACTTTGGAAAGAGAGTTAAACAGTACGTGAAATTGT
Kout_18_Ceriporia_triumphalis
GAAAGATGAAAAGCACTTTGGAAAGAGAGTTAAACAGTACGTGAAATTGT
Rivoire3701_Ceriporia_bresadol
GAAAGATGAAAAGCACTTTGGAAAGAGAGTTAAACAGTACGTGAAATTGT
VS4018 --
Ryvarden21832_Ceriporia_manzan
GAAAGATGAAAAGCACTTTGGAAAGAGAGTTAAACAGTACGTGAAATTGT
Dai24539
GAAAGATGAAAAGCACTTTGGAAAGAGAGTTAAACAGTACGTGAAATTGT
Dai24541
GAAAGATGAAAAGCACTTTGGAAAGAGAGTTAAACAGTACGTGAAATTGT
JV1105_12_Ceriporia_occidental
GAAAGATGAAAAGCACTTTGGAAAGAGAGTTAAACAGTACGTGAAATTGT
VS8558Ceriporia_occidentalis
GAAAGATGAAAAGCACTTTGGAAAGAGAGTTAAACAGTACGTGAAATTGT
Dai22445
GAAAGATGAAAAGCACTTTGGAAAGAGAGTTAAACAGTACGTGAAATTGT
846Dai16368
GAAAGATGAAAAGCACTTTGGAAAGAGAGTTAAACAGTACGTGAAATTGT
Dai17951_Ceriporia_aurantiocar
GAAAGATGAAAAGCACTTTGGAAAGAGAGTTAAACAGTACGTGAAATTGC
Miettinen_11701C_viridans
GAAAGATGAAAAGCACTTTGGAAAGAGAGTTAAACAGTACGTGAAATTGC
JV0105_10Ceriporia_aurantiocar
GAAAGATGAAAAGCACTTTGGAAAGAGAGTTAAACAGTACGTGAAATTGC
Yuan5702C_viridans --
858Dai17003 --
Yuan2747_Ceriporia_viridans --
Yuan2744C_viridans --
Li1046C_viridans --
865C_sinoviridans --
871Dai15062 --
Dai7642_Ceriporia_humilis --
Spirin4706_Ceriporia_humilis --
Spirin4944_Ceriporia_sericea

GAAAGATGAAAAGCACTTTGGAAAGAGAGTTAAACAGTACGTGAAATTGC
WCG1547Dai26044ceriporia
GAAAGATGAAAAGCACTTTGGAAAGAGAGTTAAACAGTACGTGAAATTGC
ZZW1558Dai27086
GAAAGATGAAAAGCACTTTGGAAAGAGAGTTAAACAGTACGTGAAATTGC
Miettinen14381_Ceriporia_mpuri
GAAAGATGAAAAGCACTTTGGAAAGAGAGTTAAACAGTACGTGAAATTGC
Miettinen15492_2_Ceriporia_sor
GAAAGATGAAAAGCACTTTGGAAAGAGAGTTAAACAGTACGTGAAATTGC
He6687
GAAAGATGAAAAGCACTTTGGAAAGAGAGTTAAACAGTACGTGAAATTGC
ZH53Dai24426
GAAAGATGAAAAGCACTTTGGAAAGAGAGTTAAACAGTACGTGAAATTGC
Vlasak0808_30_Ceriporia_punica
GAAAGATGAAAAGCACTTTGGAAAGAGAGTTAAACAGTACGTGAAATTGC
887Dai13376
GAAAGATGAAAAGCACTTTGGAAAGAGAGTTAAACAGTACGTGAAATTGC
WCG1443Dai24998
GAAAGATGAAAAGCACTTTGGAAAGAGAGTTAAACAGTACGTGAAATTGC
0108_6Ceriporia_spissa
GAAAGATGAAAAGCACTTTGGAAAGAGAGTTAAACAGTACGTGAAATTGC
Dai19164
GAAAGATGAAAAGCACTTTGGAAAGAGAGTTAAACAGTACGTGAAATTGC
Dai17937_Ceriporia_bubalinomar
GAAAGATGAAAAGCACTTTGGAAAGAGAGTTAAACAGTACGTGAAATTGC
903Dai12113
GAAAGATGAAAAGCACTTTGGAAAGAGAGTTAAACAGTACGTGAAATTGC
LZB929Dai25079
GAAAGATGAAAAGCACTTTGGAAAGAGAGTTAAACAGTACGTGAAATTGC
LX45Dai26988 --
LX43Dai26986
GAAAGATGAAAAGCACTTTGGAAAGAGAGTTAAACAGTACGTGAAATTGC
Dai7759Ceriporia --
Cui8012_Ceriporia_viridans --
GC1704_54Ceriporia_viridans
GAAAGATGAAAAGCACTTTGGAAAGAGAGTTAAACAGTACGTGAAATTGC
Dai23392
GAAAGATGAAAAGCACTTTGGAAAGAGAGTTAAACAGTACGTGAAATTGC
WCG1585Dai26113Ceriproia
GAAAGATGAAAAGCACTTTGGAAAGAGAGTTAAACAGTACGTGAAATTGC
Dai18675C_eucalypti
GAAAGATGAAAAGCACTTTGGAAAGAGAGTTAAACAGTACGTGAAATTGC
Dai22034
GAAAGATGAAAAGCACTTTGGAAAGAGAGTTAAACAGTACGTGAAATTGC

JV1008_41JTardaFLORIDAKeys --
Rivoire1161_Ceriporia_pierii --
Dai23499C_pierii
GAAAGATGAAAAGCACTTTGGAAAGAGAGTTAAACAGTACGTGAAATTGC
Dai23500
GAAAGATGAAAAGCACTTTGGAAAGAGAGTTAAACAGTACGTGAAATTGC
841Dai15899
GAAAGATGAAAAGCACTTTGGAAAGAGAGTTAAACAGTACGTGAAATTGC
842Dai15904 --
LZB1066xinjiang --
LZB1065xinjiang --
851Dai16779
GAAAGATGAAAAGCACTTTGGAAAGAGAGTTAAACAGTACGTGAAATTGC
RMJ119sp_Candelabrochaete_sept --
RLG9759spCandelabrochaete_sept
GAAAGATGAAAAGCACTTTGGAAAGAGAGTTAAACAGTACGTGAAATTGC
RLG10478Phanerochaete_allantos --
Dai19118_Ceriporia_spissa
GAAAGATGAAAAGCACTTTGGAAAGAGAGTTAAACAGTACGTGAAATTGC
Dai18486A
GAAAGATGAAAAGCACTTTGGAAAGAGAGTTAAACAGTACGTGAAATTGC
WEI17_024_Ceriporia_mellita
GAAAGATGAAAAGCACTTTGGAAAGAGAGTTAAACAGTACGTGAAATTGC
GC1508_71Ceriporia_mellita
GAAAGATGAAAAGCACTTTGGAAAGAGAGTTAAACAGTACGTGAAATTGC
GC1608_7_Ceriporia_mellita
GAAAGATGAAAAGCACTTTGGAAAGAGAGTTAAACAGTACGTGAAATTGC
ZZW1557Dai27085
GAAAGATGAAAAGCACTTTGGAAAGAGAGTTAAACAGTACGTGAAATTGC
ZZW1554Dai27083
GAAAGATGAAAAGCACTTTGGAAAGAGAGTTAAACAGTACGTGAAATTGC
Dai8168
GAAAGATGAAAAGCACTTTGGAAAGAGAGTTAAACAGTACGTGAAATTGC
BR4865C_mellita
GAAAGATGAAAAGCACTTTGGAAAGAGAGTTAAACAGTACGTGAAATTGC
MEL2382688Ceriporia_sp --
Dai8110
GAAAGATGAAAAGCACTTTGGAAAGAGAGTTAAACAGTACGTGAAATTGC
Cui8097 --
909Cui6740 --
W1258Dai24695
GAAAGATGAAAAGCACTTTGGAAAGAGAGTTAAACAGTACGTGAAATTGC
JV0110_26_Ceriporia_griseoviol
GAAAGATGAAAAGCACTTTGGAAAGAGAGTTAAACAGTACGTGAAATTGC

896Dai13202

GAAAGATGAAAAGCACTTTGGAAAGAGAGTTAAACAGTACGTGAAATTGC

LWY393Dai27053C_griseoviolasce

GAAAGATGAAAAGCACTTTGGAAAGAGAGTTAAACAGTACGTGAAATTGC

LWY394DAI27054

GAAAGATGAAAAGCACTTTGGAAAGAGAGTTAAACAGTACGTGAAATTGC

FP135015G_pannocinctus

GAAAGATGAAAAGCACTTTGGAAAGAGAGTTAAACAGTACGTGAAATTGC

L15726SpG_pannocinctus

GAAAGATGAAAAGCACTTTGGAAAGAGAGTTAAACAGTACGTGAAATTGC

Dai22221

GAAAGATGAAAAGCACTTTGGAAAGAGAGTTAAACAGTACGTGAAATTGC

Dai22633

GAAAGATGAAAAGCACTTTGGAAAGAGAGTTAAACAGTACGTGAAATTGC

Dai23260

GAAAGATGAAAAGCACTTTGGAAAGAGAGTTAAACAGTACGTGAAATTGC

Dai23626

GAAAGATGAAAAGCACTTTGGAAAGAGAGTTAAACAGTACGTGAAATTGC

Dai16238G_citrinoalbus GGAAGATG-AAAGCACTTTGAAGAGAG---TAAACAGTACGTGAAATGCT

1175Dai15293

GAAAGATGAAAAGCACTTTGGAAAGAGAGTTAAACAGTACGTGAAATTGC

Dai19547

GAAAGATGAAAAGCACTTTGGAAAGAGAGTTAAACAGTACGTGAAATTGC

918063G_africanus

GAAAGATGAAAAGCACTTTGGAAAGAGAGTTAAACAGTACGTGAAATTGC

918572G_africanus

GAAAGATGAAAAGCACTTTGGAAAGAGAGTTAAACAGTACGTGAAATTGC

Dai18536A --

1164Cui17922

GAAAGATGAAAAGCACTTTGGAAAGAGAGTTAAACAGTACGTGAAATTGC

Dai22225

GAAAGATGAAAAGCACTTTGGAAAGAGAGTTAAACAGTACGTGAAATTGC

1163Dai20655

GAAAGATGAAAAGCACTTTGGAAAGAGAGTTAAACAGTACGTGAAATTGC

Yuan4397G_hainanensis

GAAAGATGAAAAGCACTTTGGAAAGAGAGTTAAACAGTACGTGAAATTGC

1176Dai15268

GAAAGATGAAAAGCACTTTGGAAAGAGAGTTAAACAGTACGTGAAATTGC

1177Dai15259

GAAAGATGAAAAGCACTTTGGAAAGAGAGTTAAACAGTACGTGAAATTGC

BZ2896G_thelephoroides

GAAAGATGAAAAGCACTTTGGAAAGAGAGTTAAACAGTACGTGAAATTGC

1166JV1808_26

GAAAGATGAAAAGCACTTTGGAAAGAGAGTTAAACAGTACGTGAAATTGC

Miettinen16992Hapalopilus_ochr

GAAAGATGAAAAGCACTTTGGAAAGAGAGTTAAACAGTACGTGAAATTGC

GC1708_338_Ceriporia_arbuscula

GAAAGATGAAAAGCACTTTGGAAAGAGAGTTAAACAGTACGTGAAATTGC

WCG1555Dai26107Ceriporia

GAAAGATGAAAAGCACTTTGGAAAGAGAGTTAAACAGTACGTGAAATTGC

GC1708_340_Ceriporia_arbuscula

GAAAGATGAAAAGCACTTTGGAAAGAGAGTTAAACAGTACGTGAAATTGC

WCG1556Dai26109Ceriporia

GAAAGATGAAAAGCACTTTGGAAAGAGAGTTAAACAGTACGTGAAATTGC

883Cui11291

GAAAGATGAAAAGCACTTTGGAAAGAGAGTTAAACAGTACGTGAAATTGC

HLX320Dai26805

GAAAGATGAAAAGCACTTTGGAAAGAGAGTTAAACAGTACGTGAAATTGC

WCG1266Dai24678A

GAAAGATGAAAAGCACTTTGGAAAGAGAGTTAAACAGTACGTGAAATTGC

Dai6090_Ceriporia_sulphuricolo

GAAAGATGAAAAGCACTTTGGAAAGAGAGTTAAACAGTACGTGAAATTGC

RLG_11354_Ceriproia_reticulata

GAAAGATGAAAAGCACTTTGGAAAGAGAGTTAAACAGTACGTGAAATTGC

ZZW1543Dai27072

GAAAGATGAAAAGCACTTTGGAAAGAGAGTTAAACAGTACGTGAAATTGC

Li1316_Ceriporia_reticulata --

KHL11981Ceriporia_reticulata

GAAAGATGAAAAGCACTTTGGAAAGAGAGTTAAACAGTACGTGAAATTGC

FP110343sp_Candelabrochaete_la

GAAAGATGAAAAGCACTTTGGAAAGAGAGTTAAACAGTACGTGAAATTGC

Li1045_Ceriporia_reticulata --

ZX136Dai25794ceriporia

GAAAGATGAAAAGCACTTTGGAAAGAGAGTTAAACAGTACGTGAAATTGC

892Dai13400

GAAAGATGAAAAGCACTTTGGAAAGAGAGTTAAACAGTACGTGAAATTGC

RLG7163Leptoporus_mollis

GAAAGATGAAAAGCACTTTGGAAAGAGAGTTAAACAGTACGTGAAATTGC

Dai21062Leptoporus_mollis

GAAAGATGAAAAGCACTTTGGAAAGAGAGTTAAACAGTACGTGAAATTGC

Dai20182Leptoporus_submollis

GAAAGATGAAAAGCACTTTGGAAAGAGAGTTAAACAGTACGTGAAATTGC

Cui18379Leptoporus_submollis

GAAAGATGAAAAGCACTTTGGAAAGAGAGTTAAACAGTACGTGAAATTGC

Wu1209_46Resiniporus_pseudogil

GAAAGATGAAAAGCACTTTGGAAAGAGAGTTAAACAGTACGTGAAATTGC

BRNM710169Resiniporus_resinasc

GAAAGATGAAAAGCACTTTGGAAAGAGAGTTAAACAGTACGTGAAATTGC

Dai14516Bjerkandera_adusta

GAAAGATGAAAAGCACTTTGGAAAGAGAGTTAAACAGTACGTGAAATTGC

Dai21100Bjerkandera_fumosa

GAAAGATGAAAAGCACTTTGGAAAGAGAGTTAAACAGTACGTGAAATTGC

Miettinen16854Ceraceomyces_sp

GAAAGATGAAAAGCACTTTGGAAAGAGAGTTAAACAGTACGTGAAATTGC

Dai10477C_spissa

GAAAGATGAAAAGCACTTTGGAAAGAGAGTTAAACAGTACGTGAAATTGC

855Dai16831

GAAAGATGAAAAGCACTTTGGAAAGAGAGTTAAACAGTACGTGAAATTGC

882Cui11282

GAAAGATGAAAAGCACTTTGGAAAGAGAGTTAAACAGTACGTGAAATTGC

Dai24566

GAAAGATGAAAAGCACTTTGGAAAGAGAGTTAAACAGTACGTGAAATTGC

Yuan5965

GAAAGATGAAAAGCACTTTGGAAAGAGAGTTAAACAGTACGTGAAATTGC

Dai3204

GAAAGATGAAAAGCACTTTGGAAAGAGAGTTAAACAGTACGTGAAATTGC

1194CUI9985

GAAAGATGAAAAGCACTTTGGAAAGAGAGTTAAACAGTACGTGAAATTGC

Dai15205_Ceriporia_albomellea

TGAAAGGGAAACGCTTGAAGTCAGTCGCGTTAATTAGAACTCAGCGTTGC

Dai15223_Ceriporia_albomellea

TGAAAGGGAAACGCTTGAAGTCAGTCGCGTTAATTAGAACTCAGCCTTGC

Li1780_Ceriporia_variegata

TGAAAGGGAAACGCTTGAAGTCAGTCGCGTTAATTAGAACTCAGCCTCGC

Dai19791_Ceriporia_variegata

TGAAAGGGAAACGCTTGAAGTCAGTCGCGTTAATTAGAACTCAGCCTCGC

Dai19886

TGAAAGGGAAACGCTTGAAGTCAGTCGCGTTAATTAGAACTCAGCCTCGC

Dai10833_Ceriporia_crassitunic

TGAAAGGGAAACGCTTGAAGTCAGTCGCGTTAGTTAGAACTCAGCCTTGC

CHWC1506_46Meruliopsis_crassit

TGAAAGGGAAACGCTTGAAGTCAGTCGCGTTAGTTAGAACTCAGCCTTGC

Dai9995_Ceriporia_crassitunica --

Wu1209_58_Meruliopsis_parvispo

TGAAAGGGAAACGCTTGAAGTCAGTCGCGTTAGCTAGAACTCAGCCTTGC

CHWC1505_129_Meruliopsis_parvi

TGAAAGGGAAACGCTTGAAGTCAGTCGCGTTAGCTAGAACTCAGCCTTGC

Dai21944

TGAAAGGGAAACGCTTGAAGTCAGTCGCGTTTGCTAGAACTCAGCCTTGC

830Dai18640A
TGAAAGGGAAACGCTTGAAGTCAGTCGCGTTTGCTAGAACTCAGCCTTGC
GC1704_60_Meruliopsis_taxicola
TGAAAGGGAAACGCTTGAAGTCAGTCGCGTTAGCTAGAACTCAGCCTTGC
Dai22625
TGAAAGGGAAACGCTTGAAGTCAGTCGCGTTAGCTAGAACTCAGCCTTGC
Dai22636
TGAAAGGGAAACGCTTGAAGTCAGTCGCGTTAGCTAGAACTCAGCCTTGC
Dai21878
TGAAAGGGAAACGCTTGAAGTCAGTCGCGTTAGCTAGAACTCAGCCTTGC
1169Dai17248
TGAAAGGGAAACGCTTGAAGTCAGTCGCGTTAGCTAGAACTCAGCCTTGC
Wu1708_43_Meruliopsis_leptocys
TGAAAGGGAAACGCTTGAAGTCAGTCGCGTTAGCTAGAACTCAGCCTTGC
Li1011
TGAAAGGGAAACGCTTGAAGTCAGTCGCGTTAGCTAGAACTCAGCCTTGC
ZX95Dai25742Meruliopsis_leptoc
TGAAAGGGAAACGCTTGAAGTCAGTCGCGTTAGCTAGAACTCAGCCTTGC
WCG1306Dai24733
TGAAAGGGAAACGCTTGAAGTCAGTCGCGTTAGCTAGAACTCAGCCTTGC
LXL99Dai25816
TGAAAGGGAAACGCTTGAAGTCAGTCGCGTTAGCTAGAACTCAGCCTTGC
WCG1559Dai26052Meruliopsis
TGAAAGGGAAACGCTTGAAGTCAGTCGCGTTAGCTAGAACTCAGCCTTGC
He7477
TGAAAGGGAAACGCTTGAAGTCAGTCGCGTTAGCTAGAACTCAGCCTTGC
HLX243Dai26217
TGAAAGGGAAACGCTTGAAGTCAGTCGCGTTAGCTAGAACTCAGCCTTGC
RussiaMW673659Meruliopsis_fagi
TGAAAGGGAAACGCTTGAAGTCAGTCGCGTTAGCTAGAACTCAGCCTTGC
FD278
TGAAAGGGAAACGCTTGAAGTCAGTCGCGTTAGCTAGAACTCAGCCTTGC
Dai10226_Ceriporia_tarda --
LE247365
TGAAAGGGAAACGCTTGAAGTCAGTCGCGTTAGCTAGAACTCAGCCTTGC
Dai8173_Meruliopsis_nanlingens
TGAAAGGGAAACGCTTGAAGTCAGTCGCGTTAGCTAGAACTCAGCCTTGC
860Dai17172
TGAAAGGGAAACGCTTGAAGTCAGTCGCGTTAGCTAGAACTCAGCCTTGC
879Dai13414
TGAAAGGGAAACGCTTGAAGTCAGTCGCGTTAGCTAGAACTCAGCCTTGC
Li_1704_Meruliopsis_pseudocyst --
833Dai18405
TGAAAGGGAAACGCTTGAAGTCAGTCGCGTTAGCTAGAACTCAGCCTTGC

HHB_10729_Meruliopsis_albostra
TGAAAGGGAAACGCTTGAAGTCAGTCGCGTTAGCTAGAACTCAGCCTTGC
Cui6878_Ceriporia_pseudocystid
TGAAAGGGAAACGCTTGAAGTCAGTCGCGTCGACTAGAACTCAGCCTTGC
869Dai14737
TGAAAGGGAAACGCTTGAAGTCAGTCGCGTCGACTAGAACTCAGCCTTGC
876Cui11626
TGAAAGGGAAACGCTTGAAGTCAGTCGCGTCGACTAGAACTCAGCCTTGC
1199WEI3388 --
776308_Meruliopsis_cystidiata
TGAAAGGGAAACGCTTGAAGTCAGTCGCGTTCGCTAGAACTCAGCCTTGC
ICN139059_Meruliopsis_cystidia
TGAAAGGGAAACGCTTGAAGTCAGTCGCGTTCGCTAGAACTCAGCCTTGC
HHB15692Ceraceomyces_serpens
TGAAAGGGAAACGCTTGAAGTCAGTCGCGTCGGCTAGAACTCAGCCTTGC
HHB_15629_Sp_Ceriporiopsis_ane
TGAAAGGGAAACGCTTGAAGTCAGTCGCGTTAGTTAGAACTCAGCCAGGC
AJ185Trametopsis_cervina
TGAAAGGGAAACGCTTGAAGTCAGTCGCGTTAGCTAGAACTCAACCAGGC
FD9Irpex_lacteus
TGAAAGGGAAACGCTTGAAGTCAGTCGCGTTAGCTAGAACTCAACCAGGC
908Dai11230
TGAAAGGGAAACGCTTGAAGTCAGTCGCGTTAGCTAGAACTCAACCAGGC
FP55521TEmmia_lacerata
TGAAAGGGAAACGATTGAAGTCAGTCGCGTTAGCTAGAACTCAACCAGGC
PBU0048Ceriporia_cystidiata
TGAAAGGGAAACGATTGAAGTCAGTCGCGTTAGCTAGAACTCAACCAGGC
MZ340C_laceratаT --
Dai21940
TGAAAGGGAAACGATTGAAGTCAGTCGCGTTAGCTAGAACTCAACCAGGC
847Dai16433
TGAAAGGGAAACGATTGAAGTCAGTCGCGTTAGCTAGAACTCAACCAGGC
MarcinEmmia_latemarginatus
TGAAAGGGAAACGATTGAAGTCAGTCGCGTTAGCTAGAACTCAACCAGGC
Meijer3729Hydnopolyporus_fimbr
TGAAAGGGAAACGCTTGAAGTCAGTCGCGTTAGCTAGAACTCAACCAGGC
RLG13408Phanerochaete_sp
TGAAAGGGAAACGCTTGAAGTCAGTCGCGTTAGTTAGAACTCAGCCAGGC
WHC1381Flavodon_flavus
TGAAAGGGAAACGCTTGAAGTCAGTCGCGTTATCTAGAACTCAACCAGGC
GB1833Phlebia_albida
TGAAAGGGAAACGCTTGAAGTCAGTCGCGTTGTCTAGAACTCAGCCAGGT
T407Phlebia_nitidula --
HHB6988Phanerochaete_exilis

TGAAAGGGAAACGCTTGAAGTCAGTCGCGTTGGTTAGAGCTCAACCAGGC
HHB8509Phanerochaetella_xeroph
TGAAAGGGAAACGCTTGAAGTCAGTCGCGTTGGTTAGAACTCAACCAGGC
PBU0051Macrohyporia_dictyopora
TGAAAGGGAAACGCTTGAAGTCAGTCGCGTTGGTTAGAACTCAACCAGGC
HHB11463Phanerochaete_sp
TGAAAGGGAAACGCTTGAAGTCAGTCGCGTTGGTTAGAACTCAACCAGGC
FP102382Byssomerulius_corium
TGAAAGGGAAACGCTTGAAGTCAGTCGCGTTAGCTACAACTCAGCCAGGC
FP102165Efibula_americana
TGAAAGGGAAACGCTTGAAGTCAGTCGCGTTGTCTAGAACTCAGCCAGGC
Murdoch90Ceriporia_torpida
TGAAAGGGAAACGCTTGAAGTCAGTCGCGTTAGTCAGAACTCAGCCTTGC
Rivoire4413_Ceriporia_purpurea
TGAAAGGGAAACGCTTGAAGTCAGTCGCGTTAGTCAGAACTCAGCCTTGC
Kout_18_Ceriporia_triumphalis
TGAAAGGGAAACGCTTGAAGTCAGTCGCGTTAGTCAGAACTCAGCCTTGC
Rivoire3701_Ceriporia_bresadol
TGAAAGGGAAACGCTTGAAGTCAGTCGCGTTAGTCAGAACTCAGCCTTGC
VS4018 --
Ryvarden21832_Ceriporia_manzan
TGAAAGGGAAACGCTTGAAGTCAGTCGCGTTAGTCAGAACTCAGCCTTGC
Dai24539
TGAAAGGGAAACGCTTGAAGTCAGTCGCGTTAGTCAGAACTCAGCCTTGC
Dai24541
TGAAAGGGAAACGCTTGAAGTCAGTCGCGTTAGTCAGAACTCAGCCTTGC
JV1105_12_Ceriporia_occidental
TGAAAGGGAAACGCTTGAAGTCAGTCGCGTTAGTCAGAACTCAGCCTTGC
VS8558Ceriporia_occidentalis
TGAAAGGGAAACGCTTGAAGTCAGTCGCGTTAGTCAGAACTCAGCCTTGC
Dai22445
TGAAAGGGAAACGCTTGAAGTCAGTCGCGTTAGTCAGAACTCAGCCTTGC
846Dai16368
TGAAAGGGAAACGCTTGAAGTCAGTCGCGTTAGTCAGAACTCAGCCTTGC
Dai17951_Ceriporia_aurantiocar
TGAAAGGGAAACGCTTGAAGTCAGTCGCGTTAGTCAGAACTCAGCCTTGC
Miettinen_11701C_viridans
TGAAAGGGAAACGCTTGAAGTCAGTCGCGTTAGTCAGAACTCAGCCTTGC
JV0105_10Ceriporia_aurantiocar
TGAAAGGGAAACGCTTGAAGTCAGTCGCGTTAGTCAGAACTCAGCCTTGC
Yuan5702C_viridans --
858Dai17003 --
Yuan2747_Ceriporia_viridans --
Yuan2744C_viridans --

Li1046C_viridans --
865C_sinoviridans --
871Dai15062 --
Dai7642_Ceriporia_humilis --
Spirin4706_Ceriporia_humilis --
Spirin4944_Ceriporia_sericea
TGAAAGGGAAACACTTGAAGTCAGTCGCGTCAGTCAGAACTCAGCCTTGC
WCG1547Dai26044ceriporia
TGAAAGGGAAACACTTGAAGTCAGTCGCGTCAGTCAGAACTCAGCCTTGC
ZZW1558Dai27086
TGAAAGGGAAACACTTGAAGTCAGTCGCGTCAGTCAGAACTCAGCCTTGC
Miettinen14381_Ceriporia_mpuri
TGAAAGGGAAACGCTTGAAGTCAGTCGCGTCAGTCAGAACTCAGCCTTGC
Miettinen15492_2_Ceriporia_sor
TGAAAGGGAAACGCTTGAAGTCAGTCGCGTCAGTCAGAACTCAGCCTTGC
He6687
TGAAAGGGAAACGCTTGAAGTCAGTCGCGTCAGTCAGAACTCAGCCTTGC
ZH53Dai24426
TGAAAGGGAAACGCTTGAAGTCAGTCGCGTCAGTCAGAACTCAGCCTTGC
Vlasak0808_30_Ceriporia_punica
TGAAAGGGAAACGCTTGAAGTCAGTCGCGTTGGTCAGAATTCAGCCTTGC
887Dai13376
TGAAAGGGAAACGCTTGAAGTCAGTCGCGTTGGTCAGAATTCAGCCTTGC
WCG1443Dai24998
TGAAAGGGAAACGCTTGAAGTCAGTCGCGTTGGTCAGAATTCAGCCTTGC
0108_6Ceriporia_spissa
TGAAAGGGAAACGCTTGAAGTCAGTCGCGTTGGTCAGAACTCAGCCTTGC
Dai19164
TGAAAGGGAAACGCTTGAAGTCAGTCGCGTTGGTCAGAACTCAGCCTTGC
Dai17937_Ceriporia_bubalinomar
TGAAAGGGAAACGCTTGAAGTCAGTCGCGTTGGTCAGAACTCAGCCTTGC
903Dai12113
TGAAAGGGAAACGCTTGAAGTCAGTCGCGTTGGTCAGAACTCAGCCTTGC
LZB929Dai25079
TGAAAGGGAAACGCTTGAAGTCAGTCGCGTTAGTCAGAACTCAGCCTTGC
LX45Dai26988 --
LX43Dai26986
TGAAAGGGAAACGCTTGAAGTCAGTCGCGTTAGTCAGAACTCAGCCTTGC
Dai7759Ceriporia --
Cui8012_Ceriporia_viridans --
GC1704_54Ceriporia_viridans
TGAAAGGGAAACGCTTGAAGTCAGTCGCGTTGGTCAGAACTCAGCCTTGC
Dai23392
TGAAAGGGAAACGCTTGAAGTCAGTCGCGTTAGTCAGAACTCAGCCTTGC

WCG1585Dai26113Ceriproia
TGAAAGGGAAACGCTTGAAGTCAGTCGCGTTAGTCAGAACTCAGCCTTGC
Dai18675C_eucalypti
TGAAAGGGAAACGCTTGAAGTCAGTCGCGTTAGTCAGAACTCAGCCTTGC
Dai22034
TGAAAGGGAAACGCTTGAAGTCAGTCGCGTTAGTTAGAAATCAGCCTTGC
JV1008_41JTardaFLORIDAKeys --
Rivoire1161_Ceriporia_pierii --
Dai23499C_pierii
TGAAAGGGAAACGCTTGAAGTCAGTCGCGTCAGTCAGAACTCAGCCTTGC
Dai23500
TGAAAGGGAAACGCTTGAAGTCAGTCGCGTCAGTCAGAACTCAGCCTTGC
841Dai15899
TGAAAGGGAAACGCTTGAAGTCAGTCGCGTTAGTCAGAACTCAGCCTTGC
842Dai15904 --
LZB1066xinjiang --
LZB1065xinjiang --
851Dai16779
TGAAAGGGAAACGCTTGAAGTCAGTCGCGTCAGTCAGAACTCAGCCTTGC
RMJ119sp_Candelabrochaete_sept --
RLG9759spCandelabrochaete_sept
TGAAAGGGAAACACTTGAAGTCAGTCGCGTCGTTCAGAAATCAGCCTCGC
RLG10478Phanerochaete_allantos --
Dai19118_Ceriporia_spissa
TGAAAGGGAAACGCTTGAAGTCAGTCGTGTCAGTCAGAACTCAGCCTTGC
Dai18486A
TGAAAGGGAAACGCTTGAAGTCAGTCGTGTCAATCAGAACTCAGCCTTGC
WEI17_024_Ceriporia_mellita
TGAAAGGGAAACGCTTGAAGTCAGTCGTGTCAGTCAGAACTCAGCCTTGC
GC1508_71Ceriporia_mellita
TGAAAGGGAAACGCTTGAAGTCAGTCGTGTCAGTCAGAACTCAGCCTTGC
GC1608_7_Ceriporia_mellita
TGAAAGGGAAACGCTTGAAGTCAGTCGTGTCAGTCAGAACTCAGCCTTGC
ZZW1557Dai27085
TGAAAGGGAAACGCTTGAAGTCAGTCGTGTCAGTCAGAACTCAGCCTTGC
ZZW1554Dai27083
TGAAAGGGAAACGCTTGAAGTCAGTCGTGTCAGTCAGAACTCAGCCTTGC
Dai8168
TGAAAGGGAAACGCTTGAAGTCAGTCGTGTCAGTCAGAACTCAGCCTTGC
BR4865C_mellita
TGAAAGGGAAACGCTTGAAGTCAGTCGTGTCAGTCAGAACTCAGCCTTGC
MEL2382688Ceriporia_sp --
Dai8110
TGAAAGGGAAACGCTTGAAGTCAGTCGTGTCAGTCAGAACTCAGCCTTGC

Cui8097 ---

909Cui6740 ---

W1258Dai24695
TGAAAGGGAAACGCTTGAAGTCAGTCGTGTCAGTCAGAACTCAGCCTTGC

JV0110_26_Ceriporia_griseoviol
TGAAAGGGAAACGCTTGAAGTCAGTCGYGTCGTTCAGAAATCAGCCTTGC

896Dai13202
TGAAAGGGAAACGCTTGAAGTCAGTCGCGTCGTTCAGAAATCAGCCTTGC

LWY393Dai27053C_griseoviolasce
TGAAAGGGAAACGCTTGAAGTCAGTCGCGTCGTTCAGAAATCAGCCTTGC

LWY394DAI27054
TGAAAGGGAAACGCTTGAAGTCAGTCGCGTCGTTCAGAAATCAGCCTTGC

FP135015G_pannocinctus
TGAAAGGGAAACGATTGAAGTCAGTCGCGTTATTCAGAACTCAGCCTTGC

L15726SpG_pannocinctus
TGAAAGGGAAACGATTGAAGTCAGTCGCGTTATTCAGAACTCAGCCTTGC

Dai22221
TGAAAGGGAAACGCTTGAAGTCAGTCGCGTTATTCAGAACTCAGCCTTAC

Dai22633
TGAAAGGGAAACGATTGAAGTCAGTCGCGTTATTCAGAACTCAGCCTTAC

Dai23260
TGAAAGGGAAACGATTGAAGTCAGTCGCGTTATTCAGAACTCAGCCTTAC

Dai23626
TGAAAGGGAAACGATTGAAGTCAGTCGCGTTATTCAGAACTCAGCCTTAC

Dai16238G_citrinoalbus --GAAGGGAAACGAT--GAGTCAGTCGCGT--ATCAGAACTCAGCTTACG

1175Dai15293
TGAAAGGGAAACGATTGAAGTCAGTCGCGTTATTCAGAACTCAGCCTTAC

Dai19547
TGAAAGGGAAACGATTGAAGTCAGTCGCGTTATTCAGAACTCAGCCTTAC

918063G_africanus
TGAAAGGGAAACGATTGAAGTCAGTCGCGTTATTCAGAACTCAGCCTTAC

918572G_africanus
TGAAAGGGAAACGATTGAAGTCAGTCGCGTTATTCAGAACTCAGCCTTAC

Dai18536A ---

1164Cui17922
TGAAAGGGAAACGATTGAAGTCAGTCGCGTTATTCAGAACTCAGCCTTAC

Dai22225
TGAAAGGGAAACGATTGAAGTCAGTCGCGTTATTCAGAACTCAGCCTTAC

1163Dai20655
TGAAAGGGAAACGATTGAAGTCAGTCGCGTTATTCAGAACTCAGCCTTAC

Yuan4397G_hainanensis
TGAAAGGGAAACGCTTGAAGTCAGTCGCGTTATTCAGAACTCAGCCTTGC

1176Dai15268

TGAAAGGGAAACGCTTGAAGTCAGTCGCGTTATTCAGAACTCAGCCTTGC

1177Dai15259

TGAAAGGGAAACGCTTGAAGTCAGTCGCGTTATTCAGAACTCAGCCTTGC

BZ2896G_thelephoroides

TGAAAGGGAAACGCTTGAAGTCAGTCGCGTTATTCAGAACTCAGCCTAGC

1166JV1808_26

TGAAAGGGAAACGCTTGAAGTCAGTCGCGTTATTCAGAACTCAGCCTAGC

Miettinen16992Hapalopilus_ochr

TGAAAGGGAAACGCTTGAAGTTAGTCGCGTTAGTCAGAACTCAGCCTAGT

GC1708_338_Ceriporia_arbuscula

TGAAAGGGAAACGCTTGAAGTCAGTCGCGTCTGTCAGAAATCAGTCTTGC

WCG1555Dai26107Ceriporia

TGAAAGGGAAACGCTTGAAGTCAGTCGCGTCTGTCAGAAATCAGTCTTGC

GC1708_340_Ceriporia_arbuscula

TGAAAGGGAAACGCTTGAAGTCAGTCGCGTCTGTCAGAAATCAGTCTTGC

WCG1556Dai26109Ceriporia

TGAAAGGGAAACGCTTGAAGTCAGTCGCGTCTGTCAGAAATCAGTCTTGC

883Cui11291

TGAAAGGGAAACGCTTGAAGTCAGTCGCGTCTGTTAGAAATCAGTCTTGC

HLX320Dai26805

TGAAAGGGAAACGCTTGAAGTCAGTCGCATCTGTCAGAAATCAGTCTTGC

WCG1266Dai24678A

TGAAAGGGAAACGCTTGAAGTCAGTCGCATCTGTTAGAAATCAGTCTTGC

Dai6090_Ceriporia_sulphuricolo

TGAAAGGGAAACGCTTGAAGTCAGTCGCGTTGGCCAGAACTCAGCCTTGC

RLG_11354_Ceriproia_reticulata

TGAAAGGGAAACACTTGAAGTCAGTCGCGTTGGCCAGAACTCAGCCTTGC

ZZW1543Dai27072

TGAAAGGGAAACACTTGAAGTCAGTCGCGTTGGCCAGAACTCAGCCTTGC

Li1316_Ceriporia_reticulata --

KHL11981Ceriporia_reticulata

TGAAAGGGAAACACTTGAAGTCAGTCGCGTTGGCCAGAACTCAGCCTTGC

FP110343sp_Candelabrochaete_la

TGAAAGGGAAACGCTTGAAGTCAGTCGCGTTGTTCAGAACTCAGCCTTGC

Li1045_Ceriporia_reticulata --

ZX136Dai25794ceriporia

TGAAAGGGAAACGCTTGAAGTCAGTCGCGTTGGCTAGAACTCAGCCTTGC

892Dai13400

TGAAAGGGAAACGCTTGAAGTCAGTCGCGTTGGCTAGAACTCAGCCTTGC

RLG7163Leptoporus_mollis

TGAAAGGGAAACGCTTGAAGTCAGTCGCGTTGTTCAGAACTCAGCCTTGC

Dai21062Leptoporus_mollis

TGAAAGGGAAACGCTTGAAGTCAGTCGCGTTGTTCAGAACTCAGCCTTGC

Dai20182Leptoporus_submollis

TGAAAGGGAAACGCTTGAAGTCAGTCGCGTTGTTCAGAACTCAGCCTTGC

Cui18379Leptoporus_submollis

TGAAAGGGAAACGCTTGAAGTCAGTCGCGTTGTTCAGAACTCAGCCTTGC

Wu1209_46Resiniporus_pseudogil

TGAAAGGGAAACGCTTGAAGTCAGTCGCGTTAGTTAGAACTCAGCCAAGC

BRNM710169Resiniporus_resinasc

TGAAAGGGAAACGCTTGAAGTCAGTCGCGTTAGTTAGAACTCAGCCAAGC

Dai14516Bjerkandera_adusta

TGAAAGGGAAACGATTGAAGTCAGTCGCGTGTGCTAGAACTCAGCCTTGC

Dai21100Bjerkandera_fumosa

TGAAAGGGAAACGATTGAAGTCAGTCGCGTGTGCTAGAACTCAGCCTTGC

Miettinen16854Ceraceomyces_sp

TGAAAGGGAAACGCTTGAAGTCAGTCGTGTCAGTCAGAACTCAGCCTTGC

Dai10477C_spissa

TGAAAGGGAAACGCTTGAAGTCAGTCGCGTTGATCAGAAATCAGCCTTGC

855Dai16831

TGAAAGGGAAACGCTTGAAGTCAGTCGCGTTGATCAGAAATCAGCCTTGC

882Cui11282

TGAAAGGGAAACGCTTGAAGTCAGTCGCGTTGATCAGAAATCAGCCTTGC

Dai24566

TGAAAGGGAAACGCTTGAAGTTAGTCGCGTTGATCAGAAATCAGCCTTGC

Yuan5965

TGAAAGGGAAACGCTTGAAGTCAGTCGCGTTGATCAGAAATCAGCCTTGC

Dai3204

TGAAAGGGAAACGCTTGAAGTCAGTCGCGTTAGCTAGAACTCAGCCTTGC

1194CUI9985

TGAAAGGGAAACGCTTGAAGTCAGTCGCGTTAGCTAGAACTCAACCAGGC

Dai15205_Ceriporia_albomellea ----TTTT----GCTCGGTGCATTTTCTAA-TGAACGGGCCAGCATCAGT

Dai15223_Ceriporia_albomellea ----TTTT----GCTCGGTGCATTTTCTAA-TGAACGGGCCAGCATCAGT

Li1780_Ceriporia_variegata ----TTTT----GCTTGGTGCATTTTCTAA-TGAACGGGCCAGCATCAGT

Dai19791_Ceriporia_variegata ----TTTT----GCTTGGTGCATTTTCTAA-TGAACGGGCCAGCATCAGT

Dai19886 ----TTTT----GCTTGGTGCATTTTCTAA-TGAACGGGCCAGCATCAGT

Dai10833_Ceriporia_crassitunic ----TTTG----GCTTGGTGCACTTTCTAA-TGAACGGGCCAGCATCAGT

CHWC1506_46Meruliopsis_crassit ----TTTG----GCTTGGTGCACTTTCTAA-TGAACGGGCCAGCATCAGT

Dai9995_Ceriporia_crassitunica --

Wu1209_58_Meruliopsis_parvispo ----TTTT----GCTTGGTGCATTTTCTAG-TTGACGGGCCAGCATCAGT

CHWC1505_129_Meruliopsis_parvi ----TTTT----GCTTGGTGCATTTTCTAG-TTGACGGGCCAGCATCAGT

Dai21944 ----TTTT----GCTTGGTGCATTTTCTAG-TTAACGGGCCAGCATCAGT

830Dai18640A ----TTTT----GCTTGGTGCATTTTCTAG-TTAACGGGCCAGCATCAGT

GC1704_60_Meruliopsis_taxicola ----TTTT----GCCTGGTGCATTTTCTAG-TTGACGGGCCAGCATCAGT

Dai22625 ----TTTT----GCCTGGTGCATTTTCTAG-TTGACGGGCCAGCATCAGT

Dai22636 ----TTTT----GCCTGGTGCATTTTCTAG-TTGACGGGCCAGCATCAGT

Dai21878 ----TTTT----GCCTGGTGCATTTTCTAG-TTGACGGGCCAGCATCAGT

1169Dai17248 ----TTTT----GCCTGGTGCATTTTCTAG-TTGACGGGCCAGCATCAGT

Wu1708_43_Meruliopsis_leptocys -----TTT----GCTTGGTGCATTTTCTAG-TTGACGGGCCAGCATCAGT

Li1011 -----TTT----GCTTGGTGCATTTTCTAG-TTGACGGGCCAGCATCAGT

ZX95Dai25742Meruliopsis_leptoc -----TTT----GCTTGGTGCATTTTCTAG-TTGACGGGCCAGCATCAGT

WCG1306Dai24733 -----TTT----GCTTGGTGCATTTTCTAG-TTGACGGGCCAGCATCAGT

LXL99Dai25816 -----TTT----GCTTGGTGCATTTTCTAG-TTGACGGGCCAGCATCAGT

WCG1559Dai26052Meruliopsis -----TTT----GCTCGGTGCATTTTCTAG-TTGACGGGCCAGCATCAGT

He7477 -----TTT----GCTCGGTGCATTTTCTAG-TTGACGGGCCAGCATCAGT

HLX243Dai26217 -----TTT----GCTCGGTGCATTTTCTAG-TTGACGGGCCAGCATCAGT

RussiaMW673659Meruliopsis_fagi -----TTT----GCTCGGTGCATTTTCTAG-TTGACGGGCCAGCATCAGT

FD278 ----TTTT----GCTTGGTGCATTTTCTAG-TTGACGGGCCAGCATCAGT

Dai10226_Ceriporia_tarda --

LE247365 ATTTTTTT----GCTTGGTGCATTTTCTAG-TTGACGGGCCAGCATCAGT

Dai8173_Meruliopsis_nanlingens A--TTTTT----GCTTGGTGCACTTTCTAG-TTGACGGGCCAGCATCAGT

860Dai17172 A--TTTTT----GCTTGGTGCACTTTCTAG-

TTGACGGGCCAGCATCAGT
879Dai13414 A--TTTTT----GCTTGGTGCACTTTCTAG-
TTGACGGGCCAGCATCAGT
Li_1704_Meruliopsis_pseudocyst ---
833Dai18405 ----TTTT----GCTCGGTGCATTTTCTAG-
TTGACGGGCCAGCATCAGT
HHB_10729_Meruliopsis_albostra T--TTTTT----GCTCGGTGCATTTTCTAG-
TTGACGGGCCAGCATCAGT
Cui6878_Ceriporia_pseudocystid ----TTTT----GCTCGGTGCATTTTCTAG-
TAGACGGGCCAGCATCAGT
869Dai14737 ----TTTT----GCTCGGTGCATTTTCTAG-
TAGACGGGCCAGCATCAGT
876Cui11626 ----TTTT----GCTCGGTGCATTTTCTAG-
TAGACGGGCCAGCATCAGT
1199WEI3388 ---
776308_Meruliopsis_cystidiata ----TTTT----GCTTGGTGCATTTTCTAG-
TAAACGGGCCAGCATCAGT
ICN139059_Meruliopsis_cystidia ----TTTT----GCTTGGTGCATTTTCTAG-
TAAACGGGCCAGCATCAGT
HHB15692Ceraceomyces_serpens ----A-TT----GCTCGGTGCATTTTCTAG-
TCGACGGGCCAGCATCAGT
HHB_15629_Sp_Ceriporiopsis_ane ----TT------
GCCTGGTGCATTTTCTAATTTAACGGGCCAGCATCAAT
AJ185Trametopsis_cervina ----TT------
GCTTGGTGCATTTTCTAGTTTAACGGGCCAGCATCAGT
FD9Irpex_lacteus ----TT------GCTTGGCGTATTTTCTAG-
TTAACGGGCCAGCATCAGT
908Dai11230 ----TT------GCTTGGCGTATTTTCTAG-
TTAACGGGCCAGCATCAGT
FP55521TEmmia_lacerata ----TT------GCTTGGCGTATTTTCTAG-
TTAACGGGCCAGCATCAGT
PBU0048Ceriporia_cystidiata ----TT------GCTTGGCGTATTTTCTAG-
TTAACGGGCCAGCATCAGT
MZ340C_lacerataT ---
Dai21940 ----TT------GCTTGGCGTATTTTCTAG-
TTAACGGGCCAGCATCAGT
847Dai16433 ----TT------GCTTGGCGTATTTTCTAG-
TTAACGGGCCAGCATCAGT
MarcinEmmia_latemarginatus ----TT------GCTTGGCGTATTTTCTAG-
TTAACGGGCCAGCATCAGT
Meijer3729Hydnopolyporus_fimbr ----TT------GCTTGGCGTACTTTCTAG-
TTAACGGGCCAGCATCAGT
RLG13408Phanerochaete_sp ----TT------GCTTGGTGCATTTTCTAG-
TTAACGGGCCAACATCAGT

WHC1381Flavodon_flavus ----TT------GCTTGGCGTATTTTCTAG-TTAACGGGCCAGCATCAGT

GB1833Phlebia_albida ----TT------ACTTGGTGCATTTTCTAG-TTAACGGGCCAGCATCAGT

T407Phlebia_nitidula ---

HHB6988Phanerochaete_exilis ----TT------GCCTGGTGCATTTTCTAT-TCAACGGGCCAGCATCAGT

HHB8509Phanerochaetella_xeroph ----TT------GCTTGGTGCATTTTCTAG-TCAACGGGCCAGCATCAGT

PBU0051Macrohyporia_dictyopora ----TT------GCTTGGTGCATTTTCTTA-TCAACGGGCCAGCATCAGT

HHB11463Phanerochaete_sp ----TT------GCTTGGTGCATTTTCTAT-CTAACGGGCCAGCATCAGT

FP102382Byssomerulius_corium ----TT------GCTTGGTGCAATTTGTAG-TTGACGGGCCAGCATCAGT

FP102165Efibula_americana ----TT------GCTTGGTGCATTTTCTAG-TCAACGGGCCAGCATCAGT

Murdoch90Ceriporia_torpida ----TTTT----GCTTGGTGCATTTTCTGA-TTGACGGGCCAGCATCAGT

Rivoire4413_Ceriporia_purpurea ----TTTT----GCTTGGTGCACTTTCTGA-TTGACGGGCCAGCATCAGT

Kout_18_Ceriporia_triumphalis ----TTTT----GCTTGGTGCACTTTCTGA-TTGACGGGCCAGCATCAGT

Rivoire3701_Ceriporia_bresadol ----TTTT----GCTTGGTGCACTTTCTGA-TTGACGGGCCAGCATCAGT

VS4018 ---

Ryvarden21832_Ceriporia_manzan ----TTTT----GCTTGGTGCACTTTCTGA-TTGACGGGCCAGCATCAGT

Dai24539 ----TTTT----GCTTGGTGCACTTTCTGA-TTGACGGGCCAGCATCAGT

Dai24541 ----TTTT----GCTTGGTGCACTTTCTGA-TTGACGGGCCAGCATCAGT

JV1105_12_Ceriporia_occidental ----TTTT--TTGCTTGGTGCATTTTCTGA-TTGACGGGCCAGCATCAGT

VS8558Ceriporia_occidentalis ----TTTT--TTGCTTGGTGCATTTTCTGA-TTGACGGGCCAGCATCAGT

Dai22445 ----TTTT----GCTTGGTGCACTTTCTGA-TTGACGGGCCAGCATCAGT

846Dai16368 ----TTTT----GCTTGGTGCACTTTCTGA-TTGACGGGCCAGCATCAGT

Dai17951_Ceriporia_aurantiocar ----TTTT----GCTTGGTGCACTTTCTGG-TTGACGGGTCAGCATCAGT

Miettinen_11701C_viridans ----TTTT----GCTTGGTGCACTTTCTGG-TTGACGGGCCAGCATCAGT

JV0105_10Ceriporia_aurantiocar ----TTTT----GCTTGGTGCACTTTCTGG-
TTGACGGGTCAGCATCAGT

Yuan5702C_viridans --

858Dai17003 --

Yuan2747_Ceriporia_viridans --

Yuan2744C_viridans --

Li1046C_viridans --

865C_sinoviridans --

871Dai15062 --

Dai7642_Ceriporia_humilis --

Spirin4706_Ceriporia_humilis --

Spirin4944_Ceriporia_sericea ----CTCG----GCTCGGTGCACTTTCTGA-
TTGACGGGCCAGCATCAGT

WCG1547Dai26044ceriporia ----CTCG----GCTCGGTGCACTTTCTGA-
TTGACGGGCCAGCATCAGT

ZZW1558Dai27086 ----CTCG----GCTCGGTGCACTTTCTGA-
TTGACGGGCCAGCATCAGT

Miettinen14381_Ceriporia_mpuri ----CTCG----GCTCGGTGCACTTTCTGA-
TTGACGGGCCAGCATCAGT

Miettinen15492_2_Ceriporia_sor ----CTCG----GCTCGGTGCACTTTCTGA-
TTGACGGGCCAGCATCAGT

He6687 ----CTCG----GCTCGGTGCACTTTCTGA-
TTGACGGGCCAGCATCAGT

ZH53Dai24426 ----CTCG----GCTCGGTGCACTTTCTGA-
TTGACGGGCCAGCATCAGT

Vlasak0808_30_Ceriporia_punica ----TTCC----GCTTGGTGTACTTTCTGG-
TTAACGGGCCAGCATCAGT

887Dai13376 ----TTTC----GCTTGGTGTACTTTCTGG-
TTAACGGGCCAGCATCAGT

WCG1443Dai24998 ----TTTC----GCTTGGTGTACTTTCTGG-
TTAACGGGCCAGCATCAGT

0108_6Ceriporia_spissa ---CTTTTG---GCTTGGTGTACTTTCTGA-
TTGACGGGTCAGCGTCTTT

Dai19164 ----CTTT--TGGCTTGGTGTACTTTCTGA-
TTGACGGGTCAGCGTCAGT

Dai17937_Ceriporia_bubalinomar ----CTTT--TGGCTTGGTGTACTTTCTGA-
TTGACGGGTCAGTGTCAGT

903Dai12113 ----CTTT--TGGCTTGGTGTACTTTCTGA-
TTGACGGGTCAGTGTCAGT

LZB929Dai25079 ----TTTT----GCTTGGTGTACTTTCTGA-
CTGACGGGCCAGCATCAGT

LX45Dai26988 --

LX43Dai26986 ----TTTT----GCTTGGTGTACTTTCTGA-
CTGACGGGCCAGCATCAGT

Dai7759Ceriporia ---
Cui8012_Ceriporia_viridans ---
GC1704_54Ceriporia_viridans ----TTTT----GCTTGGTGTACTTTCTGG-
CTGACGGGCCAGCATCAGT
Dai23392 ----TTTT----GCTTGGTGTACTTTCTGG-
CTGACGGGCCAGCATCAGT
WCG1585Dai26113Ceriproia ----TTTT----GCTTGGTGTACTTTCTGG-
CTGACGGGCCAGCATCAGT
Dai18675C_eucalypti ----TTTT----GCTTGGTGTACTTTCTGG-
CTGACGGGCCAGCATCAGT
Dai22034 ----TTTT----GCTTGGTGTACTTTCTAA-
TTGACGGGCCAGCATCAGT
JV1008_41JTardaFLORIDAKeys ---
Rivoire1161_Ceriporia_pierii ---
Dai23499C_pierii ----CTCG----GCTTGGTGCATTTTCTGA-
TTGACGGGTCAGCATCAGT
Dai23500 ----CTCG----GCTTGGTGCATTTTCTGA-
TTGACGGGTCAGCATCAGT
841Dai15899 ----TTTTTTTGCCTGGTGCATTTTCTGA-
TTGACGGGCCAGCATCAGT
842Dai15904 ---
LZB1066xinjiang ---
LZB1065xinjiang ---
851Dai16779 ----CTCG----GCTCGGTGCACTTTCTGA-
TTGACGGGCCAGCATCAGT
RMJ119sp_Candelabrochaete_sept ---
RLG9759spCandelabrochaete_sept ----TTCC----GCTTGGTGTACTTTCTGA-
CTGACGGGCCAGCATCAGT
RLG10478Phanerochaete_allantos ---
Dai19118_Ceriporia_spissa T--TTTTT----GCTTGGTGCATTTTCTGA-
TGGATAGGCCAGCATCAGT
Dai18486A ----TTTT----GCTTGGTGCATTTTCTGA-
TTGATAGGCCAGCATCAGT
WEI17_024_Ceriporia_mellita ----TTTT----GCTTGGTGCATTTTCTGA-
TTGATAGGCCAGCATCAGT
GC1508_71Ceriporia_mellita ----TTTT----GCTTGGTGCATTTTCTGA-
TTGATAGGCCAGCATCAGT
GC1608_7_Ceriporia_mellita ----TTTT----GCTTGGTGCATTTTCTGA-
TTGATAGGCCAGCATCAGT
ZZW1557Dai27085 ----TTTT----GCTTGGTGCATTTTCTGA-
TTGATAGGCCAGCATCAGT
ZZW1554Dai27083 ----TTTT----GCTTGGTGCATTTTCTGA-
TTGATAGGCCAGCATCAGT
Dai8168 ----TTTT----GCTTGGTGCATTTTCTGA-

TTGATAGGCCAGCATCAGT
BR4865C_mellita ---TTTTT----GCTTGGTGCATTTTCTGA-
TTGATAGGCCAGCATCAGT
MEL2382688Ceriporia_sp ---
Dai8110 ----TTTT----GCTTGGTGCATTTTCTGA-
TTGATAGGCCAGCATCAGT
Cui8097 ---
909Cui6740 ---
W1258Dai24695 T--TTTTT----GCTTGGTGCATTTTCTGA-
TTGATAGGCCAGCATCAGT
JV0110_26_Ceriporia_griseoviol ---TTTTT----GCTTGGTGCATTTTCTGA-
CTGACGGGCCAGCATCAGT
896Dai13202 ---TTTTT----GCTTGGTGCATTTTCTGA-
CTGACGGGCCAGCATCAGT
LWY393Dai27053C_griseoviolasce ---TTTTT----GCTTGGTGCATTTTCTGA-
CTGACGGGCCAGCATCAGT
LWY394DAI27054 ---TTTTT----GCTTGGTGCATTTTCTGA-
CTGACGGGCCAGCATCAGT
FP135015G_pannocinctus ----CTTG----GCATGGTGCATTTTCTGT-
TTAACGGGCCAGCATCAGT
L15726SpG_pannocinctus ----CTTG----GCATGGTGCATTTTCTGT-
TTAACGGGCCAGCATCAGT
Dai22221 ----TTCG----GTTTGGTGCATTTTCTGT-
TTAACGGGCCAGCATCAGT
Dai22633 ----CTCG----GTATGGTGCATTTTCTGT-
TTAACGGGCCAGCATCAGT
Dai23260 ----CTCG----GTATGGTGCATTTTCTGT-
TTAACGGGCCAGCATCAGT
Dai23626 ----CTCG----GTATGGTGCATTTTCTGT-
TTAACGGGCCAGCATCAGT
Dai16238G_citrinoalbus ----------TGGTATGGTGCA-TTTCTGT-
TTAACGGGCCAGCATCAGT
1175Dai15293 ----CTTG----GTATGGTGCATTTTCTGT-
TTAACGGGCCAGCATCAGT
Dai19547 ----CTTG----GTATGGTGCATTTTCTGT-
TTAACGGGCCAGCATCAGT
918063G_africanus ----CTTG----GTATGGTGCATTTTCTGT-
TTAACGGGCCAGCATCAGT
918572G_africanus ----CTTG----GTATGGTGCATTTTCTGT-
TTAACGGGCCAGCATCAGT
Dai18536A ---
1164Cui17922 ----CTCG----GTATGGTGCATTTTCTGT-
TTAACGGGCCAGCATCAGT
Dai22225 ----CTTG----GTATGGTGCATTTTCTGT-

TTAACGGGCCAGCATCAGT
1163Dai20655 ----TTTG----GTATGGTGCATTTTCTGT-
TTAACGGGCCAGCATCAGT
Yuan4397G_hainanensis ----CTCG----GCTTGGTGCATTTTCTGT-
TTAACGGGCCAGCATCAGT
1176Dai15268 ----CTCG----GCTTGGTGCATTTTCTGT-
TTAACGGGCCAGCATCAGT
1177Dai15259 ----CTCG----GCTTGGTGCATTTTCTGT-
TTAACGGGCCAGCATCAGT
BZ2896G_thelephoroides ----CTCG----GCTTGGTGCATTTTCTGT-
TTAACGGGCCAGCATCAGT
1166JV1808_26 ----CTCG----GCTTGGTGCATTTTCTGT-
TTAACGGGCCAGCATCAGT
Miettinen16992Hapalopilus_ochr ----TTTT----GCTTGGTGCATTTTCTGA-
TTGACGGGCCAGCATCAGT
GC1708_338_Ceriporia_arbuscula ----TTTT----GCTTGATGTATTTTCTGA-
CCGACGGGCCAGCATCAGT
WCG1555Dai26107Ceriporia ----TTTT----GCTTGATGTATTTTCTGA-
CCGACGGGCCAGCATCAGT
GC1708_340_Ceriporia_arbuscula ----TTTT----GCTTGATGTATTTTCTGA-
CCGACGGGCCAGCATCAGT
WCG1556Dai26109Ceriporia ----TTTT----GCTTGATGTATTTTCTGA-
CCGACGGGCCAGCATCAGT
883Cui11291 ----TTTT----GCTTGATGTATTTTCTGA-
CTGACGGGCCAGCATCAGT
HLX320Dai26805 A--TTTTT----GCTTGATGTATTTTCTGG-
CTGATGGGCCAGCATCAGT
WCG1266Dai24678A ---TTTTT----GCTTGATGTATTTTCTGA-
CTGATGGGCCAGCATCAGT
Dai6090_Ceriporia_sulphuricolo ----TTT-----GCTAGGTGCATTTTCTGG-
TCGACGGGCCAGCATCAGT
RLG_11354_Ceriproia_reticulata ----TTTT----GCTTGGTGCACTTTCTGA-
TTAACGGGCCAGCATCAGT
ZZW1543Dai27072 ----TTTT----GCTTGGTGCACTTTCTGA-
TTAACGGGCCAGCATCAGT
Li1316_Ceriporia_reticulata --
KHL11981Ceriporia_reticulata ----TTT-----GCTTGGTGCACTTTCTGA-
TTAACGGGCCAGCATCAGT
FP110343sp_Candelabrochaete_la ----CTCG----GCTTGGTGCACTTTCTGA-
TTAACGGGCCAGCATCAGT
Li1045_Ceriporia_reticulata --
ZX136Dai25794ceriporia ----TTTT----GCTTGGTGCACTTTCTAG-
TTAACGGGCCAGCATCAGT
892Dai13400 ----TTTT----GCTTGGTGCACTTTCTAG-

TTAACGGGCCAGCATCAGT
RLG7163Leptoporus_mollis ----TTTT----GCTCGGTGCATTTTCTGT-TTAACGGGCCAGCATCAGT
Dai21062Leptoporus_mollis ----TTTT----GCTCGGTGCATTTTCTGT-TTAACGGGCCAGCATCAGT
Dai20182Leptoporus_submollis ----TTTT----GCTCGGTGCATTTTCTGT-TTAACGGGCCAGCATCAGT
Cui18379Leptoporus_submollis ----TTTT----GCTCGGTGCATTTTCTGT-TTAACGGGCCAGCATCAGT
Wu1209_46Resiniporus_pseudogil ----TT------GCTTGGTGCATTTTCTAGTTTAACGGGCCAGCATCAGT
BRNM710169Resiniporus_resinasc ----------TTGYTTGGTGCATTTTCTAGTTTAACGGGCCAGCATCAGT
Dai14516Bjerkandera_adusta ----TTTT----GCTTGGTGCATTTTCTAG-TGTACGGGCCAGCATCAGT
Dai21100Bjerkandera_fumosa ----TTTT----GCTTGGTGCATTTTCTAG-TGTACGGGCCAGCATCAGT
Miettinen16854Ceraceomyces_sp ----TTTT----GCTAGGTGCATTTTCTGA-TTGACGGGTCAGCATCAGT
Dai10477C_spissa ----TTGT----GCTTGGTGTACTTTCTGT-TTAACGGGTCAGCATCAGT
855Dai16831 ----TTGT----GCTTGGTGTACTTTCTGT-TTAACGGGTCAGCATCAGT
882Cui11282 ----TTGT----GCTTGGTGTACTTTCTGT-TTAACGGGTCAGCATCAGT
Dai24566 ----TTTT----GCTTGGTGTACTTTCTGT-TTAACGGGTCAGCATCAGT
Yuan5965 ----TTTT----GCTTGGTGTACTTTCTGT-TTAACGGGTCAGCATCAGT
Dai3204 ----TTTT----GCTCGGTGCATTTTCTAG-TTGACGGGCCAGCATCAGT
1194CUI9985 ----TT------GCTTGGTGCATTTTCTAGTTTAACGGGCCAGCATCAGT

Dai15205_Ceriporia_albomellea TTTGACTGCGAGATAAAGGTCGGAGGAATGTGGCACCTTTGGGTGTGTTA
Dai15223_Ceriporia_albomellea TTTGACTGCGAGATAAAGGTCGGAGGAATGTGGCACCTTTGGGTGTGTTA
Li1780_Ceriporia_variegata TTTGACTGCGAGATAAAGGTCGGAGGAATGTGGCACCCTTGGGTGTGTTA
Dai19791_Ceriporia_variegata TCTGACTGCGAGATAAAGGTCGGAGGAATGTGGCACCCTTGGGTGTGTTA
Dai19886

TCTGACTGCGAGATAAAGGTCGGAGGAATGTGGCACCCTTGGGTGTGTTA
Dai10833_Ceriporia_crassitunic
TTTGGCTGCGAGATAAAGGTCGGAGGAATGTGGCACCTTTGGGTGTGTTA
CHWC1506_46Meruliopsis_crassit
TTTGGCTGCGAGATAAAGGTCGGAGGAATGTGGCACCTTTGGGTGTGTTA
Dai9995_Ceriporia_crassitunica --
Wu1209_58_Meruliopsis_parvispo
TTTGACTGCGGGATAAAGGTTAGAGGAATGTGGCACCTTCGGGTGTGTTA
CHWC1505_129_Meruliopsis_parvi
TTTGACTGCGGGATAAAGGTTAGAGGAATGTGGCACCTTCGGGTGTGTTA
Dai21944
TTTGACCGCGGGATAAAGATCAGGGGAATGTGGCACCTTCGGGTGTGTTA
830Dai18640A
TTTGATCGCGGGATAAAGGTTAGGGAAATGTGGCACCTTCGGGTGTGTTA
GC1704_60_Meruliopsis_taxicola
TTTGATTGCAGGATAAAGGTCAGAGGAATGTGGCACCTTCGGGTGTGTTA
Dai22625
TTTGATTGCAGGATAAAGGTCAGAGGAATGTGGCACCTTCGGGTGTGTTA
Dai22636
TTTGATTGCAGGATAAAGGTCAGAGGAATGTGGCACCTTCGGGTGTGTTA
Dai21878
TTTGATCGCAGGATAAAGGTCAGAGGAATGTGGCACCTTCGGGTGTGTTA
1169Dai17248
TTTGATTGCAGGATAAAGGTCAGAGGAATGTGGCACCTTCGGGTGTGTTA
Wu1708_43_Meruliopsis_leptocys
TTTGATTGCGGGATAAAGGTCAGAGGAATGTGGCACCTTCGGGTGTGTTA
Li1011
TTTGATTGCGGGATAAAGGTCAGAGGAATGTGGCACCTTCGGGTGTGTTA
ZX95Dai25742Meruliopsis_leptoc
TTTGATTGCGGGATAAAGGTCAGAGGAATGTGGCACCTTCGGGTGTGTTA
WCG1306Dai24733
TTTGATTGCGGGATAAAGGTCAGAGGAATGTGGCACCTTCGGGTGTGTTA
LXL99Dai25816
TTTGATTGCGGGATAAAGGTCAGAGGAATGTGGCACCTTCGGGTGTGTTA
WCG1559Dai26052Meruliopsis
TTTGATTGCGGGATAAAGGTCCGAGGAATGTGGCACCTTCGGGTGTGTTA
He7477
TTTGATTGCGGGATAAAGGTCCGAGGAATGTGGCACCTTCGGGTGTGTTA
HLX243Dai26217
TTTGATTGCGGGATAAAGGTCCGAGGAATGTGGCACCTTCGGGTGTGTTA
RussiaMW673659Meruliopsis_fagi
TTTGATTGCGGGATAAAGGTCCGAGGAATGTGGCACCTTCGGGTGTGTTA
FD278
TTTGATTGCGGGAGAAAGGTTAGAGGAATGTGGCACCCTCGGGTGTGTTA

Dai10226_Ceriporia_tarda ---
LE247365
TTTGACTGCGGGATAAAGGTCAGAGAAATGTGGCACCTTCGGGTGTGTTA
Dai8173_Meruliopsis_nanlingens
TTTGACTGCGGGATAAAGGTCAGAGAAATGTGGCACCTTCGGGTGTGTTA
860Dai17172
TTTGACTGCGGGATAAAGGTCAGAGAAATGTGGCACCTTCGGGTGTGTTA
879Dai13414
TTTGACTGCGGGATAAAGGTCAGAGAAATGTGGCACCTTCGGGTGTGTTA
Li_1704_Meruliopsis_pseudocyst ---
833Dai18405
TTTGACTGCAGGATAAAGGTCAGAGGAATGTGGCACCTTCGGGTGTGTTA
HHB_10729_Meruliopsis_albostra
TTTGACTGCAGGATAAAGGTCAGAGGAATGTGGCACCTTCGGGTGTGTTA
Cui6878_Ceriporia_pseudocystid
TTTGATTGCAGGATAAAGGTCAGGGGAATGTGGCACCTTCGGGTGTGTTA
869Dai14737
TTTGATTGCAGGATAAAGGTCAGGGGAATGTGGCACCTTCGGGTGTGTTA
876Cui11626
TTTGATTGCAGGATAAAGGTCAGGGGAATGTGGCACCTTCGGGTGTGTTA
1199WEI3388 ---
776308_Meruliopsis_cystidiata
TTTGACCGCAGGAAAAAGGTCAGGGGAATGTGGCACCTTCGGGTGTGTTA
ICN139059_Meruliopsis_cystidia
TTTGACCGCAGGAAAAAGGTCAGGGGAATGTGGCACCTTCGGGTGTGTTA
HHB15692Ceraceomyces_serpens
TTTGACCGCGGGACAAAGGTCGGAGGAATGTGGCACCTTCGGGTGTGTTA
HHB_15629_Sp_Ceriporiopsis_ane
TTTGGCTGCGGGATAAAGGTCAGAGAAATGTGGCAGCTTCGGCTGTGTTA
AJ185Trametopsis_cervina
TTTGGCTGCAGGATAAAGGTCAGAGAAATGTGGCAGCTTCGGCTGTGTTA
FD9Irpex_lacteus
TTTGACCGCAGGAAAAGGGCCAGGGAAATGTGGCATCTTCGGATGTGTTA
908Dai11230
TTTGACCGCAGGAAAAGGGCCAGGGAAATGTGGCATCTTCGGATGTGTTA
FP55521TEmmia_lacerata
TTTGACCGCAGGAAAAAGGCCAGAGAAATGTGGCACCTTCGGGTGTGTTA
PBU0048Ceriporia_cystidiata
TTTGACTGCAGGAAAAAGGCCAGAGAAATGTGGCACCTTCGGGTGTGTTA
MZ340C_lacerataT ---
Dai21940
TTTGACTGCAGGAAAAAGGCCAGAGAAATGTGGCACCTTCGGGTGTGTTA
847Dai16433
TTTGACTGCAGGAAAAAGGCCAGAGAAATGTGGCACCTTCGGGTGTGTTA

MarcinEmmia_latemarginatus
TTTGACCGCAGGAAAAAGGCCAGAGAAATGTGGCACCTTCGGGTGTGTTA
Meijer3729Hydnopolyporus_fimbr
TTTGACCACAGGATAAAGGCCAGGGAAATGTGGCACCTTCAGGTGTGTCA
RLG13408Phanerochaete_sp
TTTGACCGCAGGAAAAAGGCCAGGGAAATGTGGCACCTTCGGGTGTGTTA
WHC1381Flavodon_flavus
TTTGACCGCAGGAAAAAGGCCAGGGAAATGTGGCACCCTCGGGTGTGTTA
GB1833Phlebia_albida
TTTGACTGCAGGAAAAAGGCCAGAGAAATGTGGCATCTTCGGATGTGTTA
T407Phlebia_nitidula --
HHB6988Phanerochaete_exilis
TTTGACTGTAGGAAAAAGATCAGAGAAATGTGGCACCTTCGGGTGTGTTA
HHB8509Phanerochaetella_xeroph
TTTGACTGTAGGAAAAAGATCAGGGAAATGTGGCACCTTCGGGTGTGTTA
PBU0051Macrohyporia_dictyopora
TTTGACTGTAGGAAAAAGATCAGAGAAATGTGGCACCTTCGGGTGTGTTA
HHB11463Phanerochaete_sp
TTTGACTGTAGGAAAAAGATCAGAGAAATGTGGCACCTTCGGGTGTGTTA
FP102382Byssomerulius_corium
TTTGACCGCAGGAAAAAGGCCAGGGAAATGTGGCACCTTCGGGTGTGTTA
FP102165Efibula_americana
TTTGACTGCGGGAAAAAGGCCGGAGAAATGTGGCACCTTCGGGTGTGTTA
Murdoch90Ceriporia_torpida
TTTGACTGCAGGAAAAAGGTTAGAGGAATGTGGCATCTTCGGATGTGTTA
Rivoire4413_Ceriporia_purpurea
TTTGACTGCAGGAGAAAGGTCAGAGGAATGTGGCATCTTCGGATGTGTTA
Kout_18_Ceriporia_triumphalis
TTTGACTGCAGGAGAAAGGTCAGGGGAATGTGGCATCTTCGGATGTGTTA
Rivoire3701_Ceriporia_bresadol
TTTGACTGCAGGAGAAAGGTCAGGGGAATGTGGCATCTTCGGATGTGTTA
VS4018 --
Ryvarden21832_Ceriporia_manzan
TTTGACTGCAGGAGAAAGGTTGGAGGAATGTGGCACCTTCGGGTGTGTTA
Dai24539
TTTGACTGCAGGAGAAAGGTCAGGGGAATGTGGCATCTTCGGATGTGTTA
Dai24541
TTTGACTGCAGGAGAAAGGTCAGGGGAATGTGGCATCTTCGGATGTGTTA
JV1105_12_Ceriporia_occidental
TTTGACTGCAGGAAAAAGGTTGGAGGAATGTGGCACCTTCGGGTGTGTTA
VS8558Ceriporia_occidentalis
TTTGACTGCAGGAAAAAGGTTGGAGGAATGTGGCACCTTCGGGTGTGTTA
Dai22445
TTTGACTGCAGGAGAAAGGTCAGAGGAATGTGGCATCTTCGGATGTGTTA

846Dai16368
TTTGACTGCAGGAAAAAGGTCAGAGGAATGTGGCATCTTCGGATGTGTTA
Dai17951_Ceriporia_aurantiocar
TTTGATTGTGGGAAAAAAGTTAGAGGAATGTGGCACCTTCGGGTGTGTTA
Miettinen_11701C_viridans
TTCGATTGTGGGAAAAAGGTTGGAGGAAGGTGGCACCTTCGGGTGTGTTA
JV0105_10Ceriporia_aurantiocar
TTTGATTGTGGGAAAAAAGTTAGAGGAATGTGGCACCTTCGGGTGTGTTA
Yuan5702C_viridans --
858Dai17003 --
Yuan2747_Ceriporia_viridans --
Yuan2744C_viridans --
Li1046C_viridans --
865C_sinoviridans --
871Dai15062 --
Dai7642_Ceriporia_humilis --
Spirin4706_Ceriporia_humilis --
Spirin4944_Ceriporia_sericea
TTCGGCTGCAGGAGAAAGGTCAGAGGAATGTGGCACCTTCGGGTGTGTTA
WCG1547Dai26044ceriporia
TTCGGCTGCAGGAGAAAGGTCAGAGGAATGTGGCACCTTCGGGTGTGTTA
ZZW1558Dai27086
TTCGGCTGCAGGAGAAAGGTCAGAGGAATGTGGCACCTTCGGGTGTGTTA
Miettinen14381_Ceriporia_mpuri
TTCGGCTGCAGGAGAAAGGTCGGGGGAATGTGGCACCTTCGGGTGTGTTA
Miettinen15492_2_Ceriporia_sor
TTCGACTGCAGGAGAAAGGTCGGAGGAATGTGGCACCTTCGGGTGTGTTA
He6687
TTCGGCTGCAGGAGAAAGGTCAGAGGAATGTGGCACCTTCGGGTGTGTTA
ZH53Dai24426
TTCGGCTGCAGGAGAAAGGTCAGGGGAATGTGGCACCTTCGGGTGTGTTA
Vlasak0808_30_Ceriporia_punica
TTTGACTGCAGGATAAAAGTCAGAGGAATGTGGCACCTTCGGGTGTGTTA
887Dai13376
TTTGACTGCAGGATAAAAGTCAGAGGAATGTGGCACCTTCGGGTGTGTTA
WCG1443Dai24998
TTCGGCTGCAGGATAAAAGTCAGAGGAATGTGGCACCTTCGGGTGTGTTA
0108_6Ceriporia_spissa
TTTGACCGCAGGAGAAAGGTCAGAGGAATGTGGCATCTTCGGATGTGTTA
Dai19164
TTTGACCGCAGGAGAAAGGTCAGAGGAATGTGGCATCTTCGGATGTGTTA
Dai17937_Ceriporia_bubalinomar
TTTGATTGCAGGAGAAAGGTCAGAGGAATGTGGCATCTTCGGGTGTGTTA
903Dai12113

TTTGATTGCAGGAGAAAGGTCAGAGGAATGTGGCATCTTCGGGTGTGTTA

LZB929Dai25079

TTTGATTGTGGGAAAAAGGTTGGGGGAATGTGGCACCTTCGGGTGTGTTA

LX45Dai26988 ---

LX43Dai26986

TTTGATTGTGGGAAAAAGGTTGGGGGAATGTGGCACCTTCGGGTGTGTTA

Dai7759Ceriporia ---

Cui8012_Ceriporia_viridans ---

GC1704_54Ceriporia_viridans

TTTGATTGTGGGAAAAAGGTCAGAGAAATGTGGCACCTTCGGGTGTGTTA

Dai23392

TTTGATTGTGGGAAAAAGGTCAGAGAAATGTGGCACCTTCGGGTGTGTTA

WCG1585Dai26113Ceriproia

TTTGATTGTGGGAAAAAGGTCAGAGAAATGTGGCACCTTCGGGTGTGTTA

Dai18675C_eucalypti

TTTGATTGTGGGAAAAAGGTCAGAGAAATGTGGCACCTTCGGGTGTGTTA

Dai22034

TTTGATTGCAGGAAAAAGGTCAGAGGAATGTGGCACCTTCGGGTGTGTTA

JV1008_41JTardaFLORIDAKeys ---

Rivoire1161_Ceriporia_pierii ---

Dai23499C_pierii

TTTGACTGCAGGAGAAAGGTCAGAGGAATGTGGCACCTTCGGGTGTGTTA

Dai23500

TTTGACTGCAGGAGAAAGGTCAGAGGAATGTGGCACCTTCGGGTGTGTTA

841Dai15899

TTTGACTGTAGGAAAAAGGTTGGAGGAATGTGGCACCTTCGGGTGTGTTA

842Dai15904 ---

LZB1066xinjiang ---

LZB1065xinjiang ---

851Dai16779

TTTGACCGCAGGAAAAAGGTCAGAGGAATGTGGCACCTTCGGGTGTGTTA

RMJ119sp_Candelabrochaete_sept ---

RLG9759spCandelabrochaete_sept

TTCGGTTGCGGGAGAAAGGTCAGGGGAATGTGGCACCTCCGGGTGTGTTA

RLG10478Phanerochaete_allantos ---

Dai19118_Ceriporia_spissa

TTTGGTCATAGGAGAAAGGTCAGAGAAATGTGGCACCCTCGGGTGTGTTA

Dai18486A

TTTGGTCATAGGAGAAAGGTCAGAGAAATGTGGCACCCTCGGGTGTGTTA

WEI17_024_Ceriporia_mellita

TTTGGTCATAGGAGAAAGGTCAGAGAAATGTGGCACCCTCGGGTGTGTTA

GC1508_71Ceriporia_mellita

TTTGGTCATAGGAGAAAGGTCAGAGAAATGTGGCACCCTCGGGTGTGTTA

GC1608_7_Ceriporia_mellita

TTTGGTCATAGGAGAAAGGTCAGAGAAATGTGGCACCCTCGGGTGTGTTA
ZZW1557Dai27085
TTTGGTCATAGGAGAAAGGTCAGAGAAATGTGGCACCCTCGGGTGTGTTA
ZZW1554Dai27083
TTTGGTCATAGGAGAAAGGTCAGAGAAATGTGGCACCCTCGGGTGTGTTA
Dai8168
TTTGGTCATAGGAGAAAGGTCAGAGAAATGTGGCACCCTCGGGTGTGTTA
BR4865C_mellita
TTTGGTCATAGGAGAAAGGTTAGAGAAATGTGGCACCCTCGGGTGTGTTA
MEL2382688Ceriporia_sp --
Dai8110
TTTGGTCGTAGGAGAAAGGTCAGAGAAATGTGGCACTCTTGAGTGTGTTA
Cui8097 --
909Cui6740 --
W1258Dai24695
TTTGATTGTAGGAGAAAGGTTAGAGAAATGTGGCACTCTTGAGTGTGTTA
JV0110_26_Ceriporia_griseoviol
TTTGACTGCGGGAGAAAGGTCAGAGGAATGTGGCACCTCCGGGTGTGTTA
896Dai13202
TTTGACTGCGGGAGAAAGGTCAGAGGAATGTGGCACCTCCGGGTGTGTTA
LWY393Dai27053C_griseoviolasce
TTTGACTGCGGGAGAAAGGTCAGAGGAATGTGGCACCTCCGGGTGTGTTA
LWY394DAI27054
TTTGACTGCGGGAGAAAGGTCAGAGGAATGTGGCACCTCCGGGTGTGTTA
FP135015G_pannocinctus
TTTGATTGCCGGAAAAAGGTCAGAGGAATGTGGCACCTTCGGGTGTGTTA
L15726SpG_pannocinctus
TTTGATTGCCGGAAAAAGGTCAGAGGAATGTGGCACCTTCGGGTGTGTTA
Dai22221
TTTGACTGCCGGAAAAAGGTCAGAGAAATGTGGCACCTTCGGGTGTGTTA
Dai22633
TTTGATTGCCGGAAAAAGGTTAGAGAAATGTGGCACCTTCGGGTGTGTTA
Dai23260
TTTGATTGCCGGAAAAAGGTTAGAGAAATGTGGCACCTTCGGGTGTGTTA
Dai23626
TTTGATTGCCGGAAAAAGGTTAGAGAAATGTGGCACCTTCGGGTGTGTTA
Dai16238G_citrinoalbus
TTTGACCGCTGGAAAAAGGTTAGAGAAATGTGGCACATTCGGGTGTGTTA
1175Dai15293
TTTGACCGCTGGAAAAAGGTTAGAGAAATGTGGCACCTTCGGGTGTGTTA
Dai19547
TTTGACCGCTGGAAAAAGGTTAGAGAAATGTGGCACCTTCGGGTGTGTTA
918063G_africanus
TTTGACCGCTGGAAAAAGGTTAGAGAAATGTGGCACCTTCGGGTGTGTTA

918572G_africanus

TTTGACCGCTGGAAAAAGGTTAGAGAAATGTGGCACCTTCGGGTGTGTTA

Dai18536A --

1164Cui17922

TTTGACCGCTGGAAAAAGGTCAGAGAAATGTGGCACCTTCGGGTGTGTTA

Dai22225

TTTGACCGCTGGAAAAAGGTTAGAGAAATGTGGCACCTTCGGGTGTGTTA

1163Dai20655

TTTGACCGCTGGAAAAAGGTTAGAGAAATGTGGCACCTTCGGGTGTGTTA

Yuan4397G_hainanensis

TTTGATTGCCGGAAAAAGATAAAGGGAATGTGGCACCCTCGGGTGTGTTA

1176Dai15268

TTTGATTGCCGGAAAAAGATAAAGGGAATGTGGCACCCTCGGGTGTGTTA

1177Dai15259

TTTGATTGCCGGAAAAAGATAAAGGGAATGTGGCACCCTCGGGTGTGTTA

BZ2896G_thelephoroides

TTTGACTGCCGGAAAAAGATGAAGGGAATGTGGCACCCTCGGGTGTGTTA

1166JV1808_26

TTTGACTGCCGGAAAAAGATGAAGGGAATGTGGCACCCTCGGGTGTGTTA

Miettinen16992Hapalopilus_ochr

TTTGATTGTGAGATAAAGGTCAGAGGAATGTGGCATCTTCGGGTGTGTTA

GC1708_338_Ceriporia_arbuscula

TTGAGCTGTAGGAAAAAGATGGGAGAAATGTGGCACCCTCGGGTGTGTTA

WCG1555Dai26107Ceriporia

TTGAGCTGTAGGAAAAAGATGGGAGAAATGTGGCACCCTCGGGTGTGTTA

GC1708_340_Ceriporia_arbuscula

TTGAGCTGTAGGAAAAAGATGGGAGAAATGTGGCACCCTCGGGTGTGTTA

WCG1556Dai26109Ceriporia

TTGAGCTGTAGGAAAAAGATGGGAGAAATGTGGCACCCTCGGGTGTGTTA

883Cui11291

TTTAGCTGTAGGAAAAAGATTAGAGAAATGTGGCACCCTTGGGTGTGTTA

HLX320Dai26805

TTTAGCTGTAGGAAAAAGGTTAGAGAAATGTGGCACCCTTGGGTGTGTTA

WCG1266Dai24678A

TTTAGTTGTAGGAAAAAGATTAGAGAAATGTGGCACCCTTGGGTGTGTTA

Dai6090_Ceriporia_sulphuricolo

TTTGACCGCGGTACAAAGGTCAGAGGAATGTGGCACCTTCGGGTGTGTTA

RLG_11354_Ceriproia_reticulata

TTTGACTACAGGAAAAAGATTAGAGGAATGTGGCACCTTCGGGTGTGTTA

ZZW1543Dai27072

TTTGACTACAGGAAAAAGATTAGAGGAATGTGGCACCTTCGGGTGTGTTA

Li1316_Ceriporia_reticulata --

KHL11981Ceriporia_reticulata

TTTGACTACAGGAAAAAGATTAGAGGAATGTGGCACCTTCGGGTGTGTTA

FP110343sp_Candelabrochaete_la
TTTGATTGCAGGAAAAAGATTAGGGGAATGTGGCACCTCCGGGTGTGTTA
Li1045_Ceriporia_reticulata --
ZX136Dai25794ceriporia
TTTGACTGCAGGAAAAAGATTAGAGGAATGTGGCACCTTCGGGTGTGTTA
892Dai13400
TTTGACTGCAGGAAAAAGATTAGAGGAATGTGGCACCTTCGGGTGTGTTA
RLG7163Leptoporus_mollis
TTTGACTGCAGGAGAAAGGTCAGAGGAATGTGGCACCTTCGGGTGTGTTA
Dai21062Leptoporus_mollis
TTTGACTGCAGGAGAAAGGTCAGAGGAACGTGGCACCTTCGGGTGTGTTA
Dai20182Leptoporus_submollis
TTTGACTGCAGGAGAAAGGTCAGGGGAATGTGGCACCTTCGGGTGTGTTA
Cui18379Leptoporus_submollis
TTTGACTGCAGGAGAAAGGTCAGGGGAATGTGGCACCTTCGGGTGTGTTA
Wu1209_46Resiniporus_pseudogil
TTTGGCTGCAGGATAAAGATTAGAGAAATGTGGCAGCCTCGGCTGTGTTA
BRNM710169Resiniporus_resinasc
TTTGGCTGCAGGATAAAGATTAGAGAAATGTGGCAGCCTCGGCTGTGTTA
Dai14516Bjerkandera_adusta
TTTGGCCGCCGGAAAAAGGCCTTGGGAATGTGGCACCTTCGGGTGTGTTA
Dai21100Bjerkandera_fumosa
TTCGGTCGCCGGAAAAAGGCCTTGGGAATGTGGCACCTTCGGGTGTGTTA
Miettinen16854Ceraceomyces_sp
TTTGATTGCGAGATAAAGGTCGGAGAAATGTGGCACCTTCGGGTGTGTTA
Dai10477C_spissa
TTTGGTTGCAGGAAAAAGATTAGAGGAACGTGGCACCTCCGGGTGTGTTA
855Dai16831
TTTGGTTGCAGGAAAAAGATTAGAGGAACGTGGCACCTCCGGGTGTGTTA
882Cui11282
TTTGGTTGCAGGAAAAAGATTAGAGGAACGTGGCACCTCCGGGTGTGTTA
Dai24566
TTCGGTTGCAGGAAAAAGATTAGAGGAATGTGGCACCTCCGGGTGTGTTA
Yuan5965
TTCGGTTGCAGGAAAAAGATTAGAGGAATGTGGCACCTCCGGGTGTGTTA
Dai3204
TTTGACTGCAGGATAAAGGTCAGAGGAATGTGGCACCTTCGGGTGTGTTA
1194CUI9985
TTTGGCTGCAGGATAAAGGTCAGAGAAATGTGGCAGCTTCGGCTGTGTTA

Dai15205_Ceriporia_albomellea
TAGCCTCTGATTGTATATCGCAGCTGGGACTGAGGATCTCAGCACGCCTT
Dai15223_Ceriporia_albomellea

TAGCCTCTGATTGTATATCGCAGCTGGGACTGAGGATCTCAGCACGCCTT

Li1780_Ceriporia_variegata

TAGCCTTTGATTGTATATCGCAGCTGGGACTGAGGATCTCAGCACGCCTT

Dai19791_Ceriporia_variegata

TAGCCTTTGATTGTATATCGCAGCTGGGACTGAGGATCTCAGCACGCCTT

Dai19886

TAGCCTTTGATTGTATATCGCAGCTGGGACTGAGGATCTCAGCACGCCTT

Dai10833_Ceriporia_crassitunic

TAGCCTTTGATTGTATATCGCAGCTGGGACTGAGGATCTCAGCACGCCTT

CHWC1506_46Meruliopsis_crassit

TAGCCTTTGATTGTATATCGCAGCTGGGACTGAGGATCTCAGCACGCCTT

Dai9995_Ceriporia_crassitunica --

Wu1209_58_Meruliopsis_parvispo

TAGCCTCTGATTGTATACCGCGGTTGGGACTGAGGATCTCAGCACGCCTT

CHWC1505_129_Meruliopsis_parvi

TAGCCTCTGATTGTATACCGCGGTTGGGACTGAGGATCTCAGAACGCCTT

Dai21944

TAGCCTCTGGTTGTATACCGTGGTTGGGACTGAGGATCTCAGTACGCCTT

830Dai18640A

TAGCCTCTGATTGTATGCCGCGGTTGGGACTGAGGATCTCAGCACGCCTT

GC1704_60_Meruliopsis_taxicola

TAGCCTTTGATTGTATACTGTGGTTGGGACTGAGGATCACAGCACGCCTT

Dai22625

TAGCCTTTGATTGTATACTGTGGTTGGGACTGAGGATCACAGCACGCCTT

Dai22636

TAGCCTTTGATTGTATACTGTGGTTGGGACTGAGGATCACAGCACGCCTT

Dai21878

TAGCCTTTGATTGTATACTGTGGTTGGGACTGAGGATCACAGCACGCCTT

1169Dai17248

TAGCCTTTGATTGTATACTGTGGTTGGGACTGAGGATCACAGCACGCCTT

Wu1708_43_Meruliopsis_leptocys

TAGCCTCAGATTGTATACCGTGGTTGGGACTGAGGATCTCAGCACGCCTT

Li1011

TAGCCTCAGATTGTATACCGTGGTTGGGACTGAGGATCTCAGCACGCCTT

ZX95Dai25742Meruliopsis_leptoc

TAGCCTCAGATTGTATACCGTGGTTGGGACTGAGGATCTCAGCACGCCTT

WCG1306Dai24733

TAGCCTCAGATTGTATACCGTGGTTGGGACTGAGGATCTCAGCACGCCTT

LXL99Dai25816

TAGCCTCAGATTGTATACCGTGGTTGGGACTGAGGATCTCAGCACGCCTT

WCG1559Dai26052Meruliopsis

TAGCCTCGGATTGTATACCGTGGTTGGGACTGAGGATCTCAGCACGCCTT

He7477

TAGCCTCGGATTGTATACCGTGGTTGGGACTGAGGATCTCAGCACGCCTT

HLX243Dai26217

TAGCCTCGGATTGTATACCGTGGTTGGGACTGAGGATCTCAGCACGCCTT

RussiaMW673659Meruliopsis_fagi

TAGCCTCGGATTGTATACCGTGGTTGGGACTGAGGATCTCAGCACGCCTT

FD278

TAGCCTCTGATTGTATACCGCAGTTGGGACTGAGGATCTCAGCACGCCTT

Dai10226_Ceriporia_tarda --

LE247365

TAGTCTCTGATTGTATACTGTGGTTGGGACTGAGGATCTCAGCACGCCTT

Dai8173_Meruliopsis_nanlingens

TAGTCTCTGATTGTATACCGTGGTTAGGACTGAGGATCTCAGCACGCCTT

860Dai17172

TAGTCTCTGATTGTATACCGTGGTTAGGACTGAGGATCTCAGCACGCCTT

879Dai13414

TAGTCTCTGATTGTATACCGTGGTTAGGACTGAGGATCTCAGCACGCCTT

Li_1704_Meruliopsis_pseudocyst --

833Dai18405

TAGCCTTTGATTGTATACTGTGGTTGGGACTGAGGATCTCAGCACGCCTT

HHB_10729_Meruliopsis_albostra

TAGCCTTTGATTGTATACTGTGGTTGGGACTGAGGATCTCAGCACGCCTT

Cui6878_Ceriporia_pseudocystid

TAGCCTCTGATTGCATACTGTGATTGGGACTGAGGATCTCAGCACGCCTT

869Dai14737

TAGCCTCTGATTGCATACTGTGATTGGGACTGAGGATCTCAGCACGCCTT

876Cui11626

TAGCCTCTGATTGCATACTGTGATTGGGACTGAGGATCTCAGCACGCCTT

1199WEI3388 --

776308_Meruliopsis_cystidiata

TAGCCTCTGATTGTATACTGTGGTTGGGACTGAGGATCTCAGCACGCCTT

ICN139059_Meruliopsis_cystidia

TAGCCTCTGATTGTATACTGTGGTTGGGACTGAGGATCTCAGCACGCCTT

HHB15692Ceraceomyces_serpens

TAGCCTCTGGCTGCATGCCGTGATTGGGACTGAGGAACTCAGCACGCATC

HHB_15629_Sp_Ceriporiopsis_ane

TAGTCTCTGGCTGCATACCGTGACCGGGATTGAGGATCTCAGCACGCATT

AJ185Trametopsis_cervina

TAGTCTCTGGCTGCATACTGTGTCCGGGACTGAGGATCTCAGCACGCATT

FD9Irpex_lacteus

TAGTCTCTGGTCGTATACTGTGATTGGGACTGAGGACCGCAGCACGCGCA

908Dai11230

TAGTCTCTGGTCGTATACTGTGATTGGGACTGAGGACCGCAGCACGCGCA

FP55521TEmmia_lacerata

TAGTCTTTGGTCATATACTGCGATTGGGACTGAGGTTCGCAGCACGCGCA

PBU0048Ceriporia_cystidiata

TAGTCTTTGGTCATATACTGCGATTGGGACTGAGGTTCGCAGCACGCGCA

MZ340C_lacerataT --

Dai21940

TAGTCTTTGGTCATATACTGCGATTGGGACTGAGGTTCGCAGCACGCGCA

847Dai16433

TAGTCTTTGGTCATATACTGCGATTGGGACTGAGGTTCGCAGCACGCGCA

MarcinEmmia_latemarginatus

TAGTCTTTGGTCATATACTGCGATTGGGACTGAGGTTCGCAGCACGCGCA

Meijer3729Hydnopolyporus_fimbr

TAGTCTCTGGTCACATACTGTGATTGGGACTGAGGATCGCAGCACGCGCA

RLG13408Phanerochaete_sp

TAGTCTCTGGTCGTATACTGTGATTGGGACTGAGGCTCTCAGCACGCATC

WHC1381Flavodon_flavus

TAGTCTCTGGTCATATACTGTGATTGGGACTGAGGACCGCAGCACGCGCA

GB1833Phlebia_albida

TAGTCTCTGGTTGCATACTGTGGTTGGGATTGAGGCTCTCAGCACGCTTT

T407Phlebia_nitidula --

HHB6988Phanerochaete_exilis

TAGTCTTTGGTTGCATGCTATGGTTGGGACTGAGGATCTCAGCACGCATC

HHB8509Phanerochaetella_xeroph

TAGTCTCTGGTTGCATGCTATGGTTGGGACTGAGGATCTCAGCACGCATC

PBU0051Macrohyporia_dictyopora

TAGTCTTTGGTTGCATACTATGGTTGGGACTGAGGATCTCAGCACGCATC

HHB11463Phanerochaete_sp

TAGTCTTTGGTTGCATACTATGGTTGGGACTGAGGATCTCAGCACGCATC

FP102382Byssomerulius_corium

TAGTCCTTGGTTGTATACTGTGATTGGGACTGAGGCTCTCAGCACGCTTT

FP102165Efibula_americana

TAGTCTCTGGTTGCATACCGTGGTTGGGACTGAGGCTCTCAGCACGCGCA

Murdoch90Ceriporia_torpida

TAGCCTCTGATTGTATACTGTGATTGGGACTGAGGAACTCAGCACGCGTA

Rivoire4413_Ceriporia_purpurea

TAGCCTCTGATTGTATACTGTGATTGGGACTGAGGAACTCAGCACGCGCA

Kout_18_Ceriporia_triumphalis

TAGACTCTGATTGTATACTGTGATTGGGACTGAGGAACTCAGCACGCGCA

Rivoire3701_Ceriporia_bresadol

TAGCCTCTGATTGTATACTGTGATTGGGACTGAGGAACTCAGCACGCGCA

VS4018 --

Ryvarden21832_Ceriporia_manzan

TAGCCTCTGATTGTATACTGTGATTGGGACTGAGGAACTCAGCACGCGCA

Dai24539

TAGCCTCTGATTGTATACTGTGATTGGGACTGAGGAACTCAGCACGCGCA

Dai24541

TAGCCTCTGATTGTATACTGTGATTGGGACTGAGGAACTCAGCACGCGCA

JV1105_12_Ceriporia_occidental
TAGCCTCTGATTGCATACTGTGATTGGGACTGAGGAACTCAGCACGCGCA
VS8558Ceriporia_occidentalis
TAGCCTCTGATTGCATACTGTGATTGGGACTGAGGAACTCAGCACGCGCA
Dai22445
TAGCCTCTGATTGTATACTGTGATTGGGACTGAGGAACTCAGCACGCGCA
846Dai16368
TAGCCTCTGATTGCATACTGTGATTGGGACTGAGGAACTCAGCACGCGCA
Dai17951_Ceriporia_aurantiocar
TAGCCTCTGATCGCATACCATGGTTGGGACTGAGGATCTCAGCACGCGTA
Miettinen_11701C_viridans
TAGCCTCTGATTGTATACCATGRTTGGGATTGAGGATCTCAGCACGCGTA
JV0105_10Ceriporia_aurantiocar
TAGCCTCTGATCGCATACCATGGTTGGGACTGAGGATCTCAGCACGCGTA
Yuan5702C_viridans --
858Dai17003 --
Yuan2747_Ceriporia_viridans --
Yuan2744C_viridans --
Li1046C_viridans --
865C_sinoviridans --
871Dai15062 --
Dai7642_Ceriporia_humilis --
Spirin4706_Ceriporia_humilis --
Spirin4944_Ceriporia_sericea
TAGCCTCCGACTGCATACTGTGTCTGGGACTGAGGAATTCAGCACGTGCA
WCG1547Dai26044ceriporia
TAGCCTCCGACTGCATACTGTGTCTGGGACTGAGGAATTCAGCACGTGCA
ZZW1558Dai27086
TAGCCTCCGACTGCATACTGTGTCTGGGACTGAGGAATTCAGCACGTGCA
Miettinen14381_Ceriporia_mpuri
TAGCCTCTGACTGCATACTGTGTCTGGGACTGAGGAATTCAGCACGTGCA
Miettinen15492_2_Ceriporia_sor
TAGCCTCTGACTGCATACTGTGGTTGGGACTGAGGAACTCAGCACGTGCA
He6687
TAGCCTCGGACTGCATACTGTGTCTGGGACTGAGGAATTCAGCACGTGCA
ZH53Dai24426
TAGCCCTCGACTGCATACTGTGTCTGGGACTGAGGAATTCAGCACGTGCA
Vlasak0808_30_Ceriporia_punica
TAACCTTTGGCTGCATACTGCGGCTGGGACTGAGGAACTCAGCACGTGTA
887Dai13376
TAACCTTTGGCTGCATACTGCGGCTGGGACTGAGGAACTCAGCACGTGCA
WCG1443Dai24998
TAACCTTTGGCTGCATACTGCGACTGGGACTGAGGAACTCAGCACGTGCA
0108_6Ceriporia_spissa

TAGCCTCTGACTGCATGCTGTGGTTGGGACTGAGGAACTCAGCACGCGCA

Dai19164

TAGCCTCTGACTGCATGCTGTGGTTGGGACTGAGGAACTCAGCACGCGCA

Dai17937_Ceriporia_bubalinomar

TAGCCTTTGACTGTATACTGTGATTGGGACTGAGGAACTCAGCACGCGCA

903Dai12113

TAGCCTTTGACTGTATACTGTGATTGGGACTGAGGAACTCAGCACGCGCA

LZB929Dai25079

TAGCCTCTGATTGCATACCATGGTTGGGACTGAGGATCTCAGCACGCGCA

LX45Dai26988 --

LX43Dai26986

TAGCCTCTGATTGCATACCATGGTTGGGACTGAGGATCTCAGCACGCGCA

Dai7759Ceriporia --

Cui8012_Ceriporia_viridans --

GC1704_54Ceriporia_viridans

TAGTCTTTGATTGTATACCATGGTTGGGACTGAGGATCTCAGCACGCGCA

Dai23392

TAGTCTTTGATTGTATACCATGGTTGGGACTGAGGATCTCAGCACGCGCA

WCG1585Dai26113Ceriproia

TAGTCTCTGATTGTATACCATGGTTGGGACTGAGGATCTCAGCACGCGCA

Dai18675C_eucalypti

TAGTCTTTGATTGTATACCATGGTTGGGACTGAGGATCTCAGCACGCGCA

Dai22034

TAGCCTCTGATTGCATACTGTGATTGGGACTGAGGAACTCAGCACGCGCA

JV1008_41JTardaFLORIDAKeys --

Rivoire1161_Ceriporia_pierii --

Dai23499C_pierii

TAGCCTCTGGCTGCATACTGTGATTGGGACTGAGGAACTCAGCACGTGCA

Dai23500

TAGCCTCTGGCTGCATACTGTGATTGGGACTGAGGAACTCAGCACGTGCA

841Dai15899

TAGCCTCTGATTGTATACTATGGTTGGGATTGAGGAACTCAGCACGCGCA

842Dai15904 --

LZB1066xinjiang --

LZB1065xinjiang --

851Dai16779

TAGCCTCTGGCTGCATACTGTGATTGGGACTGAGGAACTCAGCACGTGCA

RMJ119sp_Candelabrochaete_sept --

RLG9759spCandelabrochaete_sept

TAGCCCCTGGCTGCATGCCGTGGTCGGGACTGAGGAACTCAGCACGCCCT

RLG10478Phanerochaete_allantos --

Dai19118_Ceriporia_spissa

TAGTCTTTGACTGTATACTATGACTGAGACTGAGGAACTCAGCACGCGCA

Dai18486A

TAGTCTTTGACTGTATACTATGACTGAGACTGAGGAACTCAGCACGCGCA
WEI17_024_Ceriporia_mellita
TAGTCTTTGACTGCATACTATGACTGAGACTGAGGAACTCAGCACGCGCA
GC1508_71Ceriporia_mellita
TAGTCTTTGACTGCATACTATGACTGAGACTGAGGAACTCAGCACGCGCA
GC1608_7_Ceriporia_mellita
TAGTCTTCGATTGTATACTATGACTGAGACTGAGGAACTCAGCACGCGCA
ZZW1557Dai27085
TAGTCTTCGATTGTATACTATGACTGAGACTGAGGAACTCAGCACGCGCA
ZZW1554Dai27083
TAGTCTTCGATTGTATACTATGACTGAGACTGAGGAACTCAGCACGCGCA
Dai8168
TAGTCTTTGATTGTATACTACGACTGAGACTGAGGAACTCAGCACGCGCA
BR4865C_mellita
TAGTCTTTGACTGTATACTATGACTGAGACTGAGGAACTCAGCACGCGCA
MEL2382688Ceriporia_sp --
Dai8110
TAGTCTTTGACTGTATACTATGACTGAGACTGAGGAACTCAGCACGCGCA
Cui8097 --
909Cui6740 --
W1258Dai24695
TAGTCTTTGACTGCATACTATGATCGAGACTGAGGAACTCAGCACGCGCA
JV0110_26_Ceriporia_griseoviol
TAGCCTTTGATCGCATGCCGTGGTTGGGACTGAGGAACTCAGCACGCCTT
896Dai13202
TAGCCTTTGATCGCATGCCGTGGTTGGGACTGAGGAACTCAGCACGCCTT
LWY393Dai27053C_griseoviolasce
TAGCCTTTGATCGCATGCCGTGGTTGGGACTGAGGAACTCAGCACGCCTT
LWY394DAI27054
TAGCCTTTGATCGCATGCCGTGGTTGGGACTGAGGAACTCAGCACGCCTT
FP135015G_pannocinctus
TAGCCTCTGATTGTATACGGTGGTTGGGACTGAGGATCGCAGCACGCCTT
L15726SpG_pannocinctus
TAGCCTCTGATTGTATACGGTGGTTGGGACTGAGGATCGCAGCACGCCTT
Dai22221
TAGTCTCTGATTGTATACGGTGGTTGGGACTGAGGAACTCAGCACGCCTT
Dai22633
TAGTCTCTAATTGTATACGGTGGTTGGGACTGAGGAACTCAGTACACCTT
Dai23260
TAGTCTCTAATTGTATACGGTGGTTGGGACTGAGGAACTCAGTACACCTT
Dai23626
TAGTCTCTAATTGTATACGGTGGTTGGGACTGAGGAACTCAGTACACCTT
Dai16238G_citrinoalbus
TAGTCTCTGATTGTATACAGTGGTTGGGACTGAGGAACTCAGTACACCTT

1175Dai15293
TAGTCTCTGATTGTATACAGTGGTTGGGACTGAGGAACTCAGTACACCTT
Dai19547
TAGTCTCTGATTGTATACAGTGGTTGGGACTGAGGAACTCAGTACACCTT
918063G_africanus
TAGTCTCTGATTGTATACAGTGGTTGGGACTGAGGAACTCAGTACACCTT
918572G_africanus
TAGTCTCTGATTGTATACAGTGGTTGGGACTGAGGAACTCAGTACACCTT
Dai18536A --
1164Cui17922
TAGTCTCTGATTGTATACAGTGGTTGGGACTGAGGAACTCAGTACACCTT
Dai22225
TAGTCTCTGATTGTATACAGTGGTTGGGACTGAGGAACTCAGTACACCTT
1163Dai20655
TAGTCTCTGATTGTATACAGTGGTTGGGACTGAGGAACTCAGTACACCTT
Yuan4397G_hainanensis
TAGCCTTTTGTTGTATACGGTGATTGGGACTGAGGTTCGCAGCACGCCTT
1176Dai15268
TAGCCTTTTGTTGTATACGGTGATTGGGACTGAGGTTCGCAGCACGCCTT
1177Dai15259
TAGCCTTTTGTTGTATACGGTGATTGGGACTGAGGTTCGCAGCACGCCTT
BZ2896G_thelephoroides
TAGCCTTTTGTTGTATACGGTGGTTGGGACTGAGGTTCGCAGCACGCCTT
1166JV1808_26
TAGCCTTTTGTTGTATACGGTGGTTGGGACTGAGGTTCGCAGCACGCCTT
Miettinen16992Hapalopilus_ochr
TAGCCTTTGATCATATATCATGATTGGAACTGAGGACCGCAGCACGCCTT
GC1708_338_Ceriporia_arbuscula
TAGTCTCTTATTGTATACTACAGTTGGGACTGAGGAACTCAGCACGCGCA
WCG1555Dai26107Ceriporia
TAGTCTCTTATTGTATACTACAGTTGGGACTGAGGAACTCAGCACGCGCA
GC1708_340_Ceriporia_arbuscula
TAGTCTCTTATTGTATACTACAGTTGGGACTGAGGAACTCAGCACGCGCA
WCG1556Dai26109Ceriporia
TAGTCTCTTATTGTATACTACAGTTGGGACTGAGGAACTCAGCACGCGCA
883Cui11291
TAGTCTCTGATTGCATACTACAGGTGGGACTGAGGAACTCAGCACGCGCA
HLX320Dai26805
TAGTCTTTAATTGTATACTACAGCCGGGACTGAGGAACTCAGCACGCGCA
WCG1266Dai24678A
TAGTCTTTAATTGTATACTACAGCCAGGACTGAGGAACTCAGCACGCGCA
Dai6090_Ceriporia_sulphuricolo
TAGCCTCTGGTTGCATGCCGCGATCGGGACTGAGGAACTCAGCACGCACC
RLG_11354_Ceriproia_reticulata

TAGCCTTTAGTTGCATACTGTGGTTGGGACTGAGGATCTCAGCACGCGCA
ZZW1543Dai27072
TAGCCTTTAGTTGCATACTGTGGTTGGGACTGAGGATCTCAGCACGCGCA
Li1316_Ceriporia_reticulata --
KHL11981Ceriporia_reticulata
TAGCCTTTGGTTGCATACTGTGGTTGGGACTGAGGATCTCAGCACGCGCA
FP110343sp_Candelabrochaete_la
TAGCCTTTAATTGCATACTGTGATTGGGACTGAGGAACTCAGCACGCGCA
Li1045_Ceriporia_reticulata --
ZX136Dai25794ceriporia
TAGCCTTTGATTGCATACTGTGGTTGGGACTGAGGATCTCAGCACGCGCA
892Dai13400
TAGCCTTTGATTGCATACTGTGGTTGGGACTGAGGATCTCAGCACGCGCA
RLG7163Leptoporus_mollis
TAGCCTCTGGTTGCATGCTGTGGTTGGGACTGAGGATCTCAGCACGCGTA
Dai21062Leptoporus_mollis
TAGCCTCTGGTTGCATGCTGTGGTTGGGACTGAGGATCTCAGCACGCGTA
Dai20182Leptoporus_submollis
TAGCCTCTGGTTGCATGCTGTGGTTGGGACTGAGGATCTCAGCACGCGTA
Cui18379Leptoporus_submollis
TAGCCTCTGGTTGCATGCTGTGGTTGGGACTGAGGATCTCAGCACGCGTA
Wu1209_46Resiniporus_pseudogil
TAGTCTCTGATTGTATACTGTGACCAGGACTGAGGATCTCAGCACGCATT
BRNM710169Resiniporus_resinasc
TAGTCTCTGATTGTATACTGTGACCAGGACTGAGGATCTCAGCACGCATT
Dai14516Bjerkandera_adusta
TAGCCCTTGGTTGTATACGGTGGCTGGGACTGAGGAACATAGCATGCCTT
Dai21100Bjerkandera_fumosa
TAGCCCTCGGTTGTATACGGTGGCTGGGACTGAGGAACATAGCACGCCTT
Miettinen16854Ceraceomyces_sp
TAGTCTTTGGCTGCATGTCGTGATTGAGACTGAGGAACTCAGCACGCATT
Dai10477C_spissa
TAGCCTTTAGTCATATACTGTGGCTGGGACTGAGGAACTCAGCACGTGTA
855Dai16831
TAGCCTTTAGTCATATACTGTGGCTGGGACTGAGGAACTCAGCACGTGTA
882Cui11282
TAGCCTTTAGTCATATACTGTGGCTGGGACTGAGGAACTCAGCACGTGTA
Dai24566
TAGCCTTTAGTCACATACTGTGATTGGGACTGAGGAACTCAGCACGTGCA
Yuan5965
TAGCCTTTAGTCACATACTGTGATTGGGACTGAGGAACTCAGCACGTGCA
Dai3204
TAGCCTTTGATTGTATACTGTGGTTGGGACTGAGGATCTCAGCACGCCTT
1194CUI9985

TAGTCTCTGGCTGCATACTGTGTCCGGGACTGAGGATCTCAGCACGCATT

Dai15205_Ceriporia_albomellea TATGGCCGGGG-TTCGCCCACGTTCGTGCTTAGGATGCTGGCGTAATGGC
Dai15223_Ceriporia_albomellea TATGGCCGGGG-TTCGCCCACGTTCGTGCTTAGGATGCTGGCGTAATGGC
Li1780_Ceriporia_variegata TATGGCCGGGG-TTCGCCCACGTTCGTGCTTAGGATGCTGGCGTAATGGC
Dai19791_Ceriporia_variegata TATGGCCGGGG-TTCGCCCACGTTCGTGCTTAGGATGCTGGCGTAATGGC
Dai19886 TATGGCCGGGG-TTCGCCCACGTTCGTGCTTAGGATGCTGGCGTAATGGC
Dai10833_Ceriporia_crassitunic TACGGCCGGGG-TTCGCCCACGTTCGTGCTTAGGATGCTGGCGTAATGGC
CHWC1506_46Meruliopsis_crassit TACGGCCGGGG-TTCGCCCACGTTCGTGCTTAGGATGCTGGCGTAATGGC
Dai9995_Ceriporia_crassitunica --
Wu1209_58_Meruliopsis_parvispo TATGGCCGGGG-TTCGCCCACGTTCGTGCTTAGGATGCTGGCGTAATGGC
CHWC1505_129_Meruliopsis_parvi TATGGCCGGGG-TTCGCCCACGTTCGTGCTTAGGATGCTGGCGTAATGGC
Dai21944 TATGGCCGGGG-TTCGCCCACGTTCGTGCTTAGGATGCTGGCGTAATGGC
830Dai18640A TATGGCCGGGG-TTCGCCCACGTTCGTGCTTAGGATGCTGGCGTAATGGC
GC1704_60_Meruliopsis_taxicola TATGGCCGGGG-TTCGCCCACGTTCGTGCTTAGGATGCTGGCGTAATGGC
Dai22625 TATGGCCGGGG-TTCGCCCACGTTCGTGCTTAGGATGCTGGCGTAATGGC
Dai22636 TATGGCCGGGG-TTCGCCCACGTTCGTGCTTAGGATGCTGGCGTAATGGC
Dai21878 TATGGCCGGGG-TTCGCCCACGTTCGTGCTTAGGATGCTGGCGTAATGGC
1169Dai17248 TATGGCCGGGG-TTCGCCCACGTTCGTGCTTAGGATGCTGGCGTAATGGC
Wu1708_43_Meruliopsis_leptocys TATGGCCGGGGTTTTACCCACGTTCGTGCTTAGGATGCTGGCGTAATGGC
Li1011 TATGGCCGGGGTTTTACCCACGTTCGTGCTTAGGATGCTGGCGTAATGGC
ZX95Dai25742Meruliopsis_leptoc TATGGCCGGGGTTTTACCCACGTTCGTGCTTAGGATGCTGGCGTAATGGC
WCG1306Dai24733 TATGGCCGGGGTTTTACCCACGTTCGTGCTTAGGATGCTGGCGTAATGGC

LXL99Dai25816
TATGGCCGGGGTTTTACCCACGTTCGTGCTTAGGATGCTGGCGTAATGGC
WCG1559Dai26052Meruliopsis TATGGCCGGGG-
TTCGCCCACGTTCGTGCTTAGGATGCTGGCGTAATGGC
He7477 TATGGCCGGGG-
TTCGCCCACGTTCGTGCTTAGGATGCTGGCGTAATGGC
HLX243Dai26217 TATGGCCGGGG-
TTCGCCCACGTTCGTGCTTAGGATGCTGGCGTAATGGC
RussiaMW673659Meruliopsis_fagi TATGGCCGGGG-
TTCGCCCACGTTCGTGCTTAGGATGCTGGCGTAATGGC
FD278 TATGGCCGGGG-
TTCGCCCACGCTCGTGCTTAGGATGCTGGCGTAATGGC
Dai10226_Ceriporia_tarda ---
LE247365 TATGGCCGGGG-
TTTACCCACGTTCGTGCTTAGGATGCTGGCGTAATGGC
Dai8173_Meruliopsis_nanlingens AATGGCCGGGG-
TTCGCCCACGTTCGTGCTTAGGATGCTGGCGTAATGGC
860Dai17172 AATGGCCGGGG-
TTCGCCCACGTTCGTGCTTAGGATGCTGGCGTAATGGC
879Dai13414 AATGGCCGGGG-
TTCGCCCACGTTCGTGCTTAGGATGCTGGCGTAATGGC
Li_1704_Meruliopsis_pseudocyst ---
833Dai18405 TATGGCCGGGG-
TTCGCCCACGTTCGTGCTTAGGATGCTGGCGTAATGGC
HHB_10729_Meruliopsis_albostra TATGGCCGGGG-
TTCGCCCACGTTCGTGCTTAGGATGCTGGCGTAATGGC
Cui6878_Ceriporia_pseudocystid TATGGCCGGGG-
TTCGCCCACGTTCGTGCTTAGGATGCTGGCGTAATGGC
869Dai14737 TATGGCCGGGG-
TTCGCCCACGTTCGTGCTTAGGATGCTGGCGTAATGGC
876Cui11626 TATGGCCGGGG-
TTCGCCCACGTTCGTGCTTAGGATGCTGGCGTAATGGC
1199WEI3388 ---
776308_Meruliopsis_cystidiata TATGGCCGGGG-
TTCGCCCACGTTCGTGCTTAGGATGCTGGCGTAATGGC
ICN139059_Meruliopsis_cystidia TATGGCCGGGG-
TTCGCCCACGTTCGTGCTTAGGATGCTGGCGTAATGGC
HHB15692Ceraceomyces_serpens TGC--------------------
TGTGCTAAGGATGCTGGCGTAATGGC
HHB_15629_Sp_Ceriporiopsis_ane TGT--------------------
TGTTGCTTAGGATGCTGGCGTAATGGC
AJ185Trametopsis_cervina TGT--------------------
TGTTGCTTAGGATGCTGGCGTAATGGC
FD9Irpex_lacteus AGC--------------------

TGTGCTTAGGATGCTGGCGTAATGGC
908Dai11230 AGC--------------------
TGTGCTTAGGATGCTGGCGTAATGGC
FP55521TEmmia_lacerata AGC--------------------
TGTGCTTAGGATGCTGGCGTAATGGC
PBU0048Ceriporia_cystidiata AGC--------------------
TGTGCTTAGGATGCTGGCGTAATGGC
MZ340C_lacerataT --
Dai21940 AGC--------------------
TGTGCTTAGGATGCTGGCGTAATGGC
847Dai16433 AGC--------------------
TGTGCTTAGGATGCTGGCGTAATGGC
MarcinEmmia_latemarginatus AGC--------------------
TGTGCTTAGGATGCTGGCGTAATGGC
Meijer3729Hydnopolyporus_fimbr AGT--------------------
TGTGCTTACGATGCTGGCGTAATGGC
RLG13408Phanerochaete_sp TGT--------------------
TGTGCTTAGGATGTTGGCGTAATGGC
WHC1381Flavodon_flavus AGC--------------------
TGTGCTTAGGATGCTGGCGTAATGGC
GB1833Phlebia_albida TGT--------------------
TGTGCTTAGGATGCTGGCGTAATGGC
T407Phlebia_nitidula --
HHB6988Phanerochaete_exilis TGT--------------------
TGTGCTTAGGATGCTGGCGTAATGGC
HHB8509Phanerochaetella_xeroph TGT--------------------
TGTGCTTAGGATGCTGGCGTAATGGC
PBU0051Macrohyporia_dictyopora TGT--------------------
TGTGCTTAGGATGCTGGCGTAATGGC
HHB11463Phanerochaete_sp TGT--------------------
TGTGCTTAGGATGCTGGCGTAATGGC
FP102382Byssomerulius_corium TGT--------------------
TGTGCTTAGGATGCTGGCGTAATGGC
FP102165Efibula_americana AGC--------------------
TGTGCTTAGGATGCTGGCGTAATGGC
Murdoch90Ceriporia_torpida AGC--------------------
TGTGCTTAGGATGCTGGCGTAATGGC
Rivoire4413_Ceriporia_purpurea AGC--------------------
TGTGCTTAGGATGCTGGCGTAATGGC
Kout_18_Ceriporia_triumphalis AGC--------------------
TGTGCTTAGGATGCTGGCGTAATGGC
Rivoire3701_Ceriporia_bresadol AGC--------------------
TGTGCTTAGGATGCTGGCGTAATGGC
VS4018 --

Ryvarden21832_Ceriporia_manzan AGC--------------------
TGTGCTTAGGATGCTGGCGTAATGGC
Dai24539 AGC--------------------
TGTGCTTAGGATGCTGGCGTAATA-C
Dai24541 AGC--------------------
TGTGCTTAGGATGCTGGCGTAATGGC
JV1105_12_Ceriporia_occidental AGC--------------------
TGTGCTTAGGATGCTGGCGTAATGGC
VS8558Ceriporia_occidentalis AGC--------------------
TGTGCTTAGGATGCTGGCGTAATGGC
Dai22445 AGC--------------------
TGTGCTTAGGATGCTGGCGTAATGGC
846Dai16368 AGC--------------------
TGTGCTTAGGATGCTGGCGTAATGGC
Dai17951_Ceriporia_aurantiocar AGC--------------------
TGTGCTAAGGATGCTGGCGTAATGGC
Miettinen_11701C_viridans AGC--------------------
TGTGCTAAGGATGCTGGCGTAATGGC
JV0105_10Ceriporia_aurantiocar AGC--------------------
TGTGCTAAGGATGCTGGCGTAATGGC
Yuan5702C_viridans --
858Dai17003 --
Yuan2747_Ceriporia_viridans --
Yuan2744C_viridans --
Li1046C_viridans --
865C_sinoviridans --
871Dai15062 --
Dai7642_Ceriporia_humilis --
Spirin4706_Ceriporia_humilis --
Spirin4944_Ceriporia_sericea AAC--------------------
TGTGCTTAGGATGCTGGCGTAATGGC
WCG1547Dai26044ceriporia AAC--------------------
TGTGCTTAGGATGCTGGCGTAATGGC
ZZW1558Dai27086 AAC--------------------
TGTGCTTAGGATGCTGGCGTAATGGC
Miettinen14381_Ceriporia_mpuri AAC--------------------
TGTGCTTAGGATGCTGGCGTAATGGC
Miettinen15492_2_Ceriporia_sor AAC--------------------
TGTGCTTAGGATGCTGGCGTAATGGC
He6687 AAC--------------------
TGTGCTTAGGATGCTGGCGTAATGGC
ZH53Dai24426 AAC--------------------
TGTGCTTAGGATGCTGGCGTAATGGC
Vlasak0808_30_Ceriporia_punica AAC--------------------

TGTGCTTAGGATGCTGGCGTAATGGC

887Dai13376 AAC--------------------TGTGCTTAGGATGCTGGCGTAATGGC

WCG1443Dai24998 AAC--------------------TGTGCTTAGGATGCT-GCGTAATGTC

0108_6Ceriporia_spissa AGC--------------------TGTGCTTAGGATGCTGGCGTAATGGC

Dai19164 AGC--------------------TGTGCTTAGGATGCTGGCGTAATGGC

Dai17937_Ceriporia_bubalinomar AGC--------------------TGTGCTTAGGATGCTGGCGTAATGGC

903Dai12113 AGC--------------------TGTGCTTAGGATGCTGGCGTAATGGC

LZB929Dai25079 AGC--------------------TGTGCTTAGGATGCTGGCGTAATGGC

LX45Dai26988 --

LX43Dai26986 AGC--------------------TGTGCTTAGGATGCTGGCGTAATGGC

Dai7759Ceriporia --

Cui8012_Ceriporia_viridans --

GC1704_54Ceriporia_viridans AGC--------------------TGTGCTAAGGATGCTGGCGTAATGGC

Dai23392 AGC--------------------TGTGCTAAGGATGCTGGCGTAATGGC

WCG1585Dai26113Ceriproia AGC--------------------TGTGCTAAGGATGCTGGCGTAATGGC

Dai18675C_eucalypti AGC--------------------TGTGCTAAGGATGCTGGCGTAATGGC

Dai22034 AGC--------------------TGTGCTTAGGATGCTGGCGTAATGGC

JV1008_41JTardaFLORIDAKeys --

Rivoire1161_Ceriporia_pierii --

Dai23499C_pierii AAC--------------------TGTGCTTAGGATGCTGGCGTAATGGC

Dai23500 AAC--------------------TGTGCTTAGGATGCTGGCGTAATGGC

841Dai15899 AGC--------------------TGTGCTTAGGATGCTGGCGTAATGGC

842Dai15904 --

LZB1066xinjiang --

LZB1065xinjiang --

851Dai16779 AAC--------------------TGTGCTTAGGATGCTGGCGTAATGGC

RMJ119sp_Candelabrochaete_sept --

RLG9759spCandelabrochaete_sept CGT--------------------
TGTGCTCAGGATGCTGGCGTAATGGC
RLG10478Phanerochaete_allantos ---
Dai19118_Ceriporia_spissa AGC--------------------
TGTGCTTAGGATGTTGGCGTAATGGC
Dai18486A AGC--------------------
TGTGCTTAGGATGTTGGCGTAATGGC
WEI17_024_Ceriporia_mellita AGC--------------------
TGTGCTTAGGATGTTGGCGTAATGGC
GC1508_71Ceriporia_mellita AGC--------------------
TGTGCTTAGGATGTTGGCGTAATGGC
GC1608_7_Ceriporia_mellita AGC--------------------
TGTGCTTAGGATGTTGGCGTAATGGC
ZZW1557Dai27085 AGC--------------------
TGTGCTTAGGATGTTGGCGTAATGGC
ZZW1554Dai27083 AGC--------------------
TGTGCTTAGGATGTTGGCGTAATGGC
Dai8168 AGC--------------------
TGTGCTTAGGATGTTGGCGTAATGGC
BR4865C_mellita AGC--------------------
TGTGCTTAGGATGTTGGCGTAATGGC
MEL2382688Ceriporia_sp ---
Dai8110 AGC--------------------
TGTGCTTAGGATGCTGGCGTAATGGC
Cui8097 ---
909Cui6740 ---
W1258Dai24695 AGC--------------------
TGTGCTTAGGATGCTGGCGTAATGGC
JV0110_26_Ceriporia_griseoviol TGTGGCCGGGG-
TTCGCCCACGTTCGTGCTTAGGATGCTGGCGTAATGGC
896Dai13202 TGTGGCCGGGG-
TTCGCCCACGTTCGTGCTTAGGATGCTGGCGTAATGGC
LWY393Dai27053C_griseoviolasce TGTGGCCGGGG-
TTCGCCCACGTTCGTGCTTAGGATGCTGGCGTAATGGC
LWY394DAI27054 TGTGGCCGGGG-
TTCGCCCACGTTCGTGCTTAGGATGCTGGCGTAATGGC
FP135015G_pannocinctus TATGGCCGGGG-
TTCGCCCACGTTCGTGCTTAGGATGCTGGCGTAATGGC
L15726SpG_pannocinctus TATGGCCGGGG-
TTCGCCCACGTTCGTGCTTAGGATGCTGGCGTAATGGC
Dai22221 TATGGCCGGGG-
TTCGCCCACGTACGTGCTTAGGATGCTGGCGTAATGGC
Dai22633 TATGGTCGGGG-
TTCGCCCACGTTTGTACTTAGGATGCTGGCGTAATGGC

Dai23260 TATGGTCGGGG-TTCGCCCACGTTTGTACTTAGGATGCTGGCGTAATGGC

Dai23626 TATGGTCGGGG-CTCGCCCACGTTTGTACTCAGGATGCTGGCGTAATGGC

Dai16238G_citrinoalbus TATGGTCGGGG-TTCGCCCACGTTTGTACTTAGGATGCTGGCGTAATGGC

1175Dai15293 TATGGTCGGGG-TTCGCCCACGTTTGTACTTAGGATGCTGGCGTAATGGC

Dai19547 TATGGTCGGGG-TTCGCCCACGTTTGTACTTAGGATGCTGGCGTAATGGC

918063G_africanus TATGGTCGGGG-TTCGCCCACGTTTGTACTTAGGATGCTGGCGTAATGGC

918572G_africanus TATGGTCGGGG-TTCGCCCACGTTTGTACTTAGGATGCTGGCGTAATGGC

Dai18536A --

1164Cui17922 TATGGTCGGGG-TTCGCCCACGTTTGTACTTAGGATGCT-ACGTAATGGC

Dai22225 TATGGTCGGGG-TTCGCCCACGTTTGTACTTAGGATGCTGGCGTAATGGC

1163Dai20655 TATGGTCGGGG-TTCGCCCACGTTTGTACTTAGGATGCTGGCGTAATGGC

Yuan4397G_hainanensis TATGGCCGGGG-TTCGCCCACGTTCGTGCTTAGGATGCTGGCGTAATGGC

1176Dai15268 TATGGCCGGGG-TTCGCCCACGTTCGTGCTTAGGATGCTGGCGTAATGGC

1177Dai15259 TATGGCCGGGG-TTCGCCCACGTTCGTGCTTAGGATGCTGGCGTAATGGC

BZ2896G_thelephoroides TATGGCCGGGG-TTCGCCCACGTTCGTGCTTAGGATGCTGGCGTAATGGC

1166JV1808_26 TATGGCCGGGG-TTCGCCCACGTTCGTGCTTAGGATGCTGGCGTAATGGC

Miettinen16992Hapalopilus_ochr TTTGGCCGGGG-TTCGCCCACGTTCGTGCTTAGGATGCTGGCGTAATGGC

GC1708_338_Ceriporia_arbuscula AGC---------------------TGTGCTTAGGATGCTGGCGTAATGGC

WCG1555Dai26107Ceriporia AGC---------------------TGTGCTTAGGATGCTGGCGTAATGGC

GC1708_340_Ceriporia_arbuscula AGC---------------------TGTGCTTAGGATGCTGGCGTAATGGC

WCG1556Dai26109Ceriporia AGC---------------------TGTGCTTAGGATGCTGGCGTAATGGC

883Cui11291 AGC---------------------TGTGCTTAGGATGCTGGCGTAATGGC

HLX320Dai26805 AGC---------------------

TGTGCTTAGGATGCTGGCGTAATGGC

WCG1266Dai24678A AGC--------------------
TGTGCTTAGGATGCTGGCGTAATGGC

Dai6090_Ceriporia_sulphuricolo TGC--------------------
TGTGCTTAGGATGCCGGCGTAATGGC

RLG_11354_Ceriproia_reticulata AGT--------------------
TGTGCTTAGGATGCTGGCGTAATGGC

ZZW1543Dai27072 AGT--------------------
TGTGCTTAGGATGCTGGCGTAATGGC

Li1316_Ceriporia_reticulata --

KHL11981Ceriporia_reticulata AGT--------------------
TGTGCTTAGGATGCTGGCGTAATGGC

FP110343sp_Candelabrochaete_la AGT--------------------
TGTGCTCAGGATGCTGGCGTAATGGC

Li1045_Ceriporia_reticulata --

ZX136Dai25794ceriporia AGT--------------------
TGTGCTTAGGATGCTGGCGTAATGGC

892Dai13400 AGT--------------------
TGTGCTTAGGATGCTGGCGTAATGGC

RLG7163Leptoporus_mollis AGT--------------------
TGTGCTTAGGATGCTGGCGTAATGGC

Dai21062Leptoporus_mollis AGT--------------------
TGTGCTTAGGATGCTGGCGTAATGGC

Dai20182Leptoporus_submollis AGT--------------------
TGTGCTTAGGATGCTGGCGTAATGGC

Cui18379Leptoporus_submollis AGT--------------------
TGTGCTTAGGATGCTGGCGTAATGGC

Wu1209_46Resiniporus_pseudogil TGT-------------------
TGTTGCTTAGGATGCTGGCGTAATGGC

BRNM710169Resiniporus_resinasc TGT-------------------TGTTGCTTAGGATGCTGGC---
-----

Dai14516Bjerkandera_adusta TATGGCGGGGC-
TTCGGCCACCTTCATGCTTTGGATGCTGGCGTAATGGC

Dai21100Bjerkandera_fumosa TACGGCGGGGC-
TTCGGCCACCTTCGTGCTTTGGATGCTGGCGTAATGGC

Miettinen16854Ceraceomyces_sp TGT--------------------
TGTGTTTAGGATGCTGGCGTAATGGC

Dai10477C_spissa TAT--------------------
TGTGCTTTGGATGCTGGCTTAATGGC

855Dai16831 TAT--------------------
TGTGCTTTGGATGCTGGCTTAATGGC

882Cui11282 TAT--------------------
TGTGCTTTGGATGCTGGCTTAATGGC

Dai24566 AAT--------------------

TGTGCTTTGGATGCTGGCTTAATGGC
Yuan5965 AAT--------------------
TGTGCTTTGGATGCTGGCTTAATGGC
Dai3204 TATGGCCGGGG-
TTCGCCCACGTTCGTGCTTAGGATGCTGGCGTAATGGC
1194CUI9985 TGT-------------------
TGTTGCTTAGGATGCTGGCGTAATGGC

Dai15205_Ceriporia_albomellea -
TTAAACGACCCGTCTTGAAACACGGACCAAGGAGTCTAACATACCTGCG
Dai15223_Ceriporia_albomellea
TTTAAACGACCCGTCTTGAAACACGGACCAAGGAGTCTAACATACCTGCG
Li1780_Ceriporia_variegata
TTTAAACGACCCGTCTTGAAACACGGACCAAGGAGTCTAACATACCTGCG
Dai19791_Ceriporia_variegata
TTTAAACGACCCGTCTTGAAACACGGACCAAGGAGTCTAACATACCTGCG
Dai19886
TTTAAACGACCCGTCTTGAAACACGGACCAAGGAGTCTAACATACCTGCG
Dai10833_Ceriporia_crassitunic
TTTAAACGACCCGTCTTGAAACACGGACCAAGGAGTCTAACATACCTGCG
CHWC1506_46Meruliopsis_crassit
TTTAAACGACCCGTCTTGAAACACGGACCAAGGAGTCTAACATACCT---
Dai9995_Ceriporia_crassitunica ---
Wu1209_58_Meruliopsis_parvispo
TTTAAACGACCCGTCTTGAAACACGGACCAAGGAGTCTAACATACCTGCG
CHWC1505_129_Meruliopsis_parvi
TTTAAACGACCCGTCTTGAAACACGGACCAAGGAGTCTAACATACCTGCG
Dai21944
TTTAAACGACCCGTCTTGAAACACGGACCAAGGAGTCTAACATACCTGCG
830Dai18640A
TTTAAACGACCCGTCTTGAAACACGGACCAAGGAGTCTAACATACCTGCG
GC1704_60_Meruliopsis_taxicola
TTTAAACGACCCGTCTTGAAACACGGACCAAGGAGTCTAACATACCTGCG
Dai22625
TTTAAACGACCCGTCTTGAAACACGGACCAAGGAGTCTAACATACCTGCG
Dai22636
TTTAAACGACCCGTCTTGAAACACGGACCAAGGAGTCTAACATACCTGCG
Dai21878
TTTAAACGACCCGTCTTGAAACACGGACCAAGGAGTCTAACATACCTGCG
1169Dai17248
TTTAAACGACCCGTCTTGAAACACGGACCAAGGAGTCTAACATACCTGCG
Wu1708_43_Meruliopsis_leptocys
TTTAAACGACCCGTCTTGAAACACGGACCAAGGAGTCTAACATACCTGCG

Li1011
TTTAAACGACCCGTCTTGAAACACGGACCAAGGAGTCTAACATACCTGCG
ZX95Dai25742Meruliopsis_leptoc
TTTAAACGACCCGTCTTGAAACACGGACCAAGGAGTCTAACATACCTGCG
WCG1306Dai24733
TTTAAACGACCCGTCTTGAAACACGGACCAAGGAGTCTAACATACCTGCG
LXL99Dai25816
TTTAAACGACCCGTCTTGAAACACGGACCAAGGAGTCTAACATACCTGCG
WCG1559Dai26052Meruliopsis
TTTAAACGACCCGTCTTGAAACACGGACCAAGGAGTCTAACATACCTGCG
He7477
TTTAAACGACCCGTCTTGAAACACGGACCAAGGAGTCTAACATACCTGCG
HLX243Dai26217
TTTAAACGACCCGTCTTGAAACACGGACCAAGGAGTCTAACATACCTGCG
RussiaMW673659Meruliopsis_fagi
TTTAAACGACCCGTCTTGAAACACGGACCAAGGAGTCTAACATACCTGCG
FD278
TTTAAACGACCCGTCTTGAAACACGGACCAAGGAGTCTAACATACCTGCG
Dai10226_Ceriporia_tarda --
LE247365
TTTAAACGACCCGTCTTGAAACACGGACCAAGGAGTCTAACATACCTGCG
Dai8173_Meruliopsis_nanlingens
TTTAAACGACCCGTCTTGAAACACGGACCAAGGAGTCTAACATACCTGCG
860Dai17172
TTTAAACGACCCGTCTTGAAACACGGACCAAGGAGTCTAACATACCTGCG
879Dai13414
TTTAAACGACCCGTCTTGAAACACGGACCAAGGAGTCTAACATACCTGCG
Li_1704_Meruliopsis_pseudocyst --
833Dai18405
TTTAAACGACCCGTCTTGAAACACGGACCAAGGAGTCTAACATACCTGCG
HHB_10729_Meruliopsis_albostra
TTTAAACGACCCGTCTTGAAACACGGACCAAGGAGTCTAACATACCTGCG
Cui6878_Ceriporia_pseudocystid
TTTAAACGACCCGTCTTGAAACACGGACCAAGGAGTCTAACATACCTGCA
869Dai14737
TTTAAACGACCCGTCTTGAAACACGGACCAAGGAGTCTAACATACCTGCA
876Cui11626
TTTAAACGACCCGTCTTGAAACACGGACCAAGGAGTCTAACATACCTGCA
1199WEI3388 --
776308_Meruliopsis_cystidiata
TTTAAACGACCCGTCTTGAAACACGGACCAAGGAGTCTAACATACCTGCA
ICN139059_Meruliopsis_cystidia
TTTAAACGACCCGTCTTGAAACACGGACCAAGGAGTCTAACATACCTGCG
HHB15692Ceraceomyces_serpens

TTTAAACGACCCGTCTTGAAACACGGACCAAGGAGTCTAACAAACATGCG
HHB_15629_Sp_Ceriporiopsis_ane
TTTAAACGACCCGTCTTGAAACACGGACCAAGGAGTCTAACAAACCTGCG
AJ185Trametopsis_cervina
TTTAAACGACCCGTCTTGAAACACGGACCAAGGAGTCTAACAAACCTGCG
FD9Irpex_lacteus
TTTAAACGACCCGTCTTGAAACACGGACCAAGGAGTCTAACAAACCTGCG
908Dai11230
TTTAAACGACCCGTCTTGAAACACGGACCAAGGAGTCTAACAAACCTGCG
FP55521TEmmia_lacerata
TTTAAACGACCCGTCTTGAAACACGGACCAAGGAGTCTAACAAACCTGCG
PBU0048Ceriporia_cystidiata
TTTAAACGACCCGTCTTGAAACACGGACCAAGGAGTCTAACAAACCTGCG
MZ340C_lacerataT --
Dai21940
TTTAAACGACCCGTCTTGAAACACGGACCAAGGAGTCTAACAAACCTGCG
847Dai16433
TTTAAACGACCCGTCTTGAAACACGGACCAAGGAGTCTAACAAACCTGCG
MarcinEmmia_latemarginatus
TTTAAACGACCCGTCTTGAAACACGGACCAAGGAGTCTAACAAACCTGCG
Meijer3729Hydnopolyporus_fimbr
TTTAAACGACCCGTCTTGAAACACGGACCAAGGAGTCTAACAAACCTGCG
RLG13408Phanerochaete_sp
TTTAAACGACCCGTCTTGAAACACGGACCAAGGAGTCTAACAAACCTGCG
WHC1381Flavodon_flavus
TTTAAACGACCCGTCTTGAAACACGGACCAAGGAGTCTAACAAACCTGCG
GB1833Phlebia_albida
TTTAAACGACCCGTCTTGAAACACGGACCAAGGAGTCTAACAAATCTGCG
T407Phlebia_nitidula --
HHB6988Phanerochaete_exilis
TTTAAACGACCCGTCTTGAAACACGGACCAAGGAGTCTAACAAACCTGCG
HHB8509Phanerochaetella_xeroph
TTTAAACGACCCGTCTTGAAACACGGACCAAGGAGTCTAACAAACCTGCG
PBU0051Macrohyporia_dictyopora
TTTAAACGACCCGTCTTGAAACACGGACCAAGGAGTCTAACAAACCTGCG
HHB11463Phanerochaete_sp
TTTAAACGACCCGTCTTGAAACACGGACCAAGGAGTCTAACAAACCTGCA
FP102382Byssomerulius_corium
TTTAAACGACCCGTCTTGAAACACGGACCAAGGAGTCTAACAAACCTGCG
FP102165Efibula_americana
TTTAAACGACCCGTCTTGAAACACGGACCAAGGAGTCTAACAAACCTGCG
Murdoch90Ceriporia_torpida
TTTAAACGACCCGTCTTGAAACACGGACCAAGGAGTCTAACATGCCTGCG
Rivoire4413_Ceriporia_purpurea

TTTAAACGACCCGTCTTGAAACACGGACCAAGGAGTCTAACATGCCTGCG

Kout_18_Ceriporia_triumphalis

TTTAAACGACCCGTCTTGAAACACGGACCAAGGAGTCTAACATGCCTGCG

Rivoire3701_Ceriporia_bresadol

TTTAAACGACCCGTCTTGAAACACGGACCAAGGAGTCTAACATGCCTGCG

VS4018 --

Ryvarden21832_Ceriporia_manzan

TTTAAACGACCCGTCTTGAAACACGGACCAAGGAGTCTAACATGCCTGCG

Dai24539

TTTAAACGACCCGTCTTGAAACACGGACCAAGGAGTCTAACATGCCTGCG

Dai24541

TTTAAACGACCCGTCTTGAAACACGGACCAAGGAGTCTAACATGCCTGCG

JV1105_12_Ceriporia_occidental

TTTAAACGACCCGTCTTGAAACACGGACCAAGGAGTCTAACATGCCTGCG

VS8558Ceriporia_occidentalis

TTTAAACGACCCGTCTTGAAACACGGACCAAGGAGTCTAACATGCCTGCG

Dai22445

TTTAAACGACCCGTCTTGAAACACGGACCAAGGAGTCTAACATGCCTGCG

846Dai16368

TTTAAACGACCCGTCTTGAAACACGGACCAAGGAGTCTAACATGCCTGCG

Dai17951_Ceriporia_aurantiocar

TTTAAACGACCCGTCTTGAAACACGGACCAAGGAGTCTAACATACCTGCG

Miettinen_11701C_viridans

TTTAAACGACCCGTCTTGAAACACGGACCAAGGAGTCTAACATGCCTGCG

JV0105_10Ceriporia_aurantiocar

TTTAAACGACCCGTCTTGAAACACGGACCAAGGAGTCTAACATACCTGCG

Yuan5702C_viridans --

858Dai17003 --

Yuan2747_Ceriporia_viridans --

Yuan2744C_viridans --

Li1046C_viridans --

865C_sinoviridans --

871Dai15062 --

Dai7642_Ceriporia_humilis --

Spirin4706_Ceriporia_humilis --

Spirin4944_Ceriporia_sericea

TTTAAATGACCCGTCTTGAAACACGGACCAAGGAGTCTAACATACCTGCG

WCG1547Dai26044ceriporia

TTTAAATGACCCGTCTTGAAACACGGACCAAGGAGTCTAACATACCTGCG

ZZW1558Dai27086

TTTAAATGACCCGTCTTGAAACACGGACCAAGGAGTCTAACATACCTGCG

Miettinen14381_Ceriporia_mpuri

TTTAAACGACCCGTCTTGAAACACGGACCAAGGAGTCTAACATACCTGCG

Miettinen15492_2_Ceriporia_sor

TTTAAACGACCCGTCTTGAAACACGGACCAAGGAGTCTAACATGCCTGCG

He6687

TTTAAACGACCCGTCTTGAAACACGGACCAAGGAGTCTAACATACCTGCG

ZH53Dai24426

TTTAAACGACCCGTCTTGAAACACGGACCAAGGAGTCTAACATACCTGCG

Vlasak0808_30_Ceriporia_punica

TTTAAACGACCCGTCTTGAAACACGGACCAAGGAGTCTAACATGCCTGCG

887Dai13376

TTTAAACGACCCGTCTTGAAACACGGACCAAGGAGTCTAACATGCCTGCG

WCG1443Dai24998

TTTAAACGACCCGTCTTGAAACACGGACCAAGGAGTCTAACATGCCTGCG

0108_6Ceriporia_spissa

TTTAAGCGACCCGTCTTGAAACACGGACCAAGGAGTCTAACATGCCTGCG

Dai19164

TTTAAGCGACCCGTCTTGAAACACGGACCAAGGAGTCTAACATGCCTGCG

Dai17937_Ceriporia_bubalinomar

TTTAAGCGACCCGTCTTGAAACACGGACCAAGGAGTCTAACATGCCTGCG

903Dai12113

TTTAAGCGACCCGTCTTGAAACACGGACCAAGGAGTCTAACATGCCTGCG

LZB929Dai25079

TTTAAACGACCCGTCTTGAAACACGGACCAAGGAGTCTAACATACCTGCA

LX45Dai26988 --

LX43Dai26986

TTTAAACGACCCGTCTTGAAACACGGACCAAGGAGTCTAACATACCTGCA

Dai7759Ceriporia --

Cui8012_Ceriporia_viridans --

GC1704_54Ceriporia_viridans

TTTAAACGACCCGTCTTGAAACACGGACCAAGGAGTCTAACATGCCTGCG

Dai23392

TTTAAACGACCCGTCTTGAAACACGGACCAAGGAGTCTAACATGCCTGCG

WCG1585Dai26113Ceriproia

TTTAAACGACCCGTCTTGAAACACGGACCAAGGAGTCTAACATGCCTGCG

Dai18675C_eucalypti

TTTAAACGACCCGTCTTGAAACACGGACCAAGGAGTCTAACATGCCTGCG

Dai22034

TTTAAACGACCCGTCTTGAAACACGGACCAAGGAGTCTAACATACCTGCG

JV1008_41JTardaFLORIDAKeys --

Rivoire1161_Ceriporia_pierii --

Dai23499C_pierii

TTTAAACGACCCGTCTTGAAACACGGACCAAGGAGTCTAACATGCCTGCG

Dai23500

TTTAAACGACCCGTCTTGAAACACGGACCAAGGAGTCTAACATGCCTGCG

841Dai15899

TTTAAACGACCCGTCTTGAAACACGGACCAAGGAGTCTAACATGCCTGCG

842Dai15904 --

LZB1066xinjiang --

LZB1065xinjiang --

851Dai16779

TTTAAACGACCCGTCTTGAAACACGGACCAAGGAGTCTAACATACCTGCG

RMJ119sp_Candelabrochaete_sept --

RLG9759spCandelabrochaete_sept

TTTAAATGACCCGTCTTGAAACACGGACCAAGGAGTCTAACATGCCCGCG

RLG10478Phanerochaete_allantos --

Dai19118_Ceriporia_spissa

TTTAAACGACCCGTCTTGAAACACGGACCAAGGAGTCTAACATACCTGCG

Dai18486A

TTTAAACGACCCGTCTTGAAACACGGACCAAGGAGTCTAACATACCTGCG

WEI17_024_Ceriporia_mellita

TTTAAACGACCCGTCTTGAAACACGGACCAAGGAGTCTAACATACCTGCG

GC1508_71Ceriporia_mellita

TTTAAACGACCCGTCTTGAAACACGGACCAAGGAGTCTAACATACCTGCG

GC1608_7_Ceriporia_mellita

TTTAAACGACCCGTCTTGAAACACGGACCAAGGAGTCTAACATACCTGCG

ZZW1557Dai27085

TTTAAACGACCCGTCTTGAAACACGGACCAAGGAGTCTAACATACCTGCG

ZZW1554Dai27083

TTTAAACGACCCGTCTTGAAACACGGACCAAGGAGTCTAACATACCTGCG

Dai8168

TTTAAACGACCCGTCTTGAAACACGGACCAAGGAGTCTAACATACCTGCG

BR4865C_mellita

TTTAAACGACCCGTCTTGAAACACGGACCAAGGAGTCTAACATACCTGCG

MEL2382688Ceriporia_sp --

Dai8110

TTTAAACGACCCGTCTTGAAACACGGACCAAGGAGTCTAACATACCTGCG

Cui8097 --

909Cui6740 --

W1258Dai24695

TTTAAACGACCCGTCTTGAAACACGGACCAAGGAGTCTAACATACCTGCG

JV0110_26_Ceriporia_griseoviol

TTTAAACGACCCGTCTTGAAACACGGACCAAGGAGTCTAACATGCCTGCG

896Dai13202

TTTAAACGACCCGTCTTGAAACACGGACCAAGGAGTCTAACATGCCTGCG

LWY393Dai27053C_griseoviolasce

TTTAAACGACCCGTCTTGAAACACGGACCAAGGAGTCTAACATGCCTGCG

LWY394DAI27054

TTTAAACGACCCGTCTTGAAACACGGACCAAGGAGTCTAACATGCCTGCG

FP135015G_pannocinctus

TTTAAACGACCCGTCTTGAAACACGGACCAAGGAGTCTAACATGCCTGCG

L15726SpG_pannocinctus
TTTAAACGACCCGTCTTGAAACACGGACCAAGGAGTCTAACATGCCTGCG
Dai22221
TTTAAACGACCCGTCTTGAAACACGGACCAAGGAGTCTAACATACCTGCG
Dai22633
TTTAAGCGACCCGTCTTGAAACACGGACCAAGGAGTCTAACATGCCTGCG
Dai23260
TTTAAGCGACCCGTCTTGAAACACGGACCAAGGAGTCTAACATGCCTGCG
Dai23626
TTTAAGCGACCCGTCTTGAAACACGGACCAAGGAGTCTAACATGCCTGCA
Dai16238G_citrinoalbus
TTTAAGCGACCCGTCTTGAAACACGGACCAAGGAGTCTAACATGCCTGCG
1175Dai15293
TTTAAGCGACCCGTCTTGAAACACGGACCAAGGAGTCTAACATGCCTGCG
Dai19547
TTTAAGCGACCCGTCTTGAAACACGGACCAAGGAGTCTAACATGCCTGCG
918063G_africanus
TTTAAGCGACCCGTCTTGAAACACGGACCAAGGAGTCTAACATGCCTGCG
918572G_africanus
TTTAAGCGACCCGTCTTGAAACACGGACCAAGGAGTCTAACATGCCTGCG
Dai18536A --
1164Cui17922
TTTAAGCGACCCGTCTTGAAACACGGACCAAGGAGTCTAACATGCCTGCA
Dai22225
TTTAAGCGACCCGTCTTGAAACACGGACCAAGGAGTCTAACATGCCTGCG
1163Dai20655
TTTAAGCGACCCGTCTTGAAACACGGACCAAGGAGTCTAACATGCCTGCG
Yuan4397G_hainanensis
TTTAAACGACCCGTCTTGAAACACGGACCAAGGAGTCTAACATGCCTGCG
1176Dai15268
TTTAAACGACCCGTCTTGAAACACGGACCAAGGAGTCTAACATGCCTGCG
1177Dai15259
TTTAAACGACCCGTCTTGAAACACGGACCAAGGAGTCTAACATGCCTGCG
BZ2896G_thelephoroides
TTTAAACGACCCGTCTTGAAACACGGACCAAGGAGTCTAACATGCCTGCG
1166JV1808_26
TTTAAACGACCCGTCTTGAAACACGGACCAAGGAGTCTAACATGCCTGCG
Miettinen16992Hapalopilus_ochr
TTTAAACGACCCGTCTTGAAACACGGACCAAGGAGTCTAACATGCCTGCA
GC1708_338_Ceriporia_arbuscula
TTTAAACGACCCGTCTTGAAACACGGACCAAGGAGTCTAACATACCTGCG
WCG1555Dai26107Ceriporia
TTTAAACGACCCGTCTTGAAACACGGACCAAGGAGTCTAACATACCTGCG
GC1708_340_Ceriporia_arbuscula

TTTAAACGACCCGTCTTGAAACACGGACCAAGGAGTCTAACATACCTGCG

WCG1556Dai26109Ceriporia

TTTAAACGACCCGTCTTGAAACACGGACCAAGGAGTCTAACATACCTGCG

883Cui11291

TTTAAACGACCCGTCTTGAAACACGGACCAAGGAGTCTAACATACCTGCG

HLX320Dai26805

TTTAAACGACCCGTCTTGAAACACGGACCAAGGAGTCTAACATACCTGCG

WCG1266Dai24678A

TTTAAACGACCCGTCTTGAAACACGGACCAAGGAGTCTAACATACCTGCG

Dai6090_Ceriporia_sulphuricolo

TTTAAACGACCCGTCTTGAAACACGGACCAAGGAGTCTAACAAACCTGCG

RLG_11354_Ceriproia_reticulata

TTTAAATGACCCGTCTTGAAACACGGACCAAGGAGTCTAACATGCCTGCG

ZZW1543Dai27072

TTTAAATGACCCGTCTTGAAACACGGACCAAGGAGTCTAACATGCCTGCG

Li1316_Ceriporia_reticulata --

KHL11981Ceriporia_reticulata

TTTAAATGACCCGTCTTGAAACACGGACCAAGGAGTCTAACATGCCTGCG

FP110343sp_Candelabrochaete_la

TTTAAACGACCCGTCTTGAAACACGGACCAAGGAGTCTAACATACCTGCG

Li1045_Ceriporia_reticulata --

ZX136Dai25794ceriporia

TTTAAACGACCCGTCTTGAAACACGGACCAAGGAGTCTAACATGCCTGCG

892Dai13400

TTTAAACGACCCGTCTTGAAACACGGACCAAGGAGTCTAACATGCCTGCG

RLG7163Leptoporus_mollis

TTTAAACGACCCGTCTTGAAACACGGACCAAGGAGTCTAACATGCCTGCA

Dai21062Leptoporus_mollis

TTTAAACGACCCGTCTTGAAACACGGACCAAGGAGTCTAACATGCCTGCA

Dai20182Leptoporus_submollis

TTTAAACGACCCGTCTTGAAACACGGACCAAGGAGTCTAACATGCCTGCA

Cui18379Leptoporus_submollis

TTTAAACGACCCGTCTTGAAACACGGACCAAGGAGTCTAACATGCCTGCA

Wu1209_46Resiniporus_pseudogil

TTTAAACGACCCGTCTTGAAACACGGACCAAGGAGTCTAACAAACCTGCG

BRNM710169Resiniporus_resinasc --

Dai14516Bjerkandera_adusta

TTTAAACGACCCGTCTTGAAACACGGACCAAGGAGTCTAACATGCCTGCG

Dai21100Bjerkandera_fumosa

TTTAAACGACCCGTCTTGAAACACGGACCAAGGAGTCTAACATGCCTGCG

Miettinen16854Ceraceomyces_sp

TTTAAACGACCCGTCTTGAAACACGGACCAAGGAGTCTAACAAACCTGCG

Dai10477C_spissa

TTTAAACGACCCGTCTTGAAACACGGACCAAGGAGTCTAACGTGCATGCG

855Dai16831
TTTAAATGACCCGTCTTGAAACACGGACCAAGGAGTCTAACGTGCATGCG
882Cui11282
TTTAAACGACCCGTCTTGAAACACGGACCAAGGAGTCTAACGTGCATGCG
Dai24566
TTTAAACGACCCGTCTTGAAACACGGACCAAGGAGTCTAACATGCATGCG
Yuan5965
TTTAAACGACCCGTCTTGAAACACGGACCAAGGAGTCTAACATGCATGCG
Dai3204
TTTAAACGACCCGTCTTGAAACACGGACCAAGGAGTCTAACATACCTGCG
1194CUI9985
TTTAAACGACCCGTCTTGAAACACGGACCAAGGAGTCTAACAAACCTGCG

Dai15205_Ceriporia_albomellea AGTGTTTGGGTGG-
TAAACCCGAGCGCATAATGAAAGTGAAAGTTGGGAT
Dai15223_Ceriporia_albomellea AGTGTTTGGGTGG-
TAAACCCGAGCGCATAATGAAAGTGAAAGTTGGGAT
Li1780_Ceriporia_variegata AGTGTTTGGGTGG-
TAAACCCGAGCGCATAATGAAAGTGAAAGTTGGGAT
Dai19791_Ceriporia_variegata AGTGTTTGGGTGG-
TAAACCCGAGCGCATAATGAAAGTGAAAGTTGGGAT
Dai19886 AGTGTTTGGGTGG-
TAAACCCGAGCGCATAATGAAAGTGAAAGTTGGGAT
Dai10833_Ceriporia_crassitunic AGTGTTTGGGTGG-
TAAACCCGAGCGCATAATGAAAGTGAAAGTTGGGAT
CHWC1506_46Meruliopsis_crassit --
Dai9995_Ceriporia_crassitunica --
Wu1209_58_Meruliopsis_parvispo AGTATTTGGGTGG-
CAAACCCGAGTGCGTAATGAAAGTGATAGTTGGGAT
CHWC1505_129_Meruliopsis_parvi AGTATTTGGGTGG-
TAAACCCGAGTGCGCAATGAAAGTGATAGTTGGGAT
Dai21944 AGTATTTGGGTGG-
TAAACCCGAGTGCGCAATGAAAGTGATAGTTGGGAT
830Dai18640A AGTATTTGGGTGG-
TAAACCCGAGTGCGCAATGAAAGTGATAGTTGGGAT
GC1704_60_Meruliopsis_taxicola AGTATTTGGGTGG-
TAAACCCGAGTGCGTAATGAAAGTGAAAGTTGGGAT
Dai22625 AGTATTTGGGTGG-
TAAACCCGAGTGCGTAATGAAAGTGAAAGTTGGGAT
Dai22636 AGTAT-TGGGTGG-
TAAACCCGAGTGCGTAATGAAAGTGAAAGTTGGGAT
Dai21878 AGTATTTGGGTGG-
TAAACCCGAGTGCGTAATGAAAGTGAAAGTTGGGAT

1169Dai17248 AGTATTTGGGTGG-TAAACCCGAGTGCGTAATGAAAGTGAAAGTTGGGAT

Wu1708_43_Meruliopsis_leptocys AGTGTTTGGGTGG-TAAACCCGAGCGCGTAATGAAAGTGATAGTTGGGAT

Li1011 AGTGTTTGGGTGG-TAAACCCGAGCGCGTAATGAAAGTGATAGTTGGGAT

ZX95Dai25742Meruliopsis_leptoc AGTATTTGGGTGG-TAAACCCGAGTGCGTAATGAAAGTGATAGTTGGGAT

WCG1306Dai24733 AGTATTTGGGTGG-TAAACCCGAGTGCGTAATGAAAGTGATAGTTGGGAT

LXL99Dai25816 AGTATTTGGGTGG-TAAACCCGAGTGCGTAATGAAAGTGATAGTTGGGAT

WCG1559Dai26052Meruliopsis AGTATTTGGGTGG-TAAACCCGAGTGCGTAATGAAAGTGATAGTTGGGAT

He7477 AGTATTTGGGTGG-TAAACCCGAGTGCGTAATGAAAGTGATAGTTGGGAT

HLX243Dai26217 AGTATTTGGGTGG-TAAACCCGAGTGCGTAATGAAAGTGATAGTTGGGAT

RussiaMW673659Meruliopsis_fagi AGTATTTGGGTGG-TAAACCCGAGTGCGTAATGAAAGTGATAGTTGGGAT

FD278 AGTGTTTGGGTGG-TAAACCCGAGCGCGTAATGAAAGTGAAAGTTGGGAT

Dai10226_Ceriporia_tarda --

LE247365 AGTGTTTGGGTGG-TAAACCCGAGCGCGTAATGAAAGTGATAGTTGGGAT

Dai8173_Meruliopsis_nanlingens AGTGTTTGGGTAG-CAAACCCGAGCGCGTAATGAAAGTGATAGTTGGGAT

860Dai17172 AGTGTTTGGGTGG-CAAACCCGAGCGCGTAATGAAAGTGATAGTTGGGAT

879Dai13414 AGTGTTTGGGTGG-CAAACCCGAGCGCGTAATGAAAGTGATAGTTGGGAT

Li_1704_Meruliopsis_pseudocyst --

833Dai18405 AGTGTTTGGGTGG-TAAACCCGAGCGCGTAATGAAAGTGATAGTTGGGAT

HHB_10729_Meruliopsis_albostra AGTGTTTGGGTGG-TAAACCCGAGCGCGTAATGAAAGTGATAGTTGGGAT

Cui6878_Ceriporia_pseudocystid AGTGTTTGGGTGG-TAAACCCGAGCGCGTAATGAAAGTGATAGTTGGGAT

869Dai14737 AGTGTTTGGGTGG-TAAACCCGAGCGCGTAATGAAAGTGATAGTTGGGAT

876Cui11626 AGTGTTTGGGTGG-TAAACCCGAGCGCGTAATGAAAGTGATAGTTGGGAT

1199WEI3388 --

776308_Meruliopsis_cystidiata AGTGTTTGGGTGG-

TAAACCCGAGCGCGTAATGAAAGTGAAAGTTGGGAT

ICN139059_Meruliopsis_cystidia AGTGTTTGGGTGG-TAAACCCGAGCGCGTAATGAAAGTGAAAGTTGGGAT

HHB15692Ceraceomyces_serpens AGTGTTTGGGTGG-AAAACCCGAGCGCGCAATGAAAGTGAAAGTTGGGAT

HHB_15629_Sp_Ceriporiopsis_ane AGTATTTGGGTGATTAAACCCGAGTGCGCAATGAAAGTGAAAGTTGGGAT

AJ185Trametopsis_cervina AGTATTTGGGTGATTAAACCCGAGTGCGTAATGAAAGTGAAAGTTGGGAT

FD9Irpex_lacteus AGTATTTGGGTGG-TAAACCCGAGTGCGCAATGAAAGTGAAAGTTGGGAT

908Dai11230 AGTATTTGGGTGG-TAAACCCGAGTGCGCAATGAAAGTGAAAGTTGGGAT

FP55521TEmmia_lacerata AGTATTTGGGTGG-TAAACCCGAGTGCGTAATGAAAGTAAAAGTTGGGAT

PBU0048Ceriporia_cystidiata AGTATTTGGGTGG-TAAACCCGAGTGCGTAATGAAAGTAAAAGTTGGGAT

MZ340C_lacerataT --

Dai21940 AGTATTTGGGTGG-TAAACCCGAGTGCGTAATGAAAGTAAAAGTTGGGAT

847Dai16433 AGTATTTGGGTGG-TAAACCCGAGTGCGTAATGAAAGTAAAAGTTGGGAT

MarcinEmmia_latemarginatus AGTATTTGGGTAG-TAAACCCGAGTGCGTAATGAAAGTAAAAGTTGGGAT

Meijer3729Hydnopolyporus_fimbr AGTATTTGGGTGG-TAAACCCGAGTGCGYAATGAAAGTGAAAGTTGGGAT

RLG13408Phanerochaete_sp AGTGTTTGGGTGG-TAAACCCGAGCGCGTAATGAAAGTGAAAGTTGGGAT

WHC1381Flavodon_flavus AGTATTTGGGTGG-TAAACCCGAGTGCGTAATGAAAGTGAAAGTTGGGAT

GB1833Phlebia_albida AGTATTTGGGTGG-TAAACCCGAGTGCGCAATGAAAGTGAAAGTTGGGAT

T407Phlebia_nitidula --

HHB6988Phanerochaete_exilis AGTGTTTGGGTGG-TAAACCCGAGCGCGTAATGAAAGTGAAAGTTGGGAT

HHB8509Phanerochaetella_xeroph AGTGTTTGGGTGG-TAAACTCGAACGCGCAATGAAAGTGAAAGTTGGGAT

PBU0051Macrohyporia_dictyopora AGTGTTTGGGTGG-TAAACCCGAGCGCGTAATGAAAGTGAAAGTTGGGAT

HHB11463Phanerochaete_sp AGTGTTTGGGTGG-TAAACCCGAGCGCGTAATGAAAGTGAAAGTTGGGAT

FP102382Byssomerulius_corium AGTGTTTGGGTGG-TAAACCCGAGCGCGTAATGAAAGTGAAAGTTGGGAT

FP102165Efibula_americana AGTGTTTGGGTGG-

TAAACTCGAGCGCGAAATGAAAGTGAAAGTTGGGAT

Murdoch90Ceriporia_torpida AGTATTTGGGTGG-
TAAACCCGAGTGCGTAATGAAAGTGAAAGTTGGGAT

Rivoire4413_Ceriporia_purpurea AGTATTTGGGTGG-
TAAACCCGAGTGCGCAATGAAAGTGAAAGTTGGGAT

Kout_18_Ceriporia_triumphalis AGTATTTGGGTGG-
TAAACCCGAGTGCGCAATGAAAGTGAAAGTTGGGAT

Rivoire3701_Ceriporia_bresadol AGTATTTGGGTGG-
TAAACCCGAGTGCGTAATGAAAGTGAAAGTTGGGAT

VS4018 --

Ryvarden21832_Ceriporia_manzan AGTATTTGGGTGG-
TAAACCCGAGTGCGCAATTAAAGTGAAAGTTGGGAT

Dai24539 AGTATTTGGGTGG-
TAAACCCGAGTGCGCAATGAAAGTGAAAGTTGGGAT

Dai24541 AGTATTTGGGTGG-
TAAACCCGAGTGCGCAATGAAAGTGAAAGTTGGGAT

JV1105_12_Ceriporia_occidental AGTATTTGGGTGG-
TAAACCCGAGTGCGCAATTAAAGTGAAAGTTGGGAT

VS8558Ceriporia_occidentalis AGTATTTGGGTGG-
TAAACCCGAGTGCGCAATTAAAGTGAAAGTTGGGAT

Dai22445 AGTATTTGGGTGG-
TAAACTCGAGTGCGTAATGAAAGTGAAAGTTGGGAT

846Dai16368 AGTATTTGGGTGG-
TAAACCCGAGTGCGTAATGAAAGTGAAAGTTGGGAT

Dai17951_Ceriporia_aurantiocar AGTGTTTGGGTGG-
AAAACCCGAGCGCGTAATGAAAGTGAAAGTTGGGAT

Miettinen_11701C_viridans AGTGTTTGGGTGG-
AAAACCCGAGCGCGTAATGAAAGTGAAAGTTGGGAT

JV0105_10Ceriporia_aurantiocar AGTGTTTGGGTGG-
AAAACCCGAGCGCGTAATGAAAGTGAAAGTTGGGAT

Yuan5702C_viridans --

858Dai17003 --

Yuan2747_Ceriporia_viridans --

Yuan2744C_viridans --

Li1046C_viridans --

865C_sinoviridans --

871Dai15062 --

Dai7642_Ceriporia_humilis --

Spirin4706_Ceriporia_humilis --

Spirin4944_Ceriporia_sericea AGTGTTTGGGTGG-
AAAACCCGAGCGCGTAATGAAAGTGAAAGTTGGGAT

WCG1547Dai26044ceriporia AGTGTTTGGGTGG-
AAAACCCGAGCGCGTAATGAAAGTGAAAGTTGGGAT

ZZW1558Dai27086 AGTGTTTGGGTGG-

AAAACCCGAGCGCGTAATGAAAGTGAAAGTTGGGAT
Miettinen14381_Ceriporia_mpuri AGTGTTTGGGTGG-
AAAACCCGAGCGCGTAATGAAAGTGAAAGTTGGGAT
Miettinen15492_2_Ceriporia_sor AGTGTTTGGGTGG-
CAAACCCGAGCGCGTAATGAAAGTGAAAGTTGGGAT
He6687 AGTGTTTGGGTGG-
AAAACCCGAGCGCGTAATGAAAGTGAAAGTTGGGAT
ZH53Dai24426 AGTGTTTGGGTGG-
AAAACCCGAGCGCGTAATGAAAGTGAAAGTTGGGAT
Vlasak0808_30_Ceriporia_punica AGTATTTGGGTGG-
CAAACCCGAGTGCGCAATGAAAGTGAAAGTTGGGAT
887Dai13376 AGTATTTGGGTGG-
CAAACCCGAGTGCGTAATGAAAGTGAAAGTTGGGAT
WCG1443Dai24998 AGTATTTGGGTGG-
TAAACCCGAGTGCGCAATGAAAGTGAAAGTTGGGAT
0108_6Ceriporia_spissa AGTGTTTGGGTGG-
AAAACCCGATCGCGCAATGAAAGTGAAAGTTGGGAT
Dai19164 AGTGTTTGGGTGG-
AAAACCCGAGCGCGCAATGAAAGTGAAAGTTGGGAT
Dai17937_Ceriporia_bubalinomar AGTGTTTGGGTGG-
AAAACCCGAGCGCGCAATGAAAGTGAAAGTTGGGAT
903Dai12113 AGTGTTTGGGTGG-
AAAACTCGAGCGCGCAATGAAAGTGAAAGTTGGGAT
LZB929Dai25079 AGTGTTTGGGTGG-
AAAACCCGAGCGCGTAATGAAAGTGAAAGTTGGGAT
LX45Dai26988 --
LX43Dai26986 AGTGTTTGGGTGG-
AAAACCCGAGCGCGTAATGAAAGTGAAAGTTGGGAT
Dai7759Ceriporia --
Cui8012_Ceriporia_viridans --
GC1704_54Ceriporia_viridans AGTGTTTGGGTGG-
AAAACCTGAGCGCGTAATGAAAGTGAAAGTTGGGAT
Dai23392 AGTGTTTGGGTGG-
AAAACCCGAGCGCGTAATGAAAGTGAAAGTTGGGAT
WCG1585Dai26113Ceriproia AGTGTTTGGGTGG-
AAAACCCGAGCGCGTAATGAAAGTGAAAGTTGGGAT
Dai18675C_eucalypti AGTGTTTGGGTGG-
AAAACCCGAGCGCGTAATGAAAGTGAAAGTTGGGAT
Dai22034 AGTGTTTGGGTGG-
AAAACCCGAGCGCGTAATGAAAGTGAAAGTTGGGAT
JV1008_41JTardaFLORIDAKeys --
Rivoire1161_Ceriporia_pierii --
Dai23499C_pierii AGTGTTTGGGTGG-
AAAACCCGAGCGCGTAATGAAAGTGAAAGTTGGGAT

Dai23500 AGTGTTTGGGTGG-AAAACCCGAGCGCGTAATGAAAGTGAAAGTTGGGAT

841Dai15899 AGTGTTTGGGTGG-TAAACCCGAGCGCGTAATGAAAGTGAAAGTTGGGAT

842Dai15904 ---

LZB1066xinjiang ---

LZB1065xinjiang ---

851Dai16779 AGTGTTTGGGTGG-AAAACCCGAGCGCGTAATGAAAGTGAAAGTTGGGAT

RMJ119sp_Candelabrochaete_sept ---

RLG9759spCandelabrochaete_sept AGTGTTTGGGTGG-TAAACCCGAGCGCGTAATGAAAGTGAAAGTTGGGAT

RLG10478Phanerochaete_allantos ---

Dai19118_Ceriporia_spissa AGTGTTTGAGTGG-CAAACTCGAGCGCGTAATGAAAGTGAAAGTTGGGAT

Dai18486A AGTGTTTGAGTGG-CAAACTCGAGCGCGTAATGAAAGTGAAAGTTGGGAT

WEI17_024_Ceriporia_mellita AGTGTTTGAGTGG-CAAACTCGAGCGCGTAATGAAAGTGAAAGTTGGGAT

GC1508_71Ceriporia_mellita AGTGTTTGAGTGG-CAAACTCGAGCGCGTAATGAAAGTGAAAGTTGGGAT

GC1608_7_Ceriporia_mellita AGTGTTTGAGTGG-CAAACTCGAGCGCGTAATGAAAGTGAAAGTTGGGAT

ZZW1557Dai27085 AGTGTTTGAGTGG-CAAACTCGAGCGCGTAATGAAAGTGAAAGTTGGGAT

ZZW1554Dai27083 AGTGTTTGAGTGG-CAAACTCGAGCGCGTAATGAAAGTGAAAGTTGGGAT

Dai8168 AGTGTTTGAGTGG-CAAACTCGAGCGCGTAATGAAAGTGAAAGTTGGGAT

BR4865C_mellita AGTGTTTGAGTGG-CAAACTCGAGCGCGTAATGAAAGTGAAAGTTGGGAT

MEL2382688Ceriporia_sp ---

Dai8110 AGTGTTTGAGTGG-CAAACTCGAGCGCGTAATGAAAGTGAAAGTTGGGAT

Cui8097 ---

909Cui6740 ---

W1258Dai24695 AGTGTTTGAGTGG-TAAACTCGAGCGCGCAATGAAAGTGAAAGTTGGGAT

JV0110_26_Ceriporia_griseoviol AGTGTTTGGGTGG-CAAACCCGAGCGCGCAATGAAAGTGATAGTTGGGAT

896Dai13202 AGTGTTTGGGTGG-CAAACCCGAGCGCGCAATGAAAGTGATAGTTGGGAT

LWY393Dai27053C_griseoviolasce AGTGTTTGGGTGG-CAAACCCGAGCGCGCAATGAAAGTGATAGTTGGGAT

LWY394DAI27054 AGTGTTTGGGTGG-CAAACCCGAGCGCGCAATGAAAGTGATAGTTGGGAT

FP135015G_pannocinctus AGTGTTTGGGTGG-AAAACCCGAGCGCGTAATTAAAGTGAAAGTTGGGAT

L15726SpG_pannocinctus AGTGTTTGGGTGG-AAAACCCGAGCGCGTAATTAAAGTGAAAGTTGGGAT

Dai22221 AGTGTTTGGGTGG-TAAACCCGAGCGCGTAATTAAAGTGAAAGTTGGGAT

Dai22633 AGTGTTTGGGTGG-TAAACCCGAGCGCGTAATGAAAGTGAAAGTTGGGAT

Dai23260 AGTGTTTGGGTGG-TAAACCCGAGCGCGTAATGAAAGTGAAAGTTGGGAT

Dai23626 AGTGTTTGGGTGG-TAAACCCGAGCGCGTAATGAAAGTGAAAGTTGGGAT

Dai16238G_citrinoalbus AGTGTTTGGGTGG-TAAACCCGAGCGCATAATGAAAGTGAAAGTTGGGAT

1175Dai15293 AGTGTTTGGGTGG-TAAACCCGAGCGCATAATGAAAGTGAAAGTTGGGAT

Dai19547 AGTGTTTGGGTGG-TAAACCCGAGCGCATAATGAAAGTGAAAGTTGGGAT

918063G_africanus AGTGTTTGGGTGG-TAAACCCGAGCGCGTAATGAAAGTGAAAGTTGGGAT

918572G_africanus AGTGTTTGGGTGG-TAAACCCGAGCGCGTAATGAAAGTGAAAGTTGGGAT

Dai18536A --

1164Cui17922 AGTGTTTGGGTGG-TAAACCCGAGCGCGTAATGAAAGTGAAAGTTGGGAT

Dai22225 AGTGTTTGGGTGG-TAAACCCGAGCGCGTAATGAAAGTGAAAGTTGGGAT

1163Dai20655 AGTGTTTGGGTGG-TAAACCCGAGCGCGTAATGAAAGTGAAAGTTGGGAT

Yuan4397G_hainanensis AGTATTTGGGTGG-AAAACCCGAGTGCGCAATGAAAGTGAAAGTTGGGAT

1176Dai15268 AGTATTTGGGTGG-AAAACCCGAGTGCGTAATGAAAGTGAAAGTTGGGAT

1177Dai15259 AGTATTTGGGTGG-AAAACCCGAGTGCGCAATGAAAGTGAAAGTTGGGAT

BZ2896G_thelephoroides AGTATTTGGGTGG-AAAACCCGAGTGCGTAATTAAAGTGAAAGTTGGGAT

1166JV1808_26 AGTATTTGGGTGG-AAAACCCGAGTGCGTAATTAAAGTGAAAGTTGGGAT

Miettinen16992Hapalopilus_ochr AGTGTTTGGGTGG-AAAACTCGAGCGCGTAATGAAAGTGAAAGTTGGGAT

GC1708_338_Ceriporia_arbuscula AGTATTTGGGTGG-

TAAACCCGAGTGCGCAATGAAAGTGAAAGTTGGGAT

WCG1555Dai26107Ceriporia AGTATTTGGGTGG-
TAAACCCGAGTGCGCAATGAAAGTGAAAGTTGGGAT

GC1708_340_Ceriporia_arbuscula AGTATTTGGGTGG-
TAAACCCGAGTGCGCAATGAAAGTGA-AGTTGGGAT

WCG1556Dai26109Ceriporia AGTATTTGGGTGG-
TAAACCCGAGTGCGCAATGAAAGTGAAAGTTGGGAT

883Cui11291 AGTATTTGGGTGG-
TAAACCCGAGTGCGCAATGAAAGTGAAAGTTGGGAT

HLX320Dai26805 AGTATTTGGGTGG-
CAAACCCGAGTGCGTAATGAAAGTGAAAGTTGGGAT

WCG1266Dai24678A AGTATTTGGGTGG-
TAAACCCGAGTGCGCAATGAAAGTGAAAGTTGGGAT

Dai6090_Ceriporia_sulphuricolo AGTGTTTGGGTGG-
AAAACCCGAGCGCGCAATGAAAGTGAAAGTTGGGAT

RLG_11354_Ceriproia_reticulata AGTATTTGGGTGG-
AAAACTCGAATGCGCAATGAAAGTGAAAGTTGGGAT

ZZW1543Dai27072 AGTATTTGGGTGG-
AAAACTCGAATGCGTAATGAAAGTGAAAGTTGGGAT

Li1316_Ceriporia_reticulata --

KHL11981Ceriporia_reticulata AGTATTTGAGTGG-
AAAACTCGAGTGCGTAATGAAAGTGAAAGTTGGGAT

FP110343sp_Candelabrochaete_la AGTATTTGGGTGG-
AAAACCCGAGTGCGTAATTAAAGTGAAAGTTGGGAT

Li1045_Ceriporia_reticulata --

ZX136Dai25794ceriporia AGTATTTGGGTGT-
AAAACCCGAGTGCGCAATGAAAGTGATAGTTGGGAT

892Dai13400 AGTATTTGGGTGT-
AAAACCCGAGTGCGTAATGAAAGTGATAGTTGGGAT

RLG7163Leptoporus_mollis AGTATTTGGGTGG-
TAAACCCGAGTGCGTAATGAAAGTGAAAGTTGGGAT

Dai21062Leptoporus_mollis AGTATCTGGGTGG-
TAAACCCGAGTGCGTAATGAAAGTGAAAGTTGGGAT

Dai20182Leptoporus_submollis AGTATTTGGGTGG-
TAAACTCGAGTGCGTAATGAAAGTGAAAGTTGGGAT

Cui18379Leptoporus_submollis AGTATTTGGGTGG-
TAAACTCGAGTGCGTAATGAAAGTGAAAGTTGGGAT

Wu1209_46Resiniporus_pseudogil
AGTATTTGGGTGATTAAACCCGAGTGCGTAATGAAAGTGAAAGTTGGGAT

BRNM710169Resiniporus_resinasc --

Dai14516Bjerkandera_adusta AGTGTTTGGGTGG-
AAAACCCGAGCGCGTAATGAAAGTGAAAGTTGGGAC

Dai21100Bjerkandera_fumosa AGTGTTTGGGTGG-
AAAACCCGAGCGCGCAATGAAAGTGAAAGTTGGGAC

Miettinen16854Ceraceomyces_sp AGTGTTTGGGTGG-CAAACCCGAGCGCGCAACGAAAGTGAAAGTTGGGAT

Dai10477C_spissa AGTATTTGGGTGG-TAAACCCGAATGCGCAATGAAAGTGAAAGTTGGGAT

855Dai16831 AGTATTTGGGTGG-TAAACCCGAATGCGCAATGAAAGTGAAAGTTGGGAT

882Cui11282 AGTATTTGGGTGG-TAAACCCGAATGCGCAATGAAAGTGAAAGTTGGGAT

Dai24566 AGTATTTGGGTGG-TAAACCCGAGTGCGTAATGAAAGTGAAAGTTGGGAT

Yuan5965 AGTATTTGGGTGG-TAAACCCGAGTGCGTAATGAAAGTGAAAGTTGGGAT

Dai3204 AGTGTTTGGGTGG-TAAACCCGAGCGCGTAATGAAAGTGATAGTTGGGAT

1194CUI9985 AGTATTTGGGTGATTAAACCCGAGTGCGTAATGAAAGTGAAAGTTGGGAT

Dai15205_Ceriporia_albomellea CTCTGTCATGGAGAGCACCGACGCCCAGACCAGACCTTCTGTGACGGATC

Dai15223_Ceriporia_albomellea CTCTGTCATGGAGAGCACCGACGCCCAGACCAGACCTTCTGTGACGGATC

Li1780_Ceriporia_variegata CTCTGTCATGGAGAGCACCGACGCCCAGACCAGACCTTCTGTGACGGATC

Dai19791_Ceriporia_variegata CTCTGTCATGGAGAGCACCGACGCCCAGACCAGACCTTCTGTGACGGATC

Dai19886 CTCTGTCATGGAGAGCACCGACGCCCAGACCAGACCTTCTGTGACGGATC

Dai10833_Ceriporia_crassitunic CTCTGTCATGGAGAGCACCGACGCCCAGACCAGACCTTCTGTGACGGATC

CHWC1506_46Meruliopsis_crassit --

Dai9995_Ceriporia_crassitunica --

Wu1209_58_Meruliopsis_parvispo CTCTGTCGTGGAGAGCACCGACGCCCGGACCAGACCTTTTGTGACGGCTC

CHWC1505_129_Meruliopsis_parvi CTCTGTCGTGGAGAGCACCGACGCCCGGACCAGACCTTTTGTGACGGCTC

Dai21944 CTCTGTCGTGGAGAGCACCGACGCCCGGACCAGACCTTTTGTGACGGCTC

830Dai18640A CTCTGTCGTGGAGAGCACCGACGCCCGGACCAGACCTTTTGTGACGGCTC

GC1704_60_Meruliopsis_taxicola CCCTGTCGTGGGGAGCACCGACGCCCGGACCAGACCTTCTGTGACGGCTC

Dai22625 CCCTGTCGTGGGGAGCACCGACGCCCGGACCAGACCTTCTGTGACGGCTC

Dai22636
CCCTGTCGTGGGGAGCACCGACGCCCGGACCAGACCTTCTGTGACGGCTC
Dai21878
CCCTGTCGTGGGGAGCACCGACGCCCGGACCAGACCTTCTGTGACGGCTC
1169Dai17248
CCCTGTCGTGGGGAGCACCGACGCCCGGACCAGACCTTCTGTGACGGCTC
Wu1708_43_Meruliopsis_leptocys
CTCTGTCGTGGAGAGCACCGACGCCCGGACCAGACCTTCTGTGACGGCTC
Li1011
CTCTGTCGTGGAGAGCACCGACGCCCGGACCAGACCTTCTGTGACGGCTC
ZX95Dai25742Meruliopsis_leptoc
CTCTGTCGTGGAGAGCACCGACGCCCGGACCAGACCTTCTGTGACGGCTC
WCG1306Dai24733
CTCTGTCGTGGAGAGCACCGACGCCCGGACCAGACCTTCTGTGACGGCTC
LXL99Dai25816
CTCTGTCGTGGAGAGCACCGACGCCCGGACCAGACCTTCTGTGACGGCTC
WCG1559Dai26052Meruliopsis
CTCTGTCGTGGAGAGCACCGACGCCCGGACCAGACCTTCTGTGACGGCTC
He7477
CTCTGTCGTGGAGAGCACCGACGCCCGGACCAGACCTTCTGTGACGGCTC
HLX243Dai26217
CTCTGTCGTGGAGAGCACCGACGCCCGGACCAGACCTTCTGTGACGGCTC
RussiaMW673659Meruliopsis_fagi
CTCTGTCGTGGAGAGCACCGACGCCCGGACCAGACCTTCTGTGACGGCTC
FD278
CTCTGTCGTGGAGAGCACCGACGCCCGGACCAGACCTTCTGTGACGGCTC
Dai10226_Ceriporia_tarda --
LE247365
CTCTGTCGTGGAGAGCACCGACGCCCGGACCAGACCTTCTGTGACGGCTC
Dai8173_Meruliopsis_nanlingens
CTCTGTCGTGGAGAGCACCGACGCCCGGACCAGACCTTCTGTGACGGCTC
860Dai17172
CTCTGTCGTGGAGAGCACCGACGCCCGGACCAGACCTTCTGTGACGGCTC
879Dai13414
CTCTGTCGTGGAGAGCACCGACGCCCGGACCAGACCTTCTGTGACGGCTT
Li_1704_Meruliopsis_pseudocyst --
833Dai18405
CTCTGTCGTGGAGAGCACCGACGCCCGGACCAGACCTTCTGTGACGGCTC
HHB_10729_Meruliopsis_albostra
CTCTGTCGTGGAGAGCACCGACGCCCGGACCAGACCTTCTGTGACGGCTC
Cui6878_Ceriporia_pseudocystid
CTCTGTCGTGGAGAGCACCGACGCCCGGACCAGACCTTCTGTGACGGCTC
869Dai14737
CTCTGTCGTGGAGAGCACCGACGCCCGGACCAGACCTTCTGTGACGGCTC

876Cui11626
CTCTGTCGTGGAGAGCACCGACGCCCGGACCAGACCTTCTGTGACGGCTC
1199WEI3388 ---
776308_Meruliopsis_cystidiata
CTCTGTCGTGGAGAGCACCGACGCCCGGACCAGACCTTCTGTGACGGCTC
ICN139059_Meruliopsis_cystidia
CTCTGTCGTGGAGAGCACCGACGCCCGGACCAGACCTTCTGTGACGGCTC
HHB15692Ceraceomyces_serpens
CCCTGTCGTGGGGAGCACCGACGCCCGGGCCAGACCTTCTGTGACGGCCC
HHB_15629_Sp_Ceriporiopsis_ane
CCCTGTCGTGGGGAGCACCGACGCCCGGACCAGACCTTTTGCGACGGCTC
AJ185Trametopsis_cervina
CCCTGTCGTGGGGAGCACCGACGCCCGGACCAGACCTTCTGTGACGGATC
FD9Irpex_lacteus
CCCTGTCGTGGGGAGCACCGACGCCCGGACCAGACCTTCTGTGACGGATC
908Dai11230
TCCTGTCGTGGGAAGCACCGACGCCCGGACCAGACCTTCTGTGACGGATC
FP55521TEmmia_lacerata
CCCTGTCGTGGGGAGCACCGACGCCCGGACCAGACCTTCTGTGACGGATC
PBU0048Ceriporia_cystidiata
CCCTGTCGTGGGGAGCACCGACGCCCGGACCAGACCTTCTGTGACGGATC
MZ340C_laceratаT ---
Dai21940
CCCTGTCGTGGGGAGCACCGACGCCCGGACCAGACCTTCTGTGACGGATC
847Dai16433
CCCTGTCGTGGGGAGCACCGACGCCCGGACCAGACCTTCTGTGACGGATC
MarcinEmmia_latemarginatus
CCCTGTCGTGGGGAGCACCGACGCCCGGACCAGACCTTCTGTGACGGATC
Meijer3729Hydnopolyporus_fimbr
CTCTGTCGTGGAGAGCACCGACGCCCGGACCAGACCTTCTGTGACGGCTC
RLG13408Phanerochaete_sp
CCCTGTCGTGGGGAGCACCGACGCCCGGACCAGACCTTCTGTGACGGATC
WHC1381Flavodon_flavus
CTCTGTCGTGGAGAGCACCGACGCCCGGACCAGACCTTCTGTGACGGATC
GB1833Phlebia_albida
CTCTGTCGTGGAGAGCACCGACGCCCGGACCAGACCTTTTGTGACGGATC
T407Phlebia_nitidula ---
HHB6988Phanerochaete_exilis
CCCTGTCGCGGGGAGCACCGACGCCCGGACCAGACCTTTTGTGACGGATC
HHB8509Phanerochaetella_xeroph
CCCTGTCGCGGGGAGCACCGACGCCCGGACCAGACCTTCTGTGACGGATC
PBU0051Macrohyporia_dictyopora
CTCTGTCGTGGAGAGCACCGACGCCCGGACCAGACCTTCTGTGACGGATC
HHB11463Phanerochaete_sp

CCCTGTCGCGGGGAGCACCGACGCCCGGACCAGACCTTTTGTGACGGATC
FP102382Byssomerulius_corium
CTCTGTCGCGGAGAGCACCGACGCCCGGACCAGACCTTCTGTGACGGATC
FP102165Efibula_americana
CCCTGTCGCGGGGAGCACCGACGCCCGGACCAGACCTTCTGTGACGGATC
Murdoch90Ceriporia_torpida
CTCTGTCGTGGAGAGCACCGACGCCCGGACCAGACCTTCTGTGACGGATC
Rivoire4413_Ceriporia_purpurea
CTCTGTCGTGGAGAGCACCGACGCCCGGACCAGACCTTCTGTGACGGATC
Kout_18_Ceriporia_triumphalis
CTCTGTCGTGGAGAGCACCGACGCCCGGACCAGACCTTTTGTGACGGATC
Rivoire3701_Ceriporia_bresadol
CTCTGTCGTGGAGAGCACCGACGCCCGGACCAGACCTTCTGTGACGGATC
VS4018 --
Ryvarden21832_Ceriporia_manzan
CTCTGTCGTGGAGAGCACCGACGCCCGGACCAGACCTTCTGTGACGGATC
Dai24539
CTCTGTCGTGGAGAGCACCGACGCCCGGACCAGACCTTCTGTGACGGATC
Dai24541
CTCTGTCGTGGAGAGCACCGACGCCCGGACCAGACCTTCTGTGACGGATC
JV1105_12_Ceriporia_occidental
CTCTGTCGTGGAGAGCACCGACGCCCGGACCAGACCTTCTGTGACGGATC
VS8558Ceriporia_occidentalis
CTCTGTCGTGGAGAGCACCGACGCCCGGACCAGACCTTCTGTGACGGATC
Dai22445
CTCTGTCGTGGAGAGCACCGACGCCCGGACCAGACCTTCTGTGACGGATC
846Dai16368
CTCTGTCGTGGAGAGCACCGACGCCCGGACCAGACCTTCTGTGACGGATC
Dai17951_Ceriporia_aurantiocar
CTCTGTCGTGGAGAGCACCGACGCCCGGACCAGACCTTCTGTGACGGATC
Miettinen_11701C_viridans
CTTCGTCGTGGAGAGCACCGACGCCCGGACCAGACCTTCTGTGACGGATC
JV0105_10Ceriporia_aurantiocar
CTCTGTCGTGGAGAGCACCGACGCCCGGACCAGACCTTCTGTGACGGATC
Yuan5702C_viridans --
858Dai17003 --
Yuan2747_Ceriporia_viridans --
Yuan2744C_viridans --
Li1046C_viridans --
865C_sinoviridans --
871Dai15062 --
Dai7642_Ceriporia_humilis --
Spirin4706_Ceriporia_humilis --
Spirin4944_Ceriporia_sericea

CTCTGTCGTGGAGAGCACCGACGCCCGGACCAGACCTTCTGTGACGGATC

WCG1547Dai26044ceriporia

CTCTGTCGTGGAGAGCACCGACGCCCGGACCAGACCTTCTGTGACGGATC

ZZW1558Dai27086

CTCTGTCGTGGAGAGCACCGACGCCCGGACCAGACCTTCTGTGACGGATC

Miettinen14381_Ceriporia_mpuri

CTCTGTCGTGGAGAGCACCGACGCCCGGACCAGACCTTCTGTGACGGATC

Miettinen15492_2_Ceriporia_sor

CTCTGTCGTGGAGAGCACYGACGCCCGGACCAGACCTTCTGTGACGGATC

He6687

CTCTGTCGTGGAGAGCACCGACGCCCGGACCAGACCTTCTGTGACGGATC

ZH53Dai24426

CTCTGTCGTGGAGAGCACCGACGCCCGGACCAGACCTTCTGTGACGGATC

Vlasak0808_30_Ceriporia_punica

CTCTGTCGTGGAGAGCACCGACGCCCGGACCAGACCTTTTGTGACGGCTC

887Dai13376

CTCTGTCGTGGAGAGCACCGACGCCCGGACCAGACCTTCTGTGACGGATC

WCG1443Dai24998

CTCTGTCGTGGAGAGCACCGACGCCCGGACCAGACCTTCTGTGACGGATC

0108_6Ceriporia_spissa

CTCTGTCGCGGAGAGCACCGACGCCCGGACCAGACCTTCTGTGACGGATC

Dai19164

CTCTGTCGCGGAGAGCACCGACGCCCGGACCAGACCTTCTGTGACGGATC

Dai17937_Ceriporia_bubalinomar

CTCTGTCGTGGAGAGCACCGACGCCCGGACCAGACCTTCTGTGACGGATC

903Dai12113

CTCTGTCGTGGAGAGCACCGACGCCCGGACCAGACCTTCTGTGACGGATC

LZB929Dai25079

CTCTGTCGTGGAGAGCACCGACGCCCGGACCAGACCTTCTGTGACGGATC

LX45Dai26988 --

LX43Dai26986

CTCTGTCGTGGAGAGCACCGACGCCCGGACCAGACCTTCTGTGACGGATC

Dai7759Ceriporia --

Cui8012_Ceriporia_viridans --

GC1704_54Ceriporia_viridans

CTCTGTCGTGGAGAGCACCGACGCCCGGACCAGACCTTCTGTGACGGATC

Dai23392

CTCTGTCGTGGAGAGCACCGACGCCCGGACCAGACCTTCTGTGACGGATC

WCG1585Dai26113Ceriproia

CTCTGTCGTGGAGAGCACCGACGCCCGGACCAGACCTTCTGTGACGGATC

Dai18675C_eucalypti

CTCTGTCGTGGAGAGCACCGACGCCCGGACCAGACCTTCTGTGACGGATC

Dai22034

CTCTGTCGTGGAGAGCACCGACGCCCGGACCAGACCTATTGTGACGGCTC

JV1008_41JTardaFLORIDAKeys ---
Rivoire1161_Ceriporia_pierii ---
Dai23499C_pierii
CTCTGTCGTGGAGAGCACCGACGCCCGGACCAGACCTTCTGTGACGGATC
Dai23500
CTCTGTCGTGGAGAGCACCGACGCCCGGACCAGACCTTCTGTGACGGATC
841Dai15899
CTCTGTCGCGGAGAGCACCGACGCCCGGACCAGACCTTCTGTGACGGATC
842Dai15904 ---
LZB1066xinjiang ---
LZB1065xinjiang ---
851Dai16779
CTCTGTCGTGGAGAGCACCGACGCCCGGACCAGACCTTCTGTGACGGCTC
RMJ119sp_Candelabrochaete_sept ---
RLG9759spCandelabrochaete_sept
CTCTGTCGTGGAGAGCACCGACGCCCGGACCAGACCTTCTGTGACGGCTC
RLG10478Phanerochaete_allantos ---
Dai19118_Ceriporia_spissa
CTCTGTCGCGGAGAGCACCGACGCCCGGACCAGACCTTCTGTGACGGATC
Dai18486A
CTCTGTCGCGGAGAGCACCGACGCCCGGACCAGACCTTCTGTGACGGATC
WEI17_024_Ceriporia_mellita
CTCTGTCGCGGAGAGCACCGACGCCCGGACCAGACCTTCTGTGACGGATC
GC1508_71Ceriporia_mellita
CTCTGTCGCGGAGAGCACCGACGCCCGGACCAGACCTTCTGTGACGGATC
GC1608_7_Ceriporia_mellita
CTCTGTCGTAGAGAGCACCGACGCCCGGACCAGACCTTCTGTGACGGATC
ZZW1557Dai27085
CTCTGTCGTAGAGAGCACCGACGCCCGGACCAGACCTTCTGTGACGGATC
ZZW1554Dai27083
CTCTGTCGTAGAGAGCACCGACGCCCGGACCAGACCTTCTGTGACGGATC
Dai8168
CTCTGTCGTAGAGAGCACCGACGCCCGGACCAGACCTTCTGTGACGGATC
BR4865C_mellita
CTCTGTCGCGGAGAGCACCGACGCCCGGACCAGACCTTCTGTGACGGATC
MEL2382688Ceriporia_sp ---
Dai8110
CTCTGTCGCGGAGAGCACCGACGCCCGGACCAGACCTTCTGTGACGGATC
Cui8097 ---
909Cui6740 ---
W1258Dai24695
CTCTGTCGCGGAGAGCACCGACGCCCGGACCAGACCTTCTGTGACGGATC
JV0110_26_Ceriporia_griseoviol
CCTTGTCATGGGGAGCACCGACGCCCGGGCCAGACCTTCTGTGACGGCTC

896Dai13202
CCTTGTCATGGGGAGCACCGACGCCCGGGCCAGACCTTCTGTGACGGCTC
LWY393Dai27053C_griseoviolasce
CCCTGTCATGGGGAGCACCGACGCCCGGGCCAGACCTTCTGTGACGGCTC
LWY394DAI27054
CCCTGTCATGGGGAGCACCGACGCCCGGGCCAGACCTTCTGTGACGGCTC
FP135015G_pannocinctus
CCCTGTCGTGGGGAGCACCGACGCCCGGACCAGACCTTCTGTGACGGATC
L15726SpG_pannocinctus
CCCTGTCGTGGGGAGCACCGACGCCCGGACCAGACCTTCTGTGACGGATC
Dai22221
CTCTGTCGTGGAGAGCACCGACGCCCGGACCAGACCTTCTGTGACGGATC
Dai22633
CTCTGTCGTGGAGAGCACCGACGCCCGGACCAGAACTTCTGTGACGGCCC
Dai23260
CTCTGTCGTGGAGAGCACCGACGCCCGGACCAGAACTTCTGTGACGGCCC
Dai23626
CTCTGTCGTGGAGAGCACCGACGCCCGGACCAGAACTTCTGTGACGGCCC
Dai16238G_citrinoalbus
CTCTGTCGTGGAGAGCACCGACGCCCGGCCCTGAACTTCTGTGACGGACC
1175Dai15293
CTCTGTCGTGGAGAGCACCGACGCCCGGCCCTGAACTTCTGTGACGGACC
Dai19547
CTCTGTCGTGGAGAGCACCGACGCCCGGCCCTGAACTTCTGTGACGGACC
918063G_africanus
CTCTGTCGTGGAGAGCACCGACGCCCGGCCCTGAACTTCTGTGACGGACC
918572G_africanus
CTCTGTCGTGGAGAGCACCGACGCCCGGCCCTGAACTTCTGTGACGGACC
Dai18536A --
1164Cui17922
CTCTGTCGTGGAGAGCACCGACGCCCGGCCCTGAACTTCTGTGACGGACC
Dai22225
CTCTGTCGTGGAGAGCACCGACGCCCGGCCCTGAACTTCTGTGACGGACC
1163Dai20655
CTCTGTCGTGGAGAGCACCGACGCCCGGCCCTGAACTTCTGTGACGGACC
Yuan4397G_hainanensis
CCCTGTCGCGGGGAGCACCGACGCCCGGACCAGACCTTCTGTGACGGATC
1176Dai15268
CCCTGTCGCGGGGAGCACCGACGCCCGGACCAGACCTTCTGTGACGGATC
1177Dai15259
CCCTGTCGCGGGGAGCACCGACGCCCGGACCAGACCTTCTGTGACGGATC
BZ2896G_thelephoroides
CCCTGTCGTGGGGAGCACCGACGCCCGGACCAGACCTTCTGTGACGGATC
1166JV1808_26

CCCTGTCGTGGGGAGCACCGACGCCCGGACCAGACCTTCTGTGACGGATC
Miettinen16992Hapalopilus_ochr
CTCTGTCATGGAGAGCACCGACGCCCGGGCTTGATCTACGGTGACGGACC
GC1708_338_Ceriporia_arbuscula
CTCTGTCGTGGAGAGCACCGACGCCCGGACCAGACCTTCTGTGACGGCTC
WCG1555Dai26107Ceriporia
CTCTGTCGTGGAGAGCACCGACGCCCGGACCAGACCTTCTGTGACGGCTC
GC1708_340_Ceriporia_arbuscula
CTCTGTCGTGGAGAGCACCGACGCCCGGACCAGACCTTCTGTGACGGCTC
WCG1556Dai26109Ceriporia
CTCTGTCGTGGAGAGCACCGACGCCCGGACCAGACCTTCTGTGACGGCTC
883Cui11291
CTCTGTCGTGGAGAGCACCGACGCCCGGACCAGACCTTCTGTGACGGCTC
HLX320Dai26805
CTCTGTCGTGGAGAGCACCGACGCCCGGACCAGACCTTCTGTGACGGCTC
WCG1266Dai24678A
CTCTGTCGTGGAGAGCACCGACGCCCGGACCAGACCTTCTGTGACGGCTC
Dai6090_Ceriporia_sulphuricolo
CTCTGTCGCGGGGAGCACCGACGCCCGGACCAGACCTTCTGTGACGGCTC
RLG_11354_Ceriproia_reticulata
CTCTGTCGTGGGGAGCACCGACGCCCGGACCAGACCTTCTGTGACGGCTC
ZZW1543Dai27072
CTCTGTCGTGGGGAGCACCGACGCCCGGACCAGACCTTCTGTGACGGCTC
Li1316_Ceriporia_reticulata --
KHL11981Ceriporia_reticulata
CTCTGTCGTGGAGAGCACCAATGCCCGGACCAGACCTTCTGTGACGGCTC
FP110343sp_Candelabrochaete_la
CTCTGTCGTGGAGAGCACCGACGCCCGGACCAGACCTTCTGTGACGGCTC
Li1045_Ceriporia_reticulata --
ZX136Dai25794ceriporia
CTCTGTCGTGGAGAGCACCGACGCCCGGACCAGACCTTCTGTGACGGCCC
892Dai13400
CTCTGTCGTGGAGAGCACCGACGCCCGGACCAGACCTTTTGTGACGGCCC
RLG7163Leptoporus_mollis
CTCTGTCGCGGAGAGCACCGACGCCCGGACCAGACCTTTTGTGACGGATC
Dai21062Leptoporus_mollis
CTCTGTCGCGGAGAGCACCGACGCCCGGACCAGACCTTCTGTGACGGATC
Dai20182Leptoporus_submollis
CTCTGTCGCGGAGAGCACCGACGCCCGGACCAGACCTTCTGTGACGGATC
Cui18379Leptoporus_submollis
CTCTGTCGCGGAGAGCACCGACGCCCGGACCAGACCTTCTGTGACGGATC
Wu1209_46Resiniporus_pseudogil
CCCTGTCGTGGGGAGCACCGACGCCCGGACCAGACCTTCTGTGACGGATC
BRNM710169Resiniporus_resinasc --

Dai14516Bjerkandera_adusta
TTCTGTCGTGGAAGGCACCGACGCCCGGACCAGACCTTCTGTGACGGATC
Dai21100Bjerkandera_fumosa
TTCTATCATGGAAGGCACCGACGCCCGGACCAGACCTTCTGTGACGGATC
Miettinen16854Ceraceomyces_sp
CTCTGTCGCGGAGAGCACCGACGCCCAGACCAGAGCTTTGTGACGGATC
Dai10477C_spissa
CTCTGTCATGGAGAGCACCGACGCCCGGACCAGACCTTCTGTGACGGATC
855Dai16831
CTCTGTCATGGAGAGCACCGACGCCCGGACCAGACCTTCTGTGACGGATC
882Cui11282
CTCTGTCATGGAGAGCACCGACGCCCGGACCAGACCTTCTGTGACGGATC
Dai24566
CTCTGTCATGGAGAGCACCGACGCCCGGACCAGACCTTCTGTGACGGATC
Yuan5965
CTCTGTCATGGAGAGCACCGACGCCCGGACCAGACCTTCTGTGACGGATC
Dai3204
CTCTGTCGTGGAGAGCACCGACGCCCGGACCAGACCTTCTGTGACGGCTC
1194CUI9985
CCCTGTCGTGGGGAGCACCGACGCCCGGACCAGACCTTCTGTGACGGATC

Dai15205_Ceriporia_albomellea
TGCGGTAGAGCATGTATGCTGGGACCCGAAAGATGGTGAACTATGCCTGA
Dai15223_Ceriporia_albomellea
TGCGGTAGAGCATGTATGCTGGGACCCGAAAGATGGTGAACTATGCCTGA
Li1780_Ceriporia_variegata
TGCGGTAGAGCATGTATGCTGGGACCCGAAAGATGGTGAACTATGCCTGA
Dai19791_Ceriporia_variegata
TGCGGTAGAGCATGTATGCTGGGACCCGAAAGATGGTGAACTATGCCTGA
Dai19886
TGCGGTAGAGCATGTATGCTGGGACCCGAAAGATGGTGAACTATGCCTGA
Dai10833_Ceriporia_crassitunic
TGCGGTAGAGCATGTATGCTGGGACCCGAAAGATGGTGAACTATGCCTGA
CHWC1506_46Meruliopsis_crassit --
Dai9995_Ceriporia_crassitunica --
Wu1209_58_Meruliopsis_parvispo
CGCGGTAGAGCAAGTATGCTGGGACCCGAAAGATGGTGAACTATGCCTGA
CHWC1505_129_Meruliopsis_parvi
CGCGGTAGAGCAAGTATGCTGGGACCCGAAAGATGGTGAACTATGCCTGA
Dai21944
CGCGGTAGAGCATGTATGCTGGGACCCGAAAGATGGTGAACTATGCCTGA
830Dai18640A
CGCGGTAGAGCAAGTATGCTGGGACCCGAAAGATGGTGAACTATGCCTGA

GC1704_60_Meruliopsis_taxicola
CGCGGTAGAGCATGTATGCTGGGACCCGAAAGATGGTGAACTATGCCTGA
Dai22625
CGCGGTAGAGCATGTATGCTGGGACCCGAAAGATGGTGAACTATGCCTGA
Dai22636
CGCGGTAGAGCATGTATGCTGGGACCCGAAAGATGGTGAACTATGCCTGA
Dai21878
CGCGGTAGAGCATGTATGCTGGGACCCGAAAGATGGTGAACTATGCCTGA
1169Dai17248
CGCGGTAGAGCATGTATGCTGGGACCCGAAAGATGGTGAACTATGCCTGA
Wu1708_43_Meruliopsis_leptocys
CGCGGTAGAGCATGTATGCTGGGACCCGAAAGATGGTGAACTATGCCTGA
Li1011
CGCGGTAGAGCATGTATGCTGGGACCCGAAAGATGGTGAACTATGCCTGA
ZX95Dai25742Meruliopsis_leptoc
CGCGGTAGAGCATGTATGCTGGGACCCGAAAGATGGTGAACTATGCCTGA
WCG1306Dai24733
CGCGGTAGAGCATGTATGCTGGGACCCGAAAGATGGTGAACTATGCCTGA
LXL99Dai25816
CGCGGTAGAGCATGTATGCTGGGACCCGAAAGATGGTGAACTATGCCTGA
WCG1559Dai26052Meruliopsis
CGCGGTAGAGCATGTATGCTGGGACCCGAAAGATGGTGAACTATGCCTGA
He7477
CGCGGTAGAGCATGTATGCTGGGACCCGAAAGATGGTGAACTATGCCTGA
HLX243Dai26217
CGCGGTAGAGCATGTATGCTGGGACCCGAAAGATGGTGAACTATGCCTGA
RussiaMW673659Meruliopsis_fagi
CGCGGTAGAGCATGTATGCTGGGACCCGAAAGATGGTGAACTATGCCTGA
FD278
CGCGGTAGAGCAAGTATGCTGGGACCCGAAAGATGGTGAACTATGCCTGA
Dai10226_Ceriporia_tarda --
LE247365
CGCGGTAGAGCATGTATGCTGGGACCCGAAAGATGGTGAACTATGCCTGA
Dai8173_Meruliopsis_nanlingens
CGCGGTAGAGCATGTATGCTGGGACCCGAAAGATGGTGAACTATGCCTGA
860Dai17172
CGCGGTAGAGCATGTATGCTGGGACCCGAAAGATGGTGAACTATGCCTGA
879Dai13414
CGCGGTAGAGCATGTATGCTGGGACCCGAAAGATGGTGAACTATGCCTGA
Li_1704_Meruliopsis_pseudocyst --
833Dai18405
CGCGGTAGAGCATGTATGCTGGGACCCGAAAGATGGTGAACTATGCCTGA
HHB_10729_Meruliopsis_albostra
CGCGGTAGAGCATGTATGCTGGGACCCGAAAGATGGTGAACTATGCCTGA

Cui6878_Ceriporia_pseudocystid
CGCGGTAGAGCATGTATGCTGGGACCCGAAAGATGGTGAACTATGCCTGA
869Dai14737
CGCGGTAGAGCATGTATGCTGGGACCCGAAAGATGGTGAACTATGCCTGA
876Cui11626
CGCGGTAGAGCATGTATGCTGGGACCCGAAAGATGGTGAACTATGCCTGA
1199WEI3388 --
776308_Meruliopsis_cystidiata
CGCGGTAGAGCATGTATGCTGGGACCCGAAAGATGGTGAACTATGCCTGA
ICN139059_Meruliopsis_cystidia
CGCGGTAGAGCATGTATGCTGGGACCCGAAAGATGGTGAACTATGCCTGA
HHB15692Ceraceomyces_serpens
CGCGGTAGAGCAGGTTTGTTGGGACCCGAAAGATGGTGAACTATGCCTGA
HHB_15629_Sp_Ceriporiopsis_ane
TGCGGTAGAGCATGTTTGTTGGGACCCGAAAGATGGTGAACTATGCCTGA
AJ185Trametopsis_cervina
TGCGGTAGAGCATGTTTGTTGGGACCCGAAAGATGGTGAACTATGCCTGA
FD9Irpex_lacteus
TGCGGTAGAGCATGTTTGTTGGGACCCGAAAGATGGTGAACTATGCCTGA
908Dai11230
TGCGGTAGAGCATGTTTGTTGGGACCCGAAAGATGGTGAACTATGCCTGA
FP55521TEmmia_lacerata
TGCGGTAGAGCATGTTTGTTGGGACCCGAAAGATGGTGAACTATGCCTGA
PBU0048Ceriporia_cystidiata
TGCGGTAGAGCATGTTTGTTGGGACCCGAAAGATGGTGAACTATGCCTGA
MZ340C_lacerataT --
Dai21940
TGCGGTAGAGCATGTTTGTTGGGACCCGAAAGATGGTGAACTATGCCTGA
847Dai16433
TGCGGTAGAGCATGTTTGTTGGGACCCGAAAGATGGTGAACTATGCCTGA
MarcinEmmia_latemarginatus
TGCGGTAGAGCATGTTTGTTGGGACCCGAAAGATGGTGAACTATGCCTGA
Meijer3729Hydnopolyporus_fimbr
TGCGGTAGAGCATGTTTGTTGGGACCCGAAAGATGGTGAACTATGCCTGA
RLG13408Phanerochaete_sp
TGCGGTAGAGCATGTTTGTTGGGACCCGAAAGATGGTGAACTATGCCTGA
WHC1381Flavodon_flavus
TGCGGTAGAGCATGTTTGTTGGGACCCGAAAGATGGTGAACTATGCCTGA
GB1833Phlebia_albida
TGCGGTAGAGCATATTTGTTGGGACCCGAAAGATGGTGAACTATGCCTGA
T407Phlebia_nitidula --
HHB6988Phanerochaete_exilis
TGCGGTAGAGCATGTTTGTTGGGACCCGAAAGATGGTGAACTATGCCTGA
HHB8509Phanerochaetella_xeroph

TGCGGTAGAGCATGTTTGTTGGGACCCGAAAGATGGTGAACTATGCCTGA
PBU0051Macrohyporia_dictyopora
TGCGGTAGAGCATGTTTGTTGGGACCCGAAAGATGGTGAACTATGCCTGA
HHB11463Phanerochaete_sp
TGCGGTAGAGCATGTTTGTTGGGACCCGAAAGATGGTGAACTATGCCTGA
FP102382Byssomerulius_corium
TGCGGTAGAGCATGTTTGTTGGGACCCGAAAGATGGTGAACTATGCCTGA
FP102165Efibula_americana
TGCGGTAGAGCATGTTTGTTGGGACCCGAAAGATGGTGAACTATGCCTGA
Murdoch90Ceriporia_torpida
TGCGGTAGAGCATGTATGTTGGGACCCGAAAGATGGTGAACTATGCCTGA
Rivoire4413_Ceriporia_purpurea
TGCGGTAGAGCATGTATGTTGGGACCCGAAAGATGGTGAACTATGCCTGA
Kout_18_Ceriporia_triumphalis
TGCGGTAGAGCATGTATGTTGGGACCCGAAAGATGGTGAACTATGCCTGA
Rivoire3701_Ceriporia_bresadol
TGCGGTAGAGCATGTATGTTGGGACCCGAAAGATGGTGAACTATGCCTGA
VS4018 --
Ryvarden21832_Ceriporia_manzan
TGCGGTAGAGCATGTATGTTGGGACCCGAAAGATGGTGAACTATGCCTGA
Dai24539
TGCGGTAGAGCATGTATGTTGGGACCCGAAAGATGGTGAACTATGCCTGA
Dai24541
TGCGGTAGAGCATGTATGTTGGGACCCGAAAGATGGTGAACTATGCCTGA
JV1105_12_Ceriporia_occidental
TGCGGTAGAGCATGTATGTTGGGACCCGAAAGATGGTGAACTATGCCTGA
VS8558Ceriporia_occidentalis
TGCGGTAGAGCATGTATGTTGGGACCCGAAAGATGGTGAACTATGCCTGA
Dai22445
TGCGGTAGAGCATGTATGTTGGGACCCGAAAGATGGTGAACTATGCCTGA
846Dai16368
TGCGGTAGAGCATGTATGTTGGGACCCGAAAGATGGTGAACTATGCCTGA
Dai17951_Ceriporia_aurantiocar
TGCGGTAGAGCATGTATGTTGGGACCCGAAAGATGGTGAACTATGCCTGA
Miettinen_11701C_viridans
TGCGGTAGAGCATGTATGTTGGGACCCGAAAGATGGTGAACTATGCCTGA
JV0105_10Ceriporia_aurantiocar
TGCGGTAGAGCATGTATGTTGGGACCCGAAAGATGGTGAACTATGCCTGA
Yuan5702C_viridans --
858Dai17003 --
Yuan2747_Ceriporia_viridans --
Yuan2744C_viridans --
Li1046C_viridans --
865C_sinoviridans --

871Dai15062 --
Dai7642_Ceriporia_humilis --
Spirin4706_Ceriporia_humilis --
Spirin4944_Ceriporia_sericea
TGCGGTAGAGCACGTATGTTGGGACCCGAAAGATGGTGAACTATGCCTGA
WCG1547Dai26044ceriporia
TGCGGTAGAGCACGTATGTTGGGACCCGAAAGATGGTGAACTATGCCTGA
ZZW1558Dai27086
TGCGGTAGAGCACGTATGTTGGGACCCGAAAGATGGTGAACTATGCCTGA
Miettinen14381_Ceriporia_mpuri
TGCGGTAGAGCACGTATGTTGGGACCCGAAAGATGGTGAACTATGCCTGA
Miettinen15492_2_Ceriporia_sor
TGCGGTAGAGCATGTATGTTGGGACCCGAAAGATGGTGAACTATGCCTGA
He6687
TGCGGTAGAGCACGTATGTTGGGACCCGAAAGATGGTGAACTATGCCTGA
ZH53Dai24426
TGCGGTAGAGCACGTATGTTGGGACCCGAAAGATGGTGAACTATGCCTGA
Vlasak0808_30_Ceriporia_punica
CGCGGTAGAGCATGTATGTTGGGACCCGAAAGATGGTGAACTATGCCTGA
887Dai13376
TGCGGTAGAGCATGTATGTTGGGACCCGAAAGATGGTGAACTATGCCTGA
WCG1443Dai24998
TGCGGTAGAGCATGTATGTTGGGACCCGAAAGATGGTGAACTATGCCTGA
0108_6Ceriporia_spissa
TGCGGTAGAGCATGTATGTTGGGACCCGAAAGATGGTGAACTATGCCTGA
Dai19164
TGCGGTAGAGCATGTATGTTGGGACCCGAAAGATGGTGAACTATGCCTGA
Dai17937_Ceriporia_bubalinomar
TGCGGTAGAGCATGTATGTTGGGACCCGAAAGATGGTGAACTATGCCTGA
903Dai12113
TGCGGTAGAGCATGTATGTTGGGACCCGAAAGATGGTGAACTATGCCTGA
LZB929Dai25079
TGCGGTAGAGCATGTATGTTGGGACCCGAAAGATGGTGAACTATGCCTGA
LX45Dai26988 --
LX43Dai26986
TGCGGTAGAGCATGTATGTTGGGACCCGAAAGATGGTGAACTATGCCTGA
Dai7759Ceriporia --
Cui8012_Ceriporia_viridans --
GC1704_54Ceriporia_viridans
TGCGGTAGAGCATGTATGTTGGGACCCGAAAGATGGTGAACTATGCCTGA
Dai23392
TGCGGTAGAGCATGTATGTTGGGACCCGAAAGATGGTGAACTATGCCTGA
WCG1585Dai26113Ceriproia
TGCGGTAGAGCATGTATGTTGGGACCCGAAAGATGGTGAACTATGCCTGA

Dai18675C_eucalypti
TGCGGTAGAGCATGTATGTTGGGACCCGAAAGATGGTGAACTATGCCTGA
Dai22034
TGCGGTAGAGCATGTATGTTGGGACCCGAAAGATGGTGAACTATGCCTGA
JV1008_41JTardaFLORIDAKeys --
Rivoire1161_Ceriporia_pierii --
Dai23499C_pierii
TGCGGTGGAGCATGTATGTTGGGACCCGAAAGATGGTGAACTATGCCTGA
Dai23500
TGCGGTGGAGCATGTATGTTGGGACCCGAAAGATGGTGAACTATGCCTGA
841Dai15899
TGCGGTAGAGCATGTATGTTGGGACCCGAAAGATGGTGAACTATGCCTGA
842Dai15904 --
LZB1066xinjiang --
LZB1065xinjiang --
851Dai16779
TGCGGTCGAGCATGTATGTTGGGACCCGAAAGATGGTGAACTATGCCTGA
RMJ119sp_Candelabrochaete_sept --
RLG9759spCandelabrochaete_sept
CGCGGTAGAGCGTGTATGTTGGGACCCGAAAGATGGTGAACTATGCCTGA
RLG10478Phanerochaete_allantos --
Dai19118_Ceriporia_spissa
TGAGGTAGAGCATGTATGTTGGGACCCGAAAGATGGTGAACTATGCCTGA
Dai18486A
TGAGGTAGAGCATGTATGTTGGGACCCGAAAGATGGTGAACTATGCCTGA
WEI17_024_Ceriporia_mellita
TGAGGTAGAGCATGTATGTTGGGACCCGAAAGATGGTGAACTATGCCTGA
GC1508_71Ceriporia_mellita
TGAGGTAGAGCATGTATGTTGGGACCCGAAAGATGGTGAACTATGCCTGA
GC1608_7_Ceriporia_mellita
TGAGGTAGAGCATGTATGTTGGGACCCGAAAGATGGTGAACTATGCCTGA
ZZW1557Dai27085
TGAGGTAGAGCATGTATGTTGGGACCCGAAAGATGGTGAACTATGCCTGA
ZZW1554Dai27083
TGAGGTAGAGCATGTATGTTGGGACCCGAAAGATGGTGAACTATGCCTGA
Dai8168
TGAGGTAGAGCATGTATGTTGGGACCCGAAAGATGGTGAACTATGCCTGA
BR4865C_mellita
TGAGGTAGAGCATGTATGTTGGGACCCGAAAGATGGTGAACTATGCCTGA
MEL2382688Ceriporia_sp --
Dai8110
TGAGGTAGAGCATGTATGTTGGGACCCGAAAGATGGTGAACTATGCCTGA
Cui8097 --
909Cui6740 --

W1258Dai24695

TGAGGTAGAGCATGTATGTTGGGACCCGAAAGATGGTGAACTATGCCTGA

JV0110_26_Ceriporia_griseoviol

CGCGGTAGAGCAAGCATGTTGGGACCCGAAAGATGGTGAACTATGCCTGA

896Dai13202

CGCGGTAGAGCAAGCATGTTGGGACCCGAAAGATGGTGAACTATGCCTGA

LWY393Dai27053C_griseoviolasce

CGCGGTAGAGCAAGCATGTTGGGACCCGAAAGATGGTGAACTATGCCTGA

LWY394DAI27054

CGCGGTAGAGCAAGCATGTTGGGACCCGAAAGATGGTGAACTATGCCTGA

FP135015G_pannocinctus

TGCGGTAGAGCATGTATGTTGGGACCCGAAAGATGGTGAACTATGCCTGA

L15726SpG_pannocinctus

TGCGGTAGAGCATGTATGTTGGGACCCGAAAGATGGTGAACTATGCCTGA

Dai22221

TGCGGTAGAGCATGTATGTTGGGACCCGAAAGATGGTGAACTATGCCTGA

Dai22633

TGCGGTAGAGCATGTATGTTGGGACCCGAAAGATGGTGAACTATGCCTGA

Dai23260

TGCGGTAGAGCATGTATGTTGGGACCCGAAAGATGGTGAACTATGCCTGA

Dai23626

TGCGGTAGAGCATGTATGTTGGGACCCGAAAGATGGTGAACTATGCCTGA

Dai16238G_citrinoalbus

TGCGGTAGAGCATGTATGTTGGGACCCGAAAGATGGTGAACTATGCCTGA

1175Dai15293

TGCGGTAGAGCATGTATGTTGGGACCCGAAAGATGGTGAACTATGCCTGA

Dai19547

TGCGGTAGAGCATGTATGTTGGGACCCGAAAGATGGTGAACTATGCCTGA

918063G_africanus

TGCGGTAGAGCATGTATGTTGGGACCCGAAAGATGGTGAACTATGCCTGA

918572G_africanus

TGCGGTAGAGCATGTATGTTGGGACCCGAAAGATGGTGAACTATGCCTGA

Dai18536A --

1164Cui17922

TGCGGTAGAGCATGTATGTTGGGACCCGAAAGATGGTGAACTATGCCTGA

Dai22225

TGCGGTAGAGCATGTATGTTGGGACCCGAAAGATGGTGAACTATGCCTGA

1163Dai20655

TGCGGTAGAGCATGTATGTTGGGACCCGAAAGATGGTGAACTATGCCTGA

Yuan4397G_hainanensis

TGCGGTAGAGCATGTATGTTGGGACCCGAAAGATGGTGAACTATGCCTGA

1176Dai15268

TGCGGTAGAGCATGTATGTTGGGACCCGAAAGATGGTGAACTATGCCTGA

1177Dai15259

TGCGGTAGAGCATGTATGTTGGGACCCGAAAGATGGTGAACTATGCCTGA
BZ2896G_thelephoroides
TGCGGTAGAGCATGTATGTTGGGACCCGAAAGATGGTGAACTATGCCTGA
1166JV1808_26
TGCGGTAGAGCATGTATGTTGGGACCCGAAAGATGGTGAACTATGCCTGA
Miettinen16992Hapalopilus_ochr
TGCGGTAGAGCATGTATGTTGGGACCCGAAAGATGGTGAACTATGCCTGA
GC1708_338_Ceriporia_arbuscula
TGCGGTAGAGCATGTATGTTGGGACCCGAAAGATGGTGAACTATGCCTGA
WCG1555Dai26107Ceriporia
TGCGGTAGAGCATGTATGTTGGGACCCGAAAGATGGTGAACTATGCCTGA
GC1708_340_Ceriporia_arbuscula
TGCGGTAGAGCATGTATGTTGGGACCCGAAAGATGGTGAACTATGCCTGA
WCG1556Dai26109Ceriporia
TGCGGTAGAGCATGTATGTTGGGACCCGAAAGATGGTGAACTATGCCTGA
883Cui11291
TGCGGTAGAGCATGTATGTTGGGACCCGAAAGATGGTGAACTATGCCTGA
HLX320Dai26805
TGCGGTAGAGCATGTATGTTGGGACCCGAAAGATGGTGAACTATGCCTGA
WCG1266Dai24678A
TGCGGTAGAGCATGTATGTTGGGACCCGAAAGATGGTGAACTATGCCTGA
Dai6090_Ceriporia_sulphuricolo
CGCGGTAGAGCATGTTTGTTGGGACCCGAAAGATGGTGAACTATGCCTGA
RLG_11354_Ceriproia_reticulata
CGAGGTAGAGCATGTATGTTGGGACCCGAAAGATGGTGAACTATGCCTGA
ZZW1543Dai27072
CGAGGTAGAGCATGTATGTTGGGACCCGAAAGATGGTGAACTATGCCTGA
Li1316_Ceriporia_reticulata --
KHL11981Ceriporia_reticulata
CGAGGTAGAGCATGTATGTTGGGACCCGAAAGATGGTGAACTATGCCTGA
FP110343sp_Candelabrochaete_la
TGCGGTAGAGCATGTATGTTGGGACCCGAAAGATGGTGAACTATGCCTGA
Li1045_Ceriporia_reticulata --
ZX136Dai25794ceriporia
TGCGGTAGAGCATGTATGTTGGGACCCGAAAGATGGTGAACTATGCCTGA
892Dai13400
TGCGGTAGAGCATGTATGTTGGGACCCGAAAGATGGTGAACTATGCCTGA
RLG7163Leptoporus_mollis
TGCGGTAGAGCATGTATGTTGGGACCCGAAAGATGGTGAACTATGCCTGA
Dai21062Leptoporus_mollis
TGCGGTAGAGCATGTATGTTGGGACCCGAAAGATGGTGAACTATGCCTGA
Dai20182Leptoporus_submollis
TGCGGTAGAGCATGTATGTTGGGACCCGAAAGATGGTGAACTATGCCTGA
Cui18379Leptoporus_submollis

TGCGGTAGAGCATGTATGTTGGGACCCGAAAGATGGTGAACTATGCCTGA

Wu1209_46Resiniporus_pseudogil
TGCGGTAGAGCATGTTTGTTGGGACCCGAAAGATGGTGAACTATGCCTGA

BRNM710169Resiniporus_resinasc --

Dai14516Bjerkandera_adusta
CGCGGTAGAGCATGTATGTTGGGACCCGAAAGATGGTGAACTATGCCTGA

Dai21100Bjerkandera_fumosa
CGCGGTAGAGCATGTATGTTGGGACCCGAAAGATGGTGAACTATGCCTGA

Miettinen16854Ceraceomyces_sp
TGCGGTAGAGCATGTTTGTTGGGACCCGAAAGATGGTGAACTATGCCTGA

Dai10477C_spissa
TGCGGTTGAGCATGTATGTTGGGACCCGAAAGATGGTGAACTATGCCTGA

855Dai16831
TGCGGTTGAGCATGTATGTTGGGACCCGAAAGATGGTGAACTATGCCTGA

882Cui11282
TGCGGTTGAGCATGTATGTTGGGACCCGAAAGATGGTGAACTATGCCTGA

Dai24566
TGCGGTTGAGCATGTATGTTGGGACCCGAAAGATGGTGAACTATGCCTGA

Yuan5965
TGCGGTTGAGCATGTATGTTGGGACCCGAAAGATGGTGAACTATGCCTGA

Dai3204
CGCGGTAGAGCATGTATGCTGGGACCCGAAAGATGGTGAACTATGCCTGA

1194CUI9985
TGCGGTAGAGCATGTTTGTTGGGACCCGAAAGATGGTGAACTATGCCTGA

Dai15205_Ceriporia_albomellea
ATAGGGTGAAGCCAGAGGAAACCCTGGTGGAGGCTCGTAGCGATTCTGAC

Dai15223_Ceriporia_albomellea ATAGGGTGAAGCCAGAGGAAACTCTGGTGGA-GCTCGTAGCGATTCTGAC

Li1780_Ceriporia_variegata
ATAGGGTGAAGCCAGAGGAAACTCTGGTGGAGGCTCGTAGCGATTCTGAC

Dai19791_Ceriporia_variegata
ATAGGGTGAAGCCAGAGGAAACTCTGGTGGAGGCTCGTAGCGATTCTGAC

Dai19886
ATAGGGTGAAGCCAGAGGAAACTCTGGTGGAGGCTCGTAGCGATTCTGAC

Dai10833_Ceriporia_crassitunic
ATAGGGTGAAGCCAGAGGAAACTCTGGTGGAGGCTCGTAGCGATTCTGAC

CHWC1506_46Meruliopsis_crassit --

Dai9995_Ceriporia_crassitunica --

Wu1209_58_Meruliopsis_parvispo -TAGGGTGAAGCCAGAGGAAACTCTGGTGGAGGCTCGTAGCGATTCTGAC

CHWC1505_129_Meruliopsis_parvi
ATAGGGTGAAGCCAGAGGAAACTCTGGTGGA-GCTCGTAGCGATTCTGAC

Dai21944
ATAGGGTGAAGCCAGAGGAAACTCTGGTGGAGGCTCGTAGCGATTCTGAC
830Dai18640A
ATAGGGTGAAGCCAGAGGAAACTCTGGTGGAGGCTCGTAGCGATTCTGAC
GC1704_60_Meruliopsis_taxicola
ATAGGGTGAAGCCAGAGGAAACTCTGGTGGAGGCTCGTAGCGATTCTGAC
Dai22625
ATAGGGTGAAGCCAGAGGAAACTCTGGTGGAGGCTCGTAGCGATTCTGAC
Dai22636
ATAGGGTGAAGCCAGAGGAAACTCTGGTGGAGGCTCGTAGCGATTCTGAC
Dai21878
ATAGGGTGAAGCCAGAGGAAACTCTGGTGGAGGCTCGTAGCGATTCTGAC
1169Dai17248
ATAGGGTGAAGCCAGAGGAAACTCTGGTGGAGGCTCGTAGCGATTCTGAC
Wu1708_43_Meruliopsis_leptocys
ATAGGGTGAAGCCAGAGGAAACTCTGGTGGAGGCTCGTAGCGATTCTGAC
Li1011
ATAGGGTGAAGCCAGAGGAAACTCTGGTGGAGGCTCGTAGCGATTCTGAC
ZX95Dai25742Meruliopsis_leptoc
ATAGGGTGAAGCCAGAGGAAACTCTGGTGGAGGCTCGTAGCGATTCTGAC
WCG1306Dai24733
ATAGGGTGAAGCCAGAGGAAACTCTGGTGGAGGCTCGTAGCGATTCTGAC
LXL99Dai25816
ATAGGGTGAAGCCAGAGGAAACTCTGGTGGAGGCTCGTAGCGATTCTGAC
WCG1559Dai26052Meruliopsis
ATAGGGTGAAGCCAGAGGAAACTCTGGTGGAGGCTCGTAGCGATTCTGAC
He7477
ATAGGGTGAAGCCAGAGGAAACTCTGGTGGAGGCTCGTAGCGATTCTGAC
HLX243Dai26217
ATAGGGTGAAGCCAGAGGAAACTCTGGTGGAGGCTCGTAGCGATTCTGAC
RussiaMW673659Meruliopsis_fagi
ATAGGGTGAAGCCAGAGGAAACTCTGGTGGAGGCTCGTAGCGATTCTGAC
FD278
ATAGGGTGAAGCCAGAGGAAACTCTGGTGGAGGCTCGTAGCGATTCTGAC
Dai10226_Ceriporia_tarda --
LE247365
ATAGGGTGAAGCCAGAGGAAACTCTGGTGGAGGCTCGTAGCGATTCTGAC
Dai8173_Meruliopsis_nanlingens
ATAGGGTGAAGCCAGAGGAAACTCTGGTGGAGGCTCGTAGCGATTCTGAC
860Dai17172
ATAGGGTGAAGCCAGAGGAAACTCTGGTGGAGGCTCGTAGCGATTCTGAC
879Dai13414
ATAGGGTGAAGCCAGAGGAAACTCTGGTGGAGGCTCGTAGCGATTCTGAC
Li_1704_Meruliopsis_pseudocyst --

833Dai18405
ATAGGGTGAAGCCAGAGGAAACTCTGGTGGAGGCTCGTAGCGATTCTGAC
HHB_10729_Meruliopsis_albostra
ATAGGGTGAAGCCAGAGGAAACTCTGGTGGAGGCTCGTAGCGATTCTGAC
Cui6878_Ceriporia_pseudocystid
ATAGGGTGAAGCCAGAGGAAACTCTGGTGGAGGCTCGTAGCGATTCTGAC
869Dai14737
ATAGGGTGAAGCCAGAGGAAACTCTGGTGGAGGCTCGTAGCGATTCTGAC
876Cui11626
ATAGGGTGAAGCCAGAGGAAACTCTGGTGGAGGCTCGTAGCGATTCTGAC
1199WEI3388 --
776308_Meruliopsis_cystidiata --
ICN139059_Meruliopsis_cystidia --
HHB15692Ceraceomyces_serpens
ATAGGGTGAAGCCAGAGGAAACTCTGGTGGAGGCTCGTAGCGATTCTGAC
HHB_15629_Sp_Ceriporiopsis_ane
ATAGGGTGAAGCCAGAGGAAACTCTGGTGGAGGCTCGTAGCGATTCTGAC
AJ185Trametopsis_cervina
ATAGGGTGAAGCCAGAGGAAACTCTGGTGGAGGCTCGTAGCGATTCTGAC
FD9Irpex_lacteus
ATAGGGTGAAGCCAGAGGAAACTCTGGTGGAGGCTCGTAGCGATTCTGAC
908Dai11230
ATAGGGTGAAGCCAGAGGAAACTCTGGTGGAGGCTCGTAGCGATTCTGAC
FP55521TEmmia_lacerata
ATAGGGTGAAGCCAGAGGAAACTCTGGTGGAGGCTCGTAGCGATTCTGAC
PBU0048Ceriporia_cystidiata
ATAGGGTGAAGCCAGAGGAAACTCTGGTGGAGGCTCGTAGCGATTCTGAC
MZ340C_lacerataT --
Dai21940
ATAGGGTGAAGCCAGAGGAAACTCTGGTGGAGGCTCGTAGCGATTCTGAC
847Dai16433
ATAGGGTGAAGCCAGAGGAAACTCTGGTGGAGGCTCGTAGCGATTCTGAC
MarcinEmmia_latemarginatus
ATAGGGTGAAGCCAGAGGAAACTCTGGTGGAGGCTCGTAGCGATTCTGAC
Meijer3729Hydnopolyporus_fimbr
ATAGGGTGAAGCCAGAGGAAACTCTGGTGGAGGCTCGTAGCGATTCTGAC
RLG13408Phanerochaete_sp
ATAGGGTGAAGCCAGAGGAAACTCTGGTGGAGGCTCGTAGCGATTCTGAC
WHC1381Flavodon_flavus
ATAGGGTGAAGCCAGAGGAAACTCTGGTGGAGGCTCGTAGCGATTCTGAC
GB1833Phlebia_albida
ATAGGGTGAAGCCAGAGGAAACTCTGGTGGAGGCTCGTAGCGATTCTGAC
T407Phlebia_nitidula --
HHB6988Phanerochaete_exilis

ATAGGGTGAAGCCAGAGGAAACTCTGGTGGAGGCTCGTAGCGATTCTGAC
HHB8509Phanerochaetella_xeroph
ATAGGGTGAAGCCAGAGGAAACTCTGGTGGAGGCTCGTAGCGATTCTGAC
PBU0051Macrohyporia_dictyopora
ATAGGGTGAAGCCAGAGGAAACTCTGGTGGAGGCTCGTAGCGATTCTGAC
HHB11463Phanerochaete_sp
ATAGGGTGAAGCCAGAGGAAACTCTGGTGGAGGCTCGTAGCGATTCTGAC
FP102382Byssomerulius_corium
ATAGGGTGAAGCCAGAGGAAACTCTGGTGGAGGCTCGTAGCGATTCTGAC
FP102165Efibula_americana
ATAGGGTGAAGCCAGAGGAAACTCTGGTGGAGGCTCGTAGCGATTCTGAC
Murdoch90Ceriporia_torpida
ATAGGGTGAAGCCAGAGGAAACTCTGGTGGAGGCTCGTAGCGATTCTGAC
Rivoire4413_Ceriporia_purpurea
ATAGGGTGAAGCCAGAGGAAACTCTGGTGGAGGCTCGTAGCGATTCTGAC
Kout_18_Ceriporia_triumphalis
ATAGGGTGAAGCCAGAGGAAACTCTGGTGGAGGCTCGTAGCGATTCTGAC
Rivoire3701_Ceriporia_bresadol
ATAGGGTGAAGCCAGAGGAAACTCTGGTGGAGGCTCGTAGCGATTCTGAC
VS4018 --
Ryvarden21832_Ceriporia_manzan
ATAGGGTGAAGCCAGAGGAAACTCTGGTGGAGGCTCGTAGCGATTCTGAC
Dai24539
ATAGGGTGAAGCCAGAGGAAACTCTGGTGGAGGCTCGTAGCGATTCTGAC
Dai24541
ATAGGGTGAAGCCAGAGGAAACTCTGGTGGAGGCTCGTAGCGATTCTGAC
JV1105_12_Ceriporia_occidental
ATAGGGTGAAGCCAGAGGAAACTCTGGTGGAGGCTCGTAGCGATTCTGAC
VS8558Ceriporia_occidentalis
ATAGGGTGAAGCCAGAGGAAACTCTGGTGGAGGCTCGTAGCGATTCTGAC
Dai22445
ATAGGGTGAAGCCAGAGGAAACTCTGGTGGAGGCTCGTAGCGATTCTGAC
846Dai16368
ATAGGGTGAAGCCAGAGGAAACTCTGGTGGAGGCTCGTAGCGATTCTGAC
Dai17951_Ceriporia_aurantiocar
ATAGGGTGAAGCCAGAGGAAACTCTGGTGGAAGCTCGTAGCGATTCTGAC
Miettinen_11701C_viridans
ATAGGGTGAAGCCAGAGGAAACTCTGGTGGAAGCTCGTAGCGATTCTGAC
JV0105_10Ceriporia_aurantiocar
ATAGGGTGAAGCCAGAGGAAACTCTGGTGGAAGCTCGTAGCGATTCTGAC
Yuan5702C_viridans --
858Dai17003 --
Yuan2747_Ceriporia_viridans --
Yuan2744C_viridans --

Li1046C_viridans --

865C_sinoviridans --

871Dai15062 --

Dai7642_Ceriporia_humilis --

Spirin4706_Ceriporia_humilis --

Spirin4944_Ceriporia_sericea

ATAGGGTGAAGCCAGAGGAAACTCTGGTGGAGGCTCGTAGCGATTCTGAC

WCG1547Dai26044ceriporia

ATAGGGTGAAGCCAGAGGAAACTCTGGTGGAGGCTCGTAGCGATTCTGAC

ZZW1558Dai27086

ATAGGGTGAAGCCAGAGGAAACTCTGGTGGAGGCTCGTAGCGATTCTGAC

Miettinen14381_Ceriporia_mpuri

ATAGGGTGAAGCCAGAGGAAACTCTGGTGGAGGCTCGTAGCGATTCTGAC

Miettinen15492_2_Ceriporia_sor

ATAGGGTGAAGCCAGAGGAAACTCTGGTGGAGGCTCGTAGCGATTCTGAC

He6687

ATAGGGTGAAGCCAGAGGAAACTCTGGTGGAGGCTCGTAGCGATTCTGAC

ZH53Dai24426

ATAGGGTGAAGCCAGAGGAAACTCTGGTGGAGGCTCGTAGCGATTCTGAC

Vlasak0808_30_Ceriporia_punica

ATAGGGTGAAGCCWGAGGAAACTCTGGTGGAGGCTCGTAGCGATTCTGAC

887Dai13376

ATAGGGTGAAGCCAGAGGAAACTCTGGTGGAGGCTCGTAGCGATTCTGAC

WCG1443Dai24998

ATAGGGCGAAGCCAGAGGAAACTCTGGTGGAGGCTCGTAGCGATTCTGAC

0108_6Ceriporia_spissa

ATAGGGTGAAGCCAGAGGAAACTCTGGTGGAGGCTCGTAGCGATTCTGAC

Dai19164

ATAGGGTGAAGCCAGAGGAAACTCTGGTGGAGGCTCGTAGCGATTCTGAC

Dai17937_Ceriporia_bubalinomar

ATAGGGTGAAGCCAGAGGAAACTCTGGTGGAGGCTCGTAGCGATTCTGAC

903Dai12113

ATAGGGTGAAGCCAGAGGAAACTCTGGTGGAGGCTCGTAGCGATTCTGAC

LZB929Dai25079

ATAGGGTGAAGCCAGAGGAAACTCTGGTGGAGGCTCGTAGCGATTCTGAC

LX45Dai26988 --

LX43Dai26986

ATAGGGTGAAGCCAGAGGAAACTCTGGTGGAGGCTCGTAGCGATTCTGAC

Dai7759Ceriporia --

Cui8012_Ceriporia_viridans --

GC1704_54Ceriporia_viridans

ATAGGGTGAAGCCAGAGGAAACTCTGGTGGAAGCTCGTAGCGATTCTGAC

Dai23392

ATAGGGTGAAGCCAGAGGAAACTCTGGTGGAAGCTCGTAGCGATTCTGAC

WCG1585Dai26113Ceriproia
ATAGGGTGAAGCCAGAGGAAACTCTGGTGGAAGCTCGTAGCGATTCTGAC
Dai18675C_eucalypti
ATAGGGTGAAGCCAGAGGAAACTCTGGTGGAAGCTCGTAGCGATTCTGAC
Dai22034
ATAGGGTGAAGCCAGAGGAAACTCTGGTGGAGGCTCGTAGCGATTCTGAC
JV1008_41JTardaFLORIDAKeys --
Rivoire1161_Ceriporia_pierii --
Dai23499C_pierii
ATAGGGTGAAGCCAGAGGAAACTCTGGTGGAGGCTCGTAGCGATTCTGAC
Dai23500
ATAGGGTGAAGCCAGAGGAAACTCTGGTGGAGGCTCGTAGCGATTCTGAC
841Dai15899
ATAGGGTGAAGCCAGAGGAAACTCTGGTGGAGGCTCGTAGCGATTCTGAC
842Dai15904 --
LZB1066xinjiang --
LZB1065xinjiang --
851Dai16779
ATAGGGTGAAGTCAGAGGAAACTCTGATGGAGGCTCGTAGCGATTCTGAC
RMJ119sp_Candelabrochaete_sept --
RLG9759spCandelabrochaete_sept
ATAGGGTGAAGCCAGAGGAAACTCTGGTGGAGGCTCGTAGCGATTCTGAC
RLG10478Phanerochaete_allantos --
Dai19118_Ceriporia_spissa
ATAGGGTGAAGCCAGAGGAAACTCTGGTGGAGGCTCGTAGCGATTCTGAC
Dai18486A
ATAGGGTGAAGCCAGAGGAAACTCTGGTGGAGGCTCGTAGCGATTCTGAC
WEI17_024_Ceriporia_mellita ATAGGGTGAAGCCAGAGGAAACTCTGGTGGA-GCTCGTAGCGATTCTGAC
GC1508_71Ceriporia_mellita ATAGGGTGAAGCCAGAGGAAACTCTGGTGGA-GCTCGTAGCGATTCTGAC
GC1608_7_Ceriporia_mellita
ATAGGGTGAAGCCAGAGGAAACTCTGGTGGAGGCTCGTAGCGATTCTGAC
ZZW1557Dai27085
ATAGGGTGAAGCCAGAGGAAACTCTGGTGGAGGCTCGTAGCGATTCTGAC
ZZW1554Dai27083
ATAGGGTGAAGCCAGAGGAAACTCTGGTGGAGGCTCGTAGCGATTCTGAC
Dai8168
ATAGGGTGAAGCCAGAGGAAACTCTGGTGGAGGCTCGTAGCGATTCTGAC
BR4865C_mellita
ATAGGGTGAAGCCAGAGGAAACTCTGGTGGAGGCTCGTAGCGATTCTGAC
MEL2382688Ceriporia_sp --
Dai8110
ATAGGGTGAAGCCAGAGGAAACTCTGGTGGAGGCTCGTAGCGATTCTGAC

Cui8097 ---

909Cui6740 ---

W1258Dai24695

ATAGGGTGAAGCCAGAGGAAACTCTGGTGGAGGCTCGTAGCGATTCTGAC

JV0110_26_Ceriporia_griseoviol

ATAGGGTGAAGCCAGAGGAAACTCTGGTGGAGGCTCGYAGCGATTCTGAC

896Dai13202

ATAGGGTGAAGCCAGAGGAAACTCTGGTGGAGGCTCGTAGCGATTCTGAC

LWY393Dai27053C_griseoviolasce

ATAGGGTGAAGCCAGAGGAAACTCTGGTGGAGGCTCGTAGCGATTCTGAC

LWY394DAI27054

ATAGGGTGAAGCCAGAGGAAACTCTGGTGGAGGCTCGTAGCGATTCTGAC

FP135015G_pannocinctus ---

L15726SpG_pannocinctus

ATAGGGTGAAGCCAGAGGAAACTCTGGTGGAGGCTCGTAGCGATTCTGAC

Dai22221

ATAGGGTGAAGCCAGAGGAAACTCTGGTGGAGGCTCGTAGCGATTCTGAC

Dai22633

ATAGGGTGAAGCCAGAGGAAACTCTGGTGGAGGCTCGTAGCGATTCTGAC

Dai23260

ATAGGGTGAAGCCAGAGGAAACTCTGGTGGAGGCTCGTAGCGATTCTGAC

Dai23626

ATAGGGTGAAGCCAGAGGAAACTCTGGTGGAGGCTCGTAGCGATTCTGAC

Dai16238G_citrinoalbus

ATAGGGTGAAGCCAGAGGAAACTCTGGTGGAGGCTCGTAGCGATTCTGAC

1175Dai15293

ATAGGGTGAAGCCAGAGGAAACTCTGGTGGAGGCTCGTAGCGATTCTGAC

Dai19547

ATAGGGTGAAGCCAGAGGAAACTCTGGTGGAGGCTCGTAGCGATTCTGAC

918063G_africanus ---

918572G_africanus ---

Dai18536A ---

1164Cui17922

ATAGGGTGAAGCCAGAGGAAACTCTGGTGGAGGCTCGTAGCGATTCTGAC

Dai22225

ATAGGGTGAAGCCAGAGGAAACTCTGGTGGAGGCTCGTAGCGATTCTGAC

1163Dai20655 ATAGGGTGAAG-CAGAGGAAACTCTGGTGGAGGCTCGTAGCGATTCTGAC

Yuan4397G_hainanensis

ATAGGGTGAAGCCAGAGGAAACTCTGGTGGAGGCTCGTAGCGATTCTGAC

1176Dai15268

ATAGGGTGAAGCCAGAGGAAACTCTGGTGGAGGCTCGTAGCGATTCTGAC

1177Dai15259

ATAGGGTGAAGCCAGAGGAAACTCTGGTGGAGGCTCGTAGCGATTCTGAC

BZ2896G_thelephoroides ---
1166JV1808_26
ATAGGGTGAAGCCAGAGGAAACTCTGGTGGAGGCTCGTAGCGATTCTGAC
Miettinen16992Hapalopilus_ochr
ATAGGGTGAAGCCAGAGGAAACTCTGGTGGAGGCTCGTAGCGATTCTGAC
GC1708_338_Ceriporia_arbuscula
ATAGGGTGAAGCCAGAGGAAACTCTGGTGGAGGCTCGTAGCGATTCTGAC
WCG1555Dai26107Ceriporia
ATAGGGTGAAGCCAGAGGAAACTCTGGTGGAGGCTCGTAGCGATTCTGAC
GC1708_340_Ceriporia_arbuscula -TAGGGTGAAGCCAGAGG-AACTCTGGTGGA-GCTCGTAGCGATTCTGAC
WCG1556Dai26109Ceriporia
ATAGGGTGAAGCCAGAGGAAACTCTGGTGGAGGCTCGTAGCGATTCTGAC
883Cui11291
ATAGGGTGAAGCCAGAGGAAACTCTGGTGGAGGCTCGTAGCGATTCTGAC
HLX320Dai26805
ATAGGGTGAAGCCAGAGGAAACTCTGGTGGAGGCTCGTAGCGATTCTGAC
WCG1266Dai24678A
ATAGGGTGAAGCCAGAGGAAACTCTGGTGGAGGCTCGTAGCGATTCTGAC
Dai6090_Ceriporia_sulphuricolo
ATAGGGTGAAGCCAGAGGAAACTCTGGTGGAGGCTCGTAGCGATTCTGAC
RLG_11354_Ceriproia_reticulata
ATAGGGTGAAGCCAGAGGAAACTCTGGTGGAGGCTCGTAGCGATTCTGAC
ZZW1543Dai27072
ATAGGGTGAAGCCAGAGGAAACTCTGGTGGAGGCTCGTAGCGATTCTGAC
Li1316_Ceriporia_reticulata ---
KHL11981Ceriporia_reticulata
ATAGGGTGAAGCCAGAGGAAACTCTGGTGGAGGCTCGTAGCGATTCTGAC
FP110343sp_Candelabrochaete_la
ATAGGGTGAAGCCAGAGGAAACTCTGGTGGAGGCTCGTAGCGATTCTGAC
Li1045_Ceriporia_reticulata ---
ZX136Dai25794ceriporia
ATAGGGTGAAGCCAGAGGAAACTCTGGTGGAGGCTCGTAGCGATTCTGAC
892Dai13400
ATAGGGTGAAGCCAGAGGAAACTCTGGTGGAGGCTCGTAGCGATTCTGAC
RLG7163Leptoporus_mollis
ATAGGGTGAAGCCAGAGGAAACTCTGGTGGAAGCTCGTAGCGATTCTGAC
Dai21062Leptoporus_mollis
ATAGGGTGAAGCCAGAGGAAACTCTGGTGGAAGCTCGTAGCGATTCTGAC
Dai20182Leptoporus_submollis
ATAGGGTGAAGCCAGAGGAAACTCTGGTGGAAGCTCGTAGCGATTCTGAC
Cui18379Leptoporus_submollis
ATAGGGTGAAGCCAGAGGAAACTCTGGTGGAAGCTCGTAGCGATTCTGAC
Wu1209_46Resiniporus_pseudogil ATAGGGTGAAGCCAGAGGAAA-----------------------

------

BRNM710169Resiniporus_resinasc --

Dai14516Bjerkandera_adusta

ATAGGGTGAAGCCAGAGGAAACTCTGGTGGAGGCTCGTAGCGATTCTGAC

Dai21100Bjerkandera_fumosa

ATAGGGTGAAGCCAGAGGAAACTCTGGTGGAGGCTCGTAGCGATTCTGAC

Miettinen16854Ceraceomyces_sp

ATAGGGTGAAGCCAGAGGAAACTCTGGTGGAGGCTCGTAGCGATTCTGAC

Dai10477C_spissa

ATAGGGTGAAGCCAGAGGAAACTCTGGTGGAGGCTCGTAGCGATTCTGAC

855Dai16831

ATAGGGTGAAGCCAGAGGAAACTCTGGTGGAGGCTCGTAGCGATTCTGAC

882Cui11282

ATAGGGTGAAGCCAGAGGAAACTCTGGTGGAGGCTCGTAGCGATTCTGAC

Dai24566

ATAGGGTGAAGCCAGAGGAAACTCTGGTGGAGGCTCGTAGCGATTCTGAC

Yuan5965

ATAGGGTGAAGCCAGAGGAAACTCTGGTGGAGGCTCGTAGCGATTCTGAC

Dai3204

ATAGGGTGAAGCCAGAGGAAACTCTGGTGGAGGCTCGTAGCGATTCTGAC

1194CUI9985

ATAGGGTGAAGCCAGAGGAAACTCTGGTGGAGGCTCGTAGCGATTCTGAC

Dai15205_Ceriporia_albomellea

GTGCAAATCGATCGTCAAATTTGGGTATAGGGGCGAAAGACTAATCGAAC

Dai15223_Ceriporia_albomellea

GTGCAAATCGATCGTCAAATTTGGGTATAGGGGCGAAAGACTAATCGAAC

Li1780_Ceriporia_variegata

GTGCAAATCGATCGTCAAATTTGGGTATAGGGGCGAAAGACTAATCGAAC

Dai19791_Ceriporia_variegata

GTGCAAATCGATCGTCAAATTTGGGTATAGGGGCGAAAGACTAATCGAAC

Dai19886

GTGCAAATCGATCGTCAAATTTGGGTATAGGGGCGAAAGACTAATCGAAC

Dai10833_Ceriporia_crassitunic

GTGCAAATCGATCGTCAAATTTGGGTATAGGGGCGAAAGACTAATCGAAC

CHWC1506_46Meruliopsis_crassit --

Dai9995_Ceriporia_crassitunica --

Wu1209_58_Meruliopsis_parvispo GTGCAAATCGATCGTC----------------------------------

CHWC1505_129_Meruliopsis_parvi GTGCAAATCGATCGTCAAATTTGGGTATAGGG--CGAAGACTAATCGAAC

Dai21944

GTGCAAATCGATCGTCAAATTTGGGTATAGGGGCGAAAGACTAATCGAAC

830Dai18640A

GTGCAAATCGATCGTCAAATTTGGGTATAGGGGCGAAAGACTAATCGAAC

GC1704_60_Meruliopsis_taxicola
GTGCAAATCGATCGTCAAATTTGGGTATAGGGGCGAAAGACTAATCGAAC

Dai22625
GTGCAAATCGATCGTCAAATTTGGGTATAGGGGCGAAAGACTAATCGAAC

Dai22636
GTGCAAATCGATCGTCAAATTTGGGTATAGGGGCGAAAGACTAATCGAAC

Dai21878
GTGCAAATCGATCGTCAAATTTGGGTATAGGGGCGAAAGACTAATCGAAC

1169Dai17248
GTGCAAATCGATCGTCAAATTTGGGTATAGGGGCGAAAGACTAATCGAAC

Wu1708_43_Meruliopsis_leptocys GTGCAAATCGATCGTCAAATTTGGGTATAGGG-CGAAAGACTAATCGAAC

Li1011
GTGCAAATCGATCGTCAAATTTGGGTATAGGGGCGAAAGACTAATCGAAC

ZX95Dai25742Meruliopsis_leptoc
GTGCAAATCGATCGTCAAATTTGGGTATAGGGGCGAAAGACTAATCGAAC

WCG1306Dai24733
GTGCAAATCGATCGTCAAATTTGGGTATAGGGGCGAAAGACTAATCGAAC

LXL99Dai25816
GTGCAAATCGATCGTCAAATTTGGGTATAGGGGCGAAAGACTAATCGAAC

WCG1559Dai26052Meruliopsis
GTGCAAATCGATCGTCAAATTTGGGTATAGGGGCGAAAGACTAATCGAAC

He7477
GTGCAAATCGATCGTCAAATTTGGGTATAGGGGCGAAAGACTAATCGAAC

HLX243Dai26217
GTGCAAATCGATCGTCAAATTTGGGTATAGGGGCGAAAGACTAATCGAAC

RussiaMW673659Meruliopsis_fagi
GTGCAAATCGATCGTCAAATTTGGGTATAGGGGCGA--------------

FD278
GTGCAAATCGATCGTCAAATTTGGGTATAGGGGCGAAAGACTAATCGAAC

Dai10226_Ceriporia_tarda --

LE247365
GTGCAAATCGATCGTCAAATTTGGGTATAGGGGCGAAAGACTAATCGAAC

Dai8173_Meruliopsis_nanlingens
GTGCAAATCGATCGTCAAATTTGGGTATAGGGGCGAAAGACTAATCGAAC

860Dai17172
GTGCAAATCGATCGTCAAATTTGGGTATAGGGGCGAAAGACTAATCGAAC

879Dai13414
GTGCAAATCGATCGTCAAATTTGGGTATAGGGGCGAAAGACTAATCGAAC

Li_1704_Meruliopsis_pseudocyst --

833Dai18405
GTGCAAATCGATCGTCAAATTTGGGTATAGGGGCGAAAGACTAATCGAAC

HHB_10729_Meruliopsis_albostra

GTGCAAATCGATCGTCAAATTTGGGTATAGGGGCGAAAGACTAATCGAAC
Cui6878_Ceriporia_pseudocystid
GTGCAAATCGATCGTCAAATTTGGGTATAGGGGCGAAAGACTAATCGAAC
869Dai14737
GTGCAAATCGATCGTCAAATTTGGGTATAGGGGCGAAAGACTAATCGAAC
876Cui11626
GTGCAAATCGATCGTCAAATTTGGGTATAGGGGCGAAAGACTAATCGAAC
1199WEI3388 --
776308_Meruliopsis_cystidiata --
ICN139059_Meruliopsis_cystidia --
HHB15692Ceraceomyces_serpens
GTGCAAATCGATCGTCAAATTTGGGTATAGGGGCGAAAGACTAATCGAAC
HHB_15629_Sp_Ceriporiopsis_ane
GTGCAAATCGATCGTCAAATTTGGGTATAGGGGCGAAAGACTAATCGAAC
AJ185Trametopsis_cervina
GTGCAAATCGATCGTCAAATTTGGGTATAGGGGCGAAAGACTAATCGAAC
FD9Irpex_lacteus
GTGCAAATCGATCGTCAAATTTGGGTATAGGGGCGAAAGACTAATCGAAC
908Dai11230
GTGCAAATCGATCGTCAAATTTGGGTATAGGGGCGAAAGACTAATCGAAC
FP55521TEmmia_lacerata
GTGCAAATCGATCGTCAAATTTGGGTATAGGGGCGAAAGACTAATCGAAC
PBU0048Ceriporia_cystidiata
GTGCAAATCGATCGTCAAATTTGGGTATAGGGGCGAAAGACTAATCGAAC
MZ340C_lacerataT --
Dai21940
GTGCAAATCGATCGTCAAATTTGGGTATAGGGGCGAAAGACTAATCGAAC
847Dai16433
GTGCAAATCGATCGTCAAATTTGGGTATAGGGGCGAAAGACTAATCGAAC
MarcinEmmia_latemarginatus
GTGCAAATCGATCGTCAAATTTGGGTATAGGGGCGAAAGACTAATCGAAC
Meijer3729Hydnopolyporus_fimbr
GTGCAAATCGATCGTCAAATTTGGGTATAGGGGCGAAAGACTAATCGAAC
RLG13408Phanerochaete_sp
GTGCAAATCGATCGTCAAATTTGGGTATAGGGGCGAAAGACTAATCGAAC
WHC1381Flavodon_flavus
GTGCAAATCGATCGTCAAATTTGGGTATAGGGGCGAAAGACTAATCGAAC
GB1833Phlebia_albida
GTGCAAATCGATCGTCAAATTTGGGTATAGGGGCGAAAGACTAATCGAAC
T407Phlebia_nitidula --
HHB6988Phanerochaete_exilis
GTGCAAATCGATCGTCAAATTTGGGTATAGGGGCGAAAGACTAATCGAAC
HHB8509Phanerochaetella_xeroph
GTGCAAATCGATCGTCAAATTTGGGTATAGGGGCGAAAGACTAATCGAAC

PBU0051Macrohyporia_dictyopora
GTGCAAATCGATCGTCAAATTTGGGTATAGGGGCGAAAGACTAATCGAAC
HHB11463Phanerochaete_sp
GTGCAAATCGATCGTCAAATTTGGGTATAGGGGCGAAAGACTAATCGAAC
FP102382Byssomerulius_corium
GTGCAAATCGATCGTCAAATTTGGGTATAGGGGCGAAAGACTAATCGAAC
FP102165Efibula_americana
GTGCAAATCGATCGTCAAATTTGGGTATAGGGGCGAAAGACTAATCGAAC
Murdoch90Ceriporia_torpida
GTGCAAATCGATCGTCAAATTTGGGTATAGGGGCGAAAGACTAATCGAAC
Rivoire4413_Ceriporia_purpurea
GTGCAAATCGATCGTCAAATTTGGGTATAGGGGCGAAAGACTAATCGAAC
Kout_18_Ceriporia_triumphalis
GTGCAAATCGATCGTCAAATTTGGGTATAGGGGCGAAAGACTAATCGAAC
Rivoire3701_Ceriporia_bresadol
GTGCAAATCGATCGTCAAATTTGGGTATAGGGGCGAAAGACTAATCGAAC
VS4018 --
Ryvarden21832_Ceriporia_manzan
GTGCAAATCGATCGTCAAATTTGGGTATAGGGGCGAAAGACTAATCGAAC
Dai24539
GTGCAAATCGATCGTCAAATTTGGGTATAGGGGCGAAAGACTAATCGAAC
Dai24541
GTGCAAATCGATCGTCAAATTTGGGTATAGGGGCGAAAGACTAATCGAAC
JV1105_12_Ceriporia_occidental
GTGCAAATCGATCGTCAAATTTGGGTATAGGGGCGAAAGACTAATCGAAC
VS8558Ceriporia_occidentalis
GTGCAAATCGATCGTCAAATTTGGGTATAGGGGCGAAAGACTAATCGAAC
Dai22445
GTGCAAATCGATCGTCAAATTTGGGTATAGGGGCGAAAGACTAATCGAAC
846Dai16368
GTGCAAATCGATCGTCAAATTTGGGTATAGGGGCGAAAGACTAATCGAAC
Dai17951_Ceriporia_aurantiocar
GTGCAAATCGATCGTCAAATTTGGGTATAGGGGCGAAAGACTAATCGAAC
Miettinen_11701C_viridans
GTGCAAATCGATCGTCAAATTTGGGTATAGGGGCGAAAGACTAATCGAAC
JV0105_10Ceriporia_aurantiocar
GTGCAAATCGATCGTCAAATTTGGGTATAGGGGCGAAAGACTAATCGAAC
Yuan5702C_viridans --
858Dai17003 --
Yuan2747_Ceriporia_viridans --
Yuan2744C_viridans --
Li1046C_viridans --
865C_sinoviridans --
871Dai15062 --

Dai7642_Ceriporia_humilis --
Spirin4706_Ceriporia_humilis --
Spirin4944_Ceriporia_sericea
GTGCAAATCGATCGTCAAATTTGGGTATAGGGGCGAAAGACTAATCGAA-
WCG1547Dai26044ceriporia
GTGCAAATCGATCGTCAAATTTGGGTATAGGGGCGAAAGACTAATCGAAC
ZZW1558Dai27086
GTGCAAATCGATCGTCAAATTTGGGTATAGGGGCGAAAGACTAATCGAAC
Miettinen14381_Ceriporia_mpuri
GTGCAAATCGATCGTCAAATTTGGGTATAGGGGCGAAAGACTAATCGAAC
Miettinen15492_2_Ceriporia_sor
GTGCAAATCGATCGTCAAATTTGGGTATAGGGGCGAAAGACTAATCGAAC
He6687
GTGCAAATCGATCGTCAAATTTGGGTATAGGGGCGAAAGACTAATCGAAC
ZH53Dai24426
GTGCAAATCGATCGTCAAATTTGGGTATAGGGGCGAAAGACTAATCGAAC
Vlasak0808_30_Ceriporia_punica
GTGCAAATCGATCGTCAAATTTGGGTATAGGGGCGAAAGACTAATCGAAC
887Dai13376
GTGCAAATCGATCGTCAAATTTGGGTATAGGGGCGAAAGACTAATCGAAC
WCG1443Dai24998
GTGCAAATCGATCGTCAAATTTGGGTATAGGGGCGAAAGACTAATCGAAC
0108_6Ceriporia_spissa
GTGCAAATCGATCGTCAAATTTGGGTATAGGGGCGAAAGACTAATCGAAC
Dai19164
GTGCAAATCGATCGTCAAATTTGGGTATAGGGGCGAAAGACTAATCGAAC
Dai17937_Ceriporia_bubalinomar
GTGCAAATCGATCGTCAAATTTGGGTATAGGGGCGAAAGACTAATCGAAC
903Dai12113
GTGCAAATCGATCGTCAAATTTGGGTATAGGGGCGAAAGACTAATCGAAC
LZB929Dai25079
GTGCAAATCGATCGTCAAATTTGGGTATAGGGGCGAAAGACTAATCGAAC
LX45Dai26988 --
LX43Dai26986
GTGCAAATCGATCGTCAAATTTGGGTATAGGGGCGAAAGACTAATCGAAC
Dai7759Ceriporia --
Cui8012_Ceriporia_viridans --
GC1704_54Ceriporia_viridans GTGCAAATCGATCGTCAAATTTGGGTATA----------
----
Dai23392
GTGCAAATCGATCGTCAAATTTGGGTATAGGGGCGAAAGACTAATCGAAC
WCG1585Dai26113Ceriproia
GTGCAAATCGATCGTCAAATTTGGGTATAGGGGCGAAAGACTAATCGAAC
Dai18675C_eucalypti

GTGCAAATCGATCGTCAAATTTGGGTATAGGGGCGAAAGACTAATCGAAC

Dai22034

GTGCAAATCGATCGTCAAATTTGGGTATAGGGGCGAAAGACTAATCGAAC

JV1008_41JTardaFLORIDAKeys --

Rivoire1161_Ceriporia_pierii --

Dai23499C_pierii

GTGCAAATCGATCGTCAAATTTGGGTATAGGGGCGAAAGACTAATCGAAC

Dai23500

GTGCAAATCGATCGTCAAATTTGGGTATAGGGGCGAAAGACTAATCGAAC

841Dai15899

GTGCAAATCGATCGTCAAATTTGGGTATAGGGGCGAAAGACTAATCGAAC

842Dai15904 --

LZB1066xinjiang --

LZB1065xinjiang --

851Dai16779

GTGCAAATCGATCGTCAAATTTGGGTATAGGGGCGAAAGACTAATCGAAC

RMJ119sp_Candelabrochaete_sept --

RLG9759spCandelabrochaete_sept

GTGCAAATCGATCGTCAAATTTGGGTATAGGGGCGAAAGACTAATCGAAC

RLG10478Phanerochaete_allantos --

Dai19118_Ceriporia_spissa

GTGCAAATCGATCGTCAAATTTGGGTATAGGGGCGAAAGACTAATCGAAC

Dai18486A

GTGCAAATCGATCGTCAAATTTGGGTATAGGGGCGAAAGACTAATCGAAC

WEI17_024_Ceriporia_mellita GTGCAAATCGATCGTC-AATTTGGGTATA-----------
----

GC1508_71Ceriporia_mellita

GTGCAAATCGATCGTCAAATTTGGGTATAGGGGCGAAAGACTAATCGAAC

GC1608_7_Ceriporia_mellita

GTGCAAATCGATCGTCAAATTTGGGTATAGGGGCGAAAGACTAATCGAAC

ZZW1557Dai27085

GTGCAAATCGATCGTCAAATTTGGGTATAGGGGCGAAAGACTAATCGAAC

ZZW1554Dai27083

GTGCAAATCGATCGTCAAATTTGGGTATAGGGGCGAAAGACTAATCGAAC

Dai8168

GTGCAAATCGATCGTCAAATTTGGGTATAGGGGCGAAAGACTAATCGAAC

BR4865C_mellita

GTGCAAATCGATCGTCAAATTTGGGTATAGGGGCGAAAGACTAATCGAAC

MEL2382688Ceriporia_sp --

Dai8110

GTGCAAATCGATCGTCAAATTTGGGTATAGGGGCGAAAGACTAATCGAAC

Cui8097 --

909Cui6740 --

W1258Dai24695

GTGCAAATCGATCGTCAAATTTGGGTATAGGGGCGAAAGACTAATCGAAC
JV0110_26_Ceriporia_griseoviol
GTGCAAATCGATCGTCAAATTTGGGTATAGGGGCGAAAGACTAATCGAAC
896Dai13202
GTGCAAATCGATCGTCAAATTTGGGTATAGGGGCGAAAGACTAATCGAAC
LWY393Dai27053C_griseoviolasce
GTGCAAATCGATCGTCAAATTTGGGTATAGGGGCGAAAGACTAATCGAAC
LWY394DAI27054
GTGCAAATCGATCGTCAAATTTGGGTATAGGGGCGAAAGACTAATCGAAC
FP135015G_pannocinctus --
L15726SpG_pannocinctus
GTGCAAATCGATCGTCAAATTTGGGTATAGGGGCGAAAGACTAATCGAAC
Dai22221
GTGCAAATCGATCGTCAAATTTGGGTATAGGGGCGAAAGACTAATCGAAC
Dai22633
GTGCAAATCGATCGTCAAATTTGGGTATAGGGGCGAAAGACTAATCGAAC
Dai23260
GTGCAAATCGATCGTCAAATTTGGGTATAGGGGCGAAAGACTAATCGAAC
Dai23626
GTGCAAATCGATCGTCAAATTTGGGTATAGGGGCGAAAGACTAATCGAAC
Dai16238G_citrinoalbus
GTGCAAATCGATCGTCAAATTTGGGTATAGGGGCGAAAGACTAATCGAAC
1175Dai15293
GTGCAAATCGATCGTCAAATTTGGGTATAGGGGCGAAAGACTAATCGAAC
Dai19547
GTGCAAATCGATCGTCAAATTTGGGTATAGGGGCGAAAGACTAATCGAAC
918063G_africanus --
918572G_africanus --
Dai18536A --
1164Cui17922
GTGCAAATCGATCGTCAAATTTGGGTATAGGGGCGAAAGACTAATCGAAC
Dai22225
GTGCAAATCGATCGTCAAATTTGGGTATAGGGGCGAAAGACTAATCGAAC
1163Dai20655
GTGCAAATCGATCGTCAAATTTGGGTATAGGGGCGAAAGACT-ATCGAA-
Yuan4397G_hainanensis
GTGCAAATCGATCGTCAAATTTGGGTATAGGGGCGAAAGACTAATCGAAC
1176Dai15268
GTGCAAATCGATCGTCAAATTTGGGTATAGGGGCGAAAGACTAATCGAAC
1177Dai15259
GTGCAAATCGATCGTCAAATTTGGGTATAGGGGCGAAAGACTAATCGAAC
BZ2896G_thelephoroides --
1166JV1808_26
GTGCAAATCGATCGTCAAATTTGGGTATAGGGGCGAAAGACTAATCGAAC

Miettinen16992Hapalopilus_ochr
GTGCAAATCGATCGTCAAATTTGGGTATAGGGGCGAAAGACTAATCGAAC
GC1708_338_Ceriporia_arbuscula
GTGCAAATCGATCGTCAAATTTGGGTATAGGGGCGAAAGACTAATCGAAC
WCG1555Dai26107Ceriporia
GTGCAAATCGATCGTCAAATTTGGGTATAGGGGCGAAAGACTAATCGAAC
GC1708_340_Ceriporia_arbuscula GTGC-AATCGATCGTCAAATTTGGGTATA-----------
----
WCG1556Dai26109Ceriporia
GTGCAAATCGATCGTCAAATTTGGGTATAGGGGCGAAAGACTAATCGAAC
883Cui11291
GTGCAAATCGATCGTCAAATTTGGGTATAGGGGCGAAAGACTAATCGAAC
HLX320Dai26805
GTGCAAATCGATCGTCAAATTTGGGTATAGGGGCGAAAGACTAATCGAAC
WCG1266Dai24678A
GTGCAAATCGATCGTCAAATTTGGGTATAGGGGCGAAAGACTAATCGAAC
Dai6090_Ceriporia_sulphuricolo
GTGCAAATCGATCGTCAAATTTGGGTATAGGGGCGAAAGACTAATCGAAC
RLG_11354_Ceriproia_reticulata
GTGCAAATCGATCGTCAAATTTGGGTATAGGGGCGAAAGACTAATCGAAC
ZZW1543Dai27072
GTGCAAATCGATCGTCAAATTTGGGTATAGGGGCGAAAGACTAATCGAAC
Li1316_Ceriporia_reticulata --
KHL11981Ceriporia_reticulata
GTGCAAATCGATCGTCAAATTTGGGTATAGGGGCGAAAGACTAATCGAAC
FP110343sp_Candelabrochaete_la
GTGCAAATCGATCGTCAAATTTGGGTATAGGGGCGAAAGACTAATCGAAC
Li1045_Ceriporia_reticulata --
ZX136Dai25794ceriporia
GTGCAAATCGATCGTCAAATTTGGGTATAGGGGCGAAAGACTAATCGAAC
892Dai13400
GTGCAAATCGATCGTCAAATTTGGGTATAGGGGCGAAAGACTAATCGAAC
RLG7163Leptoporus_mollis
GTGCAAATCGATCGTCAAATTTGGGTATAGGGGCGAAAGACTAATCGAAC
Dai21062Leptoporus_mollis
GTGCAAATCGATCGTCAAATTTGGGTATAGGGGCGAAAGACTAATCGAAC
Dai20182Leptoporus_submollis
GTGCAAATCGATCGTCAAATTTGGGTATAGGGGCGAAAGACTAATCGAAC
Cui18379Leptoporus_submollis
GTGCAAATCGATCGTCAAATTTGGGTATAGGGGCGAAAGACTAATCGAAC
Wu1209_46Resiniporus_pseudogil --
BRNM710169Resiniporus_resinasc --
Dai14516Bjerkandera_adusta
GTGCAAATCGATCGTCAAATTTGGGTATAGGGGCGAAAGACTAATCGAAC

Dai21100Bjerkandera_fumosa
GTGCAAATCGATCGTCAAATTTGGGTATAGGGGCGAAAGACTAATCGAAC
Miettinen16854Ceraceomyces_sp
GTGCAAATCGATCGTCAAATTTGGGTATAGGGGCGAAAGACTAATCGAAC
Dai10477C_spissa
GTGCAAATCGATCGTCAAATTTGGGTATAGGGGCGAAAGACTAATCGAAC
855Dai16831
GTGCAAATCGATCGTCAAATTTGGGTATAGGGGCGAAAGACTAATCGAAC
882Cui11282
GTGCAAATCGATCGTCAAATTTGGGTATAGGGGCGAAAGACTAATCGAAC
Dai24566
GTGCAAATCGATCGTCAAATTTGGGTATAGGGGCGAAAGACTAATCGAAC
Yuan5965
GTGCAAATCGATCGTCAAATTTGGGTATAGGGGCGAAAGACTAATCGAAC
Dai3204
GTGCAAATCGATCGTCAAATTTGGGTATAGGGGCGAAAGACTAATCGAAC
1194CUI9985
GTGCAAATCGATCGTCAAATTTGGGTATAGGGGCGAAAGACTAATCGAAC

Dai15205_Ceriporia_albomellea
CATCTAGTAGCTGGTTCCTGCCGAAGTTTCCCTCAGGATAGCAGAAACTC
Dai15223_Ceriporia_albomellea
CATCTAGTAGCTGGTTCCTGCCGAAGTTTCCCTCA-GATAGCAGAAACTC
Li1780_Ceriporia_variegata
CATCTAGTAGCTGGTTCCTGCCGAAGTTTCCCTCAGGATAGCAGAAACTC
Dai19791_Ceriporia_variegata
CATCTAGTAGCTGGTTCCTGCCGAAGTTTCCCTCAGGATAGCAGAAACTC
Dai19886
CATCTAGTAGCTGGTTCCTGCCGAAGTTTCCCTCAGGATAGCAGAAACTC
Dai10833_Ceriporia_crassitunic
CATCTAGTAGCTGGTTCCTGCCGAAGTTTCCCTCAGGATAGCAGAAACTC
CHWC1506_46Meruliopsis_crassit --
Dai9995_Ceriporia_crassitunica --
Wu1209_58_Meruliopsis_parvispo --
CHWC1505_129_Meruliopsis_parvi CATCTAGT--
Dai21944
CATCTAGTAGCTGGTTCCTGCCGAAGTTTCCCTCAGGATAGCAGAAACTC
830Dai18640A
CATCTAGTAGCTGGTTCCTGCCGAAGTTTCCCTCAGGATAGCAGAAACTC
GC1704_60_Meruliopsis_taxicola CATCTAGTAGCTGGTTCC--------------------------------
Dai22625
CATCTAGTAGCTGGTTCCTGCCGAAGTTTCCCTCAGGATAGCAGAAACTC
Dai22636

CATCTAGTAGCTGGTTCCTGCCGAAGTTTCCCTCAGGATAGCAGAAACTC
Dai21878
CATCTAGTAGCTGGTTCCTGCCGAAGTTTCCCTCAGGATAGCAGAAACTC
1169Dai17248
CATCTAGTAGCTGGTTCCTGCCGAAGTTTCCCTCAGGATAGCAGAAACTC
Wu1708_43_Meruliopsis_leptocys CATCTAGTAGCTGGTTCCT-----------------------------
-
Li1011
CATCTAGTAGCTGGTTCCTGCCGAAGTTTCCCTCAGGATAGCAGAAACTC
ZX95Dai25742Meruliopsis_leptoc
CATCTAGTAGCTGGTTCCTGCCGAAGTTTCCCTCAGGATAGCAGAAACTC
WCG1306Dai24733
CATCTAGTAGCTGGTTCCTGCCGAAGTTTCCCTCAGGATAGCAGAAACTC
LXL99Dai25816
CATCTAGTAGCTGGTTCCTGCCGAAGTTTCCCTCAGGATAGCAGAAACTC
WCG1559Dai26052Meruliopsis
CATCTAGTAGCTGGTTCCTGCCGAAGTTTCCCTCAGGATAGCAGAAACTC
He7477
CATCTAGTAGCTGGTTCCTGCCGAAGTTTCCCTCAGGATAGCAGAAACTC
HLX243Dai26217
CATCTAGTAGCTGGTTCCTGCCGAAGTTTCCCTCAGGATAGCAGAAACTC
RussiaMW673659Meruliopsis_fagi --
FD278
CATCTAGTAGCTGGTTCCTGCCGAAGTTTCCCTCAGGATAGCAGAAACTC
Dai10226_Ceriporia_tarda --
LE247365
CATCTAGTAGCTGGTTCCTGCCGAAGTTTCCCTCAGGATAGCAGAAACTC
Dai8173_Meruliopsis_nanlingens
CATCTAGTAGCTGGTTCCTGCCGAAGTTTCCCTCAGGATAGCAGAAACTC
860Dai17172
CATCTAGTAGCTGGTTCCTGCCGAAGTTTCCCTCAGGATAGCAGAAACTC
879Dai13414
CATCTAGTAGCTGGTTCCTGCCGAAGTTTCCCTCAGGATAGCAGAAACTC
Li_1704_Meruliopsis_pseudocyst --
833Dai18405
CATCTAGTAGCTGGTTCCTGCCGAAGTTTCCCTCAGGATAGCAGAAACTC
HHB_10729_Meruliopsis_albostra
CATCTAGTAGCTGGTTCCTGCCGAAGTTTCCCTCAGGATAGCAGAAACTC
Cui6878_Ceriporia_pseudocystid
CATCTAGTAGCTGGTTCCTGCCGAAGTTTCCCTCAGGATAGCAGAAACTC
869Dai14737
CATCTAGTAGCTGGTTCCTGCCGAAGTTTCCCTCAGGATAGCAGAAACTC
876Cui11626
CATCTAGTAGCTGGTTCCTGCCGAAGTTTCCCTCAGGATAGCAGAAACTC

1199WEI3388 --
776308_Meruliopsis_cystidiata --
ICN139059_Meruliopsis_cystidia --
HHB15692Ceraceomyces_serpens
CATCTAGTAGCTGGTTCCTGCCGAAGTTTCCCTCAGGATAGCAGAAGCTC
HHB_15629_Sp_Ceriporiopsis_ane
CATCTAGTAGCTGGTTCCTGCCGAAGTTTCCCTCAGGATAGCAGAAACTC
AJ185Trametopsis_cervina
CATCTAGTAGCTGGTTCCTGCCGAAGTTTCCCTCAGGATAGCAGAAACTC
FD9Irpex_lacteus
CATCTAGTAGCTGGTTCCTGCCGAAGTTTCCCTCAGGATAGCAGAAACTC
908Dai11230
CATCTAGTAGCTGGTTCCTGCCGAAGTTTCCCTCAGGATAGCAGAAACTC
FP55521TEmmia_lacerata
CATCTAGTAGCTGGTTCCTGCCGAAGTTTCCCTCAGGATAGCAGAAACTC
PBU0048Ceriporia_cystidiata
CATCTAGTAGCTGGTTCCTGCCGAAGTTTCCCTCAGGATAGCAGAAACTC
MZ340C_lacerataT --
Dai21940
CATCTAGTAGCTGGTTCCTGCCGAAGTTTCCCTCAGGATAGCAGAAACTC
847Dai16433
CATCTAGTAGCTGGTTCCTGCCGAAGTTTCCCTCAGGATAGCAGAAACTC
MarcinEmmia_latemarginatus CATCTAGTAGCTGGTTCCTGCCGAA-----------------
----
Meijer3729Hydnopolyporus_fimbr
CATCTAGTAGCTGGTTCCTGCCGAAGTTTCCCTCAGGATAGCAGAAACTC
RLG13408Phanerochaete_sp
CATCTAGTAGCTGGTTCCTGCCGAAGTTTCCCTCAGGATAGCAGAAACTC
WHC1381Flavodon_flavus CATCTCAT--
GB1833Phlebia_albida
CATCTAGTAGCTGGTTCCTGCCGAAGTTTCCCTCAGGATAGCAGAAACTC
T407Phlebia_nitidula --
HHB6988Phanerochaete_exilis
CATCTAGTAGCTGGTTCCTGCCGAAGTTTCCCTCAGGATAGCAGAAACTC
HHB8509Phanerochaetella_xeroph
CATCTAGTAGCTGGTTCCTGCCGAAGTTTCCCTCAGGATAGCAGAAACTC
PBU0051Macrohyporia_dictyopora
CATCTAGTAGCTGGTTCCTGCCGAAGTTTCCCTCAGGATAGCAGAAACTC
HHB11463Phanerochaete_sp
CATCTAGTAGCTGGTTCCTGCCGAAGTTTCCCTCAGGATAGCAGAAACTC
FP102382Byssomerulius_corium
CATCTAGTAGCTGGTTCCTGCCGAAGTTTCCCTCAGGATAGCAGAAACTC
FP102165Efibula_americana
CATCTAGTAGCTGGTTCCTGCCGAAGTTTCCCTCAGGATAGCAGAAACTC

Murdoch90Ceriporia_torpida CATCTAGTAGCTGGTTCCTGC---------------------------
--
Rivoire4413_Ceriporia_purpurea CATCTAGTAGCTGGTTCCTGC----------------------------
-
Kout_18_Ceriporia_triumphalis CATCTAGTAG--
Rivoire3701_Ceriporia_bresadol
CATCTAGTAGCTGGTTCCTGCCGAAGTTTCCCTCAGGATAGCAGAAACTC
VS4018 --
Ryvarden21832_Ceriporia_manzan CATCTAGTAGCTGGTTCCTGC-------------------------
----
Dai24539
CATCTAGTAGCTGGTTCCTGCCGAAGTTTCCCTCAGGATAGCAGAAACTC
Dai24541
CATCTAGTAGCTGGTTCCTGCCGAAGTTTCCCTCAGGATAGCAGAAACTC
JV1105_12_Ceriporia_occidental CATCTAGTAGCTGGTTCCTGC---------------------------
--
VS8558Ceriporia_occidentalis CATCTAGTAGCTGGTTCCTGC----------------------------
-
Dai22445
CATCTAGTAGCTGGTTCCTGCCGAAGTTTCCCTCAGGATAGCAGAAACTC
846Dai16368
CATCTAGTAGCTGGTTCCTGCCGAAGTTTCCCTCAGGATAGCAGAAACTC
Dai17951_Ceriporia_aurantiocar
CATCTAGTAGCTGGTTCCTGCCGAAGTTTCCCTCAGGATAGCAGAAACTC
Miettinen_11701C_viridans CATCTAGTAGCTGGTTCCTGCCGA--------------------
------
JV0105_10Ceriporia_aurantiocar CATCTAGTAGCTGGTTCCTGC----------------------------
-
Yuan5702C_viridans --
858Dai17003 --
Yuan2747_Ceriporia_viridans --
Yuan2744C_viridans --
Li1046C_viridans --
865C_sinoviridans --
871Dai15062 --
Dai7642_Ceriporia_humilis --
Spirin4706_Ceriporia_humilis --
Spirin4944_Ceriporia_sericea --
WCG1547Dai26044ceriporia
CATCTAGTAGCTGGTTCCTGCCGAAGTTTCCCTCAGGATAGCAGAAACTC
ZZW1558Dai27086
CATCTAGTAGCTGGTTCCTGCCGAAGTTTCCCTCAGGATAGCAGAAACTC
Miettinen14381_Ceriporia_mpuri
CATCTAGTAGCTGGTTCCTGCCGAAGTTTCCCTCAGGATAGCAGAAACTC

Miettinen15492_2_Ceriporia_sor
CATCTAGTAGCTGGTTCCTGCCGAAGTTTCCCTCAGGATAGCAGAAACTC
He6687
CATCTAGTAGCTGGTTCCTGCCGAAGTTTCCCTCAGGATAGCAGAAACTC
ZH53Dai24426
CATCTAGTAGCTGGTTCCTGCCGAAGTTTCCCTCAGGATAGCAGAAACTC
Vlasak0808_30_Ceriporia_punica CATCTAGTAGCTGGTTCCTGC----------------------------
887Dai13376
CATCTAGTAGCTGGTTCCTGCCGAAGTTTCCCTCAGGATAGCAGAAACTC
WCG1443Dai24998
CATCTAGTAGCTGGTTCCTGCCGAAGTTTCCCTCAGGATAGCAGAAACTC
0108_6Ceriporia_spissa CATCTAGTAACTGGTTCCTGC----------------------------
Dai19164
CATCTAGTAGCTGGTTCCTGCCGAAGTTTCCCTCAGGATAGCAGAAACTC
Dai17937_Ceriporia_bubalinomar
CATCTAGTAGCTGGTTCCTGCCGAAGTTTCCCTCAGGATAGCAGAAACTC
903Dai12113
CATCTAGTAGCTGGTTCCTGCCGAAGTTTCCCTCAGGATAGCAGAAACTC
LZB929Dai25079
CATCTAGTAGCTGGTTCCTGCCGAAGTTTCCCTCAGGATAGCAGAAACTC
LX45Dai26988 --
LX43Dai26986
CATCTAGTAGCTGGTTCCTGCCGAAGTTTCCCTCAGGATAGCAGAAACTC
Dai7759Ceriporia --
Cui8012_Ceriporia_viridans --
GC1704_54Ceriporia_viridans --
Dai23392
CATCTAGTAGCTGGTTCCTGCCGAAGTTTCCCTCAGGATAGCAGAAACTC
WCG1585Dai26113Ceriproia
CATCTAGTAGCTGGTTCCTGCCGAAGTTTCCCTCAGGATAGCAGAAACTC
Dai18675C_eucalypti
CATCTAGTAGCTGGTTCCTGCCGAAGTTTCCCTCAGGATAGCAGAAACTC
Dai22034
CATCTAGTAGCTGGTTCCTGCCGAAGTTTCCCTCAGGATAGCAGAAACTC
JV1008_41JTardaFLORIDAKeys --
Rivoire1161_Ceriporia_pierii --
Dai23499C_pierii
CATCTAGTAGCTGGTTCCTGCCGAAGTTTCCCTCAGGATAGCAGAAACTC
Dai23500
CATCTAGTAGCTGGTTCCTGCCGAAGTTTCCCTCAGGATAGCAGAAACTC
841Dai15899
CATCTAGTAGCTGGTTCCTGCCGAAGTTTCCCTCAGGATAGCAGAAACTC

842Dai15904 --
LZB1066xinjiang --
LZB1065xinjiang --
851Dai16779
CATCTAGTAGCTGGTTCCTGCCGAAGTTTCCCTCAGGATAGCAGAAACTC
RMJ119sp_Candelabrochaete_sept --
RLG9759spCandelabrochaete_sept CATCTAGTAGCT--------------------------------------
RLG10478Phanerochaete_allantos --
Dai19118_Ceriporia_spissa
CATCTAGTAGCTGGTTCCTGCCGAAGTTTCCCTCAGGATAGCAGAAACTC
Dai18486A
CATCTAGTAGCTGGTTCCTGCCGAAGTTTCCCTCAGGATAGCAGAAACTC
WEI17_024_Ceriporia_mellita --
GC1508_71Ceriporia_mellita CATCTAGTA---
GC1608_7_Ceriporia_mellita CATCTAGTAGCTGGTTCCTGC--------------------------
---
ZZW1557Dai27085
CATCTAGTAGCTGGTTCCTGCCGAAGTTTCCCTCAGGATAGCAGAAACTC
ZZW1554Dai27083
CATCTAGTAGCTGGTTCCTGCCGAAGTTTCCCTCAGGATAGCAGAAACTC
Dai8168
CATCTAGTAGCTGGTTCCTGCCGAAGTTTCCCTCAGGATAGCAGAAACTC
BR4865C_mellita CATCTAGTAGCTGGTTCCTGC-----------------------

MEL2382688Ceriporia_sp --
Dai8110
CATCTAGTAGCTGGTTCCTGCCGAAGTTTCCCTCAGGATAGCAGAAACTC
Cui8097 --
909Cui6740 --
W1258Dai24695
CATCTAGTAGCTGGTTCCTGCCGAAGTTTCCCTCAGGATAGCAGAAACTC
JV0110_26_Ceriporia_griseoviol CATCTAGTAGCTGGTTCCTGC----------------------------
-
896Dai13202
CATCTAGTAGCTGGTTCCTGCCGAAGTTTCCCTCAGGATAGCAGAAACTC
LWY393Dai27053C_griseoviolasce
CATCTAGTAGCTGGTTCCTGCCGAAGTTTCCCTCAGGATAGCAGAAACTC
LWY394DAI27054
CATCTAGTAGCTGGTTCCTGCCGAAGTTTCCCTCAGGATAGCAGAAACTC
FP135015G_pannocinctus --
L15726SpG_pannocinctus
CATCTAGTAGCTGGTTCCTGCCGAAGTTTCCCTCAGGATAGCAGAAACTC
Dai22221
CATCTAGTAGCTGGTTCCTGCCGAAGTTTCCCTCAGGATAGCAGAAACTC

Dai22633
CATCTAGTAGCTGGTTCCTGCCGAAGTTTCCCTCAGGATAGCAGAAACTC
Dai23260
CATCTAGTAGCTGGTTCCTGCCGAAGTTTCCCTCAGGATAGCAGAAACTC
Dai23626
CATCTAGTAGCTGGTTCCTGCCGAAGTTTCCCTCAGGATAGCAGAAACTC
Dai16238G_citrinoalbus
CATCTAGTAGCTGGTTCCTGCCGAAGTTTCCCTCAGGATAGCAGAAACTC
1175Dai15293
CATCTAGTAGCTGGTTCCTGCCGAAGTTTCCCTCAGGATAGCAGAAACTC
Dai19547
CATCTAGTAGCTGGTTCCTGCCGAAGTTTCCCTCAGGATAGCAGAAACTC
918063G_africanus --
918572G_africanus --
Dai18536A --
1164Cui17922
CATCTAGTAGCTGGTTCCTGCCGAAGTTTCCCTCAGGATAGCAGAAACTC
Dai22225
CATCTAGTAGCTGGTTCCTGCCGAAGTTTCCCTCAGGATAGCAGAAACTC
1163Dai20655 CATCTAGTAGCT-GCTCCTGC-GAAGTTTCCCTCA-GATAGCAGAAACTC
Yuan4397G_hainanensis
CATCTAGTAGCTGGTTCCTGCCGAAGTTTCCCTCAGGATAGCAGAAACTC
1176Dai15268
CATCTAGTAGCTGGTTCCTGCCGAAGTTTCCCTCAGGATAGCAGAAACTC
1177Dai15259
CATCTAGTAGCTGGTTCCTGCCGAAGTTTCCCTCAGGATAGCAGAAACTC
BZ2896G_thelephoroides --
1166JV1808_26 CATCTAGTAGCTGGT-CCTGCCGAAGTTTCCCTCA-GATAGCAGAAACTC
Miettinen16992Hapalopilus_ochr
CATCTAGTAGCTGGTTCCTGCCGAAGTTTCCCTCAGGATAGCAGAAACTC
GC1708_338_Ceriporia_arbuscula CATCTAGTAGCTGGT-----------------------------------
WCG1555Dai26107Ceriporia
CATCTAGTAGCTGGTTCCTGCCGAAGTTTCCCTCAGGATAGCAGAAACTC
GC1708_340_Ceriporia_arbuscula --
WCG1556Dai26109Ceriporia
CATCTAGTAGCTGGTTCCTGCCGAAGTTTCCCTCAGGATAGCAGAAACTC
883Cui11291
CATCTAGTAGCTGGTTCCTGCCGAAGTTTCCCTCAGGATAGCAGAAACTC
HLX320Dai26805
CATCTAGTAGCTGGTTCCTGCCGAAGTTTCCCTCAGGATAGCAGAAACTC
WCG1266Dai24678A
CATCTAGTAGCTGGTTCCTGCCGAAGTTTCCCTCAGGATAGCAGAAACTC

Dai6090_Ceriporia_sulphuricolo

CATCTAGTAGCTGGTTCCTGCCGAAGTTTCCCTCAGGATAGCAGAAACTC

RLG_11354_Ceriproia_reticulata

CATCTAGTAGCTGGTTCCTGCCGAAGTTTCCCTCAGGATAGCAGAAACTC

ZZW1543Dai27072

CATCTAGTAGCTGGTTCCTGCCGAAGTTTCCCTCAGGATAGCAGAAACTC

Li1316_Ceriporia_reticulata ---

KHL11981Ceriporia_reticulata

CATCTAGTAGCTGGTTCCTGCCGAAGTTTCCCTCAGGATAGCAGAAACTC

FP110343sp_Candelabrochaete_la

CATCTAGTAGCTGGTTCCTGCCGAAGTTTCCCTCAGGATAGCAGAAACTC

Li1045_Ceriporia_reticulata ---

ZX136Dai25794ceriporia

CATCTAGTAGCTGGTTCCTGCCGAAGTTTCCCTCAGGATAGCAGAAACTT

892Dai13400

CATCTAGTAGCTGGTTCCTGCCGAAGTTTCCCTCAGGATAGCAGAAACTC

RLG7163Leptoporus_mollis

CATCTAGTAGCTGGTTCCTGCCGAAGTTTCCCTCAGGATAGCAGAAACTC

Dai21062Leptoporus_mollis

CATCTAGTAGCTGGTTCCTGCCGAAGTTTCCCTCAGGATAGCAGAAACTC

Dai20182Leptoporus_submollis

CATCTAGTAGCTGGTTCCTGCCGAAGTTTCCCTCAGGATAGCAGAAACTC

Cui18379Leptoporus_submollis

CATCTAGTAGCTGGTTCCTGCCGAAGTTTCCCTCAGGATAGCAGAAACTC

Wu1209_46Resiniporus_pseudogil ---

BRNM710169Resiniporus_resinasc ---

Dai14516Bjerkandera_adusta

CATCTAGTAGCTGGTTCCTGCCGAAGTTTCCCTCAGGATAGCAGAAACTC

Dai21100Bjerkandera_fumosa

CATCTAGTAGCTGGTTCCTGCCGAAGTTTCCCTCAGGATAGCAGAAACTC

Miettinen16854Ceraceomyces_sp

CATCTAGTAGCTGGTTCCTGCCGAAGTTTCCCTCAGGATAGCAGAAACTC

Dai10477C_spissa

CATCTAGTAGCTGGTTCCTGCCGAAGTTTCCCTCAGGATAGCAGAAACTC

855Dai16831

CATCTAGTAGCTGGTTCCTGCCGAAGTTTCCCTCAGGATAGCAGAAACTC

882Cui11282

CATCTAGTAGCTGGTTCCTGCCGAAGTTTCCCTCAGGATAGCAGAAACTC

Dai24566

CATCTAGTAGCTGGTTCCTGCCGAAGTTTCCCTCAGGATAGCAGAAACTC

Yuan5965

CATCTAGTAGCTGGTTCCTGCCGAAGTTTCCCTCAGGATAGCAGAAACTC

Dai3204

CATCTAGTAGCTGGTTCCTGCCGAAGTTTCCCTCAGGATAGCAGAAACTC

1194CUI9985
CATCTAGTAGCTGGTTCCTGCCGAAGTTTCCCTCAGGATAGCAGAAACTC

Dai15205_Ceriporia_albomellea ATA--
TCAGATTTATGTGGTAAAGCGAATGATTAGAGGCCTTGGGGTTGA
Dai15223_Ceriporia_albomellea ATA--
TCAGATTTATGTGGTAAAGCGAATGATTAGAGGCCTTGGGGTTGA
Li1780_Ceriporia_variegata ATA--
TCAGATTTATGTGGTAAAGCGAATGATTAGAGGCCTTGGGGTTGA
Dai19791_Ceriporia_variegata ATA--
TCAGATTTATGTGGTAAAGCGAATGATTAGAGGCCTTGGGGTTGA
Dai19886 ATA--
TCAGATTTATGTGGTAAAGCGAATGATTAGAGGCCTTGGGGTTGA
Dai10833_Ceriporia_crassitunic ATA--
TCAGATTTATGTGGTAAAGCGAATGATTAGAGGCCTTGGGGTTGA
CHWC1506_46Meruliopsis_crassit --
Dai9995_Ceriporia_crassitunica --
Wu1209_58_Meruliopsis_parvispo --
CHWC1505_129_Meruliopsis_parvi --
Dai21944 GTA--
TCAGATTTATGTGGTAAAGCGAATGATTAGAGGCCTTGGGGTTGA
830Dai18640A GTA--
TCAGATTTATGTGGTAAAGCGAATGATTAGAGGCCTTGGGGTTGA
GC1704_60_Meruliopsis_taxicola --
Dai22625 GTA--
TCAGATTTATGTGGTAAAGCGAATGATTAGAGGCCTTGGGGTTGA
Dai22636 GTA--
TCAGATTTATGTGGTAAAGCGAATGATTAGAGGCCTTGGGGTTGA
Dai21878 GTA--
TCAGATTTATGTGGTAAAGCGAATGATTAGAGGCCTTGGGGTTGA
1169Dai17248 GTA--
TCAGATTTATGTGGTAAAGCGAATGATTAGAGGCCTTGGGGTTGA
Wu1708_43_Meruliopsis_leptocys --
Li1011 GTA--
TCAGATTTATGTGGTAAAGCGAATGATTAGAGGCCTTGGGGTTGA
ZX95Dai25742Meruliopsis_leptoc GTA--
TCAGATTTATGTGGTAAAGCGAATGATTAGAGGCCTTGGGGTTGA
WCG1306Dai24733 GTA--
TCAGATTTATGTGGTAAAGCGAATGATTAGAGGCCTTGGGGTTGA
LXL99Dai25816 GTA--
TCAGATTTATGTGGTAAAGCGAATGATTAGAGGCCTTGGGGTTGA
WCG1559Dai26052Meruliopsis GTA--
TCAGATTTATGTGGTAAAGCGAATGATTAGAGGCCTTGGGGTTGA

He7477 GTA--
TCAGATTTATGTGGTAAAGCGAATGATTAGAGGCCTTGGGGTTGA
HLX243Dai26217 GTA--
TCAGATTTATGTGGTAAAGCGAATGATTAGAGGCCTTGGGGTTGA
RussiaMW673659Meruliopsis_fagi ---
FD278 GTA--
TCAGATTTATGTGGTAAAGCGAATGATTAGAGGCCTTGGGGTTGA
Dai10226_Ceriporia_tarda ---
LE247365 GTA--
TCAGATTTATGTGGTAAAGCGAATGATTAGAGGCCTTGGGGTTGA
Dai8173_Meruliopsis_nanlingens GTA--
TCAGATTTATGTGGTAAAGCGAATGATTAGAGGCCTTGGGGTTGA
860Dai17172 GTG--
TCAGATTTATGTGGTAAAGCGAATGATTAGAGGCCTTGGGGTTGA
879Dai13414 GTA--
TCAGATTTATGTGGTAAAGCGAATGATTAGAGGCCTTGGGGTTGA
Li_1704_Meruliopsis_pseudocyst ---
833Dai18405
ATATCTCAGATTTATGTGGTAAAGCGAATGATTAGAGGCCTTGGGGTTGA
HHB_10729_Meruliopsis_albostra
ATATCTCAGATTTATGTGGTAAAGCGAATGATTAGAGGCCTTGGGGTTGA
Cui6878_Ceriporia_pseudocystid GTA--
TCAGATTTATGTGGTAAAGCGAATGATTAGAGGCCTTGGGGTTGA
869Dai14737 GTA--
TCAGATTTATGTGGTAAAGCGAATGATTAGAGGCCTTGGGGTTGA
876Cui11626 GTA--
TCAGATTTATGTGGTAAAGCGAATGATTAGAGGCCTTGGGGTTGA
1199WEI3388 ---
776308_Meruliopsis_cystidiata ---
ICN139059_Meruliopsis_cystidia ---
HHB15692Ceraceomyces_serpens ATA--
TCAGATTTATGTGGTAAAGCGAATGATTAGAGGCCTTGGGGTTGA
HHB_15629_Sp_Ceriporiopsis_ane ATA--
TCAGATTTATGTGGTAAAGCGAATGATTAGAGGCCTTGGGGTTGA
AJ185Trametopsis_cervina ATA--
TCAGATTTATGTGGTAAAGCGAATGATTAGAGGCCTTGGGGTTGA
FD9Irpex_lacteus ATA--
TCAGATTTATGTGGTAAAGCGAATGATTAGAGGCCTTGGGGTTGA
908Dai11230 ATA--
TCAGATTTATGTGGTAAAGCGAATGATTAGAGGCCTTGGGGTTGA
FP55521TEmmia_lacerata GTA--
TCAGATTTATGTGGTAAAGCGAATGATTAGAGGCCTTGGGGTTGA
PBU0048Ceriporia_cystidiata GTA--
TCAGATTTATGTGGTAAAGCGAATGATTAGAGGCCTTGGGGTTGA

MZ340C_lacerataT --
Dai21940 GTA--
TCAGATTTATGTGGTAAAGCGAATGATTAGAGGCCTTGGGGTTGA
847Dai16433 GTA--
TCAGATTTATGTGGTAAAGCGAATGATTAGAGGCCTTGGGGTTGA
MarcinEmmia_latemarginatus --
Meijer3729Hydnopolyporus_fimbr ATT--
TCAGATTTATGTGGTAAAGCGAATGATTAGAGGCCTTGGGGTTGA
RLG13408Phanerochaete_sp GTA--
TCAGATTTATGTGGTAAAGCGAATGATTAGAGGCCTTGGGGTTGA
WHC1381Flavodon_flavus --
GB1833Phlebia_albida ATA--
TCAGATTTATGTGGTAAAGCGAATGATTAGAGGCCTTGGGGTTGA
T407Phlebia_nitidula --
HHB6988Phanerochaete_exilis ATA--
TCAGATTTATGTGGTAAAGCGAATGATTAGAGGCCTTGGGGTTGA
HHB8509Phanerochaetella_xeroph ATA--
TCAGATTTATGTGGTAAAGCGAATGATTAGAGGCCTTGGGGTTGA
PBU0051Macrohyporia_dictyopora ATA--
TCAGATTTATGTGGTAAAGCGAATGATTAGAGGCCTTGGGGTTGA
HHB11463Phanerochaete_sp ATA--
TCAGATTTATGTGGTAAAGCGAATGATTAGAGGCCTTGGGGTTGA
FP102382Byssomerulius_corium ATA--
TCAGATTTATGTGGTAAAGCGAATGATTAGAGGCCTTGGGGTTGA
FP102165Efibula_americana GTA--
TCAGATTTATGTGGTAAAGCGAATGATTAGAGGCCTTGGGGTTGA
Murdoch90Ceriporia_torpida --
Rivoire4413_Ceriporia_purpurea --
Kout_18_Ceriporia_triumphalis --
Rivoire3701_Ceriporia_bresadol GTA--
TCAGATTTATGTGGTAAAGCGAATGATTAGAGGCCTTGGGGTTGA
VS4018 --
Ryvarden21832_Ceriporia_manzan --
Dai24539 GTA--
TCAGATTTATGTGGTAAAGCGAATGATTAGAGGCCTTGGGGTTGA
Dai24541 GTA--
TCAGATTTATGTGGTAAAGCGAATGATTAGAGGCCTTGGGGTTGA
JV1105_12_Ceriporia_occidental --
VS8558Ceriporia_occidentalis --
Dai22445 GTA--
TCAGATTTATGTGGTAAAGCGAATGATTAGAGGCCTTGGGGTTGA
846Dai16368 GTA--
TCAGATTTATGTGGTAAAGCGAATGATTAGAGGCCTTGGGGTTGA
Dai17951_Ceriporia_aurantiocar ATA--

TCAGATTTATGTGGTAAAGCGAATGATTAGAGGCCTTGGGGTTGA
Miettinen_11701C_viridans --
JV0105_10Ceriporia_aurantiocar --
Yuan5702C_viridans --
858Dai17003 --
Yuan2747_Ceriporia_viridans --
Yuan2744C_viridans --
Li1046C_viridans --
865C_sinoviridans --
871Dai15062 --
Dai7642_Ceriporia_humilis --
Spirin4706_Ceriporia_humilis --
Spirin4944_Ceriporia_sericea --
WCG1547Dai26044ceriporia GTA--
TCAGATTTATGTGGTAAAGCGAATGATTAGAGGCCTTGGGGTTGA
ZZW1558Dai27086 GTA--
TCAGATTTATGTGGTAAAGCGAATGATTAGAGGCCTTGGGGTTGA
Miettinen14381_Ceriporia_mpuri GTA--
TCAGATTTATGTGGTAAAGCGAATGATTAGAGGCCTTGGGGTTGA
Miettinen15492_2_Ceriporia_sor GTA--
TCAGATTTATGTGGTAAAGCGAATGATTAGAGGCCTTGGGGTTGA
He6687 GTA--
TCAGATTTATGTGGTAAAGCGAATGATTAGAGGCCTTGGGGTTGA
ZH53Dai24426 GTA--
TCAGATTTATGTGGTAAAGCGAATGATTAGAGGCCTTGGGGTTGA
Vlasak0808_30_Ceriporia_punica --
887Dai13376 GTA--
TCAGATTTATGTGGTAAAGCGAATGATTAGAGGCCTTGGGGTTGA
WCG1443Dai24998 GTA--
TCAGATTTATGTGGTAAAGCGAATGATTAGAGGCCTTGGGGTTGA
0108_6Ceriporia_spissa --
Dai19164 ATA--
TCAGATTTATGTGGTAAAGCGAATGATTAGAGGCCTTGGGGTTGA
Dai17937_Ceriporia_bubalinomar ATA--
TCAGATTTATGTGGTAAAGCGAATGATTAGAGGCCTTGGGGTTGA
903Dai12113 ATA--
TCAGATTTATGTGGTAAAGCGAATGATTAGAGGCCTTGGGGTTGA
LZB929Dai25079 ATA--
TCAGATTTATGTGGTAAAGCGAATGATTAGAGGCCTTGGGGTTGA
LX45Dai26988 --
LX43Dai26986 ATA--
TCAGATTTATGTGGTAAAGCGAATGATTAGAGGCCTTGGGGTTGA
Dai7759Ceriporia --
Cui8012_Ceriporia_viridans --

GC1704_54Ceriporia_viridans --
Dai23392 ATA--
TCAGATTTATGTGGTAAAGCGAATGATTAGAGGCCTTGGGGTTGA
WCG1585Dai26113Ceriproia ATA--
TCAGATTTATGTGGTAAAGCGAATGATTAGAGGCCTTGGGGTTGA
Dai18675C_eucalypti ATA--
TCAGATTTATGTGGTAAAGCGAATGATTAGAGGCCTTGGGGTTGA
Dai22034 ATA--
TCAGATTTATGTGGTAAAGCGAATGATTAGAGGCCTTGGGGTTGA
JV1008_41JTardaFLORIDAKeys --
Rivoire1161_Ceriporia_pierii --
Dai23499C_pierii GTA--
TCAGATTTATGTGGTAAAGCGAATGATTAGAGGCCTTGGGGTTGA
Dai23500 GTA--
TCAGATTTATGTGGTAAAGCGAATGATTAGAGGCCTTGGGGTTGA
841Dai15899 ATA--
TCAGATTTATGTGGTAAAGCGAATGATTAGAGGCCTTGGGGTTGA
842Dai15904 --
LZB1066xinjiang --
LZB1065xinjiang --
851Dai16779 GTA--
TCAGATTTATGTGGTAAAGCGAATGATTAGAGGCCTTGGGGTTGA
RMJ119sp_Candelabrochaete_sept --
RLG9759spCandelabrochaete_sept --
RLG10478Phanerochaete_allantos --
Dai19118_Ceriporia_spissa ATA--
TCAGATTTATGTGGTAAAGCGAATGATTAGAGGCCTTGGGGTTGA
Dai18486A ATA--
TCAGATTTATGTGGTAAAGCGAATGATTAGAGGCCTTGGGGTTGA
WEI17_024_Ceriporia_mellita --
GC1508_71Ceriporia_mellita --
GC1608_7_Ceriporia_mellita --
ZZW1557Dai27085 ATA--
TCAGATTTATGTGGTAAAGCGAATGATTAGAGGCCTTGGGGTTGA
ZZW1554Dai27083 ATA--
TCAGATTTATGTGGTAAAGCGAATGATTAGAGGCCTTGGGGTTGA
Dai8168 ATA--
TCAGATTTATGTGGTAAAGCGAATGATTAGAGGCCTTGGGGTTGA
BR4865C_mellita --
MEL2382688Ceriporia_sp --
Dai8110 ATA--
TCAGATTTATGTGGTAAAGCGAATGATTAGAGGCCTTGGGGTTGA
Cui8097 --
909Cui6740 --

W1258Dai24695 ATA--
TCAGATTTATGTGGTAAAGCGAATGATTAGAGGCCTTGGGGTTGA
JV0110_26_Ceriporia_griseoviol --
896Dai13202 GTA--
TCAGATTTATGTGGTAAAGCGAATGATTAGAGGCCTTGGGGTTGA
LWY393Dai27053C_griseoviolasce GTA--
TCAGATTTATGTGGTAAAGCGAATGATTAGAGGCCTTGGGGTTGA
LWY394DAI27054 GTA--
TCAGATTTATGTGGTAAAGCGAATGATTAGAGGCCTTGGGGTTGA
FP135015G_pannocinctus --
L15726SpG_pannocinctus ATA--
TCAGATTTATGTGGTAAAGCGAATGATTAGAGGCCTTGGGGTTGA
Dai22221 ATA--
TCAGATTTATGTGGTAAAGCGAATGATTAGAGGCCTTGGGGTTGA
Dai22633 ATA--
TCAGATTTATGTGGTAAAGCGAATGATTAGAGGCCTTGGGGTTGA
Dai23260 ATA--
TCAGATTTATGTGGTAAAGCGAATGATTAGAGGCCTTGGGGTTGA
Dai23626 ATA--
TCAGATTTATGTGGTAAAGCGAATGATTAGAGGCCTTGGGGTTGA
Dai16238G_citrinoalbus ATA--
TCAGATTTATGTGGTAAAGCGAATGATTAGAGGCCTTGGGGTTGA
1175Dai15293 ATA--
TCAGATTTATGTGGTAAAGCGAATGATTAGAGGCCTTGGGGTTGA
Dai19547 ATA--
TCAGATTTATGTGGTAAAGCGAATGATTAGAGGCCTTGGGGTTGA
918063G_africanus --
918572G_africanus --
Dai18536A --
1164Cui17922 ATA--
TCAGATTTATGTGGTAAAGCGAATGATTAGAGGCCTTGGGGTTGA
Dai22225 ATA--
TCAGATTTATGTGGTAAAGCGAATGATTAGAGGCCTTGGGGTTGA
1163Dai20655 ATA--
TCAGATTTATGTGGTAAAGCGAATGATTAGAGGGCTTGGGGTTGA
Yuan4397G_hainanensis ATA--
TCAGATTTATGTGGTAAAGCGAATGATTAGAGGCCTTGGGGTTGA
1176Dai15268 ATA--
TCAGATTTATGTGGTAAAGCGAATGATTAGAGGCCTTGGGGTTGA
1177Dai15259 ATA--
TCAGATTTATGTGGTAAAGCGAATGATTAGAGGCCTTGGGGTTGA
BZ2896G_thelephoroides --
1166JV1808_26 ATA--TCAGATTTATGTGGTAAAGCGA--
TGATAGAGGCCTTGGGGTTG-

Miettinen16992Hapalopilus_ochr ATA--
TCAGATTTATGTGGTAAAGCGAATGATTAGAGGCCTTGGGGTTGA
GC1708_338_Ceriporia_arbuscula --
WCG1555Dai26107Ceriporia GTA--
TCAGATTTATGTGGTAAAGCGAATGATTAGAGGCCTTGGGGTTGA
GC1708_340_Ceriporia_arbuscula --
WCG1556Dai26109Ceriporia GTA--
TCAGATTTATGTGGTAAAGCGAATGATTAGAGGCCTTGGGGTTGA
883Cui11291 GTA--
TCAGATTTATGTGGTAAAGCGAATGATTAGAGGCCTTGGGGTTGA
HLX320Dai26805 GTA--
TCAGATTTATGTGGTAAAGCGAATGATTAGAGGCCTTGGGGTTGA
WCG1266Dai24678A GTA--
TCAGATTTATGTGGTAAAGCGAATGATTAGAGGCCTTGGGGTTGA
Dai6090_Ceriporia_sulphuricolo GTA--
TCAGATTTATGTGGTAAAGCGAATGATTAGAGGCCTTGGGGTTGA
RLG_11354_Ceriproia_reticulata GTA--
TCAGATTTATGTGGTAAAGCGAATGATTAGAGGCCTTGGGGTTGA
ZZW1543Dai27072 GTA--
TCAGATTTATGTGGTAAAGCGAATGATTAGAGGCCTTGGGGTTGA
Li1316_Ceriporia_reticulata --
KHL11981Ceriporia_reticulata GTA--
TCAGATTTATGTGGTAAAGCGAATGATTAGAGGCCTTGGGGTTGA
FP110343sp_Candelabrochaete_la GTC--
TCAGATTTATGTGGTAAAGCGAATGATTAGAGGCCTTGGGGTTGA
Li1045_Ceriporia_reticulata --
ZX136Dai25794ceriporia ATA--
TCAGATTTATGTGGTAAAGCGAATGATTAGAGGCCTTGGGGTTGT
892Dai13400 GTA--
TCAGATTTATGTGGTAAAGCGAATGATTAGAGGCCTTGGGGTTGT
RLG7163Leptoporus_mollis ATA--
TCAGATTTATGTGGTAAAGCGAATGATTAGAGGCCTTGGGGTTGA
Dai21062Leptoporus_mollis ATA--
TCAGATTTATGTGGTAAAGCGAATGATTAGAGGCCTTGGGGTTGA
Dai20182Leptoporus_submollis ATA--
TCAGATTTATGTGGTAAAGCGAATGATTAGAGGCCTTGGGGTTGA
Cui18379Leptoporus_submollis ATA--
TCAGATTTATGTGGTAAAGCGAATGATTAGAGGCCTTGGGGTTGA
Wu1209_46Resiniporus_pseudogil --
BRNM710169Resiniporus_resinasc --
Dai14516Bjerkandera_adusta GTA--
TCAGATTTATGTGGTAAAGCGAATGATTAGAGGCCTTGGGGTTGA
Dai21100Bjerkandera_fumosa GTA--
TCAGATTTATGTGGTAAAGCGAATGATTAGAGGCCTTGGGGTTGA

Miettinen16854Ceraceomyces_sp ATA--TCAGATTTATGTGGTAAAGCGAATGATTAGAGGCCTTGGGGTTGA

Dai10477C_spissa ATA--TCAGATTTATGTGGTAAAGCGAATGATTAGAGGCCTTGGGGTTGA

855Dai16831 ATA--TCAGATTTATGTGGTAAAGCGAATGATTAGAGGCCTTGGGGTTGA

882Cui11282 ATA--TCAGATTTATGTGGTAAAGCGAATGATTAGAGGCCTTGGGGTTGA

Dai24566 ATA--TCAGATTTATGTGGTAAAGCGAATGATTAGAGGCCTTGGGGTTGA

Yuan5965 ATA--TCAGATTTATGTGGTAAAGCGAATGATTAGAGGCCTTGGGGTTGA

Dai3204 ATATCTCAGATTTATGTGGTAAAGCGAATGATTAGAGGCCTTGGGGTTGA

1194CUI9985 ATA--TCAGATTTATGTGGTAAAGCGAATGATTAGAGGCCTTGGGGTTGA

Dai15205_Ceriporia_albomellea AACAACCTTAACCTATTCTCAAACTTTAAATATGTAAGAACAAGCCGTCA

Dai15223_Ceriporia_albomellea AACAACCTTAACCTATTCTCAAACTTTAAATATGTAAGAACAAGCCGTCA

Li1780_Ceriporia_variegata AACAACCTTAACCTATTCTCAAACTTTAAATATGTAAGAACAAGCCGTCA

Dai19791_Ceriporia_variegata AACAACCTTAACCTATTCTCAAACTTTAAATATGTAAGAACAAGCCGTCA

Dai19886 AACAACCTTAACCTATTCTCAAACTTTAAATATGTAAGAACAAGCCGTCA

Dai10833_Ceriporia_crassitunic AACAACCTTAACCTATTCTCAAACTTTAAATATGTAAGAACAAGCCGTCA

CHWC1506_46Meruliopsis_crassit --

Dai9995_Ceriporia_crassitunica --

Wu1209_58_Meruliopsis_parvispo --

CHWC1505_129_Meruliopsis_parvi --

Dai21944 AACAACCTTAACCTATTCTCAAACTTTAAATATGTAAGAACAAGCCGTCA

830Dai18640A AACAACCTTAACCTATTCTCAAACTTTAAATATGTAAGAACAAGCCGTCA

GC1704_60_Meruliopsis_taxicola --

Dai22625 AACAACCTTAACCTATTCTCAAACTTTAAATATGTAAGAACAAGCCGTCA

Dai22636 AACAACCTTAACCTATTCTCAAACTTTAAATATGTAAGAACAAGCCGTCA

Dai21878

AACAACCTTAACCTATTCTCAAACTTTAAATATGTAAGAACAAGCCGTCA

1169Dai17248

AACAACCTTAACCTATTCTCAAACTTTAAATATGTAAGAACAAGCCGTCA

Wu1708_43_Meruliopsis_leptocys --

Li1011

AACAACCTTAACCTATTCTCAAACTTTAAATATGTAAGAACAAGCCGTCA

ZX95Dai25742Meruliopsis_leptoc

AACAACCTTAACCTATTCTCAAACTTTAAATATGTAAGAACAAGCCGTCA

WCG1306Dai24733

AACAACCTTAACCTATTCTCAAACTTTAAATATGTAAGAACAAGCCGTCA

LXL99Dai25816

AACAACCTTAACCTATTCTCAAACTTTAAATATGTAAGAACAAGCCGTCA

WCG1559Dai26052Meruliopsis

AACAACCTTAACCTATTCTCAAACTTTAAATATGTAAGAACAAGCCGTCA

He7477

AACAACCTTAACCTATTCTCAAACTTTAAATATGTAAGAACAAGCCGTCA

HLX243Dai26217

AACAACCTTAACCTATTCTCAAACTTTAAATATGTAAGAACAAGCCGTCA

RussiaMW673659Meruliopsis_fagi --

FD278

AACAACCTTAACCTATTCTCAAACTTTAAATATGTAAGAACAAGCCGTCA

Dai10226_Ceriporia_tarda --

LE247365

AACAACCTTAAC?TATTCTCAAACTTTAAATATGTAAGAACAAGCCGTCA

Dai8173_Meruliopsis_nanlingens

AACAACCTTAACCTATTCTCAAACTTTAAATATGTAAGAACAAGCCGTCA

860Dai17172

AACAACCTTAACCTATTCTCAAACTTTAAATATGTAAGAACAAGCCGTCA

879Dai13414

AACAACCTTAACCTATTCTCAAACTTTAAATATGTAAGAACAAGCCGTCA

Li_1704_Meruliopsis_pseudocyst --

833Dai18405

AACAACCTTAACCTATTCTCAAACTTTAAATATGTAAGAACAAGCCGTCA

HHB_10729_Meruliopsis_albostra

AACAACCTTAACCTATTCTCAAACTTTAAATATGTAAGAACAAGCCGTCA

Cui6878_Ceriporia_pseudocystid

AACAACCTTAACCTATTCTCAAACTTTAAATATGTAAGAACAAGCCGTCA

869Dai14737

AACAACCTTAACCTATTCTCAAACTTTAAATATGTAAGAACAAGCCGTCA

876Cui11626

AACAACCTTAACCTATTCTCAAACTTTAAATATGTAAGAACAAGCCGTCA

1199WEI3388 --

776308_Meruliopsis_cystidiata --

ICN139059_Meruliopsis_cystidia --

HHB15692Ceraceomyces_serpens
AACAACCTTAACCTATTCTCAAACTTTAAATATGTAAGAACGAGCCGTCA
HHB_15629_Sp_Ceriporiopsis_ane
AACAACCTTAACCTATTCTCAAACTTTAAATATGTAAGAACAAGCCGTCA
AJ185Trametopsis_cervina
AACAACCTTAACCTATTCTCAAACTTTAAATATGTAAGAACAAGCCGTCA
FD9Irpex_lacteus
AACAACCTTAACCTATTCTCAAACTTTAAATATGTAAGAACGAGCCGTCA
908Dai11230
AACAACCTTAACCTATTCTCAAACTTTAAATATGTAAGAACGAGCCGTCA
FP55521TEmmia_lacerata
AACAACCTTAACCTATTCTCAAACTTTAAATATGTAAGAACGAGCCGTCA
PBU0048Ceriporia_cystidiata
AACAACCTTAACCTATTCTCAAACTTTAAATATGTAAGAACGAGCCGTCA
MZ340C_lacerataT --
Dai21940
AACAACCTTAACCTATTCTCAAACTTTAAATATGTAAGAACGAGCCGTCA
847Dai16433
AACAACCTTAACCTATTCTCAAACTTTAAATATGTAAGAACGAGCCGTCA
MarcinEmmia_latemarginatus --
Meijer3729Hydnopolyporus_fimbr
AACAACCTTAACCTATTCTCAAACTTTAAATATGTAAGAACAAGCCGTCA
RLG13408Phanerochaete_sp
AACAACCTTAACCTATTCTCAAACTTTAAATATGTAAGAACGAGCCGTCA
WHC1381Flavodon_flavus --
GB1833Phlebia_albida
AACAACCTTAACCTATTCTCAAACTTTAAATATGTAAGAACGAGCCGTCA
T407Phlebia_nitidula --
HHB6988Phanerochaete_exilis
AACAACCTTAACCTATTCTCAAACTTTAAATATGTAAGAACGAGCCGTCA
HHB8509Phanerochaetella_xeroph
AACAACCTTAACCTATTCTCAAACTTTAAATATGTAAGAACGAGCCGTCA
PBU0051Macrohyporia_dictyopora
AACAACCTTAACCTATTCTCAAACTTTAAATATGTAAGAACGAGCCGTCA
HHB11463Phanerochaete_sp
AACAACCTTAACCTATTCTCAAACTTTAAATATGTAAGAACGAGCCGTCA
FP102382Byssomerulius_corium
AACAACCTTAACCTATTCTCAAACTTTAAATATGTAAGAACGAGCCGTCA
FP102165Efibula_americana
AACAACCTTAACCTATTCTCAAACTTTAAATATGTAAGAACAAGCCGTCA
Murdoch90Ceriporia_torpida --
Rivoire4413_Ceriporia_purpurea --
Kout_18_Ceriporia_triumphalis --
Rivoire3701_Ceriporia_bresadol

AACAACCTTAACCTATTCTCAAACTTTAAATATGTAAGAACGAGCCGTCA
VS4018 ---
Ryvarden21832_Ceriporia_manzan ---
Dai24539
AACAACCTTAACCTATTCTCAAACTTTAAATATGTAAGAACGAGCCGTCA
Dai24541
AACAACCTTAACCTATTCTCAAACTTTAAATATGTAAGAACGAGCCGTCA
JV1105_12_Ceriporia_occidental ---
VS8558Ceriporia_occidentalis ---
Dai22445
AACAACCTTAACCTATTCTCAAACTTTAAATATGTAAGAACGAGCCGTCA
846Dai16368
AACAACCTTAACCTATTCTCAAACTTTAAATATGTAAGAACGAGCCGTCA
Dai17951_Ceriporia_aurantiocar
AACAACCTTAACCTATTCTCAAACTTTAAATATGTAAGAACGAGCCGTCA
Miettinen_11701C_viridans ---
JV0105_10Ceriporia_aurantiocar ---
Yuan5702C_viridans ---
858Dai17003 ---
Yuan2747_Ceriporia_viridans ---
Yuan2744C_viridans ---
Li1046C_viridans ---
865C_sinoviridans ---
871Dai15062 ---
Dai7642_Ceriporia_humilis ---
Spirin4706_Ceriporia_humilis ---
Spirin4944_Ceriporia_sericea ---
WCG1547Dai26044ceriporia
AACAACCTTAACCTATTCTCAAACTTTAAATATGTAAGAACGAGCCGTCA
ZZW1558Dai27086
AACAACCTTAACCTATTCTCAAACTTTAAATATGTAAGAACGAGCCGTCA
Miettinen14381_Ceriporia_mpuri
AACAACCTTAACCTATTCTCAAACTTTAAATATGTAAGAACGAGCCGTCA
Miettinen15492_2_Ceriporia_sor
AACAACCTTAACCTATTCTCAAACTTTAAATATGTAAGAACGAGCCGTCA
He6687
AACAACCTTAACCTATTCTCAAACTTTAAATATGTAAGAACGAGCCGTCA
ZH53Dai24426
AACAACCTTAACCTATTCTCAAACTTTAAATATGTAAGAACGAGCCGTCA
Vlasak0808_30_Ceriporia_punica ---
887Dai13376
AACAACCTTAACCTATTCTCAAACTTTAAATATGTAAGAACGAGCCGTCA
WCG1443Dai24998
AACAACCTTAACCTATTCTCAAACTTTAAATATGTAAGAACGAGCCGTCA

0108_6Ceriporia_spissa --
Dai19164
AACAACCTTAACCTATTCTCAAACTTTAAATATGTAAGAACGAGCCGTCA
Dai17937_Ceriporia_bubalinomar
AACAACCTTAACCTATTCTCAAACTTTAAATATGTAAGAACGAGCCGTCA
903Dai12113
AACAACCTTAACCTATTCTCAAACTTTAAATATGTAAGAACGAGCCGTCA
LZB929Dai25079
AACAACCTTAACCTATTCTCAAACTTTAAATATGTAAGAACGAGCCGTCA
LX45Dai26988 --
LX43Dai26986
AACAACCTTAACCTATTCTCAAACTTTAAATATGTAAGAACGAGCCGTCA
Dai7759Ceriporia --
Cui8012_Ceriporia_viridans --
GC1704_54Ceriporia_viridans --
Dai23392
AACAACCTTAACCTATTCTCAAACTTTAAATATGTAAGAACGAGCCGTCA
WCG1585Dai26113Ceriproia
AACAACCTTAACCTATTCTCAAACTTTAAATATGTAAGAACGAGCCGTCA
Dai18675C_eucalypti
AACAACCTTAACCTATTCTCAAACTTTAAATATGTAAGAACGAGCCGTCA
Dai22034
AACAACCTTAACCTATTCTCAAACTTTAAATATGTAAGAACAAGCCGTCA
JV1008_41JTardaFLORIDAKeys --
Rivoire1161_Ceriporia_pierii --
Dai23499C_pierii
AACAACCTTAACCTATTCTCAAACTTTAAATATGTAAGAACGAGCCGTCA
Dai23500
AACAACCTTAACCTATTCTCAAACTTTAAATATGTAAGAACGAGCCGTCA
841Dai15899
AACAACCTTAACCTATTCTCAAACTTTAAATATGTAAGAACGAGCCGTCA
842Dai15904 --
LZB1066xinjiang --
LZB1065xinjiang --
851Dai16779
AACAACCTTAACCTATTCTCAAACTTTAAATATGTAAGAACGAGCCGTCA
RMJ119sp_Candelabrochaete_sept --
RLG9759spCandelabrochaete_sept --
RLG10478Phanerochaete_allantos --
Dai19118_Ceriporia_spissa
AACAACCTTAACCTATTCTCAAACTTTAAATATGTAAGAACGAGCCGTCA
Dai18486A
AACAACCTTAACCTATTCTCAAACTTTAAATATGTAAGAACGAGCCGTCA
WEI17_024_Ceriporia_mellita --

GC1508_71Ceriporia_mellita --
GC1608_7_Ceriporia_mellita --
ZZW1557Dai27085
AACAACCTTAACCTATTCTCAAACTTTAAATATGTAAGAACGAGCCGTCA
ZZW1554Dai27083
AACAACCTTAACCTATTCTCAAACTTTAAATATGTAAGAACGAGCCGTCA
Dai8168
AACAACCTTAACCTATTCTCAAACTTTAAATATGTAAGAACGAGCCGTCA
BR4865C_mellita --
MEL2382688Ceriporia_sp --
Dai8110
AACAACCTTAACCTATTCTCAAACTTTAAATATGTAAGAACGAGCCGTCA
Cui8097 --
909Cui6740 --
W1258Dai24695
AACAACCTTAACCTATTCTCAAACTTTAAATATGTAAGAACGAGCCGTCA
JV0110_26_Ceriporia_griseoviol --
896Dai13202
AACAACCTTAACCTATTCTCAAACTTTAAATATGTAAGAACGAGCCGTCA
LWY393Dai27053C_griseoviolasce
AACAACCTTAACCTATTCTCAAACTTTAAATATGTAAGAACGAGCCGTCA
LWY394DAI27054
AACAACCTTAACCTATTCTCAAACTTTAAATATGTAAGAACGAGCCGTCA
FP135015G_pannocinctus --
L15726SpG_pannocinctus
AACAACCTTAACCTATTCTCAAACTTTAAATATGTAAGAACGAGCCGTCA
Dai22221
AACAACCTTAACCTATTCTCAAACTTTAAATATGTAAGAACGAGCCGTCA
Dai22633
AACAACCTTAACCTATTCTCAAACTTTAAATATGTAAGAACGAGCCGTCT
Dai23260
AACAACCTTAACCTATTCTCAAACTTTAAATATGTAAGAACGAGCCGTCT
Dai23626
AACAACCTTAACCTATTCTCAAACTTTAAATATGTAAGAACGAGCCGTCT
Dai16238G_citrinoalbus
AACAACCTTAACCTATTCTCAAACTTTAAATATGTAAGAACGAGCCGTCT
1175Dai15293
AACAACCTTAACCTATTCTCAAACTTTAAATATGTAAGAACGAGCCGTCT
Dai19547
AACAACCTTAACCTATTCTCAAACTTTAAATATGTAAGAACGAGCCGTCT
918063G_africanus --
918572G_africanus --
Dai18536A --
1164Cui17922

AACAACCTTAACCTATTCTCAAACTTTAAATATGTAAGAACGAGCCGTCT
Dai22225
AACAACCTTAACCTATTCTCAAACTTTAAATATGTAAGAACGAGCCGTCT
1163Dai20655 A---
Yuan4397G_hainanensis
AACAACCTTAACCTATTCTCAAACTTTAAATATGTAAGAACAAGCCGTCA
1176Dai15268
AACAACCTTAACCTATTCTCAAACTTTAAATATGTAAGAACAAGCCGTCA
1177Dai15259
AACAACCTTAACCTATTCTCAAACTTTAAATATGTAAGAACAAGCCGTCA
BZ2896G_thelephoroides --
1166JV1808_26 --
Miettinen16992Hapalopilus_ochr
AACAACCTTAACCTATTCTCAAACTTTAAATATGTAAGAACGAGCTGTCA
GC1708_338_Ceriporia_arbuscula --
WCG1555Dai26107Ceriporia
AACAACCTTAACCTATTCTCAAACTTTAAATATGTAAGAACAAGCCGTCA
GC1708_340_Ceriporia_arbuscula --
WCG1556Dai26109Ceriporia
AACAACCTTAACCTATTCTCAAACTTTAAATATGTAAGAACAAGCCGTCA
883Cui11291
AACAACCTTAACCTATTCTCAAACTTTAAATATGTAAGAACAAGCCGTCA
HLX320Dai26805
AACAACCTTAACCTATTCTCAAACTTTAAATATGTAAGAACAAGCCGTCA
WCG1266Dai24678A
AACAACCTTAACCTATTCTCAAACTTTAAATATGTAAGAACAAGCCGTCA
Dai6090_Ceriporia_sulphuricolo
AACAACCTTAACCTATTCTCAAACTTTAAATATGTAAGAACGAGCCGTCA
RLG_11354_Ceriproia_reticulata
AACAACCTTAACCTATTCTCAAACTTTAAATATGTAAGAACAAGCCGTCA
ZZW1543Dai27072
AACAACCTTAACCTATTCTCAAACTTTAAATATGTAAGAACAAGCCGTCA
Li1316_Ceriporia_reticulata --
KHL11981Ceriporia_reticulata
AACAACCTTAACCTATTCTCAAACTTTAAATATGTAAGAACAAGCCGTCA
FP110343sp_Candelabrochaete_la
AACAACCTTAACCTATTCTCAAACTTTAAATATGTAAGAACAAGCCGTCA
Li1045_Ceriporia_reticulata --
ZX136Dai25794ceriporia
AACAACCTTAACCTATTCTCAAACTTTAAATATGTAAGAACAAGCCGTCA
892Dai13400
AACAACCTTAACCTATTCTCAAACTTTAAATATGTAAGAACAAGCCGTCA
RLG7163Leptoporus_mollis
AACAACCTTAACCTATTCTCAAACTTTAAATATGTAAGAACGAGCCGTCA

Dai21062Leptoporus_mollis
AACAACCTTAACCTATTCTCAAACTTTAAATATGTAAGAACGAGCCGTCA
Dai20182Leptoporus_submollis
AACAACCTTAACCTATTCTCAAACTTTAAATATGTAAGAACGAGCCGTCA
Cui18379Leptoporus_submollis
AACAACCTTAACCTATTCTCAAACTTTAAATATGTAAGAACGAGCCGTCA
Wu1209_46Resiniporus_pseudogil --
BRNM710169Resiniporus_resinasc --
Dai14516Bjerkandera_adusta
AACAACCTTAACCTATTCTCAAACTTTAAATATGTAAGAACGAGCCGTCA
Dai21100Bjerkandera_fumosa
AACAACCTTAACCTATTCTCAAACTTTAAATATGTAAGAACGAGCCGTCA
Miettinen16854Ceraceomyces_sp
AACAACCTTAACCTATTCTCAAACTTTAAATATGTAAGAACGAGCTGTCA
Dai10477C_spissa
AACAACCTTAACCTATTCTCAAACTTTAAATATGTAAGAACGAGCCGTCA
855Dai16831
AACAACCTTAACCTATTCTCAAACTTTAAATATGTAAGAACGAGCCGTCA
882Cui11282
AACAACCTTAACCTATTCTCAAACTTTAAATATGTAAGAACGAGCCGTCA
Dai24566
AACAACCTTAACCTATTCTCAAACTTTAAATATGTAAGAACGAGCCGTCA
Yuan5965
AACAACCTTAACCTATTCTCAAACTTTAAATATGTAAGAACGAGCCGTCA
Dai3204
AACAACCTTAACCTATTCTCAAACTTTAAATATGTAAGAACAAGCCGTCA
1194CUI9985
AACAACCTTAACCTATTCTCAAACTTTAAATATGTAAGAACAAGCCGTCA

Dai15205_Ceriporia_albomellea
CTTGATTGGACCGCTTGGTGATTGAGAGTTTCTAGTGGGCCATTTTTGGT
Dai15223_Ceriporia_albomellea
CTTGATTGGACCGCTTGGTGATTGAGAGTTTCTAGTGGGCCATTTTTGGT
Li1780_Ceriporia_variegata
CTTGATTGGACCGCTTGGCGATTGAGAGTTTCTAGTGGGCCATTTTTGGT
Dai19791_Ceriporia_variegata
CTTGATTGGACCGCTTGGCGATTGAGAGTTTCTAGTGGGCCATTTTTGGT
Dai19886
CTTGATTGGACCGCTTGGCGATTGAGAGTTTCTAGTGGGCCATTTTTGGT
Dai10833_Ceriporia_crassitunic
CTTGATTGGACCGCTTGGTGATTGAGAGTTTCTAGTGGGCCATTTTTGGT
CHWC1506_46Meruliopsis_crassit --
Dai9995_Ceriporia_crassitunica --

Wu1209_58_Meruliopsis_parvispo --
CHWC1505_129_Meruliopsis_parvi --
Dai21944
CTTGATTGGACCGCTTGGCGATTGAGAGTTTCTAGTGGGCCATTTTTGGT
830Dai18640A
CTTAATTGGACCGCTTGGCGATTGAGAGTTTCTAGTGGGCCATTTTTGGT
GC1704_60_Meruliopsis_taxicola --
Dai22625
CTTGATTGGACCGCTTGGCGATTGAGAGTTTCTAGTGGGCCATTTTTGGT
Dai22636
CTTGATTGGACCGCTTGGCGATTGAGAGTTTCTAGTGGGCCATTTTTGGT
Dai21878
CTTGATTGGACCGCTTGGCGATTGAGAGTTTCTAGTGGGCCATTTTTGGT
1169Dai17248
CTTGATTGGACCGCTTGGCGATTGAGAGTTTCTAGTGGGCCATTTTTGGT
Wu1708_43_Meruliopsis_leptocys --
Li1011
CTTGATTGGACCGCTTGGCGATTGAGAGTTTCTAGTGGGCCATTTTTGGT
ZX95Dai25742Meruliopsis_leptoc
CTTGATTGGACCGCTTGGCGATTGAGAGTTTCTAGTGGGCCATTTTTGGT
WCG1306Dai24733
CTTGATTGGACCGCTTGGCGATTGAGAGTTTCTAGTGGGCCATTTTTGGT
LXL99Dai25816
CTTGATTGGACCGCTTGGCGATTGAGAGTTTCTAGTGGGCCATTTTTGGT
WCG1559Dai26052Meruliopsis
CTTGATTGGACCGCTTGGCGATTGAGAGTTTCTAGTGGGCCATTTTTGGT
He7477
CTTGATTGGACCGCTTGGCGATTGAGAGTTTCTAGTGGGCCATTTTTGGT
HLX243Dai26217
CTTGATTGGACCGCTTGGCGATTGAGAGTTTCTAGTGGGCCATTTTTGGT
RussiaMW673659Meruliopsis_fagi --
FD278
CTTAATTGGACCGCTTGGCGATTGAGAGTTTCTAGTGGGCCATTTTTGGT
Dai10226_Ceriporia_tarda --
LE247365
CTTAATTGGACCGCTTGGCGATTGAGAGTTTCTAGTGGGCCATTTTTGGT
Dai8173_Meruliopsis_nanlingens
CTTAATTGGACCGCTTGGCGATTGAGAGTTTCTAGTGGGCCATTTTTGGT
860Dai17172
CTTAATTGGACCGCTTGGCGATTGAGAGTTTCTAGTGGGCCATTTTTGGT
879Dai13414
CTTAATTGGACCGCTTGGCGATTGAGAGTTTCTAGTGGGCCATTTTTGGT
Li_1704_Meruliopsis_pseudocyst --
833Dai18405

CTTGATTGGACCGCTTGGCGATTGAGAGTTTCTAGTGGGCCATTTTTGGT

HHB_10729_Meruliopsis_albostra

CTTGATTGGACCGCTTGGCGATTGAGAGTTTCTAGTGGGCCATTTTTGGT

Cui6878_Ceriporia_pseudocystid

CTTGATTGGACCGCTTGGCGATTGAGAGTTTCTAGTGGGCCATTTTTGGT

869Dai14737

CTTGATTGGACCGCTTGGCGATTGAGAGTTTCTAGTGGGCCATTTTTGGT

876Cui11626

CTTGATTGGACCGCTTGGCGATTGAGAGTTTCTAGTGGGCCATTTTTGGT

1199WEI3388 --

776308_Meruliopsis_cystidiata --

ICN139059_Meruliopsis_cystidia --

HHB15692Ceraceomyces_serpens

CTTGATTGGACCGCTCGGCGATTGAGAGTTTCTAGTGGGCCATTTTTGGT

HHB_15629_Sp_Ceriporiopsis_ane

CTTGATTGGACCGCTTGGCGATTGAGAGTTTCTAGTGGGCCATTTTTGGT

AJ185Trametopsis_cervina

CTTTGTTGGACCGCTTGGCGATTGAGAGTTTCTAGTGGGCCATTTTTGGT

FD9Irpex_lacteus

CTTAGTTGGACCGCTCGGCGATTGAGAGTTTCTAGTGGGCCATTTTTGGT

908Dai11230

CTTAGTTGGACCGCTCGGCGATTGAGAGTTTCTAGTGGGCCATTTTTGGT

FP55521TEmmia_lacerata

CTTAATTGGACCGCTCGGCGATTGAGAGTTTCTAGTGGGCCATTTTTGGT

PBU0048Ceriporia_cystidiata

CTTAATTGGACCGCTCGGCGATTGAGAGTTTCTAGTGGGCCATTTTTGGT

MZ340C_lacerataT --

Dai21940

CTTAATTGGACCGCTCGGCGATTGAGAGTTTCTAGTGGGCCATTTTTGGT

847Dai16433

CTTAATTGGACCGCTCGGCGATTGAGAGTTTCTAGTGGGCCATTTTTGGT

MarcinEmmia_latemarginatus --

Meijer3729Hydnopolyporus_fimbr

CTTAATTGGACCGCTTGGCGATTGAGAGTTTCTAGTGGGCCATTTTTGGT

RLG13408Phanerochaete_sp

CTTGATTGGACCGCTCGGCGATTGAGAGTTTCTAGTGGGCCATTTTTGGT

WHC1381Flavodon_flavus --

GB1833Phlebia_albida

CTTGATTGGACCGCTCGGCGATTGAGAGTTTCTAGTGGGCCATTTTTGGT

T407Phlebia_nitidula --

HHB6988Phanerochaete_exilis

CTTGATTGGACCGCTCGGCGATTGAGAGTTTCTAGTGGGCCATTTTTGGT

HHB8509Phanerochaetella_xeroph

CTTGATTGGACCGCTCGGCGATTGAGAGTTTCTAGTGGGCCATTTTTGGT

PBU0051Macrohyporia_dictyopora
CTTGATTGGACCGCTCGGCGATTGAGAGTTTCTAGTGGGCCATTTTGGT
HHB11463Phanerochaete_sp
CTTGATTGGACCGCTCGGCGATTGAGAGTTTCTAGTGGGCCATTTTGGT
FP102382Byssomerulius_corium
CTTGATTGGACCGCTCGGCGATTGAGAGTTTCTAGTGGGCCATTTTGGT
FP102165Efibula_americana
CTTGATTGGACCGCTTGGCGATTGAGAGTTTCTAGTGGGCCATTTTGGT
Murdoch90Ceriporia_torpida ---
Rivoire4413_Ceriporia_purpurea ---
Kout_18_Ceriporia_triumphalis ---
Rivoire3701_Ceriporia_bresadol
CTTGATTGGACCGCTCGGCGATTGAGAGTTTCTAGTGGGCCATTTTGGT
VS4018 ---
Ryvarden21832_Ceriporia_manzan ---
Dai24539
CTTGATTGGACCGCTCGGCGATTGAGAGTTTCTAGTGGGCCATTTTGGT
Dai24541
CTTGATTGGACCGCTCGGCGATTGAGAGTTTCTAGTGGGCCATTTTGGT
JV1105_12_Ceriporia_occidental ---
VS8558Ceriporia_occidentalis ---
Dai22445
CTTGATTGGACCGCTCGGCGATTGAGAGTTTCTAGTGGGCCATTTTGGT
846Dai16368
CTTGATTGGACCGCTCGGCGATTGAGAGTTTCTAGTGGGCCATTTTGGT
Dai17951_Ceriporia_aurantiocar
CTTGATTGGACCGCTCGGCGATTGAGAGTTTCTAGTGGGCCATTTTGGT
Miettinen_11701C_viridans ---
JV0105_10Ceriporia_aurantiocar ---
Yuan5702C_viridans ---
858Dai17003 ---
Yuan2747_Ceriporia_viridans ---
Yuan2744C_viridans ---
Li1046C_viridans ---
865C_sinoviridans ---
871Dai15062 ---
Dai7642_Ceriporia_humilis ---
Spirin4706_Ceriporia_humilis ---
Spirin4944_Ceriporia_sericea ---
WCG1547Dai26044ceriporia
CTTGATTGGACCGCTCGGCGATTGAGAGTTTCTAGTGGGCCATTTTGGT
ZZW1558Dai27086
CTTGATTGGACCGCTCGGCGATTGAGAGTTTCTAGTGGGCCATTTTGGT
Miettinen14381_Ceriporia_mpuri

CTTGATTGGACCGCTCGGCGATTGAGAGTTTCTAGTGGGCCATTTTTGGT
Miettinen15492_2_Ceriporia_sor
CTTGATTGGACCGCTCGGCGATTGAGAGTTTCTAGTGGGCCATTTTTGGT
He6687
CTTGATTGGACCGCTCGGCGATTGAGAGTTTCTAGTGGGCCATTTTTGGT
ZH53Dai24426
CTTGATTGGACCGCTCGGCGATTGAGAGTTTCTAGTGGGCCATTTTTGGT
Vlasak0808_30_Ceriporia_punica --
887Dai13376
CTTGATTGGACCGCTCGGCGATTGAGAGTTTCTAGTGGGCCATTTTTGGT
WCG1443Dai24998
CTTGATTGGACCGCTCGGCGATTGAGAGTTTCTAGTGGGCCATTTTTGGT
0108_6Ceriporia_spissa --
Dai19164
CTTGATTGGACCGCTCGGCGATTGAGAGTTTCTAGTGGGCCATTTTTGGT
Dai17937_Ceriporia_bubalinomar
CTTGATTGGACCGCTCGGCGATTGAGAGTTTCTAGTGGGCCATTTTTGGT
903Dai12113
CTTGATTGGACCGCTCGGCGATTGAGAGTTTCTAGTGGGCCATTTTTGGT
LZB929Dai25079
CTTGATTGGACCGCTCGGCGATTGAGAGTTTCTAGTGGGCCATTTTTGGT
LX45Dai26988 --
LX43Dai26986
CTTGATTGGACCGCTCGGCGATTGAGAGTTTCTAGTGGGCCATTTTTGGT
Dai7759Ceriporia --
Cui8012_Ceriporia_viridans --
GC1704_54Ceriporia_viridans --
Dai23392
CTTGATTGGACCGCTCGGCGATTGAGAGTTTCTAGTGGGCCATTTTTGGT
WCG1585Dai26113Ceriproia
CTTGATTGGACCGCTCGGCGATTGAGAGTTTCTAGTGGGCCATTTTTGGT
Dai18675C_eucalypti
CTTGATTGGACCGCTCGGCGATTGAGAGTTTCTAGTGGGCCATTTTTGGT
Dai22034
CTTGATTGGACCGCTTGGTGATTGAGAGTTTCTAGTGGGCCATTTTTGGT
JV1008_41JTardaFLORIDAKeys --
Rivoire1161_Ceriporia_pierii --
Dai23499C_pierii
CTTGATTGGACCGCTCGGCGATTGAGAGTTTCTAGTGGGCCATTTTTGGT
Dai23500
CTTGATTGGACCGCTCGGCGATTGAGAGTTTCTAGTGGGCCATTTTTGGT
841Dai15899
CTTGATTGGACCGCTCGGCGATTGAGAGTTTCTAGTGGGCCATTTTTGGT
842Dai15904 --

LZB1066xinjiang --
LZB1065xinjiang --
851Dai16779
CTTGATTGGACCGCTCGGCGAATGAGAGTTTCTAGTGGGCCATTTTTGGT
RMJ119sp_Candelabrochaete_sept --
RLG9759spCandelabrochaete_sept --
RLG10478Phanerochaete_allantos --
Dai19118_Ceriporia_spissa
CTTGATTGGACCGCTCGGTGATTGAGAGTTTCTAGTGGGCCATTTTTGGT
Dai18486A
CTTGATTGGACCGCTCGGTGATTGAGAGTTTCTAGTGGGCCATTTTTGGT
WEI17_024_Ceriporia_mellita --
GC1508_71Ceriporia_mellita --
GC1608_7_Ceriporia_mellita --
ZZW1557Dai27085
CTTGATTGGACCGCTCGGTGATTGAGAGTTTCTAGTGGGCCATTTTTGGT
ZZW1554Dai27083
CTTGATTGGACCGCTCGGTGATTGAGAGTTTCTAGTGGGCCATTTTTGGT
Dai8168
CTTGATTGGACCGCTCGGTGATTGAGAGTTTCTAGTGGGCCATTTTTGGT
BR4865C_mellita --
MEL2382688Ceriporia_sp --
Dai8110
CTTGATTGGACCGCTCGGTGATTGAGAGTTTCTAGTGGGCCATTTTTGGT
Cui8097 --
909Cui6740 --
W1258Dai24695
CTTGATTGGACCGCTCGGTGATTGAGAGTTTCTAGTGGGCCATTTTTGGT
JV0110_26_Ceriporia_griseoviol --
896Dai13202
CTTGATTGGACCGCTCGGCGATTGAGAGTTTCTAGTGGGCCATTTTTGGT
LWY393Dai27053C_griseoviolasce
CTTGATTGGACCGCTCGGCGATTGAGAGTTTCTAGTGGGCCATTTTTGGT
LWY394DAI27054
CTTGATTGGACCGCTCGGCGATTGAGAGTTTCTAGTGGGCCATTTTTGGT
FP135015G_pannocinctus --
L15726SpG_pannocinctus
CTTGATTGGACCGCTCGGCGATTGAGAGTTTCTAGTGGGCCATTTTTGGT
Dai22221
CTTGATTGGACCGCTCGGCGATTGAGAGTTTCTAGTGGGCCATTTTTGGT
Dai22633
CTTGATTGGACCGCTCGGCGATTGAGAGTTTCTAGTGGGCCATTTTTGGT
Dai23260
CTTGATTGGACCGCTCGGCGATTGAGAGTTTCTAGTGGGCCATTTTTGGT

Dai23626
CTTGATTGGACCGCTCGGCGATTGAGAGTTTCTAGTGGGCCATTTTTGGT
Dai16238G_citrinoalbus
CTTGATTGGACCGCTCGGCGATTGAGAGTTTCTAGTGGGCCATTTTTGGT
1175Dai15293
CTTGATTGGACCGCTCGGCGATTGAGAGTTTCTAGTGGGCCATTTTTGGT
Dai19547
CTTGATTGGACCGCTCGGCGATTGAGAGTTTCTAGTGGGCCATTTTTGGT
918063G_africanus --
918572G_africanus --
Dai18536A --
1164Cui17922
CTTGATTGGACCGCTCGGCGATTGAGAGTTTCTAGTGGGCCATTTTTGGT
Dai22225
CTTGATTGGACCGCTCGGCGATTGAGAGTTTCTAGTGGGCCATTTTTGGT
1163Dai20655 --
Yuan4397G_hainanensis
CTTGATTGGACCGCTTGGCGATTGAGAGTTTCTAGTGGGCCATTTTTGGT
1176Dai15268
CTTGATTGGACCGCTTGGCGATTGAGAGTTTCTAGTGGGCCATTTTTGGT
1177Dai15259
CTTGATTGGACCGCTTGGCGATTGAGAGTTTCTAGTGGGCCATTTTTGGT
BZ2896G_thelephoroides --
1166JV1808_26 --
Miettinen16992Hapalopilus_ochr
CTTGATTGGACTGCTCGGTGATTGAGAGTTTCTAGTGGGCCATTTTTGGT
GC1708_338_Ceriporia_arbuscula --
WCG1555Dai26107Ceriporia
CTTGATTGGACCGCTTGGCGATTGAGAGTTTCTAGTGGGCCATTTTTGGT
GC1708_340_Ceriporia_arbuscula --
WCG1556Dai26109Ceriporia
CTTGATTGGACCGCTTGGCGATTGAGAGTTTCTAGTGGGCCATTTTTGGT
883Cui11291
CTTGATTGGACCGCTTGGCGATTGAGAGTTTCTAGTGGGCCATTTTTGGT
HLX320Dai26805
CTTGATTGGACCGCTTGGCGATTGAGAGTTTCTAGTGGGCCATTTTTGGT
WCG1266Dai24678A
CTTGATTGGACCGCTTGGCGATTGAGAGTTTCTAGTGGGCCATTTTTGGT
Dai6090_Ceriporia_sulphuricolo
CTTGATTGGACCGCTCGGCGATTGAGAGTTTCTAGTGGGCCATTTTTGGT
RLG_11354_Ceriproia_reticulata
CTTGATTGGACCGCTTGGCGATTGAGAGTTTCTAGTGGGCCATTTTTGGT
ZZW1543Dai27072
CTTGATTGGACCGCTTGGCGATTGAGAGTTTCTAGTGGGCCATTTTTGGT

Li1316_Ceriporia_reticulata ---

KHL11981Ceriporia_reticulata

CTTGATTGGACCGCTTGGCGATTGAGAGTTTCTAGTGGGCCATTTTTGGT

FP110343sp_Candelabrochaete_la

CTTAGTTGGACCGCTTGGCGATTGAGAGTTTCTAGTGGGCCATTTTTGGT

Li1045_Ceriporia_reticulata ---

ZX136Dai25794ceriporia

CTTGATTGGACCGCTTGGCGATTGAGAGTTTCTAGTGGGCCATTTTTGGT

892Dai13400

CTTGATTGGACCGCTTGGCGATTGAGAGTTTCTAGTGGGCCATTTTTGGT

RLG7163Leptoporus_mollis

CTTGATTGGACCGCTCGGCGATTGAGAGTTTCTAGTGGGCCATTTTTGGT

Dai21062Leptoporus_mollis

CTTGATTGGACCGCTCGGCGATTGAGAGTTTCTAGTGGGCCATTTTTGGT

Dai20182Leptoporus_submollis

CTTGATTGGACCGCTCGGCGATTGAGAGTTTCTAGTGGGCCATTTTTGGT

Cui18379Leptoporus_submollis

CTTGATTGGACCGCTCGGCGATTGAGAGTTTCTAGTGGGCCATTTTTGGT

Wu1209_46Resiniporus_pseudogil ---

BRNM710169Resiniporus_resinasc ---

Dai14516Bjerkandera_adusta

CTTGATTGGACCGCTCGGCGATTGAGAGTTTCTAGTGGGCCATTTTTGGT

Dai21100Bjerkandera_fumosa

CTTAATTGGACCGCTCGGCGATTGAGAGTTTCTAGTGGGCCATTTTTGGT

Miettinen16854Ceraceomyces_sp

CTTGATTGGACTGCTCGGCGATTGAGAGTTTCTAGTGGGCCATTTTTGGT

Dai10477C_spissa

CTTGATTGGACCGCTCGGCGATTGAGAGTTTCTAGTGGGCCATTTTTGGT

855Dai16831

CTTGATTGGACCGCTCGGCGATTGAGAGTTTCTAGTGGGCCATTTTTGGT

882Cui11282

CTTGATTGGACCGCTCGGCGATTGAGAGTTTCTAGTGGGCCATTTTTGGT

Dai24566

CTTGATTGGACCGCTCGGCGATTGAGAGTTTCTAGTGGGCCATTTTTGGT

Yuan5965

CTTGATTGGACCGCTCGGCGATTGAGAGTTTCTAGTGGGCCATTTTTGGT

Dai3204

CTTGATTGGACCGCTTGGCGATTGAGAGTTTCTAGTGGGCCATTTTTGGT

1194CUI9985

CTTTGTTGGACCGCTTGGCGATTGAGAGTTTCTAGTGGGCCATTTTTGGT

Dai15205_Ceriporia_albomellea

AAGCAGAACTGGCGATGCGGGATGAACCGAACGCAAGGTTAAGGTGCCGG

Dai15223_Ceriporia_albomellea
AAGCAGAACTGGCGATGCGGGATGAACCGAACGCAAGGTTAAGGTGCCGG
Li1780_Ceriporia_variegata
AAGCAGAACTGGCGATGCGGGATGAACCGAACGCAAGGTTAAGGTGCCGG
Dai19791_Ceriporia_variegata
AAGCAGAACTGGCGATGCGGGATGAACCGAACGCAAGGTTAAGGTGCCGG
Dai19886
AAGCAGAACTGGCGATGCGGGATGAACCGAACGCAAGGTTAAGGTGCCGG
Dai10833_Ceriporia_crassitunic
AAGCAGAACTGGCGATGCGGGATGAACCGAACGCAAGGTTAAGGTGCCGG
CHWC1506_46Meruliopsis_crassit --
Dai9995_Ceriporia_crassitunica --
Wu1209_58_Meruliopsis_parvispo --
CHWC1505_129_Meruliopsis_parvi --
Dai21944
AAGCAGAACTGGCGATGCGGGATGAACCGAACGCGAGGTTAAGGTGCCGG
830Dai18640A
AAGCAGAACTGGCGATGCGGGATGAACCGAACGCGAGGTTAAGGTGCCGG
GC1704_60_Meruliopsis_taxicola --
Dai22625
AAGCAGAACTGGCGATGCGGGATGAACCGAACGCGAGGTTAAGGTGCCGG
Dai22636
AAGCAGAACTGGCGATGCGGGATGAACCGAACGCGAGGTTAAGGTGCCGG
Dai21878
AAGCAGAACTGGCGATGCGGGATGAACCGAACGCGAGGTTAAGGTGCCGG
1169Dai17248
AAGCAGAACTGGCGATGCGGGATGAACCGAACGCGAGGTTAAGGTGCCGG
Wu1708_43_Meruliopsis_leptocys --
Li1011
AAGCAGAACTGGCGATGCGGGATGAACCGAACGTGAGGTTAAGGTGCCGG
ZX95Dai25742Meruliopsis_leptoc
AAGCAGAACTGGCGATGCGGGATGAACCGAACGCGAGGTTAAGGTGCCGG
WCG1306Dai24733
AAGCAGAACTGGCGATGCGGGATGAACCGAACGCGAGGTTAAGGTGCCGG
LXL99Dai25816
AAGCAGAACTGGCGATGCGGGATGAACCGAACGCGAGGTTAAGGTGCCGG
WCG1559Dai26052Meruliopsis
AAGCAGAACTGGCGATGCGGGATGAACCGAACGTGAGGTTAAGGTGCCGG
He7477
AAGCAGAACTGGCGATGCGGGATGAACCGAACGTGAGGTTAAGGTGCCGG
HLX243Dai26217
AAGCAGAACTGGCGATGCGGGATGAACCGAACGTGAGGTTAAGGTGCCGG
RussiaMW673659Meruliopsis_fagi --
FD278

AAGCAGAACTGGCGATGCGGGATGAACCGAACGCGAGGTTAAGGTGCCGG
Dai10226_Ceriporia_tarda --
LE247365
AAGCAGAAC?GGCGATGCGGGATGAACCGAACGCGAGGTTAAGGTGCCGG
Dai8173_Meruliopsis_nanlingens
AAGCAGAACTGGCGATGCGGGATGAACCGAACGCGAGGTTAAGGTGCCGG
860Dai17172
AAGCAGAACTGGCGATGCGGGATGAACCGAACGCGAGGTTAAGGTGCCGG
879Dai13414
AAGCAGAACTGGCGATGCGGGATGAACCGAACGCGAGGTTAAGGTGCCGG
Li_1704_Meruliopsis_pseudocyst --
833Dai18405
AAGCAGAACTGGCGATGCGGGATGAACCGAACGCGAGGTTAAGGTGCCGG
HHB_10729_Meruliopsis_albostra
AAGCAGAACTGGCGATGCGGGATGAACCGAACGCGAGGTTAAGGTGCCGG
Cui6878_Ceriporia_pseudocystid
AAGCAGAACTGGCGATGCGGGATGAACCGAACGCGAGGTTAAGGTGCCGG
869Dai14737
AAGCAGAACTGGCGATGCGGGATGAACCGAACGCGAGGTTAAGGTGCCGG
876Cui11626
AAGCAGAACTGGCGATGCGGGATGAACCGAACGCGAGGTTAAGGTGCCGG
1199WEI3388 --
776308_Meruliopsis_cystidiata --
ICN139059_Meruliopsis_cystidia --
HHB15692Ceraceomyces_serpens
AAGCAGAACTGGCGATGCGGGATGAACCGAACGCGAGGTTAAGGTGCCGG
HHB_15629_Sp_Ceriporiopsis_ane
AAGCAGAACTGGCGATGCGGGATGAACCGAACGTGAGGTTAAGGTGCCGG
AJ185Trametopsis_cervina
AAGCAGAACTGGCGATGCGGGATGAACCGAACGTGAGGTTAAGGTGCCGG
FD9Irpex_lacteus
AAGCAGAACTGGCGATGCGGGATGAACCGAACGTGAGGTTAAGGTGCCGG
908Dai11230
AAGCAGAACTGGCGATGCGGGATGAACCGAACGTGAGGTTAAGGTGCCGG
FP55521TEmmia_lacerata
AAGCAGAACTGGCGATGCGGGATGAACCGAACGTGAGGTTAAGGTGCCGG
PBU0048Ceriporia_cystidiata
AAGCAGAACTGGCGATGCGGGATGAACCGAACGTGAGGTTAAGGTGCCGG
MZ340C_lacerataT --
Dai21940
AAGCAGAACTGGCGATGCGGGATGAACCGAACGTGAGGTTAAGGTGCCGG
847Dai16433
AAGCAGAACTGGCGATGCGGGATGAACCGAACGTGAGGTTAAGGTGCCGG
MarcinEmmia_latemarginatus --

Meijer3729Hydnopolyporus_fimbr
AAGCAGAACTGGCGATGCGGGATGAACCGAACGTGAGGTTAAGGTGCCGG
RLG13408Phanerochaete_sp
AAGCAGAACTGGCGATGCGGGATGAACCGAACGCGAGGTTAAGGTGCCGG
WHC1381Flavodon_flavus --
GB1833Phlebia_albida
AAGCAGAACTGGCGATGCGGGATGAACCGAACGCGAGGTTAAGGTGCCGG
T407Phlebia_nitidula --
HHB6988Phanerochaete_exilis
AAGCAGAACTGGCGATGCGGGATGAACCGAACGCGAGGTTAAGGTGCCGG
HHB8509Phanerochaetella_xeroph
AAGCAGAACTGGCGATGCGGGATGAACCGAACGCGAGGTTAAGGTGCCGG
PBU0051Macrohyporia_dictyopora
AAGCAGAACTGGCGATGCGGGATGAACCGAACGCGAGGTTAAGGTGCCGG
HHB11463Phanerochaete_sp
AAGCAGAACTGGCGATGCGGGATGAACCGAACGCGAGGTTAAGGTGCCGG
FP102382Byssomerulius_corium
AAGCAGAACTGGCGATGCGGGATGAACCGAACGCGAGGTTAAGGTGCCGG
FP102165Efibula_americana
AAGCAGAACTGGCGATGCGGGATGAACCGAACGCGAGGTTAAGGTGCCGG
Murdoch90Ceriporia_torpida --
Rivoire4413_Ceriporia_purpurea --
Kout_18_Ceriporia_triumphalis --
Rivoire3701_Ceriporia_bresadol AAGCAGAACTGGCGATGCGGGATGAA----------------
----
VS4018 --
Ryvarden21832_Ceriporia_manzan --
Dai24539
AAGCAGAACTGGCGATGCGGGATGAACCGAACGCGAGGTTAAGGTGCCGG
Dai24541
AAGCAGAACTGGCGATGCGGGATGAACCGAACGCGAGGTTAAGGTGCCGG
JV1105_12_Ceriporia_occidental --
VS8558Ceriporia_occidentalis --
Dai22445
AAGCAGAACTGGCGATGCGGGATGAACCGAACGCGAGGTTAAGGTGCCGG
846Dai16368
AAGCAGAACTGGCGATGCGGGATGAACCGAACGCGAGGTTAAGGTGCCGG
Dai17951_Ceriporia_aurantiocar
AAGCAGAACTGGCGATGCGGGATGAACCGAACGCGAGGTTAAGGTGCCGG
Miettinen_11701C_viridans --
JV0105_10Ceriporia_aurantiocar --
Yuan5702C_viridans --
858Dai17003 --
Yuan2747_Ceriporia_viridans --

Yuan2744C_viridans --
Li1046C_viridans --
865C_sinoviridans --
871Dai15062 --
Dai7642_Ceriporia_humilis --
Spirin4706_Ceriporia_humilis --
Spirin4944_Ceriporia_sericea --
WCG1547Dai26044ceriporia
AAGCAGAACTGGCGATGCGGGATGAACCGAACGCGAGGTTAAGGTGCCGG
ZZW1558Dai27086
AAGCAGAACTGGCGATGCGGGATGAACCGAACGCGAGGTTAAGGTGCCGG
Miettinen14381_Ceriporia_mpuri
TAGCAGAACTGGCGATGCGGGATGAACCGAACGCGAGGTTAAGGTGCCGG
Miettinen15492_2_Ceriporia_sor
AAGCAGAACTGGCGATGCGGGATGAACCGAACGCGAGGTTAAGGTGCCGG
He6687
AAGCAGAACTGGCGATGCGGGATGAACCGAACGCGAGGTTAAGGTGCCGG
ZH53Dai24426
AAGCAGAACTGGCGATGCGGGATGAACCGAACGCGAGGTTAAGGTGCCGG
Vlasak0808_30_Ceriporia_punica --
887Dai13376
AAGCAGAACTGGCGATGCGGGATGAACCGAACGCGAGGTTAAGGTGCCGG
WCG1443Dai24998
AAGCAGAACTGGCGATGCGGGATGAACCGAACGCGAGGTTAAGGTGCCGG
0108_6Ceriporia_spissa --
Dai19164
AAGCAGAACTGGCGATGCGGGATGAACCGAACGTGAGGTTAAGGTGCCGG
Dai17937_Ceriporia_bubalinomar
AAGCAGAACTGGCGATGCGGGATGAACCGAACGTGAGGTTAAGGTGCCGG
903Dai12113
AAGCAGAACTGGCGATGCGGGATGAACCGAACGTGAGGTTAAGGTGCCGG
LZB929Dai25079
AAGCAGAACTGGCGATGCGGGATGAACCGAACGCGAGGTTAAGGTGCCGG
LX45Dai26988 --
LX43Dai26986
AAGCAGAACTGGCGATGCGGGATGAACCGAACGCGAGGTTAAGGTGCCGG
Dai7759Ceriporia --
Cui8012_Ceriporia_viridans --
GC1704_54Ceriporia_viridans --
Dai23392
AAGCAGAACTGGCGATGCGGGATGAACCGAACGCAAGGTTAAGGTGCCGG
WCG1585Dai26113Ceriproia
AAGCAGAACTGGCGATGCGGGATGAACCGAACGCAAGGTTAAGGTGCCGG
Dai18675C_eucalypti

AAGCAGAACTGGCGATGCGGGATGAACCGAACGCAAGGTTAAGGTGCCGG
Dai22034
AAGCAGAACTGGCGATGCGGGATGAACCGAACGCGAGGTTAAGGTGCCGG
JV1008_41JTardaFLORIDAKeys --
Rivoire1161_Ceriporia_pierii --
Dai23499C_pierii
AAGCAGAACTGGCGATGCGGGATGAACCGAACGCGAGGTTAAGGTGCCGG
Dai23500
AAGCAGAACTGGCGATGCGGGATGAACCGAACGCGAGGTTAAGGTGCCGG
841Dai15899
AAGCAGAACTGGCGATGCGGGATGAACCGAACGCGAGGTTAAGGTGCCGG
842Dai15904 --
LZB1066xinjiang --
LZB1065xinjiang --
851Dai16779
AAGCAGAACTGGCGATGCGGGATGAACCGAACGCGAGGTTAAGGTGCCGG
RMJ119sp_Candelabrochaete_sept --
RLG9759spCandelabrochaete_sept --
RLG10478Phanerochaete_allantos --
Dai19118_Ceriporia_spissa
AAGCAGAACTGGCGATGCGGGATGAACCGAACGCGAGGTTAAGGTGCCGG
Dai18486A
AAGCAGAACTGGCGATGCGGGATGAACCGAACGCGAGGTTAAGGTGCCGG
WEI17_024_Ceriporia_mellita --
GC1508_71Ceriporia_mellita --
GC1608_7_Ceriporia_mellita --
ZZW1557Dai27085
AAGCAGAACTGGCGATGCGGGATGAACCGAACGCGAGGTTAAGGTGCCGG
ZZW1554Dai27083
AAGCAGAACTGGCGATGCGGGATGAACCGAACGCGAGGTTAAGGTGCCGG
Dai8168
AAGCAGAACTGGCGATGCGGGATGAACCGAACGCGAGGTTAAGGTGCCGG
BR4865C_mellita --
MEL2382688Ceriporia_sp --
Dai8110
AAGCAGAACTGGCGATGCGGGATGAACCGAACGCGAGGTTAAGGTGCCGG
Cui8097 --
909Cui6740 --
W1258Dai24695
AAGCAGAACTGGCGATGCGGGATGAACCGAACGCGAGGTTAAGGTGCCGG
JV0110_26_Ceriporia_griseoviol --
896Dai13202
AAGCAGAACTGGCGATGCGGGATGAACCGAACGCGAGGTTAAGGTGCCGG
LWY393Dai27053C_griseoviolasce

AAGCAGAACTGGCGATGCGGGATGAACCGAACGCGAGGTTAAGGTGCCGG

LWY394DAI27054

AAGCAGAACTGGCGATGCGGGATGAACCGAACGCGAGGTTAAGGTGCCGG

FP135015G_pannocinctus --

L15726SpG_pannocinctus

AAGCAGAACTGGCGATGCGGGATGAACCGAACGTGAGGTTAAGGTGCCGG

Dai22221

AAGCAGAACTGGCGATGCGGGATGAACCGAACGTGAGGTTAAGGTGCCGG

Dai22633

AAGCAGAACTGGCGATGCGGGATGAACCGAACGTGAGGTTAAGGTGCCGG

Dai23260

AAGCAGAACTGGCGATGCGGGATGAACCGAACGTGAGGTTAAGGTGCCGG

Dai23626

AAGCAGAACTGGCGATGCGGGATGAACCGAACGTGAGGTTAAGGTGCCGG

Dai16238G_citrinoalbus

AAGCAGAACTGGCGATGCGGGATGAACCGAACGTGAGGTTAAGGTGCCGG

1175Dai15293

AAGCAGAACTGGCGATGCGGGATGAACCGAACGTGAGGTTAAGGTGCCGG

Dai19547

AAGCAGAACTGGCGATGCGGGATGAACCGAACGTGAGGTTAAGGTGCCGG

918063G_africanus --

918572G_africanus --

Dai18536A --

1164Cui17922

AAGCAGAACTGGCGATGCGGGATGAACCGAACGTGAGGTTAAGGTGCCGG

Dai22225

AAGCAGAACTGGCGATGCGGGATGAACCGAACGTGAGGTTAAGGTGCCGG

1163Dai20655 --

Yuan4397G_hainanensis

AAGCAGAACTGGCGATGCGGGATGAACCGAACGTGAGGTTAAGGTGCCGG

1176Dai15268

AAGCAGAACTGGCGATGCGGGATGAACCGAACGTGAGGTTAAGGTGCCGG

1177Dai15259

AAGCAGAACTGGCGATGCGGGATGAACCGAACGTGAGGTTAAGGTGCCGG

BZ2896G_thelephoroides --

1166JV1808_26 --

Miettinen16992Hapalopilus_ochr

AAGCAGAACTGGCGATGCGGGATGAACCGAACGCAAGGTTAAGGTGCCGG

GC1708_338_Ceriporia_arbuscula --

WCG1555Dai26107Ceriporia

AAGCAGAACTGGCGATGCGGGATGAACCGAACGCAAGGTTAAGGTGCCGG

GC1708_340_Ceriporia_arbuscula --

WCG1556Dai26109Ceriporia

AAGCAGAACTGGCGATGCGGGATGAACCGAACGCAAGGTTAAGGTGCCGG

883Cui11291
AAGCAGAACTGGCGATGCGGGATGAACCGAACGCAAGGTTAAGGTGCCGG
HLX320Dai26805
AAGCAGAACTGGCGATGCGGGATGAACCGAACGCAAGGTTAAGGTGCCGG
WCG1266Dai24678A
AAGCAGAACTGGCGATGCGGGATGAACCGAACGCAAGGTTAAGGTGCCGG
Dai6090_Ceriporia_sulphuricolo
AAGCAGAACTGGCGATGCGGGATGAACCGAACGCGAGGTTGAGGTGCCGG
RLG_11354_Ceriproia_reticulata
AAGCAGAACTGGCGATGCGGGATGAACCGAACGCGAGGTTAAGGTGCCGG
ZZW1543Dai27072
AAGCAGAACTGGCGATGCGGGATGAACCGAACGCGAGGTTAAGGTGCCGG
Li1316_Ceriporia_reticulata --
KHL11981Ceriporia_reticulata
AAGCAGAACTGGCGATGCGGGATGAACCGAACGCGAGGTTAAGGTGCCGG
FP110343sp_Candelabrochaete_la
AAGCAGAACTGGCGATGCGGGATGAACCGAACGTGAGGTTAAGGTGCCGG
Li1045_Ceriporia_reticulata --
ZX136Dai25794ceriporia
AAGCAGAACTGGCGATGCGGGATGAACCGAACGTGAGGTTAAGGTGCCGG
892Dai13400
AAGCAGAACTGGCGATGCGGGATGAACCGAACGTGAGGTTAAGGTGCCGG
RLG7163Leptoporus_mollis
AAGCAGAACTGGCGATGCGGGATGAACCGAACGCGAGGTTAAGGTGCCGG
Dai21062Leptoporus_mollis
AAGCAGAACTGGCGATGCGGGATGAACCGAACGCGAGGTTAAGGTGCCGG
Dai20182Leptoporus_submollis
AAGCAGAACTGGCGATGCGGGATGAACCGAACGCGAGGTTAAGGTGCCGG
Cui18379Leptoporus_submollis
AAGCAGAACTGGCGATGCGGGATGAACCGAACGCGAGGTTAAGGTGCCGG
Wu1209_46Resiniporus_pseudogil --
BRNM710169Resiniporus_resinasc --
Dai14516Bjerkandera_adusta
AAGCAGAACTGGCGATGCGGGATGAACCGAACGCGAGGTTAAGGTGCCGG
Dai21100Bjerkandera_fumosa
AAGCAGAACTGGCGATGCGGGATGAACCGAACGCGAGGTTAAGGTGCCGG
Miettinen16854Ceraceomyces_sp
AAGCAGAACTGGCGATGCGGGATGAACCGAACGCGAGGTTAAGGTGCCGG
Dai10477C_spissa
AAGCAGAACTGGCGATGCGGGATGAACCGAACGTGAGGTTAAGGTGCCGG
855Dai16831
AAGCAGAACTGGCGATGCGGGATGAACCGAACGTGAGGTTAAGGTGCCGG
882Cui11282
AAGCAGAACTGGCGATGCGGGATGAACCGAACGTGAGGTTAAGGTGCCGG

Dai24566
AAGCAGAACTGGCGATGCGGGATGAACCGAACGTGAGGTTAAGGTGCCGG
Yuan5965
AAGCAGAACTGGCGATGCGGGATGAACCGAACGTGAGGTTAAGGTGCCGG
Dai3204
AAGCAGAACTGGCGATGCGGGATGAACCGAACGCGAGGTTAAGGTGCCGG
1194CUI9985
AAGCAGAACTGGCGATGCGGGATGAACCGAACGTGAGGTTAAGGTGCCGG

Dai15205_Ceriporia_albomellea
AATACACGCTCATCAGACACCACAAAAGGTGTTAGTTCATCTAGACAGCA
Dai15223_Ceriporia_albomellea
AATACACGCTCATCAGACACCACAAAAGGTGTTAGTTCATCTAGACAGCA
Li1780_Ceriporia_variegata
AATACACGCTCATCAGACACCACAAAAGGTGTTAGTTCATCTAGACAGCA
Dai19791_Ceriporia_variegata
AATACACGCTCATCAGACACCACAAAAGGTGTTAGTTCATCTAGACAGCA
Dai19886
AATACACGCTCATCAGACACCACAAAAGGTGTTAGTTCATCTAGACAGCA
Dai10833_Ceriporia_crassitunic
AATACACGCTCATCAGACACCACAAAAGGTGTTAGTTCATCTAGACAGCA
CHWC1506_46Meruliopsis_crassit --
Dai9995_Ceriporia_crassitunica --
Wu1209_58_Meruliopsis_parvispo --
CHWC1505_129_Meruliopsis_parvi --
Dai21944
AATACACGCTCATCAGACACCACAAAAGGTGTTAGTTCATCTAGACAGCA
830Dai18640A
AATACACGCTCATCAGACACCACAAAAGGTGTTAGTTCATCTAGACAGCA
GC1704_60_Meruliopsis_taxicola --
Dai22625
AATACACGCTCATCAGACACCACAAAAGGTGTTAGTTCATCTAGACAGCA
Dai22636
AATACACGCTCATCAGACACCACAAAAGGTGTTAGTTCATCTAGACAGCA
Dai21878
AATACACGCTCATCAGACACCACAAAAGGTGTTAGTTCATCTAGACAGCA
1169Dai17248
AATACACGCTCATCAGACACCACAAAAGGTGTTAGTTCATCTAGACAGCA
Wu1708_43_Meruliopsis_leptocys --
Li1011
AATACACGCTCATCAGACACCACAAAAGGTGTTAGTTCATCTAGACAGCA
ZX95Dai25742Meruliopsis_leptoc
AATACACGCTCATCAGACACCACAAAAGGTGTTAGTTCATCTAGACAGCA

WCG1306Dai24733
AATACACGCTCATCAGACACCACAAAAGGTGTTAGTTCATCTAGACAGCA
LXL99Dai25816
AATACACGCTCATCAGACACCACAAAAGGTGTTAGTTCATCTAGACAGCA
WCG1559Dai26052Meruliopsis
AATACACGCTCATCAGACACCACAAAAGGTGTTAGTTCATCTAGACAGCA
He7477
AATACACGCTCATCAGACACCACAAAAGGTGTTAGTTCATCTAGACAGCA
HLX243Dai26217
AATACACGCTCATCAGACACCACAAAAGGTGTTAGTTCATCTAGACAGCA
RussiaMW673659Meruliopsis_fagi --
FD278
AATACACGCTCATCAGACACCACAAAAGGTGTTAGTTCATCTAGACAGCA
Dai10226_Ceriporia_tarda --
LE247365
AATACACGCTCATCAGACACCACAAAAGGTGTTAGTTCATCTAGACAGCA
Dai8173_Meruliopsis_nanlingens
AATACACGCTCATCAGACACCACAAAAGGTGTTAGTTCATCTAGACAGCA
860Dai17172
AATACACGCTCATCAGACACCACAAAAGGTGTTAGTTCATCTAGACAGCA
879Dai13414
AATACACGCTCATCAGACACCACAAAAGGTGTTAGTTCATCTAGACAGCA
Li_1704_Meruliopsis_pseudocyst --
833Dai18405
AATACACGCTCATCAGACACCACAAAAGGTGTTAGTTCATCTAGACAGCA
HHB_10729_Meruliopsis_albostra
AATACACGCTCATCAGACACCACAAAAGGTGTTAGTTCATCTAGACAGCA
Cui6878_Ceriporia_pseudocystid
AATACACGCTCATCAGACACCACAAAAGGTGTTAGTTCATCTAGACAGCA
869Dai14737
AATACACGCTCATCAGACACCACAAAAGGTGTTAGTTCATCTAGACAGCA
876Cui11626
AATACACGCTCATCAGACACCACAAAAGGTGTTAGTTCATCTAGACAGCA
1199WEI3388 --
776308_Meruliopsis_cystidiata --
ICN139059_Meruliopsis_cystidia --
HHB15692Ceraceomyces_serpens
AATACACGCTCATCAGACACCACAAAAGGTGTTAGTTCATCTAGACAGCA
HHB_15629_Sp_Ceriporiopsis_ane
AATACACGCTCATCAGACACCACAAAAGGTGTTAGTTCATCTAGACAGCA
AJ185Trametopsis_cervina
AATACACGCTCATCAGACACCACAAAAGGTGTTAGTTCATCTAGACAGCA
FD9Irpex_lacteus
AATACACGCTCATCAGACACCACAAAAGGTGTTAGTTCATCTAGACAGCA

908Dai11230
AATACACGCTCATCAGACACCACAAAAGGTGTTAGTTCATCTAGACAGCA
FP55521TEmmia_lacerata
AATACACGCTCATCAGACACCACAAAAGGTGTTAGTTCATCTAGACAGCA
PBU0048Ceriporia_cystidiata
AATACACGCTCATCAGACACCACAAAAGGTGTTAGTTCATCTAGACAGCA
MZ340C_lacerataT --
Dai21940
AATACACGCTCATCAGACACCACAAAAGGTGTTAGTTCATCTAGACAGCA
847Dai16433
AATACACGCTCATCAGACACCACAAAAGGTGTTAGTTCATCTAGACAGCA
MarcinEmmia_latemarginatus --
Meijer3729Hydnopolyporus_fimbr
AATACACGCTCATCAGACACCACAAAAGGTGTTAGTTCATCTAGACAGCA
RLG13408Phanerochaete_sp
AATACACGCTCATCAGACACCACAAAAGGTGTTAGTTCATTTAGACAGCA
WHC1381Flavodon_flavus --
GB1833Phlebia_albida
AATACACGCTCATCAGACACCACAAAAGGTGTTAGTTCATCTAGACAGCA
T407Phlebia_nitidula --
HHB6988Phanerochaete_exilis
AATACACGCTCATCAGACACCACAAAAGGTGTTAGTTCATCTAGACAGCA
HHB8509Phanerochaetella_xeroph
AATACACGCTCATCAGACACCACAAAAGGTGTTAGTTCATCTAGACAGCA
PBU0051Macrohyporia_dictyopora
AATACACGCTCATCAGACACCACAAAAGGTGTTAGTTCATCTAGACAGCA
HHB11463Phanerochaete_sp
AATACACGCTCATCAGACACCACAAAAGGTGTTAGTTCATCTAGACAGCA
FP102382Byssomerulius_corium
AATACACGCTCATCAGACACCACAAAAGGTGTTAGTTCATCTAGACAGCA
FP102165Efibula_americana
AATACACGCTCATCAGACACCACAAAAGGTGTTAGTTCATCTAGACAGCA
Murdoch90Ceriporia_torpida --
Rivoire4413_Ceriporia_purpurea --
Kout_18_Ceriporia_triumphalis --
Rivoire3701_Ceriporia_bresadol --
VS4018 --
Ryvarden21832_Ceriporia_manzan --
Dai24539
AATACACGCTCATCAGACACCACAAAAGGTGTTAGTTCATCTAGACAGCA
Dai24541
AATACACGCTCATCAGACACCACAAAAGGTGTTAGTTCATCTAGACAGCA
JV1105_12_Ceriporia_occidental --
VS8558Ceriporia_occidentalis --

Dai22445
AATACACGCTCATCAGACACCACAAAAGGTGTTAGTTCATCTAGACAGCA
846Dai16368
AATACACGCTCATCAGACACCACAAAAGGTGTTAGTTCATCTAGACAGCA
Dai17951_Ceriporia_aurantiocar
AATACACGCTCATCAGACACCACAAAAGGTGTTAGTTCATCTAGACAGCA
Miettinen_11701C_viridans --
JV0105_10Ceriporia_aurantiocar --
Yuan5702C_viridans --
858Dai17003 --
Yuan2747_Ceriporia_viridans --
Yuan2744C_viridans --
Li1046C_viridans --
865C_sinoviridans --
871Dai15062 --
Dai7642_Ceriporia_humilis --
Spirin4706_Ceriporia_humilis --
Spirin4944_Ceriporia_sericea --
WCG1547Dai26044ceriporia
AATACACGCTCATCAGACACCACAAAAGGTGTTAGTTCATCTAGACAGCA
ZZW1558Dai27086
AATACACGCTCATCAGACACCACAAAAGGTGTTAGTTCATCTAGACAGCA
Miettinen14381_Ceriporia_mpuri
AATACACGCTCATCAGACACCACAAAAGGTGTTAGTTCATCTAGACAGCA
Miettinen15492_2_Ceriporia_sor
AATACACGCTCATCAGACACCACAAAAGGTGTTAGTTCATCTAGACAGCA
He6687
AATACACGCTCATCAGACACCACAAAAGGTGTTAGTTCATCTAGACAGCA
ZH53Dai24426
AATACACGCTCATCAGACACCACAAAAGGTGTTAGTTCATCTAGACAGCA
Vlasak0808_30_Ceriporia_punica --
887Dai13376
AATACACGCTCATCAGACACCACAAAAGGTGTTAGTTCATCTAGACAGCA
WCG1443Dai24998
AATACACGCTCATCAGACACCACAAAAGGTGTTAGTTCATCTAGACAGCA
0108_6Ceriporia_spissa --
Dai19164
AATACACGCTCATCAGACACCACAAAAGGTGTTAGTTCATCTAGACAGCA
Dai17937_Ceriporia_bubalinomar
AATACACGCTCATCAGACACCACAAAAGGTGTTAGTTCATCTAGACAGCA
903Dai12113
AATACACGCTCATCAGACACCACAAAAGGTGTTAGTTCATCTAGACAGCA
LZB929Dai25079
AATACACGCTCATCAGACACCACAAAAGGTGTTAGTTCATCTAGACAGCA

LX45Dai26988 --
LX43Dai26986
AATACACGCTCATCAGACACCACAAAAGGTGTTAGTTCATCTAGACAGCA
Dai7759Ceriporia --
Cui8012_Ceriporia_viridans --
GC1704_54Ceriporia_viridans --
Dai23392
AATACACGCTCATCAGACACCACAAAAGGTGTTAGTTCATCTAGACAGCA
WCG1585Dai26113Ceriproia
AATACACGCTCATCAGACACCACAAAAGGTGTTAGTTCATCTAGACAGCA
Dai18675C_eucalypti
AATACACGCTCATCAGACACCACAAAAGGTGTTAGTTCATCTAGACAGCA
Dai22034
AATACACGCTCATCAGACACCACAAAAGGTGTTAGTTCATCTAGACAGCA
JV1008_41JTardaFLORIDAKeys --
Rivoire1161_Ceriporia_pierii --
Dai23499C_pierii
AATACACGCTCATCAGACACCACAAAAGGTGTTAGTTCATCTAGACAGCA
Dai23500
AATACACGCTCATCAGACACCACAAAAGGTGTTAGTTCATCTAGACAGCA
841Dai15899
AATACACGCTCATCAGACACCACAAAAGGTGTTAGTTCATCTAGACAGCA
842Dai15904 --
LZB1066xinjiang --
LZB1065xinjiang --
851Dai16779
AATACACGCTCATCAGACACCACAAAAGGTGTTAGTTCATCTAGACAGCA
RMJ119sp_Candelabrochaete_sept --
RLG9759spCandelabrochaete_sept --
RLG10478Phanerochaete_allantos --
Dai19118_Ceriporia_spissa
AATACACGCTCATCAGACACCACAAAAGGTGTTAGTTCATCTAGACAGCA
Dai18486A
AATACACGCTCATCAGACACCACAAAAGGTGTTAGTTCATCTAGACAGCA
WEI17_024_Ceriporia_mellita --
GC1508_71Ceriporia_mellita --
GC1608_7_Ceriporia_mellita --
ZZW1557Dai27085
AATACACGCTCATCAGACACCACAAAAGGTGTTAGTTCATCTAGACAGCA
ZZW1554Dai27083
AATACACGCTCATCAGACACCACAAAAGGTGTTAGTTCATCTAGACAGCA
Dai8168
AATACACGCTCATCAGACACCACAAAAGGTGTTAGTTCATCTAGACAGCA
BR4865C_mellita --

MEL2382688Ceriporia_sp --
Dai8110
AATACACGCTCATCAGACACCACAAAAGGTGTTAGTTCATCTAGACAGCA
Cui8097 --
909Cui6740 --
W1258Dai24695
AATATACGCTCATCAGACACCACAAAAGGTGTTAGTTCATCTAGACAGCA
JV0110_26_Ceriporia_griseoviol --
896Dai13202
AATACACGCTCATCAGACACCACAAAAGGTGTTAGTTCATCTAGACAGCA
LWY393Dai27053C_griseoviolasce
AATACACGCTCATCAGACACCACAAAAGGTGTTAGTTCATCTAGACAGCA
LWY394DAI27054
AATACACGCTCATCAGACACCACAAAAGGTGTTAGTTCATCTAGACAGCA
FP135015G_pannocinctus --
L15726SpG_pannocinctus
AATACACGCTCATCAGACACCACAAAAGGTGTTAGTTCATCTAGACAGCA
Dai22221
AATACACGCTCATCAGACACCACAAAAGGTGTTAGTTCATCTAGACAGCA
Dai22633
AATACACGCTCATCAGACACCACAAAAGGTGTTAGTTCATCTAGACAGCA
Dai23260
AATACACGCTCATCAGACACCACAAAAGGTGTTAGTTCATCTAGACAGCA
Dai23626
AATACACGCTCATCAGACACCACAAAAGGTGTTAGTTCATCTAGACAGCA
Dai16238G_citrinoalbus
AATACACGCTCATCAGACACCACAAAAGGTGTTAGTTCATCTAGACAGCA
1175Dai15293
AATACACGCTCATCAGACACCACAAAAGGTGTTAGTTCATCTAGACAGCA
Dai19547
AATACACGCTCATCAGACACCACAAAAGGTGTTAGTTCATCTAGACAGCA
918063G_africanus --
918572G_africanus --
Dai18536A --
1164Cui17922
AATACACGCTCATCAGACACCACAAAAGGTGTTAGTTCATCTAGACAGCA
Dai22225
AATACACGCTCATCAGACACCACAAAAGGTGTTAGTTCATCTAGACAGCA
1163Dai20655 --
Yuan4397G_hainanensis
AATACACGCTCATCAGACACCACAAAAGGTGTTAGTTCATCTAGACAGCA
1176Dai15268
AATACACGCTCATCAGACACCACAAAAGGTGTTAGTTCATCTAGACAGCA
1177Dai15259

AATACACGCTCATCAGACACCACAAAAGGTGTTAGTTCATCTAGACAGCA
BZ2896G_thelephoroides --
1166JV1808_26 --
Miettinen16992Hapalopilus_ochr
AATACACGCTCATCAGACACCACAAAAGGTGTTAGTTCATCTAGACAGCA
GC1708_338_Ceriporia_arbuscula --
WCG1555Dai26107Ceriporia
AATACACGCTCATCAGACACCACAAAAGGTGTTAGTTCATCTAGACAGCA
GC1708_340_Ceriporia_arbuscula --
WCG1556Dai26109Ceriporia
AATACACGCTCATCAGACACCACAAAAGGTGTTAGTTCATCTAGACAGCA
883Cui11291
AATACACGCTCATCAGACACCACAAAAGGTGTTAGTTCATCTAGACAGCA
HLX320Dai26805
AATACACGCTCATCAGACACCACAAAAGGTGTTAGTTCATCTAGACAGCA
WCG1266Dai24678A
AATACACGCTCATCAGACACCACAAAAGGTGTTAGTTCATCTAGACAGCA
Dai6090_Ceriporia_sulphuricolo
AATACACGCTCATCAGACACCACAAAAGGTGTTAGTTCATCTAGACAGCA
RLG_11354_Ceriproia_reticulata
AATACACGCTCATCAGACACCACAAAAGGTGTTAGTTCATCTAGACAGCA
ZZW1543Dai27072
AATACACGCTCATCAGACACCACAAAAGGTGTTAGTTCATCTAGACAGCA
Li1316_Ceriporia_reticulata --
KHL11981Ceriporia_reticulata
AATACACGCTCATCAGACACCACAAAAGGTGTTAGTTCATCTAGACAGCA
FP110343sp_Candelabrochaete_la
AATACACGCTCATCAGACACCACAAAAGGTGTTAGTTCATCTAGACAGCA
Li1045_Ceriporia_reticulata --
ZX136Dai25794ceriporia
AATACACGCTCATCAGACACCACAAAAGGTGTTAGTTCATCTAGACAGCA
892Dai13400
AATACACGCTCATCAGACACCACAAAAGGTGTTAGTTCATCTAGACAGCA
RLG7163Leptoporus_mollis
AATACACGCTCATCAGACACCACAAAAGGTGTTAGTTCATCTAGACAGCA
Dai21062Leptoporus_mollis
AATACACGCTCATCAGACACCACAAAAGGTGTTAGTTCATCTAGACAGCA
Dai20182Leptoporus_submollis
AATACACGCTCATCAGACACCACAAAAGGTGTTAGTTCATCTAGACAGCA
Cui18379Leptoporus_submollis
AATACACGCTCATCAGACACCACAAAAGGTGTTAGTTCATCTAGACAGCA
Wu1209_46Resiniporus_pseudogil --
BRNM710169Resiniporus_resinasc --
Dai14516Bjerkandera_adusta

AATACACGCTCATCAGACACCACAAAAGGTGTTAGTTCATCTAGACAGCA
Dai21100Bjerkandera_fumosa
AATACACGCTCATCAGACACCACAAAAGGTGTTAGTTCATCTAGACAGCA
Miettinen16854Ceraceomyces_sp
AATACACGCTCATCAGACACCACAAAAGGTGTTAGTTCATCTAGACAGCA
Dai10477C_spissa
AATACACGCTCATCAGACACCACAAAAGGTGTTAGTTCATCTAGACAGCA
855Dai16831
AATACACGCTCATCAGACACCACAAAAGGTGTTAGTTCATCTAGACAGCA
882Cui11282
AATACACGCTCATCAGACACCACAAAAGGTGTTAGTTCATCTAGACAGCA
Dai24566
AATACACGCTCATCAGACACCACAAAAGGTGTTAGTTCATCTAGACAGCA
Yuan5965
AATACACGCTCATCAGACACCACAAAAGGTGTTAGTTCATCTAGACAGCA
Dai3204
AATACACGCTCATCAGACACCACAAAAGGTGTTAGTTCATCTAGACAGCA
1194CUI9985
AATACACGCTCATCAGACACCACAAAAGGTGTTAGTTCATCTAGACAGCA

Dai15205_Ceriporia_albomellea
GGACGGTGGCCATGGAAGTCGGAACCCGCTAAGGAGTGTGTAACAACTCA
Dai15223_Ceriporia_albomellea
GGACGGTGGCCATGGAAGTCGGAACCCGCTAAGGAGTGTGTAACAACTCA
Li1780_Ceriporia_variegata
GGACGGTGGCCATGGAAGTCGGAACCCGCTAAGGAGTGTGTAACAACTCA
Dai19791_Ceriporia_variegata
GGACGGTGGCCATGGAAGTCGGAACCCGCTAAGGAGTGTGTAACAACTCA
Dai19886
GGACGGTGGCCATGGAAGTCGGAACCCGCTAAGGAGTGTGTAACAACTCA
Dai10833_Ceriporia_crassitunic
GGACGGTGGCCATGGAAGTCGGAACCCGCTAAGGAGTGTGTAACAACTCA
CHWC1506_46Meruliopsis_crassit --
Dai9995_Ceriporia_crassitunica --
Wu1209_58_Meruliopsis_parvispo --
CHWC1505_129_Meruliopsis_parvi --
Dai21944
GGACGGTGGCCATGGAAGTCGGAACCCGCTAAGGAGTGTGTAACAACTCA
830Dai18640A
GGACGGTGGCCATGGAAGTCGGAACCCGCTAAGGAGTGTGTAACAACTCA
GC1704_60_Meruliopsis_taxicola --
Dai22625
GGACGGTGGCCATGGAAGTCGGAATCCGCTAAGGAGTGTGTAACAACTCA

Dai22636
GGACGGTGGCCATGGAAGTCGGAATCCGCTAAGGAGTGTGTAACAACTCA
Dai21878
GGACGGTGGCCATGGAAGTCGGAATCCGCTAAGGAGTGTGTAACAACTCA
1169Dai17248
GGACGGTGGCCATGGAAGTCGGAATCCGCTAAGGAGTGTGTAACAACTCA
Wu1708_43_Meruliopsis_leptocys --
Li1011
GGACGGTGGCCATGGAAGTCGGAATCCGCTAAGGAGTGTGTAACAACTCA
ZX95Dai25742Meruliopsis_leptoc
GGACGGTGGCCATGGAAGTCGGAATCCGCTAAGGAGTGTGTAACAACTCA
WCG1306Dai24733
GGACGGTGGCCATGGAAGTCGGAATCCGCTAAGGAGTGTGTAACAACTCA
LXL99Dai25816
GGACGGTGGCCATGGAAGTCGGAATCCGCTAAGGAGTGTGTAACAACTCA
WCG1559Dai26052Meruliopsis
GGACGGTGGCCATGGAAGTCGGAACCCGCTAAGGAGTGTGTAACAACTCA
He7477
GGACGGTGGCCATGGAAGTCGGAACCCGCTAAGGAGTGTGTAACAACTCA
HLX243Dai26217
GGACGGTGGCCATGGAAGTCGGAACCCGCTAAGGAGTGTGTAACAACTCA
RussiaMW673659Meruliopsis_fagi --
FD278
GGACGGTGGCCATGGAAGTCGGAACCCGCTAAGGAGTGTGTAACAACTCA
Dai10226_Ceriporia_tarda --
LE247365
GGAC?GTGGCC?TGGAAGTCGGAATCCG?TAAGGAGTGTGTAACAACTCA
Dai8173_Meruliopsis_nanlingens
GGACGGTGGCCATGGAAGTCGGAATCCGCTAAGGAGTGTGTAACAACTCA
860Dai17172
GGACGGTGGCCATGGAAGTCGGAATCCGCTAAGGAGTGTGTAACAACTCA
879Dai13414
GGACGGTGGCCATGGAAGTCGGAATCCGCTAAGGAGTGTGTAACAACTCA
Li_1704_Meruliopsis_pseudocyst --
833Dai18405
GGACGGTGGCCATGGAAGTCGGAACCCGCTAAGGAGTGTGTAACAACTCA
HHB_10729_Meruliopsis_albostra
GGACGGTGGCCATGGAAGTCGGAACCCGCTAAGGAGTGTGTAACAACTCA
Cui6878_Ceriporia_pseudocystid
GGACGGTGGCCATGGAAGTCGGAACCCGCTAAGGAGTGTGTAACAACTCA
869Dai14737
GGACGGTGGCCATGGAAGTCGGAACCCGCTAAGGAGTGTGTAACAACTCA
876Cui11626
GGACGGTGGCCATGGAAGTCGGAACCCGCTAAGGAGTGTGTAACAACTCA

1199WEI3388 ---

776308_Meruliopsis_cystidiata ---

ICN139059_Meruliopsis_cystidia ---

HHB15692Ceraceomyces_serpens

GGACGGTGGCCATGGAAGTCGGAACCCGCTAAGGAGTGTGTAACAACTCA

HHB_15629_Sp_Ceriporiopsis_ane

GGACGGTGGCCATGGAAGTCGGAATCCGCTAAGGAGTGTGTAACAACTCA

AJ185Trametopsis_cervina

GGACGGTGGCCATGGAAGTCGGAATCCGCTAAGGAGTGTGTAACAACTCA

FD9Irpex_lacteus

GGACGGTGGCCATGGAAGTCGGAACCCGCTAAGGAGTGTGTAACAACTCA

908Dai11230

GGACGGTGGCCATGGAAGTCGGAACCCGCTAAGGAGTGTGTAACAACTCA

FP55521TEmmia_lacerata

GGACGGTGGCCATGGAAGTCGGAATCCGCTAAGGAGTGTGTAACAACTCA

PBU0048Ceriporia_cystidiata

GGACGGTGGCCATGGAAGTCGGAATCCGCTAAGGAGTGTGTAACAACTCA

MZ340C_laceratаT ---

Dai21940

GGACGGTGGCCATGGAAGTCGGAATCCGCTAAGGAGTGTGTAACAACTCA

847Dai16433

GGACGGTGGCCATGGAAGTCGGAATCCGCTAAGGAGTGTGTAACAACTCA

MarcinEmmia_latemarginatus ---

Meijer3729Hydnopolyporus_fimbr

GGACGGTGGCCATGGAAGTCGGAATCCGCTAAGGAGTGTGTAACAACTCA

RLG13408Phanerochaete_sp

GGACGGTGGCCATGGAAGTCGGAACCCGCTAAGGAGTGTGTAACAACTCA

WHC1381Flavodon_flavus ---

GB1833Phlebia_albida

GGACGGTGGCCATGGAAGTCGGAATCCGCTAAGGAGTGTGTAACAACTCA

T407Phlebia_nitidula ---

HHB6988Phanerochaete_exilis

GGACGGTGGCCATGGAAGTCGGAACCCGCTAAGGAGTGTGTAACAACTCA

HHB8509Phanerochaetella_xeroph

GGACGGTGGCCATGGAAGTCGGAACCCGCTAAGGAGTGTGTAACAACTCA

PBU0051Macrohyporia_dictyopora

GGACGGTGGCCATGGAAGTCGGAACCCGCTAAGGAGTGTGTAACAACTCA

HHB11463Phanerochaete_sp

GGACGGTGGCCATGGAAGTCGGAACCCGCTAAGGAGTGTGTAACAACTCA

FP102382Byssomerulius_corium

GGACGGTGGCCATGGAAGTCGGAATCCGCTAAGGAGTGTGTAACAACTCA

FP102165Efibula_americana

GGACGGTGGCCATGGAAGTCGGAACCCGCTAAGGAGTGTGTAACAACTCA

Murdoch90Ceriporia_torpida ---

Rivoire4413_Ceriporia_purpurea --
Kout_18_Ceriporia_triumphalis --
Rivoire3701_Ceriporia_bresadol --
VS4018 --
Ryvarden21832_Ceriporia_manzan --
Dai24539
GGACGGTGGCCATGGAAGTCGGAACCCGCTAAGGAGTGTGTAACAACTCA
Dai24541
GGACGGTGGCCATGGAAGTCGGAACCCGCTAAGGAGTGTGTAACAACTCA
JV1105_12_Ceriporia_occidental --
VS8558Ceriporia_occidentalis --
Dai22445
GGACGGTGGCCATGGAAGTCGGAACCCGCTAAGGAGTGTGTAACAACTCA
846Dai16368
GGACGGTGGCCATGGAAGTCGGAACCCGCTAAGGAGTGTGTAACAACTCA
Dai17951_Ceriporia_aurantiocar
GGACGGTGGCCATGGAAGTCGGAACCCGCTAAGGAGTGTGTAACAACTCA
Miettinen_11701C_viridans --
JV0105_10Ceriporia_aurantiocar --
Yuan5702C_viridans --
858Dai17003 --
Yuan2747_Ceriporia_viridans --
Yuan2744C_viridans --
Li1046C_viridans --
865C_sinoviridans --
871Dai15062 --
Dai7642_Ceriporia_humilis --
Spirin4706_Ceriporia_humilis --
Spirin4944_Ceriporia_sericea --
WCG1547Dai26044ceriporia
GGACGGTGGCCATGGAAGTCGGAACCCGCTAAGGAGTGTGTAACAACTCA
ZZW1558Dai27086
GGACGGTGGCCATGGAAGTCGGAACCCGCTAAGGAGTGTGTAACAACTCA
Miettinen14381_Ceriporia_mpuri
GGACGGTGGCCATGGAAGTCGGAACCCGCTAAGGAGTGTGTAACAACTCA
Miettinen15492_2_Ceriporia_sor
GGACGGTGGCCATGGAAGTCGGAACCCGCTAAGGAGTGTGTAACAACTCA
He6687
GGACGGTGGCCATGGAAGTCGGAACCCGCTAAGGAGTGTGTAACAACTCA
ZH53Dai24426
GGACGGTGGCCATGGAAGTCGGAACCCGCTAAGGAGTGTGTAACAACTCA
Vlasak0808_30_Ceriporia_punica --
887Dai13376
GGACGGTGGCCATGGAAGTCGGAACCCGCTAAGGAGTGTGTAACAACTCA

WCG1443Dai24998
GGACGGTGGCCATGGAAGTCGGAACCCGCTAAGGAGTGTGTAACAACTCA
0108_6Ceriporia_spissa --
Dai19164
GGACGGTGGCCATGGAAGTCGGAACCCGCTAAGGATTGTGTAACAACTCA
Dai17937_Ceriporia_bubalinomar
GGACGGTGGCCATGGAAGTCGGAACCCGCTAAGGATTGTGTAACAACTCA
903Dai12113
GGACGGTGGCCATGGAAGTCGGAACCCGCTAAGGATTGTGTAACAACTCA
LZB929Dai25079
GGACGGTGGCCATGGAAGTCGGAACCCGCTAAGGAGTGTGTAACAACTCA
LX45Dai26988 --
LX43Dai26986
GGACGGTGGCCATGGAAGTCGGAACCCGCTAAGGAGTGTGTAACAACTCA
Dai7759Ceriporia --
Cui8012_Ceriporia_viridans --
GC1704_54Ceriporia_viridans --
Dai23392
GGACGGTGGCCATGGAAGTCGGAACCCGCTAAGGAGTGTGTAACAACTCA
WCG1585Dai26113Ceriproia
GGACGGTGGCCATGGAAGTCGGAACCCGCTAAGGAGTGTGTAACAACTCA
Dai18675C_eucalypti
GGACGGTGGCCATGGAAGTCGGAACCCGCTAAGGAGTGTGTAACAACTCA
Dai22034
GGACGGTGGCCATGGAAGTCGGAACCCGCTAAGGAGTGTGTAACAACTCA
JV1008_41JTardaFLORIDAKeys --
Rivoire1161_Ceriporia_pierii --
Dai23499C_pierii
GGACGGTGGCCATGGAAGTCGGAACCCGCTAAGGAGTGTGTAACAACTCA
Dai23500
GGACGGTGGCCATGGAAGTCGGAACCCGCTAAGGAGTGTGTAACAACTCA
841Dai15899
GGACGGTGGCCATGGAAGTCGGAACCCGCTAAGGAGTGTGTAACAACTCA
842Dai15904 --
LZB1066xinjiang --
LZB1065xinjiang --
851Dai16779
GGACGGTGGCCATGGAAGTCGGAACCCGCTAAGGAGTGTGTAACAACTCA
RMJ119sp_Candelabrochaete_sept --
RLG9759spCandelabrochaete_sept --
RLG10478Phanerochaete_allantos --
Dai19118_Ceriporia_spissa
GGACGGTGGCCATGGAAGTCGGAACCCGCTAAGGAGTGTGTAACAACTCA
Dai18486A

GGACGGTGGCCATGGAAGTCGGAACCCGCTAAGGAGTGTGTAACAACTCA

WEI17_024_Ceriporia_mellita --

GC1508_71Ceriporia_mellita --

GC1608_7_Ceriporia_mellita --

ZZW1557Dai27085

GGACGGTGGCCATGGAAGTCGGAACCCGCTAAGGAGTGTGTAACAACTCA

ZZW1554Dai27083

GGACGGTGGCCATGGAAGTCGGAACCCGCTAAGGAGTGTGTAACAACTCA

Dai8168

GGACGGTGGCCATGGAAGTCGGAACCCGCTAAGGAGTGTGTAACAACTCA

BR4865C_mellita --

MEL2382688Ceriporia_sp --

Dai8110

GGACGGTGGCCATGGAAGTCGGAACCCGCTAAGGAGTGTGTAACAACTCA

Cui8097 --

909Cui6740 --

W1258Dai24695

GGACGGTGGCCATGGAAGTCGGAATCCGCTAAGGAGTGTGTAACAACTCA

JV0110_26_Ceriporia_griseoviol --

896Dai13202

GGACGGTGGCCATGGAAGTCGGAACCCGCTAAGGAGTGTGTAACAACTCA

LWY393Dai27053C_griseoviolasce

GGACGGTGGCCATGGAAGTCGGAACCCGCTAAGGAGTGTGTAACAACTCA

LWY394DAI27054

GGACGGTGGCCATGGAAGTCGGAACCCGCTAAGGAGTGTGTAACAACTCA

FP135015G_pannocinctus --

L15726SpG_pannocinctus

GGACGGTGGCCATGGAAGTCGGAATCCGCTAAGGAGTGTGTAACAACTCA

Dai22221

GGACGGTGGCCATGGAAGTCGGAACCCGCTAAGGAGTGTGTAACAACTCA

Dai22633

GGACGGTGGCCATGGAAGTCGGAACCCGCTAAGGAGTGTGTAACAACTCA

Dai23260

GGACGGTGGCCATGGAAGTCGGAACCCGCTAAGGAGTGTGTAACAACTCA

Dai23626

GGACGGTGGCCATGGAAGTCGGAACCCGCTAAGGAGTGTGTAACAACTCA

Dai16238G_citrinoalbus

GGACGGTGGCCATGGAAGTCGGAACCCGCTAAGGAGTGTGTAACAACTCA

1175Dai15293

GGACGGTGGCCATGGAAGTCGGAACCCGCTAAGGAGTGTGTAACAACTCA

Dai19547

GGACGGTGGCCATGGAAGTCGGAACCCGCTAAGGAGTGTGTAACAACTCA

918063G_africanus --

918572G_africanus --

Dai18536A --
1164Cui17922
GGACGGTGGCCATGGAAGTCGGAACCCGCTAAGGAGTGTGTAACAACTCA
Dai22225
GGACGGTGGCCATGGAAGTCGGAACCCGCTAAGGAGTGTGTAACAACTCA
1163Dai20655 --CA
Yuan4397G_hainanensis
GGACGGTGGCCATGGAAGTCGGAACCCGCTAAGGAGTGTGTAACAACTCA
1176Dai15268
GGACGGTGGCCATGGAAGTCGGAACCCGCTAAGGAGTGTGTAACAACTCA
1177Dai15259
GGACGGTGGCCATGGAAGTCGGAACCCGCTAAGGAGTGTGTAACAACTCA
BZ2896G_thelephoroides --
1166JV1808_26 --
Miettinen16992Hapalopilus_ochr
GGACGGTGGCCATGGAAGTCGGAATCCGCTAAGGAGTGTGTAACAACTCA
GC1708_338_Ceriporia_arbuscula --
WCG1555Dai26107Ceriporia
GGACGGTGGCCATGGAAGTCGGAACCCGCTAAGGAGTGTGTAACAACTCA
GC1708_340_Ceriporia_arbuscula --
WCG1556Dai26109Ceriporia
GGACGGTGGCCATGGAAGTCGGAACCCGCTAAGGAGTGTGTAACAACTCA
883Cui11291
GGACGGTGGCCATGGAAGTCGGAACCCGCTAAGGAGTGTGTAACAACTCA
HLX320Dai26805
GGACGGTGGCCATGGAAGTCGGAACCCGCTAAGGAGTGTGTAACAACTCA
WCG1266Dai24678A
GGACGGTGGCCATGGAAGTCGGAACCCGCTAAGGAGTGTGTAACAACTCA
Dai6090_Ceriporia_sulphuricolo
GGACGGTGGCCATGGAAGTCGGAACCCGCTAAGGAGTGTGTAACAACTCA
RLG_11354_Ceriproia_reticulata
GGACGGTGGCCATGGAAGTCGGAACCCGCTAAGGAGTGTGTAACAACTCA
ZZW1543Dai27072
GGACGGTGGCCATGGAAGTCGGAACCCGCTAAGGAGTGTGTAACAACTCA
Li1316_Ceriporia_reticulata --
KHL11981Ceriporia_reticulata
GGACGGTGGCCATGGAAGTCGGAACCCGCTAAGGAGTGTGTAACAACTCA
FP110343sp_Candelabrochaete_la
GGACGGTGGCCATGGAAGTCGGAACCCGCTAAGGAGTGTGTAACAACTCA
Li1045_Ceriporia_reticulata --
ZX136Dai25794ceriporia
GGACGGTGGCCATGGAAGTCGGAACCCGCTAAGGAGTGTGTAACAACTCA
892Dai13400
GGACGGTGGCCATGGAAGTCGGAACCCGCTAAGGAGTGTGTAACAACTCA

RLG7163Leptoporus_mollis
GGACGGTGGCCATGGAAGTCGGAACCCGCTAAGGAGTGTGTAACAACTCA
Dai21062Leptoporus_mollis
GGACGGTGGCCATGGAAGTCGGAACCCGCTAAGGAGTGTGTAACAACTCA
Dai20182Leptoporus_submollis
GGACGGTGGCCATGGAAGTCGGAACCCGCTAAGGAGTGTGTAACAACTCA
Cui18379Leptoporus_submollis
GGACGGTGGCCATGGAAGTCGGAACCCGCTAAGGAGTGTGTAACAACTCA
Wu1209_46Resiniporus_pseudogil --
BRNM710169Resiniporus_resinasc --
Dai14516Bjerkandera_adusta
GGACGGTGGCCATGGAAGTCGGAATCCGCTAAGGAGTGTGTAACAACTCA
Dai21100Bjerkandera_fumosa
GGACGGTGGCCATGGAAGTCGGAATCCGCTAAGGAGTGTGTAACAACTCA
Miettinen16854Ceraceomyces_sp
GGACGGTGGCCATGGAAGTCGGAATCCGCTAAGGAGTGTGTAACAACTCA
Dai10477C_spissa
GGACGGTGGCCATGGAAGTCGGAACCCGCTAAGGAGTGTGTAACAACTCA
855Dai16831
GGACGGTGGCCATGGAAGTCGGAACCCGCTAAGGAGTGTGTAACAACTCA
882Cui11282
GGACGGTGGCCATGGAAGTCGGAACCCGCTAAGGAGTGTGTAACAACTCA
Dai24566
GGACGGTGGCCATGGAAGTCGGAACCCGCTAAGGAGTGTGTAACAACTCA
Yuan5965
GGACGGTGGCCATGGAAGTCGGAACCCGCTAAGGAGTGTGTAACAACTCA
Dai3204
GGACGGTGGCCATGGAAGTCGGAACCCGCTAAGGAGTGTGTAACAACTCA
1194CUI9985
GGACGGTGGCCATGGAAGTCGGAATCCGCTAAGGAGTGTGTAACAACTCA

Dai15205_Ceriporia_albomellea
CCTGCCGAATGAACTAGCCCTGAAAATGGATGGCGCTCAAGCGTGTTACC
Dai15223_Ceriporia_albomellea
CCTGCCGAATGAACTAGCCCTGAAAATGGATGGCGCTCAAGCGTGTTACC
Li1780_Ceriporia_variegata
CCTGCCGAATGAACTAGCCCTGAAAATGGATGGCGCTCAAGCGTGTTACC
Dai19791_Ceriporia_variegata
CCTGCCGAATGAACTAGCCCTGAAAATGGATGGCGCTCAAGCGTGTTACC
Dai19886
CCTGCCGAATGAACTAGCCCTGAAAATGGATGGCGCTCAAGCGTGTTACC
Dai10833_Ceriporia_crassitunic
CCTGCCGAATGAACTAGCCCTGAAAATGGATGGCGCTCAAGCGTGTTACC

CHWC1506_46Meruliopsis_crassit --
Dai9995_Ceriporia_crassitunica --
Wu1209_58_Meruliopsis_parvispo --
CHWC1505_129_Meruliopsis_parvi --
Dai21944
CCTGCCGAATGAACTAGCCCTGAAAATGGATGGCGCTCAAGCGTGTTACC
830Dai18640A
CCTGCCGAATGAACTAGCCCTGAAAATGGATGGCGCTCAAGCGTGTTACC
GC1704_60_Meruliopsis_taxicola --
Dai22625
CCTGCCGAATGAACTAGCCCTGAAAATGGATGGCGCTCAAGCGTGTTACC
Dai22636
CCTGCCGAATGAACTAGCCCTGAAAATGGATGGCGCTCAAGCGTGTTACC
Dai21878
CCTGCCGAATGAACTAGCCCTGAAAATGGATGGCGCTCAAGCGTGTTACC
1169Dai17248
CCTGCCGAATGAACTAGCCCTGAAAATGGATGGCGCTCAAGCGTGTTACC
Wu1708_43_Meruliopsis_leptocys --
Li1011
CCTGCCGAATGAACTAGCCCTGAAAATGGATGGCGCTCAAGCGTGTTACC
ZX95Dai25742Meruliopsis_leptoc
CCTGCCGAATGAACTAGCCCTGAAAATGGATGGCGCTCAAGCGTGTTACC
WCG1306Dai24733
CCTGCCGAATGAACTAGCCCTGAAAATGGATGGCGCTCAAGCGTGTTACC
LXL99Dai25816
CCTGCCGAATGAACTAGCCCTGAAAATGGATGGCGCTCAAGCGTGTTACC
WCG1559Dai26052Meruliopsis
CCTGCCGAATGAACTAGCCCTGAAAATGGATGGCGCTCAAGCGTGTTACC
He7477
CCTGCCGAATGAACTAGCCCTGAAAATGGATGGCGCTCAAGCGTGTTACC
HLX243Dai26217
CCTGCCGAATGAACTAGCCCTGAAAATGGATGGCGCTCAAGCGTGTTACC
RussiaMW673659Meruliopsis_fagi --
FD278
CCTGCCGAATGAACTAGCCCTGAAAATGGATGGCGCTCAAGCGTGTTACC
Dai10226_Ceriporia_tarda --
LE247365 CCTGCCGAATGAACTAGCCCTGAA--------------

Dai8173_Meruliopsis_nanlingens
CCTGCCGAATGAACTAGCCCTGAAAATGGATGGCGCTCAAGCGTGTTACC
860Dai17172
CCTGCCGAATGAACTAGCCCTGAAAATGGATGGCGCTCAAGCGTGTTACC
879Dai13414
CCTGCCGAATGAACTAGCCCTGAAAATGGATGGCGCTCAAGCGTGTTACC

Li_1704_Meruliopsis_pseudocyst --
833Dai18405
CCTGCCGAATGAACTAGCCCTGAAAATGGATGGCGCTCAAGCGTGTTACC
HHB_10729_Meruliopsis_albostra
CCTGCCGAATGAACTAGCCCTGAAAATGGATGGCGCTCAAGCGTGTTACC
Cui6878_Ceriporia_pseudocystid
CCTGCCGAATGAACTAGCCCTGAAAATGGATGGCGCTCAAGCGTGTTACC
869Dai14737
CCTGCCGAATGAACTAGCCCTGAAAATGGATGGCGCTCAAGCGTGTTACC
876Cui11626
CCTGCCGAATGAACTAGCCCTGAAAATGGATGGCGCTCAAGCGTGTTACC
1199WEI3388 --
776308_Meruliopsis_cystidiata --
ICN139059_Meruliopsis_cystidia --
HHB15692Ceraceomyces_serpens
CCTGCCGAATGAACTAGCCCTGAAAATGGATGGCGCTCAAGCGTGTTACC
HHB_15629_Sp_Ceriporiopsis_ane
CCTGCCGAATGAACTAGCCCTGAAAATGGATGGCGCTCAAGCGTGTTACC
AJ185Trametopsis_cervina
CCTGCCGAATGAACTAGCCCTGAAAATGGATGGCGCTCAAGCGTGTTACC
FD9Irpex_lacteus
CCTGCCGAATGAACTAGCCCTGAAAATGGATGGCGCTCAAGCGTGTTACC
908Dai11230
CCTGCCGAATGAACTAGCCCTGAAAATGGATGGCGCTCAAGCGTGTTACC
FP55521TEmmia_lacerata
CCTGCCGAATGAACTAGCCCTGAAAATGGATGGCGCTCAAGCGTGTTACC
PBU0048Ceriporia_cystidiata
CCTGCCGAATGAACTAGCCCTGAAAATGGATGGCGCTCAAGCGTGTTACC
MZ340C_laceratаT --
Dai21940
CCTGCCGAATGAACTAGCCCTGAAAATGGATGGCGCTCAAGCGTGTTACC
847Dai16433
CCTGCCGAATGAACTAGCCCTGAAAATGGATGGCGCTCAAGCGTGTTACC
MarcinEmmia_latemarginatus --
Meijer3729Hydnopolyporus_fimbr
CCTGCCGAATGAACTAGCCCTGAAAATGGATGGCGCTCAAGCGTGTTACC
RLG13408Phanerochaete_sp
CCTGCCGAATGAACTAGCCCTGAAAATGGATGGCGCTCAAGCGTGTTACC
WHC1381Flavodon_flavus --
GB1833Phlebia_albida
CCTGCCGAATGAACTAGCCCTGAAAATGGATGGCGCTCAAGCGTGTTACC
T407Phlebia_nitidula --
HHB6988Phanerochaete_exilis
CCTGCCGAATGAACTAGCCCTGAAAATGGATGGCGCTCAAGCGTGTTACC

HHB8509Phanerochaetella_xeroph
CCTGCCGAATGAACTAGCCCTGAAAATGGATGGCGCTCAAGCGTGTTACC
PBU0051Macrohyporia_dictyopora
CCTGCCGAATGAACTAGCCCTGAAAATGGATGGCGCTCAAGCGTGTTACC
HHB11463Phanerochaete_sp
CCTGCCGAATGAACTAGCCCTGAAAATGGATGGCGCTCAAGCGTGTTACC
FP102382Byssomerulius_corium
CCTGCCGAATGAACTAGCCCTGAAAATGGATGGCGCTCAAGCGTGTTACC
FP102165Efibula_americana
CCTGCCGAATGAACTAGCCCTGAAAATGGATGGCGCTCAAGCGTGTTACC
Murdoch90Ceriporia_torpida --
Rivoire4413_Ceriporia_purpurea --
Kout_18_Ceriporia_triumphalis --
Rivoire3701_Ceriporia_bresadol --
VS4018 --
Ryvarden21832_Ceriporia_manzan --
Dai24539
CCTGCCGAATGAACTAGCCCTGAAAATGGATGGCGCTCAAGCGTGTTACC
Dai24541
CCTGCCGAATGAACTAGCCCTGAAAATGGATGGCGCTCAAGCGTGTTACC
JV1105_12_Ceriporia_occidental --
VS8558Ceriporia_occidentalis --
Dai22445
CCTGCCGAATGAACTAGCCCTGAAAATGGATGGCGCTCAAGCGTGTTACC
846Dai16368
CCTGCCGAATGAACTAGCCCTGAAAATGGATGGCGCTCAAGCGTGTTACC
Dai17951_Ceriporia_aurantiocar
CCTGCCGAATGAACTAGCCCTGAAAATGGATGGCGCTCAAGCGTGTTACC
Miettinen_11701C_viridans --
JV0105_10Ceriporia_aurantiocar --
Yuan5702C_viridans --
858Dai17003 --
Yuan2747_Ceriporia_viridans --
Yuan2744C_viridans --
Li1046C_viridans --
865C_sinoviridans --
871Dai15062 --
Dai7642_Ceriporia_humilis --
Spirin4706_Ceriporia_humilis --
Spirin4944_Ceriporia_sericea --
WCG1547Dai26044ceriporia
CCTGCCGAATGAACTAGCCCTGAAAATGGATGGCGCTCAAGCGTGTTACC
ZZW1558Dai27086
CCTGCCGAATGAACTAGCCCTGAAAATGGATGGCGCTCAAGCGTGTTACC

Miettinen14381_Ceriporia_mpuri
CCTGCCGAATGAACTAGCCCTGAAAATGGATGGCGCTCAAGCGTGTTACC
Miettinen15492_2_Ceriporia_sor
CCTGCCGAATGAACTAGCCCTGAAAATGGATGGCGCTCAAGCGTGTTACC
He6687
CCTGCCGAATGAACTAGCCCTGAAAATGGATGGCGCTCAAGCGTGTTACC
ZH53Dai24426
CCTGCCGAATGAACTAGCCCTGAAAATGGATGGCGCTCAAGCGTGTTACC
Vlasak0808_30_Ceriporia_punica --
887Dai13376
CCTGCCGAATGAACTAGCCCTGAAAATGGATGGCGCTCAAGCGTGTTACC
WCG1443Dai24998
CCTGCCGAATGAACTAGCCCTGAAAATGGATGGCGCTCAAGCGTGTTACC
0108_6Ceriporia_spissa --
Dai19164
CCTGCCGAATGAACTAGCCCTGAAAATGGATGGCGCTCAAGCGTGTTACC
Dai17937_Ceriporia_bubalinomar
CCTGCCGAATGAACTAGCCCTGAAAATGGATGGCGCTCAAGCGTGTTACC
903Dai12113
CCTGCCGAATGAACTAGCCCTGAAAATGGATGGCGCTCAAGCGTGTTACC
LZB929Dai25079
CCTGCCGAATGAACTAGCCCTGAAAATGGATGGCGCTCAAGCGTGTTACC
LX45Dai26988 --
LX43Dai26986
CCTGCCGAATGAACTAGCCCTGAAAATGGATGGCGCTCAAGCGTGTTACC
Dai7759Ceriporia --
Cui8012_Ceriporia_viridans --
GC1704_54Ceriporia_viridans --
Dai23392
CCTGCCGAATGAACTAGCCCTGAAAATGGATGGCGCTCAAGCGTGTTACC
WCG1585Dai26113Ceriproia
CCTGCCGAATGAACTAGCCCTGAAAATGGATGGCGCTCAAGCGTGTTACC
Dai18675C_eucalypti
CCTGCCGAATGAACTAGCCCTGAAAATGGATGGCGCTCAAGCGTGTTACC
Dai22034
CCTGCCGAATGAACTAGCCCTGAAAATGGATGGCGCTCAAGCGTGTTACC
JV1008_41JTardaFLORIDAKeys --
Rivoire1161_Ceriporia_pierii --
Dai23499C_pierii
CCTGCCGAATGAACTAGCCCTGAAAATGGATGGCGCTCAAGCGTGTTACC
Dai23500
CCTGCCGAATGAACTAGCCCTGAAAATGGATGGCGCTCAAGCGTGTTACC
841Dai15899
CCTGCCGAATGAACTAGCCCTGAAAATGGATGGCGCTCAAGCGTGTTACC

842Dai15904 ---
LZB1066xinjiang ---
LZB1065xinjiang ---
851Dai16779
CCTGCCGAATGAACTAGCCCTGAAAATGGATGGCGCTCAAGCGTGTTACC
RMJ119sp_Candelabrochaete_sept ---
RLG9759spCandelabrochaete_sept ---
RLG10478Phanerochaete_allantos ---
Dai19118_Ceriporia_spissa
CCTGCCGAATGAACTAGCCCTGAAAATGGATGGCGCTCAAGCGTGTTACC
Dai18486A
CCTGCCGAATGAACTAGCCCTGAAAATGGATGGCGCTCAAGCGTGTTACC
WEI17_024_Ceriporia_mellita ---
GC1508_71Ceriporia_mellita ---
GC1608_7_Ceriporia_mellita ---
ZZW1557Dai27085
CCTGCCGAATGAACTAGCCCTGAAAATGGATGGCGCTCAAGCGTGTTACC
ZZW1554Dai27083
CCTGCCGAATGAACTAGCCCTGAAAATGGATGGCGCTCAAGCGTGTTACC
Dai8168
CCTGCCGAATGAACTAGCCCTGAAAATGGATGGCGCTCAAGCGTGTTACC
BR4865C_mellita ---
MEL2382688Ceriporia_sp ---
Dai8110
CCTGCCGAATGAACTAGCCCTGAAAATGGATGGCGCTCAAGCGTGTTACC
Cui8097 ---
909Cui6740 ---
W1258Dai24695
CCTGCCGAATGAACTAGCCCTGAAAATGGATGGCGCTCAAGCGTGTTACC
JV0110_26_Ceriporia_griseoviol ---
896Dai13202
CCTGCCGAATGAACTAGCCCTGAAAATGGATGGCGCTCAAGCGTGTTACC
LWY393Dai27053C_griseoviolasce
CCTGCCGAATGAACTAGCCCTGAAAATGGATGGCGCTCAAGCGTGTTACC
LWY394DAI27054
CCTGCCGAATGAACTAGCCCTGAAAATGGATGGCGCTCAAGCGTGTTACC
FP135015G_pannocinctus ---
L15726SpG_pannocinctus
CCTGCCGAATGAACTAGCCCTGAAAATGGATGGCGCTCAAGCGTGTTACC
Dai22221
CCTGCCGAATGAACTAGCCCTGAAAATGGATGGCGCTCAAGCGTGTTACC
Dai22633
CCTGCCGAATGAACTAGCCCTGAAAATGGATGGCGCTCAAGCGTGTTACC
Dai23260

CCTGCCGAATGAACTAGCCCTGAAAATGGATGGCGCTCAAGCGTGTTACC
Dai23626
CCTGCCGAATGAACTAGCCCTGAAAATGGATGGCGCTCAAGCGTGTTACC
Dai16238G_citrinoalbus
CCTGCCGAATGAACTAGCCCTGAAAATGGATGGCGCTCAAGCGTGTTACC
1175Dai15293
CCTGCCGAATGAACTAGCCCTGAAAATGGATGGCGCTCAAGCGTGTTACC
Dai19547
CCTGCCGAATGAACTAGCCCTGAAAATGGATGGCGCTCAAGCGTGTTACC
918063G_africanus --
918572G_africanus --
Dai18536A --
1164Cui17922
CCTGCCGAATGAACTAGCCCTGAAAATGGATGGCGCTCAAGCGTGTTACC
Dai22225
CCTGCCGAATGAACTAGCCCTGAAAATGGATGGCGCTCAAGCGTGTTACC
1163Dai20655 CCT---
Yuan4397G_hainanensis
CCTGCCGAATGAACTAGCCCTGAAAATGGATGGCGCTCAAGCGTGTTACC
1176Dai15268
CCTGCCGAATGAACTAGCCCTGAAAATGGATGGCGCTCAAGCGTGTTACC
1177Dai15259
CCTGCCGAATGAACTAGCCCTGAAAATGGATGGCGCTCAAGCGTGTTACC
BZ2896G_thelephoroides --
1166JV1808_26 --
Miettinen16992Hapalopilus_ochr
CCTGCCGAATGAACTAGCCCTGAAAATGGATGGCGCTCAAGCGTGTTACC
GC1708_338_Ceriporia_arbuscula CCTGC---
WCG1555Dai26107Ceriporia
CCTGCCGAATGAACTAGCCCTGAAAATGGATGGCGCTCAAGCGTGTTACC
GC1708_340_Ceriporia_arbuscula --
WCG1556Dai26109Ceriporia
CCTGCCGAATGAACTAGCCCTGAAAATGGATGGCGCTCAAGCGTGTTACC
883Cui11291
CCTGCCGAATGAACTAGCCCTGAAAATGGATGGCGCTCAAGCGTGTTACC
HLX320Dai26805
CCTGCCGAATGAACTAGCCCTGAAAATGGATGGCGCTCAAGCGTGTTACC
WCG1266Dai24678A
CCTGCCGAATGAACTAGCCCTGAAAATGGATGGCGCTCAAGCGTGTTACC
Dai6090_Ceriporia_sulphuricolo
CCTGCCGAATGAACTAGCCCTGAAAATGGATGGCGCTCAAGCGTGTTACC
RLG_11354_Ceriproia_reticulata
CCTGCCGAATGAACTAGCCCTGAAAATGGATGGCGCTCAAGCGTGTTACC
ZZW1543Dai27072

CCTGCCGAATGAACTAGCCCTGAAAATGGATGGCGCTCAAGCGTGTTACC

Li1316_Ceriporia_reticulata --

KHL11981Ceriporia_reticulata

CCTGCCGAATGAACTAGCCCTGAAAATGGATGGCGCTCAAGCGTGTTACC

FP110343sp_Candelabrochaete_la

CCTGCCGAATGAACTAGCCCTGAAAATGGATGGCGCTCAAGCGTGTTACC

Li1045_Ceriporia_reticulata --

ZX136Dai25794ceriporia

CCTGCCGAATGAACTAGCCCTGAAAATGGATGGCGCTCAAGCGTGTTACC

892Dai13400

CCTGCCGAATGAACTAGCCCTGAAAATGGATGGCGCTCAAGCGTGTTACC

RLG7163Leptoporus_mollis

CCTGCCGAATGAACTAGCCCTGAAAATGGATGGCGCTCAAGCGTGTTACC

Dai21062Leptoporus_mollis

CCTGCCGAATGAACTAGCCCTGAAAATGGATGGCGCTCAAGCGTGTTACC

Dai20182Leptoporus_submollis

CCTGCCGAATGAACTAGCCCTGAAAATGGATGGCGCTCAAGCGTGTTACC

Cui18379Leptoporus_submollis

CCTGCCGAATGAACTAGCCCTGAAAATGGATGGCGCTCAAGCGTGTTACC

Wu1209_46Resiniporus_pseudogil --

BRNM710169Resiniporus_resinasc --

Dai14516Bjerkandera_adusta

CCTGCCGAATGAACTAGCCCTGAAAATGGATGGCGCTCAAGCGTGTTACC

Dai21100Bjerkandera_fumosa

CCTGCCGAATGAACTAGCCCTGAAAATGGATGGCGCTCAAGCGTGTTACC

Miettinen16854Ceraceomyces_sp

CCTGCCGAATGAACTAGCCCTGAAAATGGATGGCGCTCAAGCGTGTTACC

Dai10477C_spissa

CCTGCCGAATGAACTAGCCCTGAAAATGGATGGCGCTCAAGCGTGTTACC

855Dai16831

CCTGCCGAATGAACTAGCCCTGAAAATGGATGGCGCTCAAGCGTGTTACC

882Cui11282

CCTGCCGAATGAACTAGCCCTGAAAATGGATGGCGCTCAAGCGTGTTACC

Dai24566

CCTGCCGAATGAACTAGCCCTGAAAATGGATGGCGCTCAAGCGTGTTACC

Yuan5965

CCTGCCGAATGAACTAGCCCTGAAAATGGATGGCGCTCAAGCGTGTTACC

Dai3204

CCTGCCGAATGAACTAGCCCTGAAAATGGATGGCGCTCAAGCGTGTTACC

1194CUI9985

CCTGCCGAATGAACTAGCCCTGAAAATGGATGGCGCTCAAGCGTGTTACC

Dai15205_Ceriporia_albomellea CATACCTTGCCGT-

CAGTGTTGAAGTGACGCACTG-ACGAGTAGGCAGGC

Dai15223_Ceriporia_albomellea CATACCTTGCCGT-CAGTGTTGAAGTGACGCACTG-ACGAGTAGGCAGGC

Li1780_Ceriporia_variegata CATACCTTGCCGT-CAGTGTTGAAGTGACGCATTG-ACGAGTAGGCAGGC

Dai19791_Ceriporia_variegata CATACCTTGCCGT-CAGTGTTGAAGTGACGCATTG-ACGAGTAGGCAGGC

Dai19886 CATACCTTGCCGT-CAGTGTTGAAGTGACGCACTG-ACGAGTAGGCAGGC

Dai10833_Ceriporia_crassitunic CATACCTTGCCGT-CAGTGTTGAAGTGATGCACTG-ACGAGTAGGCAGGC

CHWC1506_46Meruliopsis_crassit ---

Dai9995_Ceriporia_crassitunica ---

Wu1209_58_Meruliopsis_parvispo ---

CHWC1505_129_Meruliopsis_parvi ---

Dai21944 CATACCTCGCCGT-CAGTGTTGAAGTGAAGCATTG-ACGAGTAGGCAGGC

830Dai18640A CATACCTCGCCGT-CAGTGTTGAAGTGAAGCACTG-ACGAGTAGGCAGGC

GC1704_60_Meruliopsis_taxicola ---

Dai22625 CATACCTCGCCGT-CAGTGTTTAAGTGATGCATTG-ACGAGTAGGCAGGC

Dai22636 CATACCTCGCCGT-CAGTGTTTAAGTGATGCATTG-ACGAGTAGGCAGGC

Dai21878 CATACCTCGCCGT-CAGTGTTTAAGTGATGCATTG-ACGAGTAGGCAGGC

1169Dai17248 CATACCTCGCCGT-CAGTGTTTAAGTGATGCATTG-ACGAGTAGGCAGGC

Wu1708_43_Meruliopsis_leptocys ---

Li1011 CATACCTCACCGT-CAGTGTTTAAGTGATGCACTG-ACGAGTAGGCAGGC

ZX95Dai25742Meruliopsis_leptoc CATACCTCGCCGT-CAGTGTTTAAGTGATGCACTG-ACGAGTAGGCAGGC

WCG1306Dai24733 CATACCTCGCCGT-CAGTGTTTAAGTGATGCACTG-ACGAGTAGGCAGGC

LXL99Dai25816 CATACCTCGCCGT-CAGTGTTTAAGTGATGCACTG-ACGAGTAGGCAGGC

WCG1559Dai26052Meruliopsis CATACCTCACCGT-CAGTGTTTAAGTGATGCACTG-ACGAGTAGGCAGGC

He7477 CATACCTCACCGT-CAGTGTTTAAGTGATGCACTG-ACGAGTAGGCAGGC

HLX243Dai26217 CATACCTCACCGT-CAGTGTTTAAGTGATGCACTG-ACGAGTAGGCAGGC

RussiaMW673659Meruliopsis_fagi ---

FD278 CATACCTCGCCGT-CAGTGTTGAAGTGAAGCATTG-ACGAGTAGGCAGGC

Dai10226_Ceriporia_tarda ---

LE247365 ---

Dai8173_Meruliopsis_nanlingens CATACCTCGCCGT-CAGTGTTTAAGTGATGCACTG-ACGAGTAGGCAGGC

860Dai17172 CATACCTCGCCGT-CAGTGTTTAAGTGATGCACTG-ACGAGTAGGCAGGC

879Dai13414 CATACCTCGCCGT-CAGTGTTTAAGTGATGCACTG-ACGAGTAGGCAGGC

Li_1704_Meruliopsis_pseudocyst ---

833Dai18405 CATACCTCGCCGT-CAGTGTTGAAGTGACGCACTG-ACGAGTAGGCAGGC

HHB_10729_Meruliopsis_albostra CATACCTCGCCGT-CAGTGTTGAAGTGACGCACTG-ACGAGTAGGCAGGC

Cui6878_Ceriporia_pseudocystid CATACCTCGCCGT-CAGTGTTGAAGTGATACATTG-ACGAGTAGGCAGGC

869Dai14737 CATACCTCGCCGT-CAGTGTTGAAGTGATACATTG-ACGAGTAGGCAGGC

876Cui11626 CATACCTCGCCGT-CAGTGTTGAAGTGATACATTG-ACGAGTAGGCAGGC

1199WEI3388 ---

776308_Meruliopsis_cystidiata ---

ICN139059_Meruliopsis_cystidia ---

HHB15692Ceraceomyces_serpens CATACCTCGCCGT-CA-TGTTGAAGTGACGCACTG-ACGAGTAGGCAGGC

HHB_15629_Sp_Ceriporiopsis_ane CATACCTCACCGT-CAGTGTTAAAGTGATGCACTG-ACGAGTAGGCAGGC

AJ185Trametopsis_cervina CATACCTCACCGT-CAGTGTTTAAGTGATGCACTG-ACGAGTAGGCAGGC

FD9Irpex_lacteus CATACCTCACCGT-CAGTGTTTAAGTGATACACTG-ACGAGTAGGCAGGC

908Dai11230 CATACCTCACCGT-CAGTGTTTAAGTGATACACTG-ACGAGTAGGCAGGC

FP55521TEmmia_lacerata CATACCTCACCGT-CAGTGTTTAAGTGAAGCATTG-ACGAGTAGGCAGGC

PBU0048Ceriporia_cystidiata CATACCTCACCGT-CAGTGTTTAAGTGAAGCATTG-ACGAGTAGGCAGGC

MZ340C_lacerataT ---

Dai21940 CATACCTCACCGT-CAGTGTTTAAGTGAAGCATTG-ACGAGTAGGCAGGC

847Dai16433 CATACCTCACCGT-CAGTGTTTAAGTGAAGCATTG-ACGAGTAGGCAGGC

MarcinEmmia_latemarginatus ---

Meijer3729Hydnopolyporus_fimbr CATACCTCACCGT-CAGTGTT-AAGTGAAGCACTG-ACGAGTAGGCAGGC

RLG13408Phanerochaete_sp CATACCT--

WHC1381Flavodon_flavus --

GB1833Phlebia_albida CATACCTCGCCGT-CAGTGTTTAAGTGATGCACTG-ACGAGTAGGCAGGC

T407Phlebia_nitidula --

HHB6988Phanerochaete_exilis CATACCTCGCCGT-CAGTGTTTAAGTGATGCACTG-ACGAGTAGGCAGGC

HHB8509Phanerochaetella_xeroph CATACCTCGCCGT-CAGTGTTTAAGTGATGCACTG-ACGAGTAGGCAGGC

PBU0051Macrohyporia_dictyopora CATACCTCGCCGTACAGTGTTTAAGTGATGCACTG-ACGAGTAGGCAGGC

HHB11463Phanerochaete_sp CATACCTCGCCGT-CAGTGTTTAAGTGATGCACTG-ACGAGTAGGCAGGC

FP102382Byssomerulius_corium CATACCTCGCCGT-CAGTGTTTAAGTGATGCACTG-ACGAGTAGGCAGGC

FP102165Efibula_americana CATACCTCGCCGT-CAGTGTTGAAGTGACGCACTG-ACGAGTAGGCAGGC

Murdoch90Ceriporia_torpida --

Rivoire4413_Ceriporia_purpurea --

Kout_18_Ceriporia_triumphalis --

Rivoire3701_Ceriporia_bresadol --

VS4018 --

Ryvarden21832_Ceriporia_manzan --

Dai24539 CATACCTCGCCGT-CAGTGTTAAAGTGATGCACTG-ACGAGTAGGCAGGC

Dai24541 CATACCTCGCCGT-CAGTGTTAAAGTGATGCACTG-ACGAGTAGGCAGGC

JV1105_12_Ceriporia_occidental --

VS8558Ceriporia_occidentalis --

Dai22445 CATACCTCGCCGT-CAGTGTTAAAGTGATGCACTG-ACGAGTAGGCAGGC

846Dai16368 CATACCTCGCCGT-CAGTGTTAAAGTGATGCACTG-ACGAGTAGGCAGGC

Dai17951_Ceriporia_aurantiocar CATACCTCGCCGT-CAGTGTTTAAGTGACACACTG-ACGAGTAGGCAGGC

Miettinen_11701C_viridans --

JV0105_10Ceriporia_aurantiocar --

Yuan5702C_viridans --

858Dai17003 --

Yuan2747_Ceriporia_viridans --

Yuan2744C_viridans --

Li1046C_viridans --

865C_sinoviridans ---
871Dai15062 ---
Dai7642_Ceriporia_humilis ---
Spirin4706_Ceriporia_humilis ---
Spirin4944_Ceriporia_sericea ---
WCG1547Dai26044ceriporia CATACCTCGCCGT-CAGTGTTTAAGTGACGCACTG-ACGAGTAGGCAGGC
ZZW1558Dai27086 CATACCTCGCCGT-CAGTGTTTAAGTGACGCACTG-ACGAGTAGGCAGGC
Miettinen14381_Ceriporia_mpuri CATACCTCGCCGT-CAGTGTTTAAGTGACGCACTG-ACGAGTAGGCAGGC
Miettinen15492_2_Ceriporia_sor CATACCTCGCCGT-CAGTGTTTAAGTGACGCACTG-ACGAGTAGGCAGGC
He6687 CATACCTCGCCGT-CAGTGTTTAAGTGACGCACTG-ACGAGTAGGCAGGC
ZH53Dai24426 CATACCTCGCCGT-CAGTGTTTAAGTGACGCACTG-ACGAGTAGGCAGGC
Vlasak0808_30_Ceriporia_punica ---
887Dai13376 CATACCTCGCCGT-CAGTGTTTAAGTGATGCACTG-ACGAGTAGGCAGGC
WCG1443Dai24998 CATACCTCGCCGT-CAGTGTTTAAGTGATGCACTG-ACGAGTAGGCAGGC
0108_6Ceriporia_spissa ---
Dai19164 CATACCTCACCGT-CAGTGTTAAAGTGATGCACTG-ACGAGTAGGCAGGC
Dai17937_Ceriporia_bubalinomar CATACCTCACCGT-CAGTGTTAAAGTGATGCACTG-ACGAGTAGGCAGGC
903Dai12113 CATACCTCACCGT-CAGTGGTAAAGTGATGCACTG-ACGAGTAGGCAGGC
LZB929Dai25079 CATACCTCGCCGT-CAGTGTTTAAGTGATGCACTG-ACGAGTAGGCAGGC
LX45Dai26988 ---
LX43Dai26986 CATACCTCGCCGT-CAGTGTTTAAGTGATGCACTG-ACGAGTAGGCAGGC
Dai7759Ceriporia ---
Cui8012_Ceriporia_viridans ---
GC1704_54Ceriporia_viridans ---
Dai23392 CATACCTTGCCGT-CAGTGTTTAAGTGATGCACTG-ACGAGTAGGCAGGC
WCG1585Dai26113Ceriproia CATACCTTGCCGT-CAGTGTTTAAGTGATGCACTG-ACGAGTAGGCAGGC
Dai18675C_eucalypti CATACCTTGCCGT-CAGTGTTTAAGTGATGCACTG-ACGAGTAGGCAGGC
Dai22034 CATACCTCGCCGT-

CAGTGTTTAAGTGATGCACTG-ACGAGTAGGCAGGC
JV1008_41JTardaFLORIDAKeys ---
Rivoire1161_Ceriporia_pierii ---
Dai23499C_pierii CATACCTCGCCGT-
CAGTGTTTAAGTGATGCATTG-ACGAGTAGGCAGGC
Dai23500 CATACCTCGCCGT-
CAGTGTTTAAGTGATGCATTG-ACGAGTAGGCAGGC
841Dai15899 CATACCTCGCCGT-
CAGTGTTTAAGTGACGCACTG-ACGAGTAGGCAGGC
842Dai15904 ---
LZB1066xinjiang ---
LZB1065xinjiang ---
851Dai16779 CATACCTCGCCGT-
CAGTGTTTAAGTGACGCACTG-ACGAGTAGGCAGGC
RMJ119sp_Candelabrochaete_sept ---
RLG9759spCandelabrochaete_sept ---
RLG10478Phanerochaete_allantos ---
Dai19118_Ceriporia_spissa CATACCTCGCCGT-
CAGTGTTGAAGTGACGCACTG-ACGAGTAGGCAGGC
Dai18486A CATACCTCGCCGT-
CAGTGTTGAAGTGACGCACTG-ACGAGTAGGCAGGC
WEI17_024_Ceriporia_mellita ---
GC1508_71Ceriporia_mellita ---
GC1608_7_Ceriporia_mellita ---
ZZW1557Dai27085 CATACCTCGCCGT-
CAGTGTTGAAGTGACGCACTG-ACGAGTAGGCAGGC
ZZW1554Dai27083 CATACCTCGCCGT-
CAGTGTTGAAGTGACGCACTG-ACGAGTAGGCAGGC
Dai8168 CATACCTCGCCGT-
CAGTGTTGAAGTGACGCACTG-ACGAGTAGGCAGGC
BR4865C_mellita ---
MEL2382688Ceriporia_sp ---
Dai8110 CATACCTCGCCGT-
CAGTGTTGAAGTGAAGCACTG-ACGAGTAGGCAGGC
Cui8097 ---
909Cui6740 ---
W1258Dai24695 CATACCTCGCTGT-
CAGTGTTGAAGTGATGCACTG-ACGAGTAGGCAGGC
JV0110_26_Ceriporia_griseoviol ---
896Dai13202 CATACCTCGCCGT-
CAGTGTTTTAGTGACGCACTG-ACGAGTAGGCAGGC
LWY393Dai27053C_griseoviolasce CATACCTCGCCGT-
CAGTGTTTTAGTGACGCACTG-ACGAGTAGGCAGGC
LWY394DAI27054 CATACCTCGCCGT-

CAGTGTTTAGTGACGCACTG-ACGAGTAGGCAGGC
FP135015G_pannocinctus --
L15726SpG_pannocinctus CATACCTCACCGT-
CAGTGTTTAAGTGATGCACTG-ACGAGTAGGCAGGC
Dai22221 CATACCTCACCGT-
CAGTGTTCAAGTGATGCATTG-ACGAGTAGGCAGGC
Dai22633 CATACCTCACCGT-
CAGTGTTTAAGTGATGCATTG-ACGAGTAGGCAGGC
Dai23260 CATACCTCACCGT-
CAGTGTTTAAGTGATGCATTG-ACGAGTAGGCAGGC
Dai23626 CATACCTCACCGT-C----------------------------------
Dai16238G_citrinoalbus CATACCTCACCGT-
CAGTGTTTAAGTGATGCATTG-ACGAGTAGGCAGGC
1175Dai15293 CATACCTCACCGT-
CAGTGTTTAAGTGATGCATTG-ACGAGTAGGCAGGC
Dai19547 CATACCTCACCGT-
CAGTGTTTAAGTGATGCATTG-ACGAGTAGGCAGGC
918063G_africanus --
918572G_africanus --
Dai18536A --
1164Cui17922 CATACCTCACCGT-CAGTGTTTAAGTGATGCA--

Dai22225 CATACCTCACCGT-
CAGTGTTTAAGTGATGCATTG-ACGAGTAGGCAGGC
1163Dai20655 --
Yuan4397G_hainanensis CATACCTCACCGT-
CAGTGTTTAAGTGATGCATTG-ACGAGTAGGCAGGC
1176Dai15268 CATACCTCACCGT-
CAGTGTTTAAGTGATGCATTG-ACGAGTAGGCAGGC
1177Dai15259 CATACCTCACCGT-
CAGTGTTTAAGTGATGCATTG-ACGAGTAGGCAGGC
BZ2896G_thelephoroides --
1166JV1808_26 --
Miettinen16992Hapalopilus_ochr CATACCTTGCCGT-
CAGTGTTTAAGTGATGCACTG-ACGAGTAGGCAGGC
GC1708_338_Ceriporia_arbuscula --
WCG1555Dai26107Ceriporia CATACCTTGCCGT-
CAGTGTTAAAGTGATACATTG-ACGAGTAGGCAGGC
GC1708_340_Ceriporia_arbuscula --
WCG1556Dai26109Ceriporia CATACCTTGCCGT-
CAGTGTTAAAGTGATACATTG-ACGAGTAGGCAGGC
883Cui11291 CATACCTTGCCGT-
CAGTGTTAAAGTGATGCATTG-ACGAGTAGGCAGGC
HLX320Dai26805 CATACCTTGCCGT-

CAGTGTTAAAGTGATACATTG-ACGAGTAGGCAGGC

WCG1266Dai24678A CATACCTTGCCGT-
CAGTGTTAAAGTGATACATTG-ACGAGTAGGCAGGC

Dai6090_Ceriporia_sulphuricolo CATACCTCGCCGT-
CAGTGTTGAAGTGACGCACTG-ACGAGTAGGCAGGC

RLG_11354_Ceriproia_reticulata CATACCTCGCCGT-
CAGTGTTAAAGTGATGCACTG-ACGAGTAGGCAGGC

ZZW1543Dai27072 CATACCTCGCCGT-
CAGTGTTAAAGTGATGCACTG-ACGAGTAGGCAGGC

Li1316_Ceriporia_reticulata --

KHL11981Ceriporia_reticulata GATACCTCGCCGT-
CAGTGTTTAAGTGATACACTG-ACGAGTAGGCAGGC

FP110343sp_Candelabrochaete_la CATACCTCACCGT-
CAGTGTTTAAGTGATGCACTG-ACGAGTAGGCAGGC

Li1045_Ceriporia_reticulata --

ZX136Dai25794ceriporia CATACCTCACCGT-
CAGTGTTTAAGTGAAGCACTGCACGAGTAGGCAGGC

892Dai13400 CATACCTCACCGT-
CAGTGTTTAAGTGAAGCACTG-ACGAGTAGGCAGGC

RLG7163Leptoporus_mollis CATACCTCGCCGT-
CAGTGTTTAAGTGATGCACTG-ACGAGTAGGCAGGC

Dai21062Leptoporus_mollis CATACCTCGCCGT-
CAGTGTTTAAGTGATGCACTG-ACGAGTAGGCAGGC

Dai20182Leptoporus_submollis CATACCTCGCCGT-
CAGTGTTTAAGTGATGCACTG-ACGAGTAGGCAGGC

Cui18379Leptoporus_submollis CATACCTCGCCGT-
CAGTGTTTAAGTGATGCACTG-ACGAGTAGGCAGGC

Wu1209_46Resiniporus_pseudogil --

BRNM710169Resiniporus_resinasc --

Dai14516Bjerkandera_adusta CATACCTCGCCGT-
CAGCGTTGAAGTGACGCGTTG-ACGAGTAGGCAGGC

Dai21100Bjerkandera_fumosa CATACCTCGCCGT-
CAGCGTTGAAGTGACGCGTTG-ACGAGTAGGCAGGC

Miettinen16854Ceraceomyces_sp CATACCTCGCCGT-
CAGTGTTTAAGTGACGCACTG-ACGAGTAGGCAGGC

Dai10477C_spissa CATACCTCACCGT-
CAGTGTTTAAGTGATGCACTG-ACGAGTAGGCAGGC

855Dai16831 CATACCTCACCGT-
CAGTGTTTAAGTGATGCACTG-ACGAGTAGGCAGGC

882Cui11282 CATACCTCACCGT-
CAGTGTTTAAGTGATGCACTG-ACGAGTAGGCAGGC

Dai24566 CATACCTCACCGT-
CAGTGTTTAAGTGATGCACTG-ACGAGTAGGCAGGC

Yuan5965 CATACCTCACCGT-

CAGTGTTTAAGTGATGCACTG-ACGAGTAGGCAGGC

Dai3204 CATACCTCGCCGT-CAGTGTTGAAGTGACGCACTG-ACGAGTAGGCAGGC

1194CUI9985 CATACCTCACCGT-CAGTGTTTAAGTGATGCACTG-ACGAGTAGGCAGGC

Dai15205_Ceriporia_albomellea GTGGAGGTTTGT------------------------------------

Dai15223_Ceriporia_albomellea GTGGAGGTTTGT------------------------------------

Li1780_Ceriporia_variegata GTGGAGGTTTGT------------------------------------

Dai19791_Ceriporia_variegata GTGGAGGTTTGT------------------------------------

Dai19886 GTGGAGGTTTGTCCTTATCATTGCTGGTGGTACTGGTGAGTTCGAGGCTG

Dai10833_Ceriporia_crassitunic GTGGAGGTTTGT------------------------------------

CHWC1506_46Meruliopsis_crassit --

Dai9995_Ceriporia_crassitunica --

Wu1209_58_Meruliopsis_parvispo --

CHWC1505_129_Meruliopsis_parvi --

Dai21944 GTGGAGGT----CCTTATCATTGCCGGCGGTACTGGTGAATTCGAGGCTG

830Dai18640A GTGGAGGT----CCTTATCATTGCCGGCGGTACTGGTGAGTTCGAGGCTG

GC1704_60_Meruliopsis_taxicola --

Dai22625 GTGGAGGTTTGTCCTTATCATTGCCGGCGGTACTGGTGAGTTCGAGGCTG

Dai22636 GTGGAGGTTTGTCCTTATCATTGCCGGCGGTACTGGTGAGTTCGAGGCTG

Dai21878 GTGGAGG-----CCTTATCATTGCCGGCGGTACTGGTGAGTTCGAGGCTG

1169Dai17248 GTGGAGGTTTGTCCTTATCATTGCCGGCGGTACTGGTGAGTTCGAGGCTG

Wu1708_43_Meruliopsis_leptocys --

Li1011 GTGGAGGTTTGT------------------------------------

ZX95Dai25742Meruliopsis_leptoc GTGGAGGTTTGT------------------------------------

WCG1306Dai24733 GTGGAGGTTTGTCCTTATCATTGCCGGCGGTACTGGTGAGTTTGAGGCTG

LXL99Dai25816 GTGGAGGTTTGTCCTTATCATTGCTGGCGGTACTGGTGAGTTTGAGGCTG

WCG1559Dai26052Meruliopsis GTGGAGGTTTGTCCTTATCATTGCCGGGGGCACTGGTGAGTTCGAGGCTG

He7477 GTGGAGGTTTGT------------------------------------

HLX243Dai26217 GTGGAGGTTTGTCCTTATCATTGCCGGGGGCACTGGTGAGTTCGAGGCTG

RussiaMW673659Meruliopsis_fagi --

FD278 GTGGAGGTTTGT-------------------------------------
Dai10226_Ceriporia_tarda ---
LE247365 ---
Dai8173_Meruliopsis_nanlingens GTGGAGGTTTGT-------------------------------------
860Dai17172
GTGGAGGTTTGTCCTTATCATTGCCGGCGGTACTGGTGAGTTCGAGGCTG
879Dai13414 GTGGAGGT----
CCTTATCATTGCCGGTGGTACTGGTGAGTTCGAGGCTG
Li_1704_Meruliopsis_pseudocyst ---
833Dai18405 GTGGAGGT----
CCTCATCATTGCCGGCGGTACCGGTGAGTTCGAGGCTG
HHB_10729_Meruliopsis_albostra GTGGAGGTTTGT-------------------------------------
Cui6878_Ceriporia_pseudocystid GTGGAGGTTTGT-------------------------------------
869Dai14737 GTGGAGGT----
CCTCATCATCGCTGCCGGTACTGGTGAGTTCGAGGCCG
876Cui11626 GTGGAGGT---
1199WEI3388 ---
776308_Meruliopsis_cystidiata ---
ICN139059_Meruliopsis_cystidia ---
HHB15692Ceraceomyces_serpens GTGGAGGTTTGT-------------------------------------
HHB_15629_Sp_Ceriporiopsis_ane GTGGAGGTTTGT-------------------------------------
AJ185Trametopsis_cervina GTGGAGGTTTGT-------------------------------------
FD9Irpex_lacteus GTGGAGGTTTGT-------------------------------------
908Dai11230
GTGGAGGTTTGTCCTCATCATCGCCGGTGGTACCGGTGAGTTCGAGGCTG
FP55521TEmmia_lacerata GTGGAGGTTTGT-------------------------------------
PBU0048Ceriporia_cystidiata GTGGAGGTTTGT-------------------------------------
MZ340C_lacerataT ---
Dai21940 GTGGAGG-----
CCTCATCATCGCTGGTGGTACTGGTGAGTTCGAGGCCG
847Dai16433
GTGGAGGTTTGTCCTCATCATCGCCGGTGGTACTGGTGAGTTCGAGGCCG
MarcinEmmia_latemarginatus ---
Meijer3729Hydnopolyporus_fimbr GTGAGGTTGT---------------------------------------
RLG13408Phanerochaete_sp ---
WHC1381Flavodon_flavus ---
GB1833Phlebia_albida
GTGGAGGTTTGTCCTCATCATTGCGGGTGGTACTGGTGAGTTCGAGGCCG
T407Phlebia_nitidula ------------
CCTCATCATTGCGGGTGGTACTGGTGAGTTCGAGGCCG
HHB6988Phanerochaete_exilis GTGGAGGTTTGT-------------------------------------
HHB8509Phanerochaetella_xeroph GTGGAGGTTTGT-------------------------------------
PBU0051Macrohyporia_dictyopora GTGGAGGTTTGT-------------------------------------
HHB11463Phanerochaete_sp GTGGAGGTTTGT-------------------------------------

FP102382Byssomerulius_corium GTGGAGGTTTG-------------------------------------
FP102165Efibula_americana
GTGGAGGTTTGTCCTCATCATCGCCGGTGGTACTGGTGAGTTCGAGGCTG
Murdoch90Ceriporia_torpida --
Rivoire4413_Ceriporia_purpurea --
Kout_18_Ceriporia_triumphalis --
Rivoire3701_Ceriporia_bresadol --
VS4018 --
Ryvarden21832_Ceriporia_manzan --
Dai24539 GTGGAGGTTTG-
CCTCATCATCGCCTCTGGTACTGGTGAGTTCGAGGCCG
Dai24541 GTGGAGGTTTG-
CCTCATCATCGCCTCTGGTACTGGTGAGTTCGAGGCCG
JV1105_12_Ceriporia_occidental --
VS8558Ceriporia_occidentalis --
Dai22445
GTGGAGGTTTGTTCTCATCATCGCCTCTGGTACTGGTGAGTTCGAGGCCG
846Dai16368
GTGGAGGTTTGTTCTCATCATCGCCTCCGGTACTGGTGAGTTCGAGGCCG
Dai17951_Ceriporia_aurantiocar GTGGAGGTTTGT-------------------------------------
Miettinen_11701C_viridans --
JV0105_10Ceriporia_aurantiocar --
Yuan5702C_viridans --
858Dai17003 --
Yuan2747_Ceriporia_viridans --
Yuan2744C_viridans --
Li1046C_viridans --
865C_sinoviridans --
871Dai15062 --
Dai7642_Ceriporia_humilis -----------
CCTCATCATTGCCTCCGGCACTGGTGAGTTCGAAGCTG
Spirin4706_Ceriporia_humilis --
Spirin4944_Ceriporia_sericea --
WCG1547Dai26044ceriporia GTGGAGGTTTGT-------------------------------------
ZZW1558Dai27086
GTGGAGGTTTGTCTCAGTCATTGACTCTGGAACTGGTGAGTTCGAAGCCG
Miettinen14381_Ceriporia_mpuri GTGGAGGTTTGT-------------------------------------
Miettinen15492_2_Ceriporia_sor GTGGAGGTTTGT-------------------------------------
He6687
GTGGAGGTTTGTCCTCATCATTGCCTCCGGCACTGGTGAGTTCGAAGCTG
ZH53Dai24426 GTGGAGGTTTGT-------------------------------------
Vlasak0808_30_Ceriporia_punica --
887Dai13376 GTGGAGGT----
CCTCATCATTGCCTCTGGTACTGGTGAATTCGAAGCTG

WCG1443Dai24998 GTGGAGGTTTGTCCTCATCATTGCCTCTGGTACTGGTGAATTCGAAGCTG
0108_6Ceriporia_spissa --
Dai19164 GTGGAGGTTTGTTCTTATCGTCGCTAGTGGTACGGGTGAATTCGAAGCCG
Dai17937_Ceriporia_bubalinomar GTGGAGGTTTGT-------------------------------------
903Dai12113 GTGGAGGTTTGT-------------------------------------
LZB929Dai25079 GTGGAGGTTTGTTCTCATCATTGCCAGCGGTACTGGTGAGTTCGAGGCTG
LX45Dai26988 ------------ TCTCATCATTGCCAGCGGTACTGGTGAGTTCGAGGCTG
LX43Dai26986 GTGGAGGTTTGTTCTCATCATTGCCAGCGGTACTGGTGAGTTCGAGGCTG
Dai7759Ceriporia --
Cui8012_Ceriporia_viridans --
GC1704_54Ceriporia_viridans --
Dai23392 GTGGAGGT---- TCTCATCATTGCCAGCGGTACTGGTGAGTTCGAGGCTG
WCG1585Dai26113Ceriproia GTGGAGGTTTGTTCTCATCATTGCCAGCGGTACTGGTGAGTTCGAGGCTG
Dai18675C_eucalypti GTGGAGGTTTGTTCTCATCATTGCCAGTGGTACTGGTGAGTTCGAGGCTG
Dai22034 GTGGAGG----- CCTCATCATTGCCAGCGGTACTGGTGAGTTCGAGGCTG
JV1008_41JTardaFLORIDAKeys --
Rivoire1161_Ceriporia_pierii --
Dai23499C_pierii GTGGAGGTTTGTCCTTATCATTGCCGGCGGTACTGGTGAGTTCGAAGCCG
Dai23500 GTGGAGGTTTGTCCTTATCATTGCCGGCGGTACTGGTGAGTTCGAAGCCG
841Dai15899 GTGGAGG----- CCTCATCATCGCCAGTGGTACTGGTGAGTTCGAGGCGG
842Dai15904 ------------ CTCATCATCGCCAGTGGTACTGGTGAGTTCGAGGCGG
LZB1066xinjiang --
LZB1065xinjiang --
851Dai16779 GTGGAGGT--
RMJ119sp_Candelabrochaete_sept --
RLG9759spCandelabrochaete_sept --
RLG10478Phanerochaete_allantos --
Dai19118_Ceriporia_spissa GTGGAGGTTTGT-------------------------------------
Dai18486A GTGGAGGTTTGT-------------------------------------
WEI17_024_Ceriporia_mellita --
GC1508_71Ceriporia_mellita --

GC1608_7_Ceriporia_mellita --
ZZW1557Dai27085 GTGGAGGTTTGT------------------------------------
ZZW1554Dai27083 GTGGAGGTTTGT------------------------------------
Dai8168
GTGGAGGTTTGTTCTCATAATTGCTGGTGGTACCGGTGAATTCGAGGCTG
BR4865C_mellita --
MEL2382688Ceriporia_sp --
Dai8110 GTGGAGGTTTGT------------------------------------
Cui8097 --
909Cui6740 --
W1258Dai24695 GTGGAGGTTTGT------------------------------------
JV0110_26_Ceriporia_griseoviol --
896Dai13202
GTGGAGGTTTGTCCTTATCATCGCCTCTGGTACCGGTGAGTTCGAGGCTG
LWY393Dai27053C_griseoviolasce
GTGGAGGTTTGTCCTCATCATCGCCTCTGGTACCGGTGAGTTCGAGGCTG
LWY394DAI27054
GTGGAGGTTTGTCCTCATCATCGCCTCTGGTACCGGTGAGTTCGAGGCTG
FP135015G_pannocinctus --
L15726SpG_pannocinctus GTGGAGGTTTGT------------------------------------
Dai22221 GTGGAGGT----
CCTCATCATTGCTGGTGGCACTGGTGAGTTCGAGGCCG
Dai22633
GTGGAGGTTTGTCCTCATCATCGCTGGTGGTACTGGTGAATTCGAGGCTG
Dai23260 GTGGAGG-----
CCTCATCATCGCTGGTGGTACTGGTGAATTCGAGGCTG
Dai23626 ------------
CCTCATCATCGCTGGTGGTACTGGTGAATTCGAGGCTG
Dai16238G_citrinoalbus GTGGAGGTTTGT------------------------------------
1175Dai15293 GTGGAGGT----
CCTCATCATCGCTGGTGGCACTGGTGAGTTTGAGGCTG
Dai19547 GTGGAGGT----
CCTCATCATCGCTGGTGGCACTGGTGAGTTCGAGGCTG
918063G_africanus --
918572G_africanus --
Dai18536A --
1164Cui17922 ------------
CCTCATCATCGCTGGTGGCACTGGTGAGTTCGAGGCTG
Dai22225
GTGGAGGTTTGTCCTCATCATCGCTGGTGGCACTGGTGAGTTCGAGGCTG
1163Dai20655 ------------
CCTCATCATCGCTGGTGGCACTGGTGAGTTCGAGGCTG
Yuan4397G_hainanensis GTGGAGGTTTGT------------------------------------
1176Dai15268

GTGGAGGTTTGTCCTCATCATTGCTGGTGGTACCGGTGAGTTCGAGGCTG
1177Dai15259 GTGGAGGTTT--
CCTCATCATTGCTGGTGGTACCGGTGAGTTCGAGGCTG
BZ2896G_thelephoroides ---
1166JV1808_26 -----------
CCTCATTATTGCTGGTGGTACCGGTGAGTTCGAGGCTG
Miettinen16992Hapalopilus_ochr GTGGAGGTTTGT------------------------------------
GC1708_338_Ceriporia_arbuscula ---
WCG1555Dai26107Ceriporia
GTGGAGGTTTGTCCTCATCATTGCCAGCGGTACTGGTGAGTTTGAGGCCG
GC1708_340_Ceriporia_arbuscula ---
WCG1556Dai26109Ceriporia
GTGGAGGTTTGTCCTCATCATTGCCAGCGGTACTGGTGAGTTTGAGGCCG
883Cui11291 GTGGAGG-----
CCTCATCATTGCCAGCGGTACTGGTGAGTTCGAGGCCG
HLX320Dai26805
GTGGAGGTTTGTCCTCATCATTGCCAGCGGTACTGGTGAGTTCGAGGCCG
WCG1266Dai24678A
GTGGAGGTTTGTCCTCATCATTGCCAGCGGTACTGGTGAGTTCGAGGCCG
Dai6090_Ceriporia_sulphuricolo GTGGAGGTTTGT------------------------------------
RLG_11354_Ceriproia_reticulata GTGGAGGTTTGT------------------------------------
ZZW1543Dai27072
GTGGAGGTTTGTCCTCATCATCGCCTCCGGCACTGGTGAGTTCGAGGCCG
Li1316_Ceriporia_reticulata ---
KHL11981Ceriporia_reticulata GTGGAGGTTTGT------------------------------------
FP110343sp_Candelabrochaete_la GTGGAGGTTTGT------------------------------------
Li1045_Ceriporia_reticulata ---
ZX136Dai25794ceriporia
GTGGAGGTTTGTCCTCATCATCGCCTCCGGTACTGGTGAGTTCGAGGCCG
892Dai13400
GTGGAGGTTTGTCCTCATCATCGCCTCCGGTACTGGTGAGTTCGAGGCCG
RLG7163Leptoporus_mollis GTGGAGGTT---------------------------------------
Dai21062Leptoporus_mollis GTGGAGGTTTGT-------
ATTGCTAGTGGTACTGGTGAGTTTGAGGCCG
Dai20182Leptoporus_submollis
GTGGAGGTTTGTCCTCATCATTGCCAGTGGTACTGGTGAGTTTGAGGCCG
Cui18379Leptoporus_submollis
GTGGAGGTTTGTCCTCATCATTGCCAGTGGTACTGGTGAGTTTGAGGCCG
Wu1209_46Resiniporus_pseudogil ---
BRNM710169Resiniporus_resinasc ---
Dai14516Bjerkandera_adusta
GTGGAGGTTTGTCCTCATCATTGCCGGCGGTACCGGTGAGTTCGAGGCTG
Dai21100Bjerkandera_fumosa GTGGAGGT----
CCTCATCATTGCCGGCGGTACCGGTGAGTTCGAGGCCG

Miettinen16854Ceraceomyces_sp GTGGAGGTTGGT--------------------------------------
Dai10477C_spissa GTGGAGGTTG--
855Dai16831
GTGGAGGTTTGTCCTCATCATTGCCTCTGGTACTGGTGAATTCGAAGCTG
882Cui11282 GTGGAGGT----
CCTCATCATTGCCTCTGGTACTGGTGAATTCGAAGCTG
Dai24566
GTGGAGGTTTGTCCTCATCATTGCCTCCGGTACTGGTGAATTCGAGGCTG
Yuan5965 GTGGAGGTTTGT--------------------------------------
Dai3204 GTGGAGGTTTGT--------------------------------------
1194CUI9985 GTGGAGGTTTGT--------------------------------------

Dai15205_Ceriporia_albomellea --
Dai15223_Ceriporia_albomellea --
Li1780_Ceriporia_variegata --
Dai19791_Ceriporia_variegata --
Dai19886
GTATCTCCAAAGATGGCCAGACCCGTGAGCACGCTCTGCTCGCCTTTACC
Dai10833_Ceriporia_crassitunic --
CHWC1506_46Meruliopsis_crassit --
Dai9995_Ceriporia_crassitunica --
Wu1209_58_Meruliopsis_parvispo --
CHWC1505_129_Meruliopsis_parvi --
Dai21944
GTATCTCCAAGGATGGCCAGACTCGTGAGCACGCTCTGCTCGCCTTCACC
830Dai18640A
GTATCTCCAAGGATGGCCAGACTCGTGAGCACGCCCTGCTTGCCTTCACC
GC1704_60_Meruliopsis_taxicola --
Dai22625
GTATCTCCAAGGATGGCCAGACTCGCGAGCACGCTCTGCTTGCCTTCACC
Dai22636
GTATCTCCAAGGATGGCCAGACTCGCGAGCACGCTCTGCTTGCCTTCACC
Dai21878
GTATCTCCAAGGATGGCCAGACTCGCGAGCACGCTCTGCTTGCCTTCACC
1169Dai17248
GTATCTCCAAGGATGGCCAGACTCGCGAGCACGCTCTGCTTGCCTTCACC
Wu1708_43_Meruliopsis_leptocys --
Li1011 --
ZX95Dai25742Meruliopsis_leptoc --
WCG1306Dai24733
GTATCTCCAAGGATGGCCAGACTCGTGAGCACGCTTTGCTCGCCTTCACC
LXL99Dai25816
GTATCTCCAAGGATGGCCAGACTCGTGAGCACGCTTTGCTCGCCTTCACC

WCG1559Dai26052Meruliopsis
GTATCTCCAAGGATGGCCAGACTCGTGAGCACGCTTTGCTCGCCTTCACC

He7477 --

HLX243Dai26217
GTATCTCCAAGGATGGCCAGACTCGTGAGCACGCTTTGCTCGCCTTCACC

RussiaMW673659Meruliopsis_fagi --

FD278 --

Dai10226_Ceriporia_tarda --

LE247365 --

Dai8173_Meruliopsis_nanlingens --

860Dai17172
GTATCTCCAAGGATGGCCAGACTCGTGAGCACGCCTTGCTCGCCTTCACC

879Dai13414
GTATCTCCAAGGATGGCCAGACTCGTGAGCACGCCTTGCTCGCCTTCACC

Li_1704_Meruliopsis_pseudocyst --

833Dai18405
GTATCTCTAAGGATGGCCAGACTCGTGAGCACGCTTTGCTCGCCTTCACC

HHB_10729_Meruliopsis_albostra --

Cui6878_Ceriporia_pseudocystid --

869Dai14737
GTATCTCCAAGGTCGGTCAGACTCGCGAGCACGCCCTCCTCGCCTTCACC

876Cui11626 --

1199WEI3388 --

776308_Meruliopsis_cystidiata --

ICN139059_Meruliopsis_cystidia --

HHB15692Ceraceomyces_serpens --

HHB_15629_Sp_Ceriporiopsis_ane --

AJ185Trametopsis_cervina --

FD9Irpex_lacteus --

908Dai11230
GTATCTCCAAGGATGGTCAGACTCGCGAGCACGCTCTCCTTGCCTTCACC

FP55521TEmmia_lacerata --

PBU0048Ceriporia_cystidiata --

MZ340C_lacerataT --

Dai21940
GTATCTCCAAGGATGGTCAGACTCGCGAGCACGCTCTCCTCGCCTTCACT

847Dai16433
GTATCTCCAAGGATGGTCAGACTCGCGAGCACGCCCTCCTCGCCTTCACT

MarcinEmmia_latemarginatus --

Meijer3729Hydnopolyporus_fimbr --

RLG13408Phanerochaete_sp --

WHC1381Flavodon_flavus --

GB1833Phlebia_albida
GCATTTCCAAGGATGGTCAGACCCGCGAGCACGCTCTCTTGGCTTTCACT

T407Phlebia_nitidula
GTATTTCCAAGGACGGTCAGACCCGCGAGCACGCTCTCTTGGCTTTCACT
HHB6988Phanerochaete_exilis --
HHB8509Phanerochaetella_xeroph --
PBU0051Macrohyporia_dictyopora --
HHB11463Phanerochaete_sp --
FP102382Byssomerulius_corium --
FP102165Efibula_americana
GTATTTCTAAGGATGGCCAGACTCGCGAGCACGCTCTCCTTGCCTTCACC
Murdoch90Ceriporia_torpida --
Rivoire4413_Ceriporia_purpurea --
Kout_18_Ceriporia_triumphalis --
Rivoire3701_Ceriporia_bresadol --
VS4018 --
Ryvarden21832_Ceriporia_manzan --
Dai24539
GTATCTCAAAGGATGGTCAGACTCGCGAGCACGCTCTGCTCGCCTTCACT
Dai24541
GTATCTCAAAGGATGGTCAGACTCGCGAGCACGCTCTGCTCGCCTTCACT
JV1105_12_Ceriporia_occidental --
VS8558Ceriporia_occidentalis --
Dai22445
GTATCTCCAAGGACGGTCAGACTCGCGAGCACGCTCTCCTCGCCTTCACC
846Dai16368
GTATCTCCAAGGACGGTCAGACTCGCGAGCACGCTCTCCTCGCCTTCACC
Dai17951_Ceriporia_aurantiocar --
Miettinen_11701C_viridans --
JV0105_10Ceriporia_aurantiocar --
Yuan5702C_viridans --
858Dai17003 --
Yuan2747_Ceriporia_viridans --
Yuan2744C_viridans --
Li1046C_viridans --
865C_sinoviridans --
871Dai15062 --
Dai7642_Ceriporia_humilis
GTATCTCGAAGGACGGTCAGACTCGCGAGCACGCTCTCCTTGCCTTCACT
Spirin4706_Ceriporia_humilis --
Spirin4944_Ceriporia_sericea --
WCG1547Dai26044ceriporia --
ZZW1558Dai27086
GTATCTCGAAGGACGGTCAGACTCGCGAGCACGCTCTCCTTGCCTTCACT
Miettinen14381_Ceriporia_mpuri --
Miettinen15492_2_Ceriporia_sor --

He6687
GTATCTCGAAGGACGGTCAGACTCGCGAGCACGCTCTCCTTGCCTTCACT
ZH53Dai24426 ---
Vlasak0808_30_Ceriporia_punica ---
887Dai13376
GTATCTCGAAGGACGGTCAGACTCGCGAGCACGCCCTCCTCGCGTTCACT
WCG1443Dai24998
GTATCTCGAAGGATGGTCAGACTCGCGAGCACGCCCTCCTCGCGTTCACT
0108_6Ceriporia_spissa ---
Dai19164
GCATTAGCTCGGCAGGTCAGACACTCGAGCACGCACTTCTTGCCCACACC
Dai17937_Ceriporia_bubalinomar ---
903Dai12113 ---
LZB929Dai25079
GTATCTCCAAGGATGGCCAGACCCGCGAGCACGCTCTTCTCGCGTTCACT
LX45Dai26988
GTATCTCCAAGGATGGCCAGACCCGCGAGCACGCTCTCCTCGCGTTCACT
LX43Dai26986
GTATCTCCAAGGATGGCCAGACCCGCGAGCACGCTCTCCTCGCGTTCACT
Dai7759Ceriporia ---
Cui8012_Ceriporia_viridans ---
GC1704_54Ceriporia_viridans ---
Dai23392
GTATCTCCAAGGATGGACAGACTCGCGAGCACGCTCTCCTTGCATTCACT
WCG1585Dai26113Ceriproia
GTATCTCCAAGGATGGTCAGACTCGCGAGCACGCTCTCCTTGCATTCACT
Dai18675C_eucalypti
GTATCTCCAAGGATGGTCAGACCCGCGAGCACGCTCTCCTTGCGTTCACT
Dai22034
GTATCTCCAAGGATGGCCAGACTCGCGAGCACGCTCTCCTCGCCTTCACC
JV1008_41JTardaFLORIDAKeys ---
Rivoire1161_Ceriporia_pierii ---
Dai23499C_pierii
GCATCTCCAAGGATGGTCAGACCCGCGAGCACGCTCTTCTCGCCTTCACT
Dai23500
GCATCTCCAAGGATGGTCAGACCCGCGAGCACGCTCTTCTCGCCTTCACT
841Dai15899
GTATCTCCAAGGATGGTCAGACTCGCGAGCACGCTCTCCTCGCTTTCACC
842Dai15904
GTATCTCCAAGGATGGTCAGACTCGCGAGCACGCTCTCCTCGCTTTCACC
LZB1066xinjiang ---
LZB1065xinjiang ---
851Dai16779 ---
RMJ119sp_Candelabrochaete_sept ---

RLG9759spCandelabrochaete_sept --
RLG10478Phanerochaete_allantos --
Dai19118_Ceriporia_spissa --
Dai18486A --
WEI17_024_Ceriporia_mellita --
GC1508_71Ceriporia_mellita --
GC1608_7_Ceriporia_mellita --
ZZW1557Dai27085 --
ZZW1554Dai27083 --
Dai8168
GTATCTCGAAGGACGGTCAGACTCGCGAGCACGCTCTCCTTGCGTTCACC
BR4865C_mellita --
MEL2382688Ceriporia_sp --
Dai8110 --
Cui8097 --
909Cui6740 --
W1258Dai24695 --
JV0110_26_Ceriporia_griseoviol --
896Dai13202
GTATCTCCAAGGACGGTCAGACTCGCGAGCACGCTCTCCTCGCCTTCACC
LWY393Dai27053C_griseoviolasce
GTATCTCCAAGGACGGTCAGACTCGCGAGCACGCTCTCCTCGCCTTCACC
LWY394DAI27054
GTATCTCCAAGGACGGTCAGACTCGCGAGCACGCTCTCCTCGCCTTCACC
FP135015G_pannocinctus --
L15726SpG_pannocinctus --
Dai22221
GTATCTCCAAGGATGGCCAGACCCGTGAACACGCTCTCCTTGCCTTCACC
Dai22633
GTATCTCCAAGGATGGTCAGACCCGTGAACACGCTCTCCTTGCCTTCACC
Dai23260
GTATCTCCAAGGATGGTCAGACCCGTGAACACGCTCTCCTTGCCTTCACC
Dai23626
GTATCTCCAAGGATGGTCAGACCCGTGAACACGCTCTCCTTGCCTTCACC
Dai16238G_citrinoalbus --
1175Dai15293
GTATCTCCAAGGATGGCCAGACCCGTGAACACGCTCTCCTCGCCTTCACC
Dai19547
GTATCTCCAAGGATGGCCAGACCCGTGAACACGCTCTCCTCGCCTTCACC
918063G_africanus --
918572G_africanus --
Dai18536A --
1164Cui17922
GTATCTCCAAGGATGGCCAGACCCGTGAACACGCTCTCCTCGCCTTCACT

Dai22225
GTATCTCCAAGGATGGCCAGACCCGTGAACACGCTCTCCTCGCCTTCACT
1163Dai20655
GTATCTCCAAGGATGGCCAGACCCGTGAACACGCTCTCCTCGCCTTCACT
Yuan4397G_hainanensis --
1176Dai15268
GTATCTCCAAGGATGGCCAGACCCGTGAACACGCTCTCCTCGCCTTCACC
1177Dai15259
GTATCTCCAAGGATGGCCAGACCCGTGAACACGCTCTCCTCGCCTTCACC
BZ2896G_thelephoroides --
1166JV1808_26
GTATCTCCAAGGATGGCCAGACCCGTGAACACGCCCTCCTCGCGTTCACC
Miettinen16992Hapalopilus_ochr --
GC1708_338_Ceriporia_arbuscula --
WCG1555Dai26107Ceriporia
GTATCTCCAAGGATGGTCAAACTCGCGAGCACGCTCTCCTCGCCTTCACT
GC1708_340_Ceriporia_arbuscula --
WCG1556Dai26109Ceriporia
GTATCTCCAAGGATGGTCAAACTCGCGAGCACGCTCTCCTCGCCTTCACT
883Cui11291
GTATTTCCAAGGATGGCCAGACCCGCGAGCACGCCCTCCTCGCCTTCACT
HLX320Dai26805
GTATCTCCAAGGATGGTCAAACTCGCGAGCACGCCCTTCTCGCCTTCACT
WCG1266Dai24678A
GTATTTCCAAGGATGGTCAAACTCGCGAGCACGCTCTCCTCGCCTTCACT
Dai6090_Ceriporia_sulphuricolo --
RLG_11354_Ceriproia_reticulata --
ZZW1543Dai27072
GTATCTCCAAGGACGGTCAGACTCGCGAGCACGCCCTCCTCGCCTTCACC
Li1316_Ceriporia_reticulata --
KHL11981Ceriporia_reticulata --
FP110343sp_Candelabrochaete_la --
Li1045_Ceriporia_reticulata --
ZX136Dai25794ceriporia
GTATCTCCAAGGACGGTCAGACTCGCGAGCACGCCCTCCTCGCCTTCACT
892Dai13400
GTATCTCCAAGGACGGTCAGACTCGCGAGCACGCCCTCCTCGCCTTCACT
RLG7163Leptoporus_mollis --
Dai21062Leptoporus_mollis
GTATCTCCAAAGATGGTCAGACTCGCGAGCACGCTCTCCTCGCCTTCACC
Dai20182Leptoporus_submollis
GTATCTCTAAGGATGGTCAGACTCGCGAGCACGCTCTCCTCGCCTTCACC
Cui18379Leptoporus_submollis
GTATCTCTAAGGATGGTCAGACTCGCGAGCACGCTCTCCTCGCCTTCACC

Wu1209_46Resiniporus_pseudogil --C
BRNM710169Resiniporus_resinasc ---
Dai14516Bjerkandera_adusta
GTATCTCCAAGGATGGCCAGACCCGCGAGCACGCCCTCCTTGCCTTCACC
Dai21100Bjerkandera_fumosa
GTATCTCCAAGGATGGCCAGACCCGAGAGCACGCTCTCCTTGCCTTCACC
Miettinen16854Ceraceomyces_sp ---
Dai10477C_spissa ---
855Dai16831
GTATTTCCAAAGACGGTCAGACTCGCGAGCATGCTCTCCTCGCCTTCACC
882Cui11282
GTATTTCCAAAGACGGTCAGACTCGCGAGCATGCTCTCCTCGCCTTCACC
Dai24566
GTATCTCCAAAGACGGTCAGACTCGCGAGCATGCTCTCCTCGCCTTCACC
Yuan5965 ---
Dai3204 ---
1194CUI9985 ---

Dai15205_Ceriporia_albomellea ---
Dai15223_Ceriporia_albomellea ---
Li1780_Ceriporia_variegata ---
Dai19791_Ceriporia_variegata ---
Dai19886
CTCGGTGTCAGGCAAATCATTATTGCCGTCAACAAAATGGACACTACGAA
Dai10833_Ceriporia_crassitunic ---
CHWC1506_46Meruliopsis_crassit ---
Dai9995_Ceriporia_crassitunica ---
Wu1209_58_Meruliopsis_parvispo ---
CHWC1505_129_Meruliopsis_parvi ---
Dai21944
CTCGGTGTCAGGCAGCTCATCGTTGCCGTCAACAAGATGGACACCACCAA
830Dai18640A
CTCGGTGTCAGGCAGCTTATCGTTGCCGTCAACAAGATGGACACCACCAA
GC1704_60_Meruliopsis_taxicola ---
Dai22625
CTCGGTGTCAGGCAGCTCATCGTTGCCGTCAACAAGATGGACACCACCAA
Dai22636
CTCGGTGTCAGGCAGCTCATCGTTGCCGTCAACAAGATGGACACCACCAA
Dai21878
CTCGGTGTCAGGCAGCTCATCGTTGCCGTCAACAAGATGGACACCACCAA
1169Dai17248
CTCGGTGTCAGGCAGCTCATCGTTGCCGTCAACAAGATGGACACCACCAA
Wu1708_43_Meruliopsis_leptocys ---

Li1011 --

ZX95Dai25742Meruliopsis_leptoc --

WCG1306Dai24733

CTCGGTGTCAGGCAGCTCATCGTTGCCGTCAACAAGATGGACACCACTAA

LXL99Dai25816

CTCGGTGTCAGGCAGCTCATCGTTGCCGTCAACAAGATGGACACCACTAA

WCG1559Dai26052Meruliopsis

CTCGGCGTTAGGCAGCTCATCGTTGCCGTCAACAAGATGGACACCACCAA

He7477 --

HLX243Dai26217

CTCGGCGTTAGGCAGCTCATCGTTGCCGTCAACAAGATGGACACCACCAA

RussiaMW673659Meruliopsis_fagi --

FD278 --

Dai10226_Ceriporia_tarda --

LE247365 --

Dai8173_Meruliopsis_nanlingens --

860Dai17172

CTCGGTGTCAGGCAGCTCATCGTTGCCGTCAACAAGATGGACACCACGAA

879Dai13414

CTCGGTGTCAGGCAGCTCATCGTTGCCGTCAACAAGATGGACACCACGAA

Li_1704_Meruliopsis_pseudocyst --

833Dai18405

CTCGGTGTTAGGCAGCTCATCGTCGCCGTCAACAAGATGGACACCACCAA

HHB_10729_Meruliopsis_albostra --

Cui6878_Ceriporia_pseudocystid --

869Dai14737

CTTGGTGTCAGGCAGCTCATCGTCGCCATCAACAAGATGGACACCACCAA

876Cui11626 --

1199WEI3388 --

776308_Meruliopsis_cystidiata --

ICN139059_Meruliopsis_cystidia --

HHB15692Ceraceomyces_serpens --

HHB_15629_Sp_Ceriporiopsis_ane --

AJ185Trametopsis_cervina --

FD9Irpex_lacteus --

908Dai11230

CTCGGTGTCAGGCAGCTCATCGTCGCCGTCAACAAGATGGACACCACCAA

FP55521TEmmia_lacerata --

PBU0048Ceriporia_cystidiata --

MZ340C_laceratаT --

Dai21940

CTCGGTGTCAGGCAACTCATCGTTGCCGTCAACAAGATGGACACTGCCGG

847Dai16433

CTCGGTGTCAGGCAACTCATCGTCGCCGTCAACAAGATGGACACTGCTGG

MarcinEmmia_latemarginatus --

Meijer3729Hydnopolyporus_fimbr --

RLG13408Phanerochaete_sp --

WHC1381Flavodon_flavus --

GB1833Phlebia_albida

CTCGGTGTCAGGCAGCTCATTGTTGCCGTTAACAAGATGGATACGACCAA

T407Phlebia_nitidula

CTCGGTGTCAGGCAGCTCATTGTTGCCGTCAACAAGATGGATACCACCAA

HHB6988Phanerochaete_exilis --

HHB8509Phanerochaetella_xeroph --

PBU0051Macrohyporia_dictyopora --

HHB11463Phanerochaete_sp --

FP102382Byssomerulius_corium --

FP102165Efibula_americana

CTCGGTGTCAGGCAGCTCATCGTCGCCGTCAACAAGATGGACACCACCAA

Murdoch90Ceriporia_torpida --

Rivoire4413_Ceriporia_purpurea --

Kout_18_Ceriporia_triumphalis --

Rivoire3701_Ceriporia_bresadol --

VS4018 --

Ryvarden21832_Ceriporia_manzan --

Dai24539

CTCGGTGTCAGGCAGCTCATTGTCGCCGTCAACAAGATGGATACCACCAA

Dai24541

CTCGGTGTCAGGCAGCTCATTGTCGCCGTCAACAAGATGGATACCACCAA

JV1105_12_Ceriporia_occidental --

VS8558Ceriporia_occidentalis --

Dai22445

CTCGGTGTCAGGCAGCTCATCGTCGCCGTCAACAAGATGGATACCACCAA

846Dai16368

CTCGGTGTCAGGCAGCTCATCGTCGCCGTCAACAAGATGGATACCACCAA

Dai17951_Ceriporia_aurantiocar --

Miettinen_11701C_viridans --

JV0105_10Ceriporia_aurantiocar --

Yuan5702C_viridans --

858Dai17003 --

Yuan2747_Ceriporia_viridans --

Yuan2744C_viridans --

Li1046C_viridans --

865C_sinoviridans --

871Dai15062 --

Dai7642_Ceriporia_humilis

CTCGGTGTCAGGCAGCTGATCGTCGCCGTCAACAAGATGGACACCACCAA

Spirin4706_Ceriporia_humilis --

Spirin4944_Ceriporia_sericea --
WCG1547Dai26044ceriporia --
ZZW1558Dai27086
CTCGGCGTCAGGCAACTGATCGTCGCCGTCAACAAGATGGACACCTGCAA
Miettinen14381_Ceriporia_mpuri --
Miettinen15492_2_Ceriporia_sor --
He6687
CTCGGTGTCAGGCAGCTGATTGTCGCCGTCAACAAGATGGACACCACCAA
ZH53Dai24426 --
Vlasak0808_30_Ceriporia_punica --
887Dai13376
CTCGGTGTCAGGCAACTCATCGTCGCGGTCAACAAGATGGACACCACTAA
WCG1443Dai24998
CTCGGTGTCAGGCAGCTCATCGTCGCGGTCAACAAGATGGACACCACTAA
0108_6Ceriporia_spissa --
Dai19164
CTGGGCATCAAACAGCTCATTGTTGCCGTCAACAAGATGGATACCGTTCA
Dai17937_Ceriporia_bubalinomar --
903Dai12113 --
LZB929Dai25079
CTCGGTGTCAGGCAGCTCATCGTCGCCGTCAACAAGATGGACACCACCAA
LX45Dai26988
CTCGGTGTCAGGCAGCTCATCGTCGCCGTCAACAAGATGGACACCACCAA
LX43Dai26986
CTCGGTGTCAGGCAGCTCATCGTCGCCGTCAACAAGATGGACACCACCAA
Dai7759Ceriporia --
Cui8012_Ceriporia_viridans --
GC1704_54Ceriporia_viridans --
Dai23392
CTCGGTGTGAGGCAGCTCATCGTCGCCGTCAACAAGATGGACACCACCAA
WCG1585Dai26113Ceriproia
CTCGGTGTGAGGCAGCTCATCGTCGCCGTCAACAAGATGGACACCACCAA
Dai18675C_eucalypti
CTCGGTGTCAGGCAGCTCATCGTCGCCGTCAACAAGATGGACACCACCAA
Dai22034
CTCGGTGTCAGGCAGCTCATCGTCGCCGTCAACAAGATGGACACCACCAA
JV1008_41JTardaFLORIDAKeys --
Rivoire1161_Ceriporia_pierii --
Dai23499C_pierii
CTCGGTGTTAGGCAACTCATCGTTGCCGTCAACAAAATGGACACTACCAA
Dai23500
CTCGGTGTTAGGCAACTCATCGTTGCCGTCAACAAAATGGACACTACCAA
841Dai15899
CTCGGTGTCAGGCAGCTCATCGTTGCCGTCAACAAGATGGACACCACGAA

842Dai15904
CTCGGTGTCAGGCAGCTCATCGTTGCCGTCAACAAGATGGACACCACGAA
LZB1066xinjiang --
LZB1065xinjiang --
851Dai16779 --
RMJ119sp_Candelabrochaete_sept --
RLG9759spCandelabrochaete_sept --
RLG10478Phanerochaete_allantos --
Dai19118_Ceriporia_spissa --
Dai18486A --
WEI17_024_Ceriporia_mellita --
GC1508_71Ceriporia_mellita --
GC1608_7_Ceriporia_mellita --
ZZW1557Dai27085 --
ZZW1554Dai27083 --
Dai8168
CTTGGCGTGCGTCAGCTAATCGTCGCCGTCAACAAAATGGACACCACCAA
BR4865C_mellita --
MEL2382688Ceriporia_sp --
Dai8110 --
Cui8097 --
909Cui6740 --
W1258Dai24695 --
JV0110_26_Ceriporia_griseoviol --
896Dai13202
CTTGGTGTCAGGCAGCTCATCGTTGCTGTCAACAAGATGGATACCACGAA
LWY393Dai27053C_griseoviolasce
CTTGGTGTCAGGCAGCTCATCGTTGCTGTCAACAAGATGGATACCACGAA
LWY394DAI27054
CTTGGTGTCAGGCAGCTCATCGTTGCTGTCAACAAGATGGATACCACGAA
FP135015G_pannocinctus --
L15726SpG_pannocinctus --
Dai22221
CTCGGTGTCAGGCAGCTCATTGTCGCCGTCAACAAGATGGACACCACTAA
Dai22633
CTCGGTGTCAGGCAACTCATCGTCGCCGTCAACAAGATGGACACCACCA-
Dai23260
CTCGGTGTCAGGCAACTCATCGTCGCCGTCAACAAGATGGACACCACCA-
Dai23626
CTCGGTGTCAGGCAACTCATCGTCGCCGTCAACAAGATGGACACCACCA-
Dai16238G_citrinoalbus --
1175Dai15293
CTCGGTGTCAGGCAACTCATCGTCGCCGTCAACAAGATGGACACCACCA-
Dai19547

CTCGGTGTCAGGCAACTCATCGTCGCCGTCAACAAGATGGACACCACCA-
918063G_africanus --
918572G_africanus --
Dai18536A --
1164Cui17922
CTCGGTGTTAGGCAACTCATCGTCGCCGTCAATAAGATGGACACCACCA-
Dai22225
CTCGGTGTCAGGCAACTCATCGTCGCCGTCAACAAGATGGACACCACCA-
1163Dai20655
CTCGGTGTCAGGCAACTCATCGTCGCCGTCAACAAGATGGACACCACCA-
Yuan4397G_hainanensis --
1176Dai15268
CTCGGTGTCAGGCAGCTCATCGTTGCCGTCAACAAGATGGACACCACCAA
1177Dai15259
CTCGGTGTCAGGCAGCTCATCGTTGCCGTCAACAAGATGGACACCACCAA
BZ2896G_thelephoroides --
1166JV1808_26
CTCGGTGTCAGGCAGCTCATTGTCGCTGTCAACAAGATGGATACCACCAA
Miettinen16992Hapalopilus_ochr --
GC1708_338_Ceriporia_arbuscula --
WCG1555Dai26107Ceriporia
CTTGGTGTCAGGCAGCTCATCGTCGCCGTCAACAAGATGGATACCACCAA
GC1708_340_Ceriporia_arbuscula --
WCG1556Dai26109Ceriporia
CTTGGTGTCAGGCAGCTCATCGTCGCCGTCAACAAGATGGATACCACCAA
883Cui11291
CTCGGTGTCAGGCAGCTCATCGTCGCCGTCAACAAGATGGACACCACCAA
HLX320Dai26805
CTCGGCGTCAGGCAGCTCATCGTCGCCGTCAACAAGATGGACACCACCAA
WCG1266Dai24678A
CTTGGTGTCAGGCAGCTCATCGTCGCCGTCAACAAGATGGACACCACCAA
Dai6090_Ceriporia_sulphuricolo --
RLG_11354_Ceriproia_reticulata --
ZZW1543Dai27072
CTCGGTGTCAGGCAGATCATCATCGCTGTCAACAAGATGGACACCACCAA
Li1316_Ceriporia_reticulata --
KHL11981Ceriporia_reticulata --
FP110343sp_Candelabrochaete_la --
Li1045_Ceriporia_reticulata --
ZX136Dai25794ceriporia
CTTGGTGTCAGGCAGCTCATCGTTGCTGTCAACAAGATGGACACTACCAA
892Dai13400
CTTGGTGTCAGGCAGCTCATCGTTGCTGTCAACAAAATGGACACTACCAA
RLG7163Leptoporus_mollis --

Dai21062Leptoporus_mollis
CTCGGTGTCAGGCAGCTCATCGTCGCTGTCAACAAGATGGATACCACCAA
Dai20182Leptoporus_submollis
CTTGGTGTCAGGCAGCTCATCGTCGCCGTCAATAAGATGGATACCACCAA
Cui18379Leptoporus_submollis
CTTGGTGTCAGGCAGCTCATCGTCGCCGTCAATAAGATGGATACCACCAA
Wu1209_46Resiniporus_pseudogil
CTCGGTGTCAGGCAACTTATTGTTGCCGTCAACAAGATGGACACGACCAA
BRNM710169Resiniporus_resinasc --
Dai14516Bjerkandera_adusta
CTCGGTGTCAGGCAGCTCATCGTCGCCGTCAACAAGATGGACACCACCAA
Dai21100Bjerkandera_fumosa
CTCGGTGTCAGGCAGCTCATCGTCGCCGTCAACAAGATGGATACCACCAA
Miettinen16854Ceraceomyces_sp --
Dai10477C_spissa --
855Dai16831
CTCGGTGTCAGGCAACTCATCGTCGCTGTCAACAAGATGGACACCACCAA
882Cui11282
CTCGGTGTCAGGCAACTCATCGTCGCTGTCAACAAGATGGACACCACCAA
Dai24566
CTCGGTGTCAGGCAACTCATCGTCGCCGTCAACAAGATGGACACCACCAA
Yuan5965 --
Dai3204 --
1194CUI9985 --

Dai15205_Ceriporia_albomellea --
Dai15223_Ceriporia_albomellea --
Li1780_Ceriporia_variegata --
Dai19791_Ceriporia_variegata --
Dai19886 GGTATATT--------
CCTTTACGGTATGTGGAGCTG------AGTCTAG
Dai10833_Ceriporia_crassitunic --
CHWC1506_46Meruliopsis_crassit --
Dai9995_Ceriporia_crassitunica --
Wu1209_58_Meruliopsis_parvispo --
CHWC1505_129_Meruliopsis_parvi --
Dai21944 GGTTCGTAT-------CCTTCATAGCGTGAGAAA-
TCAG-----ATCCAG
830Dai18640A GGTACGTAT-------
CCTCTGTAGCGCGGGAAACTTAA-----ATCTGG
GC1704_60_Meruliopsis_taxicola --
Dai22625 GGTTTGTCTT------CCTTCGTGGCGTGGGAAT-
CGAT-----CTCTAG

Dai22636 GGTTTGTCTT------CCTTCGTGGCGTGGGAAT-CGAT-----CTCTAG
Dai21878 GGTTTGTCTT------CCTTCGTGGCGTGGGAAT-CAAT-----CTCTAG
1169Dai17248 GGTTTGTCTT------CCTTCGTGGCGTGGGAAT-CAAT-----CTCTAG
Wu1708_43_Meruliopsis_leptocys --
Li1011 --
ZX95Dai25742Meruliopsis_leptoc --
WCG1306Dai24733 GGTTCGTTC-------GCTTCGTGGCGTGGGAAC-CTTC-----GTCTAA
LXL99Dai25816 GGTTCGTTC-------GCTTCGTGGCGTGGGAAC-CTTC-----GTCTAA
WCG1559Dai26052Meruliopsis GGTTCGTCC-------CTTTTGTGGCGTAGGAAC-CGTA-----GTTTAG
He7477 --
HLX243Dai26217 GGTTCGTCC-------CTTTTGTGGCGTAGGAAC-CGTA-----GTTTAG
RussiaMW673659Meruliopsis_fagi --
FD278 --
Dai10226_Ceriporia_tarda --
LE247365 --
Dai8173_Meruliopsis_nanlingens --
860Dai17172 GGTTAGTTTT---CTGTCTTCGTGGCGTGGGAAT-TTAC-----ATTTAG
879Dai13414 GGTTAGTTTT---CTGTCTTCGTGGCGTGGGAAT-TTAC-----ATTTAG
Li_1704_Meruliopsis_pseudocyst --
833Dai18405 GGTTCGTTGC------TCTTCGTGGCGTGGGAGT-CAAA-----A--TAG
HHB_10729_Meruliopsis_albostra --
Cui6878_Ceriporia_pseudocystid --
869Dai14737 GGTACATAAATATGCACACCGTCATCGGATGAAT---------------
876Cui11626 --
1199WEI3388 --
776308_Meruliopsis_cystidiata --
ICN139059_Meruliopsis_cystidia --
HHB15692Ceraceomyces_serpens --
HHB_15629_Sp_Ceriporiopsis_ane --
AJ185Trametopsis_cervina --
FD9Irpex_lacteus --
908Dai11230 GGTTTG-------TCGACTTT-----TTACGAATTTC----ACTCGATTG

FP55521TEmmia_lacerata --
PBU0048Ceriporia_cystidiata --
MZ340C_lacerataT --
Dai21940 GGTACGTTCT------TTTGGTTAGTCTGATATCATC-------GAGCTA
847Dai16433 GGTACGTTCT------TTTGGTTAGTCTGATATTATT-------GAGCTA
MarcinEmmia_latemarginatus --
Meijer3729Hydnopolyporus_fimbr --
RLG13408Phanerochaete_sp --
WHC1381Flavodon_flavus --
GB1833Phlebia_albida GGTTTGTG---------TTTCGTGGCGTGTACATATC-------GGCTTG
T407Phlebia_nitidula GGTTTGTG---------TTTCGTGGCGTGTGTATATC-------GAGCTG
HHB6988Phanerochaete_exilis --
HHB8509Phanerochaetella_xeroph --
PBU0051Macrohyporia_dictyopora --
HHB11463Phanerochaete_sp --
FP102382Byssomerulius_corium --
FP102165Efibula_americana GGTCGGTG--------ATTTAGTATTGCGTGCGTTC--------GAACTG
Murdoch90Ceriporia_torpida --
Rivoire4413_Ceriporia_purpurea --
Kout_18_Ceriporia_triumphalis --
Rivoire3701_Ceriporia_bresadol --
VS4018 --
Ryvarden21832_Ceriporia_manzan --
Dai24539 GGTTAGCA---------TCATCTGGCTTGAGAGTATT-------GCGTAG
Dai24541 GGTCAGCA---------TCATCTGGCTTGAGAGTATT-------GCGTAG
JV1105_12_Ceriporia_occidental --
VS8558Ceriporia_occidentalis --
Dai22445 GGTTAGTCA-------TTAATATGATGTGTGAATATC-------GCGTGG
846Dai16368 GGTTAGTAA-------TCAATACGATGTGCGAATATC-------GCGTGG
Dai17951_Ceriporia_aurantiocar --
Miettinen_11701C_viridans --
JV0105_10Ceriporia_aurantiocar --
Yuan5702C_viridans --
858Dai17003 --
Yuan2747_Ceriporia_viridans --

Yuan2744C_viridans ---
Li1046C_viridans ---
865C_sinoviridans ---
871Dai15062 ---
Dai7642_Ceriporia_humilis GGTTGGTA------CCTTTTTGTGGCGTGCGAAACTG-------GTATAG
Spirin4706_Ceriporia_humilis ---
Spirin4944_Ceriporia_sericea ---
WCG1547Dai26044ceriporia -----------------TTTGTGGCGTG-GAAAATG-------GTATAG
ZZW1558Dai27086 GGTTGGTA--------AATTTGTGGCGTG-GAAAATG-------GTATAG
Miettinen14381_Ceriporia_mpuri ---
Miettinen15492_2_Ceriporia_sor ---
He6687 GGTTGGTA------CCTTTTTGTGGCGTGCGAAACTG-------GTATAG
ZH53Dai24426 ---
Vlasak0808_30_Ceriporia_punica ---
887Dai13376 GGTACAGCAAA--TCTTCATCTCGCTTCG--AATTTG-------TCGTTG
WCG1443Dai24998 GGTACAGCAA---ACTTCATTTTGACGTC--AATTTT-------TCGTTG
0108_6Ceriporia_spissa ---
Dai19164 GGTTCGCAGATCTTGCATTTTGCCGCATAGAAAGTAT-------TCATCG
Dai17937_Ceriporia_bubalinomar ---
903Dai12113 ---
LZB929Dai25079 GGTCAGCGG-------CATCCCTGGCATGGGTGATCG-------CCTTGG
LX45Dai26988 GGTCAGCGG-------CATCCCTGGCATGGGTGATCG-------CCTTGG
LX43Dai26986 GGTCAGCGG-------CATCCCTGGCATGGGTGATCG-------CCTTGG
Dai7759Ceriporia ---
Cui8012_Ceriporia_viridans ---
GC1704_54Ceriporia_viridans ---
Dai23392 GGTTAGTGG-------CGT--CTGTCGC-AGAAATGA-------GCCTAG
WCG1585Dai26113Ceriproia GGTCAGTGG-------CGT--CTGTCGC-AGAAATGA-------GCCTAG
Dai18675C_eucalypti GGTCAGTGG-------CGT--TTGATGCGAGAGATTA-------GCCTAG
Dai22034 GGTCAGTCG-------ATCTATTGATGTGGGAATTTT-------AGATTG

JV1008_41JTardaFLORIDAKeys --
Rivoire1161_Ceriporia_pierii --
Dai23499C_pierii GGCTAG-------T-
ATTCTTGTAGCGCATCAAATCAG------TCGAAG
Dai23500 GGCTAG-------T-
ATTCTTGTAGCGCATCAAATCAG------TCGAAG
841Dai15899 GGTACATA--------AGCGCATAGCATGTGAAT-----
----TATCTAG
842Dai15904 GGTACATAA-------AGCGCATAGCATGTGAAT----
-----TATCTAG
LZB1066xinjiang --
LZB1065xinjiang --
851Dai16779 --
RMJ119sp_Candelabrochaete_sept --
RLG9759spCandelabrochaete_sept --
RLG10478Phanerochaete_allantos --
Dai19118_Ceriporia_spissa --
Dai18486A --
WEI17_024_Ceriporia_mellita --
GC1508_71Ceriporia_mellita --
GC1608_7_Ceriporia_mellita --
ZZW1557Dai27085 --
ZZW1554Dai27083 --
Dai8168
GGTTCGCTTACATTGTTGGTCATATTTCAAGAAA--------------AA
BR4865C_mellita --
MEL2382688Ceriporia_sp --
Dai8110 --
Cui8097 --
909Cui6740 --
W1258Dai24695 --
JV0110_26_Ceriporia_griseoviol --
896Dai13202 GGTTCGTGC-------
AATCTCCGGCGTCGGAGGACC-------TCAGAA
LWY393Dai27053C_griseoviolasce GGTTCGTGC-------
AATCTCCGGCGTCGGAGGACC-------TCAGAA
LWY394DAI27054 GGTTCGTGC-------
AATCTCCGGCGTCGGAGGACC-------TCAGAA
FP135015G_pannocinctus --
L15726SpG_pannocinctus --
Dai22221
GGTTAGTTCAAGACAACTGTTTTAGCGCCTGAAC----------------
Dai22633 --
Dai23260 --

Dai23626 --
Dai16238G_citrinoalbus --
1175Dai15293 --
Dai19547 --
918063G_africanus --
918572G_africanus --
Dai18536A --
1164Cui17922 --
Dai22225 --
1163Dai20655 --
Yuan4397G_hainanensis --
1176Dai15268 GGTTA-------------
GTCTCAATCAATGCGCCGATATC----CTCTG
1177Dai15259 GGTTA-------------
GTCTCAATCAATGCGCCGATATCG--TCTCTG
BZ2896G_thelephoroides --
1166JV1808_26 GGTTAG-----------
TGTTTTAATCCCAGCGCTCATGTACAGCCTCTA
Miettinen16992Hapalopilus_ochr --
GC1708_338_Ceriporia_arbuscula --
WCG1555Dai26107Ceriporia GGTTAGTA-------------
GTGACGTGGGAATATTCG-GCGTGGATAG
GC1708_340_Ceriporia_arbuscula --
WCG1556Dai26109Ceriporia GGTTAGTA-------------
GTGACGTGGGAATATTCG-GCGTGGATAG
883Cui11291 GGTCAGCA-------------
ATGACGTGGGAATCTTCG-AAATGAATTG
HLX320Dai26805 GGTCAGTA-------------
GTGACGTGGGAATTTTCC-AAAGGAATAG
WCG1266Dai24678A GGTCAGTA-------------
CTGACGTGGGAATCCTCG-AAAGCAATAG
Dai6090_Ceriporia_sulphuricolo --
RLG_11354_Ceriproia_reticulata --
ZZW1543Dai27072 GGTCAG-------T-
GGATTTGTGGCGTGGAAAATA--------TAGAG
Li1316_Ceriporia_reticulata --
KHL11981Ceriporia_reticulata --
FP110343sp_Candelabrochaete_la --
Li1045_Ceriporia_reticulata --
ZX136Dai25794ceriporia GGTTGG-------
TCAAGTTTGTGGCGTGAGAATTTA--------TGCTG
892Dai13400 GGTTGG-------
TCAAGTTTGTGGCGTGAGAATTTA--------TGCTG
RLG7163Leptoporus_mollis --

Dai21062Leptoporus_mollis GGTCAG-------CGGAAT---CGGCGTGCGAAGTT--------GTACAG

Dai20182Leptoporus_submollis GGTCAG-------CGGATTTTGTGGCGTGCGAAGTT--------ATACAG

Cui18379Leptoporus_submollis GGTCAG-------CGGATTTTGTGGCGTGCGAAGTT--------ATACAG

Wu1209_46Resiniporus_pseudogil GGTTAG---------TATTTGTGCTGTGTTGCAATTGTG---AAATTTG

BRNM710169Resiniporus_resinasc --

Dai14516Bjerkandera_adusta AGTCAGCGC----TTTTTTTCGAGTCATTAAAA-----------------

Dai21100Bjerkandera_fumosa GGTTGGTTC----TCTTCCGCCGTTTATTGAAAATCC-------------

Miettinen16854Ceraceomyces_sp --

Dai10477C_spissa --

855Dai16831 GGTAAG---------ATCACCTCATCGCGCCGTTCTC-------GCAATA

882Cui11282 GGTAAG---------ATCACCTCGTCGCGCCGTTCTC-------GCAATA

Dai24566 GGTAAG---------ATTATCCCATCGCGCCGTTCTC-------TCAATG

Yuan5965 --

Dai3204 --

1194CUI9985 --

Dai15205_Ceriporia_albomellea --

Dai15223_Ceriporia_albomellea --

Li1780_Ceriporia_variegata --

Dai19791_Ceriporia_variegata --

Dai19886 ACGTTGACTGAGGTT--------ATAGTGGTCCGAGGATCGCTTCAACGA

Dai10833_Ceriporia_crassitunic --

CHWC1506_46Meruliopsis_crassit --

Dai9995_Ceriporia_crassitunica --

Wu1209_58_Meruliopsis_parvispo --

CHWC1505_129_Meruliopsis_parvi --

Dai21944 CCGCTGACTGAAT-----CACT-ACAGTGGTCTGAGGATCGCTTCAACGA

830Dai18640A CCACTGACCGAAT--------T-ACAGTGGTCTGAGGATCGTTTCAACGA

GC1704_60_Meruliopsis_taxicola --

Dai22625 ACGCTGATTAATCATTATCTGT-GTAGTGGTCCGAGGATCGTTTCAACGA

Dai22636 ACGCTGATTAATCATTATCTGT-
GTAGTGGTCCGAGGATCGTTTCAACGA
Dai21878 ACGCTGATTAATCATTATCTGT-
GTAGTGGTCCGAGGATCGTTTCAACGA
1169Dai17248 ACGCTGATTAATCATTATCTGT-
GTAGTGGTCCGAGGATCGTTTCAACGA
Wu1708_43_Meruliopsis_leptocys --
Li1011 --
ZX95Dai25742Meruliopsis_leptoc --
WCG1306Dai24733 ACGCTGACCGGAT--------T-
GCAGTGGTCTGAGGATCGTTTCAACGA
LXL99Dai25816 ACGCTGACCGGAT--------T-
GCAGTGGTCTGAGGATCGTTTCAACGA
WCG1559Dai26052Meruliopsis ACGCTAACAGAAC--------T-
GCAGTGGTCTGAGGATCGCTTCAACGA
He7477 --
HLX243Dai26217 ACGCTAACAGAAC--------T-
GCAGTGGTCTGAGGATCGCTTCAACGA
RussiaMW673659Meruliopsis_fagi --
FD278 --
Dai10226_Ceriporia_tarda --
LE247365 --
Dai8173_Meruliopsis_nanlingens --
860Dai17172 ACGCTGAATGCAT--------T-
TTAGTGGTCTGAGGACCGTTTCAACGA
879Dai13414 ACGCTGAATGCAT--------T-
TTAGTGGTCTGAGGACCGTTTCAACGA
Li_1704_Meruliopsis_pseudocyst --
833Dai18405 ACGCTGATTGGAT--------C-
GCAGTGGTCTGAGGACCGATTCAGCGA
HHB_10729_Meruliopsis_albostra --
Cui6878_Ceriporia_pseudocystid --
869Dai14737 -CACTCACTATTGAT--------
GCAGTGGAGCGAGGACCGTTTCAACGA
876Cui11626 --
1199WEI3388 --
776308_Meruliopsis_cystidiata --
ICN139059_Meruliopsis_cystidia --
HHB15692Ceraceomyces_serpens --
HHB_15629_Sp_Ceriporiopsis_ane --
AJ185Trametopsis_cervina --
FD9Irpex_lacteus --
908Dai11230 GCGCTAATAGGTA------TAT-
GCAGTGGTCTGAGGATCGTTTCAACGA

FP55521TEmmia_lacerata ---
PBU0048Ceriporia_cystidiata ---
MZ340C_laceratаT ---
Dai21940 GTACTGACTGCGA------TCT-GTAGTGGTCCGAGTCTCGTTTCAACGA
847Dai16433 GTACTGACTGCGA------TCT-GTAGTGGTCCGAGTCTCGTTTCAACGA
MarcinEmmia_latemarginatus ---
Meijer3729Hydnopolyporus_fimbr ---
RLG13408Phanerochaete_sp ---
WHC1381Flavodon_flavus ---
GB1833Phlebia_albida GCGCTGATTATGA------TAT-GCAGTGGTCCGAGGACCGTTTCAACGA
T407Phlebia_nitidula GCGCTGACTATGG------TAT-GCAGTGGTCCGAGGACCGTTTCAACGA
HHB6988Phanerochaete_exilis ---
HHB8509Phanerochaetella_xeroph ---
PBU0051Macrohyporia_dictyopora ---
HHB11463Phanerochaete_sp ---
FP102382Byssomerulius_corium ---
FP102165Efibula_americana AGGCTGAACCTTT------TTT-TCAGTGGTCTGAAGACCGTTTCAACGA
Murdoch90Ceriporia_torpida ---
Rivoire4413_Ceriporia_purpurea ---
Kout_18_Ceriporia_triumphalis ---
Rivoire3701_Ceriporia_bresadol ---
VS4018 ---
Ryvarden21832_Ceriporia_manzan ---
Dai24539 GTGCTGACTTCTC------TGC-ACAGTGGTCTGAGGATCGCTTCAACGA
Dai24541 GTGCTGACTTCTC------TGC-ACAGTGGTCTGAGGATCGCTTCAACGA
JV1105_12_Ceriporia_occidental ---
VS8558Ceriporia_occidentalis ---
Dai22445 ATGCTGACTTCTC------TAT-GCAGTGGTCTGAGGATCGTTTCAACGA
846Dai16368 ATGCTGACTTCTC------TAT-GCAGTGGTCTGAGGATCGTTTCAACGA
Dai17951_Ceriporia_aurantiocar ---
Miettinen_11701C_viridans ---
JV0105_10Ceriporia_aurantiocar ---
Yuan5702C_viridans ---
858Dai17003 ---
Yuan2747_Ceriporia_viridans ---

Yuan2744C_viridans --
Li1046C_viridans --
865C_sinoviridans --
871Dai15062 --
Dai7642_Ceriporia_humilis ACGCTGATTGTTC------CGT-ACAGTGGTCCGAGGACCGCTTCAATGA
Spirin4706_Ceriporia_humilis --
Spirin4944_Ceriporia_sericea --
WCG1547Dai26044ceriporia ACGCTGACTGTTC------CGT-GCAGTGGTCCGAGGACCGCTTCAACGA
ZZW1558Dai27086 ACGCTGACTGTTC------CGT-GCAGTGGTCCGAGGACCGCTTCAACGA
Miettinen14381_Ceriporia_mpuri --
Miettinen15492_2_Ceriporia_sor --
He6687 ACGCTGACTGTTC------CGT-ACAGTGGTCCGAGGACCGCTTCAATGA
ZH53Dai24426 --
Vlasak0808_30_Ceriporia_punica --
887Dai13376 ATACTGAACGTAT------TTC-ATAGTGGTCAGAGGATCGATTCAACGA
WCG1443Dai24998 ATACTGACCGTCT------TGA-ATAGTGGTCAGAGGACCGATTCAACGA
0108_6Ceriporia_spissa --
Dai19164 ATACTAATTAATG------TGT-ATAGTGGTCCAAGTCCCGTTACGACGA
Dai17937_Ceriporia_bubalinomar --
903Dai12113 --
LZB929Dai25079 GTGCTGACTAGTT------TGT-GCAGTGGTCTGAGGACCGTTTCAACGA
LX45Dai26988 GTGCTGACTAGTT------TGT-GCAGTGGTCTGAGGACCGTTTCAACGA
LX43Dai26986 GTGCTGACTAGTT------TGT-GCAGTGGTCTGAGGACCGTTTCAACGA
Dai7759Ceriporia --
Cui8012_Ceriporia_viridans --
GC1704_54Ceriporia_viridans --
Dai23392 ACGCTGACCAACC------GCA-ACAGTGGTCTGAAGACCGTTTCAACGA
WCG1585Dai26113Ceriproia ACGCTGATAAACC------GCA-ACAGTGGTCTGAAGACCGTTTCAACGA
Dai18675C_eucalypti ACGCTGATCAATC------GGA-ACAGTGGTCTGAAGACCGTTTCAACGA
Dai22034 GCGCTGACCGGTC------TCT-GCAGTGGTCTGAGGACCGTTTCAACGA

JV1008_41JTardaFLORIDAKeys --
Rivoire1161_Ceriporia_pierii --
Dai23499C_pierii ACGCTGACTA-TC------TGT-ACAGTGGTCTGAGGACCGATTCAACGA
Dai23500 ACGCTGACTA-TC------TGT-ACAGTGGTCTGAGGACCGATTCAACGA
841Dai15899 ACGCTGACTGGTA------TGC-ACAGTGGTCTGAGGACCGTTTCAACGA
842Dai15904 ACGCTGACTGGGTA-----TGC-ACAGTGGTCTGAGGACCGTTTCAACGA
LZB1066xinjiang --
LZB1065xinjiang --
851Dai16779 --
RMJ119sp_Candelabrochaete_sept --
RLG9759spCandelabrochaete_sept --
RLG10478Phanerochaete_allantos --
Dai19118_Ceriporia_spissa --
Dai18486A --
WEI17_024_Ceriporia_mellita --
GC1508_71Ceriporia_mellita --
GC1608_7_Ceriporia_mellita --
ZZW1557Dai27085 --
ZZW1554Dai27083 --
Dai8168 ACATTGATCGACA------TTTCTCAGTGGTCCGAGGATCGTTTCATTGA
BR4865C_mellita --
MEL2382688Ceriporia_sp --
Dai8110 --
Cui8097 --
909Cui6740 --
W1258Dai24695 --
JV0110_26_Ceriporia_griseoviol --
896Dai13202 GCGCTAACTGCTC------TATCACAGTGGTCTGAGGACCGTTTCAATGA
LWY393Dai27053C_griseoviolasce GCGCTAACTGCTC------TATCACAGTGGTCTGAGGACCGTTTCAATGA
LWY394DAI27054 GCGCTAACTGCTC------TATCACAGTGGTCTGAGGACCGTTTCAATGA
FP135015G_pannocinctus --
L15726SpG_pannocinctus --
Dai22221 -TGCTTATTAACTTT----TTT-GCAGTGGAGCGAGGACCGTTTCAACGA
Dai22633 ------------------------AGTGGAGCGAGGACCGTTTCAACGA

Dai23260 ------------------------
AGTGGAGCGAGGACCGTTTCAACGA
Dai23626 ------------------------
AGTGGAGCGAGGACCGTTTCAACGA
Dai16238G_citrinoalbus --
1175Dai15293 ------------------------
AGTGGAGCGAAGACCGTTTCAACGA
Dai19547 ------------------------
AGTGGAGCGAAGACCGTTTCAACGA
918063G_africanus --
918572G_africanus --
Dai18536A --
1164Cui17922 ------------------------
AATGGAGCGAAGACCGTTTCAATGA
Dai22225 ------------------------
AGTGGAGCGAAGACCGTTTCAACGA
1163Dai20655 ------------------------
AGTGGAGCGAAGACCGTTTCAACGA
Yuan4397G_hainanensis --
1176Dai15268 ATGTTGAGTTTAA------CGT-
GTAGTGGAGTGAGGACCGTTTCAACGA
1177Dai15259 ATGTTGAGTTTAA------CGT-
GTAGTGGAGTGAGGACCGTTTCAACGA
BZ2896G_thelephoroides --
1166JV1808_26 ATGGTGTGTACTA------TAC--
TAGTGGAGTGAGGACCGTTTCAATGA
Miettinen16992Hapalopilus_ochr --
GC1708_338_Ceriporia_arbuscula --
WCG1555Dai26107Ceriporia ACGCTGACTGGTG------TGT-
GCAGTGGTCTGAGGACCGGTTCAATGA
GC1708_340_Ceriporia_arbuscula --
WCG1556Dai26109Ceriporia ACGCTGACTGGTG------TGT-
GCAGTGGTCTGAGGACCGGTTCAATGA
883Cui11291 ACGCTGACTGGCC------TGT-
GCAGTGGTCTGAGGACCGTTTCAATGA
HLX320Dai26805 ACGCTGACTGGTG------TGT-
TCAGTGGTCTGAGGACCGCTTCAACGA
WCG1266Dai24678A ACGCTGACTGGTG------TGT-
GCAGTGGTCTGAGGACCGCTTCAACGA
Dai6090_Ceriporia_sulphuricolo --
RLG_11354_Ceriproia_reticulata --
ZZW1543Dai27072 ATGCTCAATT-TC------AAC-
ACAGTGGTCCGAGGACCGATTCAACGA
Li1316_Ceriporia_reticulata --

KHL11981Ceriporia_reticulata --
FP110343sp_Candelabrochaete_la --
Li1045_Ceriporia_reticulata --
ZX136Dai25794ceriporia ACAACAAAAT-CT------CGT-GCAGTGGTCTGAGGATCGATTCAACGA
892Dai13400 ACAACAAAAT-CT------CGT-GCAGTGGTCTGAGGATCGATTCAACGA
RLG7163Leptoporus_mollis --
Dai21062Leptoporus_mollis ACGCTGATTGGTC------TGA-GTAGTGGTCTGAGGATCGGTTCAACGA
Dai20182Leptoporus_submollis ACGCTGATTGGTC------TGA-GTAGTGGTCTGAGGATCGATTCAACGA
Cui18379Leptoporus_submollis ACGCTGATTGGTC------TGA-GTAGTGGTCTGAGGATCGATTCAACGA
Wu1209_46Resiniporus_pseudogil TCGCTGACCTCCGTT--------ACAGTGGAGTGAGGACCGTTTCAACGA
BRNM710169Resiniporus_resinasc --
Dai14516Bjerkandera_adusta ----TAAATCTCACC--CCTAT-GTAGTGGAGCGAGGACCGTTTCAACGA
Dai21100Bjerkandera_fumosa -CATCAAATTTACCT--TTTGT-ATAGTGGAGCGAGGACCGTTTCAACGA
Miettinen16854Ceraceomyces_sp --
Dai10477C_spissa --
855Dai16831 ACGCTGACTGGTG------TGT-CTAGTGGTCCGAGGATCGCTTCAACGA
882Cui11282 ACGCTGACTGGTG------TGT-CTAGTGGTCCGAGGATCGCTTCAACGA
Dai24566 TCGCTGACTGGTG------TGT-TTAGTGGTCTGAGGATCGCTTCAACGA
Yuan5965 --
Dai3204 --
1194CUI9985 --

Dai15205_Ceriporia_albomellea --
Dai15223_Ceriporia_albomellea --
Li1780_Ceriporia_variegata --
Dai19791_Ceriporia_variegata --
Dai19886 AATCATCAAGGAGACATCCACTTTCATCAAGAAGGTTGGCTACAACCCTA
Dai10833_Ceriporia_crassitunic --
CHWC1506_46Meruliopsis_crassit --
Dai9995_Ceriporia_crassitunica --
Wu1209_58_Meruliopsis_parvispo --

CHWC1505_129_Meruliopsis_parvi --
Dai21944
AATTATCAAGGAGACGTCTACCTTCATCAAGAAGGTCGGCTACAACCCCA
830Dai18640A
AATTATCAAGGAGACGTCTACCTTCATCAAGAAGGTCGGTTACAACCCCA
GC1704_60_Meruliopsis_taxicola --
Dai22625
AATCATCAAGGAGACGTCCACCTTCATCAAGAAGGTTGGTTACAACCCCA
Dai22636
AATCATCAAGGAGACGTCCACCTTCATCAAGAAGGTTGGTTACAACCCCA
Dai21878
AATCATCAAGGAGACGTCCACCTTCATCAAGAAGGTTGGTTACAACCCCA
1169Dai17248
AATCATCAAGGAGACGTCCACCTTCATCAAGAAGGTTGGTTACAACCCCA
Wu1708_43_Meruliopsis_leptocys --
Li1011 --
ZX95Dai25742Meruliopsis_leptoc --
WCG1306Dai24733
AATCATCAAGGAGACGTCCACCTTCATCAAGAAAGTCGGCTACAACCCCA
LXL99Dai25816
AATCATCAAGGAGACGTCCACCTTCATCAAGAAAGTCGGCTACAACCCCA
WCG1559Dai26052Meruliopsis
AATCGTCAAGGAGACGTCCACCTTCATCAAGAAGGTCGGCTACAACCCCA
He7477 --
HLX243Dai26217
AATCGTCAAGGAGACGTCCACCTTCATCAAGAAGGTCGGCTACAACCCCA
RussiaMW673659Meruliopsis_fagi --
FD278 --
Dai10226_Ceriporia_tarda --
LE247365 --
Dai8173_Meruliopsis_nanlingens --
860Dai17172
AATCATCAAGGAGACGTCCACCTTCATCAAGAAGGTCGGTTACAACCCCA
879Dai13414
AATCATCAAGGAGACGTCCACCTTCATCAAGAAGGTCGGTTACAACCCCA
Li_1704_Meruliopsis_pseudocyst --
833Dai18405
AATCGTCAAGGAGACGTCCACCTTCATCAAGAAGGTCGGCTACAACCCCA
HHB_10729_Meruliopsis_albostra --
Cui6878_Ceriporia_pseudocystid --
869Dai14737
AATCGTCAAGGAGACGTCCACTTTCATCAAGAAGGTCGGCTACAACCCGA
876Cui11626 --
1199WEI3388 --

776308_Meruliopsis_cystidiata --
ICN139059_Meruliopsis_cystidia --
HHB15692Ceraceomyces_serpens --
HHB_15629_Sp_Ceriporiopsis_ane --
AJ185Trametopsis_cervina --
FD9Irpex_lacteus --
908Dai11230
AATCGTTAAGGAGACGTCCACCTTCATCAAAAAGGTTGGTTACAACCCCA
FP55521TEmmia_lacerata --
PBU0048Ceriporia_cystidiata --
MZ340C_laceratаT --
Dai21940
AATCGTTAAGGAGACTTCCACCTTCATCAAAAAGGTTGGTTACAACCCCA
847Dai16433
AATCGTTAAGGAGACTTCCACCTTCATCAAAAAGGTTGGTTACAACCCCA
MarcinEmmia_latemarginatus --
Meijer3729Hydnopolyporus_fimbr --
RLG13408Phanerochaete_sp --
WHC1381Flavodon_flavus --
GB1833Phlebia_albida
AATCATCAAGGAAACGTCCACCTTCATCAAAAAGGTTGGTTACAACCCCA
T407Phlebia_nitidula
AATCATCAAGGAGACGTCCACCTTCATCAAAAAGGTTGGTTACAACCCCA
HHB6988Phanerochaete_exilis --
HHB8509Phanerochaetella_xeroph --
PBU0051Macrohyporia_dictyopora --
HHB11463Phanerochaete_sp --
FP102382Byssomerulius_corium --
FP102165Efibula_americana
AATTATCAAGGAGACGTCTACCTTCATCAAAAAGGTTGGCTACAACCCCA
Murdoch90Ceriporia_torpida --
Rivoire4413_Ceriporia_purpurea --
Kout_18_Ceriporia_triumphalis --
Rivoire3701_Ceriporia_bresadol --
VS4018 --
Ryvarden21832_Ceriporia_manzan --
Dai24539
AATCGTCAAGGAGACGTCCACCTTCATCAAGAAGGTCGGTTATAACCCCA
Dai24541
AATCGTCAAGGAGACGTCCACCTTCATCAAGAAGGTCGGTTACAACCCCA
JV1105_12_Ceriporia_occidental --
VS8558Ceriporia_occidentalis --
Dai22445
AATTGTCAAGGAGACGTCCACCTTCATCAAGAAGGTCGGTTACAACCCCA

846Dai16368
AATCGTCAAGGAGACGTCCACCTTCATCAAGAAGGTCGGTTACAACCCCA
Dai17951_Ceriporia_aurantiocar --
Miettinen_11701C_viridans --
JV0105_10Ceriporia_aurantiocar --
Yuan5702C_viridans --
858Dai17003 --
Yuan2747_Ceriporia_viridans --
Yuan2744C_viridans --
Li1046C_viridans --
865C_sinoviridans --
871Dai15062 --
Dai7642_Ceriporia_humilis
AATCGTCAAGGAGACGTCCACCTTCATTAAGAAGGTCGGCTACAACCCCA
Spirin4706_Ceriporia_humilis --
Spirin4944_Ceriporia_sericea --
WCG1547Dai26044ceriporia
AATCATCAAGGAGACGTCCACCTTCATTAAGAAGGTCGGCTATAACCCCA
ZZW1558Dai27086
AATCATCAAGGAGACGTCCACCTTCATTAAGAAGGTCGGCTATAACCCCA
Miettinen14381_Ceriporia_mpuri --
Miettinen15492_2_Ceriporia_sor --
He6687
AATCGTCAAGGAGACGTCCACCTTCATTAAGAAGGTCGGCTACAACCCCA
ZH53Dai24426 --
Vlasak0808_30_Ceriporia_punica --
887Dai13376
AATCGTCAAGGAGACGTCCACCTTCATCAAGAAGGTCGGTTACAACCCGA
WCG1443Dai24998
AATCGTCAAGGAAACGTCCACCTTCATCAAGAAGGTCGGTTACAACCCGA
0108_6Ceriporia_spissa --
Dai19164 AATCGTCACGGAAA----------
GCTTAAAAGGTTCGGTTACAACTTGG
Dai17937_Ceriporia_bubalinomar --
903Dai12113 --
LZB929Dai25079
AATCGTCAAGGAGACGTCTACCTTCATCAAGAAGGTCGGTTACAACCCGA
LX45Dai26988
AATCGTCAAGGAGACGTCTACCTTCATCAAGAAGGTCGGTTACAACCCGA
LX43Dai26986
AATCGTCAAGGAGACGTCTACCTTCATCAAGAAGGTCGGTTACAACCCGA
Dai7759Ceriporia --
Cui8012_Ceriporia_viridans --
GC1704_54Ceriporia_viridans --

Dai23392
AATCATCAAGGAGACGTCGACCTTCATCAAGAAGGTCGGTTACAACCCCA
WCG1585Dai26113Ceriproia
AATCATCAAGGAGACGTCGACCTTCATCAAGAAGGTCGGTTACAACCCCA
Dai18675C_eucalypti
AATCATCAAGGAGACGTCGACCTTCATCAAGAAGGTCGGCTACAACCCCA
Dai22034
AATCGTCAAGGAGACGTCCACCTTCATCAAGAAGGTCGGTTACAACCCCA
JV1008_41JTardaFLORIDAKeys --
Rivoire1161_Ceriporia_pierii --
Dai23499C_pierii
GATCGTCAAGGAGACGTCCACCTTCATTAAGAAGGTCGGTTACAACCCCA
Dai23500
GATCGTCAAGGAGACGTCCACCTTCATTAAGAAGGTCGGTTACAACCCCA
841Dai15899
AATCGTTAAGGAGACGTCTACCTTCATCAAGAAGGTTGGTTACAACCCCA
842Dai15904
AATCGTTAAGGAGACGTCTACCTTCTTCAGAAGGGTGGGTTCCACCCCCA
LZB1066xinjiang --
LZB1065xinjiang --
851Dai16779 --
RMJ119sp_Candelabrochaete_sept --
RLG9759spCandelabrochaete_sept --
RLG10478Phanerochaete_allantos --
Dai19118_Ceriporia_spissa --
Dai18486A --
WEI17_024_Ceriporia_mellita --
GC1508_71Ceriporia_mellita --
GC1608_7_Ceriporia_mellita --
ZZW1557Dai27085 --
ZZW1554Dai27083 --
Dai8168
GATTGTCAAGGAAACGTCCAACTTCATCAAGAAGGTTGGTTACAACCCGA
BR4865C_mellita --
MEL2382688Ceriporia_sp --
Dai8110 --
Cui8097 --
909Cui6740 --
W1258Dai24695 --
JV0110_26_Ceriporia_griseoviol --
896Dai13202
AATCGTCAAAGAGACGTCCACCTTCATCAAGAAGGTCGGTTACAACCCCA
LWY393Dai27053C_griseoviolasce
AATCGTCAAAGAGACGTCCACCTTCATCAAGAAGGTCGGTTACAACCCCA

LWY394DAI27054
AATCGTCAAAGAGACGTCCACCTTCATCAAGAAGGTCGGTTACAACCCCA
FP135015G_pannocinctus --
L15726SpG_pannocinctus --
Dai22221
AATCATCAAGGAAACCTCCACTTTCATCAAGAAGGTCGGTTACAACCCCA
Dai22633
AATCGTCAAGGAAACCTCCACCTTCATCAAGAAGGTCGGCTACAACCCCA
Dai23260
AATCGTCAAGGAAACCTCCACCTTCATCAAGAAGGTCGGCTACAACCCCA
Dai23626
AATCGTCAAGGAAACCTCCACCTTCATCAAGAAGGTCGGCTACAACCCCA
Dai16238G_citrinoalbus --
1175Dai15293
AATCGTCAAGGAAACCTCCACCTTCATCAAGAAGGTCGGTTACAACCCCA
Dai19547
AATCGTCAAGGAAACCTCCACCTTCATCAAGAAGGTCGGTTACAACCCCA
918063G_africanus --
918572G_africanus --
Dai18536A --
1164Cui17922
AATTGTCAAGGAAACCTCCACCTTCATCAAGAAGGTCGGTTACAACCCCA
Dai22225
AATCGTCAAGGAAACCTCCACTTTCATCAAGAAGGTCGGTTACAACCCCA
1163Dai20655
AATCGTCAAGGAAACCTCCACTTTCATCAAGAAGGTCGGTTACAACCCCA
Yuan4397G_hainanensis --
1176Dai15268
AATCGTCAAGGAGACGTCCACCTTCATCAAGAAGGTCGGCTACAACCCCA
1177Dai15259
AATCGTCAAGGAGACGTCCACCTTCATCAAGAAGGTCGGCTACAACCCCA
BZ2896G_thelephoroides --
1166JV1808_26
AATCGTCAAGGAGACATCCACCTTCATCAAGAAGGTTGGTTACAACCCCA
Miettinen16992Hapalopilus_ochr --
GC1708_338_Ceriporia_arbuscula --
WCG1555Dai26107Ceriporia
AATCGTCAAGGAGACGTCTACCTTCATCAAGAAGGTCGGTTACAACCCTA
GC1708_340_Ceriporia_arbuscula --
WCG1556Dai26109Ceriporia
AATCGTCAAGGAGACGTCTACCTTCATCAAGAAGGTCGGTTACAACCCCA
883Cui11291
AATCGTCAAGGAGACGTCTACCTTCATCAAGAAGGTCGGCTACAACCCCA
HLX320Dai26805

AATCGTCAAGGAGACGTCCACCTTCATCAAGAAGGTCGGCTACAACCCCA
WCG1266Dai24678A
AATCGTCAAGGAGACGTCCACCTTCATCAAGAAGGTCGGCTACAACCCCA
Dai6090_Ceriporia_sulphuricolo --
RLG_11354_Ceriproia_reticulata --
ZZW1543Dai27072
GATTGTTAAGGAGACGTCTACCTTCATCAAGAAGGTTGGTTACAACCCCA
Li1316_Ceriporia_reticulata --
KHL11981Ceriporia_reticulata --
FP110343sp_Candelabrochaete_la --
Li1045_Ceriporia_reticulata --
ZX136Dai25794ceriporia
AATCGTCAAGGAGACGTCCACCTTCATCAAGAAGGTCGGCTACAACCCCA
892Dai13400
AATCGTCAAGGAGACGTCCACCTTCATCAAGAAGGTCGGCTACAACCCCA
RLG7163Leptoporus_mollis --
Dai21062Leptoporus_mollis
AATCGTCAAGGAGACGTCCACCTTCATCAAGAAGGTCGGATATAACCCCA
Dai20182Leptoporus_submollis
AATCGTCAAGGAGACGTCCACCTTCATCAAGAAGGTCGGATATAACCCTA
Cui18379Leptoporus_submollis
AATCGTCAAGGAGACGTCCACCTTCATCAAGAAGGTCGGATATAACCCTA
Wu1209_46Resiniporus_pseudogil
GATTGTCAAGGAAACGTCTACCTTCATCAAGAAGGTTGGCTACAACCCGA
BRNM710169Resiniporus_resinasc --
Dai14516Bjerkandera_adusta
AATCGTCAAGGAGACCTCCACCTTCATCAAGAAGGTCGGCTACAACCCCA
Dai21100Bjerkandera_fumosa
AATCGTCAAGGAGACCTCCACCTTCATCAAGAAGGTCGGCTACAACCCCA
Miettinen16854Ceraceomyces_sp --
Dai10477C_spissa --
855Dai16831
AATCGTCAAGGAGACGTCTACCTTCATCAAGAAGGTCGGTTACAACCCGA
882Cui11282
AATCGTCAAGGAGACGTCTACCTTCATCAAGAAGGTCGGTTACAACCCGA
Dai24566
AATCGTCAAGGAGACGTCGACCTTCATCAAGAAGGTCGGTTACAACCCGA
Yuan5965 --
Dai3204 --
1194CUI9985 --

Dai15205_Ceriporia_albomellea --
Dai15223_Ceriporia_albomellea --

Li1780_Ceriporia_variegata --

Dai19791_Ceriporia_variegata --

Dai19886

AGAGTGTTGCCTTCGTGCCTATCTCTGGCTGGCACGGCGACAACATGTTG

Dai10833_Ceriporia_crassitunic --

CHWC1506_46Meruliopsis_crassit --

Dai9995_Ceriporia_crassitunica --

Wu1209_58_Meruliopsis_parvispo --

CHWC1505_129_Meruliopsis_parvi --

Dai21944

AGTCCGTCGCTTTCGTCCCCATCTCTGGCTGGCACGGTGACAACATGTTG

830Dai18640A

AGGCCGTCGCCTTCGTGCCCATCTCTGGCTGGCACGGTGATAACATGTTG

GC1704_60_Meruliopsis_taxicola --

Dai22625

AGGCCGTCGCCTTCGTGCCCATCTCTGGCTGGCACGGAGACAACATGTTG

Dai22636

AGGCCGTCGCCTTCGTGCCCATCTCTGGCTGGCACGGAGACAACATGTTG

Dai21878

AGGCCGTCGCCTTCGTGCCCATCTCTGGCTGGCACGGTGACAACATGTTG

1169Dai17248

AGGCCGTCGCCTTCGTGCCCATCTCTGGCTGGCACGGTGACAACATGTTG

Wu1708_43_Meruliopsis_leptocys --

Li1011 --

ZX95Dai25742Meruliopsis_leptoc --

WCG1306Dai24733

AGGCCGTCGCTTTCGTGCCCATTTCTGGCTGGCACGGTGACAACATGTTG

LXL99Dai25816

AGGCCGTCGCTTTCGTGCCCATTTCTGGCTGGCACGGTGACAACATGTTG

WCG1559Dai26052Meruliopsis

AGGCCGTCGCCTTCGTGCCCATCTCTGGCTGGCACGGTGACAACATGTTG

He7477 --

HLX243Dai26217

AGGCCGTCGCCTTCGTGCCCATCTCTGGCTGGCACGGTGACAACATGTTG

RussiaMW673659Meruliopsis_fagi --

FD278 --

Dai10226_Ceriporia_tarda --

LE247365 --

Dai8173_Meruliopsis_nanlingens --

860Dai17172

AGGCCGTCGCCTTCGTGCCCATCTCTGGCTGGCATGGTGACAACATGTTG

879Dai13414

AGGCCGTCGCCTTCGTGCCCATCTCTGGCTGGCATGGTGACAACATGTTG

Li_1704_Meruliopsis_pseudocyst --

833Dai18405
AGGCTGTCGCCTTCGTGCCCATTTCTGGCTGGCATGGCGACAACATGTTG
HHB_10729_Meruliopsis_albostra --
Cui6878_Ceriporia_pseudocystid --
869Dai14737
AGGCGGTCGCTTTCGTGCCCATCTCCGGCTGGCATGGCGACAACATGTTG
876Cui11626 --
1199WEI3388 --
776308_Meruliopsis_cystidiata --
ICN139059_Meruliopsis_cystidia --
HHB15692Ceraceomyces_serpens --
HHB_15629_Sp_Ceriporiopsis_ane --
AJ185Trametopsis_cervina --
FD9Irpex_lacteus --
908Dai11230
AGGCCGTTGCCTTCGTCCCCATCTCCGGCTGGCACGGTGACAACATGTTG
FP55521TEmmia_lacerata --
PBU0048Ceriporia_cystidiata --
MZ340C_lacerataT --
Dai21940
AGGCCGTCGCCTTCGTCCCCATCTCCGGCTGGCACGGTGATAACATGTTG
847Dai16433
AGGCCGTCGCCTTCGTCCCCATCTCCGGCTGGCACGGTGATAACATGTTG
MarcinEmmia_latemarginatus --
Meijer3729Hydnopolyporus_fimbr --
RLG13408Phanerochaete_sp --
WHC1381Flavodon_flavus --
GB1833Phlebia_albida
AGGCCGTCGCTTTCGTCCCGATTTCTGGCTGGCACGGCGACAACATGTTG
T407Phlebia_nitidula
AGGCCGTTGCTTTCGTCCCGATTTCCGGCTGGCACGGTGACAACATGTTG
HHB6988Phanerochaete_exilis --
HHB8509Phanerochaetella_xeroph --
PBU0051Macrohyporia_dictyopora --
HHB11463Phanerochaete_sp --
FP102382Byssomerulius_corium --
FP102165Efibula_americana
AGGCTGTTGCCTTCGTTCCCATCTCCGGCTGGCACGGTGACAACATGTTG
Murdoch90Ceriporia_torpida --
Rivoire4413_Ceriporia_purpurea --
Kout_18_Ceriporia_triumphalis --
Rivoire3701_Ceriporia_bresadol --
VS4018 --
Ryvarden21832_Ceriporia_manzan --

Dai24539
AGGCCGTCGCCTTCGTGCCCATCTCTGGCTGGCACGGTGACAACATGTTG
Dai24541
AGGCCGTCGCCTTCGTGCCCATCTCTGGCTGGCACGGTGACAACATGTTG
JV1105_12_Ceriporia_occidental --
VS8558Ceriporia_occidentalis --
Dai22445
AGGCTGTCGCCTTCGTGCCCATCTCTGGCTGGCACGGAGACAACATGTTG
846Dai16368
AGGCTGTCGCCTTCGTGCCCATCTCTGGCTGGCACGGAGACAACATGTTG
Dai17951_Ceriporia_aurantiocar --
Miettinen_11701C_viridans --
JV0105_10Ceriporia_aurantiocar --
Yuan5702C_viridans --
858Dai17003 --
Yuan2747_Ceriporia_viridans --
Yuan2744C_viridans --
Li1046C_viridans --
865C_sinoviridans --
871Dai15062 --
Dai7642_Ceriporia_humilis
AGGCCGTCGCCTTCGTGCCTATCTCTGGGTGGCATGGTGACAACATGTTG
Spirin4706_Ceriporia_humilis --
Spirin4944_Ceriporia_sericea --
WCG1547Dai26044ceriporia
AGGCCGTCGCCTTCGTGCCTATCTCTGGCTGGCACGGTGACAACATGTTG
ZZW1558Dai27086
AGGCCGTCGCCTTCGTGCCTATCTCTGGCTGGCACGGTGACAACATGTTG
Miettinen14381_Ceriporia_mpuri --
Miettinen15492_2_Ceriporia_sor --
He6687
AGGCCGTCGCCTTCGTGCCCATCTCTGGCTGGCATGGTGACAACATGTTG
ZH53Dai24426 --
Vlasak0808_30_Ceriporia_punica --
887Dai13376
AGGCCGTCGCTTTCGTCCCTATCTCTGGCTGGCATGGTGACAACATGTTG
WCG1443Dai24998
AGGCCGTCGCTTTCGTCCCCATCTCTGGCTGGCATGGTGACAACATGTTG
0108_6Ceriporia_spissa --
Dai19164
ACTCCGTTCAATTCTTGCCTATTTCTGGCTTCCACGGTGACAACATGCTG
Dai17937_Ceriporia_bubalinomar --
903Dai12113 --
LZB929Dai25079

AGGCCGTCGCCTTCGTGCCCATCTCTGGCTGGCACGGTGACAACATGTTG
LX45Dai26988
AGGCCGTCGCCTTCGTGCCCATCTCTGGCTGGCACGGTGACAACATGTTG
LX43Dai26986
AGGCCGTCGCCTTCGTGCCCATCTCTGGCTGGCACGGTGACAACATGTTG
Dai7759Ceriporia --
Cui8012_Ceriporia_viridans --
GC1704_54Ceriporia_viridans --
Dai23392
AGGCTGTCGCCTTCGTCCCCATCTCCGGCTGGCACGGTGACAACATGTTG
WCG1585Dai26113Ceriproia
AGGCTGTCGCCTTCGTGCCCATCTCCGGCTGGCACGGTGACAACATGTTG
Dai18675C_eucalypti
AGGCTGTCGCCTTCGTGCCCATCTCCGGCTGGCACGGTGACAACATGTTG
Dai22034
AGGCTGTCGCCTTCGTGCCCATCTCTGGCTGGCACGGTGACAACATGTTG
JV1008_41JTardaFLORIDAKeys --
Rivoire1161_Ceriporia_pierii --
Dai23499C_pierii
AGGCTGTCGCCTTCGTGCCCATTTCTGGCTGGCACGGTGATAACATGTTG
Dai23500
AGGCTGTCGCCTTCGTGCCCATTTCTGGCTGGCACGGTGATAACATGTTG
841Dai15899
AGGCCGTCGCATTTGTGCCCATCTCCGGCTGGCACGGTGACAACATGTTG
842Dai15904
GGGCCGTCGCATTTGTGCCCATCTCCGGCTGGGACGGGGGACACATGTTG
LZB1066xinjiang --
LZB1065xinjiang --
851Dai16779 --
RMJ119sp_Candelabrochaete_sept --
RLG9759spCandelabrochaete_sept --
RLG10478Phanerochaete_allantos --
Dai19118_Ceriporia_spissa --
Dai18486A --
WEI17_024_Ceriporia_mellita --
GC1508_71Ceriporia_mellita --
GC1608_7_Ceriporia_mellita --
ZZW1557Dai27085 --
ZZW1554Dai27083 --
Dai8168
AGGCGGTGGCTTTCGTTCCGATTTCTGGCTGGCACGGTGATAACATGTTG
BR4865C_mellita --
MEL2382688Ceriporia_sp --
Dai8110 --

Cui8097 ---
909Cui6740 ---
W1258Dai24695 ---
JV0110_26_Ceriporia_griseoviol ---
896Dai13202
AGGCTGTCGCATTCGTGCCGATCTCAGGCTGGCACGGTGACAACATGTTG
LWY393Dai27053C_griseoviolasce
AGGCTGTCGCATTCGTGCCGATCTCAGGCTGGCACGGTGACAACATGTTG
LWY394DAI27054
AGGCTGTCGCATTCGTGCCGATCTCAGGCTGGCACGGTGACAACATGTTG
FP135015G_pannocinctus ---
L15726SpG_pannocinctus ---
Dai22221
AGAGCGTCGCCTTCGTCCCCATCTCTGGCTGGCACGGTGATAACATGTTG
Dai22633
AGGCCGTCGCTTTCGTCCCCATCTCTGGCTGGCACGGTGACAACATGTTG
Dai23260
AGGCCGTCGCTTTCGTCCCCATCTCTGGCTGGCACGGTGACAACATGTTG
Dai23626
AGGCCGTCGCTTTCGTCCCCATCTCTGGCTGGCACGGTGACAACATGTTG
Dai16238G_citrinoalbus ---
1175Dai15293
AGGCCGTCGCTTTCGTCCCCATCTCTGGCTGGCACGGTGACAACATGTTG
Dai19547
AGGCCGTCGCTTTCGTCCCCATCTCTGGCTGGCACGGTGACAACATGTTG
918063G_africanus ---
918572G_africanus ---
Dai18536A ---
1164Cui17922
AGGCCGTCGCTTTCGTCCCCATCTCTGGCTGGCACGGTGACAACATGTTG
Dai22225
AGGCTGTCGCTTTCGTCCCCATCTCTGGCTGGCACGGTGACAACATGTTG
1163Dai20655
AGGCTGTCGCTTTCGTCCCCATCTCTGGCTGGCACGGTGACAACATGTTG
Yuan4397G_hainanensis ---
1176Dai15268
AGGCTGTTGCCTTCGTGCCTATCTCCGGCTGGCACGGTGACAACATGTTG
1177Dai15259
AGGCTGTTGCCTTCGTGCCTATCTCCGGCTGGCACGGTGACAACATGTTG
BZ2896G_thelephoroides ---
1166JV1808_26
AGGCTGTTGCCTTCGTGCCCATTTCCGGCTGGCACGGTGACAACATGTTG
Miettinen16992Hapalopilus_ochr ---
GC1708_338_Ceriporia_arbuscula ---

WCG1555Dai26107Ceriporia
AGGCCGTCGCTTTCGTGCCCATCTCTGGCTGGCACGGTGACAACATGTTG
GC1708_340_Ceriporia_arbuscula --
WCG1556Dai26109Ceriporia
AGGCCGTCGCTTTCGTGCCCATCTCTGGCTGGCACGGTGACAACATGTTG
883Cui11291
AGGCCGTCGCCTTCGTGCCCATCTCTGGCTGGCACGGTGACAACATGTTG
HLX320Dai26805
AGGCTGTCGCCTTCGTGCCCATCTCCGGCTGGCACGGTGACAACATGTTG
WCG1266Dai24678A
AGGCTGTTGCCTTTGTGCCCATCTCCGGCTGGCACGGTGACAACATGTTG
Dai6090_Ceriporia_sulphuricolo --
RLG_11354_Ceriproia_reticulata --
ZZW1543Dai27072
AGGCCGTCGCTTTCGTGCCCATCTCTGGCTGGCACGGCGACAACATGTTG
Li1316_Ceriporia_reticulata --
KHL11981Ceriporia_reticulata --
FP110343sp_Candelabrochaete_la --
Li1045_Ceriporia_reticulata --
ZX136Dai25794ceriporia
AGGCCGTTGCCTTCGTCCCCATTTCTGGCTGGCACGGTGACAACATGCTG
892Dai13400
AGGCCGTTGCCTTCGTCCCCATTTCTGGCTGGCACGGTGACAACATGCTG
RLG7163Leptoporus_mollis --
Dai21062Leptoporus_mollis
AGGCCGTCGCCTTCGTGCCCATCTCTGGCTGGCACGGTGACAACATGTTG
Dai20182Leptoporus_submollis
AGGCAGTCGCCTTCGTGCCCATCTCTGGCTGGCACGGTGACAACATGTTG
Cui18379Leptoporus_submollis
AGGCAGTCGCCTTCGTGCCCATCTCTGGCTGGCACGGTGACAACATGTTG
Wu1209_46Resiniporus_pseudogil
AGACTGTTGCCTTCGTCCCCATTTCAGGCTGGCACGGCGACAACATGTTG
BRNM710169Resiniporus_resinasc --
Dai14516Bjerkandera_adusta
AGGCCGTCGCCTTCGTCCCCATCTCCGGCTGGCACGGTGACAACATGTTG
Dai21100Bjerkandera_fumosa
AGGCCGTCGCCTTCGTCCCCATCTCCGGCTGGCACGGTGACAACATGTTG
Miettinen16854Ceraceomyces_sp --
Dai10477C_spissa --
855Dai16831
AGGCTGTCGCTTTCGTCCCTATCTCTGGCTGGCACGGTGACAACATGTTG
882Cui11282
AGGCTGTCGCTTTCGTCCCTATCTCTGGCTGGCACGGTGACAACATGTTG
Dai24566

AGGCTGTCGCTTTCGTCCCTATCTCTGGCTGGCACGGCGACAACATGTTG
Yuan5965 ---
Dai3204 ---
1194CUI9985 ---

Dai15205_Ceriporia_albomellea ---
Dai15223_Ceriporia_albomellea ---
Li1780_Ceriporia_variegata ---
Dai19791_Ceriporia_variegata ---
Dai19886
GAGGAGTCACCCAAGTTAGTATATATATCTATAT---------------A
Dai10833_Ceriporia_crassitunic ---
CHWC1506_46Meruliopsis_crassit ---
Dai9995_Ceriporia_crassitunica ---
Wu1209_58_Meruliopsis_parvispo ---
CHWC1505_129_Meruliopsis_parvi ---
Dai21944 GAGGAGTCCGTTAAGTGAGTACTTAGTT------
TTTACGT------AAA
830Dai18640A GAGGAGTCCGCCAAGTGAGTATATGCCT------
CTTTGGTAC---AAAT
GC1704_60_Meruliopsis_taxicola ---
Dai22625 GAGGAGTCTGCCAAGTGAGTTTGCGC-T------
CCCATCC---------
Dai22636 GAGGAGTCTGCCAAGTGAGTTTGCGC-T------
CCCATCC---------
Dai21878 GAGGAGTCTGCCAAGTGAGTTTGCGC-T------
CCCATCT---------
1169Dai17248 GAGGAGTCTGCCAAGTGAGTTTGCGC-T------
CCCATCT---------
Wu1708_43_Meruliopsis_leptocys ---
Li1011 ---
ZX95Dai25742Meruliopsis_leptoc ---
WCG1306Dai24733 GAGGAGTCCGCCAAGTGCGTATATACTT------
TTTGTGC---------
LXL99Dai25816 GAGGAGTCCGCCAAGTGCGTATATACTT------
TTTGTGC---------
WCG1559Dai26052Meruliopsis GAGGAGTCCGCCAAGTGCGTACATGCTC------
TTTGTGC---------
He7477 ---
HLX243Dai26217 GAGGAGTCCGCCAAGTGCGTACATGCTC------
TTTGTGC---------
RussiaMW673659Meruliopsis_fagi ---
FD278 ---

Dai10226_Ceriporia_tarda --
LE247365 --
Dai8173_Meruliopsis_nanlingens --
860Dai17172 GAGGAGTCCGCCAAGTGAGTACATGTGT------
TCTATGC---------
879Dai13414 GAGGAGTCCGCCAAGTGAGTACATGTGT------
TCTATGC---------
Li_1704_Meruliopsis_pseudocyst --
833Dai18405 GAGGAGTCCCCCAAGTGCGTTCCACGAT------
TTCATGC---------
HHB_10729_Meruliopsis_albostra --
Cui6878_Ceriporia_pseudocystid --
869Dai14737 GAGGAGTCTGCCAAGTAAGCGCATCTTC------
AGGATGTCTCTGAGCT
876Cui11626 --
1199WEI3388 --
776308_Meruliopsis_cystidiata --
ICN139059_Meruliopsis_cystidia --
HHB15692Ceraceomyces_serpens --
HHB_15629_Sp_Ceriporiopsis_ane --
AJ185Trametopsis_cervina --
FD9Irpex_lacteus --
908Dai11230 GAGGCCTCCACCAAGTGAGTATATGC-----------
GTTCGGATCGA--
FP55521TEmmia_lacerata --
PBU0048Ceriporia_cystidiata --
MZ340C_lacerataT --
Dai21940
GAGGCTTCCACCAAGTGAGTATATGCGGCCTCTT----------------
847Dai16433
GAGGCTTCCACCAAGTGAGTATATGCGGCCTCTT----------------
MarcinEmmia_latemarginatus --
Meijer3729Hydnopolyporus_fimbr --
RLG13408Phanerochaete_sp --
WHC1381Flavodon_flavus --
GB1833Phlebia_albida GAGCCATCCGCCAAGTGAGTATATGC--------------
------ACTT
T407Phlebia_nitidula GAACCATCCGCCAAGTGAGTATATGC----------------
----GCTT
HHB6988Phanerochaete_exilis --
HHB8509Phanerochaetella_xeroph --
PBU0051Macrohyporia_dictyopora --
HHB11463Phanerochaete_sp --
FP102382Byssomerulius_corium --

FP102165Efibula_americana GAGGCTTCCGTCAAGTGAGTATATGC---------
CGGTACTGAGAGCCT
Murdoch90Ceriporia_torpida --
Rivoire4413_Ceriporia_purpurea --
Kout_18_Ceriporia_triumphalis --
Rivoire3701_Ceriporia_bresadol --
VS4018 --
Ryvarden21832_Ceriporia_manzan --
Dai24539 GAGGAGTCCGCGAAGTGAGTATATGC----------
ACGTCGCTACAAT
Dai24541 GAGGAGTCCGCGAAGTGAGTATATGC----------
ACGTCGCTACAAT
JV1105_12_Ceriporia_occidental --
VS8558Ceriporia_occidentalis --
Dai22445 GAGGAGTCCGCCAAGTGAGTATATGC----------
GAGTCGCTGCGTT
846Dai16368 GAGGAGTCCGCCAAGTGAGTATATGC----------
GAGTCGCTGCGTT
Dai17951_Ceriporia_aurantiocar --
Miettinen_11701C_viridans --
JV0105_10Ceriporia_aurantiocar --
Yuan5702C_viridans --
858Dai17003 --
Yuan2747_Ceriporia_viridans --
Yuan2744C_viridans --
Li1046C_viridans --
865C_sinoviridans --
871Dai15062 --
Dai7642_Ceriporia_humilis GAGGAGTCCGACAAGTGAGTATATGC----------
GCTTGTGCCGAGT
Spirin4706_Ceriporia_humilis --
Spirin4944_Ceriporia_sericea --
WCG1547Dai26044ceriporia GAGGGGTCCCCAAGTGAGTATATGC----------
GCTTCTGTCGAGT
ZZW1558Dai27086 GAGGGGTCCCCAAGTGAGTATATGC---------
-GCTTCTGTCGAGT
Miettinen14381_Ceriporia_mpuri --
Miettinen15492_2_Ceriporia_sor --
He6687 GAGGAGTCCGACAAGTGAGTATATGC----------
GCTTGTGTTGAGT
ZH53Dai24426 --
Vlasak0808_30_Ceriporia_punica --
887Dai13376 GAGGAGTCCAGCAAGTGAATATATGC----------
GCTTCGTCTGCG-

WCG1443Dai24998 GAGGAGTCCAGCAAGTGAGTATATGC---------
--GCTTCGTATACG-
0108_6Ceriporia_spissa --
Dai19164 GAACCTTCCGAAAAGTGAGTAACCTT------------

Dai17937_Ceriporia_bubalinomar --
903Dai12113 --
LZB929Dai25079
GAGGAGTCCGCCAAGTGAGTATATGCGTGACGA-----------------
LX45Dai26988
GAGGAGTCCGCCAAGTGAGTATATGCGTGACGA-----------------
LX43Dai26986
GAGGAGTCCGCCAAGTGAGTATATGCGTGACGA-----------------
Dai7759Ceriporia --
Cui8012_Ceriporia_viridans --
GC1704_54Ceriporia_viridans --
Dai23392
GAGGAGTCCGTCAAGTGCGTATATGCGTGGTGA-----------------
WCG1585Dai26113Ceriproia
GAGGAGTCCGTCAAGTGCGTATATGCGTGGTGA-----------------
Dai18675C_eucalypti
GAGGAGTCGCCCAAGTGCGTATATGCGTGGTGA-----------------
Dai22034
GAGGAGTCCACCAAGTGAGTGTATGCGCGCCAA-----------------
JV1008_41JTardaFLORIDAKeys --
Rivoire1161_Ceriporia_pierii --
Dai23499C_pierii GAGGAGTCCGACAAGTGAGCTTGTGT-----------
GCATCAGCTGA-G
Dai23500 GAGGAGTCCGACAAGTGAGCTTGTGT-----------
GCATCAGCTGA-G
841Dai15899 GAGGAGTCTGCCAAGTGAGTATATACGCTT-
AGT----------------
842Dai15904
GAGGAGTCTGCCCAGTGAGTATATACGCTTTAGT----------------
LZB1066xinjiang --
LZB1065xinjiang --
851Dai16779 --
RMJ119sp_Candelabrochaete_sept --
RLG9759spCandelabrochaete_sept --
RLG10478Phanerochaete_allantos --
Dai19118_Ceriporia_spissa --
Dai18486A --
WEI17_024_Ceriporia_mellita --
GC1508_71Ceriporia_mellita --

GC1608_7_Ceriporia_mellita --
ZZW1557Dai27085 --
ZZW1554Dai27083 --
Dai8168
GAGGAATCCCCTAAGTAAGTCAAAAAATCCTATT----------------
BR4865C_mellita --
MEL2382688Ceriporia_sp --
Dai8110 --
Cui8097 --
909Cui6740 --
W1258Dai24695 --
JV0110_26_Ceriporia_griseoviol --
896Dai13202
GAGGAGTCCAGCAAGTGAGTGTTTGCGCGCC------------------
LWY393Dai27053C_griseoviolasce GAGGAGTCCAGCAAGTGAGTGTTTGCGCGCC--
----
LWY394DAI27054
GAGGAGTCCAGCAAGTGAGTGTTTGCGCGCC------------------
FP135015G_pannocinctus --
L15726SpG_pannocinctus --
Dai22221
GAGGAGTCTGCCAAGTACGTAGTTCTCCACTGTC----------------
Dai22633 GAAGAGTCCGCCAAGTATGTCGT------------------
-----GCTG
Dai23260 GAGGAGTCCGCCAAGTATGTCGT------------------
-----GCTG
Dai23626 GAGGAGTCCGCCAAGTATGTCGT------------------
-----GCTG
Dai16238G_citrinoalbus --
1175Dai15293 GAGGAGTCCGCCAAGTATGTCCTTCC------------
-ACAA--AGCTC
Dai19547 GAGGAGTCCGCCAAGTATGTCCTTCC------------
-ACAA--AGCTC
918063G_africanus --
918572G_africanus --
Dai18536A --
1164Cui17922 GAGGAGTCCGCCAAGTATGTCCTTCC------------
-------CAGAT
Dai22225 GAGGAGTCCGCCAAGTATGTCCTTCC------------
-AAGG----CAC
1163Dai20655 GAGGAGTCCGCCAAGTATGTCCTTCC------------
-AAGG----CAC
Yuan4397G_hainanensis --
1176Dai15268 GAGGAGTCCTCTAA-----------------------------------

1177Dai15259 GAGGAGTCCTCCAA---------------------------------
-

BZ2896G_thelephoroides ---

1166JV1808_26 GAGGAGTCCTCTAA---------------------------------
-

Miettinen16992Hapalopilus_ochr ---

GC1708_338_Ceriporia_arbuscula ---

WCG1555Dai26107Ceriporia GAGGAGTCCGCCAAGTGAGTATATGC-----------
GCGCTGCTTATCA

GC1708_340_Ceriporia_arbuscula ---

WCG1556Dai26109Ceriporia GAGGAGTCCGCCAAGTGAGTATATGC-----------
GTGCTGCTTATCA

883Cui11291 GAGGAGTCGGCCAAGTGAGTATATGC-----------
CCTTTAACGATGA

HLX320Dai26805 GAGGAGTCCTCCAAGTGAGTATATG------------
-CGTCAATGATCA

WCG1266Dai24678A GAGGAGTCCAGCAAGTGAGTATATG----------
---CGTCAATGATCA

Dai6090_Ceriporia_sulphuricolo ---

RLG_11354_Ceriproia_reticulata ---

ZZW1543Dai27072 GAGGAGTCCCCCAAGTGAGTGTCTGC---------
--GCGCTACGTCT-G

Li1316_Ceriporia_reticulata ---

KHL11981Ceriporia_reticulata ---

FP110343sp_Candelabrochaete_la ---

Li1045_Ceriporia_reticulata ---

ZX136Dai25794ceriporia GAGGAGTCCGCCAAGTGAGTATATGC-----------
GCGCCAAAAGT-G

892Dai13400 GAGGAGTCCGCCAAGTGAGTATATGC-----------
GCGCCAAAAGT-G

RLG7163Leptoporus_mollis ---

Dai21062Leptoporus_mollis GAGGAGTCCGTGAAGTGAG--TATGC-----------
GCGCCAACAAA-G

Dai20182Leptoporus_submollis GAGGAGTCCGCGAAGTGAGTATATGC-----------
GCGTCAACAAACC

Cui18379Leptoporus_submollis GAGGAGTCCGCGAAGTGAGTATATGC-----------
GCGTCAACAAACC

Wu1209_46Resiniporus_pseudogil GAAGAGTCTGCAAAGTACGTTTGTGT-------------
----

BRNM710169Resiniporus_resinasc ---

Dai14516Bjerkandera_adusta GAGGAGTCCCCCAAGTAAGTTCCTCTATGACGT-
----

Dai21100Bjerkandera_fumosa
GAGGAGTCCCCCAAGTCAGTTGCTCTGCGACGT-----------------

Miettinen16854Ceraceomyces_sp --
Dai10477C_spissa --
855Dai16831 GAGGAGTCTACCAAGTGAGTATATGCGCT------

882Cui11282 GAGGAGTCTACCAAGTGAGTATATGCGCT------

Dai24566 GAGGAGTCTTCCAAGTGAGTATATGCGCT------

Yuan5965 --
Dai3204 --
1194CUI9985 --

Dai15205_Ceriporia_albomellea --
Dai15223_Ceriporia_albomellea --
Li1780_Ceriporia_variegata --
Dai19791_Ceriporia_variegata --
Dai19886 TTTTCAAACATTTATTATCAGCCAGAC-----------
GTTGACGTCG--
Dai10833_Ceriporia_crassitunic --
CHWC1506_46Meruliopsis_crassit --
Dai9995_Ceriporia_crassitunica --
Wu1209_58_Meruliopsis_parvispo --
CHWC1505_129_Meruliopsis_parvi --
Dai21944 CTCTTATTCAGGGGCTC--------------------
ATGGCTGT----
830Dai18640A TTGTTGTTCAGGTGCTG--------------------
ATCACTGT----
GC1704_60_Meruliopsis_taxicola --
Dai22625 CTTAGGAATGTGTACCG--------------------
GTACTGACGC--
Dai22636 CTTAGGAATGTGTACCG--------------------
GTACTGACGC--
Dai21878 CTTAGGAATGTGTACCG--------------------
GTACTGACGC--
1169Dai17248 CTTAGGAATGTGTACCG--------------------
GTACTGACGC--
Wu1708_43_Meruliopsis_leptocys --
Li1011 --
ZX95Dai25742Meruliopsis_leptoc --
WCG1306Dai24733 CTGTTGTCCGGTTATTG--------------------
ATCGTTCT----
LXL99Dai25816 CTGTTGTCCGGTTATTG--------------------
ATCGTTCT----

WCG1559Dai26052Meruliopsis TGGTTATCTGGTTTCTT--------------------
ACTTTTCC----
He7477 --
HLX243Dai26217 TGGTTATCTGGTTTCTT--------------------
ACTTTTCC----
RussiaMW673659Meruliopsis_fagi --
FD278 --
Dai10226_Ceriporia_tarda --
LE247365 --
Dai8173_Meruliopsis_nanlingens --
860Dai17172 TTGCTCTCTAGATATTA--------------------
ATCATTCCAT--
879Dai13414 TTGCTCTCTAGATATTA--------------------
ACCATTCCAT--
Li_1704_Meruliopsis_pseudocyst --
833Dai18405 TCTTTGCCTGGGTGCTG--------------------
ACTGTTCT----
HHB_10729_Meruliopsis_albostra --
Cui6878_Ceriporia_pseudocystid --
869Dai14737 TATTTGCTGACTGTATGCGCGC--------------------

876Cui11626 --
1199WEI3388 --
776308_Meruliopsis_cystidiata --
ICN139059_Meruliopsis_cystidia --
HHB15692Ceraceomyces_serpens --
HHB_15629_Sp_Ceriporiopsis_ane --
AJ185Trametopsis_cervina --
FD9Irpex_lacteus --
908Dai11230 ---TTAATCGCATGTAA-------------
CTGACGTATCACC------
FP55521TEmmia_lacerata --
PBU0048Ceriporia_cystidiata --
MZ340C_laceratаT --
Dai21940 ---------------------------
ACATGAGTTTACGAAGTTTAAC
847Dai16433 ---------------------------
ACATGAGTTTACGAAGTTGAAC
MarcinEmmia_latemarginatus --
Meijer3729Hydnopolyporus_fimbr --
RLG13408Phanerochaete_sp --
WHC1381Flavodon_flavus --
GB1833Phlebia_albida CTCCCACGTCCAAGT-------------
GATTAACAGATCATCACTT--

T407Phlebia_nitidula CTCCTACCTCTAAGT-------------GACTAACAGRTCATCACTT--
HHB6988Phanerochaete_exilis ---
HHB8509Phanerochaetella_xeroph ---
PBU0051Macrohyporia_dictyopora ---
HHB11463Phanerochaete_sp ---
FP102382Byssomerulius_corium ---
FP102165Efibula_americana TTTCGGATCTGACGGTATTC-----------------------------
Murdoch90Ceriporia_torpida ---
Rivoire4413_Ceriporia_purpurea ---
Kout_18_Ceriporia_triumphalis ---
Rivoire3701_Ceriporia_bresadol ---
VS4018 ---
Ryvarden21832_Ceriporia_manzan ---
Dai24539 GATTTATCTGATTGCTTA------------------TATTACTTTC---
Dai24541 GATTTATCTGATTGCTTA------------------TATTACTTTC---
JV1105_12_Ceriporia_occidental ---
VS8558Ceriporia_occidentalis ---
Dai22445 ATTATGTCTGGTTGCTTA------------------TATTACTTT----
846Dai16368 ATTATGTCTGGTTGCTTA------------------TATTACTTT----
Dai17951_Ceriporia_aurantiocar ---
Miettinen_11701C_viridans ---
JV0105_10Ceriporia_aurantiocar ---
Yuan5702C_viridans ---
858Dai17003 ---
Yuan2747_Ceriporia_viridans ---
Yuan2744C_viridans ---
Li1046C_viridans ---
865C_sinoviridans ---
871Dai15062 ---
Dai7642_Ceriporia_humilis CATTTAACAAGGCATTAAAC-----------------ATCG-TTTGG--
Spirin4706_Ceriporia_humilis ---
Spirin4944_Ceriporia_sericea ---
WCG1547Dai26044ceriporia CATTGAACAAGGCATTAAGC-----------------ATTG-TTTGG--
ZZW1558Dai27086 CATTGAACAAGGCATTAAGC-----------------ATTG-TTTGG--
Miettinen14381_Ceriporia_mpuri ---

Miettinen15492_2_Ceriporia_sor --
He6687 CATC-AATAAGGCATTAAGC------------------
ATCG-TTTGG--
ZH53Dai24426 --
Vlasak0808_30_Ceriporia_punica --
887Dai13376 -----ATTAACGCGGTGCTA------------------
ATGTCTTTT---
WCG1443Dai24998 -----AGATATGCGATGCTA------------------
ATGTCTGT----
0108_6Ceriporia_spissa --
Dai19164 ------------CGTCATCAACAAG---
CCCACAGCTCATTGTCATCGGT
Dai17937_Ceriporia_bubalinomar --
903Dai12113 --
LZB929Dai25079 ------------TTTCATAGTAGCGACAATGCTAA---
CTCGTTCT----
LX45Dai26988 ------------TTTCATAGTAGCGACAATGCTAA---
CTCGTTCT----
LX43Dai26986 ------------TTTCATAGTAGCGACAATGCTAA---
CTCGTTCT----
Dai7759Ceriporia --
Cui8012_Ceriporia_viridans --
GC1704_54Ceriporia_viridans --
Dai23392 ------------TGTCAGATCACGCACCTGACTGA-
CGCTCGTGTTTG--
WCG1585Dai26113Ceriproia ------------TGTCAGATCACGCACCTGACTGA-
CGTTTGTGTTTG--
Dai18675C_eucalypti ------------TGTCAAAATATGCACCTCACTGA-
CGTTTGTTTATG--
Dai22034 ------------
TTTGAAGTGACTTGCGGGTCTAATCTCTAATCTT----
JV1008_41JTardaFLORIDAKeys --
Rivoire1161_Ceriporia_pierii --
Dai23499C_pierii TTTCTAGCAAAGTAATG---------------------
AGCATCATTGCC
Dai23500 TTTCTAGCAAAGTAATG---------------------
AGCATCATTGCC
841Dai15899 ------------TGTAATCCGTC-----------
TCTCATCGCCCTTT--
842Dai15904 ------------TGGAATC----------
CCGTCTCTCATCGCCCTTT--
LZB1066xinjiang --
LZB1065xinjiang --
851Dai16779 --

RMJ119sp_Candelabrochaete_sept --
RLG9759spCandelabrochaete_sept --
RLG10478Phanerochaete_allantos --
Dai19118_Ceriporia_spissa --
Dai18486A --
WEI17_024_Ceriporia_mellita --
GC1508_71Ceriporia_mellita --
GC1608_7_Ceriporia_mellita --
ZZW1557Dai27085 --
ZZW1554Dai27083 --
Dai8168 ------------TGTTATCCGA-----------
AACTAATTGCGCTTG--
BR4865C_mellita --
MEL2382688Ceriporia_sp --
Dai8110 --
Cui8097 --
909Cui6740 --
W1258Dai24695 --
JV0110_26_Ceriporia_griseoviol --
896Dai13202 ------------TGTAACCGGTT-------------
TTGTTGGTCTTGAC
LWY393Dai27053C_griseoviolasce ------------TGTAACCGGTT-------------
TTGTTGGTCTTGAC
LWY394DAI27054 ------------TGTAACCGGTT-------------
TTGTTGGTCTTGAC
FP135015G_pannocinctus --
L15726SpG_pannocinctus --
Dai22221 ----------------------------
CGCAAACTAAATGGATCCTTG
Dai22633 CTATGATCGATTCGGCGCG----------
CGTTTACTGATAGCGCTTTCT
Dai23260 CTATGATCGATTCGGTGCG----------
CGTTTACTGATAGCGCTTTCT
Dai23626 CTACGATCGATTCGGCGCG----------
CGTTTACTGATAGCGCTTTCT
Dai16238G_citrinoalbus --
1175Dai15293 ATATGATCAATAT-----------------
CTTACTGATTGCGGCTC--
Dai19547 ATATGATCAATAT-----------------
CTTACTGATTGTGGCTC--
918063G_africanus --
918572G_africanus --
Dai18536A --
1164Cui17922 TCATAATCAATAT-----------------

TTTACTGATTGCGGTTCGT

Dai22225 ATATAATCAATAT-----------------
TTTACTGATTGCGGCTCAT

1163Dai20655 ATATAATCAATAT-----------------
TTTACTGATTGCGGCTCAT

Yuan4397G_hainanensis --
1176Dai15268 --
1177Dai15259 --
BZ2896G_thelephoroides --
1166JV1808_26 --
Miettinen16992Hapalopilus_ochr --
GC1708_338_Ceriporia_arbuscula --
WCG1555Dai26107Ceriporia TTTTCGATCTGGTATGA--------------------
ATTGTCGTC-TC

GC1708_340_Ceriporia_arbuscula --
WCG1556Dai26109Ceriporia TTTTCGATCTGGTATGA--------------------
ATTGTCGTC-TC

883Cui11291 TTTCTGTACCGGTGTGA--------------------
ATTGTTATCCTC

HLX320Dai26805 TTCTTGATCCGGTATGA--------------------
ATTGTTATG--C

WCG1266Dai24678A TT-TTGATTCGGTATGA--------------------
ATTGTCATC--C

Dai6090_Ceriporia_sulphuricolo --
RLG_11354_Ceriproia_reticulata --
ZZW1543Dai27072 ACGTCATCGAGATGTTG--------------------
ATGATTGCCGCC

Li1316_Ceriporia_reticulata --
KHL11981Ceriporia_reticulata --
FP110343sp_Candelabrochaete_la --
Li1045_Ceriporia_reticulata --
ZX136Dai25794ceriporia ACGTCATCTGGATGTTG--------------------
ATGATTCCTGTC

892Dai13400 ACGTCATCTGGATGTTG--------------------
ATGATTCCTGTC

RLG7163Leptoporus_mollis --
Dai21062Leptoporus_mollis ATTTCGCCTCGATCCTA--------------------
ACAAACGTCACT

Dai20182Leptoporus_submollis ATTTCACCTCGGTCTTT-----------------------
AACGTCATC

Cui18379Leptoporus_submollis ATTTCACCTCGGTCTTT-----------------------
AACGTCATC

Wu1209_46Resiniporus_pseudogil ------------CAG-------------
CAGACAAGTGTTACTTCGCAT

BRNM710169Resiniporus_resinasc --
Dai14516Bjerkandera_adusta ------------CGCGAAAAGC----------------
GTAGATCCTAAC
Dai21100Bjerkandera_fumosa ------------CGTGAAAAAA----------------
CTAGACATTGAC
Miettinen16854Ceraceomyces_sp --
Dai10477C_spissa --
855Dai16831 ------------TGGTATGC------------------
GTCAGTGTTGCT
882Cui11282 ------------TGGTATGC------------------
GTCAGTGTTGCT
Dai24566 ------------TGATATGC------------------
GTCAGTGTTGCT
Yuan5965 --
Dai3204 --
1194CUI9985 --

Dai15205_Ceriporia_albomellea --
Dai15223_Ceriporia_albomellea --
Li1780_Ceriporia_variegata --
Dai19791_Ceriporia_variegata --
Dai19886 ---------
TCTACAGCATGCCATGGTATAAGGGCTGGACCAAAGAGACT
Dai10833_Ceriporia_crassitunic --
CHWC1506_46Meruliopsis_crassit --
Dai9995_Ceriporia_crassitunica --
Wu1209_58_Meruliopsis_parvispo --
CHWC1505_129_Meruliopsis_parvi --
Dai21944 ----------
CTATAGCATGCCCTGGTACAAGGGCTGGACGAAGGAGACT
830Dai18640A ----------
CTATAGCATGCCCTGGTACAAGGGCTGGACAAAGGAGACC
GC1704_60_Meruliopsis_taxicola --
Dai22625 ---------
AAGGTAGCATGCCATGGTACAAGGGCTGGACCAAGGAGACC
Dai22636 ---------
AAGGTAGCATGCCATGGTACAAGGGCTGGACCAAGGAGACC
Dai21878 ---------
AAGGTAGCATGCCATGGTACAAGGGCTGGACCAAGGAGACC
1169Dai17248 ---------
AAGGTAGCATGCCATGGTACAAGGGCTGGACCAAGGAGACC
Wu1708_43_Meruliopsis_leptocys --
Li1011 --

ZX95Dai25742Meruliopsis_leptoc --
WCG1306Dai24733 ----------
TTCTAGCATGCCATGGTACAAGGGCTGGACCAAGGAAACC
LXL99Dai25816 ----------
TTCTAGCATGCCATGGTACAAGGGCTGGACCAAGGAAACC
WCG1559Dai26052Meruliopsis ----------
TTTTAGCATGCCATGGTATAAGGGCTGGACCAAGGAGACC
He7477 --
HLX243Dai26217 ----------
TTTTAGCATGCCATGGTATAAGGGCTGGACCAAGGAGACC
RussiaMW673659Meruliopsis_fagi --
FD278 --
Dai10226_Ceriporia_tarda --
LE247365 --
Dai8173_Meruliopsis_nanlingens --
860Dai17172 ---------
ATTATAGCATGCCATGGTATAAGGGTTGGACCAAGGAGACC
879Dai13414 ---------
ATTATAGCATGCCATGGTATAAGGGTTGGACCAAGGAGACC
Li_1704_Meruliopsis_pseudocyst --
833Dai18405 ---------
TTTACAGCATGCCATGGTACAAGGGCTGGACCAAGGAGACC
HHB_10729_Meruliopsis_albostra --
Cui6878_Ceriporia_pseudocystid --
869Dai14737 --------
GTTTAAAGCATGCCGTGGTACAAGGGCTGGTCGAGGGAGACC
876Cui11626 --
1199WEI3388 --
776308_Meruliopsis_cystidiata --
ICN139059_Meruliopsis_cystidia --
HHB15692Ceraceomyces_serpens --
HHB_15629_Sp_Ceriporiopsis_ane --
AJ185Trametopsis_cervina --
FD9Irpex_lacteus --
908Dai11230 ------------
TTAGCATGCCATGGTACAAGGGCTGGACCAAGGAGACC
FP55521TEmmia_lacerata --
PBU0048Ceriporia_cystidiata --
MZ340C_lacerataT --
Dai21940 GATA---
TGTCTGCAGCATGCCGTGGTACAAGGGCTGGACCAAGGAGACC
847Dai16433 GATA---
TGTCTGCAGCATGCCGTGGTACAAGGGCTGGACCAAGGAGACC
MarcinEmmia_latemarginatus --

Meijer3729Hydnopolyporus_fimbr --
RLG13408Phanerochaete_sp --
WHC1381Flavodon_flavus --
GB1833Phlebia_albida ------------
ACAGCATGCCATGGTACAAGGGCTGGACTAAGGAGAAC
T407Phlebia_nitidula ------------
TTAGCATGCCATGGTACAAGGGCTGGACCAAGGAGAAC
HHB6988Phanerochaete_exilis --
HHB8509Phanerochaetella_xeroph --
PBU0051Macrohyporia_dictyopora --
HHB11463Phanerochaete_sp --
FP102382Byssomerulius_corium --
FP102165Efibula_americana -------
GTTCGCTAGCATGCCATGGTACAAGGGCTGGACCAAGGAGAAC
Murdoch90Ceriporia_torpida --
Rivoire4413_Ceriporia_purpurea --
Kout_18_Ceriporia_triumphalis --
Rivoire3701_Ceriporia_bresadol --
VS4018 --
Ryvarden21832_Ceriporia_manzan --
Dai24539 ---G----
TCGTGCAGCATGCCCTGGTACAAGGGCTGGACCAAGGAGACC
Dai24541 ---G----
TCGTGCAGCATGCCCTGGTACAAGGGCTGGACCAAGGAGACC
JV1105_12_Ceriporia_occidental --
VS8558Ceriporia_occidentalis --
Dai22445 ---G----
TCATACAGCATGCCCTGGTACAAGGGCTGGACCAAGGAGACC
846Dai16368 ---G----
TCATACAGCATGCCCTGGTACAAGGGTTGGACCAAGGAGACC
Dai17951_Ceriporia_aurantiocar --
Miettinen_11701C_viridans --
JV0105_10Ceriporia_aurantiocar --
Yuan5702C_viridans --
858Dai17003 --
Yuan2747_Ceriporia_viridans --
Yuan2744C_viridans --
Li1046C_viridans --
865C_sinoviridans --
871Dai15062 --
Dai7642_Ceriporia_humilis ---------
CTTTCAGCATGACATGGTATAAGGGCTGGACGAAGGAGACT
Spirin4706_Ceriporia_humilis --
Spirin4944_Ceriporia_sericea --

WCG1547Dai26044ceriporia ---------
CATTCAGCATGACATGGTATAAGGGCTGGACGAAGGAGACG

ZZW1558Dai27086 ---------
CATTCAGCATGACATGGTATAAGGGCTGGACGAAGGAGACG

Miettinen14381_Ceriporia_mpuri ---

Miettinen15492_2_Ceriporia_sor ---

He6687 ---------
CTTTCAGCATGCCGTGGTATAAGGGTTGGACAAAGGAGACC

ZH53Dai24426 ---

Vlasak0808_30_Ceriporia_punica ---

887Dai13376 ---------
TTCCTAGCATGCCGTGGTACAAGGGCTGGACTAAAGAGACC

WCG1443Dai24998 ---------
CTTTCAGCATGCCATGGTACAAGGGCTGGTCTAAGGAGACC

0108_6Ceriporia_spissa ---

Dai19164 CGTG----TAATGCAGCCTTAAATGGCACA----
TTCGAATAAG------

Dai17937_Ceriporia_bubalinomar ---

903Dai12113 ---

LZB929Dai25079 ------------
ATAGCATGCCATGGTACAAGGGCTGGACCAAGGAGACC

LX45Dai26988 ------------
ATAGCATGCCATGGTACAAGGGCTGGACCAAGGAGACC

LX43Dai26986 ------------
ATAGCATGCCATGGTACAAGGGCTGGACCAAGGAGACC

Dai7759Ceriporia ---

Cui8012_Ceriporia_viridans ---

GC1704_54Ceriporia_viridans ---

Dai23392 ------
ACGTACATAGCATGCCATGGTACAAGGGCTGGACCAAGGAGACC

WCG1585Dai26113Ceriproia ------
ACGTACATAGCATGCCATGGTACAAGGGCTGGACCAAGGAGACC

Dai18675C_eucalypti ------
ACGTACGTAGCATGCCATGGTACAAGGGCTGGACCAAGGAGACC

Dai22034 ------------
CCAGCATGCCATGGTACAAGGGCTGGACCAAGGAGACC

JV1008_41JTardaFLORIDAKeys ---

Rivoire1161_Ceriporia_pierii ---

Dai23499C_pierii TT----------
TCAGCATGCCATGGTACAAGGGCTGGACTAAGGAGACA

Dai23500 TT----------
TCAGCATGCCATGGTACAAGGGCTGGACTAAGGAGACA

841Dai15899 ---------
TCTCCAGCATGCCATGGTACAAGGGCTGGACCAAGGAGACC

842Dai15904 ---------
TCTCCAGCATGCCATGGTACAAGGGCTGGACCAGGGAGACC
LZB1066xinjiang ---
LZB1065xinjiang ---
851Dai16779 ---
RMJ119sp_Candelabrochaete_sept ---
RLG9759spCandelabrochaete_sept ---
RLG10478Phanerochaete_allantos ---
Dai19118_Ceriporia_spissa ---
Dai18486A ---
WEI17_024_Ceriporia_mellita ---
GC1508_71Ceriporia_mellita ---
GC1608_7_Ceriporia_mellita ---
ZZW1557Dai27085 ---
ZZW1554Dai27083 ---
Dai8168 -------
CTTGTACAGTATGCCGTGGTACAAGGGCTGGACCAAGGAGACG
BR4865C_mellita ---
MEL2382688Ceriporia_sp ---
Dai8110 ---
Cui8097 ---
909Cui6740 ---
W1258Dai24695 ---
JV0110_26_Ceriporia_griseoviol ---
896Dai13202 CTTG-----
ATAATAGCATGCCGTGGTATAAGGGCTGGACCAAGGAGACC
LWY393Dai27053C_griseoviolasce CTTG-----
ATAATAGCATGCCGTGGTATAAGGGCTGGACCAAGGAGACC
LWY394DAI27054 CTTG-----
ATAATAGCATGCCGTGGTATAAGGGCTGGACCAAGGAGACC
FP135015G_pannocinctus ---
L15726SpG_pannocinctus ---
Dai22221 CTT-------
TTACAGCATGCCATGGTACAAGGGCTGGACCAGGGAGACC
Dai22633 TCC--------
TATAGCATGCCATGGTACAAGGGCTGGACTAAGGAGACC
Dai23260 TCC--------
TATAGCATGCCATGGTACAAGGGCTGGACTAAGGAGACC
Dai23626 TCC--------
TATAGCATGCCATGGTACAAGGGCTGGACTAAGGAGACC
Dai16238G_citrinoalbus ---
1175Dai15293 -----------
AATAGCATGCCATGGTACAAGGGCTGGACCAAGGAGACC
Dai19547 -----------

AATAGCATGCCATGGTACAAGGGCTGGACCAAGGAGACC
918063G_africanus --
918572G_africanus --
Dai18536A --
1164Cui17922 CTC--------
AATAGCATGCCATGGTACAAGGGCTGGACCAAGGAGACC
Dai22225 CTT--------
AATAGCATGCCATGGTACAAGGGCTGGACCAAGGAGACC
1163Dai20655 CTT--------
AATAGCATGCCATGGTACAAGGGCTGGACCAAGGAGACC
Yuan4397G_hainanensis --
1176Dai15268 ----------------
CATGCCGTGGTACAAGGGCTGGACCAAGGAGACC
1177Dai15259 ----------------
CATGCCGTGGTACAAGGGCTGGACCAAGGAGACC
BZ2896G_thelephoroides --
1166JV1808_26 ----------------
CATGCCATGGTACAAGGGCTGGACCAAGGAGACC
Miettinen16992Hapalopilus_ochr --
GC1708_338_Ceriporia_arbuscula --
WCG1555Dai26107Ceriporia GC-------
CTTATAGCATGCCATGGTACAAGGGCTGGACTAAGGAGACC
GC1708_340_Ceriporia_arbuscula --
WCG1556Dai26109Ceriporia GC-------
CTTATAGCATGCCATGGTACAAGGGCTGGACTAAGGAGACC
883Cui11291 GC-------
ATTTTAGCATGCCATGGTACAAGGGCTGGACCAAGGAGACG
HLX320Dai26805 AA-------
CCTTTAGCATGCCATGGTACAAGGGATGGACCAAGGAGACC
WCG1266Dai24678A AC-------
CATTTAGCATGCCATGGTACAAGGGATGGACCAAGGAGACC
Dai6090_Ceriporia_sulphuricolo --
RLG_11354_Ceriproia_reticulata --
ZZW1543Dai27072 AT----------
CCAGCATGCCATGGTACAAGGGTTGGACCAAGGAGACA
Li1316_Ceriporia_reticulata --
KHL11981Ceriporia_reticulata --
FP110343sp_Candelabrochaete_la --
Li1045_Ceriporia_reticulata --
ZX136Dai25794ceriporia AT----------
GTAGCATGCCATGGTACAAGGGCTGGACGAAGGAGACC
892Dai13400 AT----------
GTAGCATGCCGTGGTACAAGGGCTGGACGAAGGAGACC
RLG7163Leptoporus_mollis --

Dai21062Leptoporus_mollis TGT------
CACATAGCATGCCATGGTACAAGGGCTGGACTAAGGAGACC
Dai20182Leptoporus_submollis TGT------
CACATAGCATGCCATGGTACAAGGGCTGGACCAAAGAGACC
Cui18379Leptoporus_submollis TGT------
CACATAGCATGCCATGGTACAAGGGCTGGACCAAAGAGACC
Wu1209_46Resiniporus_pseudogil
GTTAACGTCACCTCAGCATGCCATGGTACAAAGGCTGGCAGAGGGAGACG
BRNM710169Resiniporus_resinasc --
Dai14516Bjerkandera_adusta TCT------
GTAATAGCATGCCGTGGTACAAGGGCTGGACCAAGGAGACC
Dai21100Bjerkandera_fumosa TCT------
ACAATAGCATGCAGTGGTACAAGGGCTGGACTAAGGAGACC
Miettinen16854Ceraceomyces_sp --
Dai10477C_spissa --
855Dai16831
CATCTATTCTGTCTAGCATGCCATGGTACAAGGGCTGGACTAAGGAGACG
882Cui11282
CATCTATTCTGTCTAGCATGCCATGGTACAAGGGCTGGACTAAGGAGACG
Dai24566
TATCAACCCTCTTCAGCATGCCATGGTACAAGGGCTGGACCAAGGAGACG
Yuan5965 --
Dai3204 --
1194CUI9985 --

Dai15205_Ceriporia_albomellea --
Dai15223_Ceriporia_albomellea --
Li1780_Ceriporia_variegata --
Dai19791_Ceriporia_variegata --
Dai19886
AAGGCTGGTGTCGTCAAGGGCAAGACCCTTCTTGATGCTATCGACGCTAT
Dai10833_Ceriporia_crassitunic --
CHWC1506_46Meruliopsis_crassit --
Dai9995_Ceriporia_crassitunica --
Wu1209_58_Meruliopsis_parvispo --
CHWC1505_129_Meruliopsis_parvi --
Dai21944
AAAGGTGGTGTCGTCAAGGGTAAGACACTCCTCGATGCCATCGACGCCAT
830Dai18640A
AAGTCTGGTGTTGTCAAGGGTAAGACGCTCCTCGATGCCATCGATGCCAT
GC1704_60_Meruliopsis_taxicola --
Dai22625
AAGGGTGGTGTTGTCAAGGGTAAGACCTTGCTCGACGCCATCGACGCCAT

Dai22636
AAGGGTGGTGTTGTCAAGGGTAAGACCTTGCTCGACGCCATCGACGCCAT
Dai21878
AAGGGTGGTGTTGTCAAGGGTAAGACCTTGCTCGACGCCATCGACGCCAT
1169Dai17248
AAGGGTGGTGTTGTCAAGGGTAAGACCTTGCTCGACGCCATCGACGCCAT
Wu1708_43_Meruliopsis_leptocys ---
Li1011 ---
ZX95Dai25742Meruliopsis_leptoc ---
WCG1306Dai24733
AAGGCTGGTGTCGTTAAGGGTAAGACCCTCCTCGATGCCATCGACGCTAT
LXL99Dai25816
AAGGCTGGTGTCGTTAAGGGTAAGACCCTCCTCGATGCTATCGACGCTAT
WCG1559Dai26052Meruliopsis
AAGGCTGGTGTCGTGAAGGGTAAGACCCTGCTCGATGCCATCGACGCTAT
He7477 ---
HLX243Dai26217
AAGGCTGGTGTCGTGAAGGGTAAGACCCTGCTCGATGCCATCGACGCTAT
RussiaMW673659Meruliopsis_fagi ---
FD278 ---
Dai10226_Ceriporia_tarda ---
LE247365 ---
Dai8173_Meruliopsis_nanlingens ---
860Dai17172
AAGGCTGGTGTTGTCAAGGGTAAGACCCTCCTCGACGCCATCGATGCTAT
879Dai13414
AAGGCTGGTGTTGTCAAGGGTAAGACCCTCCTCGACGCCATCGATGCTAT
Li_1704_Meruliopsis_pseudocyst ---
833Dai18405
AAGGCTGGTGTCGTGAAGGGTAAGACCCTGCTCGACGCTATTGACGCTAT
HHB_10729_Meruliopsis_albostra ---
Cui6878_Ceriporia_pseudocystid ---
869Dai14737
AAGGCCGGTCCTGTCAAGGGCAAGACCCTCCTCGACGCCATCGATGCCAT
876Cui11626 ---
1199WEI3388 ---
776308_Meruliopsis_cystidiata ---
ICN139059_Meruliopsis_cystidia ---
HHB15692Ceraceomyces_serpens ---
HHB_15629_Sp_Ceriporiopsis_ane ---
AJ185Trametopsis_cervina ---
FD9Irpex_lacteus ---
908Dai11230
AAGGCTGGTGTCGTCAAGGGTATTACTCTCCTCGACGCCATCGATGCCAT

FP55521TEmmia_lacerata --
PBU0048Ceriporia_cystidiata --
MZ340C_lacerataT --
Dai21940
AAGGGTGGTGTTGTCAAGGGTGTCACTCTCCTCGACGCCATCGACGCCAT
847Dai16433
AAGGGTGGTGTTGTCAAGGGTGTCACTCTCCTCGACGCCATCGACGCCAT
MarcinEmmia_latemarginatus --
Meijer3729Hydnopolyporus_fimbr --
RLG13408Phanerochaete_sp --
WHC1381Flavodon_flavus --
GB1833Phlebia_albida
AAGTCTGGTGCCGTCAAGGGTGTTACTCTTCTCGACGCCATCGATGCTAT
T407Phlebia_nitidula
AAGTCTGGTGCCGTCAAGGGTGTYACTCTCCTCGACGCCATCGATGCTAT
HHB6988Phanerochaete_exilis --
HHB8509Phanerochaetella_xeroph --
PBU0051Macrohyporia_dictyopora --
HHB11463Phanerochaete_sp --
FP102382Byssomerulius_corium --
FP102165Efibula_americana
AAGAGTGGCCCGGTTAAGGGTATGACTCTCCTCGACGCCATCGACGCCAT
Murdoch90Ceriporia_torpida --
Rivoire4413_Ceriporia_purpurea --
Kout_18_Ceriporia_triumphalis --
Rivoire3701_Ceriporia_bresadol --
VS4018 --
Ryvarden21832_Ceriporia_manzan --
Dai24539
AAGGCTGGTGTCGTCAAGGGCAAGACCCTCCTCGATGCCATCGATGCCAT
Dai24541
AAGGCTGGTGTCGTCAAGGGTAAGACCCTTCTCGATGCCATCGATGCCAT
JV1105_12_Ceriporia_occidental --
VS8558Ceriporia_occidentalis --
Dai22445
AAGGCTGGTGTCGTCAAGGGCAAGACCCTCCTCGATGCCATCGATGCCAT
846Dai16368
AAGGCTGGTGTCGTCAAGGGCAAGACCCTCCTCGATGCCATCGATGCCAT
Dai17951_Ceriporia_aurantiocar --
Miettinen_11701C_viridans --
JV0105_10Ceriporia_aurantiocar --
Yuan5702C_viridans --
858Dai17003 --
Yuan2747_Ceriporia_viridans --

Yuan2744C_viridans --
Li1046C_viridans --
865C_sinoviridans --
871Dai15062 --
Dai7642_Ceriporia_humilis
AAGGCTGGTGTCGTCAAGGGCAAGACTCTCCTCGATGCCATCGATGCCAT
Spirin4706_Ceriporia_humilis --
Spirin4944_Ceriporia_sericea --
WCG1547Dai26044ceriporia
AAGGCTGGTGTCGTCAAGGGCGTGACTCTCCTCGATGCCATTGATGCCAT
ZZW1558Dai27086
AAGGCTGGTGTCGTCAAGGGCGTGACTCTCCTCGATGCCATTGATGCCAT
Miettinen14381_Ceriporia_mpuri --
Miettinen15492_2_Ceriporia_sor --
He6687
AAGGCTGGTGTCGTCAAGGGCAAGACTCTCCTCGATGCCATCGATGCCAT
ZH53Dai24426 --
Vlasak0808_30_Ceriporia_punica --
887Dai13376
AAGGCTGGTGTCGTCAAGGGCAAGACCCTCCTCGATGCCATTGATGCCAT
WCG1443Dai24998
AAGGCTGGTGTTGTCAAGGGCAAGACCCTCCTCGATGCCATCGATGCTAT
0108_6Ceriporia_spissa --
Dai19164 ------------------
GGAAAGACTCTTCTTGAAGCTATTGACAATGT
Dai17937_Ceriporia_bubalinomar --
903Dai12113 --
LZB929Dai25079
AAGGCTGGTGTCGTCAAAGGCAAGACTCTGCTCGACGCCATTGACGCCAT
LX45Dai26988
AAGGCTGGTGTCGTCAAAGGCAAGACTCTGCTCGACGCCATTGACGCCAT
LX43Dai26986
AAGGCTGGTGTCGTCAAAGGCAAGACTCTGCTCGACGCCATTGACGCCAT
Dai7759Ceriporia --
Cui8012_Ceriporia_viridans --
GC1704_54Ceriporia_viridans --
Dai23392
AAGGCTGGTGTCGTCAAGGGCAAGACCCTCCTCGACGCCATCGACGCCAT
WCG1585Dai26113Ceriproia
AAGGCTGGTGTCGTCAAGGGCAAGACCCTCCTCGACGCCATCGATGCCAT
Dai18675C_eucalypti
AAGGCTGGTGTCGTCAAGGGCAAGACCCTCCTCGATGCCATTGACGCTAT
Dai22034
AAGGCTGGTGTCGTCAAGGGCAAGACCCTCCTCGATGCTATTGATGCCAT

JV1008_41JTardaFLORIDAKeys --
Rivoire1161_Ceriporia_pierii --
Dai23499C_pierii
AAGGCTGGTGTCGTTAAGGGCAAGACCCTTCTCGATGCCATTGATGCCAT
Dai23500
AAGGCTGGTGTCGTTAAGGGCAAGACCCTTCTCGATGCCATTGATGCCAT
841Dai15899
AAGTCTGGTGTCGTCAAAGGCAAGACTCTCCTCGATGCCATTGATGCCAT
842Dai15904
AAGTCTGGTGTCGTCAAGGGCAAGACTCTCCTCGATGCCATTGATGCCAT
LZB1066xinjiang --
LZB1065xinjiang --
851Dai16779 --
RMJ119sp_Candelabrochaete_sept --
RLG9759spCandelabrochaete_sept --
RLG10478Phanerochaete_allantos --
Dai19118_Ceriporia_spissa --
Dai18486A --
WEI17_024_Ceriporia_mellita --
GC1508_71Ceriporia_mellita --
GC1608_7_Ceriporia_mellita --
ZZW1557Dai27085 --
ZZW1554Dai27083 --
Dai8168
AAGGCTGGTGTTGTGAAAGGTAAGACCCTCCTCGACGCAATCGATGCCAT
BR4865C_mellita --
MEL2382688Ceriporia_sp --
Dai8110 --
Cui8097 --
909Cui6740 --
W1258Dai24695 --
JV0110_26_Ceriporia_griseoviol --
896Dai13202
AAGTCCGGTGTCGTCAAGGGCAAGACTCTCCTCGATGCCATTGATGCCAT
LWY393Dai27053C_griseoviolasce
AAGTCCGGTGTCGTCAAGGGCAAGACTCTCCTCGATGCCATTGATGCCAT
LWY394DAI27054
AAGTCCGGTGTCGTCAAGGGCAAGACTCTCCTCGATGCCATTGATGCCAT
FP135015G_pannocinctus --
L15726SpG_pannocinctus --
Dai22221
AAGGCTGGTGTCGTCAAGGGTAAGACCCTCCTCGATGCCATCGATGCCAT
Dai22633
AAGGCCGGTGTTGTCAAGGGTAAGACCCTCCTCGACGCCATTGACGCCAT

Dai23260
AAGGCCGGTGTTGTCAAGGGTAAGACCCTCCTCGACGCCATTGACGCCAT
Dai23626
AAGGCCGGTGTTGTCAAGGGTAAGACCCTCCTCGACGCCATTGACGCCAT
Dai16238G_citrinoalbus --
1175Dai15293
AAGGCCGGTGTTGTCAAGGGTAAGACCCTCCTCGACGCCATTGATGCCAT
Dai19547
AAGGCCGGTGTTGTCAAGGGTAAGACCCTCCTCGACGCCATTGACGCCAT
918063G_africanus --
918572G_africanus --
Dai18536A --
1164Cui17922
AAGGCCGGCGTTGTCAAGGGTAAGACCCTCCTCGACGCCATTGACGCCAT
Dai22225
AAGGCCGGTGTCGTCAAGGGCAAGACCCTCCTCGACGCCATTGACGCCAT
1163Dai20655
AAGGCCGGTGTCGTCAAGGGCAAGACCCTCCTCGACGCCATTGACGCCAT
Yuan4397G_hainanensis --
1176Dai15268
AAGGCTGGTGTCGTCAAGGGCAAGACCCTCCTCGATGCTATCGATGCCAT
1177Dai15259
AAGGCTGGTGTCGTCAAGGGCAAGACCCTCCTCGATGCTATCGATGCCAT
BZ2896G_thelephoroides --
1166JV1808_26
AAGGCTGGTGTCGTTAAGGGTAAGACCCTCCTCGATGCCATCGATGCTAT
Miettinen16992Hapalopilus_ochr --
GC1708_338_Ceriporia_arbuscula --
WCG1555Dai26107Ceriporia
AAGGCTGGTGTCGTCAAGGGCAAGACCCTCCTCGATGCCATTGATGCCAT
GC1708_340_Ceriporia_arbuscula --
WCG1556Dai26109Ceriporia
AAGGCTGGTGTCGTCAAGGGCAAGACCCTCCTCGATGCCATTGATGCCAT
883Cui11291
AAGGCTGGTGTTGTCAAGGGCAAGACCCTCCTCGATGCCATTGATGCCAT
HLX320Dai26805
AAGGCTGGTGTTGTCAAGGGCAAGACTCTCCTTGATGCGATCGATGCGAT
WCG1266Dai24678A
AAGGCTGGCGTTGTCAAGGGCAAGACTCTCCTCGATGCCATTGATGCTAT
Dai6090_Ceriporia_sulphuricolo --
RLG_11354_Ceriproia_reticulata --
ZZW1543Dai27072
AAGACTGGTGTCGTCAAGGGCAAGACTCTCCTCGATGCCATTGATGCCAT
Li1316_Ceriporia_reticulata --

KHL11981Ceriporia_reticulata --
FP110343sp_Candelabrochaete_la --
Li1045_Ceriporia_reticulata --
ZX136Dai25794ceriporia
AAGGCTGGTGTCGTCAAGGGCAAGACCCTTCTCGATGCTATCGATGCCAT
892Dai13400
AAGGCTGGTGTCGTCAAGGGCAAGACCCTTCTCGATGCTATCGACGCCAT
RLG7163Leptoporus_mollis --
Dai21062Leptoporus_mollis
AAGGGTGGTGTCGTCAAGGGCAAGACTCTCCTCGATGCTATTGATGCCAT
Dai20182Leptoporus_submollis
AAGGCCGGTGTCGTCAAGGGGAAGACTCTCCTCGATGCTATTGACGCCAT
Cui18379Leptoporus_submollis
AAGGCCGGTGTCGTCAAGGGGAAGACTCTCCTCGATGCTATTGACGCCAT
Wu1209_46Resiniporus_pseudogil
AAGGCCGGCGTTGTCAAGGGTAAGACCCTGCTTGATGCCATCGACGCCAT
BRNM710169Resiniporus_resinasc --
Dai14516Bjerkandera_adusta
AAGGCCGGTGTCGTCAAGGGTAAGACCCTCCTCGACGCCATCGATGCCAT
Dai21100Bjerkandera_fumosa
AAGGCTGGTGTAGTCAAGGGCAAGACCCTCCTCGATGCCATCGACGCTAT
Miettinen16854Ceraceomyces_sp --
Dai10477C_spissa --
855Dai16831
AAGGCCGGTGTCGTCAAGGGCAAGACTTTGCTCGATGCCATCGATGCCAT
882Cui11282
AAGGCCGGTGTCGTCAAGGGCAAGACTTTGCTCGATGCCATCGATGCCAT
Dai24566
AAGGCTGGTGTTGTCAAGGGCAAGACTTTGCTCGATGCCATCGATGCCAT
Yuan5965 --
Dai3204 --
1194CUI9985 --

Dai15205_Ceriporia_albomellea --
Dai15223_Ceriporia_albomellea --
Li1780_Ceriporia_variegata --
Dai19791_Ceriporia_variegata --
Dai19886
TGAGCCCCCTGTCCGTCCCTCCGACAAGCCTCTCCGTCTGCCCCTCCAAG
Dai10833_Ceriporia_crassitunic --
CHWC1506_46Meruliopsis_crassit --
Dai9995_Ceriporia_crassitunica --
Wu1209_58_Meruliopsis_parvispo --

CHWC1505_129_Meruliopsis_parvi --
Dai21944
CGAGCCCCCGTCCGTCCATCCGACAAGCCTCTCCGTCTCCCCCTCCAGG
830Dai18640A
CGAGCCCCCGTCCGTCCCTCCGACAAGCCCCTCCGTCTTCCCCTCCAGG
GC1704_60_Meruliopsis_taxicola --
Dai22625
CGAACCCCCCTCCCGTCCCTCCGACAAGCCTCTTCGTCTTCCCCTCCAGG
Dai22636
CGAACCCCCCTCCCGTCCCTCCGACAAGCCTCTTCGTCTTCCCCTCCAGG
Dai21878
CGAACCCCCCTCCCGTCCCTCCGACAAGCCTCTTCGTCTTCCCCTCCAGG
1169Dai17248
CGAACCCCCCTCCCGTCCCTCCGACAAGCCTCTTCGTCTTCCCCTCCAGG
Wu1708_43_Meruliopsis_leptocys --
Li1011 --
ZX95Dai25742Meruliopsis_leptoc --
WCG1306Dai24733
CGAGCCCCCCGTCCGTCCCTCCGACAAGCCTCTGCGTCTCCCTCTCCAGG
LXL99Dai25816
CGAGCCCCCCGTCCGTCCCTCCGACAAGCCTCTGCGTCTCCCTCTCCAGG
WCG1559Dai26052Meruliopsis
CGAACCCCCCGTCCGTCCCTCCGACAAGCCCCTTCGTCTCCCCCTCCAGG
He7477 --
HLX243Dai26217
CGAACCCCCCGTCCGTCCCTCCGACAAGCCCCTTCGTCTCCCCCTCCAGG
RussiaMW673659Meruliopsis_fagi --
FD278 --
Dai10226_Ceriporia_tarda --
LE247365 --
Dai8173_Meruliopsis_nanlingens --
860Dai17172
CGAACCCCCTGTCCGTCCGTCCGACAAGCCCCTTCGTCTTCCTCTCCAGG
879Dai13414
CGAACCCCCTGTCCGTCCGTCCGACAAGCCCCTTCGTCTTCCTCTCCAGG
Li_1704_Meruliopsis_pseudocyst --
833Dai18405
CGAGCCACCTGTCCGTCCCTCCGACAAGCCCCTCCGTCTCCCCCTCCAGG
HHB_10729_Meruliopsis_albostra --
Cui6878_Ceriporia_pseudocystid --
869Dai14737
CGAACCCCCAGTCCGTCCCTCCGACAAGCCCCTCCGTCGCCCCCCAGCG-
876Cui11626 --
1199WEI3388 --

776308_Meruliopsis_cystidiata ---
ICN139059_Meruliopsis_cystidia ---
HHB15692Ceraceomyces_serpens ---
HHB_15629_Sp_Ceriporiopsis_ane ---
AJ185Trametopsis_cervina ---
FD9Irpex_lacteus ---
908Dai11230
CGAGCCCCCGTTCGTCCCTCCGACAAGCCCCTCCGTCTCCCCCTCCAGG
FP55521TEmmia_lacerata ---
PBU0048Ceriporia_cystidiata ---
MZ340C_lacerataT ---
Dai21940
CGAGCCCCCGTTCGTCCCGTCGAGAAGCCCCTCCGTCTTCCCCTCCAGG
847Dai16433
CGAGCCCCCGTTCGTCCCGTCGAGAAGCCCCTCCGTCTTCCCCTCCAGG
MarcinEmmia_latemarginatus ---
Meijer3729Hydnopolyporus_fimbr ---
RLG13408Phanerochaete_sp ---
WHC1381Flavodon_flavus ---
GB1833Phlebia_albida
CGAGCCTCCCGTCCGCCCGTCCGACAGACCCCTTCGTCTTCCTCTCCAGG
T407Phlebia_nitidula
CGAGCCCCCTGTCCGTCCCTCCGACAGGCCTCTTCGTCTTCCTCTCCAGG
HHB6988Phanerochaete_exilis ---
HHB8509Phanerochaetella_xeroph ---
PBU0051Macrohyporia_dictyopora ---
HHB11463Phanerochaete_sp ---
FP102382Byssomerulius_corium ---
FP102165Efibula_americana
CGAGCCTCCTTCCCGTCCCTCAGACCGTCCCCTCCGTCTTCCTCTCCAGG
Murdoch90Ceriporia_torpida ---
Rivoire4413_Ceriporia_purpurea ---
Kout_18_Ceriporia_triumphalis ---
Rivoire3701_Ceriporia_bresadol ---
VS4018 ---
Ryvarden21832_Ceriporia_manzan ---
Dai24539
TGAGCCCCCGTCCCGTCCCTCCGACAAGCCCCTCCGTCTCCCCCTCCAGG
Dai24541
TGAGCCCCCGTCCCGTCCCTCCGACAAGCCCCTCCGTCTCCCCCTCCAGG
JV1105_12_Ceriporia_occidental ---
VS8558Ceriporia_occidentalis ---
Dai22445
CGAGCCCCCGTCCCGTCCCTCCGACAAGCCCCTCCGTCTCCCCCTCCAGG

846Dai16368
CGAGCCCCGTCCCGTCCCTCCGACAAGCCCCTCCGTCTCCCCCTCCAGG
Dai17951_Ceriporia_aurantiocar ---
Miettinen_11701C_viridans ---
JV0105_10Ceriporia_aurantiocar ---
Yuan5702C_viridans ---
858Dai17003 ---
Yuan2747_Ceriporia_viridans ---
Yuan2744C_viridans ---
Li1046C_viridans ---
865C_sinoviridans ---
871Dai15062 ---
Dai7642_Ceriporia_humilis
CGAGCCCCCCGTTCGTCCCTCCGACAAGCCCCTCCGTCTCCCCCTTCAGG
Spirin4706_Ceriporia_humilis ---
Spirin4944_Ceriporia_sericea ---
WCG1547Dai26044ceriporia
TGAACCCCCCTCGCGTCCCTCCGACAAGCCCCTCCGTCTCCGCCTCAGGG
ZZW1558Dai27086
TGAACCCCCCTCGCGTCCCTCCGACAAGCCCCTCCGTCTCCCCCTTCAGG
Miettinen14381_Ceriporia_mpuri ---
Miettinen15492_2_Ceriporia_sor ---
He6687
CGAGCCCCCCGCTCGTCCCTCCGACAAGCCCCTCCGTCTTCCCCTTCAGG
ZH53Dai24426 ---
Vlasak0808_30_Ceriporia_punica ---
887Dai13376
TGAACCCCCAGTTCGTCCCTCCGACAAGCCCCTCCGTCTTCCCCTTCAGG
WCG1443Dai24998
TGAACCCCCGGTTCGTCCCTCCGACAAGCCGCTCCGTCTTCCCCTTCAGG
0108_6Ceriporia_spissa ---
Dai19164
CGTTCCACCGCAACGCCCCTTCGACAAGCCCCTACGACTTCCCCTCCAAG
Dai17937_Ceriporia_bubalinomar ---
903Dai12113 ---
LZB929Dai25079
CGAGCCTCCCGTCCGTCCGTCCGACAAGCCCCTCCGTCTGCCCCTCCAGG
LX45Dai26988
CGAGCCTCCCGTCCGTCCGTCCGACAAGCCCCTCCGTCTGCCCTGTCAGG
LX43Dai26986
CGAGCCTCCCGTCCGTCCGTCCGACAAGCCCCTCCGTCTGCCCCTCCAGG
Dai7759Ceriporia ---
Cui8012_Ceriporia_viridans ---
GC1704_54Ceriporia_viridans ---

Dai23392
CGAGCCCCCGGTCCGTCCCTCCGACAAGCCTCTGCGACTCCCTCTGCAGG
WCG1585Dai26113Ceriproia
CGAGCCCCCGGTCCGTCCCTCCGACAAGCCTCTGCGACTCCCTCTGCAGG
Dai18675C_eucalypti
CGAGCCCCCGTCCCGTCCGTCCGACAAGCCTCTGCGTCTTCCTTTGCAGG
Dai22034 TGAGCCTCCCGTTCGTCC-TCCGACAAGCCCCTCCTCTCACTGC------
JV1008_41JTardaFLORIDAKeys --
Rivoire1161_Ceriporia_pierii --
Dai23499C_pierii
CGAGCCCCCGGTTCGTCCATCCGACAAACCCCTCCGTCTCCCCCTGCAGG
Dai23500
CGAGCCCCCGGTTCGTCCATCCGACAAACCCCTCCGTCTCCCCCTGCAGG
841Dai15899
CGAGCCCCCTTCCCGTCCTTCCGACAAGCCCCTCCGTCTTCCCCTTCAGG
842Dai15904
CGAGCCCCCTTCCCGTCCTTCCGACAAGCCCCTCCGTCTTCCCCTTCAGG
LZB1066xinjiang --
LZB1065xinjiang --
851Dai16779 --
RMJ119sp_Candelabrochaete_sept --
RLG9759spCandelabrochaete_sept --
RLG10478Phanerochaete_allantos --
Dai19118_Ceriporia_spissa --
Dai18486A --
WEI17_024_Ceriporia_mellita --
GC1508_71Ceriporia_mellita --
GC1608_7_Ceriporia_mellita --
ZZW1557Dai27085 --
ZZW1554Dai27083 --
Dai8168
CGAGCCCCCCGTTCGTCCCGAGAACAAGCCCCTCCGCCTTCCTCTCCAGG
BR4865C_mellita --
MEL2382688Ceriporia_sp --
Dai8110 --
Cui8097 --
909Cui6740 --
W1258Dai24695 --
JV0110_26_Ceriporia_griseoviol --
896Dai13202
CGAGCCCCCTTCTCGTCCCTCCGACAAGCCCCTCCGTCTCCCTCTCCAGG
LWY393Dai27053C_griseoviolasce
CGAGCCCCCTTCTCGTCCCTCCGACAAGCCCCTCCGTCTCCCTCTCCAGG

LWY394DAI27054

CGAGCCCCCTTCTCGTCCCTCCGACAAGCCCCTCCGTCTCCCTCTCCAGG

FP135015G_pannocinctus --

L15726SpG_pannocinctus --

Dai22221

CGAACCCCCTGTCCGTCCCTCCGACAAGCCCCTCCGCCTCCCCCTTCAGG

Dai22633

CGAACCCCCCGTCCGACCCTCCGACAAGCCCCTCCGTCTCCCTCTCCAGG

Dai23260

CGAACCCCCCGTCCGTCCCTCCGACAAGCCTCTCCGTCTCCCTCTCCAGG

Dai23626

CGAACCCCCCGTCCGTCCCTCCGACAAGCCCCTCCGTCTCCCTCTCCAGG

Dai16238G_citrinoalbus --

1175Dai15293

CGAACCCCCCGTCCGTCCCTCCGACAAGCCCCTCCGTCTACCTCTCCAGG

Dai19547

CGAACCCCCCGTCCGTCCCTCCGACAAGCCCCTCCGTCTACCTCTCCAGG

918063G_africanus --

918572G_africanus --

Dai18536A --

1164Cui17922

CGAACCCCCCGTCCGTCCCTCCGACAAGCCCCTCCGTCTCCCTCTCCAGG

Dai22225

TGAACCCCCCGTCCGTCCCTCCGACAAGCCCCTCCGTCTCCCTCTCCAGG

1163Dai20655

TGAACCCCCCGTCCGTCCCTCCGACAAGCCCCTCCGTCTCCCTCTCCAGG

Yuan4397G_hainanensis --

1176Dai15268

TGAGCCTCCCGTCCGTCCCTCCGACAAGCCCCTCCGTCTCCCCCTCCAGG

1177Dai15259

TGAGCCTCCCGTCCGTCCCTCTGACAAGCCCCTCCGTCTCCCCCTCCAGG

BZ2896G_thelephoroides --

1166JV1808_26

TGAGCCCCCCGTCCGTCCCTCCGACAAGCCCCTCCGTCTCCCCCTCCAGG

Miettinen16992Hapalopilus_ochr --

GC1708_338_Ceriporia_arbuscula --

WCG1555Dai26107Ceriporia

CGAGCCCCCCGTTCGTCCCTCCGACAAGCCTCTCCGTCTCCCTCTCCAGG

GC1708_340_Ceriporia_arbuscula --

WCG1556Dai26109Ceriporia

CGAGCCCCCCGTTCGTCCCTCCGACAAGCCTCTCCGTCTCCCTCTCCAGG

883Cui11291

TGAGCCCCCGGTTCGTCCTTCCGACAAGCCCCTCCGTCTCCCCCTCCAGG

HLX320Dai26805

CGAGCCCCGGTTCGTCCCTCCGACAAGCCCCTCCGTCTCCCTCTTCAGG

WCG1266Dai24678A

CGAGCCCCCGTTCGTCCCTCCGACAAGCCCCTTCGTCTCCCCCTTCAGG

Dai6090_Ceriporia_sulphuricolo --

RLG_11354_Ceriproia_reticulata --

ZZW1543Dai27072

CGAACCCCCATCCCGTCCCTCCGACAAGCCCCTCCGTCTCCCCCTTCAGG

Li1316_Ceriporia_reticulata --

KHL11981Ceriporia_reticulata --

FP110343sp_Candelabrochaete_la --

Li1045_Ceriporia_reticulata --

ZX136Dai25794ceriporia

CGAACCCCCCGTCCGTCCCTCCGACAAGCCCCTCCGTCTTCCCCTTCAGG

892Dai13400

CGAACCCCCCGTCCGTCCCTCCGACAAGCCCCTCCGTCTTCCCCTTCAGG

RLG7163Leptoporus_mollis --

Dai21062Leptoporus_mollis

CGAGCCCCCTTCTCGCCCCTCCGACAAGCCGCTTCGTCTTCCTCTGCAGG

Dai20182Leptoporus_submollis

CGAGCCCCCTTCTCGCCCCTCCGACAAGCCCCTTCGTCTTCCTCTGCAGG

Cui18379Leptoporus_submollis

CGAGCCCCCTTCTCGCCCCTCCGACAAGCCCCTTCGTCTTCCTCTGCAGG

Wu1209_46Resiniporus_pseudogil

CGAGCCTCCCGTCCGTCCGTCCGACAAGCCTCTTCGTCTTCCCCTCCAGG

BRNM710169Resiniporus_resinasc --

Dai14516Bjerkandera_adusta

CGAGCCCCCCGTCCGTCCCTCCGACAAGCCCCTCCTCTCCCCCTTCCCCG

Dai21100Bjerkandera_fumosa

CGAACCTCCCGTCCGTCCGTCCGACAAGCCCCTCCGCCTTCCCCTCCAGG

Miettinen16854Ceraceomyces_sp --

Dai10477C_spissa --

855Dai16831

CGAACCCCCCGTCCGACCCTCCGACAAGCCCCTTCGTCTTCCCCTTCAGG

882Cui11282

CGAACCCCCCGTCCGACCCTCCGACAAGCCCCTTCGTCTCCCCCTTCAGG

Dai24566

CGAACCCCCCGTTCGTCCTAGCGACAAGCACCTGCGAGTGGGCTGCCAGG

Yuan5965 --

Dai3204 --

1194CUI9985 --

Dai15205_Ceriporia_albomellea --

Dai15223_Ceriporia_albomellea --

Li1780_Ceriporia_variegata --
Dai19791_Ceriporia_variegata --
Dai19886 --
Dai10833_Ceriporia_crassitunic --
CHWC1506_46Meruliopsis_crassit
AGGCTTTATCGTGAAAGTGAAGAAGATCCTGGAATGTATATGTGTAAATT
Dai9995_Ceriporia_crassitunica --
Wu1209_58_Meruliopsis_parvispo --
CHWC1505_129_Meruliopsis_parvi --
Dai21944
AGGCTTCATCGTGAAAGTGAAGAAGATCCTGGAGTGTATCTGTGTAAATT
830Dai18640A
AGGTTTCATCGTGAAAGTGAAGAAGATCCTGGAGTGTATATGTGTAAATT
GC1704_60_Meruliopsis_taxicola --
Dai22625 --
Dai22636 --
Dai21878 --
1169Dai17248 --
Wu1708_43_Meruliopsis_leptocys --
Li1011 --
ZX95Dai25742Meruliopsis_leptoc --
WCG1306Dai24733 --
LXL99Dai25816
AGGCTTCATCGTGAAAGTGAAGAAGATCCTGGAGTGTATATGTGTAAATT
WCG1559Dai26052Meruliopsis
AGGCTTTATCGTGAAAGTGAAGAAGATCCTGGAGTGTATATGTGTAAATT
He7477 --
HLX243Dai26217
AGGCTTTATCGTGAAAGTGAAGAAGATCCTGGAGTGTATATGTGTAAATT
RussiaMW673659Meruliopsis_fagi --
FD278
AGGCTTCATCGTGAAAGTAAAGAAGATTCTGGAGTGTGTATGTGTTAATT
Dai10226_Ceriporia_tarda --
LE247365 --
Dai8173_Meruliopsis_nanlingens --
860Dai17172 --
879Dai13414
AGGCTTCATCGTGAAAGTGAAGAAGATTCTGGAGTGTATATGTGTAAATT
Li_1704_Meruliopsis_pseudocyst --
833Dai18405
AGGCTTCATCGTGAAAGTGAAGAAAATCCTGGAGTGTGTATGTGTTAATT
HHB_10729_Meruliopsis_albostra
AGGCTTCATCGTGAAAGTGAAGAAAATCCTGGAGTGTGTATGTGTTAATT
Cui6878_Ceriporia_pseudocystid --

869Dai14737 --
876Cui11626 --
1199WEI3388 --
776308_Meruliopsis_cystidiata --
ICN139059_Meruliopsis_cystidia --
HHB15692Ceraceomyces_serpens
AGGCTTCATCGTAAAGGTCAAGAAGATATTGGAGTGTATCTGCGTAAATT
HHB_15629_Sp_Ceriporiopsis_ane
AGGCTTCATTGTGAAAGTCAAGAAGATTTTGGAGTGTGTTTGCGTAAACT
AJ185Trametopsis_cervina
AGGCTTCATTGTGAAAGTCAAGAAGATTCTGGAATGTGTTTGCGTAAATT
FD9Irpex_lacteus
AGGCTTCATCGTGAAAGTGAAGAAAATATTGGAGTGCATCTGTGTAAATT
908Dai11230
AGGCTTCATCGTGAAAGTGAAGAAGATATTGGAGTGTATCTGTGTAAATT
FP55521TEmmia_lacerata
AGGCTTCATTGTGAAAGTGAAGAAGATACTGGAGTGTATCTGCGTAAATT
PBU0048Ceriporia_cystidiata --
MZ340C_lacerataT --
Dai21940
AGGCTTCATCGTGAAAGTGAAGAAGATACTGGAGTGTATCTGTGTAAATT
847Dai16433 --
MarcinEmmia_latemarginatus --
Meijer3729Hydnopolyporus_fimbr --
RLG13408Phanerochaete_sp
AGGGTTTATCGTGAAAGTGAAGAAGATTCTGGAGTGTGTCTGCGTAAATT
WHC1381Flavodon_flavus --
GB1833Phlebia_albida
AGGCTTCATCGTGAAAGTGAAGAAAATTTTGGAATGTATCTGCGTAAATT
T407Phlebia_nitidula
AGGCTTCATCGTGAAAGTGAAGAAAATTTTGGAATGCATCTGCGTAAATT
HHB6988Phanerochaete_exilis
AGGCTTCATCGTGAAAGTGAAGAAGATTTTGGAATGTATTTGCGTGAACT
HHB8509Phanerochaetella_xeroph
AGGATTCATCGTGAAAGTGAAGAAGATCTTGGAATGTATTTGCGTAAACT
PBU0051Macrohyporia_dictyopora --
HHB11463Phanerochaete_sp
AGGCTTCATCGTAAAAGTCAAGAAGATCTTGGAGTGTATTTGCGTAAACT
FP102382Byssomerulius_corium
AGGCTTCATCATAAAAGTGAAGAAGATCCTGGAGTGTATTTGCGTAAATT
FP102165Efibula_americana
AGGCTTCATCGTGAAAGTGAAGAAGATCTTGGAATGCATTTGCGTAAATT
Murdoch90Ceriporia_torpida --
Rivoire4413_Ceriporia_purpurea --

Kout_18_Ceriporia_triumphalis ---
Rivoire3701_Ceriporia_bresadol ---
VS4018 ---
Ryvarden21832_Ceriporia_manzan ---
Dai24539 ---
Dai24541
AGGCTTCATTGAGAAAGTGAAGAAGATTCTGGAGTGTATATGTGTCAACT
JV1105_12_Ceriporia_occidental ---
VS8558Ceriporia_occidentalis ---
Dai22445 ---
846Dai16368 ---
Dai17951_Ceriporia_aurantiocar ---
Miettinen_11701C_viridans ---
JV0105_10Ceriporia_aurantiocar ---
Yuan5702C_viridans ---
858Dai17003 ---
Yuan2747_Ceriporia_viridans ---
Yuan2744C_viridans ---
Li1046C_viridans ---
865C_sinoviridans ---
871Dai15062 ---
Dai7642_Ceriporia_humilis ---
Spirin4706_Ceriporia_humilis ---
Spirin4944_Ceriporia_sericea ---
WCG1547Dai26044ceriporia
AGGCTTCATCGTGAAGGTGAAGAAGATTCTGGAGTGCGTATGTGTAAATT
ZZW1558Dai27086 ---
Miettinen14381_Ceriporia_mpuri ---
Miettinen15492_2_Ceriporia_sor ---
He6687
AGGCTTCATCGTGAAGGTGAAGAAGATTCTGGAGTGCGTATGTGTAAACT
ZH53Dai24426 ---
Vlasak0808_30_Ceriporia_punica ---
887Dai13376 -----------------------------
GGAGTGCATATGTGTAAACT
WCG1443Dai24998
AGGCTTCATTGTGAAGGTGAAGAAAATCCTGGAGTGCATATGTGTAAACT
0108_6Ceriporia_spissa ---
Dai19164 ---
Dai17937_Ceriporia_bubalinomar ---
903Dai12113 ---
LZB929Dai25079 ---
LX45Dai26988
AGGCTTCATCGTGAAGGTGAAGAAGATCCTGGAGTGTATATGTGTAAACT

LX43Dai26986
AGGCTTCATCGTGAAGGTGAAGAAGATCCTGGAGTGTATATGTGTAAACT
Dai7759Ceriporia --
Cui8012_Ceriporia_viridans --
GC1704_54Ceriporia_viridans --
Dai23392
AGGCTTCATCGTGAAGGTGAAGAAGATTTTGGAGTGTATATGTGTAAACT
WCG1585Dai26113Ceriproia
AGGCTTCATCGTGAAGGTGAAGAAGATTTTGGAGTGTATATGTGTAAACT
Dai18675C_eucalypti --
Dai22034
AGGCTTCATTGTGAAAGTGAAGAAAATCCTGGAGTGTATCTGCGTGAATT
JV1008_41JTardaFLORIDAKeys --
Rivoire1161_Ceriporia_pierii --
Dai23499C_pierii
AGGCTTCATCGTGAAGGTGAAGAAGATTCTGGAGTGCGTATGCGTAAATT
Dai23500 --
841Dai15899
AGGCTTCATTGTGAAAGTGAAGAAGATTTTGGAGTGTATATGCGTAAATT
842Dai15904
AGGCTTCATTGTGAAAGTGAAGAAGATTTTGGAGTGTATATGCGTAAATT
LZB1066xinjiang --
LZB1065xinjiang --
851Dai16779 --
RMJ119sp_Candelabrochaete_sept
AGGTTTCCTTGTGAAAGTGAAGAAGATCCTGGAGTGTATATGTGTAAATT
RLG9759spCandelabrochaete_sept --
RLG10478Phanerochaete_allantos --
Dai19118_Ceriporia_spissa --
Dai18486A --
WEI17_024_Ceriporia_mellita --
GC1508_71Ceriporia_mellita --
GC1608_7_Ceriporia_mellita ------------------------------
GGAGTGMATATGTGTAAATT
ZZW1557Dai27085 --
ZZW1554Dai27083 --
Dai8168 --
BR4865C_mellita --
MEL2382688Ceriporia_sp --
Dai8110 --
Cui8097 --
909Cui6740 --
W1258Dai24695
AGGGTTTCTTGTGAAAGTGAAGAAGATCCTGGAGTGCATATGTGTAAACT

JV0110_26_Ceriporia_griseoviol --
896Dai13202 --
LWY393Dai27053C_griseoviolasce
AGGCTTCATCGTGAAAGTCAAGAAGATCCTGGAGTGTATCTGTGTAAACT
LWY394DAI27054 --
FP135015G_pannocinctus --
L15726SpG_pannocinctus --
Dai22221
AGGATTCCTCACGAAAGTGAAGAAGATCTTGGAGTGCATATGTGTCAACT
Dai22633
AGGTTTCCTTACGAAAGTGAAGAAGATCTTGGAATGTATCTGTGTAAACT
Dai23260
AGGTTTCCTGACGAAAGTGAAGAAGATCTTGGAATGTATCTGTGTAAACT
Dai23626
AGGTTTCCTGACGAAAGTGAAGAAGATCTTGGAATGTATCTGTGTAAACT
Dai16238G_citrinoalbus --
1175Dai15293 --
Dai19547 --
918063G_africanus --
918572G_africanus --
Dai18536A --
1164Cui17922 --
Dai22225
AGGTTTCCTAACGAAAGTGAAGAAGATCTTGGAATGTATTTGTGTAAATT
1163Dai20655
AGGTTTCCTGACGAAAGTTAAGAAGATCTTGGAATGTATTTGCGTAAACT
Yuan4397G_hainanensis --
1176Dai15268
AGGTTTCCTGACGAAAGTGAAGAAAATTTTGGAATGCATATGTGTCAATT
1177Dai15259 --
BZ2896G_thelephoroides --
1166JV1808_26 --
Miettinen16992Hapalopilus_ochr
AGGCTTCATCGTGAAAGTGAAGAAGATTTTGGAGTGCATATGTGTAAATT
GC1708_338_Ceriporia_arbuscula -------------------------------------TATGTGTAAATT
WCG1555Dai26107Ceriporia
AGGCTTCATCGTGAAAGTGAAGAAGATCTTGGAGTGTGTATGTGTAAATT
GC1708_340_Ceriporia_arbuscula --
WCG1556Dai26109Ceriporia
AGGCTTCATCGTGAAAGTGAAGAAGATCTTGGAGTGTGTATGTGTAAATT
883Cui11291
AGGCTTCATCGTGAAAGTGAAGAAGATCCTGGAGTGCATATGTGTAAATT
HLX320Dai26805
AGGCTTCATCGTGAAAGTGAAGAAGATCCTGGAGTGTATATGTGTAAATT

WCG1266Dai24678A
AGGCTTCATCGTGAAAGTGAAGAAGATCCTGGAGTGTATATGTGTAAATT
Dai6090_Ceriporia_sulphuricolo ---
RLG_11354_Ceriproia_reticulata
AGGCTTCATCGTGAAGGTGAAGAAGATCCTGGAGTGCATCTGTGTGAACT
ZZW1543Dai27072
AGGCTTCATCGTGAAGGTGAAGAAGATCCTGGAGTGCATCTGTGTGAACT
Li1316_Ceriporia_reticulata ---
KHL11981Ceriporia_reticulata ---
FP110343sp_Candelabrochaete_la ---
Li1045_Ceriporia_reticulata ---
ZX136Dai25794ceriporia ---
892Dai13400
AGGCTTCATCGTGAAGGTGAAGAAGATCCTGGAGTGCATCTGTGTGAACT
RLG7163Leptoporus_mollis
AGGCTTCATAGTGAAAGTCAAGAAGATCCTGGAGTGTATCTGCGTAAATT
Dai21062Leptoporus_mollis ---
Dai20182Leptoporus_submollis
AGGCTTTATAGTGAAAGTGAAGAAGATCCTGGAGTGTATCTGCGTAAATT
Cui18379Leptoporus_submollis
AGGCTTTATAGTGAAAGTGAAGAAGATCCTGGAGTGTATCTGCGTAAATT
Wu1209_46Resiniporus_pseudogil ---
BRNM710169Resiniporus_resinasc ---
Dai14516Bjerkandera_adusta
AGGCTTCATTGTGAAAGTGAAGAAGATCCTGGAGTGCATCTGTGTAAACT
Dai21100Bjerkandera_fumosa
AGGCTTCATTGTGAAGGTCAAGAAGATCTTGGAGTGCATCTGTGTAAACT
Miettinen16854Ceraceomyces_sp
AGGCTTCATCGTGAAAGTGAAGAAGATCCTGGAATGTATTTGCGTAAATT
Dai10477C_spissa ---
855Dai16831 ---
882Cui11282 ---
Dai24566 ---
Yuan5965 ---
Dai3204 ---
1194CUI9985 ---

Dai15205_Ceriporia_albomellea ---
Dai15223_Ceriporia_albomellea ---
Li1780_Ceriporia_variegata ---
Dai19791_Ceriporia_variegata ---
Dai19886 ---
Dai10833_Ceriporia_crassitunic ---

CHWC1506_46Meruliopsis_crassit
GTGGAAAGCTAAAGGCGGATATCGTGAGTTTGTCTCCCTTTCGTAAACC
Dai9995_Ceriporia_crassitunica --
Wu1209_58_Meruliopsis_parvispo --
CHWC1505_129_Meruliopsis_parvi --
Dai21944
GTGGAAGACTAAAGGCGGATATCGTGAGTTTTGTCTCCTTTTCGTAAACC
830Dai18640A
GCGGAAGACTAAAGGCGGATATCGTGAGTTTTGTCTCCTTTTCGTAAACC
GC1704_60_Meruliopsis_taxicola --
Dai22625 --
Dai22636 --
Dai21878 --
1169Dai17248 --
Wu1708_43_Meruliopsis_leptocys ------------
GGCGGATATCGTGAGTTTTGTCTCCCTTTCGTAAACT
Li1011 --
ZX95Dai25742Meruliopsis_leptoc --
WCG1306Dai24733 --
LXL99Dai25816
GTGGGAGACTCAAGGCGGATATCGTGAGTTTTGTCTCCCTTTCGTAAACT
WCG1559Dai26052Meruliopsis
GTGGGAGACTCAAGGCGGATATCGTGAGTTTTGTCTCCCTTTCGTAAACT
He7477 --
HLX243Dai26217
GTGGGAGACTCAAGGCGGATATCGTGAGTTTTGTCTCCCTTTCGTAAACT
RussiaMW673659Meruliopsis_fagi --
FD278
GTGGGAGGCTCAAAGCGGATATCGTGAGTTCTGTCTCCYTTTTGTAAACT
Dai10226_Ceriporia_tarda --
LE247365 --
Dai8173_Meruliopsis_nanlingens --
860Dai17172 --
879Dai13414
GCGGGAGACTAAAGGCGGATATCGTGAGTTTTGTCTCCCTTTCGTAAACT
Li_1704_Meruliopsis_pseudocyst --
833Dai18405
GTGGGAGACTCAAGGCGGATATCGTGAGTTCTGTCTCCTTTTCGTAAACC
HHB_10729_Meruliopsis_albostra
GTGGGAGACTCAAGGCGGATATCGTGAGTTCTGTCTCCTTTTCGTAAACC
Cui6878_Ceriporia_pseudocystid --
869Dai14737 --
876Cui11626 --
1199WEI3388 --

776308_Meruliopsis_cystidiata --
ICN139059_Meruliopsis_cystidia --
HHB15692Ceraceomyces_serpens
GCGGGAGGCTGAAGGCGGACATCGTGAGTCTTGTCTCCCTTTCGTATTCT
HHB_15629_Sp_Ceriporiopsis_ane
GCGGGAGACTGAAGGCCGATATCGTGAGTTTGTCTCCCTTTCGTATACT
AJ185Trametopsis_cervina
GTGGGAGACTGAAGGCCGACATCGTGAGTTTGTCTCCCTTTCGTATACT
FD9Irpex_lacteus
GTGGAAGACTGAAGGCGGATACCGTGAGTTTTGCCTCCTTTTCGTAAACT
908Dai11230
GTGGAAGACTGAAGGCGGACACCGTGAGTTTTGCCTCCTTTTCGTAAACT
FP55521TEmmia_lacerata
GTGGGAAACTCAAGGCGGATATCGTGAGTTTTGCCTCTTTTTCGTATACT
PBU0048Ceriporia_cystidiata --
MZ340C_lacerataT --
Dai21940
GTGGGAAACTCAAGGCGGATATCGTGAGTTTTGCCTCTTTTTCGTATACT
847Dai16433 --
MarcinEmmia_latemarginatus --
Meijer3729Hydnopolyporus_fimbr --
RLG13408Phanerochaete_sp
GTGGAAGGCTCAAGGCGGATACYGTGAGTTTTGCGTCCTTTTCGTATACT
WHC1381Flavodon_flavus --
GB1833Phlebia_albida
GTGGAAGACTGAAGGCGGATATCGTGAGTTTTGCCTCCCTTTCGTAAACT
T407Phlebia_nitidula
GTGGAAGACTGAAGGCGGATATCGTGAGTTTTGCCTCCCTTTCGTAAACT
HHB6988Phanerochaete_exilis
GCGGAAGGCTGAAGGCGGATACCGTGAGTTATGCGTCCTTTTCGTAAACT
HHB8509Phanerochaetella_xeroph
GCGGAAGGCTGAAGGCGGATACCGTGAGTTATGCGTCCTTTTCGTAAACT
PBU0051Macrohyporia_dictyopora --
HHB11463Phanerochaete_sp
GCGGAAGGCTAAAGGCGGATATCGTGAGTTATGCGTCCTTTTCGTAAACT
FP102382Byssomerulius_corium GCGGAAGACTGAAGGCCGATACCGTGAG-TTTGCCTCCCTTTCGTATACT
FP102165Efibula_americana
GCGGGAAATTGAAGGCGGATATCGTGAGTTTTGCCTCCCTTTCGTAAACT
Murdoch90Ceriporia_torpida --
Rivoire4413_Ceriporia_purpurea --
Kout_18_Ceriporia_triumphalis --
Rivoire3701_Ceriporia_bresadol --
VS4018 --

Ryvarden21832_Ceriporia_manzan --
Dai24539 --
Dai24541
GTGGGAGACTCAAGGCGGATATCGTGAGTTTTGTCTCCCTTTCGTAAACC
JV1105_12_Ceriporia_occidental --
VS8558Ceriporia_occidentalis --
Dai22445 --
846Dai16368 --
Dai17951_Ceriporia_aurantiocar --
Miettinen_11701C_viridans --
JV0105_10Ceriporia_aurantiocar --
Yuan5702C_viridans --
858Dai17003 --
Yuan2747_Ceriporia_viridans --
Yuan2744C_viridans --
Li1046C_viridans --
865C_sinoviridans --
871Dai15062 --
Dai7642_Ceriporia_humilis --
Spirin4706_Ceriporia_humilis --
Spirin4944_Ceriporia_sericea --
WCG1547Dai26044ceriporia
GTGGAAGACTGAAAGCAGATATCGTGAGTTTTGTCTCCATTTCGTAAACC
ZZW1558Dai27086 --
Miettinen14381_Ceriporia_mpuri --
Miettinen15492_2_Ceriporia_sor --
He6687
GTGGGAGACTGAAAGCAGATATCGTGAGTTTCGTCTCCATTTCGTAAACT
ZH53Dai24426 --
Vlasak0808_30_Ceriporia_punica --
887Dai13376
GCGGGAGACTCAAGGCGGATATCGTGAGTTTTGTCTCCCTTTCGTAAACT
WCG1443Dai24998
GCGGGAGACTCAAGGCGGATATCGTGAGTTTTGTCTCCCTTTCGTAAACT
0108_6Ceriporia_spissa --
Dai19164 --
Dai17937_Ceriporia_bubalinomar --
903Dai12113 --
LZB929Dai25079 --
LX45Dai26988
GTGGCAGACTCAAAGCGGATATCGTGAGTTTTATCCCCCTTTCGTAAACC
LX43Dai26986
GTGGCAGACTCAAAGCGGATATCGTGAGTTTTATCCCCCTTTCGTAAACC
Dai7759Ceriporia --

Cui8012_Ceriporia_viridans --
GC1704_54Ceriporia_viridans --
Dai23392
GTGGGAGACTCAAAGCGGATATCGTGAGTTTTGTCTCCCTTTCGTAAACC
WCG1585Dai26113Ceriproia
GTGGGAGACTCAAAGCGGATATCGTGAGTTTTGTCTCCCTTTCGTAAACC
Dai18675C_eucalypti --
Dai22034
GCGGGAGACTGAAGGCGGATATCGTGAGTTCTGTCTCCCTTTCGTAGCCC
JV1008_41JTardaFLORIDAKeys --
Rivoire1161_Ceriporia_pierii --
Dai23499C_pierii
GTGGGCGACTCAAAGCAGATATCGTGAGTTTCGTCTTCATTTCGTAAACC
Dai23500 --
841Dai15899
GCGGGAGACTCAAAGCGGATACCGTGAGTCTCGTCTTCCTTTCGTAAACC
842Dai15904
GCGGGAGACTCAAAGCGGATACCGTGAGTCTCGTCTTCCTTTCGTAAACC
LZB1066xinjiang --
LZB1065xinjiang --
851Dai16779 --
RMJ119sp_Candelabrochaete_sept
GCGGGAGATTGAAGGCGGATATCGTGAGTTTTGTCCCTCTTTCGTAAACC
RLG9759spCandelabrochaete_sept --
RLG10478Phanerochaete_allantos --
Dai19118_Ceriporia_spissa --
Dai18486A --
WEI17_024_Ceriporia_mellita --
GC1508_71Ceriporia_mellita --
GC1608_7_Ceriporia_mellita
GTGGGAGACTCAATGCGGATATCGTGAGTTTTGTCTCCCTTTCGTAAACC
ZZW1557Dai27085 --
ZZW1554Dai27083 --
Dai8168 --
BR4865C_mellita --
MEL2382688Ceriporia_sp --
Dai8110 --
Cui8097 --
909Cui6740 --
W1258Dai24695
GTGGCAGACTCAAAGCGGATACCGTGAGTTTCGTCTCCCTTTCGTAAACT
JV0110_26_Ceriporia_griseoviol --
896Dai13202 --
LWY393Dai27053C_griseoviolasce

GCGGAAGGCTCAAAGCGGATATCGTGAGTTTTGTCTCCCTTTCGTAAACC
LWY394DAI27054 --
FP135015G_pannocinctus --
L15726SpG_pannocinctus --
Dai22221 GTGGGAAACTGAAGGCGGATATCGTGAGTT---
GTCCCTTTTCCTCTACT
Dai22633 GCGGAAAGTTGAAGGCGGATATCGTGAGTT---
GTCTTTTTTCCCTTACT
Dai23260 GCGGAAAGTTGAAGGCGGATATCGTGAGTT---
GTCTTTTTTCCCTTACT
Dai23626 GCGGAAAGTTGAAGGCGGATATCGTGAGTT---
GTCTTTTTTCCCTTACT
Dai16238G_citrinoalbus --
1175Dai15293 --
Dai19547 --
918063G_africanus --
918572G_africanus --
Dai18536A --
1164Cui17922 --
Dai22225 GCGGAAAGTTGAAGGCGGATATCGTGAGTT---
GTCTTTTTTCCTCTACT
1163Dai20655 GCGGAAAGTTGAAGGCGGATATCGTGAGTT---
GTCTTTTTTCCTCTACT
Yuan4397G_hainanensis --
1176Dai15268 GCGGAAGATTGAAGGCGGATATCGTGAGTT-
TGTCTTCTATTCGTTCACT
1177Dai15259 --
BZ2896G_thelephoroides --
1166JV1808_26 --
Miettinen16992Hapalopilus_ochr
GCGGGAGACTCAAAGCGGATACCGTGAGTCTTGTCTCCCTTTCGTTCACT
GC1708_338_Ceriporia_arbuscula
GTGGGAGACTGATTGCGGACATCGTGAGTTTTGTCTCCCTTT-GWAAACC
WCG1555Dai26107Ceriporia
GCGGGAGACTGAAGGCGGACATCGTGAGTTTTGTCTCCCTTTCGTAAACC
GC1708_340_Ceriporia_arbuscula ---AAACC
WCG1556Dai26109Ceriporia
GCGGGAGACTGAAGGCGGACATCGTGAGTTTTGTCTCCCTTTCGTAAACC
883Cui11291
GTGGGAGACTGAAGGCGGACATCGTGAGTTTTGTCTTCCTTTCGTAAACC
HLX320Dai26805
GTGGCAGACTGAAGGCGGACATCGTGAGTTTTGTCTCCCTTTCGTAAACC
WCG1266Dai24678A
GCGGGAGACTGAAGGCGGACATCGTGAGTTTTGTCTCCCTTTCGTAAACC

Dai6090_Ceriporia_sulphuricolo --

RLG_11354_Ceriproia_reticulata

GTGGGAGACTGAAAGCGGATACCGTGAGTCCCGCCTTCCTTTCGTAAACT

ZZW1543Dai27072

GTGGGAGACTGAAAGCGGATACCGTGAGTCCCGCCTTCCTTTCGTAAACT

Li1316_Ceriporia_reticulata --

KHL11981Ceriporia_reticulata --

FP110343sp_Candelabrochaete_la --

Li1045_Ceriporia_reticulata --

ZX136Dai25794ceriporia --

892Dai13400

GTGGGAGACTGAAAGCGGATATCGTGAGTTCCGTCTCCCTTTCGTAAACC

RLG7163Leptoporus_mollis

GTGGGAGACTCAAAGCGGATATCGTGAGTCTTGTCTCCCCTTCGTAAACT

Dai21062Leptoporus_mollis --

Dai20182Leptoporus_submollis

GTGGGAGACTCAAAGCGGATATCGTGAGTCTTGTCTCCCCTTCGTAAACT

Cui18379Leptoporus_submollis

GTGGGAGACTCAAAGCGGATATCGTGAGTCTTGTCTCCCCTTCGTAAACT

Wu1209_46Resiniporus_pseudogil --

BRNM710169Resiniporus_resinasc --

Dai14516Bjerkandera_adusta

GTGGGAGACTGAAGGCGGATATTGTGAGTTCCGTCTCCTTTTCCTCTACT

Dai21100Bjerkandera_fumosa

GTGGGAGACTGAAGGCGGATATTGTGAGTTCCGTCTCCTTTTCCTCTACT

Miettinen16854Ceraceomyces_sp

GTGGCAGACTGAAAGCGGATATCGTGAGTTTTGTCTCCTTTTCGTATTCT

Dai10477C_spissa --

855Dai16831 --

882Cui11282 --

Dai24566 --

Yuan5965 --

Dai3204 --

1194CUI9985 --

Dai15205_Ceriporia_albomellea --

Dai15223_Ceriporia_albomellea --

Li1780_Ceriporia_variegata --

Dai19791_Ceriporia_variegata --

Dai19886 --

Dai10833_Ceriporia_crassitunic --

CHWC1506_46Meruliopsis_crassit

CGTTTCGCACCCCACCACCCAATTTTCTGGGGGCTCTCTCCTTTGGAGAA

Dai9995_Ceriporia_crassitunica --
Wu1209_58_Meruliopsis_parvispo --
CHWC1505_129_Meruliopsis_parvi --
Dai21944
CGTTTCGCACCCCACCACCCAATTTTCTGGGGGCTCTCTCCTTTGGAGAA
830Dai18640A
CGTTTCGCACCCCACCACCCAATTTTCTGGGGGCTCTCTCCTTTGGAGAA
GC1704_60_Meruliopsis_taxicola --
Dai22625 --
Dai22636 --
Dai21878 --
1169Dai17248 --
Wu1708_43_Meruliopsis_leptocys
CGTTTCGCACCCCACTACCCAATTTTCTGGGGGCTCTCTCCTTTGGAGAA
Li1011 --
ZX95Dai25742Meruliopsis_leptoc --
WCG1306Dai24733 --
LXL99Dai25816
CGTTTCGCACCCCACCACCCAATTTTCTGGGGGCTCTCTCCCTTGGAGAA
WCG1559Dai26052Meruliopsis
CGTTTCGCACCCCACCACCCAATTTTCTGGGGGCTCTCTCCCTTGGAGAA
He7477 --
HLX243Dai26217
CGTTTCGCACCCCACCACCCAATTTTCTGGGGGCTCTCTCCCTTGGAGAA
RussiaMW673659Meruliopsis_fagi --
FD278
CGTTTCGCACCCCACCACCCAATTTTCTGGGGGCTCTCTCCGTTGGAGAA
Dai10226_Ceriporia_tarda --
LE247365 --
Dai8173_Meruliopsis_nanlingens --
860Dai17172 --
879Dai13414
CGTTTCGCACCCCACCACCCAATTTTCTGGGGGCTCTCTCCTTTGGAGAA
Li_1704_Meruliopsis_pseudocyst --
833Dai18405
CGTTTCGCACCCCACCACCCAATTTTCTGGGGGCTCTCTCCCTTGGAGAA
HHB_10729_Meruliopsis_albostra
CGTTTCGCACCCCACCACCCAATTTTCTGGGGGCTCTCTCCCTTGGAGAA
Cui6878_Ceriporia_pseudocystid --
869Dai14737 --
876Cui11626 --
1199WEI3388 --
776308_Meruliopsis_cystidiata --
ICN139059_Meruliopsis_cystidia --

HHB15692Ceraceomyces_serpens
CGTTTCGCACCCCACCACCCAATTTTCTGGGGGCTCTCCGGAGGAGAA
HHB_15629_Sp_Ceriporiopsis_ane
CGTTTCGCACCCCACCACCCAATTTTCTGGGGGCTCTCCATAGGAGAA
AJ185Trametopsis_cervina
CGTTTCGCACCCCACCACCCAATTTTCTGGGGGCTCTCCATTGGAGAA
FD9Irpex_lacteus
CGTTTCGCACCCCACCACCCAATTTTCTGGGGGCTCTCCATGGGAGAA
908Dai11230
CGTTTCGCACCCCACCACCCAATTTTCTGGGGGCTCTCCATGGGAGAA
FP55521TEmmia_lacerata
CGTTTCGCACCCCACCATCCAATTTTCTGGGGGCTCTCCATGGGAGAA
PBU0048Ceriporia_cystidiata --
MZ340C_lacerataT --
Dai21940
CGTTTCGCACCCCACCATCCAATTTTCTGGGGGCTCTCCATGGGAGAA
847Dai16433 --
MarcinEmmia_latemarginatus --
Meijer3729Hydnopolyporus_fimbr --
RLG13408Phanerochaete_sp
CGTTTCGCACCCCACCACCCAATTTTCTGGGGGCTCTCCTGCGGAGAA
WHC1381Flavodon_flavus ---------------------ATTTCTGGGGCTCTCT-
CATGGGAGAA
GB1833Phlebia_albida
CGTTTCGCACCCCACCACCCAATTTTCTGGGGGCTCTCCATTGGAGAA
T407Phlebia_nitidula
CGTTTCGCACCCCACCACCCAATTTTCTGGGGGCTCTCCATCGGAGAA
HHB6988Phanerochaete_exilis
CGTTTCGCACCCCACCACCCAATTTTCTGGGGGCTCTCCATT-GAGAA
HHB8509Phanerochaetella_xeroph
CGTTTCGCACCCCACCACCCAATTTTCTGGGGGCTCTCCAGTGGAGAA
PBU0051Macrohyporia_dictyopora --
HHB11463Phanerochaete_sp
CGTTTCGCACCCCACCACCCAATTTTCTGGGGGCTCTCCAATGGAGAA
FP102382Byssomerulius_corium
CGTTTCGCACCCCACCACCCAATTTTCTGGGGGCTCTCCGATGGAGAA
FP102165Efibula_americana
CGTTTCGCACCCCACCACCCAATTTTCTGGGGGCTCTCCATCGGAGAA
Murdoch90Ceriporia_torpida --
Rivoire4413_Ceriporia_purpurea --
Kout_18_Ceriporia_triumphalis --
Rivoire3701_Ceriporia_bresadol --
VS4018 --
Ryvarden21832_Ceriporia_manzan --

Dai24539 --
Dai24541
CGTTTCGCACCCCACCACCCAATTTTCTGGGGGCTCTCTCCGTTGGAGAA
JV1105_12_Ceriporia_occidental --
VS8558Ceriporia_occidentalis --
Dai22445 --
846Dai16368 --
Dai17951_Ceriporia_aurantiocar --
Miettinen_11701C_viridans --
JV0105_10Ceriporia_aurantiocar --
Yuan5702C_viridans --
858Dai17003 --
Yuan2747_Ceriporia_viridans --
Yuan2744C_viridans --
Li1046C_viridans --
865C_sinoviridans --
871Dai15062 --
Dai7642_Ceriporia_humilis --
Spirin4706_Ceriporia_humilis --
Spirin4944_Ceriporia_sericea --
WCG1547Dai26044ceriporia
CGTTTCGCACCCCACCACCCAATTTTCTGGGGGCTCTCTCCGCTGGAGAA
ZZW1558Dai27086 --
Miettinen14381_Ceriporia_mpuri --
Miettinen15492_2_Ceriporia_sor --
He6687
CGTTTCGCACCCCACCACCCAATTTTCTGGGGGCTCTCTCCACTGGAGAA
ZH53Dai24426 --
Vlasak0808_30_Ceriporia_punica --
887Dai13376
CGTTTCGCACCCCACCACCCAATTTTCTGGGGGCTCTCTCCGCTGGAGAA
WCG1443Dai24998
CGTTTCGCACCCCACCACCCAATTTTCTGGGGGCTCTCTCCGCTGGAGAA
0108_6Ceriporia_spissa --
Dai19164 --
Dai17937_Ceriporia_bubalinomar --
903Dai12113 --
LZB929Dai25079 --
LX45Dai26988
CGTTTCGCACCCCACCACCCAATTTTCTGGGGGCTCTCTCCGCTGGAGAA
LX43Dai26986
CGTTTCGCACCCCACCACCCAATTTTCTGGGGGCTCTCTCCGCTGGAGAA
Dai7759Ceriporia --
Cui8012_Ceriporia_viridans --

GC1704_54Ceriporia_viridans --
Dai23392
CGTTTCGCACCCCACTACCCAATTTTCTGGGGGCTCTCCGATGGAGAA
WCG1585Dai26113Ceriproia
CGTTTCGCACCCCACTACCCAATTTTCTGGGGGCTCTCCGATGGAGAA
Dai18675C_eucalypti --
Dai22034
CGTTTCGCACCCCACCACCCAATTTTCTGGGGGCTCTCCTCTGGAGAA
JV1008_41JTardaFLORIDAKeys --
Rivoire1161_Ceriporia_pierii --
Dai23499C_pierii
CGTTTCGCACCCCACCACCCAATTTTCTAGGGGCTCTCCTTTGGAGAA
Dai23500 --
841Dai15899
CGTTTCGCACCCCACCACCCAATTTTCTGGGGGCTCTCCATTGGAGAA
842Dai15904
CGTTTCGCACCCCACCACCCAATTTTCTGGGGGCTCTCCATTGGAGAA
LZB1066xinjiang --
LZB1065xinjiang --
851Dai16779 --
RMJ119sp_Candelabrochaete_sept
CGTTTCGCACCCCACCACCCAATTTTCTGGGGGCTCTCCTCTGGAGAA
RLG9759spCandelabrochaete_sept --
RLG10478Phanerochaete_allantos --
Dai19118_Ceriporia_spissa --
Dai18486A --
WEI17_024_Ceriporia_mellita --
GC1508_71Ceriporia_mellita --
GC1608_7_Ceriporia_mellita
CGTTTCGCACCCCACCACCCAATTTTCTGGGGGCTCTCCTTTGGAGAA
ZZW1557Dai27085 --
ZZW1554Dai27083 --
Dai8168 --
BR4865C_mellita --
MEL2382688Ceriporia_sp --
Dai8110 --
Cui8097 --
909Cui6740 --
W1258Dai24695
CGTTTCGCACCCCACCGCCCAATTTTCTGGGGGCTCTCCTTTGGAGAA
JV0110_26_Ceriporia_griseoviol --
896Dai13202 --
LWY393Dai27053C_griseoviolasce
CGTTTCGCACCCCACCACCCAATTTTCTGGGGGCTCTCCTTTGGAGAA

LWY394DAI27054 --
FP135015G_pannocinctus --
L15726SpG_pannocinctus --
Dai22221
CGTTTTGCACCCCACCACCCAATTTTCTGGGGGCTCTCTCCTTTGGAGAA
Dai22633
CGTTTCGCACCCCACCACCCAATTTTCTGGGGGCTCTCTCCGTTGGAGAA
Dai23260
CGTTTCGCACCCCACCACCCAATTTTCTGGGGGCTCTCTCCATTGGAGAA
Dai23626
CGTTTCGCACCCCACCACCCAATTTTCTGGGGGCTCTCTCCGTTGGAGAA
Dai16238G_citrinoalbus --
1175Dai15293 --
Dai19547 --
918063G_africanus --
918572G_africanus --
Dai18536A --
1164Cui17922 --
Dai22225
CGTTTCGCACCCCACCACCCAATTTTCTGGGGGCTCTCTCCGTTGGAGAA
1163Dai20655
CGTTTCGCACCCCACCACCCAATTTTCTGGGGGCTCTCTCCGTTGGAGAA
Yuan4397G_hainanensis --
1176Dai15268
CGTTTCGCACCCCACCACCCAATTTTCTGGGGGCTCTCTCCATTGGAGAA
1177Dai15259 --
BZ2896G_thelephoroides --
1166JV1808_26 --
Miettinen16992Hapalopilus_ochr
CGTTTCGCACCCCACTACCCAATTTTCTGGGGGCTCTCTCCATCGGAGAA
GC1708_338_Ceriporia_arbuscula
CGTTTCGCACCCCACCACCCAATTTTCTGGGGGCTCTCTCCTTTGGAGAA
WCG1555Dai26107Ceriporia
CGTTTCGCACCCCACCACCCAATTTTCTGGGGGCTCTCTCCTTTGGAGAA
GC1708_340_Ceriporia_arbuscula
CGTTTCGCACCCCACCACCCAATTTTCTGGGGGCTCTCTCCTTTGGAGAA
WCG1556Dai26109Ceriporia
CGTTTCGCACCCCACCACCCAATTTTCTGGGGGCTCTCTCCTTTGGAGAA
883Cui11291
CGTTTCGCACCCCACCACCCAATTTTCTGGGGGCTCTCTCCTTTGGAGAA
HLX320Dai26805
CGTTTCGCACCCCACCACCCAATTTTCTGGGGGCTCTCTCCTTTGGAGAA
WCG1266Dai24678A
CGTTTCGCACCCCACCACCCAATTTTCTGGGGGCTCTCTCCTTTGGAGAA

Dai6090_Ceriporia_sulphuricolo ---
RLG_11354_Ceriproia_reticulata
CGTTTCGCACCCCACCACCCAATTTCTGGGGGCTCTCCTATGGAGAA
ZZW1543Dai27072
CGTTTCGCACCCCACCACCCAATTTCTGGGGGCTCTCCTTTGGAGAA
Li1316_Ceriporia_reticulata ---
KHL11981Ceriporia_reticulata ---
FP110343sp_Candelabrochaete_la ---
Li1045_Ceriporia_reticulata ---
ZX136Dai25794ceriporia ---
892Dai13400
CGTTTCGCACCCCACCACCCAATTTCTGGGGGCTCTCCTTTGGAGAA
RLG7163Leptoporus_mollis
CGTTTCGCACCCCACCGTCCAATTTCTGGGGGCTCTCCTTTGGAGAA
Dai21062Leptoporus_mollis ---
Dai20182Leptoporus_submollis
CGTTTCGCACCCCACCACCCAATTTCTGGGGGCTCTCCTTTGGAGAA
Cui18379Leptoporus_submollis
CGTTTCGCACCCCACCACCCAATTTCTGGGGGCTCTCCTTTGGAGAA
Wu1209_46Resiniporus_pseudogil ---
BRNM710169Resiniporus_resinasc ---
Dai14516Bjerkandera_adusta
CGTTTCGCACCCCACCACCCAATTTCTGGGGGCTCTCCTCTGGAGAA
Dai21100Bjerkandera_fumosa
CGTTTCGCACCCCACCACCCAATTTCTGGGGGCTCTCCTCTGGAGAA
Miettinen16854Ceraceomyces_sp
CGTTTCGCACCCCACCACCCAATTTCTGGGGGCTCTCCACAGGAGAA
Dai10477C_spissa ---
855Dai16831 ---
882Cui11282 ---
Dai24566 ---
Yuan5965 ---
Dai3204 ---
1194CUI9985 ---

Dai15205_Ceriporia_albomellea --
Dai15223_Ceriporia_albomellea --
Li1780_Ceriporia_variegata --
Dai19791_Ceriporia_variegata --
Dai19886 --
Dai10833_Ceriporia_crassitunic --
CHWC1506_46Meruliopsis_crassit
CCCCAAGCCCGCACTACGTTTGCTCTCGATAATCGAGACGTTCTTCCACC

Dai9995_Ceriporia_crassitunica --
Wu1209_58_Meruliopsis_parvispo --
CHWC1505_129_Meruliopsis_parvi --
Dai21944
CCCCAAGCCCGCACTGCGTCTACTCTCGATAATCGAGGCGTTCTTCCACC
830Dai18640A
CCCCAAGCCCGCACTGCGTTTACTCTCGATAATCGAGGCGTTCTTCCACC
GC1704_60_Meruliopsis_taxicola --
Dai22625 --
Dai22636 --
Dai21878 --
1169Dai17248 --
Wu1708_43_Meruliopsis_leptocys
CCCCAAGCCCGCACTGCGTTAGTTCTCGAAAATCGAG-CGTTCTTCCACC
Li1011 --
ZX95Dai25742Meruliopsis_leptoc --
WCG1306Dai24733 --
LXL99Dai25816
CCCCAAGCCCGCACTGCGTTAGTTCTCGACAATCGAGACGTTCTTCCACC
WCG1559Dai26052Meruliopsis
CCCCAAGCCCGCACTGCGTTAGTTCTCGACAATCGAGGCGTTCTTCCACC
He7477 --
HLX243Dai26217
CCCCAAGCCCGCACTGCGTTAGTTCTCGACAATCGAGGCGTTCTTCCACC
RussiaMW673659Meruliopsis_fagi --
FD278
CCCCAAGCCCGCGCTGCGTTTGTTCTCGACCATCGAGACGTTCTTCCGCC
Dai10226_Ceriporia_tarda --
LE247365 --
Dai8173_Meruliopsis_nanlingens --
860Dai17172 --
879Dai13414
CCCCAAGCCCGCACTGCGGTCGTTCTCGATAATCGAGGCGTTCTTCCACC
Li_1704_Meruliopsis_pseudocyst --
833Dai18405 CCCCAAGCCCACACTGCGTT------------------
CGTTCTTCCACT
HHB_10729_Meruliopsis_albostra CCCCAAGCCCACACTGCGTT------------------
CGTTCTTCCACT
Cui6878_Ceriporia_pseudocystid --
869Dai14737 --
876Cui11626 --
1199WEI3388 --
776308_Meruliopsis_cystidiata --
ICN139059_Meruliopsis_cystidia --

HHB15692Ceraceomyces_serpens
CCCCAAGCCCGCTCTGCGGCAGTCCTCGATAATCGAGGCGTTCTTCCACC
HHB_15629_Sp_Ceriporiopsis_ane
CCCCAAGCCCGCACTGTGGTCGTTCTCGATAATCGAGGCGTTCTTCCACC
AJ185Trametopsis_cervina
CCCCAAGCCCGCATTGTGGTCGTCCTCGATAATCGAGGCGTTCTTCCACC
FD9Irpex_lacteus
CCCCAAGCCCGCTCTGCGTTCGTTCTCGACAATCGAGACGTTCTTCCACC
908Dai11230
CCCCAAGCCCGCTCTGCGTTCGTTCTCGACAATCGAGACGTTCTTCCACC
FP55521TEmmia_lacerata
CCCCAAGCCCGCTCTACGGTCGTTCTCGACAATCGAGGCGTTCTTCCACC
PBU0048Ceriporia_cystidiata --
MZ340C_lacerataT --
Dai21940
CCCCAAGCCCGCTCTACGGTCGTTCTCGACAATCGAGGCGTTCTTCCACC
847Dai16433 --
MarcinEmmia_latemarginatus --
Meijer3729Hydnopolyporus_fimbr --
RLG13408Phanerochaete_sp
CCCCAAGCCCGCACTACGGTCGTTCTCGACAATCGAGACGTTCTTCCACC
WHC1381Flavodon_flavus
CCCCAAGCCCGCTCTGCGTTCGTTCTCGATAATCGAGGCGTTCTTCCACC
GB1833Phlebia_albida
CCCCAAGCCCGCTCTACGGTCGTTCTCGACAATCGAGGCGTTCTTCCACC
T407Phlebia_nitidula
CCCCAAGCCTGCTCTACGGTCGTTCTCGACAATCGAGGCGTTCTTCCACC
HHB6988Phanerochaete_exilis
CCCCAAGCCCACACTACGGCCGTTCTCGACAATCGAGGCGTTCTTCCACC
HHB8509Phanerochaetella_xeroph
CCCCAAGCCCGCACTACGGTCGTTCTCGACAATCGAGGCGTTCTTCCACC
PBU0051Macrohyporia_dictyopora --
HHB11463Phanerochaete_sp
CCCCAAGCCCGCACTACGGTCGTTCTCGACAATCGAGGCGTTCTTCCACC
FP102382Byssomerulius_corium
CCCCAAGCCCGCGCTACGGTCGTTCTCGACAATCGAGACGTTCTTCTACC
FP102165Efibula_americana
CCCCAAGCCCGCTCTATGGTAGTCCTCGACAATCGAGGCGTTCTTCCACC
Murdoch90Ceriporia_torpida --
Rivoire4413_Ceriporia_purpurea --
Kout_18_Ceriporia_triumphalis --
Rivoire3701_Ceriporia_bresadol --
VS4018 --
Ryvarden21832_Ceriporia_manzan --

Dai24539 --
Dai24541
CCCCAAGCCCGCGCTGCGTTCGTTCTCGAAAATCGAGGCGTTCTTCCACC
JV1105_12_Ceriporia_occidental --
VS8558Ceriporia_occidentalis --
Dai22445 --
846Dai16368 --
Dai17951_Ceriporia_aurantiocar --
Miettinen_11701C_viridans --
JV0105_10Ceriporia_aurantiocar --
Yuan5702C_viridans --
858Dai17003 --
Yuan2747_Ceriporia_viridans --
Yuan2744C_viridans --
Li1046C_viridans --
865C_sinoviridans --
871Dai15062 --
Dai7642_Ceriporia_humilis --
Spirin4706_Ceriporia_humilis --
Spirin4944_Ceriporia_sericea --
WCG1547Dai26044ceriporia
CCCCAAGCCCGCATTGCGTTCGTTCTCGAGAATCGAGGCGTTCTTCCACC
ZZW1558Dai27086 --
Miettinen14381_Ceriporia_mpuri --
Miettinen15492_2_Ceriporia_sor --
He6687
CCCCAAGCCCGCATTGCGTTCGTTCTCGAGAATCGAGGCGTTCTTCCACC
ZH53Dai24426 --
Vlasak0808_30_Ceriporia_punica --
887Dai13376
CCCCAAGCCCGTATTGCGGTCGTTCTCGACTACCGAGGCGTTCTTCCACC
WCG1443Dai24998
CCCCAAGCCCGTACTGCGGTCGTTCTCGACTACCGAGGCGTTCTTCCACC
0108_6Ceriporia_spissa --
Dai19164 --
Dai17937_Ceriporia_bubalinomar --
903Dai12113 --
LZB929Dai25079 --
LX45Dai26988
CCCCAAGCCCGCACTGCGTTCGTTCTCGACATTCGAGGCGTTCTTCCACC
LX43Dai26986
CCCCAAGCCCGCACTGCGTTCGTTCTCGACATTCGAGGCGTTCTTCCACC
Dai7759Ceriporia --
Cui8012_Ceriporia_viridans --

GC1704_54Ceriporia_viridans --
Dai23392
CCCCAAGCCCGCACTGCGTTCGTTCTCGAGAATCGAGGCGTTCTTCCACC
WCG1585Dai26113Ceriproia
CCCCAAGCCCGCACTGCGTTCGTTCTCGAGAATCGAGGCGTTCTTCCACC
Dai18675C_eucalypti --
Dai22034
CCCCAAGCCCGCACTGCGGTAGTTCTCGAGTATCGAGGCGTTCTTCTGCC
JV1008_41JTardaFLORIDAKeys --
Rivoire1161_Ceriporia_pierii --
Dai23499C_pierii
CCCCAAGCCCGCACTGCGTTCGTTCTCGAGAATCGAGGCGTTCTTCCACC
Dai23500 --
841Dai15899
CCCCAAGCCCGCACTGCGGTCGTTCTCGATAATCGAGGCGTTCTTCCACC
842Dai15904
CCCCAAGCCCGCACTGCGGTCGTTCTCGATAATCGAGGCGTTCTTCCACC
LZB1066xinjiang --
LZB1065xinjiang --
851Dai16779 --
RMJ119sp_Candelabrochaete_sept
CCCCAAGCCCGCACTGCGTTCGTTCTCGGTCTTCGAGGCGTTCTTCTACC
RLG9759spCandelabrochaete_sept --
RLG10478Phanerochaete_allantos --
Dai19118_Ceriporia_spissa --
Dai18486A --
WEI17_024_Ceriporia_mellita --
GC1508_71Ceriporia_mellita --
GC1608_7_Ceriporia_mellita
CCCCAAGCCCGCACTGCGTTCRTTCTCGAGAATCGAGGCGTTCTTCCACC
ZZW1557Dai27085 --
ZZW1554Dai27083 --
Dai8168 --
BR4865C_mellita --
MEL2382688Ceriporia_sp --
Dai8110 --
Cui8097 --
909Cui6740 --
W1258Dai24695
CCCCAAGCCCGCGCTGCGTTCGTTCTCGATAATCGAGGCGTTCTTCCACC
JV0110_26_Ceriporia_griseoviol --
896Dai13202 --
LWY393Dai27053C_griseoviolasce
CCCCAAGCCCGTACTGCGTTCGTTCTCGAAAATCGAGGCGTTCTTCCACC

LWY394DAI27054 --
FP135015G_pannocinctus --
L15726SpG_pannocinctus --
Dai22221
CCCCAAGCCCGCTCTGCGTTCGTTCTCGATTATCGAGGCGTTCTTCCACC
Dai22633
CCCCAAGCCCGCTCTGCGTTCGTTCTCGAGAATCGAGGCGTTCTTCCACC
Dai23260
CCCCAAGCCCGCTCTGCGTTCGTTCTCGAGAATCGAGGCGTTCTTCCACC
Dai23626
CCCCAAGCCCGCTCTGCGTTCGTTCTCGAGAATCGAGGCGTTCTTCCACC
Dai16238G_citrinoalbus --
1175Dai15293 --
Dai19547 --
918063G_africanus --
918572G_africanus --
Dai18536A --
1164Cui17922 --
Dai22225
CCCCAAGCCCGCTCTGCGTTCGTTCTCGAGAATCGAGGCGTTCTTCCACC
1163Dai20655
CCCCAAGCCCGCTCCGCGTTCGTTCTCGAGAATCGAGGCGTTCTTCCACC
Yuan4397G_hainanensis --
1176Dai15268
CCCCAAGCCCGCTCTGCGTTCGTTCTCGAAAATCGAGGCGTTCTTCTGCC
1177Dai15259 --
BZ2896G_thelephoroides --
1166JV1808_26 --
Miettinen16992Hapalopilus_ochr CCCCAAGCCCGCTCCACG-
TAGTGCTCGACAATCGAGCCGTTCTTCCACC
GC1708_338_Ceriporia_arbuscula
CCCCAAGCCCGCACTGCGTTCATTCTCGATACTCGAGGTGTTCTTCTACC
WCG1555Dai26107Ceriporia
CCCCAAGCCCGCACTGCGTTCATTCTCGATACTCGAGGTGTTCTTCTACC
GC1708_340_Ceriporia_arbuscula
CCCCAAGCCCGCACTGCGTTCATTCTCGATACTCGAGGTGTTCTTCTACC
WCG1556Dai26109Ceriporia
CCCCAAGCCCGCACTGCGTTCATTCTCGATACTCGAGGTGTTCTTCTACC
883Cui11291
CCCCAAGCCCGCACTGCGTTCATTCTCGATAATCGAGGTGTTCTTCTACC
HLX320Dai26805
CCCCAAGCCCGCACTGCGTTCATTCTCGATAATCGAGGTGTTCTTCTACC
WCG1266Dai24678A
CCCCAAGCCCGCACTGCGTTCATTCTCGATAATCGAGGTGTTCTTCTACC

Dai6090_Ceriporia_sulphuricolo ---
RLG_11354_Ceriproia_reticulata
CCCCAAGCCCGCACTGCGTTAGTTCTCGAGTATCGAGGCGTTCTTCTGCC
ZZW1543Dai27072
CCCCAAGCCCGCACTGCGTTAGTTCTCGAGTATCGAGGCGTTCTTCTGCC
Li1316_Ceriporia_reticulata ---
KHL11981Ceriporia_reticulata ---
FP110343sp_Candelabrochaete_la ---
Li1045_Ceriporia_reticulata ---
ZX136Dai25794ceriporia ---
892Dai13400
CCCCAAGCCCGCACTGCGTTAGTTCTCGAGTATCGAGACGTTCTTCTGCC
RLG7163Leptoporus_mollis
CCCCAAGCCCGCACTGCGTTCGTTCTCGAAAATCGAGGCGTTCTTCCACC
Dai21062Leptoporus_mollis ---
Dai20182Leptoporus_submollis
CCCCAAGCCCGCACTGCGTTCGTTCTCGAAAATCGAGGCGTTCTTCCACC
Cui18379Leptoporus_submollis
CCCCAAGCCCGCACTGCGTTCGTTCTCGAAAATCGAGGCGTTCTTCCACC
Wu1209_46Resiniporus_pseudogil ---
BRNM710169Resiniporus_resinasc ---
Dai14516Bjerkandera_adusta
CCCCAAGCCCGCTCTGCGTTCGCTCTCGTTATTCGAGACGTTCTTCTACC
Dai21100Bjerkandera_fumosa
CCCCAAGCCCGCTCTGCGTTTGCTCTCGTTCCTCGAGACGTTCTTCTACC
Miettinen16854Ceraceomyces_sp
CCCCAAACCCGCTCTGCGGTAGTTCTCGATAATCGAGACGTTCTTCCACC
Dai10477C_spissa ---
855Dai16831 ---
882Cui11282 ---
Dai24566 ---
Yuan5965 ---
Dai3204 ---
1194CUI9985 ---

Dai15205_Ceriporia_albomellea ---
Dai15223_Ceriporia_albomellea ---
Li1780_Ceriporia_variegata ---
Dai19791_Ceriporia_variegata ---
Dai19886 ---
Dai10833_Ceriporia_crassitunic ---
CHWC1506_46Meruliopsis_crassit
CAGACGCGGACTGCGCGCGTTTTCGATCGCTTGATCGCCGYGTGGTCCCC

Dai9995_Ceriporia_crassitunica --
Wu1209_58_Meruliopsis_parvispo --
CHWC1505_129_Meruliopsis_parvi --
Dai21944
CAGACGCAGACTGCGCGCGTTTTCGATCGCTTGATCGCCGCGCGGTCC-T
830Dai18640A
CAGACGCGGACTGCGCGCGTTTTCGATCGCTTGATCGCCGCGTGGTCC-T
GC1704_60_Meruliopsis_taxicola --
Dai22625 --
Dai22636 --
Dai21878 --
1169Dai17248 --
Wu1708_43_Meruliopsis_leptocys
CAACCGCGGACCGCGCGCGTTTTCGATCGCTTGATCGCCGCGTGGGCCAC
Li1011 --
ZX95Dai25742Meruliopsis_leptoc --
WCG1306Dai24733 --
LXL99Dai25816
CAGACGCGGACCGCGCGCGTTTTCGATCGCTTGATCGCCGCGTGGACCAC
WCG1559Dai26052Meruliopsis
CAAATGCGGACCGCGCGCGTTTTCGATCGCTTGATCGCCGCGTGGACCAC
He7477 --
HLX243Dai26217
CAAATGCGGACCGCGCGCGTTTTCGATCGCTTGATCGCCGCGTGGACCAC
RussiaMW673659Meruliopsis_fagi --
FD278
CAGACGTAGATCACGCGCGTTTTCGGTCGCTTGATCGCCGCGTGA-CCAT
Dai10226_Ceriporia_tarda --
LE247365 --
Dai8173_Meruliopsis_nanlingens --
860Dai17172 --
879Dai13414
CAGACGCGGACTGCGCGCGTTTTCGATCGCTTGATCGCCGCGTGGGCCAC
Li_1704_Meruliopsis_pseudocyst --
833Dai18405
CGGACGCGGGCCACGCGCGTTTTCGATCGCTTGATCGCCGCGTGGACCAC
HHB_10729_Meruliopsis_albostra
CGGACGCGGGCCACGCGCGTTTTCGATCGCTTGATCGCCGCGTGGACCAC
Cui6878_Ceriporia_pseudocystid --
869Dai14737 --
876Cui11626 --
1199WEI3388 --
776308_Meruliopsis_cystidiata --
ICN139059_Meruliopsis_cystidia --

HHB15692Ceraceomyces_serpens
CGGACGCGGACTGCGCGCGTTTTCGATCGCTTGATCGCCGCGCGGTCCAT
HHB_15629_Sp_Ceriporiopsis_ane
TGGACGCGGTCTGCGCGCGTTTTCGATCGCTTGATCGCCGCGCGGACCAC
AJ185Trametopsis_cervina
TGGACGCGGTCTGCGCGCGTTTTCGATCGCTTGATCGCCGCGCGGACTAC
FD9Irpex_lacteus
CGGACGTGGTCTGCGCGCGTTTTCGATCGCTTGATCGCCGCGTGGGCCCT
908Dai11230
CGGACGTGGTCTGCGCGCGTTTTCGATCGCTTGATCGCCGCGTGGGCCAT
FP55521TEmmia_lacerata
CGGACGCGGTCTGCGCGCGTTTTCGATCGCTTGATCGCCGCGCCGGCCCT
PBU0048Ceriporia_cystidiata --
MZ340C_lacerataT --
Dai21940
CGGACGTGGTCTGCGCGCGTTTTCGATCGCTTGATCGCCGCGCGGGCCCT
847Dai16433 --
MarcinEmmia_latemarginatus --
Meijer3729Hydnopolyporus_fimbr --
RLG13408Phanerochaete_sp
TGGGCGTGGCTTGCACGCGTTTTCGATCGCTTGATCGCCGTGCGGGCCAT
WHC1381Flavodon_flavus
CCGACGTGGTCTGCGCGCGTTTTCGGTCGCTTGATCGCCGCGTGGGCCAT
GB1833Phlebia_albida
CGGGCGAGGTCTGCACGCGTTTTCGGTCGCTTGATCGCCGTGTGGGCCAT
T407Phlebia_nitidula
CGGACGAGGCCTGCACGCGTTTTCGGTCGCTTGATCGCCGTGCGGGCCAT
HHB6988Phanerochaete_exilis
CGGACGTGGTCCGCGCGCGTTTTCGGTCGCTTGATCGCCGCGTGGGCCGT
HHB8509Phanerochaetella_xeroph
CGGACGTGGTCCGCGCGCGTTTTCGGTCGCTTGATCGCCGCGTGGGCCAT
PBU0051Macrohyporia_dictyopora --
HHB11463Phanerochaete_sp
CGGACCTGGTCCGCGCGCGTTTTCGGTCGCTTGATCGCCGCGTGGGCCAT
FP102382Byssomerulius_corium
CGGACGTGGACCGCGCGCGTTTTCGATCGCTTGATCGCCGCGTGGGCCAT
FP102165Efibula_americana
CAGACGTGGTCTGCGCGCGTTTTCGGTCGCTTGATCGCCGCGT-GGCCAT
Murdoch90Ceriporia_torpida --
Rivoire4413_Ceriporia_purpurea --
Kout_18_Ceriporia_triumphalis --
Rivoire3701_Ceriporia_bresadol --
VS4018 --
Ryvarden21832_Ceriporia_manzan --

Dai24539 --
Dai24541
CGGATGCGGACTGCGCGCGTTTTCGATCGCTTGATCGCCGCGTGGACCAT
JV1105_12_Ceriporia_occidental --
VS8558Ceriporia_occidentalis --
Dai22445 --
846Dai16368 --
Dai17951_Ceriporia_aurantiocar --
Miettinen_11701C_viridans --
JV0105_10Ceriporia_aurantiocar --
Yuan5702C_viridans --
858Dai17003 --
Yuan2747_Ceriporia_viridans --
Yuan2744C_viridans --
Li1046C_viridans --
865C_sinoviridans --
871Dai15062 --
Dai7642_Ceriporia_humilis --
Spirin4706_Ceriporia_humilis --
Spirin4944_Ceriporia_sericea --
WCG1547Dai26044ceriporia
CGGACGCGGACCACGCGCGTTTTCGATCGCTTGATCGCCGCGTGGGCCAC
ZZW1558Dai27086 --
Miettinen14381_Ceriporia_mpuri --
Miettinen15492_2_Ceriporia_sor --
He6687
TGGACGCGGACCACGCGCGTTTTCGATCGCTTGATCGCCGCGTGGGCCAC
ZH53Dai24426 --
Vlasak0808_30_Ceriporia_punica --
887Dai13376
CGGACGCGGTCTGCGCGCGTTTTCGATCGCTTGATCGCCGCGCGGGCCAC
WCG1443Dai24998
CGGACGCGGTCTGCGCGCGTTTTCGATCGCTTGATCGCCGCGCGGGCCAC
0108_6Ceriporia_spissa --
Dai19164 --
Dai17937_Ceriporia_bubalinomar --
903Dai12113 --
LZB929Dai25079 --
LX45Dai26988
CAGATGCGGACTGCGCGCGTTTTCGATCGCTTGATCGCCGCGTGGACCAC
LX43Dai26986
CAGATGCGGACTGCGCGCGTTTTCGATCGCTTGATCGCCGCGTGGACCAC
Dai7759Ceriporia --
Cui8012_Ceriporia_viridans --

GC1704_54Ceriporia_viridans --
Dai23392
CAGACGCGGACCGCGCGCGTTTTCGATCGCTTGATCGCCGCGTGGACCAT
WCG1585Dai26113Ceriproia
CAGACGCGGACCGCGCGCGTTTTCGATCGCTTGATCGCCGCGTGGACCAT
Dai18675C_eucalypti --
Dai22034
CGGACGCGGACCGCGCGCGTTTTCGATCGCTTGATCGCCGCGTGGACCAC
JV1008_41JTardaFLORIDAKeys --
Rivoire1161_Ceriporia_pierii --
Dai23499C_pierii
TGGACGCAGGCCACGCGCGTTTTCGATCGCTTGATCGCCGCGTGGGCCAC
Dai23500 --
841Dai15899
CAGACGCGGGCCACGCGCGTTTTCGATCGCTTGATCGCCGCGTGGACTAT
842Dai15904
CAGACGCGGGCCACGCGCGTTTTCGATCGCTTGATCGCCGCGTGGACTAT
LZB1066xinjiang --
LZB1065xinjiang --
851Dai16779 --
RMJ119sp_Candelabrochaete_sept
CGGACGCGGACCGCGCGCGTTTTCGATCGCTTGATCGCCGCGTGGGCCCC
RLG9759spCandelabrochaete_sept --
RLG10478Phanerochaete_allantos --
Dai19118_Ceriporia_spissa --
Dai18486A --
WEI17_024_Ceriporia_mellita --
GC1508_71Ceriporia_mellita --
GC1608_7_Ceriporia_mellita
CAAACGCGGACCGCGCGCGTTTTCGATCGCTTGATCGCCGCGCGGACCAC
ZZW1557Dai27085 --
ZZW1554Dai27083 --
Dai8168 --
BR4865C_mellita --
MEL2382688Ceriporia_sp --
Dai8110 --
Cui8097 --
909Cui6740 --
W1258Dai24695
CGGATGCGGACCGCGCGCGTTTTCGATCGCTTGATCGCCGCGCGGACGAC
JV0110_26_Ceriporia_griseoviol --
896Dai13202 --
LWY393Dai27053C_griseoviolasce
CGGATGCGGACTGCGCGCGTTTTCGATCGCTTGATCGCCGCGTGGACCAC

LWY394DAI27054 --
FP135015G_pannocinctus --
L15726SpG_pannocinctus --
Dai22221
CGGATGCGGTCTGCGCGCGTTTTCGGTCGCTTGATCGCCGCGTGGACTCT
Dai22633
CGGATGCGGTCTGCGCGCGTTTTCGGTCGCTTGATCGCCGCACGGACCAC
Dai23260
CGGATGCGGTCTGCGCGCGTTTTCGGTCGCTTGATCGCCGCACGGACCAC
Dai23626
CGGATGCGGTCTGCGCGCGTTTTCGGTCGCTTGATCGCCGCACGGACCAC
Dai16238G_citrinoalbus --
1175Dai15293 --
Dai19547 --
918063G_africanus --
918572G_africanus --
Dai18536A --
1164Cui17922 --
Dai22225
CGGATGCGGTCTGCGCGCGTTTTCGGTCGCTTGATCGCCGCACGGACCAC
1163Dai20655
CGGATGCGGTCTGCGCGCGTTTTCGGTCGCTTGATCGCCGCACGGACCAC
Yuan4397G_hainanensis --
1176Dai15268
CGGATGCGATTTGCGCGCGTTTTCGATCGCTTGATCGCCGCGCGAGTCAC
1177Dai15259 --
BZ2896G_thelephoroides --
1166JV1808_26 --
Miettinen16992Hapalopilus_ochr
CAGACGCGAGTTGCGCGCGTTTTCGATCGCTTGATCGCCGCGTGATTCAT
GC1708_338_Ceriporia_arbuscula
CGGACGCGGACCACGCGCGTTTTCGATCGCTTGATCGCCGCGTGGGCTAC
WCG1555Dai26107Ceriporia
CGGACGCGGACCACGCGCGTTTTCGATCGCTTGATCGCCGCGTGGGCTAC
GC1708_340_Ceriporia_arbuscula
CGGACGCGGACCACGCGCGTTTTCGATCGCTTGATCGCCGCGTGGGCTAC
WCG1556Dai26109Ceriporia
CGGACGCGGACCACGCGCGTTTTCGATCGCTTGATCGCCGCGTGGGCTAC
883Cui11291
CGGACGCGGACCACGCGCGTTTTCGATCGCTTGATCGCCGCGTGGGCTAC
HLX320Dai26805
CGGACGCGGACCACGCGCGTTTTCGATCGCTTGATCGCCGCGTGGGCCAC
WCG1266Dai24678A
CAGACGCGGACCACGCGCGTTTTCGATCGCTTGATCGCCGCGTGGGCTAC

Dai6090_Ceriporia_sulphuricolo --
RLG_11354_Ceriproia_reticulata
CGGACGCGGACCACGCGCGTTTTCGATCGCTTGATCGCCGCGAGGACCCC
ZZW1543Dai27072
CGGACGCGGACCACGCGCGTTTTCGATCGCTTGATCGCCGCGAGGACCCC
Li1316_Ceriporia_reticulata --
KHL11981Ceriporia_reticulata --
FP110343sp_Candelabrochaete_la --
Li1045_Ceriporia_reticulata --
ZX136Dai25794ceriporia --
892Dai13400
CGGACGCGGACCACGCGCGTTTTCGATCGCTTGATCGCCGCGAGGACCCC
RLG7163Leptoporus_mollis
CGGACGCGGACTGCGCGCGTTTTCGATCGCTTGATCGCCGCGTGGGCCAC
Dai21062Leptoporus_mollis --
Dai20182Leptoporus_submollis
CGGACGCGGACTGCGCGCGTTTTCGATCGCTTGATCGCCGCGTGGGCCAC
Cui18379Leptoporus_submollis
CGGACGCGGACTGCGCGCGTTTTCGATCGCTTGATCGCCGCGTGGGCCAC
Wu1209_46Resiniporus_pseudogil --
BRNM710169Resiniporus_resinasc --
Dai14516Bjerkandera_adusta
CGGACTTGGCCTGCGCGCGTTTTCGGTCGCTTGATCGCCGCGTGGGCTAT
Dai21100Bjerkandera_fumosa
CGGACATGGTCTGCGCGCGTTTTCGGTCGCTTGATCGCCGCGTGGGCTAT
Miettinen16854Ceraceomyces_sp
CGGACGCGGACTGTGCGCGTTTTCGATCGCTTGATCGCCGCGCGGTCCAC
Dai10477C_spissa --
855Dai16831 --
882Cui11282 --
Dai24566 --
Yuan5965 --
Dai3204 --
1194CUI9985 --

Dai15205_Ceriporia_albomellea --
Dai15223_Ceriporia_albomellea --
Li1780_Ceriporia_variegata --
Dai19791_Ceriporia_variegata --
Dai19886 --
Dai10833_Ceriporia_crassitunic --
CHWC1506_46Meruliopsis_crassit
TGYACACGTCTGGAGTTGGTTGGACACGTGGTTGCGTGGTGAAATGGGGG

Dai9995_Ceriporia_crassitunica --

Wu1209_58_Meruliopsis_parvispo --------------------------
GTGGTTGCGTGGTGAAATGGGGG

CHWC1505_129_Meruliopsis_parvi --

Dai21944
CGCATACGTCTGGAGTTGGTTGGACACGTGGTTGCGTGGTGAAATGGGGG

830Dai18640A
CACATACGTCTGGAGCTGGTTGGACACGTGGTTGCGTGGTGAAATGGGGG

GC1704_60_Meruliopsis_taxicola ----------
TGGTGTTGGTTGGACACGTGGTTGCGTGGTGAAAT-GGGG

Dai22625 --

Dai22636 -
GTATACGTTCGGTGTTGGTTGGACACGTGGTTGCGTGGTGAAAT--GGG

Dai21878 --

1169Dai17248 --

Wu1708_43_Meruliopsis_leptocys
TGCATACGTTTGGTGTTGGTTGGACACGTGGCTGCGTGGTGAAATGGGGG

Li1011 --

ZX95Dai25742Meruliopsis_leptoc --

WCG1306Dai24733 --

LXL99Dai25816
TGCATACGTTTGGTGTTGGTTGGACACGTGGCTGCGTGGTGAAATGGGGG

WCG1559Dai26052Meruliopsis
TGCATACGTTTGGTGTTGGTTGGACACGTGGCTGCGTGGTGAAATGGGGG

He7477 --

HLX243Dai26217
TGCATACGTTTGGTGTTGGTTGGACACGTGGCTGCGTGGTGAAATGGGGG

RussiaMW673659Meruliopsis_fagi --

FD278
CACATACGTCTGGTGTCGGTTGGACATGTGGCTGCGTGGTGAAAAGGGGG

Dai10226_Ceriporia_tarda --

LE247365 --

Dai8173_Meruliopsis_nanlingens --

860Dai17172 --

879Dai13414
TATGTACGTTTGGTGTTGGTTGGACACGTGGCTGCGTGGTGAAATGGGGG

Li_1704_Meruliopsis_pseudocyst --

833Dai18405
TGTTTACGTCTGGTGTTGGTTGGACACGCGGCTGTGTGGTGAAATGGGGG

HHB_10729_Meruliopsis_albostra
TGTTTACGTCTGGTGTTGGTTGGACACGCGGCTGTGTGGTGAAATGGGGG

Cui6878_Ceriporia_pseudocystid --

869Dai14737 --

876Cui11626 --

1199WEI3388 --
776308_Meruliopsis_cystidiata --
ICN139059_Meruliopsis_cystidia --
HHB15692Ceraceomyces_serpens
TGCGTACGTCCGGTGCTGGTTGGACACGCAGTTGCGCGGTGAAATGGGGG
HHB_15629_Sp_Ceriporiopsis_ane
TGCACACGTCCGGTGTTGGTTGGACCCGCAGTTGCGTGGTGAAATGGGGG
AJ185Trametopsis_cervina
TGCATACGTCCAGTGCTGGTTGGACACACATTTGCGTGGTGAAATGGGGG
FD9Irpex_lacteus
TGCATGCGTTCGGTGATGGTTGGACACGTAGTCGCGTGGTGAAAAGGGGG
908Dai11230
TGCATGCGTCAGGTGATGGTTGGACACGTAGTTGCGTGGTGAAAAGGGGG
FP55521TEmmia_lacerata
TGCATGCGTCAGGTGATGGTTGGACCCGTAGTTGCGTGGTGAAAAGGGGG
PBU0048Ceriporia_cystidiata --
MZ340C_laceratаT --
Dai21940
TGCATGCGTCAGGTGATGGTTGGACCCGTAGTTGCGTGGTGAAAAGGGGG
847Dai16433 --
MarcinEmmia_latemarginatus --
Meijer3729Hydnopolyporus_fimbr --
RLG13408Phanerochaete_sp
TGCATGCGTCCGGTGTTGGTTGGACACGTAGCTGCGTGGTGAAAAGGGGG
WHC1381Flavodon_flavus
TGCATACGTCAGGTGATGGTTGGACACGTAGATGCGTGGTGAAAAGGGGG
GB1833Phlebia_albida
TGCATGCGTCCGGTGTTGGTTGGACACGTAGTTGCGTGGTGAAAAGGGGG
T407Phlebia_nitidula
TGCATGCGTCCGGTGTTGGTTGGACACGTAGTTGCGTGGTGAAAAGGGGG
HHB6988Phanerochaete_exilis
TGCATGCGTCCGGTGCTGGTTGGACACGTAGTTGTGTGGTGAAAAGGGGG
HHB8509Phanerochaetella_xeroph
TGCATGCGTCCGGTGCTGGTTGGACACGTAGTTGCGTGGTGAAAAGGGGG
PBU0051Macrohyporia_dictyopora --
HHB11463Phanerochaete_sp
TGCATGCGTCCGGAGCTGGTTGGACACGTAGTTGCGTGGTGAAAAGGGGG
FP102382Byssomerulius_corium
CGCATACGTCCGGTGTTGGTTGGACACGGAGCCGCGTGGTGAAAAGGGGG
FP102165Efibula_americana
CACAAACGTCTGGTGTTGGTTGGACCCATAGTGGCGTGGTGAAAAGGGGG
Murdoch90Ceriporia_torpida --
Rivoire4413_Ceriporia_purpurea --
Kout_18_Ceriporia_triumphalis --

Rivoire3701_Ceriporia_bresadol --
VS4018 --
Ryvarden21832_Ceriporia_manzan --
Dai24539 --
Dai24541
TGCATACCTCCGGTGTTGGTTGGACACGCGGTCGTGTGGTGAAATGGGGG
JV1105_12_Ceriporia_occidental --
VS8558Ceriporia_occidentalis --
Dai22445 --
846Dai16368 --
Dai17951_Ceriporia_aurantiocar --
Miettinen_11701C_viridans --
JV0105_10Ceriporia_aurantiocar --
Yuan5702C_viridans --
858Dai17003 --
Yuan2747_Ceriporia_viridans --
Yuan2744C_viridans --
Li1046C_viridans --
865C_sinoviridans --
871Dai15062 --
Dai7642_Ceriporia_humilis --
Spirin4706_Ceriporia_humilis --
Spirin4944_Ceriporia_sericea --
WCG1547Dai26044ceriporia
TACACACGTCTGGTGTTGGTTGGACACGCGGTTGCGTGGTGAAATGGGGG
ZZW1558Dai27086 --
Miettinen14381_Ceriporia_mpuri --
Miettinen15492_2_Ceriporia_sor --
He6687
TACACACGTCCGGTGTTGGTTGGACACGTGGTTGCGTGGTGAAATGGGGG
ZH53Dai24426 --
Vlasak0808_30_Ceriporia_punica --
887Dai13376 ATTGCACGTCCGGTGCTGGTTGGACACGCAG-
TGCGCGGTGAAATGGGGG
WCG1443Dai24998
ATTGCACGTCCGGTGATGGTTGGACACGCAG-TGCGCGGTGAAATGGGGG
0108_6Ceriporia_spissa --
Dai19164 --
Dai17937_Ceriporia_bubalinomar --
903Dai12113 --
LZB929Dai25079 --
LX45Dai26988
TGCATACGTCTGGTGTTGGTTGGACACGCGGTTGCGTGGTGAAATGGGGG
LX43Dai26986

TGCATACGTCTGGTGTTGGTTGGACACGCGGTTGCGTGGTGAAATGGGGG
Dai7759Ceriporia --
Cui8012_Ceriporia_viridans --
GC1704_54Ceriporia_viridans --
Dai23392
TACATACGTCTGGTGTTGGTTGGACACGCGGCTGCGTGGTGAAATGGGGG
WCG1585Dai26113Ceriproia
TACATACGTCTGGTGTTGGTTGGACACGCGGCTGCGTGGTGAAATGGGGG
Dai18675C_eucalypti --
Dai22034 TGCA-
ACGTCTGGTGTCGGTTGGACACGCAGCTGCGCGGTGAAATGGGGG
JV1008_41JTardaFLORIDAKeys --
Rivoire1161_Ceriporia_pierii --
Dai23499C_pierii
TGCACACGTCTGGTGTTGGTTGGACACGTGGCTGCGTGGTGAAATGGGGG
Dai23500 --
841Dai15899
CGCATACGTCTGGTGTTGGTTGGACACGCGGTTGCGTGGTGAAATGGGGG
842Dai15904
CGCATACGTCTGGTGTTGGTTGGACACGCGGTTGCGTGGTGAAATGGGGG
LZB1066xinjiang --
LZB1065xinjiang --
851Dai16779 --
RMJ119sp_Candelabrochaete_sept TGCA-
ACGTCCGGTGTTGGTTGGACACGCGGTTGCGCGGTGAAATGGGGG
RLG9759spCandelabrochaete_sept --
RLG10478Phanerochaete_allantos --
Dai19118_Ceriporia_spissa --
Dai18486A --
WEI17_024_Ceriporia_mellita --
GC1508_71Ceriporia_mellita ---GGGGG
GC1608_7_Ceriporia_mellita
TGCACACGTTTGGTGTTGGTTGGACACGTAGTTGCGTGGTGAAATGGGGG
ZZW1557Dai27085 --
ZZW1554Dai27083 --
Dai8168 --
BR4865C_mellita --
MEL2382688Ceriporia_sp --
Dai8110 --
Cui8097 --
909Cui6740 --
W1258Dai24695 TGCACA-
ATTCGGTGTTGGTTGGACACGTAGTCGCGTGGTGAAATGGGGG
JV0110_26_Ceriporia_griseoviol --

896Dai13202 --

LWY393Dai27053C_griseoviolasce TGCAGGCATCTGGTGTCGGTTGGACACGTAGCTGCG-GGTGAAATGGGGG

LWY394DAI27054 --

FP135015G_pannocinctus --

L15726SpG_pannocinctus --

Dai22221 A--TCACATTCGGTGTTGGTTGGACACGTGGAAGCGTGGTGAAATGGGGG

Dai22633 T--ACACATCTGGTGTTGGTTGGACACGTGGTTGCGTGGTGAAATGGGGG

Dai23260 T--ACACATCTGGTGTTGGTTGGACACGTGGTTGCGTGGTGAAATGGGGG

Dai23626 T--ACACATCTGGTGTTGGTTGGACACGTGGTTGCGTGGTGAAATGGGGG

Dai16238G_citrinoalbus --

1175Dai15293 --

Dai19547 --

918063G_africanus --

918572G_africanus --

Dai18536A --

1164Cui17922 --

Dai22225 T--ACACATCTGGTGTTGGTTGGACACGTGGCTGCGTGGTGAAATGGGGG

1163Dai20655 T--ACACATCTGGTGTTGGTTGGACACGTGGCTGCGTGGTGAAATGGGGG

Yuan4397G_hainanensis --

1176Dai15268 A-CATACATCCGGTGTTGGTTGGACACGTGGTTGCGTGGTGAAATGGGGG

1177Dai15259 --

BZ2896G_thelephoroides --

1166JV1808_26 --

Miettinen16992Hapalopilus_ochr TGCATACGTCTGGTGTTGGTTGGACGCGAGGTTGCGCGGTGAAATGGGGG

GC1708_338_Ceriporia_arbuscula TGCATACGTCTGG-GCTGGTTGGACGCGCAGTTGCGTGGTGAAATGGGGG

WCG1555Dai26107Ceriporia TGCATACGTCTGG-GCTGGTTGGACGCGCAGTTGCGTGGTGAAATGGGGG

GC1708_340_Ceriporia_arbuscula TGCATACGTCTGG-GCTGGTTGGACGCGCAGTTGCGTGGTGAAATGGGGG

WCG1556Dai26109Ceriporia TGCATACGTCTGG-GCTGGTTGGACGCGCAGTTGCGTGGTGAAATGGGGG

883Cui11291 TGCATACGTCCGG-GCTGGTTGGACGCGCGGTTGCGTGGTGAAATGGGGG

HLX320Dai26805 TGCACACGTCTGG-

GCTGGTTGGACGCGCGGTTGCGTGGTGAAATGGGGG

WCG1266Dai24678A TGCATACGTCTGG-GCTGGTTGGACGCGCGGTTGCGTGGTGAAATGGGGG

Dai6090_Ceriporia_sulphuricolo --

RLG_11354_Ceriproia_reticulata TGCACATGTCCGGTGTCGGTTGGGCACGC-GCTGCGTGGTGAAATGGGGG

ZZW1543Dai27072 TGCACATGTCCGGTGTCGGTTGGGCACGC-GCTGCGTGGTGAAATGGGGG

Li1316_Ceriporia_reticulata --

KHL11981Ceriporia_reticulata --

FP110343sp_Candelabrochaete_la ---AAATGGGGG

Li1045_Ceriporia_reticulata --

ZX136Dai25794ceriporia --

892Dai13400 TGCACATGTCCGGTGTCGGTTGGGCACGC-GCTGCGTGGTGAAATGGGGG

RLG7163Leptoporus_mollis TGCATACGTCTGGGGTTGGTTGGACACGTGGTTGCGTGGTGAAATGGGGG

Dai21062Leptoporus_mollis --

Dai20182Leptoporus_submollis TGCATACGTCTGGTGTTGGTTGGACACGTGGTTGTGTGGTGAAATGGGGG

Cui18379Leptoporus_submollis TGCATACGTCTGGTGTTGGTTGGACACGTGGTTGTGTGGTGAAATGGGGG

Wu1209_46Resiniporus_pseudogil -----------GGTGCTGGTTGGACACGCAGCTGTGTGGTGAAAT-GGGG

BRNM710169Resiniporus_resinasc --

Dai14516Bjerkandera_adusta TGCAAATGTCCGGTGTTAGTTGGACGCGCGGTGGCGTGGTGAAATGGGGG

Dai21100Bjerkandera_fumosa TGCAAATGTCCGGTGTTGGTTGGACGCGCGGTGGCGTGGTGAAATGGGGG

Miettinen16854Ceraceomyces_sp TAC--ACGTCCGGTGCTGGTTGGACCCGTGGTAGCGTGGTGAAAT-GGGG

Dai10477C_spissa --

855Dai16831 --

882Cui11282 --

Dai24566 --

Yuan5965 --

Dai3204 --

1194CUI9985 --

Dai15205_Ceriporia_albomellea --

Dai15223_Ceriporia_albomellea --

Li1780_Ceriporia_variegata --

Dai19791_Ceriporia_variegata --

Dai19886 --
Dai10833_Ceriporia_crassitunic --
CHWC1506_46Meruliopsis_crassit
GCTGAACGAGGAGTGCGGAAAACCAGAATGTTT-GGTGTGGTGAAGCCTC
Dai9995_Ceriporia_crassitunica --
Wu1209_58_Meruliopsis_parvispo GCTGAACGAGGAGTGCGG-
AAATCAGAATGTTC-AGTGTGGTGAAGCCTC
CHWC1505_129_Meruliopsis_parvi --
Dai21944
GCTGAACGAGGAGTGCGGAAAATCAGAATGTTC-GGTGTGGTCAAGCCTC
830Dai18640A
GCTGAACGAGGAGTGCGGAAAATCAGAATGTTC-AGTGTGGTCAAGCCTC
GC1704_60_Meruliopsis_taxicola
GCTGAACGAGGAGTGCGAGAAATTAGAATGTKCAGGTGTGGTTAAGCCTC
Dai22625 --
Dai22636 GCTGAACGAGGAGTGCGG-
AAATTAGAATGTTTAGGTGTGGTTAAGCCTC
Dai21878 --
1169Dai17248 --
Wu1708_43_Meruliopsis_leptocys
GCTGAACGAGGAGTGCGGAAAATCAGAATGTTC-GGTGTGGTCAAGCCTC
Li1011 --
ZX95Dai25742Meruliopsis_leptoc --
WCG1306Dai24733 --
LXL99Dai25816 GCTGAACGAGGAGTGCGG-
AAATCAGGATGTTC-GGTGTGGTCAAGCCTC
WCG1559Dai26052Meruliopsis GCTGAACGAGGAGTGCGG-
AAATCAGAATGTTC-GGTGTGGTCAAGCCTC
He7477 --
HLX243Dai26217 GCTGAACGAGGAGTGCGG-
AAATCAGAATGTTC-GGTGTGGTCAAGCCTC
RussiaMW673659Meruliopsis_fagi --
FD278
GCTGAACGAGGAGTGCGGAAAACTAGAGTGTTC-GGTGTGGTCAAGCCTC
Dai10226_Ceriporia_tarda --
LE247365 --
Dai8173_Meruliopsis_nanlingens --
860Dai17172 --
879Dai13414
GCTGAACGAGGAGTGCGGAAATCTAGTATGTTC-GGCGTGGTTAAGCCTC
Li_1704_Meruliopsis_pseudocyst --
833Dai18405
GCTGAACGAGGAGTGCGGAAAATCAGAATGTCC-GGTGTGGTCAAGCCTC
HHB_10729_Meruliopsis_albostra

GCTGAACGAGGAGTGCGAGAAATCAGAATGTCC-GGTGTGGTTAAGCCTC
Cui6878_Ceriporia_pseudocystid --
869Dai14737 --
876Cui11626 --
1199WEI3388 --
776308_Meruliopsis_cystidiata --
ICN139059_Meruliopsis_cystidia --
HHB15692Ceraceomyces_serpens
GCTGAGCGAGGAGTGCGGAAAATTGGACTATCTGGGCGTGGTTAAGCCTC
HHB_15629_Sp_Ceriporiopsis_ane
GCTGAGCGAGGAGTGCGGAAACACGGAAAATC-TAGCGTGGTCAAGCCTC
AJ185Trametopsis_cervina GCTGAGCGAGGAGTGCGGAAAGCTGCAAAATC-TGGGGTGGTGAAGCCTC
FD9Irpex_lacteus
GCTGAGCGAGGAGTGCGGAAAAAAGGAATATC-CGGCGTGGTTAAGCCTC
908Dai11230
GCTGAGCGAGGAGTGCGGAAAAAAGGAATATC-CGGCGTGGTTAAGCCTC
FP55521TEmmia_lacerata
GCTGAGCGAGGAGTGCGGAAAATACGACTATCTCGGCGTGGTGAAGCCTC
PBU0048Ceriporia_cystidiata --
MZ340C_lacerataT --
Dai21940
GCTGAGCGAGGAGTGCGGAAAATACGACTATCTCGGTGTGGTAAAGCCTC
847Dai16433 --
MarcinEmmia_latemarginatus --
Meijer3729Hydnopolyporus_fimbr --
RLG13408Phanerochaete_sp
GCTGAGCGAGGAGTGCGGAAAATTGGAATATC-CGGTGCGGTGAAGCCCC
WHC1381Flavodon_flavus
GCTGAGCGAGGAGTGCGGAAAACAGGAATATC-GGACGAGGTTAAGCCTC
GB1833Phlebia_albida
GCTGAGCGAGGAGTGCGGAAAATTGGGATCTC-CAGTGGGGTAACGCCTC
T407Phlebia_nitidula GCTGAGCGAGGAGTGCGGAAAATTGGGATCTC-CAGTGGGGTAACGCCTC
HHB6988Phanerochaete_exilis GCTGAGCGAGGAGTGCGGAAA-CTAGAAGATC-CGGTGTGGTGAAGCCAT
HHB8509Phanerochaetella_xeroph GCTGAGCGAGGAGTGCGGAAA-ATAGAAGATC-CGGCGTGGTGAAGCCAT
PBU0051Macrohyporia_dictyopora --
HHB11463Phanerochaete_sp GCTGAGCGAGGAGTGCGGAAA-CTAAAAGATT-CGGCGTGGTGAAGCCAC
FP102382Byssomerulius_corium GCTGAGCGAGGAGTGCGGAAA-TCAGAATATC-CGGTATGGTGACGCCTT
FP102165Efibula_americana GCTGAGCGAGGAGTGCGGAAAA--CTGAAATC-

TGGCGTGGTAAAGCCTC
Murdoch90Ceriporia_torpida --
Rivoire4413_Ceriporia_purpurea --
Kout_18_Ceriporia_triumphalis --
Rivoire3701_Ceriporia_bresadol --
VS4018 --
Ryvarden21832_Ceriporia_manzan --
Dai24539 ------------------------
GAGATCTGGATGTGGTAAAGCCAC
Dai24541
GCTGAACGAGGAGTGCGGAAATCTTAGAGATCTGGATGTGGTAAAGCCAC
JV1105_12_Ceriporia_occidental --
VS8558Ceriporia_occidentalis --
Dai22445 --
846Dai16368 --
Dai17951_Ceriporia_aurantiocar --
Miettinen_11701C_viridans --
JV0105_10Ceriporia_aurantiocar --
Yuan5702C_viridans --
858Dai17003 --
Yuan2747_Ceriporia_viridans --
Yuan2744C_viridans --
Li1046C_viridans --
865C_sinoviridans --
871Dai15062 --
Dai7642_Ceriporia_humilis --
Spirin4706_Ceriporia_humilis --
Spirin4944_Ceriporia_sericea --
WCG1547Dai26044ceriporia GCTGAACGAGGAGTGCGGAAA-
CTCTGAAATCCAGACGTGGTAACGCCAC
ZZW1558Dai27086 --
Miettinen14381_Ceriporia_mpuri --
Miettinen15492_2_Ceriporia_sor --
He6687 GCTGAACGAGGAGTGCGGAAA-
CTCTGAGATCCAGATGTGGTGACGCCAC
ZH53Dai24426 --
Vlasak0808_30_Ceriporia_punica --
887Dai13376 GCCGAACGAGGAGTGCGGAAAG-
CTGGAAATCTGGGTGTGGTCAAGCCAT
WCG1443Dai24998 GCCGAACGAGGAGTGCGGAAAG-
TTGGAAATCTGGGTGTGGTCAAGCCAT
0108_6Ceriporia_spissa --
Dai19164 --
Dai17937_Ceriporia_bubalinomar --

903Dai12113 --
LZB929Dai25079 --
LX45Dai26988
GCCGAACGAGGAGTGCGGAACTCTTGGATATCTGGATGTGGTTACGCCTC
LX43Dai26986
GCCGAACGAGGAGTGCGGAACTCTTGGATATCTGGATGTGGTTACGCCTC
Dai7759Ceriporia --
Cui8012_Ceriporia_viridans --
GC1704_54Ceriporia_viridans --
Dai23392
GCCGAACGAGGAGTGCGGAACTCTTGGAAATCTGGATGTGGTAACGCCTC
WCG1585Dai26113Ceriproia
GCCGAACGAGGAGTGCGGAACTCTTGGAAATCTGGATGTGGTAACGCCTC
Dai18675C_eucalypti --
Dai22034
GCTGAACGAGGAGTGCGGAAACTTCGGACATCCGGATGTGGTGCAGCCTC
JV1008_41JTardaFLORIDAKeys --
Rivoire1161_Ceriporia_pierii --
Dai23499C_pierii
GCTGAACGAGGAGTGCGGAAATTTTTGAGATCTGGGTGTGGTGACGCCAC
Dai23500 --
841Dai15899
GCCGAACGAGGAGTGCGGAATTGATTGATACTCGGACAAGGTAACGCCTT
842Dai15904
GCCGAACGAGGAGTGCGGAATTGATTGATACTCGGACAAGGTAACGCCTT
LZB1066xinjiang --
LZB1065xinjiang --
851Dai16779 --
RMJ119sp_Candelabrochaete_sept
GCTGAACGAGGAGTGCGGAAACTACGGAGATCTGGATGTGGTCAAGCCAC
RLG9759spCandelabrochaete_sept --
RLG10478Phanerochaete_allantos --
Dai19118_Ceriporia_spissa --
Dai18486A --
WEI17_024_Ceriporia_mellita --
GC1508_71Ceriporia_mellita
GCTGAACGAGGAGTGCGGAAACCTGGAAAATCTGGATGTGGTGACGCCTC
GC1608_7_Ceriporia_mellita
GCTGAACGAGGAGTGCGGAAACCTGGAAAATCTGGATGTGGTGACGCCTC
ZZW1557Dai27085 --
ZZW1554Dai27083 --
Dai8168 --
BR4865C_mellita --
MEL2382688Ceriporia_sp --

Dai8110 ---
Cui8097 ---
909Cui6740 ---
W1258Dai24695
GCTGAACGAGGAGTGCGGAAATCTGGAAAATCCGGATGTGGTGACGCCAC
JV0110_26_Ceriporia_griseoviol ---
896Dai13202 ---
LWY393Dai27053C_griseoviolasce
GCTGAACGAGGAGTGCGGAAATCTCGAGACTCGGGATGCGGTCAAGCCAC
LWY394DAI27054 ---
FP135015G_pannocinctus ---
L15726SpG_pannocinctus ---
Dai22221
GCTGAGCGAGGAGTGCGGAAAATTAGAATCCCAAGGTGTGGTGACGCCAT
Dai22633
GCTGAGCGAGGAGTGCGGAAAACCAGAATCCTAAGGCGTGGTCAAGCCAC
Dai23260
GCTGAGCGAGGAGTGCGGAAAACCAGAATCCTAAGGCGTGGTTAAGCCAC
Dai23626
GCTGAGCGAGGAGTGCGGAAAACCAGAATCCTAAGGCGTGGTTAAGCCAC
Dai16238G_citrinoalbus ---
1175Dai15293 ---
Dai19547 -----
GCGAGGAGTGCGGAAAATCAGAATCCTGAGGTGTGGTCAAGCCAT
918063G_africanus ---
918572G_africanus ---
Dai18536A ---
1164Cui17922 ---
Dai22225
GCTGAGCGAGGAGTGCGGAAAATCAGAATCCTAAGGTGTGGTCAAGCCAC
1163Dai20655
GCTGAGCGAGGAGTGCGGAAAATCAGAATCCTAAGGTGTGGTCAAGCCAC
Yuan4397G_hainanensis ---
1176Dai15268 GCTGAGCGAGGAGTGCGGAAAA-
AAGAAATCCTGAGCGTGGTCAAGCCTC
1177Dai15259 ---
BZ2896G_thelephoroides ---
1166JV1808_26 ---
Miettinen16992Hapalopilus_ochr
GCTGAGCGAGGAGTGCGGAAAAAGTGAACCTAGTGGCGTGGTCAATCCGC
GC1708_338_Ceriporia_arbuscula
GCCGAACGAGGAGTGCGGAAACTTTGGAAATCTGGATGTGGTCAAGCCTC
WCG1555Dai26107Ceriporia
GCCGAACGAGGAGTGCGGAAACTTTGGAAATCTGGATGTGGTCAAGCCTC

GC1708_340_Ceriporia_arbuscula
GCCGAACGAGGAGTGCGGAAACTTTGGAAATCTGGATGTGGTCAAGCCTC
WCG1556Dai26109Ceriporia
GCCGAACGAGGAGTGCGGAAACTTTGGAAATCTGGATGTGGTCAAGCCTC
883Cui11291
GCCGAACGAGGAGTGCGGAAACTTTGAAAATCTGGATGTGGTCAAGCCTC
HLX320Dai26805
GCCGAACGAGGAGTGCGGAAATTTCGGAAATCTGGATGTGGTCAAGCCTC
WCG1266Dai24678A
GCCGAACGAGGAGTGCGGAAATCTTGGAAATCTGGATGTGGTTAAGCCTC
Dai6090_Ceriporia_sulphuricolo --
RLG_11354_Ceriproia_reticulata
GCTGAACGAGGAGTGCGGAAATTGCGGAAATCTCGATGTGGTTACGCCAC
ZZW1543Dai27072
GCTGAACGAGGAGTGCGGAAATTGCGGAAATCTCGATGTGGTTACGCCAC
Li1316_Ceriporia_reticulata --
KHL11981Ceriporia_reticulata --
FP110343sp_Candelabrochaete_la
GCTGAACGAGGAGTGCGGAAATCGTGGAAATCTTGATGTGGTGACGCCAC
Li1045_Ceriporia_reticulata --
ZX136Dai25794ceriporia --
892Dai13400
GCTGAACGAGGAGTGCGGAAATCGTGGAAATCTTGATGTGGTGACGCCAC
RLG7163Leptoporus_mollis GCTGAACGAGGAGTGCGGAAGT-TCGAAAATCTGAGTGTGGTTAAGCCTC
Dai21062Leptoporus_mollis --
Dai20182Leptoporus_submollis GCTGAACGAGGAGTGCGGAAGT-TCGAAAATCTGGGTGTGGTTAAGCCTC
Cui18379Leptoporus_submollis GCTGAACGAGGAGTGCGGAAGT-TCGAAAATCTGGGTGTGGTTAAGCCTC
Wu1209_46Resiniporus_pseudogil GCTGAGCGAGGAGTGCGGAAAGCCG-AAAATC-TGACGAGGTGAAGCCTC
BRNM710169Resiniporus_resinasc --
Dai14516Bjerkandera_adusta GCTGAGCGAGGAGTGCGAAAAC-AAGGATAAGCCCGCGTGGTGACGCCAT
Dai21100Bjerkandera_fumosa GCTGAGCGAGGAGTGCGAAAAC-AA-GATAAGCCCGTGTGGTGACGCCAT
Miettinen16854Ceraceomyces_sp
GCTGAGCGAGGAGTGCGGAAAATAAGAATATCCAGGTGTGGTTAAGCCTC
Dai10477C_spissa --
855Dai16831 --
882Cui11282 --
Dai24566 --
Yuan5965 --

Dai3204 --
1194CUI9985 --

Dai15205_Ceriporia_albomellea --
Dai15223_Ceriporia_albomellea --
Li1780_Ceriporia_variegata --
Dai19791_Ceriporia_variegata --
Dai19886 --
Dai10833_Ceriporia_crassitunic --
CHWC1506_46Meruliopsis_crassit AC-
TAGACAAGTGACCTAGGATGCTCGCTGTGAAGCAGACTGAGTGATCC
Dai9995_Ceriporia_crassitunica --
Wu1209_58_Meruliopsis_parvispo
ATCTGAACAAGTGGCCTGAGACTCTCGCTGTCAAGCAGATTGAGTGCCTC
CHWC1505_129_Meruliopsis_parvi --
Dai21944
ACCTGAACAAGTGGCCCGAGACTTTCACTGTGAAGCAGTACGAATGGTCT
830Dai18640A
AACTGAACAAGTGGCCTGAGACCTTCACTGTGAAGCAGTACGAATGGTCC
GC1704_60_Meruliopsis_taxicola
ACCTGGACAAATGGCCCGAAGCCCTCGCTGTGAAGCAGACTGAGTGGTTT
Dai22625 ----------------------------------
CAGACTGAGTAATTT
Dai22636
ACCTGGACAAATGGCCCGAAGCCCTCGCTGTGAAGCAGACTGAGTGGTTT
Dai21878 -----------------------------
TGAAGCAGACTGAGTGGTTT
1169Dai17248 --
Wu1708_43_Meruliopsis_leptocys
ACTTGAACGAATGGCCCGAGACCCTCGCTGTGAAGCAGATTGAGTGGTTC
Li1011 --
ZX95Dai25742Meruliopsis_leptoc --
WCG1306Dai24733 --
LXL99Dai25816
GCTTGAACGAATGGCCCGAGACCCTCACTGTAAAGCAGATTGAGTGGTTC
WCG1559Dai26052Meruliopsis
ACTTGAACGAATGGCCCGAGACCCTCGCTGTGAAGCAGATTGAGTGGTTC
He7477 --
HLX243Dai26217
ACTTGAACGAATGGCCCGAGACCCTCGCTGTGAAGCAGATTGAGTGGTTC
RussiaMW673659Meruliopsis_fagi --
FD278
ACCTGAACAAATGGCCCAAGACCCTCGCTGTAAAGCAGAACGAGCGGTGC

Dai10226_Ceriporia_tarda --
LE247365 --
Dai8173_Meruliopsis_nanlingens --
860Dai17172 --
879Dai13414
GCCTGGACAAATGGCCCGAGACCCTCGCTGTAAAGCAGACGGAGTGGTTC
Li_1704_Meruliopsis_pseudocyst --
833Dai18405
ACTTGGACAAATGGCCCGAGACCCTCGCTGTGAAGCAGATTGAGTGGTTC
HHB_10729_Meruliopsis_albostra
ACTTGGACAATTGGCCCGAGACCCTCGCTGTGAAGCAGATTGAGTGGTTC
Cui6878_Ceriporia_pseudocystid --
869Dai14737 --
876Cui11626 --
1199WEI3388 --
776308_Meruliopsis_cystidiata --
ICN139059_Meruliopsis_cystidia --
HHB15692Ceraceomyces_serpens
GCTCGGATAATTGGCCCAAAACCCCTGCTGTGGCGCAGACTAGGTGGT--
HHB_15629_Sp_Ceriporiopsis_ane
GCCTAGATAAGTGGCCCAAGACCCTCTCTGTGACGCAGGACGAGTGGTGC
AJ185Trametopsis_cervina
CCTAAGATAAGTGGCCCAGGATGCTCCCTGTCGCGCAGGAAGA---GATC
FD9Irpex_lacteus
GCCGCGATAACTGGCCCCAGATGCTCCCTGTGACGCAGGTCGAGTGAATC
908Dai11230
GCCGCGATAACTGGCCCCAGATGCTCCCTGTGACGCAGGTCGAGTGATTC
FP55521TEmmia_lacerata
GCCGTGGTAACTGGCCCAAGATGYTCCCTGTAAAGCAGGTTGAGTACATC
PBU0048Ceriporia_cystidiata --
MZ340C_lacerataT --
Dai21940
GCCGAGGTCGCTGGCCCAAGATGCTCCCTGTAAAGCAGGTTGAGTATATC
847Dai16433 --
MarcinEmmia_latemarginatus --
Meijer3729Hydnopolyporus_fimbr --
RLG13408Phanerochaete_sp
GCCTGGATAAGTGGCCCAAGGCGCTCTCTGTAAMGCAGAACGAGTGGTTT
WHC1381Flavodon_flavus
GCCGCGATAACTGGCCCAAGATGCTCCCTGTGACGCAGGTCGAGTGAATC
GB1833Phlebia_albida
GCTTGGATCAATGGCCCAAGACGTTCCCTGTGACGCAGGATGAGCGATTC
T407Phlebia_nitidula
GCTTGGATCAATGGCCCAAGACGCTCCCTGTGACGCAGGATGAGCGATTC

HHB6988Phanerochaete_exilis
GCTCGGATAAATGGCCTAAGACGCTCTCTGTGACGCAGCACGAGTGGTTC
HHB8509Phanerochaetella_xeroph
GCTTGGATGAATGGCCTAAGACGCTCCCTGTGACGCAGCACGAGTGGTTC
PBU0051Macrohyporia_dictyopora --
HHB11463Phanerochaete_sp
GCCCGAATAAACGGCCTAAGACGCTCCCTGTGACGCAGCACGAGTGGTTC
FP102382Byssomerulius_corium
ACCTGGATAAGTGGCCCAAGACGCTCCCTGTGGCGCAGGACGAGTCGTTC
FP102165Efibula_americana
GCTTGGATAATTGGCCCAAGACTCTCCCTGTGACGCAGGGTTGGGAGTTC
Murdoch90Ceriporia_torpida --
Rivoire4413_Ceriporia_purpurea --
Kout_18_Ceriporia_triumphalis --
Rivoire3701_Ceriporia_bresadol --
VS4018 --
Ryvarden21832_Ceriporia_manzan --
Dai24539
ATCTGGATGAGTGCCCAAAGACCCTCTCTGTGACGCAGAACGAGT-GCTC
Dai24541
ATCTGGATGAGTGGCCAAAGACCCTCTCTGTGACGCAGAACGAGT-GCTC
JV1105_12_Ceriporia_occidental --
VS8558Ceriporia_occidentalis --
Dai22445 --
846Dai16368 --
Dai17951_Ceriporia_aurantiocar --
Miettinen_11701C_viridans --
JV0105_10Ceriporia_aurantiocar --
Yuan5702C_viridans --
858Dai17003 --
Yuan2747_Ceriporia_viridans --
Yuan2744C_viridans --
Li1046C_viridans --
865C_sinoviridans --
871Dai15062 --
Dai7642_Ceriporia_humilis --
Spirin4706_Ceriporia_humilis --
Spirin4944_Ceriporia_sericea --
WCG1547Dai26044ceriporia
GTCCGGATGTGTGGCCAAAGATCCTCACTGTAACGCAGAATGAGTGGTTC
ZZW1558Dai27086 --
Miettinen14381_Ceriporia_mpuri --
Miettinen15492_2_Ceriporia_sor --
He6687

ATCCGGATATGCGGCCAAAGACCCTCGCTGTAACGCAGAATGAGTGGTCC

ZH53Dai24426 ---

Vlasak0808_30_Ceriporia_punica ---

887Dai13376

ACCCGGATACGTGGCCCAAGACGCTCGCTGTAACGCAGGCGGAGCAGCTC

WCG1443Dai24998

ACCCGGATACATGGCCGAAGACGCTCGCTGTGACGCAGGCGGAGCAGCTC

0108_6Ceriporia_spissa ---

Dai19164 ---

Dai17937_Ceriporia_bubalinomar ---

903Dai12113 ---

LZB929Dai25079 ---

LX45Dai26988

ATCCGGATGAGTGGCCGAAGACCCTCTCTGTAACGCAGAGCAAGCGGTCC

LX43Dai26986

ATCCGGATGAGTGGCCGAAGACCCTCTCTGTAACGCAGAGCAAGCGGTCC

Dai7759Ceriporia ---

Cui8012_Ceriporia_viridans ---

GC1704_54Ceriporia_viridans ---

Dai23392

ATCGAGATGAGTGGCCAAAGACCCTCTCTGTGACGCAGAATGAGTGGTTC

WCG1585Dai26113Ceriproia

ATCGAGATGAGTGGCCAAAGACCCTCTCTGTGACGCAGAATGAGTGGTTC

Dai18675C_eucalypti ---

Dai22034

ATCCGGATGAGTGGCTCAAGACCCTCGCTGTTACGCAGAACGAGTGGTCC

JV1008_41JTardaFLORIDAKeys ---

Rivoire1161_Ceriporia_pierii ---

Dai23499C_pierii

ATTCAGGTATATGGTCAAAGACCCTCTCTGTGACGCAGAATGAGCGGTTC

Dai23500 ---

841Dai15899

GTCCGGATAATTGGCCAAAAACCCTCCCTGTATCGCAGCGTGAGCGGTTC

842Dai15904

GTCCGGATAATTGGCCAAAAACCCTCCCTGTATCGCAGCGTGAGCGGTTC

LZB1066xinjiang ---

LZB1065xinjiang ---

851Dai16779 ---

RMJ119sp_Candelabrochaete_sept

ATCCGGATAAGTGGCCAAAGACCCTCGCTGTCACGCAGAGAGAGTGGTTC

RLG9759spCandelabrochaete_sept ---

RLG10478Phanerochaete_allantos ---

Dai19118_Ceriporia_spissa ---

Dai18486A ---

WEI17_024_Ceriporia_mellita --
GC1508_71Ceriporia_mellita
ATCTGGATATGTGGTCAAAGACCCTCGCTGTGACGCAGTACGAGTGGCTC
GC1608_7_Ceriporia_mellita
ATCTGGATATGTGGTCAAAGACCCTCGCTGTGACGCAGTACGAGTGGCTC
ZZW1557Dai27085 --
ZZW1554Dai27083 --
Dai8168 --
BR4865C_mellita --
MEL2382688Ceriporia_sp --
Dai8110 --
Cui8097 --
909Cui6740 --
W1258Dai24695
ATCTGGATATGTGGTCAAAGACCCTCGCTGTGACGCAGAACGGGTGGCTC
JV0110_26_Ceriporia_griseoviol --
896Dai13202 --
LWY393Dai27053C_griseoviolasce
ATCCGGGTAACTGGCCAAAGACACTCTCTGTTACGCAGAGCGAGCGGTTC
LWY394DAI27054 --
FP135015G_pannocinctus --
L15726SpG_pannocinctus --
Dai22221
GCCGGGATACCTGGTCCACCTCCCTCACTGTGAAGCAGATCGAGAGGATG
Dai22633
GCCGGGATACCTGGCCCACCTCCCTCACTGTGACGCAGATCGAGTGGATG
Dai23260
GCCGGGATACCTGGCCCACCTCCCTCACTGTGACGCAGATCGAGTGGATG
Dai23626
GCCGGGATACCTGGCCCACCTCCCTCACTGTGACGCAGATCGAGTGGATG
Dai16238G_citrinoalbus --
1175Dai15293 --
Dai19547
GCCGGGATACCTGGTCCACCTCCCTCACTGTGACGCAGATCGAGTGGATG
918063G_africanus --
918572G_africanus --
Dai18536A --
1164Cui17922 --
Dai22225
GCCGGGATACCTGGTCCACCTCCCTCACTGTGACGCAGATCGAGTGGATG
1163Dai20655
GTCGGGATACCTGGTCCACCTCCCTCACTGTGACGCAGATCGAGTGGATG
Yuan4397G_hainanensis --
1176Dai15268 GC--

GGGATACTGGCCCACCTCCCTCACTGTGACGCAGAAAGAGTGGATG

1177Dai15259 ---

BZ2896G_thelephoroides ---

1166JV1808_26 ---

Miettinen16992Hapalopilus_ochr

GCCTGGGTGACAGGTCCAGGGCCCTCGCTGTGACGCAGTTCGAGCGGTAC

GC1708_338_Ceriporia_arbuscula

ATCCGGATGAATGGCCCAAGACCCTCTCTGTGGCGCAGAATGAGTGGTTC

WCG1555Dai26107Ceriporia

ATCCGGATGAATGGCCCAAGACCCTCTCTGTGGCGCAGAATGAGTGGTTC

GC1708_340_Ceriporia_arbuscula

ATCCGGATGAATGGCCCAAGACCCTCTCTGTGGCGCAGAATGAGTGGTTC

WCG1556Dai26109Ceriporia

ATCCGGATGAATGGCCCAAGACCCTCTCTGTGGCGCAGAATGAGTGGTTC

883Cui11291

ATCCGGATGAACGGCCTGAGACCCTCTCTGTGGCGCAGAACGAGTGGTTC

HLX320Dai26805

ATCTGGATAAATGGCCCAAGACCCTCTCTGTGACGCAGAATGAGCGGATC

WCG1266Dai24678A

ATCTGGATGAATGGCCCAAGACCCTCTCTGTGACGCAGAATGAGTGGTTC

Dai6090_Ceriporia_sulphuricolo ---

RLG_11354_Ceriproia_reticulata

GTTGAGATAAATGGCCAAAGACCCCCGCTGTGACGCAGAATGGGCGGTTC

ZZW1543Dai27072

GTTGAGATAAATGGCCAAAGACCCCCGCTGTGACGCAGAATGGGCGGTTC

Li1316_Ceriporia_reticulata ---

KHL11981Ceriporia_reticulata ---

FP110343sp_Candelabrochaete_la

GTTAAGAAGGATGGCCAAAGACCCTCACTGTGACGCAGTAAGAGTGGTTC

Li1045_Ceriporia_reticulata ---

ZX136Dai25794ceriporia ---

892Dai13400

GTTGAGAAGGATGGCTTAAAACCCTCGCTGTGACGCAGCACGAG-GGTTC

RLG7163Leptoporus_mollis

ACTCGGATGAGTGGCCAAAGACCGTCGCTGTGACGCAGAACGGGCGGATC

Dai21062Leptoporus_mollis ---

Dai20182Leptoporus_submollis

ACTCGGATGAGTGGCCAAAGATCGTCGCTGTGACGCAGAACGGGCGGATC

Cui18379Leptoporus_submollis

ACTCGGATGAGTGGCCAAAGATCGTCGCTGTGACGCAGAACGGGCGGATC

Wu1209_46Resiniporus_pseudogil

GTTCAGATAAGTGGCCCAAGACCCTCTCTGTAACGCAGGAAGAGTGGTCC

BRNM710169Resiniporus_resinasc ---

Dai14516Bjerkandera_adusta

TGCGGGCTATATAACCAACCGCTCTCGCTGTGAAGCAGATCGAGCG----

Dai21100Bjerkandera_fumosa

TACGGGCTTTATAACCAGCCGCTCTCGCTGTGAAGCAGTTCAAGAG----

Miettinen16854Ceraceomyces_sp

GCTTGGATAAGTGGCTTAGGACCCTCGCTGTGACGCAGACAGAGTGGTTC

Dai10477C_spissa --

855Dai16831 --

882Cui11282 --

Dai24566 --

Yuan5965 --

Dai3204 --

1194CUI9985 --

Dai15205_Ceriporia_albomellea --

Dai15223_Ceriporia_albomellea --

Li1780_Ceriporia_variegata --

Dai19791_Ceriporia_variegata --

Dai19886 --

Dai10833_Ceriporia_crassitunic --

CHWC1506_46Meruliopsis_crassit

TTGCACGGTCGGCACTTGTTACTCGATACAATTGCCCCCTTTTTCGTCG

Dai9995_Ceriporia_crassitunica --

Wu1209_58_Meruliopsis_parvispo

TTGCACGGTCGGCACTTGGTACTCGACACAATTGCCCCCTTTCTCTGTTG

CHWC1505_129_Meruliopsis_parvi --

Dai21944

CTGCATGGCCGGCACTTGTTACTCGATGCAACTGCCCCCTTTTTCTGTTG

830Dai18640A

TTACATGGCCGGCACTTGTTACTCGATACAATTGCCCCCTTTTTCTGTCG

GC1704_60_Meruliopsis_taxicola

TCGCGTGGCCGGCACTTGTTACTCGATACCGTTGCCCCCTTTTT--GCTA

Dai22625 TCGCGT-

GCCCGCACTTGTTACTCGATACCGTTGCCCCCTTTTT--GCTA

Dai22636 TCGCGT-

GCCGGCACTTGTTACTCGATACCGTTGCCCCCTTTTT--GCTA

Dai21878 TCACGT-

GCCGGCACTTGTTACTCGATACCGTTGCCCCCTTTTT--GCTA

1169Dai17248 --

Wu1708_43_Meruliopsis_leptocys

TTGCATGGCCGGCACTTGTTACTCGATACTATTGCCCCCTTTTTCTGTCG

Li1011 --

ZX95Dai25742Meruliopsis_leptoc --

WCG1306Dai24733 --

LXL99Dai25816
TTGCATGGCCGGCACTTGTTACTCGATACTATTGCCCCCTTTTTCTGTTG

WCG1559Dai26052Meruliopsis
TTGCATGGCCGGCACTTGTTACTCGATACTATTGCCCCCTTTTTCTGTCG

He7477 --

HLX243Dai26217
TTGCATGGCCGGCACTTGTTACTCGATACTATTGCCCCCTTTTTCTATCG

RussiaMW673659Meruliopsis_fagi --

FD278
TTGCATGGCCTGCACTTTCTACTCGATAGACCTGCCCCCTTTT---GTTG

Dai10226_Ceriporia_tarda --

LE247365 --

Dai8173_Meruliopsis_nanlingens --

860Dai17172 --

879Dai13414
TTGCATGGCCTGCACTTGTTACTCGATACTATTGCCCCCTTTTTCTGTTG

Li_1704_Meruliopsis_pseudocyst --

833Dai18405
TTGCATGGCCGGCACTTATTACTCGATGCTATTGCCCCCTTTTTCTGTCG

HHB_10729_Meruliopsis_albostra
TTGCATGGCCGGCACTTATTACTCGATGCTATTGCCCCCTTTTTCTGTCG

Cui6878_Ceriporia_pseudocystid --

869Dai14737 --

876Cui11626 --

1199WEI3388 --

776308_Meruliopsis_cystidiata --

ICN139059_Meruliopsis_cystidia --

HHB15692Ceraceomyces_serpens
TTTTTAGGCCTGCACTTGATACTCGATTTAGCTGCCCCCTGTTTATGTTG

HHB_15629_Sp_Ceriporiopsis_ane TTGT-TGGCCTGCACTTTATGCGC-
AAATGGTTGCCCCCTGCTTGTGTTA

AJ185Trametopsis_cervina TTGT-
TGGCCTGCACTTTATACGCGAATTGTTTGCCCCCTGTTTGTGTTA

FD9Irpex_lacteus TGGT-
GGGCCTGCACTTTGTACGCGACTTCATTGCCCCCTGTTTATTTGG

908Dai11230 TGGT-
GGGCCTGCACTTTGTACGCGACTTCATTGCCCCCTGTTTATTTGG

FP55521TEmmia_lacerata TGGT-
GGGCCTGCACTTTGTACGCGATTTGATTGCCCCC-GTTCATTTGA

PBU0048Ceriporia_cystidiata --

MZ340C_lacerataT --

Dai21940 TGGT-
GGGCCTGCACTTTGTACGCGATTTGATTGCCCCC-GGTCATTTGA

847Dai16433 --

MarcinEmmia_latemarginatus --
Meijer3729Hydnopolyporus_fimbr --
RLG13408Phanerochaete_sp TTGT-TGGCCTGCACTTTATACGCGATTCTATTGCCCCCTG-TTTTATTC
WHC1381Flavodon_flavus TGGT-GGGCCTGCACTTTGTACGCGACTTCATTGCCCCCTGTTTGTTTTG
GB1833Phlebia_albida TTGT-TGGCCTGC-------GCGCGATTTCATTGCCCCCTGCTTGTCTTC
T407Phlebia_nitidula TTGT-TGGCCTGC-------GCGCGATTTCATTGCCCCCTACCGGTCTTC
HHB6988Phanerochaete_exilis TTAT-TGGCCTGCAC-TTATGCGCGACTTCATCGCCCCCTATTTATGTTT
HHB8509Phanerochaetella_xeroph TTAT-TGGCCTGCAC-TTATACGCGACTTCAGTGCCCCCTA-TTATGTTT
PBU0051Macrohyporia_dictyopora --
HHB11463Phanerochaete_sp TTAT-TGGCCTGCACTTTATACGCGATTTCATTGCCCCCTATTTGTGTTC
FP102382Byssomerulius_corium TTGCGTGGCCTGCACTTTATACGCGATTTCATTGCCCCCTCATTGTCTTC
FP102165Efibula_americana TTGTTTGGCCTGCACTTTATACGCGATTTCATTG-CCCCCATTTGTCTTT
Murdoch90Ceriporia_torpida --
Rivoire4413_Ceriporia_purpurea --
Kout_18_Ceriporia_triumphalis --
Rivoire3701_Ceriporia_bresadol --
VS4018 --
Ryvarden21832_Ceriporia_manzan --
Dai24539 TTGTATGGCCTGCACTTGATACTCGATACGATCGCCCCCTGTTAT-GTTG
Dai24541 TTGTATGGCCTGCACTTGATACTCGATACGATCGCCCCCTGTTATTTGT-
JV1105_12_Ceriporia_occidental --
VS8558Ceriporia_occidentalis --
Dai22445 --
846Dai16368 --
Dai17951_Ceriporia_aurantiocar --
Miettinen_11701C_viridans --
JV0105_10Ceriporia_aurantiocar --
Yuan5702C_viridans --
858Dai17003 --
Yuan2747_Ceriporia_viridans --
Yuan2744C_viridans --
Li1046C_viridans --
865C_sinoviridans --

871Dai15062 --
Dai7642_Ceriporia_humilis --
Spirin4706_Ceriporia_humilis --
Spirin4944_Ceriporia_sericea --
WCG1547Dai26044ceriporia TTTTGTGGCCAGCACTTATTACTCGAAATGATTGCCCCCTGT---TCTTG
ZZW1558Dai27086 --
Miettinen14381_Ceriporia_mpuri --
Miettinen15492_2_Ceriporia_sor --
He6687 TTTTGTGGCCAGCACTTATTACTCGAAATGATTGCCCCCTGT---TCTTG
ZH53Dai24426 ---------------------------------------TGT---TCTTG
Vlasak0808_30_Ceriporia_punica --
887Dai13376 TT-CATGGCCAGCACTTCATGCTCGTTACGATCGCCCTTTGTTACTGTTA
WCG1443Dai24998 TT-CATGGCCAGCACTTCATGCTCGTTACGATCGCCCTTTATTACTGTTG
0108_6Ceriporia_spissa --
Dai19164 --
Dai17937_Ceriporia_bubalinomar --
903Dai12113 --
LZB929Dai25079 --
LX45Dai26988 TTCTACGGCCTGCACTTGGTACTCGATACGATCGCCCCCTATTGTTGTTG
LX43Dai26986 TTCTACGGCCTGCACTTGGTACTCGATACGATCGCCCCCTATTGTTGTTG
Dai7759Ceriporia --
Cui8012_Ceriporia_viridans --
GC1704_54Ceriporia_viridans --
Dai23392 TTGCTTGGCCTGCACTTGATACTCGATACGATCGCCCCCTGTGGTTGCTG
WCG1585Dai26113Ceriproia TTGCTTGGCCTGCACTTGATACTCGATACGATCGCCCCCTGTGGTTGCTG
Dai18675C_eucalypti --
Dai22034 TTGCACGGCCTGCACTTGATACTCGGGACGATCGCCCCCATTTGTTACTG
JV1008_41JTardaFLORIDAKeys --
Rivoire1161_Ceriporia_pierii --
Dai23499C_pierii TTTTTTGGCCAGCACTTGATACTCGATATGATTGCCCCCTGT---TCTTG
Dai23500 ----------AGCACTTGATACTCGATATGATTGCCCCCTGT---TCTTG
841Dai15899 TTATGTGGCCTGCACTTGATACTCGATACGATCGCCCCCTATTATACCCT

842Dai15904
TTATGTGGCCTGCACTTGATACTCGATACGATCGCCCCCTATTATACCCT
LZB1066xinjiang --
LZB1065xinjiang --
851Dai16779 --
RMJ119sp_Candelabrochaete_sept
TTATGTGGCCTGCACTTGATACTCGGCATGATCGCCCCCTGTTGTTTTT-
RLG9759spCandelabrochaete_sept --
RLG10478Phanerochaete_allantos --
Dai19118_Ceriporia_spissa --
Dai18486A --
WEI17_024_Ceriporia_mellita --
GC1508_71Ceriporia_mellita TTGCATGGCCAGCACTT-
ACACTCGATACGGTCGCCCCCTGTTGTCGATG
GC1608_7_Ceriporia_mellita TTGCATGGCCAGCACTT-
ACACTCGATACGGTCGCCCCCTGTTGTCGATG
ZZW1557Dai27085 --
ZZW1554Dai27083 --
Dai8168 --
BR4865C_mellita --
MEL2382688Ceriporia_sp --
Dai8110 --
Cui8097 --
909Cui6740 --
W1258Dai24695 TTGCATGGCCAGCACTT-
ATACTCGATACGATCGCCCCCTGTTGTCGATG
JV0110_26_Ceriporia_griseoviol --
896Dai13202 --
LWY393Dai27053C_griseoviolasce
TTTTGTGGCCTGCACTTGATTCTCGATACCACTGCCCCC------TGTTG
LWY394DAI27054 --
FP135015G_pannocinctus --
L15726SpG_pannocinctus --
Dai22221
GTGTCTGGCCTGCACTTACTACTCGATTTGATCGCCCCCTATTGT-TTTA
Dai22633
GTGTCTGGCCTGCACTTGATACTCGATTTGACTGCCCCCTATTG--TTTA
Dai23260
GTGTCTGGCCTGCACTTGATACTCGATTTGACTGCCCCCTATTG--TTTA
Dai23626
GTGTCTGGCCTGCACTTGATACTCGATTTGACTGCCCCCTATTG--TTTA
Dai16238G_citrinoalbus --
1175Dai15293 --
Dai19547 GTGTCTGGCCTGCAC-

TGATACTCGATTTGATTGCCCCCTATTGTT----
918063G_africanus --
918572G_africanus --
Dai18536A --
1164Cui17922 --
Dai22225
GTGTCTGGCCTGCACTTGATACTCGATTTGATTGCCCCCTATTG--TTTA
1163Dai20655
GTGTCTGGCCTGCACTTGATACTCGATTTGATTGCCCCCTATTG--TTTA
Yuan4397G_hainanensis --
1176Dai15268
GTGTTAGGCCTGCACTTAATACTCGATTGAACCGCCCCCTGTTGT-CTTG
1177Dai15259 --
BZ2896G_thelephoroides --
1166JV1808_26 --
Miettinen16992Hapalopilus_ochr
TTGTTTGGCCAGCACTTATTTCTCGGTTCTTCTGCCCCCTGTTTG-----
GC1708_338_Ceriporia_arbuscula
TTGTCCGGCCTGCACTTGATACTCGATACGCTTGCCCCCTGTTGATCTTG
WCG1555Dai26107Ceriporia
TTGTCCGGCCTGCACTTGATACTCGATACGCTTGCCCCCTGTTGATCTTG
GC1708_340_Ceriporia_arbuscula
TTGTCCGGCCTGCACTTGATACTCGATACGCTTGCCCCCTGTTGATCTTG
WCG1556Dai26109Ceriporia
TTGTCCGGCCTGCACTTGATACTCGATACGCTTGCCCCCTGTTGATCTTG
883Cui11291 TCTCAC-
GCCTGCACTTGATACTCGATACGCTTGCCCCCTGTTGATATTG
HLX320Dai26805
TTGCATGGCCTGCACTTGATACTCGATACGCTTGCCCCCTGTTGATGTTG
WCG1266Dai24678A
TTGCATGGCCTGCACTTGATACTCGATACGCTTGCCCCCTGTTGATGTTG
Dai6090_Ceriporia_sulphuricolo --
RLG_11354_Ceriproia_reticulata
TTGCATGGCCAGCACTTGATACTCGATACCGTCGCCCCCTGTTATTATT-
ZZW1543Dai27072
TTGCATGGCCAGCACTTGATACTCGATACCGTCGCCCCCTGTTATTATT-
Li1316_Ceriporia_reticulata --
KHL11981Ceriporia_reticulata --
FP110343sp_Candelabrochaete_la
TTGCGTGGCCAGCACTTAATACTCGATACGACCGCCCCCTGTTGATATT-
Li1045_Ceriporia_reticulata --
ZX136Dai25794ceriporia --
892Dai13400
TTGCATGGCCAGCACTTGATACTCGATAAGGTCGCCCCCTGTTGATATT-

RLG7163Leptoporus_mollis
TTGCATGACCAGCACTTGATGTTCGCTATAATTGCCCCCTGTTGTTGTT-

Dai21062Leptoporus_mollis --

Dai20182Leptoporus_submollis
TTGCATGGCCAGCACTTGATGTTCGCTATAATTGCCCCCTGTTGTTGTT-

Cui18379Leptoporus_submollis
TTGCATGGCCAGCACTTGATGTTCGCTATAATTGCCCCCTGTTGTTGTT-

Wu1209_46Resiniporus_pseudogil TTGT-TGGCCTGCACTTTATACGCGAGTTGACTG-CCCCTGTTCATATTA

BRNM710169Resiniporus_resinasc --

Dai14516Bjerkandera_adusta TTGGTTGGCTTGCACTTTTTTTGGCGTT-----GCCCCCTGTTGT-----

Dai21100Bjerkandera_fumosa TTGGTTGGCTTGCAC-TTTTTTGGCGTTGCCCCGCCCCCTATCGT-----

Miettinen16854Ceraceomyces_sp
TTTCTAGGCCAGCACTTGATACTCGATTTAACTGCCCCCTGTTTGCGTTG

Dai10477C_spissa --

855Dai16831 --

882Cui11282 --

Dai24566 --

Yuan5965 --

Dai3204 --

1194CUI9985 --

Dai15205_Ceriporia_albomellea --

Dai15223_Ceriporia_albomellea --

Li1780_Ceriporia_variegata --

Dai19791_Ceriporia_variegata --

Dai19886 --

Dai10833_Ceriporia_crassitunic --

CHWC1506_46Meruliopsis_crassit
TTGGTGTTGTTGTTGGAGGGAACTTTGTTTGTCTAGTCGCATTTGCTAAC

Dai9995_Ceriporia_crassitunica --

Wu1209_58_Meruliopsis_parvispo
TCGGTGTTGTTGCTGGAGATGATCTTGTTCTTTTAGTCGCATTTGCTAAC

CHWC1505_129_Meruliopsis_parvi --

Dai21944
TTGGTGTTGTCGCTGGAGGGAATTTTGTTTGTTTAGTTCCACTTGCTAAC

830Dai18640A
TTGGTGTTGTCGTTGGAGGGAATCCTGTTCGTTTAGTTGCACTTGCTAAC

GC1704_60_Meruliopsis_taxicola
TTGGTGTTGCTGTTGGAGTGGTTTTTGTTTGTCTRGATGCATTTACTAAC

Dai22625

TTGGTGTTGCTGTTGGAGTGGTTTTGTTTGTCTAGATGCATTTACTAAC
Dai22636
TTGGTGTTGCTGTTGGAGTGGTTTTGTTTGTCTAGATGCATTTACTAAC
Dai21878
TTGGTGTTGCTGTTGGAGTGGTTTTGTTTGTCTAGATGCATTTACTAAC
1169Dai17248 ---
Wu1708_43_Meruliopsis_leptocys
TTGGTGTTGTTGTCGGAGGGGATTTGTTCGTCTAGTTGCATTTGCTAAC
Li1011 ---
ZX95Dai25742Meruliopsis_leptoc ---
WCG1306Dai24733 ---
LXL99Dai25816
TTGGTGTTGTTGTCGGAGGGGATTTGTTCGTCTAGTTGCATTTGCTAAC
WCG1559Dai26052Meruliopsis
TTGGTGTTGTTGTCGGAGGGGATTTGTTCGTCTAGTTGCATTTACTAAC
He7477 ---
HLX243Dai26217
TTGGTGTTGTTGTCGGAGGGGATTTGTTCGTCTAGTTGCATTTACTAAC
RussiaMW673659Meruliopsis_fagi ---
FD278
TTGGTGTCGTTGTTGGAGGAAAGTTTGTTCGTCTAGTCGCATTTGCTAAC
Dai10226_Ceriporia_tarda ---
LE247365 ---
Dai8173_Meruliopsis_nanlingens ---
860Dai17172 ---
879Dai13414
TTGGTGTTGTTGTCGGAGGAGATTCTGTTCGTCTAGTTGCAGTTGCTAAC
Li_1704_Meruliopsis_pseudocyst ---
833Dai18405
TTGGTGTTGCTGTCGGAGGGGATTTGTGCTTTTAGTCGCATTTACTAAC
HHB_10729_Meruliopsis_albostra
TTGGTGTCGTTGTCGGAGGGGATTTGTGCTTTTAGTCGCATTTACTAAC
Cui6878_Ceriporia_pseudocystid ---
869Dai14737 ---
876Cui11626 ---
1199WEI3388 ---
776308_Meruliopsis_cystidiata ---
ICN139059_Meruliopsis_cystidia ---
HHB15692Ceraceomyces_serpens GTGCTGTTGTTGTTGGG-
GGAGTTGTGTTCTTCTAGCCACATTTACTAAC
HHB_15629_Sp_Ceriporiopsis_ane C----GTTTTATTGGAA-GCAGTT-
AATCCCTTTAGTAACATTTGCTAAC
AJ185Trametopsis_cervina T----GTTGTTATTGGA-GGTTCT-
TGTTTTCTTAGCAGCATTTGCTAAC

FD9Irpex_lacteus T----GTTGTCACCGCAAGGAGTTATACTC--
TTAGTAACATTTGCTAAC
908Dai11230 T----GTTGTCACCGCAAGGAGTTATACTC--
TTAGTAACATTTGCTAAC
FP55521TEmmia_lacerata C----GTCAT-TGTCCATGGAGTTATGCCC--
TTAGTACCATTTGCTAAC
PBU0048Ceriporia_cystidiata --
MZ340C_laceratаT --
Dai21940 C---GTTCATCCGTCCATGGAGTTATGCCC--
TTAGTACCATTTGCTAAC
847Dai16433 -------------------AGTTATGCCC--
TTAGTACCATTTGCTAAC
MarcinEmmia_latemarginatus --
Meijer3729Hydnopolyporus_fimbr --
RLG13408Phanerochaete_sp G----
ATCGTTACCGGACAGGATCCATGACTGTTAGCAACATTTGCTAAC
WHC1381Flavodon_flavus T----GTCATAACCGCAAGCTCCATGTTC---
TCAGTAACATTTGCTAAC
GB1833Phlebia_albida G----GTCATCGCCAGACAGTATTTGTCT---
TGATAAGCGTTTGCTAAC
T407Phlebia_nitidula G----GTCATCGCTAGACGGCATTTGTCT---
TGAAAAGCGTTTGCTAAC
HHB6988Phanerochaete_exilis ------TAACCACTGGATGGGACTTGCGATT-
TCATTAACATTTGCTAAC
HHB8509Phanerochaetella_xeroph ------TTACCACTGGATGG-------GATT-
TCATTAACAGTTGCTAAC
PBU0051Macrohyporia_dictyopora --
HHB11463Phanerochaete_sp A----GTCGTCACTGGATGGGATTCATGATT-
TCAGCAACTTTTGCTAAC
FP102382Byssomerulius_corium G----
GACGTCGGCGGATAGATTATGGCCCTATGGCAACACCTTGCTAAC
FP102165Efibula_americana A----------
GTCGCATGGGATTTATTGTTTGATCTAACATTTGCTAAC
Murdoch90Ceriporia_torpida --
Rivoire4413_Ceriporia_purpurea --
Kout_18_Ceriporia_triumphalis --
Rivoire3701_Ceriporia_bresadol --
VS4018 --
Ryvarden21832_Ceriporia_manzan --
Dai24539
TCGGTGTTGTTGTTGGAGGGGGTCATGTCTCTCTAGTCATATTTGCTAAC
Dai24541 -
CGGTGTTGTTGTTGGAGGGGGTCATGTCTCTCTAGTCATATTTGCTAAC
JV1105_12_Ceriporia_occidental --

VS8558Ceriporia_occidentalis --
Dai22445 --
846Dai16368 --------------------------------
TAGTCATATTTGCTAAC
Dai17951_Ceriporia_aurantiocar --
Miettinen_11701C_viridans --
JV0105_10Ceriporia_aurantiocar --
Yuan5702C_viridans --
858Dai17003 --
Yuan2747_Ceriporia_viridans --
Yuan2744C_viridans --
Li1046C_viridans --
865C_sinoviridans --
871Dai15062 --
Dai7642_Ceriporia_humilis --
Spirin4706_Ceriporia_humilis --
Spirin4944_Ceriporia_sericea --
WCG1547Dai26044ceriporia
ACCGTATCGTTATTGGAGGGGGTTGTGTTGCTTTAGTCATATTCGCTAAC
ZZW1558Dai27086 --
Miettinen14381_Ceriporia_mpuri --
Miettinen15492_2_Ceriporia_sor --
He6687 ACCGTATCGTTA-
TGGAGGGGGTTGTGTTGCTTTAGTCATATTCGCTAAC
ZH53Dai24426 ACCGTATCGTTA-
TGGAGGGGGTTGTGTTGCTTTAGTCATATTCGCTAAC
Vlasak0808_30_Ceriporia_punica --
887Dai13376 T------CGTTGTTCGAGGTGATTTCGG----
CAAGCCATATTTGCTAAC
WCG1443Dai24998 T------CGTTGTTCGAGGTGATTTTGAT--
TCAAGCCATATTTGCTAAC
0108_6Ceriporia_spissa --
Dai19164 --
Dai17937_Ceriporia_bubalinomar --
903Dai12113 --
LZB929Dai25079 --
LX45Dai26988 TCGCTG-CGTTATTGGCGGGGGTTGT--
TTCTTTAGTCATATTTGCTAAC
LX43Dai26986 TCGCTG-CGTTATTGGCGGGGGTTGT--
TTCTTTAGTCATATTTGCTAAC
Dai7759Ceriporia --
Cui8012_Ceriporia_viridans --
GC1704_54Ceriporia_viridans --
Dai23392 CTGGTGTCCTTATTCGAGGGGGTCGTG-

TCTTTTAGTCATATTTGCTAAC
WCG1585Dai26113Ceriproia CTGGTGTCCTTATTCGAGGGGGTCGTG-TCTTTTAGTCATATTTGCTAAC
Dai18675C_eucalypti --
Dai22034 GTGAAGTTGTTGTTGGAGGGGCTCGTGTTACATTATCCAATTTTGCTAAC
JV1008_41JTardaFLORIDAKeys --
Rivoire1161_Ceriporia_pierii --
Dai23499C_pierii ACCGTATTGCTATTGGAGGGGGTTGTGTTGCTTTAGTCATATTTACTAAC
Dai23500 ACCGTATTGCTATTGGAGGGGGTTGTGTTGCTTTAGTCATATTTACTAAC
841Dai15899 TTGCTGCTGTTGTTGGAGGGGGTTGTGTGTCTCTAGCCCTATTCACTAAC
842Dai15904 TTGCTGCTGTTGTTGGAGGGGGTTGTGTGTCTCTAGCCCTATTCACTAAC
LZB1066xinjiang --
LZB1065xinjiang --
851Dai16779 --
RMJ119sp_Candelabrochaete_sept --AGTGGTCTTGTTGGAAGGGGATTTATTGGTTGAAGCCATATTACTAAC
RLG9759spCandelabrochaete_sept --
RLG10478Phanerochaete_allantos --
Dai19118_Ceriporia_spissa --
Dai18486A --
WEI17_024_Ceriporia_mellita --
GC1508_71Ceriporia_mellita GTGTTGTTGCCGTTGGACGGGGTTGTGACTGTTTATGCAAATTTGCTAAC
GC1608_7_Ceriporia_mellita GTGTTGTTGCCGTTGGACGGGGTTGTGACTGTTTATGCAAATTTGCTAAC
ZZW1557Dai27085 --
ZZW1554Dai27083 --
Dai8168 --
BR4865C_mellita --
MEL2382688Ceriporia_sp --
Dai8110 --
Cui8097 --
909Cui6740 --
W1258Dai24695 GTGTTGTTGCCGTTGGACGGGGTTGTGACTGTTTATGCAAATTTGCTAAC
JV0110_26_Ceriporia_griseoviol --
896Dai13202 --
LWY393Dai27053C_griseoviolasce TCGTTGTTGTTGCCGGAGGGGGTTGT--CTGTTTAGTCACATCTGCTAAC

LWY394DAI27054 ---

FP135015G_pannocinctus ---

L15726SpG_pannocinctus ---

Dai22221 TTGGTGTTGTTGTC-GGGGGTGCTGGC----TATAGTCACAGTCGCTGAC

Dai22633 TTGGTGTTGTTGTTGGGGGGAGTTGCCCCCGTAGAGTCACATTCACTAAC

Dai23260 TTGGTGTTGTTGTTGGGGGGAGTTGCCCCCGTAGAGTCACATTCACTAAC

Dai23626 TTGGTGTTGTTGTTGGGGGGAGTCGCCCCCGTAGAGTCACATTCACTAAC

Dai16238G_citrinoalbus ---

1175Dai15293 ---

Dai19547 -TATTGGTGTTGTTGGGGGGAATTGTCCCCGTAGAGTCACATTCACTAAC

918063G_africanus ---

918572G_africanus ---

Dai18536A ---

1164Cui17922 ---

Dai22225 TTGGTGTTGTTGTTGGGGGGAGTTGCCCCCGTAGAGTCACATTCACTAAC

1163Dai20655 TTGGTGTTGTTGTTGGGGGGAGTTGCCCCCGTAGAGTCACATTCACTAAC

Yuan4397G_hainanensis ---

1176Dai15268 CCATTTCTGTGGCTGGAGAAGCTTG---CCTTATAGAGTCATTTACTAAC

1177Dai15259 ---

BZ2896G_thelephoroides ---

1166JV1808_26 ---

Miettinen16992Hapalopilus_ochr -TGTTATTGTTGTTTGAGCAGATCG----TTCCTAGCAACACGTACTAAC

GC1708_338_Ceriporia_arbuscula TCGCTGTCGCCGTTGGAAGGGGGTCTGTTGCATTAGCCATATTTACTAAC

WCG1555Dai26107Ceriporia TCGCTGTCGCCGTTGGAAGGGGGTCTGTTGCATTAGCCATATTTACTAAC

GC1708_340_Ceriporia_arbuscula TCGCTGTCGCCGTTGGAAGGGGGTCTGTTGCATTAGCCATATTTACTAAC

WCG1556Dai26109Ceriporia TCGCTGTCGCCGTTGGAAGGGGGTCTGTTGCATTAGCCATATTTACTAAC

883Cui11291 TCGTTGTCGCCGTTGGAAGGGGGTCTGTTGCATTAGCCATATTTACTAAC

HLX320Dai26805 TCGTTGTCGCCGTTGGAAGGGGGTTTGTTGCATTAGCCATATTTACTAAC

WCG1266Dai24678A

TCGTTGTCGCCGTTGGAAGGGGGTTTGTTGCATTAGCCATATTTACTAAC
Dai6090_Ceriporia_sulphuricolo --
RLG_11354_Ceriproia_reticulata ----TGGTGTCGTTGGAGGGGGTTGTGTTCTTCTAGTCATATTTGCTAAC
ZZW1543Dai27072 ----TGGTGTCGTTGGAGGGGGTTGTGTTCTTCTAGTCATATTTGCTAAC
Li1316_Ceriporia_reticulata --
KHL11981Ceriporia_reticulata --
FP110343sp_Candelabrochaete_la ----TGGTGTCGTTGGAGGGGGTTATGTTCTTTAGTCATATTCGCTAAC
Li1045_Ceriporia_reticulata --
ZX136Dai25794ceriporia --
892Dai13400 ----TGGTGTCATTGGAGGGGGTTGTGTTTTTCTAGT--AACTTGCTAAC
RLG7163Leptoporus_mollis -TGGTGTTGTTGCTGGA-GGGATTATGTCTCGATAGTCATATTTACTAAC
Dai21062Leptoporus_mollis --
Dai20182Leptoporus_submollis -TGGTGTTGTTGCTGGA-GGTATTATGTCTCGATAGTCATATTTACTAAC
Cui18379Leptoporus_submollis -TGGTGTTGTTGCTGGA-GGTATTATGTCTCGATAGTCATATTTACTAAC
Wu1209_46Resiniporus_pseudogil T----GTCGTTATTGGA-GGCATCGCTTTCCCCCCGCAGCACTTGCTAAC
BRNM710169Resiniporus_resinasc --
Dai14516Bjerkandera_adusta -CGGTGTTATTGTTTGATGGAAGTGCA---CCGTAGCCGCGTTTGCTAAC
Dai21100Bjerkandera_fumosa -CGGTGTTACTATTTGATGGAAGTGCGTCCCCGTAGTCGCATTTGCTAAC
Miettinen16854Ceraceomyces_sp G------TGTTGTTGGA-GGAGTTATGTTCCATTACTAACGTTTGCTAAC
Dai10477C_spissa --
855Dai16831 --
882Cui11282 --
Dai24566 --
Yuan5965 --
Dai3204 --
1194CUI9985 --

Dai15205_Ceriporia_albomellea --
Dai15223_Ceriporia_albomellea --
Li1780_Ceriporia_variegata --
Dai19791_Ceriporia_variegata --
Dai19886 --

Dai10833_Ceriporia_crassitunic ---
CHWC1506_46Meruliopsis_crassit GTCTCGTTTGCCATGTTTACATCACTCATGAACGTGCCAATTTTGTGTGA
Dai9995_Ceriporia_crassitunica ---
Wu1209_58_Meruliopsis_parvispo GTCTCGTTTGCCATGTTCACATCACT-ATGAACGTGCCTCCCTTGTGTGA
CHWC1505_129_Meruliopsis_parvi ---
Dai21944 GTCTCGTTTGCCATGTTCACATCACTCATGAACGTGCCATCCTTGTGTGA
830Dai18640A GTTTCGTTTGCCATGTTCGCATCACTCATGAACGTGCCATCCTTGTGTGA
GC1704_60_Meruliopsis_taxicola RKCTCGTCTGCCATGTATTCTTCACCCATGAACGTGCCATCGTCGTGTGA
Dai22625 ATCTCGTCTGCCATGTATTCTTCACCCATGAACGTGCCATCGTCGTGTGA
Dai22636 ATCTCGTCTGCCATGTATTCTTCACCCATGAACGTGCCATCGTCGTGTGA
Dai21878 ATCTCGTCTGCCATGTTCTCTTCACCCATGAATGTGCCATCATCGTGTGA
1169Dai17248 ---
Wu1708_43_Meruliopsis_leptocys GTTTCGTTTGCCATGTTCACATCACTCATGAACCTGCCATCTTTGTGTGA
Li1011 ---
ZX95Dai25742Meruliopsis_leptoc ---
WCG1306Dai24733 ---
LXL99Dai25816 GTTTCGTTTGCCATGTTCACATCACTCATGAACGTGCCATCATTGTGTGA
WCG1559Dai26052Meruliopsis GTCTCGTTTGCCATATTCACATCACTCATGAACGTGCCATCCTTGTGTGA
He7477 ---
HLX243Dai26217 GTCTCGTTTGCCATATTCACATCACTCATGAACGTGCCATCCTTGTGTGA
RussiaMW673659Meruliopsis_fagi ---
FD278 GTTTCATTTGCCATATTCACATCACTCATGAACGTGCCGTGCT-GTGTGA
Dai10226_Ceriporia_tarda ---
LE247365 ---
Dai8173_Meruliopsis_nanlingens ---
860Dai17172 ---
879Dai13414 GTCTCGTTTGCCATATTCACATCACTCATGAACGTACCATCCTTGTGTGA
Li_1704_Meruliopsis_pseudocyst ---
833Dai18405 GCCTCGTTTGCCATATT--CTTCACTCATGAACGTGCCATCCTCGTGTGA

HHB_10729_Meruliopsis_albostra GCCTCGTTTACCATATT--
CTTCACTCATGAACGTGCCATCCTCGTGTGA
Cui6878_Ceriporia_pseudocystid --
869Dai14737 --
876Cui11626 --
1199WEI3388 --
776308_Meruliopsis_cystidiata --
ICN139059_Meruliopsis_cystidia --
HHB15692Ceraceomyces_serpens GTTCTTATTCCC-
TATATCCTCTTATCCTGAACTTGCTTCGTCTGTGTGA
HHB_15629_Sp_Ceriporiopsis_ane
GTCTTGATCTCCATATCCTTGTCCCACCCGGGCATACTCAACTTGTGTGA
AJ185Trametopsis_cervina
GTCTTGATCTACATATTCTCATCCCGCCCGGACCTTCTCAACTTGTGTGA
FD9Irpex_lacteus GTCTTGATCTGTATGTT---CTCAC---------
TACTACGCTTGTGTGA
908Dai11230 GTCTTGATCTGTATGTT---CTCAC---------
TACTACGCTTGTGTGA
FP55521TEmmia_lacerata GTCTTGATCTCTATATTC-CCTCAC---------
TGCTTCCCTTGTGTGA
PBU0048Ceriporia_cystidiata --
MZ340C_lacerataT --
Dai21940 GTCTTGATCTCTATATTC-CCTCAC---------
TGCTTCGCTTGTGTGA
847Dai16433 GTCTTGATCTCTATATTC-CCTCAC---------
TGCTTCGCTTGTGTGA
MarcinEmmia_latemarginatus --
Meijer3729Hydnopolyporus_fimbr --
RLG13408Phanerochaete_sp
GCCTTGATCTTTATGCTCTCCTCATGGCTCAATGTACTACCCTTGTGTGA
WHC1381Flavodon_flavus GTCTTGATCTGTATGCTC---TCAC---------
TACTGCCCTTGTGTGA
GB1833Phlebia_albida
GTCTTGATCTCTATATCCTCATCACGCCTGAACCTGCTTCACTTGTGTGA
T407Phlebia_nitidula
GTCTTGATCTCTATATCCTCATCACGCCTGAACGTGCTTCACTCGTGTGA
HHB6988Phanerochaete_exilis GTCATGATCTATATCTCC---
TCACGCCTCCACGTGCTTTGCCTGTGTGA
HHB8509Phanerochaetella_xeroph ATCATGATCTATATCTTC---
TCACGCCTCCTTGTGCTTTGCCTGTGTGA
PBU0051Macrohyporia_dictyopora --
HHB11463Phanerochaete_sp GTCCTGATCTATATCTCC---
TCACCCCTCCACGTGCTTTGCCTGTGTGA
FP102382Byssomerulius_corium

GTCTTCATCTCTATGTTGCCACCACGTCTGAACATGCTCCACCTGCGTGA
FP102165Efibula_americana GTCTTGATCTCCATATCT---TCACGCATATA-----TATACTTGTGTGC
Murdoch90Ceriporia_torpida --
Rivoire4413_Ceriporia_purpurea --
Kout_18_Ceriporia_triumphalis --
Rivoire3701_Ceriporia_bresadol --
VS4018 --
Ryvarden21832_Ceriporia_manzan --
Dai24539 ATCCTTACCTCCATATCCTCTC-ACCCCTGAACGTGCTTCGAATGTGTGA
Dai24541 ATCCTTACCTCCATATCCTCTC-ACCCCTGAACGTGCTTCGAATGTGTGA
JV1105_12_Ceriporia_occidental --
VS8558Ceriporia_occidentalis --
Dai22445 --
846Dai16368 GTCCTTACCTCCATATCCTCCC-ACCCCTGAATGCG-TTCGAATGTGTGA
Dai17951_Ceriporia_aurantiocar --
Miettinen_11701C_viridans --
JV0105_10Ceriporia_aurantiocar --
Yuan5702C_viridans --
858Dai17003 --
Yuan2747_Ceriporia_viridans --
Yuan2744C_viridans --
Li1046C_viridans --
865C_sinoviridans --
871Dai15062 --
Dai7642_Ceriporia_humilis --
Spirin4706_Ceriporia_humilis --
Spirin4944_Ceriporia_sericea --
WCG1547Dai26044ceriporia GGCCTTACCTCCATATTCTCCACATCCCTAAATGTGCTTTGCTTGTGTGA
ZZW1558Dai27086 --
Miettinen14381_Ceriporia_mpuri --
Miettinen15492_2_Ceriporia_sor --
He6687 GGTCTTACCTCCATATTCTCCACATCCCTGAACGTGCTTTGCTTGTGTGA
ZH53Dai24426 GGTCTTACCTCCATATTCTCCACATCCCTGAACGTGCTTTGCTTGTGTGA
Vlasak0808_30_Ceriporia_punica --
887Dai13376 GGCCTTACCTTCATACTCTCTCC----CTGACCGTTTCCGG--TGTGTGA
WCG1443Dai24998 GGCCTTACCCTCATACTCTCTCC----

CTGACCGTTTCCGG--TGTGTGA
0108_6Ceriporia_spissa --
Dai19164 --
Dai17937_Ceriporia_bubalinomar --
903Dai12113 --
LZB929Dai25079 --
LX45Dai26988
GCCCTTACCCCCATATCTCTCCCACTCCTGATCG--CTTCGTCCGTGTGA
LX43Dai26986
GCCCTTACCCCCATATCTCTCCCACTCCTGATCG--CTTCGTCCGTGTGA
Dai7759Ceriporia --
Cui8012_Ceriporia_viridans --
GC1704_54Ceriporia_viridans --
Dai23392 GCCCTCACTCCCATATCTTTCCCACT-
CTGATCGTACTGCGACCGTGTGA
WCG1585Dai26113Ceriproia GCCCTCACTCCCATATCTTTCCCACT-
CTGATCGTACTGCGACCGTGTGA
Dai18675C_eucalypti --
Dai22034 GTTCCTATCCCCATATTCCCTT-
ACCTCCGAACTTGCAATAACTGTGTGA
JV1008_41JTardaFLORIDAKeys --
Rivoire1161_Ceriporia_pierii --
Dai23499C_pierii
GGCCTTACCCCCATATTCCCCACATTCCTAATTGTGC-CTGTCTGTGTGA
Dai23500
GGCCTTACCCCCATATTCCCCACATTCCTAATTGTGC-CTGTCTGTGTGA
841Dai15899
GCCCTTACCCCCATATCTTTCCCACCCCTGATCTCGTGCTTCGCGTGTGA
842Dai15904
GCCCTTACCCCCATATCTTTCCCACCCCTGATCTCGTGCTTCGCGTGTGA
LZB1066xinjiang --
LZB1065xinjiang --
851Dai16779 --
RMJ119sp_Candelabrochaete_sept GTCCTTACCTCTATGTCCGTCCC-
TCTCTGAATGTGCTTCGCTTGCGTGA
RLG9759spCandelabrochaete_sept --
RLG10478Phanerochaete_allantos --
Dai19118_Ceriporia_spissa --
Dai18486A --
WEI17_024_Ceriporia_mellita --
GC1508_71Ceriporia_mellita
GCTCTTATCTCCTCATGTCTCCTGCCTGTGATCTTCCTTTGCTTGCGTGT
GC1608_7_Ceriporia_mellita
GCTCTTATCTCCTCATGTCTCCTGCCTGTGATCTTCCTTTGCTTGCGTGT

ZZW1557Dai27085 ---
ZZW1554Dai27083 ---
Dai8168 ---
BR4865C_mellita ---
MEL2382688Ceriporia_sp ---
Dai8110 ---
Cui8097 ---
909Cui6740 ---
W1258Dai24695
GCTCTCATCTCCTCATACTCCCTGCCTGTGATCTTCCTCTGTTTGCGTGT
JV0110_26_Ceriporia_griseoviol ---
896Dai13202 ---
LWY393Dai27053C_griseoviolasce GCCTTGATCTCCATATCCTCCTA----
TTAAACGTACTTCTCCTGTGTGA
LWY394DAI27054 ---
FP135015G_pannocinctus ---
L15726SpG_pannocinctus ---
Dai22221 GTTTGAACCCGCATGTCTCTTCAA--ATTGA--
TTTCATCCTTCGCGTGA
Dai22633 GTTTGAACCCGCATGTCTTCTCCATCCATGA--
TCTTATCGCTCGTGTGA
Dai23260 GTTTGAACCCGCATGTCTTCTCCATCCTTGA--
TCTTATCGCTCGTGTGA
Dai23626 GTTTGAACCCGCATGTCTTCTCCATCCTTGA--
TCTTATCGCTCGTGTGA
Dai16238G_citrinoalbus ---
1175Dai15293 ---
Dai19547
GTTTGAACCCGCATGTCCCCATCACTCATGATCTTGTCGCTCTCGTGTGA
918063G_africanus ---
918572G_africanus ---
Dai18536A ---
1164Cui17922 ---
Dai22225 GTTTGAACCCGCATGTCTTCCCCATCCATGA--
TCTTATCGCTCGTGTGA
1163Dai20655 GTTTGAACCCGCATGTCTTCCCCATCCATGA--
TCTTATCGCTCGTGTGA
Yuan4397G_hainanensis ---
1176Dai15268
GTTTGAACCCGCATATTCCATCAATCCTCGACGTCTCTGCCCTTGTGTGA
1177Dai15259 ---
BZ2896G_thelephoroides ---
1166JV1808_26 ---
Miettinen16992Hapalopilus_ochr GAGTTTATCTCCATATTTCCTCACAC-------------

TCTGTGTGA

GC1708_338_Ceriporia_arbuscula GTTCTTACCCCCATATTCCTCCCACCACTGAACATATAACGCCTGTGTGA

WCG1555Dai26107Ceriporia GTTCTTACCCCCATATTCCTCCC--------------------------

GC1708_340_Ceriporia_arbuscula GTTCTTACCCCCATATTCCTCCCACCACTGAACATATAACGCCTGTGTGA

WCG1556Dai26109Ceriporia GTTCTTACCCCCATATTCCTCCCACCACTGAACATATAACGCCTGTGTGA

883Cui11291 GTCCTCACCCCCATATTCCTCCCACCACTGAACATATGATGCCTGTGTGA

HLX320Dai26805 GTTCTTATCCCCATATTCCTCCCACCACTGAACATATGACGCTTGTGTGA

WCG1266Dai24678A GTTCTTACCCCCATATTCCTCCCACCACTGAACATATGACGCTTGTGTGA

Dai6090_Ceriporia_sulphuricolo --

RLG_11354_Ceriproia_reticulata GGTCTTACCCCCATGTTCTTCCCATCTCGGAACGTGCATCGTCTGTGTGA

ZZW1543Dai27072 GGCCTTACCCCCATGTTCTTCCCATCTCGGAACGTGCATCGTCTGTGTGA

Li1316_Ceriporia_reticulata --

KHL11981Ceriporia_reticulata --

FP110343sp_Candelabrochaete_la GACCCTACCCCCATGTTCTTCCC----CGTAACGTGCATCGGCTGTGTGA

Li1045_Ceriporia_reticulata --

ZX136Dai25794ceriporia --

892Dai13400 GGCCTTGCCCCCATATTCTTCCCACCTCGGAACGTGTATCGCCTGTGTGA

RLG7163Leptoporus_mollis GTCCTCACCTCCATATCCCTCT-GTCACTGAATGTGCTTCGCTTGTGTGA

Dai21062Leptoporus_mollis --

Dai20182Leptoporus_submollis GTCCTCACCTCCATATCCCTCT-GCCACTGAATGTGCTTCGCTTGTGTGA

Cui18379Leptoporus_submollis GTCCTCACCTCCATATCCCTCT-GCCACTGAATGTGCTTCGCTTGTGTGA

Wu1209_46Resiniporus_pseudogil GTCTTGATCTACATATCCTYATTCCACCCGGACGCTCCGAATTTGCGTGA

BRNM710169Resiniporus_resinasc --

Dai14516Bjerkandera_adusta GTTCGAATTTGCATGTCCTCCTCACCT-----------------GTGTGA

Dai21100Bjerkandera_fumosa GATTGAATTTCCATGTCCTCCTCACCT-----------------GTGTGA

Miettinen16854Ceraceomyces_sp GTCCTTATTCCCATGTTTCTTCATCAATGAACGTGCTTCGCCTGTGTGA

Dai10477C_spissa --
855Dai16831 --
882Cui11282 --
Dai24566 --
Yuan5965 --
Dai3204 --
1194CUI9985 --

Dai15205_Ceriporia_albomellea --
Dai15223_Ceriporia_albomellea --
Li1780_Ceriporia_variegata --
Dai19791_Ceriporia_variegata --
Dai19886 --
Dai10833_Ceriporia_crassitunic --
CHWC1506_46Meruliopsis_crassit
CCGTTTACCTTTGTAAGTCGGACCCTACATTCGCTGAAAAGATCAGGCAC
Dai9995_Ceriporia_crassitunica --
Wu1209_58_Meruliopsis_parvispo
CCATTTCCCGTTGTAAGTCGGACCCCACATTTGCTGACAAGATCAGGCAC
CHWC1505_129_Meruliopsis_parvi --
Dai21944
CTATTTGCTATTGTAAGTCGGACCCCACCTTCGCTGACAAGATCCGGCAC
830Dai18640A
CCATTTGCCTTTGTAAGTCGGACCCCACCTTCGCTGACAAGATCAGGCAC
GC1704_60_Meruliopsis_taxicola
ATATTTGCCTTCGTAAGTCGGACCCTGCCTTCGCTGACAAGATCAGGCAC
Dai22625
ATATTTGCCTTCGTAAGTCGGACCCTGCCTTCGCTGACAAGATCAGGCAC
Dai22636
ATATTTGCCTTCGTAAGTCGGACCCTGCCTTCGCTGACAAGATCAGGCAC
Dai21878
CTATTTGCCTTCGTAAGTCGGACCCCGCCTTCGCTGACAAGATCAGGCAC
1169Dai17248 --
Wu1708_43_Meruliopsis_leptocys
CCATTTACCTTTGTAAGTCGGATCCCGCCTTCGCTGACAAGATCAGGCAC
Li1011 --
ZX95Dai25742Meruliopsis_leptoc --
WCG1306Dai24733 --
LXL99Dai25816
CCATTTGTCCTTGTAAGTCGGATCCCGCCTTCGCTGACAAGATCAGGCAC
WCG1559Dai26052Meruliopsis
CCATCTGCTTTTGTAAGTCGGACCCTGCCTTCGCTGACAAGATCAGGCAC
He7477 --

HLX243Dai26217
CCATTTGCTTTTGTAAGTCGGACCCTGCCTTCGCTGACAAGATCAGGCAC
RussiaMW673659Meruliopsis_fagi ---
FD278 CAATTTGTATT--
TAAGTCGGATCCCACCTTCGCTGACAAGATCAGGCGC
Dai10226_Ceriporia_tarda ---
LE247365 ---
Dai8173_Meruliopsis_nanlingens ---
860Dai17172 ---
879Dai13414
CCATTTATCTTTGTAAGTCGGACCCCTCCTTTGCTGACAAGATCAGGCAC
Li_1704_Meruliopsis_pseudocyst ---
833Dai18405
CTGTCAACTTGCGTAAGTCGGACCCCTCCTTCGCTGACAAGATCAGGCAC
HHB_10729_Meruliopsis_albostra
CTGTCAACTTGCGTAAGTCGGATCCCTCCTTCGCTGACAAGATCAGGCAC
Cui6878_Ceriporia_pseudocystid ---
869Dai14737 ---
876Cui11626 ---
1199WEI3388 ---
776308_Meruliopsis_cystidiata ---
ICN139059_Meruliopsis_cystidia ---
HHB15692Ceraceomyces_serpens
CTATTGCCATATGTAAGTCGGACCCAGCGTTTGCTGACAAGATCAGGCAC
HHB_15629_Sp_Ceriporiopsis_ane
TCATTGTGCCATGTAAGTCGGACCCTGCTTTCGCCGAAAAAATCAGGCAT
AJ185Trametopsis_cervina
CCATTGTGCGATGTAAGTCGGACCCCGCCTTTGCTGAGAAGATCAGACAT
FD9Irpex_lacteus
CCGAAAATGCGTGTAAGTCGGACTCAGCGTTTGCTGACAAGATCAGACAC
908Dai11230
CCGAAAATGCGTGTAAGTCGGACTCAGCGTTTGCTGACAAGATCAGACAC
FP55521TEmmia_lacerata
TCGGACATGCGTGTAAGTCGGACGCCGCGTTTGCTGACAAGATCAGACAC
PBU0048Ceriporia_cystidiata ---
MZ340C_lacerataT ---
Dai21940
TCGGAAATGCGTGTAAGTCGGACGCAGCGTTTGCTGACAAGATCAGACAC
847Dai16433
TCGGAAATGCGTGTAAGTCGGACGCAGCGTTTGCTGACAAGATCAGACAC
MarcinEmmia_latemarginatus ---
Meijer3729Hydnopolyporus_fimbr ---
RLG13408Phanerochaete_sp
CCGAATCGCCGCGTAAGTCGGACCCTGCTTTCGCTGACAAGATTAGGCAT

WHC1381Flavodon_flavus
CCGAAAATACACGTAAGTCGGACACAGCGTTTGCTGACAAGATCAGACAC
GB1833Phlebia_albida CCGA--
TTGATTGTAAGTCGGACCCAGCGTTTGCAGACAAGATCAGGCAT
T407Phlebia_nitidula CCGA--
TTGATTGTAAGTCGGACCCAGCGTTTGCAGACAAAATCAGGCAT
HHB6988Phanerochaete_exilis
TAATATCCGTACGTAAGTCGGACCCGGCCTTCGCTGACAAGATCAGGCAT
HHB8509Phanerochaetella_xeroph TGAAATTCGTACGTAAGTCGGACCC----------------
----
PBU0051Macrohyporia_dictyopora --
HHB11463Phanerochaete_sp
CGAAATTCGTGCGTAAGTCGGACCCCGCCTTTGCTGACAAGATTAGGCAT
FP102382Byssomerulius_corium CTGA--
TTGCGTGTAAGTCGGACCCAGCCTTTGCTGACAAGATCAGGCAC
FP102165Efibula_americana CCAA--
CCGCTTGTAAGTCGGACCCTCCATTTGCTGACAAGATCAGGCAT
Murdoch90Ceriporia_torpida --
Rivoire4413_Ceriporia_purpurea --
Kout_18_Ceriporia_triumphalis --
Rivoire3701_Ceriporia_bresadol --
VS4018 --
Ryvarden21832_Ceriporia_manzan --
Dai24539
ATCTTCGACTGCGTAAGTCGGATCCCGCGTTTGCTGATAAAATCAGGCAC
Dai24541
ATCTTCGACTGCGTAAGTCGGATCCCGCGTTTGCTGATAAAATCAGGCAC
JV1105_12_Ceriporia_occidental --
VS8558Ceriporia_occidentalis --
Dai22445 --
846Dai16368
ATCTTTGACCGCGTAAGTCGGATCCCGCGTTCGCTGATAAAATTCGGCAC
Dai17951_Ceriporia_aurantiocar --
Miettinen_11701C_viridans --
JV0105_10Ceriporia_aurantiocar --
Yuan5702C_viridans --
858Dai17003 --
Yuan2747_Ceriporia_viridans --
Yuan2744C_viridans --
Li1046C_viridans --
865C_sinoviridans --
871Dai15062 --
Dai7642_Ceriporia_humilis --
Spirin4706_Ceriporia_humilis --

Spirin4944_Ceriporia_sericea --
WCG1547Dai26044ceriporia
ATGTTTGACATCGTAAGTCGGACCCCTCCTTCGCTGACAAGATCAGGCAC
ZZW1558Dai27086 --
Miettinen14381_Ceriporia_mpuri --
Miettinen15492_2_Ceriporia_sor --
He6687
ATGTTTGATACCGTAAGTCGGACCCCTCCTTCGCTGACAAGATCAGGCAC
ZH53Dai24426
ATGTTTGATACCGTAAGTCGGACCCCTCCTTCGCTGACAAGATCAGGCAC
Vlasak0808_30_Ceriporia_punica --
887Dai13376
ACTGTTCCCACCGTAAGTCGGACCCCGCTTTCGCTGACAAGATCAGGCAC
WCG1443Dai24998
ACTGTTCCCACCGTAAGTCGGACCCCGCTTTCGCTGACAAGATCAGGCAC
0108_6Ceriporia_spissa --
Dai19164 --
Dai17937_Ceriporia_bubalinomar --
903Dai12113 --
LZB929Dai25079 --
LX45Dai26988 AT-
TTGTTCTGCGTAAGTCGGACCCCTCCTTCGCTGACAAGATCCGGCAT
LX43Dai26986 AT-
TTGTTCTGCGTAAGTCGGACCCCTCCTTCGCTGACAAGATCCGGCAT
Dai7759Ceriporia --
Cui8012_Ceriporia_viridans --
GC1704_54Ceriporia_viridans --
Dai23392
ATCCTCTCCTGCGTAAGTCGGACCCCAGTTTCGCTGACAAGATCAGACAC
WCG1585Dai26113Ceriproia
ATCCTCTCCTGCGTAAGTCGGACCCCAGTTTCGCTGACAAGATCAGACAC
Dai18675C_eucalypti --
Dai22034 ATG--
TGGCTACGTAAGTCGGACCCCGCTTTCGCTGACAAGATTCGGCAT
JV1008_41JTardaFLORIDAKeys --
Rivoire1161_Ceriporia_pierii --
Dai23499C_pierii
ATATTTGATTCCGTAAGTCGGACCCCGCCTTCGCTGACAAGATCAGGCAC
Dai23500
ATATTTGATTCCGTAAGTCGGACCCCGCCTTCGCTGACAAGATCAGGCAC
841Dai15899 AT-
TTTTACCACGTAAGTCGGATCCTGCATTCGCTGACAAGATCAGGCAC
842Dai15904 AT-
TTTTACCACGTAAGTCGGATCCTGCATTCGCTGACAAGATCAGGCAC

LZB1066xinjiang --
LZB1065xinjiang --
851Dai16779 --
RMJ119sp_Candelabrochaete_sept
AATTCTCATGATGTAAGTCCGACCCCGCTTTCGCTGACAAGATCAGACAC
RLG9759spCandelabrochaete_sept --
RLG10478Phanerochaete_allantos --
Dai19118_Ceriporia_spissa --
Dai18486A --
WEI17_024_Ceriporia_mellita --
GC1508_71Ceriporia_mellita
CTCTGAAATCATGTAAGTCGGACCCCGCGTTCACTGATAAGATCAGGCAC
GC1608_7_Ceriporia_mellita
CTGTGACATGATGTAAGTCGGACCCCGCTTTCACTGACAAGATCAGGCAC
ZZW1557Dai27085 --
ZZW1554Dai27083 --
Dai8168 --
BR4865C_mellita --
MEL2382688Ceriporia_sp --
Dai8110 --
Cui8097 --
909Cui6740 --
W1258Dai24695
TTCTGTAACTATGTAAGTCGGACCCCGCTTTCGCTGACAAGATCAGGCAC
JV0110_26_Ceriporia_griseoviol --
896Dai13202 --
LWY393Dai27053C_griseoviolasce
ATCGTCTATTGCGTAAGTCGGACCCTGGTTTCGCTGAGAAGATCAGACAT
LWY394DAI27054 --
FP135015G_pannocinctus --
L15726SpG_pannocinctus --
Dai22221 ACGTTTGGTGT-
GTAAGTCCGACCCTGCCTTCTCTGACAAGATCAGACAC
Dai22633
ACGTTCCGTATCGTAAGTCGGACCCTGCCTTCGCTGACAAGATCAGGCAT
Dai23260
ACGTTCCGTATCGTAAGTCGGACCCTGCCTTCGCTGACAAGATCAGGCAT
Dai23626
ACGTTCCGTATCGTAAGTCGGACTCTGCCTTCGCTGACAAGATCAGGCAT
Dai16238G_citrinoalbus --
1175Dai15293 --
Dai19547
ACGTTTGCGTACGTAAGTCGGACCCTGCCTTTGCTGACAAGATCAGGCAT
918063G_africanus --

918572G_africanus --
Dai18536A --
1164Cui17922 --
Dai22225
ACGTTCCGTATCGTAAGTCGGACCCTGCCTTCGCTGACAAGATCAGGCAT
1163Dai20655
ACGTTCCGTATCGTAAGTCGGACCCTGCCTTTGCTGACAAGATCAGGCAT
Yuan4397G_hainanensis --
1176Dai15268
ACAATTGATGGCGTAAGTCGGACCCTGCCTTTGCTGACAAGATCCGGCAT
1177Dai15259 --
BZ2896G_thelephoroides --
1166JV1808_26 --
Miettinen16992Hapalopilus_ochr
ATATCTACGAACGTAAGTCGGACCCTGCCTTCGCTGACAAAATTAGGCAC
GC1708_338_Ceriporia_arbuscula
ATTCTTTGCTACGTAAGTCGGACCCCGCATTCGCTGAGAAGATCAGGCAC
WCG1555Dai26107Ceriporia --
GC1708_340_Ceriporia_arbuscula
ATTCTTTGCTACGTAAGTCGGACCCCGCATTCGCTGAGAAGATCAGGCAC
WCG1556Dai26109Ceriporia
ATTCTTTGCTACGTAAGTCGGACCCAGCATTCGCTGAGAAGATCAGGCAC
883Cui11291
TTTTTATGCTACGTAAGTCGGACCCCACATTCGCTGAGAAGATCAGGCAC
HLX320Dai26805
ATCTAATGCTACGTAAGTCGGACCCCGCATTCGCTGAGAAGATCAGGCAC
WCG1266Dai24678A
ATTCCATTCTACGTAAGTCGGACCCTGCATTCGCTGAGAAGATCAGGCAC
Dai6090_Ceriporia_sulphuricolo --
RLG_11354_Ceriproia_reticulata
ACCTTTCGCTATGTAAGTCGGACCCAGGGTTCGCTGACAAGATCAGACAC
ZZW1543Dai27072
ACCTTTCGCTATGTAAGTCGGACCCAGGGTTCGCTGACAAGATCAGACAC
Li1316_Ceriporia_reticulata --
KHL11981Ceriporia_reticulata --
FP110343sp_Candelabrochaete_la ATC-
CTCACTATGTAAGTCGGACCCAGCGTTCGCTGACAAGATCAGACAC
Li1045_Ceriporia_reticulata --
ZX136Dai25794ceriporia --
892Dai13400
ACCTTTCCCTATGTAAGTCGGACCCAGCGTTCGCTGACAAGATCAGACAC
RLG7163Leptoporus_mollis
CTATTTGGATTTGTAAGTCGGACCCTGCTTTTGCTGACAAGATCAGGCAC
Dai21062Leptoporus_mollis --

Dai20182Leptoporus_submollis
CTATTTGGATTTGTAAGTCGGACCCTGCTTTTGCTGACAAAATCAGGCAC
Cui18379Leptoporus_submollis
CTATTTGGATTTGTAAGTCGGACCCTGCTTTTGCTGACAAAATCAGGCAC
Wu1209_46Resiniporus_pseudogil
CCATTGTGAAATGTAAGTCGGATCCTGCCTTCGCTGAAAAGATTAGGCAT
BRNM710169Resiniporus_resinasc --
Dai14516Bjerkandera_adusta TTTGTTCTG--
CGTAAGTCGGACCCCACGTTCGCAGACAGGATTCGTCAT
Dai21100Bjerkandera_fumosa TTTGCTCTA--
CGTAAGTCGGACCCCACGTTCGCAGACAGAATTCGACAT
Miettinen16854Ceraceomyces_sp
CCTTTACGACATGTAAGTCCGACCCAGCCTTCGCTGAGAAGATTAGGCAC
Dai10477C_spissa --
855Dai16831 --
882Cui11282 --
Dai24566 --
Yuan5965 --
Dai3204 --
1194CUI9985 --

Dai15205_Ceriporia_albomellea --
Dai15223_Ceriporia_albomellea --
Li1780_Ceriporia_variegata --
Dai19791_Ceriporia_variegata --
Dai19886 --
Dai10833_Ceriporia_crassitunic --
CHWC1506_46Meruliopsis_crassit
GTTCGCGATCCGAAAGCGCGCATGCAAGTCGTATGGAGTTTTTGCAAAAG
Dai9995_Ceriporia_crassitunica --
Wu1209_58_Meruliopsis_parvispo
GTTCGCGATCCGAAAGCGCGCATGCAAGTCGTATGGAATTTTTGCAAGAG
CHWC1505_129_Meruliopsis_parvi --
Dai21944
GTTCGAGATCCAAAAGCGCGCATGCAAGTCGTGTGGAACTTTTGCAAAAG
830Dai18640A
GTTCGAGATCCAAAAGCGCGCATGCAAGTCGTGTGGAACTTTTGCAAAAG
GC1704_60_Meruliopsis_taxicola
GTTCGCGATCCTAAAGCACGCATGCAAGTCGTGTGGAGCTTTTGCAAGAG
Dai22625
GTTCGCGATCCTAAAGCACGCATGCAAGTCGTGTGGAGCTTTTGCAAGAG
Dai22636
GTTCGCGATCCTAAAGCACGCATGCAAGTCGTGTGGAGCTTTTGCAAGAG

Dai21878
GTTCGCGATCCTAAAGCACGCATGCAAGTCGTGTGGAGCTTTTGCAAGAG
1169Dai17248 ---
Wu1708_43_Meruliopsis_leptocys
GTTCGCGACCCGAAAGCGCGCATGCAAGTCGTATGGGGTTTTTGCAAGAG
Li1011 ---
ZX95Dai25742Meruliopsis_leptoc ---
WCG1306Dai24733 ---
LXL99Dai25816
GTTCGCGATCCGAAAGCTCGCATGCAAGTCGTATGGGGTTTTTGCAAGAG
WCG1559Dai26052Meruliopsis
GTTCGCGATCCGAAAGCGCGCATGCAAGTCGTATGGGGTTTTTGCAAGAG
He7477 ---
HLX243Dai26217
GTTCGCGATCCGAAAGCGCGCATGCAAGTCGTATGGGGTTTTTGCAAGAG
RussiaMW673659Meruliopsis_fagi ---
FD278
GTTCGCGATCCGAAAGCACGCATGCAAGTAGTATGGAATTTTTGCAAAGG
Dai10226_Ceriporia_tarda ---
LE247365 ---
Dai8173_Meruliopsis_nanlingens ---
860Dai17172 ---
879Dai13414
GTTCGCGATCCCAAAGCGCGTATGCAAGTCGTGTGGGGCTTTTGCAAGAG
Li_1704_Meruliopsis_pseudocyst ---
833Dai18405
GTTCGCGATCCGAAAGCGCGCATGCAAGTCGTGTGGAATTTTTGCAAGAG
HHB_10729_Meruliopsis_albostra
GTTCGCGATCCGAAAGCGCGCATGCAAGTCGTGTGGAATTTTTGCAAGAG
Cui6878_Ceriporia_pseudocystid ---
869Dai14737 ---
876Cui11626 ---
1199WEI3388 ---
776308_Meruliopsis_cystidiata ---
ICN139059_Meruliopsis_cystidia ---
HHB15692Ceraceomyces_serpens
GTTCGTGATCCCAAAACGCGCATGCAAGTGGTTTGGGGCTTTTGCAAGGG
HHB_15629_Sp_Ceriporiopsis_ane
GTCCGTGATCCGAAAGCGCGTATGCAAGTGGTATGGAACTTTTGCAAGGG
AJ185Trametopsis_cervina
GTCCGTGACCCGAAAGCACGTATGCAAGTTGTGTGGAACTTCTGCAAGGG
FD9Irpex_lacteus
GTCCGGGACCCGAAGGCACGTATGCAGGTGGTATGGAACTATTGCAAAGG
908Dai11230

GTCCGGGACCCGAAGGCACGTATGCAGGTGGTATGGAACTATTGCAAAGG
FP55521TEmmia_lacerata
GTCCGGGACCCCAAGGCACGCATGCAGGTAGTATGGAACTACTGCAAGGG
PBU0048Ceriporia_cystidiata ---
MZ340C_lacerataT ---
Dai21940
GTCCGGGACCCCAAGGCACGCATGCAGGTAGTATGGAACTACTGCAAAGG
847Dai16433
GTCCGGGACCCCAAGGCACGCATGCAGGTAGTATGGAACTACTGCAAAGG
MarcinEmmia_latemarginatus ---
Meijer3729Hydnopolyporus_fimbr ---
RLG13408Phanerochaete_sp
GTTCGGGATCCAAAAGCGCGGATGCAAGTAGTATGGAACTACTGCAAAGG
WHC1381Flavodon_flavus
GTTCGGGACCCGAAAGCACGTATGCAGGTGGTCTGGAACTATTGCAAAGG
GB1833Phlebia_albida
GTCCGTGATCCAAAGGCACGCATGCAAGTGGTATGGGGCTTCTGCAAGGG
T407Phlebia_nitidula
GTCCGTGATCCAAAGGCACGCATGCAGGTGGTATGGGGCTTCTGCAAGGG
HHB6988Phanerochaete_exilis
GTACGGGATCCCAAAGCACGCATGCAAGTGGTATGGAATTACTGCAAAGG
HHB8509Phanerochaetella_xeroph ---
PBU0051Macrohyporia_dictyopora ---
HHB11463Phanerochaete_sp
GTTCGGGATCCCAAAGCACGCATGCAGGTGGTATGGAATTACTGCAAAGG
FP102382Byssomerulius_corium
GTCCGTGATCCGAAAGCACGAATGCAAGTTGTGTGGAATTACTGCAAGGG
FP102165Efibula_americana
GTTCGGGATCCGAAAGCTCGTATGCAAGTTGTGTGGAGCTTCTGCAAGGG
Murdoch90Ceriporia_torpida ---
Rivoire4413_Ceriporia_purpurea ---
Kout_18_Ceriporia_triumphalis ---
Rivoire3701_Ceriporia_bresadol ---
VS4018 ---
Ryvarden21832_Ceriporia_manzan ---
Dai24539
GTCCGTGATCCCAAAGCGCGCATGCAAGTCGTCTGGGGCTTTTGCAAGAG
Dai24541
GTCCGTGATCCCAAAGCGCGCATGCAAGTCGTCTGGGGCTTTTGCAAGAG
JV1105_12_Ceriporia_occidental ---
VS8558Ceriporia_occidentalis ---
Dai22445 ---
846Dai16368
GTTCGTGATCCCAAAGCGCGTATGCAAGTCGTCTGGGGCTTTTGCAAGAG

Dai17951_Ceriporia_aurantiocar --
Miettinen_11701C_viridans --
JV0105_10Ceriporia_aurantiocar --
Yuan5702C_viridans --
858Dai17003 --
Yuan2747_Ceriporia_viridans --
Yuan2744C_viridans --
Li1046C_viridans --
865C_sinoviridans --
871Dai15062 --
Dai7642_Ceriporia_humilis --
Spirin4706_Ceriporia_humilis --
Spirin4944_Ceriporia_sericea --
WCG1547Dai26044ceriporia
GTTCGTGATCCGAAAGCGCGCATGCAAGTCGTCTGGGGCTTCTGCAAGGG
ZZW1558Dai27086 --
Miettinen14381_Ceriporia_mpuri --
Miettinen15492_2_Ceriporia_sor --
He6687
GTCCGTGATCCGAAAGCGCGCATGCAAGTCGTCTGGGGCTTCTGCAAGGG
ZH53Dai24426
GTCCGTGATCCGAAAGCGCGCATGCAAGTCGTCTGGGGCTTCTGCAAGGG
Vlasak0808_30_Ceriporia_punica --
887Dai13376
GTTCGTGACCGAAAAGCGCGCATGCAAGTCGTTTGGAATTTCTGCAAGAG
WCG1443Dai24998
GTTCGTGACCGAAAAGCGCGCATGCAAGTCGTCTGGAATTTCTGCAAGAG
0108_6Ceriporia_spissa --
Dai19164 --
Dai17937_Ceriporia_bubalinomar --
903Dai12113 --
LZB929Dai25079 --
LX45Dai26988
GTCCGAGATCCCAAAGCTCGCATGCAAGTCGTTTGGAACTTCTGCAAGAG
LX43Dai26986
GTCCGAGATCCCAAAGCTCGCATGCAAGTCGTTTGGAACTTCTGCAAGAG
Dai7759Ceriporia --
Cui8012_Ceriporia_viridans --
GC1704_54Ceriporia_viridans --
Dai23392
GTCCGTGATCCCAAAGCTCGCATGCAAGTCGTTTGGAACTTCTGCAAGAG
WCG1585Dai26113Ceriproia
GTCCGTGATCCCAAAGCTCGCATGCAAGTCGTTTGGAACTTCTGCAAGAG
Dai18675C_eucalypti --

Dai22034
GTTCGTGATCCGAAAGCGCGCATGCAAGTTGTCTGGAATTTCTGCAAGAG
JV1008_41JTardaFLORIDAKeys --
Rivoire1161_Ceriporia_pierii --
Dai23499C_pierii
GTTCGTGATCCAAAAGCGCGTATGCAAGTAGTCTGGAACTTCTGCAAAGG
Dai23500
GTTCGTGATCCAAAAGCGCGTATGCAAGTAGTCTGGAACTTCTGCAAAGG
841Dai15899
GTCCGCGACCCTAAAGCTCGTATGCAAGTCGTATGGAACTTCTGCAAAAG
842Dai15904
GTCCGCGACCCTAAAGCTCGTATGCAAGTCGTATGGAACTTCTGCAAAAG
LZB1066xinjiang --
LZB1065xinjiang --
851Dai16779 --
RMJ119sp_Candelabrochaete_sept
GTCCGCGATCCGAAAGCACGAATGCAAGTCGTCTGGAACTTCTGCAAGAG
RLG9759spCandelabrochaete_sept --
RLG10478Phanerochaete_allantos --
Dai19118_Ceriporia_spissa --
Dai18486A --
WEI17_024_Ceriporia_mellita --
GC1508_71Ceriporia_mellita
GTTCGCGATCCCAAGGCGCGCATGCAAGTCGTCTGGAATTTCTGCAAGAG
GC1608_7_Ceriporia_mellita
GTTCGCGATCCCAAGGCGCGCATGCAAGTCGTCTGGAATTTCTGCAAGAG
ZZW1557Dai27085 --
ZZW1554Dai27083 --
Dai8168 --
BR4865C_mellita --
MEL2382688Ceriporia_sp --
Dai8110 --
Cui8097 --
909Cui6740 --
W1258Dai24695
GTTCGCGATCCCAAAGCGCGCATGCAAGTCGTCTGGAACTTCTGCAAAAA
JV0110_26_Ceriporia_griseoviol --
896Dai13202 --
LWY393Dai27053C_griseoviolasce
GTTCGAGACCCGAAAGCGCGCATGCAAGTCGTTTGGAACTTTTGCAAGAG
LWY394DAI27054 --
FP135015G_pannocinctus --
L15726SpG_pannocinctus --
Dai22221

GTCCGTGACCCAAAAGCACGAATGCAAGTTGTGTGGGGTTTTGCAAGAG

Dai22633

GTTCGTGATCCGAAGGCGCGGATGCAAGTTGTGTGGGGATTCTGCAAGAG

Dai23260

GTTCGTGATCCGAAGGCGCGGATGCAAGTTGTGTGGGGATTCTGCAAGAG

Dai23626

GTTCGTGATCCGAAGGCGCGGATGCAAGTTGTGTGGGGATTCTGCAAGAG

Dai16238G_citrinoalbus --

1175Dai15293 --

Dai19547

GTTCGTGATCCGAAGGCGCGGATGCAAGTTGTGTGGGGATTCTGCAAGAG

918063G_africanus --

918572G_africanus --

Dai18536A --

1164Cui17922 --

Dai22225

GTTCGTGATCCGAAGGCGCGGATGCAGGTTGTGTGGGGATTCTGCAAGAG

1163Dai20655

GTCCGTGATCCGAAGGCGCGGATGCAGGTTGTGTGGGGATTCTGCAAGAG

Yuan4397G_hainanensis --

1176Dai15268

GTTCGTGACCCGAAAGCACGTATGCAAGTAGTATGGGCTTTTGCAAGAG

1177Dai15259 --

BZ2896G_thelephoroides --

1166JV1808_26 --

Miettinen16992Hapalopilus_ochr

GTACGAGATCCGAAAGCGCGCATGCAAGTCGTCTGGAACTACTGCAAAGG

GC1708_338_Ceriporia_arbuscula

GTTCGTGATCCCAAAGCCCGCATGCAAGTCGTGTGGAACTTTTGCAAGAG

WCG1555Dai26107Ceriporia --

GC1708_340_Ceriporia_arbuscula

GTTCGTGATCCCAAAGCCCGCATGCAAGTCGTGTGGAACTTTTGCAAGAG

WCG1556Dai26109Ceriporia

GTTCGTGATCCCAAAGCCCGCATGCAAGTCGTGTGGAACTTTTGCAAGAG

883Cui11291

GTTCGTGATCCCAAAGCCCGCATGCAAGTTGTATGGAACTTTTGCAAGAG

HLX320Dai26805

GTTCGTGATCCCAAAGCCCGTATGCAAGTTGTATGGAACTTTTGCAAGAG

WCG1266Dai24678A

GTTCGTGATCCCAAAGCCCGTATGCAAGTTGTATGGAACTTTTGCAAGAG

Dai6090_Ceriporia_sulphuricolo --

RLG_11354_Ceriproia_reticulata

GTTCGTGATCCCAAAGCGCGCATGCAAGTCGTCTGGGGTTTCTGCAAAGC

ZZW1543Dai27072

GTTCGTGATCCCAAAGCGCGCATGCAAGTCGTTTGGGCTTTCTGCAAAGC
Li1316_Ceriporia_reticulata --
KHL11981Ceriporia_reticulata --
FP110343sp_Candelabrochaete_la
GTTCGTGATCCCAAAGCGCGCATGCAAGTTGTCTGGAACTTCTGCAAAGC
Li1045_Ceriporia_reticulata --
ZX136Dai25794ceriporia --
892Dai13400
GTTCGTGATCCCAAAGCGCGCATGCAAGTCGTTTGGGGCTTCTGCAAAGC
RLG7163Leptoporus_mollis
GTTCGCGACCCCAAAGCTCGGATGCAAGTCGTTTGGAACTTCTGCAAGAG
Dai21062Leptoporus_mollis --
Dai20182Leptoporus_submollis
GTTCGCGATCCCAAAGCTCGGATGCAAGTCGTTTGGAACTTCTGCAAGAG
Cui18379Leptoporus_submollis
GTTCGCGATCCCAAAGCTCGGATGCAAGTCGTTTGGAACTTCTGCAAGAG
Wu1209_46Resiniporus_pseudogil
GTCCGTGATCCGAAAGCGCGTATGCAAGTTGTTTGGAACTTCTGCAAGGG
BRNM710169Resiniporus_resinasc --
Dai14516Bjerkandera_adusta
GTACGGGATCCGAAAGCGCGGATGCAAGTGGTTTGGAATTACTGCAAGGG
Dai21100Bjerkandera_fumosa
GTCAGGGATCCGAAAGCGCGGATGCAAGTAGTTTGGAATTACTGCAAGGG
Miettinen16854Ceraceomyces_sp
GTTCGTGATCCCAAAGCGCGTATGCAAGTAGTGTGGAACTACTCCAAAGG
Dai10477C_spissa --
855Dai16831 --
882Cui11282 --
Dai24566 --
Yuan5965 --
Dai3204 --
1194CUI9985 --

Dai15205_Ceriporia_albomellea --
Dai15223_Ceriporia_albomellea --
Li1780_Ceriporia_variegata --
Dai19791_Ceriporia_variegata --
Dai19886 --
Dai10833_Ceriporia_crassitunic --
CHWC1506_46Meruliopsis_crassit
CAAAATGAACTGCGAACCGGACGAACCCAGGGATGAAAATGATGGCATGG
Dai9995_Ceriporia_crassitunica --
Wu1209_58_Meruliopsis_parvispo

CAAAATGACCTGCGAACCGGACGAGCCAAGGGATGAAAATGACGGCATGG
CHWC1505_129_Meruliopsis_parvi --
Dai21944
CAAAATGATTTGCGATCCCGACGAGCCTAGGGACGAAAATGACGGCATGG
830Dai18640A
CAAAATGATTTGCGATCCGGACGAGCCCAGGGACGAAAATGACGGCATGG
GC1704_60_Meruliopsis_taxicola
CAAAATGGTCTGCGAACCGGACGAGCCTAAGGATGAA---GACGGCATGG
Dai22625
CAAAATGGTCTGCGAACCGGACGAGCCTAAGGATGAA---GACGGCATGG
Dai22636
CAAAATGGTCTGCGAACCGGACGAGCCTAAGGATGAA---GACGGCATGG
Dai21878
CAAAATGATCTGCGAACCGGACGAGCCTAAGGATGAA---GACGGCATGG
1169Dai17248 --
Wu1708_43_Meruliopsis_leptocys
CAAAATGATTTGCGAACCGGACGAGCCCAAGGATGAACATGACGGTATGG
Li1011 --
ZX95Dai25742Meruliopsis_leptoc --
WCG1306Dai24733 --
LXL99Dai25816
CAAAATGACTTGCGAACCGGACGAACCCAAGGATGAACATGACGGCATGG
WCG1559Dai26052Meruliopsis
CAAAATGACTTGCGAGCCGGACGAGCCCAAGGATGAACATGATGGTATGG
He7477 --
HLX243Dai26217
CAAAATGACTTGCGAGCCGGACGAGCCCAAGGATGAACATGATGGTATGG
RussiaMW673659Meruliopsis_fagi --
FD278
CAAAATGACCTGCGAACCGGACGAGCCAAAGGATGAAAATGACGGCATGG
Dai10226_Ceriporia_tarda --
LE247365 --
Dai8173_Meruliopsis_nanlingens --
860Dai17172 --
879Dai13414
CAAAATGATCTGCGAGCCGGACGAGCCCAAGGATGAAGCTGACGGCATGG
Li_1704_Meruliopsis_pseudocyst --
833Dai18405
CAAAATGATTTGCGAACCGGACGAGCCCAAGGATGACAGTGACGGCATGG
HHB_10729_Meruliopsis_albostra
CAAAATGATTTGCGAACCGGACGAGCCCAAGGATGACAGTGACGGCATGG
Cui6878_Ceriporia_pseudocystid --
869Dai14737 --
876Cui11626 --

1199WEI3388 --
776308_Meruliopsis_cystidiata --
ICN139059_Meruliopsis_cystidia --
HHB15692Ceraceomyces_serpens
CAAGATGATCTGCGAGCCGGACGAGCCCAAGGACGAGAATGATGGCATGG
HHB_15629_Sp_Ceriporiopsis_ane
CAAGATGATTTGCGAGCCGGACGAGCCGAAGGACGAGAATGATGGAATGG
AJ185Trametopsis_cervina
CAAAATGATATGTGAGCCGGACGAGCCGAAGGACGAGACAGATGGAGTGG
FD9Irpex_lacteus
CAAGATGGTCTGCGAACCGGACGAGCCCAAGGAGGAG---GATGGAGCGG
908Dai11230
CAAAATGGTCTGCGAACCGGACGAGCCCAAGGAAGAG---GATGGAGCGG
FP55521TEmmia_lacerata
CAAGATGATCTGTGACCCGGATGAGCCCAAAGACGAG---GACGGCGGCG
PBU0048Ceriporia_cystidiata --
MZ340C_laceratаT --
Dai21940
CAAGATGATCTGTGAGCCGGATGAGCCCAAAGACGAG---GATGGTGGTG
847Dai16433
CAAGATGATCTGTGAGCCGGATGAGCCCAAAGACGAG---GATGGTGGTG
MarcinEmmia_latemarginatus --
Meijer3729Hydnopolyporus_fimbr --
RLG13408Phanerochaete_sp
CAAAATGATTTGCGAACCAGACGAGCCCAAGGACGAGAATGACGGCGGTG
WHC1381Flavodon_flavus
CAAGATGGTATGCGAACCAGACGACCCGAAGGACGAG---GATGGCGGAG
GB1833Phlebia_albida
CAAGATGGTTTGCGAACCGGACGAACCTAAGGACGAGACCGATGGTGCTG
T407Phlebia_nitidula
CAAGATGGTCTGCGAACCGGACGAGCCTAAGGACGAGACCGATGGTGCTG
HHB6988Phanerochaete_exilis
CAAGATGATATGCGAGCCAGACGAGCCCAAGGACGAGACAGACGGCGGCG
HHB8509Phanerochaetella_xeroph --
PBU0051Macrohyporia_dictyopora --
HHB11463Phanerochaete_sp
CAAGATGATTTGCGAACCAGATGAGCCCAAGGACGAGACAGAAGGTGGCG
FP102382Byssomerulius_corium
CAAGATGATCTGCGAGCCAGACGAACCTAAGGACGAG---GACGGTGGTG
FP102165Efibula_americana
CAAGATGGTCTGCGAGCCGGATGAGCCAAGGGACGAGAGCGACAACGCTG
Murdoch90Ceriporia_torpida --
Rivoire4413_Ceriporia_purpurea --
Kout_18_Ceriporia_triumphalis --

Rivoire3701_Ceriporia_bresadol --
VS4018 --
Ryvarden21832_Ceriporia_manzan --
Dai24539
CAAAATGGTTTGCGAACCGGACGAGCCTAAGGACGAAAATGATGGTGCGG
Dai24541
CAAAATGGTTTGCGAACCGGACGAGCCTAAGGACGAAAATGATGGTGCGG
JV1105_12_Ceriporia_occidental --
VS8558Ceriporia_occidentalis --
Dai22445 --
846Dai16368
CAAGATGGTTTGCGAACCGGACGAGCCCAAGGACGAAAATGACGGTGCGG
Dai17951_Ceriporia_aurantiocar --
Miettinen_11701C_viridans --
JV0105_10Ceriporia_aurantiocar --
Yuan5702C_viridans --
858Dai17003 --
Yuan2747_Ceriporia_viridans --
Yuan2744C_viridans --
Li1046C_viridans --
865C_sinoviridans --
871Dai15062 --
Dai7642_Ceriporia_humilis --
Spirin4706_Ceriporia_humilis --
Spirin4944_Ceriporia_sericea --
WCG1547Dai26044ceriporia
CAAGATGATTTGCGAGCCGGACGAACCCAAGGATGAAAATGACGGAATGG
ZZW1558Dai27086 --
Miettinen14381_Ceriporia_mpuri --
Miettinen15492_2_Ceriporia_sor --
He6687
CAAGATGATTTGCGAGCCGGACGAGCCCAAGGATGAAAACGACGGAATGG
ZH53Dai24426
CAAGATGATTTGCGAGCCGGACGAGCCCAAGGATGAAAACGACGGAATGG
Vlasak0808_30_Ceriporia_punica --
887Dai13376
CAAGATGTTTTGCGAACCGGACGAACCAAAGGACGATAATGACGGGATGG
WCG1443Dai24998
CAAGATGAATTGCGAGCCGGACGAGGTCAAGGACGACAATGACGGGATGG
0108_6Ceriporia_spissa --
Dai19164 --
Dai17937_Ceriporia_bubalinomar --
903Dai12113 --
LZB929Dai25079 --

LX45Dai26988
CAAAATGGTGTGCGAACCGGACGAGCCAAAGGACGAAAATGACGGTATGG
LX43Dai26986
CAAAATGGTGTGCGAACCGGACGAGCCAAAGGACGAAAATGACGGTATGG
Dai7759Ceriporia --
Cui8012_Ceriporia_viridans --
GC1704_54Ceriporia_viridans --
Dai23392
CAAAATGGTCTGCGAACCCGACGAGCCAAAGGACGAGGATGGTGGTGTGG
WCG1585Dai26113Ceriproia
CAAAATGGTCTGCGAACCCGACGAGCCAAAGGACGAGGATGGTGGTGTGG
Dai18675C_eucalypti --
Dai22034
CAAGATGGTTTGCGAACCGGACGAGCCGAAGGATGAGACTGATGGTATGG
JV1008_41JTardaFLORIDAKeys --
Rivoire1161_Ceriporia_pierii --
Dai23499C_pierii
CAAAATGATTTGCGAGCCGGACGAACCAAAGGATGAAAATGACGGGATGG
Dai23500
CAAAATGATTTGCGAGCCGGACGAACCAAAGGATGAAAATGACGGGATGG
841Dai15899
CAAAATGATTTGCGAGCCGGACGAGCCTAAAGACGAGAACGACGGCGCGG
842Dai15904
CAAAATGATTTGCGAGCCGGACGAGCCTAAAGACGAGAACGACGGCGCGG
LZB1066xinjiang --
LZB1065xinjiang --
851Dai16779 --
RMJ119sp_Candelabrochaete_sept
CAAGATGATCTGCGAGCCAGACGAGCCCAAGGACGAAAATGATGGCATGG
RLG9759spCandelabrochaete_sept --
RLG10478Phanerochaete_allantos --
Dai19118_Ceriporia_spissa --
Dai18486A --
WEI17_024_Ceriporia_mellita --
GC1508_71Ceriporia_mellita
TAAAATGATATGCGAACCGGACGAGATCAAAGACGAAAATGATGGTTTGG
GC1608_7_Ceriporia_mellita
TAAAATGATATGCGAACCGGACGAGATCAAAGACGAAAATGATGGTTTGG
ZZW1557Dai27085 --
ZZW1554Dai27083 --
Dai8168 --
BR4865C_mellita --
MEL2382688Ceriporia_sp --
Dai8110 --

Cui8097 --
909Cui6740 --
W1258Dai24695
CAAAATGATATGCGAACCGGACGAGGTCAAGGACGAAAATGATGCGTTGG
JV0110_26_Ceriporia_griseoviol --
896Dai13202 --
LWY393Dai27053C_griseoviolasce
CAAAATGATTTGCGAGCCGGACGATCCCAAGGATGAAAACGATGGCGTGG
LWY394DAI27054 --
FP135015G_pannocinctus --
L15726SpG_pannocinctus --
Dai22221
CAAGATGGTCTGCGAGCCAGACGAGCCGAAGGACGAGAACGATGGTATGG
Dai22633
CAAAATGATTTGCGAACCAGACGAGCCGAAGGACGAGAACGATGGTATGG
Dai23260
CAAAATGATCTGCGAACCAGACGAGCCGAAGGACGAGAACGATGGTATGG
Dai23626 CAAAATGATCTGCGAACCAGACGAG-CGAAGGACGAGAACGATGGTATGG
Dai16238G_citrinoalbus --
1175Dai15293 --
Dai19547
CAAAATGATTTGCGAGCCTGACGAGCCGAAGGACGAGAACGATGGTATGG
918063G_africanus --
918572G_africanus --
Dai18536A --
1164Cui17922 --
Dai22225 CAAAATGATCTGCGAACCTGACGA-CCGAAGGACGAGAACGATGGTATGG
1163Dai20655
CAAAATGATCTGCGAACCTGACGAGCCGAAGGACGAGAACGATGGTATGG
Yuan4397G_hainanensis --
1176Dai15268
CAAAATGGTCTGTGAACCCGACGAACCGAAGGATGAG---GACGGGATGG
1177Dai15259 --
BZ2896G_thelephoroides --
1166JV1808_26 --
Miettinen16992Hapalopilus_ochr
AAAGATGATCTGCGAGTCGGATGAGCCTAAAGATGAGACTGATGGCATTG
GC1708_338_Ceriporia_arbuscula
CAAAATGATTTGCGAGCCGGACGAGCCCAAGGATGACGCTGATGGCATGG
WCG1555Dai26107Ceriporia --
GC1708_340_Ceriporia_arbuscula
CAAAATGATTTGCGAGCCGGACGAGCCCAAGGATGACGCTGATGGCATGG

WCG1556Dai26109Ceriporia
CAAAATGATTTGCGAGCCGGACGAGCCCAAGGATGACGCTGATGGCATGG
883Cui11291
CAAAATGATTTGCGAGCCGGACGAGCCCAAGGATGACGCTGATGGTATGG
HLX320Dai26805
CAAAATGACTTGCGAGCCGGACGAGCCCAAGGATGACGCTGACGGTATGG
WCG1266Dai24678A
CAAAATGATTTGCGAGCCGGACGAGCCCAAGGATGACGCTGATGGCATGG
Dai6090_Ceriporia_sulphuricolo --
RLG_11354_Ceriproia_reticulata
GAAGATGATTTGCGAACCGGACGATCCCAAGGACGAAACCGATGGTATGG
ZZW1543Dai27072
GAAGATGATTTGCGAACCGGACGATCCCAAGGACGAAACCGATGGTATGG
Li1316_Ceriporia_reticulata --
KHL11981Ceriporia_reticulata --
FP110343sp_Candelabrochaete_la
GAAGATGGTTTGCGAACCGGATGACCCCAAGGACGAAACCGATGGGATGG
Li1045_Ceriporia_reticulata --
ZX136Dai25794ceriporia --
892Dai13400
GAAGATGATTTGCGAGCCGGACGATCCCAAGGACGAAACCGACGGGATGG
RLG7163Leptoporus_mollis
CAAAATGATTTGCGAACCTGACGAGCCGAAGGACGAGAATGACGGTATGG
Dai21062Leptoporus_mollis --
Dai20182Leptoporus_submollis
CAAAATGATTTGCGAACCTGACGAGCCGAAGGACGAGAATGACGGTATGG
Cui18379Leptoporus_submollis
CAAAATGATTTGCGAACCTGACGAGCCGAAGGACGAGAATGACGGTATGG
Wu1209_46Resiniporus_pseudogil
CAAAATGGTATGCGAACCGGACGAGCCAAAGGATGAGAATGATGGCGGGG
BRNM710169Resiniporus_resinasc --
Dai14516Bjerkandera_adusta
CAAAATGGTCTGTGAGCCGGACGAGCCCAAGGATGAAACAGACAACATGG
Dai21100Bjerkandera_fumosa
CAAGATGGTCTGCGAGCCGGACGAGCCCAAGGACGAAACAGAGAACATGG
Miettinen16854Ceraceomyces_sp
GAAGATGATTTGCGAGCCGGACGAGCCCAAGGACGAGAATGATGGTGTTG
Dai10477C_spissa --
855Dai16831 --
882Cui11282 --
Dai24566 --
Yuan5965 --
Dai3204 --
1194CUI9985 --

Dai15205_Ceriporia_albomellea --
Dai15223_Ceriporia_albomellea --
Li1780_Ceriporia_variegata --
Dai19791_Ceriporia_variegata --
Dai19886 --
Dai10833_Ceriporia_crassitunic --
CHWC1506_46Meruliopsis_crassit
AGAATGAGGAGTCGAAACGCGGTCATGGTGGTTGTGGCGCAGCGCAGCCG
Dai9995_Ceriporia_crassitunica --
Wu1209_58_Meruliopsis_parvispo
AAAATGAGGAACCTAAGCGCGGTCATGGTGGCTGCGGTGCAGCGCAGCCA
CHWC1505_129_Meruliopsis_parvi --
Dai21944
AGAACGAGGAACCCAAGCACAGTCATGGTGGTTGCGGCGCAGCTCAACCA
830Dai18640A
ACAACGAGGAACCCAAGCATAGTCATGGTGGTTGCGGGGCAGCGCAGCCG
GC1704_60_Meruliopsis_taxicola
ACGGTGAAGAGGCCAAGCGCGGACACGGTGGGTGTGGCGCCGCACAGCCA
Dai22625
ACGGTGAAGAAGCCAAGCGCGGACACGGTGGGTGTGGCGCCGCACAGCCA
Dai22636
ACGGTGAAGAAGCCAAGCGCGGACACGGTGGGTGTGGCGCCGCACAGCCA
Dai21878
ACGGTGAAGAAGCCAAGCGCGGACACGGTGGGTGTGGCGCCGCACAGCCA
1169Dai17248 --
Wu1708_43_Meruliopsis_leptocys
ATAATGAGGAACCCAAGCGCGGTCATGGTGGGTGTGGCGCCGCACAGCCA
Li1011 --
ZX95Dai25742Meruliopsis_leptoc --
WCG1306Dai24733 --
LXL99Dai25816
ATAATGAGGAACCCAAGCGTGGTCATGGTGGGTGTGGCGCTGCACAGCCA
WCG1559Dai26052Meruliopsis
ATAATGAGGAACCCAAGCGCGGTCATGGTGGGTGTGGCGCCGGACAGCCA
He7477 --
HLX243Dai26217
ATAATGAGGAACCCAAGCGCGGTCATGGTGGGTGTGGCGCCGGACAGCCA
RussiaMW673659Meruliopsis_fagi --
FD278
AGAACGAGGAACCCAAGCGGGGTCACGGAGGTTGTGGCGCAGCACGGCCG
Dai10226_Ceriporia_tarda --
LE247365 --

Dai8173_Meruliopsis_nanlingens ---
860Dai17172 ---
879Dai13414
ATAATGAGGAGCCCAAGCGTGGTCATGGTGGGTGTGGCGCTGCACAGCCA
Li_1704_Meruliopsis_pseudocyst ---
833Dai18405
ACAATGAGGAGCCCAAGCGCGGTCATGGTGGCTGTGGCGCTGCACAGCCA
HHB_10729_Meruliopsis_albostra
ATAATGAGGAGCCCAAGCGCGGTCATGGTGGCTGTGGCGCTGCACAGCCA
Cui6878_Ceriporia_pseudocystid ---
869Dai14737 ---
876Cui11626 ---
1199WEI3388 ---
776308_Meruliopsis_cystidiata ---
ICN139059_Meruliopsis_cystidia ---
HHB15692Ceraceomyces_serpens
ACAATGACGAACCAAAGCGCGGCCATGGTGGCTGTGGCGCTGCGCAGCCC
HHB_15629_Sp_Ceriporiopsis_ane
ACAACGAGGAACGGAAACCTGGTCACGGCGGCTGTGGTGCCGCCCAGCCC
AJ185Trametopsis_cervina
ACAACGACGAGCGGAAACCGGGTCACGGAGGCTGCGGTGCCGCGCAGCCC
FD9Irpex_lacteus
ATGGCGACGAGCCCAAACGTGGCCATGGTGGATGTGGCGCAGCGCAGCCG
908Dai11230
ATGGTGACGAGCCCAAACGTGGCCATGGTGGATGTGGCGCAGCGCAGCCG
FP55521TEmmia_lacerata
ACGGCGATGAAACTAAACACGGTCATGGTGGTTGTGGCGCAGCACAGCCA
PBU0048Ceriporia_cystidiata ---
MZ340C_lacerataT ---
Dai21940
ACGGCGACGAAACCAAACACGGTCATGGTGGCTGTGGCGCAGCACAGCCG
847Dai16433
ACGGCGACGAACCCAAACACGGTCATGGTGGCTGTGGCGCAGCACAGCCG
MarcinEmmia_latemarginatus ---
Meijer3729Hydnopolyporus_fimbr ---
RLG13408Phanerochaete_sp
ACAACGAGGAACCCAAACGTGGACACGGCGGYTGCGGTGCGGCACAACCA
WHC1381Flavodon_flavus
ACGGTGACGAGCCCAAACGTGGTCATGGTGGCTGTGGCGCGGCGCAGCCA
GB1833Phlebia_albida
ACAACGATGAACCGAAACGCGGTCACGGTGGCTGTGGTGCCGCGCAGCCA
T407Phlebia_nitidula
ACAACGATGAACCGAAACGTGGTCACGGTGGTTGTGGTGCCGCGCAGCCA
HHB6988Phanerochaete_exilis

ATGGTGAGGAACCCAAGCGAGGGCATGGTGGTTGCGGTGCAGCTCAGCCA
HHB8509Phanerochaetella_xeroph --
PBU0051Macrohyporia_dictyopora --
HHB11463Phanerochaete_sp
ACGGCGATGAACCCAACCGCGGGCACGGCGGTTGCGGTGCCGCCCAGCCA
FP102382Byssomerulius_corium
ATGGTGAGGAACCCAAGCGTGGCCACGGAGGCTGTGGCGCGGCACAACCA
FP102165Efibula_americana
ACAACGATGAACCGAAGCGTGGGCATGGCGGCTGTGGCGCAGCGCAGCCT
Murdoch90Ceriporia_torpida --
Rivoire4413_Ceriporia_purpurea --
Kout_18_Ceriporia_triumphalis --
Rivoire3701_Ceriporia_bresadol --
VS4018 --
Ryvarden21832_Ceriporia_manzan --
Dai24539
ATAACGAGGAACCAAAGCGCGGCCATGGTGGTTGTGGCGCCGCACAACCG
Dai24541
ATAACGAGGAACCAAAGCGCGGCCATGGTGGTTGTGGCGCCGCACAACCG
JV1105_12_Ceriporia_occidental --
VS8558Ceriporia_occidentalis --
Dai22445 --
846Dai16368
ATAACGAGGAACCGAAGCGCGGTCATGGTGGCTGTGGCGCCGCACAACCG
Dai17951_Ceriporia_aurantiocar --
Miettinen_11701C_viridans --
JV0105_10Ceriporia_aurantiocar --
Yuan5702C_viridans --
858Dai17003 --
Yuan2747_Ceriporia_viridans --
Yuan2744C_viridans --
Li1046C_viridans --
865C_sinoviridans --
871Dai15062 --
Dai7642_Ceriporia_humilis --
Spirin4706_Ceriporia_humilis --
Spirin4944_Ceriporia_sericea --
WCG1547Dai26044ceriporia
ATAATGAGGAGCCGAAACGCGGTCATGGCGGCTGTGGAGCTGCTCAACCG
ZZW1558Dai27086 --
Miettinen14381_Ceriporia_mpuri --
Miettinen15492_2_Ceriporia_sor --
He6687
ACAATGAGGAGCCGAAGCGCGGTCATGGCGGCTGTGGAGCTGCTCAACCG

ZH53Dai24426
ACAATGAGGAGCCGAAGCGCGGTCATGGCGGCTGTGGAGCTGCTCAACCG
Vlasak0808_30_Ceriporia_punica --
887Dai13376
ACAATGAGGAGCCAAAGCGTGGCCACGGTGGCTGTGGTGCCGCGCAGCCG
WCG1443Dai24998
ACAATGAGGAGCCAAAGCGCGGCCACGGTGGCTGTGGTGCCGCGCAGCCG
0108_6Ceriporia_spissa --
Dai19164 --
Dai17937_Ceriporia_bubalinomar --
903Dai12113 --
LZB929Dai25079 --
LX45Dai26988
ACAACGAGGAGCCGAAGCGTGGCCACGGCGGTTGTGGCGCACCACAGCCA
LX43Dai26986
ACAACGAGGAGCCGAAGCGTGGCCACGGCGGTTGTGGCGCACCACAGCCA
Dai7759Ceriporia --
Cui8012_Ceriporia_viridans --
GC1704_54Ceriporia_viridans --
Dai23392
ATAGTGAGGAGCCAAAGCGTGGTCACGGCGGTTGTGGCGCACCTCAGCCC
WCG1585Dai26113Ceriproia
ATAGTGAGGAGCCAAAGCGTGGTCACGGCGGTTGTGGCGCACCTCAGCCC
Dai18675C_eucalypti --
Dai22034
ACCAGGAGGAACCGAAACGCGGTCATGGTGGATGTGGTGCCGCTCAGCCG
JV1008_41JTardaFLORIDAKeys --
Rivoire1161_Ceriporia_pierii --
Dai23499C_pierii
ATAATGAGGAGCCGAAACGTGGTCATGGAGGCTGCGGAGC-GCTCAG-CG
Dai23500
ATAATGAGGAGCCGAAACGTGGTCATGGAGGCTGCGGAGCCGCTCAGCCG
841Dai15899
ACAACGAGGAGCCGAAGCGCGGTCATGGCGGTTGCGGTGCCGCCCAACCG
842Dai15904
ACAACGAGGAGCCGAAGCGCGGTCATGGCGGTTGCGGTGCCGCCCAACCG
LZB1066xinjiang --
LZB1065xinjiang --
851Dai16779 --
RMJ119sp_Candelabrochaete_sept
ATAACGACGAGCCGAAGCGTGGTCATGGCGGGTGTGGTGCCGCTCAACCG
RLG9759spCandelabrochaete_sept --
RLG10478Phanerochaete_allantos --
Dai19118_Ceriporia_spissa --

Dai18486A --
WEI17_024_Ceriporia_mellita --
GC1508_71Ceriporia_mellita
ATAATGATGAGCCGAAGCGTGGTCATGGCGGCTGTGGGGCCCCTCAACCT
GC1608_7_Ceriporia_mellita
ATAATGATGAGCCGAAGCGTGGTCATGGCGGCTGTGGGGCCCCTCAACCT
ZZW1557Dai27085 --
ZZW1554Dai27083 --
Dai8168 --
BR4865C_mellita --
MEL2382688Ceriporia_sp --
Dai8110 --
Cui8097 --
909Cui6740 --
W1258Dai24695
ATAATGACGAGCCGAAGCGTGGTCATGGTGGTTGTGGGGCCGCTCAACCT
JV0110_26_Ceriporia_griseoviol --
896Dai13202 --
LWY393Dai27053C_griseoviolasce
AGAACGAGGAACCAAAGCGCGGTCACGGGGGCTGCGGTGCTGCCCAGCCT
LWY394DAI27054 --
FP135015G_pannocinctus --
L15726SpG_pannocinctus --
Dai22221
ATAATGAGGAACCGAAACGCGGTCACGGCGGATGTGGTGCTGCCCAGTCT
Dai22633
ATAATGAGGAGCCCAAGCGTGGTCACGGTGGATGCGGCGCTGCTCAACCG
Dai23260
ATAATGAGGAGCACAAGCGTGGTCACGGTGGATGCGGTGCTGCTCAACCG
Dai23626
ATAATGAGGAGCACAAGCGTGGTCACGGTGGATGCGGTGCTGCTCAACCG
Dai16238G_citrinoalbus --
1175Dai15293 --
Dai19547
ATAATGAGGAGCCCAAGCGTGGTCACGGTGGATGCGGTGCTGCTCAACCG
918063G_africanus --
918572G_africanus --
Dai18536A --
1164Cui17922 --
Dai22225 ATAATGAGGAGCCC-
AGCGTGGTCACGGTGGATGCGGTGCTGCTCAACCG
1163Dai20655
ATAATGAGGAGCCCAAGCGTGGTCACGGTGGATGCGGTGCTGCTCAACCG
Yuan4397G_hainanensis --

1176Dai15268 AAAATGAGGAGACGAAGCGTGGT-ACGGTGGTTGCGGGGC-GTTCAACCG
1177Dai15259 --
BZ2896G_thelephoroides --
1166JV1808_26 --
Miettinen16992Hapalopilus_ochr ACAATGACGAGCCGAGACGTGGTCATGGTGGCTGTGGCGCTCCTCAGCCG
GC1708_338_Ceriporia_arbuscula ATCATGAGGAGCCGAAGCGCGGTCATGGCGGTTGTGGTGCCGCTCAACCG
WCG1555Dai26107Ceriporia --
GC1708_340_Ceriporia_arbuscula ATCATGAGGAGCCGAAGCGCGGTCATGGCGGTTGTGGTGCCGCTCAACCG
WCG1556Dai26109Ceriporia ATCATGAGGAGCCGAAGCGCGGTCATGGCGGTTGTGGTGCCGCTCAACCG
883Cui11291 ATCATGAGGAGCCGAAGCGTGGCCATGGCGGCTGTGGCGCTGCTCAACCG
HLX320Dai26805 ATCATGAGGAGCCGAAGCGTGGTCATGGCGGTTGTGGCGCTGCTCAACCG
WCG1266Dai24678A ATAATGAGGAGCCGAAGCGTGGTCATGGCGGTTGTGGCGCCGCTCAACCG
Dai6090_Ceriporia_sulphuricolo --
RLG_11354_Ceriproia_reticulata AGAATGAGGAGCCGAAGCGTGGCCACGGCGGCTGCGGTGCTCCTCAGCCG
ZZW1543Dai27072 AGAATGAGGAGCCGAAGCGTGGCCACGGCGGCTGCGGTGCTCCTCAGCCG
Li1316_Ceriporia_reticulata --
KHL11981Ceriporia_reticulata --
FP110343sp_Candelabrochaete_la AGAATGAGGAGCCCAAACGTGGCCACGGCGGCTGCGGCGCTGCTCAGCCG
Li1045_Ceriporia_reticulata --
ZX136Dai25794ceriporia --
892Dai13400 AGAATGAGGAGCCA------------------------------------
RLG7163Leptoporus_mollis ATGCCGAGGAACCAAAGCGCGGCCATGGAGGATGTGGCGCCGCGCAACCG
Dai21062Leptoporus_mollis --
Dai20182Leptoporus_submollis ATGCCGAGGAACCAAAGCGCGGCCATGGAGGATGTGGCGCCGCGCAACCT
Cui18379Leptoporus_submollis ATGCCGAGGAACCAAAGCGCGGCCATGGAGGATGTGGCGCCGCGCAACCT
Wu1209_46Resiniporus_pseudogil ACAATGATGAACGGAAAACTGGTCACGGCGGCTGTGGTGCCGCGCAGCCC
BRNM710169Resiniporus_resinasc --
Dai14516Bjerkandera_adusta

ACAACGACGAGCCGAAGCGAGGGCATGGTGGCTGCGGTGCTGCACAGCCG

Dai21100Bjerkandera_fumosa ATAACGACGAG-CGAAGCGAGGGCATGGCGGTTGCGGTGCTGCACAACCG

Miettinen16854Ceraceomyces_sp ACAATGAGGAACCGAAGCGCGGCCATGGTGGTTGTGGTGCGGCACAGCCC

Dai10477C_spissa --

855Dai16831 --

882Cui11282 --

Dai24566 --

Yuan5965 --

Dai3204 --

1194CUI9985 --

Dai15205_Ceriporia_albomellea --

Dai15223_Ceriporia_albomellea --

Li1780_Ceriporia_variegata --

Dai19791_Ceriporia_variegata --

Dai19886 --

Dai10833_Ceriporia_crassitunic --

CHWC1506_46Meruliopsis_crassit CAGATACGGAAGGAAGGCCTCAAGCTCTTCGTTCAGTACAAGCGCTCGAA

Dai9995_Ceriporia_crassitunica --

Wu1209_58_Meruliopsis_parvispo CAAATACGGAAGGAAGGCCTCAAGCTCTTCGTTCAGTACAAGCGTTCGAA

CHWC1505_129_Meruliopsis_parvi --

Dai21944 CAGATACGGAAAGAAGGCCTCAAGCTCTTCGTTCAGTACAAGCGTTCGAA

830Dai18640A CAGATACGAAAGGAAGGCCTCAAGCTCTTCGTTCAGTACAAGCGTTCGAA

GC1704_60_Meruliopsis_taxicola CAGATTCGGAAAGAGGGCCTCAAACTCTTCGTTCAGTACAAGCGTTCGAA

Dai22625 CAGATTCGGAAAGAGGGCCTCAAACTCTTCGTTCAGTACAAGCGTTCGAA

Dai22636 CAGATTCGGAAAGAGGGCCTCAAACTCTTCGTTCAGTACAAGCGTTCGAA

Dai21878 CAGATTCGGAAAGAGGGCCTCAAGCTCTTCGTTCAGTACAAGCGTTCGAA

1169Dai17248 --

Wu1708_43_Meruliopsis_leptocys CAGATACGGAAGGAAGGCCTCAAGCTCTTCGTTCAGTATAAACGTTCGAA

Li1011 --

ZX95Dai25742Meruliopsis_leptoc --

WCG1306Dai24733 --

LXL99Dai25816
CAGATACGGAAGGAAGGCCTCAAGCTCTTCGTTCAGTATAAACGTTCGAA
WCG1559Dai26052Meruliopsis
CAGATACGGAAGGAAGGCCTCAAGCTCTTCGTTCAGTATAAACGTTCGAA
He7477 ---
HLX243Dai26217
CAGATACGGAAGGAAGGCCTCAAGCTCTTCGTTCAGTATAAACGTTCGAA
RussiaMW673659Meruliopsis_fagi ---
FD278
CAGGTACGGAAGGAAGGTCTCAAGCTCTTCGTTCAGTACAAGCGCTTGAA
Dai10226_Ceriporia_tarda ---
LE247365 ---
Dai8173_Meruliopsis_nanlingens ---
860Dai17172 ---
879Dai13414
CAGATTCGGAAAGAAGGCCTCAAACTTTTCGTTCAGTATAAGCGTTCGAA
Li_1704_Meruliopsis_pseudocyst ---
833Dai18405
CAAATCCGGAAAGAAGGCCTCAAGCTCTTTGTCCAGTACAAGCGTTCGAA
HHB_10729_Meruliopsis_albostra
CAAATCCGGAAAGAAGGCCTCAAGCTCTTTGTCCAGTACAAGCGTTCGAA
Cui6878_Ceriporia_pseudocystid ---
869Dai14737 ---
876Cui11626 ---
1199WEI3388 ---
776308_Meruliopsis_cystidiata ---
ICN139059_Meruliopsis_cystidia ---
HHB15692Ceraceomyces_serpens
CAGATCCGGAAAGAGGGCCTTAAGCTCTTCGTTCAGTACAAGCGCTCGAA
HHB_15629_Sp_Ceriporiopsis_ane
CAAGTTCGCAAAGAAGGCCTCAAACTCTTCGTTCAGTACAAGCGTTCGAA
AJ185Trametopsis_cervina
CAGATTCGCAAAGAAGGCCTCAAACTCTTTGTCCAGTACAAGCGTTCGAA
FD9Irpex_lacteus
CAGATTCGGAAAGAGGGCCTCAAGCTCTTTGTCCAATACAAGCGATCCAA
908Dai11230
CAGATTCGGAAAGAGGGCCTCAAGCTCTTTGTCCAATACAAGCGATCCAA
FP55521TEmmia_lacerata
CAGATTCGAAAAGAGGGTCTCAAACTCTTCGTCCAATACAAGCGTTCAAA
PBU0048Ceriporia_cystidiata ---
MZ340C_laceratаT ---
Dai21940
CAGATTCGAAAAGAGGGTCTCAAACTCTTCGTCCAATACAAGCGTTCGAA
847Dai16433

CAGATTCGAAAAGAGGGTCTCAAACTCTTCGTCCAATACAAGCGTTCGAA
MarcinEmmia_latemarginatus --
Meijer3729Hydnopolyporus_fimbr --
RLG13408Phanerochaete_sp
CAAATCCGGAAAGAAGGCCTCAAGCTCTTTGTTCAATACAAGCGTTCGAA
WHC1381Flavodon_flavus
CAGATCCGCAAGGAGGGTCTGAAGCTGTTCGTCCAATACAAGCGATCTAA
GB1833Phlebia_albida
CAAATCCGGAAGGAAGGTCTGAAGCTCTTCGTTCAATACAAGCGTTCGAA
T407Phlebia_nitidula
CAAGTACGGAAAGAAGGTCTGAAGCTCTTCGTTCAATACAAGCGTTCGAA
HHB6988Phanerochaete_exilis
CAGATAAGGAAAGAGGGTCTCAAACTTTTCGTCCAATACAAGCGTTCGAA
HHB8509Phanerochaetella_xeroph --
PBU0051Macrohyporia_dictyopora --
HHB11463Phanerochaete_sp
CAAGTACGGAAGGAGGGTCTCAAACTTTTCGTTCAATACAAGCGTTCGAA
FP102382Byssomerulius_corium
CAAATTCGTAAGGAGGGCCTCAAGCTGTTTGTCCAATACAAGCGTTCGAA
FP102165Efibula_americana
CAGATCCGCAAAGAAGGTTTAAAACTGTTTGTTCAATATAAACGTTCGAA
Murdoch90Ceriporia_torpida --
Rivoire4413_Ceriporia_purpurea --
Kout_18_Ceriporia_triumphalis --
Rivoire3701_Ceriporia_bresadol --
VS4018 --
Ryvarden21832_Ceriporia_manzan --
Dai24539
CAAATCCGGAAGGAGGGTCTCAAGCTCTTCGTTCAGTACAAGCGTTCGAA
Dai24541
CAAATCCGGAAGGAGGGTCTCAAGCTCTTCGTTCAGTACAAGCGTTCGAA
JV1105_12_Ceriporia_occidental --
VS8558Ceriporia_occidentalis --
Dai22445 --
846Dai16368
CAAATCCGGAAAGAGGGACTCAAGCTCTTCGTTCAGTACAAGCGTTCGAA
Dai17951_Ceriporia_aurantiocar --
Miettinen_11701C_viridans --
JV0105_10Ceriporia_aurantiocar --
Yuan5702C_viridans --
858Dai17003 --
Yuan2747_Ceriporia_viridans --
Yuan2744C_viridans --
Li1046C_viridans --

865C_sinoviridans --
871Dai15062 --
Dai7642_Ceriporia_humilis --
Spirin4706_Ceriporia_humilis --
Spirin4944_Ceriporia_sericea --
WCG1547Dai26044ceriporia
CAAATCCGGAAAGAGGGCCTTAAACTCTTCGTTCAGTACAAGCGTTCGAA
ZZW1558Dai27086 --
Miettinen14381_Ceriporia_mpuri --
Miettinen15492_2_Ceriporia_sor --
He6687
CAAATCCGGAAAGAGGGCCTCAAACTCTTCGTTCAGTACAAGCGTTCGAA
ZH53Dai24426
CAAATCCGGAAAGAGGGCCTCAAACTCTTCGTTCAGTACAAGCGTTCGAA
Vlasak0808_30_Ceriporia_punica --
887Dai13376
CAAATTCGGAAGGAAGGCCTCAAACTCTTCGTTCAGTACAAGCGTTCGAA
WCG1443Dai24998
CAAATTCGGAAGGAAGGCCTTAAACTCTTCGTTCAGTACAAGCGTTCGAA
0108_6Ceriporia_spissa --
Dai19164 --
Dai17937_Ceriporia_bubalinomar --
903Dai12113 --
LZB929Dai25079 --
LX45Dai26988
CAGATTCGAAAGGAAGGCCTCAAGCTCTTCGTTCAGTACAAGCGTTCGAA
LX43Dai26986
CAGATTCGAAAGGAAGGCCTCAAGCTCTTCGTTCAGTACAAGCGTTCGAA
Dai7759Ceriporia --
Cui8012_Ceriporia_viridans --
GC1704_54Ceriporia_viridans --
Dai23392
CAAATCCGAAAGGAAGGCCTCAAACTCTTCGTTCAGTACAAGCGTTCGAA
WCG1585Dai26113Ceriproia
CAAATCCGAAAGGAAGGCCTCAAACTCTTCGTTCAGTACAAGCGTTCGAA
Dai18675C_eucalypti --
Dai22034
CAGATTCGAAAAGAAGGCCTCAAACTCTTCGTTCAGTACAAGCGTTCGAA
JV1008_41JTardaFLORIDAKeys --
Rivoire1161_Ceriporia_pierii --
Dai23499C_pierii
CAGATTCGGAAAGAAGGGCTCAAACTCTTCGTTCAGTACAAGCGTTCGAA
Dai23500
CAGATTCGGAAAGAAGGCCTCAAACTCTTCGTTCAGTACAAGCGTTCGAA

841Dai15899
CAAATCCGCAAGGAAGGCCTCAAGCTCTTTGTTCAGTACAAGCGTTCGAA
842Dai15904
CAAATCCGCAAGGAAGGCCTCAAGCTCTTTGTTCAGTACAAGCGTTCGAA
LZB1066xinjiang --
LZB1065xinjiang --
851Dai16779 --
RMJ119sp_Candelabrochaete_sept
CAAATCCGCAAGGAAGGCCTCAAGCTCTTCGTCCAGTACAAACGATCGAA
RLG9759spCandelabrochaete_sept --
RLG10478Phanerochaete_allantos --
Dai19118_Ceriporia_spissa --
Dai18486A --
WEI17_024_Ceriporia_mellita --
GC1508_71Ceriporia_mellita
CAAATTCGGAAGGAGGGGCTGAAACTCTTCGTTCAGTACAAGCGTTCGAA
GC1608_7_Ceriporia_mellita
CAAATTCGGAAGGAGGGGCTGAAACTCTTCGTTCAGTACAAGCGTTCGAA
ZZW1557Dai27085 --
ZZW1554Dai27083 --
Dai8168 --
BR4865C_mellita --
MEL2382688Ceriporia_sp --
Dai8110 --
Cui8097 --
909Cui6740 --
W1258Dai24695
CAAATCCGCAAGGAAGGGCTGAAACTCTTTGTTCAGTACAAGCGCTCGAA
JV0110_26_Ceriporia_griseoviol --
896Dai13202 --
LWY393Dai27053C_griseoviolasce
CAAATTCGTAAAGAAGGCCTCAAGCTCTTCGTCCAGTATAAACGTTCGAA
LWY394DAI27054 --
FP135015G_pannocinctus --
L15726SpG_pannocinctus --
Dai22221 CAGATTCGTAAGGAAGGTCTCAAACTCTTTG-TCAGTACAAGCGTTCGAA
Dai22633 CAGATCCGT-AGGGAGGGCTCAAGCTCTTTGTTCAGTACAAGCGTTCGAA
Dai23260
CAGATCCGTAAGGAAGGGCTCAAGCTCTTTGTTCAGTACAAGCGTTCGAA
Dai23626 CAGATCCGT-AAGGAAGGCTCAAGCTCTTTGTTCAGTACAAGCGTTCGAA
Dai16238G_citrinoalbus --

1175Dai15293 --
Dai19547
CAGATCCGCAAGGAAGGCCTCAAACTCTTCGTTCAGTACAAGCGTTCGAA
918063G_africanus --
918572G_africanus --
Dai18536A --
1164Cui17922 --
Dai22225 CAGATCCGT-AAGGAAGGCCTCA-------------------------
1163Dai20655 CAGATCCGC-AAGGAAGGCTCA--------------------------
Yuan4397G_hainanensis --
1176Dai15268 CAGATTCGC-AAGGAGGTCTCAAACTCTTCGTTCAGTACAAGCGTTCGAA
1177Dai15259 --
BZ2896G_thelephoroides --
1166JV1808_26 --
Miettinen16992Hapalopilus_ochr
CAAATCCGGAAGGAAGGGCTCAAACTCTTCGTCCAGTATAAACGGTCAAA
GC1708_338_Ceriporia_arbuscula
CAAATACGGAAGGAAGGCCTCAAGCTATTTGTTCAATACAAGCGTTCGAA
WCG1555Dai26107Ceriporia --
GC1708_340_Ceriporia_arbuscula
CAAATACGGAAGGAAGGCCTCAAGCTATTTGTTCAATACAAGCGTTCGAA
WCG1556Dai26109Ceriporia
CAAATACGGAAGGAAGGCCTCAAGCTATTTGTTCAATACAAGCGTTCGAA
883Cui11291
CAGATCCGGAAGGAAGGCCTCAAGCTCTTCGTTCAGTACAAGCGTTCGAA
HLX320Dai26805
CAAATTCGGAAAGAAGGCCTCAAGCTCTTCGTTCAGTACAAGCGTTCGAA
WCG1266Dai24678A
CAAATTCGGAAGGAAGGCCTCAAGCTCTTCGTTCAGTACAAGCGTTCGAA
Dai6090_Ceriporia_sulphuricolo --
RLG_11354_Ceriproia_reticulata
CAAATCCGGAAAGAAGGCCTCAAACTCTTCGTTCAGTACAAGCGTTCGAA
ZZW1543Dai27072
CAAATCCGGAAAGAAGGCCTCAAACTCTTCGTTCAGTACAAGCGTTCGAA
Li1316_Ceriporia_reticulata --
KHL11981Ceriporia_reticulata --
FP110343sp_Candelabrochaete_la
CAAATCCGGAAAGAAGGCCTCAAACTCTTCGTTCAGTACAAGCGTTCGAA
Li1045_Ceriporia_reticulata --
ZX136Dai25794ceriporia --
892Dai13400 --

RLG7163Leptoporus_mollis
CAGATTCGCAAGGAAGGTCTCAAACTCTTCGTTCAGTACAAGCGTTCGAA
Dai21062Leptoporus_mollis --
Dai20182Leptoporus_submollis
CAGGTCCGCAAGGAAGGTCTCAAACTCTTCGTTCAGTACAAGCGTTCGAA
Cui18379Leptoporus_submollis
CAGGTCCGCAAGGAAGGTCTCAAACTCTTCGTTCAGTACAAGCGTTCGAA
Wu1209_46Resiniporus_pseudogil
CAAATTCGGAAGGAAGGCCTTAAACTCTTCGTTCAGTACAAGCGTTCGAA
BRNM710169Resiniporus_resinasc --
Dai14516Bjerkandera_adusta
CAAATACGGAAAGAGGGTTTGAAGTTATTCGTGCAGTACAAGCGGTCGAA
Dai21100Bjerkandera_fumosa
CAAATACGGAAAGAAGGTTTGAAGTTATTCGTGCAGTACAAGCGGTCGAA
Miettinen16854Ceraceomyces_sp
CAGATCCGAAAAGAGGGTCTCAAGCTCTTCGTTCAGTACAAGCGTTCGAA
Dai10477C_spissa --
855Dai16831 --
882Cui11282 --
Dai24566 --
Yuan5965 --
Dai3204 --
1194CUI9985 --

Dai15205_Ceriporia_albomellea --
Dai15223_Ceriporia_albomellea --
Li1780_Ceriporia_variegata --
Dai19791_Ceriporia_variegata --
Dai19886 --
Dai10833_Ceriporia_crassitunic --
CHWC1506_46Meruliopsis_crassit
AGACGAAGAAGAGGTTTGTATGGTATGGGCCTATTTGTCACATAGTTCTA
Dai9995_Ceriporia_crassitunica --
Wu1209_58_Meruliopsis_parvispo
AGACGAAGACGAGGTCTGTATGACATGGGCCTGTT-ATCACATGGTTCTA
CHWC1505_129_Meruliopsis_parvi --
Dai21944
AGACGAAGACGAGGTCTGTATGATATAAGCTTGTCTATTGCATGATTCTA
830Dai18640A
AGACGAAGACGAGGTCTGTATGGTATAGGCCTGTTTATTGCATGCTTCTA
GC1704_60_Meruliopsis_taxicola
AGACGAAGACGAGGTTTGTATCACGTGGAGTCGTTCRTAATACAGTTCTG
Dai22625

AGACGAAGACGAGGTTTGTATCACGTGGAGTCGTTCATAATACAGTTCTA

Dai22636

AGACGAAGACGAGGTTTGTATCACGTGGAGTCGTTCATAATACAGTTCTA

Dai21878

AGACGAAGACGAGGTTTGTATCACATGGACTCGTTCATAATACAGTTCTA

1169Dai17248 --

Wu1708_43_Meruliopsis_leptocys

AGACGAAGACGAGGTTTGTATAACATGGGCCTATTCGTCACACCATTCTA

Li1011 --

ZX95Dai25742Meruliopsis_leptoc --

WCG1306Dai24733 --

LXL99Dai25816

AGACGATGAAGAGGTTTGTATAACACGGGCATATTCGTCACACCGTTCTA

WCG1559Dai26052Meruliopsis

AGACGACGAGGAGGTTTGTATAACATAGGCCTATTCGTCACACCGTTCTA

He7477 --

HLX243Dai26217

AGACGACGAGGAGGTTTGTATAACATAGGCCTATTCGTCACACCGTTCTA

RussiaMW673659Meruliopsis_fagi --

FD278 AGACAAAGACGAGGT-TGTATAGCATGGACATGTTTGCTTAACGGTTCTA

Dai10226_Ceriporia_tarda --

LE247365 --

Dai8173_Meruliopsis_nanlingens --

860Dai17172 --

879Dai13414

AGACGAAGACGAGGTTTGTATACCACGGACCTGTTCGTCGCGCAGTTCTG

Li_1704_Meruliopsis_pseudocyst --

833Dai18405

AGACGAAGATGAGGTTTGTATGGTGTGGGCCTGTTCGTCACACAGTTCTG

HHB_10729_Meruliopsis_albostra

AGACGAAGATGAGGTCTGTATGGTGTGGGCCTGTTCGTCACACAGTTCTG

Cui6878_Ceriporia_pseudocystid --

869Dai14737 --

876Cui11626 --

1199WEI3388 --

776308_Meruliopsis_cystidiata --

ICN139059_Meruliopsis_cystidia --

HHB15692Ceraceomyces_serpens AGACGAAGACGAGGTTTGTATCGTGTAGATAT--TGACTTCGGAGTTCTA

HHB_15629_Sp_Ceriporiopsis_ane

AGACGAAGACGAGGTTTGTTTTGTGTGGGTATGACCTTCACA--GTTCTG

AJ185Trametopsis_cervina

AGACGAAGACGAGGTTTGTTTTGAGTAGGAATGACCTTCGCTTCGTTCTG

FD9Irpex_lacteus
GGATGACGACGAGGTTTGTATCGTCATAGCTTTTC----ACA--ATTCTG
908Dai11230
GGATGACGACGAGGTTTGTATCGTCATAGCTTTTC----ACA--GTTCTG
FP55521TEmmia_lacerata
GGACGACGATGAGGTTTGTATAGTCATGACTTTACAACAACA--GTTCTG
PBU0048Ceriporia_cystidiata --
MZ340C_lacerataT --
Dai21940 GGACGATGATGAGGTTTGTATAATCATGAC-
TTACAACAACA--GTTCTG
847Dai16433 GGACGATGATGAGGTTTGTATAATCATGAC-
TTACAACAACA--GTTCTG
MarcinEmmia_latemarginatus --
Meijer3729Hydnopolyporus_fimbr --
RLG13408Phanerochaete_sp AGATGATGACGAGGTTTGTATCATGTTAAC-
GTCC----ACA--GTTCTG
WHC1381Flavodon_flavus AGACGATGATGAGGTCTGTA---
CCGTCGTTCTTC----ACA--ATTCTG
GB1833Phlebia_albida AGACGACGACGAGGTTTGTA-AACAGTGAC--
TTC----ACA--TTTCTG
T407Phlebia_nitidula AGACGACGACGAGGTTTGTATTACAGTGAC--
TTC----ACA--TTTCTG
HHB6988Phanerochaete_exilis AGATGACGACGAGGTTTGCGAAACATTGAC--
TTC----ACA--TGTCTG
HHB8509Phanerochaetella_xeroph --
PBU0051Macrohyporia_dictyopora --
HHB11463Phanerochaete_sp AGATGATGACGAGGTTTGCAATACATTGAC--
TTC----ACA--TGTATG
FP102382Byssomerulius_corium AGACGACGATGAGGTCTGTATGACGTTGAC-
GTTT----ACA--TTTCTG
FP102165Efibula_americana GGACGACGATGAGGTTTGTACGACCTTAGC------
---GCA--TTACTA
Murdoch90Ceriporia_torpida --
Rivoire4413_Ceriporia_purpurea --
Kout_18_Ceriporia_triumphalis --
Rivoire3701_Ceriporia_bresadol --
VS4018 --
Ryvarden21832_Ceriporia_manzan --
Dai24539
AGACGAAGACGAGGTTTGTATTATGTGGACATGATAGCC-CATAGTTCTG
Dai24541
AGACGAAGACGAGGTTTGTATTATGTGGACATGATAGCC-CATAGTTCTG
JV1105_12_Ceriporia_occidental --
VS8558Ceriporia_occidentalis --

Dai22445 --
846Dai16368
AGACGAAGACGAGGTTTGTATCATGTGGATATGATAACC-CACAGTTCTG
Dai17951_Ceriporia_aurantiocar --
Miettinen_11701C_viridans --
JV0105_10Ceriporia_aurantiocar --
Yuan5702C_viridans --
858Dai17003 --
Yuan2747_Ceriporia_viridans --
Yuan2744C_viridans --
Li1046C_viridans --
865C_sinoviridans --
871Dai15062 --
Dai7642_Ceriporia_humilis --
Spirin4706_Ceriporia_humilis --
Spirin4944_Ceriporia_sericea --
WCG1547Dai26044ceriporia AGACGAAGACGAGGTTTGTATTATGTGAAT-----
TGCCACACAGTTCTG
ZZW1558Dai27086 --
Miettinen14381_Ceriporia_mpuri --
Miettinen15492_2_Ceriporia_sor --
He6687 AGACGAAGACGAGGTTTGTATTATACGAAT----
-TGCCACACAGTTCTG
ZH53Dai24426 AGACGAAGACGAGGTTTGTATTATACGAAT---
--TGCCACACAGTTCTG
Vlasak0808_30_Ceriporia_punica --
887Dai13376
AGACGAAGATGAGGTTCGTAATACATTGAGAT----GTCATCTGATTCTG
WCG1443Dai24998
AGACGAAGATGAGGTTCGTAATGCACTGAGAT----GTCATTTGATTTA
0108_6Ceriporia_spissa --
Dai19164 --
Dai17937_Ceriporia_bubalinomar --
903Dai12113 --
LZB929Dai25079 --
LX45Dai26988 AGATGACGACGAGGTTTGTATCGTGT-
GACAGTGTGGCCACGCGATTTTG
LX43Dai26986 AGATGACGACGAGGTTTGTATCGTGT-
GACAGTGTGGCCACGCGATTTTG
Dai7759Ceriporia --
Cui8012_Ceriporia_viridans --
GC1704_54Ceriporia_viridans --
Dai23392
AGACGAAGACGAGGTTTGTATTGTGTGGACAATGTGGCCACACAGTTCTA

WCG1585Dai26113Ceriproia
AGACGAAGACGAGGTTTGTATTGTGTGGACAATGTGGCCACACAGTTCTA
Dai18675C_eucalypti --
Dai22034
AGACGAAGACGAGGTTTGTAACGTATGCGCACGATGTTCGCATAGTTCTG
JV1008_41JTardaFLORIDAKeys --
Rivoire1161_Ceriporia_pierii --
Dai23499C_pierii AGACGAAGACGAGGTTTGTATCATGTGGACAT-GGTGGCACACAGTTCTA
Dai23500
AGACGAAGACGAGGTTTGTATCATGTGGACATGGTGGCCACACAGTTCTA
841Dai15899
AGACGAAGACGAGGTTTGTAACGTGTAGACACTCGTGTCTGACCGTTCTG
842Dai15904
AGACGAAGACGAGGTTTGTAACGTGTAGACACTCGTGTCTGACCGTTCTG
LZB1066xinjiang --
LZB1065xinjiang --
851Dai16779 --
RMJ119sp_Candelabrochaete_sept GGACGAGGATGAGGTTTGTATCGTGTGGACAA-TTCGCCACACATTTCTG
RLG9759spCandelabrochaete_sept --
RLG10478Phanerochaete_allantos --
Dai19118_Ceriporia_spissa --
Dai18486A --
WEI17_024_Ceriporia_mellita --
GC1508_71Ceriporia_mellita AGACGATGACGAGGTTTGTATCGTGT-GGCCTCACCGTTTCACAGTTCTG
GC1608_7_Ceriporia_mellita AGACGATGACGAGGTTTGTATTGTGT-GGCCTCACCGTCTCACAGCTCTG
ZZW1557Dai27085 --
ZZW1554Dai27083 --
Dai8168 --
BR4865C_mellita --
MEL2382688Ceriporia_sp --
Dai8110 --
Cui8097 --
909Cui6740 --
W1258Dai24695
AGATGAGGACGAGGTTTGTATTGTGTAGACCTCGCCGTCTCACAGTTCTG
JV0110_26_Ceriporia_griseoviol --
896Dai13202 --
LWY393Dai27053C_griseoviolasce
AGACGAGGACGAGGTTTGTATCGTGTGGACAAGACCGCCACACATATCTG
LWY394DAI27054 --

FP135015G_pannocinctus --
L15726SpG_pannocinctus --
Dai22221
AGACGAAGACGAGGTTTGTATCATCCGATCGCAAATACCGTATCGTTCTG
Dai22633
AGACGAAGACGAGGTCTGTATCATCTGAGAAC------------------
Dai23260
AGACGAAGACGAGGTCTGTATCATCTGAAAACTAACGTGCAATTTTTCTG
Dai23626
AGACGAAGATGAGGTCTGTATCACCTGAAACT-AACGTGCAATTTTTCTG
Dai16238G_citrinoalbus --
1175Dai15293 --
Dai19547 AGATGAAGATGAGGTCTGTATCACAATGATCT-
---------TTGTTCTG
918063G_africanus --
918572G_africanus --
Dai18536A --
1164Cui17922 --
Dai22225 --
1163Dai20655 --
Yuan4397G_hainanensis --
1176Dai15268 AGACGAAGATGAGGTTTGCGTC-------------------

1177Dai15259 --
BZ2896G_thelephoroides --
1166JV1808_26 --
Miettinen16992Hapalopilus_ochr
AGATGATGACGAGGTTTGTATCACGCGTGTGCATCCGCCTCTGCCATCTA
GC1708_338_Ceriporia_arbuscula
AGACGACGATGAGGTTTGTATCATGTGGACAGCGCCTTCAC-CAGTTCTG
WCG1555Dai26107Ceriporia --
GC1708_340_Ceriporia_arbuscula
AGACGACGATGAGGTTTGTATCATGTGGACAGCGCCTTCAC-CAGTTCTG
WCG1556Dai26109Ceriporia
AGACGACGATGAGGTTTGTATCATGTGGAAAGCGCCTTCAC-CAGTTCTG
883Cui11291
AGACGATGATGAGGTTTGTATCACATGGATAGGGTTTTCAC-CGGTTCTG
HLX320Dai26805
AGACGAGGATGAGGTTTGTATCGTGTGAGCAAGGTCTTCAC-CAGTTCTG
WCG1266Dai24678A
AGACGAGGATGAGGTTTGTATCATATGGACCAGGTCTTCAC-CAGTTCTA
Dai6090_Ceriporia_sulphuricolo --
RLG_11354_Ceriproia_reticulata
AGACGAAGATGAGGTTCGTATCCTGCGGGCTGTTCAGTCTCGGAGTTCTG

ZZW1543Dai27072
AGACGAAGATGAGGTTCGTATCCTGCGGGCTATTCAGTCTCGGAGTTCTG
Li1316_Ceriporia_reticulata --
KHL11981Ceriporia_reticulata --
FP110343sp_Candelabrochaete_la
AGACGAAGATGAGGTTCGTATCCTGCAGGCTGTTCAGTCTCGGAGTTCTG
Li1045_Ceriporia_reticulata --
ZX136Dai25794ceriporia --
892Dai13400 --
RLG7163Leptoporus_mollis
AGACGAAGACGAGGTTTGTAGCATGTGGACATGGAGGTGACACAACTCTA
Dai21062Leptoporus_mollis --
Dai20182Leptoporus_submollis
AGACGACGAAGAGGTTTGTAGCATGTGGACATGGAGGTCACACAACTCTA
Cui18379Leptoporus_submollis
AGACGACGAAGAGGTTTGTAGCATGTGGACATGGAGGTCACACAACTCTA
Wu1209_46Resiniporus_pseudogil AGACGAAGACGAGGTTCG-TCCATGTGKGGATGACTTTCAGA--ATTCTC
BRNM710169Resiniporus_resinasc --
Dai14516Bjerkandera_adusta
GGACGAAGACGAGGTTTGTAGCACGAGCGTAAAGCAGCCAAGAATTTCTA
Dai21100Bjerkandera_fumosa
GGACGAAGACGAGGTTTGTAGCACGAGCGCAAAGCAGCCAAAATATTCTA
Miettinen16854Ceraceomyces_sp AGACGAAGATGAGGTTTGTATCGTGCGAACAT--TGACTT-ACAGTTCTA
Dai10477C_spissa --
855Dai16831 --
882Cui11282 --
Dai24566 --
Yuan5965 --
Dai3204 --
1194CUI9985 --

Dai15205_Ceriporia_albomellea --
Dai15223_Ceriporia_albomellea --
Li1780_Ceriporia_variegata --
Dai19791_Ceriporia_variegata --
Dai19886 --
Dai10833_Ceriporia_crassitunic --
CHWC1506_46Meruliopsis_crassit
ACGCTTAGGCGTAGGAGGTCAAGAGCTTACAGCCAGATAAGAGGCCGTTC
Dai9995_Ceriporia_crassitunica --
Wu1209_58_Meruliopsis_parvispo

ACGCTTAAGCGTAGGACGTCAAGAGTTTACAGCCAGATAAGAGGCTGTTC
CHWC1505_129_Meruliopsis_parvi --
Dai21944 ACGCTGAAGCGTAGGATGTCAAGAGT-----------

830Dai18640A
ACGCTTAAGCGTAGGACATCAAGAGTTTACAGCCAGATAAGAGGCTGTTC
GC1704_60_Meruliopsis_taxicola
ACGGTTCAGCGTAGGAGGTTAAGAGTCTACAGCCCGATAAGAGGCTGTTC
Dai22625
ACGGTTCAGCGTAGGAGGTTAAGAGTCTACAGCCCGATAAAAGGCTGTTC
Dai22636
ACGGTTCAGCGTAGGAGGTTAAGAGTCTACAGCCCGATAAAAGGCTGTTC
Dai21878
ACGGCTCAGCGTAGGAGGTTAAGAGTTTACAGCCCGATAAAAGGCTGTTT
1169Dai17248 --
Wu1708_43_Meruliopsis_leptocys
ATAGTTGAGCGTAGGAGACCAAGAGTTTGCAGCCGGATAAGAGGCTATTC
Li1011 --
ZX95Dai25742Meruliopsis_leptoc --
WCG1306Dai24733 --
LXL99Dai25816
ATGGTTGAGCGTAGGAGGTCAAGAGTTTACAGCCGGATAAGAGGCTGTTC
WCG1559Dai26052Meruliopsis
ATAGTTGAGCGTAGGAGGTCAAGAGTTTGCAGCCGGATAAGAGGCTATTC
He7477 --
HLX243Dai26217
ATAGTTGAGCGTAGGAGGTCAAGAGTTTGCAGCCGGATAAGAGGCTATTC
RussiaMW673659Meruliopsis_fagi --
FD278
ACAGCTGAGCGTAGGAGTCCAAGAGCTTGCAGCCAGATAAGAGGCTTTTC
Dai10226_Ceriporia_tarda --
LE247365 --
Dai8173_Meruliopsis_nanlingens --
860Dai17172 --
879Dai13414
ACGGTTAAGTGTAGGAGGTCAAAAGCCTACAGCCGGATAAGAGGCTGTTC
Li_1704_Meruliopsis_pseudocyst --
833Dai18405
ACAGCTAAGCGTAGGAGGTGAAGAGTTTGCAGCCGGATAAGAGGCTGTTC
HHB_10729_Meruliopsis_albostra
ACAGCTAAGCGTAGGAGGTGAAGAGTTTGCAGCCGGATAAGAGGCTGTTC
Cui6878_Ceriporia_pseudocystid --
869Dai14737 --
876Cui11626 --

1199WEI3388 ---
776308_Meruliopsis_cystidiata ---
ICN139059_Meruliopsis_cystidia ---
HHB15692Ceraceomyces_serpens
ATGTTGGAGTATAGGAGGTCAAGAGCTTACAGCCGGATAAGAGGCTTTTC
HHB_15629_Sp_Ceriporiopsis_ane ACTTTC---
CTCAGGAGGTTAAGAGCTTGCAACCTGACAAGAGGCTTTTC
AJ185Trametopsis_cervina
ATCTTTGTGGGCAGGAGGTTAAGAGCCTGCAACCAGACAAGAGGCTATTT
FD9Irpex_lacteus
ATTCCTCAGCATAGGAGGTGAAGAGCTTGCAACCAGACAAGAGGCTATTC
908Dai11230
ATTCCTCAGCATAGGAGGTGAAGAGCTTGCAACCAGAC-AGAGGCTATTC
FP55521TEmmia_lacerata
ACTCCTCAGCATAGGAGGTGAAGAGCTTACAACCTGACAAGAGGCTGTTC
PBU0048Ceriporia_cystidiata ---
MZ340C_laceratаT ---
Dai21940
ACTCCTCAGCATAGGAGGTGAAGAGCTTACAACCTGACAAGAGGCTGTTC
847Dai16433
ACTCCTCAGCATAGGAGGTGAAGAGCTTACAACCTGACAAGAGGCTGTTC
MarcinEmmia_latemarginatus ---
Meijer3729Hydnopolyporus_fimbr ---
RLG13408Phanerochaete_sp
ATCCTTAAGCATAGGAGGTCAAGAGCTTGCAACCAGACAAGAGATTATTC
WHC1381Flavodon_flavus
ACTCCTCAGCATAGGAGGTGAAGAGCTTGCAACCAGACAAGAGGCTATTC
GB1833Phlebia_albida
ATCCTTGAGCACAGGAGGTGAAGAGCCTACAACCGGACAAGAGGCTGTTC
T407Phlebia_nitidula
ATCCTTGAGCACAGGAGGTGAAGAGCTTACAACCGGACAAGAGGCTATTC
HHB6988Phanerochaete_exilis
ATGTTTAAGCATAGGATGTCAAGAGCTTACAGCCGGACAAGAGACTGTTC
HHB8509Phanerochaetella_xeroph ---
PBU0051Macrohyporia_dictyopora ---
HHB11463Phanerochaete_sp
ATGTTTAAGCGTAGGATATCAAGAGCTTACAACCGGACAAGAGGCTGTTC
FP102382Byssomerulius_corium
ATCCTTCAGCATAGGAGGTGAAGAGCTTGCAACCGGACAAAAGGCTGTTC
FP102165Efibula_americana
ACAAGTTAAGATAGGAGGTGAAAAGCTTACAACCAGACAAAAGGCTGTTC
Murdoch90Ceriporia_torpida ---
Rivoire4413_Ceriporia_purpurea ---
Kout_18_Ceriporia_triumphalis ---

Rivoire3701_Ceriporia_bresadol --
VS4018 --
Ryvarden21832_Ceriporia_manzan --
Dai24539
ATGGTTAAGCATAGGAGACTAAGAGCTTACAGCCGGATAAGAGGCTTTTC
Dai24541
ATGGTTAAGCATAGGAGACTAAGAGCTTACAGCCGGATAAGAGGCTTTTC
JV1105_12_Ceriporia_occidental --
VS8558Ceriporia_occidentalis --
Dai22445 --
846Dai16368
ATGGTTGAGTATAGGAGGTTAAAAGCCTACAGCCGGATAAGAGGCTCTTC
Dai17951_Ceriporia_aurantiocar --
Miettinen_11701C_viridans --
JV0105_10Ceriporia_aurantiocar --
Yuan5702C_viridans --
858Dai17003 --
Yuan2747_Ceriporia_viridans --
Yuan2744C_viridans --
Li1046C_viridans --
865C_sinoviridans --
871Dai15062 --
Dai7642_Ceriporia_humilis --
Spirin4706_Ceriporia_humilis --
Spirin4944_Ceriporia_sericea --
WCG1547Dai26044ceriporia
ATACATCAGTGTAGGAGGTCAAGAGTCTGCAGCCGGATAAGAGGCTTTTC
ZZW1558Dai27086 --
Miettinen14381_Ceriporia_mpuri --
Miettinen15492_2_Ceriporia_sor --
He6687
ATACATCAGCATAGGAGGTCAAGAGTCTACAACCGGATAAGAGGCTTTTT
ZH53Dai24426
ATACATCAGCATAGGAGGTCAAGAGTCTACAACCGGATAAGAGGCTTTTC
Vlasak0808_30_Ceriporia_punica --
887Dai13376
ACAGCTGCTCGTAGGAGACCAAGAGTCTGCAGCCGGATAAAAGGCTCTTT
WCG1443Dai24998
ACAGCTGCCTGTAGGAGACCAAGAGTTTGCAGCCCGATAAACGGCTCTTC
0108_6Ceriporia_spissa --
Dai19164 --
Dai17937_Ceriporia_bubalinomar --
903Dai12113 --
LZB929Dai25079 --

LX45Dai26988
ACGAGTCAGTATAGGAGAGCAAAAGCTTGCAGCCGGATAAGAGGCTATTC
LX43Dai26986
ACGAGTCAGTATAGGAGAGCAAAAGCTTGCAGCCGGATAAGAGGCTATTC
Dai7759Ceriporia --
Cui8012_Ceriporia_viridans --
GC1704_54Ceriporia_viridans --
Dai23392
ACAATTCAATCTAGGAGGTCAAGAGCTTGCAGCCGGATAAACGGCTTTTC
WCG1585Dai26113Ceriproia
ACAATTCAATCTAGGAGGTCAAGAGCTTGCAGCCGGATAAACGGCTTTTC
Dai18675C_eucalypti --
Dai22034
ATGGCTCAGCATAGGAGGTCAAGAGTCTGCAGCCGGATAAGAGGCTTTTC
JV1008_41JTardaFLORIDAKeys --
Rivoire1161_Ceriporia_pierii --
Dai23499C_pierii
ATACTTCAGAGTAGGAGGTCAAGAGTCTACAGCC----------------
Dai23500
ATACTTCAGAGTAGGAGGTCAAGAGTCTACAGCCGGATAAAAGGCTTTTT
841Dai15899
ATAGCTCAGAATAGGAGGTGAAGAGCCTACAGCCCGATAAGAGGCTATTT
842Dai15904
ATAGCTCAGAATAGGAGGTGAAGAGCCTACAGCCCGATAAGAGGCTATTT
LZB1066xinjiang --
LZB1065xinjiang --
851Dai16779 --
RMJ119sp_Candelabrochaete_sept
ATGGTTGAGAACAGGAGGTTAAAAGCCTGCAGCCGGATAAGAGGCTGTTC
RLG9759spCandelabrochaete_sept --
RLG10478Phanerochaete_allantos --
Dai19118_Ceriporia_spissa --
Dai18486A --
WEI17_024_Ceriporia_mellita --
GC1508_71Ceriporia_mellita
ATAGTTAAGATCAGGAAATCAAAAGCCTGCAGCCTGACAAAAGACTGTTT
GC1608_7_Ceriporia_mellita
ATAGTTAAGATCAGGAAATCAAAAGCCTGCAGCCTGACAAAAGACTGTTT
ZZW1557Dai27085 --
ZZW1554Dai27083 --
Dai8168 --
BR4865C_mellita --
MEL2382688Ceriporia_sp --
Dai8110 --

Cui8097 ---
909Cui6740 ---
W1258Dai24695
ATGGTTAAGATCAGGAGATCAAAAGCCTGCAGCCTGACAAAAGACTCTTC
JV0110_26_Ceriporia_griseoviol ---
896Dai13202 ---
LWY393Dai27053C_griseoviolasce
ACAGCTCAGCTTAGGAGATTAAAAGCCTCCAACCGGATAAGAGGCTTTTT
LWY394DAI27054 ---
FP135015G_pannocinctus ---
L15726SpG_pannocinctus ---
Dai22221 ACACGT-CACACAGGAGATCAAATCCC----------

Dai22633 ---
Dai23260 ACGTG--AGTACAGGAGGTCAAGTCCCTG-------

Dai23626 AAGTGTAAGTACCG------------------------------------
Dai16238G_citrinoalbus ---
1175Dai15293 ---
Dai19547
ACGTCCTGGCACAGGAGGTCAAATCTTTGCAGCCCGATAAGAGGCTGTTT
918063G_africanus ---
918572G_africanus ---
Dai18536A ---
1164Cui17922 ---
Dai22225 ---
1163Dai20655 ---
Yuan4397G_hainanensis ---
1176Dai15268 ----------ACATGAG--------------------------------
1177Dai15259 ---
BZ2896G_thelephoroides ---
1166JV1808_26 ---
Miettinen16992Hapalopilus_ochr ATTTTTG---
GCAGGAAGCGAAAAGCTTGCAGCCTGATAAGCGACTGATT
GC1708_338_Ceriporia_arbuscula
ATGGTCGAGTATAGGAGACCAAAAGCTTACAGCCGGACAAGAGACTCTTC
WCG1555Dai26107Ceriporia ---
GC1708_340_Ceriporia_arbuscula
ATGGTCGAGTATAGGAGACCAAAAGCTTACAGCCGGACAAGAGACTCTTC
WCG1556Dai26109Ceriporia
ATGGTCGAGTATAGGAGACCAAAAGCTTACAGCCGGACAAGAGACTCTTC
883Cui11291
ATGGTCGAGCATAGGAGGTCAAAAGCCTACAGCCGGATAAGAGACTGTTT
HLX320Dai26805

ATGGTCGAGTTTAGGAGGTCAAAAGCCTACAGCCGGATAAGAGACTCTTT

WCG1266Dai24678A

ATGGTCGAGTTTAGGAGGTCAAAAGCTTACAGCCGGATAAGAGACTCTTT

Dai6090_Ceriporia_sulphuricolo --

RLG_11354_Ceriproia_reticulata

ATAGTCGAGTGTAGGAGGTGAAGAGTTTGCAGCCGGATAAGAGGCTTTTC

ZZW1543Dai27072

ATAGTCGAGTGTAGGAGGTGAAGAGTTTGCAGCCGGATAAGAGGCTTTTC

Li1316_Ceriporia_reticulata --

KHL11981Ceriporia_reticulata --

FP110343sp_Candelabrochaete_la

ATAGTCGAGTGTAGGAGGTGAAGAGCTTGCAGCCAGATAAGAGGCTTTTC

Li1045_Ceriporia_reticulata --

ZX136Dai25794ceriporia --

892Dai13400 --

RLG7163Leptoporus_mollis

ACAGCTAAGTTTAGGAGGTCAAGAGCTTGCAGCCGGATAAGAGGCTTTTT

Dai21062Leptoporus_mollis --

Dai20182Leptoporus_submollis

ACAGCTAAGTTTAGGAGGTCAAGAGCTTGCAGCCGGATAAGAGGCTTTTC

Cui18379Leptoporus_submollis

ACAGCTAAGTTTAGGAGGTCAAGAGCTTGCAGCCGGATAAGAGGCTTTTC

Wu1209_46Resiniporus_pseudogil ATTTTC--

CCTCAGGAGGTCAAGAGTCTGCAACCCGACAAGAGATTATTC

BRNM710169Resiniporus_resinasc --

Dai14516Bjerkandera_adusta

ACAGCTGCTTGTAGGAGGTCAAGGCTTTGCAGCCTGATAA----------

Dai21100Bjerkandera_fumosa

ACAGCTGGTTGTAGGAGGTCAAGGCTTTGCAGCCTGATAAACGTTTGTTT

Miettinen16854Ceraceomyces_sp

ATATTCGAGCGTAGGAGGTCAAGAGCTTACAACCAGATAAAAGACTTTTC

Dai10477C_spissa --

855Dai16831 --

882Cui11282 --

Dai24566 --

Yuan5965 --

Dai3204 --

1194CUI9985 --

Dai15205_Ceriporia_albomellea --

Dai15223_Ceriporia_albomellea --

Li1780_Ceriporia_variegata --

Dai19791_Ceriporia_variegata --

Dai19886 --

Dai10833_Ceriporia_crassitunic --

CHWC1506_46Meruliopsis_crassit
CCACCACATGAGGTCTATACGGCGTTGAAGAAGATGTCCGATTCTGACCT

Dai9995_Ceriporia_crassitunica --

Wu1209_58_Meruliopsis_parvispo
CCGCCACACGAGGTCTATACGGCGCTGAAGAAAATGTCAGATTCTGACCT

CHWC1505_129_Meruliopsis_parvi --

Dai21944 --

830Dai18640A
CCTCCACACGAGGTCTACACAGCGTTGAAGAAGATGTCAGATTCAGATCT

GC1704_60_Meruliopsis_taxicola
CCGCCTCATGAAGTCTACACGGCATTGAAGAAGATGTCAGACTCCGACCT

Dai22625
CCACCTCATGAAGTCTACACGGCATTGAAGAAGATGTCAGACTCCGACCT

Dai22636
CCACCTCATGAAGTCTACACGGCATTGAAGAAGATGTCAGACTCCGACCT

Dai21878
CCACCTCATGAAGTCTACACAGCATTGAAGAAGATGTCAGACTCCGACCT

1169Dai17248 --

Wu1708_43_Meruliopsis_leptocys
CCACCACACGAAGTCTACACCGCGCTGAAGAAGATGTCGGATTCTGACCT

Li1011 --

ZX95Dai25742Meruliopsis_leptoc --

WCG1306Dai24733 --

LXL99Dai25816
CCGCCACATGAAGTCTACACCGCGCTGAAGAAGATGTCGGATTCTGACCT

WCG1559Dai26052Meruliopsis
CCACCACACGAAGTTTACACCGCGCTGAAGAAGATGTCGGATTCTGACCT

He7477 --

HLX243Dai26217
CCACCACACGAAGTTTACACCGCGCTGAAGAAGATGTCGGATTCTGACCT

RussiaMW673659Meruliopsis_fagi --

FD278
CCGCCACATGAAGTCTACACAGCGCTGAAGAAGATGTCGGATTCTGATCT

Dai10226_Ceriporia_tarda --

LE247365 --

Dai8173_Meruliopsis_nanlingens --

860Dai17172 --

879Dai13414
CCGCCACATGAGGTCTACACGGCCCTCAAGAAGATGTCGGACTCCGACCT

Li_1704_Meruliopsis_pseudocyst --

833Dai18405
CCGCCACATGAAGTCTATACGGCGTTGAAGAAAATGTCGGATTCCGATCT

HHB_10729_Meruliopsis_albostra
CCGCCACATGAAGTCTATACGGCGTTGAAGAAAATGTCGGATTCCGATCT
Cui6878_Ceriporia_pseudocystid --
869Dai14737 --
876Cui11626 --
1199WEI3388 --
776308_Meruliopsis_cystidiata --
ICN139059_Meruliopsis_cystidia --
HHB15692Ceraceomyces_serpens
CCTCCTCATGAAGTCTATACTGCTCTCAAGAAGATGTCGGATTCTGATCT
HHB_15629_Sp_Ceriporiopsis_ane
CCCCCACATGAGGTCTACACCGCGCTCAAGAAAATGTCAGATTCCGACCT
AJ185Trametopsis_cervina
CCCCCGCACGAAGTTTACACTGCGCTGAAGAAGATGTCGGACTCCGACTT
FD9Irpex_lacteus
CCTCCACATGAAGTGTACACCGCTCTGAAGAAGATGTCAGACTCAGATCT
908Dai11230
CCTCCACATGAAGTGTACACCGCCCTGAAGAAGATGTCAGA---------
FP55521TEmmia_lacerata
CCTCCTCATGAAGTATACACGGCGTTGAAGAAGATGTCAGACTCTGATCT
PBU0048Ceriporia_cystidiata --
MZ340C_laceratаT --
Dai21940
CCTCCTCATGAAGTATATACGGCATTGAAGAAGATGTCAGATTCTGATCT
847Dai16433
CCTCCTCATGAAGTATATACGGCATTGAAGAAGATGTCAGACTCTGATCT
MarcinEmmia_latemarginatus --
Meijer3729Hydnopolyporus_fimbr --
RLG13408Phanerochaete_sp
CCTCCGCAGGAAGTCTACACCACTCTGAAGAAGATGGTCGATGCAGACCT
WHC1381Flavodon_flavus
CCTCCACACGAAGTGTATACGGCACTGAAGAAGATGTCAGACTCAGATCT
GB1833Phlebia_albida
CCTCCACATGAAGTTTACACTGCTCTGAAGAAGATGTCAGATGCAGATCT
T407Phlebia_nitidula
CCTCCACATGAAGTTTACACTGCTCTGAAGAAGATGTCAGATGCAGATCT
HHB6988Phanerochaete_exilis
CCCCCAACAGAAGTCTATACCGCGTTGAAGAAGATGTCGGACGGCGATCT
HHB8509Phanerochaetella_xeroph --
PBU0051Macrohyporia_dictyopora --
HHB11463Phanerochaete_sp
CCTCCGACAGAAGTGTATACCGCGTTGAAGAAGATGTCGGACGGCGATTT
FP102382Byssomerulius_corium
CCTCCTCACGAGGTCTACACGGCTCTCAAGAAGATGTCAGATGCGGATCT

FP102165Efibula_americana
CCTCCCCACGAAGTTTACACCGCTCTCAAGAAGATGTCGGATGCAGATCT
Murdoch90Ceriporia_torpida ---
Rivoire4413_Ceriporia_purpurea ---
Kout_18_Ceriporia_triumphalis ---
Rivoire3701_Ceriporia_bresadol ---
VS4018 ---
Ryvarden21832_Ceriporia_manzan ---
Dai24539
CCCCCACACGAAGTCTATACTGCGCTGAAGAAGATGTCCGATTCTGACCT
Dai24541
CCCCCACACGAAGTCTATACTGCGCTGAAGAAGATGTCCGATTCTGACCT
JV1105_12_Ceriporia_occidental ---
VS8558Ceriporia_occidentalis ---
Dai22445 ---
846Dai16368
CCTCCGCACGAGGTCTATACTGCGCTGAAGAAGATGTCCGATTCTGACCT
Dai17951_Ceriporia_aurantiocar ---
Miettinen_11701C_viridans ---
JV0105_10Ceriporia_aurantiocar ---
Yuan5702C_viridans ---
858Dai17003 ---
Yuan2747_Ceriporia_viridans ---
Yuan2744C_viridans ---
Li1046C_viridans ---
865C_sinoviridans ---
871Dai15062 ---
Dai7642_Ceriporia_humilis ---
Spirin4706_Ceriporia_humilis ---
Spirin4944_Ceriporia_sericea ---
WCG1547Dai26044ceriporia
CCTCCACATGAAGTCTACACGACGCTGAAGAAGATGTCCGATTCTGATCT
ZZW1558Dai27086 ---
Miettinen14381_Ceriporia_mpuri ---
Miettinen15492_2_Ceriporia_sor ---
He6687
CCTCCCCATGAAGTCTACACGACGCTGAAGAAGATGTCCGATTCTGATCT
ZH53Dai24426
CCTCCCCATGAAGTCTACACGACGCTGAAGAAGATGTCCGATTCTGATCT
Vlasak0808_30_Ceriporia_punica ---
887Dai13376
CCCCCGCACGAGGTCTATACTGCCTTGAAGAAGATGTCGGAGGCTGACTT
WCG1443Dai24998
CCCCCGCACGAGGTTTACACTGCCTTGAAGAAGATGTCGGAGGCTGACTT

0108_6Ceriporia_spissa --
Dai19164 --
Dai17937_Ceriporia_bubalinomar --
903Dai12113 --
LZB929Dai25079 --
LX45Dai26988
CCTCCGCAAGAGGTCTATACCGCATTGAAGAAGATGTCTGACGCCGATCT
LX43Dai26986
CCTCCGCAAGAGGTCTATACCGCATTGAAGAAGATGTCTGACGCCGATCT
Dai7759Ceriporia --
Cui8012_Ceriporia_viridans --
GC1704_54Ceriporia_viridans --
Dai23392
CCTCCGCAAGAAGTCTATACTGCGCTCAAGAAGATGTCTGATGCCGACCT
WCG1585Dai26113Ceriproia
CCTCCGCAAGAAGTCTATACCGCGCTCAAGAAGATGTCTGATGCCGACCT
Dai18675C_eucalypti --
Dai22034
CCTCCGCATGAGGTCTACACTGCGCTGAAGAAAATGTCTGACTCTGACCT
JV1008_41JTardaFLORIDAKeys --
Rivoire1161_Ceriporia_pierii --
Dai23499C_pierii --
Dai23500
CCTCCACAGGAAGTCTACACGACATTGAAGAAGATGTCCGATTCCGACCT
841Dai15899
CCCCCCCACGAAGTCTATACCGCGCTGAAGAAGATGTCCGACGCTGACCT
842Dai15904
CCCCCCCACGAAGTCTATACCGCGCTGAAGAAGATGTCCGACGCTGACCT
LZB1066xinjiang --
LZB1065xinjiang --
851Dai16779 --
RMJ119sp_Candelabrochaete_sept
CCTCCGCACGAGGTGTATACAGCACTGAAGAAAATGTCTGATGCTGACCT
RLG9759spCandelabrochaete_sept --
RLG10478Phanerochaete_allantos --
Dai19118_Ceriporia_spissa --
Dai18486A --
WEI17_024_Ceriporia_mellita --
GC1508_71Ceriporia_mellita
CCTCCGCATGAAGTTTACACCGCGCTGAAGAAGATGTCAGATTCCGACTT
GC1608_7_Ceriporia_mellita
CCTCCGCATGAAGTTTACACAGCGCTGAAGAAGATGTCAGATTCCGACTT
ZZW1557Dai27085 --
ZZW1554Dai27083 --

Dai8168 --
BR4865C_mellita --
MEL2382688Ceriporia_sp --
Dai8110 --
Cui8097 --
909Cui6740 --
W1258Dai24695
CCTCCGCATGAAGTTTACACTGCGCTTAAGAAGATGTCAGATTCCGACTT
JV0110_26_Ceriporia_griseoviol --
896Dai13202 --
LWY393Dai27053C_griseoviolasce
CCTCCACATGAAGTCTACACTGCTTTGAAGAAGATGTCCGATGCTGATCT
LWY394DAI27054 --
FP135015G_pannocinctus --
L15726SpG_pannocinctus --
Dai22221 --
Dai22633 --
Dai23260 --
Dai23626 --
Dai16238G_citrinoalbus --
1175Dai15293 --
Dai19547
CCACCACATGAAGTGTACACGGCTCTGAAGAAGATGTCAGACGGTGATCT
918063G_africanus --
918572G_africanus --
Dai18536A --
1164Cui17922 --
Dai22225 --
1163Dai20655 --
Yuan4397G_hainanensis --
1176Dai15268 --
1177Dai15259 --
BZ2896G_thelephoroides --
1166JV1808_26 --
Miettinen16992Hapalopilus_ochr
CCCCCGCACGAAGTATACACCGCGCTTAAAAAGATATCAGACTCTGACCT
GC1708_338_Ceriporia_arbuscula
CCTCCACATGAGGTTTACACCGCTCTGAAGAAGATGTCTGATTCCGATCT
WCG1555Dai26107Ceriporia --
GC1708_340_Ceriporia_arbuscula
CCTCCACATGAGGTTTACACCGCTCTGAAGAAGATGTCTGATTCCGATCT
WCG1556Dai26109Ceriporia
CCTCCACATGAGGTTTACACCGCTCTGAAGAAGATGTCTGATTCCGATCT
883Cui11291

CCTCCGCATGAGGTTTACACCGCTTTGAAGAAGATGTCAGATTCAGATTT
HLX320Dai26805
CCTCCACATGAGGTTTACACCGCTTTGAAGAAGATGTCCGATTCCGATTT
WCG1266Dai24678A
CCTCCACATGAGGTTTACACCGCTTTGAAGAAGATGTCCGATTCCGATTT
Dai6090_Ceriporia_sulphuricolo ---
RLG_11354_Ceriproia_reticulata
CCTCCGCATGAGGTCTACACCGCATTAAAGAAGATGTCGGACTCTGACCT
ZZW1543Dai27072
CCTCCGCATGAGGTCTACACCGCATTAAAGAAGATGTCGGACTCTGACCT
Li1316_Ceriporia_reticulata ---
KHL11981Ceriporia_reticulata ---
FP110343sp_Candelabrochaete_la
CCTCCGCATGAGGTCTACACCACATTGAAGAAAATGTCGGATTCCGACCT
Li1045_Ceriporia_reticulata ---
ZX136Dai25794ceriporia ---
892Dai13400 ---
RLG7163Leptoporus_mollis
CCTCCTCATGAAGTCTACACTGCATTGAAGAAAATGTCTGACTCTGACCT
Dai21062Leptoporus_mollis ---
Dai20182Leptoporus_submollis
CCTCCTCATGAAGTCTACACTGCATTGAAGAAAATGTCTGACTCTGACCT
Cui18379Leptoporus_submollis
CCTCCTCATGAAGTCTACACTGCATTGAAGAAAATGTCTGACTCTGACCT
Wu1209_46Resiniporus_pseudogil
CCACCTCATGAAGTCTACACTGCCCTGAAGAAGATGTCTGATTCCGACCT
BRNM710169Resiniporus_resinasc ---
Dai14516Bjerkandera_adusta ---------GAAGACTAT-------------------------------
Dai21100Bjerkandera_fumosa CCTCCCCACGAGGTCTACCACG----------------------
------
Miettinen16854Ceraceomyces_sp
CCTCCTCACGAAGTCTACACTGCTCTCAAGAAGATGTCGGACGCCGATCT
Dai10477C_spissa ---
855Dai16831 ---
882Cui11282 ---
Dai24566 ---
Yuan5965 ---
Dai3204 ---
1194CUI9985 ---

Dai15205_Ceriporia_albomellea ---
Dai15223_Ceriporia_albomellea ---
Li1780_Ceriporia_variegata ---

Dai19791_Ceriporia_variegata --
Dai19886 --
Dai10833_Ceriporia_crassitunic --
CHWC1506_46Meruliopsis_crassit
CCATCTCCTTGGCCTCTCTGATGAGTATGCGCGCCCTGAATGGATGATCC
Dai9995_Ceriporia_crassitunica --
Wu1209_58_Meruliopsis_parvispo
TCATCTCCTCGGTCTCTCCGACGAGTATGCGCGGCCTGAGTGGATGATCC
CHWC1505_129_Meruliopsis_parvi --
Dai21944 --
830Dai18640A
TCACCTTCTCGGCCTTTCCGATGAGTACGCGCGGCCTGAGTGGATGATCC
GC1704_60_Meruliopsis_taxicola
TCATCTCCTCGGCCTTTCCGASGAGTATGCGCGACCGGAGTGGATGATTC
Dai22625
TCATCTCCTCGGCCTTTCCGACGAGTATGCGCGACCGGAGTGGATGATTC
Dai22636
TCATCTCCTCGGCCTTTCCGACGAGTATGCGCGACCGGAGTGGATGATTC
Dai21878
TCATCTCCTCGGCCTTTCCGACGAGTATGCGCGACCGGAGTGGATGATTC
1169Dai17248 --
Wu1708_43_Meruliopsis_leptocys
TCACCTCCTTGGTCTTTCCGATGAGTACGCGCGGCCTGAATGGATGATCC
Li1011 --
ZX95Dai25742Meruliopsis_leptoc --
WCG1306Dai24733 --
LXL99Dai25816
TCACCTCCTTGGTCTGTCCGATGAGTACGCGCGGCCCGAATGGATGATCC
WCG1559Dai26052Meruliopsis
TCATCTCCTTGGTCTTTCCGATGAGTATGCGCGGCCTGAATGGATGATCC
He7477 --
HLX243Dai26217
CCATCTCCTTGGTCTTTCCGATGAGTATGCGCGGCCTGAATGGATGATCC
RussiaMW673659Meruliopsis_fagi --
FD278
ACATCTTCTTGGACTCTCCGACGAGTACGCACGCCCTGAGTGGATGATCC
Dai10226_Ceriporia_tarda --
LE247365 --
Dai8173_Meruliopsis_nanlingens --
860Dai17172 --
879Dai13414
TCATCTCCTTGGCCTCTCCGATGAATACGCGCGTCCTGAATGGATGATCC
Li_1704_Meruliopsis_pseudocyst --
833Dai18405

CCATCTCCTTGGTCTTTCCGATGAGTACGCGAGGCCGGAGTGGATGATCC

HHB_10729_Meruliopsis_albostra

CCATCTCCTTGGTCTTTCCGATGAGTACGCGAGGCCGGAGTGGATGATCC

Cui6878_Ceriporia_pseudocystid --

869Dai14737 --

876Cui11626 --

1199WEI3388 --

776308_Meruliopsis_cystidiata --

ICN139059_Meruliopsis_cystidia --

HHB15692Ceraceomyces_serpens

TCACTTACTCGGCCTTTCGGATGAGTACGCTCGGCCGGAGTGGATGATCC

HHB_15629_Sp_Ceriporiopsis_ane

TCACCTTCTTGGGCTCTCAGACGAATATGCTCGCCCGGAGTGGATGATTC

AJ185Trametopsis_cervina

ACACCTTCTAGGGCTTTCGGATGAGTATGCCCGCCCTGAGTGGATGATTC

FD9Irpex_lacteus

TCATCTTCTCGGCCTCTCAGACGAGTACGCACGTCCAGAGTGGATGATCC

908Dai11230 --

FP55521TEmmia_lacerata

TCATCTTCTCGGTCTATCAGACGAGTATGCACGACCTGAATGGATGATCT

PBU0048Ceriporia_cystidiata --

MZ340C_lacerataT --

Dai21940

TCATCTTCTTGGTCTATCAGACGAGTATGCACGTCCTGAATGGATGATCC

847Dai16433

TCATCTTCTCGGTCTATCAGACGAGTATGCACGTCCTGAATGGATGATCC

MarcinEmmia_latemarginatus --

Meijer3729Hydnopolyporus_fimbr --

RLG13408Phanerochaete_sp

TCACCTTTTGGGTCTATCTGATGAATATGCTCGTCCGGAGTGGATGATCT

WHC1381Flavodon_flavus

TCATCTCCTTGGTCTTTCGGACGAATATGCACGTCCGGAGTGGATGATCC

GB1833Phlebia_albida

TCATCTCCTTGGCCTCTCGGATGAGTATGCTCGCCCGGAGTGGATGATCT

T407Phlebia_nitidula

TCATCTCCTTGGCCTCTCAGATGAGTATGCTCGCCCGGAGTGGATGATCT

HHB6988Phanerochaete_exilis

CCACCTCTTGGGTCTCTCGGACGAGTATGCACGTCCAGAGTGGATGATTT

HHB8509Phanerochaetella_xeroph --

PBU0051Macrohyporia_dictyopora --

HHB11463Phanerochaete_sp

TCATCTCTTGGGTCTCTCAGACGAGTATGCTCGCCCGGAGTGGATGATTT

FP102382Byssomerulius_corium

CCATCTGCTCGGGCTTTCAGATGAATATGCTCGCCCCGAGTGGATGATCC

FP102165Efibula_americana
CCACCTTCTAGGCCTGTCGGATGAGTATGCTCGTCCTGAGTGGATGATTC
Murdoch90Ceriporia_torpida --
Rivoire4413_Ceriporia_purpurea --
Kout_18_Ceriporia_triumphalis --
Rivoire3701_Ceriporia_bresadol --
VS4018 --
Ryvarden21832_Ceriporia_manzan --
Dai24539
TCATGTCCTTGGCCTCTCTGACGAGTATGCGCGCCCCGAATGGATGATCC
Dai24541
TCATGTCCTTGGCCTCTCTGACGAGTATGCGCGCCCCGAATGGATGATCC
JV1105_12_Ceriporia_occidental --
VS8558Ceriporia_occidentalis --
Dai22445 --
846Dai16368
CCATGTTCTTGGCCTCTCTGACGAATATGCACGCCCCGAGTGGATGATTC
Dai17951_Ceriporia_aurantiocar --
Miettinen_11701C_viridans --
JV0105_10Ceriporia_aurantiocar --
Yuan5702C_viridans --
858Dai17003 --
Yuan2747_Ceriporia_viridans --
Yuan2744C_viridans --
Li1046C_viridans --
865C_sinoviridans --
871Dai15062 --
Dai7642_Ceriporia_humilis --
Spirin4706_Ceriporia_humilis --
Spirin4944_Ceriporia_sericea --
WCG1547Dai26044ceriporia
TCATCTCCTTGGCCTCTCAGACGAATACGCGCGTCCCGAATGGATGATCT
ZZW1558Dai27086 --
Miettinen14381_Ceriporia_mpuri --
Miettinen15492_2_Ceriporia_sor --
He6687
TCATCTCCTTGGCCTCTCAGACGAATACGCGCGTCCCGAATGGATGATTT
ZH53Dai24426
TCATCTCCTTGGCCTCTCAGACGAATACGCGCGTCCCGAATGGATGATTT
Vlasak0808_30_Ceriporia_punica --
887Dai13376
GCACCTCCTCGGTCTCTCTGATGAATACGCCCGTCCCGAGTGGATGATCC
WCG1443Dai24998
GCATCTCCTCGGTCTCTCTGATGAATACGCCCGTCCCGAGTGGATGATCC

0108_6Ceriporia_spissa --
Dai19164 --
Dai17937_Ceriporia_bubalinomar --
903Dai12113 --
LZB929Dai25079 --
LX45Dai26988
TCACCTTCTCGGTCTCTCCGACGAATATGCGCGACCTGAGTGGATGATTT
LX43Dai26986
TCACCTTCTCGGTCTCTCCGACGAATATGCGCGACCTGAGTGGATGATTT
Dai7759Ceriporia --
Cui8012_Ceriporia_viridans --
GC1704_54Ceriporia_viridans --
Dai23392
TCATCTTCTTGGTCTCTCTGACGAGTATGCGCGCCCGGAATGGATGATCT
WCG1585Dai26113Ceriproia
TCATCTTCTTGGTCTCTCTGACGAGTATGCGCGCCCGGAATGGATGATCT
Dai18675C_eucalypti --
Dai22034
CCATCTTCTCGGTCTCTCCGACGAATACGCCCGACCAGAGTGGATGATTC
JV1008_41JTardaFLORIDAKeys --
Rivoire1161_Ceriporia_pierii --
Dai23499C_pierii --
Dai23500
CCATTTGCTTGGTCTCTCTGACGAATACGCACGACCGGAGTGGATGATCT
841Dai15899
GCACCTACTCGGACTTTCTGATGAGTATGCGCGCCCCGAATGGATGATCC
842Dai15904
GCACCTACTCGGACTTTCTGATGAGTATGCGCGCCCCGAATGGATGATCC
LZB1066xinjiang --
LZB1065xinjiang --
851Dai16779 --
RMJ119sp_Candelabrochaete_sept
GCATCTCCTCGGCCTCTCTGACGAGTACGCGCGTCCCGAGTGGATGATTC
RLG9759spCandelabrochaete_sept --
RLG10478Phanerochaete_allantos --
Dai19118_Ceriporia_spissa --
Dai18486A --
WEI17_024_Ceriporia_mellita --
GC1508_71Ceriporia_mellita
ACATCTGCTTGGCCTTTCTGACGAATATGCGCGCCCCGAGTGGATGATTC
GC1608_7_Ceriporia_mellita
ACATCTGCTTGGCCTTTCTGACGATTACGCGCGCCCCGAGTGGATGATTT
ZZW1557Dai27085 --
ZZW1554Dai27083 --

Dai8168 --
BR4865C_mellita --
MEL2382688Ceriporia_sp --
Dai8110 --
Cui8097 --
909Cui6740 --
W1258Dai24695
ACATCTACTTGGCCTTTCTGACGAGTATGCGCGCCCCGAGTGGATGATCC
JV0110_26_Ceriporia_griseoviol --
896Dai13202 --
LWY393Dai27053C_griseoviolasce
GCATCTATTGGGCCTTTCTGACGAATATGCGCGACCGGAATGGATGATTC
LWY394DAI27054 --
FP135015G_pannocinctus --
L15726SpG_pannocinctus --
Dai22221 --
Dai22633 --
Dai23260 --
Dai23626 --
Dai16238G_citrinoalbus --
1175Dai15293 --
Dai19547
CCATCTCCTCGGCCTCTCGGACGAGTATGCTCGTCCCGAGTGGATGATCC
918063G_africanus --
918572G_africanus --
Dai18536A --
1164Cui17922 --
Dai22225 --
1163Dai20655 --
Yuan4397G_hainanensis --
1176Dai15268 --
1177Dai15259 --
BZ2896G_thelephoroides --
1166JV1808_26 --
Miettinen16992Hapalopilus_ochr
ACATTTACTGGGCTTGTCTGACGAGTATGCTCGTCCAGAGTGGATGATTC
GC1708_338_Ceriporia_arbuscula
GCACCTCCTTGGGCTGTCTGACGAATATGCACGTCCCGAGTGGATGATCT
WCG1555Dai26107Ceriporia --
GC1708_340_Ceriporia_arbuscula
GCACCTCCTTGGGCTGTCTGACGAATATGCACGTCCCGAGTGGATGATCT
WCG1556Dai26109Ceriporia
GCACCTCCTTGGGCTGTCTGACGAATATGCACGTCCCGAGTGGATGATCT
883Cui11291

GCACCTTCTTGGTCTCTCTGACGAGTACGCACGCCCCGAGTGGATGATCT
HLX320Dai26805
GCACCTCCTCGGGCTGTCTGACGAGTATGCACGCCCCGAGTGGATGATAT
WCG1266Dai24678A
GCACCTTCTTGGCCTGTCTGACGAGTATGCACGCCCCGAGTGGATGATAT
Dai6090_Ceriporia_sulphuricolo --
RLG_11354_Ceriproia_reticulata
GCACCTCCTCGGTCTCTCTGATGAGTACGCGCGCCCGGAATGGATGATCT
ZZW1543Dai27072
GCACCTCCTCGGTCTCTCTGATGAGTACGCGCGCCCGGAATGGATGATCT
Li1316_Ceriporia_reticulata --
KHL11981Ceriporia_reticulata --
FP110343sp_Candelabrochaete_la ACATCTCCTCGG?CTCTCTGATGAGTAC-------------
----
Li1045_Ceriporia_reticulata --
ZX136Dai25794ceriporia --
892Dai13400 --
RLG7163Leptoporus_mollis
CCACCTTCTTGGCCTCTCTGACGAGTACGCGCGCCCGGAGTGGATGATTC
Dai21062Leptoporus_mollis --
Dai20182Leptoporus_submollis
CCACCTGCTTGGCCTCTCTGACGAGTACGCGCGCCCGGAGTGGATGATCC
Cui18379Leptoporus_submollis
CCACCTGCTTGGCCTCTCTGACGAGTACGCGCGCCCGGAGTGGATGATCC
Wu1209_46Resiniporus_pseudogil
CCACCTTTTGGGGCTTTCGGATGAGTATGCTCGGCCTGAGTGGATGATCC
BRNM710169Resiniporus_resinasc --
Dai14516Bjerkandera_adusta --
Dai21100Bjerkandera_fumosa --
Miettinen16854Ceraceomyces_sp
GCATCTACTTGGACTTTCAGACGAGTATGCTCGCCCGGAATGGATGATCT
Dai10477C_spissa --
855Dai16831 --
882Cui11282 --
Dai24566 --
Yuan5965 --
Dai3204 --
1194CUI9985 --

Dai15205_Ceriporia_albomellea --
Dai15223_Ceriporia_albomellea --
Li1780_Ceriporia_variegata --
Dai19791_Ceriporia_variegata --

Dai19886 ---
Dai10833_Ceriporia_crassitunic ---
CHWC1506_46Meruliopsis_crassit
TTACAGTTCTCCCTGTTCCCCCGCCTCCCGTACGACCTAGTATAGCAGTG
Dai9995_Ceriporia_crassitunica ---
Wu1209_58_Meruliopsis_parvispo
TCACTGTACTCCCTGTCCCCCCGCCTCCGGTGCGACCCAGTATAGCAGTC
CHWC1505_129_Meruliopsis_parvi ---
Dai21944 ---
830Dai18640A
TCACGGTCCTTCCTGTTCCCCCGCCTCCCGTGCGACCTAGTATAGCAGTG
GC1704_60_Meruliopsis_taxicola
TGACTGTCCTCCCTGTACCTCCTCCTCCCGTGCGACCCAGTATTGCAGTG
Dai22625
TGACTGTCCTCCCTGTACCTCCTCCTCCCGTGCGACCCAGTATTGCAGTG
Dai22636
TGACTGTCCTCCCTGTACCTCCTCCTCCCGTGCGACCCAGTATTGCAGTG
Dai21878
TGACAGTCCTCCCTGTACCTCCTCCTCCCGTGCGACCCAGTATTGCAGTC
1169Dai17248 ---
Wu1708_43_Meruliopsis_leptocys
TCACAGTCCTTCCCGTCCCTCCCCCTCCTGTGCGACCCAGTATAGCGGTG
Li1011 ---
ZX95Dai25742Meruliopsis_leptoc ---
WCG1306Dai24733 ---
LXL99Dai25816
TCACGGTCCTTCCTGTTCCTCCTCCTCCTGTGCGACCCAGCATAGCAGTG
WCG1559Dai26052Meruliopsis
TCACGGTTCTCCCCGTTCCTCCTCCTCCTGTGCGACCCAGCATAGCAGTG
He7477 ---
HLX243Dai26217
TCACGGTTCTCCCCGTTCCTCCTCCTCCTGTGCGACCCAGCATAGCAGTG
RussiaMW673659Meruliopsis_fagi ---
FD278
TCACGGTTCTCCCTGTCCCACCGCCTCCTGTTCGACCCAGTATCGCAGTG
Dai10226_Ceriporia_tarda ---
LE247365 ---
Dai8173_Meruliopsis_nanlingens ---
860Dai17172 ---
879Dai13414
TGACGGTCCTCCCTGTTCCTCCTCCTCCTGTGCGACCCAGTATAGCGGTG
Li_1704_Meruliopsis_pseudocyst ---
833Dai18405
TGACGGTCCTCCCTGTACCTCCCCCTCCTGTGCGACCCAGTATAGCGGTG

HHB_10729_Meruliopsis_albostra
TGACGGTCCTCCCTGTACCTCCCCCTCCTGTGCGACCCAGTATAGCGGTG
Cui6878_Ceriporia_pseudocystid --
869Dai14737 --
876Cui11626 --
1199WEI3388 --
776308_Meruliopsis_cystidiata --
ICN139059_Meruliopsis_cystidia --
HHB15692Ceraceomyces_serpens
TGACTGTCCTTCCCGTGCCCCCTCCACCTGTGCGTCCTAGTATAGCTGTA
HHB_15629_Sp_Ceriporiopsis_ane
TCACCGTTTTACCAGTCCCGCCTCCGCCTGTTCGTCCTAGTATCGCGGTG
AJ185Trametopsis_cervina
TGACGGTTCTCCCCGTCCCGCCCCCTCCTGTGCGCCCCAGTATTGCGGTG
FD9Irpex_lacteus TCACCGTTCTACCCGTGC-------------------------------
-
908Dai11230 --
FP55521TEmmia_lacerata
TGACCGTCCTCCCTGTTCCCCCTCCTCCCGTACGCCCGAGTATAGCGGTG
PBU0048Ceriporia_cystidiata --
MZ340C_lacerataT --
Dai21940
TGACCGTCCTCCCCGTTCCCCCTCCACCCGTACGCCCGAGTATAGCGGTC
847Dai16433
TGACCGTCCTCCCCGTTCCTCCTCCACCCGTACGCCCGAGTATAGCGGTC
MarcinEmmia_latemarginatus --
Meijer3729Hydnopolyporus_fimbr --
RLG13408Phanerochaete_sp
TGACTGTTCTTCCTGTACCGCCTCCTCCTGTGCGCCCAAGTATAGCTGTG
WHC1381Flavodon_flavus
TTACGGTCCTTCCTGTGCCTCCACCTCCCGTTCGCCCCAGTATAGCGGTC
GB1833Phlebia_albida
TGACTGTGCTCCCCGTACCTCCTCCTCCGGTCCGTCCG-----------
T407Phlebia_nitidula
TGACTGTGCTCCCCGTACCTCCTCCCCCGGTCCGTCCGAGCATAGCCGTG
HHB6988Phanerochaete_exilis
TGACTGTTCTGCCCGTTCCCCCGCCTCCTGTCCGCCCAAGTATAGCTGTC
HHB8509Phanerochaetella_xeroph --
PBU0051Macrohyporia_dictyopora --
HHB11463Phanerochaete_sp
TGACCGTTTTGCCGGTCCCTCCCCCTCCTGTCCGCCCAAGTATAGCTGTC
FP102382Byssomerulius_corium
TGACAGTCCTTCCTGTCCCACCCCCGCCTGTGCGCCCTAGTATAGCCGTG
FP102165Efibula_americana

TTACCGTTCTCCCCGTGCCTCCTCCTCCTGTGCGACCAAGCATAGCGGTA
Murdoch90Ceriporia_torpida --
Rivoire4413_Ceriporia_purpurea --
Kout_18_Ceriporia_triumphalis --
Rivoire3701_Ceriporia_bresadol --
VS4018 --
Ryvarden21832_Ceriporia_manzan --
Dai24539
TGACGGTATTGCCTGTTCCTCCCCACCCGTGCGCCCGAGTATAGCTGTT
Dai24541
TGACGGTATTGCCTGTTCCTCCCCACCCGTGCGCCCGAGTATAGCTGTT
JV1105_12_Ceriporia_occidental --
VS8558Ceriporia_occidentalis --
Dai22445 --
846Dai16368
TAACGGTATTGCCTGTTCCTCCACCACCCGTGCGCCCCAGTATAGCTGTT
Dai17951_Ceriporia_aurantiocar --
Miettinen_11701C_viridans --
JV0105_10Ceriporia_aurantiocar --
Yuan5702C_viridans --
858Dai17003 --
Yuan2747_Ceriporia_viridans --
Yuan2744C_viridans --
Li1046C_viridans --
865C_sinoviridans --
871Dai15062 --
Dai7642_Ceriporia_humilis --
Spirin4706_Ceriporia_humilis --
Spirin4944_Ceriporia_sericea --
WCG1547Dai26044ceriporia
TGACCGTCTTGCCTGTTCCACCGCCCCCTGTGCGCCCTAGTATAGCCGTG
ZZW1558Dai27086 --
Miettinen14381_Ceriporia_mpuri --
Miettinen15492_2_Ceriporia_sor --
He6687
TGACCGTCTTGCCCGTTCCACCGCCCCCCGTGCGTCCCAGTATCGCTGTG
ZH53Dai24426
TGACCGTCTTGCCCGTTCCACCGCCCCCCGTGCGTCCCAGTATCGCTGTG
Vlasak0808_30_Ceriporia_punica --
887Dai13376
TGACCGTTCTACCCGTCCCGCCGCCACCCGTTCGTCCTAGCATAGCCGTG
WCG1443Dai24998
TGACCGTTTTACCCGTCCCGCCGCCACCCGTTCGTCCTAGCATAGCGGTG
0108_6Ceriporia_spissa --

Dai19164 --
Dai17937_Ceriporia_bubalinomar --
903Dai12113 --
LZB929Dai25079 --
LX45Dai26988
TGACGGTCCTGCCAGTTCCCCCTCCTCCCGTTCGTCCGAGTATCGCTGTC
LX43Dai26986
TGACGGTCCTGCCAGTTCCCCCTCCTCCCGTTCGTCCGAGTATCGCTGTC
Dai7759Ceriporia --
Cui8012_Ceriporia_viridans --
GC1704_54Ceriporia_viridans --
Dai23392
TGACAGTTCTGCCAGTACCCCCTCCTCCTGTGCGGCCCAGTATCGCTGTT
WCG1585Dai26113Ceriproia
TGACAGTTCTGCCAGTACCCCCTCCTCCTGTGCGGCCCAGTATCGCTGTT
Dai18675C_eucalypti --
Dai22034
TGACTGTGTTACCAGTTCCTCCTCCACCCGTACGCCCTAGTATAGCTGTT
JV1008_41JTardaFLORIDAKeys --
Rivoire1161_Ceriporia_pierii --
Dai23499C_pierii --
Dai23500
TGACCGTCTTGCCCGTGCCTCCGCCTCCTGTGCGACCTAGTATAGCTGTA
841Dai15899
TCACAGTCCTGCCTGTTCCCCCTCCACCAGTGCGTCCAAGTATAGCTGTC
842Dai15904
TCACAGTCCTGCCTGTTCCCCCTCCACCAGTGCGTCCAAGTATAGCTGTC
LZB1066xinjiang --
LZB1065xinjiang --
851Dai16779 --
RMJ119sp_Candelabrochaete_sept
TGACCGTACTACCCGTCCCTCCTCCTCCCGTGCGTCCC------------
RLG9759spCandelabrochaete_sept --
RLG10478Phanerochaete_allantos --
Dai19118_Ceriporia_spissa --
Dai18486A --
WEI17_024_Ceriporia_mellita --
GC1508_71Ceriporia_mellita
TGACTGTTCTCCCTGTCCCTCCCCCTCCTGTCCGTCCGAGTATAGCTGTT
GC1608_7_Ceriporia_mellita
TGACYGTTCTCCCTGTCCCTCCCCCTCCTGTCCGTCCGAGTATAGCTGTT
ZZW1557Dai27085 --
ZZW1554Dai27083 --
Dai8168 --

BR4865C_mellita --
MEL2382688Ceriporia_sp --
Dai8110 --
Cui8097 --
909Cui6740 --
W1258Dai24695
TGACTGTTCTCCCTGTCCCTCCCCCTCCCGTTCGTCCCAGTATAGCTGTT
JV0110_26_Ceriporia_griseoviol --
896Dai13202 --
LWY393Dai27053C_griseoviolasce
TGACCGTTTTGCCCGTTCCACCTCCTCCCGTGCGGCCGAGCATAGCCGTA
LWY394DAI27054 --
FP135015G_pannocinctus --
L15726SpG_pannocinctus --
Dai22221 --
Dai22633 --
Dai23260 --
Dai23626 --
Dai16238G_citrinoalbus --
1175Dai15293 --
Dai19547
TTACAGTCCTTCCTGTTCCACCACCTCCTGTTCGTCCCAGTATAGCGGTT
918063G_africanus --
918572G_africanus --
Dai18536A --
1164Cui17922 --
Dai22225 --
1163Dai20655 --
Yuan4397G_hainanensis --
1176Dai15268 --
1177Dai15259 --
BZ2896G_thelephoroides --
1166JV1808_26 --
Miettinen16992Hapalopilus_ochr
TGMCCGTGCTTCCAGTGCCACCTCCGCCGGTTCGCCCGAGTATCGCTGTG
GC1708_338_Ceriporia_arbuscula
TGACGGTCCTACCGGTACCGCCTCCGCCCGTGCGCCCGAGTATAGCAGTG
WCG1555Dai26107Ceriporia --
GC1708_340_Ceriporia_arbuscula
TGACGGTCCTACCGGTACCGCCTCCGCCCGTGCGCCCGAGTATAGCAGTG
WCG1556Dai26109Ceriporia
TGACGGTCCTACCGGTACCGCCTCCGCCCGTGCGCCCGAGTATAGCAGTG
883Cui11291
TAACGGTCCTGCCGGTACCACCACCGCCTGTGCGCCCGAGTATAGCAGTC

HLX320Dai26805
TGACGGTCTTACCCGTACCGCCTCCGCCCGTGCGCCCGAGTATAGCAGTC
WCG1266Dai24678A
TGACGGTCTTACCCGTACCGCCTCCACCCGTGCGCCCGAGTATAGCAGTC
Dai6090_Ceriporia_sulphuricolo --
RLG_11354_Criproia_reticulata
TGACCGTCTTGCCGGTACCTCCTCCGCCCGTGCGACCCAGTATAGCCGTC
ZZW1543Dai27072
TGACCGTCTTGCCAGTACCTCCTCCGCCCGTGCGACCCAGTATAGCCGTC
Li1316_Ceriporia_reticulata --
KHL11981Ceriporia_reticulata --
FP110343sp_Candelabrochaete_la --
Li1045_Ceriporia_reticulata --
ZX136Dai25794ceriporia --
892Dai13400 --
RLG7163Leptoporus_mollis TGACCGTCTTGCCTGTTCCGCCTCCACCC--------
----
Dai21062Leptoporus_mollis --
Dai20182Leptoporus_submollis
TGACCGTCTTGCCTGTTCCGCCTCCACCCGTGCGCCCGAGTATAGCTGTC
Cui18379Leptoporus_submollis
TGACCGTCTTGCCTGTTCCGCCTCCACCCGTGCGCCCGAGTATAGCTGTC
Wu1209_46Resiniporus_pseudogil
TGACGGTTTTACCCGTCCCACCTCCTCCTGTTCGCCCCAGTATAGCGGTA
BRNM710169Resiniporus_resinasc --
Dai14516Bjerkandera_adusta --
Dai21100Bjerkandera_fumosa --
Miettinen16854Ceraceomyces_sp
TGACTGTACTCCCCGTTCCCCCACCGCCCGTACGCCCCATTATAGCCGTA
Dai10477C_spissa --
855Dai16831 --
882Cui11282 --
Dai24566 --
Yuan5965 --
Dai3204 --
1194CUI9985 --

Dai15205_Ceriporia_albomellea --
Dai15223_Ceriporia_albomellea --
Li1780_Ceriporia_variegata --
Dai19791_Ceriporia_variegata --
Dai19886 --
Dai10833_Ceriporia_crassitunic --

CHWC1506_46Meruliopsis_crassit
GATGGTGGTACGATGCGTAGTGAGGATGATCTGACTTACAAGCTCGGCGA
Dai9995_Ceriporia_crassitunica --
Wu1209_58_Meruliopsis_parvispo
GATGGTGGCACGATGCGTAGCGAGGACGATCTAACCTACAAGCTGGGTGA
CHWC1505_129_Meruliopsis_parvi --
Dai21944 --
830Dai18640A
GACGGTGGTACGATGCGCAGCGAAGATGACTTAACCTATAAGCTGGGTGA
GC1704_60_Meruliopsis_taxicola
GATGGTGGTACTATGCGCAGCGAGGATGACTTAACATATAAGCTAGGCGA
Dai22625
GATGGTGGTACTATGCGCAGCGAGGATGACTTAACATATAAGCTAGGCGA
Dai22636
GATGGTGGTACTATGCGCAGCGAGGATGACTTAACATATAAGCTAGGCGA
Dai21878
GATGGTGGCACTATGCGCAGCGAGGATGATTTAACATATAAGCTAGGCGA
1169Dai17248 --
Wu1708_43_Meruliopsis_leptocys GACGGTGGTACTATGCGCAGCGAGGATGATT-----
----
Li1011 --
ZX95Dai25742Meruliopsis_leptoc --
WCG1306Dai24733 --
LXL99Dai25816
GACGGTGGTACTATGCGCAGTGAGGATGATTTGACATACAAATTAGGTGA
WCG1559Dai26052Meruliopsis
GACGGTGGTACTATGCGCAGCGAAGATGATTTGACATACAAGCTAGGTGA
He7477 --
HLX243Dai26217
GACGGTGGTACTATGCGCAGCGAAGATGATTTGACATACAAGCTAGGTGA
RussiaMW673659Meruliopsis_fagi --
FD278
GATGGCGGTACCATGCGTAGCGAGGATGACTTGACGTACAAGCTAGGCGA
Dai10226_Ceriporia_tarda --
LE247365 --
Dai8173_Meruliopsis_nanlingens --
860Dai17172 --
879Dai13414
GATGGTGGCACCATGCGCAGTGAGGATGACTTGACGTATAAGTTGGGCGA
Li_1704_Meruliopsis_pseudocyst --
833Dai18405
GATGGCGGTACCATGCGTAGCGAGGATGATTTGACATACAAGCTGGGTGA
HHB_10729_Meruliopsis_albostra
GATGGCGGTACCATGCGTAGCGAGGATGACTTGACATACAAGCTGGGTGA

Cui6878_Ceriporia_pseudocystid --

869Dai14737 --

876Cui11626 --

1199WEI3388 --

776308_Meruliopsis_cystidiata --

ICN139059_Meruliopsis_cystidia --

HHB15692Ceraceomyces_serpens GATGGCGGCACGATGCGGAGTGAGGACGACTTGACGTACAAGTTGGGGA

HHB_15629_Sp_Ceriporiopsis_ane GACGGCGGAACTATGCGTAGTGAGGATGATTTGACGTACAAGTTGGGCGA

AJ185Trametopsis_cervina GACGGCGGGACAATGCGCAGTGAAGATGATTTGACGTACAAGTTAGGCGA

FD9Irpex_lacteus --

908Dai11230 --

FP55521TEmmia_lacerata GACGGTGGAACCATGCGTAGTGAGGATGACTTGACCTACAAACTTGGTGA

PBU0048Ceriporia_cystidiata --

MZ340C_lacerataT --

Dai21940 GATGGTGGGACAATGCGCAGCGAGGATGATTTGACATACAAACTTGGTGA

847Dai16433 GATGGTGGAACAATGCGCAGCGAGGATGATTTGACATATAAGCTTGGTGA

MarcinEmmia_latemarginatus --

Meijer3729Hydnopolyporus_fimbr --

RLG13408Phanerochaete_sp GACGGTGGCACCATGCGGAGCGAGGATGATTTAACTTACAAACTGGGAGA

WHC1381Flavodon_flavus GATGGTGGCACCATGCGCAGCGAAGATGACCTGACATATAAGTTGGGTGA

GB1833Phlebia_albida --

T407Phlebia_nitidula GATGGTGGGACGATGCGCAGTGAAGATGATCTGACATACAAGTTGGGCGA

HHB6988Phanerochaete_exilis GACGGTGGAACCATGCGCAGCGAAGATGATTTAACCTACAAGTTGGGTGA

HHB8509Phanerochaetella_xeroph --

PBU0051Macrohyporia_dictyopora --

HHB11463Phanerochaete_sp GATGGTGGAACTATGCGCAGTGAGGATGACTTGACCTACAAGCTGGGCGA

FP102382Byssomerulius_corium GACGGTGGAACCATGCGCAGCGAGGATGACTTGACATACAAGCTGGGTGA

FP102165Efibula_americana GATGGTGGAACCATGCGGAGCGAAGATGATCTCACCTATAAGTTGGGCGA

Murdoch90Ceriporia_torpida --

Rivoire4413_Ceriporia_purpurea --

Kout_18_Ceriporia_triumphalis --

Rivoire3701_Ceriporia_bresadol --
VS4018 --
Ryvarden21832_Ceriporia_manzan --
Dai24539
GACGGCGGTACAATGCGGAGTGAGGACGATCTCACGTATAAGCTGGGCGA
Dai24541
GACGGCGGTACAATGCGGAGTGAGGACGATCTCACGTATAAGCTGGGCGA
JV1105_12_Ceriporia_occidental --
VS8558Ceriporia_occidentalis --
Dai22445 --
846Dai16368
GACGGCGGTACAATGCGGAGTGAGGACGATCTCACGTATAAGCTGGGCGA
Dai17951_Ceriporia_aurantiocar --
Miettinen_11701C_viridans --
JV0105_10Ceriporia_aurantiocar --
Yuan5702C_viridans --
858Dai17003 --
Yuan2747_Ceriporia_viridans --
Yuan2744C_viridans --
Li1046C_viridans --
865C_sinoviridans --
871Dai15062 --
Dai7642_Ceriporia_humilis --
Spirin4706_Ceriporia_humilis --
Spirin4944_Ceriporia_sericea --
WCG1547Dai26044ceriporia
GATGGCGGTACTATGCGCAGCGAAGATGACTTGACATACAAGCTTGGCGA
ZZW1558Dai27086 --
Miettinen14381_Ceriporia_mpuri --
Miettinen15492_2_Ceriporia_sor --
He6687
GATGGCGGTACCATGCGCAGTGAAGATGACTTGACATACAAGCTTGGCGA
ZH53Dai24426
GATGGCGGTACCATGCGCAGTGAAGATGACTTGACATACAAGCTTGGCGA
Vlasak0808_30_Ceriporia_punica --
887Dai13376
GATGGCGGTACGATGCGCAGTGAAGATGACTTGACCTACAAGCTAGGCGA
WCG1443Dai24998
GATGGCGGTACGATGCGGAGTGAAGATGACTTGACCTACAAGCTGGGTGA
0108_6Ceriporia_spissa --
Dai19164 --
Dai17937_Ceriporia_bubalinomar --
903Dai12113 --
LZB929Dai25079 --

LX45Dai26988
GATGGCGGTACAATGCGGAGTGAAGATGACTTGACCTATAAGCTGGGCGA
LX43Dai26986
GATGGCGGTACAATGCGGAGTGAAGATGACTTGACCTATAAGCTGGGCGA
Dai7759Ceriporia --
Cui8012_Ceriporia_viridans --
GC1704_54Ceriporia_viridans --
Dai23392
GATGGCGGTACGATGCGGAGTGAAGATGATTTAACCTACAAGTTAGGCGA
WCG1585Dai26113Ceriproia
GATGGCGGTACGATGCGGAGTGAAGATGATTTAACCTACAAGTTAGGCGA
Dai18675C_eucalypti --
Dai22034
GATGGCGGGACAATGCGCAGCGAGGACGATTTGACCTACAAGCTCGGTGA
JV1008_41JTardaFLORIDAKeys --
Rivoire1161_Ceriporia_pierii --
Dai23499C_pierii --
Dai23500
GACGGCGGTACTATGCGCAGCGAAGACGACCTGACATACAAGCTCGGCGA
841Dai15899
GACGGCGGGACAATGCGGAGCGAGGACGATCTAACCTACAAGTTGGGCGA
842Dai15904
GACGGCGGGACAATGCGGAGCGAGGACGATCTAACCTACAAGTTGGGCGA
LZB1066xinjiang --
LZB1065xinjiang --
851Dai16779 --
RMJ119sp_Candelabrochaete_sept --
RLG9759spCandelabrochaete_sept --
RLG10478Phanerochaete_allantos --
Dai19118_Ceriporia_spissa --
Dai18486A --
WEI17_024_Ceriporia_mellita --
GC1508_71Ceriporia_mellita
GATGGTGGTACTATGCGCAGCGAGGATGACTTGACGTATAAGCTTGGCGA
GC1608_7_Ceriporia_mellita GATGGGGG--
ZZW1557Dai27085 --
ZZW1554Dai27083 --
Dai8168 --
BR4865C_mellita --
MEL2382688Ceriporia_sp --
Dai8110 --
Cui8097 --
909Cui6740 --
W1258Dai24695

GACGGCGGTACCATGCGCAGCGAGGACGACTTGACGTACAAGCTCGGCGA

JV0110_26_Ceriporia_griseoviol --

896Dai13202 --

LWY393Dai27053C_griseoviolasce

GATGGCGGCACAATGCGGAGCGAAGATGATTTGACGTACAAGCTCGGCGA

LWY394DAI27054 --

FP135015G_pannocinctus --

L15726SpG_pannocinctus --

Dai22221 --

Dai22633 --

Dai23260 --

Dai23626 --

Dai16238G_citrinoalbus --

1175Dai15293 --

Dai19547

GATGGCGGCACCATGCGAAGTGAAGATGATTTGACCTACAAGTTGGGTGA

918063G_africanus --

918572G_africanus --

Dai18536A --

1164Cui17922 --

Dai22225 --

1163Dai20655 --

Yuan4397G_hainanensis --

1176Dai15268 --

1177Dai15259 --

BZ2896G_thelephoroides --

1166JV1808_26 --

Miettinen16992Hapalopilus_ochr

GATGGAGGTACTATGCGTAGTGAAGACGATCTAACATACAAGTTAGGCGA

GC1708_338_Ceriporia_arbuscula GACGGCGGGACTATGCGCAGCGAGGACGA-------
----

WCG1555Dai26107Ceriporia --

GC1708_340_Ceriporia_arbuscula GACGGCGGGACTATGCGCAGCGAGGACGA-------
----

WCG1556Dai26109Ceriporia

GACGGCGGGACTATGCGCAGCGAGGACGACTTGACGTACAAGCTTGGGGA

883Cui11291

GATGGCGGGACTATGCGAAGCGAGGATGACTTGACGTACAAACTTGGTGA

HLX320Dai26805

GATGGCGGGACTATGCGGAGCGAGGATGACTTGACCTACAAACTGGGTGA

WCG1266Dai24678A

GATGGCGGGACTATGCGCAGCGAGGACGACTTGACATACAAGCTGGGTGA

Dai6090_Ceriporia_sulphuricolo --

RLG_11354_Ceriproia_reticulata

GATGGCGGCACGATGCGGAGCGAAGACGACTTGACCTACAAGTTGGGTGA

ZZW1543Dai27072

GATGGCGGCACGATGCGGAGCGAAGACGACTTGACCTACAAGCTGGGTGA

Li1316_Ceriporia_reticulata --

KHL11981Ceriporia_reticulata --

FP110343sp_Candelabrochaete_la --

Li1045_Ceriporia_reticulata --

ZX136Dai25794ceriporia --

892Dai13400 --

RLG7163Leptoporus_mollis --

Dai21062Leptoporus_mollis --

Dai20182Leptoporus_submollis

GATGGTGGCACAATGCGTAGCGAAGACGACCTGACGTATAAATTGGGCGA

Cui18379Leptoporus_submollis

GATGGTGGCACAATGCGTAGCGAAGACGACCTGACGTATAAATTGGGCGA

Wu1209_46Resiniporus_pseudogil

GACGGTGGAACGATGCGTAGTGAAGATGACTTGACGTACAAACTGGGCGA

BRNM710169Resiniporus_resinasc --

Dai14516Bjerkandera_adusta --

Dai21100Bjerkandera_fumosa --

Miettinen16854Ceraceomyces_sp

GATGGCGGTACAATGCGCAGTGAAGATGACTTGACGTATAAGTTGGGAGA

Dai10477C_spissa --

855Dai16831 --

882Cui11282 --

Dai24566 --

Yuan5965 --

Dai3204 --

1194CUI9985 --

Dai15205_Ceriporia_albomellea --

Dai15223_Ceriporia_albomellea --

Li1780_Ceriporia_variegata --

Dai19791_Ceriporia_variegata --

Dai19886 --

Dai10833_Ceriporia_crassitunic --

CHWC1506_46Meruliopsis_crassit

TATCATCAAAGCGTCGGCAAATGTCCGTCGATGTGAGCAAGAAGGTGCAC

Dai9995_Ceriporia_crassitunica --

Wu1209_58_Meruliopsis_parvispo

TATCATCAAAGCTTCAGCAAATGTGCGTCGATGTGAGCAGGAGGGTGCAC

CHWC1505_129_Meruliopsis_parvi --

Dai21944 --

830Dai18640A
TATCATCAAGGCTTCCGCGAATGTCCGCCGCTGTGAACAGGAGGGTGCAC
GC1704_60_Meruliopsis_taxicola
TATTATCAAGGCTTCCGCGAATGTGCGTCGTTGTGAGCAGGAGGGCGCGC
Dai22625
TATTATCAAAGCCTCCGCGAATGTGCGTCGTTGTGAGCAGGAGGGCGCAC
Dai22636
TATTATCAAAGCCTCCGCGAATGTGCGTCGTTGTGAGCAGGAGGGCGCAC
Dai21878
TATCATCAAAGCCTCCGCGAATGTGCGTCGTTGTGAGCAGGAGGGCGCAC
1169Dai17248 --
Wu1708_43_Meruliopsis_leptocys --
Li1011 --
ZX95Dai25742Meruliopsis_leptoc --
WCG1306Dai24733 --
LXL99Dai25816
TATCATCAAAGCCTCGGCAAACGTGCGCCGATGTGAGCAAGAGGGTGCAC
WCG1559Dai26052Meruliopsis
TATCATCAAAGCCTCGGCAAATGTGCGTCGGTGTGAGCAAGAGGGTGCAC
He7477 --
HLX243Dai26217
TATCATCAAAGCCTCGGCAAATGTGCGTCGGTGTGAGCAAGAGGGTGCAC
RussiaMW673659Meruliopsis_fagi --
FD278
CATCATCAAAGCCTCAGCAAATGTCCGCCGGTGTGAGCAAGAGGGCGCAC
Dai10226_Ceriporia_tarda --
LE247365 --
Dai8173_Meruliopsis_nanlingens --
860Dai17172 --
879Dai13414
TATCATCAAGGCCTCAGCGAACGTGCGTCGATGCGAGCAAGAGGGTGCGC
Li_1704_Meruliopsis_pseudocyst --
833Dai18405
CATCATCAAGGCTTCAGCGAATGTGCGCCGATGTGAGCAGGAGGGTGCGC
HHB_10729_Meruliopsis_albostra
CATCATCAAGGCTTCAGCGAATGTGCGCCGATGTGAGCAGGAGGGTGCGC
Cui6878_Ceriporia_pseudocystid --
869Dai14737 --
876Cui11626 --
1199WEI3388 --
776308_Meruliopsis_cystidiata --
ICN139059_Meruliopsis_cystidia --
HHB15692Ceraceomyces_serpens
TATCATTAAAGCGTCAGCGAATGTGCGTCGATGCGAGCAGGAAGGCGCGC

HHB_15629_Sp_Ceriporiopsis_ane
TATCATCAAGGCGTCTGCCAACGTGCGTCGATGTGAACAGGAGGGTGCCC

AJ185Trametopsis_cervina
TATCATCAAGGCGTCTGCCAACGTGCGTCGATGTGAACAAGAGGGTGCCC

FD9Irpex_lacteus --

908Dai11230 --

FP55521TEmmia_lacerata
TATCATCAAGGCTTCCGCAAACGTTCGCCGATGTGAACAAGAAGGTGCTC

PBU0048Ceriporia_cystidiata --

MZ340C_lacerataT --

Dai21940
TATCATCAAAGCCTCTGCAAACGTTCGTCGATGTGAACAGGAAGGTGCTC

847Dai16433
TATCATCAAGGCCTCTGCAAACGTTCGTCGATGTGAACAGGAAGGTGCTC

MarcinEmmia_latemarginatus --

Meijer3729Hydnopolyporus_fimbr --

RLG13408Phanerochaete_sp
CATCATCAAGGCGTCTGCCAACGTGCGTCGATGCGAGCAAGAAGGTGCTC

WHC1381Flavodon_flavus
TATTATCAAAGCTTCCGCCAATGTTCGTCGATGTGAGCAAGAGGGCGCTC

GB1833Phlebia_albida --

T407Phlebia_nitidula
CATCATCAAAGCATCCGCTAATGTGCGTCGATGTGAGCAAGAGGGTGCAC

HHB6988Phanerochaete_exilis
TATCATTAAAGCTTCCGCCAACGTGCGTCGCTGTGAGCAAGAGGGAGCTC

HHB8509Phanerochaetella_xeroph --

PBU0051Macrohyporia_dictyopora --

HHB11463Phanerochaete_sp TATAATTAAAGCCTCTGCT------------------
CAAGAGGGAGCTC

FP102382Byssomerulius_corium
CATCATCAAGGCATCCGCCAACGTGCGTCGGTGTGAGCAAGAGGGGGCAC

FP102165Efibula_americana
TATCATCAAAGCGTCTGCCAACGTGCGCCGTTGTGAGCAAGAAGGAGCTC

Murdoch90Ceriporia_torpida --

Rivoire4413_Ceriporia_purpurea --

Kout_18_Ceriporia_triumphalis --

Rivoire3701_Ceriporia_bresadol --

VS4018 --

Ryvarden21832_Ceriporia_manzan --

Dai24539
TATTATCAAAGCGTCCGCAAACGTACGTCGGTGTGAGCAAGAAGGAGCAC

Dai24541
TATTATCAAAGCGTCCGCAAACGTACGTCGGTGTGAGCAAGAAGGAGCAC

JV1105_12_Ceriporia_occidental --

VS8558Ceriporia_occidentalis --
Dai22445 --
846Dai16368
TATCATCAAAGCATCCGCAAACGTTCGTCGGTGTGAGCAAGAAGGGGCAC
Dai17951_Ceriporia_aurantiocar --
Miettinen_11701C_viridans --
JV0105_10Ceriporia_aurantiocar --
Yuan5702C_viridans --
858Dai17003 --
Yuan2747_Ceriporia_viridans --
Yuan2744C_viridans --
Li1046C_viridans --
865C_sinoviridans --
871Dai15062 --
Dai7642_Ceriporia_humilis --
Spirin4706_Ceriporia_humilis --
Spirin4944_Ceriporia_sericea --
WCG1547Dai26044ceriporia
TATCATCAAGGCTTCTGCGAATGTACGGCGCTGTGAGCAAGAGGGCGCAC
ZZW1558Dai27086 --
Miettinen14381_Ceriporia_mpuri --
Miettinen15492_2_Ceriporia_sor --
He6687
TATCATCAAGGCCTCTGCGAATGTACGGCGCTGTGAGCAAGAGGGTGCAC
ZH53Dai24426
TATCATCAAGGCCTCTGCGAATGTACGGCGCTGTGAGCAAGAGGGTGCAC
Vlasak0808_30_Ceriporia_punica --
887Dai13376
CATCATCAAGGCCTCTGCGAATGTGCGACGATGCGAGCAGGAGGGGGCGC
WCG1443Dai24998
CATCATCAAGGCCTCTGCGAATGTACGACGATGCGAGCAGGAGGGCGCGC
0108_6Ceriporia_spissa --
Dai19164 --
Dai17937_Ceriporia_bubalinomar --
903Dai12113 --
LZB929Dai25079 --
LX45Dai26988
CATCATCAAGGCTTCCGCAAATGTCCGTCGATGTGAACAGGAAGGAGCTC
LX43Dai26986
CATCATCAAGGCTTCCGCAAATGTCCGTCGATGTGAACAGGAAGGAGCTC
Dai7759Ceriporia --
Cui8012_Ceriporia_viridans --
GC1704_54Ceriporia_viridans --
Dai23392

CATCATTAAGGCCTCCGCTAATGTCCGTCGTTGCGAACAGGAGGGAGCTC

WCG1585Dai26113Ceriproia

CATCATCAAGGCCTCCGCTAATGTCCGTCGTTGCGAACAGGAGGGAGCTC

Dai18675C_eucalypti --

Dai22034

TATCATTAAGGCATCTGCAAATGTCCGTCGGTGCGAACAGGAAGGAGCTC

JV1008_41JTardaFLORIDAKeys --

Rivoire1161_Ceriporia_pierii --

Dai23499C_pierii --

Dai23500

TATAATAAAAGCTTCTGCGAATGTGCGACGGTGTGAGCAAGAGGGTGCAC

841Dai15899

CATAATCAAAGCTTCCGCGAACGTCCGGCGGTGCGAACAGGAAGGTGCTC

842Dai15904

CATAATCAAAGCTTCCGCGAACGTCCGGCGGTGCGAACAGGAAGGTGCTC

LZB1066xinjiang --

LZB1065xinjiang --

851Dai16779 --

RMJ119sp_Candelabrochaete_sept --

RLG9759spCandelabrochaete_sept --

RLG10478Phanerochaete_allantos --

Dai19118_Ceriporia_spissa --

Dai18486A --

WEI17_024_Ceriporia_mellita --

GC1508_71Ceriporia_mellita

CATCATTAAAGCGTCCGCGAACGTACGTCGATGTGAGCAGGAGGGTGCAC

GC1608_7_Ceriporia_mellita --

ZZW1557Dai27085 --

ZZW1554Dai27083 --

Dai8168 --

BR4865C_mellita --

MEL2382688Ceriporia_sp --

Dai8110 --

Cui8097 --

909Cui6740 --

W1258Dai24695

CATTATCAAAGCGTCCGCAAATGTCCGTCGATGTGAGCAGGAGGGTGCAC

JV0110_26_Ceriporia_griseoviol --

896Dai13202 --

LWY393Dai27053C_griseoviolasce

TATTATCAAGGCTTCTGCCAACGTACGACGCTGTGAGCAAGAGGGGGCTC

LWY394DAI27054 --

FP135015G_pannocinctus --

L15726SpG_pannocinctus --

Dai22221 --
Dai22633 --
Dai23260 --
Dai23626 --
Dai16238G_citrinoalbus --
1175Dai15293 --
Dai19547
CATCATCAAGGCTTCTGCGAATGTCCGTAGATGCGAACAGGAGGGTGCTC
918063G_africanus --
918572G_africanus --
Dai18536A --
1164Cui17922 --
Dai22225 --
1163Dai20655 --
Yuan4397G_hainanensis --
1176Dai15268 --
1177Dai15259 --
BZ2896G_thelephoroides --
1166JV1808_26 --
Miettinen16992Hapalopilus_ochr
CATCATCAAGGCTTCCGCAAACGTGCGTCGCTGTGAACAGGAAGGCGCCC
GC1708_338_Ceriporia_arbuscula --
WCG1555Dai26107Ceriporia --
GC1708_340_Ceriporia_arbuscula --
WCG1556Dai26109Ceriporia
CATCATCAAGGCATCCGCAAATGTACGGCGATGTGAGCAGGAAGGAGCTC
883Cui11291
CATCATCAAGGCATCTGCAAATGTACGACGGTGCGAGCAGGAAGGAGCAC
HLX320Dai26805
CATTATCAAGGCATCCGCAAATGTGCGACGATGCGAGCAGGAAGGAGCTC
WCG1266Dai24678A
CATTATCAAGGCATCCGCAAATGTACGACGATGCGAGCAGGAAGGAGCTC
Dai6090_Ceriporia_sulphuricolo --
RLG_11354_Ceriproia_reticulata
TATTATCAAGGCGTCCGCAAATGTACGGCGATGTGAGCAGGAAGGTGCCC
ZZW1543Dai27072
TATTATCAAGGCGTCCGCAAATGTACGGCGATGTGAGCAGGAAGGTGCCC
Li1316_Ceriporia_reticulata --
KHL11981Ceriporia_reticulata --
FP110343sp_Candelabrochaete_la --
Li1045_Ceriporia_reticulata --
ZX136Dai25794ceriporia --
892Dai13400 --
RLG7163Leptoporus_mollis --

Dai21062Leptoporus_mollis --
Dai20182Leptoporus_submollis
TATCATTAAAGCGTCTGCAAACGTGCGTCGATGTGAGCAGGAAGGAGCCC
Cui18379Leptoporus_submollis
TATCATTAAAGCGTCTGCAAACGTGCGTCGATGTGAGCAGGAAGGAGCCC
Wu1209_46Resiniporus_pseudogil
TATCATCAAAGCGTCTGCCAATGTGCGTCGGTGCGAGCAGGAGGGTGCCC
BRNM710169Resiniporus_resinasc --
Dai14516Bjerkandera_adusta --
Dai21100Bjerkandera_fumosa --
Miettinen16854Ceraceomyces_sp
TATTATCAAAGCGTCTGCGAATGTGCGACGATGTGAGCAGGAGGGTGCAC
Dai10477C_spissa --
855Dai16831 --
882Cui11282 --
Dai24566 --
Yuan5965 --
Dai3204 --
1194CUI9985 --

Dai15205_Ceriporia_albomellea --
Dai15223_Ceriporia_albomellea --
Li1780_Ceriporia_variegata --
Dai19791_Ceriporia_variegata --TGAAACTGCG
Dai19886 --TGAAACTGCG
Dai10833_Ceriporia_crassitunic --
CHWC1506_46Meruliopsis_crassit
CTGCTCATGTGATCACGGAGTTTGAACAACTGCTTCAGGT----------
Dai9995_Ceriporia_crassitunica --
Wu1209_58_Meruliopsis_parvispo
CCGCTCATGTGATTACGGAGTTCGAGCAATTACTTCAGGT----------
CHWC1505_129_Meruliopsis_parvi --
Dai21944 --TGAAACTGCG
830Dai18640A
CGGCACATGTGATCACGGAGTTCGAGCAGCTGCTTCAGGTTGAAACTGCG
GC1704_60_Meruliopsis_taxicola
CTGCCCATGTGATCACGGAGTTCGAGCAGTTGCTCCAGCT----------
Dai22625
CTGCTCATGTGATCACGGAGTTCGAGCAGTTGCTGCAGGTTGAAACTGCG
Dai22636
CTGCTCATGTGATCACGGAGTTCGAGCAGTTGCTGCAGGTTGAAACTGCG
Dai21878
CTGCTCATGTGATTACGGAGTTCGAGCAGTTGCTGCAGGTTGAAACTGCG

1169Dai17248 --TGAAACTGCG
Wu1708_43_Meruliopsis_leptocys --
Li1011 --
ZX95Dai25742Meruliopsis_leptoc --TGAAACTGCG
WCG1306Dai24733 --TGAAACTGCG
LXL99Dai25816
CCGCACATGTAATCACAGAGTTCGAGCAACTACTTCAGGTTGAAACTGCG
WCG1559Dai26052Meruliopsis
CCGCACATGTAATCACAGAGTTTGAGCAGTTACTTCAGGTTGAAACTGCG
He7477 --
HLX243Dai26217
CCGCACATGTAATCACAGAGTTTGAGCAGTTACTTCAGGTTGAAACTGCG
RussiaMW673659Meruliopsis_fagi --
FD278
CCGCACATGTAATCACTGAGTTTGAGCAACTGCTCCAGGT----------
Dai10226_Ceriporia_tarda --
LE247365 --
Dai8173_Meruliopsis_nanlingens --
860Dai17172 --TGAAACTGCG
879Dai13414
CTGCACATGTAATCACGGAATTTGAACAATTACTTCAGGTTGAAACTGCG
Li_1704_Meruliopsis_pseudocyst --
833Dai18405
CCGCCCATGTGATCACGGAGTTCGAGCAATTACTCCAGGTTGAAACTGCG
HHB_10729_Meruliopsis_albostra
CCGCCCATGTGATCACGGAGTTCGAGCAATTGCTCCAGGT----------
Cui6878_Ceriporia_pseudocystid --
869Dai14737 --TGAAACTGCG
876Cui11626 --TGAAACTGCG
1199WEI3388 --
776308_Meruliopsis_cystidiata --
ICN139059_Meruliopsis_cystidia --
HHB15692Ceraceomyces_serpens CCGCGCACGTCATAACAGAGTT--------------------
----
HHB_15629_Sp_Ceriporiopsis_ane
CGGCACACGTTATCACCGAGTTCGAACAGTTACTCCAGGT----------
AJ185Trametopsis_cervina
CGGCACATGTTATCACAGAGTTCGAGCAGCTGCTACAGGT----------
FD9Irpex_lacteus --
908Dai11230 --TGAAACTGCG
FP55521TEmmia_lacerata
CAGCGCACGTAATCACGGAGTTTGAGCAGCTTCTACAAGT----------
PBU0048Ceriporia_cystidiata --
MZ340C_lacerataT --

Dai21940
CAGCGCACGTCATCACAGAGTTTGAGCAACTTCTACAAGTTGAAACTGCG
847Dai16433
CAGCGCATGTCATTACAGAGTTTGAGCAACTTCTACAAGTTGAAACTGCG
MarcinEmmia_latemarginatus --
Meijer3729Hydnopolyporus_fimbr --
RLG13408Phanerochaete_sp
CTGCACATGTCATCACCGAGTTCGAACAGTTGCTTCAGGT----------
WHC1381Flavodon_flavus
CGGCTCATGTCATCACAGAGTTCGAACAGTTGCTCCAGGT----------
GB1833Phlebia_albida --
T407Phlebia_nitidula
CTGCGCATGTGATCACAGAGTTCGAGCAATTGCTACAGGT----------
HHB6988Phanerochaete_exilis
CTGCACACGTAATCACGGAGTTCGAGCAGTTGCTCCAGGT----------
HHB8509Phanerochaetella_xeroph --
PBU0051Macrohyporia_dictyopora --
HHB11463Phanerochaete_sp CTGCGCATGTAA-------------------GCTTCAGGT----
------
FP102382Byssomerulius_corium
CCGCACACGTTATTACGGAATTTGAGCAGCTGCTCCAGGT----------
FP102165Efibula_americana
CTGCCCACGTTATCACTGAATTCGAACAATTGCTTCAGGT----------
Murdoch90Ceriporia_torpida --
Rivoire4413_Ceriporia_purpurea --
Kout_18_Ceriporia_triumphalis --
Rivoire3701_Ceriporia_bresadol --
VS4018 --
Ryvarden21832_Ceriporia_manzan --
Dai24539
CAGCGCATGTCATTACAGAGTTCGAGCAGTTACTGCAGGTTGAAACTGCG
Dai24541
CAGCGCATGTCATTACAGAGTTCGAGCAGTTACTGCAGGTTGAAACTGCG
JV1105_12_Ceriporia_occidental --
VS8558Ceriporia_occidentalis --
Dai22445 --TGAAACTGCG
846Dai16368
CAGCACATGTCATTACGGAGTTCGAGCAGCTACTACAGGTTGAAACTGCG
Dai17951_Ceriporia_aurantiocar --
Miettinen_11701C_viridans --
JV0105_10Ceriporia_aurantiocar --
Yuan5702C_viridans --
858Dai17003 --TGAAACTGCG
Yuan2747_Ceriporia_viridans --

Yuan2744C_viridans --
Li1046C_viridans --
865C_sinoviridans --
871Dai15062 --
Dai7642_Ceriporia_humilis --TGAAACTGCG
Spirin4706_Ceriporia_humilis --
Spirin4944_Ceriporia_sericea --
WCG1547Dai26044ceriporia
CCGCGCACGTCATCACAGAATTTGAGCAGCTACTTCAGGTTGAAACTGCG
ZZW1558Dai27086 --TGAAACTGCG
Miettinen14381_Ceriporia_mpuri --
Miettinen15492_2_Ceriporia_sor --
He6687
CTGCGCACGTCATCACAGAGTTTGAGCAACTGCTTCAGGTTGAAACTGCG
ZH53Dai24426
CCGCGCACGTCATCACAGAGTTTGAGCAACTGCTTCAGGTTGAAACTGCG
Vlasak0808_30_Ceriporia_punica --
887Dai13376
CGGCGCACGTCATCACCGAGTTTGAGCATCTGCTTCAGGTTGAAACTGCG
WCG1443Dai24998
CGGCGCATGTCATAACCGAGTTTGAACAACTGCTCCAGGT----------
0108_6Ceriporia_spissa --
Dai19164 --
Dai17937_Ceriporia_bubalinomar --
903Dai12113 --TGAAACTGCG
LZB929Dai25079 --TGAAACTGCG
LX45Dai26988
CTGCGCACGTGATCACTGAGTTCGAACAGCTGCTCCAGGT----------
LX43Dai26986
CTGCGCACGTGATCACTGAGTTCGAACAGCTGCTCCAGGTTGAAACTGCG
Dai7759Ceriporia --
Cui8012_Ceriporia_viridans --
GC1704_54Ceriporia_viridans --
Dai23392
CAGCGCATGTCATCACTGAATTCGAACAGTTGCTCCAGGTTGAAACTGCG
WCG1585Dai26113Ceriproia
CAGCGCATGTCATCACTGAATTCGAACAGTTGCTCCAGGTTGAAACTGCG
Dai18675C_eucalypti --
Dai22034
CCGCGCATGTCATCACGGAGTTCGAGCAACTCCTACAGGTTGAAACTGCG
JV1008_41JTardaFLORIDAKeys --
Rivoire1161_Ceriporia_pierii --
Dai23499C_pierii --TGAAACTGCG
Dai23500

CTGCGCATGTCATTACAGAGTTTGAGCAGTTGCTTCAGGTTGAAACTGCG
841Dai15899
CAGCCCATGTCATCACGGAGTTCGAGCAGCTTCTTCAGGTTGAAACTGCG
842Dai15904
CAGCCCATGTCATCACGGAGTTCGAGCAGCTTCTTCAGGTTGAAACTGCG
LZB1066xinjiang --
LZB1065xinjiang --
851Dai16779 --
RMJ119sp_Candelabrochaete_sept --
RLG9759spCandelabrochaete_sept --
RLG10478Phanerochaete_allantos --
Dai19118_Ceriporia_spissa --TGAAACTGCG
Dai18486A --TGAAACTGCG
WEI17_024_Ceriporia_mellita --
GC1508_71Ceriporia_mellita
CGGCACAMGTCATCACGGAGTTCGAACAACTCCTCCAGGT----------
GC1608_7_Ceriporia_mellita --
ZZW1557Dai27085 --
ZZW1554Dai27083 --
Dai8168 --
BR4865C_mellita --
MEL2382688Ceriporia_sp --
Dai8110 --
Cui8097 --
909Cui6740 --
W1258Dai24695
CGGCGCACGTCATCACGGAGTTCGAACAACTCCTCCAGGTTGAAACTGCG
JV0110_26_Ceriporia_griseoviol --
896Dai13202 --TGAAACTGCG
LWY393Dai27053C_griseoviolasce
CTGCTCATGTCATCACCGAGTTTGAGCAGTTGCTGCAGGTTGAAACTGCG
LWY394DAI27054 --TGAAACTGCG
FP135015G_pannocinctus --
L15726SpG_pannocinctus --
Dai22221 --TGAAACTGCG
Dai22633 --TGAAACTGCG
Dai23260 ----------------------------------GCAGGTTG-
AACTGCG
Dai23626 --TGAAACTGCG
Dai16238G_citrinoalbus --TGAAACTGCG
1175Dai15293 --TGAAACTGCG
Dai19547
CTGCACACGTCATCACGGAGTTTGAGCAGTTGCTTCAGGTTGAAACTGCG
918063G_africanus --

918572G_africanus --
Dai18536A --
1164Cui17922 --TGAAACTGCG
Dai22225 --TGAAACTGCG
1163Dai20655 --TGAAACTGCG
Yuan4397G_hainanensis --
1176Dai15268 --TGAAACTGCG
1177Dai15259 --TGAAACTGCG
BZ2896G_thelephoroides --
1166JV1808_26 --TGAAACTGCG
Miettinen16992Hapalopilus_ochr
CCGCGCATGTCATAACGGAATTTGAGCAGTTGCTCCAGGT----------
GC1708_338_Ceriporia_arbuscula --
WCG1555Dai26107Ceriporia --TGAAACTGCG
GC1708_340_Ceriporia_arbuscula --
WCG1556Dai26109Ceriporia
CTGCCCATGTCATTACGGAGTTCGAGCAGCTGCTTCAGGTTGAAACTGCG
883Cui11291 CTGCGCATGTCATCACGGAGTTCGA--------------
-TGAAACTGCG
HLX320Dai26805
CTGCTCATGTCATCACGGAATTCGAGCAATTACTCCAGGTTGAAACTGCG
WCG1266Dai24678A
CTGCCCATGTCATCACGGAGTTTGAGCAGTTACTCCAGGT----------
Dai6090_Ceriporia_sulphuricolo --
RLG_11354_Ceriproia_reticulata
CTGCGCACGTCATCACGGAGTTCGAACAGCTGCTTCAGGT----------
ZZW1543Dai27072
CTGCGCACGTCATCACGGAGTTCGAACAGCTGCTTCAGGTTGAAACTGCG
Li1316_Ceriporia_reticulata --
KHL11981Ceriporia_reticulata --
FP110343sp_Candelabrochaete_la --
Li1045_Ceriporia_reticulata --
ZX136Dai25794ceriporia --
892Dai13400 --TGAAACTGCG
RLG7163Leptoporus_mollis --
Dai21062Leptoporus_mollis --
Dai20182Leptoporus_submollis
CTGCGCATGTCATCACGGAATTCGAGCAACTGCTACA-------------
Cui18379Leptoporus_submollis
CTGCGCATGTCATCACGGAATTCGAGCAACTGCTACAG------------
Wu1209_46Resiniporus_pseudogil
CGGCCCATGTTATCACCGAATTTGAACAGTTGCTTCAGGT----------
BRNM710169Resiniporus_resinasc --
Dai14516Bjerkandera_adusta --CG

Dai21100Bjerkandera_fumosa --TGAAACTGCG
Miettinen16854Ceraceomyces_sp
CGGCTCATGTCATCACCGAATTTGAGCAATTGCTTCAGGT----------
Dai10477C_spissa --
855Dai16831 --TGAAACTGCG
882Cui11282 --TGAAACTGCG
Dai24566 --TGAAACTGCG
Yuan5965 --
Dai3204 --
1194CUI9985 --

Dai15205_Ceriporia_albomellea --
Dai15223_Ceriporia_albomellea --
Li1780_Ceriporia_variegata --
Dai19791_Ceriporia_variegata
AATGGCTCATTAAATCAGTTATAGTTTATTTGATGGTGCTTTGCTACATG
Dai19886
AATGGCTCATTAAATCAGTTATAGTTTATTTGATGGTGCTTTGCTACATG
Dai10833_Ceriporia_crassitunic -------------------------------------ACCTTGCTACATG
CHWC1506_46Meruliopsis_crassit --
Dai9995_Ceriporia_crassitunica --
Wu1209_58_Meruliopsis_parvispo --
CHWC1505_129_Meruliopsis_parvi --
Dai21944
AATGGCTCATTAAATCAGTTATAGTTTATTTGATGGTGCTTTGCTACATG
830Dai18640A
AATGGCTCATTAAATCAGTTATAGTTTATTTGATGGTGCTTTGCTACATG
GC1704_60_Meruliopsis_taxicola --
Dai22625
AATGGCTCATTAAATCAGTTATAGTTTATTTGATGGTGCTTTGCTACATG
Dai22636
AATGGCTCATTAAATCAGTTATAGTTTATTTGATGGTGCTTTGCTACATG
Dai21878
AATGGCTCATTAAATCAGTTATAGTTTATTTGATGGTGCTTTGCTACATG
1169Dai17248
AATGGCTCATTAAATCAGTTATAGTTTATTTGATGGTGCTTTGCTACATG
Wu1708_43_Meruliopsis_leptocys --
Li1011 --
ZX95Dai25742Meruliopsis_leptoc
AATGGCTCATTAAATCAGTTATAGTTTATTTGATGGTGCTTTGCTACATG
WCG1306Dai24733
AATGGCTCATTAAATCAGTTATAGTTTATTTGATGGTGCTTTGCTACATG
LXL99Dai25816

AATGGCTCATTAAATCAGTTATAGTTTATTTGATGGTGCTTTGCTACATG

WCG1559Dai26052Meruliopsis

AATGGCTCATTAAATCAGTTATAGTTTATTTGATGGTGCTTTGCTACATG

He7477 ---

HLX243Dai26217

AATGGCTCATTAAATCAGTTATAGTTTATTTGATGGTGCTTTGCTACATG

RussiaMW673659Meruliopsis_fagi ---

FD278 ---

Dai10226_Ceriporia_tarda ---

LE247365 ---

Dai8173_Meruliopsis_nanlingens ---

860Dai17172

AATGGCTCATTAAATCAGTTATAGTTTATTTGATGGTGCTTTGCTACATG

879Dai13414

AATGGCTCATTAAATCAGTTATAGTTTATTTGATGGTGCTTTGCTACATG

Li_1704_Meruliopsis_pseudocyst ---

833Dai18405

AATGGCTCATTAAATCAGTTATAGTTTATTTGATGGTGCTTTGCTACATG

HHB_10729_Meruliopsis_albostra ---

Cui6878_Ceriporia_pseudocystid ---

869Dai14737

AATGGCTCATTAAATCAGTTATAGTTTATTTGATGGTGCTTTGCTACATG

876Cui11626

AATGGCTCATTAAATCAGTTATAGTTTATTTGATGGTGCTTTGCTACATG

1199WEI3388 ---

776308_Meruliopsis_cystidiata ---

ICN139059_Meruliopsis_cystidia ---

HHB15692Ceraceomyces_serpens ---

HHB_15629_Sp_Ceriporiopsis_ane ---

AJ185Trametopsis_cervina ---

FD9Irpex_lacteus ---

908Dai11230

AATGGCTCATTAAATCAGTTATAGTTTATTTGATGGTGCTTTGCTACATG

FP55521TEmmia_lacerata ---

PBU0048Ceriporia_cystidiata ---

MZ340C_laceratаT ---

Dai21940

AATGGCTCATTAAATCAGTTATAGTTTATTTGATGGTGCTTTGCTACATG

847Dai16433

AATGGCTCATTAAATCAGTTATAGTTTATTTGATGGTGCTTTGCTACATG

MarcinEmmia_latemarginatus ---

Meijer3729Hydnopolyporus_fimbr ---

RLG13408Phanerochaete_sp ---

WHC1381Flavodon_flavus ---

GB1833Phlebia_albida --
T407Phlebia_nitidula --
HHB6988Phanerochaete_exilis --
HHB8509Phanerochaetella_xeroph --
PBU0051Macrohyporia_dictyopora --
HHB11463Phanerochaete_sp --
FP102382Byssomerulius_corium --
FP102165Efibula_americana --
Murdoch90Ceriporia_torpida --
Rivoire4413_Ceriporia_purpurea --
Kout_18_Ceriporia_triumphalis --
Rivoire3701_Ceriporia_bresadol --
VS4018 --
Ryvarden21832_Ceriporia_manzan --
Dai24539
AATGGCTCATTAAATCAGTTATAGTTTATTTGATGGTGCTTTGCTACATG
Dai24541
AATGGCTCATTAAATCAGTTATAGTTTATTTGATGGTGCTTTGCTACATG
JV1105_12_Ceriporia_occidental --
VS8558Ceriporia_occidentalis --
Dai22445
AATGGCTCATTAAATCAGTTATAGTTTATTTGATGGTGCTTTGCTACATG
846Dai16368
AATGGCTCATTAAATCAGTTATAGTTTATTTGATGGTGCTTTGCTACATG
Dai17951_Ceriporia_aurantiocar --
Miettinen_11701C_viridans --
JV0105_10Ceriporia_aurantiocar --
Yuan5702C_viridans --
858Dai17003
AATGGCTCATTAAATCAGTTATAGTTTATTTGATGGTGCTTTGCTACATG
Yuan2747_Ceriporia_viridans --
Yuan2744C_viridans --
Li1046C_viridans --
865C_sinoviridans --
871Dai15062 --
Dai7642_Ceriporia_humilis
AATGGCTCATTAAATCAGTTATAGTTTATTTGATGGTGCTTTGCTACATG
Spirin4706_Ceriporia_humilis --
Spirin4944_Ceriporia_sericea --
WCG1547Dai26044ceriporia
AATGGCTCATTAAATCAGTTATAGTTTATTTGATGGTGCTTTGCTACATG
ZZW1558Dai27086
AATGGCTCATTAAATCAGTTATAGTTTATTTGATGGTGCTTTGCTACATG
Miettinen14381_Ceriporia_mpuri --

Miettinen15492_2_Ceriporia_sor --
He6687
AATGGCTCATTAAATCAGTTATAGTTTATTTGATGGTGCTTTGCTACATG
ZH53Dai24426
AATGGCTCATTAAATCAGTTATAGTTTATTTGATGGTGCTTTGCTACATG
Vlasak0808_30_Ceriporia_punica --
887Dai13376
AATGGCTCATTAAATCAGTTATAGTTTATTTGATGGTGCTTTGCTACATG
WCG1443Dai24998 --
0108_6Ceriporia_spissa --
Dai19164 --
Dai17937_Ceriporia_bubalinomar --
903Dai12113
AATGGCTCATTAAATCAGTTATAGTTTATTTGATGGTGCTTTGCTACATG
LZB929Dai25079
AATGGCTCATTAAATCAGTTATAGTTTATTTGATGGTGCTTTGCTACATG
LX45Dai26988 --
LX43Dai26986
AATGGCTCATTAAATCAGTTATAGTTTATTTGATGGTGCTTTGCTACATG
Dai7759Ceriporia --
Cui8012_Ceriporia_viridans --
GC1704_54Ceriporia_viridans --
Dai23392
AATGGCTCATTAAATCAGTTATAGTTTATTTGATGGTGCTTTGCTACATG
WCG1585Dai26113Ceriproia
AATGGCTCATTAAATCAGTTATAGTTTATTTGATGGTACTTTGCTACATG
Dai18675C_eucalypti --
Dai22034
AATGGCTCATTAAATCAGTTATAGTTTATTTGATGGTGCTTTGCTACATG
JV1008_41JTardaFLORIDAKeys --
Rivoire1161_Ceriporia_pierii --
Dai23499C_pierii
AATGGCTCATTAAATCAGTTATAGTTTATTTGATGGTGCTTTGCTACATG
Dai23500
AATGGCTCATTAAATCAGTTATAGTTTATTTGATGGTGCTTTGCTACATG
841Dai15899
AATGGCTCATTAAATCAGTTATAGTTTATTTGATGGTGCTTTGCTACATG
842Dai15904
AATGGCTCATTAAATCAGTTATAGTTTATTTGATGGTGCTTTGCTACATG
LZB1066xinjiang --
LZB1065xinjiang --
851Dai16779 --
RMJ119sp_Candelabrochaete_sept --
RLG9759spCandelabrochaete_sept --

RLG10478Phanerochaete_allantos --
Dai19118_Ceriporia_spissa
AATGGCTCATTAAATCAGTTATAGTTTATTTGATGGTGCTTTGCTACATG
Dai18486A
AATGGCTCATTAAATCAGTTATAGTTTATTTGATGGTGCTTTGCTACATG
WEI17_024_Ceriporia_mellita --
GC1508_71Ceriporia_mellita --
GC1608_7_Ceriporia_mellita --
ZZW1557Dai27085 --
ZZW1554Dai27083 --
Dai8168 --
BR4865C_mellita --
MEL2382688Ceriporia_sp --
Dai8110 --
Cui8097 --
909Cui6740 --
W1258Dai24695
AATGGCTCATTAAATCAGTTATAGTTTATTTGATGGTGCTTTGCTACATG
JV0110_26_Ceriporia_griseoviol --
896Dai13202
AATGGCTCATTAAATCAGTTATAGTTTATTTGATGGTGCTTTGCTACATG
LWY393Dai27053C_griseoviolasce
AATGGCTCATTAAATCAGTTATAGTTTATTTGATGGTGCTTTGCTACATG
LWY394DAI27054
AATGGCTCATTAAATCAGTTATAGTTTATTTGATGGTGCTTTGCTACATG
FP135015G_pannocinctus --
L15726SpG_pannocinctus --
Dai22221
AATGGCTCATTAAATCAGTTATAGTTTATTTGATGGTGCTTTGCTACATG
Dai22633
AATGGCTCATTAAATCAGTTATAGTTTATTTGATGGTGCTTTGCTACATG
Dai23260
AATGGCTCATTAAATCAGTTATAGTTTATTTGATGGTGCTTTGCTACATG
Dai23626
AATGGCTCATTAAATCAGTTATAGTTTATTTGATGGTGCTTTGCTACATG
Dai16238G_citrinoalbus
AATGGCTCATTAAATCAGTTATAGTTTATTTGATGGTTCATTGCTACATG
1175Dai15293
AATGGCTCATTAAATCAGTTATAGTTTATTTGATGGTGCTTTGCTACATG
Dai19547
AATGGCTCATTAAATCAGTTATAGTTTATTTGATGGTGCTTTGCTACATG
918063G_africanus --
918572G_africanus --
Dai18536A --

1164Cui17922
AATGGCTCATTAAATCAGTTATAGTTTATTTGATGGTGCTTTGCTACATG
Dai22225
AATGGCTCATTAAATCAGTTATAGTTTATTTGATGGTGCTTTGCTACATG
1163Dai20655
AATGGCTCATTAAATCAGTTATAGTTTATTTGATGGTGCTTTGCTACATG
Yuan4397G_hainanensis --
1176Dai15268
AATGGCTCATTAAATCAGTTATAGTTTATTTGATGGTGCTTTGCTACATG
1177Dai15259
AATGGCTCATTAAATCAGTTATAGTTTATTTGATGGTGCTTTGCTACATG
BZ2896G_thelephoroides --
1166JV1808_26
AATGGCTCATTAAATCAGTTATAGTTTATTTGATGGTGCTTTGCTACATG
Miettinen16992Hapalopilus_ochr --
GC1708_338_Ceriporia_arbuscula --
WCG1555Dai26107Ceriporia
AATGGCTCATTAAATCAGTTATAGTTTATTTGATGGTGCTTTGCTACATG
GC1708_340_Ceriporia_arbuscula --
WCG1556Dai26109Ceriporia
AATGGCTCATTAAATCAGTTATAGTTTATTTGATGGTGCTTTGCTACATG
883Cui11291
AATGGCTCATTAAATCAGTTATAGTTTATTTGATGGTGCTTTGCTACATG
HLX320Dai26805
AATGGCTCATTAAATCAGTTATAGTTTATTTGATGGTGCTTTGCTACATG
WCG1266Dai24678A --
Dai6090_Ceriporia_sulphuricolo --
RLG_11354_Ceriproia_reticulata --
ZZW1543Dai27072
AATGGCTCATTAAATCAGTTATAGTTTATTTGATGGTGCTTTGCTACATG
Li1316_Ceriporia_reticulata --
KHL11981Ceriporia_reticulata --
FP110343sp_Candelabrochaete_la --
Li1045_Ceriporia_reticulata --
ZX136Dai25794ceriporia --
892Dai13400
AATGGCTCATTAAATCAGTTATAGTTTATTTGATGGTGCTTTGCTACATG
RLG7163Leptoporus_mollis --
Dai21062Leptoporus_mollis --
Dai20182Leptoporus_submollis --
Cui18379Leptoporus_submollis --
Wu1209_46Resiniporus_pseudogil --
BRNM710169Resiniporus_resinasc --
Dai14516Bjerkandera_adusta

AATGGCTCATTAAATCAGTTATAGTTTATTTGATGGTGCTTTACTACATG

Dai21100Bjerkandera_fumosa

AATGGCTCATTAAATCAGTTATAGTTTATTTGATGGTACTTTACTACATG

Miettinen16854Ceraceomyces_sp --

Dai10477C_spissa --

855Dai16831

AATGGCTCATTAAATCAGTTATAGTTTATTTGATGGTGCTTTGCTACATG

882Cui11282

AATGGCTCATTAAATCAGTTATAGTTTATTTGATGGTGCTTTGCTACATG

Dai24566

AATGGCTCATTAAATCAGTTATAGTTTATTTGATGGTGCTTTGCTACATG

Yuan5965 --

Dai3204 --

1194CUI9985 --

Dai15205_Ceriporia_albomellea --

Dai15223_Ceriporia_albomellea --

Li1780_Ceriporia_variegata --

Dai19791_Ceriporia_variegata

GATAACTGTGGTAATTCTAGAGCTAATACATGCAATCAAGCCCTGACTTC

Dai19886

GATAACTGTGGTAATTCTAGAGCTAATACATGCAATCAAGCCCTGACTTC

Dai10833_Ceriporia_crassitunic

GATAACTGTGGTAATTCTAGAGCTAATACATGCAATCAAGCCCCGACTTC

CHWC1506_46Meruliopsis_crassit --

Dai9995_Ceriporia_crassitunica --

Wu1209_58_Meruliopsis_parvispo --

CHWC1505_129_Meruliopsis_parvi --

Dai21944

GATAACTGTGGTAATTCTAGAGCTAATACATGCAATCAAGCCCCAACTTC

830Dai18640A

GATAACTGTGGTAATTCTAGAGCTAATACATGCAATCAAGCCCCGACTTC

GC1704_60_Meruliopsis_taxicola --

Dai22625

GATAACTGTGGTAATTCTAGAGCTAATACATGCAATCAAGCCCCGACTTC

Dai22636

GATAACTGTGGTAATTCTAGAGCTAATACATGCAATCAAGCCCCGACTTC

Dai21878

GATAACTGTGGTAATTCTAGAGCTAATACATGCAATCAAGCCCCGACTTC

1169Dai17248

GATAACTGTGGTAATTCTAGAGCTAATACATGCAATCAAGCCCCGACTTC

Wu1708_43_Meruliopsis_leptocys --

Li1011 --

ZX95Dai25742Meruliopsis_leptoc
GATAACTGTGGTAATTCTAGAGCTAATACATGCAATCAAGCCCCAACTTC
WCG1306Dai24733
GATAACTGTGGTAATTCTAGAGCTAATACATGCAATCAAGCCCCAACTTC
LXL99Dai25816
GATAACTGTGGTAATTCTAGAGCTAATACATGCAATCAAGCCCCAACTTC
WCG1559Dai26052Meruliopsis
GATAACTGTGGTAATTCTAGAGCTAATACATGCAATCAAGCCCCGACTTC
He7477 --
HLX243Dai26217
GATAACTGTGGTAATTCTAGAGCTAATACATGCAATCAAGCCCCGACTTC
RussiaMW673659Meruliopsis_fagi --
FD278 --
Dai10226_Ceriporia_tarda --
LE247365 --
Dai8173_Meruliopsis_nanlingens --
860Dai17172
GATAACTGTGGTAATTCTAGAGCTAATACATGCAATCAAGCCCCGACTTC
879Dai13414
GATAACTGTGGTAATTCTAGAGCTAATACATGCAATCAAGCCCCGACTTC
Li_1704_Meruliopsis_pseudocyst --
833Dai18405
GATAACTGTGGTAATTCTAGAGCTAATACATGCAATCAAGCCCCGACTTC
HHB_10729_Meruliopsis_albostra --
Cui6878_Ceriporia_pseudocystid --
869Dai14737
GATAACTGTGGTAATTCTAGAGCTAATACATGCAATCAAGCCCCGACTTC
876Cui11626
GATAACTGTGGTAATTCTAGAGCTAATACATGCAATCAAGCCCCGACTTC
1199WEI3388 --
776308_Meruliopsis_cystidiata --
ICN139059_Meruliopsis_cystidia --
HHB15692Ceraceomyces_serpens --
HHB_15629_Sp_Ceriporiopsis_ane --
AJ185Trametopsis_cervina --
FD9Irpex_lacteus --
908Dai11230
GATAACTGTGGTAATTCTAGAGCTAATACATGCAATCAAGCCCCGACTTC
FP55521TEmmia_lacerata --
PBU0048Ceriporia_cystidiata --
MZ340C_lacerataT --
Dai21940
GATAACTGTGGTAATTCTAGAGCTAATACATGCAATCAAGCCCCGACTTC
847Dai16433

GATAACTGTGGTAATTCTAGAGCTAATACATGCAATCAAGCCCCGACTTC
MarcinEmmia_latemarginatus --
Meijer3729Hydnopolyporus_fimbr --
RLG13408Phanerochaete_sp --
WHC1381Flavodon_flavus --
GB1833Phlebia_albida --
T407Phlebia_nitidula --
HHB6988Phanerochaete_exilis --
HHB8509Phanerochaetella_xeroph --
PBU0051Macrohyporia_dictyopora --
HHB11463Phanerochaete_sp --
FP102382Byssomerulius_corium --
FP102165Efibula_americana --
Murdoch90Ceriporia_torpida --
Rivoire4413_Ceriporia_purpurea --
Kout_18_Ceriporia_triumphalis --
Rivoire3701_Ceriporia_bresadol --
VS4018 --
Ryvarden21832_Ceriporia_manzan --
Dai24539
GATAACTGTGGTAATTCTAGAGCTAATACATGCAATCAAGCCCCGACTTC
Dai24541
GATAACTGTGGTAATTCTAGAGCTAATACATGCAATCAAGCCCCGACTTC
JV1105_12_Ceriporia_occidental --
VS8558Ceriporia_occidentalis --
Dai22445
GATAACTGTGGTAATTCTAGAGCTAATACATGCAATCAAGCCCCGACTTC
846Dai16368
GATAACTGTGGTAATTCTAGAGCTAATACATGCAATCAAGCCCCGACTTC
Dai17951_Ceriporia_aurantiocar --
Miettinen_11701C_viridans --
JV0105_10Ceriporia_aurantiocar --
Yuan5702C_viridans --
858Dai17003
GATAACTGTGGTAATTCTAGAGCTAATACATGCAATCAAGCCCCGACTTC
Yuan2747_Ceriporia_viridans --
Yuan2744C_viridans --
Li1046C_viridans --
865C_sinoviridans --
871Dai15062 --
Dai7642_Ceriporia_humilis
GATAACTGTGGTAATTCTAGAGCTAATACGTGCAATCAAGCCCCGACTTC
Spirin4706_Ceriporia_humilis --
Spirin4944_Ceriporia_sericea --

WCG1547Dai26044ceriporia
GATAACTGTGGTAATTCTAGAGCTAATACGTGCAATCAAGCCCCGACTTC
ZZW1558Dai27086
GATAACTGTGGTAATTCTAGAGCTAATACGTGCAATCAAGCCCCGACTTC
Miettinen14381_Ceriporia_mpuri --
Miettinen15492_2_Ceriporia_sor --
He6687
GATAACTGTGGTAATTCTAGAGCTAATACGTGCAATCAAGCCCCGACTTC
ZH53Dai24426
GATAACTGTGGTAATTCTAGAGCTAATACGTGCAATCAAGCCCCGACTCC
Vlasak0808_30_Ceriporia_punica --
887Dai13376
GATAACTGTGGTAATTCTAGAGCTAATACATGCAATCAAGCCCC------
WCG1443Dai24998 --GCCC
0108_6Ceriporia_spissa --
Dai19164 ---------------------
GCTAATACATGCAATCAAGCCCT------
Dai17937_Ceriporia_bubalinomar --
903Dai12113
GATAACTGTGGTAATTCTAGAGCTAATACATGCAATCAAGCCCT------
LZB929Dai25079
GATAACTGTGGTAATTCTAGAGCTAATACATGCAATCAAGCCCTGACTTC
LX45Dai26988 --
LX43Dai26986
GATAACTGTGGTAATTCTAGAGCTAATACATGCAATCAAGCCCTGACTTC
Dai7759Ceriporia --
Cui8012_Ceriporia_viridans --
GC1704_54Ceriporia_viridans --
Dai23392
GATAACTGTGGTAATTCTAGAGCTAATACATGCAATCAAGCCCTGACTTC
WCG1585Dai26113Ceriproia
GATAACTGTGGTAATTCTAGAGCTAATACATGCAATCAAGCCCCGACTTC
Dai18675C_eucalypti --
Dai22034
GATAACTGTGGTAATTCTAGAGCTAATACATGCAATCAAGCCCCGACTTC
JV1008_41JTardaFLORIDAKeys --
Rivoire1161_Ceriporia_pierii --
Dai23499C_pierii
GATAACTGTGGTAATTCTAGAGCTAATACATGCAATCAAGCCCCAACTTC
Dai23500
GATAACTGTGGTAATTCTAGAGCTAATACATGCAATCAAGCCCCAACTTC
841Dai15899
GATAACTGTGGTAATTCTAGAGCTAATACATGCAATCAAGCCCCGACTTC
842Dai15904

GATAACTGTGGTAATTCTAGAGCTAATACATGCAATCAAGCCCCGACTTC
LZB1066xinjiang --
LZB1065xinjiang --
851Dai16779 --
RMJ119sp_Candelabrochaete_sept --
RLG9759spCandelabrochaete_sept --
RLG10478Phanerochaete_allantos --
Dai19118_Ceriporia_spissa
GATAACTGTGGTAATTCTAGAGCTAATACATGCAATCAAGCCCC------
Dai18486A
GATAACTGTGGTAATTCTAGAGCTAATACATGCAATCAAGCCCC------
WEI17_024_Ceriporia_mellita --
GC1508_71Ceriporia_mellita --
GC1608_7_Ceriporia_mellita --
ZZW1557Dai27085 --
ZZW1554Dai27083 --
Dai8168 --
BR4865C_mellita --
MEL2382688Ceriporia_sp --
Dai8110 --
Cui8097 --
909Cui6740 --
W1258Dai24695
GATAACTGTGGTAATTCTAGAGCTAATACATGCAATCAAGCCCC------
JV0110_26_Ceriporia_griseoviol --
896Dai13202
GATAACTGTGGTAATTCTAGAGCTAATACATGCAATCAAGCCCCGACTTC
LWY393Dai27053C_griseoviolasce
GATAACTGTGGTAATTCTAGAGCTAATACATGCAATCAAGCCCCGACTTC
LWY394DAI27054
GATAACTGTGGTAATTCTAGAGCTAATACATGCAATCAAGCCCCGACTTC
FP135015G_pannocinctus --
L15726SpG_pannocinctus --
Dai22221
GATAACTGTGGTAATTCTAGAGCTAATACATGCAATCAAGCCCCGACTTC
Dai22633
GATAACTGTGGTAATTCTAGAGCTAATACATGCAATCAAGCCCCGACTTC
Dai23260
GATAACTGTGGTAATTCTAGAGCTAATACATGCAATCAAGCCCCGACTTC
Dai23626
GATAACTGTGGTAATTCTAGAGCTAATACATGCAATCAAGCCCCGACTTC
Dai16238G_citrinoalbus
GATAACTGTGGTAATTCTAGAGCTAATACATGCAATCAAGCCCCGACTTC
1175Dai15293

GATAACTGTGGTAATTCTAGAGCTAATACATGCAATCAAGCCCCAACTTC

Dai19547

GATAACTGTGGTAATTCTAGAGCTAATACATGCAATCAAGCCCCAACTTC

918063G_africanus --

918572G_africanus --

Dai18536A --

1164Cui17922

GATAACTGTGGTAATTCTAGAGCTAATACATGCAATCAAGCCCCGACTTC

Dai22225

GATAACTGTGGTAATTCTAGAGCTAATACATGCAATCAAGCCCCGACTTC

1163Dai20655

GATAACTGTGGTAATTCTAGAGCTAATACATGCAATCAAGCCCCGACTTC

Yuan4397G_hainanensis --

1176Dai15268

GATAACTGTGGTAATTCTAGAGCTAATACATGCAATCAAGCCCCGACTTC

1177Dai15259

GATAACTGTGGTAATTCTAGAGCTAATACATGCAATCAAGCCCCGACTTC

BZ2896G_thelephoroides --

1166JV1808_26

GATAACTGTGGTAATTCTAGAGCTAATACATGCAATCAAGCCCCGACTTC

Miettinen16992Hapalopilus_ochr --

GC1708_338_Ceriporia_arbuscula --

WCG1555Dai26107Ceriporia

GATAACTGTGGTAATTCTAGAGCTAATACATGCAATCAAGCCCCGACTTC

GC1708_340_Ceriporia_arbuscula --

WCG1556Dai26109Ceriporia

GATAACTGTGGTAATTCTAGAGCTAATACATGCAATCAAGCCCCGACTTC

883Cui11291

GATAACTGTGGTAATTCTAGAGCTAATACATGCAATCAAGCCCCGACTTC

HLX320Dai26805

GATAACTGTGGTAATTCTAGAGCTAATACATGCAATCAAGCCCCAACTTC

WCG1266Dai24678A --------------------------------------GCCCCGACTTC

Dai6090_Ceriporia_sulphuricolo --

RLG_11354_Ceriproia_reticulata --

ZZW1543Dai27072

GATAACTGTGGTAATTCTAGAGCTAATACATGCAATCAAGCCCCGACTTC

Li1316_Ceriporia_reticulata --

KHL11981Ceriporia_reticulata --

FP110343sp_Candelabrochaete_la --

Li1045_Ceriporia_reticulata --

ZX136Dai25794ceriporia ---C

892Dai13400

GATAACTGTGGTAATTCTAGAGCTAATACATGCAATCAAGCCCCGACTCC

RLG7163Leptoporus_mollis --

Dai21062Leptoporus_mollis --
Dai20182Leptoporus_submollis --
Cui18379Leptoporus_submollis --
Wu1209_46Resiniporus_pseudogil --
BRNM710169Resiniporus_resinasc --
Dai14516Bjerkandera_adusta
GATAACTGTGGTAATTCTAGAGCTAATACATGCAATCAAGCCCCGACTTC
Dai21100Bjerkandera_fumosa
GATAACTGTGGTAATTCTAGAGCTAATACATGCAATCAAGCCCCGACTTC
Miettinen16854Ceraceomyces_sp --
Dai10477C_spissa --
855Dai16831
GATAACTGTGGTAATTCTAGAGCTAATACATGCAATCAAGCCCCAACTTC
882Cui11282
GATAACTGTGGTAATTCTAGAGCTAATACATGCAATCAAGCCCCAACTTC
Dai24566
GATAACTGTGGTAATTCTAGAGCTAATACATGCAATCAAGCCCCAACTTC
Yuan5965 --
Dai3204 --
1194CUI9985 --

Dai15205_Ceriporia_albomellea --
Dai15223_Ceriporia_albomellea --
Li1780_Ceriporia_variegata --
Dai19791_Ceriporia_variegata
TGGAAGGGGTGTATTTATTAGATAAAAAACCAACGCGGTTCGCCGCTCCC
Dai19886
TGGAAGGGGTGTATTTATTAGATAAAAAACCAACGCGGTTCGCCGCTCCC
Dai10833_Ceriporia_crassitunic
CGGGAGGGGTGTATTTATTAGATAAAAAACCAACGCGGCTCGCCGCTCCT
CHWC1506_46Meruliopsis_crassit --
Dai9995_Ceriporia_crassitunica --
Wu1209_58_Meruliopsis_parvispo --
CHWC1505_129_Meruliopsis_parvi --
Dai21944
TGGAAGGGGTGTATTTATTAGATAAAAAACCAACGCGGCTCGCCGCTCCC
830Dai18640A
TGGAAGGGGTGTATTTATTAGATAAAAAACCAACGCGGTTCGCCGCTCCC
GC1704_60_Meruliopsis_taxicola --
Dai22625
TGGAAGGGGTGTATTTATTAGATAAAAAATCAACGCGGTTCGCCGCTCCC
Dai22636
TGGAAGGGGTGTATTTATTAGATAAAAAATCAACGCGGTTCGCCGCTCCC

Dai21878
TGGAAGGGGTGTATTTATTAGATAAAAAACCAACGCGGTTCGCCGCTCCC
1169Dai17248
TGGAAGGGGTGTATTTATTAGATAAAAAACCAACGCGGTTCGCCGCTCCC
Wu1708_43_Meruliopsis_leptocys --
Li1011 --
ZX95Dai25742Meruliopsis_leptoc
TGGAAGGGGTGTATTTATTAGATAAAAAACCAACGCGGTTCGCCGCTCCC
WCG1306Dai24733
TGGAAGGGGTGTATTTATTAGATAAAAAACCAACGCGGTTCGCCGCTCCC
LXL99Dai25816
TGGAAGGGGTGTATTTATTAGATAAAAAACCAACGCGGTTCGCCGCTCCC
WCG1559Dai26052Meruliopsis
TGGAAGGGGTGTATTTATTAGATAAAAAACCAACGCGGTTCGCCGCTCCC
He7477 --
HLX243Dai26217
TGGAAGGGGTGTATTTATTAGATAAAAAACCAACGCGGTTCGCCGCTCCC
RussiaMW673659Meruliopsis_fagi --
FD278 --
Dai10226_Ceriporia_tarda --
LE247365 --
Dai8173_Meruliopsis_nanlingens --
860Dai17172
TGGAAGGGGTGTATTTATTAGATAAAAAACCAACGCGGTTCGCCGCTCCC
879Dai13414
TGGAAGGGGTGTATTTATTAGATAAAAAACCAACGCGGTTCGCCGCTCCC
Li_1704_Meruliopsis_pseudocyst --
833Dai18405
TGGAAGGGGTGTATTTATTAGATAAAAAACCAACGCGGTTCGCCGCTCCC
HHB_10729_Meruliopsis_albostra --
Cui6878_Ceriporia_pseudocystid --
869Dai14737
TGGAAGGGGTGTATTTATTAGATAAAAAACCAACGCGGTTCGCCGCTCCC
876Cui11626
TGGAAGGGGTGTATTTATTAGATAAAAAACCAACGCGGTTCGCCGCTCCC
1199WEI3388 --
776308_Meruliopsis_cystidiata --
ICN139059_Meruliopsis_cystidia --
HHB15692Ceraceomyces_serpens --
HHB_15629_Sp_Ceriporiopsis_ane --
AJ185Trametopsis_cervina --
FD9Irpex_lacteus --
908Dai11230
TGGAAGGGGTGTATTTATTAGATAAAAAACCAACGCGGTTCGCCGCTCCC

FP55521TEmmia_lacerata --
PBU0048Ceriporia_cystidiata --
MZ340C_lacerataT --
Dai21940
TGGAAGGGGTGTATTTATTAGATAAAAAACCAATGCGGTTCGCCGCTCCC
847Dai16433
TGGAAGGGGTGTATTTATTAGATAAAAAACCAATGCGGTTCGCCGCTCCC
MarcinEmmia_latemarginatus --
Meijer3729Hydnopolyporus_fimbr --
RLG13408Phanerochaete_sp --
WHC1381Flavodon_flavus --
GB1833Phlebia_albida --
T407Phlebia_nitidula --
HHB6988Phanerochaete_exilis --
HHB8509Phanerochaetella_xeroph --
PBU0051Macrohyporia_dictyopora --
HHB11463Phanerochaete_sp --
FP102382Byssomerulius_corium --
FP102165Efibula_americana --
Murdoch90Ceriporia_torpida --
Rivoire4413_Ceriporia_purpurea --
Kout_18_Ceriporia_triumphalis --
Rivoire3701_Ceriporia_bresadol --
VS4018 --
Ryvarden21832_Ceriporia_manzan --
Dai24539
TGGAAGGGGTGTATTTATTAGATAAAAAACCAACGCGGTTCGCCGCTCCC
Dai24541
TGGAAGGGGTGTATTTATTAGATAAAAAACCAACGCGGTTCGCCGCTCCC
JV1105_12_Ceriporia_occidental --
VS8558Ceriporia_occidentalis --
Dai22445
TGGAAGGGGTGTATTTATTAGATAAAAAACCAACGCGGTTCGCCGCTCCC
846Dai16368
TGGAAGGGGTGTATTTATTAGATAAAAAACCAACGCGGTTCGCCGCTCCC
Dai17951_Ceriporia_aurantiocar --
Miettinen_11701C_viridans --
JV0105_10Ceriporia_aurantiocar --
Yuan5702C_viridans --
858Dai17003
TGGAAGGGGTGTATTTATTAGATAAAAAACCAACGCGGTTCGCCGCTCCC
Yuan2747_Ceriporia_viridans --
Yuan2744C_viridans --
Li1046C_viridans --

865C_sinoviridans --
871Dai15062 --
Dai7642_Ceriporia_humilis
TGGAAGGGGTGTATTTATTAGATAAAAAACCAACGCGGTTCGCCGCTCCC
Spirin4706_Ceriporia_humilis --
Spirin4944_Ceriporia_sericea --
WCG1547Dai26044ceriporia
TGGAAGGGGTGTATTTATTAGATAAAAAACCAACGCGGTTCGCCGCTCCC
ZZW1558Dai27086
TGGAAGGGGTGTATTTATTAGATAAAAAACCAACGCGGTTCGCCGCTCCC
Miettinen14381_Ceriporia_mpuri --
Miettinen15492_2_Ceriporia_sor --
He6687
TGGAAGGGGTGTATTTATTAGATAAAAAACCAACGCGGTTCGCCGCTCCC
ZH53Dai24426
TGGAAGGGGTGTATTTATTAGATAAAAAACCAACGCGGTTCGCCGCTCCC
Vlasak0808_30_Ceriporia_punica --
887Dai13376 -
GCAAGGGGTGTATTTATTAGATAAAAAACCAATGCGGTTCGCCGCTCCC
WCG1443Dai24998
CGCAAGGGGTGTATTTATTAGATAAAAAACCAATGCGGTTCGCCGCTCCC
0108_6Ceriporia_spissa --
Dai19164 -
GCAAAGGGTGTATTTATTAGATAAAAAACCAATGCGGTTCGCCGCTCCC
Dai17937_Ceriporia_bubalinomar --
903Dai12113 -
GCAAAGGGTGTATTTATTAGATAAAAAACCAATGCGGTTCGCCGCTCCC
LZB929Dai25079
TGGAAGGGGTGTATTTATTAGATAAAAAACCAACGCGGTTCGCCGCTCCC
LX45Dai26988 --
LX43Dai26986
TGGAAGGGGTGTATTTATTAGATAAAAAACCAACGCGGTTCGCCGCTCCC
Dai7759Ceriporia --
Cui8012_Ceriporia_viridans --
GC1704_54Ceriporia_viridans --
Dai23392
TGGAAGGGGTGTATTTATTAGATAAAAAACCAACGCGGTTCGCCGCTCCC
WCG1585Dai26113Ceriproia
CGGAAGGGGTGTATTTATTAGATAAAAAACCAACGCGGTTCGCCGCTCCC
Dai18675C_eucalypti --
Dai22034
TGGAAGGGGTGTATTTATTAGATAAAAAACCAACGCGGTTCGCCGCTCCC
JV1008_41JTardaFLORIDAKeys --
Rivoire1161_Ceriporia_pierii --

Dai23499C_pierii
TGGAAGGGGTGTATTTATTAGATAAAAAACCAACGCGGTTCGCCGCTCCC
Dai23500
TGGAAGGGGTGTATTTATTAGATAAAAAACCAACGCGGTTCGCCGCTCCC
841Dai15899
TGGAAGGGGTGTATTTATTAGATAAAAAACCAACGCGGTTCGCCGCTCCC
842Dai15904
TGGAAGGGGTGTATTTATTAGATAAAAAACCAACGCGGTTCGCCGCTCCC
LZB1066xinjiang --
LZB1065xinjiang --
851Dai16779 --GGCGGCTCCT
RMJ119sp_Candelabrochaete_sept --
RLG9759spCandelabrochaete_sept --
RLG10478Phanerochaete_allantos --
Dai19118_Ceriporia_spissa -
GCAAGGGGTGTATTTATTAGATAAAAAACCAATGCGGCTCGCCGCTCCC
Dai18486A -
GCAAGGGGTGTATTTATTATATAAAAAACCAATGCGGCTCGCCGCTCCC
WEI17_024_Ceriporia_mellita --
GC1508_71Ceriporia_mellita --
GC1608_7_Ceriporia_mellita --
ZZW1557Dai27085 --
ZZW1554Dai27083 --
Dai8168 --
BR4865C_mellita --
MEL2382688Ceriporia_sp --
Dai8110 --
Cui8097 --
909Cui6740 --
W1258Dai24695 -
GCAAGGGGTGTATTTATTAGATAAAAAACCAATGCGGTTCGCCGCTCCC
JV0110_26_Ceriporia_griseoviol --
896Dai13202
TGGAAGGGGTGTATTTATTAGATAAAAAACCAACGCGGTTCGCCGCTCCC
LWY393Dai27053C_griseoviolasce
TGGAAGGGGTGTATTTATTAGATAAAAAACCAACGCGGTTCGCCGCTCCC
LWY394DAI27054
TGGAAGGGGTGTATTTATTAGATAAAAAACCAACGCGGTTCGCCGCTCCC
FP135015G_pannocinctus --
L15726SpG_pannocinctus --
Dai22221
TGGAAGGGGTGTATTTATTAGATAAAAAACCAACGCGGTTCGCCGCTCCC
Dai22633
TGGAAGGGGTGTATTTATTAGATAAAAAACCAACGCGGTTTACCGCTCCC

Dai23260
TGGAAGGGGTGTATTTATTAGATAAAAAACCAACGCGGTTTACCGCTCCC
Dai23626
TGGAAGGGGTGTATTTATTAGATAAAAAACCAACGCGGTTTACCGCTCCC
Dai16238G_citrinoalbus
TGGAAGGGGTGTATTTATTAGATAAAAAGCCAACGCGGTTCGCCGCTCCT
1175Dai15293
TGGAAGGGGTGTATTTATTAGATAAAAAACCAACGCGGTTCGCCGCTCCC
Dai19547
TGGAAGGGGTGTATTTATTAGATAAAAAACCAACGCGGTTCGCCGCTCCC
918063G_africanus --
918572G_africanus --
Dai18536A --
1164Cui17922
TGGAAGGGGTGTATTTATTAGATAAAAAACCAACGCGGTTCGCCGCTCCC
Dai22225
TGGAAGGGGTGTATTTATTAGATAAAAAACCAACGCGGTTCGCCGCTCCC
1163Dai20655
TGGAAGGGGTGTATTTATTAGATAAAAAACCAACGCGGTTCGCCGCTCCC
Yuan4397G_hainanensis --
1176Dai15268
TGGAAGGGGTGTATTTATTAGATAAAAAACCAACGCGGTTCGCCGCTCCC
1177Dai15259
TGGAAGGGGTGTATTTATTAGATAAAAAACCAACGCGGTTCGCCGCTCCC
BZ2896G_thelephoroides --
1166JV1808_26
TGGAAGGGGTGTATTTATTAGATAAAAAACCAACGCGGTTCGCCGCTCCC
Miettinen16992Hapalopilus_ochr --
GC1708_338_Ceriporia_arbuscula --
WCG1555Dai26107Ceriporia
TGGAAGGGGTGTATTTATTAGATAAAAGACCAATGCGGTTCGCCGCTCCC
GC1708_340_Ceriporia_arbuscula --
WCG1556Dai26109Ceriporia
TGGAAGGGGTGTATTTATTAGATAAAAGACCAATGCGGTTCGCCGCTCCC
883Cui11291
TGGAAGGGGTGTATTTATTAGATAAAAAACCAATGCGGTTCGCCGCTCCC
HLX320Dai26805
AGGAAGGGGTGTATTTATTAGATAAAAAACCAATGCGGTTCGCCGCTCCC
WCG1266Dai24678A
TGGAAGGGGTGTATTTATTAGATAAAAAACCAATGCGGTTCGCCGCTCCT
Dai6090_Ceriporia_sulphuricolo --
RLG_11354_Ceriproia_reticulata --
ZZW1543Dai27072
TGGAAGGGGTGTATTTATTAGATAAAAAACCAACGCGGTTCGCCGCTCCC

Li1316_Ceriporia_reticulata --
KHL11981Ceriporia_reticulata --
FP110343sp_Candelabrochaete_la --
Li1045_Ceriporia_reticulata --
ZX136Dai25794ceriporia
TGGAAGGGGTGTATTTATTAGATAAAAAACCAACGCGGTTCGCCGCTCCC
892Dai13400
TGGAAGGGGTGTATTTATTAGATAAAAAACCAACGCGGTTCGCCGCTCCC
RLG7163Leptoporus_mollis --
Dai21062Leptoporus_mollis --
Dai20182Leptoporus_submollis --
Cui18379Leptoporus_submollis --
Wu1209_46Resiniporus_pseudogil --
BRNM710169Resiniporus_resinasc --
Dai14516Bjerkandera_adusta
TGGAAGGGGTGTATTTATTAGATAAAAAACCAACGCGGTTCGCCGCTCCC
Dai21100Bjerkandera_fumosa
TGGAAGGGGTGTATTTATTAGATAAAAAACCAACGCGGTTCGCCGCTCCC
Miettinen16854Ceraceomyces_sp --
Dai10477C_spissa --
855Dai16831
TGGAAGGGGTGTATTTATTAGATAAAAAGCCAACGCGGTTCGCTGCTCTC
882Cui11282
TGGAAGGGGTGTATTTATTAGATAAAAAGCCAACGCGGTTCGCCGCTCTC
Dai24566
TGGAAGGGGTGTATTTATTAGATAAAAAGCCAACGCGGTTCGCCGCTCTC
Yuan5965 --
Dai3204 --
1194CUI9985 --

Dai15205_Ceriporia_albomellea --
Dai15223_Ceriporia_albomellea --
Li1780_Ceriporia_variegata --
Dai19791_Ceriporia_variegata TTGGTGATTCATAATAACT-TCTCGAATCGCATGG-CCTTGTGCCGGCGA
Dai19886 TTGGTGATTCATAATAACT-TCTCGAATCGCATGG-CCTTGTGCCGGCGA
Dai10833_Ceriporia_crassitunic TTGGTGATTCATAATAACT-CGTCGAATCGCATGG-CCTTGTGCCGGCGA
CHWC1506_46Meruliopsis_crassit --
Dai9995_Ceriporia_crassitunica --
Wu1209_58_Meruliopsis_parvispo --
CHWC1505_129_Meruliopsis_parvi --

Dai21944 TTGGTGATTCATAATAACT-TTTCGAATCGCATGG-CCTTGTGCCGGCGA

830Dai18640A TTGGTGATTCATAATAACT-TTTCGAATCGCATGG-CCTTGTGCCGGCGA

GC1704_60_Meruliopsis_taxicola --

Dai22625 TTGGTGATTCATAATAACT-TCACGAATCGCATGG-CCTTGTGCCGGCGA

Dai22636 TTGGTGATTCATAATAACT-TCACGAATCGCATGG-CCTTGTGCCGGCGA

Dai21878 TTGGTGATTCATAATAACT-TCTCGAATCGCATGG-CCTTGTGCCGGCGA

1169Dai17248 TTGGTGATTCATAATAACT-TCTCGAATCGCATGG-CCTTGTGCCGGCGA

Wu1708_43_Meruliopsis_leptocys --

Li1011 --

ZX95Dai25742Meruliopsis_leptoc TTGGTGATTCATAATAACT-TCTCGAATCGCATGG-CCTTGTGCCGGCGA

WCG1306Dai24733 TTGGTGATTCATAATAACT-TCTCGAATCGCATGG-CCTTGTGCCGGCGA

LXL99Dai25816 TTGGTGATTCATAATAACT-TCTCGAATCGCATGG-CCTTGTGCCGGCGA

WCG1559Dai26052Meruliopsis TTGGTGATTCATAATAACT-TCTCGAATCGCATGG-CCTTGTGCCGGCGA

He7477 --

HLX243Dai26217 TTGGTGATTCATAATAACT-TCTCGAATCGCATGG-CCTTGTGCCGGCGA

RussiaMW673659Meruliopsis_fagi --

FD278 --

Dai10226_Ceriporia_tarda --

LE247365 --

Dai8173_Meruliopsis_nanlingens --

860Dai17172 TTGGTGATTCATAATAACT-TCTCGAATCGCATGG-CCTTGTGCCGGCGA

879Dai13414 TTGGTGATTCATAATAACT-TCTCGAATCGCATGG-CCTTGTGCCGGCGA

Li_1704_Meruliopsis_pseudocyst --

833Dai18405 TTGGTGATTCATAATAACT-TCTCGAATCGCATGG-CCTTGTGCCGGCGA

HHB_10729_Meruliopsis_albostra --

Cui6878_Ceriporia_pseudocystid --

869Dai14737 TTGGTGATTCATAATAACT-TCTCGAATCGCATGG-CCTTGTGCCGGCGA

876Cui11626 TTGGTGATTCATAATAACT-TCTCGAATCGCATGG-CCTTGTGCCGGCGA

1199WEI3388 --
776308_Meruliopsis_cystidiata --
ICN139059_Meruliopsis_cystidia --
HHB15692Ceraceomyces_serpens --
HHB_15629_Sp_Ceriporiopsis_ane --
AJ185Trametopsis_cervina --
FD9Irpex_lacteus --
908Dai11230 TTGGTGATTCATAATAACT-TCTCGAATCGCATGG-CCTTGTGCCGGCGA
FP55521TEmmia_lacerata --
PBU0048Ceriporia_cystidiata --
MZ340C_lacerataT --
Dai21940 TTGGTGATTCATAATAACT-TCTCGAATCGCATGG-CCTTGTGCCGGCGA
847Dai16433 TTGGTGATTCATAATAACT-TCTCGAATCGCATGG-CCTTGTGCCGGCGA
MarcinEmmia_latemarginatus --
Meijer3729Hydnopolyporus_fimbr --
RLG13408Phanerochaete_sp --
WHC1381Flavodon_flavus --
GB1833Phlebia_albida --
T407Phlebia_nitidula --
HHB6988Phanerochaete_exilis --
HHB8509Phanerochaetella_xeroph --
PBU0051Macrohyporia_dictyopora --
HHB11463Phanerochaete_sp --
FP102382Byssomerulius_corium --
FP102165Efibula_americana --
Murdoch90Ceriporia_torpida --
Rivoire4413_Ceriporia_purpurea --
Kout_18_Ceriporia_triumphalis --
Rivoire3701_Ceriporia_bresadol --
VS4018 --
Ryvarden21832_Ceriporia_manzan --
Dai24539 TTGGTGATTCATAATAACT-TCTCGAATCGCATGG-CCTTGTGCCGGCGA
Dai24541 TTGGTGATTCATAATAACT-TCTCGAATCGCATGG-CCTTGTGCCGGCGA
JV1105_12_Ceriporia_occidental --
VS8558Ceriporia_occidentalis --
Dai22445 TTGGTGATTCATAATAACT-TCTCGAATCGCATGG-CCTTGTGCCGGCGA
846Dai16368 TTGGTGATTCATAATAACT-TCTCGAATCGCATGG-CCTTGTGCCGGCGA

Dai17951_Ceriporia_aurantiocar --
Miettinen_11701C_viridans --
JV0105_10Ceriporia_aurantiocar --
Yuan5702C_viridans --
858Dai17003 TTGGTGATTCATAATAACT-TGTCGAATCGTATGG-CCTTGTGCCGGCGA
Yuan2747_Ceriporia_viridans --
Yuan2744C_viridans --
Li1046C_viridans --
865C_sinoviridans --
871Dai15062 --
Dai7642_Ceriporia_humilis TTGGTGATTCATAATAACT-TCTCGAATCGCATGG-CCTTGCGCCGGCGA
Spirin4706_Ceriporia_humilis --
Spirin4944_Ceriporia_sericea --
WCG1547Dai26044ceriporia TTGGTGATTCATAATAACT-TCTCGAATCGCATGG-CCTTGCGCCGGCGA
ZZW1558Dai27086 TTGGTGATTCATAATAACT-TCTCGAATCGCATGG-CCTTGCGCCGGCGA
Miettinen14381_Ceriporia_mpuri --
Miettinen15492_2_Ceriporia_sor --
He6687 TTGGTGATTCATAATAACT-TCTCGAATCGCATGG-CCTTGCGCCGGCGA
ZH53Dai24426 TTGGTGATTCATAATAACT-TCTCGAATCGCATGG-CCTTGCGCCGGCGA
Vlasak0808_30_Ceriporia_punica --
887Dai13376 TTGGTGATTCATAATAACT-TCTCGAATCGCATGG-CCTTGTGCCGGCGA
WCG1443Dai24998 TTGGTGATTCATAATAACT-TCTCGAATCGCATGG-CCTTGTGCCGGCGA
0108_6Ceriporia_spissa --
Dai19164 TTGGTGATTCATAATAACT-TCTCGAATCGCACGG-CCTTGCGCTGGCGA
Dai17937_Ceriporia_bubalinomar --
903Dai12113 TTGGTGATTCATAATAACT-TCTCGAATCGCATGG-CCTTGTGCCGGCGA
LZB929Dai25079 TTGGTGATTCATAATAACT-TCTCGAATCGCATGG-CCTTGTGCCGGCGA
LX45Dai26988 --
LX43Dai26986 TTGGTGATTCATAATAACT-TCTCGAATCGCATGG-CCTTGTGCCGGCGA
Dai7759Ceriporia --
Cui8012_Ceriporia_viridans --
GC1704_54Ceriporia_viridans --

Dai23392 TTGGTGATTCATAATAACT-TCTCGAATCGTATGG-CCTTGTGCCGGCGA

WCG1585Dai26113Ceriproia TTGGTGATTCATAATAACT-TCTCGAATCGCATGG-CCTTGCGCCGGCGA

Dai18675C_eucalypti --

Dai22034 TTGGTGATTCATAATAACT-TCTCGAATCGCATGG-CCTTGTGCTGGCGA

JV1008_41JTardaFLORIDAKeys --

Rivoire1161_Ceriporia_pierii --

Dai23499C_pierii TTGGTGATTCATAATAACT-TCTCGAATCGCATGG-CCTTGCGCCGGCGA

Dai23500 TTGGTGATTCATAATAACT-TCTCGAATCGCATGG-CCTTGCGCCGGCGA

841Dai15899 TTGGTGATTCATAATAACT-TCTCGAATCGCATGG-CCTTGTGCCGGCGA

842Dai15904 TTGGTGATTCATAATAACT-TCTCGAATCGCATGG-CCTTGTGCCGGCGA

LZB1066xinjiang --

LZB1065xinjiang --

851Dai16779 TTGGTGATTCATAATAACCGTCTCGAATCGCATGGCCCTTGCGCCGGCGA

RMJ119sp_Candelabrochaete_sept --

RLG9759spCandelabrochaete_sept --

RLG10478Phanerochaete_allantos --

Dai19118_Ceriporia_spissa TTGGTGATTCATAATAACT-TTTCGAATCGCATGG-CCTTGCGCCGGCGA

Dai18486A TTGGTGATTCATAATAACT-TTTCGAATCGCATGG-CCTTGCGCCGGGGA

WEI17_024_Ceriporia_mellita --

GC1508_71Ceriporia_mellita --

GC1608_7_Ceriporia_mellita --

ZZW1557Dai27085 --

ZZW1554Dai27083 --

Dai8168 --

BR4865C_mellita --

MEL2382688Ceriporia_sp --

Dai8110 --

Cui8097 --

909Cui6740 --

W1258Dai24695 TTGGTGATTCATAATAACT-TTTCGAATCGCATGG-CCTTGCGCCGGCGA

JV0110_26_Ceriporia_griseoviol --

896Dai13202 TTGGTGATTCATAATAACT-TCTCGAATCGCATGG-CCTTGTGCCGGCGA

LWY393Dai27053C_griseoviolasce TTGGTGATTCATAATAACT-TCTCGAATCGCATGG-CCTTGTGCCGGCGA

LWY394DAI27054 TTGGTGATTCATAATAACT-TCTCGAATCGCATGG-CCTTGTGCCGGCGA

FP135015G_pannocinctus --

L15726SpG_pannocinctus --

Dai22221 TTGGTGATTCATAATAACT-TCTCGAATCGTATGG-CCTTGTGCCGGCGA

Dai22633 TTGGTGATTCATAATAACT-TCTCGAATCGTATGG-CCTTGTGCCGGCGA

Dai23260 TTGGTGATTCATAATAACT-TCTCGAATCGTATGG-CCTTGTGCCGGCGA

Dai23626 TTGGTGATTCATAATAACT-TCTCGAATCGTATGG-CCTTGTGCCGGCGA

Dai16238G_citrinoalbus TTGGTGATTCATAATAACT-TCTCGAATCGCATGG-CCTTGCGCCGGCGA

1175Dai15293 TTGGTGATTCATAATAACT-TCTCGAATCGTATGG-CCTTGTGCCGGCGA

Dai19547 TTGGTGATTCATAATAACT-TCTCGAATCGTATGG-CCTTGTGCCGGCGA

918063G_africanus --

918572G_africanus --

Dai18536A --

1164Cui17922 TTGGTGATTCATAATAACT-TCTCGAATCGTATGG-CCTTGTGCCGGCGA

Dai22225 TTGGTGATTCATAATAACT-TCTCGAATCGTATGG-CCTTGTGCCGGCGA

1163Dai20655 TTGGTGATTCATAATAACT-TCTCGAATCGTATGG-CCTTGTGCCGGCGA

Yuan4397G_hainanensis --

1176Dai15268 TTGGTGATTCATAATAACT-TCTCGAATCGCATGG-CCTTGTGCCGGCGA

1177Dai15259 TTGGTGATTCATAATAACT-TCTCGAATCGCATGG-CCTTGTGCCGGCGA

BZ2896G_thelephoroides --

1166JV1808_26 TTGGTGATTCATAATAACT-TCTCGAATCGCATGG-CCTTGTGCTGGCGA

Miettinen16992Hapalopilus_ochr --

GC1708_338_Ceriporia_arbuscula --

WCG1555Dai26107Ceriporia TTGGTGATTCATAATAACT-TCTCGAATCGCATGG-CCTTGTGCCGGCGA

GC1708_340_Ceriporia_arbuscula --

WCG1556Dai26109Ceriporia TTGGTGATTCATAATAACT-TCTCGAATCGCATGG-CCTTGTGCCGGCGA

883Cui11291 TTGGTGATTCATAATAACT-TCTCGAATCGTATGG-CCTTGTGCCGGCGA

HLX320Dai26805 TTGGTGATTCATAATAACT-TCTCGAATCGCATGG-CCTTGTGCCGGCGA

WCG1266Dai24678A TTGGTGATTCATAATAACT-TGTCGAATCGCATGG-CCTTGTGCCGGCGA

Dai6090_Ceriporia_sulphuricolo --

RLG_11354_Ceriproia_reticulata --

ZZW1543Dai27072 TTGGTGATTCATAATAACT-TCTCGAATCGTATGG-CCTTGCGCCGGCGA

Li1316_Ceriporia_reticulata --

KHL11981Ceriporia_reticulata --

FP110343sp_Candelabrochaete_la --

Li1045_Ceriporia_reticulata ---GGCGA

ZX136Dai25794ceriporia TTGGTGATTCATAATAACT-TCTCGAATCGCATGG-CCTTGTGCTGGCGA

892Dai13400 TTGGTGATTCATAATAACT-TCTCGAATCGCATGG-CCTTGTGCTGGCGA

RLG7163Leptoporus_mollis --

Dai21062Leptoporus_mollis --

Dai20182Leptoporus_submollis --

Cui18379Leptoporus_submollis --

Wu1209_46Resiniporus_pseudogil --

BRNM710169Resiniporus_resinasc --

Dai14516Bjerkandera_adusta TTGGTGATTCATAATAACT-TCTCGAATCGCATGG-CCTTGTGCCGGCGA

Dai21100Bjerkandera_fumosa TTGGTGATTCATAATAACT-TCTCGAATCGCATGG-CCTTGTGCCGGCGA

Miettinen16854Ceraceomyces_sp --

Dai10477C_spissa --

855Dai16831 TTGGTGATTCATAATAACT-TCTCGAATCGCATGG-CCTTGTGCTGGCGA

882Cui11282 TTGGTGATTCATAATAACT-TCTCGAATCGCATGG-CCTTGTGCTGGCGA

Dai24566 TTGGTGATTCATAATAACT-TCTCGAATCGCATGG-CCTTGTGCTGGCGA

Yuan5965 --

Dai3204 --

1194CUI9985 --

Dai15205_Ceriporia_albomellea --

Dai15223_Ceriporia_albomellea --

Li1780_Ceriporia_variegata --

Dai19791_Ceriporia_variegata
TGCTTCATTCAAATATCTGCCCTATCAACTTTCGATGGTAGGATAGAGGC
Dai19886
TGCTTCATTCAAATATCTGCCCTATCAACTTTCGATGGTAGGATAGAGGC
Dai10833_Ceriporia_crassitunic
TGCTTCATTCAAATATCTGCCCTATCAACTTTCGATGGTAGGATAGAGGC
CHWC1506_46Meruliopsis_crassit --
Dai9995_Ceriporia_crassitunica --
Wu1209_58_Meruliopsis_parvispo --
CHWC1505_129_Meruliopsis_parvi --
Dai21944
TGCTTCATTCAAATATCTGCCCTATCAACTTTCGATGGTAGGATAGAGGC
830Dai18640A
TGCTTCATTCAAATATCTGCCCTATCAACTTTCGATGGTAGGATAGAGGC
GC1704_60_Meruliopsis_taxicola --
Dai22625
TGCTTCATTCAAATATCTGCCCTATCAACTTTCGATGGTAGGATAGAGGC
Dai22636
TGCTTCATTCAAATATCTGCCCTATCAACTTTCGATGGTAGGATAGAGGC
Dai21878
TGCTTCATTCAAATATCTGCCCTATCAACTTTCGATGGTAGGATAGAGGC
1169Dai17248
TGCTTCATTCAAATATCTGCCCTATCAACTTTCGATGGTAGGATAGAGGC
Wu1708_43_Meruliopsis_leptocys --
Li1011 --
ZX95Dai25742Meruliopsis_leptoc
TGCTTCATTCAAATATCTGCCCTATCAACTTTCGATGGTAGGATAGAGGC
WCG1306Dai24733
TGCTTCATTCAAATATCTGCCCTATCAACTTTCGATGGTAGGATAGAGGC
LXL99Dai25816
TGCTTCATTCAAATATCTGCCCTATCAACTTTCGATGGTAGGATAGAGGC
WCG1559Dai26052Meruliopsis
TGCTTCATTCAAATATCTGCCCTATCAACTTTCGATGGTAGGATAGAGGC
He7477 --
HLX243Dai26217
TGCTTCATTCAAATATCTGCCCTATCAACTTTCGATGGTAGGATAGAGGC
RussiaMW673659Meruliopsis_fagi --
FD278 --
Dai10226_Ceriporia_tarda --
LE247365 --
Dai8173_Meruliopsis_nanlingens --
860Dai17172
TGCTTCATTCAAATATCTGCCCTATCAACTTTCGATGGTAGGATAGAGGC
879Dai13414

TGCTTCATTCAAATATCTGCCCTATCAACTTTCGATGGTAGGATAGAGGC
Li_1704_Meruliopsis_pseudocyst --
833Dai18405
TGCTTCATTCAAATATCTGCCCTATCAACTTTCGATGGTAGGATAGAGGC
HHB_10729_Meruliopsis_albostra --
Cui6878_Ceriporia_pseudocystid --
869Dai14737
TGCTTCATTCAAATATCTGCCCTATCAACTTTCGATGGTAGGATAGAGGC
876Cui11626
TGCTTCATTCAAATATCTGCCCTATCAACTTTCGATGGTAGGATAGAGGC
1199WEI3388 --
776308_Meruliopsis_cystidiata --
ICN139059_Meruliopsis_cystidia --
HHB15692Ceraceomyces_serpens --
HHB_15629_Sp_Ceriporiopsis_ane --
AJ185Trametopsis_cervina --
FD9Irpex_lacteus --
908Dai11230
TGCTTCATTCAAATATCTGCCCTATCAACTTTCGATGGTAGGATAGAGGC
FP55521TEmmia_lacerata --
PBU0048Ceriporia_cystidiata --
MZ340C_lacerataT --
Dai21940
TGCTTCATTCAAATATCTGCCCTATCAACTTTCGATGGTAGGATAGAGGC
847Dai16433
TGCTTCATTCAAATATCTGCCCTATCAACTTTCGATGGTAGGATAGAGGC
MarcinEmmia_latemarginatus --
Meijer3729Hydnopolyporus_fimbr --
RLG13408Phanerochaete_sp --
WHC1381Flavodon_flavus --
GB1833Phlebia_albida --
T407Phlebia_nitidula --
HHB6988Phanerochaete_exilis --
HHB8509Phanerochaetella_xeroph --
PBU0051Macrohyporia_dictyopora --
HHB11463Phanerochaete_sp --
FP102382Byssomerulius_corium --
FP102165Efibula_americana --
Murdoch90Ceriporia_torpida --
Rivoire4413_Ceriporia_purpurea --
Kout_18_Ceriporia_triumphalis --
Rivoire3701_Ceriporia_bresadol --
VS4018 --
Ryvarden21832_Ceriporia_manzan --

Dai24539
TGCTTCATTCAAATATCTGCCCTATCAACTTTCGATGGTAGGATAGAGGC
Dai24541
TGCTTCATTCAAATATCTGCCCTATCAACTTTCGATGGTAGGATAGAGGC
JV1105_12_Ceriporia_occidental --
VS8558Ceriporia_occidentalis --
Dai22445
TGCTTCATTCAAATATCTGCCCTATCAACTTTCGATGGTAGGATAGAGGC
846Dai16368
TGCTTCATTCAAATATCTGCCCTATCAACTTTCGATGGTAGGATAGAGGC
Dai17951_Ceriporia_aurantiocar --
Miettinen_11701C_viridans --
JV0105_10Ceriporia_aurantiocar --
Yuan5702C_viridans --
858Dai17003
TGCTTCATTCAAATATCTGCCCTATCAACTTTCGATGGTAGGATAGAGGC
Yuan2747_Ceriporia_viridans --
Yuan2744C_viridans --
Li1046C_viridans --
865C_sinoviridans --
871Dai15062 --
Dai7642_Ceriporia_humilis
TGCTTCATTCAAATATCTGCCCTATCAACTTTCGATGGTAGGATAGAGGC
Spirin4706_Ceriporia_humilis --
Spirin4944_Ceriporia_sericea --
WCG1547Dai26044ceriporia
TGCTTCATTCAAATATCTGCCCTATCAACTTTCGATGGTAGGATAGAGGC
ZZW1558Dai27086
TGCTTCATTCAAATATCTGCCCTATCAACTTTCGATGGTAGGATAGAGGC
Miettinen14381_Ceriporia_mpuri --
Miettinen15492_2_Ceriporia_sor --
He6687
TGCTTCATTCAAATATCTGCCCTATCAACTTTCGATGGTAGGATAGAGGC
ZH53Dai24426
TGCTTCATTCAAATATCTGCCCTATCAACTTTCGATGGTAGGATAGAGGC
Vlasak0808_30_Ceriporia_punica --
887Dai13376
TGCTTCATTCAAATATCTGCCCTATCAACTTTCGATGGTAGGATAGAGGC
WCG1443Dai24998
TGCTTCATTCAAATATCTGCCCTATCAACTTTCGATGGTAGGATAGAGGC
0108_6Ceriporia_spissa --
Dai19164
TGCTTCATTCAAATATCTGCCCTATCAACTTTCGATGGTAGGATAGAGGC
Dai17937_Ceriporia_bubalinomar --

903Dai12113
TGCTTCATTCAAATATCTGCCCTATCAACTTTCGATGGTAGGATAGAGGC
LZB929Dai25079
TGCTTCATTCAAATATCTGCCCTATCAACTTTCGATGGTAGGATAGAGGC
LX45Dai26988 ---
LX43Dai26986
TGCTTCATTCAAATATCTGCCCTATCAACTTTCGATGGTAGGATAGAGGC
Dai7759Ceriporia ---
Cui8012_Ceriporia_viridans ---
GC1704_54Ceriporia_viridans ---
Dai23392
TGCTTCATTCAAATATCTGCCCTATCAACTTTCGATGGTAGGATAGAGGC
WCG1585Dai26113Ceriproia
TGCTTCATTCAAATATCTGCCCTATCAACTTTCGATGGTAGGATAGAGGC
Dai18675C_eucalypti ---
Dai22034
TGCTTCATTCAAATATCTGCCCTATCAACTTTCGATGGTAGGATAGAGGC
JV1008_41JTardaFLORIDAKeys ---
Rivoire1161_Ceriporia_pierii ---
Dai23499C_pierii
TGCTTCATTCAAATATCTGCCCTATCAACTTTCGATGGTAGGATAGAGGC
Dai23500
TGCTTCATTCAAATATCTGCCCTATCAACTTTCGATGGTAGGATAGAGGC
841Dai15899
TGCTTCATTCAAATATCTGCCCTATCAACTTTCGATGGTAGGATAGAGGC
842Dai15904
TGCTTCATTCAAATATCTGCCCTATCAACTTTCGATGGTAGGATAGAGGC
LZB1066xinjiang ---
LZB1065xinjiang ---
851Dai16779
TGCTTCATTCAAATATCTGCCCTATCAACTTTCGATGGTAGGATAGAGGC
RMJ119sp_Candelabrochaete_sept ---
RLG9759spCandelabrochaete_sept ---
RLG10478Phanerochaete_allantos ---
Dai19118_Ceriporia_spissa
TGCTTCATTCAAATATCTGCCCTATCAACTTCCGATGGTAGGATAGAGGC
Dai18486A
TGCTTCATTCAAATATCTGCCCTATCAACTTCCGATGGTAGGATAGAGGC
WEI17_024_Ceriporia_mellita ---
GC1508_71Ceriporia_mellita ---
GC1608_7_Ceriporia_mellita ---
ZZW1557Dai27085 ---
ZZW1554Dai27083 ----------------------
TATCAACTCCCGATGGTAGGATAGAGGC

Dai8168 ---
BR4865C_mellita ---
MEL2382688Ceriporia_sp ---
Dai8110 ---
Cui8097 ---
909Cui6740 ---
W1258Dai24695
TGCTTCATTCAAATATCTGCCCTATCAACTTCCGATGGTAGGATAGAGGC
JV0110_26_Ceriporia_griseoviol ---
896Dai13202
TGCTTCATTCAAATATCTGCCCTATCAACTTTCGATGGTAGGATAGAGGC
LWY393Dai27053C_griseoviolasce
TGCTTCATTCAAATATCTGCCCTATCAACTTTCGATGGTAGGATAGAGGC
LWY394DAI27054
TGCTTCATTCAAATATCTGCCCTATCAACTTTCGATGGTAGGATAGAGGC
FP135015G_pannocinctus ---
L15726SpG_pannocinctus ---
Dai22221
TGCTTCATTCAAATATCTGCCCTATCAACTTTCGATGGTAGGATAGAGGC
Dai22633
TGCTTCATTCAAATATCTGCCCTATCAACTTTCGATGGTAGGATAGAGGC
Dai23260
TGCTTCATTCAAATATCTGCCCTATCAACTTTCGATGGTAGGATAGAGGC
Dai23626
TGCTTCATTCAAATATCTGCCCTATCAACTTTCGATGGTAGGATAGAGGC
Dai16238G_citrinoalbus
TGCTTCATTCAAATATCTGCCCTATCAACTTTCGATGGTAGGATAGAGGC
1175Dai15293
TGCTTCATTCAAATATCTGCCCTATCAACTTTCGATGGTAGGATAGAGGC
Dai19547
TGCTTCATTCAAATATCTGCCCTATCAACTTTCGATGGTAGGATAGAGGC
918063G_africanus ---
918572G_africanus ---
Dai18536A ---
1164Cui17922
TGCTTCATTCAAATATCTGCCCTATCAACTTTCGATGGTAGGATAGAGGC
Dai22225
TGCTTCATTCAAATATCTGCCCTATCAACTTTCGATGGTAGGATAGAGGC
1163Dai20655
TGCTTCATTCAAATATCTGCCCTATCAACTTTCGATGGTAGGATAGAGGC
Yuan4397G_hainanensis ---
1176Dai15268
TGCTTCATTCAAATATCTGCCCTATCAACTTTCGATGGTAGGATAGAGGC
1177Dai15259

TGCTTCATTCAAATATCTGCCCTATCAACTTTCGATGGTAGGATAGAGGC
BZ2896G_thelephoroides --
1166JV1808_26
TGCTTCATTCAAATATCTGCCCTATCAACTTTCGATGGTAGGATAGAGGC
Miettinen16992Hapalopilus_ochr --
GC1708_338_Ceriporia_arbuscula --
WCG1555Dai26107Ceriporia
TGCTTCATTCAAATATCTGCCCTATCAACTTTCGATGGTAGGATAGAGGC
GC1708_340_Ceriporia_arbuscula --
WCG1556Dai26109Ceriporia
TGCTTCATTCAAATATCTGCCCTATCAACTTTCGATGGTAGGATAGAGGC
883Cui11291
TGCTTCATTCAAATATCTGCCCTATCAACTTTCGATGGTAGGATAGAGGC
HLX320Dai26805
TGCTTCATTCAAATATCTGCCCTATCAACTTTCGATGGTAGGATAGAGGC
WCG1266Dai24678A
TGCTTCATTCAAATATCTGCCCTATCAACTTTCGATGGTAGGATAGAGGC
Dai6090_Ceriporia_sulphuricolo --
RLG_11354_Ceriproia_reticulata --
ZZW1543Dai27072
TGCTTCATTCAAATATCTGCCCTATCAACTTTCGATGGTAGGATAGAGGC
Li1316_Ceriporia_reticulata --
KHL11981Ceriporia_reticulata --
FP110343sp_Candelabrochaete_la --
Li1045_Ceriporia_reticulata
TGCTTCATTCAAATATCTGCCCTATCAACTTTCGATGGTAGGATAGAGGC
ZX136Dai25794ceriporia
TGCTTCATTCAAATATCTGCCCTATCAACTTTCGATGGTAGGATAGAGGC
892Dai13400
TGCTTCATTCAAATATCTGCCCTATCAACTTTCGATGGTAGGATAGAGGC
RLG7163Leptoporus_mollis --
Dai21062Leptoporus_mollis --
Dai20182Leptoporus_submollis --
Cui18379Leptoporus_submollis --
Wu1209_46Resiniporus_pseudogil --
BRNM710169Resiniporus_resinasc --
Dai14516Bjerkandera_adusta
TGCTTCATTCAAATATCTGCCCTATCAACTTTCGATGGTAGGATAGAGGC
Dai21100Bjerkandera_fumosa
TGCTTCATTCAAATATCTGCCCTATCAACTTTCGATGGTAGGATAGAGGC
Miettinen16854Ceraceomyces_sp --
Dai10477C_spissa --
855Dai16831
TGCTTCATTCAAATATCTGCCCTATCAACTTTCGATGGTAGGATAGAGGC

882Cui11282
TGCTTCATTCAAATATCTGCCCTATCAACTTTCGATGGTAGGATAGAGGC
Dai24566
TGCTTCATTCAAATATCTGCCCTATCAACTTTCGATGGTAGGATAGAGGC
Yuan5965 --
Dai3204 --
1194CUI9985 --

Dai15205_Ceriporia_albomellea --
Dai15223_Ceriporia_albomellea --
Li1780_Ceriporia_variegata --
Dai19791_Ceriporia_variegata
CTACCATGGTTTCAACGGGTAACGGGGAATAAGGGTTCGATTCCGGAGAG
Dai19886
CTACCATGGTTTCAACGGGTAACGGGGAATAAGGGTTCGATTCCGGAGAG
Dai10833_Ceriporia_crassitunic
CTACCATGGTTTCAACGGGTAACGGGGAATAAGGGTTCGATTCCGGAGAG
CHWC1506_46Meruliopsis_crassit --
Dai9995_Ceriporia_crassitunica --
Wu1209_58_Meruliopsis_parvispo --
CHWC1505_129_Meruliopsis_parvi --
Dai21944
CTACCATGGTTTCAACGGGTAACGGGGAATAAGGGTTCGATTCCGGAGAG
830Dai18640A
CTACCATGGTTTCAACGGGTAACGGGGAATAAGGGTTCGATTCCGGAGAG
GC1704_60_Meruliopsis_taxicola --
Dai22625
CTACCATGGTTTCAACGGGTAACGGGGAATAAGGGTTCGATTCCGGAGAG
Dai22636
CTACCATGGTTTCAACGGGTAACGGGGAATAAGGGTTCGATTCCGGAGAG
Dai21878
CTACCATGGTTTCAACGGGTAACGGGGAATAAGGGTTCGATTCCGGAGAG
1169Dai17248
CTACCATGGTTTCAACGGGTAACGGGGAATAAGGGTTCGATTCCGGAGAG
Wu1708_43_Meruliopsis_leptocys --
Li1011 --
ZX95Dai25742Meruliopsis_leptoc
CTACCATGGTTTCAACGGGTAACGGGGAATAAGGGTTCGATTCCGGAGAG
WCG1306Dai24733
CTACCATGGTTTCAACGGGTAACGGGGAATAAGGGTTCGATTCCGGAGAG
LXL99Dai25816
CTACCATGGTTTCAACGGGTAACGGGGAATAAGGGTTCGATTCCGGAGAG
WCG1559Dai26052Meruliopsis

CTACCATGGTTTCAACGGGTAACGGGGAATAAGGGTTCGATTCCGGAGAG
He7477 ---
HLX243Dai26217
CTACCATGGTTTCAACGGGTAACGGGGAATAAGGGTTCGATTCCGGAGAG
RussiaMW673659Meruliopsis_fagi ---
FD278 ---
Dai10226_Ceriporia_tarda ---
LE247365 ---
Dai8173_Meruliopsis_nanlingens -------------------------
AATAAGGGTTCGATTCCGGAGAG
860Dai17172
CTACCATGGTTTCAACGGGTAACGGGGAATAAGGGTTCGATTCCGGAGAG
879Dai13414
CTACCATGGTTTCAACGGGTAACGGGGAATAAGGGTTCGATTCCGGAGAG
Li_1704_Meruliopsis_pseudocyst ---
833Dai18405
CTACCATGGTTTCAACGGGTAACGGGGAATAAGGGTTCGATTCCGGAGAG
HHB_10729_Meruliopsis_albostra ---
Cui6878_Ceriporia_pseudocystid ---
869Dai14737
CTACCATGGTTTCAACGGGTAACGGGGAATAAGGGTTCGATTCCGGAGAG
876Cui11626
CTACCATGGTTTCAACGGGTAACGGGGAATAAGGGTTCGATTCCGGAGAG
1199WEI3388 ---
776308_Meruliopsis_cystidiata ---
ICN139059_Meruliopsis_cystidia ---
HHB15692Ceraceomyces_serpens ---
HHB_15629_Sp_Ceriporiopsis_ane ---
AJ185Trametopsis_cervina ---
FD9Irpex_lacteus ---
908Dai11230
CTACCATGGTTTCAACGGGTAACGGGGAATAAGGGTTCGATTCCGGAGAG
FP55521TEmmia_lacerata ---
PBU0048Ceriporia_cystidiata ---
MZ340C_lacerataT ---
Dai21940
CTACCATGGTTTCAACGGGTAACGGGGAATAAGGGTTCGATTCCGGAGAG
847Dai16433
CTACCATGGTTTCAACGGGTAACGGGGAATAAGGGTTCGATTCCGGAGAG
MarcinEmmia_latemarginatus ---
Meijer3729Hydnopolyporus_fimbr ---
RLG13408Phanerochaete_sp ---
WHC1381Flavodon_flavus ---
GB1833Phlebia_albida ---

T407Phlebia_nitidula --
HHB6988Phanerochaete_exilis --
HHB8509Phanerochaetella_xeroph --
PBU0051Macrohyporia_dictyopora --
HHB11463Phanerochaete_sp --
FP102382Byssomerulius_corium --
FP102165Efibula_americana --
Murdoch90Ceriporia_torpida --
Rivoire4413_Ceriporia_purpurea --
Kout_18_Ceriporia_triumphalis --
Rivoire3701_Ceriporia_bresadol --
VS4018 --
Ryvarden21832_Ceriporia_manzan --
Dai24539 CTACCATGGTTTCAACGGGTAACGGGGAATAAGGGTTCGATTCCGGAGAG
Dai24541 CTACCATGGTTTCAACGGGTAACGGGGAATAAGGGTTCGATTCCGGAGAG
JV1105_12_Ceriporia_occidental --
VS8558Ceriporia_occidentalis --
Dai22445 CTACCATGGTTTCAACGGGTAACGGGGAATAAGGGTTCGATTCCGGAGAG
846Dai16368 CTACCATGGTTTCAACGGGTAACGGGGAATAAGGGTTCGATTCCGGAGAG
Dai17951_Ceriporia_aurantiocar --
Miettinen_11701C_viridans --
JV0105_10Ceriporia_aurantiocar --
Yuan5702C_viridans --
858Dai17003 CTACCATGGTTTCAACGGGTAACGGGGAATAAGGGTTCGATTCCGGAGAG
Yuan2747_Ceriporia_viridans --
Yuan2744C_viridans --
Li1046C_viridans --
865C_sinoviridans -------------------------------------CGATTCCGGAGAG
871Dai15062 --
Dai7642_Ceriporia_humilis CTACCATGGTTTCAACGGGTAACGGGGAATAAGGGTTCGATTCCGGAGAG
Spirin4706_Ceriporia_humilis --
Spirin4944_Ceriporia_sericea --
WCG1547Dai26044ceriporia CTACCATGGTTTCAACGGGTAACGGGGAATAAGGGTTCGATTCCGGAGAG
ZZW1558Dai27086 CTACCATGGTTTCAACGGGTAACGGGGAATAAGGGTTCGATTCCGGAGAG
Miettinen14381_Ceriporia_mpuri --
Miettinen15492_2_Ceriporia_sor --

He6687
CTACCATGGTTTCAACGGGTAACGGGGAATAAGGGTTCGATTCCGGAGAG
ZH53Dai24426
CTACCATGGTTTCAACGGGTAACGGGGAATAAGGGTTCGATTCCGGAGAG
Vlasak0808_30_Ceriporia_punica --
887Dai13376
CTACCATGGTTTCAACGGGTAACGGGGAATAAGGGTTCGATTCCGGAGAG
WCG1443Dai24998
CTACCATGGTTTCAACGGGTAACGGGGAATAAGGGTTCGATTCCGGAGAG
0108_6Ceriporia_spissa --
Dai19164
CTACCATGGTTTCAACGGGTAACGGGGAATAAGGGTTCGATTCCGGAGAG
Dai17937_Ceriporia_bubalinomar --
903Dai12113
CTACCATGGTTTCAACGGGTAACGGGGAATAAGGGTTCGATTCCGGAGAG
LZB929Dai25079
CTACCATGGTTTCAACGGGTAACGGGGAATAAGGGTTCGATTCCGGAGAG
LX45Dai26988 --
LX43Dai26986
CTACCATGGTTTCAACGGGTAACGGGGAATAAGGGTTCGATTCCGGAGAG
Dai7759Ceriporia --
Cui8012_Ceriporia_viridans --
GC1704_54Ceriporia_viridans --
Dai23392
CTACCATGGTTTCAACGGGTAACGGGGAATAAGGGTTCGATTCCGGAGAG
WCG1585Dai26113Ceriproia
CTACCATGGTTTCAACGGGTAACGGGGAATAAGGGTTCGATTCCGGAGAG
Dai18675C_eucalypti --
Dai22034
CTACCATGGTTTCAACGGGTAACGGGGAATAAGGGTTCGATTCCGGAGAG
JV1008_41JTardaFLORIDAKeys --
Rivoire1161_Ceriporia_pierii --
Dai23499C_pierii
CTACCATGGTTTCAACGGGTAACGGGGAATAAGGGTTCGATTCCGGAGAG
Dai23500
CTACCATGGTTTCAACGGGTAACGGGGAATAAGGGTTCGATTCCGGAGAG
841Dai15899
CTACCATGGTTTCAACGGGTAACGGGGAATAAGGGTTCGATTCCGGAGAG
842Dai15904
CTACCATGGTTTCAACGGGTAACGGGGAATAAGGGTTCGATTCCGGAGAG
LZB1066xinjiang --
LZB1065xinjiang --
851Dai16779
CTACCATGGTTTTAACGGGTAACGGGGAATAAGGGTTCGATTCCGGAGAG

RMJ119sp_Candelabrochaete_sept --
RLG9759spCandelabrochaete_sept --
RLG10478Phanerochaete_allantos --
Dai19118_Ceriporia_spissa
CTACCATGGTGACAACGGGTAACGGGGAATAAGGGTTCGATTCCGGAGAG
Dai18486A
CTACCATGGTGACAACGGGTAACGGGGAATAAGGGTTCGATTCCGGAGAG
WEI17_024_Ceriporia_mellita --
GC1508_71Ceriporia_mellita --
GC1608_7_Ceriporia_mellita --
ZZW1557Dai27085 --
ZZW1554Dai27083
CTACCATGGTGACAACGGGTAACGGGGAATAAGGGTTCGATTCCGGAGAG
Dai8168 --
BR4865C_mellita --
MEL2382688Ceriporia_sp --
Dai8110 --
Cui8097 --
909Cui6740 --
W1258Dai24695
CTACCATGGTGACAACGGGTAACGGGGAATAAGGGTTCGATTCCGGAGAG
JV0110_26_Ceriporia_griseoviol --
896Dai13202
CTACCATGGTTTCAACGGGTAACGGGGAATAAGGGTTCGATTCCGGAGAG
LWY393Dai27053C_griseoviolasce
CTACCATGGTTTCAACGGGTAACGGGGAATAAGGGTTCGATTCCGGAGAG
LWY394DAI27054
CTACCATGGTTTCAACGGGTAACGGGGAATAAGGGTTCGATTCCGGAGAG
FP135015G_pannocinctus --
L15726SpG_pannocinctus --
Dai22221
CTACCATGGTTTCAACGGGTAACGGGGAATAAGGGTTCGATTCCGGAGAG
Dai22633
CTACCATGGTTTCAACGGGTAACGGGGAATAAGGGTTCGATTCCGGAGAG
Dai23260
CTACCATGGTTTCAACGGGTAACGGGGAATAAGGGTTCGATTCCGGAGAG
Dai23626
CTACCATGGTTTCAACGGGTAACGGGGAATAAGGGTTCGATTCCGGAGAG
Dai16238G_citrinoalbus
CTACCATGGTTTCAACGGGTAACGGGGAATAAGGGTTCGATTCCGGAGAG
1175Dai15293
CTACCATGGTTTCAACGGGTAACGGGGAATAAGGGTTCGATTCCGGAGAG
Dai19547
CTACCATGGTTTCAACGGGTAACGGGGAATAAGGGTTCGATTCCGGAGAG

918063G_africanus --
918572G_africanus --
Dai18536A --
1164Cui17922
CTACCATGGTTTCAACGGGTAACGGGGAATAAGGGTTCGATTCCGGAGAG
Dai22225
CTACCATGGTTTCAACGGGTAACGGGGAATAAGGGTTCGATTCCGGAGAG
1163Dai20655
CTACCATGGTTTCAACGGGTAACGGGGAATAAGGGTTCGATTCCGGAGAG
Yuan4397G_hainanensis --
1176Dai15268
CTACCATGGTTTCAACGGGTAACGGGGAATAAGGGTTCGATTCCGGAGAG
1177Dai15259
CTACCATGGTTTCAACGGGTAACGGGGAATAAGGGTTCGATTCCGGAGAG
BZ2896G_thelephoroides --
1166JV1808_26
CTACCATGGTTTCAACGGGTAACGGGGAATAAGGGTTCGATTCCGGAGAG
Miettinen16992Hapalopilus_ochr --
GC1708_338_Ceriporia_arbuscula --
WCG1555Dai26107Ceriporia
CTACCATGGTTTCAACGGGTAACGGGGAATAAGGGTTCGATTCCGGAGAG
GC1708_340_Ceriporia_arbuscula --
WCG1556Dai26109Ceriporia
CTACCATGGTTTCAACGGGTAACGGGGAATAAGGGTTCGATTCCGGAGAG
883Cui11291
CTACCATGGTTTCAACGGGTAACGGGGAATAAGGGTTCGATTCCGGAGAG
HLX320Dai26805
CTACCATGGTTTCAACGGGTAACGGGGAATAAGGGTTCGATTCCGGAGAG
WCG1266Dai24678A
CTACCATGGTTTCAACGGGTAACGGGGAATAAGGGTTCGATTCCGGAGAG
Dai6090_Ceriporia_sulphuricolo --
RLG_11354_Ceriproia_reticulata --
ZZW1543Dai27072
CTACCATGGTTTCAACGGGTAACGGGGAATAAGGGTTCGATTCCGGAGAG
Li1316_Ceriporia_reticulata ---------------------------------TTCGATTCCGGAGAG
KHL11981Ceriporia_reticulata --
FP110343sp_Candelabrochaete_la --
Li1045_Ceriporia_reticulata
CTACCATGGTTTCAACGGGTAACGGGGAATAAGGGTTCGATTCCGGAGAG
ZX136Dai25794ceriporia
CTACCATGGTTTCAACGGGTAACGGGGAATAAGGGTTCGATTCCGGAGAG
892Dai13400
CTACCATGGTTTCAACGGGTAACGGGGAATAAGGGTTCGATTCCGGAGAG
RLG7163Leptoporus_mollis --

Dai21062Leptoporus_mollis --

Dai20182Leptoporus_submollis --

Cui18379Leptoporus_submollis --

Wu1209_46Resiniporus_pseudogil --

BRNM710169Resiniporus_resinasc --

Dai14516Bjerkandera_adusta
CTACCATGGTTTCAACGGGTAACGGGGAATAAGGGTTCGATTCCGGAGAG

Dai21100Bjerkandera_fumosa
CTACCATGGTTTCAACGGGTAACGGGGAATAAGGGTTCGATTCCGGAGAG

Miettinen16854Ceraceomyces_sp --

Dai10477C_spissa --

855Dai16831
CTACCATGGTTTCAACGGGTAACGGGGAATAAGGGTTCGATTCCGGAGAG

882Cui11282
CTACCATGGTTTCAACGGGTAACGGGGAATAAGGGTTCGATTCCGGAGAG

Dai24566
CTACCATGGTTTCAACGGGTAACGGGGAATAAGGGTTCGATTCCGGAGAG

Yuan5965 --

Dai3204 --

1194CUI9985 --

Dai15205_Ceriporia_albomellea --

Dai15223_Ceriporia_albomellea --

Li1780_Ceriporia_variegata --

Dai19791_Ceriporia_variegata
GGAGCCTGAGAAACGGCTACCACATCCAAGGAAGGCAGCAGGCGCGCAAA

Dai19886
GGAGCCTGAGAAACGGCTACCACATCCAAGGAAGGCAGCAGGCGCGCAAA

Dai10833_Ceriporia_crassitunic
GGAGCCTGAGAAACGGCTACCACATCCAAGGAAGGCAGCAGGCGCGCAAA

CHWC1506_46Meruliopsis_crassit --

Dai9995_Ceriporia_crassitunica --

Wu1209_58_Meruliopsis_parvispo --

CHWC1505_129_Meruliopsis_parvi --

Dai21944
GGAGCCTGAGAAACGGCTACCACATCCAAGGAAGGCAGCAGGCGCGCAAA

830Dai18640A
GGAGCCTGAGAAACGGCTACCACATCCAAGGAAGGCAGCAGGCGCGCAAA

GC1704_60_Meruliopsis_taxicola --

Dai22625
GGAGCCTGAGAAACGGCTACCACATCCAAGGAAGGCAGCAGGCGCGCAAA

Dai22636
GGAGCCTGAGAAACGGCTACCACATCCAAGGAAGGCAGCAGGCGCGCAAA

Dai21878
GGAGCCTGAGAAACGGCTACCACATCCAAGGAAGGCAGCAGGCGCGCAAA
1169Dai17248
GGAGCCTGAGAAACGGCTACCACATCCAAGGAAGGCAGCAGGCGCGCAAA
Wu1708_43_Meruliopsis_leptocys --
Li1011 --
ZX95Dai25742Meruliopsis_leptoc
GGAGCCTGAGAAACGGCTACCACATCCAAGGAAGGCAGCAGGCGCGCAAA
WCG1306Dai24733
GGAGCCTGAGAAACGGCTACCACATCCAAGGAAGGCAGCAGGCGCGCAAA
LXL99Dai25816
GGAGCCTGAGAAACGGCTACCACATCCAAGGAAGGCAGCAGGCGCGCAAA
WCG1559Dai26052Meruliopsis
GGAGCCTGAGAAACGGCTACCACATCCAAGGAAGGCAGCAGGCGCGCAAA
He7477 --
HLX243Dai26217
GGAGCCTGAGAAACGGCTACCACATCCAAGGAAGGCAGCAGGCGCGCAAA
RussiaMW673659Meruliopsis_fagi --
FD278 --
Dai10226_Ceriporia_tarda --
LE247365 --
Dai8173_Meruliopsis_nanlingens
GGAGCCTGAGAAACGGCTACCACATCCAAGGAAGGCAGCAGGCGCGCAAA
860Dai17172
GGAGCCTGAGAAACGGCTACCACATCCAAGGAAGGCAGCAGGCGCGCAAA
879Dai13414
GGAGCCTGAGAAACGGCTACCACATCCAAGGAAGGCAGCAGGCGCGCAAA
Li_1704_Meruliopsis_pseudocyst --
833Dai18405
GGAGCCTGAGAAACGGCTACCACATCCAAGGAAGGCAGCAGGCGCGCAAA
HHB_10729_Meruliopsis_albostra --
Cui6878_Ceriporia_pseudocystid --
869Dai14737
GGAGCCTGAGAAACGGCTACCACATCCAAGGAAGGCAGCAGGCGCGCAAA
876Cui11626
GGAGCCTGAGAAACGGCTACCACATCCAAGGAAGGCAGCAGGCGCGCAAA
1199WEI3388 --
776308_Meruliopsis_cystidiata --
ICN139059_Meruliopsis_cystidia --
HHB15692Ceraceomyces_serpens --
HHB_15629_Sp_Ceriporiopsis_ane --
AJ185Trametopsis_cervina --
FD9Irpex_lacteus --
908Dai11230

GGAGCCTGAGAAACGGCTACCACATCCAAGGAAGGCAGCAGGCGCAAA
FP55521TEmmia_lacerata --
PBU0048Ceriporia_cystidiata --
MZ340C_lacerataT --
Dai21940
GGAGCCTGAGAAACGGCTACCACATCCAAGGAAGGCAGCAGGCGCAAA
847Dai16433
GGAGCCTGAGAAACGGCTACCACATCCAAGGAAGGCAGCAGGCGCAAA
MarcinEmmia_latemarginatus --
Meijer3729Hydnopolyporus_fimbr --
RLG13408Phanerochaete_sp --
WHC1381Flavodon_flavus --
GB1833Phlebia_albida --
T407Phlebia_nitidula --
HHB6988Phanerochaete_exilis --
HHB8509Phanerochaetella_xeroph --
PBU0051Macrohyporia_dictyopora --
HHB11463Phanerochaete_sp --
FP102382Byssomerulius_corium --
FP102165Efibula_americana --
Murdoch90Ceriporia_torpida --
Rivoire4413_Ceriporia_purpurea --
Kout_18_Ceriporia_triumphalis --
Rivoire3701_Ceriporia_bresadol --
VS4018 --
Ryvarden21832_Ceriporia_manzan --
Dai24539
GGAGCCTGAGAAACGGCTACCACATCCAAGGAAGGCAGCAGGCGCAAA
Dai24541
GGAGCCTGAGAAACGGCTACCACATCCAAGGAAGGCAGCAGGCGCAAA
JV1105_12_Ceriporia_occidental --
VS8558Ceriporia_occidentalis --
Dai22445
GGAGCCTGAGAAACGGCTACCACATCCAAGGAAGGCAGCAGGCGCAAA
846Dai16368
GGAGCCTGAGAAACGGCTACCACATCCAAGGAAGGCAGCAGGCGCAAA
Dai17951_Ceriporia_aurantiocar --
Miettinen_11701C_viridans --
JV0105_10Ceriporia_aurantiocar --
Yuan5702C_viridans --
858Dai17003
GGAGCCTGAGAAACGGCTACCACATCCAAGGAAGGCAGCAGGCGCAAA
Yuan2747_Ceriporia_viridans --
Yuan2744C_viridans --

Li1046C_viridans --
865C_sinoviridans
GGAGCCTGAGAAACGGCTACCACATCCAAGGAAGGCAGCAGGCGCGCAAA
871Dai15062 --
Dai7642_Ceriporia_humilis
GGAGCCTGAGAAACGGCTACCACATCCAAGGAAGGCAGCAGGCGCGCAAA
Spirin4706_Ceriporia_humilis --
Spirin4944_Ceriporia_sericea --
WCG1547Dai26044ceriporia
GGAGCCTGAGAAACGGCTACCACATCCAAGGAAGGCAGCAGGCGCGCAAA
ZZW1558Dai27086
GGAGCCTGAGAAACGGCTACCACATCCAAGGAAGGCAGCAGGCGCGCAAA
Miettinen14381_Ceriporia_mpuri --
Miettinen15492_2_Ceriporia_sor --
He6687
GGAGCCTGAGAAACGGCTACCACATCCAAGGAAGGCAGCAGGCGCGCAAA
ZH53Dai24426
GGAGCCTGAGAAACGGCTACCACATCCAAGGAAGGCAGCAGGCGCGCAAA
Vlasak0808_30_Ceriporia_punica --
887Dai13376
GGAGCCTGAGAAACGGCTACCACATCCAAGGAAGGCAGCAGGCGCGCAAA
WCG1443Dai24998
GGAGCCTGAGAAACGGCTACCACATCCAAGGAAGGCAGCAGGCGCGCAAA
0108_6Ceriporia_spissa --
Dai19164
GGAGCCTGAGAAACGGCTACCACATCCAAGGAAGGCAGCAGGCGCGCAAA
Dai17937_Ceriporia_bubalinomar --
903Dai12113
GGAGCCTGAGAAACGGCTACCACATCCAAGGAAGGCAGCAGGCGCGCAAA
LZB929Dai25079
GGAGCCTGAGAAACGGCTACCACATCCAAGGAAGGCAGCAGGCGCGCAAA
LX45Dai26988 --
LX43Dai26986
GGAGCCTGAGAAACGGCTACCACATCCAAGGAAGGCAGCAGGCGCGCAAA
Dai7759Ceriporia --
Cui8012_Ceriporia_viridans --
GC1704_54Ceriporia_viridans --
Dai23392
GGAGCCTGAGAAACGGCTACCACATCCAAGGAAGGCAGCAGGCGCGCAAA
WCG1585Dai26113Ceriproia
GGAGCCTGAGAAACGGCTACCACATCCAAGGAAGGCAGCAGGCGCGCAAA
Dai18675C_eucalypti --
Dai22034
GGAGCCTGAGAAACGGCTACCACATCCAAGGAAGGCAGCAGGCGCGCAAA

JV1008_41JTardaFLORIDAKeys --
Rivoire1161_Ceriporia_pierii --
Dai23499C_pierii
GGAGCCTGAGAAACGGCTACCACATCCAAGGAAGGCAGCAGGCGCGCAAA
Dai23500
GGAGCCTGAGAAACGGCTACCACATCCAAGGAAGGCAGCAGGCGCGCAAA
841Dai15899
GGAGCCTGAGAAACGGCTACCACATCCAAGGAAGGCAGCAGGCGCGCAAA
842Dai15904
GGAGCCTGAGAAACGGCTACCACATCCAAGGAAGGCAGCAGGCGCGCAAA
LZB1066xinjiang --
LZB1065xinjiang --
851Dai16779
GGAGCCTGAGAAACGGCTACCACATCCAAGGAAGGCAGCAGGCGCGCAAA
RMJ119sp_Candelabrochaete_sept --
RLG9759spCandelabrochaete_sept --
RLG10478Phanerochaete_allantos --
Dai19118_Ceriporia_spissa
GGAGCCTGAGAAACGGCTACCACATCCAAGGAAGGCAGCAGGCGCGCAAA
Dai18486A
GGAGCCTGAGAAACGGCTACCACATCCAAGGAAGGCAGCAGGCGCGCAAA
WEI17_024_Ceriporia_mellita --
GC1508_71Ceriporia_mellita --
GC1608_7_Ceriporia_mellita --
ZZW1557Dai27085 --
ZZW1554Dai27083
GGAGCCTGAGAAACGGCTACCACATCCAAGGAAGGCAGCAGGCGCGCAAA
Dai8168 --
BR4865C_mellita --
MEL2382688Ceriporia_sp --
Dai8110 --
Cui8097 --
909Cui6740 --
W1258Dai24695
GGAGCCTGAGAAACGGCTACCACATCCAAGGAAGGCAGCAGGCGCGCAAA
JV0110_26_Ceriporia_griseoviol --
896Dai13202
GGAGCCTGAGAAACGGCTACCACATCCAAGGAAGGCAGCAGGCGCGCAAA
LWY393Dai27053C_griseoviolasce
GGAGCCTGAGAAACGGCTACCACATCCAAGGAAGGCAGCAGGCGCGCAAA
LWY394DAI27054
GGAGCCTGAGAAACGGCTACCACATCCAAGGAAGGCAGCAGGCGCGCAAA
FP135015G_pannocinctus --
L15726SpG_pannocinctus --

Dai22221
GGAGCCTGAGAAACGGCTACCACATCCAAGGAAGGCAGCAGGCGCGCAAA
Dai22633
GGAGCCTGAGAAACGGCTACCACATCCAAGGAAGGCAGCAGGCGCGCAAA
Dai23260
GGAGCCTGAGAAACGGCTACCACATCCAAGGAAGGCAGCAGGCGCGCAAA
Dai23626
GGAGCCTGAGAAACGGCTACCACATCCAAGGAAGGCAGCAGGCGCGCAAA
Dai16238G_citrinoalbus
GGAGCCTGAGAAACGGCTGCCACATCCAAGGAAGGCAGCAGGCGCGCAAA
1175Dai15293
GGAGCCTGAGAAACGGCTACCACATCCAAGGAAGGCAGCAGGCGCGCAAA
Dai19547
GGAGCCTGAGAAACGGCTACCACATCCAAGGAAGGCAGCAGGCGCGCAAA
918063G_africanus --
918572G_africanus --
Dai18536A --
1164Cui17922
GGAGCCTGAGAAACGGCTACCACATCCAAGGAAGGCAGCAGGCGCGCAAA
Dai22225
GGAGCCTGAGAAACGGCTACCACATCCAAGGAAGGCAGCAGGCGCGCAAA
1163Dai20655
GGAGCCTGAGAAACGGCTACCACATCCAAGGAAGGCAGCAGGCGCGCAAA
Yuan4397G_hainanensis --
1176Dai15268
GGAGCCTGAGAAACGGCTACCACATCCAAGGAAGGCAGCAGGCGCGCAAA
1177Dai15259
GGAGCCTGAGAAACGGCTACCACATCCAAGGAAGGCAGCAGGCGCGCAAA
BZ2896G_thelephoroides --
1166JV1808_26
GGAGCCTGAGAAACGGCTACCACATCCAAGGAAGGCAGCAGGCGCGCAAA
Miettinen16992Hapalopilus_ochr --
GC1708_338_Ceriporia_arbuscula --
WCG1555Dai26107Ceriporia
GGAGCCTGAGAAACGGCTACCACATCCAAGGAAGGCAGCAGGCGCGCAAA
GC1708_340_Ceriporia_arbuscula --
WCG1556Dai26109Ceriporia
GGAGCCTGAGAAACGGCTACCACATCCAAGGAAGGCAGCAGGCGCGCAAA
883Cui11291
GGAGCCTGAGAAACGGCTACCACATCCAAGGAAGGCAGCAGGCGCGCAAA
HLX320Dai26805
GGAGCCTGAGAAACGGCTACCACATCCAAGGAAGGCAGCAGGCGCGCAAA
WCG1266Dai24678A
GGAGCCTGAGAAACGGCTACCACATCCAAGGAAGGCAGCAGGCGCGCAAA

Dai6090_Ceriporia_sulphuricolo --

RLG_11354_Ceriproia_reticulata --

ZZW1543Dai27072

GGAGCCTGAGAAACGGCTACCACATCCAAGGAAGGCAGCAGGCGCGCAAA

Li1316_Ceriporia_reticulata

GGAGCCTGAGAAACAGCTACCACATCCAAGGAAGGCAGCAGGCGCGCAAA

KHL11981Ceriporia_reticulata --

FP110343sp_Candelabrochaete_la --

Li1045_Ceriporia_reticulata

GGAGCCTGAGAAACGGCTACCACATCCAAGGAAGGCAGCAGGCGCGCAAA

ZX136Dai25794ceriporia

GGAGCCTGAGAAACGGCTACCACATCCAAGGAAGGCAGCAGGCGCGCAAA

892Dai13400

GGAGCCTGAGAAACGGCTACCACATCCAAGGAAGGCAGCAGGCGCGCAAA

RLG7163Leptoporus_mollis --

Dai21062Leptoporus_mollis --

Dai20182Leptoporus_submollis --

Cui18379Leptoporus_submollis --

Wu1209_46Resiniporus_pseudogil --

BRNM710169Resiniporus_resinasc --

Dai14516Bjerkandera_adusta

GGAGCCTGAGAAACGGCTACCACATCCAAGGAAGGCAGCAGGCGCGCAAA

Dai21100Bjerkandera_fumosa

GGAGCCTGAGAAACGGCTACCACATCCAAGGAAGGCAGCAGGCGCGCAAA

Miettinen16854Ceraceomyces_sp --

Dai10477C_spissa --

855Dai16831

GGAGCCTGAGAAACGGCTACCACATCCAAGGAAGGCAGCAGGCGCGCAAA

882Cui11282

GGAGCCTGAGAAACGGCTACCACATCCAAGGAAGGCAGCAGGCGCGCAAA

Dai24566

GGAGCCTGAGAAACGGCTACCACATCCAAGGAAGGCAGCAGGCGCGCAAA

Yuan5965 --

Dai3204 --

1194CUI9985 --

Dai15205_Ceriporia_albomellea --

Dai15223_Ceriporia_albomellea --

Li1780_Ceriporia_variegata --

Dai19791_Ceriporia_variegata

TTACCCAATCCCGATACGGGGAGGTAGTGACAATAAATAACGATATAGGG

Dai19886

TTACCCAATCCCGACACGGGGAGGTAGTGACAATAAATAACGATATAGGG

Dai10833_Ceriporia_crassitunic
TTACCCAATCCCGACACGGGGAGGTAGTGACAATAAATAACAATATAGGG
CHWC1506_46Meruliopsis_crassit --
Dai9995_Ceriporia_crassitunica --
Wu1209_58_Meruliopsis_parvispo --
CHWC1505_129_Meruliopsis_parvi --
Dai21944
TTACCCAATCCCGACACGGGGAGGTAGTGACAATAAATAACGATATAGGG
830Dai18640A
TTACCCAATCCCGACACGGGGAGGTAGTGACAATAAATAACGATATAGGG
GC1704_60_Meruliopsis_taxicola --
Dai22625
TTACCCAATCCCGACACGGGGAGGTAGTGACAATAAATAACGATATAGGG
Dai22636
TTACCCAATCCCGACACGGGGAGGTAGTGACAATAAATAACGATATAGGG
Dai21878
TTACCCAATCCCGACACGGGGAGGTAGTGACAATAAATAACGATATAGGG
1169Dai17248
TTACCCAATCCCGACACGGGGAGGTAGTGACAATAAATAACGATATAGGG
Wu1708_43_Meruliopsis_leptocys --
Li1011 --
ZX95Dai25742Meruliopsis_leptoc
TTACCCAATCCCGACACGGGGAGGTAGTGACAATAAATAACGATATAGGG
WCG1306Dai24733
TTACCCAATCCCGACACGGGGAGGTAGTGACAATAAATAACGATATAGGG
LXL99Dai25816
TTACCCAATCCCGACACGGGGAGGTAGTGACAATAAATAACGATATAGGG
WCG1559Dai26052Meruliopsis
TTACCCAATCCCGACACGGGGAGGTAGTGACAATAAATAACGATATAGGG
He7477 --
HLX243Dai26217
TTACCCAATCCCGACACGGGGAGGTAGTGACAATAAATAACGATATAGGG
RussiaMW673659Meruliopsis_fagi --
FD278 --
Dai10226_Ceriporia_tarda --
LE247365 --
Dai8173_Meruliopsis_nanlingens
TTACCCAATCCCGACACGGGGAGGTAGTGACAATAAATAACAATACAGGG
860Dai17172
TTACCCAATCCCGACACGGGGAGGTAGTGACAATAAATAACGATATAGGG
879Dai13414
TTACCCAATCCCGACACGGGGAGGTAGTGACAATAAATAACGATATAGGG
Li_1704_Meruliopsis_pseudocyst --
833Dai18405

TTACCCAATCCCGACACGGGGAGGTAGTGACAATAAATAACGATATAGGG

HHB_10729_Meruliopsis_albostra --

Cui6878_Ceriporia_pseudocystid --

869Dai14737

TTACCCAATCCCGACACGGGGAGGTAGTGACAATAAATAACGATATAGGG

876Cui11626

TTACCCAATCCCGACACGGGGAGGTAGTGACAATAAATAACGATATAGGG

1199WEI3388 --

776308_Meruliopsis_cystidiata --

ICN139059_Meruliopsis_cystidia --

HHB15692Ceraceomyces_serpens --

HHB_15629_Sp_Ceriporiopsis_ane --

AJ185Trametopsis_cervina --

FD9Irpex_lacteus --

908Dai11230

TTACCCAATCCCGACACGGGGAGGTAGTGACAATAAATAACGATATAGGG

FP55521TEmmia_lacerata --

PBU0048Ceriporia_cystidiata --

MZ340C_lacerataT --

Dai21940

TTACCCAATCCCGACACGGGGAGGTAGTGACAATAAATAACGATATAGGG

847Dai16433

TTACCCAATCCCGACACGGGGAGGTAGTGACAATAAATAACGATATAGGG

MarcinEmmia_latemarginatus --

Meijer3729Hydnopolyporus_fimbr --

RLG13408Phanerochaete_sp --

WHC1381Flavodon_flavus --

GB1833Phlebia_albida --

T407Phlebia_nitidula --

HHB6988Phanerochaete_exilis --

HHB8509Phanerochaetella_xeroph --

PBU0051Macrohyporia_dictyopora --

HHB11463Phanerochaete_sp --

FP102382Byssomerulius_corium --

FP102165Efibula_americana --

Murdoch90Ceriporia_torpida --

Rivoire4413_Ceriporia_purpurea --

Kout_18_Ceriporia_triumphalis --

Rivoire3701_Ceriporia_bresadol --

VS4018 --

Ryvarden21832_Ceriporia_manzan --

Dai24539

TTACCCAATCCCGACACGGGGAGGTAGTGACAATAAATAACGATATAGGG

Dai24541

TTACCCAATCCCGACACGGGGAGGTAGTGACAATAAATAACGATATAGGG
JV1105_12_Ceriporia_occidental --
VS8558Ceriporia_occidentalis --
Dai22445
TTACCCAATCCCGACACGGGGAGGTAGTGACAATAAATAACGATATAGGG
846Dai16368
TTACCCAATCCCGACACGGGGAGGTAGTGACAATAAATAACGATATAGGG
Dai17951_Ceriporia_aurantiocar --
Miettinen_11701C_viridans --
JV0105_10Ceriporia_aurantiocar --
Yuan5702C_viridans --
858Dai17003
TTACCCAATCCCGACACGGGGAGGTAGTGACAATAAATAACGATATAGGG
Yuan2747_Ceriporia_viridans --
Yuan2744C_viridans --
Li1046C_viridans --
865C_sinoviridans
TTACCCAATCCCGACACGGGGAGGTAGTGACAATAAATAACAATATAGGG
871Dai15062 --
Dai7642_Ceriporia_humilis
TTACCCAATCCCGATACGGGGAGGTAGTGACAATAAATAACGATATAGGG
Spirin4706_Ceriporia_humilis --
Spirin4944_Ceriporia_sericea --
WCG1547Dai26044ceriporia
TTACCCAATCCCGATACGGGGAGGTAGTGACAATAAATAACGATATAGGG
ZZW1558Dai27086
TTACCCAATCCCGATACGGGGAGGTAGTGACAATAAATAACGATATAGGG
Miettinen14381_Ceriporia_mpuri --
Miettinen15492_2_Ceriporia_sor --
He6687
TTACCCAATCCCGATACGGGGAGGTAGTGACAATAAATAACGATATAGGG
ZH53Dai24426
TTACCCAATCCCGATACGGGGAGGTAGTGACAATAAATAACGATATAGGG
Vlasak0808_30_Ceriporia_punica --
887Dai13376
TTACCCAATCCCGACACGGGGAGGTAGTGACAATAAATAACGATATAGGG
WCG1443Dai24998
TTACCCAATCCCGACACGGGGAGGTAGTGACAATAAATAACGATATAGGG
0108_6Ceriporia_spissa --
Dai19164
TTACCCAATCCCGACACGGGGAGGTAGTGACAATAAATAACGATATAGGG
Dai17937_Ceriporia_bubalinomar --
903Dai12113
TTACCCAATCCCGACACGGGGAGGTAGTGACAATAAATAACGATATAGGG

LZB929Dai25079

TTACCCAATCCCGACACGGGGAGGTAGTGACAATAAATAACGATATAGGG

LX45Dai26988 --

LX43Dai26986

TTACCCAATCCCGACACGGGGAGGTAGTGACAATAAATAACGATATAGGG

Dai7759Ceriporia --

Cui8012_Ceriporia_viridans --

GC1704_54Ceriporia_viridans --

Dai23392

TTACCCAATCCCGACACGGGGAGGTAGTGACAATAAATAACGATATAGGG

WCG1585Dai26113Ceriproia

TTACCCAATCCCGACACGGGGAGGTAGTGACAATAAATAACAATATAGGG

Dai18675C_eucalypti --

Dai22034

TTACCCAATCCCGACACGGGGAGGTAGTGACAATAAATAACGATATAGGG

JV1008_41JTardaFLORIDAKeys --

Rivoire1161_Ceriporia_pierii --

Dai23499C_pierii

TTACCCAATCCCGACACGGGGAGGTAGTGACAATAAATAACGATATAGGG

Dai23500

TTACCCAATCCCGACACGGGGAGGTAGTGACAATAAATAACGATATAGGG

841Dai15899

TTACCCAATCCCGACACGGGGAGGTAGTGACAATAAATAACGATATAGGG

842Dai15904

TTACCCAATCCCGACACGGGGAGGTAGTGACAATAAATAACGATATAGGG

LZB1066xinjiang --

LZB1065xinjiang --

851Dai16779

TTACCCAATCCCGACACGGGGAGGTAGTGACAATAAATAACAATATAGGG

RMJ119sp_Candelabrochaete_sept --

RLG9759spCandelabrochaete_sept --

RLG10478Phanerochaete_allantos --

Dai19118_Ceriporia_spissa

TTACCCAATCCCGACACGGGGAGGTAGTGACAATAAATAACGATATAGGG

Dai18486A

TTACCCAATCCCGACACGGGGAGGTAGTGACAATAAATAACGATATAGGG

WEI17_024_Ceriporia_mellita --

GC1508_71Ceriporia_mellita --

GC1608_7_Ceriporia_mellita --

ZZW1557Dai27085 --

ZZW1554Dai27083

TTACCCAATCCCGACACGGGGAGGTAGTGACAATAAATAACGATATAGGG

Dai8168 --

BR4865C_mellita --

MEL2382688Ceriporia_sp ---
Dai8110 ---
Cui8097 ---
909Cui6740 ---
W1258Dai24695
TTACCCAATCCCGACACGGGGAGGTAGTGACAATAAATAACGATATAGGG
JV0110_26_Ceriporia_griseoviol ---
896Dai13202
TTACCCAATCCCGACACGGGGAGGTAGTGACAATAAATAACGATATAGGG
LWY393Dai27053C_griseoviolasce
TTACCCAATCCCGACACGGGGAGGTAGTGACAATAAATAACGATATAGGG
LWY394DAI27054
TTACCCAATCCCGACACGGGGAGGTAGTGACAATAAATAACGATATAGGG
FP135015G_pannocinctus ---
L15726SpG_pannocinctus ---
Dai22221
TTACCCAATCCCGACACGGGGAGGTAGTGACAATAAATAACGATATAGGG
Dai22633
TTACCCAATCCCGACACGGGGAGGTAGTGACAATAAATAACGATATAGGG
Dai23260
TTACCCAATCCCGACACGGGGAGGTAGTGACAATAAATAACGATATAGGG
Dai23626
TTACCCAATCCCGACACGGGGAGGTAGTGACAATAAATAACGATATAGGG
Dai16238G_citrinoalbus
TTACCCAATCCCGACACGGGGAGGTAGTGACAATAAATAACAATATAGGG
1175Dai15293
TTACCCAATCCCGACACGGGGAGGTAGTGACAATAAATAACGATATAGGG
Dai19547
TTACCCAATCCCGACACGGGGAGGTAGTGACAATAAATAACGATATAGGG
918063G_africanus ---
918572G_africanus ---
Dai18536A ---
1164Cui17922
TTACCCAATCCCGACACGGGGAGGTAGTGACAATAAATAACGATATAGGG
Dai22225
TTACCCAATCCCGACACGGGGAGGTAGTGACAATAAATAACGATATAGGG
1163Dai20655
TTACCCAATCCCGACACGGGGAGGTAGTGACAATAAATAACGATATAGGG
Yuan4397G_hainanensis ---
1176Dai15268
TTACCCAATCCCGACACGGGGAGGTAGTGACAATAAATAACGATATAGGG
1177Dai15259
TTACCCAATCCCGACACGGGGAGGTAGTGACAATAAATAACGATATAGGG
BZ2896G_thelephoroides ---

1166JV1808_26
TTACCCAATCCCGACACGGGGAGGTAGTGACAATAAATAACGATATAGGG
Miettinen16992Hapalopilus_ochr --
GC1708_338_Ceriporia_arbuscula --
WCG1555Dai26107Ceriporia
TTACCCAATCCCGACACGGGGAGGTAGTGACAATAAATAACGATATAGGG
GC1708_340_Ceriporia_arbuscula --
WCG1556Dai26109Ceriporia
TTACCCAATCCCGACACGGGGAGGTAGTGACAATAAATAACGATATAGGG
883Cui11291
TTACCCAATCCCGACACGGGGAGGTAGTGACAATAAATAACGATATAGGG
HLX320Dai26805
TTACCCAATCCCGACACGGGGAGGTAGTGACAATAAATAACGATATAGGG
WCG1266Dai24678A
TTACCCAATCCCGACACGGGGAGGTAGTGACAATAAATAACGATATAGGG
Dai6090_Ceriporia_sulphuricolo --
RLG_11354_Ceriproia_reticulata --
ZZW1543Dai27072
TTACCCAATCCCGACACGGGGAGGTAGTGACAATAAATAACGATATAGGG
Li1316_Ceriporia_reticulata
TTACCCAATCCCGACACGGGGAGGTAGTGACAATAAATAACAATACAGGG
KHL11981Ceriporia_reticulata --
FP110343sp_Candelabrochaete_la --
Li1045_Ceriporia_reticulata
TTACCCAATCCCGACACGGGGAGGTAGTGACAATAAATAACGATATAGGG
ZX136Dai25794ceriporia
TTACCCAATCCCGACACGGGGAGGTAGTGACAATAAATAACGATATAGGG
892Dai13400
TTACCCAATCCCGACACGGGGAGGTAGTGACAATAAATAACGATATAGGG
RLG7163Leptoporus_mollis --
Dai21062Leptoporus_mollis --
Dai20182Leptoporus_submollis --
Cui18379Leptoporus_submollis --
Wu1209_46Resiniporus_pseudogil --
BRNM710169Resiniporus_resinasc --
Dai14516Bjerkandera_adusta
TTACCCAATCCCGACACGGGGAGGTAGTGACAATAAATAACAATATAGGG
Dai21100Bjerkandera_fumosa
TTACCCAATCCCGACACGGGGAGGTAGTGACAATAAATAACAATATAGGG
Miettinen16854Ceraceomyces_sp --
Dai10477C_spissa --
855Dai16831
TTACCCAATCCCGACACGGGGAGGTAGTGACAATAAATAACGATATAGGG
882Cui11282

TTACCCAATCCCGACACGGGGAGGTAGTGACAATAAATAACGATATAGGG

Dai24566
TTACCCAATCCCGACACGGGGAGGTAGTGACAATAAATAACGATATAGGG

Yuan5965 --

Dai3204 --

1194CUI9985 --

Dai15205_Ceriporia_albomellea --

Dai15223_Ceriporia_albomellea --

Li1780_Ceriporia_variegata --

Dai19791_Ceriporia_variegata -
CTCTTTTGGGTCTTATAATTGGAATGAGTACAATTTAAATCTCTTAACG

Dai19886 -
CTCTTTTGGGTCTTATAATTGGAATGAGTACAATTTAAATCTCTTAACG

Dai10833_Ceriporia_crassitunic -
CTCTTTTGGGTCTTATAATTGGAATGAGTACAATTTAAATCCCTTAACG

CHWC1506_46Meruliopsis_crassit --

Dai9995_Ceriporia_crassitunica --

Wu1209_58_Meruliopsis_parvispo --

CHWC1505_129_Meruliopsis_parvi --

Dai21944 -
CTCTTTTGGGTCTTATAATTGGAATGAGTACAATTTAAATCTCTTAACG

830Dai18640A -
CTCTTTTGGGTCTTATAATTGGAATGAGTACAATTTAAATCTCTTAACG

GC1704_60_Meruliopsis_taxicola --

Dai22625 -
CTCTTTTGGGTCTTATAATTGGAATGAGTACAATTTAAATCTCTTAACG

Dai22636 -
CTCTTTTGGGTCTTATAATTGGAATGAGTACAATTTAAATCTCTTAACG

Dai21878 -
CTCTTTTGGGTCTTATAATTGGAATGAGTACAATTTAAATCTCTTAACG

1169Dai17248 -
CTCTTTTGGGTCTTATAATTGGAATGAGTACAATTTAAATCTCTTAACG

Wu1708_43_Meruliopsis_leptocys --

Li1011 --

ZX95Dai25742Meruliopsis_leptoc -
CTCTTTTGGGTCTTATAATTGGAATGAGTACAATTTAAATCTCTTAACG

WCG1306Dai24733 -
CTCTTTTGGGTCTTATAATTGGAATGAGTACAATTTAAATCTCTTAACG

LXL99Dai25816 -
CTCTTTTGGGTCTTATAATTGGAATGAGTACAATTTAAATCTCTTAACG

WCG1559Dai26052Meruliopsis -
CTCTTTTGGGTCTTATAATTGGAATGAGTACAATTTAAATCTCTTAACG

He7477 --
HLX243Dai26217 -
CTCTTTTGGGTCTTATAATTGGAATGAGTACAATTTAAATCTCTTAACG
RussiaMW673659Meruliopsis_fagi --
FD278 --
Dai10226_Ceriporia_tarda --
LE247365 --
Dai8173_Meruliopsis_nanlingens
CCTCTTTTGGGTCCTGTAATTGGAATGAGTACAATTTAAATCCCTTAACG
860Dai17172 -
CTCTTTTGGGTCTTATAATTGGAATGAGTACAATTTAAATCTCTTAACG
879Dai13414 -
CTCTTTTGGGTCTTATAATTGGAATGAGTACAATTTAAATCTCTTAACG
Li_1704_Meruliopsis_pseudocyst --
833Dai18405 -
CTCTTTTGGGTCTTATAATTGGAATGAGTACAATTTAAATCTCTTAACG
HHB_10729_Meruliopsis_albostra --
Cui6878_Ceriporia_pseudocystid --
869Dai14737 -
CTCTTTTGGGTCTTATAATTGGAATGAGTACAATTTAAATCTCTTAACG
876Cui11626 -
CTCTTTTGGGTCTTATAATTGGAATGAGTACAATTTAAATCTCTTAACG
1199WEI3388 --
776308_Meruliopsis_cystidiata --
ICN139059_Meruliopsis_cystidia --
HHB15692Ceraceomyces_serpens --
HHB_15629_Sp_Ceriporiopsis_ane --
AJ185Trametopsis_cervina --
FD9Irpex_lacteus --
908Dai11230 -
CTCTTTTGGGTCTTATAATTGGAATGAGTACAATTTAAATCTCTTAACG
FP55521TEmmia_lacerata --
PBU0048Ceriporia_cystidiata --
MZ340C_laceratаT --
Dai21940 -
CTCTTTTGGGTCTTATAATTGGAATGAGTACAATTTAAATCTCTTAACG
847Dai16433 -
CTCTTTTGGGTCTTATAATTGGAATGAGTACAATTTAAATCTCTTAACG
MarcinEmmia_latemarginatus --
Meijer3729Hydnopolyporus_fimbr --
RLG13408Phanerochaete_sp --
WHC1381Flavodon_flavus --
GB1833Phlebia_albida --
T407Phlebia_nitidula --

HHB6988Phanerochaete_exilis --
HHB8509Phanerochaetella_xeroph --
PBU0051Macrohyporia_dictyopora --
HHB11463Phanerochaete_sp --
FP102382Byssomerulius_corium --
FP102165Efibula_americana --
Murdoch90Ceriporia_torpida --
Rivoire4413_Ceriporia_purpurea --
Kout_18_Ceriporia_triumphalis --
Rivoire3701_Ceriporia_bresadol --
VS4018 --
Ryvarden21832_Ceriporia_manzan --
Dai24539 -
CTCTTTTGGGTCTTATAATTGGAATGAGTACAATTTAAATCTCTTAACG
Dai24541 -
CTCTTTTGGGTCTTATAATTGGAATGAGTACAATTTAAATCTCTTAACG
JV1105_12_Ceriporia_occidental --
VS8558Ceriporia_occidentalis --
Dai22445 -
CTCTTTTGGGTCTTATAATTGGAATGAGTACAATTTAAATCTCTTAACG
846Dai16368 -
CTCTTTTGGGTCTTATAATTGGAATGAGTACAATTTAAATCTCTTAACG
Dai17951_Ceriporia_aurantiocar --
Miettinen_11701C_viridans --
JV0105_10Ceriporia_aurantiocar --
Yuan5702C_viridans --
858Dai17003 -
CTCTTTTGGGTCTTATAATTGGAATGAGTACAATTTAAATCTCTTAACG
Yuan2747_Ceriporia_viridans --
Yuan2744C_viridans --
Li1046C_viridans --
865C_sinoviridans -
CTCTTTTGGGTCTTATAATTGGAATGAGTACAATTTAAATCCCTTAACG
871Dai15062 --
Dai7642_Ceriporia_humilis -
CTCTTTTGGGTCTTATAATTGGAATGAGTACAATTTAAATCTCTTAACG
Spirin4706_Ceriporia_humilis --
Spirin4944_Ceriporia_sericea --
WCG1547Dai26044ceriporia -
CTCTTTTGGGTCTTATAATTGGAATGAGTACAATTTAAATCTCTTAACG
ZZW1558Dai27086 -
CTCTTTTGGGTCTTATAATTGGAATGAGTACAATTTAAATCTCTTAACG
Miettinen14381_Ceriporia_mpuri --
Miettinen15492_2_Ceriporia_sor --

He6687 -
CTCTTTTGGGTCTTATAATTGGAATGAGTACAATTTAAATCTCTTAACG
ZH53Dai24426 -
CTCTTTTGGGTCTTATAATTGGAATGAGTACAATTTAAATCTCTTAACG
Vlasak0808_30_Ceriporia_punica ---
887Dai13376 -
CTCTTTTGGGTCTTATAATTGGAATGAGTACAATTTAAATCTCTTAACG
WCG1443Dai24998 -
CTCTTTTGGGTCTTATAATTGGAATGAGTACAATTTAAATCTCTTAACG
0108_6Ceriporia_spissa ---
Dai19164 -
CTCTTTTGGGTCTTATAATTGGAATGAGTACAATTTAAATCTCTTAACG
Dai17937_Ceriporia_bubalinomar ---
903Dai12113 -
CTCTTTTGGGTCTTATAATTGGAATGAGTACAATTTAAATCTCTTAACG
LZB929Dai25079 -
CTCTTTTGGGTCTTATAATTGGAATGAGTACAATTTAAATCTCTTAACG
LX45Dai26988 ---
LX43Dai26986 -
CTCTTTTGGGTCTTATAATTGGAATGAGTACAATTTAAATCTCTTAACG
Dai7759Ceriporia ---
Cui8012_Ceriporia_viridans ---
GC1704_54Ceriporia_viridans ---
Dai23392 -
CTCTTTTGGGTCTTATAATTGGAATGAGTACAATTTAAATCTCTTAACG
WCG1585Dai26113Ceriproia -
CTCTTTTGGGTCTTATAATTGGAATGAGTACAATTTAAATCCCTTAACG
Dai18675C_eucalypti ---
Dai22034 -
CTCTTTTGGGTCTTATAATTGGAATGAGTACAATTTAAATCTCTTAACG
JV1008_41JTardaFLORIDAKeys ---
Rivoire1161_Ceriporia_pierii ---
Dai23499C_pierii -
CTCTTTTGGGTCTTATAATTGGAATGAGTACAATTTAAATCTCTTAACG
Dai23500 -
CTCTTTTGGGTCTTATAATTGGAATGAGTACAATTTAAATCTCTTAACG
841Dai15899 -
CTCTTTTGGGTCTTATAATTGGAATGAGTACAATTTAAATCTCTTAACG
842Dai15904 -
CTCTTTTGGGTCTTATAATTGGAATGAGTACAATTTAAATCTCTTAACG
LZB1066xinjiang ---
LZB1065xinjiang ---
851Dai16779 -
CTCTTTCGGGTCTTATAATTGGAATGAGTACAATTTAAATCTCTTAACG

RMJ119sp_Candelabrochaete_sept --
RLG9759spCandelabrochaete_sept --
RLG10478Phanerochaete_allantos --
Dai19118_Ceriporia_spissa -
CTCTTTTGGGTCTTATAATTGGAATGAGTACAATTTAAATCTCTTAACG
Dai18486A -
CTCTTTTGGGTCTTATAATTGGAATGAGTACAATTTAAATCTCTTAACG
WEI17_024_Ceriporia_mellita --
GC1508_71Ceriporia_mellita --
GC1608_7_Ceriporia_mellita --
ZZW1557Dai27085 --
ZZW1554Dai27083 -
CTCTTTTGGGTCTTATAATTGGAATGAGTACAATTTAAATCTCTTAACG
Dai8168 --
BR4865C_mellita --
MEL2382688Ceriporia_sp --
Dai8110 --
Cui8097 --
909Cui6740 --
W1258Dai24695 -
CTCTTTTGGGTCTTATAATTGGAATGAGTACAATTTAAATCTCTTAACG
JV0110_26_Ceriporia_griseoviol --
896Dai13202 -
CTCTTTTGGGTCTTATAATTGGAATGAGTACAATTTAAATCTCTTAACG
LWY393Dai27053C_griseoviolasce -
CTCTTTTGGGTCTTATAATTGGAATGAGTACAATTTAAATCTCTTAACG
LWY394DAI27054 -
CTCTTTTGGGTCTTATAATTGGAATGAGTACAATTTAAATCTCTTAACG
FP135015G_pannocinctus --
L15726SpG_pannocinctus --
Dai22221 -
CTCTTTTGGGTCTTATAATTGGAATGAGTACAATTTAAATCTCTTAACG
Dai22633 -
CTCTTTTGGGTCTTATAATTGGAATGAGTACAATTTAAATCTCTTAACG
Dai23260 -
CTCTTTTGGGTCTTATAATTGGAATGAGTACAATTTAAATCTCTTAACG
Dai23626 -
CTCTTTTGGGTCTTATAATTGGAATGAGTACAATTTAAATCTCTTAACG
Dai16238G_citrinoalbus -
CCCTTTTGGGTCTTATAATTGGAATGAGTACAATTTAAATCCCTTAACG
1175Dai15293 -
CTCTTTCGGGTCTTATAATTGGAATGAGTACAATTTAAATCTCTTAACG
Dai19547 -
CTCTTTCGGGTCTTATAATTGGAATGAGTACAATTTAAATCTCTTAACG

918063G_africanus --
918572G_africanus --
Dai18536A --
1164Cui17922 -
CTCTTTCGGGTCTTATAATTGGAATGAGTACAATTTAAATCTCTTAACG
Dai22225 -
CTCTTTCGGGTCTTATAATTGGAATGAGTACAATTTAAATCTCTTAACG
1163Dai20655 -
CTCTTTCGGGTCTTATAATTGGAATGAGTACAATTTAAATCTCTTAACG
Yuan4397G_hainanensis --
1176Dai15268 -
CTCTTTTGGGTCTTATAATTGGAATGAGTACAATTTAAATCTCTTAACG
1177Dai15259 -
CTCTTTTGGGTCTTATAATTGGAATGAGTACAATTTAAATCTCTTAACG
BZ2896G_thelephoroides --
1166JV1808_26 -
CTCTTTTGGGTCTTATAATTGGAATGAGTACAATTTAAATCTCTTAACG
Miettinen16992Hapalopilus_ochr --
GC1708_338_Ceriporia_arbuscula --
WCG1555Dai26107Ceriporia -
CTCTTTTGGGTCTTATAATTGGAATGAGTACAATTTAAATCTCTTAACG
GC1708_340_Ceriporia_arbuscula --
WCG1556Dai26109Ceriporia -
CTCTTTTGGGTCTTATAATTGGAATGAGTACAATTTAAATCTCTTAACG
883Cui11291 -
CTCTTTTGGGTCTTATAATTGGAATGAGTACAATTTAAATCTCTTAACG
HLX320Dai26805 -
CTCTTTTGGGTCTTATAATTGGAATGAGTACAATTTAAATCTCTTAACG
WCG1266Dai24678A -
CTCTTTTGGGTCTTATAATTGGAATGAGTACAATTTAAATCTCTTAACG
Dai6090_Ceriporia_sulphuricolo --
RLG_11354_Ceriproia_reticulata --
ZZW1543Dai27072 -
CTCTTTTGGGTCTTATAATTGGAATGAGTACAATTTAAATCTCTTAACG
Li1316_Ceriporia_reticulata -
CTCTTTTGGGTCCTGTAATTGGAATGAGTACAATTTAAATCCCTTAACG
KHL11981Ceriporia_reticulata --
FP110343sp_Candelabrochaete_la --
Li1045_Ceriporia_reticulata -
CTCTTTTGGGTCTTATAATTGGAATGAGTACAATTTAAATCTCTTAACG
ZX136Dai25794ceriporia -
CTCTTTTGGGTCTTATAATTGGAATGAGTACAATTTAAATCTCTTAACG
892Dai13400 -
CTCTTTTGGGTCTTATAATTGGAATGAGTACAATTTAAATCTCTTAACG

RLG7163Leptoporus_mollis --
Dai21062Leptoporus_mollis --
Dai20182Leptoporus_submollis --
Cui18379Leptoporus_submollis --
Wu1209_46Resiniporus_pseudogil --
BRNM710169Resiniporus_resinasc --
Dai14516Bjerkandera_adusta -
CTCTTTCGGGTCTTATAATTGGAATGAGTACAATTTAAATCTCTTAACG
Dai21100Bjerkandera_fumosa -
CTCTTTCGGGTCTTATAATTGGAATGAGTACAATTTAAATCTCTTAACG
Miettinen16854Ceraceomyces_sp --
Dai10477C_spissa --
855Dai16831 -
CTCTTTTGGGTCTTATAATTGGAATGAGTACAATTTAAATCTCTTAACG
882Cui11282 -
CTCTTTTGGGTCTTATAATTGGAATGAGTACAATTTAAATCTCTTAACG
Dai24566 -
CTCTTTTGGGTCTTATAATTGGAATGAGTACAATTTAAATCTCTTAACG
Yuan5965 --
Dai3204 --
1194CUI9985 --

Dai15205_Ceriporia_albomellea --
Dai15223_Ceriporia_albomellea --
Li1780_Ceriporia_variegata --
Dai19791_Ceriporia_variegata
AGGAACAATTGGAGGGCAAGTCTGGTGCCAGCAGCCGCGGTAATTCCAGC
Dai19886
AGGAACAATTGGAGGGCAAGTCTGGTGCCAGCAGCCGCGGTAATTCCAGC
Dai10833_Ceriporia_crassitunic
AGGAACGATTGGAGGGCAAGTCTGGTGCCAGCAGCCGCGGTAATTCCAGC
CHWC1506_46Meruliopsis_crassit --
Dai9995_Ceriporia_crassitunica --
Wu1209_58_Meruliopsis_parvispo --
CHWC1505_129_Meruliopsis_parvi --
Dai21944
AGGAACAATTGGAGGGCAAGTCTGGTGCCAGCAGCCGCGGTAATTCCAGC
830Dai18640A
AGGAACAATTGGAGGGCAAGTCTGGTGCCAGCAGCCGCGGTAATTCCAGC
GC1704_60_Meruliopsis_taxicola --
Dai22625
AGGAACAATTGGAGGGCAAGTCTGGTGCCAGCAGCCGCGGTAATTCCAGC
Dai22636

AGGAACAATTGGAGGGCAAGTCTGGTGCCAGCAGCCGCGGTAATTCCAGC
Dai21878
AGGAACAATTGGAGGGCAAGTCTGGTGCCAGCAGCCGCGGTAATTCCAGC
1169Dai17248
AGGAACAATTGGAGGGCAAGTCTGGTGCCAGCAGCCGCGGTAATTCCAGC
Wu1708_43_Meruliopsis_leptocys --
Li1011 --
ZX95Dai25742Meruliopsis_leptoc
AGGAACAATTGGAGGGCAAGTCTGGTGCCAGCAGCCGCGGTAATTCCAGC
WCG1306Dai24733
AGGAACAATTGGAGGGCAAGTCTGGTGCCAGCAGCCGCGGTAATTCCAGC
LXL99Dai25816
AGGAACAATTGGAGGGCAAGTCTGGTGCCAGCAGCCGCGGTAATTCCAGC
WCG1559Dai26052Meruliopsis
AGGAACAATTGGAGGGCAAGTCTGGTGCCAGCAGCCGCGGTAATTCCAGC
He7477 --
HLX243Dai26217
AGGAACAATTGGAGGGCAAGTCTGGTGCCAGCAGCCGCGGTAATTCCAGC
RussiaMW673659Meruliopsis_fagi --
FD278 --
Dai10226_Ceriporia_tarda --
LE247365 --
Dai8173_Meruliopsis_nanlingens
AGGAACAATTGGAGGGCAAGTCTGGTGCCAGCAGCCGCGGTAATTCCAGC
860Dai17172
AGGAACAATTGGAGGGCAAGTCTGGTGCCAGCAGCCGCGGTAATTCCAGC
879Dai13414
AGGAACAATTGGAGGGCAAGTCTGGTGCCAGCAGCCGCGGTAATTCCAGC
Li_1704_Meruliopsis_pseudocyst --
833Dai18405
AGGAACAATTGGAGGGCAAGTCTGGTGCCAGCAGCCGCGGTAATTCCAGC
HHB_10729_Meruliopsis_albostra --
Cui6878_Ceriporia_pseudocystid --
869Dai14737
AGGAACAATTGGAGGGCAAGTCTGGTGCCAGCAGCCGCGGTAATTCCAGC
876Cui11626
AGGAACAATTGGAGGGCAAGTCTGGTGCCAGCAGCCGCGGTAATTCCAGC
1199WEI3388 --
776308_Meruliopsis_cystidiata --
ICN139059_Meruliopsis_cystidia --
HHB15692Ceraceomyces_serpens --
HHB_15629_Sp_Ceriporiopsis_ane --
AJ185Trametopsis_cervina --
FD9Irpex_lacteus --

908Dai11230
AGGAACAATTGGAGGGCAAGTCTGGTGCCAGCAGCCGCGGTAATTCCAGC
FP55521TEmmia_lacerata --
PBU0048Ceriporia_cystidiata --
MZ340C_laceratаT --
Dai21940
AGGAACAATTGGAGGGCAAGTCTGGTGCCAGCAGCCGCGGTAATTCCAGC
847Dai16433
AGGAACAATTGGAGGGCAAGTCTGGTGCCAGCAGCCGCGGTAATTCCAGC
MarcinEmmia_latemarginatus --
Meijer3729Hydnopolyporus_fimbr --
RLG13408Phanerochaete_sp --
WHC1381Flavodon_flavus --
GB1833Phlebia_albida --
T407Phlebia_nitidula --
HHB6988Phanerochaete_exilis --
HHB8509Phanerochaetella_xeroph --
PBU0051Macrohyporia_dictyopora --
HHB11463Phanerochaete_sp --
FP102382Byssomerulius_corium --
FP102165Efibula_americana --
Murdoch90Ceriporia_torpida --
Rivoire4413_Ceriporia_purpurea --
Kout_18_Ceriporia_triumphalis --
Rivoire3701_Ceriporia_bresadol --
VS4018 --
Ryvarden21832_Ceriporia_manzan --
Dai24539
AGGAACAATTGGAGGGCAAGTCTGGTGCCAGCAGCCGCGGTAATTCCAGC
Dai24541
AGGAACAATTGGAGGGCAAGTCTGGTGCCAGCAGCCGCGGTAATTCCAGC
JV1105_12_Ceriporia_occidental --
VS8558Ceriporia_occidentalis --
Dai22445
AGGAACAATTGGAGGGCAAGTCTGGTGCCAGCAGCCGCGGTAATTCCAGC
846Dai16368
AGGAACAATTGGAGGGCAAGTCTGGTGCCAGCAGCCGCGGTAATTCCAGC
Dai17951_Ceriporia_aurantiocar --
Miettinen_11701C_viridans --
JV0105_10Ceriporia_aurantiocar --
Yuan5702C_viridans --
858Dai17003
AGGAACAATTGGAGGGCAAGTCTGGTGCCAGCAGCCGCGGTAATTCCAGC
Yuan2747_Ceriporia_viridans --

Yuan2744C_viridans --
Li1046C_viridans --
865C_sinoviridans
AGGAACAATTGGAGGGCAAGTCTGGTGCCAGCAGCCGCGGTAATTCCAGC
871Dai15062 --
Dai7642_Ceriporia_humilis
AGGAACAATTGGAGGGCAAGTCTGGTGCCAGCAGCCGCGGTAATTCCAGC
Spirin4706_Ceriporia_humilis --
Spirin4944_Ceriporia_sericea --
WCG1547Dai26044ceriporia
AGGAACAATTGGAGGGCAAGTCTGGTGCCAGCAGCCGCGGTAATTCCAGC
ZZW1558Dai27086
AGGAACAATTGGAGGGCAAGTCTGGTGCCAGCAGCCGCGGTAATTCCAGC
Miettinen14381_Ceriporia_mpuri --
Miettinen15492_2_Ceriporia_sor --
He6687
AGGAACAATTGGAGGGCAAGTCTGGTGCCAGCAGCCGCGGTAATTCCAGC
ZH53Dai24426
AGGAACAATTGGAGGGCAAGTCTGGTGCCAGCAGCCGCGGTAATTCCAGC
Vlasak0808_30_Ceriporia_punica --
887Dai13376
AGGAACAATTGGAGGGCAAGTCTGGTGCCAGCAGCCGCGGTAATTCCAGC
WCG1443Dai24998
AGGAACAATTGGAGGGCAAGTCTGGTGCCAGCAGCCGCGGTAATTCCAGC
0108_6Ceriporia_spissa --
Dai19164
AGGACCAATTGGAGGGCAAGTCTGGTGCCAGCAGCCGCGGTAATTCCAGC
Dai17937_Ceriporia_bubalinomar --
903Dai12113
AGGACCAATTGGAGGGCAAGTCTGGTGCCAGCAGCCGCGGTAATTCCAGC
LZB929Dai25079
AGGAACAATTGGAGGGCAAGTCTGGTGCCAGCAGCCGCGGTAATTCCAGC
LX45Dai26988 --
LX43Dai26986
AGGAACAATTGGAGGGCAAGTCTGGTGCCAGCAGCCGCGGTAATTCCAGC
Dai7759Ceriporia --
Cui8012_Ceriporia_viridans --
GC1704_54Ceriporia_viridans --
Dai23392
AGGAACAATTGGAGGGCAAGTCTGGTGCCAGCAGCCGCGGTAATTCCAGC
WCG1585Dai26113Ceriproia
AGGAACAATTGGAGGGCAAGTCTGGTGCCAGCAGCCGCGGTAATTCCAGC
Dai18675C_eucalypti --
Dai22034

AGGAACAATTGGAGGGCAAGTCTGGTGCCAGCAGCCGCGGTAATTCCAGC
JV1008_41JTardaFLORIDAKeys --
Rivoire1161_Ceriporia_pierii --
Dai23499C_pierii
AGGAACAATTGGAGGGCAAGTCTGGTGCCAGCAGCCGCGGTAATTCCAGC
Dai23500
AGGAACAATTGGAGGGCAAGTCTGGTGCCAGCAGCCGCGGTAATTCCAGC
841Dai15899
AGGAACAATTGGAGGGCAAGTCTGGTGCCAGCAGCCGCGGTAATTCCAGC
842Dai15904
AGGAACAATTGGAGGGCAAGTCTGGTGCCAGCAGCCGCGGTAATTCCAGC
LZB1066xinjiang --
LZB1065xinjiang --
851Dai16779
AGGAACAATTGGAGGGCAAGTCTGGTGCCAGCAGCCGCGGTAATTCCAGC
RMJ119sp_Candelabrochaete_sept --
RLG9759spCandelabrochaete_sept --
RLG10478Phanerochaete_allantos --
Dai19118_Ceriporia_spissa
AGGATCAATTGGAGGGCAAGTCTGGTGCCAGCAGCCGCGGTAATTCCAGC
Dai18486A
AGGATCAATTGGAGGGCAAGTCTGGTGCCAGCAGCCGCGGTAATTCCAGC
WEI17_024_Ceriporia_mellita --
GC1508_71Ceriporia_mellita --
GC1608_7_Ceriporia_mellita --
ZZW1557Dai27085 --
ZZW1554Dai27083
AGGATCAATTGGAGGGCAAGTCTGGTGCCAGCAGCCGCGGTAATTCCAGC
Dai8168 --
BR4865C_mellita --
MEL2382688Ceriporia_sp --
Dai8110 --
Cui8097 --
909Cui6740 --
W1258Dai24695
AGGAACAATTGGAGGGCAAGTCTGGTGCCAGCAGCCGCGGTAATTCCAGC
JV0110_26_Ceriporia_griseoviol --
896Dai13202
AGGAACAATTGGAGGGCAAGTCTGGTGCCAGCAGCCGCGGTAATTCCAGC
LWY393Dai27053C_griseoviolasce
AGGAACAATTGGAGGGCAAGTCTGGTGCCAGCAGCCGCGGTAATTCCAGC
LWY394DAI27054
AGGAACAATTGGAGGGCAAGTCTGGTGCCAGCAGCCGCGGTAATTCCAGC
FP135015G_pannocinctus --

L15726SpG_pannocinctus --
Dai22221
AGGAACAATTGGAGGGCAAGTCTGGTGCCAGCAGCCGCGGTAATTCCAGC
Dai22633
AGGAACAATTGGAGGGCAAGTCTGGTGCCAGCAGCCGCGGTAATTCCAGC
Dai23260
AGGAACAATTGGAGGGCAAGTCTGGTGCCAGCAGCCGCGGTAATTCCAGC
Dai23626
AGGAACAATTGGAGGGCAAGTCTGGTGCCAGCAGCCGCGGTAATTCCAGC
Dai16238G_citrinoalbus
AGGAACAATTGGAGGGCAAGTCTGGTGCCAGCAGCCGCGGTAATTCCAGC
1175Dai15293
AGGAACAATTGGAGGGCAAGTCTGGTGCCAGCAGCCGCGGTAATTCCAGC
Dai19547
AGGAACAATTGGAGGGCAAGTCTGGTGCCAGCAGCCGCGGTAATTCCAGC
918063G_africanus --
918572G_africanus --
Dai18536A --
1164Cui17922
AGGAACAATTGGAGGGCAAGTCTGGTGCCAGCAGCCGCGGTAATTCCAGC
Dai22225
AGGAACAATTGGAGGGCAAGTCTGGTGCCAGCAGCCGCGGTAATTCCAGC
1163Dai20655
AGGAACAATTGGAGGGCAAGTCTGGTGCCAGCAGCCGCGGTAATTCCAGC
Yuan4397G_hainanensis --
1176Dai15268
AGGAACAATTGGAGGGCAAGTCTGGTGCCAGCAGCCGCGGTAATTCCAGC
1177Dai15259
AGGAACAATTGGAGGGCAAGTCTGGTGCCAGCAGCCGCGGTAATTCCAGC
BZ2896G_thelephoroides --
1166JV1808_26
AGGAACAATTGGAGGGCAAGTCTGGTGCCAGCAGCCGCGGTAATTCCAGC
Miettinen16992Hapalopilus_ochr --
GC1708_338_Ceriporia_arbuscula --
WCG1555Dai26107Ceriporia
AGGAACAATTGGAGGGCAAGTCTGGTGCCAGCAGCCGCGGTAATTCCAGC
GC1708_340_Ceriporia_arbuscula --
WCG1556Dai26109Ceriporia
AGGAACAATTGGAGGGCAAGTCTGGTGCCAGCAGCCGCGGTAATTCCAGC
883Cui11291
AGGAACAATTGGAGGGCAAGTCTGGTGCCAGCAGCCGCGGTAATTCCAGC
HLX320Dai26805
AGGAACAATTGGAGGGCAAGTCTGGTGCCAGCAGCCGCGGTAATTCCAGC
WCG1266Dai24678A

AGGAACAATTGGAGGGCAAGTCTGGTGCCAGCAGCCGCGGTAATTCCAGC
Dai6090_Ceriporia_sulphuricolo ---
RLG_11354_Ceriproia_reticulata ---
ZZW1543Dai27072
AGGAACAATTGGAGGGCAAGTCTGGTGCCAGCAGCCGCGGTAATTCCAGC
Li1316_Ceriporia_reticulata
AGGAACAATTGGAGGGCAAGTCTGGTGCCAGCAGCCGCGGTAATTCCAGC
KHL11981Ceriporia_reticulata ---
FP110343sp_Candelabrochaete_la ---
Li1045_Ceriporia_reticulata
AGGAACAATTGGAGGGCAAGTCTGGTGCCAGCAGCCGCGGTAATTCCAGC
ZX136Dai25794ceriporia
AGGAACAATTGGAGGGCAAGTCTGGTGCCAGCAGCCGCGGTAATTCCAGC
892Dai13400
AGGAACAATTGGAGGGCAAGTCTGGTGCCAGCAGCCGCGGTAATTCCAGC
RLG7163Leptoporus_mollis ---
Dai21062Leptoporus_mollis ---
Dai20182Leptoporus_submollis ---
Cui18379Leptoporus_submollis ---
Wu1209_46Resiniporus_pseudogil ---
BRNM710169Resiniporus_resinasc ---
Dai14516Bjerkandera_adusta
AGGAACAATTGGAGGGCAAGTCTGGTGCCAGCAGCCGCGGTAATTCCAGC
Dai21100Bjerkandera_fumosa
AGGAACAATTGGAGGGCAAGTCTGGTGCCAGCAGCCGCGGTAATTCCAGC
Miettinen16854Ceraceomyces_sp ---
Dai10477C_spissa ---
855Dai16831
AGGAACAATTGGAGGGCAAGTCTGGTGCCAGCAGCCGCGGTAATTCCAGC
882Cui11282
AGGAACAATTGGAGGGCAAGTCTGGTGCCAGCAGCCGCGGTAATTCCAGC
Dai24566
AGGAACAATTGGAGGGCAAGTCTGGTGCCAGCAGCCGCGGTAATTCCAGC
Yuan5965 ---
Dai3204 ---
1194CUI9985 ---

Dai15205_Ceriporia_albomellea ---
Dai15223_Ceriporia_albomellea ---
Li1780_Ceriporia_variegata ---
Dai19791_Ceriporia_variegata
TCCAATAGCGTATATTAAAGTTGTTGCAGTTAAAAAGCTCGTAGTTGAAC
Dai19886

TCCAATAGCGTATATTAAAGTTGTTGCAGTTAAAAAGCTCGTAGTTGAAC

Dai10833_Ceriporia_crassitunic

TCCAATAGCGTATATTAAAGTTGTTGCAGTTAAAAAGCTCGTAGTTGAAC

CHWC1506_46Meruliopsis_crassit ---

Dai9995_Ceriporia_crassitunica ---

Wu1209_58_Meruliopsis_parvispo ---

CHWC1505_129_Meruliopsis_parvi ---

Dai21944

TCCAATAGCGTATATTAAAGTTGTTGCAGTTAAAAAGCTCGTAGTTGAAC

830Dai18640A

TCCAATAGCGTATATTAAAGTTGTTGCAGTTAAAAAGCTCGTAGTTGAAC

GC1704_60_Meruliopsis_taxicola ---

Dai22625

TCCAATAGCGTATATTAAAGTTGTTGCAGTTAAAAAGCTCGTAGTTGAAC

Dai22636

TCCAATAGCGTATATTAAAGTTGTTGCAGTTAAAAAGCTCGTAGTTGAAC

Dai21878

TCCAATAGCGTATATTAAAGTTGTTGCAGTTAAAAAGCTCGTAGTTGAAC

1169Dai17248

TCCAATAGCGTATATTAAAGTTGTTGCAGTTAAAAAGCTCGTAGTTGAAC

Wu1708_43_Meruliopsis_leptocys ---

Li1011 ---

ZX95Dai25742Meruliopsis_leptoc

TCCAATAGCGTATATTAAAGTTGTTGCAGTTAAAAAGCTCGTAGTTGAAC

WCG1306Dai24733

TCCAATAGCGTATATTAAAGTTGTTGCAGTTAAAAAGCTCGTAGTTGAAC

LXL99Dai25816

TCCAATAGCGTATATTAAAGTTGTTGCAGTTAAAAAGCTCGTAGTTGAAC

WCG1559Dai26052Meruliopsis

TCCAATAGCGTATATTAAAGTTGTTGCAGTTAAAAAGCTCGTAGTTGAAC

He7477 ---

HLX243Dai26217

TCCAATAGCGTATATTAAAGTTGTTGCAGTTAAAAAGCTCGTAGTTGAAC

RussiaMW673659Meruliopsis_fagi ---

FD278 ---

Dai10226_Ceriporia_tarda ---

LE247365 ---

Dai8173_Meruliopsis_nanlingens

TCCAATAGCGTATATTAAAGTTGTTGCAGTTAAAAAGCTCGTAGTTGAAC

860Dai17172

TCCAATAGCGTATATTAAAGTTGTTGCAGTTAAAAAGCTCGTAGTTGAAC

879Dai13414

TCCAATAGCGTATATTAAAGTTGTTGCAGTTAAAAAGCTCGTAGTTGAAC

Li_1704_Meruliopsis_pseudocyst ---

833Dai18405
TCCAATAGCGTATATTAAAGTTGTTGCAGTTAAAAAGCTCGTAGTTGAAC
HHB_10729_Meruliopsis_albostra --
Cui6878_Ceriporia_pseudocystid --
869Dai14737
TCCAATAGCGTATATTAAAGTTGTTGCAGTTAAAAAGCTCGTAGTTGAAC
876Cui11626
TCCAATAGCGTATATTAAAGTTGTTGCAGTTAAAAAGCTCGTAGTTGAAC
1199WEI3388 --
776308_Meruliopsis_cystidiata --
ICN139059_Meruliopsis_cystidia --
HHB15692Ceraceomyces_serpens --
HHB_15629_Sp_Ceriporiopsis_ane --
AJ185Trametopsis_cervina --
FD9Irpex_lacteus --
908Dai11230
TCCAATAGCGTATATTAAAGTTGTTGCAGTTAAAAAGCTCGTAGTTGAAC
FP55521TEmmia_lacerata --
PBU0048Ceriporia_cystidiata --
MZ340C_lacerataT --
Dai21940
TCCAATAGCGTATATTAAAGTTGTTGCAGTTAAAAAGCTCGTAGTTGAAC
847Dai16433
TCCAATAGCGTATATTAAAGTTGTTGCAGTTAAAAAGCTCGTAGTTGAAC
MarcinEmmia_latemarginatus --
Meijer3729Hydnopolyporus_fimbr --
RLG13408Phanerochaete_sp --
WHC1381Flavodon_flavus --
GB1833Phlebia_albida --
T407Phlebia_nitidula --
HHB6988Phanerochaete_exilis --
HHB8509Phanerochaetella_xeroph --
PBU0051Macrohyporia_dictyopora --
HHB11463Phanerochaete_sp --
FP102382Byssomerulius_corium --
FP102165Efibula_americana --
Murdoch90Ceriporia_torpida --
Rivoire4413_Ceriporia_purpurea --
Kout_18_Ceriporia_triumphalis --
Rivoire3701_Ceriporia_bresadol --
VS4018 --
Ryvarden21832_Ceriporia_manzan --
Dai24539
TCCAATAGCGTATATTAAAGTTGTTGCAGTTAAAAAGCTCGTAGTTGAAC

Dai24541
TCCAATAGCGTATATTAAAGTTGTTGCAGTTAAAAAGCTCGTAGTTGAAC
JV1105_12_Ceriporia_occidental ---
VS8558Ceriporia_occidentalis ---
Dai22445
TCCAATAGCGTATATTAAAGTTGTTGCAGTTAAAAAGCTCGTAGTTGAAC
846Dai16368
TCCAATAGCGTATATTAAAGTTGTTGCAGTTAAAAAGCTCGTAGTTGAAC
Dai17951_Ceriporia_aurantiocar ---
Miettinen_11701C_viridans ---
JV0105_10Ceriporia_aurantiocar ---
Yuan5702C_viridans ---
858Dai17003
TCCAATAGCGTATATTAAAGTTGTTGCAGTTAAAAAGCTCGTAGTTGAAC
Yuan2747_Ceriporia_viridans ---
Yuan2744C_viridans ---
Li1046C_viridans ---
865C_sinoviridans
TCCAATAGCGTATATTAAAGTTGTTGCAGTTAAAAAGCTCGTAGTTGAAC
871Dai15062 ---
Dai7642_Ceriporia_humilis
TCCAATAGCGTATATTAAAGTTGTTGCAGTTAAAAAGCTCGTAGTTGAAC
Spirin4706_Ceriporia_humilis ---
Spirin4944_Ceriporia_sericea ---
WCG1547Dai26044ceriporia
TCCAATAGCGTATATTAAAGTTGTTGCAGTTAAAAAGCTCGTAGTTGAAC
ZZW1558Dai27086
TCCAATAGCGTATATTAAAGTTGTTGCAGTTAAAAAGCTCGTAGTTGAAC
Miettinen14381_Ceriporia_mpuri ---
Miettinen15492_2_Ceriporia_sor ---
He6687
TCCAATAGCGTATATTAAAGTTGTTGCAGTTAAAAAGCTCGTAGTTGAAC
ZH53Dai24426
TCCAATAGCGTATATTAAAGTTGTTGCAGTTAAAAAGCTCGTAGTTGAAC
Vlasak0808_30_Ceriporia_punica ---
887Dai13376
TCCAATAGCGTATATTAAAGTTGTTGCAGTTAAAAAGCTCGTAGTTGAAC
WCG1443Dai24998
TCCAATAGCGTATATTAAAGTTGTTGCAGTTAAAAAGCTCGTAGTTGAAC
0108_6Ceriporia_spissa ---
Dai19164
TCCAATAGCGTATATTAAAGTTGTTGCAGTTAAAAAGCTCGTAGTTGAAC
Dai17937_Ceriporia_bubalinomar ---
903Dai12113

TCCAATAGCGTATATTAAAGTTGTTGCAGTTAAAAAGCTCGTAGTTGAAC

LZB929Dai25079

TCCAATAGCGTATATTAAAGTTGTTGCAGTTAAAAAGCTCGTAGTTGAAC

LX45Dai26988 --

LX43Dai26986

TCCAATAGCGTATATTAAAGTTGTTGCAGTTAAAAAGCTCGTAGTTGAAC

Dai7759Ceriporia --

Cui8012_Ceriporia_viridans --

GC1704_54Ceriporia_viridans --

Dai23392

TCCAATAGCGTATATTAAAGTTGTTGCAGTTAAAAAGCTCGTAGTTGAAC

WCG1585Dai26113Ceriproia

TCCAATAGCGTATATTAAAGTTGTTGCAGTTAAAAAGCTCGTAGTTGAAC

Dai18675C_eucalypti --

Dai22034

TCCAATAGCGTATATTAAAGTTGTTGCAGTTAAAAAGCTCGTAGTTGAAC

JV1008_41JTardaFLORIDAKeys --

Rivoire1161_Ceriporia_pierii --

Dai23499C_pierii

TCCAATAGCGTATATTAAAGTTGTTGCAGTTAAAAAGCTCGTAGTTGAAC

Dai23500

TCCAATAGCGTATATTAAAGTTGTTGCAGTTAAAAAGCTCGTAGTTGAAC

841Dai15899

TCCAATAGCGTATATTAAAGTTGTTGCAGTTAAAAAGCTCGTAGTTGAAC

842Dai15904

TCCAATAGCGTATATTAAAGTTGTTGCAGTTAAAAAGCTCGTAGTTGAAC

LZB1066xinjiang --

LZB1065xinjiang --

851Dai16779

TCCAATAGCGTATATTAAAGTTGTTGCAGTTAAAAAGCTCGTAGTTGAAC

RMJ119sp_Candelabrochaete_sept --

RLG9759spCandelabrochaete_sept --

RLG10478Phanerochaete_allantos --

Dai19118_Ceriporia_spissa

TCCAATAGCGTATATTAAAGTTGTTGCAGTTAAAACGCTCGTAGTTGAAC

Dai18486A

TCCAATAGCGTATATTAAAGTTGTTGCAGTTAAAACGCTCGTAGTTGAAC

WEI17_024_Ceriporia_mellita --

GC1508_71Ceriporia_mellita --

GC1608_7_Ceriporia_mellita --

ZZW1557Dai27085 --

ZZW1554Dai27083

TCCAATAGCGTATATTAAAGTTGTTGCAGTTAAAACGCTCGTAGTTGAAC

Dai8168 --

BR4865C_mellita --
MEL2382688Ceriporia_sp --
Dai8110 --
Cui8097 --
909Cui6740 --
W1258Dai24695
TCCAATAGCGTATATTAAAGTTGTTGCAGTTAAAACGCTCGTAGTTGAAC
JV0110_26_Ceriporia_griseoviol --
896Dai13202
TCCAATAGCGTATATTAAAGTTGTTGCAGTTAAAAAGCTCGTAGTTGAAC
LWY393Dai27053C_griseoviolasce
TCCAATAGCGTATATTAAAGTTGTTGCAGTTAAAAAGCTCGTAGTTGAAC
LWY394DAI27054
TCCAATAGCGTATATTAAAGTTGTTGCAGTTAAAAAGCTCGTAGTTGAAC
FP135015G_pannocinctus --
L15726SpG_pannocinctus --
Dai22221
TCCAATAGCGTATATTAAAGTTGTTGCAGTTAAAAAGCTCGTAGTTGAAC
Dai22633
TCCAATAGCGTATATTAAAGTTGTTGCAGTTAAAAAGCTCGTAGTTGAAC
Dai23260
TCCAATAGCGTATATTAAAGTTGTTGCAGTTAAAAAGCTCGTAGTTGAAC
Dai23626
TCCAATAGCGTATATTAAAGTTGTTGCAGTTAAAAAGCTCGTAGTTGAAC
Dai16238G_citrinoalbus
TCCAATAGCGTATATTAAAGTTGTTGCAGTTAAAAAGCTCGTAGTTGAAC
1175Dai15293
TCCAATAGCGTATATTAAAGTTGTTGCAGTTAAAAAGCTCGTAGTTGAAC
Dai19547
TCCAATAGCGTATATTAAAGTTGTTGCAGTTAAAAAGCTCGTAGTTGAAC
918063G_africanus --
918572G_africanus --
Dai18536A --
1164Cui17922
TCCAATAGCGTATATTAAAGTTGTTGCAGTTAAAAAGCTCGTAGTTGAAC
Dai22225
TCCAATAGCGTATATTAAAGTTGTTGCAGTTAAAAAGCTCGTAGTTGAAC
1163Dai20655
TCCAATAGCGTATATTAAAGTTGTTGCAGTTAAAAAGCTCGTAGTTGAAC
Yuan4397G_hainanensis --
1176Dai15268
TCCAATAGCGTATATTAAAGTTGTTGCAGTTAAAAAGCTCGTAGTTGAAC
1177Dai15259
TCCAATAGCGTATATTAAAGTTGTTGCAGTTAAAAAGCTCGTAGTTGAAC

BZ2896G_thelephoroides --
1166JV1808_26
TCCAATAGCGTATATTAAAGTTGTTGCAGTTAAAAAGCTCGTAGTTGAAC
Miettinen16992Hapalopilus_ochr --
GC1708_338_Ceriporia_arbuscula --
WCG1555Dai26107Ceriporia
TCCAATAGCGTATATTAAAGTTGTTGCAGTTAAAAAGCTCGTAGTTGAAC
GC1708_340_Ceriporia_arbuscula --
WCG1556Dai26109Ceriporia
TCCAATAGCGTATATTAAAGTTGTTGCAGTTAAAAAGCTCGTAGTTGAAC
883Cui11291
TCCAATAGCGTATATTAAAGTTGTTGCAGTTAAAAAGCTCGTAGTTGAAC
HLX320Dai26805
TCCAATAGCGTATATTAAAGTTGTTGCAGTTAAAAAGCTCGTAGTTGAAC
WCG1266Dai24678A
TCCAATAGCGTATATTAAAGTTGTTGCAGTTAAAAAGCTCGTAGTTGAAC
Dai6090_Ceriporia_sulphuricolo --
RLG_11354_Ceriproia_reticulata --
ZZW1543Dai27072
TCCAATAGCGTATATTAAAGTTGTTGCAGTTAAAAAGCTCGTAGTTGAAC
Li1316_Ceriporia_reticulata
TCCAATAGCGTATATTAAAGTTGTTGCAGTTAAAAAGCTCGTAGTTGAAC
KHL11981Ceriporia_reticulata --
FP110343sp_Candelabrochaete_la --
Li1045_Ceriporia_reticulata
TCCAATAGCGTATATTAAAGTTGTTGCAGTTAAAAAGCTCGTAGTTGAAC
ZX136Dai25794ceriporia
TCCAATAGCGTATATTAAAGTTGTTGCAGTTAAAAAGCTCGTAGTTGAAC
892Dai13400
TCCAATAGCGTATATTAAAGTTGTTGCAGTTAAAAAGCTCGTAGTTGAAC
RLG7163Leptoporus_mollis --
Dai21062Leptoporus_mollis --
Dai20182Leptoporus_submollis --
Cui18379Leptoporus_submollis --
Wu1209_46Resiniporus_pseudogil --
BRNM710169Resiniporus_resinasc --
Dai14516Bjerkandera_adusta
TCCAATAGCGTATATTAAAGTTGTTGCAGTTAAAAAGCTCGTAGTTGAAC
Dai21100Bjerkandera_fumosa
TCCAATAGCGTATATTAAAGTTGTTGCAGTTAAAAAGCTCGTAGTTGAAC
Miettinen16854Ceraceomyces_sp --
Dai10477C_spissa --
855Dai16831
TCCAATAGCGTATATTAAAGTTGTTGCAGTTAAAAAGCTCGTAGTTGAAC

882Cui11282
TCCAATAGCGTATATTAAAGTTGTTGCAGTTAAAAAGCTCGTAGTTGAAC
Dai24566
TCCAATAGCGTATATTAAAGTTGTTGCAGTTAAAAAGCTCGTAGTTGAAC
Yuan5965 --
Dai3204 --
1194CUI9985 --

Dai15205_Ceriporia_albomellea --
Dai15223_Ceriporia_albomellea --
Li1780_Ceriporia_variegata --
Dai19791_Ceriporia_variegata TTCAGACCTGGCTGGGCGGTCCGCC-TCACGGTGTG-TACTGTCTGGCTG
Dai19886 TTCAGACCTGGCTGGGCGGTCCGCC-TCACGGTGTG-TACTGTCTGGCTG
Dai10833_Ceriporia_crassitunic TTCAGACCTGGCCGGGCGGTCTGCC-TAACGGTATG-TACTGTCTGGCCG
CHWC1506_46Meruliopsis_crassit --
Dai9995_Ceriporia_crassitunica --
Wu1209_58_Meruliopsis_parvispo --
CHWC1505_129_Meruliopsis_parvi --
Dai21944 TTCAGACCTGGCTGGGCGGTCCGCC-TCACGGTGTG-TACTGTCTGGCTG
830Dai18640A TTCAGACCTGGCCGGGCGGTCCGCC-TCACGGTGTG-TACTGTCTGGCTG
GC1704_60_Meruliopsis_taxicola --
Dai22625 TTCAGACCTGGCTGGGCGGTCCGCC-TCACGGTGTG-TACTGTCTGGCTG
Dai22636 TTCAGACCTGGCTGGGCGGTCCGCC-TCACGGTGTG-TACTGTCTGGCTG
Dai21878 TTCAGACCTGGCTGGGCGGTCCGCC-TCACGGTGTG-TACTGTCTGGCTG
1169Dai17248 TTCAGACCTGGCTGGGCGGTCCGCC-TCACGGTGTG-TACTGTCTGGCTG
Wu1708_43_Meruliopsis_leptocys --
Li1011 --
ZX95Dai25742Meruliopsis_leptoc TTCAGACCTGGCTGGGCGGTCCGCC-TCACGGTGTG-TACTGTCTGGCTG
WCG1306Dai24733 TTCAGACCTGGCTGGGCGGTCCGCC-TCACGGTGTG-TACTGTCTGGCTG
LXL99Dai25816 TTCAGACCTGGCTGGGCGGTCCGCC-TCACGGTGTG-TACTGTCTGGCTG
WCG1559Dai26052Meruliopsis TTCAGACCTGGCTGGGCGGTCCGCC-

TCACGGTGTG-TACTGTCTGGCTG
He7477 --
HLX243Dai26217 TTCAGACCTGGCTGGGCGGTCCGCC-
TCACGGTGTG-TACTGTCTGGCTG
RussiaMW673659Meruliopsis_fagi --
FD278 --
Dai10226_Ceriporia_tarda --
LE247365 --
Dai8173_Meruliopsis_nanlingens
TTCAGGCCTGGCCGGGCGGTCCGCCTTCACGGTGTG-TACTGTCTGGCTG
860Dai17172 TTCAGACCTGGCTGGGCGGTCCGCC-
TCACGGTGTG-TACTGTCTGGCTG
879Dai13414 TTCAGACCTGGCTGGGCGGTCCGCC-
TCACGGTGTG-TACTGTCTGGCTG
Li_1704_Meruliopsis_pseudocyst --
833Dai18405 TTCAGACCTGGCTGGGCGGTCCGCC-
TCACGGTGTG-TACTGTCTGGCTG
HHB_10729_Meruliopsis_albostra --
Cui6878_Ceriporia_pseudocystid --
869Dai14737 TTCAGACCTGGCTGGGCGGTCCGCC-
TCACGGTGTG-TACTGTCTGGCTG
876Cui11626 TTCAGACCTGGCTGGGCGGTCCGCC-
TCACGGTGTG-TACTGTCTGGCTG
1199WEI3388 --
776308_Meruliopsis_cystidiata --
ICN139059_Meruliopsis_cystidia --
HHB15692Ceraceomyces_serpens --
HHB_15629_Sp_Ceriporiopsis_ane --
AJ185Trametopsis_cervina --
FD9Irpex_lacteus --
908Dai11230 TTCAGACCTGGCTGGGCGGTCCGCC-
TAACGGTGTG-TACTGTCTGGCTG
FP55521TEmmia_lacerata --
PBU0048Ceriporia_cystidiata --
MZ340C_laceratаT --
Dai21940 TTCAGACCTGGCTGGGCGGTCCGCC-
TAACGGTGTG-TACTGTCTGGCTG
847Dai16433 TTCAGACCTGGCTGGGCGGTCCGCC-
TAACGGTGTG-TACTGTCTGGCTG
MarcinEmmia_latemarginatus --
Meijer3729Hydnopolyporus_fimbr --
RLG13408Phanerochaete_sp --
WHC1381Flavodon_flavus --
GB1833Phlebia_albida --

T407Phlebia_nitidula --
HHB6988Phanerochaete_exilis --
HHB8509Phanerochaetella_xeroph --
PBU0051Macrohyporia_dictyopora --
HHB11463Phanerochaete_sp --
FP102382Byssomerulius_corium --
FP102165Efibula_americana --
Murdoch90Ceriporia_torpida --
Rivoire4413_Ceriporia_purpurea --
Kout_18_Ceriporia_triumphalis --
Rivoire3701_Ceriporia_bresadol --
VS4018 --
Ryvarden21832_Ceriporia_manzan --
Dai24539 TTCAGACCTGGCTGGGCGGTCCGCC-TCACGGTGTG-TACTGTCCGGCTG
Dai24541 TTCAGACCTGGCTGGGCGGTCCGCC-TCACGGTGTG-TACTGTCCGGCTG
JV1105_12_Ceriporia_occidental --
VS8558Ceriporia_occidentalis --
Dai22445 TTCAGACCTGGCTGGGCGGTCCGCC-TCACGGTGTG-TACTGTCCGGCTG
846Dai16368 TTCAGACCTGGCTGGGCGGTCCGCC-TCACGGTGTG-TACTGTCCGGCTG
Dai17951_Ceriporia_aurantiocar --
Miettinen_11701C_viridans --
JV0105_10Ceriporia_aurantiocar --
Yuan5702C_viridans --
858Dai17003 TTCAGACCTGGCTGGGCGGTCCGCC-TCACGGTGTG-TACTGTCTGGCTG
Yuan2747_Ceriporia_viridans --
Yuan2744C_viridans --
Li1046C_viridans --
865C_sinoviridans TTCAGACCTGGCCGGGCGGTCCGCC-TCACGGTATG-TACTGTCTGGCCG
871Dai15062 --
Dai7642_Ceriporia_humilis TTCAGGCCTGGCTGGGCGGTCCGCC-TAACGGTGTG-TACTGTCCGGCTG
Spirin4706_Ceriporia_humilis --
Spirin4944_Ceriporia_sericea --
WCG1547Dai26044ceriporia TTCAGGCCTGGCTGGGCGGTCCGCC-TCACGGTGTG-TACTGTCCGGCTG
ZZW1558Dai27086 TTCAGGCCTGGCTGGGCGGTCCGCC-TCACGGTGTG-TACTGTCCGGCTG
Miettinen14381_Ceriporia_mpuri --

Miettinen15492_2_Ceriporia_sor ---
He6687 TTCAGGCCTGGCTGGGCGGTCCGCC-TAACGGTGTG-TACTGTCCGGCTG
ZH53Dai24426 TTCAGGCCTGGCTGGGCGGTCCGCC-TAACGGTGTG-TACTGTCCGGCTG
Vlasak0808_30_Ceriporia_punica ---
887Dai13376 TTCAGACCTGGCTGGGCGGTCCGCC-TCACGGTGTG-TACTGTCTGGCTG
WCG1443Dai24998 TTCAGACCTGGCTGGGCGGTCCGCC-TCACGGTGTG-TACTGTCTGGCTG
0108_6Ceriporia_spissa ---
Dai19164 TTCAGACCTGGCTGGGCGGTCCGCC-TCACGGCGTG-TACTGTTCGGCTG
Dai17937_Ceriporia_bubalinomar ---
903Dai12113 TTCAGACCTGGCTGGGTGGTCCGCC-TCACGGCGTG-TACTGTTCGGCTG
LZB929Dai25079 TTCAGACCTGGCTGGGCGGTCCGCC-TCACGGTGTG-TACTGTCTGGCTG
LX45Dai26988 ---
LX43Dai26986 TTCAGACCTGGCTGGGCGGTCCGCC-TCACGGTGTG-TACTGTCTGGCTG
Dai7759Ceriporia ---
Cui8012_Ceriporia_viridans ---
GC1704_54Ceriporia_viridans ---
Dai23392 TTCAGATCTGGCTGGGCGGTCCGCC-TCACGGTGTG-TACTGTTCGGCTG
WCG1585Dai26113Ceriproia TTCAGACCTGGCCGGGCGGTCTGCC-TAACGGTATG-TACTGTCTGGCCG
Dai18675C_eucalypti ---
Dai22034 TTCAGACCTGGCTGGGCGGTCCGCC-TCACGGTGTG-TACTGTCCGGCTG
JV1008_41JTardaFLORIDAKeys ---
Rivoire1161_Ceriporia_pierii ---
Dai23499C_pierii TTCAGGCCTGGCTGGGCGGTCCGCC-TCACGGTGTG-TACTGTTCGGCTG
Dai23500 TTCAGGCCTGGCTGGGCGGTCCGCC-TCACGGTGTG-TACTGTTCGGCTG
841Dai15899 TTCAGACCTGGCTGGGCGGTCCGCC-TCACGGTGTG-TACTGTCCGGCTG
842Dai15904 TTCAGACCTGGCTGGGCGGTCCGCC-TCACGGTGTG-TACTGTCCGGCTG
LZB1066xinjiang ---
LZB1065xinjiang ---
851Dai16779 TTCAGGCCTGGCCGGGCGGTCTGCC-

TAACGGTATG-TACTGTCTGGCTG
RMJ119sp_Candelabrochaete_sept --
RLG9759spCandelabrochaete_sept --
RLG10478Phanerochaete_allantos --
Dai19118_Ceriporia_spissa TTCAGACCTGGCTGGGCGGTCCGCC-
TCACGGTGTG-TACTGTCTGGCTG
Dai18486A TTCAGACCTGGCTGGGCGGTCCGCC-
TCACGGTGTG-TACTGTCTGGCTG
WEI17_024_Ceriporia_mellita --
GC1508_71Ceriporia_mellita --
GC1608_7_Ceriporia_mellita --
ZZW1557Dai27085 --
ZZW1554Dai27083 TTCAGACCTGGCTGGGCGGTCCGCC-
TCACGGTGTG-TACTGTCTGGCTG
Dai8168 --
BR4865C_mellita --
MEL2382688Ceriporia_sp --
Dai8110 --
Cui8097 --
909Cui6740 --
W1258Dai24695 TTCAGACCTGGCTGGGCGGTCCGCC-
TCACGGTGTG-TACTGTCTGGCTG
JV0110_26_Ceriporia_griseoviol --
896Dai13202 TTCAGACCTGGCTGGGCGGTCCGCT-
TCACGGCGTG-TACTGTTCGGCTG
LWY393Dai27053C_griseoviolasce TTCAGACCTGGCTGGGCGGTCCGCT-
TCACGGCGTG-TACTGTTCGGCTG
LWY394DAI27054 TTCAGACCTGGCTGGGCGGTCCGCT-
TCACGGCGTG-TACTGTTCGGCTG
FP135015G_pannocinctus --
L15726SpG_pannocinctus --
Dai22221 TTCAGATCCGGCTGGGCGGTCTGCC-
TCACGGTATG-TACTGTCTGGCTG
Dai22633 TTCAGACCTGGCTGGGCGGTCTGCC-
TCACGGTATG-TACTGTCTGGCTG
Dai23260 TTCAGACCTGGCTGGGCGGTCTGCC-
TCACGGTATG-TACTGTCTGGCTG
Dai23626 TTCAGACCTGGCTGGGCGGTCTGCC-
TCACGGTATG-TACTGTCTGGCTG
Dai16238G_citrinoalbus TTCAGACCTGTCCGGGTGGTCCCCT-
TTATGGCGTGTTACTGTCTGGCCG
1175Dai15293 TTCAGATCTGGCTGGGCGGTCTGCC-
TCACGGTATG-TACTGTCTGGCTG
Dai19547 TTCAGATCTGGCTGGGCGGTCTGCC-

TCACGGTATG-TACTGTCTGGCTG
918063G_africanus ---
918572G_africanus ---
Dai18536A ---
1164Cui17922 TTCAGATCTGGCTGGGCGGTCTGCC-
TCACGGTATG-TACTGTCTGGCTG
Dai22225 TTCAGATCTGGCTGGGCGGTCTGCC-
TCACGGTATG-TACTGTCTGGCTG
1163Dai20655 TTCAGATCTGGCTGGGCGGTCTGCC-
TCACGGTATG-TACTGTCTGGCTG
Yuan4397G_hainanensis ---
1176Dai15268 TTCAGACCTGGCCGGGCGGTCTGCC-
TCACGGTATG-TACTGTCTGGCTG
1177Dai15259 TTCAGACCTGGCCGGGCGGTCTGCC-
TCACGGTATG-TACTGTCTGGCTG
BZ2896G_thelephoroides ---
1166JV1808_26 TTCAGACCTGGCCGGGCGGTCTGCC-
TCACGGTATG-TACTGTCTGGCTG
Miettinen16992Hapalopilus_ochr ---
GC1708_338_Ceriporia_arbuscula ---
WCG1555Dai26107Ceriporia TTCAGACCTGGCTGGGCGGTCCGCC-
TCACGGTGTG-TACTGTTCGGCTG
GC1708_340_Ceriporia_arbuscula ---
WCG1556Dai26109Ceriporia TTCAGACCTGGCTGGGCGGTCCGCC-
TCACGGTGTG-TACTGTTCGGCTG
883Cui11291 TTCAGACCTGGCTGGGCGGTCCGCC-
TCACGGTGTG-TACTGTTCGGCTG
HLX320Dai26805 TTCAGACCTGGCTGGGCGGTCCGCC-
TCACGGTGTG-TACTGTTCGGCTG
WCG1266Dai24678A TTCAGACCTGGCTGGGCGGTCCGCC-
TCACGGTGTG-TACTGTTCGGCTG
Dai6090_Ceriporia_sulphuricolo ---
RLG_11354_Ceriproia_reticulata ---
ZZW1543Dai27072 TTCAGACCTGGCTGGGTGGTCCGCC-
TCACGGTGTG-TACTGTTCGGCTG
Li1316_Ceriporia_reticulata TTCAGGCCTGGCCGGGCGGTCCGCC-
TCACGGTGTG-TACTGTCTGGCTG
KHL11981Ceriporia_reticulata ---
FP110343sp_Candelabrochaete_la ---
Li1045_Ceriporia_reticulata TTCAGACCTGGCTGGGCGGTCCGCC-
TCACGGTGAG-TACTGTCCGGCTG
ZX136Dai25794ceriporia TTCAGACCTGGCTGGGCGGTCCGCC-
TCACGGTGAG-TACTGTCTGGCTG
892Dai13400 TTCAGACCTGGCTGGGCGGTCCGCC-

TCACGGTGAG-TACTGTCCGGCTG
RLG7163Leptoporus_mollis ---
Dai21062Leptoporus_mollis ---
Dai20182Leptoporus_submollis ---
Cui18379Leptoporus_submollis ---
Wu1209_46Resiniporus_pseudogil ---
BRNM710169Resiniporus_resinasc ---
Dai14516Bjerkandera_adusta TTCAGACCTGGCTGGGCGGTCTGCC-
TAACGGTATG-TACTGTCCGGCTG
Dai21100Bjerkandera_fumosa TTCAGACCTGGCTGGGCGGTCTGCC-
TAACGGTATG-TACTGTTCGGCTG
Miettinen16854Ceraceomyces_sp ---
Dai10477C_spissa ---
855Dai16831 TTCAGACCTGGCTGGGTGGTCCGCC-
TCACGGTGTG-TACTGTCTGGCTG
882Cui11282 TTCAGACCTGGCTGGGTGGTCCGCC-
TCACGGTGTG-TACTGTCTGGCTG
Dai24566 TTCAGACCTGGCTGGGTGGTCCGCC-
TCACGGTGTG-TACTGTCTGGCTG
Yuan5965 ---
Dai3204 ---
1194CUI9985 ---

Dai15205_Ceriporia_albomellea ---
Dai15223_Ceriporia_albomellea ---
Li1780_Ceriporia_variegata ---
Dai19791_Ceriporia_variegata
GGTCTTACCTCTTGGTGAGCCGGCATGCCCTTCATTGGGTGTGTCGGGGA
Dai19886
GGTCTTACCTCTTGGTGAGCCGGCATGCCCTTCATTGGGTGTGTCGGGGA
Dai10833_Ceriporia_crassitunic
GGCCTTACCTCTTGGTGAGCCGGCATGCCCTTCACTGGGTGCGTCGGGGA
CHWC1506_46Meruliopsis_crassit ---
Dai9995_Ceriporia_crassitunica ---
Wu1209_58_Meruliopsis_parvispo ---
CHWC1505_129_Meruliopsis_parvi ---
Dai21944
GGTCTTACCTCTTGGTGAGCCGGCATGCCCTTTACTGGGTGTGTCGGGGA
830Dai18640A
GGTCTTACCTCCTGGTGAGCCGGCATGCCCTTTACTGGGTGTGTCGGGGA
GC1704_60_Meruliopsis_taxicola ---
Dai22625
GGTCTTACCTCTTGGTGAGCCGGCATGCCCTTTACTGGGTGTGTCGGGGA

Dai22636
GGTCTTACCTCTTGGTGAGCCGGCATGCCCTTTACTGGGTGTGTCGGGGA
Dai21878
GGTCTTACCTCTTGGTGAGCCGGCATGCCCTTTACTGGGTGTGTCGGGGA
1169Dai17248
GGTCTTACCTCTTGGTGAGCCGGCATGCCCTTTACTGGGTGTGTCGGGGA
Wu1708_43_Meruliopsis_leptocys ---
Li1011 ---
ZX95Dai25742Meruliopsis_leptoc
GGTCTTACCTCTTGGTGAGCCGGCATGCCCTTCACTGGGTGTGTCGGGGA
WCG1306Dai24733
GGTCTTACCTCTTGGTGAGCCGGCATGCCCTTCACTGGGTGTGTCGGGGA
LXL99Dai25816
GGTCTTACCTCTTGGTGAGCCGGCATGCCCTTCACTGGGTGTGTCGGGGA
WCG1559Dai26052Meruliopsis
GGTCTTACCTCTTGGTGAGCCGGCATGCCCTTCACTGGGTGTGTCGGGGA
He7477 ---
HLX243Dai26217
GGTCTTACCTCTTGGTGAGCCGGCATGCCCTTCACTGGGTGTGTCGGGGA
RussiaMW673659Meruliopsis_fagi ---
FD278 ---
Dai10226_Ceriporia_tarda ---
LE247365 ---
Dai8173_Meruliopsis_nanlingens
GGTCTTACCTCTTGGTGAACCAGCATGCCCTTCACTGGGTGTGTTGGGGA
860Dai17172
GGTCTTACCTCTTGGTGAGCCGGCATGCCCTTCACTGGGTGTGTCGGGGA
879Dai13414
GGTCTTACCTCTTGGTGAGCCGGCATGCCCTTCACTGGGTGTGTCGGGGA
Li_1704_Meruliopsis_pseudocyst ---
833Dai18405
GGTCTTACCTCTTGGTGAGCCGGCATGCTCTTTACTGGGTGTGTCGGGGA
HHB_10729_Meruliopsis_albostra ---
Cui6878_Ceriporia_pseudocystid ---
869Dai14737
GGTCTTACCTCTTGGTGAGCCGGCATGCCCTTTACTGGGTGTGTCGGGGA
876Cui11626
GGTCTTACCTCTTGGTGAGCCGGCATGCCCTTTACTGGGTGTGTCGGGGA
1199WEI3388 ---
776308_Meruliopsis_cystidiata ---
ICN139059_Meruliopsis_cystidia ---
HHB15692Ceraceomyces_serpens ---
HHB_15629_Sp_Ceriporiopsis_ane ---
AJ185Trametopsis_cervina ---

FD9Irpex_lacteus --
908Dai11230
GGTCTTACCTCTTGGTGAGCCGGCATGCCCTTCACTGGGTGTGTCGGGGA
FP55521TEmmia_lacerata --
PBU0048Ceriporia_cystidiata --
MZ340C_lacerataT --
Dai21940
GGTCTTACCTCTTGGTGAGCCGGTATGCCCTTCACTGGGTGTATCGGGGA
847Dai16433
GGTCTTACCTCTTGGTGAGCCGGTATGCCCTTCACTGGGTGTATCGGGGA
MarcinEmmia_latemarginatus --
Meijer3729Hydnopolyporus_fimbr --
RLG13408Phanerochaete_sp --
WHC1381Flavodon_flavus --
GB1833Phlebia_albida --
T407Phlebia_nitidula --
HHB6988Phanerochaete_exilis --
HHB8509Phanerochaetella_xeroph --
PBU0051Macrohyporia_dictyopora --
HHB11463Phanerochaete_sp --
FP102382Byssomerulius_corium --
FP102165Efibula_americana --
Murdoch90Ceriporia_torpida --
Rivoire4413_Ceriporia_purpurea --
Kout_18_Ceriporia_triumphalis --
Rivoire3701_Ceriporia_bresadol --
VS4018 --
Ryvarden21832_Ceriporia_manzan --
Dai24539
GGTCTTACCTCTTGGTGAGCCAGCATGCCCTTTACTGGGTGTGTTGGGGA
Dai24541
GGTCTTACCTCTTGGTGAGCCAGCATGCCCTTTACTGGGTGTGTTGGGGA
JV1105_12_Ceriporia_occidental --
VS8558Ceriporia_occidentalis --
Dai22445
GGTCTTACCTCTTGGTGAGCCGGCATGCCCTTTACTGGGTGTGTCGGGGA
846Dai16368
GGTCTTACCTCTTGGTGAGCCGGCATGCCCTTTACTGGGTGTGTCGGGGA
Dai17951_Ceriporia_aurantiocar --
Miettinen_11701C_viridans --
JV0105_10Ceriporia_aurantiocar --
Yuan5702C_viridans --
858Dai17003
GGTCTTACCTCTTGGTGAGCCGGCATGCCCTTCACTGGGTGTGTCGGGGA

Yuan2747_Ceriporia_viridans --
Yuan2744C_viridans --
Li1046C_viridans --
865C_sinoviridans
GATCTTACCTCTTGGTGAGCCGGCATGCCCTTTACTGGGTGTGTCGGGGA
871Dai15062 --
Dai7642_Ceriporia_humilis
GGTCTTACCTCTTGGTGAGCCGGCATGCCCTTCACTGGGTGTGTCGGGGA
Spirin4706_Ceriporia_humilis --
Spirin4944_Ceriporia_sericea --
WCG1547Dai26044ceriporia
GGTCTTACCTCTTGGTGAGCCGGCATGCCCTTCACTGGGTGTGTCGGGGA
ZZW1558Dai27086
GGTCTTACCTCTTGGTGAGCCGGCATGCCCTTCACTGGGTGTGTCGGGGA
Miettinen14381_Ceriporia_mpuri --
Miettinen15492_2_Ceriporia_sor --
He6687
GGTCTTACCTCTTGGTGAGCCGGCATGCCCTTCACTGGGTGTGTCGGGGA
ZH53Dai24426
GGTCTTACCTCTTGGTGAGCCGGCATGCCCTTCACTGGGTGTGTCGGGGA
Vlasak0808_30_Ceriporia_punica --
887Dai13376
GGTCTTACCTCTTGGTGAGCCGGCATGCCCTTTACTGGGTGTGTCGAGGA
WCG1443Dai24998
GGTCTTACCTCTTGGTGAGCCGGCATGCCCTTTACTGGGTGTGTCGAGGA
0108_6Ceriporia_spissa --
Dai19164
GGCCTTACCTCTTGGTGAGCCGGCATGCCCTTAATTGGGTGTGTCGGTGA
Dai17937_Ceriporia_bubalinomar --
903Dai12113
GGTCTTACCTCTTGGTGAGCTGGCATGCCCTTAATTGGGTGTGTCAGTGA
LZB929Dai25079
GGTCTTACCTCTTGGTGAGCCAGCATGCCCTTAACTGGGTGTGTTGGGGA
LX45Dai26988 --
LX43Dai26986
GGTCTTACCTCTTGGTGAGCCAGCATGCCCTTAACTGGGTGTGTTGGGGA
Dai7759Ceriporia --
Cui8012_Ceriporia_viridans --
GC1704_54Ceriporia_viridans --
Dai23392
GATCTTACCTCTTGGTGAGCCGGCATGCCCTTTACTGGGTGTGTCGGGGA
WCG1585Dai26113Ceriproia
GGCCTTACCTCTTGGTGAGCCGGCATGCCCTTCACTGGGTGTGTCGGGGA
Dai18675C_eucalypti --

Dai22034
GGCCTTACCTCTTGGTGAGCTAGCATGCCCTTTACTGGGTGTGTTGGGGA
JV1008_41JTardaFLORIDAKeys --
Rivoire1161_Ceriporia_pierii --
Dai23499C_pierii
GGTCTTACCTCTTGGTGAGCCGGCATGCCCTTCACTGGGTGTGTCGGGGA
Dai23500
GGTCTTACCTCTTGGTGAGCCGGCATGCCCTTCACTGGGTGTGTCGGGGA
841Dai15899
AGTCTTACCTCTTGGTGAGCCGGCATGCCCTTCACTGGGTGTGTCGGGGA
842Dai15904
AGTCTTACCTCTTGGTGAGCCGGCATGCCCTTCACTGGGTGTGTCGGGGA
LZB1066xinjiang --
LZB1065xinjiang --
851Dai16779
GGTCTTACCTCTTGGTGAGCCAGCATGCCCTTTACTGGGTGTGTTGGGGA
RMJ119sp_Candelabrochaete_sept --
RLG9759spCandelabrochaete_sept --
RLG10478Phanerochaete_allantos --
Dai19118_Ceriporia_spissa
AGTCTTACCTCTTGGTGCGCCGGCATGCCCTTAACTGGGTGTGTTGGGGA
Dai18486A
AGTCTTACCTCTTGGTGAGCCGGCATGCCCTTAACTGGGTGTGTTGGGGA
WEI17_024_Ceriporia_mellita --
GC1508_71Ceriporia_mellita --
GC1608_7_Ceriporia_mellita --
ZZW1557Dai27085 --
ZZW1554Dai27083
AGTCTTACCTCTTGGTGCGCCGGCATGCCCTTAACTGGGTGTGTTGGGGA
Dai8168 --
BR4865C_mellita --
MEL2382688Ceriporia_sp --
Dai8110 --
Cui8097 --
909Cui6740 --
W1258Dai24695
GGTCTTACCTCTTGGTGCGCCGGCATGCCCTTAACTGGGTGTGTTGGGGA
JV0110_26_Ceriporia_griseoviol --
896Dai13202
GGTCTTACCTCTTGGTGAGCCGGCATGCCCTTCATTGGGTGTGTCGGGGA
LWY393Dai27053C_griseoviolasce
GGTCTTACCTCTTGGTGAGCCGGCATGCCCTTCATTGGGTGTGTCGGGGA
LWY394DAI27054
GGTCTTACCTCTTGGTGAGCCGGCATGCCCTTCATTGGGTGTGTCGGGGA

FP135015G_pannocinctus --
L15726SpG_pannocinctus --
Dai22221 GATCTTACCTCTTGGTGAGCC-GCATGCCCTTTACTGGGTGTGTTGGGGA
Dai22633 GGTCTTACCTCTTGGTGAGCC-GCATGCCCTTTACTGGGTGTGTTGGGGA
Dai23260 GGTCTTACCTCTTGGTGAGCC-GCATGCCCTTTACTGGGTGTGTTGGGGA
Dai23626 GGTCTTACCTCTTGGTGAGCC-GCATGCCCTTTACTGGGTGTGTTGGGGA
Dai16238G_citrinoalbus GGCCTTTCCTCTTGGTGAGCCTGCATGCCCTTTACTGGACGTGTAGGAGA
1175Dai15293 GGTCTTACCTCTTGGTGAGCC-GCATGCCCTTTACTGGGTGTGTTGGGGA
Dai19547 GGTCTTACCTCTTGGTGAGCC-GCATGCCCTTTACTGGGTGTGTTGGGGA
918063G_africanus --
918572G_africanus --
Dai18536A --
1164Cui17922 GGTCTTACCTCTTGGTGAGCC-GCATGCCCTTTACTGGGTGTGTTGGGGA
Dai22225 GGTCTTACCTCTTGGTGAGCC-GCATGCCCTTTACTGGGTGTGTTGGGGA
1163Dai20655 GGTCTTACCTCTTGGTGAGCC-GCATGCCCTTTACTGGGTGTGTTGGGGA
Yuan4397G_hainanensis --
1176Dai15268 GGTCTTACCTCTTGGTGAGCTGGCATGCCCTTTACTGGGTGTGTCAGGGA
1177Dai15259 GGTCTTACCTCTTGGTGAGCTGGCATGCCCTTTACTGGGTGTGTCAGGGA
BZ2896G_thelephoroides --
1166JV1808_26 GGTCTTACCTCTTGGTGAGCTGGCATGCCCTTTACTGGGTGTGTCAGGGA
Miettinen16992Hapalopilus_ochr --
GC1708_338_Ceriporia_arbuscula --
WCG1555Dai26107Ceriporia GGTCTTACCTCTTGGTGAGCCAGCATGCCCTTAACTGGGTGTGTTGGGGA
GC1708_340_Ceriporia_arbuscula --
WCG1556Dai26109Ceriporia GGTCTTACCTCTTGGTGAGCCAGCATGCCCTTAACTGGGTGTGTTGGGGA
883Cui11291 GGTCTTACCTCTTGGTGAGCCAGCATGCCCTTAACTGGGTGTGTTGGGGA
HLX320Dai26805 GGTCTTACCTCTTGGTGAGCCAGCATGCCCTTAACTGGGTGTGTTGGAGA

WCG1266Dai24678A
GGTCTTACCTCTTGGTGAGCCAGCATGCCCTTTACTGGGTGTGTTGGGGA
Dai6090_Ceriporia_sulphuricolo --
RLG_11354_Ceriproia_reticulata --
ZZW1543Dai27072
GGTCTTACCTCTTGGTGAGCTGGCATGTCCTTCACTGGGTGTGTCAGGGA
Li1316_Ceriporia_reticulata
GGTCTTACCTCTTGGTGAACCGGCATGCCCTTAACGGGGTGTGTCGGGGA
KHL11981Ceriporia_reticulata --
FP110343sp_Candelabrochaete_la --
Li1045_Ceriporia_reticulata
GGTCTTACCTCTTGGTGAGCTGGCATGTCCTTAACTGGGTGTGTCAGGGA
ZX136Dai25794ceriporia
GGTCTTACCTCTTGGTGAGCTGGCATGTCCTTAACTGGGTGTGTCAGGGA
892Dai13400
GGTCTTACCTCTTGGTGAGCTGGCATGTCCTTAACTGGGTGTGTCAGGGA
RLG7163Leptoporus_mollis --
Dai21062Leptoporus_mollis --
Dai20182Leptoporus_submollis --
Cui18379Leptoporus_submollis --
Wu1209_46Resiniporus_pseudogil --
BRNM710169Resiniporus_resinasc --
Dai14516Bjerkandera_adusta
GGTCTTACCTCTTGGTGAGCCGGCATGCCCTTCACTGGGTGTGTCGGGGA
Dai21100Bjerkandera_fumosa
GGTCTTACCTCTTGGTGAGCCGGCATGCCCTTCATTGGGTGTGTCGGGGA
Miettinen16854Ceraceomyces_sp --
Dai10477C_spissa --
855Dai16831
GGTCTTACCTCTTGGTGAGCCGGCATGCCCTTAACTGGGTGTGTCGGGGA
882Cui11282
GGTCTTACCTCTTGGTGAGCCGGCATGCCCTTAACTGGGTGTGTCGGGGA
Dai24566
GGTCTTACCTCTTGGTGAGCCGGCATGCCCTTCACTGGGTGTGTCGGGGA
Yuan5965 --
Dai3204 --
1194CUI9985 --

Dai15205_Ceriporia_albomellea --
Dai15223_Ceriporia_albomellea --
Li1780_Ceriporia_variegata --
Dai19791_Ceriporia_variegata
ACCAGGACTTTTACCTTGAGAAAATTAGAGTGTTCAAAGCAGGCCTGCGC

Dai19886
ACCAGGACTTTTACCTTGAGAAAATTAGAGTGTTCAAAGCAGGCCTGCGC
Dai10833_Ceriporia_crassitunic
ACCAGGACCTTTACCTTGAGAAAATTAGAGTGTTCAAAGCAGGCCTATGC
CHWC1506_46Meruliopsis_crassit --
Dai9995_Ceriporia_crassitunica --
Wu1209_58_Meruliopsis_parvispo --
CHWC1505_129_Meruliopsis_parvi --
Dai21944
ACCAGGACTTTTACCTTGAGAAAATTAGAGTGTTCAAAGCAGGCCTGTGC
830Dai18640A
ACCAGGACTTTTACCTTGAGAAAATTAGAGTGTTCAAAGCAGGCCTGTGC
GC1704_60_Meruliopsis_taxicola --
Dai22625
ACCAGGACTTTTACCTTGAGAAAATTAGAGTGTTCAAAGCAGGCCTGCGC
Dai22636
ACCAGGACTTTTACCTTGAGAAAATTAGAGTGTTCAAAGCAGGCCTGCGC
Dai21878
ACCAGGACTTTTACCTTGAGAAAATTAGAGTGTTCAAAGCAGGCCTGCGC
1169Dai17248
ACCAGGACTTTTACCTTGAGAAAATTAGAGTGTTCAAAGCAGGCCTGCGC
Wu1708_43_Meruliopsis_leptocys --
Li1011 --
ZX95Dai25742Meruliopsis_leptoc
ACCAGGACTTTTACCTTGAGAAAATTAGAGTGTTCAAAGCAGGCCTGCGC
WCG1306Dai24733
ACCAGGACTTTTACCTTGAGAAAATTAGAGTGTTCAAAGCAGGCCTGCGC
LXL99Dai25816
ACCAGGACTTTTACCTTGAGAAAATTAGAGTGTTCAAAGCAGGCCTGCGC
WCG1559Dai26052Meruliopsis
ACCAGGACTTTTACCTTGAGAAAATTAGAGTGTTCAAAGCAGGCCTGCGC
He7477 --
HLX243Dai26217
ACCAGGACTTTTACCTTGAGAAAATTAGAGTGTTCAAAGCAGGCCTGCGC
RussiaMW673659Meruliopsis_fagi --
FD278 --
Dai10226_Ceriporia_tarda --
LE247365 --
Dai8173_Meruliopsis_nanlingens
ACCAGGACTTTTACCTTGAGAAAATTAGAGTGCTCAAAGCAGGCCTACGC
860Dai17172
ACCAGGACTTTTACCTTGAGAAAATTAGAGTGTTCAAAGCAGGCCTGCGC
879Dai13414
ACCAGGACTTTTACCTTGAGAAAATTAGAGTGTTCAAAGCAGGCCTGCGC

Li_1704_Meruliopsis_pseudocyst --

833Dai18405 ACCAGGACTTTTACCTTGAGAAAATTAGAGTGTTCAAAGCAGGCCTGCGC

HHB_10729_Meruliopsis_albostra --

Cui6878_Ceriporia_pseudocystid --

869Dai14737 ACCAGGACTTTTACCTTGAGAAAATTAGAGTGTTCAAAGCAGGCCTGCGC

876Cui11626 ACCAGGACTTTTACCTTGAGAAAATTAGAGTGTTCAAAGCAGGCCTGCGC

1199WEI3388 --

776308_Meruliopsis_cystidiata --

ICN139059_Meruliopsis_cystidia --

HHB15692Ceraceomyces_serpens --

HHB_15629_Sp_Ceriporiopsis_ane --

AJ185Trametopsis_cervina --

FD9Irpex_lacteus --

908Dai11230 ACCAGGACTTTTACCTTGAGAAAATTAGAGTGTTCAAAGCAGGCCTGCGC

FP55521TEmmia_lacerata --

PBU0048Ceriporia_cystidiata --

MZ340C_laceratаT --

Dai21940 ACCAGGACTTTTACCTTGAGAAAATTAGAGTGTTCAAAGCAGGCCTGCGC

847Dai16433 ACCAGGACTTTTACCTTGAGAAAATTAGAGTGTTCAAAGCAGGCCTGCGC

MarcinEmmia_latemarginatus --

Meijer3729Hydnopolyporus_fimbr --

RLG13408Phanerochaete_sp --

WHC1381Flavodon_flavus --

GB1833Phlebia_albida --

T407Phlebia_nitidula --

HHB6988Phanerochaete_exilis --

HHB8509Phanerochaetella_xeroph --

PBU0051Macrohyporia_dictyopora --

HHB11463Phanerochaete_sp --

FP102382Byssomerulius_corium --

FP102165Efibula_americana --

Murdoch90Ceriporia_torpida --

Rivoire4413_Ceriporia_purpurea --

Kout_18_Ceriporia_triumphalis --

Rivoire3701_Ceriporia_bresadol --

VS4018 --

Ryvarden21832_Ceriporia_manzan --

Dai24539

ACCAGGACTTTTACCTTGAGAAAATTAGAGTGTTCAAAGCAGGCCTGCGC

Dai24541

ACCAGGACTTTTACCTTGAGAAAATTAGAGTGTTCAAAGCAGGCCTGCGC

JV1105_12_Ceriporia_occidental --

VS8558Ceriporia_occidentalis --

Dai22445

ACCAGGACTTTTACCTTGAGAAAATTAGAGTGTTCAAAGCAGGCCTGCGC

846Dai16368

ACCAGGACTTTTACCTTGAGAAAATTAGAGTGTTCAAAGCAGGCCTGCGC

Dai17951_Ceriporia_aurantiocar --

Miettinen_11701C_viridans --

JV0105_10Ceriporia_aurantiocar --

Yuan5702C_viridans --

858Dai17003

ACCAGGACTTTTACCTTGAGAAAATTAGAGTGTTCAAAGCAGGCCTGCGC

Yuan2747_Ceriporia_viridans --

Yuan2744C_viridans --

Li1046C_viridans --

865C_sinoviridans

ACCAGGACTTTTACCTTGAGAAAATTAGAGTGTTCAAAGCAGGCCTATGC

871Dai15062 --

Dai7642_Ceriporia_humilis

ACCAGGACTTTTACCTTGAGAAAATTAGAGTGTTCAAAGCAGGCCTGCGC

Spirin4706_Ceriporia_humilis --

Spirin4944_Ceriporia_sericea --

WCG1547Dai26044ceriporia

ACCAGGACTTTTACCTTGAGAAAATTAGAGTGTTCAAAGCAGGCCTGCGC

ZZW1558Dai27086

ACCAGGACTTTTACCTTGAGAAAATTAGAGTGTTCAAAGCAGGCCTGCGC

Miettinen14381_Ceriporia_mpuri --

Miettinen15492_2_Ceriporia_sor --

He6687

ACCAGGACTTTTACCTTGAGAAAATTAGAGTGTTCAAAGCAGGCCTGCGC

ZH53Dai24426

ACCAGGACTTTTACCTTGAGAAAATTAGAGTGTTCAAAGCAGGCCTGCGC

Vlasak0808_30_Ceriporia_punica --

887Dai13376

ACCAGGACTTTTACCTTGAGAAAATTAGAGTGTTCAAAGCAGGCCTACGC

WCG1443Dai24998

ACCAGGACTTTTACCTTGAGAAAATTAGAGTGTTCAAAGCAGGCCTATGC

0108_6Ceriporia_spissa --

Dai19164

ACCAGGACCTTTACCTTGAGAAAATTAGAGTGTTCAAAGCAGGCCTGTGC

Dai17937_Ceriporia_bubalinomar --

903Dai12113
ACCAGGACCTTTACCTTGAGAAAATTAGAGTGTTCAAAGCAGGCCTGTGC
LZB929Dai25079
ACCAGGACTTTTACCTTGAGAAAATTAGAGTGTTCAAAGCAGGCCTGCGC
LX45Dai26988 --
LX43Dai26986
ACCAGGACTTTTACCTTGAGAAAATTAGAGTGTTCAAAGCAGGCCTGCGC
Dai7759Ceriporia --
Cui8012_Ceriporia_viridans --
GC1704_54Ceriporia_viridans --
Dai23392
ACCAGGACTTTTACCTTGAGAAAATTAGAGTGTTCAAAGCAGGCCTGCGC
WCG1585Dai26113Ceriproia
ACCAGGACCTTTACCTTGAGAAAATTAGAGTGTTCAAAGCAGGCCTATGC
Dai18675C_eucalypti --
Dai22034
ACCAGGACTTTTACCTTGAGAAAATTAGAGTGTTCAAAGCAGGCCTGTGC
JV1008_41JTardaFLORIDAKeys --
Rivoire1161_Ceriporia_pierii --
Dai23499C_pierii
ACCAGGACTTTTACCTTGAGAAAATTAGAGTGTTCAAAGCAGGCCTATGC
Dai23500
ACCAGGACTTTTACCTTGAGAAAATTAGAGTGTTCAAAGCAGGCCTATGC
841Dai15899
ACCAGGACTTTTACCTTGAGAAAATTAGAGTGTTCAAAGCAGGCCTGCGC
842Dai15904
ACCAGGACTTTTACCTTGAGAAAATTAGAGTGTTCAAAGCAGGCCTGCGC
LZB1066xinjiang --
LZB1065xinjiang --
851Dai16779
ACCAGGATTTTTACCTTGAGAAAATTAGAGTGTTCAAAGCAGGCTTATGC
RMJ119sp_Candelabrochaete_sept --
RLG9759spCandelabrochaete_sept --
RLG10478Phanerochaete_allantos --
Dai19118_Ceriporia_spissa
ACCAGGACTTTTACCTTGAGAAAATTAGAGTGTTCAAAGCAGGCCTATGC
Dai18486A
ACCAGGACTTTTACCTTGAGAAAATTAGAGTGTTCAAAGCAGGCCTATGC
WEI17_024_Ceriporia_mellita --
GC1508_71Ceriporia_mellita --
GC1608_7_Ceriporia_mellita --
ZZW1557Dai27085 --
ZZW1554Dai27083
ACCAGGACTTTTACCTTGAGAAAATTAGAGTGTTCAAAGCAGGCCTATGC

Dai8168 --
BR4865C_mellita --
MEL2382688Ceriporia_sp --
Dai8110 --
Cui8097 --
909Cui6740 --
W1258Dai24695
ACCAGGACTTTTACCTTGAGAAAATTAGAGTGTTCAAAGCAGGCCTATGC
JV0110_26_Ceriporia_griseoviol --
896Dai13202
ACCAGGACTTTTACCTTGAGAAAATTAGAGTGTTCAAAGCAGGCCTGCGC
LWY393Dai27053C_griseoviolasce
ACCAGGACTTTTACCTTGAGAAAATTAGAGTGTTCAAAGCAGGCCTGTGC
LWY394DAI27054
ACCAGGACTTTTACCTTGAGAAAATTAGAGTGTTCAAAGCAGGCCTGTGC
FP135015G_pannocinctus --
L15726SpG_pannocinctus --
Dai22221
ACCAGGACTTTTACCTTGAGAAAATTAGAGTGTTCAAAGCAGGCTTATGC
Dai22633
ACCAGGACTTTTACCTTGAGAAAATTAGAGTGTTCAAAGCAGGCTTATGC
Dai23260
ACCAGGACTTTTACCTTGAGAAAATTAGAGTGTTCAAAGCAGGCTTATGC
Dai23626
ACCAGGACTTTTACCTTGAGAAAATTAGAGTGTTCAAAGCAGGCTTATGC
Dai16238G_citrinoalbus
ACCAGGACCTTTACCTTGAGAAAATTAGAGTGTTCAAAGCAGGCATATGC
1175Dai15293
ACCAGGACTTTTACCTTGAGAAAATTAGAGTGTTCAAAGCAGGCTTATGC
Dai19547
ACCAGGACTTTTACCTTGAGAAAATTAGAGTGTTCAAAGCAGGCTTATGC
918063G_africanus --
918572G_africanus --
Dai18536A --
1164Cui17922
ACCAGGACTTTTACCTTGAGAAAATTAGAGTGTTCAAAGCAGGCTTATGC
Dai22225
ACCAGGACTTTTACCTTGAGAAAATTAGAGTGTTCAAAGCAGGCTTATGC
1163Dai20655
ACCAGGACTTTTACCTTGAGAAAATTAGAGTGTTCAAAGCAGGCTTATGC
Yuan4397G_hainanensis --
1176Dai15268
ACCAGGACTTTTACCTTGAGAAAATTAGAGTGTTCAAAGCAGGCTTATGC
1177Dai15259

ACCAGGACTTTTACCTTGAGAAAATTAGAGTGTTCAAAGCAGGCTTATGC
BZ2896G_thelephoroides ---
1166JV1808_26
ACCAGGACTTTTACCTTGAGAAAATTAGAGTGTTCAAAGCAGGCTTATGC
Miettinen16992Hapalopilus_ochr ---
GC1708_338_Ceriporia_arbuscula ---
WCG1555Dai26107Ceriporia
ACCAGGACTTTTACCTTGAGAAAATTAGAGTGTTCAAAGCAGGCCTGCGC
GC1708_340_Ceriporia_arbuscula ---
WCG1556Dai26109Ceriporia
ACCAGGACTTTTACCTTGAGAAAATTAGAGTGTTCAAAGCAGGCCTGCGC
883Cui11291
ACCAGGACTTTTACCTTGAGAAAATTAGAGTGTTCAAAGCAGGCCTATGC
HLX320Dai26805
ACCAGGACTTTTACCTTGAGAAAATTAGAGTGTTCAAAGCAGGCCTATGC
WCG1266Dai24678A
ACCAGGACTTTTACCTTGAGAAAATTAGAGTGTTCAAAGCAGGCCTATGC
Dai6090_Ceriporia_sulphuricolo ---
RLG_11354_Ceriproia_reticulata ---
ZZW1543Dai27072
ACCAGGACTTTTACCTTGAGAAAATTAGAGTGTTCAAAGCAGGCCTGCGC
Li1316_Ceriporia_reticulata
ACCAGGACTTTTACCTTGAGAAAATTAGAGTGCTCAAAGCAGGCCTATGC
KHL11981Ceriporia_reticulata ---
FP110343sp_Candelabrochaete_la ---
Li1045_Ceriporia_reticulata
ACCAGGACTTTTACCTTGAGAAAATTAGAGTGTTCAAAGCAGGCGTTTGC
ZX136Dai25794ceriporia
ACCAGGACTTTTACCTTGAGAAAATTAGAGTGTTCAAAGCAGGCGTTTGC
892Dai13400
ACCAGGACTTTTACCTTGAGAAAATTAGAGTGTTCAAAGCAGGCGTTTGC
RLG7163Leptoporus_mollis ---
Dai21062Leptoporus_mollis ---
Dai20182Leptoporus_submollis ---
Cui18379Leptoporus_submollis ---
Wu1209_46Resiniporus_pseudogil ---
BRNM710169Resiniporus_resinasc ---
Dai14516Bjerkandera_adusta
ACCAGGACTTTTACCTTGAGAAAATTAGAGTGTTCAAAGCAGGCTTATGC
Dai21100Bjerkandera_fumosa
ACCAGGACTTTTACCTTGAGAAAATTAGAGTGTTCAAAGCAGGCTTATGC
Miettinen16854Ceraceomyces_sp ---
Dai10477C_spissa ---
855Dai16831

ACCAGGACTTTTACCTTGAGAAAATTAGAGTGTTCAAAGCAGGCCTATGC

882Cui11282

ACCAGGACTTTTACCTTGAGAAAATTAGAGTGTTCAAAGCAGGCCTATGC

Dai24566

ACCAGGACTTTTACCTTGAGAAAATTAGAGTGTTCAAAGCAGGCCTATGC

Yuan5965 --

Dai3204 --

1194CUI9985 --

Dai15205_Ceriporia_albomellea --

Dai15223_Ceriporia_albomellea --

Li1780_Ceriporia_variegata --

Dai19791_Ceriporia_variegata

CTGAATACATTAGCATGGAATAATAAAATAGGACGTGCGGTTCTATTTTG

Dai19886

CTGAATACATTAGCATGGAATAATAAAATAGGACGTGCGGTTCTATTTTG

Dai10833_Ceriporia_crassitunic

CCGAATACATTAGCATGGAATAATAGAATAGGACGCGC------------

CHWC1506_46Meruliopsis_crassit --

Dai9995_Ceriporia_crassitunica --

Wu1209_58_Meruliopsis_parvispo --

CHWC1505_129_Meruliopsis_parvi --

Dai21944

CTGAATACATTAGCATGGAATAATAAAATAGGATGTGCGGTTCTATTTTG

830Dai18640A

CTGAATACATTAGCATGGAATAATAAAATAGGACGTGCGGTTCTATTTTG

GC1704_60_Meruliopsis_taxicola --

Dai22625

CTGAATACATTAGCATGGAATAATAAAATAGGACGTGTGATTCTATTTTG

Dai22636

CTGAATACATTAGCATGGAATAATAAAATAGGACGTGTGATTCTATTTTG

Dai21878

CTGAATACATTAGCATGGAATAATAAAATAGGACGTGTGATTCTATTTTG

1169Dai17248

CTGAATACATTAGCATGGAATAATAAAATAGGACGTGTGATTCTATTTTG

Wu1708_43_Meruliopsis_leptocys --

Li1011 --

ZX95Dai25742Meruliopsis_leptoc

CTGAATACATTAGCATGGAATAATAAAATAGGACGTGCGGTTCTATTTTG

WCG1306Dai24733

CTGAATACATTAGCATGGAATAATAAAATAGGACGTGCGG----------

LXL99Dai25816

CTGAATACATTAGCATGGAATAATAAAATAGGACGTGCGGTTCTATTTTG

WCG1559Dai26052Meruliopsis
CTGAATACATTAGCATGGAATAATAAAATAGGACGTGCGGTTCTATTTG
He7477 --
HLX243Dai26217
CTGAATACATTAGCATGGAATAATAAAATAGGACGTGCGGTTCTATTTG
RussiaMW673659Meruliopsis_fagi --
FD278 --
Dai10226_Ceriporia_tarda --
LE247365 --
Dai8173_Meruliopsis_nanlingens
TTGAATACATTAGCATGGAATAATAGAATAGGACGTGCGGTCCTATTTG
860Dai17172
CTGAATACATTAGCATGGAATAATAAAATAGGACGTGCGGTTCTATTTG
879Dai13414
CTGAATACATTAGCATGGAATAATAAAATAGGACGTGCGGTTCTATTTG
Li_1704_Meruliopsis_pseudocyst --
833Dai18405
CTGAATACATTAGCATGGAATAATAAAATAGGACGTGCGGTTCTATTTG
HHB_10729_Meruliopsis_albostra --
Cui6878_Ceriporia_pseudocystid --
869Dai14737
CTGAATACATTAGCATGGAATAATAAAATAGGACGTGCGGTTCTATTTG
876Cui11626
CTGAATACATTAGCATGGAATAATAAAATAGGACGTGCGGTTCTATTTG
1199WEI3388 --
776308_Meruliopsis_cystidiata --
ICN139059_Meruliopsis_cystidia --
HHB15692Ceraceomyces_serpens --
HHB_15629_Sp_Ceriporiopsis_ane --
AJ185Trametopsis_cervina --
FD9Irpex_lacteus --
908Dai11230
CTGAATACATTAGCATGGAATAATAAAATAGGACGTGCGGTTCTATTTG
FP55521TEmmia_lacerata --
PBU0048Ceriporia_cystidiata --
MZ340C_lacerataT --
Dai21940
CTGAATACATTAGCATGGAATAATAAAATAGGACGTGCGGTTCTATTTG
847Dai16433
CTGAATACATTAGCATGGAATAATAAAATAGGACGTGCGGTTCTATTTG
MarcinEmmia_latemarginatus --
Meijer3729Hydnopolyporus_fimbr --
RLG13408Phanerochaete_sp --
WHC1381Flavodon_flavus --

GB1833Phlebia_albida ---
T407Phlebia_nitidula ---
HHB6988Phanerochaete_exilis ---
HHB8509Phanerochaetella_xeroph ---
PBU0051Macrohyporia_dictyopora ---
HHB11463Phanerochaete_sp ---
FP102382Byssomerulius_corium ---
FP102165Efibula_americana ---
Murdoch90Ceriporia_torpida ---
Rivoire4413_Ceriporia_purpurea ---
Kout_18_Ceriporia_triumphalis ---
Rivoire3701_Ceriporia_bresadol ---
VS4018 ---
Ryvarden21832_Ceriporia_manzan ---
Dai24539
CTGAATACATTAGCATGGAATAATAAAATAGGACGTGCGGTTCTATTTG
Dai24541
CTGAATACATTAGCATGGAATAATAAAATAGGACGTGCGGTTCTATTTG
JV1105_12_Ceriporia_occidental ---
VS8558Ceriporia_occidentalis ---
Dai22445
CTGAATACATTAGCATGGAATAATAAAATAGGACGTGCGGTTCTATTTG
846Dai16368
CTGAATACATTAGCATGGAATAATAAAATAGGACGTGCGGTTCTATTTG
Dai17951_Ceriporia_aurantiocar ---
Miettinen_11701C_viridans ---
JV0105_10Ceriporia_aurantiocar ---
Yuan5702C_viridans ---
858Dai17003
CTGAATACATTAGCATGGAATAATAAAATAGGACGTGCGGTTCTATTTG
Yuan2747_Ceriporia_viridans ---
Yuan2744C_viridans ---
Li1046C_viridans ---
865C_sinoviridans
CCGAATACATTAGCATGGAATAATAAAATAGGACGTGCGGTTCTATTTG
871Dai15062 ---
Dai7642_Ceriporia_humilis
CTGAATACATTAGCATGGAATAATAAAATAGGACGTGCGGTTCTATTTG
Spirin4706_Ceriporia_humilis ---
Spirin4944_Ceriporia_sericea ---
WCG1547Dai26044ceriporia
CTGAATACATTAGCATGGAATAATAAAATAGGACGTGCGGTTCTATTTG
ZZW1558Dai27086
CTGAATACATTAGCATGGAATAATAAAATAGGACGTGCGGTTCTATTTG

Miettinen14381_Ceriporia_mpuri ---
Miettinen15492_2_Ceriporia_sor ---
He6687
CTGAATACATTAGCATGGAATAATAAAATAGGACGTGCGGTTCTATTTTG
ZH53Dai24426
CTGAATACATTAGCATGGAATAATAAAATAGGACGTGCGGTTCTATTTTG
Vlasak0808_30_Ceriporia_punica ---
887Dai13376
CTGAATACATTAGCATGGAATAATAAAATAGGACGAGCGGTTCTATTTTG
WCG1443Dai24998
CTGAATACATTAGCATGGAATAATAAAATAGGACGAGCGGTTCTATTTTG
0108_6Ceriporia_spissa ---
Dai19164
CTGAATACATTAGCATGGAATAATAAAATAGGACGTGCGGTTCTATTTTG
Dai17937_Ceriporia_bubalinomar ---
903Dai12113
CTGAATACATTAGCATGGAATAATAAAATAGGATGTGCGGTTCTATTTTG
LZB929Dai25079
CTGAATACATTAGCATGGAATAATAAAATAGGACGTGCGGTTCTATTTTG
LX45Dai26988 ---
LX43Dai26986
CTGAATACATTAGCATGGAATAATAAAATAGGACGTGCGGTTCTATTTTG
Dai7759Ceriporia ---
Cui8012_Ceriporia_viridans ---
GC1704_54Ceriporia_viridans ---
Dai23392
CTGAATACATTAGCATGGAATAATAAAATAGGACGTGCGGTTCTATTTTG
WCG1585Dai26113Ceriproia
CCGAATACATTAGCATGGAATAATAAAATAGGACGTGCGGTTCTATTTTG
Dai18675C_eucalypti ---
Dai22034
CTGAATACATTAGCATGGAATAATAAAATAGGACGTGCGGTTCTATTTTG
JV1008_41JTardaFLORIDAKeys ---
Rivoire1161_Ceriporia_pierii ---
Dai23499C_pierii
CTGAATACATTAGCATGGAATAATAAAATAGGATGTGCGGTTCTATTTTG
Dai23500
CTGAATACATTAGCATGGAATAATAAAATAGGATGTGCGGTTCTATTTTG
841Dai15899
CTGAATACATTAGCATGGAATAATAAAATAGGACGTGCGGTTCTATTTTG
842Dai15904
CTGAATACATTAGCATGGAATAATAAAATAGGACGTGCGGTTCTATTTTG
LZB1066xinjiang ---
LZB1065xinjiang ---

851Dai16779
CCGAATACATTAGCATGGAATAATAAAATAGGACGTGCGGTTCTATTTG
RMJ119sp_Candelabrochaete_sept ---
RLG9759spCandelabrochaete_sept ---
RLG10478Phanerochaete_allantos ---
Dai19118_Ceriporia_spissa
CTGAATACATTAGCATGGAATAATAAAATAGGACGTGCGGTTCTATTTG
Dai18486A
CTGAATACATTAGCATGGAATAATAAAATAGGACGTGCGGTTCTATTTG
WEI17_024_Ceriporia_mellita ---
GC1508_71Ceriporia_mellita ---
GC1608_7_Ceriporia_mellita ---
ZZW1557Dai27085 ---
ZZW1554Dai27083
CTGAATACATTAGCATGGAATAATAAAATAGGACGTGCGGTTCTATTTG
Dai8168 ---
BR4865C_mellita ---
MEL2382688Ceriporia_sp ---
Dai8110 ---
Cui8097 ---
909Cui6740 ---
W1258Dai24695
CTGAATACATTAGCATGGAATAATAAAATAGGACGTGCGGTTCTATTTG
JV0110_26_Ceriporia_griseoviol ---
896Dai13202
CTGAATACATTAGCATGGAATAATAAAATAGGACGTGCGGTTCTATTTG
LWY393Dai27053C_griseoviolasce
CTGAATACATTAGCATGGAATAATAAAATAGGACGTGCGGTTCTATTTG
LWY394DAI27054
CTGAATACATTAGCATGGAATAATAAAATAGGACGTGCGGTTCTATTTG
FP135015G_pannocinctus ---
L15726SpG_pannocinctus ---
Dai22221
CCGAATACATTAGCATGGAATAATAAAATAGGACGTGCGGTTCTATTTG
Dai22633
CCGAATACATTAGCATGGAATAATAAAATAGGACGTGCGGTTCTATTTG
Dai23260
CCGAATACATTAGCATGGAATAATAAAATAGGACGTGCGGCTCTATTTG
Dai23626
CCGAATACATTAGCATGGAATAATAAAATAGGACGTGCGGTTCTATTTG
Dai16238G_citrinoalbus
CCGAATACATTAGCATGGAATAATAAAATAGGACGTGCGGTTCTATTTG
1175Dai15293
CCGAATACATTAGCATGGAATAATAAAATAGGACGTGCGGTTCTATTTG

Dai19547
CCGAATACATTAGCATGGAATAATAAAATAGGACGTGCGGTTCTATTTG
918063G_africanus --
918572G_africanus --
Dai18536A --
1164Cui17922
CCGAATACATTAGCATGGAATAATAAAATAGGACGTGCGGTTCTATTTG
Dai22225
CCGAATACATTAGCATGGAATAATAAAATAGGACGTGCGGTTCTATTTG
1163Dai20655
CCGAATACATTAGCATGGAATAATAAAATAGGACGTGCGGTTCTATTTG
Yuan4397G_hainanensis --
1176Dai15268
CCGAATACATTAGCATGGAATAATAAAATAGGACGTGCGGTTCTATTTG
1177Dai15259
CCGAATACATTAGCATGGAATAATAAAATAGGACGTGCGGTTCTATTTG
BZ2896G_thelephoroides --
1166JV1808_26
CCGAATACATTAGCATGGAATAATAAAATAGGACGTGCGGTTCTATTTG
Miettinen16992Hapalopilus_ochr --
GC1708_338_Ceriporia_arbuscula --
WCG1555Dai26107Ceriporia
CTGAATACATTAGCATGGAATAATAAAATAGGACGTGCGGTTCTATTTG
GC1708_340_Ceriporia_arbuscula --
WCG1556Dai26109Ceriporia
CTGAATACATTAGCATGGAATAATAAAATAGGACGTGCGGTTCTATTTG
883Cui11291
CTGAATACATTAGCATGGAATAATAAAATAGGACGTGCGGTTCTATTTG
HLX320Dai26805
CTGAATACATTAGCATGGAATAATAAAATAGGACGTGCGGTTCTATTTG
WCG1266Dai24678A
CTGAATACATTAGCATGGAATAATAAAATAGGACGTGCGGTTCTATTTG
Dai6090_Ceriporia_sulphuricolo --
RLG_11354_Ceriproia_reticulata --
ZZW1543Dai27072
CTGAATACATTAGCATGGAATAATAAAATAGGACGTGCGGTTCTATTTG
Li1316_Ceriporia_reticulata
CCGAATACATTAGCATGGAATAATGAAATAGGACGTGCGGTTCTATTTG
KHL11981Ceriporia_reticulata --
FP110343sp_Candelabrochaete_la --
Li1045_Ceriporia_reticulata
CTGAATACATTAGCATGGAATAATAAAATAGGACGTGCGGTTCTATTTG
ZX136Dai25794ceriporia
CTGAATACATTAGCATGGAATAATAAAATAGGACGTGCGGTTCTATTTG

892Dai13400
CTGAATACATTAGCATGGAATAATAAAATAGGACGTGCGGTTCTATTTG
RLG7163Leptoporus_mollis --
Dai21062Leptoporus_mollis --
Dai20182Leptoporus_submollis --
Cui18379Leptoporus_submollis --
Wu1209_46Resiniporus_pseudogil --
BRNM710169Resiniporus_resinasc --
Dai14516Bjerkandera_adusta
CCGAATACATTAGCATGGAATAATAAAATAGGACGTGCGGTTCTATTTG
Dai21100Bjerkandera_fumosa
CCGAATACATTAGCATGGAATAATAAAATAGGACGTGCGGTTCTATTTG
Miettinen16854Ceraceomyces_sp --
Dai10477C_spissa --
855Dai16831
CTGAATACATTAGCATGGAATAATAAAATAGGACGTGCGGTTCTATTTG
882Cui11282
CTGAATACATTAGCATGGAATAATAAAATAGGACGTGCGGTTCTATTTG
Dai24566
CTGAATACATTAGCATGGAATAATAAAATAGGACGTGCGGTTCTATTTG
Yuan5965 --
Dai3204 --
1194CUI9985 --

Dai15205_Ceriporia_albomellea --
Dai15223_Ceriporia_albomellea --
Li1780_Ceriporia_variegata --
Dai19791_Ceriporia_variegata
TTGGTTTCTAGAGTCGCCGTAATGATTAATAGGGATAGTTGGGGGCATTA
Dai19886
TTGGTTTCTAGAGTCGCCGTAATGATTAATAGGGATAGTTGGGGGCATTA
Dai10833_Ceriporia_crassitunic --
CHWC1506_46Meruliopsis_crassit --
Dai9995_Ceriporia_crassitunica --
Wu1209_58_Meruliopsis_parvispo --
CHWC1505_129_Meruliopsis_parvi --
Dai21944
TTGGTTTCTAGAGTCGCCGTAATGATTAATAGGGATAGTTGGGGGCATTA
830Dai18640A
TTGGTTTCTAGAGTCGCCGTAATGATTAATAGGGATAGTTGGGGGCATTA
GC1704_60_Meruliopsis_taxicola --
Dai22625
TTGGTTTCTAGAGTCGCCGTAATGATTAATAGGGATAGTTGGGGGCATTA

Dai22636
TTGGTTTCTAGAGTCGCCGTAATGATTAATAGGGATAGTTGGGGGCATTA
Dai21878
TTGGTTTCTAGAGTCGCCGTAATGATTAATAGGGATAGTTGGGGGCATTA
1169Dai17248
TTGGTTTCTAGAGTCGCCGTAATGATTAATAGGGATAGTTGGGGGCATTA
Wu1708_43_Meruliopsis_leptocys --
Li1011 --
ZX95Dai25742Meruliopsis_leptoc
TTGGTTTCTAGAGTCGCCGTAATGATTAATAGGGATAGTTGGGGGCATTA
WCG1306Dai24733 --
LXL99Dai25816
TTGGTTTCTAGAGTCGCCGTAATGATTAATAGGGATAGTTGGGGGCATTA
WCG1559Dai26052Meruliopsis
TTGGTTTCTAGAGTCGCCGTAATGATTAATAGGGATAGTTGGGGGCATTA
He7477 --
HLX243Dai26217
TTGGTTTCTAGAGTCGCCGTAATGATTAATAGGGATAGTTGGGGGCATTA
RussiaMW673659Meruliopsis_fagi --
FD278 --
Dai10226_Ceriporia_tarda --
LE247365 --
Dai8173_Meruliopsis_nanlingens
TTGGTTTCTAGGATCGCCGTAATGATCAATAGGGACAGTTGGGGGCATTT
860Dai17172
TTGGTTTCTAGAGTCGCCGTAATGATTAATAGGGATAGTTGGGGGCATTA
879Dai13414
TTGGTTTCTAGAGTCGCCGTAATGATTAATAGGGATAGTTGGGGGCATTA
Li_1704_Meruliopsis_pseudocyst --
833Dai18405
TTGGTTTCTAGAGTCGCCGTAATGATTAATAGGGATAGTTGGGGGCATTA
HHB_10729_Meruliopsis_albostra --
Cui6878_Ceriporia_pseudocystid --
869Dai14737
TTGGTTTCTAGAGTCGCCGTAATGATTAATAGGGATAGTTGGGGGCATTA
876Cui11626
TTGGTTTCTAGAGTCGCCGTAATGATTAATAGGGATAGTTGGGGGCATTA
1199WEI3388 --
776308_Meruliopsis_cystidiata --
ICN139059_Meruliopsis_cystidia --
HHB15692Ceraceomyces_serpens --
HHB_15629_Sp_Ceriporiopsis_ane --
AJ185Trametopsis_cervina --
FD9Irpex_lacteus --

908Dai11230
TTGGTTTCTAGAGTCGCCGTAATGATTAATAGGGATAGTTGGGGGCATTA
FP55521TEmmia_lacerata --
PBU0048Ceriporia_cystidiata --
MZ340C_lacerataT --
Dai21940
TTGGTTTCTAGAGTCGCCGTAATGATTAATAGGGATAGTTGGGGGCATTA
847Dai16433
TTGGTTTCTAGAGTCGCCGTAATGATTAATAGGGATAGTTGGGGGCATTA
MarcinEmmia_latemarginatus --
Meijer3729Hydnopolyporus_fimbr --
RLG13408Phanerochaete_sp --
WHC1381Flavodon_flavus --
GB1833Phlebia_albida --
T407Phlebia_nitidula --
HHB6988Phanerochaete_exilis --
HHB8509Phanerochaetella_xeroph --
PBU0051Macrohyporia_dictyopora --
HHB11463Phanerochaete_sp --
FP102382Byssomerulius_corium --
FP102165Efibula_americana --
Murdoch90Ceriporia_torpida --
Rivoire4413_Ceriporia_purpurea --
Kout_18_Ceriporia_triumphalis --
Rivoire3701_Ceriporia_bresadol --
VS4018 --
Ryvarden21832_Ceriporia_manzan --
Dai24539
TTGGTTTCTAGAGTCGCCGTAATGATTAATAGGGATAGTTGGGGGCATTA
Dai24541
TTGGTTTCTAGAGTCGCCGTAATGATTAATAGGGATAGTTGGGGGCATTA
JV1105_12_Ceriporia_occidental --
VS8558Ceriporia_occidentalis --
Dai22445
TTGGTTTCTAGAGTCGCCGTAATGATTAATAGGGATAGTTGGGGGCATTA
846Dai16368
TTGGTTTCTAGAGTCGCCGTAATGATTAATAGGGATAGTTGGGGGCATTA
Dai17951_Ceriporia_aurantiocar --
Miettinen_11701C_viridans --
JV0105_10Ceriporia_aurantiocar --
Yuan5702C_viridans --
858Dai17003
TTGGTTTCTAGAGTCGCCGTAATGATTAATAGGGATAGTTGGGGGCATTA
Yuan2747_Ceriporia_viridans --

Yuan2744C_viridans --
Li1046C_viridans --
865C_sinoviridans
TTGGTTTCTAGAGTCGCCGTAATGATTAATAGGGATAGTTGGGGGCATTA
871Dai15062 --
Dai7642_Ceriporia_humilis
TTGGTTTCTAGAGTCGCCGTAATGATTAATAGGGATAGTTGGGGGCATTA
Spirin4706_Ceriporia_humilis --
Spirin4944_Ceriporia_sericea --
WCG1547Dai26044ceriporia
TTGGTTTCTAGAGTCGCCGTAATGATTAATAGGGATAGTTGGGGGCATTA
ZZW1558Dai27086
TTGGTTTCTAGAGTCGCCGTAATGATTAATAGGGATAGTTGGGGGCATTA
Miettinen14381_Ceriporia_mpuri --
Miettinen15492_2_Ceriporia_sor --
He6687
TTGGTTTCTAGAGTCGCCGTAATGATTAATAGGGATAGTTGGGGGCATTA
ZH53Dai24426
TTGGTTTCTAGAGTCGCCGTAATGATTAATAGGGATAGTTGGGGGCATTA
Vlasak0808_30_Ceriporia_punica --
887Dai13376
TTGGTTTCTAGAGTCGCCGTAATGATTAATAGGGATAGTTGGGGGCATTA
WCG1443Dai24998
TTGGTTTCTAGAGTCGCCGTAATGATTAATAGGGATAGTTGGGGGCATTA
0108_6Ceriporia_spissa --
Dai19164
TTGGTTTCTAGAGTCGCCGTAATGATTAATAGGGATAGTTGGGGGCATTA
Dai17937_Ceriporia_bubalinomar --
903Dai12113
TTGGTTTCTAGAGTCGCCGTAATGATTAATAGGGATAGTTGGGGGCATTA
LZB929Dai25079
TTGGTTTCTAGAGTCGCCGTAATGATTAATAGGGATAGTTGGGGGCATTA
LX45Dai26988 --
LX43Dai26986
TTGGTTTCTAGAGTCGCCGTAATGATTAATAGGGATAGTTGGGGGCATTA
Dai7759Ceriporia --
Cui8012_Ceriporia_viridans --
GC1704_54Ceriporia_viridans --
Dai23392
TTGGTTTCTAGAGTCGCCGTAATGATTAATAGGGATAGTTGGGGGCATTA
WCG1585Dai26113Ceriproia
TTGGTTTCTAGAGTCGCCGTAATGATTAATAGGGATAGTTGGGGGCATTA
Dai18675C_eucalypti --
Dai22034

TTGGTTTCTAGAGTCGCCGTAATGATTAATAGGGATAGTTGGGGGCATTA
JV1008_41JTardaFLORIDAKeys --
Rivoire1161_Ceriporia_pierii --
Dai23499C_pierii
TTGGTTTCTAGAGTCGCCGTAATGATTAATAGGGATAGTTGGGGGCATTA
Dai23500
TTGGTTTCTAGAGTCGCCGTAATGATTAATAGGGATAGTTGGGGGCATTA
841Dai15899
TTGGTTTCTAGAGTCGCCGTAATGATTAATAGGGATAGTTGGGGGCATTA
842Dai15904
TTGGTTTCTAGAGTCGCCGTAATGATTAATAGGGATAGTTGGGGGCATTA
LZB1066xinjiang --
LZB1065xinjiang --
851Dai16779
TTGGTTTCTAGAGTCGCCGTAATGATTAATAGGGATAGTTGGGGGCATTA
RMJ119sp_Candelabrochaete_sept --
RLG9759spCandelabrochaete_sept --
RLG10478Phanerochaete_allantos --
Dai19118_Ceriporia_spissa
TTGGTTTCTAGAGTCGCCGTAATGATTAATAGGGATAGTTGGGGGCATTA
Dai18486A
TTGGTTTCTAGAGTCGCCGTAATGATTAATAGGGATAGTTGGGGGCATTA
WEI17_024_Ceriporia_mellita --
GC1508_71Ceriporia_mellita --
GC1608_7_Ceriporia_mellita --
ZZW1557Dai27085 --
ZZW1554Dai27083
TTGGTTTCTAGAGTCGCCGTAATGATTAATAGGGATAGTTGGGGGCATTA
Dai8168 --
BR4865C_mellita --
MEL2382688Ceriporia_sp --
Dai8110 --
Cui8097 --
909Cui6740 --
W1258Dai24695
TTGGTTTCTAGAGTCGCCGTAATGATTAATAGGGATAGTTGGGGGCATTA
JV0110_26_Ceriporia_griseoviol --
896Dai13202
TTGGTTTCTAGAGTCGCCGTAATGATTAATAGGGATAGTTGGGGGCATTA
LWY393Dai27053C_griseoviolasce
TTGGTTTCTAGAGTCGCCGTAATGATTAATAGGGATAGTTGGGGGCATTA
LWY394DAI27054
TTGGTTTCTAGAGTCGCCGTAATGATTAATAGGGATAGTTGGGGGCATTA
FP135015G_pannocinctus --

L15726SpG_pannocinctus --
Dai22221 TTGGTTTCTAGAGTCG-
CGTAATGATTAATAGGGATAGTTGGGGGCATTA
Dai22633 TTGGTTTCTAGAATCG-
CGTAATGATTAATAGGGATAGTTGGGGGCATTA
Dai23260 TTGGTTTCTAGAATCG-
CGTAATGATTAATAGGGATAGTTGGGGGCATTA
Dai23626 TTGGTTTCTAGAATCG-
CGTAATGATTAATAGGGATAGTTGGGGGCATTA
Dai16238G_citrinoalbus
TTGGTTTCTAGAGTCGCCGTAATGATTAATAGGGATAGTTGGGGGCATTA
1175Dai15293
TTGGTTTCTAGAGTCGCCGTAATGATTAATAGGGATAGTTGGGGGCATTA
Dai19547
TTGGTTTCTAGAGTCGCCGTAATGATTAATAGGGATAGTTGGGGGCATTA
918063G_africanus --
918572G_africanus --
Dai18536A --
1164Cui17922
TTGGTTTCTAGAGTCGCCGTAATGATTAATAGGGATAGTTGGGGGCATTA
Dai22225 TTGGTTTCTAGAGTCG-
CGTAATGATTAATAGGGATAGTTGGGGGCATTA
1163Dai20655
TTGGTTTCTAGAGTCGCCGTAATGATTAATAGGGATAGTTGGGGGCATTA
Yuan4397G_hainanensis --
1176Dai15268
TTGGTTTCTAGAGTCGCCGTAATGATTAATAGGGATAGTTGGGGGCATTA
1177Dai15259
TTGGTTTCTAGAGTCGCCGTAATGATTAATAGGGATAGTTGGGGGCATTA
BZ2896G_thelephoroides --
1166JV1808_26
TTGGTTTCTAGAGTCGCCGTAATGATTAATAGGGATAGTTGGGGGCATTA
Miettinen16992Hapalopilus_ochr --
GC1708_338_Ceriporia_arbuscula --
WCG1555Dai26107Ceriporia
TTGGTTTTTAGAGTCGCCGTAATGATTAATAGGGATAGTTGGGGGCATTA
GC1708_340_Ceriporia_arbuscula --
WCG1556Dai26109Ceriporia
TTGGTTTTTAGAGTCGCCGTAATGATTAATAGGGATAGTTGGGGGCATTA
883Cui11291
TTGGTTTCTAGAGTCGCCGTAATGATTAATAGGGATAGTTGGGGGCATTA
HLX320Dai26805
TTGGTTTCTAGAGTCGCCGTAATGATTAATAGGGATAGTTGGGGGCATTA
WCG1266Dai24678A

TTGGTTTCTAGAGTCGCCGTAATGATTAATAGGGATAGTTGGGGGCATTA
Dai6090_Ceriporia_sulphuricolo --
RLG_11354_Ceriproia_reticulata --
ZZW1543Dai27072
TTGGTTTCTAGAGTCGCCGTAATGATTAATAGGGATAGTTGGGGGCATTA
Li1316_Ceriporia_reticulata
TTGGTTTCTAGGATCGCCGTAATGATCAATAGGGACAGTTGGGGGCATTT
KHL11981Ceriporia_reticulata --
FP110343sp_Candelabrochaete_la --
Li1045_Ceriporia_reticulata
TTGGTTTCTAGAGTCGCCGTAATGATTAATAGGGATAGTTGGGGGCATTA
ZX136Dai25794ceriporia
TTGGTTTCTAGAGTCGCCGTAATGATTAATAGGGATAGTTGGGGGCATTA
892Dai13400
TTGGTTTCTAGAGTCGCCGTAATGATTAATAGGGATAGTTGGGGGCATTA
RLG7163Leptoporus_mollis --
Dai21062Leptoporus_mollis --
Dai20182Leptoporus_submollis --
Cui18379Leptoporus_submollis --
Wu1209_46Resiniporus_pseudogil --
BRNM710169Resiniporus_resinasc --
Dai14516Bjerkandera_adusta
TTGGTTTCTAGAGTCGCCGTAATGATTAATAGGGATAGTTGGGGGCATTA
Dai21100Bjerkandera_fumosa
TTGGTTTCTAGAGTCGCCGTAATGATTAATAGGGATAGTTGGGGGCATTA
Miettinen16854Ceraceomyces_sp --
Dai10477C_spissa --
855Dai16831
TTGGTTTCTAGAGTCGCCGTAATGATTAATAGGGATAGTTGGGGGCATTA
882Cui11282
TTGGTTTCTAGAGTCGCCGTAATGATTAATAGGGATAGTTGGGGGCATTA
Dai24566
TTGGTTTCTAGAGTCGCCGTAATGATTAATAGGGATAGTTGGGGGCATTA
Yuan5965 --
Dai3204 --
1194CUI9985 --

Dai15205_Ceriporia_albomellea --
Dai15223_Ceriporia_albomellea --
Li1780_Ceriporia_variegata --
Dai19791_Ceriporia_variegata
GTATTCCGTTGCTAGAGGTGAAATTCTTGGATTTACGGAAGACTAACTAT
Dai19886

GTATTCCGTTGCTAGAGGTGAAATTCTTGGATTTACGGAAGACTAACTAT

Dai10833_Ceriporia_crassitunic ---

CHWC1506_46Meruliopsis_crassit ---

Dai9995_Ceriporia_crassitunica ---

Wu1209_58_Meruliopsis_parvispo ---

CHWC1505_129_Meruliopsis_parvi ---

Dai21944

GTATTCCGTTGCTAGAGGTGAAATTCTTGGATTTACGGAAGACTAACTAC

830Dai18640A

GTATTCCGTTGCTAGAGGTGAAATTCTTGGATTTACGGAAGACTAACTAC

GC1704_60_Meruliopsis_taxicola ---

Dai22625

GTATTCCGTTGCTAGAGGTGAAATTCTTGGATTTACGGAAGACTAACTAC

Dai22636

GTATTCCGTTGCTAGAGGTGAAATTCTTGGATTTACGGAAGACTAACTAC

Dai21878

GTATTCCGTTGCTAGAGGTGAAATTCTTGGATTTACGGAAGACTAACTAC

1169Dai17248

GTATTCCGTTGCTAGAGGTGAAATTCTTGGATTTACGGAAGACTAACTAC

Wu1708_43_Meruliopsis_leptocys ---

Li1011 ---

ZX95Dai25742Meruliopsis_leptoc

GTATTCCGTTGCTAGAGGTGAAATTCTTGGATTTACGGAAGACTAACTAT

WCG1306Dai24733 ---

LXL99Dai25816

GTATTCCGTTGCTAGAGGTGAAATTCTTGGATTTACGGAAGACTAACTAT

WCG1559Dai26052Meruliopsis

GTATTCCGTTGCTAGAGGTGAAATTCTTGGATTTACGGAAGACTAACTAT

He7477 ---

HLX243Dai26217

GTATTCCGTTGCTAGAGGTGAAATTCTTGGATTTACGGAAGACTAACTAT

RussiaMW673659Meruliopsis_fagi ---

FD278 ---

Dai10226_Ceriporia_tarda ---

LE247365 ---

Dai8173_Meruliopsis_nanlingens

GTATTGCGTCGCTAGAGGTGAAATTCTTGGATTGACGCAAGACAAACTAT

860Dai17172

GTATTCCGTTGCTAGAGGTGAAATTCTTGGATTTACGGAAGACTAACTAT

879Dai13414

GTATTCCGTTGCTAGAGGTGAAATTCTTGGATTTACGGAAGACTAACTAT

Li_1704_Meruliopsis_pseudocyst ---

833Dai18405

GTATTCCGTTGCTAGAGGTGAAATTCTTGGATTTACGGAAGACTAACTAC

HHB_10729_Meruliopsis_albostra --
Cui6878_Ceriporia_pseudocystid --
869Dai14737
GTATTCCGTTGCTAGAGGTGAAATTCTTGGATTTACGGAAGACTAACTAT
876Cui11626
GTATTCCGTTGCTAGAGGTGAAATTCTTGGATTTACGGAAGACTAACTAT
1199WEI3388 --
776308_Meruliopsis_cystidiata --
ICN139059_Meruliopsis_cystidia --
HHB15692Ceraceomyces_serpens --
HHB_15629_Sp_Ceriporiopsis_ane --
AJ185Trametopsis_cervina --
FD9Irpex_lacteus --
908Dai11230
GTATTCCGTTGCTAGAGGTGAAATTCTTGGATTTACGGAAGACTAACTAC
FP55521TEmmia_lacerata --
PBU0048Ceriporia_cystidiata --
MZ340C_lacerataT --
Dai21940
GTATTCCGTTGCTAGAGGTGAAATTCTTGGATTTACGGAAGACTAACTAC
847Dai16433
GTATTCCGTTGCTAGAGGTGAAATTCTTGGATTTACGGAAGACTAACTAC
MarcinEmmia_latemarginatus --
Meijer3729Hydnopolyporus_fimbr --
RLG13408Phanerochaete_sp --
WHC1381Flavodon_flavus --
GB1833Phlebia_albida --
T407Phlebia_nitidula --
HHB6988Phanerochaete_exilis --
HHB8509Phanerochaetella_xeroph --
PBU0051Macrohyporia_dictyopora --
HHB11463Phanerochaete_sp --
FP102382Byssomerulius_corium --
FP102165Efibula_americana --
Murdoch90Ceriporia_torpida --
Rivoire4413_Ceriporia_purpurea --
Kout_18_Ceriporia_triumphalis --
Rivoire3701_Ceriporia_bresadol --
VS4018 --
Ryvarden21832_Ceriporia_manzan --
Dai24539
GTATTCCGTTGCTAGAGGTGAAATTCTTGGATTTACGGAAGACTAACTAT
Dai24541
GTATTCCGTTGCTAGAGGTGAAATTCTTGGATTTACGGAAGACTAACTAT

JV1105_12_Ceriporia_occidental --
VS8558Ceriporia_occidentalis --
Dai22445
GTATTCCGTTGCTAGAGGTGAAATTCTTGGATTTACGGAAGACTAACTAT
846Dai16368
GTATTCCGTTGCTAGAGGTGAAATTCTTGGATTTACGGAAGACTAACTAT
Dai17951_Ceriporia_aurantiocar --
Miettinen_11701C_viridans --
JV0105_10Ceriporia_aurantiocar --
Yuan5702C_viridans --
858Dai17003
GTATTCCGTTGCTAGAGGTGAAATTCTTGGATTTACGGAAGACTAACTAC
Yuan2747_Ceriporia_viridans --
Yuan2744C_viridans --
Li1046C_viridans --
865C_sinoviridans
GTATTCCGTTGCTAGAGGTGAAATTCTTGGATTTACGCAAGACTAACTAC
871Dai15062 --
Dai7642_Ceriporia_humilis
GTATTCCGTTGCTAGAGGTGAAATTCTTGGATTTACGGAAGACTAACTAT
Spirin4706_Ceriporia_humilis --
Spirin4944_Ceriporia_sericea --
WCG1547Dai26044ceriporia
GTATTCCGTTGCTAGAGGTGAAATTCTTGGATTTACGGAAGACTAACTAT
ZZW1558Dai27086
GTATTCCGTTGCTAGAGGTGAAATTCTTGGATTTACGGAAGACTAACTAT
Miettinen14381_Ceriporia_mpuri --
Miettinen15492_2_Ceriporia_sor --
He6687
GTATTCCGTTGCTAGAGGTGAAATTCTTGGATTTACGGAAGACTAACTAT
ZH53Dai24426
GTATTCCGTTGCTAGAGGTGAAATTCTTGGATTTACGGAAGACTAACTAT
Vlasak0808_30_Ceriporia_punica --
887Dai13376
GTATTCCGTTGCTAGAGGTGAAATTCTTGGATTTACGGAAGACTAACTAT
WCG1443Dai24998
GTATTCCGTTGCTAGAGGTGAAATTCTTGGATTTACGGAAGACTAACTAC
0108_6Ceriporia_spissa --
Dai19164
GTATTCCGTTGCTAGAGGTGAAATTCTTGGATTTACGGAAGACTAACTAC
Dai17937_Ceriporia_bubalinomar --
903Dai12113
GTATTCCGTTGCTAGAGGTGAAATTCTTGGATTTACGGAAGACTAACTAC
LZB929Dai25079

GTATTCCGTTGCTAGAGGTGAAATTCTTGGATTTACGGAAGACTAACTAT

LX45Dai26988 --

LX43Dai26986

GTATTCCGTTGCTAGAGGTGAAATTCTTGGATTTACGGAAGACTAACTAT

Dai7759Ceriporia --

Cui8012_Ceriporia_viridans --

GC1704_54Ceriporia_viridans --

Dai23392

GTATTCCGTTGCTAGAGGTGAAATTCTTGGATTTACGGAAGACTAACTAT

WCG1585Dai26113Ceriproia

GTATTCAGTTGCTAGAGGTGAAATTCTTGGATTTACTGAAGACTAACTAC

Dai18675C_eucalypti --

Dai22034

GTATTCCGTTGCTAGAGGTGAAATTCTTGGATTTACGGAAGACTAACTAT

JV1008_41JTardaFLORIDAKeys --

Rivoire1161_Ceriporia_pierii --

Dai23499C_pierii

GTATTCCGTTGCTAGAGGTGAAATTCTTGGATTTACGGAAGACTAACTAT

Dai23500

GTATTCCGTTGCTAGAGGTGAAATTCTTGGATTTACGGAAGACTAACTAT

841Dai15899

GTATTCCGTTGCTAGAGGTGAAATTCTTGGATTTACGGAAGACTAACTAC

842Dai15904

GTATTCCGTTGCTAGAGGTGAAATTCTTGGATTTACGGAAGACTAACTAC

LZB1066xinjiang --

LZB1065xinjiang --

851Dai16779

GTATTCCGTTGCTAGAGGTGAAATTCTTGGATTTACGGAAGACTAACTAT

RMJ119sp_Candelabrochaete_sept --

RLG9759spCandelabrochaete_sept --

RLG10478Phanerochaete_allantos --

Dai19118_Ceriporia_spissa

GTATTCCGTTGCTAGAGGTGAAATTCTTGGATTTACGGAAGACTAACTAT

Dai18486A

GTATTCCGTTGCTAGAGGTGAAATTCTTGGATTTACGGAAGACTAACTAT

WEI17_024_Ceriporia_mellita --

GC1508_71Ceriporia_mellita --

GC1608_7_Ceriporia_mellita --

ZZW1557Dai27085 --

ZZW1554Dai27083

GTATTCCGTTGCTAGAGGTGAAATTCTTGGATTTACGGAAGACTAACTAT

Dai8168 --

BR4865C_mellita --

MEL2382688Ceriporia_sp --

Dai8110 ---
Cui8097 ---
909Cui6740 ---
W1258Dai24695
GTATTCCGTTGCTAGAGGTGAAATTCTTGGATTTACGGAAGACTAACTAT
JV0110_26_Ceriporia_griseoviol ---
896Dai13202
GTATTCCGTTGCTAGAGGTGAAATTCTTGGATTTACGGAAGACTAACTAC
LWY393Dai27053C_griseoviolasce
GTATTCCGTTGCTAGAGGTGAAATTCTTGGATTTACGGAAGACTAACTAC
LWY394DAI27054
GTATTCCGTTGCTAGAGGTGAAATTCTTGGATTTACGGAAGACTAACTAT
FP135015G_pannocinctus ---
L15726SpG_pannocinctus ---
Dai22221 GTATTCCG-
TGCTAGAGGTGAAATTCTTGGATTTACGGAAGACTAACTAC
Dai22633 GTATTCCG-
TGCTAGAGGTGAAATTCTTGGATTTACGGAAGACTAACTAC
Dai23260 GTATTCCG-
TGCTAGAGGTGAAATTCTTGGATTTACGGAAGACTAACTAC
Dai23626
GTATTCCGTTGCTAGAGGTGAAATTCTTGGATTTACGGAAGACTAACTAC
Dai16238G_citrinoalbus
GTATTCAGTTGCTAGAGGTGAAATTCTTGGATTTACTGAAGACTAACTAC
1175Dai15293
GTATTCCGTTGCTAGAGGTGAAATTCTTGGATTTACGGAAGACTAACTAC
Dai19547
GTATTCCGTTGCTAGAGGTGAAATTCTTGGATTTACGGAAGACTAACTAC
918063G_africanus ---
918572G_africanus ---
Dai18536A ---
1164Cui17922
GTATTCCGTTGCTAGAGGTGAAATTCTTGGATTTACGGAAGACTAACTAC
Dai22225 GTATTCCG-
TGCTAGAGGTGAAATTCTTGGATTTACGGAAGACTAACTAC
1163Dai20655
GTATTCCGTTGCTAGAGGTGAAATTCTTGGATTTACGGAAGACTAACTAC
Yuan4397G_hainanensis ---
1176Dai15268
GTATTCCGTTGCTAGAGGTGAAATTCTTGGATTTACGGAAGACTAACTAC
1177Dai15259
GTATTCCGTTGCTAGAGGTGAAATTCTTGGATTTACGGAAGACTAACTAC
BZ2896G_thelephoroides ---
1166JV1808_26

GTATTCCGTTGCTAGAGGTGAAATTCTTGGATTTACGGAAGACTAACTAC
Miettinen16992Hapalopilus_ochr --
GC1708_338_Ceriporia_arbuscula --
WCG1555Dai26107Ceriporia
GTATTCCGTTGCTAGAGGTGAAATTCTTGGATTTACGGAAGACTAACTAC
GC1708_340_Ceriporia_arbuscula --
WCG1556Dai26109Ceriporia
GTATTCCGTTGCTAGAGGTGAAATTCTTGGATTTACGGAAGACTAACTAC
883Cui11291
GTATTCCGTTGCTAGAGGTGAAATTCTTGGATTTACGGAAGACTAACTAT
HLX320Dai26805
GTATTCCGTTGCTAGAGGTGAAATTCTTGGATTTACGGAAGACTAACTAC
WCG1266Dai24678A
GTATTCCGTTGCTAGAGGTGAAATTCTTGGATTTACGGAAGACTAACTAC
Dai6090_Ceriporia_sulphuricolo --
RLG_11354_Ceriproia_reticulata --
ZZW1543Dai27072
GTATTCCGTTGCTAGAGGTGAAATTCTTGGATTTACGGAAGACTAACTAC
Li1316_Ceriporia_reticulata
GTATTGCGTCGCTAGAGGTGAAATTCTTGGATTGACGCAAGACAAACTAT
KHL11981Ceriporia_reticulata --
FP110343sp_Candelabrochaete_la --
Li1045_Ceriporia_reticulata
GTATTCCGTTGCTAGAGGTGAAATTCTTGGATTTACGGAAGACTAACTAT
ZX136Dai25794ceriporia
GTATTCCGTTGCTAGAGGTGAAATTCTTGGATTTACGGAAGACTAACTAT
892Dai13400
GTATTCCGTTGCTAGAGGTGAAATTCTTGGATTTACGGAAGACTAACTAT
RLG7163Leptoporus_mollis --
Dai21062Leptoporus_mollis --
Dai20182Leptoporus_submollis --
Cui18379Leptoporus_submollis --
Wu1209_46Resiniporus_pseudogil --
BRNM710169Resiniporus_resinasc --
Dai14516Bjerkandera_adusta
GTATTCCGTTGCTAGAGGTGAAATTCTTGGATTTACGGAAGACTAACTAT
Dai21100Bjerkandera_fumosa
GTATTCCGTTGCTAGAGGTGAAATTCTTGGATTTACGGAAGACTAACTAC
Miettinen16854Ceraceomyces_sp --
Dai10477C_spissa --
855Dai16831
GTATTCCGTTGCTAGAGGTGAAATTCTTGGATTTACGGAAGACTAACTAC
882Cui11282
GTATTCCGTTGCTAGAGGTGAAATTCTTGGATTTACGGAAGACTAACTAC

Dai24566
GTATTCCGTTGCTAGAGGTGAAATTCTTGGATTTACGGAAGACTAACTAC
Yuan5965 --
Dai3204 --
1194CUI9985 --

Dai15205_Ceriporia_albomellea --
Dai15223_Ceriporia_albomellea --
Li1780_Ceriporia_variegata --
Dai19791_Ceriporia_variegata
TGCGAAAGCATTTGCCAAGGATGTTTTCATTAATCAAGAACGAAGGTTAG
Dai19886
TGCGAAAGCATTTGCCAAGGATGTTTTCATTAATCAAGAACGAAGGTTAG
Dai10833_Ceriporia_crassitunic --
CHWC1506_46Meruliopsis_crassit --
Dai9995_Ceriporia_crassitunica --
Wu1209_58_Meruliopsis_parvispo --
CHWC1505_129_Meruliopsis_parvi --
Dai21944
TGCGAAAGCATTTGCCAAGGATGTTTTCATTAATCAAGAACGAAGGTTAG
830Dai18640A
TGCGAAAGCATTTGCCAAGGATGTTTTCATTAATCAAGAACGAAGGTTAG
GC1704_60_Meruliopsis_taxicola --
Dai22625
TGCGAAAGCATTTGCCAAGGATGTTTTCATTAATCAAGAACGAAGGTTAG
Dai22636
TGCGAAAGCATTTGCCAAGGATGTTTTCATTAATCAAGAACGAAGGTTAG
Dai21878
TGCGAAAGCATTTGCCAAGGATGTTTTCATTAATCAAGAACGAAGGTTAG
1169Dai17248
TGCGAAAGCATTTGCCAAGGATGTTTTCATTAATCAAGAACGAAGGTTAG
Wu1708_43_Meruliopsis_leptocys --
Li1011 --
ZX95Dai25742Meruliopsis_leptoc
TGCGAAAGCATTTGCCAAGGATGTTTTCATTAATCAAGAACGAAGGTTAG
WCG1306Dai24733 --
LXL99Dai25816
TGCGAAAGCATTTGCCAAGGATGTTTTCATTAATCAAGAACGAAGGTTAG
WCG1559Dai26052Meruliopsis
TGCGAAAGCATTTGCCAAGGATGTTTTCATTAATCAAGAACGAAGGTTAG
He7477 --
HLX243Dai26217
TGCGAAAGCATTTGCCAAGGATGTTTTCATTAATCAAGAACGAAGGTTAG

RussiaMW673659Meruliopsis_fagi --
FD278 --
Dai10226_Ceriporia_tarda --
LE247365 --
Dai8173_Meruliopsis_nanlingens
TGCGAAAGCATTTGCCAAGGATGTTTTCATTAATCAAGAACGAAGGTTAG
860Dai17172
TGCGAAAGCATTTGCCAAGGATGTTTTCATTAATCAAGAACGAAGGTTAG
879Dai13414
TGCGAAAGCATTTGCCAAGGATGTTTTCATTAATCAAGAACGAAGGTTAG
Li_1704_Meruliopsis_pseudocyst --
833Dai18405
TGCGAAAGCATTTGCCAAGGATGTTTTCATTAATCAAGAACGAAGGTTAG
HHB_10729_Meruliopsis_albostra --
Cui6878_Ceriporia_pseudocystid --
869Dai14737
TGCGAAAGCATTTGCCAAGGATGTTTTCATTAATCAAGAACGAAGGTTAG
876Cui11626
TGCGAAAGCATTTGCCAAGGATGTTTTCATTAATCAAGAACGAAGGTTAG
1199WEI3388 --
776308_Meruliopsis_cystidiata --
ICN139059_Meruliopsis_cystidia --
HHB15692Ceraceomyces_serpens --
HHB_15629_Sp_Ceriporiopsis_ane --
AJ185Trametopsis_cervina --
FD9Irpex_lacteus --
908Dai11230
TGCGAAAGCATTTGCCAAGGATGTTTTCATTAATCAAGAACGAAGGTTAG
FP55521TEmmia_lacerata --
PBU0048Ceriporia_cystidiata --
MZ340C_lacerataT --
Dai21940
TGCGAAAGCATTTGCCAAGGATGTTTTCATTAATCAAGAACGAAGGTTAG
847Dai16433
TGCGAAAGCATTTGCCAAGGATGTTTTCATTAATCAAGAACGAAGGTTAG
MarcinEmmia_latemarginatus --
Meijer3729Hydnopolyporus_fimbr --
RLG13408Phanerochaete_sp --
WHC1381Flavodon_flavus --
GB1833Phlebia_albida --
T407Phlebia_nitidula --
HHB6988Phanerochaete_exilis --
HHB8509Phanerochaetella_xeroph --
PBU0051Macrohyporia_dictyopora --

HHB11463Phanerochaete_sp --
FP102382Byssomerulius_corium --
FP102165Efibula_americana --
Murdoch90Ceriporia_torpida --
Rivoire4413_Ceriporia_purpurea --
Kout_18_Ceriporia_triumphalis --
Rivoire3701_Ceriporia_bresadol --
VS4018 --
Ryvarden21832_Ceriporia_manzan --
Dai24539
TGCGAAAGCATTTGCCAAGGATGTTTTCATTAATCAAGAACGAAGGTTAG
Dai24541
TGCGAAAGCATTTGCCAAGGATGTTTTCATTAATCAAGAACGAAGGTTAG
JV1105_12_Ceriporia_occidental --
VS8558Ceriporia_occidentalis --
Dai22445
TGCGAAAGCATTTGCCAAGGATGTTTTCATTAATCAAGAACGAAGGTTAG
846Dai16368
TGCGAAAGCATTTGCCAAGGATGTTTTCATTAATCAAGAACGAAGGTTAG
Dai17951_Ceriporia_aurantiocar --
Miettinen_11701C_viridans --
JV0105_10Ceriporia_aurantiocar --
Yuan5702C_viridans --
858Dai17003
TGCGAAAGCATTTGCCAAGGATGTTTTCATTAATCAAGAACGAAGGTTAG
Yuan2747_Ceriporia_viridans --
Yuan2744C_viridans --
Li1046C_viridans --
865C_sinoviridans
TGCGAAAGCATTTGCCAAGGATGTTTTCATTAATCAAGAACGAAGGTTAG
871Dai15062 --
Dai7642_Ceriporia_humilis
TGCGAAAGCATTTGCCAAGGATGTTTTCATTAATCAAGAACGAAGGTTAG
Spirin4706_Ceriporia_humilis --
Spirin4944_Ceriporia_sericea --
WCG1547Dai26044ceriporia
TGCGAAAGCATTTGCCAAGGATGTTTTCATTAATCAAGAACGAAGGTTAG
ZZW1558Dai27086
TGCGAAAGCATTTGCCAAGGATGTTTTCATTAATCAAGAACGAAGGTTAG
Miettinen14381_Ceriporia_mpuri --
Miettinen15492_2_Ceriporia_sor --
He6687
TGCGAAAGCATTTGCCAAGGATGTTTTCATTAATCAAGAACGAAGGTTAG
ZH53Dai24426

TGCGAAAGCATTTGCCAAGGATGTTTTCATTAATCAAGAACGAAGGTTAG
Vlasak0808_30_Ceriporia_punica --
887Dai13376
TGCGAAAGCATTTGCCAAGGATGTTTTCATTAATCAAGAACGAAGGTTAG
WCG1443Dai24998
TGCGAAAGCATTTGCCAAGGATGTTTTCATTAATCAAGAACGAAGGTTAG
0108_6Ceriporia_spissa --
Dai19164
TGCGAAAGCATTTGCCAAGGATGTTTTCATTAATCAAGAACGAAGGTTAG
Dai17937_Ceriporia_bubalinomar --
903Dai12113
TGCGAAAGCATTTGCCAAGGATGTTTTCATTAATCAAGAACGAAGGTTAG
LZB929Dai25079
TGCGAAAGCATTTGCCAAGGATGTTTTCATTAATCAAGAACGAAGGTTAG
LX45Dai26988 --
LX43Dai26986
TGCGAAAGCATTTGCCAAGGATGTTTTCATTAATCAAGAACGAAGGTTAG
Dai7759Ceriporia --
Cui8012_Ceriporia_viridans --
GC1704_54Ceriporia_viridans --
Dai23392
TGCGAAAGCATTTGCCAAGGATGTTTTCATTAATCAAGAACGAAGGTTAG
WCG1585Dai26113Ceriproia
TGCGAAAGCATTTGCCAAGGATGTTTTCATTAATCAAGAACGAAGGTTAG
Dai18675C_eucalypti --
Dai22034
TGCGAAAGCATTTGCCAAGGATGTTTTCATTAATCAAGAACGAAGGTTAG
JV1008_41JTardaFLORIDAKeys --
Rivoire1161_Ceriporia_pierii --
Dai23499C_pierii
TGCGAAAGCATTTGCCAAGGATGTTTTCATTAATCAAGAACGAAGGTTAG
Dai23500
TGCGAAAGCATTTGCCAAGGATGTTTTCATTAATCAAGAACGAAGGTTAG
841Dai15899
TGCGAAAGCATTTGCCAAGGATGTTTTCATTAATCAAGAACGAAGGTTAG
842Dai15904
TGCGAAAGCATTTGCCAAGGATGTTTTCATTAATCAAGAACGAAGGTTAG
LZB1066xinjiang --
LZB1065xinjiang --
851Dai16779
TGCGAAAGCATTTGCCAAGGATGTTTTCATTAATCAAGAACGAAGGTTAG
RMJ119sp_Candelabrochaete_sept --
RLG9759spCandelabrochaete_sept --
RLG10478Phanerochaete_allantos --

Dai19118_Ceriporia_spissa
TGCGAAAGCATTTGCCAAGGATGTTTTCATTAATCAAGAACGAAGGTTAG
Dai18486A
TGCGAAAGCATTTGCCAAGGATGTTTTCATTAATCAAGAACGAAGGTTAG
WEI17_024_Ceriporia_mellita --
GC1508_71Ceriporia_mellita --
GC1608_7_Ceriporia_mellita --
ZZW1557Dai27085 --
ZZW1554Dai27083
TGCGAAAGCATTTGCCAAGGATGTTTTCATTAATCAAGAACGAAGGTTAG
Dai8168 --
BR4865C_mellita --
MEL2382688Ceriporia_sp --
Dai8110 --
Cui8097 --
909Cui6740 --
W1258Dai24695
TGCGAAAGCATTTGCCAAGGATGTTTTCATTAATCAAGAACGAAGGTTAG
JV0110_26_Ceriporia_griseoviol --
896Dai13202
TGCGAAAGCATTTGCCAAGGATGTTTTCATTAATCAAGAACGAAGGTTAG
LWY393Dai27053C_griseoviolasce
TGCGAAAGCATTTGCCAAGGATGTTTTCATTAATCAAGAACGAAGGTTAG
LWY394DAI27054
TGCGAAAGCATTTGCCAAGGATGTTTTCATTAATCAAGAACGAAGGTTAG
FP135015G_pannocinctus --
L15726SpG_pannocinctus --
Dai22221 TGCGAAAGCATTTG-
CCAGGATGTTTTCATTAATCAAGAACGAAGGTTA-
Dai22633 TGCGAAAGCATTTG-
CAAGGATGTTTTCATTAATCAAGAACGAAGGTTA-
Dai23260 TGCGAAAGCATTTG-
CCAGGATGTTTTCATTAATCAAGAACGAAGGTTAG
Dai23626 TGCGAAAGCATTTG-
CAAGGATGTTTTCATTAATCAAGAACGAGGGTA--
Dai16238G_citrinoalbus
TGCGAAAGCATTTGCCAAGGATGTTTTCATTAATCAAGAACGAAGGTTAG
1175Dai15293
TGCGAAAGCATTTGCCAAGGATGTTTTCATTAATCAAGAACGAAGGTTAG
Dai19547
TGCGAAAGCATTTGCCAAGGATGTTTTCATTAATCAAGAACGAAGGTTAG
918063G_africanus --
918572G_africanus --
Dai18536A --

1164Cui17922
TGCGAAAGCATTTGCCAAGGATGTTTTCATTAATCAAGAACGAAGGTTAG
Dai22225 TGCGAAAGCATTTG-
CAAGGATGTTTTCATTAATCAAGAACGAAGGTTAG
1163Dai20655
TGCGAAAGCATTTGCCAAGGATGTTTTCATTAATCAAGAACGAAGGTTAG
Yuan4397G_hainanensis --
1176Dai15268
TGCGAAAGCATTTGCCAAGGATGTTTTCATTAATCAAGAACGAAGGTTAG
1177Dai15259
TGCGAAAGCATTTGCCAAGGATGTTTTCATTAATCAAGAACGAAGGTTAG
BZ2896G_thelephoroides --
1166JV1808_26
TGCGAAAGCATTTGCCAAGGATGTTTTCATTAATCAAGAACGAAGGTTAG
Miettinen16992Hapalopilus_ochr --
GC1708_338_Ceriporia_arbuscula --
WCG1555Dai26107Ceriporia
TGCGAAAGCATTTGCCAAGGATGTTTTCATTAATCAAGAACGAAGGTTAG
GC1708_340_Ceriporia_arbuscula --
WCG1556Dai26109Ceriporia
TGCGAAAGCATTTGCCAAGGATGTTTTCATTAATCAAGAACGAAGGTTAG
883Cui11291
TGCGAAAGCATTTGCCAAGGATGTTTTCATTAATCAAGAACGAAGGTTAG
HLX320Dai26805
TGCGAAAGCATTTGCCAAGGATGTTTTCATTAATCAAGAACGAAGGTTAG
WCG1266Dai24678A
TGCGAAAGCATTTGCCAAGGATGTTTTCATTAATCAAGAACGAAGGTTAG
Dai6090_Ceriporia_sulphuricolo --
RLG_11354_Ceriproia_reticulata --
ZZW1543Dai27072
TGCGAAAGCATTTGCCAAGGATGTTTTCATTAATCAAGAACGAAGGTTAG
Li1316_Ceriporia_reticulata
TGCGAAAGCATTTGCCAAGGATGTTTTCATTAATCAAGAACGAAGGTTAG
KHL11981Ceriporia_reticulata --
FP110343sp_Candelabrochaete_la --
Li1045_Ceriporia_reticulata
TGCGAAAGCATTTGCCAAGGATGTTTTCATTAATCAAGAACGAAGGTTAG
ZX136Dai25794ceriporia
TGCGAAAGCATTTGCCAAGGATGTTTTCATTAATCAAGAACGAAGGTTAG
892Dai13400
TGCGAAAGCATTTGCCAAGGATGTTTTCATTAATCAAGAACGAAGGTTAG
RLG7163Leptoporus_mollis --
Dai21062Leptoporus_mollis --
Dai20182Leptoporus_submollis --

Cui18379Leptoporus_submollis --
Wu1209_46Resiniporus_pseudogil --
BRNM710169Resiniporus_resinasc --
Dai14516Bjerkandera_adusta
TGCGAAAGCATTTGCCAAGGATGTTTTCATTAATCAAGAACGAAGGTTAG
Dai21100Bjerkandera_fumosa
TGCGAAAGCATTTGCCAAGGATGTTTTCATTAATCAAGAACGAAGGTTAG
Miettinen16854Ceraceomyces_sp --
Dai10477C_spissa --
855Dai16831
TGCGAAAGCATTTGCCAAGGATGTTTTCATTAATCAAGAACGAAGGTTAG
882Cui11282
TGCGAAAGCATTTGCCAAGGATGTTTTCATTAATCAAGAACGAAGGTTAG
Dai24566
TGCGAAAGCATTTGCCAAGGATGTTTTCATTAATCAAGAACGAAGGTTAG
Yuan5965 --
Dai3204 --
1194CUI9985 --

Dai15205_Ceriporia_albomellea --
Dai15223_Ceriporia_albomellea --
Li1780_Ceriporia_variegata --
Dai19791_Ceriporia_variegata
GGGATCGAAAACGATCAGATACCGTTGTAGTCTTAACAGTAAACTATGCC
Dai19886
GGGATCGAAAACGATCAGATACCGTTGTAGTCTTAACAGTAAACTATGCC
Dai10833_Ceriporia_crassitunic --
CHWC1506_46Meruliopsis_crassit --
Dai9995_Ceriporia_crassitunica --
Wu1209_58_Meruliopsis_parvispo --
CHWC1505_129_Meruliopsis_parvi --
Dai21944
GGGATCGAAAACGATCAGATACCGTTGTAGTCTTAACAGTAAACTATGCC
830Dai18640A
GGGATCGAAAACGATCAGATACCGTTGTAGTCTTAACAGTAAACTATGCC
GC1704_60_Meruliopsis_taxicola --
Dai22625
GGGATCGAAAACGATCAGATACCGTTGTAGTCTTAACAGTAAACTATGCC
Dai22636
GGGATCGAAAACGATCAGATACCGTTGTAGTCTTAACAGTAAACTATGCC
Dai21878
GGGATCGAAAACGATCAGATACCGTTGTAGTCTTAACAGTAAACTATGCC
1169Dai17248

GGGATCGAAAACGATCAGATACCGTTGTAGTCTTAACAGTAAACTATGCC

Wu1708_43_Meruliopsis_leptocys --

Li1011 --

ZX95Dai25742Meruliopsis_leptoc

GGGATCGAAAACGATCAGATACCGTTGTAGTCTTAACAGTAAACTATGCC

WCG1306Dai24733 --

LXL99Dai25816

GGGATCGAAAACGATCAGATACCGTTGTAGTCTTAACAGTAAACTATGCC

WCG1559Dai26052Meruliopsis

GGGATCGAAAACGATCAGATACCGTTGTAGTCTTAACAGTAAACTATGCC

He7477 --

HLX243Dai26217

GGGATCGAAAACGATCAGATACCGTTGTAGTCTTAACAGTAAACTATGCC

RussiaMW673659Meruliopsis_fagi --

FD278 --

Dai10226_Ceriporia_tarda --

LE247365 --

Dai8173_Meruliopsis_nanlingens

GGGATCGAAAACGATCAGATACCGTTGTAGTCTTAACAGTAAACTATGCC

860Dai17172

GGGATCGAAAACGATCAGATACCGTTGTAGTCTTAACAGTAAACTATGCC

879Dai13414

GGGATCGAAAACGATCAGATACCGTTGTAGTCTTAACAGTAAACTATGCC

Li_1704_Meruliopsis_pseudocyst --

833Dai18405

GGGATCGAAAACGATCAGATACCGTTGTAGTCTTAACAGTAAACTATGCC

HHB_10729_Meruliopsis_albostra --

Cui6878_Ceriporia_pseudocystid --

869Dai14737

GGGATCGAAAACGATCAGATACCGTTGTAGTCTTAACAGTAAACTATGCC

876Cui11626

GGGATCGAAAACGATCAGATACCGTTGTAGTCTTAACAGTAAACTATGCC

1199WEI3388 --

776308_Meruliopsis_cystidiata --

ICN139059_Meruliopsis_cystidia --

HHB15692Ceraceomyces_serpens --

HHB_15629_Sp_Ceriporiopsis_ane --

AJ185Trametopsis_cervina --

FD9Irpex_lacteus --

908Dai11230

GGGATCGAAAACGATCAGATACCGTTGTAGTCTTAACAGTAAACTATGCC

FP55521TEmmia_lacerata --

PBU0048Ceriporia_cystidiata --

MZ340C_lacerataT --

Dai21940
GGGATCGAAAACGATCAGATACCGTTGTAGTCTTAACAGTAAACTATGCC
847Dai16433
GGGATCGAAAACGATCAGATACCGTTGTAGTCTTAACAGTAAACTATGCC
MarcinEmmia_latemarginatus --
Meijer3729Hydnopolyporus_fimbr --
RLG13408Phanerochaete_sp --
WHC1381Flavodon_flavus --
GB1833Phlebia_albida --
T407Phlebia_nitidula --
HHB6988Phanerochaete_exilis --
HHB8509Phanerochaetella_xeroph --
PBU0051Macrohyporia_dictyopora --
HHB11463Phanerochaete_sp --
FP102382Byssomerulius_corium --
FP102165Efibula_americana --
Murdoch90Ceriporia_torpida --
Rivoire4413_Ceriporia_purpurea --
Kout_18_Ceriporia_triumphalis --
Rivoire3701_Ceriporia_bresadol --
VS4018 --
Ryvarden21832_Ceriporia_manzan --
Dai24539
GGGATCGAAAACGATCAGATACCGTTGTAGTCTTAACAGTAAACTATGCC
Dai24541
GGGATCGAAAACGATCAGATACCGTTGTAGTCTTAACAGTAAACTATGCC
JV1105_12_Ceriporia_occidental --
VS8558Ceriporia_occidentalis --
Dai22445
GGGATCGAAAACGATCAGATACCGTTGTAGTCTTAACAGTAAACTATGCC
846Dai16368
GGGATCGAAAACGATCAGATACCGTTGTAGTCTTAACAGTAAACTATGCC
Dai17951_Ceriporia_aurantiocar --
Miettinen_11701C_viridans --
JV0105_10Ceriporia_aurantiocar --
Yuan5702C_viridans --
858Dai17003
GGGATCGAAAACGATCAGATACCGTTGTAGTCTTAACAGTAAACTATGCC
Yuan2747_Ceriporia_viridans --
Yuan2744C_viridans --
Li1046C_viridans --
865C_sinoviridans
GGGATCGAAAACGATCAGATACCGTTGTAGTCTTAACAGTAAACTATGCC
871Dai15062 --

Dai7642_Ceriporia_humilis
GGGATCGAAAACGATCAGATACCGTTGTAGTCTTAACAGTAAACTATGCC
Spirin4706_Ceriporia_humilis --
Spirin4944_Ceriporia_sericea --
WCG1547Dai26044ceriporia
GGGATCGAAAACGATCAGATACCGTTGTAGTCTTAACAGTAAACTATGCC
ZZW1558Dai27086
GGGATCGAAAACGATCAGATACCGTTGTAGTCTTAACAGTAAACTATGCC
Miettinen14381_Ceriporia_mpuri --
Miettinen15492_2_Ceriporia_sor --
He6687
GGGATCGAAAACGATCAGATACCGTTGTAGTCTTAACAGTAAACTATGCC
ZH53Dai24426
GGGATCGAAAACGATCAGATACCGTTGTAGTCTTAACAGTAAACTATGCC
Vlasak0808_30_Ceriporia_punica --
887Dai13376
GGGATCGAAAACGATCAGATACCGTTGTAGTCTTAACAGTAAACTATGCC
WCG1443Dai24998
GGGATCGAAAACGATCAGATACCGTTGTAGTCTTAACAGTAAACTATGCC
0108_6Ceriporia_spissa --
Dai19164
GGGATCGAAAACGATCAGATACCGTTGTAGTCTTAACAGTAAACTATGCC
Dai17937_Ceriporia_bubalinomar --
903Dai12113
GGGATCGAAAACGATCAGATACCGTTGTAGTCTTAACAGTAAACTATGCC
LZB929Dai25079
GGGATCGAAAACGATCAGATACCGTTGTAGTCTTAACAGTAAACTATGCC
LX45Dai26988 --
LX43Dai26986
GGGATCGAAAACGATCAGATACCGTTGTAGTCTTAACAGTAAACTATGCC
Dai7759Ceriporia --
Cui8012_Ceriporia_viridans --
GC1704_54Ceriporia_viridans --
Dai23392
GGGATCGAAAACGATCAGATACCGTTGTAGTCTTAACAGTAAACTATGCC
WCG1585Dai26113Ceriproia
GGGATCGAAAACGATCAGATACCGTTGTAGTCTTAACAGTAAACTATGCC
Dai18675C_eucalypti --
Dai22034
GGGATCGAAAACGATCAGATACCGTTGTAGTCTTAACAGTAAACTATGCC
JV1008_41JTardaFLORIDAKeys --
Rivoire1161_Ceriporia_pierii --
Dai23499C_pierii
GGGATCGAAAACGATCAGATACCGTTGTAGTCTTAACAGTAAACTATGCC

Dai23500
GGGATCGAAAACGATCAGATACCGTTGTAGTCTTAACAGTAAACTATGCC
841Dai15899
GGGATCGAAAACGATCAGATACCGTTGTAGTCTTAACAGTAAACTATGCC
842Dai15904
GGGATCGAAAACGATCAGATACCGTTGTAGTCTTAACAGTAAACTATGCC
LZB1066xinjiang --
LZB1065xinjiang --
851Dai16779
GGGATCGAAAACGATCAGATACCGTTGTAGTCTTAACAGTAAACTATGCC
RMJ119sp_Candelabrochaete_sept --
RLG9759spCandelabrochaete_sept --
RLG10478Phanerochaete_allantos --
Dai19118_Ceriporia_spissa
GGGATCGAAAACGATTAGATACCGTTGTAGTCTTAACAGTAAACTATGCC
Dai18486A
GGGATCGAAAACGATTAGATACCGTTGTAGTCTTAACAGTAAACTATGCC
WEI17_024_Ceriporia_mellita --
GC1508_71Ceriporia_mellita --
GC1608_7_Ceriporia_mellita --
ZZW1557Dai27085 --
ZZW1554Dai27083
GGGATCGAAAACGCTTAGATACCGTTGTAGTCTTAACAGTAAACTATGCC
Dai8168 --
BR4865C_mellita --
MEL2382688Ceriporia_sp --
Dai8110 --
Cui8097 --
909Cui6740 --
W1258Dai24695
GGGATCGAAAACGATTAGATACCGTTGTAGTCTTAACAGTAAACTATGCC
JV0110_26_Ceriporia_griseoviol --
896Dai13202
GGGATCGAAAACGATCAGATACCGTTGTAGTCTTAACAGTAAACTATGCC
LWY393Dai27053C_griseoviolasce
GGGATCGAAAACGATCAGATACCGTTGTAGTCTTAACAGTAAACTATGCC
LWY394DAI27054
GGGATCGAAAACGCTTAGATACCGTTGTAGTCTTAACAGTAAACTATGCC
FP135015G_pannocinctus --
L15726SpG_pannocinctus --
Dai22221 --
Dai22633 GGGATCG-AAACGATCAGATACCGTT------------

Dai23260 GGGATCG-AAACGATCAGATAC--------------------

Dai23626 --
Dai16238G_citrinoalbus
GGGATCGAAAACGATCAGATACCGTTGTAGTCTTAACAGTAAACTATGCC
1175Dai15293
GGGATCGAAAACGATCAGATACCGTTGTAGTCTTAACAGTAAACTATGCC
Dai19547
GGGATCGAAAACGATCAGATACCGTTGTAGTCTTAACAGTAAACTATGCC
918063G_africanus --
918572G_africanus --
Dai18536A --
1164Cui17922
GGGATCGAAAACGATCAGATACCGTTGTAGTCTTAACAGTAAACTATGCC
Dai22225 GGGATCGAAAACGATCAG--------------------------

1163Dai20655
GGGATCGAAAACGATCAGATACCGTTGTAGTCTTAACAGTAAACTATGCC
Yuan4397G_hainanensis --
1176Dai15268
GGGATCGAAAACGATCAGATACCGTTGTAGTCTTAACAGTAAACTATGCC
1177Dai15259
GGGATCGAAAACGATCAGATACCGTTGTAGTCTTAACAGTAAACTATGCC
BZ2896G_thelephoroides --
1166JV1808_26
GGGATCGAAAACGATCAGATACCGTTGTAGTCTTAACAGTAAACTATGCC
Miettinen16992Hapalopilus_ochr --
GC1708_338_Ceriporia_arbuscula --
WCG1555Dai26107Ceriporia
GGGATCGAAAACGATCAGATACCGTTGTAGTCTTAACAGTAAACTATGCC
GC1708_340_Ceriporia_arbuscula --
WCG1556Dai26109Ceriporia
GGGATCGAAAACGATCAGATACCGTTGTAGTCTTAACAGTAAACTATGCC
883Cui11291
GGGATCGAAAACGATCAGATACCGTTGTAGTCTTAACAGTAAACTATGCC
HLX320Dai26805
GGGATCGAAAACGATCAGATACCGTTGTAGTCTTAACAGTAAACTATGCC
WCG1266Dai24678A
GGGATCGAAAACGATCAGATACCGTTGTAGTCTTAACAGTAAACTATGCC
Dai6090_Ceriporia_sulphuricolo --
RLG_11354_Ceriproia_reticulata --
ZZW1543Dai27072
GGGATCGAAAACGATCAGATACCGTTGTAGTCTTAACAGTAAACTATGCC
Li1316_Ceriporia_reticulata
GGGATCGAAAACGATCAGATACCGTTGTAGTCTTAACAGTAAACTATGCC

KHL11981Ceriporia_reticulata --
FP110343sp_Candelabrochaete_la --
Li1045_Ceriporia_reticulata
GGGATCGAAAACGATCAGATACCGTTGTAGTCTTAACAGTAAACTATGCC
ZX136Dai25794ceriporia
GGGATCGAAAACGATCAGATACCGTTGTAGTCTTAACAGTAAACTATGCC
892Dai13400
GGGATCGAAAACGATCAGATACCGTTGTAGTCTTAACAGTAAACTATGCC
RLG7163Leptoporus_mollis --
Dai21062Leptoporus_mollis --
Dai20182Leptoporus_submollis --
Cui18379Leptoporus_submollis --
Wu1209_46Resiniporus_pseudogil --
BRNM710169Resiniporus_resinasc --
Dai14516Bjerkandera_adusta
GGGATCGAAAACGATCAGATACCGTTGTAGTCTTAACAGTAAACTATGCC
Dai21100Bjerkandera_fumosa
GGGATCGAAAACGATCAGATACCGTTGTAGTCTTAACAGTAAACTATGCC
Miettinen16854Ceraceomyces_sp --
Dai10477C_spissa --
855Dai16831
GGGATCGAAAACGATCAGATACCGTTGTAGTCTTAACAGTAAACTATGCC
882Cui11282
GGGATCGAAAACGATCAGATACCGTTGTAGTCTTAACAGTAAACTATGCC
Dai24566
GGGATCGAAAACGATCAGATACCGTTGTAGTCTTAACAGTAAACTATGCC
Yuan5965 --
Dai3204 --
1194CUI9985 --

Dai15205_Ceriporia_albomellea --
Dai15223_Ceriporia_albomellea --
Li1780_Ceriporia_variegata --
Dai19791_Ceriporia_variegata GACTAGGGATCGGGCGAACTC-AATTTGATGTGT-CGCTCGGCACCTTAC
Dai19886 GACTAGGGATCGGGCGAACTC-AATTTGATGTGT-CGCTCGGCACCTTAC
Dai10833_Ceriporia_crassitunic --
CHWC1506_46Meruliopsis_crassit --
Dai9995_Ceriporia_crassitunica --
Wu1209_58_Meruliopsis_parvispo --
CHWC1505_129_Meruliopsis_parvi --
Dai21944 GACTAGGGATCGGGCGAACTC-

ACTTTTATGTGT-CGCTCGGCACCTTAC

830Dai18640A GACTAGGGATCGGGCGAACTC-ACTTTTATGTGT-CGCTCGGCACCTTAC

GC1704_60_Meruliopsis_taxicola --

Dai22625 GACTAGGGATCGGGCGAACTC-AATTTGATGTGT-CGCTCGGCACCTTAC

Dai22636 GACTAGGGATCGGGCGAACTC-AATTTGATGTGT-CGCTCGGCACCTTAC

Dai21878 GACTAGGGATCGGGCGAACTC-AATTTGATGTGT-CGCTCGGCACCTTAC

1169Dai17248 GACTAGGGATCGGGCGAACTC-AATTTGATGTGT-CGCTCGGCACCTTAC

Wu1708_43_Meruliopsis_leptocys --

Li1011 --

ZX95Dai25742Meruliopsis_leptoc GACTAGGGATCGGGCGAACTC-AATTTTATGTGT-CGCTCGGCACCTTAC

WCG1306Dai24733 --

LXL99Dai25816 GACTAGGGATCGGGCGAACTC-AATTTTATGTGT-CGCTCGGCACCTTAC

WCG1559Dai26052Meruliopsis GACTAGGGATCGGGCGAACTC-AATTTGATGTGT-CGCTCGGCACCTTAC

He7477 --

HLX243Dai26217 GACTAGGGATCGGGCGAACTC-AATTTGATGTGT-CGCTCGGCACCTTAC

RussiaMW673659Meruliopsis_fagi --

FD278 --

Dai10226_Ceriporia_tarda --

LE247365 --

Dai8173_Meruliopsis_nanlingens GACTAGGGATCGGACAACCTCGATATTTATGTGTCTGTTCGGCACCTTAC

860Dai17172 GACTAGGGATCGGGCGAACTC-AATTTTATGTGT-CGCTCGGCACCTTAC

879Dai13414 GACTAGGGATCGGGCGAACTC-AATTTTATGTGT-CGCTCGGCACCTTAC

Li_1704_Meruliopsis_pseudocyst --

833Dai18405 GACTAGGGATCGGGCGAACTC-AATTTGATGTGT-CGCTCGGCACCTTAC

HHB_10729_Meruliopsis_albostra --

Cui6878_Ceriporia_pseudocystid --

869Dai14737 GACTAGGGATCGGGCGAACTC-AATTTTATGTGT-CGCTCGGCACCTTAC

876Cui11626 GACTAGGGATCGGGCGAACTC-AATTTTATGTGT-CGCTCGGCACCTTAC

1199WEI3388 --

776308_Meruliopsis_cystidiata ---
ICN139059_Meruliopsis_cystidia ---
HHB15692Ceraceomyces_serpens ---
HHB_15629_Sp_Ceriporiopsis_ane ---
AJ185Trametopsis_cervina ---
FD9Irpex_lacteus ---
908Dai11230 GACTAGGGATCGGGCGAACTC-
AATTTGATGTGT-CGCTCGGCACCTTAC
FP55521TEmmia_lacerata ---
PBU0048Ceriporia_cystidiata ---
MZ340C_laceratаT ---
Dai21940 GACTAGGGATCGGGCGAACTC-
AATATGATGTGT-CGCTCGGCACCTTAC
847Dai16433 GACTAGGGATCGGGCGAACTC-
AATATGATGTGT-CGCTCGGCACCTTAC
MarcinEmmia_latemarginatus ---
Meijer3729Hydnopolyporus_fimbr ---
RLG13408Phanerochaete_sp ---
WHC1381Flavodon_flavus ---
GB1833Phlebia_albida ---
T407Phlebia_nitidula ---
HHB6988Phanerochaete_exilis ---
HHB8509Phanerochaetella_xeroph ---
PBU0051Macrohyporia_dictyopora ---
HHB11463Phanerochaete_sp ---
FP102382Byssomerulius_corium ---
FP102165Efibula_americana ---
Murdoch90Ceriporia_torpida ---
Rivoire4413_Ceriporia_purpurea ---
Kout_18_Ceriporia_triumphalis ---
Rivoire3701_Ceriporia_bresadol ---
VS4018 ---
Ryvarden21832_Ceriporia_manzan ---
Dai24539 GACTAGGGATCGGGCGAACTC-
AATTTGATGTGT-CGCTCGGCACCTTAC
Dai24541 GACTAGGGATCGGGCGAACTC-
AATTTGATGTGT-CGCTCGGCACCTTAC
JV1105_12_Ceriporia_occidental ---
VS8558Ceriporia_occidentalis ---
Dai22445 GACTAGGGATCGGGCGAACTC-
AATTTGATGTGT-CGCTCGGCACCTTAC
846Dai16368 GACTAGGGATCGGGCGAACTC-
AATTTGATGTGT-CGCTCGGCACCTTAC
Dai17951_Ceriporia_aurantiocar ---

Miettinen_11701C_viridans --
JV0105_10Ceriporia_aurantiocar --
Yuan5702C_viridans --
858Dai17003 GACTAGGGATCGGGCGAACTC-AATTTGATGTGT-CGCTCGGCACCTTAC
Yuan2747_Ceriporia_viridans --
Yuan2744C_viridans --
Li1046C_viridans --
865C_sinoviridans GACTAGGGATCGGGCGAACTC-AATTTTATGTGT-CGCTCGGCACCTTAC
871Dai15062 --
Dai7642_Ceriporia_humilis GACTAGGGATCGGGCGAACTCGAATTTGATGTGT-CGCTCGGCACCTTAC
Spirin4706_Ceriporia_humilis --
Spirin4944_Ceriporia_sericea --
WCG1547Dai26044ceriporia GACTAGGGATCGGGCGAACTC-AATTTGATGTGT-CGCTCGGCACCTTAC
ZZW1558Dai27086 GACTAGGGATCGGGCGAACTC-AATTTGATGTGT-CGCTCGGCACCTTAC
Miettinen14381_Ceriporia_mpuri --
Miettinen15492_2_Ceriporia_sor --
He6687 GACTAGGGATCGGGCGAACTC-AATTTGATGTGT-CGCTCGGCACCTTAC
ZH53Dai24426 GACTAGGGATCGGGCGAACTC-AATTTGATGTGT-CGCTCGGCACCTTAC
Vlasak0808_30_Ceriporia_punica --
887Dai13376 GACTAGGGATCGGGCGAACTC-AATTTTATGTGT-CGCTCGGCACCTTAC
WCG1443Dai24998 GACTAGGGATCGGGCGAACTC-AATTTTATGTGT-CGCTCGGCACCTTAC
0108_6Ceriporia_spissa --
Dai19164 GACTAGGGATCGGGCGAACTC-AATTTGATGTGT-CGCTCGGCACCTTAC
Dai17937_Ceriporia_bubalinomar --
903Dai12113 GACTAGGGATCGGGCGAACTC-AATTTGATGTGT-CGCTCGGCACCTTAC
LZB929Dai25079 GACTAGGGATCGGGCGAACTC-AATTTGATGTGT-CGCTCGGCACCTTAC
LX45Dai26988 --
LX43Dai26986 GACTAGGGATCGGGCGAACTC-AATTTGATGTGT-CGCTCGGCACCTTAC
Dai7759Ceriporia --
Cui8012_Ceriporia_viridans --
GC1704_54Ceriporia_viridans --

Dai23392 GACTAGGGATCGGGCGAACTC-AATTTGATGTGT-CGCTCGGCACCTTAC
WCG1585Dai26113Ceriproia GACTAGGGATCGGGCGACCTC-ACTATTATGTGT-CGCTCGGCACCTTAC
Dai18675C_eucalypti --
Dai22034 GACTAGGGATCGGGCGAACTC-AATTTGATGTGT-CGCTCGGCACCTTAC
JV1008_41JTardaFLORIDAKeys --
Rivoire1161_Ceriporia_pierii --
Dai23499C_pierii GACTAGGGATCGGGCGAACTC-AATCTGATGTGT-CGCTCGGCACCTTAC
Dai23500 GACTAGGGATCGGGCGAACTC-AATCTGATGTGT-CGCTCGGCACCTTAC
841Dai15899 GACTAGGGATCGGGCGAACTC-AATTTGATGTGT-CGCTCGGCACCTTAC
842Dai15904 GACTAGGGATCGGGCGAACTC-AATTTGATGTGT-CGCTCGGCACCTTAC
LZB1066xinjiang --
LZB1065xinjiang --
851Dai16779 GACTAGGGATCGGGCGAACTC-AATTTGATGTGT-CGCTCGGCACCTTAC
RMJ119sp_Candelabrochaete_sept --
RLG9759spCandelabrochaete_sept --
RLG10478Phanerochaete_allantos --
Dai19118_Ceriporia_spissa GACTAGGGATCGGGCGAACTC-AATTTCATGTGT-CGCTCGGCACCTTAC
Dai18486A GACTAGGGATCGGGCGAACTC-AATTTCATGTGT-CGCTCGGCACCTTAC
WEI17_024_Ceriporia_mellita --
GC1508_71Ceriporia_mellita --
GC1608_7_Ceriporia_mellita --
ZZW1557Dai27085 --
ZZW1554Dai27083 GACTAGGGATCGGGCGAACTC-AATTTCATGTGT-CGCTCGGCACCTTAC
Dai8168 --
BR4865C_mellita --
MEL2382688Ceriporia_sp --
Dai8110 --
Cui8097 --
909Cui6740 --
W1258Dai24695 GACTAGGGATCGGGCGAACTC-AATTTCATGTGT-CGCTCGGCACCTTAC
JV0110_26_Ceriporia_griseoviol --
896Dai13202 GACTAGGGATCGGGCGAACTC-

AATCTGATGTGT-CGCTCGGCACCTTAC

LWY393Dai27053C_griseoviolasce GACTAGGGATCGGGCGAACTC-AATCTGATGTGT-CGCTCGGCACCTTAC

LWY394DAI27054 GACTAGGGATCGGGCGAACTC-AATTTCATGTGT-CGCTCGGCACCTTAC

FP135015G_pannocinctus --

L15726SpG_pannocinctus --

Dai22221 --

Dai22633 --

Dai23260 --

Dai23626 --

Dai16238G_citrinoalbus GACTAGGGATCGGGCGACCTC-ACTTTTATGTGT-CGCTCGGCACCTTAC

1175Dai15293 GACTAGGGATCGGGCGATCTC-AAATTTATGTGT-CGCTCGGCACCTTAC

Dai19547 GACTAGGGATCGGGCGATCTC-AAATTTATGTGT-CGCTCGGCACCTTAC

918063G_africanus --

918572G_africanus --

Dai18536A --

1164Cui17922 GACTAGGGATCGGGCGATCTC-AAATTTATGTGT-CGCTCGGCACCTTAC

Dai22225 --

1163Dai20655 GACTAGGGATCGGGCGATCTC-AAATTTATGTGT-CGCTCGGCACCTTAC

Yuan4397G_hainanensis --

1176Dai15268 GACTAGGGATCGGGCGAACTC-ACTTTTATGTGT-CGCTCGGCACCTTAC

1177Dai15259 GACTAGGGATCGGGCGAACTC-ACTTTTATGTGT-CGCTCGGCACCTTAC

BZ2896G_thelephoroides --

1166JV1808_26 GACTAGGGATCGGGCGAACTC-ACTTTTATGTGT-CGCTCGGCACCTTAC

Miettinen16992Hapalopilus_ochr --

GC1708_338_Ceriporia_arbuscula --

WCG1555Dai26107Ceriporia GACTAGGGATCGGGCGAACTC-AATTTTATGTGT-CGCTCGGCACCTTAC

GC1708_340_Ceriporia_arbuscula --

WCG1556Dai26109Ceriporia GACTAGGGATCGGGCGAACTC-AATTTTATGTGT-CGCTCGGCACCTTAC

883Cui11291 GACTAGGGATCGGGCGAACTC-AATTTTATGTGT-CGCTCGGCACCTTAC

HLX320Dai26805 GACTAGGGATCGGGCGAACTC-AATTTTATGTGT-CGCTCGGCACCTTAC

WCG1266Dai24678A GACTAGGGATCGGGCGAACTC-AATTTTATGTGT-CGCTCGGCACCTTAC
Dai6090_Ceriporia_sulphuricolo ---
RLG_11354_Ceriproia_reticulata ---
ZZW1543Dai27072 GACTAGGGATCGGGCGAACTC-AATTTGATGTGT-CGCTCGGCACCTTAC
Li1316_Ceriporia_reticulata GACTAGGGATCGGACAACCTC-AAATTTATGTGT-TGTTCGGCACCTTAC
KHL11981Ceriporia_reticulata ---
FP110343sp_Candelabrochaete_la ---
Li1045_Ceriporia_reticulata GACTAGGGATCGGGCGAACTC-AATTTGATGTGT-CGCTCGGCACCTTAC
ZX136Dai25794ceriporia GACTAGGGATCGGGCGAACTC-AATTTGATGTGT-CGCTCGGCACCTTAC
892Dai13400 GACTAGGGATCGGGCGAACTC-AATTTGATGTGT-CGCTCGGCACCTTAC
RLG7163Leptoporus_mollis ---
Dai21062Leptoporus_mollis ---
Dai20182Leptoporus_submollis ---
Cui18379Leptoporus_submollis ---
Wu1209_46Resiniporus_pseudogil ---
BRNM710169Resiniporus_resinasc ---
Dai14516Bjerkandera_adusta GACTAGGGATCGGGCGAACTC-AATTTGATGTGT-CGCTCGGCACCTTAC
Dai21100Bjerkandera_fumosa GACTAGGGATCGGGCGAACTC-AATTTGATGTGT-CGCTCGGCACCTTAC
Miettinen16854Ceraceomyces_sp ---
Dai10477C_spissa ---
855Dai16831 GACTAGGGATCGGGCGAACTC-AATTTGATGTGT-CGCTCGGCACCTTAC
882Cui11282 GACTAGGGATCGGGCGAACTC-AATTTGATGTGT-CGCTCGGCACCTTAC
Dai24566 GACTAGGGATCGGGCGAACTC-AATTTGATGTGT-CGCTCGGCACCTTAC
Yuan5965 ---
Dai3204 ---
1194CUI9985 ---

Dai15205_Ceriporia_albomellea -------
Dai15223_Ceriporia_albomellea -------
Li1780_Ceriporia_variegata -------
Dai19791_Ceriporia_variegata -GAGAAA
Dai19886 -GAGAAA

Dai10833_Ceriporia_crassitunic	-------
CHWC1506_46Meruliopsis_crassit	-------
Dai9995_Ceriporia_crassitunica	-------
Wu1209_58_Meruliopsis_parvispo	-------
CHWC1505_129_Meruliopsis_parvi	-------
Dai21944	-GAGAAA
830Dai18640A	-GAGAAA
GC1704_60_Meruliopsis_taxicola	-------
Dai22625	-GAGAAA
Dai22636	-GAGAAA
Dai21878	-GAGAAA
1169Dai17248	-GAGAAA
Wu1708_43_Meruliopsis_leptocys	-------
Li1011	-------
ZX95Dai25742Meruliopsis_leptoc	-GAGAAA
WCG1306Dai24733	-------
LXL99Dai25816	-GAGAAA
WCG1559Dai26052Meruliopsis	-GAGAAA
He7477	-------
HLX243Dai26217	-GAGAAA
RussiaMW673659Meruliopsis_fagi	-------
FD278	-------
Dai10226_Ceriporia_tarda	-------
LE247365	-------
Dai8173_Meruliopsis_nanlingens	-GAGAAA
860Dai17172	-GAGAAA
879Dai13414	-GAGAAA
Li_1704_Meruliopsis_pseudocyst	-------
833Dai18405	-GAGAAA
HHB_10729_Meruliopsis_albostra	-------
Cui6878_Ceriporia_pseudocystid	-------
869Dai14737	-GAGAAA
876Cui11626	-GAGAAA
1199WEI3388	-------
776308_Meruliopsis_cystidiata	-------
ICN139059_Meruliopsis_cystidia	-------
HHB15692Ceraceomyces_serpens	-------
HHB_15629_Sp_Ceriporiopsis_ane	-------
AJ185Trametopsis_cervina	-------
FD9Irpex_lacteus	-------
908Dai11230	-GAGAAA
FP55521TEmmia_lacerata	-------
PBU0048Ceriporia_cystidiata	-------
MZ340C_lacerataT	-------

Dai21940 -GAGAAA
847Dai16433 -GAGAAA
MarcinEmmia_latemarginatus -------
Meijer3729Hydnopolyporus_fimbr -------
RLG13408Phanerochaete_sp -------
WHC1381Flavodon_flavus -------
GB1833Phlebia_albida -------
T407Phlebia_nitidula -------
HHB6988Phanerochaete_exilis -------
HHB8509Phanerochaetella_xeroph -------
PBU0051Macrohyporia_dictyopora -------
HHB11463Phanerochaete_sp -------
FP102382Byssomerulius_corium -------
FP102165Efibula_americana -------
Murdoch90Ceriporia_torpida -------
Rivoire4413_Ceriporia_purpurea -------
Kout_18_Ceriporia_triumphalis -------
Rivoire3701_Ceriporia_bresadol -------
VS4018 -------
Ryvarden21832_Ceriporia_manzan -------
Dai24539 -GAGAAA
Dai24541 -GAGAAA
JV1105_12_Ceriporia_occidental -------
VS8558Ceriporia_occidentalis -------
Dai22445 -GAGAAA
846Dai16368 -GAGAAA
Dai17951_Ceriporia_aurantiocar -------
Miettinen_11701C_viridans -------
JV0105_10Ceriporia_aurantiocar -------
Yuan5702C_viridans -------
858Dai17003 -GAGAAA
Yuan2747_Ceriporia_viridans -------
Yuan2744C_viridans -------
Li1046C_viridans -------
865C_sinoviridans -GAGAAA
871Dai15062 -------
Dai7642_Ceriporia_humilis AGAGAAA
Spirin4706_Ceriporia_humilis -------
Spirin4944_Ceriporia_sericea -------
WCG1547Dai26044ceriporia -GAGAAA
ZZW1558Dai27086 -GAGAAA
Miettinen14381_Ceriporia_mpuri -------
Miettinen15492_2_Ceriporia_sor -------
He6687 -GAGAAA

ZH53Dai24426 -GAGAAA
Vlasak0808_30_Ceriporia_punica -------
887Dai13376 -GAGAAA
WCG1443Dai24998 -GAGAAA
0108_6Ceriporia_spissa -------
Dai19164 -GAGAAA
Dai17937_Ceriporia_bubalinomar -------
903Dai12113 -GAGAAA
LZB929Dai25079 -GAGAAA
LX45Dai26988 -------
LX43Dai26986 -GAGAAA
Dai7759Ceriporia -------
Cui8012_Ceriporia_viridans -------
GC1704_54Ceriporia_viridans -------
Dai23392 -GAGAAA
WCG1585Dai26113Ceriproia -GAGAAA
Dai18675C_eucalypti -------
Dai22034 -GAGAAA
JV1008_41JTardaFLORIDAKeys -------
Rivoire1161_Ceriporia_pierii -------
Dai23499C_pierii -GAGAAA
Dai23500 -GAGAAA
841Dai15899 -GAGAAA
842Dai15904 -GAGAAA
LZB1066xinjiang -------
LZB1065xinjiang -------
851Dai16779 -GAGAAA
RMJ119sp_Candelabrochaete_sept -------
RLG9759spCandelabrochaete_sept -------
RLG10478Phanerochaete_allantos -------
Dai19118_Ceriporia_spissa -GAGAAA
Dai18486A -GAGAAA
WEI17_024_Ceriporia_mellita -------
GC1508_71Ceriporia_mellita -------
GC1608_7_Ceriporia_mellita -------
ZZW1557Dai27085 -------
ZZW1554Dai27083 -GAGAAA
Dai8168 -------
BR4865C_mellita -------
MEL2382688Ceriporia_sp -------
Dai8110 -------
Cui8097 -------
909Cui6740 -------
W1258Dai24695 -GAGAAA

JV0110_26_Ceriporia_griseoviol -------
896Dai13202 -GAGAAA
LWY393Dai27053C_griseoviolasce -GAGAAA
LWY394DAI27054 -GAGAAA
FP135015G_pannocinctus -------
L15726SpG_pannocinctus -------
Dai22221 -------
Dai22633 -------
Dai23260 -------
Dai23626 -------
Dai16238G_citrinoalbus -GAGAAA
1175Dai15293 -GAGAAA
Dai19547 -GAGAAA
918063G_africanus -------
918572G_africanus -------
Dai18536A -------
1164Cui17922 -GAGAAA
Dai22225 -------
1163Dai20655 -GAGAAA
Yuan4397G_hainanensis -------
1176Dai15268 -GAGAAA
1177Dai15259 -GAGAAA
BZ2896G_thelephoroides -------
1166JV1808_26 -GAGAAA
Miettinen16992Hapalopilus_ochr -------
GC1708_338_Ceriporia_arbuscula -------
WCG1555Dai26107Ceriporia -GAGAAA
GC1708_340_Ceriporia_arbuscula -------
WCG1556Dai26109Ceriporia -GAGAAA
883Cui11291 -GAGAAA
HLX320Dai26805 -GAGAAA
WCG1266Dai24678A -GAGAAA
Dai6090_Ceriporia_sulphuricolo -------
RLG_11354_Ceriproia_reticulata -------
ZZW1543Dai27072 -GAGAAA
Li1316_Ceriporia_reticulata -GAGAAA
KHL11981Ceriporia_reticulata -------
FP110343sp_Candelabrochaete_la -------
Li1045_Ceriporia_reticulata -GAGAAA
ZX136Dai25794ceriporia -GAGAAA
892Dai13400 -GAGAAA
RLG7163Leptoporus_mollis -------
Dai21062Leptoporus_mollis -------
Dai20182Leptoporus_submollis -------

Cui18379Leptoporus_submollis	-------
Wu1209_46Resiniporus_pseudogil	-------
BRNM710169Resiniporus_resinasc	-------
Dai14516Bjerkandera_adusta	-GAGAAA
Dai21100Bjerkandera_fumosa	-GAGAAA
Miettinen16854Ceraceomyces_sp	-------
Dai10477C_spissa	-------
855Dai16831	-GAGAAA
882Cui11282	-GAGAAA
Dai24566	-GAGAAA
Yuan5965	-------
Dai3204	-------
1194CUI9985	-------
