# Supplementary material for: ﻿Studies in phylogeny and divergence times of Irpicaceae and Meripilaceae (Polyporales, Basidiomycota), with an emphasis on Ceriporia and Meripilus including ten new species
Source: IMA Fungus. 2025 Oct 15;16:e161336. doi: 10.3897/imafungus.16.161336 (PMC12547424; doi:10.3897/imafungus.16.161336)
Supplement: Supplementary material 2 — Alignment for Fig. 2 [file imafungus-16-e161336-s002.pdf]

matrix
JV1310_11SanguinolentusCernys TGAATTC--CAATTGGATG-AGA-TAGCTGCT-
GGCTTTC----------
MJ39_00_SK TGAATTC--CAATTGGATG-AGA-TAGCTGCT-
GGCTTTC----------
MJ111_04_CZ TGAATTC--CAATTGGATG-AGA-TAGCTGCT-
GGCTTTC----------
JV1610_BOKYsmrk TGAATTC---AATTGGATG-AGA-TAGCTGCT-
GGCTTTC----------
Dai21030 TGAATTC--CAATTGGATG-AGA-TAGCTGCT-
GGCTTTC----------
Dai20976P_furcatus TG-ATTC--CAATTGGATG-AGA-TAGCTGCT-
GGCTTTC----------
Dai2105 TGAATTC--CAATTGGATG-AGA-TAGCTGCT-
GGCTTTC----------
Dai2544 TGAATTC--CAATTGGATG-AGA-TAGCTGCT-
GGCTTTC----------
Dai11313 TGAATTC--CAATTGGATG-AGA-TAGCTGCT-
GGCTTTC----------
WCG1611Dai26167 TGAATTC--CAATTGGATG-AGA-TAGCTGCT-
GGCTTTC----------
WCG1518Dai25999Physisporinus TGAATTC--C-ATTGGATG-AGA-TAGCTGCT-
GGCTTTC----------
TAA15097 TTGATTC--CAATTGGATG-AGA-TAGCTGCT-
GGCTTTC----------
JV8909_19_CZ TGAATTC--CAATTGGATG-AGA-TAGCTGCT-
GGCTTTC----------
JV1310_15_P_sanguinolentus2_CZ TGAATTC--CAATTGGATG-AGA-TAGCTGCT-
GGCTTTC----------
MJ53_02_CZ TGAATTC--CAATTGGATG-AGA-TAGCTGCT-
GGCTTTC----------
CLZhao21647P_yunnanensis TGAATTC--CAATTGGATG-AGA-TAGCTGCT-
GGCTTTC----------
CLZhao21583P_yunnanensis TGAATTC--CAATTGGATG-AGA-TAGCTGCT-
GGCTTTC----------
Dai22272 TGAATCC--CAATTGGATG-AGA-TAGCTGCT-
GGCTTTC----------
Dai22279 TGAATCC--CAATTGGATG-AGA-TAGCTGCT-
GGCTTTC----------
MJ332_94_CZ TGAATTC--CAACTGGGTG-AGA-TTGCTGCT-
GGCTCTC----------
MJ642_94_CZ_Expallescens TGAATTC--CAACTGGGTG-AGA-TTGCTGCT-
GGCTCTC----------
Dai21060P_vinctus TGAATTC--CAACTGGGTG-AGA-TTGCTGCT-

GGCTCTC----------
JV0511_23LRP_pouzarii TGAATTC--CAACTGGGTG-AGA-TTGCTGCT-
GGCTCTC----------
JQ409462_R_pouzarii_PRM899856_ TGAATTC--CAACTGGGTG-AGA-TTGCTGCT-
GGCTCTC----------
JV0308_66_WA TGAATTC--CAACTGGRTG-AGA-TTGCTGCT-
GGCTCTC----------
JV0309_45_WA_USA TGAATTC--CAACTGGRTG-AGA-TTGCTGCT-
GGCTCTC----------
JV0709_83_CA_USA TGAATTC--CAACTGGRTG-AGA-TTGCTGCT-
GGCTCTC----------
Dai21043P_pouzarii TGA-TTC--CAACTGGGTG-AGA-TTGCTGCT-
GGCTCTC----------
MJ144_95_CZ TGAATTC--CAATTGGGTG-AGA-TTGCTGCT-
GGCTCTC----------
JV0909_3_CZ TGAATTC--CAATTGGGTG-AGA-TTGCTGCT-
GGCTCTC----------
JV0609_1_K TGAATTC--CAATTGGGTG-AGA-TTGCTGCT-
GGCTCTC----------
Dai20396Physisporinus_castanop TGAATTC--CAATTGGGTG-AGA-TTGCTGCT-
GGCTCTC----------
Dai20397Physisporinus_castanop TGAATTC--CAATTGGGTG-AGA-TTGCTGCT-
GGCTCTC----------
MJ19_09_SK_Abies TGAATTC--CAACTGGGTG-AGA-TTGATGCT-
GGCTCTC----------
JV0509_40_J_TN_USA_Betula TGAATTC--CAACTGGGTG-AGA-TTGATGCT-
GGCTCTC----------
JV0808_33crocatus_PA_USAlist TGAATTC--CAACTGGGTG-AGA-TTGATGCT-
GGCTCTC----------
DLL2009_061P_crocatus TGAATTC--CAACTGGGTG-AGA-TTGATGCT-
GGCTCTC----------
Dai12800P_subcrocatus -GGCTTCGATCACTGGGTG-AGA-TTGATGCT-
GGCTCTC----------
Dai15917P_subcrocatus GGGCTTCGATCACTGGGTG-AGA-TTGATGCT-
GGCTCTC----------
Cui16620 TGAATTC--CAACTGGGTG-AGA-TTGCTGCT-
GGCTTTC----------
HCFC1088Meripilus_stillicidior ------------------------TTGCTGCT-GGCTTTC----------
MCW590Meripilus_obsrcurus TGAATTC--CAACTGGGTG-AGA-TTGCTGCT-
GGCTCTC----------
MCW722Meripilus_obsrcurus TGAATTC--CAACTGGGTG-AGA-TTGCTGCT-
GGCTCTC----------
Cui9381P_tibeticus TGAATTC--CAACTGGGTG-AGA-TTGCTGCT-
GGCTCTC----------

Cui9588P_tibeticus TTGATTC--CAACTGGGTG-AGA-TTGCTGCT-
GGCTCTC----------
Va2_Beneschova TGAATTC--CAACTGGGTG-AGA-TTGCTGCT-
GGCTCTC----------
CWU3874_Ukraine_Alnus TGAATTC--CAACTGGGTG-AGA-TTGCTGCT-
GGCTCTC----------
WCG1293Dai24718Physisporinus TGAATTC--CAACTGGATG-AGA-TTGCTGCT-
GGCTTTC----------
WCG1268Dai24682A TGAATTC--C-AACGGATG-AGA-TTGCTGCT-
GGCTTTT----------
WCG1269Dai24683A TGAATTC--C-AACGGATG-AGA-TTGCTGCT-
GGCTTTT----------
WCG1279Dai24694A TGAATTC--CAAACGGATG-AGA-TTGCTGCT-
GGCTTTT----------
Dai16971 TGAATTC--CAAACGGATG-AGA-TTGCTGCT-
GGCTTTT----------
ZQY1043Dai26696 TGAATTC--CAAACGGATG-AGA-TTGCTGCT-
GGCTTTT----------
Doll880 TGAATTC--AAACTGGATG-TGA-TAGCTGCT-
GGCTTTC----------
Doll1000 TGAATTC-AAAACTGGATG-TGA-TAGCTGCT-
GGCTTTC----------
1DAI18529 TGAATTC--AAATTGGATG-AGA-TTGCTGCT-
GGCTTCC----------
Dai19535 TGAATTC--CAACTGGGTGCGGA-TTGTTGCT-
GGCTTTC----------
1704_79_hnedyVillaLaPaz TGAATTC--CACTTGGTTG-AGA-TTGCTGCT-
GGCCTTT----------
F2061 TGA--TT--CACTTGGGTG-AGA-TTGCTGCT-
GGCCTTT----------
1DAI18268 TGAATTC--CACTTGGGTG-AGA-TTGCTGCT-
GGCCTCT----------
1DAI18540A TGAATTC--CATTTGGGTG-AGA-TTGCTGCT-
GGCCTCT----------
Dai17695 TGAATTC--CACTTGGGTG-AGA-TTGCTGCT-
GGCCTCT----------
LKY18Dai26373 TGAATTC--CACTTGGGTG-AGA-TTGCTGCT-
GGCCTCT----------
Dai17839P_sulphureus TGAGTTC--CAATCGGGTG-AGA-TTGTTGCT-
GGCCTCT---------T
Dai17841P_sulphureus TGAGTTC--CAATCGGGTG-AGA-TTGTTGCT-
GGCCTCT---------T
Dai19877P_roseus TGA--TT--CCACCGGGCG-AGA-TTGTTGCT-
GGCCTTT---------C

1508_18_1_Kout TGAATTC--CAACTGGGTG-AGG-TTGTTGCT-GGTCTTTT-------AT
KP859303_R_vinctus_RP185_BRAZI TGAATTC--CAACTGGGTG-AGG-TTGTTGCT-GGTCTTTT-------AT
JK1807_15Rigidoporus_sp_Puerto TGAATTC--CAACTGGGTG-AGG-TTGTTGCT-GGTCTTTT-------AT
JV0509_47_J_TN_USA TGAATTC--CAACCGGGTG-AGG-TTG-TGCT-GGCCCTC--------AG
JV0709_188 TGAATTC--CAACCGGGTG-AGG-TTG-TGCT-GGCCCTC--------AG
JV0509_127_PA_USA TGAATTC--CAACCGGGTG-AGG-TTG-TGCT-GGCCCTC--------AG
JV1009_59_NJ_USA TGAATTC--CAACCGGGTG-AGG-TTG-TGCT-GGCCCTC--------AG
Dai15497P_crataegi TGAATTC--CAACCGGGTG-AGG-TTG-TGCT-GGCCCTC--------AC
Dai15499P_crataegi TGAATTC--CAACCGGGTG-AGG-TTG-TGCT-GGCCCTC--------AC
Cui3266P_cinereus TGAATTC--CAATCGGGTG-AGG-TTG-TGCT-GGCTCTCTC------GG
WCG1256Dai24690 TGAATTC--CAATCGGGTG-AGG-TTG-TGCT-GGCTCTCTC------GG
1DAI17581 --
WCG1255Dai24688 TGAATTC--CAATCGGGTG-AGG-TTG-TGCT-GGCTCTCTC------GG
Dai22427 TGATTCC----ATCGGGTG-AGG-TTG-TGCT-GGCTCTCTC------GG
MV690Meripilus_concrescens TGAATTC--CAATCGGGTG-AGG-TTG-TGCT-GGCTCTCTC------GG
MV513Meripilus_galapagensis TGAATTC--CAATCGGGTG-AGG-TTG-TGCT-GGCTCTCTC-----GGG
Dai19793 TGAATTC--CAACCGGGTG-AGG-TTG-TGCT-GGCCCTT--------AG
OQ553780P_tamilnaduensis TGAATCC--CAA--GGGTG-AGG-TTG-TGCT-GGCTCTTGT-----TAG
OQ553779P_tamilnaduensis TGAATCC--CAA--GGGTG-AGG-TTG-TGCT-GGCTCTTGT-----TAG
A164FB3Meripilus_giganteus TGAATCC--CAATTGGGTG-AGG-TTG-TGCT-GGCTCTTGT-----TAG
JV1407_36_Vinctus_Meandrica TGAATCA--TAGT-------AGG-TTG-TGCT-GGCTCTAT-------CT
1807_3K_Rigidoporus_PuertoRico TGAATCA--TAGT-------AGG-TTG-TGCT-GGCTCTAT-------CT
Cui16903P_vinctus TGAATCA--TAGT-------AGG-TTG-TGCT-GGCTCTAT---

----CT
JV1008_18R_Lineatus TGAATTC--CAATTGGGTC-AGG-TTG-TGCT-
GGCTGAA----------
JV1407_37_1_Vinctus_Carara TGAATTC--CAATTGGGTC-AGG-TTG-TGCT-
GGCTGAA----------
Dai17986P_lineatus TGAATTC--CAATTGGGTC-AGG-TTG-TGCT-
GGCTGAA----------
Dai18281 TGAATTC--CAATTGGGTC-AGG-TTG-TGCT-
GGCTGAA----------
1DAI19796 AGAATTC--CAATTGGGTC-AGG-TTG-TGCT-
GGCTAAA----------
ZQY797Dai25241 AGAATTC--CAATTGGGTC-AGG-TTG-TGCT-
GGCTAAA----------
WCG1289Dai24711 --GATTC--CAATTGGGTC-AGG-TTG-
TGCTCGGCTGAG----------
Dai22598 AGAATTC--CAATTGGGTC-AGG-TTG-TGCT-
GGCTGAA----------
Dai20523 AGAATTC--C-ATTGGGTC-AGG-TTG-TGCT-
GGCTGAA----------
Dai17885 AGAATTC--CAATTGGGTC-AGG-TTG-TGCT-
GGCTGAA----------
Dai17553 AGAATTC--CAATTGGGTC-AGG-TTG-TGCT-
GGCTGAA----------
Dai19639 AGAATTC--CAATTGGGTC-AGG-TTG-TGCT-
GGCTGAA----------
JV0110_48_CZ TGAATTT--AAACTGGGTG-AGG-TTGCTGCT-
GGCTCCTTC-----GC-
MJ129_04 TGAATTT--AAACTGGGTG-AGG-TTGCTGCT-
GGCTCCTTC-----GC-
Cui10340P_eminens TGAATTT--AAACTGGGTG-AGG-TTGCTGCT-
GGCTCCTTC-----GC-
Cui10341P_eminens TTGATTT--AAACTGGGTG-AGG-TTGCTGCT-
GGCTCCTTC-----GC-
Dai12685P_eminens TGAATTT--AAACTGGGTG-AGG-TTGCTGCT-
GGCTCCTTC-----GC-
Miettinen_13591Rigidoporus_und ----------AACTGGGTG-AGG-TTGCTGCT-GGCTCCTTC--
---GC-
Dai20868 TGATTT---AAACTGGGTG-AGG-TTGCTGCT-
GGCTCCTTT-----GCG
Dai20832 TGATTT---AAACTGGGTG-AGG-TTGCTGCT-
GGCTCCTTT-----GCG
Dai11400 TGAATTT--AAACTGGGTG-AGG-TTGCTGCT-
GGCTCCTTT-----GCG
Dai22472 TGATTT----AACTGGGTG-AGG-TTGCTGCT-

GGCTCCTTT-----GCG
1CUI10475 TGAATTT--GAACTGGGTG-AGG-TTGCTGCT-GGCTCCTTC-----GT-
1CUI10491 TGAATTT--GAACTGGGTG-AGG-TTGCTGCT-GGCTCCTTC-----GT-
HCFC1095Meripilus_robledoi -----------ATTGGGTG-AGG-TTGCTGCT-GGCTCCTTT-----GN-
MCW702Meripilus_revolubilis TGAATTC-TAAATTGGGTG-AGG-TTGCTGCT-GGCTCCCTT-----GT-
1704_83_zluty_HaciendaBaru TGAATTC--CAACTGGGTG-AAATTTGTTGCT-GGCCTTTT------GTG
Dai9925P_lavendulus TGATTCC--AAACTGGGCG-AAA-TTGTTGCT-GGTCTCT--------CG
Dai13587AP_lavendulus TGATTCC--AAACTGGGCG-AAA-TTGTTGCT-GGTCTCT--------CG
PDD70600P_longicystidius TGAATTC--CAACCGGGCG-AGA-TTGTTGCT-GGTCCCT--------CG
Cui16630 TGAATTC--CAACCGGGCG-AGA-TTGTTGCT-GGTCCCT--------CG
FP_135344Meripilus_giganteus TGAACTCCCAACTTGGGTG-AGG-TTGTTGCT-GGCCCTGAG--------
FP_100460_Sp TGAACTCCCAACTTGGGTG-AGG-TTGTTGCT-GGCCCTGAG--------
CBS421_48Meripilus_giganteus TGAACTCCCAACTTGGGTGAAGG-TTGTTGCT-GGCCCTGAG--------
Cui9203 TGAACTCCCAACTTGGGTG-AGG-TTGTTGCT-GGCCCTGAG--------
Cui9202 TGAACTCCCAACTTGGGTG-AGG-TTGTTGCT-GGCCCTGAG--------
TUFC100564Japan TGAATTCTAAACTTGGGTG-AGG-TTGTTGCT-GGCCCCTTG--------
Russell5913Meripilus_sumstinei TGAATTCCAAACTTGGGTG-AGG-TTGTTGCT-GGCCCCTTG--------
RP215Meripilus_brasiliensis ----------------------------TGCT-GGCTCCTTC-----CCG
RP200Meripilus_brasiliensis ----------CCTTGGGGT-AGA-TTGTTGCT-GGCTCCTTC-----CCG
JV1712_13J_R_vinctus2_LSUPuert --
Dai10503R_hypobrunneus TGAATTT-----TATGGCG-GAA-TTGTAGCT-GGCCCCAA------CC-
Dai10569Rigidoporus_hypobrunne TGAATTT-----TATGGCG-GAA-TTGTAGCT-GGCCCCAA------CC-
1DAI19451 TGAATTT-----TATGGCG-GAA-TTGTAGCT-GGCCCCAA------CC-
CM108bRigidoporus_hypobrunneus TGAATTT-----TATGGCG-GAA-TTGTAGCT-

GGCCCCAA------CC-
1CUI16874 TGAATTT-----TATGGCG-GAA-TTGTAGCT-GGCCCCAA------CC-
FD299Cerrena_unicolor TGAATTT-----TATGGCA-GAG-TTGTAGCT-GGCCCCAA------TC-
KHL_GB_Cerrena_uniclor TGAATTT-----TATGGCA-GAG-TTGTAGCT-GGCCCCAA------TC-
Dai12892Cerrena_albocinnamomea TGAATTT-----TATGGCA-GAG-TTGTAGCT-GGTCCTAA------TC-
Dai12955C_albocinnamomea TGAATTT-----TATGGCA-GAG-TTGTAGCT-GGTCCTAA------TC-
SNUm03110102C_aurantiopora ---------------GGCA-GAG-TTGTAGCT-GGTCCTAA------TC-
NIBRFG0000102423C_aurantiopora ---------------GGCA-GAG-TTGTAGCT-GGTCCTAA------TC-
Dai7359Antrodiella_zonata TGAATTT-----TATGGCA-GAG-TTGTAGCT-GGTCCTAA------TC-
F20080702KCM29C_consors ---------------GGCA-GAG-TTGTAGCT-GGTCCTAA------TC-
F20080208LYW10Cerrena_consors ---------------GGCA-GAG-TTGTAGCT-GGTCCTAA------TC-
Dai7821Antrodiella_zonata ---------------------------TAGCT-GGTCCTAA------TC-
CFMR_DCL04_31Pseudolagarobasid ------------TATGACA-GGG-TTGTAGCT-GGCCCCAA------TC-
VPB197Pseudolagarobasidium_bel TGAATTT-----TATGACA-GGG-TTGTAGCT-GGCCCCAA------TC-
CBS115543Pseudolagarobasidium_ TGAATTT-----TATGACA-GGG-TTGTTGCT-GGCCCTTA------CT-
CBS115544 TGAATTT-----TATGACA-GGG-TTGTTGCT-GGCCCTTA------CT-
Han405Pseudolagarobasidium_bai --TGATC-----TATGAAG-GGG-TTGTAGCT-GGCCTCAA------TTG
Han406Pseudolagarobasidium_bai --TGATC-----TATGAAG-GGG-TTGTAGCT-GGCCTCAA------TTG
MUcc838Spongipellis_delectans TGAATT------TATGACA-AGG-TTGTCGCT-GGCTCTAA------TT-
BRNM686401S_delectans TGAATT------TATGACA-AGG-TTGTCGCT-GGCTCTAA------TT-
OSM_F925S_delectans TGAATT------TATGACA-AGG-TTGTCGCT-GGCTCTAA------TT-
BRNM67093Spongipellis_litschau TGAATT------CATGACA-AGG-TTGTCGCT-GGCCTCAC------AC-
CFMRccFP59199TS_unicolor TGAATT------CATGACA-AGG-TTGTCGCT-GGCCCCAC------AC-

CFMRccFP71791TS_unicolor TGAATT------CATGACA-AGG-TTGTCGCT-
GGCCCCAC------AC-
Dai13845P_lischaueri TGAATT------CATGACA-AGG-TTGTCGCT-GGCCCCAC-
-----AC-
Dai20266P_lischaueri TGAATT------CATGACA-AGG-TTGTCGCT-GGCCCCAC-
-----AC-
CFMR_HHB11240Radulodon_america TGATTG------------G-GAG-ACTGTGCT-GGCACTAT-
-----CT-
RLG6350Radulodon_americanus TGATTG------------G-GAG-ACTGTGCT-GGCACTAT----
--CT-
KY415963Radulodon_erikssonii TGATTG------------G-GAG-ACTGTGCT-GGCACTAT------
CT-
HHB9567spRadulodon_casearius TGATTG------------G-GAG-ACTGTGCT-GGCACTAT-----
-CT-
KRT_Iso_26Radulodon_casearius TGATTG------------G-GAG-ACTGTGCT-GGCACTAT------
CT-
CBS126044Radulodon_erikssonii TGATTG------------G-GAG-ACTGTGCT-GGCACTAT------
CT-
He6183YUNNANENSIS TGATTG------------G-GAG-ACTGTGCT-
GGCACTAT------CT-
Cui17979YUNNANENSIS TGATTG------------G-GAG-ACTGTGCT-
GGCACTAT------CT-
Miettinen2091Junghuhnia_fimbri ---------------AGTG-GGG-TTGTAGCT-GGCCTTTC------
ATC
KHL12316S_tenue TGAAAT-------AGAGTG-GGG-TTGTAGCT-
GGCCTTTC------ATC
PRM846564S_pachyodon TGAATT------TATGACA-GGG-TTGTAGCT-
GGCCTCAA------TTG
SP_Lgt_S_pachyodon TGAATTT-----TATGACA-GGG-TTGTAGCT-
GGCCTCAA------TTG
Ryvarden44669Tyromyces_xuchile TGAATT------TATGACA-GGG-TTGTAGCT-
GGCCTCAA------TCG
PW17_171sinuosus --
W53Dai12234 TGAA-TT-----TATGACA-AGG-TTGTAGCT-
GGCTCTAAAT--ATTGG
HHB4100SpAntella_americana TGAATT-------TGGATG-GAG-TTGTAGCT-
GGTCTCAG----AAATG
W3Dai20901spumeus TGAATTT--CAATGTGTGG-GGGCTTGCTGCT-
GGTTCTCTCTCCCGGGA
He6736 TGAATTT--CAATGTGGTG-GGGCTTGCTGCT-
GGTTCTCTCTCCCGGGA
BRNM734877S_spumeus CGAATTT--CAATGTG-TG-GGGCTTGCTGCT-
GGTCCTCTCTTCCGGGA
BRNM712630S_spumeus CGAATTT--CAATGTG-TG-GGGCTTGCTGCT-

GGTTCTCTCTYCCGGGA
Dai1723Loweomyces_sibiricus TGAATTT--CAACGCG-TG-GGATTTGCTGCT-
GGTTCTCTCTCCCGGGA
W54Cui10009 TGAATTT--CAATGTG-TG-GGGCTTGCTGCT-
GGTCCTCTCTTTCGGGA
W1Dai20899 CGAATTT--CAATGTG-TG-GGGCTTGCTGCT-
GGTCCTCTCTTTTGGGA
HHB13445Trametes_ochracea CGAGTTTTGAAAC-------GGG-TTGTAGCT-
GGCCTTCCG--------
Dai16222 TGA-----TGAACTGAGTG-AGG-TTGTAGCT-
GGCTTCAGT---CATTG
Dai16240 TGA-----TGAACTGAGTG-AGG-TTGTAGCT-
GGCTTCAGT---CATTG

JV1310_11SanguinolentusCernys GG-GCATGTGCACA-TCTTGTTCA--------TTTACCT--
CTCACCTCT
MJ39_00_SK GG-GCATGTGCGCA-TCTTGTTCA--------TTTACCT-
-CTCACCTCT
MJ111_04_CZ GG-GCATGTGCRCA-TCTTGTTCA--------TTTACCT--
CTCACCTCT
JV1610_BOKYsmrk GG-GCATGTGCGCA-TCTTGTTCA--------
TTTACCT--CTCACCTCT
Dai21030 GG-GCATGTGCGCA-TCTTGTTCA--------TTTACCT--
CTCACCTCT
Dai20976P_furcatus GG-GCATGTGCGCA-TCTTGTTCA--------TTTACCT--
CTCACCTCT
Dai2105 GG-GCATGTGCACA-TCTTGTTCA--------TTTACCT--
CTCACCTCT
Dai2544 GG-GCATGTGCACA-TCTTGTTCA--------TTTACCT--
CTCACCTCT
Dai11313 GG-GCATGTGCGCA-TCTTGTTCA--------TTTACCT--
CTCACCTCT
WCG1611Dai26167 GG-GCATGTGCGCA-TCTTGTTCA--------TTTACCT-
-CTCACCTCT
WCG1518Dai25999Physisporinus GG-GCATGTGCGCA-TCTTGTTCA--------TTTACCT--
CTCACCTCT
TAA15097 GG-GCATGTGCGCA-TCTTGTTCA--------TTTACCT--
CCCACCTCT
JV8909_19_CZ GG-GCATGTGCGCA-TCTCGTTCA--------TTTGCCT-
-CTCACCTCT
JV1310_15_P_sanguinolentus2_CZ GG-GCATGTGCGCA-TCTCGTTCA--------TTTGCCT--
CTCACCTCT
MJ53_02_CZ GG-GCATGTGCGCA-TCTCGTTCA--------TTTGCCT-
-CTCACCTCT

CLZhao21647P_yunnanensis GG-GCATGTGCGCA-TCTTGTTCA--------TTTGCCT--
CTCACCTCT
CLZhao21583P_yunnanensis GG-GCATGTGCGCA-TCTTGTTCA--------TTTGCCT--
CTCACCTCT
Dai22272 GA-GCATGTGCGCA-TCTTGTTCA--------TTTACCT--
CTCACCTCT
Dai22279 GAGGCATGTGCGCA-TCTTGTTCA--------TTTACCT--
CTCACCTCT
MJ332_94_CZ GG-GCATGTGCACA-TCTCATTCA--------TTTACCT--
CTCACCTCT
MJ642_94_CZ_Expallescens GG-GCATGTGCACA-TCTCATTCA--------TTTACCT--
CTCACCTCT
Dai21060P_vinctus GG-GCATGTGCACA-TCTCATTCA--------TTTACCT--
CTCACCTCT
JV0511_23LRP_pouzarii GG-GCATGTGCACA-TCTCATTCA--------TTTACCT--
CTCACCTCT
JQ409462_R_pouzarii_PRM899856_ GG-GCATGTGCACA-TCTCATTCA--------TTTACCT--
CTCACCTCT
JV0308_66_WA GG-GCATGTGCACA-TCTCATTCA--------TTTACCT-
-CTCACCTCT
JV0309_45_WA_USA GG-GCATGTGCACA-TCTCATTCA--------
TTTACCT--CTCACCTCT
JV0709_83_CA_USA GG-GCATGTGCACA-TCTCATTCA--------
TTTACCT--CTCACCTCT
Dai21043P_pouzarii GA-GCATGTGCACA-TCTCATTCA--------TTTACCT--
CTCACCTCT
MJ144_95_CZ GG-GCATGTGCACA-TCTCATTCA--------TTTACCT--
CTCACCTCT
JV0909_3_CZ GG-GCATGTGCACA-TCTCATTCA--------TTTACCT--
CTCACCTCT
JV0609_1_K GG-GCATGTGCACA-TCTCATTCA--------TTTACCT--
CTCACCTCT
Dai20396Physisporinus_castanop GG-GCATGTGCACA-TCTCATTCA--------TTTACCT--
CTCACCTCT
Dai20397Physisporinus_castanop GG-GCATGTGCACA-TCTCATTCA--------TTTACCT--
CTCACCTCT
MJ19_09_SK_Abies GA-GCATGTGCACG-TCTCATTCA--------TTTACCT--
CTCACCTCT
JV0509_40_J_TN_USA_Betula GA-GCATGTGCACG-TCTCATTCA--------TTTACCT--
CTCACCTCT
JV0808_33crocatus_PA_USAlist GA-GCATGTGCACG-TCTCATTCA--------TTTACCT--
CTCACCTCT
DLL2009_061P_crocatus GA-GCATGTGCACG-TCTCATTCA--------TTTACCT--
CTCACCTCT

Dai12800P_subcrocatus GA-GCATGTGCACG-TCTCATTCA--------TTTACCT--
CTCACCTCT
Dai15917P_subcrocatus GA-GCATGTGCACG-TCTCATTCA--------TTTACCT--
CTCACCTCT
Cui16620 GG-GCATGTGCACG-TCTCATTCA--------TTTACCT--
CTCACCTCT
HCFC1088Meripilus_stillicidior GG-GCATGTGCACG-TCTCATTCA--------TTTACCT--
CTCACCTCT
MCW590Meripilus_obsrcurus GG-GCATGTGCACG-TCTCATTCA--------TTTACCT--
CTCACCTCT
MCW722Meripilus_obsrcurus GG-GCATGTGCACG-TCTCATTCA--------TTTACCT--
CTCACCTCT
Cui9381P_tibeticus GG-GCATGTGCACA-TCTCATTCA--------TTTACCT--
CTCACCTCT
Cui9588P_tibeticus GG-GCATGTGCACA-TCTCATTCA--------TTTACCT--
CTCACCTCT
Va2_Beneschova GA-GCATGTGCACA-TCTCATTCA--------TTTACCT--
CTCACCTCT
CWU3874_Ukraine_Alnus GA-GCATGTGCACA-TCTCATTCA--------TTTACCT--
CTCACCTCT
WCG1293Dai24718Physisporinus GG-GCATGTGCGCA-TCTTGTTCA--------TTTACCT--
TTCACCTCT
WCG1268Dai24682A GG-GCATGTGCGCA-TCTCATTCA-------TTCTTAT-
-CTCACCTCT
WCG1269Dai24683A GG-GCATGTGCGCA-TCTCATTCA-------TTCTTAT-
-CTCACCTCT
WCG1279Dai24694A GG-GCATGTGCGCA-TCTCATTCA-------TTCTTAT-
-CTCACCTCT
Dai16971 GG-GCATGTGCGCA-TCTCATTCA--------TTCTTAT--
CTCACCTCT
ZQY1043Dai26696 GG-GCATGTGCGCA-TCTCATTCA--------TTCTTAT--
CTCACCTCT
Doll880 GG-GCATGTGCACA-TCTCATTCA--------TTCATCT--
CATACCTCT
Doll1000 GG-GCATGTGCACA-TCTCATTCA--------TTCATCT--
CATACCTCT
1DAI18529 GA-GCATGTGCACG-TCTCATTCA--------TTCATCT--
CTCACCTCT
Dai19535 GA-GCATGTGCACG-TCTCACTCAT-----AATATCTTC-
-CATACCTCT
1704_79_hnedyVillaLaPaz GG-GCATGTGCGCA-TCTCTCTCA--------TTTACCT--
CATACCTCT
F2061 GG-GCATGTGCGCA-TCTCTCTCA--------TTTACCT--
CATACCTCT

1DAI18268 GG-GCATGTGCACG-TCTCTCTCA--------TTTACCT--
CTCACCTCT
1DAI18540A GG-GCATGTGCACG-TCTCTCTCA--------TTTACCT--
CTCACCTCT
Dai17695 GG-GCATGTGCACA-TCTCTCTCA--------TTTACCT--
CTCACCTCT
LKY18Dai26373 GG-GCATGTGCACG-TCTCTCTCA--------TTTACCT--
CTCACCTCT
Dai17839P_sulphureus TG-GCATGTGCACG-TCTCTCTCG--------TTTTCTT--
CTCACCTCT
Dai17841P_sulphureus TG-GCATGTGCACG-TCTCTCTCG--------TTTTCTT--
CTCACCTCT
Dai19877P_roseus CG-GCATGTGCACA-TTTCTCTCG--------TTGTCTT--
CTCACCTCT
1508_18_1_Kout GG-ACATGTGCACA-TCTCTCTCA--------TTTACCT--
TTCACCTCT
KP859303_R_vinctus_RP185_BRAZI GG-ACATGTGCACA-TCTCTCTCA--------TTTACCT-
-TTCACCTCT
JK1807_15Rigidoporus_sp_Puerto GG-ACATGTGCACA-TCTCTCTCA--------TTTACCT--
TTCACCTCT
JV0509_47_J_TN_USA GG-GCATGTGCACA-CCTCGCTCA------
TCAATATCT--CATACCTCT
JV0709_188 GG-GCATGTGCACA-CCTYGCTCA------
TCAATATCT--CATACCTCT
JV0509_127_PA_USA GG-GCATGTGCACA-CCTTGCTCA------
TCAATATCT--CATACCTCT
JV1009_59_NJ_USA GG-GCATGTGCACA-CCTTGCTCA------
TCAATATCT--CATACCTCT
Dai15497P_crataegi GG-GTATGTGCACG-CCTTGCTCA------TCAACCTCT--
CACACCTCT
Dai15499P_crataegi GG-GTATGTGCACG-CCTTGCTCA------TCAACCTCT--
CACACCTCT
Cui3266P_cinereus GG-GCATGTGCACA-CCTTGCTCA------TTAATACCT--
TCCACCTCT
WCG1256Dai24690 GG-GCATGTGCACA-CCTTGCTCA------
TTAATACCT--TCCACCTCT
1DAI17581 ---------------------------------ATACCT--TCCACCTCT
WCG1255Dai24688 GG-GCATGTGCACA-CCTTGCTCA------
TTAATACCT--TCCACCTCT
Dai22427 GG-GCATGTGCACA-CCTTGCTCA------TTAATACCT-
-TCCACCTCT
MV690Meripilus_concrescens GG-GCATGTGCACA-CCTTGCTCA------TTAATACCT--
TCCACCTCT
MV513Meripilus_galapagensis GG-GCATGTGCACA-TCTTGCTCA------TTAATACCT--

TCCACCTCT
Dai19793 GG-GCATGTGCACG-CCTTGCTCA------TCAATATCT-
---CACCTCT
OQ553780P_tamilnaduensis GA-GCATGTGCACA-CCTTGCTCA------TCAATATTC--
CACACCTCT
OQ553779P_tamilnaduensis GA-GCATGTGCACA-CCTTGCTCA------TCAATATTC--
CACACCTCT
A164FB3Meripilus_giganteus GA-GCATGTGCACA-CCTTGCTCA------TCAATATTC--
CACACCTCT
JV1407_36_Vinctus_Meandrica GA-ACAAGTGCACG--CCTGCTCC--------AAAAYATT-
CAAACCTCT
1807_3K_Rigidoporus_PuertoRico GA-ACAAGTGCACG--CCTGCTCC--------AAAACATT-
CAAACCTCT
Cui16903P_vinctus GA-ACAAGTGCACG--CCTGCTCC--------AAAACATT-
CAAACCTCT
JV1008_18R_Lineatus AA-GCAAGTGCACG--CCTGCTCA--------TAAGCATT-
CAAACCTCT
JV1407_37_1_Vinctus_Carara AA-GCAAGTGCACG--CCTGCTCA--------TAAGCATT-
CAAACCTCT
Dai17986P_lineatus AA-GCAAGTGCACG--CCTGCTCA--------TAAGCATT-
CAAACCTCT
Dai18281 AA-GCAAGTGCACG--CCTGCTCA--------TAAGCATT-
CAAACCTCT
1DAI19796 AA-GCAAGTGCACG--CCTGCTCA--------
TAAGTATT-CAAACCTCT
ZQY797Dai25241 AA-GCAAGTGCACG--CCTGCTCA--------
TAAGTATT-CAAACCTCT
WCG1289Dai24711 AA-GCAAGTGCACG--CCTGCTCA--------
TAAGTATT-CAAACCTCT
Dai22598 AA-GCAAGTGCACG--CCTGCTCA--------TAAGCATT-
CAAACCTCT
Dai20523 AA-GCAAGTGCACG--CCTGCTCA--------TAAGCATT-
CAAACCTCT
Dai17885 AA-GCAAGTGCACG--CCTGCTCA--------TAAGCATT-
CAAACCTCT
Dai17553 AA-GCAAGTGCACG--CCTGCTCA--------TAAGCATT-
CAAACCTCT
Dai19639 AA-GCAAGTGCACG--CCTGCTCA--------TAAGCATT-
CAAACCTCT
JV0110_48_CZ GA-GCATGTGCACA-TCTCACTCA------
TCCAACCTT--CACACCCCT
MJ129_04 GA-GCATGTGCACA-TCTCACTCA------
TCCAACCTT--CACACCCCT
Cui10340P_eminens GA-GCATGTGCACA-TCTCACTCA------TCCAACCTT-

-CACACCCCT
Cui10341P_eminens GA-GCATGTGCACA-TCTCACTCA------TCCAACCTT-
-CACACCCCT
Dai12685P_eminens GA-GCATGTGCACA-TCTCACTCA------TCCAACCTT-
-CACACCCCT
Miettinen_13591Rigidoporus_und GA-GCATGTGCACA-TCTCACTCA------TCCAACCTT--
CACACCCCT
Dai20868 GG-GCATGTGCACA-CCTCACTCA------
TCCAACCTT--CACACCCCT
Dai20832 GG-GCATGTGCACA-CCTCACTCA------
TCCAACCTT--CACACCCCT
Dai11400 GG-GCATGTGCACA-CCTCACTCA------
TCCAACCTT--CACACCCCT
Dai22472 GG-GCATGTGCACA-CCTCACTCA------
TCCAACCTT--CACACCCCT
1CUI10475 GA-GCATGTGCACA-TCTCACTCA------
TCCAACCTT--CACACCCCT
1CUI10491 GA-GCATGTGCACA-TCTCACTCA------
TCCAACCTT--CACACCCCT
HCFC1095Meripilus_robledoi GG-GCATGTGCACA-TCTCACTCAT-----TCCAACCTT--
TACACCCCT
MCW702Meripilus_revolubilis GG-GCATGTGCACA-TCTCACTCAT-----TCCAACCTT--
TACACCCCT
1704_83_zluty_HaciendaBaru AG-GCATGTGCACA-TTTCATCTATA----TAAACCTTT--
TATACCCCT
Dai9925P_lavendulus GG-ACATGTGCACG-TTTCGTTCG------CAAACCTCT--
CATACCCCT
Dai13587AP_lavendulus GG-ACATGTGCACG-TTTCGTTCG------CAAACCTCT-
-CATACCCCT
PDD70600P_longicystidius GG-GCATGTGCACG-TCTTGTCCA------CAAACCTCT--
CATACCCCT
Cui16630 GG-GCATGTGCACG-TCTTGTCCA------
CAAACCTCT--CATACCCCT
FP_135344Meripilus_giganteus GG-GCATGTGCACG-TCTCGCTCAT-----TTTATCTCT--
CACACCCCT
FP_100460_Sp GG-GCATGTGCACG-TCTCGCTCAT-----TTTATCTCT-
-CACACCCCT
CBS421_48Meripilus_giganteus GG-GCATGTGCACG-TCTCGCTCAT-----TTTATCTCT--
CACACCCCT
Cui9203 GG-GCATGTGCACG-TCTCGCTCAT-----TTTATCTCT-
-CACACCCCT
Cui9202 GG-GCATGTGCACG-TCTCGCTCAT-----TTTATCTCT-
-CACACCCCT
TUFC100564Japan GG-GTATGTGCACA-TCTCGCTCA------TTCATTTCT-

-TATACCCCT
Russell5913Meripilus_sumstinei GG-GTATGTGCACA-TCTTGCTCA------TTCATTCCT--TATACCTCT
RP215Meripilus_brasiliensis GG-GTATGTGCACG-TCTTGCTCA------TTCATTTCT--TATACCTCT
RP200Meripilus_brasiliensis GG-GTATGTGCACG-TCTTGCTCA------TTCATTTCT--TATACCTCT
JV1712_13J_R_vinctus2_LSUPuert --
Dai10503R_hypobrunneus GG-GCATGTGCACA-TTCTGTTCAT-----TCCATTCTC-ATACACCTCT
Dai10569Rigidoporus_hypobrunne GG-GCATGTGCACA-TTCTGTTCAT-----TCCATTCTC-ATACACCTCT
1DAI19451 GG-GCATGTGCACA-TTCTGTTCAT-----TCCATTCTC-ATACACCTCT
CM108bRigidoporus_hypobrunneus GG-GCATGTGCACA-TTCTGTTCAT-----TCCATTCTC-ATACACCTCT
1CUI16874 GG-GCATGTGCACA-TTCTGTTCAT-----TCCATTCTC-ATACACCTCT
FD299Cerrena_unicolor GG-GTATGTGCACA-CTTTGTTCAT-----TCCATTCTC-ATACACCTCT
KHL_GB_Cerrena_uniclor GG-GTATGTGCACA-CTTTGTTCAT-----TCCATTCTC-ATACACCTCT
Dai12892Cerrena_albocinnamomea GG-ACATGTGCACA-CTTTGTTCAT-----TCCAATTCTCATATACCTCT
Dai12955C_albocinnamomea GG-ACATGTGCACA-CTTTGTTCAT-----TCCAATTCTCATATACCTCT
SNUm03110102C_aurantiopora GG-ACATGTGCACA-CTTTGTTCAT-----TCCAATTCTCATATACCTCT
NIBRFG0000102423C_aurantiopora GG-ACATGTGCACA-CTTTGTTCAT-----TCCAATTCTCATATACCTCT
Dai7359Antrodiella_zonata GG-ACATGTGCACA-CTTTGTTCAT-----TCCAATTCTCATACACCTCT
F20080702KCM29C_consors GG-ACATGTGCACA-CTTTGTTCAT-----TCCAATTCTCATACACCTCT
F20080208LYW10Cerrena_consors GG-ACATGTGCACA-CTTTGTTCAT-----TCCAATTCTCATACACCTCT
Dai7821Antrodiella_zonata GG-ACATGTGCACA-CTTTGTTCAT-----TCCAATTCTCATACACCTCT
CFMR_DCL04_31Pseudolagarobasid GG-GCATGTGCACACCTTTGTACAT-----TCCAATTCT--TACACCTCT
VPB197Pseudolagarobasidium_bel GG-GCATGTGCACACCTTTGTACAT-----TCCAATTCT--TACACCTCT
CBS115543Pseudolagarobasidium_ GG-GCATGTGCACA-CTTTGTACAT-----TCCAATTCT--TACACCTCT

CBS115544 GG-GCATGTGCACA-CTTTGTACAT-----
TCCAATTCT--TACACCTCT
Han405Pseudolagarobasidium_bai GG-GCATGTGCACGCCTTTGTACAT-----TCCAATTCT--
TACACCTCT
Han406Pseudolagarobasidium_bai GG-GCATGTGCACGCCTTTGTACAT-----TCCAATTCT--
TACACCTCT
MUcc838Spongipellis_delectans GG-GCATGTGCACG-CCTTGCTCATT---CTCCAATTCT--
TACACCTCT
BRNM686401S_delectans GG-GCATGTGCACG-CCTTGCTCATT---
CTCCAATTCT--TACACCTCT
OSM_F925S_delectans GG-GCATGTGCACG-CCTTGCTCATT---
CTCCAATTCT--TACACCTCT
BRNM67093Spongipellis_litschau GG-GCATGTGCACA-CCTTGCTCAT-----TCCAATTCT--
TACACCTCT
CFMRccFP59199TS_unicolor GG-GCATGTGCACG-CCTTGCTCAT-----
TCCAATTCT--TACACCTCT
CFMRccFP71791TS_unicolor GG-GCATGTGCACG-CCTTGCTCAT-----
TCCAATTCT--TACACTTCT
Dai13845P_lischaueri GG-GCATGTGCACA-CTTTGCTCAT-----TCCAATTCT--
TACACCTCT
Dai20266P_lischaueri GG-GCATGTGCACA-CCTTGCTCAT-----TCCAATTCT--
TACACCTCT
CFMR_HHB11240Radulodon_america GG-GTATGTGCACGTCTCTATAAAC-----
TCCAATTTC--TACACCCCT
RLG6350Radulodon_americanus GG-GTATGTGCACGTCTCTATAAAC-----
TCCAATTTC--TACACCCCT
KY415963Radulodon_erikssonii GG-GTATGTGCACGTCTCTATAAAC-----TCCAATTTC--
TACACCCCT
HHB9567spRadulodon_casearius GG-GTATGTGCACGTCTCTATAAAC-----TCCAATTTC-
-TACACCCCT
KRT_Iso_26Radulodon_casearius GG-GTATGTGCACGTCTCTATAAAC-----TCCAATTTC--
TACACCCCT
CBS126044Radulodon_erikssonii GG-GTATGTGCACGTCTCTATAAAC-----TCCAATTTC--
TACACCCCT
He6183YUNNANENSIS GG-GTATGTGCACGTCTCTATAAAC-----
TCCAATTTC--TACACCCCT
Cui17979YUNNANENSIS GG-GTATGTGCACGTCTCTATAAAC-----
TCCAATTTC--TACACCCCT
Miettinen2091Junghuhnia_fimbri GG-GCATGTGCACA-CTTTATTCG------ACCACCTC---
TACACCTCT
KHL12316S_tenue AG-GCATGTGCACA-CCTCATTCG------
ACCACCTTC--TACACCTCT
PRM846564S_pachyodon GG-GCATGTGCACG-CCTTGCTCAT-----
TCCAACCTTT-TACACCTCT

SP_Lgt_S_pachyodon GG-GCATGTGCACG-CCTTGCTCAT----
TCCAACCTTT-TACACCTCT
Ryvarden44669Tyromyces_xuchile GG-GCATGTGCACG-CCTTGCTCATT----TCCAACCTT-
-TACACCTCT
PW17_171sinuosus ---CCTCT
W53Dai12234 GG-CAATGTGCACG-CCTTGCTCATT---
TTCCAACCTT--TACACCTCT
HHB4100SpAntella_americana GG-GCATGTGCACA-CTCTGTTCA------TTCACCCTT-
-CACACCTCT
W3Dai20901spumeus GG-ACATGTGCACGGTCTCATTCAT----
TCCAACCTTC-AAACCCTCT
He6736 GG-ACATGTGCACGGTCTCATTCAT----
TCCAACCTTC-AAACCCTCT
BRNM734877S_spumeus GG-ACATGTGCACGGTCTCATTCAT----
YCCAACCTTC-AAACCCTCT
BRNM712630S_spumeus GG-ACATGTGCACGGTCTCATTCAT----
TCCAACCTTC-AAACCCTCT
Dai1723Loweomyces_sibiricus GG-ACATGTGCACGGTCTCATGCGT----
TCCAACCTTC-AAACCCTCT
W54Cui10009 GG-ACATGTGCACGGTCTCATTCAT----
TCCAACCTTC-AAACCCTCT
W1Dai20899 GG-ACATGTGCACGGTCTCATTCAT----
TCCAACCTTC-AAACCCTCC
HHB13445Trametes_ochracea AG-GCATGTGCACG-CCCTGCTCATCCAC-----------
TCTACCCCT
Dai16222 GA-GCATGTGCACA-CTTTGCTTGTT--
CTTCCAACCTT--TACACTGTT
Dai16240 GA-GCATGTGCACA-CTTTGCTTGTT--
CTTCCAACCTT--TACACTGTT

JV1310_11SanguinolentusCernys GTGCACTTTTCATGGAACGGTCGG---
TGGTTGTTGGC-TTCACGG---
MJ39_00_SK GTGCACTTTTCATGGAACGGTCGG---
TGGTTGTTGGC-TTCAYGG---
MJ111_04_CZ GTGCACTTTTCATGGAACGGTCGG---
TGGTTGTTGGC-TTCAYGG---
JV1610_BOKYsmrk GTGCACTTTTCATGGAACGGTCGG---
TGGTTGTTGGC-TTCAYGG---
Dai21030 GTGCACTTTTCATGGAACGGTCGG---
TGGTTGTTGGC-TTCATGG---
Dai20976P_furcatus GTGCACTTTTCATGGAACGGTCGG---
TGGTTGTTGGC-TTCATGG---
Dai2105 GTGCACTTTTCATGGAACAATCAG---
TCGTCGTTGGC-TTTATGG---

Dai2544 GTGCACTTTTCATGGAACAATCAG----
TCGTCGTTGGC-TTTATGG---
Dai11313 GTGCACTTTTCATGGAACAATCAG----
TGGTTGTTGGC-TTTATGG---
WCG1611Dai26167 GTGCACTTTTCATGGAACAATCAG----
TGGTTGTTGGC-TTTATGG---
WCG1518Dai25999Physisporinus GTGCACTTTTCATGGAACAATCAG----
TGGTTGTTGGC-TTTATGG---
TAA15097 GTGCACTTTTCATGGAACAATCGG----
TGGTTGTTGGC-TTCATGG---
JV8909_19_CZ GTGCACTTTTCATGGAACAATCGG----
TGGCTGTTGGC-TTCACGG---
JV1310_15_P_sanguinolentus2_CZ GTGCACTTTTCATGGAACAATCGG----
TGGCTGTTGGC-TTCACGG---
MJ53_02_CZ GTGCACTTTTCATGGAACAATCGG----
TGGCTGTTGGC-TTCACGG---
CLZhao21647P_yunnanensis GTGCACTTTTCATGGAACAATCAG----
TGGTTGTTGGC-TTCACGG---
CLZhao21583P_yunnanensis GTGCACTTTTCATGGAACAATCAG----
TGGTTGTTGGC-TTCACGG---
Dai22272 GTGCACTTTTCATGGAACAATCAG----
TGGTTGTTGGC-TTCATGG---
Dai22279 GTGCACTTTTCATGGAACAATCAG----
TGGTTGTTGGC-TTCATGG---
MJ332_94_CZ GTGCACTTTTCACAGGACAGCTGG----
TGGTTGTAGGC-TTCATTG---
MJ642_94_CZ_Expallescens GTGCACTTTTCACAGGACAGCTGG----
TGGTTGTAGGC-TTCATTG---
Dai21060P_vinctus GTGCACTTTTCACAGGACAGCTGG----
TGGTTGTAGGC-TTCATTG---
JV0511_23LRP_pouzarii GTGCACTTTTCACAGGACAACAGG----
TGGTTGTGGGC-TTTATTG---
JQ409462_R_pouzarii_PRM899856_ GTGCACTTTTCACAGGACAACAGG----
TGGTTGTGGGC-TTTATTG---
JV0308_66_WA GTGCACTTTTCACACGACAGCTGG----
TGGTTGTGGGC-TTCATTG---
JV0309_45_WA_USA GTGCACTTTTCACACGACAGCTGG----
TGGTTGTGGGC-TTCATTG---
JV0709_83_CA_USA GTGCACTTTTCACACGACAGCTGG----
TGGTTGTGGGC-TTCATTG---
Dai21043P_pouzarii GTGCACTTTTCACAGGACAACAGG----
TGGTTGTGGGC-TTTATTG---
MJ144_95_CZ GTGCACTTTTCACAGGACAGCTGG----
TGGCTGTGGGC-TTCATTG---

JV0909_3_CZ GTGCACTTTTCACAGGACAGCTGG----
TGGCTGTGGGC-TTCATTG---
JV0609_1_K GTGCACTTTTCACAGGACAGCTGG----
TGGCTGTGGGC-TTCATTG---
Dai20396Physisporinus_castanop GTGCACTTTTCACAGGACAGCTGG----
TGGCTGTGGGC-TTCATTG---
Dai20397Physisporinus_castanop GTGCACTTTTCACAGGACAGCTGG----
TGGCTGTGGGC-TTCATTG---
MJ19_09_SK_Abies GTGCACCTTTCACAGGACAGCTGG----
TGGCCATTGGC-TTCATTG---
JV0509_40_J_TN_USA_Betula GTGCACCTTTCACAGGACAGCTGG----
TGGCCATTGGC-TTCATTG---
JV0808_33crocatus_PA_USAlist GTGCACCTTTCACAGGACAGCTGG----
TGGCCATTGGC-TTCATTG---
DLL2009_061P_crocatus GTGCACCTTTCACAGGACAGCTGG----
TGGCCATTGGC-TTCATTG---
Dai12800P_subcrocatus GTGCACCTTTCACAGGACAGCTGG----
TGGCCATTGGC-TTCATTG---
Dai15917P_subcrocatus GTGCACCTTTCACAGGACAGCTGG----
TGGCCATTGGC-TTCATTG---
Cui16620 GTGCACTTTTCACAGGACAGCTAG----
TGGCTGTGGAC-TTCATTG---
HCFC1088Meripilus_stillicidior GTGCACTTTTCACAGGACAGCTAG----TGGCTGTGGAC-
TTCATTG---
MCW590Meripilus_obsrcurus GTGCACTTTTCACAGGACAGCTGG----
TGGCTGTGGAC-TTTATTG---
MCW722Meripilus_obsrcurus GTGCACTTTTCACAGGACAGCTGG----
TGGCTGTGGAC-TTTATTG---
Cui9381P_tibeticus GTGCACTTTTCACAGGACAGCTGG----
TGGCTGTTGGC-TTCATTG---
Cui9588P_tibeticus GTGCACTTTTCACAGGACAGCTGG----
TGGCTGTTGGC-TTCATTG---
Va2_Beneschova GTGCACTTTTCACAGGACAACCGG----
TGGCTGTTGGC-TTCATTG---
CWU3874_Ukraine_Alnus GTGCACTTTTCACAGGACAACCGG----
TGGCTGTTGGC-TTCATTG---
WCG1293Dai24718Physisporinus GTGCACCTTTCGCAGAACGATCAG----
TGATTGTTGGCTTTCATGG---
WCG1268Dai24682A GTGCACTTTTCACAGGAATGG------------------
CTTGTGG---
WCG1269Dai24683A GTGCACTTTTCACAGGAATGG------------------
CTTGTGG---
WCG1279Dai24694A GTGCACTTTTCACAGGAATGG------------------
CTTGTGG---

Dai16971 GTGCACTTTTCACAGGAATGG-----------------
CTTGTGG---
ZQY1043Dai26696 GTGCACTTTTCACAGGAATGG-----------------
CTTGTGG---
Doll880 GTGCACTTTTCATGGAATGGCTGG---
TCGCTGAGGGC-GTTGTTG---
Doll1000 GTGCACTTTTCATGGAATGGCTGG---
TCGCTGAGGGC-GTTGTTG---
1DAI18529 GTGCACTTTTCATGGAATGGCTGG---
TGGCTGAGGGT-TTTATTA---
Dai19535 GTGCACTTTTCGCAGGACAGCTGG----CGG----------

1704_79_hnedyVillaLaPaz GTGCACATTTCACAGGACAGCTCG----CAG------------
------
F2061 GTGCACATTTCACAGGACAGCTCG----CGG----------

1DAI18268 GTGCACTTCTCACAGGACAGCTCG----TGG---------

1DAI18540A GTGCACTTCTCACAGGACAGCTCG----TGG---------

Dai17695 GTGCACTTCTCACAGGACAGCTCG----TGG----------

LKY18Dai26373 GTGCACTTCTCACAGGACAGCTCG----TGG---------

Dai17839P_sulphureus GTGCACTTTTCACAGGACAGGTGG---
TGGCTGTGGGG-CCCTTGTGC-
Dai17841P_sulphureus GTGCACTTTTCACAGGACAGGTGG---
TGGCTGTGGGG-CCCTTGTGC-
Dai19877P_roseus GTGCACTTTTCACAGGACAGGTGG---
TGGCTGTGAGT-TTCTTG----
1508_18_1_Kout GTGCACTTTTCACAGGACAGCTGG---
TGGTCATGGAC-TTCTTG----
KP859303_R_vinctus_RP185_BRAZI GTGCACTTTTCACAGGACAGCTGG---
TGGTCATGGAC-TTCTTG----
JK1807_15Rigidoporus_sp_Puerto GTGCACTTTTCACAGGACAGCTGG---
TGGTCATGGAC-TTCTTG----
JV0509_47_J_TN_USA GTGCACTTTTCATAGGATAGCTTG---
TGGCTGCTGAT-TTTATAT---
JV0709_188 GTGCACTTTTCATAGGATAGCTTG---
TGGCTGYTGAT-TTTWTAT---
JV0509_127_PA_USA GTGCACTTTTCATAGGATAGCTTG---
TGGCTGCTGAT-TTTATAT---
JV1009_59_NJ_USA GTGCACTTTTCATAGGATAGCTTG---
TGGCTGCTGAT-TTTATAT---

Dai15497P_crataegi GTGCACCTTTCATAGGATGGCTTG----
CGGTGGTTGAT-TTTATAT---
Dai15499P_crataegi GTGCACCTTTCATAGGATGGCTTG----
CGGTGGTTGAT-TTTATAT---
Cui3266P_cinereus GTGCACTTTTCATAGGATGACTGG---
TTGGCTGTTGAT-CTTGTAT---
WCG1256Dai24690 GTGCACTTTTCATAGGATGACTGG---
TTGGCTGTTGAT-CTTGTAT---
1DAI17581 GTGCACTTTTCATAGGATGACTGG---
TTGGCTGTTGAT-CTTGTAT---
WCG1255Dai24688 GTGCACTTTTCATAGGATGACTGG---
TTGGCTGTTGAT-CTTGTAT---
Dai22427 GTGCACTTTTCATAGGATGACTGG---
TTGGCTGTTGAT-CTTGTAT---
MV690Meripilus_concrescens GTGCACTTTTCATAGGATGGCTTG---
TTGGCTGTTGAT-CTTGTAT---
MV513Meripilus_galapagensis GTGCACTTTTCATAGGATGGCTTT---
TTGGTCGTTGATCCTTGTAT---
Dai19793 GTGCACTTTTCATAGGATAGCTTG----
TGGCTGTTGAT-TTTATAT---
OQ553780P_tamilnaduensis GTGCACTTTTCATAGGATGACTTG---
TTGGCTGTTGTTTATTTCAT---
OQ553779P_tamilnaduensis GTGCACTTTTCATAGGATGACTTG---
TTGGCTGTTGTTTATTTCAT---
A164FB3Meripilus_giganteus GTGCACTTTTCATAGGATGACTTG---
TTGGCTGTTGTTTATTTCAT---
JV1407_36_Vinctus_Meandrica GTGCACTTTTCATAGAATAGCTTG----
TAAATTGAGTGACTTGYGT---
1807_3K_Rigidoporus_PuertoRico GTGCACTTTTCATAGAATAGCTTG----
TAAATTGAGTGACTTGTGT---
Cui16903P_vinctus GTGCACTTTTCATAGAATAGCTTG----
TAAATTGAGTGACTTGTGT---
JV1008_18R_Lineatus GTGCACCTTTCATAGAATAGCTTG----
TAGATTGGGTGACCTGTGT---
JV1407_37_1_Vinctus_Carara GTGCACCTTTCATAGAATAGCTTG----
TAGATTGGGTGACATGTGT---
Dai17986P_lineatus GTGCACCTTTCATAGAATAGCTTG----
TAGATTGGGTGACCTGTGT---
Dai18281 GTGCACCTTTCATAGAATAGCTTG----
TAGATTGGGTGACCTGTGT---
1DAI19796 GTGCACCTTTCATAGAATAGCTTG----
TAGATTAGGTGACCTGTGT---
ZQY797Dai25241 GTGCACCTTTCATAGAATAGCTTG----
TAGATTAGGTGACCTGTGT---

WCG1289Dai24711 GTGCACCTTTCATAGAATAGCTTG----
TAGATTAGGTGACCTGTGT---
Dai22598 GTGCACCTTTCATGGAATAGCTTG----
TGGATTGGGTGACCTATGT---
Dai20523 GTGCACCTTTCATGGAATAGCTTG----
TGGATTGGGTGACCTATGT---
Dai17885 GTGCACCTTTCATGGAATAGCTTG----
TGGATTGGGTGACCTATGT---
Dai17553 GTGCACCTTTCATGGAATAGCTTG----
TGGATTGGGTGACCTATGT---
Dai19639 GTGCACCTTTCATGGAATAGCTTG----
TGGATTGGGTGACCTATGT---
JV0110_48_CZ GTGCACTCTTCATGGAACGGATCC--------
TGCGGTTTCTCTTCT---
MJ129_04 GTGCACTCTTCATGGAACGGATCC--------
TGCGGTTTCTCTTCT---
Cui10340P_eminens GTGCACTCTTCATGGAACGGACCC--------
TGCGGTTTCTCTTCT---
Cui10341P_eminens GTGCACTCTTCATGGAACGGACCC--------
TGCGGTTTCTCTTCT---
Dai12685P_eminens GTGCACTCTTCATGGAACGGATCC--------
TGCGGTTTCTCTTCT---
Miettinen_13591Rigidoporus_und GTGCACTCTTCATGGAACGGATCC--------
TGCGGTTTCTCTTCT---
Dai20868 GTGCACTCTTCATAGAATGGATCC--------
TGCGGTTCCTCTTCT---
Dai20832 GTGCACTCTTCATAGAATGGATCC--------
TGCGGTTCCTCTTCT---
Dai11400 GTGCACTCTTCATAGAATGGATCC--------
TGCGGTTCCTCTTCT---
Dai22472 GTGCACTCTTCATAGAATGGATCC--------
TGCGGTTCCTCTTCT---
1CUI10475 GTGCACTTTTCATGGAACGGATCC--------
TGCGGTCCCTCATCT---
1CUI10491 GTGCACTTTTCATGGAACGGATCC--------
TGCGGTCCCTCATCT---
HCFC1095Meripilus_robledoi GTGCACTTTTCATGGAATGGATCC--------
TGCGATCTCTCATCT---
MCW702Meripilus_revolubilis GTGCACTTTTCATGGAATGGATCC--------
TGCGGTCTCTCATCT---
1704_83_zluty_HaciendaBaru GTGCACTTTTCATAGAACAGCTTG----
CAGTCATTGACCTCTTTGG---
Dai9925P_lavendulus GTGCACTTTTCATAGGATCGCTCG----
CGGCTGTTGGCCTCCCCGG---

Dai13587AP_lavendulus GTGCACTTTTCATAGGATCGCTCG----
CGGCTGTTGGCCTCCCCGG---
PDD70600P_longicystidius GTGCACTTTTCATAGGATAGCTCG----
CGGCTGTCAACCTCCTCGG---
Cui16630 GTGCACTTTTCATAGGATAGCTCG----
CGGCTGTCAACCTCCTCGG---
FP_135344Meripilus_giganteus
GTGCACCTTTCATGGGATGGCTTGCGGCCGTCCGTCGGCCTTTGCGT---
FP_100460_Sp
GTGCACCTTTCATGGGATGGCTTGCGGCCGTCCGTCGGCCTTTGCGT---
CBS421_48Meripilus_giganteus
GTGCACCTTTCATGGGATGGCTTGCGGCCGTCCGTCGGCCTTTGCGT---
Cui9203
GTGCACCTTTCATGGGATGGCTTGCGGCCGTCCGTCGGCCTTTGCGT---
Cui9202
GTGCACCTTTCATGGGATGGCTTGCGGCCGTCCGTCGGCCTTTGCGT---
TUFC100564Japan GTGCACCTTTCATAGAATGGCTTG---
CAGGCCTTCGGCCTTTGTGC---
Russell5913Meripilus_sumstinei GTGCACCTTTCATAGGATGGCTTG----
CGGCTGCTGGCCTTTGTGC---
RP215Meripilus_brasiliensis GTGCACCTTTCATAGAATGGCTTG----
TTGCTGTTGGCCCCTCCTTGGG
RP200Meripilus_brasiliensis GTGCACCTTTCATAGAATGGCTTG----
TTGCTGTTGGCCCCTCCTTGGG
JV1712_13J_R_vinctus2_LSUPuert ----------CATAGGTTTGG-TA----TAGAAAAGGTCTTT---
-----
Dai10503R_hypobrunneus GTGCACTTTACATAGGTTTGG-TA----
TAGAAAAGGTCTTT--------
Dai10569Rigidoporus_hypobrunne GTGCACTTTACATAGGTTTGG-TA----
TAGAAAAGGTCTTT--------
1DAI19451 GTGCACTTTACATAGGTTTGG-TA----
TAGAAAAGGTCTTT--------
CM108bRigidoporus_hypobrunneus GTGCACTTTACATAGGTTTGG-TA----
TAGAAAAGGTCTTT--------
1CUI16874 GTGCACTTTACATAGGTTTGG-TA----
TAGAAAAGGTCTTT--------
FD299Cerrena_unicolor GTGCACTTTTCATAGGTTTAGTTA----
TGGATGAGGCACTTTTAT----
KHL_GB_Cerrena_uniclor GTGCACTTTTCATAGGTTTAGTTA----
TGGATGAGGCACTTTTAT----
Dai12892Cerrena_albocinnamomea GTGCACTTTTCATAGGTTTAA-TG----
TGGAAAAGGTCTCT--------
Dai12955C_albocinnamomea GTGCACTTTTCATAGGTTTAA-TG----
TGGAAAAGGTCTCT--------

SNUm03110102C_aurantiopora GTGCACTTTTCATAGGTTTAA-TG----
TGGAAAAGGTCTCT--------
NIBRFG0000102423C_aurantiopora GTGCACTTTTCATAGGTTTAA-TG----
TGGAAAAGGTCTCT--------
Dai7359Antrodiella_zonata GTGCACTTTTCATAGGTTTAA-TG----
TGGAAAAGGTCTCT--------
F20080702KCM29C_consors GTGCACTTTTCATAGGTTTAA-TG----
TGGAAAAGGTCTCT--------
F20080208LYW10Cerrena_consors GTGCACTTTTCATAGGTTTAA-TG----
TGGAAAAGGTCTCT--------
Dai7821Antrodiella_zonata GTGCACTTTTCATAGGTTTAA-TG----
TGGAAAAGGTCTCT--------
CFMR_DCL04_31Pseudolagarobasid GTGCACTTTTCATAGATTTAG-TG----
TGGAAAAGGTCATATT------
VPB197Pseudolagarobasidium_bel GTGCACTTTTCATAGATTTAG-TG----
TGGAAAAGGTCATTTT------
CBS115543Pseudolagarobasidium_ GTGCACTTTTCATAGATTTAG-TG----
TGGAAGRGATCTTTTT------
CBS115544 GTGCACTTTTCATAGATTTAG-TG----
TGGAAGRGATCTTTTT------
Han405Pseudolagarobasidium_bai GTGCACTTTTCATAGATTTAG-TA----
TGGAAAAGGTTATACT------
Han406Pseudolagarobasidium_bai GTGCACTTTTCATAGATTTAG-TA----
TGGAAAAGGTTATACT------
MUcc838Spongipellis_delectans GTGCACTTTTCATAGGTTGGT-TG----TGG-----------------
-
BRNM686401S_delectans GTGCACTTTTCATAGGTTGGT-TG----TGG------------
------
OSM_F925S_delectans GTGCACTTTTCATAGGTTGGT-TG----TGG-------------
-----
BRNM67093Spongipellis_litschau GTGCACTTTTCATAGGTTGGT-TG----TGG----------------
--
CFMRccFP59199TS_unicolor GTGCACTTTTCATAGGTTGGT-TT----TGG-------------
-----
CFMRccFP71791TS_unicolor GTGCACTTTTCATAGGTTGGT-TT----TGG-------------
-----
Dai13845P_lischaueri GTGCACTTTTCATAGGTTGGT-TG----TGG----------------
--
Dai20266P_lischaueri GTGCACTTTTCATAGGTTGGT-TG----TGG----------------
--
CFMR_HHB11240Radulodon_america GTGCACCTTACATAGGTATAGG---------------
TTTTTTTGTG-G
RLG6350Radulodon_americanus GTGCACCTTACATAGGTATAGG---------------
TTTTTTTGTG-G

KY415963Radulodon_erikssonii GTGCACCTTACATAGGTATAGGY-------------
TTTTTTTTGTG-G
HHB9567spRadulodon_casearius GTGCACCTTACATAGGTATAGG---------------
TTTTTTTGTG-G
KRT_Iso_26Radulodon_casearius GTGCACCTTACATAGGTATAGG---------------
TTTTTTTGTG-G
CBS126044Radulodon_erikssonii GTGCACCTTACATAGGTATAGGT--------------
TTTTTTTGTG-G
He6183YUNNANENSIS GTGCACCTTACATAGGTATAGG---------------
TTTTTTTGTG-G
Cui17979YUNNANENSIS GTGCACCTTACATAGGTATAGG---------------
TTTTTTTGTG-G
Miettinen2091Junghuhnia_fimbri GTGCACTTCTCATGGGTTGGATTG----TGTCTGA--------
------
KHL12316S_tenue GTGCACTTCTCATGGGTTGGGTCA----AGTCTGA--

PRM846564S_pachyodon GTGCACTTTACATAGGCTTGGTTT----GGGCTGT---

SP_Lgt_S_pachyodon GTGCACTTTACATAGGCTTGGTTT----GGGCTGT----

Ryvarden44669Tyromyces_xuchile GTGCACTTTACATAGGCTTGGTCT---
GGGCTGTGTCCATTTATA----
PW17_171sinuosus GTGCACTTTTCATAGGTTTGGCTT----GGGCTGT----

W53Dai12234 GTGCACTTTTCATAGGTTTGGCTT----GGGCCGT---

HHB4100SpAntella_americana GTGCACTTCTCATGGGGTTGG----------------------------
W3Dai20901spumeus GTGCACTTTTCATAGGAACGGTTGA--G--------------

He6736 GTGCACTTTTCATAGGAACGGTTGA--G--------------

BRNM734877S_spumeus GTGCACYTTTCATAGGAACGGTTGA--G-----------

BRNM712630S_spumeus GTGCACCTTTCATAGGAACGGTTGA--G------------

Dai1723Loweomyces_sibiricus GTGCACTTTTCATAGGAACAGCTGA--G-----------------
----
W54Cui10009 GTGCACTTTTCATAGGAACGGTTGA--G--------------

W1Dai20899 GTGCACTTTTCATAGGAACGGTTGA--G--------------

HHB13445Trametes_ochracea GTGCACTTACTGTAGGTTGGCGTGG--G-----------
CTCCTTC---
Dai16222 GTGCACTTCTCATGGGATCAAGTT-------------------------

Dai16240 GTGCACTTCTCATGGGATCAAGTT----------------------

JV1310_11SanguinolentusCernys -----------CTGACGACCGCAGGTT-GCTTCTGTGTA----
CT---TT
MJ39_00_SK -----------CTGACGACYGCAGGTT-GCTTCTGTGTA--
--CT---TT
MJ111_04_CZ -----------CTGACGACCGCAGGTT-GCTTCTGTGTA---
-CT---TT
JV1610_BOKYsmrk -----------CTGACGACCGCAGGTT-GCTTCTGTGTA-
---CT---TT
Dai21030 -----------CTGACGACCGCAGGTT-GCTTCTGTGTA----
CT---TT
Dai20976P_furcatus -----------CTGACGACCGCAGGTT-GCTTCTGTGTA----
CT---TT
Dai2105 -----------CTGATGACTGCAGGTT-GCTTCTGTGTG----
CT---TC
Dai2544 -----------CTGATGACTGCAGGTT-GCTTCTGTGTG----
CT---TC
Dai11313 -----------CTGATGACTGCAGGTT-GCTTCTGTGTG----
CT---TC
WCG1611Dai26167 -----------CTGATGACTGCAGGTT-GCTTCTGTGTG--
--CT---TC
WCG1518Dai25999Physisporinus -----------CTGATGACTGCAGGTT-GCTTCTGTGTG----
CT---TC
TAA15097 -----------CTGATGACTGCAGGTT-GCTTCTGTGTA---
-CT---TT
JV8909_19_CZ -----------CCGATGACCGCAGGTT-GCTTCTGTGTG---
-CC---TC
JV1310_15_P_sanguinolentus2_CZ -----------CCGATGACCGCAGGTT-GCTTCTGTGTG----
CC---TC
MJ53_02_CZ -----------CCGATGACCGCAGGTT-GCTTCTGTGTG--
--CC---TC
CLZhao21647P_yunnanensis -----------CCGATGACCACAGGTT-GCTTCTGTGTG----
CC---TC
CLZhao21583P_yunnanensis -----------CCGATGACCACAGGTT-GCTTCTGTGTG----
CC---TC
Dai22272 -----------CTGATAACTGCAGGTT-GCTTCTGTGTA----
TT---TT
Dai22279 -----------CTGATAACTGCAGGTT-GCTTCTGTGTA----
TT---TT
MJ332_94_CZ -----------TCTATGGCTACAGGTT-GCTT-TGTGTG----
CT---TT

MJ642_94_CZ_Expallescens -----------TCTATGGCTACAGGTT-GCTT-TGTGTG----
CT---TT
Dai21060P_vinctus -----------TCTATGGCTACAGGTT-GCTT-TGTGTG----CT-
--TT
JV0511_23LRP_pouzarii -----------TCCATAGCTGCAGGTT-GCTT-CGTGTG----
CT---TT
JQ409462_R_pouzarii_PRM899856_ -----------YCCATAGCTGCAKGTT-GCTT-CGTGTG----
CT---TT
JV0308_66_WA -----------TCCATAGCTGCAGGTT-GCTT-TGTGTG----
CT---TT
JV0309_45_WA_USA -----------TCCATAGCTGCAGGTT-GCTT-TGTGTG---
-CT---TT
JV0709_83_CA_USA -----------TCCATAGCTGCAGGTT-GCTT-TGTGTG---
-CT---TT
Dai21043P_pouzarii -----------TCCATAGCTGCAGGTT-GCTT-CGTGTG----CT-
--TT
MJ144_95_CZ -----------TCCATGGCTGCAGGTT-GCCT-TGTGTG----
CT---TT
JV0909_3_CZ -----------TCCATGGCTGCAGGTT-GCCT-TGTGTG----
CT---TT
JV0609_1_K -----------TCCATGGCTGCAGGTT-GCCT-TGTGTG----
CT---TT
Dai20396Physisporinus_castanop -----------ACCATGGCTGCAGGTT-GCCT-TGTGTG----CT--
-TT
Dai20397Physisporinus_castanop -----------ACCATGGCTGCAGGTT-GCCT-TGTGTG----CT--
-TT
MJ19_09_SK_Abies -----------CTYATGACTACAGGTT-GCTT-TGTGTG----
CT---TT
JV0509_40_J_TN_USA_Betula -----------CTCATGACTACAGGTT-GCTT-TGTGTG----
CT---TT
JV0808_33crocatus_PA_USAlist -----------CTCATGACTACAGGTT-GCTT-TGTGTG----CT-
--TT
DLL2009_061P_crocatus -----------CTCATGACTACAGGTT-GCTT-TGTGTG----
CT---TT
Dai12800P_subcrocatus -----------CTCATGACTACAGGTT-GCTT-TGTGTG----CT-
--TT
Dai15917P_subcrocatus -----------CTTATGACTACAGGTT-GCTT-TGTGTG----CT-
--TT
Cui16620 -----------GCCATGGCTGCAGGTT-GCCT-TGTGTG----
CT---TT
HCFC1088Meripilus_stillicidior -----------GCCATGGCTGCAGGTT-GCCT-TGTGTG----CT---
TT
MCW590Meripilus_obsrcurus -----------GCCATGGCTGCAGGTT-GCCT-TGTGTG----
CT---TT

MCW722Meripilus_obsrcurus -----------GCCATGGCTGCAGGTT-GCCT-TGTGTG----
CT---TT
Cui9381P_tibeticus -----------CCTACGGCTGCAAGTT-GCTT-CGTGTG----CT-
--TC
Cui9588P_tibeticus -----------CCTATGGCTGCAAGTT-GCTT-CGTGTG----CT--
-TC
Va2_Beneschova -----------CCTATGGCTGCAAGTT-GCTT-TGTGTG----
CT---TT
CWU3874_Ukraine_Alnus -----------CCTATGGCTGCAAGTT-GCTT-TGTGTG----
CT---TT
WCG1293Dai24718Physisporinus -----------CTGATGATTGCAGATT-GCTTTTGCGTG----
CT---TT
WCG1268Dai24682A -----------CTGATGGCTGCAGGTT-GTTTCTGTGTG--
--CT---TT
WCG1269Dai24683A -----------CTGATGGCTGCAGGTT-GTTTCTGTGTG--
--CT---TT
WCG1279Dai24694A -----------CTGATGGCTGCAGGTT-GTTTCTGTGTG--
--CT---TT
Dai16971 -----------CTGATGGCTGCAGGTT-GTTTCTGTGTG----
CT---TT
ZQY1043Dai26696 -----------CTGATGGCTGCAGGTT-GTTTCTGTGTG---
-CT---TT
Doll880 -----------CCCAAGGCAGGATGCT-GTTT-CATGTA----
CT---TC
Doll1000 -----------CCCAAGGCAGGATGCT-GTTT-CATGTA----
CT---TC
1DAI18529 -----------CCCAAGGTCGGATGCT-GTTT-CATGTG----
CT---TT
Dai19535 -----------CCGATGGCTGCAGGTT-GCTC-TGTGTG----
CT---TC
1704_79_hnedyVillaLaPaz -----------CCTCTAGCTGCAGGCT-GCTCCTGTGTA----
TT---TT
F2061 -----------CCTCTAGCTGCAGGCT-GCTTCTGTGTA----
TT---TT
1DAI18268 -----------CCTCTGGCTGCAGGCT-GCTTCTGTGTA----
CT---TT
1DAI18540A -----------CCTCTGGCTGCAGGCT-GCTTCTGTGTA---
-CT---TT
Dai17695 -----------CCTCTGGCTGCAGGCT-GCTTCTGTGTA----
CT---TT
LKY18Dai26373 -----------CCTCTGGCTGCAGGCT-GCTTCTGTGTA---
-CT---TT
Dai17839P_sulphureus -----------CTTTTGGCTGCAGCTT-GTTTTTGTGTG----
CT---TC

Dai17841P_sulphureus -----------CTTTTGGCTGCAGCTT-GTTTTGTGTG----
CT---TC
Dai19877P_roseus -----------CCTATGGCTGCAGCTT-GTTTCTGTGTG----
CT---TC
1508_18_1_Kout -----------TCTTTGATTGCAGGTT-GTTTCTGTGTG----
AT---TT
KP859303_R_vinctus_RP185_BRAZI -----------TCTTTGATTGCAGGTT-GTTTCTGTGTG----
AT---TT
JK1807_15Rigidoporus_sp_Puerto -----------TCTTTGATTGCAGGTT-GTTTCTGTGTG----AT-
--TT
JV0509_47_J_TN_USA -----------CAACAG--TTACAGTT-GTTC-TGTGCT----
TT---TT
JV0709_188 -----------CAACAG--TTACAGTT-GTTC-TGTGCT----
TT---TT
JV0509_127_PA_USA -----------CAACAG--TTACAGTT-GTTC-TGTGCT----
TT---TT
JV1009_59_NJ_USA -----------CAACAG--TTACAGTT-GTTC-TGTGCT----
TT---TT
Dai15497P_crataegi -----------CAATGG--CTGCAGTT-GTTC-TGTGC------T---
TT
Dai15499P_crataegi -----------CAATGG--CTGCAGTT-GTTC-TGTGC------T---
TT
Cui3266P_cinereus -----------CAATGG--CTGCAGTT-GTTC-TGTGT------T---
TT
WCG1256Dai24690 -----------CAATGA--CTGCAGTT-GTTC-TGTGT------
T---TT
1DAI17581 -----------CAATGG--CTGCAGTT-GTTC-TGTGT------T--
-TT
WCG1255Dai24688 -----------TAATGG--CTGCAGTT-GTTC-TGTGT------
T---TT
Dai22427 -----------CAATGG--CTGCAGTT-GTTC-TGTGT------T---
TT
MV690Meripilus_concrescens -----------CAATGG--CTGCAGTT-GTTC-TGTGT------T---
TT
MV513Meripilus_galapagensis -----------CAATGG--CTGCAGCT-GTTC-TGTGT------T---
TT
Dai19793 -----------TGATAG--CTGCAGTT-GTTC-TGTGC-----TT--
-TT
OQ553780P_tamilnaduensis -----------CAATGG--CCACAGTT-GTTC-TGTGTTT---TT-
--TC
OQ553779P_tamilnaduensis -----------CAATGG--CCACAGTT-GTTC-TGTGTTT---TT-
--TC
A164FB3Meripilus_giganteus -----------CAATGG--CCACAGTT-GTTC-TGTGTTT---TT--
-TC

JV1407_36_Vinctus_Meandrica -----------CATTTGGCTTGCAGCT-ACTC-TGTGTCT---TT---TT
1807_3K_Rigidoporus_PuertoRico -----------CATTTGGCTTGCAGCT-ACTC-TGTGTCT---TT---TT
Cui16903P_vinctus -----------CATTTGGCTTGCAGCT-ACTC-TGTGTCT---TT---TT
JV1008_18R_Lineatus -----------CACTTRGTTTGCAGCT-GCTC-TGTGT-----TT---TT
JV1407_37_1_Vinctus_Carara -----------CACTTGGTTTGCAGCT-GCTC-TGTGT-----TT---TT
Dai17986P_lineatus -----------CACTTGGTTTGCAGCT-GCTC-TGTGT-----TT---TT
Dai18281 -----------CACTTGGTTTGCAGCT-GCTC-TGTGT-----TT---TT
1DAI19796 -----------CACTTGGTTTGCAGCT-GCTC-TGTGT-----TT---TT
ZQY797Dai25241 -----------CACTTGGTTTGCAGCT-GCTC-TGTGT-----TT---TT
WCG1289Dai24711 -----------CACTTGGTTTGCAGCT-GCTC-TGTGT-----TT---TT
Dai22598 -----------TGCTTGATTTACAGCT-GCTC-TGTGT-----TT---TT
Dai20523 -----------TGCTTGATTTACAGCT-GCTC-TGTGT-----TT---TT
Dai17885 -----------TGCTTGATTTACAGCT-GCTC-TGTGT-----TT---TT
Dai17553 -----------TGCTTGATTTACAGCT-GCTC-TGTGT-----TT---TT
Dai19639 -----------TGCTTGATTTACAGCT-GCTC-TGTGT-----TT---TT
JV0110_48_CZ -----------GAGAAATTTGCAGACC-GCTC-TGTGTTTA--TT---TT
MJ129_04 -----------GAGAAATTTGCAGACC-GCTC-TGTGTTTA--TT---TT
Cui10340P_eminens -----------GAGAAATTTGCAGACC-GCTC-TGTGTTTA--TT---TT
Cui10341P_eminens -----------GAGAAATTTGCAGACC-GCTC-TGTGTTTA--TT---TT
Dai12685P_eminens -----------GAGAAATTTGCAGACC-GCTC-TGTGTTTA--TT---TT
Miettinen_13591Rigidoporus_und -----------GAGAAATTTGCAGACC-GCTC-TGTGTTTA--TT---TT
Dai20868 -----------GAGGAATTTGCAGACC-ACTC-TGTGTTAA--TT---TT

Dai20832 -----------GAGGAATTTGCAGACC-ACTC-TGTGTTAA-
-TT---TT
Dai11400 -----------GAGGAATTTGCAGACC-ACTC-TGTGTTAA-
-TT---TT
Dai22472 -----------GAGGAATTTGCAGACC-ACTC-TGTGTTAA-
-TT---TT
1CUI10475 -----------GAGGGATTTGCAGACC-GCTC-
TGTGTATA--TT---TT
1CUI10491 -----------GAGGGATTTGCAGACC-GCTC-
TGTGTATA--TT---TT
HCFC1095Meripilus_robledoi -----------GAGGGATTTGCAGACC-ACTC-TGTGTGT---
TT---TT
MCW702Meripilus_revolubilis -----------GGGGGATTTGCAGACC-ACTC-TGTGCAT---
TT---TC
1704_83_zluty_HaciendaBaru -----------TTGATATCGGCAGTTT-GTTC-
TGTGTCAATTCT---TT
Dai9925P_lavendulus -----------CTAGTGGCTGCAG-CT-GTCC-
TGTGTTATATCT---GT
Dai13587AP_lavendulus -----------CTAGTGGCTGCAG-CT-GTCC-
TGTGTTATATCT---GT
PDD70600P_longicystidius -----------TCGATGACTGCAG-CT-GTTC-TGTGTCAT--
TT---TT
Cui16630 -----------TCGATGACTGCAG-CT-GTTC-TGTGTCAT--
TT---TT
FP_135344Meripilus_giganteus -----------CGATGGCTCTGCAGCT-GCCT-CGTGTTT--------
TT
FP_100460_Sp -----------CGATGGCTCTGCAGCT-GCCT-CGTGTTT----
----TT
CBS421_48Meripilus_giganteus -----------CGATGGCTCTGCAGCT-GCCT-CGTGTTT-------
-TT
Cui9203 -----------CGATGGCTCTGCAGCT-GCCT-CGTGTTT----
----TT
Cui9202 -----------CGATGGCTCTGCAGCT-GCCT-CGTGTTT----
----TT
TUFC100564Japan -----------TGATGGCTGCGCAGCC-GTTC-TGTGTTT---
-----TT
Russell5913Meripilus_sumstinei -----------CAATGGTTCTGCAGCT-GTTC-TGTGTTT--------
TT
RP215Meripilus_brasiliensis TGGGTGTC---CAAGGTTTTGAAACCG-GTCC-AAGGCT--
--TT---TT
RP200Meripilus_brasiliensis TGGGTGTC---CAAGGGTTTGATAGCT-GTCC-TAGGCT---
-TT---TT
JV1712_13J_R_vinctus2_LSUPuert ATTGACTT---------TGGATTTACT-GACC-TATGCT----
TT---TA

Dai10503R_hypobrunneus ATTGACTT---------TGGAAATACT-GACC-TATGCT----
TT---T-
Dai10569Rigidoporus_hypobrunne ATTGACTT---------TGGAAATACT-GACC-TATGCT----
TT---T-
1DAI19451 ATTGACTT---------TGGAAATACT-GACC-TATGCT----
TT---T-
CM108bRigidoporus_hypobrunneus ATTGACTT---------TGGAAATACT-GACC-TATGCT----
TT---T-
1CUI16874 ATTGACTT---------TGGAAATACT-GACC-TATGCT----
TT---T-
FD299Cerrena_unicolor AGTGTCTT---------GGAAGTGACT-ATCC-TATGTA----
TT---TT
KHL_GB_Cerrena_uniclor AGTGTCTT---------GGAAGTGACT-ATCC-TATGTA----
TT---TT
Dai12892Cerrena_albocinnamomea --GGCCTT---------GGAAAT-ATT-ATCC-TATGTA-----T---
TT
Dai12955C_albocinnamomea --GGCCTT---------GGAAAT-ATT-ATCC-TATGTA-----T--
-TT
SNUm03110102C_aurantiopora --GGCCTT---------GGAAAT-ATT-ATCC-TATGTA-----T--
-TT
NIBRFG0000102423C_aurantiopora --GGCCTT---------GGAAAT-ATT-ATCC-TATGTA-----T--
-TT
Dai7359Antrodiella_zonata --GGCCTT---------GGAAAT-ATT-AGCC-TATGTA-----T---
TT
F20080702KCM29C_consors --GGCCTT---------GGAAAT-ATT-AGCC-TATGTA-----
T---TT
F20080208LYW10Cerrena_consors --GGCCTT---------GGAAAT-ATT-AGCC-TATGTA-----T-
--TT
Dai7821Antrodiella_zonata --GGCCTT---------GGAAAT-ATT-AGCC-TATGTA-----T---
TT
CFMR_DCL04_31Pseudolagarobasid -TGATCTT---------GGAAATACTG-AATC-TATGTA-----
T---TT
VPB197Pseudolagarobasidium_bel -TGATCTT---------GGAAATACTG-AATC-TATGTA-----T--
-CT
CBS115543Pseudolagarobasidium_ --GATCTT---------GGAAATGCTG-GATC-TGTGTT-----T-
--TT
CBS115544 --GATCTT---------GGAAATGCTG-GATC-TGTGTT-----
T---TT
Han405Pseudolagarobasidium_bai --GGCCTT---------GAAAATACTA-AGTC-TATGTT-----T---
TT
Han406Pseudolagarobasidium_bai --GGCCTT---------GAAAATACTA-AGTC-TATGTT-----T---
TT
MUcc838Spongipellis_delectans -CTGTCTT---CGCGGACGGTTCAGCC-TGCC-TATGCT---
--T---TT

BRNM686401S_delectans -CTGTCTT---CGCGGACGGTTCAGCC-TGCC-TATGCT-----T---TT
OSM_F925S_delectans -CTGTCTT---CGCGGACGGTTCAGCC-TGCC-TATGCT-----T---TT
BRNM67093Spongipellis_litschau -CTGTCTT-----CGGATGGCTTGACC-TGCC-TATGCT-----T---TT
CFMRccFP59199TS_unicolor -CTGTCTT-----CGGATGGCTTGACC-TGCC-TATGCT-----T---TT
CFMRccFP71791TS_unicolor -CTGTCTT-----CGGATGGCTTGACC-TGCC-TATGCT-----T---TT
Dai13845P_lischaueri -CTGTCTT-----CGGATGGCTTGACC-TGCC-TATGCT-----T---TT
Dai20266P_lischaueri -CTGTCTT-----CGGATGGCTTGACC-TGCC-TATGCT-----T---TT
CFMR_HHB11240Radulodon_america GCTGWCTCTG-GATGGMTTGCTGGCCT-TGCC-TATGCA----TT---TC
RLG6350Radulodon_americanus GCTGTCTCCG-GATGGCTTGCTGGCCT-TGCC-TATGCA----TT---TC
KY415963Radulodon_erikssonii GCTGTCTCTG-GATGGCTTGCTGGCCT-TGCC-TATGCA----TT---TC
HHB9567spRadulodon_casearius GCTGTCTCTG-GATGGCTTGCTGGCCT-TGCC-TATGCA----TT---TT
KRT_Iso_26Radulodon_casearius GCTGTCTCTG-GATGGCTTGCTGGCCT-TGCC-TATGCA----TT---TT
CBS126044Radulodon_erikssonii GCTGTCTCTG-GATGGCTTGCTGGCCT-TGCC-TATGCA----TT---TC
He6183YUNNANENSIS GCTGTCTCTG-GATGGCTTGCTGGCCT-TGCC-TATGCA----TT---TC
Cui17979YUNNANENSIS GCTGTCTCTG-GATGGCTTGCTGGCCT-TGCC-TATGCA----TT---TC
Miettinen2091Junghuhnia_fimbri --AATATT-------GACAGGATCCCT-CTCA-TGTGTT----TT---AT
KHL12316S_tenue --AATATT-------GATGAAGCCCTT-CTCG-TGTGTT----TT---AT
PRM846564S_pachyodon ----CCTT---TATTGGATGGCTGGCC-GGCC-TATGCA----TC---TT
SP_Lgt_S_pachyodon ----CCTT---TATTGGATGGCTGGCC-GGCC-TATGCA----TC---TT
Ryvarden44669Tyromyces_xuchile ------------ATGGGATGGCTGGCT-GGCC-TATGCA----TCCTATT
PW17_171sinuosus ---CCCTTTGTTGTGGATGGTCTGGCT-GGCC-TATGCC--ATCT---TT
W53Dai12234 ---CCCTTTGTTGTGGATGGTCTGGCT-GGCC-TATGCC--ATCT---TT

HHB4100SpAntella_americana GCTGCGTCTGAAATATGGCGAGGCACT-CTCT-CATGTG----TT-TATA
W3Dai20901spumeus ----------------------AAACTGGTCC-TATGCAT---TT---TT
He6736 ----------------------AAACCGGTCC-CATGCAT---TT---TT
BRNM734877S_spumeus ----------------------AAACT-GTCC-TATGCRT---TT---TT
BRNM712630S_spumeus ----------------------AARCY-GTCC-TATGCAT---TT---TT
Dai1723Loweomyces_sibiricus ----------------------AAACT-GTCC-TATGCAT---TT---TT
W54Cui10009 ----------------------AAGCT-GTCC-TATGCAT---TT---TT
W1Dai20899 ----------------------AAGCT-GTCC-TATGCAT---TT---TT
HHB13445Trametes_ochracea -----------ACGGGAGCATTCTGCC-GGCC-TATGTATA------CT
Dai16222 GTGGTATGA--AATACAACCAACAGCT-GTTCTCGTGTGTT-TTT---TT
Dai16240 GTGGTATGA--AATACAACCAACAGCT-GTTCTCGTGTGTT-TTT---TT

JV1310_11SanguinolentusCernys AT-AAACCTCAGAATCA----GTTTAGAATG-TATA--TCGTGTA--CCC
MJ39_00_SK AT-AAACCTCAGAATCA----GTTTAGAATG-TATA--TCGTGTA--CCC
MJ111_04_CZ AT-AAACCTCAGAATCA----GTTTAGAATG-TATA--TCGTGTA--CCC
JV1610_BOKYsmrk AT-AAACCTCAGAATCA----GTTTAGAATG-TATA--TCGTGTA--YCC
Dai21030 AT-AAACCTCAGAATCA----GTTTAGAATG-TATA--TCGTGTA--CCC
Dai20976P_furcatus AT-AAACCTCAGAATCA----GTTTAGAATG-TATA--TCGTGTA--CCC
Dai2105 AT-AAACCTCAGAATCA----GTTTAGAATG-TATA--TCGTGTA--CCT
Dai2544 AT-AAACCTCAGAATCA----GTTTAGAATG-TATA--TCGTGTA--CCT
Dai11313 AT-AAACCTCAGAATCA----GTTTAGAATG-TATA--TCGTGTA--CCT
WCG1611Dai26167 AT-AAACCTCAGAATCA----GTTTAGAATG-TATA--TCGTGTA--CCT
WCG1518Dai25999Physisporinus AT-AAACCTCAGAATCA----GTTTAGAATG-TATA--TCGTGTA--CCT
TAA15097 AT-AAACCTCAGAATCA----GTTTAGAATG-TATA--TCATGTA--CCT
JV8909_19_CZ AT-AAACTTCAGAATCA----GGTTAGAATG-TATA--CCGTGTA--CCT

JV1310_15_P_sanguinolentus2_CZ AT-AAACTTCAGAATCA----GTTTAGAATG-TATA--
CCGTGTA--CCT
MJ53_02_CZ AT-AAACTTCAGAATCA----GTTTAGAATG-TATA--
CCGTGTA--CCT
CLZhao21647P_yunnanensis AT-AAACTTCAGAATCA----GTTTAGAATG-TATA--
CCGTGTA--CCT
CLZhao21583P_yunnanensis AT-AAACTTCAGAATCA----GTTTAGAATG-TATA--
CCGTGTA--CCT
Dai22272 AT-AAACCTCGGAATCA----GTTTAGAATG-TATA--
TCGTGTA--CCT
Dai22279 AT-AAACCTCGGAATCA----GTTTAGAATG-TATA--
TCGTGTA--CCT
MJ332_94_CZ AT-AAACTCATAATCAA----GTTCAGAATG-TCTA--
TTGTGTA--TCC
MJ642_94_CZ_Expallescens AT-AAACTCATAATCAA----GTTCAGAATG-TCTA--
TTGTGTA--TCC
Dai21060P_vinctus AT-AAACTCATAATCAA----GTTCAGAATG-TCTA--
TTGTGTA--TCC
JV0511_23LRP_pouzarii AC-AAACTCATAATCAA----GTTTAGAATG-TTCA--
TTGTGTA--TCC
JQ409462_R_pouzarii_PRM899856_ AC-AAACTCATAATCAA----GTTTAGAATG-TTCA--
TTGTGTA--TCC
JV0308_66_WA AT-AAACTCATGATCAA----GTTTAGAATG-TTTA--
TTGTGTA--TCC
JV0309_45_WA_USA AT-AAACTCATGATCAA----GTTTAGAATG-TTTA--
TTGTGTA--TCC
JV0709_83_CA_USA AT-AAACTCATGATCAA----GTTTAGAATG-TTTA--
TTGTGTA--TCC
Dai21043P_pouzarii AC-AAACTCATAATCAA----GTTTAGAATG-TTCA--
TTGTGTA--TCC
MJ144_95_CZ AA-AAACTCAAAAATAA----GTTTAGAATG-TTCA--
TTGTGTA--TCT
JV0909_3_CZ AA-AAACTCAAAAATAA----GTTTAGAATG-TTCA--
TTGTGTA--TCT
JV0609_1_K AA-AAACTCAAAAATAA----GTTTAGAATG-TTCA--
TTGTGTA--TCT
Dai20396Physisporinus_castanop AA-AAACTCAAAAATAA----GTTTAGAATG-TTTA--
TTGTGTA--TCC
Dai20397Physisporinus_castanop AA-AAACTCAAAAATAA----GTTTAGAATG-TTTA--
TTGTGTA--TCC
MJ19_09_SK_Abies AT-AAACTCAAAATTAA----GTTTAGAATG-TTTA--
CTGTGTA--TCC
JV0509_40_J_TN_USA_Betula AT-AAACTCAAAATTAA----GTTTAGAATG-TTTA--
CTGTGTA--TCC

JV0808_33crocatus_PA_USAlist AY-AAACTCAAAATTAA----GTTTAGAATG-TTTA--
CTGTGTA--TCC
DLL2009_061P_crocatus AC-AAACTCAAAATTAA----GTTTAGAATG-TTTA--
CTGTGTA--TCC
Dai12800P_subcrocatus AC-AAACTCAAAATTAA----GTTTAGAATG-TTTA--
CTGTGTA--TCC
Dai15917P_subcrocatus AT-AAACTCAAAATTAA----GTTTAGAATG-TTTA--
CTGTGTA--TCC
Cui16620 AC-AAACTCAAAAATAA----GTTTAGAATG-TCTA--
TTGTGTA--TCC
HCFC1088Meripilus_stillicidior AC-AAACTCAAGAATAA----GTTTAGAATG-TCTA--
TTGTGTA--TCC
MCW590Meripilus_obsrcurus AC-AAACTCAAAAATAA----GTTCAGAATG-TTCA--
TTGTGTA--TCC
MCW722Meripilus_obsrcurus AC-AAACTCAAAAATAA----GTTCAGAATG-TTCA--
TTGTGTA--TCC
Cui9381P_tibeticus AT-AAACTCAAAGTTAA----GTTTAGAATG-TTCA--
TTGTGTA--TTC
Cui9588P_tibeticus AT-AAACTCAAAGTTAA----GTTTAGAATG-TTCA--
TTGTGTA--TTC
Va2_Beneschova AA-AAACTCAAAATCAA----GTTCAGAATG-TTCA--
TTGTGTA--TTT
CWU3874_Ukraine_Alnus AA-AAACTCAAAATCAA----GTTCAGAATG-TTCA--
TTGTGTA--TTT
WCG1293Dai24718Physisporinus AC-AAACATGATTATTG----GTTTAGAATG-TTTAC-
TTGTGTAT-TCC
WCG1268Dai24682A AC-AAACCCTCATATT-----GTTTAGAATG-TATA--
TCGTGTA--TTT
WCG1269Dai24683A AC-AAACCCTCATATT-----GTTTAGAATG-TATA--
TCGTGTA--TTT
WCG1279Dai24694A AC-AAACCCTCATATT-----GTTTAGAATG-TATA--
TCGTGTA--TTT
Dai16971 AC-AAACCCTCATATT-----GTTTAGAATG-TATA--
TCGTGTA--TTT
ZQY1043Dai26696 AC-AAACCCTCATATT-----GTTTAGAATG-TATA--
TCGTGTA--TTT
Doll880 AT-AAACCACAACATA-----GTTTAGAATG-TATA--
CTGTGTG--TCT
Doll1000 AT-AAACCACAACATA-----GTTTAGAATG-TATA--
CTGTGTG--TCT
1DAI18529 AT-AAA-CACAACATA-----GTTTAGAATG-TATA--
CTGTGTC--TTT
Dai19535 AT-AAACTTCAAAGTCG----GTTTAGAATG-TTTA--
TCGTGTA--TAC

1704_79_hnedyVillaLaPaz AC-AAACCTTTTATAAT--AGTCGAGAATG-TTT---
CTGTGTA--TTT
F2061 AC-AAACCTTTTATAAT--AGTTGAGAATG-TTT---
CTGTGTA--TTT
1DAI18268 AC-AAACTTTTGATTAT---GGTTAAGAATG-TTT---
CTGTGTA--TCC
1DAI18540A AC-AAACTTTTAATTAT---GGTTAAGAATG-TTT---
CTGTGTA--TCC
Dai17695 AC-AAACTTTTAATCAT---GGTTTAGAATG-TTC---
CTGTGTA--TCC
LKY18Dai26373 AC-AAACTTTTAATTAT---GGTTAAGAATG-TTT---
CTGTGTA--TCC
Dai17839P_sulphureus AC-AAACCTATGAATCA----GTTTAGAATG-TCTC--
TTGTGTA--TTG
Dai17841P_sulphureus AC-AAACCTATGAATCA----GTTTAGAATG-TCTC--
TTGTGTA--TTG
Dai19877P_roseus AC-AAACCTATGCATCA----GTTTAGAATG-TTC---
TTGTGTA--TCT
1508_18_1_Kout TA-TAAACCCTTTATAAC--AGTCAAGAATG-TTTA---
TGTGTA--TCT
KP859303_R_vinctus_RP185_BRAZI TA-TAAACCCTTTATAAC--AGTCAAGAATG-TTTA--
-TGTGTA--TCT
JK1807_15Rigidoporus_sp_Puerto TA-TAAACCCTTTATAAC--AGTCAAGAATG-TTTA---
TGTGTA--TCT
JV0509_47_J_TN_USA AT-AAACTTTTAAACCA----GTCAAGAATG-TTCA--
TTGTGTG--TAT
JV0709_188 AT-AAACTTTTAAACCA----GTCAAGAATG-TTCA--
TTGTGTG--TAT
JV0509_127_PA_USA AT-AAACTTTTAAACCA----GTCAAGAATG-TTCA--
TTGTGTG--TAT
JV1009_59_NJ_USA AT-AAACTTTTAAACCA----GTCAAGAATG-TTCA--
TTGTGTG--TAT
Dai15497P_crataegi AC-AAACTTTGAAACTA----GTCAAGAATG-TTTA--
TTGTGTG--TAT
Dai15499P_crataegi AC-AAACTTTGAAACTA----GTCAAGAATG-TTTA--
TTGTGTG--TAT
Cui3266P_cinereus AC-AAACCTTGAAATCA----GTCTAGAATG-TTTT--
TTGTGTG--TAT
WCG1256Dai24690 AC-AAACCTTGAAATCA----GTCTAGAATG-TTTT--
TTGTGTG--TAT
1DAI17581 AC-AAACCTTGAAATCA----GTCTAGAATG-TTTT--
TTGTGTG--TAT
WCG1255Dai24688 AC-AAACCTTGAAATCA----GTCTAGAATGTTTTT-
-TTGTGTG--TAT

Dai22427 AC-AAACCTTGAAATCA----GTCTAGAATG-TTTT--
TTGTGTG--TAT
MV690Meripilus_concrescens AC-AAACCTTGAAATCA----GTTTAGAATGTTTTT--
TTGTGTG--TAT
MV513Meripilus_galapagensis AC-AAACCTTGAAATCA----
GTTTAGAATGTTTTTCTTGTGTG--CAT
Dai19793 AT-AAACTTTTAAACCA----GTCAAGAATG-TTTA--
TTGTGTG--TAT
OQ553780P_tamilnaduensis AT-AAACCTATAAAACA----GTTTAGAATG-TTTA--
TTGTGTG--TAT
OQ553779P_tamilnaduensis AT-AAACCTATAAAACA----GTTTAGAATG-TTTA--
TTGTGTG--TAT
A164FB3Meripilus_giganteus AT-AAACCTATAAAACA----GTTTAGAATG-TTTA--
TTGTGTG--TAT
JV1407_36_Vinctus_Meandrica ATAAACTTTTATTATCA----GTTTAGAATG-TCT---
TTGTGTA--AAT
1807_3K_Rigidoporus_PuertoRico ATAAACCTTTATTATCA----GTTTAGAATG-TCT---
TTGTGTA--AAT
Cui16903P_vinctus ATAAACCTTTATTATCA----GTTTAGAATG-TCT---
TTGTGTA--AAT
JV1008_18R_Lineatus AC-AAACTTATAAATCA----GTTCAGAATG-TTTA--
TTGTGTA--AAT
JV1407_37_1_Vinctus_Carara AC-AAACTTATAAATCA----GTTCAGAATG-TTTA--
TTGTGTA--AAT
Dai17986P_lineatus AC-AAACTTATAAATTA----GTTCAGAATG-TTTA--
TTGTGTA--AAT
Dai18281 AC-AAACTTATAAATCA----GTTCAGAATG-TTTA--
TTGTGTA--AAT
1DAI19796 AC-AAACTTTAAAATCA----GTTTAGAATG-TTTA--
TTGTGTA--AAT
ZQY797Dai25241 AC-AAACTTTAAAATCA----GTTTAGAATG-TTTA--
TTGTGTA--AAT
WCG1289Dai24711 AC-AAACTTTAAAATCA----GTTTAGAATG-TTTA--
TTGTGTA--AAT
Dai22598 AT-AAACTTCTTAATCA----GTTCAGAATG-TCTA--
TTGTGTT--AAT
Dai20523 AT-AAACTTCTTAATCA----GTTCAGAATG-TCTA--
TTGTGTT--AAT
Dai17885 AT-AAACTTCTTAATCA----GTTCAGAATG-TCTA--
TTGTGTT--AAT
Dai17553 AT-AAACTTTTTAATCA----GTTCAGAATG-TCTA--
TTGTGTT--AAT
Dai19639 AT-AAACTTCTTAATCA----GTTCAGAATG-TCTA--
TTGTGTT--AAT

JV0110_48_CZ AT-AAACTCTGAAACA-----GTCTTGAATG-TCATC-
TTGCGCAT-AAT
MJ129_04 AT-AAACTCTGAAACA-----GTCTTGAATG-TCATC-
TTGCGCAT-AAT
Cui10340P_eminens AC-AAACTCTGAAACA-----GTCTTGAATG-TCATC-
TTGCGCAT-AAT
Cui10341P_eminens AC-AAACTCTGAAACA-----GTCTTGAATG-TCATC-
TTGCGCAT-AAT
Dai12685P_eminens AT-AAACTCTGAAACA-----GTCTTGAATG-TCATC-
TTGCGCAT-AAT
Miettinen_13591Rigidoporus_und AT-AAACTCTGAAACA-----GTCTTGAATG-TCATC-
TTGCGCAT-AAT
Dai20868 AC-AAACTCTGAAACA-----GTCTTGAATG-TCATC-
TTGCGCAT-AAT
Dai20832 AC-AAACTCTGAAACA-----GTCTTGAATG-TCATC-
TTGCGCAT-AAT
Dai11400 AC-AAACTCTGAAACA-----GTCTTGAATG-TCATC-
TTGCGCAT-AAT
Dai22472 AC-AAACTCTGAAACA-----GTCTTGAATG-TCATC-
TTGCGCAT-AAT
1CUI10475 AC-AAACTCTGAAACA-----GTCTTGAATG-TCACC-
TTGCGCAT-AAT
1CUI10491 AC-AAACTCTGAAACA-----GTCTTGAATG-TCACC-
TTGCGCAT-AAT
HCFC1095Meripilus_robledoi AC-AAACTCTGAAACA-----GTCTTGAATG-TCATC-
TTGCGCAT-AAT
MCW702Meripilus_revolubilis AC-AAACTCCGAAACA-----GTCTTGAATG-TCATC-
TTGCGCAT-AAT
1704_83_zluty_HaciendaBaru AC-AAACTCATTGTCA-----GTTATGAATG-
TTCATTATGTGTGCGCAT
Dai9925P_lavendulus AT-AAACTCGTTGTCA-----GTTTAGAATG-
TCTATCTTGTGTA--TAT
Dai13587AP_lavendulus AT-AAACTCGTTGTCA-----GTTTAGAATG-
TCTATCTTGTGTA--TAT
PDD70600P_longicystidius AT-AAACTTGTTGTCA-----GTTTTGAATG-
TCTATTTTGTGTA--TAT
Cui16630 AT-AAACTTGTTGTCA-----GTTTTGAATG-
TCTATTTTGTGTA--TAT
FP_135344Meripilus_giganteus AC-AAACCTTTTAATCA----GTCTTGAATG-TTTA--
TCGCGCG--CAT
FP_100460_Sp AC-AAACCTTTTAATCA----GTCTTGAATG-TTTA--
TCGCGCG--CAT
CBS421_48Meripilus_giganteus AC-AAACCTTTTAATCA----GTCTTGAATG-TTTA--
TCGCGCG--CAT

Cui9203 AC-AAACCTTTTAATCA----GTCTTGAATG-TTTA--
TCGCGCG--CAT
Cui9202 AC-AAACCTTTTAATCA----GTCTTGAATG-TTTA--
TCGCGCG--CAT
TUFC100564Japan AC-AAACCTTTAAATCA----GTCTTGAATGTTTTA--
TCGCGCG--CAT
Russell5913Meripilus_sumstinei AC-AAACCTTTGAATCA----GTTTTGAATG-TTTA--
TCGTGCA--TAT
RP215Meripilus_brasiliensis AC-AAACTTTTAAATCA----GTCTTGAATG-TTAT--
TCGCACAT-GAT
RP200Meripilus_brasiliensis AC-AAACTTTTAAATCA----GTCTTGAATG-TTAT--
TCGCACAT-GAT
JV1712_13J_R_vinctus2_LSUPuert AC-AAACGCTTCAG-------TTTTAGAATG-TCAT--
CCGCGTAT---A
Dai10503R_hypobrunneus AT-AAACGCTTCAG-------TTTTAGAATG-TCAT--
CCGCGTAT---A
Dai10569Rigidoporus_hypobrunne AT-AAACGCTTCAG-------TTTTAGAATG-TCAT--
CCGCGTAT---A
1DAI19451 AT-AAACGCTTCAG-------TTTTAGAATG-TCAT--
CCGCGTAT---A
CM108bRigidoporus_hypobrunneus AC-AAACGCTTCAG-------TTTTAGAATG-TCAT--
CCGCGTAT---A
1CUI16874 AC-AAACGCTTCAG-------TTTTAGAATG-TCAT--
CCGCGTAT---A
FD299Cerrena_unicolor AC-AAACGCTTCAG-------TTTTAGAATG-TCAT--
TCGCGTAT---A
KHL_GB_Cerrena_uniclor AC-AAACGCTTCAG-------TTTTAGAATG-TCAT--
TCGCGTAT---A
Dai12892Cerrena_albocinnamomea AC-AAACGCTTTAG-------TTTTAGAATG-TCAT--
CCGCGTAT---A
Dai12955C_albocinnamomea AC-AAACGCTTTAG-------TTTTAGAATG-TCAT--
CCGCGTAT---A
SNUm03110102C_aurantiopora AC-AAACGCTTTAG-------TTTTAGAATG-TCAT--
CCGCGTAT---A
NIBRFG0000102423C_aurantiopora AC-AAACGCTTTAG-------TTTTAGAATG-TCAT--
CCGCGTAT---A
Dai7359Antrodiella_zonata AC-AAACGCTTTAG-------TTTTAGAATG-TCAA--
TCGCGTAT---A
F20080702KCM29C_consors AC-AAACGCTTTAG-------TTTTAGAATG-TCAA--
TCGCGTAT---A
F20080208LYW10Cerrena_consors AC-AAACGCTTTAG-------TTTTAGAATG-TCAA--
TCGCGTAT---A
Dai7821Antrodiella_zonata AC-AAACGCTTTAG-------TTTTAGAATG-TCAA--
TCGCGTAT---A

CFMR_DCL04_31Pseudolagarobasid AC-AAACGCTTTAG------TTATTAGAATG-TCAT--
CCGCGTAT---A
VPB197Pseudolagarobasidium_bel AC-AAACGCTTTAG------TTATTAGAATG-TCAT--
CCGCGTAT---A
CBS115543Pseudolagarobasidium_ AC-AAACGCTTTAG-------TATTAGAATG-TCAT--
CTGCGAAT---A
CBS115544 AC-AAACGCTTTAG-------TATTAGAATG-TCAT--
CTGCGAAT---A
Han405Pseudolagarobasidium_bai AC-AAACGCTTTAG------TAATCAGAATG-TCAT--
CTGCGAAT---A
Han406Pseudolagarobasidium_bai AC-AAACGCTTTAG------TAATCAGAATG-TCAT--
CTGCGAAT---A
MUcc838Spongipellis_delectans AC-AAACGCTTCAG------TTATAGAATG-TATC--
TCGCGTAT---A
BRNM686401S_delectans AC-AAACGCTTCAG------TTATAGAATG-TATC--
TCGCGTAT---A
OSM_F925S_delectans AC-AAACGCTTCAG------TTATAGAATG-TATC--
TCGCGTAT---A
BRNM67093Spongipellis_litschau AC-AAAYGCTTCAG------TTATAGAATG-TATC--
TCGCGTAT---A
CFMRccFP59199TS_unicolor AC-AAACGCTTCAG------TTATAGAATG-TATY--
TCGCGTAT---A
CFMRccFP71791TS_unicolor AC-AAACGCTTCAG------TTATAGAATG-TATC--
TCGCGTAT---A
Dai13845P_lischaueri AC-AAACGCTTCAG------TTATAGAATG-TATC--
TCGCGTAT---A
Dai20266P_lischaueri AC-AAACGCTTCAG------TTATAGAATG-TATC--
TCGCGTAT---A
CFMR_HHB11240Radulodon_america AC-AAACGCTTCAG-------TTTTAGAATG-TCATA-
TCGCATAC---A
RLG6350Radulodon_americanus AC-AAACGCTTCAG-------TTTTAGAATG-TCATA-
TCGCATAC---A
KY415963Radulodon_erikssonii AC-AAACGCTTCAG-------TTTTAGAATG-TCATG-
TCGCATAC---A
HHB9567spRadulodon_casearius AC-AAACGCTTCAG-------TTTTAGAATG-TCATA-
TCGCATAC---A
KRT_Iso_26Radulodon_casearius AC-AAACGCTTCAG-------TTTTAGAATG-TCATA-
TCGCATAC---A
CBS126044Radulodon_erikssonii AC-AAACGCTTCAG-------TTTTAGAATG-TCATG-
TCGCATAC---A
He6183YUNNANENSIS AC-AAACGCTTCAG-------TTTTAGAATG-
TCATA-TCGCATAC---A
Cui17979YUNNANENSIS AC-AAACGCTTCAG-------TTTTAGAATG-TCATA-
TCGCATAC---A

Miettinen2091Junghuhnia_fimbri AC-ACACGATTATG-------TTTAAGAATG-TCAA--
TACAATGCGTAA
KHL12316S_tenue AC-AAACAATCATG-------TTATAGAATG-TCAT--
TATCATGTGTAA
PRM846564S_pachyodon AC-AAACGCTTCAG-------TTTTAGAATG-TCAT--
CTGCGTAT---A
SP_Lgt_S_pachyodon AC-AAACGCTTCAG-------TTTTAGAATG-TCAT--
CTGCGTAT---A
Ryvarden44669Tyromyces_xuchile AC-AAACGCTTTAG-------TTTTAGAATG-TTAAT-
CTGCGCAT---A
PW17_171sinuosus AC-AAACGCTTCAG------TTTTAGAATG-TGAAT-
CTGCATAT---A
W53Dai12234 AC-AAACGCTTCAG------TTTTAGAATG-TGAAT-
CTGCATAT---A
HHB4100SpAntella_americana CC-ACACACTTTGAAAAG--TTTTTAGAATG-TAAC--
AATCATGCATCA
W3Dai20901spumeus AC-AAACCCATGTATAG----TTTTTGAATG-TCCAC-
ATGCTCAC-GAA
He6736 AC-AAACCCATGTATAG----TTTTTGAATG-TCCAC-
ATGCTCAC-GAA
BRNM734877S_spumeus AC-AAACCCATGTATAG----TTTTTGAATG-
TCGAY-ATGCTCAC-GAA
BRNM712630S_spumeus AC-AAACCCATGTATAG----TTTTTGAATG-
TCGAC-ATGCTCAY-GAA
Dai1723Loweomyces_sibiricus AC-AAACCCATGTATAG----TTTTTGAATG-TCGAC-
ATGCTCAT-GAA
W54Cui10009 AC-AAACCCATGTATAG----TTTTTGAATG-TCAAC-
ATGCTCAT-GAA
W1Dai20899 AC-AAACCCATGTATAG----TTTTTGAATG-TCAAC-
ATGCTCAT-GAA
HHB13445Trametes_ochracea AC-AAACACTTTAAAG-----TATCAGAATG----
TAAACGCGT---CTA
Dai16222 AAAAAACATACATCTGTTTGTTTAAGGAATG-
TTATTCATTTGTGCATCA
Dai16240 AAAAAACATACATCTGTTTGTTTAAGGAATG-
TTATTCATTTGTGCATCA

JV1310_11SanguinolentusCernys ACACA---
TTTAAATACAACTTTCAGCAACGGATCTCTTGGCTCTCGCA
MJ39_00_SK ACACA---
TTTAAATACAACTTTCAGCAACGGATCTCTTGGCTCTCGCA
MJ111_04_CZ ACACA---
TTTAAATACAACTTTCAGCAACGGATCTCTTGGCTCTCGCA
JV1610_BOKYsmrk ACACA---

TTTAAATACAACTTTCAGCAACGGATCTCTTGGCTCTCGCA
Dai21030 ACACA---
TTTAAATACAACTTTCAGCAACGGATCTCTTGGCTCTCGCA
Dai20976P_furcatus ACACA---
TTTAAATACAACTTTCAGCAACGGATCTCTTGGCTCTCGCA
Dai2105 ACACA---
TTTAAATACAACTTTCAGCAACGGATCTCTTGGCTCTCGCA
Dai2544 ACACA---
TTTAAATACAACTTTCAGCAACGGATCTCTTGGCTCTCGCA
Dai11313 ACACA---
TTTAAATACAACTTTCAGCAACGGATCTCTTGGCTCTCGCA
WCG1611Dai26167 ACACA---
TTTAAATACAACTTTCAGCAACGGATCTCTTGGCTCTCGCA
WCG1518Dai25999Physisporinus ACACA---
TTTAAATACAACTTTCAGCAACGGATCTCTTGGCTCTCGCA
TAA15097 ACACA---
TTTAAATACAACTTTCAGCAACGGATCTCTTGGCTCTCGCA
JV8909_19_CZ ACACA---
TTTAAATACAACTTTCAGCAACGGATCTCTTGGCTCTCGCA
JV1310_15_P_sanguinolentus2_CZ ACACA---
TTTAAATACAACTTTCAGCAACGGATCTCTTGGCTCTCGCA
MJ53_02_CZ ACACA---
TTTAAATACAACTTTCAGCAACGGATCTCTTGGCTCTCGCA
CLZhao21647P_yunnanensis ACACA---
TTTAAATACAACTTTCAGCAACGGATCTCTTGGCTCTCGCA
CLZhao21583P_yunnanensis ACACA---
TTTAAATACAACTTTCAGCAACGGATCTCTTGGCTCTCGCA
Dai22272 ACACA---
TTTAAATACAACTTTCAGCAACGGATCTCTTGGCTCTCGCA
Dai22279 ACACA---
TTTAAATACAACTTTCAGCAACGGATCTCTTGGCTCTCGCA
MJ332_94_CZ ACACA---
TTTAAATACAACTTTCAGCAACGGATCTCTTGGCTCTCGCA
MJ642_94_CZ_Expallescens ACACA---
TTTAAATACAACTTTCAGCAACGGATCTCTTGGCTCTCGCA
Dai21060P_vinctus ACACA---
TTTAAATACAACTTTCAGCAACGGATCTCTTGGCTCTCGCA
JV0511_23LRP_pouzarii ACACA---
TTTAAATACAACTTTCAGCAACGGATCTCTTGGCTCTCGCA
JQ409462_R_pouzarii_PRM899856_ ACACA---
TTTAAATACAACTTTCAGCAACGGATCTCTTGGCTCTCGCA
JV0308_66_WA ACACA---
TTTAATTACAACTTTCAGCAACGGATCTCTTGGCTCTCGCA
JV0309_45_WA_USA ACACA---

TTTAATTACAACTTTCAGCAACGGATCTCTTGGCTCTCGCA
JV0709_83_CA_USA ACACA----
TTTAATTACAACTTTCAGCAACGGATCTCTTGGCTCTCGCA
Dai21043P_pouzarii ACACA----
TTTAAATACAACTTTCAGCAACGGATCTCTTGGCTCTCGCA
MJ144_95_CZ ACACA----
TTTAAATACAACTTTCAGCAACGGATCTCTTGGCTCTCGCA
JV0909_3_CZ ACACA----
TTTAAATACAACTTTCAGCAACGGATCTCTTGGCTCTCGCA
JV0609_1_K ACACA----
TTTAAATACAACTTTCAGCAACGGATCTCTTGGCTCTCGCA
Dai20396Physisporinus_castanop ACACA----
TTTAAATACAACTTTCAGCAACGGATCTCTTGGCTCTCGCA
Dai20397Physisporinus_castanop ACACA----
TTTAAATACAACTTTCAGCAACGGATCTCTTGGCTCTCGCA
MJ19_09_SK_Abies ACACA----
TTTAAATACAACTTTCAGCAACGGATCTCTTGGCTCTCGCA
JV0509_40_J_TN_USA_Betula ACACA----
TTTAAATACAACTTTCAGCAACGGATCTCTTGGCTCTCGCA
JV0808_33crocatus_PA_USAlist ACACA----
TTTAAATACAACTTTCAGCAACGGATCTCTTGGCTCTCGCA
DLL2009_061P_crocatus ACACA----
TTTAAATACAACTTTCAGCAACGGATCTCTTGGCTCTCGCA
Dai12800P_subcrocatus ACACA----
TTTAAATACAACTTTCAGCAACGGATCTCTTGGCTCTCGCA
Dai15917P_subcrocatus ACACA----
TTTAAATACAACTTTCAGCAACGGATCTCTTGGCTCTCGCA
Cui16620 ACACA----
TTTAAATACAACTTTCAGCAACGGATCTCTTGGCTCTCGCA
HCFC1088Meripilus_stillicidior ACACA----
TTTAAATACAACTTTCAGCAACGGATCTCTTGGCTCTCGCA
MCW590Meripilus_obsrcurus ACACA----
TTTAAATACAACTTTCAGCAACGGATCTCTTGGCTCTCGCA
MCW722Meripilus_obsrcurus ACACA----
TTTAAATACAACTTTCAGCAACGGATCTCTTGGCTCTCGCA
Cui9381P_tibeticus ACACA----
TTTAAATACAACTTTCAGCAACGGATCTCTTGGCTCTCGCA
Cui9588P_tibeticus ACACA----
TTTAAATACAACTTTCAGCAACGGATCTCTTGGCTCTCGCA
Va2_Beneschova ACACA----
TTTAAATACAACTTTCAGCAACGGATCTCTTGGCTCTCGCA
CWU3874_Ukraine_Alnus ACACA----
TTTAAATACAACTTTCAGCAACGGATCTCTTGGCTCTCGCA
WCG1293Dai24718Physisporinus ACACA----

TTGAAATACAACTTTCAGCAACGGATCTCTTGGCTCTCGCA
WCG1268Dai24682A ACACA----
ATTTAATACAACTTTCAGCAACGGATCTCTTGGCTCTCGCA
WCG1269Dai24683A ACACA----
ATTTAATACAACTTTCAGCAACGGATCTCTTGGCTCTCGCA
WCG1279Dai24694A ACACA----
ATTTAATACAACTTTCAGCAACGGATCTCTTGGCTCTCGCA
Dai16971 ACACA----
ATTTAATACAACTTTCAGCAACGGATCTCTTGGCTCTCGCA
ZQY1043Dai26696 ACACA----
ATTTAATACAACTTTCAGCAACGGATCTCTTGGCTCTCGCA
Doll880 ACACA----
TTTAAATACAACTTTCAGCAACGGATCTCTTGGCTCTCGCA
Doll1000 ACACA----
TTTAAATACAACTTTCAGCAACGGATCTCTTGGCTCTCGCA
1DAI18529 ACACA----
TTTAAATACAACTTTCAGCAACGGATCTCTTGGCTCTCGCA
Dai19535 GCACA----
TTTATATACAACTTTCAGCAACGGATCTCTTGGCTCTCGCA
1704_79_hnedyVillaLaPaz ACACA----
ATGAAATACAACTTTCAGCAACGGATCTCTTGGCTCTCGCA
F2061 ACACA----
ATGAAATACAACTTTCAGCAACGGATCTCTTGGCTCTCGCA
1DAI18268 ACACA----
ATGAAATACAACTTTCAGCAACGGATCTCTTGGCTCTCGCA
1DAI18540A ACACA----
ATGAAATACAACTTTCAGCAACGGATCTCTTGGCTCTCGCA
Dai17695 ACACA----
ATGAAATACAACTTTCAGCAACGGATCTCTTGGCTCTCGCA
LKY18Dai26373 ACACA----
ATGAAATACAACTTTCAGCAACGGATCTCTTGGCTCTCGCA
Dai17839P_sulphureus ACACAT---
TTGAAATACAACTTTCAGCAACGGATCTCTTGGCTCTCGCA
Dai17841P_sulphureus ACACAT---
TTGAAATACAACTTTCAGCAACGGATCTCTTGGCTCTCGCA
Dai19877P_roseus ACACAT---
TTGAAATACAACTTTCAGCAACGGATCTCTTGGCTCTCGCA
1508_18_1_Kout GCACA----
ATTAAATACAACTTTCAGCAACGGATCTCTTGGCTCTCGCA
KP859303_R_vinctus_RP185_BRAZI GCACA----
ATTAAATACAACTTTCAGCAACGGATCTCTTGGCTCTCGCA
JK1807_15Rigidoporus_sp_Puerto GCACA----
ATTAAATACAACTTTCAGCAACGGATCTCTTGGCTCTCGCA
JV0509_47_J_TN_USA ACACA----

TTTTAATACAACTTTCAGCAACGGATCTCTTGGCTCTCGCA
JV0709_188 ACACA----
TTTTAATACAACTTTCAGCAACGGATCTCTTGGCTCTCGCA
JV0509_127_PA_USA ACACA----
TTTTAATACAACTTTCAGCAACGGATCTCTTGGCTCTCGCA
JV1009_59_NJ_USA ACACA----
TTTTAATACAACTTTCAGCAACGGATMTCTTAGGGCTCGCA
Dai15497P_crataegi ACACA----
TTTTAATACAACTTTCAGCAACGGATCTCTTGGCTCTCGCA
Dai15499P_crataegi ACACA----
TTTTAATACAACTTTCAGCAACGGATCTCTTGGCTCTCGCA
Cui3266P_cinereus ACACA----
TCTTAATACAACTTTCAGCAACGGATCTCTTGGCTCTCGCA
WCG1256Dai24690 ACACA----
TCTTAATACAACTTTCAGCAACGGATCTCTTGGCTCTCGCA
1DAI17581 ACACA----
TCTTAATACAACTTTCAGCAACGGATCTCTTGGCTCTCGCA
WCG1255Dai24688 ACACA----
TCTTAATACAACTTTCAGCAACGGATCTCTTGGCTCTCGCA
Dai22427 ACACA----
TCTTAATACAACTTTCAGCAACGGATCTCTTGGCTCTCGCA
MV690Meripilus_concrescens ACACAT---
TTTTAATACAACTTTCAGCAACGGATCTCTTGGCTCTCGCA
MV513Meripilus_galapagensis ACACA----
TTTTAATACAACTTTCAGCAACGGATCTCTTGGCTCTCGCA
Dai19793 GCACA----
TTTAAATACAACTTTCAGCAACGGATCTCTTGGCTCTCGCA
OQ553780P_tamilnaduensis ACACA-----
TTTAATACAACTTTCAGCAACGGATCTCTTGGCTCTCGCA
OQ553779P_tamilnaduensis ACACA-----
TTTAATACAACTTTCAGCAACGGATCTCTTGGCTCTCGCA
A164FB3Meripilus_giganteus ACACA-----
TTTAATACAACTTTCAGCAACGGATCTCTTGGCTCTCGCA
JV1407_36_Vinctus_Meandrica ACACA----
TTTTTATACAACTTTCAGCAACGGATCTCTTGGCTCTCGCA
1807_3K_Rigidoporus_PuertoRico ACACA----
TTTTTATACAACTTTCAGCAACGGATCTCTTGGCTCTCGCA
Cui16903P_vinctus ACACA----
TTTTTATACAACTTTCAGCAACGGATCTCTTGGCTCTCGCA
JV1008_18R_Lineatus ACACA----
TTTTTATACAACTTTCAGCAACGGATCTCTTGGCTCTCGCA
JV1407_37_1_Vinctus_Carara ACACA----
TTTTTATACAACTTTCAGCAACGGATCTCTTGGCTCTCGCA
Dai17986P_lineatus ACACA----

TTTTTATACAACTTTCAGCAACGGATCTCTTGGCTCTCGCA
Dai18281 ACACA----
TTTTTATACAACTTTCAGCAACGGATCTCTTGGCTCTCGCA
1DAI19796 ACACA----
TTTTTATACAACTTTCAGCAACGGATCTCTTGGCTCTCGCA
ZQY797Dai25241 ACACA----
TTTTTATACAACTTTCAGCAACGGATCTCTTGGCTCTCGCA
WCG1289Dai24711 ACACA----
TTTTTATACAACTTTCAGCAACGGATCTCTTGGCTCTCGCA
Dai22598 ACACA----
TCTTTATACAACTTTCAGCAACGGATCTCTTGGCTCTCGCA
Dai20523 ACACA----
TCTTTATACAACTTTCAGCAACGGATCTCTTGGCTCTCGCA
Dai17885 ACACA----
TCTTTATACAACTTTCAGCAACGGATCTCTTGGCTCTCGCA
Dai17553 ACACA----
TCTTTATACAACTTTCAGCAACGGATCTCTTGGCTCTCGCA
Dai19639 ACACA----
TCTTTATACAACTTTCAGCAACGGATCTCTTGGCTCTCGCA
JV0110_48_CZ ACGCA----
TTTAAATACAACTTTCAGCAACGGATCTCTTGGCTCTCGCA
MJ129_04 ACGCA----
TTTAAATACAACTTTCAGCAACGGATCTCTTGGCTCTCGCA
Cui10340P_eminens ACGCA----
TTTAAATACAACTTTCAGCAACGGATCTCTTGGCTCTCGCA
Cui10341P_eminens ACGCA----
TTTAAATACAACTTTCAGCAACGGATCTCTTGGCTCTCGCA
Dai12685P_eminens ACGCA----
TTTAAATACAACTTTCAGCAACGGATCTCTTGGCTCTCGCA
Miettinen_13591Rigidoporus_und ACGCA----
TTTAAATACAACTTTCAGCAACGGATCTCTTGGCTCTCGCA
Dai20868 ACGCA----
TTTAAATACAACTTTCAGCAACGGATCTCTTGGCTCTCGCA
Dai20832 ACGCA----
TTTAAATACAACTTTCAGCAACGGATCTCTTGGCTCTCGCA
Dai11400 ACGCA----
TTTAAATACAACTTTCAGCAACGGATCTCTTGGCTCTCGCA
Dai22472 ACGCA----
TTTAAATACAACTTTCAGCAACGGATCTCTTGGCTCTCGCA
1CUI10475 ACGCA----
TTTAAATACAACTTTCAGCAACGGATCTCTTGGCTCTCGCA
1CUI10491 ACGCA----
TTTAAATACAACTTTCAGCAACGGATCTCTTGGCTCTCGCA
HCFC1095Meripilus_robledoi ACGCA----

TTTAAATACAACTTTCAGCAACGGATCTCTTGGCTCTCGCA
MCW702Meripilus_revolubilis ACGCA----
TCTAAATACAACTTTCAGCAACGGATCTCTTGGCTCTCGCA
1704_83_zluty_HaciendaBaru GCGCA----
TTGAAATACAACTTTCAGCAACGGATCTCTTGGCTCTCGCA
Dai9925P_lavendulus GCACA----
TTGAAATACAACTTTCAGCAACGGATCTCTTGGCTCTCGCA
Dai13587AP_lavendulus GCACA----
TTGAAATACAACTTTCAGCAACGGATCTCTTGGCTCTCGCA
PDD70600P_longicystidius GCACA----
TTGAAATACAACTTTCAGCAACGGATCTCTTGGCTCTCGCA
Cui16630 GCACA----
TTGAAATACAACTTTCAGCAACGGATCTCTTGGCTCTCGCA
FP_135344Meripilus_giganteus
GCGCATTTAAATCAATACAACTTTCAGCAACGGATCTCTTGGCTCTCGCA
FP_100460_Sp
GCGCATTTAAATCAATACAACTTTCAGCAACGGATCTCTTGGCTCTCGCA
CBS421_48Meripilus_giganteus
GCGCATTTAAATCAATACAACTTTCAGCAACGGATCTCTTGGCTCTCGCA
Cui9203
GCGCATTTAAATCAATACAACTTTCAGCAACGGATCTCTTGGCTCTCGCA
Cui9202
GCGCATTTAAATCAATACAACTTTCAGCAACGGATCTCTTGGCTCTCGCA
TUFC100564Japan
GCGCATTTAAATCAATACAACTTTCAGCAACGGATCTCTTGGCTCTCGCA
Russell5913Meripilus_sumstinei
GCACATTTAAATTAATACAACTTTCAGCAACGGATCTCTTGGCTCTCGCA
RP215Meripilus_brasiliensis GTGCATAT-
AAATAATACAACTTTCAGCAACGGATCTCTTGGCTCTCGCA
RP200Meripilus_brasiliensis GTGCATAT-
AAATAATACAACTTTCAGCAACGGATCTCTTGGCTCTCGCA
JV1712_13J_R_vinctus2_LSUPuert ACGCA----A-
TAAATACAACTTTCAGCAACGGATCTCTTGGCTCTCGCA
Dai10503R_hypobrunneus ACGCA----A-
TAAATACAACTTTCAGCAACGGATCTCTTGGCTCTCGCA
Dai10569Rigidoporus_hypobrunne ACGCA----A-
TAAATACAACTTTCAGCAACGGATCTCTTGGCTCTCGCA
1DAI19451 ACGCA----A-
TAAATACAACTTTCAGCAACGGATCTCTTGGCTCTCGCA
CM108bRigidoporus_hypobrunneus ACGCA----A-
TAAATACAACTTTCAGCAACGGATCTCTTGGCTCTCGCA
1CUI16874 ACGCA----A-
TAAATACAACTTTCAGCAACGGATCTCTTGGCTCTCGCA
FD299Cerrena_unicolor ACGCA----A-

TAAATACAACTTTCAGCAACGGATCTCTTGGCTCTCGCA
KHL_GB_Cerrena_uniclor ACGCA----A-
TAAATACAACTTTCAGCAACGGATCTCTTGGCTCTCGCA
Dai12892Cerrena_albocinnamomea ACGCA----A-
TAAATACAACTTTCAGCAACGGATCTCTTGGCTCTCGCA
Dai12955C_albocinnamomea ACGCA----A-
TAAATACAACTTTCAGCAACGGATCTCTTGGCTCTCGCA
SNUm03110102C_aurantiopora ACGCA----A-
TAAATACAACTTTCAGCAACGGATCTCTTGGCTCTCGCA
NIBRFG0000102423C_aurantiopora ACGCA----A-
TAAATACAACTTTCAGCAACGGATCTCTTGGCTCTCGCA
Dai7359Antrodiella_zonata ACGCA----A-
TAAATACAACTTTTAGCAACGGATCTCTTGGCTCTCGCA
F20080702KCM29C_consors ACGCA----A-
TAAATACAACTTTTAGCAACGGATCTCTTGGCTCTCGCA
F20080208LYW10Cerrena_consors ACGCA----A-
TAAATACAACTTTTAGCAACGGATCTCTTGGCTCTCGCA
Dai7821Antrodiella_zonata ACGCA----A-
TAAATACAACTTTTAGCAACGGATCTCTTGGCTCTCGCA
CFMR_DCL04_31Pseudolagarobasid ACGCA----A-
TAAATACAACTTTCAGCAACGGATCTCTTGGCTCTCGCA
VPB197Pseudolagarobasidium_bel ACGCA----A-
TAAATACAACTTTCAGCAACGGATCTCTTGGCTCTCGCA
CBS115543Pseudolagarobasidium_ ACGCA----A-
TAAATACAACTTTCAGCAACGGATCTCTTGGCTCTCGCA
CBS115544 ACGCA----A-
TAAATACAACTTTCAGCAACGGATCTCTTGGCTCTCGCA
Han405Pseudolagarobasidium_bai ACGCA----A-
TAAATACAACTTTCAGCAACGGATCTCTTGGCTCTCGCA
Han406Pseudolagarobasidium_bai ACGCA----A-
TAAATACAACTTTCAGCAACGGATCTCTTGGCTCTCGCA
MUcc838Spongipellis_delectans ACGCA----T-
TATATACAACTTTCAGCAACGGATCTCTTGGCTCTCGCA
BRNM686401S_delectans ACGCA----T-
TATATACAACTTTCAGCAACGGATCTCTTGGCTCTCGCA
OSM_F925S_delectans ACGCA----T-
TATATACAACTTTCAGCAACGGATCTCTTGGCTCTCGCA
BRNM67093Spongipellis_litschau ACGCA----T-
TATATACAACTTTCAGCAACGGATCTCTTGGCTCTCGCA
CFMRccFP59199TS_unicolor ACGCA----T-
TATATACAACTTTCAGCAACGGATCTCTTGGCTCTCGCA
CFMRccFP71791TS_unicolor ACGCA----T-
TATATACAACTTTCAGCAACGGATCTCTTGGCTCTCGCA
Dai13845P_lischaueri ACGCA----T-

TATATACAACTTTCAGCAACGGATCTCTTGGCTCTCGCA
Dai20266P_lischaueri ACGCA----T-
TATATACAACTTTCAGCAACGGATCTCTTGGCTCTCGCA
CFMR_HHB11240Radulodon_america ATGCA----A-
TATATACAACTTTCAGCAACGGATCTCTTGGCTCTCGCA
RLG6350Radulodon_americanus ATGCA----A-
TATATACAACTTTCAGCAACGGATCTCTTGGCTCTCGCA
KY415963Radulodon_erikssonii ATGCA----A-
TATATACAACTTTCAGCAACGGATCTCTTGGCTCTCGCA
HHB9567spRadulodon_casearius ATGCA----A-
TATATACAACTTTCAGCAACGGATCTCTTGGCTCTCGCA
KRT_Iso_26Radulodon_casearius ATGCA----A-
TATATACAACTTTCAGCAACGGATCTCTTGGCTCTCGCA
CBS126044Radulodon_erikssonii ATGCA----A-
TATATACAACTTTCAGCAACGGATCTCTTGGCTCTCGCA
He6183YUNNANENSIS ATGCA----A-
TATATACAACTTTCAGCAACGGATCTCTTGGCTCTCGCA
Cui17979YUNNANENSIS ATGCA----A-
TATATACAACTTTCAGCAACGGATCTCTTGGCTCTCGCA
Miettinen2091Junghuhnia_fimbri ATGCA----T-
ATAATACAACTTTCAACAACGGATCTCTTGGCTCTCGCA
KHL12316S_tenue AAGCA----T-
ATAATACAACTTTCAGCAACGGATCTCTTGGCTCTCGCA
PRM846564S_pachyodon ACGCA----
ACTATATACAACTTTCAGCAACGGATCTCTTGGCTCTCGCA
SP_Lgt_S_pachyodon ACGCA----
ACTATATACAACTTTCAGCAACGGATCTCTTGGCTCTCGCA
Ryvarden44669Tyromyces_xuchile ACGCA----
ACTATATACAACTTTCAGCAACGGATCTCTTGGCTCTCGCA
PW17_171sinuosus ATGCA----
ACAATATACAACTTTCAGCAACGGATCTCTTGGCTCTCGCA
W53Dai12234 ATGCA----
ACAGTATACAACTTTCAGCAACGGATCTCTTGGCTCTCGCA
HHB4100SpAntella_americana ATGCA----T-
ATAATACAACTTTCAGCAACGGATCTCTTGGCTCTCGCA
W3Dai20901spumeus GCATT----
TTATAATACAACTTTCAGCAACGGATCTCTTGGCTCTCGCA
He6736 GCATT----
TTATAATACAACTTTCAGCAACGGATCTCTTGGCTCTCGCA
BRNM734877S_spumeus GCATT----
TWATAATACAACTTTCAGCAACGGATCTCTTGGCTCTCGCA
BRNM712630S_spumeus GCATT----
TWATAATACAACTTTCAGCAACGGATCTCTTGGCTCTCGCA
Dai1723Loweomyces_sibiricus GCATT----T-

ATAATACAACTTTCAGCAACGGATCTCTTGGCTCTCGCA
W54Cui10009 GCATC----T-
ATAATACAACTTTCAGCAACGGATCTCTTGGCTCTCGCA
W1Dai20899 GCATC----T-
ATAATACAACTTTCAGCAACGGATCTCTTGGCTCTCGCA
HHB13445Trametes_ochracea ACGCAT--
CTATAATACAACTTTTAGCAACGGATCTCTTGGCTCTCGCA
Dai16222 TTGCA---
TATAAATACAACTTTCAGCAACGGATCTCTTGGCTCTCGCA
Dai16240 TTGCA---
TATAAATACAACTTTCAGCAACGGATCTCTTGGCTCTCGCA

JV1310_11SanguinolentusCernys
TCGATGAAGAACGCAGCGAAATGCGATAAGTAATGTGAATTGCAGAATTC
MJ39_00_SK
TCGATGAAGAACGCAGCGAAATGCGATAAGTAATGTGAATTGCAGAATTC
MJ111_04_CZ
TCGATGAAGAACGCAGCGAAATGCGATAAGTAATGTGAATTGCAGAATTC
JV1610_BOKYsmrk
TCGATGAAGAACGCAGCGAAATGCGATAAGTAATGTGAATTGCAGAATTC
Dai21030
TCGATGAAGAACGCAGCGAAATGCGATAAGTAATGTGAATTGCAGAATTC
Dai20976P_furcatus
TCGATGAAGAACGCAGCGAAATGCGATAAGTAATGTGAATTGCAGAATTC
Dai2105
TCGATGAAGAACGCAGCGAAATGCGATAAGTAATGTGAATTGCAGAATTC
Dai2544
TCGATGAAGAACGCAGCGAAATGCGATAAGTAATGTGAATTGCAGAATTC
Dai11313
TCGATGAAGAACGCAGCGAAATGCGATAAGTAATGTGAATTGCAGAATTC
WCG1611Dai26167
TCGATGAAGAACGCAGCGAAATGCGATAAGTAATGTGAATTGCAGAATTC
WCG1518Dai25999Physisporinus
TCGATGAAGAACGCAGCGAAATGCGATAAGTAATGTGAATTGCAGAATTC
TAA15097
TCGATGAAGAACGCAGCGAAATGCGATAAGTAATGTGAATTGCAGAATTC
JV8909_19_CZ
TCGATGAAGAACGCAGCGAAATGCGATAAGTAATGTGAATTGCAGAATTC
JV1310_15_P_sanguinolentus2_CZ
TCGATGAAGAACGCAGCGAAATGCGATAAGTAATGTGAATTGCAGAATTC
MJ53_02_CZ
TCGATGAAGAACGCAGCGAAATGCGATAAGTAATGTGAATTGCAGAATTC
CLZhao21647P_yunnanensis
TCGATGAAGAACGCAGCGAAATGCGATAAGTAATGTGAATTGCAGAATTC

CLZhao21583P_yunnanensis
TCGATGAAGAACGCAGCGAAATGCGATAAGTAATGTGAATTGCAGAATTC
Dai22272
TCGATGAAGAACGCAGCGAAATGCGATAAGTAATGTGAATTGCAGAATTC
Dai22279
TCGATGAAGAACGCAGCGAAATGCGATAAGTAATGTGAATTGCAGAATTC
MJ332_94_CZ
TCGATGAAGAACGCAGCGAAATGCGATAAGTAATGTGAATTGCAGAATTC
MJ642_94_CZ_Expallescens
TCGATGAAGAACGCAGCGAAATGCGATAAGTAATGTGAATTGCAGAATTC
Dai21060P_vinctus
TCGATGAAGAACGCAGCGAAATGCGATAAGTAATGTGAATTGCAGAATTC
JV0511_23LRP_pouzarii
TCGATGAAGAACGCAGCGAAATGCGATAAGTAATGTGAATTGCAGAATTC
JQ409462_R_pouzarii_PRM899856_
TCGATGAAGAACGCAGCGAAATGCGATAAGTAATGTGAATTGCAGAATTC
JV0308_66_WA
TCGATGAAGAACGCAGCGAAATGCGATAAGTAATGTGAATTGCAGAATTC
JV0309_45_WA_USA
TCGATGAAGAACGCAGCGAAATGCGATAAGTAATGTGAATTGCAGAATTC
JV0709_83_CA_USA
TCGATGAAGAACGCAGCGAAATGCGATAAGTAATGTGAATTGCAGAATTC
Dai21043P_pouzarii
TCGATGAAGAACGCAGCGAAATGCGATAAGTAATGTGAATTGCAGAATTC
MJ144_95_CZ
TCGATGAAGAACGCAGCGAAATGCGATAAGTAATGTGAATTGCAGAATTC
JV0909_3_CZ
TCGATGAAGAACGCAGCGAAATGCGATAAGTAATGTGAATTGCAGAATTC
JV0609_1_K
TCGATGAAGAACGCAGCGAAATGCGATAAGTAATGTGAATTGCAGAATTC
Dai20396Physisporinus_castanop
TCGATGAAGAACGCAGCGAAATGCGATAAGTAATGTGAATTGCAGAATTC
Dai20397Physisporinus_castanop
TCGATGAAGAACGCAGCGAAATGCGATAAGTAATGTGAATTGCAGAATTC
MJ19_09_SK_Abies
TCGATGAAGAACGCAGCGAAATGCGATAAGTAATGTGAATTGCAGAATTC
JV0509_40_J_TN_USA_Betula
TCGATGAAGAACGCAGCGAAATGCGATAAGTAATGTGAATTGCAGAATTC
JV0808_33crocatus_PA_USAlist
TCGATGAAGAACGCAGCGAAATGCGATAAGTAATGTGAATTGCAGAATTC
DLL2009_061P_crocatus
TCGATGAAGAACGCAGCGAAATGCGATAAGTAATGTGAATTGCAGAATTC
Dai12800P_subcrocatus
TCGATGAAGAACGCAGCGAAATGCGATAAGTAATGTGAATTGCAGAATTC

Dai15917P_subcrocatus
TCGATGAAGAACGCAGCGAAATGCGATAAGTAATGTGAATTGCAGAATTC
Cui16620
TCGATGAAGAACGCAGCGAAATGCGATAAGTAATGTGAATTGCAGAATTC
HCFC1088Meripilus_stillicidior
TCGATGAAGAACGCAGCGAAATGCGATAAGTAATGTGAATTGCAGAATTC
MCW590Meripilus_obsrcurus
TCGATGAAGAACGCAGCGAAATGCGATAAGTAATGTGAATTGCAGAATTC
MCW722Meripilus_obsrcurus
TCGATGAAGAACGCAGCGAAATGCGATAAGTAATGTGAATTGCAGAATTC
Cui9381P_tibeticus
TCGATGAAGAACGCAGCGAAATGCGATAAGTAATGTGAATTGCAGAATTC
Cui9588P_tibeticus
TCGATGAAGAACGCAGCGAAATGCGATAAGTAATGTGAATTGCAGAATTC
Va2_Beneschova
TCGATGAAGAACGCAGCGAAATGCGATAAGTAATGTGAATTGCAGAATTC
CWU3874_Ukraine_Alnus
TCGATGAAGAACGCAGCGAAATGCGATAAGTAATGTGAATTGCAGAATTC
WCG1293Dai24718Physisporinus
TCGATGAAGAACGCAGCGAAATGCGATAAGTAATGTGAATTGCAGAATTC
WCG1268Dai24682A
TCGATGAAGAACGCAGCGAAATGCGATAAGTAATGTGAATTGCAGAATTC
WCG1269Dai24683A
TCGATGAAGAACGCAGCGAAATGCGATAAGTAATGTGAATTGCAGAATTC
WCG1279Dai24694A
TCGATGAAGAACGCAGCGAAATGCGATAAGTAATGTGAATTGCAGAATTC
Dai16971
TCGATGAAGAACGCAGCGAAATGCGATAAGTAATGTGAATTGCAGAATTC
ZQY1043Dai26696
TCGATGAAGAACGCAGCGAAATGCGATAAGTAATGTGAATTGCAGAATTC
Doll880
TCGATGAAGAACGCAGCGAAATGCGATAAGTAATGTGAATTGCAGAATTC
Doll1000
TCGATGAAGAACGCAGCGAAATGCGATAAGTAATGTGAATTGCAGAATTC
1DAI18529
TCGATGAAGAACGCAGCGAAATGCGATAAGTAATGTGAATTGCAGAATTC
Dai19535
TCGATGAAGAACGCAGCGAAATGCGATAAGTAATGTGAATTGCAGAATTC
1704_79_hnedyVillaLaPaz
TCGATGAAGAACGCAGCGAAATGCGATAAGTAATGTGAATTGCAGAATTC
F2061
TCGATGAAGAACGCAGCGAAATGCGATAAGTAATGTGAATTGCAGAATTC
1DAI18268
TCGATGAAGAACGCAGCGAAATGCGATAAGTAATGTGAATTGCAGAATTC

1DAI18540A
TCGATGAAGAACGCAGCGAAATGCGATAAGTAATGTGAATTGCAGAATTC
Dai17695
TCGATGAAGAACGCAGCGAAATGCGATAAGTAATGTGAATTGCAGAATTC
LKY18Dai26373
TCGATGAAGAACGCAGCGAAATGCGATAAGTAATGTGAATTGCAGAATTC
Dai17839P_sulphureus
TCGATGAAGAACGCAGCGAAATGCGATAAGTAATGTGAATTGCAGAATTC
Dai17841P_sulphureus
TCGATGAAGAACGCAGCGAAATGCGATAAGTAATGTGAATTGCAGAATTC
Dai19877P_roseus
TCGATGAAGAACGCAGCGAAATGCGATAAGTAATGTGAATTGCAGAATTC
1508_18_1_Kout
TCGATGAAGAACGCAGCGAAATGCGATAAGTAATGTGAATTGCAGAATTC
KP859303_R_vinctus_RP185_BRAZI
TCGATGAAGAACGCAGCGAAATGCGATAAGTAATGTGAATTGCAGAATTC
JK1807_15Rigidoporus_sp_Puerto
TCGATGAAGAACGCAGCGAAATGCGATAAGTAATGTGAATTGCAGAATTC
JV0509_47_J_TN_USA
TCGATGAAGAACGCAGCGAAATGCGATAAGTAATGTGAATTGCAGAATTC
JV0709_188
TCGATGAAGAACGCAGCGAAATGCGATAAGTAATGTGAATTGCAGAATTC
JV0509_127_PA_USA
TCGATGAAGAACGCAGCGAAATGCGATAAGTAATGTGAATTGCAGAATTC
JV1009_59_NJ_USA
TCGATGAAGAACGCAGCGAAATGCGATAAGTAATGTGAATTGCAGAATTC
Dai15497P_crataegi
TCGATGAAGAACGCAGCGAAATGCGATAAGTAATGTGAATTGCAGAATTC
Dai15499P_crataegi
TCGATGAAGAACGCAGCGAAATGCGATAAGTAATGTGAATTGCAGAATTC
Cui3266P_cinereus
TCGATGAAGAACGCAGCGAAATGCGATAAGTAATGTGAATTGCAGAATTC
WCG1256Dai24690
TCGATGAAGAACGCAGCGAAATGCGATAAGTAATGTGAATTGCAGAATTC
1DAI17581
TCGATGAAGAACGCAGCGAAATGCGATAAGTAATGTGAATTGCAGAATTC
WCG1255Dai24688
TCGATGAAGAACGCAGCGAAATGCGATAAGTAATGTGAATTGCAGAATTC
Dai22427
TCGATGAAGAACGCAGCGAAATGCGATAAGTAATGTGAATTGCAGAATTC
MV690Meripilus_concrescens
TCGATGAAGAACGCAGCGAAATGCGATAAGTAATGTGAATTGCAGAATTC
MV513Meripilus_galapagensis
TCGATGAAGAACGCAGCGAAATGCGATAAGTAATGTGAATTGCAGAATTC

Dai19793
TCGATGAAGAACGCAGCGAAATGCGATAAGTAATGTGAATTGCAGAATTC
OQ553780P_tamilnaduensis
TCGATGAAGAACGCAGCGAAATGCGATAAGTAATGTGAATTGCAGAATTC
OQ553779P_tamilnaduensis
TCGATGAAGAACGCAGCGAAATGCGATAAGTAATGTGAATTGCAGAATTC
A164FB3Meripilus_giganteus
TCGATGAAGAACGCAGCGAAATGCGATAAGTAATGTGAATTGCAGAATTC
JV1407_36_Vinctus_Meandrica
TCGATGAAGAACGCAGCGAAATGCGATAAGTAATGTGAATTGCAGAATTC
1807_3K_Rigidoporus_PuertoRico TCGAT---
Cui16903P_vinctus
TCGATGAAGAACGCAGCGAAATGCGATAAGTAATGTGAATTGCAGAATTC
JV1008_18R_Lineatus
TCGATGAAGAACGCAGCGAAATGCGATAAGTAATGTGAATTGCAGAATTC
JV1407_37_1_Vinctus_Carara
TCGATGAAGAACGCAGCGAAATGCGATAAGTAATGTGAATTGCAGAATTC
Dai17986P_lineatus
TCGATGAAGAACGCAGCGAAATGCGATAAGTAATGTGAATTGCAGAATTC
Dai18281
TCGATGAAGAACGCAGCGAAATGCGATAAGTAATGTGAATTGCAGAATTC
1DAI19796
TCGATGAAGAACGCAGCGAAATGCGATAAGTAATGTGAATTGCAGAATTC
ZQY797Dai25241
TCGATGAAGAACGCAGCGAAATGCGATAAGTAATGTGAATTGCAGAATTC
WCG1289Dai24711
TCGATGAAGAACGCAGCGAAATGCGATAAGTAATGTGAATTGCAGAATTC
Dai22598
TCGATGAAGAACGCAGCGAAATGCGATAAGTAATGTGAATTGCAGAATTC
Dai20523
TCGATGAAGAACGCAGCGAAATGCGATAAGTAATGTGAATTGCAGAATTC
Dai17885
TCGATGAAGAACGCAGCGAAATGCGATAAGTAATGTGAATTGCAGAATTC
Dai17553
TCGATGAAGAACGCAGCGAAATGCGATAAGTAATGTGAATTGCAGAATTC
Dai19639
TCGATGAAGAACGCAGCGAAATGCGATAAGTAATGTGAATTGCAGAATTC
JV0110_48_CZ
TCGATGAAGAACGCAGCGAAATGCGATAAGTAATGTGAATTGCAGAATTC
MJ129_04
TCGATGAAGAACGCAGCGAAATGCGATAAGTAATGTGAATTGCAGAATTC
Cui10340P_eminens
TCGATGAAGAACGCAGCGAAATGCGATAAGTAATGTGAATTGCAGAATTC
Cui10341P_eminens

TCGATGAAGAACGCAGCGAAATGCGATAAGTAATGTGAATTGCAGAATTC
Dai12685P_eminens
TCGATGAAGAACGCAGCGAAATGCGATAAGTAATGTGAATTGCAGAATTC
Miettinen_13591Rigidoporus_und
TCGATGAAGAACGCAGCGAAATGCGATAAGTAATGTGAATTGCAGAATTC
Dai20868
TCGATGAAGAACGCAGCGAAATGCGATAAGTAATGTGAATTGCAGAATTC
Dai20832
TCGATGAAGAACGCAGCGAAATGCGATAAGTAATGTGAATTGCAGAATTC
Dai11400
TCGATGAAGAACGCAGCGAAATGCGATAAGTAATGTGAATTGCAGAATTC
Dai22472
TCGATGAAGAACGCAGCGAAATGCGATAAGTAATGTGAATTGCAGAATTC
1CUI10475
TCGATGAAGAACGCAGCGAAATGCGATAAGTAATGTGAATTGCAGAATTC
1CUI10491
TCGATGAAGAACGCAGCGAAATGCGATAAGTAATGTGAATTGCAGAATTC
HCFC1095Meripilus_robledoi
TCGATGAAGAACGCAGCGAAATGCGATAAGTAATGTGAATTGCAGAATTC
MCW702Meripilus_revolubilis
TCGATGAAGAACGCAGCGAAATGCGATAAGTAATGTGAATTGCAGAATTC
1704_83_zluty_HaciendaBaru
TCGATGAAGAACGCAGCGAAATGCGATAAGTAATGTGAATTGCAGAATTC
Dai9925P_lavendulus
TCGATGAAGAACGCAGCGAAATGCGATAAGTAATGTGAATTGCAGAATTC
Dai13587AP_lavendulus
TCGATGAAGAACGCAGCGAAATGCGATAAGTAATGTGAATTGCAGAATTC
PDD70600P_longicystidius
TCGATGAAGAACGCAGCGAAATGCGATAAGTAATGTGAATTGCAGAATTC
Cui16630
TCGATGAAGAACGCAGCGAAATGCGATAAGTAATGTGAATTGCAGAATTC
FP_135344Meripilus_giganteus
TCGATGAAGAACGCAGCGAAATGCGATAAGTAATGTGAATTGCAGAATTC
FP_100460_Sp
TCGATGAAGAACGCAGCGAAATGCGATAAGTAATGTGAATTGCAGAATTC
CBS421_48Meripilus_giganteus
TCGATGAAGAACGCAGCGAAATGCGATAAGTAATGTGAATTGCAGAATTC
Cui9203
TCGATGAAGAACGCAGCGAAATGCGATAAGTAATGTGAATTGCAGAATTC
Cui9202
TCGATGAAGAACGCAGCGAAATGCGATAAGTAATGTGAATTGCAGAATTC
TUFC100564Japan
TCGATGAAGAACGCAGCGAAATGCGATAAGTAATGTGAATTGCAGAATTC
Russell5913Meripilus_sumstinei

TCGATGAAGAACGCAGCGAAATGCGATAAGTAATGTGAATTGCAGAATTC
RP215Meripilus_brasiliensis
TCGATGAAGAACGCAGCGAAATGCGATAAGTAATGTGAATTGCAGAATTC
RP200Meripilus_brasiliensis
TCGATGAAGAACGCAGCGAAATGCGATAAGTAATGTGAATTGCAGAATTC
JV1712_13J_R_vinctus2_LSUPuert
TCGATGAAGAACGCAGCGAAATGCGATAAGTAATGTGAATTGCAGAATTC
Dai10503R_hypobrunneus
TCGATGAAGAACGCAGCGAAATGCGATAAGTAATGTGAATTGCAGAATTC
Dai10569Rigidoporus_hypobrunne
TCGATGAAGAACGCAGCGAAATGCGATAAGTAATGTGAATTGCAGAATTC
1DAI19451
TCGATGAAGAACGCAGCGAAATGCGATAAGTAATGTGAATTGCAGAATTC
CM108bRigidoporus_hypobrunneus
TCGATGAAGAACGCAGCGAAATGCGATAAGTAATGTGAATTGCAGAATTC
1CUI16874
TCGATGAAGAACGCAGCGAAATGCGATAAGTAATGTGAATTGCAGAATTC
FD299Cerrena_unicolor
TCGATGAAGAACGCAGCGAAATGCGATAAGTAATGTGAATTGCAGAATTC
KHL_GB_Cerrena_uniclor
TCGATGAAGAACGCAGCGAAATGCGATAAGTAATGTGAATTGCAGAATTC
Dai12892Cerrena_albocinnamomea
TCGATGAAGAACGCAGCGAAATGCGATAAGTAATGTGAATTGCAGAATTC
Dai12955C_albocinnamomea
TCGATGAAGAACGCAGCGAAATGCGATAAGTAATGTGAATTGCAGAATTC
SNUm03110102C_aurantiopora
TCGATGAAGAACGCAGCGAAATGCGATAAGTAATGTGAATTGCAGAATTC
NIBRFG0000102423C_aurantiopora
TCGATGAAGAACGCAGCGAAATGCGATAAGTAATGTGAATTGCAGAATTC
Dai7359Antrodiella_zonata
TCGATGAAGAACGCAGCGAAATGCGATAAGTAATGTGAATTGCAGAATTC
F20080702KCM29C_consors
TCGATGAAGAACGCAGCGAAATGCGATAAGTAATGTGAATTGCAGAATTC
F20080208LYW10Cerrena_consors
TCGATGAAGAACGCAGCGAAATGCGATAAGTAATGTGAATTGCAGAATTC
Dai7821Antrodiella_zonata
TCGATGAAGAACGCAGCGAAATGCGATAAGTAATGTGAATTGCAGAATTC
CFMR_DCL04_31Pseudolagarobasid
TCGATGAAGAACGCAGCGAAATGCGATAAGTAATGTGAATTGCAGAATTC
VPB197Pseudolagarobasidium_bel
TCGATGAAGAACGCAGCGAAATGCGATAAGTAATGTGAATTGCAGAATTC
CBS115543Pseudolagarobasidium_
TCGATGAAGAACGCAGCGAAATGCGATAAGTAATGTGAATTGCAGAATTC
CBS115544

TCGATGAAGAACGCAGCGAAATGCGATAAGTAATGTGAATTGCAGAATTC
Han405Pseudolagarobasidium_bai
TCGATGAAGAACGCAGCGAAATGCGATAAGTAATGTGAATTGCAGAATTC
Han406Pseudolagarobasidium_bai
TCGATGAAGAACGCAGCGAAATGCGATAAGTAATGTGAATTGCAGAATTC
MUcc838Spongipellis_delectans
TCGATGAAGAACGCAGCGAAATGCGATAAGTAATGTGAATTGCAGAATTC
BRNM686401S_delectans
TCGATGAAGAACGCAGCGAAATGCGATAAGTAATGTGAATTGCAGAATTC
OSM_F925S_delectans
TCGATGAAGAACGCAGCGAAATGCGATAAGTAATGTGAATTGCAGAATTC
BRNM67093Spongipellis_litschau
TCGATGAAGAACGCAGCGAAATGCGATAAGTAATGTGAATTGCAGAATTC
CFMRccFP59199TS_unicolor
TCGATGAAGAACGCAGCGAAATGCGATAAGTAATGTGAATTGCAGAATTC
CFMRccFP71791TS_unicolor
TCGATGAAGAACGCAGCGAAATGCGATAAGTAATGTGAATTGCAGAATTC
Dai13845P_lischaueri
TCGATGAAGAACGCAGCGAAATGCGATAAGTAATGTGAATTGCAGAATTC
Dai20266P_lischaueri
TCGATGAAGAACGCAGCGAAATGCGATAAGTAATGTGAATTGCAGAATTC
CFMR_HHB11240Radulodon_america
TCGATGAAGAACGCAGCGAAATGCGATAAGTAATGTGAATTGCAGAATTC
RLG6350Radulodon_americanus
TCGATGAAGAACGCAGCGAAATGCGATAAGTAATGTGAATTGCAGAATTC
KY415963Radulodon_erikssonii
TCGATGAAGAACGCAGCGAAATGCGATAAGTAATGTGAATTGCAGAATTC
HHB9567spRadulodon_casearius
TCGATGAAGAACGCAGCGAAATGCGATAAGTAATGTGAATTGCAGAATTC
KRT_Iso_26Radulodon_casearius
TCGATGAAGAACGCAGCGAAATGCGATAAGTAATGTGAATTGCAGAATTC
CBS126044Radulodon_erikssonii
TCGATGAAGAACGCAGCGAAATGCGATAAGTAATGTGAATTGCAGAATTC
He6183YUNNANENSIS
TCGATGAAGAACGCAGCGAAATGCGATAAGTAATGTGAATTGCAGAATTC
Cui17979YUNNANENSIS
TCGATGAAGAACGCAGCGAAATGCGATAAGTAATGTGAATTGCAGAATTC
Miettinen2091Junghuhnia_fimbri
TCGATGAAGAACGCAGCGAAATGCGATAAGTAATGTGAATTGCAGAATTC
KHL12316S_tenue
TCGATGAAGAACGCAGCGAAATGCGATAAGTAATGTGAATTGCAGAATTC
PRM846564S_pachyodon
TCGATGAAGAACGCAGCGAAATGCGATAAGTAATGTGAATTGCAGAATTC
SP_Lgt_S_pachyodon

TCGATGAAGAACGCAGCGAAATGCGATAAGTAATGTGAATTGCAGAATTC
Ryvarden44669Tyromyces_xuchile
TCGATGAAGAACGCAGCGAAATGCGATAAGTAATGTGAATTGCAGAATTC
PW17_171sinuosus
TCGATGAAGAACGCAGCGAAATGCGATAAGTAATGTGAATTGCAGAATTC
W53Dai12234
TCGATGAAGAACGCAGCGAAATGCGATAAGTAATGTGAATTGCAGAATTC
HHB4100SpAntella_americana
TCGATGAAGAACGCAGCGAAATGCGATAAGTAATGTGAATTGCAGAATTC
W3Dai20901spumeus
TCGATGAAGAACGCAGCGAAATGCGATAAGTAATGTGAATTGCAGAATTC
He6736
TCGATGAAGAACGCAGCGAAATGCGATAAGTAATGTGAATTGCAGAATTC
BRNM734877S_spumeus
TCGATGAAGAACGCAGCGAAATGCGATAAGTAATGTGAATTGCAGAATTC
BRNM712630S_spumeus
TCGATGAAGAACGCAGCGAAATGCGATAAGTAATGTGAATTGCAGAATTC
Dai1723Loweomyces_sibiricus
TCGATGAAGAACGCAGCGAAATGCGATAAGTAATGTGAATTGCAGAATTC
W54Cui10009
TCGATGAAGAACGCAGCGAAATGCGATAAGTAATGTGAATTGCAGAATTC
W1Dai20899
TCGATGAAGAACGCAGCGAAATGCGATAAGTAATGTGAATTGCAGAATTC
HHB13445Trametes_ochracea
TCGATGAAGAACGCAGCGAAATGCGATAAGTAATGTGAATTGCAGAATTC
Dai16222
TCGATGAAGAACGCAGCGAAATGCGATAAGTAATGTGAATTGCAGAATTC
Dai16240
TCGATGAAGAACGCAGCGAAATGCGATAAGTAATGTGAATTGCAGAATTC

JV1310_11SanguinolentusCernys
AGTGAATCATCGAATCTTTGAACGCACCTTGCGCTCCTTGGTATTCCGAG
MJ39_00_SK
AGTGAATCATCGAATCTTTGAACGCACCTTGCGCTCCTTGGTATTCCGAG
MJ111_04_CZ
AGTGAATCATCGAATCTTTGAACGCACCTTGCGCTCCTTGGTATTCCGAG
JV1610_BOKYsmrk
AGTGAATCATCGAATCTTTGAACGCACCTTGCGCTCCTTGGTATTCCGAG
Dai21030
AGTGAATCATCGAATCTTTGAACGCACCTTGCGCTCCTTGGTATTCCGAG
Dai20976P_furcatus
AGTGAATCATCGAATCTTTGAACGCACCTTGCGCTCCTTGGTATTCCGAG
Dai2105
AGTGAATCATCGAATCTTTGAACGCACCTTGCGCTCCTTGGTATTCCGAG

Dai2544
AGTGAATCATCGAATCTTTGAACGCACCTTGCGCTCCTTGGTATTCCGAG
Dai11313
AGTGAATCATCGAATCTTTGAACGCACCTTGCGCTCCTTGGTATTCCGAG
WCG1611Dai26167
AGTGAATCATCGAATCTTTGAACGCACCTTGCGCTCCTTGGTATTCCGAG
WCG1518Dai25999Physisporinus
AGTGAATCATCGAATCTTTGAACGCACCTTGCGCTCCTTGGTATTCCGAG
TAA15097
AGTGAATCATCGAATCTTTGAACGCATCTTGCGCTCCTTGGTATTCCGAG
JV8909_19_CZ
AGTGAATCATCGAATCTTTGAACGCATCTTGCGCTCCTTGGCATTCCGAG
JV1310_15_P_sanguinolentus2_CZ
AGTGAATCATCGAATCTTTGAACGCATCTTGCGCTCCTTGGCATTCCGAG
MJ53_02_CZ
AGTGAATCATCGAATCTTTGAACGCATCTTGCGCTCCTTGGCATTCCGAG
CLZhao21647P_yunnanensis
AGTGAATCATCGAATCTTTGAACGCATCTTGCGCTCCTTGGCATTCCGAG
CLZhao21583P_yunnanensis
AGTGAATCATCGAATCTTTGAACGCATCTTGCGCTCCTTGGCATTCCGAG
Dai22272
AGTGAATCATCGAATCTTTGAACGCATCTTGCGCTCCTTGGCATTCCGAG
Dai22279
AGTGAATCATCGAATCTTTGAACGCATCTTGCGCTCCTTGGCATTCCGAG
MJ332_94_CZ
AGTGAATCATCGAATCTTTGAACGCACCTTGCGCTCCTTGGTATTCCGAG
MJ642_94_CZ_Expallescens
AGTGAATCATCGAATCTTTGAACGCACCTTGCGCTCCTTGGTATTCCGAG
Dai21060P_vinctus
AGTGAATCATCGAATCTTTGAACGCACCTTGCGCTCCTTGGTATTCCGAG
JV0511_23LRP_pouzarii
AGTGAATCATCGAATCTTTGAACGCACCTTGCGCTCCTTGGTATTCCGAG
JQ409462_R_pouzarii_PRM899856_
AGTGAATCATCGAATCTTTGAACGCACCTTGCGCTCCTTGGTATTCCGAG
JV0308_66_WA
AGTGAATCATCGAATCTTTGAACGCACCTTGCGCTCCTTGGTATTCCGAG
JV0309_45_WA_USA
AGTGAATCATCGAATCTTTGAACGCACCTTGCGCTCCTTGGTATTCCGAG
JV0709_83_CA_USA
AGTGAATCATCGAATCTTTGAACGCACCTTGCGCTCCTTGGTATTCCGAG
Dai21043P_pouzarii
AGTGAATCATCGAATCTTTGAACGCACCTTGCGCTCCTTGGTATTCCGAG
MJ144_95_CZ
AGTGAATCATCGAATCTTTGAACGCACCTTGCGCTCCTTGGTATTCCGAG

JV0909_3_CZ
AGTGAATCATCGAATCTTTGAACGCACCTTGCGCTCCTTGGTATTCCGAG
JV0609_1_K
AGTGAATCATCGAATCTTTGAACGCACCTTGCGCTCCTTGGTATTCCGAG
Dai20396Physisporinus_castanop
AGTGAATCATCGAATCTTTGAACGCACCTTGCGCTCCTTGGTATTCCGAG
Dai20397Physisporinus_castanop
AGTGAATCATCGAATCTTTGAACGCACCTTGCGCTCCTTGGTATTCCGAG
MJ19_09_SK_Abies
AGTGAATCATCGAATCTTTGAACGCACCTTGCGCTCCTTGGTATTCCGAG
JV0509_40_J_TN_USA_Betula
AGTGAATCATCGAATCTTTGAACGCACCTTGCGCTCCTTGGTATTCCGAG
JV0808_33crocatus_PA_USAlist
AGTGAATCATCGAATCTTTGAACGCACCTTGCGCTCCTTGGTATTCCGAG
DLL2009_061P_crocatus
AGTGAATCATCGAATCTTTGAACGCACCTTGCGCTCCTTGGTATTCCGAG
Dai12800P_subcrocatus
AGTGAATCATCGAATCTTTGAACGCACCTTGCGCTCCTTGGTATTCCGAG
Dai15917P_subcrocatus
AGTGAATCATCGAATCTTTGAACGCACCTTGCGCTCCTTGGTATTCCGAG
Cui16620
AGTGAATCATCGAATCTTTGAACGCACCTTGCGCTCCTTGGTATTCCGAG
HCFC1088Meripilus_stillicidior
AGTGAATCATCGAATCTTTGAACGCACCTTGCGCTCCTTGGTATTCCGAG
MCW590Meripilus_obsrcurus
AGTGAATCATCGAATCTTTGAACGCACCTTGCGCTCCTTGGTATTCCGAG
MCW722Meripilus_obsrcurus
AGTGAATCATCGAATCTTTGAACGCACCTTGCGCTCCTTGGTATTCCGAG
Cui9381P_tibeticus
AGTGAATCATCGAATCTTTGAACGCACCTTGCACTCCTTGGTATTCCGAG
Cui9588P_tibeticus
AGTGAATCATCGAATCTTTGAACGCACCTTGCACTCCTTGGTATTCCGAG
Va2_Beneschova
AGTGAATCATCGAATCTTTGAACGCACCTTGCGCTCCTTGGTATTCCGAG
CWU3874_Ukraine_Alnus
AGTGAATCATCGAATCTTTGAACGCACCTTGCGCTCCTTGGTATTCCGAG
WCG1293Dai24718Physisporinus
AGTGAATCATCGAATCTTTGAACGCATCTTGCGCTCCTTGGTATTCCGAG
WCG1268Dai24682A
AGTGAATCATCGAATCTTTGAACGCACCTTGCGCTCCTTGGTATTCCGAG
WCG1269Dai24683A
AGTGAATCATCGAATCTTTGAACGCACCTTGCGCTCCTTGGTATTCCGAG
WCG1279Dai24694A
AGTGAATCATCGAATCTTTGAACGCACCTTGCGCTCCTTGGTATTCCGAG

Dai16971
AGTGAATCATCGAATCTTTGAACGCACCTTGCGCTCCTTGGTATTCCGAG
ZQY1043Dai26696
AGTGAATCATCGAATCTTTGAACGCACCTTGCGCTCCTTGGTATTCCGAG
Doll880
AGTGAATCATCGAATCTTTGAACGCACCTTGCGCTCCTTGGTATTCCGAG
Doll1000
AGTGAATCATCGAATCTTTGAACGCACCTTGCGCTCCTTGGTATTCCGAG
1DAI18529
AGTGAATCATCGAATCTTTGAACGCACCTTGCGCTCCTTGGTATTCCGAG
Dai19535
AGTGAATCATCGAATCTTTGAACGCACCTTGCGCTCCTTGGTATTCTGAG
1704_79_hnedyVillaLaPaz
AGTGAATCATCGAATCTTTGAACGCATCTTGCGCTCCTTGGTATTCCGAG
F2061
AGTGAATCATCGAATCTTTGAACGCATCTTGCGCTCCTTGGTATTCCGAG
1DAI18268
AGTGAATCATCGAATCTTTGAACGCATCTTGCGCTCCTTGGTATTCCGAG
1DAI18540A
AGTGAATCATCGAATCTTTGAACGCATCTTGCGCTCCTTGGTATTCCGAG
Dai17695
AGTGAATCATCGAATCTTTGAACGCATCTTGCGCTCCTTGGTATTCCGAG
LKY18Dai26373
AGTGAATCATCGAATCTTTGAACGCATCTTGCGCTCCTTGGTATTCCGAG
Dai17839P_sulphureus
AGTGAATCATCGAATCTTTGAACGCACCTTGCGCTCCTTGGTATTCCGAG
Dai17841P_sulphureus
AGTGAATCATCGAATCTTTGAACGCACCTTGCGCTCCTTGGTATTCCGAG
Dai19877P_roseus
AGTGAATCATCGAATCTTTGAACGCACCTTGCGCCCCTTGGTATTCCGAG
1508_18_1_Kout
AGTGAATCATCGAATCTTTGAACGCACCTTGCGCTCCTTGGTATTCCGAG
KP859303_R_vinctus_RP185_BRAZI
AGTGAATCATCGAATCTTTGAACGCACCTTGCGCTCCTTGGTATTCCGAG
JK1807_15Rigidoporus_sp_Puerto
AGTGAATCATCGAATCTTTGAACGCACCTTGCGCTCCTTGGTATTCCGAG
JV0509_47_J_TN_USA
AGTGAATCATCGAATCTTTGAACGCACCTTGCGCTCCTTGGTATTCCGAG
JV0709_188
AGTGAATCATCGAATCTTTGAACGCACCTTGCGCTCCTTGGTATTCCGAG
JV0509_127_PA_USA
AGTGAATCATCGAATCTTTGAACGCACCTTGCGCTCCTTGGTATTCCGAG
JV1009_59_NJ_USA
AGTGAATCATCGAATCTTTGAACGCACCTTGCGCTCCTTGGTATTCCGAG

Dai15497P_crataegi
AGTGAATCATCGAATCTTTGAACGCACCTTGCGCTCCTTGGTATTCCGAG
Dai15499P_crataegi
AGTGAATCATCGAATCTTTGAACGCACCTTGCGCTCCTTGGTATTCCGAG
Cui3266P_cinereus
AGTGAATCATCGAATCTTTGAACGCACCTTGCGCTCCTTGGTATTCCGAG
WCG1256Dai24690
AGTGAATCATCGAATCTTTGAACGCACCTTGCGCTCCTTGGTATTCCGAG
1DAI17581
AGTGAATCATCGAATCTTTGAACGCACCTTGCGCTCCTTGGTATTCCGAG
WCG1255Dai24688
AGTGAATCATCGAATCTTTGAACGCACCTTGCGCTCCTTGGTATTCCGAG
Dai22427
AGTGAATCATCGAATCTTTGAACGCACCTTGCGCTCCTTGGTATTCCGAG
MV690Meripilus_concrescens
AGTGAATCATCGAATCTTTGAACGCACCTTGCGCTCCTTGGTATTCCGAG
MV513Meripilus_galapagensis
AGTGAATCATCGAATCTTTGAACGCACCTTGCGCTCCTTGGTATTCCGAG
Dai19793
AGTGAATCATCGAATCTTTGAACGCACCTTGCGCTCCTTGGTATTCCGAG
OQ553780P_tamilnaduensis
AGTGAATCATCGAATCTTTGAACGCACCTTGCGCTCCTTGGTATTCCGAG
OQ553779P_tamilnaduensis
AGTGAATCATCGAATCTTTGAACGCACCTTGCGCTCCTTGGTATTCCGAG
A164FB3Meripilus_giganteus
AGTGAATCATCGAATCTTTGAACGCACCTTGCGCTCCTTGGTATTCCGAG
JV1407_36_Vinctus_Meandrica
AGTGAATCATCGAATCTTTGAACGCACCTTGCGCTCCTTGGTATTCCGAG
1807_3K_Rigidoporus_PuertoRico --
Cui16903P_vinctus
AGTGAATCATCGAATCTTTGAACGCACCTTGCGCTCCTTGGTATTCCGAG
JV1008_18R_Lineatus
AGTGAATCATCGAATCTTTGAACGCACCTTGCGCTCCTTGGTATTCCGAG
JV1407_37_1_Vinctus_Carara
AGTGAATCATCGAATCTTTGAACGCACCTTGCGCTCCTTGGTATTCCGAG
Dai17986P_lineatus
AGTGAATCATCGAATCTTTGAACGCACCTTGCGCTCCTTGGTATTCCGAG
Dai18281
AGTGAATCATCGAATCTTTGAACGCACCTTGCGCTCCTTGGTATTCCGAG
1DAI19796
AGTGAATCATCGAATCTTTGAACGCACCTTGCGCTCCTTGGTATTCCGAG
ZQY797Dai25241
AGTGAATCATCGAATCTTTGAACGCACCTTGCGCTCCTTGGTATTCCGAG
WCG1289Dai24711

AGTGAATCATCGAATCTTTGAACGCACCTTGCGCTCCTTGGTATTCCGAG
Dai22598
AGTGAATCATCGAATCTTTGAACGCACCTTGCGCTCCTTGGTATTCCGAG
Dai20523
AGTGAATCATCGAATCTTTGAACGCACCTTGCGCTCCTTGGTATTCCGAG
Dai17885
AGTGAATCATCGAATCTTTGAACGCACCTTGCGCTCCTTGGTATTCCGAG
Dai17553
AGTGAATCATCGAATCTTTGAACGCACCTTGCGCTCCTTGGTATTCCGAG
Dai19639
AGTGAATCATCGAATCTTTGAACGCACCTTGCGCTCCTTGGTATTCCGAG
JV0110_48_CZ
AGTGAATCATCGAATCTTTGAACGCACCTTGCGCTCCTTGGTATTCCGAG
MJ129_04
AGTGAATCATCGAATCTTTGAACGCACCTTGCGCTCCTTGGTATTCCGAG
Cui10340P_eminens
AGTGAATCATCGAATCTTTGAACGCACCTTGCGCTCCTTGGTATTCCGAG
Cui10341P_eminens
AGTGAATCATCGAATCTTTGAACGCACCTTGCGCTCCTTGGTATTCCGAG
Dai12685P_eminens
AGTGAATCATCGAATCTTTGAACGCACCTTGCGCTCCTTGGTATTCCGAG
Miettinen_13591Rigidoporus_und
AGTGAATCATCGAATCTTTGAACGCACCTTGCGCTCCTTGGTATTCCGAG
Dai20868
AGTGAATCATCGAATCTTTGAACGCACCTTGCGCTCCTTGGTATTCCGAG
Dai20832
AGTGAATCATCGAATCTTTGAACGCACCTTGCGCTCCTTGGTATTCCGAG
Dai11400
AGTGAATCATCGAATCTTTGAACGCACCTTGCGCTCCTTGGTATTCCGAG
Dai22472
AGTGAATCATCGAATCTTTGAACGCACCTTGCGCTCCTTGGTATTCCGAG
1CUI10475
AGTGAATCATCGAATCTTTGAACGCACCTTGCGCTCCTTGGTATTCCGAG
1CUI10491
AGTGAATCATCGAATCTTTGAACGCACCTTGCGCTCCTTGGTATTCCGAG
HCFC1095Meripilus_robledoi
AGTGAATCATCGAATCTTTGAACGCACCTTGCGCTCCTTGGTATTCCGAG
MCW702Meripilus_revolubilis
AGTGAATCATCGAATCTTTGAACGCACCTTGCGCTCCTTGGTATTCCGAG
1704_83_zluty_HaciendaBaru
AGTGAATCATCGAATCTTTGAACGCACCTTGCGCTCCTTGGTATTCCGAG
Dai9925P_lavendulus
AGTGAATCATCGAATCTTTGAACGCACCTTGCGCTCCTTGGTATTCCGAG
Dai13587AP_lavendulus

AGTGAATCATCGAATCTTTGAACGCACCTTGCGCTCCTTGGTATTCCGAG
PDD70600P_longicystidius
AGTGAATCATCGAATCTTTGAACGCACCTTGCGCTCCTTGGTATTCCGAG
Cui16630
AGTGAATCATCGAATCTTTGAACGCACCTTGCGCTCCTTGGTATTCCGAG
FP_135344Meripilus_giganteus
AGTGAATCATCGAATCTTTGAACGCACCTTGCGCTCCTTGGTATTCCGAG
FP_100460_Sp
AGTGAATCATCGAATCTTTGAACGCACCTTGCGCTCCTTGGTATTCCGAG
CBS421_48Meripilus_giganteus
AGTGAATCATCGAATCTTTGAACGCACCTTGCGCTCCTTGGTATTCCGAG
Cui9203
AGTGAATCATCGAATCTTTGAACGCACCTTGCGCTCCTTGGTATTCCGAG
Cui9202
AGTGAATCATCGAATCTTTGAACGCACCTTGCGCTCCTTGGTATTCCGAG
TUFC100564Japan
AGTGAATCATCGAATCTTTGAACGCACCTTGCGCTCCTTGGTATTCCGAG
Russell5913Meripilus_sumstinei
AGTGAAYCATCGAATCTTTGAACGCACCTTGCGCTCCTTGGTATTCCGAG
RP215Meripilus_brasiliensis
AGTGAATCATCGAATCTTTGAACGCACCTTGCGCTCCTTGGTATTCCGAG
RP200Meripilus_brasiliensis
AGTGAATCATCGAATCTTTGAACGCACCTTGCGCTCCTTGGTATTCCGAG
JV1712_13J_R_vinctus2_LSUPuert
AGTGAATCATCGAATCTTTGAACGCATCTTGCGCCCTTTGGTATTCCGAA
Dai10503R_hypobrunneus
AGTGAATCATCGAATCTTTGAACGCATCTTGCGCCCTTTGGTATTCCGAA
Dai10569Rigidoporus_hypobrunne
AGTGAATCATCGAATCTTTGAACGCATCTTGCGCCCTTTGGTATTCCGAA
1DAI19451
AGTGAATCATCGAATCTTTGAACGCATCTTGCGCCCTTTGGTATTCCGAA
CM108bRigidoporus_hypobrunneus
AGTGAATCATCGAATCTTTGAACGCATCTTGCGCCCTTTGGTATTCCGAA
1CUI16874
AGTGAATCATCGAATCTTTGAACGCATCTTGCGCCCTTTGGTATTCCGAA
FD299Cerrena_unicolor
AGTGAATCATCGAATCTTTGAACGCACCTTGCGCCCCTTGGTATTCCGAG
KHL_GB_Cerrena_uniclor
AGTGAATCATCGAATCTTTGAACGCACCTTGCGCCCCTTGGTATTCCGAG
Dai12892Cerrena_albocinnamomea
AGTGAATCATCGAATCTTTGAACGCACCTTGCGCCCCTTGGTATTCCGAG
Dai12955C_albocinnamomea
AGTGAATCATCGAATCTTTGAACGCACCTTGCGCCCCTTGGTATTCCGAG
SNUm03110102C_aurantiopora

AGTGAATCATCGAATCTTTGAACGCACCTTGCGCCCCTTGGTATTCCGAG
NIBRFG0000102423C_aurantiopora
AGTGAATCATCGAATCTTTGAACGCACCTTGCGCCCCTTGGTATTCCGAG
Dai7359Antrodiella_zonata
AGTGAATCATCGAATCTTTGAACGCACCTTGCGCCCCTTGGTATTCCGAG
F20080702KCM29C_consors
AGTGAATCATCGAATCTTTGAACGCACCTTGCGCCCCTTGGTATTCCGAG
F20080208LYW10Cerrena_consors
AGTGAATCATCGAATCTTTGAACGCACCTTGCGCCCCTTGGTATTCCGAG
Dai7821Antrodiella_zonata
AGTGAATCATCGAATCTTTGAACGCACCTTGCGCCCCTTGGTATTCCGAG
CFMR_DCL04_31Pseudolagarobasid
AGTGAATCATCGAATCTTTGAACGCACCTTGCGCCCCTTGGTATTCCGAG
VPB197Pseudolagarobasidium_bel
AGTGAATCATCGAATCTTTGAACGCACCTTGCGCCCCTTGGTATTCCGAG
CBS115543Pseudolagarobasidium_
AGTGAATCATCGAATCTTTGAACGCACCTTGCGCCCCTTGGTATTCCGAG
CBS115544
AGTGAATCATCGAATCTTTGAACGCACCTTGCGCCCCTTGGTATTCCGAG
Han405Pseudolagarobasidium_bai
AGTGAATCATCGAATCTTTGAACGCATCTTGCGCCCCTTGGTATTCCGAG
Han406Pseudolagarobasidium_bai
AGTGAATCATCGAATCTTTGAACGCATCTTGCGCCCCTTGGTATTCCGAG
MUcc838Spongipellis_delectans
AGTGAATCATCGAATCTTTGAACGCACCTTGCGCCCTTTGGTATTCCGAA
BRNM686401S_delectans
AGTGAATCATCGAATCTTTGAACGCACCTTGCGCCCTTTGGTATTCCGAA
OSM_F925S_delectans
AGTGAATCATCGAATCTTTGAACGCACCTTGCGCCCTTTGGTATTCCGAA
BRNM67093Spongipellis_litschau
AGTGAATCATCGAATCTTTGAACGCACCTTGCGCCCTTTGGTATTCCGAA
CFMRccFP59199TS_unicolor
AGTGAATCATCGAATCTTTGAACGCACCTTGCGCCCTTTGGTATTCCGAA
CFMRccFP71791TS_unicolor
AGTGAATCATCGAATCTTTGAACGCACCTTGCGCCCTTTGGTATTCCGAA
Dai13845P_lischaueri
AGTGAATCATCGAATCTTTGAACGCACCTTGCGCCCTTTGGTATTCCGAA
Dai20266P_lischaueri
AGTGAATCATCGAATCTTTGAACGCACCTTGCGCCCTTTGGTATTCCGAA
CFMR_HHB11240Radulodon_america
AGTGAATCATCGAATGTTTGAACGCATCTGGCGCCCCCTGGCATTCCGGG
RLG6350Radulodon_americanus
AGTGAATCATCGAATGTTTGAACGCATCTGGCGCCCCCTGGCATTCCGGG
KY415963Radulodon_erikssonii

AGTGAATCATCGAATGTTTGAACGCATCTGGCGCCCCCTGGCATTCCGGG
HHB9567spRadulodon_casearius
AGTGAATCATCGAATGTTTGAACGCATCTGGCGCCCCCTGGCATTCCGGG
KRT_Iso_26Radulodon_casearius
AGTGAATCATCGAATGTTTGAACGCATCTGGCGCCCCCTGGCATTCCGGG
CBS126044Radulodon_erikssonii
AGTGAATCATCGAATGTTTGAACGCATCTGGCGCCCCCTGGCATTCCGGG
He6183YUNNANENSIS
AGTGAATCATCGAATGTTTGAACGCATCTGGCGCCCCCTGGCATTCCGGG
Cui17979YUNNANENSIS
AGTGAATCATCGAATGTTTGAACGCATCTGGCGCCCCCTGGCATTCCGGG
Miettinen2091Junghuhnia_fimbri
AGTGAATCATCGAATCTTTGAACGCACCTTGCGCTCCTTGGTATTCCGAG
KHL12316S_tenue
AGTGAATCATCGAATCTTTGAACGCACCTTGCGCTCCTTGGTATTCCGAG
PRM846564S_pachyodon
AGTGAATCATCGAATCTTTGAATGCACCTTGCGCCTCCCGGTATTCCGGG
SP_Lgt_S_pachyodon
AGTGAATCATCGAATCTTTGAATGCACCTTGCGCCTCCCGGTATTCCGGG
Ryvarden44669Tyromyces_xuchile
AGTGAATCATCGAATCTTTGAATGCACCTTGCGCCTCCCGGTATTCCGGG
PW17_171sinuosus
AGTGAATCATCGAATCTTTGAATGCACCTTGCGCCTCCCGGTATTCCGGG
W53Dai12234
AGTGAATCATCGAATCTTTGAATGCATCTTGCGCCTCCCGGTATTCCGGG
HHB4100SpAntella_americana
AGTGAATCATCGAATCTTTGAACGCACCTTGCGCTCCTTGGTATTCCGAG
W3Dai20901spumeus
AGTGAATCATCGAATCTTTGAACGCACCTTGCGCTCCTTGGTATTCCGAG
He6736
AGTGAATCATCGAATCTTTGAACGCACCTTGCGCTCCTTGGTATTCCGAG
BRNM734877S_spumeus
AGTGAATCATCGAATCTTTGAACGCACCTTGCGCTCCTTGGTATTCCGAG
BRNM712630S_spumeus
AGTGAATCATCGAATCTTTGAACGCACCTTGCGCTCCTTGGTATTCCGAG
Dai1723Loweomyces_sibiricus
AGTGAATCATCGAATCTTTGAACGCACCTTGCGCTCCTTGGTATTCCGAG
W54Cui10009
AGTGAATCATCGAATCTTTGAACGCACCTTGCGCTCCTTGGTATTCCGAG
W1Dai20899
AGTGAATCATCGAATCTTTGAACGCACCTTGCGCTCCTTGGTATTCCGAG
HHB13445Trametes_ochracea
AGTGAATCATCGAATCTTTGAACGCACCTTGCGCTCCTTGGTATTCCGAG
Dai16222

AGTGAATCATCGAATCTTTGAACGCACCTTGCGCTCCTTGGTATTCCGAG
Dai16240
AGTGAATCATCGAATCTTTGAACGCACCTTGCGCTCCTTGGTATTCCGAG

JV1310_11SanguinolentusCernys
GAGCATGCCTGTTTGAGTGTCATGGTATTCTCAATTC-TCTTGG-T----
MJ39_00_SK
GAGCATGCCTGTTTGAGTGTCATGGTATTCTCAATTC-YCTTGG-T----
MJ111_04_CZ
GAGCATGCCTGTTTGAGTGTCATGGTATTCTCAATTC-TCTTGG-T----
JV1610_BOKYsmrk
GAGCATGCCTGTTTGAGTGTCATGGTATTCTCAATTC-TCTTGG-T----
Dai21030
GAGCATGCCTGTTTGAGTGTCATGGTATTCTCAATTC-TCTTGG-T----
Dai20976P_furcatus
GAGCATGCCTGTTTGAGTGTCATGGTATTCTCAATTC-TCTTGG-T----
Dai2105
GAGCATGCCTGTTTGAGTGTCATGGTATTCTCAATTC-TCTTGG-T----
Dai2544
GAGCATGCCTGTTTGAGTGTCATGGTATTCTCAATTC-TCTTGG-T----
Dai11313
GAGCATGCCTGTTTGAGTGTCATGGTATTCTCAATTC-TCTTGG-T----
WCG1611Dai26167
GAGCATGCCTGTTTGAGTGTCATGGTATTCTCAATTC-TCTTGG-T----
WCG1518Dai25999Physisporinus
GAGCATGCCTGTTTGAGTGTCATGGTATTCTCAATTC-TCTTGG-T----
TAA15097
GAGCATGCCTGTTTGAGTGTCATGGTATTCTCAATTC-TCTTGG-T----
JV8909_19_CZ
GAGCATGCCTGTTTGAGTGTCATGGTATTCTCAATTC-TCTTGG-T----
JV1310_15_P_sanguinolentus2_CZ
GAGCATGCCTGTTTGAGTGTCATGGTATTCTCAATTC-TCTTGG-T----
MJ53_02_CZ
GAGCATGCCTGTTTGAGTGTCATGGTATTCTCAATTC-TCTTGG-T----
CLZhao21647P_yunnanensis
GAGCATGCCTGTTTGAGTGTCATGGTATTCTCAATTC-TCTTGG-T----
CLZhao21583P_yunnanensis
GAGCATGCCTGTTTGAGTGTCATGGTATTCTCAATTC-TCTTGG-T----
Dai22272
GAGCATGCCTGTTTGAGTGTCATGGTATTCTCAATTC-TCTTGG-T----
Dai22279
GAGCATGCCTGTTTGAGTGTCATGGTATTCTCAATTC-TCTTGG-T----
MJ332_94_CZ
GAGCATGCCTGTTTGAGTGTCATGGTATTCTCAATTC-TCTCAG-T----

MJ642_94_CZ_Expallescens
GAGCATGCCTGTTTGAGTGTCATGGTATTCTCAATTC-TCTCAG-T----
Dai21060P_vinctus
GAGCATGCCTGTTTGAGTGTCATGGTATTCTCAATTC-TCTCAG-T----
JV0511_23LRP_pouzarii
GAGCATGCCTGTTTGAGTGTCATGGTATTCTCAATTC-TCTCAG-T----
JQ409462_R_pouzarii_PRM899856_
GAGCATGCCTGTTTGAGTGTCATGGTATTCTCAATTC-TCTCAG-T----
JV0308_66_WA
GAGCATGCCTGTTTGAGTGTCAYGGTATTCTCAATTC-TCTCAG-T----
JV0309_45_WA_USA
GAGCATGCCTGTTTGAGTGTCATGGTATTCTCAATTC-TCTCAG-T----
JV0709_83_CA_USA
GAGCATGCCTGTTTGAGTGTCATGGTATTCTCAATTC-TCTCAG-T----
Dai21043P_pouzarii
GAGCATGCCTGTTTGAGTGTCATGGTATTCTCAATTC-TCTCAG-T----
MJ144_95_CZ
GAGCATGCCTGTTTGAGTGTCATGGTATTCTCAATTC-TCTCAG-C----
JV0909_3_CZ
GAGCATGCCTGTTTGAGTGTCATGGTATTCTCAATTC-TCTCAG-C----
JV0609_1_K
GAGCATGCCTGTTTGAGTGTCATGGTATTCTCAATTC-TCTCAG-C----
Dai20396Physisporinus_castanop GAGCATGCCTGTTTGAGTGTCATGGTATTCTCAATTC-TCTCAG------
Dai20397Physisporinus_castanop GAGCATGCCTGTTTGAGTGTCATGGTATTCTCAATTC-TCTCAG------
MJ19_09_SK_Abies
GAGCATGCCTGTTTGAGTGTCATGGTATTCTCAATTC-CTTCAG-T----
JV0509_40_J_TN_USA_Betula
GAGCATGCCTGTTTGAGTGTCATGGTATTCTCAATTC-CTTCAG-T----
JV0808_33crocatus_PA_USAlist
GAGCATGCCTGTTTGAGTGTCATGGTATTCTCAATTC-CTTCAG-T----
DLL2009_061P_crocatus
GAGCATGCCTGTTTGAGTGTCATGGTATTCTCAATTC-CTTCAG-T----
Dai12800P_subcrocatus
GAGCATGCCTGTTTGAGTGTCATGGTATTCTCAATTC-CTTCAG-T----
Dai15917P_subcrocatus
GAGCATGCCTGTTTGAGTGTCATGGTATTCTCAATTC-CTTCAG-T----
Cui16620
GAGCATGCCTGTTTGAGTGTCATGGTATTCTCAATTC-TCTCAGTT----
HCFC1088Meripilus_stillicidior GAGCATGCCTGTTTGAGTGTCATGGTATTCTCAATTC-TCTCAGTT----
MCW590Meripilus_obsrcurus
GAGCATGCCTGTTTGAGTGTCATGGTATTCTCAATTC-TCTCAG-T----

MCW722Meripilus_obsrcurus
GAGCATGCCTGTTTGAGTGTCATGGTATTCTCAATTC-TCTCAG-T----
Cui9381P_tibeticus GAGTATGCCTGTTTGAGTGTCATGGTATTCTCAATTC-
TCTCAG-C----
Cui9588P_tibeticus GAGTATGCCTGTTTGAGTGTCATGGTATTCTCAATTC-
TCTCAG-C----
Va2_Beneschova
GAGCATGCCTGTTTGAGTGTCATGGTATTCTCAATTA-TCTCAG-T----
CWU3874_Ukraine_Alnus
GAGCATGCCTGTTTGAGTGTCATGGTATTCTCAATTA-TCTCAG-T----
WCG1293Dai24718Physisporinus
GAGCATGCCTGTTTGAGTGTCATGGTATTCTCAATTC-CCTTGG-T----
WCG1268Dai24682A
GAGCATGCCTGTTTGAGTGTCACGGTATTCTCAATTC-CCTTAG-T----
WCG1269Dai24683A
GAGCATGCCTGTTTGAGTGTCACGGTATTCTCAATTC-CCTTAG-T----
WCG1279Dai24694A
GAGCATGCCTGTTTGAGTGTCATGGTATTCTCAATTC-CCTTAG-T----
Dai16971
GAGCATGCCTGTTTGAGTGTCATGGTATTCTCAATTC-CCTTAG-T----
ZQY1043Dai26696
GAGCATGCCTGTTTGAGTGTCATGGTATTCTCAATTC-CCTTAG-T----
Doll880
GAGCATGCCTGTTTGAGTGTCATGGTATTCTCAATTC-CCTTGGAT----
Doll1000
GAGCATGCCTGTTTGAGTGTCATGGTATTCTCAATTC-CCTTGGAT----
1DAI18529
GAGCATGCCTGTTTGAGTGTCATGGTATTCTCAATTC-CCTTGGGT----
Dai19535
GAGCATGCCTGTTTGAGTGTCATGGTATTCTCAATTC-TCTCAG-T----
1704_79_hnedyVillaLaPaz
GAGCATGCCTGTTTGAGTGTCATGATATTCTCAATTC-CTTCAG-C----
F2061
GAGCATGCCTGTTTGAGTGTCATGATATTCTCAATTC-CTTCAG-C----
1DAI18268
GAGCATGCCTGTTTGAGTGTCATGATATTCTCAATTC-CTTCAG-C----
1DAI18540A
GAGCATGCCTGTTTGAGTGTCATGATATTCTCAATTC-CTTCAG-C----
Dai17695
GAGCATGCCTGTTTGAGTGTCATGATATTCTCAATTC-CTTCAG-T----
LKY18Dai26373
GAGCATGCCTGTTTGAGTGTCATGATATTCTCAATTC-CTTCAG-C----
Dai17839P_sulphureus
GAGCATGCCTGTTTGAGTGTCATGGTATTCTCAATCC-CTCTGG-T----

Dai17841P_sulphureus
GAGCATGCCTGTTTGAGTGTCATGGTATTCTCAATCC-CTCTGG-T----
Dai19877P_roseus
GAGCATGCCTGTTTGAGTGTCATGGTATTCTCAATTT-CTCTGG-T----
1508_18_1_Kout
GAGCATGTCTGTTTGAGTGTCATGGTATTCTCAATTC-CTTCAG-C----
KP859303_R_vinctus_RP185_BRAZI
GAGCATGCCTGTTTGAGTGTCATGGTATTCTCAATTC-CTTCAG-C----
JK1807_15Rigidoporus_sp_Puerto
GAGCATGTCTGTTTGAGTGTCATGGTATTCTCAATTC-CTTCAG-C----
JV0509_47_J_TN_USA
GAGCATGCCTGTTTGAGTGTCATGGTATTCTCAATTC--CCAAGTC----
JV0709_188
GAGCATGCCTGTTTGAGTGTCATGGTATTCTCAATTC--CCAAGTC----
JV0509_127_PA_USA
GAGCATGCCTGTTTGAGTGTCATGGTATTCTCAATTC--CCAAGTC----
JV1009_59_NJ_USA
GAGCATGCCTGTTTGAGTGTCATGGTATTCTCAATTC--CCAAGTC----
Dai15497P_crataegi
GAGCATGCCTGTTTGAGTGTCATGGTATTCTCAATTC--CCAAGTC----
Dai15499P_crataegi
GAGCATGCCTGTTTGAGTGTCATGGTATTCTCAATTC--CCAAGTC----
Cui3266P_cinereus
GAGCATGCCTGTTTGAGTGTCATGGTATTCTCAATTC-ATCAGGTC----
WCG1256Dai24690
GAGCATGCCTGTTTGAGTGTCATGGTATTCTCAATTC-ATCAGGTC----
1DAI17581
GAGCATGCCTGTTTGAGTGTCATGGTATTCTCAATTC-ATCAGGTC----
WCG1255Dai24688
GAGCATGCCTGTTTGAGTGTCATGGTATTCTCAATTC-ATCAGGTC----
Dai22427
GAGCATGCCTGTTTGAGTGTCATGGTATTCTCAATTC-ATCAGGTC----
MV690Meripilus_concrescens
GAGCATGCCTGTTTGAGTGTCATGGTATTCTCAATTC-ACCAGGTC----
MV513Meripilus_galapagensis
GAGCATGCCTGTTTGAGTGTCATGGTATTCTCAATTC-ATTAGGTC----
Dai19793
GAGCATGCCTGTTTGAGTGTCATGGTATTCTCAATTC--CCAAGTC----
OQ553780P_tamilnaduensis
GAGCATGCCTGTTTGAGTGTCATGGTATTCTCAATTC-ACCAAGTC----
OQ553779P_tamilnaduensis
GAGCATGCCTGTTTGAGTGTCATGGTATTCTCAATTC-ACCAAGTC----
A164FB3Meripilus_giganteus
GAGCATGCCTGTTTGAGTGTCATGGTATTCTCAATTC-ACCAAGTC----

JV1407_36_Vinctus_Meandrica
GAGCATGCCTGTTTGAGTGTCATGGTATTCTCAATTC-TCCAAGTC----
1807_3K_Rigidoporus_PuertoRico --
Cui16903P_vinctus
GAGCATGCCTGTTTGAGTGTCATGGTATTCTCAATTC-TCCAAGTC----
JV1008_18R_Lineatus
GAGCATGCCTGTTTGAGTGTCATGGTATTCTCAATTC-TCCAGGTC----
JV1407_37_1_Vinctus_Carara
GAGCATGCCTGTTTGAGTGTCATGGTATTCTCAATTC-TCCAGGTC----
Dai17986P_lineatus
GAGCATGCCTGTTTGAGTGTCATGGTATTCTCAATTC-TCTAGGTC----
Dai18281
GAGCATGCCTGTTTGAGTGTCATGGTATTCTCAATTC-TCTAGGTC----
1DAI19796
GAGCATGCCTGTTTGAGTGTCATGGTATTCTCAATTC-TCCAGGTC----
ZQY797Dai25241
GAGCATGCCTGTTTGAGTGTCATGGTATTCTCAATTC-TCCAGGTC----
WCG1289Dai24711
GAGCATGCCTGTTTGAGTGTCATGGTATTCTCAATTC-TCCAGGTC----
Dai22598
GAGCATGCCTGTTTGAGTGTCATGGTATTCTCAATTC-TTCAGGTC----
Dai20523
GAGCATGCCTGTTTGAGTGTCATGGTATTCTCAATTC-TTCAGGTC----
Dai17885
GAGCATGCCTGTTTGAGTGTCATGGTATTCTCAATTC-TTCAGGTC----
Dai17553
GAGCATGCCTGTTTGAGTGTCATGGTATTCTCAATTC-TTCAGGTC----
Dai19639
GAGCATGCCTGTTTGAGTGTCATGGTATTCTCAATTC-TTCAGGTC----
JV0110_48_CZ
GAGCATGCCTGTTTGAGTGTCATGGTATTCTCAATTC-CCTCAA-C----
MJ129_04
GAGCATGCCTGTTTGAGTGTCATGGTATTCTCAATTC-CCTCAA-C----
Cui10340P_eminens
GAGCATGCCTGTTTGAGTGTCATGGTATTCTCAATTC-CCTCAA-C----
Cui10341P_eminens
GAGCATGCCTGTTTGAGTGTCATGGTATTCTCAATTC-CCTCAA-C----
Dai12685P_eminens
GAGCATGCCTGTTTGAGTGTCATGGTATTCTCAATTC-CCTCAA-C----
Miettinen_13591Rigidoporus_und GAGCATGCCTGTTTGAGTGTCATGGTATTCTCAATTC-CCTCAA-C----
Dai20868
GAGCATGCCTGTTTGAGTGTCATGGTATTCTCAATTC-CCTCAA-C----
Dai20832

GAGCATGCCTGTTTGAGTGTCATGGTATTCTCAATTC-CCTCAA-C----
Dai11400
GAGCATGCCTGTTTGAGTGTCATGGTATTCTCAATTC-CCTCAA-C----
Dai22472
GAGCATGCCTGTTTGAGTGTCATGGTATTCTCAATTC-CCTCAA-C----
1CUI10475
GAGCATGCCTGTTTGAGTGTCATGGTATTCTCAATCC-CCTCAA-C----
1CUI10491
GAGCATGCCTGTTTGAGTGTCATGGTATTCTCAATCC-CCTCAA-C----
HCFC1095Meripilus_robledoi
GAGCATGCCTGTTTGAGTGTCATGGTATTCTCAATTC-CCTCAA-C----
MCW702Meripilus_revolubilis
GAGCATGCCTGTTTGAGTGTCATGGTATTCTCAATTC-CCTCAA-C----
1704_83_zluty_HaciendaBaru
GAGCATGCCTGTTTGAGTGTCATGGTATTCTCAATTC-CTTTGT-T----
Dai9925P_lavendulus
GAGCATGCCTGTTTGAGTGTCATGTTATTCTCGATTC-CTCTGT-T----
Dai13587AP_lavendulus
GAGCATGCCTGTTTGAGTGTCATGTTATTCTCGATTC-CTCTGT-T----
PDD70600P_longicystidius
GAGCATGCCTGTTTGAGTGTCATGGTTATTTCAATTC-CCTCGT-T----
Cui16630
GAGCATGCCTGTTTGAGTGTCATGGTTATTTCAATTC-CCTCGT-T----
FP_135344Meripilus_giganteus GAGCATGCCTGTTTGAGTGTCATGGTATTCTCAATTC-GCTCTCAT----
FP_100460_Sp
GAGCATGCCTGTTTGAGTGTCATGGTATTCTCAATTC-GCTCTCAT----
CBS421_48Meripilus_giganteus
GAGCATGCCTGTTTGAGTGTCATGGTATTCTCAATTC-GCTCTCAT----
Cui9203
GAGCATGCCTGTTTGAGTGTCATGGTATTCTCAATTC-GCTCTCAT----
Cui9202
GAGCATGCCTGTTTGAGTGTCATGGTATTCTCAATTC-GCTCTCAT----
TUFC100564Japan
GAGCATGCCTGTTTGAGTGTCATGGTATTCTCAATCC-ACTCTCAT----
Russell5913Meripilus_sumstinei GAGCATGCCTGTTTGAGTGTCATGGTATTCTCAATTC-ACCTTAAT----
RP215Meripilus_brasiliensis GAGCATGCCTGTTTGAGTGTCATGGTATTCTCAATTC-ACTTTCAATT--
RP200Meripilus_brasiliensis GAGCATGCCTGTTTGAGTGTCATGGTATTCTCAATTC-ACTTTCAAT---
JV1712_13J_R_vinctus2_LSUPuert
GGGCATGCCTGTTTGAGTGTCATGGTATTCTCAATAC-CCCAAA-T----
Dai10503R_hypobrunneus

GGGCATGCCTGTTTGAGTGTCATGGTATTCTCAATAC-CCCAAA-T----
Dai10569Rigidoporus_hypobrunne
GGGCATGCCTGTTTGAGTGTCATGGTATTCTCAATAC-CCCAAA-T----
1DAI19451
GGGCATGCCTGTTTGAGTGTCATGGTATTCTCAATAC-CCCAAA-T----
CM108bRigidoporus_hypobrunneus
GGGCATGCCTGTTTGAGTGTCATGGTATTCTCAATAC-CCCAAA-T----
1CUI16874
GGGCATGCCTGTTTGAGTGTCATGGTATTCTCAATAC-CCCAAA-T----
FD299Cerrena_unicolor
GGGCATGCCTGTTTGAGTGTCATGGTATTCTCAATAC-CCTAAA-T----
KHL_GB_Cerrena_uniclor
GGGCATGCCTGTTTGAGTGTCATGGTATTCTCAATAC-CCTAAA-T----
Dai12892Cerrena_albocinnamomea
GGGCATGCCTGTTTGAGTGTCATGGTATTCTCAATAC-CCTAAA-T----
Dai12955C_albocinnamomea
GGGCATGCCTGTTTGAGTGTCATGGTATTCTCAATAC-CCTAAA-T----
SNUm03110102C_aurantiopora
GGGCATGCCTGTTTGAGTGTCATGGTATTCTCAATAC-CCTAAA-T----
NIBRFG0000102423C_aurantiopora
GGGCATGCCTGTTTGAGTGTCATGGTATTCTCAATAC-CCTAAA-T----
Dai7359Antrodiella_zonata GGGCATGCCTGTTTGAGTGTCATGGTATTCTCAATAC-CCTAAA-T----
F20080702KCM29C_consors
GGGCATGCCTGTTTGAGTGTCATGGTATTCTCAATAC-CCTAAA-T----
F20080208LYW10Cerrena_consors
GGGCATGCCTGTTTGAGTGTCATGGTATTCTCAATAC-CCTAAA-T----
Dai7821Antrodiella_zonata GGGCATGCCTGTTTGAGTGTCATGGTATTCTCAATAC-CCTAAA-T----
CFMR_DCL04_31Pseudolagarobasid
GGGCATGCCTGTTTGAGTGTCATGGTATTCTCAATAC-CCTAAA-T----
VPB197Pseudolagarobasidium_bel
GGGCATGCCTGTTTGAGTGTCATGGTATTCTCAATAC-CCTAAA-T----
CBS115543Pseudolagarobasidium_
GGGCATGCCTGTTTGAGTGTCATGGTATTCTCAATAC-CCTACA-T----
CBS115544
GGGCATGCCTGTTTGAGTGTCATGGTATTCTCAATAC-CCTACA-T----
Han405Pseudolagarobasidium_bai
GGGCATGCCTGTTTGAGTGTCATGGTATTCTCAATAC-CCTAAA-T----
Han406Pseudolagarobasidium_bai
GGGCATGCCTGTTTGAGTGTCATGGTATTCTCAATAC-CCTAAA-T----
MUcc838Spongipellis_delectans GGGCATGCCTGTTTGAGTGTCATGGTATTCTCAATAC-CCCAAG-T----
BRNM686401S_delectans

GGGCATGCCTGTTTGAGTGTCATGGTATTCTCAATAC-CCCAAG-T----
OSM_F925S_delectans
GGGCATGCCTGTTTGAGTGTCATGGTATTCTCAATAC-CCCAAG-T----
BRNM67093Spongipellis_litschau
GGGCATGCCTGTTTGAGTGTCATGGTATTCTCAATAC-CCCAAA-T----
CFMRccFP59199TS_unicolor
GGGCATGCCTGTTTGAGTGTCATGGTATTCTCAATAC-YCCAAA-T----
CFMRccFP71791TS_unicolor
GGGCATGCCTGTTTGAGTGTCATGGTATTCTCAATAC-TCCAAA-T----
Dai13845P_lischaueri GGGCATGCCTGTTTGAGTGTCATGGTATTCTCAATAC-CCCAAA-T----
Dai20266P_lischaueri GGGCATGCCTGTTTGAGTGTCATGGTATTCTCAATAC-CCCAAA-T----
CFMR_HHB11240Radulodon_america
GGGCACGCCCGTTTGAGTGTCATGGTATTCTCAATAC-TCCAAA-T----
RLG6350Radulodon_americanus
GGGCACGCCCGTTTGAGTGTCATGGTATTCTCAATAC-TCCACA-T----
KY415963Radulodon_erikssonii
GGGCACGCCCGTTTGAGTGTCATGGTATTCTCAATAC-TCCAAA-T----
HHB9567spRadulodon_casearius
GGGCACGCCCGTTTGAGTGTCATGGTATTCTCAATAC-TTCAAA-T----
KRT_Iso_26Radulodon_casearius
GGGCACGCCCGTTTGAGTGTCATGGTATTCTCAATAC-TTCAAA-T----
CBS126044Radulodon_erikssonii
GGGCACGCCCGTTTGAGTGTCATGGTATTCTCAATAC-TCCAAA-T----
He6183YUNNANENSIS
GGGCACGCCCGTTTGAGTGTCATGGTATTCTCAATAC-TCCAAA-T----
Cui17979YUNNANENSIS
GGGCACGCCCGTTTGAGTGTCATGGTATTCTCAATAC-TCCAAA-T----
Miettinen2091Junghuhnia_fimbri GAGCATGCTTGTTTGAGTGTCATGGTATTCTCAACCC-TTCTGT-T----
KHL12316S_tenue
GAGCATGCTTGTTTGAGTGTCATGGTATTCTCAACCC-TTCTGT-A----
PRM846564S_pachyodon
AGGCATGCCTGTTTGAGTGTCATGGTATTCTCAATAC-CCTAAA-T----
SP_Lgt_S_pachyodon
AGGCATGCCTGTTTGAGTGTCATGGTATTCTCAATAC-CCTAAA-T----
Ryvarden44669Tyromyces_xuchile
AGGCATGCCTGTTTGAGTGTCATGGTATTCTCAATAC-CCTAAA-T----
PW17_171sinuosus
AGGCATGCCTGTTTGAGTGTCATGGTATTCTCAATAC-CCTAAA-T----
W53Dai12234
AGGCATGCCTGTTTGAGTGTCATGGTATTCTCAATAC-CCTAAA-T----
HHB4100SpAntella_americana

GAGCATGCCTGTTTGAGTGTCATGGTATTCTCAACCC-TCTCACAT----
W3Dai20901spumeus
GAGCATGCCTGTTTGAGTGTCATGGTATTCTCAATTC-CTTTGATT----
He6736
GAGCATGCCTGTTTGAGTGTCATGGTATTCTCAATTC-CTTTGATT----
BRNM734877S_spumeus
GAGCATGCCTGTTTGAGTGTCATGGTATTCTCAATTC-CTTTGATT----
BRNM712630S_spumeus
GAGCATGCCTGTTTGAGTGTCATGGTATTCTCAATTC-CTTTGATT----
Dai1723Loweomyces_sibiricus
GAGCATGCCTGTTTGAGTGTCATGGTATTCTCAATTCTCTTTGATT----
W54Cui10009
GAGCATGCCTGTTTGAGTGTCATGGTATTCTCAATTC-CTTTGATT----
W1Dai20899
GAGCATGCCTGTTTGAGTGTCATGGTATTCTCAATTC-CTTTGATT----
HHB13445Trametes_ochracea
GAGCATGCCTGTTTGAGTGTCATGGAATTCTCAACCT---------ATAA
Dai16222
GAGCATGCCTGTTTGAGTGTCATGGTATTCTCAAATC-TCTTGTGT----
Dai16240
GAGCATGCCTGTTTGAGTGTCATGGTATTCTCAAATC-TCTTGTGT----

JV1310_11SanguinolentusCernys --TTCTGC------------CAA-----
GGTGATATTGGACTTGGAGGCT
MJ39_00_SK --TTCTGC------------CAA-----
GGTGATATTGGACTTGGAGGCT
MJ111_04_CZ --TTCTGC------------CAA-----
GGTGATATTGGACTTGGAGGCT
JV1610_BOKYsmrk --TTCTGC------------CAA-----
GGTGATATTGGACTTGGAGGCT
Dai21030 --TTCTGC------------CAA-----
GGTGATATTGGACTTGGAGGCT
Dai20976P_furcatus --TTCTGC------------CAA-----
GGTGATATTGGACTTGGAGGCT
Dai2105 --TTCTGC------------CAA-----
GGTGATATTGGATTTGGAGGCT
Dai2544 --TTCTGC------------CAA-----
GGTGATATTGGATTTGGAGGCT
Dai11313 --TTTTGC------------CAA-----
GGTGATATTGGATTTGGAGGCT
WCG1611Dai26167 --TTCTGC------------CAA-----
GGTGATATTGGATTTGGAGGCT
WCG1518Dai25999Physisporinus --TTCTGC------------CAA-----
GGTGATATTGGATTTGGAGGCT

TAA15097 --TTCTGC-----------CAA----
GGTGATATTGGACTTGGAGGCT
JV8909_19_CZ --TTCTGC-----------CAA----
GATGATATTGGACTTGGAGGCT
JV1310_15_P_sanguinolentus2_CZ --TTCTGC-----------CAA----
GATGATATTGGACTTGGAGGCT
MJ53_02_CZ --TTCTGC-----------CAA----
GATGATATTGGACTTGGAGGCT
CLZhao21647P_yunnanensis --TTCTGC-----------TGA----
GATGATATTGGACTTGGAGGCC
CLZhao21583P_yunnanensis --TTCTGC-----------TGA----
GATGATATTGGACTTGGAGGCC
Dai22272 --TTCTGC-----------CAA----
GATGATATTGGACTTGGAGGCT
Dai22279 --TTCTGC-----------CAA----
GATGATATTGGACTTGGAGGCT
MJ332_94_CZ --TTTTGC-----------TGG----
GATGATATTGGACTTGGAGGTT
MJ642_94_CZ_Expallescens --TTTTGC-----------TGG----
GATGATATTGGACTTGGAGGTT
Dai21060P_vinctus --TTTTGC-----------TGG----
GATGATATTGGACTTGGAGGTT
JV0511_23LRP_pouzarii --TTTTGC-----------TGA----
GATGATATTGGACTTGGAGGTT
JQ409462_R_pouzarii_PRM899856_ --TTTTGC-----------TGA----
GATGATATTGGACTTGGAGGTT
JV0308_66_WA --TTTTGC-----------TGA----
GATGATATTGGACTTGGAGGTT
JV0309_45_WA_USA --TTTTGC-----------TGA----
GATGATATTGGACTTGGAGGTT
JV0709_83_CA_USA --TTTTGC-----------TGA----
GATGATATTGGACTTGGAGGTT
Dai21043P_pouzarii --TTTTGC-----------TGA----
GATGATATTGGACTTGGAGGTT
MJ144_95_CZ --TTTTGC-----------TGG----
GATGATATTGGACTTGGAGGTT
JV0909_3_CZ --TTTTGC-----------TGG----
GATGATATTGGACTTGGAGGTT
JV0609_1_K --TTTTGC-----------TGG----
GATGATATTGGACTTGGAGGTT
Dai20396Physisporinus_castanop --CTTTGC-----------TGA----
GATGATATTGGACTTGGAGGTT
Dai20397Physisporinus_castanop --CTTTGC-----------TGA----
GATGATATTGGACTTGGAGGTT

MJ19_09_SK_Abies --TTTTGC-----------TGA----
GGTGATATTGGACTTGGAGGTT
JV0509_40_J_TN_USA_Betula --TTTTGC-----------TGA----
GGTGATATTGGACTTGGAGGTT
JV0808_33crocatus_PA_USAlist --TTTTGC-----------TGA----
GGTGATATTGGACTTGGAGGTT
DLL2009_061P_crocatus --TTTTGC-----------TGA----
GGTGATATTGGACTTGGAGGTT
Dai12800P_subcrocatus --TTTTGC-----------TGA----
GGTGATATTGGACTTGGAGGTT
Dai15917P_subcrocatus --TTTTGC-----------TGA----
GGTGATATTGGACTTGGAGGTT
Cui16620 --TTTTGC-----------TGG----
GATGATATTGGATTTGGAGGTT
HCFC1088Meripilus_stillicidior --TTTTGC-----------TGG----
GATGATATTGGATTTGGAGGTT
MCW590Meripilus_obsrcurus --TTTTGC-----------TGG----
GATGATATTGGACTTGGAGGTT
MCW722Meripilus_obsrcurus --TTTTGC-----------TGG----
GATGATATTGGACTTGGAGGTT
Cui9381P_tibeticus --TTTTGT-----------TGG----
GATGATATTGGACTTGGAGGTT
Cui9588P_tibeticus --TTTTGT-----------TGG----
GATGATATTGGACTTGGAGGTT
Va2_Beneschova --TTTTTC-----------TGG-----GATGA-
ATTGGATTTGGAGGTT
CWU3874_Ukraine_Alnus --TTTTTC-----------TGG-----GATGA-
ATTGGATTTGGAGGTT
WCG1293Dai24718Physisporinus --TTCTGC-----------TGA----
GGTGATATTGGACTTGGAGGCT
WCG1268Dai24682A --GTTTTC-----------TAA----
GGTGATATTGGACTTGGAGGTT
WCG1269Dai24683A --GTTTTC-----------TAA----
GGTGATATTGGACTTGGAGGTT
WCG1279Dai24694A --GTTTTC-----------TAA----
GGTGATATTGGACTTGGAGGTT
Dai16971 --GTTTTC-----------TAA----
GGTGATATTGGACTTGGAGGTT
ZQY1043Dai26696 --GTTTGC-----------TAA----
GGTGATATTGGACTTGGAGGTT
Doll880 --TTTT-C-----------TAA----
GGTGATATTGGATTTGGAGGTT
Doll1000 --TTTT-C-----------TAA----
GGTGATATTGGATTTGGAGGTT

1DAI18529 --TTTTCC------------TGA-----
GGTGATATTGGATTTGGAGGTT
Dai19535 --GTTTGC------------TGG-----
GATGATATTGGACTTGGAGGTA
1704_79_hnedyVillaLaPaz --TTCTGC------------TGG-----
AGTGACATTGGACTTGGAGGCT
F2061 --TTCTGC------------TGG-----
AGTGACATTGGACTTGGAGGCT
1DAI18268 --TCTTGC------------TGG-----
AGTGACATTGGATTTGGAGGCT
1DAI18540A --TCTTGC------------TGG-----
AGTGACATTGGATTTGGAGGCT
Dai17695 --TCTTGC------------TGG-----
AGTGATATTGGATTTGGAGGCT
LKY18Dai26373 --TCTTGC------------TGG-----
AGTGACATTGGATTTGGAGGCT
Dai17839P_sulphureus --TCTTGC------------TGG-----
AGCGGCATTGGATTTGGAGGCT
Dai17841P_sulphureus --TCTTGC------------TGG-----
AGCGGCATTGGATTTGGAGGCT
Dai19877P_roseus --TCTTGC------------CAG-----
AGTAATATTGGACTTGGAGGCT
1508_18_1_Kout --TTTTGT------------TGA-----
AGAGATATTGGACTTGGAGGTA
KP859303_R_vinctus_RP185_BRAZI --TTTTGT------------TGA-----
AGAGATATTGGACTTGGAGGTA
JK1807_15Rigidoporus_sp_Puerto --TTTTGT------------TGA-----
AGAGATATTGGACTTGGAGGTA
JV0509_47_J_TN_USA --TTTTRT------------
GAGACTCTGGTGATATTGGATTTGGAGGC-
JV0709_188 --TTTTRT------------
GAGACTCTGGTGATATTGGATTTGGAGGC-
JV0509_127_PA_USA --TTTTAT------------
GAGACTCTGGTGATATTGGATTTGGAGGC-
JV1009_59_NJ_USA --TTTTAT------------
GAGACTCTGGTGATATTGGATTTGGAGGC-
Dai15497P_crataegi --TTTTGT------------
GAGACTCTGGTGATATTGGACTTGGAGGT-
Dai15499P_crataegi --TTTTGT------------
GAGACTCTGGTGATATTGGACTTGGAGGT-
Cui3266P_cinereus --TTTTGT------------
TAGACTCTGGTGGTATTGGACTTGGAGGC-
WCG1256Dai24690 --TTTTGT------------
TAGACTCTGGTGGTATTGGACTTGGAGGC-

1DAI17581 --TTTTGT-----------
TAGACTCTGGTGGTATTGGACTTGGAGGC-
WCG1255Dai24688 --TTTTGT-----------
TAGACTCTGGTGGTATTGGACTTGGAGGC-
Dai22427 --TTTTGT-----------
TAGACTCTGGTGGTATTGGACTTGGAGGC-
MV690Meripilus_concrescens --TTTTGT-----------
GAGACTCTGGTGGTATTGGACTTGGAGGC-
MV513Meripilus_galapagensis -TTTTTGT-----------
GAGACTCTGGTGGTATTGGACTTGGAGGC-
Dai19793 --TTTTGT-----------
GAGACTCTGGTGATATTGGACTTGGAGGCT
OQ553780P_tamilnaduensis --TTTTGT-----------
AAGACTTTGGTGATATTGGACTTGGAGGT-
OQ553779P_tamilnaduensis --TTTTGT-----------
AAGACTTTGGTGATATTGGACTTGGAGGT-
A164FB3Meripilus_giganteus --TTTTGT-----------
AAGACTTTGGTGATATTGGACTTGGAGGT-
JV1407_36_Vinctus_Meandrica TTTTTTGT-----------
GAGACTCTGGTGATATTGGACTTGGAGG-A
1807_3K_Rigidoporus_PuertoRico --
Cui16903P_vinctus TTTTTTGT-----------
GAGACTCTGGTGATATTGGACTTGGAGG-A
JV1008_18R_Lineatus ---TTTGT-----------
AAGACTCTGGTGATATTGGACTTGGAGG-C
JV1407_37_1_Vinctus_Carara ---TTTGT-----------
AAGACTCTGGTGATATTGGACTTGGAGG-C
Dai17986P_lineatus --TTTTGT-----------
AAGACTCTGGTGATATTGGACTTGGAGG-C
Dai18281 --TTTTGT-----------
AAGACTCTGGTGATATTGGACTTGGAGG-C
1DAI19796 --TTTTGT-----------
AAGACTCTGGTGATATTGGACTTGGAGG-C
ZQY797Dai25241 --TTTTGT-----------
AAGACTCTGGTGATATTGGACTTGGAGG-C
WCG1289Dai24711 --TTTTGT-----------
AAGACTCTGGTGATATTGGACTTGGAGG-C
Dai22598 -TTTTTGT-----------
GAGACTCTGATGATATTGGACTTGGAGGTT
Dai20523 -TTTTTGT-----------
GAGACTCTGATGATATTGGACTTGGAGGTT
Dai17885 -TTTTTGT-----------
GAGACTCTGATGATATTGGACTTGGAGGTT
Dai17553 -TTTTTGT-----------

GAGACTCTGATGATATTGGACTTGGAGGTT
Dai19639 TTTTTTGT-----------
GAGACTCTGATGATATTGGACTTGGAGGTT
JV0110_48_CZ --CTTTGT------------TGGATGG-
GGTGATATTGGACTTGGAGGCT
MJ129_04 --CTTTGT------------TGGATGG-
GGTGATATTGGACTTGGAGGCT
Cui10340P_eminens --CTTTGT------------TGGATGG-
GGTGATATTGGACTTGGAGGCT
Cui10341P_eminens --CTTTGT------------TGGATGG-
GGTGATATTGGACTTGGAGGCT
Dai12685P_eminens --CTTTGT------------TGGATGG-
GGTGATATTGGACTTGGAGGCT
Miettinen_13591Rigidoporus_und --CTTTGT------------TGGATGG-
GGTGATATTGGACTTGGAGGCT
Dai20868 --CTTTGT------------TGGATGA-
GGTGATATTGGACTTGGAGGTT
Dai20832 --CTTTGT------------TGGAAGA-
GGTGATATTGGACTTGGAGGTT
Dai11400 --CTTTGT------------TGGAAGA-
GGTGATATTGGACTTGGAGGTT
Dai22472 --CTTTGT------------TGGAAGA-
GGTGATATTGGACTTGGAGGTT
1CUI10475 --CTTTGT------------TGGATGA-
GGTGGTATTGGATTTGGAGGTT
1CUI10491 --CTTTGT------------TGGATGA-
GGTGGTATTGGATTTGGAGGTT
HCFC1095Meripilus_robledoi --CTTTGT------------TGGATGG-
GGTGATATTGGACTTGGAGGTT
MCW702Meripilus_revolubilis --CTTTGC------------TGGATGG-
GGTGATATTGGACTTGGAGGTT
1704_83_zluty_HaciendaBaru --CTTGTT------------CGAACAA-
AGTGATATTGGACTTGGAGGCA
Dai9925P_lavendulus --CTCTTT------------TGAACGG-
GGTGATATCGGACTTGGAGGTA
Dai13587AP_lavendulus --CTCTTT------------TGAACGG-
GGTGATATCGGACTTGGAGGTA
PDD70600P_longicystidius --CTCTTT------------TGAACGG-
AGTGATATTGGACTTGGAGGCA
Cui16630 --CTCTTT------------TGAACGG-
AGTGATATTGGACTTGGAGGCA
FP_135344Meripilus_giganteus ---TTTAT------------TGAG----
GGCGGCATTGGATGTGGAGGCT
FP_100460_Sp ---TTTAT------------TGAG----

GGTGGCATTGGATGTGGAGGCT
CBS421_48Meripilus_giganteus ---TTTAT-----------TGAG----
GGCGGCATTGGATGTGGAGGCT
Cui9203 ---TTTAT-----------TGAG----
GGCGGCATTGGATGTGGAGGCT
Cui9202 ---TTTAT-----------TGAG----
GGCGGCATTGGATGTGGAGGCT
TUFC100564Japan ---TTTAT-----------TGAG----
GGTGGCATTGGATGTGGAGGCT
Russell5913Meripilus_sumstinei ---TTTGA-------------------
GGTGGCATTGGATTTGGAGGAC
RP215Meripilus_brasiliensis TTTTTTTT-----------TGAA----
AGGGATATTGGATTTGGAGGAT
RP200Meripilus_brasiliensis TTTTTTTT-----------TKAA----
AGGGATATTGGATTTGGAGGAT
JV1712_13J_R_vinctus2_LSUPuert --CTTTGC-------------GGATAA-
GGGTGTGTTGGACTTGGAGG-T
Dai10503R_hypobrunneus --CTTTGC-------------GGATAA-
GGGTGTGTTGGACTTGGAGG-T
Dai10569Rigidoporus_hypobrunne --CTTTGC-------------GGATAA-
GGGTGTGTTGGACTTGGAGG-T
1DAI19451 --CTTTGC-------------GGATAA-
GGGTGTGTTGGACTTGGAGG-T
CM108bRigidoporus_hypobrunneus --CTTTGC-------------GGATAA-
GGGTGTGTTGGACTTGGAGG-T
1CUI16874 --CTTTGC-------------GGATAA-
GGGTGTGTTGGACTTGGAGG-T
FD299Cerrena_unicolor --CTTTGC-------------GGATGA-
GGGTGTATTGGATTTGGAGG-T
KHL_GB_Cerrena_uniclor --CTTTGC-------------GGATGA-
GGGTGTATTGGATTTGGAGG-T
Dai12892Cerrena_albocinnamomea --CTTTGC-------------GGATGA-
AGGTGTATTGGACTTGGAGG-T
Dai12955C_albocinnamomea --CTTTGC-------------GGATGA-
AGGTGTATTGGACTTGGAGG-T
SNUm03110102C_aurantiopora --CTTTGC-------------GGATGA-
AGGTGTATTGGACTTGGAGG-T
NIBRFG0000102423C_aurantiopora --CTTTGC-------------GGATGA-
AGGTGTATTGGACTTGGAGG-T
Dai7359Antrodiella_zonata --CTTTGC-------------GGATGA-
GGGTGTATTGGACTTGGAGG-T
F20080702KCM29C_consors --CTTTGC-------------GGATGA-
GGGTGTATTGGACTTGGAGG-T
F20080208LYW10Cerrena_consors --CTTTGC-------------GGATGA-

GGGTGTATTGGACTTGGAGG-T
Dai7821Antrodiella_zonata --CTTTGC------------GGATGA-
GGGTGTATTGGACTTGGAGG-T
CFMR_DCL04_31Pseudolagarobasid --CTTTAT------------GGATGA-
AGGTGTATTGGATTTGGAGG-T
VPB197Pseudolagarobasidium_bel --CTTTGC------------GGATGA-
AGGTGTATTGGATTTGGAGG-T
CBS115543Pseudolagarobasidium_ --CTTTGC------------GGATGA-
AGGTGTATTGGATTTGGAGG-T
CBS115544 --CTTTGC------------GGATGA-
AGGTGTATTGGATTTGGAGG-T
Han405Pseudolagarobasidium_bai --CTTTGT------------GGATGC-
AGGTGTATTGGATTTGGAGG-T
Han406Pseudolagarobasidium_bai --CTTTGT------------GGATGC-
AGGTGTATTGGATTTGGAGG-T
MUcc838Spongipellis_delectans --CTTTGC------------GGATGA-
GGGTGTATTGGATTTGGAGG-T
BRNM686401S_delectans --CTTTGC------------GGATGA-
GGGTGTATTGGATTTGGAGG-T
OSM_F925S_delectans --CTTTGC------------GGATGA-
GGGTGTATTGGATTTGGAGG-T
BRNM67093Spongipellis_litschau --CTTTGC------------GGATGA-
GGGTGTGTTGGACTTGGAGG-T
CFMRccFP59199TS_unicolor --CTTTGC------------GGATGA-
GGGTGTGTTGGACTTGGAGG-T
CFMRccFP71791TS_unicolor --CTTTGC------------GGATGA-
GGGTGTGTTGGACTTGGAGG-T
Dai13845P_lischaueri --CTTTGC------------GGATGA-
GGGTGTGTTGGACTTGGAGG-T
Dai20266P_lischaueri --CTTTGC------------GGATGA-
GGGTGTGTTGGACTTGGAGG-T
CFMR_HHB11240Radulodon_america --CTTTGC------------GGATGA-
GGATGTATTGGACTTGGAGG-T
RLG6350Radulodon_americanus --CTTTGC------------GGATGA-
GGATGTATTGGACTTGGAGG-T
KY415963Radulodon_erikssonii --CTTTGC------------GGATGA-
GGATGTATTGGACTTGGAGG-T
HHB9567spRadulodon_casearius --CTTTGC------------GGATGA-
GGATGTATTGGACTTGGAGG-T
KRT_Iso_26Radulodon_casearius --CTTTGC------------GGATGA-
GGATGTATTGGACTTGGAGG-T
CBS126044Radulodon_erikssonii --CTTTGC------------GGATGA-
GGATGTATTGGACTTGGAGG-T
He6183YUNNANENSIS --CTTTGC------------GGATGA-

GGATGTATTGGATTTGGAGGCC
Cui17979YUNNANENSIS --CTTTGC------------GGATGA-
GGATGTATTGGATTTGGAGGCC
Miettinen2091Junghuhnia_fimbri --TCTTTT------------GAAGCA-
GTTGGGCTTGGACTTGGAGG-C
KHL12316S_tenue --TTTTTTTATTTAAAAAAAAGGCA-
GCTGGGCTTGGACTTGGAGG-C
PRM846564S_pachyodon --CTTTGC------------GGATGA-
GGGTGTATTGGACTTGGAGGTT
SP_Lgt_S_pachyodon --CTTTGC------------GGATGA-
GGGTGTATTGGACTTGGAGGTT
Ryvarden44669Tyromyces_xuchile --CTTTGC------------AGATGA-
GGGTGTATTGGACTTGGAGGTT
PW17_171sinuosus --CTTTGC------------GGATGA-
GGGTGTATTGGACTTGGAGGTA
W53Dai12234 --CTTTGC------------GGATGA-
GGGTGTATTGGACTTGGAGGTA
HHB4100SpAntella_americana TTTTTTGTG------------------
GTTGGGCTTGGACTTGGAGGTA
W3Dai20901spumeus -TCTTTCC---------
ACGGGATCAAAGAGATATTGGATTTGGAGGCT
He6736 -TCTTTCC---------
ATGGGATCAAAGAGATATTGGATTTGGAGGCT
BRNM734877S_spumeus -TCTTTCC---------
AYGGGAYCAAAGAGAYATTGGATTTGGAGGC-
BRNM712630S_spumeus -TCTTTCC---------
ATGGGATCAAAGAGAYATTGGATTTGGAGGC-
Dai1723Loweomyces_sibiricus -TCTTTCC---------
ATGGGATCAAAGAGATATTGGATTTGGAGGC-
W54Cui10009 -TCTTTCC---------
ATGGGATCAGAGAGATATTGGATTTGGAGGC-
W1Dai20899 -TCTTTCC---------
ATGGGATCAGAGAGATATTGGATTTGGAGGC-
HHB13445Trametes_ochracea ATCCTTGTGATC--------------
TATAGGCTTGGACTTGGAGGCT
Dai16222
TTCTTTTTTTTTTAAAAAAAAAAATACAAGCAGTTTTGGACTTGGAGGTA
Dai16240
TTCTTTTTTTTTTAAAAAAAAAAATACAAGCAGTTTTGGACTTGGAGGTA

JV1310_11SanguinolentusCernys A-T----TGCCGGCATC-----------
ATCTCTGAAGCCGGCTCCTCT
MJ39_00_SK A-T----TGCCGGCATC-----------
ATCTCTGAAGCCGGCTCCTCT

MJ111_04_CZ A-T----TGCCGGCATC-----------
ATCTCTGAAGCCGGCTCCTCT
JV1610_BOKYsmrk A-T----TGCCGGCATC-----------
ATCTCTGAAGCCGGCTCCTCT
Dai21030 A-T----TGCCGGCATC-----------
ATCTCTGAAGCCGGCTCCTCT
Dai20976P_furcatus A-T----TGCCGGCATC-----------
ATCTCTGAAGCCGGCTCCTCT
Dai2105 A-T----TGCTGGCATC-----------
ATCTCAGAAGCCGGCTCCTCT
Dai2544 A-T----TGCTGGCATC-----------
ATCTCAGAAGCCGGCTCCTCT
Dai11313 A-T----TGCTGGCATC-----------
ATCTTAGAAGCCAGCTCCTCT
WCG1611Dai26167 A-T----TGCTGGCATC-----------
ATCTTAGAAGCCAGCTCCTCT
WCG1518Dai25999Physisporinus A-T----TGCTGGCATC-----------
ATCTTAGAAGCCAGCTCCTCT
TAA15097 A-T----TGCTGGCATC-----------
ATCTCTGAAGCCGGCTCCTCT
JV8909_19_CZ A-T----TGCTGGCATC-----------
GTTTCAGAAGCCAGCTCCTCT
JV1310_15_P_sanguinolentus2_CZ A-T----TGCTGGCATC-----------
GTTTCAGAAGCCAGCTCCTCT
MJ53_02_CZ A-T----TGCTGGCATC-----------
GTTTCAGAAGCCAGCTCCTCT
CLZhao21647P_yunnanensis A-T----TGCTGGCATC-----------
GTTCCAGAAGCCAGCTCCTCT
CLZhao21583P_yunnanensis A-T----TGCTGGCATC-----------
GTTCCAGAAGCCAGCTCCTCT
Dai22272 A-T----TGCTGGCATC-----------
GTTTCTGAAGCCAGCTCCTCT
Dai22279 A-T----TGCTGGCATC-----------
GTTTCTGAAGCCAGCTCCTCT
MJ332_94_CZ A-T----TGCCGGCATC------------
CTTGTGAAACCGGCTCCTCT
MJ642_94_CZ_Expallescens A-T----TGCCGGCATC------------
CTTGTGAAACCGGCTCCTCT
Dai21060P_vinctus A-T----TGCCGGCATC------------
CTTGTGAAACCGGCTCCTCT
JV0511_23LRP_pouzarii A-T----TGCTGGCATC------------
CTTGTGAAACCAGCTCCTCT
JQ409462_R_pouzarii_PRM899856_ A-T----TGCTGGCATC------------
CTTGTGAAACCAGCTCCTCT

JV0308_66_WA A-T----TGCCGGTATT------------
CTTGTGAAACCGGCTCCTCT
JV0309_45_WA_USA A-T----TGCCGGTATT------------
CTTGTGAAACCGGCTCCTCT
JV0709_83_CA_USA A-T----TGCCGGTATT------------
CTTGTGAAACCGGCTCCTCT
Dai21043P_pouzarii A-T----TGCTGGCATC------------
CTTGTGAAACCAGCTCCTCT
MJ144_95_CZ A-T----TGCCGGTATC------------
CTTGTGARATYGGCTCCTCT
JV0909_3_CZ A-T----TGCCGGTATC------------
CTTGTGAGATYGGCTCCTCT
JV0609_1_K A-T----TGCCGGTATC------------
CTTGTGAGATCGGCTCCTCT
Dai20396Physisporinus_castanop A-T----TGCCGATGTT------------
CTCGTAAAATCGGCTCCTCT
Dai20397Physisporinus_castanop A-T----TGCCGATGTT------------
CTCGTAAAATCGGCTCCTCT
MJ19_09_SK_Abies A-T----TGCTGGCATC------------
CTCGTGAAACCAGCTCCTCT
JV0509_40_J_TN_USA_Betula A-T----TGCTGGCATC------------
CTCGTGAAACCAGCTCCTCT
JV0808_33crocatus_PA_USAlist A-T----TGCTGGCATC------------
CTCGTGAAACCAGCTCCTCT
DLL2009_061P_crocatus A-T----TGCTGGCATC------------
CTCGTGAAACCAGCTCCTCT
Dai12800P_subcrocatus A-T----TGCTGGCATC------------
CTCGTGAAACCAGCTCCTCT
Dai15917P_subcrocatus A-T----TGCTGGCATC------------
CTCGTGAAACCAGCTCCTCT
Cui16620 A-T----TGCCGGCATC------------
CTCGTGAAACCGGCTCCTCT
HCFC1088Meripilus_stillicidior A-T----TGCCGGCATC------------
CTCGTGAAACCGGCTCCTCT
MCW590Meripilus_obsrcurus A-T----TGCCGGCATC------------
CTTGTGAAACCGGCTCCTCT
MCW722Meripilus_obsrcurus A-T----TGCCGGCATC------------
CTTGTGAAACCGGCTCCTCT
Cui9381P_tibeticus A-T----TGCTGGCATC------------
CTTGTGAAACCAGCTCCTCT
Cui9588P_tibeticus A-T----TGCTGGCATC------------
CTTGTGAAACCAGCTCCTCT
Va2_Beneschova ATT----TGCTGGCATC------------
CTTGTGAAACCGGCTCCTCT

CWU3874_Ukraine_Alnus ATT----TGCTGGCATC------------
CTTGTGAAAYCRGCTCCTCT
WCG1293Dai24718Physisporinus A-T----TGCTGGCATC-----------
ATTTTTGAAGCCGGCTCCTCT
WCG1268Dai24682A G-T----TGCTGGCATC-----------
ATTGTGAAAGTCAGCTCCTCT
WCG1269Dai24683A G-T----TGCTGGCATC-----------
ATTGTGAAAGTCAGCTCCTCT
WCG1279Dai24694A G-T----TGCTGGCATC-----------
ATTGTGAAAGTCAGCTCCTCT
Dai16971 G-T----TGCTGGCATC-----------
ATTGTGAAAGTCAGCTCCTCT
ZQY1043Dai26696 G-T----TGCTGGCATC-----------
ATTGTGAAAGTCAGCTCCTCT
Doll880 GCT----TGCTGGACTC-----------
TGTTTGGAAACCAGCTCCTCT
Doll1000 GCT----TGCTGGACTC-----------
TGTTTGGAAACCAGCTCCTCT
1DAI18529 CTT----TGCTGGACTCTT---------
TTTTTGGAAACCAGCTCCTCT
Dai19535 C-T----TGCTGGGTGCT----------
CTTCGGAAACCCGGCTCCTCT
1704_79_hnedyVillaLaPaz G-T----TGCAGGAATC-----------
CTTCTGGATGCCAGCTCCTCT
F2061 G-T----TGCAGGAATC-----------
CTTCTGGATGCCAGCTCCTCT
1DAI18268 G-T----TGCAGGAATC-----------
CCTCTGGATACCGGCTCCTCT
1DAI18540A G-T----TGCAGGAATC-----------
CCTCTGGATACCGGCTCCTCT
Dai17695 G-T----TGCAGGAATC-----------
CCTCTGGATACCGGCTCCTCT
LKY18Dai26373 G-T----TGCAGGAATC-----------
CCTCTGGATACCGGCTCCTCT
Dai17839P_sulphureus T------TGCTGGAATT------------
TTTGAAATATCAGCTTCTCT
Dai17841P_sulphureus T------TGCTGGAATT------------
TTTGAAATATCAGCTCCTCT
Dai19877P_roseus T------TGCTGGAATC------------
TTCAGAATGCCAGCTCCTCT
1508_18_1_Kout A------TGCTGGCATT-----------
GTTCTGAATGCCAGCTCCTCT
KP859303_R_vinctus_RP185_BRAZI A------TGCTGGCATT-----------
GTTCTGAATGCCAGCTCCTCT

JK1807_15Rigidoporus_sp_Puerto A------TGCTGGCATT-----------
GTTCTGAATGCCAGCTCCTCT
JV0509_47_J_TN_USA TTT----TGCTGGCATCT-----------
GTTAAGGAGCCAGCTCCTCT
JV0709_188 TTT----TGCTGGCATCT-----------
GTTAAGGAGCCAGCTCCTCT
JV0509_127_PA_USA TTT----TGCTGGCATCT-----------
GTTAAGGAGCCAGCTCCTCT
JV1009_59_NJ_USA TTT----TGCTGGCATCT-----------
GTTAAGGAGCCAGCTCCTCT
Dai15497P_crataegi TTT----TGCTGGCATCT------------
TACAGAAGCCAGCTCCTCT
Dai15499P_crataegi TTT----TGCTGGCATCT------------
TACAGAAGCCAGCTCCTCT
Cui3266P_cinereus AAT----TGCTGGCATCT------------
CTCAGAAGCCAGCTCCTCT
WCG1256Dai24690 AAT----TGCTGGCATCT------------
CTCAGAAGCCAGCTCCTCT
1DAI17581 AAT----TGCTGGCATCT------------
CTCAGAAGCCAGCTCCTCT
WCG1255Dai24688 AAT----TGCTGGCATCT------------
CTCAGAAGCCAGCTCCTCT
Dai22427 AAT----TGCTGGCATCT------------
CTCAGAAGCCAGCTCCTCT
MV690Meripilus_concrescens AAT----TGCTGGCATCT------------
CTCAGAAGCCAGCTCCTCT
MV513Meripilus_galapagensis TAT----TGCTGGTATCT------------
CTCAGAAGCCAGCTCCTCT
Dai19793 TTT----TGCTGGCATCT------------
CTTAGAAGCCAGCTCCTCT
OQ553780P_tamilnaduensis TCT----TGCTGGCATCT-----------
TGCTTGAAACCAGCTCCTCT
OQ553779P_tamilnaduensis TCT----TGCTGGCATCT-----------
TGCTTGAAACCAGCTCCTCT
A164FB3Meripilus_giganteus TCT----TGCTGGCATCT-----------
TGCTTGAAACCAGCTCCTCT
JV1407_36_Vinctus_Meandrica TAT----TGCTGGAATCTT----------GC---
AGGTGCCAGCTCCTCT
1807_3K_Rigidoporus_PuertoRico --
Cui16903P_vinctus TAT----TGCTGGAATCTT----------GC---
AGGTGCCAGCTCCTCT
JV1008_18R_Lineatus TAT----TGCTGGAACCTT----------GC---
AGGTACCTGCTCCTCT
JV1407_37_1_Vinctus_Carara TAT----TGCTGGAACCTT----------GC---

AGGTACCTGCTCCTCT
Dai17986P_lineatus TAT----TGCTGGAACCTT---------GC--
AGGTACCTGCTCCTCT
Dai18281 TAT----TGCTGGAACCTT---------GC--
AGGTGCCTGCTCCTCT
1DAI19796 TAT----TGCTGGAACCTT---------
GCAAAAGGTACCTGCTCCTCT
ZQY797Dai25241 TAT----TGCTGGAACCTT---------
GCAAAAGGTACCTGCTCCTCT
WCG1289Dai24711 TAT----TGCTGGAACCTT---------
GCAAAAGGTACCTGCTCCTCT
Dai22598 TAT----TGCTGGCATCTT---------GC--
AGGTGCCCGCTCCTCT
Dai20523 TAT----TGCTGGCATCTT---------GC--
AGGTGCCTGCTCCTCT
Dai17885 TAT----TGCTGGCATCTT---------GC--
AGGTGCCTGCTCCTCT
Dai17553 TAT----TGCTGGCATCTT---------GC--
AGGTGCCTGCTCCTCT
Dai19639 TAT----TGCTGGCATCTT---------GC--
AGGTGCCTGCTCCTCT
JV0110_48_CZ T-T----TGCTGGCATC------------
TTAGTGAAGTCAGCTCCTCT
MJ129_04 T-T----TGCTGGCATC------------
TTAGTGAAGTCAGCTCCTCT
Cui10340P_eminens T-T----TGCTGGCATC------------
TTAGTGAAGTCAGCTCCTCT
Cui10341P_eminens T-T----TGCTGGCATC------------
TTAGTGAAGTCAGCTCCTCT
Dai12685P_eminens T-T----TGCTGGCATC------------
TTAGTGAAGTCAGCTCCTCT
Miettinen_13591Rigidoporus_und T-T----TGCTGGCATC------------
TTAGTGAAGTCAGCTCCTCT
Dai20868 T-T----TGCTGGCATC------------
CTAGGGAAGTCAGCTCCTCT
Dai20832 T-T----TGCTGGCATC------------
CTAGTGAAGTCAGCTCCTCT
Dai11400 T-T----TGCTGGCATC------------
CTAGTGAAGTCAGCTCCTCT
Dai22472 T-T----TGCTGGCATC------------
CTAGTGAAGTCAGCTCCTCT
1CUI10475 T-T----TGCTGGCATC------------
TCAGTGAGGTCGGCTCCTCT
1CUI10491 T-T----TGCTGGCATC------------

TCAGTGAGGTCGGCTCCTCT
HCFC1095Meripilus_robledoi C-T----TGCTGGCATC------------
CCTGTGAAGTCAGCTCCTCT
MCW702Meripilus_revolubilis C-T----TGCTGGCACC------------
CCTGCGGAGTCAGCTCCTCT
1704_83_zluty_HaciendaBaru TTT----TGCTGGCGAT--------------
AACAAGTCAGCTCCTCT
Dai9925P_lavendulus TAT----TGCTGGCGAT--------------
ACCGAGTCAGCTCCTCT
Dai13587AP_lavendulus TAT----TGCTGGCGAT--------------
ACCGAGTCAGCTCCTCT
PDD70600P_longicystidius TTT----TGCTGGTGAT--------------
ACCAAGCCAGCTCCTCT
Cui16630 TTT----TGCTGGTGAT--------------
ACCAAGCCAGCTCCTCT
FP_135344Meripilus_giganteus CTT----TGCTGGCCAT--------------
TTTGTGCCAGCTCCTCT
FP_100460_Sp CTT----TGCTGGCCAT--------------
TTTGTGCCAGCTCCTCT
CBS421_48Meripilus_giganteus CTT----TGCTGGCCAT--------------
TTTGTGCCAGCTCCTCT
Cui9203 CTT----TGCTGGCCAT--------------
TTTGTGCCAGCTCCTCT
Cui9202 CTT----TGCTGGCCAT--------------
TTTGTGCCAGCTCCTCT
TUFC100564Japan CTT----TGCTGGCCAT--------------
TTTGTGCCAGCTCCTCT
Russell5913Meripilus_sumstinei ATC----TGCTGGCGGC-------------------GCCAGCTCCTCT
RP215Meripilus_brasiliensis CAT----TGCTGGCATTAT----------------TGTCAGCTCCTCT
RP200Meripilus_brasiliensis CAT----TGCTGGCATTAT----------------TGTCAGCTCCTCT
JV1712_13J_R_vinctus2_LSUPuert TTT----TGCAGGTAATG-----------
ATTGTATTACCAGCTCCTCT
Dai10503R_hypobrunneus TTT----TGCAGGTAATG-----------
ATTGTATTACCAGCTCCTCT
Dai10569Rigidoporus_hypobrunne TTT----TGCAGGTAATG-----------
ATTGTGTTACCAGCTCCTCT
1DAI19451 TTT----TGCAGGTAATG-----------
ATTGTGTTACCAGCTCCTCT
CM108bRigidoporus_hypobrunneus TTT----TGCAGGTAATG-----------
ATTGTATTACCAGCTCCTCT
1CUI16874 TTT----TGCAGGTAATG-----------
ATTGTATTACCAGCTCCTCT
FD299Cerrena_unicolor TTT----TGCAGGCAAT------------ATT-
CATTGTCAGCTCCTCT

KHL_GB_Cerrena_uniclor TTT----TGCAGGCAAT------------ATT-
CATTGTCAGCTCCTCT
Dai12892Cerrena_albocinnamomea TTT----TGCAGGCACT------------
ATTACTTTGTCTGCTCCTCT
Dai12955C_albocinnamomea TTT----TGCAGGCACT------------
ATTACTTTGTCTGCTCCTCT
SNUm03110102C_aurantiopora TTT----TGCAGGCACT------------
ATTACTTTGTCTGCTCCTCT
NIBRFG0000102423C_aurantiopora TTT----TGCAGGCACT------------
ATTACTTTGTCTGCTCCTCT
Dai7359Antrodiella_zonata TTT----TGCAGGCAAT------------
ATTACTTTGTCGGCTCCTCT
F20080702KCM29C_consors TTT----TGCAGGCAAT------------
ATTACTTTGTCGGCTCCTCT
F20080208LYW10Cerrena_consors TTT----TGCAGGCAAT------------
ATTACTTTGTCGGCTCCTCT
Dai7821Antrodiella_zonata TTT----TGCAGGCAAT------------
ATTACTTTGTCGGCTCCTCT
CFMR_DCL04_31Pseudolagarobasid TTA----TGTTGGCTCT-------------
TTCAAGTGTCAGCTCCTCT
VPB197Pseudolagarobasidium_bel TTA----TGCTGGCTCT-------------
TTCAAGAGTCAGCTCCTCT
CBS115543Pseudolagarobasidium_ TTA----TGTTGGCTCT-------------
CTTGAGAGTCAGCTCCTCT
CBS115544 TTA----TGTTGGCTCT-------------
CTTGAGAGTCAGCTCCTCT
Han405Pseudolagarobasidium_bai CTA----TGCTGGCTCT-------------
TTCAAGTGTTAGCTCCTCT
Han406Pseudolagarobasidium_bai CTA----TGCTGGCTCT-------------
TTCAAGTGTTAGCTCCTCT
MUcc838Spongipellis_delectans TTA----TGCTGGCGTT-------------------
TGTCGGCTCCTCT
BRNM686401S_delectans TTA----TGCTGGCGTT-------------------
TGTCGGCTCCTCT
OSM_F925S_delectans TTA----TGCTGGCGTT-------------------
TGTCGGCTCCTCT
BRNM67093Spongipellis_litschau TTA----TGCCGGCAGT------------------
CTGTCGGCTCCTCT
CFMRccFP59199TS_unicolor TTA----TGCCGGCAGT------------------
TTGTCGGCTCCTCT
CFMRccFP71791TS_unicolor TTA----TGCCGGCAGT------------------
TTGTCGGCTCCTCT
Dai13845P_lischaueri TTA----TGCCGGCAGT------------------
CTGTCGGCTCCTCT

Dai20266P_lischaueri TTA----TGCCGGCAGT-----------------
CTGTCGGCTCCTCT
CFMR_HHB11240Radulodon_america TTA----TGCCGGCTTTT----------
AATTATTTATCGGCTCCTCT
RLG6350Radulodon_americanus TTA----TGCCGGCTTTT----------
AATTATTTMTCGGCTCCTCT
KY415963Radulodon_erikssonii TTA----TGCCGGCTTTT----------
AATTATTTGTTGGCTCCTCT
HHB9567spRadulodon_casearius TTA----TGCCGGCTTTT----------
TATTATTTGTCGGCTCCTCT
KRT_Iso_26Radulodon_casearius TTA----TGCCGGCTTTT----------
TATTATTTGTCGGCTCCTCT
CBS126044Radulodon_erikssonii TTA----TGCCGGCTTTT----------
AATTATTTGTTGGCTCCTCT
He6183YUNNANENSIS TTA----TGCCGGCTTTA----------
TATTATTTGTCGGCTCCTCT
Cui17979YUNNANENSIS TTA----TGCCGGCTTTA----------
TATTATTTGTCGGCTCCTCT
Miettinen2091Junghuhnia_fimbri -TT---
TGCTGGTGTCTGAGTAATTTATTACTCAGATGTGGGCTCCTCT
KHL12316S_tenue TTT----TGCTGGTGATTGAA----
TTTTTATTCAAGCATCAGCTCCTCT
PRM846564S_pachyodon TTTCTT-TGCTGGCTTCA----------------
TTGTCGGCTCCTCT
SP_Lgt_S_pachyodon TTTCTT-TGCTGGCTTCA----------------
TTGTCGGCTCCTCT
Ryvarden44669Tyromyces_xuchile TTTATCATGCTGGCTTCA----------------
CTGTCGGCTCCTCT
PW17_171sinuosus TTTTT--TGCTGGCTTCA----------------
TTGTCGGCTCCTCT
W53Dai12234 TTTTT--TGCTGGCTTCA----------------
TTGTCGGCTCCTCT
HHB4100SpAntella_americana TCA---CTGCTGGTGTTCA--------
TTTTTGAACGCGGGCTCCTCT
W3Dai20901spumeus TTCGT--TGCCGGCGTATC-----------------
GCCGGCTCCTCT
He6736 TTCAT--TGCCGGCGTATC-----------------
GCCGGCTCCTCT
BRNM734877S_spumeus TTCRT--TGCCGGCGTATC-----------------
GCCGGCTCCTCT
BRNM712630S_spumeus TTCAT--TGCCGGCGTATC-----------------
GCCGGCTCCTCT
Dai1723Loweomyces_sibiricus TTCAT--TGCCGGCATAGC-----------------
GCCGGCTCCTCT

W54Cui10009 TTCGT--TGTCGGCGCGTC-----------------
GCCGGCTCCTCT
W1Dai20899 TTCGT--TGTCGGCGTGTC-----------------
GCCGGCTCCTCT
HHB13445Trametes_ochracea -------TGCTGGCCCTTGCG----------------
GTCGGCTCCTCT
Dai16222 AAT----TGTTGGCTTTT-------------------
GTCAGCTCCTCT
Dai16240 AAT----TGTTGGCTTTT-------------------
GTCAGCTCCTCT

JV1310_11SanguinolentusCernys TAAATGAATCAG-TGTG-AAC-AT------
TCACTTCATACTTGGTGTGA
MJ39_00_SK TAAWTGAATCAG-TGTG-AAC-AT------
TCACTTCATACTTGGTGTGA
MJ111_04_CZ TAAWTGAATCAG-TGTG-AAC-AT------
TCACTTCATACTTGGTGTGA
JV1610_BOKYsmrk TAAWTGAATCAG-TGTG-AAC-AT------
TCACTTCATACTTGGTGTGA
Dai21030 TAATTGAATCAG-TGTG-AAC-AT------
TCACTTCATACTTGGTGTGA
Dai20976P_furcatus TAATTGAATCAG-TGTG-AAC-AT------
TCACTTCATACTTGGTGTGA
Dai2105 TAAATGAATCAG-TGTG-AAC-AT------
TCACTTCATGCTTGGTGTGA
Dai2544 TAAATGAATCAG-TGTG-AAC-AT------
TCACTTCATGCTTGGTGTGA
Dai11313 TAAATGAATCAG-TGTG-AAC-AT------
TCACTTCATACTTGGTGTGA
WCG1611Dai26167 TAAATGAATCAG-TGTG-AAC-AT------
TCACTTCATACTTGGTGTGA
WCG1518Dai25999Physisporinus TAAATGAATCAG-TGTG-AAC-AT------
TCACTTCATACTTGGTGTGA
TAA15097 TAAATGAATCAG-TGTG-AAC-AT------
TCACTTCATACTTGGTGTGA
JV8909_19_CZ TAAATGAATCAG-TGTG-AAC-AT------
TCACTTCATACTTGGTGTGA
JV1310_15_P_sanguinolentus2_CZ TAAATGAATCAG-TGTG-AAC-AT------
TCACTTCATACTTGGTGTGA
MJ53_02_CZ TAAATGAATCAG-TGTG-AAC-AT------
TCACTTCATACTTGGTGTGA
CLZhao21647P_yunnanensis TAAATGAATCAG-TGTG-AAC-AT------
TCACTTCATACTTGGTGTGA
CLZhao21583P_yunnanensis TAAATGAATCAG-TGTG-AAC-AT------

TCACTTCATACTTGGTGTGA
Dai22272 TAAATGAATCAG-TGTG-AAC-AT-----
TCACTTCATACTTGGTGTGA
Dai22279 TAAATGAATCAG-TGTG-AAC-AT-----
TCACTTCATACTTGGTGTGA
MJ332_94_CZ TAAATGTATCAG-TGTG-AAT-AT-----
TCACTTCATACTCAGTGTGA
MJ642_94_CZ_Expallescens TAAATGTATCAG-TGTG-AAT-AT-----
TCACTTCATACTCAGTGTGA
Dai21060P_vinctus TAAATGTATCAG-TGTG-AAT-AT-----
TCACTTCATACTCAGTGTGA
JV0511_23LRP_pouzarii TAAATGCATCAG-TGTG-AAT-AT-----
TCACTTCATATTCAGTGTGA
JQ409462_R_pouzarii_PRM899856_ TAAATGCATCAG-TGTG-AAT-AT-----
TCACTTCATATTCAGTGTGA
JV0308_66_WA TAAATGCATCAG-TGTG-AAT-AT-----
TCACTTCATACTCAGTGTGA
JV0309_45_WA_USA TAAATGCATCAG-TGTG-AAT-AT-----
TCACTTCATACTCAGTGTGA
JV0709_83_CA_USA TAAATGCATCAG-TGTG-AAT-AT-----
TCACTTCATACTCAGTGTGA
Dai21043P_pouzarii TAAATGCATCAG-TGTG-AAT-AT-----
TCACTTCATATTCAGTGTGA
MJ144_95_CZ CAAATGCATCAG-TGTG-AAT-AT-----
TCACTTCATACTCAGTGTGA
JV0909_3_CZ CAAATGCATCAG-TGTG-AAT-AT-----
TCACTTCATACTCAGTGTGA
JV0609_1_K CAAATGCATCAG-TGTG-AAT-AT-----
TCACTTCATACTCAGTGTGA
Dai20396Physisporinus_castanop CAAATGCATCAG-TGTG-AAT-AT-----
TCACTTCATACTCAGTGTGA
Dai20397Physisporinus_castanop CAAATGCATCAG-TGTG-AAT-AT-----
TCACTTCATACTCAGTGTGA
MJ19_09_SK_Abies TAAATGCATCAG-TGTG-AAT-SC------
TCACTTCATACTCAGTGTGA
JV0509_40_J_TN_USA_Betula TAAATGCATCAG-TGTG-AAT-GC------
TCACTTCATACTCAGTGTGA
JV0808_33crocatus_PA_USAlist TAAATGCATCAG-TGTG-AAT-GC------
TCACTTCATACTCAGTGTGA
DLL2009_061P_crocatus TAAATGCATCAG-TGTG-AAT-GC------
TCACTTCATACTCAGTGTGA
Dai12800P_subcrocatus TAAATGCATCAG-TGTG-AAT-GC------
TCACTTCATACTCAGTGTGA
Dai15917P_subcrocatus TAAATGCATCAG-TGTG-AAT-CC------

TCACTTCATACTCAGTGTGA
Cui16620 TAAATGCATCAG-TGTG-AAT-AT------
TCACTTCATACTCAGTGTGA
HCFC1088Meripilus_stillicidior TAAATGCATCAG-TGTG-AAT-AT------
TCACTTCATACTCAGTGTGA
MCW590Meripilus_obsrcurus TAAATACATCAG-TGTG-AAT-AT------
TCACTTCATACTCAGTGTGA
MCW722Meripilus_obsrcurus TAAATACATCAG-TGTG-AAT-AT------
TCACTTCATACTCAGTGTGA
Cui9381P_tibeticus TAAATGCATCAG-TGTG-AAT-AC------
TCACTTCATACTCAGTGTGA
Cui9588P_tibeticus TAAATGCATCAG-TGTG-AAT-AC------
TCACTTCATACTCAGTGTGA
Va2_Beneschova TAAATGTATCAG-TGTG-AAT-GC------
TCACTTCATACTCAGTGTGA
CWU3874_Ukraine_Alnus TAAATGTATCAG-TGTG-AAT-GC------
TCACTTCATACTCAGTGTGA
WCG1293Dai24718Physisporinus CAAATGCATTAG-TGTG-AAC-AT------
TTATCTCATGCTTGGTGTGA
WCG1268Dai24682A TAAATGCATTAG-TGTG-AAT-GT------
TCATTTCATACTCAGTGTGA
WCG1269Dai24683A TAAATGCATTAG-TGTG-AAT-GT------
TCATTTCATACTCAGTGTGA
WCG1279Dai24694A TAAATGCATTAG-TGTG-AAT-GT------
TCATTTCATACTCAGTGTGA
Dai16971 TAAATGCATTAG-TGTG-AAT-GT------
TCATTTCATACTCAGTGTGA
ZQY1043Dai26696 TAAATGCATTAG-TGTG-AAT-GT------
TCATTTCATACTCAGTGTGA
Doll880 TAAATACATTAG-TGTG-AAC-AT------
TCACTTCATACTCGGTGTGA
Doll1000 TAAATACATTAG-TGTG-AAC-AT------
TCACTTCATACTCGGTGTGA
1DAI18529 TAAATGCATTAG-TGTG-AAC-AT------
TCACTTCATACTCGGTGTGA
Dai19535 TAAATGCATTAG-TGTG-AAT-GC------
TCACTTCATACTCGGTGTGA
1704_79_hnedyVillaLaPaz TAATTGCATTAG-TGTG-AAT-GT------
CCACTTTATGCTCAGTGTGA
F2061 TAATTGCATTAG-TGTG-AAT-GT------
CCACTTTATGCTCAGTGTGA
1DAI18268 CAATTGCATTAG-TGTG-AAT-GT------
CCACTTCATGCTTGGTGTGA
1DAI18540A CAATTGCATTAG-TGTG-AAT-GT------

CCACTTCATGCTTGGTGTGA
Dai17695 CAATTGCATTAG-TGTG-AAT-AT------
CCACTTCATGCTTGGTGTGA
LKY18Dai26373 CAATTGCATTAG-TGTG-AAT-GT------
CCACTTCATGCTTGGTGTGA
Dai17839P_sulphureus CAATTGCATTAG-TGTG-AAT-GT------
TTGCTTCATGCTTGATGTGA
Dai17841P_sulphureus CAATTGCATTAG-TGTG-AAT-GT------
TTGCTTCATGCTTGATGTGA
Dai19877P_roseus GAATTGCATTAG-TGTG-AAT-GT------
TTGCTTCATACTTGATGTGA
1508_18_1_Kout TAATTGCATTAG-TGTG-AAT-GT------
GCACTTTATACTCAATGTGA
KP859303_R_vinctus_RP185_BRAZI WAATTGCATTAG-TGTG-AAT-GT------
GCACTTTATACTCAATGTGA
JK1807_15Rigidoporus_sp_Puerto TAATTGCATTAG-TGTG-AAT-GT------
GCACTTTATACTCAATGTGA
JV0509_47_J_TN_USA TAAATGAATTAG-TGTG-AAY-GT-----
CTCACTTCATGCTCAGTGTGA
JV0709_188 TAAATGAATTAG-TGTG-AAY-GT-----
CTCACTTCATGCTCAGTGTGA
JV0509_127_PA_USA TAAATGAATTAG-TGTG-AAC-GT-----
CTCACTTCATGCTCAGTGTGA
JV1009_59_NJ_USA TAAATGAATTAG-TGTG-AAC-GT-----
CTCACTTCATGCTCAGTGTGA
Dai15497P_crataegi TAAATAAATTAG-TGTG-GAT-AT------
TCACTTCATGCTCAGTGTGA
Dai15499P_crataegi TAAATAAATTAG-TATG-GAT-AT------
TCACTTCATGCTCAGTGTGA
Cui3266P_cinereus CAAATGCATTAG-TGTG-AAT-GT------
TCACTTCATGCTCAGTGTGA
WCG1256Dai24690 CAAATGCATTAG-TGTG-AAT-GT------
TCACTTCATGCTCAGTGTGA
1DAI17581 CAAATGCATTAG-TGTG-AAT-GT------
TCACTTCATGCTCAGTGTGA
WCG1255Dai24688 CAAATGCATTAG-TGTG-AAT-GT------
TCACTTCATGCTCAGTGTGA
Dai22427 CAAATGCATTAG-TGTG-AAT-GT------
TCACTTCATGCTCAGTGTGA
MV690Meripilus_concrescens CAAATGCATTAG-TGTG-AAT-GT------
TCACTTCATGCTCAGTGTGA
MV513Meripilus_galapagensis CAAATGCATTAG-TGTG-AAT-GT------
TCACTTCATGCTCAGTGTGA
Dai19793 CAAATGCATCAGTTATG-AAT-GT------

TCACTTCATGCTCAGTGTGA
OQ553780P_tamilnaduensis TAAATGCATTAG-TGTG-AAT-GG------
TCACTTCATACTCAGTGTGA
OQ553779P_tamilnaduensis TAAATGCATTAG-TGTG-AAT-GG------
TCACTTCATACTCAGTGTGA
A164FB3Meripilus_giganteus TAAATGCATTAG-TGTG-AAT-GG------
TCACTTCATACTCAGTGTGA
JV1407_36_Vinctus_Meandrica CAAATGTATCAG-TATG-AAT-GT------
TCACTTCATGCTCAGTGTGA
1807_3K_Rigidoporus_PuertoRico ---
Cui16903P_vinctus CAAATGTATCAG-TATG-AAT-GT------
TCACTTCATGCTCAGTGTGA
JV1008_18R_Lineatus CAAATGTATCAG-TATG-AAT-GT------
TCACTTCATGCTCAGTGTGA
JV1407_37_1_Vinctus_Carara CAAATGTATCAG-TATG-AAT-GT------
TCACTTCATGCTCAGTGTGA
Dai17986P_lineatus CAAATGTATCAG-TATG-AAT-GT------
TCACTTCATGCTCAGTGTGA
Dai18281 CAAATGCATCAG-TATG-AAT-GT------
TCACTTCATGCTCAGTGTGA
1DAI19796 CAAATGCATCAG-TATG-AAT-GT------
TCACTTCATGCTCAGTGTGA
ZQY797Dai25241 CAAATGCATCAG-TATG-AAT-GT------
TCACTTCATGCTCAGTGTGA
WCG1289Dai24711 CAAATGCATCAG-TATG-AAT-GT------
TCACTTCATGCTCAGTGTGA
Dai22598 CAAATGCATCAG-TATG-AAT-GT------
TCACTTCATGCTCAGTGTGA
Dai20523 CAAATGCATCAG-TATG-AAT-GT------
TCACTTCATGCTCAGTGTGA
Dai17885 CAAATGCATCAG-TATG-AAT-GT------
TCACTTCATGCTCAGTGTGA
Dai17553 CAAATGCATCAG-TATG-AAT-GT------
TCACTTCATGCTCAGTGTGA
Dai19639 CAAATGCATCAG-TATG-AAT-GT------
TCACTTCATGCTCAGTGTGA
JV0110_48_CZ TAAATGCATCAG-TGTG-AGT-CC-------
CACTTCATGCTCAGTGTGA
MJ129_04 TAAATGCATCAG-TGTG-AGT-CC-------
CACTTCATGCTCAGTGTGA
Cui10340P_eminens TAAATGCATCAG-TGTG-AGT-CC-------
CACTTCATGCTCAGTGTGA
Cui10341P_eminens TAAATGCATCAG-TGTG-AGT-CC-------
CACTTCATGCTCAGTGTGA

Dai12685P_eminens TAAATGCATCAG-TGTG-AGT-CC-------
CACTTCATGCTCAGTGTGA
Miettinen_13591Rigidoporus_und TAAATGCATCAG-TGTG-AGT-CC-------
CACTTCATGCTCAGTGTGA
Dai20868 TAAATGCATCAG-TGTG-AGT-CC-------
CACTTCATGCTCAGTGTGA
Dai20832 TAAATGCATCAG-TGTG-AGT-CC-------
CACTTCATGCTCAGTGTGA
Dai11400 TAAATGCATCAG-TGTG-AGT-CC-------
CACTTCATGCTCAGTGTGA
Dai22472 TAAATGCATCAG-TGTG-AGT-CC-------
CACTTCATGCTCAGTGTGA
1CUI10475 TAAATGCATCAG-TGTG-AAT-CC-------
CGCTTCATGCTCAGTGTGA
1CUI10491 TAAATGCATCAG-TGTG-AAT-CC-------
CGCTTCATGCTCAGTGTGA
HCFC1095Meripilus_robledoi TAAATGCATCAG-TGTG-AAT-CC-------
CGCTTCATGCTCAGTGTGA
MCW702Meripilus_revolubilis CAAATGCATCAG-TGTG-AAT-CC-------
CGCTTCATGCTCAGTGTGA
1704_83_zluty_HaciendaBaru CAAAACTATCAG-CGTG-AAG-GG------
TCACCTTATGCTCAGTGTGA
Dai9925P_lavendulus CAAAAGTATCAG-TGTG-AAG-GG------
TCGCTTTATGCTCAATGTGA
Dai13587AP_lavendulus CAAAAGTATCAG-TGTG-AAG-GG------
TCGCTTTATGCTCAATGTGA
PDD70600P_longicystidius CAAAAACATCAG-TGTG-AA--GG------
TCACTTTATGCTCAGTGTGA
Cui16630 CAAAAACATCAG-TGTG-AA--GG------
TCACTTTATGCTCAGTGTGA
FP_135344Meripilus_giganteus TAAAAATATTAG-TGTG-AAT-GC------
TCACTTCATGCTCAGTGTGA
FP_100460_Sp TAAAAATATTAG-TGTG-AAT-GC------
TCAGTTCATGCTCAGTGTGA
CBS421_48Meripilus_giganteus TAAAAATATTAG-TGTG-AAT-GC------
TCACTTCATGCTCAGTGTGA
Cui9203 TAAAAATATTAG-TGTG-AAT-GC------
TCACTTCATGCTCAGTGTGA
Cui9202 TAAAAATATTAG-TGTG-AAT-GC------
TCACTTCATGCTCAGTGTGA
TUFC100564Japan TAAAGATATTAG-TGTG-AAT-GC------
TCACTTCATGCTCAGTGTGA
Russell5913Meripilus_sumstinei TAAAGATATTAG-TGTG-AAT-GC------
TCACTTCATGCTCAGTGTGA

RP215Meripilus_brasiliensis TAAAGGTATTAG-TGTGAAAT-GC------
TCACTTCATGCTCAGTGTGA
RP200Meripilus_brasiliensis TAAAGGTATTAG-TGTGAAAT-GC------
TCACTTCATGCTCAGTGTGA
JV1712_13J_R_vinctus2_LSUPuert TAAATGCATTAG-CAGA-GAT-AAT----ACTGCTAC-
TCTCCAGTGTGA
Dai10503R_hypobrunneus TAAATGCATTAG-CAGA-GAT-AAT----ACTGCTAC-
TCTCCAGTGTGA
Dai10569Rigidoporus_hypobrunne TAAATGCATTAG-CAGA-GAT-AAT----ACTGCTAC-
TCTCCAGTGTGA
1DAI19451 TAAATGCATTAG-CAGA-GAT-AAT----ACTGCTAC-
TCTCCAGTGTGA
CM108bRigidoporus_hypobrunneus TAAATGCATTAG-CAGA-GAT-AAT----ACTGCTAC-
TCTCCAGTGTGA
1CUI16874 TAAATGCATTAG-CAGA-GAT-AAT----ACTGCTAC-
TCTCCAGTGTGA
FD299Cerrena_unicolor TAAATACATTAG-CAGA-GAT-ATT----ACTGCTAC-
TCTCCAGTGTGA
KHL_GB_Cerrena_uniclor TAAATACATTAG-CAGA-GAT-ATT----ACTGCTAC-
TCTCCAGTGTGA
Dai12892Cerrena_albocinnamomea TAAATACATTAG-CAGA-GAT-ATT----ACTGCTAC-
TCTCCAGTGTGA
Dai12955C_albocinnamomea TAAATACATTAG-CAGA-GAT-ATT----ACTGCTAC-
TCTCCAGTGTGA
SNUm03110102C_aurantiopora TAAATACATTAG-CAGA-GAT-ATT----ACTGCTAC-
TCTCCAGTGTGA
NIBRFG0000102423C_aurantiopora TAAATACATTAG-CAGA-GAT-ATT----ACTGCTAC-
TCTCCAGTGTGA
Dai7359Antrodiella_zonata TAAATACATTAG-CAGA-GAT-ATT----ACTGCTAC-
TCTCCAGCGTGA
F20080702KCM29C_consors TAAATACATTAG-CAGA-GAT-ATT----ACTGCTAC-
TCTCCAGCGTGA
F20080208LYW10Cerrena_consors TAAATACATTAG-CAGA-GAT-ATT----ACTGCTAC-
TCTCCAGCGTGA
Dai7821Antrodiella_zonata TAAATACATTAG-CAGA-GAT-ATT----ACTGCTAC-
TCTCCAGCGTGA
CFMR_DCL04_31Pseudolagarobasid TAAATATATTAG-CAGA-GAT-GTT----ACTGCTAC-
TCTCCAGTGTGA
VPB197Pseudolagarobasidium_bel TAAATATATTAG-CAGA-GAT-GTT----ACTGCTAC-
TCTCCAGTGTGA
CBS115543Pseudolagarobasidium_ TAAATGTATTAG-CAGA-GAT-GTT----ACTGCTAC-
TCTCCAGTGTGA
CBS115544 TAAATGTATTAG-CAGA-GAT-GTT----ACTGCTAC-
TCTCCAGTGTGA

Han405Pseudolagarobasidium_bai TAAATGTATTAG-CAGA-GAT-GTT----ACTGCTAC-TCTCCAGTGTGA
Han406Pseudolagarobasidium_bai TAAATGTATTAG-CAGA-GAT-GTT----ACTGCTAC-TCTCCAGTGTGA
MUcc838Spongipellis_delectans TAAATGCATTAG-CAAA-GAT-GTT----ACTGCTAC-TCTTCAGCGTGA
BRNM686401S_delectans TAAATGCATTAG-CAAA-GAT-GTT----ACTGCTAC-TCTTCAGCGTGA
OSM_F925S_delectans TAAATGCATTAG-CAAA-GAT-GTT----ACTGCTAC-TCTTCAGCGTGA
BRNM67093Spongipellis_litschau TAAATGCATTAG-CAAA-GAT-GTT----ACTGCTAC-TCTTCGGCGTGA
CFMRccFP59199TS_unicolor TAAATGCATTAG-CAAA-GAT-GTT----ACTGCTAC-TCTTCGGCGTGA
CFMRccFP71791TS_unicolor TAAATGCATTAG-CAAA-GAT-GTT----ACTGCTAC-TCTTCGGCGTGA
Dai13845P_lischaueri TAAATGCATTAG-CAAA-GAT-GTT----ACTGCTAC-TCTTCGGCGTGA
Dai20266P_lischaueri TAAATGCATTAG-CAAA-GAT-GTT----ACTGCTAC-TCTTCGGCGTGA
CFMR_HHB11240Radulodon_america TAAATGCATTAG-CAGA-GAT-GTT----ACTGCTAC-TCTCCAGCGTGA
RLG6350Radulodon_americanus TAAATGCATTAG-CAGA-GAT-GTT----ACTGCTAC-TCTCCAGCGTGA
KY415963Radulodon_erikssonii TAAATGCATTAG-CAGA-GAT-GTT----ACTGCTAC-TCTCCAGCGTGA
HHB9567spRadulodon_casearius TAAATGCATTAG-CAGA-GAT-GTT----ACTGCTAC-TCTCCAGTGTGA
KRT_Iso_26Radulodon_casearius TAAATGCATTAG-CAGA-GAT-GTT----ACTGCTAC-TCTCCAGTGTGA
CBS126044Radulodon_erikssonii TAAATGCATTAG-CAGA-GAT-GTT----ACTGCTAC-TCTCCAGCGTGA
He6183YUNNANENSIS TAAATGCATTAG-CAGA-GATGGTT----ACTGCTAC-TCTCCAGCGTGA
Cui17979YUNNANENSIS TAAATGCATTAG-CAGA-GATGGTT----ACTGCTAC-TCTCCAGCGTGA
Miettinen2091Junghuhnia_fimbri GAAATACATTAG-CTTG-AATAAAA----TCAAGTAT-GATTCAGCGTGA
KHL12316S_tenue GAAATGCATYAG-CTTG-AATAGAA----CCAAGTAT-GATTCAACGTGA
PRM846564S_pachyodon GAAATACATTAG-TGCA-GAT-ATT----GCTGTTAC-TCTTCAGTGTGA
SP_Lgt_S_pachyodon GAAATACATTAG-TGCA-GAT-ATT----GCTGTTAC-TCTTCAGTGTGA

Ryvarden44669Tyromyces_xuchile GAAATATATTAG-CAGA-GAT-ATT----GCTGTTACTTCTCCAGTGTGA
PW17_171sinuosus GAAATAAATTAG-CAGA-GAT-ATT----GCTGTTAC-TCTCCAGTGTGA
W53Dai12234 GAAATAAATTAG-CAGA-GAT-ATT----GCTGTTAC-TCTCCAGTGTGA
HHB4100SpAntella_americana GAAATGCATTAG-CTGG-AAT-TTTA---CTTGGCAT-GATTCAATGTGA
W3Dai20901spumeus TAAATGCATTAG-CGTG-AATAGACATA-CCCGATAT-TCTCCAGCGTGA
He6736 TAAATGCATTAG-CGTG-AATAGACATA-CCCGATAT-TCTCCAGCGTGA
BRNM734877S_spumeus YAAATGCATTAG-CRTG-AATAGACATA-CCCGATAT-TCTCCAGCGTGA
BRNM712630S_spumeus CAAATGCATTAG-CGTG-AATAGACATA-CCCGATAT-TCTCCAGCGTGA
Dai1723Loweomyces_sibiricus TAAATGCATTAG-CGCG-AATAGACGCATCCCGATAT-TCTCCAGCGTGA
W54Cui10009 TGAATGCATTAG-CGTG-AATAGACATA-CCCGATAT-TCTCCAGCGTGA
W1Dai20899 TGAATGCATTAG-CGTG-AATAGACATA-CCCGATAT-TCTCCGGCGTGA
HHB13445Trametes_ochracea TGAATGCATTAG-CTCG-----ATTCCGTACGGATCGGCTCTCAGTGTGA
Dai16222 GAAATGCATTAG-CTTGAAACTGT-----ACCAAGCACAATCCAGCGTGA
Dai16240 GAAATGCATTAG-CTTGAAACTGT-----ACCAAGCACAATCCAGCGTGA

JV1310_11SanguinolentusCernys TAATTATC-TACACTATG---TTTGAGTGATA-CAG--------CTTCAT
MJ39_00_SK TAATTATC-TACACTATG---TTTGAGTGATA-CAG--------CTTCAT
MJ111_04_CZ TAATTATC-TACACTATG---TTTGAGTGATA-CAG--------CTTCAT
JV1610_BOKYsmrk TAATTATC-TACACTATG---TTTGAGTGATA-CAG--------CTTCAT
Dai21030 TAATTATC-TACACTATG---TTTGAGTGATA-CAG--------CTTCAT
Dai20976P_furcatus TAATTATC-TACACTATG---TTTGAGTGATA-CAG--------CTTCAT
Dai2105 TAATTATC-TACACTATG---TTTGAGTGATA-GAG--------CTTCAT
Dai2544 TAATTATC-TACACTATG---TTTGAGTGATA-GAG----

---CTTCAT
Dai11313 TAATTATC-TACACTATG---TTTGAGTGATA-GAG-----
---CTTCAT
WCG1611Dai26167 TAATTATC-TACACTATG---TTTGAGTGATA-GAG---
-----CTTCAT
WCG1518Dai25999Physisporinus TAATTATC-TACACTATG---TTTGAGTGATA-GAG-----
---CTTCAT
TAA15097 TAATTATC-TACACTATG---TTTGAGTGATA-CGG----
----CTTCAT
JV8909_19_CZ TAATTATC-TGCACTATG---TCTGAGTGATA-GAG---
-----TTTCAT
JV1310_15_P_sanguinolentus2_CZ TAATTATC-TGCACTATG---TCTGAGTGATA-GAG------
--TTTCAT
MJ53_02_CZ TAATTATC-TGCACTATG---TCTGAGTGATA-GAG---
-----TTTCAT
CLZhao21647P_yunnanensis TAATTATC-TGCACTATG---TCTGAGTGATA-GAG-----
---TTTCAT
CLZhao21583P_yunnanensis TAATTATC-TGCACTATG---TCTGAGTGATA-GAG-----
---TTTCAT
Dai22272 TAATTATC-TACACTATG---TTGGAGTGATA-GAG-----
---CTTCAT
Dai22279 TAATTATC-TACACTATG---TTGGAGTGATA-GAG-----
---CTTCAT
MJ332_94_CZ TAATTATC-TACGCTGTG---CATGAGTGATG-CAA---
-----CTTCAT
MJ642_94_CZ_Expallescens TAATTATC-TACGCTGTG---CATGAGTGATG-CAA-----
---CTTCAT
Dai21060P_vinctus TAATTATC-TACGCTGTG---CATGAGTGATG-CAA------
--CTTCAT
JV0511_23LRP_pouzarii TAATTATC-TACGCTGTG---TGTGAGTGATG-CAA-----
---TTTCAT
JQ409462_R_pouzarii_PRM899856_ TAATTATC-TACGCTGTG---TGTGAGTGATG-CAA----
----TTTCAT
JV0308_66_WA TAATTATC-TATGCTGTG---TGTGAGTGATG-CAA---
-----CTTCAT
JV0309_45_WA_USA TAATTATC-TATGCTGTG---TGTGAGTGATG-CAA-
-------CTTCAT
JV0709_83_CA_USA TAATTATC-TATGCTGTG---TGTGAGTGATG-CAA--
------CTTCAT
Dai21043P_pouzarii TAATTATC-TACGCTGTG---TGTGAGTGATG-CAA------
--TTTCAT
MJ144_95_CZ TAATTATC-TACGCTGTG---CRTGAGTGATG-GGA--
------CTTCAT
JV0909_3_CZ TAATTATC-TACGCTGTG---CGTGAGTGATG-GGA---

-----CTTCAT
JV0609_1_K TAATTATC-TACGCTGTG---CGTGAGTGATG-GGA---
-----CTTCAT
Dai20396Physisporinus_castanop TAATTATC-TACGCTGTG---TGTGAGTGATG-AGA--------
CTTCAT
Dai20397Physisporinus_castanop TAATTATC-TACGCTGTG---TGTGAGTGATG-AGA--------
CTTCAT
MJ19_09_SK_Abies TAATTATC-TATGCTGTG---TGTGAGTGATA-CAA----
----CTTCAT
JV0509_40_J_TN_USA_Betula TAATTATC-TATGCTGTG---TGTGAGTGATA-CAA---
-----CTTCAT
JV0808_33crocatus_PA_USAlist TAATTATC-TATGCTGTG---TGTGAGTGATA-CAA-------
-CTTCAT
DLL2009_061P_crocatus TAATTATC-TATGCTGTG---TGTGAGTGATA-CAA-----
---CTTCAT
Dai12800P_subcrocatus TAATTATC-TATGCTGTG---TGTGAGTGATA-CAA-------
-CTTCAT
Dai15917P_subcrocatus TAATTATC-TATGCTGTG---TGTGAGTGATA-CAA-------
-CTTCAT
Cui16620 TAATTATC-TACGCTGTG---CATGAGTGATG-CGA----
----CTTCAT
HCFC1088Meripilus_stillicidior TAATTATC-TACGCTGTG---CATGAGTGATG-CGA--------
CTTCAT
MCW590Meripilus_obsrcurus TAATTATC-TACGCTGTG---CATGAGTGATG-TGA-----
---CTTCAT
MCW722Meripilus_obsrcurus TAATTATC-TACGCTGTG---CATGAGTGATG-TGA-----
---CTTCAT
Cui9381P_tibeticus TAATTATC-TACGCTGTG---CATGAGTGATA-CAA--------
CTTCAT
Cui9588P_tibeticus TAATTATC-TACGCTGTG---CATGAGTGATA-CAA--------
CTTCAT
Va2_Beneschova TAATTATC-TACGCTGTG---CATGAGTGATA-CAA-----
---CTTCAT
CWU3874_Ukraine_Alnus TAATTATC-TACGCTGTG---CATGAGTGATA-CAA---
-----CTTCAT
WCG1293Dai24718Physisporinus TAATTATC-TACGCCATG---TTTGAGTGATG-CGA-----
---TTTCAT
WCG1268Dai24682A TAATTATC-TGCACTGTG---TATAAGTGATA-AAG--
------CTTCAT
WCG1269Dai24683A TAATTATC-TGCACTGTG---TATAAGTGATA-AAG--
------CTTCAT
WCG1279Dai24694A TAATTATC-TGCACTGTG---CATAAGTGATA-AAG-
-------CTTCAT
Dai16971 TAATTATC-TGCACTGTG---TATAAGTGATA-AAG-----

---CTTCAT
ZQY1043Dai26696 TAATTATC-TGCACTGTG---TTTGAGTGATA-AAG---
-----CTTCAT
Doll880 TAAATATC-TACGCTGTG---TTTGTGTGATA-AAG-----
---CTTCAT
Doll1000 TAAATATC-TACGCTGTG---TTTGTGTGATA-AAG-----
---CTTCAT
1DAI18529 TAAATATC-TACACTGTG---TTTGGGTGATA-GAG---
-----CTTCAT
Dai19535 TAATTATC-TACGCCGTG---CTTGAGTGATA-TAG-----
---CTTCAT
1704_79_hnedyVillaLaPaz TAATTATC-TGCACTGTG---CTTGGGTGATA-CAG------
--CTTCAT
F2061 TAATTATC-TGCACTGTG---CTTGGGTGATA-CAG----
----CTTCAT
1DAI18268 TAATTATC-TGCATCGTG---CTTGAGTGATA-CAG----
----CTTCAT
1DAI18540A TAATTATC-TGCATCGTG---CTTGAGTGATA-CAG---
-----CTTCAT
Dai17695 TAATTATC-TACATCATG---CTTGAGTGATA-CAG-----
---CTTCAT
LKY18Dai26373 TAATTATC-TGCATCGTG---CTTGAGTGATA-CAG---
-----CTTCAT
Dai17839P_sulphureus TAATTATC-TGCATCGTG---CTTGGGCAGCG-TGA------
--CTTCAT
Dai17841P_sulphureus TAATTATC-TGCATCGTG---CTTGGGCAGCG-TGA------
--CTTCAT
Dai19877P_roseus TAATTATC-TGCATCATA---TTTGAGCAGCA-CTG------
--CTTCAT
1508_18_1_Kout TAATTATC-TGCATTGTG---TTAGAG-GGTA-TAG------
--ATTCAT
KP859303_R_vinctus_RP185_BRAZI TAATTATC-TGCATTGTG---TTAGAG-GGTA-TAG----
----ATTCAT
JK1807_15Rigidoporus_sp_Puerto TAATTATC-TGCATTGTG---TTAGAG-GGTA-TAG--------
ATTCAT
JV0509_47_J_TN_USA TAATTATC-TGCACTGTG---TTTGAGTGTTRWTRG-
-------TTTCAT
JV0709_188 TAATTATC-TGCACTGTG---TTTGAGTGTTRWTAG---
-----TTTCAT
JV0509_127_PA_USA TAATTATC-TGCACTGTG---TTTGAGTGTTAATAG--
------TTTCAT
JV1009_59_NJ_USA TAATTATC-TGCACTGTG---TTTGAGTGTTAATAG--
------TTTCAT
Dai15497P_crataegi TAATTATC-TGCACTGTG---TTTGAGTGGTA-TAG-------

-GTTCAT
Dai15499P_crataegi TAATTATC-TGCACTGTG---TTTGAGTGGTA-TAG-------
-GTTCAT
Cui3266P_cinereus TAATTATC-TGCACTGTG---TTTGGGTGTTA-TAG-------
-GTTCAT
WCG1256Dai24690 TAATTATC-TGCACTGTG---TTTGGGTGTTA-TAG--
------GTTCAT
1DAI17581 TAATTATC-TGCACTGTG---TTTGGGTGTTA-TAG---
----GTTCAT
WCG1255Dai24688 TAATTATC-TGCACTGTG---TTTGGGTGTTA-TAG--
------GTTCAT
Dai22427 TAATTATC-TGCACTGTG---TTTGGGTGTTA-TAG----
---GTTCAT
MV690Meripilus_concrescens TAATTATC-TGCACTGTG---TTTGGGTGTTA-TAG-------
-GTTCAT
MV513Meripilus_galapagensis TAATTATC-TGCACTGTG---TTTGGGTGTTA-TAG-------
-GTTCAT
Dai19793 TAATTATC-TGCACTGTG--TTTTGAGTGTTAATAA---
-----GTTCAT
OQ553780P_tamilnaduensis TAATTCTT-TGCACTGTG---TTTGGGTGTTA-TAT-------
-ATTCAT
OQ553779P_tamilnaduensis TAATTCTT-TGCACTGTG---TTTGGGTGTTA-TAT-------
-ATTCAT
A164FB3Meripilus_giganteus TAATTCTT-TGCACTGTG---TTTGGGTGTTA-TAT--------
ATTCAT
JV1407_36_Vinctus_Meandrica TAATTATC-TGCACTGTG---TCTGAGTGTCA-TAA------
--ATTCAT
1807_3K_Rigidoporus_PuertoRico --
Cui16903P_vinctus TAATTATC-TGCACTGTG---TCTGAGTGTCA-TAA------
--ATTCAT
JV1008_18R_Lineatus TAATTATC-TGCACTGTG---TCTGAGTGTCA-TAG-----
---ATTCAT
JV1407_37_1_Vinctus_Carara TAATTATC-TGCACTGTG---TCTGAGTGTCA-TAG------
--ATTCAT
Dai17986P_lineatus TAATTATC-TGCACTGTG---TCTGAGTGTCA-TAA-------
-ATTCAT
Dai18281 TAATTATC-TGCACTGTG---TCTGAGTGTCA-TAA---
----ATTCAT
1DAI19796 TAATTATC-TGCACTGTG---TCCGAGTGTCA-TAA---
-----ATTCAT
ZQY797Dai25241 TAATTATC-TGCACTGTG---TCCGAGTGTCA-TAA---
-----ATTCAT
WCG1289Dai24711 TAATTATC-TGCACTGTG---TCTGAGTGTCA-TAA--
------ATTCAT

Dai22598 TAATTATC-TGCACTGTG---TCTGAGTGTTA-TGG-----
---ATTCAT
Dai20523 TAATTATC-TGCACTGTG---TCTGAGTGTTA-TGG-----
---ATTCAT
Dai17885 TAATTATC-TGCACTGTG---TCTGAGTGTTA-TGG-----
---ATTCAT
Dai17553 TAATTATC-TGCACTGTG---TCTGAGTGTTA-TGG-----
---ATTCAT
Dai19639 TAATTATC-TGCACTGTG---TCTGAGTGTTA-TGG-----
---ATTCAT
JV0110_48_CZ TAATTATC-TGCACTGTT---TTTGGGTGTGG-CAA---
-----CTTCAT
MJ129_04 TAATTATC-TGCACTGTT---TTTGGGTGTGG-CAA----
----CTTCAT
Cui10340P_eminens TAATTATC-TGCACTGTT---TTTGGGTGTGG-CAA-----
---CTTCAT
Cui10341P_eminens TAATTATC-TGCACTGTT---TTTGGGTGTGG-CAA-----
---CTTCAT
Dai12685P_eminens TAATTATC-TGCACTGTT---TTTGGGTGTGG-CAA-----
---CTTCAT
Miettinen_13591Rigidoporus_und TAATTATC-TGCACTGTT---TTTGGGTGTGG-CAA--------
CTTCAT
Dai20868 TAATTATC-TGCACTGTT---TTTGGGTGTGG-CAA-----
---TCTCAT
Dai20832 TAATTATC-TGCACTGTT---TTTGGGTGTGG-CAA-----
---TCTCAT
Dai11400 TAATTATC-TGCACTGTT---TTTGGGTGTGG-CAA-----
---TCTCAT
Dai22472 TAATTATC-TGCACTGTT---TTTGGGTGTGG-CAA-----
---TCTCAT
1CUI10475 TAATTATC-TGCACTGTT---TTTGGGCGTGG-CAA----
----CTTCAT
1CUI10491 TAATTATC-TGCACTGTT---TTTGGGCGTGG-CAA----
----CTTCAT
HCFC1095Meripilus_robledoi TAATTATC-TGCACTGTT---TTTGGGCGCGG-AAA------
--CTTCAT
MCW702Meripilus_revolubilis TAATTATT-TGCACTGTT---TTTGGGCGCGG-AAA-------
-CTTCAT
1704_83_zluty_HaciendaBaru TAATTATC-TGCATTGCG----CTCGGTGCTA-CTT--------
TTTCAG
Dai9925P_lavendulus TAATTATC-TGCATTGCG----CGGAGCGATG-TTG-------
-TTTCAT
Dai13587AP_lavendulus TAATTATC-TGCATTGCG----CGGAGCGATG-TTG------
--TTTCAT

PDD70600P_longicystidius TAATTATC-TGCATTGTG----CCGAGTGTTA-CTA--------
TCTCAT
Cui16630 TAATTATC-TGCATTGTG----CCGAGTGTTA-CTA------
--TCTCAT
FP_135344Meripilus_giganteus TAATTATCTTGCATTGTG---CTTGGGTGTGG-CGA-------
-CTTCAT
FP_100460_Sp TAATTATCTTGCATTGTG---CTTGGGTGTGG-CAA---
-----CTTCAT
CBS421_48Meripilus_giganteus TAATTATCTTGCATTGTG---CTTGGGTGTGG-CGA------
--CTTCAT
Cui9203 TAATTATCTTGCATTGTG---CTTGGGTGTGG-CGA---
-----CTTCAT
Cui9202 TAATTATCTTGCATTGTG---CTTGGGTGTGG-CGA---
-----CTTCAT
TUFC100564Japan TAATTATCTTGCATTGTG---CTTGGGTGTGG-CGA--
------CTTCAT
Russell5913Meripilus_sumstinei TAATTATCTTGCATTGTG---CTTGGGTGTGG-CGG--------
CTTCAT
RP215Meripilus_brasiliensis TAATTATCTTGCATTGTG---CTTGGGTGTGG-CGG--------
CTTCAT
RP200Meripilus_brasiliensis TAATTATCTTGCATTGTG---CTTGGGTGTGG-CGG--------
CTTCAT
JV1712_13J_R_vinctus2_LSUPuert TAATTGTC-TACACTG-T---TAGTAGTGCGG-
TATAACAAAATGTCTAT
Dai10503R_hypobrunneus TAATTGTC-TACACTG-T---TAGTAGTGCGG-
TATAACAAAATGTCTAT
Dai10569Rigidoporus_hypobrunne TAATTGTC-TACACTG-T---TAGTAGTGCGG-
TATAACAAAATGTCTAT
1DAI19451 TAATTGTC-TACACTG-T---TAGTAGTGCGG-
TATAACAAAATGTCTAT
CM108bRigidoporus_hypobrunneus TAATTGTC-TACACTG-T---TAGTAGTGCGG-
TATAACAAAATGTCTAT
1CUI16874 TAATTGTC-TACACTG-T---TAGTAGTGCGG-
TATAACAAAATGTCTAT
FD299Cerrena_unicolor TAATTGTC-TACACTGTT---TAGTAGTGCGG-
TATAATCAAA-GTCTTT
KHL_GB_Cerrena_uniclor TAATTGTC-TACACTG-T---TAGTAGTGCGG-
TATAATCAAA-GTCTTT
Dai12892Cerrena_albocinnamomea TAATTGTC-TACGCTG-T---TAGTAGTGCGG-
TATATAAAC--ATCTAT
Dai12955C_albocinnamomea TAATTGTC-TACGCTG-T---TAGTAGTGCGG-
TATATAAAC--ATCTAT
SNUm03110102C_aurantiopora TAATTGTC-TACGCTG-T---TAGTAGTGCGG-
TATATAAAC--ATCTAT

NIBRFG0000102423C_aurantiopora TAATTGTC-TACGCTG-T---TAGTAGTGCGG-TATATAAAC--ATCTAT
Dai7359Antrodiella_zonata TAATTGTC-TACGCTG-T---TAGTAGTGCGG-TATATAAAC--ATCTAT
F20080702KCM29C_consors TAATTGTC-TACGCTG-T---TAGTAGTGCGG-TATATAAAC--ATCTAT
F20080208LYW10Cerrena_consors TAATTGTC-TACGCTG-T---TAGTAGTGCGG-TATATAAAC--ATCTAT
Dai7821Antrodiella_zonata TAATTGTC-TACGCTG-Y---TAGTAGTGCGG-TATATAAAC--ATCTAT
CFMR_DCL04_31Pseudolagarobasid TAATTGTC-TACACTG-T---TAGTAGTGCAG-TAAAAATAAA-GTCTAT
VPB197Pseudolagarobasidium_bel TAATTGTC-TACACTG-T---TAGTAGTGCAG-TAAAAATAAA-GTCTAT
CBS115543Pseudolagarobasidium_ TAATTGTC-TACACTG-T---TAGTAGTGCAG-TRAAAATTAA-GTCTAT
CBS115544 TAATTGTC-TACACTG-T---TAGTAGTGCAG-TAAAAATYAA-GTCTAT
Han405Pseudolagarobasidium_bai TAATTGTC-TACACTG-C---TAGTAGTGCAG-T-AAATTGAA-GTCTAT
Han406Pseudolagarobasidium_bai TAATTGTC-TACACTG-C---TAGTAGTGCAG-T-AAATTGAA-GTCTAT
MUcc838Spongipellis_delectans TAATTGTC-TACGCTG-----CCGTTGTACGG-TATAAATAA--GTCTCT
BRNM686401S_delectans TAATTGTC-TACGCTG-----CCGTTGTACGG-TATAAATAA--GTCTCT
OSM_F925S_delectans TAATTGTC-TACGCTG-----CCGTTGTACGG-TATAAATAA--GTCTCT
BRNM67093Spongipellis_litschau TAATTGTC-TACGCTG-----CTGTGGTACGG-TATATTGAA--GTCTCT
CFMRccFP59199TS_unicolor TAATTGTC-TACGCTG-----CTGTGGTACGG-TATATTGAA--GTCTCT
CFMRccFP71791TS_unicolor TAATTGTC-TACGCTG-----CTGTGGTACGG-TATATTGAA--GTCTCT
Dai13845P_lischaueri TAATTGTC-TACGCTG-----CTGTGGTACGG-TATATTGAA--GTCTCT
Dai20266P_lischaueri TAATTGTC-TACGCTG-----CTGTGGTACGG-TATATTGAA--GTCTCT
CFMR_HHB11240Radulodon_america TAATTGTC-TACGCTG-----TAGTAGTGCGG-TATATTGAA--GTCTCC
RLG6350Radulodon_americanus TAATTGTC-TACGCTG-----TAGTAGTGCGG-TATATTGAA--GTCTCC
KY415963Radulodon_erikssonii TAATTGTC-TACGCTG-----TAGTAGTGCGG-TATGTTGAA--GTCTCC

HHB9567spRadulodon_casearius TAATTGTC-TACGCTG-----TAGTAGTGCGG-TATATTGAA--GTCTCT
KRT_Iso_26Radulodon_casearius TAATTGTC-TACGCTG-----TAGTAGTGCGG-TATATTGAA--GTCTCT
CBS126044Radulodon_erikssonii TAATTGTC-TACGCTG-----TAGTAGTGCGG-TATGTTGAA--GTCTCC
He6183YUNNANENSIS TAATTGTC-TACGCTG-----CAGCAGTGCGG-TATAATTGAA-GTCTCT
Cui17979YUNNANENSIS TAATTGTC-TACGCTG-----CAGCAGTGCGG-TATAATTGAA-GTCTCT
Miettinen2091Junghuhnia_fimbri TAATTGTC-TACGTTG-----CTTTGTCTTGG-TGAATAAAA--ATTCTA
KHL12316S_tenue TAATTGTC-TACGTTG-----CTTCATCTCGG-TGAAAAAAAT-ATTCTA
PRM846564S_pachyodon TAATTGTC-TACGCTG-----CAGATGTATGG-CGAATTGAA--GTCTGT
SP_Lgt_S_pachyodon TAATTGTC-TACGCTG-----CAGATGTATGG-CGAATTGAA--GTCTGT
Ryvarden44669Tyromyces_xuchile TAATTGTC-TACGCTG-C---AAGATGTACGG-CGAACTGAA--GTCTGT
PW17_171sinuosus TAATTGTC-TACGCTG-----CAGATGTACGG-TGAACTGAA--GTCTAT
W53Dai12234 TAATTGTC-TACGCTG-----CAGATGTACGG-TGAACTGAA--GTCTAT
HHB4100SpAntella_americana TAATTGTC-TACGTTG-----TCTCAGCTTGG-TATTAAATTT-GTTTCA
W3Dai20901spumeus TAATTATC-TGCGCTGT----TGAAGGGGTTG-CGTATA-----TTGCAT
He6736 TAATTATC-TGCGCTGT----TGAAGGGGTTG-CATATA-----TCGCAT
BRNM734877S_spumeus TAATTATC-TGCGCTGT----TGAAGGGGYTG-CATATA-----TCGCAT
BRNM712630S_spumeus TAATTATC-TGCGCTGT----TGAAGGGGTTG-CATATA-----TCGCAT
Dai1723Loweomyces_sibiricus TAATTATC-TGCGCTGT----TGAAGGAGCTG-CATATA-----TTTCAT
W54Cui10009 TAATTATC-TGCGCTGTT---GAAGGGATTTG-CATATA-----TTTCAT
W1Dai20899 TAATTATC-TGCGCTGTT---GAAGGGATTTG-CATATA-----TTTCAT
HHB13445Trametes_ochracea TAATTGTC-TACGCTGTGACCGTGAAGTGTTT-----------TGGCGA
Dai16222 TAAACAC--TGCGCTGCT---TTGTATTGGTTTTTATTTTTATGTTTCAA

Dai16240 TAAACAC--TGCGCTGCT--
TTGTATTGGTTTTTATTTTTATGTTTCAA

JV1310_11SanguinolentusCernys GC-TTCGAATCGTCGCA-------AGAC--A-CC--
GCTTTGA-C-TTCG
MJ39_00_SK GC-TTCGAATCGTCGCA------AGAC--A-CY--
GCTTTGA-C-TTCG
MJ111_04_CZ GC-TTCGAATCGTCGCA------AGAC--A-CC--
GCTTTGA-C-TTC-
JV1610_BOKYsmrk GC-TTCGAATCGTCGCA------AGAC--A-CC--
GCTTTGA-C-TTCG
Dai21030 GC-TTCGAATCGTCGCA------AGAC--A-CC--
GCTTTGA-C-TTC-
Dai20976P_furcatus GC-TTCGAATCGTCGCA-------AGAC--A-CC--
GCTTTGA-C-TTCG
Dai2105 GC-TTCGAATCGTCGCA-------AGAC--A-CT--ACT-----
-----G
Dai2544 GC-TTCGAATCGTCGCA-------AGAC--A-CT--ACT-----
-----G
Dai11313 GC-TTCGAATCGTTGCA-------AGAC--A-CT--ACT------
----G
WCG1611Dai26167 GC-TTCGAATCGTCGCA-------AGAC--A-CT--
ACTTTGA-C-TTCG
WCG1518Dai25999Physisporinus GC-TTCGAATCGTCGCA-------AGAC--A-CT--
ACTTTGA-C-TTC-
TAA15097 GC-TTCGAATCGTCGCA-------AGAC--A-CT--ACT----
------G
JV8909_19_CZ GC-TTCGAATCGTCGCG-------AGAC--A-CT--
ATTTTGA-C-TTC-
JV1310_15_P_sanguinolentus2_CZ GC-TTCGAATCGTCGCR-------AGAC--A-CT--
ATTTTGA-C-TTCG
MJ53_02_CZ GC-TTCGAATCGTCGCR-------AGAC--A-CT--
ATTTTGA-C-TTCG
CLZhao21647P_yunnanensis GC-TTCAAATTGTCGCG-------AGAC--A-CT--
ATTTTGA-C-TTCG
CLZhao21583P_yunnanensis GC-TTCAAATTGTCGCG-------AGAC--A-CT--
ATTTTGA-C-TTCG
Dai22272 GC-TTCAAACTGTCGCA-------AGAC--A-CT--
ACTTTGA-C-TTCG
Dai22279 GC-TTCAAACTGTCGCA-------AGAC--A-CT--
ACTTTGA-C-TTCG
MJ332_94_CZ GC-TTCGAACTGTCGCG-------AGAC-----C--
ACTTTGA-C-TTC-
MJ642_94_CZ_Expallescens GC-TTCGAACTGTCGCG-------AGAC-----C--

ACTTTGA-C-TTC-
Dai21060P_vinctus GC-TTCGAACTGTCGCG-------AGAC-----C--
ACTTTGA-C-TTCG
JV0511_23LRP_pouzarii GC-TTCTAACCGTCGCA-------AGAC--A-CT--
ACTTTGA-C-TTCG
JQ409462_R_pouzarii_PRM899856_ GC-TTCTAACYGTCGCA-------AGAC--A-CT--
ACTTTGA-C-TTC-
JV0308_66_WA GC-TTCAAACTGTCGCG-------AGAC--A-TT--
ACTTTGA-C-TTC-
JV0309_45_WA_USA GC-TTCAAACTGTCGCG-------AGAC--A-TT--
ACTTTGA-C-TTC-
JV0709_83_CA_USA GC-TTCRAACTGTCGCG-------AGAC--A-TT--
ACTTTGA-C-TTCG
Dai21043P_pouzarii GC-TTCTAACCGTCGCA-------AGAC--A-CT--
ACTTTGA-C-TTCG
MJ144_95_CZ GC-TTCAAACTGTCGCG-------AGAC--AATC--
TCTTTGA-C-TTCG
JV0909_3_CZ GC-TTCAAACTGTCGCG-------AGAC--AATC--
TCTTTGA-C-TTCG
JV0609_1_K GC-TTCAAACTGTCGCG-------AGAC--AATC--
TCTTTGA-C-TTC-
Dai20396Physisporinus_castanop GC-TTCGAACTGTCGCA-------AGAC--AATA--
TCTTTGA-C-TTCG
Dai20397Physisporinus_castanop GC-TTCGAACTGTCGCA-------AGAC--AATA--
TCTTTGA-C-TTCG
MJ19_09_SK_Abies GC-TTCGAACCGTCGCA-------AGAC--A-TA--
CTTTTGA-C-TTCG
JV0509_40_J_TN_USA_Betula GC-TTCGAACCGTCGCA-------AGAC--A-TA--
CTTTTGA-C-TTC-
JV0808_33crocatus_PA_USAlist GC-TTCGAACCGTCGCA-------AGAC--A-TA--
CTTTTGA-C-TTCG
DLL2009_061P_crocatus GC-TTCGAACCGTCGCA-------AGAC--A-TA--
CTTTTGA-C-TTC-
Dai12800P_subcrocatus GC-TTCGAACTGTCGCA-------AGAC--A-TA--
CTTTTGA-C-TTCG
Dai15917P_subcrocatus GC-TTCGAACCGTCGCA-------AGAC--A-TA--
CTTTTGA-C-TTCG
Cui16620 GC-TTCAAACTGTCGTG-------AGAC--A-TC--
TCTTTGA-C-TTCG
HCFC1088Meripilus_stillicidior GC-TTCAAACTGTCGCG-------AGAC--A-TT--TCTTTGA-
C-TTCG
MCW590Meripilus_obsrcurus GC-TTCAAACCGTCGCA-------AGAC--A-AT--
TCTTTGA-C-TTC-
MCW722Meripilus_obsrcurus GC-TTCAAACCGTCGCA-------AGAC--A-WT--

TCTTTGA-C-TTC-
Cui9381P_tibeticus GC-TTCAAACTGTCGCA------AGAC--A-TC--ATT--------
--G
Cui9588P_tibeticus GC-TTCAAACTGTCGCA------AGAC--A-TC--ATT--------
--G
Va2_Beneschova GC-TTCGAACCGTCGCA------AGAC--A-TC--
ACTTTGA-C-TTC-
CWU3874_Ukraine_Alnus GC-TTCGAACCGTCGCA------AGAC--A-TC--
ACTTTGA-C-TTCG
WCG1293Dai24718Physisporinus GC-TTCGAATTGTCGTG-------AGAC--A-TA--
ACTTTGA-C-TTC-
WCG1268Dai24682A GC-TTCTAATCGTCGCA-------AGAC--AATT--
CCTTTGA-C-TTCG
WCG1269Dai24683A GC-TTCTAATCGTCGCA-------AGAC--AATT--
CCTTTGA-C-TTCG
WCG1279Dai24694A GC-TTCTAATCGTCGCA-------AGAC--AATT--
CCTTTGA-C-TTCG
Dai16971 GC-TTCTAATCGTCGCA-------AGAC--AATT--
CCTTTGA-C-TTCG
ZQY1043Dai26696 GC-TTCTAATCGTCGCA-------AGAC--AATT--
CCTTTGA-C-TTCG
Doll880 GC-TTCAAATTGTCGCA-------AGAC--A-CT--
ATCTTGA-C-TTC-
Doll1000 GC-TTCAAATTGTCGCA-------AGAC--A-CT--
ATCTTGA-C-TTCG
1DAI18529 GC-TTCAAATTGTCGTG-------AGAC--A-CT--
ATCTTGA-C-TTCG
Dai19535 GC-TTCGAACCGTCGCG-------AGAC--A-AT--
GCTTTGA-C-TTCG
1704_79_hnedyVillaLaPaz GC-TTCAAACTGTCGCA-------AGAC--A-AT--
CTATTGA-C-TTCG
F2061 GC-TTCAAACTGTCGCA-------AGAC--A-AT--
CTATTGA-C-TTC-
1DAI18268 GC-TTCAAACTGTCGCA-------AGAC--A-TT--
TTCTTGA-C-TTCG
1DAI18540A GC-TTCAAACTGTCGCA-------AGAC--A-TT--
TTATTGA-C-TTCG
Dai17695 GC-TTCAAACTGTCGCA-------AGAC--A-TT--
TTATTGA-C-TTCG
LKY18Dai26373 GC-TCCAAACTGTCGCA-------AGAC--A-TT--
TTCTTGA-C-TTC-
Dai17839P_sulphureus GC-TTCAAACTGTCGCA-------AGAC--A-AT--
TCTTTGA-C-TTCG
Dai17841P_sulphureus GC-TTCAAACTGTCGCA-------AGAC--A-AT--

TCTTTGA-C-TTCG
Dai19877P_roseus GC-TTCGAATCGTCGCA-------AGAC--A-TT--
CCTTTGA-C-TTCG
1508_18_1_Kout GC-TTCCAATTGTCACA-------TGAC--A-TT--
TCATTGA-C-TTC-
KP859303_R_vinctus_RP185_BRAZI GC-TTCCAATTGTCACA-------TGAC--A-TT--
TYATTGA-C-TTC-
JK1807_15Rigidoporus_sp_Puerto GC-TTCCAATTGTCACA-------TGAC--A-TT--TCATTGA-
C-TTCG
JV0509_47_J_TN_USA GC-TTCAAACTGTCGAA-------AGAC--A-AT--
WTTTTGA-C-TTCG
JV0709_188 GC-TTCAAACTGTCGAA-------AGAC--A-AT--
WTTTTGA-C-TTCG
JV0509_127_PA_USA GC-TTCAAACTGTCGAA-------AGAC--A-AT--
ATTTTGA-C-TTCG
JV1009_59_NJ_USA GC-TTCAAACTGTCGAA-------AGAC--A-AT--
ATTTTGA-C-TTCG
Dai15497P_crataegi GC-TTCAAACTGTCGCG-------AGAC--A-AC--ACT-------
---G
Dai15499P_crataegi GC-TTCAAACTGTCGCG-------AGAC--A-AC--ACT-------
---G
Cui3266P_cinereus GC-TTCAAACTGTCGCG-------AGAC--AAAC--ACT-----
-----G
WCG1256Dai24690 GC-TTCAAACTGTCGCG-------AGAC--AAAC--
ACTTTGA-C-TTCG
1DAI17581 GC-TTCAAACTGTCGCG-------AGAC--AAAC--
ACTTTGA-C-TTCG
WCG1255Dai24688 GC-TTCAAACTGTCGCG-------AGAC--AAAC--
ACTTTGA-C-TTCG
Dai22427 GC-TTCAAACTGTCGCG-------AGAC--AAAC--ACT-
TGA-C-TTCG
MV690Meripilus_concrescens GC-TTCAAACTGTCGCG-------AGAC-AAAAC--
ACTTTGA-C-TTCG
MV513Meripilus_galapagensis GC-TTCAAACTGTCGCG-------AGAC-AAAAC--
ACTTTGA-C-TTCG
Dai19793 GC-TTCAAACCGTCGCA-------AGAC--A-AT--
ATTTTGA-C-TTCG
OQ553780P_tamilnaduensis GC-TTCTAATTGTCGCG-------AGAC--A-TT--
ACTTTGA-C-TTGA
OQ553779P_tamilnaduensis GC-TTCTAATTGTCGCG-------AGAC--A-TT--
ACTTTGA-C-TTGA
A164FB3Meripilus_giganteus GC-TTCTAATTGTCGCG-------AGAC--A-TT--
ACTTTGA-C-TTC-
JV1407_36_Vinctus_Meandrica GC-TTCAAACTGTCGCA-------AGAC--AATTC-

TATTTGA-C-TTCG
1807_3K_Rigidoporus_PuertoRico --G
Cui16903P_vinctus GC-TTCAAACTGTCGCA-------AGAC--AATTC-
TATTTGA-C-TTCG
JV1008_18R_Lineatus GC-TTCAAACTGTCGTC------AAGAC--AATT--
CATTTGA-C-TTCG
JV1407_37_1_Vinctus_Carara GC-TTCAAACTGTCGTC------AAGAC--AATT--
CATTTGA-C-TTCG
Dai17986P_lineatus GC-TTCAAACTGTCGTC------AAGAC--AATT--
CATTTGA-C-TTCG
Dai18281 GC-TTCAAACTGTCGTC------AAGAC--AATT--
CATTTGA-C-TTCG
1DAI19796 GC-TTCAAACTGTCGTC------AAGAC--AATC--
CATTTGA-C-TTC-
ZQY797Dai25241 GC-TTCAAACTGTCGTC------AAGAC--AATC--
CATTTGA-C-TTCG
WCG1289Dai24711 GC-TTCAAACTGTCGTC------AAGAC--AATC--
CATTTGA-C-TTC-
Dai22598 GC-TTCAAACTGTCGTC------AAGAC--AATT--
CATTTGA-C-TTCG
Dai20523 GC-TTCAAACTGTCGTC------AAGAC--AATT--
CATTTGA-C-TTCG
Dai17885 GC-TTCAAACTGTCGTC------AAGAC--AATT--
CATTTGA-C-TTCG
Dai17553 GC-TTCAAACTGTCGTC------AAGAC--AATT--
TATTTGA-C-TTCG
Dai19639 GC-TTCAAACTGTCGTC------AAGAC--AATT--
CATTTGA-C-TTCG
JV0110_48_CZ GC-TTCCAACTGTCGCA-------AGAC--AAAC--
CTTTTGA-C-ATCG
MJ129_04 GC-TTCCAACTGTCGCA-------AGAC--AAAC--
CTTTTGA-C-ATCG
Cui10340P_eminens GC-TTCCAACCGTCGCA-------AGAC--AAAC--CTT----
----
Cui10341P_eminens GC-TTCCAACCGTCGCA-------AGAC--AAAC--CTT----
------G
Dai12685P_eminens GC-TTCCAACTGTCGCA-------AGAC--AAAC--
CTTTTGA-C-ATCG
Miettinen_13591Rigidoporus_und GC-TTCCAACTGTCGCA-------AGAC--AAAC--
CTTTTGA-C-ATCG
Dai20868 GC-TTCCAATCGTCGCA-------AGAC--AAAC--
CTTTTGA-C-ATCG
Dai20832 GC-TTCTAATCGTCGCA-------AGAC--AAAC--
CTTTTGA-C-ATCG

Dai11400 GC-TTCCAATCGTCGCA-------AGAC--AAAC--CTT----
------G
Dai22472 GC-TTCTAATTGTCGCA-------AGAC--AAAC--
CTTTTGA-C-ATCG
1CUI10475 GC-TTCCAACCGTCGCG-------AGAC--AAAC--
CTTTTGA-C-ATCG
1CUI10491 GC-TTCCAACCGTCGCG-------AGAC--AAAC--
CTTTTGA-C-ATC-
HCFC1095Meripilus_robledoi GC-TTCCAATCGTCGCG-------AGAC--AAAC--
CCTTTGA------G
MCW702Meripilus_revolubilis GC-TTCCAACTGTCGCG-------AGAC--AAACC-
CTTTTGA------G
1704_83_zluty_HaciendaBaru GC-TTCGAATCGTCTTC-------GGAC--AATT--
TTATTGA-C-ATCG
Dai9925P_lavendulus GC-TTCCAATCGTCTTC-------GGAC--AAATG-
CAATCAA-C-ATCG
Dai13587AP_lavendulus GC-TTCCAATCGTCTTC-------GGAC--AAATG-
CAATCAA-C-ATCG
PDD70600P_longicystidius GC-TTCGAACCGTCTTC-------GGAC--AATC--
TGATCAA-C-ATC-
Cui16630 GC-TTCGAACCGTCTTC-------GGAC--AATC--
TGATCAA-C-ATCG
FP_135344Meripilus_giganteus GC-TTCTAATCGTCGCA-------AGAC--A-AC--CT-
TTGACA-ATCG
FP_100460_Sp GC-TTCTAATCGTCGCA-------AGAC--A-AC--CT-
TTGACA-ATC-
CBS421_48Meripilus_giganteus GC-TTCTAATCGTCGCA-------AGAC--A-AC--CT-
TTGACA-ATC-
Cui9203 GC-TTCTAATCGTCGCA-------AGAC--A-AC--CT-
TTGACA-ATC-
Cui9202 GC-TTCTAATCGTCGCA-------AGAC--A-AC--CT-
TTGACA-ATC-
TUFC100564Japan GC-TTCTAATCGTCGCA-------AGAC--A-AC--CT-
TTGACA-ATC-
Russell5913Meripilus_sumstinei GC-TTCCAACCGTCGCA-------AGAC--A-AC--
CTCTTGACA-ATC-
RP215Meripilus_brasiliensis GC-TTCCAACCG------------------------------------G
RP200Meripilus_brasiliensis GC-TTCCAACCGTCGCA-------AGAC--A------------------G
JV1712_13J_R_vinctus2_LSUPuert GC-TTCTAATCGTCTTC-------GGAC--AAC---
TTTTGAC-A-ATCG
Dai10503R_hypobrunneus GC-TTCTAATCGTCTTC-------GGAC--AAC---TT---------
--G
Dai10569Rigidoporus_hypobrunne GC-TTCTAATCGTCTTC-------GGAC--AAC---TT----------
G

1DAI19451 GC-TTCTAATCGTCTTC-------GGAC--AAC---
TTTTGAC-A-ATC-
CM108bRigidoporus_hypobrunneus GC-TTCTAATCGTCTTT-------GGAC--AAC---
TTTTGAC-A-ATC-
1CUI16874 GC-TTCTAATCGTCTTC-------GGAC--AAC---
TTTTGAC-A-ATCG
FD299Cerrena_unicolor GC-TTCTAATCGTCTTC-------GGAC--AAT---TCTTTGA-
C-ATCG
KHL_GB_Cerrena_uniclor GC-TTCTAATCGTCTTC-------GGAC--AAT---
TCTTTGA-C-ATCG
Dai12892Cerrena_albocinnamomea GC-TTCTAATCGTCTTC-------GGAC--AA----
CTTTTGA-C-ATCG
Dai12955C_albocinnamomea GC-TTCTAATCGTCTTC-------GGAC--AA----
CTTTTGA-C-ATCG
SNUm03110102C_aurantiopora GC-TTCTAATCGTCTTC-------GGAC--AA----
CTTTTGA-C-ATCG
NIBRFG0000102423C_aurantiopora GC-TTCTAATCGTCTTC-------GGAC--AA----
CTTTTGA-C-ATCG
Dai7359Antrodiella_zonata GC-TTCTAATCGTCTTC-------GGAC--AAT---TCTTTGA-
C-ATCG
F20080702KCM29C_consors GC-TTCTAATCGTCTTC-------GGAC--AAT---
TCTTTGA-C-ATC-
F20080208LYW10Cerrena_consors GC-TTCTAATCGTCTTC-------GGAC--AAT---
TCTTTGA-C-ATCG
Dai7821Antrodiella_zonata GC-TTCTAATCGTCTTC-------G-----------------------G
CFMR_DCL04_31Pseudolagarobasid GC-TTCTAAATGTCCTT-------GGAC--AAC---
TTTTTGA-C-ATC-
VPB197Pseudolagarobasidium_bel GC-TTCTAAAT--------------------------------------
CBS115543Pseudolagarobasidium_ GC-TTCTAATCGTCTTC-------GGAC--AGT---
TTTTTGA-C-ATC-
CBS115544 GC-TTCTAATCGTCTTC-------GGAC--AGT---
TTTTTGA-C-ATC-
Han405Pseudolagarobasidium_bai GC-TTCTAATTGTCCTA-------GGAC--AATC--TTTTTGA-
C-ATCG
Han406Pseudolagarobasidium_bai GC-TTCTAATTGTCCTA-------GGAC--AATC--TTTTTGA-
C-ATCG
MUcc838Spongipellis_delectans GC-TTCTAATCGTCTTC-------GGAC--AAT---TTCTTGA-
C-ATCG
BRNM686401S_delectans GC-TTCTAATCGTCTTC-------GGAC--AAT---
TTCTTGA-C-ATCG
OSM_F925S_delectans GC-TTCTAATCGTCTTC-------GGAC--AAT---
TTCTTGA-C-ATCG
BRNM67093Spongipellis_litschau GC-TTCTAATCGTCTTC-------GGAC--AATT--TCTTTGA-
C-ATCG

CFMRccFP59199TS_unicolor GC-TTCTAATCGTCTTC-------GGAC--AATT--
TCTTTGA-C-ATCG
CFMRccFP71791TS_unicolor GC-TTCTAATCGTCTTC-------GGAC--AATT--
TCTTTGA-C-ATCG
Dai13845P_lischaueri GC-TTCTAATCGTCTTC-------GGAC--AATT--TCTTTGA-
C-ATCG
Dai20266P_lischaueri GC-TTCTAATCGTCTTC-------GGAC--AATT--TCTTTGA-
C-ATCG
CFMR_HHB11240Radulodon_america GC-TTCTAATTGTCTTT-------GGAC--
AAATCTTATTGAC-T-ATT-
RLG6350Radulodon_americanus GC-TTCTAATTGTCTTT-------GGAC--
AAATCTTATTGAC-T-ATT-
KY415963Radulodon_erikssonii GC-TTCTAATTGTCTTT-------GGAC--
AAATCTTATTGAC-T-ATTG
HHB9567spRadulodon_casearius GC-TTCTAATTGTCTTT-------GGAC--AAATC-
TATTGAC-T-ATTG
KRT_Iso_26Radulodon_casearius GC-TTCTAATTGTCTTT-------GGAC--AAATC-
TATTGAC-T-ATT-
CBS126044Radulodon_erikssonii GC-TTCTAATTGTCTTT-------GGAC--
AAATCTCATTGAC-T-ATTG
He6183YUNNANENSIS GC-TTCTAATTGTCTTT-------GGAC--AAATC-
TATTGAC-T-ATTG
Cui17979YUNNANENSIS GC-TTCTAATTGTCTTT-------GGAC--AAATC-
TATTGAC-T-ATTG
Miettinen2091Junghuhnia_fimbri GC-TTCAAACCGTCCCTC-----GGGAC--AAT---
TTTTGAA-A-ATCG
KHL12316S_tenue GC-TTCCAACTGTCCTTT-----GGGAC--AAT---
CTCTGAA-C-ATCG
PRM846564S_pachyodon GC-TTCTAATTGTCTTT-------GGAC--AA----
TTTTTGA-A-ATTG
SP_Lgt_S_pachyodon GC-TTCTAATCGTCTTT-------GGAC--AA----
TTTTTGA-A-ATT-
Ryvarden44669Tyromyces_xuchile GCTTTCTAATTGTCTTT-------GGAC--AAT---TTTTTAA-
A-ATTG
PW17_171sinuosus GC-TTCTAACTGTCTTT-------GGAC--AA----
TTTTTGA-A-ATT-
W53Dai12234 GC-TTCTAACTGTCTTT-------GGAC--AA----
TTTTTGA-A-ATTG
HHB4100SpAntella_americana GC-TTCTAAACGTCTTTG-----CAGAC--AAT--------A-
T-ATCG
W3Dai20901spumeus GC-TTCAAATGGGTCTCTTCATTGAGAC--
AATTTCATTCTGA-C-ATCG
He6736 GC-TTCAAATGGGTCTCTTCATTGAGAC--
AATTTCATTCTGA-C-ATCG

BRNM734877S_spumeus GC-TTCAAATGGGTCTCTTCATTGAGAC--AATTTYATTYTGA-C-ATCG
BRNM712630S_spumeus GC-TTCAAATGGGTCTCTTCATTGAGAC--ARTTTTATTTTGA-C-ATCG
Dai1723Loweomyces_sibiricus GC-TTCAAACGGGTCTCTTTATTGAGAC--AATTTCATCTTGA-C-ATC-
W54Cui10009 GC-TTCAAACGGGTCTTTTCATTGAGAC--AATTCCATCTTGA-CTATCG
W1Dai20899 GC-TTCAAACGGGTCTCTTCATTGAGAC--AATTCCATCTTGA-C-ATC-
HHB13445Trametes_ochracea GC-TTCTAACCGTCCAC-----TAGGAC--AAA---TTTTTAA-C-ATCG
Dai16222 GC-TTAAAACTGTTCCTGGCA-AGGAACTCA-----TTTTGGA-C-ATCG
Dai16240 GC-TTAAAACTGTTCCTGGCA-AGGAACTCA-----TTTTGGA-C-ATCG

JV1310_11SanguinolentusCernys TAACTGCGAGTGAAGCGGGAAAAGCTCAAATTTAAAATCTGGCAGTCTTT
MJ39_00_SK TAACTGCGAGTGAAGCGGGAAAAGCTCAAATTTAAAATCTGGCAGTCTTT
MJ111_04_CZ --
JV1610_BOKYsmrk TAACTGCGAGTGAAGCGGGAAAAGCTCAAATTTAAAATCTGGCAGTCTTT
Dai21030 --
Dai20976P_furcatus TAACTGCGAGTGAAGCGGGAAAAGCTCAAATTTAAAATCTGGCAGTCTTT
Dai2105 TAACTGCGAGTGAAGCGGGAAAAGCTCAAATTTAAAATCTGGCAGTCTTC
Dai2544 TAACTGCGAGTGAAGCGGGAAAAGCTCAAATTTAAAATCTGGCAGTCTTC
Dai11313 TAACTGCGAGTGAAGCGGGAAAAGCTCAAATTTAAAATCTGGCAGTCTTC
WCG1611Dai26167 TAACTGCGAGTGAAGCGGGAAAAGCTCAAATTTAAAATCTGGCAGTCTTC
WCG1518Dai25999Physisporinus -------GAGTGAAGCGGGAAAAGCTCAAATTTAAAATCTGGCAGTCTTC
TAA15097 TAACTGCGAGTGAAGCGGGAAAAGCTCAAATTTAAAATCTGGCAGTCTTT
JV8909_19_CZ --
JV1310_15_P_sanguinolentus2_CZ TAACTGCGAGTGAAGCGGGAAAAGCTCAAATTTAAAATCTGGCAGTCTTT
MJ53_02_CZ TAACTGCGAGTGAAGCGGGAAAAGCTCAAATTTAAAATCTGGCAGTCTTT

CLZhao21647P_yunnanensis
TAACTGCGAGTGAAGCGGGAAAAGCTCAAATTTAAAATCTGGCAGTCTTC
CLZhao21583P_yunnanensis
TAACTGCGAGTGAAGCGGGAAAAGCTCAAATTTAAAATCTGGCAGTCTTC
Dai22272
TAACTGCGAGTGAAGCGGGAAAAGCTCAAATTTAAAATCTGGCAGTCTTT
Dai22279
TAACTGCGAGTGAAGCGGGAAAAGCTCAAATTTAAAATCTGGCAGTCTTT
MJ332_94_CZ --
MJ642_94_CZ_Expallescens --
Dai21060P_vinctus
TAACTGCGAGTGAAGCGGGAAAAGCTCAAATTTAAAATCTAGCGGTCTTT
JV0511_23LRP_pouzarii
TAACTGCGAGTGAAGCGGGAAAAGCTCAAATTTAAAATCTGGTAGTCTTT
JQ409462_R_pouzarii_PRM899856_ --
JV0308_66_WA --
JV0309_45_WA_USA --
JV0709_83_CA_USA
TAACTGCGAGTGAAGCGGGAAAAGCTCAAATTTAAAATCTGGCAGTCTTT
Dai21043P_pouzarii
TAACTGCGAGTGAAGCGGGAAAAGCTCAAATTTAAAATCTGGTAGTCTTT
MJ144_95_CZ
TAACTGCGAGTGAAGCGCGAAAAGCTCAAATTTAAAATCTGGCGGTCTTT
JV0909_3_CZ
TAACTGCGAGTGAAGCGGGAAAAGCTCAAATTTAAAATCTGGCGGTCTTT
JV0609_1_K --
Dai20396Physisporinus_castanop
TAACTGCGAGTGAAGCGGGAAAAGCTCAAATTTAAAATCTGGCGGTCTTT
Dai20397Physisporinus_castanop
TAACTGCGAGTGAAGCGGGAAAAGCTCAAATTTAAAATCTGGCGGTCTTT
MJ19_09_SK_Abies
TAACTGCGAGTGAAGCGGGAAAAGCTCAAATTTAAAATCTGGTGGTCTTT
JV0509_40_J_TN_USA_Betula --
JV0808_33crocatus_PA_USAlist
TAACTGCGAGTGAAGCGGGAAAAGCTCAAATTTAAAATCTGGTGGTCTTT
DLL2009_061P_crocatus --
Dai12800P_subcrocatus
TAACTGCGAGTGAAGCGGGAAAAGCTCAAATTTAAAATCTGGTGGTCTTT
Dai15917P_subcrocatus
TAACTGCGAGTGAAGCGGGAAAAGCTCAAATTTAAAATCTGGTGGTCTTT
Cui16620
TAACTGCGAGTGAAGCGGGAAAAGCTCAAATTTAAAATCTGGCGGTCTTT
HCFC1088Meripilus_stillicidior
TAACTGCGAGTGAAGCGGGAAAAGCTCAAATTTAAAATCTGSCGGTYTTT

MCW590Meripilus_obsrcurus --
MCW722Meripilus_obsrcurus --
Cui9381P_tibeticus
TAACTGCGAGTGAAGCGGGAAAAGCTCAAATTTAAAATCTGGCGGTCTTT
Cui9588P_tibeticus
TAACTGCGAGTGAAGCGGGAAAAGCTCAAATTTAAAATCTGGCGGTCTTT
Va2_Beneschova --
CWU3874_Ukraine_Alnus
TAACTGCGAGTGAAGCGGGAAAAGCTCAAATTTAAAATCTGGCGGTCTTT
WCG1293Dai24718Physisporinus --
WCG1268Dai24682A
TAACTGCGAGTGAAGCGGGAAAAGCTCAAATTTAAAATCTGGCAGT-TTT
WCG1269Dai24683A
TAACTGCGAGTGAAGCGGGAAAAGCTCAAATTTAAAATCTGGCAGT-TTT
WCG1279Dai24694A
TAACTGCGAGTGAAGCGGGAAAAGCTCAAATTTAAAATCTGGCAGT-TTT
Dai16971
TAACTGCGAGTGAAGCGGGAAAAGCTCAAATTTAAAATCTGGCAGT-TTT
ZQY1043Dai26696
TAACTGCGAGTGAAGCGGGAAAAGCTCAAATTTAAAATCTGGCAGT-TTT
Doll880 --
Doll1000
TAACTGCGAGTGAAGCGGGAAAAGCTCAAATATAAAATCTGGCAGTC-TT
1DAI18529
TAACTGCGAGTGAAGCGGGAAAAGCTCAAATTTAAAATCTGGCAGTCTT-
Dai19535
TAACTGCGAGTGAAGCGGGAAAAGCTCAAATTTAAAATCTGGCAGTCTTT
1704_79_hnedyVillaLaPaz
TAACTGCGAGTGAAGCGGGAAAAGCTCAAATTTAAAATCTGGCTGTCTTT
F2061 --
1DAI18268
TAACTGCGAGTGAAGCGGGAAAAGCTCAAATTTAAAATCTGGCTGTCTTT
1DAI18540A
TAACTGCGAGTGAAGCGGGAAAAGCTCAAATTTAAAATCTGGCTGTCTTT
Dai17695
TAACTGCGAGTGAAGCGGGAAAAGCTCAAATTTAAAATCTGGCTGTCTTT
LKY18Dai26373 --
Dai17839P_sulphureus
TAACTGCGAGTGAAGCGGGAAAAGCTCAAATTTAAAATCTGGCAGTCTTT
Dai17841P_sulphureus
TAACTGCGAGTGAAGCGGGAAAAGCTCAAATTTAAAATCTGGCAGTCTTT
Dai19877P_roseus
TAACTGCGAGTGAAGCGGGAAAAGCTCAAATTTAAAATCTGGCAGTCTTT
1508_18_1_Kout --

KP859303_R_vinctus_RP185_BRAZI ---
JK1807_15Rigidoporus_sp_Puerto
TAACTGCGAGTGAAGCGGGAAGAGCTCAAATTTAAAATCTGGCAG-CTTT
JV0509_47_J_TN_USA
TAACTGCGAGTGAAGCGGGAAAAGCTCAAATTTAAAATCTGGCAGTCTTT
JV0709_188
TAACTGCGAGTGAAGCGGGAAAAGCTCAAATTTAAAATCTGGCAGTCTTT
JV0509_127_PA_USA
TAACTGCGAGTGAAGCGGGAAAAGCTCAAATTTAAAATCTGGCAGTCTTT
JV1009_59_NJ_USA
TAACTGCGAGTGAAGCGGGAAAAGCTCAAATTTAAAATCTGGCAGTCTTT
Dai15497P_crataegi
TAACTGCGAGTGAAGCGGGAAAAGCTCAAATTTAAAATCTGGCAGTCTTT
Dai15499P_crataegi
TAACTGCGAGTGAAGCGGGAAAAGCTCAAATTTAAAATCTGGCAGTCTTT
Cui3266P_cinereus
TAACTGCGAGTGAAGCGGGAAAAGCTCAAATTTAAAATCTGGCGGTCTTT
WCG1256Dai24690
TAACTGCGAGTGAAGCGGGAAAAGCTCAAATTTAAAATCTGGCGGTCTTT
1DAI17581
TAACTGCGAGTGAAGCGGGAAAAGCTCAAATTTAAAATCTGGCGGTCTTT
WCG1255Dai24688
TAACTGCGAGTGAAGCGGGAAAAGCTCAAATTTAAAATCTGGCGGTCTTT
Dai22427
TAACTGCGAGTGAAGCGGGAAAAGCTCAAATTTAAAATCTGGCGGTCTTT
MV690Meripilus_concrescens
TAACTGCGAGTGAAGCGGGAAAAGCTCAAATTTAAAATCTGGCGGTCTTT
MV513Meripilus_galapagensis
TAACTGCGAGTGAAGCGGGAAAAGCTCAAATTTAAAATCTGGCGGTCTTT
Dai19793
TAACTGCGAGTGAAGCGGGAAAAGCTCAAATTTAAAATCTGGCAGTCTTT
OQ553780P_tamilnaduensis
TAACTGCGAGTGAAGCGGGAAAAGCTCAAATTTAAAATCTGGCAGTCTTT
OQ553779P_tamilnaduensis
TAACTGCGAGTGAAGCGGGAAAAGCTCAAATTTAAAATCTGGCAGTCTTT
A164FB3Meripilus_giganteus ---
JV1407_36_Vinctus_Meandrica
TAACTGCGAGTGAAGCGGGAAAAGCTCAAATTTAAAATCTGGCAGTCTTT
1807_3K_Rigidoporus_PuertoRico
TAACTGCGAGTGAAGCGGGAAAAGCTCAAATTTAAAATCTGGCAGTCTTT
Cui16903P_vinctus
TAACTGCGAGTGAAGCGGGAAAAGCTCAAATTTAAAATCTGGCAGTCTTT
JV1008_18R_Lineatus
TAACTGCGAGTGAAGCGGGAAAAGCTCAAATTTAAAATCTGGCAGTCTTT

JV1407_37_1_Vinctus_Carara
TAACTGCGAGTGAAGCGGGAAAAGCTCAAATTTAAAATCTGGCAGTCTTT
Dai17986P_lineatus
TAACTGCGAGTGAAGCGGGAAAAGCTCAAATTTAAAATCTGGCAGTCTTT
Dai18281
TAACTGCGAGTGAAGCGGGAAAAGCTCAAATTTAAAATCTGGCAGTCTTT
1DAI19796 ------------------------------TTTAAAATCTGGCAGTCTTT
ZQY797Dai25241
TAACTGCGAGTGAAGCGGGAAAAGCTCAAATTTAAAATCTGGCAGTCTTT
WCG1289Dai24711 --
Dai22598
TAACTGCGAGTGAAGCGGGAAAAGCTCAAATTTAAAATCTGGCAGTCTTT
Dai20523
TAACTGCGAGTGAAGCGGGAAAAGCTCAAATTTAAAATCTGGCAGTCTTT
Dai17885
TAACTGCGAGTGAAGCGGGAAAAGCTCAAATTTAAAATCTGGCAGTCTTT
Dai17553
TAACTGCGAGTGAAGCGGGAAAAGCTCAAATTTAAAATCTGGCAGTCTTT
Dai19639
TAACTGCGAGTGAAGCGGGAAAAGCTCAAATTTAAAATCTGGCAGTCTTT
JV0110_48_CZ
TAACTGCGAGTGAAGCGGGAAAAGCTCAAATTTAAAATCTGGCAGTCTTT
MJ129_04
TAACTGCGAGTGAAGCGGGAAAAGCTCAAATTTAAAATCTGGCAGTCTTT
Cui10340P_eminens --
Cui10341P_eminens
TAACTGCGAGTGAAGCGGGAAAAGCTCAAATTTAAAATCTGGCAGTCTTT
Dai12685P_eminens
TAACTGCGAGTGAAGCGGGAAAAGCTCAAATTTAAAATCTGGCAGTCTTT
Miettinen_13591Rigidoporus_und
TAACTGCGAGTGAAGCGGGAAAAGCTCAAATTTAAAATCTGGCAGTCTTT
Dai20868
TAACTGCGAGTGAAGCGGGAAAAGCTCAAATTTAAAATCTGGCAGTCTTT
Dai20832
TAACTGCGAGTGAAGCGGGAAAAGCTCAAATTTAAAATCTGGCAGTCTTT
Dai11400
TAACTGCGAGTGAAGCGGGAAAAGCTCAAATTTAAAATCTGGCAGTCTTT
Dai22472
TAACTGCGAGTGAAGCGGGAAAAGCTCAAATTTAAAATCTGGCAGTCTTT
1CUI10475
TAACTGCGAGTGAAGCGGGAAAAGCTCAAATTTAAAATCTGGCAGTCTTT
1CUI10491 --
HCFC1095Meripilus_robledoi
TAACTGCGAGTGAAGCGGGAAAAGCTCAAATTTAAAATCTGGCGGTCTTT

MCW702Meripilus_revolubilis
TAACTGCGAGTGAAGCGGGAAAAGCTCAAATTTAAAATCTGGCAGTCTTT
1704_83_zluty_HaciendaBaru
TAACTGCGAGTGAAGCGGGAAAAGCTCAAATTTAAAATCTGGCAGTCTTT
Dai9925P_lavendulus
TAACTGCGAGTGAAGCGGGAAAAGCTCAAATTTAAAATCTGGCAGTCTTC
Dai13587AP_lavendulus
TAACTGCGAGTGAAGCGGGAAAAGCTCAAATTTAAAATCTGGCAGTCTTC
PDD70600P_longicystidius --
Cui16630
TAACTGCGAGTGAAGCGGGAAAAGCTCAAATTTAAAATCTGGCAGTCTTT
FP_135344Meripilus_giganteus
TAACTGCGAGTGAAGCGGGAAAAGCTCAAATTTAAAATCTGGCAGTCTTC
FP_100460_Sp --
CBS421_48Meripilus_giganteus --
Cui9203 --
Cui9202 -------
GAGTGAAGCGGGAAAAGCTCAAATTTAAAATCTGGCGGTCTTC
TUFC100564Japan --ACTGCGAGTG-
AGCGGGAAAAGCTCAAATTTAAAATCTGGCGGCCTTC
Russell5913Meripilus_sumstinei --
RP215Meripilus_brasiliensis
TAACTGCGAGTGAAGCGGGAAAAGCTCAAATTTAAAATCTGGCAGTCTTT
RP200Meripilus_brasiliensis
TAACTGCGAGTGAAGCGGGAAAAGCTCAAATTTAAAATCTGGCAGTCTTT
JV1712_13J_R_vinctus2_LSUPuert
TAACTGCGAGTGAAGCGGGAAAAGCTCAAATTTAAAATCTGGCAGTCTTT
Dai10503R_hypobrunneus
TAACTGCGAGTGAAGCGGGAAAAGCTCAAATTTAAAATCTGGCAGTCTTT
Dai10569Rigidoporus_hypobrunne
TAACTGCGAGTGAAGCGGGAAAAGCTCAAATTTAAAATCTGGCAGTCTTT
1DAI19451 -----------------
GAAAAGCTCAAATTTAAAATCTGGCAGTCTTT
CM108bRigidoporus_hypobrunneus --
1CUI16874
TAACTGCGAGTGAAGCGGGAAAAGCTCAAATTTAAAATCTGGCAGTCTTT
FD299Cerrena_unicolor
TAACTGCGAGTGAAGCGGGAAAAGCTCAAATTTAAAATCTGGCAGCCTTC
KHL_GB_Cerrena_uniclor
TAACTGCGAGTGAAGCGGGAAAAGCTCAAATTTAAAATCTGGCAGCCTTC
Dai12892Cerrena_albocinnamomea
TAACTGCGAGTGAAGCGGGAAAAGCTCAAATTTAAAATCTGGCGGTCTTT
Dai12955C_albocinnamomea
TAACTGCGAGTGAAGCGGGAAAAGCTCAAATTTAAAATCTGGCGGTCTTT

SNUm03110102C_aurantiopora
TAACTGCGAGTGAAGCGGGAAAAGCTCAAATTTAAAATCTGGCGGTCTTT
NIBRFG0000102423C_aurantiopora
TAACTGCGAGTGAAGCGGGAAAAGCTCAAATTTAAAATCTGGCGGTCTTT
Dai7359Antrodiella_zonata
TAACTGCGAGTGAAGCGGGAAAAGCTCAAATTTAAAATCTGGCGGTCTTT
F20080702KCM29C_consors -
AACTGCGAGTGAAGCGGGAAAAGCTCAAATTTAAAATCTGGCGGTCTTT
F20080208LYW10Cerrena_consors
TAACTGCGAGTGAAGCGGGAAAAGCTCAAATTTAAAATCTGGCGGTCTTT
Dai7821Antrodiella_zonata
TAACTGCGAGTGAAGCGGGAAAAGCTCAAATTTAAAATCTGGCGGTCTTT
CFMR_DCL04_31Pseudolagarobasid --
VPB197Pseudolagarobasidium_bel --
CBS115543Pseudolagarobasidium_ --
CBS115544 --
Han405Pseudolagarobasidium_bai
TAACTGCGAGTGAAGCGGGAAAAGCTCAAATTTAAAATCTGATAGTCTTT
Han406Pseudolagarobasidium_bai
TAACTGCGAGTGAAGCGGGAAAAGCTCAAATTTAAAATCTGATAGTCTTT
MUcc838Spongipellis_delectans
TAACTGCGAGTGAAGCGGGAAAAGCTCAAATTTAAAATCTGGCAGTCTTT
BRNM686401S_delectans
TAACTGCGAGTGAAGCGGGAAAAGCTCAAATTTAAAATCTGGCAGTCTTT
OSM_F925S_delectans
TAACTGCGAGTGAAGCGGGAAAAGCTCAAATTTAAAATCTGGCAGTCTTT
BRNM67093Spongipellis_litschau
TAACTGCGAGTGAAGCGGGAAAAGCTCAAATTTAAAATCTGGCAGCCTTT
CFMRccFP59199TS_unicolor
TAACTGCGAGTGAAGCGGGAAAAGCTCAAATTTAAAATCTGGCAGCCTTT
CFMRccFP71791TS_unicolor
TAACTGCGAGTGAAGCGGGAAAAGCTCAAATTTAAAATCTGGCAGCCTTT
Dai13845P_lischaueri
TAACTGCGAGTGAAGCGGGAAAAGCTCAAATTTAAAATCTGGCAGCCTTT
Dai20266P_lischaueri
TAACTGCGAGTGAAGCGGGAAAAGCTCAAATTTAAAATCTGGCAGCCTTT
CFMR_HHB11240Radulodon_america --
RLG6350Radulodon_americanus --
KY415963Radulodon_erikssonii
TAACTGCGAGTGAACCGGGAAAAGCTCAAATTTAAAATCTGGCAGTCTTT
HHB9567spRadulodon_casearius
TAACTGCGAGTGAACCGGGAAAAGCTCAAATTTAAAATCTGGCAGTCTTT
KRT_Iso_26Radulodon_casearius --
CBS126044Radulodon_erikssonii

TAACTGCGAGTGAACCGGGAAAAGCTCAAATTTAAAATCTGGCAGTCTTT
He6183YUNNANENSIS
TAACTGCGAGTGAACCGGGAAAAGCTCAAATTTAAAATCTGGCGGTCTTT
Cui17979YUNNANENSIS
TAACTGCGAGTGAACCGGGAAAAGCTCAAATTTAAAATCTGGCGGTCTTT
Miettinen2091Junghuhnia_fimbri
TAACTGCGAGTGAAGCGGGAAAAGCTCAAATTTAAAATCTGGCAG-CTTT
KHL12316S_tenue
TAACTGCGAGTGAAGCGGGAAAAGCTCAAATTTAAAATCTGGCAG-CTTT
PRM846564S_pachyodon
TAACTGCGAGTGAAGCGGGAAAAGCTCAAATTTAAAATCTGGCAGTCTTT
SP_Lgt_S_pachyodon --
Ryvarden44669Tyromyces_xuchile
TAACTGCGAGTGAAGCGGGAAAAGCTCAAATTTAAAATCTGGCAGTCTTT
PW17_171sinuosus --
W53Dai12234
TAACTGCGAGTGAAGCGGGAAAAGCTCAAATTTAAAATCTGGCAGTCTTT
HHB4100SpAntella_americana
TAACTGCGAGTGAAGCGGGAAAAGCTCAAATTTAAAATCTGGCAG-CTTT
W3Dai20901spumeus
TAACTGCGAGTGAAGCGGGAAAAGCTCAAATTTAAAATCTGGCGGTCTCT
He6736
TAACTGCGAGTGAAGCGGGAAAAGCTCAAATTTAAAATCTGGCGGTCTCT
BRNM734877S_spumeus
TAACTGCGAGTGAAGCGGGAAAAGCTCAAATTTAAAATCTGGCGGTCTCT
BRNM712630S_spumeus
TAACTGCGAGTGAAGCGGGAAAAGCTCAAATTTAAAATCTGGCGGTCTCT
Dai1723Loweomyces_sibiricus --
W54Cui10009
TAACTGCGAGTGAAGCGGGAAAAGCTCAAATTTAAAATCTGGCGGTCTCT
W1Dai20899 --
HHB13445Trametes_ochracea
TAACTGCGAGTGAAGCGGGAAAAGCTCAAATTTAAAATCTGGCGGTCTTT
Dai16222
TAACTGCGAGTGAAGCGGGAAAAGCTCAAATTTAAAATCTGGCA--TCTT
Dai16240
TAACTGCGAGTGAAGCGGGAAAAGCTCAAATTTAAAATCTGGCA--TCTT

JV1310_11SanguinolentusCernys
GATTGTCCGAGTTGTAATCTGGAGAAGTGCTTTCCGCGCCGGACCGTGTA
MJ39_00_SK
GATTGTCCGAGTTGTAATCTGGAGAAGTGCTTTCCGCGCCGGACCGTGTA
MJ111_04_CZ --
JV1610_BOKYsmrk

GATTGTCCGAGTTGTAATCTGGAGAAGTGCTTTCCGCGCCGGACCGTGTA
Dai21030 ---
Dai20976P_furcatus
GATTGTCCGAGTTGTAATCTGGAGAAGTGCTTTCCGCGCCGGACCGTGTA
Dai2105
GATTGTCCGAGTTGTAATCTGGAGAAGTGCTTTCCGCGCTGGACCGTGTA
Dai2544
GATTGTCCGAGTTGTAATCTGGAGAAGTGCTTTCCGCGCTGGACCGTGTA
Dai11313
GATTGTCCGAGTTGTAATCTGGAGAAGTGCTTTCCGCGCTGGACCGTGTA
WCG1611Dai26167
GATTGTCCGAGTTGTAATCTGGAGAAGTGCTTTCCGCGCTGGACCGTGTA
WCG1518Dai25999Physisporinus
GATTGTCCGAGTTGTAATCTGGAGAAGTGCTTTCTGCGCTGGACCGTGTA
TAA15097
GATTGTCCGAGTTGTAATCTGGAGAAGTGCTTTCCGCGCTGGACCGTGTA
JV8909_19_CZ ---
JV1310_15_P_sanguinolentus2_CZ
GATTGTCCGAGTTGTAATCTGGAGAAGTGCTTTCCGCGCTGGACCGTGTA
MJ53_02_CZ
GATTGTCCGAGTTGTAATCTGGAGAAGTGCTTTCCGCGCTGGACCGTGTA
CLZhao21647P_yunnanensis
GATTGTCCGAGTTGTAATCTGGAGAAGTGCTTTCCGTGCTGGACCGTGTA
CLZhao21583P_yunnanensis
GATTGTCCGAGTTGTAATCTGGAGAAGTGCTTTCCGTGCTGGACCGTGTA
Dai22272
GATTGTCCGAGTTGTAATCTGGAGAAGTGCTTTCCGCGCTGGACCGTGTA
Dai22279
GATTGTCCGAGTTGTAATCTGGAGAAGTGCTTTCCGCGCTGGACCGTGTA
MJ332_94_CZ ---
MJ642_94_CZ_Expallescens ---
Dai21060P_vinctus
GGCCGTTCGAGTTGTAATCTGGAGAAGTGCTTTCCGCGCTGGACCGTGTA
JV0511_23LRP_pouzarii
GGCTGCCCGAGTTGTAATCTGGAGAAGTGCTTTCCGCGCTGGACCGTGTA
JQ409462_R_pouzarii_PRM899856_ ---
JV0308_66_WA ---
JV0309_45_WA_USA ---
JV0709_83_CA_USA
GGCTGTCCGAGTTGTAATCTGGAGAAGTGCTTTCTGCGCTGGACCGTGTA
Dai21043P_pouzarii
GGCTGCCCGAGTTGTAATCTGGAGAAGTGCTTTCCGCGCTGGACCGTGTA
MJ144_95_CZ
GACCGTCCGAGTTGTAATCTGGAGAAGTGCTTTCCGCGCTGGACCGTGTA

JV0909_3_CZ

GGCCGTCCGAGTTGTAATCTGGAGAAGTGCTTTCCGCGCTGGACCGTGTA

JV0609_1_K --

Dai20396Physisporinus_castanop

GGCTGTCCGAGTTGTAATCTGGAGAAGTGCTTTCCGCGCTGGACCGTGTA

Dai20397Physisporinus_castanop

GGCCGTCCGAGTTGTAATCTGGAGAAGTGCTTTCCGCGCTGGACCGTGTA

MJ19_09_SK_Abies

GGCCATCCGAGTTGTAATCTGGAGAAGTGCTTTCCGCTCTGGACCGTGTA

JV0509_40_J_TN_USA_Betula --

JV0808_33crocatus_PA_USAlist

GGCCATCCGAGTTGTAATCTGGAGAAGTGCTTTCCGCTCTGGACCGTGTA

DLL2009_061P_crocatus --

Dai12800P_subcrocatus

GGCCATCCGAGTTGTAATCTGGAGAAGTGCTTTCCGCTCTGGACCGTGTA

Dai15917P_subcrocatus

GGCCATCCGAGTTGTAATCTGGAGAAGTGCTTTCCGCTCTGGACCGTGTA

Cui16620

GGCCGTCCGAGTTGTAATCTGGAGAAGTGCTTTCCGCGCTGGACCGTGTA

HCFC1088Meripilus_stillicidior

GGCCGTCCGAGTTGTAATCTGGAGAAGTGCTTTCCSCGCTGGACCGTGTA

MCW590Meripilus_obsrcurus --

MCW722Meripilus_obsrcurus --

Cui9381P_tibeticus

GGCTGTCCGAGTTGTAATCTGGAGAAGTGCTTTCCGCTCTGGACCGTGTA

Cui9588P_tibeticus

GGCTGTCCGAGTTGTAATCTGGAGAAGTGCTTTCCGCTCTGGACCGTGTA

Va2_Beneschova --

CWU3874_Ukraine_Alnus

GGCCGTCCGAGTTGTAATCTGGAGAAGTGCTTTCCGCTCTGGACCGTGTA

WCG1293Dai24718Physisporinus --

WCG1268Dai24682A

GGCTGTCCGAGTTGTAATCTGGAGAAGTGCTTTCTGTGCTGGACCGTGTA

WCG1269Dai24683A

GGCTGTCCGAGTTGTAATCTGGAGAAGTGCTTTCTGTGCTGGACCGTGTA

WCG1279Dai24694A

GGCTGTCCGAGTTGTAATCTGGAGAAGTGCTTTCTGTGCTGGACCGTGTA

Dai16971

GGCTGTCCGAGTTGTAATCTGGAGAAGTGCTTTCTGTGCTGGACCGTGTA

ZQY1043Dai26696

GACTGTCCGAGTTGTAATCTGGAGAAGTGCTTTCTGTGCTGGACCGTGTA

Doll880 --

Doll1000

GGCTGTCCGAGTTGTAATCTGGAGAAGTGCTTTCTGCGCTGGACCGTGTA

1DAI18529
GGCTGTCCGAGTTGTAATCTGGAGAAGTGCTTTCTGCGCTGGACCGTGTA
Dai19535
GGCTGTCCGAGTTGTAATCTGGAGAAGTGCTTTCCGCGCCGGACCGTGTA
1704_79_hnedyVillaLaPaz
GGCTGCCCGAGTTGTAATCTGGAGAAGTGCTTTCTGTGCTGGACCGTGTA
F2061 --
1DAI18268
GGCTGCCCGAGTTGTAATCTGGAGAAGTGCTTTCTGCGCTGGACCGTGTA
1DAI18540A
GGCTGCCCGAGTTGTAATCTGGAGAAGTGCTTTCTGCGCTGGACCGTGTA
Dai17695
GGCTGCCCGAGTTGTAATCTGGAGAAGTGCTTTCTGCGCTGGACCGTGTA
LKY18Dai26373 --
Dai17839P_sulphureus
GATTGTCCGAGTTGTAATCTGGAGAAGTGCTTTCTGTGCCGGACCGTGTA
Dai17841P_sulphureus
GATTGTCCGAGTTGTAATCTGGAGAAGTGCTTTCTGTGCCGGACCGTGTA
Dai19877P_roseus
GGCTGTCCGAGTTGTAATCTGGAGAAGTGCTTTCTGTGCCGGACCGTGTA
1508_18_1_Kout --
KP859303_R_vinctus_RP185_BRAZI --
JK1807_15Rigidoporus_sp_Puerto
GGCTGTCCGAATTGTAATCTGGAGAAGTGCTTTCTGCTCTGGACCGTGTA
JV0509_47_J_TN_USA
GATTGTCCGAGTTGTAATCTGGAGAAGTGCTTTCCGCGCTGGACCGTGTA
JV0709_188
GATTGTCCGAGTTGTAATCTGGAGAAGTGCTTTCCGCGCTGGACCGTGTA
JV0509_127_PA_USA
GATTGTCCGAGTTGTAATCTGGAGAAGTGCTTTCCGCGCTGGACCGTGTA
JV1009_59_NJ_USA
GATTGTCCGAGTTGTAATCTGGAGAAGTGCTTTCCGCGCTGGACCGTGTA
Dai15497P_crataegi
GGCTGTCCGAGTTGTAATCTGGAGAAGTGCTTTCCGCGCTGGACCGTGTA
Dai15499P_crataegi
GGCTGTCCGAGTTGTAATCTGGAGAAGTGCTTTCCGCGCTGGACCGTGTA
Cui3266P_cinereus
GACTGTCCGAGTTGTAATCTGGAGAAGTGCTTTCCGCGCTGGACCGTGTA
WCG1256Dai24690
GACTGTCCGAGTTGTAATCTGGAGAAGTGCTTTCCGCGCTGGACCGTGTA
1DAI17581
GACTGTCCGAGTTGTAATCTGGAGAAGTGCTTTCCGCGCTGGACCGTGTA
WCG1255Dai24688
GACTGTCCGAGTTGTAATCTGGAGAAGTGCTTTCCGCGCTGGACCGTGTA

Dai22427
GACTGTCCGAGTTGTAATCTGGAGAAGTGCTTTCCGCGCTGGACCGTGTA
MV690Meripilus_concrescens
GACTGTCCGAGTTGTAATCTGGAGAAGTGCTTTCCGCGCTGGACCGTGTA
MV513Meripilus_galapagensis
GGCTGTCCGAGTTGTAATCTGGAGAAGTGCTTTCCGCGCCGGACCGTGTA
Dai19793
GATTGTCCGAGTTGTAATCTGGAGAAGTGCTTTCTGCGCTGGACCGTGTA
OQ553780P_tamilnaduensis
GATTGTCCGAGTTGTAATCTGGAGAAGTGCTTTCCGCGCTGGACCGTGTA
OQ553779P_tamilnaduensis
GATTGTCCGAGTTGTAATCTGGAGAAGTGCTTTCCGCGCTGGACCGTGTA
A164FB3Meripilus_giganteus --
JV1407_36_Vinctus_Meandrica
GGCTGTCCGAGTTGTAATCTGGAGAAGTGCTTTCCGCGCTGGACCGTGTA
1807_3K_Rigidoporus_PuertoRico
GGCTGTCCGAGTTGTAATCTGGAGAAGTGCTTTCCGCGCTGGACCGTGTA
Cui16903P_vinctus
GGCTGTCCGAGTTGTAATCTGGAGAAGTGCTTTCCGCGCTGGACCGTGTA
JV1008_18R_Lineatus
GGCTGTCCGAGTTGTAATCTGGAGAAGTGCTTTCCGTGCTGGACCGTGTA
JV1407_37_1_Vinctus_Carara
GGCTGTCCGAGTTGTAATCTGGAGAAGTGCTTTCCGTGCTGGACCGTGTA
Dai17986P_lineatus
GGCTGTCCGAGTTGTAATCTGGAGAAGTGCTTTCCGCGCTGGACCGTGTA
Dai18281
GGCTGTCCGAGTTGTAATCTGGAGAAGTGCTTTCCGCGCTGGACCGTGTA
1DAI19796
GGCTGTCCGAGTTGTAATCTGGAGAAGTGTTTTCCGCGCTGGACCGTGTA
ZQY797Dai25241
GGCTGTCCGAGTTGTAATCTGGAGAAGTGTTTTCCGCGCTGGACCGTGTA
WCG1289Dai24711 --
Dai22598
GGCTGTCCGAGTTGTAATCTGGAGAAGTGTTTTCCGCGCTGGACCGTGTA
Dai20523
GGCTGTCCGAGTTGTAATCTGGAGAAGTGTTTTCCGCGCTGGACCGTGTA
Dai17885
GGCTGTCCGAGTTGTAATCTGGAGAAGTGTTTTCCGCGCTGGACCGTGTA
Dai17553
GGCTGTCCGAGTTGTAATCTGGAGAAGTGTTTTCCGCGCTGGACCGTGTA
Dai19639
GGCTGTCCGAGTTGTAATCTGGAGAAGTGTTTTCCGCGCTGGACCGTGTA
JV0110_48_CZ
GGCTGTCCGAGTTGTATTCTAGAGAAGCGCTTTCCGTGCCGGACCGTGTA

MJ129_04
GGCTGTCCGAGTTGTATTCTAGAGAAGCGCTTTCCGTGCCGGACCGTGTA
Cui10340P_eminens ---
Cui10341P_eminens
GGCTGTCCGAGTTGTATTCTAGAGAAGCGCTTTCCGTGCCGGACCGTGTA
Dai12685P_eminens
GGCTGTCCGAGTTGTATTCTAGAGAAGCGCTTTCCGTGCCGGACCGTGTA
Miettinen_13591Rigidoporus_und
GGCTGTCCGAGTTGTATTCTAGAGAAGTGCTTTCCGTGCCGGACCGTGTA
Dai20868
GGCTGTCCGAGTTGTATTCTAGAGAAGCGCTTTCCGTGCCGGACCGTGTA
Dai20832
GGCTGTCCGAGTTGTATTCTAGAGAAGCGCTTTCCGTGCCGGACCGTGTA
Dai11400
GGCTGTCCGAGTTGTATTCTAGAGAAGCGCTTTCCGTGCCGGACCGTGTA
Dai22472
GGCTGTCCGAGTTGTATTCTAGAGAAGCGCTTTCCGTGCCGGACCGTGTA
1CUI10475
GGCTGTCCGAGTTGTATTCTAGAGAAGCGCTTTCCGTGCCGGACCGTGTA
1CUI10491 ---
HCFC1095Meripilus_robledoi
GGCTGTCCGAGTTGTAATCTGGAGAAGCGCTTTCCGTGCCGGACCGTGTA
MCW702Meripilus_revolubilis
GGCTGTCCGAGTTGTAATCTGGAGAAGCGCTTTCCGTGCCGGACCGTGTA
1704_83_zluty_HaciendaBaru
GATTGTCCGAGTTGTAATCTGGAGAAGTGCTTTCCGCGCCGGACCATGTA
Dai9925P_lavendulus
GATTGTCCGAGTTGTAATCTGGAGAAGCGCTTTCCGCGCCGGACCATGTA
Dai13587AP_lavendulus
GATTGTCCGAGTTGTAATCTGGAGAAGCGCTTTCCGCGCCGGACCATGTA
PDD70600P_longicystidius ---
Cui16630
GATTGTCCGAGTTGTAATCTGGAGAAGTGCTTTCCGCGCCGGACCATGTA
FP_135344Meripilus_giganteus
GGCTGTCCGAGTTGTAATCTGGAGAAGTGCCTTCCGCGCCGGACCGTGTA
FP_100460_Sp ---
CBS421_48Meripilus_giganteus ---
Cui9203 ---
Cui9202
GGCCGTCCGAGTTGTAATCTGGAGAAGTGCCTTCCGCGCCGGACCGTGTA
TUFC100564Japan
GGCCGTCCGAGTTGTAATCTGGAGAAGTGCCTTCCGCGCCGGACCGTGTA
Russell5913Meripilus_sumstinei ---
RP215Meripilus_brasiliensis

GGCTGTCCGAGTTGTAATCTGGAGAAGTGCTTTCCGCGCTGGACCGTGTA
RP200Meripilus_brasiliensis
GGCTGTCCGAGTTGTAATCTGGAGAAGTGCTTTCCGCGCTGGACCGTGTA
JV1712_13J_R_vinctus2_LSUPuert
GGCTGTCCGAGTTGTAGTCTGGAGAAGCGTTTTCCGCGCTGGACCGTGTA
Dai10503R_hypobrunneus
GGCTGTCCGAGTTGTAGTCTGGAGAAGCGTTTTCCGCGCTGGACCGTGTA
Dai10569Rigidoporus_hypobrunne
GGCTGTCCGAGTTGTAGTCTGGAGAAGCGTTTTCCGCGCTGGACCGTGTA
1DAI19451
GGCTGTCCGAGTTGTAGTCTGGAGAAGCGTTTTCCGCGCTGGACCGTGTA
CM108bRigidoporus_hypobrunneus --
1CUI16874
GGCTGTCCGAGTTGTAGTCTGGAGAAGTGTTTTCCGCGCTGGACCGTGTA
FD299Cerrena_unicolor
GGTTGTCCGAGTTGTAGTCTGGAGAAGCGTTTTCCGCGCTGGACCGTGTA
KHL_GB_Cerrena_uniclor
GGTTGTCCGAGTTGTAGTCTGGAGAAGCGTTTTCCGCGCTGGACCGTGTA
Dai12892Cerrena_albocinnamomea
GGCTGTCCGAGTTGTAGTCTGGAGAAGCGTTTTCCGCGCTGGACCGTGTA
Dai12955C_albocinnamomea
GGCTGTCCGAGTTGTAGTCTGGAGAAGCGTTTTCCGCGCTGGACCGTGTA
SNUm03110102C_aurantiopora
GGCTGTCCGAGTTGTAGTCTGGAGAAGCGTTTTCCGCGCTGGACCGTGTA
NIBRFG0000102423C_aurantiopora
GGCTGTCCGAGTTGTAGTCTGGAGAAGCGTTTTCCGCGCTGGACCGTGTA
Dai7359Antrodiella_zonata
GGCTGTCCGAGTTGTAGTCTGGAGAAGCGTTTTCCGCGCTGGACCGTGTA
F20080702KCM29C_consors
GGCTGTCCGAGTTGTAGTCTGGAGAAGCGTTTTCCGCGCTGGACCGTGTA
F20080208LYW10Cerrena_consors
GGCTGTCCGAGTTGTAGTCTGGAGAAGCGTTTTCCGCGCTGGACCGTGTA
Dai7821Antrodiella_zonata
GGCTGTCCGAGTTGTAGTCTGGAGAAGCGTTTTCCGCGCTGGACCGTGTA
CFMR_DCL04_31Pseudolagarobasid --
VPB197Pseudolagarobasidium_bel --
CBS115543Pseudolagarobasidium_ --
CBS115544 --
Han405Pseudolagarobasidium_bai
GGCTGTCCGAGTTGTAGTCTGGAGAAGCGTTTTCTGTGCTGGACCGTGTA
Han406Pseudolagarobasidium_bai
GGCTGTCCGAGTTGTAGTCTGGAGAAGCGTTTTCTGTGCTGGACCGTGTA
MUcc838Spongipellis_delectans
GGCTGTCCGAGTTGTAGTCTGGAGAAGTGTTTTCCGCGCTGGACCGTGTA

BRNM686401S_delectans
GGCTGTCCGAGTTGTAGTCTGGAGAAGTGTTTTCCGCGCTGGACCGTGTA
OSM_F925S_delectans
GGCTGTCCGAGTTGTAGTCTGGAGAAGTGTTTTCCGCGCTGGACCGTGTA
BRNM67093Spongipellis_litschau
GGTTGTCCGAGTTGTAGTCTGGAGAAGCGTCTTCCGCGCTGGACCGTGTA
CFMRccFP59199TS_unicolor
GGTTGTCCGAGTTGTAGTCTGGAGAAGCGTCTTCCGCGCTGGACCGTGTA
CFMRccFP71791TS_unicolor
GGTTGTCCGAGTTGTAGTCTGGAGAAGCGTCTTCCGCGCTGGACCGTGTA
Dai13845P_lischaueri
GGTTGTCCGAGTTGTAGTCTGGAGAAGCGTCTTCCGCGCTGGACCGTGTA
Dai20266P_lischaueri
GGTTGTCCGAGTTGTAGTCTGGAGAAGCGTCTTCCGCGCTGGACCGTGTA
CFMR_HHB11240Radulodon_america --
RLG6350Radulodon_americanus --
KY415963Radulodon_erikssonii
GGCTGTCCGAGTTGTAGTCTGGAGAAGCGTCTTCCGCGCTGGACCGTGTA
HHB9567spRadulodon_casearius
GGCTGTCCGAGTTGTAGTCTGGAGAAGCGTCTTCCGCGCTGGACCGTGTA
KRT_Iso_26Radulodon_casearius --
CBS126044Radulodon_erikssonii
GGCTGTCCGAGTTGTAGTCTGGAGAAGCGTCTTCCGCGCTGGACCGTGTA
He6183YUNNANENSIS
GGCTGTCCGAGTTGTAGTCTGGAGAAGCGTCTTCCGCGCTGGACCGTGTA
Cui17979YUNNANENSIS
GGCTGTCCGAGTTGTAGTCTGGAGAAGCGTCTTCCGCGCTGGACCGTGTA
Miettinen2091Junghuhnia_fimbri
GGCTGTCCGAGTTGTAGTCTGGAGAAGCGCTTTCTGTGCTGGACCGTGTA
KHL12316S_tenue
GGTTGTCCGAGTTGTAGTCTGGAGAAGCGTTTTCTGTGCTGGACCGTGTA
PRM846564S_pachyodon
GGCTGTCCGAGTTGTAGTCTGGAGAAGTGTTTTCCGCGCCGGACCGTGTA
SP_Lgt_S_pachyodon --
Ryvarden44669Tyromyces_xuchile
GGCTGTCCGAGTTGTAGTCTGGAGAAGTGTTTTCCGCGCCGGACCGTGTA
PW17_171sinuosus --
W53Dai12234
GGCTGTCCGAGTTGTAGTCTGGAGAAGCGTTTTCCGTGCCGGACCGTGTA
HHB4100SpAntella_americana
GGTTGTCCGAGTTGTAGTCTGGAGAAGCGTTTTCTGTGCTGGACCGTGTA
W3Dai20901spumeus
GGCCGTCCGAGTTGTAGTCTGGAGAAGTGCTTTCCGCGCCGGACCGTGTA
He6736

GGCCGTCCGAGTTGTAGTCTGGAGAAGTGCTTTCCGCGCCGGACCGTGTA
BRNM734877S_spumeus
GGCCGTCCGAGTTGTAGTCTGGAGAAGTGCTTTCCGCGCCGGACCGTGTA
BRNM712630S_spumeus
GGCCGTCCGAGTTGTAGTCTGGAGAAGTGCTTTCCGCGCCGGACCGTGTA
Dai1723Loweomyces_sibiricus --
W54Cui10009
GGCCGTCCGAGTTGTAGTCTGGAGAAGTGCTTTCCGCGCTGGACCGTGTA
W1Dai20899 --
HHB13445Trametes_ochracea
GGCCGTCCGAGTTGTAGTCTGGAGAAGCGTCTTCCGCGTTGGACCGTGTA
Dai16222
TGTTGCCCGAGTTGTAGTCTGGAGAAGCGTTTTCTGTGCTGGACCGTGTA
Dai16240
TGTTGCCCGAGTTGTAGTCTGGAGAAGCGTTTTCTGTGCTGGACCGTGTA

JV1310_11SanguinolentusCernys
CAAGTCTCTTGGAATAGAGCGTCATAGAGGGTGAGAATCCCGTCTTTGAC
MJ39_00_SK
CAAGTCTCTTGGAATAGAGCGTCATAGAGGGTGAGAATCCCGTCTTTGAC
MJ111_04_CZ --
JV1610_BOKYsmrk
CAAGTCTCTTGGAATAGAGCGTCATAGAGGGTGAGAATCCCGTCTTTGAC
Dai21030 --
Dai20976P_furcatus
CAAGTCTCTTGGAATAGAGCGTCATAGAGGGTGAGAATCCCGTCTTTGAC
Dai2105
CAAGTCTCTTGGAATAGAGCGTCATAGAGGGTGAGAATCCCGTCTTTGAC
Dai2544
CAAGTCTCTTGGAATAGAGCGTCATAGAGGGTGAGAATCCCGTCTTTGAC
Dai11313
CAAGTCTCTTGGAATAGAGCGTCATAGAGGGTGAGAATCCCGTCTTTGAC
WCG1611Dai26167
CAAGTCTCTTGGAATAGAGCGTCATAGAGGGTGAGAATCCCGTCTTTGAC
WCG1518Dai25999Physisporinus
CAAGTCTCTTGGAATAGAGCGTCATAGAGGGTGAGAATCCCGTCTTTGAC
TAA15097
CAAGTCTCTTGGAATAGAGCGTCATAGAGGGTGAGAATCCCGTCTTTGAC
JV8909_19_CZ --
JV1310_15_P_sanguinolentus2_CZ
CAAGTCTCTTGGAATAGAGCGTCATAGAGGGTGAGAATCCCGTCTTTGAC
MJ53_02_CZ
CAAGTCTCTTGGAATAGAGCGTCATAGAGGGTGAGAATCCCGTCTTTGAC
CLZhao21647P_yunnanensis

CAAGTCTCTTGGAATAGAGCGTCATAGAGGGTGAGAATCCCGTCTTTGAC
CLZhao21583P_yunnanensis
CAAGTCTCTTGGAATAGAGCGTCATAGAGGGTGAGAATCCCGTCTTTGAC
Dai22272
CAAGTCTCTTGGAATAGAGCGTCATAGAGGGTGAGAATCCCGTCTTTGAC
Dai22279
CAAGTCTCTTGGAATAGAGCGTCATAGAGGGTGAGAATCCCGTCTTTGAC
MJ332_94_CZ --
MJ642_94_CZ_Expallescens --
Dai21060P_vinctus
CAAGTCTCTTGGAATAGAGCGTCATAGAGGGTGAGAATCCCGTCTTTGAC
JV0511_23LRP_pouzarii
CAAGTCTCTTGGAATAGAGCGTCATAGAGGGTGAGAATCCCGTCTTTGAC
JQ409462_R_pouzarii_PRM899856_ --
JV0308_66_WA --
JV0309_45_WA_USA --
JV0709_83_CA_USA
CAAGTCTCTTGGAATAGAGCGTCATAGAGGGTGAGAATCCCGTCTTTGAC
Dai21043P_pouzarii
CAAGTCTCTTGGAATAGAGCGTCATAGAGGGTGAGAATCCCGTCTTTGAC
MJ144_95_CZ
CAAGTCTCTTGGAATAGAGCGTCATAGAGGGTGAGAATCCCGTCTTTGAC
JV0909_3_CZ
CAAGTCTCTTGGAATAGAGCGTCATAGAGGGTGAGAATCCCGTCTCTGAC
JV0609_1_K --
Dai20396Physisporinus_castanop
CAAGTCTCTTGGAATAGAGCGTCATAGAGGGTGAGAATCCCGTCTTTGAC
Dai20397Physisporinus_castanop
CAAGTCTCTTGGAATAGAGCGTCATAGAGGGTGAGAATCCCGTCTTTGAC
MJ19_09_SK_Abies
CAAGTCTCTTGGAATAGAGCGTCATAGAGGGTGAGAATCCCGTCTCTGAC
JV0509_40_J_TN_USA_Betula --
JV0808_33crocatus_PA_USAlist
CAAGTCTCTTGGAATAGAGCGTCATAGAGGGTGAGAATCCCGTCTTTGAC
DLL2009_061P_crocatus --
Dai12800P_subcrocatus
CAAGTCTCTTGGAATAGAGCGTCATAGAGGGTGAGAATCCCGTCTTTGAC
Dai15917P_subcrocatus
CAAGTCTCTTGGAATAGAGCGTCATAGAGGGTGAGAATCCCGTCTTTGAC
Cui16620
CAAGTCTCTTGGAATAGAGCGTCATAGAGGGTGAGAATCCCGTCTTTGAC
HCFC1088Meripilus_stillicidior
CAAGTCTCTTGGAATAGAGCGTCATAGAGGGTGAGAATCCCGTCTTTGAC
MCW590Meripilus_obsrcurus --

MCW722Meripilus_obsrcurus --
Cui9381P_tibeticus
CAAGTCTCTTGGAATAGAGCGTCATAGAGGGTGAGAATCCCGTCTTTGAC
Cui9588P_tibeticus
CAAGTCTCTTGGAATAGAGCGTCATAGAGGGTGAGAATCCCGTCTTTGAC
Va2_Beneschova --
CWU3874_Ukraine_Alnus
CAAGTCTCTTGGAATAGAGCGTCATAGAGGGTGAGAATCCCGTCTTTGAC
WCG1293Dai24718Physisporinus --
WCG1268Dai24682A
CAAGTCTCTTGGAATAGAGCGTCATAGAGGGTGAGAATCCCGTCTTTGAC
WCG1269Dai24683A
CAAGTCTCTTGGAATAGAGCGTCATAGAGGGTGAGAATCCCGTCTTTGAC
WCG1279Dai24694A
CAAGTCTCTTGGAATAGAGCGTCATAGAGGGTGAGAATCCCGTCTTTGAC
Dai16971
CAAGTCTCTTGGAATAGAGCGTCATAGAGGGTGAGAATCCCGTCTTTGAC
ZQY1043Dai26696
CAAGTCTCTTGGAATAGAGCGTCATAGAGGGTGAGAATCCCGTCTTTGAC
Doll880 --
Doll1000
CAAGTCTCTTGGAATAGAGCGTCATAGAGGGTGAGAATCCCGTCTTTGAC
1DAI18529
CAAGTCTCTTGGAATAGAGCGTCATAGAGGGTGAGAATCCCGTCTTTGAC
Dai19535
CAAGTCTCTTGGAATAGAGCGTCATAGAGGGTGAGAATCCCGTCTTTGAC
1704_79_hnedyVillaLaPaz
CAAGTCTCTTGGAATAGAGCGTCATAGAGGGTGAGAATCCCGTCTTTGAC
F2061 --
1DAI18268
CAAGTCTCTTGGAATAGAGCGTCATAGAGGGTGAGAATCCCGTCTTTGAC
1DAI18540A
CAAGTCTCTTGGAATAGAGCGTCATAGAGGGTGAGAATCCCGTCTTTGAC
Dai17695
CAAGTCTCTTGGAATAGAGCGTCATAGAGGGTGAGAATCCCGTCTTTGAC
LKY18Dai26373 --
Dai17839P_sulphureus
CAAGTCTCTTGGAATAGAGCGTCATAGAGGGTGAGAATCCCGTCTTTGAC
Dai17841P_sulphureus
CAAGTCTCTTGGAATAGAGCGTCATAGAGGGTGAGAATCCCGTCTTTGAC
Dai19877P_roseus
CAAGTCTCTTGGAATAGGGCGTCATAGAGGGTGAGAATCCCGTCTTTGAC
1508_18_1_Kout --
KP859303_R_vinctus_RP185_BRAZI --

JK1807_15Rigidoporus_sp_Puerto
CAAGTCTCCTGGAATGGAGCGTCATAGAGGGTGAGAATCCCGTCTTTGAC
JV0509_47_J_TN_USA
CAAGTCTCTTGGAATAGAGCGTCATAGAGGGTGAGAATCCCGTCTTTGAC
JV0709_188
CAAGTCTCTTGGAATAGAGCGTCATAGAGGGTGAGAATCCCGTCTTTGAC
JV0509_127_PA_USA
CAAGTCTCTTGGAATAGAGCGTCATAGAGGGTGAGAATCCCGTCTTTGAC
JV1009_59_NJ_USA
CAAGTCTCTTGGAATAGAGCGTCATAGAGGGTGAGAATCCCGTCTTTGAC
Dai15497P_crataegi
CAAGTCTCTTGGAATAGAGCGTCATAGAGGGTGAGAATCCCGTCTTTGAC
Dai15499P_crataegi
CAAGTCTCTTGGAATAGAGCGTCATAGAGGGTGAGAATCCCGTCTTTGAC
Cui3266P_cinereus
CAAGTCTCTTGGAATAGAGCGTCATAGAGGGTGAGAATCCCGTCTTTGAC
WCG1256Dai24690
CAAGTCTCTTGGAATAGAGCGTCATAGAGGGTGAGAATCCCGTCTTTGAC
1DAI17581
CAAGTCTCTTGGAATAGAGCGTCATAGAGGGTGAGAATCCCGTCTTTGAC
WCG1255Dai24688
CAAGTCTCTTGGAATAGAGCGTCATAGAGGGTGAGAATCCCGTCTTTGAC
Dai22427
CAAGTCTCTTGGAATAGAGCGTCATAGAGGGTGAGAATCCCGTCTTTGAC
MV690Meripilus_concrescens
CAAGTCTCTTGGAATAGAGCGTCATAGAGGGTGAGAATCCCGTCTTTGAC
MV513Meripilus_galapagensis
CAAGTCTCTTGGAATAGGGCGTCATAGAGGGTGAGAATCCCGTCTTTGAC
Dai19793
CAAGTCTCTTGGAATAGAGCGTCATAGAGGGTGAGAATCCCGTCTTTGAC
OQ553780P_tamilnaduensis
CAAGTCTCTTGGAATAGAGCGTCATAGAGGGTGAGAATCCCGTCTTTGAC
OQ553779P_tamilnaduensis
CAAGTCTCTTGGAATAGAGCGTCATAGAGGGTGAGAATCCCGTCTTTGAC
A164FB3Meripilus_giganteus --
JV1407_36_Vinctus_Meandrica
CAAGTCTCTTGGAATAGAGCGTCRTAGAGGGTGAGAATCCCGTCTTTGAC
1807_3K_Rigidoporus_PuertoRico
CAAGTCTCTTGGAATAGAGCGTCATAGAGGGTGAGAATCCCGTCTTTGAC
Cui16903P_vinctus
CAAGTCTCTTGGAATAGAGCGTCATAGAGGGTGAGAATCCCGTCTTTGAC
JV1008_18R_Lineatus
CAAGTCTCTTGGAATAGAGCGTCATAGAGGGTGAGAATCCCGTCTTTGAC
JV1407_37_1_Vinctus_Carara

CAAGTCTCTTGGAATAGAGCGTCATAGAGGGTGAGAATCCCGTCTTTGAC
Dai17986P_lineatus
CAAGTCTCTTGGAATAGAGCGTCATAGAGGGTGAGAATCCCGTCTTTGAC
Dai18281
CAAGTCTCTTGGAATAGAGCGTCATAGAGGGTGAGAATCCCGTCTTTGAC
1DAI19796
CAAGTCTCTTGGAATAGAGCGTCATAGAGGGTGAGAATCCCGTCTTTGAC
ZQY797Dai25241
CAAGTCTCTTGGAATAGAGCGTCATAGAGGGTGAGAATCCCGTCTTTGAC
WCG1289Dai24711 --
Dai22598
CAAGTCTCTTGGAATAGAGCGTCATAGAGGGTGAGAATCCCGTCTTTGAC
Dai20523
CAAGTCTCTTGGAATAGAGCGTCATAGAGGGTGAGAATCCCGTCTTTGAC
Dai17885
CAAGTCTCTTGGAATAGAGCGTCATAGAGGGTGAGAATCCCGTCTTTGAC
Dai17553
CAAGTCTCTTGGAATAGAGCGTCATAGAGGGTGAGAATCCCGTCTTTGAC
Dai19639
CAAGTCTCTTGGAATAGAGCGTCATAGAGGGTGAGAATCCCGTCTTTGAC
JV0110_48_CZ
CAAGTCTCTTGGAACAGAGCGTCATAGAGGGTGAGAATCCCGTCTTTGAC
MJ129_04
CAAGTCTCTTGGAACAGAGCGTCATAGAGGGTGAGAATCCCGTCTTTGAC
Cui10340P_eminens --
Cui10341P_eminens
CAAGTCTCTTGGAACAGAGCGTCATAGAGGGTGAGAATCCCGTCTTTGAC
Dai12685P_eminens
CAAGTCTCTTGGAACAGAGCGTCATAGAGGGTGAGAATCCCGTCTTTGAC
Miettinen_13591Rigidoporus_und
CAAGTCTCTTGGAACAGAGCGTCATAGAGGGTGAGAATCCCGTCTTTGAC
Dai20868
CAAGTCTCTTGGAACAGAGCATCATAGAGGGTGAGAATCCCGTCTTTGAC
Dai20832
CAAGTCTCTTGGAACAGAGCGTCATAGAGGGTGAGAATCCCGTCTTTGAC
Dai11400
CAAGTCTCTTGGAACAGAGCGTCATAGAGGGTGAGAATCCCGTCTTTGAC
Dai22472
CAAGTCTCTTGGAACAGAGCGTCATAGAGGGTGAGAATCCCGTCTTTGAC
1CUI10475
CAAGTCTCTTGGAACAGAGCGTCATAGAGGGTGAGAATCCCGTCTTTGAC
1CUI10491 --
HCFC1095Meripilus_robledoi
CAAGTCTCTTGGAACAGAGCGTCATAGAGGGTGAGAATCCCGTCTTTGAC

MCW702Meripilus_revolubilis
CAAGTCTCTTGGAACAGAGCGTCATAGAGGGTGAGAATCCCGTCTTTGAC
1704_83_zluty_HaciendaBaru
TAAGTCTCTTGGAATAGAGCGTCATAGAGGGTGAGAATCCCGTCTTTGAC
Dai9925P_lavendulus
TAAGTCTCTTGGAATAGAGCGTCACAGAGGGTGAGAATCCCGTCTTTGAC
Dai13587AP_lavendulus
TAAGTCTCTTGGAATAGAGCGTCACAGAGGGTGAGAATCCCGTCTTTGAC
PDD70600P_longicystidius --
Cui16630
TAAGTCTCTTGGAATAGAGCGTCATAGAGGGTGAGAATCCCGTCTTTGAC
FP_135344Meripilus_giganteus
CAAGTCTCTTGGAATAGAGCGTCATAGAGGGTGAGAATCCCGTCTTTGAC
FP_100460_Sp --
CBS421_48Meripilus_giganteus --
Cui9203 --
Cui9202
CAAGTCTCTTGGAATAGAGCGTCATAGAGGGTGAGAATCCCGTCTTTGAC
TUFC100564Japan
CAAGTCTCTTGGAATAGAGCGTCATAGAGGGTGAGAATCCCGTCTTTGAC
Russell5913Meripilus_sumstinei --
RP215Meripilus_brasiliensis
CAAGTCTCTTGGAATAGAGCGTCATAGAGGGTGAGAATCCCGTCTTTGAC
RP200Meripilus_brasiliensis
CAAGTCTCTTGGAATAGAGCGTCATAGAGGGTGAGAATCCCGTCTTTGAC
JV1712_13J_R_vinctus2_LSUPuert
TAAGTCCCTTGGAATAGGGCGTCATAGAGGGTGAGAATCCCGTCTTTGAC
Dai10503R_hypobrunneus
TAAGTCCCTTGGAATAGGGCGTCATAGAGGGTGAGAATCCCGTCTTTGAC
Dai10569Rigidoporus_hypobrunne
TAAGTCCCTTGGAATAGGGCGTCATAGAGGGTGAGAATCCCGTCTTTGAC
1DAI19451
TAAGTCCCTTGGAATAGGGCGTCATAGAGGGTGAGAATCCCGTCTTTGAC
CM108bRigidoporus_hypobrunneus --
1CUI16874
TAAGTCCCTTGGAATAGGGCGTCATAGAGGGTGAGAATCCCGTCTTTGAC
FD299Cerrena_unicolor
TAAGTCCCTTGGAATAGGGCGTCATAGAGGGTGAGAATCCCGTCTTTGAC
KHL_GB_Cerrena_uniclor
TAAGTCCCTTGGAATAGGGCGTCATAGAGGGTGAGAATCCCGTCTTTGAC
Dai12892Cerrena_albocinnamomea
CAAGTCCCTTGGAATAGGGCGTCATAGAGGGTGAGAATCCCGTCTTTGAC
Dai12955C_albocinnamomea
CAAGTCCCTTGGAATAGGGCGTCATAGAGGGTGAGAATCCCGTCTTTGAC

SNUm03110102C_aurantiopora
CAAGTCCCTTGGAATAGGGCGTCATAGAGGGTGAGAATCCCGTCTTTGAC
NIBRFG0000102423C_aurantiopora
CAAGTCCCTTGGAATAGGGCGTCATAGAGGGTGAGAATCCCGTCTTTGAC
Dai7359Antrodiella_zonata
CAAGTCCCTTGGAATAGGGCGTCATAGAGGGTGAGAATCCCGTCTTTGAC
F20080702KCM29C_consors
CAAGTCCCTTGGAATAGGGCGTCATAGAGGGTGAGAATCCCGTCTTTGAC
F20080208LYW10Cerrena_consors
CAAGTCCCTTGGAATAGGGCGTCATAGAGGGTGAGAATCCCGTCTTTGAC
Dai7821Antrodiella_zonata
CAAGTCCCTTGGAATAGGGCGTCATAGAGGGTGAGAATCCCGTCTTTGAC
CFMR_DCL04_31Pseudolagarobasid --
VPB197Pseudolagarobasidium_bel --
CBS115543Pseudolagarobasidium_ --
CBS115544 --
Han405Pseudolagarobasidium_bai
TAAGTTCCTTGGAATAGGGCGTCATAGAGGGTGAGAATCCCGTCTTTGAC
Han406Pseudolagarobasidium_bai
TAAGTTCCTTGGAATAGGGCGTCATAGAGGGTGAGAATCCCGTCTTTGAC
MUcc838Spongipellis_delectans
CAAGTCCCTTGGAATAGGGCGTCATAGAGGGTGAGAATCCCGTCTTTGAC
BRNM686401S_delectans
CAAGTCCCTTGGAATAGGGCGTCATAGAGGGTGAGAATCCCGTCTTTGAC
OSM_F925S_delectans
CAAGTCCCTTGGAATAGGGCGTCATAGAGGGTGAGAATCCCGTCTTTGAC
BRNM67093Spongipellis_litschau
CAAGTCCCTTGGAATAGGGCGTCATAGAGGGTGAGAATCCCGTCTTTGAC
CFMRccFP59199TS_unicolor
CAAGTCCCTTGGAATAGGGCGTCATAGAGGGTGAGAATCCCGTCTTTGAC
CFMRccFP71791TS_unicolor
CAAGTCCCTTGGAATAGGGCGTCATAGAGGGTGAGAATCCCGTCTTTGAC
Dai13845P_lischaueri
CAAGTCCCTTGGAATAGGGCGTCATAGAGGGTGAGAATCCCGTCTTTGAC
Dai20266P_lischaueri
CAAGTCCCTTGGAATAGGGCGTCATAGAGGGTGAGAATCCCGTCTTTGAC
CFMR_HHB11240Radulodon_america --
RLG6350Radulodon_americanus --
KY415963Radulodon_erikssonii
AAAGTCCCCTGGAATGGGGCCTCATAGAGGGTGAGAAGCCCGTCTTTGAC
HHB9567spRadulodon_casearius
AAAGTCCCCTGGAATGGGGCCTCATAGAGGGTGAGAAGCCCGTCTTTGAC
KRT_Iso_26Radulodon_casearius --
CBS126044Radulodon_erikssonii

AAAGTCCCCTGGAATGGGGCCTCATAGAGGGTGAGAAGCCCGTCTTTGAC
He6183YUNNANENSIS
AAAGTCCCCTGGAATGGGGCCTCATAGAGGGTGAGAAGCCCGTCTTTGAC
Cui17979YUNNANENSIS
AAAGTCCCCTGGAATGGGGCCTCATAGAGGGTGAGAAGCCCGTCTTTGAC
Miettinen2091Junghuhnia_fimbri
CAAGTCTCTTGGAATAGAGCGTCATAGAGGGTGAGAATCCCGTCTTTGAC
KHL12316S_tenue
CAAGTCTCTTGGAATAGAGCGTCATAGAGGGTGAGAATCCCGTCTTTGAC
PRM846564S_pachyodon
TAAGTCCCCTGGAATGGGGCGTCGTAGAGGGTGACAATCCCGTCTTTGAC
SP_Lgt_S_pachyodon --
Ryvarden44669Tyromyces_xuchile
TAAGTCCCCTGGAATGGGGCGTCATAGAGGGTGACAATCCCGTCTTTGAC
PW17_171sinuosus --
W53Dai12234
TAAGTCCCCTGGAATGGGGCGTCATAGAGGGTGACAATCCCGTCTTTGAC
HHB4100SpAntella_americana
CAAGTCTCTTGGAACAGAGCGTCGTAGAGGGTGAGAATCCCGTCTTTGAC
W3Dai20901spumeus
CAAGTCTCTTGGAACAGAGCGTCATAGAGGGTGAGAATCCCGTCTTTGAC
He6736
CAAGTCTCTTGGAACAGAGCGTCATAGAGGGTGAGAATCCCGTCTTTGAC
BRNM734877S_spumeus
CAAGTCTCTTGGAACAGAGCGTCATAGAGGGTGAGAATCCCGTCTTTGAC
BRNM712630S_spumeus
CAAGTCTCTTGGAACAGAGCGTCATAGAGGGTGAGAATCCCGTCTTTGAC
Dai1723Loweomyces_sibiricus --
W54Cui10009
CAAGTCTCTTGGAACAGAGCGTCATAGAGGGTGAGAATCCCGTCTTTGAC
W1Dai20899 --
HHB13445Trametes_ochracea
CAAGTCTCTTGGAACAGAGCGTCATAGAGGGTGAGAATCCCGTCTTTGAC
Dai16222
CAAGTCTCTTGGAACAGAGCGTCGTAGAGGGTGAGAATCCCGTCTTTGAC
Dai16240
CAAGTCTCTTGGAACAGAGCGTCGTAGAGGGTGAGAATCCCGTCTTTGAC

JV1310_11SanguinolentusCernys ACGGACTACCGGTGC-
TTTGTGATGCGCTTTCAAAGAGTCGAGTTGTTTG
MJ39_00_SK ACGGACTACCGGTGC-
TTTGTGATGCGCTTTCAAAGAGTCGAGTTGTTTG
MJ111_04_CZ --
JV1610_BOKYsmrk ACGGACTACCGGTGC-

TTTGTGATGCGCTTTCAAAGAGTCGAGTTGTTTG
Dai21030 ---
Dai20976P_furcatus ACGGACTACCGGTGC-
TTTGTGATGCGCTTTCAAAGAGTCGAGTTGTTTG
Dai2105 ACGGACTACCGGTGC-
TTTGTGATGCGCTTTCAAAGAGTCGAGTTGTTTG
Dai2544 ACGGACTACCGGTGC-
TTTGTGATGCGCTTTCAAAGAGTCGAGTTGTTTG
Dai11313 ACGGACTACTGGTGC-
TTTGTGATGCGCTTTCAAAGAGTCGAGTTGTTTG
WCG1611Dai26167 ACGGACTACCGGTGC-
TTTGTGATGCGCTTTCAAAGAGTCGAGTTGTTTG
WCG1518Dai25999Physisporinus ACGGACTACCGGTGC-
TTTGTGATGCGCTTTCAAAGAGTCGAGTTGTTTG
TAA15097 ACGGACTACCGGTGC-
TTTGTGATGCGCTTTCAAAGAGTCGAGTTGTTTG
JV8909_19_CZ ---
JV1310_15_P_sanguinolentus2_CZ ACGGACTACCGGTGC-
TTTGTGATGCGCTTTCAAAGAGTCGAGTTGTTTG
MJ53_02_CZ ACGGACTACCGGTGC-
TTTGTGATGCGCTTTCAAAGAGTCGAGTTGTTTG
CLZhao21647P_yunnanensis ACGGACTACCGGTGC-
TTTGTGATGCGCTTTCAAAGAGTCGAGTTGTTTG
CLZhao21583P_yunnanensis ACGGACTACCGGTGC-
TTTGTGATGCGCTTTCAAAGAGTCGAGTTGTTTG
Dai22272 ACGGACTACCGGTGC-
TTTGTGATGTGCTTTCAAAGAGTCGAGTTGTTTG
Dai22279 ACGGACTACCGGTGC-
TTTGTGATGTGCTTTCAAAGAGTCGAGTTGTTTG
MJ332_94_CZ ---
MJ642_94_CZ_Expallescens ---
Dai21060P_vinctus ACGGACTACCAGTGC-
TTTGTGATGTGCTTTCAAAGAGTCGAGTTGTTTG
JV0511_23LRP_pouzarii ACGGACTACCAGTGC-
TTTGTGATGTGCTTTCAAAGAGTCGAGTTGTTTG
JQ409462_R_pouzarii_PRM899856_ ---
JV0308_66_WA ---
JV0309_45_WA_USA ---
JV0709_83_CA_USA ACGGACTACCAGTGC-
TTTGTGATGTGCTTTCAAAGAGTCGAGTTGTTTG
Dai21043P_pouzarii ACGGACTACCAGTGC-
TTTGTGATGTGCTTTCAAAGAGTCGAGTTGTTTG
MJ144_95_CZ ACGGACTACCAGTGC-
TTTGTGATGTGCTTTCAAAGAGTCGAGTTGTTTG

JV0909_3_CZ ACGGACTACCAGTGC-
TATGTGATGTGCTTTCAAAGAGTCGAGTTGTTTG
JV0609_1_K --
Dai20396Physisporinus_castanop ACGGACTACCAGTGC-
TTTGTGATGCGCTTTCAAAGAGTCGAGTTGTTTG
Dai20397Physisporinus_castanop ACGGACTACCAGTGC-
TTTGTGATGCGCTTTCAAAGAGTCGAGTTGTTTG
MJ19_09_SK_Abies ACGGACTACCAGTGC-
TTTGTGATGTGCTTTCAAAGAGTCGAGTTGTTTG
JV0509_40_J_TN_USA_Betula --
JV0808_33crocatus_PA_USAlist ACGGACTACCAGTGC-
TTTGTGATGTGCTTTCAAAGAGTCGAGTTGTTTG
DLL2009_061P_crocatus --
Dai12800P_subcrocatus ACGGACTACCAGTGC-
TTTGTGATGTGCTTTCAAAGAGTCGAGTTGTTTG
Dai15917P_subcrocatus ACGGACTACCAGTGC-
TTTGTGATGTGCTTTCAAAGAGTCGAGTTGTTTG
Cui16620 ACGGACTACCAGTGC-
TTTGTGATGTGCTTTCAAAGAGTCGAGTTGTTTG
HCFC1088Meripilus_stillicidior ACGGACTACCAGTGC-
TTTGTGATGTGCTTTCAAAGAGTCGAGTTGTTTG
MCW590Meripilus_obsrcurus --
MCW722Meripilus_obsrcurus --
Cui9381P_tibeticus ACGGACTACCAGTGC-
TTTGTGATGTGCTTTCAAAGAGTCGAGTTGTTTG
Cui9588P_tibeticus ACGGACTACCAGTGC-
TTTGTGATGTGCTTTCAAAGAGTCGAGTTGTTTG
Va2_Beneschova --
CWU3874_Ukraine_Alnus ACGGACTACCAGTGC-
TTTGTGATGTGCTTTCAAAGAGTCGAGTTGTTTG
WCG1293Dai24718Physisporinus --
WCG1268Dai24682A ACGGACTACCAGTGC-
TTTGTGATGCGCTTTCAAAGAGTCGAGTTGTTTG
WCG1269Dai24683A ACGGACTACCAGTGC-
TTTGTGATGCGCTTTCAAAGAGTCGAGTTGTTTG
WCG1279Dai24694A ACGGACTACCAGTGC-
TTTGTGATGCGCTTTCAAAGAGTCGAGTTGTTTG
Dai16971 ACGGACTACCAGTGC-
TTTGTGATGCGCTTTCAAAGAGTCGAGTTGTTTG
ZQY1043Dai26696 ACGGACTACCAGTGC-
TTTGTGATGCGCTTTCAAAGAGTCGAGTTGTTTG
Doll880 --
Doll1000 ACGGACTACCAGTGC-
TTTGTGATGCGCTTTCAAAGAGTCGAGTTGTTTG

1DAI18529 ACGGACTACCAGTGC-
TTTGTGATGCGCTTTCAAAGAGTCGAGTTGTTTG
Dai19535 ACGGACTACCGGTGC-
TTTGTGATGCGCTTTCAAAGAGTCGAGTTGTTTG
1704_79_hnedyVillaLaPaz ACGGACTGCCAGTGC-
TTTGTGATGCGCTTTCAAAGAGTCGAGTTGTTTG
F2061 --
1DAI18268 ACGGACCGCCAGTGC-
TTTGTGATGCGCTTTCAAAGAGTCGAGTTGTTTG
1DAI18540A ACGGACCGCCAGTGC-
TTTGTGATGCGCTTTCAAAGAGTCGAGTTGTTTG
Dai17695 ACGGACCGCCAGTGC-
TTTGTGATGCGCTTTCAAAGAGTCGAGTTGTTTG
LKY18Dai26373 --
Dai17839P_sulphureus ACGGACTACCGGTGC-
TTTGTGATGCGCTTTCAAAGAGTCGAGTTGTTTG
Dai17841P_sulphureus ACGGACTACCGGTGC-
TTTGTGATGCGCTTTCAAAGAGTCGAGTTGTTTG
Dai19877P_roseus ACGGACTACCGGTGC-
TTTGTGATGCGCTTTCAAAGAGTCGAGTTGTTTG
1508_18_1_Kout --
KP859303_R_vinctus_RP185_BRAZI --
JK1807_15Rigidoporus_sp_Puerto ACGGACTACCAGTGC-
TTTGTGATGTGCTTTCAAAGAGTCGAGTTGTTTG
JV0509_47_J_TN_USA ACGGACTACCAGTGC-
TTTGTGATGCGCTTTCAAAGAGTCGAGTTGTTTG
JV0709_188 ACGGACTACCAGTGC-
TTTGTGATGCGCTTTCAAAGAGTCGAGTTGTTTG
JV0509_127_PA_USA ACGGACTACCAGTGC-
TTTGTGATGCGCTTTCAAAGAGTCGAGTTGTTTG
JV1009_59_NJ_USA ACGGACTACCAGTGC-
TTTGTGATGCGCTTTCAAAGAGTCGAGTTGTTTG
Dai15497P_crataegi ACGGACTACCAGTGC-
TTTGTGATGCGCTTTCAAAGAGTCGAGTTGTTTG
Dai15499P_crataegi ACGGACTACCAGTGC-
TTTGTGATGCGCTTTCAAAGAGTCGAGTTGTTTG
Cui3266P_cinereus ACGGACTACCAGTGC-
TTTGTGATGCGCTTTCAAAGAGTCGAGTTGTTTG
WCG1256Dai24690 ACGGACTACCAGTGC-
TTTGTGATGCGCTTTCAAAGAGTCGAGTTGTTTG
1DAI17581 ACGGACTACCAGTGC-
TTTGTGATGCGCTTTCAAAGAGTCGAGTTGTTTG
WCG1255Dai24688 ACGGACTACCAGTGC-
TTTGTGATGCGCTTTCAAAGAGTCGAGTTGTTTG

Dai22427 ACGGACTACCAGTGC-TTTGTGATGCGCTTTCAAAGAGTCGAGTTGTTTG
MV690Meripilus_concrescens ACGGACTACCGGTGC-TTTGTGATGCGCTTTCAAAGAGTCGAGTTGTTTG
MV513Meripilus_galapagensis ACGGACTACCGGTGC-TTTGTGATGCGCTTTCAAAGAGTCGAGTTGTTTG
Dai19793 ACGGACTACCAGTGC-TTTGTGATGCGCTTTCAAAGAGTCGAGTTGTTTG
OQ553780P_tamilnaduensis ACGGACTACCAGTGC-TTTGTGATGCGCTTTCAAAGAGTCGAGTTGTTTG
OQ553779P_tamilnaduensis ACGGACTACCAGTGC-TTTGTGATGCGCTTTCAAAGAGTCGAGTTGTTTG
A164FB3Meripilus_giganteus --
JV1407_36_Vinctus_Meandrica ACGGACTACCAGTGC-TTTGTGATGCGCTTTCAAAGAGTCGAGTTGTTTG
1807_3K_Rigidoporus_PuertoRico ACGGACTACCAGTGC-TTTGTGATGCGCTTTCAAAGAGTCGAGTTGTTTG
Cui16903P_vinctus ACGGACTACCAGTGC-TTTGTGATGCGCTTTCAAAGAGTCGAGTTGTTTG
JV1008_18R_Lineatus ACGGACTACCAGTGC-TTTGTGATGCGCTTTCAAAGAGTCGAGTTGTTTG
JV1407_37_1_Vinctus_Carara ACGGACTACCAGTGC-TTTGTGATGCGCTTTCAAAGAGTCGAGTTGTTTG
Dai17986P_lineatus ACGGACTACCAGTGC-TTTGTGATGCGCTTTCAAAGAGTCGAGTTGTTTG
Dai18281 ACGGACTACCAGTGC-TTTGTGATGCGCTTTCAAAGAGTCGAGTTGTTTG
1DAI19796 ACGGACTACCAGTGC-TTTGTGATGCGCTTTCAAAGAGTCGAGTTGTTTG
ZQY797Dai25241 ACGGACTACCAGTGC-TTTGTGATGCGCTTTCAAAGAGTCGAGTTGTTTG
WCG1289Dai24711 --
Dai22598 ACGGACTACCAGTGC-TTTGTGATGCGCTTTCAAAGAGTCGAGTTGTTTG
Dai20523 ACGGACTACCAGTGC-TTTGTGATGCGCTTTCAAAGAGTCGAGTTGTTTG
Dai17885 ACGGACTACCAGTGC-TTTGTGATGCGCTTTCAAAGAGTCGAGTTGTTTG
Dai17553 ACGGACTACCAGTGC-TTTGTGATGCGCTTTCAAAGAGTCGAGTTGTTTG
Dai19639 ACGGACTACCAGTGC-TTTGTGATGCGCTTTCAAAGAGTCGAGTTGTTTG
JV0110_48_CZ ACGGACTACCGGTGC-TTTGTGATGCGTTTTCAAAGAGTCGAGTTGTTTG

MJ129_04 ACGGACTACCGGTGC-
TTTGTGATGCGTTTTCAAAGAGTCGAGTTGTTTG
Cui10340P_eminens --
Cui10341P_eminens ACGGACTACCGGTGC-
TTTGTGATGCGTTTTCAAAGAGTCGAGTTGTTTG
Dai12685P_eminens ACGGACTACCGGTGC-
TTTGTGATGCGTTTTCAAAGAGTCGAGTTGTTTG
Miettinen_13591Rigidoporus_und ACGGACTACCGGTGC-
TTTGTGATGCGTTTTCAAAGAGTCGAGTTGTTTG
Dai20868 ACGGACTACCGGTGC-
TTTGTGATGCGTTTTCAAAGAGTCGAGTTGTTTG
Dai20832 ACGGACTACCGGTGC-
TTTGTGATGCGTTTTCAAAGAGTCGAGTTGTTTG
Dai11400 ACGGACTACCGGTGC-
TTTGTGATGCGTTTTCAAAGAGTCGAGTTGTTTG
Dai22472 ACGGACTACCGGTGC-
TTTGTGATGCGTTTTCAAAGAGTCGAGTTGTTTG
1CUI10475 ACGGACTACCGGTGC-
TTTGTGATGCGTTTTCAAAGAGTCGAGTTGTTTG
1CUI10491 --
HCFC1095Meripilus_robledoi ACGGACTACCGGTGC-
TTTGTGATGCGTTTTCAAAGAGTCGAGTTGTTTG
MCW702Meripilus_revolubilis ACGGACTACCGGTGC-
TTTGTGATGCGTTTTCAAAGAGTCGAGTTGTTTG
1704_83_zluty_HaciendaBaru ATGGACTACCGGTGC-
TTTGTGAGGCGCTTTCAAAGAGTCGAGTTGTTTG
Dai9925P_lavendulus ATGGACTCCCGGTGC-
TTTGCGATGCGTTTTCAAAGAGTCGAGTTGTTTG
Dai13587AP_lavendulus ATGGACTCCCGGTGC-
TTTGCGATGCGTTTTCAAAGAGTCGAGTTGTTTG
PDD70600P_longicystidius --
Cui16630 ATGGACTCCCGGTGC-
TTTGCGATGCGCTTTCAAAGAGTCGAGTTGTTTG
FP_135344Meripilus_giganteus ACGGACCGCCGGTGC-
TTTGCGATGCGCTTTCAAAGAGTCGAGTTGTTTG
FP_100460_Sp --
CBS421_48Meripilus_giganteus --
Cui9203 --
Cui9202 ACGGACCGCCGGTGC-
TTTGCGATGCGCTTTCAAAGAGTCGAGTTGTTTG
TUFC100564Japan ACGGACCGCCGGTGC-
TTTGCGATGCGCTTTCAAAGAGTCGAGTTGTTTG
Russell5913Meripilus_sumstinei --
RP215Meripilus_brasiliensis ACGGACCGCCAGTGC-

TTTGCGATGCGCTTTCAAAGAGTCGAGTTGTTTG
RP200Meripilus_brasiliensis ACGGACCGCCAGTGC-
TTTGCGATGCGCTTTCAAAGAGTCGAGTTGTTTG
JV1712_13J_R_vinctus2_LSUPuert ACGGACTACCAGTGC-
TTTGTGATGCGCTCTCAAAGAGTCGAGTTGTTTG
Dai10503R_hypobrunneus ACGGACTACCAGTGC-
TTTGTGATGCGCTCTCAAAGAGTCGAGTTGTTTG
Dai10569Rigidoporus_hypobrunne ACGGACTACCAGTGC-
TTTGTGATGCGCTCTCAAAGAGTCGAGTTGTTTG
1DAI19451 ACGGACTACCAGTGC-
TTTGTGATGCGCTCTCAAAGAGTCGAGTTGTTTG
CM108bRigidoporus_hypobrunneus ---
1CUI16874 ACGGACTACCAGTGC-
TTTGTGATGCGCTCTCAAAGAGTCGAGTTGTTTG
FD299Cerrena_unicolor ACGGACTACCAGTGC-
TTTGTGATGCGCTCTCAAAGAGTCGAGTTGTTTG
KHL_GB_Cerrena_uniclor ACGGACTACCAGTGC-
TTTGTGATGCGCTCTCAAAGAGTCGAGTTGTTTG
Dai12892Cerrena_albocinnamomea ACGGACTACCAGTGC-
TTTGTGATGCGCTCTCAAAGAGTCGAGTTGTTTG
Dai12955C_albocinnamomea ACGGACTACCAGTGC-
TTTGTGATGCGCTCTCAAAGAGTCGAGTTGTTTG
SNUm03110102C_aurantiopora ACGGACTACCAGTGC-
TTTGTGATGCGCTCTCAAAGAGTCGAGTTGTTTG
NIBRFG0000102423C_aurantiopora ACGGACTACCAGTGC-
TTTGTGATGCGCTCTCAAAGAGTCGAGTTGTTTG
Dai7359Antrodiella_zonata ACGGACTACCAGTGC-
TTTGTGATGCGCTCTCAAAGAGTCGAGTTGTTTG
F20080702KCM29C_consors ACGGACTACCAGTGC-
TTTGTGATGCGCTCTCAAAGAGTCGAGTTGTTTG
F20080208LYW10Cerrena_consors ACGGACTACCAGTGC-
TTTGTGATGCGCTCTCAAAGAGTCGAGTTGTTTG
Dai7821Antrodiella_zonata ACGGACTACCAGTGC-
TTTGTGATGCGCTCTCAAAGAGTCGAGTTGTTTG
CFMR_DCL04_31Pseudolagarobasid ---
VPB197Pseudolagarobasidium_bel ---
CBS115543Pseudolagarobasidium_ ---
CBS115544 ---
Han405Pseudolagarobasidium_bai ACGGACTACCAGTGC-
TCTGTGATGCGCTCTCAAAGAGTCGAGTTGTTTG
Han406Pseudolagarobasidium_bai ACGGACTACCAGTGC-
TCTGTGATGCGCTCTCAAAGAGTCGAGTTGTTTG
MUcc838Spongipellis_delectans ACGGACTACCAGTGC-
TTTGTGATGCGCTCTCAAAGAGTCGAGTTGTTTG

BRNM686401S_delectans ACGGACTACCAGTGC-
TTTGTGATGCGCTCTCAAAGAGTCGAGTTGTTTG
OSM_F925S_delectans ACGGACTACCAGTGC-
TTTGTGATGCGCTCTCAAAGAGTCGAGTTGTTTG
BRNM67093Spongipellis_litschau ACGGACTCCCAGTGC-
TTTGTGATGCGCTCTCAAAGAGTCGAGTTGTTTG
CFMRccFP59199TS_unicolor ACGGACTCCCAGTGC-
TTTGTGATGCGCTCTCAAAGAGTCGAGTTGTTTG
CFMRccFP71791TS_unicolor ACGGACTCCCAGTGC-
TTTGTGATGCGCTCTCAAAGAGTCGAGTTGTTTG
Dai13845P_lischaueri ACGGACTCCCAGTGC-
TTTGTGATGCGCTCTCAAAGAGTCGAGTTGTTTG
Dai20266P_lischaueri ACGGACTCCCAGTGC-
TTTGTGATGCGCTCTCAAAGAGTCGAGTTGTTTG
CFMR_HHB11240Radulodon_america --
RLG6350Radulodon_americanus --
KY415963Radulodon_erikssonii ATGGACTGCCAGTGC-
TTTGTGATGCGCTCTCGAAGAGTCGCGTTGTTTG
HHB9567spRadulodon_casearius
ACGGACTGCCAGTGCTTTTGTGATGCGCTCTCAAAGAGTCGCGTTGTTTG
KRT_Iso_26Radulodon_casearius --
CBS126044Radulodon_erikssonii ATGGACTGCCAGTGC-
TTTGTGATGCGCTCTCGAAGAGTCGCGTTGTTTG
He6183YUNNANENSIS ACGGACTGCCAGTGC-
TTTGTGATGCGCTCTCGAAGAGTCGCGTTGTTTG
Cui17979YUNNANENSIS ACGGACTGCCAGTGC-
TTTGTGATGCGCTCTCGAAGAGTCGCGTTGTTTG
Miettinen2091Junghuhnia_fimbri ACGGACTGCCAGTGC-
TATGTGATGCGCTCTCAAAGAGTCGAGTTGTTTG
KHL12316S_tenue ACGGACTGCCAGTGC-
TTTGTGATGCGCTCTCAAAGAGTCGAGTTGTTTG
PRM846564S_pachyodon ACGGACTCCCGGTGC-
TTTGTGATGCGCTCTCAAAGAGTCGAGTTGTTTG
SP_Lgt_S_pachyodon --
Ryvarden44669Tyromyces_xuchile ACGGACTACCGGTGC-
TTTGTGATGCGCTCTCAAAGAGTCGAGTTGTTTG
PW17_171sinuosus --
W53Dai12234 ACGGACTACCGGTGC-
TTTGTGATGCGCTCTCAAAGAGTCGAGTTGTTTG
HHB4100SpAntella_americana ACGGACTGCCAGTGC-
TTTGTGATGCGCTCTCAAAGAGTCGAGTTGTTTG
W3Dai20901spumeus ACGGACTACCGGTGC-
TTTGTGATGCGCTCTCAAAGAGTCGAGTTGTTTG
He6736 ACGGACTACCGGTGC-

TTTGTGATGCGCTCTCAAAGAGTCGAGTTGTTTG
BRNM734877S_spumeus ACGGACTACCGGTGC-
TTTGTGATGCGCTCTCAAAGAGTCGAGTTGTTTG
BRNM712630S_spumeus ACGGACTACCGGTGC-
TTTGTGATGCGCTCTCAAAGAGTCGAGTTGTTTG
Dai1723Loweomyces_sibiricus --
W54Cui10009 ACGGACTACCAGTGC-
TTTGTGATGCGCTCTCAAAGAGTCGAGTTGTTTG
W1Dai20899 --
HHB13445Trametes_ochracea ACGGACTACCAATGC-
TTTGTGATGCGCTCTCAAAGAGTCGCGTTGTTTG
Dai16222 ACGGACTGCCGGTGC-
TTTGTGATGCGCTCTCAAAGAGTCGAGTTGTTTG
Dai16240 ACGGACTGCCGGTGC-
TTTGTGATGCGCTCTCAAAGAGTCGAGTTGTTTG

JV1310_11SanguinolentusCernys
GGAATGCAGCTCAAAATGGGTGGTAAATTCCATCTAAAGCTAAATATTGG
MJ39_00_SK
GGAATGCAGCTCAAAATGGGTGGTAAATTCCATCTAAAGCTAAATATTGG
MJ111_04_CZ --
JV1610_BOKYsmrk
GGAATGCAGCTCAAAATGGGTGGTAAATTCCATCTAAAGCTAAATATTGG
Dai21030 --
Dai20976P_furcatus
GGAATGCAGCTCAAAATGGGTGGTAAATTCCATCTAAAGCTAAATATTGG
Dai2105
GGAATGCAGCTCAAAATGGGTGGTAAATTCCATCTAAAGCTAAATATTGG
Dai2544
GGAATGCAGCTCAAAATGGGTGGTAAATTCCATCTAAAGCTAAATATTGG
Dai11313
GGAATGCAGCTCAAAATGGGTGGTAAATTCCATCTAAAGCTAAATATTGG
WCG1611Dai26167
GGAATGCAGCTCAAAATGGGTGGTAAATTCCATCTAAAGCTAAATATTGG
WCG1518Dai25999Physisporinus
GGAATGCAGCTCAAAATGGGTGGTAAATTCCATCTAAAGCTAAATATTGG
TAA15097
GGAATGCAGCTCAAAATGGGTGGTAAATTCCATCTAAAGCTAAATATTGG
JV8909_19_CZ --
JV1310_15_P_sanguinolentus2_CZ
GGAATGCAGCTCAAAATGGGTGGTAAATTCCATCTAAAGCTAAATATTGG
MJ53_02_CZ
GGAATGCAGCTCAAAATGGGTGGTAAATTCCATCTAAAGCTAAATATTGG
CLZhao21647P_yunnanensis

GGAATGCAGCTCAAAATGGGTGGTAAATTCCATCTAAAGCTAAATATTGG
CLZhao21583P_yunnanensis
GGAATGCAGCTCAAAATGGGTGGTAAATTCCATCTAAAGCTAAATATTGG
Dai22272
GGAATGCAGCTCAAAATGGGTGGTAAATTCCATCTAAAGCTAAATATTGG
Dai22279
GGAATGCAGCTCAAAATGGGTGGTAAATTCCATCTAAAGCTAAATATTGG
MJ332_94_CZ --
MJ642_94_CZ_Expallescens --
Dai21060P_vinctus
GGAATGCAGCTCAAAATGGGTGGTAAATTCCATCTAAAGCTAAATATTGG
JV0511_23LRP_pouzarii
GGAATGCAGCTCAAAATGGGTGGTAAATTCCATCTAAAGCTAAATATTGG
JQ409462_R_pouzarii_PRM899856_ --
JV0308_66_WA --
JV0309_45_WA_USA --
JV0709_83_CA_USA
GGAATGCAGCTCAAAATGGGTGGTAAATTCCATCTAAAGCTAAATATTGG
Dai21043P_pouzarii
GGAATGCAGCTCAAAATGGGTGGTAAATTCCATCTAAAGCTAAATATTGG
MJ144_95_CZ
GGAATGCAGCTCAAAATGGGTGGTAAATTCCATCTAAAGCTAAATATTGG
JV0909_3_CZ
GGAATGCAGCTCAAAATGGGTGGTAAATTCCATCTAAAGCTAAATATTGG
JV0609_1_K --
Dai20396Physisporinus_castanop
GGAATGCAGCTCAAAATGGGTGGTAAATTCCATCTAAAGCTAAATATTGG
Dai20397Physisporinus_castanop
GGAATGCAGCTCAAAATGGGTGGTAAATTCCATCTAAAGCTAAATATTGG
MJ19_09_SK_Abies
GGAATGCAGCTCAAAATGGGTGGTAAATTCCATCTAAAGCTAAATATTGG
JV0509_40_J_TN_USA_Betula --
JV0808_33crocatus_PA_USAlist
GGAATGCAGCTCAAAATGGGTGGTAAATTCCATCTAAAGCTAAATATTGG
DLL2009_061P_crocatus --
Dai12800P_subcrocatus
GGAATGCAGCTCAAAATGGGTGGTAAATTCCATCTAAAGCTAAATATTGG
Dai15917P_subcrocatus
GGAATGCAGCTCAAAATGGGTGGTAAATTCCATCTAAAGCTAAATATTGG
Cui16620
GGAATGCAGCTCAAAATGGGTGGTAAATTCCATCTAAAGCTAAATATTGG
HCFC1088Meripilus_stillicidior
GGAATGCAGCTCAAAATGGGTGGTAAATTCCATCTAAAGCTAAATATTGG
MCW590Meripilus_obsrcurus --

MCW722Meripilus_obsrcurus ---
Cui9381P_tibeticus
GGAATGCAGCTCAAAATGGGTGGTAAATTCCATCTAAAGCTAAATATTGG
Cui9588P_tibeticus
GGAATGCAGCTCAAAATGGGTGGTAAATTCCATCTAAAGCTAAATATTGG
Va2_Beneschova ---
CWU3874_Ukraine_Alnus
GGAATGCAGCTCAAAATGGGTGGTAAATTCCATCTAAAGCTAAATATTGG
WCG1293Dai24718Physisporinus ---
WCG1268Dai24682A
GGAATGCAGCTCAAAATGGGTGGTAAATTCCATCTAAAGCTAAATATTGG
WCG1269Dai24683A
GGAATGCAGCTCAAAATGGGTGGTAAATTCCATCTAAAGCTAAATATTGG
WCG1279Dai24694A
GGAATGCAGCTCAAAATGGGTGGTAAATTCCATCTAAAGCTAAATATTGG
Dai16971
GGAATGCAGCTCAAAATGGGTGGTAAATTCCATCTAAAGCTAAATATTGG
ZQY1043Dai26696
GGAATGCAGCTCAAAATGGGTGGTAAATTCCATCTAAAGCTAAATATTGG
Doll880 ---
Doll1000
GGAATGCAGCTCAAAATGGGTGGTAAATTCCATCTAAAGCTAAATATTGG
1DAI18529
GGAATGCAGCTCAAAATGGGTGGTAAATTCCATCTAAAGCTAAATATTGG
Dai19535
GGAATGCAGCTCAAAATGGGTGGTAAATTCCATCTAAAGCTAAATATTGG
1704_79_hnedyVillaLaPaz
GGAATGCAGCTCAAAATGGGTGGTAAATTCCATCTAAAGCTAAATATTGG
F2061 ---
1DAI18268
GGAATGCAGCTCAAAATGGGTGGTAAATTCCATCTAAAGCTAAATATTGG
1DAI18540A
GGAATGCAGCTCAAAATGGGTGGTAAATTCCATCTAAAGCTAAATATTGG
Dai17695
GGAATGCAGCTCAAAATGGGTGGTAAATTCCATCTAAAGCTAAATATTGG
LKY18Dai26373 ---
Dai17839P_sulphureus
GGAATGCAGCTCAAAATGGGTGGTAAATTCCATCTAAAGCTAAATATTGG
Dai17841P_sulphureus
GGAATGCAGCTCAAAATGGGTGGTAAATTCCATCTAAAGCTAAATATTGG
Dai19877P_roseus
GGAATGCAGCTCAAAATGGGTGGTAAATTCCATCTAAAGCTAAATATTGG
1508_18_1_Kout ---
KP859303_R_vinctus_RP185_BRAZI ---

JK1807_15Rigidoporus_sp_Puerto
GGAATGCAGCTCAAAATGGGTGGTAAATTCCATCTAAAGCTAAATATTGG
JV0509_47_J_TN_USA
GGAATGCAGCTCAAAATGGGTGGTAAATTCCATCTAAAGCTAAATATTGG
JV0709_188
GGAATGCAGCTCAAAATGGGTGGTAAATTCCATCTAAAGCTAAATATTGG
JV0509_127_PA_USA
GGAATGCAGCTCAAAATGGGTGGTAAATTCCATCTAAAGCTAAATATTGG
JV1009_59_NJ_USA
GGAATGCAGCTCAAAATGGGTGGTAAATTCCATCTAAAGCTAAATATTGG
Dai15497P_crataegi
GGAATGCAGCTCAAAATGGGTGGTAAATTCCATCTAAAGCTAAATATTGG
Dai15499P_crataegi
GGAATGCAGCTCAAAATGGGTGGTAAATTCCATCTAAAGCTAAATATTGG
Cui3266P_cinereus
GGAATGCAGCTCAAAATGGGTGGTAAATTCCATCTAAAGCTAAATATTGG
WCG1256Dai24690
GGAATGCAGCTCAAAATGGGTGGTAAATTCCATCTAAAGCTAAATATTGG
1DAI17581
GGAATGCAGCTCAAAATGGGTGGTAAATTCCATCTAAAGCTAAATATTGG
WCG1255Dai24688
GGAATGCAGCTCAAAATGGGTGGTAAATTCCATCTAAAGCTAAATATTGG
Dai22427
GGAATGCAGCTCAAAATGGGTGGTAAATTCCATCTAAAGCTAAATATTGG
MV690Meripilus_concrescens
GGAATGCAGCTCAAAATGGGTGGTAAATTCCATCTAAAGCTAAATATTGG
MV513Meripilus_galapagensis
GGAATGCAGCTCAAAATGGGTGGTAAATTCCATCTAAAGCTAAATATTGG
Dai19793
GGAATGCAGCTCAAAATGGGTGGTAAATTCCATCTAAAGCTAAATATTGG
OQ553780P_tamilnaduensis
GGAATGCAGCTCAAAATGGGTGGTAAATTCCATCTAAAGCTAAATATTGG
OQ553779P_tamilnaduensis
GGAATGCAGCTCAAAATGGGTGGTAAATTCCATCTAAAGCTAAATATTGG
A164FB3Meripilus_giganteus --
JV1407_36_Vinctus_Meandrica
GGAATGCAGCTCAAAATGGGTGGTAAATTCCATCTAAAGCTAAATATTGG
1807_3K_Rigidoporus_PuertoRico
GGAATGCAGCTCAAAATGGGTGGTAAATTCCATCTAAAGCTAAATATTGG
Cui16903P_vinctus
GGAATGCAGCTCAAAATGGGTGGTAAATTCCATCTAAAGCTAAATATTGG
JV1008_18R_Lineatus
GGAATGCAGCTCAAAATGGGTGGTAAATTCCATCTAAAGCTAAATATTGG
JV1407_37_1_Vinctus_Carara

GGAATGCAGCTCAAAATGGGTGGTAAATTCCATCTAAAGCTAAATATTGG
Dai17986P_lineatus
GGAATGCAGCTCAAAATGGGTGGTAAATTCCATCTAAAGCTAAATATTGG
Dai18281
GGAATGCAGCTCAAAATGGGTGGTAAATTCCATCTAAAGCTAAATATTGG
1DAI19796
GGAATGCAGCTCAAAATGGGTGGTAAATTCCATCTAAAGCTAAATATTGG
ZQY797Dai25241
GGAATGCAGCTCAAAATGGGTGGTAAATTCCATCTAAAGCTAAATATTGG
WCG1289Dai24711 --
Dai22598
GGAATGCAGCTCAAAATGGGTGGTAAATTCCATCTAAAGCTAAATATTGG
Dai20523
GGAATGCAGCTCAAAATGGGTGGTAAATTCCATCTAAAGCTAAATATTGG
Dai17885
GGAATGCAGCTCAAAATGGGTGGTAAATTCCATCTAAAGCTAAATATTGG
Dai17553
GGAATGCAGCTCAAAATGGGTGGTAAATTCCATCTAAAGCTAAATATTGG
Dai19639
GGAATGCAGCTCAAAATGGGTGGTAAATTCCATCTAAAGCTAAATATTGG
JV0110_48_CZ
GGAATGCAGCTCAAAATGGGTGGTAAATTCCATCTAAAGCTAAATATTGG
MJ129_04
GGAATGCAGCTCAAAATGGGTGGTAAATTCCATCTAAAGCTAAATATTGG
Cui10340P_eminens --
Cui10341P_eminens
GGAATGCAGCTCAAAATGGGTGGTAAATTCCATCTAAAGCTAAATATTGG
Dai12685P_eminens
GGAATGCAGCTCAAAATGGGTGGTAAATTCCATCTAAAGCTAAATATTGG
Miettinen_13591Rigidoporus_und
GGAATGCAGCTCAAAATGGGTGGTAAATTCCATCTAAAGCTAAATATTGG
Dai20868
GGAATGCAGCTCAAAATGGGTGGTAAATTCCATCTAAAGCTAAATATTGG
Dai20832
GGAATGCAGCTCAAAATGGGTGGTAAATTCCATCTAAAGCTAAATATTGG
Dai11400
GGAATGCAGCTCAAAATGGGTGGTAAATTCCATCTAAAGCTAAATATTGG
Dai22472
GGAATGCAGCTCAAAATGGGTGGTAAATTCCATCTAAAGCTAAATATTGG
1CUI10475
GGAATGCAGCTCAAAATGGGTGGTAAATTCCATCTAAAGCTAAATATTGG
1CUI10491 --
HCFC1095Meripilus_robledoi
GGAATGCAGCTCAAAATGGGTGGTAAATTCCATCTAAAGCTAAATATTGG

MCW702Meripilus_revolubilis
GGAATGCAGCTCAAAATGGGTGGTAAATTCCATCTAAAGCTAAATATTGG
1704_83_zluty_HaciendaBaru
GGAATGCAGCTCAAAATGGGTGGTAAATTCCATCTAAAGCTAAATATTGG
Dai9925P_lavendulus
GGAATGCAGCTCAAAATGGGTGGTAAATTCCATCTAAAGCTAAATATTGG
Dai13587AP_lavendulus
GGAATGCAGCTCAAAATGGGTGGTAAATTCCATCTAAAGCTAAATATTGG
PDD70600P_longicystidius --
Cui16630
GGAATGCAGCTCAAAATGGGTGGTAAATTCCATCTAAAGCTAAATATTGG
FP_135344Meripilus_giganteus
GGAATGCAGCTCAAAATGGGTGGTAAATTCCATCTAAAGCTAAATATTGG
FP_100460_Sp --
CBS421_48Meripilus_giganteus --
Cui9203 --
Cui9202
GGAATGCAGCTCAAAATGGGTGGTAAATTCCATCTAAAGCTAAATATTGG
TUFC100564Japan
GGAATGCAGCTCAAAATGGGTGGTAAATTCCATCTAAAGCTAAATATTGG
Russell5913Meripilus_sumstinei --
RP215Meripilus_brasiliensis
GGAATGCAGCTCAAAATGGGTGGTAAATTCCATCTAAAGCTAAATATTGG
RP200Meripilus_brasiliensis
GGAATGCAGCTCAAAATGGGTGGTAAATTCCATCTAAAGCTAAATATTGG
JV1712_13J_R_vinctus2_LSUPuert
GGAATGCAGCTCAAAATGGGTGGTAAATTCCATCTAAAGCTAAATATTGG
Dai10503R_hypobrunneus
GGAATGCAGCTCAAAATGGGTGGTAAATTCCATCTAAAGCTAAATATTGG
Dai10569Rigidoporus_hypobrunne
GGAATGCAGCTCAAAATGGGTGGTAAATTCCATCTAAAGCTAAATATTGG
1DAI19451
GGAATGCAGCTCAAAATGGGTGGTAAATTCCATCTAAAGCTAAATATTGG
CM108bRigidoporus_hypobrunneus --
1CUI16874
GGAATGCAGCTCAAAATGGGTGGTAAATTCCATCTAAAGCTAAATATTGG
FD299Cerrena_unicolor
GGAATGCAGCTCAAAATGGGTGGTAAATTCCATCTAAAGCTAAATATTGG
KHL_GB_Cerrena_uniclor
GGAATGCAGCTCAAAATGGGTGGTAAATTCCATCTAAAGCTAAATATTGG
Dai12892Cerrena_albocinnamomea
GGAATGCAGCTCAAAATGGGTGGTAAATTCCATCTAAAGCTAAATATTGG
Dai12955C_albocinnamomea
GGAATGCAGCTCAAAATGGGTGGTAAATTCCATCTAAAGCTAAATATTGG

SNUm03110102C_aurantiopora
GGAATGCAGCTCAAAATGGGTGGTAAATTCCATCTAAAGCTAAATATTGG
NIBRFG0000102423C_aurantiopora
GGAATGCAGCTCAAAATGGGTGGTAAATTCCATCTAAAGCTAAATATTGG
Dai7359Antrodiella_zonata
GGAATGCAGCTCAAAATGGGTGGTAAATTCCATCTAAAGCTAAATATTGG
F20080702KCM29C_consors
GGAATGCAGCTCAAAATGGGTGGTAAATTCCATCTAAAGCTAAATATTGG
F20080208LYW10Cerrena_consors
GGAATGCAGCTCAAAATGGGTGGTAAATTCCATCTAAAGCTAAATATTGG
Dai7821Antrodiella_zonata
GGAATGCAGCTCAAAATGGGTGGTAAATTCCATCTAAAGCTAAATATTGG
CFMR_DCL04_31Pseudolagarobasid --
VPB197Pseudolagarobasidium_bel --
CBS115543Pseudolagarobasidium_ --
CBS115544 --
Han405Pseudolagarobasidium_bai
GGAATGCAGCTCAAAATGGGTGGTAAATTCCATCTAAAGCTAAATATTGG
Han406Pseudolagarobasidium_bai
GGAATGCAGCTCAAAATGGGTGGTAAATTCCATCTAAAGCTAAATATTGG
MUcc838Spongipellis_delectans
GGAATGCAGCTCAAAATGGGTGGTAAATTCCATCTAAAGCTAAATATTGG
BRNM686401S_delectans
GGAATGCAGCTCAAAATGGGTGGTAAATTCCATCTAAAGCTAAATATTGG
OSM_F925S_delectans
GGAATGCAGCTCAAAATGGGTGGTAAATTCCATCTAAAGCTAAATATTGG
BRNM67093Spongipellis_litschau
GGAATGCAGCTCAAAATGGGTGGTAAATTCCATCTAAAGCTAAATATTGG
CFMRccFP59199TS_unicolor
GGAATGCAGCTCAAAATGGGTGGTAAATTCCATCTAAAGCTAAATATTGG
CFMRccFP71791TS_unicolor
GGAATGCAGCTCAAAATGGGTGGTAAATTCCATCTAAAGCTAAATATTGG
Dai13845P_lischaueri
GGAATGCAGCTCAAAATGGGTGGTAAATTCCATCTAAAGCTAAATATTGG
Dai20266P_lischaueri
GGAATGCAGCTCAAAATGGGTGGTAAATTCCATCTAAAGCTAAATATTGG
CFMR_HHB11240Radulodon_america --
RLG6350Radulodon_americanus --
KY415963Radulodon_erikssonii
GGAATGCAGCGCAAAATGGGTGGTAAATTCCATCTAAAGCTAAATATAGG
HHB9567spRadulodon_casearius
GGAATGCAGCGCAAAATGGGTGGTAAATTCCATCTAAAGCTAAATATAGG
KRT_Iso_26Radulodon_casearius --
CBS126044Radulodon_erikssonii

GGAATGCAGCGCAAAATGGGTGGTAAATTCCATCTAAAGCTAAATATAGG
He6183YUNNANENSIS
GGAATGCAGCGCAAAATGGGTGGTAAATTCCATCTAAAGCTAAATATAGG
Cui17979YUNNANENSIS
GGAATGCAGCGCAAAATGGGTGGTAAATTCCATCTAAAGCTAAATATAGG
Miettinen2091Junghuhnia_fimbri
GGAATGCAGCTCAAAATGGGTGGTAAATTCCATCTAAAGCTAAATATTGG
KHL12316S_tenue
GGAATGCAGCTCAAAATGGGTGGTAAATTCCATCTAAAGCTAAATATTGG
PRM846564S_pachyodon
GGAATGCAGCTCAAAATGGGTGGTAAATTCCATCTAAAGCTAAATATTGG
SP_Lgt_S_pachyodon --
Ryvarden44669Tyromyces_xuchile
GGAATGCAGCTCAAAATGGGTGGTAAATTCCATCTAAAGCTAAATATTGG
PW17_171sinuosus --
W53Dai12234
GGAATGCAGCTCAAAATGGGTGGTAAATTCCATCTAAAGCTAAATATTGG
HHB4100SpAntella_americana
GGAATGCAGCTCAAAATGGGTGGTAAATTCCATCTAAAGCTAAATATTGG
W3Dai20901spumeus
GGAATGCAGCTCAAAATGGGTGGTAAATTCCATCTAAAGCTAAATATTGG
He6736
GGAATGCAGCTCAAAATGGGTGGTAAATTCCATCTAAAGCTAAATATTGG
BRNM734877S_spumeus
GGAATGCAGCTCAAAATGGGTGGTAAATTCCATCTAAAGCTAAATATTGG
BRNM712630S_spumeus
GGAATGCAGCTCAAAATGGGTGGTAAATTCCATCTAAAGCTAAATATTGG
Dai1723Loweomyces_sibiricus --
W54Cui10009
GGAATGCAGCTCAAAATGGGTGGTAAATTCCATCTAAAGCTAAATATTGG
W1Dai20899 --
HHB13445Trametes_ochracea
GGAATGCAGCGCAAAATGGGAGGTGAATTCCTTCTAAAGCTAAATATTGG
Dai16222
GGAATGCAGCTCAAAATGGGTGGTAAATTCCATCTAAAGCTAAATATTGG
Dai16240
GGAATGCAGCTCAAAATGGGTGGTAAATTCCATCTAAAGCTAAATATTGG

JV1310_11SanguinolentusCernys
CGAGAGACCGATAGCGAACAAGTACCGTGAGGGAAAGATGAAAAGCACTT
MJ39_00_SK
CGAGAGACCGATAGYGAACAAGTACCGTGAGGGAAAGATGAAAAGCACTT
MJ111_04_CZ --
JV1610_BOKYsmrk

CGAGAGACCGATAGCGAACAAGTACCGTGAGGGAAAGATGAAAAGCACTT
Dai21030 ---
Dai20976P_furcatus
CGAGAGACCGATAGCGAACAAGTACCGTGAGGGAAAGATGAAAAGCACTT
Dai2105
CGAGAGACCGATAGCGAACAAGTACCGTGAGGGAAAGATGAAAAGCACTT
Dai2544
CGAGAGACCGATAGCGAACAAGTACCGTGAGGGAAAGATGAAAAGCACTT
Dai11313
CGAGAGACCGATAGCGAACAAGTACCGTGAGGGAAAGATGAAAAGCACTT
WCG1611Dai26167
CGAGAGACCGATAGCGAACAAGTACCGTGAGGGAAAGATGAAAAGCACTT
WCG1518Dai25999Physisporinus
CGAGAGACCGATAGCGAACAAGTACCGTGAGGGAAAGATGAAAAGCACTT
TAA15097
CGAGAGACCGATAGCGAACAAGTACCGTGAGGGAAAGATGAAAAGCACTT
JV8909_19_CZ ---
JV1310_15_P_sanguinolentus2_CZ
CGAGAGACCGATAGCGAACAAGTACCGTGAGGGAAAGATGAAAAGCACTT
MJ53_02_CZ
CGAGAGACCGATAGCGAACAAGTACCGTGAGGGAAAGATGAAAAGCACTT
CLZhao21647P_yunnanensis
CGAGAGACCGATAGCGAACAAGTACCGTGAGGGAAAGATGAAAAGCACTT
CLZhao21583P_yunnanensis
CGAGAGACCGATAGCGAACAAGTACCGTGAGGGAAAGATGAAAAGCACTT
Dai22272
CGAGAGACCGATAGCGAACAAGTACCGTGAGGGAAAGATGAAAAGCACTT
Dai22279
CGAGAGACCGATAGCGAACAAGTACCGTGAGGGAAAGATGAAAAGCACTT
MJ332_94_CZ ---
MJ642_94_CZ_Expallescens ---
Dai21060P_vinctus
CGAGAGACCGATAGCGAACAAGTACCGTGAGGGAAAGATGAAAAGCACTT
JV0511_23LRP_pouzarii
CGAGAGACCGATAGCGAACAAGTACCGTGAGGGAAAGATGAAAAGCACTT
JQ409462_R_pouzarii_PRM899856_ ---
JV0308_66_WA ---
JV0309_45_WA_USA ---
JV0709_83_CA_USA
CGAGAGACCGATAGCGAACAAGTACCGTGAGGGAAAGATGAAAAGCACTT
Dai21043P_pouzarii
CGAGAGACCGATAGCGAACAAGTACCGTGAGGGAAAGATGAAAAGCACTT
MJ144_95_CZ
CGAGAGACCGATAGCGAACAAGTACCGTGAGGGAAAGATGAAAAGCACTT

JV0909_3_CZ
CGAGAGACCGATAGCGAACAAGTACCGTGAGGGAAAGATGAAAAGCACTT
JV0609_1_K --
Dai20396Physisporinus_castanop
CGAGAGACCGATAGCGAACAAGTACCGTGAGGGAAAGATGAAAAGCACTT
Dai20397Physisporinus_castanop
CGAGAGACCGATAGCGAACAAGTACCGTGAGGGAAAGATGAAAAGCACTT
MJ19_09_SK_Abies
CGAGAGACCGATAGCGAACAAGTACCGTGAGGGAAAGATGAAAAGCACTT
JV0509_40_J_TN_USA_Betula --
JV0808_33crocatus_PA_USAlist
CGAGAGACCGATAGCGAACAAGTACCGTGAGGGAAAGATGAAAAGCACTT
DLL2009_061P_crocatus --
Dai12800P_subcrocatus
CGAGAGACCGATAGCGAACAAGTACCGTGAGGGAAAGATGAAAAGCACTT
Dai15917P_subcrocatus
CGAGAGACCGATAGCGAACAAGTACCGTGAGGGAAAGATGAAAAGCACTT
Cui16620
CGAGAGACCGATAGCGAACAAGTACCGTGAGGGAAAGATGAAAAGCACTT
HCFC1088Meripilus_stillicidior
CGAGAGACCGATAGCGAACAAGTACCGTGAGGGAAAGATGAAAAGCACTT
MCW590Meripilus_obsrcurus --
MCW722Meripilus_obsrcurus --
Cui9381P_tibeticus
CGAGAGACCGATAGCGAACAAGTACCGTGAGGGAAAGATGAAAAGCACTT
Cui9588P_tibeticus
CGAGAGACCGATAGCGAACAAGTACCGTGAGGGAAAGATGAAAAGCACTT
Va2_Beneschova --
CWU3874_Ukraine_Alnus
CGAGAGACCGATAGCGAACAAGTACCGTGAGGGAAAGATGAAAAGCACTT
WCG1293Dai24718Physisporinus --
WCG1268Dai24682A
CGAGAGACCGATAGCGAACAAGTACCGTGAGGGAAAGATGAAAAGTACTT
WCG1269Dai24683A
CGAGAGACCGATAGCGAACAAGTACCGTGAGGGAAAGATGAAAAGTACTT
WCG1279Dai24694A
CGAGAGACCGATAGCGAACAAGTACCGTGAGGGAAAGATGAAAAGTACTT
Dai16971
CGAGAGACCGATAGCGAACAAGTACCGTGAGGGAAAGATGAAAAGTACTT
ZQY1043Dai26696
CGAGAGACCGATAGCGAACAAGTACCGTGAGGGAAAGATGAAAAGTACTT
Doll880 --
Doll1000
CGAGAGACCGATAGCGAACAAGTACCGTGAGGGAAAGATGAAAAGCACTT

1DAI18529
CGAGAGACCGATAGCGAACAAGTACCGTGAGGGAAAGATGAAAAGCACTT
Dai19535
CGAGAGACCGATAGCGAACAAGTACCGTGAGGGAAAGATGAAAAGCACTT
1704_79_hnedyVillaLaPaz
CGAGAGACCGATAGCGAACAAGTACCGTGAGGGAAAGATGAAAAGCACTT
F2061 --
1DAI18268
CGAGAGACCGATAGCGAACAAGTACCGTGAGGGAAAGATGAAAAGCACTT
1DAI18540A
CGAGAGACCGATAGCGAACAAGTACCGTGAGGGAAAGATGAAAAGCACTT
Dai17695
CGAGAGACCGATAGCGAACAAGTACCGTGAGGGAAAGATGAAAAGCACTT
LKY18Dai26373 --
Dai17839P_sulphureus
CGAGAGACCGATAGCGAACAAGTACCGTGAGGGAAAGATGAAAAGCACTT
Dai17841P_sulphureus
CGAGAGACCGATAGCGAACAAGTACCGTGAGGGAAAGATGAAAAGCACTT
Dai19877P_roseus
CGAGAGACCGATAGCGAACAAGTACCGTGAGGGAAAGATGAAAAGCACTT
1508_18_1_Kout --
KP859303_R_vinctus_RP185_BRAZI --
JK1807_15Rigidoporus_sp_Puerto
CGAGAGACCGATAGCGAACAAGTACCGTGAGGGAAAGATGAAAAGCACTT
JV0509_47_J_TN_USA
CGAGAGACCGATAGCGAACAAGTACCGTGAGGGAAAGATGAAAAGCACTT
JV0709_188
CGAGAGACCGATAGCGAACAAGTACCGTGAGGGAAAGATGAAAAGCACTT
JV0509_127_PA_USA
CGAGAGACCGATAGCGAACAAGTACCGTGAGGGAAAGATGAAAAGCACTT
JV1009_59_NJ_USA
CGAGAGACCGATAGCGAACAAGTACCGTGAGGGAAAGATGAAAAGCACTT
Dai15497P_crataegi
CGAGAGACCGATAGCGAACAAGTACCGTGAGGGAAAGATGAAAAGCACTT
Dai15499P_crataegi
CGAGAGACCGATAGCGAACAAGTACCGTGAGGGAAAGATGAAAAGCACTT
Cui3266P_cinereus
CGAGAGACCGATAGCGAACAAGTACCGTGAGGGAAAGATGAAAAGCACTT
WCG1256Dai24690
CGAGAGACCGATAGCGAACAAGTACCGTGAGGGAAAGATGAAAAGCACTT
1DAI17581
CGAGAGACCGATAGCGAACAAGTACCGTGAGGGAAAGATGAAAAGCACTT
WCG1255Dai24688
CGAGAGACCGATAGCGAACAAGTACCGTGAGGGAAAGATGAAAAGCACTT

Dai22427
CGAGAGACCGATAGCGAACAAGTACCGTGAGGGAAAGATGAAAAGCACTT
MV690Meripilus_concrescens
CGAGAGACCGATAGCGAACAAGTACCGTGAGGGAAAGATGAAAAGCACTT
MV513Meripilus_galapagensis
CGAGAGACCGATAGCGAACAAGTACCGTGAGGGAAAGATGAAAAGCACTT
Dai19793
CGAGAGACCGATAGCGAACAAGTACCGTGAGGGAAAGATGAAAAGCACTT
OQ553780P_tamilnaduensis
CGAGAGACCGATAGCGAACAAGTACCGTGAGGGAAAGATGAAAAGCACTT
OQ553779P_tamilnaduensis
CGAGAGACCGATAGCGAACAAGTACCGTGAGGGAAAGATGAAAAGCACTT
A164FB3Meripilus_giganteus --
JV1407_36_Vinctus_Meandrica
CGAGAGACCGATAGCGAACAAGTACCGTGAGGGAAAGATGAAAAGCACTT
1807_3K_Rigidoporus_PuertoRico
CGAGAGACCGATAGCGAACAAGTACCGTGAGGGAAAGATGAAAAGCACTT
Cui16903P_vinctus
CGAGAGACCGATAGCGAACAAGTACCGTGAGGGAAAGATGAAAAGCACTT
JV1008_18R_Lineatus
CGAGAGACCGATAGCGAACAAGTACCGTGAGGGAAAGATGAAAAGCACTT
JV1407_37_1_Vinctus_Carara
CGAGAGACCGATAGCGAACAAGTACCGTGAGGGAAAGATGAAAAGCACTT
Dai17986P_lineatus
CGAGAGACCGATAGCGAACAAGTACCGTGAGGGAAAGATGAAAAGCACTT
Dai18281
CGAGAGACCGATAGCGAACAAGTACCGTGAGGGAAAGATGAAAAGCACTT
1DAI19796
CGAGAGACCGATAGCGAACAAGTACCGTGAGGGAAAGATGAAAAGCACTT
ZQY797Dai25241
CGAGAGACCGATAGCGAACAAGTACCGTGAGGGAAAGATGAAAAGCACTT
WCG1289Dai24711 --
Dai22598
CGAGAGACCGATAGCGAACAAGTACCGTGAGGGAAAGATGAAAAGCACTT
Dai20523
CGAGAGACCGATAGCGAACAAGTACCGTGAGGGAAAGATGAAAAGCACTT
Dai17885
CGAGAGACCGATAGCGAACAAGTACCGTGAGGGAAAGATGAAAAGCACTT
Dai17553
CGAGAGACCGATAGCGAACAAGTACCGTGAGGGAAAGATGAAAAGCACTT
Dai19639
CGAGAGACCGATAGCGAACAAGTACCGTGAGGGAAAGATGAAAAGCACTT
JV0110_48_CZ
CGAGAGACCGATAGCGAACAAGTACCGTGAGGGAAAGATGAAAAGCACTT

MJ129_04
CGAGAGACCGATAGCGAACAAGTACCGTGAGGGAAAGATGAAAAGCACTT
Cui10340P_eminens ---
Cui10341P_eminens
CGAGAGACCGATAGCGAACAAGTACCGTGAGGGAAAGATGAAAAGCACTT
Dai12685P_eminens
CGAGAGACCGATAGCGAACAAGTACCGTGAGGGAAAGATGAAAAGCACTT
Miettinen_13591Rigidoporus_und
CGAGAGACCGATAGCGAACAAGTACCGTGAGGGAAAGATGAAAAGCACTT
Dai20868
CGAGAGACCGATAGCGAACAAGTACCGTGAGGGAAAGATGAAAAGCACTT
Dai20832
CGAGAGACCGATAGCGAACAAGTACCGTGAGGGAAAGATGAAAAGCACTT
Dai11400
CGAGAGACCGATAGCGAACAAGTACCGTGAGGGAAAGATGAAAAGCACTT
Dai22472
CGAGAGACCGATAGCGAACAAGTACCGTGAGGGAAAGATGAAAAGCACTT
1CUI10475
CGAGAGACCGATAGCGAACAAGTACCGTGAGGGAAAGATGAAAAGCACTT
1CUI10491 ---
HCFC1095Meripilus_robledoi
CGAGAGACCGATAGCGAACAAGTACCGTGAGGGAAAGATGAAAAGCACTT
MCW702Meripilus_revolubilis
CGAGAGACCGATAGCGAACAAGTACCGTGAGGGAAAGATGAAAAGCACTT
1704_83_zluty_HaciendaBaru
CGAGAGACCGATAGCGAACAAGTACCGTGAGGGAAAGATGAAAAGCACTT
Dai9925P_lavendulus
CGAGAGACCGATAGCGAACAAGTACCGTGAGGGAAAGATGAAAAGCACTT
Dai13587AP_lavendulus
CGAGAGACCGATAGCGAACAAGTACCGTGAGGGAAAGATGAAAAGCACTT
PDD70600P_longicystidius ---
Cui16630
CGAGAGACCGATAGCGAACAAGTACCGTGAGGGAAAGATGAAAAGCACTT
FP_135344Meripilus_giganteus
CGAGAGACCGATAGCGAACAAGTACCGTGAGGGAAAGATGAAAAGCACTT
FP_100460_Sp ---
CBS421_48Meripilus_giganteus ---
Cui9203 ---
Cui9202
CGAGAGACCGATAGCGAACAAGTACCGTGAGGGAAAGATGAAAAGCACTT
TUFC100564Japan
CGAGAGACCGATAGCGAACAAGTACCGTGAGGGAAAGATGAAAAGCACTT
Russell5913Meripilus_sumstinei ---
RP215Meripilus_brasiliensis

CGAGAGACCGATAGCGAACAAGTACCGTGAGGGAAAGATGAAAAGCACTT
RP200Meripilus_brasiliensis
CGAGAGACCGATAGCGAACAAGTACCGTGAGGGAAAGATGAAAAGCACTT
JV1712_13J_R_vinctus2_LSUPuert
CGAGAGACCGATAGCGAACAAGTACCGTGAGGGAAAGATGAAAAGCACTT
Dai10503R_hypobrunneus
CGAGAGACCGATAGCGAACAAGTACCGTGAGGGAAAGATGAAAAGCACTT
Dai10569Rigidoporus_hypobrunne
CGAGAGACCGATAGCGAACAAGTACCGTGAGGGAAAGATGAAAAGCACTT
1DAI19451
CGAGAGACCGATAGCGAACAAGTACCGTGAGGGAAAGATGAAAAGCACTT
CM108bRigidoporus_hypobrunneus --
1CUI16874
CGAGAGACCGATAGCGAACAAGTACCGTGAGGGAAAGATGAAAAGCACTT
FD299Cerrena_unicolor
CGAGAGACCGATAGCGAACAAGTACCGTGAGGGAAAGATGAAAAGCACTT
KHL_GB_Cerrena_uniclor
CGAGAGACCGATAGCGAACAAGTACCGTGAGGGAAAGATGAAAAGCACTT
Dai12892Cerrena_albocinnamomea
CGAGAGACCGATAGCGAACAAGTACCGTGAGGGAAAGATGAAAAGCACTT
Dai12955C_albocinnamomea
CGAGAGACCGATAGCGAACAAGTACCGTGAGGGAAAGATGAAAAGCACTT
SNUm03110102C_aurantiopora
CGAGAGACCGATAGCGAACAAGTACCGTGAGGGAAAGATGAAAAGCACTT
NIBRFG0000102423C_aurantiopora
CGAGAGACCGATAGCGAACAAGTACCGTGAGGGAAAGATGAAAAGCACTT
Dai7359Antrodiella_zonata
CGAGAGACCGATAGCGAACAAGTACCGTGAGGGAAAGATGAAAAGCACTT
F20080702KCM29C_consors
CGAGAGACCGATAGCGAACAAGTACCGTGAGGGAAAGATGAAAAGCACTT
F20080208LYW10Cerrena_consors
CGAGAGACCGATAGCGAACAAGTACCGTGAGGGAAAGATGAAAAGCACTT
Dai7821Antrodiella_zonata
CGAGAGACCGATAGCGAACAAGTACCGTGAGGGAAAGATGAAAAGCACTT
CFMR_DCL04_31Pseudolagarobasid --
VPB197Pseudolagarobasidium_bel --
CBS115543Pseudolagarobasidium_ --
CBS115544 --
Han405Pseudolagarobasidium_bai
CGAGAGACCGATAGCGAACAAGTACCGTGAGGGAAAGATGAAAAGCACTT
Han406Pseudolagarobasidium_bai
CGAGAGACCGATAGCGAACAAGTACCGTGAGGGAAAGATGAAAAGCACTT
MUcc838Spongipellis_delectans
CGAGAGACCGATAGCGAACAAGTACCGTGAGGGAAAGATGAAAAGCACTT

BRNM686401S_delectans
CGAGAGACCGATAGCGAACAAGTACCGTGAGGGAAAGATGAAAAGCACTT
OSM_F925S_delectans
CGAGAGACCGATAGCGAACAAGTACCGTGAGGGAAAGATGAAAAGCACTT
BRNM67093Spongipellis_litschau
CGAGAGACCGATAGCGAACAAGTACCGTGAGGGAAAGATGAAAAGCACTT
CFMRccFP59199TS_unicolor
CGAGAGACCGATAGCGAACAAGTACCGTGAGGGAAAGATGAAAAGCACTT
CFMRccFP71791TS_unicolor
CGAGAGACCGATAGCGAACAAGTACCGTGAGGGAAAGATGAAAAGCACTT
Dai13845P_lischaueri
CGAGAGACCGATAGCGAACAAGTACCGTGAGGGAAAGATGAAAAGCACTT
Dai20266P_lischaueri
CGAGAGACCGATAGCGAACAAGTACCGTGAGGGAAAGATGAAAAGCACTT
CFMR_HHB11240Radulodon_america --
RLG6350Radulodon_americanus --
KY415963Radulodon_erikssonii
CGAGAGACCGATAGCGAACAAGTACCGTGAGGGAAAGATGAAAAGCACTT
HHB9567spRadulodon_casearius
CGAGAGACCGATAGCGAACAAGTACCGTGAGGGAAAGATGAAAAGCACTT
KRT_Iso_26Radulodon_casearius --
CBS126044Radulodon_erikssonii
CGAGAGACCGATAGCGAACAAGTACCGTGAGGGAAAGATGAAAAGCACTT
He6183YUNNANENSIS
CGAGAGACCGATAGCGAACAAGTACCGTGAGGGAAAGATGAAAAGCACTT
Cui17979YUNNANENSIS
CGAGAGACCGATAGCGAACAAGTACCGTGAGGGAAAGATGAAAAGCACTT
Miettinen2091Junghuhnia_fimbri
CGAGAGACCGATAGCGAACAAGTACCGTGAGGGAAAGATGAAAAGCACTT
KHL12316S_tenue
CGAGAGACCGATAGCGAACAAGTACCGTGAGGGAAAGATGAAAAGCACTT
PRM846564S_pachyodon
CGAGAGACCGATAGCGAACAAGTACCGTGAGGGAAAGATGAAAAGCACTT
SP_Lgt_S_pachyodon --
Ryvarden44669Tyromyces_xuchile
CGAGAGACCGATAGCGAACAAGTACCGTGAGGGAAAGATGAAAAGCACTT
PW17_171sinuosus --
W53Dai12234
CGAGAGACCGATAGCGAACAAGTACCGTGAGGGAAAGATGAAAAGCACTT
HHB4100SpAntella_americana
CGAGAGACCGATAGCGAACAAGTACCGTGAGGGAAAGATGAAAAGCACTT
W3Dai20901spumeus
CGAGAGACCGATAGCGAACAAGTACCGTGAGGGAAAGATGAAAAGCACTT
He6736

CGAGAGACCGATAGCGAACAAGTACCGTGAGGGAAAGATGAAAAGCACTT
BRNM734877S_spumeus
CGAGAGACCGATAGCGAACAAGTACCGTGAGGGAAAGATGAAAAGCACTT
BRNM712630S_spumeus
CGAGAGACCGATAGCGAACAAGTACCGTGAGGGAAAGATGAAAAGCACTT
Dai1723Loweomyces_sibiricus --
W54Cui10009
CGAGAGACCGATAGCGAACAAGTACCGTGAGGGAAAGATGAAAAGCACTT
W1Dai20899 --
HHB13445Trametes_ochracea
CGAGAGACCGATAGCGAACAAGTACCGTGAGGGAAAGATGAAAAGCACTT
Dai16222
CGAGAGACCGATAGCGAACAAGTACCGTGAGGGAAAGATGAAAAGCACTT
Dai16240
CGAGAGACCGATAGCGAACAAGTACCGTGAGGGAAAGATGAAAAGCACTT

JV1310_11SanguinolentusCernys
TGGAAAGAGAGTTAAACAGTACGTGAAATTGCTGAAAGGGAAACACTTGA
MJ39_00_SK
TGGAAAGAGAGTTAAACAGTACGTGAAATTGCTGAAAGGGAAACACTTGA
MJ111_04_CZ --
JV1610_BOKYsmrk
TGGAAAGAGAGTTAAACAGTACGTGAAATTGCTGAAAGGGAAACACTTGA
Dai21030 --
Dai20976P_furcatus
TGGAAAGAGAGTTAAACAGTACGTGAAATTGCTGAAAGGGAAACACTTGA
Dai2105
TGGAAAGAGAGTTAAACAGTACGTGAAATTGCTGAAAGGGAAACACTTGA
Dai2544
TGGAAAGAGAGTTAAACAGTACGTGAAATTGCTGAAAGGGAAACACTTGA
Dai11313
TGGAAAGAGAGTTAAACAGTACGTGAAATTGCTGAAAGGGAAACACTTGA
WCG1611Dai26167
TGGAAAGAGAGTTAAACAGTACGTGAAATTGCTGAAAGGGAAACACTTGA
WCG1518Dai25999Physisporinus
TGGAAAGAGAGTTAAACAGTACGTGAAATTGCTGAAAGGGAAACACTTGA
TAA15097
TGGAAAGAGAGTTAAACAGTACGTGAAATTGCTGAAAGGGAAACACTTGA
JV8909_19_CZ --
JV1310_15_P_sanguinolentus2_CZ
TGGAAAGAGAGTTAAACAGTACGTGAAATTGCTGAAAGGGAAACACTTGA
MJ53_02_CZ
TGGAAAGAGAGTTAAACAGTACGTGAAATTGCTGAAAGGGAAACACTTGA
CLZhao21647P_yunnanensis

TGGAAAGAGAGTTAAACAGTACGTGAAATTGCTGAAAGGGAAACACTTGA
CLZhao21583P_yunnanensis
TGGAAAGAGAGTTAAACAGTACGTGAAATTGCTGAAAGGGAAACACTTGA
Dai22272
TGGAAAGAGAGTTAAACAGTACGTGAAATTGCTGAAAGGGAAACACTTGA
Dai22279
TGGAAAGAGAGTTAAACAGTACGTGAAATTGCTGAAAGGGAAACACTTGA
MJ332_94_CZ --
MJ642_94_CZ_Expallescens --
Dai21060P_vinctus
TGGAAAGAGAGTTAAACAGTACGTGAAATTGCTGAAAGGGAAACACTTGA
JV0511_23LRP_pouzarii
TGGAAAGAGAGTTAAACAGTACGTGAAATTGCTGAAAGGGAAACACTTGA
JQ409462_R_pouzarii_PRM899856_ --
JV0308_66_WA --
JV0309_45_WA_USA --
JV0709_83_CA_USA
TGGAAAGAGAGTTAAACAGTACGTGAAATTGCTGAAAGGGAAACACTTGA
Dai21043P_pouzarii
TGGAAAGAGAGTTAAACAGTACGTGAAATTGCTGAAAGGGAAACACTTGA
MJ144_95_CZ
TGGAAAGAGAGTTAAACAGTACGTGAAATTGCTGAAAGGGAAACACTTGA
JV0909_3_CZ
TGGAAAGAGAGTTAAACAGTACGTGAAATTGCTGAAAGGGAAACACTTGA
JV0609_1_K --
Dai20396Physisporinus_castanop
TGGAAAGAGAGTTAAACAGTACGTGAAATTGCTGAAAGGGAAACACTTGA
Dai20397Physisporinus_castanop
TGGAAAGAGAGTTAAACAGTACGTGAAATTGCTGAAAGGGAAACACTTGA
MJ19_09_SK_Abies
TGGAAAGAGAGTTAAACAGTACGTGAAATTGCTGAAAGGGAAACACTTGA
JV0509_40_J_TN_USA_Betula --
JV0808_33crocatus_PA_USAlist
TGGAAAGAGAGTTAAACAGTACGTGAAATTGCTGAAAGGGAAACACTTGA
DLL2009_061P_crocatus --
Dai12800P_subcrocatus
TGGAAAGAGAGTTAAACAGTACGTGAAATTGCTGAAAGGGAAACACTTGA
Dai15917P_subcrocatus
TGGAAAGAGAGTTAAACAGTACGTGAAATTGCTGAAAGGGAAACACTTGA
Cui16620
TGGAAAGAGAGTTAAACAGTACGTGAAATTGCTGAAAGGGAAACACTTGA
HCFC1088Meripilus_stillicidior
TGGAAAGAGAGTTAAACAGTACGTGAAATTGCTGAAAGGGAAACACTTGA
MCW590Meripilus_obsrcurus --

MCW722Meripilus_obsrcurus --
Cui9381P_tibeticus
TGGAAAGAGAGTTAAACAGTACGTGAAATTGCTGAAAGGGAAACACTTGA
Cui9588P_tibeticus
TGGAAAGAGAGTTAAACAGTACGTGAAATTGCTGAAAGGGAAACACTTGA
Va2_Beneschova --
CWU3874_Ukraine_Alnus
TGGAAAGAGAGTTAAACAGTACGTGAAATTGCTGAAAGGGAAACACTTGA
WCG1293Dai24718Physisporinus --
WCG1268Dai24682A
TGGAAAGAGAGTTAAACAGTACGTGAAATTGCTGAAAGGGAAACACTTGA
WCG1269Dai24683A
TGGAAAGAGAGTTAAACAGTACGTGAAATTGCTGAAAGGGAAACACTTGA
WCG1279Dai24694A
TGGAAAGAGAGTTAAACAGTACGTGAAATTGCTGAAAGGGAAACACTTGA
Dai16971
TGGAAAGAGAGTTAAACAGTACGTGAAATTGCTGAAAGGGAAACACTTGA
ZQY1043Dai26696
TGGAAAGAGAGTTAAACAGTACGTGAAATTGCTGAAAGGGAAACACTTGA
Doll880 --
Doll1000
TGGAAAGAGAGTTAAACAGTACGTGAAATTGCTGAAAGGGAAACGCTTGA
1DAI18529
TGGAAAGAGAGTTAAACAGTACGTGAAATTGCTGAAAGGGAAACGCTTGA
Dai19535
TGGAAAGAGAGTTAAACAGTACGTGAAATTGCTGAAAGGGAAACGCTTGA
1704_79_hnedyVillaLaPaz
TGGAAAGAGAGTTAAACAGTACGTGAAATTGCTGAAAGGGAAACGCTTGA
F2061 --
1DAI18268
TGGAAAGAGAGTTAAACAGTACGTGAAATTGCTGAAAGGGAAACGCTTGA
1DAI18540A
TGGAAAGAGAGTTAAACAGTACGTGAAATTGCTGAAAGGGAAACGCTTGA
Dai17695
TGGAAAGAGAGTTAAACAGTACGTGAAATTGCTGAAAGGGAAACGCTTGA
LKY18Dai26373 --
Dai17839P_sulphureus
TGGAAAGAGAGTTAAACAGTACGTGAAATTGCTGAAAGGGAAACGCTTGA
Dai17841P_sulphureus
TGGAAAGAGAGTTAAACAGTACGTGAAATTGCTGAAAGGGAAACGCTTGA
Dai19877P_roseus
TGGAAAGAGAGTTAAACAGTACGTGAAATTGCTGAAAGGGAAACGCTTGA
1508_18_1_Kout --
KP859303_R_vinctus_RP185_BRAZI --

JK1807_15Rigidoporus_sp_Puerto
TGGAAAGAGAGTTAAACAGTACGTGAAATTGCTGAAAGGGAAACGCTTGA
JV0509_47_J_TN_USA
TGGAAAGAGAGTTAAACAGTACGTGAAATTGCTGAAAGGGAAACACTTGA
JV0709_188
TGGAAAGAGAGTTAAACAGTACGTGAAATTGCTGAAAGGGAAACACTTGA
JV0509_127_PA_USA
TGGAAAGAGAGTTAAACAGTACGTGAAATTGCTGAAAGGGAAACACTTGA
JV1009_59_NJ_USA
TGGAAAGAGAGTTAAACAGTACGTGAAATTGCTGAAAGGGAAACACTTGA
Dai15497P_crataegi
TGGAAAGAGAGTTAAACAGTACGTGAAATTGCTGAAAGGGAAACACTTGA
Dai15499P_crataegi
TGGAAAGAGAGTTAAACAGTACGTGAAATTGCTGAAAGGGAAACACTTGA
Cui3266P_cinereus
TGGAAAGAGAGTTAAACAGTACGTGAAATTGCTGAAAGGGAAACACTTGA
WCG1256Dai24690
TGGAAAGAGAGTTAAACAGTACGTGAAATTGCTGAAAGGGAAACACTTGA
1DAI17581
TGGAAAGAGAGTTAAACAGTACGTGAAATTGCTGAAAGGGAAACACTTGA
WCG1255Dai24688
TGGAAAGAGAGTTAAACAGTACGTGAAATTGCTGAAAGGGAAACACTTGA
Dai22427
TGGAAAGAGAGTTAAACAGTACGTGAAATTGCTGAAAGGGAAACACTTGA
MV690Meripilus_concrescens
TGGAAAGAGAGTTAAACAGTACGTGAAATTGCTGAAAGGGAAACACTTGA
MV513Meripilus_galapagensis
TGGAAAGAGAGTTAAACAGTACGTGAAATTGCTGAAAGGGAAACACTTGA
Dai19793
TGGAAAGAGAGTTAAACAGTACGTGAAATTGCTGAAAGGGAAACACTTGA
OQ553780P_tamilnaduensis
TGGAAAGAGAGTTAAACAGTACGTGAAATTGCTGAAAGGGAAACGCTTGA
OQ553779P_tamilnaduensis
TGGAAAGAGAGTTAAACAGTACGTGAAATTGCTGAAAGGGAAACGCTTGA
A164FB3Meripilus_giganteus --
JV1407_36_Vinctus_Meandrica
TGGAAAGAGAGTTAAACAGTACGTGAAATTGCTGAAAGGGAAACGCTTGA
1807_3K_Rigidoporus_PuertoRico
TGGAAAGAGAGTTAAACAGTACGTGAAATTGCTGAAAGGGAAACGCTTGA
Cui16903P_vinctus
TGGAAAGAGAGTTAAACAGTACGTGAAATTGCTGAAAGGGAAACGCTTGA
JV1008_18R_Lineatus
TGGAAAGAGAGTTAAACAGTACGTGAAATTGCTGAAAGGGAAACGCTTGA
JV1407_37_1_Vinctus_Carara

TGGAAAGAGAGTTAAACAGTACGTGAAATTGCTGAAAGGAAACGCTTGA
Dai17986P_lineatus
TGGAAAGAGAGTTAAACAGTACGTGAAATTGCTGAAAGGAAACGCTTGA
Dai18281
TGGAAAGAGAGTTAAACAGTACGTGAAATTGCTGAAAGGAAACGCTTGA
1DAI19796
TGGAAAGAGAGTTAAACAGTACGTGAAATTGCTGAAAGGAAACGCTTGA
ZQY797Dai25241
TGGAAAGAGAGTTAAACAGTACGTGAAATTGCTGAAAGGAAACGCTTGA
WCG1289Dai24711 ---
Dai22598
TGGAAAGAGAGTTAAACAGTACGTGAAATTGCTGAAAGGAAACGCTTGA
Dai20523
TGGAAAGAGAGTTAAACAGTACGTGAAATTGCTGAAAGGAAACGCTTGA
Dai17885
TGGAAAGAGAGTTAAACAGTACGTGAAATTGCTGAAAGGAAACGCTTGA
Dai17553
TGGAAAGAGAGTTAAACAGTACGTGAAATTGCTGAAAGGAAACGCTTGA
Dai19639
TGGAAAGAGAGTTAAACAGTACGTGAAATTGCTGAAAGGAAACGCTTGA
JV0110_48_CZ
TGGAAAGAGAGTTAAACAGTACGTGAAATTGCTGAAAGGAAACGCTTGA
MJ129_04
TGGAAAGAGAGTTAAACAGTACGTGAAATTGCTGAAAGGAAACGCTTGA
Cui10340P_eminens ---
Cui10341P_eminens
TGGAAAGAGAGTTAAACAGTACGTGAAATTGCTGAAAGGAAACGCTTGA
Dai12685P_eminens
TGGAAAGAGAGTTAAACAGTACGTGAAATTGCTGAAAGGAAACGCTTGA
Miettinen_13591Rigidoporus_und
TGGAAAGAGAGTTAAACAGTACGTGAAATTGCTGAAAGGAAACGCTTGA
Dai20868
TGGAAAGAGAGTTAAACAGTACGTGAAATTGCTGAAAGGAAACGCTTGA
Dai20832
TGGAAAGAGAGTTAAACAGTACGTGAAATTGCTGAAAGGAAACGCTTGA
Dai11400
TGGAAAGAGAGTTAAACAGTACGTGAAATTGCTGAAAGGAAACGCTTGA
Dai22472
TGGAAAGAGAGTTAAACAGTACGTGAAATTGCTGAAAGGAAACGCTTGA
1CUI10475
TGGAAAGAGAGTTAAACAGTACGTGAAATTGCTGAAAGGAAACGCTTGA
1CUI10491 ---
HCFC1095Meripilus_robledoi
TGGAAAGAGAGTTAAACAGTACGTGAAATTGCTGAAAGGAAACGCTTGA

MCW702Meripilus_revolubilis
TGGAAAGAGAGTTAAACAGTACGTGAAATTGCTGAAAGGGAAACGCTTGA
1704_83_zluty_HaciendaBaru
TGGAAAGAGAGTTAAACAGTACGTGAAATTGCTGAAAGGGAAACGCTTGA
Dai9925P_lavendulus
TGGAAAGAGAGTTAAACAGTACGTGAAATTGCTGAAAGGGAAACGCTTGA
Dai13587AP_lavendulus
TGGAAAGAGAGTTAAACAGTACGTGAAATTGCTGAAAGGGAAACGCTTGA
PDD70600P_longicystidius --
Cui16630
TGGAAAGAGAGTTAAACAGTACGTGAAATTGCTGAAAGGGAAACGCTTGA
FP_135344Meripilus_giganteus
TGGAAAGAGAGTTAAACAGTACGTGAAATTGCTGAAAGGGAAACACTTGA
FP_100460_Sp --
CBS421_48Meripilus_giganteus --
Cui9203 --
Cui9202
TGGAAAGAGAGTTAAACAGTACGTGAAATTGCTGAAAGGGAAACACTTGA
TUFC100564Japan
TGGAAAGAGAGTTAAACAGTACGTGAAATTGCTGAAAGGGAAACACTTGA
Russell5913Meripilus_sumstinei --
RP215Meripilus_brasiliensis
TGGAAAGAGAGTTAAACAGTACGTGAAATTGCTGAAAGGGAAACACTTGA
RP200Meripilus_brasiliensis
TGGAAAGAGAGTTAAMCAGTACGTGAAATTGCTGAAAGGGAAACACTTGA
JV1712_13J_R_vinctus2_LSUPuert
TGGAAAGAGAGTTAAACAGTACGTGAAATTGCTGAAAGGGAAACGCTTGA
Dai10503R_hypobrunneus
TGGAAAGAGAGTTAAACAGTACGTGAAATTGCTGAAAGGGAAACGCTTGA
Dai10569Rigidoporus_hypobrunne
TGGAAAGAGAGTTAAACAGTACGTGAAATTGCTGAAAGGGAAACGCTTGA
1DAI19451
TGGAAAGAGAGTTAAACAGTACGTGAAATTGCTGAAAGGGAAACGCTTGA
CM108bRigidoporus_hypobrunneus --
1CUI16874
TGGAAAGAGAGTTAAACAGTACGTGAAATTGCTGAAAGGGAAACGCTTGA
FD299Cerrena_unicolor
TGGAAAGAGAGTTAAACAGTACGTGAAATTGCTGAAAGGGAAACGCTTGA
KHL_GB_Cerrena_uniclor
TGGAAAGAGAGTTAAACAGTACGTGAAATTGCTGAAAGGGAAACGCTTGA
Dai12892Cerrena_albocinnamomea
TGGAAAGAGAGTTAAACAGTACGTGAAATTGCTGAAAGGGAAACGATTGA
Dai12955C_albocinnamomea
TGGAAAGAGAGTTAAACAGTACGTGAAATTGCTGAAAGGGAAACGATTGA

SNUm03110102C_aurantiopora
TGGAAAGAGAGTTAAACAGTACGTGAAATTGCTGAAAGGGAAACGATTGA
NIBRFG0000102423C_aurantiopora
TGGAAAGAGAGTTAAACAGTACGTGAAATTGCTGAAAGGGAAACGATTGA
Dai7359Antrodiella_zonata
TGGAAAGAGAGTTAAACAGTACGTGAAATTGCTAAAAGGGAAACGCTTGA
F20080702KCM29C_consors
TGGAAAGAGAGTTAAACAGTACGTGAAATTGCTAAAAGGGAAACGCTTGA
F20080208LYW10Cerrena_consors
TGGAAAGAGAGTTAAACAGTACGTGAAATTGCTAAAAGGGAAACGCTTGA
Dai7821Antrodiella_zonata
TGGAAAGAGAGTTAAACAGTACGTGAAATTGCTAAAAGGGAAACGCTTGA
CFMR_DCL04_31Pseudolagarobasid --
VPB197Pseudolagarobasidium_bel --
CBS115543Pseudolagarobasidium_ --
CBS115544 --
Han405Pseudolagarobasidium_bai
TGGAAAGAGAGTTAAACAGTACGTGAAATTGCTGAAAGGGAAACGCTTGA
Han406Pseudolagarobasidium_bai
TGGAAAGAGAGTTAAACAGTACGTGAAATTGCTGAAAGGGAAACGCTTGA
MUcc838Spongipellis_delectans
TGGAAAGAGAGTTAAACAGTACGTGAAATTGCTGAAAGGGAAACGATTGA
BRNM686401S_delectans
TGGAAAGAGAGTTAAACAGTACGTGAAATTGCTGAAAGGGAAACGATTGA
OSM_F925S_delectans
TGGAAAGAGAGTTAAACAGTACGTGAAATTGCTGAAAGGGAAACGATTGA
BRNM67093Spongipellis_litschau
TGGAAAGAGAGTTAAACAGTACGTGAAATTGCTGAAAGGGAAACGCTTGA
CFMRccFP59199TS_unicolor
TGGAAAGAGAGTTAAACAGTACGTGAAATTGCTGAAAGGGAAACGCTTGA
CFMRccFP71791TS_unicolor
TGGAAAGAGAGTTAAACAGTACGTGAAATTGCTGAAAGGGAAACGCTTGA
Dai13845P_lischaueri
TGGAAAGAGAGTTAAACAGTACGTGAAATTGCTGAAAGGGAAACGCTTGA
Dai20266P_lischaueri
TGGAAAGAGAGTTAAACAGTACGTGAAATTGCTGAAAGGGAAACGCTTGA
CFMR_HHB11240Radulodon_america --
RLG6350Radulodon_americanus --
KY415963Radulodon_erikssonii
TGGAAAGAGAGTTAAACAGTACGTGAAATTGCTGAAAGGGAAACGCTTGA
HHB9567spRadulodon_casearius
TGGAAAGAGAGTTAAACAGTACGTGAAATTGCTGAAAGGGAAACGCTTGA
KRT_Iso_26Radulodon_casearius --
CBS126044Radulodon_erikssonii

TGGAAAGAGAGTTAAACAGTACGTGAAATTGCTGAAAGGGAAACGCTTGA
He6183YUNNANENSIS
TGGAAAGAGAGTTAAACAGTACGTGAAATTGCTGAAAGGGAAACGCTTGA
Cui17979YUNNANENSIS
TGGAAAGAGAGTTAAACAGTACGTGAAATTGCTGAAAGGGAAACGCTTGA
Miettinen2091Junghuhnia_fimbri
TGGAAAGAGAGTTAAACAGTACGTGAAATTGTTGAAAGGGAAACGATTGA
KHL12316S_tenue
TGGAAAGAGAGTTAAACAGTACGTGAAATTGCTGAAAGGGAAACGCTTGA
PRM846564S_pachyodon
TGGAAAGAGAGTTAAACAGTACGTGAAATTGCTGAAAGGGAAACGCTTGA
SP_Lgt_S_pachyodon --
Ryvarden44669Tyromyces_xuchile
TGGAAAGAGAGTTAAACAGTACGTGAAATTGCTGAAAGGGAAACGCTTGA
PW17_171sinuosus --
W53Dai12234
TGGAAAGAGAGTTAAACAGTACGTGAAATTGCTGAAAGGGAAACGCTTGA
HHB4100SpAntella_americana
TGGAAAGAGAGTTAAACAGTACGTGAAATTGCTGAAAGGGAAACGCTTGA
W3Dai20901spumeus
TGGAAAGAGAGTTAAACAGTACGTGAAATTGCTGAAAGGGAAACACTTGA
He6736
TGGAAAGAGAGTTAAACAGTACGTGAAATTGCTGAAAGGGAAACACTTGA
BRNM734877S_spumeus
TGGAAAGAGAGTTAAACAGTACGTGAAATTGCTGAAAGGGAAACACTTGA
BRNM712630S_spumeus
TGGAAAGAGAGTTAAACAGTACGTGAAATTGCTGAAAGGGAAACACTTGA
Dai1723Loweomyces_sibiricus --
W54Cui10009
TGGAAAGAGAGTTAAACAGTACGTGAAATTGCTGAAAGGGAAACACTTGA
W1Dai20899 --
HHB13445Trametes_ochracea
TGGAAAGAGAGTTAAACAGTACGTGAAATTGCTAAAAGGGAAACGCTTGA
Dai16222
TGGAAAGAGAGTTAAACAGTACGTGAAATTGCTGAAAGGGAAACGCTTGA
Dai16240
TGGAAAGAGAGTTAAACAGTACGTGAAATTGCTGAAAGGGAAACGCTTGA

JV1310_11SanguinolentusCernys AGTCAGTCGCGTTGTTCAGAACTCAGCCTGG---
CCTTG-GCCTGGTGCA
MJ39_00_SK AGTCAGTCGCGTTGTTCAGAACTCAGCCTGG---
CCTTG-GCCTGGTGCA
MJ111_04_CZ --
JV1610_BOKYsmrk AGTCAGTCGCGTTGTTCAGAACTCAGCCTGG---

CCTTG-GCCTGGTGCA
Dai21030 --
Dai20976P_furcatus AGTCAGTCGCGTTGTTCAGAACTCAGCCTGG---
CCTTG-GCCTGGTGCA
Dai2105 AGTCAGTCGCGTTGTTCAGAACTCAGCCTGG---
CCTTG-GCCTGGTGCA
Dai2544 AGTCAGTCGCGTTGTTCAGAACTCAGCCTGG---
CCTTG-GCCTGGTGCA
Dai11313 AGTCAGTCGCGTTGTTCAGAACTCAGCCTGG---
CCTTG-GCCTGGTGCA
WCG1611Dai26167 AGTCAGTCGCGTTGTTCAGAACTCAGCCTGG---
CCTTG-GCCTGGTGCA
WCG1518Dai25999Physisporinus AGTCAGTCGCGTTGTTCAGAACTCAGCCTGG---
CCTTG-GCCTGGTGCA
TAA15097 AGTCAGTCGCGTTGTTCAGAACTCAGCCTGG---
CCTTG-GCCCGGTGCA
JV8909_19_CZ --
JV1310_15_P_sanguinolentus2_CZ AGTCAGTCGCGTTGTTCAGAACTCAGCCTGG---
CCTTG-GCCTGGTGCA
MJ53_02_CZ AGTCAGTCGCGTTGTTCAGAACTCAGCCTGG---
CCTTG-GCCTGGTGCA
CLZhao21647P_yunnanensis AGTCAGTCGCGTTGTTCAGAACTCAGCCTGG---
CCTTG-GCCTGGTGCA
CLZhao21583P_yunnanensis AGTCAGTCGCGTTGTTCAGAACTCAGCCTGG---
CCTTG-GCCTGGTGCA
Dai22272 AGTCAGTCGCGTTGTTCAGAACTCAGCCTGG---
CCTTG-GCCTGGTGCA
Dai22279 AGTCAGTCGCGTTGTTCAGAACTCAGCCTGG---
CCTTG-GCCTGGTGCA
MJ332_94_CZ --
MJ642_94_CZ_Expallescens --
Dai21060P_vinctus AGTCAGTCGCGTTGTTCAGAACTCAGCCTGG---
CCTCG-GCTTGGTGCA
JV0511_23LRP_pouzarii AGTCAGTCGCGTCGTTCAGAACTCAGCCTGG---
CCTTG-GCTTGGTGCA
JQ409462_R_pouzarii_PRM899856_ --
JV0308_66_WA --
JV0309_45_WA_USA --
JV0709_83_CA_USA AGTCAGTCGCGTTGTTCAGAACTCAGCCTGG---
CCTTG-GCTTGGTGCA
Dai21043P_pouzarii AGTCAGTCGCGTCGTTCAGAACTCAGCCTGG---
CCTTG-GCTTGGTGCA
MJ144_95_CZ AGTCAGTCGCGTTGTTCAGAACTCAGCCTGG---
CCTCG-GCTTGGTGCA

JV0909_3_CZ AGTCAGTCGCGTTGTTCAGAACTCAGCCTGG---
CCTCG-GCTTGGTGCA
JV0609_1_K ---
Dai20396Physisporinus_castanop AGTCAGTCGCGTTGTTCAGAACTCAGCCTGG---
CCTCG-GCTTGGTGCA
Dai20397Physisporinus_castanop AGTCAGTCGCGTTGTTCAGAACTCAGCCTGG---
CCTCG-GCTTGGTGCA
MJ19_09_SK_Abies AGTCAGTCGCGTTGTTCAGAACTCAGCCTGG---
CTTTG-GCTTGGTGCA
JV0509_40_J_TN_USA_Betula ---
JV0808_33crocatus_PA_USAlist AGTCAGTCGCGTTGTTCAGAACTCAGCCTGG---
CTTTG-GCTTGGTGCA
DLL2009_061P_crocatus ---
Dai12800P_subcrocatus AGTCAGTCGCGTTGTTCAGAACTCAGCCTGG---
CTTTG-GCTTGGTGCA
Dai15917P_subcrocatus AGTCAGTCGCGTTGTTCAGAACTCAGCCTGG---
CTTTG-GCTTGGTGCA
Cui16620 AGTCAGTCGCGTTGTTCAGAACTCAGCCTGG---
CCTTG-GCTTGGTGCA
HCFC1088Meripilus_stillicidior AGTCAGTCGCGTTGTTCAGAACTCAGCCTGG---
CCTTG-GCTTGGTGCA
MCW590Meripilus_obsrcurus ---
MCW722Meripilus_obsrcurus ---
Cui9381P_tibeticus AGTCAGTCGCGTTGTTCAGAACTCAGCCTGG---
CCTCG-GCTTGGTGCA
Cui9588P_tibeticus AGTCAGTCGCGTTGTTCAGAACTCAGCCTGG---
CCTCG-GCTTGGTGCA
Va2_Beneschova ---
CWU3874_Ukraine_Alnus AGTCAGTCGCGTTGTTCAGAACTCAGCCTGG---
CCTTG-GCTTGGTGCA
WCG1293Dai24718Physisporinus ---
WCG1268Dai24682A AGTCAGTCGCGTTGTTCAGAACTCAGCCTGG---
CCTTG-GCTTGGTGCA
WCG1269Dai24683A AGTCAGTCGCGTTGTTCAGAACTCAGCCTGG---
CCTTG-GCTTGGTGCA
WCG1279Dai24694A AGTCAGTCGCGTTGTTCAGAACTCAGCCTGG---
CCTTG-GCTTGGTGCA
Dai16971 AGTCAGTCGCGTTGTTCAGAACTCAGCCTGG---
CCTTG-GCTTGGTGCA
ZQY1043Dai26696 AGTCAGTCGCGTTGTTCAGAACTCAGCCTGG---
CCTTG-GCTTGGTGCA
Doll880 ---
Doll1000 AGTCAGTCGCGTTGTTCAGAACTCAGCCTGG---
CCTTG-GCTTGGTGCA

1DAI18529 AGTCAGTCGCGTTGTTCAGAACTCAGCCTGG---
CCTTG-GCTTGGTGCA
Dai19535 AGTCAGTCGCGTTGTTCAGAACTCAGCCTGG---
CCTTG-GCTCGGTGCA
1704_79_hnedyVillaLaPaz AGTCAGTCGCGTTGTTCAGAACTCAGCCTGG---
CCTTG-GCTTGGTGCA
F2061 --
1DAI18268 AGTCAGTCGCGTTGTTCAGAACTCAGCCTGG---
CCTTG-GCTTGGTGCA
1DAI18540A AGTCAGTCGCGTTGTTCAGAACTCAGCCTGG---
CCTTG-GCTTGGTGCA
Dai17695 AGTCAGTCGCGTTGTTCAGAACTCAGCCTGG---
CCTTG-GCTTGGTGCA
LKY18Dai26373 --
Dai17839P_sulphureus AGTCAGTCGCGTTGTTCAGAACTCAGCTTGG---
CCTTG-GCTTGGTGCA
Dai17841P_sulphureus AGTCAGTCGCGTTGTTCAGAACTCAGCTTGG---
CCTTG-GCTTGGTGCA
Dai19877P_roseus AGTCAGTCGCGTTGTTTAGAACTCAGCCTGG---
CCTTG-GCTTGGTGCA
1508_18_1_Kout --
KP859303_R_vinctus_RP185_BRAZI --
JK1807_15Rigidoporus_sp_Puerto AGTCAGTCGCGTTGTTCAGAACTCAGCCTGG---
CTTTG-GCTTGGTGCA
JV0509_47_J_TN_USA AGTCAGTCGCGTTGTTCAGAACTCAGCCTAG---
CCTTG-GCTTGGTGCA
JV0709_188 AGTCAGTCGCGTTGTTCAGAACTCAGCCTAG---
CCTTG-GCTTGGTGCA
JV0509_127_PA_USA AGTCAGTCGCGTTGTTCAGAACTCAGCCTAG---
CCTTG-GCTTGGTGCA
JV1009_59_NJ_USA AGTCAGTCGCGTTGTTCAGAACTCAGCCTAG---
CCTTG-GCTTGGTGCA
Dai15497P_crataegi AGTCAGTCGCGTTGTTCAGAACTCAGCCTGG---
TCTTG-GCCTGGTGCA
Dai15499P_crataegi AGTCAGTCGCGTTGTTCAGAACTCAGC-TGG---
TCTTG-GCCTGGTGCA
Cui3266P_cinereus AGTCAGTCGCGTTGTTCAGAACTCAGCCTAG---
CCTTG-GCTTGGTGCA
WCG1256Dai24690 AGTCAGTCGCGTTGTTCAGAACTCAGCCTAG---
CCTTG-GCTTGGTGCA
1DAI17581 AGTCAGTCGCGTTGTTCAGAACTCAGCCTAG---
CCTTG-GCTTGGTGCA
WCG1255Dai24688 AGTCAGTCGCGTTGTTCAGAACTCAGCCTAG---
CCTTG-GCTTGGTGCA

Dai22427 AGTCAGTCGCGTTGTTCAGAACTCAGCCTAG---
CCTTG-GCTTGGTGCA
MV690Meripilus_concrescens AGTCAGTCGCGTTGTTCAGAACTCAGCCTGG---
CCTCG-GCTTGGTGCA
MV513Meripilus_galapagensis AGTCAGTCGCGTTGTTCAGAACTCAGCCTGG---
CCTTG-GCTTGGTGCA
Dai19793 AGTCAGTCGCGTTGTTCAGAACTCAGCCTGG---
CCTTG-GCTTGGTGCA
OQ553780P_tamilnaduensis AGTCAGTCGCGTTGTTCAGAACTCAGCCTGG---
CTTTG-GCTTGGTGCA
OQ553779P_tamilnaduensis AGTCAGTCGCGTTGTTCAGAACTCAGCCTGG---
CTTTG-GCTTGGTGCA
A164FB3Meripilus_giganteus --
JV1407_36_Vinctus_Meandrica AGTCAGTCGCGTTGTTCAGAACTCAGCCTGG---
CTTTG-GCTTGGTGCA
1807_3K_Rigidoporus_PuertoRico AGTCAGTCGCGTTGTTCAGAACTCAGCCTGG---
CTTTG-GCTTGGTGCA
Cui16903P_vinctus AGTCAGTCGCGTTGTTCAGAACTCAGCCTGG---
CTTTG-GCTTGGTGCA
JV1008_18R_Lineatus AGTCAGTCGCGTTGTTCAGAACTCAGCCTGG---
CCTTG-GCTTGGTGCA
JV1407_37_1_Vinctus_Carara AGTCAGTCGCGTTGTTCAGAACTCAGCCTGG---
CYTTG-GCTTGGTGCA
Dai17986P_lineatus AGTCAGTCGCGTTGTTCAGAACTCAGCCTGG---
CTTTG-GCTTGGTGCA
Dai18281 AGTCAGTCGCGTTGTTCAGAACTCAGCCTGG---
CTTTG-GCTTGGTGCA
1DAI19796 AGTCAGTCGCGTTGTTCAGAACTCAGCCTGG---
CTTTG-GCTTGGTGCA
ZQY797Dai25241 AGTCAGTCGCGTTGTTCAGAACTCAGCCTGG---
CTTTG-GCTTGGTGCA
WCG1289Dai24711 --
Dai22598 AGTCAGTCGCGTTGTTCAGAACTCAGCCTGG---
CTTTG-GCTTGGTGCA
Dai20523 AGTCAGTCGCGTTGTTCAGAACTCAGCCTGG---
CTTTG-GCTTGGTGCA
Dai17885 AGTCAGTCGCGTTGTTCAGAACTCAGCCTGG---
CTTTG-GCTTGGTGCA
Dai17553 AGTCAGTCGCGTTGTTCAGAACTCAGCCTGG---
CTTTG-GCTTGGTGCA
Dai19639 AGTCAGTCGCGTTGTTCAGAACTCAGCCTGG---
CTTTG-GCTTGGTGCA
JV0110_48_CZ AGTCAGTCGCGTCGTTCAGAACTCAGCCTGG---
CTTTT-GCTTGGTGCA

MJ129_04 AGTCAGTCGCGTCGTTCAGAACTCAGCCTGG--
CTTTT-GCTTGGTGCA
Cui10340P_eminens --
Cui10341P_eminens AGTCAGTCGCGTCGTTCAGAACTCAGCCTGG--
CTTTT-GCTTGGTGCA
Dai12685P_eminens AGTCAGTCGCGTCGTTCAGAACTCAGCCTGG--
CTTTT-GCTTGGTGCA
Miettinen_13591Rigidoporus_und AGTCAGTCGCGTCGTTCAGAACTCAGCCTGG--
CTTTT-GCTTGGTGCA
Dai20868 AGTCAGTCGCGTTGTTCAGAACTCAGCCTGG--
CTTTT-GCTTGGTGCA
Dai20832 AGTCAGTCGCGTTGTTCAGAACTCAGCCTGG--
CTTTT-GCTTGGTGCA
Dai11400 AGTCAGTCGCGTTGTTCAGAACTCAGCCTGG--
CTTTT-GCTTGGTGCA
Dai22472 AGTCAGTCGCGTTGTTCAGAACTCAGCCTGG--
CTTTT-GCTTGGTGCA
1CUI10475 AGTCAGTCGCGTCGTTCAGAACTCAGCCTGG--
CTTTT-GCTCGGTGCA
1CUI10491 --
HCFC1095Meripilus_robledoi AGTCAGTCGCGTTGTTCANAACTCANNCTGG--
CTTTT-GCTCGGTGCA
MCW702Meripilus_revolubilis AGTCAGTCGCGTTGTTCAGAACTCAGCCTGG--
CTTTT-GCTCGGTGCA
1704_83_zluty_HaciendaBaru AGTCAGTCGCGTCATTCAGAACTCAGCCTGG--
CTTTT-GCTTGGTGCA
Dai9925P_lavendulus AGTCAGTCGCGTCATTCAGAACTCAGCCTGG--
CTTTC-GCTTGGTGCA
Dai13587AP_lavendulus AGTCAGTCGCGTCATTCAGAACTCAGCCTGG--
CTTTC-GCTTGGTGCA
PDD70600P_longicystidius --
Cui16630 AGTCAGTCGCGTCATTCAGAACTCAGCCTGG--
CTTTT-GCTTGGTGCA
FP_135344Meripilus_giganteus AGTCAGTCGCGTTGGTCAGAACTCAGCCCGG--
CCATGCGCTTGGTGCA
FP_100460_Sp --
CBS421_48Meripilus_giganteus --
Cui9203 --
Cui9202 AGTCAGTCGCGTTGGTCAGAACTCAGCCCGG--
CCATGCGCTTGGTGCA
TUFC100564Japan AGTCAGTCGCGTTGGTCAGAACTCAGCCCGG--
CCATGCGCTTGGTGCA
Russell5913Meripilus_sumstinei --
RP215Meripilus_brasiliensis AGTCAGTCGCGTTGGTCAGAACTCAGCCCGG--

CCTTGTGCTTGGTGCA
RP200Meripilus_brasiliensis AGTCAGTCGCGTTGGTCAGAACTCAGCCCGG---
CCTTGTGCTTGGTGCA
JV1712_13J_R_vinctus2_LSUPuert AGTCAGTCGCGTTATKCAGAACTCAGCCTGG---
CTTTT-GCTTGGTGTA
Dai10503R_hypobrunneus AGTCAGTCGCGTTATGCAGAACTCAGCCTGG---
CTTTT-GCTTGGTGTA
Dai10569Rigidoporus_hypobrunne AGTCAGTCGCGTTATTCAGAACTCAGCCTGG---
CTTTT-GCTTGGTGTA
1DAI19451 AGTCAGTCGCGTTATTCAGAACTCAGCCTGG---
CTTTT-GCTTGGTGTA
CM108bRigidoporus_hypobrunneus --
1CUI16874 AGTCAGTCGCGTTATGCAGAACTCAGCCTGG---
CTTTT-GCTTGGTGTA
FD299Cerrena_unicolor AGTCAGTCGCGTTATGCAGAACTCAGCCTGG---
CTTTT-GCTTGGTGTA
KHL_GB_Cerrena_uniclor AGTCAGTCGCGTTATGCAGAACTCAGCCTGG---
CTTTT-GCTTGGTGTA
Dai12892Cerrena_albocinnamomea AGTCAGTCGCGTTGTACAGAACTCAGCCTGG---
CTTTT-GCTTGGTGTA
Dai12955C_albocinnamomea AGTCAGTCGCGTTGTACAGAACTCAGCCTGG---
CTTTT-GCTTGGTGTA
SNUm03110102C_aurantiopora AGTCAGTCGCGTTGTACAGAACTCAGCCTGG---
CTTTT-GCTTGGTGTA
NIBRFG0000102423C_aurantiopora AGTCAGTCGCGTTGTACAGAACTCAGCCTGG---
CTTTT-GCTTGGTGTA
Dai7359Antrodiella_zonata AGTCAGTCGCGTTATGCAGAACTCAGCCTGG---
CTTTT-GCTTGGTGTA
F20080702KCM29C_consors AGTCAGTCGCGTTATGCAGAACTCAGCCTGG---
CTTTT-GCTTGGTGTA
F20080208LYW10Cerrena_consors AGTCAGTCGCGTTATGCAGAACTCAGCCTGG---
CTTTT-GCTTGGTGTA
Dai7821Antrodiella_zonata AGTCAGTCGCGTTATGCAGAACTCAGCCTGG---
CTTTT-GCTTGGTGTA
CFMR_DCL04_31Pseudolagarobasid --
VPB197Pseudolagarobasidium_bel --
CBS115543Pseudolagarobasidium_ --
CBS115544 --
Han405Pseudolagarobasidium_bai AGTCAGTCGCGTTGTACAGAACTCAGCCTGG---
CTTTT-GCTCGGTGTA
Han406Pseudolagarobasidium_bai AGTCAGTCGCGTTGTACAGAACTCAGCCTGG---
CTTTT-GCTCGGTGTA
MUcc838Spongipellis_delectans AGTCAGTCGCGTCGTGCAGAACTCAGCCTGG---
CTTTT-GCTTGGTGTA

BRNM686401S_delectans AGTCAGTCGCGTCGTGCAGAACTCAGCCTGG---
CTTTT-GCTTGGTGTA
OSM_F925S_delectans AGTCAGTCGCGTCGTGCAGAACTCAGCCTGG---
CTTTT-GCTTGGTGTA
BRNM67093Spongipellis_litschau AGTCAGTCGCGTCATGCAGAACTCAGCCTGG---
CTTTTTGCTTGGTGTA
CFMRccFP59199TS_unicolor AGTCAGTCGCGTCATGCAGAACTCAGCCTGG---
CTTTT-GCTCGGTGTA
CFMRccFP71791TS_unicolor AGTCAGTCGCGTCATGCAGAACTCAGCCTGG---
CTTTT-GCTCGGTGTA
Dai13845P_lischaueri AGTCAGTCGCGTCATGCAGAACTCAGCCTGG---
CTTTT-GCTCGGTGTA
Dai20266P_lischaueri AGTCAGTCGCGTCATGCAGAACTCAGCCTGG---
CTTTT-GCTCGGTGTA
CFMR_HHB11240Radulodon_america --
RLG6350Radulodon_americanus --
KY415963Radulodon_erikssonii AGTCAGTCGCGTTATGCAGAACTCAGCCCGG---
CTTGG-GCTAGGTGTA
HHB9567spRadulodon_casearius AGTCAGTCGCGTTATGCAGAACTCAGCCTGG---
CTTTG-GCCAGGTGTA
KRT_Iso_26Radulodon_casearius --
CBS126044Radulodon_erikssonii AGTCAGTCGCGTTATGCAGAACTCAGCCCGG---
CTTTG-GCTAGGTGTA
He6183YUNNANENSIS AGTCAGTCGCGTTATGCAGAACTCAGCCCGG--
-CTTTG-GCCAGGTGTA
Cui17979YUNNANENSIS AGTCAGTCGCGTTATGCAGAACTCAGCCTGG---
CTTTG-GCCAGGTGTA
Miettinen2091Junghuhnia_fimbri AGTCAGTCGCGTTGATCAGAACTCAGCCTGG---
CTTTT-GCTTGGTGCA
KHL12316S_tenue AGTCAGTCGCGTTGACCAGAACTCAGCCTGG---
CTTTT-GCTTGGTGCA
PRM846564S_pachyodon AGTCAGTCGCGTCGTGCAGAACTCAGCCTGG---
CCTTG-GCCTGGTGTA
SP_Lgt_S_pachyodon --
Ryvarden44669Tyromyces_xuchile AGTCAGTCGCGTCGTGCAGAACTCAGCCTGG---
CCTTG-GCCTGGTGTA
PW17_171sinuosus --
W53Dai12234 AGTCAGTCGCGTCGTGCAGAACTCAGCCTGG---
CCTTG-GCCTGGTGTA
HHB4100SpAntella_americana AGTCAGTCGCGTTGGCCAGAACTCAGCCTGG---
CTTTT-GCCTGGTGCA
W3Dai20901spumeus AGTCAGTCGCGTTGTCCAGAACTCAGCCTGGCA-
TTTTTTGCCTGGTGCA
He6736 AGTCAGTCGCGTTGTCCAGAACTCAGCCTGGCA-

TTTTTTGCCCGGTGCA
BRNM734877S_spumeus
AGTCAGTCGCGTTGTCCAGAACTCAGCCTGGCATTTTYTTGCCTGGTGCA
BRNM712630S_spumeus
AGTCAGTCGCGTTGTCCAGAACTCAGCCTGGCA-TTTYTTGCCTGGTGCA
Dai1723Loweomyces_sibiricus --
W54Cui10009 AGTCAGTCGCGTTGTCCAGAACTCAGCCTGGCA-TTTTTTGCCCGGTGCA
W1Dai20899 --
HHB13445Trametes_ochracea AGTCAGTCGCGTCGTCCGGAACTCAGCTTTG---CTTCG-GCTTAGTGCA
Dai16222 AGTCAGTCGCGTTGGTCAGAACTCAGCCTGG---CTTTT-GCCTGGTGCA
Dai16240 AGTCAGTCGCGTTGGTCAGAACTCAGCCTGG---CTTTT-GCCTGGTGCA

JV1310_11SanguinolentusCernys TTTTCTGAAC-GACGGGCCAGCATCAATTTTGACTGTTGGATAAAGGTTA
MJ39_00_SK TTTTCTGAAC-GACGGGCCAGCATCAATTTTGACTGTTGGATAAAGGTTA
MJ111_04_CZ --
JV1610_BOKYsmrk TTTTCTGAAC-GACGGGCCAGCATCAATTTTGACTGTTGGATAAAGGTTA
Dai21030 --
Dai20976P_furcatus TTTTCTGAAT-GACGGGCCAGCATCAATTTTGACTGTTGGATAAAGGTTA
Dai2105 TTTTCTGAAT-GACGGGCCAGCATCAATTTTGACTGTTGGATAAAGGTCG
Dai2544 TTTTCTGAAT-GACGGGCCAGCATCAATTTTGACTGTTGGATAAAGGTCG
Dai11313 TTTTCTGAAT-GACGGGCCAGCATCAATTTTGACTGTTGGATAAAGGTTA
WCG1611Dai26167 TTTTCTGAAC-GACGGGCCAGCATCAATTTTGACTGTTGGATAAAGGTTA
WCG1518Dai25999Physisporinus TTTTCTGAAT-GACGGGCCAGCATCAATTTTGACTGTTGGATAAAGGTTA
TAA15097 TTTTCTGAAC-GACGGGCCAGCATCAATTTTGACTGTTGGATAAAGGTTA
JV8909_19_CZ --
JV1310_15_P_sanguinolentus2_CZ TTTTCTGAAT-GACGGGCCAGCATCAATTTCGACTGTTGGATAAAGGTTG
MJ53_02_CZ TTTTCTGAAT-GACGGGCCAGCATCAATTTCGACTGTTGGATAAAGGTTG
CLZhao21647P_yunnanensis TTTTCTGAAT-

GACGGGCCAGCATCAATTTTGACTGTTGGATAAAGGTTG
CLZhao21583P_yunnanensis TTTTCTGAAT-
GACGGGCCAGCATCAATTTTGACTGTTGGATAAAGGTTG
Dai22272 TTTTCTGAAT-
GACGGGCCAGCATCAATTTCGACTGTTGGATAAAGGTTA
Dai22279 TTTTCTGAAT-
GACGGGCCAGCATCAATTTCGACTGTTGGATAAAGGTTA
MJ332_94_CZ ---
MJ642_94_CZ_Expallescens ---
Dai21060P_vinctus TTTTCTGAAT-
GACGGGCCAGCATCAATTTTGACTGTTGGATAAAGGCCA
JV0511_23LRP_pouzarii TTTTCTGAAT-
GACGGGCCAGCATCAATTTTGACTGTTGGATAAAGGTTA
JQ409462_R_pouzarii_PRM899856_ ---
JV0308_66_WA ---
JV0309_45_WA_USA ---
JV0709_83_CA_USA TTTTCTGAAT-
GACGGGCCAGCATCAATTTTGACTGTTGGATAAAGGCCA
Dai21043P_pouzarii TTTTCTGAAT-
GACGGGCCAGCATCAATTTTGACTGTTGGATAAAGGTTA
MJ144_95_CZ TTTTCTGAAC-
GACGGGCCAGCATCAATTTTGAYCGTTGGATAAAGGCCA
JV0909_3_CZ TTTTCTGAAC-
GACGGGCCAGCATCAATTTTGACCGTTGGATAAAGGCCA
JV0609_1_K ---
Dai20396Physisporinus_castanop TTTTCTGAAT-
GACGGGCCAGCATCAATTTTGACCGTTGGATAAAGGCCA
Dai20397Physisporinus_castanop TTTTCTGAAT-
GACGGGCCAGCATCAATTTTGACCGTTGGATAAAGGCCA
MJ19_09_SK_Abies TTTTCTGAAT-
GACGGGCCAGCATCAATTTTGACTGTTGGATAAAGGCCA
JV0509_40_J_TN_USA_Betula ---
JV0808_33crocatus_PA_USAlist TTTTCTGAAT-
GACGGGCCAGCATCAATTTTGACTGTTGGATAAAGGCCA
DLL2009_061P_crocatus ---
Dai12800P_subcrocatus TTTTCTGAAT-
GACGGGCCAGCATCAATTTTGACTGTTGGATAAAGGCCA
Dai15917P_subcrocatus TTTTCTGAAT-
GACGGGCCAGCATCAATTTTGACTGTTGGATAAAGGCCA
Cui16620 TTTTCTGAAT-
AACGGGCCAGCATCAATTTTGACTGTTGGATAAAGGCCA
HCFC1088Meripilus_stillicidior TTTTCTGAAT-
AACGGGCCAGCATCAATTTTGACTGTTGGATAAAGGCCA
MCW590Meripilus_obsrcurus ---

MCW722Meripilus_obsrcurus --
Cui9381P_tibeticus TTTTCTGAAT-GACGGGCCAGCATCAATTTTGACTGTTGGATAAAGGCCA
Cui9588P_tibeticus TTTTCTGAAT-GACGGGCCAGCATCAATTTTGACTGTTGGATAAAGGCCA
Va2_Beneschova --
CWU3874_Ukraine_Alnus TTTTCTGAAT-GACGGGCCAGCATCAATTTTGACCGTTGGATAAAGGCCA
WCG1293Dai24718Physisporinus --
WCG1268Dai24682A TTTTCTGAAT-GACGGGCCAGCATCAATTTTGACTGTTGGATAAAGATCA
WCG1269Dai24683A TTTTCTGAAT-GACGGGCCAGCATCAATTTTGACTGTTGGATAAAGATCA
WCG1279Dai24694A TTTTCTGAAT-GACGGGCCAGCATCAATTTTGACTGTTGGATAAAGATCA
Dai16971 TTTTCTGAAT-GACGGGCCAGCATCAATTTTGACTGTTGGATAAAGATCA
ZQY1043Dai26696 TTTTCTGAAT-GACGGGCCAGCATCAATTTTGACTGTTGGATAAAGGTCA
Doll880 --
Doll1000 TTTTCTGAAT-AACGGGCCAGCATCAATTTTGACTGTTGGATAAAGGCCA
1DAI18529 TTTTCTGAAT-AACGGGCCAGCATCAATTTTGACTGTTGGATAAAGGCTA
Dai19535 TTTTCTGAAT-AACGGGCCAGCATCAATTTCGACCGTTGGATAAAGGCTG
1704_79_hnedyVillaLaPaz TTTTCTGAAT-GACGGGCCAGCATCAATTTTGACTGTTGGATAAAGACTG
F2061 --
1DAI18268 TTTTCTGAAT-GACGGGCCAGCATCAATTTTGACTGTTGGATAAAGGCTG
1DAI18540A TTTTCTGAAT-GACGGGCCAGCATCAATTTTGACTGTTGGATAAAGGCTG
Dai17695 TTTTCTGAAT-GACGGGCCAGCATCAATTTTGACTGTTGGATAAAGGCTG
LKY18Dai26373 --
Dai17839P_sulphureus TTTTCTGAAT-GACGGGCCAGCATCAATTTTGACTGTCGGAAAAAGGCTG
Dai17841P_sulphureus TTTTCTGAAT-GACGGGCCAGCATCAATTTTGACTGTCGGAAAAAGGCTG
Dai19877P_roseus TTTTCTGAAT-GACGGGCCAGCATCAATTTTGACTGTCGGATAAAGGCTG
1508_18_1_Kout --
KP859303_R_vinctus_RP185_BRAZI --

JK1807_15Rigidoporus_sp_Puerto CTTTCTGAAT-AACGGGCCAGCATCAATTTCGACTGTTGGATAAAGGCCA
JV0509_47_J_TN_USA TTTTCTGAAT-AACGGGCCAGCATCAATTTTGACTGTTGGATAAAGGCCG
JV0709_188 TTTTCTGAAT-AACGGGCCAGCATCAATTTTGACTGTTGGATAAAGGCCG
JV0509_127_PA_USA TTTTCTGAAT-AACGGGCCAGCATCAATTTTGACTGTTGGATAAAGGCCG
JV1009_59_NJ_USA TTTTCTGAAT-AACGGGCCAGCATCAATTTTGACTGTTGGATAAAGGCCG
Dai15497P_crataegi TTTTCTGAAT-AACGGGCCAGCATCAATTTTGACTGTTGGATAAAGGCTG
Dai15499P_crataegi TTTTCTGAAT-AACGGGCCAGCATCAATTTTGACTGTTGGATAAAGGCTG
Cui3266P_cinereus TTTTCTGAAT-AACGGGCCAGCATCAATTTTGACTGTTGGATAAAGGCTG
WCG1256Dai24690 TTTTCTGAAT-AACGGGCCAGCATCAATTTTGACTGTTGGATAAAGGCTG
1DAI17581 TTTTCTGAAT-AACGGGCCAGCATCAATTTTGACTGTTGGATAAAGGCTG
WCG1255Dai24688 TTTTCTGAAT-AACGGGCCAGCATCAATTTTGACTGTTGGATAAAGGCTG
Dai22427 TTTTCTGAAT-AACGGGCCAGCATCAATTTTGACTGTTGGATAAAGGCTG
MV690Meripilus_concrescens TTTTCTGAAT-AACGGGCCAGCATCAATTTTGACTGTTGGATAAAGGCTG
MV513Meripilus_galapagensis TTTTCTGAAT-AACGGGCCAGCATCAATTTTGACTGTTGGATAAAGGCTG
Dai19793 TTTTCTGAAT-AACGGGCCAGCATCAATTTTGACTGTTGGATAAAGGCCA
OQ553780P_tamilnaduensis TTTTCTGAAT-AACGGGCCAGCATCAATTTTGACTATTGGATAAAGGCTG
OQ553779P_tamilnaduensis TTTTCTGAAT-AACGGGCCAGCATCAATTTTGACTATTGGATAAAGGCTG
A164FB3Meripilus_giganteus ---
JV1407_36_Vinctus_Meandrica TTTTCTGAGT-AACGGGCCAGCATCAATTTTGACTGTTGGATAAAGGCTG
1807_3K_Rigidoporus_PuertoRico TTTTCTGAGT-AACGGGCCAGCATCAATTTTGACTGTTGGATAAAGGCTG
Cui16903P_vinctus TTTTCTGAGT-AACGGGCCAGCATCAATTTTGACTGTTGGATAAAGGCTG
JV1008_18R_Lineatus TTTTCTGAAT-AACGGGCCAGCATCAATTTTGACTGTTGGATAAAGGCTA
JV1407_37_1_Vinctus_Carara TTTTCTGAAT-

AACGGGCCAGCATCAATTTTGACTGTTGGATAAAGGCTA
Dai17986P_lineatus TTTTCTGAAT-
AACGGGCCAGCATCAATTTTGACTGTTGGATAAAGGCCA
Dai18281 TTTTCTGAAT-
AACGGGCCAGCATCAATTTTGACTGTTGGATAAAGGCTA
1DAI19796 TTTTCTGAAT-
AACGGGCCAGCATCAATTTTGACTGTTGGATAAAGGCCA
ZQY797Dai25241 TTTTCTGAAT-
AACGGGCCAGCATCAATTTTGACTGTTGGATAAAGGCCA
WCG1289Dai24711 --
Dai22598 TTTTCTGAAT-
AACGGGCCAGCATCAATTTTGACTGTTGGATAAAGGCTA
Dai20523 TTTTCTGAAT-
AACGGGCCAGCATCAATTTTGACTGTTGGATAAAGGCTA
Dai17885 TTTTCTGAAT-
AACGGGCCAGCATCAATTTTGACTGTTGGATAAAGGCTA
Dai17553 TTTTCTGAAT-
AACGGGCCAGCATCAATTTTGACTGTTGGATAAAGGCTA
Dai19639 TTTTCTGAAT-
AACGGGCCAGCATCAATTTTGACTGTTGGATAAAGGCTA
JV0110_48_CZ CTTTCTGAAT-
GACGGGCCAGCATCAATTTTGACTGTTGGATAAAGGCCA
MJ129_04 CTTTCTGAAT-
GACGGGCCAGCATCAATTTTGACTGTTGGATAAAGGCCA
Cui10340P_eminens --
Cui10341P_eminens CTTTCTGAAT-
GACGGGCCAGCATCAATTTTGACTGTTGGATAAAGGCCA
Dai12685P_eminens CTTTCTGAAT-
GACGGGCCAGCATCAATTTTGACTGTTGGATAAAGGCCA
Miettinen_13591Rigidoporus_und CTTTCTGAAT-
GACGGGCCAGCATCAATTTTGACTGTTGGATAAAGGCCA
Dai20868 CTTTCTGAAT-
GACGGGCCAGCATCAATTTTGACTGTTGGATAAAGGCCA
Dai20832 CTTTCTGAAT-
GACGGGCCAGCATCAATTTCGACTGTTGGATAAAGGCCA
Dai11400 CTTTCTGAAT-
GACGGGCCAGCATCAATTTCAACTGTTGGATAAAGGCCA
Dai22472 CTTTCTGAAT-
GACGGGCCAGCATCAATTTTGACTGTTGGATAAAGGCTA
1CUI10475 CTTTCTGAAT-
GACGGGCCAGCATCAATTTTGACTGTTGGATAAAGGCTG
1CUI10491 --
HCFC1095Meripilus_robledoi CTTTCTGAAT-
GACGGGCCAGCATCAATTTTGAVTGTCGGATAAAGGTVA

MCW702Meripilus_revolubilis CTTTCTGAAT-GACGGGCCAGCATCAATTTTGACTGTCGGATAAAGGCCA
1704_83_zluty_HaciendaBaru TTTTCTGATTCGACGGGCCAGCATGAATTTTGACTGTTGGATAAAGGCTG
Dai9925P_lavendulus TTTTCTGATT-GACGGGCCAGCATCAATTTTGACCGTCGGATAAAGGCTG
Dai13587AP_lavendulus TTTTCTGATT-GACGGGCCAGCATCAATTTTGACCGTCGGATAAAGGCTG
PDD70600P_longicystidius --
Cui16630 TTTTCTGAGT-GACGGGCCAGCATCAATTTTGACCGTCGGATAAAGGCTG
FP_135344Meripilus_giganteus TTTTCTGATC-GACGGGCCAGCATCAATTTCGACCGTGGGATAAAGGCTG
FP_100460_Sp --
CBS421_48Meripilus_giganteus --
Cui9203 --
Cui9202 TTTTCTGATC-GACGGGCCAGCATCAATTTCAACCGTGGGATAAAGGCTG
TUFC100564Japan TTTTCTGATC-GACGGGCCAGCATCAATTTTGACCATGGGATAAAGGCCG
Russell5913Meripilus_sumstinei --
RP215Meripilus_brasiliensis TTTTCTGGCG-GACGGGCCAGCATCAATTTTGACCGTTGGATAAAGGCCA
RP200Meripilus_brasiliensis TTTTCTGGCG-GACGGGCCAGCATCAATTTTGACCGTTGGATAAAGGCCA
JV1712_13J_R_vinctus2_LSUPuert TTTTCTGTAT-GACGGGCCAGCATCAATTTTGGCCGCTGGAAAAAGGCTC
Dai10503R_hypobrunneus TTTTCTGTAT-GACGGGCCAGCATCAATTTTGGCCGCTGGAAAAAGGCTC
Dai10569Rigidoporus_hypobrunne TTTTCTGTAT-GACGGGCCAGCATCAATTTTGGCCGCTGGAAAAAGGCTC
1DAI19451 TTTTCTGTAT-GACGGGCCAGCATCAATTTTGGCCGCTGGAAAAAGGCTC
CM108bRigidoporus_hypobrunneus --
1CUI16874 TTTTCTGTAT-GACGGGCCAGCATCAATTTTGGCCGCTGGAAAAAGGCTC
FD299Cerrena_unicolor TTTTCTGTAT-GACGGGTCAGCATCAATTTTGATCGCTGGAAAAAGGCCT
KHL_GB_Cerrena_uniclor TTTTCTGTAT-GACGGGTCAGCATCAATTTTGATCGCTGGAAAAAGGTCT
Dai12892Cerrena_albocinnamomea TTTTCTGTAT-GACGGGCCAGCATCAATTTTGGCCGCTGGAAAAAGGCTT
Dai12955C_albocinnamomea TTTTCTGTAT-GACGGGCCAGCATCAATTTTGGCCGCTGGAAAAAGGCTT

SNUm03110102C_aurantiopora TTTTCTGTAT-GACGGGCCAGCATCAATTTTGGCCGCTGGAAAAAGGCTT
NIBRFG0000102423C_aurantiopora TTTTCTGTAT-GACGGGCCAGCATCAATTTTGGCCGCTGGAAAAAGGCTT
Dai7359Antrodiella_zonata TTTTTTGTAT-GACGGGCCAGCATCAATTTTGGCCGCTGGAAAAAGGCCT
F20080702KCM29C_consors TTTTCTGTAT-GACGGGCCAGCATCAATTTTGGCCGCTGGAAAAAGGCCT
F20080208LYW10Cerrena_consors TTTTCTGTAT-GACGGGCCAGCATCAATTTTGGCCGCTGGAAAAAGGCCT
Dai7821Antrodiella_zonata TTTTTTGTAT-GACGGGCCAGCATCAATTTTGGCCGCTGGAAAAAGGCCT
CFMR_DCL04_31Pseudolagarobasid --
VPB197Pseudolagarobasidium_bel --
CBS115543Pseudolagarobasidium_ --
CBS115544 --
Han405Pseudolagarobasidium_bai TTTTCTGTAT-AACGGGCCAGCATCAATTTTGGCCGCTGGAAAAAGGCTT
Han406Pseudolagarobasidium_bai TTTTCTGTAT-AACGGGCCAGCATCAATTTTGGCCGCTGGAAAAAGGCTT
MUcc838Spongipellis_delectans TTTTCTGCAT-GACGGGCCAGCATCAGTTTTGGCCGCTGGAAAAGGGTTT
BRNM686401S_delectans TTTTCTGCAT-GACGGGCCAGCATCAGTTTTGGCCGCTGGAAAAGGGTTT
OSM_F925S_delectans TTTTCTGCAT-GACGGGCCAGCATCAGTTTTGGCCGCTGGAAAAGGGTTT
BRNM67093Spongipellis_litschau TTTTCTGCAT-GACGGGCCAGCATCAGTTTTGGTCGCTGGAAAAAGTCTT
CFMRccFP59199TS_unicolor TTTTCTGCAT-GACGGGCCAGCATCAGTTTTGGTCGCTGGAAAAAGTCTC
CFMRccFP71791TS_unicolor TTTTCTGCAT-GACGGGCCAGCATCAGTTTTGGTCGCTGGAAAAAGTCTC
Dai13845P_lischaueri TTTTCTGCAT-GACGGGCCAGCATCAGTTTTGGTCGCTGGAAAAAGTCTT
Dai20266P_lischaueri TTTTCTGCAT-GACGGGCCAGCATCAGTTTTGGTCGCTGGAAAAAGTCTT
CFMR_HHB11240Radulodon_america --
RLG6350Radulodon_americanus --
KY415963Radulodon_erikssonii TTTTCTGTAT-GACGGGCCAGCATCAGTTTCGGCCGCTGGAAAAGGGCTC
HHB9567spRadulodon_casearius TTTTCTGCAT-GACGGGCCAGCATCAGTTTTGGCCGCTGGAAAAGGGCCC
KRT_Iso_26Radulodon_casearius --
CBS126044Radulodon_erikssonii TTTTCTGTAT-

GACGGGCCAGCATCAGTTTCGGCCGCTGGAAAAGGGCTC
He6183YUNNANENSIS TTTTCTGCAT-
GACGGGCCAGCATCAGTTTCTGCCGCTGGAAAAGGGCCC
Cui17979YUNNANENSIS TTTTCTGCAT-
GACGGGCCAGCATCAGTTTCGGCCGCTGGAAAAGGGCCC
Miettinen2091Junghuhnia_fimbri TTTTCTGATT-
GACGGGCCAGCATCAGTTTTGACCGCTGGATAAAGGTCT
KHL12316S_tenue TTTTCTGGTT-
GACGGGCCAGCATCGGTTTTGACCGCTGGATAAAGGCCT
PRM846564S_pachyodon TTTTCTGCGC-
GACGGGCCAGCATCGATTTCGGTCGCTGGAAAAGGGCCT
SP_Lgt_S_pachyodon ---
Ryvarden44669Tyromyces_xuchile TTTTCTGCAT-
GACGGGCCAGCATCGATTTCGGTCGCTGGAAAAGGGTCT
PW17_171sinuosus ---
W53Dai12234 TTTTCTGCAT-
GACGGGCCAGCATCAATTTCGGTCGCTGGAAAAGGGCCT
HHB4100SpAntella_americana TTTTCTGGTT-
GACGGGCCAGCATCAATTTTGACCGCTGGATAAAGGCCT
W3Dai20901spumeus
TTTTCTGGTTCGACGGGCCAGCATCAATTTTGACCGTTGGATAAAGGCCT
He6736
TTTTCTGGTTCGACGGGCCAGCATCAATTTTGACCGTTGGATAAAGGCCT
BRNM734877S_spumeus
TTTTCTGGTTCGACGGGCCAGCATCAATTTTGACCGTTGGATAAAGGCCT
BRNM712630S_spumeus
TTTTCTGGTTCGACGGGCCAGCATCAATTTTGACCGTTGGATAAAGGCCT
Dai1723Loweomyces_sibiricus ---
W54Cui10009
TTTTCTGGTTCGACGGGCCAGCATCAATTTTGACCGTTGGATAAAGGCCT
W1Dai20899 ---
HHB13445Trametes_ochracea CTTTCCGGTT-
GACGGGCCAGCATCGATTTTGACCGCTGGAAAAGGGCTG
Dai16222 TTTTCTGGTT-
GACGGGCCAGCATCAATTTTGACTGCTGGATAAAGGCCT
Dai16240 TTTTCTGGTT-
GACGGGCCAGCATCAATTTTGACTGCTGGATAAAGGCCT

JV1310_11SanguinolentusCernys
GGGAAATGTGGCTTCTTCGGGAGTGTTATAGTCCTTGGCTGCATACAATG
MJ39_00_SK
GGGAAATGTGGCTTCTTCGGGAGTGTTATAGTCCTTGGCTGCATACAATG
MJ111_04_CZ ---
JV1610_BOKYsmrk

GGGAAATGTGGCTTCTTCGGGAGTGTTATAGTCCTTGGCTGCATACAATG
Dai21030 ---
Dai20976P_furcatus
GGGAAATGTGGCTTCTTCGGGAGTGTTATAGTCCTTGGCTGCATACAATG
Dai2105
GGGAAATGTGGCTTCTTCGGGAGTGTTATAGTCCTTGGCTGCATACAATG
Dai2544
GGGAAATGTGGCTTCTTCGGGAGTGTTATAGTCCTTGGCTGCATACAATG
Dai11313
GGGAAATGTGGCTTCTTCGGGAGTGTTATAGTCCTTGGCTGCATACAATG
WCG1611Dai26167
GGGAAATGTGGCTTCTTTGGGAGTGTTATAGTCCTTGGCTGCATACAATG
WCG1518Dai25999Physisporinus
GGGAAATGTGGCTTCTTCGGGAGTGTTATAGTCCTTGGCTGCATACAATG
TAA15097
GGGAAATGTGGCTTCTTCGGGAGTGTTATAGTCCTTGGCTGCATACAATG
JV8909_19_CZ ---
JV1310_15_P_sanguinolentus2_CZ
GGGAAATGTGGCTTCTTYGGGAGTGTTATAGTCCTTGGCTGCATACAATG
MJ53_02_CZ
GGGAAATGTGGCTTCTTCGGGAGTGTTATAGTCCTTGGCTGCATACAATG
CLZhao21647P_yunnanensis
GGGAAATGTGGCTTCTTCGGGAGTGTTATAGTCCTCGGCTGCATACGATG
CLZhao21583P_yunnanensis
GGGAAATGTGGCTTCTTCGGGAGTGTTATAGTCCTCGGCTGCATACGATG
Dai22272
GGGAAATGTGGCTTCTTCGGGAGTGTTATAGTCCTTGGCTGCATACAATG
Dai22279
GGGAAATGTGGCTTCTTCGGGAGTGTTATAGTCCTTGGCTGCATACAATG
MJ332_94_CZ ---
MJ642_94_CZ_Expallescens ---
Dai21060P_vinctus
GAGAAATGTGGCTCCTTTGGGAGTGTTATAGTCTCTGGTTGCATACAATG
JV0511_23LRP_pouzarii
GAGAAATGTGGCTTCTTTGGGAGTGTTATAGTCTCTGATTGCATACAATG
JQ409462_R_pouzarii_PRM899856_ ---
JV0308_66_WA ---
JV0309_45_WA_USA ---
JV0709_83_CA_USA
GAGAAATGTGGCTCCTTTGGGAGTGTTATAGTCTCTGGTTGCATACAATG
Dai21043P_pouzarii
GAGAAATGTGGCTTCTTTGGGAGTGTTATAGTCTCTGATTGCATACAATG
MJ144_95_CZ
GAGAAATGTGGCTCCTTTGGGAGTGTTATAGTCTTTGGTTGCATACAATG

JV0909_3_CZ
GAGAAATGTGGCTCCTTTGGGAGTGTTATAGTCTTTGGTTGCATACAATG
JV0609_1_K --
Dai20396Physisporinus_castanop
GAGAAATGTGGCTCCTTTGGGAGTGTTATAGTCTTTGGTTGCATACAATG
Dai20397Physisporinus_castanop
GAGAAATGTGGCTCCTTTGGGAGTGTTATAGTCTTTGGTTGCATACAATG
MJ19_09_SK_Abies
GAGAAATGTGGCTCCTTTGGGAGTGTTATAGTCTTTGGTTGCATACAATG
JV0509_40_J_TN_USA_Betula ---------------------
TGTTATAGTCTTTGGTTGCATACAATG
JV0808_33crocatus_PA_USAlist
GAGAAATGTGGCTCCTTTGGGAGTGTTATAGTCTTTGGTTGCATACAATG
DLL2009_061P_crocatus --
Dai12800P_subcrocatus
GAGAAATGTGGCTCCTTTGGGAGTGTTATAGTCTTTGGTTGCATACAATG
Dai15917P_subcrocatus
GAGAAATGTGGCTCCTTTGGGAGTGTTATAGTCTTTGGTTGCATACAATG
Cui16620
GAGAAATGTGGCTCCTTTGGGAGTGTTATAGTCTCTGGTTGCATACAATG
HCFC1088Meripilus_stillicidior
GAGAAATGTGGCTCCTTTGGGAGTGTTATAGTCTCTGGTTGCATACAATG
MCW590Meripilus_obsrcurus --
MCW722Meripilus_obsrcurus --
Cui9381P_tibeticus
GAGAAATGTGGCTCCTTCGGGAGTGTTATAGTCTTTGGTTGCATACAATG
Cui9588P_tibeticus
GAGAAATGTGGCTCCTTCGGGAGTGTTATAGTCTTTGGTTGCATACAATG
Va2_Beneschova --
CWU3874_Ukraine_Alnus
GAGAAATGTGGCTCCTTTGGGAGTGTTATAGTCTTTGGTTGCATGCAATG
WCG1293Dai24718Physisporinus --
WCG1268Dai24682A
AGGAAATGTGGCTCCTTTGGGAGTGTTATAGTCCTTGGTTGCATACAATG
WCG1269Dai24683A
AGGAAATGTGGCTCCTTTGGGAGTGTTATAGTCCTTGGTTGCATACAATG
WCG1279Dai24694A
AGGAAATGTGGCTCCTTTGGGAGTGTTATAGTCCTTGGTTGCATACAATG
Dai16971
AGGAAATGTGGCTCCTTTGGGAGTGTTATAGTCCTTGGTTGCATACAATG
ZQY1043Dai26696
AGGAAATGTGGCTCCTTTGGGAGTGTTATAGTCCTTGGTTGCATACAATG
Doll880 --
Doll1000

GGGAAATGTGGCTCCTCCGGGAGTGTTATAGTCCTTGGTTGCATACAATG
1DAI18529
GGGAAATGTGGCTCCTCCGGGAGTGTTATAGTCCTTGGTTGCATACAATG
Dai19535
GAGAAATGTGGCTCCCTTGGGAGTGTTATAGTCTCTGGTTGCATACGATG
1704_79_hnedyVillaLaPaz
AGGAAATGTGGCTCCTTCGGGAGTGTTATAGTCTTCAGTTGCATACAATG
F2061 --
1DAI18268
GGGAAATGTGGCTCCTTTGGGAGTGTTATAGTCTTCAGTTGCATACAATG
1DAI18540A
GGGAAATGTGGCTCCTTTGGGAGTGTTATAGTCTTCAGTTGCATACAATG
Dai17695
GGGAAATGTGGCTCCTTTGGGAGTGTTATAGTCTTCAGTTGCATACAATG
LKY18Dai26373 --
Dai17839P_sulphureus
GAGAAATGTGGCTCCTTTGGGAGTGTTATAGTCTCTGGTTGCATACGATG
Dai17841P_sulphureus
GAGAAATGTGGCTCCTTTGGGAGTGTTATAGTCTCTGGTTGCATACGATG
Dai19877P_roseus
GGGAAATGTGGCTCCTTTGGGAGTGTTATAGTCTCTGGTTGCATACGATG
1508_18_1_Kout --
KP859303_R_vinctus_RP185_BRAZI --
JK1807_15Rigidoporus_sp_Puerto
GTGAAATGTGGCTCCTTTGGGAGTGTTATAGTCTCTGGTTGCATACAATG
JV0509_47_J_TN_USA
AGGAAATGTGGCTCCTTTGGGAGTGTTATAGTCCTTGGTTGCATACAATG
JV0709_188
AGGAAATGTGGCTCCTTTGGGAGTGTTATAGTCCTTGGTTGCATACAATG
JV0509_127_PA_USA
AGGAAATGTGGCTCCTTTGGGAGTGTTATAGTCCTTGGTTGCATACAATG
JV1009_59_NJ_USA
AGGAAATGTGGCTCCTTTGGGAGTGTTATAGTCCTTGGTTGCATACAATG
Dai15497P_crataegi
GGGAAATGTGGCTCCTTTGGGAGTGTTATAGTCCCTAGTTGCATACAATG
Dai15499P_crataegi
GGGAAATGTGGCTCCTTTGGGAGTGTTATAGTCCCTAGTTGCATACAATG
Cui3266P_cinereus
AGGAAATGTGGCTCCTTTGGGAGTGTTATAGTCCTCAGTTGCATACAATG
WCG1256Dai24690
AGGAAATGTGGCTCCTTTGGGAGTGTTATAGTCCTCAGTTGCATACAATG
1DAI17581
AGGAAATGTGGCTCCTTTGGGAGTGTTATAGTCCTCAGTTGCATACAATG
WCG1255Dai24688

AGGAAATGTGGCTCCTTTGGGAGTGTTATAGTCCTCAGTTGCATACAATG
Dai22427
AGGAAATGTGGCTCCTTTGGGAGTGTTATAGTCCTCAGTTGCATACAATG
MV690Meripilus_concrescens
GGGAAATGTGGCTCCTTTGGGAGTGTTATAGTCCTCAGTTGCATACAATG
MV513Meripilus_galapagensis
GGGAAATGTGGCTCCTTTGGGAGTGTTATAGTCCTCAGTTGCATACAATG
Dai19793
GGGAAATGTGGCTCCCTTGGGAGTGTTATAGTCCTTGGTTGCATACAATG
OQ553780P_tamilnaduensis
AGGAAATGTGGCTCCTTTGGGAGTGTTATAGTCCTTGGTTGCATACAATG
OQ553779P_tamilnaduensis
AGGAAATGTGGCTCCTTTGGGAGTGTTATAGTCCTTGGTTGCATACAATG
A164FB3Meripilus_giganteus --
JV1407_36_Vinctus_Meandrica
AGGAAATGTGGCTCCTTTGGGAGTGTTATAGTCCTTGGTTGCATACAATG
1807_3K_Rigidoporus_PuertoRico
AGGAAATGTGGCTCCTTTGGGAGTGTTATAGTCCTTGGTTGCATACAATG
Cui16903P_vinctus
AGGAAATGTGGCTCCTTTGGGAGTGTTATAGTCCTTGGTTGCATACAATG
JV1008_18R_Lineatus
GGGAAATGTGGCTCCCTTGGGAGTGTTATAGTCCTTGGTTGCATACAATG
JV1407_37_1_Vinctus_Carara
GGGAAATGTGGCTCCCTTGGGAGTGTTATAGTCCTTGGTTGCATACAATG
Dai17986P_lineatus
GGGAAATGTGGCTCCCTTGGGAGTGTTATAGTCCTTGGTTGCATACAATG
Dai18281
GGGAAATGTGGCTCCCTTGGGAGTGTTATAGTCCTTGGTTGCATACAATG
1DAI19796
GGGAAATGTGGCTCCCTTGGGAGTGTTATAGTCCTTGGTTGCATACAATG
ZQY797Dai25241
GGGAAATGTGGCTCCCTTGGGAGTGTTATAGTCCTTGGTTGCATACAATG
WCG1289Dai24711 --
Dai22598
GGGAAATGTGGCTCCTTTGGGAGTGTTATAGTCCTTGGTTGCATACAATG
Dai20523
GGGAAATGTGGCTCCTTTGGGAGTGTTATAGTCCTTGGTTGCATACAATG
Dai17885
GGGAAATGTGGCTCCTTTGGGAGTGTTATAGTCCTTGGTTGCATACAATG
Dai17553
GGGAAATGTGGCTCCTTTGGGAGTGTTATAGTCCTTGGTTGCATACAATG
Dai19639
GGGAAATGTGGCTCCTTTGGGAGTGTTATAGTCCTTGGTTGCATACAATG
JV0110_48_CZ

GAGAAATGTGGCTCCTTTGGGAGTGTTATAGTCTCCGGTTGCATACAATG
MJ129_04
GAGAAATGTGGCTCCTTTGGGAGTGTTATAGTCTCCGGTTGCATACAATG
Cui10340P_eminens --
Cui10341P_eminens
GAGAAATGTGGCTCCTTTGGGAGTGTTATAGTCTCCGGTTGCATACAATG
Dai12685P_eminens
GAGAAATGTGGCTCCTTTGGGAGTGTTATAGTCTCCGGTTGCATACAATG
Miettinen_13591Rigidoporus_und
GAGAAATGTGGCTCCTTTGGGAGTGTTATAGTCTCCGGTTGCATACAATG
Dai20868
GAGAAATGTGGCTCCTTTGGGAGTGTTATAGTCTCTGGTTGCATACAATG
Dai20832
GAGAAATGTGGCTCCTTTGGGAGTGTTATAGTCTCTGGTTGCATACAATG
Dai11400
GAGAAATGTGGCTCCTTTGGGAGTGTTATAGTCTCTGGTTGCATACAATG
Dai22472
GAGAAATGTGGCTCCTTTGGGAGTGTTATAGTCTCTGGTTGCATACAATG
1CUI10475
GAGAAATGTGGCTCCTTTGGGAGTGTTATAGTCTCCGGTTGCATACGATG
1CUI10491 --
HCFC1095Meripilus_robledoi
GAGAAATGTGGCTCCTTTGGGAGTGTTATAGTCTCTGGTTGCATGCAATG
MCW702Meripilus_revolubilis
GAGAAATGTGGCTCCTTCGGGAGTGTTATAGTCTCTGGTTGCATGCGATG
1704_83_zluty_HaciendaBaru
GAGAAATGTGGCTCCCTTGGGAGTGTTATAGTCTCTGGTCACATACAATG
Dai9925P_lavendulus
GAGAAATGTGGCTCTTTCGGGAGTGTTATAGTCTCTGGTCGCATGCGATG
Dai13587AP_lavendulus
GAGAAATGTGGCTCTTTCGGGAGTGTTATAGTCTCTGGTCGCATGCGATG
PDD70600P_longicystidius --
Cui16630
GAGAAATGTGGCTCTTTCGGGAGTGTTATAGTCTCTGGTTGCATACGATG
FP_135344Meripilus_giganteus
GAGAAAGGTGGCTCCCTTGGGAGTGTTATAGTCTCCGGTTGCATGCCACG
FP_100460_Sp --
CBS421_48Meripilus_giganteus --
Cui9203 --
Cui9202
GAGAAAGGTGGCTCCCTTGGGAGTGTTATAGTCTCCGGTTGCATGCCACG
TUFC100564Japan
GAGAAAGGTGGCTCCCTTGGGAGTGTTATAGTCTCTGGTTGCATGCCATG
Russell5913Meripilus_sumstinei --

RP215Meripilus_brasiliensis
GAGAAAGGTGGCTCTTTCGGGAGTGTTATAGTCTTTGGTTGCATACAATG
RP200Meripilus_brasiliensis
GAGAAAGGTGGCTCTTTCGGGAGTGTTATAGTCTTTGGTTGCATACAATG
JV1712_13J_R_vinctus2_LSUPuert
TAGAAATGTGGCACCTCCGGGTGTGTTATAGTCTAGGGTCATATACAGCG
Dai10503R_hypobrunneus
TAGAAATGTGGCACCTCCGGGTGTGTTATAGTCTAGGGTCATATACAGCG
Dai10569Rigidoporus_hypobrunne
TAGAAATGTGGCACCTCCGGGTGTGTTATAGTCTAGGGTCATATACAGCG
1DAI19451
TAGAAATGTGGCACCTCCGGGTGTGTTATAGTCTAGGGTCATATACAGCG
CM108bRigidoporus_hypobrunneus --
1CUI16874
TAGAAATGTGGCACCTCCGGGTGTGTTATAGTCTAGGGTCATATACAGCG
FD299Cerrena_unicolor
CAGAAATGTGGCACCTTCGGGTGTGTTATAGTCTGGGGTCATATACAGCG
KHL_GB_Cerrena_uniclor
CAGAAATGTGGCACCTTCGGGTGTGTTATAGTCTGGGGTCATATACAGCG
Dai12892Cerrena_albocinnamomea
CAGAAATGTGGCACCTTCGGGTGTGTTATAGTCTGAGGTCATATACAGCG
Dai12955C_albocinnamomea
CAGAAATGTGGCACCTTCGGGTGTGTTATAGTCTGAGGTCATATACAGCG
SNUm03110102C_aurantiopora
CAGAAATGTGGCACCTTCGGGTGTGTTATAGTCTGAGGTCATATACAGCG
NIBRFG0000102423C_aurantiopora
CAGAAATGTGGCACCTTCGGGTGTGTTATAGTCTGAGGTCATATACAGCG
Dai7359Antrodiella_zonata
CAGAAATGTGGCACCTTCGGGTGTGTTATAGTCTGAGGTCATATACAGCG
F20080702KCM29C_consors
CAGAAATGTGGCACCTTCGGGTGTGTTATAGTCTGAGGTCATATACAGCG
F20080208LYW10Cerrena_consors
CAGAAATGTGGCACCTTCGGGTGTGTTATAGTCTGAGGTCATATACAGCG
Dai7821Antrodiella_zonata
CAGAAATGTGGCACCTTCGGGTGTGTTATAGTCTGAGGTCATATACAGCG
CFMR_DCL04_31Pseudolagarobasid --
VPB197Pseudolagarobasidium_bel --
CBS115543Pseudolagarobasidium_ --
CBS115544 --
Han405Pseudolagarobasidium_bai
TAGAAATGTGGCACCTTCGGGTGTGTTATAGTCTAGAGTCATATACAGCG
Han406Pseudolagarobasidium_bai
TAGAAATGTGGCACCTTCGGGTGTGTTATAGTCTAGAGTCATATACAGCG
MUcc838Spongipellis_delectans

CAGGAATGTGGCACCTTCGGGTGTGTTATAGCCTGGGGTCGTATACAGCG
BRNM686401S_delectans
CAGGAATGTGGCACCTTCGGGTGTGTTATAGCCTGGGGTCGTATACAGCG
OSM_F925S_delectans
CAGGAATGTGGCACCTTCGGGTGTGTTATAGCCTGGGGTCGTATACAGCG
BRNM67093Spongipellis_litschau
CAGGAATGTGGCACCTTCGGGTGTGTTATAGCCTGGAGTCGTATACAGCG
CFMRccFP59199TS_unicolor
CAGGAATGTGGCACCTTCGGGTGTGTTATAGCCTGGAGTCGTATACAGCG
CFMRccFP71791TS_unicolor
CAGGAATGTGGCACCTTCGGGTGTGTTATAGCCTGGAGTCGTATACAGCG
Dai13845P_lischaueri
CAGGAATGTGGCACCTTCGGGTGTGTTATAGCCTGGAGTCGTATACAGCG
Dai20266P_lischaueri
CAGGAATGTGGCACCTTCGGGTGTGTTATAGCCTGGAGTCGTATACAGCG
CFMR_HHB11240Radulodon_america --
RLG6350Radulodon_americanus --
KY415963Radulodon_erikssonii
CGGGAATGTGGCACCTTYGGGTGTGTTATAGCCCGGGGTCGCATGCAGTG
HHB9567spRadulodon_casearius
CGGGAATGTGGCACCTTCGGGTGTGTTATAGCCTGGGGTCACATACGGCG
KRT_Iso_26Radulodon_casearius --
CBS126044Radulodon_erikssonii
CGGGAATGTGGCACCTTCGGGTGTGTTATAGCCCGGGGTCGCATGCAGTG
He6183YUNNANENSIS
CGGGAATGTGGCACCTTCGGGTGTGTTATAGCCCGGGGTCGCATGCGGCG
Cui17979YUNNANENSIS
CGGGAATGTGGCACCTTCGGGTGTGTTATAGCCCGGGGTCGCATGCGGCG
Miettinen2091Junghuhnia_fimbri
TAGGAATGTGGCACCTTCGGGTGTGTTATAGCCTAGGGTTGTATACAGTG
KHL12316S_tenue
TGGGAATGTGGCACCTTCGGGTGTGTTATAGCCCAGGGTTGTATACAGTG
PRM846564S_pachyodon
CGGGAATGTGGCACCCTCGGGTGTGTTATAGCCCGGGGTCGTATGCAGTG
SP_Lgt_S_pachyodon --
Ryvarden44669Tyromyces_xuchile
CGGGAATGTGGCACCTTCGGGTGTGTTATAGCCCGGGGTCGTATACAGTG
PW17_171sinuosus --
W53Dai12234
CGGGAATGTGGCACCTTCGGGTGTGTTATAGCCCGGGGTCGTATACAGTG
HHB4100SpAntella_americana
TGGGAATGTGGCACCTTCGGGTGTGTTATAGCCCTTGGTTGTATACAGTG
W3Dai20901spumeus
CGGAAATGTGGCACCTTCGGGTGTGTTATAGTCCTTGGTCGCATACAACG

He6736
CGGAAATGTGGCACCTTCGGGTGTGTTATAGTCCTTGGTCGCATACAACG
BRNM734877S_spumeus
CGGAAATGTGGCACCTTCGGGTGTGTTATAGTCCTTGGTCGCATACAACG
BRNM712630S_spumeus
CGGAAATGTGGCACCTTCGGGTGTGTTATAGTCCTTGGTCGCATACAACG
Dai1723Loweomyces_sibiricus --
W54Cui10009
CGGAAATGTGGCACCTTCGGGTGTGTTATAGTCCCTGGTCGCATGCAACG
W1Dai20899 --
HHB13445Trametes_ochracea
GAGGAATGTGGCACCTTCGGGTGTGTTATAGCCTTCAGTCGCATACAGCG
Dai16222
AAGGAATGTGGCACCTTCGGGTGTGTTATAGCCTTTGGTCACATGCAGTG
Dai16240
AAGGAATGTGGCACCTTCGGGTGTGTTATAGCCTTTGGTCACATGCAGTG

JV1310_11SanguinolentusCernys GTTGGGATTGAGGAACTCAGCATGCC-
TTTATGGTCGGGGTTCG-CCCAC
MJ39_00_SK GTTGGGATTGAGGAACTCAGCATGCC-
TTTATGGTCGGGGTTCG-CCCAC
MJ111_04_CZ --
JV1610_BOKYsmrk GTTGGGATTGAGGAACTCAGCATGCC-
TTTATGGTCGGGGTTCG-CCCAC
Dai21030 --
Dai20976P_furcatus GTTGGGATTGAGGAACTCAGCATGCC-
TTTATGGTCGGGGTTCG-CCCAC
Dai2105 GTTGGGATTGAGGAACTCAGCATGCC-
TTTATGGTCGGGGTTCG-CCCAC
Dai2544 GTTGGGATTGAGGAACTCAGCATGCC-
TTTATGGTCGGGGTTCG-CCCAC
Dai11313 GTTGGGATTGAGGAACTCAGCATGCC-
TTTATGGTCGGGGTTCG-CCCAC
WCG1611Dai26167 GTTGGGATTGAGGAACTCAGCATGCC-
TTTATGGTCGGGGTTCG-CCCAC
WCG1518Dai25999Physisporinus GTTGGGATTGAGGAACTCAGCATGCC-
TTTATGGTCGGGGTTCG-CCCAC
TAA15097 GTTGGGATTGAGGAACTCAGCATGCC-
TTTATGGTCGGGGTTCG-CCCAC
JV8909_19_CZ --
JV1310_15_P_sanguinolentus2_CZ GTTGGGATTGAGGAACTCAGCATGCC-
TTTATGGTCGGGGTTCG-CCCAC
MJ53_02_CZ GTTGGGATTGAGGAACTCAGCATGCC-
TTTATGGTCGGGGTTCG-CCCAC

CLZhao21647P_yunnanensis GTTGGGATTGAGGAACTCAGCATGCC-TTTATGGTCGGGGTTTG-CCCAC
CLZhao21583P_yunnanensis GTTGGGATTGAGGAACTCACCATCCC-TTTATGGTCGGGGTTTG-CCCAC
Dai22272 GTTGGGATTGAGGAACTCAGCATGCC-TTTATGGTCGGGGTTCG-CCCAC
Dai22279 GTTGGGATTGAGGAACTCAGCATGCC-TTTATGGTCGGGGTTCG-CCCAC
MJ332_94_CZ --
MJ642_94_CZ_Expallescens --
Dai21060P_vinctus GTTGGGATTGAGGAACTCAGCATGCC-TTTATGGTCGGGGTTCG-CCCAC
JV0511_23LRP_pouzarii GTTGGGATTGAGGAACTCAGCATGCC-TTTATGGTCGGGGTTCG-CCCAC
JQ409462_R_pouzarii_PRM899856_ --
JV0308_66_WA --
JV0309_45_WA_USA --
JV0709_83_CA_USA GTTGGGATTGAGGAACTCAGCATGCC-TTTACGGTCGGGGTTCG-CCCAC
Dai21043P_pouzarii GTTGGGATTGAGGAACTCAGCATGCC-TTTATGGTCGGGGTTCG-CCCAC
MJ144_95_CZ GTTGGGATTGAGGTACTCAGCATGCC-TTTATGGTCGGGGTTCG-CCCAC
JV0909_3_CZ GTTGGGATTGAGGTACTCAGCATGCC-TTTATGGTCGGGGTTCG-CCCAC
JV0609_1_K --
Dai20396Physisporinus_castanop GTTGGGATTGAGGAACTCAGCATGCC-TTTATGGTCGGGGTTCG-CCCAC
Dai20397Physisporinus_castanop GTTGGGATTGAGGAACTCAGCATGCC-TTTATGGTCGGGGTTCG-CCCAC
MJ19_09_SK_Abies GTTGGGATTGAGGAACTCAGCATGCC-TTTATGGTCGGGGTTCG-CCCAC
JV0509_40_J_TN_USA_Betula GTTGGGATTGAGGAACTCAGCATGCC-TTTATGGTCGGGGTTCG-CCCAC
JV0808_33crocatus_PA_USAlist GTTGGGATTGAGGAACTCAGCATGCC-TTTATGGTCGGGGTTCG-CCCAC
DLL2009_061P_crocatus --
Dai12800P_subcrocatus GTTGGGATTGAGGAACTCAGCATGCC-TTTATGGTCGGGGTTCG-CCCAC
Dai15917P_subcrocatus GTTGGGATTGAGGAACTCAGCATGCC-TTTATGGTCGGGGTTCG-CCCAC
Cui16620 GTTGGGATTGAGGTACTCAGCATGCCTTTTATGGTTGGGGTTCG-CCCAC
HCFC1088Meripilus_stillicidior GTTGGGATTGAGGAACTCAGCATGCC-

TTTATGGTTGGGGTTCG-CCCAC
MCW590Meripilus_obsrcurus --
MCW722Meripilus_obsrcurus --
Cui9381P_tibeticus GTTGGGATTGAGGAACTCAGCATGCC-
TTTATGGTCGGGGTTCG-CCCAC
Cui9588P_tibeticus GTTGGGATTGAGGAACTCAGCATGCC-
TTTATGGTCGGGGTTCG-CCCAC
Va2_Beneschova --
CWU3874_Ukraine_Alnus GTTGGGATTGAGGAACTCAGCATGCC-
TTTATGGTCGGGGTTCG-CCCAC
WCG1293Dai24718Physisporinus --
WCG1268Dai24682A GTTGGGATTGAGGATCTCAGCATGCC-
TTTATGGTCGGGGTTCG-CCCAC
WCG1269Dai24683A GTTGGGATTGAGGATCTCAGCATGCC-
TTTATGGTCGGGGTTCG-CCCAC
WCG1279Dai24694A GTTGGGATTGAGGATCTCAGCATGCC-
TTTATGGTCGGGGTTCG-CCCAC
Dai16971 GTTGGGATTGAGGATCTCAGCATGCC-
TTTATGGTCGGGGTTCG-CCCAC
ZQY1043Dai26696 GTTGGGATTGAGGATCTCAGCATGCC-
TTTATGGTCGGGGTTCG-CCCAC
Doll880 --
Doll1000 GTTGGGATTGAGGAACTCAGCATGCC-
TTTATGGTCGGGGTTCG-CCCAC
1DAI18529 GTTGGGATTGAGGAACTCAGCATGCC-
TTTATGGTCGGGGTTCG-CCCAC
Dai19535 GTTGGGATTGAGGAACTCAGCATGCC-
TTTATGGTCGGGGTTCG-CCCAC
1704_79_hnedyVillaLaPaz GTTGGGATTGAGGAATTCAGCATGCC-
TTTATGGTCGGGGTTCG-CCCAC
F2061 --
1DAI18268 GTTGGGATTGAGGAACTCAGCATGCC-
TTTATGGTCGGGGTTCG-CCCAC
1DAI18540A GTTGGGATTGAGGAACTCAGCATGCC-
TTTATGGTCGGGGTTCG-CCCAC
Dai17695 GTTGGGATTGAGGAACTCAGCATGCC-
TTTATGGTCGGGGTTCG-CCCAC
LKY18Dai26373 --
Dai17839P_sulphureus GTTGGGATTGAGGATCTCAGCATGCC-
TTTATGGTCGGGGTTCG-CCCAC
Dai17841P_sulphureus GTTGGGATTGAGGATCTCAGCATGCC-
TTTATGGTCGGGGTTCG-CCCAC
Dai19877P_roseus GTTGGGATTGAGGAACTCAGCATGCC-
TTTATGGTCGGGGTTCG-CCCAC

1508_18_1_Kout --
KP859303_R_vinctus_RP185_BRAZI --
JK1807_15Rigidoporus_sp_Puerto GTTGAGATTGAGGAACTCAGCATGCC-TTTATGGTCGGGGTTCG-CCCAC
JV0509_47_J_TN_USA GTTGGGATTGAGGAACTCAGCATGCC-TTTATGGTCGGGGTTCG-CCCAC
JV0709_188 GTTGGGATTGAGGAACTCAGCATGCC-TTTATGGTCGGGGTTCG-CCCAC
JV0509_127_PA_USA GTTGGGATTGAGGAACTCAGCATGCC-TTTATGGTCGGGGTTCG-CCCAC
JV1009_59_NJ_USA GTTGGGATTGA-GAACTCAGCATGC--TTTATGGTCGGGGTTCG-CCCAC
Dai15497P_crataegi GTTGGGATTGAGGAACTCAGCATGCC-TTTATGGTCGGGGTTTTACCCAC
Dai15499P_crataegi GTTGGGATTGAGGAACTCAGCATGCC-TTTATGGTCGGGGTTTT-CCCAC
Cui3266P_cinereus GTTGGGATTGAGGAACTCAGCATGCC-TTTATGGTCGGGGTTCG-CCCAC
WCG1256Dai24690 GTTGGGATTGAGGAACTCAGCATGCC-TTTATGGTCGGGGTTCG-CCCAC
1DAI17581 GTTGGGATTGAGGAACTCAGCATGCC-TTTATGGTCGGGGTTCG-CCCAC
WCG1255Dai24688 GTTGGGATTGAGGAACTCAGCATGCC-TTTATGGTCGGGGTTCG-CCCAC
Dai22427 GTTGGGATTGAGGAACTCAGCATGCC-TTTATGGTCGGGGTTCG-CCCAC
MV690Meripilus_concrescens GTTGGGATTGAGGAACTCAGCATGCC-TTTATGGTCGGGGTTCG-CCCAC
MV513Meripilus_galapagensis GTTGGGATTGAGGAACTCAGCATGCC-TTTATGGNNNNNNNNNN-NNNNN
Dai19793 GTTGGGATTGAGGAACTCAGCATGCC-TTTATGGTCGGGGTTCG-CCCAC
OQ553780P_tamilnaduensis GTTGGGATTGAGGAACTCAGCATGCC-TTTATGGTCGGGGTTCG-CCCAC
OQ553779P_tamilnaduensis GTTGGGATTGAGGAACTCAGCATGCC-TTTATGGTCGGGGTTCG-CCCAC
A164FB3Meripilus_giganteus --
JV1407_36_Vinctus_Meandrica GTTGGGATTGAGGAACTCAGCATGCC-TTTATGGTCGGGGTTCG-CCCAC
1807_3K_Rigidoporus_PuertoRico GTTGGGATTGAGGAACTCAGCATGCC--TTATGGTCGGGGTTCG-CCCAC
Cui16903P_vinctus GTTGGGATTGAGGAACTCAGCATGCC-TTTATGGTCGGGGTTCG-CCCAC
JV1008_18R_Lineatus GTTGGGATTGAGGAACTCAGCATGCC-

TTTATGGTCGGGGTTCG-CCCAC
JV1407_37_1_Vinctus_Carara GTTGGGATTGAGGAACTCAGCATGCC-
TTTATGGTCGGGGTTCG-CCCAC
Dai17986P_lineatus GTTGGGATTGAGGAACTCAGCATGCC-
TTTATGGTCGGGGTTCG-CCCAC
Dai18281 GTTGGGATTGAGGAACTCAGCATGCC-
TTTATGGTCGGGGTTCG-CCCAC
1DAI19796 GTTGGGATTGAGGAACTCAGCATGCC-
TTTATGGTCGGGGTTCG-CCCAC
ZQY797Dai25241 GTTGGGATTGAGGAACTCAGCATGCC-
TTTATGGTCGGGGTTCG-CCCAC
WCG1289Dai24711 --
Dai22598 GTTGGGATTGAGGAACTCAGCATGCC-
TTTATGGTCGGGGTTCG-CCCAC
Dai20523 GTTGGGATTGAGGAACTCAGCATGCC-
TTTATGGTCGGGGTTCG-CCCAC
Dai17885 GTTGGGATTGAGGAACTCAGCATGCC-
TTTATGGTCGGGGTTCG-CCCAC
Dai17553 GTTGGGATTGAGGAACTCAGCATGCC-
TTTATGGTCGGGGTTCG-CCCAC
Dai19639 GTTGGGATTGAGGAACTCAGCATGCC-
TTTATGGTCGGGGTTCG-CCCAC
JV0110_48_CZ GTTAGGATTGAGGAACTCAGCATGCC-
TTTATGGTCGGGGTTCR-CCCAC
MJ129_04 GTTAGGATTGAGGAACTCAGCATGCC-
TTTATGGTCGGGGTTCG-CCCAC
Cui10340P_eminens --
Cui10341P_eminens GTTAGGATTGAGGAACTCAGCATGCC-
TTTATGGTCGGGGTTCG-CCCAC
Dai12685P_eminens GTTAGGATTGAGGAACTCAGCATGCC-
TTTATGGTCGGGGTTCG-CCCAC
Miettinen_13591Rigidoporus_und GTTAGGATTGAGGAACTCAGCATGCC-
TTTATGGTCGGGGTTCG-CCCAC
Dai20868 GTTGGGATTGAGGAACTCAGCATGCC-
TTTATGGTCGGGGTTCG-CCCAC
Dai20832 GTTGGGATTGAGGAACTCAGCATGCC-
TTTATGGTCGGGGTTCG-CCCAC
Dai11400 GTTGGGATTGAGGAACTCAGCATGCC-
TTTATGGTCGGGGTTCG-CCCAC
Dai22472 GTTGGGATTGAGGAACTCAGCATGCC-
TTTATGGTCGGGGTTCG-CCCAC
1CUI10475 GTTGGGATTGAGGAACTCAGCATGCC-
TTTATGGTCGGGGTTCG-CCCAC
1CUI10491 --

HCFC1095Meripilus_robledoi GTTGGGATTGAGGAACTCAGCA---------------------------
-
MCW702Meripilus_revolubilis GTTGGGATTGAGGAACTCAGCATGCC-
TTTATGGTCGGGGTTCG-CCCAC
1704_83_zluty_HaciendaBaru GTTGGGATTGAGGAACTCAGCATGCC--
TCACGGCCGGGGTTCG-CCCAC
Dai9925P_lavendulus GTTGGGATTGAGGAACTCAGCATGCC--
TCACGGTCGGGGTTCG-CCCAC
Dai13587AP_lavendulus GTTGGGATTGAGGAACTCAGCATGCC--
TCACGGTCGGGGTTCG-CCCAC
PDD70600P_longicystidius --
Cui16630 GTTGGGATTGAGGAACTCAGCATGCC--
TCACGGTCGGGGTTCG-CCCAC
FP_135344Meripilus_giganteus GTTGGGATTGAGGAACTCAGCATGCC-
TTTACGGCCGGGGTTCG-CCCAC
FP_100460_Sp --
CBS421_48Meripilus_giganteus --
Cui9203 --
Cui9202 GTTGGGATTGAGGAACTCAGCATGCC-
TTTACGGCCGGGGTTCG-CCCAC
TUFC100564Japan GTTGGGATTGAGGAACTCAGCATGCC-
TTTATGGCCGGGGTTCG-CCCAC
Russell5913Meripilus_sumstinei --
RP215Meripilus_brasiliensis GTTGGGATTGAGGAACTCAGCATGCC-
TTTACGGTCGGGGTTCG-CCCAC
RP200Meripilus_brasiliensis GTTGGGATTGAGGAACTCAGCATGCC-
TTTACGGTCGGGGTTCG-CCCAC
JV1712_13J_R_vinctus2_LSUPuert GCTGGGATTGAGGTCTGCAGCACGCC-
TTTATGGCTGGGGTTCG-CCCAC
Dai10503R_hypobrunneus GCTGGGATTGAGGTCTGCAGCACGCC-
TTTATGGCTGGGGTTCG-CCCAC
Dai10569Rigidoporus_hypobrunne GCTGGGATTGAGGTCTGCAGCACGCC-
TTTATGGCTGGGGTTCG-CCCAC
1DAI19451 GCTGGGATTGAGGTCTGCAGCACGCC-
TTTATGGCTGGGGTTCG-CCCAC
CM108bRigidoporus_hypobrunneus --
1CUI16874 GCTGGGATTGAGGTCTGCAGCACGCC-
TTTATGGCTGGGGTTCG-CCCAC
FD299Cerrena_unicolor GTTGGGATTGAGGTCTGCAGCACGCC-
TTTATGGCTGGGGTTCG-CCCAC
KHL_GB_Cerrena_uniclor GTTGGGATTGAGGTCTGCAGCACGCC-
TTTATGGCTGGGGTTCG-CCCAC
Dai12892Cerrena_albocinnamomea GCTGGGATTGAGGTCTGCAGCACGCC-
TTTATGGCTGGGGTTCG-CCCAC

Dai12955C_albocinnamomea GCTGGGATTGAGGTCTGCAGCACGCC-
TTTATGGCTGGGGTTCG-CCCAC
SNUm03110102C_aurantiopora GCTGGGATTGAGGTCTGCAGCACGCC-
TTTATGGCTGGGGTTCG-CCCAC
NIBRFG0000102423C_aurantiopora GCTGGGATTGAGGTCTGCAGCACGCC-
TTTATGGCTGGGGTTCG-CCCAC
Dai7359Antrodiella_zonata GCTGGGATTGAGGTCTGCAGCACGCC-
TTTATGGCTGGGGTTCG-CCCAC
F20080702KCM29C_consors GCTGGGATTGAGGTCTGCAGCACGCC-
TTTATGGCTGGGGTTCG-CCCAC
F20080208LYW10Cerrena_consors GCTGGGATTGAGGTCTGCAGCACGCC-
TTTATGGCTGGGGTTCG-CCCAC
Dai7821Antrodiella_zonata GCTGGGATTGAGGTCTGCAGCACGCC-
TTTATGGCTGGGGTTCG-CCCAC
CFMR_DCL04_31Pseudolagarobasid --
VPB197Pseudolagarobasidium_bel --
CBS115543Pseudolagarobasidium_ --
CBS115544 --
Han405Pseudolagarobasidium_bai GCTGGGATTGAGGTCTGCAGCACGCC-
TTTATGGCTGGGGTTCG-CCCAC
Han406Pseudolagarobasidium_bai GCTGGGATTGAGGTCTGCAGCACGCC-
TTTATGGCTGGGGTTCG-CCCAC
MUcc838Spongipellis_delectans GCTGGGACTGAGGATCGCAGTGCGCC-
TTTATGGCTGGGGTTCG-CCCAC
BRNM686401S_delectans GCTGGGACTGAGGATCGCAGTGCGCC-
TTTATGGCTGGGGTTCG-CCCAC
OSM_F925S_delectans GCTGGGACTGAGGATCGCAGTGCGCC-
TTTATGGCTGGGGTTCG-CCCAC
BRNM67093Spongipellis_litschau GCTGGGACTGAGGATCGCAGCGCGCC-
TTTATGGCTGGGGTTCG-CCCAC
CFMRccFP59199TS_unicolor GCYGGGACTGAGGATCGCAGCGCGCC-
TTTATGGCTGGGGTTCG-CCCAC
CFMRccFP71791TS_unicolor GCAGGGACTGAGGATCGCAGCGCGCC-
TTTATGGCTGGGGTTCG-CCCAC
Dai13845P_lischaueri GCTGGGACTGAGGATCGCAGCGCGCC-
TTTATGGCTGGGGTTCG-CCCAC
Dai20266P_lischaueri GCTGGGACTGAGGATCGCAGCGCGCC-
TTTATGGCTGGGGTTCG-CCCAC
CFMR_HHB11240Radulodon_america --
RLG6350Radulodon_americanus --
KY415963Radulodon_erikssonii GCTGGGACTGAGGTCTGCAGCACGCC-
TTTATGGCTGGGGTTCG-CCCAC
HHB9567spRadulodon_casearius GCTGGGACTGAGGTCTGCAGCACGCC-
TTTATGGCTGGGGTTCG-CCCAC

KRT_Iso_26Radulodon_casearius ---
CBS126044Radulodon_erikssonii GCTGGGACTGAGGTCTGCAGCACGCC-TTTATGGCTGGGGTTCG-CCCAC
He6183YUNNANENSIS GCTGGGACTGAGGTCTGCAGCACGCC-TTTATGGCTGGGGTTCG-CCCAC
Cui17979YUNNANENSIS GCTGGGACTGAGGTCTGCAGCACGCC-TTTATGGCTGGGGTTCG-CCCAC
Miettinen2091Junghuhnia_fimbri GTTGGGACTGAGGATCACAGCATGCC-TTTACGGCCGGGGTTCG-CCCAC
KHL12316S_tenue GTTGGGACTGAGGATCTCAGCATGCC-TTTATGGTCGGGGTTCG-CCCAC
PRM846564S_pachyodon GCTGGGATCGAGGAATGCAGCACGCC-TTTATGGCTGGGGTTCG-CCCAC
SP_Lgt_S_pachyodon ---
Ryvarden44669Tyromyces_xuchile GCTGGGATTGAGGAATGCAGCACGCC-TTTATGGCTGGGGTTCG-CCCAC
PW17_171sinuosus ---
W53Dai12234 GCTGGGATTGAGGAATGCAGCACGCC-TTCACGGCTGGGATTCG-CCCAC
HHB4100SpAntella_americana GTTGGGATTGAGGATCTCAGCATGCC-TTTATGGTCGGGGTTCG-CCCAC
W3Dai20901spumeus GTTGGGATTGAGGATCTCAGCATGCC-TTTATGGTCGGGGTTCG-CCCAC
He6736 GTTGGGATTGAGGATCTCAGCATGCC-TTTATGGTCGGGGTTCG-CCCAC
BRNM734877S_spumeus GTTGGGATTGAGGATCTCAGCATGCC-TTTATGGTCGGGGTTCG-CCCAC
BRNM712630S_spumeus GTTGGGATTGAGGATCTCAGCATGCC-TTTATGGTCGGGGTTCG-CCCAC
Dai1723Loweomyces_sibiricus ---
W54Cui10009 GTTGGGATTGAGGATCTCAGCATGCC-TTTATGGTCGGGGTTCG-CCCAC
W1Dai20899 ---
HHB13445Trametes_ochracea GTTGGGATCGAGGAACGCAGCGCGCC--TTATGGCTGGGGTTCG-CCCAC
Dai16222 GTTGGGATTGAGGATCTCAGCATGCC-TTTATGGTCGGGGTTCG-CCCAC
Dai16240 GTTGGGATTGAGGATCTCAGCATGCC-TTTATGGTCGGGGTTCG-CCCAC

JV1310_11SanguinolentusCernys GT-TCATGCTTAGGATGCTGGCGTAATGGCTTTAAATGACCCGTCTTGAA
MJ39_00_SK GT-TCATGCTTAGGATGCTGGCGTAATGGCTTTAAATGACCCGTCTTGAA

MJ111_04_CZ --
JV1610_BOKYsmrk GT-
TCATGCTTAGGATGCTGGCGTAATGGCTTTAAATGACCCGTCTTGAA
Dai21030 --
Dai20976P_furcatus GT-
TCATGCTTAGGATGCTGGCGTAATGGCTTTAAATGACCCGTCTTGAA
Dai2105 GT-
TCATGCTTAGGATGCTGGCGTAATGGCTTTAAATGACCCGTCTTGAA
Dai2544 GT-
TCATGCTTAGGATGCTGGCGTAATGGCTTTAAATGACCCGTCTTGAA
Dai11313 GT-
TCATGCTTAGGATGCTGGCGTAATGGCTTTAAATGACCCGTCTTGAA
WCG1611Dai26167 GT-
TCATGCTTAGGATGCTGGCGTAATGGCTTTAAATGACCCGTCTTGAA
WCG1518Dai25999Physisporinus GT-
TCATGCTTAGGATGCTGGCGTAATGGCTTTAAATGACCCGTCTTGAA
TAA15097 GT-
TCATGCTTAGGATGCTGGCGTAATGGCTTTAAATGACCCGTCTTGAA
JV8909_19_CZ --
JV1310_15_P_sanguinolentus2_CZ GT-
TCATGCTTAGGATGCTGGCGTAATGGCTTTAAATGACCCGTCTTGAA
MJ53_02_CZ GT-
TCATGCTTAGGATGCTGGCGTAATGGCTTTAAATGACCCGTCTTGAA
CLZhao21647P_yunnanensis GT-
TCATGCTTAGGATGCTGGCGTAATGGCTTTAAATGACCCGTCTTGAA
CLZhao21583P_yunnanensis GT-
TCATGCTTAGGATGCTGGCGTAATGGCTTTAAATGACCCGTCTTGAA
Dai22272 GT-
TCATGCTTAGGATGCTGGCGTAATGGCTTTAAATGACCCGTCTTGAA
Dai22279 GT-
TCATGCTTAGGATGCTGGCGTAATGGCTTTAAATGACCCGTCTTGAA
MJ332_94_CZ --
MJ642_94_CZ_Expallescens --
Dai21060P_vinctus GT-
TCATGCTTAGGATGCTGGCGTAATGGCTTTAAATGACCCGTCTTGAA
JV0511_23LRP_pouzarii GT-
TCATGCTTAGGATGCTGGCGTAATGGCTTTAAATGACCCGTCTTGAA
JQ409462_R_pouzarii_PRM899856_ --
JV0308_66_WA --
JV0309_45_WA_USA --
JV0709_83_CA_USA GT-
TCATGCTTAGGATGCTGGCGTAATGGCTTTAAATGACCCGTCTTGAA
Dai21043P_pouzarii GT-
TCATGCTTAGGATGCTGGCGTAATGGCTTTAAATGACCCGTCTTGAA

MJ144_95_CZ GT-
TCATGCTTAGGATGCTGGCGTAATGGCTTTAAATGACCCGTCTTGAA
JV0909_3_CZ GT-
TCATGCTTAGGATGCTGGCGTAATGGCTTTAAATGACCCGTCTTGAA
JV0609_1_K ---
Dai20396Physisporinus_castanop GT-
TCATGCTTAGGATGCTGGCGTAATGGCTTTAAATGACCCGTCTTGAA
Dai20397Physisporinus_castanop GT-
TCATGCTTAGGATGCTGGCGTAATGGCTTTAAATGACCCGTCTTGAA
MJ19_09_SK_Abies GT-
TCATGCTTAGGATGCTGGCGTAATGGCTTTAAATGACCCGTCTTGAA
JV0509_40_J_TN_USA_Betula GT-
TCATGCTTAGGATGCTGGCGTAATGGCTTTAAATGACCCGTCTTGAA
JV0808_33crocatus_PA_USAlist GT-
TCATGCTTAGGATGCTGGCGTAATGGCTTTAAATGACCCGTCTTGAA
DLL2009_061P_crocatus ---
Dai12800P_subcrocatus GT-
TCATGCTTAGGATGCTGGCGTAATGGCTTTAAATGACCCGTCTTGAA
Dai15917P_subcrocatus GT-
TCATGCTTAGGATGCTGGCGTAATGGCTTTAAATGACCCGTCTTGAA
Cui16620 AT-
TCATGCTTAGGATGCTGGCGTAATGGCTTTAAATGACCCGTCTTGAA
HCFC1088Meripilus_stillicidior AT-
TCATGCTTAGGATGCTGGCGTAATGGCTTTAAATGACCCGTCTTGAA
MCW590Meripilus_obsrcurus ---
MCW722Meripilus_obsrcurus ---
Cui9381P_tibeticus GT-
TCATGCTTAGGATGCTGGCGTAATGGCTTTAAATGACCCGTCTTGAA
Cui9588P_tibeticus GT-
TCATGCTTAGGATGCTGGCGTAATGGCTTTAAATGACCCGTCTTGAA
Va2_Beneschova ---
CWU3874_Ukraine_Alnus GT-
TCATGCTTAGGATGCTGGCGTAATGGCTTTAAATGACCCGTCTTGAA
WCG1293Dai24718Physisporinus ---
WCG1268Dai24682A GT-
TCATGCTTAGGATGCTGGCGTAATGGCTTTAAATGACCCGTCTTGAA
WCG1269Dai24683A GT-
TCATGCTTAGGATGCTGGCGTAATGGCTTTAAATGACCCGTCTTGAA
WCG1279Dai24694A GT-
TCATGCTTAGGATGCTGGCGTAATGGCTTTAAATGACCCGTCTTGAA
Dai16971 GT-
TCATGCTTAGGATGCTGGCGTAATGGCTTTAAATGACCCGTCTTGAA
ZQY1043Dai26696 GT-
TCATGCTTAGGATGCTGGCGTAATGGCTTTAAATGACCCGTCTTGAA

Doll880 --
Doll1000 GT-
TCATGCTTAGGATGCTGGCGTAATGGCTTTAAACGACCCGTCTTGAA
1DAI18529 GT-
TCATGCTTAGGATGCTGGCGTAATGGCTTTAAACGACCCGTCTTGAA
Dai19535 GT-
TCATGCTTAGGATGCTGGCGTAATGGCTTTAAACGACCCGTCTTGAA
1704_79_hnedyVillaLaPaz GT-
TCATGCTTAGGATGCTGGCATAATGGCTTTAAACGACCCGTCTTGAA
F2061 --
1DAI18268 GT-
TCATGCTTAGGATGCTGGCGTAATGGCTTTAAACGACCCGTCTTGAA
1DAI18540A GT-
TCATGCTTAGGATGCTGGCGTAATGGCTTTAAACGACCCGTCTTGAA
Dai17695 GT-
TCATGCTTAGGATGCTGGCGTAATGGCTTTAAACGACCCGTCTTGAA
LKY18Dai26373 --
Dai17839P_sulphureus GT-
TCATGCTTAGGATGCTGGCGTAATGGCTTTAAACGACCCGTCTTGAA
Dai17841P_sulphureus GT-
TCATGCTTAGGATGCTGGCGTAATGGCTTTAAACGACCCGTCTTGAA
Dai19877P_roseus GT-
TCATGCTTAGGATGCTGGCATAATGGCTTTAAACGACCCGTCTTGAA
1508_18_1_Kout --
KP859303_R_vinctus_RP185_BRAZI --
JK1807_15Rigidoporus_sp_Puerto GT-
TCATGCTTAGGATGCTGGCGTAATGGCTTTAAACGACCCGTCTTGAA
JV0509_47_J_TN_USA GT-
TCATGCTTAGGATGCTGGCGTAATGGCTTTAAATGACCCGTCTTGAA
JV0709_188 GT-
TCATGCTTAGGATGCTGGCGTAATGGCTTTAAATGACCCGTCTTGAA
JV0509_127_PA_USA GT-
TCATGCTTAGGATGCTGGCGTAATGGCTTTAAATGACCCGTCTTGAA
JV1009_59_NJ_USA GT-TCATGC-
TAGGATGCTGGCGTAATGGCTTTAAATGACCCGTCTTGAA
Dai15497P_crataegi GT-
TCATGCTTAGGATGCTGGCGTAATGGCTTTAAATGACCCGTCTTGAA
Dai15499P_crataegi GT-
TCATGCTTAGGATGCTGGCGTAATGGCTTTAAATGACCCGTCTTGAA
Cui3266P_cinereus GT-
TCATGCTTAGGATGCTGGCGTAATGGCTTTAAATGACCCGTCTTGAA
WCG1256Dai24690 GT-
TCATGCTTAGGATGCTGGCGTAATGGCTTTAAATGACCCGTCTTGAA
1DAI17581 GT-

TCATGCTTAGGATGCTGGCGTAATGGCTTTAAATGACCCGTCTTGAA
WCG1255Dai24688 GT-
TCATGCTTAGGATGCTGGCGTAATGGCTTTAAATGACCCGTCTTGAA
Dai22427 GT-
TCATGCTTAGGATGCTGGCGTAATGGCTTTAAATGACCCGTCTTGAA
MV690Meripilus_concrescens GT-
TCATGCTTAGGATGCTGGCGTAATGGCTTTAAATGACCCGTCTTGAA
MV513Meripilus_galapagensis NN-
NNNNNNNNNNNNNNNNNNNNNNNNNNNNNNNNNNNNNNNNNNNNNNN
Dai19793 GT-
TCATGCTTAGGATGCTGGCGTAATGGCTTTAAATGACCCGTCTTGAA
OQ553780P_tamilnaduensis GT-
TCATGCTTAGGATGCTGGCGTAATGGCTTTAAACGACCCGTCTTGAA
OQ553779P_tamilnaduensis GT-
TCATGCTTAGGATGCTGGCGTAATGGCTTTAAACGACCCGTCTTGAA
A164FB3Meripilus_giganteus ---
JV1407_36_Vinctus_Meandrica GT-
TCATGCTTAGGATGCTGGCGTAATGGCTTTAAACGACCCGTCTTGAA
1807_3K_Rigidoporus_PuertoRico GT-
TCATGCTTAGGATGCTGGCGTAATGGCTTTAAACGACCCGTCTTGAA
Cui16903P_vinctus GT-
TCATGCTTAGGATGCTGGCGTAATGGCTTTAAACGACCCGTCTTGAA
JV1008_18R_Lineatus GT-
TCATGCTTAGGATGCTGGCGTAATGGCTTTAAACGACCCGTCTTGAA
JV1407_37_1_Vinctus_Carara GT-
TCATGCTTAGGATGCTGGCGTAATGGCTTTAAACGACCCGTCTTGAA
Dai17986P_lineatus GT-
TCATGCTTAGGATGCTGGCGTAATGGCTTTAAACGACCCGTCTTGAA
Dai18281 GT-
TCATGCTTAGGATGCTGGCGTAATGGCTTTAAACGACCCGTCTTGAA
1DAI19796 GT-
TCATGCTTAGGATGCTGGCGTAATGGCTTTAAACGACCCGTCTTGAA
ZQY797Dai25241 GT-
TCATGCTTAGGATGCTGGCGTAATGGCTTTAAACGACCCGTCTTGAA
WCG1289Dai24711 ---
Dai22598 GT-
TCATGCTTAGGATGCTGGCGTAATGGCTTTAAACGACCCGTCTTGAA
Dai20523 GT-
TCATGCTTAGGATGCTGGCGTAATGGCTTTAAACGACCCGTCTTGAA
Dai17885 GT-
TCATGCTTAGGATGCTGGCGTAATGGCTTTAAACGACCCGTCTTGAA
Dai17553 GT-
TCATGCTTAGGATGCTGGCGTAATGGCTTTAAACGACCCGTCTTGAA
Dai19639 GT-

TCATGCTTAGGATGCTGGCGTAATGGCTTTAAACGACCCGTCTTGAA
JV0110_48_CZ GT-
TCATGCTTAGGATGCTGGCGTAATGGCTTTAAACGACCCGTCTTGAA
MJ129_04 GT-
TCATGCTTAGGATGCTGGCGTAATGGCTTTAAACGACCCGTCTTGAA
Cui10340P_eminens ---
Cui10341P_eminens GT-
TCATGCTTAGGATGCTGGCGTAATGGCTTTAAACGACCCGTCTTGAA
Dai12685P_eminens GT-
TCATGCTTAGGATGCTGGCGTAATGGCTTTAAACGACCCGTCTTGAA
Miettinen_13591Rigidoporus_und GT-
TCATGCTTAGGATGCTGGCGTAATGGCTTTAAACGACCCGTCTTGAA
Dai20868 GT-
TCATGCTTAGGATGCTGGCGTAATGGCTTTAAACGACCCGTCTTGAA
Dai20832 GT-
TCATGCTTAGGATGCTGGCGTAATGGCTTTAAACGACCCGTCTTGAA
Dai11400 GT-
TCATGCTTAGGATGCTGGCGTAATGGCTTTAAACGACCCGTCTTGAA
Dai22472 GT-
TCATGCTTAGGATGCTGGCGTAATGGCTTTAAACGACCCGTCTTGAA
1CUI10475 GT-
TCATGCTTAGGATGCTGGCGTAATGGCTTTAAACGACCCGTCTTGAA
1CUI10491 ---
HCFC1095Meripilus_robledoi ---
MCW702Meripilus_revolubilis GT-
TCATGCTTAGGATGCTGGCGTAATGGCTTTAAACGACCCGTCTTGAA
1704_83_zluty_HaciendaBaru GT-
TCATGCTTAGGATGCTGGCGTAATGGCTTTAAACGACCCGTCTTGAA
Dai9925P_lavendulus GT-
TCATGCTTAGGATGCTGGCGTAATGGCTTTAAACGACCCGTCTTGAA
Dai13587AP_lavendulus GT-
TCATGCTTAGGATGCTGGCGTAATGGCTTTAAACGACCCGTCTTGAA
PDD70600P_longicystidius ---
Cui16630 GT-
TCATGCTTAGGATGCTGGCGTAATGGCTTTAAACGACCCGTCTTGAA
FP_135344Meripilus_giganteus GT-
TCATGCTTAGGATGCTGGCGTAATGGCTTTAAATGACCCGTCTTGAA
FP_100460_Sp ---
CBS421_48Meripilus_giganteus ---
Cui9203 ---
Cui9202 GT-
TCATGCTTAGGATGCTGGCGTAATGGCTTTAAATGACCCGTCTTGAA
TUFC100564Japan GT-
TCATGCTTAGGATGCTGGCGTAATGGCTTTAAATGACCCGTCTTGAA

Russell5913Meripilus_sumstinei ---
RP215Meripilus_brasiliensis GT-TCATGCTTAGGATGCTGGCGTAATGGCTTTAAATGACCCGTCTTGAA
RP200Meripilus_brasiliensis GT-TCATGCTTAGGATGCTGGCGTAATGGCTTTAAATGACCCGTCTTGAA
JV1712_13J_R_vinctus2_LSUPuert AT-TCGTGCTTAGGATGCTGGCGTAATGGCTTTAAACGACCCGTCTTGAA
Dai10503R_hypobrunneus AT-TCGTGCTTAGGATGCTGGCGTAATGGCTTTAAACGACCCGTCTTGAA
Dai10569Rigidoporus_hypobrunne AT-TCGTGCTTAGGATGCTGGCGTAATGGCTTTAAACGACCCGTCTTGAA
1DAI19451 AT-TCGTGCTTAGGATGCTGGCGTAATGGCTTTAAACGACCCGTCTTGAA
CM108bRigidoporus_hypobrunneus ---
1CUI16874 AT-TCGTGCTTAGGATGCTGGCGTAATGGCTTTAAACGACCCGTCTTGAA
FD299Cerrena_unicolor AT-TCGTGCTTAGGATGCTGGCGTAATGGCTTTAAACGACCCGTCTTGAA
KHL_GB_Cerrena_uniclor AT-TYSYGCTTAGGATGCTGGCGTAATGGCTTTAAACGACCCGTCTTGAA
Dai12892Cerrena_albocinnamomea AT-TCGTGCTTAGGATGCTGGCGTAATGGCTTTAAACGACCCGTCTTGAA
Dai12955C_albocinnamomea AT-TCGTGCTTAGGATGCTGGCGTAATGGCTTTAAACGACCCGTCTTGAA
SNUm03110102C_aurantiopora AT-TCGTGCTTAGGATGCTGGCGTAATGGCTTTAAACGACCCGTCTTGAA
NIBRFG0000102423C_aurantiopora AT-TCGTGCTTAGGATGCTGGCGTAATGGCTTTAAACGACCCGTCTTGAA
Dai7359Antrodiella_zonata AT-TCGTGCTTAGGATGCTGGCGTAATGGCTTTAAACGACCCGTCTTGAA
F20080702KCM29C_consors AT-TCGTGCTTAGGATGCTGGCGTAATGGCTTTAAACGACCCGTCTTGAA
F20080208LYW10Cerrena_consors AT-TCGTGCTTAGGATGCTGGCGTAATGGCTTTAAACGACCCGTCTTGAA
Dai7821Antrodiella_zonata AT-TCGTGCTTAGGATGCTGGCGTAATGGCTTTAAACGACCCGTCTTGAA
CFMR_DCL04_31Pseudolagarobasid ---
VPB197Pseudolagarobasidium_bel ---
CBS115543Pseudolagarobasidium_ ---
CBS115544 ---
Han405Pseudolagarobasidium_bai AT-TCGTGCTTAGGATGCTGGCGTAATGGCTTTAAACGACCCGTCTTGAA
Han406Pseudolagarobasidium_bai AT-TCGTGCTTAGGATGCTGGCGTAATGGCTTTAAACGACCCGTCTTGAA

MUcc838Spongipellis_delectans --
BRNM686401S_delectans --
OSM_F925S_delectans --
BRNM67093Spongipellis_litschau --
CFMRccFP59199TS_unicolor AT-TCGCGCTTAGGATGCTGGCGTAATGGCTTTAAACGACCCGTCTTGAA
CFMRccFP71791TS_unicolor AT-TCGCGCTTAGGATGCTGGCGTAATGGCTTTAAACGACCCGTCTTGAA
Dai13845P_lischaueri AT-TCGCGCTTAGGATGCTGGCGTAATGGCTTTAAACGACCCGTCTTGAA
Dai20266P_lischaueri AT-TCGCGCTTAGGATGCTGGCGTAATGGCTTTAAACGACCCGTCTTGAA
CFMR_HHB11240Radulodon_america --
RLG6350Radulodon_americanus --
KY415963Radulodon_erikssonii AT-TCGTGCTTAGGATGCTGGCGTAATGGCTTTAAGCGACCCGTCTTGAA
HHB9567spRadulodon_casearius AT-TCGTGCTTAGGATGCTGGCGTAATGGCTTTAAGCGACCCGTCTTGAA
KRT_Iso_26Radulodon_casearius --
CBS126044Radulodon_erikssonii AT-TCGTGCTTAGGATGCTGGCGTAATGGCTTTAAGCGACCCGTCTTGAA
He6183YUNNANENSIS AT-TCGTGCTTAGGATGCTGGCGTAATGGCTTTAAGCGACCCGTCTTGAA
Cui17979YUNNANENSIS AT-TCGTGCTTAGGATGCTGGCGTAATGGCTTTAAGCGACCCGTCTTGAA
Miettinen2091Junghuhnia_fimbri GT-TCATGCTTAGGATGCTGGCGTAATGGCTTTAAACGACCCGTCTTGAA
KHL12316S_tenue GT-TCATGCTTAGGATGCTGGCGTAATGGCTTTAAACGACCCGTCTTGAA
PRM846564S_pachyodon --
SP_Lgt_S_pachyodon --
Ryvarden44669Tyromyces_xuchile AT-TCGTGCTTAGGATGCTGGCGTAATGGCTTTAATCGACCCGTCTTGAA
PW17_171sinuosus --
W53Dai12234 AT-TCGTGCTTAGGATGCTGGCGTAATGGCTTTAATCGACCCGTCTTGAA
HHB4100SpAntella_americana GT-TCATGCTTAGGATGCTGGCGTAATGGCTTTAAACGACCCGTCTTGAA
W3Dai20901spumeus GTCTCATGCTTAGGATGCTGGCGTAATGGCTTTAAATGACCCGTCTTGAA
He6736 GTCTCATGCTTAGGATGCTGGCGTAATGGCTTTAAATGACCCGTCTTGAA
BRNM734877S_spumeus --
BRNM712630S_spumeus --

Dai1723Loweomyces_sibiricus --
W54Cui10009
GTCTCATGCTTAGGATGCTGGCGTAATGGCTTTAAATGACCCGTCTTGAA
W1Dai20899 --
HHB13445Trametes_ochracea AT-
TCGCGCTTAGGATGCTGGCATAATGGCTTTAAACGACCCGTCTTGAA
Dai16222 GT-
TCATGCTTAGGATGCTGGCGTAATGGCTTTAAACGACCCGTCTTGAA
Dai16240 GT-
TCATGCTTAGGATGCTGGCGTAATGGCTTTAAACGACCCGTCTTGAA

JV1310_11SanguinolentusCernys ACACGGA-
CCAAGGAGTCTAACATGCCTGCAAGTGTTTGGGTGGAAAACC
MJ39_00_SK ACACGGA-
CCAAGGAGTCTAACATGCCTGCAAGTGTTTGGGTGGAAAACC
MJ111_04_CZ --
JV1610_BOKYsmrk ACACGGA-
CCAAGGAGTCTAACATGCCTGCAAGTGTTTGGGTGGAAAACC
Dai21030 --
Dai20976P_furcatus ACACGGA-
CCAAGGAGTCTAACATGCCTGCAAGTGTTTGGGTGGAAAACC
Dai2105 ACACGGA-
CCAAGGAGTCTAACATGCCTGCAAGTGTTTGGGTGGAAAACC
Dai2544 ACACGGA-
CCAAGGAGTCTAACATGCCTGCAAGTGTTTGGGTGGAAAACC
Dai11313 ACACGGA-
CCAAGGAGTCTAACATGCCTGCAAGTGTTTGGGTGGAAAACC
WCG1611Dai26167 ACACGGA-
CCAAGGAGTCTAACATGCCTGCAAGTGTTTGGGTGGAAAACC
WCG1518Dai25999Physisporinus ACACGGA-
CCAAGGAGTCTAACATGCCTGCAAGTGTTTGGGTGGAAAACC
TAA15097 ACACGGA-
CCAAGGAGTCTAACATGCCTGCAAGTGTTTGGGTGGAAAACC
JV8909_19_CZ --
JV1310_15_P_sanguinolentus2_CZ ACACGGA-
CCAAGGAGTCTAACATGCCTGCAAGTGTTTGGGTGGAAAACC
MJ53_02_CZ ACACGGA-
CCAAGGAGTCTAACATGCCTGCAAGTGTTTGGGTGGAAAACC
CLZhao21647P_yunnanensis ACACGGA-
CCAAGGAGTCTAACATGCCTGCAAGTGTTTGGGTGGAAAACC
CLZhao21583P_yunnanensis ACACGGA-
CCAAGGAGTCTAACATGCCTGCAAGTGTTTGGGTGGAAAACC
Dai22272 ACACGGA-
CCAAGGAGTCTAACATGCCTGCAAGTGTTTGGGTGGAAAACC

Dai22279 ACACGGA-
CCAAGGAGTCTAACATGCCTGCAAGTGTTTGGGTGGAAAACC
MJ332_94_CZ --
MJ642_94_CZ_Expallescens --
Dai21060P_vinctus ACACGGA-
CCAAGGAGTCTAACATGCCTGCGAGTATTTGGGTGGAAAACC
JV0511_23LRP_pouzarii ACACGGA-
CCAAGGAGTCTAACATGCCTGCGAGTATTTGGGTGGAAAACC
JQ409462_R_pouzarii_PRM899856_ --
JV0308_66_WA --
JV0309_45_WA_USA --
JV0709_83_CA_USA ACACGGA-
CCAAGGAGTCTAACATGCCTGCGAGTATTTGGGTGGAAAACC
Dai21043P_pouzarii ACACGGA-
CCAAGGAGTCTAACATGCCTGCGAGTATTTGGGTGGAAAACC
MJ144_95_CZ ACACGGA-
CCAAGGAGTCTAACATGCCTGCGAGTGTTTGGGTGGAAAACC
JV0909_3_CZ ACACGGA-
CCAAGGAGTCTAACATGCCTGCGAGTGTTTGGGTGGAAAACC
JV0609_1_K --
Dai20396Physisporinus_castanop ACACGGA-
CCAAGGAGTCTAACATGCCTGCGAGTGTTTGGGTGGAAAACC
Dai20397Physisporinus_castanop ACACGGA-
CCAAGGAGTCTAACATGCCTGCGAGTGTTTGGGTGG-AAACC
MJ19_09_SK_Abies ACACGGA-
CCAAGGAGTCTAACATGCCTGCGAGTGTTTGGGTGGAAAACC
JV0509_40_J_TN_USA_Betula ACACGGA-
CCAAGGAGTCTAACATGCCTGCGAGTGTTTGGGTGGAAAACC
JV0808_33crocatus_PA_USAlist ACACGGA-
CCAAGGAGTCTAACATGCCTGCGAGTGTTTGGGTGGAAAACC
DLL2009_061P_crocatus --
Dai12800P_subcrocatus ACACGGA-
CCAAGGAGTCTAACATGCCTGCGAGTGTTTGGGTGGAAAACC
Dai15917P_subcrocatus ACACGGA-
CCAAGGAGTCTAACATGCCTGCGAGTGTTTGGGTGGAAAACC
Cui16620 ACACGGA-
CCAAGGAGTCTAACATGCCTGCGAGTGTTTGGGTGGAAAACC
HCFC1088Meripilus_stillicidior ACACGGA-
CCAAGGAGTCTAACATGCCTGCGAGTGTTTGGGTGGAAAACC
MCW590Meripilus_obsrcurus --
MCW722Meripilus_obsrcurus --
Cui9381P_tibeticus ACACGGA-
CCAAGGAGTCTAACATGCCTGCGAGTGTTTGGGTGGAAAACC
Cui9588P_tibeticus ACACGGA-

CCAAGGAGTCTAACATGCCTGCGAGTGTTTGGGTGGAAAACC
Va2_Beneschova --
CWU3874_Ukraine_Alnus ACACGGA-
CCAAGGAGTCTAACATGCCTGCGAGTGTTTGGGTGGAAAACC
WCG1293Dai24718Physisporinus --
WCG1268Dai24682A ACACGGA-
CCAAGGAGTCTAACATGCCTGCGAGTGTTTGGGTGGAAAACC
WCG1269Dai24683A ACACGGA-
CCAAGGAGTCTAACATGCCTGCGAGTGTTTGGGTGGAAAACC
WCG1279Dai24694A ACACGGA-
CCAAGGAGTCTAACATGCCTGCGAGTGTTTGGGTGGAAAACC
Dai16971 ACACGGA-
CCAAGGAGTCTAACATGCCTGCGAGTGTTTGGGTGGAAAACC
ZQY1043Dai26696 ACACGGA-
CCAAGGAGTCTAACATGCCTGCGAGTGTTTGGGTGGAAAACC
Doll880 --
Doll1000 ACACGGA-
CCAAGGAGTCTAACATGCCTGCGAGTGTTTGGGTGGAAAACC
1DAI18529 ACACGGA-
CCAAGGAGTCTAACATGCCTGCGAGTGTTTGGGTGGAAAACC
Dai19535 ACACGGA-
CCAAGGAGTCTAACATGCCTGCGAGTGTTTGGGTGGAAAACC
1704_79_hnedyVillaLaPaz ACACGGA-
CCAAGGAGTCTAACATGCCTGCAAGTGTTTGGGTGGAAAACC
F2061 --
1DAI18268 ACACGGA-
CCAAGGAGTCTAACATGCCTGCAAGTGTTTGGGTGGAAAACC
1DAI18540A ACACGGA-
CCAAGGAGTCTAACATGCCTGCAAGTGTTTGGGTGGAAAACC
Dai17695 ACACGGA-
CCAAGGAGTCTAACATGCCTGCAAGTGTTTGGGTGGAAAACC
LKY18Dai26373 --
Dai17839P_sulphureus ACACGGA-
CCAAGGAGTCTAACATGCCTGCGAGTGTTTGGGTGGAAAACC
Dai17841P_sulphureus ACACGGA-
CCAAGGAGTCTAACATGCCTGCGAGTGTTTGGGTGGAAAACC
Dai19877P_roseus ACACGGA-
CCAAGGAGTCTAACATGCCTGCAAGTGTTTGGGTGGAAAACC
1508_18_1_Kout --
KP859303_R_vinctus_RP185_BRAZI --
JK1807_15Rigidoporus_sp_Puerto ACACGGA-
CCAAGGAGTCTAACATGCCTGCGAGTGTTTGGGTGGAAAACC
JV0509_47_J_TN_USA ACACGGA-
CCAAGGAGTCTAACATGCCTGCGAGTGTTTGGGTGGAAAACC

JV0709_188 ACACGGA-
CCAAGGAGTCTAACATGCCTGCGAGTGTTTGGGTGGAAAACC
JV0509_127_PA_USA ACACGGA-
CCAAGGAGTCTAACATGCCTGCGAGTGTTTGGGTGGAAAACC
JV1009_59_NJ_USA ACACGGA-
CCAAGGAGTCTAACATGCCTGCGAGTGTTTGGGTGGAAAACC
Dai15497P_crataegi ACACGGA-
CCAAGGAGTCTAACATGCCTGCGAGTGTTTGGGTGGAAAACC
Dai15499P_crataegi ACACGGA-
CCAAGGAGTCTAACATGCCTGCGAGTGTTTGGGTGGAAAACC
Cui3266P_cinereus ACACGGA-
CCAAGGAGTCTAACATGCCTGCGAGTGTTTGGGTGGAAAACC
WCG1256Dai24690 ACACGGA-
CCAAGGAGTCTAACATGCCTGCGAGTGTTTGGGTGGAAAACC
1DAI17581 ACACGGA-CCAAGGAGTCTAACATG-
CTGCGAGTGTTTGGGTGGAAAACC
WCG1255Dai24688 ACACGGA-
CCAAGGAGTCTAACATGCCTGCGAGTGTTTGGGTGGAAAACC
Dai22427 ACACGGA-
CCAAGGAGTCTAACATGCCTGCGAGTGTTTGGGTGGAAAACC
MV690Meripilus_concrescens ACACGGA-
CCAAGGAGTCTAACATGCCTGCGAGTGTTTGGGTGGAAAACC
MV513Meripilus_galapagensis NNNNNNN-
NNNNNNNNNNNNNNNNNNNNNNNNNNNNNNNNNNNNNNNNNN
Dai19793 ACACGGA-
CCAAGGAGTCTAACATGCCTGCGAGTGTTTGGGTGGAAAACC
OQ553780P_tamilnaduensis ACACGGA-
CCAAGGAGTCTAACATGCCTGCGAGTGTTTGGGTGGAAAACC
OQ553779P_tamilnaduensis ACACGGA-
CCAAGGAGTCTAACATGCCTGCGAGTGTTTGGGTGGAAAACC
A164FB3Meripilus_giganteus --
JV1407_36_Vinctus_Meandrica ACACGGA-
CCAAGGAGTCTAACATGCCTGCGAGTGTTTGGGTGGAAAACC
1807_3K_Rigidoporus_PuertoRico
ACACGGACCCAAGGAGTCTAACATGCCTGCGAGTGTTTGGGTGGAAAACC
Cui16903P_vinctus ACACGGA-
CCAAGGAGTCTAACATGCCTGCGAGTGTTTGGGTGGAAAACC
JV1008_18R_Lineatus ACACGGA-
CCAAGGAGTCTAACATGCCTGCGAGTGTTTGGGTGGAAAACC
JV1407_37_1_Vinctus_Carara ACACGGA-
CCAAGGAGTCTAACNTGCCTGCGAGTGTTTGGGTGGAAAACC
Dai17986P_lineatus ACACGGA-
CCAAGGAGTCTAACATGCCTGCGAGTGTTTGGGTGGAAAACC
Dai18281 ACACGGA-

CCAAGGAGTCTAACATGCCTGCGAGTGTTTGGGTGGAAAACC
1DAI19796 -CACGGA-
CCAAGGAGTCTAACATGCCTGCGAGTGTTTGGGTGGAAAACC
ZQY797Dai25241 ACACGGA-
CCAAGGAGTCTAACATGCCTGCGAGTGTTTGGGTGGAAAACC
WCG1289Dai24711 ---
Dai22598 ACACGGA-
CCAAGGAGTCTAACATGCCTGCGAGTGTTTGGGTGGAAAACC
Dai20523 ACACGGA-
CCAAGGAGTCTAACATGCCTGCGAGTGTTTGGGTGGAAAACC
Dai17885 ACACGGA-
CCAAGGAGTCTAACATGCCTGCGAGTGTTTGGGTGGAAAACC
Dai17553 ACACGGA-
CCAAGGAGTCTAACATGCCTGCGAGTGTTTGGGTGGAAAACC
Dai19639 ACACGGA-CCAAGGAGTCTAACATG-
CTGCGAGTGTTTGGGTGGAAAACC
JV0110_48_CZ ACACGGA-
CCAAGGAGTCTAACATGCCTGCGAGTGTTTGGGTGGAAAACC
MJ129_04 ACACGGA-
CCAAGGAGTCTAACATGCCTGCGAGTGTTTGGGTGGAAAACC
Cui10340P_eminens ---
Cui10341P_eminens ACACGGA-
CCAAGGAGTCTAACATGCCTGCGAGTGTTTGGGTGGAAAACC
Dai12685P_eminens ACACGGA-
CCAAGGAGTCTAACATGCCTGCGAGTGTTTGGGTGGAAAACC
Miettinen_13591Rigidoporus_und ACACGGA-
CCAAGGAGTCTAACATGCCTGCGAGTGTTTGGGTGGAAAACC
Dai20868 ACACGGA-
CCAAGGAGTCTAACATGCCTGCGAGTGTTTGGGTGGAAAACC
Dai20832 ACACGGA-
CCAAGGAGTCTAACATGCCTGCGAGTGTTTGGGTGGAAAACC
Dai11400 ACACGGA-
CCAAGGAGTCTAACATGCCTGCGAGTGTTTGGGTGGAAAACC
Dai22472 ACACGGA-
CCAAGGAGTCTAACATGCCTGCGAGTGTTTGGGTGGAAAACC
1CUI10475 ACACGGA-
CCAAGGAGTCTAACATGCCTGCGAGTGTTTGGGTGGAAAACC
1CUI10491 ---
HCFC1095Meripilus_robledoi ---
MCW702Meripilus_revolubilis ACACGGA-
CCAAGGAGTCTAACATGCCTGCGAGTGTTTGGGTGGAAAACC
1704_83_zluty_HaciendaBaru ACACGGA-
CCAAGGAGTCTAACATGCCTGCGAGTGTTTGGGTGTAAAACC
Dai9925P_lavendulus ACACGGA-

CCAAGGAGTCTAACATGCCTGCGAGTGTTTGGGTGGAAAACC
Dai13587AP_lavendulus ACACGGA-
CCAAGGAGTCTAACATGCCTGCGAGTGTTTGGGTGGAAAACC
PDD70600P_longicystidius --
Cui16630 ACACGGA-
CCAAGGAGTCTAACATGCCTGCGAGTGTTTGGGTGGAAAACC
FP_135344Meripilus_giganteus ACACGGA-
CCAAGGAGTCTAACATGCCTGCGAGTGTTTGGGTGGAAAACC
FP_100460_Sp --
CBS421_48Meripilus_giganteus --
Cui9203 --
Cui9202 ACACGGA-
CCAAGGAGTCTAACATGCCTGCGAGTGTTTGGGTGGAAAACC
TUFC100564Japan ACACGGA-
CCAAGGAGTCTAACATGCCTGCGAGTGTTTGGGTGGAAAACC
Russell5913Meripilus_sumstinei --
RP215Meripilus_brasiliensis ACACGGA-
CCAAGGAGTCTAACATGCCTGCGAGTGTTTGGGTGGAAAACC
RP200Meripilus_brasiliensis ACACGGA-
CCAAGGAGTCTAACATGCCTGCGAGTGTTTGGGTGGAAAACC
JV1712_13J_R_vinctus2_LSUPuert ACACGGA-
CCAAGGAGTCTAACATGCCTGCGAGTGTTTGGGTGGAAAACC
Dai10503R_hypobrunneus ACACGGA-
CCAAGGAGTCTAACATGCCTGCGAGTGTTTGGGTGGAAAACC
Dai10569Rigidoporus_hypobrunne ACACGGA-
CCAAGGAGTCTAACATGCCTGCGAGTGTTTGGGTGGAAAACC
1DAI19451 ACACGGA-
CCAAGGAGTCTAACATGCCTGCGAGTGTTTGGGTGGAAAACC
CM108bRigidoporus_hypobrunneus --
1CUI16874 ACACGGA-
CCAAGGAGTCTAACATGCCTGCGAGTGTTTGGGTGGAAAACC
FD299Cerrena_unicolor ACACGGA-
CCAAGGAGTCTAACATGCCTGCGAGTGTTTGGGTGGAAAACC
KHL_GB_Cerrena_uniclor ACACGGA-
CCAAGGAGTCTAACATGCCTGCGAGTGTTTGGGTGGAAAACC
Dai12892Cerrena_albocinnamomea ACACGGA-
CCAAGGAGTCTAACATGCCTGCGAGTGTTTGGGTGGAAAACC
Dai12955C_albocinnamomea ACACGGA-
CCAAGGAGTCTAACATGCCTGCGAGTGTTTGGGTGGAAAACC
SNUm03110102C_aurantiopora ACACGGA-
CCAAGGAGTCTAACATGCCTGCGAGTGTTTGGGTGGAAAACC
NIBRFG0000102423C_aurantiopora ACACGGA-
CCAAGGAGTCTAACATGCCTGCGAGTGTTTGGGTGGAAAACC
Dai7359Antrodiella_zonata ACACGGA-

CCAAGGAGTCTAACATGCCTGCGAGTGTTTGGGTGGAAAACC
F20080702KCM29C_consors ACACGGA-
CCAAGGAGTCTAACATGCCTGCGAGTGTTTGGGTGGAAAACC
F20080208LYW10Cerrena_consors ACACGGA-
CCAAGGAGTCTAACATGCCTGCGAGTGTTTGGGTGGAAAACC
Dai7821Antrodiella_zonata ACACGGA-
CCAAGGAGTCTAACATGCCTGCGAGTGTTTGGGTGGAAAACC
CFMR_DCL04_31Pseudolagarobasid --
VPB197Pseudolagarobasidium_bel --
CBS115543Pseudolagarobasidium_ --
CBS115544 --
Han405Pseudolagarobasidium_bai ACACGGA-
CCAAGGAGTCTAACATGCCTGCGAGTGTTTGGGTGGAAAACC
Han406Pseudolagarobasidium_bai ACACGGA-
CCAAGGAGTCTAACATGCCTGCGAGTGTTTGGGTGGAAAACC
MUcc838Spongipellis_delectans --
BRNM686401S_delectans --
OSM_F925S_delectans --
BRNM67093Spongipellis_litschau --
CFMRccFP59199TS_unicolor --
CFMRccFP71791TS_unicolor ACACGGA-
CCAAGGAGTCTAACACACCTGCGAGTGTTTGGGTGGAAAACC
Dai13845P_lischaueri ACACGGA-
CCAAGGAGTCTAACACACCTGCGAGTGTTTGGGTGGAAAACC
Dai20266P_lischaueri ACACGGA-
CCAAGGAGTCTAACACACCTGCGAGTGTTTGGGTGGAAAACC
CFMR_HHB11240Radulodon_america --
RLG6350Radulodon_americanus --
KY415963Radulodon_erikssonii ACACGGA-
CCAAGGAGTCTAACATGCCTGCGAGTGTTTGGGTGGAAAACC
HHB9567spRadulodon_casearius ACACGGA-
CCAAGGAGTCTAACATGCCTGCGAGTGTTTGGGTGGAAAACC
KRT_Iso_26Radulodon_casearius --
CBS126044Radulodon_erikssonii ACACGGA-
CCAAGGAGTCTAACATGCCTGCGAGTGTTTGGGTGGAAAACC
He6183YUNNANENSIS ACACGGA-
CCAAGGAGTCTAACATGCCTGCGAGTGTTTGGGTGGAAAACC
Cui17979YUNNANENSIS ACACGGA-
CCAAGGAGTCTAACATGCCTGCGAGTGTTTGGGTGGAAAACC
Miettinen2091Junghuhnia_fimbri ACACGGA-
CCAAGGAGTCTAACATGCCTGCGAGTGTTTGGGTGGAAAACC
KHL12316S_tenue ACACGGA-
CCAAGGAGTCTAACATGCCTGCGAGTGTTTGGGTGGAAAACC
PRM846564S_pachyodon --

SP_Lgt_S_pachyodon --
Ryvarden44669Tyromyces_xuchile ACACGGA-CCAAGGAGTCTAACATACCTGCAAGTGTTTGGGTGGCAAACC
PW17_171sinuosus --
W53Dai12234 ACACGGA-CCAAGGAGTCTAACATGCCTGCGAGTGTTTGGGTGGAAAACC
HHB4100SpAntella_americana ACACGGA-CCAAGGAGTCTAACATGCCTGCGAGTGTTTGGGTGGAAAACC
W3Dai20901spumeus ACACGGA-CCAAGGAGTCTAACATGCCTGCGAGTGTTTGGGTGGAAAACC
He6736 ACACGGA-CCAAGGAGTCTAACATGCCTGCGAGTGTTTGGGTGGAAAACC
BRNM734877S_spumeus --
BRNM712630S_spumeus --
Dai1723Loweomyces_sibiricus --
W54Cui10009 ACACGGA-CCAAGGAGTCTAACATGCCTGCGAGTATTTGGGTGGAAAACC
W1Dai20899 --
HHB13445Trametes_ochracea ACACGGA-CCAAGGAGTCTAACATGCCTGCGAGTGTTTGGGTGGAAAACC
Dai16222 ACACGGA-CCAAGGAGTCTAACATGCCTGCGAGTGTTTGGGTGGAAAACC
Dai16240 ACACGGA-CCAAGGAGTCTAACATGCCTGCGAGTGTTTGGGTGGAAAACC

JV1310_11SanguinolentusCernys CGAGCGCGCAATGAAAGTGAAAGTTGGGATCCCT-GTCGTGGGGAGCACC
MJ39_00_SK CGAGCGCGCAATGAAAGTGAAAGTTGGGATCCCT-GTCGTGGGGAGCACC
MJ111_04_CZ --
JV1610_BOKYsmrk CGAGCGCGCAATGAAAGTGAAAGTTGGGATCCCT-GTCGTGGGGAGCACC
Dai21030 --
Dai20976P_furcatus CGAGCGCGCAATGAAAGTGAAAGTTGGGATCCCT-GTCGTGGGGAGCACC
Dai2105 CGAGCGCGCAATGAAAGTGAAAGTTGGGATCCCT-GTCGTGGGGAGCACC
Dai2544 CGAGCGCGCAATGAAAGTGAAAGTTGGGATCCCT-GTCGTGGGGAGCACC
Dai11313 CGAGCGCGCAATGAAAGTGAAAGTTGGGATCCCT-GTCGTGGGGAGCACC
WCG1611Dai26167 CGAGCGCGCAATGAAAGTGAAAGTTGGGATCCCT-GTCGTGGGGAGCACC
WCG1518Dai25999Physisporinus CGAGCGCGCAATGAAAGTGAAAGTTGGGATCCCT-

GTCGTGGGGAGCACC
TAA15097
CGAGCGCGCAATGAAAGTGAAAGTTGGGATCCCT-GTCGTGGGGAGCACC
JV8909_19_CZ --
JV1310_15_P_sanguinolentus2_CZ CGAGCGCGCAATGAAAGTGAAAGTTGGGATCCCT-GTCGTGGGGAGCACC
MJ53_02_CZ
CGAGCGCGCAATGAAAGTGAAAGTTGGGATCCCT-GTCGTGGGGAGCACC
CLZhao21647P_yunnanensis
CGAGCGCGCAATGAAAGTGAAAGTTGGGATCCCTCGTCGTGGGGAGCACC
CLZhao21583P_yunnanensis CGAGCGCGCAATGAAAGTGAAAGTTGGGATCCTC-GTCGTGGGGAGCACC
Dai22272 CGAGCGCGCAATGAAAGTGAAAGTTGGGATCCCT-GTCGTGGGGAGCACC
Dai22279 CGAGCGCGCAATGAAAGTGAAAGTTGGGATCCCT-GTCGTGGGGAGCACC
MJ332_94_CZ --
MJ642_94_CZ_Expallescens --
Dai21060P_vinctus CGAGTGCACAATGAAAGTGAAAGTTGGGATCTCT-GTCGTGGGGAGCACC
JV0511_23LRP_pouzarii CGAGTGCGCAATGAAAGTGAAAGTTGGGATCCCT-GTCGTGGGGAGCACC
JQ409462_R_pouzarii_PRM899856_ --
JV0308_66_WA --
JV0309_45_WA_USA --
JV0709_83_CA_USA
CGAGTGCGCAATGAAAGTGAAAGTTGGGATCTCT-GTCGTGGGGAGCACC
Dai21043P_pouzarii CGAGTGCGCAATGAAAGTGAAAGTTGGGATCCCT-GTCGTGGGGAGCACC
MJ144_95_CZ
CGAGCGCGCAATGAAAGTGAAAGTTGGGATCCCT-GTCGTGGGGAGCACC
JV0909_3_CZ
CGAGCGCGCAATGAAAGTGAAAGTTGGGATCCCT-GTCGTGGGGAGCACC
JV0609_1_K --
Dai20396Physisporinus_castanop CGAGCGCGCAATGAAAGTGAAAGTTGGGATCCCT-GTCGTGGGGAGCACC
Dai20397Physisporinus_castanop CGAGCGCGCAATGAAAGTGAAAGTTGGGATCCCT-GTCGTGGGGAGCACC
MJ19_09_SK_Abies
CGAGCGCGCAATGAAAGTGAAAGTTGGGATCTCT-GTCGTGGGGAGCACC
JV0509_40_J_TN_USA_Betula
CGAGCGCGCAATGAAAGTGAAAGTTGGGATCTCT-GTCGTGGGGAGCACC
JV0808_33crocatus_PA_USAlist CGAGCGCGCAATGAAAGTGAAAGTTGGGATCTCT-GTCGTGGGGAGCACC

DLL2009_061P_crocatus --
Dai12800P_subcrocatus CGAGCGCGCAATGAAAGTGAAAGTTGGGATCTCT-
GTCGTGGGGAGCACC
Dai15917P_subcrocatus CGAGCGCGCAATGAAAGTGAAAGTTGGGATCTCT-
GTTGTGGGGAGCACC
Cui16620 CGAGCGCGCAATGAAAGTGAAAGTTGGGATCTCT-
GTCGTGGGGAGCACC
HCFC1088Meripilus_stillicidior CGAGCGCGCAATGAAAGTGAAAGTTGGGATCTCT-
GTCGTGGGGAGCACC
MCW590Meripilus_obsrcurus --
MCW722Meripilus_obsrcurus --
Cui9381P_tibeticus CGAGTGCGCAATGAAAGTGAAAGTTGGGATCTCT-
GTCGTGGGGAGCACC
Cui9588P_tibeticus CGAGTGCGCAATGAAAGTGAAAGTTGGGATCTCT-
GTCGTGGGGAGCACC
Va2_Beneschova --
CWU3874_Ukraine_Alnus
CGAGCGCGCAATGAAAGTGAAAGTTGGGATCTCT-GTCGTGGGGAGCACC
WCG1293Dai24718Physisporinus --
WCG1268Dai24682A
CGAGCGCGCAATGAAAGTGAAAGTTGGGATCTCT-GTCGTGGGGAGCACC
WCG1269Dai24683A
CGAGCGCGCAATGAAAGTGAAAGTTGGGATCTCT-GTCGTGGGGAGCACC
WCG1279Dai24694A
CGAGCGCGCAATGAAAGTGAAAGTTGGGATCTCT-GTCGTGGGGAGCACC
Dai16971 CGAGCGCGCAATGAAAGTGAAAGTTGGGATCTCT-
GTCGTGGGGAGCACC
ZQY1043Dai26696
CGAGCGCGCAATGAAAGTGAAAGTTGGGATCTCT-GTCGTGGGGAGCACC
Doll880 --
Doll1000 CGAGCGCGAAATGAAAGTGAAAGTTGGGATCCCT-
GTCGTGGGGAGCACC
1DAI18529
CGAGCGCGAAATGAAAGTGAAAGTTGGGATCCCT-GTCGTGGGGAGCACC
Dai19535 CGAGCGCGCAATGAAAGTGAAAGTTGGGATCTCT-
GTCGTGGGGAGCACC
1704_79_hnedyVillaLaPaz CGAGCGCGCAATGAAAGTGAAAGTTGGGATCTCT-
GTCGTGGAGAGCACC
F2061 --
1DAI18268
CGAGCGCGCAATGAAAGTGAAAGTTGGGATCTCT-GTCGTGGAGAGCACC
1DAI18540A
CGAGCGCGCAATGAAAGTGAAAGTTGGGATCTCT-GTCGTGGAGAGCACC
Dai17695 CGAGCGCGCAATGAAAGTGAAAGTTGGGATCTCT-

GTCGTGGAGAGCACC
LKY18Dai26373 ---
Dai17839P_sulphureus CGAGCGCGCAATGAAAGTGAAAGTTGGGATCTCT-GTCGTGGAGAGCACC
Dai17841P_sulphureus CGAGCGCGCAATGAAAGTGAAAGTTGGGATCTCT-GTCGTGGAGAGCACC
Dai19877P_roseus CGAGCGCGCAATGAAAGTGAAAGTTGGGATCTCT-GTCGTGGAGAGCACC
1508_18_1_Kout ---
KP859303_R_vinctus_RP185_BRAZI ---
JK1807_15Rigidoporus_sp_Puerto CGAGCGCACAATGAAAGTGAAAGTTGGGATCTCT-GTCGTGGAGAGCACC
JV0509_47_J_TN_USA CGAGCGCGCAATGAAAGTGAAAGTTGGGATCCCT-GTCGTGGGGAGCACC
JV0709_188 CGAGCGCGCAATGAAAGTGAAAGTTGGGATCCCT-GTCGTGGGGAGCACC
JV0509_127_PA_USA CGAGCGCGCAATGAAAGTGAAAGTTGGGATCCCT-GTCGTGGGGAGCACC
JV1009_59_NJ_USA CGAGCGCGCAATGAAAGTGAAAGTTGGGATCCCT-GTCGTGGGGAGCACC
Dai15497P_crataegi CGAGCGCGCAATGAAAGTGAAAGTTGGGATCCCT-GTCGTGGGGAGCACC
Dai15499P_crataegi CGAGCGCGCAATGAAAGTGAAAGTTGGGATCCCT-GTCGTGGGGAGCACC
Cui3266P_cinereus CGAGCGCGCAATGAAAGTGAAAGTTGGGATCCCT-GTCGTGGGGAGCACT
WCG1256Dai24690 CGAGCGCGCAATGAAAGTGAAAGTTGGGATCCCT-GTCGTGGGGAGCACT
1DAI17581 CGAGCGCGCAATGAAAGTGAAAGTTGGGATCCCT-GTCGTGGGGAGCACT
WCG1255Dai24688 CGAGCGCGCAATGAAAGTGAAAGTTGGGATCCCT-GTCGTGGGGAGCACT
Dai22427 CGAGCGCGCAATGAAAGTGAAAGTTGGGATCCCT-GTCGTGGGGAGCACT
MV690Meripilus_concrescens CGAGCGCGAAATGAAAGTGAAAGTTGGGATCCCT-GTCGTGGGGAGCACT
MV513Meripilus_galapagensis NNNNNNNNNNNNNNNNNNNNNNNNNNNNNNNNNN-NNNNNNNNNNNNNNN
Dai19793 CGAGCGCGTAATGAAAGTGAAAGTTGGGATCCCT-GTCGTGGGGAGCACC
OQ553780P_tamilnaduensis CGAGCGCGCAATGAAAGTGAAAGTTGGGATCCTT-GTCGTGAGGAGCACC
OQ553779P_tamilnaduensis CGAGCGCGCAATGAAAGTGAAAGTTGGGATCCTT-GTCGTGAGGAGCACC

A164FB3Meripilus_giganteus --
JV1407_36_Vinctus_Meandrica CGAGCGCGCAATGAAAGTGAAAGTTGGGATCTCT-GTCGCGGAGAGCACC
1807_3K_Rigidoporus_PuertoRico CGAGCGCGCAATGAAAGTGAAAGTTGGGATCTCT-GTCGCGGAGAGCACC
Cui16903P_vinctus CGAGCGCGCAATGAAAGTGAAAGTTGGGATCTCT-GTCGCGGAGAGCACC
JV1008_18R_Lineatus CGAGCGCGCAATGAAAGTGAAAGTTGGGATCTCT-GTCGTGGAGAGCACC
JV1407_37_1_Vinctus_Carara CGAGCGCGCAATGAAAGTGAAAGTTGGGATCTCT-GTCGTGGAGAGCACC
Dai17986P_lineatus CGAGCGCGCAATGAAAGTGAAAGTTGGGATCTCT-GTCGTGGAGAGCACC
Dai18281 CGAGCGCGCAATGAAAGTGAAAGTTGGGATCTCT-GTCGTGGAGAGCACC
1DAI19796
CGAGCGCGCAATGAAAGTGAAAGTTGGGATCTCT-GTCGTGGAGAGCACC
ZQY797Dai25241
CGAGCGCGCAATGAAAGTGAAAGTTGGGATCTCT-GTCGTGGAGAGCACC
WCG1289Dai24711 --
Dai22598 CGAGCGCGCAATGAAAGTGAAAGTTGGGATCTCT-GTCGTGGAGAGCACC
Dai20523 CGAGCGCGCAATGAAAGTGAAAGTTGGGATCTCT-GTCGTGGAGAGCACC
Dai17885 CGAGCGCGCAATGAAAGTGAAAGTTGGGATCTCT-GTCGTGGAGAGCACC
Dai17553 CGAGCGCGCAATGAAAGTGAAAGTTGGGATCTCT-GTCGTGGAGAGCACC
Dai19639 CGAGCGCGCAATGAAAGTGAAAGTTGGGATCTCT-GTCGTGGAGAGCACC
JV0110_48_CZ
CGAGCGCGCAATGAAAGTGAAAGTTGGGATCCCT-GTCGTGGGGAGCACT
MJ129_04
CGAGCGCGCAATGAAAGTGAAAGTTGGGATCCCT-GTCGTGGGGAGCACT
Cui10340P_eminens --
Cui10341P_eminens CGAGCGCGCAATGAAAGTGAAAGTTGGGATCCCT-GTCGTGGGGAGCACT
Dai12685P_eminens CGAGCGCGCAATGAAAGTGAAAGTTGGGATCCCT-GTCGTGGGGAGCACT
Miettinen_13591Rigidoporus_und CGAGCGCGCAATGAAAGTGAAAGTTGGGATCCCT-GTCGTGGGGAGCACT
Dai20868 CGAGCGCGCAATGAAAGTGAAAGTTGGGATCCCT-GTCGTGGGGAGCACT
Dai20832 CGAGCGCGCAATGAAAGTGAAAGTTGGGATCCCT-

GTCGTGGGGAGCACT
Dai11400 CGAGCGCGCAATGAAAGTGAAAGTTGGGATCCCT-
GTCGTGGGGAGCACT
Dai22472 CGAGCGCGCAATGAAAGTGAAAGTTGGGATCCCT-
GTCGTGGGGAGCACT
1CUI10475
CGAGCGCGCAATGAAAGTGAAAGTTGGGATCCCT-GTCGTGGGGAGCACT
1CUI10491 --
HCFC1095Meripilus_robledoi --
MCW702Meripilus_revolubilis CGAGCGCGCAATGAAAGTGAAAGTTGGGATCCCT-
GTCGTGGGGAGCACT
1704_83_zluty_HaciendaBaru CGAGCGCGCAATGAAAGTGAAAGTTGGGATCTCT-
GTCGTGGAGAGCACC
Dai9925P_lavendulus CGAGCGCGCAATGAAAGTGAAAGTTGGGATCTCT-
GTCGCGGAGAGCACC
Dai13587AP_lavendulus CGAGCGCGCAATGAAAGTGAAAGTTGGGATCTCT-
GTCGCGGAGAGCACC
PDD70600P_longicystidius --
Cui16630 CGAGCGCGCAATGAAAGTGAAAGTTGGGATCTCT-
GTCGTGGAGAGCACC
FP_135344Meripilus_giganteus CGAGCGCGCAATGAAAGTGAAAGTTGGGATCCCT-
GTCGCGGGGAGCACC
FP_100460_Sp --
CBS421_48Meripilus_giganteus --
Cui9203 --
Cui9202 CGAGCGCGCAATGAAAGTGAAAGTTGGGATCCCT-
GTCGCGGGGAGCACC
TUFC100564Japan
CGAGCGCGCAATGAAAGTGAAAGTTGGGATCCCT-GTCGCGGGGAGCACC
Russell5913Meripilus_sumstinei --
RP215Meripilus_brasiliensis CGAGCGCGCAATGAAAGTGAAAGTTGGGATCTCT-
GTCGTGGGGAGCACC
RP200Meripilus_brasiliensis CGAGCGCGCAATGAAAGTGAAAGTTGGGATCTCT-
GTCGTGGGGAGCACC
JV1712_13J_R_vinctus2_LSUPuert CGAGCGCGTAATGAAAGTGAAAGTTGAGATCCCT-
GTCGTGGGGAGCATC
Dai10503R_hypobrunneus CGAGCGCGTAATGAAAGTGAAAGTTGAGATCCCT-
GTCGTGGGGAGCATC
Dai10569Rigidoporus_hypobrunne CGAGCGCGTAATGAAAGTGAAAGTTGAGATCCCT-
GTCGTGGGGAGCATC
1DAI19451
CGAGCGCGTAATGAAAGTGAAAGTTGAGATCCCT-GTCGTGGGGAGCATC
CM108bRigidoporus_hypobrunneus --
1CUI16874

CGAGCGCGTAATGAAAGTGAAAGTTGAGATCCCT-GTCGTGGGGAGCATC
FD299Cerrena_unicolor CGAGCGCGTAATGAAAGTGAAAGTTGAGATCCCT-GTCGTGGGGAGCATC
KHL_GB_Cerrena_uniclor CGAGCGCGTAATGAAAGTGAAAGTTGAGATCCCT-GTCGTGGGGAGCATC
Dai12892Cerrena_albocinnamomea CGAGCGCGCAATGAAAGTGAAAGTTGAGATCCCT-GTCGTGGGGAGCATC
Dai12955C_albocinnamomea CGAGCGCGCAATGAAAGTGAAAGTTGAGATCCCT-GTCGTGGGGAGCATC
SNUm03110102C_aurantiopora CGAGCGCGCAATGAAAGTGAAAGTTGAGATCCCT-GTCGTGGGGAGCATC
NIBRFG0000102423C_aurantiopora CGAGCGCGCAATGAAAGTGAAAGTTGAGATCCCT-GTCGTGGGGAGCATC
Dai7359Antrodiella_zonata CGAGCGCGCAATGAAAGTGAAAGTTGAGATCCCT-GTCGTGGGGAGCATC
F20080702KCM29C_consors
CGAGCGCGCAATGAAAGTGAAAGTTGAGATCCCT-GTCGTGGGGAGCATC
F20080208LYW10Cerrena_consors CGAGCGCGCAATGAAAGTGAAAGTTGAGATCCCT-GTCGTGGGGAGCATC
Dai7821Antrodiella_zonata CGAGCGCGCAATGAAAGTGAAAGTTGAGATCCCT-GTCGTGGGGAGCATC
CFMR_DCL04_31Pseudolagarobasid --
VPB197Pseudolagarobasidium_bel --
CBS115543Pseudolagarobasidium_ --
CBS115544 --
Han405Pseudolagarobasidium_bai CGAGCGCGTAATGAAAGTGAAAGTTGAGATCCCT-GTCGTGGGGAGCATC
Han406Pseudolagarobasidium_bai CGAGCGCGTAATGAAAGTGAAAGTTGAGATCCCT-GTCGTGGGGAGCATC
MUcc838Spongipellis_delectans --
BRNM686401S_delectans --
OSM_F925S_delectans --
BRNM67093Spongipellis_litschau --
CFMRccFP59199TS_unicolor --
CFMRccFP71791TS_unicolor
CGAGCGCGCAATGAAAGTGAAAGTTGGGATCCCT-GTCGTGGGGAGCACC
Dai13845P_lischaueri CGAGCGCGTAATGAAAGTGAAAGTCGGGATCCCT-GTCGTGGGGAGCACC
Dai20266P_lischaueri CGAGCGCGTAATGAAAGTGAAAGTCGGGATCCCT-GTCGTGGGGAGCACC
CFMR_HHB11240Radulodon_america --
RLG6350Radulodon_americanus --
KY415963Radulodon_erikssonii CGAGCGCGCAATGAAAGTGAAAGTCGGGAACCCT-GTCGCGGGGTGCACC

HHB9567spRadulodon_casearius CGAGCGCGCAATGAAAGTGAAAGTCGGGAACCCT-GTCGCGGGGTGCACC
KRT_Iso_26Radulodon_casearius --
CBS126044Radulodon_erikssonii CGAGCGCGCAATGAAAGTGAAAGTCGGGAACCCT-GTCGCGGGGTGCACC
He6183YUNNANENSIS
CGAGCGCGCAATGAAAGTGAAAGTCGGGAACCCT-GTCGCGGGGTGCACC
Cui17979YUNNANENSIS
CGAGCGCGCAATGAAAGTGAAAGTCGGGAACCCT-GTCGCGGGGTGCACC
Miettinen2091Junghuhnia_fimbri CGAGCGCGTAATGAAAGTGAAAGTTGGGATCCCT-GTCGTAGGGAGCACC
KHL12316S_tenue
CGAGCGCGTAATGAAAGTGAAAGTTGGGATCCCT-GTCGTGGGGAGCACC
PRM846564S_pachyodon --
SP_Lgt_S_pachyodon --
Ryvarden44669Tyromyces_xuchile CGAGCGCGCAATGAAAGTGAAAGTCGGGATCCTC-GCCA-GGGGAGCACC
PW17_171sinuosus --
W53Dai12234
CGAGCGCGCAATGAAAGTGAAAGTCGGGATCCTC-GCCA-GGGGAGCACC
HHB4100SpAntella_americana CGAGCGCGTAATGAAAGTGAAAGTTGGGATCCCT-GTCGTGGGGAGCACC
W3Dai20901spumeus
CGAGCGCGCAATGAAAGTGATAGTTGGGATCCCT-GTCGTGGGGAGCACT
He6736 CGAGCGCGCAATGAAAGTGATAGTTGGGATCCCT-GTCGTGGGGAGCACT
BRNM734877S_spumeus --
BRNM712630S_spumeus --
Dai1723Loweomyces_sibiricus --
W54Cui10009 CGAGTGCGCAATGAAAGTGATAGTTGGGATCCCT-GTCGTGGGGAGCACT
W1Dai20899 --
HHB13445Trametes_ochracea CGAGCGCGTAATGAAAGTGAAAGTTGAGACCTCT-GTCGTGGAGGGCATC
Dai16222 CGAGCGCACAATGAAAGTGAAAGTTGGGATCTCT-GTCGTGGAGAGCACC
Dai16240 CGAGCGCACAATGAAAGTGAAAGTTGGGATCTCT-GTCGTGGAGAGCACC

JV1310_11SanguinolentusCernys GACG-CCCGGACCAGACCTTCTGTGACGGATCCGCGGTGGAGCATGTATG
MJ39_00_SK GACG-CCCGGACCAGACCTTCTGTGACGGATCCGCGGTGGAGCATGTATG
MJ111_04_CZ --

JV1610_BOKYsmrk GACG-CCCGGACCAGACCTTCTGTGACGGATCCGCGGTGGAGCATGTATG
Dai21030 --
Dai20976P_furcatus GACG-CCCGGACCAGACCTTCTGTGACGGATCCGCGGTGGAGCATGTATG
Dai2105 GACG-CCCGGACCAGACCTTCTGTGACGGATCCGCGGTGGAGCATGTATG
Dai2544 GACG-CCCGGACCAGACCTTCTGTGACGGATCCGCGGTGGAGCATGTATG
Dai11313 GACG-CCCGGACCAGACCTTCTGTGACGGATCCGCGGTGGAGCATGTATG
WCG1611Dai26167 GACG-CCCGGACCAGACCTTCTGTGACGGATCCGCGGTGGAGCATGTATG
WCG1518Dai25999Physisporinus GACG-CCCGGACCAGACCTTCTGTGACGGATCCGCGGTGGAGCATGTATG
TAA15097 GACG-CCCGGACCAGACCTTCTGTGACGGATCCGCGGTGGAGCATGTATG
JV8909_19_CZ --
JV1310_15_P_sanguinolentus2_CZ GACG-CCCRGACCAGACCTTCTGTGACGGATCCGCGGTGGAGCATGTATG
MJ53_02_CZ GACG-CCCGGACCAGACCTTCTGTGACGGATCCGCGGTGGAGCATGTATG
CLZhao21647P_yunnanensis GACG-CCCGGACCAGACCTTCTGCGACGGATCCGCGGTGGAGCATGTATG
CLZhao21583P_yunnanensis GACG-CCCGGACCAGACCTTCTGCGACGGATCCGCGGTGGAGCATGTATG
Dai22272 GACG-CCCGGACCAGACCTTCTGTGACGGATCCGCGGTAGAGCATGTATG
Dai22279 GACG-CCCGGACCAGACCTTCTGTGACGGATCCGCGGTAGAGCATGTATG
MJ332_94_CZ --
MJ642_94_CZ_Expallescens --
Dai21060P_vinctus GACG-CCCGGACCAGACCTTCTGTGACGGATCCGCGGTAGAGCATGTATG
JV0511_23LRP_pouzarii GACG-CCCGGACCAGACCTTCTGTGACGGATCCGCGGTAGAGCATGTATG
JQ409462_R_pouzarii_PRM899856_ --
JV0308_66_WA --
JV0309_45_WA_USA --
JV0709_83_CA_USA GACG-CCCGGACCAGACCTTCTGTGACGGATCCGCGGTAGAGCATGTATG
Dai21043P_pouzarii GACG-CCCGGACCAGACCTTCTGTGACGGATCCGCGGTAGAGCATGTATG
MJ144_95_CZ GACG-

CCCGGACCAGACCTTCTGTGACGGATCCGCGGTAGAGCATGTATG
JV0909_3_CZ GACG-
CCCGGACCAGACCTTCTGTGACGGATCCGCGGTAGAGCATGTATG
JV0609_1_K ---
Dai20396Physisporinus_castanop GACG-
CCCGGACCAGACCTTCTGTGACGGATCCGCGGTAGAGCATGTATG
Dai20397Physisporinus_castanop GACG-
CCCGGACCAGACCTTCTGTGACGGATCCGCGGTAGAGCATGTATG
MJ19_09_SK_Abies GACG-
CCCGGACCAGACCTTCTGTGACGGATCCGCGGTAGAGCATGTATG
JV0509_40_J_TN_USA_Betula GACG-
CCCGGACCAGACCTTCTGTGACGGATCCGCGGTAGAGCATGTATG
JV0808_33crocatus_PA_USAlist GACG-
CCCGGACCAGACCTTCTGTGACGGATCCGCGGTAGAGCATGTATG
DLL2009_061P_crocatus ---
Dai12800P_subcrocatus GACG-
CCCGGACCAGACCTTCTGTGACGGATCCGCGGTAGAGCATGTATG
Dai15917P_subcrocatus GACG-
CCCGGACCAGACCTTCTGTGACGGATCCGCGGTAGAGCATGTATG
Cui16620 GACG-
CCCGGACCAGACCTTCTGTGACGGATCCGCGGTAGAGCATGTATG
HCFC1088Meripilus_stillicidior GACG-
CCCGGACCAGACCTTCTGTGACGGATCCGCGGTAGAGCATGTATG
MCW590Meripilus_obsrcurus ---
MCW722Meripilus_obsrcurus ---
Cui9381P_tibeticus GACG-
CCCGGACCAGAACTTCTGTGACGGATCCGCGGTAGAGCATGTATG
Cui9588P_tibeticus GACG-
CCCGGACCAGAACTTCTGTGACGGATCCGCGGTAGAGCATGTATG
Va2_Beneschova ---
CWU3874_Ukraine_Alnus GACG-
CCCGGACCAGACCTTCTGTGACGGATCCGCGGTAGAGCTTGTATG
WCG1293Dai24718Physisporinus ---
WCG1268Dai24682A GACG-
CCCGGACCAGACCTTCTGTGACGGATCCGCGGTAGAGCATGTATG
WCG1269Dai24683A GACG-
CCCGGACCAGACCTTCTGTGACGGATCCGCGGTAGAGCATGTATG
WCG1279Dai24694A GACG-
CCCGGACCAGACCTTCTGTGACGGATCCGCGGTAGAGCATGTATG
Dai16971 GACG-
CCCGGACCAGACCTTCTGTGACGGATCCGCGGTAGAGCATGTATG
ZQY1043Dai26696 GACG-
CCCGGACCAGACCTTCTGTGACGGATCCGCGGTAGAGCATGTATG
Doll880 ---

Doll1000 GACG-
CCCGGACCAGACCTTCTGTGACGGATCCGCGGTAGAGCATGTATG
1DAI18529 GACG-
CCCGGACCAGACCTTCTGTGACGGATCCGCGGTAGAGCATGTATG
Dai19535 GACG-
CCCGGACCAGACCTTCTGTGACGGATCTGCGGTAGAGCATGTATG
1704_79_hnedyVillaLaPaz GACG-
CCCGGACCAGACCTTCTGTGACGGATCCGCGGTAGAGCATGTATG
F2061 --
1DAI18268 GACG-
CCCGGACCAGACCTTCTGTGACGGATCCGCGGTAGAGCATGTATG
1DAI18540A GACG-
CCCGGACCAGACCTTCTGTGACGGATCCGCGGTAGAGCATGTATG
Dai17695 GACG-
CCCGGACCAGACCTTCTGTGACGGATCCGCGGTAGAGCATGTATG
LKY18Dai26373 --
Dai17839P_sulphureus GACG-
CCCGGACCAGACCTTCTGTGACGGATCCGCGGTAGAGCATGTATG
Dai17841P_sulphureus GACG-
CCCGGACCAGACCTTCTGTGACGGATCCGCGGTAGAGCATGTATG
Dai19877P_roseus GACG-
CCCGGACCAGACCTTCTGTGACGGATCCGCGGTAGAGCATGTATG
1508_18_1_Kout --
KP859303_R_vinctus_RP185_BRAZI --
JK1807_15Rigidoporus_sp_Puerto GACG-
CCCGGACCAGACCTTCTGTGACGGATCCGCGGTAGAGCCTGTATG
JV0509_47_J_TN_USA
GACGCCCCGGACCAGACCTTCTGTGACGGATCCGCGGTAGAGCATGTATG
JV0709_188 GACG-
CCCGGACCAGACCTTCTGTGACGGATCCGCGGTAGAGCATGTATG
JV0509_127_PA_USA GACG-
CCCGGACCAGACCTTCTGTGACGGATCCGCGGTAGAGCATGTATG
JV1009_59_NJ_USA
GACGCCCCGGACCAGACCTTCTGTGACGGATCCGCGGTAGAGCATGTATG
Dai15497P_crataegi GACG-
CCCGGACCAGACCTTCTGTGACGGTTCCGCGGTAGAGCATGTATG
Dai15499P_crataegi GACG-
CCCGGACCAGACCTTCTGTGACGGTTCCGCGGTAGAGCATGTATG
Cui3266P_cinereus GACG-
CCCGGACCAGACCTTCTGTGACGGATCCGCGGTAGAGCATGTATG
WCG1256Dai24690 GACG-
CCCGGACCAGACCTTCTGTGACGGATCCGCGGTAGAGCATGTATG
1DAI17581 GACG-
CCCGGACCAGACCTTCTGTGACGGATCCGCGGTAGAGCATGTATG

WCG1255Dai24688 GACG-
CCCGGACCAGACCTTCTGTGACGGATCCGCGGTAGAGCATGTATG
Dai22427 GACG-
CCCGGACCAGACCTTCTGTGACGGATCCGCGGTAGAGCATGTATG
MV690Meripilus_concrescens GACG-
CCCGGACCAGACCTTCTGTGACGGATCCGCGGTAGAGCATGTATG
MV513Meripilus_galapagensis GACG-
CCCGGACCAGACCTTGTGTGACGGATCCGCGGTAGAGCATGTATG
Dai19793 GACG-
CCCGGACCAGACCTTCTGTGACGGATCCGCGGTAGAGCATGTATG
OQ553780P_tamilnaduensis GACG-
CCCGGACCAGACCTTCTGTGACGGATCCACGGTAGAGCATGTATG
OQ553779P_tamilnaduensis GACG-
CCCGGACCAGACCTTCTGTGACGGATCCACGGTAGAGCATGTATG
A164FB3Meripilus_giganteus ---
JV1407_36_Vinctus_Meandrica GACG-
CCCGGACCAGACCTTCTGTGACGGATCCGCGGTAGAGCATGTATG
1807_3K_Rigidoporus_PuertoRico
GACGCCCCGGACCAGACCTTCTGTGACGGATCCGCGGTAGAGCATGTATG
Cui16903P_vinctus GACG-
CCCGGACCAGACCTTCTGTGACGGATCCGCGGTAGAGCATGTATG
JV1008_18R_Lineatus AATG-
CCCGGACCAGACCTTCTGTGACGGTTCCGCGGTAGAGCATGTATG
JV1407_37_1_Vinctus_Carara AATG-
CCCGGACCAGACCTTCTGTGACGGTTCCGCGGTAGAGCATGTATG
Dai17986P_lineatus AATG-
CCCGGACCAGACCTTCTGTGACGGTTCCGCGGTAGAGCATGTATG
Dai18281 AATG-
CCCGGACCAGACCTTCTGTGACGGTTCCGCGGTAGAGCATGTATG
1DAI19796 AATG-
CCCGGACCAGACCTTCTGTGACGGCTCCGCGGTAGAGCATGTATG
ZQY797Dai25241 AATG-
CCCGGACCAGACCTTCTGTGACGGCTCCGCGGTAGAGCATGTATG
WCG1289Dai24711 ---
Dai22598 AATG-
CCCGGACCAGACCTTCTGTGACGGTTCCGCGGTAGAGCATGTATG
Dai20523 AATG-
CCCGGACCAGACCTTCTGTGACGGTTCCGCGGTAGAGCATGTATG
Dai17885 AATG-
CCCGGACCAGACCTTCTGTGACGGTTCCGCGGTAGAGCATGTATG
Dai17553 AATG-
CCCGGACCAGACCTTCTGTGACGGTTCCGCGGTAGAGCATGTATG
Dai19639 AATG-
CCCGGACCAGACCTTCTGTGACGGTTCCGCGGTAGAGCATGTATG

JV0110_48_CZ GACG-
CCCGGACCAGACCTTCTGTGACGGATCCGCGGTAGAGCATGTATG
MJ129_04 GACG-
CCCGGACCAGACCTTCTGTGACGGATCCGCGGTAGAGCATGTATG
Cui10340P_eminens ---
Cui10341P_eminens GACG-
CCCGGACCAGACCTTCTGTGACGGATCCGCGGTAGAGCATGTATG
Dai12685P_eminens GACG-
CCCGGACCAGACCTTCTGTGACGGATCCGCGGTAGAGCATGTATG
Miettinen_13591Rigidoporus_und GACG-
CCCGGACCAGACCTTCTGTGACGGATCCGCGGTAGAGCATGTATG
Dai20868 GACG-
CCCGGACCAGACCTTCTGTGACGGATCCGCGGTAGAGCATGTATG
Dai20832 GACG-
CCCGGACCAGACCTTCTGTGACGGATCCGCGGTAGAGCATGTATG
Dai11400 GACG-
CCCGGACCAGACCTTCTGTGACGGATCCGCGGTAGAGCATGTATG
Dai22472 GACG-
CCCGGACCAGACCTTCTGTGACGGATCCGCGGTAGAGCATGTATG
1CUI10475 GACG-
CCCGGACCAGACCTTTTGTGACGGATCCGCGGTAGAGCATGTATG
1CUI10491 ---
HCFC1095Meripilus_robledoi ---
MCW702Meripilus_revolubilis GACG-
CCCGGACCAGACCTTTTGTGACGGATCCGCGGTAGAGCATGTATG
1704_83_zluty_HaciendaBaru GACG-
CCCGGACCAGACCTTTTGTGACGGATCCGCGGTAGAGCATGTATG
Dai9925P_lavendulus GACG-
CCCGGACCAGACCTTCTGTGACGGATCCGCGGTAGAGCATGTATG
Dai13587AP_lavendulus GACG-
CCCGGACCAGACCTTCTGTGACGGATCCGCGGTAGAGCATGTATG
PDD70600P_longicystidius ---
Cui16630 GACG-
CCCGGACCAGACCTTCTGTGACGGATCCGCGGTAGAGCATGTATG
FP_135344Meripilus_giganteus GACG-
CCCGGACCAGACCTTCTGTGACGGATCCGCGGTAGAGCATGTATG
FP_100460_Sp ---
CBS421_48Meripilus_giganteus ---
Cui9203 ---
Cui9202 GACG-
CCCGGACCAGACCTTCTGTGACGGATCCGCGGTAGAGCATGTATG
TUFC100564Japan GACG-
CCCGGACCAGACCTTCTGTGACGGATCCGCGGTAGAGCATGTATG
Russell5913Meripilus_sumstinei ---

RP215Meripilus_brasiliensis GACG-
CCCGGACCAGACCTTCTGTGACGGTTCCGCGGTAGAGCATGTATG
RP200Meripilus_brasiliensis GACG-
CCCGGACCAGACCTTCTGTGACGGTTCCGCGGTAGAGCATGTATG
JV1712_13J_R_vinctus2_LSUPuert GACG-
CCCGGACCAGACCTTTTGTGACGGATCTGCGGTAGAGCATGTATG
Dai10503R_hypobrunneus GACG-
CCCGGACCAGACCTTTTGTGACGGATCTGCGGTAGAGCATGTATG
Dai10569Rigidoporus_hypobrunne GACG-
CCCGGACCAGACCTTTTGTGACGGATCTGCGGTAGAGCATGTATG
1DAI19451 GACG-
CCCGGACCAGACCTTTTGTGACGGATCTGCGGTAGAGCATGTATG
CM108bRigidoporus_hypobrunneus ---
1CUI16874 GACG-
CCCGGACCAGACCTTTTGTGACGGATCTGCGGTAGAGCATGTATG
FD299Cerrena_unicolor GACG-
CCCGGACCAGACCTTTTGTGACGGATCTGCGGTAGAGCATGTATG
KHL_GB_Cerrena_uniclor GACG-
CCCGGACCAGACCTTTTGTGACGGATCTGCGGTAGAGCATGTATG
Dai12892Cerrena_albocinnamomea GACG-
CCCGGACCAGACCTTTTGTGACGGATCTGCGGTAGAGCATGTATG
Dai12955C_albocinnamomea GACG-
CCCGGACCAGACCTTTTGTGACGGATCTGCGGTAGAGCATGTATG
SNUm03110102C_aurantiopora GACG-
CCCGGACCAGACCTTTTGTGACGGATCTGCGGTAGAGCATGTATG
NIBRFG0000102423C_aurantiopora GACG-
CCCGGACCAGACCTTTTGTGACGGATCTGCGGTAGAGCATGTATG
Dai7359Antrodiella_zonata GACG-
CCCGGACCAGACCTTTTGTGACGGATCTGCGGTAGAGCATGTATG
F20080702KCM29C_consors GACG-
CCCGGACCAGACCTTTTGTGACGGATCTGCGGTAGAGCATGTATG
F20080208LYW10Cerrena_consors GACG-
CCCGGACCAGACCTTTTGTGACGGATCTGCGGTAGAGCATGTATG
Dai7821Antrodiella_zonata GACG-
CCCGGACCAGACCTTTTGTGACGGATCTGCGGTAGAGCATGTATG
CFMR_DCL04_31Pseudolagarobasid ---
VPB197Pseudolagarobasidium_bel ---
CBS115543Pseudolagarobasidium_ ---
CBS115544 ---
Han405Pseudolagarobasidium_bai GACG-
CCCGGACCAGACCTTTTGTGACGGATCTGCGGTAGAGCATGTATG
Han406Pseudolagarobasidium_bai GACG-
CCCGGACCAGACCTTTTGTGACGGATCTGCGGTAGAGCATGTATG
MUcc838Spongipellis_delectans ---

BRNM686401S_delectans --
OSM_F925S_delectans --
BRNM67093Spongipellis_litschau --
CFMRccFP59199TS_unicolor --
CFMRccFP71791TS_unicolor GACG-CCCGGACCAGACCTTCTGTGACGGCTCCGCGGTAGAGCATGTGTG
Dai13845P_lischaueri GACG-CCCGGACCAGACCTTCTGTGACGGCTCCGCGGTAGAGCATGTGTG
Dai20266P_lischaueri GACG-CCCGGACCAGACCTTCTGTGACGGCTCCGCGGTAGAGCATGTGTG
CFMR_HHB11240Radulodon_america --
RLG6350Radulodon_americanus --
KY415963Radulodon_erikssonii GATG-CCCGGCCCAGAGCTTTTGTGACGGTGCTGCGGTGGAGCATGTATG
HHB9567spRadulodon_casearius GATG-CCCGGCCCAGAGCTTTTGTGACGGTGCTGCGGTGGAGCATGTATG
KRT_Iso_26Radulodon_casearius --
CBS126044Radulodon_erikssonii GATG-CCCGGCCCAGAGCTTTTGTGACGGTGCTGCGGTGGAGCATGTATG
He6183YUNNANENSIS GATG-CCCGGCCCAGAGCTTTTGTGACGGTGCTGCGGTGGAGCATGTATG
Cui17979YUNNANENSIS GATG-CCCGGCCCAGAGCTTTTGTGACGGTGCTGCGGTGGAGCATGTATG
Miettinen2091Junghuhnia_fimbri GACG-CCCGGACCAGACCTTCTGTGACGGTTCCGCGGTAGAGCATGTATG
KHL12316S_tenue GACG-CCCGGACCAGACCTTCTGTGACGGATCCGCGGTAGAGCATGTATG
PRM846564S_pachyodon --
SP_Lgt_S_pachyodon --
Ryvarden44669Tyromyces_xuchile GACG-CCCGGCCCAGACCTTTTGCGACGGTGCTGCGGTGGAGCATGTATG
PW17_171sinuosus --
W53Dai12234 GACG-CCCGGCCCAGACCTTTTGCGACGGTGCTGCGGTGGAGCATGTATG
HHB4100SpAntella_americana GACG-CCCGGACCAGACCTTCTGTGACGGCTCCGCGGTAGAGCATGTATG
W3Dai20901spumeus GACG-CCCGGACCAGACCTTCTGTGACGGATCCGCGGTAGAGCATGTATG
He6736 GACG-CCCGGACCAGACCTTCTGTGACGGATCCGCGGTAGAGCATGTATG
BRNM734877S_spumeus --
BRNM712630S_spumeus --
Dai1723Loweomyces_sibiricus --
W54Cui10009 GACG-

CCCGGACCAGACCTTCTGTGACGGATCCGCGGTAGAGCATGTATG
W1Dai20899 --
HHB13445Trametes_ochracea GACG-
CCCGGACCTGACGTTTCTGAAGGATCCGCGGTAGAGCATGTATG
Dai16222 GACG-
CCCGGACCAGACCTTTTGTGACGGATCCGCGGTAGAGCATGTATG
Dai16240 GACG-
CCCGGACCAGACCTTTTGTGACGGATCCGCGGTAGAGCATGTATG

JV1310_11SanguinolentusCernys TTGGGACCCG-
AAAGATGGTGAACTATGCCTGAATAGGGTGAAGCCAGAG
MJ39_00_SK TTGGGACCCG-
AAAGATGGTGAACTATGCCTGAATAGGGTGAAGCCAGAG
MJ111_04_CZ --
JV1610_BOKYsmrk TTGGGACCCG-
AAAGATGGTGAACTATGCCTGAATAGGGTGAAGCCAGAG
Dai21030 --
Dai20976P_furcatus TTGGGACCCG-
AAAGATGGTGAACTATGCCTGAATAGGGTGAAGCCAGAG
Dai2105 TTGGGACCCG-
AAAGATGGTGAACTATGCCTGAATAGGGTGAAGCCAGAG
Dai2544 TTGGGACCCG-
AAAGATGGTGAACTATGCCTGAATAGGGTGAAGCCAGAG
Dai11313 TTGGGACCCG-
AAAGATGGTGAACTATGCCTGAATAGGGTGAAGCCAGAG
WCG1611Dai26167 TTGGGACCCG-
AAAGATGGTGAACTATGCCTGAATAGGGTGAAGCCAGAG
WCG1518Dai25999Physisporinus TTGGGACCCG-
AAAGATGGTGAACTATGCCTGAATAGGGTGAAGCCAGAG
TAA15097 TTGGGACCCG-
AAAGATGGTGAACTATGCCTGAATAGGGTGAAGCCAGAG
JV8909_19_CZ --
JV1310_15_P_sanguinolentus2_CZ TTGGGACCCG-
AAAGATGGTGAACTATGCCTGAATAGGGTGAAGCCAGAG
MJ53_02_CZ TTGGGACCCG-
AAAGATGGTGAACTATGCCTGAATAGGGTGAAGCCAGAG
CLZhao21647P_yunnanensis TTGGGACCCC-
AAAAATGGTGAACTATGCCTGAATAGGGTGAAGCCAGAG
CLZhao21583P_yunnanensis TTGGGACCCG-
AAAGATGGTGAACTATGCCTGAATAGGGTGAAGCCAGAG
Dai22272 TTGGGACCCG-
AAAGATGGTGAACTATGCCTGAATAGGGTGAAGCCAGAG
Dai22279 TTGGGACCCG-
AAAGATGGTGAACTATGCCTGAATAGGGTGAAGCCAGAG

MJ332_94_CZ ---
MJ642_94_CZ_Expallescens ---
Dai21060P_vinctus TTGGGACCCG-
AAAGATGGTGAACTATGCCTGAATAGGGTGAAGCCAGAG
JV0511_23LRP_pouzarii TTGGGACCCG-
AAAGATGGTGAACTATGCCTGAATAGGGTGAAGCCAGAG
JQ409462_R_pouzarii_PRM899856_ ---
JV0308_66_WA ---
JV0309_45_WA_USA ---
JV0709_83_CA_USA TTGGGACCCG-
AAAGATGGTGAACTATGCCTGAATAGGGTGAAGCCAGAG
Dai21043P_pouzarii TTGGGACCCG-
AAAGATGGTGAACTATGCCTGAATAGGGTGAAGCCAGAG
MJ144_95_CZ TTGGGACCCG-
AAAGATGGTGAACTATGCCTGAATAGGGTGAAGCCAGAG
JV0909_3_CZ TTGGGACCCG-
AAAGATGGTGAACTATGCCTGAATAGGGTGAAGCCAGAG
JV0609_1_K ---
Dai20396Physisporinus_castanop TTGGGACCCG-
AAAGATGGTGAACTATGCCTGAATAGGGTGAAGCCAGAG
Dai20397Physisporinus_castanop TTGGGACCCG-
AAAGATGGTGAACTATGCCTGAATAGGGTGAAGCCAGAG
MJ19_09_SK_Abies TTGGGACCCG-
AAAGATGGTGAACTATGCCTGAATAGGGTGAAGCCAGAG
JV0509_40_J_TN_USA_Betula TTGGGACCCG-
AAAGATGGTGAACTATGCCTGAATAGGGTGAAGCCAGAG
JV0808_33crocatus_PA_USAlist TTGGGACCCG-
AAAGATGGTGAACTATGCCTGAATAGGGTGAAGCCAGAG
DLL2009_061P_crocatus ---
Dai12800P_subcrocatus TTGGGACCCG-
AAAGATGGTGAACTATGCCTGAATAGGGTGAAGCCAGAG
Dai15917P_subcrocatus TTGGGACCCG-
AAAGATGGTGAACTATGCCTGAATAGGGTGAAGCCAGAG
Cui16620 TTGGGACCCG-
AAAGATGGTGAACTATGCCTGAATAGGGTGAAGCCAGAG
HCFC1088Meripilus_stillicidior TTGGGACCCG-
AAAGATGGTGAACTATGCCTGAATAGGGTGAAGCCAGAG
MCW590Meripilus_obsrcurus ---
MCW722Meripilus_obsrcurus ---
Cui9381P_tibeticus TTGGGACCCG-
AAAGATGGTGAACTATGCCTGAATAGGGTGAAGCCAGAG
Cui9588P_tibeticus TTGGGACCCG-
AAAGATGGTGAACTATGCCTGAATAGGGTGAAGCCAGAG
Va2_Beneschova ---

CWU3874_Ukraine_Alnus TTGGGACCCG-AAAGATGGTGAACTATGCCTGAATAGGGTGAAGCCAGAG
WCG1293Dai24718Physisporinus ---
WCG1268Dai24682A TTGGGACCCG-AAAGATGGTGAACTATGCCTGAATAGGGTGAAGCCAGAG
WCG1269Dai24683A TTGGGACCCG-AAAGATGGTGAACTATGCCTGAATAGGGTGAAGCCAGAG
WCG1279Dai24694A TTGGGACCCG-AAAGATGGTGAACTATGCCTGAATAGGGTGAAGCCAGAG
Dai16971 TTGGGACCCG-AAAGATGGTGAACTATGCCTGAATAGGGTGAAGCCAGAG
ZQY1043Dai26696 TTGGGACCCG-AAAGATGGTGAACTATGCCTGAATAGGGTGAAGCCAGAG
Doll880 ---
Doll1000 TTGGGACCCG-AAAGATGGTGAACTATGCCTGAATAGGGTGAAGCCAGAG
1DAI18529 TTGGGACCCG-AAAGATGGTGAACTATGCCTGAATAGGGTGAAGCCAGAG
Dai19535 TTGGGACCCG-AAAGATGGTGAACTATGCCTGAATAGGGTGAAGCCAGAG
1704_79_hnedyVillaLaPaz TTGGGACCCG-AAAGATGGTGAACTATGCCTGAATAGGGTGAAGCCAGAG
F2061 ---
1DAI18268 TTGGGACCCG-AAAGATGGTGAACTATGCCTGAATAGGGTGAAGCCAGAG
1DAI18540A TTGGGACCCG-AAAGATGGTGAACTATGCCTGAATAGGGTGAAGCCAGAG
Dai17695 TTGGGACCCG-AAAGATGGTGAACTATGCCTGAATAGGGTGAAGCCAGAG
LKY18Dai26373 ---
Dai17839P_sulphureus TTGGGACCCG-AAAGATGGTGAACTATGCCTGAATAGGGTGAAGCCAGAG
Dai17841P_sulphureus TTGGGACCCG-AAAGATGGTGAACTATGCCTGAATAGGGTGAAGCCAGAG
Dai19877P_roseus TTGGGACCCG-AAAGATGGTGAACTATGCCTGAATAGGGTGAAGCCAGAG
1508_18_1_Kout ---
KP859303_R_vinctus_RP185_BRAZI ---
JK1807_15Rigidoporus_sp_Puerto TTGGGACCCG-AAAGATGGTGAACTATGCCTGAATAGGGTGAAGCCAGAG
JV0509_47_J_TN_USA TTGGGACCCG-AAAGATGGTGAACTATGCCTGAATAGGGTGAAGCCAGAG
JV0709_188 TTGGGACCCG-AAAGATGGTGAACTATGCCTGAATAGGGTGAAGCCAGAG

JV0509_127_PA_USA TTGGGACCCG-
AAAGATGGTGAACTATGCCTGAATAGGGTGAAGCCAGAG
JV1009_59_NJ_USA TTGGGACCCG-
AAAGATGGTGAACTATGCCTGAATAGGGTGAAGCCAGAG
Dai15497P_crataegi TTGGGACCCG-
AAAGATGGTGAACTATGCCTGAATAGGGTGAAGCCAGAG
Dai15499P_crataegi TTGGGACCCG-
AAAGATGGTGAACTATGCCTGAATAGGGTGAAGCCAGAG
Cui3266P_cinereus TTGGGACCCG-
AAAGATGGTGAACTATGCCTGAATAGGGTGAAGCCAGAG
WCG1256Dai24690 TTGGGACCCG-
AAAGATGGTGAACTATGCCTGAATAGGGTGAAGCCAGAG
1DAI17581 TTGGGACCCG-
AAAGATGGTGAACTATGCCTGAATAGGGTGAAGCCAGAG
WCG1255Dai24688 TTGGGACCCG-
AAAGATGGTGAACTATGCCTGAATAGGGTGAAGCCAGAG
Dai22427 TTGGGACCCG-
AAAGATGGTGAACTATGCCTGAATAGGGTGAAGCCAGAG
MV690Meripilus_concrescens TTGGGACCCG-
AAAGATGGTGAACTATGCCTGAATAGGGTGAAGCCAGAG
MV513Meripilus_galapagensis TTGGGACCCG-
AAAGATGGTGAAATATGCCTGAATAGGGTGAAGCCAGAG
Dai19793 TTGGGACCCG-
AAAGATGGTGAACTATGCCTGAATAGGGTGAAGCCAGAG
OQ553780P_tamilnaduensis TTGGGACGCG-
AAAGATGGTGAATTATGCCTGAATAGGGTGAAGCCAGAG
OQ553779P_tamilnaduensis TTGGGACGCG-
AAAGATGGTGAATTATGCCTGAATAGGGTGAAGCCAGAG
A164FB3Meripilus_giganteus ---
JV1407_36_Vinctus_Meandrica TTGGGACCCG-
AAAGATGGTGAACTATGCCTGAATAGGGTGAAGCCAGAG
1807_3K_Rigidoporus_PuertoRico
TTGGGACCCGAAAAGATGGTGAACTATGCCTGAATAGGGTGAAGCCAGAG
Cui16903P_vinctus TTGGGACCCG-
AAAGATGGTGAACTATGCCTGAATAGGGTGAAGCCAGAG
JV1008_18R_Lineatus TTGGGACCCG-
AAAGATGGTGAACTATGCCTGAATAGGGTGAAGCCAGAG
JV1407_37_1_Vinctus_Carara TTGGGACCCG-
AAAGATGGTGAACTATGCCTGAATAGGGTGAAGCCAGAG
Dai17986P_lineatus TTGGGACCCG-
AAAGATGGTGAACTATGCCTGAATAGGGTGAAGCCAGAG
Dai18281 TTGGGACCCG-
AAAGATGGTGAACTATGCCTGAATAGGGTGAAGCCAGAG
1DAI19796 TTGGGACCCG-

AAAGATGGTGAACTATGCCTGAATAGGGTGAAGCCAGAG
ZQY797Dai25241 TTGGGACCCG-
AAAGATGGTGAACTATGCCTGAATAGGGTGAAGCCAGAG
WCG1289Dai24711 --
Dai22598 TTGGGACCCG-
AAAGATGGTGAACTATGCCTGAATAGGGTGAAGCCAGAG
Dai20523 TTGGGACCCG-
AAAGATGGTGAACTATGCCTGAATAGGGTGAAGCCAGAG
Dai17885 TTGGGACCCG-
AAAGATGGTGAACTATGCCTGAATAGGGTGAAGCCAGAG
Dai17553 TTGGGACCCG-
AAAGATGGTGAACTATGCCTGAATAGGGTGAAGCCAGAG
Dai19639 TTGGGACCCG-
AAAGATGGTGAACTATGCCTGAATAGGGTGAAGCCAGAG
JV0110_48_CZ TTGGGACCCG-
AAAGATGGTGAACTATGCCTGAATAGGGTGAAGCCAGAG
MJ129_04 TTGGGACCCG-
AAAGATGGTGAACTATGCCTGAATAGGGTGAAGCCAGAG
Cui10340P_eminens --
Cui10341P_eminens TTGGGACCCG-
AAAGATGGTGAACTATGCCTGAATAGGGTGAAGCCAGAG
Dai12685P_eminens TTGGGACCCG-
AAAGATGGTGAACTATGCCTGAATAGGGTGAAGCCAGAG
Miettinen_13591Rigidoporus_und TTGGGACCCG-
AAAGATGGTGAACTATGCCTGAATAGGGTGAAGCCAGAG
Dai20868 TTGGGACCCG-
AAAGATGGTGAACTATGCCTGAATAGGGTGAAGCCAGAG
Dai20832 TTGGGACCCG-
AAAGATGGTGAACTATGCCTGAATAGGGTGAAGCCAGAG
Dai11400 TTGGGACCCG-
AAAGATGGTGAACTATGCCTGAATAGGGTGAAGCCAGAG
Dai22472 TTGGGACCCG-
AAAGATGGTGAACTATGCCTGAATAGGGTGAAGCCAGAG
1CUI10475 TTGGGACCCG-
AAAGATGGTGAACTATGCCTGAATAGGGTGAAGCCAGAG
1CUI10491 --
HCFC1095Meripilus_robledoi --
MCW702Meripilus_revolubilis TTGGGACCCG-
AAAGATGGTGAACTATGCCTGAATAGGGTGAAGCCAGAG
1704_83_zluty_HaciendaBaru TTGGGACCCG-
AAAGATGGTGAACTATGCCTGAATAGGGTGAAGCCAGAG
Dai9925P_lavendulus TTGGGACCCG-
AAAGATGGTGAACTATGCCTGAATAGGGTGAAGCCAGAG
Dai13587AP_lavendulus TTGGGACCCG-

AAAGATGGTGAACTATGCCTGAATAGGGTGAAGCCAGAG
PDD70600P_longicystidius --
Cui16630 TTGGGACCCG-
AAAGATGGTGAACTATGCCTGAATAGGGTGAAGCCAGAG
FP_135344Meripilus_giganteus TTGGGACCCG-
AAAGATGGTGAACTATGCCTGAATAGGGTGAAGCCAGAG
FP_100460_Sp --
CBS421_48Meripilus_giganteus --
Cui9203 --
Cui9202 TTGGGACCCG-
AAAGATGGTGAACTATGCCTGAATAGGGTGAAGCCAGAG
TUFC100564Japan TTGGGACCCG-
AAAGATGGTGAACTATGCCTGAATAGGGTGAAGCCAGAG
Russell5913Meripilus_sumstinei --
RP215Meripilus_brasiliensis TTGGGACCCG-
AAAGATGGTGAACTATGCCTGAATAGGGTGAAGCCAGAG
RP200Meripilus_brasiliensis TTGGGACCCG-
AAAGATGGTGAACTATGCCTGAATAGGGTGAAGCCAGAG
JV1712_13J_R_vinctus2_LSUPuert TTGGGACCCG-
AAAGATGGTGAACTATGCCTGAATAGGGTGAAGCCAGAG
Dai10503R_hypobrunneus TTGGGACCCG-
AAAGATGGTGAACTATGCCTGAATAGGGTGAAGCCAGAG
Dai10569Rigidoporus_hypobrunne TTGGGACCCG-
AAAGATGGTGAACTATGCCTGAATAGGGTGAAGCCAGAG
1DAI19451 TTGGGACCCG-
AAAGATGGTGAACTATGCCTGAATAGGGTGAAGCCAGAG
CM108bRigidoporus_hypobrunneus --
1CUI16874 TTGGGACCCG-
AAAGATGGTGAACTATGCCTGAATAGGGTGAAGCCAGAG
FD299Cerrena_unicolor TTGGGACCCG-
AAAGATGGTGAACTATGCCTGAATAGGGTGAAGCCAGAG
KHL_GB_Cerrena_uniclor TTGGGACCCG-
AAAGATGGTGAACTATGCCTGAATAGGGTGAAGCCAGAG
Dai12892Cerrena_albocinnamomea TTGGGACCCG-
AAAGATGGTGAACTATGCCTGAATAGGGTGAAGCCAGAG
Dai12955C_albocinnamomea TTGGGACCCG-
AAAGATGGTGAACTATGCCTGAATAGGGTGAAGCCAGAG
SNUm03110102C_aurantiopora TTGGGACCCG-
AAAGATGGTGAACTATGCCTGAATAGGGTGAAGCCAGAG
NIBRFG0000102423C_aurantiopora TTGGGACCCG-
AAAGATGGTGAACTATGCCTGAATAGGGTGAAGCCAGAG
Dai7359Antrodiella_zonata TTGGGACCCG-
AAAGATGGTGAACTATGCCTGAATAGGGTGAAGCCAGAG
F20080702KCM29C_consors TTGGGACCCG-

AAAGATGGTGAACTATGCCTGAATAGGGTGAAGCCAGAN
F20080208LYW10Cerrena_consors TTGGGACCCG-
AAAGATGGTGAACTATGCCTGAATAGGGTGAAGCCAGAG
Dai7821Antrodiella_zonata TTGGGACCCG-
AAAGATGGTGAACTATGCCTGAATAGGGTGAAGCCAGAG
CFMR_DCL04_31Pseudolagarobasid --
VPB197Pseudolagarobasidium_bel --
CBS115543Pseudolagarobasidium_ --
CBS115544 --
Han405Pseudolagarobasidium_bai TTGGGACCCG-
AAAGATGGTGAACTATGCCTGAATAGGGTGAAGCCAGAG
Han406Pseudolagarobasidium_bai TTGGGACCCG-
AAAGATGGTGAACTATGCCTGAATAGGGTGAAGCCAGAG
MUcc838Spongipellis_delectans --
BRNM686401S_delectans --
OSM_F925S_delectans --
BRNM67093Spongipellis_litschau --
CFMRccFP59199TS_unicolor --
CFMRccFP71791TS_unicolor T---
Dai13845P_lischaueri TTGGGACCCG-
AAAGATGGTGAACTATGCCTGAATAGGGTGAAGCCAGAG
Dai20266P_lischaueri TTGGGACCCG-
AAAGATGGTGAACTATGCCTGAATAGGGTGAAGCCAGAG
CFMR_HHB11240Radulodon_america --
RLG6350Radulodon_americanus --
KY415963Radulodon_erikssonii TTGGGACCCG-
AAAGATGGTGAACTATGCCTGAATAGGGTGAAGCCAGAG
HHB9567spRadulodon_casearius TTGGGACCCG-
AAAGATGGTGAACTATGCCTGAATAGGGTGAAGCCAGAG
KRT_Iso_26Radulodon_casearius --
CBS126044Radulodon_erikssonii TTGGGACCCG-
AAAGATGGTGAACTATGCCTGAATAGGGTGAAGCCAGAG
He6183YUNNANENSIS TTGGGACCCG-
AAAGATGGTGAACTATGCCTGAATAGGGTGAAGCCAGAG
Cui17979YUNNANENSIS TTGGGACCCG-
AAAGATGGTGAACTATGCCTGAATAGGGTGAAGCCAGAG
Miettinen2091Junghuhnia_fimbri TTGGGACCCG-
AAAGATGGTGAACTATGCCTGAATAGGGTGAAGCCAGAG
KHL12316S_tenue TTGGGACCCG-
AAAGATGGTGAACTATGCCTGAATAGGGTGAAGCCAGAG
PRM846564S_pachyodon --
SP_Lgt_S_pachyodon --
Ryvarden44669Tyromyces_xuchile TTGGGACCCG-
AAAGATGGTGAACTATGCCTGAATAGGGTGAAGCCAGAG

PW17_171sinuosus --
W53Dai12234 TTGGGACCCG-
AAAGATGGTGAACTATGCCTGAATAGGGTGAAGCCAGAG
HHB4100SpAntella_americana TTGGGACCCG-
AAAGATGGTGAACTATGCCTGAATAGGGTGAAGCCAGAG
W3Dai20901spumeus TTGGGACCCG-
AAAGATGGTGAACTATGCCTGAATAGGGTGAAGCCAGAG
He6736 TTGGGACCCG-
AAAGATGGTGAACTATGCCTGAATAGGGTGAAGCCAGAG
BRNM734877S_spumeus --
BRNM712630S_spumeus --
Dai1723Loweomyces_sibiricus --
W54Cui10009 TTGGGACCCG-
AAAGATGGTGAACTATGCCTGAATAGGGTGAAGCCAGAG
W1Dai20899 --
HHB13445Trametes_ochracea TTGGGACCCG-
AAAGATGGTGAACTATGCCTGAATAGGGTGAAGCCAGAG
Dai16222 TTGGGACCCG-
AAAGATGGTGAACTATGCCTGAATAGGGTGAAGCCAGAG
Dai16240 TTGGGACCCG-
AAAGATGGTGAACTATGCCTGAATAGGGTGAAGCCAGAG

JV1310_11SanguinolentusCernys
GAAACTCTGGTGGAGGCTCGTAGCGATTCTGACGTGCAAATCGATCGTCA
MJ39_00_SK
GAAACTCTGGTGGAGGCTCGTAGCGATTCTGACGTGCAAATCGATCGTCA
MJ111_04_CZ --
JV1610_BOKYsmrk
GAAACTCTGGTGGAGGCTCGTAGCGATTCTGACGTGCAAATCGATCGTCA
Dai21030 --
Dai20976P_furcatus
GAAACTCTGGTGGAGGCTCGTAGCGATTCTGACGTGCAAATCGATCGTCA
Dai2105
GAAACTCTGGTGGAGGCTCGTAGCGATTCTGACGTGCAAATCGATCGTCA
Dai2544
GAAACTCTGGTGGAGGCTCGTAGCGATTCTGACGTGCAAATCGATCGTCA
Dai11313
GAAACTCTGGTGGAGGCTCGTAGCGATTCTGACGTGCAAATCGATCGTCA
WCG1611Dai26167
GAAACTCTGGTGGAGGCTCGTAGCGATTCTGACGTGCAAATCGATCGTCA
WCG1518Dai25999Physisporinus
GAAACTCTGGTGGAGGCTCGTAGCGATTCTGACGTGCAAATCGATCGTCA
TAA15097
GAAACTCTGGTGGAGGCTCGTAGCGATTCTGACGTGCAAATCGATCGTCA

JV8909_19_CZ ---
JV1310_15_P_sanguinolentus2_CZ
GAAACTCTGGTGGAGGCTCGTAGCGATTCTGACGTGCAAATCGATCGTCA
MJ53_02_CZ
GAAACTCTGGTGGAGGCTCGTAGCGATTCTGACGTGCAAATCGATCGTCA
CLZhao21647P_yunnanensis
GAAACTCTGGTGGAGGCTCGTAGCGATTCTGACGTGCAAATCGATCGTCA
CLZhao21583P_yunnanensis
GAAACTCTGGTGGAGGCTCGTAGCGATTCTGACGTGCAAATCGATCGTCA
Dai22272
GAAACTCTGGTGGAGGCTCGTAGCGATTCTGACGTGCAAATCGATCGTCA
Dai22279
GAAACTCTGGTGGAGGCTCGTAGCGATTCTGACGTGCAAATCGATCGTCA
MJ332_94_CZ ---
MJ642_94_CZ_Expallescens ---
Dai21060P_vinctus
GAAACTCTGGTGGAGGCTCGTAGCGATTCTGACGTGCAAATCGATCGTCA
JV0511_23LRP_pouzarii
GAAACTCTGGTGGAGGCTCGTAGCGATTCTGACGTGCAAATCGATCGTCA
JQ409462_R_pouzarii_PRM899856_ ---
JV0308_66_WA ---
JV0309_45_WA_USA ---
JV0709_83_CA_USA
GAAACTCTGGTGGAGGCTCGTAGCGATTCTGACGTGCAAATCGATCGTCA
Dai21043P_pouzarii
GAAACTCTGGTGGAGGCTCGTAGCGATTCTGACGTGCAAATCGATCGTCA
MJ144_95_CZ
GAAACTCTGGTGGAGGCTCGTAGCGATTCTGACGTGCAAATCGATCGTCA
JV0909_3_CZ
GAAACTCTGGTGGAGGCTCGTAGCGATTCTGACGTGCAAATCGATCGTCA
JV0609_1_K ---
Dai20396Physisporinus_castanop
GAAACTCTGGTGGAGGCTCGTAGCGATTCTGACGTGCAAATCGATCGTCA
Dai20397Physisporinus_castanop
GAAACTCTGGTGGAGGCTCGTAGCGATTCTGACGTGCAAATCGATCGTCA
MJ19_09_SK_Abies
GAAACTCTGGTGGAGGCTCGTAGCGATTCTGACGTGCAAATCGATCGTCA
JV0509_40_J_TN_USA_Betula
GAAACTCTGGTGGAGGCTCGTAGCGATTCTGACGTGCAAATCGATCGTCA
JV0808_33crocatus_PA_USAlist
GAAACTCTGGTGGAGGCTCGTAGCGATTCTGACGTGCAAATCGATCGTCA
DLL2009_061P_crocatus ---
Dai12800P_subcrocatus
GAAACTCTGGTGGAGGCTCGTAGCGATTCTGACGTGCAAATCGATCGTCA

Dai15917P_subcrocatus
GAAACTCTGGTGGAGGCTCGTAGCGATTCTGACGTGCAAATCGATCGTCA
Cui16620
GAAACTCTGGTGGAGGCTCGTAGCGATTCTGACGTGCAAATCGATCGTCA
HCFC1088Meripilus_stillicidior
GAAACTCTGGTGGAGGCTCGTAGCGATTCTGACGTGCAAATCGATCGTCA
MCW590Meripilus_obsrcurus --
MCW722Meripilus_obsrcurus --
Cui9381P_tibeticus
GAAACTCTGGTGGAGGCTCGTAGCGATTCTGACGTGCAAATCGATCGTCA
Cui9588P_tibeticus
GAAACTCTGGTGGAGGCTCGTAGCGATTCTGACGTGCAAATCGATCGTCA
Va2_Beneschova --
CWU3874_Ukraine_Alnus
GAAACTCTGGTGGAGGCTCGTAGCGATTCTGACGTGCAAATCGATCGTCA
WCG1293Dai24718Physisporinus --
WCG1268Dai24682A
GAAACTCTGGTGGAGGCTCGTAGCGATTCTGACGTGCAAATCGATCGTCA
WCG1269Dai24683A
GAAACTCTGGTGGAGGCTCGTAGCGATTCTGACGTGCAAATCGATCGTCA
WCG1279Dai24694A
GAAACTCTGGTGGAGGCTCGTAGCGATTCTGACGTGCAAATCGATCGTCA
Dai16971
GAAACTCTGGTGGAGGCTCGTAGCGATTCTGACGTGCAAATCGATCGTCA
ZQY1043Dai26696
GAAACTCTGGTGGAGGCTCGTAGCGATTCTGACGTGCAAATCGATCGTCA
Doll880 --
Doll1000
GAAACTCTGGTGGAGGCTCGTAGCGATTCTGACGTGCAAATCGATCGTCA
1DAI18529
GAAACTCTGGTGGAGGCTCGTAGCGATTCTGACGTGCAAATCGATCGTCA
Dai19535
GAAACTCTGGTGGAGGCTCGTAGCGATTCTGACGTGCAAATCGATCGTCA
1704_79_hnedyVillaLaPaz
GAAACTCTGGTGGAGGCTCGTAGCGATTCTGACGTGCAAATCGATCGTCA
F2061 --
1DAI18268
GAAACTCTGGTGGAGGCTCGTAGCGATTCTGACGTGCAAATCGATCGTCA
1DAI18540A
GAAACTCTGGTGGAGGCTCGTAGCGATTCTGACGTGCAAATCGATCGTCA
Dai17695
GAAACTCTGGTGGAGGCTCGTAGCGATTCTGACGTGCAAATCGATCGTCA
LKY18Dai26373 --
Dai17839P_sulphureus

GAAACTCTGGTGGAGGCTCGTAGCGATTCTGACGTGCAAATCGATCGTCA
Dai17841P_sulphureus
GAAACTCTGGTGGAGGCTCGTAGCGATTCTGACGTGCAAATCGATCGTCA
Dai19877P_roseus
GAAACTCTGGTGGAGGCTCGTAGCGATTCTGACGTGCAAATCGATCGTCA
1508_18_1_Kout --
KP859303_R_vinctus_RP185_BRAZI --
JK1807_15Rigidoporus_sp_Puerto
GAAACTCTGGTGGAGGCTCGTAGCGATTCTGACGTGCAAATCGATCGTCA
JV0509_47_J_TN_USA
GAAACTCTGGTGGAGGCTCGTAGCGATTCTGACGTGCAAATCGATCGTCA
JV0709_188
GAAACTCTGGTGGAGGCTCGTAGCGATTCTGACGTGCAAATCGATCGTCA
JV0509_127_PA_USA
GAAACTCTGGTGGAGGCTCGTAGCGATTCTGACGTGCAAATCGATCGTCA
JV1009_59_NJ_USA
GAAACTCTGGTGGAGGCTCGTAGCGATTCTGACGTGCAAATCGATCGTCA
Dai15497P_crataegi
GAAACTCTGGTGGAGGCTCGTAGCGATTCTGACGTGCAAATCGATCGTCA
Dai15499P_crataegi
GAAACTCTGGTGGAGGCTCGTAGCGATTCTGACGTGCAAATCGATCGTCA
Cui3266P_cinereus
GAAACTCTGGTGGAGGCTCGTAGCGATTCTGACGTGCAAATCGATCGTCA
WCG1256Dai24690
GAAACTCTGGTGGAGGCTCGTAGCGATTCTGACGTGCAAATCGATCGTCA
1DAI17581
GAAACTCTGGTGGAGGCTCGTAGCGATTCTGACGTGCAAATCGATCGTCA
WCG1255Dai24688
GAAACTCTGGTGGAGGCTCGTAGCGATTCTGACGTGCAAATCGATCGTCA
Dai22427
GAAACTCTGGTGGAGGCTCGTAGCGATTCTGACGTGCAAATCGATCGTCA
MV690Meripilus_concrescens
GAAACTCTGGTGGAGGCTCGTAGCGATTCTGACGTGCAAATCGATCGTCA
MV513Meripilus_galapagensis
GAAACTCTGGTGGAGGCTAGTAGCGATTCTGACGTGCAAATCGATCGTCA
Dai19793
GAAACTCTGGTGGAGGCTCGTAGCGATTCTGACGTGCAAATCGATCGTCA
OQ553780P_tamilnaduensis
GAAACTATGGTGGAAGCTAGTAGCGATTGTGATGTGCAAATCGATCGTGA
OQ553779P_tamilnaduensis
GAAACTATGGTGGAAGCTAGTAGCGATTGTGATGTGCAAATCGATCGTGA
A164FB3Meripilus_giganteus --
JV1407_36_Vinctus_Meandrica
GAAACTCTGGTGGAGGCTCGTAGCGATTCTGACGTGCAAATCGATCGTCA

1807_3K_Rigidoporus_PuertoRico
GAAACTCTGGTGGAGGCTCGTAGCGATTCTGACGTGCAAATCGATCGTCA
Cui16903P_vinctus
GAAACTCTGGTGGAGGCTCGTAGCGATTCTGACGTGCAAATCGATCGTCA
JV1008_18R_Lineatus
GAAACTCTGGTGGAGGCTCGTAGCGATTCTGACGTGCAAATCGATCGTCA
JV1407_37_1_Vinctus_Carara
GAAACTCTGGTGGAGGCTCGTAGCGATTCTGACGTGCAAATCGATCGTCA
Dai17986P_lineatus
GAAACTCTGGTGGAGGCTCGTAGCGATTCTGACGTGCAAATCGATCGTCA
Dai18281
GAAACTCTGGTGGAGGCTCGTAGCGATTCTGACGTGCAAATCGATCGTCA
1DAI19796
GAAACTCTGGTGGAGGCTCGTAGCGATTCTGACGTGCAAATCGATCGTCA
ZQY797Dai25241
GAAACTCTGGTGGAGGCTCGTAGCGATTCTGACGTGCAAATCGATCGTCA
WCG1289Dai24711 --
Dai22598
GAAACTCTGGTGGAGGCTCGTAGCGATTCTGACGTGCAAATCGATCGTCA
Dai20523
GAAACTCTGGTGGAGGCTCGTAGCGATTCTGACGTGCAAATCGATCGTCA
Dai17885
GAAACTCTGGTGGAGGCTCGTAGCGATTCTGACGTGCAAATCGATCGTCA
Dai17553
GAAACTCTGGTGGAGGCTCGTAGCGATTCTGACGTGCAAATCGATCGTCA
Dai19639
GAAACTCTGGTGGAGGCTCGTAGCGATTCTGACGTGCAAATCGATCGTCA
JV0110_48_CZ
GAAACTCTGGTGGAGGCTCGTAGCGATTCTGACGTGCAAATCGATCGTCA
MJ129_04
GAAACTCTGGTGGAGGCTCGTAGCGATTCTGACGTGCAAATCGATCGTCA
Cui10340P_eminens --
Cui10341P_eminens
GAAACTCTGGTGGAGGCTCGTAGCGATTCTGACGTGCAAATCGATCGTCA
Dai12685P_eminens
GAAACTCTGGTGGAGGCTCGTAGCGATTCTGACGTGCAAATCGATCGTCA
Miettinen_13591Rigidoporus_und
GAAACTCTGGTGGAGGCTCGTAGCGATTCTGACGTGCAAATCGATCGTCA
Dai20868
GAAACTCTGGTGGAGGCTCGTAGCGATTCTGACGTGCAAATCGATCGTCA
Dai20832
GAAACTCTGGTGGAGGCTCGTAGCGATTCTGACGTGCAAATCGATCGTCA
Dai11400
GAAACTCTGGTGGAGGCTCGTAGCGATTCTGACGTGCAAATCGATCGTCA

Dai22472
GAAACTCTGGTGGAGGCTCGTAGCGATTCTGACGTGCAAATCGATCGTCA
1CUI10475
GAAACTCTGGTGGAGGCTCGTAGCGATTCTGACGTGCAAATCGATCGTCA
1CUI10491 --
HCFC1095Meripilus_robledoi --
MCW702Meripilus_revolubilis
GATACTCTGGTGGAGGCTCGTAGCGATTCTGACGTGCAAATCGATCGTCA
1704_83_zluty_HaciendaBaru
GAAACTCTGGTGGAGGCTCGTAGCGATTCTGACGTGCAAATCGATCGTCA
Dai9925P_lavendulus
GAAACTCTGGTGGAGGCTCGTAGCGATTCTGACGTGCAAATCGATCGTCA
Dai13587AP_lavendulus
GAAACTCTGGTGGAGGCTCGTAGCGATTCTGACGTGCAAATCGATCGTCA
PDD70600P_longicystidius --
Cui16630
GAAACTCTGGTGGAGGCTCGTAGCGATTCTGACGTGCAAATCGATCGTCA
FP_135344Meripilus_giganteus
GAAACTCTGGTGGAGGCTCGTAGCGATTCTGACGTGCAAATCGATCGTCA
FP_100460_Sp --
CBS421_48Meripilus_giganteus --
Cui9203 --
Cui9202
GAAACTCTGGTGGAGGCTCGTAGCGATTCTGACGTGCAAATCGATCGTCA
TUFC100564Japan
GAAACTCTGGTGGAGGCTCGTAGCGATTCTGACGTGCAAATCGATCGTCA
Russell5913Meripilus_sumstinei --
RP215Meripilus_brasiliensis
GAAACTCTGGTGGAGGCTCGTAGCGATTCTGACGTGCAAATCGATCGTCA
RP200Meripilus_brasiliensis
GAAACTCTGGTGGAGGCTCGTAGCGATTCTGACGTGCAAATCGATCGTCA
JV1712_13J_R_vinctus2_LSUPuert
GAAACTCTGGTGGAGGCTCGTAGCGATTCTGACGTGCAAATCGATCGTCA
Dai10503R_hypobrunneus
GAAACTCTGGTGGAGGCTCGTAGCGATTCTGACGTGCAAATCGATCGTCA
Dai10569Rigidoporus_hypobrunne
GAAACTCTGGTGGAGGCTCGTAGCGATTCTGACGTGCAAATCGATCGTCA
1DAI19451
GAAACTCTGGTGGAGGCTCGTAGCGATTCTGACGTGCAAATCGATCGTCA
CM108bRigidoporus_hypobrunneus --
1CUI16874
GAAACTCTGGTGGAGGCTCGTAGCGATTCTGACGTGCAAATCGATCGTCA
FD299Cerrena_unicolor
GAAACTCTGGTGGAGGCTCGTAGCGATTCTGACGTGCAAATCGATCGTCA

KHL_GB_Cerrena_uniclor
GAAACTCTGGTGGAGGCTCGTAGCGATTCTGACGTGCAAATCGATCGTCA
Dai12892Cerrena_albocinnamomea
GAAACTCTGGTGGAGGCTCGTAGCGATTCTGACGTGCAAATCGATCGTCA
Dai12955C_albocinnamomea
GAAACTCTGGTGGAGGCTCGTAGCGATTCTGACGTGCAAATCGATCGTCA
SNUm03110102C_aurantiopora
GAAACTCTGGTGGAGGCTCGTAGCGATTCTGACGTGCAAATCGATCGTCA
NIBRFG0000102423C_aurantiopora
GAAACTCTGGTGGAGGCTCGTAGCGATTCTGACGTGCAAATCGATCGTCA
Dai7359Antrodiella_zonata
GAAACTCTGGTGGAGGCTCGTAGCGATTCTGACGTGCAAATCGATCGTCA
F20080702KCM29C_consors
GAAACTCTGGTGGAGGCTCGTAGCGATTCTGACGTGCAAATCGATCGTCA
F20080208LYW10Cerrena_consors
GAAACTCTGGTGGAGGCTCGTANCGATTCTGACGTGCAAATCGATCGTCA
Dai7821Antrodiella_zonata
GAAACTCTGGTGGAGGCTCGTAGCGATTCTGACGTGCAAATCGATCGTCA
CFMR_DCL04_31Pseudolagarobasid --
VPB197Pseudolagarobasidium_bel --
CBS115543Pseudolagarobasidium_ --
CBS115544 --
Han405Pseudolagarobasidium_bai
GAAACTCTGGTGGAGGCTCGTAGCGATTCTGACGTGCAAATCGATCGTCA
Han406Pseudolagarobasidium_bai
GAAACTCTGGTGGAGGCTCGTAGCGATTCTGACGTGCAAATCGATCGTCA
MUcc838Spongipellis_delectans --
BRNM686401S_delectans --
OSM_F925S_delectans --
BRNM67093Spongipellis_litschau --
CFMRccFP59199TS_unicolor --
CFMRccFP71791TS_unicolor --
Dai13845P_lischaueri
GAAACTCTGGTGGAGGCTCGTAGCGATTCTGACGTGCAAATCGATCGTCA
Dai20266P_lischaueri
GAAACTCTGGTGGAGGCTCGTAGCGATTCTGACGTGCAAATCGATCGTCA
CFMR_HHB11240Radulodon_america --
RLG6350Radulodon_americanus --
KY415963Radulodon_erikssonii
GAAACTCTGGTGGAGGCTCGTAGCGATTCTGACGTGCAAATCGATCGTCA
HHB9567spRadulodon_casearius
GAAACTCTGGTGGAGGCTCGTAGCGATTCTGACGTGCAAATCGATCGTCA
KRT_Iso_26Radulodon_casearius --
CBS126044Radulodon_erikssonii

GAAACTCTGGTGGAGGCTCGTAGCGATTCTGACGTGCAAATCGATCGTCA
He6183YUNNANENSIS
GAAACTCTGGTGGAGGCTCGTAGCGATTCTGACGTGCAAATCGATCGTCA
Cui17979YUNNANENSIS
GAAACTCTGGTGGAGGCTCGTAGCGATTCTGACGTGCAAATCGATCGTCA
Miettinen2091Junghuhnia_fimbri
GAAACTCTGGTGGAGGCTCGTAGCGATTCTGACGTGCAAATCGATCGTCA
KHL12316S_tenue
GAAACTCTGGTGGAGGCTCGTAGCGATTCTGACGTGCAAATCGATCGTCA
PRM846564S_pachyodon --
SP_Lgt_S_pachyodon --
Ryvarden44669Tyromyces_xuchile
GAAACTCTGGTGGAGGCTCGTAGCGATTCTGACGTGCAAATCGATCGTCA
PW17_171sinuosus --
W53Dai12234
GAAACTCTGGTGGAGGCTCGTAGCGATTCTGACGTGCAAATCGATCGTCA
HHB4100SpAntella_americana
GAAACTCTGGTGGAGGCTCGTAGCGATTCTGACGTGCAAATCGATCGTCA
W3Dai20901spumeus
GAAACTCTGGTGGAGGCTCGTAGCGATTCTGACGTGCAAATCGATCGTCA
He6736
GAAACTCTGGTGGAGGCTCGTAGCGATTCTGACGTGCAAATCGATCGTCA
BRNM734877S_spumeus --
BRNM712630S_spumeus --
Dai1723Loweomyces_sibiricus --
W54Cui10009
GAAACTCTGGTGGAGGCTCGTAGCGATTCTGACGTGCAAATCGATCGTCA
W1Dai20899 --
HHB13445Trametes_ochracea
GAAACTCTGGTGGAGGCTCGTAGCGATTCTGACGTGCAAATCGATCGTCA
Dai16222
GAAACTCTGGTGGAGGCTCGTAGCGATTCTGACGTGCAAATCGATCGTCA
Dai16240
GAAACTCTGGTGGAGGCTCGTAGCGATTCTGACGTGCAAATCGATCGTCA

JV1310_11SanguinolentusCernys AATTTGGGTATAGGGGCGAAAGA-CTAA-TCGAACCATCTAGTAGCTGGT
MJ39_00_SK AATTTGGGTATAGGGGCGAAAGA-CTAA-TCGAACCTTGC----------
MJ111_04_CZ --
JV1610_BOKYsmrk AATTTGGGTATAGGGGCGAAAGA-CTAA-TCGAACCTTTTCG--------
Dai21030 --
Dai20976P_furcatus AATTTGGGTATAGGGGCGAAAGA-CTAA-

TCGAACCATCTAGTAGCTGGT
Dai2105 AATTTGGGTATAGGGGCGAAAGA-CTAA-
TCGAACCATCTAGTAGCTGGT
Dai2544 AATTTGGGTATAGGGGCGAAAGA-CTAA-
TCGAACCATCTAGTAGCTGGT
Dai11313 AATTTGGGTATAGGGGCGAAAGA-CTAA-
TCGAACCATCTAGTAGCTGGT
WCG1611Dai26167 AATTTGGGTATAGGGGCGAAAGA-CTAA-
TCGAACCATCTAGTAGCTGGT
WCG1518Dai25999Physisporinus AATTTGGGTATAGGGGCGAAAGA-CTAA-
TCGAACCATCTAGTAGCTGGT
TAA15097 AATTTGGGTATAGGGGCGAAAGA-CTAA-
TCGAACCATCTAGTAGCTGGT
JV8909_19_CZ ---
JV1310_15_P_sanguinolentus2_CZ AATTTGGGTATAGGGGCGAAAGACCTAA-
TCCGTCTATTTCGG-------
MJ53_02_CZ AATTTGGGTATAGGGGCGAAAGA-CTAA-
TCGAACCCTTTTGCCC-----
CLZhao21647P_yunnanensis AATTTGGGTATAGGGGCGAAAGA-CTAA-
TCGAACCATCTAGTAGCTGGT
CLZhao21583P_yunnanensis AATTTGGGTATAGGGGCGAAAGA-CTAA-
TCGAACCATCTAGTAGCTGGT
Dai22272 AATTTGGGTATAGGGGCGAAAGA-CTAA-
TCGAACCATCTAGTAGCTGGT
Dai22279 AATTTGGGTATAGGGGCGAAAGA-CTAA-
TCGAACCATCTAGTAGCTGGT
MJ332_94_CZ ---
MJ642_94_CZ_Expallescens ---
Dai21060P_vinctus AATTTGGGTATAGGGGCGAAAGA-CTAA-
TCGAACCATCTAGTAGCTGGT
JV0511_23LRP_pouzarii AATTTGGGTATAGGGGCGAAAGA-CTAA-
TCGAACCATCTAGTAGCTGGT
JQ409462_R_pouzarii_PRM899856_ ---
JV0308_66_WA ---
JV0309_45_WA_USA ---
JV0709_83_CA_USA AATTTGGGTATAGGGGCGAAAGA-CTAA-
TCGACCTTTTGTCC-------
Dai21043P_pouzarii AATTTGGGTATAGGGGCGAAAGA-CTAA-
TCGAACCATCTAGTAGCTGGT
MJ144_95_CZ AATTTGGGTATAGGGGCGAAAGA-CTAA-
TCGAACCTTTTGGTCC-----
JV0909_3_CZ AATTTGGGTATAGGGGCGAAAGA-CTAA-
TCGACCATTTGTCC-------
JV0609_1_K ---

Dai20396Physisporinus_castanop AATTTGGGTATAGGGGCGAAAGA-CTAA-TCGAACCATCTAGTAGCTGGT
Dai20397Physisporinus_castanop AATTTGGGTATAGGGGCGAAAGA-CTAA-TCGAACCATCTAGTAGCTGGT
MJ19_09_SK_Abies AATTTGGGTATAGGGGCGAAAGA-CTAAATCGAACATCTTTGCCC-----
JV0509_40_J_TN_USA_Betula AATTTGGGTATAGGGGCGAAAGA-CTAA-TCGAACCATCTAGTAGCTGGT
JV0808_33crocatus_PA_USAlist AATTTGGGTATAGGGGCGAAAGA-CTAA-TCGGAACCTTTTTTCT-----
DLL2009_061P_crocatus --
Dai12800P_subcrocatus AATTTGGGTATAGGGGCGAAAGA-CTAA-TCGAACCATCTAGTAGCTGGT
Dai15917P_subcrocatus AATTTGGGTATAGGGGCGAAAGA-CTAA-TCGAACCATCTAGTAGCTGGT
Cui16620 AATTTGGGTATAGGGGCGAAAGA-CTAA-TCGAACCATCTAGTAGCTGGT
HCFC1088Meripilus_stillicidior AATTTGGGTATAGGGGCGAAAGA-CTAA-TCGAACCATCTAGTAGCTGGT
MCW590Meripilus_obsrcurus --
MCW722Meripilus_obsrcurus --
Cui9381P_tibeticus AATTTGGGTATAGGGGCGAAAGA-CTAA-TCGAACCATCTAGTAGCTGGT
Cui9588P_tibeticus AATTTGGGTATAGGGGCGAAAGA-CTAA-TCGAACCATCTAGTAGCTGGT
Va2_Beneschova --
CWU3874_Ukraine_Alnus AATTTGGGTATAGGGGCGAAAGA-CTAA-TCGACCCTCTC--------G-
WCG1293Dai24718Physisporinus --
WCG1268Dai24682A AATTTGGGTATAGGGGCGAAAGA-CTAA-TCGAACCATCTAGTAGCTGGT
WCG1269Dai24683A AATTTGGGTATAGGGGCGAAAGA-CTAA-TCGAACCATCTAGTAGCTGGT
WCG1279Dai24694A AATTTGGGTATAGGGGCGAAAGA-CTAA-TCGAACCATCTAGTAGCTGGT
Dai16971 AATTTGGGTATAGGGGCGAAAGA-CTAA-TCGAACCATCTAGTAGCTGGT
ZQY1043Dai26696 AATTTGGGTATAGGGGCGAAAGA-CTAA-TCGAACCATCTAGTAGCTGGT
Doll880 --
Doll1000 AATTTGGGTATAGGGGCGAAAGA-CTAA-TCGAACCCTTTTGGTCC----
1DAI18529 AATTTGGGTATAGGGGCGAAAGA-CTAA-TCGAACCATCTAGTAGCTGGT

Dai19535 AATTTGGGTATAGGGGCGAAAGA-CTAA-TCGAACCATCTAGTAGCTGGT
1704_79_hnedyVillaLaPaz AATTTGGGTATAGGGTAGAAGCA-TTAG-CCGAAATCTTTCGAATTTC--
F2061 ---
1DAI18268 AATTTGGGTATAGGGGCGAAAGA-CTAA-TCGAACCATCTAGTAGCTGGT
1DAI18540A AATTTGGGTATAGGGGCGAAAGA-CTAA-TCGAACCATCTAGTAGCTGGT
Dai17695 AATTTGGGTATAGGGGCGAAAGA-CTAA-TCGAACCATCTAGTAGCTGGT
LKY18Dai26373 ---
Dai17839P_sulphureus AATTTGGGTATAGGGGCGAAAGA-CTAA-TCGAACCATCTAGTAGCTGGT
Dai17841P_sulphureus AATTTGGGTATAGGGGCGAAAGA-CTAA-TCGAACCATCTAGTAGCTGGT
Dai19877P_roseus AATTTGGGTATAGGGGCGAAAGA-CTAA-TCGAACCATCTAGTAGCTGGT
1508_18_1_Kout ---
KP859303_R_vinctus_RP185_BRAZI ---
JK1807_15Rigidoporus_sp_Puerto AATTTGGGTATAGGGGCGAAAGA-CTAA-TCGAACCCTCTTGCCC-----
JV0509_47_J_TN_USA AATTTGGGTATAGGGGCGAAAGA-CTAA-TCGAACCATCTAGTAGCTGGT
JV0709_188 AATTTGGGTATAGGGGCGAAAGA-CTAAATCGAACCCTTTTGGCCC----
JV0509_127_PA_USA AATTTGGGTATAGGGGCGAAAGA-CTAA--TCGACCCTTTTG--------
JV1009_59_NJ_USA AATTTGGGTATAGGGGCGAAAGA-CTAA-TCGAACCATCTAGTAGCTGGT
Dai15497P_crataegi AATTTGGGTATAGGGGCGAAAGA-CTAA-TCGAACCATCTAGTAGCTGGT
Dai15499P_crataegi AATTTGGGTATAGGGGCGAAAGA-CTAA-TCGAACCATCTAGTAGCTGGT
Cui3266P_cinereus AATTTGGGTATAGGGGCGAAAGA-CTAA-TCGAACCATCTAGTAGCTGGT
WCG1256Dai24690 AATTTGGGTATAGGGGCGAAAGA-CTAA-TCGAACCATCTAGTAGCTGGT
1DAI17581 AATTTGGGTATAGGGGCGAAAGA-CTAA-TCGAACCATCTAGTAGCTGGT
WCG1255Dai24688 AATTTGGGTATAGGGGCGAAAGA-CTAA-TCGAACCATCTAGTAGCTGGT
Dai22427 AATTTGGGTATAGGGGCGAAAGA-CTAA-TCGAACCATCTAGTAGCTGGT

MV690Meripilus_concrescens AATTTGGGTATAGGGGCGAAAGA-CTAA-TCGAACCATCTAGTAGCTGGT
MV513Meripilus_galapagensis AATTTGGGTATAGGGGCGAAAGA-CTAA-TCGAACCATCTAGTAGCTGGT
Dai19793 AATTTGGGTATAGGGGCGAAAGA-CTAA-TCGAACCATCTAGTAGCTGGT
OQ553780P_tamilnaduensis AATTTGGGTATAGGGGGGAAAGA-CTAA-TCGAAGCATCTAATAGCTGG-
OQ553779P_tamilnaduensis AATTTGGGTATAGGGGGGAAAGA-CTAA-TCGAAGCATCTAATAGCTGG-
A164FB3Meripilus_giganteus --
JV1407_36_Vinctus_Meandrica AATTTGGG--
1807_3K_Rigidoporus_PuertoRico AATTTGGGTATAGGGGCGAAAGA-CTAA-TCGAACCATCTAGTAGCTGGT
Cui16903P_vinctus AATTTGGGTATAGGGGCGAAAGA-CTAA-TCGAACCATCTAGTAGCTGGT
JV1008_18R_Lineatus AATTTGGGTATAGGGGCGAAAGA-CTAA-TCAAGACTTCTTGCC------
JV1407_37_1_Vinctus_Carara AATTTGGGTATAGGGGCGAAAGA-CTAA-TCGACCCTCTTGCC-------
Dai17986P_lineatus AATTTGGGTATAGGGGCGAAAGA-CTAA-TCGAACCATCTAGTAGCTGGT
Dai18281 AATTTGGGTATAGGGGCGAAAGA-CTAA-TCGAACCATCTAGTAGCTGGT
1DAI19796 AATTTGGGTATAGGGGCGAAAGA-CTAA-TCGAACCATCTAGTAGCTGGT
ZQY797Dai25241 AATTTGGGTATAGGGGCGAAAGA-CTAA-TCGAACCATCTAGTAGCTGGT
WCG1289Dai24711 --
Dai22598 AATTTGGGTATAGGGGCGAAAGA-CTAA-TCGAACCATCTAGTAGCTGGT
Dai20523 AATTTGGGTATAGGGGCGAAAGA-CTAA-TCGAACCATCTAGTAGCTGGT
Dai17885 AATTTGGGTATAGGGGCGAAAGA-CTAA-TCGAACCATCTAGTAGCTGGT
Dai17553 AATTTGGGTATAGGGGCGAAAGA-CTAA-TCGAACCATCTAGTAGCTGGT
Dai19639 AATTTGGGTATAGGGGCGAAAGA-CTAA-TCGAACCATCTAGTAGCTGGT
JV0110_48_CZ AATTTGGGTATAGGGGCGAAAGA-CTAA-TCGAACCTCTTTGCCC-----
MJ129_04 AATTTGGGTATAGGGGCGAAAGA-CTAA-TCGAACCCATTTTGTCC----
Cui10340P_eminens --

Cui10341P_eminens AATTTGGGTATAGGGGCGAAAGA-CTAA-TCGAACCATCTAGTAGCTGGT
Dai12685P_eminens AATTTGGGTATAGGGGCGAAAGA-CTAA-TCGAACCATCTAGTAGCTGGT
Miettinen_13591Rigidoporus_und AATTTGGGTATAGGGGCGAAAGA-CTAA-TCGAACCATCTAGTAGCTGGT
Dai20868 AATTTGGGTATAGGGGCGAAAGA-CTAA-TCGAACCATCTAGTAGCTGGT
Dai20832 AATTTGGGTATAGGGGCGAAAGA-CTAA-TCGAACCATCTAGTAGCTGGT
Dai11400 AATTTGGGTATAGGGGCGAAAGA-CTAA-TCGAACCATCTAGTAGCTGGT
Dai22472 AATTTGGGTATAGGGGCGAAAGA-CTAA-TCGAACCATCTAGTAGCTGGT
1CUI10475 AATTTGGGTATAGGGGCGAAAGA-CTAA-TCGAACCATCTAGTAGCTGGT
1CUI10491 --
HCFC1095Meripilus_robledoi --
MCW702Meripilus_revolubilis AATTTGGGTATAGG------------------------------------
1704_83_zluty_HaciendaBaru AATTTGGGTATAGGGGCGAAAGA-CTAA-TCGACCCTTTTGGC-------
Dai9925P_lavendulus AATTTGGGTATAGGGGCGAAAGA-CTAA-TCGAACCATCTAGTAGCTGGT
Dai13587AP_lavendulus AATTTGGGTATAGGGGCGAAAGA-CTAA-TCGAACCATCTAGTAGCTGGT
PDD70600P_longicystidius --
Cui16630 AATTTGGGTATAGGGGCGAAAGA-CTAA-TCGAACCATCTAGTAGCTGGT
FP_135344Meripilus_giganteus AATTTGGGTATAGGGGCGAAAGA-CTAA-TCGAACCATCTAGTAGCTGGT
FP_100460_Sp --
CBS421_48Meripilus_giganteus --
Cui9203 --
Cui9202 AATTTGGGTATAGGGGCGAAAGA-CTAA-TCGAACCATCTAGTAGCTGGT
TUFC100564Japan AATTTGGGTATAGGG-----------------------------------
Russell5913Meripilus_sumstinei --
RP215Meripilus_brasiliensis AATTTGGGTATAGGGGCGAAAGA-CTAA-TCGAACCATCTAGTAGCTGGT
RP200Meripilus_brasiliensis AATTTGGGTATAGGGGCGAAAGA-CTAA-TCGAACCATCTAGTAGCTGGT
JV1712_13J_R_vinctus2_LSUPuert AATTTGGGTATAGGGGCGAAAGA-CTAATTCGAACCCTTTTGCCC-----
Dai10503R_hypobrunneus AATTTGGGTATAGGGGCGAAAGA-CTAA-

TCGAACCATCTAGTAGCTGGT
Dai10569Rigidoporus_hypobrunne AATTTGGGTATAGGGGCGAAAGA-CTAA-TCGAACCATCTAGTAGCTGGT
1DAI19451 AATTTGGGTATAGGGGCGAAAGA-CTAA-TCGAACCATCTAGTAGCTGGT
CM108bRigidoporus_hypobrunneus --
1CUI16874 AATTTGGGTATAGGGGCGAAAGA-CTAA-TCGAACCATCTAGTAGCTGGT
FD299Cerrena_unicolor AATTTGGGTATAGGGGCGAAAGA-CTAA-TCGAACCATCTAGTAGCTGGT
KHL_GB_Cerrena_uniclor AATTTGGGTATAGGGGCGAAAGA-CTAA-TCGAACCATCTAGTAGCTGGT
Dai12892Cerrena_albocinnamomea AATTTGGGTATAGGGGCGAAAGA-CTAA-TCGAACCATCTAGTAGCTGGT
Dai12955C_albocinnamomea AATTTGGGTATAGGGGCGAAAGA-CTAA-TCGAACCATCTAGTAGCTGGT
SNUm03110102C_aurantiopora AATTTGGGTATAGGGGCGAAAGA-CTAA-TCGAACCAT------------
NIBRFG0000102423C_aurantiopora AATTTGGGTATAGGGGCGAAAGA-CTAA-TC-------------------
Dai7359Antrodiella_zonata AATTTGGGTATAGGGGCGAAAGA-CTAA-TCGAACCATCTAGTAGCTGGT
F20080702KCM29C_consors AATTTGGGTATAGGGGCGAAAGA-CTAA-TCGAACCATCTAGTAGCTGGT
F20080208LYW10Cerrena_consors AATTTGGGTATAGGGGCGAAAGA-CTAA-TCGAACCATCTAGTAGCTGGT
Dai7821Antrodiella_zonata AATTTGGGTATAGGGGCGAAAGA-CTAA-TCGAACCATCTAGTAGCTGGT
CFMR_DCL04_31Pseudolagarobasid --
VPB197Pseudolagarobasidium_bel --
CBS115543Pseudolagarobasidium_ --
CBS115544 --
Han405Pseudolagarobasidium_bai AATTTGGGTATAGGGGCGAAAGA-CTAA-TCGAACCATCTAGTAGCTGGT
Han406Pseudolagarobasidium_bai AATTTGGGTATAGGGGCGAAAGA-CTAA-TCGAACCATCTAGTAGCTGGT
MUcc838Spongipellis_delectans --
BRNM686401S_delectans --
OSM_F925S_delectans --
BRNM67093Spongipellis_litschau --
CFMRccFP59199TS_unicolor --
CFMRccFP71791TS_unicolor --
Dai13845P_lischaueri AATTTGGGTATAGGGGCGAAAGA-CTAA-TCGAACCATCTAGTAGCTGGT

Dai20266P_lischaueri AATTTGGGTATAGGGGCGAAAGA-CTAA-
TCGAACCATCTAGTAGCTGGT
CFMR_HHB11240Radulodon_america --
RLG6350Radulodon_americanus --
KY415963Radulodon_erikssonii AATTTGGGTATAGGGGCGAAAGA-CTAA-
TCGAACCATCTAGTAGCTGGT
HHB9567spRadulodon_casearius AATTTGGGTATAGGGGCGAAAGA-CTAA-
TCGAACCATCTAGTAGCTGGT
KRT_Iso_26Radulodon_casearius --
CBS126044Radulodon_erikssonii AATTTGGGTATAGGGGCGAAAGA-CTAA-
TCGAACCATCTAGTAGCTGGT
He6183YUNNANENSIS AATTTGGGTATAGGGGCGAAAGA-CTAA-
TCGAACCATCTAGTAGCTGGT
Cui17979YUNNANENSIS AATTTGGGTATAGGGGCGAAAGA-CTAA-
TCGAACCATCTAGTAGCTGGT
Miettinen2091Junghuhnia_fimbri AATTTGGGTATAGGGGCGAAAGA-CTAA-
TCGAACCATCTAGTAGCTGGT
KHL12316S_tenue AATTTGGGTATAGGGGCGAAAGA-CTAA-
TCGAACCATCTAGTAGCTGGT
PRM846564S_pachyodon --
SP_Lgt_S_pachyodon --
Ryvarden44669Tyromyces_xuchile AATTTGGGTATAGGGGCGAAAGA-CTAA-
TCGAACCATCTAGTAGCTGGT
PW17_171sinuosus --
W53Dai12234 AATTTGGGTATAGGGGCGAAAGA-CTAA-
TCGAACCATCTAGTAGCTGGT
HHB4100SpAntella_americana AATTTGGGTATAGGGGCGAAAGA-CTAA-
TCGAACCATCTAGTAGCTGGT
W3Dai20901spumeus AATTTGGGTATAGGGGCGAAAGA-CTAA-
TCGAACCATCTAGTAGCTGGT
He6736 AATTTGGGTATAGGGGCGAAAGA-CTAA-
TCGAACCATCTAGTAGCTGGT
BRNM734877S_spumeus --
BRNM712630S_spumeus --
Dai1723Loweomyces_sibiricus --
W54Cui10009 AATTTGGGTATAGGGGCGAAAGA-CTAA-
TCGAACCATCTAGTAGCTGGT
W1Dai20899 --
HHB13445Trametes_ochracea AATTTGGGTATAGGGGCGAAAGA-CTAA-
TCGAACCATCTAGTAGCTGGT
Dai16222 AATTTGGGTATAGGGGCGAAAGA-CTAA-
TCGAACCATCTAGTAGCTGGT
Dai16240 AATTTGGGTATAGGGGCGAAAGA-CTAA-
TCGAACCATCTAGTAGCTGGT

JV1310_11SanguinolentusCernys
TCCTGCCGAAGTTTCCCTCAGGATAGCAGAAACTCGTCTCAGATTTATGT
MJ39_00_SK --
MJ111_04_CZ --
JV1610_BOKYsmrk --
Dai21030 --
Dai20976P_furcatus
TCCTGCCGAAGTTTCCCTCAGGATAGCAGAAACTCGTCTCAGATTTATGT
Dai2105
TCCTGCCGAAGTTTCCCTCAGGATAGCAGAAACTCGTATCAGATTTATGT
Dai2544
TCCTGCCGAAGTTTCCCTCAGGATAGCAGAAACTCGTATCAGATTTATGT
Dai11313
TCCTGCCGAAGTTTCCCTCAGGATAGCAGAAACTCGTATCAGATTTATGT
WCG1611Dai26167
TCCTGCCGAAGTTTCCCTCAGGATAGCAGAAACTCGTATCAGATTTATGT
WCG1518Dai25999Physisporinus
TCCTGCCGAAGTTTCCCTCAGGATAGCAGAAACTCGTATCAGATTTATGT
TAA15097
TCCTGCCGAAGTTTCCCTCAGGATAGCAGAAACTCGTCTCAGATTTATGT
JV8909_19_CZ --
JV1310_15_P_sanguinolentus2_CZ --
MJ53_02_CZ --
CLZhao21647P_yunnanensis
TCCTGCCGAAGTTTCCCTCAGGATAGCAGAAACTCGTCTCAGATTTATGT
CLZhao21583P_yunnanensis
TCCTGCCGAAGTTTCCCTCAGGATAGCAGAAACTCGTCTCAGATTTATGT
Dai22272
TCCTGCCGAAGTTTCCCTCAGGATAGCAGAAACTCGTCTCAGATTTATGT
Dai22279
TCCTGCCGAAGTTTCCCTCAGGATAGCAGAAACTCGTCTCAGATTTATGT
MJ332_94_CZ --
MJ642_94_CZ_Expallescens --
Dai21060P_vinctus
TCCTGCCGAAGTTTCCCTCAGGATAGCAGAAACTCGTATCAGATTTATGT
JV0511_23LRP_pouzarii
TCCTGCCGAAGTTTCCCTCAGGATAGCAGAAACTCGTATCAGATTTATGT
JQ409462_R_pouzarii_PRM899856_ --
JV0308_66_WA --
JV0309_45_WA_USA --
JV0709_83_CA_USA --
Dai21043P_pouzarii
TCCTGCCGAAGTTTCCCTCAGGATAGCAGAAACTCGTATCAGATTTATGT

MJ144_95_CZ --
JV0909_3_CZ --
JV0609_1_K --
Dai20396Physisporinus_castanop
TCCTGCCGAAGTTTCCCTCAGGATAGCAGAAACTCGTATCAGATTTATGT
Dai20397Physisporinus_castanop
TCCTGCCGAAGTTTCCCTCAGGATAGCAGAAACTCGTATCAGATTTATGT
MJ19_09_SK_Abies --
JV0509_40_J_TN_USA_Betula
TCCTGCCGAAGTTTCCCTCAGGATAGCAGAAACTCGTATCAGATTTATGT
JV0808_33crocatus_PA_USAlist --
DLL2009_061P_crocatus --
Dai12800P_subcrocatus
TCCTGCCGAAGTTTCCCTCAGGATAGCAGAAACTCGTATCAGATTTATGT
Dai15917P_subcrocatus
TCCTGCCGAAGTTTCCCTCAGGATAGCAGAAACTCGTATCAGATTTATGT
Cui16620
TCCTGCCGAAGTTTCCCTCAGGATAGCAGAAACTCGTATCAGATTTATGT
HCFC1088Meripilus_stillicidior
TCCTGCCGAAGTTTCCCTCAGGATAGCAGAAACTCGTATCAGATTTATGT
MCW590Meripilus_obsrcurus --
MCW722Meripilus_obsrcurus --
Cui9381P_tibeticus
TCCTGCCGAAGTTTCCCTCAGGATAGCAGAAACTCGTATCAGATTTATGT
Cui9588P_tibeticus
TCCTGCCGAAGTTTCCCTCAGGATAGCAGAAACTCGTATCAGATTTATGT
Va2_Beneschova --
CWU3874_Ukraine_Alnus --
WCG1293Dai24718Physisporinus --
WCG1268Dai24682A
TCCTGCCGAAGTTTCCCTCAGGATAGCAGAAACTCGTCTCAGATTTATGT
WCG1269Dai24683A
TCCTGCCGAAGTTTCCCTCAGGATAGCAGAAACTCGTCTCAGATTTATGT
WCG1279Dai24694A
TCCTGCCGAAGTTTCCCTCAGGATAGCAGAAACTCGTCTCAGATTTATGT
Dai16971
TCCTGCCGAAGTTTCCCTCAGGATAGCAGAAACTCGTCTCAGATTTATGT
ZQY1043Dai26696
TCCTGCCGAAGTTTCCCTCAGGATAGCAGAAACTCGTCTCAGATTTATGT
Doll880 --
Doll1000 --
1DAI18529
TCCTGCCGAAGTTTCCCTCAGGATAGCAGAAACTCGTCTCAGATTTATGT
Dai19535

TCCTGCCGAAGTTTCCCTCAGGATAGCAGAAACTCGTCTCAGATTTATGT
1704_79_hnedyVillaLaPaz --
F2061 --
1DAI18268
TCCTGCCGAAGTTTCCCTCAGGATAGCAGAAACTCGTCTCAGATTTATGT
1DAI18540A
TCCTGCCGAAGTTTCCCTCAGGATAGCAGAAACTCGTCTCAGATTTATGT
Dai17695
TCCTGCCGAAGTTTCCCTCAGGATAGCAGAAACTCGTCTCAGATTTATGT
LKY18Dai26373 --
Dai17839P_sulphureus
TCCTGCCGAAGTTTCCCTCAGGATAGCAGAAACTCGTCTCAGATTTATGT
Dai17841P_sulphureus
TCCTGCCGAAGTTTCCCTCAGGATAGCAGAAACTCGTCTCAGATTTATGT
Dai19877P_roseus
TCCTGCCGAAGTTTCCCTCAGGATAGCAGAAACTCGTCTCAGATTTATGT
1508_18_1_Kout --
KP859303_R_vinctus_RP185_BRAZI --
JK1807_15Rigidoporus_sp_Puerto --
JV0509_47_J_TN_USA
TCCTGCCGAAGTTTCCCTCAGGATAGCAGAAACTCGTCTCAGATTTATGT
JV0709_188 --
JV0509_127_PA_USA --
JV1009_59_NJ_USA
TCCTGCCGAAGTTTCCCTCAGGATAGCAGAAACTCGTCTCAGATTTATGT
Dai15497P_crataegi
TCCTGCCGAAGTTTCCCTCAGGATAGCAGAAACTCGTCTCAGATTTATGT
Dai15499P_crataegi
TCCTGCCGAAGTTTCCCTCAGGATAGCAGAAACTCGTCTCAGATTTATGT
Cui3266P_cinereus
TCCTGCCGAAGTTTCCCTCAGGATAGCAGAAACTCGTCTCAGATTTATGT
WCG1256Dai24690
TCCTGCCGAAGTTTCCCTCAGGATAGCAGAAACTCGTCTCAGATTTATGT
1DAI17581
TCCTGCCGAAGTTTCCCTCAGGATAGCAGAAACTCGTCTCAGATTTATGT
WCG1255Dai24688
TCCTGCCGAAGTTTCCCTCAGGATAGCAGAAACTCGTCTCAGATTTATGT
Dai22427
TCCTGCCGAAGTTTCCCTCAGGATAGCAGAAACTCGTCTCAGATTTATGT
MV690Meripilus_concrescens
TCCTGCCGAAGTTTCCCTCAGGATAGCAGAAACTCGTCTCAGATTTATGT
MV513Meripilus_galapagensis
TCCTGCCGAAGTTTCCCTCAGGATAGCAGAAACTCGTCTCAGATTTATGT
Dai19793

TCCTGCCGAAGTTTCCCTCAGGATAGCAGAAACTCGTCTCAGATTTATGT
OQ553780P_tamilnaduensis TGCTGCAGAAGTTGCC-TAAAGATAGCAGAAAAT-GTTTTAAATTTATGT
OQ553779P_tamilnaduensis TGCTGCAGAAGTTGCC-TAAAGATAGCAGAAAAT-GTTTTAAATTTATGT
A164FB3Meripilus_giganteus --
JV1407_36_Vinctus_Meandrica --
1807_3K_Rigidoporus_PuertoRico TCCTGCCGAAGTTTCCCTCAGGATAGCAGAAACTCGTCTCAGATTTATGT
Cui16903P_vinctus TCCTGCCGAAGTTTCCCTCAGGATAGCAGAAACTCGTCTCAGATTTATGT
JV1008_18R_Lineatus --
JV1407_37_1_Vinctus_Carara --
Dai17986P_lineatus TCCTGCCGAAGTTTCCCTCAGGATAGCAGAAACTCGTCTCAGATTTATGT
Dai18281 TCCTGCCGAAGTTTCCCTCAGGATAGCAGAAACTCGTCTCAGATTTATGT
1DAI19796 TCCTGCCGAAGTTTCCCTCAGGATAGCAGAAACTCGTCTCAGATTTATGT
ZQY797Dai25241 TCCTGCCGAAGTTTCCCTCAGGATAGCAGAAACTCGTCTCAGATTTATGT
WCG1289Dai24711 --
Dai22598 TCCTGCCGAAGTTTCCCTCAGGATAGCAGAAACTCGTCTCAGATTTATGT
Dai20523 TCCTGCCGAAGTTTCCCTCAGGATAGCAGAAACTCGTCTCAGATTTATGT
Dai17885 TCCTGCCGAAGTTTCCCTCAGGATAGCAGAAACTCGTCTCAGATTTATGT
Dai17553 TCCTGCCGAAGTTTCCCTCAGGATAGCAGAAACTCGTCTCAGATTTATGT
Dai19639 TCCTGCCGAAGTTTCCCTCAGGATAGCAGAAACTCGTCTCAGATTTATGT
JV0110_48_CZ --
MJ129_04 --
Cui10340P_eminens --
Cui10341P_eminens TCCTGCCGAAGTTTCCCTCAGGATAGCAGAAACTCATGTCAGATTTATGT
Dai12685P_eminens TCCTGCCGAAGTTTCCCTCAGGATAGCAGAAACTCATGTCAGATTTATGT
Miettinen_13591Rigidoporus_und TCCTGCCGAAGTTTCCCTCAGGATAGCAGAAACTCATGTCAGATTTATGT
Dai20868 TCCTGCCGAAGTTTCCCTCAGGATAGCAGAAACTCATGTCAGATTTATGT
Dai20832

TCCTGCCGAAGTTTCCCTCAGGATAGCAGAAACTCATGTCAGATTTATGT
Dai11400
TCCTGCCGAAGTTTCCCTCAGGATAGCAGAAACTCATGTCAGATTTATGT
Dai22472
TCCTGCCGAAGTTTCCCTCAGGATAGCAGAAACTCATGTCAGATTTATGT
1CUI10475
TCCTGCCGAAGTTTCCCTCAGGATAGCAGAAACTCATGTCAGATTTATGT
1CUI10491 --
HCFC1095Meripilus_robledoi --
MCW702Meripilus_revolubilis --
1704_83_zluty_HaciendaBaru --
Dai9925P_lavendulus
TCCTGCCGAAGTTTCCCTCAGGATAGCAGAAACTCGTCTCAGATTTATGT
Dai13587AP_lavendulus
TCCTGCCGAAGTTTCCCTCAGGATAGCAGAAACTCGTCTCAGATTTATGT
PDD70600P_longicystidius --
Cui16630
TCCTGCCGAAGTTTCCCTCAGGATAGCAGAAACTCGTCTCAGATTTATGT
FP_135344Meripilus_giganteus
TCCTGCCGAAGTTTCCCTCAGGATAGCAGAAACTCGTCTCAGATTTATGT
FP_100460_Sp --
CBS421_48Meripilus_giganteus --
Cui9203 --
Cui9202
TCCTGCCGAAGTTTCCCTCAGGATAGCAGAAACTCGTCTCAGATTTATGT
TUFC100564Japan --
Russell5913Meripilus_sumstinei --
RP215Meripilus_brasiliensis
TCCTGCCGAAGTTTCCCTCAGGATAGCAGAAACTCGTCTCAGATTTATGT
RP200Meripilus_brasiliensis
TCCTGCCGAAGTTTCCCTCAGGATAGCAGAAACTCGTCTCAGATTTATGT
JV1712_13J_R_vinctus2_LSUPuert --
Dai10503R_hypobrunneus
TCCTGCCGAAGTTTCCCTCAGGATAGCAGAAACTCGTATCAGATTTATGT
Dai10569Rigidoporus_hypobrunne
TCCTGCCGAAGTTTCCCTCAGGATAGCAGAAACTCGTATCAGATTTATGT
1DAI19451
TCCTGCCGAAGTTTCCCTCAGGATAGCAGAAACTCGTATCAGATTTATGT
CM108bRigidoporus_hypobrunneus --
1CUI16874
TCCTGCCGAAGTTTCCCTCAGGATAGCAGAAACTCGTATCAGATTTATGT
FD299Cerrena_unicolor
TCCTGCCGAAGTTTCCCTCAGGATAGCAGAAACTCGTATCAGATTTATGT
KHL_GB_Cerrena_uniclor

TCCTGCCGAAGTTTCCCTCAGGATAGCAGAAACTCGTATCAGATTTATGT
Dai12892Cerrena_albocinnamomea
TCCTGCCGAAGTTTCCCTCAGGATAGCAGAAACTCGTATCAGATTTATGT
Dai12955C_albocinnamomea
TCCTGCCGAAGTTTCCCTCAGGATAGCAGAAACTCGTATCAGATTTATGT
SNUm03110102C_aurantiopora --
NIBRFG0000102423C_aurantiopora --
Dai7359Antrodiella_zonata
TCCTGCCGAAGTTTCCCTCAAGATAGCAGAACTTCGTATCAGATTTATGT
F20080702KCM29C_consors TCCTGCCGA---
F20080208LYW10Cerrena_consors TCCTGCCGAAGTTCCC----------------------------------
Dai7821Antrodiella_zonata
TCCTGCCGAAGTTTCCCTCAAGATAGCAGAACTTCGTATCAGATTTATGT
CFMR_DCL04_31Pseudolagarobasid --
VPB197Pseudolagarobasidium_bel --
CBS115543Pseudolagarobasidium_ --
CBS115544 --
Han405Pseudolagarobasidium_bai
TCCTGCCGAAGTTTCCCTCAGGATAGCAGAAACTCGTATCAGATTTATGT
Han406Pseudolagarobasidium_bai
TCCTGCCGAAGTTTCCCTCAGGATAGCAGAAACTCGTATCAGATTTATGT
MUcc838Spongipellis_delectans --
BRNM686401S_delectans --
OSM_F925S_delectans --
BRNM67093Spongipellis_litschau --
CFMRccFP59199TS_unicolor --
CFMRccFP71791TS_unicolor --
Dai13845P_lischaueri
TCCTGCCGAAGTTTCCCTCAGGATAGCAGAAACTCATATCAGATTTATGT
Dai20266P_lischaueri
TCCTGCCGAAGTTTCCCTCAGGATAGCAGAAACTCATATCAGATTTATGT
CFMR_HHB11240Radulodon_america --
RLG6350Radulodon_americanus --
KY415963Radulodon_erikssonii
TCCTGCCGAAGTTTCCCTCAGGATAGCAGAAACTCGTATCAGATTTATGT
HHB9567spRadulodon_casearius
TCCTGCCGAAGTTTCCCTCAGGATAGCAGAAACTCGTATCAGATTTATGT
KRT_Iso_26Radulodon_casearius --
CBS126044Radulodon_erikssonii TCCTGC--
He6183YUNNANENSIS
TCCTGCCGAAGTTTCCCTCAGGATAGCAGAAACTCGTATCAGATTTATGT
Cui17979YUNNANENSIS
TCCTGCCGAAGTTTCCCTCAGGATAGCAGAAACTCGTATCAGATTTATGT
Miettinen2091Junghuhnia_fimbri

TCCTGCCGAAGTTTCCCTCAGGATAGCAGAAACTCATGTCAGATTTATGT
KHL12316S_tenue
TCCTGCCGAAGTTTCCCTCAGGATAGCAGAAACTCATGTCAGATTTATGT
PRM846564S_pachyodon --
SP_Lgt_S_pachyodon --
Ryvarden44669Tyromyces_xuchile
TCCTGCCGAAGTTTCCCTCAGGATAGCAGAAACTCGTATCAGATTTATGT
PW17_171sinuosus --
W53Dai12234
TCCTGCCGAAGTTTCCCTCAGGATAGCAGAAACTCGTATCAGATTTATGT
HHB4100SpAntella_americana
TCCTGCCGAAGTTTCCCTCAGGATAGCAGAAACTCATGTCAGATTTATGT
W3Dai20901spumeus
TCCTGCCGAAGTTTCCCTCAGGATAGCAGAAACTCATGTCAGATTTATGT
He6736
TCCTGCCGAAGTTTCCCTCAGGATAGCAGAAACTCATGTCAGATTTATGT
BRNM734877S_spumeus --
BRNM712630S_spumeus --
Dai1723Loweomyces_sibiricus --
W54Cui10009
TCCTGCCGAAGTTTCCCTCAGGATAGCAGAAACTCATGTCAGATTTATGT
W1Dai20899 --
HHB13445Trametes_ochracea
TCCTGCCGAAGTTTCCCTCAGGATAGCAGAAACTCATATCAGATTTATGT
Dai16222
TCCTGCCGAAGTTTCCCTCAGGATAGCAGAAACTCATGTCAGATTTATGT
Dai16240
TCCTGCCGAAGTTTCCCTCAGGATAGCAGAAACTCATGTCAGATTTATGT

JV1310_11SanguinolentusCernys
GGTAAAGCGAATGATTAGAGGCCTTGGGGTTGAAACAACCTTAACCTATT
MJ39_00_SK --
MJ111_04_CZ --
JV1610_BOKYsmrk --
Dai21030 --
Dai20976P_furcatus
GGTAAAGCGAATGATTAGAGGCCTTGGGGTTGAAACAACCTTAACCTATT
Dai2105
GGTAAAGCGAATGATTAGAGGCCTTGGGGTTGAAACAACCTTAACCTATT
Dai2544
GGTAAAGCGAATGATTAGAGGCCTTGGGGTTGAAACAACCTTAACCTATT
Dai11313
GGTAAAGCGAATGATTAGAGGCCTTGGGGTTGAAACAACCTTAACCTATT
WCG1611Dai26167

GGTAAAGCGAATGATTAGAGGCCTTGGGGTTGAAACAACCTTAACCTATT
WCG1518Dai25999Physisporinus
GGTAAAGCGAATGATTAGAGGCCTTGGGGTTGAAACAACCTTAACCTATT
TAA15097
GGTAAAGCGAATGATTAGAGGCCTTGGGGTTGAAACAACCTTAACCTATT
JV8909_19_CZ --
JV1310_15_P_sanguinolentus2_CZ --
MJ53_02_CZ --
CLZhao21647P_yunnanensis
GGTAAAGCGAATGATTAGAGGCCTTGGGGTTGAAACAACCTTAACCTATT
CLZhao21583P_yunnanensis
GGTAAAGCGAATGATTAGAGGCCTTGGGGTTGAAACAACCTTAACCTATT
Dai22272
GGTAAAGCGAATGATTAGAGGCCTTGGGGTTGAAACAACCTTAACCTATT
Dai22279
GGTAAAGCGAATGATTAGAGGCCTTGGGGTTGAAACAACCTTAACCTATT
MJ332_94_CZ --
MJ642_94_CZ_Expallescens --
Dai21060P_vinctus
GGTAAAGCGAATGATTAGAGGCCTTGGGGTTGAAACAACCTTAACCTATT
JV0511_23LRP_pouzarii
GGTAAAGCGAATGATTAGAGGCCTTGGGGTTGAAACAACCTTAACCTATT
JQ409462_R_pouzarii_PRM899856_ --
JV0308_66_WA --
JV0309_45_WA_USA --
JV0709_83_CA_USA --
Dai21043P_pouzarii
GGTAAAGCGAATGATTAGAGGCCTTGGGGTTGAAACAACCTTAACCTATT
MJ144_95_CZ --
JV0909_3_CZ --
JV0609_1_K --
Dai20396Physisporinus_castanop
GGTAAAGCGAATGATTAGAGGCCTTGGGGTTGAAACAACCTTAACCTATT
Dai20397Physisporinus_castanop
GGTAAAGCGAATGATTAGAGGCCTTGGGGTTGAAACAACCTTAACCTATT
MJ19_09_SK_Abies --
JV0509_40_J_TN_USA_Betula
GGTAAAGCGAATGATTAGAGGCCTTGGGGTTGAAACAACCTTAACCTATT
JV0808_33crocatus_PA_USAlist --
DLL2009_061P_crocatus --
Dai12800P_subcrocatus
GGTAAAGCGAATGATTAGAGGCCTTGGGGTTGAAACAACCTTAACCTATT
Dai15917P_subcrocatus
GGTAAAGCGAATGATTAGAGGCCTTGGGGTTGAAACAACCTTAACCTATT

Cui16620
GGTAAAGCGAATGATTAGAGGCCTTGGGGTTGAAACAACCTTAACCTATT
HCFC1088Meripilus_stillicidior
GGTAAAGCGAATGATTAGAGGCCTTGGGGTTGAAACAACCTTAACCTATT
MCW590Meripilus_obsrcurus --
MCW722Meripilus_obsrcurus --
Cui9381P_tibeticus
GGTAAAGCGAATGATTAGAGGCCTTGGGGTTGAAACAACCTTAACCTATT
Cui9588P_tibeticus
GGTAAAGCGAATGATTAGAGGCCTTGGGGTTGAAACAACCTTAACCTATT
Va2_Beneschova --
CWU3874_Ukraine_Alnus --
WCG1293Dai24718Physisporinus --
WCG1268Dai24682A
GGTAAAGCGAATGATTAGAGGCCTTGGGGTTGAAACAACCTTAACCTATT
WCG1269Dai24683A
GGTAAAGCGAATGATTAGAGGCCTTGGGGTTGAAACAACCTTAACCTATT
WCG1279Dai24694A
GGTAAAGCGAATGATTAGAGGCCTTGGGGTTGAAACAACCTTAACCTATT
Dai16971
GGTAAAGCGAATGATTAGAGGCCTTGGGGTTGAAACAACCTTAACCTATT
ZQY1043Dai26696
GGTAAAGCGAATGATTAGAGGCCTTGGGGTTGAAACAACCTTAACCTATT
Doll880 --
Doll1000 --
1DAI18529
GGTAAAGCGAATGATTAGAGGCCTTGGGGTTGAAACAACCTTAACCTATT
Dai19535
GGTAAAGCGAATGATTAGAGGCCTTGGGGTTGAAACAACCTTAACCTATT
1704_79_hnedyVillaLaPaz --
F2061 --
1DAI18268
GGTAAAGCGAATGATTAGAGGCCTTGGGGTTGAAACAACCTTAACCTATT
1DAI18540A
GGTAAAGCGAATGATTAGAGGCCTTGGGGTTGAAACAACCTTAACCTATT
Dai17695
GGTAAAGCGAATGATTAGAGGCCTTGGGGTTGAAACAACCTTAACCTATT
LKY18Dai26373 --
Dai17839P_sulphureus
GGTAAAGCGAATGATTAGAGGCCTTGGGGTTGAAACAACCTTAACCTATT
Dai17841P_sulphureus
GGTAAAGCGAATGATTAGAGGCCTTGGGGTTGAAACAACCTTAACCTATT
Dai19877P_roseus
GGTAAAGCGAATGATTAGAGGCCTTGGGGTTGAAACAACCTTAACCTATT

1508_18_1_Kout --
KP859303_R_vinctus_RP185_BRAZI --
JK1807_15Rigidoporus_sp_Puerto --
JV0509_47_J_TN_USA
GGTAAAGCGAATGATTAGAGGCCTTGGGGTTGAAACAACCTTAACCTATT
JV0709_188 --
JV0509_127_PA_USA --
JV1009_59_NJ_USA
GGTAAAGCGAATGATTAGAGGCCTTGGGGTTGAAACAACCTTAACCTATT
Dai15497P_crataegi
GGTAAAGCGAATGATTAGAGGCCTTGGGGTTGAAACAACCTTAACCTATT
Dai15499P_crataegi
GGTAAAGCGAATGATTAGAGGCCTTGGGGTTGAAACAACCTTAACCTATT
Cui3266P_cinereus
GGTAAAGCGAATGATTAGAGGCCTTGGGGTTGAAACAACCTTAACCTATT
WCG1256Dai24690
GGTAAAGCGAATGATTAGAGGCCTTGGGGTTGAAACAACCTTAACCTATT
1DAI17581
GGTAAAGCGAATGATTAGAGGCCTTGGGGTTGAAACAACCTTAACCTATT
WCG1255Dai24688
GGTAAAGCGAATGATTAGAGGCCTTGGGGTTGAAACAACCTTAACCTATT
Dai22427
GGTAAAGCGAATGATTAGAGGCCTTGGGGTTGAAACAACCTTAACCTATT
MV690Meripilus_concrescens
GGTAAAGCGAATGATTAGAGGCCTTGGGGTTGAAACAACCTTAACCTATT
MV513Meripilus_galapagensis
GGTAAAGCGAATGATTAGAGGCCTTGGGGTTGAAACAACCTTAACCTATT
Dai19793
GGTAAAGCGAATGATTAGAGGCCTTGGGGTTGAAACAACCTTAACCTATT
OQ553780P_tamilnaduensis
GGTAAGGCGAATGATGAAAGGTCTTGGAGTTGAAACAACCTTAACCTATT
OQ553779P_tamilnaduensis
GGTAAGGCGAATGATGAAAGGTCTTGGAGTTGAAACAACCTTAACCTATT
A164FB3Meripilus_giganteus --
JV1407_36_Vinctus_Meandrica --
1807_3K_Rigidoporus_PuertoRico
GGTAAAGCGAATGATTAGAGGCCTTGGGGTTGAAACAACCTTAACCTATT
Cui16903P_vinctus
GGTAAAGCGAATGATTAGAGGCCTTGGGGTTGAAACAACCTTAACCTATT
JV1008_18R_Lineatus --
JV1407_37_1_Vinctus_Carara --
Dai17986P_lineatus
GGTAAAGCGAATGATTAGAGGCCTTGGGGTTGAAACAACCTTAACCTATT
Dai18281

GGTAAAGCGAATGATTAGAGGCCTTGGGGTTGAAACAACCTTAACCTATT
1DAI19796
GGTAAAGCGAATGATTAGAGGCCTTGGGGTTGAAACAACCTTAACCTATT
ZQY797Dai25241
GGTAAAGCGAATGATTAGAGGCCTTGGGGTTGAAACAACCTTAACCTATT
WCG1289Dai24711 --
Dai22598
GGTAAAGCGAATGATTAGAGGCCTTGGGGTTGAAACAACCTTAACCTATT
Dai20523
GGTAAAGCGAATGATTAGAGGCCTTGGGGTTGAAACAACCTTAACCTATT
Dai17885
GGTAAAGCGAATGATTAGAGGCCTTGGGGTTGAAACAACCTTAACCTATT
Dai17553
GGTAAAGCGAATGATTAGAGGCCTTGGGGTTGAAACAACCTTAACCTATT
Dai19639
GGTAAAGCGAATGATTAGAGGCCTTGGGGTTGAAACAACCTTAACCTATT
JV0110_48_CZ --
MJ129_04 --
Cui10340P_eminens --
Cui10341P_eminens
GGTAAAGCGAATGATTAGAGGCCTTGGGGTTGAAACAACCTTAACCTATT
Dai12685P_eminens
GGTAAAGCGAATGATTAGAGGCCTTGGGGTTGAAACAACCTTAACCTATT
Miettinen_13591Rigidoporus_und
GGTAAAGCGAATGATTAGAGGCCTTGGGGTTGAAACAACCTTAACCTATT
Dai20868
GGTAAAGCGAATGATTAGAGGCCTTGGGGTTGAAACAACCTTAACCTATT
Dai20832
GGTAAAGCGAATGATTAGAGGCCTTGGGGTTGAAACAACCTTAACCTATT
Dai11400
GGTAAAGCGAATGATTAGAGGCCTTGGGGTTGAAACAACCTTAACCTATT
Dai22472
GGTAAAGCGAATGATTAGAGGCCTTGGGGTTGAAACAACCTTAACCTATT
1CUI10475
GGTAAAGCGAATGATTAGAGGCCTTGGGGTTGAAACAACCTTAACCTATT
1CUI10491 --
HCFC1095Meripilus_robledoi --
MCW702Meripilus_revolubilis --
1704_83_zluty_HaciendaBaru --
Dai9925P_lavendulus
GGTAAAGCGAATGATTAGAGGCCTTGGGGTTGAAACAACCTTAACCTATT
Dai13587AP_lavendulus
GGTAAAGCGAATGATTAGAGGCCTTGGGGTTGAAACAACCTTAACCTATT
PDD70600P_longicystidius --

Cui16630
GGTAAAGCGAATGATTAGAGGCCTTGGGGTTGAAACAACCTTAACCTATT
FP_135344Meripilus_giganteus
GGTAAAGCGAATGATTAGAGGCCTTGGGGTTGAAACAACCTTAACCTATT
FP_100460_Sp --
CBS421_48Meripilus_giganteus --
Cui9203 --
Cui9202
GGTAAAGCGAATGATTAGAGGCCTTGGGGTTGAAACAACCTTAACCTATT
TUFC100564Japan --
Russell5913Meripilus_sumstinei --
RP215Meripilus_brasiliensis
GGTAAAGCGAATGATTAGAGGCCTTGGGGTTGAAACAACCTTAACCTATT
RP200Meripilus_brasiliensis
GGTAAAGCGAATGATTAGAGGCCTTGGGGTTGAAACAACCTTAACCTATT
JV1712_13J_R_vinctus2_LSUPuert --
Dai10503R_hypobrunneus
GGTAAAGCGAATGATTAGAGGCCTTGGGGTTGAAACAACCTTAACCTATT
Dai10569Rigidoporus_hypobrunne
GGTAAAGCGAATGATTAGAGGCCTTGGGGTTGAAACAACCTTAACCTATT
1DAI19451
GGTAAAGCGAATGATTAGAGGCCTTGGGGTTGAAACAACCTTAACCTATT
CM108bRigidoporus_hypobrunneus --
1CUI16874
GGTAAAGCGAATGATTAGAGGCCTTGGGGTTGAAACAACCTTAACCTATT
FD299Cerrena_unicolor
GGTAAAGCGAATGATTAGAGGCCTTGGGGTTGAAACAACCTTAACCTATT
KHL_GB_Cerrena_uniclor
GGTAAAGCGAATGATTAGAGGCCTTGGGGTTGAAACAACCTTAACCTATT
Dai12892Cerrena_albocinnamomea
GGTAAAGCGAATGATTAGAGGCCTTGGGGTTGAAACAACCTTAACCTATT
Dai12955C_albocinnamomea
GGTAAAGCGAATGATTAGAGGCCTTGGGGTTGAAACAACCTTAACCTATT
SNUm03110102C_aurantiopora --
NIBRFG0000102423C_aurantiopora --
Dai7359Antrodiella_zonata
GTTAAAGCGAATGATTAGAGGCCTTGGGGTTGAAACAACCTTAACCTATT
F20080702KCM29C_consors --
F20080208LYW10Cerrena_consors --
Dai7821Antrodiella_zonata
GTTAAAGCGAATGATTAGAGGCCTTGGGGTTGAAACAACCTTAACCTATT
CFMR_DCL04_31Pseudolagarobasid --
VPB197Pseudolagarobasidium_bel --
CBS115543Pseudolagarobasidium_ --

CBS115544 --
Han405Pseudolagarobasidium_bai
GGTAAAGCGAATGATTAGAGGCCTTGGGGTTGAAACAACCTTAACCTATT
Han406Pseudolagarobasidium_bai
GGTAAAGCGAATGATTAGAGGCCTTGGGGTTGAAACAACCTTAACCTATT
MUcc838Spongipellis_delectans --
BRNM686401S_delectans --
OSM_F925S_delectans --
BRNM67093Spongipellis_litschau --
CFMRccFP59199TS_unicolor --
CFMRccFP71791TS_unicolor --
Dai13845P_lischaueri
GGTAAAGCGAATGATTAGAGGCCTTGGGGTTGAAACAACCTTAACCTATT
Dai20266P_lischaueri
GGTAAAGCGAATGATTAGAGGCCTTGGGGTTGAAACAACCTTAACCTATT
CFMR_HHB11240Radulodon_america --
RLG6350Radulodon_americanus --
KY415963Radulodon_erikssonii
GGTAAAGCGAATGATTAGAGGCCTTGGGGTTGAAACAACCTTAACCTATT
HHB9567spRadulodon_casearius
GGTAAAGCGAATGATTAGAGGCCTTGGGGTTGAAACAACCTTAACCTATT
KRT_Iso_26Radulodon_casearius --
CBS126044Radulodon_erikssonii --
He6183YUNNANENSIS
GGTAAAGCGAATGATTAGAGGCCTTGGGGTTGAAACAACCTTAACCTATT
Cui17979YUNNANENSIS
GGTAAAGCGAATGATTAGAGGCCTTGGGGTTGAAACAACCTTAACCTATT
Miettinen2091Junghuhnia_fimbri
GGTAAAGCGAATGATTAGAGGATTTGGGGTTGAAACAACCTCAACCTATT
KHL12316S_tenue
GGTAAAGCGAATGATTAGAGGCCTTGGGGTTGAAACAACCTTAACCTATT
PRM846564S_pachyodon --
SP_Lgt_S_pachyodon --
Ryvarden44669Tyromyces_xuchile
GGTAAAGCGAATGATTAGAGGCCTTGGGGTTGAAACAACCTTAACCTATT
PW17_171sinuosus --
W53Dai12234
GGTAAAGCGAATGATTAGAGGCCTTGGGGTTGAAACAACCTTAACCTATT
HHB4100SpAntella_americana
GGTAAAGCGAATGATTAGAGGCCTTGGGGTTGAAACAACCTTAACCTATT
W3Dai20901spumeus
GGTAAAGCGAATGATTAGAGGCCTTGGGGTTGAAACAACCTTAACCTATT
He6736
GGTAAAGCGAATGATTAGAGGCCTTGGGGTTGAAACAACCTTAACCTATT

BRNM734877S_spumeus ---
BRNM712630S_spumeus ---
Dai1723Loweomyces_sibiricus ---
W54Cui10009
GGTAAAGCGAATGATTAGAGGCCTTGGGGTTGAAACAACCTTAACCTATT
W1Dai20899 ---
HHB13445Trametes_ochracea
GGTAAAGCGAATGATTAGAGGCCTTGGGGTTGAAACAACCTTAACCTATT
Dai16222
GGTAAAGCGAATGATTAGAGGCCTTGGGGTTGAAACAACCTTAACCTATT
Dai16240
GGTAAAGCGAATGATTAGAGGCCTTGGGGTTGAAACAACCTTAACCTATT

JV1310_11SanguinolentusCernys
CTCAAACTTTAAATATGTAAGAACGAGCCGTCACTTGATTGGACCGCTCG
MJ39_00_SK ---
MJ111_04_CZ ---
JV1610_BOKYsmrk ---
Dai21030 ---
Dai20976P_furcatus
CTCAAACTTTAAATATGTAAGAACGAGCCGTCACTTGATTGGACCGCTCG
Dai2105
CTCAAACTTTAAATATGTAAGAACGAGCCGTCACTTGATTGGACCGCTCG
Dai2544
CTCAAACTTTAAATATGTAAGAACGAGCCGTCACTTGATTGGACCGCTCG
Dai11313
CTCAAACTTTAAATATGTAAGAACGAGCCGTCACTTGATTGGACCGCTCG
WCG1611Dai26167
CTCAAACTTTAAATATGTAAGAACGAGCCGTCACTTGATTGGACCGCTCG
WCG1518Dai25999Physisporinus
CTCAAACTTTAAATATGTAAGAACGAGCCGTCACTTGATTGGACCGCTCG
TAA15097
CTCAAACTTTAAATATGTAAGAACGAGCCGTCACTTGATTGGACCGCTCG
JV8909_19_CZ ---
JV1310_15_P_sanguinolentus2_CZ ---
MJ53_02_CZ ---
CLZhao21647P_yunnanensis
CTCAAACTTTAAATATGTAAGAACGAGCCGTCACTTGATTGGACCGCTCG
CLZhao21583P_yunnanensis
CTCAAACTTTAAATATGTAAGAACGAGCCGTCACTTGATTGGACCGCTCG
Dai22272
CTCAAACTTTAAATATGTAAGAACGAGCCGTCACTTGATTGGACCGCTCG
Dai22279
CTCAAACTTTAAATATGTAAGAACGAGCCGTCACTTGATTGGACCGCTCG

MJ332_94_CZ --
MJ642_94_CZ_Expallescens --
Dai21060P_vinctus
CTCAAACTTTAAATATGTAAGAACGAGCCGTCACTTGATTGGACCGCTCG
JV0511_23LRP_pouzarii CTC---
JQ409462_R_pouzarii_PRM899856_ --
JV0308_66_WA --
JV0309_45_WA_USA --
JV0709_83_CA_USA --
Dai21043P_pouzarii
CTCAAACTTTAAATATGTAAGAACGAGCCGTCACTTGATTGGACCGCTCG
MJ144_95_CZ --
JV0909_3_CZ --
JV0609_1_K --
Dai20396Physisporinus_castanop
CTCAAACTTTAAATATGTAAGAACGAGCCGTCACTTGATTGGACCGCTCG
Dai20397Physisporinus_castanop
CTCAAACTTTAAATATGTAAGAACGAGCCGTCACTTGATTGGACCGCTCG
MJ19_09_SK_Abies --
JV0509_40_J_TN_USA_Betula
CTCAAACTTTAAATATGTAAGAACGAGCCGTCACTTGATTGGACCGCTCG
JV0808_33crocatus_PA_USAlist --
DLL2009_061P_crocatus --
Dai12800P_subcrocatus
CTCAAACTTTAAATATGTAAGAACGAGCCGTCACTTGATTGGACCGCTCG
Dai15917P_subcrocatus
CTCAAACTTTAAATATGTAAGAACGAGCCGTCACTTGATTGGACCGCTCG
Cui16620
CTCAAACTTTAAATATGTAAGAACGAGCCGTCACTTGATTGGACCGCTCG
HCFC1088Meripilus_stillicidior
CTCAAACTTTAAATATGTAAGAACGAGCCGTCACTTGATTGGACCGCTCG
MCW590Meripilus_obsrcurus --
MCW722Meripilus_obsrcurus --
Cui9381P_tibeticus
CTCAAACTTTAAATATGTAAGAACGAGCCGTCACTTGATTGGACCGCTCG
Cui9588P_tibeticus
CTCAAACTTTAAATATGTAAGAACGAGCCGTCACTTGATTGGACCGCTCG
Va2_Beneschova --
CWU3874_Ukraine_Alnus --
WCG1293Dai24718Physisporinus --
WCG1268Dai24682A
CTCAAACTTTAAATATGTAAGAACGAGCCGTCACTTGATTGGACCGCTCG
WCG1269Dai24683A
CTCAAACTTTAAATATGTAAGAACGAGCCGTCACTTGATTGGACCGCTCG

WCG1279Dai24694A
CTCAAACTTTAAATATGTAAGAACGAGCCGTCACTTGATTGGACCGCTCG
Dai16971
CTCAAACTTTAAATATGTAAGAACGAGCCGTCACTTGATTGGACCGCTCG
ZQY1043Dai26696
CTCAAACTTTAAATATGTAAGAACGAGCCGTCACTTGATTGGACCGCTCG
Doll880 --
Doll1000 --
1DAI18529
CTCAAACTTTAAATATGTAAGAACGAGCCGTCACTTGATTGGACCGCTCG
Dai19535
CTCAAACTTTAAATATGTAAGAACGAGCCGTCTCTTGATTGGACCGCTCG
1704_79_hnedyVillaLaPaz --
F2061 --
1DAI18268
CTCAAACTTTAAATATGTAAGAACGAGCCGTCACTTGATTGGACCGCTCG
1DAI18540A
CTCAAACTTTAAATATGTAAGAACGAGCCGTCACTTGATTGGACCGCTCG
Dai17695
CTCAAACTTTAAATATGTAAGAACGAGCCGTCACTTGATTGGACCGCTCG
LKY18Dai26373 --
Dai17839P_sulphureus
CTCAAACTTTAAATATGTAAGAACGAGCCGTCACTTGATTGGACCGCTCG
Dai17841P_sulphureus
CTCAAACTTTAAATATGTAAGAACGAGCCGTCACTTGATTGGACCGCTCG
Dai19877P_roseus
CTCAAACTTTAAATATGTAAGAACGAGCCGTCACTTGATTGGACCGCTCG
1508_18_1_Kout --
KP859303_R_vinctus_RP185_BRAZI --
JK1807_15Rigidoporus_sp_Puerto --
JV0509_47_J_TN_USA
CTCAAACTTTAAATATGTAAGAACGAGCCGTCACTTGATTGGACCGCTCG
JV0709_188 --
JV0509_127_PA_USA --
JV1009_59_NJ_USA
CTCAAACTTTAAATATGTAAGAACGAGCCGTCACTTGATTGGACCGCTCG
Dai15497P_crataegi
CTCAAACTTTAAATATGTAAGAACGAGCCGTCACTTGATTGGACCGCTCG
Dai15499P_crataegi
CTCAAACTTTAAATATGTAAGAACGAGCCGTCACTTGATTGGACCGCTCG
Cui3266P_cinereus
CTCAAACTTTAAATATGTAAGAACGAGCCGTCACTTGATTGGACCGCTCG
WCG1256Dai24690
CTCAAACTTTAAATATGTAAGAACGAGCCGTCACTTGATTGGACCGCTCG

1DAI17581
CTCAAACTTTAAATATGTAAGAACGAGCCGTCACTTGATTGGACCGCTCG
WCG1255Dai24688
CTCAAACTTTAAATATGTAAGAACGAGCCGTCACTTGATTGGACCGCTCG
Dai22427
CTCAAACTTTAAATATGTAAGAACGAGCCGTCACTTGATTGGACCGCTCG
MV690Meripilus_concrescens
CTCAAACTTTAAATATGTAAGAACGAGCCGTCACTTGATTGGACCGCTCG
MV513Meripilus_galapagensis
CTCAAACTTTAAATATGTAAGAACGAGCCGTCACTTGATTGGACCGCTCG
Dai19793
CTCAAACTTTAAATATGTAAGAACGAGCCGTCACTTGATTGGACCGCTCG
OQ553780P_tamilnaduensis CTCAAACTTTAAATATGTAAGAAGAG-----------------------
OQ553779P_tamilnaduensis CTCAAACTTTAAATATGTAAGAAGAG-----------------------
A164FB3Meripilus_giganteus --
JV1407_36_Vinctus_Meandrica --
1807_3K_Rigidoporus_PuertoRico
CTCAAACTTTAAATATGTAAGAACGAGCCGTCACTTGATTGGACCGCTCG
Cui16903P_vinctus
CTCAAACTTTAAATATGTAAGAACGAGCCGTCACTTGATTGGACCGCTCG
JV1008_18R_Lineatus --
JV1407_37_1_Vinctus_Carara --
Dai17986P_lineatus
CTCAAACTTTAAATATGTAAGAACGAGCCGTCACTTGATTGGACCGCTCG
Dai18281
CTCAAACTTTAAATATGTAAGAACGAGCCGTCACTTGATTGGACCGCTCG
1DAI19796
CTCAAACTTTAAATATGTAAGAACGAGCCGTCACTTGATTGGACCGCTCG
ZQY797Dai25241
CTCAAACTTTAAATATGTAAGAACGAGCCGTCACTTGATTGGACCGCTCG
WCG1289Dai24711 --
Dai22598
CTCAAACTTTAAATATGTAAGAACGAGCCGTCACTTGATTGGACCGCTCG
Dai20523
CTCAAACTTTAAATATGTAAGAACGAGCCGTCACTTGATTGGACCGCTCG
Dai17885
CTCAAACTTTAAATATGTAAGAACGAGCCGTCACTTGATTGGACCGCTCG
Dai17553
CTCAAACTTTAAATATGTAAGAACGAGCCGTCACTTGATTGGACCGCTCG
Dai19639
CTCAAACTTTAAATATGTAAGAACGAGCCGTCACTTGATTGGACCGCTCG
JV0110_48_CZ --

MJ129_04 --
Cui10340P_eminens --
Cui10341P_eminens
CTCAAACTTTAAATATGTAAGAACGAGCCGTCACTTGATTGGACCGCTCG
Dai12685P_eminens
CTCAAACTTTAAATATGTAAGAACGAGCCGTCACTTGATTGGACCGCTCG
Miettinen_13591Rigidoporus_und
CTCAAACTTTAAATATGTAAGAACGAGCCGTCACTTGATTGGACCGCTCG
Dai20868
CTCAAACTTTAAATATGTAAGAACGAGCCGTCACTTGATTGGACCGCTCG
Dai20832
CTCAAACTTTAAATATGTAAGAACGAGCCGTCACTTGATTGGACCGCTCG
Dai11400
CTCAAACTTTAAATATGTAAGAACGAGCCGTCACTTGATTGGACCGCTCG
Dai22472
CTCAAACTTTAAATATGTAAGAACGAGCCGTCACTTGATTGGACCGCTCG
1CUI10475
CTCAAACTTTAAATATGTAAGAACGAGCCGTCACTTGATTGGACCGCTCG
1CUI10491 --
HCFC1095Meripilus_robledoi --
MCW702Meripilus_revolubilis --
1704_83_zluty_HaciendaBaru --
Dai9925P_lavendulus
CTCAAACTTTAAATATGTAAGAACGAGCCGTCACTTGATTGGACCGCTCG
Dai13587AP_lavendulus
CTCAAACTTTAAATATGTAAGAACGAGCCGTCACTTGATTGGACCGCTCG
PDD70600P_longicystidius --
Cui16630
CTCAAACTTTAAATATGTAAGAACGAGCCGTCACTTGATTGGACCGCTCG
FP_135344Meripilus_giganteus
CTCAAACTTTAAATATGTAAGAACGAGCCGTCACTTGATTGGACCGCTCG
FP_100460_Sp --
CBS421_48Meripilus_giganteus --
Cui9203 --
Cui9202
CTCAAACTTTAAATATGTAAGAACGAGCCGTCACTTGATTGGACCGCTCG
TUFC100564Japan --
Russell5913Meripilus_sumstinei --
RP215Meripilus_brasiliensis
CTCAAACTTTAAATATGTAAGAACGAGCCGTCACTTGATTGGACCGCTCG
RP200Meripilus_brasiliensis
CTCAAACTTTAAATATGTAAGAACGAGCCGTCACTTGATTGGACCGCTCG
JV1712_13J_R_vinctus2_LSUPuert --
Dai10503R_hypobrunneus

CTCAAACTTTAAATATGTAAGAACAAGCCGTCTCTTAATTGGACCGCTTG
Dai10569Rigidoporus_hypobrunne
CTCAAACTTTAAATATGTAAGAACAAGCCGTCTCTTAATTGGACCGCTTG
1DAI19451
CTCAAACTTTAAATATGTAAGAACCAGCCGTCTCTTAATTGGACCGCTTG
CM108bRigidoporus_hypobrunneus --
1CUI16874
CTCAAACTTTAAATATGTAAGAACAAGCCGTCTCTTAATTGGACCGCTTG
FD299Cerrena_unicolor
CTCAAACTTTAAATATGTAAGAACAAGCCGTCTCTTAATTGGACCGCTTG
KHL_GB_Cerrena_uniclor
CTCAAACTTTAAATATGTAAGAACAAGCCGTCTCTTAATTGGACCGCTTG
Dai12892Cerrena_albocinnamomea
CTCAAACTTTAAATATGTAAGAACAAGCCGTCTCTTAATTGGACCGCTTG
Dai12955C_albocinnamomea
CTCAAACTTTAAATATGTAAGAACAAGCCGTCTCTTAATTGGACCGCTTG
SNUm03110102C_aurantiopora --
NIBRFG0000102423C_aurantiopora --
Dai7359Antrodiella_zonata
CTCAAACTTTAAATATGTAAGAACAAGCCGTCTCTTAATT-GACCGCTTG
F20080702KCM29C_consors --
F20080208LYW10Cerrena_consors --
Dai7821Antrodiella_zonata
CTCAAACTTTAAATATGTAAGAACAAGCCGTCTCTTAATT-GACCGCTTG
CFMR_DCL04_31Pseudolagarobasid --
VPB197Pseudolagarobasidium_bel --
CBS115543Pseudolagarobasidium_ --
CBS115544 --
Han405Pseudolagarobasidium_bai
CTCAAACTTTAAATATGTAAGAACAAGCCGTCTCTTAATTGGACCGCTTG
Han406Pseudolagarobasidium_bai
CTCAAACTTTAAATATGTAAGAACAAGCCGTCTCTTAATTGGACCGCTTG
MUcc838Spongipellis_delectans --
BRNM686401S_delectans --
OSM_F925S_delectans --
BRNM67093Spongipellis_litschau --
CFMRccFP59199TS_unicolor --
CFMRccFP71791TS_unicolor --
Dai13845P_lischaueri
CTCAAACTTTAAATATGTAAGAACAAGCCGTCTCTTAATTGGACCGCTTG
Dai20266P_lischaueri
CTCAAACTTTAAATATGTAAGAACAAGCCGTCTCTTAATTGGACCGCTTG
CFMR_HHB11240Radulodon_america --
RLG6350Radulodon_americanus --

KY415963Radulodon_erikssonii
CTCAAACTTTAAATATGTAAGAACAAGCCGTCTCTTAATTGGACCGCTTG
HHB9567spRadulodon_casearius
CTCAAACTTTAAATATGTAAGAACAAGCCGTCTCTTAATTGGACCGCTTG
KRT_Iso_26Radulodon_casearius --
CBS126044Radulodon_erikssonii --
He6183YUNNANENSIS
CTCAAACTTTAAATATGTAAGAACAAGCCGTCTCTTAATTGGACCGCTTG
Cui17979YUNNANENSIS
CTCAAACTTTAAATATGTAAGAACAAGCCGTCTCTTAATTGGACCGCTTG
Miettinen2091Junghuhnia_fimbri
CTCAAACTTTAAATATGTAAGAACGAGCCGTCACTTGATTGGACCGCTCG
KHL12316S_tenue
CTCAAACTTTAAATATGTAAGAACGAGCCGTCACTTGATTGGACCGCTCG
PRM846564S_pachyodon --
SP_Lgt_S_pachyodon --
Ryvarden44669Tyromyces_xuchile
CTCAAACTTTAAATATGTAAGAACAAGCCGTCTCTTAGTTGGACCGCTTG
PW17_171sinuosus --
W53Dai12234
CTCAAACTTTAAATATGTAAGAACAAGCCGTCTCTTAATTGGACCGCTTG
HHB4100SpAntella_americana
CTCAAACTTTAAATATGTAAGAACGAGCCGTCACTTGATTGGACCGCTCG
W3Dai20901spumeus
CTCAAACTTTAAATATGTAAGAACGAGCCGTCACTTGATTGGACCGCTCG
He6736
CTCAAACTTTAAATATGTAAGAACGAGCCGTCACTTGATTGGACCGCTCG
BRNM734877S_spumeus --
BRNM712630S_spumeus --
Dai1723Loweomyces_sibiricus --
W54Cui10009
CTCAAACTTTAAATATGTAAGAACGAGCCGTCACTTGATTGGACCGCTCG
W1Dai20899 --
HHB13445Trametes_ochracea
CTCAAACTTTAAATATGTAAGAACGAGCCGTCGCTTGATTGGACCGCTCG
Dai16222
CTCAAACTTTAAATATGTAAGAACGAGCCGTCACTTGATTGGACCGCTCG
Dai16240
CTCAAACTTTAAATATGTAAGAACGAGCCGTCACTTGATTGGACCGCTCG

JV1310_11SanguinolentusCernys
GCGATTGAGAGTTTCTAGTGGGCCATTTTTGGTAAGCAGAACTGGCGATG
MJ39_00_SK --
MJ111_04_CZ --

JV1610_BOKYsmrk --
Dai21030 --
Dai20976P_furcatus
GCGATTGAGAGTTTCTAGTGGGCCATTTTTGGTAAGCAGAACTGGCGATG
Dai2105
GCGATTGAGAGTTTCTAGTGGGCCATTTTTGGTAAGCAGAACTGGCGATG
Dai2544
GCGATTGAGAGTTTCTAGTGGGCCATTTTTGGTAAGCAGAACTGGCGATG
Dai11313
GCGATTGAGAGTTTCTAGTGGGCCATTTTTGGTAAGCAGAACTGGCGATG
WCG1611Dai26167
GCGATTGAGAGTTTCTAGTGGGCCATTTTTGGTAAGCAGAACTGGCGATG
WCG1518Dai25999Physisporinus
GCGATTGAGAGTTTCTAGTGGGCCATTTTTGGTAAGCAGAACTGGCGATG
TAA15097
GCGATTGAGAGTTTCTAGTGGGCCATTTTTGGTAAGCAGAACTGGCGATG
JV8909_19_CZ --
JV1310_15_P_sanguinolentus2_CZ --
MJ53_02_CZ --
CLZhao21647P_yunnanensis
GCGATTGAGAGTTTCTAGTGGGCCATTTTTGGTAAGCAGAACTGGCGATG
CLZhao21583P_yunnanensis
GCGATTGAGAGTTTCTAGTGGGCCATTTTTGGTAAGCAGAACTGGCGATG
Dai22272
GCGATTGAGAGTTTCTAGTGGGCCATTTTTGGTAAGCAGAACTGGCGATG
Dai22279
GCGATTGAGAGTTTCTAGTGGGCCATTTTTGGTAAGCAGAACTGGCGATG
MJ332_94_CZ --
MJ642_94_CZ_Expallescens --
Dai21060P_vinctus
GCGATTGGGAGTTTCTAGTGGGCCATTTTTGGTAAGCAGAACTGGCGATG
JV0511_23LRP_pouzarii --
JQ409462_R_pouzarii_PRM899856_ --
JV0308_66_WA --
JV0309_45_WA_USA --
JV0709_83_CA_USA --
Dai21043P_pouzarii
GTGATTGGGAGTTTCTAGTGGGCCATTTTTGGTAAGCAGAACTGGCGATG
MJ144_95_CZ --
JV0909_3_CZ --
JV0609_1_K --
Dai20396Physisporinus_castanop
GCGATTGGGAGTTTCTAGTGGGCCATTTTTGGTAAGCAGAACTGGCGATG
Dai20397Physisporinus_castanop

GCGATTGGGAGTTTCTAGTGGGCCATTTTGGTAAGCAGAACTGGCGATG
MJ19_09_SK_Abies --
JV0509_40_J_TN_USA_Betula
GCGATTGGGAGTTTCTAGTGGGCCATTTTGGTAAGCAGAACTGGCGATG
JV0808_33crocatus_PA_USAlist --
DLL2009_061P_crocatus --
Dai12800P_subcrocatus
GCGATTGGGAGTTTCTAGTGGGCCATTTTGGTAAGCAGAACTGGCGATG
Dai15917P_subcrocatus
GCGATTGGGAGTTTCTAGTGGGCCATTTTGGTAAGCAGAACTGGCGATG
Cui16620
GCGATTGGGAGTTTCTAGTGGGCCATTTTGGTAAGCAGAACTGGCGATG
HCFC1088Meripilus_stillicidior
GCGATTGGGAGTTTCTAGTGGGCCATTTTGGTAAGCAGAACTGGCGATG
MCW590Meripilus_obsrcurus --
MCW722Meripilus_obsrcurus --
Cui9381P_tibeticus
GCGATTGGGAGTTTCTAGTGGGCCATTTTGGTAAGCAGAACTGGCGATG
Cui9588P_tibeticus
GTGATTGGGAGTTTCTAGTGGGCCATTTTGGTAAGCAGAACTGGCGATG
Va2_Beneschova --
CWU3874_Ukraine_Alnus --
WCG1293Dai24718Physisporinus --
WCG1268Dai24682A
GCGATTGAGAGTTTCTAGTGGGCCATTTTGGTAAGCAGAACTGGCGATG
WCG1269Dai24683A
GCGATTGAGAGTTTCTAGTGGGCCATTTTGGTAAGCAGAACTGGCGATG
WCG1279Dai24694A
GCGATTGAGAGTTTCTAGTGGGCCATTTTGGTAAGCAGAACTGGCGATG
Dai16971
GCGATTGAGAGTTTCTAGTGGGCCATTTTGGTAAGCAGAACTGGCGATG
ZQY1043Dai26696
GCGATTGAGAGTTTCTAGTGGGCCATTTTGGTAAGCAGAACTGGCGATG
Doll880 --
Doll1000 --
1DAI18529
GCGATTGAGAGTTTCTAGTGGGCCATTTTGGTAAGCAGAACTGGCGATG
Dai19535
GCGATTGAGAGTTTCTAGTGGGCCATTTTGGTAAGCAGAACTGGCGATG
1704_79_hnedyVillaLaPaz --
F2061 --
1DAI18268
GCGATTGAGAGTTTCTAGTGGGCCATTTTGGTAAGCAGAACTGGCGATG
1DAI18540A

GCGATTGAGAGTTTCTAGTGGGCCATTTTTGGTAAGCAGAACTGGCGATG
Dai17695
GCGATTGAGAGTTTCTAGTGGGCCATTTTTGGTAAGCAGAACTGGCGATG
LKY18Dai26373 --
Dai17839P_sulphureus
GCGATTGAGAGTTTCTAGTGGGCCATTTTTGGTAAGCAGAACTGGCGATG
Dai17841P_sulphureus
GCGATTGAGAGTTTCTAGTGGGCCATTTTTGGTAAGCAGAACTGGCGATG
Dai19877P_roseus
GCGATTGAGAGTTTCTAGTGGGCCATTTTTGGTAAGCAGAACTGGCGATG
1508_18_1_Kout --
KP859303_R_vinctus_RP185_BRAZI --
JK1807_15Rigidoporus_sp_Puerto --
JV0509_47_J_TN_USA
GCGATTGAGAGTTTCTAGTGGGCCATTTTTGGTAAGCAGAACTGGCGATG
JV0709_188 --
JV0509_127_PA_USA --
JV1009_59_NJ_USA
GCGATTGAGAGTTTCTAGTGGGCCATTTTTGGTAAGCAGAACTGGCGATG
Dai15497P_crataegi
GCGATTGAGAGTTTCTAGTGGGCCATTTTTGGTAAGCAGAACTGGCGATG
Dai15499P_crataegi
GCGATTGAGAGTTTCTAGTGGGCCATTTTTGGTAAGCAGAACTGGCGATG
Cui3266P_cinereus
GCGATTGAGAGTTTCTAGTGGGCCATTTTTGGTAAGCAGAACTGGCGATG
WCG1256Dai24690
GCGATTGAGAGTTTCTAGTGGGCCATTTTTGGTAAGCAGAACTGGCGATG
1DAI17581
GCGATTGAGAGTTTCTAGTGGGCCATTTTTGGTAAGCAGAACTGGCGATG
WCG1255Dai24688
GCGATTGAGAGTTTCTAGTGGGCCATTTTTGGTAAGCAGAACTGGCGATG
Dai22427
GCGATTGAGAGTTTCTAGTGGGCCATTTTTGGTAAGCAGAACTGGCGATG
MV690Meripilus_concrescens
GCGATTGAGAGTTTCTAGTGGGCCATTTTTGGTAAGCAGAACTGGCGATG
MV513Meripilus_galapagensis
GCGATTGAGAGTTTCTAGTGGGCCATTTTTGGTAAGCAGAACTGGCGATG
Dai19793
GCGATTGAGAGTTTCTAGTGGGCCATTTTTGGTAAGCAGAACTGGCGATG
OQ553780P_tamilnaduensis --
OQ553779P_tamilnaduensis --
A164FB3Meripilus_giganteus --
JV1407_36_Vinctus_Meandrica --
1807_3K_Rigidoporus_PuertoRico

GCGATTGAGAGTTTCTAGTGGGCCATTTTTGGTAAGCAGAACTGGCGATG
Cui16903P_vinctus
GCGATTGAGAGTTTCTAGTGGGCCATTTTTGGTAAGCAGAACTGGCGATG
JV1008_18R_Lineatus --
JV1407_37_1_Vinctus_Carara --
Dai17986P_lineatus
GCGATTGAGAGTTTCTAGTGGGCCATTTTTGGTAAGCAGAACTGGCGATG
Dai18281
GCGATTGAGAGTTTCTAGTGGGCCATTTTTGGTAAGCAGAACTGGCGATG
1DAI19796
GCGATTGAGAGTTTCTAGTGGGCCATTTTTGGTAAGCAGAACTGGCGATG
ZQY797Dai25241
GCGATTGAGAGTTTCTAGTGGGCCATTTTTGGTAAGCAGAACTGGCGATG
WCG1289Dai24711 --
Dai22598
GCGATTGAGAGTTTCTAGTGGGCCATTTTTGGTAAGCAGAACTGGCGATG
Dai20523
GCGATTGAGAGTTTCTAGTGGGCCATTTTTGGTAAGCAGAACTGGCGATG
Dai17885
GCGATTGAGAGTTTCTAGTGGGCCATTTTTGGTAAGCAGAACTGGCGATG
Dai17553
GCGATTGAGAGTTTCTAGTGGGCCATTTTTGGTAAGCAGAACTGGCGATG
Dai19639
GCGATTGAGAGTTTCTAGTGGGCCATTTTTGGTAAGCAGAACTGGCGATG
JV0110_48_CZ --
MJ129_04 --
Cui10340P_eminens --
Cui10341P_eminens
GCGATTGAGAGTTTCTAGTGGGCCATTTTTGGTAAGCAGAACTGGCGATG
Dai12685P_eminens
GCGATTGAGAGTTTCTAGTGGGCCATTTTTGGTAAGCAGAACTGGCGATG
Miettinen_13591Rigidoporus_und
GCGATTGAGAGTTTCTAGTGGGCCATTTTTGGTAAGCAGAACTGGCGATG
Dai20868
GCGATTGAGAGTTTCTAGTGGGCCATTTTTGGTAAGCAGAACTGGCGATG
Dai20832
GCGATTGAGAGTTTCTAGTGGGCCATTTTTGGTAAGCAGAACTGGCGATG
Dai11400
GCGATTGAGAGTTTCTAGTGGGCCATTTTTGGTAAGCAGAACTGGCGATG
Dai22472
GCGATTGAGAGTTTCTAGTGGGCCATTTTTGGTAAGCAGAACTGGCGATG
1CUI10475
GCGATTGAGAGTTTCTAGTGGGCCATTTTTGGTAAGCAGAACTGGCGATG
1CUI10491 --

HCFC1095Meripilus_robledoi ---
MCW702Meripilus_revolubilis ---
1704_83_zluty_HaciendaBaru ---
Dai9925P_lavendulus
GCGATTGAGAGTTTCTAGTGGGCCATTTTGGTAAGCAGAACTGGCGATG
Dai13587AP_lavendulus
GCGATTGAGAGTTTCTAGTGGGCCATTTTGGTAAGCAGAACTGGCGATG
PDD70600P_longicystidius ---
Cui16630
GCGATTGAGAGTTTCTAGTGGGCCATTTTGGTAAGCAGAACTGGCGATG
FP_135344Meripilus_giganteus
GCGATTGAGAGTTTCTAGTGGGCCATTTTGGTAAGCAGAACTGGCGATG
FP_100460_Sp ---
CBS421_48Meripilus_giganteus ---
Cui9203 ---
Cui9202
GCGATTGAGAGTTTCTAGTGGGCCATTTTGGTAAGCAGAACTGGCGATG
TUFC100564Japan ---
Russell5913Meripilus_sumstinei ---
RP215Meripilus_brasiliensis
GCGATTGAGAGTTTCTAGTGGGCCATTTTGGTAAGCAGAACTGGCGATG
RP200Meripilus_brasiliensis
GCGATTGAGAGTTTCTAGTGGGCCATTTTGGTAAGCAGAACTGGCGATG
JV1712_13J_R_vinctus2_LSUPuert ---
Dai10503R_hypobrunneus
GCGATTGAGAGTTTCTAGTGGGCCATTTTGGTAAGCAGAACTGGCGATG
Dai10569Rigidoporus_hypobrunne
GCGATTGAGAGTTTCTAGTGGGCCATTTTGGTAAGCAGAACTGGCGATG
1DAI19451
GCGATTGAGAGTTTCTAGTGGGCCATTTTGGTAAGCAGAACTGGCGATG
CM108bRigidoporus_hypobrunneus ---
1CUI16874
GCGATTGAGAGTTTCTAGTGGGCCATTTTGGTAAGCAGAACTGGCGATG
FD299Cerrena_unicolor
GCGATTGAGAGTTTCTAGTGGGCCATTTTGGTAAGCAGAACTGGCGATG
KHL_GB_Cerrena_uniclor
GCGATTGAGAGTTTCTAGTGGGCCATTTTGGTAAGCAGAACTGGCGATG
Dai12892Cerrena_albocinnamomea
GCGATTGAGAGTTTCTAGTGGGCCATTTTGGTAAGCAGAACTGGCGATG
Dai12955C_albocinnamomea
GCGATTGAGAGTTTCTAGTGGGCCATTTTGGTAAGCAGAACTGGCGATG
SNUm03110102C_aurantiopora ---
NIBRFG0000102423C_aurantiopora ---
Dai7359Antrodiella_zonata TCGATTGAGAGTTTCTAGT------------------------------

F20080702KCM29C_consors ---
F20080208LYW10Cerrena_consors ---
Dai7821Antrodiella_zonata TCGATTGAGAGTTTCTAGT------------------------------
CFMR_DCL04_31Pseudolagarobasid ---
VPB197Pseudolagarobasidium_bel ---
CBS115543Pseudolagarobasidium_ ---
CBS115544 ---
Han405Pseudolagarobasidium_bai
GCGATTGAGAGTTTCTAGTGGGCCATTTTGGTAAGCAGAACTGGCGATG
Han406Pseudolagarobasidium_bai
GCGATTGAGAGTTTCTAGTGGGCCATTTTGGTAAGCAGAACTGGCGATG
MUcc838Spongipellis_delectans ---
BRNM686401S_delectans ---
OSM_F925S_delectans ---
BRNM67093Spongipellis_litschau ---
CFMRccFP59199TS_unicolor ---
CFMRccFP71791TS_unicolor ---
Dai13845P_lischaueri
GCGATTGAGAGTTTCTAGTGGGCCATTTTGGTAAGCAGAACTGGCGATG
Dai20266P_lischaueri
GCGATTGAGAGTTTCTAGTGGGCCATTTTGGTAAGCAGAACTGGCGATG
CFMR_HHB11240Radulodon_america ---
RLG6350Radulodon_americanus ---
KY415963Radulodon_erikssonii
GCGATTGAGAGTTTCTAGTGGGCCATTTTGGTAAGCAGAACTGGCGATG
HHB9567spRadulodon_casearius
GCGATTGAGAGTTTCTAGTGGGCCATTTTGGTAAGCAGAACTGGCGATG
KRT_Iso_26Radulodon_casearius ---
CBS126044Radulodon_erikssonii ---
He6183YUNNANENSIS
GCGATTGAGAGTTTCTAGTGGGCCATTTTGGTAAGCAGAACTGGCGATG
Cui17979YUNNANENSIS
GCGATTGAGAGTTTCTAGTGGGCCATTTTGGTAAGCAGAACTGGCGATG
Miettinen2091Junghuhnia_fimbri
GCGATTGAGAGTTTCTAGTGGGCCATTTTGGTAAGCAGAACTGGCGATG
KHL12316S_tenue
GCGATTGAGAGTTTCTAGTGGGCCATTTTGGTAAGCAGAACTGGCGATG
PRM846564S_pachyodon ---
SP_Lgt_S_pachyodon ---
Ryvarden44669Tyromyces_xuchile
GCGATTGAGAGTTTCTAGTGGGCCATTTTGGTAAGCAGAACTGGCGATG
PW17_171sinuosus ---
W53Dai12234
GCGATTGAGAGTTTCTAGTGGGCCATTTTGGTAAGCAGAACTGGCGATG

HHB4100SpAntella_americana
GCGATTGAGAGTTTCTAGTGGGCCATTTTTGGTAAGCAGAACTGGCGATG
W3Dai20901spumeus
GCGATTGAGAGTTTCTAGTGGGCCATTTTTGGTAAGCAGAACTGGCGATG
He6736
GCGATTGAGAGTTTCTAGTGGGCCATTTTTGGTAAGCAGAACTGGCGATG
BRNM734877S_spumeus --
BRNM712630S_spumeus --
Dai1723Loweomyces_sibiricus --
W54Cui10009
GCGATTGAGAGTTTCTAGTGGGCCATTTTTGGTAAGCAGAACTGGCGATG
W1Dai20899 --
HHB13445Trametes_ochracea
GCGATTGAGAGTTTCTAGTGGGCCATTTTTGGTAAGCAGAACTGGCGATG
Dai16222
GTGATTGAGAGTTTCTAGTGGGCCATTTTTGGTAAGCAGAACTGGCGATG
Dai16240
GTGATTGAGAGTTTCTAGTGGGCCATTTTTGGTAAGCAGAACTGGCGATG

JV1310_11SanguinolentusCernys
CGGGATGAACCGAACGCGAGGTTAAGGTGCCGGAATGCACGCTCATCAGA
MJ39_00_SK --
MJ111_04_CZ --
JV1610_BOKYsmrk --
Dai21030 --
Dai20976P_furcatus
CGGGATGAACCGAACGCGAGGTTAAGGTGCCGGAATGCACGCTCATCAGA
Dai2105
CGGGATGAACCGAACGCGAGGTTAAGGTGCCGGAATGCACGCTCATCAGA
Dai2544
CGGGATGAACCGAACGCGAGGTTAAGGTGCCGGAATGCACGCTCATCAGA
Dai11313
CGGGATGAACCGAACGCGAGGTTAAGGTGCCGGAATGCACGCTCATCAGA
WCG1611Dai26167
CGGGATGAACCGAACGCGAGGTTAAGGTGCCGGAATGCACGCTCATCAGA
WCG1518Dai25999Physisporinus
CGGGATGAACCGAACGCGAGGTTAAGGTGCCGGAATGCACGCTCATCAGA
TAA15097
CGGGATGAACCGAACGCGAGGTTAAGGTGCCGGAATGCACGCTCATCAGA
JV8909_19_CZ --
JV1310_15_P_sanguinolentus2_CZ --
MJ53_02_CZ --
CLZhao21647P_yunnanensis
CGGGATGAACCGAACGCGAGGTTAAGGTGCCGGAATGCACGCTCATCAGA

CLZhao21583P_yunnanensis
CGGGATGAACCGAACGCGAGGTTAAGGTGCCGGAATGCACGCTCATCAGA
Dai22272
CGGGATGAACCGAACGCGAGGTTAAGGTGCCGGAATGCACGCTCATCAGA
Dai22279
CGGGATGAACCGAACGCGAGGTTAAGGTGCCGGAATGCACGCTCATCAGA
MJ332_94_CZ --
MJ642_94_CZ_Expallescens --
Dai21060P_vinctus
CGGGATGAACCGAACGCGAGGTTAAGGTGCCGGAATGCACGCTCATCAGA
JV0511_23LRP_pouzarii --
JQ409462_R_pouzarii_PRM899856_ --
JV0308_66_WA --
JV0309_45_WA_USA --
JV0709_83_CA_USA --
Dai21043P_pouzarii
CGGGATGAACCGAACGCGAGGTTAAGGTGCCGGAATGCACGCTCATCAGA
MJ144_95_CZ --
JV0909_3_CZ --
JV0609_1_K --
Dai20396Physisporinus_castanop
CGGGATGAACCGAACGCGAGGTTAAGGTGCCGGAATGCACGCTCATCAGA
Dai20397Physisporinus_castanop
CGGGATGAACCGAACGCGAGGTTAAGGTGCCGGAATGCACGCTCATCAGA
MJ19_09_SK_Abies --
JV0509_40_J_TN_USA_Betula
CGGGATGAACCGAACGCGAGGTTAAGGTGCCGGAATGCACGCTCATCAGA
JV0808_33crocatus_PA_USAlist --
DLL2009_061P_crocatus --
Dai12800P_subcrocatus
CGGGATGAACCGAACGCGAGGTTAAGGTGCCGGAATGCACGCTCATCAGA
Dai15917P_subcrocatus
CGGGATGAACCGAACGCGAGGTTAAGGTGCCGGAATGCACGCTCATCAGA
Cui16620
CGGGATGAACCGAACGCGAGGTTAAGGTGCCGGAATGCACGCTCATCAGA
HCFC1088Meripilus_stillicidior
CGGGATGAACCGAACGCGAGGTTAAGGTGCCGGAATGCACGCTCATCAGA
MCW590Meripilus_obsrcurus --
MCW722Meripilus_obsrcurus --
Cui9381P_tibeticus
CGGGATGAACCGAACGCGAGGTTAAGGTGCCGGAATGCACGCTCATCAGA
Cui9588P_tibeticus
CGGGATGAACCGAACGCGAGGTTAAGGTGCCGGAATGCACGCTCATCAGA
Va2_Beneschova --

CWU3874_Ukraine_Alnus --
WCG1293Dai24718Physisporinus --
WCG1268Dai24682A
CGGGATGAACCGAACGCGAGGTTAAGGTGCCGGAATGCACGCTCATCAGA
WCG1269Dai24683A
CGGGATGAACCGAACGCGAGGTTAAGGTGCCGGAATGCACGCTCATCAGA
WCG1279Dai24694A
CGGGATGAACCGAACGCGAGGTTAAGGTGCCGGAATGCACGCTCATCAGA
Dai16971
CGGGATGAACCGAACGCGAGGTTAAGGTGCCGGAATGCACGCTCATCAGA
ZQY1043Dai26696
CGGGATGAACCGAACGCGAGGTTAAGGTGCCGGAATGCACGCTCATCAGA
Doll880 --
Doll1000 --
1DAI18529
CGGGATGAACCGAACGCGAGGTTAAGGTGCCGGAATGCACGCTCATCAGA
Dai19535
CGGGATGAACCGAACGCGAGGTTAAGGTGCCGGAATGCACGCTCATCAGA
1704_79_hnedyVillaLaPaz --
F2061 --
1DAI18268
CGGGATGAACCGAACGCGAGGTTAAGGTGCCGGAATGCACGCTCATCAGA
1DAI18540A
CGGGATGAACCGAACGCGAGGTTAAGGTGCCGGAATGCACGCTCATCAGA
Dai17695
CGGGATGAACCGAACGCGAGGTTAAGGTGCCGGAATGCACGCTCATCAGA
LKY18Dai26373 --
Dai17839P_sulphureus
CGGGATGAACCGAACGCGAGGTTAAGGTGCCGGAATGCACGCTCATCAGA
Dai17841P_sulphureus
CGGGATGAACCGAACGCGAGGTTAAGGTGCCGGAATGCACGCTCATCAGA
Dai19877P_roseus
CGGGATGAACCGAACGCGAGGTTAAGGTGCCGGAATGCACGCTCATCAGA
1508_18_1_Kout --
KP859303_R_vinctus_RP185_BRAZI --
JK1807_15Rigidoporus_sp_Puerto --
JV0509_47_J_TN_USA
CGGGATGAACCGAACGCGAGGTTAAGGTGCCGGAATGCACGCTCATCAGA
JV0709_188 --
JV0509_127_PA_USA --
JV1009_59_NJ_USA
CGGGATGAACCGAACGCGAGGTTAAGGTGCCGGAATGCACGCTCATCAGA
Dai15497P_crataegi
CGGGATGAACCGAACGCGAGGTTAAGGTGCCGGAATGCACGCTCATCAGA

Dai15499P_crataegi
CGGGATGAACCGAACGCGAGGTTAAGGTGCCGGAATGCACGCTCATCAGA
Cui3266P_cinereus
CGGGATGAACCGAACGCGAGGTTAAGGTGCCGGAATGCACGCTCATCAGA
WCG1256Dai24690
CGGGATGAACCGAACGCGAGGTTAAGGTGCCGGAATGCACGCTCATCAGA
1DAI17581
CGGGATGAACCGAACGCGAGGTTAAGGTGCCGGAATGCACGCTCATCAGA
WCG1255Dai24688
CGGGATGAACCGAACGCGAGGTTAAGGTGCCGGAATGCACGCTCATCAGA
Dai22427
CGGGATGAACCGAACGCGAGGTTAAGGTGCCGGAATGCACGCTCATCAGA
MV690Meripilus_concrescens
CGGGATGAACCGAACGCGAGGTTAAGGTGCCGGAATGCACGCTCATCAGA
MV513Meripilus_galapagensis
CGGGATGAACCGAACGCGAGGTTAAGGTGCCGGAATGCACGCTCATCAGA
Dai19793
CGGGATGAACCGAACGCGAGGTTAAGGTGCCGGAATGCACGCTCATCAGA
OQ553780P_tamilnaduensis --
OQ553779P_tamilnaduensis --
A164FB3Meripilus_giganteus --
JV1407_36_Vinctus_Meandrica --
1807_3K_Rigidoporus_PuertoRico
CGGGATGAACCGAACGCGAGGTTAAGGTGCCGGAATGCACGCTCATCAGA
Cui16903P_vinctus
CGGGATGAACCGAACGCGAGGTTAAGGTGCCGGAATGCACGCTCATCAGA
JV1008_18R_Lineatus --
JV1407_37_1_Vinctus_Carara --
Dai17986P_lineatus
CGGGATGAACCGAACGCGAGGTTAAGGTGCCGGAATGCACGCTCATCAGA
Dai18281
CGGGATGAACCGAACGCGAGGTTAAGGTGCCGGAATGCACGCTCATCAGA
1DAI19796
CGGGATGAACCGAACGCGAGGTTAAGGTGCCGGAATGCACGCTCATCAGA
ZQY797Dai25241
CGGGATGAACCGAACGCGAGGTTAAGGTGCCGGAATGCACGCTCATCAGA
WCG1289Dai24711 --
Dai22598
CGGGATGAACCGAACGCGAGGTTAAGGTGCCGGAATGCACGCTCATCAGA
Dai20523
CGGGATGAACCGAACGCGAGGTTAAGGTGCCGGAATGCACGCTCATCAGA
Dai17885
CGGGATGAACCGAACGCGAGGTTAAGGTGCCGGAATGCACGCTCATCAGA
Dai17553

CGGGATGAACCGAACGCGAGGTTAAGGTGCCGGAATGCACGCTCATCAGA
Dai19639
CGGGATGAACCGAACGCGAGGTTAAGGTGCCGGAATGCACGCTCATCAGA
JV0110_48_CZ --
MJ129_04 --
Cui10340P_eminens --
Cui10341P_eminens
CGGGATGAACCGAACGCGAGGTTAAGGTGCCGGAATGCACGCTCATCAGA
Dai12685P_eminens
CGGGATGAACCGAACGCGAGGTTAAGGTGCCGGAATGCACGCTCATCAGA
Miettinen_13591Rigidoporus_und
CGGGATGAACCGAACGCGAGGTTAAGGTGCCGGAATGCACGCTCATCAGA
Dai20868
CGGGATGAACCGAACGCGAGGTTAAGGTGCCGGAATGCACGCTCATCAGA
Dai20832
CGGGATGAACCGAACGCGAGGTTAAGGTGCCGGAATGCACGCTCATCAGA
Dai11400
CGGGATGAACCGAACGCGAGGTTAAGGTGCCGGAATGCACGCTCATCAGA
Dai22472
CGGGATGAACCGAACGCGAGGTTAAGGTGCCGGAATGCACGCTCATCAGA
1CUI10475
CGGGATGAACCGAACGCGAGGTTAAGGTGCCGGAATGCACGCTCATCAGA
1CUI10491 --
HCFC1095Meripilus_robledoi --
MCW702Meripilus_revolubilis --
1704_83_zluty_HaciendaBaru --
Dai9925P_lavendulus
CGGGATGAACCGAACGCGAGGTTAAGGTGCCGGAATGCACGCTCATCAGA
Dai13587AP_lavendulus
CGGGATGAACCGAACGCGAGGTTAAGGTGCCGGAATGCACGCTCATCAGA
PDD70600P_longicystidius --
Cui16630
CGGGATGAACCGAACGCGAGGTTAAGGTGCCGGAATGCACGCTCATCAGA
FP_135344Meripilus_giganteus
CGGGATGAACCGAACGCGAGGTTAAGGTGCCGGAATGCACGCTCATCAGA
FP_100460_Sp --
CBS421_48Meripilus_giganteus --
Cui9203 --
Cui9202
CGGGATGAACCGAACGCGAGGTTAAGGTGCCGGAATGCACGCTCATCAGA
TUFC100564Japan --
Russell5913Meripilus_sumstinei --
RP215Meripilus_brasiliensis
CGGGATGAACCGAACGCGAGGTTAAGGTGCCGGAATGCACGCTCATCAGA

RP200Meripilus_brasiliensis
CGGGATGAACCGAACGCGAGGTTAAGGTGCCGGAATGCACGCTCATCAGA
JV1712_13J_R_vinctus2_LSUPuert --
Dai10503R_hypobrunneus
CGGGATGAACCGAACGCGAGGTTAAGGTGCCGGAATACACGCTCATCAGA
Dai10569Rigidoporus_hypobrunne
CGGGATGAACCGAACGCGAGGTTAAGGTGCCGGAATACACGCTCATCAGA
1DAI19451
CGGGATGAACCGAACGCGAGGTTAAGGTGCCGGAATACACGCTCATCAGA
CM108bRigidoporus_hypobrunneus --
1CUI16874
CGGGATGAACCGAACGCGAGGTTAAGGTGCCGGAATACACGCTCATCAGA
FD299Cerrena_unicolor
CGGGATGAACCGAACGCGAGGTTAAGGTGCCGGAATACACGCTCATCAGA
KHL_GB_Cerrena_uniclor
CGGGATGAACCGAACGCGAGGTTAAGGTGCCGGAATACACGCTCATCAGA
Dai12892Cerrena_albocinnamomea
CGGGATGAACCGAACGCGAGGTTAAGGTGCCGGAATACACGCTCATCAGA
Dai12955C_albocinnamomea
CGGGATGAACCGAACGCGAGGTTAAGGTGCCGGAATACACGCTCATCAGA
SNUm03110102C_aurantiopora --
NIBRFG0000102423C_aurantiopora --
Dai7359Antrodiella_zonata --
F20080702KCM29C_consors --
F20080208LYW10Cerrena_consors --
Dai7821Antrodiella_zonata --
CFMR_DCL04_31Pseudolagarobasid --
VPB197Pseudolagarobasidium_bel --
CBS115543Pseudolagarobasidium_ --
CBS115544 --
Han405Pseudolagarobasidium_bai
CGGGATGAACCGAACGCGAGGTTAAGGTGCCGGAATACACGCTCATCAGA
Han406Pseudolagarobasidium_bai
CGGGATGAACCGAACGCGAGGTTAAGGTGCCGGAATACACGCTCATCAGA
MUcc838Spongipellis_delectans --
BRNM686401S_delectans --
OSM_F925S_delectans --
BRNM67093Spongipellis_litschau --
CFMRccFP59199TS_unicolor --
CFMRccFP71791TS_unicolor --
Dai13845P_lischaueri
CGGGATGAACCGAACGCGAGGTTAAGGTGCCGGAATACACGCTCATCAGA
Dai20266P_lischaueri
CGGGATGAACCGAACGCGAGGTTAAGGTGCCGGAATACACGCTCATCAGA

CFMR_HHB11240Radulodon_america --
RLG6350Radulodon_americanus --
KY415963Radulodon_erikssonii
CGGGATGAACCGAACGCGAGGTTAAGGTGCCGGAATGCACGCTCATCAGA
HHB9567spRadulodon_casearius
CGGGATGAACCGAACGCGAGGTTAAGGTGCCGGAATGCACGCTCATCAGA
KRT_Iso_26Radulodon_casearius --
CBS126044Radulodon_erikssonii --
He6183YUNNANENSIS
CGGGATGAACCGAACGCGAGGTTAAGGTGCCGGAATGCACGCTCATCAGA
Cui17979YUNNANENSIS
CGGGATGAACCGAACGCGAGGTTAAGGTGCCGGAATGCACGCTCATCAGA
Miettinen2091Junghuhnia_fimbri
CGGGATGAACCGAACGCGAGGTTAAGGTGCCGGAATACACGCTCATCAGA
KHL12316S_tenue
CGGGATGAACCGAACGCGAGGTTAAGGTGCCGGAATACACGCTCATCAGA
PRM846564S_pachyodon --
SP_Lgt_S_pachyodon --
Ryvarden44669Tyromyces_xuchile
CGGGATGAACCGAACGCGAGGTTAAGGTGCCGGAATGCACGCTCATCAGA
PW17_171sinuosus --
W53Dai12234
CGGGATGAACCGAACGCGAGGTTAAGGTGCCGGAATGCACGCTCATCAGA
HHB4100SpAntella_americana
CGGGATGAACCGAACGCGAGGTTAAGGTGCCGGAATACACGCTCATCAGA
W3Dai20901spumeus
CGGGATGAACCGAACGCGAGGTTAAGGTGCCGGAATACGCACTCATCAGA
He6736
CGGGATGAACCGAACGCGAGGTTAAGGTGCCGGAATACGCACTCATCAGA
BRNM734877S_spumeus --
BRNM712630S_spumeus --
Dai1723Loweomyces_sibiricus --
W54Cui10009
CGGGATGAACCGAACGCGAGGTTAAGGTGCCGGAATACGCACTCATCAGA
W1Dai20899 --
HHB13445Trametes_ochracea
CGGGATGAACCGAACGCGAGGTTAAGGTGCCGGAATACACGCTCATCAGA
Dai16222
CGGGATGAACCGAACGCGAGGTTAAGGTGCCGGAATGCACGCTCATCAGA
Dai16240
CGGGATGAACCGAACGCGAGGTTAAGGTGCCGGAATGCACGCTCATCAGA

JV1310_11SanguinolentusCernys
CACCACAAAAGGTGTTAGTTCATCTAGACAGCAGGACGGTGGCCATGGAA

MJ39_00_SK --
MJ111_04_CZ --
JV1610_BOKYsmrk --
Dai21030 --
Dai20976P_furcatus
CACCACAAAAGGTGTTAGTTCATCTAGACAGCAGGACGGTGGCCATGGAA
Dai2105
CACCACAAAAGGTGTTAGTTCATCTAGACAGCAGGACGGTGGCCATGGAA
Dai2544
CACCACAAAAGGTGTTAGTTCATCTAGACAGCAGGACGGTGGCCATGGAA
Dai11313
CACCACAAAAGGTGTTAGTTCATCTAGACAGCAGGACGGTGGCCATGGAA
WCG1611Dai26167
CACCACAAAAGGTGTTAGTTCATCTAGACAGCAGGACGGTGGCCATGGAA
WCG1518Dai25999Physisporinus
CACCACAAAAGGTGTTAGTTCATCTAGACAGCAGGACGGTGGCCATGGAA
TAA15097
CACCACAAAAGGTGTTAGTTCATCTAGACAGCAGGACGGTGGCCATGGAA
JV8909_19_CZ --
JV1310_15_P_sanguinolentus2_CZ --
MJ53_02_CZ --
CLZhao21647P_yunnanensis
CACCACAAAAGGTGTTAGTTCATCTAGACAGCAGGACGGTGGCCATGGAA
CLZhao21583P_yunnanensis
CACCACAAAAGGTGTTAGTTCATCTAGACAGCAGGACGGTGGCCATGGAA
Dai22272
CACCACAAAAGGTGTTAGTTCATCTAGACAGCAGGACGGTGGCCATGGAA
Dai22279
CACCACAAAAGGTGTTAGTTCATCTAGACAGCAGGACGGTGGCCATGGAA
MJ332_94_CZ --
MJ642_94_CZ_Expallescens --
Dai21060P_vinctus
CACCACAAAAGGTGTTAGTTCATCTAGACAGCAGGACGGTGGCCATGGAA
JV0511_23LRP_pouzarii --
JQ409462_R_pouzarii_PRM899856_ --
JV0308_66_WA --
JV0309_45_WA_USA --
JV0709_83_CA_USA --
Dai21043P_pouzarii
CACCACAAAAGGTGTTAGTTCATCTAGACAGCAGGACGGTGGCCATGGAA
MJ144_95_CZ --
JV0909_3_CZ --
JV0609_1_K --
Dai20396Physisporinus_castanop

CACCACAAAAGGTGTTAGTTCATCTAGACAGCAGGACGGTGGCCATGGAA
Dai20397Physisporinus_castanop
CACCACAAAAGGTGTTAGTTCATCTAGACAGCAGGACGGTGGCCATGGAA
MJ19_09_SK_Abies --
JV0509_40_J_TN_USA_Betula
CACCACAAAAGGTGTTAGTTCATCTAGACAGCAGGACGGTGGCCATGGAA
JV0808_33crocatus_PA_USAlist --
DLL2009_061P_crocatus --
Dai12800P_subcrocatus
CACCACAAAAGGTGTTAGTTCATCTAGACAGCAGGACGGTGGCCATGGAA
Dai15917P_subcrocatus
CACCACAAAAGGTGTTAGTTCATCTAGACAGCAGGACGGTGGCCATGGAA
Cui16620
CACCACAAAAGGTGTTAGTTCATCTAGACAGCAGGACGGTGGCCATGGAA
HCFC1088Meripilus_stillicidior
CACCACAAAAGGTGTTAGTTCATCTAGACAGCAGGACGGTGGCCATGGAA
MCW590Meripilus_obsrcurus --
MCW722Meripilus_obsrcurus --
Cui9381P_tibeticus
CACCACAAAAGGTGTTAGTTCATCTAGACAGCAGGACGGTGGCCATGGAA
Cui9588P_tibeticus
CACCACAAAAGGTGTTAGTTCATCTAGACAGCAGGACGGTGGCCATGGAA
Va2_Beneschova --
CWU3874_Ukraine_Alnus --
WCG1293Dai24718Physisporinus --
WCG1268Dai24682A
CACCACAAAAGGTGTTAGTTCATCTAGACAGCAGGACGGTGGCCATGGAA
WCG1269Dai24683A
CACCACAAAAGGTGTTAGTTCATCTAGACAGCAGGACGGTGGCCATGGAA
WCG1279Dai24694A
CACCACAAAAGGTGTTAGTTCATCTAGACAGCAGGACGGTGGCCATGGAA
Dai16971
CACCACAAAAGGTGTTAGTTCATCTAGACAGCAGGACGGTGGCCATGGAA
ZQY1043Dai26696
CACCACAAAAGGTGTTAGTTCATCTAGACAGCAGGACGGTGGCCATGGAA
Doll880 --
Doll1000 --
1DAI18529
CACCACAAAAGGTGTTAGTTCATCTAGACAGCAGGACGGTGGCCATGGAA
Dai19535
CACCACAAAAGGTGTTAGTTCATCTAGACAGCAGGACGGTGGCCATGGAA
1704_79_hnedyVillaLaPaz --
F2061 --
1DAI18268

CACCACAAAAGGTGTTAGTTCATCTAGACAGCAGGACGGTGGCCATGGAA
1DAI18540A
CACCACAAAAGGTGTTAGTTCATCTAGACAGCAGGACGGTGGCCATGGAA
Dai17695
CACCACAAAAGGTGTTAGTTCATCTAGACAGCAGGACGGTGGCCATGGAA
LKY18Dai26373 --
Dai17839P_sulphureus
CACCACAAAAGGTGTTAGTTCATCTAGACAGCAGGACGGTGGCCATGGAA
Dai17841P_sulphureus
CACCACAAAAGGTGTTAGTTCATCTAGACAGCAGGACGGTGGCCATGGAA
Dai19877P_roseus
CACCACAAAAGGTGTTAGTTCATCTAGACAGCAGGACGGTGGCCATGGAA
1508_18_1_Kout --
KP859303_R_vinctus_RP185_BRAZI --
JK1807_15Rigidoporus_sp_Puerto --
JV0509_47_J_TN_USA
CACCACAAAAGGTGTTAGTTCATCTAGACAGCAGGACGGTGGCCATGGAA
JV0709_188 --
JV0509_127_PA_USA --
JV1009_59_NJ_USA
CACCACAAAAGGTGTTAGTTCATCTAGACAGCAGGACGGTGGCCATGGAA
Dai15497P_crataegi
CACCACAAAAGGTGTTAGTTCATCTAGACAGCAGGACGGTGGCCATGGAA
Dai15499P_crataegi
CACCACAAAAGGTGTTAGTTCATCTAGACAGCAGGACGGTGGCCATGGAA
Cui3266P_cinereus
CACCACAAAAGGTGTTAGTTCATCTAGACAGCAGGACGGTGGCCATGGAA
WCG1256Dai24690
CACCACAAAAGGTGTTAGTTCATCTAGACAGCAGGACGGTGGCCATGGAA
1DAI17581
CACCACAAAAGGTGTTAGTTCATCTAGACAGCAGGACGGTGGCCATGGAA
WCG1255Dai24688
CACCACAAAAGGTGTTAGTTCATCTAGACAGCAGGACGGTGGCCATGGAA
Dai22427
CACCACAAAAGGTGTTAGTTCATCTAGACAGCAGGACGGTGGCCATGGAA
MV690Meripilus_concrescens
CACCACAAAAGGTGTTAGTTCATCTAGACAGCAGGACGGTGGCCATGGAA
MV513Meripilus_galapagensis
CACCACAAAAGGTGTTAGTTCATCTAGACAGCAGGACGGTGGCCATGGAA
Dai19793
CACCACAAAAGGTGTTAGTTCATCTAGACAGCAGGACGGTGGCCATGGAA
OQ553780P_tamilnaduensis --
OQ553779P_tamilnaduensis --
A164FB3Meripilus_giganteus --

JV1407_36_Vinctus_Meandrica --
1807_3K_Rigidoporus_PuertoRico
CACCACAAAAGGTGTTAGTTCATCTAGACAGCAGGACGGTGGCCATGGAA
Cui16903P_vinctus
CACCACAAAAGGTGTTAGTTCATCTAGACAGCAGGACGGTGGCCATGGAA
JV1008_18R_Lineatus --
JV1407_37_1_Vinctus_Carara --
Dai17986P_lineatus
CACCACAAAAGGTGTTAGTTCATCTAGACAGCAGGACGGTGGCCATGGAA
Dai18281
CACCACAAAAGGTGTTAGTTCATCTAGACAGCAGGACGGTGGCCATGGAA
1DAI19796
CACCACAAAAGGTGTTAGTTCATCTAGACAGCAGGACGGTGGCCATGGAA
ZQY797Dai25241
CACCACAAAAGGTGTTAGTTCATCTAGACAGCAGGACGGTGGCCATGGAA
WCG1289Dai24711 --
Dai22598
CACCACAAAAGGTGTTAGTTCATCTAGACAGCAGGACGGTGGCCATGGAA
Dai20523
CACCACAAAAGGTGTTAGTTCATCTAGACAGCAGGACGGTGGCCATGGAA
Dai17885
CACCACAAAAGGTGTTAGTTCATCTAGACAGCAGGACGGTGGCCATGGAA
Dai17553
CACCACAAAAGGTGTTAGTTCATCTAGACAGCAGGACGGTGGCCATGGAA
Dai19639
CACCACAAAAGGTGTTAGTTCATCTAGACAGCAGGACGGTGGCCATGGAA
JV0110_48_CZ --
MJ129_04 --
Cui10340P_eminens --
Cui10341P_eminens
CACCACAAAAGGTGTTAGTTCATCTAGACAGCAGGACGGTGGCCATGGAA
Dai12685P_eminens
CACCACAAAAGGTGTTAGTTCATCTAGACAGCAGGACGGTGGCCATGGAA
Miettinen_13591Rigidoporus_und
CACCACAAAAGGTGTTAGTTCATCTAGACAGCAGGACGGTGGCCATGGAA
Dai20868
CACCACAAAAGGTGTTAGTTCATCTAGACAGCAGGACGGTGGCCATGGAA
Dai20832
CACCACAAAAGGTGTTAGTTCATCTAGACAGCAGGACGGTGGCCATGGAA
Dai11400
CACCACAAAAGGTGTTAGTTCATCTAGACAGCAGGACGGTGGCCATGGAA
Dai22472
CACCACAAAAGGTGTTAGTTCATCTAGACAGCAGGACGGTGGCCATGGAA
1CUI10475

CACCACAAAAGGTGTTAGTTCATCTAGACAGCAGGACGGTGGCCATGGAA
1CUI10491 --
HCFC1095Meripilus_robledoi --
MCW702Meripilus_revolubilis --
1704_83_zluty_HaciendaBaru --
Dai9925P_lavendulus
CACCACAAAAGGTGTTAGTTCATCTAGACAGCAGGACGGTGGCCATGGAA
Dai13587AP_lavendulus
CACCACAAAAGGTGTTAGTTCATCTAGACAGCAGGACGGTGGCCATGGAA
PDD70600P_longicystidius --
Cui16630
CACCACAAAAGGTGTTAGTTCATCTAGACAGCAGGACGGTGGCCATGGAA
FP_135344Meripilus_giganteus
CACCACAAAAGGTGTTAGTTCATCTAGACAGCAGGACGGTGGCCATGGAA
FP_100460_Sp --
CBS421_48Meripilus_giganteus --
Cui9203 --
Cui9202
CACCACAAAAGGTGTTAGTTCATCTAGACAGCAGGACGGTGGCCATGGAA
TUFC100564Japan --
Russell5913Meripilus_sumstinei --
RP215Meripilus_brasiliensis
CACCACAAAAGGTGTTAGTTCATCTAGACAGCAGGACGGTGGCCATGGAA
RP200Meripilus_brasiliensis
CACCACAAAAGGTGTTAGTTCATCTAGACAGCAGGACGGTGGCCATGGAA
JV1712_13J_R_vinctus2_LSUPuert --
Dai10503R_hypobrunneus
CACCACAAAAGGTGTTAGTTCATCTAGACAGCAGGACGGTGGCCATGGAA
Dai10569Rigidoporus_hypobrunne
CACCACAAAAGGTGTTAGTTCATCTAGACAGCAGGACGGTGGCCATGGAA
1DAI19451
CACCACAAAAGGTGTTAGTTCATCTAGACAGCAGGACGGTGGCCATGGAA
CM108bRigidoporus_hypobrunneus --
1CUI16874
CACCACAAAAGGTGTTAGTTCATCTAGACAGCAGGACGGTGGCCATGGAA
FD299Cerrena_unicolor
CACCACAAAAGGTGTTAGTTCATCTAGACAGCAGGACGGTGGCCATGGAA
KHL_GB_Cerrena_uniclor
CACCACAAAATGTGTTAGTTCATCTAGACAGCAGGACGGTGGCCATGGAA
Dai12892Cerrena_albocinnamomea
CACCACAAAAGGTGTTAGTTCATCTAGACAGCAGGACGGTGGCCATGGAA
Dai12955C_albocinnamomea
CACCACAAAAGGTGTTAGTTCATCTAGACAGCAGGACGGTGGCCATGGAA
SNUm03110102C_aurantiopora --

NIBRFG0000102423C_aurantiopora --
Dai7359Antrodiella_zonata --
F20080702KCM29C_consors --
F20080208LYW10Cerrena_consors --
Dai7821Antrodiella_zonata --
CFMR_DCL04_31Pseudolagarobasid --
VPB197Pseudolagarobasidium_bel --
CBS115543Pseudolagarobasidium_ --
CBS115544 --
Han405Pseudolagarobasidium_bai
CACCACAAAAGGTGTTAGTTCATCTAGACAGCAGGACGGTGGCCATGGAA
Han406Pseudolagarobasidium_bai
CACCACAAAAGGTGTTAGTTCATCTAGACAGCAGGACGGTGGCCATGGAA
MUcc838Spongipellis_delectans --
BRNM686401S_delectans --
OSM_F925S_delectans --
BRNM67093Spongipellis_litschau --
CFMRccFP59199TS_unicolor --
CFMRccFP71791TS_unicolor --
Dai13845P_lischaueri
CACCACAAAAGGTGTTAGTTCATCTAGACAGCAGGACGGTGGCCATGGAA
Dai20266P_lischaueri
CACCACAAAAGGTGTTAGTTCATCTAGACAGCAGGACGGTGGCCATGGAA
CFMR_HHB11240Radulodon_america --
RLG6350Radulodon_americanus --
KY415963Radulodon_erikssonii
CACCACAAAAGGTGTTAGTTCATCTAGACAGCAGGACGGTGGCCATGGAA
HHB9567spRadulodon_casearius
CACCACAAAAGGTGTTAGTTCATCTAGACAGCAGGACGGTGGCCATGGAA
KRT_Iso_26Radulodon_casearius --
CBS126044Radulodon_erikssonii --
He6183YUNNANENSIS
CACCACAAAAGGTGTTAGTTCATCTAGACAGCAGGACGGTGGCCATGGAA
Cui17979YUNNANENSIS
CACCACAAAAGGTGTTAGTTCATCTAGACAGCAGGACGGTGGCCATGGAA
Miettinen2091Junghuhnia_fimbri
CACCACAAAAGGTGTTAGTTCATCTAGACAGCAGGACGGTGGCCATGGAA
KHL12316S_tenue
CACCACAAAAGGTGTTAGTTCATCTAGACAGCAGGACGGTGGCCATGGAA
PRM846564S_pachyodon --
SP_Lgt_S_pachyodon --
Ryvarden44669Tyromyces_xuchile
CACCACAAAAGGTGTTAGTTCATCTAGACAGCAGGACGGTGGCCATGGAA
PW17_171sinuosus --

W53Dai12234
CACCACAAAAGGTGTTAGTTCATCTAGACAGCAGGACGGTGGCCATGGAA
HHB4100SpAntella_americana
CACCACAAAAGGTGTTAGTTCATCTAGACAGCAGGACGGTGGCCATGGAA
W3Dai20901spumeus
CACCACAAAAGGTGTTAGTTCATCTAGACAGCAGGACGGTGGCCATGGAA
He6736
CACCACAAAAGGTGTTAGTTCATCTAGACAGCAGGACGGTGGCCATGGAA
BRNM734877S_spumeus --
BRNM712630S_spumeus --
Dai1723Loweomyces_sibiricus --
W54Cui10009
CACCACAAAAGGTGTTAGTTCATCTAGACAGCAGGACGGTGGCCATGGAA
W1Dai20899 --
HHB13445Trametes_ochracea
CACCACAAAAGGTGTTAGTTCATCTAGACAGCAGGACGGTGGCCATGGAA
Dai16222
CACCACAAAAGGTGTTAGTTCATCTAGACAGCAGGACGGTGGCCATGGAA
Dai16240
CACCACAAAAGGTGTTAGTTCATCTAGACAGCAGGACGGTGGCCATGGAA

JV1310_11SanguinolentusCernys
GTCGGAATCCGCTAAGGAGTGTGTAACAACTCACCTGCCGAATGAACTAG
MJ39_00_SK --
MJ111_04_CZ --
JV1610_BOKYsmrk --
Dai21030 --
Dai20976P_furcatus
GTCGGAATCCGCTAAGGAGTGTGTAACAACTCACCTGCCGAATGAACTAG
Dai2105
GTCGGAATCCGCTAAGGAGTGTGTAACAACTCACCTGCCGAATGAACTAG
Dai2544
GTCGGAATCCGCTAAGGAGTGTGTAACAACTCACCTGCCGAATGAACTAG
Dai11313
GTCGGAATCCGCTAAGGAGTGTGTAACAACTCACCTGCCGAATGAACTAG
WCG1611Dai26167
GTCGGAATCCGCTAAGGAGTGTGTAACAACTCACCTGCCGAATGAACTAG
WCG1518Dai25999Physisporinus
GTCGGAATCCGCTAAGGAGTGTGTAACAACTCACCTGCCGAATGAACTAG
TAA15097
GTCGGAATCCGCTAAGGAGTGTGTAACAACTCACCTGCCGAATGAACTAG
JV8909_19_CZ --
JV1310_15_P_sanguinolentus2_CZ --
MJ53_02_CZ --

CLZhao21647P_yunnanensis
GTCGGAATCCGCTAAGGAGTGTGTAACAACTCACCTGCCGAATGAACTAG
CLZhao21583P_yunnanensis
GTCGGAATCCGCTAAGGAGTGTGTAACAACTCACCTGCCGAATGAACTAG
Dai22272
GTCGGAATCCGCTAAGGAGTGTGTAACAACTCACCTGCCGAATGAACTAG
Dai22279
GTCGGAATCCGCTAAGGAGTGTGTAACAACTCACCTGCCGAATGAACTAG
MJ332_94_CZ --
MJ642_94_CZ_Expallescens --
Dai21060P_vinctus
GTCGGAATCCGCTAAGGAATGTGTAACAACTCACCTGCCGAATGAACTAG
JV0511_23LRP_pouzarii --
JQ409462_R_pouzarii_PRM899856_ --
JV0308_66_WA --
JV0309_45_WA_USA --
JV0709_83_CA_USA --
Dai21043P_pouzarii
GTCGGAATCCGCTAAGGAGTGTGTAACAACTCACCTGCCGAATGAACTAG
MJ144_95_CZ --
JV0909_3_CZ --
JV0609_1_K --
Dai20396Physisporinus_castanop
GTCGGAATCCGCTAAGGAGTGTGTAACAACTCACCTGCCGAATGAACTAG
Dai20397Physisporinus_castanop
GTCGGAATCCGCTAAGGAGTGTGTAACAACTCACCTGCCGAATGAACTAG
MJ19_09_SK_Abies --
JV0509_40_J_TN_USA_Betula
GTCGGAATCCGCTAAGGAGTGTGTAACAACTCACCTGCCGAATGAACTAG
JV0808_33crocatus_PA_USAlist --
DLL2009_061P_crocatus --
Dai12800P_subcrocatus
GTCGGAATCCGCTAAGGAGTGTGTAACAACTCACCTGCCGAATGAACTAG
Dai15917P_subcrocatus
GTCGGAATCCGCTAAGGAGTGTGTAACAACTCACCTGCCGAATGAACTAG
Cui16620
GTCGGAATCCGCTAAGGAATGTGTAACAACTCACCTGCCGAATGAACTAG
HCFC1088Meripilus_stillicidior
GTCGGAATCCGCTAAGGAATGTGTAACAACTCACCTGCCGAATGAACTAG
MCW590Meripilus_obsrcurus --
MCW722Meripilus_obsrcurus --
Cui9381P_tibeticus
GTCGGAATCCGCTAAGGAGTGTGTAACAACTCACCTGCCGAATGAACTAG
Cui9588P_tibeticus

GTCGGAATCCGCTAAGGAGTGTGTAACAACTCACCTGCCGAATGAACTAG
Va2_Beneschova --
CWU3874_Ukraine_Alnus --
WCG1293Dai24718Physisporinus --
WCG1268Dai24682A
GTCGGAATCCGCTAAGGAGTGTGTAACAACTCACCTGCCGAATGAACTAG
WCG1269Dai24683A
GTCGGAATCCGCTAAGGAGTGTGTAACAACTCACCTGCCGAATGAACTAG
WCG1279Dai24694A
GTCGGAATCCGCTAAGGAGTGTGTAACAACTCACCTGCCGAATGAACTAG
Dai16971
GTCGGAATCCGCTAAGGAGTGTGTAACAACTCACCTGCCGAATGAACTAG
ZQY1043Dai26696
GTCGGAATCCGCTAAGGAGTGTGTAACAACTCACCTGCCGAATGAACTAG
Doll880 --
Doll1000 --
1DAI18529
GTCGGAATCCGCTAAGGAGTGTGTAACAACTCACCTGCCGAATGAACTAG
Dai19535
GTCGGAATCCGCTAAGGAGTGTGTAACAACTCACCTGCCGAATGAACTAG
1704_79_hnedyVillaLaPaz --
F2061 --
1DAI18268
GTCGGAATCCGCTAAGGAGTGTGTAACAACTCACCTGCCGAATGAACTAG
1DAI18540A
GTCGGAATCCGCTAAGGAGTGTGTAACAACTCACCTGCCGAATGAACTAG
Dai17695
GTCGGAATCCGCTAAGGAGTGTGTAACAACTCACCTGCCGAATGAACTAG
LKY18Dai26373 --
Dai17839P_sulphureus
GTCGGAATCCGCTAAGGAGTGTGTAACAACTCACCTGCCGAATGAACTAG
Dai17841P_sulphureus
GTCGGAATCCGCTAAGGAGTGTGTAACAACTCACCTGCCGAATGAACTAG
Dai19877P_roseus
GTCGGAATCCGCTAAGGAGTGTGTAACAACTCACCTGCCGAATGAACTAG
1508_18_1_Kout --
KP859303_R_vinctus_RP185_BRAZI --
JK1807_15Rigidoporus_sp_Puerto --
JV0509_47_J_TN_USA
GTCGGAATCCGCTAAGGAGTGTGTAACAACTCACCTGCCGAATGAACTAG
JV0709_188 --
JV0509_127_PA_USA --
JV1009_59_NJ_USA
GTCGGAATCCGCTAAGGAGTGTGTAACAACTCACCTGCCGAATGAACTAG

Dai15497P_crataegi
GTCGGAATCCGCTAAGGAGTGTGTAACAACTCACCTGCCGAATGAACTAG
Dai15499P_crataegi
GTCGGAATCCGCTAAGGAGTGTGTAACAACTCACCTGCCGAATGAACTAG
Cui3266P_cinereus
GTCGGAATCCGCTAAGGAATGTGTAACAACTCACCTGCCGAATGAACTAG
WCG1256Dai24690
GTCGGAATCCGCTAAGGAATGTGTAACAACTCACCTGCCGAATGAACTAG
1DAI17581
GTCGGAATCCGCTAAGGAATGTGTAACAACTCACCTGCCGAATGAACTAG
WCG1255Dai24688
GTCGGAATCCGCTAAGGAATGTGTAACAACTCACCTGCCGAATGAACTAG
Dai22427
GTCGGAATCCGCTAAGGAATGTGTAACAACTCACCTGCCGAATGAACTAG
MV690Meripilus_concrescens
GTCGGAATCCGCTAAGGAATGTGTAACAACTCACCTGCCGAATGAACTAG
MV513Meripilus_galapagensis
GTCGGAATCCGCTAAGGAATGTGTAACAACTCACCTGCCGAATGAACTAG
Dai19793
GTCGGAATCCGCTAAGGAGTGTGTAACAACTCACCTGCCGAATGAACTAG
OQ553780P_tamilnaduensis --
OQ553779P_tamilnaduensis --
A164FB3Meripilus_giganteus --
JV1407_36_Vinctus_Meandrica --
1807_3K_Rigidoporus_PuertoRico
GTCGGAATCCGCTAAGGAGTGTGTAACAACTCACCTGCCGAATGAACTAG
Cui16903P_vinctus
GTCGGAATCCGCTAAGGAGTGTGTAACAACTCACCTGCCGAATGAACTAG
JV1008_18R_Lineatus --
JV1407_37_1_Vinctus_Carara --
Dai17986P_lineatus
GTCGGAATCCGCTAAGGAGTGTGTAACAACTCACCTGCCGAATGAACTAG
Dai18281
GTCGGAATCCGCTAAGGAGTGTGTAACAACTCACCTGCCGAATGAACTAG
1DAI19796
GTCGGAATCCGCTAAGGAGTGTGTAACAACTCACCTGCCGAATGAACTAG
ZQY797Dai25241
GTCGGAATCCGCTAAGGAGTGTGTAACAACTCACCTGCCGAATGAACTAG
WCG1289Dai24711 --
Dai22598
GTCGGAATCCGCTAAGGAGTGTGTAACAACTCACCTGCCGAATGAACTAG
Dai20523
GTCGGAATCCGCTAAGGAGTGTGTAACAACTCACCTGCCGAATGAACTAG
Dai17885

GTCGGAATCCGCTAAGGAGTGTGTAACAACTCACCTGCCGAATGAACTAG
Dai17553
GTCGGAATCCGCTAAGGAGTGTGTAACAACTCACCTGCCGAATGAACTAG
Dai19639
GTCGGAATCCGCTAAGGAGTGTGTAACAACTCACCTGCCGAATGAACTAG
JV0110_48_CZ --
MJ129_04 --
Cui10340P_eminens --
Cui10341P_eminens
GTCGGAATCCGCTAAGGAGTGTGTAACAACTCACCTGCCGAATGAACTAG
Dai12685P_eminens
GTCGGAATCCGCTAAGGAGTGTGTAACAACTCACCTGCCGAATGAACTAG
Miettinen_13591Rigidoporus_und
GTCGGAATCCGCTAAGGAGTGTGTAACAACTCACCTGCCGAATGAACTAG
Dai20868
GTCGGAATCCGCTAAGGAGTGTGTAACAACTCACCTGCCGAATGAACTAG
Dai20832
GTCGGAATCCGCTAAGGAGTGTGTAACAACTCACCTGCCGAATGAACTAG
Dai11400
GTCGGAATCCGCTAAGGAGTGTGTAACAACTCACCTGCCGAATGAACTAG
Dai22472
GTCGGAATCCGCTAAGGAGTGTGTAACAACTCACCTGCCGAATGAACTAG
1CUI10475
GTCGGAATCCGCTAAGGAGTGTGTAACAACTCACCTGCCGAATGAACTAG
1CUI10491 --
HCFC1095Meripilus_robledoi --
MCW702Meripilus_revolubilis --
1704_83_zluty_HaciendaBaru --
Dai9925P_lavendulus
GTCGGAATCCGCTAAGGAGTGTGTAACAACTCACCTGCCGAATGAACTAG
Dai13587AP_lavendulus
GTCGGAATCCGCTAAGGAGTGTGTAACAACTCACCTGCCGAATGAACTAG
PDD70600P_longicystidius --
Cui16630
GTCGGAATCCGCTAAGGAGTGTGTAACAACTCACCTGCCGAATGAACTAG
FP_135344Meripilus_giganteus
GTCGGAATCCGCTAAGGAGTGTGTAACAACTCACCTGCCGAATGAACTAG
FP_100460_Sp --
CBS421_48Meripilus_giganteus --
Cui9203 --
Cui9202
GTCGGAATCCGCTAAGGAGTGTGTAACAACTCACCTGCCGAATGAACTAG
TUFC100564Japan --
Russell5913Meripilus_sumstinei --

RP215Meripilus_brasiliensis
GTCGGAATCCGCTAAGGAGTGTGTAACAACTCACCTGCCGAATGAACTAG
RP200Meripilus_brasiliensis
GTCGGAATCCGCTAAGGAGTGTGTAACAACTCACCTGCCGAATGAACTAG
JV1712_13J_R_vinctus2_LSUPuert --
Dai10503R_hypobrunneus
GTCGGAATCCGCTAAGGAGTGTGTAACAACTCACCTGCCGAATGAACTAG
Dai10569Rigidoporus_hypobrunne
GTCGGAATCCGCTAAGGAGTGTGTAACAACTCACCTGCCGAATGAACTAG
1DAI19451
GTCGGAATCCGCTAAGGAGTGTGTAACAACTCACCTGCCGAATGAACTAG
CM108bRigidoporus_hypobrunneus --
1CUI16874
GTCGGAATCCGCTAAGGAGTGTGTAACAACTCACCTGCCGAATGAACTAG
FD299Cerrena_unicolor
GTCGGAATCCGCTAAGGAGTGTGTAACAACTCACCTGCCGAATGAACTAG
KHL_GB_Cerrena_uniclor
GTCGGAATCCGCTAAGGAGTGTGTAACAACTCACCTGCCGAATGAACTAG
Dai12892Cerrena_albocinnamomea
GTCGGAATCCGCTAAGGAGTGTGTAACAACTCACCTGCCGAATGAACTAG
Dai12955C_albocinnamomea
GTCGGAATCCGCTAAGGAGTGTGTAACAACTCACCTGCCGAATGAACTAG
SNUm03110102C_aurantiopora --
NIBRFG0000102423C_aurantiopora --
Dai7359Antrodiella_zonata --
F20080702KCM29C_consors --
F20080208LYW10Cerrena_consors --
Dai7821Antrodiella_zonata --
CFMR_DCL04_31Pseudolagarobasid --
VPB197Pseudolagarobasidium_bel --
CBS115543Pseudolagarobasidium_ --
CBS115544 --
Han405Pseudolagarobasidium_bai
GTCGGAATCCGCTAAGGAGTGTGTAACAACTCACCTGCCGAATGAACTAG
Han406Pseudolagarobasidium_bai
GTCGGAATCCGCTAAGGAGTGTGTAACAACTCACCTGCCGAATGAACTAG
MUcc838Spongipellis_delectans --
BRNM686401S_delectans --
OSM_F925S_delectans --
BRNM67093Spongipellis_litschau --
CFMRccFP59199TS_unicolor --
CFMRccFP71791TS_unicolor --
Dai13845P_lischaueri
GTCGGAATCCGCTAAGGAGTGTGTAACAACTCACCTGCCGAATGAACTAG

Dai20266P_lischaueri
GTCGGAATCCGCTAAGGAGTGTGTAACAACTCACCTGCCGAATGAACTAG
CFMR_HHB11240Radulodon_america --
RLG6350Radulodon_americanus --
KY415963Radulodon_erikssonii
GTCGGAATCCGCTAAGGAGTGTGTAACAACTCACCTGCCGAATGAACTAG
HHB9567spRadulodon_casearius
GTCGGAATCCGCTAAGGAGTGTGTAACAACTCACCTGCCGAATGAACTAG
KRT_Iso_26Radulodon_casearius --
CBS126044Radulodon_erikssonii --
He6183YUNNANENSIS
GTCGGAATCCGCTAAGGAGTGTGTAACAACTCACCTGCCGAATGAACTAG
Cui17979YUNNANENSIS
GTCGGAATCCGCTAAGGAGTGTGTAACAACTCACCTGCCGAATGAACTAG
Miettinen2091Junghuhnia_fimbri
GTCGGAATCCGCTAAGGACTGTGTAACAACTCACCTGCCGAATGAACTAG
KHL12316S_tenue
GTCGGAACCCGCTAAGGAGTGTGTAACAACTCACCTGCCGAATGAACTAG
PRM846564S_pachyodon --
SP_Lgt_S_pachyodon --
Ryvarden44669Tyromyces_xuchile
GTCGGAATCCGCTAAGGAGTGTGTAACAACTCACCTGCCGAATGAACTAG
PW17_171sinuosus --
W53Dai12234
GTCGGAATCCGCTAAGGAGTGTGTAACAACTCACCTGCCGAATGAACTAG
HHB4100SpAntella_americana
GTCGGAATCCGCTAAGGAGTGTGTAACAACTCACCTGCCGAATGAACTAG
W3Dai20901spumeus
GTCGGAATCCGCTAAGGAGTGTGTAACAACTCACCTGCCGAATGAACTAG
He6736
GTCGGAATCCGCTAAGGAGTGTGTAACAACTCACCTGCCGAATGAACTAG
BRNM734877S_spumeus --
BRNM712630S_spumeus --
Dai1723Loweomyces_sibiricus --
W54Cui10009
GTCGGAATCCGCTAAGGAGTGTGTAACAACTCACCTGCCGAATGAACTAG
W1Dai20899 --
HHB13445Trametes_ochracea
GTCGGAATCCGCTAAGGAGTGTGTAACAACTCACCTGCCGAATGAACTAG
Dai16222
GTCGGAACCCGCTAAGGAGTGTGTAACAACTCACCTGCCGAATGAACTAG
Dai16240
GTCGGAACCCGCTAAGGAGTGTGTAACAACTCACCTGCCGAATGAACTAG

JV1310_11SanguinolentusCernys
CCCTGAAAATGGATGGCGCTCAAGCGTGCTACCCATACCTCGCCGTCAGT
MJ39_00_SK --
MJ111_04_CZ --
JV1610_BOKYsmrk --
Dai21030 --
Dai20976P_furcatus
CCCTGAAAATGGATGGCGCTCAAGCGTGCTACCCATACCTCGCCGTCAGT
Dai2105
CCCTGAAAATGGATGGCGCTCAAGCGTGCTACCCATACCTCGCCGTCAGT
Dai2544
CCCTGAAAATGGATGGCGCTCAAGCGTGCTACCCATACCTCGCCGTCAGT
Dai11313
CCCTGAAAATGGATGGCGCTCAAGCGTGCTACCCATACCTCGCCGTCAGT
WCG1611Dai26167
CCCTGAAAATGGATGGCGCTCAAGCGTGCTACCCATACCTCGCCGTCAGT
WCG1518Dai25999Physisporinus
CCCTGAAAATGGATGGCGCTCAAGCGTGCTACCCATACCTCGCCGTCAGT
TAA15097
CCCTGAAAATGGATGGCGCTCAAGCGTGCTACCCATACCTCGCCGTCAGT
JV8909_19_CZ --
JV1310_15_P_sanguinolentus2_CZ --
MJ53_02_CZ --
CLZhao21647P_yunnanensis
CCCTGAAAATGGATGGCGCTCAAGCGTGCTACCCATACCTCGCCGTCAGT
CLZhao21583P_yunnanensis
CCCTGAAAATGGATGGCGCTCAAGCGTGCTACCCATACCTCGCCGTCAGT
Dai22272
CCCTGAAAATGGATGGCGCTCAAGCGTGCTACCCATACCTCGCCGTCAGT
Dai22279
CCCTGAAAATGGATGGCGCTCAAGCGTGCTACCCATACCTCGCCGTCAGT
MJ332_94_CZ --
MJ642_94_CZ_Expallescens --
Dai21060P_vinctus
CCCTGAAAATGGATGGCGCTCAAGCGTGCTACCCATACCTCGCCGTCAGT
JV0511_23LRP_pouzarii --
JQ409462_R_pouzarii_PRM899856_ --
JV0308_66_WA --
JV0309_45_WA_USA --
JV0709_83_CA_USA --
Dai21043P_pouzarii
CCCTGAAAATGGATGGCGCTCAAGCGTGCTACCCATACCTCGCCGTCAGT
MJ144_95_CZ --
JV0909_3_CZ --

JV0609_1_K --
Dai20396Physisporinus_castanop
CCCTGAAAATGGATGGCGCTCAAGCGTGCTACCCATACCTCGCCGTCAGT
Dai20397Physisporinus_castanop
CCCTGAAAATGGATGGCGCTCAAGCGTGCTACCCATACCTCGCCGTCAGG
MJ19_09_SK_Abies --
JV0509_40_J_TN_USA_Betula
CCCTGAAAATGGATGGCGCTCAAGCGTGCTACCCATACCTCGCCGTCAGT
JV0808_33crocatus_PA_USAlist --
DLL2009_061P_crocatus --
Dai12800P_subcrocatus
CCCTGAAAATGGATGGCGCTCAAGCGTGCTACCCATACCTCGCCGTCAGT
Dai15917P_subcrocatus
CCCTGAAAATGGATGGCGCTCAAGCGTGCTACCCATACCTCGCCGTCAGT
Cui16620
CCCTGAAAATGGATGGCGCTCAAGCGTGCTACCCATACCTCGCCGTCAGT
HCFC1088Meripilus_stillicidior
CCCTGAAAATGGATGGCGCTCAAGCGTGCTACCCATACCTCGCCGTCAGT
MCW590Meripilus_obsrcurus --
MCW722Meripilus_obsrcurus --
Cui9381P_tibeticus
CCCTGAAAATGGATGGCGCTCAAGCGTGCTACCCATACCTCGCCGTCAGT
Cui9588P_tibeticus
CCCTGAAAATGGATGGCGCTCAAGCGTGCTACCCATACCTCGCCGTCAGT
Va2_Beneschova --
CWU3874_Ukraine_Alnus --
WCG1293Dai24718Physisporinus --
WCG1268Dai24682A
CCCTGAAAATGGATGGCGCTCAAGCGTGCTACCCATACCTCGCCGTCAGT
WCG1269Dai24683A
CCCTGAAAATGGATGGCGCTCAAGCGTGCTACCCATACCTCGCCGTCAGT
WCG1279Dai24694A
CCCTGAAAATGGATGGCGCTCAAGCGTGCTACCCATACCTCGCCGTCAGT
Dai16971
CCCTGAAAATGGATGGCGCTCAAGCGTGCTACCCATACCTCGCCGTCAGT
ZQY1043Dai26696
CCCTGAAAATGGATGGCGCTCAAGCGTGCTACCCATACCTCGCCGTCAGT
Doll880 --
Doll1000 --
1DAI18529
CCCTGAAAATGGATGGCGCTCAAGCGTGCTACCCATACCTCGCCGTCAGT
Dai19535
CCCTGAAAATGGATGGCGCTCAAGCGTGCTACCCATACCTCGCCGTCAGT
1704_79_hnedyVillaLaPaz --

F2061 --
1DAI18268
CCCTGAAAATGGATGGCGCTCAAGCGTGCTACCCATACCTCGCCGTCAGT
1DAI18540A
CCCTGAAAATGGATGGCGCTCAAGCGTGCTACCCATACCTCGCCGTCAGT
Dai17695
CCCTGAAAATGGATGGCGCTCAAGCGTGCTACCCATACCTCGCCGTCAGT
LKY18Dai26373 --
Dai17839P_sulphureus
CCCTGAAAATGGATGGCGCTCAAGCGTGCTACCCATACCTCGCCGTCAGT
Dai17841P_sulphureus
CCCTGAAAATGGATGGCGCTCAAGCGTGCTACCCATACCTCGCCGTCAGT
Dai19877P_roseus
CCCTGAAAATGGATGGCGCTCAAGCGTGCTACCCATACCTCGCCGTCAGT
1508_18_1_Kout --
KP859303_R_vinctus_RP185_BRAZI --
JK1807_15Rigidoporus_sp_Puerto --
JV0509_47_J_TN_USA
CCCTGAAAATGGATGGCGCTCAAGCGTGCTACCCATACCTCGCCGTCAGT
JV0709_188 --
JV0509_127_PA_USA --
JV1009_59_NJ_USA
CCCTGAAAATGGATGGCGCTCAAGCGTGCTACCCATACCTCGCCGTCAGT
Dai15497P_crataegi
CCCTGAAAATGGATGGCGCTCAAGCGTGCTACCCATACCTCGCCGTCAGT
Dai15499P_crataegi
CCCTGAAAATGGATGGCGCTCAAGCGTGCTACCCATACCTCGCCGTCAGT
Cui3266P_cinereus
CCCTGAAAATGGATGGCGCTCAAGCGTGCTACCCATACCTCGCCGTCAGT
WCG1256Dai24690
CCCTGAAAATGGATGGCGCTCAAGCGTGCTACCCATACCTCGCCGTCAGT
1DAI17581
CCCTGAAAATGGATGGCGCTCAAGCGTGCTACCCATACCTCGCCGTCAGT
WCG1255Dai24688
CCCTGAAAATGGATGGCGCTCAAGCGTGCTACCCATACCTCGCCGTCAGT
Dai22427
CCCTGAAAATGGATGGCGCTCAAGCGTGCTACCCATACCTCGCCGTCAGT
MV690Meripilus_concrescens
CCCTGAAAATGGATGGCGCTCAAGCGTGCTACCCATACCTCGCCGTCAGT
MV513Meripilus_galapagensis
CCCTGAAAATGGATGGCGCTCAAGCGTGCTACCCATACCTCGCCGTCAGT
Dai19793
CCCTGAAAATGGATGGCGCTCAAGCGTGCTACCCATACCTCGCCGTCAGT
OQ553780P_tamilnaduensis --

OQ553779P_tamilnaduensis --
A164FB3Meripilus_giganteus --
JV1407_36_Vinctus_Meandrica --
1807_3K_Rigidoporus_PuertoRico
CCCTGAAAATGGATGGCGCTCAAGCGTGCTACCCATACCTCGCCGTCAGT
Cui16903P_vinctus
CCCTGAAAATGGATGGCGCTCAAGCGTGCTACCCATACCTCGCCGTCAGT
JV1008_18R_Lineatus --
JV1407_37_1_Vinctus_Carara --
Dai17986P_lineatus
CCCTGAAAATGGATGGCGCTCAAGCGTGCTACCCATACCTCGCCGTCAGT
Dai18281
CCCTGAAAATGGATGGCGCTCAAGCGTGCTACCCATACCTCGCCGTCAGT
1DAI19796
CCCTGAAAATGGATGGCGCTCAAGCGTGCTACCCATACCTCGCCGTCAGT
ZQY797Dai25241
CCCTGAAAATGGATGGCGCTCAAGCGTGCTACCCATACCTCGCCGTCAGT
WCG1289Dai24711 --
Dai22598
CCCTGAAAATGGATGGCGCTCAAGCGTGCTACCCATACCTCGCCGTCAGT
Dai20523
CCCTGAAAATGGATGGCGCTCAAGCGTGCTACCCATACCTCGCCGTCAGT
Dai17885
CCCTGAAAATGGATGGCGCTCAAGCGTGCTACCCATACCTCGCCGTCAGT
Dai17553
CCCTGAAAATGGATGGCGCTCAAGCGTGCTACCCATACCTCGCCGTCAGT
Dai19639
CCCTGAAAATGGATGGCGCTCAAGCGTGCTACCCATACCTCGCCGTCAGT
JV0110_48_CZ --
MJ129_04 --
Cui10340P_eminens --
Cui10341P_eminens
CCCTGAAAATGGATGGCGCTCAAGCGTGCTACCCATACCTCGCCGTCAGT
Dai12685P_eminens
CCCTGAAAATGGATGGCGCTCAAGCGTGCTACCCATACCTCGCCGTCAGT
Miettinen_13591Rigidoporus_und
CCCTGAAAATGGATGGCGCTCAAGCGTGCTACCCATACCTCGCCGTCAGT
Dai20868
CCCTGAAAATGGATGGCGCTCAAGCGTGCTACCCATACCTCGCCGTCAGT
Dai20832
CCCTGAAAATGGATGGCGCTCAAGCGTGCTACCCATACCTCGCCGTCAGT
Dai11400
CCCTGAAAATGGATGGCGCTCAAGCGTGCTACCCATACCTCGCCGTCAGT
Dai22472

CCCTGAAAATGGATGGCGCTCAAGCGTGCTACCCATACCTCGCCGTCAGT
1CUI10475
CCCTGAAAATGGATGGCGCTCAAGCGTGCTACCCATACCTCGCCGTCAGT
1CUI10491 --
HCFC1095Meripilus_robledoi --
MCW702Meripilus_revolubilis --
1704_83_zluty_HaciendaBaru --
Dai9925P_lavendulus
CCCTGAAAATGGATGGCGCTCAAGCGTGCTACCCATACCTCGCCGTCAGT
Dai13587AP_lavendulus
CCCTGAAAATGGATGGCGCTCAAGCGTGCTACCCATACCTCGCCGTCAGT
PDD70600P_longicystidius --
Cui16630
CCCTGAAAATGGATGGCGCTCAAGCGTGCTACCCATACCTCGCCGTCAGT
FP_135344Meripilus_giganteus
CCCTGAAAATGGATGGCGCTCAAGCGTGCTACCCATACCTCGCCGTCAGC
FP_100460_Sp --
CBS421_48Meripilus_giganteus --
Cui9203 --
Cui9202
CCCTGAAAATGGATGGCGCTCAAGCGTGCTACCCATACCTCGCCGTCAGC
TUFC100564Japan --
Russell5913Meripilus_sumstinei --
RP215Meripilus_brasiliensis
CCCTGAAAATGGATGGCGCTCAAGCGTGCTACCCATACCTCGCCGTCAGT
RP200Meripilus_brasiliensis
CCCTGAAAATGGATGGCGCTCAAGCGTGCTACCCATACCTCGCCGTCAGT
JV1712_13J_R_vinctus2_LSUPuert --
Dai10503R_hypobrunneus
CCCTGAAAATGGATGGCGCTCAAGCGTGTTACCCATACCTCGCCGTTAGT
Dai10569Rigidoporus_hypobrunne
CCCTGAAAATGGATGGCGCTCAAGCGTGTTACCCATACCTCGCCGTTAGT
1DAI19451
CCCTGAAAATGGATGGCGCTCAAGCGTGTTACCCATACCTCGCCGTTAGT
CM108bRigidoporus_hypobrunneus --
1CUI16874
CCCTGAAAATGGATGGCGCTCAAGCGTGTTACCCATACCTCGCCGTTAGT
FD299Cerrena_unicolor
CCCTGAAAATGGATGGCGCTCAAGCGTGTTACCCATACCTCGCCGTTAGT
KHL_GB_Cerrena_uniclor
CCCTGAAAATGGATGGCGCTCAAGCGTGTTACCCATACCTCGCCGTTAGT
Dai12892Cerrena_albocinnamomea
CCCTGAAAATGGATGGCGCTCAAGCGTGTTACCCATACCTCGCCGTTAGT
Dai12955C_albocinnamomea

CCCTGAAAATGGATGGCGCTCAAGCGTGTTACCCATACCTCGCCGTTAGT
SNUm03110102C_aurantiopora --
NIBRFG0000102423C_aurantiopora --
Dai7359Antrodiella_zonata --
F20080702KCM29C_consors --
F20080208LYW10Cerrena_consors --
Dai7821Antrodiella_zonata --
CFMR_DCL04_31Pseudolagarobasid --
VPB197Pseudolagarobasidium_bel --
CBS115543Pseudolagarobasidium_ --
CBS115544 --
Han405Pseudolagarobasidium_bai
CCCTGAAAATGGATGGCGCTCAAGCGTGTTACCCATACCTCGCCGTTAGT
Han406Pseudolagarobasidium_bai
CCCTGAAAATGGATGGCGCTCAAGCGTGTTACCCATACCTCGCCGTTAGT
MUcc838Spongipellis_delectans --
BRNM686401S_delectans --
OSM_F925S_delectans --
BRNM67093Spongipellis_litschau --
CFMRccFP59199TS_unicolor --
CFMRccFP71791TS_unicolor --
Dai13845P_lischaueri
CCCTGAAAATGGATGGCGCTCAAGCGTGTTACCCATACCTCGCCGTTAGT
Dai20266P_lischaueri
CCCTGAAAATGGATGGCGCTCAAGCGTGTTACCCATACCTCGCCGTTAGT
CFMR_HHB11240Radulodon_america --
RLG6350Radulodon_americanus --
KY415963Radulodon_erikssonii
CCCTGAAAATGGATGGCGCTCAAGCGTGCTACCCATACCTCGCCGTTAGT
HHB9567spRadulodon_casearius
CCCTGAAAATGGATGGCGCTCAAGCATGCTACCCATACCTCGCCGTTAGT
KRT_Iso_26Radulodon_casearius --
CBS126044Radulodon_erikssonii --
He6183YUNNANENSIS
CCCTGAAAATGGATGGCGCTCAAGCGTGCTACCCATACCTCGCCGTTAGT
Cui17979YUNNANENSIS
CCCTGAAAATGGATGGCGCTCAAGCGTGCTACCCATACCTCGCCGTTAGT
Miettinen2091Junghuhnia_fimbri
CCCTGAAAATGGATGGCGCTCAAGCGTGTTACCCATACCTCGCCGTCAGT
KHL12316S_tenue
CCCTGAAAATGGATGGCGCTCAAGCGTGTTACCCATACCTCGCCGTCAGT
PRM846564S_pachyodon --
SP_Lgt_S_pachyodon --
Ryvarden44669Tyromyces_xuchile

CCCTGAAAATGGATGGCGCTCAAGCGTGCTACCCATACCTCGCCGTTAGT
PW17_171sinuosus --
W53Dai12234
CCCTGAAAATGGATGGCGCTCAAGCGTGCTACCCATACCTCGCCGTTAGT
HHB4100SpAntella_americana
CCCTGAAAATGGATGGCGCTCAAGCGTGTTACCCATACCTCGCCGTCAGT
W3Dai20901spumeus
CCCTGAAAATGGATGGCGCTCAAGTGTGTTACCCATACCTCGCCGTCAGT
He6736
CCCTGAAAATGGATGGCGCTCAAGTGTGTTACCCATACCTCGCCGTCAGT
BRNM734877S_spumeus --
BRNM712630S_spumeus --
Dai1723Loweomyces_sibiricus --
W54Cui10009
CCCTGAAAATGGATGGCGCTCAAGTGTGTTACCCATACCTCGCCGTCAGT
W1Dai20899 --
HHB13445Trametes_ochracea
CCCTGAAAATGGATGGCGCTCAAGCGTGTTACCCATACCTCGCCGTCAGC
Dai16222
CCCTGAAAATGGATGGCGCTCAAGCGTGCTACCCATACCTCGCCGTCAGT
Dai16240
CCCTGAAAATGGATGGCGCTCAAGCGTGCTACCCATACCTCGCCGTCAGT

JV1310_11SanguinolentusCernys GTTGAAGTGATGCACTGACGAGTAGGCAGGC-
GTGGAGGTCC-GTGATTG
MJ39_00_SK --
MJ111_04_CZ ---GATTG
JV1610_BOKYsmrk ---GATTG
Dai21030 --
Dai20976P_furcatus GTTGAAGTGATGCACTGACGAGTAGGCAGGC-
GTGGAGGTCC-GTGATTG
Dai2105 GTTGAAGTGATGCACTGACGAGTAGGCAGGC-
GTGGAGGTCC-GT-----
Dai2544 GTTGAAGTGATGCACTGACGAGTAGGCAGGC-
GTGGAGGTCC-GT-----
Dai11313 GTTGAAGTGATGCACTGACGAGTAGGCAGGC-
GTGGAGGTCC-GT-----
WCG1611Dai26167 GTTGAAGTGATGCACTGACGAGTAGGCAGGC-
GTGGAGGTCC-GTGATTG
WCG1518Dai25999Physisporinus GTTGAAGTGATGCACTGACGAGTAGGCAGGC-
GTGGAGGTCC-GT-----
TAA15097 GTTGAAGTGATGCACTGACGAGTAGGCAGGC-
GTGGAGGTCC-GT-----
JV8909_19_CZ --

JV1310_15_P_sanguinolentus2_CZ --
MJ53_02_CZ --
CLZhao21647P_yunnanensis GTTGAAGTGATGCACTGACGAGTAGGCAGGC-
GTGGAGGTCC-GT-----
CLZhao21583P_yunnanensis GTTGAAGTGATGCACTGACGAGTAGGCAGGC-
GTGGAGGTCC-GT-----
Dai22272 GTTGAAGTGATGCACTGACGAGTAGGCAGGC-
GTGGAGGTCC-GTGATTG
Dai22279 GTTGAAGTGATGCACTGACGAGTAGGCAGGC-
GTGGAGGTCC-GT-----
MJ332_94_CZ --
MJ642_94_CZ_Expallescens --
Dai21060P_vinctus GTTAAAGTGATGCACTGACGAGTAGGCAGGC-
GTGGAGGTCC-GT-----
JV0511_23LRP_pouzarii ---GATTG
JQ409462_R_pouzarii_PRM899856_ --
JV0308_66_WA ---GATTG
JV0309_45_WA_USA ---GATTG
JV0709_83_CA_USA ---GATTG
Dai21043P_pouzarii GTTGAAGTGATGCACTGACGAGTAGGCAGGC-
GTGGAGGTCC-GTGATTG
MJ144_95_CZ --
JV0909_3_CZ --
JV0609_1_K ---GATTG
Dai20396Physisporinus_castanop GTTGAAGTGATGCACTGACGAGTAGGCAGGC-
GTGGAGGTCC-GTGATTG
Dai20397Physisporinus_castanop GTTGAAGTGATGCACTGACGAGTAGGCAGGC-
GTGGAGGTCC-GTGATTG
MJ19_09_SK_Abies --
JV0509_40_J_TN_USA_Betula GTTGAAGTGATGCACTGACGAGTAGGCAGGC-
GTGGAGGTCC-GTGATTG
JV0808_33crocatus_PA_USAlist ---GATTG
DLL2009_061P_crocatus --
Dai12800P_subcrocatus GTTGAAGTGATGCACTGACGAGTAGGCAGGC-
GTGGAGGTCCTGT-----
Dai15917P_subcrocatus GTTGAAGTGATGCACTGACGAGTAGGCAGGC-
GTGGAGGTCC-GTGATTG
Cui16620 GTTGAAGTGATGCACTGACGAGTAGGCAGGC-
GTGGAGGTCC-GT-----
HCFC1088Meripilus_stillicidior GTKGAAGTGATGCACTGACGAGTAGGCAGGC-
GTGGAGGTCC-GT-----
MCW590Meripilus_obsrcurus --
MCW722Meripilus_obsrcurus --
Cui9381P_tibeticus GTTGAAGTGATGCACTGACGAGTAGGCAGGC-

GTGGAGGTCC-GT-----
Cui9588P_tibeticus GTTGAAGTGATGCACTGACGAGTAGGCAGGC-
GTGGAGGTCC-GTGATTG
Va2_Beneschova ---
CWU3874_Ukraine_Alnus ---
WCG1293Dai24718Physisporinus ---
WCG1268Dai24682A GTTGAAGTGATGCACTGACGAGTAGGCAGGC-
GTGGAGGTCC-GT-----
WCG1269Dai24683A GTTGAAGTGATGCACTGACGAGTAGGCAGGC-
GTGGAGGTCC-GT-----
WCG1279Dai24694A GTTGAAGTGATGCACTGACGAGTAGGCAGGC-
GTGGAGGTCC-GTGATTG
Dai16971 GTTGAAGTGATGCACTGACGAGTAGGCAGGC-
GTGGAGGTCC-GT-----
ZQY1043Dai26696 GTTGAAGTGATGCACTGACGAGTAGGCAGGC-
GTGGAGGTCC-GT-----
Doll880 --GATTG
Doll1000 ---
1DAI18529 GTTGAAGTGATGCACTGACGAGTAGGCAGGC-
GTGGAGGTCC-GT-----
Dai19535 GTTGAAGTGATGCACTGACGAGTAGGCAGGC-
GTGGGGGTCC-GTGATTG
1704_79_hnedyVillaLaPaz --GATTG
F2061 ---
1DAI18268 GTTGAAGTGATGCACTGACGAGTAGGCAGGC-
GTGGAGGTCC-GT-----
1DAI18540A GTTGAAGTGATGCACTGACGAGTAGGCAGGC-
GTGGAGGTCC-GTGATTG
Dai17695 GTTGAAGTGATGCACTGACGAGTAGGCAGGC-
GTGGAGGTCC-GT-----
LKY18Dai26373 ---
Dai17839P_sulphureus GTTGAAGTGATGCACTGACGAGTAGGCAGGC-
GTGGAGGTTT-GTGATTG
Dai17841P_sulphureus GTTGAAGTGATGCACTGACGAGTAGGCAGGC-
GTGGAGGTTT-GTGATTG
Dai19877P_roseus GTTGAAGTGATGCACTGACGAGTAGGCAGGC-
GTGGAGGTCC-GT-----
1508_18_1_Kout ---
KP859303_R_vinctus_RP185_BRAZI ---
JK1807_15Rigidoporus_sp_Puerto ---
JV0509_47_J_TN_USA GTTGAAGTGATACACTGACGAGTAGGCAGGC-
GTGGAGGTCC-GTGATTG
JV0709_188 ---
JV0509_127_PA_USA --GATTG

JV1009_59_NJ_USA GTTGAAGTGATACACTGACGAGTAGGCAGGC-GTGGAGGTCC-GTGATTG
Dai15497P_crataegi GTTGAAGTGATGCACTGACGAGTAGGCAGGC-GTGGAGGTCC-GTGATTG
Dai15499P_crataegi GTTGAAGTGATGCACTGACGAGTAGGCAGGC-GTGGAGGTCC-GTGATTG
Cui3266P_cinereus GTTGAAGTGATGCACTGACGAGTAGGCAGGC-GTGGAGGTCC-GT-----
WCG1256Dai24690 GTTGAAGTGATGCACTGACGAGTAGGCAGGC-GTGGAGGTCC-GTGATTG
1DAI17581 GTTGAAGTGATGCACTGACGAGTAGGCAGGC-GTGGAGGTCC-GT-----
WCG1255Dai24688 GTTGAAGTGATGCACTGACGAGTAGGCAGGC-GTGGAGGTCC-GTGATTG
Dai22427 GTTGAAGTGATGCACTGACGAGTAGGCAGGC-GTGGAGGTCC-GT-----
MV690Meripilus_concrescens GTTGAAGTGATGCACTGACGAGTAGGCAGGC-GT----------------
MV513Meripilus_galapagensis GTTGAAGTGATGCACTGACGAGTAGGCAGGC-GTGGAGGTCC-GT-----
Dai19793 GTTGAAGTGATGCACTGACGAGTAGGCAGGC-GTGGAGGTCC-GTGATTG
OQ553780P_tamilnaduensis --
OQ553779P_tamilnaduensis --
A164FB3Meripilus_giganteus --
JV1407_36_Vinctus_Meandrica ---GATTG
1807_3K_Rigidoporus_PuertoRico GTTAAAGTGATGCACTGACGAGTAGGCAGGC-GTGGAGGTCC-GTGATTG
Cui16903P_vinctus GTTAAAGTGATGCACTGACGAGTAGGCAGGC-GTGGAGGTCC-GTGATTG
JV1008_18R_Lineatus --
JV1407_37_1_Vinctus_Carara ---GATTG
Dai17986P_lineatus GTTTAAGTGATGCACTGACGAGTAGGCAGGC-GTGGAGGTCC-GTGATTG
Dai18281 GTTTAAGTGATGCACTGACGAGTAGGCAGGC-GTGGAGGTCC-GTGATTG
1DAI19796 GTTAAAGTGATGCACTGACGAGTAGGCAGGC-GTGGAGGTCC-GTGATTG
ZQY797Dai25241 GTTAAAGTGATGCACTGACGAGTAGGCAGGC-GTGGAGGTCC-GTGATTG
WCG1289Dai24711 --
Dai22598 GTTAAAGTGATGCACTGACGAGTAGGCAGGC-GTGGAGGTCC-GTGATTG
Dai20523 GTTAAAGTGATGCACTGACGAGTAGGCAGGC-

GTGGAGGTCC-GTGATTG
Dai17885 GTTAAAGTGATGCACTGACGAGTAGGCAGGC-
GTGGAGGTCC-GTGATTG
Dai17553 GTTAAAGTGATGCACTGACGAGTAGGCAGGC-
GTGGAGGTCC-GT-----
Dai19639 GTTAAAGTGATGCACTGACGAGTAGGCAGGC-
GTGGAGGTCC-GTGATTG
JV0110_48_CZ ---GATTG
MJ129_04 --
Cui10340P_eminens ---GATTG
Cui10341P_eminens GTTGAAGTGATGCACTGACGAGTAGGCAGGC-
GTGGAGGTCC-GT-----
Dai12685P_eminens GTTGAAGTGATGCACTGACGAGTAGGCAGGC-
GTGGAGGTCC-GTGATTG
Miettinen_13591Rigidoporus_und GTTGAAGTGATGCACTGACGAGTAGGCAGGC-
GTGGAGGTCC-GT-----
Dai20868 GTTGAAGTGATGCACTGACGAGTAGGCAGGC-
GTGGAGGTCC-GTGATTG
Dai20832 GTTGAAGTGATGCACTGACGAGTAGGCAGGC-
GTGGAGGTCC-GTGATTG
Dai11400 GTTGAAGTGATGCACTGACGAGTAGGCAGGC-
GTGGAGGTCC-GTGATTG
Dai22472 GTTGAAGTGATGCACTGACGAGTAGGCAGGC-
GTGGAGGTCC-GTGATTG
1CUI10475 GTTGAAGTGATGCACTGACGAGTAGGCAGGC-
GTGGAGGTCC-GT-----
1CUI10491 --
HCFC1095Meripilus_robledoi --
MCW702Meripilus_revolubilis --
1704_83_zluty_HaciendaBaru --
Dai9925P_lavendulus GTTGAAGTGATGCACTGACGAGTAGGCAGGC-
GTGGAGGTCA-GT-----
Dai13587AP_lavendulus GTTGAAGTGATGCACTGACGAGTAGGCAGGC-
GTGGAGGTCA-GTGATTG
PDD70600P_longicystidius --
Cui16630 GTTGAAGTGACGCACTGACGAGTAGGCAGGC-
GTGGAGGTCA-GT-----
FP_135344Meripilus_giganteus GTTGAAGTGACGCGCTGACGAGTAGGCAGGC-
GTGGAGGTCC-GT-----
FP_100460_Sp --
CBS421_48Meripilus_giganteus --
Cui9203 --
Cui9202 GTTGAAGTGACGCGCTGACGAGTAGGCAGGC-
GTGGAGGTCC-GT-----

TUFC100564Japan --

Russell5913Meripilus_sumstinei --

RP215Meripilus_brasiliensis GTTGAAGTGACGCACTGACGAGTAGGCAGGC-GTGGAGGTCC-GT-----

RP200Meripilus_brasiliensis GTTGAAGTGACGCACTGACGAGTAGGCAGGC-GTGGAGGTCC-GT-----

JV1712_13J_R_vinctus2_LSUPuert --

Dai10503R_hypobrunneus GTTGAAGTGATGCACTAACGAGTAGGCAGGC-GTGGAGGTTT-GT-----

Dai10569Rigidoporus_hypobrunne GTTGAAGTGATGCACTAACGAGTAGGCAGGC-GTGGAGGTTT-GT-----

1DAI19451 GTTGAAGTGATGCACTAACGAGTAGGCAGGC-GTGGAGGT----------

CM108bRigidoporus_hypobrunneus --

1CUI16874 GTTGAAGTGATGCACTAACGAGTAGGCAGGC-GTGGAGGTTT-GT-----

FD299Cerrena_unicolor GTTGAAGTGATGCACTAACGAGTAGGCAGGC-GTGGAGGTTT-GT-----

KHL_GB_Cerrena_uniclor GTGAACGTGATGCACTAACGAGTAGGCAGGCGGTGGAGG-----------

Dai12892Cerrena_albocinnamomea GTTGAAGTGATGCACTAACGAGTAGGCAGGC-GTGGAGGTTT-GT-----

Dai12955C_albocinnamomea GTTGAAGTGATGCACTAACGAGTAGGCAGGC-GTGGAGGTTT-GT-----

SNUm03110102C_aurantiopora --

NIBRFG0000102423C_aurantiopora --

Dai7359Antrodiella_zonata --

F20080702KCM29C_consors --

F20080208LYW10Cerrena_consors --

Dai7821Antrodiella_zonata --

CFMR_DCL04_31Pseudolagarobasid --

VPB197Pseudolagarobasidium_bel --

CBS115543Pseudolagarobasidium_ --

CBS115544 --

Han405Pseudolagarobasidium_bai GTTGAAGTGATGCACTAACGAGTAGGCAGGC-GTGGAGGTTT-GT-----

Han406Pseudolagarobasidium_bai GTTGAAGTGATGCACTAACGAGTAGGCAGGC-GTGGAGGTTT-GT-----

MUcc838Spongipellis_delectans --

BRNM686401S_delectans --

OSM_F925S_delectans --

BRNM67093Spongipellis_litschau --

CFMRccFP59199TS_unicolor --

CFMRccFP71791TS_unicolor --

Dai13845P_lischaueri GTTGAAGTGAAGCACTAACGAGTAGGCAGGC-GTGGAGGTTT-GTGATTG
Dai20266P_lischaueri GTTGAAGTGAAGCACTAACGAGTAGGCAGGC-GTGGAGGT----------
CFMR_HHB11240Radulodon_america ---
RLG6350Radulodon_americanus ---
KY415963Radulodon_erikssonii GTTGAAGTGACGCACTAACGAGTAGGCAGGC-GTGGAGGTTT-GT-----
HHB9567spRadulodon_casearius GTTGAAGTGACGCACTAACGAGTAGGCAGGC-GTGGAGGTTT-GT-----
KRT_Iso_26Radulodon_casearius ---
CBS126044Radulodon_erikssonii ---
He6183YUNNANENSIS GTTGAAGTGATGCACTAACGAGTAGGCAGGC-GTGGAGGTTT-GTGACTG
Cui17979YUNNANENSIS GTTGAAGTGATGCACTAACGAGTAGGCAGGC-GTGGAGGTTT-GTGACTG
Miettinen2091Junghuhnia_fimbri GTTGAAGTGACACACTGACGAGTAGGCAGGC-GTGGAGGTTT-GT-----
KHL12316S_tenue GTTGAAGTGATACACTGACGAGTAGGCAGGC-GTG---------------
PRM846564S_pachyodon ---
SP_Lgt_S_pachyodon ---
Ryvarden44669Tyromyces_xuchile GTTAAAGTGATGCACTAACGAGTAGGCAGGC-GTGGAGGTTT-GT-----
PW17_171sinuosus ---
W53Dai12234 GTTAAAGTGATGCACTAACGAGTAGGCAGGC-GTGGAGGTTT-GTGACTG
HHB4100SpAntella_americana GTTGAAGTGACACACTGACGAGTAGGCAGGC-GTGGAGGTTT-GT-----
W3Dai20901spumeus GTTTAAGTGATGCACTGACGAGTAGGCAGGC-GTGGAGGTCA-GTGGATG
He6736 GTTTAAGTGATGCACTGACGAGTAGGCAGGC-GTGGAGGTCA-GT-----
BRNM734877S_spumeus ---
BRNM712630S_spumeus ---
Dai1723Loweomyces_sibiricus ---
W54Cui10009 GTTTAAGTGATGCACTGACGAGTAGGCAGGC-GTGGAGGTCA-GTGATTG
W1Dai20899 --GATTG
HHB13445Trametes_ochracea GTTTAAGTGACGCGCTGACGAGTAGGCAGGC-GTGGAGGTCA-GT-----
Dai16222 GTTGAAGTGACACACTGACGAGTAGGCAGGC-GTGGAGGT----------
Dai16240 GTTGAAGTGACACACTGACGAGTAGGCAGGC-

GTGGAGGT---------

JV1310_11SanguinolentusCernys CGCTATTCTCATCAT-
CGCCGCTGGTACTGGTGAGTTCGAGGCCGGTATC
MJ39_00_SK --
MJ111_04_CZ CGCTATTCTCATCAT-
CGCCGCTGGTACTGGTGAGTTCGAGGCCGGTATC
JV1610_BOKYsmrk CGCTATTCTCATCAT-
CGCCGCTGGTACTGGTGAGTTCGAGGCCGGTATC
Dai21030 --
Dai20976P_furcatus CGCTATTCTCATCAT-
CGCCGCTGGTACTGGTGAGTTCGAGGCCGGTATC
Dai2105 --
Dai2544 --
Dai11313 --
WCG1611Dai26167 CGCTATCCTCATCAT-
CGCCGCTGGTACTGGTGAGTTCGAGGCCGGTATA
WCG1518Dai25999Physisporinus --
TAA15097 --
JV8909_19_CZ --
JV1310_15_P_sanguinolentus2_CZ --
MJ53_02_CZ --
CLZhao21647P_yunnanensis --
CLZhao21583P_yunnanensis --
Dai22272 CGCTATTCTCATCAT-
CGCCGCTGGTACTGGTGAGTTCGAGGAAGGAATT
Dai22279 --
MJ332_94_CZ --
MJ642_94_CZ_Expallescens --
Dai21060P_vinctus --
JV0511_23LRP_pouzarii CGCTATCCTCATCAT-
CGCCGCTGGTACTGGTGAGTTCGAGGCCGGTATC
JQ409462_R_pouzarii_PRM899856_ --
JV0308_66_WA CGCTATCCTCATCAT-
CGCCGCTGGTACTGGTGAGTTCGAGGCCGGTATC
JV0309_45_WA_USA CGCTATCCTCATCAT-
CGCCGCTGGTACTGGTGAGTTCGAGGCCGGTATC
JV0709_83_CA_USA CGCTATCCTCATCAT-
CGCCGCTGGTACTGGTGAGTTCGAGGCCGGTATC
Dai21043P_pouzarii CGCTATCCTCATCAT-
CGCCGCTGGTACTGGTGAGTTCGAGGCCGGTATC
MJ144_95_CZ --
JV0909_3_CZ --
JV0609_1_K CGCTATCCTCATCAT-

CGCCGCTGGTACTGGTGAGTTCGAGGCCGGTATC
Dai20396Physisporinus_castanop CGCTATCCTCATCAT-
CGCCGCTGGTACTGGTGAGTTCGAGGCCGGTATC
Dai20397Physisporinus_castanop CGCTATCCTCATCAT-
CGCCGCTGGTACTGGTGAGTTCGAGGCCGGTATC
MJ19_09_SK_Abies --
JV0509_40_J_TN_USA_Betula CGCTATCCTAATCAT-
CGCCGCTGGTACTGGTGAGTTCGAGGCCGGTATC
JV0808_33crocatus_PA_USAlist CGCTATCCTAATCAT-
CGCCGCTGGTACTGGTGAGTTCGAGGCCGGTATC
DLL2009_061P_crocatus --
Dai12800P_subcrocatus --
Dai15917P_subcrocatus CGCTATCCTAATCAT-
CGCCGCTGGTACTGGTGAGTTCGAGGCCGGTATC
Cui16620 --
HCFC1088Meripilus_stillicidior --
MCW590Meripilus_obsrcurus --
MCW722Meripilus_obsrcurus --
Cui9381P_tibeticus --
Cui9588P_tibeticus CGCTATCCTCATCAT-
CGCCGCTGGTACTGGTGAGTTCGAGGCCGGTATC
Va2_Beneschova --
CWU3874_Ukraine_Alnus --
WCG1293Dai24718Physisporinus --
WCG1268Dai24682A --
WCG1269Dai24683A --
WCG1279Dai24694A CGCTATCCTCATCAT-
TGCCGCTGGTACTGGTGAGTTCGAGGCCGGTATC
Dai16971 --
ZQY1043Dai26696 --
Doll880 CGCTATCCTCATCAT-
CGCCGCTGGTACTGGTGAGTTCGAGGCCGGTATC
Doll1000 --
1DAI18529 --
Dai19535 CGCTATCCTCATCAT-
CGCCGCTGGTACTGGTGAGTTCGAGGCGGGTATC
1704_79_hnedyVillaLaPaz CGCTATCCTCATCAT-
CGCCGCTGGTACTGGTGAGTTCGAGGCCGGTATC
F2061 --
1DAI18268 --
1DAI18540A CGCTATCCTCATCAT-
CGCCGCTGGTACTGGTGAGTTCGAGGCCGGTATC
Dai17695 --
LKY18Dai26373 --

Dai17839P_sulphureus CGCTATCCTCATCAT-CGCCGCTGGTACTGGTGAGTTCGAGGCCGGTATC
Dai17841P_sulphureus CGCTATCCTCATCAT-CGCCGCTGGTACTGGTGAGTTCGAGGCCGGTATC
Dai19877P_roseus --
1508_18_1_Kout --
KP859303_R_vinctus_RP185_BRAZI --
JK1807_15Rigidoporus_sp_Puerto --
JV0509_47_J_TN_USA CGCTATCCTCATTAT-CGCTGCTGGTACTGGTGAGTTCGAAGCGGGTATC
JV0709_188 --
JV0509_127_PA_USA CGCTATCCTCATTAT-CGCTGCTGGTACTGGTGAGTTCGAAGCGGGTATC
JV1009_59_NJ_USA CGCTATCCTCATTAT-CGCTGCTGGTACTGGTGAGTTCGAAGCGGGTATC
Dai15497P_crataegi CGCTATCCTCATCAT-CGCCGCTGGTACTGGTGAGTTCGAAGCCGGTATC
Dai15499P_crataegi CGCTATCCTCATCAT-CGCCGCTGGTACTGGTGAGTTCGAAGCCGGTATC
Cui3266P_cinereus --
WCG1256Dai24690 CGCTATTCTCATCAT-CGCCGCTGGTACCGGTGAGTTCGAAGCTGGTATC
1DAI17581 --
WCG1255Dai24688 CGCTATTCTCATCAT-CGCCGCTGGTACCGGTGAGTTCGAAGCTGGTATC
Dai22427 --
MV690Meripilus_concrescens --
MV513Meripilus_galapagensis --
Dai19793 CGCTATTCTCATCAT-CGCCGCTGGTACTGGTGAGTTCGAAGCCGGTATC
OQ553780P_tamilnaduensis --
OQ553779P_tamilnaduensis --
A164FB3Meripilus_giganteus --
JV1407_36_Vinctus_Meandrica CGCTATCCTCATCAT-CGCCGCTGGTACTGGTGAGTTCGAAGCTGGTATC
1807_3K_Rigidoporus_PuertoRico CGCTATCCTCATCAT-CGCCGCTGGTACTGGTGAGTTCGAAGCTGGTATC
Cui16903P_vinctus CGCTATCCTCATCAT-CGCCGCTGGTACTGGTGAGTTCGAAGCTGGTATC
JV1008_18R_Lineatus --
JV1407_37_1_Vinctus_Carara CGCTATCCTCATCAT-CGCCGCTGGTACTGGTGAGTTCGAAGCTGGTATC
Dai17986P_lineatus CGCTATTCTCATCAT-CGCCGCTGGTACTGGTGAGTTCGAAGCCGGTATC

Dai18281 CGCTATTCTCATCAT-
CGCCGCTGGTACTGGTGAGTTCGAAGCCGGTATC
1DAI19796 CGCTATCCTCATCAT-
CGCCGCTGGTACTGGTGAGTTCGAAGCCGGTATC
ZQY797Dai25241 CGCTATCCTCATCAT-
CGCCGCTGGTACTGGTGAGTTCGAAGCCGGTATC
WCG1289Dai24711 --
Dai22598 CGCTATCCTCATCAT-
CGCCGCTGGTACTGGTGAGTTCGAAGCCGGTATC
Dai20523
CGCTATCCTCATCATAAGCCGCTGGTACTGGTGAGTTCGAAGCCGGTATC
Dai17885 CGCTATCCTCATCAT-
CGCCGCTGGTACTGGTGAGTTCGAAGCCGGTATC
Dai17553 --
Dai19639 CGCTATCCTCATCAT-
CGCCGCTGGTACTGGTGAGTTCGAAGCCGGTATC
JV0110_48_CZ CGCTGTTCTTATCAT-
CGCCGCTGGTACCGGAGAGTTCGAGGCCGGTATC
MJ129_04 --
Cui10340P_eminens CGCTGTTCTTATCAT-
CGCCGCTGGTACTGGAGAGTTCGAGGCCGGTATC
Cui10341P_eminens --
Dai12685P_eminens CGCTGTTCTTATCAT-
CGCCGCTGGTACCGGAGAGTTCGAGGCCGGTATC
Miettinen_13591Rigidoporus_und --
Dai20868 CGCTATCCTCATCAT-
CGCCGCTGGTACTGGTGAGTTCGAGGCCGGTATC
Dai20832 CGCTATCCTCATCAT-
CGCCGCTGGTACTGGTGAGTTCGAGGCCGGTATC
Dai11400 CGCTATCCTCATCAT-
TGCCGCTGGTACTGGTGAGTTCGAGGCCGGTATC
Dai22472 CGCTATCCTCATCAT-
CGCCGCTGGTACTGGTGAGTTCGAGGCCGGTATC
1CUI10475 --
1CUI10491 --
HCFC1095Meripilus_robledoi --
MCW702Meripilus_revolubilis --
1704_83_zluty_HaciendaBaru --
Dai9925P_lavendulus --
Dai13587AP_lavendulus CGCTATTCTCATCAT-
TGCCGCTGGTACTGGTGAGTTCGAAGCCGGTATC
PDD70600P_longicystidius --
Cui16630 --
FP_135344Meripilus_giganteus --

FP_100460_Sp ---
CBS421_48Meripilus_giganteus ---
Cui9203 ---
Cui9202 ---
TUFC100564Japan ---
Russell5913Meripilus_sumstinei ---
RP215Meripilus_brasiliensis ---
RP200Meripilus_brasiliensis ---
JV1712_13J_R_vinctus2_LSUPuert ---
Dai10503R_hypobrunneus ---
Dai10569Rigidoporus_hypobrunne ---
1DAI19451 ---
CM108bRigidoporus_hypobrunneus ---
1CUI16874 ---
FD299Cerrena_unicolor ---
KHL_GB_Cerrena_uniclor ---
Dai12892Cerrena_albocinnamomea ---
Dai12955C_albocinnamomea ---
SNUm03110102C_aurantiopora ---
NIBRFG0000102423C_aurantiopora ---
Dai7359Antrodiella_zonata ---
F20080702KCM29C_consors ---
F20080208LYW10Cerrena_consors ---
Dai7821Antrodiella_zonata ---
CFMR_DCL04_31Pseudolagarobasid ---
VPB197Pseudolagarobasidium_bel ---
CBS115543Pseudolagarobasidium_ ---
CBS115544 ---
Han405Pseudolagarobasidium_bai ---
Han406Pseudolagarobasidium_bai ---
MUcc838Spongipellis_delectans ---
BRNM686401S_delectans ---
OSM_F925S_delectans ---
BRNM67093Spongipellis_litschau ---
CFMRccFP59199TS_unicolor ---
CFMRccFP71791TS_unicolor ---
Dai13845P_lischaueri CGCTATTCTCATCAT-
TGCTGCCGGTACTGGTGAGTTCGAGGCCGGAATT
Dai20266P_lischaueri ---
CFMR_HHB11240Radulodon_america ---
RLG6350Radulodon_americanus ---
KY415963Radulodon_erikssonii ---
HHB9567spRadulodon_casearius ---
KRT_Iso_26Radulodon_casearius ---

CBS126044Radulodon_erikssonii ---
He6183YUNNANENSIS CGCTATCCTCATCAT-
CGCCGCCGGTACTGGTGAGTTCGAGGCCGGTATC
Cui17979YUNNANENSIS CGCTATCCTCATCAT-
CGCCGCCGGTACTGGTGAGTTCGAGGCCGGTATC
Miettinen2091Junghuhnia_fimbri ---
KHL12316S_tenue ---
PRM846564S_pachyodon ---
SP_Lgt_S_pachyodon ---
Ryvarden44669Tyromyces_xuchile ---
PW17_171sinuosus ---
W53Dai12234 CGCTATCCTTATCAT-
CGCTGCCGGTACTGGTGAGTTCGAGGCTGGTATC
HHB4100SpAntella_americana ---
W3Dai20901spumeus TGCTATTCTCGTCAT-
CGCAGCCGGTACTGGTGAGTTCGAGGCTGGAATC
He6736 ---
BRNM734877S_spumeus ---
BRNM712630S_spumeus ---
Dai1723Loweomyces_sibiricus ---
W54Cui10009 TGCTATTCTCATCAT-
CGCAGCTGGTACTGGTGAGTTCGAGGCTGGAATT
W1Dai20899 TGCTATTCTCATCAT-
CGCAGCTGGTACTGGTGAGTTCGAGGCTGGAATT
HHB13445Trametes_ochracea ---
Dai16222 ---
Dai16240 ---

JV1310_11SanguinolentusCernys
TCCAAGGATGGTCAGACTCGCGAGCACGCCCTCCTTGCCTTCACCCTTGG
MJ39_00_SK ---
MJ111_04_CZ
TCCAAGGATGGTCAGACTCGCGAGCACGCCCTCCTTGCCTTCACCCTTGG
JV1610_BOKYsmrk
TCCAAGGATGGTCAGACTCGCGAGCACGCCCTCCTTGCCTTCACCCTTGG
Dai21030 ---
Dai20976P_furcatus
TCCAAGGATGGTCAGACTCGCGAGCACGCCCTCCTTGCCTTCACCCTTGG
Dai2105 ---
Dai2544 ---
Dai11313 ---
WCG1611Dai26167
TCCAAGGATGGTCAGACTCGCGAGCACGCCCTCCTTGCCTTCACCCTTGG
WCG1518Dai25999Physisporinus ---

TAA15097 --
JV8909_19_CZ --
JV1310_15_P_sanguinolentus2_CZ --
MJ53_02_CZ --
CLZhao21647P_yunnanensis --
CLZhao21583P_yunnanensis --
Dai22272
TCCAAGGATGGTCAGACTCGCGAGCACGCCCTCCTTGCCTTCACCCTTGG
Dai22279 --
MJ332_94_CZ --
MJ642_94_CZ_Expallescens --
Dai21060P_vinctus --
JV0511_23LRP_pouzarii
TCCAAGGACGGTCAAACTCGCGAGCACGCTCTCCTTGCCTTCACTCTCGG
JQ409462_R_pouzarii_PRM899856_ --
JV0308_66_WA
TCCAAGGACGGTCAAACTCGCGAGCACGCCCTCCTTGCCTTCACTCTTGG
JV0309_45_WA_USA
TCCAAGGACGGTCAAACTCGCGAGCACGCCCTCCTTGCCTTCACTCTTGG
JV0709_83_CA_USA
TCCAAGGACGGTCAAACTCGCGAGCACGCCCTCCTTGCCTTCACTCTTGG
Dai21043P_pouzarii
TCCAAGGACGGTCAAACTCGCGAGCACGCTCTCCTTGCCTTCACTCTCGG
MJ144_95_CZ --
JV0909_3_CZ --
JV0609_1_K
TCCAAGGATGGTCAGACTCGCGAGCACGCTCTCCTTGCCTTCACCCTCGG
Dai20396Physisporinus_castanop
TCCAAGGACGGTCAGACTCGCGAGCACGCTCTCCTTGCCTTCACCCTCGG
Dai20397Physisporinus_castanop
TCCAAGGACGGTCAGACTCGCGAGCACGCTCTCCTTGCCTTCACCCTCGG
MJ19_09_SK_Abies --
JV0509_40_J_TN_USA_Betula
TCCAAGGACGGCCAAACTCGCGAGCATGCTCTCCTTGCCTTCACTCTTGG
JV0808_33crocatus_PA_USAlist
TCCAAGGACGGTCAAACTCGCGAGCATGCTCTCCTTGCCTTCACTCTTGG
DLL2009_061P_crocatus --
Dai12800P_subcrocatus --
Dai15917P_subcrocatus
TCCAAGGACGGTCAAACTCGCGAGCATGCTCTCCTTGCCTTCACTCTTGG
Cui16620 --
HCFC1088Meripilus_stillicidior --
MCW590Meripilus_obsrcurus --
MCW722Meripilus_obsrcurus --

Cui9381P_tibeticus --
Cui9588P_tibeticus
TCCAAGGACGGTCAAACTCGCGAGCACGCCCTCCTTGCCTTCACTCTTGG
Va2_Beneschova --
CWU3874_Ukraine_Alnus --
WCG1293Dai24718Physisporinus --
WCG1268Dai24682A --
WCG1269Dai24683A --
WCG1279Dai24694A
TCCAAGGACGGTCAGACTCGCGAGCACGCCCTCCTTGCTTTCACTCTCGG
Dai16971 --
ZQY1043Dai26696 --
Doll880
TCCAAGGACGGTCAGACTCGCGAGCACGCTCTCCTTGCCTTCACTCTTGG
Doll1000 --
1DAI18529 --
Dai19535
TCCAAGGACGGTCAAACTCGCGAGCACGCCCTCCTTGCCTTCACTCTTGG
1704_79_hnedyVillaLaPaz
TCCAAGGACGGTCAGACTCGCGAGCATGCTCTCCTTGCCTTCACCCTCGG
F2061 --
1DAI18268 --
1DAI18540A
TCCAAGGACGGTCAGACTCGCGAGCACGCTCTCCTTGCCTTCACCCTCGG
Dai17695 --
LKY18Dai26373 --
Dai17839P_sulphureus
TCCAAGGATGGTCAGACCCGCGAGCACGCTCTCCTCGCCTTCACCCTTGG
Dai17841P_sulphureus
TCCAAGGATGGTCAGACCCGCGAGCACGCTCTCCTCGCCTTCACCCTTGG
Dai19877P_roseus --
1508_18_1_Kout --
KP859303_R_vinctus_RP185_BRAZI --
JK1807_15Rigidoporus_sp_Puerto --
JV0509_47_J_TN_USA
TCCAAGGACGGTCAGACTCGCGAGCACGCCCTCCTTGCCTTCACCCTCGG
JV0709_188 --
JV0509_127_PA_USA
TCCAAGGACGGTCAGACTCGCGAGCACGCCCTCCTTGCCTTCACCCTCGG
JV1009_59_NJ_USA
TCCAAGGACGGTCAGACTCGCGAGCACGCCCTCCTTGCCTTCACCCTCGG
Dai15497P_crataegi
TCAAAGGACGGCCAGACTCGCGAGCACGCTCTCCTTGCCTTCACCCTTGG
Dai15499P_crataegi

TCAAAGGACGGCCAGACTCGCGAGCACGCTCTCCTTGCCTTCACCCTTGG
Cui3266P_cinereus --
WCG1256Dai24690
TCCAAGGATGGTCAGACTCGCGAGCACGCTCTCCTCGCCTTCACCCTCGG
1DAI17581 --
WCG1255Dai24688
TCCAAGGATGGTCAGACTCGCGAGCACGCTCTCCTCGCCTTCACCCTCGG
Dai22427 --
MV690Meripilus_concrescens --
MV513Meripilus_galapagensis --
Dai19793
TCCAAGGATGGTCAGACTCGCGAGCACGCTCTCCTTGCCTTCACCCTCGG
OQ553780P_tamilnaduensis --
OQ553779P_tamilnaduensis --
A164FB3Meripilus_giganteus --
JV1407_36_Vinctus_Meandrica
TCCAAGGACGGTCAAACCCGAGAGCACGCTCTCCTTGCCTTCACCCTTGG
1807_3K_Rigidoporus_PuertoRico
TCCAAGGACGGTCAAACCCGAGAGCACGCTCTCCTTGCCTTCACCCTTGG
Cui16903P_vinctus
TCCAAGGACGGTCAAACCCGAGAGCACGCTCTCCTTGCCTTCACCCTTGG
JV1008_18R_Lineatus --
JV1407_37_1_Vinctus_Carara
TCCAAGGACGGCCAAACCCGAGAGCACGCTCTCCTTGCCTTCACCCTCGG
Dai17986P_lineatus
TCCAAGGACGGCCAAACCCGAGAGCACGCTCTCCTTGCCTTCACCCTCGG
Dai18281
TCCAAGGACGGCCAAACCCGAGAGCACGCTCTCCTTGCCTTCACCCTCGG
1DAI19796
TCCAAGGACGGCCAAACCCGAGAGCACGCTCTCCTTGCCTTCACCCTCGG
ZQY797Dai25241
TCCAAGGACGGCCAAACCCGAGAGCACGCTCTCCTTGCCTTCACCCTCGG
WCG1289Dai24711 --
Dai22598
TCCAAGGACGGCCAAACCCGAGAGCACGCTCTCCTTGCCTTCACCCTCGG
Dai20523
TCCAAGGACGGCCAAACCCGAGAGCACGCTCTCCTTGCCTTCACCCTCGG
Dai17885
TCCAAGGACGGCCAAACCCGAGAGCACGCTCTCCTTGCCTTCACCCTCGG
Dai17553 --
Dai19639
TCCAAGGACGGCCAAACCCGAGAGCACGCTCTCCTTGCCTTCACCCTCGG
JV0110_48_CZ
TCCAAGGACGGTCAAACTCGCGAGCACGCTCTCCTTGCGTTCACTCTTGG

MJ129_04 --
Cui10340P_eminens
TCCAAGGACGGTCAAACTCGCGAGCACGCTCTCCTTGCATTCACTCTTGG
Cui10341P_eminens --
Dai12685P_eminens
TCCAAGGACGGTCAAACTCGCGAGCACGCTCTCCTTGCGTTCACTCTTGG
Miettinen_13591Rigidoporus_und --
Dai20868
TCAAAGGATGGTCAAACTCGCGAGCACGCCCTCCTTGCCTTCACTCTTGG
Dai20832
TCAAAGGATGGTCAAACTCGCGAGCACGCCCTCCTTGCCTTCACTCTTGG
Dai11400
TCAAAGGATGGTCAAACTCGCGAGCACGCCCTCCTTGCCTTCACTCTTGG
Dai22472
TCAAAGGATGGTCAAACTCGCGAGCACGCCCTCCTTGCCTTCACTCTTGG
1CUI10475 --
1CUI10491 --
HCFC1095Meripilus_robledoi --
MCW702Meripilus_revolubilis --
1704_83_zluty_HaciendaBaru --
Dai9925P_lavendulus --
Dai13587AP_lavendulus
TCCAAGGACGGTCAGACTCGCGAGCACGCCCTCCTCGCCTTCACTCTCGG
PDD70600P_longicystidius --
Cui16630 --
FP_135344Meripilus_giganteus --
FP_100460_Sp --
CBS421_48Meripilus_giganteus --
Cui9203 --
Cui9202 --
TUFC100564Japan --
Russell5913Meripilus_sumstinei --
RP215Meripilus_brasiliensis --
RP200Meripilus_brasiliensis --
JV1712_13J_R_vinctus2_LSUPuert --
Dai10503R_hypobrunneus --
Dai10569Rigidoporus_hypobrunne --
1DAI19451 --
CM108bRigidoporus_hypobrunneus --
1CUI16874 --
FD299Cerrena_unicolor --
KHL_GB_Cerrena_uniclor --
Dai12892Cerrena_albocinnamomea --
Dai12955C_albocinnamomea --

SNUm03110102C_aurantiopora --
NIBRFG0000102423C_aurantiopora --
Dai7359Antrodiella_zonata --
F20080702KCM29C_consors --
F20080208LYW10Cerrena_consors --
Dai7821Antrodiella_zonata --
CFMR_DCL04_31Pseudolagarobasid --
VPB197Pseudolagarobasidium_bel --
CBS115543Pseudolagarobasidium_ --
CBS115544 --
Han405Pseudolagarobasidium_bai --
Han406Pseudolagarobasidium_bai --
MUcc838Spongipellis_delectans --
BRNM686401S_delectans --
OSM_F925S_delectans --
BRNM67093Spongipellis_litschau --
CFMRccFP59199TS_unicolor --
CFMRccFP71791TS_unicolor --
Dai13845P_lischaueri
TCCAAGGATGGTCAGACTCGCGAGCACGCACTCCTTGCCTTCACCCTCGG
Dai20266P_lischaueri --
CFMR_HHB11240Radulodon_america --
RLG6350Radulodon_americanus --
KY415963Radulodon_erikssonii --
HHB9567spRadulodon_casearius --
KRT_Iso_26Radulodon_casearius --
CBS126044Radulodon_erikssonii --
He6183YUNNANENSIS
TCCAAGGACGGTCAGACTCGCGAGCACGCCCTTCTTGCCTTCACCCTCGG
Cui17979YUNNANENSIS
TCCAAGGACGGTCAAACTCGCGAGCACGCCCTTCTTGCCTTCACCCTCGG
Miettinen2091Junghuhnia_fimbri --
KHL12316S_tenue --
PRM846564S_pachyodon --
SP_Lgt_S_pachyodon --
Ryvarden44669Tyromyces_xuchile --
PW17_171sinuosus --
W53Dai12234
TCCAAGGATGGCCAGACCCGAGAGCACGCCCTCCTTGCCTTCACCCTCGG
HHB4100SpAntella_americana --
W3Dai20901spumeus
TCCAAGGACGGTCAAACTCGCGAGCATGCCCTCCTTGCATTCACTCTTGG
He6736 --
BRNM734877S_spumeus --

BRNM712630S_spumeus --
Dai1723Loweomyces_sibiricus --
W54Cui10009
TCCAAGGACGGTCAAACTCGCGAGCATGCCCTCCTTGCGTTCACTCTTGG
W1Dai20899
TCCAAGGACGGTCAAACTCGCGAGCATGCCCTCCTTGCGTTCACTCTTGG
HHB13445Trametes_ochracea --
Dai16222 --
Dai16240 --

JV1310_11SanguinolentusCernys
TGTCAGGCAACTTATTGTTGCCATCAACAAGATGGACACTACCAAGGTAT
MJ39_00_SK --
MJ111_04_CZ
TGTCAGGCAACTTATTGTTGCCATCAACAAGATGGACACTACCAAGGTAT
JV1610_BOKYsmrk
TGTCAGGCAACTTATTGTTGCCATCAACAAGATGGACACTACCAAGGTAT
Dai21030 --
Dai20976P_furcatus
TGTCAGGCAACTTATTGTTGCCATCAACAAGATGGACACCACCAAGGTAT
Dai2105 --
Dai2544 --
Dai11313 --
WCG1611Dai26167
AGTCAGGCAACTTATCGTTGCCATCAACAAGATGGACACTACCAAGGTAT
WCG1518Dai25999Physisporinus --
TAA15097 --
JV8909_19_CZ --
JV1310_15_P_sanguinolentus2_CZ --
MJ53_02_CZ --
CLZhao21647P_yunnanensis --
CLZhao21583P_yunnanensis --
Dai22272
TGTCAGGCAACTTATCGTTGCCATCAACAAGATGGATACTACCAAGGTAT
Dai22279 --
MJ332_94_CZ --
MJ642_94_CZ_Expallescens --
Dai21060P_vinctus --
JV0511_23LRP_pouzarii
TGTCAGGCAGCTTATCGTTGCCATCAACAAGATGGATACCACCAAGGTAT
JQ409462_R_pouzarii_PRM899856_ --
JV0308_66_WA
TGTCAGGCAACTCATTGTTGCCATCAACAAGATGGATACCACCAAGGTAT
JV0309_45_WA_USA

TGTCAGGCAACTCATTGTTGCCATCAACAAGATGGATACTACCAAGGTAT
JV0709_83_CA_USA
TGTCAGGCAACTCATTGTTGCCATCAACAAGATGGATACCACCAAGGTAT
Dai21043P_pouzarii
TGTCAGGCAGCTTATCGTTGCCATCAACAAGATGGATACCACCAAGGTAT
MJ144_95_CZ --
JV0909_3_CZ --
JV0609_1_K
TGTCAGGCAACTCATCGTTGCCATCAACAAGATGGACACCACCAAGGTAT
Dai20396Physisporinus_castanop
TGTCAGGCAACTCATCGTTGCCATCAACAAGATGGACACCACCAAGGTAT
Dai20397Physisporinus_castanop
TGTCAGGCAACTCATCGTTGCCATCAACAAGATGGACACCACCAAGGTAT
MJ19_09_SK_Abies --
JV0509_40_J_TN_USA_Betula
TGTCAGGCAACTCATTGTTGCCATCAACAAGATGGACACCACCAAGGTAT
JV0808_33crocatus_PA_USAlist
TGTCAGGCAACTCATTGTTGCCATCAACAAGATGGACACCACCAAGGTAT
DLL2009_061P_crocatus --
Dai12800P_subcrocatus --
Dai15917P_subcrocatus
TGTCAGGCAACTCATTGTTGCCATCAACAAGATGGACACCACCAAGGTAT
Cui16620 --
HCFC1088Meripilus_stillicidior --
MCW590Meripilus_obsrcurus --
MCW722Meripilus_obsrcurus --
Cui9381P_tibeticus --
Cui9588P_tibeticus
TGTCAGGCAACTCATTGTCGCCATCAACAAGATGGACACTACCAAGGTAT
Va2_Beneschova --
CWU3874_Ukraine_Alnus --
WCG1293Dai24718Physisporinus --
WCG1268Dai24682A --
WCG1269Dai24683A --
WCG1279Dai24694A
TGTCAGGCAACTCATCGTTGCCATCAACAAGATGGATACCACCAAGGTAT
Dai16971 --
ZQY1043Dai26696 --
Doll880
TGTCAGGCAACTTATTGTTGCCATCAACAAGATGGACACCACTAAGGTAG
Doll1000 --
1DAI18529 --
Dai19535
TGTCAGGCAGCTCATTGTTGCCATCAACAAGATGGATACCACCAAGGCAC

1704_79_hnedyVillaLaPaz
TGTCAGGCAACTTATCGTTGCCATCAACAAGATGGACACCACCAAGGTAC
F2061 --
1DAI18268 --
1DAI18540A
TGTCAGGCAACTTATCGTTGCCATCAACAAGATGGACACCACCAAGGTAC
Dai17695 --
LKY18Dai26373 --
Dai17839P_sulphureus
TGTCAGGCAACTCATCGTTGCCATCAACAAGATGGACACCACCAAGGTAT
Dai17841P_sulphureus
TGTCAGGCAACTCATCGTTGCCATCAACAAGATGGACACCACCAAGGTAT
Dai19877P_roseus --
1508_18_1_Kout --
KP859303_R_vinctus_RP185_BRAZI --
JK1807_15Rigidoporus_sp_Puerto --
JV0509_47_J_TN_USA
TGTCAGGCAACTTATCGTTGCCATCAACAAGATGGACACCACCAAGGTAC
JV0709_188 --
JV0509_127_PA_USA
TGTCAGGCAACTTATTGTTGCCATCAACAAGATGGACACCACCAAGGTAC
JV1009_59_NJ_USA
TGTCAGGCAACTTATCGTTGCCATCAACAAGATGGACACCACCAAGGTAC
Dai15497P_crataegi
TGTCAGGCAACTTATCGTTGCCATCAACAAGATGGACACCACCAAGGTAT
Dai15499P_crataegi
TGTCAGGCAACTTATCGTTGCCATCAACAAGATGGACACCACCAAGGTAT
Cui3266P_cinereus --
WCG1256Dai24690
TGTCAGGCAACTTATCGTTGCCATCAACAAGATGGATACCACCAAGGTTC
1DAI17581 --
WCG1255Dai24688
TGTCAGGCAACTTATCGTTGCCATCAACAAGATGGATACCACCAAGGTAC
Dai22427 --
MV690Meripilus_concrescens --
MV513Meripilus_galapagensis --
Dai19793
TGTCAGGCAACTTATCGTTGCCATCAACAAGATGGACACCACCAAGGTAC
OQ553780P_tamilnaduensis --
OQ553779P_tamilnaduensis --
A164FB3Meripilus_giganteus --
JV1407_36_Vinctus_Meandrica
TGTCAGGCAGCTCATCGTTGCCATCAACAAGATGGATACCACCAAGGTAG
1807_3K_Rigidoporus_PuertoRico

TGTCAGGCAGCTCATCGTTGCCATCAACAAGATGGATACCACCAAGGTAG
Cui16903P_vinctus
TGTCAGGCAGCTCATCGTTGCCATCAACAAGATGGATACCACCAAGGTAG
JV1008_18R_Lineatus --
JV1407_37_1_Vinctus_Carara
TGTCAGGCAGCTCATCGTTGCCATCAACAAGATGGATACCACCAAGGTCA
Dai17986P_lineatus
TGTCAGGCAGCTCATCGTTGCCATCAACAAGATGGATACCACCAAGGTCA
Dai18281
TGTCAGGCAGCTCATCGTTGCCATCAACAAGATGGATACCACCAAGGTCA
1DAI19796
TGTCAGGCAGCTCATCGTTGCCATCAACAAGATGGATACCACCAAGGTCA
ZQY797Dai25241
TGTCAGGCAGCTCATCGTTGCCATCAACAAGATGGATACCACCAAGGTCA
WCG1289Dai24711 --
Dai22598
TGTCAGGCAGCTCATCGTTGCCATCAACAAGATGGATACCACCAAGGTCA
Dai20523
TGTCAGGCAGCTCATCGTTGCCATCAACAAGATGGATACCACCAAGGTCA
Dai17885
TGTCAGGCAGCTCATCGTTGCCATCAACAAGATGGATACCACCAAGGTCA
Dai17553 --
Dai19639
TGTCAGGCAGCTCATCGTTGCCATCAACAAGATGGATACCACCAAGGTCA
JV0110_48_CZ
CGTCAGGCAACTCATCGTTGCCATCAACAAGATGGATACCACCAAGGTAT
MJ129_04 --
Cui10340P_eminens
CGTCAGGCAACTTATCGTTGCCATCAACAAGATGGATACCACCAAAGTAT
Cui10341P_eminens --
Dai12685P_eminens
CGTCAGGCAACTCATCGTTGCCATCAACAAGATGGATACCACCAAGGTAT
Miettinen_13591Rigidoporus_und --
Dai20868
TGTCAGGCAACTCATTGTTGCCATTAACAAGATGGATACCACCAAGGTAC
Dai20832
TGTCAGGCAACTCATCGTTGCCATCAACAAGATGGATACCACCAAGGTAC
Dai11400
TGTCAGGCAACTCATCGTTGCCATCAACAAGATGGATACCACCAAGGTAC
Dai22472
TGTCAGGCAACTCATCGTTGCCATCAACAAGATGGATACCACCAAGGTAC
1CUI10475 --
1CUI10491 --
HCFC1095Meripilus_robledoi --

MCW702Meripilus_revolubilis --
1704_83_zluty_HaciendaBaru --
Dai9925P_lavendulus --
Dai13587AP_lavendulus
TGTCAGGCAGCTCATCGTTGCCATCAACAAGATGGACACTACTAAGGTGA
PDD70600P_longicystidius --
Cui16630 --
FP_135344Meripilus_giganteus --
FP_100460_Sp --
CBS421_48Meripilus_giganteus --
Cui9203 --
Cui9202 --
TUFC100564Japan --
Russell5913Meripilus_sumstinei --
RP215Meripilus_brasiliensis --
RP200Meripilus_brasiliensis --
JV1712_13J_R_vinctus2_LSUPuert --
Dai10503R_hypobrunneus --
Dai10569Rigidoporus_hypobrunne --
1DAI19451 --
CM108bRigidoporus_hypobrunneus --
1CUI16874 --
FD299Cerrena_unicolor --
KHL_GB_Cerrena_uniclor --
Dai12892Cerrena_albocinnamomea --
Dai12955C_albocinnamomea --
SNUm03110102C_aurantiopora --
NIBRFG0000102423C_aurantiopora --
Dai7359Antrodiella_zonata --
F20080702KCM29C_consors --
F20080208LYW10Cerrena_consors --
Dai7821Antrodiella_zonata --
CFMR_DCL04_31Pseudolagarobasid --
VPB197Pseudolagarobasidium_bel --
CBS115543Pseudolagarobasidium_ --
CBS115544 --
Han405Pseudolagarobasidium_bai --
Han406Pseudolagarobasidium_bai --
MUcc838Spongipellis_delectans --
BRNM686401S_delectans --
OSM_F925S_delectans --
BRNM67093Spongipellis_litschau --
CFMRccFP59199TS_unicolor --
CFMRccFP71791TS_unicolor --

Dai13845P_lischaueri
TGTCAGGCAGCTCATCGTCGCCATCAACAAGATGGATACCACCAAGGTAC
Dai20266P_lischaueri --
CFMR_HHB11240Radulodon_america --
RLG6350Radulodon_americanus --
KY415963Radulodon_erikssonii --
HHB9567spRadulodon_casearius --
KRT_Iso_26Radulodon_casearius --
CBS126044Radulodon_erikssonii --
He6183YUNNANENSIS
TGTCAGACAGCTCATCGTCGCCATCAACAAGATGGACACTACCAAGGTTA
Cui17979YUNNANENSIS
TGTCAGGCAGCTCATCGTCGCCATCAACAAGATGGATACCACAAAGGTTA
Miettinen2091Junghuhnia_fimbri --
KHL12316S_tenue --
PRM846564S_pachyodon --
SP_Lgt_S_pachyodon --
Ryvarden44669Tyromyces_xuchile --
PW17_171sinuosus --
W53Dai12234
TGTTAGGCAACTCATCGTCGCCATCAACAAGATGGATACCACCAAGGTTA
HHB4100SpAntella_americana --
W3Dai20901spumeus
TGTCAGGCAACTGATCGTTGCCATCAACAAGATGGACACCACCAAGGTCA
He6736 --
BRNM734877S_spumeus --
BRNM712630S_spumeus --
Dai1723Loweomyces_sibiricus --
W54Cui10009
TGTCAGGCAACTGATCGTTGCCATCAACAAGATGGACACCACCAAGGTCG
W1Dai20899
TGTCAGGCAACTGATCGTTGCCATCAACAAGATGGACACCACCAAGGTCG
HHB13445Trametes_ochracea --
Dai16222 --
Dai16240 --

JV1310_11SanguinolentusCernys GA-----TAAGGCTGTTCTC-CTG--GTTCCCATCTG--
CCGCTCACCG-
MJ39_00_SK --
MJ111_04_CZ GA-----TAAGGCTGTTCTC-CTG--GTTCCCATCTG--
CCGCTCACCG-
JV1610_BOKYsmrk GA-----TAAGGCTGTTCTC-CTG--GTTCCCATCTG--
CCGCTCACCG-
Dai21030 --

Dai20976P_furcatus GA-----TAAGGCTGTTCTC-CTG--GTTCCCATCTG--
CCGCTCACCG-
Dai2105 --
Dai2544 --
Dai11313 --
WCG1611Dai26167 GA-----TAAGGCTGTTCTC-CTG--GTTCCCATCTA--
CCGCTCACCG-
WCG1518Dai25999Physisporinus --
TAA15097 --
JV8909_19_CZ --
JV1310_15_P_sanguinolentus2_CZ --
MJ53_02_CZ --
CLZhao21647P_yunnanensis --
CLZhao21583P_yunnanensis --
Dai22272 GA-----TAAGGCTGTTCTG-CTG--GTTCCCATCTA--
CCGCTCACCG-
Dai22279 --
MJ332_94_CZ --
MJ642_94_CZ_Expallescens --
Dai21060P_vinctus --
JV0511_23LRP_pouzarii GA-----TTGACTTGATCCAGCTA--ATCACGAATTG--
TCCCTAAATG-
JQ409462_R_pouzarii_PRM899856_ --
JV0308_66_WA GA-----TTAATTTGATCAC-TTA--GTTATGAATCG--
TCCCTTCATA-
JV0309_45_WA_USA GA-----TTAATTTGATCAC-TTA--GTTATGAATCG--
TCCCTTCATA-
JV0709_83_CA_USA GA-----TTAATTTGATCAC-TTA--GTTATGAATCG--
TCCCTTCATA-
Dai21043P_pouzarii GA-----TTGACTTGATCCAGCTA--ATCACGAATTG--
TCCCTAAATG-
MJ144_95_CZ --
JV0909_3_CZ --
JV0609_1_K GA-----TTGATTTGATCCAGCTA--GTTACAAATCG--
CCCCTTAATG-
Dai20396Physisporinus_castanop GA-----TTGATTTGATCCAGTTG--GTTACAAATCG--
CCCCTTAATG-
Dai20397Physisporinus_castanop GA-----TTGATTTGATCCAGTTG--GTTACAAATCG--
CCCCTTAATG-
MJ19_09_SK_Abies --
JV0509_40_J_TN_USA_Betula GG-----CTCATTTGATCTTATTA--CTTATGGATCG--
TCTCTTCATG-
JV0808_33crocatus_PA_USAlist GG-----CTCATTTGATCTTATTA--CTTATGGATCG--
TCTCTTCATG-

DLL2009_061P_crocatus --
Dai12800P_subcrocatus --
Dai15917P_subcrocatus GG-----CTCATTTGATCTTATTA--CTTATGGATCG--
TCTCTTCATG-
Cui16620 --
HCFC1088Meripilus_stillicidior --
MCW590Meripilus_obsrcurus --
MCW722Meripilus_obsrcurus --
Cui9381P_tibeticus --
Cui9588P_tibeticus GA-----TTAATTTGA-----TTA--ATTAAGACTTG--
TCTCTTAATG-
Va2_Beneschova --
CWU3874_Ukraine_Alnus --
WCG1293Dai24718Physisporinus --
WCG1268Dai24682A --
WCG1269Dai24683A --
WCG1279Dai24694A GA-----TAACTCTGTCTCC-CCG--ATCATACATAT---
CACTCAATA-
Dai16971 --
ZQY1043Dai26696 --
Doll880 GC-----ATATGCTATTCTG-CTA--GCAATAAATTA--
CCGCTTAATG-
Doll1000 --
1DAI18529 --
Dai19535 GT-----ATAGTCGACCCTA-TCA----TATGAATCA--
CCCCTTACTG-
1704_79_hnedyVillaLaPaz GC-----TTAGATATCTCC--TTG--
ATCGTGCATCGCCCCTCATAATT-
F2061 --
1DAI18268 --
1DAI18540A GT-----TTCGATATCACC--CTG--
ATCGTGCATCGCCCCTCTTAATT-
Dai17695 --
LKY18Dai26373 --
Dai17839P_sulphureus GT-----GTACCCTGTACTC-TCGAATGTGCAGATCA--
TCACTCAATG-
Dai17841P_sulphureus GT-----GTACCCTGTACTC-TCGAATGTGCAGATCA--
TCACTCAATG-
Dai19877P_roseus --
1508_18_1_Kout --
KP859303_R_vinctus_RP185_BRAZI --
JK1807_15Rigidoporus_sp_Puerto --
JV0509_47_J_TN_USA GA-----TCGTAGTACCCTC-ACA--GTGATAGCATA--
-TACTCAACTG

JV0709_188 ---
JV0509_127_PA_USA GA-----TCGTAGTACCCTC-ACA--GTGATAGCATA--
-TACTCAACTG
JV1009_59_NJ_USA GA-----TCGTAGTACCCTC-ACA--GTGATAGCATA--
-TACTCAACTG
Dai15497P_crataegi GA-----CTGTTGTACCCTC-ACA--GTGATAGCATA---
TACTCAACT-
Dai15499P_crataegi GA-----CTGTTGTACCCTC-ACA--GTGATAGCATA---
TACTCAACC-
Cui3266P_cinereus ---
WCG1256Dai24690 GA-----TCGTTCTACCCTC-ACA--GTGATAGCATA--
-TACTCAATT-
1DAI17581 ---
WCG1255Dai24688 GA-----TCGTACTACCCTC-ACA--GTGATAGCATA--
-TACTCAATT-
Dai22427 ---
MV690Meripilus_concrescens ---
MV513Meripilus_galapagensis ---
Dai19793 GA-----TCGCAGTACCCTC-ACA--GTGATAGCATA---
TACTCAACC-
OQ553780P_tamilnaduensis ---
OQ553779P_tamilnaduensis ---
A164FB3Meripilus_giganteus ---
JV1407_36_Vinctus_Meandrica ATAAATCCCGTACACCCTCG-
CAGTCATGGCATATAC--TCATTTAAAC-
1807_3K_Rigidoporus_PuertoRico ATAAATCCCGTACACCCTCG-CAGTCATGGCATATAC-
-TCATTTAAAC-
Cui16903P_vinctus ATAAATCCCGTACACCCTCG-
CAGTCATGGCATATAC--TCATTTAAAC-
JV1008_18R_Lineatus ---
JV1407_37_1_Vinctus_Carara GT-----TTGAACACCCTCG-CAGTGATCGCATATAC--
TTATTCAATT-
Dai17986P_lineatus GT-----TTGAACACCCTCG-CAGTGATCGCATATAT--
TTATTCAATT-
Dai18281 GT-----TTGAACACCCTCG-CAGTGATCGCATATAC--
TTATTCAATT-
1DAI19796 GT-----TTGAACACCCTCG-CAGTGATCGCATATAC--
TTATCCAATC-
ZQY797Dai25241 GT-----TTGAACACCCTCG-CAGTGATCGCATATAC-
-TTATCCAATC-
WCG1289Dai24711 ---
Dai22598 GT-----TCGAACACCCTCG-CAGTTATCGCATATAC--
TTATCCAATT-
Dai20523 GT-----TTGAACACCCTCG-CAGTTATCGCATATAC--

TTATCCAATT-
Dai17885 GT-----TTGAATACCCTCG-CAGTTATCGCATATAC--
TTATCCAATC-
Dai17553 --
Dai19639 GT-----TTGAACACCCTCG-CAGTTATCGCATATAC--
TTATCCAATT-
JV0110_48_CZ GT-----TTCGAGGCACTTA-TCA--ATCTCTA-----
CTTTATCTCA-
MJ129_04 --
Cui10340P_eminens GT-----TTCGGGGCACTTA-TCA--ATCTCTA-----
CTTTATCTCA-
Cui10341P_eminens --
Dai12685P_eminens GT-----TTCGAGGCACTTA-TCA--ATCTCTA-----
CTTTATCTCA-
Miettinen_13591Rigidoporus_und --
Dai20868 GT-----TTCAATACACTT--CAA--ATTTGAA-----
CTTTATCTCA-
Dai20832 GT-----TTCAATACACTT--CAA--ATCTGAA-----
CTTTATCTCA-
Dai11400 GT-----TTCAATACCCTT--CAA--ATCTGAA-----
CTTTATCTCA-
Dai22472 GT-----TTCAATACAGTT--CAA--ATTTGAA-----
CTTTATCTCA-
1CUI10475 --
1CUI10491 --
HCFC1095Meripilus_robledoi --
MCW702Meripilus_revolubilis --
1704_83_zluty_HaciendaBaru --
Dai9925P_lavendulus --
Dai13587AP_lavendulus GG-----ACTGTGCTTTCTC-GAG--CCTTCAGTATA--
TCTC-------
PDD70600P_longicystidius --
Cui16630 --
FP_135344Meripilus_giganteus --
FP_100460_Sp --
CBS421_48Meripilus_giganteus --
Cui9203 --
Cui9202 --
TUFC100564Japan --
Russell5913Meripilus_sumstinei --
RP215Meripilus_brasiliensis --
RP200Meripilus_brasiliensis --
JV1712_13J_R_vinctus2_LSUPuert --
Dai10503R_hypobrunneus --

Dai10569Rigidoporus_hypobrunne --
1DAI19451 --
CM108bRigidoporus_hypobrunneus --
1CUI16874 --
FD299Cerrena_unicolor --
KHL_GB_Cerrena_uniclor --
Dai12892Cerrena_albocinnamomea --
Dai12955C_albocinnamomea --
SNUm03110102C_aurantiopora --
NIBRFG0000102423C_aurantiopora --
Dai7359Antrodiella_zonata --
F20080702KCM29C_consors --
F20080208LYW10Cerrena_consors --
Dai7821Antrodiella_zonata --
CFMR_DCL04_31Pseudolagarobasid --
VPB197Pseudolagarobasidium_bel --
CBS115543Pseudolagarobasidium_ --
CBS115544 --
Han405Pseudolagarobasidium_bai --
Han406Pseudolagarobasidium_bai --
MUcc838Spongipellis_delectans --
BRNM686401S_delectans --
OSM_F925S_delectans --
BRNM67093Spongipellis_litschau --
CFMRccFP59199TS_unicolor --
CFMRccFP71791TS_unicolor --
Dai13845P_lischaueri GT-----TTAACCTCCTCTC-----TATATGCA------
GTCACAACTA-
Dai20266P_lischaueri --
CFMR_HHB11240Radulodon_america --
RLG6350Radulodon_americanus --
KY415963Radulodon_erikssonii --
HHB9567spRadulodon_casearius --
KRT_Iso_26Radulodon_casearius --
CBS126044Radulodon_erikssonii --
He6183YUNNANENSIS GT-----AAAATTTA------CTA--ATTTTCTGCGA--
ACGATTACTA-
Cui17979YUNNANENSIS GT-----AAATATTCTCTTTCCTA--ATTTTATGTGA--
ACGATTACTA-
Miettinen2091Junghuhnia_fimbri --
KHL12316S_tenue --
PRM846564S_pachyodon --
SP_Lgt_S_pachyodon --
Ryvarden44669Tyromyces_xuchile --

PW17_171sinuosus --
W53Dai12234 GT-----ATCCATTTTCATG-TTT--ATAGGCCGTGA--ATGATAATTA-
HHB4100SpAntella_americana --
W3Dai20901spumeus GT-----TTTGATTATTTCT-TTT--ATCATTGT-----TGTGTAGCTG-
He6736 --
BRNM734877S_spumeus --
BRNM712630S_spumeus --
Dai1723Loweomyces_sibiricus --
W54Cui10009 GT-----TTTGATCATTTCT-TTT--ATCATTGC-----TGTGTAGCTG-
W1Dai20899 GT-----TTTGATCATTTCT-TTT--ATCATTGC-----TGTGTAGCTG-
HHB13445Trametes_ochracea --
Dai16222 --
Dai16240 --

JV1310_11SanguinolentusCernys TTATCCTATGTAGTGGAGCGAGGACCGTTTCAATGAAATCGTCAAGGAAA
MJ39_00_SK --
MJ111_04_CZ TTATCCTATGTAGTGGAGCGAGGACCGTTTCAATGAAATCGTCAAGGAAA
JV1610_BOKYsmrk TTATCCTATGTAGTGGAGCGAGGACCGTTTCAATGAAATCGTCAAGGAAA
Dai21030 --
Dai20976P_furcatus TTATCCTATGTAGTGGAGCGAGGACCGTTTCAATGAAATCGTCAAGGAAA
Dai2105 --
Dai2544 --
Dai11313 --
WCG1611Dai26167 TTACCCTATGTAGTGGAGCGAGGACCGTTTCAATGAAATCGTCAAGGAAA
WCG1518Dai25999Physisporinus --
TAA15097 --
JV8909_19_CZ --
JV1310_15_P_sanguinolentus2_CZ --
MJ53_02_CZ --
CLZhao21647P_yunnanensis --
CLZhao21583P_yunnanensis --
Dai22272 TTACCCTATGTAGTGGAGCGAGGACCGTTTCAATGAAATCGTCAAGGAAA
Dai22279 --
MJ332_94_CZ --

MJ642_94_CZ_Expallescens ---
Dai21060P_vinctus ---
JV0511_23LRP_pouzarii
TCTCTGTCTGCAGTGGAGTGAGGACCGTTTCAACGAAATCGTCAAGGAAA
JQ409462_R_pouzarii_PRM899856_ ---
JV0308_66_WA
CCTCTGTCTGCAGTGGAGTGAGGACCGTTTCAACGAAATCGTCAAGGAAA
JV0309_45_WA_USA
CCTCTGTCTGCAGTGGAGTGAGGACCGTTTCAACGAAATCGTCAAGGAAA
JV0709_83_CA_USA
CCTCTGTCTGCAGTGGAGTGAGGACCGTTTCAACGAAATCGTCAAGGAAA
Dai21043P_pouzarii
TCTCTGTCTGCAGTGGAGTGAGGACCGTTTCAACGAAATCGTCAAGGAAA
MJ144_95_CZ ---
JV0909_3_CZ ---
JV0609_1_K --
TCTGTTTGCAGTGGAGCGAGGACCGTTTCAATGAAATCGTCAAGGAAA
Dai20396Physisporinus_castanop --
TCTGTTTGCAGTGGAGCGAGGACCGTTTCAATGAAATCGTCAAGGAAA
Dai20397Physisporinus_castanop --
TCTGTTTGCAGTGGAGCGAGGACCGTTTCAATGAAATCGTCAAGGAAA
MJ19_09_SK_Abies ---
JV0509_40_J_TN_USA_Betula
TCTCTATGCGCAGTGGAGTGAGGACCGTTTCAACGAAATCGTCAAGGAAA
JV0808_33crocatus_PA_USAlist
TCTCTATGCGCAGTGGAGTGAGGACCGTTTCAACGAAATCGTCAAGGAAA
DLL2009_061P_crocatus ---
Dai12800P_subcrocatus ---
Dai15917P_subcrocatus
TCTCTATGCGCAGTGGAGTGAGGACCGTTTCAACGAAATCGTCAAGGAAA
Cui16620 ---
HCFC1088Meripilus_stillicidior ---
MCW590Meripilus_obsrcurus ---
MCW722Meripilus_obsrcurus ---
Cui9381P_tibeticus ---
Cui9588P_tibeticus
TCTCTGTCTGCAGTGGAGTGAGGACCGTTTCAACGAAATCGTCAAGGAAA
Va2_Beneschova ---
CWU3874_Ukraine_Alnus ---
WCG1293Dai24718Physisporinus ---
WCG1268Dai24682A --
CCCCTATGCAGTGGAGCGAGGACCGTTTCAATGAAATTGTCAAGGAAA
WCG1269Dai24683A --
CCCCTATGCAGTGGAGCGAGGACCGTTTCAATGAAATTGTCAAGGAAA

WCG1279Dai24694A
TACCCCTATGCAGTGGAGCGAGGACCGTTTCAATGAAATTGTCAAGGAAA
Dai16971 --
ZQY1043Dai26696 --
Doll880
TTCCCCTATATAGTGGAGCGAGGACCGTTTCAATGAAATCGTCAAGGAAA
Doll1000 --
1DAI18529 --
Dai19535
ATTTTCCTTGCAGTGGAGTGAGGACCGTTTCAATGAAATCGTCAAGGAAA
1704_79_hnedyVillaLaPaz
TCTTGCTATGCAGTGGAGTGAGGACCGTTTCAATGAAATCGTCAAGGAAA
F2061 --
1DAI18268 --
1DAI18540A
TCTCGCTATGTAGTGGAGTGAGGACCGTTTCAATGAAATCGTCAAGGAAA
Dai17695 --
LKY18Dai26373 --
Dai17839P_sulphureus
TCCTTCTATGCAGTGGAGTGAGGACCGTTTCAACGAAATTGTTAAGGAAA
Dai17841P_sulphureus
TCCTTCTATGCAGTGGAGTGAGGACCGTTTCAACGAAATTGTTAAGGAAA
Dai19877P_roseus --
1508_18_1_Kout --
KP859303_R_vinctus_RP185_BRAZI --
JK1807_15Rigidoporus_sp_Puerto --
JV0509_47_J_TN_USA
GACTCCTATGTAGTGGAGTGAGGACCGTTTCAACGAAATCGTTAAGGAAA
JV0709_188 --
JV0509_127_PA_USA
GACTCCTATGTAGTGGAGTGAGGACCGTTTCAACGAAATCGTTAAGGAAA
JV1009_59_NJ_USA
GACTCCTATGTAGTGGAGTGAGGACCGTTTCAACGAAATCGTTAAGGAAA
Dai15497P_crataegi
CTCTCCTATGCAGTGGAGTGAGGACCGTTTCAATGAAATCGTTAAGGAAA
Dai15499P_crataegi
CTCTCCTATGCAGTGGAGTGAGGACCGTTTCAATGAAATCGTTAAGGAAA
Cui3266P_cinereus --
WCG1256Dai24690
GGCTCCTATATAGTGGAGTGAGGACCGTTTCAATGAAATCGTCAAGGAAA
1DAI17581 --
WCG1255Dai24688
GGCTCCTATATAGTGGAGTGAGGACCGTTTCAATGAAATCGTCAAGGAAA
Dai22427 --

MV690Meripilus_concrescens --
MV513Meripilus_galapagensis --
Dai19793
GACTCCTATGTAGTGGAGTGAGGACCGTTTCAATGAAATCGTCAAGGAAA
OQ553780P_tamilnaduensis --
OQ553779P_tamilnaduensis --
A164FB3Meripilus_giganteus --
JV1407_36_Vinctus_Meandrica
TGTAATTTAATAGTGGAGCGAGGATCGTTTCAATGAAATCGTCAAGGAAA
1807_3K_Rigidoporus_PuertoRico
TGTAATTTAACAGTGGAGCGAGGATCGTTTCAATGAAATCGTCAAGGAAA
Cui16903P_vinctus
TGTAATTTAATAGTGGAGCGAGGATCGTTTCAATGAAATCGTCAAGGAAA
JV1008_18R_Lineatus --
JV1407_37_1_Vinctus_Carara
CGTCATCCCATAGTGGAGCGAGGACCGTTTCAATGAAATCGTCAAGGAAA
Dai17986P_lineatus
CGTCATCCCGTAGTGGAGCGAGGACCGTTTCAATGAAATCGTTAAGGAAA
Dai18281
CGTCATCCCATAGTGGAGCGAGGACCGTTTCAATGAAATCGTCAAGGAAA
1DAI19796
CGTCATCTCATAGTGGAGCGAGGACCGTTTCAATGAAATCGTCAAGGAAA
ZQY797Dai25241
CGTCATCTCATAGTGGAGCGAGGACCGTTTCAATGAAATCGTCAAGGAAA
WCG1289Dai24711 --
Dai22598
CGTCAACCAACAGTGGAGCGAGGACCGTTTCAATGAAATCGTCAAGGAAA
Dai20523
CGTCAACCAACAGTGGAGCGAGGACCGTTTCAATGAAATCGTCAAGGAAA
Dai17885
CGTCAACCAACAGTGGAGCGAGGACCGTTTCAATGAAATCGTCAAGGAAA
Dai17553 --
Dai19639
CGTCAACCAACAGTGGAGCGAGGACCGTTTCAATGAAATCGTCAAGGAAA
JV0110_48_CZ
TTGGAACATGCAGTGGAGCGAAGACCGTTTCAATGAAATTGTCAAGGAAA
MJ129_04 --
Cui10340P_eminens
TTGGAATATGCAGTGGAGCGAGGACCGTTTCAATGAAATCGTCAAGGAAA
Cui10341P_eminens --
Dai12685P_eminens
TTGGAACATGCAGTGGAGCGAAGACCGTTTCAATGAAATTGTCAAGGAAA
Miettinen_13591Rigidoporus_und --
Dai20868

TTTTTGCATGCAGTGGAGCGAGGACCGTTTCAACGAAATTGTCAAGGAAA

Dai20832

TTTTTGCATGCAGTGGAGCGAGGACCGTTTCAACGAAATTGTCAAGGAAA

Dai11400

TTTTTGCATGCAGTGGAGCGAGGACCGTTTCAACGAAATTGTCAAGGAAA

Dai22472

TTTTTCCATACAGTGGAGCGAGGACCGTTTCAACGAAATTGTCAAGGAAA

1CUI10475 --

1CUI10491 --

HCFC1095Meripilus_robledoi --

MCW702Meripilus_revolubilis --

1704_83_zluty_HaciendaBaru --

Dai9925P_lavendulus --

Dai13587AP_lavendulus

ATTTTGTTTGCAGTGGAGTGAAGACCGTTTCAATGAAATCGTCAAGGAAA

PDD70600P_longicystidius --

Cui16630 --

FP_135344Meripilus_giganteus --

FP_100460_Sp --

CBS421_48Meripilus_giganteus --

Cui9203 --

Cui9202 --

TUFC100564Japan --

Russell5913Meripilus_sumstinei --

RP215Meripilus_brasiliensis --

RP200Meripilus_brasiliensis --

JV1712_13J_R_vinctus2_LSUPuert --

Dai10503R_hypobrunneus --

Dai10569Rigidoporus_hypobrunne --

1DAI19451 --

CM108bRigidoporus_hypobrunneus --

1CUI16874 --

FD299Cerrena_unicolor --

KHL_GB_Cerrena_uniclor --

Dai12892Cerrena_albocinnamomea --

Dai12955C_albocinnamomea --

SNUm03110102C_aurantiopora --

NIBRFG0000102423C_aurantiopora --

Dai7359Antrodiella_zonata --

F20080702KCM29C_consors --

F20080208LYW10Cerrena_consors --

Dai7821Antrodiella_zonata --

CFMR_DCL04_31Pseudolagarobasid --

VPB197Pseudolagarobasidium_bel --

CBS115543Pseudolagarobasidium_ --
CBS115544 --
Han405Pseudolagarobasidium_bai --
Han406Pseudolagarobasidium_bai --
MUcc838Spongipellis_delectans --
BRNM686401S_delectans --
OSM_F925S_delectans --
BRNM67093Spongipellis_litschau --
CFMRccFP59199TS_unicolor --
CFMRccFP71791TS_unicolor --
Dai13845P_lischaueri
AGACTATGTGCAGTGGAGCGAGGACCGTTACAACGAAATCGTTAAGGAAA
Dai20266P_lischaueri --
CFMR_HHB11240Radulodon_america --
RLG6350Radulodon_americanus --
KY415963Radulodon_erikssonii --
HHB9567spRadulodon_casearius --
KRT_Iso_26Radulodon_casearius --
CBS126044Radulodon_erikssonii --
He6183YUNNANENSIS
ACTTGATCTACAGTGGAGCGAGGACCGTTACAACGAAATCGTTAAGGAGA
Cui17979YUNNANENSIS
ACTTGATCTATAGTGGAGCGAGGACCGTTACAACGAAATCGTTAAGGAGA
Miettinen2091Junghuhnia_fimbri --
KHL12316S_tenue --
PRM846564S_pachyodon --
SP_Lgt_S_pachyodon --
Ryvarden44669Tyromyces_xuchile --
PW17_171sinuosus --
W53Dai12234
ATTATGCGTACAGTGGAGCGAGGACCGTTACAACGAAATCGTTAAGGAAA
HHB4100SpAntella_americana --
W3Dai20901spumeus
ACGTCGTATGCAGTGGAGTGAAGATCGTTACAACGAAATTGTCAAGGAAA
He6736 --
BRNM734877S_spumeus --
BRNM712630S_spumeus --
Dai1723Loweomyces_sibiricus --
W54Cui10009
ACGTCGTATGCAGTGGAGTGAAGACCGTTTCAACGAAATTGTCAAGGAAA
W1Dai20899
ACGTCGTATGCAGTGGAGTGAAGACCGTTTCAACGAAATTGTCAAGGAAA
HHB13445Trametes_ochracea --
Dai16222 --

Dai16240 --

JV1310_11SanguinolentusCernys
CCTCTACCTTCATCAAGAAGGTCGGTTACAACCCCAAGGCCGTAGCTTTC
MJ39_00_SK --
MJ111_04_CZ
CCTCTACCTTCATCAAGAAGGTCGGTTACAACCCCAAGGCCGTAGCTTTC
JV1610_BOKYsmrk
CCTCTACCTTCATCAAGAAGGTCGGTTACAACCCCAAGGCCGTAGCTTTC
Dai21030 --
Dai20976P_furcatus
CCTCTACCTTCATCAAGAAGGTCGGTTACAACCCCAAGGCCGTAGCTTTC
Dai2105 --
Dai2544 --
Dai11313 --
WCG1611Dai26167
CCTCTACCTTCATCAAGAAGGTCGGTTACAACCCCAAGGCCGTCGCTTTC
WCG1518Dai25999Physisporinus --
TAA15097 --
JV8909_19_CZ --
JV1310_15_P_sanguinolentus2_CZ --
MJ53_02_CZ --
CLZhao21647P_yunnanensis --
CLZhao21583P_yunnanensis --
Dai22272
CCTCTACCTTCATCAAGAAGGTCGGTTACAACCCAAAGGCCGTCGCTTTC
Dai22279 --
MJ332_94_CZ --
MJ642_94_CZ_Expallescens --
Dai21060P_vinctus --
JV0511_23LRP_pouzarii
CCTCCACCTTCATCAAGAAGGTCGGTTACAACCCCAAGTCCGTTGCTTTC
JQ409462_R_pouzarii_PRM899856_ --
JV0308_66_WA
CCTCCACCTTCATCAAGAAGGTCGGTTACAATCCCAAGTCCGTCGCTTTC
JV0309_45_WA_USA
CCTCCACCTTCATCAAGAAGGTCGGTTACAATCCCAAGTCCGTCGCTTTC
JV0709_83_CA_USA
CCTCCACCTTCATCAAGAAGGTCGGTTACAATCCCAAGTCCGTCGCTTTC
Dai21043P_pouzarii
CCTCCACCTTCATCAAGAAGGTCGGTTACAACCCCAAGTCCGTTGCTTTC
MJ144_95_CZ --
JV0909_3_CZ --
JV0609_1_K

CCTCCACCTTCATCAAGAAGGTCGGTTACAACCCCAAGTCTGTCGCTTTC
Dai20396Physisporinus_castanop
CGTCCACCTTCATCAAGAAGGTCGGTTACAACCCCAAGTCTGTTGCTTTC
Dai20397Physisporinus_castanop
CGTCCACCTTCATCAAGAAGGTCGGTTACAACCCCAAGTCTGTTGCTTTC
MJ19_09_SK_Abies --
JV0509_40_J_TN_USA_Betula
CCTCCACTTTCATCAAGAAGGTCGGTTACAACCCCAAAGCCGTTGCTTTC
JV0808_33crocatus_PA_USAlist
CCTCCACTTTCATCAAGAAGGTCGGTTACAACCCCAAAGCCGTTGCTTTC
DLL2009_061P_crocatus --
Dai12800P_subcrocatus --
Dai15917P_subcrocatus
CCTCCACTTTCATCAAGAAGGTCGGTTACAACCCCAAAGCCGTTGCTTTC
Cui16620 --
HCFC1088Meripilus_stillicidior --
MCW590Meripilus_obsrcurus --
MCW722Meripilus_obsrcurus --
Cui9381P_tibeticus --
Cui9588P_tibeticus
CCTCCACCTTCATCAAGAAGGTCGGTTACAACCCCAAGTCCGTCGCTTTC
Va2_Beneschova --
CWU3874_Ukraine_Alnus --
WCG1293Dai24718Physisporinus --
WCG1268Dai24682A
CCTCTACCTTCATCAAGAAGGTCGGTTACAACCCCAAGGCCGTCGCTTTC
WCG1269Dai24683A
CCTCTACCTTCATCAAGAAGGTCGGTTACAACCCCAAGGCCGTCGCTTTC
WCG1279Dai24694A
CCTCTACCTTCATCAAGAAGGTAGGTTACAACCCCAAGGCCGTCGCTTTC
Dai16971 --
ZQY1043Dai26696 --
Doll880
CCTCCACCTTCATCAAGAAGGTCGGTTACAACCCCAAGTCCGTTGCTTTC
Doll1000 --
1DAI18529 --
Dai19535
CCTCCACCTTCATTAAGAAGGTCGGTTACAACCCCAAGGGTGTCGCTTTC
1704_79_hnedyVillaLaPaz
CCTCCACCTTCATCAAGAAGGTCGGTTACAACCCCAAGGGTGTCGCATTT
F2061 --
1DAI18268 --
1DAI18540A
CCTCCACCTTCATCAAGAAGGTCGGTTACAACCCCAAGGGTGTCGCATTC

Dai17695 --
LKY18Dai26373 --
Dai17839P_sulphureus
CCTCCACCTTCATCAAGAAGGTCGGTTACAACCCCAAGGCCGTCGCATTC
Dai17841P_sulphureus
CCTCCACCTTCATCAAGAAGGTCGGTTACAACCCCAAGGCCGTCGCATTC
Dai19877P_roseus --
1508_18_1_Kout --
KP859303_R_vinctus_RP185_BRAZI --
JK1807_15Rigidoporus_sp_Puerto --
JV0509_47_J_TN_USA
CCTCCACCTTCATTAAGAAGGTCGGTTACAACCCCAAGTCTGTTGCCTTC
JV0709_188 --
JV0509_127_PA_USA
CCTCCACCTTCATTAAGAAGGTCGGTTACAACCCCAAGTCTGTTGCCTTC
JV1009_59_NJ_USA
CCTCCACCTTCATTAAGAAGGTCGGTTACAACCCCAAGTCTGTTGCCTTC
Dai15497P_crataegi
CCTCCACCTTCATCAAAAAGGTCGGTTACAACCCCAAGTCCGTTGCCTTC
Dai15499P_crataegi
CCTCCACCTTCATCAAGAAGGTCGGTTACAACCCCAAGTCCGTTGCCTTC
Cui3266P_cinereus --
WCG1256Dai24690
CCTCCACCTTCATCAAGAAGGTTGGTTACAACCCCAAGTCCGTTGCTTTC
1DAI17581 --
WCG1255Dai24688
CCTCCACCTTCATCAAGAAGGTCGGTTACAACCCCAAGTCCGTTGCTTTC
Dai22427 --
MV690Meripilus_concrescens --
MV513Meripilus_galapagensis --
Dai19793
CCTCCACCTTCATCAAGAAGGTCGGCTACAACCCCAAGTCCGTTGCCTTC
OQ553780P_tamilnaduensis --
OQ553779P_tamilnaduensis --
A164FB3Meripilus_giganteus --
JV1407_36_Vinctus_Meandrica
CTTCCACCTTCATCAAGAAGGTTGGTTACAACCCCAAGTCCGTTGCTTTC
1807_3K_Rigidoporus_PuertoRico
CTTCCACCTTCATCAAGAAGGTCGGTTACAACCCCAAGTCCGTTGCTTTC
Cui16903P_vinctus
CTTCCACCTTCATCAAGAAGGTCGGTTACAACCCCAAGTCCGTTGCTTTC
JV1008_18R_Lineatus --
JV1407_37_1_Vinctus_Carara
CCTCCACCTTCATCAAGAAGGTCGGTTACAACCCCAAGGCCGTCGCTTTC

Dai17986P_lineatus
CCTCCACCTTCATCAAGAAGGTCGGTTACAACCCCAAGGCTGTCGCTTTC
Dai18281
CCTCCACCTTCATCAAGAAGGTCGGTTACAACCCCAAGGCCGTCGCTTTC
1DAI19796
CTTCCACCTTCATCAAGAAGGTCGGTTACAACCCCAAGGCCGTCGCTTTC
ZQY797Dai25241
CTTCCACCTTCATCAAGAAGGTCGGTTACAACCCCAAGGCCGTCGCTTTC
WCG1289Dai24711 --
Dai22598
CTTCCACCTTCATCAAGAAGGTCGGTTACAACCCCAAGGCTGTCGCTTTC
Dai20523
CTTCCACCTTCATCAAGAAGGTCGGTTACAACCCCAAGGCTGTCGCTTTC
Dai17885
CTTCCACCTTCATCAAGAAGGTCGGTTACAACCCCAAGGCTGTCGCTTTC
Dai17553 --
Dai19639
CTTCCACCTTCATCAAGAAGGTCGGTTACAACCCCAAGGCTGTCGCTTTC
JV0110_48_CZ
CCTCCACCTTCATCAAGAAGGTCGGTTATAACCCCAAAGCCGTTGCATTC
MJ129_04 --
Cui10340P_eminens
CCTCCACCTTCATCAAGAAGGTCGGTTATAACCCCAAAGCCGTTGCATTC
Cui10341P_eminens --
Dai12685P_eminens
CCTCCACCTTCATCAAGAAGGTCGGTTATAACCCCAAAGCCGTTGCATTC
Miettinen_13591Rigidoporus_und --
Dai20868
CCTCCACCTTCATCAAGAAGGTCGGTTACAACCCCAAATCCGTTGCTTTC
Dai20832
CCTCCACCTTCATCAAGAAGGTCGGTTACAACCCCAAATCCGTTGCATTC
Dai11400
CCTCCACCTTCATCAAGAAGGTCGGTTACAACCCCAAATCCGTTGCATTC
Dai22472
CCTCCACCTTCATCAAGAAGGTCGGTTACAACCCCAAATCCGTTGCTTTC
1CUI10475 --
1CUI10491 --
HCFC1095Meripilus_robledoi --
MCW702Meripilus_revolubilis --
1704_83_zluty_HaciendaBaru --
Dai9925P_lavendulus --
Dai13587AP_lavendulus
CATCTACTTTCATCAAGAAGGTCGGTTACAACCCCAAGGCTGTCGCATTC
PDD70600P_longicystidius --

Cui16630 --
FP_135344Meripilus_giganteus --
FP_100460_Sp --
CBS421_48Meripilus_giganteus --
Cui9203 --
Cui9202 --
TUFC100564Japan --
Russell5913Meripilus_sumstinei --
RP215Meripilus_brasiliensis --
RP200Meripilus_brasiliensis --
JV1712_13J_R_vinctus2_LSUPuert --
Dai10503R_hypobrunneus --
Dai10569Rigidoporus_hypobrunne --
1DAI19451 --
CM108bRigidoporus_hypobrunneus --
1CUI16874 --
FD299Cerrena_unicolor --
KHL_GB_Cerrena_uniclor --
Dai12892Cerrena_albocinnamomea --
Dai12955C_albocinnamomea --
SNUm03110102C_aurantiopora --
NIBRFG0000102423C_aurantiopora --
Dai7359Antrodiella_zonata --
F20080702KCM29C_consors --
F20080208LYW10Cerrena_consors --
Dai7821Antrodiella_zonata --
CFMR_DCL04_31Pseudolagarobasid --
VPB197Pseudolagarobasidium_bel --
CBS115543Pseudolagarobasidium_ --
CBS115544 --
Han405Pseudolagarobasidium_bai --
Han406Pseudolagarobasidium_bai --
MUcc838Spongipellis_delectans --
BRNM686401S_delectans --
OSM_F925S_delectans --
BRNM67093Spongipellis_litschau --
CFMRccFP59199TS_unicolor --
CFMRccFP71791TS_unicolor --
Dai13845P_lischaueri
CCTCCACCTTCATCAAAAAGGTCGGCTACAACCCCAAGGCTGTTCCTTTC
Dai20266P_lischaueri --
CFMR_HHB11240Radulodon_america --
RLG6350Radulodon_americanus --
KY415963Radulodon_erikssonii --

HHB9567spRadulodon_casearius --
KRT_Iso_26Radulodon_casearius --
CBS126044Radulodon_erikssonii --
He6183YUNNANENSIS
CCTCCACCTTCATTAAGAAGGTCGGTTACAACCCCAAGGCCGTTCCCTTT
Cui17979YUNNANENSIS
CCTCCACCTTCATCAAGAAGGTCGGTTATAACCCCAAGGCCGTTCCCTTT
Miettinen2091Junghuhnia_fimbri --
KHL12316S_tenue --
PRM846564S_pachyodon --
SP_Lgt_S_pachyodon --
Ryvarden44669Tyromyces_xuchile --
PW17_171sinuosus --
W53Dai12234
CCTCCACCTTCATCAAGAAGGTCGGTTACAACCCCAAGAACGTCCCATTC
HHB4100SpAntella_americana --
W3Dai20901spumeus
CCTCCGCCTTCATCAAAAAGGTCGGCTATAACCCGAAGAATGTTGCTTTC
He6736 --
BRNM734877S_spumeus --
BRNM712630S_spumeus --
Dai1723Loweomyces_sibiricus --
W54Cui10009
CCTCCGCCTTCATCAAAAAGGTTGGCTATAACCCGAAGAATGTTGCTTTC
W1Dai20899
CCTCCGCCTTCATCAAAAAGGTTGGCTATAACCCGAAGAATGTCGCTTTC
HHB13445Trametes_ochracea --
Dai16222 --
Dai16240 --

JV1310_11SanguinolentusCernys
GTCCCAATCTCCGGCTGGCACGGCGACAACATGTTGGAGGAGTCTGCCAA
MJ39_00_SK --
MJ111_04_CZ
GTCCCAATCTCCGGCTGGCACGGCGACAACATGTTGGAGGAGTCTGCCAA
JV1610_BOKYsmrk
GTCCCAATCTCCGGCTGGCACGGCGACAACATGTTGGAGGAGTCTGCCAA
Dai21030 --
Dai20976P_furcatus
GTCCCAATCTCCGGCTGGCACGGCGACAACATGTTGGAGGAGTCTGCCAA
Dai2105 --
Dai2544 --
Dai11313 --
WCG1611Dai26167

GTCCCAATTTCCGGCTGGCACGGCGACAACATGTTGGAGGAGTCTCCCAA
WCG1518Dai25999Physisporinus --
TAA15097 --
JV8909_19_CZ --
JV1310_15_P_sanguinolentus2_CZ --
MJ53_02_CZ --
CLZhao21647P_yunnanensis --
CLZhao21583P_yunnanensis --
Dai22272
GTCCCAATTTCCGGCTGGCACGGCGACAACATGTTGGAGGAGTCTGCCAA
Dai22279 --
MJ332_94_CZ --
MJ642_94_CZ_Expallescens --
Dai21060P_vinctus --
JV0511_23LRP_pouzarii
GTCCCCATCTCTGGCTGGCACGGTGACAACATGTTGGAGGAGTCCGCCAA
JQ409462_R_pouzarii_PRM899856_ --
JV0308_66_WA
GTCCCCATTTCCGGCTGGCACGGCGACAACATGTTGGAGGAGTCCCCCAA
JV0309_45_WA_USA
GTCCCCATTTCCGGCTGGCACGGCGACAACATGTTGGAGGAGTCCCCCAA
JV0709_83_CA_USA
GTCCCCATTTCCGGCTGGCACGGCGACAACATGTTGGAGGAGTCCCCCAA
Dai21043P_pouzarii
GTCCCCATCTCTGGCTGGCACGGTGACAACATGTTGGAGGAGTCCGCCAA
MJ144_95_CZ --
JV0909_3_CZ --
JV0609_1_K
GTCCCCATTTCTGGCTGGCACGGTGACAACATGTTGGAAGAGTCCGCCAA
Dai20396Physisporinus_castanop
GTCCCCATTTCTGGCTGGCACGGTGACAACATGTTGGAGGAGTCCGCCAA
Dai20397Physisporinus_castanop
GTCCCCATTTCTGGCTGGCACGGTGACAACATGTTGGAGGAGTCCGCCAA
MJ19_09_SK_Abies --
JV0509_40_J_TN_USA_Betula
GTCCCCATCTCCGGCTGGCACGGTGACAACATGTTGGAGGAGTCCCCCAA
JV0808_33crocatus_PA_USAlist
GTCCCCATCTCCGGCTGGCACGGTGACAACATGTTGGAGGAGTCCCCCAA
DLL2009_061P_crocatus --
Dai12800P_subcrocatus --
Dai15917P_subcrocatus
GTCCCCATCTCCGGCTGGCACGGTGACAACATGTTGGAGGAGTCCCCCAA
Cui16620 --
HCFC1088Meripilus_stillicidior --

MCW590Meripilus_obsrcurus --
MCW722Meripilus_obsrcurus --
Cui9381P_tibeticus --
Cui9588P_tibeticus
GTCCCCATATCCGGCTGGCACGGTGACAACATGTTGGAGGAGTCCGCCAA
Va2_Beneschova --
CWU3874_Ukraine_Alnus --
WCG1293Dai24718Physisporinus --
WCG1268Dai24682A
GTCCCCATCTCTGGCTGGCACGGTGATAACATGTTGGAGGAGTCCGCCAA
WCG1269Dai24683A
GTCCCCATCTCTGGCTGGCACGGTGATAACATGTTGGAGGAGTCCGCCAA
WCG1279Dai24694A
GTCCCCATCTCTGGCTGGCACGGTGATAACATGTTGGAGGAGTCCGCCAA
Dai16971 --
ZQY1043Dai26696 --
Doll880
GTCCCCATCTCTGGCTGGCACGGTGACAACATGTTGGAGGAGTCCTCCAA
Doll1000 --
1DAI18529 --
Dai19535
GTCCCCATTTCCGGCTGGCACGGTGACAACATGTTGGAGGAGTCCTCCAA
1704_79_hnedyVillaLaPaz
GTCCCCATCTCTGGCTGGCACGGTGACAACATGTTGGAAGAGTCCGCCAA
F2061 --
1DAI18268 --
1DAI18540A
GTCCCCATCTCTGGCTGGCACGGTGACAACATGTTGGAGGAGTCCGCCAA
Dai17695 --
LKY18Dai26373 --
Dai17839P_sulphureus
GTCCCCATCTCTGGTTGGCACGGTGACAACATGTTGGAGGAGTCCGCCAA
Dai17841P_sulphureus
GTCCCCATCTCTGGTTGGCACGGTGACAACATGTTGGAGGAGTCCGCCAA
Dai19877P_roseus --
1508_18_1_Kout --
KP859303_R_vinctus_RP185_BRAZI --
JK1807_15Rigidoporus_sp_Puerto --
JV0509_47_J_TN_USA
GTCCCCATCTCCGGCTGGCACGGTGACAACATGTTGGAGGAGTCCGCCAA
JV0709_188 --
JV0509_127_PA_USA
GTCCCCATCTCCGGCTGGCACGGTGACAACATGTTGGAGGAGTCCGCCAA
JV1009_59_NJ_USA

GTCCCCATCTCCGGCTGGCACGGTGACAACATGTTGGAGGAGTCCGCCAA
Dai15497P_crataegi
GTCCCCATCTCCGGCTGGCACGGTGACAACATGTTGGAGGAGTCAAGCAA
Dai15499P_crataegi
GTCCCCATCTCCGGCTGGCACGGTGACAACATGTTGGAGGAGTCAAGCAA
Cui3266P_cinereus --
WCG1256Dai24690
GTCCCCATCTCTGGCTGGCACGGCGACAACATGTTGGAGGAGTCCGCCAA
1DAI17581 --
WCG1255Dai24688
GTCCCCATCTCTGGCTGGCACGGTGACAACATGTTGGAGGAGTCCGCCAA
Dai22427 --
MV690Meripilus_concrescens --
MV513Meripilus_galapagensis --
Dai19793
GTCCCCATCTCCGGCTGGCACGGTGACAACATGTTGGAGGAGTCTGCCAA
OQ553780P_tamilnaduensis --
OQ553779P_tamilnaduensis --
A164FB3Meripilus_giganteus --
JV1407_36_Vinctus_Meandrica
GTCCCCATCTCTGGCTGGCACGGTGACAACATGTTGGAGGAGTCCACTAA
1807_3K_Rigidoporus_PuertoRico
GTCCCCATCTCTGGCTGGCACGGTGACAACATGTTGGAGGAGTCCACTAA
Cui16903P_vinctus
GTCCCCATCTCTGGCTGGCACGGTGACAACATGTTGGAGGAGTCCACTAA
JV1008_18R_Lineatus --
JV1407_37_1_Vinctus_Carara
GTCCCCATCTCTGGCTGGCACGGTGACAACATGTTGGAGGAGTCCTCCAA
Dai17986P_lineatus
GTCCCCATCTCTGGCTGGCACGGTGACAACATGTTGGAGGAGTCCTCCAA
Dai18281
GTCCCCATCTCTGGCTGGCACGGTGACAACATGTTGGAGGAGTCCTCCAA
1DAI19796
GTCCCCATCTCTGGCTGGCACGGTGACAACATGTTGGAGGAATCCGCCAA
ZQY797Dai25241
GTCCCCATCTCTGGCTGGCACGGTGACAACATGTTGGAGGAATCCGCCAA
WCG1289Dai24711 --
Dai22598
GTCCCCATCTCTGGCTGGCACGGTGACAACATGTTGGAGGAGTCCGCCAA
Dai20523
GTCCCCATCTCTGGCTGGCACGGTGACAACATGTTGGAGGAGTCCGCCAA
Dai17885
GTCCCCATCTCTGGCTGGCACGGTGACAACATGTTGGAGGAGTCCGCCAA
Dai17553 --

Dai19639
GTCCCCATCTCTGGCTGGCACGGTGACAACATGTTGGAGGAGTCCGCCAA
JV0110_48_CZ
GTCCCTATTTCCGGCTGGCACGGTGACAACATGTTGGAGGAGTCCGCCAA
MJ129_04 --
Cui10340P_eminens
GTCCCTATCTCCGGCTGGCACGGTGACAACATGTTGGAGGAGTCCCCCAA
Cui10341P_eminens --
Dai12685P_eminens
GTCCCTATTTCCGGCTGGCACGGTGACAACATGTTGGAGGAGTCCGCCAA
Miettinen_13591Rigidoporus_und --
Dai20868
GTCCCCATCTCCGGCTGGCACGGTGACAACATGTTGGAGGAGTCCAGCAA
Dai20832
GTCCCCATCTCCGGCTGGCACGGTGACAACATGTTGGAGGAGTCCAGCAA
Dai11400
GTCCCCATCTCCGGCTGGCACGGTGACAACATGTTGGAGGAGTCCAGCAA
Dai22472
GTCCCCATTTCCGGCTGGCACGGTGACAACATGTTGGAGGAGTCCAGCAA
1CUI10475 --
1CUI10491 --
HCFC1095Meripilus_robledoi --
MCW702Meripilus_revolubilis --
1704_83_zluty_HaciendaBaru --
Dai9925P_lavendulus --
Dai13587AP_lavendulus
GTTCCCATCTCTGGTTGGCACGGTGACAACATGTTGGAGGAGTCATCAAA
PDD70600P_longicystidius --
Cui16630 --
FP_135344Meripilus_giganteus --
FP_100460_Sp --
CBS421_48Meripilus_giganteus --
Cui9203 --
Cui9202 --
TUFC100564Japan --
Russell5913Meripilus_sumstinei --
RP215Meripilus_brasiliensis --
RP200Meripilus_brasiliensis --
JV1712_13J_R_vinctus2_LSUPuert --
Dai10503R_hypobrunneus --
Dai10569Rigidoporus_hypobrunne --
1DAI19451 --
CM108bRigidoporus_hypobrunneus --
1CUI16874 --

FD299Cerrena_unicolor --
KHL_GB_Cerrena_uniclor --
Dai12892Cerrena_albocinnamomea --
Dai12955C_albocinnamomea --
SNUm03110102C_aurantiopora --
NIBRFG0000102423C_aurantiopora --
Dai7359Antrodiella_zonata --
F20080702KCM29C_consors --
F20080208LYW10Cerrena_consors --
Dai7821Antrodiella_zonata --
CFMR_DCL04_31Pseudolagarobasid --
VPB197Pseudolagarobasidium_bel --
CBS115543Pseudolagarobasidium_ --
CBS115544 --
Han405Pseudolagarobasidium_bai --
Han406Pseudolagarobasidium_bai --
MUcc838Spongipellis_delectans --
BRNM686401S_delectans --
OSM_F925S_delectans --
BRNM67093Spongipellis_litschau --
CFMRccFP59199TS_unicolor --
CFMRccFP71791TS_unicolor --
Dai13845P_lischaueri
GTCCCCATCTCTGGCTGGCATGGTGACAACATGTTGGAGGAGTCTGCCAA
Dai20266P_lischaueri --
CFMR_HHB11240Radulodon_america --
RLG6350Radulodon_americanus --
KY415963Radulodon_erikssonii --
HHB9567spRadulodon_casearius --
KRT_Iso_26Radulodon_casearius --
CBS126044Radulodon_erikssonii --
He6183YUNNANENSIS
GTCCCGATCTCTGGCTGGCACGGTGATAACATGTTGGAGGAATCCGCTAA
Cui17979YUNNANENSIS
GTCCCGATCTCTGGCTGGCACGGTGACAACATGTTGGAGGAGTCCGCTAA
Miettinen2091Junghuhnia_fimbri --
KHL12316S_tenue --
PRM846564S_pachyodon --
SP_Lgt_S_pachyodon --
Ryvarden44669Tyromyces_xuchile --
PW17_171sinuosus --
W53Dai12234
GTCCCCATCTCTGGCTGGCACGGTGACAACATGTTGGAGGAGTCCTCTAA
HHB4100SpAntella_americana --

W3Dai20901spumeus
GTTCCTATCTCCGGCTGGCATGGTGACAACATGTTGGAGGAGTCCCCAAA
He6736 --
BRNM734877S_spumeus --
BRNM712630S_spumeus --
Dai1723Loweomyces_sibiricus --
W54Cui10009
GTTCCTATCTCCGGCTGGCATGGTGACAACATGTTGGAGGAGTCCCCAAA
W1Dai20899
GTTCCTATCTCCGGCTGGCATGGTGACAACATGTTGGAGGAGTCCCCAAA
HHB13445Trametes_ochracea --
Dai16222 --
Dai16240 --

JV1310_11SanguinolentusCernys GTGAGTATTGATTTGATT-CGTTTATCG---AACTGA-T--
----
MJ39_00_SK --
MJ111_04_CZ GTGAGTATTTATTTGATT-CGTTTATCG---AACTGA-
T-----------
JV1610_BOKYsmrk GTGAGTATTTATTTGATT-CGTTTATCG---
AACTGA-T-----------
Dai21030 --
Dai20976P_furcatus GTGAGTATTGATTTGATT-CGTTTATCG---AACTGA-T--

Dai2105 --
Dai2544 --
Dai11313 --
WCG1611Dai26167 GTGGGCATTTATCTGATT-CGTTTATCG---
AACTGA-T-----------
WCG1518Dai25999Physisporinus --
TAA15097 --
JV8909_19_CZ --
JV1310_15_P_sanguinolentus2_CZ --
MJ53_02_CZ --
CLZhao21647P_yunnanensis --
CLZhao21583P_yunnanensis --
Dai22272 GTGAGCATTTATTTGATT-CTTTTATCG---AATTGA-T-

Dai22279 --
MJ332_94_CZ --
MJ642_94_CZ_Expallescens --
Dai21060P_vinctus --
JV0511_23LRP_pouzarii GTAAGTAATAACTTAGTT-C-TTTGCCG---AACTGC-
C-----------

JQ409462_R_pouzarii_PRM899856_ --
JV0308_66_WA GTAAGTAAATACTTCGTT-TATTCGACG---
AACTGT-T-----------
JV0309_45_WA_USA GTAAGTAAATACTTCGTT-TATTCGACG---
AACTGT-T-----------
JV0709_83_CA_USA GTAAGTAAATACTTCGTT-TATTCGACG---
AACTGT-T-----------
Dai21043P_pouzarii GTAAGTAATAACTTAGTT-C-TTTGCCG---AACTGC-C-

MJ144_95_CZ --
JV0909_3_CZ --
JV0609_1_K GTAAGTAAACACCTTGTT-TGTTCGTCG---
AAGCGC-T-----------
Dai20396Physisporinus_castanop GTAAGTAAACACCTTGTT-TGTTCGCCG---AAGCGC-T--
----
Dai20397Physisporinus_castanop GTAAGTAAACACCTTGTT-TGTTCGCCG---AAGCGC-T--
----
MJ19_09_SK_Abies --
JV0509_40_J_TN_USA_Betula GTATGTAATCACTTTGTT-TATTCGTCG---AACTGC-
T-----------
JV0808_33crocatus_PA_USAlist GTATGTAATCACTTTGTT-TATTCGTCG---AACTGC-T--
----
DLL2009_061P_crocatus --
Dai12800P_subcrocatus --
Dai15917P_subcrocatus GTATGTAATCACTTTGTT-TATTCGTCG---AACTGC-T--
----
Cui16620 --
HCFC1088Meripilus_stillicidior --
MCW590Meripilus_obsrcurus --
MCW722Meripilus_obsrcurus --
Cui9381P_tibeticus --
Cui9588P_tibeticus GTAAGTAAACACTTTGTC-CATTCGCCG---AACTGC-T-

Va2_Beneschova --
CWU3874_Ukraine_Alnus --
WCG1293Dai24718Physisporinus --
WCG1268Dai24682A GTAAGTCAATATATGTT--TTCTCGTGA---
CACAAT-A-----------
WCG1269Dai24683A GTAAGTCAATATATGTT--TTCTCGTGA---
CACAAT-A-----------
WCG1279Dai24694A GTAAGTCAATATATATT--TTCTCGTGA---CACAAT-
A-----------
Dai16971 --
ZQY1043Dai26696 --

Doll880 GTAAGCGGTGTATCTTCTTTTTCACCAG---
AATGGC-T-----------
Doll1000 ---
1DAI18529 ---
Dai19535 GTAAGTATATGGCAGTG--CTCCCGTTA---CATTCC-
C-----------
1704_79_hnedyVillaLaPaz GTACGTAATAATTTGTCCTCGCGCATCG---AGTAAG-
T-----------
F2061 ---
1DAI18268 ---
1DAI18540A GTACGTCACAATTTGTCTATACGCACCA---
AATAAG-T-----------
Dai17695 ---
LKY18Dai26373 ---
Dai17839P_sulphureus GTGGGTCGTTTTTTTTGTCTAACAGTCG---GGCATC-
T-----------
Dai17841P_sulphureus GTGGGTCGTTTTTTTTGTCTAACAGTCG---GGCATC-
T-----------
Dai19877P_roseus ---
1508_18_1_Kout ---
KP859303_R_vinctus_RP185_BRAZI ---
JK1807_15Rigidoporus_sp_Puerto ---
JV0509_47_J_TN_USA GTAAGCGGAGGTCTCATCTTCTCACTCG---
GTTTGC-C-----------
JV0709_188 ---
JV0509_127_PA_USA GTAAGCGGAGGTCTCATCTTCTCACTCG---
GTTTGC-C-----------
JV1009_59_NJ_USA GTAAGCGGAGGTCTCATCTTCTCACTCG---
GTTTGC-C-----------
Dai15497P_crataegi GTAAGTGTAGACTTTGTTTGTTCACTCG---GATTGC-
CT----------
Dai15499P_crataegi GTAAGTGTAGACTTTGTTTGTTCACTCG---GATTGC-
CT----------
Cui3266P_cinereus ---
WCG1256Dai24690 GTAAGTAAAGATCTTAGC-GATCACCTG---
GATAGC-T-----------
1DAI17581 ---
WCG1255Dai24688 GTAAGTAAAGATCTTAGC-GATCACCTG---
GATTGC-T-----------
Dai22427 ---
MV690Meripilus_concrescens ---
MV513Meripilus_galapagensis ---
Dai19793 GTAAGCGAAGGCTTCGTTTCATTGTTCC---
GTTCAC-C-----------

OQ553780P_tamilnaduensis --
OQ553779P_tamilnaduensis --
A164FB3Meripilus_giganteus --
JV1407_36_Vinctus_Meandrica GTAAGTTTGGACCTTATC-AGGGACTCG---ATCAGC-
G-----------
1807_3K_Rigidoporus_PuertoRico GTAAGTTTGGACCTTATC-AGGGACTCG---ATCAGC-
G-----------
Cui16903P_vinctus GTAAGTTTGGACCTTATC-AGGGACTCG---ATCAGC-
G-----------
JV1008_18R_Lineatus --
JV1407_37_1_Vinctus_Carara GTACGTTAAGACCTTATG-AGCGACTTG---ACCAAA-
G-----------
Dai17986P_lineatus GTACGTTAGGACCTTATG-AGGGACTTG---ACCAAA-
G-----------
Dai18281 GTACGTTAGGACCTTATG-AGCGACTTG---
ACCAAA-G-----------
1DAI19796 GTATGTTTGGACCTTATC-AGAGACTTG---
ATCACA-G-----------
ZQY797Dai25241 GTATGTTTGGACCTTATC-AGAGACTTG---
ATCACA-G-----------
WCG1289Dai24711 --
Dai22598 GTAAGTTTGTACCTTATC-AGAGACTTG---
ATCAGATA-----------
Dai20523 GTAAGTTTGTACCTTATC-CGAGACTTG---
ATCAGATA-----------
Dai17885 GTAAGTTTGTACCTTATC-AGAGACTTG---
ATCAGATT-----------
Dai17553 --
Dai19639 GTAAGTTTGTACCTTATC-AGAGACTTG---
ATCAGATA-----------
JV0110_48_CZ GTGAGTGTATGCGCCTAT-TTTATCATA---ATACAC-
C-----------
MJ129_04 --
Cui10340P_eminens GTGAGTGTATGCGCCTAT-TTTATCATA---ATACAC-
C-----------
Cui10341P_eminens --
Dai12685P_eminens GTGAGTGTATGCGCCTAT-TTTATCATA---ATACAC-
C-----------
Miettinen_13591Rigidoporus_und --
Dai20868 GTGAGTGTATGCGCCCAT-TTTATTATC---AGATGT-
C-----------
Dai20832 GTGAGTGTATGCGCCCGT-TTTATTATC---AGATGT-
C-----------
Dai11400 GTGAGTGTATGCGCCCGT-TTTATTATC---AGATGT-

C----------
Dai22472 GTGAGTGTATGCGCCCAT-TTTATTATC---AGATGT-
C----------
1CUI10475 --
1CUI10491 --
HCFC1095Meripilus_robledoi --
MCW702Meripilus_revolubilis --
1704_83_zluty_HaciendaBaru --
Dai9925P_lavendulus --
Dai13587AP_lavendulus GTGAGTGGACATTTATTT-CACTGATTT---
TTTTCCGTGAAAAGAATTC
PDD70600P_longicystidius --
Cui16630 --
FP_135344Meripilus_giganteus --
FP_100460_Sp --
CBS421_48Meripilus_giganteus --
Cui9203 --
Cui9202 --
TUFC100564Japan --
Russell5913Meripilus_sumstinei --
RP215Meripilus_brasiliensis --
RP200Meripilus_brasiliensis --
JV1712_13J_R_vinctus2_LSUPuert --
Dai10503R_hypobrunneus --
Dai10569Rigidoporus_hypobrunne --
1DAI19451 --
CM108bRigidoporus_hypobrunneus --
1CUI16874 --
FD299Cerrena_unicolor --
KHL_GB_Cerrena_uniclor --
Dai12892Cerrena_albocinnamomea --
Dai12955C_albocinnamomea --
SNUm03110102C_aurantiopora --
NIBRFG0000102423C_aurantiopora --
Dai7359Antrodiella_zonata --
F20080702KCM29C_consors --
F20080208LYW10Cerrena_consors --
Dai7821Antrodiella_zonata --
CFMR_DCL04_31Pseudolagarobasid --
VPB197Pseudolagarobasidium_bel --
CBS115543Pseudolagarobasidium_ --
CBS115544 --
Han405Pseudolagarobasidium_bai --
Han406Pseudolagarobasidium_bai --

MUcc838Spongipellis_delectans --
BRNM686401S_delectans --
OSM_F925S_delectans --
BRNM67093Spongipellis_litschau --
CFMRccFP59199TS_unicolor --
CFMRccFP71791TS_unicolor --
Dai13845P_lischaueri GTGAGTAAATGCGGTTCA-GTTTCATAA---
CGTCCGTCT----------
Dai20266P_lischaueri --
CFMR_HHB11240Radulodon_america --
RLG6350Radulodon_americanus --
KY415963Radulodon_erikssonii --
HHB9567spRadulodon_casearius --
KRT_Iso_26Radulodon_casearius --
CBS126044Radulodon_erikssonii --
He6183YUNNANENSIS GTAAGTAACTTAATGTCG-AAATGTCTC---
CGTTCA-------------
Cui17979YUNNANENSIS GTAAGTAACTTAATGTCG-AAATGTCTT---
CGTTCA-------------
Miettinen2091Junghuhnia_fimbri --
KHL12316S_tenue --
PRM846564S_pachyodon --
SP_Lgt_S_pachyodon --
Ryvarden44669Tyromyces_xuchile --
PW17_171sinuosus --
W53Dai12234 GTATGTACTTGTGTTATC-GCTTTATTG---TTTTCG--

HHB4100SpAntella_americana --
W3Dai20901spumeus GTCCGTATATGCGCTATT-
TAAAGTTTTCACAACGAT-T-----------
He6736 --
BRNM734877S_spumeus --
BRNM712630S_spumeus --
Dai1723Loweomyces_sibiricus --
W54Cui10009 GTCCGTATATGCACTTTT-TAGAGTTTT---AACAAT-
T-----------
W1Dai20899 GTCCGTATATGCACTTTT-TAGAGTTTT---AACAAT-
T-----------
HHB13445Trametes_ochracea --
Dai16222 --
Dai16240 --

JV1310_11SanguinolentusCernys ---CTTAACGCC------CTCATCCTTAGCA-
TGACATGGTACAAGGGCT

MJ39_00_SK --
MJ111_04_CZ ---CTTAACGCC------CTCATCCTTAGCA-
TGACATGGTACAAGGGCT
JV1610_BOKYsmrk ---CTTAACGCC------CTCATCCTTAGCA-
TGACATGGTACAAGGGCT
Dai21030 --
Dai20976P_furcatus ---CTTAACGCC------CTCATCCTTAGCA-
TGACATGGTACAAGGGCT
Dai2105 --
Dai2544 --
Dai11313 --
WCG1611Dai26167 ---CTTAACGCC------CTCATCCATAGCA-
TGACATGGTACAAGGGCT
WCG1518Dai25999Physisporinus --
TAA15097 --
JV8909_19_CZ --
JV1310_15_P_sanguinolentus2_CZ --
MJ53_02_CZ --
CLZhao21647P_yunnanensis --
CLZhao21583P_yunnanensis --
Dai22272 ---CTTAACGCC------
CTTATCCTTAGCATTTTCATGGTACAAGGGCT
Dai22279 --
MJ332_94_CZ --
MJ642_94_CZ_Expallescens --
Dai21060P_vinctus --
JV0511_23LRP_pouzarii ---CTTAATGTCGATGTTCGAACCTGTAGCA-
TGCCATGGTACAAGGGTT
JQ409462_R_pouzarii_PRM899856_ --
JV0308_66_WA ---CTTAATGTC------CAAATTTGTAGCA-
TGACATGGTACAAGGGCT
JV0309_45_WA_USA ---CTTAATGTC------CAAATTTGTAGCA-
TGACATGGTACAAGGGCT
JV0709_83_CA_USA ---CTTAATGTC------CAAATTTGTAGCA-
TGACATGGTACAAGGGCT
Dai21043P_pouzarii ---CTTAATGTCGATGTTCGAACCTGTAGCA-
TGCCATGGTACAAGGGTT
MJ144_95_CZ --
JV0909_3_CZ --
JV0609_1_K ---CTCAATATT------GAAATCTGTAGCA-
TGACATGGTACAAGGGCT
Dai20396Physisporinus_castanop ---CTAAAAATT------GAAATCTGTAGCA-
TGCCATGGTACAAGGGAT
Dai20397Physisporinus_castanop ---CTAAAAATT------GAAATCTGTAGCA-

TGCCATGGTACAAGGGAT
MJ19_09_SK_Abies --
JV0509_40_J_TN_USA_Betula ---TTTGAAGTG------TAAATCTGTAGCA-
TGCCATGGTACAAGGGCT
JV0808_33crocatus_PA_USAlist ---TTTAAAGTG------TAAATCTGTAGCA-
TGCCATGGTACAAGGGCT
DLL2009_061P_crocatus --
Dai12800P_subcrocatus --
Dai15917P_subcrocatus ---TATAAAGTG------TAAATCTGTAGCA-
TGCCATGGTACAAGGGCT
Cui16620 --
HCFC1088Meripilus_stillicidior --
MCW590Meripilus_obsrcurus --
MCW722Meripilus_obsrcurus --
Cui9381P_tibeticus --
Cui9588P_tibeticus ---CTAAATGTC------CAAATCTCTAGCA-
TGACATGGTACAAGGGCT
Va2_Beneschova --
CWU3874_Ukraine_Alnus --
WCG1293Dai24718Physisporinus --
WCG1268Dai24682A ---TTTAACATT------CGCAACTTTAGCA-
TGCCATGGTATAAGGGCT
WCG1269Dai24683A ---TTTAACATT------CGCAACTTTAGCA-
TGCCATGGTATAAGGGCT
WCG1279Dai24694A ---TTTAACATT------CGCAACTTTAGCA-
TGCCATGGTATAAGGGCT
Dai16971 --
ZQY1043Dai26696 --
Doll880 ---TCTGAACCT------CAAATTTCTAGCA-
TGCCATGGTATAAGGGTT
Doll1000 --
1DAI18529 --
Dai19535 ---CTTAATCCTGACA--TGCACCCTTAGCA-
TGCCATGGTACAAGGGCT
1704_79_hnedyVillaLaPaz ---TTTAACATT------TAACCTTTCAGCA-
TGCCATGGTACAAGGGCT
F2061 --
1DAI18268 --
1DAI18540A ---TTTAACATT------TAAACTTTCAGCA-
TGCCATGGTACAAGGGCT
Dai17695 --
LKY18Dai26373 --
Dai17839P_sulphureus ---CTTGACACC-----TAATTTTTCAGCA-
TGACATGGTACAAGGGCT

Dai17841P_sulphureus ---CTTGACACC-----TAATTTTTCAGCA-
TGACATGGTACAAGGGCT
Dai19877P_roseus --
1508_18_1_Kout --
KP859303_R_vinctus_RP185_BRAZI --
JK1807_15Rigidoporus_sp_Puerto --
JV0509_47_J_TN_USA ----CCTAATGC------TCTATTTAAAGCA-
TGCCATGGTACAAGGGCT
JV0709_188 --
JV0509_127_PA_USA ----CCTAATGC------TCTATTTAAAGCA-
TGCCATGGTACAAGGGCT
JV1009_59_NJ_USA ----CCTAATGC------TCTATTTAAAGCA-
TGCCATGGTACAAGGGCT
Dai15497P_crataegi ---TTTTGACGC------ATCATCTTCAGCA-
TGCCATGGTACAAGGGCT
Dai15499P_crataegi ---TTTTGACGC------ATCATCTTCAGCA-
TGCCATGGTACAAGGGCT
Cui3266P_cinereus --
WCG1256Dai24690 ----CTTCATTC------GCTATCTTTAGCA-
TGCCATGGTACAAGGGTT
1DAI17581 --
WCG1255Dai24688 ----CTTAATTC------GCTATATTTAGCA-
TGCCATGGTACAAGGGCT
Dai22427 --
MV690Meripilus_concrescens --
MV513Meripilus_galapagensis --
Dai19793 ---TTTTGACAC------TCAATTTATAGCA-
TGCCATGGTACAAGGGTT
OQ553780P_tamilnaduensis --
OQ553779P_tamilnaduensis --
A164FB3Meripilus_giganteus --
JV1407_36_Vinctus_Meandrica ---ATTAACATT---------GTCTTCAGCA-
TGCCATGGTACAAGGGCT
1807_3K_Rigidoporus_PuertoRico ---ATTAACATT---------GTCTTCAGCA-
TGCCATGGTACAAGGGCT
Cui16903P_vinctus ---ATTAACATT---------GTCTTCAGCA-
TGCCATGGTACAAGGGCT
JV1008_18R_Lineatus --
JV1407_37_1_Vinctus_Carara ---ATTAACACC---------ACTTTCAGCA-
TGCCATGGTACAAGGGCT
Dai17986P_lineatus ---ATTGACCCC---------ACTTTCAGCA-
TGCCATGGTACAAGGGCT
Dai18281 ---ATTAACACC---------ACTTTCAGCA-
TGCCATGGTACAAGGGCT

1DAI19796 ---ATTAACAAC---------ACTTTCAGCA-
TGCCATGGTACAAGGGCT
ZQY797Dai25241 ---ATTAACAAC---------ACTTTCAGCA-
TGCCATGGTACAAGGGCT
WCG1289Dai24711 --
Dai22598 ---ATTAACATC---------GCTTTCAGCA-
TGCCATGGTACAAGGGCT
Dai20523 ---ATTAACATC---------GCTTTCAGCA-
TGCCATGGTACAAGGGCT
Dai17885 ---ATTAACATC---------GCTTTCAGCA-
TGCCATGGTATAAGGGCT
Dai17553 --
Dai19639 ---ATTAACATC---------GCTTTCAGCA-
TGCCATGGTACAAGGGCT
JV0110_48_CZ ---CTAATTGCG---------CGCTGTAGTA-
TGCCATGGTACAAGGGCT
MJ129_04 --
Cui10340P_eminens ---CTAATTGTG---------CGCTGTAGTA-
TGCCATGGTACAAGGGCT
Cui10341P_eminens --
Dai12685P_eminens ---CTAATTGCG---------CGCTGTAGTA-
TGCCATGGTACAAGGGCT
Miettinen_13591Rigidoporus_und --
Dai20868 ---CTAAACAAG---------CGCCATAGCA-
TGCCATGGTACAAGGGTT
Dai20832 ---CTAAACAAG---------CGCCGTAGCA-
TGCCATGGTACAAGGGTT
Dai11400 ---CTAAACAAG---------TGCCGTAGCA-
TGCCATGGTACAAGGGTT
Dai22472 ---CTAAACAAG---------CGCCATAGCA-
TGCCATGGTACAAGGGTT
1CUI10475 --
1CUI10491 --
HCFC1095Meripilus_robledoi --
MCW702Meripilus_revolubilis --
1704_83_zluty_HaciendaBaru --
Dai9925P_lavendulus --
Dai13587AP_lavendulus GTACTCATCAAC---------ATTTGTAGTA-
TGCCATGGTACAAGGGCT
PDD70600P_longicystidius --
Cui16630 --
FP_135344Meripilus_giganteus --
FP_100460_Sp --
CBS421_48Meripilus_giganteus --

Cui9203 --
Cui9202 --
TUFC100564Japan --
Russell5913Meripilus_sumstinei --
RP215Meripilus_brasiliensis --
RP200Meripilus_brasiliensis --
JV1712_13J_R_vinctus2_LSUPuert --
Dai10503R_hypobrunneus --
Dai10569Rigidoporus_hypobrunne --
1DAI19451 --
CM108bRigidoporus_hypobrunneus --
1CUI16874 --
FD299Cerrena_unicolor --
KHL_GB_Cerrena_uniclor --
Dai12892Cerrena_albocinnamomea --
Dai12955C_albocinnamomea --
SNUm03110102C_aurantiopora --
NIBRFG0000102423C_aurantiopora --
Dai7359Antrodiella_zonata --
F20080702KCM29C_consors --
F20080208LYW10Cerrena_consors --
Dai7821Antrodiella_zonata --
CFMR_DCL04_31Pseudolagarobasid --
VPB197Pseudolagarobasidium_bel --
CBS115543Pseudolagarobasidium_ --
CBS115544 --
Han405Pseudolagarobasidium_bai --
Han406Pseudolagarobasidium_bai --
MUcc838Spongipellis_delectans --
BRNM686401S_delectans --
OSM_F925S_delectans --
BRNM67093Spongipellis_litschau --
CFMRccFP59199TS_unicolor --
CFMRccFP71791TS_unicolor --
Dai13845P_lischaueri ---CCTAACCAC--------GCTCTACAGCA-
TGCCATGGTACAAGGGCT
Dai20266P_lischaueri --
CFMR_HHB11240Radulodon_america --
RLG6350Radulodon_americanus --
KY415963Radulodon_erikssonii --
HHB9567spRadulodon_casearius --
KRT_Iso_26Radulodon_casearius --
CBS126044Radulodon_erikssonii --
He6183YUNNANENSIS ---GTAATTGAC------CATGATCGTAGCA-

TGCCCTGGTACAAGGGCT
Cui17979YUNNANENSIS ---GTAATTGAC------CATGATCGTAGCA-TGCCCTGGTACAAGGGCT
Miettinen2091Junghuhnia_fimbri --
KHL12316S_tenue --
PRM846564S_pachyodon --
SP_Lgt_S_pachyodon --
Ryvarden44669Tyromyces_xuchile --
PW17_171sinuosus --
W53Dai12234 ---TGCACTAAT------CACGTCTACAGCA-TGCCTTGGTACAAGGGCT
HHB4100SpAntella_americana --
W3Dai20901spumeus ---TCTAATTAG-AAA--GCATCATTTAGTA-TGACATGGTATAAGGGCT
He6736 --
BRNM734877S_spumeus --
BRNM712630S_spumeus --
Dai1723Loweomyces_sibiricus --
W54Cui10009 ---TTTAAGTTG-AAA--TCATTTTTTAGTA-TGACATGGTACAAGGGCT
W1Dai20899 ---TTCAAGTTG-AAA--TCCTCTTTTAGTA-TGACATGGTACAAGGGCT
HHB13445Trametes_ochracea --
Dai16222 --
Dai16240 --

JV1310_11SanguinolentusCernys GGACCAAGGAGACTAAGGCCGGTGTCACCAAAGGCAAGACCCTCCTCGAT
MJ39_00_SK --
MJ111_04_CZ GGACCAAGGAGACTAAGGCCGGTGTCACCAAAGGCAAGACCCTCCTCGAT
JV1610_BOKYsmrk GGACCAAGGAGACTAAGGCCGGTGTCACCAAAGGCAAGACCCTCCTCGAT
Dai21030 --
Dai20976P_furcatus GGACCAAGGAGACTAAGGCCGGTGTCACCAAAGGCAAGACCCTCCTCGAT
Dai2105 --
Dai2544 --
Dai11313 --
WCG1611Dai26167 GGACCAAGGAGACCAAGGCCGGTGTCACCAAGGGCAAGACCCTCCTCGAT
WCG1518Dai25999Physisporinus --
TAA15097 --
JV8909_19_CZ --

JV1310_15_P_sanguinolentus2_CZ --
MJ53_02_CZ --
CLZhao21647P_yunnanensis --
CLZhao21583P_yunnanensis --
Dai22272
GGACCAAGGAGACCAAGGCCGGTGTCACCAAGGGTAAGACCCTCCTCGAT
Dai22279 --
MJ332_94_CZ --
MJ642_94_CZ_Expallescens --
Dai21060P_vinctus --
JV0511_23LRP_pouzarii
GGACCAAGGAGACCAAGGCCGGTGTTACCAAGGGCAAGACTCTCCTTGAC
JQ409462_R_pouzarii_PRM899856_ --
JV0308_66_WA
GGACCAAGGAAACCAAGGCCGGTGTTACCAAGGGCAAGACTCTCCTCGAC
JV0309_45_WA_USA
GGACCAAGGAAACCAAGGCCGGTGTTACCAAGGGCAAGACTCTCCTCGAC
JV0709_83_CA_USA
GGACCAAGGAAACCAAGGCCGGTGTTACCAAGGGCAAGACTCTCCTCGAC
Dai21043P_pouzarii
GGACCAAGGAGACCAAGGCCGGTGTTACCAAGGGCAAGACTCTCCTTGAC
MJ144_95_CZ --
JV0909_3_CZ --
JV0609_1_K
GGACAAAGGAAACCAAGGCCGGTGTTACCAAGGGCAAGACTCTCCTCGAC
Dai20396Physisporinus_castanop
GGACAAAGGAAACCAAGGCCGGTGTCACCAAGGGCAAGACTCTCCTCGAC
Dai20397Physisporinus_castanop
GGACAAAGGAAACCAAGGCCGGTGTCACCAAGGGCAAGACTCTCCTCGAC
MJ19_09_SK_Abies --
JV0509_40_J_TN_USA_Betula
GGACCAAGGAAACCAAGGCCGGTGTTACCAAGGGCAAGACTCTCCTCGAC
JV0808_33crocatus_PA_USAlist
GGACCAAGGAAACCAAGGCCGGTGTTACCAAGGGCAAGACTCTCCTCGAC
DLL2009_061P_crocatus --
Dai12800P_subcrocatus --
Dai15917P_subcrocatus
GGACCAAGGAAACCAAGGCCGGTGTTACCAAGGGCAAGACTCTCCTCGAC
Cui16620 --
HCFC1088Meripilus_stillicidior --
MCW590Meripilus_obsrcurus --
MCW722Meripilus_obsrcurus --
Cui9381P_tibeticus --
Cui9588P_tibeticus

GGACCAAGGAAACAAAGGCCGGTGTTACCAAGGGCAAGACTCTCCTCGAC
Va2_Beneschova --
CWU3874_Ukraine_Alnus --
WCG1293Dai24718Physisporinus --
WCG1268Dai24682A
GGACCAAGGATACCAAGGCCGGTTCTACCAAGGGAAAGACTCTCCTCGAT
WCG1269Dai24683A
GGACCAAGGATACCAAGGCCGGTTCTACCAAGGGAAAGACTCTTCTCGAT
WCG1279Dai24694A
GGACCAAGGATACCAAGGCCGGTTCTACCAAGGGAAAGACTCTCCTCGAT
Dai16971 --
ZQY1043Dai26696 --
Doll880
GGACCAAGGATACCAAGGCCGGTTCCACCAAGGGAAAGACTCTTCTCGAC
Doll1000 --
1DAI18529 --
Dai19535
GGACCAAGGAGACCAAGGCTGGTGTTACCAAGGGCAAGACCCTCCTCGAT
1704_79_hnedyVillaLaPaz
GGACCAAGGAGACCAAGGCTGGTGTTACCAAGGGCAAGACCCTCCTCGAT
F2061 --
1DAI18268 --
1DAI18540A
GGACCAAGGAGACTAAGGCTGGTGTTACCAAGGGCAAGACCCTCCTCGAC
Dai17695 --
LKY18Dai26373 --
Dai17839P_sulphureus
GGACCAAGGAGACCAAGGCTGGTGTCACCAAGGGAAAGACTCTCCTTGAT
Dai17841P_sulphureus
GGACCAAGGAGACCAAGGCTGGTGTCACCAAGGGAAAGACTCTCCTTGAT
Dai19877P_roseus --
1508_18_1_Kout --
KP859303_R_vinctus_RP185_BRAZI --
JK1807_15Rigidoporus_sp_Puerto --
JV0509_47_J_TN_USA
GGACCAAGGAGACCAAGGCTGGTTCTTCCAAGGGCAAGACCCTCCTCGAT
JV0709_188 --
JV0509_127_PA_USA
GGACCAAGGAGACCAAGGCTGGTTCTTCCAAGGGCAAGACCCTCCTCGAT
JV1009_59_NJ_USA
GGACCAAGGAGACCAAGGCTGGTTCTTCCAAGGGCAAGACCCTCCTCGAT
Dai15497P_crataegi
GGACCAAGGAGACCAAGGCCGGTTCATCCAAGGGAAAGACTCTTCTCGAT
Dai15499P_crataegi

GGACCAAGGAGACCAAGGCCGGTTCATCCAAGGGAAAGACTCTTCTCGAT
Cui3266P_cinereus --
WCG1256Dai24690
GGACCAAGGAGACCAAGGCTGGTGCCTCCAAGGGCAAGACTCTCCTTGAT
1DAI17581 --
WCG1255Dai24688
GGACCAAGGAGACCAAGGCTGGTGCCTCCAAGGGCAAGACTCTCCTTGAT
Dai22427 --
MV690Meripilus_concrescens --
MV513Meripilus_galapagensis --
Dai19793
GGACCAAGGAGACCAAGGGTGGTTCCTCCAAGGGCAAGACCCTCCTCGAT
OQ553780P_tamilnaduensis --
OQ553779P_tamilnaduensis --
A164FB3Meripilus_giganteus --
JV1407_36_Vinctus_Meandrica
GGACCAAGGAGACCAAGGCTGGCGCCACCAAGGGCAAGACCCTCCTCGAT
1807_3K_Rigidoporus_PuertoRico
GGACCAAGGAGACCAAGGCTGGCGCCACCAAGGGCAAGACCCTCCTCGAT
Cui16903P_vinctus
GGACCAAGGAGACCAAGGCTGGCGCCACCAAGGGCAAGACCCTCCTCGAT
JV1008_18R_Lineatus --
JV1407_37_1_Vinctus_Carara
GGACCAAGGATACCAAGGCTGGCTCCACCAAGGGCAAGACCCTCCTCGAC
Dai17986P_lineatus
GGACCAAGGATACCAAGGCTGGTTCCACCAAGGGCAAGACCCTCCTCGAC
Dai18281
GGACCAAGGATACCAAGGCCGGCTCCACCAAGGGCAAGACCCTCCTCGAC
1DAI19796
GGACCAAGGATACCAAGGCTGGCTCCACCAAGGGCAAGACCCTTCTCGAT
ZQY797Dai25241
GGACCAAGGATACCAAGGCTGGCTCCACCAAGGGCAAGACCCTTCTCGAT
WCG1289Dai24711 --
Dai22598
GGACCAAGGACACCAAGGCTGGTTCCACCAAGGGCAAGACTCTCCTCGAT
Dai20523
GGACCAAGGACACCAAGGCTGGTTCCACCAAGGGCAAGACTCTCCTCGAT
Dai17885
GGACCAAGGACACTAAGGCTGGCTCCACCAAGGGCAAGACTCTCCTCGAT
Dai17553 --
Dai19639
GGACCAAGGACACCAAGGCTGGTTCCACCAAGGGCAAGACTCTCCTCGAT
JV0110_48_CZ
GGACTAAGGAGACCAAGGCTGGTGCCACCAAGGGCAAGACGCTCCTTGAT

MJ129_04 --
Cui10340P_eminens
GGACTAAGGAGACCAAGGCTGGTGCCACCAAGGGCAAGACGCTCCTTGAT
Cui10341P_eminens --
Dai12685P_eminens
GGACTAAGGAGACCAAGGCTGGTGCCACCAAGGGCAAGACGCTCCTTGAT
Miettinen_13591Rigidoporus_und --
Dai20868
GGACCAAGGAGACCAAGGCTGGTGCCACCAAGGGCAAGACCCTCCTTGAT
Dai20832
GGACCAAGGAGACCAAGGCCGGTGCCACCAAGGGCAAGACCCTCCTTGAT
Dai11400
GGACCAAGGAGACCAAGGCTGGTGCCACCAAGGGCAAGACCCTCCTTGAT
Dai22472
GGACCAAGGAGACCAAGGCTGGTGCCACCAAGGGCAAGACCCTCCTTGAT
1CUI10475 --
1CUI10491 --
HCFC1095Meripilus_robledoi --
MCW702Meripilus_revolubilis --
1704_83_zluty_HaciendaBaru --
Dai9925P_lavendulus --
Dai13587AP_lavendulus
GGACCAAGGAGACTAAGGCTGGTGTCACCAAGGGAAAGACTCTCCTCGAT
PDD70600P_longicystidius --
Cui16630 --
FP_135344Meripilus_giganteus --
FP_100460_Sp --
CBS421_48Meripilus_giganteus --
Cui9203 --
Cui9202 --
TUFC100564Japan --
Russell5913Meripilus_sumstinei --
RP215Meripilus_brasiliensis --
RP200Meripilus_brasiliensis --
JV1712_13J_R_vinctus2_LSUPuert --
Dai10503R_hypobrunneus --
Dai10569Rigidoporus_hypobrunne --
1DAI19451 --
CM108bRigidoporus_hypobrunneus --
1CUI16874 --
FD299Cerrena_unicolor --
KHL_GB_Cerrena_uniclor --
Dai12892Cerrena_albocinnamomea --
Dai12955C_albocinnamomea --

SNUm03110102C_aurantiopora --

NIBRFG0000102423C_aurantiopora --

Dai7359Antrodiella_zonata --

F20080702KCM29C_consors --

F20080208LYW10Cerrena_consors --

Dai7821Antrodiella_zonata --

CFMR_DCL04_31Pseudolagarobasid --

VPB197Pseudolagarobasidium_bel --

CBS115543Pseudolagarobasidium_ --

CBS115544 --

Han405Pseudolagarobasidium_bai --

Han406Pseudolagarobasidium_bai --

MUcc838Spongipellis_delectans --

BRNM686401S_delectans --

OSM_F925S_delectans --

BRNM67093Spongipellis_litschau --

CFMRccFP59199TS_unicolor --

CFMRccFP71791TS_unicolor --

Dai13845P_lischaueri
GGACCAAGGAGACCAAGGCTGGTGTTGTCAAGGGTAAAACCCTCCTTGAT

Dai20266P_lischaueri --

CFMR_HHB11240Radulodon_america --

RLG6350Radulodon_americanus --

KY415963Radulodon_erikssonii --

HHB9567spRadulodon_casearius --

KRT_Iso_26Radulodon_casearius --

CBS126044Radulodon_erikssonii --

He6183YUNNANENSIS
GGACCAAGGAGACCAAGGCTGGTGTCGTCAAGGGCAAGACTCTCCTCGAT

Cui17979YUNNANENSIS
GGACCAAGGAGACCAAGGCTGGTGTCATCAAGGGCAAGACTCTCCTCGAT

Miettinen2091Junghuhnia_fimbri --

KHL12316S_tenue --

PRM846564S_pachyodon --

SP_Lgt_S_pachyodon --

Ryvarden44669Tyromyces_xuchile --

PW17_171sinuosus --

W53Dai12234
GGACTAAGGAGACCAAAGCTGGTGTCGTCAAGGGCAAGACCCTTCTTGAT

HHB4100SpAntella_americana --

W3Dai20901spumeus
GGACCAAGGAGACCAAGGCTGGTGTCACCAAGGGAAAGACTCTCCTTGAC

He6736 --

BRNM734877S_spumeus --

BRNM712630S_spumeus --
Dai1723Loweomyces_sibiricus --
W54Cui10009
GGACTAAGGAGACCAAGGCTGGTGTCACCAAGGGAAAGACTCTCCTTGAT
W1Dai20899
GGACTAAGGAGACCAAGGCTGGTGTCACCAAGGGAAAGACTCTCCTTGAT
HHB13445Trametes_ochracea --
Dai16222 --
Dai16240 --

JV1310_11SanguinolentusCernys
GCCATCGATGCCATCGAACCCCCAGCTCGTCCCTCGGACAAGCCT-CTCC
MJ39_00_SK --
MJ111_04_CZ
GCCATCGATGCCATCGAACCCCCAGCCCGTCCCTCGGACAAGCCT-CTCC
JV1610_BOKYsmrk
GCCATCGATGCCATCGAACCCCCAGCTCGTCCCTCGGACAAGCCT-CTCC
Dai21030 --
Dai20976P_furcatus
GCCATCGATGCCATCGAACCCCCAGCTCGTCCCTCGGACAAGCCT-CTCC
Dai2105 --
Dai2544 --
Dai11313 --
WCG1611Dai26167
GCCATCGATGCCATCGAACCCCCAGCCCGTCCCTCGGACAAGCCT-CTCC
WCG1518Dai25999Physisporinus --
TAA15097 --
JV8909_19_CZ --
JV1310_15_P_sanguinolentus2_CZ --
MJ53_02_CZ --
CLZhao21647P_yunnanensis --
CLZhao21583P_yunnanensis --
Dai22272
GCCATCGATGCCATCGAACCCCCAGCCCGTCCCTCGGACAAGCCT-CTCC
Dai22279 --
MJ332_94_CZ --
MJ642_94_CZ_Expallescens --
Dai21060P_vinctus --
JV0511_23LRP_pouzarii
GCCATCGATGCCATCGAGCCCCCAGCACGTCCCTCCGACAAGCCT-CTCC
JQ409462_R_pouzarii_PRM899856_ --
JV0308_66_WA
GCCATTGATGCCATCGAGCCCCCAGCCCGTCCCTCCGACAAGCCT-CTCC
JV0309_45_WA_USA

GCCATTGATGCCATCGAGCCCCCAGCCCGTCCCTCCGACAAGCCT-CTCC
JV0709_83_CA_USA
GCCATTGATGCCATCGAGCCCCCAGCCCGTCCCTCCGACAAGCCT-CTCC
Dai21043P_pouzarii
GCCATCGATGCCATCGAGCCCCCAGCACGTCCCTCCGACAAGCCT-CTCC
MJ144_95_CZ --
JV0909_3_CZ --
JV0609_1_K
GCCATTGATGCCATCGAGCCCCCAGCTCGTCCCTCCGATAAGCCT-CTCC
Dai20396Physisporinus_castanop
GCCATTGATGCCATCGAGCCCCCAGCTCGTCCCTCCGATAAGCCT-CTCC
Dai20397Physisporinus_castanop
GCCATTGATGCCATCGAGCCCCCAGCTCGTCCCTCCGATAAGCCT-CTCC
MJ19_09_SK_Abies --
JV0509_40_J_TN_USA_Betula
GCCATTGATGCCATCGAGCCCCCAGCCCGTCCCTCCGACAAGCCT-CTCC
JV0808_33crocatus_PA_USAlist
GCCATTGATGCCATCGAGCCCCCAGCCCGTCCCTCCGACAAGCCT-CTCC
DLL2009_061P_crocatus --
Dai12800P_subcrocatus --
Dai15917P_subcrocatus
GCCATTGATGCCATCGAGCCCCCAGCCCGTCCCTCCGACAAGCCT-CTCC
Cui16620 --
HCFC1088Meripilus_stillicidior --
MCW590Meripilus_obsrcurus --
MCW722Meripilus_obsrcurus --
Cui9381P_tibeticus --
Cui9588P_tibeticus
GCCATCGACGCCATCGAGCCCCCAGCCCGTCCCTCCGACAAGCCC-CTCC
Va2_Beneschova --
CWU3874_Ukraine_Alnus --
WCG1293Dai24718Physisporinus --
WCG1268Dai24682A
GCTATCGATGCCATCGAACCCCCAGTCCGACCCTCGGACAAGCCCTCTCC
WCG1269Dai24683A
GCTATCGATGCCATCGAACCCCCAGTCCGACCCTCGGACAAGCCC-CTCC
WCG1279Dai24694A
GCTATCGATGCTATCGAACCCCCAGTCCGACCCTCGGACAAGCCC-CTCC
Dai16971 --
ZQY1043Dai26696 --
Doll880
GCTATCGATGCTATCGAGCCCCCAGTCCGACCCTCCGACAAGCCC-CTCC
Doll1000 --
1DAI18529 --

Dai19535
GCTATCGATGCCATCGAACCCCCAGTTCGTCCTTCCGACAAGCCT-CTCC
1704_79_hnedyVillaLaPaz
GCCATCGATGCCATCGAGCCCCCAGTCCGACCCTCCGACAAACCT-CTTC
F2061 --
1DAI18268 --
1DAI18540A
GCCATCGATGCCATCGAGCCCCCAGTCCGACCCTCCGACAAGCCC-CTCC
Dai17695 --
LKY18Dai26373 --
Dai17839P_sulphureus
GCCATCGATGCTATTGAGCCCCCAGTTCGTCCTTCCGACAAGCCT-CTCC
Dai17841P_sulphureus
GCCATCGATGCTATTGAGCCCCCAGTTCGTCCTTCCGACAAGCCT-CTCC
Dai19877P_roseus --
1508_18_1_Kout --
KP859303_R_vinctus_RP185_BRAZI --
JK1807_15Rigidoporus_sp_Puerto --
JV0509_47_J_TN_USA
GCTATCGACGCTATTGACCCCCCAGTCCGTCCCTCTGACAAGCCC-CTCC
JV0709_188 --
JV0509_127_PA_USA
GCTATCGACGCTATTGACCCCCCAGTCCGTCCCTCTGACAAGCCC-CTCC
JV1009_59_NJ_USA
GCTATCGACGCTATTGACCCCCCAGTCCGTCCCTCTGACAAGCCC-CTCC
Dai15497P_crataegi
GCTATCGATGCCATCGAGCCCCCAGTACGTCCCTCCGACAAGCCC-CTCC
Dai15499P_crataegi
GCTATCGATGCCATCGAGCCCCCAGTACGTCCCTCCGACAAGCCC-CTCC
Cui3266P_cinereus --
WCG1256Dai24690
GCCATCGACTCCATCGACCCCCCAGTTCGACCCTCCGACAAGCCC-CTCC
1DAI17581 --
WCG1255Dai24688
GCCATCGACTCCATCGACCCCCCAGTTCGACCCTCCGACAAGCCC-CTCC
Dai22427 --
MV690Meripilus_concrescens --
MV513Meripilus_galapagensis --
Dai19793
GCCATCGACGCCATTGAGCCCCCAGTCCGTCCCTCTGACAAGCCC-CTCC
OQ553780P_tamilnaduensis --
OQ553779P_tamilnaduensis --
A164FB3Meripilus_giganteus --
JV1407_36_Vinctus_Meandrica

GCCATCGATGCCATCGAGCCCCCAGTCCGTCCCTCTGACAAGCCT-CTCC
1807_3K_Rigidoporus_PuertoRico
GCCATCGATGCCATCGAGCCCCCAGTCCGTCCCTCTGACAAGCCT-CTCC
Cui16903P_vinctus
GCCATCGATGCCATCGAGCCCCCAGTCCGTCCCTCTGACAAGCCT-CTCC
JV1008_18R_Lineatus --
JV1407_37_1_Vinctus_Carara
GCCATCGACGCCATCGAGCCCCCAGTCCGTCCCTCCGACAAGCCC-CTCC
Dai17986P_lineatus
GCCATCGACGCCATCGAGCCCCCAGTCCGTCCCTCTGACAAGCCC-CTCC
Dai18281
GCCATCGACGCCATCGAGCCCCCAGTCCGTCCCTCCGACAAGCCC-CTCC
1DAI19796
GCCATCGATGCCATCGAGCCCCCAGTCCGTCCCTCTGACAAGCCT-CTCC
ZQY797Dai25241
GCCATCGATGCCATCGAGCCCCCAGTCCGTCCCTCTGACAAGCCT-CTCC
WCG1289Dai24711 --
Dai22598
GCCATCGACGCCATCGAGCCCCCCGTCCGTCCCTCTGACAAGCCC-CTCC
Dai20523
GCCATCGACGCCATCGAGCCCCCCGTCCGTCCCTCTGACAAGCCC-CTCC
Dai17885
GCCATCGACGCCATCGAGCCCCCCGTCCGTCCCTCTGACAAGCCC-CTCC
Dai17553 --
Dai19639
GCCATCGACGCCATCGAGCCCCCCGTCCGTCCCTCTGACAAGCCC-CTCC
JV0110_48_CZ
GCAATTGACGCCATCGAGCCTCCATCTCGCCCCTCCGACAAGCCC-CTCC
MJ129_04 --
Cui10340P_eminens
GCAATTGACGCCATCGAGCCTCCATCTCGCCCCTCTGACAAGCCC-CTCC
Cui10341P_eminens --
Dai12685P_eminens
GCAATTGACGCCATCGAGCCTCCATCTCGCCCCTCCGACAAGCCC-CTCC
Miettinen_13591Rigidoporus_und --
Dai20868
GCCATTGATGCCATCGAGCCCCCAGCTCGTCCCTCCGACAAGCCC-CTCC
Dai20832
GCCATTGACGCCATTGAGCCCCCAGCTCGTCCCTCCGACAAGCCC-CTCC
Dai11400
GCCATTGACGCCATTGAGCCCCCAGCTCGTCCCTCCGACAAGCCC-CTCC
Dai22472
GCCATTGATGCCATTGAGCCCCCAGCTCGTCCCTCCGACAAGCCC-CTCC
1CUI10475 --

1CUI10491 --
HCFC1095Meripilus_robledoi --
MCW702Meripilus_revolubilis --
1704_83_zluty_HaciendaBaru --
Dai9925P_lavendulus --
Dai13587AP_lavendulus
GCCATTGATGCCATTGAACCCCCAATCCGACCCTCCGATAAGCCC-CTCC
PDD70600P_longicystidius --
Cui16630 --
FP_135344Meripilus_giganteus --
FP_100460_Sp --
CBS421_48Meripilus_giganteus --
Cui9203 --
Cui9202 --
TUFC100564Japan --
Russell5913Meripilus_sumstinei --
RP215Meripilus_brasiliensis --
RP200Meripilus_brasiliensis --
JV1712_13J_R_vinctus2_LSUPuert --
Dai10503R_hypobrunneus --
Dai10569Rigidoporus_hypobrunne --
1DAI19451 --
CM108bRigidoporus_hypobrunneus --
1CUI16874 --
FD299Cerrena_unicolor --
KHL_GB_Cerrena_uniclor --
Dai12892Cerrena_albocinnamomea --
Dai12955C_albocinnamomea --
SNUm03110102C_aurantiopora --
NIBRFG0000102423C_aurantiopora --
Dai7359Antrodiella_zonata --
F20080702KCM29C_consors --
F20080208LYW10Cerrena_consors --
Dai7821Antrodiella_zonata --
CFMR_DCL04_31Pseudolagarobasid --
VPB197Pseudolagarobasidium_bel --
CBS115543Pseudolagarobasidium_ --
CBS115544 --
Han405Pseudolagarobasidium_bai --
Han406Pseudolagarobasidium_bai --
MUcc838Spongipellis_delectans --
BRNM686401S_delectans --
OSM_F925S_delectans --
BRNM67093Spongipellis_litschau --

CFMRccFP59199TS_unicolor --
CFMRccFP71791TS_unicolor --
Dai13845P_lischaueri
GCCATTGATGCCATCGAGCCTCCCGTCCGTCCGTCGGACAAGCCT-CTTC
Dai20266P_lischaueri --
CFMR_HHB11240Radulodon_america --
RLG6350Radulodon_americanus --
KY415963Radulodon_erikssonii --
HHB9567spRadulodon_casearius --
KRT_Iso_26Radulodon_casearius --
CBS126044Radulodon_erikssonii --
He6183YUNNANENSIS
GCCATCGATGCCATCGAGCCCCCCGTCCGTCCCTCCGACAAGCCC-CTCC
Cui17979YUNNANENSIS
GCCATCGATGCCATCGAGCCCCCAGTCCGTCCCTCCGACAAGCCC-CTCC
Miettinen2091Junghuhnia_fimbri --
KHL12316S_tenue --
PRM846564S_pachyodon --
SP_Lgt_S_pachyodon --
Ryvarden44669Tyromyces_xuchile --
PW17_171sinuosus --
W53Dai12234
GCCATCGATGCCATCGAGCCTCCTGTTCGTCCTTCCGACAAGCCT-CTCC
HHB4100SpAntella_americana --
W3Dai20901spumeus
GCCATTGACGCCATCGAGCCCCCAGTCCGCCCCTCCGACAGGCCC-CTTC
He6736 --
BRNM734877S_spumeus --
BRNM712630S_spumeus --
Dai1723Loweomyces_sibiricus --
W54Cui10009
GCCATTGATGCCATCGAACCCCCACTCCGCCCCTCCGACAAGCTC-TCTC
W1Dai20899
GCCATTGATGCCATCGAACCCCCACTCCGCCCCTCCGACAAGCCC-CTTC
HHB13445Trametes_ochracea --
Dai16222 --
Dai16240 --

JV1310_11SanguinolentusCernys GACTCCCTCTCCAGGATGTTTTGGCCA-
CCTGTTCCGAATGGTTGTTCAG
MJ39_00_SK --
MJ111_04_CZ GACTCCCTCTCCAGGATGTTT---------------------------
--
JV1610_BOKYsmrk GACTCCCTCTCCAGGATGTTT---------------------------

---
Dai21030 --
Dai20976P_furcatus GACTCCCTCTCCAGGATGTTTTGGCCA-
CCTGTTCCGAAT-GTTGTTCAG
Dai2105 --
Dai2544 --
Dai11313 --
WCG1611Dai26167 GACTTCCTCTCCAGGATGTTTTGGCGA-
CCTGTTTCGAAT-GTTGTTCAG
WCG1518Dai25999Physisporinus --
TAA15097 --
JV8909_19_CZ --
JV1310_15_P_sanguinolentus2_CZ --
MJ53_02_CZ --
CLZhao21647P_yunnanensis --
CLZhao21583P_yunnanensis --
Dai22272 GACTTCCTCTCCAGGATGTTTTGGCGA-
CCTGTTCCGAAT-GTTGTTCAG
Dai22279 --
MJ332_94_CZ --
MJ642_94_CZ_Expallescens --
Dai21060P_vinctus --------------------TGGCGA-TCTGTTCCGAAT-
GTTGTTTAG
JV0511_23LRP_pouzarii GACTTCCTCTCCAGGATGTCT----------------------------
JQ409462_R_pouzarii_PRM899856_ --
JV0308_66_WA GACTTCCTCTCCAGGATGTCT---------------------------
--
JV0309_45_WA_USA GACTTCCTCTCCAGGATGTCT-------------------------
----
JV0709_83_CA_USA GACTTCCTCTCCAGGATGTCT--------------------------
---
Dai21043P_pouzarii GACTTCCTCTCCAGGATGTCTTGGCGA-
TCTGTTCCGAAT-GTTGTTCAG
MJ144_95_CZ --
JV0909_3_CZ --
JV0609_1_K GACTTCCTCTCCAGGATGTCT----------------------------
-
Dai20396Physisporinus_castanop GACTTCCTCTCCAGGATGTCTTGGCAA-
TCTGTTCCGAAT-GTTGTTCAG
Dai20397Physisporinus_castanop GACTTCCTCTCCAGGATGTCTTGGCAA-TCTGTTCCG-
AT-GTTGTTCAG
MJ19_09_SK_Abies --
JV0509_40_J_TN_USA_Betula GACTTCCTCTCCAGGATGTCT---------------------------
--

JV0808_33crocatus_PA_USAlist GACTTCCTCTCCAGGATGTCT-----------------------------
DLL2009_061P_crocatus --
Dai12800P_subcrocatus --
Dai15917P_subcrocatus GACTTCCTCTCCAGGATGTCT-----------------------------
Cui16620 --
HCFC1088Meripilus_stillicidior --
MCW590Meripilus_obsrcurus --
MCW722Meripilus_obsrcurus --
Cui9381P_tibeticus --
Cui9588P_tibeticus GACTTCCTCTCCAGGATGTCT-----------------------------
Va2_Beneschova --
CWU3874_Ukraine_Alnus --
WCG1293Dai24718Physisporinus --
WCG1268Dai24682A GACTAACTATTTCT--
TCCCTTCGCGAACCTGTTCCGAAT-GTTGTTTAG
WCG1269Dai24683A
GACTCCTCTGGCATGGTTTGCTCGCGAACCTGTTCCGAAT-GTTGTTTAG
WCG1279Dai24694A
GACTTCCTCTCCAGGATGTCTTCGCGAACTTGTTCCGAAT-GTTGTTCAG
Dai16971 --
ZQY1043Dai26696 --
Doll880 GACTTCCCCTCCAGGATGTCTTGGCGA-
CTTGTTCCGAAT-GTTGTTTAG
Doll1000 ---------------------TGGCGA-CTTGTTCCGAAT-
GTTGTTTAG
1DAI18529 --
Dai19535 GACTTCCTCTCCAGGATGTCTTGGCGA-
CCTGTTCCGAAT-GTTGTTCAG
1704_79_hnedyVillaLaPaz GACTTCCTCTCCAGGATGTCTTGGCGA-
CTTGTTCCGAT--GCTGTTTAG
F2061 --
1DAI18268 --
1DAI18540A GACTTCCTCTCCAGGATGTCT---------------------------
--
Dai17695 --
LKY18Dai26373 --
Dai17839P_sulphureus GACTTCCTCTCCAGGATGTCTTGGCGA-
CTTGTTCCGAAT-GTTGTTCAG
Dai17841P_sulphureus GACTTCCTCTCCAGGATGTCTTGGCGA-
CTTGTTCCGAAT-GTTGTTTAG
Dai19877P_roseus ---------------------TGGCGA-TCTGTTCCGAAG-
GTTGTTCAG
1508_18_1_Kout --
KP859303_R_vinctus_RP185_BRAZI --

JK1807_15Rigidoporus_sp_Puerto ---------------------TGGCGA-CTTGTTCCGAAT-
GTTGTTCAG
JV0509_47_J_TN_USA GACTTCCTCTCCAGGATGTCTTGGCGA-
CTTGTTCCGAAT-GTTGTTCAG
JV0709_188 --
JV0509_127_PA_USA GACTTCCTCTCCAGGATGTCTTGGCGA-
CTTGTTCCGAAT-GTTGTTCAG
JV1009_59_NJ_USA GACTTCCTCTCCAGGATGTCT---------------------------
--
Dai15497P_crataegi GACTTCCTCTCCAGGATGTCTTGGCA--
CTTGTTCCGAAT-GTTGTTCAG
Dai15499P_crataegi GACTTCCTCTCCAGGATGTCTTGGCCA-
CTTGTTCCGAAT-GTTGTTCAG
Cui3266P_cinereus --
WCG1256Dai24690 GACTTCCTCTCCAGGATGTCT--------------------------
---
1DAI17581 --
WCG1255Dai24688 GACTTCCTCTCCAGGATGTCTTGGCGA-
CTTGTTCCGAAT-GTTGTTCAG
Dai22427 --
MV690Meripilus_concrescens --
MV513Meripilus_galapagensis --
Dai19793 GACTTCCTCTCCAGGATGTCTTGGCAA-
CTTGTTCCGAAT-GTTGTTCAG
OQ553780P_tamilnaduensis --
OQ553779P_tamilnaduensis --
A164FB3Meripilus_giganteus --
JV1407_36_Vinctus_Meandrica GACTTCCTCTCCAGGATGTCT-----------------------------
1807_3K_Rigidoporus_PuertoRico GACTTCCTCTCCAGGATGTCTTGGCGA-
CTTATTCCGGAT-GTTGTTCAG
Cui16903P_vinctus GACTTCCTCTCCAGGATGTCTTGGCGA-
CTTATTCCGGAT-GTTGTTCAG
JV1008_18R_Lineatus --
JV1407_37_1_Vinctus_Carara GACTTCCTCTCCAGGATGTCTTGGCAA-
CTTGTTCCGGAT-GTTATTCAG
Dai17986P_lineatus GACTTCCTCTCCAGGATGTCTTGGCAA-
CTTGTTCCGGAT-GTTATTTAG
Dai18281 GACTTCCTCTCCAGGATGTCTTGGCAA-
CTTGTTCCGGAT-GTTATTCAG
1DAI19796 GACTTCCTCTCCAGGATGTCT----------------------------
-
ZQY797Dai25241 GACTTCCTCTCCAGGATGTCTTGGCAA-
CTTGTTCCGGAT-GTTATTCAG
WCG1289Dai24711 --

Dai22598 GACTTCCTCTCCAGGATGTCT--------------------------

Dai20523 GACTTCCTCTCCAGGATGTCT--------------------------

Dai17885 GACTTCCTCTCCAGGATGTCT--------------------------

Dai17553 ---

Dai19639 GACTTCCTCTCCAGGATGTCTTGGCAA-CTTGTTCCGGAT-GTTATTCAG

JV0110_48_CZ GACTTCCTCTCCAGGATGTCT---------------------------

MJ129_04 ---

Cui10340P_eminens GACTTCCTCTCCAGGATGTCT--------------------------

Cui10341P_eminens ---

Dai12685P_eminens GACTTCCTCTCCAGGATGTCT--------------------------

Miettinen_13591Rigidoporus_und ---

Dai20868 GACTTCCTCTCCAGGATGTCT--------------------------

Dai20832 GACTTCCTCTCCAGGATGTCTTGGGAG-ACGACCTCGTGG-GGGGGGGAA

Dai11400 GACTTCCTCTCCAGGATGTCT--------------------------

Dai22472 GACTTCCTCTCCAGGATGTCT--------------------------

1CUI10475 ---

1CUI10491 ---

HCFC1095Meripilus_robledoi ---

MCW702Meripilus_revolubilis ---

1704_83_zluty_HaciendaBaru ---

Dai9925P_lavendulus ---

Dai13587AP_lavendulus GACTTCCTCTACAGGATGTCT--------------------------

PDD70600P_longicystidius ---

Cui16630 ---

FP_135344Meripilus_giganteus ------------------TGGCAAATTTGTTCCGTAT-GCTCTTCCG

FP_100460_Sp ---

CBS421_48Meripilus_giganteus ---

Cui9203 ---

Cui9202 ---

TUFC100564Japan ---

Russell5913Meripilus_sumstinei ---

RP215Meripilus_brasiliensis ---

RP200Meripilus_brasiliensis ---

JV1712_13J_R_vinctus2_LSUPuert ---

Dai10503R_hypobrunneus ---

Dai10569Rigidoporus_hypobrunne ---

1DAI19451 ---

CM108bRigidoporus_hypobrunneus ---

1CUI16874 ---

FD299Cerrena_unicolor ---

KHL_GB_Cerrena_uniclor --
Dai12892Cerrena_albocinnamomea --
Dai12955C_albocinnamomea --
SNUm03110102C_aurantiopora --
NIBRFG0000102423C_aurantiopora --
Dai7359Antrodiella_zonata --
F20080702KCM29C_consors --
F20080208LYW10Cerrena_consors --
Dai7821Antrodiella_zonata --
CFMR_DCL04_31Pseudolagarobasid --
VPB197Pseudolagarobasidium_bel --
CBS115543Pseudolagarobasidium_ --
CBS115544 --
Han405Pseudolagarobasidium_bai --
Han406Pseudolagarobasidium_bai --
MUcc838Spongipellis_delectans --
BRNM686401S_delectans --
OSM_F925S_delectans --
BRNM67093Spongipellis_litschau --
CFMRccFP59199TS_unicolor --
CFMRccFP71791TS_unicolor --
Dai13845P_lischaueri GTCTTCCTCTCCAGGATGTTTTGGCAA-
TCTTTTCCGCAT-GTTGTTCCG
Dai20266P_lischaueri --
CFMR_HHB11240Radulodon_america --
RLG6350Radulodon_americanus --
KY415963Radulodon_erikssonii --
HHB9567spRadulodon_casearius --
KRT_Iso_26Radulodon_casearius --
CBS126044Radulodon_erikssonii --
He6183YUNNANENSIS GTCTTCCTCTCCAGGATGTCT----------------------
-----
Cui17979YUNNANENSIS GTCTTCCTCTTCAGGATGTCT-----------------------
----
Miettinen2091Junghuhnia_fimbri --
KHL12316S_tenue --
PRM846564S_pachyodon --
SP_Lgt_S_pachyodon --
Ryvarden44669Tyromyces_xuchile --
PW17_171sinuosus --
W53Dai12234 GTCTTCCTCTCCAGGATGTTTTAGCCA-
TCTCTTCCGTAT-GCTCTTCCG
HHB4100SpAntella_americana --
W3Dai20901spumeus GTCTTCCACTCCAGGATGTCT---------------------------

-
He6736 --
BRNM734877S_spumeus --
BRNM712630S_spumeus --
Dai1723Loweomyces_sibiricus --
W54Cui10009 GTCTACAATCGCAGGATTCC-----------------------------
-
W1Dai20899 GTCTTCCACTCCAGGATGTCTTGGCAA-
CTTGTTCCGAAT-GTTGTTCCG
HHB13445Trametes_ochracea --
Dai16222 --
Dai16240 --

JV1310_11SanguinolentusCernys
GAAATTGACGAAGGATGTTTACCGTTACTTGCAAAAGGTATGCTTG-TGA
MJ39_00_SK --
MJ111_04_CZ --
JV1610_BOKYsmrk --
Dai21030 --
Dai20976P_furcatus
GAAATTGACGAAGGATGTTTACCGTTACTTGCAAAAGGTATGCTTG-TGA
Dai2105 --
Dai2544 --
Dai11313 --
WCG1611Dai26167
GAAATTGACGAAGGATGTTTACCGTTACTTGCAAAAGGTATGCTTC-TGA
WCG1518Dai25999Physisporinus --
TAA15097 --
JV8909_19_CZ --
JV1310_15_P_sanguinolentus2_CZ --
MJ53_02_CZ --
CLZhao21647P_yunnanensis --
CLZhao21583P_yunnanensis --
Dai22272
GAAATTGACGAAGGATGTTTACCGTTACTTGCAAAAGGTATGCTTC-TGA
Dai22279 --
MJ332_94_CZ --
MJ642_94_CZ_Expallescens --
Dai21060P_vinctus
GAAGTTGACAAAGGATGTTTACCGCTACTTGCAAAAGGTATGTACC-TTA
JV0511_23LRP_pouzarii --
JQ409462_R_pouzarii_PRM899856_ --
JV0308_66_WA --
JV0309_45_WA_USA --

JV0709_83_CA_USA --
Dai21043P_pouzarii
GAAGTTGACAAAGGATGTCTATCGCTACTTGCAAAAGGTATGTGCC-TTC
MJ144_95_CZ --
JV0909_3_CZ --
JV0609_1_K --
Dai20396Physisporinus_castanop
GAAGTTGACAAAAGACGTTTACCGCTACTTGCAAAAGGTATGTGCT-TTA
Dai20397Physisporinus_castanop
GAAGTTGACAAAAGACGTTTACCGCTACTTGCAAAAGGTATGTGCT-TTA
MJ19_09_SK_Abies --
JV0509_40_J_TN_USA_Betula --
JV0808_33crocatus_PA_USAlist --
DLL2009_061P_crocatus --
Dai12800P_subcrocatus --
Dai15917P_subcrocatus --
Cui16620 --
HCFC1088Meripilus_stillicidior --
MCW590Meripilus_obsrcurus --
MCW722Meripilus_obsrcurus --
Cui9381P_tibeticus --
Cui9588P_tibeticus --
Va2_Beneschova --
CWU3874_Ukraine_Alnus --
WCG1293Dai24718Physisporinus --
WCG1268Dai24682A
GAAGTTGACAAAAGACGTTTACCGCTATCTGCAAAAGGTATAATCC-CCC
WCG1269Dai24683A
GAAGTTGACAAAAGACGTTTACCGCTATCTGCAAAAGGTATAATCC-CCC
WCG1279Dai24694A
GAAGTTGACAAAAGACGTTTACCGCTATCTGCAAAAGGTATAATTC-CCC
Dai16971 --
ZQY1043Dai26696 --
Doll880
GAAGCTGACAAAGGATGTCTATCGCTACTTGCAAAAGGTATACTCC-TTC
Doll1000
GAAGCTGACAAAGGATGTCTATCGCTACCTGCAAAAGGTATACTCC-CTC
1DAI18529 --
Dai19535
GAAGTTGACAAAAGATGTCTACCGCTACCTCCAAAAGGTACGTTTC-CTC
1704_79_hnedyVillaLaPaz
GAAGCTGACAAAGGACGTGTATCGTTACTTGCAAAAGGTATTTCAG-TGG
F2061 --
1DAI18268 --

1DAI18540A --
Dai17695 --
LKY18Dai26373 --
Dai17839P_sulphureus
GAAGCTCACAAAGGATGTATATCGCTATTTGCAGAAGGTGAGCACC-TTA
Dai17841P_sulphureus
GAAGCTCACAAAGGATGTATATCGCTATTTGCAGAAGGTGTGCACC-TTA
Dai19877P_roseus
GAAGCTCACGAAGGATGTTTATCGCTACTTGCAGAAGGTGTGTACC-TTA
1508_18_1_Kout --
KP859303_R_vinctus_RP185_BRAZI --
JK1807_15Rigidoporus_sp_Puerto
GAAGTTGACAAAGGATGTTTACCGTTATCTGCAAAAGGTACTCACC-CTT
JV0509_47_J_TN_USA
GAAGTTGACGAAGGATGTGTATCGCTACTTACAAAAGGTATAATCT-TTC
JV0709_188 --
JV0509_127_PA_USA
GAAGTTGACGAAGGACGTGTATCGCTACTTACAAAAGGTATAATCT-TTC
JV1009_59_NJ_USA --
Dai15497P_crataegi
AAAGTTGACAAAGGACGTGTACCGCTACTTACAAAAGGTATAACCT-TTC
Dai15499P_crataegi
AAAGTTGACAAAGGACGTGTACCGCTACTTACAAAAGGTATAACCT-TTC
Cui3266P_cinereus --
WCG1256Dai24690 --
1DAI17581 --
WCG1255Dai24688
GAAATTGACAAAGGACGTATATCGCTATCTACAAAAGGTCTGGCCT-TCT
Dai22427 --
MV690Meripilus_concrescens --
MV513Meripilus_galapagensis --
Dai19793
AAAGTTGACGAAGGACGTGTACCGTTACCTACAAAAGGTATGATCT-CTT
OQ553780P_tamilnaduensis --
OQ553779P_tamilnaduensis --
A164FB3Meripilus_giganteus --
JV1407_36_Vinctus_Meandrica --
1807_3K_Rigidoporus_PuertoRico
GAAACTGACAAAGGACGTATATCGCTACTTACAAAAGGTAAGAATCAGTC
Cui16903P_vinctus
GAAACTGACAAAGGACGTATATCGCTACTTACAAAAGGTAAGAATCAGTC
JV1008_18R_Lineatus --
JV1407_37_1_Vinctus_Carara
GAAACTGACAAAGGATGTGTACCGATACTTACAAAAGGTGGGAGTC-GTA

Dai17986P_lineatus
GAAACTGACAAAGGATGTATACCGTTACTTACAAAAGGTGGGATTC-GTC
Dai18281
GAAACTGACAAAGGATGTATACCGTTACTTACAAAAGGTGGGATTC-GTC
1DAI19796 --
ZQY797Dai25241
GAAACTGACAAAGGATGTGTACCGTTACTTACAAAAGGTAGGAGTC-GTC
WCG1289Dai24711 --
Dai22598 --
Dai20523 --
Dai17885 --
Dai17553 --
Dai19639
GAAACTGACAAAGGATGTGTACCGTTACTTACAAAAGGTAAGAATC-GTT
JV0110_48_CZ --
MJ129_04 --
Cui10340P_eminens --
Cui10341P_eminens --
Dai12685P_eminens --
Miettinen_13591Rigidoporus_und --
Dai20868 --
Dai20832
GAAAGTTGAGAAAGATGTGTATCGATACCTCCAAAAGGTACGCTGTTCTC
Dai11400 --
Dai22472 --
1CUI10475 --
1CUI10491 --
HCFC1095Meripilus_robledoi --
MCW702Meripilus_revolubilis --
1704_83_zluty_HaciendaBaru --
Dai9925P_lavendulus --
Dai13587AP_lavendulus --
PDD70600P_longicystidius --
Cui16630 --
FP_135344Meripilus_giganteus
GAAGCTCACAAAAGATGTTTATCGATACTTGCAAAAGGTGGGCTCG-TAT
FP_100460_Sp --
CBS421_48Meripilus_giganteus --
Cui9203 --
Cui9202 --
TUFC100564Japan --
Russell5913Meripilus_sumstinei --
RP215Meripilus_brasiliensis --
RP200Meripilus_brasiliensis --

JV1712_13J_R_vinctus2_LSUPuert --
Dai10503R_hypobrunneus --
Dai10569Rigidoporus_hypobrunne --
1DAI19451 --
CM108bRigidoporus_hypobrunneus --
1CUI16874 --
FD299Cerrena_unicolor --
KHL_GB_Cerrena_uniclor --
Dai12892Cerrena_albocinnamomea --
Dai12955C_albocinnamomea --
SNUm03110102C_aurantiopora --
NIBRFG0000102423C_aurantiopora --
Dai7359Antrodiella_zonata --
F20080702KCM29C_consors --
F20080208LYW10Cerrena_consors --
Dai7821Antrodiella_zonata --
CFMR_DCL04_31Pseudolagarobasid --
VPB197Pseudolagarobasidium_bel --
CBS115543Pseudolagarobasidium_ --
CBS115544 --
Han405Pseudolagarobasidium_bai --
Han406Pseudolagarobasidium_bai --
MUcc838Spongipellis_delectans --
BRNM686401S_delectans --
OSM_F925S_delectans --
BRNM67093Spongipellis_litschau --
CFMRccFP59199TS_unicolor --
CFMRccFP71791TS_unicolor --
Dai13845P_lischaueri
CAAGCTGACCAAAGATGTCTATCGCTACTTGCAAAAGGTAACAGCT-TTG
Dai20266P_lischaueri --
CFMR_HHB11240Radulodon_america --
RLG6350Radulodon_americanus --
KY415963Radulodon_erikssonii --
HHB9567spRadulodon_casearius --
KRT_Iso_26Radulodon_casearius --
CBS126044Radulodon_erikssonii --
He6183YUNNANENSIS --
Cui17979YUNNANENSIS --
Miettinen2091Junghuhnia_fimbri --
KHL12316S_tenue --
PRM846564S_pachyodon --
SP_Lgt_S_pachyodon --
Ryvarden44669Tyromyces_xuchile --

PW17_171sinuosus --
W53Dai12234
TAAGCTCACAAAAGACGTCTATCGCTATCTGCAGAAGGTAATATTG----
HHB4100SpAntella_americana --
W3Dai20901spumeus ------------------------------GAAGGTGAGATCTCGCG
He6736 --
BRNM734877S_spumeus --
BRNM712630S_spumeus --
Dai1723Loweomyces_sibiricus --
W54Cui10009 --
W1Dai20899
GAAGTTGACAAAAGATGTTTACCGATACCTGCAGAAGGTGAGATCACGCG
HHB13445Trametes_ochracea --
Dai16222 --
Dai16240 --

JV1310_11SanguinolentusCernys TGG------TTTGGACTGAA---
CTTTCGCGCTCATCCCACTGTC-----
MJ39_00_SK --
MJ111_04_CZ --
JV1610_BOKYsmrk --
Dai21030 --
Dai20976P_furcatus TGG------TTTGGACTGAA---
CTTTCGCGCTCATCCCACTGTC-----
Dai2105 --
Dai2544 --
Dai11313 --
WCG1611Dai26167 TGA------TTTGGACTGAA---
CTTTCGCGCTCATCTCACTGTC-----
WCG1518Dai25999Physisporinus --
TAA15097 --
JV8909_19_CZ --
JV1310_15_P_sanguinolentus2_CZ --
MJ53_02_CZ --
CLZhao21647P_yunnanensis --
CLZhao21583P_yunnanensis --
Dai22272 TGG------TTTGGAATGAA---
CTTCCGCGCTCATCCCACTGTC-----
Dai22279 --
MJ332_94_CZ --
MJ642_94_CZ_Expallescens --
Dai21060P_vinctus TGC----TACCTGGTATGG-------
TCGAGTTCACTTATCCGTC-----
JV0511_23LRP_pouzarii --

JQ409462_R_pouzarii_PRM899856_ --
JV0308_66_WA --
JV0309_45_WA_USA --
JV0709_83_CA_USA --
Dai21043P_pouzarii TGCA---TATCTGGTGTGA----
CGTCGAGCTCATCTATCCGTC-----
MJ144_95_CZ --
JV0909_3_CZ --
JV0609_1_K --
Dai20396Physisporinus_castanop TGC----TATTTGGTATGA----
CGTCGAGTTCATTGACCCGTC-----
Dai20397Physisporinus_castanop TGC----TATTTGGTATGA----
CGTCGAGTTCATTGACCCGTC-----
MJ19_09_SK_Abies --
JV0509_40_J_TN_USA_Betula --
JV0808_33crocatus_PA_USAlist --
DLL2009_061P_crocatus --
Dai12800P_subcrocatus --
Dai15917P_subcrocatus --
Cui16620 --
HCFC1088Meripilus_stillicidior --
MCW590Meripilus_obsrcurus --
MCW722Meripilus_obsrcurus --
Cui9381P_tibeticus --
Cui9588P_tibeticus --
Va2_Beneschova --
CWU3874_Ukraine_Alnus --
WCG1293Dai24718Physisporinus --
WCG1268Dai24682A TAT-----
CTCAGAGTAAGGGTTTCTTGGGTTGATCTC----TT-----
WCG1269Dai24683A TAT-----
CTCAGAGTAAGGGTTTCTTGGGTTGATCTC----TT-----
WCG1279Dai24694A TAT-----
TATCGAGTAAAGGTTCCTTGGGTTGATCTC----TT-----
Dai16971 --
ZQY1043Dai26696 --
Doll880 TCTA--ATCGTTGACATACC---
TCCTGTAGCTCATGTTTTCATT-----
Doll1000 TAGA--ATCGTTGACATCCC---
TCTTGTAGCTCATGTTTTCATT-----
1DAI18529 --
Dai19535 GCTA--CCCCTTGCCACGG----
TTATGGGCTCATTCGTCCCGTA----
1704_79_hnedyVillaLaPaz TTT---TCTCCTTGTCTGC-----TACTAAGCTAACGCA--

CGTTC----
F2061 --
1DAI18268 --
1DAI18540A --
Dai17695 --
LKY18Dai26373 --
Dai17839P_sulphureus CCT----GACTTGACATAT---
TCTTAAGACTCATCTGCTCGTCG----
Dai17841P_sulphureus CCT----GACTTGACATAC---
CCTTCAGACTCATCTGCTCGTCG----
Dai19877P_roseus CCT----AATTTGACATAC---
CCATGAAACTCATCTGCTTGTCG----
1508_18_1_Kout --
KP859303_R_vinctus_RP185_BRAZI --
JK1807_15Rigidoporus_sp_Puerto ATTGCTACTCTTGATATGA-----
CACTGACATCACACGCACTTC-----
JV0509_47_J_TN_USA AAAA--AGCTTC-CTATGTT------
TCGCGCTGATGATCAATCC-----
JV0709_188 --
JV0509_127_PA_USA AAAA--GGCTTC-TTATGTT------
TCGCGCTGATGATCAATCC-----
JV1009_59_NJ_USA --
Dai15497P_crataegi TCGA--TATTCTGTTATGCT------
CCTCACTTATCATCGTTCT-----
Dai15499P_crataegi TCGA--TATTCTGTTATGCT------
CCTCACTTATCATCGTTCT-----
Cui3266P_cinereus --
WCG1256Dai24690 --
1DAI17581 --
WCG1255Dai24688 GAGA--CCCCGTGGTACAATG-----
CCGTGCTGACTTTTTCTCC-----
Dai22427 --
MV690Meripilus_concrescens --
MV513Meripilus_galapagensis --
Dai19793 ATAA--CACCAT-AATGCTT------
TTGCGCTGACCATCACTTT-----
OQ553780P_tamilnaduensis --
OQ553779P_tamilnaduensis --
A164FB3Meripilus_giganteus --
JV1407_36_Vinctus_Meandrica --
1807_3K_Rigidoporus_PuertoRico ACCA----TCCCAGTATGTG---
AAATCGTGCTGATTTGTTGAGT-----
Cui16903P_vinctus ACCA----TCCCAGTATGTG---
AAATCGTGCTGATTTGTTGAGT-----

JV1008_18R_Lineatus ---
JV1407_37_1_Vinctus_Carara TCCA--GCGCTTAGTATGTG---
AATCGTTACTGATACCTTGGGC-----
Dai17986P_lineatus GCCA--GCCCTTAGCATTTG---
TATCGTTACTGATACCTTGGGC-----
Dai18281 GCCA--GCGCTTAGCATTTG---
TATCGTTACTGATACCTTGGGC-----
1DAI19796 ---
ZQY797Dai25241 ACCA--GCGCTCAGCACGTG---
AATCGGTACTGATACCTTGGAC-----
WCG1289Dai24711 ---
Dai22598 ---
Dai20523 ---
Dai17885 ---
Dai17553 ---
Dai19639 GCCA--ACGCTTCGTTTATG---
AATCGGTACTGATATCTTGGAC-----
JV0110_48_CZ ---
MJ129_04 ---
Cui10340P_eminens ---
Cui10341P_eminens ---
Dai12685P_eminens ---
Miettinen_13591Rigidoporus_und ---
Dai20868 ---
Dai20832 CGCCT----------------
GCTTCAGAGCTAACTCTCACGATCACAT
Dai11400 ---
Dai22472 ---
1CUI10475 ---
1CUI10491 ---
HCFC1095Meripilus_robledoi ---
MCW702Meripilus_revolubilis ---
1704_83_zluty_HaciendaBaru ---
Dai9925P_lavendulus ---
Dai13587AP_lavendulus ---
PDD70600P_longicystidius ---
Cui16630 ---
FP_135344Meripilus_giganteus AGTA--ACTTTTTGTTGGA----
GCTGTGGGCTGATGGTTCATTC-----
FP_100460_Sp ---
CBS421_48Meripilus_giganteus ---
Cui9203 ---
Cui9202 ---
TUFC100564Japan ---

Russell5913Meripilus_sumstinei --
RP215Meripilus_brasiliensis --
RP200Meripilus_brasiliensis --
JV1712_13J_R_vinctus2_LSUPuert --
Dai10503R_hypobrunneus --
Dai10569Rigidoporus_hypobrunne --
1DAI19451 --
CM108bRigidoporus_hypobrunneus --
1CUI16874 --
FD299Cerrena_unicolor --
KHL_GB_Cerrena_uniclor --
Dai12892Cerrena_albocinnamomea --
Dai12955C_albocinnamomea --
SNUm03110102C_aurantiopora --
NIBRFG0000102423C_aurantiopora --
Dai7359Antrodiella_zonata --
F20080702KCM29C_consors --
F20080208LYW10Cerrena_consors --
Dai7821Antrodiella_zonata --
CFMR_DCL04_31Pseudolagarobasid --
VPB197Pseudolagarobasidium_bel --
CBS115543Pseudolagarobasidium_ --
CBS115544 --
Han405Pseudolagarobasidium_bai --
Han406Pseudolagarobasidium_bai --
MUcc838Spongipellis_delectans --
BRNM686401S_delectans --
OSM_F925S_delectans --
BRNM67093Spongipellis_litschau --
CFMRccFP59199TS_unicolor --
CFMRccFP71791TS_unicolor --
Dai13845P_lischaueri AATAC-CTTTATGAAACGCT---------
TACTGACACCACTTAC-----
Dai20266P_lischaueri --
CFMR_HHB11240Radulodon_america --
RLG6350Radulodon_americanus --
KY415963Radulodon_erikssonii --
HHB9567spRadulodon_casearius --
KRT_Iso_26Radulodon_casearius --
CBS126044Radulodon_erikssonii --
He6183YUNNANENSIS --
Cui17979YUNNANENSIS --
Miettinen2091Junghuhnia_fimbri --
KHL12316S_tenue --

PRM846564S_pachyodon --
SP_Lgt_S_pachyodon --
Ryvarden44669Tyromyces_xuchile --
PW17_171sinuosus --
W53Dai12234 -----CTCTCATGGGCTTTT---
TTTGCGGTATAATTACTTACCTATATC
HHB4100SpAntella_americana --
W3Dai20901spumeus TGCACTCTGCTTGGATTGACC--------
ATCTGATTGCTTGGCT-----
He6736 --
BRNM734877S_spumeus --
BRNM712630S_spumeus --
Dai1723Loweomyces_sibiricus --
W54Cui10009 --
W1Dai20899 TGTACTCTGCTTGGATTGACC--------
AGCTGATTGCTTGGCT-----
HHB13445Trametes_ochracea --
Dai16222 --
Dai16240 --

JV1310_11SanguinolentusCernys CC-
CAGTGCGTCGAGACCCACAAGGAATTCAACTTGGCTCTGGCGGTGAA
MJ39_00_SK --
MJ111_04_CZ --
JV1610_BOKYsmrk --
Dai21030 --
Dai20976P_furcatus CC-
CAGTGCGTCGAGACCCACAAGGAATTCAACTTGGCTCTGGCGGTGAA
Dai2105 --
Dai2544 --
Dai11313 --
WCG1611Dai26167 CC-
CAGTGCGTCGAGACCCACAAGGATTTCAACTTGGCTCTGGCGGTGAA
WCG1518Dai25999Physisporinus --
TAA15097 --
JV8909_19_CZ --
JV1310_15_P_sanguinolentus2_CZ --
MJ53_02_CZ --
CLZhao21647P_yunnanensis --
CLZhao21583P_yunnanensis --
Dai22272 CC-
CAGTGCGTCGAGACCCACAAGGAATTCAACTTGGCTCTGGCGGTGAA
Dai22279 --
MJ332_94_CZ --

MJ642_94_CZ_Expallescens ---
Dai21060P_vinctus
CCGCAGTGCGTCGAGACACACAAGGAGTTCAACTTGGCGCTGGCTGTGAA
JV0511_23LRP_pouzarii ---
JQ409462_R_pouzarii_PRM899856_ ---
JV0308_66_WA ---
JV0309_45_WA_USA ---
JV0709_83_CA_USA ---
Dai21043P_pouzarii
AACCAGTGCGTCGAGACACACAAAGAGTTCAACTTGGCGCTGGCTGTGAA
MJ144_95_CZ ---
JV0909_3_CZ ---
JV0609_1_K ---
Dai20396Physisporinus_castanop
CCGCAGTGCGTCGAGACACACAAGGAGTTTAACTTGGCGCTGGCTGTGAA
Dai20397Physisporinus_castanop
CCGCAGTGCGTCGAGACACACAAGGAGTTTAACTTGGCGCTGGCTGTGAA
MJ19_09_SK_Abies ---
JV0509_40_J_TN_USA_Betula ---
JV0808_33crocatus_PA_USAlist ---
DLL2009_061P_crocatus ---
Dai12800P_subcrocatus ---
Dai15917P_subcrocatus ---
Cui16620 ---
HCFC1088Meripilus_stillicidior ---
MCW590Meripilus_obsrcurus ---
MCW722Meripilus_obsrcurus ---
Cui9381P_tibeticus ---
Cui9588P_tibeticus ---
Va2_Beneschova ---
CWU3874_Ukraine_Alnus ---
WCG1293Dai24718Physisporinus ---
WCG1268Dai24682A
CCATAGTGCGTCGAGACTCACAAGGAGTTCAACCTGGCTTTGGCTGTGAA
WCG1269Dai24683A
CCATAGTGCGTCGAGACTCACAAGGAGTTCAACCTGGCTTTGGCTGTGAA
WCG1279Dai24694A
CCATAGTGCGTCGAGACCCACAAGGAGTTCAACCTGGCCTTGGCTGTGAA
Dai16971 ---
ZQY1043Dai26696 ---
Doll880 -
TTTAGTGTGTCGAGACCCATAAGGACTTCAATTTGGCCCTGGCCGTGAA
Doll1000 -
TCTAGTGTGTCGAGACCCACAAGGACTTCAATCTGGCCCTGGCCGTGAA

1DAI18529 --
Dai19535
CTTTAGTGCGTCGAGACACACAAGGAGTTTAATTTGGCCCTAGCCGTGAA
1704_79_hnedyVillaLaPaz
TATAAGTGCGTCGAGACCCACAAGGAATTCAACTTAGCCTTGGCCGTGAA
F2061 --
1DAI18268 --
1DAI18540A --
Dai17695 --
LKY18Dai26373 --
Dai17839P_sulphureus
GCGTAGTGTGTCGAGACTCACAAGGAGTTTAACCTGGCGCTAGCTGTGAA
Dai17841P_sulphureus
GTGTAGTGTGTCGAGACTCACAAGGAGTTTAACCTGGCGCTAGCTGTGAA
Dai19877P_roseus
ACGTAGTGTGTCGAGACTCACAAGGAGTTCAACCTGGCGCTAGCTGTGAA
1508_18_1_Kout --
KP859303_R_vinctus_RP185_BRAZI --
JK1807_15Rigidoporus_sp_Puerto
TTCCAGTGCGTCGAGACTCACAAAGATTTCAACTTGGCGTTGGCCGTGAA
JV0509_47_J_TN_USA
CTTCAGTGCGTTGAGACGCACAAGGAGTTCAACTTGGCTCTGGCCGTTAA
JV0709_188 --
JV0509_127_PA_USA
CTTCAGTGCGTTGAGACGCACAAGGAGTTCAACTTGGCTCTGGCCGTTAA
JV1009_59_NJ_USA --
Dai15497P_crataegi
CTCCAGTGCGTTGAGACGCACAAGGAGTTCAACTTGGCTCTGGCCGTCAA
Dai15499P_crataegi
CTCCAGTGCGTTGAGACGCACAAGGAGTTCAACTTGGCTCTGGCCGTCAA
Cui3266P_cinereus --
WCG1256Dai24690 --
1DAI17581 --
WCG1255Dai24688
CTTTAGTGTGTCGAGACGCACAAGGAGTTCAATTTGGCGTTGGCTGTTAA
Dai22427 --
MV690Meripilus_concrescens --
MV513Meripilus_galapagensis --
Dai19793
CTGCAGTGTGTTGAAACGCACAAAGAGTTCAACTTGGCGTTGGCCGTCAA
OQ553780P_tamilnaduensis --
OQ553779P_tamilnaduensis --
A164FB3Meripilus_giganteus --
JV1407_36_Vinctus_Meandrica --

1807_3K_Rigidoporus_PuertoRico
TCTCAGTGCGTCGAGACGCACAAGGAGTTCAATCTGGCATTGGCTGTCAA
Cui16903P_vinctus
TCTCAGTGCGTCGAGACGCACAAGGAGTTCAATCTGGCATTGGCTGTCAA
JV1008_18R_Lineatus --
JV1407_37_1_Vinctus_Carara
TCTCAGTGCGTCGAGACGCACAAGGAGTTCAATTTAGCATTGGCTGTCAA
Dai17986P_lineatus
TCTCAGTGCGTCGAGACGCACAAGGAGTTCAATTTGGCATTGGCTGTCAA
Dai18281
TCTCAGTGCGTCGAGACGCACAAGGAGTTCAATTTGGCATTGGCTGTCAA
1DAI19796 --
ZQY797Dai25241
ACTCAGTGCGTGGAGACGCACAAGGAGTTCAATTTGGCGTTGGCTGTCAA
WCG1289Dai24711 --
Dai22598 --
Dai20523 --
Dai17885 --
Dai17553 --
Dai19639
CCTCAGTGCGTGGAGACGCACAAGGAGTTCAATTTGGCGTTGGCCGTCAA
JV0110_48_CZ --
MJ129_04 --
Cui10340P_eminens --
Cui10341P_eminens --
Dai12685P_eminens --
Miettinen_13591Rigidoporus_und --
Dai20868 --
Dai20832
TTATAGTGCGTTGAGACTCACAAAGAGTTCAACTTGGCCTTAGCTGTCAA
Dai11400 --
Dai22472 --
1CUI10475 --
1CUI10491 --
HCFC1095Meripilus_robledoi --
MCW702Meripilus_revolubilis --
1704_83_zluty_HaciendaBaru --
Dai9925P_lavendulus --
Dai13587AP_lavendulus --
PDD70600P_longicystidius --
Cui16630 --
FP_135344Meripilus_giganteus
CGATAGTGTGTCGAGACTCACAAGGAGTTCAATTTGGCCTTAGCTGTGAA
FP_100460_Sp --

CBS421_48Meripilus_giganteus --
Cui9203 --
Cui9202 --
TUFC100564Japan --
Russell5913Meripilus_sumstinei --
RP215Meripilus_brasiliensis --
RP200Meripilus_brasiliensis --
JV1712_13J_R_vinctus2_LSUPuert --
Dai10503R_hypobrunneus --
Dai10569Rigidoporus_hypobrunne --
1DAI19451 --
CM108bRigidoporus_hypobrunneus --
1CUI16874 --
FD299Cerrena_unicolor --
KHL_GB_Cerrena_uniclor --
Dai12892Cerrena_albocinnamomea --
Dai12955C_albocinnamomea --
SNUm03110102C_aurantiopora --
NIBRFG0000102423C_aurantiopora --
Dai7359Antrodiella_zonata --
F20080702KCM29C_consors --
F20080208LYW10Cerrena_consors --
Dai7821Antrodiella_zonata --
CFMR_DCL04_31Pseudolagarobasid --
VPB197Pseudolagarobasidium_bel --
CBS115543Pseudolagarobasidium_ --
CBS115544 --
Han405Pseudolagarobasidium_bai --
Han406Pseudolagarobasidium_bai --
MUcc838Spongipellis_delectans --
BRNM686401S_delectans --
OSM_F925S_delectans --
BRNM67093Spongipellis_litschau --
CFMRccFP59199TS_unicolor --
CFMRccFP71791TS_unicolor --
Dai13845P_lischaueri
TTCCAGTGCGTTGAGACGCACAAGGAGTTCAATCTGTCCTTGGCCGTCAA
Dai20266P_lischaueri --
CFMR_HHB11240Radulodon_america --
RLG6350Radulodon_americanus --
KY415963Radulodon_erikssonii --
HHB9567spRadulodon_casearius --
KRT_Iso_26Radulodon_casearius --
CBS126044Radulodon_erikssonii --

He6183YUNNANENSIS --
Cui17979YUNNANENSIS --
Miettinen2091Junghuhnia_fimbri --
KHL12316S_tenue --
PRM846564S_pachyodon --
SP_Lgt_S_pachyodon --
Ryvarden44669Tyromyces_xuchile --
PW17_171sinuosus --
W53Dai12234
ATCAAGTGTGTCGAGACACACAAGGAATTCAACTTGTCCTTGGCAGTAAA
HHB4100SpAntella_americana --
W3Dai20901spumeus -
TCCAGTGTGTGGAGACTCATAAGGAGTTCAATCTATCGCTTGCTGTGAA
He6736 --
BRNM734877S_spumeus --
BRNM712630S_spumeus --
Dai1723Loweomyces_sibiricus --
W54Cui10009 --
W1Dai20899 -
TCCAGTGTGTGGAGACTCATAAGGAGTTCAATCTATCGCTTGCTGTGAA
HHB13445Trametes_ochracea --
Dai16222 --
Dai16240 --

JV1310_11SanguinolentusCernys
ACACAACACAATCACGAACGGGTTGAAATATTCACTCGCCACTGGAAACT
MJ39_00_SK --
MJ111_04_CZ --
JV1610_BOKYsmrk --
Dai21030 --
Dai20976P_furcatus
ACACAACACAATCACGAACGGGTTGAAATATTCACTCGCCACTGGAAACT
Dai2105 --
Dai2544 --
Dai11313 --
WCG1611Dai26167
ACACAACACAATCACGAACGGGTTGAAATACTCACTCGCCACTGGGAACT
WCG1518Dai25999Physisporinus --
TAA15097 --
JV8909_19_CZ --
JV1310_15_P_sanguinolentus2_CZ --
MJ53_02_CZ --
CLZhao21647P_yunnanensis --
CLZhao21583P_yunnanensis --

Dai22272
ACATAACACAATCACGAACGGGTTGAAATATTCACTCGCCACTGGAAACT
Dai22279 --
MJ332_94_CZ --
MJ642_94_CZ_Expallescens --
Dai21060P_vinctus
GCACAATACGATCACAAACGGGCTCAAGTATTCGCTCGCCACCGGGAATT
JV0511_23LRP_pouzarii --
JQ409462_R_pouzarii_PRM899856_ --
JV0308_66_WA --
JV0309_45_WA_USA --
JV0709_83_CA_USA --
Dai21043P_pouzarii
ACATAATACGATCACGAACGGGCTCAAGTATTCGCTCGCCACCGGAAACT
MJ144_95_CZ --
JV0909_3_CZ --
JV0609_1_K --
Dai20396Physisporinus_castanop
GCACAACACGATCACAAACGGGCTCAAGTACTCGCTCGCCACCGGAAACT
Dai20397Physisporinus_castanop
GCACAACACGATCACAAACGGGCTCAAGTACTCGCTCGCCACCGGAAACT
MJ19_09_SK_Abies --
JV0509_40_J_TN_USA_Betula --
JV0808_33crocatus_PA_USAlist --
DLL2009_061P_crocatus --
Dai12800P_subcrocatus --
Dai15917P_subcrocatus --
Cui16620 --
HCFC1088Meripilus_stillicidior --
MCW590Meripilus_obsrcurus --
MCW722Meripilus_obsrcurus --
Cui9381P_tibeticus --
Cui9588P_tibeticus --
Va2_Beneschova --
CWU3874_Ukraine_Alnus --
WCG1293Dai24718Physisporinus --
WCG1268Dai24682A
GCACAACACGATCACAAACGGCCTGAAGTACTCCCTTGCCACCGGTAATT
WCG1269Dai24683A
GCACAACACGATCACAAACGGCCTGAAGTACTCCCTTGCCACCGGTAATT
WCG1279Dai24694A
GCACAACACGATCACAAACGGCCTGAAGTACTCCCTTGCCACCGGTAATT
Dai16971 --
ZQY1043Dai26696 --

Doll880
GCATAACACAATTACAAACGGGTTGAAGTACTCCCTTGCTACTGGAAATT
Doll1000
GCACAACACAATCACAAACGGGTTGAAGTACTCCCTTGCCACTGGAAATT
1DAI18529 --
Dai19535
ACATAACACGATTACCAATGGTCTCAAATATTCCCTTGCCACCGGAAACT
1704_79_hnedyVillaLaPaz
GCACAACACCATCACTAACGGTCTAAAATATTCTCTCGCCACTGGCAACT
F2061 --
1DAI18268 --
1DAI18540A --
Dai17695 --
LKY18Dai26373 --
Dai17839P_sulphureus
ACACAATACCATCACAAACGGCCTGAAGTACTCGCTCGCCACCGGTAACT
Dai17841P_sulphureus
ACACAATACCATCACAAACGGCCTGAAGTACTCGCTCGCCACCGGTAACT
Dai19877P_roseus
ACATAACACCATCACAAACGGCCTGAAGTACTCTCTTGCCACCGGTAATT
1508_18_1_Kout --
KP859303_R_vinctus_RP185_BRAZI --
JK1807_15Rigidoporus_sp_Puerto
ACATAATACCATTACCAATGGCCTCAAATATTCTCTCGCTACTGGGAACT
JV0509_47_J_TN_USA
ACACAATACGATTACAAATGGTCTCAAGTATTCGCTCGCCACCGGGAACT
JV0709_188 --
JV0509_127_PA_USA
ACACAATACGATTACAAATGGTCTCAAGTATTCGCTCGCCACCGGGAACT
JV1009_59_NJ_USA --
Dai15497P_crataegi
GCACAATACGATCACGAATGGTCTCAAGTATTCGCTCGCCACCGGAAACT
Dai15499P_crataegi
GCACAATACGATCACGAATGGTCTCAAGTATTCGCTCGCCACCGGAAACT
Cui3266P_cinereus --
WCG1256Dai24690 --
1DAI17581 --
WCG1255Dai24688
GCACAATACGATTACCAATGGTCTCAAATACTCACTCGCTACCGGGAACT
Dai22427 --
MV690Meripilus_concrescens --
MV513Meripilus_galapagensis --
Dai19793
ACACAATACGATTACAAATGGGCTCAAGTATTCGCTGGCCACTGGAAACT

OQ553780P_tamilnaduensis --
OQ553779P_tamilnaduensis --
A164FB3Meripilus_giganteus --
JV1407_36_Vinctus_Meandrica --
1807_3K_Rigidoporus_PuertoRico
ACACAACACTATCACCAATGGACTCAAGTATTCGCTCGCCACTGGGAATT
Cui16903P_vinctus
ACACAACACTATCACCAATGGACTCAAGTATTCGCTCGCCACTGGGAATT
JV1008_18R_Lineatus --
JV1407_37_1_Vinctus_Carara
ACACAATACCATCACCAATGGACTCAAGTATTCGCTCGCCACTGGGAACT
Dai17986P_lineatus
ACACAATACCATCACCAATGGACTCAAGTATTCGCTCGCCACTGGGAACT
Dai18281
ACACAATACCATCACCAATGGACTCAAGTATTCGCTCGCCACTGGGAACT
1DAI19796 --
ZQY797Dai25241
GCACAATACCATCACCAATGGACTCAAGTATTCGCTCGCCACTGGGAACT
WCG1289Dai24711 --
Dai22598 --
Dai20523 --
Dai17885 --
Dai17553 --
Dai19639
ACACAATACCATTACCAATGGACTCAAATATTCGCTCGCCACTGGAAACT
JV0110_48_CZ --
MJ129_04 --
Cui10340P_eminens --
Cui10341P_eminens --
Dai12685P_eminens --
Miettinen_13591Rigidoporus_und --
Dai20868 --
Dai20832
GCACAACACAATTACCAATGGTCTCAAGTATTCGCTTGCTACTGGTAACT
Dai11400 --
Dai22472 --
1CUI10475 --
1CUI10491 --
HCFC1095Meripilus_robledoi --
MCW702Meripilus_revolubilis --
1704_83_zluty_HaciendaBaru --
Dai9925P_lavendulus --
Dai13587AP_lavendulus --
PDD70600P_longicystidius --

Cui16630 --
FP_135344Meripilus_giganteus
GCACAACACTATCACAAATGGCTTGAAATACTCTCTGGCCACTGGAAACT
FP_100460_Sp --
CBS421_48Meripilus_giganteus --
Cui9203 --
Cui9202 --
TUFC100564Japan --
Russell5913Meripilus_sumstinei --
RP215Meripilus_brasiliensis --
RP200Meripilus_brasiliensis --
JV1712_13J_R_vinctus2_LSUPuert --
Dai10503R_hypobrunneus --
Dai10569Rigidoporus_hypobrunne --
1DAI19451 --
CM108bRigidoporus_hypobrunneus --
1CUI16874 --
FD299Cerrena_unicolor --
KHL_GB_Cerrena_uniclor --
Dai12892Cerrena_albocinnamomea --
Dai12955C_albocinnamomea --
SNUm03110102C_aurantiopora --
NIBRFG0000102423C_aurantiopora --
Dai7359Antrodiella_zonata --
F20080702KCM29C_consors --
F20080208LYW10Cerrena_consors --
Dai7821Antrodiella_zonata --
CFMR_DCL04_31Pseudolagarobasid --
VPB197Pseudolagarobasidium_bel --
CBS115543Pseudolagarobasidium_ --
CBS115544 --
Han405Pseudolagarobasidium_bai --
Han406Pseudolagarobasidium_bai --
MUcc838Spongipellis_delectans --
BRNM686401S_delectans --
OSM_F925S_delectans --
BRNM67093Spongipellis_litschau --
CFMRccFP59199TS_unicolor --
CFMRccFP71791TS_unicolor --
Dai13845P_lischaueri
GCATAACACTATCACCAACGGTCTGAAATACTCGCTGGCGACGGGTAATT
Dai20266P_lischaueri --
CFMR_HHB11240Radulodon_america --
RLG6350Radulodon_americanus --

KY415963Radulodon_erikssonii --
HHB9567spRadulodon_casearius --
KRT_Iso_26Radulodon_casearius --
CBS126044Radulodon_erikssonii --
He6183YUNNANENSIS --
Cui17979YUNNANENSIS --
Miettinen2091Junghuhnia_fimbri --
KHL12316S_tenue --
PRM846564S_pachyodon --
SP_Lgt_S_pachyodon --
Ryvarden44669Tyromyces_xuchile --
PW17_171sinuosus --
W53Dai12234
GCACAATACCATCACAAATGGTCTGAAGTATTCACTGGCTACCGGAAACT
HHB4100SpAntella_americana --
W3Dai20901spumeus
GCACAATACCATTACTAATGGTCTCAAGTACTCTCTTGCCACCGGTAACT
He6736 --
BRNM734877S_spumeus --
BRNM712630S_spumeus --
Dai1723Loweomyces_sibiricus --
W54Cui10009 --
W1Dai20899
GCACAATACCATTACCAATGGTCTCAAGTATTCTCTTGCCACCGGTAACT
HHB13445Trametes_ochracea --
Dai16222 --
Dai16240 --

JV1310_11SanguinolentusCernys
GGGGAGATCAGAAGAAGTCCATGGCTTCTAAGGCCGGTGTCTCTCAAGTA
MJ39_00_SK --
MJ111_04_CZ --
JV1610_BOKYsmrk --
Dai21030 --
Dai20976P_furcatus
GGGGAGATCAGAAGAAGTCCATGGCTTCTAAGGCCGGTGTCTCTCAAGTA
Dai2105 --
Dai2544 --
Dai11313 --
WCG1611Dai26167
GGGGAGATCAAAAGAAGTCCATGGCTTCTAAGGCCGGTGTCTCTCAAGTA
WCG1518Dai25999Physisporinus --
TAA15097 --
JV8909_19_CZ --

JV1310_15_P_sanguinolentus2_CZ --
MJ53_02_CZ --
CLZhao21647P_yunnanensis --
CLZhao21583P_yunnanensis --
Dai22272
GGGGAGATCAAAAGAAGTCCATGGCTTCTAAGGCCGGTGTCTCCCAAGTA
Dai22279 --
MJ332_94_CZ --
MJ642_94_CZ_Expallescens --
Dai21060P_vinctus
GGGGAGACCAAAAGAAGTCCATGGCTTCTAAAGCTGGTGTCTCCCAAGTG
JV0511_23LRP_pouzarii --
JQ409462_R_pouzarii_PRM899856_ --
JV0308_66_WA --
JV0309_45_WA_USA --
JV0709_83_CA_USA --
Dai21043P_pouzarii
GGGGAGACCAAAAGAAGTCCATGGCTTCCAAAGCTGGTGTCTCCCAAGTG
MJ144_95_CZ --
JV0909_3_CZ --
JV0609_1_K --
Dai20396Physisporinus_castanop
GGGGAGACCAAAAGAAGTCCATGGCTTCCAAAGCTGGTGTCTCCCAAGTA
Dai20397Physisporinus_castanop
GGGGAGACCAAAAGAAGTCCATGGCTTCCAAAGCTGGTGTCTCCCAAGTA
MJ19_09_SK_Abies --
JV0509_40_J_TN_USA_Betula --
JV0808_33crocatus_PA_USAlist --
DLL2009_061P_crocatus --
Dai12800P_subcrocatus --
Dai15917P_subcrocatus --
Cui16620 --
HCFC1088Meripilus_stillicidior --
MCW590Meripilus_obsrcurus --
MCW722Meripilus_obsrcurus --
Cui9381P_tibeticus --
Cui9588P_tibeticus --
Va2_Beneschova --
CWU3874_Ukraine_Alnus --
WCG1293Dai24718Physisporinus --
WCG1268Dai24682A
GGGGCGACCAAAAGAAGTCTATGGCTTCTAAGGCCGGTGTCTCTCAAGTG
WCG1269Dai24683A
GGGGCGACCAAAAGAAGTCTATGGCTTCTAAGGCCGGTGTCTCTCAAGTG

WCG1279Dai24694A
GGGGCGACCAAAAGAAGTCTATGGCTTCTAAGGCCGGTGTCTCTCAAGTG
Dai16971 --
ZQY1043Dai26696 --
Doll880
GGGGAGACCAAAAGAAGTCCATGGCTTCCAAGGCAGGCGTCTCCCAAGTG
Doll1000
GGGGAGACCAAAAGAAGTCCATGGCTTCCAAGGCAGGCGTCTCCCAAGTG
1DAI18529 --
Dai19535
GGGGAGACCAGAAGAAGACCATGGCTTCCAAAGCAGGTGTCTCCCAAGTG
1704_79_hnedyVillaLaPaz
GGGGGGACCAAAAGAAATCTATGGCTTCGAAAGCCGGTGTGTCTCAAGTG
F2061 --
1DAI18268 --
1DAI18540A --
Dai17695 --
LKY18Dai26373 --
Dai17839P_sulphureus
GGGGAGACCAGAAGAAGTCGATGGCGTCAAAGGCCGGTGTCTCACAAGTG
Dai17841P_sulphureus
GGGGAGACCAGAAGAAGTCGATGGCGTCGAAGGCCGGTGTCTCACAAGTG
Dai19877P_roseus
GGGGAGACCAGAAGAAGTCGATGGCGTCGAAGGCCGGTGTCTCACAAGTG
1508_18_1_Kout --
KP859303_R_vinctus_RP185_BRAZI --
JK1807_15Rigidoporus_sp_Puerto
GGGGAGACCAAAAGAAGTCTATGGCTTCGAAGGCCGGTGTGTCCCAGGTG
JV0509_47_J_TN_USA
GGGGAGACCAGAAGAAGTCGATGGCCTCCAAAGCTGGCGTGTCTCAAGTG
JV0709_188 --
JV0509_127_PA_USA
GGGGAGACCAGAAGAAGTCGATGGCCTCCAAGGCTGGCGTGTCTCAAGTG
JV1009_59_NJ_USA --
Dai15497P_crataegi
GGGGAGACCAGAAGAAGTCGATGGCCTCCAAAGCTGGCGTGTCCCAAGTA
Dai15499P_crataegi
GGGGAGACCAGAAGAAGTCGATGGCCTCCAAAGCTGGCGTGTCCCAAGTA
Cui3266P_cinereus --
WCG1256Dai24690 --
1DAI17581 --
WCG1255Dai24688
GGGGAGACCAGAAGAAGTCGATGGCCTCAAAGGCCGGTGTGTCCCAAGTG
Dai22427 --

MV690Meripilus_concrescens --
MV513Meripilus_galapagensis --
Dai19793
GGGGAGACCAGAAGAAGTCGATGGCCTCCAAAGCTGGAGTGTCTCAAGTG
OQ553780P_tamilnaduensis --
OQ553779P_tamilnaduensis --
A164FB3Meripilus_giganteus --
JV1407_36_Vinctus_Meandrica --
1807_3K_Rigidoporus_PuertoRico
GGGGAGACCAGAAGAAGTCGATGGCGTCGAAAGCAGGTGTCTCACAAGTT
Cui16903P_vinctus
GGGGAGACCAGAAGAAGTCGATGGCGTCGAAAGCAGGTGTCTCACAAGTT
JV1008_18R_Lineatus --
JV1407_37_1_Vinctus_Carara
GGGGAGACCAGAAGAAGTCGATGGCGTCAAAGGCCGGTGTCTCCCAAGTG
Dai17986P_lineatus
GGGGAGACCAGAAGAAGTCGATGGCGTCGAAGGCCGGTGTCTCTCAAGTG
Dai18281
GGGGAGACCAGAAGAAGTCGATGGCGTCGAAGGCCGGTGTCTCTCAAGTG
1DAI19796 --
ZQY797Dai25241
GGGGAGACCAGAAGAAGTCGATGGCGTCGAAGGCCGGTGTCTCTCAAGTG
WCG1289Dai24711 --
Dai22598 --
Dai20523 --
Dai17885 --
Dai17553 --
Dai19639
GGGGAGACCAGAAGAAGTCAATGGCGTCGAAGGCCGGTGTCTCTCAAGTG
JV0110_48_CZ --
MJ129_04 --
Cui10340P_eminens --
Cui10341P_eminens --
Dai12685P_eminens --
Miettinen_13591Rigidoporus_und --
Dai20868 --
Dai20832
GGGGTGACCAGAAGAAATCTATGGCGTCAAAGGCTGGTGTCTCTCAGGTG
Dai11400 --
Dai22472 --
1CUI10475 --
1CUI10491 --
HCFC1095Meripilus_robledoi --
MCW702Meripilus_revolubilis --

1704_83_zluty_HaciendaBaru --
Dai9925P_lavendulus --
Dai13587AP_lavendulus --
PDD70600P_longicystidius --
Cui16630 --
FP_135344Meripilus_giganteus
GGGGAGACCAGAAGAAATCCATGGCGTCTAAAGCCGGTGTCTCTCAAGTG
FP_100460_Sp --
CBS421_48Meripilus_giganteus --
Cui9203 --
Cui9202 --
TUFC100564Japan --
Russell5913Meripilus_sumstinei --
RP215Meripilus_brasiliensis --
RP200Meripilus_brasiliensis --
JV1712_13J_R_vinctus2_LSUPuert --
Dai10503R_hypobrunneus --
Dai10569Rigidoporus_hypobrunne --
1DAI19451 --
CM108bRigidoporus_hypobrunneus --
1CUI16874 --
FD299Cerrena_unicolor --
KHL_GB_Cerrena_uniclor --
Dai12892Cerrena_albocinnamomea --
Dai12955C_albocinnamomea --
SNUm03110102C_aurantiopora --
NIBRFG0000102423C_aurantiopora --
Dai7359Antrodiella_zonata --
F20080702KCM29C_consors --
F20080208LYW10Cerrena_consors --
Dai7821Antrodiella_zonata --
CFMR_DCL04_31Pseudolagarobasid --
VPB197Pseudolagarobasidium_bel --
CBS115543Pseudolagarobasidium_ --
CBS115544 --
Han405Pseudolagarobasidium_bai --
Han406Pseudolagarobasidium_bai --
MUcc838Spongipellis_delectans --
BRNM686401S_delectans --
OSM_F925S_delectans --
BRNM67093Spongipellis_litschau --
CFMRccFP59199TS_unicolor --
CFMRccFP71791TS_unicolor --
Dai13845P_lischaueri

GGGGTGACCAGAAGAAATCCATGGCTTCCAAAGCTGGCGTCTCACAGGTG
Dai20266P_lischaueri --
CFMR_HHB11240Radulodon_america --
RLG6350Radulodon_americanus --
KY415963Radulodon_erikssonii --
HHB9567spRadulodon_casearius --
KRT_Iso_26Radulodon_casearius --
CBS126044Radulodon_erikssonii --
He6183YUNNANENSIS --
Cui17979YUNNANENSIS --
Miettinen2091Junghuhnia_fimbri --
KHL12316S_tenue --
PRM846564S_pachyodon --
SP_Lgt_S_pachyodon --
Ryvarden44669Tyromyces_xuchile --
PW17_171sinuosus --
W53Dai12234
GGGGTGACCAAAAGAAGTCGATGGCATCCAAAGCTGGTGTTTCGCAGGTG
HHB4100SpAntella_americana --
W3Dai20901spumeus
GGGGTGACCAGAAGAAGTCCATGGCCTCAAAGGCCGGTGTGTCGCAGGTG
He6736 --
BRNM734877S_spumeus --
BRNM712630S_spumeus --
Dai1723Loweomyces_sibiricus --
W54Cui10009 --
W1Dai20899
GGGGTGACCAGAAGAAGTCCATGGCCTCAAAGGCCGGCGTGTCGCAGGTG
HHB13445Trametes_ochracea --
Dai16222 --
Dai16240 --

JV1310_11SanguinolentusCernys
TTGAACAGGTACACGTACGCCTCAACGCTTTCCCATCTTCGTCGCTGCAA
MJ39_00_SK --
MJ111_04_CZ --
JV1610_BOKYsmrk --
Dai21030 --
Dai20976P_furcatus
TTGAACAGGTACACGTACGCCTCAACGCTTTCCCATCTTCGTCGCTGCAA
Dai2105 --
Dai2544 --
Dai11313 --
WCG1611Dai26167

TTGAACAGGTACACGTACGCCTCAACGCTTTCCCATCTTCGTCGCTGCAA
WCG1518Dai25999Physisporinus --
TAA15097 --
JV8909_19_CZ --
JV1310_15_P_sanguinolentus2_CZ --
MJ53_02_CZ --
CLZhao21647P_yunnanensis --
CLZhao21583P_yunnanensis --
Dai22272
TTGAACAGGTACACGTACGCCTCAACGCTTTCTCATCTTCGTCGCTGCAA
Dai22279 --
MJ332_94_CZ --
MJ642_94_CZ_Expallescens --
Dai21060P_vinctus
TTGAATCGGTACACGTATGCTTCGACGCTTTCTCATCTTCGTCGTTGCAA
JV0511_23LRP_pouzarii --
JQ409462_R_pouzarii_PRM899856_ --
JV0308_66_WA --
JV0309_45_WA_USA --
JV0709_83_CA_USA --
Dai21043P_pouzarii
TTAAATCGGTACACCTATGCTTCGACCCTTTCTCATCTCCGTCGTTGCAA
MJ144_95_CZ --
JV0909_3_CZ --
JV0609_1_K --
Dai20396Physisporinus_castanop
TTGAATCGGTACACGTATGCGTCAACCCTTTCTCATCTCCGTCGTTGCAA
Dai20397Physisporinus_castanop
TTGAATCGGTACACGTATGCGTCAACCCTTTCTCATCTCCGTCGTTGCAA
MJ19_09_SK_Abies --
JV0509_40_J_TN_USA_Betula --
JV0808_33crocatus_PA_USAlist --
DLL2009_061P_crocatus --
Dai12800P_subcrocatus --
Dai15917P_subcrocatus --
Cui16620 --
HCFC1088Meripilus_stillicidior --
MCW590Meripilus_obsrcurus --
MCW722Meripilus_obsrcurus --
Cui9381P_tibeticus --
Cui9588P_tibeticus --
Va2_Beneschova --
CWU3874_Ukraine_Alnus --
WCG1293Dai24718Physisporinus --

WCG1268Dai24682A
CTGAATCGGTACACCTATGCCTCTACTCTTTCCCATCTTCGTCGTTGCAA
WCG1269Dai24683A
CTGAATCGGTACACCTATGCCTCTACTCTTTCCCATCTTCGTCGTTGCAA
WCG1279Dai24694A
CTGAATCGGTACACCTATGCCTCTACTCTTTCCCATCTTCGTCGTTGCAA
Dai16971 --
ZQY1043Dai26696 --
Doll880
TTGAACCGATACACATATGCCTCTACACTTTCCCATCTTCGTCGTTGTAA
Doll1000
TTGAACCGATACACATATGCCTCTACACTTTCCCATCTTCGTCGTTGTAA
1DAI18529 --
Dai19535
TTGAATCGATATACCTACGCCTCCACACTTTCCCATCTCCGTCGTTGCAA
1704_79_hnedyVillaLaPaz
CTGAACCGGTATACTTACGCTTCTACACTCTCCCACCTTCGTCGTTGCAA
F2061 --
1DAI18268 --
1DAI18540A --
Dai17695 --
LKY18Dai26373 --
Dai17839P_sulphureus
TTGAATCGATACACCTACGCCTCCACACTTTCCCATCTCCGCCGTTGCAA
Dai17841P_sulphureus
TTGAATCGATACACCTACGCCTCCACACTTTCCCATCTCCGCCGTTGTAA
Dai19877P_roseus
CTGAACCGCTACACTTACGCTTCCACACTTTCCCATCTCCGTCGTTGCAA
1508_18_1_Kout --
KP859303_R_vinctus_RP185_BRAZI --
JK1807_15Rigidoporus_sp_Puerto
CTGAACCGTTACACCTACGCCTCCACACTCTCCCACCTTCGTCGCTGCAA
JV0509_47_J_TN_USA
CTGAATCGATACACTTACGCCTCCACACTTTCCCATCTCCGTCGTTGCAA
JV0709_188 --
JV0509_127_PA_USA
CTGAATCGATACACTTACGCCTCCACACTTTCCCATCTCCGTCGTTGCAA
JV1009_59_NJ_USA --
Dai15497P_crataegi
CTGAATCGATACACTTACGCCTCCACACTTTCCCATCTCCGTCGTTGTAA
Dai15499P_crataegi
CTGAATCGATACACTTATGCCTCCACACTTTCCCATCTCCGTCGTTGTAA
Cui3266P_cinereus --
WCG1256Dai24690 --

1DAI17581 --
WCG1255Dai24688
CTGAATCGGTACACTTATGCCTCTACACTTTCCCATCTTCGCCGATGCAA
Dai22427 --
MV690Meripilus_concrescens --
MV513Meripilus_galapagensis --
Dai19793
CTGAATCGATACACTTACGCCTCTACCCTTTCCCATCTTCGTCGTTGCAA
OQ553780P_tamilnaduensis --
OQ553779P_tamilnaduensis --
A164FB3Meripilus_giganteus --
JV1407_36_Vinctus_Meandrica --
1807_3K_Rigidoporus_PuertoRico
TTGAACCGATACACTTACGCTTCGACACTTTCCCATCTTCGCCGATGCAA
Cui16903P_vinctus
TTGAACCGATACACTTACGCTTCGACACTTTCCCATCTTCGCCGATGCAA
JV1008_18R_Lineatus --
JV1407_37_1_Vinctus_Carara
TTGAATCGGTACACATACGCTTCGACACTTTCCCATCTTCGCCGATGCAA
Dai17986P_lineatus
TTGAATCGGTACACATACGCTTCGACACTTTCCCATCTCCGCCGATGCAA
Dai18281
TTGAATCGGTATACATACGCTTCGACACTTTCCCATCTCCGCCGATGCAA
1DAI19796 --
ZQY797Dai25241
TTGAATCGATACACGTACGCTTCGACACTTTCCCATCTTCGACGATGCAA
WCG1289Dai24711 --
Dai22598 --
Dai20523 --
Dai17885 --
Dai17553 --
Dai19639
TTGAACCGATACACTTACGCTTCGACACTTTCCCATCTTCGCCGATGTAA
JV0110_48_CZ --
MJ129_04 --
Cui10340P_eminens --
Cui10341P_eminens --
Dai12685P_eminens --
Miettinen_13591Rigidoporus_und --
Dai20868 --
Dai20832
TTGAATAGGTACACATACGCCTCCACACTCTCCCACTTGCGTCGATGCAA
Dai11400 --
Dai22472 --

1CUI10475 --
1CUI10491 --
HCFC1095Meripilus_robledoi --
MCW702Meripilus_revolubilis --
1704_83_zluty_HaciendaBaru --
Dai9925P_lavendulus --
Dai13587AP_lavendulus --
PDD70600P_longicystidius --
Cui16630 --
FP_135344Meripilus_giganteus
CTGAATCGATACACCTACGCCTCCACCCTTTCTCACCTTCGGCGTTGCAA
FP_100460_Sp --
CBS421_48Meripilus_giganteus --
Cui9203 --
Cui9202 --
TUFC100564Japan --
Russell5913Meripilus_sumstinei --
RP215Meripilus_brasiliensis --
RP200Meripilus_brasiliensis --
JV1712_13J_R_vinctus2_LSUPuert --
Dai10503R_hypobrunneus --
Dai10569Rigidoporus_hypobrunne --
1DAI19451 --
CM108bRigidoporus_hypobrunneus --
1CUI16874 --
FD299Cerrena_unicolor --
KHL_GB_Cerrena_uniclor --
Dai12892Cerrena_albocinnamomea --
Dai12955C_albocinnamomea --
SNUm03110102C_aurantiopora --
NIBRFG0000102423C_aurantiopora --
Dai7359Antrodiella_zonata --
F20080702KCM29C_consors --
F20080208LYW10Cerrena_consors --
Dai7821Antrodiella_zonata --
CFMR_DCL04_31Pseudolagarobasid --
VPB197Pseudolagarobasidium_bel --
CBS115543Pseudolagarobasidium_ --
CBS115544 --
Han405Pseudolagarobasidium_bai --
Han406Pseudolagarobasidium_bai --
MUcc838Spongipellis_delectans --
BRNM686401S_delectans --
OSM_F925S_delectans --

BRNM67093Spongipellis_litschau ---
CFMRccFP59199TS_unicolor ---
CFMRccFP71791TS_unicolor ---
Dai13845P_lischaueri
CTCAATCGTTATACCTATGCCTCCACACTGTCCCATTTGCGTCGGTGCAA
Dai20266P_lischaueri ---
CFMR_HHB11240Radulodon_america ---
RLG6350Radulodon_americanus ---
KY415963Radulodon_erikssonii ---
HHB9567spRadulodon_casearius ---
KRT_Iso_26Radulodon_casearius ---
CBS126044Radulodon_erikssonii ---
He6183YUNNANENSIS ---
Cui17979YUNNANENSIS ---
Miettinen2091Junghuhnia_fimbri ---
KHL12316S_tenue ---
PRM846564S_pachyodon ---
SP_Lgt_S_pachyodon ---
Ryvarden44669Tyromyces_xuchile ---
PW17_171sinuosus ---
W53Dai12234
TTGAACCGATATACTTATGCTTCGACATTATCCCACTTGCGACGGTGTAA
HHB4100SpAntella_americana ---
W3Dai20901spumeus
CTGAATAGGTACACTTACGCCTCCACGTTGTCCCATCTGCGTCGATGCAA
He6736 ---
BRNM734877S_spumeus ---
BRNM712630S_spumeus ---
Dai1723Loweomyces_sibiricus ---
W54Cui10009 ---
W1Dai20899
CTGAATAGGTACACTTACGCCTCCACGTTGTCCCATTTGCGTCGATGCAA
HHB13445Trametes_ochracea ---
Dai16222 ---
Dai16240 ---

JV1310_11SanguinolentusCernys
TACCCCGCTCGGTCGAGAAGGAAAGATCGCGAAACCTCGTCAGCTTCACA
MJ39_00_SK ---
MJ111_04_CZ ---
JV1610_BOKYsmrk ---
Dai21030 ---
Dai20976P_furcatus
TACCCCGCTCGGTCGAGAAGGAAAGATCGCGAAACCTCGTCAGCTTCACA

Dai2105 --
Dai2544 --
Dai11313 --
WCG1611Dai26167
TACCCCACTCGGTCGAGAAGGAAAGATTGCGAAACCTCGTCAGCTTCACA
WCG1518Dai25999Physisporinus --
TAA15097 --
JV8909_19_CZ --
JV1310_15_P_sanguinolentus2_CZ --
MJ53_02_CZ --
CLZhao21647P_yunnanensis --
CLZhao21583P_yunnanensis --
Dai22272
TACCCCGCTCGGTCGAGAAGGAAAAATCGCGAAACCTCGTCAGCTTCACA
Dai22279 --
MJ332_94_CZ --
MJ642_94_CZ_Expallescens --
Dai21060P_vinctus
CACCCCGCTTGGTCGAGAAGGAAAGATCGCCAAACCCCGTCAGCTACACA
JV0511_23LRP_pouzarii --
JQ409462_R_pouzarii_PRM899856_ --
JV0308_66_WA --
JV0309_45_WA_USA --
JV0709_83_CA_USA --
Dai21043P_pouzarii
CACCCCGCTCGGTCGAGAAGGAAAGATCGCCAAACCCCGTCAGTTACACA
MJ144_95_CZ --
JV0909_3_CZ --
JV0609_1_K --
Dai20396Physisporinus_castanop
CACCCCGCTTGGTCGAGAAGGAAAGATCGCCAAACCCCGTCAGCTACACA
Dai20397Physisporinus_castanop
CACCCCGCTTGGTCGAGAAGGAAAGATCGCCAAACCCCGTCAGCTACACA
MJ19_09_SK_Abies --
JV0509_40_J_TN_USA_Betula --
JV0808_33crocatus_PA_USAlist --
DLL2009_061P_crocatus --
Dai12800P_subcrocatus --
Dai15917P_subcrocatus --
Cui16620 --
HCFC1088Meripilus_stillicidior --
MCW590Meripilus_obsrcurus --
MCW722Meripilus_obsrcurus --
Cui9381P_tibeticus --

Cui9588P_tibeticus --
Va2_Beneschova --
CWU3874_Ukraine_Alnus --
WCG1293Dai24718Physisporinus --
WCG1268Dai24682A
TACGCCGCTTGGTCGAGAAGGGAAGATCGCGAAGCCTCGTCAACTACATA
WCG1269Dai24683A
TACGCCGCTTGGTCGAGAAGGGAAGATCGCGAAGCCTCGTCAACTACATA
WCG1279Dai24694A
TACACCGCTTGGTCGAGAAGGGAAGATCGCGAAACCTCGTCAACTACATA
Dai16971 --
ZQY1043Dai26696 --
Doll880
TACCCCATTGGGTCGAGAAGGAAAGATTGCCAAACCTCGTCAACTACACA
Doll1000
TACCCCGTTGGGTCGAGAAGGAAAGATTGCCAAACCTCGTCAACTACACA
1DAI18529 --
Dai19535
CACACCGCTTGGTCGAGAAGGAAAGATTGCCAAACCCCGTCAGCTGCACA
1704_79_hnedyVillaLaPaz
TACCCCACTTGGACGAGAAGGAAAGATCGCCAAACCACGTCAGCTACATA
F2061 --
1DAI18268 --
1DAI18540A --
Dai17695 --
LKY18Dai26373 --
Dai17839P_sulphureus
TACTCCACTTGGTCGAGAAGGAAAGATTGCCAAGCCCCGTCAACTCCATA
Dai17841P_sulphureus
TACTCCACTTGGTCGAGAAGGAAAGATTGCCAAGCCGCGTCAACTTCATA
Dai19877P_roseus
TACTCCGCTTGGTCGAGAAGGAAAGATTGCCAAGCCCCGTCAGCTTCATA
1508_18_1_Kout --
KP859303_R_vinctus_RP185_BRAZI --
JK1807_15Rigidoporus_sp_Puerto
TACTCCGCTTGGACGAGAAGGAAAAATCGCGAAACCTCGTCAACTTCATA
JV0509_47_J_TN_USA
CACTCCGCTTGGACGAGAAGGAAAGATTGCAAAACCCCGTCAATTGCACA
JV0709_188 --
JV0509_127_PA_USA
CACTCCGCTTGGACGAGAAGGAAAGATTGCAAAACCCCGTCAATTGCACA
JV1009_59_NJ_USA --
Dai15497P_crataegi
CACTCCACTTGGGCGAGAAGGGAAAATCGCGAAACCACGTCAGTTGCACA

Dai15499P_crataegi
CACTCCTCTTGGGCGAGAAGGGAAAATCGCGAAACCACGTCAGTTGCACA
Cui3266P_cinereus --
WCG1256Dai24690 --
1DAI17581 --
WCG1255Dai24688
TACTCCACTTGGACGAGAAGGGAAGATCGCCAAACCCCGTCAGCTACACA
Dai22427 --
MV690Meripilus_concrescens --
MV513Meripilus_galapagensis --
Dai19793
CACTCCACTCGGGCGAGAAGGAAAGATCGCTAAACCCCGTCAATTGCACA
OQ553780P_tamilnaduensis --
OQ553779P_tamilnaduensis --
A164FB3Meripilus_giganteus --
JV1407_36_Vinctus_Meandrica --
1807_3K_Rigidoporus_PuertoRico
TACCCCACTTGGACGAGAGGGAAAGATCGCCAAACCTCGACAATTGCACA
Cui16903P_vinctus
TACCCCACTTGGACGAGAGGGAAAGATCGCCAAACCTCGACAATTGCACA
JV1008_18R_Lineatus --
JV1407_37_1_Vinctus_Carara
TACCCCCCTGGGGCGAGAAGGAAAGATAGCCAAACCTCGACAATTACACA
Dai17986P_lineatus
TACCCCCCTCGGGCGAGAAGGAAAGATCGCCAAACCTCGACAATTGCACA
Dai18281
TACCCCCCTCGGGCGAGAAGGAAAGATCGCCAAACCTCGACAATTGCACA
1DAI19796 --
ZQY797Dai25241
TACTCCCCTTGGGCGAGAAGGAAAGATCGCCAAACCTCGACAATTGCACA
WCG1289Dai24711 --
Dai22598 --
Dai20523 --
Dai17885 --
Dai17553 --
Dai19639
TACTCCCCTCGGACGAGAAGGAAAGATCGCCAAACCTCGACAATTGCACA
JV0110_48_CZ --
MJ129_04 --
Cui10340P_eminens --
Cui10341P_eminens --
Dai12685P_eminens --
Miettinen_13591Rigidoporus_und --
Dai20868 --

Dai20832
CACCCCACTGGGTCGTGAGGGTAAAATCGCGAAGCCTCGTCAGCTTCATA
Dai11400 --
Dai22472 --
1CUI10475 --
1CUI10491 --
HCFC1095Meripilus_robledoi --
MCW702Meripilus_revolubilis --
1704_83_zluty_HaciendaBaru --
Dai9925P_lavendulus --
Dai13587AP_lavendulus --
PDD70600P_longicystidius --
Cui16630 --
FP_135344Meripilus_giganteus
TACCCCTCTTGGTCGTGAAGGGAAGATTGCCAAACCCCGTCAACTACACA
FP_100460_Sp --
CBS421_48Meripilus_giganteus --
Cui9203 --
Cui9202 --
TUFC100564Japan --
Russell5913Meripilus_sumstinei --
RP215Meripilus_brasiliensis --
RP200Meripilus_brasiliensis --
JV1712_13J_R_vinctus2_LSUPuert --
Dai10503R_hypobrunneus --
Dai10569Rigidoporus_hypobrunne --
1DAI19451 --
CM108bRigidoporus_hypobrunneus --
1CUI16874 --
FD299Cerrena_unicolor --
KHL_GB_Cerrena_uniclor --
Dai12892Cerrena_albocinnamomea --
Dai12955C_albocinnamomea --
SNUm03110102C_aurantiopora --
NIBRFG0000102423C_aurantiopora --
Dai7359Antrodiella_zonata --
F20080702KCM29C_consors --
F20080208LYW10Cerrena_consors --
Dai7821Antrodiella_zonata --
CFMR_DCL04_31Pseudolagarobasid --
VPB197Pseudolagarobasidium_bel --
CBS115543Pseudolagarobasidium_ --
CBS115544 --
Han405Pseudolagarobasidium_bai --

Han406Pseudolagarobasidium_bai --
MUcc838Spongipellis_delectans --
BRNM686401S_delectans --
OSM_F925S_delectans --
BRNM67093Spongipellis_litschau --
CFMRccFP59199TS_unicolor --
CFMRccFP71791TS_unicolor --
Dai13845P_lischaueri
CACACCTCTTGGTCGTGAGGGCAAGATCGCTAAACCCCGTCAACTGCATA
Dai20266P_lischaueri --
CFMR_HHB11240Radulodon_america --
RLG6350Radulodon_americanus --
KY415963Radulodon_erikssonii --
HHB9567spRadulodon_casearius --
KRT_Iso_26Radulodon_casearius --
CBS126044Radulodon_erikssonii --
He6183YUNNANENSIS --
Cui17979YUNNANENSIS --
Miettinen2091Junghuhnia_fimbri --
KHL12316S_tenue --
PRM846564S_pachyodon --
SP_Lgt_S_pachyodon --
Ryvarden44669Tyromyces_xuchile --
PW17_171sinuosus --
W53Dai12234
CACACCCCTTGGTCGTGAGGGGAAAATTGCAAAACCTCGACAGTTGCATA
HHB4100SpAntella_americana --
W3Dai20901spumeus
TACCCCTCTTGGTCGTGAAGGCAAGATCGCCAAGCCGCGTCAACTCCACA
He6736 --
BRNM734877S_spumeus --
BRNM712630S_spumeus --
Dai1723Loweomyces_sibiricus --
W54Cui10009 --
W1Dai20899
TACCCCCCTTGGTCGTGAAGGCAAGATCGCCAAGCCGCGTCAACTCCACA
HHB13445Trametes_ochracea --
Dai16222 --
Dai16240 --

JV1310_11SanguinolentusCernys
ACACTCATTGGGGGATGGTGTGCCCTGCAGAGACACCAGAAGGGCAAGCT
MJ39_00_SK --
MJ111_04_CZ --

JV1610_BOKYsmrk --
Dai21030 --
Dai20976P_furcatus
ATACTCATTGGGGGATGGTGTGCCCTGCAGAGACACCAGAAGGGCAAGCT
Dai2105 --
Dai2544 --
Dai11313 --
WCG1611Dai26167
ACACTCATTGGGGGATGGTGTGCCCTGCAGAGACACCAGAAGGGCAAGCT
WCG1518Dai25999Physisporinus --
TAA15097 --
JV8909_19_CZ --
JV1310_15_P_sanguinolentus2_CZ --
MJ53_02_CZ --
CLZhao21647P_yunnanensis --
CLZhao21583P_yunnanensis --
Dai22272
ACACTCATTGGGGGATGGTGTGCCCTGCAGAGACACCGGAAGGGCAAGCT
Dai22279 --
MJ332_94_CZ --
MJ642_94_CZ_Expallescens --
Dai21060P_vinctus
ATACTCATTGGGGAATGGTGTGTCCTGCAGAGACACCCGAAGGACAAGCT
JV0511_23LRP_pouzarii --
JQ409462_R_pouzarii_PRM899856_ --
JV0308_66_WA --
JV0309_45_WA_USA --
JV0709_83_CA_USA --
Dai21043P_pouzarii
ATACTCATTGGGGGATGGTGTGCCCGGCGGAGACGCCCGAAGGACAAGCT
MJ144_95_CZ --
JV0909_3_CZ --
JV0609_1_K --
Dai20396Physisporinus_castanop
ATACTCATTGGGGAATGGTGTGTCCGGCAGAGACACCCGAAGGACAAGCT
Dai20397Physisporinus_castanop
ATACTCATTGGGGAATGGTGTGTCCGGCAGAGACACCCGAAGGACAAGCT
MJ19_09_SK_Abies --
JV0509_40_J_TN_USA_Betula --
JV0808_33crocatus_PA_USAlist --
DLL2009_061P_crocatus --
Dai12800P_subcrocatus --
Dai15917P_subcrocatus --
Cui16620 --

HCFC1088Meripilus_stillicidior --
MCW590Meripilus_obsrcurus --
MCW722Meripilus_obsrcurus --
Cui9381P_tibeticus --
Cui9588P_tibeticus --
Va2_Beneschova --
CWU3874_Ukraine_Alnus --
WCG1293Dai24718Physisporinus --
WCG1268Dai24682A
ATACTCATTGGGGAATGGTGTGTCCTGCAGAGACACCCGAAGGCCAAGCT
WCG1269Dai24683A
ATACTCATTGGGGAATGGTGTGTCCTGCAGAGACACCCGAAGGCCAAGCT
WCG1279Dai24694A
ACACCCATTGGGGAATGGTGTGTCCTGCAGAGACACCCGAAGGCCAAGCT
Dai16971 --
ZQY1043Dai26696 --
Doll880
ATACGCATTGGGGGATGGTGTGTCCTGCGGAGACACCCGAAGGTCAAGCT
Doll1000
ATACGCATTGGGGGATGGTGTGTCCTGCGGAGACACCTGAAGGTCAAGCT
1DAI18529 --
Dai19535
ATACTCACTGGGGTATGGTGTGTCCCGCCGAAACTCCCGAAGGGCAAGCT
1704_79_hnedyVillaLaPaz
ATACCCATTGGGGAATGGTTTGCCCTGCTGAGACGCCCGAAGGCCAAGCT
F2061 --
1DAI18268 --
1DAI18540A --
Dai17695 --
LKY18Dai26373 --
Dai17839P_sulphureus
ACACGCATTGGGGAATGGTGTGTCCAGCAGAGACACCCGAAGGTCAAGCG
Dai17841P_sulphureus
ACACGCATTGGGGAATGGTGTGTCCAGCAGAGACACCCGAAGGTCAAGCG
Dai19877P_roseus
ATACACACTGGGGGATGGTATGCCCAGCAGAGACACCCGAAGGTCAAGCT
1508_18_1_Kout --
KP859303_R_vinctus_RP185_BRAZI --
JK1807_15Rigidoporus_sp_Puerto
ATACGCATTGGGGGATGGTCTGCCCGGCAGAGACCCCCGAAGGCCAAGCT
JV0509_47_J_TN_USA
ATACTCACTGGGGAATGGTATGCCCTGCCGAAACGCCGGAAGGACAGGCT
JV0709_188 --
JV0509_127_PA_USA

ATACTCACTGGGGAATGGTATGCCCTGCCGAAACGCCGGAAGGACAGGCT
JV1009_59_NJ_USA --
Dai15497P_crataegi
ATACCCACTGGGGAATGGTATGTCCTGCAGAAACGCCAGAAGGACAGGCT
Dai15499P_crataegi
ATACCCACTGGGGAATGGTATGCCCTGCAGAAACGCCAGAAGGACAGGCT
Cui3266P_cinereus --
WCG1256Dai24690 --
1DAI17581 --
WCG1255Dai24688
ACACTCACTGGGGGATGGTGTGCCCTGCGGAAACGCCAGAAGGACAGGCT
Dai22427 --
MV690Meripilus_concrescens --
MV513Meripilus_galapagensis --
Dai19793
ATACCCACTGGGGAATGGTATGTCCTGCCGAAACGCCGGAAGGACAAGCT
OQ553780P_tamilnaduensis --
OQ553779P_tamilnaduensis --
A164FB3Meripilus_giganteus --
JV1407_36_Vinctus_Meandrica --
1807_3K_Rigidoporus_PuertoRico
ATACCCACTGGGGTATGGTGTGCCCCGCCGAGACTCCAGAAGGACAAGCT
Cui16903P_vinctus
ATACCCACTGGGGTATGGTGTGCCCCGCCGAGACTCCAGAAGGACAAGCT
JV1008_18R_Lineatus --
JV1407_37_1_Vinctus_Carara
ATACCCACTGGGGCATGGTATGTCCCGCCGAGACGCCAGAAGGGCAAGCT
Dai17986P_lineatus
ATACCCACTGGGGCATGGTGTGTCCCGCCGAGACGCCAGAAGGACAAGCT
Dai18281
ATACCCACTGGGGTATGGTATGTCCCGCCGAGACGCCAGAAGGACAAGCT
1DAI19796 --
ZQY797Dai25241
ATACCCACTGGGGCATGGTATGTCCCGCCGAGACGCCAGAAGGACAAGCT
WCG1289Dai24711 --
Dai22598 --
Dai20523 --
Dai17885 --
Dai17553 --
Dai19639
ATACCCACTGGGGCATGGTGTGTCCCGCCGAGACGCCAGAAGGACAAGCT
JV0110_48_CZ --
MJ129_04 --
Cui10340P_eminens --

Cui10341P_eminens --
Dai12685P_eminens --
Miettinen_13591Rigidoporus_und --
Dai20868 --------------------------------------GAAGGACAAGCT
Dai20832
ACACTCATTGGGGAATGGTATGTCCTGCGGAGACTCCGGAAGGACAAGCT
Dai11400 --
Dai22472 --
1CUI10475 --
1CUI10491 --
HCFC1095Meripilus_robledoi --
MCW702Meripilus_revolubilis --
1704_83_zluty_HaciendaBaru --
Dai9925P_lavendulus --
Dai13587AP_lavendulus --
PDD70600P_longicystidius --
Cui16630 --
FP_135344Meripilus_giganteus
ACACCCATTGGGGAATGGTGTGCCCCGCCGAAACTCCAGAAGGGCAGGCA
FP_100460_Sp --
CBS421_48Meripilus_giganteus --
Cui9203 --
Cui9202 --
TUFC100564Japan --
Russell5913Meripilus_sumstinei --
RP215Meripilus_brasiliensis --
RP200Meripilus_brasiliensis --
JV1712_13J_R_vinctus2_LSUPuert --
Dai10503R_hypobrunneus --
Dai10569Rigidoporus_hypobrunne --
1DAI19451 --
CM108bRigidoporus_hypobrunneus --
1CUI16874 --
FD299Cerrena_unicolor --
KHL_GB_Cerrena_uniclor --
Dai12892Cerrena_albocinnamomea --
Dai12955C_albocinnamomea --
SNUm03110102C_aurantiopora --
NIBRFG0000102423C_aurantiopora --
Dai7359Antrodiella_zonata --
F20080702KCM29C_consors --
F20080208LYW10Cerrena_consors --
Dai7821Antrodiella_zonata --
CFMR_DCL04_31Pseudolagarobasid --

VPB197Pseudolagarobasidium_bel --
CBS115543Pseudolagarobasidium_ --
CBS115544 --
Han405Pseudolagarobasidium_bai --
Han406Pseudolagarobasidium_bai --
MUcc838Spongipellis_delectans --
BRNM686401S_delectans --
OSM_F925S_delectans --
BRNM67093Spongipellis_litschau --
CFMRccFP59199TS_unicolor --
CFMRccFP71791TS_unicolor --
Dai13845P_lischaueri
ACACTCATTGGGGCATGGTTTGTCCTGCGGAGACCCCCGAAGGTCAAGCA
Dai20266P_lischaueri --
CFMR_HHB11240Radulodon_america --
RLG6350Radulodon_americanus --
KY415963Radulodon_erikssonii --
HHB9567spRadulodon_casearius --
KRT_Iso_26Radulodon_casearius --
CBS126044Radulodon_erikssonii --
He6183YUNNANENSIS --
Cui17979YUNNANENSIS --
Miettinen2091Junghuhnia_fimbri --
KHL12316S_tenue --
PRM846564S_pachyodon --
SP_Lgt_S_pachyodon --
Ryvarden44669Tyromyces_xuchile --
PW17_171sinuosus --
W53Dai12234
ATACTCACTGGGGTATGGTTTGCCCTGCGGAGACCCCGGAAGGACAGGCT
HHB4100SpAntella_americana --
W3Dai20901spumeus
ATACCCACTGGGGAATGGTGTGTCCTGCAGAGACACCGGAAGGTCAAGCT
He6736 --
BRNM734877S_spumeus --
BRNM712630S_spumeus --
Dai1723Loweomyces_sibiricus --
W54Cui10009 --
W1Dai20899
ATACCCACTGGGGAATGGTGTGTCCTGCAGAGACACCGGAAGGTCAAGCT
HHB13445Trametes_ochracea --
Dai16222 --
Dai16240 --

JV1310_11SanguinolentusCernys
TGCGGTCTCGTGAAGAACCTGTCGCTCATGTCCTGTATCTCCGTCGGTTC
MJ39_00_SK --
MJ111_04_CZ --
JV1610_BOKYsmrk --
Dai21030 --
Dai20976P_furcatus
TGCGGTCTCGTGAAGAACCTGTCGCTCATGTCCTGTATCTCCGTCGGTTC
Dai2105 --
Dai2544 --
Dai11313 --
WCG1611Dai26167
TGCGGTCTCGTGAAGAACCTGTCGCTCATGTCCTGTATCTCCGTCGGCTC
WCG1518Dai25999Physisporinus --
TAA15097 --
JV8909_19_CZ --
JV1310_15_P_sanguinolentus2_CZ --
MJ53_02_CZ --
CLZhao21647P_yunnanensis --
CLZhao21583P_yunnanensis --
Dai22272
TGCGGTCTCGTGAAGAACCTGTCGCTCATGTCCTGTATCTCCGTCGGTTC
Dai22279 --
MJ332_94_CZ --
MJ642_94_CZ_Expallescens --
Dai21060P_vinctus
TGCGGTCTCGTCAAGAACTTGTCGCTGATGTCATGTATCTCCGTTGGCTC
JV0511_23LRP_pouzarii --
JQ409462_R_pouzarii_PRM899856_ --
JV0308_66_WA --
JV0309_45_WA_USA --
JV0709_83_CA_USA --
Dai21043P_pouzarii
TGCGGTCTCGTCAAAAACCTGTCACTCATGTCGTGTATCTCCGTCGGCTC
MJ144_95_CZ --
JV0909_3_CZ --
JV0609_1_K --
Dai20396Physisporinus_castanop
TGCGGTCTCGTCAAGAACTTATCGCTCATGTCGTGTATATCCGTTGGCTC
Dai20397Physisporinus_castanop
TGCGGTCTCGTCAAGAACTTATCGCTCATGTCGTGTATATCCGTTGGCTC
MJ19_09_SK_Abies --
JV0509_40_J_TN_USA_Betula --
JV0808_33crocatus_PA_USAlist --

DLL2009_061P_crocatus --
Dai12800P_subcrocatus --
Dai15917P_subcrocatus --
Cui16620 --
HCFC1088Meripilus_stillicidior --
MCW590Meripilus_obsrcurus --
MCW722Meripilus_obsrcurus --
Cui9381P_tibeticus --
Cui9588P_tibeticus --
Va2_Beneschova --
CWU3874_Ukraine_Alnus --
WCG1293Dai24718Physisporinus --
WCG1268Dai24682A
TGTGGTCTCGTCAAGAACCTGTCGCTCATGTCCTGTATCTCCGTCGGTTC
WCG1269Dai24683A
TGTGGTCTCGTCAAGAACCTGTCGCTCATGTCCTGTATCTCCGTCGGTTC
WCG1279Dai24694A
TGTGGTCTCGTCAAGAACCTGTCGCTCATGTCCTGTATCTCCGTCGGTTC
Dai16971 --
ZQY1043Dai26696 --
Doll880
TGCGGTCTCGTCAAGAACCTGTCGCTAATGTCATGTATCTCCGTTGGTTC
Doll1000
TGCGGTCTTGTCAAGAACCTATCGCTAATGTCATGTATCTCCGTTGGTTC
1DAI18529 --
Dai19535
TGTGGACTTGTCAAGAACTTATCGCTCATGTCGTGTATTTCCGTCGGCTC
1704_79_hnedyVillaLaPaz
TGCGGTCTTGTCAAGAACTTGTCGCTTATGTCGTGTATCTCTGTTGGCTC
F2061 --
1DAI18268 --
1DAI18540A --
Dai17695 --
LKY18Dai26373 --
Dai17839P_sulphureus
TGCGGTCTCGTCAAGAACCTGTCACTCATGTCGTGCATCTCTGTCGGCTC
Dai17841P_sulphureus
TGCGGTCTCGTCAAGAACCTGTCACTCATGTCGTGCATCTCTGTCGGCTC
Dai19877P_roseus
TGTGGTCTCGTCAAGAACCTATCACTCATGTCGTGTATTTCCGTCGGTTC
1508_18_1_Kout --
KP859303_R_vinctus_RP185_BRAZI --
JK1807_15Rigidoporus_sp_Puerto
TGTGGTCTCGTCAAGAACCTGTCACTCATGTCGTGTATCTCAGTCGGTTC

JV0509_47_J_TN_USA
TGTGGCCTTGTCAAGAACTTGTCACTTATGTCATGTATTTCCGTCGGTTC
JV0709_188 --
JV0509_127_PA_USA
TGTGGCCTTGTCAAGAACTTGTCACTTATGTCATGTATTTCCGTCGGTTC
JV1009_59_NJ_USA -------------
GAACTTGTCACTTATGTCATGTATTTCCGTCGGTTC
Dai15497P_crataegi
TGCGGTCTTGTCAAGAACTTGTCGCTTATGTCTTGTATTTCCGTTGGGTC
Dai15499P_crataegi
TGCGGTCTTGTCAAGAACTTGTCGCTTATGTCTTGTATTTCCGTTGGGTC
Cui3266P_cinereus --
WCG1256Dai24690 --
1DAI17581 --
WCG1255Dai24688
TGCGGTCTCGTCAAGAACTTGTCGCTTATGTCATGTATCTCCGTCGGTTC
Dai22427 --
MV690Meripilus_concrescens --
MV513Meripilus_galapagensis --
Dai19793
TGTGGTCTTGTCAAGAACTTGTCGCTTATGTCATGTATCTCTGTCGGCTC
OQ553780P_tamilnaduensis --
OQ553779P_tamilnaduensis --
A164FB3Meripilus_giganteus --
JV1407_36_Vinctus_Meandrica --
1807_3K_Rigidoporus_PuertoRico
TGTGGTCTCGTCAAAAATCTATCGCTTATGTCTTGTATTTCTGTCGGTTC
Cui16903P_vinctus
TGTGGTCTCGTCAAAAATCTATCGCTTATGTCTTGTATTTCTGTCGGTTC
JV1008_18R_Lineatus --
JV1407_37_1_Vinctus_Carara
TGTGGTCTCGTCAAGAACCTGTCCCTGATGTCTTGCATCTCCGTCGGTTC
Dai17986P_lineatus
TGTGGTCTCGTCAAGAACCTGTCCCTGATGTCTTGCATCTCTGTCGGTTC
Dai18281
TGTGGTCTCGTCAAGAACCTGTCCCTGATGTCTTGCATCTCTGTCGGTTC
1DAI19796 --
ZQY797Dai25241
TGTGGTCTTGTCAAGAACCTGTCCCTGATGTCTTGTATCTCCGTCGGCTC
WCG1289Dai24711 --
Dai22598 --
Dai20523 --
Dai17885 --
Dai17553 --

Dai19639
TGTGGTCTCGTCAAAATCTGTCTCTGATGTCTTGCATCTCCGTCGGTTC
JV0110_48_CZ --
MJ129_04 --
Cui10340P_eminens --
Cui10341P_eminens --
Dai12685P_eminens --
Miettinen_13591Rigidoporus_und --
Dai20868
TGTGGTCTGGTCAAGAACTTGTCGCTCATGTCCTGTATCTCTGTCGGTTC
Dai20832
TGCGGTCTGGTCAAGAACTTGTCGCTCATGTCCTGTATTTCGGTCGGTTC
Dai11400 --
Dai22472 --
1CUI10475 --
1CUI10491 --
HCFC1095Meripilus_robledoi --
MCW702Meripilus_revolubilis --
1704_83_zluty_HaciendaBaru --
Dai9925P_lavendulus --
Dai13587AP_lavendulus --
PDD70600P_longicystidius --
Cui16630 --
FP_135344Meripilus_giganteus
TGTGGTCTCGTCAAGAACTTGTCCCTCATGTCATGTATCTCCGTCGGTTC
FP_100460_Sp --
CBS421_48Meripilus_giganteus --
Cui9203 --
Cui9202 --
TUFC100564Japan --
Russell5913Meripilus_sumstinei --
RP215Meripilus_brasiliensis --
RP200Meripilus_brasiliensis --
JV1712_13J_R_vinctus2_LSUPuert --
Dai10503R_hypobrunneus --
Dai10569Rigidoporus_hypobrunne --
1DAI19451 --
CM108bRigidoporus_hypobrunneus --
1CUI16874 --
FD299Cerrena_unicolor --
KHL_GB_Cerrena_uniclor --
Dai12892Cerrena_albocinnamomea --
Dai12955C_albocinnamomea --
SNUm03110102C_aurantiopora --

NIBRFG0000102423C_aurantiopora --

Dai7359Antrodiella_zonata --

F20080702KCM29C_consors --

F20080208LYW10Cerrena_consors --

Dai7821Antrodiella_zonata --

CFMR_DCL04_31Pseudolagarobasid --

VPB197Pseudolagarobasidium_bel --

CBS115543Pseudolagarobasidium_ --

CBS115544 --

Han405Pseudolagarobasidium_bai --

Han406Pseudolagarobasidium_bai --

MUcc838Spongipellis_delectans --

BRNM686401S_delectans --

OSM_F925S_delectans --

BRNM67093Spongipellis_litschau --

CFMRccFP59199TS_unicolor --

CFMRccFP71791TS_unicolor --

Dai13845P_lischaueri
TGTGGTCTGGTCAAGAACCTTTCCCTTATGTCGTGTATTTCTGTCGGTTC

Dai20266P_lischaueri --

CFMR_HHB11240Radulodon_america --

RLG6350Radulodon_americanus --

KY415963Radulodon_erikssonii --

HHB9567spRadulodon_casearius --

KRT_Iso_26Radulodon_casearius --

CBS126044Radulodon_erikssonii --

He6183YUNNANENSIS --

Cui17979YUNNANENSIS --

Miettinen2091Junghuhnia_fimbri --

KHL12316S_tenue --

PRM846564S_pachyodon --

SP_Lgt_S_pachyodon --

Ryvarden44669Tyromyces_xuchile --

PW17_171sinuosus --

W53Dai12234
TGCGGTCTCGTCAAAAACCTGTCACTCATGTCGTGCATCTCCGTCGGTTC

HHB4100SpAntella_americana --

W3Dai20901spumeus
TGTGGACTTGTCAAGAACTTATCACTCATGGCATGCATATCTGTCGGCTC

He6736 --

BRNM734877S_spumeus --

BRNM712630S_spumeus --

Dai1723Loweomyces_sibiricus --

W54Cui10009 --

W1Dai20899
TGTGGACTTGTGAAGAACTTATCACTCATGGCATGCATATCAGTCGGCTC
HHB13445Trametes_ochracea --
Dai16222 --
Dai16240 --

JV1310_11SanguinolentusCernys
CCTCTCAGCGCCCGTCATCGAGTTCTTGGAGGAGTGGGGTCTGGAATCAC
MJ39_00_SK --
MJ111_04_CZ --
JV1610_BOKYsmrk --
Dai21030 --
Dai20976P_furcatus
CCTCTCAGCGCCCGTCATCGAGTTCTTGGAGGAGTGGGGTCTGGAATCAC
Dai2105 --
Dai2544 --
Dai11313 --
WCG1611Dai26167
CCTCTCAGCGCCCGTCATCGAGTTCTTGGAGGAGTGGGGTCTGGAATCAC
WCG1518Dai25999Physisporinus --
TAA15097 --
JV8909_19_CZ --
JV1310_15_P_sanguinolentus2_CZ --
MJ53_02_CZ --
CLZhao21647P_yunnanensis --
CLZhao21583P_yunnanensis --
Dai22272
CCTCTCAGCACCCGTCATCGAGTTCTTAGAGGAATGGGGTCTGGAATCAC
Dai22279 --
MJ332_94_CZ --
MJ642_94_CZ_Expallescens --
Dai21060P_vinctus
CCTCTCAGCACCCGTCATCGAGTTCTTGGAGGAATGGGGTTTGGAATCAT
JV0511_23LRP_pouzarii --
JQ409462_R_pouzarii_PRM899856_ --
JV0308_66_WA --
JV0309_45_WA_USA --
JV0709_83_CA_USA --
Dai21043P_pouzarii
CCTCTCAGCGCCCGTCATCGAGTTCTTGGAGGAATGGGGCCTGGAATCAC
MJ144_95_CZ --
JV0909_3_CZ --
JV0609_1_K --
Dai20396Physisporinus_castanop

CCTCTCAGCGCCCGTTATCGAGTTCTTGGAGGAGTGGGGTTTGGAATCAT
Dai20397Physisporinus_castanop
CCTCTCAGCGCCCGTTATCGAGTTCTTGGAGGAGTGGGGTTTGGAATCAT
MJ19_09_SK_Abies --
JV0509_40_J_TN_USA_Betula --
JV0808_33crocatus_PA_USAlist --
DLL2009_061P_crocatus --
Dai12800P_subcrocatus --
Dai15917P_subcrocatus --
Cui16620 --
HCFC1088Meripilus_stillicidior --
MCW590Meripilus_obsrcurus --
MCW722Meripilus_obsrcurus --
Cui9381P_tibeticus --
Cui9588P_tibeticus --
Va2_Beneschova --
CWU3874_Ukraine_Alnus --
WCG1293Dai24718Physisporinus --
WCG1268Dai24682A
GCTCTCGGCACCCGTCATCGAGTTCTTGGAGGAGTGGGGTCTTGAATCAC
WCG1269Dai24683A
GCTCTCGGCACCCGTCATCGAGTTCTTGGAGGAGTGGGGTCTTGAATCAC
WCG1279Dai24694A
GCTCTCGGCACCCGTCATCGAGTTCTTAGAGGAGTGGGGTCTGGAGTCAC
Dai16971 --
ZQY1043Dai26696 --
Doll880
GCTGTCGGCACCCGTCATCGAGTTCTTGGAGGAGTGGGGTTTGGAATCGT
Doll1000
GCTCTCGGCGCCCGTCATCGAGTTCTTGGAGGAATGGGGTTTGGAATCGT
1DAI18529 --
Dai19535
GCTTTCGGCGCCTGTCATCGAATTCTTGGAGGAGTGGGGTTTGGAGTCTT
1704_79_hnedyVillaLaPaz
GCTGTCGGCGCCCGTTATCGAGTTCTTGGAGGAATGGGGTTTGGAGTCAT
F2061 --
1DAI18268 --
1DAI18540A --
Dai17695 --
LKY18Dai26373 --
Dai17839P_sulphureus
GCTCTCGGCACCCGTCATCGAGTTCTTGGAGGAGTGGGGTTTGGAGTCAT
Dai17841P_sulphureus
GCTCTCGGCACCCGTCATCGAGTTCTTGGAGGAGTGGGGTTTGGAGTCAT

Dai19877P_roseus
GCTCTCGGCGCCCGTTATCGAGTTCTTGGAGGAGTGGGGTCTGGAATCAT
1508_18_1_Kout ---
KP859303_R_vinctus_RP185_BRAZI ---
JK1807_15Rigidoporus_sp_Puerto
GCTTTCGGCGCCCGTCATCGAGTTCTTGGAGGAGTGGGGTCTGGAATCGC
JV0509_47_J_TN_USA
GCTCTCTGCGCCCGTCATCGAATTCTTGGAGGAGTGGGGGTTGGAGTCAT
JV0709_188 ---
JV0509_127_PA_USA
GCTCTCTGCGCCCGTCATCGAATTCTTGGAGGAGTGGGGGTTGGAGTCAT
JV1009_59_NJ_USA
GCTCTCTGCGCCCGTCATCGAATTCTTGGAGGAGTGGGGGTTGGAGTCAT
Dai15497P_crataegi
ACTTTCTGCGCCCGTCATCGAGTTCTTGGAGGAGTGGGGGTTGGAATCGC
Dai15499P_crataegi
ACTTTCTGCGCCCGTCATCGAGTTCTTGGAGGAGTGGGGGTTGGAATCGC
Cui3266P_cinereus ---
WCG1256Dai24690 ---
1DAI17581 ---
WCG1255Dai24688
CCTTTCGGCCCCCGTCATCGAGTTCTTGGAGGAGTGGGGATTGGAGTCAC
Dai22427 ---
MV690Meripilus_concrescens ---
MV513Meripilus_galapagensis ---
Dai19793
ACTCTCTGCGCCTGTCATCGAGTTCTTGGAAGAGTGGGGTTTGGAGTCAT
OQ553780P_tamilnaduensis ---
OQ553779P_tamilnaduensis ---
A164FB3Meripilus_giganteus ---
JV1407_36_Vinctus_Meandrica ---
1807_3K_Rigidoporus_PuertoRico
GCTCTCTGCCCCCGTCATCGAGTTCTTGGAGGAATGGGGCTTAGAGTCGT
Cui16903P_vinctus
GCTCTCTGCCCCCGTCATCGAGTTCTTGGAGGAATGGGGCTTAGAGTCGT
JV1008_18R_Lineatus ---
JV1407_37_1_Vinctus_Carara
GCTCTCTGCTCCTGTCATCGAGTTCTTGGAAGAGTGGGGTTTGGAGTCGT
Dai17986P_lineatus
GCTCTCTGCTCCCGTCATCGAGTTCTTGGAAGAGTGGGGTTTGGAGTCGT
Dai18281
GCTCTCTGCTCCCGTCATCGAGTTCTTGGAAGAGTGGGGTTTGGAGTCGT
1DAI19796 ---
ZQY797Dai25241

GCTCTCCGCTCCCGTCATTGAGTTCTTGGAGGAGTGGGGTCTGGAGTCGT
WCG1289Dai24711 --
Dai22598 --
Dai20523 --
Dai17885 --
Dai17553 --
Dai19639
GCTCTCTGCTCCCGTCATCGAGTTCTTGGAGGAGTGGGGTTTGGAGTCGT
JV0110_48_CZ --
MJ129_04 --
Cui10340P_eminens --
Cui10341P_eminens --
Dai12685P_eminens --
Miettinen_13591Rigidoporus_und --
Dai20868
GCTCTCCGCCCCCGTCATCGAGTTCTTGGAGGAATGGGGTTTGGAATCAT
Dai20832
ACTCTCCGCCCCTGTTATTGAGTTCTTGGAGGAGTGGGGTTTGGAGTCGT
Dai11400 --
Dai22472 --
1CUI10475 --
1CUI10491 --
HCFC1095Meripilus_robledoi --
MCW702Meripilus_revolubilis --
1704_83_zluty_HaciendaBaru --
Dai9925P_lavendulus --
Dai13587AP_lavendulus --
PDD70600P_longicystidius --
Cui16630 --
FP_135344Meripilus_giganteus
GCTCTCAGCCCCTGTTATCGAGTTCTTGGAGGAGTGGGGTTTGGAATCGC
FP_100460_Sp --
CBS421_48Meripilus_giganteus --
Cui9203 --
Cui9202 --
TUFC100564Japan --
Russell5913Meripilus_sumstinei --
RP215Meripilus_brasiliensis --
RP200Meripilus_brasiliensis --
JV1712_13J_R_vinctus2_LSUPuert --
Dai10503R_hypobrunneus --
Dai10569Rigidoporus_hypobrunne --
1DAI19451 --
CM108bRigidoporus_hypobrunneus --

1CUI16874 --
FD299Cerrena_unicolor --
KHL_GB_Cerrena_uniclor --
Dai12892Cerrena_albocinnamomea --
Dai12955C_albocinnamomea --
SNUm03110102C_aurantiopora --
NIBRFG0000102423C_aurantiopora --
Dai7359Antrodiella_zonata --
F20080702KCM29C_consors --
F20080208LYW10Cerrena_consors --
Dai7821Antrodiella_zonata --
CFMR_DCL04_31Pseudolagarobasid --
VPB197Pseudolagarobasidium_bel --
CBS115543Pseudolagarobasidium_ --
CBS115544 --
Han405Pseudolagarobasidium_bai --
Han406Pseudolagarobasidium_bai --
MUcc838Spongipellis_delectans --
BRNM686401S_delectans --
OSM_F925S_delectans --
BRNM67093Spongipellis_litschau --
CFMRccFP59199TS_unicolor --
CFMRccFP71791TS_unicolor --
Dai13845P_lischaueri
ATTCTCTGGACCTGTCATCGAGTTCTTGGAAGAGTGGGGTTTGGAGTCAT
Dai20266P_lischaueri --
CFMR_HHB11240Radulodon_america --
RLG6350Radulodon_americanus --
KY415963Radulodon_erikssonii --
HHB9567spRadulodon_casearius --
KRT_Iso_26Radulodon_casearius --
CBS126044Radulodon_erikssonii --
He6183YUNNANENSIS --
Cui17979YUNNANENSIS --
Miettinen2091Junghuhnia_fimbri --
KHL12316S_tenue --
PRM846564S_pachyodon --
SP_Lgt_S_pachyodon --
Ryvarden44669Tyromyces_xuchile --
PW17_171sinuosus --
W53Dai12234
ATTCTCCGGTCCTGTCATCGAGTTCTTGGAGGAGTGGGGTCTGGAGTCCT
HHB4100SpAntella_americana --
W3Dai20901spumeus

ACTTTCTGCACCCGTCATCGAGTTCTTGGAGGAATGGGGTCTGGAGTCTC
He6736 --
BRNM734877S_spumeus --
BRNM712630S_spumeus --
Dai1723Loweomyces_sibiricus --
W54Cui10009 --
W1Dai20899
ACTTTCTGCGCCCGTCATCGAGTTCTTGGAGGAATGGGGTCTGGAGTCTC
HHB13445Trametes_ochracea --
Dai16222 --
Dai16240 --

JV1310_11SanguinolentusCernys
TGGAAGAGAACGCACATTCGTCCACGCCGTGTACCAAAGTCTTTGTCAAT
MJ39_00_SK --
MJ111_04_CZ --
JV1610_BOKYsmrk --
Dai21030 --
Dai20976P_furcatus
TGGAAGAGAACGCACATTCGTCCACGCCGTGTACCAAAGTCTTTGTCAAT
Dai2105 --
Dai2544 --
Dai11313 --
WCG1611Dai26167
TGGAAGAGAACGCACATTCATCCACGCCGTGTACCAAAGTCTTTGTCAAT
WCG1518Dai25999Physisporinus --
TAA15097 --
JV8909_19_CZ --
JV1310_15_P_sanguinolentus2_CZ --
MJ53_02_CZ --
CLZhao21647P_yunnanensis --
CLZhao21583P_yunnanensis --
Dai22272
TGGAAGAGAACGCACACTCGTCCACGCCGTGTACCAAGGTCTTTGTCAAT
Dai22279 --
MJ332_94_CZ --
MJ642_94_CZ_Expallescens --
Dai21060P_vinctus
TGGAGGAGAACGCTCACTCAGCCACGCCTTGCACCAAAGTGTTCGTGAAT
JV0511_23LRP_pouzarii --
JQ409462_R_pouzarii_PRM899856_ --
JV0308_66_WA --
JV0309_45_WA_USA --
JV0709_83_CA_USA --

Dai21043P_pouzarii
TGGAAGAGAACGCCCACTCAGCTACGCCTTGCACCAAAGTGTTCGTGAAC
MJ144_95_CZ --
JV0909_3_CZ --
JV0609_1_K --
Dai20396Physisporinus_castanop
TGGAGGAGAATGCTCATTCGGCAACGCCTTGTACCAAAGTGTTCGTGAAC
Dai20397Physisporinus_castanop
TGGAGGAGAATGCTCATTCGGCAACGCCTTGTACCAAAGTGTTCGTGAAC
MJ19_09_SK_Abies --
JV0509_40_J_TN_USA_Betula --
JV0808_33crocatus_PA_USAlist --
DLL2009_061P_crocatus --
Dai12800P_subcrocatus --
Dai15917P_subcrocatus --
Cui16620 --
HCFC1088Meripilus_stillicidior --
MCW590Meripilus_obsrcurus --
MCW722Meripilus_obsrcurus --
Cui9381P_tibeticus --
Cui9588P_tibeticus --
Va2_Beneschova --
CWU3874_Ukraine_Alnus --
WCG1293Dai24718Physisporinus --
WCG1268Dai24682A
TAGAAGAGAACGCTCATTCTTCCACGCCTTGTACAAAAGTATTCGTGAAT
WCG1269Dai24683A
TAGAAGAGAACGCTCATTCTTCCACGCCTTGTACAAAAGTATTCGTGAAT
WCG1279Dai24694A
TAGAAGAGAACGCTCATTCTTCCACGCCTTGTACAAAAGTGTTTGTGAAC
Dai16971 --
ZQY1043Dai26696 --
Doll880
TAGAGGAGAATGCTCATTCTTCCACGCCTTGTACCAAAGTATTCGTCAAC
Doll1000
TAGAGGAGAATGCTCACTCTTCCACGCCTTGCACCAAAGTATTCGTCAAC
1DAI18529 --
Dai19535
TGGAGGAGAACGCACATTCGTCGACACCTTGCACCAAGGTGTTCGTGAAT
1704_79_hnedyVillaLaPaz
TGGAGGAGAATGCTCACTCATCGACGCCATGCACCAAGGTGTTCGTGAAC
F2061 --
1DAI18268 --
1DAI18540A --

Dai17695 --
LKY18Dai26373 --
Dai17839P_sulphureus
TGGAAGAAAACGCTCACTCTTCTACGCCTTGCACTAAGGTGTTCGTGAAC
Dai17841P_sulphureus
TGGAAGAAAACGCTCACTCTTCTACGCCTTGCACTAAGGTGTTCGTGAAC
Dai19877P_roseus
TGGAAGAGAACGCTCACTCGTCCACGCCTTGCACCAAGGTGTTCGTAAAT
1508_18_1_Kout --
KP859303_R_vinctus_RP185_BRAZI --
JK1807_15Rigidoporus_sp_Puerto
TGGAAGAGAATGCTCACTCGGCCACGCCGTGCACGAAGGTGTTCGTGAAT
JV0509_47_J_TN_USA
TGGAAGAGAATGCTCACTCGTCAACACCTTGCACTAAGGTGTTCGTGAAT
JV0709_188 --
JV0509_127_PA_USA
TGGAAGAGAATGCTCACTCGTCAACACCTTGCACTAAGGTGTTCGTGAAT
JV1009_59_NJ_USA
TGGAAGAGAATGCTCACTCGTCAACACCTTGCACTAAGGTGTTCGTGAAT
Dai15497P_crataegi
TGGAGGAGAATGCTCATTCATCCACACCTTGTACCAAGGTATTCGTTAAC
Dai15499P_crataegi
TGGAGGAGAATGCTCATTCATCCACACCTTGTACCAAGGTATTCGTTAAC
Cui3266P_cinereus --
WCG1256Dai24690 --
1DAI17581 --
WCG1255Dai24688
TGGAAGAAAACGCTCACTCGTCCACACCTTGCACAAAGGTGTTCGTGAAC
Dai22427 --
MV690Meripilus_concrescens --
MV513Meripilus_galapagensis --
Dai19793
TGGAGGAGAATGCCCACTCGTCGACGCCTTGTACCAAGGTGTTCGTGAAT
OQ553780P_tamilnaduensis --
OQ553779P_tamilnaduensis --
A164FB3Meripilus_giganteus --
JV1407_36_Vinctus_Meandrica --
1807_3K_Rigidoporus_PuertoRico
TAGAAGAGAATGCTCATTCAGCGACGCCTTGCACAAAGGTCTTCGTGAAT
Cui16903P_vinctus
TAGAAGAGAATGCTCATTCAGCGACGCCTTGCACAAAGGTCTTCGTGAAT
JV1008_18R_Lineatus --
JV1407_37_1_Vinctus_Carara
TAGAAGAGAATGCACATTCAGCCACACCTTGCACAAAGGTCTTCGTGAAC

Dai17986P_lineatus

TAGAAGAGAATGCACATTCAGCCACACCTTGCACAAAGGTCTTCGTGAAC

Dai18281

TAGAAGAGAATGCACATTCAGCCACACCTTGCACAAAGGTCTTCGTGAAC

1DAI19796 --

ZQY797Dai25241

TGGAAGAGAATGCACATTCAGCCACACCTTGCACAAAGGTCTTCGTGAAT

WCG1289Dai24711 --

Dai22598 --

Dai20523 --

Dai17885 --

Dai17553 --

Dai19639

TAGAAGAGAATGCACATTCGGCCACACCTTGCACAAAGGTCTTCGTGAAC

JV0110_48_CZ --

MJ129_04 --

Cui10340P_eminens --

Cui10341P_eminens --

Dai12685P_eminens --

Miettinen_13591Rigidoporus_und --

Dai20868

TGGAAGAAAACGCGCATTCGTCGACGCCTTGCACTAAGGTGTTCGTGAAC

Dai20832

TGGAAGAAAACGCGCATTCGTCGACGCCTTGCACCAAGGTGTTCGTGAAT

Dai11400 --

Dai22472 --

1CUI10475 --

1CUI10491 --

HCFC1095Meripilus_robledoi --

MCW702Meripilus_revolubilis --

1704_83_zluty_HaciendaBaru --

Dai9925P_lavendulus --

Dai13587AP_lavendulus --

PDD70600P_longicystidius --

Cui16630 --

FP_135344Meripilus_giganteus

TGGAAGAGAATGCCCATTCGTCCACACCTTGTACCAAGGTATTTGTGAAT

FP_100460_Sp --

CBS421_48Meripilus_giganteus --

Cui9203 --

Cui9202 --

TUFC100564Japan --

Russell5913Meripilus_sumstinei --

RP215Meripilus_brasiliensis --

RP200Meripilus_brasiliensis --
JV1712_13J_R_vinctus2_LSUPuert --
Dai10503R_hypobrunneus --
Dai10569Rigidoporus_hypobrunne --
1DAI19451 --
CM108bRigidoporus_hypobrunneus --
1CUI16874 --
FD299Cerrena_unicolor --
KHL_GB_Cerrena_uniclor --
Dai12892Cerrena_albocinnamomea --
Dai12955C_albocinnamomea --
SNUm03110102C_aurantiopora --
NIBRFG0000102423C_aurantiopora --
Dai7359Antrodiella_zonata --
F20080702KCM29C_consors --
F20080208LYW10Cerrena_consors --
Dai7821Antrodiella_zonata --
CFMR_DCL04_31Pseudolagarobasid --
VPB197Pseudolagarobasidium_bel --
CBS115543Pseudolagarobasidium_ --
CBS115544 --
Han405Pseudolagarobasidium_bai --
Han406Pseudolagarobasidium_bai --
MUcc838Spongipellis_delectans --
BRNM686401S_delectans --
OSM_F925S_delectans --
BRNM67093Spongipellis_litschau --
CFMRccFP59199TS_unicolor --
CFMRccFP71791TS_unicolor --
Dai13845P_lischaueri
TAGAAGAGAACGCACACTCGGCGACACCTTGCACTAAAGTATTCGTGAAC
Dai20266P_lischaueri --
CFMR_HHB11240Radulodon_america --
RLG6350Radulodon_americanus --
KY415963Radulodon_erikssonii --
HHB9567spRadulodon_casearius --
KRT_Iso_26Radulodon_casearius --
CBS126044Radulodon_erikssonii --
He6183YUNNANENSIS --
Cui17979YUNNANENSIS --
Miettinen2091Junghuhnia_fimbri --
KHL12316S_tenue --
PRM846564S_pachyodon --
SP_Lgt_S_pachyodon --

Ryvarden44669Tyromyces_xuchile --
PW17_171sinuosus --
W53Dai12234
TGGAAGAAAATGTTCACTCGCCCACGCCCACGACGAAAGTGTTCGTGAAT
HHB4100SpAntella_americana --
W3Dai20901spumeus
TGGAAGAGAATGCTCATTCGTCGACGCCCTGTACCAAGGTGTTCGTGAAC
He6736 --
BRNM734877S_spumeus --
BRNM712630S_spumeus --
Dai1723Loweomyces_sibiricus --
W54Cui10009 --
W1Dai20899
TGGAAGAGAATGCCCATTCGTCGACGCCTTGTACCAAGGTGTTCGTGAAC
HHB13445Trametes_ochracea --
Dai16222 --
Dai16240 --

JV1310_11SanguinolentusCernys
GGTGTCTGGATGGGCGTACATCGCGACCCTGCTAACTTGGTGAAGACGAT
MJ39_00_SK --
MJ111_04_CZ --
JV1610_BOKYsmrk --
Dai21030 --
Dai20976P_furcatus
GGTGTCTGGATGGGCGTACATCGCGACCCTGCTAACTTGGTGAAGACGAT
Dai2105 --
Dai2544 --
Dai11313 --
WCG1611Dai26167
GGTGTCTGGATGGGTGTACATCGCGACCCTGCTAACTTGGTGAAGACGAT
WCG1518Dai25999Physisporinus --
TAA15097 --
JV8909_19_CZ --
JV1310_15_P_sanguinolentus2_CZ --
MJ53_02_CZ --
CLZhao21647P_yunnanensis --
CLZhao21583P_yunnanensis --
Dai22272
GGTGTCTGGATGGGCGTACATCGCGACCCTGCTAACTTGGTGAAGACGAT
Dai22279 --
MJ332_94_CZ --
MJ642_94_CZ_Expallescens --
Dai21060P_vinctus

GGTGTTTGGATGGGTGTACATCGTGACCCAGCCAACTTGGTGAAGACTAT
JV0511_23LRP_pouzarii --
JQ409462_R_pouzarii_PRM899856_ --
JV0308_66_WA --
JV0309_45_WA_USA --
JV0709_83_CA_USA --
Dai21043P_pouzarii
GGTGTTTGGATGGGTGTACATCGCGACCCAGCCAATTTGGTGAAGACTAT
MJ144_95_CZ --
JV0909_3_CZ --
JV0609_1_K --
Dai20396Physisporinus_castanop
GGTGTTTGGATGGGTGTACATCGTGACCCAGCCAATTTGGTGAAGACTAT
Dai20397Physisporinus_castanop
GGTGTTTGGATGGGTGTACATCGTGACCCAGCCAATTTGGTGAAGACTAT
MJ19_09_SK_Abies --
JV0509_40_J_TN_USA_Betula --
JV0808_33crocatus_PA_USAlist --
DLL2009_061P_crocatus --
Dai12800P_subcrocatus --
Dai15917P_subcrocatus --
Cui16620 --
HCFC1088Meripilus_stillicidior --
MCW590Meripilus_obsrcurus --
MCW722Meripilus_obsrcurus --
Cui9381P_tibeticus --
Cui9588P_tibeticus --
Va2_Beneschova --
CWU3874_Ukraine_Alnus --
WCG1293Dai24718Physisporinus --
WCG1268Dai24682A
GGTGTCTGGATGGGAGTGCACCGTGACCCCGCCAACTTAGTGAAGACCAT
WCG1269Dai24683A
GGTGTCTGGATGGGAGTGCACCGTGACCCCGCCAACTTAGTGAAGACCAT
WCG1279Dai24694A
GGTGTCTGGATGGGAGTGCACCGTGACCCCGCCAACTTAGTGAAGACGAT
Dai16971 --
ZQY1043Dai26696 --
Doll880
GGTGTCTGGATGGGCGTGCATCGCGATCCAGACAACTTGGTGAAGACGAT
Doll1000
GGTGTCTGGATGGGCGTGCATCGCGATCCAGACAACTTGGTCAAGACGAT
1DAI18529 --
Dai19535

GGTGTCTGGATGGGTGTACATCGCGATCCAGCCAATCTGGTAAAGACTAT
1704_79_hnedyVillaLaPaz
GGTGTCTGGATGGGTGTCCATCGTGATCCGGCCAACCTGGTCAAGACCAT
F2061 --
1DAI18268 --
1DAI18540A --
Dai17695 --
LKY18Dai26373 --
Dai17839P_sulphureus
GGTGTTTGGATGGGTGTTCACCGCGACCCGGCTAACCTGGTGAAGACTAT
Dai17841P_sulphureus
GGTGTTTGGATGGGTGTTCACCGCGACCCGGCTAACCTGGTGAAGACTAT
Dai19877P_roseus
GGTGTCTGGATGGGTGTCCACCGCGACCCTGCTAATCTTGTCAAGACCAT
1508_18_1_Kout --
KP859303_R_vinctus_RP185_BRAZI --
JK1807_15Rigidoporus_sp_Puerto
GGGGTTTGGATGGGTGTTCATCGCGATCCCGCAAATTTGGTCAAGACGAT
JV0509_47_J_TN_USA
GGCGTTTGGATGGGTGTCCACCGTGATCCTGCGAATCTGGTGAAGACGAT
JV0709_188 --
JV0509_127_PA_USA
GGCGTTTGGATGGGTGTCCACCGTGATCCTGCGAATCTGGTGAAGACGAT
JV1009_59_NJ_USA
GGCGTTTGGATGGGTGTCCACCGTGATCCTGCGAATCTGGTGAAGACGAT
Dai15497P_crataegi
GGTGTTTGGATGGGTGTTCACCGTGATCCTGCGAATTTGGTCAAGACGAT
Dai15499P_crataegi
GGTGTTTGGATGGGTGTTCACCGTGATCCTGCGAATTTGGTCAAGACGAT
Cui3266P_cinereus --
WCG1256Dai24690 --
1DAI17581 --
WCG1255Dai24688
GGGGTCTGGATGGGTGTTCACCGTGATCCCGCGAACTTGGTCAAGACGAT
Dai22427 --
MV690Meripilus_concrescens --
MV513Meripilus_galapagensis --
Dai19793
GGTGTTTGGATGGGTGTCCACCGTGATCCTGCGAATTTGGTGAAGACGAT
OQ553780P_tamilnaduensis --
OQ553779P_tamilnaduensis --
A164FB3Meripilus_giganteus --
JV1407_36_Vinctus_Meandrica --
1807_3K_Rigidoporus_PuertoRico

GGCGTCTGGATGGGTGTACATCGGGATCCTGCGAATTTGGTGAAGACGAT
Cui16903P_vinctus
GGCGTCTGGATGGGTGTACATCGGGATCCTGCGAATTTGGTGAAGACGAT
JV1008_18R_Lineatus --
JV1407_37_1_Vinctus_Carara
GGTGTGTGGATGGGCGTACATCGGGATCCCGCGAATTTGGTGAAGACGAT
Dai17986P_lineatus
GGTGTGTGGATGGGCGTACATCGGGATCCTGCGAATTTGGTGAAGACGAT
Dai18281
GGTGTGTGGATGGGCGTACATCGGGATCCTGCGAATTTGGTGAAGACGAT
1DAI19796 --
ZQY797Dai25241
GGTGTCTGGATGGGCGTACATCGGGATCCCGCGAATTTGGTGAAGACGAT
WCG1289Dai24711 --
Dai22598 --
Dai20523 --
Dai17885 --
Dai17553 --
Dai19639
GGTGTGTGGATGGGCGTACATCGGGATCCTGCGAATTTGGTGAAGACGAT
JV0110_48_CZ --
MJ129_04 --
Cui10340P_eminens --
Cui10341P_eminens --
Dai12685P_eminens --
Miettinen_13591Rigidoporus_und --
Dai20868
GGTGTGTGGATGGGGGTTCACCGTGATCCCGCGAACTTGGTCAAGACGAT
Dai20832
GGTGTGTGGATGGGAGTTCACCGAGATCCCGCAAACTTGGTCAAGACGAT
Dai11400 --
Dai22472 --
1CUI10475 --
1CUI10491 --
HCFC1095Meripilus_robledoi --
MCW702Meripilus_revolubilis --
1704_83_zluty_HaciendaBaru --
Dai9925P_lavendulus --
Dai13587AP_lavendulus --
PDD70600P_longicystidius --
Cui16630 --
FP_135344Meripilus_giganteus
GGTGTTTGGATGGGTGTACATCGTGACCCCGCGAATTTGGTGAAGACGAT
FP_100460_Sp --

CBS421_48Meripilus_giganteus --
Cui9203 --
Cui9202 --
TUFC100564Japan --
Russell5913Meripilus_sumstinei --
RP215Meripilus_brasiliensis --
RP200Meripilus_brasiliensis --
JV1712_13J_R_vinctus2_LSUPuert --
Dai10503R_hypobrunneus --
Dai10569Rigidoporus_hypobrunne --
1DAI19451 --
CM108bRigidoporus_hypobrunneus --
1CUI16874 --
FD299Cerrena_unicolor --
KHL_GB_Cerrena_uniclor --
Dai12892Cerrena_albocinnamomea --
Dai12955C_albocinnamomea --
SNUm03110102C_aurantiopora --
NIBRFG0000102423C_aurantiopora --
Dai7359Antrodiella_zonata --
F20080702KCM29C_consors --
F20080208LYW10Cerrena_consors --
Dai7821Antrodiella_zonata --
CFMR_DCL04_31Pseudolagarobasid --
VPB197Pseudolagarobasidium_bel --
CBS115543Pseudolagarobasidium_ --
CBS115544 --
Han405Pseudolagarobasidium_bai --
Han406Pseudolagarobasidium_bai --
MUcc838Spongipellis_delectans --
BRNM686401S_delectans --
OSM_F925S_delectans --
BRNM67093Spongipellis_litschau --
CFMRccFP59199TS_unicolor --
CFMRccFP71791TS_unicolor --
Dai13845P_lischaueri
GGCGTTTGGATGGGCGTTCATCGCGATCCAGCCAATCTCGTGAAGACGAT
Dai20266P_lischaueri --
CFMR_HHB11240Radulodon_america --
RLG6350Radulodon_americanus --
KY415963Radulodon_erikssonii --
HHB9567spRadulodon_casearius --
KRT_Iso_26Radulodon_casearius --
CBS126044Radulodon_erikssonii --

He6183YUNNANENSIS --
Cui17979YUNNANENSIS --
Miettinen2091Junghuhnia_fimbri --
KHL12316S_tenue --
PRM846564S_pachyodon --
SP_Lgt_S_pachyodon --
Ryvarden44669Tyromyces_xuchile --
PW17_171sinuosus --
W53Dai12234
GGTGTCTGGATGGGTGTTCACCGAGACCCTGTCAACCTTGTTAAGACTAT
HHB4100SpAntella_americana --
W3Dai20901spumeus
GGCGTCTGGATGGGTGTCCATCGTGACCCAGCGAACCTCGTGAAGACGAT
He6736 --
BRNM734877S_spumeus --
BRNM712630S_spumeus --
Dai1723Loweomyces_sibiricus --
W54Cui10009 --
W1Dai20899
GGTGTCTGGATGGGTGTCCATCGTGACCCAGCAAACCTCGTGAAGACGAT
HHB13445Trametes_ochracea --
Dai16222 --
Dai16240 --

JV1310_11SanguinolentusCernys
TAAGAAGCTGCGACGGAAGGATGATATTAGTCCCGAAGTCTCGGTTGTCA
MJ39_00_SK --
MJ111_04_CZ --
JV1610_BOKYsmrk --
Dai21030 --
Dai20976P_furcatus
TAAGAAGCTGCGACGGAAGGATGATATTAGCCCCGAAGTCTCGGTTGTCA
Dai2105 --
Dai2544 --
Dai11313 --
WCG1611Dai26167
TAAGAAGCTGCGACGGAAGGATGATATTAGTCCCGAAGTCTCGGTTGTCA
WCG1518Dai25999Physisporinus --
TAA15097 --
JV8909_19_CZ --
JV1310_15_P_sanguinolentus2_CZ --
MJ53_02_CZ --
CLZhao21647P_yunnanensis --
CLZhao21583P_yunnanensis --

Dai22272
CAAGAAGCTGCGACGGAAGGATGATATTAGTCCCGAAGTCTCGGTTGTCA
Dai22279 --
MJ332_94_CZ --
MJ642_94_CZ_Expallescens --
Dai21060P_vinctus
CAAGAAATTGCGACGGAAGGACGATATCAGCCCTGAAGTTTCGGTGGTCA
JV0511_23LRP_pouzarii --
JQ409462_R_pouzarii_PRM899856_ --
JV0308_66_WA --
JV0309_45_WA_USA --
JV0709_83_CA_USA --
Dai21043P_pouzarii
CAAGAAGCTGCGACGGAAGGATGATATCAGTCCTGAGGTTTCGGTGGTCA
MJ144_95_CZ --
JV0909_3_CZ --
JV0609_1_K --
Dai20396Physisporinus_castanop
CAAGAAATTGCGACGGAAGGACGATATCAGTCCCGAAGTTTCGGTGGTCA
Dai20397Physisporinus_castanop
CAAGAAATTGCGACGGAAGGACGATATCAGTCCCGAAGTTTCGGTGGTCA
MJ19_09_SK_Abies --
JV0509_40_J_TN_USA_Betula --
JV0808_33crocatus_PA_USAlist --
DLL2009_061P_crocatus --
Dai12800P_subcrocatus --
Dai15917P_subcrocatus --
Cui16620 --
HCFC1088Meripilus_stillicidior --
MCW590Meripilus_obsrcurus --
MCW722Meripilus_obsrcurus --
Cui9381P_tibeticus --
Cui9588P_tibeticus --
Va2_Beneschova --
CWU3874_Ukraine_Alnus --
WCG1293Dai24718Physisporinus --
WCG1268Dai24682A
CAAGAAGCTTCGACGGAAGGACGATATCAGCCCCGAGGTCTCGGTTGTTA
WCG1269Dai24683A
CAAGAAGCTTCGACGGAAGGACGATATCAGCCCCGAGGTCTCGGTTGTTA
WCG1279Dai24694A
CAAGAAGCTTCGACGGAAGGACGATATCAGTCCTGAAGTCTCGGTTGTTA
Dai16971 --
ZQY1043Dai26696 --

Doll880
TAAAAAGCTTCGACGGAAGGACGATATTAGCCCTGAAGTTTCAGTTGTCA
Doll1000
CAAAAAGCTTCGACGGAAGGACGATATTAGCCCTGAAGTTTCAGTTGTCA
1DAI18529 --
Dai19535
CAAGAAGCTGAGACGAAAGGACGATATCAGTCCCGAAGTGTCGGTCGTCA
1704_79_hnedyVillaLaPaz
CAAGAAACTACGTCGCAAAGACGATCTCAGTCCCGAAGTTTCGATCGTTC
F2061 --
1DAI18268 --
1DAI18540A --
Dai17695 --
LKY18Dai26373 --
Dai17839P_sulphureus
CAAGAAATTGCGGCGGAAGGATGACATCAGTCCCGATGTTTCGGTCGTTC
Dai17841P_sulphureus
CAAGAAATTGCGGCGGAAGGATGACATCAGTCCCGATGTTTCAGTCGTTC
Dai19877P_roseus
TAAGAAATTGCGACGGAAGGACGATATCAGCCCTGAAGTTTCAGTCGTCA
1508_18_1_Kout --
KP859303_R_vinctus_RP185_BRAZI --
JK1807_15Rigidoporus_sp_Puerto
CAAGAAACTGCGTCGAAAGGACGATATCAGTCCTGACGTTTCGGTCGTCA
JV0509_47_J_TN_USA
CAAGAAACTCCGACGAAAGGATGACATCAGCCCCGAGGTCTCTGTTGTTC
JV0709_188 --
JV0509_127_PA_USA
CAAGAAACTCCGACGAAAGGATGACATCAGTCCCGAGGTCTCTGTTGTTC
JV1009_59_NJ_USA
CAAGAAACTCCGACGAAAGGATGACATCAGCCCCGAGGTCTCTGTTGTTC
Dai15497P_crataegi
TAAGAAGTTGCGACGAAAGGACGATATCAGTCCTGAAGTCTCTGTCGTTC
Dai15499P_crataegi
TAAGAAGTTGCGACGAAAGGACGATATCAGTCCTGAAGTCTCTGTCGTTC
Cui3266P_cinereus --
WCG1256Dai24690 --
1DAI17581 --
WCG1255Dai24688
CAAGAAGCTGCGACGAAAGGATGATATCAGTCCTGAAGTATCCGTTGTTC
Dai22427 --
MV690Meripilus_concrescens --
MV513Meripilus_galapagensis --
Dai19793

TAAGAAGCTGCGACGAAAGGACGACATCAGTCCTGAAGTCTCTGTTGTTC
OQ553780P_tamilnaduensis --
OQ553779P_tamilnaduensis --
A164FB3Meripilus_giganteus --
JV1407_36_Vinctus_Meandrica --
1807_3K_Rigidoporus_PuertoRico
CAAGAAGCTGCGAAGAAAGGACGATATCAGTCCTGAGGTCTCCGTCGTTC
Cui16903P_vinctus
CAAGAAGCTGCGAAGAAAGGACGATATCAGTCCTGAGGTCTCCGTCGTTC
JV1008_18R_Lineatus --
JV1407_37_1_Vinctus_Carara
CAAGAAGCTGCGAAGGAAGGACGACATCAGTCCTGAGGTCTCCGTCGTTC
Dai17986P_lineatus
CAAGAAGTTGCGAAGGAAGGACGATATCAGTCCTGAGGTATCCGTCGTTC
Dai18281
CAAGAAGTTGCGAAGGAAGGACGATATCAGTCCTGAGGTATCCGTCGTTC
1DAI19796 --
ZQY797Dai25241
CAAGAAGTTGCGAAGGAAGGACGATATCAGTCCTGAGGTCTCCGTCGTTC
WCG1289Dai24711 --
Dai22598 --
Dai20523 --
Dai17885 --
Dai17553 --
Dai19639
TAAGAAGCTGCGAAGGAAGGACGATATCAGTCCTGAGGTCTCCGTCGTTC
JV0110_48_CZ --
MJ129_04 --
Cui10340P_eminens --
Cui10341P_eminens --
Dai12685P_eminens --
Miettinen_13591Rigidoporus_und --
Dai20868
CAAGAAGTTGCGGCGGAAGGACGATATCAGTCCCGAAGTCTCTGTCGTTC
Dai20832
CAAGAAGTTGCGACGGAAGGACGATATCAGCCCCGAAGTCTCTGTCGTTC
Dai11400 --
Dai22472 --
1CUI10475 --
1CUI10491 --
HCFC1095Meripilus_robledoi --
MCW702Meripilus_revolubilis --
1704_83_zluty_HaciendaBaru --
Dai9925P_lavendulus --

Dai13587AP_lavendulus --
PDD70600P_longicystidius --
Cui16630 --
FP_135344Meripilus_giganteus
CAAGAAGCTGCGACGGAAGGACGACATTAGTCCTGAAGTATCGGTTGTCA
FP_100460_Sp --
CBS421_48Meripilus_giganteus --
Cui9203 --
Cui9202 --
TUFC100564Japan --
Russell5913Meripilus_sumstinei --
RP215Meripilus_brasiliensis --
RP200Meripilus_brasiliensis --
JV1712_13J_R_vinctus2_LSUPuert --
Dai10503R_hypobrunneus --
Dai10569Rigidoporus_hypobrunne --
1DAI19451 --
CM108bRigidoporus_hypobrunneus --
1CUI16874 --
FD299Cerrena_unicolor --
KHL_GB_Cerrena_uniclor --
Dai12892Cerrena_albocinnamomea --
Dai12955C_albocinnamomea --
SNUm03110102C_aurantiopora --
NIBRFG0000102423C_aurantiopora --
Dai7359Antrodiella_zonata --
F20080702KCM29C_consors --
F20080208LYW10Cerrena_consors --
Dai7821Antrodiella_zonata --
CFMR_DCL04_31Pseudolagarobasid --
VPB197Pseudolagarobasidium_bel --
CBS115543Pseudolagarobasidium_ --
CBS115544 --
Han405Pseudolagarobasidium_bai --
Han406Pseudolagarobasidium_bai --
MUcc838Spongipellis_delectans --
BRNM686401S_delectans --
OSM_F925S_delectans --
BRNM67093Spongipellis_litschau --
CFMRccFP59199TS_unicolor --
CFMRccFP71791TS_unicolor --
Dai13845P_lischaueri
CAAGAAGCTTCGACGAAAGGACGATATCAGTCCTGAAGTCTCCGTTGTGC
Dai20266P_lischaueri --

CFMR_HHB11240Radulodon_america --
RLG6350Radulodon_americanus --
KY415963Radulodon_erikssonii --
HHB9567spRadulodon_casearius --
KRT_Iso_26Radulodon_casearius --
CBS126044Radulodon_erikssonii --
He6183YUNNANENSIS --
Cui17979YUNNANENSIS --
Miettinen2091Junghuhnia_fimbri --
KHL12316S_tenue --
PRM846564S_pachyodon --
SP_Lgt_S_pachyodon --
Ryvarden44669Tyromyces_xuchile --
PW17_171sinuosus --
W53Dai12234
CAAGAAGCTGCGACGAAAGACGACATCAGCCCTGAAGTTTCGGTCGTAC
HHB4100SpAntella_americana --
W3Dai20901spumeus
CAAGAAATTGCGGCGAAAGGACGACATCAGCCCTGAAGTCTCTGTGGTTC
He6736 --
BRNM734877S_spumeus --
BRNM712630S_spumeus --
Dai1723Loweomyces_sibiricus --
W54Cui10009 --
W1Dai20899
CAAGAAGTTGCGGCGAAAGGACGACATCAGCCCTGAAGTCTCTGTGGTTC
HHB13445Trametes_ochracea --
Dai16222 --
Dai16240 --

JV1310_11SanguinolentusCernys
GGGATATCCGTGAAAGGGAGTTGCGGCTGTACACCGATGC-TGGTCGTGT
MJ39_00_SK --
MJ111_04_CZ --
JV1610_BOKYsmrk --
Dai21030 --
Dai20976P_furcatus
GGGATATCCGTGAAAGGGAGTTGCGGCTGTACACCGACGC-TGGTCGTGT
Dai2105 --
Dai2544 --
Dai11313 --
WCG1611Dai26167
GGGATATCCGTGAAAGGGAGTTGCGGCTGTACACCGACGC-TGGTCGTGT
WCG1518Dai25999Physisporinus --

TAA15097 --
JV8909_19_CZ --
JV1310_15_P_sanguinolentus2_CZ --
MJ53_02_CZ --
CLZhao21647P_yunnanensis --
CLZhao21583P_yunnanensis --
Dai22272
GGGATATCCGTGAAAGGGAGTTGCGGCTGTACACCGACGC-TGGTCGTGT
Dai22279 --
MJ332_94_CZ --
MJ642_94_CZ_Expallescens --
Dai21060P_vinctus
GGGACATCCGTGAAAGAGAGTTACGGCTTTATACCGATGC-TGGTCGTGT
JV0511_23LRP_pouzarii --
JQ409462_R_pouzarii_PRM899856_ --
JV0308_66_WA --
JV0309_45_WA_USA --
JV0709_83_CA_USA --
Dai21043P_pouzarii
GGGACATCCGGGAGAGAGAGTTACGGCTGTACACCGATGC-TGGTCGTGT
MJ144_95_CZ --
JV0909_3_CZ --
JV0609_1_K --
Dai20396Physisporinus_castanop
GGGATATTCGTGAAAGGGAATTACGGTTGTACACCGATGC-CGGTCGTGT
Dai20397Physisporinus_castanop
GGGATATTCGTGAAAGGGAATTACGGTTGTACACCGATGC-CGGTCGTGT
MJ19_09_SK_Abies --
JV0509_40_J_TN_USA_Betula --
JV0808_33crocatus_PA_USAlist --
DLL2009_061P_crocatus --
Dai12800P_subcrocatus --
Dai15917P_subcrocatus --
Cui16620 --
HCFC1088Meripilus_stillicidior --
MCW590Meripilus_obsrcurus --
MCW722Meripilus_obsrcurus --
Cui9381P_tibeticus --
Cui9588P_tibeticus --
Va2_Beneschova --
CWU3874_Ukraine_Alnus --
WCG1293Dai24718Physisporinus --
WCG1268Dai24682A
GGGATATTCGTGAAAGGGAGTTACGTTTGTACACTGATGC-TGGTCGTGT

WCG1269Dai24683A

GGGATATTCGTGAAAGGGAGTTACGTTTGTACACTGATGC-TGGTCGTGT

WCG1279Dai24694A

GGGATATTCGTGAAAGGGAGTTACGTTTGTACACTGATGC-TGGTCGTGT

Dai16971 --

ZQY1043Dai26696 --

Doll880

GGGATATTCGTGAAAGGGAGTTACGACTCTATACCGACGC-CGGTCGCGT

Doll1000

GGGATATTCGTGAAAGGGAGTTACGACTCTATACCGACGC-CGGTCGCGT

1DAI18529 --

Dai19535

GGGATATCCGTGAACGGGAGTTGCGCTTATACACCGATGC-TGGTCGTGT

1704_79_hnedyVillaLaPaz

GGGATATTCGTGAGAGGGAATTGCGCTTGTATACCGACGC-TGGTCGCGT

F2061 --

1DAI18268 --

1DAI18540A --

Dai17695 --

LKY18Dai26373 --

Dai17839P_sulphureus

GGGACATCCGTGAGAGGGAACTACGGTTGTATACTGACGC-TGGTCGTGT

Dai17841P_sulphureus

GGGACATCCGTGAGAGGGAACTACGGTTGTATACTGACGC-TGGTCGTGT

Dai19877P_roseus

GGGACATCCGTGAGAGGGAGCTACGGCTGTATACCGATGC-TGGTCGCGT

1508_18_1_Kout --

KP859303_R_vinctus_RP185_BRAZI --

JK1807_15Rigidoporus_sp_Puerto

GAGATATTCGCGAGCGAGAATTGCGCCTGTATACCGATGC-TGGTCGCGT

JV0509_47_J_TN_USA

GTGATATCCGTGAACGGGAGCTTCGATTGTATACCGACGC-AGGTCGTGT

JV0709_188 --

JV0509_127_PA_USA

GTGATATCCGTGAACGGGAGCTTCGATTGTATACCGACGC-AGGTCGTGT

JV1009_59_NJ_USA

GTGATATCCGTGAACGGGAGCTTCGATTGTATACCGACGC-AGGTCGTGT

Dai15497P_crataegi GGGATATCCGTGAACGGGAACTTCGATTGTAT---------

Dai15499P_crataegi

GGGATATCCGTGAACGGGAACTTCGATTGTATACCGACGC-AGGTCGTGT

Cui3266P_cinereus --

WCG1256Dai24690 --

1DAI17581 --

WCG1255Dai24688
GGGATATTCGTGAGCGGGAGCTTCGATTGTATACAGACGC-GGGCCGTGT
Dai22427 --
MV690Meripilus_concrescens --
MV513Meripilus_galapagensis --
Dai19793
GGGATATCCGTGAACGGGAACTTCGATTGTATACCGACGC-AGGTCGTGT
OQ553780P_tamilnaduensis --
OQ553779P_tamilnaduensis --
A164FB3Meripilus_giganteus --
JV1407_36_Vinctus_Meandrica --
1807_3K_Rigidoporus_PuertoRico
GGGATATTCGTGAAAGGGAACTTCGATTGTATACGGATGC-GGGTCGTGT
Cui16903P_vinctus
GGGATATTCGTGAAAGGGAACTTCGATTGTATACGGATGC-GGGTCGTGT
JV1008_18R_Lineatus --
JV1407_37_1_Vinctus_Carara
GGGATATCCGTGAAAGGGAACTTCGGTTGTATACGGATGC-GGGTCGTGT
Dai17986P_lineatus
GGGATATCCGTGAAAGGGAACTTCGGTTGTATACGGATGC-GGGTCGTGT
Dai18281
GGGATATCCGTGAAAGGGAACTTCGGTTGTATACGGATGC-GGGTCGTGT
1DAI19796 --
ZQY797Dai25241
GGGATATTCGTGAAAGGGAACTTCGATTGTATACGGATGC-GGGTCGTGT
WCG1289Dai24711 --
Dai22598 --
Dai20523 --
Dai17885 --
Dai17553 --
Dai19639
GGGATATTCGTGAAAGGGAACTCCGATTATATACGGATGCAGGGTCGTGT
JV0110_48_CZ --
MJ129_04 --
Cui10340P_eminens --
Cui10341P_eminens --
Dai12685P_eminens --
Miettinen_13591Rigidoporus_und --
Dai20868
GCGATATCCGTGAACGCGAACTTAGGTTATACACGGATGC-CGGACGTGT
Dai20832
GCGATATTCGTGAACGCGAACTGAGGTTATACACAGATGC-TGGACGTGT
Dai11400 --
Dai22472 --

1CUI10475 --
1CUI10491 --
HCFC1095Meripilus_robledoi --
MCW702Meripilus_revolubilis --
1704_83_zluty_HaciendaBaru --
Dai9925P_lavendulus --
Dai13587AP_lavendulus --
PDD70600P_longicystidius --
Cui16630 --
FP_135344Meripilus_giganteus
GGGATATCCGTGAGCGGGAACTTCGGTTATACACCGACGC-TGGCCGTGT
FP_100460_Sp --
CBS421_48Meripilus_giganteus --
Cui9203 --
Cui9202 --
TUFC100564Japan --
Russell5913Meripilus_sumstinei --
RP215Meripilus_brasiliensis --
RP200Meripilus_brasiliensis --
JV1712_13J_R_vinctus2_LSUPuert --
Dai10503R_hypobrunneus --
Dai10569Rigidoporus_hypobrunne --
1DAI19451 --
CM108bRigidoporus_hypobrunneus --
1CUI16874 --
FD299Cerrena_unicolor --
KHL_GB_Cerrena_uniclor --
Dai12892Cerrena_albocinnamomea --
Dai12955C_albocinnamomea --
SNUm03110102C_aurantiopora --
NIBRFG0000102423C_aurantiopora --
Dai7359Antrodiella_zonata --
F20080702KCM29C_consors --
F20080208LYW10Cerrena_consors --
Dai7821Antrodiella_zonata --
CFMR_DCL04_31Pseudolagarobasid --
VPB197Pseudolagarobasidium_bel --
CBS115543Pseudolagarobasidium_ --
CBS115544 --
Han405Pseudolagarobasidium_bai --
Han406Pseudolagarobasidium_bai --
MUcc838Spongipellis_delectans --
BRNM686401S_delectans --
OSM_F925S_delectans --

BRNM67093Spongipellis_litschau --
CFMRccFP59199TS_unicolor --
CFMRccFP71791TS_unicolor --
Dai13845P_lischaueri
GAGACATTCGTGAGAGGGAACTGCGGTTGTATACTGACGC-CGGCCGTGT
Dai20266P_lischaueri --
CFMR_HHB11240Radulodon_america --
RLG6350Radulodon_americanus --
KY415963Radulodon_erikssonii --
HHB9567spRadulodon_casearius --
KRT_Iso_26Radulodon_casearius --
CBS126044Radulodon_erikssonii --
He6183YUNNANENSIS --
Cui17979YUNNANENSIS --
Miettinen2091Junghuhnia_fimbri --
KHL12316S_tenue --
PRM846564S_pachyodon --
SP_Lgt_S_pachyodon --
Ryvarden44669Tyromyces_xuchile --
PW17_171sinuosus --
W53Dai12234
GAGATACTCGAGAGCGGGAACTTCGGATTTACACCGATGC-TGGTCGTGT
HHB4100SpAntella_americana --
W3Dai20901spumeus
GAGATATCCGTGAGCGAGAACTTCGGTTGTACACGGATGC-TGGCCGGGT
He6736 --
BRNM734877S_spumeus --
BRNM712630S_spumeus --
Dai1723Loweomyces_sibiricus --
W54Cui10009 --
W1Dai20899
GAGATATCCGTGAGCGAGAACTTCGGTTGTACACGGATGC-TGGTCGGGT
HHB13445Trametes_ochracea --
Dai16222 --
Dai16240 --

JV1310_11SanguinolentusCernys
GTGTCGCCCGCTTTTCATCGTCGAAGACCAACAGCTGGCGATCCAGAAGA
MJ39_00_SK --
MJ111_04_CZ --
JV1610_BOKYsmrk --
Dai21030 --
Dai20976P_furcatus GTGTCG--
Dai2105 --

Dai2544 --
Dai11313 --
WCG1611Dai26167
GTGTCGCCCGCTTTTCATTGTCGAAGACCAACAGCTGGCGATCCAGAAGA
WCG1518Dai25999Physisporinus --
TAA15097 --
JV8909_19_CZ --
JV1310_15_P_sanguinolentus2_CZ --
MJ53_02_CZ --
CLZhao21647P_yunnanensis --
CLZhao21583P_yunnanensis --
Dai22272
GTGTCGCCCGCTTTTCATCGTCGAAGACCAACAGCTGGCGATCCAGAAGA
Dai22279 --
MJ332_94_CZ --
MJ642_94_CZ_Expallescens --
Dai21060P_vinctus
CTGTCGTCCGCTCTTCATCGTCGAGGACCAACAACTGGCGATCCAGAAGA
JV0511_23LRP_pouzarii --
JQ409462_R_pouzarii_PRM899856_ --
JV0308_66_WA --
JV0309_45_WA_USA --
JV0709_83_CA_USA --
Dai21043P_pouzarii
ATGTCGACCGCTCTTCATCGTCGAGGATCAACAATTGGCGATCCAGAAGA
MJ144_95_CZ --
JV0909_3_CZ --
JV0609_1_K --
Dai20396Physisporinus_castanop
TTGTCGTCCGCTCTTCATCGTCGAGGACCAACAGCTGGCGATTCAGAAGA
Dai20397Physisporinus_castanop
TTGTCGTCCGCTCTTCATCGTCGAGGACCAACAGCTGGCGATTCAGAAGA
MJ19_09_SK_Abies --
JV0509_40_J_TN_USA_Betula --
JV0808_33crocatus_PA_USAlist --
DLL2009_061P_crocatus --
Dai12800P_subcrocatus --
Dai15917P_subcrocatus --
Cui16620 --
HCFC1088Meripilus_stillicidior --
MCW590Meripilus_obsrcurus --
MCW722Meripilus_obsrcurus --
Cui9381P_tibeticus --
Cui9588P_tibeticus --

Va2_Beneschova --
CWU3874_Ukraine_Alnus --
WCG1293Dai24718Physisporinus --
WCG1268Dai24682A
GTGTCGTCCCCTTTTCATCGTCGAGGATCAACAATTGGCAATTAAGAAGA
WCG1269Dai24683A
GTGTCGTCCCCTTTTCATCGTCGAGGATCAACAATTGGCAATTAAGAAGA
WCG1279Dai24694A
GTGTCGTCCCCTTTTCATCGTCGAGGATCAACAATTGGCGATCAAGAAGA
Dai16971 --
ZQY1043Dai26696 --
Doll880
GTGTCGTCCCCTCTTCATCGTGGAAGATCAACAACTGGCGATCCAGAAGA
Doll1000
GTGTCGTCCCCTCTTCATCGTGGAGGATCAACAACTGGCGATCCAGAAGA
1DAI18529 --
Dai19535
GTGTCGTCCCCTCTTTATCGTGGAGGATCAGCAGCTGGCGATCCAGAAGA
1704_79_hnedyVillaLaPaz
GTGTCGCCCGCTCTTCATCGTCGAGGACCAACAATTGGCGATCCAAAAGA
F2061 --
1DAI18268 --
1DAI18540A --
Dai17695 --
LKY18Dai26373 --
Dai17839P_sulphureus
CTGTCGCCCACTCTTCATCGTCGAGGACCAACAACT--------------
Dai17841P_sulphureus
CTGTCGCCCACTCTTCATCGTCGAGGACCAACAACTGGCGATTCAGAAGA
Dai19877P_roseus
CTGCCGTCCACTTTTCATCGTCGAAGACCAACAACTGGCGATTCAAAAGA
1508_18_1_Kout --
KP859303_R_vinctus_RP185_BRAZI --
JK1807_15Rigidoporus_sp_Puerto
GTGTCGTCCTCTCTTCATCGTCGAGGATCAGCAATTGGTGATTCAAAAGA
JV0509_47_J_TN_USA
ATGTCGACCGCTTTTCATCGTCGAGGACCAGCAGTTGTCAATACAAAAGA
JV0709_188 --
JV0509_127_PA_USA ATGTCGACCGCTT-------------------------------------
JV1009_59_NJ_USA
ATGTCGACCGCTTTTCATTGTCGAGGACCAGCAGTTGTCAATACAAAAGA
Dai15497P_crataegi --
Dai15499P_crataegi TTGTCGACCGC---------------------------------------
Cui3266P_cinereus --

WCG1256Dai24690 --
1DAI17581 --
WCG1255Dai24688
TTGTCGACCGCTTTTCATTGTCGAGGAACAGCAGCTGGCGATCCAGAAAA
Dai22427 --
MV690Meripilus_concrescens --
MV513Meripilus_galapagensis --
Dai19793
ATGTAGACCGCTTTTCATCGTCGAGGACCAGCAGTTGTCAATACAAAAGA
OQ553780P_tamilnaduensis --
OQ553779P_tamilnaduensis --
A164FB3Meripilus_giganteus --
JV1407_36_Vinctus_Meandrica --
1807_3K_Rigidoporus_PuertoRico
ATGCCGCCCGCTTTTCATCGTCGAGGATCAACAGTTGGCGATCCAGAAGA
Cui16903P_vinctus
ATGCCGCCCGCTTTTCATCGTCGAGGATCAACAGTTGGCGATCCAGAAGA
JV1008_18R_Lineatus --
JV1407_37_1_Vinctus_Carara
CTGTCGACCGCTCTTCATTGTCGAGGACCAACAGTTAGCGATTCAGAAGA
Dai17986P_lineatus
CTGTCGACCGCTCTTCATTGTCGAGGACCAACAGTTAGCGATTCAGAAGA
Dai18281
TTGTCGACCGCTCTTCATTGTCGAGGACCAACAGTTAGCGATTCAGAAGA
1DAI19796 --
ZQY797Dai25241
TTGTCGACCGCTCTTCATTGTCGAGGACCAACAGTTAGCGATTCAGAAGA
WCG1289Dai24711 --
Dai22598 --
Dai20523 --
Dai17885 --
Dai17553 --
Dai19639
ATGTCGACCGCTCTTCATTGTCGAGGACCAACAACTAGCGATTCAG-AGA
JV0110_48_CZ --
MJ129_04 --
Cui10340P_eminens --
Cui10341P_eminens --
Dai12685P_eminens --
Miettinen_13591Rigidoporus_und --
Dai20868
ATGCCGACCTCTTTTCATCGTCGAGAACCAAATATTGTCGGTTCGAAAGA
Dai20832
ATGCCGACCTCTTTTCATCGTCGAGAACCAAATACTGTCGGTTCGAAAGA

Dai11400 --
Dai22472 --
1CUI10475 --
1CUI10491 --
HCFC1095Meripilus_robledoi --
MCW702Meripilus_revolubilis --
1704_83_zluty_HaciendaBaru --
Dai9925P_lavendulus --
Dai13587AP_lavendulus --
PDD70600P_longicystidius --
Cui16630 --
FP_135344Meripilus_giganteus
TTGTCGACCCCTCTTTATCGTCGAGGAGCAACAACTGGCAATTCAAAAGA
FP_100460_Sp --
CBS421_48Meripilus_giganteus --
Cui9203 --
Cui9202 --
TUFC100564Japan --
Russell5913Meripilus_sumstinei --
RP215Meripilus_brasiliensis --
RP200Meripilus_brasiliensis --
JV1712_13J_R_vinctus2_LSUPuert --
Dai10503R_hypobrunneus --
Dai10569Rigidoporus_hypobrunne --
1DAI19451 --
CM108bRigidoporus_hypobrunneus --
1CUI16874 --
FD299Cerrena_unicolor --
KHL_GB_Cerrena_uniclor --
Dai12892Cerrena_albocinnamomea --
Dai12955C_albocinnamomea --
SNUm03110102C_aurantiopora --
NIBRFG0000102423C_aurantiopora --
Dai7359Antrodiella_zonata --
F20080702KCM29C_consors --
F20080208LYW10Cerrena_consors --
Dai7821Antrodiella_zonata --
CFMR_DCL04_31Pseudolagarobasid --
VPB197Pseudolagarobasidium_bel --
CBS115543Pseudolagarobasidium_ --
CBS115544 --
Han405Pseudolagarobasidium_bai --
Han406Pseudolagarobasidium_bai --
MUcc838Spongipellis_delectans --

BRNM686401S_delectans --
OSM_F925S_delectans --
BRNM67093Spongipellis_litschau --
CFMRccFP59199TS_unicolor --
CFMRccFP71791TS_unicolor --
Dai13845P_lischaueri
CTGTCGACCACTATTCATCGTCGAAGACCAGCAGTTATTGCTCAAGAAGA
Dai20266P_lischaueri --
CFMR_HHB11240Radulodon_america --
RLG6350Radulodon_americanus --
KY415963Radulodon_erikssonii --
HHB9567spRadulodon_casearius --
KRT_Iso_26Radulodon_casearius --
CBS126044Radulodon_erikssonii --
He6183YUNNANENSIS --
Cui17979YUNNANENSIS --
Miettinen2091Junghuhnia_fimbri --
KHL12316S_tenue --
PRM846564S_pachyodon --
SP_Lgt_S_pachyodon --
Ryvarden44669Tyromyces_xuchile --
PW17_171sinuosus --
W53Dai12234
CTGTCGACCTCTATTCATTGTAGAGGATGAGCAGCTCGCGATCAAAAAGA
HHB4100SpAntella_americana --
W3Dai20901spumeus
ATGTCGACCCCTCTTCATCGTCGAAAACCAACAGTTGGCCATCCAGAAGA
He6736 --
BRNM734877S_spumeus --
BRNM712630S_spumeus --
Dai1723Loweomyces_sibiricus --
W54Cui10009 --
W1Dai20899
ATGTCGACCCCTCTTCATCGTCGAAAACCAACAGTTGGCCATCCAGAAGA
HHB13445Trametes_ochracea --
Dai16222 --
Dai16240 --

JV1310_11SanguinolentusCernys
AGCACGTTCGGTGGATCAATCAGAAAGCGAACGATGAGGGAGAGGAGTAT
MJ39_00_SK --
MJ111_04_CZ --
JV1610_BOKYsmrk --
Dai21030 --

Dai20976P_furcatus --
Dai2105 --
Dai2544 --
Dai11313 --
WCG1611Dai26167
AGCACGTTCGGTGGATCAATCAGAAAGCGAACGATGAGGGAGAGGAGTAT
WCG1518Dai25999Physisporinus --
TAA15097 --
JV8909_19_CZ --
JV1310_15_P_sanguinolentus2_CZ --
MJ53_02_CZ --
CLZhao21647P_yunnanensis --
CLZhao21583P_yunnanensis --
Dai22272
AGCACGTTCGGTGGATCAATCAGAAAGCGAACGACGAGGGAGAGGAATAT
Dai22279 --
MJ332_94_CZ --
MJ642_94_CZ_Expallescens --
Dai21060P_vinctus
AGCATGTTCGGTGGATCAACCAGAAAGCCAACGATAACGGAGAGGAGTAC
JV0511_23LRP_pouzarii --
JQ409462_R_pouzarii_PRM899856_ --
JV0308_66_WA --
JV0309_45_WA_USA --
JV0709_83_CA_USA --
Dai21043P_pouzarii
AACATGTCCGGTGGATCAACCAAAAGGCGAACGACAACGGAGAGGAGTAC
MJ144_95_CZ --
JV0909_3_CZ --
JV0609_1_K --
Dai20396Physisporinus_castanop
AGCACGTGCGGTGGATCAACCAGAAAGCCAACGATAATGGAGAGGAGTAT
Dai20397Physisporinus_castanop
AGCACGTGCGGTGGATCAACCAGAAAGCCAACGATAATGGAGAGGAGTAT
MJ19_09_SK_Abies --
JV0509_40_J_TN_USA_Betula --
JV0808_33crocatus_PA_USAlist --
DLL2009_061P_crocatus --
Dai12800P_subcrocatus --
Dai15917P_subcrocatus --
Cui16620 --
HCFC1088Meripilus_stillicidior --
MCW590Meripilus_obsrcurus --
MCW722Meripilus_obsrcurus --

Cui9381P_tibeticus --
Cui9588P_tibeticus --
Va2_Beneschova --
CWU3874_Ukraine_Alnus --
WCG1293Dai24718Physisporinus --
WCG1268Dai24682A
AGCACGTTCGTTGGATCAACGAGAAAGCTAACGACGAAGGAGAGGAGTAC
WCG1269Dai24683A
AGCACGTTCGTTGGATCAACGAGAAAGCTAACGACGAAGGAGAGGAGTAC
WCG1279Dai24694A
AGCACGTTCGGTGGATCAACGAGAAATCTAATGACGAGGGAGAGGAGTAC
Dai16971 --
ZQY1043Dai26696 --
Doll880
AACATGTTCGGTGGATTATCCAGAAAACAAATGACGAGGGAGAAGAGTAC
Doll1000
AGCATGTTCGGTGGATTATCCAGAAGACAAACGACGAGGGAGAAGAGTAC
1DAI18529 --
Dai19535
AGCATGTTCGGTGGATCAACCAGAAGGCAAACGACGAGGGGGATGAGTAC
1704_79_hnedyVillaLaPaz
AGCACATACGATGGATCAACCAAAAAGCAAATGACGACGGAGAGGAATAT
F2061 --
1DAI18268 --
1DAI18540A --
Dai17695 --
LKY18Dai26373 --
Dai17839P_sulphureus --
Dai17841P_sulphureus AACACGTTCGATGGATCAACCAGAA-
GCTAACGATGATGGAGAAGAGTAT
Dai19877P_roseus
AACACGTTCGATGGATCAACCAGAAGGCTAACGATGAAGGGGACGAGTAC
1508_18_1_Kout --
KP859303_R_vinctus_RP185_BRAZI --
JK1807_15Rigidoporus_sp_Puerto
AACACATCAACTGGATCAACCAGAAAGCTAACGACGAGGGCGAGGAATAC
JV0509_47_J_TN_USA
AGCATATTCGATGGATCAATCAAAAGGTTAATGACGAGGGAGAGGAATAC
JV0709_188 --
JV0509_127_PA_USA --
JV1009_59_NJ_USA
AGCATATTCGATGGATCAATCAAAAGGTTAATGACGAGGGAGAGGAATAC
Dai15497P_crataegi --
Dai15499P_crataegi --

Cui3266P_cinereus ---
WCG1256Dai24690 ---
1DAI17581 ---
WCG1255Dai24688
AGCATATTCGATGGATCAATCAAAAAGTCAATGACGATGGAGATGAGTAT
Dai22427 ---
MV690Meripilus_concrescens ---
MV513Meripilus_galapagensis ---
Dai19793
AGCATATCCGATGGATTAATCAAAAGGTGAATGACGAGGGAGAGGAATAC
OQ553780P_tamilnaduensis ---
OQ553779P_tamilnaduensis ---
A164FB3Meripilus_giganteus ---
JV1407_36_Vinctus_Meandrica ---
1807_3K_Rigidoporus_PuertoRico
AACATATTCGATGGATCAACCAAAAAGCTACGGACGAGGGAGAAGAATAT
Cui16903P_vinctus
AACATATTCGATGGATCAACCAAAAAGCTACGGACGAGGGAGAAGAATAT
JV1008_18R_Lineatus ---
JV1407_37_1_Vinctus_Carara
AACATATCCGATGGATCAACCAAAAGGCGACCGACGAGGGAGAAGAATAT
Dai17986P_lineatus
AACATATCCGATGGATCAACCAAAAGGCGACCGACGAGGGAGAAGAATAT
Dai18281
AACATATTCGATGGATCAACCAAAAGGCGACCGACGAGGGAGAAGAATAT
1DAI19796 ---
ZQY797Dai25241
AACATATTCGATGGATCAACCAAAAGGCGACTGACGAGGGAGAAGAATAT
WCG1289Dai24711 ---
Dai22598 ---
Dai20523 ---
Dai17885 ---
Dai17553 ---
Dai19639
AACATATCCGATGGATCAACCAAAAGGCGACCGACGAGGGAGAAGAATAT
JV0110_48_CZ ---
MJ129_04 ---
Cui10340P_eminens ---
Cui10341P_eminens ---
Dai12685P_eminens ---
Miettinen_13591Rigidoporus_und ---
Dai20868
AGCATATCAGGTGGATTGACCAAAAGATGAATGATGATGGTGAAGAGTAT
Dai20832

AGCATATCAGGTGGATTGACCAGAAGATGAATGACGATGGTGAAGAATAC
Dai11400 --
Dai22472 --
1CUI10475 --
1CUI10491 --
HCFC1095Meripilus_robledoi --
MCW702Meripilus_revolubilis --
1704_83_zluty_HaciendaBaru --
Dai9925P_lavendulus --
Dai13587AP_lavendulus --
PDD70600P_longicystidius --
Cui16630 --
FP_135344Meripilus_giganteus
AACATATACGATGGCTCAATCAAAAGACGAGTGATGATGGGGAGGAATAC
FP_100460_Sp --
CBS421_48Meripilus_giganteus --
Cui9203 --
Cui9202 --
TUFC100564Japan --
Russell5913Meripilus_sumstinei --
RP215Meripilus_brasiliensis --
RP200Meripilus_brasiliensis --
JV1712_13J_R_vinctus2_LSUPuert --
Dai10503R_hypobrunneus --
Dai10569Rigidoporus_hypobrunne --
1DAI19451 --
CM108bRigidoporus_hypobrunneus --
1CUI16874 --
FD299Cerrena_unicolor --
KHL_GB_Cerrena_uniclor --
Dai12892Cerrena_albocinnamomea --
Dai12955C_albocinnamomea --
SNUm03110102C_aurantiopora --
NIBRFG0000102423C_aurantiopora --
Dai7359Antrodiella_zonata --
F20080702KCM29C_consors --
F20080208LYW10Cerrena_consors --
Dai7821Antrodiella_zonata --
CFMR_DCL04_31Pseudolagarobasid --
VPB197Pseudolagarobasidium_bel --
CBS115543Pseudolagarobasidium_ --
CBS115544 --
Han405Pseudolagarobasidium_bai --
Han406Pseudolagarobasidium_bai --

MUcc838Spongipellis_delectans --
BRNM686401S_delectans --
OSM_F925S_delectans --
BRNM67093Spongipellis_litschau --
CFMRccFP59199TS_unicolor --
CFMRccFP71791TS_unicolor --
Dai13845P_lischaueri
AACATATCCGCTGGATCGCCGACAGGAAGGACGATATGGGTGCGCCATTC
Dai20266P_lischaueri --
CFMR_HHB11240Radulodon_america --
RLG6350Radulodon_americanus --
KY415963Radulodon_erikssonii --
HHB9567spRadulodon_casearius --
KRT_Iso_26Radulodon_casearius --
CBS126044Radulodon_erikssonii --
He6183YUNNANENSIS --
Cui17979YUNNANENSIS --
Miettinen2091Junghuhnia_fimbri --
KHL12316S_tenue --
PRM846564S_pachyodon --
SP_Lgt_S_pachyodon --
Ryvarden44669Tyromyces_xuchile --
PW17_171sinuosus --
W53Dai12234
AACACATACGCATGCTCGAAGACAAGGAAAGCAAC------------TTC
HHB4100SpAntella_americana --
W3Dai20901spumeus
AACACATTCGCTGGATCAATCAGAAGGCGAGTGATGAGGGCGAGGAGTAC
He6736 --
BRNM734877S_spumeus --
BRNM712630S_spumeus --
Dai1723Loweomyces_sibiricus --
W54Cui10009 --
W1Dai20899
AACACATTCGCTGGCTCAATCAGAAGGCGAATGATGAGGGCGAGGAGTAC
HHB13445Trametes_ochracea --
Dai16222 --
Dai16240 --

JV1310_11SanguinolentusCernys AAATGGGATTCTTTAGTTCGGGGTGGT---
GTTGTCGAGTTGTTGGATGC
MJ39_00_SK --
MJ111_04_CZ --
JV1610_BOKYsmrk --

Dai21030 --
Dai20976P_furcatus --
Dai2105 --
Dai2544 --
Dai11313 --
WCG1611Dai26167 AAATGGGATTCTTTGGTTCGGGGTGGT--
GTTGTCGAGTTGTTGGATGC
WCG1518Dai25999Physisporinus --
TAA15097 --
JV8909_19_CZ --
JV1310_15_P_sanguinolentus2_CZ --
MJ53_02_CZ --
CLZhao21647P_yunnanensis --
CLZhao21583P_yunnanensis --
Dai22272 AAATGGGATTCTTTGGTTCGGGGTGGT--
GTCGTCGAGTTGTTGGATGC
Dai22279 --
MJ332_94_CZ --
MJ642_94_CZ_Expallescens --
Dai21060P_vinctus AAATGGGATTCGCTGGTCAGGGGCGGC--
GTTATCGAGTTGCTGGATGC
JV0511_23LRP_pouzarii --
JQ409462_R_pouzarii_PRM899856_ --
JV0308_66_WA --
JV0309_45_WA_USA --
JV0709_83_CA_USA --
Dai21043P_pouzarii AAATGGGATTCGCTGGTGAGGGGTGGC--
GTCATCGAGTTGTTAGACGC
MJ144_95_CZ --
JV0909_3_CZ --
JV0609_1_K --
Dai20396Physisporinus_castanop AAATGGGATTCGCTAGTCAGGGGTGGC--
GTCATCGAGTTGCTGGATGC
Dai20397Physisporinus_castanop AAATGGGATTCGCTAGTCAGGGGTGGC--
GTCATCGAGTTGCTGGATGC
MJ19_09_SK_Abies --
JV0509_40_J_TN_USA_Betula --
JV0808_33crocatus_PA_USAlist --
DLL2009_061P_crocatus --
Dai12800P_subcrocatus --
Dai15917P_subcrocatus --
Cui16620 --
HCFC1088Meripilus_stillicidior --
MCW590Meripilus_obsrcurus --

MCW722Meripilus_obsrcurus --
Cui9381P_tibeticus --
Cui9588P_tibeticus --
Va2_Beneschova --
CWU3874_Ukraine_Alnus --
WCG1293Dai24718Physisporinus --
WCG1268Dai24682A AAATGGGATTCTCTGGTTAGAGGTGGC---GTTGTCGAGCTGTTGGACGC
WCG1269Dai24683A AAATGGGATTCTCTGGTTAGAGGTGGC---GTTGTCGAGCTGTTGGACGC
WCG1279Dai24694A AAATGGGATTCTCTGGTTAGAGGTGGC---GTTGTCGAGCTGTTGGACGC
Dai16971 --
ZQY1043Dai26696 --
Doll880 AAATGGGATTCACTCGTAAGGGGTGGC---GTCGTCGAGCTGTTGGATGC
Doll1000 AAATGGGATTCACTCGTAAGGGGTGGC---GTCGTCGAGCTGTTGGATGC
1DAI18529 --
Dai19535 AAGTGGGATTCGTTGGTCAGGGGCGGT---GTCATCGAGTTGCTAGACGC
1704_79_hnedyVillaLaPaz AAGTGGGATTCCTTGGTCAAGGGCGGCGGTGTTATCGAGTTGCTGGATGC
F2061 --
1DAI18268 --
1DAI18540A --
Dai17695 --
LKY18Dai26373 --
Dai17839P_sulphureus --
Dai17841P_sulphureus AAATGGGATTCATTGGTCAGGGGTGGT---GTTATTGAGCTGTTGGATGC
Dai19877P_roseus AAATGGGATTCATTGGTCAGGGGCGGT---GTTATCGAGCTGTTGGATGC
1508_18_1_Kout --
KP859303_R_vinctus_RP185_BRAZI --
JK1807_15Rigidoporus_sp_Puerto AAATGGGATTCACTGGTCAAGGGTGGT---GTTATCGAGTTGCTGGACGC
JV0509_47_J_TN_USA AAATGGGATCAGCTGGTTAGAGGTGGT---GTTATTGAGTTGTTGGATGC
JV0709_188 --
JV0509_127_PA_USA --
JV1009_59_NJ_USA AAATGGGATCAGCTGGTTAGAGGTGGT---GTTATTGAGTTGTTGGATGC
Dai15497P_crataegi --

Dai15499P_crataegi --
Cui3266P_cinereus --
WCG1256Dai24690 --
1DAI17581 --
WCG1255Dai24688 AAGTGGACCAGCTAGTCAGAGGTGGT--
GTCATTGAACTGTTGGACGC
Dai22427 --
MV690Meripilus_concrescens --
MV513Meripilus_galapagensis --
Dai19793 AAATGGGACCAGCTCGTCAGGGGTGGC--
GTTATCGAGCTGTTGGATGC
OQ553780P_tamilnaduensis --
OQ553779P_tamilnaduensis --
A164FB3Meripilus_giganteus --
JV1407_36_Vinctus_Meandrica --
1807_3K_Rigidoporus_PuertoRico AAATGGGATCAGTTGGTGAGGGGTGGT--
GTTATTGAATTGTTGGACGC
Cui16903P_vinctus AAATGGGATCAGTTGGTGAGGGGTGGT--
GTTATTGAATTGTTGGACGC
JV1008_18R_Lineatus --
JV1407_37_1_Vinctus_Carara AAATGGGATCAGTTAGTAAGAGGTGGG--
GTAATTGAATTGTTGGACGC
Dai17986P_lineatus AAATGGGATCAGTTAGTAAGGGGTGGG--
GTAATTGAATTGTTGGACGC
Dai18281 AAATGGGATCAGTTAGTAAGGGGTGGG--
GTAATTGAATTGTTGGACGC
1DAI19796 --
ZQY797Dai25241 AAATGGGATCAGTTAGTGAGGGGTGGG--
GTAATTGAATTGTTGGACGC
WCG1289Dai24711 --
Dai22598 --
Dai20523 --
Dai17885 --
Dai17553 --
Dai19639 AA-TGGGATCAGTTAGTGAGGGGTGGA--
GTAATTGAATTGTTGG----
JV0110_48_CZ --
MJ129_04 --
Cui10340P_eminens --
Cui10341P_eminens --
Dai12685P_eminens --
Miettinen_13591Rigidoporus_und --
Dai20868 AAATGGGATCAGTTGGTAAGAGGCGGT--
GTCATCGAGCTGTTGGATGC

Dai20832 AAATGGGATCAGTTGGTAAGAGGGGGT--
GTCATCGAGCTGTTGGATGC
Dai11400 ---
Dai22472 ---
1CUI10475 ---
1CUI10491 ---
HCFC1095Meripilus_robledoi ---
MCW702Meripilus_revolubilis ---
1704_83_zluty_HaciendaBaru ---
Dai9925P_lavendulus ---
Dai13587AP_lavendulus ---
PDD70600P_longicystidius ---
Cui16630 ---
FP_135344Meripilus_giganteus AAGTGGGATCAGTTAGTCAGGGGTGGT--
GTTATCGAGTTACTCGACGC
FP_100460_Sp ---
CBS421_48Meripilus_giganteus ---
Cui9203 ---
Cui9202 ---
TUFC100564Japan ---
Russell5913Meripilus_sumstinei ---
RP215Meripilus_brasiliensis ---
RP200Meripilus_brasiliensis ---
JV1712_13J_R_vinctus2_LSUPuert ---
Dai10503R_hypobrunneus ---
Dai10569Rigidoporus_hypobrunne ---
1DAI19451 ---
CM108bRigidoporus_hypobrunneus ---
1CUI16874 ---
FD299Cerrena_unicolor ---
KHL_GB_Cerrena_uniclor ---
Dai12892Cerrena_albocinnamomea ---
Dai12955C_albocinnamomea ---
SNUm03110102C_aurantiopora ---
NIBRFG0000102423C_aurantiopora ---
Dai7359Antrodiella_zonata ---
F20080702KCM29C_consors ---
F20080208LYW10Cerrena_consors ---
Dai7821Antrodiella_zonata ---
CFMR_DCL04_31Pseudolagarobasid ---
VPB197Pseudolagarobasidium_bel ---
CBS115543Pseudolagarobasidium_ ---
CBS115544 ---
Han405Pseudolagarobasidium_bai ---

Han406Pseudolagarobasidium_bai --
MUcc838Spongipellis_delectans --
BRNM686401S_delectans --
OSM_F925S_delectans --
BRNM67093Spongipellis_litschau --
CFMRccFP59199TS_unicolor --
CFMRccFP71791TS_unicolor --
Dai13845P_lischaueri TTCTGGGACGACATAGTCAAACGAGGC---
TTGATCGAACTGCTGGATGC
Dai20266P_lischaueri --
CFMR_HHB11240Radulodon_america --
RLG6350Radulodon_americanus --
KY415963Radulodon_erikssonii --
HHB9567spRadulodon_casearius --
KRT_Iso_26Radulodon_casearius --
CBS126044Radulodon_erikssonii --
He6183YUNNANENSIS --
Cui17979YUNNANENSIS --
Miettinen2091Junghuhnia_fimbri --
KHL12316S_tenue --
PRM846564S_pachyodon --
SP_Lgt_S_pachyodon --
Ryvarden44669Tyromyces_xuchile --
PW17_171sinuosus --
W53Dai12234 GCGTGGGATGACATGATTAAGAAGGGC---
ATCATCGAGTTGTTGGATGC
HHB4100SpAntella_americana --
W3Dai20901spumeus AAGTGGGATCAGTTGGTGAGGGGTGGT---
GTGATTGAGCTGTTGGATGC
He6736 --
BRNM734877S_spumeus --
BRNM712630S_spumeus --
Dai1723Loweomyces_sibiricus --
W54Cui10009 --
W1Dai20899 AAGTGGGATCAGTTGGTGAGAGGTGGT---
GTGATCGAGCTGTTGGATGC
HHB13445Trametes_ochracea --
Dai16222 --
Dai16240 --

JV1310_11SanguinolentusCernys
CGAAGAAGAGGAAACGGTTATGATCTGTATGACGCCCGAAGATTTAGAAG
MJ39_00_SK --
MJ111_04_CZ --

JV1610_BOKYsmrk --
Dai21030 --
Dai20976P_furcatus --
Dai2105 --
Dai2544 --
Dai11313 --
WCG1611Dai26167
CGAAGAAGAGGAGACAGTTATGATCTGTATGACACCCGAAGATTTAGAGA
WCG1518Dai25999Physisporinus --
TAA15097 --
JV8909_19_CZ --
JV1310_15_P_sanguinolentus2_CZ --
MJ53_02_CZ --
CLZhao21647P_yunnanensis --
CLZhao21583P_yunnanensis --
Dai22272
CGAAGAAGAGGAGACAGTTATGATCTGTATGACGCCCGAAGATTTAGAGA
Dai22279 --
MJ332_94_CZ --
MJ642_94_CZ_Expallescens --
Dai21060P_vinctus
TGAAGAGGAGGAGACGGTGATGATCTGTATGACACCTGAAGATTTGGAGG
JV0511_23LRP_pouzarii --
JQ409462_R_pouzarii_PRM899856_ --
JV0308_66_WA --
JV0309_45_WA_USA --
JV0709_83_CA_USA --
Dai21043P_pouzarii
CGAAGAGGAGGAGACGGTGATGATTTGTATGACCCCCGAAGACTTGGAGG
MJ144_95_CZ --
JV0909_3_CZ --
JV0609_1_K --
Dai20396Physisporinus_castanop
TGAAGAAGAGGAGACAGTGATGATCTGTATGACACCCGAAGATTTGGAGG
Dai20397Physisporinus_castanop
TGAAGAAGAGGAGACAGTGATGATCTGTATGACACCCGAAGATTTGGAGG
MJ19_09_SK_Abies --
JV0509_40_J_TN_USA_Betula --
JV0808_33crocatus_PA_USAlist --
DLL2009_061P_crocatus --
Dai12800P_subcrocatus --
Dai15917P_subcrocatus --
Cui16620 --
HCFC1088Meripilus_stillicidior --

MCW590Meripilus_obsrcurus --
MCW722Meripilus_obsrcurus --
Cui9381P_tibeticus --
Cui9588P_tibeticus --
Va2_Beneschova --
CWU3874_Ukraine_Alnus --
WCG1293Dai24718Physisporinus --
WCG1268Dai24682A
CGAGGAAGAGGAAACCGTTATGATTTGTATGACACCGGAAGACCTAGAGA
WCG1269Dai24683A
CGAGGAAGAGGAAACCGTTATGATTTGTATGACACCGGAAGACCTAGAGA
WCG1279Dai24694A
CGAGGAAGAGGAAACCGTTATGATTTGTATGACACCGGAAGACCTAGAGA
Dai16971 --
ZQY1043Dai26696 --
Doll880
CGAAGAGGAGGAGACTGTCATGATATGTATGACACCCGAAGAACTGGAGA
Doll1000
CGAAGAGGAGGAGACTGTCATGATCTGTATGACACCCGAAGAGCTGGAGA
1DAI18529 --
Dai19535
TGAAGAGGAGGAGACGGTCATGATCTGTATGACACCTGAGGACTTGGAGA
1704_79_hnedyVillaLaPaz
CGAGGAAGAGGAAACCGTTATGATTTGTATGACTCCCGAAGACCTCGAGA
F2061 --
1DAI18268 --
1DAI18540A --
Dai17695 --
LKY18Dai26373 --
Dai17839P_sulphureus --
Dai17841P_sulphureus CGA--GAGGAGAGACGG-
TATGATTTGTATGACGCCCGATGATTTGGAG-
Dai19877P_roseus
CGAAGAGGAAGAGACGGTTATGATTTGTATGACGCCCGAGGATTTGGAGA
1508_18_1_Kout --
KP859303_R_vinctus_RP185_BRAZI --
JK1807_15Rigidoporus_sp_Puerto
CGAGGAAGAGGAGACCGTCATGATCTGTATGACGCCTGGGGATTTGGAGA
JV0509_47_J_TN_USA
TGAGGAAGAGGAGACGGTGATGATTTGCATGACGCCTGAAGATCTAGAGT
JV0709_188 --
JV0509_127_PA_USA --
JV1009_59_NJ_USA
TGAGGAAGAGGAGACGGTGATGATTTGCATGACGCCTGAAGATCTAGAGT

Dai15497P_crataegi --
Dai15499P_crataegi --
Cui3266P_cinereus --
WCG1256Dai24690 --
1DAI17581 --
WCG1255Dai24688
GGAGGAAGAGGAGACGGTGATGATTTGTATGACGCCTGAAGATCTCGAGA
Dai22427 --
MV690Meripilus_concrescens --
MV513Meripilus_galapagensis --
Dai19793
TGAGGAGGAGGAGACGGTGATGATCTGCATGACGCCTGAAGATCTGGAGT
OQ553780P_tamilnaduensis --
OQ553779P_tamilnaduensis --
A164FB3Meripilus_giganteus --
JV1407_36_Vinctus_Meandrica --
1807_3K_Rigidoporus_PuertoRico
AGAAGAAGAAGAGACGGTGATGATTTGCATGACGCCTGAGGACCTGGAGA
Cui16903P_vinctus
AGAAGAAGAAGAGACGGTGATGATTTGCATGACGCCTGAGGACCTGGAGA
JV1008_18R_Lineatus --
JV1407_37_1_Vinctus_Carara
AGAAGAAGAAGAGACAGTAATGATCTGTATGACGCCTGAGGACCTGGAGA
Dai17986P_lineatus
AGAAGAAGAAGAGACAGTGATGATCTGTATGACGCCTGAGGACCTGGAGA
Dai18281
AGAAGAAGAAGAGACAGTAATGATCTGTATGACACCTGAGGACCTGGAGA
1DAI19796 --
ZQY797Dai25241
AGAAGAAGAAGAGACGGTGATGATCTGTATGACGCCGGAGGACCTGGAGA
WCG1289Dai24711 --
Dai22598 --
Dai20523 --
Dai17885 --
Dai17553 --
Dai19639 --
JV0110_48_CZ --
MJ129_04 --
Cui10340P_eminens --
Cui10341P_eminens --
Dai12685P_eminens --
Miettinen_13591Rigidoporus_und --
Dai20868
CGAAGAAGAGGAGACGGTTATGATTTGTATGACACCCGAAGACTTAGAGA

Dai20832
CGAAGAAGAGGAGACGGTGATGATCTGTATGACTCCCGAAGACTTGGAAA
Dai11400 --
Dai22472 --
1CUI10475 --
1CUI10491 --
HCFC1095Meripilus_robledoi --
MCW702Meripilus_revolubilis --
1704_83_zluty_HaciendaBaru --
Dai9925P_lavendulus --
Dai13587AP_lavendulus --
PDD70600P_longicystidius --
Cui16630 --
FP_135344Meripilus_giganteus
AGAAGAAGAGGAGACAGTCATGATCTGTATGACGCCGGAAGACTTAGAGA
FP_100460_Sp --
CBS421_48Meripilus_giganteus --
Cui9203 --
Cui9202 --
TUFC100564Japan --
Russell5913Meripilus_sumstinei --
RP215Meripilus_brasiliensis --
RP200Meripilus_brasiliensis --
JV1712_13J_R_vinctus2_LSUPuert --
Dai10503R_hypobrunneus --
Dai10569Rigidoporus_hypobrunne --
1DAI19451 --
CM108bRigidoporus_hypobrunneus --
1CUI16874 --
FD299Cerrena_unicolor --
KHL_GB_Cerrena_uniclor --
Dai12892Cerrena_albocinnamomea --
Dai12955C_albocinnamomea --
SNUm03110102C_aurantiopora --
NIBRFG0000102423C_aurantiopora --
Dai7359Antrodiella_zonata --
F20080702KCM29C_consors --
F20080208LYW10Cerrena_consors --
Dai7821Antrodiella_zonata --
CFMR_DCL04_31Pseudolagarobasid --
VPB197Pseudolagarobasidium_bel --
CBS115543Pseudolagarobasidium_ --
CBS115544 --
Han405Pseudolagarobasidium_bai --

Han406Pseudolagarobasidium_bai --
MUcc838Spongipellis_delectans --
BRNM686401S_delectans --
OSM_F925S_delectans --
BRNM67093Spongipellis_litschau --
CFMRccFP59199TS_unicolor --
CFMRccFP71791TS_unicolor --
Dai13845P_lischaueri CGA---AGAGAGAACTGTCATGATCTGCATGACTCCGGATGATTTGGAGG
Dai20266P_lischaueri --
CFMR_HHB11240Radulodon_america --
RLG6350Radulodon_americanus --
KY415963Radulodon_erikssonii --
HHB9567spRadulodon_casearius --
KRT_Iso_26Radulodon_casearius --
CBS126044Radulodon_erikssonii --
He6183YUNNANENSIS --
Cui17979YUNNANENSIS --
Miettinen2091Junghuhnia_fimbri --
KHL12316S_tenue --
PRM846564S_pachyodon --
SP_Lgt_S_pachyodon --
Ryvarden44669Tyromyces_xuchile --
PW17_171sinuosus --
W53Dai12234 CGAAGAAGAGGAAACAGCCATGATCTGCATGACTCCGGAGGACTTGGAGG
HHB4100SpAntella_americana --
W3Dai20901spumeus TGAGGAAGAGGAGACGGTGATGATATGTATGACACCAGAGGACTTGGAGA
He6736 --
BRNM734877S_spumeus --
BRNM712630S_spumeus --
Dai1723Loweomyces_sibiricus --
W54Cui10009 --
W1Dai20899 TGAGGAGGAAGAGACGGTGATGATTTGTATGACACCAGAGGACTTGGAGA
HHB13445Trametes_ochracea --
Dai16222 --
Dai16240 --

JV1310_11SanguinolentusCernys ATTCCAGGCTCCAGTCGACTGGGATT---GA-------------------
MJ39_00_SK --
MJ111_04_CZ --

JV1610_BOKYsmrk --
Dai21030 --
Dai20976P_furcatus --
Dai2105 --
Dai2544 --
Dai11313 --
WCG1611Dai26167 ATTCCAGGCTCCAGTCGAGTGGGATT---
GACCCCCAAGACGATCACGAC
WCG1518Dai25999Physisporinus --
TAA15097 --
JV8909_19_CZ --
JV1310_15_P_sanguinolentus2_CZ --
MJ53_02_CZ --
CLZhao21647P_yunnanensis --
CLZhao21583P_yunnanensis --
Dai22272 ATTCCAGGCTCCAGTCGACTGGGATT---
GATCCCCAAGACGATCACGAC
Dai22279 --
MJ332_94_CZ --
MJ642_94_CZ_Expallescens --
Dai21060P_vinctus
CTTCGAGGCTGCAGTCGAGTGGTCTTGTTGACCCCCGTGACGATCACGAA
JV0511_23LRP_pouzarii --
JQ409462_R_pouzarii_PRM899856_ --
JV0308_66_WA --
JV0309_45_WA_USA --
JV0709_83_CA_USA --
Dai21043P_pouzarii
CTTCGAGGCTGCAGTCGAGCGGTGTCGTTGAGTCCCATGACGATCACGAA
MJ144_95_CZ --
JV0909_3_CZ --
JV0609_1_K --
Dai20396Physisporinus_castanop
CTTCGAGACTGCAATCGAGCGGGGTTGCAGACACCCACGACGATCAAGAA
Dai20397Physisporinus_castanop
CTTCGAGACTGCAATCGAGCGGGGTTGCAGACACCCACGACGATCAAGAA
MJ19_09_SK_Abies --
JV0509_40_J_TN_USA_Betula --
JV0808_33crocatus_PA_USAlist --
DLL2009_061P_crocatus --
Dai12800P_subcrocatus --
Dai15917P_subcrocatus --
Cui16620 --
HCFC1088Meripilus_stillicidior --

MCW590Meripilus_obsrcurus --
MCW722Meripilus_obsrcurus --
Cui9381P_tibeticus --
Cui9588P_tibeticus --
Va2_Beneschova --
CWU3874_Ukraine_Alnus --
WCG1293Dai24718Physisporinus --
WCG1268Dai24682A ACTCTAGGTTGCAATCGAGTGGTGGT---GACCCTCAGGACGAGCACGAT
WCG1269Dai24683A ACTCTAGGTTGCAATCGAGTGGTGGT---GACCCTCAGGACGAGCACGAT
WCG1279Dai24694A ACTCTAGGTTGCAA------------------------------------
Dai16971 --
ZQY1043Dai26696 --
Doll880 ATTCCAGGTCGCAAACGGGTGGGTCT---GACCCACAGGAGGAACAGGAC
Doll1000 ATTCCAGGTCGCAAACGGGTGGGTCT---GACCCACAGGAGGAGCAAGAC
1DAI18529 --
Dai19535 ATTCCAGGTTGCAATCGAGCGGTGCC---GATCCTCATACCAACGAGGAT
1704_79_hnedyVillaLaPaz ACTCCAGACTGCAGTCGAGCGGCGTA---GAATCTCAGGACGACCACGAT
F2061 --
1DAI18268 --
1DAI18540A --
Dai17695 --
LKY18Dai26373 --
Dai17839P_sulphureus --
Dai17841P_sulphureus ATTCCAGATCGCA-TCGAGCGGCATA---CACCC---AGACGATCACGA-
Dai19877P_roseus ATTCTAGACTGCAGTCGAGTGGTAAT---AACCCCCAGGACGATCACGAA
1508_18_1_Kout --
KP859303_R_vinctus_RP185_BRAZI --
JK1807_15Rigidoporus_sp_Puerto ACTCTAGGTTACAGTCGAGCGGTGCC---GATACCCAGGAGGACATTGAT
JV0509_47_J_TN_USA CTTCCCGTCTACAATCCAGTGGCGTT---GATCCTCAAACCAACGATGAG
JV0709_188 --
JV0509_127_PA_USA --
JV1009_59_NJ_USA CTTCCCGTCTACAATCCAGTGGCGTT---GATCCTCAAACCAACGATGAG
Dai15497P_crataegi --

Dai15499P_crataegi --
Cui3266P_cinereus --
WCG1256Dai24690 --
1DAI17581 --
WCG1255Dai24688 CATCGCGTCTACAATCCAGTGGCATC---
GATCCGCAAAGCAATGAGGAT
Dai22427 --
MV690Meripilus_concrescens --
MV513Meripilus_galapagensis --
Dai19793 CTTCCCGTCTACAATCCAGTGGCGTT---
GACCCTCAAACCAACGAGGAT
OQ553780P_tamilnaduensis --
OQ553779P_tamilnaduensis --
A164FB3Meripilus_giganteus --
JV1407_36_Vinctus_Meandrica --
1807_3K_Rigidoporus_PuertoRico ATTCACGTCTCCAGTCTACTGGTATG---
GAATCACAACCGAACGAAGAA
Cui16903P_vinctus ATTCACGTCTCCAGTCTACTGGTATG---
GAATCACAACCGAACGAAGAA
JV1008_18R_Lineatus --
JV1407_37_1_Vinctus_Carara ATTCACGTCTCCAGTCCAGCGGCATC---
GACCCACAATCGAACGAAGAT
Dai17986P_lineatus ATTCACGTCTCCAGTCCAGCGGCATC---
GACCCACAATCGAACGAAGAC
Dai18281 ATTCACGTCTCCAGTCCAGCGGCATC---
GACCCACAATCGAACGAAGAC
1DAI19796 --
ZQY797Dai25241 ATTCGCGTCTCCAGTCTAGCGGCATC---
GACCCACAATCGAACGAAGAT
WCG1289Dai24711 --
Dai22598 --
Dai20523 --
Dai17885 --
Dai17553 --
Dai19639 --
JV0110_48_CZ --
MJ129_04 --
Cui10340P_eminens --
Cui10341P_eminens --
Dai12685P_eminens --
Miettinen_13591Rigidoporus_und --
Dai20868
ATTCACGACTCCAATCGACAACGGGAGCTGATCCTCATGCGAACGAGGAC
Dai20832

ATTCACGACTGCAATCGACGACTGGTGCTGATCCTCACGCGAACGAGGAC
Dai11400 --
Dai22472 --
1CUI10475 --
1CUI10491 --
HCFC1095Meripilus_robledoi --
MCW702Meripilus_revolubilis --
1704_83_zluty_HaciendaBaru --
Dai9925P_lavendulus --
Dai13587AP_lavendulus --
PDD70600P_longicystidius --
Cui16630 --
FP_135344Meripilus_giganteus ATTCCCGGTTACAGTCCAGCGGTGAC--
AACACCCTCGATAATGAGGAC
FP_100460_Sp --
CBS421_48Meripilus_giganteus --
Cui9203 --
Cui9202 --
TUFC100564Japan --
Russell5913Meripilus_sumstinei --
RP215Meripilus_brasiliensis --
RP200Meripilus_brasiliensis --
JV1712_13J_R_vinctus2_LSUPuert --
Dai10503R_hypobrunneus --
Dai10569Rigidoporus_hypobrunne --
1DAI19451 --
CM108bRigidoporus_hypobrunneus --
1CUI16874 --
FD299Cerrena_unicolor --
KHL_GB_Cerrena_uniclor --
Dai12892Cerrena_albocinnamomea --
Dai12955C_albocinnamomea --
SNUm03110102C_aurantiopora --
NIBRFG0000102423C_aurantiopora --
Dai7359Antrodiella_zonata --
F20080702KCM29C_consors --
F20080208LYW10Cerrena_consors --
Dai7821Antrodiella_zonata --
CFMR_DCL04_31Pseudolagarobasid --
VPB197Pseudolagarobasidium_bel --
CBS115543Pseudolagarobasidium_ --
CBS115544 --
Han405Pseudolagarobasidium_bai --
Han406Pseudolagarobasidium_bai --

MUcc838Spongipellis_delectans --
BRNM686401S_delectans --
OSM_F925S_delectans --
BRNM67093Spongipellis_litschau --
CFMRccFP59199TS_unicolor --
CFMRccFP71791TS_unicolor --
Dai13845P_lischaueri CCTCAAGATTGCAATC---------------------------------
Dai20266P_lischaueri --
CFMR_HHB11240Radulodon_america --
RLG6350Radulodon_americanus --
KY415963Radulodon_erikssonii --
HHB9567spRadulodon_casearius --
KRT_Iso_26Radulodon_casearius --
CBS126044Radulodon_erikssonii --
He6183YUNNANENSIS --
Cui17979YUNNANENSIS --
Miettinen2091Junghuhnia_fimbri --
KHL12316S_tenue --
PRM846564S_pachyodon --
SP_Lgt_S_pachyodon --
Ryvarden44669Tyromyces_xuchile --
PW17_171sinuosus --
W53Dai12234 CCTCCCGATTAGCGGGCGCTGGTATG--
GATCAAGAAGATGAAGCAGAG
HHB4100SpAntella_americana --
W3Dai20901spumeus ATTCGAGATTGCAAGCGAGTGGAATC--
GACCCTCATGCTAATGAAGAC
He6736 --
BRNM734877S_spumeus --
BRNM712630S_spumeus --
Dai1723Loweomyces_sibiricus --
W54Cui10009 --
W1Dai20899 ATTCGAGATTGCAAGCGAGTGGAATC--
GACCCTCATGCTAACGAAGAC
HHB13445Trametes_ochracea --
Dai16222 --
Dai16240 --

JV1310_11SanguinolentusCernys --
MJ39_00_SK --
MJ111_04_CZ --
JV1610_BOKYsmrk --
Dai21030 --
Dai20976P_furcatus --

Dai2105 --
Dai2544 --
Dai11313 --
WCG1611Dai26167 TTCGATCCGGCGGCTCGTTTGAAG---
TCTGGAGTTAATGCCCACACCTG
WCG1518Dai25999Physisporinus --
TAA15097 --
JV8909_19_CZ --
JV1310_15_P_sanguinolentus2_CZ --
MJ53_02_CZ --
CLZhao21647P_yunnanensis --
CLZhao21583P_yunnanensis --
Dai22272 TTCGATCCGGCGGCTCGTTTGAAG---
TCTGGAGTTAATGCCCACACCTG
Dai22279 --
MJ332_94_CZ --
MJ642_94_CZ_Expallescens --
Dai21060P_vinctus TTCGATCCGGCGGTACGCTTGAAG---
TCTGGTGTCAATGCTCACACCTG
JV0511_23LRP_pouzarii --
JQ409462_R_pouzarii_PRM899856_ --
JV0308_66_WA --
JV0309_45_WA_USA --
JV0709_83_CA_USA --
Dai21043P_pouzarii TTCGATCCGGCGGCACGATTGAAG---
TCTGGCGTCAACGCCCACACCTG
MJ144_95_CZ --
JV0909_3_CZ --
JV0609_1_K --
Dai20396Physisporinus_castanop TTCGATCCAGCTACACGTCTGAAG---
TCTGGTGTCAATGCTCATACCTG
Dai20397Physisporinus_castanop TTCGATCCAGCTACACGTCTGAAG---
TCTGGTGTCAATGCTCATACCTG
MJ19_09_SK_Abies --
JV0509_40_J_TN_USA_Betula --
JV0808_33crocatus_PA_USAlist --
DLL2009_061P_crocatus --
Dai12800P_subcrocatus --
Dai15917P_subcrocatus --
Cui16620 --
HCFC1088Meripilus_stillicidior --
MCW590Meripilus_obsrcurus --
MCW722Meripilus_obsrcurus --
Cui9381P_tibeticus --

Cui9588P_tibeticus --
Va2_Beneschova --
CWU3874_Ukraine_Alnus --
WCG1293Dai24718Physisporinus --
WCG1268Dai24682A TTCGATCCAGCGGCACGTCTGAAG---
TCGGGTGTTAATG----------
WCG1269Dai24683A TTCGATCCAGCGGCACGTCTGAAG---
TCGGGTGTTAATGCTCACACCTG
WCG1279Dai24694A --
Dai16971 --
ZQY1043Dai26696 --
Doll880 TTCGATCCGGCGGCGCGTTTGAAG---
TCGGGCGTCAATGCCCACACCTG
Doll1000 TTCGATCCGGCGGCACGTTTGAAG---
TCGGGGGTCAATGCCCACACCTG
1DAI18529 --
Dai19535 TTCGACCCTGCGGCACGTCTGAAG---
TCTGGTGTCAACGCGCACACCTG
1704_79_hnedyVillaLaPaz TTCGACCCAGCAGCACGTCTGAAG---
TCCGGAGTAAATGCGCACACTTG
F2061 --
1DAI18268 --
1DAI18540A --
Dai17695 --
LKY18Dai26373 --
Dai17839P_sulphureus --
Dai17841P_sulphureus -TCGATC--GCGATACG-ATGAAG---TCTGGG--
TGATGCGCATACTGG
Dai19877P_roseus TTCGATCCGGCGGTACGTTTGAAG---
TCTGGGGTCAATGCGCACACCTG
1508_18_1_Kout --
KP859303_R_vinctus_RP185_BRAZI --
JK1807_15Rigidoporus_sp_Puerto TTCGATCCAGCGGCACGTTTGAAG---
TCTGGTGTCAATGCGCACACCTG
JV0509_47_J_TN_USA TTCGATCCGGCGGCTCGTCTTAAA---
TCTGGTGTCAATGCGCATACATG
JV0709_188 --
JV0509_127_PA_USA --
JV1009_59_NJ_USA TTCGATCCGGCGGCTCGTCTCAAA---
TCTGGTGTCAATGCGCATACATG
Dai15497P_crataegi --
Dai15499P_crataegi --
Cui3266P_cinereus --
WCG1256Dai24690 --

1DAI17581 ---
WCG1255Dai24688 TTTGACCCGGCGGCTCGTTTGAAG---
TCTGGGGTCAATGCGCATACATG
Dai22427 ---
MV690Meripilus_concrescens ---
MV513Meripilus_galapagensis ---
Dai19793 TTCGACCCGGCGGCTCGTCTGAAA---
TCTGGTGTCAATGCGCATACTTG
OQ553780P_tamilnaduensis ---
OQ553779P_tamilnaduensis ---
A164FB3Meripilus_giganteus ---
JV1407_36_Vinctus_Meandrica ---
1807_3K_Rigidoporus_PuertoRico TTCGATCCTGCGGCTCGTCTCAAG---
TCTGGAGTCAATGCGCATACGTG
Cui16903P_vinctus TTCGATCCTGCGGCTCGTCTCAAG---
TCTGGAGTCAATGCGCATACGTG
JV1008_18R_Lineatus ---
JV1407_37_1_Vinctus_Carara TTCGATCCCGCGGCTCGTCTCAAG---
TCTGGAGTCAACGCGCATACATG
Dai17986P_lineatus TTCGATCCCGCGGCTCGTCTCAAG---
TCTGGAGTTAACGCGCATACATG
Dai18281 TTCGATCCCGCGGCTCGTCTCAAG---
TCTGGAGTTAACGCGCATACATG
1DAI19796 ---
ZQY797Dai25241 TTTGATCCCGCGGCTCGTCTCAAG---
TCTGGAGTCAACGCGCATACATG
WCG1289Dai24711 ---
Dai22598 ---
Dai20523 ---
Dai17885 ---
Dai17553 ---
Dai19639 ---
JV0110_48_CZ ---
MJ129_04 ---
Cui10340P_eminens ---
Cui10341P_eminens ---
Dai12685P_eminens ---
Miettinen_13591Rigidoporus_und ---
Dai20868 TTCGACCCAGCAGCTCGACTTAAG---
TCTGGTGTCAATGCACACACTTG
Dai20832 TTCGACCCAGCAGCTCGACTCAAG---
TCAGGTGTCAATGCGCACACTTG
Dai11400 ---
Dai22472 ---

1CUI10475 ---
1CUI10491 ---
HCFC1095Meripilus_robledoi ---
MCW702Meripilus_revolubilis ---
1704_83_zluty_HaciendaBaru ---
Dai9925P_lavendulus ---
Dai13587AP_lavendulus ---
PDD70600P_longicystidius ---
Cui16630 ---
FP_135344Meripilus_giganteus TTCGATCCTGCCGCACGTCTCAAA--
TCTGGTGTAAATGCACATACTTG
FP_100460_Sp ---
CBS421_48Meripilus_giganteus ---
Cui9203 ---
Cui9202 ---
TUFC100564Japan ---
Russell5913Meripilus_sumstinei ---
RP215Meripilus_brasiliensis ---
RP200Meripilus_brasiliensis ---
JV1712_13J_R_vinctus2_LSUPuert ---
Dai10503R_hypobrunneus ---
Dai10569Rigidoporus_hypobrunne ---
1DAI19451 ---
CM108bRigidoporus_hypobrunneus ---
1CUI16874 ---
FD299Cerrena_unicolor ---
KHL_GB_Cerrena_uniclor ---
Dai12892Cerrena_albocinnamomea ---
Dai12955C_albocinnamomea ---
SNUm03110102C_aurantiopora ---
NIBRFG0000102423C_aurantiopora ---
Dai7359Antrodiella_zonata ---
F20080702KCM29C_consors ---
F20080208LYW10Cerrena_consors ---
Dai7821Antrodiella_zonata ---
CFMR_DCL04_31Pseudolagarobasid ---
VPB197Pseudolagarobasidium_bel ---
CBS115543Pseudolagarobasidium_ ---
CBS115544 ---
Han405Pseudolagarobasidium_bai ---
Han406Pseudolagarobasidium_bai ---
MUcc838Spongipellis_delectans ---
BRNM686401S_delectans ---
OSM_F925S_delectans ---

BRNM67093Spongipellis_litschau --
CFMRccFP59199TS_unicolor --
CFMRccFP71791TS_unicolor --
Dai13845P_lischaueri --
Dai20266P_lischaueri --
CFMR_HHB11240Radulodon_america --
RLG6350Radulodon_americanus --
KY415963Radulodon_erikssonii --
HHB9567spRadulodon_casearius --
KRT_Iso_26Radulodon_casearius --
CBS126044Radulodon_erikssonii --
He6183YUNNANENSIS --
Cui17979YUNNANENSIS --
Miettinen2091Junghuhnia_fimbri --
KHL12316S_tenue --
PRM846564S_pachyodon --
SP_Lgt_S_pachyodon --
Ryvarden44669Tyromyces_xuchile --
PW17_171sinuosus --
W53Dai12234
TTCGATCCTTCGAAGAGGCTGAGGACAACTGGTTCTAGCATCCACACATG
HHB4100SpAntella_americana --
W3Dai20901spumeus TTCGACCCTGCCTTGCGGTTGAAA--
TCTGGTGTCAATGCACATACGTG
He6736 --
BRNM734877S_spumeus --
BRNM712630S_spumeus --
Dai1723Loweomyces_sibiricus --
W54Cui10009 --
W1Dai20899 TTCGACCCTGCCTTGCGGTTGAAA--
TCTGGTGTCAATGCGCATACATG
HHB13445Trametes_ochracea --
Dai16222 --
Dai16240 --

JV1310_11SanguinolentusCernys --
MJ39_00_SK --
MJ111_04_CZ --
JV1610_BOKYsmrk --
Dai21030 --
Dai20976P_furcatus --
Dai2105 --
Dai2544 --
Dai11313 --

WCG1611Dai26167
GACTCATTGCGAAATTCATCCTAGTATGATCCTGGGTGTTTGCGCCAGTA
WCG1518Dai25999Physisporinus --
TAA15097 --
JV8909_19_CZ --
JV1310_15_P_sanguinolentus2_CZ --
MJ53_02_CZ --
CLZhao21647P_yunnanensis --
CLZhao21583P_yunnanensis --
Dai22272
GACCCATTGCGAAATTCATCCTAGTATGATCCTGGGTGTTTGCGCCAGTA
Dai22279 --
MJ332_94_CZ --
MJ642_94_CZ_Expallescens --
Dai21060P_vinctus
GACCCATTGCGAGATCCACCCTAGCATGATTTGGGTGTTTGCGCAAGTA
JV0511_23LRP_pouzarii --
JQ409462_R_pouzarii_PRM899856_ --
JV0308_66_WA --
JV0309_45_WA_USA --
JV0709_83_CA_USA --
Dai21043P_pouzarii
GACTCATTGCGAGATCCACCCCAGTATGATTCTGGGCGTTTGTGCAAGTA
MJ144_95_CZ --
JV0909_3_CZ --
JV0609_1_K --
Dai20396Physisporinus_castanop
GACTCATTGCGAGATCCACCCTAGTATGATTCTAGGCGTTTGTGCAAGTA
Dai20397Physisporinus_castanop
GACTCATTGCGAGATCCACCCTAGTATGATTCTAGGCGTTTGTGCAAGTA
MJ19_09_SK_Abies --
JV0509_40_J_TN_USA_Betula --
JV0808_33crocatus_PA_USAlist --
DLL2009_061P_crocatus --
Dai12800P_subcrocatus --
Dai15917P_subcrocatus --
Cui16620 --
HCFC1088Meripilus_stillicidior --
MCW590Meripilus_obsrcurus --
MCW722Meripilus_obsrcurus --
Cui9381P_tibeticus --
Cui9588P_tibeticus --
Va2_Beneschova --
CWU3874_Ukraine_Alnus --

WCG1293Dai24718Physisporinus --
WCG1268Dai24682A --
WCG1269Dai24683A GACACATTGCGAGATTCATCCCAGC-----------------

WCG1279Dai24694A --
Dai16971 --
ZQY1043Dai26696 --
Doll880
GACTCATTGCGAGATTCACCCAAGCATGATTTTGGGCGTTTGCGCGAGTA
Doll1000
GACTCATTGCGAGATTCACCCAAGCATGATTTTGGGTGTTTGCGCGAGTA
1DAI18529 --
Dai19535
GACCCACTGCGAAATTCACCCCAGCATGATTCTTGGTGTTTGCGCTAGCA
1704_79_hnedyVillaLaPaz
GACTCATTGCGAGATTCACCCAAGTATGATCCTTGGCGTCTGTGCAAGTA
F2061 --
1DAI18268 --
1DAI18540A --
Dai17695 --
LKY18Dai26373 --
Dai17839P_sulphureus --
Dai17841P_sulphureus ACCAT---
Dai19877P_roseus
GACCCACTGTGAAATTCACCCCAGCATGATTCTGGGCGTGTGCGCAAGTA
1508_18_1_Kout --
KP859303_R_vinctus_RP185_BRAZI --
JK1807_15Rigidoporus_sp_Puerto
GACTCACTGCGAGATTCACCCAAGTATGATCCTCGGCGTCTGTGCGAGTA
JV0509_47_J_TN_USA
GACCCATTGCGAAATTCACCCAAGTATGATCCTTGGCGTATGTGCAAGTA
JV0709_188 --
JV0509_127_PA_USA --
JV1009_59_NJ_USA
GACCCATTGCGAAATTCACCCAAGTATGATCCTTGGCGTATGTGCAAGTA
Dai15497P_crataegi --
Dai15499P_crataegi --
Cui3266P_cinereus --
WCG1256Dai24690 --
1DAI17581 --
WCG1255Dai24688
GACTCACTGCGAGATTCATCCAAGTATGATCCTCGGTGTGTGTGCGAGTA
Dai22427 --
MV690Meripilus_concrescens --

MV513Meripilus_galapagensis --
Dai19793 GACTCACTGCGAAATTCACCCAAGTATGATACTTGGCGTGTGTGCGAGTA
OQ553780P_tamilnaduensis --
OQ553779P_tamilnaduensis --
A164FB3Meripilus_giganteus --
JV1407_36_Vinctus_Meandrica --
1807_3K_Rigidoporus_PuertoRico GACTCATTGCGAGATCCATCCCAGTATGATCCTTGGTGTGTGCGCGAGTA
Cui16903P_vinctus GACTCATTGCGAAATCCATCCCAGTATGATCCTTGGTGTGTGCGCGAGTA
JV1008_18R_Lineatus --
JV1407_37_1_Vinctus_Carara GACTCATTGCGAGATTCATCCCAGTATGATCCTCGGTGTATGCGCGAGTA
Dai17986P_lineatus GACTCATTGCGAGATTCATCCCAGTATGATCCTCGGTGTATGCGCGAGTA
Dai18281 GACTCATTGCGAGATTCATCCCAGTATGATCCTCGGTGTATGCGCGAGTA
1DAI19796 --
ZQY797Dai25241 GACTCATTGCGAGATTCATCCCAGTATGATCCTCGGTGTATGCGCGAGTA
WCG1289Dai24711 --
Dai22598 --
Dai20523 --
Dai17885 --
Dai17553 --
Dai19639 --
JV0110_48_CZ --
MJ129_04 --
Cui10340P_eminens --
Cui10341P_eminens --
Dai12685P_eminens --
Miettinen_13591Rigidoporus_und --
Dai20868 GACGCACTGTGAGATTCATCCTAGCATGATTCTCGGTGTTTGTGCTAGTA
Dai20832 GACGCACTGTGAGATTCATCCCAGCATGATTCTCGGTGTTTGCGCTAGTA
Dai11400 --
Dai22472 --
1CUI10475 --
1CUI10491 --
HCFC1095Meripilus_robledoi --
MCW702Meripilus_revolubilis --
1704_83_zluty_HaciendaBaru --

Dai9925P_lavendulus --
Dai13587AP_lavendulus --
PDD70600P_longicystidius --
Cui16630 --
FP_135344Meripilus_giganteus
GACGCACTGCGAGATTCATCCAAGTATGATTCTCGGTGTTTGTGCAAGTA
FP_100460_Sp --
CBS421_48Meripilus_giganteus --
Cui9203 --
Cui9202 --
TUFC100564Japan --
Russell5913Meripilus_sumstinei --
RP215Meripilus_brasiliensis --
RP200Meripilus_brasiliensis --
JV1712_13J_R_vinctus2_LSUPuert --
Dai10503R_hypobrunneus --
Dai10569Rigidoporus_hypobrunne --
1DAI19451 --
CM108bRigidoporus_hypobrunneus --
1CUI16874 --
FD299Cerrena_unicolor --
KHL_GB_Cerrena_uniclor --
Dai12892Cerrena_albocinnamomea --
Dai12955C_albocinnamomea --
SNUm03110102C_aurantiopora --
NIBRFG0000102423C_aurantiopora --
Dai7359Antrodiella_zonata --
F20080702KCM29C_consors --
F20080208LYW10Cerrena_consors --
Dai7821Antrodiella_zonata --
CFMR_DCL04_31Pseudolagarobasid --
VPB197Pseudolagarobasidium_bel --
CBS115543Pseudolagarobasidium_ --
CBS115544 --
Han405Pseudolagarobasidium_bai --
Han406Pseudolagarobasidium_bai --
MUcc838Spongipellis_delectans --
BRNM686401S_delectans --
OSM_F925S_delectans --
BRNM67093Spongipellis_litschau --
CFMRccFP59199TS_unicolor --
CFMRccFP71791TS_unicolor --
Dai13845P_lischaueri --
Dai20266P_lischaueri --

CFMR_HHB11240Radulodon_america --
RLG6350Radulodon_americanus --
KY415963Radulodon_erikssonii --
HHB9567spRadulodon_casearius --
KRT_Iso_26Radulodon_casearius --
CBS126044Radulodon_erikssonii --
He6183YUNNANENSIS --
Cui17979YUNNANENSIS --
Miettinen2091Junghuhnia_fimbri --
KHL12316S_tenue --
PRM846564S_pachyodon --
SP_Lgt_S_pachyodon --
Ryvarden44669Tyromyces_xuchile --
PW17_171sinuosus --
W53Dai12234
GACACACTGCGAGATTCATCCAAGTATGATTCTCGGAGTTTGTGCCAGTA
HHB4100SpAntella_americana --
W3Dai20901spumeus
GACCCATTGTGAAATCCACCCGAGCATGATCCTCGGTGTTTGCGCGAGTA
He6736 --
BRNM734877S_spumeus --
BRNM712630S_spumeus --
Dai1723Loweomyces_sibiricus --
W54Cui10009 --
W1Dai20899
GACCCATTGTGAGATCCACCCAAGCATGATCCTCGGTGTATGCGCGAGTA
HHB13445Trametes_ochracea --
Dai16222 --
Dai16240 --

JV1310_11SanguinolentusCernys ----------------------------AAATTTGTACTGTGAAACTGC
MJ39_00_SK --
MJ111_04_CZ ----------------------------
AAATTTGTACTGTGAAACTGC
JV1610_BOKYsmrk ----------------------------
AAATTTGTACTGTGAAACTGC
Dai21030 --
Dai20976P_furcatus ----------------------------CAATTTGTACTGTGAAACTGC
Dai2105 --
Dai2544 --
Dai11313 --
WCG1611Dai26167
TCATCCCATTCCCCGATCATAACCAGGTAAAATTTGTACTGTGAAACTGC
WCG1518Dai25999Physisporinus --

TAA15097 --
JV8909_19_CZ --
JV1310_15_P_sanguinolentus2_CZ --
MJ53_02_CZ -----------------------------
CACTCTATACTGTGAAACTGC
CLZhao21647P_yunnanensis --
CLZhao21583P_yunnanensis --
Dai22272
TCATCCCATTCCCCGATCATAACCAGGTAAAATTTGTACTGTGAAACTGC
Dai22279 ------------------------------AATTTGTACTGTGAAACTGC
MJ332_94_CZ --
MJ642_94_CZ_Expallescens --
Dai21060P_vinctus
TTATTCCGTTTCCCGATCACAACCAGGTAAAATTTGTACTGTGAAACTGC
JV0511_23LRP_pouzarii -----------------------------AAATTTGTACTGTGAAACTGC
JQ409462_R_pouzarii_PRM899856_ --
JV0308_66_WA ------------------------------
AATTTGTACTGTGAAACTGC
JV0309_45_WA_USA -----------------------------
CAATTTGTACTGTGAAACTGC
JV0709_83_CA_USA -----------------------------
AAATTTGTACTGTGAAACTGC
Dai21043P_pouzarii
TTATTCCGTTCCCCGATCACAACCAGGTAAAATTTGTACTGTGAAACTGC
MJ144_95_CZ --
JV0909_3_CZ ------------------------------
AATTTGTACTGTGAAACTGC
JV0609_1_K --
Dai20396Physisporinus_castanop
TTATTCCGTTCCCCGATCACAACCAGGTAAAATTTGTACTGTGAAACTGC
Dai20397Physisporinus_castanop
TTATTCCGTTCCCCGATCACAACCAGGTAAAATTTGTACTGTGAAACTGC
MJ19_09_SK_Abies --
JV0509_40_J_TN_USA_Betula -----------------------------
AAATTTGTACTGTGAAACTGC
JV0808_33crocatus_PA_USAlist -----------------------------AAATTTGTACTGTGAAACTGC
DLL2009_061P_crocatus --
Dai12800P_subcrocatus --
Dai15917P_subcrocatus ------------------------------AATTTGTACTGTGAAACTGC
Cui16620 --
HCFC1088Meripilus_stillicidior --
MCW590Meripilus_obsrcurus --
MCW722Meripilus_obsrcurus --
Cui9381P_tibeticus -----------------------------AAATTTGTACTGTG-AACTGC

Cui9588P_tibeticus -----------------------------AAATTTGTACTGTGAAACTGC
Va2_Beneschova --
CWU3874_Ukraine_Alnus --
WCG1293Dai24718Physisporinus --
WCG1268Dai24682A -----------------------------
AAATTTGTACTGTGAAACTGC
WCG1269Dai24683A --
WCG1279Dai24694A -----------------------------
AAATTTGTACTGTGAAACTGC
Dai16971 --
ZQY1043Dai26696 --
Doll880
TCATCCCGTTCCCCGATCATAACCAGGTGAAATTTGTACTGTGAAACTGC
Doll1000
TCATCCCGTTCCCAGATCATAATCAGGTGCTATTTGTACTGAGAAACTGC
1DAI18529 -----------------------------
AAATTTGTACTGTGAAACTGC
Dai19535
TCATCCCATTCCCTGATCATAACCAGGTAAAATTTGTACTGTGAAACTGC
1704_79_hnedyVillaLaPaz
TCATTCCATTCCCTGATCATAACCAGGTACAATTTGTACTGTGAAACTGC
F2061 --
1DAI18268 --
1DAI18540A -----------------------------
AAATTTGTACTGTGAAACTGC
Dai17695 -----------------------------
AAATTTGTACTGTGAAACTGC
LKY18Dai26373 --
Dai17839P_sulphureus -----------------------------AAATTTGTACTGTGAAACTGC
Dai17841P_sulphureus -----------------------------AAATTTGTACTGTGAAACTGC
Dai19877P_roseus
TCATTCCATTCCCCGATCACAACCAGGTAAAATTTGTACTGTG-AACTGC
1508_18_1_Kout --
KP859303_R_vinctus_RP185_BRAZI --
JK1807_15Rigidoporus_sp_Puerto
TCATTCCATTCCCCGACCACAACCAGGTACAATTTGTACTGTGAAACTGC
JV0509_47_J_TN_USA
TTATTCCCTTCCCAGATCACAACCAGGTGAAATTTGTACTGTGAAACTGC
JV0709_188 --
JV0509_127_PA_USA -----------------------------
AAATTTGTACTGTGAAACTGC
JV1009_59_NJ_USA TTATTCCCTTCCCAGATCACAACCAGGTG-
AATTTGTACTGTGAAACTGC
Dai15497P_crataegi ------------------------------AATTTGTACTGTGAAACTGC

Dai15499P_crataegi -----------------------------AAATTTGTACTGTGAAACTGC
Cui3266P_cinereus --
WCG1256Dai24690 --
1DAI17581 --
WCG1255Dai24688 TCATCCCTTTCCCAGATCACAATCAGGTG---------

Dai22427 --
MV690Meripilus_concrescens --
MV513Meripilus_galapagensis --
Dai19793
TCATTCCTTTCCCGGATCATAACCAGGTGAAATTTGTACTGTG-AACTGC
OQ553780P_tamilnaduensis --
OQ553779P_tamilnaduensis --
A164FB3Meripilus_giganteus --
JV1407_36_Vinctus_Meandrica -----------------------------AAATTTGTACTGTGAAACTGC
1807_3K_Rigidoporus_PuertoRico
TTATTCCGTTCCCAGATCACAACCAGGTAAAATTTGTACTGTGAAACTGC
Cui16903P_vinctus
TTATTCCGTTCCCAGATCACAACCAGGTAAAATTTGTACTGTGAAACTGC
JV1008_18R_Lineatus -----------------------------AAATTTGTACTGTGAAACTGC
JV1407_37_1_Vinctus_Carara
TCATTCCTTTCCCAGATCACAACCAGGTAAAATTTGTACTGTGAAACTGC
Dai17986P_lineatus
TCATTCCTTTCCCAGATCATAACCAGGTAAAATTTGTACTGTGAAACTGC
Dai18281
TCATTCCTTTCCCAGATCATAACCAGGTAAAATTTGTACTGTGAAACTGC
1DAI19796 -----------------------------
AAATTTGTACTGTGAAACTGC
ZQY797Dai25241
TCATTCCTTTCCCAGATCACAACCAGGTAAAATTTGTACTGTGAAACTGC
WCG1289Dai24711 --
Dai22598 -----------------------------
CAATTTGTACTGTGAAACTGC
Dai20523 --
Dai17885 --
Dai17553 -----------------------------
AAATTTGTACTGTGAAACTGC
Dai19639 -----------------------------
CAATTTGTACTGTGAAACTGC
JV0110_48_CZ -----------------------------
AAATTTGTACTGTGAAACTGC
MJ129_04 --
Cui10340P_eminens --
Cui10341P_eminens -----------------------------AAATTTGTACTGTG-AACTGC

Dai12685P_eminens -----------------------------AAATTTGTACTGTGAAACTGC
Miettinen_13591Rigidoporus_und --
Dai20868
TCATCCCGTTCCCCGATCACAACCAGGTGAAATTTGTACTGTGAAACTGC
Dai20832 TCATCCCATTCCCCGATCACAACCAGGTG-
AATTTGTACTGTGAAACTGC
Dai11400 -----------------------------
AAATTTGTACTGTGAAACTGC
Dai22472 -----------------------------
AAATTTGTACTGTGAAACTGC
1CUI10475 --
1CUI10491 --
HCFC1095Meripilus_robledoi --
MCW702Meripilus_revolubilis --
1704_83_zluty_HaciendaBaru -----------------------------AAATTTGTACTGTGAAACTGC
Dai9925P_lavendulus --
Dai13587AP_lavendulus -----------------------------AAATTTGTACTGTG-AACTGC
PDD70600P_longicystidius --
Cui16630 --
FP_135344Meripilus_giganteus TCATTCCCTTCCCAGATCATAACCAGGTA-----------------
----
FP_100460_Sp --
CBS421_48Meripilus_giganteus --
Cui9203 -----------------------------
AAATTTGTACTGTGAAACTGC
Cui9202 -----------------------------
AAATTTGTACTGTGAAACTGC
TUFC100564Japan --
Russell5913Meripilus_sumstinei --
RP215Meripilus_brasiliensis --
RP200Meripilus_brasiliensis --
JV1712_13J_R_vinctus2_LSUPuert -----------------------------------GTGTACTTGAACTGC
Dai10503R_hypobrunneus --
Dai10569Rigidoporus_hypobrunne --
1DAI19451 --
CM108bRigidoporus_hypobrunneus --
1CUI16874 --
FD299Cerrena_unicolor --
KHL_GB_Cerrena_uniclor --
Dai12892Cerrena_albocinnamomea --
Dai12955C_albocinnamomea --
SNUm03110102C_aurantiopora --
NIBRFG0000102423C_aurantiopora --
Dai7359Antrodiella_zonata --

F20080702KCM29C_consors --
F20080208LYW10Cerrena_consors --
Dai7821Antrodiella_zonata --
CFMR_DCL04_31Pseudolagarobasid --
VPB197Pseudolagarobasidium_bel --
CBS115543Pseudolagarobasidium_ --
CBS115544 --
Han405Pseudolagarobasidium_bai --
Han406Pseudolagarobasidium_bai --
MUcc838Spongipellis_delectans --
BRNM686401S_delectans --
OSM_F925S_delectans --
BRNM67093Spongipellis_litschau --
CFMRccFP59199TS_unicolor --
CFMRccFP71791TS_unicolor --
Dai13845P_lischaueri --
Dai20266P_lischaueri ----------------------------CAGTTTGTACTGTGAAACTGC
CFMR_HHB11240Radulodon_america --
RLG6350Radulodon_americanus --
KY415963Radulodon_erikssonii --
HHB9567spRadulodon_casearius --
KRT_Iso_26Radulodon_casearius --
CBS126044Radulodon_erikssonii --
He6183YUNNANENSIS ---------------------------
AAGTTTGTACTGTGAAACTGC
Cui17979YUNNANENSIS ----------------------------
AGTTTGTACTGTGAAACTGC
Miettinen2091Junghuhnia_fimbri --
KHL12316S_tenue --
PRM846564S_pachyodon --
SP_Lgt_S_pachyodon --
Ryvarden44669Tyromyces_xuchile --
PW17_171sinuosus --
W53Dai12234 TCATTCCTTTCCCTGACCATAACCAGGTA-----------

HHB4100SpAntella_americana --
W3Dai20901spumeus
TCATTCCTTTCCCTGACCATAATCAGGTGAAATTTGTACTGTGAAACTGC
He6736 --
BRNM734877S_spumeus --
BRNM712630S_spumeus --
Dai1723Loweomyces_sibiricus --
W54Cui10009 ---------------------------
AAATTTGTACTGTGAAACTGC

W1Dai20899
TCATTCCTTTCCCTGACCACAATCAGGTGAAATTTGTACTGTGAAACTGC
HHB13445Trametes_ochracea --
Dai16222 --
Dai16240 --

JV1310_11SanguinolentusCernys
GAATGGCTCATTAAATCAGTTATAGTTTATTTGATGGTGCTTTGCTACAT
MJ39_00_SK --
MJ111_04_CZ
GAATGGCTCATTAAATCAGTTATAGTTTATTTGATGGTGCTTTGCTACAT
JV1610_BOKYsmrk
GAATGGCTCATTAAATCAGTTATAGTTTATTTGATGGTGCTTTGCTACAT
Dai21030 --
Dai20976P_furcatus
GAATGGCTCATTAAATCAGTTATAGTTTATTTGATGGTGCTTTGCTACAT
Dai2105 --
Dai2544 --
Dai11313 --
WCG1611Dai26167
GAATGGCTCATTAAATCAGTTATAGTTTATTTGATGGTGCTTTGCTACAT
WCG1518Dai25999Physisporinus --
TAA15097 --
JV8909_19_CZ --
JV1310_15_P_sanguinolentus2_CZ --
MJ53_02_CZ
GAATGGCTCATTAAATCAGTTATAGTTTATTTGATGGTACCTTGCTACAT
CLZhao21647P_yunnanensis --
CLZhao21583P_yunnanensis --
Dai22272
GAATGGCTCATTAAATCAGTTATAGTTTATTTGATGGTGCTTTGCTACAT
Dai22279
GAATGGCTCATTAAATCAGTTATAGTTTATTTGATGGTGCTTTGCTACAT
MJ332_94_CZ --
MJ642_94_CZ_Expallescens --
Dai21060P_vinctus
GAATGGCTCATTAAATCAGTTATAGTTTATTTGATGGTGCTTTGCTACAT
JV0511_23LRP_pouzarii
GAATGGCTCATTAAATCAGTTATAGTTTATTTGATGGTGCTTTGCTACAT
JQ409462_R_pouzarii_PRM899856_ --
JV0308_66_WA
GAATGGCTCATTAAATCAGTTATAGTTTATTTGATGGTGCTTTGCTACAT
JV0309_45_WA_USA
GAATGGCTCATTAAATCAGTTATAGTTTATTTGATGGTGCTTTGCTACAT

JV0709_83_CA_USA
GAATGGCTCATTAAATCAGTTATAGTTTATTTGATGGTGCTTTGCTACAT
Dai21043P_pouzarii
GAATGGCTCATTAAATCAGTTATAGTTTATTTGATGGTGCTTTGCTACAT
MJ144_95_CZ --
JV0909_3_CZ
GAATGGCTCATTAAATCAGTTATAGTTTATTTGATGGTATCTTGCTACAT
JV0609_1_K --
Dai20396Physisporinus_castanop
GAATGGCTCATTAAATCAGTTATAGTTTATTTGATGGTGCTTTGCTACAT
Dai20397Physisporinus_castanop
GAATGGCTCATTAAATCAGTTATAGTTTATTTGATGGTGCTTTGCTACAT
MJ19_09_SK_Abies --
JV0509_40_J_TN_USA_Betula
GAATGGCTCATTAAATCAGTTATAGTTTATTTGATGGTGCTTTGCTACAT
JV0808_33crocatus_PA_USAlist
GAATGGCTCATTAAATCAGTTATAGTTTATTTGATGGTGCTTTGCTACAT
DLL2009_061P_crocatus --
Dai12800P_subcrocatus --
Dai15917P_subcrocatus
GAATGGCTCATTAAATCAGTTATAGTTTATTTGATGGTGCTTTGCTACAT
Cui16620 --
HCFC1088Meripilus_stillicidior --
MCW590Meripilus_obsrcurus --
MCW722Meripilus_obsrcurus --
Cui9381P_tibeticus
GAATGGCTCATTAAATCAGTTATAGTTTATTTGATGGTGCTTTGCTACAT
Cui9588P_tibeticus
GAATGGCTCATTAAATCAGTTATAGTTTATTTGATGGTGCTTTGCTACAT
Va2_Beneschova --
CWU3874_Ukraine_Alnus --
WCG1293Dai24718Physisporinus --
WCG1268Dai24682A
GAATGGCTCATTAAATCAGTTATAGTTTATTTGATGGTGCTTTGCTACAT
WCG1269Dai24683A --
WCG1279Dai24694A
GAATGGCTCATTAAATCAGTTATAGTTTATTTGATGGTGCTTTGCTACAT
Dai16971 --
ZQY1043Dai26696 --
Doll880
GAATGGCTCATTAAATCAGTTATAGTTTATTTGATGATACTTTGCTACAT
Doll1000
AAATGGCTCATTAAATCAGTTATAGTTTATTTGATGATACTTTGCTACAT
1DAI18529

GAATGGCTCATTAAATCAGTTATAGTTTATTTGATGGTGCTTTGCTACAT
Dai19535
GAATGGCTCATTAAATCAGTTATAGTTTATTTGATGGTGCTTTGCTACAT
1704_79_hnedyVillaLaPaz
GAATGGCTCATTAAATCAGTTATAGTTTATTTGATGGTGCTTTGCTACAT
F2061 --
1DAI18268 --
1DAI18540A
GAATGGCTCATTAAATCAGTTATAGTTTATTTGATGGTGCTTTGCTACAT
Dai17695
GAATGGCTCATTAAATCAGTTATAGTTTATTTGATGGTGCTTTGCTACAT
LKY18Dai26373 --
Dai17839P_sulphureus
GAATGGCTCATTAAATCAGTTATAGTTTATTTGATGGTGCTTTGCTACAT
Dai17841P_sulphureus
GAATGGCTCATTAAATCAGTTATAGTTTATTTGATGGTGCTTTGCTACAT
Dai19877P_roseus
GAATGGCTCATTAAATCAGTTATAGTTTATTTGATGGTGCTTTGCTACAT
1508_18_1_Kout --
KP859303_R_vinctus_RP185_BRAZI --
JK1807_15Rigidoporus_sp_Puerto
GAATGGCTCATTAAATCAGTTATAGTTTATTTGATGGTGCTTTGCTACAT
JV0509_47_J_TN_USA
GAATGGCTCATTAAATCAGTTATAGTTTATTTGATGGTGCTTTGCTACAT
JV0709_188 --
JV0509_127_PA_USA
GAATGGCTCATTAAATCAGTTATAGTTTATTTGATGGTGCTTTGCTACAT
JV1009_59_NJ_USA
GAATGGCTCATTAAATCAGTTATAGTTTATTTGATGGTGCTTTGCTACAT
Dai15497P_crataegi
GAATGGCTCATTAAATCAGTTATAGTTTATTTGATGGTGCTTTGCTACAT
Dai15499P_crataegi
GAATGGCTCATTAAATCAGTTATAGTTTATTTGATGGTGCTTTGCTACAT
Cui3266P_cinereus --
WCG1256Dai24690 --
1DAI17581 --
WCG1255Dai24688 --
Dai22427 --
MV690Meripilus_concrescens --
MV513Meripilus_galapagensis --
Dai19793
GAATGGCTCATTAAATCAGTTATAGTTTATTTGATGGTGCTTTGCTACAT
OQ553780P_tamilnaduensis --
OQ553779P_tamilnaduensis --

A164FB3Meripilus_giganteus --
JV1407_36_Vinctus_Meandrica
GAATGGCTCATTAAATCAGTTATAGTTTATTTGATGGTGCTTTGCTACAT
1807_3K_Rigidoporus_PuertoRico
GAATGGCTCATTAAATCAGTTATAGTTTATTTGATGGTGCTTTGCTACAT
Cui16903P_vinctus
GAATGGCTCATTAAATCAGTTATAGTTTATTTGATGGTGCTTTGCTACAT
JV1008_18R_Lineatus
GAATGGCTCATTAAATCAGTTATAGTTTATTTGATGGTGCTTTGCTACAT
JV1407_37_1_Vinctus_Carara
GAATGGCTCATTAAATCAGTTATAGTTTATTTGATGGTGCTTTGCTACAT
Dai17986P_lineatus
GAATGGCTCATTAAATCAGTTATAGTTTATTTGATGGTGCTTTGCTACAT
Dai18281
GAATGGCTCATTAAATCAGTTATAGTTTATTTGATGGTGCTTTGCTACAT
1DAI19796
GAATGGCTCATTAAATCAGTTATAGTTTATTTGATGGTGCTTTGCTACAT
ZQY797Dai25241
GAATGGCTCATTAAATCAGTTATAGTTTATTTGATGGTGCTTTGCTACAT
WCG1289Dai24711 --
Dai22598
GAATGGCTCATTAAATCAGTTATAGTTTATTTGATGGTGCTTTGCTACAT
Dai20523 --
Dai17885 --
Dai17553
GAATGGCTCATTAAATCAGTTATAGTTTATTTGATGGTGCTTTGCTACAT
Dai19639
GAATGGCTCATTAAATCAGTTATAGTTTATTTGATGGTGCTTTGCTACAT
JV0110_48_CZ
GAATGGCTCATTAAATCAGTTATAGTTTATTTGATGGTGCTTTGCTACAT
MJ129_04 --
Cui10340P_eminens --
Cui10341P_eminens
GAATGGCTCATTAAATCAGTTATAGTTTATTTGATGGTGCTTTGCTACAT
Dai12685P_eminens
GAATGGCTCATTAAATCAGTTATAGTTTATTTGATGGTGCTTTGCTACAT
Miettinen_13591Rigidoporus_und --
Dai20868
GAATGGCTCATTAAATCAGTTATAGTTTATTTGATGGTGCTTTGCTACAT
Dai20832
GAATGGCTCATTAAATCAGTTATAGTTTATTTGATGGTGCTTTGCTACAT
Dai11400
GAATGGCTCATTAAATCAGTTATAGTTTATTTGATGGTGCTTTGCTACAT
Dai22472

GAATGGCTCATTAAATCAGTTATAGTTTATTTGATGGTGCTTTGCTACAT
1CUI10475 --
1CUI10491 --
HCFC1095Meripilus_robledoi --
MCW702Meripilus_revolubilis --
1704_83_zluty_HaciendaBaru
GAATGGCTCATTAAATCAGTTATAGTTTATTTGATGGTGCTTTGCTACAT
Dai9925P_lavendulus --
Dai13587AP_lavendulus
GAATGGCTCATTAAATCAGTTATAGTTTATTTGATGGTGCTTTGCTACAT
PDD70600P_longicystidius --
Cui16630 --
FP_135344Meripilus_giganteus --
FP_100460_Sp --
CBS421_48Meripilus_giganteus --
Cui9203
GAATGGCTCATTAAATCAGTTATAGTTTATTTGATGGTGCTTTGCTACAT
Cui9202
GAATGGCTCATTAAATCAGTTATAGTTTATTTGATGGTGCTTTGCTACAT
TUFC100564Japan --
Russell5913Meripilus_sumstinei --
RP215Meripilus_brasiliensis --
RP200Meripilus_brasiliensis --
JV1712_13J_R_vinctus2_LSUPuert GATGCTCATAATCAGTATAGTATGATGTG-----------
CTTGCTACAT
Dai10503R_hypobrunneus --
Dai10569Rigidoporus_hypobrunne --
1DAI19451 --
CM108bRigidoporus_hypobrunneus --
1CUI16874 --
FD299Cerrena_unicolor --
KHL_GB_Cerrena_uniclor --
Dai12892Cerrena_albocinnamomea --
Dai12955C_albocinnamomea --
SNUm03110102C_aurantiopora --
NIBRFG0000102423C_aurantiopora --
Dai7359Antrodiella_zonata --
F20080702KCM29C_consors --
F20080208LYW10Cerrena_consors --
Dai7821Antrodiella_zonata --
CFMR_DCL04_31Pseudolagarobasid --
VPB197Pseudolagarobasidium_bel --
CBS115543Pseudolagarobasidium_ --
CBS115544 --

Han405Pseudolagarobasidium_bai --
Han406Pseudolagarobasidium_bai --
MUcc838Spongipellis_delectans --
BRNM686401S_delectans --
OSM_F925S_delectans --
BRNM67093Spongipellis_litschau --
CFMRccFP59199TS_unicolor --
CFMRccFP71791TS_unicolor --
Dai13845P_lischaueri --
Dai20266P_lischaueri
GAATGGCTCATTAAATCAGTTATAGTTTATTTGATGGTGCTTTGCTACAT
CFMR_HHB11240Radulodon_america --
RLG6350Radulodon_americanus --
KY415963Radulodon_erikssonii --
HHB9567spRadulodon_casearius --
KRT_Iso_26Radulodon_casearius --
CBS126044Radulodon_erikssonii --
He6183YUNNANENSIS
GAATGGCTCATTAAATCAGTTATAGTTTATTTGATGGTGCATTGCTACAT
Cui17979YUNNANENSIS
GAATGGCTCATTAAATCAGTTATAGTTTATTTGATGGTGCATTGCTACAT
Miettinen2091Junghuhnia_fimbri --
KHL12316S_tenue --
PRM846564S_pachyodon --
SP_Lgt_S_pachyodon --
Ryvarden44669Tyromyces_xuchile --
PW17_171sinuosus --
W53Dai12234 --
HHB4100SpAntella_americana --
W3Dai20901spumeus
GAATGGCTCATTAAATCAGTTATAGTTTATTTGATGGTGCTTTGCTACAT
He6736 --
BRNM734877S_spumeus --
BRNM712630S_spumeus --
Dai1723Loweomyces_sibiricus --
W54Cui10009
GAATGGCTCATTAAATCAGTTATAGTTTATTTGATGGTGCTTTGCTACAT
W1Dai20899
GAATGGCTCATTAAATCAGTTATAGTTTATTTGATGGTGCTTTGCTACAT
HHB13445Trametes_ochracea --
Dai16222 --
Dai16240 --

JV1310_11SanguinolentusCernys

GGATAACTGTGGTAATTCTAGAGCTAATACATGCAATCAAGCCCCGACTT
MJ39_00_SK --
MJ111_04_CZ
GGATAACTGTGGTAATTCTAGAGCTAATACATGCAATCAAGCCCCAACTT
JV1610_BOKYsmrk
GGATAACTGTGGTAATTCTAGAGCTAATACATGCAATCAAGCCCCAACTT
Dai21030 --
Dai20976P_furcatus
GGATAACTGTGGTAATTCTAGAGCTAATACATGCAATCAAGCCCCAACTT
Dai2105 --
Dai2544 --
Dai11313 --
WCG1611Dai26167
GGATAACTGTGGTAATTCTAGAGCTAATACATGCAATCAAGCCCCAACTT
WCG1518Dai25999Physisporinus --
TAA15097 --
JV8909_19_CZ --
JV1310_15_P_sanguinolentus2_CZ --
MJ53_02_CZ
GGATAACTGTGGTAATTCTAGAGCTAATACATGCGTCAAAGCCCCGACTC
CLZhao21647P_yunnanensis --
CLZhao21583P_yunnanensis --
Dai22272
GGATAACTGTGGTAATTCTAGAGCTAATACATGCAATCAAGCCCCAACTT
Dai22279
GGATAACTGTGGTAATTCTAGAGCTAATACATGCAATCAAGCCCCAACTT
MJ332_94_CZ --
MJ642_94_CZ_Expallescens --
Dai21060P_vinctus
GGATAACTGTGGTAATTCTAGAGCTAATACATGCAATCAAGCCCCGACTT
JV0511_23LRP_pouzarii
GGATAACTGTGGTAATTCTAGAGCTAATACATGCAATCAAGCCCCGACTT
JQ409462_R_pouzarii_PRM899856_ --
JV0308_66_WA
GGATAACTGTGGTAATTCTAGAGCTAATACATGCAATCAAGCCCCGACTT
JV0309_45_WA_USA
GGATAACTGTGGTAATTCTAGAGCTAATACATGCAATCAAGCCCCGACTT
JV0709_83_CA_USA
GGATAACTGTGGTAATTCTAGAGCTAATACATGCAATCAAGCCCCGACTT
Dai21043P_pouzarii
GGATAACTGTGGTAATTCTAGAGCTAATACATGCAATCAAGCCCCGACTT
MJ144_95_CZ --
JV0909_3_CZ
GGATAACTGTGGTAATTCTAGAGCTAATACATGCAATCAAGCCCCGACTT

JV0609_1_K --
Dai20396Physisporinus_castanop
GGATAACTGTGGTAATTCTAGAGCTAATACATGCAATCAAGCCCCGACTT
Dai20397Physisporinus_castanop
GGATAACTGTGGTAATTCTAGAGCTAATACATGCAATCAAGCCCCGACTT
MJ19_09_SK_Abies --
JV0509_40_J_TN_USA_Betula
GGATAACTGTGGTAATTCTAGAGCTAATACATGCAATCAAGCCCCGACTT
JV0808_33crocatus_PA_USAlist
GGATAACTGTGGTAATTCTAGAGCTAATACATGCAATCAAGCCCCGACTT
DLL2009_061P_crocatus --
Dai12800P_subcrocatus --
Dai15917P_subcrocatus
GGATAACTGTGGTAATTCTAGAGCTAATACATGCAATCAAGCCCCGACTT
Cui16620 --
HCFC1088Meripilus_stillicidior --
MCW590Meripilus_obsrcurus --
MCW722Meripilus_obsrcurus --
Cui9381P_tibeticus
GGATAACTGTGGTAATTCTAGAGCTAATACATGCAATCAAGCCCCGACTT
Cui9588P_tibeticus
GGATAACTGTGGTAATTCTAGAGCTAATACATGCAATCAAGCCCCGACTT
Va2_Beneschova --
CWU3874_Ukraine_Alnus --
WCG1293Dai24718Physisporinus --
WCG1268Dai24682A
GGATAACTGTGGTAATTCTAGAGCTAATACATGCAATCAAGCCCCAACTT
WCG1269Dai24683A --GCCCCAACTT
WCG1279Dai24694A
GGATAACTGTGGTAATTCTAGAGCTAATACATGCAATCAAGCCCCAACTT
Dai16971 --
ZQY1043Dai26696 --
Doll880
GGATAACTGTGGTAATTCTAGAGCTAATACATGCAATCAAGCCCCGACTT
Doll1000
GGATAACTGTGGTAATTCTAGAGCTAATACATGCAATCAAGCCCCGACTT
1DAI18529
GGATAACTGTGGTAATTCTAGAGCTAATACATGCAATCAAGCCCCGACTT
Dai19535
GGATAACTGTGGTAATTCTAGAGCTAATACATGCAATCAAGCCCCGACTT
1704_79_hnedyVillaLaPaz
GGATAACTGTGGTAATTCTAGAGCTAATACATGCAATCAAGCCCCGACTC
F2061 --
1DAI18268 --

1DAI18540A
GGATAACTGTGGTAATTCTAGAGCTAATACATGCAATCAAGCCCCGACTC
Dai17695
GGATAACTGTGGTAATTCTAGAGCTAATACATGCAATCAAGCCCCGACTC
LKY18Dai26373 --
Dai17839P_sulphureus
GGATAACTGTGGTAATTCTAGAGCTAATACATGCAATCAAGCCCCGACTT
Dai17841P_sulphureus
GGATAACTGTGGTAATTCTAGAGCTAATACATGCAATCAAGCCCCGACTT
Dai19877P_roseus
GGATAACTGTGGTAATTCTAGAGCTAATACATGCAATCAAGCCCCGACTT
1508_18_1_Kout --
KP859303_R_vinctus_RP185_BRAZI --
JK1807_15Rigidoporus_sp_Puerto
GGATAACTGTGGTAATTCTAGAGCTAATACATGCAATCAAGCCCCGACTC
JV0509_47_J_TN_USA
GGATAACTGTGGTAATTCTAGAGCTAATACATGCAATCAAGCCCCGACTT
JV0709_188 --
JV0509_127_PA_USA
GGATAACTGTGGTAATTCTAGAGCTAATACATGCAATCAAGCCCCGACTT
JV1009_59_NJ_USA
GGATAACTGTGGTAATTCTAGAGCTAATACATGCAATCAAGCCCCGACTT
Dai15497P_crataegi
GGATAACTGTGGTAATTCTAGAGCTAATACATGCAATCAAGCCCCGACTT
Dai15499P_crataegi
GGATAACTGTGGTAATTCTAGAGCTAATACATGCAATCAAGCCCCGACTT
Cui3266P_cinereus --
WCG1256Dai24690 --GCCCCGACTT
1DAI17581 --
WCG1255Dai24688 --GCCCCGACTT
Dai22427 --
MV690Meripilus_concrescens --
MV513Meripilus_galapagensis --
Dai19793
GGATAACTGTGGTAATTCTAGAGCTAATACATGCAATCAAGCCCCGACTT
OQ553780P_tamilnaduensis --
OQ553779P_tamilnaduensis --
A164FB3Meripilus_giganteus --
JV1407_36_Vinctus_Meandrica
GGATAACTGTGGTAATTCTAGAGCTAATACATGCAATCAAGCCCCGACTT
1807_3K_Rigidoporus_PuertoRico
GGATAACTGTGGTAATTCTAGAGCTAATACATGCAATCAAGCCCCGACTT
Cui16903P_vinctus
GGATAACTGTGGTAATTCTAGAGCTAATACATGCAATCAAGCCCCGACTT

JV1008_18R_Lineatus
GGATAACTGTGGTAATTCTAGAGCTAATACATGCAATCAAGCCCCGACTT
JV1407_37_1_Vinctus_Carara
GGATAACTGTGGTAATTCTAGAGCTAATACATGCAATCAAGCCCCGACTT
Dai17986P_lineatus
GGATAACTGTGGTAATTCTAGAGCTAATACATGCAATCAAGCCCCGACTT
Dai18281
GGATAACTGTGGTAATTCTAGAGCTAATACATGCAATCAAGCCCCGACTT
1DAI19796
GGATAACTGTGGTAATTCTAGAGCTAATACATGCAATCAAGCCCCGACTT
ZQY797Dai25241
GGATAACTGTGGTAATTCTAGAGCTAATACATGCAATCAAGCCCCGACTT
WCG1289Dai24711 --
Dai22598
GGATAACTGTGGTAATTCTAGAGCTAATACATGCAATCAAGCCCCGACTT
Dai20523 --
Dai17885 --
Dai17553
GGATAACTGTGGTAATTCTAGAGCTAATACATGCAATCAAGCCCCGACTT
Dai19639
GGATAACTGTGGTAATTCTAGAGCTAATACATGCAATCAAGCCCCGACTT
JV0110_48_CZ
GGATAACTGTGGTAATTCTAGAGCTAATACATGCAATCAAGCCCCGACTT
MJ129_04 --
Cui10340P_eminens --
Cui10341P_eminens
GGATAACTGTGGTAATTCTAGAGCTAATACATGCAATCAAGCCCCGACTT
Dai12685P_eminens
GGATAACTGTGGTAATTCTAGAGCTAATACATGCAATCAAGCCCCGACTT
Miettinen_13591Rigidoporus_und --
Dai20868
GGATAACTGTGGTAATTCTAGAGCTAATACATGCAATCAAGCCCCGACTT
Dai20832
GGATAACTGTGGTAATTCTAGAGCTAATACATGCAATCAAGCCCCGACTT
Dai11400
GGATAACTGTGGTAATTCTAGAGCTAATACATGCAATCAAGCCCCGACTT
Dai22472
GGATAACTGTGGTAATTCTAGAGCTAATACATGCAATCAAGCCCCGACTT
1CUI10475 --
1CUI10491 --
HCFC1095Meripilus_robledoi --
MCW702Meripilus_revolubilis --
1704_83_zluty_HaciendaBaru
GGATAACTGTGGTAATTCTAGAGCTAATACATGCAATCAAGCCCCGACTT

Dai9925P_lavendulus --
Dai13587AP_lavendulus
GGATAACTGTGGTAATTCTAGAGCTAATACATGCAATCAAGCCCCGACTT
PDD70600P_longicystidius --
Cui16630 --
FP_135344Meripilus_giganteus --
FP_100460_Sp --
CBS421_48Meripilus_giganteus --
Cui9203
GGATAACTGTGGTAATTCTAGAGCTAATACATGCAATCAAGCCCCGACTT
Cui9202
GGATAACTGTGGTAATTCTAGAGCTAATACATGCAATCAAGCCCCGACTT
TUFC100564Japan --
Russell5913Meripilus_sumstinei --
RP215Meripilus_brasiliensis --
RP200Meripilus_brasiliensis --
JV1712_13J_R_vinctus2_LSUPuert GA----
TACTGTGTATCTAGAGCTAATACATGCATCAG---CCCGACTT
Dai10503R_hypobrunneus --
Dai10569Rigidoporus_hypobrunne --
1DAI19451 --
CM108bRigidoporus_hypobrunneus --
1CUI16874 --
FD299Cerrena_unicolor --
KHL_GB_Cerrena_uniclor --
Dai12892Cerrena_albocinnamomea --
Dai12955C_albocinnamomea --
SNUm03110102C_aurantiopora --
NIBRFG0000102423C_aurantiopora --
Dai7359Antrodiella_zonata --
F20080702KCM29C_consors --
F20080208LYW10Cerrena_consors --
Dai7821Antrodiella_zonata --
CFMR_DCL04_31Pseudolagarobasid --
VPB197Pseudolagarobasidium_bel --
CBS115543Pseudolagarobasidium_ --
CBS115544 --
Han405Pseudolagarobasidium_bai --
Han406Pseudolagarobasidium_bai --
MUcc838Spongipellis_delectans --
BRNM686401S_delectans --
OSM_F925S_delectans --
BRNM67093Spongipellis_litschau --
CFMRccFP59199TS_unicolor --

CFMRccFP71791TS_unicolor --
Dai13845P_lischaueri --GCCCCGACTT
Dai20266P_lischaueri
GGATAACTGTGGTAATTCTAGAGCTAATACATGCAATCAAGCCCCGACTT
CFMR_HHB11240Radulodon_america --
RLG6350Radulodon_americanus --
KY415963Radulodon_erikssonii --
HHB9567spRadulodon_casearius --
KRT_Iso_26Radulodon_casearius --
CBS126044Radulodon_erikssonii --
He6183YUNNANENSIS
GGATAACTGTGGTAATTCTAGAGCTAATACATGCATACAAGCCCCGACTT
Cui17979YUNNANENSIS
GGATAACTGTGGTAATTCTAGAGCTAATACATGCATACAAGCCCCGACTT
Miettinen2091Junghuhnia_fimbri --
KHL12316S_tenue --
PRM846564S_pachyodon --
SP_Lgt_S_pachyodon --
Ryvarden44669Tyromyces_xuchile --
PW17_171sinuosus --
W53Dai12234 --
HHB4100SpAntella_americana --
W3Dai20901spumeus
GGATAACTGTGGTAATTCTAGAGCTAATACATGCAATCAAGCCCCGACTT
He6736 --
BRNM734877S_spumeus --
BRNM712630S_spumeus --
Dai1723Loweomyces_sibiricus --
W54Cui10009
GGATAACTGTGGTAATTCTAGAGCTAATACATGCAATCAAGCCCCGACTT
W1Dai20899
GGATAACTGTGGTAATTCTAGAGCTAATACATGCAATCAAGCCCCGACTT
HHB13445Trametes_ochracea --
Dai16222 --
Dai16240 --

JV1310_11SanguinolentusCernys
TTGGAAGGGGTGTATTTATTAGATAAAAAACCAACGCGGTTCGCCGCTCC
MJ39_00_SK --
MJ111_04_CZ
TTGGAAGGGGTGTATTTATTAGATAAAAAACCAACGCGGTTCGCCGCTCC
JV1610_BOKYsmrk
TTGGAAGGGGTGTATTTATTAGATAAAAAACCAACGCGGTTCGCCGCTCC
Dai21030 --

Dai20976P_furcatus
TTGGAAGGGGTGTATTTATTAGATAAAAAACCAACGCGGTTCGCCGCTCC
Dai2105 ---
Dai2544 ---
Dai11313 ---
WCG1611Dai26167
TTGGAAGGGGTGTATTTATTAGATAAAAAACCAACGCGGTTCGCCGCTCC
WCG1518Dai25999Physisporinus ---
TAA15097 ---
JV8909_19_CZ ---
JV1310_15_P_sanguinolentus2_CZ ---
MJ53_02_CZ
CTGGAAGGGGTGTATTTATTAGATAAAAAACCAACGCGGTTCGCCGCTCC
CLZhao21647P_yunnanensis ---
CLZhao21583P_yunnanensis ---
Dai22272
TTGGAAGGGGTGTATTTATTAGATAAAAAACCAACGCGGTTCGCCGCTCC
Dai22279
TTGGAAGGGGTGTATTTATTAGATAAAAAACCAACGCGGTTCGCCGCTCC
MJ332_94_CZ ---
MJ642_94_CZ_Expallescens ---
Dai21060P_vinctus
TTGGGAGGGGTGTATTTATTAGATAAAAAACCAACGCGGTTCGCCGCTCC
JV0511_23LRP_pouzarii
TTGGGAGGGGTGTATTTATTAGATAAAAAACCAACGCGGTTCGCCGCTCC
JQ409462_R_pouzarii_PRM899856_ ---
JV0308_66_WA
TTGGGAGGGGTGTATTTATTAGATAAAAAACCAACGCGGTTCGCCGCTCC
JV0309_45_WA_USA
TTGGGAGGGGTGTATTTATTAGATAAAAAACCAACGCGGTTCGCCGCTCC
JV0709_83_CA_USA
TTGGGAGGGGTGTATTTATTAGATAAAAAACCAACGCGGTTCGCCGCTCC
Dai21043P_pouzarii
TTGGGAGGGGTGTATTTATTAGATAAAAAACCAACGCGGTTCGCCGCTCC
MJ144_95_CZ ---
JV0909_3_CZ
CCGGGAGGGGTGTATTTATTAGATAAAAAACCAACGCGGCTCGCCGCTCC
JV0609_1_K ---
Dai20396Physisporinus_castanop
TTGGGAGGGGTGTATTTATTAGATAAAAAACCAACGCGGTTCGCCGCTCC
Dai20397Physisporinus_castanop
TTGGGAGGGGTGTATTTATTAGATAAAAAACCAACGCGGTTCGCCGCTCC
MJ19_09_SK_Abies ---
JV0509_40_J_TN_USA_Betula

TTGGGAGGGGTGTATTTATTAGATAAAAAACCAACGCGGTTCGCCGCTCC
JV0808_33crocatus_PA_USAlist
TTGGGAGGGGTGTATTTATTAGATAAAAAACCAACGCGGTTCGCCGCTCC
DLL2009_061P_crocatus --
Dai12800P_subcrocatus --
Dai15917P_subcrocatus
TTGGGAGGGGTGTATTTATTAGATAAAAAACCAACGCGGTTCGCCGCTCC
Cui16620 --
HCFC1088Meripilus_stillicidior --
MCW590Meripilus_obsrcurus --
MCW722Meripilus_obsrcurus --
Cui9381P_tibeticus
TTGGAAGGGGTGTATTTATTAGATAAAAAACCAACGCGGTTCGCCGCTCC
Cui9588P_tibeticus
TTGGAAGGGGTGTATTTATTAGATAAAAAACCAACGCGGTTCGCCGCTCC
Va2_Beneschova --
CWU3874_Ukraine_Alnus --
WCG1293Dai24718Physisporinus --
WCG1268Dai24682A
TTGGAAGGGGTGTATTTATTAGATAAAAAACCAACGCGGTTCGCCGCTCC
WCG1269Dai24683A
TTGGAAGGGGTGTATTTATTAGATAAAAAACCAACGCGGTTCGCCGCTCC
WCG1279Dai24694A
TTGGAAGGGGTGTATTTATTAGATAAAAAACCAACGCGGTTCGCCGCTCC
Dai16971 --
ZQY1043Dai26696 --
Doll880
TTGGAAGGGGTGTATTTATTAGATAAAAAACCAACGCGGTTCGCCGCTCC
Doll1000
TTGGAAGGGGTGTATTTATTAGATAAAAAACCAACGCGGTTCGCCGCTCC
1DAI18529
CTGGAAGGGGTGTATTTATTAGATAAAAAACCAACGCGGTTCGCCGCTCC
Dai19535
TTGGAAGGGGTGTATTTATTAGATAAAAAACCAACGCGGTTCGCCGCTCC
1704_79_hnedyVillaLaPaz
TTGGAAGGGGTGTATTTATTAGATAAAAAACCAACGCGGTTCGCCGCTCC
F2061 --
1DAI18268 --
1DAI18540A
TTGGAAGGGGTGTATTTATTAGATAAAAAACCAACGCGGTTCGCCGCTCC
Dai17695
TTGGAAGGGGTGTATTTATTAGATAAAAAACCAACGCGGTTCGCCGCTCC
LKY18Dai26373 --
Dai17839P_sulphureus

TTGGAAGGGGTGTATTTATTAGATAAAAAACCAACGCGGTTCGCCGCTCC
Dai17841P_sulphureus
TTGGAAGGGGTGTATTTATTAGATAAAAAACCAACGCGGTTCGCCGCTCC
Dai19877P_roseus
TTGGAAGGGGTGTATTTATTAGATAAAAAACCAACGCGGTTCGCCGCTCC
1508_18_1_Kout --
KP859303_R_vinctus_RP185_BRAZI --
JK1807_15Rigidoporus_sp_Puerto
TCGGAAGGGGTGTATTTATTAGATAAAAAACCAACGCGGTTCGCCGCTCC
JV0509_47_J_TN_USA
TTGGAAGGGGTGTATTTATTAGATAAAAAACCAACGCGGTTCGCCGCTCC
JV0709_188 --
JV0509_127_PA_USA
TTGGAAGGGGTGTATTTATTAGATAAAAAACCAACGCGGTTCGCCGCTCC
JV1009_59_NJ_USA
TTGGAAGGGGTGTATTTATTAGATAAAAAACCAACGCGGTTCGCCGCTCC
Dai15497P_crataegi
TTGGAAGGGGTGTATTTATTAGATAAAAAACCAACGCGGTTCGCCGCTCC
Dai15499P_crataegi
TTGGAAGGGGTGTATTTATTAGATAAAAAACCAACGCGGTTCGCCGCTCC
Cui3266P_cinereus --
WCG1256Dai24690
CTGGAAGGGGTGTATTTATTAGATAAAAAACCAACGCGGTTCGCCGCTCC
1DAI17581 --
WCG1255Dai24688
CTGGAAGGGGTGTATTTATTAGATAAAAAACCAACGCGGTTCGCCGCTCC
Dai22427 --
MV690Meripilus_concrescens --
MV513Meripilus_galapagensis --
Dai19793
CTGGAAGGGGTGTATTTATTAGATAAAAAACCAACGCGGTTCGCCGCTCC
OQ553780P_tamilnaduensis --
OQ553779P_tamilnaduensis --
A164FB3Meripilus_giganteus --
JV1407_36_Vinctus_Meandrica
CTGGAAGGGGTGTATTTATTAGATAAAAAACCAACGCGGTTCGCCGCTCC
1807_3K_Rigidoporus_PuertoRico
CTGGAAGGGGTGTATTTATTAGATAAAAAACCAACGCGGTTCGCCGCTCC
Cui16903P_vinctus
CTGGAAGGGGTGTATTTATTAGATAAAAAACCAACGCGGTTCGCCGCTCC
JV1008_18R_Lineatus
TTGGAAGGGGTGTATTTATTAGATAAAAAACCAACGCGGTTCGCCGCTCC
JV1407_37_1_Vinctus_Carara
TTGGAAGGGGTGTATTTATTAGATAAAAAACCAACGCGGTTCGCCGCTCC

Dai17986P_lineatus
TTGGAAGGGGTGTATTTATTAGATAAAAAACCAACGCGGTTCGCCGCTCC
Dai18281
TTGGAAGGGGTGTATTTATTAGATAAAAAACCAACGCGGTTCGCCGCTCC
1DAI19796
CTGGAAGGGGTGTATTTATTAGATAAAAAACCAACGCGGTTCGCCGCTCC
ZQY797Dai25241
CTGGAAGGGGTGTATTTATTAGATAAAAAACCAACGCGGTTCGCCGCTCC
WCG1289Dai24711 --
Dai22598
CTGGAAGGGGTGTATTTATTAGATAAAAAACCAACGCGGTTCGCCGCTCC
Dai20523 --
Dai17885 --
Dai17553
CTGGAAGGGGTGTATTTATTAGATAAAAAACCAACGCGGTTCGCCGCTCC
Dai19639
CTGGAAGGGGTGTATTTATTAGATAAAAAACCAACGCGGTTCGCCGCTCC
JV0110_48_CZ
CTGGGAGGGGTGTATTTATTAGATAAAAAACCAACGCGGTTCGCCGCTCC
MJ129_04 --
Cui10340P_eminens --
Cui10341P_eminens
CTGGGAGGGGTGTATTTATTAGATAAAAAACCAACGCGGTTCGCCGCTCC
Dai12685P_eminens
CTGGGAGGGGTGTATTTATTAGATAAAAAACCAACGCGGTTCGCCGCTCC
Miettinen_13591Rigidoporus_und --
Dai20868
CTGGGAGGGGTGTATTTATTAGATAAAAAACCAACGCGGTTCGCCGCTCC
Dai20832
CTGGGAGGGGTGTATTTATTAGATAAAAAACCAACGCGGTTCGCCGCTCC
Dai11400
CTGGGAGGGGTGTATTTATTAGATAAAAAACCAACGCGGTTCGCCGCTCC
Dai22472
CTGGGAGGGGTGTATTTATTAGATAAAAAACCAACGCGGTTCGCCGCTCC
1CUI10475 --
1CUI10491 --
HCFC1095Meripilus_robledoi --
MCW702Meripilus_revolubilis --
1704_83_zluty_HaciendaBaru
CTGGGAGGGGTGTATTTATTAGATAAAAAACCAACGCGGTTCGCCGCTCC
Dai9925P_lavendulus --
Dai13587AP_lavendulus
CTGGGAGGGGTGTATTTATTAGATAAAAAACCAACGCGGTTCGCCGCTCC
PDD70600P_longicystidius --

Cui16630 --
FP_135344Meripilus_giganteus --
FP_100460_Sp --
CBS421_48Meripilus_giganteus --
Cui9203
CTGGGAGGGGTGTATTTATTAGATAAAAAACCAACGCGGTTCGCCGCTCC
Cui9202
CTGGGAGGGGTGTATTTATTAGATAAAAAACCAACGCGGTTCGCCGCTCC
TUFC100564Japan --
Russell5913Meripilus_sumstinei --
RP215Meripilus_brasiliensis --
RP200Meripilus_brasiliensis --
JV1712_13J_R_vinctus2_LSUPuert CTGGGAGGGGTGTATTTAT-
AGATAAAAAACCAACGCGGTTCG-CGCTCC
Dai10503R_hypobrunneus --
Dai10569Rigidoporus_hypobrunne --
1DAI19451 --
CM108bRigidoporus_hypobrunneus --
1CUI16874 --
FD299Cerrena_unicolor --
KHL_GB_Cerrena_uniclor --
Dai12892Cerrena_albocinnamomea --
Dai12955C_albocinnamomea --
SNUm03110102C_aurantiopora --
NIBRFG0000102423C_aurantiopora --
Dai7359Antrodiella_zonata --
F20080702KCM29C_consors --
F20080208LYW10Cerrena_consors --
Dai7821Antrodiella_zonata --
CFMR_DCL04_31Pseudolagarobasid --
VPB197Pseudolagarobasidium_bel --
CBS115543Pseudolagarobasidium_ --
CBS115544 --
Han405Pseudolagarobasidium_bai --
Han406Pseudolagarobasidium_bai --
MUcc838Spongipellis_delectans --
BRNM686401S_delectans --
OSM_F925S_delectans --
BRNM67093Spongipellis_litschau --
CFMRccFP59199TS_unicolor --
CFMRccFP71791TS_unicolor --
Dai13845P_lischaueri CTGGAAGGGGTGTATTTATTAGAT-
AAAAACCAACGCGGCTCGCCGCTCC
Dai20266P_lischaueri

CTGGAAGGGGTGTATTTATTAGATAAAAAACCAACGCGGCTCGCCGCTCC
CFMR_HHB11240Radulodon_america --
RLG6350Radulodon_americanus --
KY415963Radulodon_erikssonii --
HHB9567spRadulodon_casearius --
KRT_Iso_26Radulodon_casearius --
CBS126044Radulodon_erikssonii --
He6183YUNNANENSIS
CTGGGAGGGGTGTATTTATTAGATAAAAAACCAACGCGGTTCGCCGCTCC
Cui17979YUNNANENSIS
CTGGGAGGGGTGTATTTATTAGATAAAAAACCAACGCGGTTCGCCGCTCC
Miettinen2091Junghuhnia_fimbri --
KHL12316S_tenue --
PRM846564S_pachyodon --
SP_Lgt_S_pachyodon --
Ryvarden44669Tyromyces_xuchile --
PW17_171sinuosus --
W53Dai12234 --
HHB4100SpAntella_americana --
W3Dai20901spumeus
CTGGAAGGGGTGTATTTATTAGATAAAAAACCAACGCGGTTCGCCGCTCC
He6736 --
BRNM734877S_spumeus --
BRNM712630S_spumeus --
Dai1723Loweomyces_sibiricus --
W54Cui10009
CTGGAAGGGGTGTATTTATTAGATAAAAAACCAACGCGGTTCGCCGCTCC
W1Dai20899
CTGGAAGGGGTGTATTTATTAGATAAAAAACCAACGCGGTTCGCCGCTCC
HHB13445Trametes_ochracea --
Dai16222 --
Dai16240 --

JV1310_11SanguinolentusCernys
ACTGGTGATTCATAATAACTTCTCGAATCGCATGGCCTTGCGCCGGCGAT
MJ39_00_SK --
MJ111_04_CZ
ACTGGTGATTCATAATAACTTCTCGAATCGCATGGCCTTGCGCCGGCGAT
JV1610_BOKYsmrk
ACTGGTGATTCATAATAACTTCTCGAATCGCATGGCCTTGCGCCGGCGAT
Dai21030 --
Dai20976P_furcatus
ACTGGTGATTCATAATAACTTCTCGAATCGCATGGCCTTGCGCCGGCGAT
Dai2105 --

Dai2544 --
Dai11313 --
WCG1611Dai26167
ACTGGTGATTCATAATAACTTCTCGAATCGCATGGCCTTGCGCCGGCGAT
WCG1518Dai25999Physisporinus
ACTGGTGATTCATAATAACTTCTCGAATCGCATGGCCTTGCGCCGGCGAT
TAA15097 --
JV8909_19_CZ --
JV1310_15_P_sanguinolentus2_CZ --
MJ53_02_CZ
CTTGGTGATTCATAATAACTTCTCGAATCGCATGGCCTTGCGCCGGCGAT
CLZhao21647P_yunnanensis --
CLZhao21583P_yunnanensis --
Dai22272
ACTGGTGATTCATAATAACTTCTCGAATCGCATGGCCTTGCGCCGGCGAT
Dai22279
ACTGGTGATTCATAATAACTTCTCGAATCGCATGGCCTTGCGCCGGCGAT
MJ332_94_CZ --
MJ642_94_CZ_Expallescens --
Dai21060P_vinctus
ACTGGTGATTCATAATAACTTCTCGAATCGCATGGCCTTGTGCCGGCGAT
JV0511_23LRP_pouzarii
ACTGGTGATTCATAATAACTTCTCGAATCGCATGGCCTTGTGCCGGCGAT
JQ409462_R_pouzarii_PRM899856_ --
JV0308_66_WA
ACTGGTGATTCATAATAACTTCTCGAATCGCATGGCCTTGTGCCGGCGAT
JV0309_45_WA_USA
ACTGGTGATTCATAATAACTTCTCGAATCGCATGGCCTTGTGCCGGCGAT
JV0709_83_CA_USA
ACTGGTGATTCATAATAACTTCTCGAATCGCATGGCCTTGTGCCGGCGAT
Dai21043P_pouzarii
ACTGGTGATTCATAATAACTTCTCGAATCGCATGGCCTTGTGCCGGCGAT
MJ144_95_CZ --
JV0909_3_CZ
TTTGGTGATTCATAATAACTTCTCGAATCGCATGGCCTTGTGCCGGCGAT
JV0609_1_K --
Dai20396Physisporinus_castanop
ACTGGTGATTCATAATAACTTCTCGAATCGCATGGCCTTGTGCCGGCGAT
Dai20397Physisporinus_castanop
ACTGGTGATTCATAATAACTTCTCGAATCGCATGGCCTTGTGCCGGCGAT
MJ19_09_SK_Abies --
JV0509_40_J_TN_USA_Betula
ACTGGTGATTCATAATAACTTCTCGAATCGCATGGCCTTGTGCCGGCGAT
JV0808_33crocatus_PA_USAlist

ACTGGTGATTCATAATAACTTCTCGAATCGCATGGCCTTGTGCCGGCGAT
DLL2009_061P_crocatus --
Dai12800P_subcrocatus --
Dai15917P_subcrocatus
ACTGGTGATTCATAATAACTTCTCGAATCGCATGGCCTTGTGCCGGCGAT
Cui16620 --
HCFC1088Meripilus_stillicidior --
MCW590Meripilus_obsrcurus --
MCW722Meripilus_obsrcurus --
Cui9381P_tibeticus
ACTGGTGATTCATAATAACTTCTCGAATCGCATGGCCTTGTGCCGGCGAT
Cui9588P_tibeticus
ACTGGTGATTCATAATAACTTCTCGAATCGCATGGCCTTGTGCCGGCGAT
Va2_Beneschova --
CWU3874_Ukraine_Alnus --
WCG1293Dai24718Physisporinus --
WCG1268Dai24682A
ACTGGTGATTCATAATAACTTCTCGAATCGCATGGCCTTGCGCCGGCGAT
WCG1269Dai24683A
ACTGGTGATTCATAATAACTTCTCGAATCGCATGGCCTTGCGCCGGCGAT
WCG1279Dai24694A
ACTGGTGATTCATAATAACTTCTCGAATCGCATGGCCTTGCGCCGGCGAT
Dai16971 --
ZQY1043Dai26696 --
Doll880
ACTGGTGATTCATAATAACTTCTCGAATCGCATGGCCTTGCGCCGGCGAT
Doll1000
ACTGGTGATTCATAATAACTTCTCGAATCGCATGGCCTTGCGCCGGCGAT
1DAI18529
ACTGGTGATTCATAATAACTTCTCGAATCGCATGGCCTTGCGCCGGCGAT
Dai19535
ACTGGTGATTCATAATAACTTCTCGAATCGCATGGCCTTGCGCCGGCGAT
1704_79_hnedyVillaLaPaz
ACTGGTGATTCATAATAACTTCTCGAATCGCATGGCCTTGTGCCGGCGAT
F2061 --
1DAI18268 --
1DAI18540A
ACTGGTGATTCATAATAACTTCTCGAATCGCATGGCCTTGCGCCGGCGAT
Dai17695
ACTGGTGATTCATAATAACTTCTCGAATCGCATGGCCTTGCGCCGGCGAT
LKY18Dai26373 --
Dai17839P_sulphureus
ACTGGTGATTCATAATAACTTCTCGAATCGCATGGCCTTGCGCCGGCGAT
Dai17841P_sulphureus

ACTGGTGATTCATAATAACTTCTCGAATCGCATGGCCTTGCGCCGGCGAT
Dai19877P_roseus
ACTGGTGATTCATAATAACTTCTCGAATCGCACGGCCTTGCGCCGGCGAT
1508_18_1_Kout --
KP859303_R_vinctus_RP185_BRAZI --
JK1807_15Rigidoporus_sp_Puerto
ACTGGTGATTCATAATAACTTCTCGAATCGCATGGCCTTGCGCCGGCGAT
JV0509_47_J_TN_USA
ACTGGTGATTCATAATAACTTCTCGAATCGCATGGCCTTGTGCTGGCGAT
JV0709_188 --
JV0509_127_PA_USA
ACTGGTGATTCATAATAACTTCTCGAATCGCATGGCCTTGTGCTGGCGAT
JV1009_59_NJ_USA
ACTGGTGATTCATAATAACTTCTCGAATCGCATGGCCTTGTGCTGGCGAT
Dai15497P_crataegi
ACTGGTGATTCATAATAACTTCTCGAATCGCATGGCCTTGTGCCGGCGAT
Dai15499P_crataegi
ACTGGTGATTCATAATAACTTCTCGAATCGCATGGCCTTGTGCCGGCGAT
Cui3266P_cinereus --
WCG1256Dai24690
ACTGGTGATTCATAATAACTTCTCGAATCGCATGGCCTTGTGCCGGCGAT
1DAI17581 --
WCG1255Dai24688
ACTGGTGATTCATAATAACTTCTCGAATCGCATGGCCTTGTGCCGGCGAT
Dai22427 --
MV690Meripilus_concrescens --
MV513Meripilus_galapagensis --
Dai19793
ACTGGTGATTCATAATAACTTCTCGAATCGCATGGCCTTGTGCCGGCGAT
OQ553780P_tamilnaduensis --
OQ553779P_tamilnaduensis --
A164FB3Meripilus_giganteus --
JV1407_36_Vinctus_Meandrica
ACTGGTGATTCATAATAACTTTTCGAATCGCATGGCCTTGTGCCGGCGAT
1807_3K_Rigidoporus_PuertoRico
ACTGGTGATTCATAATAACTTTTCGAATCGCATGGCCTTGTGCCGGCGAT
Cui16903P_vinctus
ACTGGTGATTCATAATAACTTTTCGAATCGCATGGCCTTGTGCCGGCGAT
JV1008_18R_Lineatus
ACTGGTGATTCATAATAACTTCTCGAATCGCATGGCCTTGTGCCGGCGAT
JV1407_37_1_Vinctus_Carara
ACTGGTGATTCATAATAACTTCTCGAATCGCATGGCCTTGTGCCGGCGAT
Dai17986P_lineatus
ACTGGTGATTCATAATAACTTCTCGAATCGCATGGCCTTGTGCCGGCGAT

Dai18281
ACTGGTGATTCATAATAACTTCTCGAATCGCATGGCCTTGTGCCGGCGAT
1DAI19796
ACTGGTGATTCATAATAACTTCTCGAATCGCATGGCCTTGTGCCGGCGAT
ZQY797Dai25241
ACTGGTGATTCATAATAACTTCTCGAATCGCATGGCCTTGTGCCGGCGAT
WCG1289Dai24711 --
Dai22598
ACTGGTGATTCATAATAACTTCTCGAATCGCATGGCCTTGTGCCGGCGAT
Dai20523 --
Dai17885 --
Dai17553
ACTGGTGATTCATAATAACTTCTCGAATCGCATGGCCTTGTGCCGGCGAT
Dai19639
ACTGGTGATTCATAATAACTTCTCGAATCGCATGGCCTTGTGCCGGCGAT
JV0110_48_CZ
ACTGGTGATTCATAATAACTTCTCGAATCGCATGGCCTTGTGCCGGCGAT
MJ129_04 --
Cui10340P_eminens --
Cui10341P_eminens
ACTGGTGATTCATAATAACTTCTCGAATCGCATGGCCTTGTGCCGGCGAT
Dai12685P_eminens
ACTGGTGATTCATAATAACTTCTCGAATCGCATGGCCTTGTGCCGGCGAT
Miettinen_13591Rigidoporus_und --
Dai20868
ACTGGTGATTCATAATAACTTCTCGAATCGCATGGCCTTGTGCCGGCGAT
Dai20832
ACTGGTGATTCATAATAACTTCTCGAATCGCATGGCCTTGTGCCGGCGAT
Dai11400
ACTGGTGATTCATAATAACTTCTCGAATCGCATGGCCTTGTGCCGGCGAT
Dai22472
ACTGGTGATTCATAATAACTTCTCGAATCGCATGGCCTTGTGCCGGCGAT
1CUI10475 --
1CUI10491 --
HCFC1095Meripilus_robledoi --
MCW702Meripilus_revolubilis --
1704_83_zluty_HaciendaBaru
GCTGGTGATTCATAATAACTTCTCGAATCGCATGGCCTTGTGCCGGCGAT
Dai9925P_lavendulus --
Dai13587AP_lavendulus
GCTGGTGATTCATAATAACTTCTCGAATCGCACGGCCTTGTGCCGGCGAT
PDD70600P_longicystidius --
Cui16630 --
FP_135344Meripilus_giganteus --

FP_100460_Sp --
CBS421_48Meripilus_giganteus --
Cui9203
ACTGGTGATTCATAATAACTTGTCGAATCGCATGGCCTTGTGCTGGCGAT
Cui9202
ACTGGTGATTCATAATAACTTGTCGAATCGCATGGCCTTGTGCTGGCGAT
TUFC100564Japan --
Russell5913Meripilus_sumstinei --
RP215Meripilus_brasiliensis --
RP200Meripilus_brasiliensis --
JV1712_13J_R_vinctus2_LSUPuert ATTGGTGA-TCATAATAACTTCTCGAATCGCATGG-
CTTGTGCCGGCGAT
Dai10503R_hypobrunneus --
Dai10569Rigidoporus_hypobrunne --
1DAI19451 --
CM108bRigidoporus_hypobrunneus --
1CUI16874 --
FD299Cerrena_unicolor --
KHL_GB_Cerrena_uniclor --
Dai12892Cerrena_albocinnamomea --
Dai12955C_albocinnamomea --
SNUm03110102C_aurantiopora --
NIBRFG0000102423C_aurantiopora --
Dai7359Antrodiella_zonata --
F20080702KCM29C_consors --
F20080208LYW10Cerrena_consors --
Dai7821Antrodiella_zonata --
CFMR_DCL04_31Pseudolagarobasid --
VPB197Pseudolagarobasidium_bel --
CBS115543Pseudolagarobasidium_ --
CBS115544 --
Han405Pseudolagarobasidium_bai --
Han406Pseudolagarobasidium_bai --
MUcc838Spongipellis_delectans --
BRNM686401S_delectans --
OSM_F925S_delectans --
BRNM67093Spongipellis_litschau --
CFMRccFP59199TS_unicolor --
CFMRccFP71791TS_unicolor --
Dai13845P_lischaueri
ATTGGTGAATCATAATAACTTCTCGAATCGCATGGCCTTGTGCCGGCGAT
Dai20266P_lischaueri
ATTGGTGAATCATAATAACTTCTCGAATCGCATGGCCTTGTGCCGGCGAT
CFMR_HHB11240Radulodon_america --

RLG6350Radulodon_americanus --
KY415963Radulodon_erikssonii --
HHB9567spRadulodon_casearius --
KRT_Iso_26Radulodon_casearius --
CBS126044Radulodon_erikssonii --
He6183YUNNANENSIS
ATTGGTGAGTCATAATAACTTCGCGAATCGCATGGCCTTGTGCCGGCGAT
Cui17979YUNNANENSIS
ATTGGTGAGTCATAATAACTTCGCGAATCGCATGGCCTTGTGCCGGCGAT
Miettinen2091Junghuhnia_fimbri --
KHL12316S_tenue --
PRM846564S_pachyodon --
SP_Lgt_S_pachyodon --
Ryvarden44669Tyromyces_xuchile --
PW17_171sinuosus --
W53Dai12234 --
HHB4100SpAntella_americana --
W3Dai20901spumeus
ACTGGTGATTCATAATAACTTCTCGAATCGCATGGCCTTGTGCCGGCGAT
He6736 --
BRNM734877S_spumeus --
BRNM712630S_spumeus --
Dai1723Loweomyces_sibiricus --
W54Cui10009
ACTGGTGATTCATAATAACTTCTCGAATCGCATGGCCTTGTGCCGGCGAT
W1Dai20899
ACTGGTGATTCATAATAACTTCTCGAATCGCATGGCCTTGTGCCGGCGAT
HHB13445Trametes_ochracea --
Dai16222 --
Dai16240 --

JV1310_11SanguinolentusCernys
GCTTCATTCAAATATCTGCCCTATCAACTTTCGATGGTAGGATAGAGGCC
MJ39_00_SK --
MJ111_04_CZ
GCTTCATTCAAATATCTGCCCTATCAACTTTCGATGGTAGGATAGAGGCC
JV1610_BOKYsmrk
GCTTCATTCAAATATCTGCCCTATCAACTTTCGATGGTAGGATAGAGGCC
Dai21030 --
Dai20976P_furcatus
GCTTCATTCAAATATCTGCCCTATCAACTTTCGATGGTAGGATAGAGGCC
Dai2105 --
Dai2544 --
Dai11313 --

WCG1611Dai26167
GCTTCATTCAAATATCTGCCCTATCAACTTTCGATGGTAGGATAGAGGCC
WCG1518Dai25999Physisporinus
GCTTCATTCAAATATCTGCCCTATCAACTTTCGATGGTAGGATAGAGGCC
TAA15097 --
JV8909_19_CZ --
JV1310_15_P_sanguinolentus2_CZ --
MJ53_02_CZ
GCTTCATTCAAATATCTGCCCTATCAACTTTCGATGGTAGGATAGAGGCC
CLZhao21647P_yunnanensis --
CLZhao21583P_yunnanensis --
Dai22272
GCTTCATTCAAATATCTGCCCTATCAACTTTCGATGGTAGGATAGAGGCC
Dai22279
GCTTCATTCAAATATCTGCCCTATCAACTTTCGATGGTAGGATAGAGGCC
MJ332_94_CZ --
MJ642_94_CZ_Expallescens --
Dai21060P_vinctus
GCTTCATTCAAATATCTGCCCTATCAACTTTCGATGGTAGGATAGAGGCC
JV0511_23LRP_pouzarii
GCTTCATTCAAATATCTGCCCTATCAACTTTCGATGGTAGGATAGAGGCC
JQ409462_R_pouzarii_PRM899856_ --
JV0308_66_WA
GCTTCATTCAAATATCTGCCCTATCAACTTTCGATGGTAGGATAGAGGCC
JV0309_45_WA_USA
GCTTCATTCAAATATCTGCCCTATCAACTTTCGATGGTAGGATAGAGGCC
JV0709_83_CA_USA
GCTTCATTCAAATATCTGCCCTATCAACTTTCGATGGTAGGATAGAGGCC
Dai21043P_pouzarii
GCTTCATTCAAATATCTGCCCTATCAACTTTCGATGGTAGGATAGAGGCC
MJ144_95_CZ --
JV0909_3_CZ
GCTTCATTCAAATATCTGCCCTATCAACTTTCGATGGTAGGATAGAGGCC
JV0609_1_K --
Dai20396Physisporinus_castanop
GCTTCATTCAAATATCTGCCCTATCAACTTTCGATGGTAGGATAGAGGCC
Dai20397Physisporinus_castanop
GCTTCATTCAAATATCTGCCCTATCAACTTTCGATGGTAGGATAGAGGCC
MJ19_09_SK_Abies --
JV0509_40_J_TN_USA_Betula
GCTTCATTCAAATATCTGCCCTATCAACTTTCGATGGTAGGATAGAGGCC
JV0808_33crocatus_PA_USAlist
GCTTCATTCAAATATCTGCCCTATCAACTTTCGATGGTAGGATAGAGGCC
DLL2009_061P_crocatus --

Dai12800P_subcrocatus --
Dai15917P_subcrocatus
GCTTCATTCAAATATCTGCCCTATCAACTTTCGATGGTAGGATAGAGGCC
Cui16620 --
HCFC1088Meripilus_stillicidior --
MCW590Meripilus_obsrcurus --
MCW722Meripilus_obsrcurus --
Cui9381P_tibeticus
GCTTCATTCAAATATCTGCCCTATCAACTTTCGATGGTAGGATAGAGGCC
Cui9588P_tibeticus
GCTTCATTCAAATATCTGCCCTATCAACTTTCGATGGTAGGATAGAGGCC
Va2_Beneschova --
CWU3874_Ukraine_Alnus --
WCG1293Dai24718Physisporinus --
WCG1268Dai24682A
GCTTCATTCAAATATCTGCCCTATCAACTTTCGATGGTAGGATAGAGGCC
WCG1269Dai24683A
GCTTCATTCAAATATCTGCCCTATCAACTTTCGATGGTAGGATAGAGGCC
WCG1279Dai24694A
GCTTCATTCAAATATCTGCCCTATCAACTTTCGATGGTAGGATAGAGGCC
Dai16971 --
ZQY1043Dai26696 --
Doll880
GCTTCATTCAAATATCTGCCCTATCAACTTTCGATGGTAGGATAGAGGCC
Doll1000
GCTTCATTCAAATATCTGCCCTATCAACTTTCGATGGTAGGATAGAGGCC
1DAI18529
GCTTCATTCAAATATCTGCCCTATCAACTTTCGATGGTAGGATAGAGGCC
Dai19535
GCTTCATTCAAATATCTGCCCTATCAACTTTCGATGGTAGGATAGAGGCC
1704_79_hnedyVillaLaPaz
GCTTCATTCAAATATCTGCCCTATCAACTTTCGATGGTAGGATAGAGGCC
F2061 --
1DAI18268 --
1DAI18540A
GCTTCATTCAAATATCTGCCCTATCAACTTTCGATGGTAGGATAGAGGCC
Dai17695
GCTTCATTCAAATATCTGCCCTATCAACTTTCGATGGTAGGATAGAGGCC
LKY18Dai26373 --
Dai17839P_sulphureus
GCTTCATTCAAATATCTGCCCTATCAACTTTCGATGGTAGGATAGAGGCC
Dai17841P_sulphureus
GCTTCATTCAAATATCTGCCCTATCAACTTTCGATGGTAGGATAGAGGCC
Dai19877P_roseus

GCTTCATTCAAATATCTGCCCTATCAACTTTCGATGGTAGGATAGAGGCC
1508_18_1_Kout --
KP859303_R_vinctus_RP185_BRAZI --
JK1807_15Rigidoporus_sp_Puerto
GCTTCATTCAAATATCTGCCCTATCAACTTTCGATGGTAGGATAGAGGCC
JV0509_47_J_TN_USA
GCTTCATTCAAATATCTGCCCTATCAACTTTCGATGGTAGGATAGAGGCC
JV0709_188 --
JV0509_127_PA_USA
GCTTCATTCAAATATCTGCCCTATCAACTTTCGATGGTAGGATAGAGGCC
JV1009_59_NJ_USA
GCTTCATTCAAATATCTGCCCTATCAACTTTCGATGGTAGGATAGAGGCC
Dai15497P_crataegi
GCTTCATTCAAATATCTGCCCTATCAACTTTCGATGGTAGGATAGAGGCC
Dai15499P_crataegi
GCTTCATTCAAATATCTGCCCTATCAACTTTCGATGGTAGGATAGAGGCC
Cui3266P_cinereus --
WCG1256Dai24690
GCTTCATTCAAATATCTGCCCTATCAACTTTCGATGGTAGGATAGAGGCC
1DAI17581 --
WCG1255Dai24688
GCTTCATTCAAATATCTGCCCTATCAACTTTCGATGGTAGGATAGAGGCC
Dai22427 --
MV690Meripilus_concrescens --
MV513Meripilus_galapagensis --
Dai19793
GCTTCATTCAAATATCTGCCCTATCAACTTTCGATGGTAGGATAGAGGCC
OQ553780P_tamilnaduensis --
OQ553779P_tamilnaduensis --
A164FB3Meripilus_giganteus --
JV1407_36_Vinctus_Meandrica
GCTTCATTCAAATATCTGCCCTATCAACTTTCGATGGTAGGATAGAGGCC
1807_3K_Rigidoporus_PuertoRico
GCTTCATTCAAATATCTGCCCTATCAACTTTCGATGGTAGGATAGAGGCC
Cui16903P_vinctus
GCTTCATTCAAATATCTGCCCTATCAACTTTCGATGGTAGGATAGAGGCC
JV1008_18R_Lineatus
GCTTCATTCAAATATCTGCCCTATCAACTTTCGATGGTAGGATAGAGGCC
JV1407_37_1_Vinctus_Carara
GCTTCATTCAAATATCTGCCCTATCAACTTTCGATGGTAGGATAGAGGCC
Dai17986P_lineatus
GCTTCATTCAAATATCTGCCCTATCAACTTTCGATGGTAGGATAGAGGCC
Dai18281
GCTTCATTCAAATATCTGCCCTATCAACTTTCGATGGTAGGATAGAGGCC

1DAI19796
GCTTCATTCAAATATCTGCCCTATCAACTTTCGATGGTAGGATAGAGGCC
ZQY797Dai25241
GCTTCATTCAAATATCTGCCCTATCAACTTTCGATGGTAGGATAGAGGCC
WCG1289Dai24711 --
Dai22598
GCTTCATTCAAATATCTGCCCTATCAACTTTCGATGGTAGGATAGAGGCC
Dai20523 --
Dai17885 --
Dai17553
GCTTCATTCAAATATCTGCCCTATCAACTTTCGATGGTAGGATAGAGGCC
Dai19639
GCTTCATTCAAATATCTGCCCTATCAACTTTCGATGGTAGGATAGAGGCC
JV0110_48_CZ
GCTTCATTCAAATATCTGCCCTATCAACTTTCGATGGTAGGATAGAGGCC
MJ129_04 --
Cui10340P_eminens --
Cui10341P_eminens
GCTTCATTCAAATATCTGCCCTATCAACTTTCGATGGTAGGATAGAGGCC
Dai12685P_eminens
GCTTCATTCAAATATCTGCCCTATCAACTTTCGATGGTAGGATAGAGGCC
Miettinen_13591Rigidoporus_und --
Dai20868
GCTTCATTCAAATATCTGCCCTATCAACTTTCGATGGTAGGATAGAGGCC
Dai20832
GCTTCATTCAAATATCTGCCCTATCAACTTTCGATGGTAGGATAGAGGCC
Dai11400
GCTTCATTCAAATATCTGCCCTATCAACTTTCGATGGTAGGATAGAGGCC
Dai22472
GCTTCATTCAAATATCTGCCCTATCAACTTTCGATGGTAGGATAGAGGCC
1CUI10475 --
1CUI10491 --
HCFC1095Meripilus_robledoi --
MCW702Meripilus_revolubilis --
1704_83_zluty_HaciendaBaru
GCTTCATTCAAATATCTGCCCTATCAACTTTCGATGGTAGGATAGAGGCC
Dai9925P_lavendulus --
Dai13587AP_lavendulus
GCTTCATTCAAATATCTGCCCTATCAACTTTCGATGGTAGGATAGAGGCC
PDD70600P_longicystidius --
Cui16630 --
FP_135344Meripilus_giganteus --
FP_100460_Sp --
CBS421_48Meripilus_giganteus --

Cui9203
GCTTCATTCAAATATCTGCCCTATCAACTTTCGATGGTAGGATAGAGGCC
Cui9202
GCTTCATTCAAATATCTGCCCTATCAACTTTCGATGGTAGGATAGAGGCC
TUFC100564Japan --
Russell5913Meripilus_sumstinei --
RP215Meripilus_brasiliensis --
RP200Meripilus_brasiliensis --
JV1712_13J_R_vinctus2_LSUPuert
GCTTCATTCAAATATCTGCCCTATCAACTTTCGATGGTAGGATAGAGGCC
Dai10503R_hypobrunneus --
Dai10569Rigidoporus_hypobrunne --
1DAI19451 --
CM108bRigidoporus_hypobrunneus --
1CUI16874 --
FD299Cerrena_unicolor --
KHL_GB_Cerrena_uniclor --
Dai12892Cerrena_albocinnamomea --
Dai12955C_albocinnamomea --
SNUm03110102C_aurantiopora --
NIBRFG0000102423C_aurantiopora --
Dai7359Antrodiella_zonata --
F20080702KCM29C_consors --
F20080208LYW10Cerrena_consors --
Dai7821Antrodiella_zonata --
CFMR_DCL04_31Pseudolagarobasid --
VPB197Pseudolagarobasidium_bel --
CBS115543Pseudolagarobasidium_ --
CBS115544 --
Han405Pseudolagarobasidium_bai --
Han406Pseudolagarobasidium_bai --
MUcc838Spongipellis_delectans --
BRNM686401S_delectans --
OSM_F925S_delectans --
BRNM67093Spongipellis_litschau --
CFMRccFP59199TS_unicolor --
CFMRccFP71791TS_unicolor --
Dai13845P_lischaueri
GCTTCATTCAAATATCTGCCCTATCAACTTTCGATGGTAGGATAGAGGCC
Dai20266P_lischaueri
GCTTCATTCAAATATCTGCCCTATCAACTTTCGATGGTAGGATAGAGGCC
CFMR_HHB11240Radulodon_america --
RLG6350Radulodon_americanus --
KY415963Radulodon_erikssonii --

HHB9567spRadulodon_casearius --
KRT_Iso_26Radulodon_casearius --
CBS126044Radulodon_erikssonii --
He6183YUNNANENSIS
GCTTCATTCAAATATCTGCCCTATCAACTTTCGATGGTAGGATAGAGGCC
Cui17979YUNNANENSIS
GCTTCATTCAAATATCTGCCCTATCAACTTTCGATGGTAGGATAGAGGCC
Miettinen2091Junghuhnia_fimbri --
KHL12316S_tenue --
PRM846564S_pachyodon --
SP_Lgt_S_pachyodon --
Ryvarden44669Tyromyces_xuchile --
PW17_171sinuosus --
W53Dai12234 --
HHB4100SpAntella_americana --
W3Dai20901spumeus
GCTTCATTCAAATATCTGCCCTATCAACTTTCGATGGTAGGATAGAGGCC
He6736 --
BRNM734877S_spumeus --
BRNM712630S_spumeus --
Dai1723Loweomyces_sibiricus --
W54Cui10009
GCTTCATTCAAATATCTGCCCTATCAACTTTCGATGGTAGGATAGAGGCC
W1Dai20899
GCTTCATTCAAATATCTGCCCTATCAACTTTCGATGGTAGGATAGAGGCC
HHB13445Trametes_ochracea --
Dai16222 --
Dai16240 --

JV1310_11SanguinolentusCernys
TACCATGGTTTCAACGGGTAACGGGGAATAAGGGTTCGATTCCGGAGAGG
MJ39_00_SK --
MJ111_04_CZ
TACCATGGTTTCAACGGGTAACGGGGAATAAGGGTTCGATTCCGGAGAGG
JV1610_BOKYsmrk
TACCATGGTTTCAACGGGTAACGGGGAATAAGGGTTCGATTCCGGAGAGG
Dai21030 --
Dai20976P_furcatus
TACCATGGTTTCAACGGGTAACGGGGAATAAGGGTTCGATTCCGGAGAGG
Dai2105 --
Dai2544 --
Dai11313 --
WCG1611Dai26167
TACCATGGTTTCAACGGGTAACGGGGAATAAGGGTTCGATTCCGGAGAGG

WCG1518Dai25999Physisporinus
TACCATGGTTTCAACGGGTAACGGGGAATAAGGGTTCGATTCCGGAGAGG
TAA15097 ---
JV8909_19_CZ ---
JV1310_15_P_sanguinolentus2_CZ ---
MJ53_02_CZ
TACCATGGTTTGACGGGTAACGGGGAATAAGGGTTCGATTCCGGAGAGG
CLZhao21647P_yunnanensis ---
CLZhao21583P_yunnanensis ---
Dai22272
TACCATGGTTTCAACGGGTAACGGGGAATAAGGGTTCGATTCCGGAGAGG
Dai22279
TACCATGGTTTCAACGGGTAACGGGGAATAAGGGTTCGATTCCGGAGAGG
MJ332_94_CZ ---
MJ642_94_CZ_Expallescens ---
Dai21060P_vinctus
TACCATGGTTTCAACGGGTAACGGGGAATAAGGGTTCGATTCCGGAGAGG
JV0511_23LRP_pouzarii
TACCATGGTTTCAACGGGTAACGGGGAATAAGGGTTCGATTCCGGAGAGG
JQ409462_R_pouzarii_PRM899856_ ---
JV0308_66_WA
TACCATGGTTTCAACGGGTAACGGGGAATAAGGGTTCGATTCCGGAGAGG
JV0309_45_WA_USA
TACCATGGTTTCAACGGGTAACGGGGAATAAGGGTTCGATTCCGGAGAGG
JV0709_83_CA_USA
TACCATGGTTTCAACGGGTAACGGGGAATAAGGGTTCGATTCCGGAGAGG
Dai21043P_pouzarii
TACCATGGTTTCAACGGGTAACGGGGAATAAGGGTTCGATTCCGGAGAGG
MJ144_95_CZ ---
JV0909_3_CZ
TACCATGGTTTCAACGGGTAACGGGGAATAAGGGTTCGATTCCGGAGAGG
JV0609_1_K ---
Dai20396Physisporinus_castanop
TACCATGGTTTCAACGGGTAACGGGGAATAAGGGTTCGATTCCGGAGAGG
Dai20397Physisporinus_castanop
TACCATGGTTTCAACGGGTAACGGGGAATAAGGGTTCGATTCCGGAGAGG
MJ19_09_SK_Abies ---
JV0509_40_J_TN_USA_Betula
TACCATGGTTTCAACGGGTAACGGGGAATAAGGGTTCGATTCCGGAGAGG
JV0808_33crocatus_PA_USAlist
TACCATGGTTTCAACGGGTAACGGGGAATAAGGGTTCGATTCCGGAGAGG
DLL2009_061P_crocatus ---
Dai12800P_subcrocatus ---
Dai15917P_subcrocatus

TACCATGGTTTCAACGGGTAACGGGGAATAAGGGTTCGATTCCGGAGAGG
Cui16620 --
HCFC1088Meripilus_stillicidior --
MCW590Meripilus_obsrcurus --
MCW722Meripilus_obsrcurus --
Cui9381P_tibeticus
TACCATGGTTTCAACGGGTAACGGGGAATAAGGGTTCGATTCCGGAGAGG
Cui9588P_tibeticus
TACCATGGTTTCAACGGGTAACGGGGAATAAGGGTTCGATTCCGGAGAGG
Va2_Beneschova --
CWU3874_Ukraine_Alnus --
WCG1293Dai24718Physisporinus --
WCG1268Dai24682A
TACCATGGTTTCAACGGGTAACGGGGAATAAGGGTTCGATTCCGGAGAGG
WCG1269Dai24683A
TACCATGGTTTCAACGGGTAACGGGGAATAAGGGTTCGATTCCGGAGAGG
WCG1279Dai24694A
TACCATGGTTTCAACGGGTAACGGGGAATAAGGGTTCGATTCCGGAGAGG
Dai16971 --
ZQY1043Dai26696 --
Doll880
TACCATGGTTTCAACGGGTAACGGGGAATAAGGGTTCGATTCCGGAGAGG
Doll1000
TACCATGGTTTCAACGGGTAACGGGGAATAAGGGTTCGATTCCGGAGAGG
1DAI18529
TACCATGGTTTCAACGGGTAACGGGGAATAAGGGTTCGATTCCGGAGAGG
Dai19535
TACCATGGTTTCAACGGGTAACGGGGAATAAGGGTTCGATTCCGGAGAGG
1704_79_hnedyVillaLaPaz
TACCATGGTTTCAACGGGTAACGGGGAATAAGGGTTCGATTCCGGAGAGG
F2061 --
1DAI18268 --
1DAI18540A
TACCATGGTTTCAACGGGTAACGGGGAATAAGGGTTCGATTCCGGAGAGG
Dai17695
TACCATGGTTTCAACGGGTAACGGGGAATAAGGGTTCGATTCCGGAGAGG
LKY18Dai26373 --
Dai17839P_sulphureus
TACCATGGTTTCAACGGGTAACGGGGAATAAGGGTTCGATTCCGGAGAGG
Dai17841P_sulphureus
TACCATGGTTTCAACGGGTAACGGGGAATAAGGGTTCGATTCCGGAGAGG
Dai19877P_roseus
TACCATGGTTTCAACGGGTAACGGGGAATAAGGGTTCGATTCCGGAGAGG
1508_18_1_Kout --

KP859303_R_vinctus_RP185_BRAZI --
JK1807_15Rigidoporus_sp_Puerto
TACCATGGTTTCAACGGGTAACGGGGAATAAGGGTTCGATTCCGGAGAGG
JV0509_47_J_TN_USA
TACCATGGTTTCAACGGGTAACGGGGAATAAGGGTTCGATTCCGGAGAGG
JV0709_188 --
JV0509_127_PA_USA
TACCATGGTTTCAACGGGTAACGGGGAATAAGGGTTCGATTCCGGAGAGG
JV1009_59_NJ_USA
TACCATGGTTTCAACGGGTAACGGGGAATAAGGGTTCGATTCCGGAGAGG
Dai15497P_crataegi
TACCATGGTTTCAACGGGTAACGGGGAATAAGGGTTCGATTCCGGAGAGG
Dai15499P_crataegi
TACCATGGTTTCAACGGGTAACGGGGAATAAGGGTTCGATTCCGGAGAGG
Cui3266P_cinereus --
WCG1256Dai24690
TACCATGGTTTCAACGGGTAACGGGGAATAAGGGTTCGATTCCGGAGAGG
1DAI17581 --
WCG1255Dai24688
TACCATGGTTTCAACGGGTAACGGGGAATAAGGGTTCGATTCCGGAGAGG
Dai22427 --
MV690Meripilus_concrescens --
MV513Meripilus_galapagensis --
Dai19793
TACCATGGTTTCAACGGGTAACGGGGAATAAGGGTTCGATTCCGGAGAGG
OQ553780P_tamilnaduensis --
OQ553779P_tamilnaduensis --
A164FB3Meripilus_giganteus --
JV1407_36_Vinctus_Meandrica
TACCATGGTTTCAACGGGTAACGGGGAATAAGGGTTCGATTCCGGAGAGG
1807_3K_Rigidoporus_PuertoRico
TACCATGGTTTCAACGGGTAACGGGGAATAAGGGTTCGATTCCGGAGAGG
Cui16903P_vinctus
TACCATGGTTTCAACGGGTAACGGGGAATAAGGGTTCGATTCCGGAGAGG
JV1008_18R_Lineatus
TACCATGGTTTCAACGGGTAACGGGGAATAAGGGTTCGATTCCGGAGAGG
JV1407_37_1_Vinctus_Carara
TACCATGGTTTCAACGGGTAACGGGGAATAAGGGTTCGATTCCGGAGAGG
Dai17986P_lineatus
TACCATGGTTTCAACGGGTAACGGGGAATAAGGGTTCGATTCCGGAGAGG
Dai18281
TACCATGGTTTCAACGGGTAACGGGGAATAAGGGTTCGATTCCGGAGAGG
1DAI19796
TACCATGGTTTCAACGGGTAACGGGGAATAAGGGTTCGATTCCGGAGAGG

ZQY797Dai25241
TACCATGGTTTCAACGGGTAACGGGGAATAAGGGTTCGATTCCGGAGAGG
WCG1289Dai24711 --
Dai22598
TACCATGGTTTCAACGGGTAACGGGGAATAAGGGTTCGATTCCGGAGAGG
Dai20523 --
Dai17885 --
Dai17553
TACCATGGTTTCAACGGGTAACGGGGAATAAGGGTTCGATTCCGGAGAGG
Dai19639
TACCATGGTTTCAACGGGTAACGGGGAATAAGGGTTCGATTCCGGAGAGG
JV0110_48_CZ
TACCATGGTTTCAACGGGTAACGGGGAATAAGGGTTCGATTCCGGAGAGG
MJ129_04 --
Cui10340P_eminens --
Cui10341P_eminens
TACCATGGTTTCAACGGGTAACGGGGAATAAGGGTTCGATTCCGGAGAGG
Dai12685P_eminens
TACCATGGTTTCAACGGGTAACGGGGAATAAGGGTTCGATTCCGGAGAGG
Miettinen_13591Rigidoporus_und --
Dai20868
TACCATGGTTTCAACGGGTAACGGGGAATAAGGGTTCGATTCCGGAGAGG
Dai20832
TACCATGGTTTCAACGGGTAACGGGGAATAAGGGTTCGATTCCGGAGAGG
Dai11400
TACCATGGTTTCAACGGGTAACGGGGAATAAGGGTTCGATTCCGGAGAGG
Dai22472
TACCATGGTTTCAACGGGTAACGGGGAATAAGGGTTCGATTCCGGAGAGG
1CUI10475 --
1CUI10491 --
HCFC1095Meripilus_robledoi --
MCW702Meripilus_revolubilis --
1704_83_zluty_HaciendaBaru
TACCATGGTTTCAACGGGTAACGGGGAATAAGGGTTCGATTCCGGAGAGG
Dai9925P_lavendulus --
Dai13587AP_lavendulus
TACCATGGTTTCAACGGGTAACGGGGAATAAGGGTTCGATTCCGGAGAGG
PDD70600P_longicystidius --
Cui16630 --
FP_135344Meripilus_giganteus --
FP_100460_Sp --
CBS421_48Meripilus_giganteus --
Cui9203
TACCATGGTTTCAACGGGTAACGGGGAATAAGGGTTCGATTCCGGAGAGG

Cui9202
TACCATGGTTTCAACGGGTAACGGGGAATAAGGGTTCGATTCCGGAGAGG
TUFC100564Japan --
Russell5913Meripilus_sumstinei --
RP215Meripilus_brasiliensis --
RP200Meripilus_brasiliensis --
JV1712_13J_R_vinctus2_LSUPuert
TACCATGGTTTCAACGGGTAACGGGGAATAAGGGTTCGATTCCGGAGAGG
Dai10503R_hypobrunneus --
Dai10569Rigidoporus_hypobrunne --
1DAI19451 --
CM108bRigidoporus_hypobrunneus --
1CUI16874 --
FD299Cerrena_unicolor --
KHL_GB_Cerrena_uniclor --
Dai12892Cerrena_albocinnamomea --
Dai12955C_albocinnamomea --
SNUm03110102C_aurantiopora --
NIBRFG0000102423C_aurantiopora --
Dai7359Antrodiella_zonata --
F20080702KCM29C_consors --
F20080208LYW10Cerrena_consors --
Dai7821Antrodiella_zonata --
CFMR_DCL04_31Pseudolagarobasid --
VPB197Pseudolagarobasidium_bel --
CBS115543Pseudolagarobasidium_ --
CBS115544 --
Han405Pseudolagarobasidium_bai --
Han406Pseudolagarobasidium_bai --
MUcc838Spongipellis_delectans --
BRNM686401S_delectans --
OSM_F925S_delectans --
BRNM67093Spongipellis_litschau --
CFMRccFP59199TS_unicolor --
CFMRccFP71791TS_unicolor --
Dai13845P_lischaueri
TACCATGGTTTCAACGGGTAACGGGGAATTAGGGTTCGATTCCGGAGAGG
Dai20266P_lischaueri
TACCATGGTTTCAACGGGTAACGGGGAATTAGGGTTCGATTCCGGAGAGG
CFMR_HHB11240Radulodon_america --
RLG6350Radulodon_americanus --
KY415963Radulodon_erikssonii --
HHB9567spRadulodon_casearius --
KRT_Iso_26Radulodon_casearius --

CBS126044Radulodon_erikssonii ---
He6183YUNNANENSIS
TACCATGGTTTCGACGGGTAACGGGGAATTAGGGTTCGATTCCGGAGAGG
Cui17979YUNNANENSIS
TACCATGGTTTCGACGGGTAACGGGGAATTAGGGTTCGATTCCGGAGAGG
Miettinen2091Junghuhnia_fimbri ---
KHL12316S_tenue ---
PRM846564S_pachyodon ---
SP_Lgt_S_pachyodon ---
Ryvarden44669Tyromyces_xuchile ---
PW17_171sinuosus ---
W53Dai12234 ---
HHB4100SpAntella_americana ---
W3Dai20901spumeus
TACCATGGTTTCAACGGGTAACGGGGAATAAGGGTTCGATTCCGGAGAGG
He6736 ---
BRNM734877S_spumeus ---
BRNM712630S_spumeus ---
Dai1723Loweomyces_sibiricus ---
W54Cui10009
TACCATGGTTTCAACGGGTAACGGGGAATAAGGGTTCGATTCCGGAGAGG
W1Dai20899
TACCATGGTTTCAACGGGTAACGGGGAATAAGGGTTCGATTCCGGAGAGG
HHB13445Trametes_ochracea ---
Dai16222 ---
Dai16240 ---

JV1310_11SanguinolentusCernys
GAGCCTGAGAAACGGCTACCACATCCAAGGAAGGCAGCAGGCGCGCAAAT
MJ39_00_SK ---
MJ111_04_CZ
GAGCCTGAGAAACGGCTACCACATCCAAGGAAGGCAGCAGGCGCGCAAAT
JV1610_BOKYsmrk
GAGCCTGAGAAACGGCTACCACATCCAAGGAAGGCAGCAGGCGCGCAAAT
Dai21030 ---
Dai20976P_furcatus
GAGCCTGAGAAACGGCTACCACATCCAAGGAAGGCAGCAGGCGCGCAAAT
Dai2105 ---
Dai2544 ---
Dai11313 ---
WCG1611Dai26167
GAGCCTGAGAAACGGCTACCACATCCAAGGAAGGCAGCAGGCGCGCAAAT
WCG1518Dai25999Physisporinus
GAGCCTGAGAAACGGCTACCACATCCAAGGAAGGCAGCAGGCGCGCAAAT

TAA15097 --
JV8909_19_CZ --
JV1310_15_P_sanguinolentus2_CZ --
MJ53_02_CZ
GAGCCTGAGAAACGGCTACCACATCTACGGAAGGCAGCAGGCGCGCAAAT
CLZhao21647P_yunnanensis --
CLZhao21583P_yunnanensis --
Dai22272
GAGCCTGAGAAACGGCTACCACATCCAAGGAAGGCAGCAGGCGCGCAAAT
Dai22279
GAGCCTGAGAAACGGCTACCACATCCAAGGAAGGCAGCAGGCGCGCAAAT
MJ332_94_CZ --
MJ642_94_CZ_Expallescens --
Dai21060P_vinctus
GAGCCTGAGAAACGGCTACCACATCCAAGGAAGGCAGCAGGCGCGCAAAT
JV0511_23LRP_pouzarii
GAGCCTGAGAAACGGCTACCACATCCAAGGAAGGCAGCAGGCGCGCAAAT
JQ409462_R_pouzarii_PRM899856_ --
JV0308_66_WA
GAGCCTGAGAAACGGCTACCACATCCAAGGAAGGCAGCAGGCGCGCAAAT
JV0309_45_WA_USA
GAGCCTGAGAAACGGCTACCACATCCAAGGAAGGCAGCAGGCGCGCAAAT
JV0709_83_CA_USA
GAGCCTGAGAAACGGCTACCACATCCAAGGAAGGCAGCAGGCGCGCAAAT
Dai21043P_pouzarii
GAGCCTGAGAAACGGCTACCACATCCAAGGAAGGCAGCAGGCGCGCAAAT
MJ144_95_CZ --
JV0909_3_CZ
GAGCCTGAGAAACGGCTACCACATCCAAGGAAGGCAGCAGGCGCGCAAAT
JV0609_1_K --
Dai20396Physisporinus_castanop
GAGCCTGAGAAACGGCTACCACATCCAAGGAAGGCAGCAGGCGCGCAAAT
Dai20397Physisporinus_castanop
GAGCCTGAGAAACGGCTACCACATCCAAGGAAGGCAGCAGGCGCGCAAAT
MJ19_09_SK_Abies --
JV0509_40_J_TN_USA_Betula
GAGCCTGAGAAACGGCTACCACATCCAAGGAAGGCAGCAGGCGCGCAAAT
JV0808_33crocatus_PA_USAlist
GAGCCTGAGAAACGGCTACCACATCCAAGGAAGGCAGCAGGCGCGCAAAT
DLL2009_061P_crocatus --
Dai12800P_subcrocatus --
Dai15917P_subcrocatus
GAGCCTGAGAAACGGCTACCACATCCAAGGAAGGCAGCAGGCGCGCAAAT
Cui16620 --

HCFC1088Meripilus_stillicidior --
MCW590Meripilus_obsrcurus --
MCW722Meripilus_obsrcurus --
Cui9381P_tibeticus
GAGCCTGAGAAACGGCTACCACATCCAAGGAAGGCAGCAGGCGCGCAAAT
Cui9588P_tibeticus
GAGCCTGAGAAACGGCTACCACATCCAAGGAAGGCAGCAGGCGCGCAAAT
Va2_Beneschova --
CWU3874_Ukraine_Alnus --
WCG1293Dai24718Physisporinus --
WCG1268Dai24682A
GAGCCTGAGAAACGGCTACCACATCCAAGGAAGGCAGCAGGCGCGCAAAT
WCG1269Dai24683A
GAGCCTGAGAAACGGCTACCACATCCAAGGAAGGCAGCAGGCGCGCAAAT
WCG1279Dai24694A
GAGCCTGAGAAACGGCTACCACATCCAAGGAAGGCAGCAGGCGCGCAAAT
Dai16971 --
ZQY1043Dai26696 --
Doll880
GAGCCTGAGAAACGGCTACCACATCCAAGGAAGGCAGCAGGCGCGCAAAT
Doll1000
GAGCCTGAGAAACGGCTACCACATCCAAGGAAGGCAGCAGGCGCGCAAAT
1DAI18529
GAGCCTGAGAAACGGCTACCACATCCAAGGAAGGCAGCAGGCGCGCAAAT
Dai19535
GAGCCTGAGAAACGGCTACCACATCCAAGGAAGGCAGCAGGCGCGCAAAT
1704_79_hnedyVillaLaPaz
GAGCCTGAGAAACGGCTACCACATCCAAGGAAGGCAGCAGGCGCGCAAAT
F2061 --
1DAI18268 --
1DAI18540A
GAGCCTGAGAAACGGCTACCACATCCAAGGAAGGCAGCAGGCGCGCAAAT
Dai17695
GAGCCTGAGAAACGGCTACCACATCCAAGGAAGGCAGCAGGCGCGCAAAT
LKY18Dai26373 --
Dai17839P_sulphureus
GAGCCTGAGAAACGGCTACCACATCCAAGGAAGGCAGCAGGCGCGCAAAT
Dai17841P_sulphureus
GAGCCTGAGAAACGGCTACCACATCCAAGGAAGGCAGCAGGCGCGCAAAT
Dai19877P_roseus
GAGCCTGAGAAACGGCTACCACATCCAAGGAAGGCAGCAGGCGCGCAAAT
1508_18_1_Kout --
KP859303_R_vinctus_RP185_BRAZI --
JK1807_15Rigidoporus_sp_Puerto

GAGCCTGAGAAACGGCTACCACATCCAAGGAAGGCAGCAGGCGCGCAAAT
JV0509_47_J_TN_USA
GAGCCTGAGAAACGGCTACCACATCCAAGGAAGGCAGCAGGCGCGCAAAT
JV0709_188 --
JV0509_127_PA_USA
GAGCCTGAGAAACGGCTACCACATCCAAGGAAGGCAGCAGGCGCGCAAAT
JV1009_59_NJ_USA
GAGCCTGAGAAACGGCTACCACATCCAAGGAAGGCAGCAGGCGCGCAAAT
Dai15497P_crataegi
GAGCCTGAGAAACGGCTACCACATCCAAGGAAGGCAGCAGGCGCGCAAAT
Dai15499P_crataegi
GAGCCTGAGAAACGGCTACCACATCCAAGGAAGGCAGCAGGCGCGCAAAT
Cui3266P_cinereus --
WCG1256Dai24690
GAGCCTGAGAAACGGCTACCACATCCAAGGAAGGCAGCAGGCGCGCAAAT
1DAI17581 --
WCG1255Dai24688
GAGCCTGAGAAACGGCTACCACATCCAAGGAAGGCAGCAGGCGCGCAAAT
Dai22427 --
MV690Meripilus_concrescens --
MV513Meripilus_galapagensis --
Dai19793
GAGCCTGAGAAACGGCTACCACATCCAAGGAAGGCAGCAGGCGCGCAAAT
OQ553780P_tamilnaduensis --
OQ553779P_tamilnaduensis --
A164FB3Meripilus_giganteus --
JV1407_36_Vinctus_Meandrica
GAGCCTGAGAAACGGCTACCACATCCAAGGAAGGCAGCAGGCGCGCAAAT
1807_3K_Rigidoporus_PuertoRico
GAGCCTGAGAAACGGCTACCACATCCAAGGAAGGCAGCAGGCGCGCAAAT
Cui16903P_vinctus
GAGCCTGAGAAACGGCTACCACATCCAAGGAAGGCAGCAGGCGCGCAAAT
JV1008_18R_Lineatus
GAGCCTGAGAAACGGCTACCACATCCAAGGAAGGCAGCAGGCGCGCAAAT
JV1407_37_1_Vinctus_Carara
GAGCCTGAGAAACGGCTACCACATCCAAGGAAGGCAGCAGGCGCGCAAAT
Dai17986P_lineatus
GAGCCTGAGAAACGGCTACCACATCCAAGGAAGGCAGCAGGCGCGCAAAT
Dai18281
GAGCCTGAGAAACGGCTACCACATCCAAGGAAGGCAGCAGGCGCGCAAAT
1DAI19796
GAGCCTGAGAAACGGCTACCACATCCAAGGAAGGCAGCAGGCGCGCAAAT
ZQY797Dai25241
GAGCCTGAGAAACGGCTACCACATCCAAGGAAGGCAGCAGGCGCGCAAAT

WCG1289Dai24711 --
Dai22598
GAGCCTGAGAAACGGCTACCACATCCAAGGAAGGCAGCAGGCGCGCAAAT
Dai20523 --
Dai17885 --
Dai17553
GAGCCTGAGAAACGGCTACCACATCCAAGGAAGGCAGCAGGCGCGCAAAT
Dai19639
GAGCCTGAGAAACGGCTACCACATCCAAGGAAGGCAGCAGGCGCGCAAAT
JV0110_48_CZ
GAGCCTGAGAAACGGCTACCACATCCAAGGAAGGCAGCAGGCGCGCAAAT
MJ129_04 --
Cui10340P_eminens --
Cui10341P_eminens
GAGCCTGAGAAACGGCTACCACATCCAAGGAAGGCAGCAGGCGCGCAAAT
Dai12685P_eminens
GAGCCTGAGAAACGGCTACCACATCCAAGGAAGGCAGCAGGCGCGCAAAT
Miettinen_13591Rigidoporus_und --
Dai20868
GAGCCTGAGAAACGGCTACCACATCCAAGGAAGGCAGCAGGCGCGCAAAT
Dai20832
GAGCCTGAGAAACGGCTACCACATCCAAGGAAGGCAGCAGGCGCGCAAAT
Dai11400
GAGCCTGAGAAACGGCTACCACATCCAAGGAAGGCAGCAGGCGCGCAAAT
Dai22472
GAGCCTGAGAAACGGCTACCACATCCAAGGAAGGCAGCAGGCGCGCAAAT
1CUI10475 --
1CUI10491 --
HCFC1095Meripilus_robledoi --
MCW702Meripilus_revolubilis --
1704_83_zluty_HaciendaBaru
GAGCCTGAGAAACGGCTACCACATCCAAGGAAGGCAGCAGGCGCGCAAAT
Dai9925P_lavendulus --
Dai13587AP_lavendulus
GAGCCTGAGAAACGGCTACCACATCCAAGGAAGGCAGCAGGCGCGCAAAT
PDD70600P_longicystidius --
Cui16630 --
FP_135344Meripilus_giganteus --
FP_100460_Sp --
CBS421_48Meripilus_giganteus --
Cui9203
GAGCCTGAGAAACGGCTACCACATCCAAGGAAGGCAGCAGGCGCGCAAAT
Cui9202
GAGCCTGAGAAACGGCTACCACATCCAAGGAAGGCAGCAGGCGCGCAAAT

TUFC100564Japan --
Russell5913Meripilus_sumstinei --
RP215Meripilus_brasiliensis --
RP200Meripilus_brasiliensis --
JV1712_13J_R_vinctus2_LSUPuert
GAGCGTGAGAAACGGCTACCACATCCAAGGAAGGCAGCAGGCGCGCAAAT
Dai10503R_hypobrunneus --
Dai10569Rigidoporus_hypobrunne --
1DAI19451 --
CM108bRigidoporus_hypobrunneus --
1CUI16874 --
FD299Cerrena_unicolor --
KHL_GB_Cerrena_uniclor --
Dai12892Cerrena_albocinnamomea --
Dai12955C_albocinnamomea --
SNUm03110102C_aurantiopora --
NIBRFG0000102423C_aurantiopora --
Dai7359Antrodiella_zonata --
F20080702KCM29C_consors --
F20080208LYW10Cerrena_consors --
Dai7821Antrodiella_zonata --
CFMR_DCL04_31Pseudolagarobasid --
VPB197Pseudolagarobasidium_bel --
CBS115543Pseudolagarobasidium_ --
CBS115544 --
Han405Pseudolagarobasidium_bai --
Han406Pseudolagarobasidium_bai --
MUcc838Spongipellis_delectans --
BRNM686401S_delectans --
OSM_F925S_delectans --
BRNM67093Spongipellis_litschau --
CFMRccFP59199TS_unicolor --
CFMRccFP71791TS_unicolor --
Dai13845P_lischaueri
GAGCCTGAGAAACGGCTACCACATCCAAGGAAGGCAGCAGGCGCGCAAAT
Dai20266P_lischaueri
GAGCCTGAGAAACGGCTACCACATCCAAGGAAGGCAGCAGGCGCGCAAAT
CFMR_HHB11240Radulodon_america --
RLG6350Radulodon_americanus --
KY415963Radulodon_erikssonii --
HHB9567spRadulodon_casearius --
KRT_Iso_26Radulodon_casearius --
CBS126044Radulodon_erikssonii --
He6183YUNNANENSIS

GAGCCTGAGAAACGGCTACCACATCCAAGGAAGGCAGCAGGCGCAAAT
Cui17979YUNNANENSIS
GAGCCTGAGAAACGGCTACCACATCCAAGGAAGGCAGCAGGCGCAAAT
Miettinen2091Junghuhnia_fimbri --
KHL12316S_tenue --
PRM846564S_pachyodon --
SP_Lgt_S_pachyodon --
Ryvarden44669Tyromyces_xuchile --
PW17_171sinuosus --
W53Dai12234 --
HHB4100SpAntella_americana --
W3Dai20901spumeus
GAGCCTGAGAAACGGCTACCACATCCAAGGAAGGCAGCAGGCGCAAAT
He6736 --
BRNM734877S_spumeus --
BRNM712630S_spumeus --
Dai1723Loweomyces_sibiricus --
W54Cui10009
GAGCCTGAGAAACGGCTACCACATCCAAGGAAGGCAGCAGGCGCAAAT
W1Dai20899
GAGCCTGAGAAACGGCTACCACATCCAAGGAAGGCAGCAGGCGCAAAT
HHB13445Trametes_ochracea --
Dai16222 --
Dai16240 --

JV1310_11SanguinolentusCernys
TACCCAATCCCGACACGGGGAGGTAGTGACAATAAATAACAATATAGGGC
MJ39_00_SK --
MJ111_04_CZ
TACCCAATCCCGACACGGGGAGGTAGTGACAATAAATAACAATATAGGGC
JV1610_BOKYsmrk
TACCCAATCCCGACACGGGGAGGTAGTGACAATAAATAACAATATAGGGC
Dai21030 --
Dai20976P_furcatus
TACCCAATCCCGACACGGGGAGGTAGTGACAATAAATAACAATATAGGGC
Dai2105 --
Dai2544 --
Dai11313 --
WCG1611Dai26167
TACCCAATCCCGACACGGGGAGGTAGTGACAATAAATAACAATATAGGGC
WCG1518Dai25999Physisporinus
TACCCAATCCCGACACGGGGAGGTAGTGACAATAAATAACAATATAGGGC
TAA15097 --
JV8909_19_CZ --

JV1310_15_P_sanguinolentus2_CZ ---
MJ53_02_CZ
TACCCAATCCCGACACGGGGAGGTAGTGACAATAAATAACAATACAGGGT
CLZhao21647P_yunnanensis ---
CLZhao21583P_yunnanensis ---
Dai22272
TACCCAATCCCGACACGGGGAGGTAGTGACAATAAATAACAATATAGGGC
Dai22279
TACCCAATCCCGACACGGGGAGGTAGTGACAATAAATAACAATATAGGGC
MJ332_94_CZ ---
MJ642_94_CZ_Expallescens ---
Dai21060P_vinctus
TACCCAATCCCGACACGGGGAGGTAGTGACAATAAATAACAATATAGGGC
JV0511_23LRP_pouzarii
TACCCAATCCCGACACGGGGAGGTAGTGACAATAAATAACAATATAGGGC
JQ409462_R_pouzarii_PRM899856_ ---
JV0308_66_WA
TACCCAATCCCGACACGGGGAGGTAGTGACAATAAATAACAATATAGGGC
JV0309_45_WA_USA
TACCCAATCCCGACACGGGGAGGTAGTGACAATAAATAACAATATAGGGC
JV0709_83_CA_USA
TACCCAATCCCGACACGGGGAGGTAGTGACAATAAATAACAATATAGGGC
Dai21043P_pouzarii
TACCCAATCCCGACACGGGGAGGTAGTGACAATAAATAACAATATAGGGC
MJ144_95_CZ ---
JV0909_3_CZ
TACCCAATCCCGACACGGGGAGGTAGTGACAATAAATAACAATATAGGGC
JV0609_1_K ---
Dai20396Physisporinus_castanop
TACCCAATCCCGACACGGGGAGGTAGTGACAATAAATAACAATATAGGGC
Dai20397Physisporinus_castanop
TACCCAATCCCGACACGGGGAGGTAGTGACAATAAATAACAATATAGGGC
MJ19_09_SK_Abies ---
JV0509_40_J_TN_USA_Betula
TACCCAATCCCGACACGGGGAGGTAGTGACAATAAATAACAATATAGGGC
JV0808_33crocatus_PA_USAlist
TACCCAATCCCGACACGGGGAGGTAGTGACAATAAATAACAATATAGGGC
DLL2009_061P_crocatus ---
Dai12800P_subcrocatus ---
Dai15917P_subcrocatus
TACCCAATCCCGACACGGGGAGGTAGTGACAATAAATAACAATATAGGGC
Cui16620 ---
HCFC1088Meripilus_stillicidior ---
MCW590Meripilus_obsrcurus ---

MCW722Meripilus_obsrcurus --
Cui9381P_tibeticus
TACCCAATCCCGACACGGGGAGGTAGTGACAATAAATAACAATATAGGGC
Cui9588P_tibeticus
TACCCAATCCCGACACGGGGAGGTAGTGACAATAAATAACAATATAGGGC
Va2_Beneschova --
CWU3874_Ukraine_Alnus --
WCG1293Dai24718Physisporinus --
WCG1268Dai24682A
TACCCAATCCCGACACGGGGAGGTAGTGACAATAAATAACAATATAGGGC
WCG1269Dai24683A
TACCCAATCCCGACACGGGGAGGTAGTGACAATAAATAACAATATAGGGC
WCG1279Dai24694A
TACCCAATCCCGACACGGGGAGGTAGTGACAATAAATAACAATATAGGGC
Dai16971 --
ZQY1043Dai26696 --
Doll880
TACCCAATCCCGACACGGGGAGGTAGTGACAATAAATAACAATATAGGGC
Doll1000
TACCCAATCCCGACACGGGGAGGTAGTGACAATAAATAACAATATAGGGC
1DAI18529
TACCCAATCCCGACACGGGGAGGTAGTGACAATAAATAACAATATAGGGC
Dai19535
TACCCAATCCCGACACGGGGAGGTAGTGACAATAAATAACAATATAGGGC
1704_79_hnedyVillaLaPaz
TACCCAATCCCGACACGGGGAGGTAGTGACAATAAATAACAATATAGGGC
F2061 --
1DAI18268 --
1DAI18540A
TACCCAATCCCGACACGGGGAGGTAGTGACAATAAATAACAATATAGGGC
Dai17695
TACCCAATCCCGACACGGGGAGGTAGTGACAATAAATAACAATATAGGGC
LKY18Dai26373 --
Dai17839P_sulphureus
TACCCAATCCCGACACGGGGAGGTAGTGACAATAAATAACAATATAGGGC
Dai17841P_sulphureus
TACCCAATCCCGACACGGGGAGGTAGTGACAATAAATAACAATATAGGGC
Dai19877P_roseus
TACCCAATCCCGACACGGGGAGGTAGTGACAATAAATAACAATATAGGGC
1508_18_1_Kout --
KP859303_R_vinctus_RP185_BRAZI --
JK1807_15Rigidoporus_sp_Puerto
TACCCAATCCCGACACGGGGAGGTAGTGACAATAAATAACAATATAGGGC
JV0509_47_J_TN_USA

TACCCAATCCCGACACGGGGAGGTAGTGACAATAAATAACAATATAGGGC
JV0709_188 ---
JV0509_127_PA_USA
TACCCAATCCCGACACGGGGAGGTAGTGACAATAAATAACAATATAGGGC
JV1009_59_NJ_USA
TACCCAATCCCGACACGGGGAGGTAGTGACAATAAATAACAATATAGGGC
Dai15497P_crataegi
TACCCAATCCCGACACGGGGAGGTAGTGACAATAAATAACAATATAGGGC
Dai15499P_crataegi
TACCCAATCCCGACACGGGGAGGTAGTGACAATAAATAACAATATAGGGC
Cui3266P_cinereus ---
WCG1256Dai24690
TACCCAATCCCGACACGGGGAGGTAGTGACAATAAATAACAATATAGGGC
1DAI17581 ---
WCG1255Dai24688
TACCCAATCCCGACACGGGGAGGTAGTGACAATAAATAACAATATAGGGC
Dai22427 ---
MV690Meripilus_concrescens ---
MV513Meripilus_galapagensis ---
Dai19793
TACCCAATCCCGACACGGGGAGGTAGTGACAATAAATAACAATATAGGGC
OQ553780P_tamilnaduensis ---
OQ553779P_tamilnaduensis ---
A164FB3Meripilus_giganteus ---
JV1407_36_Vinctus_Meandrica
TACCCAATCCCGACACGGGGAGGTAGTGACAATAAATAACAATATAGGGC
1807_3K_Rigidoporus_PuertoRico
TACCCAATCCCGACACGGGGAGGTAGTGACAATAAATAACAATATAGGGC
Cui16903P_vinctus
TACCCAATCCCGACACGGGGAGGTAGTGACAATAAATAACAATATAGGGC
JV1008_18R_Lineatus
TACCCAATCCCGACACGGGGAGGTAGTGACAATAAATAACAATATAGGGC
JV1407_37_1_Vinctus_Carara
TACCCAATCCCGACACGGGGAGGTAGTGACAATAAATAACAATATAGGGC
Dai17986P_lineatus
TACCCAATCCCGACACGGGGAGGTAGTGACAATAAATAACAATATAGGGC
Dai18281
TACCCAATCCCGACACGGGGAGGTAGTGACAATAAATAACAATATAGGGC
1DAI19796
TACCCAATCCCGACACGGGGAGGTAGTGACAATAAATAACAATATAGGGC
ZQY797Dai25241
TACCCAATCCCGACACGGGGAGGTAGTGACAATAAATAACAATATAGGGC
WCG1289Dai24711 ---
Dai22598

TACCCAATCCCGACACGGGGAGGTAGTGACAATAAATAACAATATAGGGC
Dai20523 --
Dai17885 --
Dai17553
TACCCAATCCCGACACGGGGAGGTAGTGACAATAAATAACAATATAGGGC
Dai19639
TACCCAATCCCGACACGGGGAGGTAGTGACAATAAATAACAATATAGGGC
JV0110_48_CZ
TACCCAATCCCGACACGGGGAGGTAGTGACAATAAATAACAATATAGGGC
MJ129_04 --
Cui10340P_eminens --
Cui10341P_eminens
TACCCAATCCCGACACGGGGAGGTAGTGACAATAAATAACAATATAGGGC
Dai12685P_eminens
TACCCAATCCCGACACGGGGAGGTAGTGACAATAAATAACAATATAGGGC
Miettinen_13591Rigidoporus_und --
Dai20868
TACCCAATCCCGACACGGGGAGGTAGTGACAATAAATAACAATATAGGGC
Dai20832
TACCCAATCCCGACACGGGGAGGTAGTGACAATAAATAACAATATAGGGC
Dai11400
TACCCAATCCCGACACGGGGAGGTAGTGACAATAAATAACAATATAGGGC
Dai22472
TACCCAATCCCGACACGGGGAGGTAGTGACAATAAATAACAATATAGGGC
1CUI10475 --
1CUI10491 --
HCFC1095Meripilus_robledoi --
MCW702Meripilus_revolubilis --
1704_83_zluty_HaciendaBaru
TACCCAATCCCGACACGGGGAGGTAGTGACAATAAATAACAATATAGGGC
Dai9925P_lavendulus --
Dai13587AP_lavendulus
TACCCAATCCCGACACGGGGAGGTAGTGACAATAAATAACAATATAGGGC
PDD70600P_longicystidius --
Cui16630 --
FP_135344Meripilus_giganteus --
FP_100460_Sp --
CBS421_48Meripilus_giganteus --
Cui9203
TACCCAATCCCGACACGGGGAGGTAGTGACAATAAATAACAATATAGGGC
Cui9202
TACCCAATCCCGACACGGGGAGGTAGTGACAATAAATAACAATATAGGGC
TUFC100564Japan --
Russell5913Meripilus_sumstinei --

RP215Meripilus_brasiliensis --
RP200Meripilus_brasiliensis --
JV1712_13J_R_vinctus2_LSUPuert
TACCCAATCCCAATACGGGGAGGTAGTGACAATAAATAACAATATAGGGC
Dai10503R_hypobrunneus --
Dai10569Rigidoporus_hypobrunne --
1DAI19451 --
CM108bRigidoporus_hypobrunneus --
1CUI16874 --
FD299Cerrena_unicolor --
KHL_GB_Cerrena_uniclor --
Dai12892Cerrena_albocinnamomea --
Dai12955C_albocinnamomea --
SNUm03110102C_aurantiopora --
NIBRFG0000102423C_aurantiopora --
Dai7359Antrodiella_zonata --
F20080702KCM29C_consors --
F20080208LYW10Cerrena_consors --
Dai7821Antrodiella_zonata --
CFMR_DCL04_31Pseudolagarobasid --
VPB197Pseudolagarobasidium_bel --
CBS115543Pseudolagarobasidium_ --
CBS115544 --
Han405Pseudolagarobasidium_bai --
Han406Pseudolagarobasidium_bai --
MUcc838Spongipellis_delectans --
BRNM686401S_delectans --
OSM_F925S_delectans --
BRNM67093Spongipellis_litschau --
CFMRccFP59199TS_unicolor --
CFMRccFP71791TS_unicolor --
Dai13845P_lischaueri
TACCCAATCCCGACACGGGGAGGTAGTGACAATAAATAACAATATAGGGC
Dai20266P_lischaueri
TACCCAATCCCGACACGGGGAGGTAGTGACAATAAATAACAATATAGGGC
CFMR_HHB11240Radulodon_america --
RLG6350Radulodon_americanus --
KY415963Radulodon_erikssonii --
HHB9567spRadulodon_casearius --
KRT_Iso_26Radulodon_casearius --
CBS126044Radulodon_erikssonii --
He6183YUNNANENSIS
TACCCAATCCCGACACGGGGAGGTAGTGACAATAAATAACAATACGGGGC
Cui17979YUNNANENSIS

TACCCAATCCCGACACGGGGAGGTAGTGACAATAAATAACAATACGGGGC
Miettinen2091Junghuhnia_fimbri --
KHL12316S_tenue --
PRM846564S_pachyodon --
SP_Lgt_S_pachyodon --
Ryvarden44669Tyromyces_xuchile --
PW17_171sinuosus --
W53Dai12234 --
HHB4100SpAntella_americana --
W3Dai20901spumeus
TACCCAATCCCGACACGGGGAGGTAGTGACAATAAATAACAATATAGGGC
He6736 --
BRNM734877S_spumeus --
BRNM712630S_spumeus --
Dai1723Loweomyces_sibiricus --
W54Cui10009
TACCCAATCCCGACACGGGGAGGTAGTGACAATAAATAACAATATAGGGC
W1Dai20899
TACCCAATCCCGACACGGGGAGGTAGTGACAATAAATAACAATATAGGGC
HHB13445Trametes_ochracea --
Dai16222 --
Dai16240 --

JV1310_11SanguinolentusCernys
TCTTTTGGGTCTTATAATTGGAATGAGTACAATTTAAATCCCTTAACGAG
MJ39_00_SK --
MJ111_04_CZ
TCTTTTGGGTCTTATAATTGGAATGAGTACAATTTAAATCCCTTAACGAG
JV1610_BOKYsmrk
TCTTTTGGGTCTTATAATTGGAATGAGTACAATTTAAATCCCTTAACGAG
Dai21030 --
Dai20976P_furcatus
TCTTTTGGGTCTTATAATTGGAATGAGTACAATTTAAATCCCTTAACGAG
Dai2105 --
Dai2544 --
Dai11313 --
WCG1611Dai26167
CCTTTCGGGTCTTATAATTGGAATGAGTACAATTTAAATCCCTTAACGAG
WCG1518Dai25999Physisporinus
TCTTTCGGGTCTTATAATTGGAATGAGTACAATTTAAATCCCTTAACGAG
TAA15097 --
JV8909_19_CZ --
JV1310_15_P_sanguinolentus2_CZ --
MJ53_02_CZ

CCTTTTGGGTCCTGTAATTGGAATGAGTACAATTTAAATACCTTAACGAG
CLZhao21647P_yunnanensis ---
CLZhao21583P_yunnanensis ---
Dai22272
TCTTTCGGGTCTTATAATTGGAATGAGTACAATTTAAATCCCTTAACGAG
Dai22279
TCTTTCGGGTCTTATAATTGGAATGAGTACAATTTAAATCCCTTAACGAG
MJ332_94_CZ ---
MJ642_94_CZ_Expallescens ---
Dai21060P_vinctus
CCTTTCGGGTCTTATAATTGGAATGAGTACAATTTAAATCCCTTAACGAG
JV0511_23LRP_pouzarii
TCTTTCGGGTCTTATAATTGGAATGAGTACAATTTAAATCCCTTAACGAG
JQ409462_R_pouzarii_PRM899856_ ---
JV0308_66_WA
CCTTTCGGGTCTTATAATTGGAATGAGTACAATTTAAATCCCTTAACGAG
JV0309_45_WA_USA
CCTTTCGGGTCTTATAATTGGAATGAGTACAATTTAAATCCCTTAACGAG
JV0709_83_CA_USA
CCTTTCGGGTCTTATAATTGGAATGAGTACAATTTAAATCCCTTAACGAG
Dai21043P_pouzarii
TCTTTCGGGTCTTATAATTGGAATGAGTACAATTTAAATCCCTTAACGAG
MJ144_95_CZ ---
JV0909_3_CZ
TCTTTTGGGTCTTATAATTGGAATGAGTACAATTTAAATCCCTTAACGAG
JV0609_1_K ---
Dai20396Physisporinus_castanop
TCTTTCGGGTCTTATAATTGGAATGAGTACAATTTAAATCCCTTAACGAG
Dai20397Physisporinus_castanop
TCTTTCGGGTCTTATAATTGGAATGAGTACAATTTAAATCCCTTAACGAG
MJ19_09_SK_Abies ---
JV0509_40_J_TN_USA_Betula
CCTTTCGGGTCTTATAATTGGAATGAGTACAATTTAAATCCCTTAACGAG
JV0808_33crocatus_PA_USAlist
CCTTTCGGGTCTTATAATTGGAATGAGTACAATTTAAATCCCTTAACGAG
DLL2009_061P_crocatus ---
Dai12800P_subcrocatus ---
Dai15917P_subcrocatus
CCTTTCGGGTCTTATAATTGGAATGAGTACAATTTAAATCCCTTAACGAG
Cui16620 ---
HCFC1088Meripilus_stillicidior ---
MCW590Meripilus_obsrcurus ---
MCW722Meripilus_obsrcurus ---
Cui9381P_tibeticus

TCTTTCGGGTCTTATAATTGGAATGAGTACAATTTAAATCCCTTAACGAG
Cui9588P_tibeticus
TCTTTCGGGTCTTATAATTGGAATGAGTACAATTTAAATCCCTTAACGAG
Va2_Beneschova --
CWU3874_Ukraine_Alnus --
WCG1293Dai24718Physisporinus --
WCG1268Dai24682A
TCTTTCGGGTCTTATAATTGGAATGAGTACAATTTAAATCCCTTAACGAG
WCG1269Dai24683A
TCTTTCGGGTCTTATAATTGGAATGAGTACAATTTAAATCCCTTAACGAG
WCG1279Dai24694A
TCTTTCGGGTCTTATAATTGGAATGAGTACAATTTAAATCCCTTAACGAG
Dai16971 --
ZQY1043Dai26696 --
Doll880
TCTTTCGGGTCTTATAATTGGAATGAGTACAATTTAAATCCCTTAACGAG
Doll1000
TCTTTCGGGTCTTATAATTGGAATGAGTACAATTTAAATCCCTTAACGAG
1DAI18529
TCTTTCGGGTCTTATAATTGGAATGAGTACAATTTAAATCCCTTAACGAG
Dai19535
TCTTTCGGGTCTTATAATTGGAATGAGTACAATTTAAATCCCTTAACGAG
1704_79_hnedyVillaLaPaz
TCTTTCGGGTCTTATAATTGGAATGAGTACAATTTAAATCCCTTAACGAG
F2061 --
1DAI18268 --
1DAI18540A
TCTTTCGGGTCTTATAATTGGAATGAGTACAATTTAAATCCCTTAACGAG
Dai17695
TCTTTCGGGTCTTATAATTGGAATGAGTACAATTTAAATCCCTTAACGAG
LKY18Dai26373 --
Dai17839P_sulphureus
TCTTTCGGGTCTTATAATTGGAATGAGTACAATTTAAATCCCTTAACGAG
Dai17841P_sulphureus
TCTTTCGGGTCTTATAATTGGAATGAGTACAATTTAAATCCCTTAACGAG
Dai19877P_roseus
TCTTTCGGGTCTTATAATTGGAATGAGTACAATTTAAATCCCTTAACGAG
1508_18_1_Kout --
KP859303_R_vinctus_RP185_BRAZI --
JK1807_15Rigidoporus_sp_Puerto
TCTTTCGGGTCTTATAATTGGAATGAGTACAATTTAAATCCCTTAACGAG
JV0509_47_J_TN_USA
TCTTTTGGGTCTTATAATTGGAATGAGTACAATTTAAATCCCTTAACGAG
JV0709_188 --

JV0509_127_PA_USA
TCTTTTGGGTCTTATAATTGGAATGAGTACAATTTAAATCCCTTAACGAG
JV1009_59_NJ_USA
TCTTTTGGGTCTTATAATTGGAATGAGTACAATTTAAATCCCTTAACGAG
Dai15497P_crataegi
TCTTTTGGGTCTTATAATTGGAATGAGTACAATTTAAATCCCTTAACGAG
Dai15499P_crataegi
TCTTTTGGGTCTTATAATTGGAATGAGTACAATTTAAATCCCTTAACGAG
Cui3266P_cinereus --
WCG1256Dai24690
CCTTTCGGGTCTTATAATTGGAATGAGTACAATTTAAATCCCTTAACGAG
1DAI17581 --
WCG1255Dai24688
CCTTTCGGGTCTTATAATTGGAATGAGTACAATTTAAATCCCTTAACGAG
Dai22427 --
MV690Meripilus_concrescens --
MV513Meripilus_galapagensis --
Dai19793
TCTTTCGGGTCTTATAATTGGAATGAGTACAATTTAAATCCCTTAACGAG
OQ553780P_tamilnaduensis --
OQ553779P_tamilnaduensis --
A164FB3Meripilus_giganteus --
JV1407_36_Vinctus_Meandrica
TCTTTCGGGTCTTATAATTGGAATGAGTACAATTTAAATCCCTTAACGAG
1807_3K_Rigidoporus_PuertoRico
TCTTTCGGGTCTTATAATTGGAATGAGTACAATTTAAATCCCTTAACGAG
Cui16903P_vinctus
TCTTTCGGGTCTTATAATTGGAATGAGTACAATTTAAATCCCTTAACGAG
JV1008_18R_Lineatus
TCTTTCGGGTCTTATAATTGGAATGAGTACAATTTAAATCCCTTAACGAG
JV1407_37_1_Vinctus_Carara
TCTTTCGGGTCTTATAATTGGAATGAGTACAATTTAAATCCCTTAACGAG
Dai17986P_lineatus
TCTTTCGGGTCTTATAATTGGAATGAGTACAATTTAAATCCCTTAACGAG
Dai18281
TCTTTCGGGTCTTATAATTGGAATGAGTACAATTTAAATCCCTTAACGAG
1DAI19796
TCTTTCGGGTCTTATAATTGGAATGAGTACAATTTAAATCCCTTAACGAG
ZQY797Dai25241
TCTTTCGGGTCTTATAATTGGAATGAGTACAATTTAAATCCCTTAACGAG
WCG1289Dai24711 --
Dai22598
TCTTTCGGGTCTTATAATTGGAATGAGTACAATTTAAATCCCTTAACGAG
Dai20523 --

Dai17885 ---
Dai17553
TCTTTCGGGTCTTATAATTGGAATGAGTACAATTTAAATCCCTTAACGAG
Dai19639
TCTTTCGGGTCTTATAATTGGAATGAGTACAATTTAAATCCCTTAACGAG
JV0110_48_CZ
TCTTTCGGGTCTTATAATTGGAATGAGTACAATTTAAATCCCTTAACGAG
MJ129_04 ---
Cui10340P_eminens ---
Cui10341P_eminens
TCTTTCGGGTCTTATAATTGGAATGAGTACAATTTAAATCCCTTAACGAG
Dai12685P_eminens
TCTTTCGGGTCTTATAATTGGAATGAGTACAATTTAAATCCCTTAACGAG
Miettinen_13591Rigidoporus_und ---
Dai20868
TCTTTCGGGTCTTATAATTGGAATGAGTACAATTTAAATCCCTTAACGAG
Dai20832
TCTTTCGGGTCTTATAATTGGAATGAGTACAATTTAAATCCCTTAACGAG
Dai11400
TCTTTCGGGTCTTATAATTGGAATGAGTACAATTTAAATCCCTTAACGAG
Dai22472
TCTTTCGGGTCTTATAATTGGAATGAGTACAATTTAAATCCCTTAACGAG
1CUI10475 ---
1CUI10491 ---
HCFC1095Meripilus_robledoi ---
MCW702Meripilus_revolubilis ---
1704_83_zluty_HaciendaBaru
TCTTTTGGGTCTTATAATTGGAATGAGTACAATTTAAATCCCTTAACGAG
Dai9925P_lavendulus ---
Dai13587AP_lavendulus
TCTTTTGGGTCTTATAATTGGAATGAGTACAATTTAAATCCCTTAACGAG
PDD70600P_longicystidius ---
Cui16630 ---
FP_135344Meripilus_giganteus ---
FP_100460_Sp ---
CBS421_48Meripilus_giganteus ---
Cui9203
TCTTTCGGGTCTTATAATTGGAATGAGTACAATTTAAATCCCTTAACGAG
Cui9202
TCTTTCGGGTCTTATAATTGGAATGAGTACAATTTAAATCCCTTAACGAG
TUFC100564Japan ---
Russell5913Meripilus_sumstinei ---
RP215Meripilus_brasiliensis ---
RP200Meripilus_brasiliensis ---

JV1712_13J_R_vinctus2_LSUPuert
TCTTTCGGGTCTTATAATTGGAATGAGTACAATTTAAATCCCTTAACGAG
Dai10503R_hypobrunneus --
Dai10569Rigidoporus_hypobrunne --
1DAI19451 --
CM108bRigidoporus_hypobrunneus --
1CUI16874 --
FD299Cerrena_unicolor --
KHL_GB_Cerrena_uniclor --
Dai12892Cerrena_albocinnamomea --
Dai12955C_albocinnamomea --
SNUm03110102C_aurantiopora --
NIBRFG0000102423C_aurantiopora --
Dai7359Antrodiella_zonata --
F20080702KCM29C_consors --
F20080208LYW10Cerrena_consors --
Dai7821Antrodiella_zonata --
CFMR_DCL04_31Pseudolagarobasid --
VPB197Pseudolagarobasidium_bel --
CBS115543Pseudolagarobasidium_ --
CBS115544 --
Han405Pseudolagarobasidium_bai --
Han406Pseudolagarobasidium_bai --
MUcc838Spongipellis_delectans --
BRNM686401S_delectans --
OSM_F925S_delectans --
BRNM67093Spongipellis_litschau --
CFMRccFP59199TS_unicolor --
CFMRccFP71791TS_unicolor --
Dai13845P_lischaueri
TCTTTCGGGTCTTATAATTGGAATGAGTACAATTTAAATCCCTTAACGAG
Dai20266P_lischaueri
TCTTTCGGGTCTTATAATTGGAATGAGTACAATTTAAATCCCTTAACGAG
CFMR_HHB11240Radulodon_america --
RLG6350Radulodon_americanus --
KY415963Radulodon_erikssonii --
HHB9567spRadulodon_casearius --
KRT_Iso_26Radulodon_casearius --
CBS126044Radulodon_erikssonii --
He6183YUNNANENSIS
TCTTTCGGGTCCCGTAATTGGAATGAGTACAATTTAAATCCCTTAACGAG
Cui17979YUNNANENSIS
TCTTTCGGGTCCCGTAATTGGAATGAGTACAATTTAAATCCCTTAACGAG
Miettinen2091Junghuhnia_fimbri --

KHL12316S_tenue --
PRM846564S_pachyodon --
SP_Lgt_S_pachyodon --
Ryvarden44669Tyromyces_xuchile --
PW17_171sinuosus --
W53Dai12234 --
HHB4100SpAntella_americana --
W3Dai20901spumeus
TCTTTCGGGTCTTATAATTGGAATGAGTACAATTTAAATCCCTTAACGAG
He6736 --
BRNM734877S_spumeus --
BRNM712630S_spumeus --
Dai1723Loweomyces_sibiricus --
W54Cui10009
TCTTTCGGGTCTTATAATTGGAATGAGTACAATTTAAATCCCTTAACGAG
W1Dai20899
TCTTTCGGGTCTTATAATTGGAATGAGTACAATTTAAATCCCTTAACGAG
HHB13445Trametes_ochracea --
Dai16222 --
Dai16240 --

JV1310_11SanguinolentusCernys
GAACAATTGGAGGGCAAGTCTGGTGCCAGCAGCCGCGGTAATTCCAGCTC
MJ39_00_SK --
MJ111_04_CZ
GAACAATTGGAGGGCAAGTCTGGTGCCAGCAGCCGCGGTAATTCCAGCTC
JV1610_BOKYsmrk
GAACAATTGGAGGGCAAGTCTGGTGCCAGCAGCCGCGGTAATTCCAGCTC
Dai21030 --
Dai20976P_furcatus
GAACAATTGGAGGGCAAGTCTGGTGCCAGCAGCCGCGGTAATTCCAGCTC
Dai2105 --
Dai2544 --
Dai11313 --
WCG1611Dai26167
GAACAATTGGAGGGCAAGTCTGGTGCCAGCAGCCGCGGTAATTCCAGCTC
WCG1518Dai25999Physisporinus
GAACAATTGGAGGGCAAGTCTGGTGCCAGCAGCCGCGGTAATTCCAGCTC
TAA15097 --
JV8909_19_CZ --
JV1310_15_P_sanguinolentus2_CZ --
MJ53_02_CZ
GAACAATTGGAGGGCAAGTCTGGTGCCAGCAGCCGCGGTAATTCCAGCTC
CLZhao21647P_yunnanensis --

CLZhao21583P_yunnanensis --
Dai22272
GAACAATTGGAGGGCAAGTCTGGTGCCAGCAGCCGCGGTAATTCCAGCTC
Dai22279
GAACAATTGGAGGGCAAGTCTGGTGCCAGCAGCCGCGGTAATTCCAGCTC
MJ332_94_CZ --
MJ642_94_CZ_Expallescens --
Dai21060P_vinctus
GAACAATTGGAGGGCAAGTCTGGTGCCAGCAGCCGCGGTAATTCCAGCTC
JV0511_23LRP_pouzarii
GAACAATTGGAGGGCAAGTCTGGTGCCAGCAGCCGCGGTAATTCCAGCTC
JQ409462_R_pouzarii_PRM899856_ --
JV0308_66_WA
GAACAATTGGAGGGCAAGTCTGGTGCCAGCAGCCGCGGTAATTCCAGCTC
JV0309_45_WA_USA
GAACAATTGGAGGGCAAGTCTGGTGCCAGCAGCCGCGGTAATTCCAGCTC
JV0709_83_CA_USA
GAACAATTGGAGGGCAAGTCTGGTGCCAGCAGCCGCGGTAATTCCAGCTC
Dai21043P_pouzarii
GAACAATTGGAGGGCAAGTCTGGTGCCAGCAGCCGCGGTAATTCCAGCTC
MJ144_95_CZ --
JV0909_3_CZ
GAACAATTGGAGGGCAAGTCTGGTGCCAGCAGCCGCGGTAATTCCAGCTC
JV0609_1_K --
Dai20396Physisporinus_castanop
GAACAATTGGAGGGCAAGTCTGGTGCCAGCAGCCGCGGTAATTCCAGCTC
Dai20397Physisporinus_castanop
GAACAATTGGAGGGCAAGTCTGGTGCCAGCAGCCGCGGTAATTCCAGCTC
MJ19_09_SK_Abies --
JV0509_40_J_TN_USA_Betula
GAACAATTGGAGGGCAAGTCTGGTGCCAGCAGCCGCGGTAATTCCAGCTC
JV0808_33crocatus_PA_USAlist
GAACAATTGGAGGGCAAGTCTGGTGCCAGCAGCCGCGGTAATTCCAGCTC
DLL2009_061P_crocatus --
Dai12800P_subcrocatus --
Dai15917P_subcrocatus
GAACAATTGGAGGGCAAGTCTGGTGCCAGCAGCCGCGGTAATTCCAGCTC
Cui16620 --
HCFC1088Meripilus_stillicidior --
MCW590Meripilus_obsrcurus --
MCW722Meripilus_obsrcurus --
Cui9381P_tibeticus
GAACAATTGGAGGGCAAGTCTGGTGCCAGCAGCCGCGGTAATTCCAGCTC
Cui9588P_tibeticus

GAACAATTGGAGGGCAAGTCTGGTGCCAGCAGCCGCGGTAATTCCAGCTC
Va2_Beneschova --
CWU3874_Ukraine_Alnus --
WCG1293Dai24718Physisporinus --
WCG1268Dai24682A
GAACAATTGGAGGGCAAGTCTGGTGCCAGCAGCCGCGGTAATTCCAGCTC
WCG1269Dai24683A
GAACAATTGGAGGGCAAGTCTGGTGCCAGCAGCCGCGGTAATTCCAGCTC
WCG1279Dai24694A
GAACAATTGGAGGGCAAGTCTGGTGCCAGCAGCCGCGGTAATTCCAGCTC
Dai16971 --
ZQY1043Dai26696 --
Doll880
GAACAATTGGAGGGCAAGTCTGGTGCCAGCAGCCGCGGTAATTCCAGCTC
Doll1000
GAACAATTGGAGGGCAAGTCTGGTGCCAGCAGCCGCGGTAATTCCAGCTC
1DAI18529
GAACAATTGGAGGGCAAGTCTGGTGCCAGCAGCCGCGGTAATTCCAGCTC
Dai19535
GAACAATTGGAGGGCAAGTCTGGTGCCAGCAGCCGCGGTAATTCCAGCTC
1704_79_hnedyVillaLaPaz
GAACAATTGGAGGGCAAGTCTGGTGCCAGCAGCCGCGGTAATTCCAGCTC
F2061 --
1DAI18268 --
1DAI18540A
GAACAATTGGAGGGCAAGTCTGGTGCCAGCAGCCGCGGTAATTCCAGCTC
Dai17695
GAACAATTGGAGGGCAAGTCTGGTGCCAGCAGCCGCGGTAATTCCAGCTC
LKY18Dai26373 --
Dai17839P_sulphureus
GAACAATTGGAGGGCAAGTCTGGTGCCAGCAGCCGCGGTAATTCCAGCTC
Dai17841P_sulphureus
GAACAATTGGAGGGCAAGTCTGGTGCCAGCAGCCGCGGTAATTCCAGCTC
Dai19877P_roseus
GAACAATTGGAGGGCAAGTCTGGTGCCAGCAGCCGCGGTAATTCCAGCTC
1508_18_1_Kout --
KP859303_R_vinctus_RP185_BRAZI --
JK1807_15Rigidoporus_sp_Puerto
GAACAATTGGAGGGCAAGTCTGGTGCCAGCAGCCGCGGTAATTCCAGCTC
JV0509_47_J_TN_USA
GAACAATTGGAGGGCAAGTCTGGTGCCAGCAGCCGCGGTAATTCCAGCTC
JV0709_188 --
JV0509_127_PA_USA
GAACAATTGGAGGGCAAGTCTGGTGCCAGCAGCCGCGGTAATTCCAGCTC

JV1009_59_NJ_USA
GAACAATTGGAGGGCAAGTCTGGTGCCAGCAGCCGCGGTAATTCCAGCTC
Dai15497P_crataegi
GAACAATTGGAGGGCAAGTCTGGTGCCAGCAGCCGCGGTAATTCCAGCTC
Dai15499P_crataegi
GAACAATTGGAGGGCAAGTCTGGTGCCAGCAGCCGCGGTAATTCCAGCTC
Cui3266P_cinereus --
WCG1256Dai24690
GAACAATTGGAGGGCAAGTCTGGTGCCAGCAGCCGCGGTAATTCCAGCTC
1DAI17581 --
WCG1255Dai24688
GAACAATTGGAGGGCAAGTCTGGTGCCAGCAGCCGCGGTAATTCCAGCTC
Dai22427 --
MV690Meripilus_concrescens --
MV513Meripilus_galapagensis --
Dai19793
GAACAATTGGAGGGCAAGTCTGGTGCCAGCAGCCGCGGTAATTCCAGCTC
OQ553780P_tamilnaduensis --
OQ553779P_tamilnaduensis --
A164FB3Meripilus_giganteus --
JV1407_36_Vinctus_Meandrica
GAACAATTGGAGGGCAAGTCTGGTGCCAGCAGCCGCGGTAATTCCAGCTC
1807_3K_Rigidoporus_PuertoRico
GAACAATTGGAGGGCAAGTCTGGTGCCAGCAGCCGCGGTAATTCCAGCTC
Cui16903P_vinctus
GAACAATTGGAGGGCAAGTCTGGTGCCAGCAGCCGCGGTAATTCCAGCTC
JV1008_18R_Lineatus
GAACAATTGGAGGGCAAGTCTGGTGCCAGCAGCCGCGGTAATTCCAGCTC
JV1407_37_1_Vinctus_Carara
GAACAATTGGAGGGCAAGTCTGGTGCCAGCAGCCGCGGTAATTCCAGCTC
Dai17986P_lineatus
GAACAATTGGAGGGCAAGTCTGGTGCCAGCAGCCGCGGTAATTCCAGCTC
Dai18281
GAACAATTGGAGGGCAAGTCTGGTGCCAGCAGCCGCGGTAATTCCAGCTC
1DAI19796
GAACAATTGGAGGGCAAGTCTGGTGCCAGCAGCCGCGGTAATTCCAGCTC
ZQY797Dai25241
GAACAATTGGAGGGCAAGTCTGGTGCCAGCAGCCGCGGTAATTCCAGCTC
WCG1289Dai24711 --
Dai22598
GAACAATTGGAGGGCAAGTCTGGTGCCAGCAGCCGCGGTAATTCCAGCTC
Dai20523 --
Dai17885 --
Dai17553

GAACAATTGGAGGGCAAGTCTGGTGCCAGCAGCCGCGGTAATTCCAGCTC
Dai19639
GAACAATTGGAGGGCAAGTCTGGTGCCAGCAGCCGCGGTAATTCCAGCTC
JV0110_48_CZ
GAACAATTGGAGGGCAAGTCTGGTGCCAGCAGCCGCGGTAATTCCAGCTC
MJ129_04 --
Cui10340P_eminens --
Cui10341P_eminens
GAACAATTGGAGGGCAAGTCTGGTGCCAGCAGCCGCGGTAATTCCAGCTC
Dai12685P_eminens
GAACAATTGGAGGGCAAGTCTGGTGCCAGCAGCCGCGGTAATTCCAGCTC
Miettinen_13591Rigidoporus_und --
Dai20868
GAACAATTGGAGGGCAAGTCTGGTGCCAGCAGCCGCGGTAATTCCAGCTC
Dai20832
GAACAATTGGAGGGCAAGTCTGGTGCCAGCAGCCGCGGTAATTCCAGCTC
Dai11400
GAACAATTGGAGGGCAAGTCTGGTGCCAGCAGCCGCGGTAATTCCAGCTC
Dai22472
GAACAATTGGAGGGCAAGTCTGGTGCCAGCAGCCGCGGTAATTCCAGCTC
1CUI10475 --
1CUI10491 --
HCFC1095Meripilus_robledoi --
MCW702Meripilus_revolubilis --
1704_83_zluty_HaciendaBaru
GAACAATTGGAGGGCAAGTCTGGTGCCAGCAGCCGCGGTAATTCCAGCTC
Dai9925P_lavendulus --
Dai13587AP_lavendulus
GAACAATTGGAGGGCAAGTCTGGTGCCAGCAGCCGCGGTAATTCCAGCTC
PDD70600P_longicystidius --
Cui16630 --
FP_135344Meripilus_giganteus --
FP_100460_Sp --
CBS421_48Meripilus_giganteus --
Cui9203
GAACAATTGGAGGGCAAGTCTGGTGCCAGCAGCCGCGGTAATTCCAGCTC
Cui9202
GAACAATTGGAGGGCAAGTCTGGTGCCAGCAGCCGCGGTAATTCCAGCTC
TUFC100564Japan --
Russell5913Meripilus_sumstinei --
RP215Meripilus_brasiliensis --
RP200Meripilus_brasiliensis --
JV1712_13J_R_vinctus2_LSUPuert
GAACAATTGGAGGGCAAGTCTGGTGCCAGCAGCCGCGGTAATTCCAGCTC

Dai10503R_hypobrunneus --
Dai10569Rigidoporus_hypobrunne --
1DAI19451 --
CM108bRigidoporus_hypobrunneus --
1CUI16874 --
FD299Cerrena_unicolor --
KHL_GB_Cerrena_uniclor --
Dai12892Cerrena_albocinnamomea --
Dai12955C_albocinnamomea --
SNUm03110102C_aurantiopora --
NIBRFG0000102423C_aurantiopora --
Dai7359Antrodiella_zonata --
F20080702KCM29C_consors --
F20080208LYW10Cerrena_consors --
Dai7821Antrodiella_zonata --
CFMR_DCL04_31Pseudolagarobasid --
VPB197Pseudolagarobasidium_bel --
CBS115543Pseudolagarobasidium_ --
CBS115544 --
Han405Pseudolagarobasidium_bai --
Han406Pseudolagarobasidium_bai --
MUcc838Spongipellis_delectans --
BRNM686401S_delectans --
OSM_F925S_delectans --
BRNM67093Spongipellis_litschau --
CFMRccFP59199TS_unicolor --
CFMRccFP71791TS_unicolor --
Dai13845P_lischaueri
GAACAATTGGAGGGCAAGTCTGGTGCCAGCAGCCGCGGTAATTCCAGCTC
Dai20266P_lischaueri
GAACAATTGGAGGGCAAGTCTGGTGCCAGCAGCCGCGGTAATTCCAGCTC
CFMR_HHB11240Radulodon_america --
RLG6350Radulodon_americanus --
KY415963Radulodon_erikssonii --
HHB9567spRadulodon_casearius --
KRT_Iso_26Radulodon_casearius --
CBS126044Radulodon_erikssonii --
He6183YUNNANENSIS
GAACAATTGGAGGGCAAGTCTGGTGCCAGCAGCCGCGGTAATTCCAGCTC
Cui17979YUNNANENSIS
GAACAATTGGAGGGCAAGTCTGGTGCCAGCAGCCGCGGTAATTCCAGCTC
Miettinen2091Junghuhnia_fimbri --
KHL12316S_tenue --
PRM846564S_pachyodon --

SP_Lgt_S_pachyodon --
Ryvarden44669Tyromyces_xuchile --
PW17_171sinuosus --
W53Dai12234 --
HHB4100SpAntella_americana --
W3Dai20901spumeus
GAACAATTGGAGGGCAAGTCTGGTGCCAGCAGCCGCGGTAATTCCAGCTC
He6736 --
BRNM734877S_spumeus --
BRNM712630S_spumeus --
Dai1723Loweomyces_sibiricus --
W54Cui10009
GAACAATTGGAGGGCAAGTCTGGTGCCAGCAGCCGCGGTAATTCCAGCTC
W1Dai20899
GAACAATTGGAGGGCAAGTCTGGTGCCAGCAGCCGCGGTAATTCCAGCTC
HHB13445Trametes_ochracea --
Dai16222 --
Dai16240 --

JV1310_11SanguinolentusCernys
CAATAGCGTATATTAAAGTTGTTGCAGTTAAAAAGCTCGTAGTTGAACTT
MJ39_00_SK --
MJ111_04_CZ
CAATAGCGTATATTAAAGTTGTTGCAGTTAAAAAGCTCGTAGTTGAACTT
JV1610_BOKYsmrk
CAATAGCGTATATTAAAGTTGTTGCAGTTAAAAAGCTCGTAGTTGAACTT
Dai21030 --
Dai20976P_furcatus
CAATAGCGTATATTAAAGTTGTTGCAGTTAAAAAGCTCGTAGTTGAACTT
Dai2105 --
Dai2544 --
Dai11313 --
WCG1611Dai26167
CAATAGCGTATATTAAAGTTGTTGCAGTTAAAAAGCTCGTAGTTGAACTT
WCG1518Dai25999Physisporinus
CAATAGCGTATATTAAAGTTGTTGCAGTTAAAAAGCTCGTAGTTGAACTT
TAA15097 --
JV8909_19_CZ --
JV1310_15_P_sanguinolentus2_CZ --
MJ53_02_CZ
CAATAGCGTATATTAAAGTTGCTGCAGTTAAAAAGCCCGTAGTCGAACTT
CLZhao21647P_yunnanensis --
CLZhao21583P_yunnanensis --
Dai22272

CAATAGCGTATATTAAAGTTGTTGCAGTTAAAAAGCTCGTAGTTGAACTT
Dai22279
CAATAGCGTATATTAAAGTTGTTGCAGTTAAAAAGCTCGTAGTTGAACTT
MJ332_94_CZ --
MJ642_94_CZ_Expallescens --
Dai21060P_vinctus
CAATAGCGTATATTAAAGTTGTTGCAGTTAAAAAGCTCGTAGTTGAACTT
JV0511_23LRP_pouzarii
CAATAGCGTATATTAAAGTTGTTGCAGTTAAAAAGCTCGTAGTTGAACTT
JQ409462_R_pouzarii_PRM899856_ --
JV0308_66_WA
CAATAGCGTATATTAAAGTTGTTGCAGTTAAAAAGCTCGTAGTTGAACTT
JV0309_45_WA_USA
CAATAGCGTATATTAAAGTTGTTGCAGTTAAAAAGCTCGTAGTTGAACTT
JV0709_83_CA_USA
CAATAGCGTATATTAAAGTTGTTGCAGTTAAAAAGCTCGTAGTTGAACTT
Dai21043P_pouzarii
CAATAGCGTATATTAAAGTTGTTGCAGTTAAAAAGCTCGTAGTTGAACTT
MJ144_95_CZ --
JV0909_3_CZ
CAATAGCGTATATTAAAGTTGTTGCAGTTAAAAAGCTCGTAGTTGAACTT
JV0609_1_K --
Dai20396Physisporinus_castanop
CAATAGCGTATATTAAAGTTGTTGCAGTTAAAAAGCTCGTAGTTGAACTT
Dai20397Physisporinus_castanop
CAATAGCGTATATTAAAGTTGTTGCAGTTAAAAAGCTCGTAGTTGAACTT
MJ19_09_SK_Abies --
JV0509_40_J_TN_USA_Betula
CAATAGCGTATATTAAAGTTGTTGCAGTTAAAAAGCTCGTAGTTGAACTT
JV0808_33crocatus_PA_USAlist
CAATAGCGTATATTAAAGTTGTTGCAGTTAAAAAGCTCGTAGTTGAACTT
DLL2009_061P_crocatus --
Dai12800P_subcrocatus --
Dai15917P_subcrocatus
CAATAGCGTATATTAAAGTTGTTGCAGTTAAAAAGCTCGTAGTTGAACTT
Cui16620 --
HCFC1088Meripilus_stillicidior --
MCW590Meripilus_obsrcurus --
MCW722Meripilus_obsrcurus --
Cui9381P_tibeticus
CAATAGCGTATATTAAAGTTGTTGCAGTTAAAAAGCTCGTAGTTGAACTT
Cui9588P_tibeticus
CAATAGCGTATATTAAAGTTGTTGCAGTTAAAAAGCTCGTAGTTGAACTT
Va2_Beneschova --

CWU3874_Ukraine_Alnus --
WCG1293Dai24718Physisporinus --
WCG1268Dai24682A
CAATAGCGTATATTAAAGTTGTTGCAGTTAAAAAGCTCGTAGTTGAACTT
WCG1269Dai24683A
CAATAGCGTATATTAAAGTTGTTGCAGTTAAAAAGCTCGTAGTTGAACTT
WCG1279Dai24694A
CAATAGCGTATATTAAAGTTGTTGCAGTTAAAAAGCTCGTAGTTGAACTT
Dai16971 --
ZQY1043Dai26696 --
Doll880
CAATAGCGTATATTAAAGTTGTTGCAGTTAAAAAGCTCGTAGTTGAACTT
Doll1000
CAATAGCGTATATTAAAGTTGTTGCAGTTAAAAAGCTCGTAGTTGAACTT
1DAI18529
CAATAGCGTATATTAAAGTTGTTGCAGTTAAAAAGCTCGTAGTTGAACTT
Dai19535
CAATAGCGTATATTAAAGTTGTTGCAGTTAAAAAGCTCGTAGTTGAACTT
1704_79_hnedyVillaLaPaz
CAATAGCGTATATTAAAGTTGTTGCAGTTAAAAAGCTCGTAGTTGAACTT
F2061 --
1DAI18268 --
1DAI18540A
CAATAGCGTATATTAAAGTTGTTGCAGTTAAAAAGCTCGTAGTTGAACTT
Dai17695
CAATAGCGTATATTAAAGTTGTTGCAGTTAAAAAGCTCGTAGTTGAACTT
LKY18Dai26373 --
Dai17839P_sulphureus
CAATAGCGTATATTAAAGTTGTTGCAGTTAAAAAGCTCGTAGTTGAACTT
Dai17841P_sulphureus
CAATAGCGTATATTAAAGTTGTTGCAGTTAAAAAGCTCGTAGTTGAACTT
Dai19877P_roseus
CAATAGCGTATATTAAAGTTGTTGCAGTTAAAAAGCTCGTAGTTGAACTT
1508_18_1_Kout --
KP859303_R_vinctus_RP185_BRAZI --
JK1807_15Rigidoporus_sp_Puerto
CAATAGCGTATATTAAAGTTGTTGCAGTTAAAAAGCTCGTAGTTGAACTT
JV0509_47_J_TN_USA
CAATAGCGTATATTAAAGTTGTTGCAGTTAAAAAGCTCGTAGTTGAACTT
JV0709_188 --
JV0509_127_PA_USA
CAATAGCGTATATTAAAGTTGTTGCAGTTAAAAAGCTCGTAGTTGAACTT
JV1009_59_NJ_USA
CAATAGCGTATATTAAAGTTGTTGCAGTTAAAAAGCTCGTAGTTGAACTT

Dai15497P_crataegi
CAATAGCGTATATTAAAGTTGTTGCAGTTAAAAAGCTCGTAGTTGAACTT
Dai15499P_crataegi
CAATAGCGTATATTAAAGTTGTTGCAGTTAAAAAGCTCGTAGTTGAACTT
Cui3266P_cinereus --
WCG1256Dai24690
CAATAGCGTATATTAAAGTTGTTGCAGTTAAAAAGCTCGTAGTTGAACTT
1DAI17581 --
WCG1255Dai24688
CAATAGCGTATATTAAAGTTGTTGCAGTTAAAAAGCTCGTAGTTGAACTT
Dai22427 --
MV690Meripilus_concrescens --
MV513Meripilus_galapagensis --
Dai19793
CAATAGCGTATATTAAAGTTGTTGCAGTTAAAAAGCTCGTAGTTGAACTT
OQ553780P_tamilnaduensis --
OQ553779P_tamilnaduensis --
A164FB3Meripilus_giganteus --
JV1407_36_Vinctus_Meandrica
CAATAGCGTATATTAAAGTTGTTGCAGTTAAAAAGCTCGTAGTTGAACTT
1807_3K_Rigidoporus_PuertoRico
CAATAGCGTATATTAAAGTTGTTGCAGTTAAAAAGCTCGTAGTTGAACTT
Cui16903P_vinctus
CAATAGCGTATATTAAAGTTGTTGCAGTTAAAAAGCTCGTAGTTGAACTT
JV1008_18R_Lineatus
CAATAGCGTATATTAAAGTTGTTGCAGTTAAAAAGCTCGTAGTTGAACTT
JV1407_37_1_Vinctus_Carara
CAATAGCGTATATTAAAGTTGTTGCAGTTAAAAAGCTCGTAGTTGAACTT
Dai17986P_lineatus
CAATAGCGTATATTAAAGTTGTTGCAGTTAAAAAGCTCGTAGTTGAACTT
Dai18281
CAATAGCGTATATTAAAGTTGTTGCAGTTAAAAAGCTCGTAGTTGAACTT
1DAI19796
CAATAGCGTATATTAAAGTTGTTGCAGTTAAAAAGCTCGTAGTTGAACTT
ZQY797Dai25241
CAATAGCGTATATTAAAGTTGTTGCAGTTAAAAAGCTCGTAGTTGAACTT
WCG1289Dai24711 --
Dai22598
CAATAGCGTATATTAAAGTTGTTGCAGTTAAAAAGCTCGTAGTTGAACTT
Dai20523 --
Dai17885 --
Dai17553
CAATAGCGTATATTAAAGTTGTTGCAGTTAAAAAGCTCGTAGTTGAACTT
Dai19639

CAATAGCGTATATTAAAGTTGTTGCAGTTAAAAAGCTCGTAGTTGAACTT
JV0110_48_CZ
CAATAGCGTATATTAAAGTTGTTGCAGTTAAAAAGCTCGTAGTTGAACTT
MJ129_04 --
Cui10340P_eminens --
Cui10341P_eminens
CAATAGCGTATATTAAAGTTGTTGCAGTTAAAAAGCTCGTAGTTGAACTT
Dai12685P_eminens
CAATAGCGTATATTAAAGTTGTTGCAGTTAAAAAGCTCGTAGTTGAACTT
Miettinen_13591Rigidoporus_und --
Dai20868
CAATAGCGTATATTAAAGTTGTTGCAGTTAAAAAGCTCGTAGTTGAACTT
Dai20832
CAATAGCGTATATTAAAGTTGTTGCAGTTAAAAAGCTCGTAGTTGAACTT
Dai11400
CAATAGCGTATATTAAAGTTGTTGCAGTTAAAAAGCTCGTAGTTGAACTT
Dai22472
CAATAGCGTATATTAAAGTTGTTGCAGTTAAAAAGCTCGTAGTTGAACTT
1CUI10475 --
1CUI10491 --
HCFC1095Meripilus_robledoi --
MCW702Meripilus_revolubilis --
1704_83_zluty_HaciendaBaru
CAATAGCGTATATTAAAGTTGTTGCAGTTAAAAAGCTCGTAGTTGAACTT
Dai9925P_lavendulus --
Dai13587AP_lavendulus
CAATAGCGTATATTAAAGTTGTTGCAGTTAAAAAGCTCGTAGTTGAACTT
PDD70600P_longicystidius --
Cui16630 --
FP_135344Meripilus_giganteus --
FP_100460_Sp --
CBS421_48Meripilus_giganteus --
Cui9203
CAATAGCGTATATTAAAGTTGTTGCAGTTAAAAAGCTCGTAGTTGAACTT
Cui9202
CAATAGCGTATATTAAAGTTGTTGCAGTTAAAAAGCTCGTAGTTGAACTT
TUFC100564Japan --
Russell5913Meripilus_sumstinei --
RP215Meripilus_brasiliensis --
RP200Meripilus_brasiliensis --
JV1712_13J_R_vinctus2_LSUPuert
CAATAGCGTATATTAAAGTTGTTGCAGTTAAAAAGCTCGTAGTTGAACTT
Dai10503R_hypobrunneus --
Dai10569Rigidoporus_hypobrunne --

1DAI19451 --
CM108bRigidoporus_hypobrunneus --
1CUI16874 --
FD299Cerrena_unicolor --
KHL_GB_Cerrena_uniclor --
Dai12892Cerrena_albocinnamomea --
Dai12955C_albocinnamomea --
SNUm03110102C_aurantiopora --
NIBRFG0000102423C_aurantiopora --
Dai7359Antrodiella_zonata --
F20080702KCM29C_consors --
F20080208LYW10Cerrena_consors --
Dai7821Antrodiella_zonata --
CFMR_DCL04_31Pseudolagarobasid --
VPB197Pseudolagarobasidium_bel --
CBS115543Pseudolagarobasidium_ --
CBS115544 --
Han405Pseudolagarobasidium_bai --
Han406Pseudolagarobasidium_bai --
MUcc838Spongipellis_delectans --
BRNM686401S_delectans --
OSM_F925S_delectans --
BRNM67093Spongipellis_litschau --
CFMRccFP59199TS_unicolor --
CFMRccFP71791TS_unicolor --
Dai13845P_lischaueri
CAATAGCGTATATTAAAGTTGTTGCAGTTAAAAAGCTCGTAGTTGAACTT
Dai20266P_lischaueri
CAATAGCGTATATTAAAGTTGTTGCAGTTAAAAAGCTCGTAGTTGAACTT
CFMR_HHB11240Radulodon_america --
RLG6350Radulodon_americanus --
KY415963Radulodon_erikssonii --
HHB9567spRadulodon_casearius --
KRT_Iso_26Radulodon_casearius --
CBS126044Radulodon_erikssonii --
He6183YUNNANENSIS
CAATAGCGTATATTAAAGTTGTTGCAGTTAAAAAGCTCGTAGTTGAACCT
Cui17979YUNNANENSIS
CAATAGCGTATATTAAAGTTGTTGCAGTTAAAAAGCTCGTAGTTGAACCT
Miettinen2091Junghuhnia_fimbri --
KHL12316S_tenue --
PRM846564S_pachyodon --
SP_Lgt_S_pachyodon --
Ryvarden44669Tyromyces_xuchile --

PW17_171sinuosus --
W53Dai12234 --
HHB4100SpAntella_americana --
W3Dai20901spumeus
CAATAGCGTATATTAAAGTTGTTGCAGTTAAAAAGCTCGTAGTTGAACTT
He6736 --
BRNM734877S_spumeus --
BRNM712630S_spumeus --
Dai1723Loweomyces_sibiricus --
W54Cui10009
CAATAGCGTATATTAAAGTTGTTGCAGTTAAAAAGCTCGTAGTTGAACTT
W1Dai20899
CAATAGCGTATATTAAAGTTGTTGCAGTTAAAAAGCTCGTAGTTGAACTT
HHB13445Trametes_ochracea --
Dai16222 --
Dai16240 --

JV1310_11SanguinolentusCernys
CAGGCCTGGCCGGGTGGTCTGCCTCACGGTATGCACTGTCTGGCTGGGTC
MJ39_00_SK --
MJ111_04_CZ
CAGGCCTGGCCGGGTGGTCTGCCTCACGGTATGCACTGTCTGGCTGGGTC
JV1610_BOKYsmrk
CAGGCCTGGCCGGGTGGTCTGCCTCACGGTATGCACTGTCTGGCTGGGTC
Dai21030 --
Dai20976P_furcatus
CAGGCCTGGCCGGGTGGTCTGCCTCACGGTATGCACTGTCTGGCTGGGTC
Dai2105 --
Dai2544 --
Dai11313 --
WCG1611Dai26167
CAGGCCTGGCCGGGCGGTCTGCCTCACGGTATGCACTGTCTGGCTGGGTC
WCG1518Dai25999Physisporinus
CAGGCCTGGCCGGGCGGTCTGCCTCACGGTATGCACTGTCTGGCTGGGTC
TAA15097 --
JV8909_19_CZ --
JV1310_15_P_sanguinolentus2_CZ --
MJ53_02_CZ
CAGACCTGGCCGGGCGGTCTGCCTCACGGTACGTACTGTCCGGCTGGGCC
CLZhao21647P_yunnanensis --
CLZhao21583P_yunnanensis --
Dai22272
CAGGCCTGGCCGGGTGGTCTGCCTCACGGTATGCACTGTCTGGCTGGGTC
Dai22279

CAGGCCTGGCCGGGTGGTCTGCCTCACGGTATGCACTGTCTGGCTGGGTC
MJ332_94_CZ ---
MJ642_94_CZ_Expallescens ---
Dai21060P_vinctus
CAGGCCTGGCCGGGTGGTCTGCCTCACGGTATGCACTGTCTGGCTGGGTC
JV0511_23LRP_pouzarii
CAGGCCTGGCCGGGTGGTCTGCCTCACGGTATGCACTGTCTGGCTGGGTC
JQ409462_R_pouzarii_PRM899856_ ---
JV0308_66_WA
CAGGCCTGGCCGGGTGGTCTGCCTCACGGTATGCACTGTCTGGCTGGGTC
JV0309_45_WA_USA
CAGGCCTGGCCGGGTGGTCTGCCTCACGGTATGCACTGTCTGGCTGGGTC
JV0709_83_CA_USA
CAGGCCTGGCCGGGTGGTCTGCCTCACGGTATGCACTGTCTGGCTGGGTC
Dai21043P_pouzarii
CAGGCCTGGCCGGGTGGTCTGCCTCACGGTATGCACTGTCTGGCTGGGTC
MJ144_95_CZ ---
JV0909_3_CZ
CAGACCTGGCCGGGCGGTCCGCCTAACGGTGTGTACTGTCTGGCTGGGCC
JV0609_1_K ---
Dai20396Physisporinus_castanop
CAGGCCTGGCCGGGTGGTCTGCCTCACGGTATGCACTGTCTGGCTGGGTC
Dai20397Physisporinus_castanop
CAGGCCTGGCCGGGTGGTCTGCCTCACGGTATGCACTGTCTGGCTGGGTC
MJ19_09_SK_Abies ---
JV0509_40_J_TN_USA_Betula
CAGGCCTGGCCGGGTGGTCTGCCTCACGGTATGCACTGTCTGGCTGGGTC
JV0808_33crocatus_PA_USAlist
CAGGCCTGGCCGGGTGGTCTGCCTCACGGTATGCACTGTCTGGCTGGGTC
DLL2009_061P_crocatus ---
Dai12800P_subcrocatus ---
Dai15917P_subcrocatus
CAGGCCTGGCCGGGTGGTCTGCCTCACGGTATGCACTGTCTGGCTGGGTC
Cui16620 ---
HCFC1088Meripilus_stillicidior ---
MCW590Meripilus_obsrcurus ---
MCW722Meripilus_obsrcurus ---
Cui9381P_tibeticus
CAGGCCTGGCCGGGTGGTCTGCCTCACGGTATGCACTGTCTGGCTGGGTC
Cui9588P_tibeticus
CAGGCCTGGCCGGGTGGTCTGCCTCACGGTATGCACTGTCTGGCTGGGTC
Va2_Beneschova ---
CWU3874_Ukraine_Alnus ---
WCG1293Dai24718Physisporinus ---

WCG1268Dai24682A
CAGGCCTGGCCGGGTGGTCTGCCTCACGGTATGCACTGTCTGGCTGGGTC
WCG1269Dai24683A
CAGGCCTGGCCGGGTGGTCTGCCTCACGGTATGCACTGTCTGGCTGGGTC
WCG1279Dai24694A
CAGGCCTGGCCGGGTGGTCTGCCTCACGGTATGCACTGTCTGGCTGGGTC
Dai16971 --
ZQY1043Dai26696 --
Doll880
CAGGCCTGGCCGGGCGGTCTGCCTCACGGTATGCACTGTCTGGCTGGGTC
Doll1000
CAGGCCTGGCCGGGCGGTCTGCCTCACGGTATGCACTGTCTGGCTGGGTC
1DAI18529
CAGGCCTGGCCGGGCGGTCTGCCTCACGGTATGCACTGTCTGGCTGGGTC
Dai19535
CAGGCCTGGCCGGGCGGTCTGCCTCACGGTATGCACTGTCTGGCTGGGTC
1704_79_hnedyVillaLaPaz
CAGGCCTGGCCGGATGGTCTGCCTCACGGCATGCACTGTCTGGCTGGGTC
F2061 --
1DAI18268 --
1DAI18540A
CAGGCCTGGCCGGATGGTCTGCCTCACGGTATGCACTGTCTGGCTGGGTC
Dai17695
CAGGCCTGGCCGGATGGTCTGCCTCACGGTATGCACTGTCTGGCTGGGTC
LKY18Dai26373 --
Dai17839P_sulphureus
CAGGCCTGGCCGGATGGTCTGCCTCACGGTACGCACTGTCTGGCTGGGTC
Dai17841P_sulphureus
CAGGCCTGGCCGGATGGTCTGCCTCACGGTACGCACTGTCTGGCTGGGTC
Dai19877P_roseus
CAGGCCTGGCCGGATGGTCTGCCTCACGGTATGCACTGTCTGGCTGGGTC
1508_18_1_Kout --
KP859303_R_vinctus_RP185_BRAZI --
JK1807_15Rigidoporus_sp_Puerto
CAGACCTGGCCGGATGGTCTGCCTCACGGTATGCACTGTCTGGCTGGGTC
JV0509_47_J_TN_USA
CAGGCCTGGCCGGGCGGTCTGCCTCACGGTATGTACTGTCTGGCTGGGTC
JV0709_188 --
JV0509_127_PA_USA
CAGGCCTGGCCGGGCGGTCTGCCTCACGGTATGTACTGTCTGGCTGGGTC
JV1009_59_NJ_USA
CAGGCCTGGCCGGGCGGTCTGCCTCACGGTATGTACTGTCTGGCTGGGTC
Dai15497P_crataegi
CAGGCCTGGCCGGGCGGTCTGCCTCACGGTATGTACTGTCTGGCTGGGTC

Dai15499P_crataegi
CAGGCCTGGCCGGGCGGTCTGCCTCACGGTATGTACTGTCTGGCTGGGTC
Cui3266P_cinereus --
WCG1256Dai24690
CAGGCCTGGCCGGGTGGTCTGCCTCACGGTATGCACTGTCTGGCTGGGTC
1DAI17581 --
WCG1255Dai24688
CAGGCCTGGCCGGGTGGTCTGCCTCACGGTATGCACTGTCTGGCTGGGTC
Dai22427 --
MV690Meripilus_concrescens --
MV513Meripilus_galapagensis --
Dai19793
CAGGCCTGGCCGGGCGGTCTGCCTCACGGTATGTACTGTCTGGCTGGGTC
OQ553780P_tamilnaduensis --
OQ553779P_tamilnaduensis --
A164FB3Meripilus_giganteus --
JV1407_36_Vinctus_Meandrica
CAGGCTCGGCCGGGCGGTCTGCCTCACGGTATGTACTGTCTGGCTGGGTC
1807_3K_Rigidoporus_PuertoRico
CAGGCTCGGCCGGGCGGTCTGCCTAACGGCATGTACTGTCTGGCTGGGTC
Cui16903P_vinctus
CAGGCTCGGCCGGGCGGTCTGCCTAACGGCATGTACTGTCTGGCTGGGTC
JV1008_18R_Lineatus
CAGGCCTGGCCGGGCGGTCTGCCTCACGGTATGTACTGTCTGGCTGGGTC
JV1407_37_1_Vinctus_Carara
CAGGCCTGGCCGGGCGGTCTGCCTCACGGTATGTACTGTCTGGCTGGGTC
Dai17986P_lineatus
CAGGCCTGGCCGGGCGGTCTGCCTCACGGTATGTACTGTCTGGCTGGGTC
Dai18281
CAGGCCTGGCCGGGCGGTCTGCCTCACGGTATGTACTGTCTGGCTGGGTC
1DAI19796
CAGGCCTGGCCGGGCGGTCTGCCTCACGGTATGTACTGTCTGGCTGGGTC
ZQY797Dai25241
CAGGCCTGGCCGGGCGGTCTGCCTCACGGTATGTACTGTCTGGCTGGGTC
WCG1289Dai24711 --
Dai22598
CAGGCCTGGCCGGGCGGTCTGCCTCACGGTATGTACTGTCTGGCTGGGTC
Dai20523 --
Dai17885 --
Dai17553
CAGGCCTGGCCGGGCGGTCTGCCTCACGGTATGTACTGTCTGGCTGGGTC
Dai19639
CAGGCCTGGCCGGGCGGTCTGCCTCACGGTATGTACTGTCTGGCTGGGTC
JV0110_48_CZ

CAGGCCTGGCTGGGCGGTCTGCCTCACGGTATGTACTGTCTGGCTGGGTC
MJ129_04 ---
Cui10340P_eminens ---
Cui10341P_eminens
CAGGCCTGGCTGGGCGGTCTGCCTCACGGTATGTACTGTCTGGCTGGGTC
Dai12685P_eminens
CAGGCCTGGCTGGGCGGTCTGCCTCACGGTATGTACTGTCTGGCTGGGTC
Miettinen_13591Rigidoporus_und ---
Dai20868
CAGGCCTGGCTGGGCGGTCTGCCTCACGGTATGTACTGTCTGGCTGGGTC
Dai20832
CAGGCCTGGCTGGGCGGTCTGCCTCACGGTATGTACTGTCTGGCTGGGTC
Dai11400
CAGGCCTGGCTGGGCGGTCTGCCTCACGGTATGTACTGTCTGGCTGGGTC
Dai22472
CAGGCCTGGCTGGGCGGTCTGCCTCACGGTATGTACTGTCTGGCTGGGTC
1CUI10475 ---
1CUI10491 ---
HCFC1095Meripilus_robledoi ---
MCW702Meripilus_revolubilis ---
1704_83_zluty_HaciendaBaru
CAGGCCTGGCCGGGCGGTCTGCCTCACGGTATGTACTGTCTGGCTGGGTC
Dai9925P_lavendulus ---
Dai13587AP_lavendulus
CAGGCCTGGCCGGGCGGTCTGCCTCACGGTATGTACTGTCTGGCTGGGTC
PDD70600P_longicystidius ---
Cui16630 ---
FP_135344Meripilus_giganteus ---
FP_100460_Sp ---
CBS421_48Meripilus_giganteus ---
Cui9203
CAGGCCTGGCCGGGCGGTCTGCCTCACGGTATGTACTGTCTGGCTGGGTC
Cui9202
CAGGCCTGGCCGGGCGGTCTGCCTCACGGTATGTACTGTCTGGCTGGGTC
TUFC100564Japan ---
Russell5913Meripilus_sumstinei ---
RP215Meripilus_brasiliensis ---
RP200Meripilus_brasiliensis ---
JV1712_13J_R_vinctus2_LSUPuert
CAGACTCGGCTGGGCGGTCTGCCTAACGGTATGTACTGTCCGGCTGGGTC
Dai10503R_hypobrunneus ---
Dai10569Rigidoporus_hypobrunne ---
1DAI19451 ---
CM108bRigidoporus_hypobrunneus ---

1CUI16874 --
FD299Cerrena_unicolor --
KHL_GB_Cerrena_uniclor --
Dai12892Cerrena_albocinnamomea --
Dai12955C_albocinnamomea --
SNUm03110102C_aurantiopora --
NIBRFG0000102423C_aurantiopora --
Dai7359Antrodiella_zonata --
F20080702KCM29C_consors --
F20080208LYW10Cerrena_consors --
Dai7821Antrodiella_zonata --
CFMR_DCL04_31Pseudolagarobasid --
VPB197Pseudolagarobasidium_bel --
CBS115543Pseudolagarobasidium_ --
CBS115544 --
Han405Pseudolagarobasidium_bai --
Han406Pseudolagarobasidium_bai --
MUcc838Spongipellis_delectans --
BRNM686401S_delectans --
OSM_F925S_delectans --
BRNM67093Spongipellis_litschau --
CFMRccFP59199TS_unicolor --
CFMRccFP71791TS_unicolor --
Dai13845P_lischaueri
CAGACCTGGCCGGGCGGTCTGCCTCACGGTATGTACTGTCTGGCTGGGTC
Dai20266P_lischaueri
CAGACCTGGCCGGGCGGTCTGCCTCACGGTATGTACTGTCTGGCTGGGTC
CFMR_HHB11240Radulodon_america --
RLG6350Radulodon_americanus --
KY415963Radulodon_erikssonii --
HHB9567spRadulodon_casearius --
KRT_Iso_26Radulodon_casearius --
CBS126044Radulodon_erikssonii --
He6183YUNNANENSIS
CGGGCCTGGCTGGGCGGTCTGCCTCACGGTATGTACTGTCCGGCTGGGTC
Cui17979YUNNANENSIS
CGGGCCTGGCTGGGCGGTCTGCCTCACGGTATGTACTGTCCGGCTGGGTC
Miettinen2091Junghuhnia_fimbri --
KHL12316S_tenue --
PRM846564S_pachyodon --
SP_Lgt_S_pachyodon --
Ryvarden44669Tyromyces_xuchile --
PW17_171sinuosus --
W53Dai12234 --

HHB4100SpAntella_americana --
W3Dai20901spumeus
CAGACCTGGCCGGGCGGTCTGCCTAACGGTATGTACTGTCTGGCTGGGTC
He6736 --
BRNM734877S_spumeus --
BRNM712630S_spumeus --
Dai1723Loweomyces_sibiricus --
W54Cui10009
CAGACCTGGCCGGGCGGTCTGCCTAACGGTATGTACTGTCTGGCTGGGTC
W1Dai20899
CAGACCTGGCCGGGCGGTCTGCCTAACGGTATGTACTGTCTGGCTGGGTC
HHB13445Trametes_ochracea --
Dai16222 --
Dai16240 --

JV1310_11SanguinolentusCernys
TTACCTCTTGGTGAGCCAGCATGCCCTTCACTGGGTGTGTTGGGGAACCA
MJ39_00_SK --
MJ111_04_CZ
TTACCTCTTGGTGAGCCAGCATGCCCTTCACTGGGTGTGTTGGGGAACCA
JV1610_BOKYsmrk
TTACCTCTTGGTGAGCCAGCATGCCCTTCACTGGGTGTGTTGGGGAACCA
Dai21030 --
Dai20976P_furcatus
TTACCTCTTGGTGAGCCAGCATGCCCTTCACTGGGTGTGTTGGGGAACCA
Dai2105 --
Dai2544 --
Dai11313 --
WCG1611Dai26167
TTACCTCTTGGTGAGCCAGCATGCCCTTCACTGGGTGTGTTGGGGAACCA
WCG1518Dai25999Physisporinus
TTACCTCTTGGTGAGCCAGCATGCCCTTCACTGGGTGTGTTGGGGAACCA
TAA15097 --
JV8909_19_CZ --
JV1310_15_P_sanguinolentus2_CZ --
MJ53_02_CZ
TTACCTCTTGGTGAGCCGGCATGCCCTTTACTGGGTGTGTCGGGGAACCA
CLZhao21647P_yunnanensis --
CLZhao21583P_yunnanensis --
Dai22272
TTACCTCTTGGTGAGCCAGCATGCCCTTCACTGGGTGTGTTGGGGAACCA
Dai22279
TTACCTCTTGGTGAGCCAGCATGCCCTTCACTGGGTGTGTTGGGGAACCA
MJ332_94_CZ --

MJ642_94_CZ_Expallescens --
Dai21060P_vinctus
TTACCTCTTGGTGAGCCAGCATGCCCTTCACTGGGTGTATTGGGGAACCA
JV0511_23LRP_pouzarii
TTACCTCTTGGTGAGCCAGTATGCCCTTCACTGGGTGTATTGGGGAACCA
JQ409462_R_pouzarii_PRM899856_ --
JV0308_66_WA
TTACCTCTTGGTGAGCCAGCATGCCCTTCACTGGGTGTATTGGGGAACCA
JV0309_45_WA_USA
TTACCTCTTGGTGAGCCAGCATGCCCTTCACTGGGTGTATTGGGGAACCA
JV0709_83_CA_USA
TTACCTCTTGGTGAGCCAGCATGCCCTTCACTGGGTGTATTGGGGAACCA
Dai21043P_pouzarii
TTACCTCTTGGTGAGCCAGTATGCCCTTCACTGGGTGTATTGGGGAACCA
MJ144_95_CZ --
JV0909_3_CZ
TTACCTCTTGGTGAGCCGGCATGCCCTTTACTGGGTGTGTCGGGGAACCA
JV0609_1_K --
Dai20396Physisporinus_castanop
TTACCTCTTGGTGAGCCAGTATGCCCTTCATTGGGTGTATTGGGGAACCA
Dai20397Physisporinus_castanop
TTACCTCTTGGTGAGCCAGTATGCCCTTCATTGGGTGTATTGGGGAACCA
MJ19_09_SK_Abies --
JV0509_40_J_TN_USA_Betula
TTACCTCTTGGTGAGCCAGTATGCCCTTCACTGGGTGTATTGGGGAACCA
JV0808_33crocatus_PA_USAlist
TTACCTCTTGGTGAGCCAGTATGCCCTTCACTGGGTGTATTGGGGAACCA
DLL2009_061P_crocatus --
Dai12800P_subcrocatus --
Dai15917P_subcrocatus
TTACCTCTTGGTGAGCCAGTATGCCCTTCACTGGGTGTATTGGGGAACCA
Cui16620 --
HCFC1088Meripilus_stillicidior --
MCW590Meripilus_obsrcurus --
MCW722Meripilus_obsrcurus --
Cui9381P_tibeticus
TTACCTCTTGGTGAGCCAGTATGCCCTTCACTGGGTGTATTGGGGAACCA
Cui9588P_tibeticus
TTACCTCTTGGTGAGCCAGTATGCCCTTCACTGGGTGTATTGGGGAACCA
Va2_Beneschova --
CWU3874_Ukraine_Alnus --
WCG1293Dai24718Physisporinus --
WCG1268Dai24682A
TTACCTCTTGGTGAGCCAGCATGCCCTTTACTGGGTGTGTTGGGGAACCA

WCG1269Dai24683A
TTACCTCTTGGTGAGCCAGCATGCCCTTTACTGGGTGTGTTGGGGAACCA
WCG1279Dai24694A
TTACCTCTTGGTGAGCCAGCATGCCCTTTACTGGGTGTGTTGGGGAACCA
Dai16971 --
ZQY1043Dai26696 --
Doll880
TTACCTCTTGGTGAGCCAGCATGCCCTTCACTGGGTGTGTTGGGGAACCA
Doll1000
TTACCTCTTGGTGAGCCAGCATGCCCTTCACTGGGTGTGTTGGGGAACCA
1DAI18529
TTACCTCTTGGTGAGCCAGCATGCCCTTCACTGGGTGTGTTGGGGAACCA
Dai19535
TTACCTCTTGGTGAGCCGGCATGCCCTTCACTGGGTGTGTCGGGGAACCA
1704_79_hnedyVillaLaPaz
TTACCTCTTGGTGATCCAGCATGCCCTTTACTGGGTGTGTTGGGGAACCA
F2061 --
1DAI18268 --
1DAI18540A
TTACCTCTTGGTGATCCAGCATGCCCTTCACTGGGTGTGTTGGGGAACCA
Dai17695
TTACCTCTTGGTGATCCAGCATGCCCTTCACTGGGTGTGTTGGGGAACCA
LKY18Dai26373 --
Dai17839P_sulphureus
TTACCTCTTGGTGAGCCGGCATGCCCTTAATTGGGTGTGTTGGGGAACCA
Dai17841P_sulphureus
TTACCTCTTGGTGAGCCGGCATGCCCTTAATTGGGTGTGTTGGGGAACCA
Dai19877P_roseus
TTACCTCTTGGTGAGCCGGCATGCCCTTCATTGGGTGTGTTGGGGAACCA
1508_18_1_Kout --
KP859303_R_vinctus_RP185_BRAZI --
JK1807_15Rigidoporus_sp_Puerto
TTACCTCTTGGTGAGCCAGCATGCCCTTTACTGGGTGTGTTGGGGAACCA
JV0509_47_J_TN_USA
TTACCTCTTGGTGAGCCGGCATGCCCTTTACTGGGTGTGTCGGGGAACCA
JV0709_188 --
JV0509_127_PA_USA
TTACCTCTTGGTGAGCCGGCATGCCCTTTACTGGGTGTGTCGGGGAACCA
JV1009_59_NJ_USA
TTACCTCTTGGTGAGCCGGCATGCCCTTTACTGGGTGTGTCGGGGAACCA
Dai15497P_crataegi
TTACCTCTTGGTGAGCCGGCATGCCCTTCACTGGGTGTGTCGGGGAACCA
Dai15499P_crataegi
TTACCTCTTGGTGAGCCGGCATGCCCTTCACTGGGTGTGTCGGGGAACCA

Cui3266P_cinereus --
WCG1256Dai24690
TTACCTCTTGGTGAGCCGGCATGCCCTTCATTGGGTGTGTCGGGGAACCA
1DAI17581 --
WCG1255Dai24688
TTACCTCTTGGTGAGCCGGCATGCCCTTCATTGGGTGTGTCGGGGAACCA
Dai22427 --
MV690Meripilus_concrescens --
MV513Meripilus_galapagensis --
Dai19793
TTACCTCTTGGTGAGCCGGCATGCCCTTCACTGGGTGTGTCGGGGAACCA
OQ553780P_tamilnaduensis --
OQ553779P_tamilnaduensis --
A164FB3Meripilus_giganteus --
JV1407_36_Vinctus_Meandrica
TTACCTCTTGGTGAGCCGGCATGTCCTTCACTGGGTGTGTCGGGGAACCA
1807_3K_Rigidoporus_PuertoRico
TTACCTCTTGGTGAGCCGGCATGTCCTTCACTGGGTGTGTCGGGGAACCA
Cui16903P_vinctus
TTACCTCTTGGTGAGCCGGCATGTCCTTCACTGGGTGTGTCGGGGAACCA
JV1008_18R_Lineatus
TTACCTCTTGGTGAGCCGGCATGCCCTTCACTGGGTGTGTCGGGGAACCA
JV1407_37_1_Vinctus_Carara
TTACCTCTTGGTGAGCCGGCATGCCCTTCACTGGGTGTGTCGGGGAACCA
Dai17986P_lineatus
TTACCTCTTGGTGAGCCGGCATGCCCTTCACTGGGTGTGTCGGGGAACCA
Dai18281
TTACCTCTTGGTGAGCCGGCATGCCCTTCACTGGGTGTGTCGGGGAACCA
1DAI19796
TTACCTCTTGGTGAGCCGGCATGCCCTTCATTGGGTGTGTCGGGGAACCA
ZQY797Dai25241
TTACCTCTTGGTGAGCCGGCATGCCCTTCATTGGGTGTGTCGGGGAACCA
WCG1289Dai24711 --
Dai22598
TTACCTCTTGGTGAGCCGGCATGCCCTTCACTGGGTGTGTCGGGGAACCA
Dai20523 --
Dai17885 --
Dai17553
TTACCTCTTGGTGAGCCGGCATGCCCTTCACTGGGTGTGTCGGGGAACCA
Dai19639
TTACCTCTTGGTGAGCCGGCATGCCCTTCACTGGGTGTGTCGGGGAACCA
JV0110_48_CZ
TTACCTCTTGGTGAGCCGGCATGTCCTTAATTGGGTGTGCCGGGGAACCA
MJ129_04 --

Cui10340P_eminens --
Cui10341P_eminens
TTACCTCTTGGTGAGCCGGCATGTCCTTAATTGGGTGTGCCGGGGAACCA
Dai12685P_eminens
TTACCTCTTGGTGAGCCGGCATGTCCTTAATTGGGTGTGCCGGGGAACCA
Miettinen_13591Rigidoporus_und --
Dai20868
TTACCTCTTGGTGAGCCGGCATGTCCTTAATTGGGTGTGCCGGGGAACCA
Dai20832
TTACCTCTTGGTGAGCCGGCATGTCCTTAATTGGGTGTGCCGGGGAACCA
Dai11400
TTACCTCTTGGTGAGCCGGCATGTCCTTAATTGGGTGTGCCGGGGAACCA
Dai22472
TTACCTCTTGGTGAGCCGGCATGTCCTTAATTGGGTGTGCCGGGGAACCA
1CUI10475 --
1CUI10491 --
HCFC1095Meripilus_robledoi --
MCW702Meripilus_revolubilis --
1704_83_zluty_HaciendaBaru
TTACCTCTTGGTGAGCCGGCATGCCCTTCATTGGGTGTATCGGGGAACCA
Dai9925P_lavendulus --
Dai13587AP_lavendulus
TTACCTCTTGGTGAGCCGGCATGCCCTTCATTGGGTGTATCGGGGAACCA
PDD70600P_longicystidius --
Cui16630 --
FP_135344Meripilus_giganteus --
FP_100460_Sp --
CBS421_48Meripilus_giganteus --
Cui9203
TTACCTCTTGGTGAGCCGGCATGCCCTTCACTGGGTGTGTCGGGGAACCA
Cui9202
TTACCTCTTGGTGAGCCGGCATGCCCTTCACTGGGTGTGTCGGGGAACCA
TUFC100564Japan --
Russell5913Meripilus_sumstinei --
RP215Meripilus_brasiliensis --
RP200Meripilus_brasiliensis --
JV1712_13J_R_vinctus2_LSUPuert
TTACCTCTTGGTGAGCCGGTATGCTCTTTACTGGGTGTGCCGGGGAACCA
Dai10503R_hypobrunneus --
Dai10569Rigidoporus_hypobrunne --
1DAI19451 --
CM108bRigidoporus_hypobrunneus --
1CUI16874 --
FD299Cerrena_unicolor --

KHL_GB_Cerrena_uniclor --
Dai12892Cerrena_albocinnamomea --
Dai12955C_albocinnamomea --
SNUm03110102C_aurantiopora --
NIBRFG0000102423C_aurantiopora --
Dai7359Antrodiella_zonata --
F20080702KCM29C_consors --
F20080208LYW10Cerrena_consors --
Dai7821Antrodiella_zonata --
CFMR_DCL04_31Pseudolagarobasid --
VPB197Pseudolagarobasidium_bel --
CBS115543Pseudolagarobasidium_ --
CBS115544 --
Han405Pseudolagarobasidium_bai --
Han406Pseudolagarobasidium_bai --
MUcc838Spongipellis_delectans --
BRNM686401S_delectans --
OSM_F925S_delectans --
BRNM67093Spongipellis_litschau --
CFMRccFP59199TS_unicolor --
CFMRccFP71791TS_unicolor --
Dai13845P_lischaueri
TTACCTCTTGGTGAGCCGGCATGCCCTTTACTGGGTGTGCCGGGGAACCA
Dai20266P_lischaueri
TTACCTCTTGGTGAGCCGGCATGCCCTTTACTGGGTGTGCCGGGGAACCA
CFMR_HHB11240Radulodon_america --
RLG6350Radulodon_americanus --
KY415963Radulodon_erikssonii --
HHB9567spRadulodon_casearius --
KRT_Iso_26Radulodon_casearius --
CBS126044Radulodon_erikssonii --
He6183YUNNANENSIS
CTACCTCCTGGTGAACCGGCATGCCCTTAACTGGGTGTGTCGGGGAACCA
Cui17979YUNNANENSIS
CTACCTCCTGGTGAACCGGCATGCCCTTAACTGGGTGTGTCGGGGAACCA
Miettinen2091Junghuhnia_fimbri --
KHL12316S_tenue --
PRM846564S_pachyodon --
SP_Lgt_S_pachyodon --
Ryvarden44669Tyromyces_xuchile --
PW17_171sinuosus --
W53Dai12234 --
HHB4100SpAntella_americana --
W3Dai20901spumeus

TTACCTCTTGGTGAGCCGGTATGTCCTTCACTGGGTGTGCCGGGGAACCA
He6736 --
BRNM734877S_spumeus --
BRNM712630S_spumeus --
Dai1723Loweomyces_sibiricus --
W54Cui10009
TTACCTCTTGGTGAGCCGGTATGTCCTTCACTGGGTGTGCCGGGGAACCA
W1Dai20899
TTACCTCTTGGTGAGCCGGTATGTCCTTCACTGGGTGTGCCGGGGAACCA
HHB13445Trametes_ochracea --
Dai16222 --
Dai16240 --

JV1310_11SanguinolentusCernys
GGACTTTTACCTTGAGAAAATTAGAGTGTTCAAAGCAGGCCTATGCCCGA
MJ39_00_SK --
MJ111_04_CZ
GGACTTTTACCTTGAGAAAATTAGAGTGTTCAAAGCAGGCCTATGCCCGA
JV1610_BOKYsmrk
GGACTTTTACCTTGAGAAAATTAGAGTGTTCAAAGCAGGCCTATGCCCGA
Dai21030 --
Dai20976P_furcatus
GGACTTTTACCTTGAGAAAATTAGAGTGTTCAAAGCAGGCCTATGCCCGA
Dai2105 --
Dai2544 --
Dai11313 --
WCG1611Dai26167
GGACTTTTACCTTGAGAAAATTAGAGTGTTCAAAGCAGGCCTATGCCCGA
WCG1518Dai25999Physisporinus
GGACTTTTACCTTGAGAAAATTAGAGTGTTCAAAGCAGGCCTATGCCCGA
TAA15097 --
JV8909_19_CZ --
JV1310_15_P_sanguinolentus2_CZ --
MJ53_02_CZ
GGACTTTTACCTTGAGAAAATTAGAGTGTTCAAAGCAGGCCTATGCCCGA
CLZhao21647P_yunnanensis --
CLZhao21583P_yunnanensis --
Dai22272
GGACTTTTACCTTGAGAAAATTAGAGTGTTCAAAGCAGGCCTATGCCCGA
Dai22279
GGACTTTTACCTTGAGAAAATTAGAGTGTTCAAAGCAGGCCTATGCCCGA
MJ332_94_CZ --
MJ642_94_CZ_Expallescens --
Dai21060P_vinctus

GGACTTTTACCTTGAGAAAATTAGAGTGTTCAAAGCAGGCCTATGCCCGA
JV0511_23LRP_pouzarii
GGACTTTTACCTTGAGAAAATTAGAGTGTTCAAAGCAGGCCTATGCCCGA
JQ409462_R_pouzarii_PRM899856_ --
JV0308_66_WA
GGACTTTTACCTTGAGAAAATTAGAGTGTTCAAAGCAGGCCTATGCCCGA
JV0309_45_WA_USA
GGACTTTTACCTTGAGAAAATTAGAGTGTTCAAAGCAGGCCTATGCCCGA
JV0709_83_CA_USA
GGACTTTTACCTTGAGAAAATTAGAGTGTTCAAAGCAGGCCTATGCCCGA
Dai21043P_pouzarii
GGACTTTTACCTTGAGAAAATTAGAGTGTTCAAAGCAGGCCTATGCCCGA
MJ144_95_CZ --
JV0909_3_CZ
GGACTTTTACCTTGAGAAAATTAGAGTGTTCAAAGCAGGCCTATGCCCGA
JV0609_1_K --
Dai20396Physisporinus_castanop
GGACTTTTACCTTGAGAAAATTAGAGTGTTCAAAGCAGGCCTATGCCCGA
Dai20397Physisporinus_castanop
GGACTTTTACCTTGAGAAAATTAGAGTGTTCAAAGCAGGCCTATGCCCGA
MJ19_09_SK_Abies --
JV0509_40_J_TN_USA_Betula
GGACTTTTACCTTGAGAAAATTAGAGTGTTCAAAGCAGGCCTATGCCCGA
JV0808_33crocatus_PA_USAlist
GGACTTTTACCTTGAGAAAATTAGAGTGTTCAAAGCAGGCCTATGCCCGA
DLL2009_061P_crocatus --
Dai12800P_subcrocatus --
Dai15917P_subcrocatus
GGACTTTTACCTTGAGAAAATTAGAGTGTTCAAAGCAGGCCTATGCCCGA
Cui16620 --
HCFC1088Meripilus_stillicidior --
MCW590Meripilus_obsrcurus --
MCW722Meripilus_obsrcurus --
Cui9381P_tibeticus
GGACTTTTACCTTGAGAAAATTAGAGTGTTCAAAGCAGGCCTATGCCCGA
Cui9588P_tibeticus
GGACTTTTACCTTGAGAAAATTAGAGTGTTCAAAGCAGGCCTATGCCCGA
Va2_Beneschova --
CWU3874_Ukraine_Alnus --
WCG1293Dai24718Physisporinus --
WCG1268Dai24682A
GGACTTTTACCTTGAGAAAATTAGAGTGTTCAAAGCAGGCCTATGCCCGA
WCG1269Dai24683A
GGACTTTTACCTTGAGAAAATTAGAGTGTTCAAAGCAGGCCTATGCCCGA

WCG1279Dai24694A
GGACTTTTACCTTGAGAAAATTAGAGTGTTCAAAGCAGGCCTATGCCCGA
Dai16971 --
ZQY1043Dai26696 --
Doll880
GGACTTTTACCTTGAGAAAATTAGAGTGTTCAAAGCAGGCCTATGCCCGA
Doll1000
GGACTTTTACCTTGAGAAAATTAGAGTGTTCAAAGCAGGCCTATGCCCGA
1DAI18529
GGACTTTTACCTTGAGAAAATTAGAGTGTTCAAAGCAGGCCTATGCCCGA
Dai19535
GGACTTTTACCTTGAGAAAATTAGAGTGTTCAAAGCAGGCCTATGCCCGA
1704_79_hnedyVillaLaPaz
GGACTTTTACCTTGAGAAAATTAGAGTGTTCAAAGCAGGCCTATGCCCGA
F2061 --
1DAI18268 --
1DAI18540A
GGACTTTTACCTTGAGAAAATTAGAGTGTTCAAAGCAGGCCTATGCCCGA
Dai17695
GGACTTTTACCTTGAGAAAATTAGAGTGTTCAAAGCAGGCCTATGCCCGA
LKY18Dai26373 --
Dai17839P_sulphureus
GGACTTTTACCTTGAGAAAATTAGAGTGTTCAAAGCAGGCCTATGCCCGA
Dai17841P_sulphureus
GGACTTTTACCTTGAGAAAATTAGAGTGTTCAAAGCAGGCCTATGCCCGA
Dai19877P_roseus
GGACTTTTACCTTGAGAAAATTAGAGTGTTCAAAGCAGGCCTATGCCCGA
1508_18_1_Kout --
KP859303_R_vinctus_RP185_BRAZI --
JK1807_15Rigidoporus_sp_Puerto
GGACTTTTACCTTGAGAAAATTAGAGTGTTCAAAGCAGGCCTATGCCCGA
JV0509_47_J_TN_USA
GGACTTTTACCTTGAGAAAATTAGAGTGTTCAAAGCAGGCCTATGCCCGA
JV0709_188 --
JV0509_127_PA_USA
GGACTTTTACCTTGAGAAAATTAGAGTGTTCAAAGCAGGCCTATGCCCGA
JV1009_59_NJ_USA
GGACTTTTACCTTGAGAAAATTAGAGTGTTCAAAGCAGGCCTATGCCCGA
Dai15497P_crataegi
GGACTTTTACCTTGAGAAAATTAGAGTGTTCAAAGCAGGCCTATGCCCGA
Dai15499P_crataegi
GGACTTTTACCTTGAGAAAATTAGAGTGTTCAAAGCAGGCCTATGCCCGA
Cui3266P_cinereus --
WCG1256Dai24690

GGACTTTTACCTTGAGAAAATTAGAGTGTTCAAAGCAGGCCTATGCCCGA
1DAI17581 --
WCG1255Dai24688
GGACTTTTACCTTGAGAAAATTAGAGTGTTCAAAGCAGGCCTATGCCCGA
Dai22427 --
MV690Meripilus_concrescens --
MV513Meripilus_galapagensis --
Dai19793
GGACTTTTACCTTGAGAAAATTAGAGTGTTCAAAGCAGGCCTATGCCCGA
OQ553780P_tamilnaduensis --
OQ553779P_tamilnaduensis --
A164FB3Meripilus_giganteus --
JV1407_36_Vinctus_Meandrica
GGACTTTTACCTTGAGAAAATTAGAGTGTTCAAAGCAGGCCTATGCCCGA
1807_3K_Rigidoporus_PuertoRico
GGACTTTTACCTTGAGAAAATTAGAGTGTTCAAAGCAGGCCTATGCCCGA
Cui16903P_vinctus
GGACTTTTACCTTGAGAAAATTAGAGTGTTCAAAGCAGGCCTATGCCCGA
JV1008_18R_Lineatus
GGACTTTTACCTTGAGAAAATTAGAGTGTTCAAAGCAGGCCTATGCCCGA
JV1407_37_1_Vinctus_Carara
GGACTTTTACCTTGAGAAAATTAGAGTGTTCAAAGCAGGCCTATGCCCGA
Dai17986P_lineatus
GGACTTTTACCTTGAGAAAATTAGAGTGTTCAAAGCAGGCCTATGCCCGA
Dai18281
GGACTTTTACCTTGAGAAAATTAGAGTGTTCAAAGCAGGCCTATGCCCGA
1DAI19796
GGACTTTTACCTTGAGAAAATTAGAGTGTTCAAAGCAGGCCTATGCCCGA
ZQY797Dai25241
GGACTTTTACCTTGAGAAAATTAGAGTGTTCAAAGCAGGCCTATGCCCGA
WCG1289Dai24711 --
Dai22598
GGACTTTTACCTTGAGAAAATTAGAGTGTTCAAAGCAGGCCTATGCCCGA
Dai20523 --
Dai17885 --
Dai17553
GGACTTTTACCTTGAGAAAATTAGAGTGTTCAAAGCAGGCCTATGCCCGA
Dai19639
GGACTTTTACCTTGAGAAAATTAGAGTGTTCAAAGCAGGCCTATGCCCGA
JV0110_48_CZ
GGACTTTTACCTTGAGAAAATTAGAGTGTTCAAAGCAGGCCTATGCCCGA
MJ129_04 --
Cui10340P_eminens --
Cui10341P_eminens

GGACTTTTACCTTGAGAAAATTAGAGTGTTCAAAGCAGGCCTATGCCCGA
Dai12685P_eminens
GGACTTTTACCTTGAGAAAATTAGAGTGTTCAAAGCAGGCCTATGCCCGA
Miettinen_13591Rigidoporus_und --
Dai20868
GGACTTTTACCTTGAGAAAATTAGAGTGTTCAAAGCAGGCCTATGCCCGA
Dai20832
GGACTTTTACCTTGAGAAAATTAGAGTGTTCAAAGCAGGCCTATGCCCGA
Dai11400
GGACTTTTACCTTGAGAAAATTAGAGTGTTCAAAGCAGGCCTATGCCCGA
Dai22472
GGACTTTTACCTTGAGAAAATTAGAGTGTTCAAAGCAGGCCTATGCCCGA
1CUI10475 --
1CUI10491 --
HCFC1095Meripilus_robledoi --
MCW702Meripilus_revolubilis --
1704_83_zluty_HaciendaBaru
GGACTTTTACCTTGAGAAAATTAGAGTGCTCAAAGCAGGCTTATGCCCGA
Dai9925P_lavendulus --
Dai13587AP_lavendulus
GGACTTTTACCTTGAGAAAATTAGAGTGCTCAAAGCAGGCTTATGCCCGA
PDD70600P_longicystidius --
Cui16630 --
FP_135344Meripilus_giganteus --
FP_100460_Sp --
CBS421_48Meripilus_giganteus --
Cui9203
GGACTTTTACCTTGAGAAAATTAGAGTGTTCAAAGCAGGCCTATGCCCGA
Cui9202
GGACTTTTACCTTGAGAAAATTAGAGTGTTCAAAGCAGGCCTATGCCCGA
TUFC100564Japan --
Russell5913Meripilus_sumstinei --
RP215Meripilus_brasiliensis --
RP200Meripilus_brasiliensis --
JV1712_13J_R_vinctus2_LSUPuert
GGACTTTTACCTTGAGAAAATTAGAGTGTTCAAAGCAGGTCTGCGCCTGA
Dai10503R_hypobrunneus --
Dai10569Rigidoporus_hypobrunne --
1DAI19451 --
CM108bRigidoporus_hypobrunneus --
1CUI16874 --
FD299Cerrena_unicolor --
KHL_GB_Cerrena_uniclor --
Dai12892Cerrena_albocinnamomea --

Dai12955C_albocinnamomea --
SNUm03110102C_aurantiopora --
NIBRFG0000102423C_aurantiopora --
Dai7359Antrodiella_zonata --
F20080702KCM29C_consors --
F20080208LYW10Cerrena_consors --
Dai7821Antrodiella_zonata --
CFMR_DCL04_31Pseudolagarobasid --
VPB197Pseudolagarobasidium_bel --
CBS115543Pseudolagarobasidium_ --
CBS115544 --
Han405Pseudolagarobasidium_bai --
Han406Pseudolagarobasidium_bai --
MUcc838Spongipellis_delectans --
BRNM686401S_delectans --
OSM_F925S_delectans --
BRNM67093Spongipellis_litschau --
CFMRccFP59199TS_unicolor --
CFMRccFP71791TS_unicolor --
Dai13845P_lischaueri
GGACTTTTACCTTGAGAAAATTAGAGTGTTCAAAGCAGGTTATCGCCTGA
Dai20266P_lischaueri
GGACTTTTACCTTGAGAAAATTAGAGTGTTCAAAGCAGGTTATCGCCTGA
CFMR_HHB11240Radulodon_america --
RLG6350Radulodon_americanus --
KY415963Radulodon_erikssonii --
HHB9567spRadulodon_casearius --
KRT_Iso_26Radulodon_casearius --
CBS126044Radulodon_erikssonii --
He6183YUNNANENSIS
GGACCTTTACCTTGAGAAAATTAGAGTGTTCAAAGCAGGTCTGCGCCTGA
Cui17979YUNNANENSIS
GGACCTTTACCTTGAGAAAATTAGAGTGTTCAAAGCAGGTCTGCGCCTGA
Miettinen2091Junghuhnia_fimbri --
KHL12316S_tenue --
PRM846564S_pachyodon --
SP_Lgt_S_pachyodon --
Ryvarden44669Tyromyces_xuchile --
PW17_171sinuosus --
W53Dai12234 --
HHB4100SpAntella_americana --
W3Dai20901spumeus
GGACTTTTACCTTGAGAAAATTAGAGTGTTCAAAGCAGGCCTATGCCCGA
He6736 --

BRNM734877S_spumeus --
BRNM712630S_spumeus --
Dai1723Loweomyces_sibiricus --
W54Cui10009
GGACTTTTACCTTGAGAAAATTAGAGTGTTCAAAGCAGGCCTATGCCCGA
W1Dai20899
GGACTTTTACCTTGAGAAAATTAGAGTGTTCAAAGCAGGCCTATGCCCGA
HHB13445Trametes_ochracea --
Dai16222 --
Dai16240 --

JV1310_11SanguinolentusCernys
ATACATTAGCATGGAATAATAAAATAGGACGTGCGGTTCTATTTTGTTGG
MJ39_00_SK --
MJ111_04_CZ
ATACATTAGCATGGAATAATAAAATAGGACGTGCGGTTCTATTTTGTTGG
JV1610_BOKYsmrk
ATACATTAGCATGGAATAATAAAATAGGACGTGCGGTTCTATTTTGTTGG
Dai21030 --
Dai20976P_furcatus
ATACATTAGCATGGAATAATAAAATAGGACGTGCGGTTCTATTTTGTTGG
Dai2105 --
Dai2544 --
Dai11313 --
WCG1611Dai26167
ATACATTAGCATGGAATAATAAAATAGGACGTGCGGTTCTATTTTGTTGG
WCG1518Dai25999Physisporinus
ATACATTAGCATGGAATAATAAAATAGGACGTGCGGTTCTATTTTGTTGG
TAA15097 --
JV8909_19_CZ --
JV1310_15_P_sanguinolentus2_CZ --
MJ53_02_CZ
ATACATTAGCATGGAATAATAGAATAGGACGTGCGGTTCTATTTTGTTGG
CLZhao21647P_yunnanensis --
CLZhao21583P_yunnanensis --
Dai22272
ATACATTAGCATGGAATAATAAAATAGGACGTGCGGTTCTATTTTGTTGG
Dai22279
ATACATTAGCATGGAATAATAAAATAGGACGTGCGGTTCTATTTTGTTGG
MJ332_94_CZ --
MJ642_94_CZ_Expallescens --
Dai21060P_vinctus
ATACATTAGCATGGAATAATAAAATAGGACGTGCGGTTCTATTTTGTTGG
JV0511_23LRP_pouzarii

ATACATTAGCATGGAATAATAAAATAGGACGTGCGGTTCTATTTTGTTGG
JQ409462_R_pouzarii_PRM899856_ --
JV0308_66_WA
ATACATTAGCATGGAATAATAAAATAGGACGTGCGGTTCTATTTTGTTGG
JV0309_45_WA_USA
ATACATTAGCATGGAATAATAAAATAGGACGTGCGGTTCTATTTTGTTGG
JV0709_83_CA_USA
ATACATTAGCATGGAATAATAAAATAGGACGTGCGGTTCTATTTTGTTGG
Dai21043P_pouzarii
ATACATTAGCATGGAATAATAAAATAGGACGTGCGGTTCTATTTTGTTGG
MJ144_95_CZ --
JV0909_3_CZ
ATACATTAGCATGGAATAATAAAATAGGACGTGCGGTTCTATTTTGTTGG
JV0609_1_K --
Dai20396Physisporinus_castanop
ATACATTAGCATGGAATAATAAAATAGGACGTGCGGTTCTATTTTGTTGG
Dai20397Physisporinus_castanop
ATACATTAGCATGGAATAATAAAATAGGACGTGCGGTTCTATTTTGTTGG
MJ19_09_SK_Abies --
JV0509_40_J_TN_USA_Betula
ATACATTAGCATGGAATAATAAAATAGGACGTGCGGTTCTATTTTGTTGG
JV0808_33crocatus_PA_USAlist
ATACATTAGCATGGAATAATAAAATAGGACGTGCGGTTCTATTTTGTTGG
DLL2009_061P_crocatus --
Dai12800P_subcrocatus --
Dai15917P_subcrocatus
ATACATTAGCATGGAATAATAAAATAGGACGTGCGGTTCTATTTTGTTGG
Cui16620 --
HCFC1088Meripilus_stillicidior --
MCW590Meripilus_obsrcurus --
MCW722Meripilus_obsrcurus --
Cui9381P_tibeticus
ATACATTAGCATGGAATAATAAAATAGGACGTGCGGTTCTATTTTGTTGG
Cui9588P_tibeticus
ATACATTAGCATGGAATAATAAAATAGGACGTGCGGTTCTATTTTGTTGG
Va2_Beneschova --
CWU3874_Ukraine_Alnus --
WCG1293Dai24718Physisporinus --
WCG1268Dai24682A
ATACATTAGCATGGAATAATAAAATAGGACGTGCGGTTCTATTTTGTTGG
WCG1269Dai24683A
ATACATTAGCATGGAATAATAAAATAGGACGTGCGGTTCTATTTTGTTGG
WCG1279Dai24694A
ATACATTAGCATGGAATAATAAAATAGGACGTGCGGTTCTATTTTGTTGG

Dai16971 --
ZQY1043Dai26696 --
Doll880
ATACATTAGCATGGAATAATAAAATAGGACGTGCGGTTCTATTTGTTGG
Doll1000
ATACATTAGCATGGAATAATAAAATAGGACGTGCGGTTCTATTTGTTGG
1DAI18529
ATACATTAGCATGGAATAATAAAATAGGACGTGCGGTTCTATTTGTTGG
Dai19535
ATACATTAGCATGGAATAATAAAATAGGACGTGCGGTTCTATTTGTTGG
1704_79_hnedyVillaLaPaz
ATACATTAGCATGGAATAATAAAATAGGACGTGCGGTTCTATTTGTTGG
F2061 --
1DAI18268 --
1DAI18540A
ATACATTAGCATGGAATAATAAAATAGGACGTGCGGTTCTATTTGTTGG
Dai17695 ATACATTAGCATG------------------------------------
LKY18Dai26373 --
Dai17839P_sulphureus
ATACATTAGCATGGAATAATAAAATAGGACGTGCGGTTCTATTTGTTGG
Dai17841P_sulphureus
ATACATTAGCATGGAATAATAAAATAGGACGTGCGGTTCTATTTGTTGG
Dai19877P_roseus
ATACATTAGCATGGAATAATAAAATAGGACGTGCGGTTCTATTTGTTGG
1508_18_1_Kout --
KP859303_R_vinctus_RP185_BRAZI --
JK1807_15Rigidoporus_sp_Puerto
ATACATTAGCATGGAATAATAAAATAGGACGTGCGGTTCTATTTGTTGG
JV0509_47_J_TN_USA
ATACATTAGCATGGAATAATAAAATAGGACGTGCGGTTCTATTTGTTGG
JV0709_188 --
JV0509_127_PA_USA
ATACATTAGCATGGAATAATAAAATAGGACGTGCGGTTCTATTTGTTGG
JV1009_59_NJ_USA
ATACATTAGCATGGAATAATAAAATAGGACGTGCGGTTCTATTTGTTGG
Dai15497P_crataegi
ATACATTAGCATGGAATAATAAAATAGGACGTGCGGTTCTATTTGTTGG
Dai15499P_crataegi
ATACATTAGCATGGAATAATAAAATAGGACGTGCGGTTCTATTTGTTGG
Cui3266P_cinereus --
WCG1256Dai24690
ATACATTAGCATGGAATAATAAAATAGGACGTGCGGTTCTATTTGTTGG
1DAI17581 --
WCG1255Dai24688

ATACATTAGCATGGAATAATAAAATAGGACGTGCGGTTCTATTTTGTTGG
Dai22427 --
MV690Meripilus_concrescens --
MV513Meripilus_galapagensis --
Dai19793
ATACATTAGCATGGAATAATAAAATAGGACGTGCGGTTCTATTTTGTTGG
OQ553780P_tamilnaduensis --
OQ553779P_tamilnaduensis --
A164FB3Meripilus_giganteus --
JV1407_36_Vinctus_Meandrica
ATACATTAGCATGGAATAATAAAATAGGACGTGCGGTTCTATTTTGTTGG
1807_3K_Rigidoporus_PuertoRico
ATACATTAGCATGGAATAATAAAATAGGACGTGCGGTTCTATTTTGTTGG
Cui16903P_vinctus
ATACATTAGCATGGAATAATAAAATAGGACGTGCGGTTCTATTTTGTTGG
JV1008_18R_Lineatus
ATACATTAGCATGGAATAATAAAATAGGACGTGCGGTTCTATTTTGTTGG
JV1407_37_1_Vinctus_Carara
ATACATTAGCATGGAATAATAAAATAGGACGTGCGGTTCTATTTTGTTGG
Dai17986P_lineatus
ATACATTAGCATGGAATAATAAAATAGGACGTGCGGTTCTATTTTGTTGG
Dai18281
ATACATTAGCATGGAATAATAAAATAGGACGTGCGGTTCTATTTTGTTGG
1DAI19796
ATACATTAGCATGGAATAATAAAATAGGACGTGCGGTTCTATTTTGTTGG
ZQY797Dai25241
ATACATTAGCATGGAATAATAAAATAGGACGTGCGGTTCTATTTTGTTGG
WCG1289Dai24711 --
Dai22598
ATACATTAGCATGGAATAATAAAATAGGACGTGCGGTTCTATTTTGTTGG
Dai20523 --
Dai17885 --
Dai17553
ATACATTAGCATGGAATAATAAAATAGGACGTGCGGTTCTATTTTGTTGG
Dai19639
ATACATTAGCATGGAATAATAAAATAGGACGTGCGGTTCTATTTTGTTGG
JV0110_48_CZ
ATACATTAGCATGGAATAATAAAATAGGACGTGCGGTTCTATTTTGTTGG
MJ129_04 --
Cui10340P_eminens --
Cui10341P_eminens
ATACATTAGCATGGAATAATAAAATAGGACGTGCGGTTCTATTTTGTTGG
Dai12685P_eminens
ATACATTAGCATGGAATAATAAAATAGGACGTGCGGTTCTATTTTGTTGG

Miettinen_13591Rigidoporus_und --
Dai20868
ATACATTAGCATGGAATAATAAAATAGGACGTGCGGTTCTATTTTGTTGG
Dai20832
ATACATTAGCATGGAATAATAAAATAGGACGTGCGGTTCTATTTTGTTGG
Dai11400
ATACATTAGCATGGAATAATAAAATAGGACGTGCGGTTCTATTTTGTTGG
Dai22472
ATACATTAGCATGGAATAATAAAATAGGACGTGCGGTTCTATTTTGTTGG
1CUI10475 --
1CUI10491 --
HCFC1095Meripilus_robledoi --
MCW702Meripilus_revolubilis --
1704_83_zluty_HaciendaBaru
ATACATTAGCATGGAATAATAAAATAGGACGTGCGGTTCTATTTTGTTGG
Dai9925P_lavendulus --
Dai13587AP_lavendulus
ATACATTAGCATGGAATAATAAAATAGGACGTGCGGTTCTATTTTGTTGG
PDD70600P_longicystidius --
Cui16630 --
FP_135344Meripilus_giganteus --
FP_100460_Sp --
CBS421_48Meripilus_giganteus --
Cui9203
ATACATTAGCATGGAATAATAAAATAGGACGTGCGGTTCTATTTTGTTGG
Cui9202
ATACATTAGCATGGAATAATAAAATAGGACGTGCGGTTCTATTTTGTTGG
TUFC100564Japan --
Russell5913Meripilus_sumstinei --
RP215Meripilus_brasiliensis --
RP200Meripilus_brasiliensis --
JV1712_13J_R_vinctus2_LSUPuert
ATACATTAGCATGGAATAATAAAATAGGACGTGCGGTTCTATTTTGTTGG
Dai10503R_hypobrunneus --
Dai10569Rigidoporus_hypobrunne --
1DAI19451 --
CM108bRigidoporus_hypobrunneus --
1CUI16874 --
FD299Cerrena_unicolor --
KHL_GB_Cerrena_uniclor --
Dai12892Cerrena_albocinnamomea --
Dai12955C_albocinnamomea --
SNUm03110102C_aurantiopora --
NIBRFG0000102423C_aurantiopora --

Dai7359Antrodiella_zonata ---
F20080702KCM29C_consors ---
F20080208LYW10Cerrena_consors ---
Dai7821Antrodiella_zonata ---
CFMR_DCL04_31Pseudolagarobasid ---
VPB197Pseudolagarobasidium_bel ---
CBS115543Pseudolagarobasidium_ ---
CBS115544 ---
Han405Pseudolagarobasidium_bai ---
Han406Pseudolagarobasidium_bai ---
MUcc838Spongipellis_delectans ---
BRNM686401S_delectans ---
OSM_F925S_delectans ---
BRNM67093Spongipellis_litschau ---
CFMRccFP59199TS_unicolor ---
CFMRccFP71791TS_unicolor ---
Dai13845P_lischaueri
ATACATTAGCATGGAATAATAAAATAGGACGTGCGGTTCTATTTGTTGG
Dai20266P_lischaueri
ATACATTAGCATGGAATAATAAAATAGGACGTGCGGTTCTATTTGTTGG
CFMR_HHB11240Radulodon_america ---
RLG6350Radulodon_americanus ---
KY415963Radulodon_erikssonii ---
HHB9567spRadulodon_casearius ---
KRT_Iso_26Radulodon_casearius ---
CBS126044Radulodon_erikssonii ---
He6183YUNNANENSIS
ATACATTAGCATGGAATAATAAAATAGGACGTGCGGTTCTATTTGTTGG
Cui17979YUNNANENSIS
ATACATTAGCATGGAATAATAAAATAGGACGTGCGGTTCTATTTGTTGG
Miettinen2091Junghuhnia_fimbri ---
KHL12316S_tenue ---
PRM846564S_pachyodon ---
SP_Lgt_S_pachyodon ---
Ryvarden44669Tyromyces_xuchile ---
PW17_171sinuosus ---
W53Dai12234 ---
HHB4100SpAntella_americana ---
W3Dai20901spumeus
ATACATTAGCATGGAATAATAAAATAGGACGTGCGGTTCTATTTGTTGG
He6736 ---
BRNM734877S_spumeus ---
BRNM712630S_spumeus ---
Dai1723Loweomyces_sibiricus ---

W54Cui10009
ATACATTAGCATGGAATAATAAAATAGGACGTGCGG-TCTATTTGTTGG
W1Dai20899
ATACATTAGCATGGAATAATAAAATAGGACGTGCGGTTCTATTTGTTGG
HHB13445Trametes_ochracea ---
Dai16222 ---
Dai16240 ---

JV1310_11SanguinolentusCernys
TTTCTAGAGTCGCCGTAATGATTAATAGGGATAGTTGGGGGCATTAGTAT
MJ39_00_SK ---
MJ111_04_CZ
TTTCTAGAGTCGCCGTAATGATTAATAGGGATAGTTGGGGGCATTAGTAT
JV1610_BOKYsmrk
TTTCTAGAGTCGCCGTAATGATTAATAGGGATAGTTGGGGGCATTAGTAT
Dai21030 ---
Dai20976P_furcatus
TTTCTAGAGTCGCCGTAATGATTAATAGGGATAGTTGGGGGCATTAGTAT
Dai2105 ---
Dai2544 ---
Dai11313 ---
WCG1611Dai26167
TTTCTAGAGTCGCCGTAATGATTAATAGGGATAGTTGGGGGCATTAGTAT
WCG1518Dai25999Physisporinus
TTTCTAGAGTCGCCGTAATGATTAATAGGGATAGTTGGGGGCATTAGTAT
TAA15097 ---
JV8909_19_CZ ---
JV1310_15_P_sanguinolentus2_CZ ---
MJ53_02_CZ
TTTCTAGAGTCGCCGTAATGATTAATAGGGACAGTCGGGGGCATTGGTAT
CLZhao21647P_yunnanensis ---
CLZhao21583P_yunnanensis ---
Dai22272
TTTCTAGAGTCGCCGTAATGATTAATAGGGATAGTTGGGGGCATTAGTAT
Dai22279
TTTCTAGAGTCGCCGTAATGATTAATAGGGATAGTTGGGGGCATTAGTAT
MJ332_94_CZ ---
MJ642_94_CZ_Expallescens ---
Dai21060P_vinctus
TTTCTAGAGTCGCCGTAATGATTAATAGGGATAGTTGGGGGCATTAGTAT
JV0511_23LRP_pouzarii
TTTCTAGAGTCGCCGTAATGATTAATAGGGATAGTTGGGGGCATTAGTAT
JQ409462_R_pouzarii_PRM899856_ ---
JV0308_66_WA

TTTCTAGAGTCGCCGTAATGATTAATAGGGATAGTTGGGGGCATTAGTAT
JV0309_45_WA_USA
TTTCTAGAGTCGCCGTAATGATTAATAGGGATAGTTGGGGGCATTAGTAT
JV0709_83_CA_USA
TTTCTAGAGTCGCCGTAATGATTAATAGGGATAGTTGGGGGCATTAGTAT
Dai21043P_pouzarii
TTTCTAGAGTCGCCGTAATGATTAATAGGGATAGTTGGGGGCATTAGTAT
MJ144_95_CZ --
JV0909_3_CZ
TTTCTAGAGTCGCCGTAATGATTAATAGGGATAGTTGGGGGCATTAGTAT
JV0609_1_K --
Dai20396Physisporinus_castanop
TTTCTAGAGTCGCCGTAATGATTAATAGGGATAGTTGGGGGCATTAGTAT
Dai20397Physisporinus_castanop
TTTCTAGAGTCGCCGTAATGATTAATAGGGATAGTTGGGGGCATTAGTAT
MJ19_09_SK_Abies --
JV0509_40_J_TN_USA_Betula
TTTCTAGAGTCGCCGTAATGATTAATAGGGATAGTTGGGGGCATTAGTAT
JV0808_33crocatus_PA_USAlist
TTTCTAGAGTCGCCGTAATGATTAATAGGGATAGTTGGGGGCATTAGTAT
DLL2009_061P_crocatus --
Dai12800P_subcrocatus --
Dai15917P_subcrocatus
TTTCTAGAGTCGCCGTAATGATTAATAGGGATAGTTGGGGGCATTAGTAT
Cui16620 --
HCFC1088Meripilus_stillicidior --
MCW590Meripilus_obsrcurus --
MCW722Meripilus_obsrcurus --
Cui9381P_tibeticus
TTTCTAGAGTCGCCGTAATGATTAATAGGGATAGTTGGGGGCATTAGTAT
Cui9588P_tibeticus
TTTCTAGAGTCGCCGTAATGATTAATAGGGATAGTTGGGGGCATTAGTAT
Va2_Beneschova --
CWU3874_Ukraine_Alnus --
WCG1293Dai24718Physisporinus --
WCG1268Dai24682A
TTTCTAGAGTCGCCGTAATGATTAATAGGGATAGTTGGGGGCATTAGTAT
WCG1269Dai24683A
TTTCTAGAGTCGCCGTAATGATTAATAGGGATAGTTGGGGGCATTAGTAT
WCG1279Dai24694A
TTTCTAGAGTCGCCGTAATGATTAATAGGGATAGTTGGGGGCATTAGTAT
Dai16971 --
ZQY1043Dai26696 --
Doll880

TTTCTAGAGTCGCCGTAATGATTAATAGGGATAGTTGGGGGCATTAGTAT
Doll1000
TTTCTAGAGTCGCCGTAATGATTAATAGGGATAGTTGGGGGCATTAGTAT
1DAI18529
TTTCTAGAGTCGCCGTAATGATTAATAGGGATAGTTGGGGGCATTAGTAT
Dai19535
TTTCTAGAGTCGCCGTAATGATTAATAGGGATAGTTGGGGGCATTAGTAT
1704_79_hnedyVillaLaPaz
TTTCTAGAGTCGCCGTAATGATTAATAGGGATAGTTGGGGGCATTAGTAT
F2061 --
1DAI18268 --
1DAI18540A
TTTCTAGAGTCGCCGTAATGATTAATAGGGATAGTTGGGGGCATTAGTAT
Dai17695 --
LKY18Dai26373 --
Dai17839P_sulphureus
TTTCTAGAGTCGCCGTAATGATTAATAGGGATAGTTGGGGGCATTAGTAT
Dai17841P_sulphureus
TTTCTAGAGTCGCCGTAATGATTAATAGGGATAGTTGGGGGCATTAGTAT
Dai19877P_roseus
TTTCTAGAGTCGCCGTAATGATTAATAGGGATAGTTGGGGGCATTAGTAT
1508_18_1_Kout --
KP859303_R_vinctus_RP185_BRAZI --
JK1807_15Rigidoporus_sp_Puerto
TTTCTAGAGTCGCCGTAATGATTAATAGGGATAGTTGGGGGCATTAGTAT
JV0509_47_J_TN_USA
TTTCTAGAGTCGCCGTAATGATTAATAGGGATAGTTGGGGGCATTAGTAT
JV0709_188 --
JV0509_127_PA_USA
TTTCTAGAGTCGCCGTAATGATTAATAGGGATAGTTGGGGGCATTAGTAT
JV1009_59_NJ_USA
TTTCTAGAGTCGCCGTAATGATTAATAGGGATAGTTGGGGGCATTAGTAT
Dai15497P_crataegi
TTTCTAGAGTCGCCGTAATGATTAATAGGGATAGTTGGGGGCATTAGTAT
Dai15499P_crataegi
TTTCTAGAGTCGCCGTAATGATTAATAGGGATAGTTGGGGGCATTAGTAT
Cui3266P_cinereus --
WCG1256Dai24690
TTTCTAGAGTCGCCGTAATGATTAATAGGGATAGTTGGGGGCATTAGTAT
1DAI17581 --
WCG1255Dai24688
TTTCTAGAGTCGCCGTAATGATTAATAGGGATAGTTGGGGGCATTAGTAT
Dai22427 --
MV690Meripilus_concrescens --

MV513Meripilus_galapagensis ---
Dai19793
TTTCTAGAGTCGCCGTAATGATTAATAGGGATAGTTGGGGGCATTAGTAT
OQ553780P_tamilnaduensis ---
OQ553779P_tamilnaduensis ---
A164FB3Meripilus_giganteus ---
JV1407_36_Vinctus_Meandrica
TTTCTAGAGTCGCCGTAATGATTAATAGGGATAGTTGGGGGCATTAGTAT
1807_3K_Rigidoporus_PuertoRico
TTTCTAGAGTCGCCGTAATGATTAATAGGGATAGTTGGGGGCATTAGTAT
Cui16903P_vinctus
TTTCTAGAGTCGCCGTAATGATTAATAGGGATAGTTGGGGGCATTAGTAT
JV1008_18R_Lineatus
TTTCTAGAGTCGCCGTAATGATTAATAGGGATAGTTGGGGGCATTAGTAT
JV1407_37_1_Vinctus_Carara
TTTCTAGAGTCGCCGTAATGATTAATAGGGATAGTTGGGGGCATTAGTAT
Dai17986P_lineatus
TTTCTAGAGTCGCCGTAATGATTAATAGGGATAGTTGGGGGCATTAGTAT
Dai18281
TTTCTAGAGTCGCCGTAATGATTAATAGGGATAGTTGGGGGCATTAGTAT
1DAI19796
TTTCTAGAGTCGCCGTAATGATTAATAGGGATAGTTGGGGGCATTAGTAT
ZQY797Dai25241
TTTCTAGAGTCGCCGTAATGATTAATAGGGATAGTTGGGGGCATTAGTAT
WCG1289Dai24711 ---
Dai22598
TTTCTAGAGTCGCCGTAATGATTAATAGGGATAGTTGGGGGCATTAGTAT
Dai20523 ---
Dai17885 ---
Dai17553
TTTCTAGAGTCGCCGTAATGATTAATAGGGATAGTTGGGGGCATTAGTAT
Dai19639
TTTCTAGAGTCGCCGTAATGATTAATAGGGATAGTTGGGGGCATTAGTAT
JV0110_48_CZ
TTTCTAGAGTCGCCGTAATGATTAATAGGGATAGTTGGGGGCATTAGTAT
MJ129_04 ---
Cui10340P_eminens ---
Cui10341P_eminens
TTTCTAGAGTCGCCGTAATGATTAATAGGGATAGTTGGGGGCATTAGTAT
Dai12685P_eminens
TTTCTAGAGTCGCCGTAATGATTAATAGGGATAGTTGGGGGCATTAGTAT
Miettinen_13591Rigidoporus_und ---
Dai20868
TTTCTAGAGTCGCCGTAATGATTAATAGGGATAGTTGGGGGCATTAGTAT

Dai20832
TTTCTAGAGTCGCCGTAATGATTAATAGGGATAGTTGGGGGCATTAGTAT
Dai11400
TTTCTAGAGTCGCCGTAATGATTAATAGGGATAGTTGGGGGCATTAGTAT
Dai22472
TTTCTAGAGTCGCCGTAATGATTAATAGGGATAGTTGGGGGCATTAGTAT
1CUI10475 --
1CUI10491 --
HCFC1095Meripilus_robledoi --
MCW702Meripilus_revolubilis --
1704_83_zluty_HaciendaBaru
TTTCTAGAGTCGCCGTAATGATTAATAGGGATAGTTGGGGGCATTAGTAT
Dai9925P_lavendulus --
Dai13587AP_lavendulus
TTTCTAGAGTCGCCGTAATGATTAATAGGGATAGTTGGGGGCATTAGTAT
PDD70600P_longicystidius --
Cui16630 --
FP_135344Meripilus_giganteus --
FP_100460_Sp --
CBS421_48Meripilus_giganteus --
Cui9203
TTTCTAGAGTCGCCGTAATGATTAATAGGGATAGTTGGGGGCATTAGTAT
Cui9202
TTTCTAGAGTCGCCGTAATGATTAATAGGGATAGTTGGGGGCATTAGTAT
TUFC100564Japan --
Russell5913Meripilus_sumstinei --
RP215Meripilus_brasiliensis --
RP200Meripilus_brasiliensis --
JV1712_13J_R_vinctus2_LSUPuert
TTTCTAGAGTCGCCGTAATGATTAATAGGGATAGTTGGGGGCATTAGTAT
Dai10503R_hypobrunneus --
Dai10569Rigidoporus_hypobrunne --
1DAI19451 --
CM108bRigidoporus_hypobrunneus --
1CUI16874 --
FD299Cerrena_unicolor --
KHL_GB_Cerrena_uniclor --
Dai12892Cerrena_albocinnamomea --
Dai12955C_albocinnamomea --
SNUm03110102C_aurantiopora --
NIBRFG0000102423C_aurantiopora --
Dai7359Antrodiella_zonata --
F20080702KCM29C_consors --
F20080208LYW10Cerrena_consors --

Dai7821Antrodiella_zonata --
CFMR_DCL04_31Pseudolagarobasid --
VPB197Pseudolagarobasidium_bel --
CBS115543Pseudolagarobasidium_ --
CBS115544 --
Han405Pseudolagarobasidium_bai --
Han406Pseudolagarobasidium_bai --
MUcc838Spongipellis_delectans --
BRNM686401S_delectans --
OSM_F925S_delectans --
BRNM67093Spongipellis_litschau --
CFMRccFP59199TS_unicolor --
CFMRccFP71791TS_unicolor --
Dai13845P_lischaueri
TTTCTAGAGTCGCCGTAATGATTAATAGGGATAGTTGGGGGCATTAGTAT
Dai20266P_lischaueri
TTTCTAGAGTCGCCGTAATGATTAATAGGGATAGTTGGGGGCATTAGTAT
CFMR_HHB11240Radulodon_america --
RLG6350Radulodon_americanus --
KY415963Radulodon_erikssonii --
HHB9567spRadulodon_casearius --
KRT_Iso_26Radulodon_casearius --
CBS126044Radulodon_erikssonii --
He6183YUNNANENSIS
TTTCTAGAGTCGCCGTAATGATTAATAGGGATAGTTGGGGGCATTAGTAT
Cui17979YUNNANENSIS
TTTCTAGAGTCGCCGTAATGATTAATAGGGATAGTTGGGGGCATTAGTAT
Miettinen2091Junghuhnia_fimbri --
KHL12316S_tenue --
PRM846564S_pachyodon --
SP_Lgt_S_pachyodon --
Ryvarden44669Tyromyces_xuchile --
PW17_171sinuosus --
W53Dai12234 --
HHB4100SpAntella_americana --
W3Dai20901spumeus
TTTCTAGAGTCGCCGTAATGATTAATAGGGATAGTTGGGGGCATTAGTAT
He6736 --
BRNM734877S_spumeus --
BRNM712630S_spumeus --
Dai1723Loweomyces_sibiricus --
W54Cui10009
TTTCTAGAGTCGCCGTAATGATTAATAGGGATAGTTGGGGGCATTAGTAT
W1Dai20899

TTTCTAGAGTCGCCGTAATGATTAATAGGGATAGTTGGGGGCATTAGTAT
HHB13445Trametes_ochracea --
Dai16222 --
Dai16240 --

JV1310_11SanguinolentusCernys
TCAGTTGCTAGAGGTGAAATTCTTGGATTTACTGAAGACTAACTACTGCG
MJ39_00_SK --
MJ111_04_CZ
TCAGTTGCTAGAGGTGAAATTCTTGGATTTACTGAAGACTAACTACTGCG
JV1610_BOKYsmrk
TCAGTTGCTAGAGGTGAAATTCTTGGATTTACTGAAGACTAACTACTGCG
Dai21030 --
Dai20976P_furcatus
TCAGTTGCTAGAGGTGAAATTCTTGGATTTACTGAAGACTAACTACTGCG
Dai2105 --
Dai2544 --
Dai11313 --
WCG1611Dai26167
TCAGTTGCTAGAGGTGAAATTCTTGGATTTACTGAAGACTAACTACTGCG
WCG1518Dai25999Physisporinus
TCAGTTGCTAGAGGTGAAATTCTTGGATTTACTGAAGACTAACTACTGCG
TAA15097 --
JV8909_19_CZ --
JV1310_15_P_sanguinolentus2_CZ --
MJ53_02_CZ
TCAGGCGCTAGAGGTGAAATTCTTGGATTGCCTGAAGACTAACTACTGCG
CLZhao21647P_yunnanensis --
CLZhao21583P_yunnanensis --
Dai22272
TCAGTTGCTAGAGGTGAAATTCTTGGATTTACTGAAGACTAACTACTGCG
Dai22279
TCAGTTGCTAGAGGTGAAATTCTTGGATTTACTGAAGACTAACTACTGCG
MJ332_94_CZ --
MJ642_94_CZ_Expallescens --
Dai21060P_vinctus
TCAGTTGCTAGAGGTGAAATTCTTGGATTTACTGAAGACTAACTATTGCG
JV0511_23LRP_pouzarii
TCAGTTGCTAGAGGTGAAATTCTTGGATTTACTGAAGACTAACTACTGCG
JQ409462_R_pouzarii_PRM899856_ --
JV0308_66_WA
TCAGTTGCTAGAGGTGAAATTCTTGGATTTACTGAAGACTAACTATTGCG
JV0309_45_WA_USA
TCAGTTGCTAGAGGTGAAATTCTTGGATTTACTGAAGACTAACTATTGCG

JV0709_83_CA_USA
TCAGTTGCTAGAGGTGAAATTCTTGGATTTACTGAAGACTAACTATTGCG
Dai21043P_pouzarii
TCAGTTGCTAGAGGTGAAATTCTTGGATTTACTGAAGACTAACTACTGCG
MJ144_95_CZ --
JV0909_3_CZ
TGCGTTGCTAGAGGTGAAATTCTTGGATTGACGCAAGACTAACTACTGCG
JV0609_1_K --
Dai20396Physisporinus_castanop
TCAGTTGCTAGAGGTGAAATTCTTGGATTTACTGAAGACTAACTACTGCG
Dai20397Physisporinus_castanop
TCAGTTGCTAGAGGTGAAATTCTTGGATTTACTGAAGACTAACTACTGCG
MJ19_09_SK_Abies --
JV0509_40_J_TN_USA_Betula
TCAGTTGCTAGAGGTGAAATTCTTGGATTTACTGAAGACTAACTACTGCG
JV0808_33crocatus_PA_USAlist
TCAGTTGCTAGAGGTGAAATTCTTGGATTTACTGAAGACTAACTATTGCG
DLL2009_061P_crocatus --
Dai12800P_subcrocatus --
Dai15917P_subcrocatus
TCAGTTGCTAGAGGTGAAATTCTTGGATTTACTGAAGACTAACTACTGCG
Cui16620 --
HCFC1088Meripilus_stillicidior --
MCW590Meripilus_obsrcurus --
MCW722Meripilus_obsrcurus --
Cui9381P_tibeticus
TCAGTTGCTAGAGGTGAAATTCTTGGATTTACTGAAGACTAACTACTGCG
Cui9588P_tibeticus
TCAGTTGCTAGAGGTGAAATTCTTGGATTTACTGAAGACTAACTACTGCG
Va2_Beneschova --
CWU3874_Ukraine_Alnus --
WCG1293Dai24718Physisporinus --
WCG1268Dai24682A
TCAGTTGCTAGAGGTGAAATTCTTGGATTTACTGAAGACTAACTACTGCG
WCG1269Dai24683A
TCAGTTGCTAGAGGTGAAATTCTTGGATTTACTGAAGACTAACTACTGCG
WCG1279Dai24694A
TCAGTTGCTAGAGGTGAAATTCTTGGATTTACTGAAGACTAACTACTGCG
Dai16971 --
ZQY1043Dai26696 --
Doll880
TCAGTTGCTAGAGGTGAAATTCTTGGATTTACTGAAGACTAACTACTGCG
Doll1000
TCAGTTGCTAGAGGTGAAATTCTTGGATTTACTGAAGACTAACTACTGCG

1DAI18529
TCAGTTGCTAGAGGTGAAATTCTTGGATTTACTGAAGACTAACTACTGCG
Dai19535
TCAGTTGCTAGAGGTGAAATTCTTGGATTTACTGAAGACTAACTACTGCG
1704_79_hnedyVillaLaPaz
TCAGTTGCTAGAGGTGAAATTCTTGGATTTACTGAAGACTAACTACTGCG
F2061 --
1DAI18268 --
1DAI18540A
TCAGTTGCTAGAGGTGAAATTCTTGGATTTACTGAAGACTAACTACTGCG
Dai17695 --
LKY18Dai26373 --
Dai17839P_sulphureus
TCAGTTGCTAGAGGTGAAATTCTTGGATTTACTGAAGACTAACTACTGCG
Dai17841P_sulphureus
TCAGTTGCTAGAGGTGAAATTCTTGGATTTACTGAAGACTAACTACTGCG
Dai19877P_roseus
TCAGTTGCTAGAGGTGAAATTCTTGGATTTACTGAAGACTAACTACTGCG
1508_18_1_Kout --
KP859303_R_vinctus_RP185_BRAZI --
JK1807_15Rigidoporus_sp_Puerto
TCAGTTGCTAGAGGTGAAATTCTTGGATTTACTGAAGACTAACTACTGCG
JV0509_47_J_TN_USA
TCAGTTGCTAGAGGTGAAATTCTTGGATTTACTGAAGACTAACTACTGCG
JV0709_188 --
JV0509_127_PA_USA
TCAGTTGCTAGAGGTGAAATTCTTGGATTTACTGAAGACTAACTACTGCG
JV1009_59_NJ_USA
TCAGTTGCTAGAGGTGAAATTCTTGGATTTACTGAAGACTAACTACTGCG
Dai15497P_crataegi
TCAGTTGCTAGAGGTGAAATTCTTGGATTTACTGAAGACTAACTACTGCG
Dai15499P_crataegi
TCAGTTGCTAGAGGTGAAATTCTTGGATTTACTGAAGACTAACTACTGCG
Cui3266P_cinereus --
WCG1256Dai24690
TCAGTTGCTAGAGGTGAAATTCTTGGATTTACTGAAGACTAACTACTGCG
1DAI17581 --
WCG1255Dai24688
TCAGTTGCTAGAGGTGAAATTCTTGGATTTACTGAAGACTAACTACTGCG
Dai22427 --
MV690Meripilus_concrescens --
MV513Meripilus_galapagensis --
Dai19793
TCAGTTGCTAGAGGTGAAATTCTTGGATTTACTGAAGACTAACTACTGCG

OQ553780P_tamilnaduensis --
OQ553779P_tamilnaduensis --
A164FB3Meripilus_giganteus --
JV1407_36_Vinctus_Meandrica
TCAGTTGCTAGAGGTGAAATTCTTGGATTTACTGAAGACTAACTACTGCG
1807_3K_Rigidoporus_PuertoRico
TCAGTTGCTAGAGGTGAAATTCTTGGATTTACTGAAGACTAACTACTGCG
Cui16903P_vinctus
TCAGTTGCTAGAGGTGAAATTCTTGGATTTACTGAAGACTAACTACTGCG
JV1008_18R_Lineatus
TCAGTTGCTAGAGGTGAAATTCTTGGATTTACTGAAGACTAACTACTGCG
JV1407_37_1_Vinctus_Carara
TCAGTTGCTAGAGGTGAAATTCTTGGATTTACTGAAGACTAACTACTGCG
Dai17986P_lineatus
TCAGTTGCTAGAGGTGAAATTCTTGGATTTACTGAAGACTAACTACTGCG
Dai18281
TCAGTTGCTAGAGGTGAAATTCTTGGATTTACTGAAGACTAACTACTGCG
1DAI19796
TCAGTTGCTAGAGGTGAAATTCTTGGATTTACTGAAGACTAACTACTGCG
ZQY797Dai25241
TCAGTTGCTAGAGGTGAAATTCTTGGATTTACTGAAGACTAACTACTGCG
WCG1289Dai24711 --
Dai22598
TCAGTTGCTAGAGGTGAAATTCTTGGATTTACTGAAGACTAACTACTGCG
Dai20523 --
Dai17885 --
Dai17553
TCAGTTGCTAGAGGTGAAATTCTTGGATTTACTGAAGACTAACTACTGCG
Dai19639
TCAGTTGCTAGAGGTGAAATTCTTGGATTTACTGAAGACTAACTACTGCG
JV0110_48_CZ
TCAGTTGCTAGAGGTGAAATTCTTGGATTTACTGAAGACTAACTATTGCG
MJ129_04 --
Cui10340P_eminens --
Cui10341P_eminens
TCAGTTGCTAGAGGTGAAATTCTTGGATTTACTGAAGACTAACTATTGCG
Dai12685P_eminens
TCAGTTGCTAGAGGTGAAATTCTTGGATTTACTGAAGACTAACTATTGCG
Miettinen_13591Rigidoporus_und --
Dai20868
TCAGTTGCTAGAGGTGAAATTCTTGGATTTACTGAAGACTAACTACTGCG
Dai20832
TCAGTTGCTAGAGGTGAAATTCTTGGATTTACTGAAGACTAACTACTGCG
Dai11400

TCAGTTGCTAGAGGTGAAATTCTTGGATTTACTGAAGACTAACTACTGCG
Dai22472
TCAGTTGCTAGAGGTGAAATTCTTGGATTTACTGAAGACTAACTACTGCG
1CUI10475 --
1CUI10491 --
HCFC1095Meripilus_robledoi --
MCW702Meripilus_revolubilis --
1704_83_zluty_HaciendaBaru
TCAGTTGCTAGAGGTGAAATTCTTGGATTTACTGAAGACTAACTATTGCG
Dai9925P_lavendulus --
Dai13587AP_lavendulus
TCAGTTGCTAGAGGTGAAATTCTTGGATTTACTGAAGACTAACTATTGCG
PDD70600P_longicystidius --
Cui16630 --
FP_135344Meripilus_giganteus --
FP_100460_Sp --
CBS421_48Meripilus_giganteus --
Cui9203
TCAGTTGCTAGAGGTGAAATTCTTGGATTTACTGAAGACTAACTACTGCG
Cui9202
TCAGTTGCTAGAGGTGAAATTCTTGGATTTACTGAAGACTAACTACTGCG
TUFC100564Japan --
Russell5913Meripilus_sumstinei --
RP215Meripilus_brasiliensis --
RP200Meripilus_brasiliensis --
JV1712_13J_R_vinctus2_LSUPuert
TCAGTTGCTAGAGGTGAAATTCTTGGATTTACTGAAGACT-ACTACTGCG
Dai10503R_hypobrunneus --
Dai10569Rigidoporus_hypobrunne --
1DAI19451 --
CM108bRigidoporus_hypobrunneus --
1CUI16874 --
FD299Cerrena_unicolor --
KHL_GB_Cerrena_uniclor --
Dai12892Cerrena_albocinnamomea --
Dai12955C_albocinnamomea --
SNUm03110102C_aurantiopora --
NIBRFG0000102423C_aurantiopora --
Dai7359Antrodiella_zonata --
F20080702KCM29C_consors --
F20080208LYW10Cerrena_consors --
Dai7821Antrodiella_zonata --
CFMR_DCL04_31Pseudolagarobasid --
VPB197Pseudolagarobasidium_bel --

CBS115543Pseudolagarobasidium_ --
CBS115544 --
Han405Pseudolagarobasidium_bai --
Han406Pseudolagarobasidium_bai --
MUcc838Spongipellis_delectans --
BRNM686401S_delectans --
OSM_F925S_delectans --
BRNM67093Spongipellis_litschau --
CFMRccFP59199TS_unicolor --
CFMRccFP71791TS_unicolor --
Dai13845P_lischaueri
TCAGTTGCTAGAGGTGAAATTCTTGGATTTACTGAAGACTAACTACTGCG
Dai20266P_lischaueri
TCAGTTGCTAGAGGTGAAATTCTTGGATTTACTGAAGACTAACTACTGCG
CFMR_HHB11240Radulodon_america --
RLG6350Radulodon_americanus --
KY415963Radulodon_erikssonii --
HHB9567spRadulodon_casearius --
KRT_Iso_26Radulodon_casearius --
CBS126044Radulodon_erikssonii --
He6183YUNNANENSIS
TCAGTTGCTAGAGGTGAAATTCTTGGATTTACTGAAGACTAACTACTGCG
Cui17979YUNNANENSIS
TCAGTTGCTAGAGGTGAAATTCTTGGATTTACTGAAGACTAACTACTGCG
Miettinen2091Junghuhnia_fimbri --
KHL12316S_tenue --
PRM846564S_pachyodon --
SP_Lgt_S_pachyodon --
Ryvarden44669Tyromyces_xuchile --
PW17_171sinuosus --
W53Dai12234 --
HHB4100SpAntella_americana --
W3Dai20901spumeus
TCAGTTGCTAGAGGTGAAATTCTTGGATTTACTGAAGACTAACTACTGCG
He6736 --
BRNM734877S_spumeus --
BRNM712630S_spumeus --
Dai1723Loweomyces_sibiricus --
W54Cui10009 TCAGTTGCTAGA-
GTGAAATTCTTGGATTTACTGAAGACTAACTACTGCG
W1Dai20899
TCAGTTGCTAGAGGTGAAATTCTTGGATTTACTGAAGACTAACTACTGCG
HHB13445Trametes_ochracea --
Dai16222 --

Dai16240 ---

JV1310_11SanguinolentusCernys
AAAGCATTTGCCAAGGATGTTTTCATTAATCAAGAACGAAGGTTAGGGGA
MJ39_00_SK ---
MJ111_04_CZ
AAAGCATTTGCCAAGGATGTTTTCATTAATCAAGAACGAAGGTTAGGGGA
JV1610_BOKYsmrk
AAAGCATTTGCCAAGGATGTTTTCATTAATCAAGAACGAAGGTTAGGGGA
Dai21030 ---
Dai20976P_furcatus
AAAGCATTTGCCAAGGATGTTTTCATTAATCAAGAACGAAGGTTAGGGGA
Dai2105 ---
Dai2544 ---
Dai11313 ---
WCG1611Dai26167
AAAGCATTTGCCAAGGATGTTTTCATTAATCAAGAACGAAGGTTAGGGGA
WCG1518Dai25999Physisporinus
AAAGCATTTGCCAAGGATGTTTTCATTAATCAAGAACGAAGGTTAGGGGA
TAA15097 ---
JV8909_19_CZ ---
JV1310_15_P_sanguinolentus2_CZ ---
MJ53_02_CZ
AAAGCATTTGCCAAGGATGTTTTCATTGATCAAGGACGAAGGTTAGGGGC
CLZhao21647P_yunnanensis ---
CLZhao21583P_yunnanensis ---
Dai22272
AAAGCATTTGCCAAGGATGTTTTCATTAATCAAGAACGAAGGTTAGGGGA
Dai22279
AAAGCATTTGCCAAGGATGTTTTCATTAATCAAGAACGAAGGTTAGGGGA
MJ332_94_CZ ---
MJ642_94_CZ_Expallescens ---
Dai21060P_vinctus
AAAGCATTTGCCAAGGATGTTTTCATTAATCAAGAACGAAGGTTAGGGGA
JV0511_23LRP_pouzarii
AAAGCATTTGCCAAGGATGTTTTCATTAATCAAGAACGAAGGTTAGGGGA
JQ409462_R_pouzarii_PRM899856_ ---
JV0308_66_WA
AAAGCATTTGCCAAGGATGTTTTCATTAATCAAGAACGAAGGTTAGGGGA
JV0309_45_WA_USA
AAAGCATTTGCCAAGGATGTTTTCATTAATCAAGAACGAAGGTTAGGGGA
JV0709_83_CA_USA
AAAGCATTTGCCAAGGATGTTTTCATTAATCAAGAACGAAGGTTAGGGGA
Dai21043P_pouzarii

AAAGCATTTGCCAAGGATGTTTTCATTAATCAAGAACGAAGGTTAGGGGA
MJ144_95_CZ --
JV0909_3_CZ
AAAGCATTTGCCAAGGATGTTTTCATTAATCAAGAACGAAGGTTAGGGGA
JV0609_1_K --
Dai20396Physisporinus_castanop
AAAGCATTTGCCAAGGATGTTTTCATTAATCAAGAACGAAGGTTAGGGGA
Dai20397Physisporinus_castanop
AAAGCATTTGCCAAGGATGTTTTCATTAATCAAGAACGAAGGTTAGGGGA
MJ19_09_SK_Abies --
JV0509_40_J_TN_USA_Betula
AAAGCATTTGCCAAGGATGTTTTCATTAATCAAGAACGAAGGTTAGGGGA
JV0808_33crocatus_PA_USAlist
AAAGCATTTGCCAAGGATGTTTTCATTAATCAAGAACGAAGGTTAGGGGA
DLL2009_061P_crocatus --
Dai12800P_subcrocatus --
Dai15917P_subcrocatus
AAAGCATTTGCCAAGGATGTTTTCATTAATCAAGAACGAAGGTTAGGGGA
Cui16620 --
HCFC1088Meripilus_stillicidior --
MCW590Meripilus_obsrcurus --
MCW722Meripilus_obsrcurus --
Cui9381P_tibeticus
AAAGCATTTGCCAAGGATGTTTTCATTAATCAAGAACGAAGGTTAGGGGA
Cui9588P_tibeticus
AAAGCATTTGCCAAGGATGTTTTCATTAATCAAGAACGAAGGTTAGGGGA
Va2_Beneschova --
CWU3874_Ukraine_Alnus --
WCG1293Dai24718Physisporinus --
WCG1268Dai24682A
AAAGCATTTGCCAAGGATGTTTTCATTAATCAAGAACGAAGGTTAGGGGA
WCG1269Dai24683A
AAAGCATTTGCCAAGGATGTTTTCATTAATCAAGAACGAAGGTTAGGGGA
WCG1279Dai24694A
AAAGCATTTGCCAAGGATGTTTTCATTAATCAAGAACGAAGGTTAGGGGA
Dai16971 --
ZQY1043Dai26696 --
Doll880
AAAGCATTTGCCAAGGATGTTTTCATTAATCAAGAACGAAGGTTAGGGGA
Doll1000
AAAGCATTTGCCAAGGATGTTTTCATTAATCAAGAACGAAGGTTAGGGGA
1DAI18529
AAAGCATTTGCCAAGGATGTTTTCATTAATCAAGAACGAAGGTTAGGGGA
Dai19535

AAAGCATTTGCCAAGGATGTTTTCATTAATCAAGAACGAAGGTTAGGGGA
1704_79_hnedyVillaLaPaz
AAAGCATTTGCCAAGGATGTTTTCATTAATCAAGAACGAAGGTTAGGGGA
F2061 --
1DAI18268 --
1DAI18540A
AAAGCATTTGCCAAGGATGTTTTCATTAATCAAGAACGAAGGTTAGGGGA
Dai17695 --
LKY18Dai26373 --
Dai17839P_sulphureus
AAAGCATTTGCCAAGGATGTTTTCATTAATCAAGAACGAAGGTTAGGGGA
Dai17841P_sulphureus
AAAGCATTTGCCAAGGATGTTTTCATTAATCAAGAACGAAGGTTAGGGGA
Dai19877P_roseus
AAAGCATTTGCCAAGGATGTTTTCATTAATCAAGAACGAAGGTTAGGGGA
1508_18_1_Kout --
KP859303_R_vinctus_RP185_BRAZI --
JK1807_15Rigidoporus_sp_Puerto
AAAGCATTTGCCAAGGATGTTTTCATTAATCAAGAACGAAGGTTAGGGGA
JV0509_47_J_TN_USA
AAAGCATTTGCCAAGGATGTTTTCATTAATCAAGAACGAAGGTTAGGGGA
JV0709_188 --
JV0509_127_PA_USA
AAAGCATTTGCCAAGGATGTTTTCATTAATCAAGAACGAAGGTTAGGGGA
JV1009_59_NJ_USA
AAAGCATTTGCCAAGGATGTTTTCATTAATCAAGAACGAAGGTTAGGGGA
Dai15497P_crataegi
AAAGCATTTGCCAAGGATGTTTTCATTAATCAAGAACGAAGGTTAGGGGA
Dai15499P_crataegi
AAAGCATTTGCCAAGGATGTTTTCATTAATCAAGAACGAAGGTTAGGGGA
Cui3266P_cinereus --
WCG1256Dai24690
AAAGCATTTGCCAAGGATGTTTTCATTAATCAAGAACGAAGGTTAGGGGA
1DAI17581 --
WCG1255Dai24688
AAAGCATTTGCCAAGGATGTTTTCATTAATCAAGAACGAAGGTTAGGGGA
Dai22427 --
MV690Meripilus_concrescens --
MV513Meripilus_galapagensis --
Dai19793
AAAGCATTTGCCAAGGATGTTTTCATTAATCAAGAACGAAGGTTAGGGGA
OQ553780P_tamilnaduensis --
OQ553779P_tamilnaduensis --
A164FB3Meripilus_giganteus --

JV1407_36_Vinctus_Meandrica
AAAGCATTTGCCAAGGATGTTTTCATTAATCAAGAACGAAGGTTAGGGGA
1807_3K_Rigidoporus_PuertoRico
AAAGCATTTGCCAAGGATGTTTTCATTAATCAAGAACGAAGGTTAGGGGA
Cui16903P_vinctus
AAAGCATTTGCCAAGGATGTTTTCATTAATCAAGAACGAAGGTTAGGGGA
JV1008_18R_Lineatus
AAAGCATTTGCCAAGGATGTTTTCATTAATCAAGAACGAAGGTTAGGGGA
JV1407_37_1_Vinctus_Carara
AAAGCATTTGCCAAGGATGTTTTCATTAATCAAGAACGAAGGTTAGGGGA
Dai17986P_lineatus
AAAGCATTTGCCAAGGATGTTTTCATTAATCAAGAACGAAGGTTAGGGGA
Dai18281
AAAGCATTTGCCAAGGATGTTTTCATTAATCAAGAACGAAGGTTAGGGGA
1DAI19796
AAAGCATTTGCCAAGGATGTTTTCATTAATCAAGAACGAAGGTTAGGGGA
ZQY797Dai25241
AAAGCATTTGCCAAGGATGTTTTCATTAATCAAGAACGAAGGTTAGGGGA
WCG1289Dai24711 --
Dai22598
AAAGCATTTGCCAAGGATGTTTTCATTAATCAAGAACGAAGGTTAGGGGA
Dai20523 --
Dai17885 --
Dai17553
AAAGCATTTGCCAAGGATGTTTTCATTAATCAAGAACGAAGGTTAGGGGA
Dai19639
AAAGCATTTGCCAAGGATGTTTTCATTAATCAAGAACGAAGGTTAGGGGA
JV0110_48_CZ
AAAGCATTTGCCAAGGATGTTTTCATTAATCAAGAACGAAGGTTAGGGGA
MJ129_04 --
Cui10340P_eminens --
Cui10341P_eminens
AAAGCATTTGCCAAGGATGTTTTCATTAATCAAGAACGAAGGTTAGGGGA
Dai12685P_eminens
AAAGCATTTGCCAAGGATGTTTTCATTAATCAAGAACGAAGGTTAGGGGA
Miettinen_13591Rigidoporus_und --
Dai20868
AAAGCATTTGCCAAGGATGTTTTCATTAATCAAGAACGAAGGTTAGGGGA
Dai20832
AAAGCATTTGCCAAGGATGTTTTCATTAATCAAGAACGAAGGTTAGGGGA
Dai11400
AAAGCATTTGCCAAGGATGTTTTCATTAATCAAGAACGAAGGTTAGGGGA
Dai22472
AAAGCATTTGCCAAGGATGTTTTCATTAATCAAGAACGAAGGTTAGGGGA

1CUI10475 --
1CUI10491 --
HCFC1095Meripilus_robledoi --
MCW702Meripilus_revolubilis --
1704_83_zluty_HaciendaBaru
AAAGCATTTGCCAAGGATGTTTTCATTAATCAAGAACGAAGGTTAGGGGA
Dai9925P_lavendulus --
Dai13587AP_lavendulus
AAAGCATTTGCCAAGGATGTTTTCATTAATCAAGAACGAAGGTTAGGGGA
PDD70600P_longicystidius --
Cui16630 --
FP_135344Meripilus_giganteus --
FP_100460_Sp --
CBS421_48Meripilus_giganteus --
Cui9203
AAAGCATTTGCCAAGGATGTTTTCATTAATCAAGAACGAAGGTTAGGGGA
Cui9202
AAAGCATTTGCCAAGGATGTTTTCATTAATCAAGAACGAAGGTTAGGGGA
TUFC100564Japan --
Russell5913Meripilus_sumstinei --
RP215Meripilus_brasiliensis --
RP200Meripilus_brasiliensis --
JV1712_13J_R_vinctus2_LSUPuert AAAGCATTTGCCAAGGATG-
TTTCATTAATCAAGAACGACG--TTAGGGA
Dai10503R_hypobrunneus --
Dai10569Rigidoporus_hypobrunne --
1DAI19451 --
CM108bRigidoporus_hypobrunneus --
1CUI16874 --
FD299Cerrena_unicolor --
KHL_GB_Cerrena_uniclor --
Dai12892Cerrena_albocinnamomea --
Dai12955C_albocinnamomea --
SNUm03110102C_aurantiopora --
NIBRFG0000102423C_aurantiopora --
Dai7359Antrodiella_zonata --
F20080702KCM29C_consors --
F20080208LYW10Cerrena_consors --
Dai7821Antrodiella_zonata --
CFMR_DCL04_31Pseudolagarobasid --
VPB197Pseudolagarobasidium_bel --
CBS115543Pseudolagarobasidium_ --
CBS115544 --
Han405Pseudolagarobasidium_bai --

Han406Pseudolagarobasidium_bai --
MUcc838Spongipellis_delectans --
BRNM686401S_delectans --
OSM_F925S_delectans --
BRNM67093Spongipellis_litschau --
CFMRccFP59199TS_unicolor --
CFMRccFP71791TS_unicolor --
Dai13845P_lischaueri
AAAGCATTTGCCAAGGATGTTTTCATTAATCAAGAACGAAGGTTAGGGGA
Dai20266P_lischaueri
AAAGCATTTGCCAAGGATGTTTTCATTAATCAAGAACGAAGGTTAGGGGA
CFMR_HHB11240Radulodon_america --
RLG6350Radulodon_americanus --
KY415963Radulodon_erikssonii --
HHB9567spRadulodon_casearius --
KRT_Iso_26Radulodon_casearius --
CBS126044Radulodon_erikssonii --
He6183YUNNANENSIS
AAAGCATTTGCCAAGGATGTTTTCATTAATCAAGAACGAAGGTTAGGGGA
Cui17979YUNNANENSIS
AAAGCATTTGCCAAGGATGTTTTCATTAATCAAGAACGAAGGTTAGGGGA
Miettinen2091Junghuhnia_fimbri --
KHL12316S_tenue --
PRM846564S_pachyodon --
SP_Lgt_S_pachyodon --
Ryvarden44669Tyromyces_xuchile --
PW17_171sinuosus --
W53Dai12234 --
HHB4100SpAntella_americana --
W3Dai20901spumeus
AAAGCATTTGCCAAGGATGTTTTCATTAATCAAGAACGAAGGTTAGGGGA
He6736 --
BRNM734877S_spumeus --
BRNM712630S_spumeus --
Dai1723Loweomyces_sibiricus --
W54Cui10009 AAAGC---
W1Dai20899
AAAGCATTTGCCAAGGATGTTTTCATTAATCAAGAACGAAGGTTAGGGGA
HHB13445Trametes_ochracea --
Dai16222 --
Dai16240 --

JV1310_11SanguinolentusCernys
TCGAAAACGATCAGATACCGTTGTAGTCTTAACAGTAAACTATGCCGACT

MJ39_00_SK --
MJ111_04_CZ
TCGAAAACGATCAGATACCGTTGTAGTCTTAACAGTAAACTATGCCGACT
JV1610_BOKYsmrk
TCGAAAACGATCAGATACCGTTGTAGTCTTAACAGTAAACTATGCCGACT
Dai21030 --
Dai20976P_furcatus
TCGAAAACGATCAGATACCGTTGTAGTCTTAACAGTAAACTATGCCGACT
Dai2105 --
Dai2544 --
Dai11313 --
WCG1611Dai26167
TCGAAAACGATCAGATACCGTTGTAGTCTTAACAGTAAACTATGCCGACT
WCG1518Dai25999Physisporinus
TCGAAAACGATCAGATACCGTTGTAGTCTTAACAGTAAACTATGCCGACT
TAA15097 --
JV8909_19_CZ --
JV1310_15_P_sanguinolentus2_CZ --
MJ53_02_CZ
TCGAAAACGATCAGATACCGTTGTAGTCTTAACAGTAAACTATGCCGACT
CLZhao21647P_yunnanensis --
CLZhao21583P_yunnanensis --
Dai22272
TCGAAAACGATCAGATACCGTTGTAGTCTTAACAGTAAACTATGCCGACT
Dai22279
TCGAAAACGATCAGATACCGTTGTAGTCTTAACAGTAAACTATGCCGACT
MJ332_94_CZ --
MJ642_94_CZ_Expallescens --
Dai21060P_vinctus
TCGAAAACGATCAGATACCGTTGTAGTCTTAACAGTAAACTATGCCGACT
JV0511_23LRP_pouzarii
TCGAAAACGATCAGATACCGTTGTAGTCTTAACAGTAAACTATGCCGACT
JQ409462_R_pouzarii_PRM899856_ --
JV0308_66_WA
TCGAAAACGATCAGATACCGTTGTAGTCTTAACAGTAAACTATGCCGACT
JV0309_45_WA_USA
TCGAAAACGATCAGATACCGTTGTAGTCTTAACAGTAAACTATGCCGACT
JV0709_83_CA_USA
TCGAAAACGATCAGATACCGTTGTAGTCTTAACAGTAAACTATGCCGACT
Dai21043P_pouzarii
TCGAAAACGATCAGATACCGTTGTAGTCTTAACAGTAAACTATGCCGACT
MJ144_95_CZ --
JV0909_3_CZ
TCGAAAACGATCAGATACCGTTGTAGTCTTAACAGTAAACTATGCCGACT

JV0609_1_K --
Dai20396Physisporinus_castanop
TCGAAAACGATCAGATACCGTTGTAGTCTTAACAGTAAACTATGCCGACT
Dai20397Physisporinus_castanop
TCGAAAACGATCAGATACCGTTGTAGTCTTAACAGTAAACTATGCCGACT
MJ19_09_SK_Abies --
JV0509_40_J_TN_USA_Betula
TCGAAAACGATCAGATACCGTTGTAGTCTTAACAGTAAACTATGCCGACT
JV0808_33crocatus_PA_USAlist
TCGAAAACGATCAGATACCGTTGTAGTCTTAACAGTAAACTATGCCGACT
DLL2009_061P_crocatus --
Dai12800P_subcrocatus --
Dai15917P_subcrocatus
TCGAAAACGATCAGATACCGTTGTAGTCTTAACAGTAAACTATGCCGACT
Cui16620 --
HCFC1088Meripilus_stillicidior --
MCW590Meripilus_obsrcurus --
MCW722Meripilus_obsrcurus --
Cui9381P_tibeticus
TCGAAAACGATCAGATACCGTTGTAGTCTTAACAGTAAACTATGCCGACT
Cui9588P_tibeticus
TCGAAAACGATCAGATACCGTTGTAGTCTTAACAGTAAACTATGCCGACT
Va2_Beneschova --
CWU3874_Ukraine_Alnus --
WCG1293Dai24718Physisporinus --
WCG1268Dai24682A
TCGAAAACGATCAGATACCGTTGTAGTCTTAACAGTAAACTATGCCGACT
WCG1269Dai24683A
TCGAAAACGATCAGATACCGTTGTAGTCTTAACAGTAAACTATGCCGACT
WCG1279Dai24694A
TCGAAAACGATCAGATACCGTTGTAGTCTTAACAGTAAACTATGCCGACT
Dai16971 --
ZQY1043Dai26696 --
Doll880
TCGAAAACGATCAGATACCGTTGTAGTCTTAACAGTAAACTATGCCGACT
Doll1000
TCGAAAACGATCAGATACCGTTGTAGTCTTAACAGTAAACTATGCCGACT
1DAI18529
TCGAAAACGATCAGATACCGTTGTAGTCTTAACAGTAAACTATGCCGACT
Dai19535
TCGAAAACGATCAGATACCGTTGTAGTCTTAACAGTAAACTATGCCGACT
1704_79_hnedyVillaLaPaz
TCGAAAACGATCAGATACCGTTGTAGTCTTAACAGTAAACTATGCCGACT
F2061 --

1DAI18268 ---
1DAI18540A
TCGAAAACGATCAGATACCGTTGTAGTCTTAACAGTAAACTATGCCGACT
Dai17695 ---
LKY18Dai26373 ---
Dai17839P_sulphureus
TCGAAAACGATCAGATACCGTTGTAGTCTTAACAGTAAACTATGCCGACT
Dai17841P_sulphureus
TCGAAAACGATCAGATACCGTTGTAGTCTTAACAGTAAACTATGCCGACT
Dai19877P_roseus
TCGAAAACGATCAGATACCGTTGTAGTCTTAACAGTAAACTATGCCGACT
1508_18_1_Kout ---
KP859303_R_vinctus_RP185_BRAZI ---
JK1807_15Rigidoporus_sp_Puerto
TCGAAAACGATCAGATACCGTTGTAGTCTTAACAGTAAACTATGCCGACT
JV0509_47_J_TN_USA
TCGAAAACGATCAGATACCGTTGTAGTCTTAACAGTAAACTATGCCGACT
JV0709_188 ---
JV0509_127_PA_USA
TCGAAAACGATCAGATACCGTTGTAGTCTTAACAGTAAACTATGCCGACT
JV1009_59_NJ_USA
TCGAAAACGATCAGATACCGTTGTAGTCTTAACAGTAAACTATGCCGACT
Dai15497P_crataegi
TCGAAAACGATCAGATACCGTTGTAGTCTTAACAGTAAACTATGCCGACT
Dai15499P_crataegi
TCGAAAACGATCAGATACCGTTGTAGTCTTAACAGTAAACTATGCCGACT
Cui3266P_cinereus ---
WCG1256Dai24690
TCGAAAACGATCAGATACCGTTGTAGTCTTAACAGTAAACTATGCCGACT
1DAI17581 ---
WCG1255Dai24688
TCGAAAACGATCAGATACCGTTGTAGTCTTAACAGTAAACTATGCCGACT
Dai22427 ---
MV690Meripilus_concrescens ---
MV513Meripilus_galapagensis ---
Dai19793
TCGAAAACGATCAGATACCGTTGTAGTCTTAACAGTAAACTATGCCGACT
OQ553780P_tamilnaduensis ---
OQ553779P_tamilnaduensis ---
A164FB3Meripilus_giganteus ---
JV1407_36_Vinctus_Meandrica
TCGAAAACGATCAGATACCGTTGTAGTCTTAACAGTAAACTATGCCGACT
1807_3K_Rigidoporus_PuertoRico
TCGAAAACGATCAGATACCGTTGTAGTCTTAACAGTAAACTATGCCGACT

Cui16903P_vinctus
TCGAAAACGATCAGATACCGTTGTAGTCTTAACAGTAAACTATGCCGACT
JV1008_18R_Lineatus
TCGAAAACGATCAGATACCGTTGTAGTCTTAACAGTAAACTATGCCGACT
JV1407_37_1_Vinctus_Carara
TCGAAAACGATCAGATACCGTTGTAGTCTTAACAGTAAACTATGCCGACT
Dai17986P_lineatus
TCGAAAACGATCAGATACCGTTGTAGTCTTAACAGTAAACTATGCCGACT
Dai18281
TCGAAAACGATCAGATACCGTTGTAGTCTTAACAGTAAACTATGCCGACT
1DAI19796
TCGAAAACGATCAGATACCGTTGTAGTCTTAACAGTAAACTATGCCGACT
ZQY797Dai25241
TCGAAAACGATCAGATACCGTTGTAGTCTTAACAGTAAACTATGCCGACT
WCG1289Dai24711 --
Dai22598
TCGAAAACGATCAGATACCGTTGTAGTCTTAACAGTAAACTATGCCGACT
Dai20523 --
Dai17885 --
Dai17553
TCGAAAACGATCAGATACCGTTGTAGTCTTAACAGTAAACTATGCCGACT
Dai19639
TCGAAAACGATCAGATACCGTTGTAGTCTTAACAGTAAACTATGCCGACT
JV0110_48_CZ
TCGAAAACGATCAGATACCGTTGTAGTCTTAACAGTAAACTATGCCGACT
MJ129_04 --
Cui10340P_eminens --
Cui10341P_eminens
TCGAAAACGATCAGATACCGTTGTAGTCTTAACAGTAAACTATGCCGACT
Dai12685P_eminens
TCGAAAACGATCAGATACCGTTGTAGTCTTAACAGTAAACTATGCCGACT
Miettinen_13591Rigidoporus_und --
Dai20868
TCGAAAACGATCAGATACCGTTGTAGTCTTAACAGTAAACTATGCCGACT
Dai20832
TCGAAAACGATCAGATACCGTTGTAGTCTTAACAGTAAACTATGCCGACT
Dai11400
TCGAAAACGATCAGATACCGTTGTAGTCTTAACAGTAAACTATGCCGACT
Dai22472
TCGAAAACGATCAGATACCGTTGTAGTCTTAACAGTAAACTATGCCGACT
1CUI10475 --
1CUI10491 --
HCFC1095Meripilus_robledoi --
MCW702Meripilus_revolubilis --

1704_83_zluty_HaciendaBaru
TCGAAAACGATCAGATACCGTTGTAGTCTTAACAGTAAACTATGCCGACT
Dai9925P_lavendulus --
Dai13587AP_lavendulus
TCGAAAACGATCAGATACCGTTGTAGTCTTAACAGTAAACTATGCCGACT
PDD70600P_longicystidius --
Cui16630 --
FP_135344Meripilus_giganteus --
FP_100460_Sp --
CBS421_48Meripilus_giganteus --
Cui9203
TCGAAAACGATCAGATACCGTTGTAGTCTTAACAGTAAACTATGCCGACT
Cui9202
TCGAAAACGATCAGATACCGTTGTAGTCTTAACAGTAAACTATGCCGACT
TUFC100564Japan --
Russell5913Meripilus_sumstinei --
RP215Meripilus_brasiliensis --
RP200Meripilus_brasiliensis --
JV1712_13J_R_vinctus2_LSUPuert TCG-
AAACGATCAGATACCGTTGTAGTCTTAACAGTAACTATTGCCGACT
Dai10503R_hypobrunneus --
Dai10569Rigidoporus_hypobrunne --
1DAI19451 --
CM108bRigidoporus_hypobrunneus --
1CUI16874 --
FD299Cerrena_unicolor --
KHL_GB_Cerrena_uniclor --
Dai12892Cerrena_albocinnamomea --
Dai12955C_albocinnamomea --
SNUm03110102C_aurantiopora --
NIBRFG0000102423C_aurantiopora --
Dai7359Antrodiella_zonata --
F20080702KCM29C_consors --
F20080208LYW10Cerrena_consors --
Dai7821Antrodiella_zonata --
CFMR_DCL04_31Pseudolagarobasid --
VPB197Pseudolagarobasidium_bel --
CBS115543Pseudolagarobasidium_ --
CBS115544 --
Han405Pseudolagarobasidium_bai --
Han406Pseudolagarobasidium_bai --
MUcc838Spongipellis_delectans --
BRNM686401S_delectans --
OSM_F925S_delectans --

BRNM67093Spongipellis_litschau --
CFMRccFP59199TS_unicolor --
CFMRccFP71791TS_unicolor --
Dai13845P_lischaueri
TCGAAAACGATCAGATACCGTTGTAGTCTTAACAGTAAACTATGCCGACT
Dai20266P_lischaueri
TCGAAAACGATCAGATACCGTTGTAGTCTTAACAGTAAACTATGCCGACT
CFMR_HHB11240Radulodon_america --
RLG6350Radulodon_americanus --
KY415963Radulodon_erikssonii --
HHB9567spRadulodon_casearius --
KRT_Iso_26Radulodon_casearius --
CBS126044Radulodon_erikssonii --
He6183YUNNANENSIS
TCGAAAACGATCAGATACCGTTGTAGTCTTAACAGTAAACTATGCCGACT
Cui17979YUNNANENSIS
TCGAAAACGATCAGATACCGTTGTAGTCTTAACAGTAAACTATGCCGACT
Miettinen2091Junghuhnia_fimbri --
KHL12316S_tenue --
PRM846564S_pachyodon --
SP_Lgt_S_pachyodon --
Ryvarden44669Tyromyces_xuchile --
PW17_171sinuosus --
W53Dai12234 --
HHB4100SpAntella_americana --
W3Dai20901spumeus
TCGAAAACGATCAGATACCGTTGTAGTCTTAACAGTAAACTATGCCGACT
He6736 --
BRNM734877S_spumeus --
BRNM712630S_spumeus --
Dai1723Loweomyces_sibiricus --
W54Cui10009 --
W1Dai20899
TCGAAAACGATCAGATACCGTTGTAGTCTTAACAGTAAACTATGCCGACT
HHB13445Trametes_ochracea --
Dai16222 --
Dai16240 --

JV1310_11SanguinolentusCernys AGGGATCGGGCGAACTCAATCTTATGTGTC-
GCTCGGCACCTTACGAGAA
MJ39_00_SK --
MJ111_04_CZ AGGGATCGGGCGAACTCAATCTTATGTGTC-
GCTCGGCACCTTACGAGAA
JV1610_BOKYsmrk AGGGATCGGGCGAACTCAATCTTATGTGTC-

GCTCGGCACCTTACGAGAA
Dai21030 --
Dai20976P_furcatus AGGGATCGGGCGAACTCAATCTTATGTGTC-
GCTCGGCACCTTACGAGAA
Dai2105 --
Dai2544 --
Dai11313 --
WCG1611Dai26167 AGGGATCGGGCGAACTCAATCTTATGTGTC-
GCTCGGCACCTTACGAGAA
WCG1518Dai25999Physisporinus AGGGATCGGGCGAACTCAATCTTATGTGTC-
GCTCGGCACCTTACGAGAA
TAA15097 --
JV8909_19_CZ --
JV1310_15_P_sanguinolentus2_CZ --
MJ53_02_CZ AGGGATCGGGCGACCTCAATATGATGTGTC-
GCTCGGCACCTTACGAGAA
CLZhao21647P_yunnanensis --
CLZhao21583P_yunnanensis --
Dai22272 AGGGATCGGGCGAACTCAATCTTATGTGTC-
GCTCGGCACCTTACGAGAA
Dai22279 AGGGATCGGGCGAACTCAATCTTATGTGTC-
GCTCGGCACCTTACGAGAA
MJ332_94_CZ --
MJ642_94_CZ_Expallescens --
Dai21060P_vinctus AGGGATCGGGCGAACTCAATCTTATGTGTC-
GCTCGGCACCTTACGAGAA
JV0511_23LRP_pouzarii AGGGATCGGGCGAACTCAATTTTATGTGTC-
GCTCGGCACCTTACGAGAA
JQ409462_R_pouzarii_PRM899856_ --
JV0308_66_WA AGGGATCGGGCGAACTCAATCTTATGTGTC-
GCTCGGCACCTTACGAGAA
JV0309_45_WA_USA AGGGATCGGGCGAACTCAATCTTATGTGTC-
GCTCGGCACCTTACGAGAA
JV0709_83_CA_USA AGGGATCGGGCGAACTCAATCTTATGTGTC-
GCTCGGCACCTTACGAGAA
Dai21043P_pouzarii AGGGATCGGGCGAACTCAATTTTATGTGTC-
GCTCGGCACCTTACGAGAA
MJ144_95_CZ --
JV0909_3_CZ AGGGATCGGGCGAACTCAATTTTATGTGTC-
GCTCGGCACCTTACGAGAA
JV0609_1_K --
Dai20396Physisporinus_castanop
AGGGATCGGGCGAACTCAATTTTATGTGTCTGTTCGGCACCTTACGAGAA
Dai20397Physisporinus_castanop

AGGGATCGGGCGAACTCAATTTTATGTGTCTGTTCGGCACCTTACGAGAA
MJ19_09_SK_Abies --
JV0509_40_J_TN_USA_Betula AGGGATCGGGCGAACTCAATCTTATGTGTC-
GCTCGGCACCTTACGAGAA
JV0808_33crocatus_PA_USAlist AGGGATCGGGCGAACTCAATCTTATGTGTC-
GCTCGGCACCTTACGAGAA
DLL2009_061P_crocatus --
Dai12800P_subcrocatus --
Dai15917P_subcrocatus AGGGATCGGGCGAACTCAATCTTATGTGTC-
GCTCGGCACCTTACGAGAA
Cui16620 --
HCFC1088Meripilus_stillicidior --
MCW590Meripilus_obsrcurus --
MCW722Meripilus_obsrcurus --
Cui9381P_tibeticus AGGGATCGGGCGAACTCAATCTTATGTGTC-
GCTCGGCACCTTACGAGAA
Cui9588P_tibeticus AGGGATCGGGCGAACTCAATCTTATGTGTC-
GCTCGGCACCTTACGAGAA
Va2_Beneschova --
CWU3874_Ukraine_Alnus --
WCG1293Dai24718Physisporinus --
WCG1268Dai24682A AGGGATCGGGCGAACTCAATCTTATGTGTC-
GCTCGGCACCTTACGAGAA
WCG1269Dai24683A AGGGATCGGGCGAACTCAATCTTATGTGTC-
GCTCGGCACCTTACGAGAA
WCG1279Dai24694A AGGGATCGGGCGAACTCAATCTTATGTGTC-
GCTCGGCACCTTACGAGAA
Dai16971 --
ZQY1043Dai26696 --
Doll880 AGGGATCGGGCGAACTCAATCTTATGTGTC-
GCTCGGCACCTTACGAGAA
Doll1000 AGGGATCGGGCGAACTCAATCTTATGTGTC-
GCTCGGCACCTTACGAGAA
1DAI18529 AGGGATCGGGCGAACTCAATCTTATGTGTC-
GCTCGGCACCTTACGAGAA
Dai19535 AGGGATCGGGCGAACTCAATCTTATGTGTC-
GCTCGGCACCTTACGAGAA
1704_79_hnedyVillaLaPaz AGGGATCGGGCGAACTCAATCTTATGTGTC-
GCTCGGCACCTTACGAGAA
F2061 --
1DAI18268 --
1DAI18540A AGGGATCGGGCGAACTCAATCTTATGTGTC-
GCTCGGCACCTTACGAGAA
Dai17695 --

LKY18Dai26373 --
Dai17839P_sulphureus AGGGATCGGGCGAACTCAATCTTATGTGTC-
GCTCGGCACCTTACGAGAA
Dai17841P_sulphureus AGGGATCGGGCGAACTCAATCTTATGTGTC-
GCTCGGCACCTTACGAGAA
Dai19877P_roseus AGGGATCGGGCGAACTCAATCTTATGTGTC-
GCTCGGCACCTTACGAGAA
1508_18_1_Kout --
KP859303_R_vinctus_RP185_BRAZI --
JK1807_15Rigidoporus_sp_Puerto AGGGATCGGGCGAACTCAATCTTATGTGTC-
GCTCGGCACCTTACGAGAA
JV0509_47_J_TN_USA AGGGATCGGGCGAACTCAATCTTATGTGTC-
GCTCGGCACCTTACGAGAA
JV0709_188 --
JV0509_127_PA_USA AGGGATCGGGCGAACTCAATCTTATGTGTC-
GCTCGGCACCTTACGAGAA
JV1009_59_NJ_USA AGGGATCGGGCGAACTCAATCTTATGTGTC-
GCTCGGCACCTTACGAGAA
Dai15497P_crataegi AGGGATCGGGCGAACTCAATCTTATGTGTC-
GCTCGGCACCTTACGAGAA
Dai15499P_crataegi AGGGATCGGGCGAACTCAATCTTATGTGTC-
GCTCGGCACCTTACGAGAA
Cui3266P_cinereus --
WCG1256Dai24690 AGGGATCGGGCGAACTCAATCTTATGTGTC-
GCTCGGCACCTTACGAGAA
1DAI17581 --
WCG1255Dai24688 AGGGATCGGGCGAACTCAATCTTATGTGTC-
GCTCGGCACCTTACGAGAA
Dai22427 --
MV690Meripilus_concrescens --
MV513Meripilus_galapagensis --
Dai19793 AGGGATCGGGCGAACTCAATCTTATGTGTC-
GCTCGGCACCTTACGAGAA
OQ553780P_tamilnaduensis --
OQ553779P_tamilnaduensis --
A164FB3Meripilus_giganteus --
JV1407_36_Vinctus_Meandrica
AGGGATCGGGCGAACTCAATTTTATGTGTCGCTCTGGCACCTTACGAGAA
1807_3K_Rigidoporus_PuertoRico AGGGATCGGGCGAACTCAATTTTATGTGTC-
GCTCGGCACCTTACGAGAA
Cui16903P_vinctus AGGGATCGGGCGAACTCAATTTTATGTGTC-
GCTCGGCACCTTACGAGAA
JV1008_18R_Lineatus AGGGATCGGGCGAACTCAATTTTATGTGTC-
GCTCGGCACCTTACGAGAA

JV1407_37_1_Vinctus_Carara AGGGATCGGGCGAACTCAATTTATGTGTC-
GCTCGGCACCTTACGAGAA
Dai17986P_lineatus AGGGATCGGGCGAACTCAATTTATGTGTC-
GCTCGGCACCTTACGAGAA
Dai18281 AGGGATCGGGCGAACTCAATTTATGTGTC-
GCTCGGCACCTTACGAGAA
1DAI19796 AGGGATCGGGCGAACTCAATTTATGTGTC-
GCTCGGCACCTTACGAGAA
ZQY797Dai25241 AGGGATCGGGCGAACTCAATTTATGTGTC-
GCTCGGCACCTTACGAGAA
WCG1289Dai24711 ---
Dai22598 AGGGATCGGGCGAACTCAATTTATGTGTC-
GCTCGGCACCTTACGAGAA
Dai20523 ---
Dai17885 ---
Dai17553 AGGGATCGGGCGAACTCAATTTATGTGTC-
GCTCGGCACCTTACGAGAA
Dai19639 AGGGATCGGGCGAACTCAATTTATGTGTC-
GCTCGGCACCTTACGAGAA
JV0110_48_CZ AGAGATCGGGCGAACTCAATTTAATGTGTC-
GCTCGGCATCTTACGAGAA
MJ129_04 ---
Cui10340P_eminens ---
Cui10341P_eminens AGAGATCGGGCGAACTCAATTTAATGTGTC-
GCTCGGCATCTTACGAGAA
Dai12685P_eminens AGAGATCGGGCGAACTCAATTTAATGTGTC-
GCTCGGCATCTTACGAGAA
Miettinen_13591Rigidoporus_und ---
Dai20868
AGGGATCGGGCGAACTCAATTTAATGTGTCTGCTCGGCACCTTACGAGAA
Dai20832 AGGGATCGGGCGAACTCAATTTAATGTGTC-
GCTCGGCACCTTACGAGAA
Dai11400 AGGGATCGGGCGAACTCAATTTAATGTGTT-
GCTCGGCACCTTACGAGAA
Dai22472 AGGGATCGGGCAAACTCAATTTAATGTGTT-
GCTCGGCACCTTACGAGAA
1CUI10475 ---
1CUI10491 ---
HCFC1095Meripilus_robledoi ---
MCW702Meripilus_revolubilis ---
1704_83_zluty_HaciendaBaru AGGGATCGGGCGAACTCAATTTGATGTGTC-
GCTCGGCACCTTACGAGAA
Dai9925P_lavendulus ---
Dai13587AP_lavendulus AGGGATCGGGCGAACTCAATTTATGTGTC-

GCTCGGCACCTTACGAGAA
PDD70600P_longicystidius --
Cui16630 --
FP_135344Meripilus_giganteus --
FP_100460_Sp --
CBS421_48Meripilus_giganteus --
Cui9203 AGGGATCGGGCGAACTCAATCTTATGTGTC-
GCTCGGCACCTTACGAGAA
Cui9202 AGGGATCGGGCGAACTCAATCTTATGTGTC-
GCTCGGCACCTTACGAGAA
TUFC100564Japan --
Russell5913Meripilus_sumstinei --
RP215Meripilus_brasiliensis --
RP200Meripilus_brasiliensis --
JV1712_13J_R_vinctus2_LSUPuert AGGGATCGGC--
Dai10503R_hypobrunneus --
Dai10569Rigidoporus_hypobrunne --
1DAI19451 --
CM108bRigidoporus_hypobrunneus --
1CUI16874 --
FD299Cerrena_unicolor --
KHL_GB_Cerrena_uniclor --
Dai12892Cerrena_albocinnamomea --
Dai12955C_albocinnamomea --
SNUm03110102C_aurantiopora --
NIBRFG0000102423C_aurantiopora --
Dai7359Antrodiella_zonata --
F20080702KCM29C_consors --
F20080208LYW10Cerrena_consors --
Dai7821Antrodiella_zonata --
CFMR_DCL04_31Pseudolagarobasid --
VPB197Pseudolagarobasidium_bel --
CBS115543Pseudolagarobasidium_ --
CBS115544 --
Han405Pseudolagarobasidium_bai --
Han406Pseudolagarobasidium_bai --
MUcc838Spongipellis_delectans --
BRNM686401S_delectans --
OSM_F925S_delectans --
BRNM67093Spongipellis_litschau --
CFMRccFP59199TS_unicolor --
CFMRccFP71791TS_unicolor --
Dai13845P_lischaueri AGGGATCGGGCGATCTCAAACTTATGTGTC-
GCTCGGCACCTTACGAGAA

Dai20266P_lischaueri AGGGATCGGGCGATCTCAAACTTATGTGTC-GCTCGGCACCTTACGAGAA
CFMR_HHB11240Radulodon_america --
RLG6350Radulodon_americanus --
KY415963Radulodon_erikssonii --
HHB9567spRadulodon_casearius --
KRT_Iso_26Radulodon_casearius --
CBS126044Radulodon_erikssonii --
He6183YUNNANENSIS AGGGATCGGGGAATCTCACATTTATGTGTT-CCTCGGCACCTTACGAGAA
Cui17979YUNNANENSIS AGGGATCGGGGAATCTCACATTTATGTGTT-CCTCGGCACCTTACGAGAA
Miettinen2091Junghuhnia_fimbri --
KHL12316S_tenue --
PRM846564S_pachyodon --
SP_Lgt_S_pachyodon --
Ryvarden44669Tyromyces_xuchile --
PW17_171sinuosus --
W53Dai12234 --
HHB4100SpAntella_americana --
W3Dai20901spumeus AGGGATCGGGCGAACTCAATTTTATGTGTC-GCTCGGCACCTTACGAGAA
He6736 --
BRNM734877S_spumeus --
BRNM712630S_spumeus --
Dai1723Loweomyces_sibiricus --
W54Cui10009 --
W1Dai20899 AGGGATCGGGCGAACTCAATTTTATGTGTC-GCTCGGCACCTTACGAGAA
HHB13445Trametes_ochracea --
Dai16222 --
Dai16240 --

JV1310_11SanguinolentusCernys ATCAAAGT
MJ39_00_SK --------
MJ111_04_CZ ATCAAAGT
JV1610_BOKYsmrk ATCAAAGT
Dai21030 --------
Dai20976P_furcatus ATCAAAGT
Dai2105 --------
Dai2544 --------
Dai11313 --------
WCG1611Dai26167 ATCAAAGT
WCG1518Dai25999Physisporinus ATCAAAGT

TAA15097	--------
JV8909_19_CZ	--------
JV1310_15_P_sanguinolentus2_CZ	--------
MJ53_02_CZ	ATCAAAGT
CLZhao21647P_yunnanensis	--------
CLZhao21583P_yunnanensis	--------
Dai22272	ATCAAAGT
Dai22279	ATCAAAGT
MJ332_94_CZ	--------
MJ642_94_CZ_Expallescens	--------
Dai21060P_vinctus	ATCAAAGT
JV0511_23LRP_pouzarii	ATCAAAGT
JQ409462_R_pouzarii_PRM899856_	--------
JV0308_66_WA	ATCAAAGT
JV0309_45_WA_USA	ATCAAAGT
JV0709_83_CA_USA	ATCAAAGT
Dai21043P_pouzarii	ATCAAAGT
MJ144_95_CZ	--------
JV0909_3_CZ	ATCAAAGT
JV0609_1_K	--------
Dai20396Physisporinus_castanop	ATCAAAGT
Dai20397Physisporinus_castanop	ATCAAAGT
MJ19_09_SK_Abies	--------
JV0509_40_J_TN_USA_Betula	ATCAAAGT
JV0808_33crocatus_PA_USAlist	ATCAAAGT
DLL2009_061P_crocatus	--------
Dai12800P_subcrocatus	--------
Dai15917P_subcrocatus	ATCAAAGT
Cui16620	--------
HCFC1088Meripilus_stillicidior	--------
MCW590Meripilus_obsrcurus	--------
MCW722Meripilus_obsrcurus	--------
Cui9381P_tibeticus	ATCAAAGT
Cui9588P_tibeticus	ATCAAAGT
Va2_Beneschova	--------
CWU3874_Ukraine_Alnus	--------
WCG1293Dai24718Physisporinus	--------
WCG1268Dai24682A	ATCAAAGT
WCG1269Dai24683A	ATCAAAGT
WCG1279Dai24694A	ATCAAAGT
Dai16971	--------
ZQY1043Dai26696	--------
Doll880	ATCAAAGT
Doll1000	ATCAAAGT

1DAI18529	ATCAAAGT
Dai19535	ATCAAAGT
1704_79_hnedyVillaLaPaz	ATCAAAGT
F2061	--------
1DAI18268	--------
1DAI18540A	ATCAAAGT
Dai17695	--------
LKY18Dai26373	--------
Dai17839P_sulphureus	ATCAAAGT
Dai17841P_sulphureus	ATCAAAGT
Dai19877P_roseus	ATCAAAGT
1508_18_1_Kout	--------
KP859303_R_vinctus_RP185_BRAZI	--------
JK1807_15Rigidoporus_sp_Puerto	ATCAAAGT
JV0509_47_J_TN_USA	ATCAAAGT
JV0709_188	--------
JV0509_127_PA_USA	ATCAAAGT
JV1009_59_NJ_USA	ATCAAAGT
Dai15497P_crataegi	ATCAAAGT
Dai15499P_crataegi	ATCAAAGT
Cui3266P_cinereus	--------
WCG1256Dai24690	ATCAAAGT
1DAI17581	--------
WCG1255Dai24688	ATCAAAGT
Dai22427	--------
MV690Meripilus_concrescens	--------
MV513Meripilus_galapagensis	--------
Dai19793	ATCAAAGT
OQ553780P_tamilnaduensis	--------
OQ553779P_tamilnaduensis	--------
A164FB3Meripilus_giganteus	--------
JV1407_36_Vinctus_Meandrica	ATCAAAGT
1807_3K_Rigidoporus_PuertoRico	ATCAAAGT
Cui16903P_vinctus	ATCAAAGT
JV1008_18R_Lineatus	ATCAAAGT
JV1407_37_1_Vinctus_Carara	ATCAAAGT
Dai17986P_lineatus	ATCAAAGT
Dai18281	ATCAAAGT
1DAI19796	ATCAAAGT
ZQY797Dai25241	ATCAAAGT
WCG1289Dai24711	--------
Dai22598	ATCAAAGT
Dai20523	--------
Dai17885	--------

Dai17553 ATCAAAGT
Dai19639 ATCAAAGT
JV0110_48_CZ ATCAAAGT
MJ129_04 --------
Cui10340P_eminens --------
Cui10341P_eminens ATCAAAGT
Dai12685P_eminens ATCAAAGT
Miettinen_13591Rigidoporus_und --------
Dai20868 ATCAAAGT
Dai20832 ATCAAAGT
Dai11400 ATCAAAGT
Dai22472 ATCAAAGT
1CUI10475 --------
1CUI10491 --------
HCFC1095Meripilus_robledoi --------
MCW702Meripilus_revolubilis --------
1704_83_zluty_HaciendaBaru ATCAAAGT
Dai9925P_lavendulus --------
Dai13587AP_lavendulus ATCAAAGT
PDD70600P_longicystidius --------
Cui16630 --------
FP_135344Meripilus_giganteus --------
FP_100460_Sp --------
CBS421_48Meripilus_giganteus --------
Cui9203 ATCAAAGT
Cui9202 ATCAAAGT
TUFC100564Japan --------
Russell5913Meripilus_sumstinei --------
RP215Meripilus_brasiliensis --------
RP200Meripilus_brasiliensis --------
JV1712_13J_R_vinctus2_LSUPuert --------
Dai10503R_hypobrunneus --------
Dai10569Rigidoporus_hypobrunne --------
1DAI19451 --------
CM108bRigidoporus_hypobrunneus --------
1CUI16874 --------
FD299Cerrena_unicolor --------
KHL_GB_Cerrena_uniclor --------
Dai12892Cerrena_albocinnamomea --------
Dai12955C_albocinnamomea --------
SNUm03110102C_aurantiopora --------
NIBRFG0000102423C_aurantiopora --------
Dai7359Antrodiella_zonata --------
F20080702KCM29C_consors --------

F20080208LYW10Cerrena_consors	--------
Dai7821Antrodiella_zonata	--------
CFMR_DCL04_31Pseudolagarobasid	--------
VPB197Pseudolagarobasidium_bel	--------
CBS115543Pseudolagarobasidium_	--------
CBS115544	--------
Han405Pseudolagarobasidium_bai	--------
Han406Pseudolagarobasidium_bai	--------
MUcc838Spongipellis_delectans	--------
BRNM686401S_delectans	--------
OSM_F925S_delectans	--------
BRNM67093Spongipellis_litschau	--------
CFMRccFP59199TS_unicolor	--------
CFMRccFP71791TS_unicolor	--------
Dai13845P_lischaueri	ATCAAAGT
Dai20266P_lischaueri	ATC-----
CFMR_HHB11240Radulodon_america	--------
RLG6350Radulodon_americanus	--------
KY415963Radulodon_erikssonii	--------
HHB9567spRadulodon_casearius	--------
KRT_Iso_26Radulodon_casearius	--------
CBS126044Radulodon_erikssonii	--------
He6183YUNNANENSIS	ATCAAAGT
Cui17979YUNNANENSIS	ATCAAAGT
Miettinen2091Junghuhnia_fimbri	--------
KHL12316S_tenue	--------
PRM846564S_pachyodon	--------
SP_Lgt_S_pachyodon	--------
Ryvarden44669Tyromyces_xuchile	--------
PW17_171sinuosus	--------
W53Dai12234	--------
HHB4100SpAntella_americana	--------
W3Dai20901spumeus	ATCAAAGT
He6736	--------
BRNM734877S_spumeus	--------
BRNM712630S_spumeus	--------
Dai1723Loweomyces_sibiricus	--------
W54Cui10009	--------
W1Dai20899	ATCAAAGT
HHB13445Trametes_ochracea	--------
Dai16222	--------
Dai16240	--------
